# YqfB protein from *Escherichia coli*: an atypical amidohydrolase active towards *N*^4^-acylcytosine derivatives

**DOI:** 10.1038/s41598-020-57664-w

**Published:** 2020-01-21

**Authors:** Rūta Stanislauskienė, Audrius Laurynėnas, Rasa Rutkienė, Agota Aučynaitė, Daiva Tauraitė, Rita Meškienė, Nina Urbelienė, Algirdas Kaupinis, Mindaugas Valius, Laura Kaliniene, Rolandas Meškys

**Affiliations:** 1 Department of Molecular Microbiology and Biotechnology, Institute of Biochemistry, Life Sciences Center, Vilnius University, Sauletekio al. 7, Vilnius, LT-10257 Lithuania; 2 Department of Bioanalysis, Institute of Biochemistry, Life Sciences Center, Vilnius University, Sauletekio al. 7, Vilnius, LT-10257 Lithuania; 3 Proteomics Centre, Institute of Biochemistry, Life Sciences Center, Vilnius University, Sauletekio al. 7, Vilnius, LT-10257 Lithuania

**Keywords:** Enzyme mechanisms, Hydrolases

## Abstract

Human activating signal cointegrator homology (ASCH) domain-containing proteins are widespread and diverse but, at present, the vast majority of those proteins have no function assigned to them. This study demonstrates that the 103-amino acid *Escherichia coli* protein YqfB, previously identified as hypothetical, is a unique ASCH domain-containing amidohydrolase responsible for the catabolism of *N*^4^-acetylcytidine (ac4C). YqfB has several interesting and unique features: i) it is the smallest monomeric amidohydrolase described to date, ii) it is active towards structurally different *N*^4^-acylated cytosines/cytidines, and iii) it has a high specificity for these substrates (k_cat_/K_m_ up to 2.8 × 10^6^ M^−1^ s^−1^). Moreover, our results suggest that YqfB contains a unique Thr-Lys-Glu catalytic triad, and Arg acting as an oxyanion hole. The mutant lacking the *yqfB* gene retains the ability to grow, albeit poorly, on *N*^4^-acetylcytosine as a source of uracil, suggesting that an alternative route for the utilization of this compound exists in *E. coli*. Overall, YqfB ability to hydrolyse various *N*^4^-acylated cytosines and cytidines not only sheds light on the long-standing mystery of how ac4C is catabolized in bacteria, but also expands our knowledge of the structural diversity within the active sites of amidohydrolases.

## Introduction

Human activating signal cointegrator 1 (ASC-1), otherwise known as the thyroid hormone receptor interactor protein 4 (ASC-1/TRIP4), interacts with a wide range of unrelated transcription factors to facilitate nuclear receptors-mediated transcription. It also plays a pivotal role in the transactivation of serum response factor (SRF), activating protein 1 (AP-1), and nuclear factor κB (NF-κB)^1,2^. In 2006 it was shown that the C-terminal domain of ASC-1 defines a large ASC-1 homology (ASCH) domain superfamily^2^. To date, the ASCH-containing proteins have been reported for a wide range of organisms representing all three kingdoms of life, and have also been found in viruses^3^. Although it has long been suggested that this domain of ~110 residues may be responsible for RNA binding during transcription coactivation, RNA processing, and regulation of translation, the vast majority of ASCH proteins are small (~140 residues) hypothetical proteins, which at present have no function assigned to them^2^. One such protein is the product of the *yqf*B gene in *Escherichia coli*. Herein we demonstrate that the 103-amino acid YqfB is a unique monomeric amidohydrolase responsible for the catabolism of the modified nucleoside, *N*^4^-acetylcytidine (ac4C), in *E. coli*.

More than 160 of differently modified nucleotides play a crucial role in various biological processes^1,2,4,5^. The biosynthetic pathways of many modified bases, nucleosides and nucleotides are well understood^5,6^, but the catabolism or salvage of those compounds are scarcely studied. Just like the ASCH proteins, the modified nucleoside ac4C is found in organisms within all three domains of life^4–9^. It prevents misreading of AUA isoleucine codons during protein synthesis^10^ and is important for tRNA stability^11^. Recently, it has been shown that ac4C plays a role in priming and activation of the NLRC4 inflammasome, which in turn induces interleukin-1β (IL-1β) production^12^. Various studies have demonstrated that different tRNA- and rRNA-specific acetyltransferases are responsible for the formation of ac4C^13–17^, yet its degradation and/or recycling remains to be clarified. Here, for the first time, we demonstrate that in *E. coli* the ASCH domain-containing protein YqfB, which seems to have a unique Thr-Lys-Glu catalytic triad, catalyses the hydrolysis of ac4C.

## Results and Discussion

### Enzyme detection and characterisation

**Figure 1:**
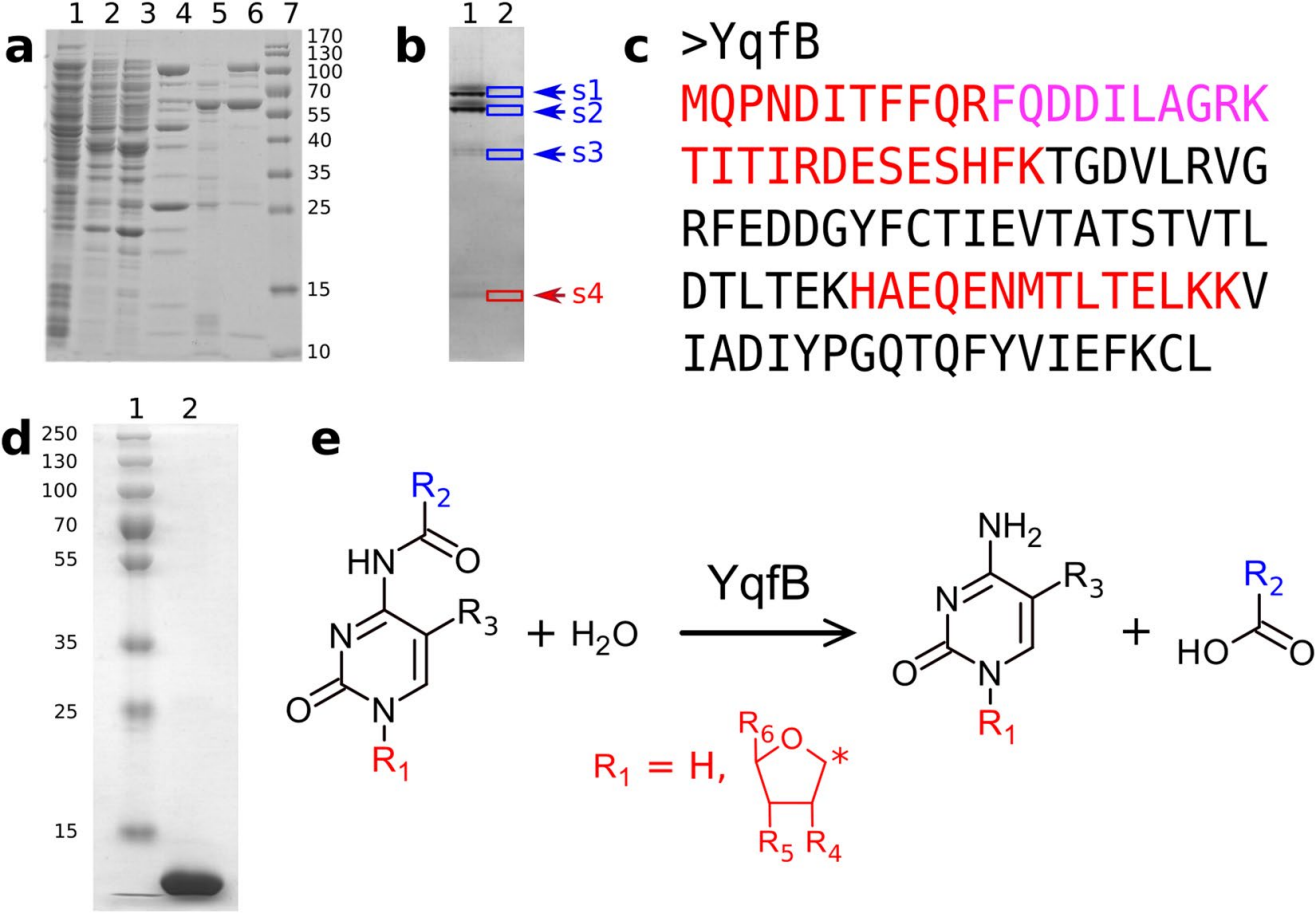
Purification and analysis of YqfB from *E. coli* DH10B. (**a**) SDS-PAGE (14%) analysis of the purification steps; lane 1 – cell-free extract, lane 2 – Q Sepharose, lane 3 – ANX Sepharose, lane 4 – Phenyl Sepharose, lane 5 – Resource ISO, lane 6 – Source 15Q, lane 7 – molecular mass marker. (**b**) Native PAGE (14%) analysis of the sample after Source 15Q chromatography (a, lane 6); lane 1 – proteins stained with Coomassie Brilliant Blue R250 (original gel is provided in Supplementary Fig. 10A), lane 2 – the corresponding unstained gel. The gel slices (s1–s4) were incubated with ac4C and the reaction products were analysed by TLC (Supplementary Fig. 1). The gel slice (s4) with hydrolase activity was applied for MS/MS analysis. (**c**) The amino acid sequence of the identified protein. The longest peptide sequences detected by MS/MS are coloured in red and purple. (**d**) SDS-PAGE analysis of the purified recombinant YqfB harbouring a C-terminal His_6_ tag (original gel is provided in Supplementary Fig. 17B); lane 1 – molecular mass marker, lane 2–13.5 µg of YqfB. (**e**) Scheme of the reaction catalysed by YqfB. An asterisk marks the carbon atom to which the group was linked. The structures of all tested substrates are listed in Table 1.

To test if an enzyme active towards ac4C exists in *Escherichia coli*, cell-free extracts of several *E. coli* strains were analysed. We observed that both the ac4C and *N*^4^-acetylcytosine were readily converted to cytidine and cytosine, respectively, in cell-free extracts of *E. coli* DH5alpha, DH10B, and BL21(DE3). To identify the enzyme involved in hydrolysis, we purified the active protein from *E. coli* DH10B cells by the application of several chromatographic steps (Fig. 1a). The MS/MS analysis of the active protein (~12 kDa) excised from the native gel (Fig. 1b, Supplementary Table 2) identified the ASHC superfamily protein (Fig. 1c) encoded by the *yqfB* gene. Although the 3D structure of YqfB has been published previously^17^, no data on its biological activity is available to date.

To analyse the catalytic properties of YqfB in more detail, the *E. coli yqfB* gene was cloned and the recombinant proteins without any tag as well as those with a His_6_ tag at either N- or C-terminus were purified to homogeneity (Fig. 1d). An *E. coli* BL21(DE3) *yqfB::Km* strain was used for YqfB production, with no interference from the chromosomal gene. Gel chromatography revealed that YqfB existed as a monomer, under tested conditions (Supplementary Fig. 2). YqfB was most active at pH 8.0 and preferred temperatures below 20 °C (Supplementary Figs. 3 and 4). Based on the substrate range of YqfB-catalysed reactions (Fig. 1e), the enzyme is active towards a wide range of *N*^4^-acylcytosines/cytidines, and the bulky groups at 5′ position are tolerated (Table 1, Supplementary Figs. 5–10). The highest k_cat_/K_m_ value (Table 2, examples of kinetic traces Supplementary Fig. 16) suggests that ac4C is likely the natural substrate of YqfB. Inhibition with the reaction product was also observed. However, the catalytic activity of YqfB was unaffected by known inhibitors of amidohydrolases: PMSF, *p*-hydroxymercuribenzoate (up to 2 mM), *p*-chloromercuribenzoate (up to 1 mM), and EDTA (up to 5 mM). Since EDTA had no influence on the reaction rate, we presumed that bivalent metal ions do not participate in the deacetylation reaction.

**Table 1:** A list of compounds tested as substrates for YqfB.

	
**Compound**	**R**_**2**_	**R**_**3**_				**Hydrolysis by YqfB**
*N*^4^-acetylcytosine (**1**)	CH_3_	H				Yes (UV, TLC, HPLC-MS)
*N*^4^-acetyl-5-fluorocytosine (**2**)	CH_3_	F				Yes (UV, TLC, HPLC-MS)
	
**Compound**	**R**_**2**_	**R**_**3**_	**R**_**4**_	**R**_**5**_	**R**_**6**_	**Hydrolysis by YqfB**
*N*^4^-acetylcytidine (**3**)	CH_3_	H	OH	OH	CH_2_OH	Yes (UV, TLC)
*N*^4^-benzoylcytidine (**4**)		H	OH	OH	CH_2_OH	Yes (UV, TLC, HPLC-MS)
*N*^4^-acetyl-2′,3′,5′-tri-*O*-acetylcytidine (**5**)	CH_3_	H	OCOCH_3_	OCOCH_3_	OCOCH_3_	No (TLC)
*N*^4^-acetyl-2′-deoxycytidine (**6**)	CH_3_	H	H	OH	CH_2_OH	Yes (UV, TLC)
*N*^4^-isobutyryl-2′-deoxycytidine (**7**)	CH(CH_3_)_2_	H	H	OH	CH_2_OH	Yes (UV, TLC)
*N*^4^-hexanoyl-2′-deoxycytidine (**8**)	C_5_H_11_	H	H	OH	CH_2_OH	Yes (UV, TLC)
*N*^4^-benzoyl-2′-deoxycytidine (**9**)		H	H	OH	CH_2_OH	Yes (UV, TLC)
*N*^4^-nicotinoyl-2′-deoxycytidine (**10**)		H	H	OH	CH_2_OH	Yes (TLC, HPLC-MS)
*N*^4^-(2-acetyl-benzoyl)-2′-deoxycytidine (**11**)		H	H	OH	CH_2_OH	No (TLC)
*N*^4^-(3-acetyl-benzoyl)-2′-deoxycytidine (**12**)		H	H	OH	CH_2_OH	Yes (TLC, HPLC-MS)
*N*^4^-(4-acetyl-benzoyl)-2′-deoxycytidine (**13**)		H	H	OH	CH_2_OH	Yes (TLC)
*N*^4^-(2-benzoyl-benzoyl)-2′-deoxycytidine (**14**)		H	H	OH	CH_2_OH	No (TLC)
*N*^4^-(3-benzoyl-benzoyl)-2′-deoxycytidine (**15**)		H	H	OH	CH_2_OH	No (TLC)
*N*^4^-(4-benzoyl-benzoyl)-2′-deoxycytidine (**16**)		H	H	OH	CH_2_OH	Yes (TLC)
*N*^4^-acetyl-2′-deoxycytidine-5′-triphosphate (**17**)	CH_3_	H	H	OH	CH_2_OP_3_O_9_^4−^	Yes (TLC)
*N*^4^-hexanoyl-2′-deoxycytidine-5′-triphosphate (**18**)	C_5_H_11_	H	H	OH	CH_2_OP_3_O_9_^4−^	Yes (UV, TLC)
*N*^4^-benzoyl-2′-deoxycytidine-5′-triphosphate (**19**)		H	H	OH	CH_2_OP_3_O_9_^4−^	Yes (TLC)
*N*^4^-nicotinoyl-2′-deoxycytidine-5′-triphosphate (**20**)		H	H	OH	CH_2_OP_3_O_9_^4−^	Yes (TLC)
*N*^4^-(2-acetyl-benzoyl)-2′-deoxycytidine-5′-triphosphate (**21**)		H	H	OH	CH_2_OP_3_O_9_^4−^	No (TLC)
*N*^4^-(3-acetyl-benzoyl)-2′-deoxycytidine-5′-triphosphate (**22**)		H	H	OH	CH_2_OP_3_O_9_^4−^	Yes (TLC)
*N*^4^-(4-acetyl-benzoyl)-2′-deoxycytidine-5′-triphosphate (**23**)		H	H	OH	CH_2_OP_3_O_9_^4–^	Yes (TLC)
*N*^4^-(2-benzoyl-benzoyl)-2′-deoxycytidine-5′-triphosphate (**24**)		H	H	OH	CH_2_OP_3_O_9_^4–^	No (TLC)
*N*^4^-(3-benzoyl-benzoyl)-2′-deoxycytidine-5′-triphosphate (**25**)		H	H	OH	CH_2_OP_3_O_9_^4–^	No (TLC)
*N*^4^-(4-benzoyl-benzoyl)-2′-deoxycytidine-5′-triphosphate (**26**)		H	H	OH	CH_2_OP_3_O_9_^4−^	Yes (TLC)
*N*^4^-acetyl-2′-deoxy-5′-*O*-DMT-cytidine (**27**)	CH_3_	H	H	OH	CH_2_O-4,4′-dimethoxy-trity	Yes (TLC)
Capecitabine (**28**)	OC_5_H_11_	F	OH	OH	CH_3_	Yes (UV, TLC)
*N*^2^-acetylisocytosine (**29**)						No (UV, TLC)
2-Acetylaminopyridine (**30**)						No (UV, TLC)
*p*-Nitroacetanilide (**31**)						No (UV, TLC)
*p*-Nitrophenyl acetate (**32**)						No (UV, TLC)

UV – spectrophotometric assay, TLC – activity analysed by thin layer chromatography, HPLC-MS – analysis using a high performance liquid chromatography system and a mass spectrometer. An asterisk marks the carbon atom to which the group was linked.

**Table 2:** Catalytic parameters of YqfB with a C-terminal His_6_ tag.

Substrate	K_m_ (M)	k_cat_ (s^-1^)	K_i_ (M)	k_cat_/K_m_ (M^−1^ s^−1^)
3^a^	(7.0 ± 0.1) × 10^−5^	196 ± 1	(7.7 ± 0.2) × 10^−5^	(2.8 ± 0.3) × 10^6^
3^b^	(7.0 ± 0.1) × 10^−5^	154 ± 3	(1.3 ± 0.1) × 10^−4^	(2.5 ± 0.2) × 10^6^
3	(6.2 ± 0.1) × 10^−5^	157 ± 1	(1.3 ± 0.2) × 10^−4^	(2.2 ± 0.1) × 10^6^
1	(7.0 ± 0.2) × 10^−5^	70 ± 3	(7 ± 3) × 10^−4^	(1 ± 0.2) × 10^5^
2	(2.1 ± 0.2) × 10^−3^	90 ± 7	(4 ± 4) × 10^−3^	(4.0 ± 0.3) × 10^4^
4	(5.1 ± 0.03) × 10^−3^	1.8 ± 0.1	(1.50 ± 0.07) × 10^−4^	(3.5 ± 0.7) × 10^3^
6	(4.00 ± 0.04) × 10^−4^	101 ± 1	N.D.	(2.30 ± 0.02) × 10^5^
7	(1.9 ± 0.08) × 10^−3^	75 ± 3	(1.60 ± 0.02) × 10^−3^	(3.75 ± 0.06) × 10^4^
8	(1.4 ± 0.1) × 10^−4^	24 ± 1	(2.80 ± 0.03) × 10^−3^	(7.2 ± 0.6) × 10^4^
9	(3.00 ± 0.01) × 10^−4^	0.07 ± 0.01	(8.5 ± 20) × 10^−7^	(2.3 ± 0.1) × 10^2^
18	N.D.	N.D.	N.D.	15 ± 0.1
28	(7.0 ± 0.2) × 10^−4^	0.016 ± 0.003	N.D.	22 ± 0.1

^a^the recombinant wild-type YqfB, ^b^the recombinant YqfB with an N-terminal His_6_ tag. N.D. – not determined. Activity was measured in 50 mM potassium phosphate buffer, pH 8.0, at 22 °C. Rate constants were calculated from experiments with at least three different initial concentrations of substrate, using statistical significance value of 0.05.

**Figure 2:**
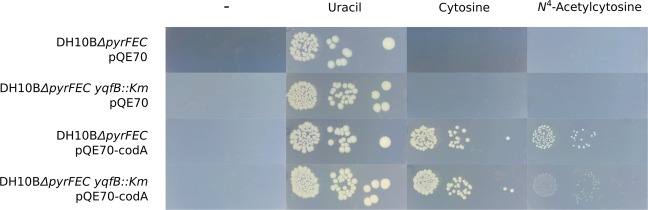
Growth of *E. coli* DH10B mutants on M9 agar medium supplemented with a source of uracil (listed on top). The cells were incubated at 37 °C for 72 hours. DH10B*ΔpyrFEC* - an *E. coli* mutant lacking the pyrimidine *de novo* biosynthesis and cytosine deaminase^18^; *DH10BΔpyrFEC yqfB::Km* - DH10B*ΔpyrFEC* mutant with the *yqfB* gene substituted for the kanamycin-resistance cassette; pQE70 - bacterial vector for protein expression (ampicillin resistance, empty vector used as negative control); pQE70-codA – pQE70 vector encoding the *E. coli* BL21(DE-3) cytosine deaminase CodA^39^.

To elucidate the function of YqfB *in vivo*, the *yqfB* gene was replaced with a kanamycin resistance cassette in the uracil auxotrophic strain *E. coli* DH10B*ΔpyrFEC*^18^. DH10B*ΔpyrFEC* and DH10B*ΔpyrFEC yqfB::Km* mutants, lacking cytosine deaminase CodA^19^, could not survive without uracil in the growth medium, and did not accept cytosine or *N*^4^-acetylcytosine as a source of uracil (Fig. 2). When these mutants were expressing either CodA or CodA and YqfB, their growth was observed on both cytosine and *N*^4^-acetylcytosine, suggesting that both compounds were converted to uracil. Although the mutant lacking the *yqfB* gene was capable of growing on *N*^4^-acetylcytosine as a source of uracil, the growth was poor compared with that of the strain with intact *yqfB* gene. These results clearly show that YqfB participates in the catabolism of *N*^4^-acetylcytosine, however an alternative route for the utilization of this compound may exist in *E. coli*.

### Analysis of the catalytic amino acids

**Figure 3:**
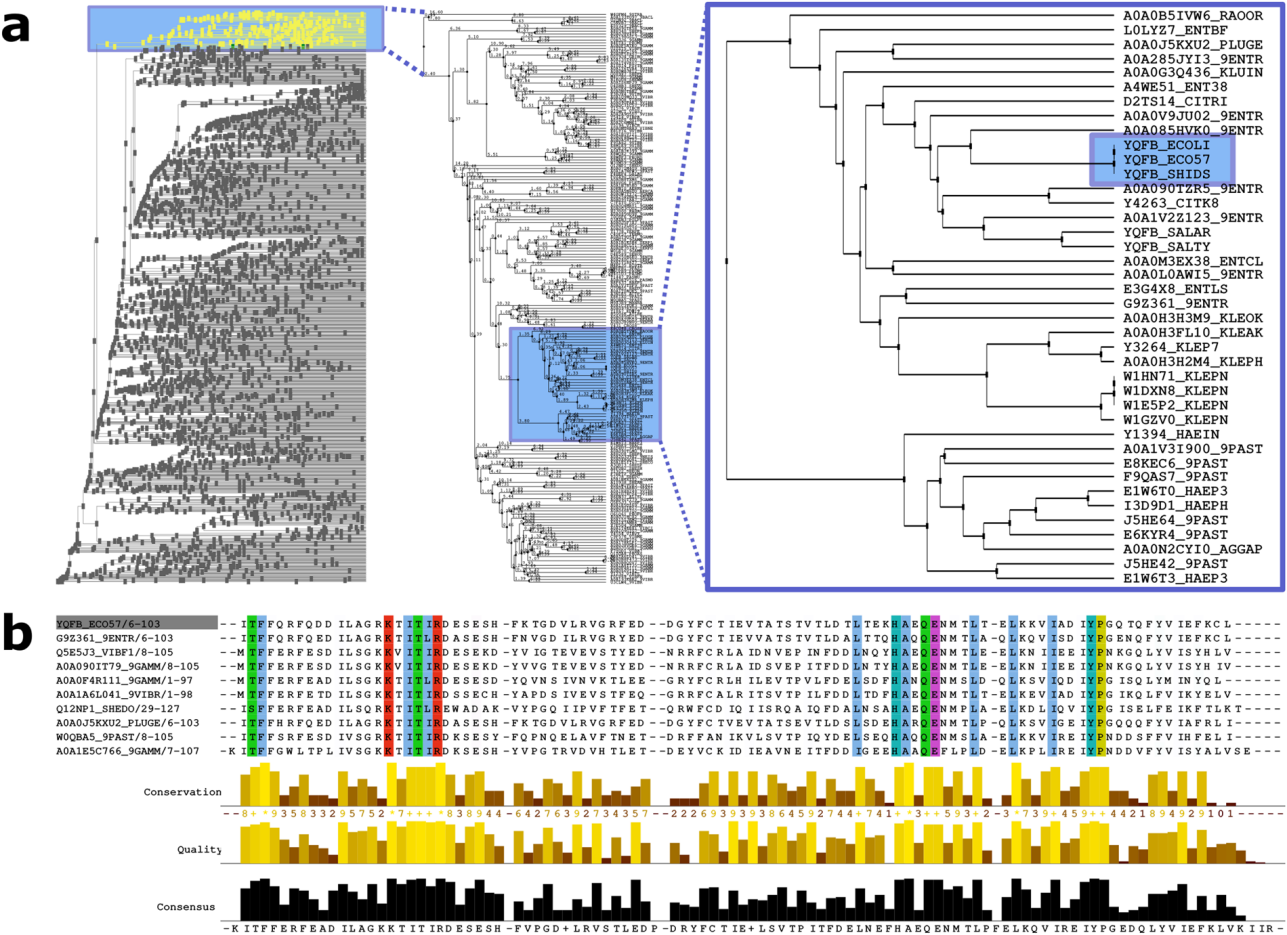
Identification of conserved amino acid residues in the YqfB-like proteins. (**a**) The phylogenetic tree of 3344 protein sequences from the ASCH superfamily; YqfB-containing branch is expanded on the right. (**b**) A representative alignment of YqfB-like proteins; 100% conserved amino acids are coloured. The full alignment is presented in Supplementary Fig. 11.

Next, to identify amino acids relevant for catalysis, we analysed multiple sequence alignments for the ASCH family. This allowed the identification of sequences closely related to YqfB, representing a separate branch in the phylogenetic tree (Supplementary Fig. 11). These sequences (Fig. 3) were realigned using MAFFT method, and the new alignment was further analysed. From the alignment we identified a set of 100% conserved amino acids (namely, Lys21, Thr24, Arg26, Asp27, His70, Glu74, and Tyr89) that were each individually replaced with alanine. The soluble mutant proteins were obtained, purified (Supplementary Fig. 12), and their catalytic properties were investigated (Table 3, Supplementary Fig. 16). The enzyme variants Arg26Ala, Thr24Ala and Lys21Ala were catalytically inactive. With ac4C and *N*^4^-acetylcytosine, the turnover number of the Glu74Ala mutant was found 1000-fold and 100-fold lower, respectively, than that of the wild-type enzyme. More than tenfold and threefold decrease in activity towards both substrates was observed for His70Ala and Tyr89Ala mutants, respectively (Table 3).

**Table 3:** Rate constants for YqfB mutants.

YqfB	Substrate	K_m_ (M)	k_cat_ (s^−1^)	K_i_ (M)	k_cat_/K_m_ (M^−1^ s^−1^)
WT	**3**	(6.2 ± 0.1) × 10^−5^	157 ± 1	(1.3 ± 0.2) × 10^−4^	(2.2 ± 0.1) × 10^6^
WT	**1**	(7.0 ± 0.2) × 10^−5^	70 ± 3	(7 ± 3) × 10^−4^	(1.0 ± 0.2) × 10^5^
E74A	**3**	(2.0 ± 0.03) × 10^−6^	(7.0 ± 0.06) × 10^−2^	(3.0 ± 2.8) × 10^−7^	(3.0 ± 0.05) × 10^4^
E74A	**1**	(3.0 ± 0.02) × 10^−4^	0.22 ± 0.001	N.D.	(7.0 ± 0.04) × 10^2^
Y89A	**3**	(2.2 ± 0.06) × 10^−3^	40 ± 1.3	N.D.	(1.9 ± 0.09) × 10^4^
Y89A	**1**	N.D.	N.D.	(7.0 ± 6) × 10^−4^	(3.2 ± 0.02) × 10^2^
H70A	**3**	(5.0 ± 0.02) × 10^−5^	23 ± 0.029	(2.0 ± 0.01) × 10^−4^	(4.0 ± 0.02) × 10^5^
H70A	**1**	N.D.	N.D.	N.D.	(7.0 ± 0.01) × 10^3^
D27A	**3**	(2.9 ± 0.11) × 10^−4^	300 ± 16.	(4.0 ± 0.2) × 10^−4^	(1.2 ± 0.01) × 10^6^
D27A	**1**	N.D.	N.D.	N.D.	(3.1 ± 0.08) × 10^4^
T24A	**1** or **3**		inactive		
R26A	**1** or **3**		inactive		
K21A	**1** or **3**		inactive		

All rate parameters were calculated from the kinetic experiments of *N*^4^-acetylcytosine (**1**) and *N*^4^-acetylcytidine (**3**) hydrolysis catalysed by YqfB. A general kinetic scheme with inhibition by product was used to describe the hydrolysis kinetics:

$$\begin{array}{c}S+E\leftrightarrow ES\to E+P\\ E+P\leftrightarrow {E}_{inh}\end{array}$$

With usual Michaelis-Menten constant, turnover number, inhibition constant, and apparent bimolecular (specificity) rate constant definitions. N.D. – not determined. Activity was measured in 50 mM potassium phosphate buffer, pH 8.0, at 22 °C. Rate constants were calculated from experiments with at least three different initial concentrations of substrate, using statistical significance value of 0.05.

From these results, a hypothesis regarding the catalysis mechanism may be inferred: Lys21 functions as a base, Thr24 as a nucleophile, and Glu74 as an acid, in a mechanism fairly typical of hydrolases. To the best of our knowledge, such catalytic triad with threonine acting as a nucleophile was observed for the first time in amidohydrolases, except for the enzymes that harbour a post-translationally formed N-terminal threonine acting as a nucleophile^20,21^. Notably, in support of our hypothesis regarding the amino acids relevant to the mechanism of catalysis, the YqfB Thr24Ser mutant that has a classical catalytic triad of amidohydrolases shows roughly the same catalytic activity as the wild-type YqfB.

### Structural basis for YqfB catalytic activity

Later, we tried to relate the observed properties of mutants with the three-dimensional structure of wild type YqfB that was published in 2005 (PDB ID:1te7)^17^. Surprisingly, the 1te7 structure failed to provide any reasonable insights into the mechanism of catalysis by YqfB. The essential Thr24 was buried deep in the structure and was not accessible through any pocket to bind the substrate. Lys21, Glu74 and His70 were far removed from Thr24 and were exposed on the surface of structure. Such discrepancy between our results and the published data might be due to the poor quality of the structure deposited to the database, as seen from the wwPDB NMR structure validation report. To investigate this issue, we carried out a series of molecular dynamics (MD) simulations of the structure with built-in disulphide bridge between Cys51 and Cys102 (only 2.07 ± 0.07% of YqfB contained free SH groups, Supplementary Table 3) and that of the disulphide bridge free YqfB variant (as in the published structure). In both cases, over the simulation duration of 2 µs, the structures rapidly unfolded and then slowly folded into rather different ones (Supplementary Fig. 6A) indicating that the published structure is not completely correct. To overcome this, we used a homology modelling approach to obtain a structure of YqfB based on its two close homologues from the same ASCH family with known good-quality structures: 1t62, a hypothetical protein EF3133 from *Enterococcus faecalis* V583, and 2z0t, a hypothetical protein PH0355 from *Pyrococcus horikoshii* OT-3 (Supplementary files 2 and 3). The model was further refined using MD simulations and was found to be of a better quality than that of the published YqfB: all amino acids important for the catalysis were found near the catalytic pocket, accessible for a substrate to bind (Supplementary Fig. 14). The model was compared with the original experimentally obtained chemical shifts (^1^H, ^13^C, and ^15^N) of 1te7 deposited in the BMRB (ID 6207). Notably, NMR experiments were carried out in a buffer solution containing 25 mM Na phosphate, 400 mM NaCl, 1 mM DTT, and 20 mM ZnCl_2_, and such a combination of high ionic strength and reducing conditions likely caused the breakage of the disulphide bond. Hence, the NMR-derived structure may significantly differ from that of a protein in its native state. Nevertheless, we analysed the compatibility of our models with the experimental data using a chemical shift prediction programme SHIFTX2^22^. The analysis showed that both the individual models and the multi-model ensemble obtained during this study are more compatible with the NMR data than those of 1te7 (Supplementary Table 4). Consequently, our models were used for further elucidation of the catalysis mechanism.

**Figure 4:**
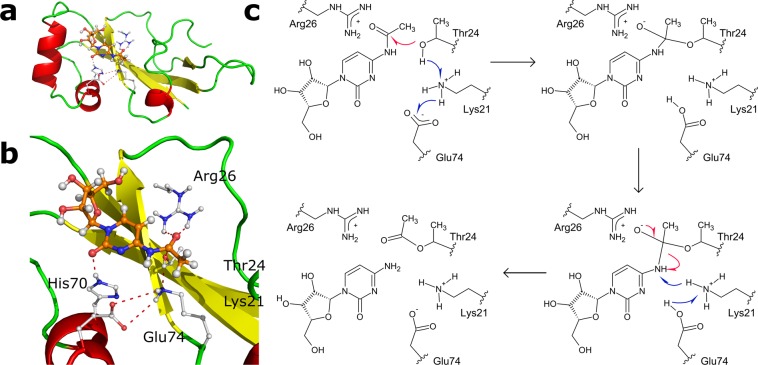
Predicted structure of the active centre of YqfB and proposed catalytic mechanism of ac4C hydrolysis. (**a**) The overall structure of the proposed enzyme-substrate complex. (**b**) A detailed view of YqfB active centre with bound ac4C, as generated by MD simulation; dashed lines correspond to hydrogen bonds. (**c**) The acylation step of ac4C hydrolysis catalysed by YqfB.

Docking studies produced a handful of structures, with ac4C residing within a pocket near catalytically important amino acids. These structures were further simulated using MD (20 ns per structure) to find substrate binding modes, which would give good quantitative and qualitative agreement with the proposed mechanism of acid-base catalysis by Lys21, Glu74 and Thr24. Two different protonation states of Lys21 were used in MD simulations, which resulted in similar substrate binding structures. However, in the case of deprotonated Lys21, the reasonable enzyme-substrate complexes were observed more frequently, in contrast to the simulations carried on protonated Lys21. This may be because deprotonated Lys21 mimics the intermediate stage of the reaction, whereas protonated Lys21 - the very beginning. During MD simulations, a set of structures explaining the catalytic properties of mutants were obtained. First, Lys21, Glu74 and Thr24 neatly aligned with the carbonyl group of ac4C, and the distances between relevant groups/atoms observed in the simulations were below 3.5 Å (Supplementary Figs. 6B and 8). Second, the aforementioned structures highlighted the role of His70, Tyr98, and Arg26. The latter functions as an atypical oxyanion hole, which, in the case of hydrolases, is usually formed by the hydrogen bond donors from two or more residues, one of which is typically a Gly. Notably, only the Obc1 protein from *Burkholderia thailandensis* and *O*-acetyltransferase PatB1 from *Bacillus cereus* possess an oxyanion hole formed by arginines^23,24^. The role of YqfB His70 and Tyr89 is to provide the framework for substrate recognition. Tyr89 forms hydrogen bonds with the ribose moiety and heterocyclic nitrogen of ac4C and *N*^4^-acetylcytosine, respectively. Thus, unsurprisingly, the Tyr89Phe and Tyr89Ala mutants showed diminished catalytic activity, and the effect was more pronounced in the case of the Tyr89Ala, probably due to the secondary effect on the structure. The role of His70 in the mechanism is rather obscure due to its close proximity to Lys21 and Glu74. The His70Ala mutant had a tenfold lower activity on both substrates, indicating the importance of this residue for catalysis, probably due to the partial bonds with carbonyl oxygen in cytidine and cytosine (Fig. 4b). The secondary function of YqfB His70 may be to provide the proper environment for Lys21 and Glu74. Based both on experimental studies and on theoretical data, the proposed mechanism of YqfB-mediated catalysis is depicted in Fig. 4c.

Our results highlight the importance of proteins belonging to an obscure ASCH superfamily. Though the ASCH proteins are widespread and diverse, their biological functions remain ambiguous because very little experimental data has been published to date. Recently, the crystal structure of an ASCH domain-containing protein from *Zymomonas mobilis* (*Zm*ASCH) has been determined revealing a ribonuclease activity. The detailed mechanism of RNA hydrolysis is yet to be elucidated, but it has been confirmed that three residues (Tyr47, Lys53, and Ser128) located in a cleft contribute to nucleic acid-binding and RNA cleavage^25^. However, the biological role of *Zm*ASCH remains unclear, and that makes YqfB the only ASCH superfamily protein with known function and mechanism of catalysis.

## Conclusions

Previously identified as hypothetical protein, the 103-amino acid protein YqfB from *E. coli* is, to the best of our knowledge, the smallest monomeric amidohydrolase described to date that has a very high specificity (k_cat_/K_m_ ~2.2 × 10^6^) for ac4C. YqfB ability to hydrolyse various *N*^4^-acylated cytosines and cytidines not only sheds light on the long-standing mystery of how ac4C is catabolized in bacteria, but may also prove useful in the development of novel biocatalysts, and should encourage further analysis of proteins belonging to the widespread ASCH superfamily.

## Methods

### Reagents

**The list of reagents** used during this work is provided in Supplementary material.

### Bacterial strains and plasmids

*E. coli* DH5alpha and DH10B, *E. coli* DH10B*ΔpyrFEC*^18^, and *E. coli* BL21(DE3) (Novagen, Germany) were used as hosts during this study. *E. coli* DH10B*ΔpyrFEC yqfB::Km* and *E. coli* BL21(DE3) *yqfB::Km* were constructed by replacing the *yqfB* gene with a kanamycin resistance cassette. The Quick and Easy *E. coli* Gene Deletion Kit (Gene Bridges) was used, following the technical protocol, Version 2.3 (June 2012). Two primers, yqfB_kn_FW and yqfB_kn_RV, were used to replace the target gene *yqfB*. The primers yqfB_US_FW and yqfB_DS_RV were used to verify gene replacement by PCR. Plasmid vectors pET21b(+), pETDuet-1 (Novagen, Germany), and pQE70 (Qiagen, Germany) were used for cloning and expression of target genes.

### Media and growth conditions

LB: 10 g l^−1^ tryptone, 5 g l^−1^ yeast extract, 5 g l^−1^ NaCl; LB agar: LB supplemented with 15 g l^−1^ agar; BHI (brain heart infusion) (Oxoid): 37 g l^−1^. All media were autoclaved for 30 min at 121 °C (1 atm). Ampicillin (0.1 g l^−1^) and kanamycin (0.015 g l^−1^) were used for selection. M9 medium was prepared as described previously^26^ with slight modifications. The M9-agarose, 5 × M9 salts, glucose, and casamino acids stock solutions were prepared separately. Casamino acids solution was filter-sterilized, all other solutions were autoclaved for 30 min at 121 °C (1 atm). M9 agarose: 0.2 g l^−1^ MgSO_4_, 0.01 g l^−1^ CaCl_2_, 15 g l^−1^ Thermo Scientific™ TopVision Agarose; 5 × M9 salts pH 7.4 stock solution: 35 g l^−1^ Na_2_HPO_4_, 15 g l^−1^ KH_2_PO_4_, 2.5 g l^−1^ NaCl, 5 g l^−1^ NH_4_Cl; 20 g l^−1^ glucose; 2 g l^−1^ casamino acids. M9 medium was supplemented with 0.1 g l^−1^ ampicillin, 0.015 g l^−1^ kanamycin, 0.023 g l^−1^ IPTG, 0.02 g l^−1^ uracil, cytosine, and *N*^4^-acetylcytosine, as required.

### Purification of *N*^4^-acetylcytosine deacetylase from *E. coli* DH10B

The *N*^4^-acetylcytosine deacetylase was purified from 1 l of *E. coli* DH10B culture. The cells were grown at 37 °C in 200 ml standard LB medium, using 1 l flasks. Each flask was inoculated with 1 ml of the overnight culture and *E. coli* was grown with shaking at 180 rpm. The cells were harvested at the late log phase (20 h after inoculation) by centrifugation for 30 min at 4,000 *g* (4 °C) and washed once with 0.9% NaCl. The cell pellet was resuspended in 2 volumes of 25 mM Tris-HCl buffer, pH 8.0, and disrupted by 4 min sonication at 22 kHz in 20 s periods with 30 s cooling intervals, using 50% amplitude. To obtain a cell-free extract, the lysate was centrifuged for 30 min at 10,000 *g* (4 °C) to remove the cell debris and undisrupted cells.

All chromatographic separations were carried out with an Äkta Purifier 100 system (GE Healthcare). At each step in the purification, the enzyme activity was analysed by thin layer chromatography (TLC) using *N*^4^-acetylcytosine as a substrate. Q-Sepharose chromatography was used as the first step in the purification of the protein. The cell-free extract was loaded onto 20 ml HiPrep Q (GE Healthcare) column equilibrated with 25 mM Tris-HCl, pH 8.0 (buffer A). The column was washed with 100 ml of buffer A, and the retained proteins were subsequently eluted with 12 column volumes of a linear salt gradient (0–0.8 M NaCl) in buffer A, at a flow rate of 2 ml min^−1^. The fractions with enzyme activity were pooled and dialyzed against 25 mM Tris-HCl buffer, pH 7.2, before loading onto the second ion exchange 5 ml HiTrap ANX column (GE Healthcare) pre-equilibrated with 25 mM Tris-HCl buffer, pH 7.2. After washing with 5 column volumes of the same buffer, deacetylase was eluted with 20 column volumes of a linear gradient (0–0.8 M NaCl) in 25 mM Tris-HCl, pH 7.2, at a flow rate of 1.2 ml min^−1^. The fractions with deacetylase activity were pooled, adjusted to 1.3 M saturation with solid ammonium sulphate, and applied to a 5 ml HiTrap Phenyl FF column (GE Healthcare) equilibrated with 1.3 M (NH_4_)_2_SO_4_ in buffer A. Proteins were initially eluted at a flow rate of 1.2 ml min^−1^ with 1.3 M (NH_4_)_2_SO_4_ in buffer A and then with 75 ml of a decreasing salt gradient (1.3–0 M (NH_4_)_2_SO_4_). Pooled fractions with deacetylase activity were again saturated with (NH_4_)_2_SO_4_ to 1.7 M, and the sample was loaded onto a second hydrophobic interaction column 1 ml Resource ISO (GE Healthcare), equilibrated with buffer A containing 1.7 M (NH_4_)_2_SO_4_. The column was washed with the equilibration buffer, and moderately bound proteins were eluted with 40 column volumes of a linear salt gradient (from 1.7 to 0.7 M (NH_4_)_2_SO_4_) in buffer A. The flow rate was maintained at 0.5 ml min^−1^. The active fractions were pooled and dialyzed against buffer A. The dialyzed enzyme solution was applied to a Source 15Q column 0.5 by 30 cm (GE Healthcare) pre-equilibrated with the same buffer. The column was well washed with buffer A, and the bound proteins were eluted with 17 column volumes of a linear gradient (0–0.4 M NaCl) in buffer A, at a flow rate of 0.5 ml min^−1^. The fractions with the highest deacetylase activity were pooled, dialyzed at 4 °C overnight against buffer A, and concentrated with carboxymethyl cellulose.

Sodium dodecyl sulphate-polyacrylamide gel electrophoresis (SDS-PAGE) was performed following the Laemmli method^27^. For protein identification (mass spectrometry), the concentrated protein solution was run on 14% native PAGE. Protein concentration was determined using the Lowry method^28^ with bovine serum albumin as a standard.

### Cloning, expression, purification, and analysis of recombinant YqfB

The *yqfB* gene was amplified using *E. coli* DH10B genomic DNA as a template and the following primers: yqfB_21b_FW, yqfB_21b_RV, yqfB_Duet_FW, yqfB_Duet_RV. A 6xHis-tag was introduced at the C-terminus of YqfB by amplifying the *E. coli* DH10B *yqfB* gene with primers yqfB_21b_FW and yqfB_21b_RV. The resulting DNA fragment was cloned into the *Nde*I and *Xho*I sites of pET21b(+). The N-terminal 6xHis-tag was fused to YqfB by amplifying the *E. coli* DH10B *yqfB* gene with primers yqfB_Duet_FW and yqfB_Duet_RV. The resulting DNA fragment was cloned into the *BamH*I and *Hind*III sites of pETDuet-1. The tag-free YqfB was obtained by amplifying the *E. coli* DH10B *yqfB* gene with primers yqfB_21b_FW and yqfB_Duet_RV, and the resulting DNA fragment was cloned into the *Nde*I and *Hind*III sites of the pET21b(+) vector.

A single colony of *E. coli* BL21(DE3) *yqfB::Km* cells transformed with either pETDuet-NHis_6_-ygfB or pET21b-ygfB-CHis_6_ was inoculated in 5 ml LB medium containing 100 µg/ml ampicillin and incubated at 37 °C with shaking (180 rpm) overnight. Two millilitres of the overnight culture were transferred to 200 ml of LB medium in a 1 l shake flask. The culture was grown under the same conditions until A_600_ reached 0.6. Then IPTG was added to a final concentration of 0.5 mM, and the incubation was continued for additional 4 h. The cells were then harvested by centrifugation for 30 min at 4,000 *g* (4 °C), resuspended in buffer A and disrupted by sonication (1 min (10 s disruption, 15 s cooling) at 22 kHz and 40% amplitude). The insoluble debris was removed by centrifugation, and the clear supernatant was used for protein purification. The cell-free extract was applied onto a 5 ml Ni^2+^ HiTrap chelating HP column (GE Healthcare), equilibrated with buffer A. After a 5-column volume wash with buffer A, the His-tagged protein was eluted with 9 column volumes of a linear imidazole (0–0.5 M) gradient in buffer A, at a flow rate of 1 ml min^−1^. To remove the imidazole, a fraction with deacetylase activity was selected, placed into a dialysis bag and dialyzed overnight at 4 °C in buffer A. The purified protein was concentrated with carboxymethyl cellulose and stored at −20 °C.

The tag-free recombinant YqfB protein was expressed from the plasmid vector pET21b-yqfB. Cell cultivation, protein overexpression, and the preparation of a cell-free extract were performed as described above. The cell-free extract was applied onto a 5 ml HiTrap ANX column (GE Healthcare) pre-equilibrated with buffer A. After washing with six column volumes of buffer A, the enzyme was eluted with 12 column volumes of a linear gradient (0–0.8 M NaCl) in 25 mM Tris-HCl, pH 8.0, at a flow rate of 1 ml min^−1^. The fractions with activity were pooled and dialyzed against 25 mM Tris-HCl buffer, pH 7.3, before loading onto the ion exchange column (1 ml HiTrap Q XL, GE Healthcare) pre-equilibrated with 25 mM Tris-HCl buffer, pH 7.3. The retained proteins were subsequently eluted with 25 column volumes of a linear salt gradient (0–0.8 M NaCl) in 25 mM Tris-HCl buffer, pH 7.3, at a flow rate of 0.5 ml min^−1^. The fractions containing deacetylase activity were pooled, adjusted to 1.5 M saturation with solid ammonium sulphate, and applied to a 1 ml HiTrap Phenyl FF column (GE Healthcare) equilibrated with 1.5 M (NH_4_)_2_SO_4_ in buffer A. Elution was performed with a linear gradient of ammonium sulphate (1.5–0 M) in buffer A, at a flow rate of 0.5 ml min^−1^. The active fractions were collected and dialyzed against buffer A. The purified protein was concentrated with carboxymethyl cellulose and stored at −20 °C.

Protein concentration was determined using the Lowry method with bovine serum albumin as a standard^29^.

### Gel-filtration chromatography

Purified YqfB (0.5 mL sample (1 mg ml^−1^)) or molecular-mass markers were loaded onto a Superdex 200 10/300 gel filtration column (GE Healthcare) and eluted (0.3 ml min^−1^) with 50 mM Tris-HCl buffer, pH 8.0, containing 100 mM NaCl. Standard protein solutions used to determine the oligomeric state of YqfB included apoferritin (443 kDa), albumin (66 kDa), and carbonic anhydrase (29 kDa) (Sigma-Aldrich).

### Determination of sulfhydryl groups

The method described previously^30^ was used to determine sulfhydryl groups. An appropriate volume of protein solution (1 mM) was mixed with the reaction buffer (100 mM Tris-HCl, pH 8.0, 1 mM EDTA) to a final volume of 1 ml. Then, 0.01 ml of DTNB (5,5′-dithio-bis-(2-nitrobenzoic acid)) solution (4 mg of DTNB dissolved in 1 ml of reaction buffer) was added. Reaction mixture was incubated at room temperature for 15 min, and the absorbance at 412 nm was measured. The absorbance is expressed as SH mol/mol protein, using the molecular extinction coefficient of 13,600 M^−1^ cm^−1^ for 5-thio-2-nitrobenzoic acid.

### YqfB activity assay

The activity of YqfB and its mutants was measured by TLC, HPLC-MS, or spectrophotometrically. For TLC analysis, the reaction mixture consisted of 50 mM potassium phosphate buffer (pH 8.0), 10 mM substrate, and an appropriate amount of protein. After the reaction, 1 µl of the mixture was spotted on silica gel-coated (60 F_254_, Merck) aluminium plate, and chloroform:methanol (9:1 v/v) was used as an eluent. The air-dried plates were visualized with UV light (254 nm). For HPLC-MS analysis, the reaction mixture consisted of 50 mM potassium phosphate buffer (pH 8.0), 2.5 mM substrate, and protein (~1 mg ml^−1^). For the spectrophotometric assay, the reaction was started by adding an appropriate amount of protein to buffer supplemented with substrate (0.1–0.5 mM). Then, a decrease in absorbance at either 310 nm (for ac4C and *N*^4^-acetyl-2′-deoxycytidine) or 295 nm (*N*^4^-acetylcytosine) was recorded at 22 °C. Rmodeler software (Ubique Calculus Ltd., Lithuania) was used for the calculation of the kinetic parameters. For all calculations, a scheme consisting of simple Michaelis-Menten kinetics with inhibition by the product was used. The dependence of YqfB activity on pH was investigated in pH range from 3 to 10, using 50 mM citrate phosphate buffer (pH 3.0–7.0), 50 mM potassium phosphate buffer (pH 6.0–8.0), 50 mM Tris-HCl buffer (pH 7.0–9.0), and 50 mM glycine-NaOH buffer (pH 9.0–10.0). The study of the dependence of YqfB activity on temperature was carried out in 50 mM potassium phosphate buffer, pH 8.0. To analyse the effect of inhibitors on the activity of YqfB, phenylmethanesulfonyl fluoride (PMSF) and *p*-hydroxymercuribenzoate (PHMB) were added to the reaction mixture to a final concentration of 1–2 mM. Also, *p*-chloromercuribenzoate (PCMB, 0.1–1 mM) and ethylenediaminetetraacetic acid (EDTA, 0.5–5 mM) were added before the assay. One unit of activity corresponded to 1 μmol of substrate converted per 1 min.

### Proteomic analysis

Proteomic analysis was carried out as described previously^31^.

### Site-directed mutagenesis of *yqfB*

Mutagenesis was performed on pET21b-ygfB-CHis_6_ with QuikChange™ Multi Site-Directed Mutagenesis Kit (Agilent Technologies, US), following the manufacturer’s protocol. Mutations were confirmed by DNA sequencing. The primers used for mutagenesis are listed in Supplemental Table 1. The mutant proteins were purified as YgfB-CHis_6_.

### Analysis of multiple sequence alignments

A representative alignment of the protein sequences from the family ASCH (the sequence of YqfB included) was downloaded from the Pfam database at the European Bioinformatics institute. The sequences were then sorted according to the topology of the tree that was built using the average distance, based on percent identity. The sequence of YqfB, together with those of YqfB-related proteins, formed a separate branch, which was realigned using MAFFT method^29^ and analysed further.

### Structure preparation

Initial structure coordinates for YqfB were taken from the structure with PDB entry code 1te7^17^. Since the crystal structure was solved under reducing conditions, the possible disulphide bridge between Cys51 and Cys102 was modelled using AMBER TLEAP program. For molecular dynamics simulations, only the first of the models was used for subsequent calculations. All models were protonated according to pH 7 using HH+ web server^32–34^. Structures for substrates (*N*^4^-acetylcytosine and ac4C) were built using Avogadro (https://avogadro.cc/) and optimized using DFT B3LYP functional with 6–31 + G(d,p) basis set with GAMESS (https://www.msg.chem.iastate.edu/gamess/).

### Homology modeling

Homology modelling was carried out using the Bioinformatics Toolkit available at the Max Planck Institute for Developmental Biology (Tübingen, Germany; https://toolkit.tuebingen.mpg.de/#/). For structure modelling, HHPRED^35,36^ was used to search for suitable templates, using default parameter settings. The known structure of YqfB (1te7) was excluded from the list of templates, and two good-quality structures PDB ID 1t62 and 2z0t, corresponding to ASCH family proteins EF3133 and PH0355, respectively, were manually selected for further modelling. MODELLER was used to build a model from templates^37^.

### Calculation of chemical shifts

**The chemical shifts** (^1^H, ^13^C, and ^15^N) were calculated using SHIFTX2 web server (http://www.shiftx2.ca/index.html) with following parameters: pH 6.5, 298 K, “Results combined with SHIFTY”, and “Protein is not deuterated”, compatible with conditions used in 1te7 structure determination. Chemical shifts were calculated for seven modelled structures and seven first structures from 1te7 separately. Prediction of chemical shifts was performed for ensembles of 7 modelled structures and all 20 models of 1te7. Differences between experimental and predicted shifts were normalized with respect to experimental shifts and averaged.

### Docking studies

Docking studies were carried out using AutodockVina^38^. The structures of the enzymes and substrates for docking were prepared using Dock Prep in USCF Chimera software. The search for enzyme-substrate complexes was carried out over the entire surface of enzyme models using the following parameters: exhaustiveness, 100; number of modes, 50; energy range, 20. Docked structures were analysed and sorted based on the distance between relevant residues and binding energy. The best structures were used in molecular dynamics simulations.

### Molecular dynamics simulation

Molecular dynamics simulations were performed using AMBER16. The files for substrates were prepared using ANTECHAMBER tool from AMBER Toolkit (http://ambermd.org/). All structures were parametrized using AMBER ff14sb and GAFF force fields for the enzyme and for the substrates, respectively. Structures were solvated with TIP3P water box of 10 Å and neutralized by adding required number of Na+ or Cl− ions. The simulations included four steps: initial minimization, system heating to 300 K for 200 ps, system equilibration, and production. For equilibration and production steps, the non-bonded interactions cut-off was set to 8 Å. The simulations were performed using SHAKE algorithm and 2 fs time-step for the integration of the trajectory. The simulations were carried out using constant volume periodic boundary conditions with isotropic pressure scaling. The temperature was maintained using Langevin dynamics with collision frequency of 2 ps-1. The duration of equilibration step was 2 ns with following 20 ns (or longer) production step. All simulations were carried out using either pmemd or sander from AMBER 16 software package. Trajectories were analysed using Cpptraj from AMBER Toolkit.

## Supplementary information

Supplementary information.

YqfB_model_Lys21_deprot.

YqfB_model_Lys21_prot.

## Data Availability

All data generated or analysed during this study are included in this article and its Supplementary information files.
