# Supplementary material for: YqfB protein from Escherichia coli: an atypical amidohydrolase active towards N4-acylcytosine derivatives
Source: Sci Rep. 2020 Jan 21;10:788. doi: 10.1038/s41598-020-57664-w (PMC6972931; doi:10.1038/s41598-020-57664-w)
Supplement: Supplementary file 1 — Supplementary information. [file 41598_2020_57664_MOESM1_ESM.pdf]

Supplementary Information

YqfB protein from *Escherichia coli*: an atypical amidohydrolase active towards *N*[4]-acylcytosine derivatives

Rūta Stanislauskienė[a,#,*], Audrius Laurynėnas[b,#], Rasa Rutkienė[a,#], Agota Aučynaitė[a], Daiva Tauraitė[a], Rita Meškienė[a], Nina Urbelienė[a], Algirdas Kaupinis[c], Mindaugas Valius[c], Laura Kaliniene[a], Rolandas Meškys[a]
[a] Department of Molecular Microbiology and Biotechnology, [b] Department of Bioanalysis, and [c] Proteomics Centre, Institute of Biochemistry, Life Sciences Center, Vilnius University, Sauletekio al. 7, Vilnius LT-10257, Lithuania
[#] Those authors contributed equally to this work

Reagents. *N*[4]-acetylcytidine, *N*[4]-acetyl-2'-deoxycytidine and *N*[4]-benzoyl-2'-deoxycytidine, *N*[4]-isobutyryl-2'-deoxycytidine were purchased from Combi-Blocks (USA). *N*[4]-acetylcytosine, *N*[4]-benzoylcytidine, *p*-nitroacetanilide, *p*-nitrophenyl acetate, *p*-hydroxymercuribenzoate, *p*-chloromercuribenzoate, capecitabine and carboxymethyl cellulose were obtained from Sigma-Aldrich (Germany). *N*[4]-acetyl-2'-deoxy-5'-*O*-DMT-cytidine and phenylmethanesulfonyl fluoride were from Carbosynth (UK) and Fluka (Switzerland), respectively. Ethylenediaminetetraacetic acid (EDTA) was from Roth (Germany). *N*[4]-hexanoyl-2'-deoxycytidine, *N*[4]-nicotinoyl-2'-deoxycytidine, *N*[4]-(2-acetyl-benzoyl)-2'-deoxycytidine, *N*[4]-(3-acetyl-benzoyl)-2'-deoxycytidine, *N*[4]-(4-acetyl-benzoyl)-2'-deoxycytidine, *N*[4]-(2-benzoyl-benzoyl)-2'-deoxycytidine, *N*[4]-(3-benzoyl-benzoyl)-2'-deoxycytidine, *N*[4]-(4-benzoyl-benzoyl)-2'-deoxycytidine, *N*[4]-acetyl-2'-deoxycytidine-5'-triphosphate, *N*[4]-hexanoyl-2'-deoxycytidine-5'-triphosphate, *N*[4]-benzoyl-2'-deoxycytidine-5'-triphosphate, *N*[4]-nicotinoyl-2'-deoxycytidine-5'-triphosphate, *N*[4]-(2-acetyl-benzoyl)-2'-deoxycytidine-5'-triphosphate, *N*[4]-(3-acetyl-benzoyl)-2'-deoxycytidine-5'-triphosphate, *N*[4]-(4-acetyl-benzoyl)-2'-deoxycytidine-5'-triphosphate, *N*[4]-(2-benzoyl-benzoyl)-2'-deoxycytidine-5'-triphosphate, *N*[4]-(3-benzoyl-benzoyl)-2'-deoxycytidine-5'-triphosphate, and *N*[4]-(4-benzoyl-benzoyl)-2'-deoxycytidine-5'-triphosphate were synthesized as described previously[1].
N[4]-acetyl-5-fluorocytosine was prepared according to the previously described method[2], with modifications. Acetic anhydride (0.19 ml, 2 mmol) and 5-fluorocytosine (0.13 g, 1 mmol) were suspended in acetic acid (0.5 mL) and heated for 30 min in a boiling water bath. After the reaction was completed (TLC), the reaction mixture was allowed to cool to room temperature and, upon cooling, the crystallization occurred. The crystalline product was centrifuged, washed with acetonitrile and dried under reduced pressure. The product was isolated as a white solid, yield 0.15 g (88%). UV (CH_3OH) λ_{max} 241; 305 nm. MS (ESI^+): *m/z* 172.05 $[M+H]^+$, 170.05 $[M-H]^-$. ^{1}H NMR (DMSO-d_6, 400 MHz): δ= 2.19 (s, 3H, CH_3), 8.12 (d, 1H, *J* = 5.1 Hz, CH=CF), 11.06 (bs, 2H, NH). ^{13}C NMR (DMSO-d_6, 100 MHz): δ= 24.91, 137.77, 140.15, 153.35, 155.69, 170.29.
Here, 2-acetylaminopyridine was prepared as reported previously[3], with modifications. To a solution of 2-aminopyridine (0.94 g, 10 mmol) and trimethylamine (2.78 ml, 20 mmol) in 10 ml of acetonitrile cooled to 0 °C, 1.89 ml (20 mmol) of acetic anhydride was added. The reaction mixture was stirred for 1–1.5 h at room temperature. After the reaction was completed (TLC), the mixture was quenched with sodium bicarbonate and extracted with ethyl acetate. The organic phase was dried (Na_2SO_4) and evaporated under reduced pressure. The residue was purified by flash column chromatography (silica gel, chloroform/methanol mixture, 10:0→10:1) twice to afford 2-acetylaminopyridine (0.36 g, 56%) as a white solid. UV (CH_3OH) λ_{max} 289 nm. MS (ESI^+): *m/z* 136.95 $[M+H]^+$. The NMR spectra were consistent with those reported previously[4].
N[4]-acetyl-2',3',5'-tri-*O*-acetylcytidine was prepared according to the reported procedure[5] with slight modifications. A mixture of acetic anhydride (0.47 ml, 5 mmol) and sodium acetate (0.04 g, 0.5 mmol) was heated for 20 min at 100 °C. Then cytidine (0.24 g, 1 mmol) was added to the hot solution and stirred for 1 h. After the reaction was completed (TLC), the mixture was

quenched with sodium bicarbonate and extracted with chloroform. The organic phase was dried (Na_2SO_4) and evaporated under reduced pressure. The residue was purified by flash column chromatography (silica gel, chloroform/methanol mixture, 10:0→10:1) to afford N^4-acetyl-2',3',5'-tri-*O*-acetylcytidine (0.4 g, 97%) as a white solid. UV (CH_3OH) λ_{max} 247; 298 nm. MS (ESI^+): *m/z* 412.05 $[M+H]^+$, 410.05 $[M-H]^-$. The NMR spectra were consistent with those reported previously[6].

N^2-acetylisocytosine was prepared by the following procedure. Isocytosine (0.17 g, 1.5 mmol) and acetic anhydride (0.28 ml, 3 mmol) were suspended in acetic acid (0.7 ml) and heated for 1 hour in a boiling water bath. After the reaction was completed (TLC), the reaction mixture was allowed to cool to room temperature and, upon cooling, the crystallization occurred. The crystalline product was centrifuged, washed with acetonitrile, and dried under reduced pressure. The product was isolated as a white solid, yield 0.17 g (75%). UV (CH_3OH) λ_{max} 283 nm. MS (ESI^+): *m/z* 154.00 $[M+H]^+$, 152.05 $[M-H]^-$. ^1H NMR (DMSO-d_6, 400 MHz): δ= 2.15 (s, 3H, CH_3), 6.02 (d, 1H, J = 6.8Hz, CH=CH), 7.78 (d, 1H, J = 6.8 Hz, CH=CH), 11.58 (bs, 2H, NH). ^{13}C NMR (DMSO-d_6, 100 MHz): δ= 24.30, 110.15, 129.70, 151.42, 173.79, 178.23.

Oligonucleotides (Supplementary Table 1) were purchased from Metabion.

Other chemicals were from Sigma-Aldrich or Alfa Aesar and used without further purification.

Supplementary Table 1. A list of oligonucleotides used in this study

**Oligonucleotide**	**Sequence, 5'–3'**
yqfB_kn_FW	cactatcataggctccagatgagtcaattcttacaggaaatagtaccatgcaattaaccctcactaaagggcg
yqfB_kn_RV	catgaaattaaatgcgaaatttcaactaacaggcgtgagttcaatttaaagtaatacgactcactatagggctc
yqfB_US_FW	caccggtcagaacgtcac
yqfB_DS_RV	actccggagaaacgtacac
yqfB_21b_FW	tatacatatgcagccaaacgacatc
yqfB_21b_RV	ttaactcgagaagacatttaaattcaatcac
yqfB_Duet_FW	tataggatccgcagccaaacgacatc
yqfB_Duet_RV	gcggaagcttttaaagacatttaaattcaatcac
codA_BL_pQ_Fw	attaagcatgccgaataacgcttta
codA_BL_pQ_Hind_Rv	taattaagctttcaacgtttgtaatcgat
**Oligonucleotides for site-specific mutagenesis**	
yqfB_K21A	cattctggctgggcgtgcaaccatcaccatccgc
yqfB_T24A	ggcgtaaaaccatcgccatccgcgacgag
yqfB_T24S	ggcgtaaaaccatcagcatccgcgacgagtc
yqfB_I25A	ggcgtaaaaccatcaccgcccgcgacgagtctgaat
yqfB_R26A	gtaaaaccatcaccatcgccgacgagtctgaatcgc
yqfB_R26K	ggcgtaaaaccatcaccatcaaggacgagtctgaatcgcactt
yqfB_D27A	catcaccatccgcgccgagtctgaatcgc
yqfB_E74A	gaaaaacatgcagagcaggcaaatatgaccctgactgaa
yqfB_Y89A	tgaaaaaagtcattgctgacatcgctcccggtcagacacaattttatg
yqfB_Y89F	gaaaaaagtcattgctgacatctttcccggtcagac

Supplementary Table 2. Detailed results of the MS/MS analysis.

A. Analysis of protein sample 6 (Fig.1, a, lane 6)

Description	mW (Da)	pI (pH)	PLGS Score	Peptides	Theoretical Peptides	Coverage (%)	Precursor RMS Mass Error (ppm)	Products	Digest Peptides	Modified Peptides	Products RMS Mass Error (ppm)	Products RMS RT Error (min)	Amount (fmol)	Amount (ngrams)
						**Replication 1**								
Aconitate hydratase A OS=Escherichia coli (strain K12) GN=acnA PE=1 SV=3	97615	5.4932	139187.5	126	69	80.9203	3.0678	2541	0	14	6.1314	0.016964	6810.517	667.9523
Glucose-6-phosphate isomerase OS=Escherichia coli (strain K12 / MC4100 / BW2952) GN=pgi PE=3 SV=1	61491	5.8257	103170.3	95	46	81.6029	1.6073	1674	0	7	6.1696	0.016173	5603.411	345.4163
UPF0267 protein YqfB OS=Escherichia coli (strain K12 / DH10B) GN=yqfB PE=3 SV=1	11897	4.4839	98996.77	15	10	64.0777	1.3417	329	0	3	5.6233	0.016787	309.7238	3.7227
Triosephosphate isomerase OS=Escherichia coli (strain K12 / DH10B) GN=tpiA PE=1 SV=1	26954	5.5679	53064.4	22	24	89.8039	2.2112	389	0	2	6.8817	0.017939	602.5045	16.3537
Pyruvate kinase I OS=Escherichia coli (strain K12) GN=pykF PE=1 SV=1	50697	5.688	21684.65	30	34	68.5106	3.0214	462	0	0	7.7795	0.016327	0	0
Periplasmic trehalase OS=Escherichia coli (strain K12 / DH10B) GN=treA PE=3 SV=1	63596	5.5005	15363.21	28	43	64.0708	2.4665	554	0	3	8.7497	0.015901	17.7382	1.1329
Oxygen-insensitive NADPH nitroreductase OS=Escherichia coli (strain K12) GN=nfsA PE=1 SV=2	26783	6.4878	10498.98	9	19	50	1.4747	151	0	0	7.9779	0.017079	74.7229	2.0239
Galactose-1-phosphate uridylyltransferase OS=Escherichia coli (strain K12) GN=galT PE=1 SV=2	39620	6.0015	5023.025	6	29	20.1149	1.7801	154	0	1	9.2648	0.016467	44.5557	1.7817
Alcohol dehydrogenase 1 OS=Saccharomyces cerevisiae (strain ATCC 204508 / S288c) GN=ADH1 PE=1 SV=5	36825	6.2168	4354.314	10	25	24.4253	5.233	141	0	1	9.6373	0.014787	50	1.8653
						**Replication 2**								
Aconitate hydratase A OS=Escherichia coli (strain K12) GN=acnA PE=1 SV=3	97615	5.4932	139187.5	126	69	80.9203	3.0678	2541	0	14	6.1314	0.016964	6810.517	667.9523
Glucose-6-phosphate isomerase OS=Escherichia coli (strain K12 / MC4100 / BW2952) GN=pgi PE=3 SV=1	61491	5.8257	103170.3	95	46	81.6029	1.6073	1674	0	7	6.1696	0.016173	5603.411	345.4163
UPF0267 protein YqfB OS=Escherichia coli (strain K12 / DH10B) GN=yqfB PE=3 SV=1	11897	4.4839	98996.77	15	10	64.0777	1.3417	329	0	3	5.6233	0.016787	309.7238	3.7227
Triosephosphate isomerase OS=Escherichia coli (strain K12 / DH10B) GN=tpiA PE=1 SV=1	26954	5.5679	53064.4	22	24	89.8039	2.2112	389	0	2	6.8817	0.017939	602.5045	16.3537
Pyruvate kinase I OS=Escherichia coli (strain K12) GN=pykF PE=1 SV=1	50697	5.688	21684.65	30	34	68.5106	3.0214	462	0	0	7.7795	0.016327	0	0
Periplasmic trehalase OS=Escherichia coli (strain K12 / DH10B) GN=treA PE=3 SV=1	63596	5.5005	15363.21	28	43	64.0708	2.4665	554	0	3	8.7497	0.015901	17.7382	1.1329

Oxygen-insensitive NADPH nitroreductase OS=Escherichia coli (strain K12) GN=nfsA PE=1 SV=2	26783	6.4878	10498.98	9	19	50	1.4747	151	0	0	7.9779	0.017079	74.7229	2.0239
Galactose-1-phosphate uridylyltransferase OS=Escherichia coli (strain K12) GN=galT PE=1 SV=2	39620	6.0015	5023.025	6	29	20.1149	1.7801	154	0	1	9.2648	0.016467	44.5557	1.7817
Alcohol dehydrogenase 1 OS=Saccharomyces cerevisiae (strain ATCC 204508 / S288c) GN=ADH1 PE=1 SV=5	36825	6.2168	4354.314	10	25	24.4253	5.233	141	0	1	9.6373	0.014787	50	1.8653
					**Replication 3**									
Aconitate hydratase A OS=Escherichia coli (strain K12) GN=acnA PE=1 SV=3	97615	5.4932	125262	132	69	80.2469	1.5812	2578	0	14	6.2281	0.017883	6678.963	655.05
Glucose-6-phosphate isomerase OS=Escherichia coli (strain K12 / MC4100 / BW2952) GN=pgi PE=3 SV=1	61491	5.8257	111544.3	91	46	80.1457	1.9027	1679	0	10	6.5688	0.017302	5711.911	352.1047
Glucose-6-phosphate isomerase OS=Shigella flexneri GN=pgi PE=3 SV=4	61507	5.8257	111193.6	91	46	80.1457	2.0138	1659	0	10	6.5607	0.016894	5711.911	352.196
UPF0267 protein YqfB OS=Escherichia coli (strain K12 / DH10B) GN=yqfB PE=3 SV=1	11897	4.4839	96167.82	15	10	69.9029	1.3932	357	0	2	6.2309	0.015493	300.8306	3.6158
Triosephosphate isomerase OS=Escherichia coli (strain K12 / DH10B) GN=tpiA PE=1 SV=1	26954	5.5679	50333.98	24	24	90.5882	1.3596	414	0	1	7.5024	0.016341	621.6895	16.8745
Pyruvate kinase I OS=Escherichia coli (strain K12) GN=pykF PE=1 SV=1	50697	5.688	23207.23	29	34	67.6596	3.3307	487	0	1	7.6815	0.015897	0	0
Periplasmic trehalase OS=Escherichia coli (strain K12 / DH10B) GN=treA PE=3 SV=1	63596	5.5005	19582.76	26	43	64.9557	1.8426	529	0	2	8.1901	0.015689	68.3676	4.3663
Oxygen-insensitive NADPH nitroreductase OS=Escherichia coli (strain K12) GN=nfsA PE=1 SV=2	26783	6.4878	11805.24	9	19	50	2.3222	145	0	0	8.192	0.017226	76.8105	2.0805
Alcohol dehydrogenase 1 OS=Saccharomyces cerevisiae (strain ATCC 204508 / S288c) GN=ADH1 PE=1 SV=5	36825	6.2168	4021.136	9	25	22.1264	6.0041	144	0	1	8.783	0.0149	50	1.8653
Galactose-1-phosphate uridylyltransferase OS=Escherichia coli (strain K12) GN=galT PE=1 SV=2	39620	6.0015	3813.007	5	29	25.2874	5.9521	170	0	0	10.103	0.018924	36.8278	1.4727

B. Analysis of the band s4 excised from the gel (Fig.1, b)

Description	mW (Da)	pI (pH)	PLGS Score	Peptides	Theoretical Peptides	Coverage (%)	Precursor RMS Mass Error (ppm)	Products	Digest Peptides	Modified Peptides	Products RMS Mass Error (ppm)	Products RMS RT Error (min)	Amount (fmol)	Amount (ngrams)
					**Replication 1**									
B1XEI7, YQFB_ECODH UPF0267 protein YqfB OS=Escherichia coli (strain K12 / DH10B) GN=yqfB PE=3 SV=1	11897	4.4839	15833.84	11	10	69.9029	1.9773	191	0	1	6.132	0.008746	171.1852	2.0576
P00330, ADH1_YEAST Alcohol dehydrogenase 1 OS=Saccharomyces	36825	6.2168	2610.897	18	25	36.2069	1.6404	179	0	1	5.1877	0.00822	50	1.8653

cerevisiae (strain ATCC 204508 / S288c) GN=ADH1 PE=1 SV=5														
							**Replication 2**							
B1XEI7, YQFB_ECODH UPF0267 protein YqfB OS=Escherichia coli (strain K12 / DH10B) GN=yqfB PE=3 SV=1	11897	4.4839	12750.51	11	10	69.9029	2.1159	189	0	1	6.2111	0.007889	169.0387	2.0318
P00330, ADH1_YEAST Alcohol dehydrogenase 1 OS=Saccharomyces cerevisiae (strain ATCC 204508 / S288c) GN=ADH1 PE=1 SV=5	36825	6.2168	2891.433	17	25	36.2069	1.6527	190	0	1	7.059	0.008662	50	1.8653
							**Replication 3**							
B1XEI7, YQFB_ECODH UPF0267 protein YqfB OS=Escherichia coli (strain K12 / DH10B) GN=yqfB PE=3 SV=1	11897	4.4839	10934.46	9	10	69.9029	1.9516	165	0	1	5.62	0.008688	172.586	2.0744
P00330, ADH1_YEAST Alcohol dehydrogenase 1 OS=Saccharomyces cerevisiae (strain ATCC 204508 / S288c) GN=ADH1 PE=1 SV=5	36825	6.2168	2609.899	17	25	36.2069	1.9335	167	0	1	5.6582	0.007159	50	1.8653

Supplementary Table 3. Free-SH group analysis of YqfB with 5,5'-dithio-*bis*-(2-nitrobenzoic acid)

**Concentration of YqfB in the reaction mixture, μM**	**ΔA_{412}**	**Concentration of SH groups in the reaction mixture, μM**	**The portion of YqfB harbouring free SH groups, %**
84	0.047	3.4	2.02
42	0.025	1.8	2.14
21	0.013	0.9	2.14
10	0.006	0.4	2.00
			**2.07±0.07** (AVERAGE±STDEV)

Supplementary Table 4. Deviation of the predicted from the experimental chemical shifts for 1te7 and the structures modelled in this study.

**Structures modelled in this study**		**1te7 models**	
Model	Averaged deviation from the experimental values in %	Model	Averaged deviation from the experimental values in %
1	3.8	1	4.0
2	4.0	2	4.3
3	4.0	3	4.1
4	4.0	4	4.4
5	4.0	5	3.8
6	4.0	6	4.1
7	4.0	7	4.2
Average	4.0	Average	4.1
Ensemble of 7 models	3.8	Ensemble of 20 models	4.2

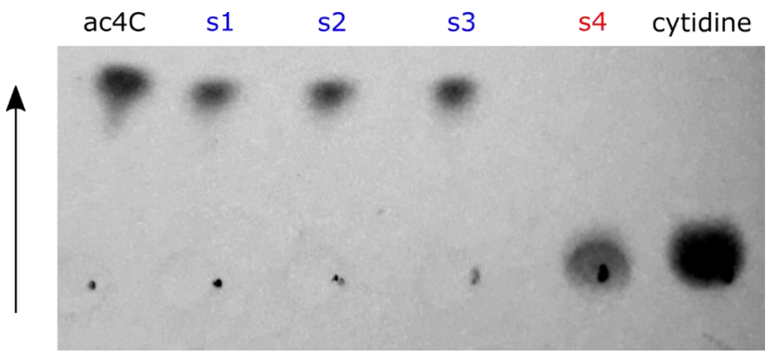

Supplementary Fig. 1. The activity of the enzyme in gel slices after the separation using the native PAGE (Fig. 1, b). The gel slices (s1–s4) from the native gel were incubated with ac4C and the reaction products were analysed by TLC; ac4C – substrate, cytidine – product control standard.

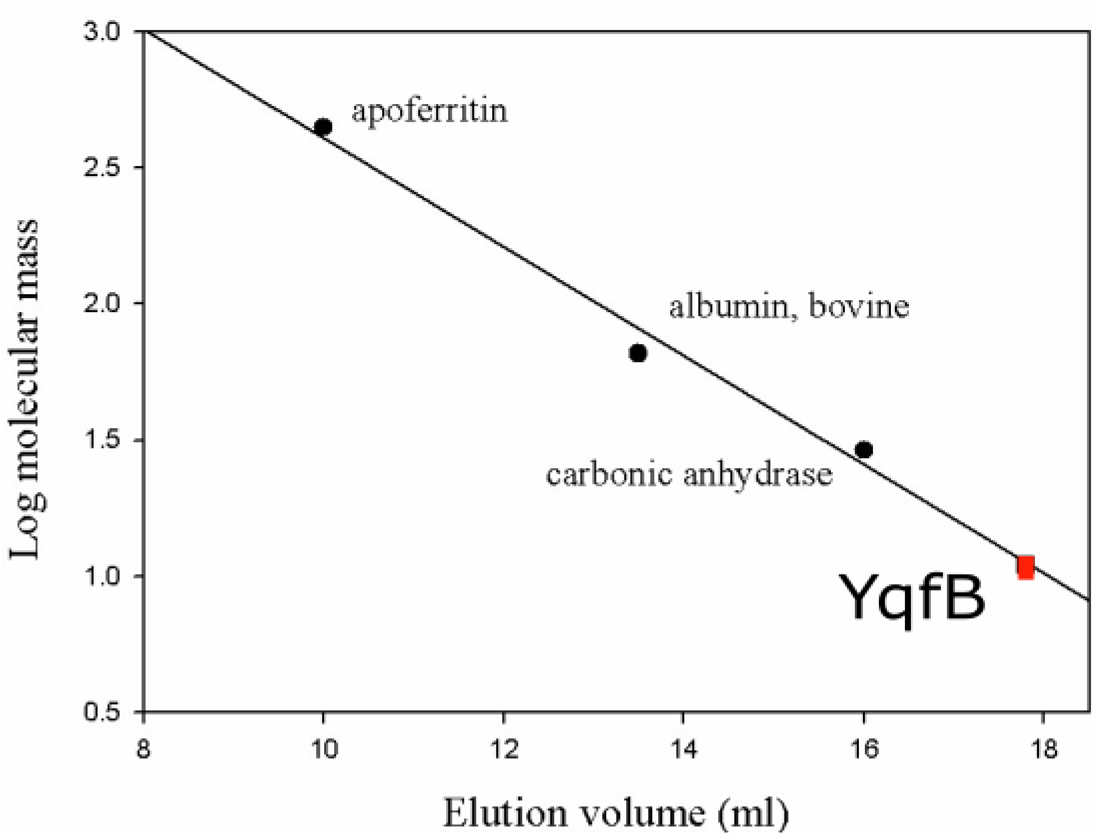

Supplementary Fig. 2 Determination of the oligomeric state of *E. coli* YqfB. The purified YqfB (0.5 ml of sample; 1 mg ml^{-1}) or standard was loaded onto a Superdex 200 10/300 gel filtration column (GE Healthcare) and eluted (0.3 ml min^{-1}) with 50 mM Tris-HCl buffer, pH 8.0, containing 100 mM NaCl. Apoferritin (443 kDa), bovine serum albumin (66 kDa) and carbonic anhydrase (29 kDa) were used as molecular mass standards.

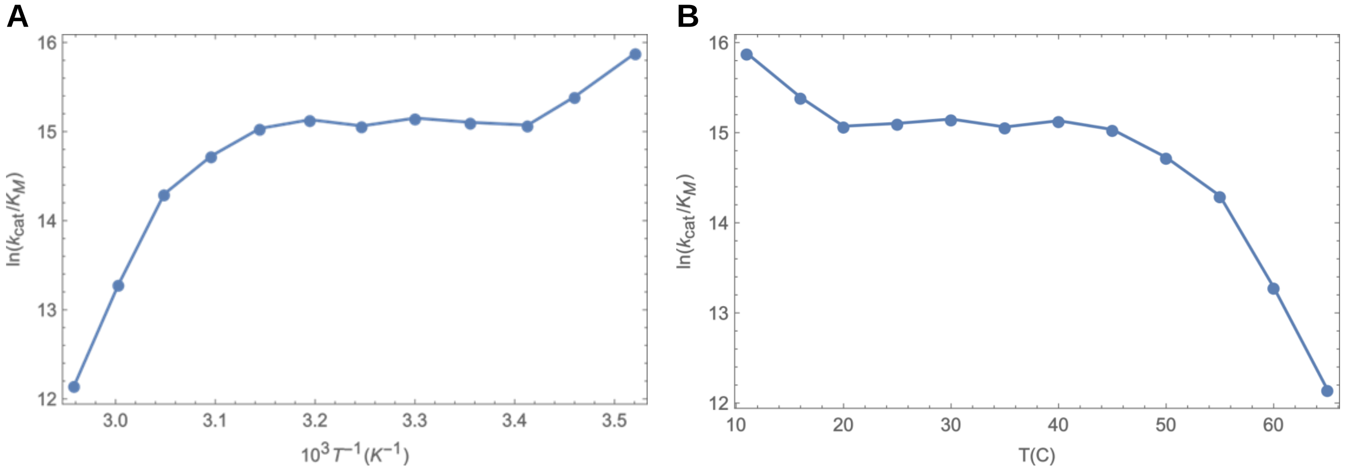

Supplementary Fig. 3 The dependence of YqfB activity on temperature. (A) Arrhenius plot and (B) $\ln(k_{cat}/K_m)$ dependence on temperature. The activity was analysed in 50 mM potassium phosphate buffer, pH 8.0, with N^4-acetylcytidine as a substrate. Rate constants were calculated from experiments with at least three different initial concentrations of substrate, using statistical significance value of 0.05.

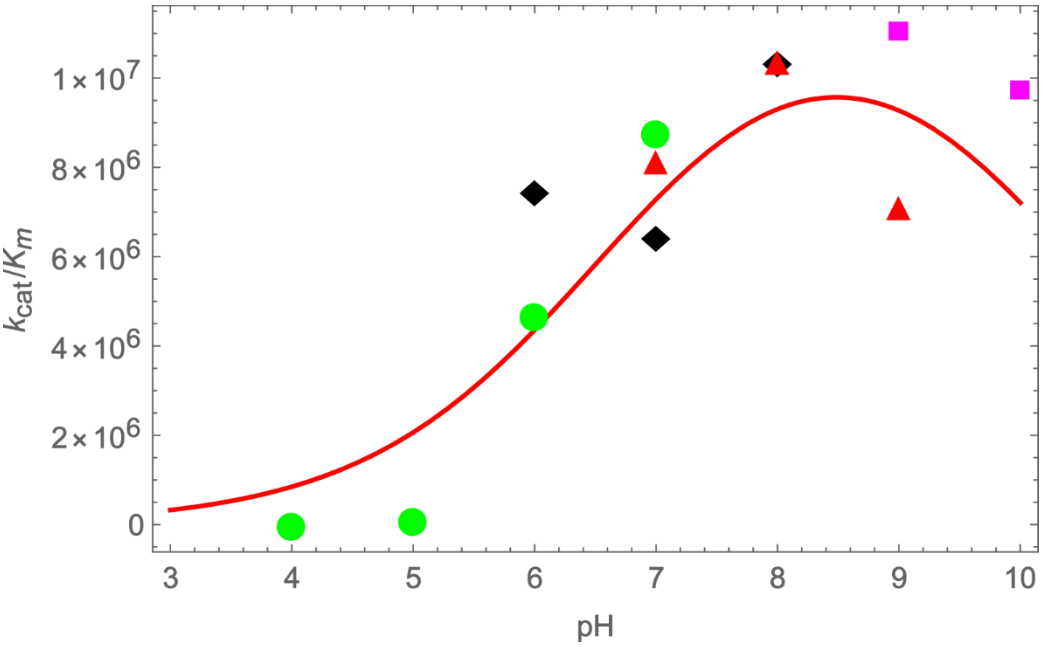

Supplementary Fig. 4 The dependence of YqfB activity on pH. The data were fitted to a model with two ionisable groups (red curve). The pKa values of those groups, as calculated from data, were 6.6 and 10.4. Green circles – 50 mM citrate-phosphate buffer, black diamonds – 50 mM potassium phosphate buffer, red triangles – 50 mM Tris-HCl buffer, magenta squares – 50 mM glycine-NaOH buffer. N^4-acetylcytidine was used as a substrate. Rate constants were calculated from experiments with at least three different initial concentrations of substrate, using statistical significance value of 0.05.

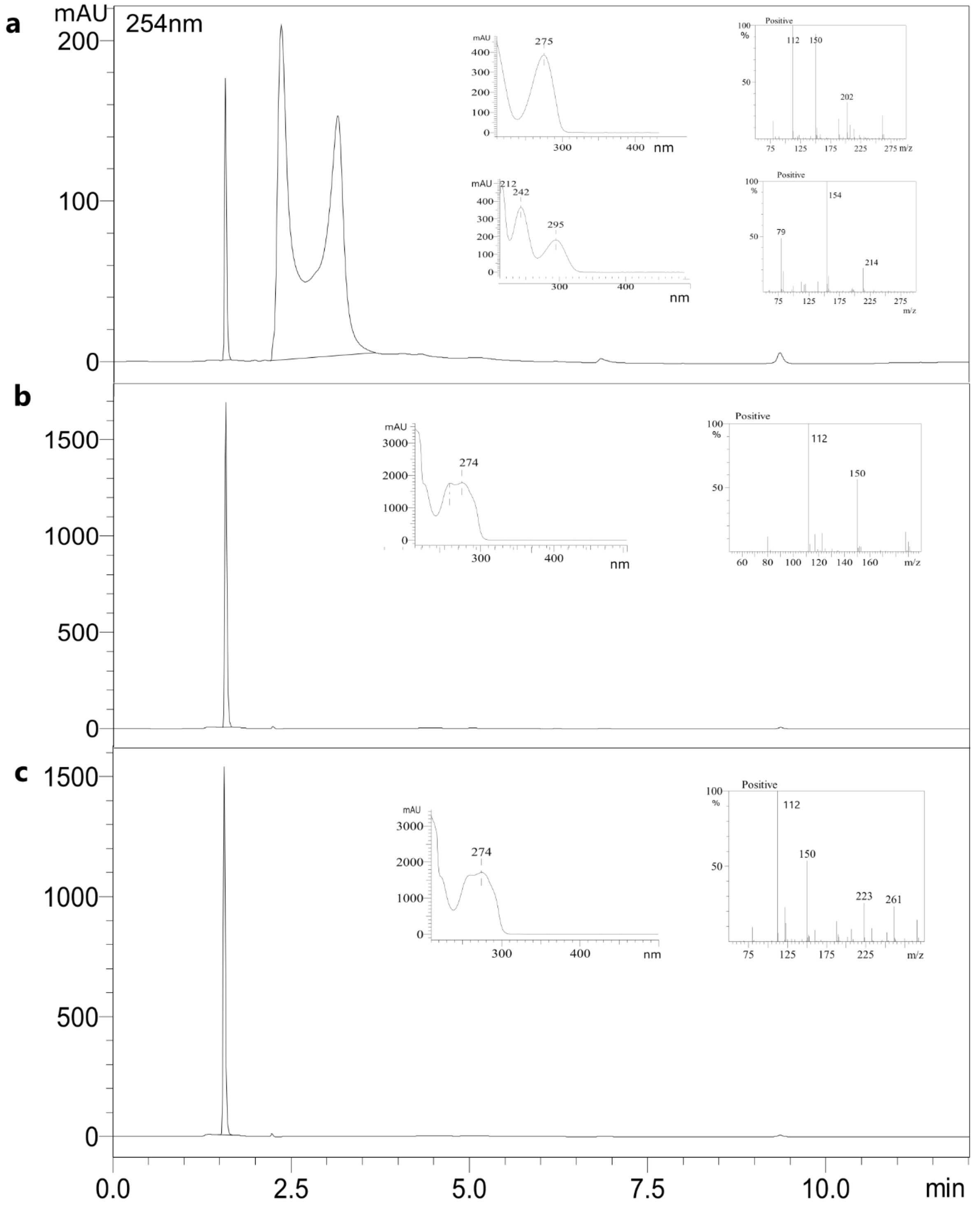

Supplementary Fig. 5. HPLC-MS analysis of the activity of YqfB. The letters on the left indicate as follows: a – *N*^{4}-acetylcytosine (**1**), b – cytosine standard, c – reaction products after incubation of *N*^{4}-acetylcytosine with YqfB.

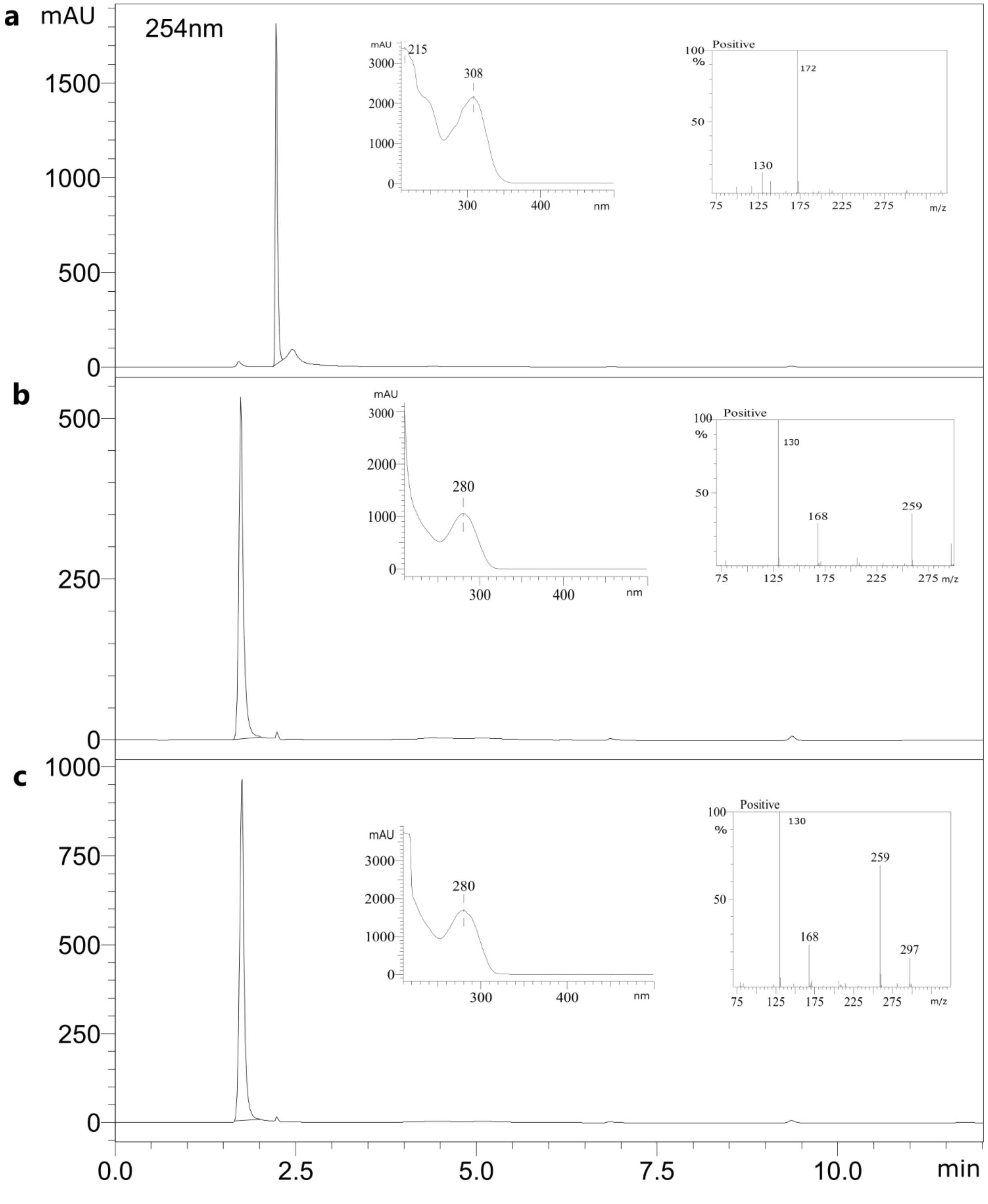

Supplementary Fig. 6. HPLC-MS analysis of the activity of YqfB. The letters on the left indicate as follows: a – *N*^{4}-acetyl-5-fluorocytosine (**2**), b – 5-fluorocytosine standard, c – reaction products after incubation of *N*^{4}-acetyl-5-fluorocytosine with YqfB.

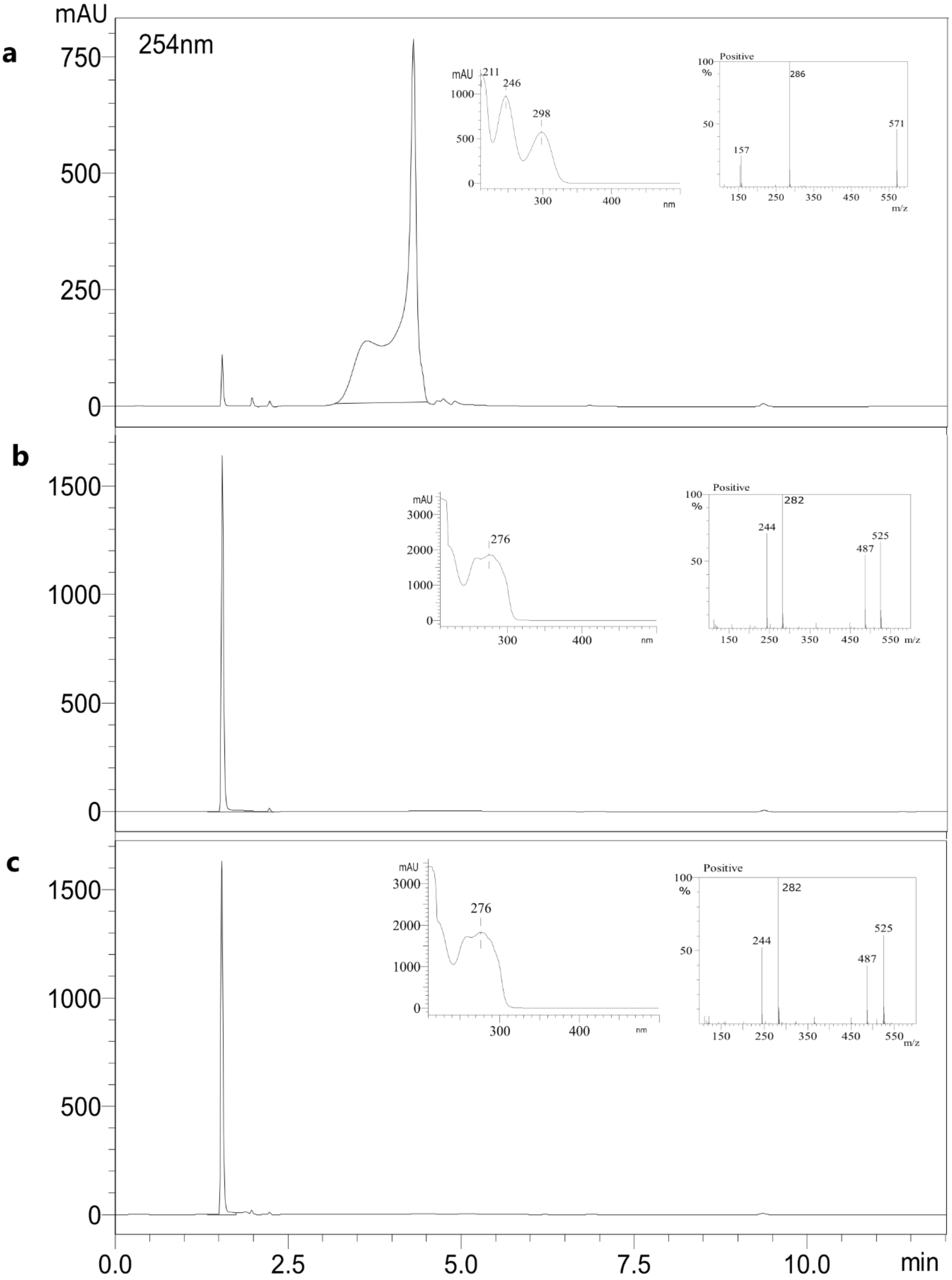

Supplementary Fig. 7. HPLC-MS analysis of the activity of YqfB. The letters on the left indicate as follows: a – N^4-acetylcytidine (**3**), b – cytidine standard, c – reaction products after incubation of N^4-acetylcytidine with YqfB.

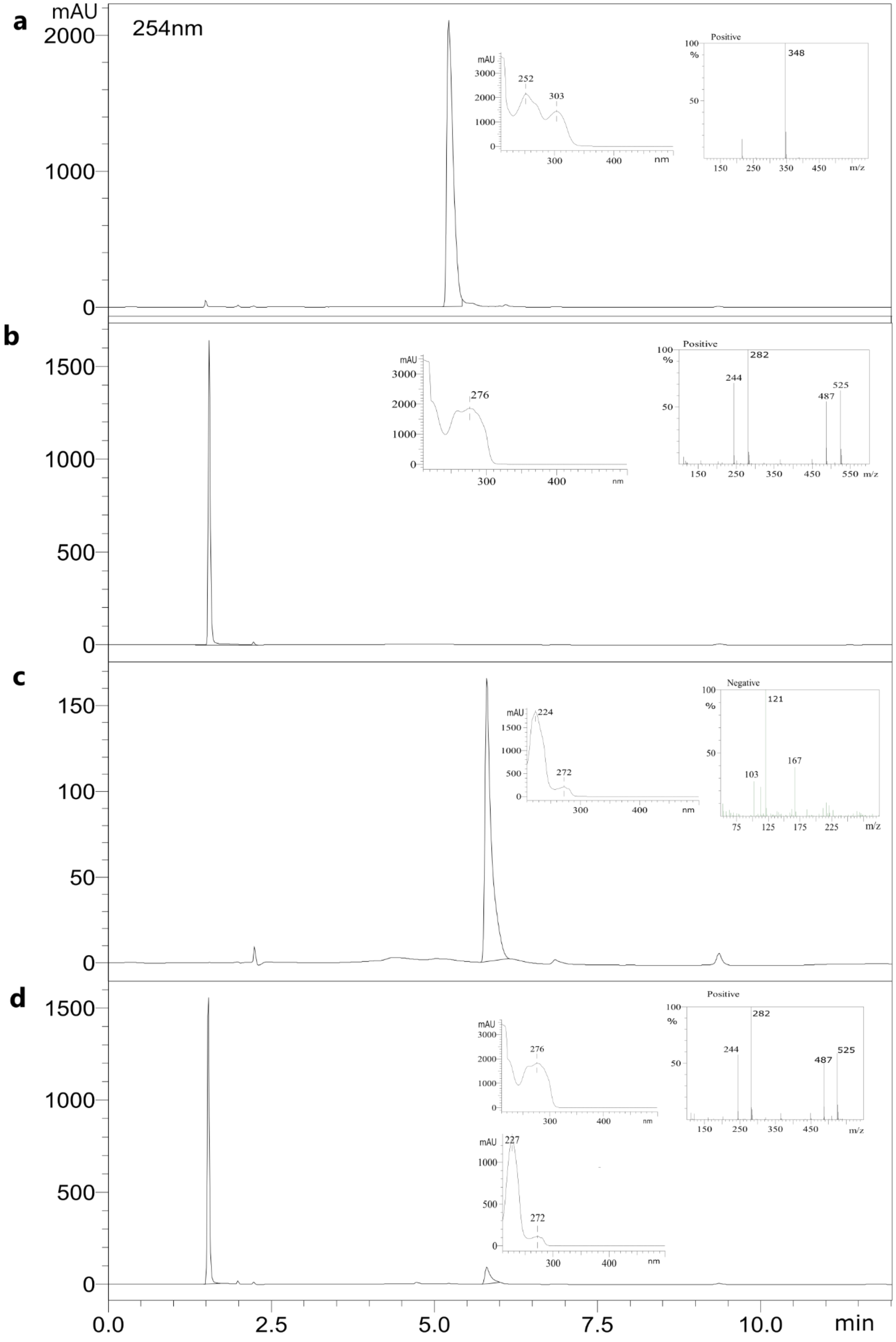

Supplementary Fig. 8. HPLC-MS analysis of the activity of YqfB. The letters on the left indicate as follows: a – *N*[4]-benzoylcytidine (**4**), b – cytidine standard, c – benzoic acid standard, d – reaction products after incubation of *N*[4]-benzoyl-cytidine with YqfB.

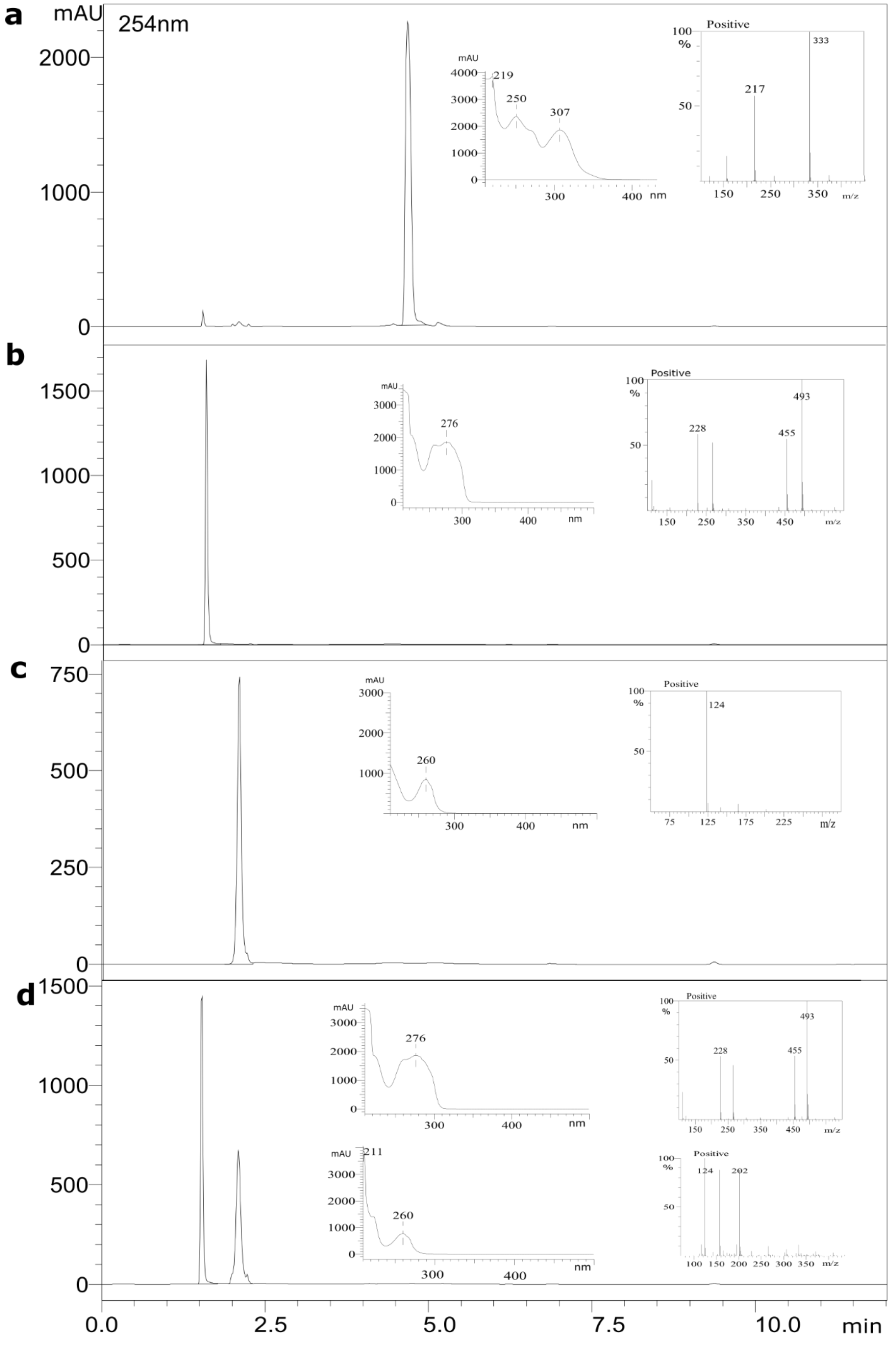

Supplementary Fig. 9. HPLC-MS analysis of the activity of YqfB. The letters on the left indicate as follows: a – *N*4-nicotinoyl-2'-deoxycytidine (**10**), b – deoxycytidine standard, c –nicotinic acid standard, d – reaction products after incubation of *N*4-nicotinoyl-2'-deoxycytidine with YqfB.

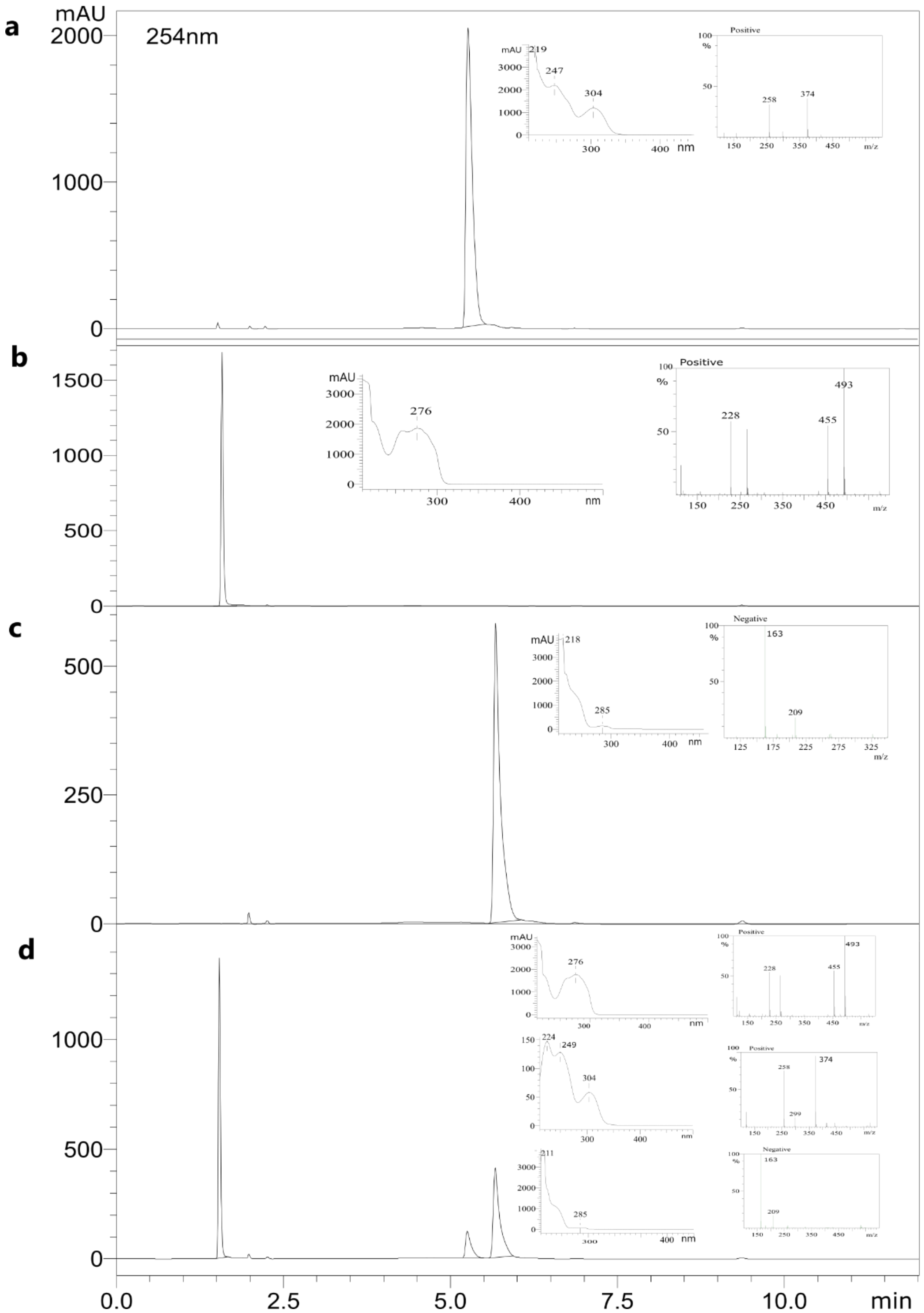

Supplementary Fig. 10. HPLC-MS analysis of the activity of YqfB. The letters on the left indicate as follows: a – *N*[4]-(3-acetyl-benzoyl)-2'-deoxycytidine (**12**), b – deoxycytidine standard, c – 3-acetylbenzoic acid standard, d – reaction products after incubation of *N*[4]-(3-acetyl-benzoyl)-2'-deoxycytidine with YqfB.

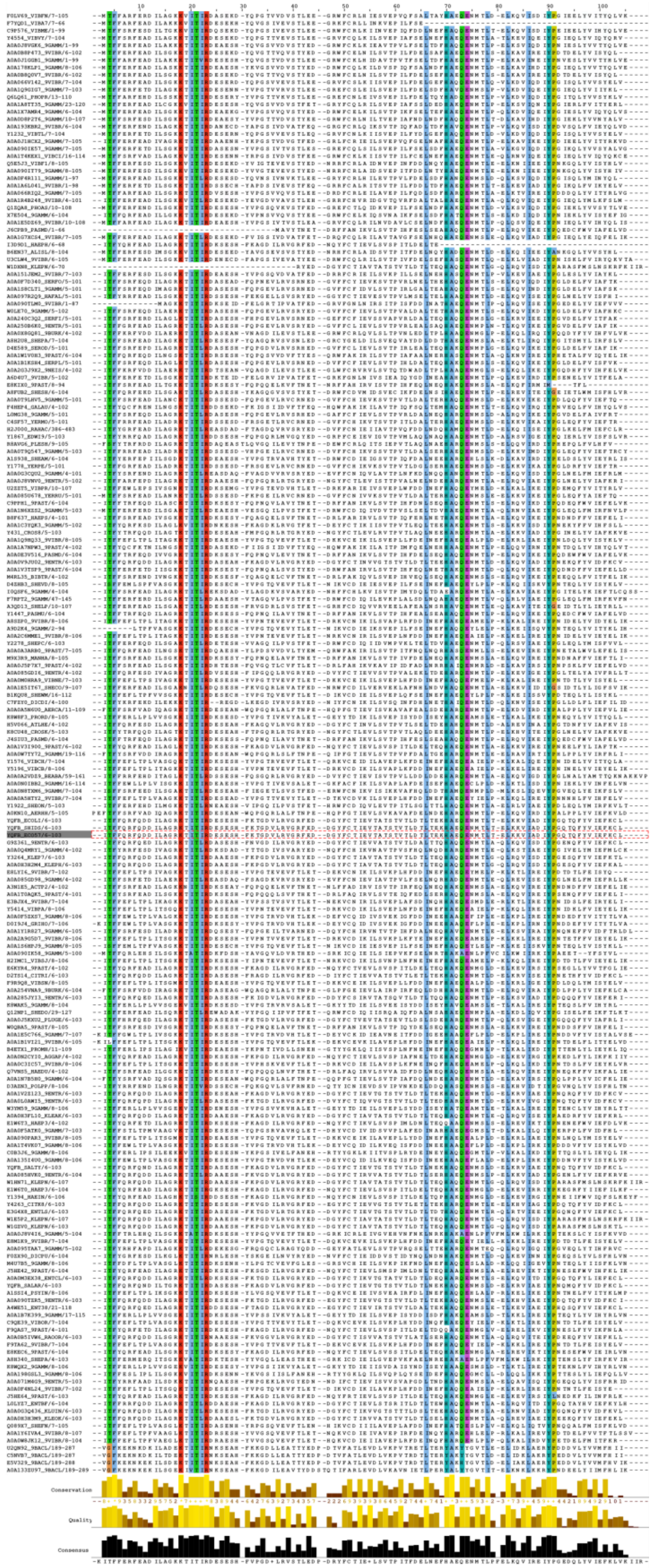

Supplementary Fig. 11. The alignment of protein sequences from YfbB-containing branch. 100% conserved amino acids are coloured.

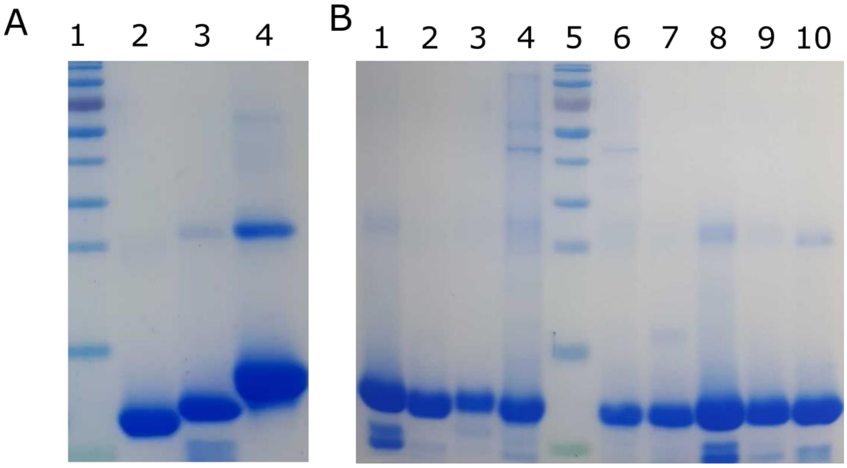

Supplementary Fig. 12. SDS-PAGE analysis of the purified recombinant YqfB variants and mutants. A. Lane 1 – molecular weight marker (from top to bottom: 170, 130, 100, 70, 55, 40, 35, 25, 15, and 10 kDa), lane 2 – tag-free recombinant YqfB, lane 3 – YqfB with a C-terminal 6xHis-tag, lane 4 –YqfB with an N-terminal 6xHis-tag. B. Lane 1 – YqfB with a C-terminal 6xHis-tag, lane 2 – R26A mutant, lane 3 – T24A mutant , lane 4 – K21A mutant, lane 5 – molecular weight marker, lane 6 – H70A mutant, lane 7 – Y89A mutant, lane 8 – T24S mutant, lane 9 – Y89F mutant, lane 10 – E74A mutant.

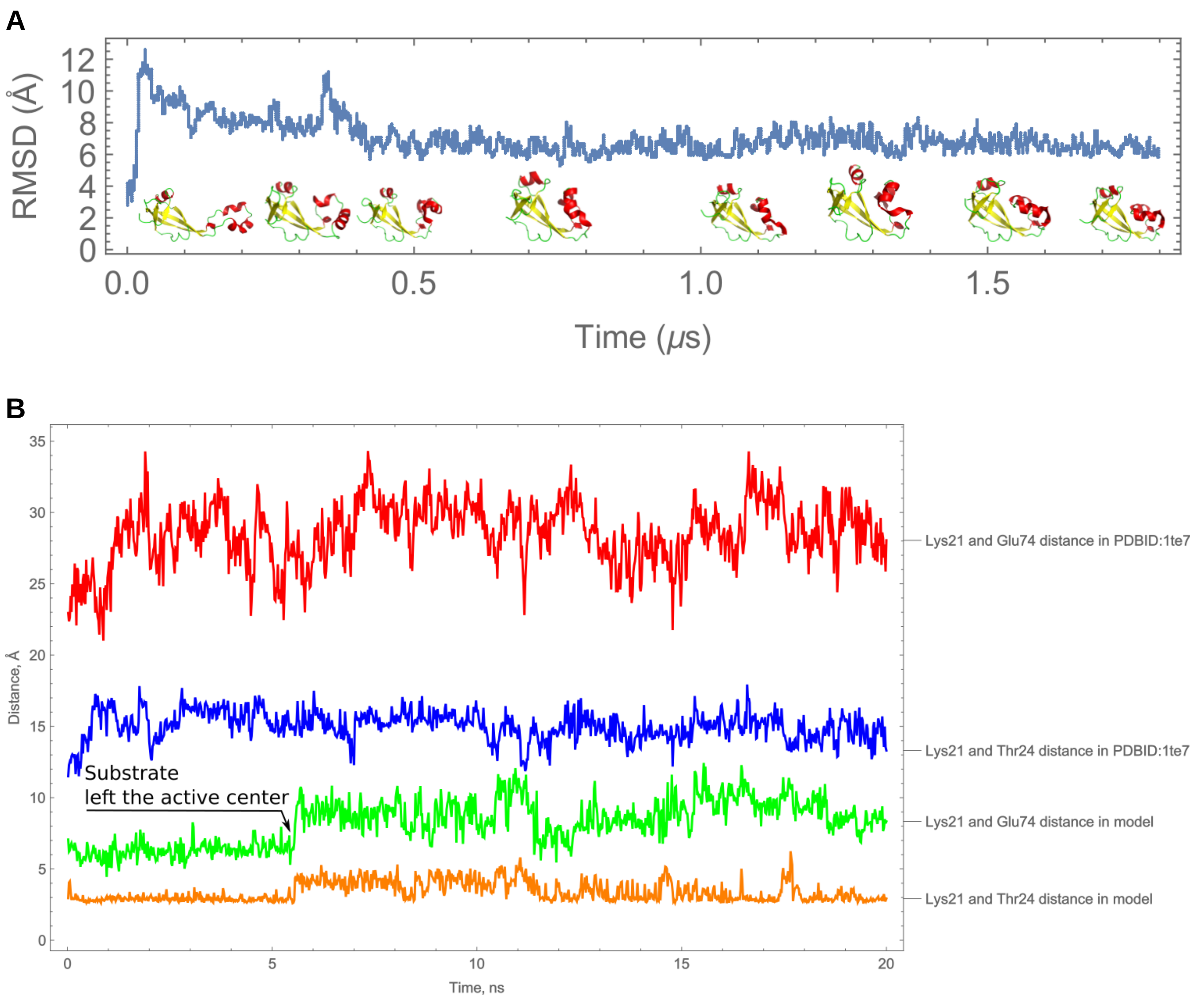

Supplementary Fig. 13. (A) Stability of the crystal structure of YqfB deposited in the PDB **(**1te7**).** RMSD from the starting structure was plotted over simulation time. It can be seen that the protein partially unfolds and then folds into a new structure rapidly. (B) **The** distance between catalytic residues in the modelled and PDBID:1te7 structures observed during molecular dynamics simulation.

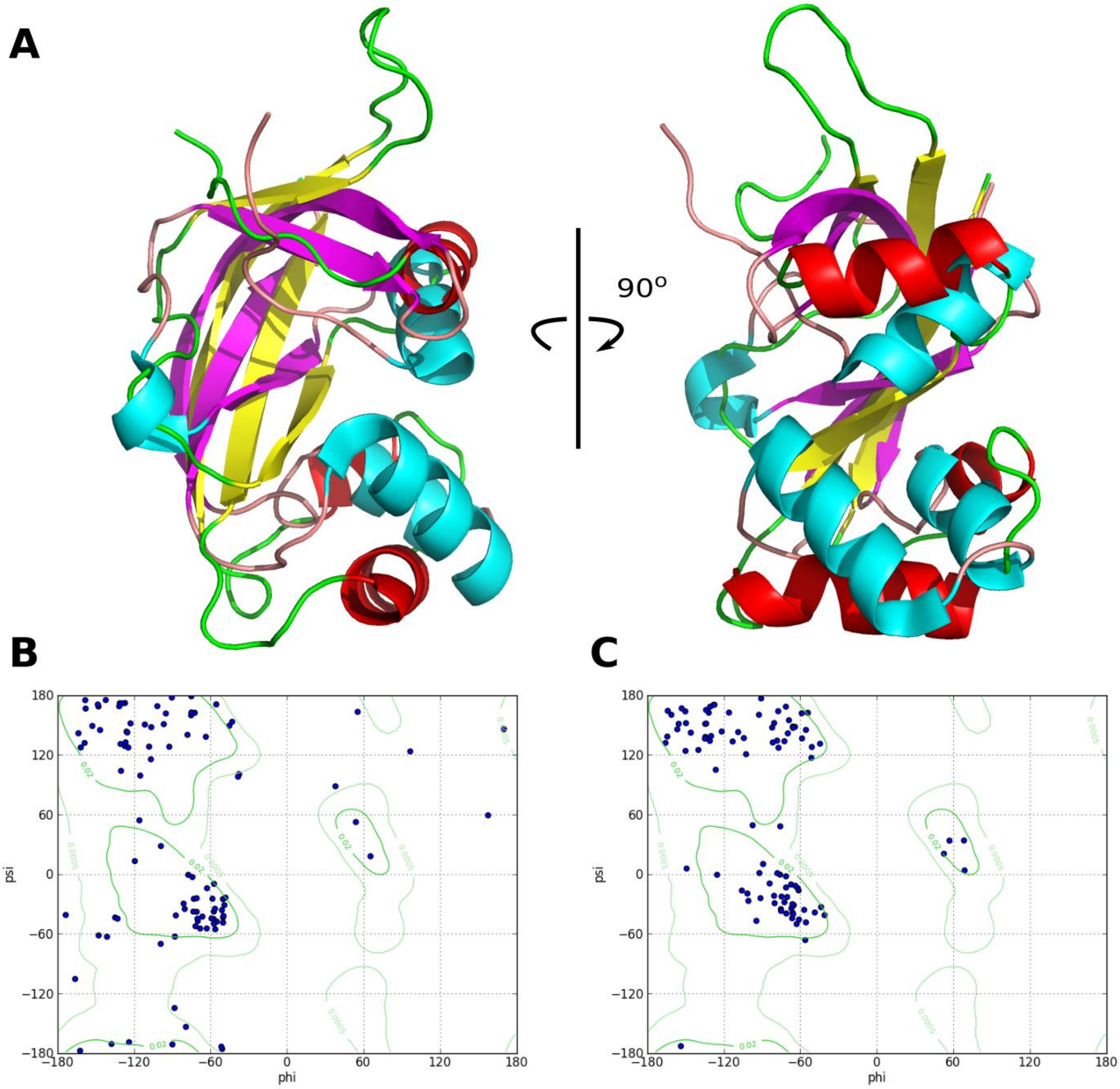

Supplementary Fig. 14. The quality of the structures of YqfB. (A) The figure illustrates the structural differences between the published structure (1te7) of YqfB and that modelled during this study, by using a homology modelling approach. It can be seen that, in contrast to 1te7, the modelled structure has an open pocket. The Ramachandran plots B and C illustrate structural qualities in terms of Ramachandran outliers, which are clearly seen in B (for 1te7) and non-existent in C (model).

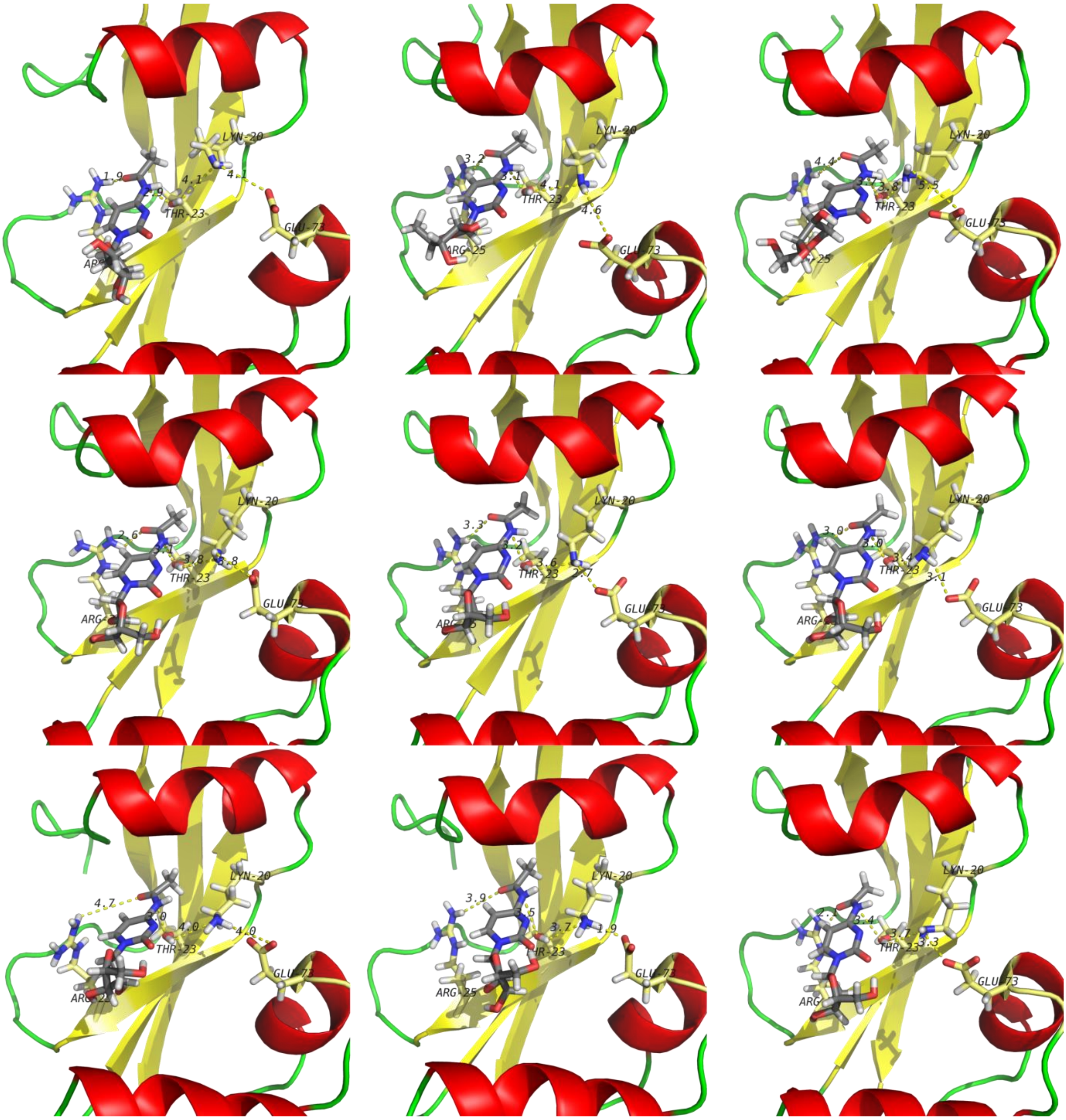

Supplementary Fig. 15. Snapshots of modelled enzyme-substrate complexes.

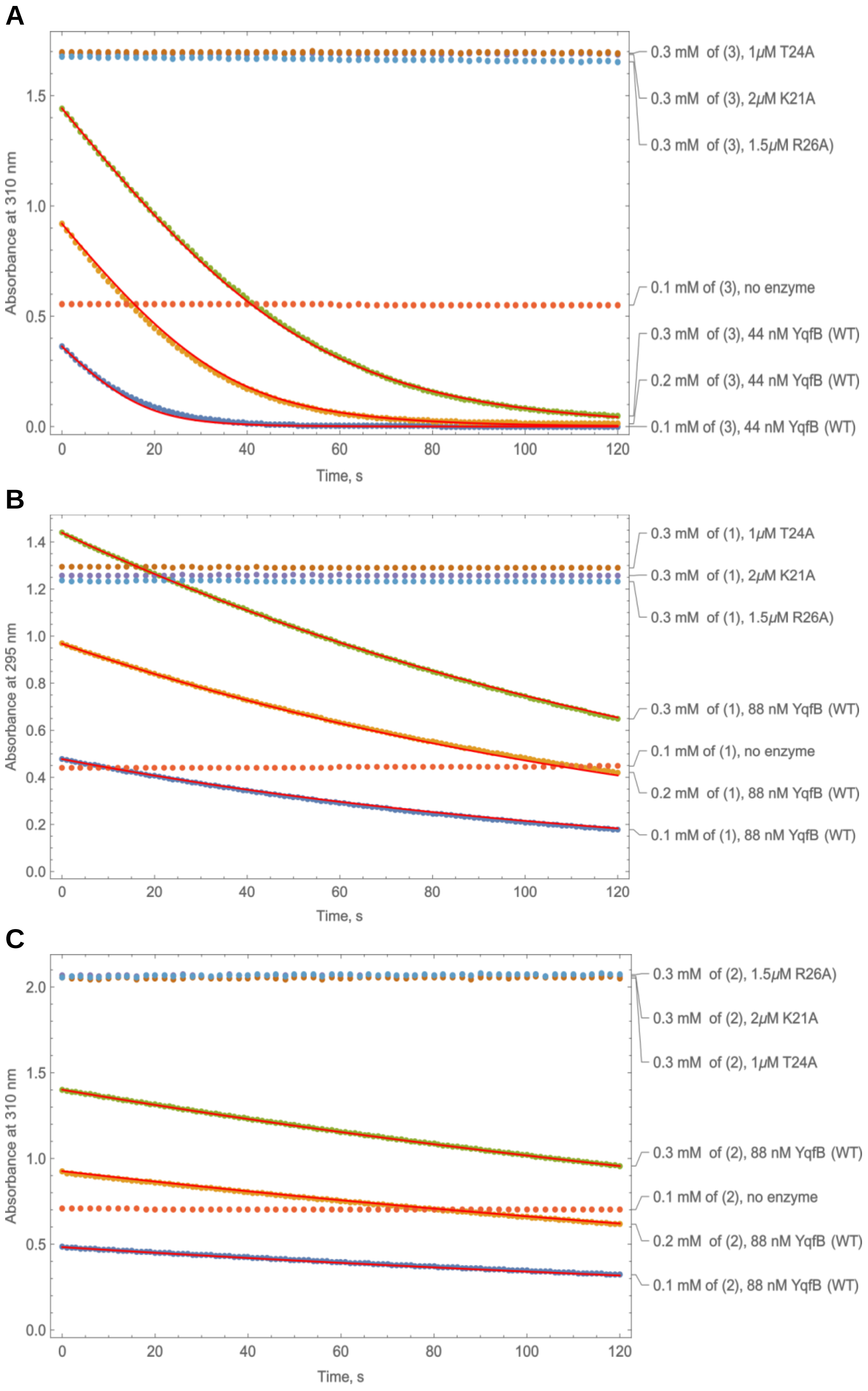

Supplementary Fig. 16. Examples of the kinetic traces of substrate hydrolysis in the presence or absence of the wild type YqfB or its inactive mutants. Red curves represent the best fits of data. (A) N^4-acetylcytidine (**3**), (B) N^4-acetylcytosine (**1**), (C) N^4-acetyl-5-fluorocytosine (**2**).

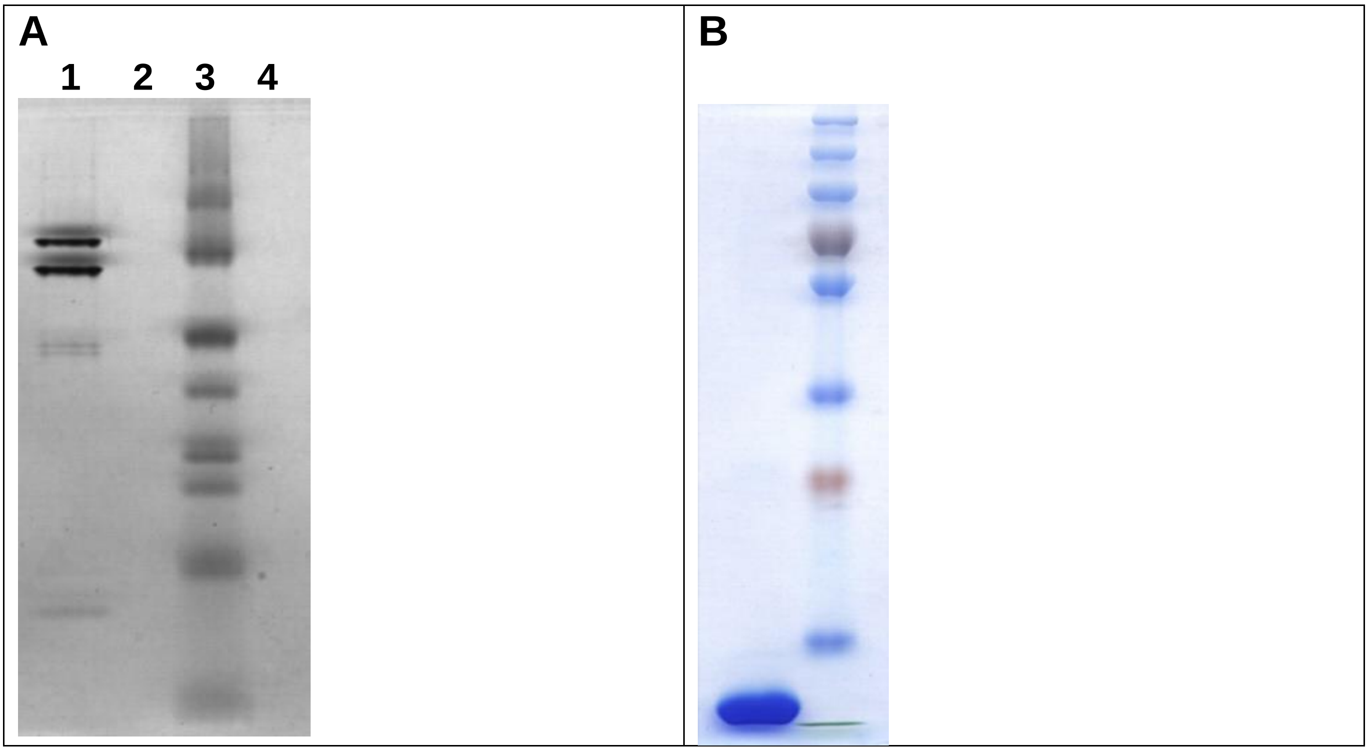

Supplementary Fig. 17. Analysis by gel electrophoresis. A – native PAGE (14%) analysis of the sample after Source 15Q chromatography; lane 1 – proteins stained with Coomassie Brilliant Blue R250, lane 2 – empty, lane 3 – molecular weight marker (from top to bottom: 170, 130, 100, 70, 55, 40, 35, 25, 15, and 10 kDa), lane 4 – empty; B – SDS-PAGE analysis of the purified recombinant YqfB harbouring a C-terminal His_6 tag.

References

1. Jakubovska, J., Tauraitė, D., Birštonas, L. & Meškys, R. *Nucleic Acids Res.* **46**, 5911–5923 (2018).
2. Duschinsky, R., Fells, E. & Hoffer, M. US3309359 (1967).
3. Bakke, J.M. & Riha, J. *J. Heterocyclic Chem.* **36**, 1143–1145 (1999).
4. Zeng, J., Tan, Y.J., Leow, M.L. & Liu, X.-W. *Org. Lett.* **14**, 4386-4389 (2012).
5. Tauraitė, D., Jakubovska, J., Dabužinskaitė, J., Bratchikov, M. & Meškys, R. *Molecules*, **22**, 72 (2017).
6. Nowak, I., Conda-Sheridan, M. & Robins M.J. *J. Org. Chem.*, **70**, 7455–7458 (2005).
