# Supplementary material for: YqfB protein from Escherichia coli: an atypical amidohydrolase active towards N4-acylcytosine derivatives
Source: Sci Rep. 2020 Jan 21;10:788. doi: 10.1038/s41598-020-57664-w (PMC6972931; doi:10.1038/s41598-020-57664-w)
Supplement: Supplementary file 2 — YqfB_model_Lys21_deprot. [file 41598_2020_57664_MOESM2_ESM.pdf]

YqfB protein from Escherichia coli: an atypical amidohydrolase active towards N4-acylcytosine derivatives

Rūta Stanislauskienėa,#,*, Audrius Laurynėnasb,#, Rasa Rutkienėa,#, Agota Aučynaitėa, Daiva Tauraitėa, Rita Meškienėa, Nina Urbelienėa, Algirdas Kaupinisc, Mindaugas Valiusc, Laura Kalinienea, Rolandas Meškysa

a Department of Molecular Microbiology and Biotechnology, b Department of Bioanalysis, and c Proteomics Centre, Institute of Biochemistry, Life Sciences Center, Vilnius University, Sauletekio al. 7, Vilnius LT-10257, Lithuania
# Those authors contributed equally to this work
* corresponding author ruta.stanislauskiene@bchi.vu.lt

The coordinates regarding protein YqfB Lys21_deprot structure are provided below.

```
MODEL      1
SHEET    1   1 1 ILE    22  ASP    26  0
SHEET    2   2 1 VAL    37  VAL    40  0
SHEET    3   3 1 CYX    50  VAL    60  0
SHEET    4   4 1 PHE    94  CYX   101  0
HELIX    1   1 ASP     13  LEU     16  1                                   4
HELIX    2   2 LYS     68  GLU     73  1                                   6
HELIX    3   3 LEU     77  ALA     85  1                                   9
ATOM      1  N   GLN     1      36.720  16.963  32.207  1.00  0.00           N
ATOM      2  H1  GLN     1      37.523  16.386  32.001  1.00  0.00           H
ATOM      3  H2  GLN     1      36.917  17.408  33.093  1.00  0.00           H
ATOM      4  H3  GLN     1      35.941  16.325  32.275  1.00  0.00           H
ATOM      5  CA  GLN     1      36.515  17.872  31.105  1.00  0.00           C
ATOM      6  HA  GLN     1      37.556  18.066  30.848  1.00  0.00           H
ATOM      7  CB  GLN     1      35.821  17.191  29.947  1.00  0.00           C
ATOM      8  HB2 GLN     1      34.913  16.691  30.283  1.00  0.00           H
ATOM      9  HB3 GLN     1      35.406  17.962  29.298  1.00  0.00           H
ATOM     10  CG  GLN     1      36.575  16.151  29.106  1.00  0.00           C
ATOM     11  HG2 GLN     1      37.012  15.428  29.794  1.00  0.00           H
ATOM     12  HG3 GLN     1      35.848  15.682  28.443  1.00  0.00           H
ATOM     13  CD  GLN     1      37.665  16.781  28.111  1.00  0.00           C
ATOM     14  OE1 GLN     1      38.237  17.799  28.496  1.00  0.00           O
ATOM     15  NE2 GLN     1      37.935  16.358  26.900  1.00  0.00           N
ATOM     16 HE21 GLN     1      37.570  15.498  26.516  1.00  0.00           H
ATOM     17 HE22 GLN     1      38.517  16.859  26.245  1.00  0.00           H
ATOM     18  C   GLN     1      35.957  19.225  31.577  1.00  0.00           C
ATOM     19  O   GLN     1      35.322  19.265  32.619  1.00  0.00           O
ATOM     20  N   PRO     2      36.187  20.326  30.845  1.00  0.00           N
ATOM     21  CD  PRO     2      37.317  20.445  29.840  1.00  0.00           C
ATOM     22  HD2 PRO     2      36.905  19.992  28.938  1.00  0.00           H
ATOM     23  HD3 PRO     2      38.249  19.978  30.157  1.00  0.00           H
ATOM     24  CG  PRO     2      37.442  21.902  29.502  1.00  0.00           C
ATOM     25  HG2 PRO     2      37.563  22.005  28.424  1.00  0.00           H
ATOM     26  HG3 PRO     2      38.342  22.192  30.043  1.00  0.00           H
ATOM     27  CB  PRO     2      36.186  22.597  30.046  1.00  0.00           C
ATOM     28  HB2 PRO     2      35.490  22.683  29.211  1.00  0.00           H
ATOM     29  HB3 PRO     2      36.453  23.593  30.398  1.00  0.00           H
ATOM     30  CA  PRO     2      35.624  21.642  31.175  1.00  0.00           C
ATOM     31  HA  PRO     2      36.015  21.915  32.155  1.00  0.00           H
ATOM     32  C   PRO     2      34.092  21.762  31.095  1.00  0.00           C
ATOM     33  O   PRO     2      33.354  21.054  30.452  1.00  0.00           O
ATOM     34  N   ASN     3      33.557  22.808  31.801  1.00  0.00           N
ATOM     35  H   ASN     3      34.195  23.430  32.276  1.00  0.00           H
ATOM     36  CA  ASN     3      32.115  23.075  31.880  1.00  0.00           C
ATOM     37  HA  ASN     3      31.603  22.122  32.012  1.00  0.00           H
ATOM     38  CB  ASN     3      31.758  24.020  33.088  1.00  0.00           C
ATOM     39  HB2 ASN     3      32.501  24.815  33.157  1.00  0.00           H
ATOM     40  HB3 ASN     3      30.717  24.323  32.986  1.00  0.00           H
```

ATOM	41	CG	ASN	3	31.866	23.323	34.416	1.00	0.00	C
ATOM	42	OD1	ASN	3	30.956	22.780	34.945	1.00	0.00	O
ATOM	43	ND2	ASN	3	33.083	23.111	34.861	1.00	0.00	N
ATOM	44	HD21	ASN	3	33.930	23.458	34.435	1.00	0.00	H
ATOM	45	HD22	ASN	3	33.123	22.339	35.511	1.00	0.00	H
ATOM	46	C	ASN	3	31.625	23.703	30.547	1.00	0.00	C
ATOM	47	O	ASN	3	32.356	24.587	30.008	1.00	0.00	O
ATOM	48	N	ASP	4	30.468	23.449	30.000	1.00	0.00	N
ATOM	49	H	ASP	4	29.780	22.892	30.486	1.00	0.00	H
ATOM	50	CA	ASP	4	30.001	24.172	28.812	1.00	0.00	C
ATOM	51	HA	ASP	4	30.772	24.633	28.195	1.00	0.00	H
ATOM	52	CB	ASP	4	29.165	23.101	27.986	1.00	0.00	C
ATOM	53	HB2	ASP	4	28.781	22.345	28.671	1.00	0.00	H
ATOM	54	HB3	ASP	4	28.357	23.614	27.465	1.00	0.00	H
ATOM	55	CG	ASP	4	30.030	22.273	27.031	1.00	0.00	C
ATOM	56	OD1	ASP	4	31.264	22.559	26.894	1.00	0.00	O
ATOM	57	OD2	ASP	4	29.535	21.305	26.384	1.00	0.00	O
ATOM	58	C	ASP	4	29.123	25.339	29.303	1.00	0.00	C
ATOM	59	O	ASP	4	28.720	26.119	28.461	1.00	0.00	O
ATOM	60	N	ILE	5	28.741	25.499	30.566	1.00	0.00	N
ATOM	61	H	ILE	5	28.981	24.799	31.253	1.00	0.00	H
ATOM	62	CA	ILE	5	28.256	26.824	31.024	1.00	0.00	C
ATOM	63	HA	ILE	5	27.429	27.170	30.403	1.00	0.00	H
ATOM	64	CB	ILE	5	27.650	26.820	32.476	1.00	0.00	C
ATOM	65	HB	ILE	5	28.454	26.673	33.198	1.00	0.00	H
ATOM	66	CG2	ILE	5	27.026	28.177	32.845	1.00	0.00	C
ATOM	67	HG21	ILE	5	26.392	28.488	32.014	1.00	0.00	H
ATOM	68	HG22	ILE	5	26.288	28.163	33.647	1.00	0.00	H
ATOM	69	HG23	ILE	5	27.817	28.902	33.033	1.00	0.00	H
ATOM	70	CG1	ILE	5	26.558	25.756	32.690	1.00	0.00	C
ATOM	71	HG12	ILE	5	25.706	25.969	32.044	1.00	0.00	H
ATOM	72	HG13	ILE	5	26.964	24.807	32.341	1.00	0.00	H
ATOM	73	CD1	ILE	5	26.088	25.546	34.167	1.00	0.00	C
ATOM	74	HD11	ILE	5	26.888	25.359	34.885	1.00	0.00	H
ATOM	75	HD12	ILE	5	25.367	26.304	34.472	1.00	0.00	H
ATOM	76	HD13	ILE	5	25.607	24.570	34.116	1.00	0.00	H
ATOM	77	C	ILE	5	29.300	27.902	31.027	1.00	0.00	C
ATOM	78	O	ILE	5	30.429	27.707	31.496	1.00	0.00	O
ATOM	79	N	THR	6	28.974	29.011	30.458	1.00	0.00	N
ATOM	80	H	THR	6	28.064	29.173	30.052	1.00	0.00	H
ATOM	81	CA	THR	6	29.873	30.255	30.529	1.00	0.00	C
ATOM	82	HA	THR	6	30.482	30.235	31.434	1.00	0.00	H
ATOM	83	CB	THR	6	30.870	30.265	29.313	1.00	0.00	C
ATOM	84	HB	THR	6	31.394	29.309	29.305	1.00	0.00	H
ATOM	85	CG2	THR	6	30.181	30.247	27.928	1.00	0.00	C
ATOM	86	HG21	THR	6	29.603	29.323	27.902	1.00	0.00	H
ATOM	87	HG22	THR	6	29.584	31.124	27.682	1.00	0.00	H
ATOM	88	HG23	THR	6	30.916	30.237	27.123	1.00	0.00	H
ATOM	89	OG1	THR	6	31.861	31.260	29.448	1.00	0.00	O
ATOM	90	HG1	THR	6	32.374	30.981	30.210	1.00	0.00	H
ATOM	91	C	THR	6	28.973	31.528	30.743	1.00	0.00	C
ATOM	92	O	THR	6	27.729	31.461	31.001	1.00	0.00	O
ATOM	93	N	PHE	7	29.667	32.672	30.481	1.00	0.00	N
ATOM	94	H	PHE	7	30.649	32.554	30.278	1.00	0.00	H
ATOM	95	CA	PHE	7	29.114	34.068	30.397	1.00	0.00	C
ATOM	96	HA	PHE	7	28.455	34.285	31.237	1.00	0.00	H
ATOM	97	CB	PHE	7	30.298	34.954	30.675	1.00	0.00	C
ATOM	98	HB2	PHE	7	30.796	35.155	29.727	1.00	0.00	H
ATOM	99	HB3	PHE	7	29.809	35.848	31.063	1.00	0.00	H
ATOM	100	CG	PHE	7	31.352	34.416	31.680	1.00	0.00	C
ATOM	101	CD1	PHE	7	32.513	33.782	31.147	1.00	0.00	C
ATOM	102	HD1	PHE	7	32.558	33.609	30.082	1.00	0.00	H
ATOM	103	CE1	PHE	7	33.409	33.192	32.094	1.00	0.00	C
ATOM	104	HE1	PHE	7	34.262	32.679	31.676	1.00	0.00	H

ATOM	105	CZ	PHE	7	33.299	33.362	33.442	1.00	0.00	C
ATOM	106	HZ	PHE	7	34.069	33.018	34.116	1.00	0.00	H
ATOM	107	CE2	PHE	7	32.182	34.090	33.932	1.00	0.00	C
ATOM	108	HE2	PHE	7	32.011	34.139	34.997	1.00	0.00	H
ATOM	109	CD2	PHE	7	31.217	34.699	33.047	1.00	0.00	C
ATOM	110	HD2	PHE	7	30.325	35.215	33.370	1.00	0.00	H
ATOM	111	C	PHE	7	28.449	34.388	29.013	1.00	0.00	C
ATOM	112	O	PHE	7	28.605	33.687	28.023	1.00	0.00	O
ATOM	113	N	PHE	8	27.835	35.589	29.031	1.00	0.00	N
ATOM	114	H	PHE	8	27.808	36.111	29.896	1.00	0.00	H
ATOM	115	CA	PHE	8	27.494	36.416	27.836	1.00	0.00	C
ATOM	116	HA	PHE	8	26.851	35.885	27.134	1.00	0.00	H
ATOM	117	CB	PHE	8	26.684	37.660	28.247	1.00	0.00	C
ATOM	118	HB2	PHE	8	27.402	38.307	28.750	1.00	0.00	H
ATOM	119	HB3	PHE	8	26.371	38.251	27.387	1.00	0.00	H
ATOM	120	CG	PHE	8	25.414	37.421	29.084	1.00	0.00	C
ATOM	121	CD1								
PHE	8	24.339	36.639	28.584	1.00	0.00			C	
ATOM	122	HD1	PHE	8	24.438	36.236	27.587	1.00	0.00	H
ATOM	123	CE1	PHE	8	23.164	36.427	29.305	1.00	0.00	C
ATOM	124	HE1	PHE	8	22.330	35.814	28.997	1.00	0.00	H
ATOM	125	CZ	PHE	8	22.994	37.091	30.537	1.00	0.00	C
ATOM	126	HZ	PHE	8	22.096	36.957	31.122	1.00	0.00	H
ATOM	127	CE2	PHE	8	24.112	37.795	31.056	1.00	0.00	C
ATOM	128	HE2	PHE	8	24.019	38.274	32.019	1.00	0.00	H
ATOM	129	CD2	PHE	8	25.288	37.998	30.362	1.00	0.00	C
ATOM	130	HD2	PHE	8	26.013	38.554	30.938	1.00	0.00	H
ATOM	131	C	PHE	8	28.860	36.695	27.128	1.00	0.00	C
ATOM	132	O	PHE	8	29.843	36.786	27.867	1.00	0.00	O
ATOM	133	N	GLN	9	28.904	36.845	25.840	1.00	0.00	N
ATOM	134	H	GLN	9	28.015	36.771	25.366	1.00	0.00	H
ATOM	135	CA	GLN	9	30.165	37.107	25.088	1.00	0.00	C
ATOM	136	HA	GLN	9	30.921	36.335	25.231	1.00	0.00	H
ATOM	137	CB	GLN	9	29.800	36.960	23.605	1.00	0.00	C
ATOM	138	HB2	GLN	9	28.940	37.607	23.430	1.00	0.00	H
ATOM	139	HB3	GLN	9	30.704	37.201	23.047	1.00	0.00	H
ATOM	140	CG	GLN	9	29.589	35.565	23.011	1.00	0.00	C
ATOM	141	HG2	GLN	9	30.488	35.092	23.405	1.00	0.00	H
ATOM	142	HG3	GLN	9	28.650	35.146	23.371	1.00	0.00	H
ATOM	143	CD	GLN	9	29.627	35.517	21.495	1.00	0.00	C
ATOM	144	OE1	GLN	9	29.559	36.516	20.782	1.00	0.00	O
ATOM	145	NE2	GLN	9	29.704	34.367	20.804	1.00	0.00	N
ATOM	146	HE21	GLN	9	29.746	34.416	19.796	1.00	0.00	H
ATOM	147	HE22	GLN	9	29.796	33.479	21.275	1.00	0.00	H
ATOM	148	C	GLN	9	30.701	38.503	25.238	1.00	0.00	C
ATOM	149	O	GLN	9	31.859	38.805	25.012	1.00	0.00	O
ATOM	150	N	ARG	10	29.815	39.452	25.611	1.00	0.00	N
ATOM	151	H	ARG	10	28.832	39.227	25.675	1.00	0.00	H
ATOM	152	CA	ARG	10	30.128	40.875	25.942	1.00	0.00	C
ATOM	153	HA	ARG	10	30.574	41.270	25.029	1.00	0.00	H
ATOM	154	CB	ARG	10	28.763	41.564	26.265	1.00	0.00	C
ATOM	155	HB2	ARG	10	28.183	41.605	25.343	1.00	0.00	H
ATOM	156	HB3	ARG	10	28.120	40.999	26.939	1.00	0.00	H
ATOM	157	CG	ARG	10	28.876	43.007	26.839	1.00	0.00	C
ATOM	158	HG2	ARG	10	29.586	42.966	27.665	1.00	0.00	H
ATOM	159	HG3	ARG	10	29.293	43.712	26.120	1.00	0.00	H
ATOM	160	CD	ARG	10	27.549	43.592	27.053	1.00	0.00	C
ATOM	161	HD2	ARG	10	26.993	43.731	26.126	1.00	0.00	H
ATOM	162	HD3	ARG	10	26.919	43.097	27.793	1.00	0.00	H
ATOM	163	NE	ARG	10	27.599	44.980	27.637	1.00	0.00	N
ATOM	164	HE	ARG	10	28.326	45.631	27.376	1.00	0.00	H
ATOM	165	CZ	ARG	10	26.677	45.570	28.371	1.00	0.00	C
ATOM	166	NH1	ARG	10	25.610	45.051	28.854	1.00	0.00	N
ATOM	167	HH11	ARG	10	24.919	45.601	29.343	1.00	0.00	H

ATOM	168	HH12	ARG	10	25.438	44.056	28.857	1.00	0.00	H
ATOM	169	NH2	ARG	10	26.778	46.891	28.584	1.00	0.00	N
ATOM	170	HH21	ARG	10	27.597	47.340	28.200	1.00	0.00	H
ATOM	171	HH22	ARG	10	26.251	47.136	29.410	1.00	0.00	H
ATOM	172	C	ARG	10	31.151	41.078	27.026	1.00	0.00	C
ATOM	173	O	ARG	10	31.819	42.076	27.246	1.00	0.00	O
ATOM	174	N	PHE	11	31.196	40.013	27.867	1.00	0.00	N
ATOM	175	H	PHE	11	30.587	39.261	27.581	1.00	0.00	H
ATOM	176	CA	PHE	11	31.925	39.911	29.111	1.00	0.00	C
ATOM	177	HA	PHE	11	32.543	40.802	29.217	1.00	0.00	H
ATOM	178	CB	PHE	11	30.910	40.061	30.275	1.00	0.00	C
ATOM	179	HB2	PHE	11	31.634	40.097	31.090	1.00	0.00	H
ATOM	180	HB3	PHE	11	30.225	39.219	30.367	1.00	0.00	H
ATOM	181	CG	PHE	11	30.152	41.360	30.274	1.00	0.00	C
ATOM	182	CD1	PHE	11	30.918	42.504	30.526	1.00	0.00	C
ATOM	183	HD1	PHE	11	31.970	42.365	30.726	1.00	0.00	H
ATOM	184	CE1	PHE	11	30.225	43.703	30.567	1.00	0.00	C
ATOM	185	HE1	PHE	11	30.720	44.646	30.742	1.00	0.00	H
ATOM	186	CZ	PHE	11	28.823	43.721	30.534	1.00	0.00	C
ATOM	187	HZ	PHE	11	28.299	44.656	30.669	1.00	0.00	H
ATOM	188	CE2	PHE	11	28.111	42.556	30.395	1.00	0.00	C
ATOM	189	HE2	PHE	11	27.039	42.603	30.517	1.00	0.00	H
ATOM	190	CD2	PHE	11	28.768	41.327	30.286	1.00	0.00	C
ATOM	191	HD2	PHE	11	28.211	40.433	30.051	1.00	0.00	H
ATOM	192	C	PHE	11	32.777	38.655	29.224	1.00	0.00	C
ATOM	193	O	PHE	11	32.287	37.649	29.771	1.00	0.00	O
ATOM	194	N	GLN	12	33.940	38.723	28.658	1.00	0.00	N
ATOM	195	H	GLN	12	34.109	39.520	28.060	1.00	0.00	H
ATOM	196	CA	GLN	12	34.964	37.583	28.449	1.00	0.00	C
ATOM	197	HA	GLN	12	34.814	36.782	29.173	1.00	0.00	H
ATOM	198	CB	GLN	12	34.780	36.840	27.129	1.00	0.00	C
ATOM	199	HB2	GLN	12	35.017	37.612	26.396	1.00	0.00	H
ATOM	200	HB3	GLN	12	35.485	36.009	27.135	1.00	0.00	H
ATOM	201	CG	GLN	12	33.425	36.202	26.797	1.00	0.00	C
ATOM	202	HG2	GLN	12	33.114	35.478	27.550	1.00	0.00	H
ATOM	203	HG3	GLN	12	32.685	36.997	26.885	1.00	0.00	H
ATOM	204	CD	GLN	12	33.356	35.574	25.397	1.00	0.00	C
ATOM	205	OE1	GLN	12	34.021	36.068	24.457	1.00	0.00	O
ATOM	206	NE2	GLN	12	32.580	34.492	25.235	1.00	0.00	N
ATOM	207	HE21	GLN	12	32.067	33.989	25.945	1.00	0.00	H
ATOM	208	HE22	GLN	12	32.621	33.969	24.373	1.00	0.00	H
ATOM	209	C	GLN	12	36.457	38.097	28.591	1.00	0.00	C
ATOM	210	O	GLN	12	37.370	37.398	29.045	1.00	0.00	O
ATOM	211	N	ASP	13	36.762	39.367	28.169	1.00	0.00	N
ATOM	212	H	ASP	13	36.078	40.095	28.021	1.00	0.00	H
ATOM	213	CA	ASP	13	38.097	39.924	27.941	1.00	0.00	C
ATOM	214	HA	ASP	13	38.875	39.238	27.604	1.00	0.00	H
ATOM	215	CB	ASP	13	38.208	40.815	26.699	1.00	0.00	C
ATOM	216	HB2	ASP	13	37.327	41.452	26.635	1.00	0.00	H
ATOM	217	HB3	ASP	13	39.075	41.471	26.777	1.00	0.00	H
ATOM	218	CG	ASP	13	38.167	40.055	25.347	1.00	0.00	C
ATOM	219	OD1	ASP	13	38.301	40.834	24.384	1.00	0.00	O
ATOM	220	OD2	ASP	13	38.165	38.802	25.199	1.00	0.00	O
ATOM	221	C	ASP	13	38.716	40.501	29.204	1.00	0.00	C
ATOM	222	O	ASP	13	39.949	40.511	29.300	1.00	0.00	O
ATOM	223	N	ASP	14	37.955	40.847	30.195	1.00	0.00	N
ATOM	224	H	ASP	14	36.989	40.619	30.008	1.00	0.00	H
ATOM	225	CA	ASP	14	38.438	41.170	31.493	1.00	0.00	C
ATOM	226	HA	ASP	14	39.055	42.061	31.382	1.00	0.00	H
ATOM	227	CB	ASP	14	37.252	41.759	32.271	1.00	0.00	C
ATOM	228	HB2	ASP	14	36.462	41.039	32.484	1.00	0.00	H
ATOM	229	HB3	ASP	14	37.783	42.047	33.178	1.00	0.00	H
ATOM	230	CG	ASP	14	36.686	43.077	31.767	1.00	0.00	C
ATOM	231	OD1	ASP	14	35.432	43.172	31.732	1.00	0.00	O

ATOM	232	OD2	ASP	14	37.404	44.010	31.385	1.00	0.00	O
ATOM	233	C	ASP	14	39.178	40.119	32.270	1.00	0.00	C
ATOM	234	O	ASP	14	39.910	40.364	33.222	1.00	0.00	O
ATOM	235	N	ILE	15	38.960	38.798	31.999	1.00	0.00	N
ATOM	236	H	ILE	15	38.280	38.696	31.259	1.00	0.00	H
ATOM	237	CA	ILE	15	39.420	37.714	32.818	1.00	0.00	C
ATOM	238	HA	ILE	15	39.181	37.887	33.868	1.00	0.00	H
ATOM	239	CB	ILE	15	38.742	36.419	32.382	1.00	0.00	C
ATOM	240	HB	ILE	15	39.051	36.153	31.370	1.00	0.00	H
ATOM	241	CG2	ILE	15	39.271	35.331	33.336	1.00	0.00	C
ATOM	242	HG21	ILE	15	38.674	34.422	33.273	1.00	0.00	H
ATOM	243	HG22	ILE	15	40.336	35.167	33.167	1.00	0.00	H
ATOM	244	HG23	ILE	15	39.183	35.693	34.360	1.00	0.00	H
ATOM	245	CG1	ILE	15	37.223	36.453	32.305	1.00	0.00	C
ATOM	246	HG12	ILE	15	36.903	36.628	33.332	1.00	0.00	H
ATOM	247	HG13	ILE	15	36.825	37.254	31.683	1.00	0.00	H
ATOM	248	CD1	ILE	15	36.639	35.195	31.730	1.00	0.00	C
ATOM	249	HD11	ILE	15	37.251	34.814	30.912	1.00	0.00	H
ATOM	250	HD12	ILE	15	36.502	34.321	32.367	1.00	0.00	H
ATOM	251	HD13	ILE	15	35.743	35.431	31.155	1.00	0.00	H
ATOM	252	C	ILE	15	40.921	37.573	32.693	1.00	0.00	C
ATOM	253	O	ILE	15	41.621	37.809	33.685	1.00	0.00	O
ATOM	254	N	LEU	16	41.453	37.331	31.489	1.00	0.00	N
ATOM	255	H	LEU	16	40.861	37.395	30.673	1.00	0.00	H
ATOM	256	CA	LEU	16	42.885	37.246	31.172	1.00	0.00	C
ATOM	257	HA	LEU	16	43.255	36.456	31.825	1.00	0.00	H
ATOM	258	CB	LEU	16	43.169	36.826	29.670	1.00	0.00	C
ATOM	259	HB2	LEU	16	42.627	35.908	29.443	1.00	0.00	H
ATOM	260	HB3	LEU	16	42.803	37.573	28.966	1.00	0.00	H
ATOM	261	CG	LEU	16	44.680	36.538	29.433	1.00	0.00	C
ATOM	262	HG	LEU	16	45.175	37.485	29.647	1.00	0.00	H
ATOM	263	CD1	LEU	16	45.338	35.366	30.298	1.00	0.00	C
ATOM	264	HD11	LEU	16	45.249	35.533	31.372	1.00	0.00	H
ATOM	265	HD12	LEU	16	44.730	34.497	30.046	1.00	0.00	H
ATOM	266	HD13	LEU	16	46.385	35.219	30.034	1.00	0.00	H
ATOM	267	CD2	LEU	16	44.931	36.223	27.973	1.00	0.00	C
ATOM	268	HD21	LEU	16	44.668	37.102	27.384	1.00	0.00	H
ATOM	269	HD22	LEU	16	46.013	36.138	27.874	1.00	0.00	H
ATOM	270	HD23	LEU	16	44.395	35.329	27.655	1.00	0.00	H
ATOM	271	C	LEU	16	43.600	38.546	31.492	1.00	0.00	C
ATOM	272	O	LEU	16	44.740	38.484	31.971	1.00	0.00	O
ATOM	273	N	ALA	17	42.937	39.713	31.279	1.00	0.00	N
ATOM	274	H	ALA	17	42.073	39.613	30.765	1.00	0.00	H
ATOM	275	CA	ALA	17	43.355	41.104	31.659	1.00	0.00	C
ATOM	276	HA	ALA	17	44.352	41.128	31.218	1.00	0.00	H
ATOM	277	CB	ALA	17	42.444	42.170	31.054	1.00	0.00	C
ATOM	278	HB1	ALA	17	41.468	42.154	31.540	1.00	0.00	H
ATOM	279	HB2	ALA	17	42.868	43.150	31.272	1.00	0.00	H
ATOM	280	HB3	ALA	17	42.171	42.046	30.006	1.00	0.00	H
ATOM	281	C	ALA	17	43.580	41.344	33.129	1.00	0.00	C
ATOM	282	O	ALA	17	43.806	42.474	33.585	1.00	0.00	O
ATOM	283	N	GLY	18	43.304	40.375	34.052	1.00	0.00	N
ATOM	284	H	GLY	18	43.046	39.469	33.688	1.00	0.00	H
ATOM	285	CA	GLY	18	43.426	40.532	35.510	1.00	0.00	C
ATOM	286	HA2	GLY	18	43.148	39.520	35.804	1.00	0.00	H
ATOM	287	HA3	GLY	18	44.419	40.881	35.795	1.00	0.00	H
ATOM	288	C	GLY	18	42.395	41.492	36.113	1.00	0.00	C
ATOM	289	O	GLY	18	42.510	41.859	37.246	1.00	0.00	O
ATOM	290	N	ARG	19	41.318	41.852	35.366	1.00	0.00	N
ATOM	291	H	ARG	19	41.276	41.427	34.451	1.00	0.00	H
ATOM	292	CA	ARG	19	40.267	42.869	35.765	1.00	0.00	C
ATOM	293	HA	ARG	19	40.588	43.470	36.616	1.00	0.00	H
ATOM	294	CB	ARG	19	39.867	43.746	34.620	1.00	0.00	C

ATOM	295	HB2	ARG	19	39.630	43.201	33.706	1.00	0.00	H
ATOM	296	HB3	ARG	19	38.967	44.205	35.029	1.00	0.00	H
ATOM	297	CG	ARG	19	40.917	44.827	34.334	1.00	0.00	C
ATOM	298	HG2	ARG	19	41.366	45.240	35.237	1.00	0.00	H
ATOM	299	HG3	ARG	19	41.635	44.316	33.692	1.00	0.00	H
ATOM	300	CD	ARG	19	40.508	45.945	33.356	1.00	0.00	C
ATOM	301	HD2	ARG	19	39.838	46.632	33.872	1.00	0.00	H
ATOM	302	HD3	ARG	19	41.328	46.582	33.021	1.00	0.00	H
ATOM	303	NE	ARG	19	39.790	45.438	32.186	1.00	0.00	N
ATOM	304	HE	ARG	19	38.844	45.095	32.262	1.00	0.00	H
ATOM	305	CZ	ARG	19	40.246	45.455	30.943	1.00	0.00	C
ATOM	306	NH1	ARG	19	41.231	46.169	30.579	1.00	0.00	N
ATOM	307	HH11	ARG	19	41.892	46.578	31.223	1.00	0.00	H
ATOM	308	HH12	ARG	19	41.417	46.274	29.592	1.00	0.00	H
ATOM	309	NH2	ARG	19	39.530	44.835	30.050	1.00	0.00	N
ATOM	310	HH21	ARG	19	39.882	44.690	29.115	1.00	0.00	H
ATOM	311	HH22	ARG	19	38.618	44.465	30.278	1.00	0.00	H
ATOM	312	C	ARG	19	39.082	42.120	36.386	1.00	0.00	C
ATOM	313	O	ARG	19	38.375	42.765	37.192	1.00	0.00	O
ATOM	335	N	THR	21	38.429	38.564	38.260	1.00	0.00	N
ATOM	336	H	THR	21	38.407	39.393	38.837	1.00	0.00	H
ATOM	337	CA	THR	21	39.070	37.340	38.844	1.00	0.00	C
ATOM	338	HA	THR	21	38.748	36.522	38.199	1.00	0.00	H
ATOM	339	CB	THR	21	40.656	37.525	38.819	1.00	0.00	C
ATOM	340	HB	THR	21	41.071	36.573	39.150	1.00	0.00	H
ATOM	341	CG2	THR	21	41.265	37.898	37.438	1.00	0.00	C
ATOM	342	HG21	THR	21	40.833	37.295	36.640	1.00	0.00	H
ATOM	343	HG22	THR	21	40.848	38.873	37.186	1.00	0.00	H
ATOM	344	HG23	THR	21	42.350	37.791	37.414	1.00	0.00	H
ATOM	345	OG1	THR	21	41.093	38.459	39.879	1.00	0.00	O
ATOM	346	HG1	THR	21	42.004	38.244	40.093	1.00	0.00	H
ATOM	347	C	THR	21	38.618	37.005	40.247	1.00	0.00	C
ATOM	348	O	THR	21	39.191	36.104	40.878	1.00	0.00	O
ATOM	349	N	ILE	22	37.621	37.718	40.724	1.00	0.00	N
ATOM	350	H	ILE	22	37.488	38.634	40.320	1.00	0.00	H
ATOM	351	CA	ILE	22	36.836	37.564	41.905	1.00	0.00	C
ATOM	352	HA	ILE	22	36.700	36.494	42.062	1.00	0.00	H
ATOM	353	CB	ILE	22	37.496	38.092	43.231	1.00	0.00	C
ATOM	354	HB	ILE	22	38.339	37.446	43.478	1.00	0.00	H
ATOM	355	CG2	ILE	22	38.042	39.513	43.239	1.00	0.00	C
ATOM	356	HG21	ILE	22	38.444	39.725	42.248	1.00	0.00	H
ATOM	357	HG22	ILE	22	37.401	40.321	43.593	1.00	0.00	H
ATOM	358	HG23	ILE	22	38.888	39.565	43.923	1.00	0.00	H
ATOM	359	CG1	ILE	22	36.619	37.783	44.444	1.00	0.00	C
ATOM	360	HG12	ILE	22	35.773	38.470	44.436	1.00	0.00	H
ATOM	361	HG13	ILE	22	36.217	36.785	44.270	1.00	0.00	H
ATOM	362	CD1	ILE	22	37.197	37.903	45.832	1.00	0.00	C
ATOM	363	HD11	ILE	22	37.943	37.118	45.956	1.00	0.00	H
ATOM	364	HD12	ILE	22	37.617	38.906	45.904	1.00	0.00	H
ATOM	365	HD13	ILE	22	36.398	37.864	46.572	1.00	0.00	H
ATOM	366	C	ILE	22	35.406	38.044	41.590	1.00	0.00	C
ATOM	367	O	ILE	22	35.245	38.942	40.733	1.00	0.00	O
ATOM	368	N	THR	23	34.402	37.470	42.131	1.00	0.00	N
ATOM	369	H	THR	23	34.545	36.628	42.669	1.00	0.00	H
ATOM	370	CA	THR	23	32.972	37.951	41.934	1.00	0.00	C
ATOM	371	HA	THR	23	33.046	39.036	41.862	1.00	0.00	H
ATOM	372	CB	THR	23	32.394	37.509	40.580	1.00	0.00	C
ATOM	373	HB	THR	23	33.141	37.612	39.793	1.00	0.00	H
ATOM	374	CG2	THR	23	31.680	36.124	40.532	1.00	0.00	C
ATOM	375	HG21	THR	23	30.638	36.199	40.843	1.00	0.00	H
ATOM	376	HG22	THR	23	31.695	35.670	39.541	1.00	0.00	H
ATOM	377	HG23	THR	23	32.270	35.519	41.221	1.00	0.00	H
ATOM	378	OG1	THR	23	31.371	38.449	40.286	1.00	0.00	O
ATOM	379	HG1	THR	23	31.742	39.220	39.850	1.00	0.00	H

```
ATOM    380  C   THR    23      32.175  37.561  43.150  1.00  0.00           C
ATOM    381  O   THR    23      32.325  36.488  43.696  1.00  0.00           O
ATOM    382  N   ILE    24      31.171  38.357  43.554  1.00  0.00           N
ATOM    383  H   ILE    24      30.949  39.208  43.057  1.00  0.00           H
ATOM    384  CA  ILE    24      30.280  38.018  44.736  1.00  0.00           C
ATOM    385  HA  ILE    24      30.645  37.140  45.269  1.00  0.00           H
ATOM    386  CB  ILE    24      30.206  39.232  45.688  1.00  0.00           C
ATOM    387  HB  ILE    24      29.964  40.130  45.120  1.00  0.00           H
ATOM    388  CG2 ILE    24      28.960  39.136  46.573  1.00  0.00           C
ATOM    389 HG21 ILE    24      29.077  38.367  47.337  1.00  0.00           H
ATOM    390 HG22 ILE    24      28.943  40.093  47.094  1.00  0.00           H
ATOM    391 HG23 ILE    24      28.052  39.219  45.976  1.00  0.00           H
ATOM    392  CG1 ILE    24      31.556  39.456  46.454  1.00  0.00
      C
ATOM    393 HG12 ILE    24      31.475  38.764  47.292  1.00  0.00           H
ATOM    394 HG13 ILE    24      32.440  39.309  45.833  1.00  0.00           H
ATOM    395  CD1 ILE    24      31.760  40.882  47.010  1.00  0.00           C
ATOM    396 HD11 ILE    24      31.000  41.241  47.705  1.00  0.00           H
ATOM    397 HD12 ILE    24      32.679  40.948  47.592  1.00  0.00           H
ATOM    398 HD13 ILE    24      31.786  41.626  46.214  1.00  0.00           H
ATOM    399  C   ILE    24      28.852  37.598  44.228  1.00  0.00           C
ATOM    400  O   ILE    24      28.257  38.183  43.262  1.00  0.00           O
ATOM    401  N   ARG    25      28.207  36.642  44.830  1.00  0.00           N
ATOM    402  H   ARG    25      28.741  36.258  45.597  1.00  0.00           H
ATOM    403  CA  ARG    25      26.873  36.074  44.586  1.00  0.00           C
ATOM    404  HA  ARG    25      26.279  36.827  44.067  1.00  0.00           H
ATOM    405  CB  ARG    25      26.955  34.723  43.861  1.00  0.00           C
ATOM    406  HB2 ARG    25      27.483  33.958  44.432  1.00  0.00           H
ATOM    407  HB3 ARG    25      25.947  34.310  43.830  1.00  0.00           H
ATOM    408  CG  ARG    25      27.470  34.824  42.437  1.00  0.00           C
ATOM    409  HG2 ARG    25      28.425  35.348  42.420  1.00  0.00           H
ATOM    410  HG3 ARG    25      27.529  33.793  42.087  1.00  0.00           H
ATOM    411  CD  ARG    25      26.477  35.513  41.528  1.00  0.00           C
ATOM    412  HD2 ARG    25      25.532  34.973  41.591  1.00  0.00           H
ATOM    413  HD3 ARG    25      26.274  36.553  41.786  1.00  0.00           H
ATOM    414  NE  ARG    25      27.012  35.613  40.156  1.00  0.00           N
ATOM    415  HE  ARG    25      26.835  34.863  39.503  1.00  0.00           H
ATOM    416  CZ  ARG    25      27.906  36.473  39.687  1.00  0.00           C
ATOM    417  NH1 ARG    25      28.446  37.402  40.451  1.00  0.00           N
ATOM    418 HH11 ARG    25      29.243  37.855  40.027  1.00  0.00           H
ATOM    419 HH12 ARG    25      28.096  37.679  41.357  1.00  0.00           H
ATOM    420  NH2 ARG    25      28.341  36.388  38.460  1.00  0.00           N
ATOM    421 HH21 ARG    25      28.990  37.088  38.129  1.00  0.00           H
ATOM    422 HH22 ARG    25      28.006  35.746  37.755  1.00  0.00           H
ATOM    423  C   ARG    25      26.116  35.861  45.982  1.00  0.00           C
ATOM    424  O   ARG    25      26.788  35.818  47.026  1.00  0.00           O
ATOM    425  N   ASP    26      24.794  35.803  45.989  1.00  0.00           N
ATOM    426  H   ASP    26      24.258  35.882  45.137  1.00  0.00           H
ATOM    427  CA  ASP    26      24.113  35.234  47.125  1.00  0.00           C
ATOM    428  HA  ASP    26      24.406  35.638  48.094  1.00  0.00           H
ATOM    429  CB  ASP    26      22.606  35.633  46.960  1.00  0.00           C
ATOM    430  HB2 ASP    26      22.101  35.164  47.805  1.00  0.00           H
ATOM    431  HB3 ASP    26      22.444  36.704  47.082  1.00  0.00           H
ATOM    432  CG  ASP    26      21.918  35.055  45.690  1.00  0.00           C
ATOM    433  OD1 ASP    26      22.379  35.390  44.587  1.00  0.00           O
ATOM    434  OD2 ASP    26      20.974  34.322  45.763  1.00  0.00           O
ATOM    435  C   ASP    26      24.486  33.691  47.119  1.00  0.00           C
ATOM    436  O   ASP    26      24.626  32.941  46.134  1.00  0.00           O
ATOM    437  N   GLU    27      24.312  33.120  48.253  1.00  0.00           N
ATOM    438  H   GLU    27      24.218  33.793  49.000  1.00  0.00           H
ATOM    439  CA  GLU    27      24.308  31.729  48.567  1.00  0.00           C
ATOM    440  HA  GLU    27      25.334  31.371  48.478  1.00  0.00           H
ATOM    441  CB  GLU    27      23.891  31.536  50.020  1.00  0.00           C
ATOM    442  HB2 GLU    27      24.543  32.147  50.645  1.00  0.00           H
```

ATOM	443	HB3	GLU	27	22.889	31.938	50.166	1.00	0.00	H
ATOM	444	CG	GLU	27	23.976	30.063	50.500	1.00	0.00	C
ATOM	445	HG2	GLU	27	24.656	29.445	49.913	1.00	0.00	H
ATOM	446	HG3	GLU	27	24.378	29.960	51.508	1.00	0.00	H
ATOM	447	CD	GLU	27	22.651	29.334	50.504	1.00	0.00	C
ATOM	448	OE1	GLU	27	21.604	29.850	51.010	1.00	0.00	O
ATOM	449	OE2	GLU	27	22.632	28.151	50.077	1.00	0.00	O
ATOM	450	C	GLU	27	23.473	30.773	47.682	1.00	0.00	C
ATOM	451	O	GLU	27	22.303	30.922	47.529	1.00	0.00	O
ATOM	452	N	SER	28	24.146	29.878	47.028	1.00	0.00	N
ATOM	453	H	SER	28	25.151	29.972	46.990	1.00	0.00	H
ATOM	454	CA	SER	28	23.534	28.707	46.456	1.00	0.00	C
ATOM	455	HA	SER	28	22.608	28.457	46.974	1.00	0.00	H
ATOM	456	CB	SER	28	23.072	28.869	45.071	1.00	0.00	C
ATOM	457	HB2	SER	28	22.329	29.661	44.974	1.00	0.00	H
ATOM	458	HB3	SER	28	23.914	29.109	44.422	1.00	0.00	H
ATOM	459	OG	SER	28	22.529	27.694	44.469	1.00	0.00	O
ATOM	460	HG	SER	28	22.247	27.999	43.604	1.00	0.00	H
ATOM	461	C	SER	28	24.607	27.601	46.389	1.00	0.00	C
ATOM	462	O	SER	28	25.725	27.757	46.010	1.00	0.00	O
ATOM	463	N	GLU	29	24.150	26.355	46.743	1.00	0.00	N
ATOM	464	H	GLU	29	23.195	26.309	47.067	1.00	0.00	H
ATOM	465	CA	GLU	29	24.975	25.176	46.642	1.00	0.00	C
ATOM	466	HA	GLU	29	25.926	25.301	47.161	1.00	0.00	H
ATOM	467	CB	GLU	29	24.213	23.993	47.255	1.00	0.00	C
ATOM	468	HB2	GLU	29	24.067	24.074	48.332	1.00	0.00	H
ATOM	469	HB3	GLU	29	23.202	24.047	46.852	1.00	0.00	H
ATOM	470	CG	GLU	29	24.744	22.568	46.947	1.00	0.00	C
ATOM	471	HG2	GLU	29	24.303	21.900	47.687	1.00	0.00	H
ATOM	472	HG3	GLU	29	24.477	22.367	45.909	1.00	0.00	H
ATOM	473	CD	GLU	29	26.250	22.404	46.916	1.00	0.00	C
ATOM	474	OE1	GLU	29	26.896	23.151	47.712	1.00	0.00	O
ATOM	475	OE2	GLU	29	26.797	21.535	46.182	1.00	0.00	O
ATOM	476	C	GLU	29	25.387	24.902	45.225	1.00	0.00	C
ATOM	477	O	GLU	29	26.506	24.439	44.974	1.00	0.00	O
ATOM	478	N	SER	30	24.444	24.984	44.226	1.00	0.00	N
ATOM	479	H	SER	30	23.530	25.368	44.419	1.00	0.00	H
ATOM	480	CA	SER	30	24.766	24.577	42.805	1.00	0.00	C
ATOM	481	HA	SER	30	25.034	23.521	42.810	1.00	0.00	H
ATOM	482	CB	SER	30	23.424	24.700	42.117	1.00	0.00	C
ATOM	483	HB2	SER	30	23.284	25.780	42.071	1.00	0.00	H
ATOM	484	HB3	SER	30	23.556	24.481	41.057	1.00	0.00	H
ATOM	485	OG	SER	30	22.302	24.036	42.713	1.00	0.00	O
ATOM	486	HG	SER	30	21.552	24.423	42.254	1.00	0.00	H
ATOM	487	C	SER	30	25.865	25.405	42.134	1.00	0.00	C
ATOM	488	O	SER	30	26.297	25.138	41.005	1.00	0.00	O
ATOM	489	N	HIE	31	26.463	26.470	42.765	1.00	0.00	N
ATOM	490	H	HIE	31	26.204	26.620	43.730	1.00	0.00	H
ATOM	491	CA	HIE	31	27.552	27.262	42.160	1.00	0.00	C
ATOM	492	HA	HIE	31	27.246	27.593	41.168	1.00	0.00	H
ATOM	493	CB	HIE	31	27.776	28.514	43.006	1.00	0.00	C
ATOM	494	HB2	HIE	31	27.947	28.249	44.050	1.00	0.00	H
ATOM	495	HB3	HIE	31	28.655	29.067	42.677	1.00	0.00	H
ATOM	496	CG	HIE	31	26.682	29.615	42.990	1.00	0.00	C
ATOM	497	ND1	HIE	31	26.124	30.257	41.875	1.00	0.00	N
ATOM	498	CE1	HIE	31	25.317	31.245	42.343	1.00	0.00	C
ATOM	499	HE1	HIE	31	24.902	32.015	41.709	1.00	0.00	H
ATOM	500	NE2	HIE	31	25.354	31.216	43.691	1.00	0.00	N
ATOM	501	HE2	HIE	31	24.932	31.889	44.315	1.00	0.00	H
ATOM	502	CD2	HIE	31	26.241	30.245	44.123	1.00	0.00	C
ATOM	503	HD2	HIE	31	26.582	30.037	45.126	1.00	0.00	H
ATOM	504	C	HIE	31	28.736	26.369	41.908	1.00	0.00	C
ATOM	505	O	HIE	31	28.869	25.293	42.421	1.00	0.00	O
ATOM	506	N	PHE	32	29.650	26.775	40.955	1.00	0.00	N

```
ATOM    507  H   PHE    32      29.411  27.607  40.436  1.00  0.00           H
ATOM    508  CA  PHE    32      30.996  26.187  40.762  1.00  0.00           C
ATOM    509  HA  PHE    32      30.814  25.148  40.488  1.00  0.00           H
ATOM    510  CB  PHE    32      31.813  27.010  39.734  1.00  0.00           C
ATOM    511  HB2 PHE    32      31.984  27.951  40.257  1.00  0.00           H
ATOM    512  HB3 PHE    32      32.818  26.591  39.680  1.00  0.00           H
ATOM    513  CG  PHE    32      31.274  27.077  38.383  1.00  0.00           C
ATOM    514  CD1 PHE    32      31.205  28.244  37.687  1.00  0.00           C
ATOM    515  HD1 PHE    32      31.486  29.186  38.136  1.00  0.00           H
ATOM    516  CE1 PHE    32      30.986  28.285  36.290  1.00  0.00           C
ATOM    517  HE1 PHE    32      30.942  29.188  35.700  1.00  0.00           H
ATOM    518  CZ  PHE
    32      30.495  27.141  35.731  1.00  0.00           C
ATOM    519  HZ  PHE    32      30.082  27.110  34.734  1.00  0.00           H
ATOM    520  CE2 PHE    32      30.523  25.892  36.453  1.00  0.00           C
ATOM    521  HE2 PHE    32      30.156  25.021  35.930  1.00  0.00           H
ATOM    522  CD2 PHE    32      30.975  25.845  37.786  1.00  0.00           C
ATOM    523  HD2 PHE    32      31.082  24.914  38.323  1.00  0.00           H
ATOM    524  C   PHE    32      31.805  26.204  42.059  1.00  0.00           C
ATOM    525  O   PHE    32      31.491  26.895  42.996  1.00  0.00           O
ATOM    526  N   LYS    33      32.841  25.332  41.973  1.00  0.00           N
ATOM    527  H   LYS    33      33.014  25.011  41.031  1.00  0.00           H
ATOM    528  CA  LYS    33      33.539  24.708  43.103  1.00  0.00           C
ATOM    529  HA  LYS    33      33.305  25.316  43.977  1.00  0.00           H
ATOM    530  CB  LYS    33      33.240  23.221  43.302  1.00  0.00           C
ATOM    531  HB2 LYS    33      33.295  22.669  42.363  1.00  0.00           H
ATOM    532  HB3 LYS    33      33.896  22.792  44.059  1.00  0.00           H
ATOM    533  CG  LYS    33      31.870  23.127  43.764  1.00  0.00           C
ATOM    534  HG2 LYS    33      31.186  23.438  42.974  1.00  0.00           H
ATOM    535  HG3 LYS    33      31.732  22.048  43.834  1.00  0.00           H
ATOM    536  CD  LYS    33      31.350  23.725  45.091  1.00  0.00           C
ATOM    537  HD2 LYS    33      31.994  23.322  45.872  1.00  0.00           H
ATOM    538  HD3 LYS    33      31.339  24.814  45.057  1.00  0.00           H
ATOM    539  CE  LYS    33      29.916  23.185  45.264  1.00  0.00           C
ATOM    540  HE2 LYS    33      29.329  23.397  44.370  1.00  0.00           H
ATOM    541  HE3 LYS    33      29.896  22.099  45.352  1.00  0.00           H
ATOM    542  NZ  LYS    33      29.177  23.857  46.365  1.00  0.00           N
ATOM    543  HZ1 LYS    33      29.146  24.810  46.034  1.00  0.00           H
ATOM    544  HZ2 LYS    33      28.230  23.518  46.458  1.00  0.00           H
ATOM    545  HZ3 LYS    33      29.682  23.810  47.239  1.00  0.00           H
ATOM    546  C   LYS    33      35.059  24.880  42.880  1.00  0.00           C
ATOM    547  O   LYS    33      35.550  24.686  41.797  1.00  0.00           O
ATOM    548  N   THR    34      35.847  25.071  43.901  1.00  0.00           N
ATOM    549  H   THR    34      35.277  25.088  44.735  1.00  0.00           H
ATOM    550  CA  THR    34      37.288  25.014  43.937  1.00  0.00           C
ATOM    551  HA  THR    34      37.672  25.997  43.667  1.00  0.00           H
ATOM    552  CB  THR    34      37.861  24.821  45.379  1.00  0.00           C
ATOM    553  HB  THR    34      37.401  23.916  45.774  1.00  0.00           H
ATOM    554  CG2 THR    34      39.395  24.649  45.412  1.00  0.00           C
ATOM    555 HG21 THR    34      39.698  24.494  46.447  1.00  0.00           H
ATOM    556 HG22 THR    34      39.658  23.753  44.851  1.00  0.00           H
ATOM    557 HG23 THR    34      39.852  25.537  44.973  1.00  0.00           H
ATOM    558  OG1 THR    34      37.625  25.987  46.130  1.00  0.00           O
ATOM    559  HG1 THR    34      36.696  25.955  46.368  1.00  0.00           H
ATOM    560  C   THR    34      37.956  24.055  42.942  1.00  0.00           C
ATOM    561  O   THR    34      37.623  22.863  42.915  1.00  0.00           O
ATOM    562  N   GLY    35      38.683  24.552  41.940  1.00  0.00           N
ATOM    563  H   GLY    35      38.791  25.543  41.781  1.00  0.00           H
ATOM    564  CA  GLY    35      39.288  23.723  40.863  1.00  0.00           C
ATOM    565  HA2 GLY    35      40.293  24.098  40.670  1.00  0.00           H
ATOM    566  HA3 GLY    35      39.547  22.743  41.262  1.00  0.00           H
ATOM    567  C   GLY    35      38.435  23.412  39.595  1.00  0.00           C
ATOM    568  O   GLY    35      38.932  22.601  38.732  1.00  0.00           O
ATOM    569  N   ASP    36      37.200  23.968  39.450  1.00  0.00           N
```

ATOM	570	H	ASP	36	36.832	24.375	40.298	1.00	0.00	H
ATOM	571	CA	ASP	36	36.331	23.788	38.238	1.00	0.00	C
ATOM	572	HA	ASP	36	36.329	22.722	38.009	1.00	0.00	H
ATOM	573	CB	ASP	36	34.903	24.228	38.448	1.00	0.00	C
ATOM	574	HB2	ASP	36	34.798	25.282	38.707	1.00	0.00	H
ATOM	575	HB3	ASP	36	34.304	24.287	37.539	1.00	0.00	H
ATOM	576	CG	ASP	36	34.056	23.427	39.465	1.00	0.00	C
ATOM	577	OD1	ASP	36	32.836	23.688	39.609	1.00	0.00	O
ATOM	578	OD2	ASP	36	34.541	22.397	39.982	1.00	0.00	O
ATOM	579	C	ASP	36	36.950	24.350	36.956	1.00	0.00	C
ATOM	580	O	ASP	36	37.384	25.535	36.967	1.00	0.00	O
ATOM	581	N	VAL	37	36.874	23.570	35.894	1.00	0.00	N
ATOM	582	H	VAL	37	36.514	22.626	35.891	1.00	0.00	H
ATOM	583	CA	VAL	37	37.516	23.997	34.646	1.00	0.00	C
ATOM	584	HA	VAL	37	38.173	24.856	34.783	1.00	0.00	H
ATOM	585	CB	VAL	37	38.506	22.993	34.040	1.00	0.00	C
ATOM	586	HB	VAL	37	38.048	22.062	33.704	1.00	0.00	H
ATOM	587	CG1	VAL	37	39.217	23.550	32.849	1.00	0.00	C
ATOM	588	HG11	VAL	37	39.847	24.426	33.007	1.00	0.00	H
ATOM	589	HG12	VAL	37	39.928	22.857	32.399	1.00	0.00	H
ATOM	590	HG13	VAL	37	38.587	23.801	31.995	1.00	0.00	H
ATOM	591	CG2	VAL	37	39.522	22.637	35.147	1.00	0.00	C
ATOM	592	HG21	VAL	37	38.936	22.128	35.912	1.00	0.00	H
ATOM	593	HG22	VAL	37	40.315	21.976	34.796	1.00	0.00	H
ATOM	594	HG23	VAL	37	39.878	23.514	35.687	1.00	0.00	H
ATOM	595	C	VAL	37	36.431	24.548	33.775	1.00	0.00	C
ATOM	596	O	VAL	37	35.430	23.848	33.495	1.00	0.00	O
ATOM	597	N	LEU	38	36.638	25.837	33.438	1.00	0.00	N
ATOM	598	H	LEU	38	37.573	26.167	33.631	1.00	0.00	H
ATOM	599	CA	LEU	38	35.805	26.623	32.538	1.00	0.00	C
ATOM	600	HA	LEU	38	34.939	26.015	32.274	1.00	0.00	H
ATOM	601	CB	LEU	38	35.484	27.946	33.235	1.00	0.00	C
ATOM	602	HB2	LEU	38	36.309	28.240	33.885	1.00	0.00	H
ATOM	603	HB3	LEU	38	35.302	28.737	32.508	1.00	0.00	H
ATOM	604	CG	LEU	38	34.245	28.151	34.175	1.00	0.00	C
ATOM	605	HG	LEU	38	33.271	27.958	33.725	1.00	0.00	H
ATOM	606	CD1	LEU	38	34.200	27.093	35.221	1.00	0.00	C
ATOM	607	HD11	LEU	38	35.076	26.979	35.860	1.00	0.00	H
ATOM	608	HD12	LEU	38	33.310	27.328	35.804	1.00	0.00	H
ATOM	609	HD13	LEU	38	33.996	26.150	34.713	1.00	0.00	H
ATOM	610	CD2	LEU	38	34.271	29.539	34.823	1.00	0.00	C
ATOM	611	HD21	LEU	38	34.375	30.176	33.945	1.00	0.00	H
ATOM	612	HD22	LEU	38	33.337	29.817	35.313	1.00	0.00	H
ATOM	613	HD23	LEU	38	35.073	29.760	35.527	1.00	0.00	H
ATOM	614	C	LEU	38	36.506	26.951	31.167	1.00	0.00	C
ATOM	615	O	LEU	38	37.721	26.861	30.949	1.00	0.00	O
ATOM	616	N	ARG	39	35.731	27.410	30.193	1.00	0.00	N
ATOM	617	H	ARG	39	34.733	27.325	30.318	1.00	0.00	H
ATOM	618	CA	ARG	39	36.105	28.079	28.879	1.00	0.00	C
ATOM	619	HA	ARG	39	37.153	28.377	28.902	1.00	0.00	H
ATOM	620	CB	ARG	39	36.051	27.112	27.692	1.00	0.00	C
ATOM	621	HB2	ARG	39	35.214	26.423	27.803	1.00	0.00	H
ATOM	622	HB3	ARG	39	35.999	27.618	26.728	1.00	0.00	H
ATOM	623	CG	ARG	39	37.249	26.079	27.667	1.00	0.00	C
ATOM	624	HG2	ARG	39	38.198	26.572	27.873	1.00	0.00	H
ATOM	625	HG3	ARG	39	37.095	25.472	28.560	1.00	0.00	H
ATOM	626	CD	ARG	39	37.330	25.215	26.384	1.00	0.00	C
ATOM	627	HD2	ARG	39	37.283	25.791	25.460	1.00	0.00	H
ATOM	628	HD3	ARG	39	38.298	24.719	26.318	1.00	0.00	H
ATOM	629	NE	ARG	39	36.227	24.250	26.316	1.00	0.00	N
ATOM	630	HE	ARG	39	35.431	24.264	26.938	1.00	0.00	H
ATOM	631	CZ	ARG	39	36.105	23.352	25.341	1.00	0.00	C
ATOM	632	NH1	ARG	39	37.014	23.167	24.439	1.00	0.00	N
ATOM	633	HH11	ARG	39	37.875	23.668	24.607	1.00	0.00	H

```
ATOM    634 HH12 ARG    39      36.926  22.404  23.783  1.00  0.00           H
ATOM    635  NH2 ARG    39      35.083  22.571  25.164  1.00  0.00           N
ATOM    636 HH21 ARG    39      35.011  22.017  24.323  1.00  0.00           H
ATOM    637 HH22 ARG    39      34.331  22.611  25.838  1.00  0.00           H
ATOM    638  C   ARG    39      35.316  29.337  28.474  1.00  0.00           C
ATOM    639  O   ARG    39      34.097  29.484  28.746  1.00  0.00           O
ATOM    640  N   VAL    40      35.983  30.279  27.784  1.00  0.00           N
ATOM    641  H   VAL    40      36.977  30.129  27.681  1.00  0.00           H
ATOM    642  CA  VAL    40      35.309  31.323  26.967  1.00  0.00           C
ATOM    643  HA  VAL    40      34.248  31.080
  27.027  1.00  0.00           H
ATOM    644  CB  VAL    40      35.665  32.709  27.556  1.00  0.00           C
ATOM    645  HB  VAL    40      35.145  33.476  26.983  1.00  0.00           H
ATOM    646  CG1 VAL    40      35.220  32.816  29.056  1.00  0.00           C
ATOM    647 HG11 VAL    40      35.358  33.877  29.267  1.00  0.00           H
ATOM    648 HG12 VAL    40      34.169  32.545  29.155  1.00  0.00           H
ATOM    649 HG13 VAL    40      35.883  32.221  29.684  1.00  0.00           H
ATOM    650  CG2 VAL    40      37.138  32.956  27.536  1.00  0.00           C
ATOM    651 HG21 VAL    40      37.430  33.869  28.054  1.00  0.00           H
ATOM    652 HG22 VAL    40      37.695  32.089  27.890  1.00  0.00           H
ATOM    653 HG23 VAL    40      37.403  33.126  26.492  1.00  0.00           H
ATOM    654  C   VAL    40      35.565  31.286  25.437  1.00  0.00           C
ATOM    655  O   VAL    40      36.719  31.055  25.078  1.00  0.00           O
ATOM    656  N   GLY    41      34.582  31.644  24.582  1.00  0.00           N
ATOM    657  H   GLY    41      33.686  31.934  24.949  1.00  0.00           H
ATOM    658  CA  GLY    41      34.804  31.780  23.163  1.00  0.00           C
ATOM    659  HA2 GLY    41      35.776  32.264  23.063  1.00  0.00           H
ATOM    660  HA3 GLY    41      34.826  30.818  22.652  1.00  0.00           H
ATOM    661  C   GLY    41      33.786  32.719  22.459  1.00  0.00           C
ATOM    662  O   GLY    41      32.634  32.854  22.941  1.00  0.00           O
ATOM    663  N   ARG    42      34.197  33.309  21.293  1.00  0.00           N
ATOM    664  H   ARG    42      35.139  33.077  21.013  1.00  0.00           H
ATOM    665  CA  ARG    42      33.359  34.124  20.394  1.00  0.00           C
ATOM    666  HA  ARG    42      32.469  33.495  20.430  1.00  0.00           H
ATOM    667  CB  ARG    42      33.092  35.427  21.007  1.00  0.00           C
ATOM    668  HB2 ARG    42      32.358  36.001  20.442  1.00  0.00           H
ATOM    669  HB3 ARG    42      32.662  35.308  22.001  1.00  0.00           H
ATOM    670  CG  ARG    42      34.286  36.298  21.125  1.00  0.00           C
ATOM    671  HG2 ARG    42      34.987  35.818  21.807  1.00  0.00           H
ATOM    672  HG3 ARG    42      34.747  36.362  20.139  1.00  0.00           H
ATOM    673  CD  ARG    42      33.921  37.622  21.657  1.00  0.00           C
ATOM    674  HD2 ARG    42      33.191  38.116  21.016  1.00  0.00           H
ATOM    675  HD3 ARG    42      33.537  37.420  22.657  1.00  0.00           H
ATOM    676  NE  ARG    42      35.072  38.515  21.584  1.00  0.00           N
ATOM    677  HE  ARG    42      35.170  38.872  20.645  1.00  0.00           H
ATOM    678  CZ  ARG    42      36.045  38.658  22.490  1.00  0.00           C
ATOM    679  NH1 ARG    42      36.013  38.091  23.717  1.00  0.00           N
ATOM    680 HH11 ARG    42      36.779  38.196  24.367  1.00  0.00           H
ATOM    681 HH12 ARG    42      35.241  37.486  23.960  1.00  0.00           H
ATOM    682  NH2 ARG    42      37.088  39.324  22.162  1.00  0.00           N
ATOM    683 HH21 ARG    42      37.061  39.834  21.291  1.00  0.00           H
ATOM    684 HH22 ARG    42      37.899  39.274  22.763  1.00  0.00           H
ATOM    685  C   ARG    42      33.780  34.156  18.935  1.00  0.00           C
ATOM    686  O   ARG    42      33.112  34.861  18.162  1.00  0.00           O
ATOM    687  N   PHE    43      34.770  33.384  18.483  1.00  0.00           N
ATOM    688  H   PHE    43      35.330  32.938  19.196  1.00  0.00           H
ATOM    689  CA  PHE    43      35.294  33.180  17.131  1.00  0.00           C
ATOM    690  HA  PHE    43      34.691  33.816  16.483  1.00  0.00           H
ATOM    691  CB  PHE    43      36.710  33.645  17.157  1.00  0.00           C
ATOM    692  HB2 PHE    43      37.307  32.879  17.652  1.00  0.00           H
ATOM    693  HB3 PHE    43      37.078  33.732  16.135  1.00  0.00           H
ATOM    694  CG  PHE    43      36.971  34.967  17.766  1.00  0.00           C
ATOM    695  CD1 PHE    43      36.792  36.173  17.006  1.00  0.00           C
ATOM    696  HD1 PHE    43      36.503  36.011  15.978  1.00  0.00           H
```

ATOM	697	CE1	PHE	43	37.000	37.470	17.584	1.00	0.00	C
ATOM	698	HE1	PHE	43	36.783	38.291	16.918	1.00	0.00	H
ATOM	699	CZ	PHE	43	37.538	37.539	18.875	1.00	0.00	C
ATOM	700	HZ	PHE	43	37.681	38.501	19.344	1.00	0.00	H
ATOM	701	CE2	PHE	43	37.740	36.374	19.649	1.00	0.00	C
ATOM	702	HE2	PHE	43	38.236	36.403	20.608	1.00	0.00	H
ATOM	703	CD2	PHE	43	37.516	35.127	19.057	1.00	0.00	C
ATOM	704	HD2	PHE	43	37.753	34.265	19.663	1.00	0.00	H
ATOM	705	C	PHE	43	35.193	31.739	16.711	1.00	0.00	C
ATOM	706	O	PHE	43	35.090	30.739	17.368	1.00	0.00	O
ATOM	707	N	GLU	44	35.097	31.589	15.358	1.00	0.00	N
ATOM	708	H	GLU	44	34.942	32.348	14.710	1.00	0.00	H
ATOM	709	CA	GLU	44	35.085	30.310	14.636	1.00	0.00	C
ATOM	710	HA	GLU	44	34.303	29.771	15.171	1.00	0.00	H
ATOM	711	CB	GLU	44	34.749	30.575	13.130	1.00	0.00	C
ATOM	712	HB2	GLU	44	33.776	31.059	13.048	1.00	0.00	H
ATOM	713	HB3	GLU	44	35.569	31.180	12.744	1.00	0.00	H
ATOM	714	CG	GLU	44	34.960	29.470	12.178	1.00	0.00	C
ATOM	715	HG2	GLU	44	35.106	29.955	11.212	1.00	0.00	H
ATOM	716	HG3	GLU	44	35.861	28.913	12.433	1.00	0.00	H
ATOM	717	CD	GLU	44	33.822	28.541	12.017	1.00	0.00	C
ATOM	718	OE1	GLU	44	33.641	27.905	10.960	1.00	0.00	O
ATOM	719	OE2	GLU	44	32.945	28.501	12.927	1.00	0.00	O
ATOM	720	C	GLU	44	36.273	29.388	14.745	1.00	0.00	C
ATOM	721	O	GLU	44	36.159	28.144	14.928	1.00	0.00	O
ATOM	722	N	ASP	45	37.485	29.986	14.802	1.00	0.00	N
ATOM	723	H	ASP	45	37.431	30.993	14.741	1.00	0.00	H
ATOM	724	CA	ASP	45	38.850	29.384	14.928	1.00	0.00	C
ATOM	725	HA	ASP	45	38.834	28.598	14.172	1.00	0.00	H
ATOM	726	CB	ASP	45	39.897	30.421	14.645	1.00	0.00	C
ATOM	727	HB2	ASP	45	39.801	30.861	13.652	1.00	0.00	H
ATOM	728	HB3	ASP	45	39.824	31.208	15.396	1.00	0.00	H
ATOM	729	CG	ASP	45	41.342	29.788	14.524	1.00	0.00	C
ATOM	730	OD1	ASP	45	41.498	28.632	14.353	1.00	0.00	O
ATOM	731	OD2	ASP	45	42.333	30.549	14.648	1.00	0.00	O
ATOM	732	C	ASP	45	39.040	28.723	16.322	1.00	0.00	C
ATOM	733	O	ASP	45	39.028	29.401	17.328	1.00	0.00	O
ATOM	734	N	ASP	46	39.204	27.411	16.391	1.00	0.00	N
ATOM	735	H	ASP	46	39.171	26.919	15.509	1.00	0.00	H
ATOM	736	CA	ASP	46	39.467	26.645	17.625	1.00	0.00	C
ATOM	737	HA	ASP	46	39.383	25.608	17.302	1.00	0.00	H
ATOM	738	CB	ASP	46	40.868	27.006	18.156	1.00	0.00	C
ATOM	739	HB2	ASP	46	40.953	28.061	18.416	1.00	0.00	H
ATOM	740	HB3	ASP	46	41.017	26.517	19.118	1.00	0.00	H
ATOM	741	CG	ASP	46	42.044	26.677	17.279	1.00	0.00	C
ATOM	742	OD1	ASP	46	42.151	25.593	16.705	1.00	0.00	O
ATOM	743	OD2	ASP	46	42.998	27.526	17.142	1.00	0.00	O
ATOM	744	C	ASP	46	38.398	26.720	18.722	1.00	0.00	C
ATOM	745	O	ASP	46	38.486	26.117	19.820	1.00	0.00	O
ATOM	746	N	GLY	47	37.266	27.354	18.412	1.00	0.00	N
ATOM	747	H	GLY	47	37.257	27.788	17.500	1.00	0.00	H
ATOM	748	CA	GLY	47	36.136	27.508	19.351	1.00	0.00	C
ATOM	749	HA2	GLY	47	35.266	27.881	18.811	1.00	0.00	H
ATOM	750	HA3	GLY	47	35.863	26.567	19.829	1.00	0.00	H
ATOM	751	C	GLY	47	36.340	28.575	20.417	1.00	0.00	C
ATOM	752	O	GLY	47	35.393	29.389	20.629	1.00	0.00	O
ATOM	753	N	TYR	48	37.515	28.555	21.084	1.00	0.00	N
ATOM	754	H	TYR	48	38.274	27.938	20.832	1.00	0.00	H
ATOM	755	CA	TYR	48	37.769	29.129	22.439	1.00	0.00	C
ATOM	756	HA	TYR	48	37.029	29.887	22.695	1.00	0.00	H
ATOM	757	CB	TYR	48	37.728	27.910	23.411	1.00	0.00	C
ATOM	758	HB2	TYR	48	38.542	27.226	23.172	1.00	0.00	H
ATOM	759	HB3	TYR	48	37.851	28.277	24.429	1.00	0.00	H
ATOM	760	CG	TYR	48	36.389	27.140	23.384	1.00	0.00	C

ATOM	761	CD1	TYR	48	35.301	27.592	24.135	1.00	0.00	C
ATOM	762	HD1	TYR	48	35.412	28.442	24.792	1.00	0.00	H
ATOM	763	CE1	TYR	48	34.071	26.888	24.025	1.00	0.00	C
ATOM	764	HE1	TYR	48	33.238	27.193	24.642	1.00	0.00	H
ATOM	765	CZ	TYR	48	34.028	25.715	23.268	1.00	0.00	C
ATOM	766	OH	TYR	48	32.834	24.938	23.301	1.00	0.00	O
ATOM	767	HH	TYR	48	32.886	24.244	22.640	1.00	0.00	H
ATOM	768	CE2	TYR	48	35.163	25.224	22.578	1.00	0.00	C
ATOM	769	HE2	TYR	48	35.071	24.332	21.976	1.00	0.00	H
ATOM	770	CD2	TYR	48	36.346	25.991	22.658	1.00	0.00	C
ATOM	771	HD2	TYR	48	37.255	25.549	22.278	1.00	0.00	H
ATOM	772	C	TYR	48	39.145	29.819	22.479	1.00	0.00	C
ATOM	773	O	TYR	48	40.099	29.287	21.774	1.00	0.00	O
ATOM	774	N	PHE	49	39.317	30.865	23.286	1.00	0.00	N
ATOM	775	H	PHE	49	38.456	31.186	23.705	1.00	0.00	H
ATOM	776	CA	PHE	49	40.616	31.605	23.432	1.00	0.00	C
ATOM	777	HA	PHE	49	41.268	31.029	22.776	1.00	0.00	H
ATOM	778	CB	PHE	49	40.519	33.056	22.942	1.00	0.00	C
ATOM	779	HB2	PHE	49	41.448	33.621	23.017	1.00	0.00	H
ATOM	780	HB3	PHE	49	40.321	33.065	21.870	1.00	0.00	H
ATOM	781	CG	PHE	49	39.526	33.908	23.704	1.00	0.00	C
ATOM	782	CD1	PHE	49	38.205	33.914	23.204	1.00	0.00	C
ATOM	783	HD1	PHE	49	37.969	33.359	22.309	1.00	0.00	H
ATOM	784	CE1	PHE	49	37.261	34.584	23.946	1.00	0.00	C
ATOM	785	HE1	PHE	49	36.204	34.559	23.729	1.00	0.00	H
ATOM	786	CZ	PHE	49	37.654	35.371	25.028	1.00	0.00	C
ATOM	787	HZ	PHE	49	36.898	35.859	25.626	1.00	0.00	H
ATOM	788	CE2	PHE	49	38.977	35.559	25.468	1.00	0.00	C
ATOM	789	HE2	PHE	49	39.253	36.207	26.287	1.00	0.00	H
ATOM	790	CD2	PHE	49	39.933	34.744	24.829	1.00	0.00	C
ATOM	791	HD2	PHE	49	40.976	34.924	25.043	1.00	0.00	H
ATOM	792	C	PHE	49	41.287	31.438	24.745	1.00	0.00	C
ATOM	793	O	PHE	49	42.361	31.946	25.031	1.00	0.00	O
ATOM	794	N	CYX	50	40.505	30.909	25.685	1.00	0.00	N
ATOM	795	H	CYX	50	39.654	30.559	25.269	1.00	0.00	H
ATOM	796	CA	CYX	50	40.752	30.784	27.108	1.00	0.00	C
ATOM	797	HA	CYX	50	41.833	30.773	27.250	1.00	0.00	H
ATOM	798	CB	CYX	50	40.398	32.131	27.854	1.00	0.00	C
ATOM	799	HB2	CYX	50	39.669	32.735	27.316	1.00	0.00	H
ATOM	800	HB3	CYX	50	40.014	31.837	28.831	1.00	0.00	H
ATOM	801	SG	CYX	50	41.755	33.352	28.072	1.00	0.00	S
ATOM	802	C	CYX	50	40.168	29.594	27.795	1.00	0.00	C
ATOM	803	O	CYX	50	38.950	29.437	27.740	1.00	0.00	O
ATOM	804	N	THR	51	41.036	28.747	28.288	1.00	0.00	N
ATOM	805	H	THR	51	42.003	29.006	28.153	1.00	0.00	H
ATOM	806	CA	THR	51	40.667	27.847	29.400	1.00	0.00	C
ATOM	807	HA	THR	51	39.622	27.560	29.282	1.00	0.00	H
ATOM	808	CB	THR	51	41.537	26.529	29.278	1.00	0.00	C
ATOM	809	HB	THR	51	42.538	26.792	29.619	1.00	0.00	H
ATOM	810	CG2	THR	51	40.869	25.442	30.143	1.00	0.00	C
ATOM	811	HG21	THR	51	41.635	24.698	30.363	1.00	0.00	H
ATOM	812	HG22	THR	51	40.425	25.721	31.098	1.00	0.00	H
ATOM	813	HG23	THR	51	40.140	24.937	29.510	1.00	0.00	H
ATOM	814	OG1	THR	51	41.452	26.164	27.928	1.00	0.00	O
ATOM	815	HG1	THR	51	41.861	25.296	27.889	1.00	0.00	H
ATOM	816	C	THR	51	40.878	28.532	30.763	1.00	0.00	C
ATOM	817	O	THR	51	41.940	29.067	31.009	1.00	0.00	O
ATOM	818	N	ILE	52	39.976	28.373	31.686	1.00	0.00	N
ATOM	819	H	ILE	52	39.438	27.553	31.448	1.00	0.00	H
ATOM	820	CA	ILE	52	39.894	28.919	33.033	1.00	0.00	C
ATOM	821	HA	ILE	52	40.826	29.410	33.311	1.00	0.00	H
ATOM	822	CB	ILE	52	38.637	29.918	33.099	1.00	0.00	C
ATOM	823	HB	ILE	52	37.814	29.366	32.645	1.00	0.00	H

ATOM	824	CG2	ILE	52	38.366	30.311	34.501	1.00	0.00	C
ATOM	825	HG21	ILE	52	38.089	29.559	35.240	1.00	0.00	H
ATOM	826	HG22	ILE	52	39.169	30.833	35.022	1.00	0.00	H
ATOM	827	HG23	ILE	52	37.626	31.111	34.509	1.00	0.00	H
ATOM	828	CG1	ILE	52	38.946	31.199	32.321	1.00	0.00	C
ATOM	829	HG12	ILE	52	39.375	31.992	32.933	1.00	0.00	H
ATOM	830	HG13	ILE	52	39.705	31.038	31.555	1.00	0.00	H
ATOM	831	CD1	ILE	52	37.699	31.754	31.649	1.00	0.00	C
ATOM	832	HD11	ILE	52	37.965	32.618	31.040	1.00	0.00	H
ATOM	833	HD12	ILE	52	37.253	31.124	30.879	1.00	0.00	H
ATOM	834	HD13	ILE	52	36.957	31.965	32.419	1.00	0.00	H
ATOM	835	C	ILE	52	39.835	27.765	34.034	1.00	0.00	C
ATOM	836	O	ILE	52	39.303	26.702	33.788	1.00	0.00	O
ATOM	837	N	GLU	53	40.296	28.029	35.297	1.00	0.00	N
ATOM	838	H	GLU	53	40.778	28.881	35.545	1.00	0.00	H
ATOM	839	CA	GLU	53	40.028	27.248	36.491	1.00	0.00	C
ATOM	840	HA	GLU	53	39.263	26.499	36.284	1.00	0.00	H
ATOM	841	CB	GLU	53	41.346	26.519	36.913	1.00	0.00	C
ATOM	842	HB2	GLU	53	41.765	26.021	36.038	1.00	0.00	H
ATOM	843	HB3	GLU	53	42.099	27.126	37.414	1.00	0.00	H
ATOM	844	CG	GLU	53	41.125	25.335	37.969	1.00	0.00	C
ATOM	845	HG2	GLU	53	40.703	25.750	38.884	1.00	0.00	H
ATOM	846	HG3	GLU	53	40.394	24.579	37.682	1.00	0.00	H
ATOM	847	CD	GLU	53	42.479	24.766	38.424	1.00	0.00	C
ATOM	848	OE1	GLU	53	43.185	24.030	37.617	1.00	0.00	O
ATOM	849	OE2	GLU	53	42.847	25.343	39.489	1.00	0.00	O
ATOM	850	C	GLU	53	39.629	28.187	37.628	1.00	0.00	C
ATOM	851	O	GLU	53	40.130	29.248	37.816	1.00	0.00	O
ATOM	852	N	VAL	54	38.610	27.868	38.378	1.00	0.00	N
ATOM	853	H	VAL	54	38.155	27.002	38.124	1.00	0.00	H
ATOM	854	CA	VAL	54	38.210	28.375	39.676	1.00	0.00	C
ATOM	855	HA	VAL	54	38.053	29.443	39.524	1.00	0.00	H
ATOM	856	CB	VAL	54	36.839	27.843	40.074	1.00	0.00	C
ATOM	857	HB	VAL	54	36.856	26.756	39.994	1.00	0.00	H
ATOM	858	CG1	VAL	54	36.349	28.266	41.499	1.00	0.00	C
ATOM	859	HG11	VAL	54	35.319	28.017	41.753	1.00	0.00	H
ATOM	860	HG12	VAL	54	37.053	27.705	42.113	1.00	0.00	H
ATOM	861	HG13	VAL	54	36.485	29.329	41.698	1.00	0.00	H
ATOM	862	CG2	VAL	54	35.727	28.316	39.203	1.00	0.00	C
ATOM	863	HG21	VAL	54	35.521	29.366	39.410	1.00	0.00	H
ATOM	864	HG22	VAL	54	36.004	28.203	38.155	1.00	0.00	H
ATOM	865	HG23	VAL	54	34.808	27.769	39.417	1.00	0.00	H
ATOM	866	C	VAL	54	39.260	28.104	40.764	1.00	0.00	C
ATOM	867	O	VAL	54	39.464	27.013	41.242	1.00	0.00	O
ATOM	868	N	THR	55	39.948	29.119	41.171	1.00	0.00	N
ATOM	869	H	THR	55	39.770	30.067	40.872	1.00	0.00	H
ATOM	870	CA	THR	55	41.051	29.049	42.105	1.00	0.00	C
ATOM	871	HA	THR	55	41.634	28.152	41.895	1.00	0.00	H
ATOM	872	CB	THR	55	41.990	30.231	41.796	1.00	0.00	C
ATOM	873	HB	THR	55	42.871	30.093	42.422	1.00	0.00	H
ATOM	874	CG2	THR	55	42.443	30.335	40.311	1.00	0.00	C
ATOM	875	HG21	THR	55	42.809	29.380	39.934	1.00	0.00	H
ATOM	876	HG22	THR	55	41.578	30.618	39.712	1.00	0.00	H
ATOM	877	HG23	THR	55	43.197	31.110	40.171	1.00	0.00	H
ATOM	878	OG1	THR	55	41.375	31.469	42.173	1.00	0.00	O
ATOM	879	HG1	THR	55	41.792	32.203	41.715	1.00	0.00	H
ATOM	880	C	THR	55	40.673	29.004	43.583	1.00	0.00	C
ATOM	881	O	THR	55	41.368	28.396	44.356	1.00	0.00	O
ATOM	882	N	ALA	56	39.499	29.565	43.831	1.00	0.00	N
ATOM	883	H	ALA	56	39.051	30.109	43.108	1.00	0.00	H
ATOM	884	CA	ALA	56	38.873	29.513	45.186	1.00	0.00	C
ATOM	885	HA	ALA	56	39.089	28.514	45.567	1.00	0.00	H
ATOM	886	CB	ALA	56	39.622	30.372	46.211	1.00	0.00	C
ATOM	887	HB1	ALA	56	39.504	29.953	47.210	1.00	0.00	H

```
ATOM    888  HB2 ALA    56      40.704  30.401  46.085  1.00  0.00            H
ATOM    889  HB3 ALA    56      39.142  31.350  46.223  1.00  0.00            H
ATOM    890  C   ALA    56      37.393  29.698  45.262  1.00  0.00            C
ATOM    891  O   ALA    56      36.826  30.428  44.419  1.00  0.00            O
ATOM    892  N   THR    57      36.753  29.182  46.354  1.00  0.00            N
ATOM    893  H   THR    57      37.381  28.677  46.962  1.00  0.00            H
ATOM    894  CA  THR
    57      35.322  29.222  46.622  1.00  0.00            C
ATOM    895  HA  THR    57      35.012  30.230  46.349  1.00  0.00            H
ATOM    896  CB  THR    57      34.514  28.187  45.766  1.00  0.00            C
ATOM    897  HB  THR    57      33.450  28.358  45.928  1.00  0.00            H
ATOM    898  CG2 THR    57      34.675  28.436  44.331  1.00  0.00            C
ATOM    899 HG21 THR    57      34.413  29.460  44.064  1.00  0.00            H
ATOM    900 HG22 THR    57      35.616  28.077  43.916  1.00  0.00            H
ATOM    901 HG23 THR    57      33.836  28.004  43.784  1.00  0.00            H
ATOM    902  OG1 THR    57      34.848  26.879  46.187  1.00  0.00            O
ATOM    903  HG1 THR    57      34.225  26.727  46.902  1.00  0.00            H
ATOM    904  C   THR    57      34.963  29.093  48.118  1.00  0.00            C
ATOM    905  O   THR    57      35.506  28.266  48.870  1.00  0.00            O
ATOM    906  N   SER    58      33.979  29.947  48.531  1.00  0.00            N
ATOM    907  H   SER    58      33.559  30.567  47.853  1.00  0.00            H
ATOM    908  CA  SER    58      33.673  30.190  49.952  1.00  0.00            C
ATOM    909  HA  SER    58      33.598  29.276  50.541  1.00  0.00            H
ATOM    910  CB  SER    58      34.879  30.958  50.571  1.00  0.00            C
ATOM    911  HB2 SER    58      35.765  30.369  50.335  1.00  0.00            H
ATOM    912  HB3 SER    58      34.881  31.967  50.157  1.00  0.00            H
ATOM    913  OG  SER    58      34.725  31.088  51.965  1.00  0.00            O
ATOM    914  HG  SER    58      35.162  30.295  52.285  1.00  0.00            H
ATOM    915  C   SER    58      32.387  30.974  50.173  1.00  0.00            C
ATOM    916  O   SER    58      31.981  31.802  49.369  1.00  0.00            O
ATOM    917  N   THR    59      31.706  30.721  51.323  1.00  0.00            N
ATOM    918  H   THR    59      32.141  30.105  51.996  1.00  0.00            H
ATOM    919  CA  THR    59      30.471  31.319  51.830  1.00  0.00            C
ATOM    920  HA  THR    59      30.187  31.895  50.949  1.00  0.00            H
ATOM    921  CB  THR    59      29.383  30.316  52.186  1.00  0.00            C
ATOM    922  HB  THR    59      29.134  29.782  51.269  1.00  0.00            H
ATOM    923  CG2 THR    59      29.749  29.447  53.322  1.00  0.00            C
ATOM    924 HG21 THR    59      30.637  28.842  53.139  1.00  0.00            H
ATOM    925 HG22 THR    59      29.994  30.102  54.158  1.00  0.00            H
ATOM    926 HG23 THR    59      29.007  28.691  53.577  1.00  0.00            H
ATOM    927  OG1 THR    59      28.221  31.082  52.545  1.00  0.00            O
ATOM    928  HG1 THR    59      28.086  31.688  51.813  1.00  0.00            H
ATOM    929  C   THR    59      30.795  32.338  52.985  1.00  0.00            C
ATOM    930  O   THR    59      31.768  32.118  53.748  1.00  0.00            O
ATOM    931  N   VAL    60      30.070  33.494  53.038  1.00  0.00            N
ATOM    932  H   VAL    60      29.454  33.768  52.286  1.00  0.00            H
ATOM    933  CA  VAL    60      30.506  34.606  53.937  1.00  0.00            C
ATOM    934  HA  VAL    60      30.797  34.111  54.864  1.00  0.00            H
ATOM    935  CB  VAL    60      31.697  35.287  53.263  1.00  0.00            C
ATOM    936  HB  VAL    60      32.455  34.520  53.102  1.00  0.00            H
ATOM    937  CG1 VAL    60      31.299  35.899  51.897  1.00  0.00            C
ATOM    938 HG11 VAL    60      31.042  35.109  51.190  1.00  0.00            H
ATOM    939 HG12 VAL    60      30.362  36.451  51.969  1.00  0.00            H
ATOM    940 HG13 VAL    60      32.111  36.343  51.321  1.00  0.00            H
ATOM    941  CG2 VAL    60      32.264  36.382  54.163  1.00  0.00            C
ATOM    942 HG21 VAL    60      32.392  35.985  55.170  1.00  0.00            H
ATOM    943 HG22 VAL    60      33.275  36.578  53.807  1.00  0.00            H
ATOM    944 HG23 VAL    60      31.698  37.305  54.040  1.00  0.00            H
ATOM    945  C   VAL    60      29.232  35.477  54.155  1.00  0.00            C
ATOM    946  O   VAL    60      28.161  35.380  53.583  1.00  0.00            O
ATOM    947  N   THR    61      29.351  36.446  55.079  1.00  0.00            N
ATOM    948  H   THR    61      30.216  36.657  55.556  1.00  0.00            H
ATOM    949  CA  THR    61      28.312  37.424  55.429  1.00  0.00            C
ATOM    950  HA  THR    61      27.359  37.316  54.911  1.00  0.00            H
```

ATOM	951	CB	THR	61	27.922	37.372	56.913	1.00	0.00	C
ATOM	952	HB	THR	61	27.030	37.991	57.011	1.00	0.00	H
ATOM	953	CG2	THR	61	27.844	36.040	57.754	1.00	0.00	C
ATOM	954	HG21	THR	61	28.840	35.599	57.766	1.00	0.00	H
ATOM	955	HG22	THR	61	27.429	36.320	58.722	1.00	0.00	H
ATOM	956	HG23	THR	61	27.228	35.334	57.197	1.00	0.00	H
ATOM	957	OG1	THR	61	28.868	38.169	57.571	1.00	0.00	O
ATOM	958	HG1	THR	61	28.440	38.639	58.291	1.00	0.00	H
ATOM	959	C	THR	61	28.795	38.832	54.967	1.00	0.00	C
ATOM	960	O	THR	61	30.016	39.147	54.993	1.00	0.00	O
ATOM	961	N	LEU	62	27.780	39.643	54.672	1.00	0.00	N
ATOM	962	H	LEU	62	26.833	39.302	54.746	1.00	0.00	H
ATOM	963	CA	LEU	62	27.843	41.075	54.356	1.00	0.00	C
ATOM	964	HA	LEU	62	28.600	41.052	53.572	1.00	0.00	H
ATOM	965	CB	LEU	62	26.510	41.647	53.799	1.00	0.00	C
ATOM	966	HB2	LEU	62	26.111	40.986	53.029	1.00	0.00	H
ATOM	967	HB3	LEU	62	25.756	41.648	54.586	1.00	0.00	H
ATOM	968	CG	LEU	62	26.693	43.085	53.152	1.00	0.00	C
ATOM	969	HG	LEU	62	26.891	43.787	53.961	1.00	0.00	H
ATOM	970	CD1	LEU	62	27.821	43.100	52.145	1.00	0.00	C
ATOM	971	HD11	LEU	62	28.783	43.086	52.657	1.00	0.00	H
ATOM	972	HD12	LEU	62	27.736	42.282	51.428	1.00	0.00	H
ATOM	973	HD13	LEU	62	27.835	44.007	51.541	1.00	0.00	H
ATOM	974	CD2	LEU	62	25.368	43.506	52.631	1.00	0.00	C
ATOM	975	HD21	LEU	62	25.261	44.376	51.983	1.00	0.00	H
ATOM	976	HD22	LEU	62	24.985	42.697	52.009	1.00	0.00	H
ATOM	977	HD23	LEU	62	24.623	43.641	53.416	1.00	0.00	H
ATOM	978	C	LEU	62	28.498	41.891	55.493	1.00	0.00	C
ATOM	979	O	LEU	62	28.995	42.989	55.231	1.00	0.00	O
ATOM	980	N	ASP	63	28.561	41.360	56.740	1.00	0.00	N
ATOM	981	H	ASP	63	28.158	40.450	56.912	1.00	0.00	H
ATOM	982	CA	ASP	63	29.404	42.008	57.800	1.00	0.00	C
ATOM	983	HA	ASP	63	29.381	43.076	57.586	1.00	0.00	H
ATOM	984	CB	ASP	63	28.647	42.097	59.149	1.00	0.00	C
ATOM	985	HB2	ASP	63	29.253	42.583	59.914	1.00	0.00	H
ATOM	986	HB3	ASP	63	27.745	42.691	58.998	1.00	0.00	H
ATOM	987	CG	ASP	63	28.256	40.704	59.719	1.00	0.00	C
ATOM	988	OD1	ASP	63	28.339	40.604	60.930	1.00	0.00	O
ATOM	989	OD2	ASP	63	27.710	39.809	59.028	1.00	0.00	O
ATOM	990	C	ASP	63	30.858	41.456	57.894	1.00	0.00	C
ATOM	991	O	ASP	63	31.687	42.118	58.565	1.00	0.00	O
ATOM	992	N	THR	64	31.264	40.322	57.287	1.00	0.00	N
ATOM	993	H	THR	64	30.565	39.827	56.754	1.00	0.00	H
ATOM	994	CA	THR	64	32.593	39.730	57.454	1.00	0.00	C
ATOM	995	HA	THR	64	33.208	40.431	58.018	1.00	0.00	H
ATOM	996	CB	THR	64	32.491	38.450	58.322	1.00	0.00	C
ATOM	997	HB	THR	64	33.531	38.143	58.431	1.00	0.00	H
ATOM	998	CG2	THR	64	32.003	38.647	59.757	1.00	0.00	C
ATOM	999	HG21	THR	64	32.065	37.758	60.384	1.00	0.00	H
ATOM	1000	HG22	THR	64	32.684	39.277	60.329	1.00	0.00	H
ATOM	1001	HG23	THR	64	30.981	39.023	59.818	1.00	0.00	H
ATOM	1002	OG1	THR	64	31.753	37.331	57.802	1.00	0.00	O
ATOM	1003	HG1	THR	64	30.835	37.610	57.775	1.00	0.00	H
ATOM	1004	C	THR	64	33.322	39.511	56.108	1.00	0.00	C
ATOM	1005	O	THR	64	34.123	38.648	56.096	1.00	0.00	O
ATOM	1006	N	LEU	65	33.076	40.223	55.074	1.00	0.00	N
ATOM	1007	H	LEU	65	32.281	40.838	55.172	1.00	0.00	H
ATOM	1008	CA	LEU	65	33.907	40.166	53.852	1.00	0.00	C
ATOM	1009	HA	LEU	65	33.799	39.157	53.455	1.00	0.00	H
ATOM	1010	CB	LEU	65	33.274	41.157	52.898	1.00	0.00	C
ATOM	1011	HB2	LEU	65	33.235	42.111	53.423	1.00	0.00	H
ATOM	1012	HB3	LEU	65	33.927	41.181	52.025	1.00	0.00	H
ATOM	1013	CG	LEU	65	31.897	40.729	52.301	1.00	0.00	C
ATOM	1014	HG	LEU	65	31.314	40.342	53.137	1.00	0.00	H

```
ATOM   1015  CD1 LEU    65       31.287  41.862  51.499  1.00  0.00            C
ATOM   1016 HD11 LEU    65       30.956  42.666  52.157  1.00  0.00            H
ATOM   1017 HD12 LEU    65       32.008  42.300  50.810  1.00  0.00            H
ATOM   1018 HD13 LEU    65       30.396  41.490  50.992  1.00  0.00            H
ATOM   1019  CD2 LEU    65       31.915  39.473
  51.456  1.00  0.00           C
ATOM   1020 HD21 LEU    65       32.498  38.674  51.915  1.00  0.00            H
ATOM   1021 HD22 LEU    65       30.914  39.077  51.285  1.00  0.00            H
ATOM   1022 HD23 LEU    65       32.392  39.640  50.490  1.00  0.00            H
ATOM   1023  C   LEU    65       35.363  40.502  54.105  1.00  0.00            C
ATOM   1024  O   LEU    65       35.626  41.232  55.092  1.00  0.00            O
ATOM   1025  N   THR    66       36.278  40.136  53.207  1.00  0.00            N
ATOM   1026  H   THR    66       35.939  39.418  52.583  1.00  0.00            H
ATOM   1027  CA  THR    66       37.605  40.645  53.066  1.00  0.00            C
ATOM   1028  HA  THR    66       37.962  41.224  53.918  1.00  0.00            H
ATOM   1029  CB  THR    66       38.624  39.529  52.671  1.00  0.00            C
ATOM   1030  HB  THR    66       38.411  39.334  51.620  1.00  0.00            H
ATOM   1031  CG2 THR    66       40.019  39.971  52.877  1.00  0.00            C
ATOM   1032 HG21 THR    66       40.264  40.296  53.889  1.00  0.00            H
ATOM   1033 HG22 THR    66       40.751  39.224  52.572  1.00  0.00            H
ATOM   1034 HG23 THR    66       40.211  40.865  52.283  1.00  0.00            H
ATOM   1035  OG1 THR    66       38.453  38.299  53.290  1.00  0.00            O
ATOM   1036  HG1 THR    66       37.504  38.263  53.431  1.00  0.00            H
ATOM   1037  C   THR    66       37.528  41.637  51.930  1.00  0.00            C
ATOM   1038  O   THR    66       36.699  41.561  51.121  1.00  0.00            O
ATOM   1039  N   GLU    67       38.359  42.676  51.888  1.00  0.00            N
ATOM   1040  H   GLU    67       39.018  42.809  52.642  1.00  0.00            H
ATOM   1041  CA  GLU    67       38.505  43.704  50.816  1.00  0.00            C
ATOM   1042  HA  GLU    67       37.514  44.141  50.692  1.00  0.00            H
ATOM   1043  CB  GLU    67       39.350  44.989  51.176  1.00  0.00            C
ATOM   1044  HB2 GLU    67       39.159  45.803  50.477  1.00  0.00            H
ATOM   1045  HB3 GLU    67       39.160  45.311  52.200  1.00  0.00            H
ATOM   1046  CG  GLU    67       40.876  44.857  50.986  1.00  0.00            C
ATOM   1047  HG2 GLU    67       41.022  44.661  49.924  1.00  0.00            H
ATOM   1048  HG3 GLU    67       41.350  45.814  51.202  1.00  0.00            H
ATOM   1049  CD  GLU    67       41.458  43.716  51.810  1.00  0.00            C
ATOM   1050  OE1 GLU    67       40.946  43.556  52.962  1.00  0.00            O
ATOM   1051  OE2 GLU    67       42.412  42.983  51.440  1.00  0.00            O
ATOM   1052  C   GLU    67       38.892  43.234  49.393  1.00  0.00            C
ATOM   1053  O   GLU    67       39.172  44.018  48.456  1.00  0.00            O
ATOM   1054  N   LYS    68       38.944  41.930  49.093  1.00  0.00            N
ATOM   1055  H   LYS    68       38.838  41.247  49.829  1.00  0.00            H
ATOM   1056  CA  LYS    68       39.404  41.334  47.850  1.00  0.00            C
ATOM   1057  HA  LYS    68       40.391  41.763  47.676  1.00  0.00            H
ATOM   1058  CB  LYS    68       39.676  39.839  48.039  1.00  0.00            C
ATOM   1059  HB2 LYS    68       40.098  39.559  49.004  1.00  0.00            H
ATOM   1060  HB3 LYS    68       38.708  39.348  47.943  1.00  0.00            H
ATOM   1061  CG  LYS    68       40.626  39.218  46.968  1.00  0.00            C
ATOM   1062  HG2 LYS    68       40.009  38.910  46.124  1.00  0.00            H
ATOM   1063  HG3 LYS    68       41.353  39.969  46.660  1.00  0.00            H
ATOM   1064  CD  LYS    68       41.224  37.859  47.407  1.00  0.00            C
ATOM   1065  HD2 LYS    68       41.871  37.935  48.281  1.00  0.00            H
ATOM   1066  HD3 LYS    68       40.473  37.107  47.649  1.00  0.00            H
ATOM   1067  CE  LYS    68       42.127  37.311  46.213  1.00  0.00            C
ATOM   1068  HE2 LYS    68       41.463  37.235  45.352  1.00  0.00            H
ATOM   1069  HE3 LYS    68       42.933  38.029  46.061  1.00  0.00            H
ATOM   1070  NZ  LYS    68       42.657  35.903  46.457  1.00  0.00            N
ATOM   1071  HZ1 LYS    68       42.835  35.693  47.428  1.00  0.00            H
ATOM   1072  HZ2 LYS    68       41.883  35.321  46.171  1.00  0.00            H
ATOM   1073  HZ3 LYS    68       43.462  35.580  45.940  1.00  0.00            H
ATOM   1074  C   LYS    68       38.578  41.609  46.566  1.00  0.00            C
ATOM   1075  O   LYS    68       39.217  41.817  45.554  1.00  0.00            O
ATOM   1076  N   HIE    69       37.224  41.797  46.661  1.00  0.00            N
ATOM   1077  H   HIE    69       36.793  41.752  47.573  1.00  0.00            H
```

ATOM	1078	CA	HIE	69	36.456	42.337	45.571	1.00	0.00	C
ATOM	1079	HA	HIE	69	36.904	42.030	44.626	1.00	0.00	H
ATOM	1080	CB	HIE	69	34.992	41.791	45.619	1.00	0.00	C
ATOM	1081	HB2	HIE	69	35.078	40.732	45.862	1.00	0.00	H
ATOM	1082	HB3	HIE	69	34.514	42.265	46.476	1.00	0.00	H
ATOM	1083	CG	HIE	69	34.249	42.204	44.355	1.00	0.00	C
ATOM	1084	ND1	HIE	69	34.350	41.549	43.120	1.00	0.00	N
ATOM	1085	CE1	HIE	69	33.401	42.123	42.324	1.00	0.00	C
ATOM	1086	HE1	HIE	69	33.224	41.843	41.296	1.00	0.00	H
ATOM	1087	NE2	HIE	69	32.605	43.006	42.999	1.00	0.00	N
ATOM	1088	HE2	HIE	69	31.806	43.516	42.650	1.00	0.00	H
ATOM	1089	CD2	HIE	69	33.170	43.156	44.261	1.00	0.00	C
ATOM	1090	HD2	HIE	69	32.805	43.819	45.032	1.00	0.00	H
ATOM	1091	C	HIE	69	36.545	43.860	45.485	1.00	0.00	C
ATOM	1092	O	HIE	69	36.873	44.347	44.370	1.00	0.00	O
ATOM	1093	N	ALA	70	36.505	44.595	46.613	1.00	0.00	N
ATOM	1094	H	ALA	70	36.182	44.185	47.478	1.00	0.00	H
ATOM	1095	CA	ALA	70	36.410	46.017	46.597	1.00	0.00	C
ATOM	1096	HA	ALA	70	35.610	46.437	45.988	1.00	0.00	H
ATOM	1097	CB	ALA	70	36.208	46.655	47.995	1.00	0.00	C
ATOM	1098	HB1	ALA	70	37.138	46.717	48.561	1.00	0.00	H
ATOM	1099	HB2	ALA	70	35.848	47.657	47.760	1.00	0.00	H
ATOM	1100	HB3	ALA	70	35.394	46.181	48.543	1.00	0.00	H
ATOM	1101	C	ALA	70	37.712	46.617	45.948	1.00	0.00	C
ATOM	1102	O	ALA	70	37.600	47.572	45.195	1.00	0.00	O
ATOM	1103	N	GLU	71	38.898	45.930	46.058	1.00	0.00	N
ATOM	1104	H	GLU	71	39.030	45.134	46.666	1.00	0.00	H
ATOM	1105	CA	GLU	71	40.154	46.273	45.363	1.00	0.00	C
ATOM	1106	HA	GLU	71	40.253	47.347	45.527	1.00	0.00	H
ATOM	1107	CB	GLU	71	41.408	45.610	45.990	1.00	0.00	C
ATOM	1108	HB2	GLU	71	41.121	44.568	46.135	1.00	0.00	H
ATOM	1109	HB3	GLU	71	42.215	45.638	45.257	1.00	0.00	H
ATOM	1110	CG	GLU	71	42.071	46.301	47.176	1.00	0.00	C
ATOM	1111	HG2	GLU	71	41.373	46.468	47.996	1.00	0.00	H
ATOM	1112	HG3	GLU	71	42.754	45.604	47.661	1.00	0.00	H
ATOM	1113	CD	GLU	71	42.795	47.630	46.946	1.00	0.00	C
ATOM	1114	OE1	GLU	71	43.336	48.212	47.912	1.00	0.00	O
ATOM	1115	OE2	GLU	71	43.050	48.007	45.756	1.00	0.00	O
ATOM	1116	C	GLU	71	40.173	46.029	43.842	1.00	0.00	C
ATOM	1117	O	GLU	71	40.527	46.931	43.129	1.00	0.00	O
ATOM	1118	N	GLN	72	39.764	44.869	43.290	1.00	0.00	N
ATOM	1119	H	GLN	72	39.426	44.162	43.928	1.00	0.00	H
ATOM	1120	CA	GLN	72	39.404	44.770	41.873	1.00	0.00	C
ATOM	1121	HA	GLN	72	40.327	44.538	41.340	1.00	0.00	H
ATOM	1122	CB	GLN	72	38.741	43.363	41.726	1.00	0.00	C
ATOM	1123	HB2	GLN	72	37.815	43.186	42.272	1.00	0.00	H
ATOM	1124	HB3	GLN	72	39.459	42.651	42.135	1.00	0.00	H
ATOM	1125	CG	GLN	72	38.566	43.053	40.196	1.00	0.00	C
ATOM	1126	HG2	GLN	72	37.986	43.809	39.667	1.00	0.00	H
ATOM	1127	HG3	GLN	72	39.545	42.878	39.752	1.00	0.00	H
ATOM	1128	CD	GLN	72	37.671	41.823	40.135	1.00	0.00	C
ATOM	1129	OE1	GLN	72	38.131	40.780	39.823	1.00	0.00	O
ATOM	1130	NE2	GLN	72	36.367	41.840	40.492	1.00	0.00	N
ATOM	1131	HE21	GLN	72	35.933	42.744	40.613	1.00	0.00	H
ATOM	1132	HE22	GLN	72	35.890	40.959	40.621	1.00	0.00	H
ATOM	1133	C	GLN	72	38.522	45.973	41.296	1.00	0.00	C
ATOM	1134	O	GLN	72	38.709	46.520	40.200	1.00	0.00	O
ATOM	1135	N	GLU	73	37.402	46.181	41.985	1.00	0.00	N
ATOM	1136	H	GLU	73	37.274	45.596	42.799	1.00	0.00	H
ATOM	1137	CA	GLU	73	36.440	47.231	41.561	1.00	0.00	C
ATOM	1138	HA	GLU	73	36.181	47.140	40.506	1.00	0.00	H
ATOM	1139	CB	GLU	73	35.082	46.960	42.331	1.00	0.00	C
ATOM	1140	HB2	GLU	73	35.244	47.000	43.408	1.00	0.00	H
ATOM	1141	HB3	GLU	73	34.405	47.759	42.032	1.00	0.00	H

ATOM	1142	CG	GLU	73	34.411	45.634	41.989	1.00	0.00	C
ATOM	1143	HG2	GLU	73	34.853	44.735	42.418	1.00	0.00	H
ATOM	1144	HG3	GLU	73	33.456	45.605	42.514	1.00	0.00	H
ATOM	1145	CD	GLU	73	34.184	45.398	40.474	1.00	0.00	C
ATOM	1146	OE1	GLU	73	34.754	44.417	39.889	1.00	0.00	O
ATOM	1147	OE2	GLU	73	33.460	46.262	39.848	1.00	0.00	O
ATOM	1148	C	GLU	73	36.914	48.684	41.909	1.00	0.00	C
ATOM	1149	O	GLU	73	36.214	49.666	41.515	1.00	0.00	O
ATOM	1150	N	ASN	74	38.077	48.903	42.543	1.00	0.00	N
ATOM	1151	H	ASN	74	38.482	48.106	43.012	1.00	0.00	H
ATOM	1152	CA	ASN	74	38.639	50.150	43.007	1.00	0.00	C
ATOM	1153	HA	ASN	74	39.454	49.864	43.671	1.00	0.00	H
ATOM	1154	CB	ASN	74	39.234	50.903	41.770	1.00	0.00	C
ATOM	1155	HB2	ASN	74	38.420	50.960	41.047	1.00	0.00	H
ATOM	1156	HB3	ASN	74	39.575	51.876	42.124	1.00	0.00	H
ATOM	1157	CG	ASN	74	40.456	50.307	41.021	1.00	0.00	C
ATOM	1158	OD1	ASN	74	41.570	50.559	41.359	1.00	0.00	O
ATOM	1159	ND2	ASN	74	40.266	49.407	40.105	1.00	0.00	N
ATOM	1160	HD21	ASN	74	40.973	48.718	39.890	1.00	0.00	H
ATOM	1161	HD22	ASN	74	39.300	49.144	39.975	1.00	0.00	H
ATOM	1162	C	ASN	74	37.714	50.973	43.888	1.00	0.00	C
ATOM	1163	O	ASN	74	37.245	52.070	43.457	1.00	0.00	O
ATOM	1164	N	MET	75	37.493	50.565	45.100	1.00	0.00	N
ATOM	1165	H	MET	75	38.015	49.819	45.538	1.00	0.00	H
ATOM	1166	CA	MET	75	36.471	51.244	46.009	1.00	0.00	C
ATOM	1167	HA	MET	75	36.775	52.290	45.976	1.00	0.00	H
ATOM	1168	CB	MET	75	35.068	51.165	45.541	1.00	0.00	C
ATOM	1169	HB2	MET	75	34.426	51.613	46.300	1.00	0.00	H
ATOM	1170	HB3	MET	75	35.013	51.754	44.626	1.00	0.00	H
ATOM	1171	CG	MET	75	34.539	49.802	45.202	1.00	0.00	C
ATOM	1172	HG2	MET	75	35.115	49.313	44.417	1.00	0.00	H
ATOM	1173	HG3	MET	75	34.552	49.197	46.108	1.00	0.00	H
ATOM	1174	SD	MET	75	32.767	49.609	44.581	1.00	0.00	S
ATOM	1175	CE	MET	75	31.906	50.442	45.980	1.00	0.00	C
ATOM	1176	HE1	MET	75	32.310	50.095	46.932	1.00	0.00	H
ATOM	1177	HE2	MET	75	32.072	51.519	45.966	1.00	0.00	H
ATOM	1178	HE3	MET	75	30.820	50.357	45.974	1.00	0.00	H
ATOM	1179	C	MET	75	36.624	50.750	47.443	1.00	0.00	C
ATOM	1180	O	MET	75	37.280	49.727	47.658	1.00	0.00	O
ATOM	1181	N	THR	76	36.148	51.483	48.446	1.00	0.00	N
ATOM	1182	H	THR	76	35.705	52.377	48.286	1.00	0.00	H
ATOM	1183	CA	THR	76	36.245	50.966	49.835	1.00	0.00	C
ATOM	1184	HA	THR	76	37.168	50.396	49.943	1.00	0.00	H
ATOM	1185	CB	THR	76	36.250	52.102	50.912	1.00	0.00	C
ATOM	1186	HB	THR	76	37.131	52.736	50.814	1.00	0.00	H
ATOM	1187	CG2	THR	76	35.038	52.950	51.037	1.00	0.00	C
ATOM	1188	HG21	THR	76	34.143	52.536	51.502	1.00	0.00	H
ATOM	1189	HG22	THR	76	35.214	53.877	51.581	1.00	0.00	H
ATOM	1190	HG23	THR	76	34.804	53.228	50.009	1.00	0.00	H
ATOM	1191	OG1	THR	76	36.315	51.649	52.226	1.00	0.00	O
ATOM	1192	HG1	THR	76	37.264	51.563	52.346	1.00	0.00	H
ATOM	1193	C	THR	76	35.172	49.856	50.093	1.00	0.00	C
ATOM	1194	O	THR	76	34.062	49.989	49.645	1.00	0.00	O
ATOM	1195	N	LEU	77	35.501	48.899	50.951	1.00	0.00	N
ATOM	1196	H	LEU	77	36.416	48.880	51.379	1.00	0.00	H
ATOM	1197	CA	LEU	77	34.605	47.802	51.399	1.00	0.00	C
ATOM	1198	HA	LEU	77	34.115	47.531	50.463	1.00	0.00	H
ATOM	1199	CB	LEU	77	35.475	46.711	52.062	1.00	0.00	C
ATOM	1200	HB2	LEU	77	36.100	46.320	51.259	1.00	0.00	H
ATOM	1201	HB3	LEU	77	36.100	47.159	52.835	1.00	0.00	H
ATOM	1202	CG	LEU	77	34.766	45.505	52.792	1.00	0.00	C
ATOM	1203	HG	LEU	77	34.169	45.839	53.640	1.00	0.00	H
ATOM	1204	CD1	LEU	77	33.939	44.721	51.719	1.00	0.00	C

ATOM	1205	HD11	LEU	77	33.366	43.898	52.146	1.00	0.00	H
ATOM	1206	HD12	LEU	77	33.147	45.391	51.384	1.00	0.00	H
ATOM	1207	HD13	LEU	77	34.663	44.631	50.910	1.00	0.00	H
ATOM	1208	CD2	LEU	77	35.700	44.533	53.487	1.00	0.00	C
ATOM	1209	HD21	LEU	77	36.075	43.855	52.720	1.00	0.00	H
ATOM	1210	HD22	LEU	77	36.498	45.035	54.033	1.00	0.00	H
ATOM	1211	HD23	LEU	77	35.105	44.017	54.241	1.00	0.00	H
ATOM	1212	C	LEU	77	33.350	48.233	52.243	1.00	0.00	C
ATOM	1213	O	LEU	77	32.357	47.574	52.093	1.00	0.00	O
ATOM	1214	N	THR	78	33.352	49.335	53.013	1.00	0.00	N
ATOM	1215	H	THR	78	34.187	49.880	53.179	1.00	0.00	H
ATOM	1216	CA	THR	78	32.066	49.931	53.585	1.00	0.00	C
ATOM	1217	HA	THR	78	31.458	49.110	53.965	1.00	0.00	H
ATOM	1218	CB	THR	78	32.386	50.942	54.688	1.00	0.00	C
ATOM	1219	HB	THR	78	31.582	51.627	54.957	1.00	0.00	H
ATOM	1220	CG2	THR	78	32.852	50.230	55.948	1.00	0.00	C
ATOM	1221	HG21	THR	78	33.211	50.940	56.693	1.00	0.00	H
ATOM	1222	HG22	THR	78	32.027	49.652	56.365	1.00	0.00	H
ATOM	1223	HG23	THR	78	33.687	49.557	55.757	1.00	0.00	H
ATOM	1224	OG1	THR	78	33.381	51.896	54.375	1.00	0.00	O
ATOM	1225	HG1	THR	78	32.982	52.765	54.287	1.00	0.00	H
ATOM	1226	C	THR	78	31.146	50.596	52.556	1.00	0.00	C
ATOM	1227	O	THR	78	29.916	50.557	52.688	1.00	0.00	O
ATOM	1228	N	GLU	79	31.713	51.153	51.489	1.00	0.00	N
ATOM	1229	H	GLU	79	32.709	51.265	51.363	1.00	0.00	H
ATOM	1230	CA	GLU	79	30.908	51.607	50.337	1.00	0.00	C
ATOM	1231	HA	GLU	79	30.000	52.097	50.687	1.00	0.00	H
ATOM	1232	CB	GLU	79	31.628	52.745	49.583	1.00	0.00	C
ATOM	1233	HB2	GLU	79	32.674	52.523	49.372	1.00	0.00	H
ATOM	1234	HB3	GLU	79	31.224	52.870	48.579	1.00	0.00	H
ATOM	1235	CG	GLU	79	31.575	54.104	50.301	1.00	0.00	C
ATOM	1236	HG2	GLU	79	32.392	53.939	51.003	1.00	0.00	H
ATOM	1237	HG3	GLU	79	31.850	54.860	49.565	1.00	0.00	H
ATOM	1238	CD	GLU	79	30.255	54.559	50.830	1.00	0.00	C
ATOM	1239	OE1	GLU	79	30.267	55.305	51.771	1.00	0.00	O
ATOM	1240	OE2	GLU	79	29.175	54.304	50.303	1.00	0.00	O
ATOM	1241	C	GLU	79	30.495	50.421	49.433	1.00	0.00	C
ATOM	1242	O	GLU	79	29.403	50.534	48.819	1.00	0.00	O
ATOM	1243	N	LEU	80	31.240	49.333	49.311	1.00	0.00	N
ATOM	1244	H	LEU	80	32.181	49.386	49.673	1.00	0.00	H
ATOM	1245	CA	LEU	80	30.733	48.187	48.712	1.00	0.00	C
ATOM	1246	HA	LEU	80	30.323	48.462	47.740	1.00	0.00	H
ATOM	1247	CB	LEU	80	31.877	47.209	48.584	1.00	0.00	C
ATOM	1248	HB2	LEU	80	32.667	47.664	47.986	1.00	0.00	H
ATOM	1249	HB3	LEU	80	32.315	47.085	49.574	1.00	0.00	H
ATOM	1250	CG	LEU	80	31.538	45.806	47.967	1.00	0.00	C
ATOM	1251	HG	LEU	80	30.757	45.351	48.575	1.00	0.00	H
ATOM	1252	CD1	LEU	80	31.000	45.902	46.524	1.00	0.00	C
ATOM	1253	HD11	LEU	80	31.727	46.417	45.896	1.00	0.00	H
ATOM	1254	HD12	LEU	80	30.777	44.906	46.142	1.00	0.00	H
ATOM	1255	HD13	LEU	80	30.154	46.586	46.469	1.00	0.00	H
ATOM	1256	CD2	LEU	80	32.711	44.874	47.919	1.00	0.00	C
ATOM	1257	HD21	LEU	80	32.511	43.892	47.489	1.00	0.00	H
ATOM	1258	HD22	LEU	80	33.430	45.365	47.264	1.00	0.00	H
ATOM	1259	HD23	LEU	80	33.240	44.764	48.865	1.00	0.00	H
ATOM	1260	C	LEU	80	29.607	47.528	49.567	1.00	0.00	C
ATOM	1261	O	LEU	80	28.712	46.795	49.009	1.00	0.00	O
ATOM	1262	N	LYS	81	29.605	47.723	50.870	1.00	0.00	N
ATOM	1263	H	LYS	81	30.426	48.181	51.240	1.00	0.00	H
ATOM	1264	CA	LYS	81	28.580	47.162	51.772	1.00	0.00	C
ATOM	1265	HA	LYS	81	28.430	46.095	51.608	1.00	0.00	H
ATOM	1266	CB	LYS	81	28.969	47.267	53.266	1.00	0.00	C
ATOM	1267	HB2	LYS	81	29.375	48.211	53.628	1.00	0.00	H
ATOM	1268	HB3	LYS	81	28.139	47.036	53.933	1.00	0.00	H

```
ATOM   1269  CG  LYS    81       30.009  46.149  53.595  1.00  0.00            C
ATOM   1270  HG2 LYS
    81       29.430  45.233  53.709  1.00  0.00            H
ATOM   1271  HG3 LYS    81       30.766  46.110  52.812  1.00  0.00            H
ATOM   1272  CD  LYS    81       30.815  46.385  54.905  1.00  0.00            C
ATOM   1273  HD2 LYS    81       31.463  47.259  54.855  1.00  0.00            H
ATOM   1274  HD3 LYS    81       30.120  46.473  55.740  1.00  0.00            H
ATOM   1275  CE  LYS    81       31.721  45.152  55.227  1.00  0.00            C
ATOM   1276  HE2 LYS    81       31.075  44.298  55.433  1.00  0.00            H
ATOM   1277  HE3 LYS    81       32.426  44.925  54.428  1.00  0.00            H
ATOM   1278  NZ  LYS    81       32.453  45.488  56.521  1.00  0.00            N
ATOM   1279  HZ1 LYS    81       31.792  45.808  57.214  1.00  0.00            H
ATOM   1280  HZ2 LYS    81       33.080  44.806  56.922  1.00  0.00            H
ATOM   1281  HZ3 LYS    81       33.230  46.104  56.331  1.00  0.00            H
ATOM   1282  C   LYS    81       27.184  47.807  51.502  1.00  0.00            C
ATOM   1283  O   LYS    81       26.170  47.130  51.277  1.00  0.00            O
ATOM   1284  N   LYS    82       27.130  49.134  51.401  1.00  0.00            N
ATOM   1285  H   LYS    82       28.021  49.599  51.502  1.00  0.00            H
ATOM   1286  CA  LYS    82       26.028  50.043  51.000  1.00  0.00            C
ATOM   1287  HA  LYS    82       25.243  49.862  51.734  1.00  0.00            H
ATOM   1288  CB  LYS    82       26.455  51.501  51.209  1.00  0.00            C
ATOM   1289  HB2 LYS    82       27.275  51.762  50.540  1.00  0.00            H
ATOM   1290  HB3 LYS    82       25.573  52.131  51.104  1.00  0.00            H
ATOM   1291  CG  LYS    82       26.827  51.740  52.658  1.00  0.00            C
ATOM   1292  HG2 LYS    82       26.002  51.630  53.363  1.00  0.00            H
ATOM   1293  HG3 LYS    82       27.597  51.002  52.880  1.00  0.00            H
ATOM   1294  CD  LYS    82       27.488  53.115  52.963  1.00  0.00            C
ATOM   1295  HD2 LYS    82       27.470  53.188  54.050  1.00  0.00            H
ATOM   1296  HD3 LYS    82       28.516  53.096  52.599  1.00  0.00            H
ATOM   1297  CE  LYS    82       26.675  54.344  52.509  1.00  0.00            C
ATOM   1298  HE2 LYS    82       26.200  54.276  51.530  1.00  0.00            H
ATOM   1299  HE3 LYS    82       25.849  54.574  53.181  1.00  0.00            H
ATOM   1300  NZ  LYS    82       27.587  55.556  52.467  1.00  0.00            N
ATOM   1301  HZ1 LYS    82       27.836  55.730  53.430  1.00  0.00            H
ATOM   1302  HZ2 LYS    82       28.486  55.461  52.017  1.00  0.00            H
ATOM   1303  HZ3 LYS    82       27.141  56.417  52.182  1.00  0.00            H
ATOM   1304  C   LYS    82       25.376  49.644  49.671  1.00  0.00            C
ATOM   1305  O   LYS    82       24.149  49.765  49.577  1.00  0.00            O
ATOM   1306  N   VAL    83       26.216  49.256  48.691  1.00  0.00            N
ATOM   1307  H   VAL    83       27.208  49.320  48.872  1.00  0.00            H
ATOM   1308  CA  VAL    83       25.669  48.874  47.330  1.00  0.00            C
ATOM   1309  HA  VAL    83       24.830  49.547  47.153  1.00  0.00            H
ATOM   1310  CB  VAL    83       26.587  49.071  46.120  1.00  0.00            C
ATOM   1311  HB  VAL    83       25.971  48.788  45.267  1.00  0.00            H
ATOM   1312  CG1 VAL    83       27.023  50.552  46.008  1.00  0.00            C
ATOM   1313 HG11 VAL    83       27.695  50.701  45.163  1.00  0.00            H
ATOM   1314 HG12 VAL    83       26.058  51.050  45.912  1.00  0.00            H
ATOM   1315 HG13 VAL    83       27.644  50.889  46.837  1.00  0.00            H
ATOM   1316  CG2 VAL    83       27.797  48.157  46.188  1.00  0.00            C
ATOM   1317 HG21 VAL    83       28.328  48.176  47.140  1.00  0.00            H
ATOM   1318 HG22 VAL    83       27.529  47.126  45.958  1.00  0.00            H
ATOM   1319 HG23 VAL    83       28.588  48.357  45.465  1.00  0.00            H
ATOM   1320  C   VAL    83       25.121  47.490  47.315  1.00  0.00            C
ATOM   1321  O   VAL    83       24.088  47.162  46.627  1.00  0.00            O
ATOM   1322  N   ILE    84       25.745  46.544  48.122  1.00  0.00            N
ATOM   1323  H   ILE    84       26.634  46.751  48.553  1.00  0.00            H
ATOM   1324  CA  ILE    84       25.175  45.258  48.290  1.00  0.00            C
ATOM   1325  HA  ILE    84       24.906  44.829  47.325  1.00  0.00            H
ATOM   1326  CB  ILE    84       26.156  44.177  48.921  1.00  0.00            C
ATOM   1327  HB  ILE    84       26.414  44.515  49.925  1.00  0.00            H
ATOM   1328  CG2 ILE    84       25.574  42.747  49.023  1.00  0.00            C
ATOM   1329 HG21 ILE    84       24.768  42.829  49.752  1.00  0.00            H
ATOM   1330 HG22 ILE    84       25.191  42.312  48.100  1.00  0.00            H
ATOM   1331 HG23 ILE    84       26.356  42.138  49.476  1.00  0.00            H
```

ATOM	1332	CG1	ILE	84	27.399	44.078	48.003	1.00	0.00	C
ATOM	1333	HG12	ILE	84	27.003	43.598	47.108	1.00	0.00	H
ATOM	1334	HG13	ILE	84	27.787	45.067	47.757	1.00	0.00	H
ATOM	1335	CD1	ILE	84	28.642	43.340	48.660	1.00	0.00	C
ATOM	1336	HD11	ILE	84	28.362	42.287	48.620	1.00	0.00	H
ATOM	1337	HD12	ILE	84	29.520	43.300	48.015	1.00	0.00	H
ATOM	1338	HD13	ILE	84	28.839	43.645	49.688	1.00	0.00	H
ATOM	1339	C	ILE	84	23.836	45.384	49.015	1.00	0.00	C
ATOM	1340	O	ILE	84	22.876	44.763	48.639	1.00	0.00	O
ATOM	1341	N	ALA	85	23.746	46.179	50.099	1.00	0.00	N
ATOM	1342	H	ALA	85	24.490	46.825	50.319	1.00	0.00	H
ATOM	1343	CA	ALA	85	22.554	46.397	50.868	1.00	0.00	C
ATOM	1344	HA	ALA	85	22.163	45.440	51.214	1.00	0.00	H
ATOM	1345	CB	ALA	85	23.058	47.297	51.997	1.00	0.00	C
ATOM	1346	HB1	ALA	85	23.289	48.285	51.598	1.00	0.00	H
ATOM	1347	HB2	ALA	85	22.230	47.367	52.702	1.00	0.00	H
ATOM	1348	HB3	ALA	85	23.988	46.911	52.413	1.00	0.00	H
ATOM	1349	C	ALA	85	21.386	47.012	50.061	1.00	0.00	C
ATOM	1350	O	ALA	85	20.218	46.771	50.463	1.00	0.00	O
ATOM	1351	N	ASP	86	21.589	47.762	48.924	1.00	0.00	N
ATOM	1352	H	ASP	86	22.547	47.867	48.622	1.00	0.00	H
ATOM	1353	CA	ASP	86	20.499	48.279	48.102	1.00	0.00	C
ATOM	1354	HA	ASP	86	19.729	48.674	48.765	1.00	0.00	H
ATOM	1355	CB	ASP	86	21.045	49.466	47.287	1.00	0.00	C
ATOM	1356	HB2	ASP	86	21.662	50.132	47.891	1.00	0.00	H
ATOM	1357	HB3	ASP	86	21.668	49.016	46.514	1.00	0.00	H
ATOM	1358	CG	ASP	86	19.934	50.386	46.695	1.00	0.00	C
ATOM	1359	OD1	ASP	86	20.010	50.712	45.510	1.00	0.00	O
ATOM	1360	OD2	ASP	86	19.030	50.719	47.511	1.00	0.00	O
ATOM	1361	C	ASP	86	19.846	47.246	47.223	1.00	0.00	C
ATOM	1362	O	ASP	86	18.932	47.590	46.469	1.00	0.00	O
ATOM	1363	N	ILE	87	20.378	46.010	47.182	1.00	0.00	N
ATOM	1364	H	ILE	87	21.274	45.861	47.624	1.00	0.00	H
ATOM	1365	CA	ILE	87	19.828	44.868	46.436	1.00	0.00	C
ATOM	1366	HA	ILE	87	18.944	45.144	45.862	1.00	0.00	H
ATOM	1367	CB	ILE	87	20.891	44.432	45.325	1.00	0.00	C
ATOM	1368	HB	ILE	87	21.807	44.133	45.835	1.00	0.00	H
ATOM	1369	CG2	ILE	87	20.352	43.360	44.494	1.00	0.00	C
ATOM	1370	HG21	ILE	87	21.128	42.984	43.827	1.00	0.00	H
ATOM	1371	HG22	ILE	87	19.991	42.565	45.146	1.00	0.00	H
ATOM	1372	HG23	ILE	87	19.539	43.676	43.840	1.00	0.00	H
ATOM	1373	CG1	ILE	87	21.448	45.613	44.448	1.00	0.00	C
ATOM	1374	HG12	ILE	87	20.664	45.717	43.697	1.00	0.00	H
ATOM	1375	HG13	ILE	87	21.474	46.524	45.046	1.00	0.00	H
ATOM	1376	CD1	ILE	87	22.825	45.405	43.820	1.00	0.00	C
ATOM	1377	HD11	ILE	87	22.890	44.503	43.211	1.00	0.00	H
ATOM	1378	HD12	ILE	87	23.052	46.278	43.208	1.00	0.00	H
ATOM	1379	HD13	ILE	87	23.644	45.395	44.539	1.00	0.00	H
ATOM	1380	C	ILE	87	19.530	43.724	47.414	1.00	0.00	C
ATOM	1381	O	ILE	87	18.533	43.032	47.197	1.00	0.00	O
ATOM	1382	N	TYR	88	20.358	43.567	48.435	1.00	0.00	N
ATOM	1383	H	TYR	88	21.218	44.094	48.489	1.00	0.00	H
ATOM	1384	CA	TYR	88	20.157	42.388	49.333	1.00	0.00	C
ATOM	1385	HA	TYR	88	19.241	41.890	49.016	1.00	0.00	H
ATOM	1386	CB	TYR	88	21.207	41.354	49.036	1.00	0.00	C
ATOM	1387	HB2	TYR	88	22.196	41.810	49.019	1.00	0.00	H
ATOM	1388	HB3	TYR	88	21.161	40.551	49.772	1.00	0.00	H
ATOM	1389	CG	TYR	88	21.214	40.685	47.623	1.00	0.00	C
ATOM	1390	CD1	TYR	88	20.629	39.425	47.463	1.00	0.00	C
ATOM	1391	HD1	TYR	88	20.101	38.918	48.256	1.00	0.00	H
ATOM	1392	CE1	TYR	88	20.699	38.769	46.261	1.00	0.00	C
ATOM	1393	HE1	TYR	88	20.336	37.767	46.081	1.00	0.00	H
ATOM	1394	CZ	TYR	88	21.384	39.396	45.171	1.00	0.00	C
ATOM	1395	OH	TYR	88	21.503	38.795				

43.931 1.00 0.00 O

ATOM	1396	HH	TYR	88	20.822	38.144	43.747	1.00	0.00	H
ATOM	1397	CE2	TYR	88	21.972	40.629	45.349	1.00	0.00	C
ATOM	1398	HE2	TYR	88	22.547	41.070	44.549	1.00	0.00	H
ATOM	1399	CD2	TYR	88	22.015	41.258	46.618	1.00	0.00	C
ATOM	1400	HD2	TYR	88	22.436	42.209	46.907	1.00	0.00	H
ATOM	1401	C	TYR	88	20.221	42.726	50.838	1.00	0.00	C
ATOM	1402	O	TYR	88	21.307	43.158	51.277	1.00	0.00	O
ATOM	1403	N	PRO	89	19.088	42.730	51.551	1.00	0.00	N
ATOM	1404	CD	PRO	89	17.728	42.570	50.902	1.00	0.00	C
ATOM	1405	HD2	PRO	89	17.469	41.522	50.751	1.00	0.00	H
ATOM	1406	HD3	PRO	89	17.728	43.174	49.995	1.00	0.00	H
ATOM	1407	CG	PRO	89	16.859	43.166	52.020	1.00	0.00	C
ATOM	1408	HG2	PRO	89	15.841	42.804	51.872	1.00	0.00	H
ATOM	1409	HG3	PRO	89	16.924	44.254	51.995	1.00	0.00	H
ATOM	1410	CB	PRO	89	17.496	42.668	53.316	1.00	0.00	C
ATOM	1411	HB2	PRO	89	17.328	41.597	53.432	1.00	0.00	H
ATOM	1412	HB3	PRO	89	17.034	43.140	54.183	1.00	0.00	H
ATOM	1413	CA	PRO	89	18.977	42.985	53.017	1.00	0.00	C
ATOM	1414	HA	PRO	89	19.328	44.011	53.133	1.00	0.00	H
ATOM	1415	C	PRO	89	19.962	42.052	53.741	1.00	0.00	C
ATOM	1416	O	PRO	89	20.154	40.893	53.358	1.00	0.00	O
ATOM	1417	N	GLY	90	20.586	42.460	54.823	1.00	0.00	N
ATOM	1418	H	GLY	90	20.455	43.452	54.960	1.00	0.00	H
ATOM	1419	CA	GLY	90	21.816	41.975	55.538	1.00	0.00	C
ATOM	1420	HA2	GLY	90	22.647	41.932	54.834	1.00	0.00	H
ATOM	1421	HA3	GLY	90	21.940	42.678	56.362	1.00	0.00	H
ATOM	1422	C	GLY	90	21.620	40.583	56.196	1.00	0.00	C
ATOM	1423	O	GLY	90	22.650	39.977	56.543	1.00	0.00	O
ATOM	1424	N	GLN	91	20.330	40.055	56.204	1.00	0.00	N
ATOM	1425	H	GLN	91	19.637	40.724	55.898	1.00	0.00	H
ATOM	1426	CA	GLN	91	19.984	38.676	56.547	1.00	0.00	C
ATOM	1427	HA	GLN	91	20.413	38.435	57.519	1.00	0.00	H
ATOM	1428	CB	GLN	91	18.466	38.570	56.867	1.00	0.00	C
ATOM	1429	HB2	GLN	91	18.212	39.431	57.484	1.00	0.00	H
ATOM	1430	HB3	GLN	91	17.904	38.648	55.936	1.00	0.00	H
ATOM	1431	CG	GLN	91	18.057	37.293	57.536	1.00	0.00	C
ATOM	1432	HG2	GLN	91	17.085	36.980	57.154	1.00	0.00	H
ATOM	1433	HG3	GLN	91	18.692	36.471	57.206	1.00	0.00	H
ATOM	1434	CD	GLN	91	18.004	37.290	59.069	1.00	0.00	C
ATOM	1435	OE1	GLN	91	18.435	38.194	59.739	1.00	0.00	O
ATOM	1436	NE2	GLN	91	17.436	36.228	59.705	1.00	0.00	N
ATOM	1437	HE21	GLN	91	16.983	35.443	59.261	1.00	0.00	H
ATOM	1438	HE22	GLN	91	17.427	36.303	60.712	1.00	0.00	H
ATOM	1439	C	GLN	91	20.554	37.581	55.601	1.00	0.00	C
ATOM	1440	O	GLN	91	20.812	36.470	56.007	1.00	0.00	O
ATOM	1441	N	THR	92	20.736	38.046	54.344	1.00	0.00	N
ATOM	1442	H	THR	92	20.626	39.010	54.064	1.00	0.00	H
ATOM	1443	CA	THR	92	21.159	37.194	53.220	1.00	0.00	C
ATOM	1444	HA	THR	92	20.376	36.470	52.994	1.00	0.00	H
ATOM	1445	CB	THR	92	21.250	38.054	52.004	1.00	0.00	C
ATOM	1446	HB	THR	92	22.010	38.810	52.199	1.00	0.00	H
ATOM	1447	CG2	THR	92	21.444	37.296	50.655	1.00	0.00	C
ATOM	1448	HG21	THR	92	22.456	36.894	50.684	1.00	0.00	H
ATOM	1449	HG22	THR	92	20.829	36.426	50.427	1.00	0.00	H
ATOM	1450	HG23	THR	92	21.448	37.992	49.815	1.00	0.00	H
ATOM	1451	OG1	THR	92	20.050	38.681	51.739	1.00	0.00	O
ATOM	1452	HG1	THR	92	20.084	39.557	52.129	1.00	0.00	H
ATOM	1453	C	THR	92	22.507	36.438	53.543	1.00	0.00	C
ATOM	1454	O	THR	92	23.285	36.980	54.332	1.00	0.00	O
ATOM	1455	N	GLN	93	22.782	35.248	52.919	1.00	0.00	N
ATOM	1456	H	GLN	93	21.993	34.680	52.645	1.00	0.00	H
ATOM	1457	CA	GLN	93	24.107	34.669	52.996	1.00	0.00	C
ATOM	1458	HA	GLN	93	24.819	35.184	53.641	1.00	0.00	H

ATOM	1459	CB	GLN	93	24.058	33.194	53.487	1.00	0.00	C
ATOM	1460	HB2	GLN	93	23.517	33.236	54.432	1.00	0.00	H
ATOM	1461	HB3	GLN	93	23.369	32.560	52.930	1.00	0.00	H
ATOM	1462	CG	GLN	93	25.285	32.314	53.702	1.00	0.00	C
ATOM	1463	HG2	GLN	93	24.907	31.327	53.967	1.00	0.00	H
ATOM	1464	HG3	GLN	93	25.837	32.198	52.770	1.00	0.00	H
ATOM	1465	CD	GLN	93	26.140	32.811	54.843	1.00	0.00	C
ATOM	1466	OE1	GLN	93	25.948	33.896	55.440	1.00	0.00	O
ATOM	1467	NE2	GLN	93	27.197	32.178	55.207	1.00	0.00	N
ATOM	1468	HE21	GLN	93	27.422	31.247	54.887	1.00	0.00	H
ATOM	1469	HE22	GLN	93	27.602	32.603	56.029	1.00	0.00	H
ATOM	1470	C	GLN	93	24.698	34.803	51.565	1.00	0.00	C
ATOM	1471	O	GLN	93	23.926	34.750	50.608	1.00	0.00	O
ATOM	1472	N	PHE	94	26.003	34.929	51.466	1.00	0.00	N
ATOM	1473	H	PHE	94	26.561	34.938	52.308	1.00	0.00	H
ATOM	1474	CA	PHE	94	26.678	35.123	50.153	1.00	0.00	C
ATOM	1475	HA	PHE	94	25.963	35.002	49.340	1.00	0.00	H
ATOM	1476	CB	PHE	94	27.150	36.602	50.184	1.00	0.00	C
ATOM	1477	HB2	PHE	94	27.867	36.685	51.001	1.00	0.00	H
ATOM	1478	HB3	PHE	94	27.746	36.886	49.316	1.00	0.00	H
ATOM	1479	CG	PHE	94	25.945	37.575	50.376	1.00	0.00	C
ATOM	1480	CD1	PHE	94	25.439	38.272	49.236	1.00	0.00	C
ATOM	1481	HD1	PHE	94	25.786	38.021	48.244	1.00	0.00	H
ATOM	1482	CE1	PHE	94	24.503	39.327	49.236	1.00	0.00	C
ATOM	1483	HE1	PHE	94	24.227	39.692	48.258	1.00	0.00	H
ATOM	1484	CZ	PHE	94	24.109	39.747	50.484	1.00	0.00	C
ATOM	1485	HZ	PHE	94	23.468	40.606	50.620	1.00	0.00	H
ATOM	1486	CE2	PHE	94	24.638	39.157	51.685	1.00	0.00	C
ATOM	1487	HE2	PHE	94	24.354	39.492	52.672	1.00	0.00	H
ATOM	1488	CD2	PHE	94	25.567	38.092	51.619	1.00	0.00	C
ATOM	1489	HD2	PHE	94	26.055	37.701	52.499	1.00	0.00	H
ATOM	1490	C	PHE	94	27.788	34.155	49.919	1.00	0.00	C
ATOM	1491	O	PHE	94	28.141	33.340	50.794	1.00	0.00	O
ATOM	1492	N	TYR	95	28.223	34.029	48.625	1.00	0.00	N
ATOM	1493	H	TYR	95	27.832	34.632	47.916	1.00	0.00	H
ATOM	1494	CA	TYR	95	29.167	33.036	48.064	1.00	0.00	C
ATOM	1495	HA	TYR	95	29.730	32.566	48.870	1.00	0.00	H
ATOM	1496	CB	TYR	95	28.286	31.992	47.444	1.00	0.00	C
ATOM	1497	HB2	TYR	95	27.601	31.695	48.238	1.00	0.00	H
ATOM	1498	HB3	TYR	95	27.679	32.452	46.664	1.00	0.00	H
ATOM	1499	CG	TYR	95	29.100	30.797	46.944	1.00	0.00	C
ATOM	1500	CD1	TYR	95	29.378	30.713	45.559	1.00	0.00	C
ATOM	1501	HD1	TYR	95	28.922	31.446	44.910	1.00	0.00	H
ATOM	1502	CE1	TYR	95	30.157	29.546	45.114	1.00	0.00	C
ATOM	1503	HE1	TYR	95	30.386	29.485	44.061	1.00	0.00	H
ATOM	1504	CZ	TYR	95	30.651	28.536	46.042	1.00	0.00	C
ATOM	1505	OH	TYR	95	31.247	27.456	45.608	1.00	0.00	O
ATOM	1506	HH	TYR	95	31.047	27.281	44.686	1.00	0.00	H
ATOM	1507	CE2	TYR	95	30.346	28.699	47.413	1.00	0.00	C
ATOM	1508	HE2	TYR	95	30.706	27.979	48.133	1.00	0.00	H
ATOM	1509	CD2	TYR	95	29.638	29.851	47.859	1.00	0.00	C
ATOM	1510	HD2	TYR	95	29.356	29.922	48.899	1.00	0.00	H
ATOM	1511	C	TYR	95	30.117	33.745	47.121	1.00	0.00	C
ATOM	1512	O	TYR	95	29.750	34.511	46.249	1.00	0.00	O
ATOM	1513	N	VAL	96	31.386	33.340	47.226	1.00	0.00	N
ATOM	1514	H	VAL	96	31.639	32.591	47.856	1.00	0.00	H
ATOM	1515	CA	VAL	96	32.507	33.871	46.516	1.00	0.00	C
ATOM	1516	HA	VAL	96	32.133	34.801	46.088	1.00	0.00	H
ATOM	1517	CB	VAL	96	33.743	34.242	47.431	1.00	0.00	C
ATOM	1518	HB	VAL	96	34.157	33.316	47.829	1.00	0.00	H
ATOM	1519	CG1	VAL	96	34.817	34.902	46.652	1.00	0.00	C
ATOM	1520	HG11	VAL	96	34.248	35.701	46.176	1.00	0.00	H
ATOM	1521	HG12	VAL	96	35.581	35.227	47.358	1.00	0.00	H

ATOM	1522	HG13	VAL	96	35.193	34.261	45.854	1.00	0.00	H
ATOM	1523	CG2	VAL	96	33.371	35.244	48.539	1.00	0.00	C
ATOM	1524	HG21	VAL	96	34.141	35.237	49.310	1.00	0.00	H
ATOM	1525	HG22	VAL	96	33.223	36.248	48.142	1.00	0.00	H
ATOM	1526	HG23	VAL	96	32.464	35.066	49.117	1.00	0.00	H
ATOM	1527	C	VAL	96	32.917	32.874	45.469	1.00	0.00	C
ATOM	1528	O	VAL	96	33.099	31.699	45.787	1.00	0.00	O
ATOM	1529	N	ILE	97	33.224	33.420	44.306	1.00	0.00	N
ATOM	1530	H	ILE	97	32.935	34.382	44.203	1.00	0.00	H
ATOM	1531	CA	ILE	97	33.980	32.745	43.210	1.00	0.00	C
ATOM	1532	HA	ILE	97	34.381	31.790	43.551	1.00	0.00	H
ATOM	1533	CB	ILE	97	33.075	32.500	41.981	1.00	0.00	C
ATOM	1534	HB	ILE	97	32.740	33.472	41.618	1.00	0.00	H
ATOM	1535	CG2	ILE	97	33.800	31.727	40.846	1.00	0.00	C
ATOM	1536	HG21	ILE	97	33.135	31.626	39.989	1.00	0.00	H
ATOM	1537	HG22	ILE	97	34.727	32.205	40.531	1.00	0.00	H
ATOM	1538	HG23	ILE	97	33.996	30.689	41.117	1.00	0.00	H
ATOM	1539	CG1	ILE	97	31.875	31.610	42.420	1.00	0.00	C
ATOM	1540	HG12	ILE	97	32.283	30.615	42.597	1.00	0.00	H
ATOM	1541	HG13	ILE	97	31.582	31.954	43.412	1.00	0.00	H
ATOM	1542	CD1	ILE	97	30.605	31.540	41.443	1.00	0.00	C
ATOM	1543	HD11	ILE	97	29.835	30.933	41.919	1.00	0.00	H
ATOM	1544	HD12	ILE	97	30.298	32.566	41.239	1.00	0.00	H
ATOM	1545	HD13	ILE	97	30.934	30.927	40.603	1.00	0.00	H
ATOM	1546	C	ILE	97	35.193	33.658	42.930	1.00	0.00	C
ATOM	1547	O	ILE	97	35.097	34.899	42.990	1.00	0.00	O
ATOM	1548	N	GLU	98	36.295	32.927	42.567	1.00	0.00	N
ATOM	1549	H	GLU	98	36.362	31.947	42.800	1.00	0.00	H
ATOM	1550	CA	GLU	98	37.567	33.439	42.076	1.00	0.00	C
ATOM	1551	HA	GLU	98	37.403	34.447	41.696	1.00	0.00	H
ATOM	1552	CB	GLU	98	38.625	33.495	43.206	1.00	0.00	C
ATOM	1553	HB2	GLU	98	38.889	32.469	43.459	1.00	0.00	H
ATOM	1554	HB3	GLU	98	39.559	33.945	42.871	1.00	0.00	H
ATOM	1555	CG	GLU	98	38.261	34.228	44.448	1.00	0.00	C
ATOM	1556	HG2	GLU	98	38.107	35.286	44.237	1.00	0.00	H
ATOM	1557	HG3	GLU	98	37.332	33.800	44.827	1.00	0.00	H
ATOM	1558	CD	GLU	98	39.344	34.292	45.549	1.00	0.00	C
ATOM	1559	OE1	GLU	98	39.088	34.025	46.739	1.00	0.00	O
ATOM	1560	OE2	GLU	98	40.467	34.710	45.140	1.00	0.00	O
ATOM	1561	C	GLU	98	38.176	32.562	40.942	1.00	0.00	C
ATOM	1562	O	GLU	98	38.256	31.325	41.070	1.00	0.00	O
ATOM	1563	N	PHE	99	38.553	33.150	39.802	1.00	0.00	N
ATOM	1564	H	PHE	99	38.565	34.158	39.734	1.00	0.00	H
ATOM	1565	CA	PHE	99	39.091	32.441	38.671	1.00	0.00	C
ATOM	1566	HA	PHE	99	39.715	31.604	38.985	1.00	0.00	H
ATOM	1567	CB	PHE	99	37.892	31.807	37.854	1.00	0.00	C
ATOM	1568	HB2	PHE	99	38.347	31.182	37.085	1.00	0.00	H
ATOM	1569	HB3	PHE	99	37.459	31.226	38.668	1.00	0.00	H
ATOM	1570	CG	PHE	99	36.760	32.677	37.275	1.00	0.00	C
ATOM	1571	CD1	PHE	99	37.043	33.801	36.463	1.00	0.00	C
ATOM	1572	HD1	PHE	99	38.036	34.021	36.099	1.00	0.00	H
ATOM	1573	CE1	PHE	99	36.052	34.621	36.020	1.00	0.00	C
ATOM	1574	HE1	PHE	99	36.333	35.420	35.350	1.00	0.00	H
ATOM	1575	CZ	PHE	99	34.675	34.378	36.375	1.00	0.00	C
ATOM	1576	HZ	PHE	99	33.892	35.046	36.047	1.00	0.00	H
ATOM	1577	CE2	PHE	99	34.354	33.294	37.216	1.00	0.00	C
ATOM	1578	HE2	PHE	99	33.360	32.983	37.502	1.00	0.00	H
ATOM	1579	CD2	PHE	99	35.410	32.448	37.595	1.00	0.00	C
ATOM	1580	HD2	PHE	99	35.165	31.573	38.178	1.00	0.00	H
ATOM	1581	C	PHE	99	40.014	33.209	37.766	1.00	0.00	C
ATOM	1582	O	PHE	99	40.156	34.469	37.907	1.00	0.00	O
ATOM	1583	N	LYS	100	40.963	32.472	37.014	1.00	0.00	N
ATOM	1584	H	LYS	100	41.125	31.482	37.132	1.00	0.00	H
ATOM	1585	CA	LYS	100	41.918	33.103	36.127	1.00	0.00	C

```
ATOM   1586  HA  LYS   100      41.553  34.117  35.962  1.00  0.00           H
ATOM   1587  CB  LYS   100      43.310  33.261  36.762  1.00  0.00           C
ATOM   1588  HB2 LYS   100      44.027  33.572  36.002  1.00  0.00           H
ATOM   1589  HB3 LYS   100      43.295  34.007  37.557  1.00  0.00           H
ATOM   1590  CG  LYS   100      43.926  31.981  37.264  1.00  0.00           C
ATOM   1591  HG2 LYS   100      43.140  31.469  37.820  1.00  0.00           H
ATOM   1592  HG3 LYS   100      44.229  31.334  36.441  1.00  0.00           H
ATOM   1593  CD  LYS   100      45.131  32.107  38.202  1.00  0.00           C
ATOM   1594  HD2 LYS   100      45.814  32.847  37.783  1.00  0.00           H
ATOM   1595  HD3 LYS   100      44.777  32.517  39.148  1.00  0.00           H
ATOM   1596  CE  LYS   100      45.894  30.792  38.518  1.00  0.00           C
ATOM   1597  HE2 LYS   100      45.589  30.370  39.476  1.00  0.00           H
ATOM   1598  HE3 LYS   100      45.699  30.058  37.736  1.00  0.00           H
ATOM   1599  NZ  LYS   100      47.315  31.005  38.646  1.00  0.00           N
ATOM   1600  HZ1 LYS   100      47.683  31.387  37.786  1.00  0.00           H
ATOM   1601  HZ2 LYS   100      47.425  31.626  39.435  1.00  0.00           H
ATOM   1602  HZ3 LYS   100      47.776  30.123  38.817  1.00  0.00           H
ATOM   1603  C   LYS   100      42.050  32.342  34.789  1.00  0.00           C
ATOM   1604  O   LYS   100      41.920  31.137  34.760  1.00  0.00           O
ATOM   1605  N   CYX   101      42.331  33.007  33.625  1.00  0.00           N
ATOM   1606  H   CYX   101      42.492  34.003  33.681  1.00  0.00           H
ATOM   1607  CA  CYX   101      42.629  32.313  32.397  1.00  0.00           C
ATOM   1608  HA  CYX   101      42.064  31.389  32.267  1.00  0.00           H
ATOM   1609  CB  CYX   101      42.220  33.159  31.190  1.00  0.00           C
ATOM   1610  HB2 CYX   101      41.145  32.981  31.165  1.00  0.00           H
ATOM   1611  HB3 CYX   101      42.432  34.210  31.388  1.00  0.00           H
ATOM   1612  SG  CYX   101      42.838  32.586  29.629  1.00  0.00           S
ATOM   1613  C   CYX   101      44.161  31.936  32.356  1.00  0.00           C
ATOM   1614  O   CYX   101      45.041  32.784  32.486  1.00  0.00           O
ATOM   1615  N   LEU   102      44.423  30.616  32.311  1.00  0.00           N
ATOM   1616  H   LEU   102      43.623  30.000  32.292  1.00  0.00           H
ATOM   1617  CA  LEU   102      45.740  29.853  32.516  1.00  0.00           C
ATOM   1618  HA  LEU   102      46.142  30.245  33.450  1.00  0.00           H
ATOM   1619  CB  LEU   102      45.372  28.372  32.640  1.00  0.00           C
ATOM   1620  HB2 LEU   102      44.913  28.079  31.696  1.00  0.00           H
ATOM   1621  HB3 LEU   102      46.301  27.831  32.818  1.00  0.00           H
ATOM   1622  CG  LEU   102      44.346  27.960  33.712  1.00  0.00           C
ATOM   1623  HG  LEU   102      43.415  28.506  33.559  1.00  0.00           H
ATOM   1624  CD1 LEU   102      44.022  26.469  33.568  1.00  0.00           C
ATOM   1625 HD11 LEU   102      44.898  25.875  33.828  1.00  0.00           H
ATOM   1626 HD12 LEU   102      43.300  26.090  34.291  1.00  0.00           H
ATOM   1627 HD13 LEU   102      43.654  26.281  32.559  1.00  0.00           H
ATOM   1628  CD2 LEU   102      44.693  28.197  35.134  1.00  0.00           C
ATOM   1629 HD21 LEU   102      44.073  27.646  35.841  1.00  0.00           H
ATOM   1630 HD22 LEU   102      45.701  27.785  35.202  1.00  0.00           H
ATOM   1631 HD23 LEU   102      44.756  29.254  35.391  1.00  0.00           H
ATOM   1632  C   LEU   102      46.725  30.002  31.349  1.00  0.00           C
ATOM   1633  O   LEU   102      46.339  30.004  30.189  1.00  0.00           O
ATOM   1634  OXT LEU   102      47.967  30.024  31.619  1.00  0.00           O
HETATM  314  N   LYN    20      38.733  40.957  35.931  1.00  0.00           N
HETATM  315  H   LYN    20      38.998  40.538  35.051  1.00  0.00           H
HETATM  316  CA  LYN    20      37.671  40.129  36.591  1.00  0.00           C
HETATM  317  HA  LYN    20      37.348  40.571  37.533  1.00  0.00           H
HETATM  318  CB  LYN    20      36.445  40.015  35.633  1.00  0.00           C
HETATM  319  HB2 LYN    20      36.674  39.412  34.754  1.00  0.00           H
HETATM  320  HB3 LYN    20      36.290  41.036  35.285  1.00  0.00           H
HETATM  321  CG  LYN    20      35.076  39.546  36.146  1.00  0.00           C
HETATM  322  HG2 LYN    20      34.380  39.843  35.362  1.00  0.00           H
HETATM  323  HG3 LYN    20      35.117  38.464  36.271  1.00  0.00           H
HETATM  324  CD  LYN    20      34.655  40.113  37.495  1.00  0.00           C
HETATM  325  HD2 LYN
    20      34.645  41.192  37.341  1.00  0.00           H
HETATM  326  HD3 LYN    20      35.338  39.829  38.295  1.00  0.00           H
HETATM  327  CE  LYN    20      33.291  39.634  37.865  1.00  0.00           C
```

```
HETATM  328  HE2 LYN    20      32.525  39.682  37.091  1.00  0.00           H
HETATM  329  HE3 LYN    20      33.319  38.577  38.130  1.00  0.00           H
HETATM  330  NZ  LYN    20      32.792  40.451  38.966  1.00  0.00           N
HETATM  331  HZ2 LYN    20      32.581  41.352  38.562  1.00  0.00           H
HETATM  332  HZ3 LYN    20      33.622  40.661  39.503  1.00  0.00           H
HETATM  333  C   LYN    20      38.125  38.729  36.983  1.00  0.00           C
HETATM  334  O   LYN    20      38.181  37.804  36.160  1.00  0.00           O
HETATM 1636  N   LIG   103      29.864  42.573  40.764  1.00  0.00           N
HETATM 1637  C   LIG   103      29.225  41.776  39.958  1.00  0.00           C
HETATM 1638  O   LIG   103      29.974  43.755  42.499  1.00  0.00           O
HETATM 1639  C5' LIG   103      25.369  40.056  45.656  1.00  0.00           C
HETATM 1640  O5' LIG   103      25.458  39.241  44.437  1.00  0.00           O
HETATM 1641  C4' LIG   103      26.287  41.317  45.414  1.00  0.00           C
HETATM 1642  O4' LIG   103      27.498  41.124  44.595  1.00  0.00           O
HETATM 1643  C3' LIG   103      25.567  42.380  44.595  1.00  0.00           C
HETATM 1644  O3' LIG   103      24.673  43.143  45.435  1.00  0.00           O
HETATM 1645  C2' LIG   103      26.618  43.286  43.926  1.00  0.00           C
HETATM 1646  O2' LIG   103      26.954  44.525  44.620  1.00  0.00           O
HETATM 1647  C1' LIG   103      27.902  42.385  43.915  1.00  0.00           C
HETATM 1648  N1  LIG   103      28.353  42.156  42.511  1.00  0.00           N
HETATM 1649  O1  LIG   103      28.715  39.605  38.076  1.00  0.00           O
HETATM 1650  N2  LIG   103      29.663  41.595  38.651  1.00  0.00           N
HETATM 1651  C6  LIG   103      29.383  42.837  41.973  1.00  0.00           C
HETATM 1652  C7  LIG   103      27.811  41.111  41.801  1.00  0.00           C
HETATM 1653  C8  LIG   103      28.184  40.973  40.446  1.00  0.00           C
HETATM 1654  C9  LIG   103      29.345  40.604  37.785  1.00  0.00           C
HETATM 1655  C10 LIG   103      29.836  40.746  36.390  1.00  0.00           C
HETATM 1656  H   LIG   103      30.349  42.300  38.408  1.00  0.00           H
HETATM 1657  H1  LIG   103      27.647  40.286  39.803  1.00  0.00           H
HETATM 1658  H4  LIG   103      28.701  42.878  44.480  1.00  0.00           H
HETATM 1659  H6  LIG   103      26.556  41.632  46.429  1.00  0.00           H
HETATM 1660  H7  LIG   103      24.879  41.973  43.845  1.00  0.00           H
HETATM 1661  H8  LIG   103      26.270  43.628  42.943  1.00  0.00           H
HETATM 1662  H9  LIG   103      24.315  40.272  45.871  1.00  0.00           H
HETATM 1663  H10 LIG   103      25.757  39.403  46.448  1.00  0.00           H
HETATM 1664  H11 LIG   103      29.667  41.728  35.933  1.00  0.00           H
HETATM 1665  H12 LIG   103      29.273  40.055  35.751  1.00  0.00           H
HETATM 1666  H13 LIG   103      30.919  40.585  36.326  1.00  0.00           H
HETATM 1667  H14 LIG   103      27.175  40.412  42.329  1.00  0.00           H
HETATM 1668  H2' LIG   103      27.322  45.173  43.994  1.00  0.00           H
HETATM 1669  H3' LIG   103      24.980  44.059  45.324  1.00  0.00           H
HETATM 1670  H5' LIG   103      26.337  38.834  44.345  1.00  0.00           H
ENDMDL
MODEL      2
SHEET    1   1 1 ILE    22  ASP    26  0
SHEET    2   2 1 VAL    37  VAL    40  0
SHEET    3   3 1 CYX    50  VAL    60  0
SHEET    4   4 1 PHE    94  CYX   101  0
HELIX    1   1 ASP     14  ALA     17  1                                  4
HELIX    2   2 LEU     62  THR     64  1                                  3
HELIX    3   3 LYS     68  GLU     73  1                                  6
HELIX    4   4 LEU     77  ALA     85  1                                  9
ATOM      1  N   GLN     1      34.523  17.746  33.012  1.00  0.00           N
ATOM      2  H1  GLN     1      35.174  18.271  33.578  1.00  0.00           H
ATOM      3  H2  GLN     1      33.697  17.782  33.592  1.00  0.00           H
ATOM      4  H3  GLN     1      34.835  16.791  32.899  1.00  0.00           H
ATOM      5  CA  GLN     1      34.190  18.453  31.704  1.00  0.00           C
ATOM      6  HA  GLN     1      35.108  18.432  31.118  1.00  0.00           H
ATOM      7  CB  GLN     1      33.079  17.721  30.921  1.00  0.00           C
ATOM      8  HB2 GLN     1      32.237  17.486  31.571  1.00  0.00           H
ATOM      9  HB3 GLN     1      32.787  18.325  30.063  1.00  0.00           H
ATOM     10  CG  GLN     1      33.667  16.439  30.366  1.00  0.00           C
ATOM     11  HG2 GLN     1      34.162  15.861  31.146  1.00  0.00           H
ATOM     12  HG3 GLN     1      32.888  15.768  30.007  1.00  0.00           H
```

ATOM	13	CD	GLN	1	34.690	16.628	29.134	1.00 0.00	C
ATOM	14	OE1	GLN	1	35.588	17.450	29.225	1.00 0.00	O
ATOM	15	NE2	GLN	1	34.536	15.842	28.154	1.00 0.00	N
ATOM	16	HE21	GLN	1	33.849	15.102	28.162	1.00 0.00	H
ATOM	17	HE22	GLN	1	35.210	15.955	27.412	1.00 0.00	H
ATOM	18	C	GLN	1	33.970	19.888	32.065	1.00 0.00	C
ATOM	19	O	GLN	1	33.597	20.251	33.181	1.00 0.00	O
ATOM	20	N	PRO	2	34.175	20.785	31.058	1.00 0.00	N
ATOM	21	CD	PRO	2	35.099	20.513	29.896	1.00 0.00	C
ATOM	22	HD2	PRO	2	34.531	20.229	29.010	1.00 0.00	H
ATOM	23	HD3	PRO	2	35.795	19.740	30.220	1.00 0.00	H
ATOM	24	CG	PRO	2	35.841	21.860	29.631	1.00 0.00	C
ATOM	25	HG2	PRO	2	36.107	22.028	28.588	1.00 0.00	H
ATOM	26	HG3	PRO	2	36.654	21.872	30.357	1.00 0.00	H
ATOM	27	CB	PRO	2	34.816	22.902	30.133	1.00 0.00	C
ATOM	28	HB2	PRO	2	34.138	23.178	29.327	1.00 0.00	H
ATOM	29	HB3	PRO	2	35.302	23.810	30.492	1.00 0.00	H
ATOM	30	CA	PRO	2	34.023	22.219	31.215	1.00 0.00	C
ATOM	31	HA	PRO	2	34.634	22.511	32.069	1.00 0.00	H
ATOM	32	C	PRO	2	32.537	22.592	31.374	1.00 0.00	C
ATOM	33	O	PRO	2	31.720	21.869	30.801	1.00 0.00	O
ATOM	34	N	ASN	3	32.208	23.562	32.186	1.00 0.00	N
ATOM	35	H	ASN	3	32.978	24.074	32.592	1.00 0.00	H
ATOM	36	CA	ASN	3	30.816	24.036	32.327	1.00 0.00	C
ATOM	37	HA	ASN	3	30.241	23.197	32.718	1.00 0.00	H
ATOM	38	CB	ASN	3	30.920	25.184	33.335	1.00 0.00	C
ATOM	39	HB2	ASN	3	31.797	25.830	33.275	1.00 0.00	H
ATOM	40	HB3	ASN	3	30.073	25.838	33.125	1.00 0.00	H
ATOM	41	CG	ASN	3	30.873	24.815	34.799	1.00 0.00	C
ATOM	42	OD1	ASN	3	29.783	24.788	35.384	1.00 0.00	O
ATOM	43	ND2	ASN	3	32.034	24.496	35.355	1.00 0.00	N
ATOM	44	HD21	ASN	3	32.838	24.419	34.748	1.00 0.00	H
ATOM	45	HD22	ASN	3	32.075	24.011	36.240	1.00 0.00	H
ATOM	46	C	ASN	3	30.276	24.633	30.997	1.00 0.00	C
ATOM	47	O	ASN	3	31.055	25.195	30.275	1.00 0.00	O
ATOM	48	N	ASP	4	28.951	24.529	30.731	1.00 0.00	N
ATOM	49	H	ASP	4	28.288	24.230	31.433	1.00 0.00	H
ATOM	50	CA	ASP	4	28.398	25.102	29.447	1.00 0.00	C
ATOM	51	HA	ASP	4	29.155	24.958	28.677	1.00 0.00	H
ATOM	52	CB	ASP	4	27.141	24.393	29.028	1.00 0.00	C
ATOM	53	HB2	ASP	4	26.426	24.839	29.719	1.00 0.00	H
ATOM	54	HB3	ASP	4	26.874	24.546	27.982	1.00 0.00	H
ATOM	55	CG	ASP	4	27.163	22.876	29.256	1.00 0.00	C
ATOM	56	OD1	ASP	4	27.919	22.096	28.623	1.00 0.00	O
ATOM	57	OD2	ASP	4	26.270	22.477	30.019	1.00 0.00	O
ATOM	58	C	ASP	4	28.090	26.598	29.676	1.00 0.00	C
ATOM	59	O	ASP	4	27.799	27.286	28.687	1.00 0.00	O
ATOM	60	N	ILE	5	27.904	27.054	30.897	1.00 0.00	N
ATOM	61	H	ILE	5	27.907	26.319	31.590	1.00 0.00	H
ATOM	62	CA	ILE	5	27.693	28.485	31.224	1.00 0.00	C
ATOM	63	HA	ILE	5	26.808	28.867	30.713	1.00 0.00	H
ATOM	64	CB	ILE	5	27.245	28.671	32.703	1.00 0.00	C
ATOM	65	HB	ILE	5	28.038	28.238	33.310	1.00 0.00	H
ATOM	66	CG2	ILE	5	27.146	30.224	33.046	1.00 0.00	C
ATOM	67	HG21	ILE	5	26.514	30.722	32.310	1.00 0.00	H
ATOM	68	HG22	ILE	5	26.804	30.332	34.075	1.00 0.00	H
ATOM	69	HG23	ILE	5	28.151	30.640	32.986	1.00 0.00	H
ATOM	70	CG1	ILE	5	25.849	27.974	32.889	1.00 0.00	C
ATOM	71	HG12	ILE	5	25.117	28.617	32.399	1.00 0.00	H
ATOM	72	HG13	ILE	5	25.833	27.002	32.395	1.00 0.00	H
ATOM	73	CD1	ILE	5	25.549	27.693	34.367	1.00 0.00	C
ATOM	74	HD11	ILE	5	26.295	27.014	34.782	1.00 0.00	H
ATOM	75	HD12	ILE	5					

25.636 28.683 34.816 1.00 0.00 H

ATOM	76	HD13	ILE	5	24.523	27.352	34.509	1.00	0.00	H
ATOM	77	C	ILE	5	28.986	29.257	30.910	1.00	0.00	C
ATOM	78	O	ILE	5	30.088	28.843	31.351	1.00	0.00	O
ATOM	79	N	THR	6	28.884	30.341	30.168	1.00	0.00	N
ATOM	80	H	THR	6	27.960	30.701	29.978	1.00	0.00	H
ATOM	81	CA	THR	6	30.027	31.193	29.773	1.00	0.00	C
ATOM	82	HA	THR	6	30.782	31.215	30.560	1.00	0.00	H
ATOM	83	CB	THR	6	30.812	30.601	28.555	1.00	0.00	C
ATOM	84	HB	THR	6	31.136	29.640	28.956	1.00	0.00	H
ATOM	85	CG2	THR	6	30.072	30.419	27.248	1.00	0.00	C
ATOM	86	HG21	THR	6	29.945	31.252	26.556	1.00	0.00	H
ATOM	87	HG22	THR	6	30.617	29.706	26.630	1.00	0.00	H
ATOM	88	HG23	THR	6	29.074	30.002	27.387	1.00	0.00	H
ATOM	89	OG1	THR	6	31.917	31.310	28.322	1.00	0.00	O
ATOM	90	HG1	THR	6	32.592	30.665	28.542	1.00	0.00	H
ATOM	91	C	THR	6	29.627	32.640	29.464	1.00	0.00	C
ATOM	92	O	THR	6	28.444	32.830	29.347	1.00	0.00	O
ATOM	93	N	PHE	7	30.531	33.612	29.510	1.00	0.00	N
ATOM	94	H	PHE	7	31.501	33.359	29.627	1.00	0.00	H
ATOM	95	CA	PHE	7	30.055	34.998	29.691	1.00	0.00	C
ATOM	96	HA	PHE	7	29.158	34.971	30.309	1.00	0.00	H
ATOM	97	CB	PHE	7	31.148	35.782	30.489	1.00	0.00	C
ATOM	98	HB2	PHE	7	31.801	36.219	29.733	1.00	0.00	H
ATOM	99	HB3	PHE	7	30.565	36.600	30.911	1.00	0.00	H
ATOM	100	CG	PHE	7	31.937	35.051	31.547	1.00	0.00	C
ATOM	101	CD1	PHE	7	33.360	34.953	31.489	1.00	0.00	C
ATOM	102	HD1	PHE	7	33.825	35.585	30.748	1.00	0.00	H
ATOM	103	CE1	PHE	7	34.062	34.083	32.368	1.00	0.00	C
ATOM	104	HE1	PHE	7	35.137	34.155	32.292	1.00	0.00	H
ATOM	105	CZ	PHE	7	33.382	33.410	33.409	1.00	0.00	C
ATOM	106	HZ	PHE	7	33.929	32.835	34.141	1.00	0.00	H
ATOM	107	CE2	PHE	7	31.974	33.531	33.504	1.00	0.00	C
ATOM	108	HE2	PHE	7	31.383	32.961	34.206	1.00	0.00	H
ATOM	109	CD2	PHE	7	31.257	34.341	32.590	1.00	0.00	C
ATOM	110	HD2	PHE	7	30.196	34.469	32.749	1.00	0.00	H
ATOM	111	C	PHE	7	29.659	35.714	28.386	1.00	0.00	C
ATOM	112	O	PHE	7	30.062	35.287	27.360	1.00	0.00	O
ATOM	113	N	PHE	8	28.874	36.773	28.362	1.00	0.00	N
ATOM	114	H	PHE	8	28.571	37.201	29.225	1.00	0.00	H
ATOM	115	CA	PHE	8	28.562	37.567	27.160	1.00	0.00	C
ATOM	116	HA	PHE	8	28.121	36.792	26.531	1.00	0.00	H
ATOM	117	CB	PHE	8	27.543	38.689	27.444	1.00	0.00	C
ATOM	118	HB2	PHE	8	27.938	39.496	28.062	1.00	0.00	H
ATOM	119	HB3	PHE	8	27.279	39.234	26.537	1.00	0.00	H
ATOM	120	CG	PHE	8	26.223	38.293	28.125	1.00	0.00	C
ATOM	121	CD1	PHE	8	25.487	39.332	28.638	1.00	0.00	C
ATOM	122	HD1	PHE	8	25.714	40.382	28.535	1.00	0.00	H
ATOM	123	CE1	PHE	8	24.299	39.098	29.348	1.00	0.00	C
ATOM	124	HE1	PHE	8	23.716	39.924	29.726	1.00	0.00	H
ATOM	125	CZ	PHE	8	23.955	37.759	29.667	1.00	0.00	C
ATOM	126	HZ	PHE	8	23.145	37.620	30.370	1.00	0.00	H
ATOM	127	CE2	PHE	8	24.759	36.682	29.272	1.00	0.00	C
ATOM	128	HE2	PHE	8	24.661	35.668	29.633	1.00	0.00	H
ATOM	129	CD2	PHE	8	25.848	36.943	28.368	1.00	0.00	C
ATOM	130	HD2	PHE	8	26.342	36.126	27.863	1.00	0.00	H
ATOM	131	C	PHE	8	29.857	38.076	26.455	1.00	0.00	C
ATOM	132	O	PHE	8	30.812	38.366	27.143	1.00	0.00	O
ATOM	133	N	GLN	9	29.834	38.436	25.192	1.00	0.00	N
ATOM	134	H	GLN	9	28.976	38.232	24.698	1.00	0.00	H
ATOM	135	CA	GLN	9	30.923	38.879	24.295	1.00	0.00	C
ATOM	136	HA	GLN	9	31.671	38.088	24.358	1.00	0.00	H
ATOM	137	CB	GLN	9	30.600	38.976	22.821	1.00	0.00	C
ATOM	138	HB2	GLN	9	29.616	39.412	22.654	1.00	0.00	H
ATOM	139	HB3	GLN	9	31.355	39.614	22.361	1.00	0.00	H

ATOM	140	CG	GLN	9	30.620	37.548	22.309	1.00	0.00	C
ATOM	141	HG2	GLN	9	31.612	37.249	21.968	1.00	0.00	H
ATOM	142	HG3	GLN	9	30.311	36.821	23.061	1.00	0.00	H
ATOM	143	CD	GLN	9	29.663	37.422	21.160	1.00	0.00	C
ATOM	144	OE1	GLN	9	29.988	37.465	20.014	1.00	0.00	O
ATOM	145	NE2	GLN	9	28.349	37.258	21.453	1.00	0.00	N
ATOM	146	HE21	GLN	9	27.708	37.333	20.677	1.00	0.00	H
ATOM	147	HE22	GLN	9	28.087	37.403	22.419	1.00	0.00	H
ATOM	148	C	GLN	9	31.684	40.121	24.746	1.00	0.00	C
ATOM	149	O	GLN	9	32.793	40.375	24.263	1.00	0.00	O
ATOM	150	N	ARG	10	31.022	40.902	25.645	1.00	0.00	N
ATOM	151	H	ARG	10	30.081	40.594	25.846	1.00	0.00	H
ATOM	152	CA	ARG	10	31.585	42.033	26.222	1.00	0.00	C
ATOM	153	HA	ARG	10	32.198	42.613	25.533	1.00	0.00	H
ATOM	154	CB	ARG	10	30.472	43.032	26.490	1.00	0.00	C
ATOM	155	HB2	ARG	10	29.841	43.172	25.612	1.00	0.00	H
ATOM	156	HB3	ARG	10	29.698	42.657	27.159	1.00	0.00	H
ATOM	157	CG	ARG	10	30.975	44.375	27.065	1.00	0.00	C
ATOM	158	HG2	ARG	10	31.320	44.349	28.099	1.00	0.00	H
ATOM	159	HG3	ARG	10	31.783	44.764	26.444	1.00	0.00	H
ATOM	160	CD	ARG	10	29.869	45.374	27.120	1.00	0.00	C
ATOM	161	HD2	ARG	10	29.664	45.768	26.125	1.00	0.00	H
ATOM	162	HD3	ARG	10	29.040	44.842	27.587	1.00	0.00	H
ATOM	163	NE	ARG	10	30.300	46.596	27.846	1.00	0.00	N
ATOM	164	HE	ARG	10	31.293	46.655	28.023	1.00	0.00	H
ATOM	165	CZ	ARG	10	29.494	47.510	28.281	1.00	0.00	C
ATOM	166	NH1	ARG	10	28.196	47.466	28.054	1.00	0.00	N
ATOM	167	HH11	ARG	10	27.554	48.021	28.602	1.00	0.00	H
ATOM	168	HH12	ARG	10	27.856	46.672	27.530	1.00	0.00	H
ATOM	169	NH2	ARG	10	29.919	48.595	28.949	1.00	0.00	N
ATOM	170	HH21	ARG	10	30.914	48.657	29.118	1.00	0.00	H
ATOM	171	HH22	ARG	10	29.178	49.223	29.224	1.00	0.00	H
ATOM	172	C	ARG	10	32.415	41.712	27.482	1.00	0.00	C
ATOM	173	O	ARG	10	33.053	42.589	28.019	1.00	0.00	O
ATOM	174	N	PHE	11	32.263	40.500	28.035	1.00	0.00	N
ATOM	175	H	PHE	11	31.494	39.921	27.730	1.00	0.00	H
ATOM	176	CA	PHE	11	32.688	40.067	29.308	1.00	0.00	C
ATOM	177	HA	PHE	11	33.371	40.837	29.667	1.00	0.00	H
ATOM	178	CB	PHE	11	31.635	40.025	30.420	1.00	0.00	C
ATOM	179	HB2	PHE	11	32.089	40.015	31.411	1.00	0.00	H
ATOM	180	HB3	PHE	11	31.099	39.087	30.271	1.00	0.00	H
ATOM	181	CG	PHE	11	30.645	41.127	30.384	1.00	0.00	C
ATOM	182	CD1	PHE	11	31.116	42.447	30.694	1.00	0.00	C
ATOM	183	HD1	PHE	11	32.175	42.583	30.854	1.00	0.00	H
ATOM	184	CE1	PHE	11	30.277	43.531	30.584	1.00	0.00	C
ATOM	185	HE1	PHE	11	30.611	44.551	30.693	1.00	0.00	H
ATOM	186	CZ	PHE	11	28.866	43.298	30.336	1.00	0.00	C
ATOM	187	HZ	PHE	11	28.138	44.096	30.302	1.00	0.00	H
ATOM	188	CE2	PHE	11	28.464	41.952	30.046	1.00	0.00	C
ATOM	189	HE2	PHE	11	27.411	41.766	29.898	1.00	0.00	H
ATOM	190	CD2	PHE	11	29.366	40.880	29.980	1.00	0.00	C
ATOM	191	HD2	PHE	11	29.010	39.881	29.778	1.00	0.00	H
ATOM	192	C	PHE	11	33.617	38.880	29.256	1.00	0.00	C
ATOM	193	O	PHE	11	33.550	38.005	30.112	1.00	0.00	O
ATOM	194	N	GLN	12	34.436	38.709	28.189	1.00	0.00	N
ATOM	195	H	GLN	12	34.444	39.407	27.458	1.00	0.00	H
ATOM	196	CA	GLN	12	35.299	37.527	28.001	1.00	0.00	C
ATOM	197	HA	GLN	12	35.085	36.833	28.814	1.00	0.00	H
ATOM	198	CB	GLN	12	34.895	36.772	26.689	1.00	0.00	C
ATOM	199	HB2	GLN	12	35.189	37.405	25.851	1.00	0.00	H
ATOM	200	HB3	GLN	12	35.554	35.904	26.688	1.00	0.00	H
ATOM	201	CG	GLN	12	33.445	36.346	26.545	1.00	0.00	C
ATOM	202	HG2	GLN	12	33.113	35.643	27.308	1.00	0.00	H

ATOM	203	HG3	GLN	12	32.871	37.266	26.656	1.00	0.00	H
ATOM	204	CD	GLN	12	33.087	35.707	25.195	1.00	0.00	C
ATOM	205	OE1	GLN	12	33.834	35.692	24.278	1.00	0.00	O
ATOM	206	NE2	GLN	12	31.843	35.117	25.117	1.00	0.00	N
ATOM	207	HE21	GLN	12	31.309	35.130	25.975	1.00	0.00	H
ATOM	208	HE22	GLN	12	31.607	34.637	24.260	1.00	0.00	H
ATOM	209	C	GLN	12	36.829	37.848	28.010	1.00	0.00	C
ATOM	210	O	GLN	12	37.620	36.891	28.101	1.00	0.00	O
ATOM	211	N	ASP	13	37.287	39.077	27.780	1.00	0.00	N
ATOM	212	H	ASP	13	36.551	39.723	27.530	1.00	0.00	H
ATOM	213	CA	ASP	13	38.648	39.569	27.598	1.00	0.00	C
ATOM	214	HA	ASP	13	39.319	38.742	27.365	1.00	0.00	H
ATOM	215	CB	ASP	13	38.788	40.683	26.473	1.00	0.00	C
ATOM	216	HB2	ASP	13	38.286	41.573	26.852	1.00	0.00	H
ATOM	217	HB3	ASP	13	39.785	41.081	26.286	1.00	0.00	H
ATOM	218	CG	ASP	13	38.024	40.418	25.141	1.00	0.00	C
ATOM	219	OD1	ASP	13	37.525	41.326	24.445	1.00	0.00	O
ATOM	220	OD2	ASP	13	37.986	39.240	24.677	1.00	0.00	O
ATOM	221	C	ASP	13	39.230	40.132	28.871	1.00	0.00	C
ATOM	222	O	ASP	13	40.483	40.316	29.028	1.00	0.00	O
ATOM	223	N	ASP	14	38.431	40.554	29.845	1.00	0.00	N
ATOM	224	H	ASP	14	37.452	40.697	29.640	1.00	0.00	H
ATOM	225	CA	ASP	14	38.758	40.965	31.219	1.00	0.00	C
ATOM	226	HA	ASP	14	39.308	41.899	31.108	1.00	0.00	H
ATOM	227	CB	ASP	14	37.492	41.185	32.049	1.00	0.00	C
ATOM	228	HB2	ASP	14	36.931	40.304	31.738	1.00	0.00	H
ATOM	229	HB3	ASP	14	37.681	41.039	33.113	1.00	0.00	H
ATOM	230	CG	ASP	14	36.623	42.454	31.770	1.00	0.00	C
ATOM	231	OD1	ASP	14	35.553	42.489	32.350	1.00	0.00	O
ATOM	232	OD2	ASP	14	37.005	43.317	30.965	1.00	0.00	O
ATOM	233	C	ASP	14	39.664	39.981	31.960	1.00	0.00	C
ATOM	234	O	ASP	14	40.481	40.478	32.781	1.00	0.00	O
ATOM	235	N	ILE	15	39.508	38.638	31.728	1.00	0.00	N
ATOM	236	H	ILE	15	38.994	38.402	30.892	1.00	0.00	H
ATOM	237	CA	ILE	15	40.054	37.623	32.599	1.00	0.00	C
ATOM	238	HA	ILE	15	39.623	37.836	33.576	1.00	0.00	H
ATOM	239	CB	ILE	15	39.417	36.280	32.186	1.00	0.00	C
ATOM	240	HB	ILE	15	39.441	36.139	31.105	1.00	0.00	H
ATOM	241	CG2	ILE	15	40.051	35.113	32.873	1.00	0.00	C
ATOM	242	HG21	ILE	15	41.108	34.988	32.639	1.00	0.00	H
ATOM	243	HG22	ILE	15	40.019	35.231	33.956	1.00	0.00	H
ATOM	244	HG23	ILE	15	39.613	34.155	32.595	1.00	0.00	H
ATOM	245	CG1	ILE	15	37.890	36.215	32.503	1.00	0.00	C
ATOM	246	HG12	ILE	15	37.613	35.336	33.087	1.00	0.00	H
ATOM	247	HG13	ILE	15	37.625	36.972	33.241	1.00	0.00	H
ATOM	248	CD1	ILE	15	36.776	36.406	31.392	1.00	0.00	C
ATOM	249	HD11	ILE	15	35.803	36.467	31.879	1.00	0.00	H
ATOM	250	HD12	ILE	15	36.997	37.273	30.771	1.00	0.00	H
ATOM	251	HD13	ILE	15	36.820	35.476	30.823	1.00	0.00	H
ATOM	252	C	ILE	15	41.559	37.646	32.638	1.00	0.00	C
ATOM	253	O	ILE	15	42.064	37.335	33.730	1.00	0.00	O
ATOM	254	N	LEU	16	42.268	37.903	31.519	1.00	0.00	N
ATOM	255	H	LEU	16	41.805	38.175	30.664	1.00	0.00	H
ATOM	256	CA	LEU	16	43.746	38.031	31.566	1.00	0.00	C
ATOM	257	HA	LEU	16	44.040	37.413	32.414	1.00	0.00	H
ATOM	258	CB	LEU	16	44.409	37.615	30.312	1.00	0.00	C
ATOM	259	HB2	LEU	16	43.936	38.063	29.438	1.00	0.00	H
ATOM	260	HB3	LEU	16	45.480	37.791	30.210	1.00	0.00	H
ATOM	261	CG	LEU	16	44.302	36.087	30.018	1.00	0.00	C
ATOM	262	HG	LEU	16	43.277	35.771	30.208	1.00	0.00	H
ATOM	263	CD1	LEU	16	44.714	35.712	28.623	1.00	0.00	C
ATOM	264	HD11	LEU	16	44.560	34.633	28.589	1.00	0.00	H
ATOM	265	HD12	LEU	16	44.106	36.261	27.904	1.00	0.00	H
ATOM	266	HD13	LEU	16	45.758	35.948	28.418	1.00	0.00	H

```
ATOM    267  CD2 LEU    16      45.213  35.236  30.908  1.00  0.00           C
ATOM    268 HD21 LEU    16      44.922  35.293  31.957  1.00  0.00           H
ATOM    269 HD22 LEU    16      45.305  34.208  30.559  1.00  0.00           H
ATOM    270 HD23 LEU    16      46.232  35.620  30.868  1.00  0.00           H
ATOM    271  C   LEU    16      44.251  39.453  31.895  1.00  0.00           C
ATOM    272  O   LEU    16      45.389  39.663  32.345  1.00  0.00           O
ATOM    273  N   ALA    17      43.434  40.500  31.629  1.00  0.00           N
ATOM    274  H   ALA    17      42.545  40.231  31.232  1.00  0.00           H
ATOM    275  CA  ALA    17      43.585  41.892  32.051  1.00  0.00           C
ATOM    276  HA  ALA    17      44.587  42.235  31.793  1.00  0.00           H
ATOM    277  CB  ALA    17      42.713  42.728  31.195  1.00  0.00           C
ATOM    278  HB1 ALA    17      42.949  43.787  31.297  1.00  0.00           H
ATOM    279  HB2 ALA    17      42.834  42.475  30.142  1.00  0.00           H
ATOM    280  HB3 ALA    17      41.665  42.616  31.473  1.00  0.00           H
ATOM    281  C   ALA    17      43.540  42.075  33.592  1.00  0.00           C
ATOM    282  O   ALA    17      43.897  43.072  34.077  1.00  0.00           O
ATOM    283  N   GLY    18      42.930  41.126  34.367  1.00  0.00           N
ATOM    284  H   GLY    18      42.511  40.352  33.872  1.00  0.00           H
ATOM    285  CA  GLY    18      42.671  41.150  35.794  1.00  0.00           C
ATOM    286  HA2 GLY    18      42.748  40.122  36.150  1.00  0.00           H
ATOM    287  HA3 GLY    18      43.480  41.671  36.305  1.00  0.00           H
ATOM    288  C   GLY    18      41.428  41.846  36.247  1.00  0.00           C
ATOM    289  O   GLY    18      41.266  42.019  37.441  1.00  0.00           O
ATOM    290  N   ARG    19      40.595  42.305  35.374  1.00  0.00           N
ATOM    291  H   ARG    19      40.900  42.184  34.419  1.00  0.00           H
ATOM    292  CA  ARG    19      39.410  43.102  35.675  1.00  0.00           C
ATOM    293  HA  ARG    19      39.541  43.801  36.501  1.00  0.00           H
ATOM    294  CB  ARG    19      39.003  43.902  34.525  1.00  0.00           C
ATOM    295  HB2 ARG    19      38.579  43.197  33.810  1.00  0.00           H
ATOM    296  HB3 ARG    19      38.153  44.555  34.725  1.00  0.00           H
ATOM    297  CG  ARG    19      40.004  44.827  33.834  1.00  0.00           C
ATOM    298  HG2 ARG    19      40.392  45.566  34.534  1.00  0.00           H
ATOM    299  HG3 ARG    19      40.817  44.255  33.386  1.00  0.00           H
ATOM    300  CD  ARG    19      39.458  45.783  32.703  1.00  0.00           C
ATOM    301  HD2 ARG    19      38.616  46.431  32.945  1.00  0.00           H
ATOM    302  HD3 ARG    19      40.183  46.513  32.345  1.00  0.00           H
ATOM    303  NE  ARG    19      39.120  44.969  31.518  1.00  0.00           N
ATOM    304  HE  ARG    19      38.168  44.629  31.522  1.00  0.00           H
ATOM    305  CZ  ARG    19      39.743  44.853  30.408  1.00  0.00           C
ATOM    306  NH1 ARG    19      40.935  45.318  30.196  1.00  0.00           N
ATOM    307 HH11 ARG    19      41.431  45.711  30.983  1.00  0.00           H
ATOM    308 HH12 ARG    19      41.382  45.307  29.290  1.00  0.00           H
ATOM    309  NH2 ARG    19      39.268  44.116  29.460  1.00  0.00           N
ATOM    310 HH21 ARG    19      39.793  44.073  28.599  1.00  0.00           H
ATOM    311 HH22 ARG    19      38.404  43.629  29.654  1.00  0.00           H
ATOM    312  C   ARG    19      38.239  42.315  36.199  1.00  0.00           C
ATOM    313  O   ARG    19      37.321  42.883  36.816  1.00  0.00           O
ATOM    335  N   THR    21      38.033  38.363  38.224  1.00  0.00           N
ATOM    336  H   THR    21      37.443  38.998  38.741  1.00  0.00           H
ATOM    337  CA  THR    21      38.711  37.317  38.927  1.00  0.00           C
ATOM    338  HA  THR    21      38.815  36.410  38.331  1.00  0.00           H
ATOM    339  CB  THR    21      40.088  37.631  39.302  1.00  0.00           C
ATOM    340  HB  THR    21      40.772  37.727  38.459  1.00  0.00           H
ATOM    341  CG2 THR    21      40.148  38.946  40.153  1.00  0.00           C
ATOM    342 HG21 THR    21      39.724  39.790  39.609  1.00  0.00           H
ATOM    343 HG22 THR    21      39.604  38.784  41.084  1.00  0.00           H
ATOM    344 HG23 THR    21      41.183  39.154  40.427  1.00  0.00           H
ATOM    345  OG1 THR    21      40.722  36.639  40.068  1.00  0.00           O
ATOM    346  HG1 THR    21      40.631  35.739  39.747  1.00  0.00           H
ATOM
    347  C   THR    21      37.966  36.955  40.224  1.00  0.00           C
ATOM    348  O   THR    21      37.938  35.760  40.511  1.00  0.00           O
ATOM    349  N   ILE    22      37.204  37.894  40.780  1.00  0.00           N
ATOM    350  H   ILE    22      37.429  38.848  40.537  1.00  0.00           H
```

ATOM	351	CA	ILE	22	36.394	37.615	42.000	1.00	0.00	C
ATOM	352	HA	ILE	22	36.426	36.540	42.176	1.00	0.00	H
ATOM	353	CB	ILE	22	37.071	38.226	43.265	1.00	0.00	C
ATOM	354	HB	ILE	22	38.048	37.744	43.310	1.00	0.00	H
ATOM	355	CG2	ILE	22	37.449	39.670	43.260	1.00	0.00	C
ATOM	356	HG21	ILE	22	38.044	39.990	42.404	1.00	0.00	H
ATOM	357	HG22	ILE	22	36.541	40.262	43.157	1.00	0.00	H
ATOM	358	HG23	ILE	22	37.954	39.885	44.201	1.00	0.00	H
ATOM	359	CG1	ILE	22	36.251	37.815	44.561	1.00	0.00	C
ATOM	360	HG12	ILE	22	35.304	38.352	44.627	1.00	0.00	H
ATOM	361	HG13	ILE	22	35.894	36.791	44.451	1.00	0.00	H
ATOM	362	CD1	ILE	22	37.166	37.870	45.806	1.00	0.00	C
ATOM	363	HD11	ILE	22	37.299	38.903	46.125	1.00	0.00	H
ATOM	364	HD12	ILE	22	36.609	37.281	46.537	1.00	0.00	H
ATOM	365	HD13	ILE	22	38.144	37.389	45.838	1.00	0.00	H
ATOM	366	C	ILE	22	34.921	38.055	41.806	1.00	0.00	C
ATOM	367	O	ILE	22	34.640	39.085	41.200	1.00	0.00	O
ATOM	368	N	THR	23	33.985	37.233	42.262	1.00	0.00	N
ATOM	369	H	THR	23	34.283	36.433	42.804	1.00	0.00	H
ATOM	370	CA	THR	23	32.506	37.517	42.132	1.00	0.00	C
ATOM	371	HA	THR	23	32.337	38.593	42.132	1.00	0.00	H
ATOM	372	CB	THR	23	31.931	36.925	40.840	1.00	0.00	C
ATOM	373	HB	THR	23	32.670	36.970	40.040	1.00	0.00	H
ATOM	374	CG2	THR	23	31.324	35.513	40.834	1.00	0.00	C
ATOM	375	HG21	THR	23	30.869	35.354	39.856	1.00	0.00	H
ATOM	376	HG22	THR	23	32.157	34.825	40.984	1.00	0.00	H
ATOM	377	HG23	THR	23	30.618	35.336	41.645	1.00	0.00	H
ATOM	378	OG1	THR	23	30.930	37.804	40.324	1.00	0.00	O
ATOM	379	HG1	THR	23	31.319	38.412	39.692	1.00	0.00	H
ATOM	380	C	THR	23	31.785	36.961	43.349	1.00	0.00	C
ATOM	381	O	THR	23	32.229	36.002	43.981	1.00	0.00	O
ATOM	382	N	ILE	24	30.688	37.652	43.782	1.00	0.00	N
ATOM	383	H	ILE	24	30.126	38.151	43.106	1.00	0.00	H
ATOM	384	CA	ILE	24	29.867	37.370	44.958	1.00	0.00	C
ATOM	385	HA	ILE	24	30.388	36.548	45.450	1.00	0.00	H
ATOM	386	CB	ILE	24	29.962	38.604	45.903	1.00	0.00	C
ATOM	387	HB	ILE	24	29.431	39.409	45.395	1.00	0.00	H
ATOM	388	CG2	ILE	24	29.192	38.243	47.247	1.00	0.00	C
ATOM	389	HG21	ILE	24	29.060	39.128	47.870	1.00	0.00	H
ATOM	390	HG22	ILE	24	28.210	37.806	47.067	1.00	0.00	H
ATOM	391	HG23	ILE	24	29.785	37.504	47.785	1.00	0.00	H
ATOM	392	CG1	ILE	24	31.386	39.005	46.213	1.00	0.00	C
ATOM	393	HG12	ILE	24	31.850	38.278	46.879	1.00	0.00	H
ATOM	394	HG13	ILE	24	32.018	38.972	45.326	1.00	0.00	H
ATOM	395	CD1	ILE	24	31.556	40.459	46.823	1.00	0.00	C
ATOM	396	HD11	ILE	24	31.045	40.488	47.785	1.00	0.00	H
ATOM	397	HD12	ILE	24	32.623	40.680	46.846	1.00	0.00	H
ATOM	398	HD13	ILE	24	31.136	41.278	46.237	1.00	0.00	H
ATOM	399	C	ILE	24	28.416	36.912	44.576	1.00	0.00	C
ATOM	400	O	ILE	24	27.876	37.414	43.563	1.00	0.00	O
ATOM	401	N	ARG	25	27.867	35.964	45.358	1.00	0.00	N
ATOM	402	H	ARG	25	28.408	35.706	46.172	1.00	0.00	H
ATOM	403	CA	ARG	25	26.604	35.170	45.200	1.00	0.00	C
ATOM	404	HA	ARG	25	25.955	35.688	44.494	1.00	0.00	H
ATOM	405	CB	ARG	25	27.039	33.844	44.436	1.00	0.00	C
ATOM	406	HB2	ARG	25	27.825	33.366	45.021	1.00	0.00	H
ATOM	407	HB3	ARG	25	26.183	33.173	44.353	1.00	0.00	H
ATOM	408	CG	ARG	25	27.547	34.165	43.026	1.00	0.00	C
ATOM	409	HG2	ARG	25	28.454	34.762	43.118	1.00	0.00	H
ATOM	410	HG3	ARG	25	27.894	33.224	42.599	1.00	0.00	H
ATOM	411	CD	ARG	25	26.580	34.775	42.014	1.00	0.00	C
ATOM	412	HD2	ARG	25	25.678	34.171	41.913	1.00	0.00	H
ATOM	413	HD3	ARG	25	26.267	35.772	42.323	1.00	0.00	H
ATOM	414	NE	ARG	25	27.196	34.821	40.695	1.00	0.00	N

ATOM	415	HE	ARG	25	27.049	34.084	40.021	1.00	0.00	H
ATOM	416	CZ	ARG	25	27.936	35.759	40.223	1.00	0.00	C
ATOM	417	NH1	ARG	25	28.292	36.811	40.895	1.00	0.00	N
ATOM	418	HH11	ARG	25	29.021	37.394	40.512	1.00	0.00	H
ATOM	419	HH12	ARG	25	28.175	36.890	41.896	1.00	0.00	H
ATOM	420	NH2	ARG	25	28.308	35.834	38.965	1.00	0.00	N
ATOM	421	HH21	ARG	25	28.573	36.748	38.627	1.00	0.00	H
ATOM	422	HH22	ARG	25	27.963	35.189	38.268	1.00	0.00	H
ATOM	423	C	ARG	25	25.722	34.975	46.403	1.00	0.00	C
ATOM	424	O	ARG	25	26.175	35.168	47.519	1.00	0.00	O
ATOM	425	N	ASP	26	24.489	34.435	46.275	1.00	0.00	N
ATOM	426	H	ASP	26	24.118	34.345	45.340	1.00	0.00	H
ATOM	427	CA	ASP	26	23.891	33.735	47.426	1.00	0.00	C
ATOM	428	HA	ASP	26	24.050	34.308	48.340	1.00	0.00	H
ATOM	429	CB	ASP	26	22.348	33.778	47.242	1.00	0.00	C
ATOM	430	HB2	ASP	26	21.921	33.640	48.236	1.00	0.00	H
ATOM	431	HB3	ASP	26	21.918	34.751	47.002	1.00	0.00	H
ATOM	432	CG	ASP	26	21.664	32.816	46.208	1.00	0.00	C
ATOM	433	OD1	ASP	26	22.270	32.425	45.205	1.00	0.00	O
ATOM	434	OD2	ASP	26	20.473	32.422	46.459	1.00	0.00	O
ATOM	435	C	ASP	26	24.468	32.285	47.627	1.00	0.00	C
ATOM	436	O	ASP	26	25.057	31.742	46.653	1.00	0.00	O
ATOM	437	N	GLU	27	24.287	31.809	48.861	1.00	0.00	N
ATOM	438	H	GLU	27	23.806	32.361	49.556	1.00	0.00	H
ATOM	439	CA	GLU	27	24.754	30.477	49.264	1.00	0.00	C
ATOM	440	HA	GLU	27	25.775	30.330	48.914	1.00	0.00	H
ATOM	441	CB	GLU	27	24.782	30.402	50.845	1.00	0.00	C
ATOM	442	HB2	GLU	27	25.260	31.360	51.047	1.00	0.00	H
ATOM	443	HB3	GLU	27	23.746	30.428	51.185	1.00	0.00	H
ATOM	444	CG	GLU	27	25.723	29.309	51.376	1.00	0.00	C
ATOM	445	HG2	GLU	27	26.266	28.869	50.541	1.00	0.00	H
ATOM	446	HG3	GLU	27	26.441	29.727	52.082	1.00	0.00	H
ATOM	447	CD	GLU	27	24.958	28.075	52.064	1.00	0.00	C
ATOM	448	OE1	GLU	27	25.574	27.016	52.396	1.00	0.00	O
ATOM	449	OE2	GLU	27	23.720	28.150	52.283	1.00	0.00	O
ATOM	450	C	GLU	27	23.943	29.325	48.695	1.00	0.00	C
ATOM	451	O	GLU	27	23.886	28.255	49.300	1.00	0.00	O
ATOM	452	N	SER	28	23.406	29.462	47.508	1.00	0.00	N
ATOM	453	H	SER	28	23.492	30.373	47.083	1.00	0.00	H
ATOM	454	CA	SER	28	22.741	28.407	46.755	1.00	0.00	C
ATOM	455	HA	SER	28	21.989	27.884	47.347	1.00	0.00	H
ATOM	456	CB	SER	28	22.120	29.081	45.494	1.00	0.00	C
ATOM	457	HB2	SER	28	21.598	29.945	45.905	1.00	0.00	H
ATOM	458	HB3	SER	28	22.973	29.311	44.856	1.00	0.00	H
ATOM	459	OG	SER	28	21.293	28.134	44.852	1.00	0.00	O
ATOM	460	HG	SER	28	20.624	28.673	44.422	1.00	0.00	H
ATOM	461	C	SER	28	23.699	27.287	46.214	1.00	0.00	C
ATOM	462	O	SER	28	24.863	27.650	45.923	1.00	0.00	O
ATOM	463	N	GLU	29	23.247	26.035	46.052	1.00	0.00	N
ATOM	464	H	GLU	29	22.355	25.847	46.486	1.00	0.00	H
ATOM	465	CA	GLU	29	24.056	25.015	45.298	1.00	0.00	C
ATOM	466	HA	GLU	29	25.044	25.013	45.757	1.00	0.00	H
ATOM	467	CB	GLU	29	23.364	23.738	45.721	1.00	0.00	C
ATOM	468	HB2	GLU	29	23.359	23.709	46.811	1.00	0.00	H
ATOM	469	HB3	GLU	29	22.285	23.788	45.576	1.00	0.00	H
ATOM	470	CG	GLU	29	23.822	22.362	45.196	1.00	0.00	C
ATOM	471	HG2	GLU	29	23.232	21.542	45.606	1.00	0.00	H
ATOM	472	HG3	GLU	29						
	23.563	22.378	44.138	1.00	0.00	H				
ATOM	473	CD	GLU	29	25.291	22.044	45.431	1.00	0.00	C
ATOM	474	OE1	GLU	29	25.924	22.546	46.425	1.00	0.00	O
ATOM	475	OE2	GLU	29	25.986	21.404	44.583	1.00	0.00	O
ATOM	476	C	GLU	29	24.187	25.258	43.761	1.00	0.00	C
ATOM	477	O	GLU	29	24.855	24.505	43.103	1.00	0.00	O

ATOM	478	N	SER	30	23.658	26.357	43.207	1.00	0.00	N
ATOM	479	H	SER	30	23.205	27.047	43.788	1.00	0.00	H
ATOM	480	CA	SER	30	23.533	26.647	41.754	1.00	0.00	C
ATOM	481	HA	SER	30	23.491	25.693	41.229	1.00	0.00	H
ATOM	482	CB	SER	30	22.283	27.489	41.487	1.00	0.00	C
ATOM	483	HB2	SER	30	22.361	28.379	42.113	1.00	0.00	H
ATOM	484	HB3	SER	30	22.188	27.817	40.452	1.00	0.00	H
ATOM	485	OG	SER	30	21.116	26.818	41.904	1.00	0.00	O
ATOM	486	HG	SER	30	20.918	26.139	41.254	1.00	0.00	H
ATOM	487	C	SER	30	24.746	27.250	41.119	1.00	0.00	C
ATOM	488	O	SER	30	24.555	27.891	40.091	1.00	0.00	O
ATOM	489	N	HIE	31	25.942	27.169	41.704	1.00	0.00	N
ATOM	490	H	HIE	31	25.992	26.589	42.529	1.00	0.00	H
ATOM	491	CA	HIE	31	27.127	27.940	41.282	1.00	0.00	C
ATOM	492	HA	HIE	31	27.042	28.347	40.273	1.00	0.00	H
ATOM	493	CB	HIE	31	27.353	29.053	42.285	1.00	0.00	C
ATOM	494	HB2	HIE	31	27.561	28.658	43.279	1.00	0.00	H
ATOM	495	HB3	HIE	31	28.172	29.716	42.004	1.00	0.00	H
ATOM	496	CG	HIE	31	26.159	29.943	42.380	1.00	0.00	C
ATOM	497	ND1	HIE	31	25.769	30.819	41.406	1.00	0.00	N
ATOM	498	CE1	HIE	31	24.771	31.544	41.914	1.00	0.00	C
ATOM	499	HE1	HIE	31	24.349	32.462	41.534	1.00	0.00	H
ATOM	500	NE2	HIE	31	24.421	31.179	43.156	1.00	0.00	N
ATOM	501	HE2	HIE	31	23.844	31.666	43.826	1.00	0.00	H
ATOM	502	CD2	HIE	31	25.404	30.185	43.511	1.00	0.00	C
ATOM	503	HD2	HIE	31	25.538	29.734	44.483	1.00	0.00	H
ATOM	504	C	HIE	31	28.384	27.080	41.109	1.00	0.00	C
ATOM	505	O	HIE	31	28.459	25.905	41.572	1.00	0.00	O
ATOM	506	N	PHE	32	29.398	27.715	40.464	1.00	0.00	N
ATOM	507	H	PHE	32	29.379	28.667	40.125	1.00	0.00	H
ATOM	508	CA	PHE	32	30.715	27.115	40.252	1.00	0.00	C
ATOM	509	HA	PHE	32	30.537	26.129	39.822	1.00	0.00	H
ATOM	510	CB	PHE	32	31.560	27.875	39.224	1.00	0.00	C
ATOM	511	HB2	PHE	32	31.928	28.729	39.794	1.00	0.00	H
ATOM	512	HB3	PHE	32	32.361	27.236	38.852	1.00	0.00	H
ATOM	513	CG	PHE	32	30.850	28.407	37.953	1.00	0.00	C
ATOM	514	CD1	PHE	32	30.408	29.754	37.913	1.00	0.00	C
ATOM	515	HD1	PHE	32	30.485	30.473	38.715	1.00	0.00	H
ATOM	516	CE1	PHE	32	29.931	30.260	36.749	1.00	0.00	C
ATOM	517	HE1	PHE	32	29.917	31.331	36.609	1.00	0.00	H
ATOM	518	CZ	PHE	32	29.753	29.370	35.629	1.00	0.00	C
ATOM	519	HZ	PHE	32	29.376	29.707	34.675	1.00	0.00	H
ATOM	520	CE2	PHE	32	30.279	28.095	35.657	1.00	0.00	C
ATOM	521	HE2	PHE	32	30.380	27.483	34.773	1.00	0.00	H
ATOM	522	CD2	PHE	32	30.833	27.608	36.847	1.00	0.00	C
ATOM	523	HD2	PHE	32	31.350	26.663	36.926	1.00	0.00	H
ATOM	524	C	PHE	32	31.386	26.828	41.592	1.00	0.00	C
ATOM	525	O	PHE	32	31.029	27.397	42.620	1.00	0.00	O
ATOM	526	N	LYS	33	32.266	25.857	41.657	1.00	0.00	N
ATOM	527	H	LYS	33	32.300	25.261	40.842	1.00	0.00	H
ATOM	528	CA	LYS	33	32.820	25.165	42.841	1.00	0.00	C
ATOM	529	HA	LYS	33	32.608	25.859	43.654	1.00	0.00	H
ATOM	530	CB	LYS	33	32.253	23.751	43.176	1.00	0.00	C
ATOM	531	HB2	LYS	33	32.732	23.074	42.470	1.00	0.00	H
ATOM	532	HB3	LYS	33	32.546	23.429	44.175	1.00	0.00	H
ATOM	533	CG	LYS	33	30.776	23.521	42.975	1.00	0.00	C
ATOM	534	HG2	LYS	33	30.419	23.828	41.991	1.00	0.00	H
ATOM	535	HG3	LYS	33	30.597	22.458	43.134	1.00	0.00	H
ATOM	536	CD	LYS	33	29.958	24.168	44.032	1.00	0.00	C
ATOM	537	HD2	LYS	33	30.453	24.105	45.000	1.00	0.00	H
ATOM	538	HD3	LYS	33	29.931	25.244	43.858	1.00	0.00	H
ATOM	539	CE	LYS	33	28.528	23.656	44.122	1.00	0.00	C
ATOM	540	HE2	LYS	33	27.947	24.572	44.228	1.00	0.00	H
ATOM	541	HE3	LYS	33	28.255	23.298	43.130	1.00	0.00	H

ATOM	542	NZ	LYS	33	28.420	22.691	45.226	1.00	0.00	N
ATOM	543	HZ1	LYS	33	29.018	21.945	44.901	1.00	0.00	H
ATOM	544	HZ2	LYS	33	28.821	23.108	46.055	1.00	0.00	H
ATOM	545	HZ3	LYS	33	27.484	22.343	45.382	1.00	0.00	H
ATOM	546	C	LYS	33	34.374	25.073	42.686	1.00	0.00	C
ATOM	547	O	LYS	33	34.906	25.149	41.587	1.00	0.00	O
ATOM	548	N	THR	34	35.172	24.827	43.731	1.00	0.00	N
ATOM	549	H	THR	34	34.667	24.750	44.602	1.00	0.00	H
ATOM	550	CA	THR	34	36.600	24.603	43.628	1.00	0.00	C
ATOM	551	HA	THR	34	36.995	25.343	42.931	1.00	0.00	H
ATOM	552	CB	THR	34	37.233	24.614	45.030	1.00	0.00	C
ATOM	553	HB	THR	34	36.851	23.823	45.675	1.00	0.00	H
ATOM	554	CG2	THR	34	38.746	24.414	44.941	1.00	0.00	C
ATOM	555	HG21	THR	34	39.171	25.250	44.385	1.00	0.00	H
ATOM	556	HG22	THR	34	39.225	24.401	45.920	1.00	0.00	H
ATOM	557	HG23	THR	34	38.935	23.418	44.539	1.00	0.00	H
ATOM	558	OG1	THR	34	37.000	25.893	45.653	1.00	0.00	O
ATOM	559	HG1	THR	34	36.079	26.163	45.672	1.00	0.00	H
ATOM	560	C	THR	34	36.801	23.225	42.995	1.00	0.00	C
ATOM	561	O	THR	34	35.971	22.319	43.226	1.00	0.00	O
ATOM	562	N	GLY	35	37.798	23.084	42.103	1.00	0.00	N
ATOM	563	H	GLY	35	38.529	23.770	41.985	1.00	0.00	H
ATOM	564	CA	GLY	35	37.971	21.939	41.135	1.00	0.00	C
ATOM	565	HA2	GLY	35	39.032	21.824	40.911	1.00	0.00	H
ATOM	566	HA3	GLY	35	37.556	21.073	41.650	1.00	0.00	H
ATOM	567	C	GLY	35	37.225	22.114	39.798	1.00	0.00	C
ATOM	568	O	GLY	35	37.348	21.248	38.955	1.00	0.00	O
ATOM	569	N	ASP	36	36.336	23.102	39.740	1.00	0.00	N
ATOM	570	H	ASP	36	36.259	23.854	40.409	1.00	0.00	H
ATOM	571	CA	ASP	36	35.588	23.270	38.446	1.00	0.00	C
ATOM	572	HA	ASP	36	35.346	22.267	38.094	1.00	0.00	H
ATOM	573	CB	ASP	36	34.265	24.035	38.600	1.00	0.00	C
ATOM	574	HB2	ASP	36	34.340	25.018	39.065	1.00	0.00	H
ATOM	575	HB3	ASP	36	33.926	24.202	37.578	1.00	0.00	H
ATOM	576	CG	ASP	36	33.100	23.293	39.373	1.00	0.00	C
ATOM	577	OD1	ASP	36	32.053	23.940	39.709	1.00	0.00	O
ATOM	578	OD2	ASP	36	33.105	22.054	39.457	1.00	0.00	O
ATOM	579	C	ASP	36	36.387	23.935	37.297	1.00	0.00	C
ATOM	580	O	ASP	36	37.274	24.788	37.605	1.00	0.00	O
ATOM	581	N	VAL	37	35.968	23.702	36.050	1.00	0.00	N
ATOM	582	H	VAL	37	35.216	23.054	35.866	1.00	0.00	H
ATOM	583	CA	VAL	37	36.630	24.356	34.870	1.00	0.00	C
ATOM	584	HA	VAL	37	37.214	25.241	35.119	1.00	0.00	H
ATOM	585	CB	VAL	37	37.475	23.312	34.106	1.00	0.00	C
ATOM	586	HB	VAL	37	36.840	22.431	34.023	1.00	0.00	H
ATOM	587	CG1	VAL	37	37.950	23.739	32.663	1.00	0.00	C
ATOM	588	HG11	VAL	37	37.127	23.952	31.981	1.00	0.00	H
ATOM	589	HG12	VAL	37	38.597	24.616	32.688	1.00	0.00	H
ATOM	590	HG13	VAL	37	38.634	22.973	32.293	1.00	0.00	H
ATOM	591	CG2	VAL	37	38.790	23.084	34.811	1.00	0.00	C
ATOM	592	HG21	VAL	37	39.377	23.995	34.928	1.00	0.00	H
ATOM	593	HG22	VAL	37	38.444	22.790	35.801	1.00	0.00	H
ATOM	594	HG23	VAL	37	39.414	22.299	34.382	1.00	0.00	H
ATOM	595	C	VAL	37	35.568	24.928	33.920	1.00	0.00	C
ATOM	596	O	VAL	37	34.533	24.301	33.765	1.00	0.00	O
ATOM	597	N	LEU	38	35.772	26.152	33.415	1.00	0.00	N
ATOM	598	H	LEU	38	36.711	26.443	33.643	1.00	0.00	H
ATOM	599	CA	LEU	38	34.949	26.940	32.534	1.00	0.00	C
ATOM	600	HA	LEU	38	34.016	26.397	32.387	1.00	0.00	H
ATOM	601	CB	LEU	38	34.676	28.287	33.169	1.00	0.00	C
ATOM	602	HB2	LEU	38	35.669	28.603	33.489	1.00	0.00	H
ATOM	603	HB3	LEU	38	34.272	29.004	32.455	1.00	0.00	H
ATOM	604	CG	LEU	38	33.714	28.155	34.367	1.00	0.00	C

ATOM	605	HG	LEU	38	32.894	27.478	34.128	1.00	0.00	H
ATOM	606	CD1	LEU	38	34.431	27.684	35.648	1.00	0.00	C
ATOM	607	HD11	LEU	38	35.443	28.082	35.708	1.00	0.00	H
ATOM	608	HD12	LEU	38	33.844	28.057	36.488	1.00	0.00	H
ATOM	609	HD13	LEU	38	34.494	26.598	35.715	1.00	0.00	H
ATOM	610	CD2	LEU	38	33.185	29.496	34.673	1.00	0.00	C
ATOM	611	HD21	LEU	38	32.629	29.780	33.780	1.00	0.00	H
ATOM	612	HD22	LEU	38	32.551	29.577	35.556	1.00	0.00	H
ATOM	613	HD23	LEU	38	33.989	30.224	34.784	1.00	0.00	H
ATOM	614	C	LEU	38	35.734	27.186	31.216	1.00	0.00	C
ATOM	615	O	LEU	38	36.953	27.022	31.147	1.00	0.00	O
ATOM	616	N	ARG	39	35.057	27.612	30.163	1.00	0.00	N
ATOM	617	H	ARG	39	34.049	27.560	30.197	1.00	0.00	H
ATOM	618	CA	ARG	39	35.774	27.987	28.904	1.00	0.00	C
ATOM	619	HA	ARG	39	36.834	28.136	29.107	1.00	0.00	H
ATOM	620	CB	ARG	39	35.738	26.853	27.844	1.00	0.00	C
ATOM	621	HB2	ARG	39	34.706	26.516	27.748	1.00	0.00	H
ATOM	622	HB3	ARG	39	35.852	27.322	26.867	1.00	0.00	H
ATOM	623	CG	ARG	39	36.715	25.655	27.974	1.00	0.00	C
ATOM	624	HG2	ARG	39	37.741	26.007	27.873	1.00	0.00	H
ATOM	625	HG3	ARG	39	36.484	25.127	28.900	1.00	0.00	H
ATOM	626	CD	ARG	39	36.480	24.566	26.888	1.00	0.00	C
ATOM	627	HD2	ARG	39	36.765	24.955	25.910	1.00	0.00	H
ATOM	628	HD3	ARG	39	37.070	23.674	27.092	1.00	0.00	H
ATOM	629	NE	ARG	39	35.086	24.173	26.807	1.00	0.00	N
ATOM	630	HE	ARG	39	34.373	24.630	27.356	1.00	0.00	H
ATOM	631	CZ	ARG	39	34.684	23.083	26.220	1.00	0.00	C
ATOM	632	NH1	ARG	39	35.513	22.194	25.584	1.00	0.00	N
ATOM	633	HH11	ARG	39	36.522	22.226	25.583	1.00	0.00	H
ATOM	634	HH12	ARG	39	35.080	21.372	25.189	1.00	0.00	H
ATOM	635	NH2	ARG	39	33.485	22.719	26.472	1.00	0.00	N
ATOM	636	HH21	ARG	39	33.182	21.843	26.070	1.00	0.00	H
ATOM	637	HH22	ARG	39	32.873	23.333	26.988	1.00	0.00	H
ATOM	638	C	ARG	39	35.156	29.332	28.366	1.00	0.00	C
ATOM	639	O	ARG	39	33.967	29.576	28.456	1.00	0.00	O
ATOM	640	N	VAL	40	36.025	30.127	27.695	1.00	0.00	N
ATOM	641	H	VAL	40	36.994	29.864	27.590	1.00	0.00	H
ATOM	642	CA	VAL	40	35.565	31.210	26.793	1.00	0.00	C
ATOM	643	HA	VAL	40	34.488	31.043	26.768	1.00	0.00	H
ATOM	644	CB	VAL	40	35.675	32.698	27.381	1.00	0.00	C
ATOM	645	HB	VAL	40	35.384	33.303	26.523	1.00	0.00	H
ATOM	646	CG1	VAL	40	34.703	32.934	28.609	1.00	0.00	C
ATOM	647	HG11	VAL	40	34.987	33.893	29.043	1.00	0.00	H
ATOM	648	HG12	VAL	40	33.739	32.936	28.102	1.00	0.00	H
ATOM	649	HG13	VAL	40	34.741	32.132	29.347	1.00	0.00	H
ATOM	650	CG2	VAL	40	37.092	32.903	27.650	1.00	0.00	C
ATOM	651	HG21	VAL	40	37.102	33.967	27.888	1.00	0.00	H
ATOM	652	HG22	VAL	40	37.380	32.293	28.507	1.00	0.00	H
ATOM	653	HG23	VAL	40	37.787	32.693	26.838	1.00	0.00	H
ATOM	654	C	VAL	40	36.093	31.151	25.315	1.00	0.00	C
ATOM	655	O	VAL	40	37.283	30.878	25.058	1.00	0.00	O
ATOM	656	N	GLY	41	35.274	31.472	24.328	1.00	0.00	N
ATOM	657	H	GLY	41	34.287	31.647	24.452	1.00	0.00	H
ATOM	658	CA	GLY	41	35.549	31.513	22.889	1.00	0.00	C
ATOM	659	HA2	GLY	41	36.565	31.898	22.808	1.00	0.00	H
ATOM	660	HA3	GLY	41	35.424	30.487	22.547	1.00	0.00	H
ATOM	661	C	GLY	41	34.567	32.439	22.086	1.00	0.00	C
ATOM	662	O	GLY	41	33.405	32.528	22.535	1.00	0.00	O
ATOM	663	N	ARG	42	34.971	32.818	20.936	1.00	0.00	N
ATOM	664	H	ARG	42	35.865	32.498	20.590	1.00	0.00	H
ATOM	665	CA	ARG	42	34.069	33.347	19.864	1.00	0.00	C
ATOM	666	HA	ARG	42	33.113	32.825	19.899	1.00	0.00	H
ATOM	667	CB	ARG	42	33.702	34.819	20.145	1.00	0.00	C
ATOM	668	HB2	ARG	42	32.933	35.082	19.420	1.00	0.00	H

ATOM	669	HB3	ARG	42	33.303	34.768	21.158	1.00	0.00	H
ATOM	670	CG	ARG	42	34.830	35.865	20.130	1.00	0.00	C
ATOM	671	HG2	ARG	42	35.650	35.561	20.782	1.00	0.00	H
ATOM	672	HG3	ARG	42	35.173	36.017	19.107	1.00	0.00	H
ATOM	673	CD	ARG	42	34.339	37.066	20.895	1.00	0.00	C
ATOM	674	HD2	ARG	42	33.535	37.519	20.313	1.00	0.00	H
ATOM	675	HD3	ARG	42	33.864	36.684	21.799	1.00	0.00	H
ATOM	676	NE	ARG	42	35.416	38.002	21.087	1.00	0.00	N
ATOM	677	HE	ARG	42	35.572	38.581	20.273	1.00	0.00	H
ATOM	678	CZ	ARG	42	36.083	38.350	22.160	1.00	0.00	C
ATOM	679	NH1	ARG	42	36.043	37.611	23.260	1.00	0.00	N
ATOM	680	HH11	ARG	42	36.544	37.927	24.078	1.00	0.00	H
ATOM	681	HH12	ARG	42	35.337	36.918	23.467	1.00	0.00	H
ATOM	682	NH2	ARG	42	36.963	39.350	22.154	1.00	0.00	N
ATOM	683	HH21	ARG	42	37.124	39.813	21.272	1.00	0.00	H
ATOM	684	HH22	ARG	42	37.445	39.534	23.023	1.00	0.00	H
ATOM	685	C	ARG	42	34.584	33.143	18.412	1.00	0.00	C
ATOM	686	O	ARG	42	33.826	33.320	17.461	1.00	0.00	O
ATOM	687	N	PHE	43	35.973	33.004	18.275	1.00	0.00	N
ATOM	688	H	PHE	43	36.573	32.840	19.071	1.00	0.00	H
ATOM	689	CA	PHE	43	36.640	32.788	16.930	1.00	0.00	C
ATOM	690	HA	PHE	43	36.381	33.589	16.237	1.00	0.00	H
ATOM	691	CB	PHE	43	38.109	33.097	17.143	1.00	0.00	C
ATOM	692	HB2	PHE	43	38.604	32.179	17.461	1.00	0.00	H
ATOM	693	HB3	PHE	43	38.572	33.358	16.191	1.00	0.00	H
ATOM	694	CG	PHE	43	38.435	34.359	18.017	1.00	0.00	C
ATOM	695	CD1	PHE	43	37.802	35.571	17.634	1.00	0.00	C
ATOM	696	HD1	PHE	43	37.245	35.642	16.711	1.00	0.00	H
ATOM	697	CE1	PHE	43	38.010	36.762	18.414	1.00	0.00	C
ATOM	698	HE1	PHE	43	37.535	37.689	18.130	1.00	0.00	H
ATOM	699	CZ	PHE	43	38.735	36.649	19.600	1.00	0.00	C
ATOM	700	HZ	PHE	43	38.783	37.500	20.263	1.00	0.00	H
ATOM	701	CE2	PHE	43	39.386	35.442	19.959	1.00	0.00	C
ATOM	702	HE2	PHE	43	39.932	35.439	20.891	1.00	0.00	H
ATOM	703	CD2	PHE	43	39.241	34.292	19.167	1.00	0.00	C
ATOM	704	HD2	PHE	43	39.477	33.342	19.626	1.00	0.00	H
ATOM	705	C	PHE	43	36.256	31.383	16.311	1.00	0.00	C
ATOM	706	O	PHE	43	35.727	30.553	17.064	1.00	0.00	O
ATOM	707	N	GLU	44	36.462	31.258	15.001	1.00	0.00	N
ATOM	708	H	GLU	44	36.922	31.989	14.477	1.00	0.00	H
ATOM	709	CA	GLU	44	36.210	30.031	14.230	1.00	0.00	C
ATOM	710	HA	GLU	44	35.319	29.515	14.590	1.00	0.00	H
ATOM	711	CB	GLU	44	35.822	30.429	12.777	1.00	0.00	C
ATOM	712	HB2	GLU	44	35.076	31.215	12.896	1.00	0.00	H
ATOM	713	HB3	GLU	44	36.654	30.934	12.284	1.00	0.00	H
ATOM	714	CG	GLU	44	35.281	29.259	11.850	1.00	0.00	C
ATOM	715	HG2	GLU	44	35.305	29.586	10.810	1.00	0.00	H
ATOM	716	HG3	GLU	44	35.905	28.376	11.995	1.00	0.00	H
ATOM	717	CD	GLU	44	33.757	28.915	12.121	1.00	0.00	C
ATOM	718	OE1	GLU	44	33.186	28.198	11.352	1.00	0.00	O
ATOM	719	OE2	GLU	44	33.208	29.607	12.989	1.00	0.00	O
ATOM	720	C	GLU	44	37.347	28.957	14.387	1.00	0.00	C
ATOM	721	O	GLU	44	36.990	27.793	14.590	1.00	0.00	O
ATOM	722	N	ASP	45	38.598	29.332	14.322	1.00	0.00	N
ATOM										
	723	H	ASP	45	38.694	30.301	14.055	1.00	0.00	H
ATOM	724	CA	ASP	45	39.757	28.469	14.487	1.00	0.00	C
ATOM	725	HA	ASP	45	39.579	27.492	14.037	1.00	0.00	H
ATOM	726	CB	ASP	45	41.009	29.112	13.834	1.00	0.00	C
ATOM	727	HB2	ASP	45	40.835	29.352	12.785	1.00	0.00	H
ATOM	728	HB3	ASP	45	40.996	30.110	14.269	1.00	0.00	H
ATOM	729	CG	ASP	45	42.311	28.276	14.064	1.00	0.00	C
ATOM	730	OD1	ASP	45	42.295	27.062	13.917	1.00	0.00	O
ATOM	731	OD2	ASP	45	43.348	28.912	14.228	1.00	0.00	O

ATOM	732	C	ASP	45	39.996	28.285	16.009	1.00	0.00	C
ATOM	733	O	ASP	45	39.935	29.198	16.815	1.00	0.00	O
ATOM	734	N	ASP	46	40.362	27.096	16.493	1.00	0.00	N
ATOM	735	H	ASP	46	40.535	26.372	15.809	1.00	0.00	H
ATOM	736	CA	ASP	46	40.642	26.753	17.904	1.00	0.00	C
ATOM	737	HA	ASP	46	41.022	25.735	17.996	1.00	0.00	H
ATOM	738	CB	ASP	46	41.863	27.431	18.487	1.00	0.00	C
ATOM	739	HB2	ASP	46	41.693	28.499	18.614	1.00	0.00	H
ATOM	740	HB3	ASP	46	42.285	27.046	19.415	1.00	0.00	H
ATOM	741	CG	ASP	46	42.981	27.306	17.458	1.00	0.00	C
ATOM	742	OD1	ASP	46	43.444	26.342	16.863	1.00	0.00	O
ATOM	743	OD2	ASP	46	43.512	28.415	17.252	1.00	0.00	O
ATOM	744	C	ASP	46	39.449	26.847	18.783	1.00	0.00	C
ATOM	745	O	ASP	46	39.371	26.118	19.795	1.00	0.00	O
ATOM	746	N	GLY	47	38.368	27.529	18.387	1.00	0.00	N
ATOM	747	H	GLY	47	38.435	28.150	17.594	1.00	0.00	H
ATOM	748	CA	GLY	47	37.069	27.563	19.084	1.00	0.00	C
ATOM	749	HA2	GLY	47	36.326	28.009	18.421	1.00	0.00	H
ATOM	750	HA3	GLY	47	36.820	26.526	19.305	1.00	0.00	H
ATOM	751	C	GLY	47	37.075	28.284	20.404	1.00	0.00	C
ATOM	752	O	GLY	47	36.158	29.078	20.649	1.00	0.00	O
ATOM	753	N	TYR	48	38.151	28.134	21.223	1.00	0.00	N
ATOM	754	H	TYR	48	39.005	27.688	20.920	1.00	0.00	H
ATOM	755	CA	TYR	48	38.285	28.731	22.543	1.00	0.00	C
ATOM	756	HA	TYR	48	37.504	29.487	22.616	1.00	0.00	H
ATOM	757	CB	TYR	48	37.952	27.694	23.626	1.00	0.00	C
ATOM	758	HB2	TYR	48	38.724	26.935	23.762	1.00	0.00	H
ATOM	759	HB3	TYR	48	38.038	28.240	24.564	1.00	0.00	H
ATOM	760	CG	TYR	48	36.642	26.938	23.521	1.00	0.00	C
ATOM	761	CD1	TYR	48	35.417	27.665	23.569	1.00	0.00	C
ATOM	762	HD1	TYR	48	35.373	28.744	23.552	1.00	0.00	H
ATOM	763	CE1	TYR	48	34.214	27.027	23.631	1.00	0.00	C
ATOM	764	HE1	TYR	48	33.372	27.702	23.655	1.00	0.00	H
ATOM	765	CZ	TYR	48	34.223	25.620	23.727	1.00	0.00	C
ATOM	766	OH	TYR	48	32.985	25.012	23.783	1.00	0.00	O
ATOM	767	HH	TYR	48	32.874	24.067	23.658	1.00	0.00	H
ATOM	768	CE2	TYR	48	35.379	24.859	23.654	1.00	0.00	C
ATOM	769	HE2	TYR	48	35.382	23.779	23.625	1.00	0.00	H
ATOM	770	CD2	TYR	48	36.649	25.528	23.562	1.00	0.00	C
ATOM	771	HD2	TYR	48	37.545	24.932	23.478	1.00	0.00	H
ATOM	772	C	TYR	48	39.627	29.492	22.701	1.00	0.00	C
ATOM	773	O	TYR	48	40.763	28.972	22.601	1.00	0.00	O
ATOM	774	N	PHE	49	39.569	30.780	23.054	1.00	0.00	N
ATOM	775	H	PHE	49	38.663	31.193	23.215	1.00	0.00	H
ATOM	776	CA	PHE	49	40.798	31.534	23.277	1.00	0.00	C
ATOM	777	HA	PHE	49	41.616	31.037	22.753	1.00	0.00	H
ATOM	778	CB	PHE	49	40.653	32.961	22.690	1.00	0.00	C
ATOM	779	HB2	PHE	49	41.582	33.527	22.761	1.00	0.00	H
ATOM	780	HB3	PHE	49	40.467	32.847	21.622	1.00	0.00	H
ATOM	781	CG	PHE	49	39.642	33.924	23.347	1.00	0.00	C
ATOM	782	CD1	PHE	49	38.295	33.941	23.000	1.00	0.00	C
ATOM	783	HD1	PHE	49	38.016	33.351	22.140	1.00	0.00	H
ATOM	784	CE1	PHE	49	37.387	34.720	23.759	1.00	0.00	C
ATOM	785	HE1	PHE	49	36.326	34.701	23.555	1.00	0.00	H
ATOM	786	CZ	PHE	49	37.879	35.506	24.864	1.00	0.00	C
ATOM	787	HZ	PHE	49	37.308	36.088	25.571	1.00	0.00	H
ATOM	788	CE2	PHE	49	39.215	35.435	25.208	1.00	0.00	C
ATOM	789	HE2	PHE	49	39.581	35.923	26.100	1.00	0.00	H
ATOM	790	CD2	PHE	49	40.141	34.634	24.463	1.00	0.00	C
ATOM	791	HD2	PHE	49	41.137	34.540	24.867	1.00	0.00	H
ATOM	792	C	PHE	49	41.207	31.479	24.800	1.00	0.00	C
ATOM	793	O	PHE	49	42.254	32.108	25.169	1.00	0.00	O
ATOM	794	N	CYX	50	40.455	30.926	25.724	1.00	0.00	N
ATOM	795	H	CYX	50	39.480	30.725	25.553	1.00	0.00	H

ATOM	796	CA	CYX	50	40.852	30.675	27.086	1.00	0.00	C
ATOM	797	HA	CYX	50	41.922	30.474	27.047	1.00	0.00	H
ATOM	798	CB	CYX	50	40.541	31.906	28.076	1.00	0.00	C
ATOM	799	HB2	CYX	50	39.722	32.426	27.578	1.00	0.00	H
ATOM	800	HB3	CYX	50	40.269	31.503	29.052	1.00	0.00	H
ATOM	801	SG	CYX	50	41.941	33.062	28.419	1.00	0.00	S
ATOM	802	C	CYX	50	40.109	29.448	27.717	1.00	0.00	C
ATOM	803	O	CYX	50	38.868	29.518	27.830	1.00	0.00	O
ATOM	804	N	THR	51	40.832	28.393	28.269	1.00	0.00	N
ATOM	805	H	THR	51	41.822	28.552	28.148	1.00	0.00	H
ATOM	806	CA	THR	51	40.277	27.556	29.403	1.00	0.00	C
ATOM	807	HA	THR	51	39.241	27.326	29.158	1.00	0.00	H
ATOM	808	CB	THR	51	40.985	26.183	29.544	1.00	0.00	C
ATOM	809	HB	THR	51	42.045	26.247	29.793	1.00	0.00	H
ATOM	810	CG2	THR	51	40.403	25.346	30.631	1.00	0.00	C
ATOM	811	HG21	THR	51	40.675	25.789	31.589	1.00	0.00	H
ATOM	812	HG22	THR	51	39.320	25.462	30.658	1.00	0.00	H
ATOM	813	HG23	THR	51	40.716	24.302	30.622	1.00	0.00	H
ATOM	814	OG1	THR	51	40.797	25.604	28.233	1.00	0.00	O
ATOM	815	HG1	THR	51	41.536	25.872	27.683	1.00	0.00	H
ATOM	816	C	THR	51	40.338	28.448	30.719	1.00	0.00	C
ATOM	817	O	THR	51	41.393	29.075	30.854	1.00	0.00	O
ATOM	818	N	ILE	52	39.386	28.399	31.611	1.00	0.00	N
ATOM	819	H	ILE	52	38.691	27.690	31.421	1.00	0.00	H
ATOM	820	CA	ILE	52	39.486	28.962	32.999	1.00	0.00	C
ATOM	821	HA	ILE	52	40.510	29.327	33.079	1.00	0.00	H
ATOM	822	CB	ILE	52	38.396	30.121	33.076	1.00	0.00	C
ATOM	823	HB	ILE	52	37.412	29.778	32.756	1.00	0.00	H
ATOM	824	CG2	ILE	52	38.186	30.591	34.478	1.00	0.00	C
ATOM	825	HG21	ILE	52	38.949	31.285	34.832	1.00	0.00	H
ATOM	826	HG22	ILE	52	37.212	31.075	34.562	1.00	0.00	H
ATOM	827	HG23	ILE	52	38.095	29.822	35.244	1.00	0.00	H
ATOM	828	CG1	ILE	52	38.762	31.312	32.181	1.00	0.00	C
ATOM	829	HG12	ILE	52	39.718	31.766	32.444	1.00	0.00	H
ATOM	830	HG13	ILE	52	38.920	30.883	31.191	1.00	0.00	H
ATOM	831	CD1	ILE	52	37.680	32.371	31.998	1.00	0.00	C
ATOM	832	HD11	ILE	52	36.822	31.964	31.463	1.00	0.00	H
ATOM	833	HD12	ILE	52	37.248	32.755	32.921	1.00	0.00	H
ATOM	834	HD13	ILE	52	38.056	33.215	31.420	1.00	0.00	H
ATOM	835	C	ILE	52	39.317	27.813	34.031	1.00	0.00	C
ATOM	836	O	ILE	52	38.391	26.941	33.906	1.00	0.00	O
ATOM	837	N	GLU	53	40.069	27.854	35.161	1.00	0.00	N
ATOM	838	H	GLU	53	40.633	28.686	35.264	1.00	0.00	H
ATOM	839	CA	GLU	53	39.881	26.923	36.328	1.00	0.00	C
ATOM	840	HA	GLU	53	39.078	26.224	36.093	1.00	0.00	H
ATOM	841	CB	GLU	53	41.221	26.202	36.485	1.00	0.00	C
ATOM	842	HB2	GLU	53	41.641	25.980	35.504	1.00	0.00	H
ATOM	843	HB3	GLU	53	41.851	26.956	36.955	1.00	0.00	H
ATOM	844	CG	GLU	53	41.186	24.975	37.412	1.00	0.00	C
ATOM	845	HG2	GLU	53	41.009	25.216	38.460	1.00	0.00	H
ATOM	846	HG3	GLU	53	40.363	24.321	37.123	1.00	0.00	H
ATOM	847	CD	GLU	53	42.461	24.085	37.387	1.00	0.00	C
ATOM	848	OE1	GLU	53	42.257	22.982	36.896	1.00	0.00	O
ATOM	849	OE2	GLU	53	43.564	24.435	37.863	1.00	0.00	O
ATOM	850	C	GLU	53	39.539	27.722	37.615	1.00	0.00	C
ATOM	851	O	GLU	53	40.144	28.710	37.873	1.00	0.00	O
ATOM	852	N	VAL	54	38.523	27.269	38.390	1.00	0.00	N
ATOM	853	H	VAL	54	38.004	26.439	38.140	1.00	0.00	H
ATOM	854	CA	VAL	54	38.203	27.824	39.647	1.00	0.00	C
ATOM	855	HA	VAL	54	37.940	28.881	39.643	1.00	0.00	H
ATOM	856	CB	VAL	54	36.964	27.231	40.321	1.00	0.00	C
ATOM	857	HB	VAL	54	37.176	26.173	40.476	1.00	0.00	H
ATOM	858	CG1	VAL	54	36.718	27.809	41.689	1.00	0.00	C

ATOM	859	HG11	VAL	54	35.862	27.333	42.167	1.00	0.00	H
ATOM	860	HG12	VAL	54	37.559	27.623	42.357	1.00	0.00	H
ATOM	861	HG13	VAL	54	36.536	28.879	41.593	1.00	0.00	H
ATOM	862	CG2	VAL	54	35.669	27.537	39.521	1.00	0.00	C
ATOM	863	HG21	VAL	54	34.822	27.381	40.189	1.00	0.00	H
ATOM	864	HG22	VAL	54	35.757	28.575	39.200	1.00	0.00	H
ATOM	865	HG23	VAL	54	35.662	26.910	38.630	1.00	0.00	H
ATOM	866	C	VAL	54	39.457	27.712	40.622	1.00	0.00	C
ATOM	867	O	VAL	54	39.827	26.609	40.891	1.00	0.00	O
ATOM	868	N	THR	55	40.031	28.729	41.203	1.00	0.00	N
ATOM	869	H	THR	55	39.553	29.617	41.151	1.00	0.00	H
ATOM	870	CA	THR	55	41.100	28.678	42.203	1.00	0.00	C
ATOM	871	HA	THR	55	41.844	27.911	41.985	1.00	0.00	H
ATOM	872	CB	THR	55	41.923	29.959	42.182	1.00	0.00	C
ATOM	873	HB	THR	55	42.636	29.999	43.006	1.00	0.00	H
ATOM	874	CG2	THR	55	42.811	30.092	40.934	1.00	0.00	C
ATOM	875	HG21	THR	55	43.794	29.645	41.083	1.00	0.00	H
ATOM	876	HG22	THR	55	42.393	29.557	40.082	1.00	0.00	H
ATOM	877	HG23	THR	55	43.077	31.135	40.760	1.00	0.00	H
ATOM	878	OG1	THR	55	41.077	31.093	42.297	1.00	0.00	O
ATOM	879	HG1	THR	55	41.412	31.606	43.035	1.00	0.00	H
ATOM	880	C	THR	55	40.619	28.414	43.604	1.00	0.00	C
ATOM	881	O	THR	55	41.287	27.653	44.296	1.00	0.00	O
ATOM	882	N	ALA	56	39.533	29.050	44.085	1.00	0.00	N
ATOM	883	H	ALA	56	39.001	29.439	43.319	1.00	0.00	H
ATOM	884	CA	ALA	56	39.098	29.019	45.444	1.00	0.00	C
ATOM	885	HA	ALA	56	39.218	27.954	45.646	1.00	0.00	H
ATOM	886	CB	ALA	56	39.950	29.951	46.398	1.00	0.00	C
ATOM	887	HB1	ALA	56	39.514	30.021	47.395	1.00	0.00	H
ATOM	888	HB2	ALA	56	40.953	29.525	46.402	1.00	0.00	H
ATOM	889	HB3	ALA	56	40.092	30.873	45.833	1.00	0.00	H
ATOM	890	C	ALA	56	37.594	29.441	45.491	1.00	0.00	C
ATOM	891	O	ALA	56	37.047	30.101	44.595	1.00	0.00	O
ATOM	892	N	THR	57	36.989	29.185	46.651	1.00	0.00	N
ATOM	893	H	THR	57	37.472	28.625	47.338	1.00	0.00	H
ATOM	894	CA	THR	57	35.574	29.509	46.899	1.00	0.00	C
ATOM	895	HA	THR	57	35.494	30.541	46.556	1.00	0.00	H
ATOM	896	CB	THR	57	34.602	28.540	46.206	1.00	0.00	C
ATOM	897	HB	THR	57	33.603	28.832	46.530	1.00	0.00	H
ATOM	898	CG2	THR	57	34.419	28.496	44.636	1.00	0.00	C
ATOM	899	HG21	THR	57	34.263	29.480	44.194	1.00	0.00	H
ATOM	900	HG22	THR	57	35.374	28.056	44.348	1.00	0.00	H
ATOM	901	HG23	THR	57	33.689	27.820	44.191	1.00	0.00	H
ATOM	902	OG1	THR	57	34.732	27.190	46.659	1.00	0.00	O
ATOM	903	HG1	THR	57	34.755	27.008	47.601	1.00	0.00	H
ATOM	904	C	THR	57	35.317	29.517	48.415	1.00	0.00	C
ATOM	905	O	THR	57	35.956	28.823	49.191	1.00	0.00	O
ATOM	906	N	SER	58	34.339	30.303	48.820	1.00	0.00	N
ATOM	907	H	SER	58	33.891	30.903	48.142	1.00	0.00	H
ATOM	908	CA	SER	58	34.163	30.540	50.212	1.00	0.00	C
ATOM	909	HA	SER	58	34.095	29.585	50.734	1.00	0.00	H
ATOM	910	CB	SER	58	35.385	31.368	50.842	1.00	0.00	C
ATOM	911	HB2	SER	58	36.294	31.049	50.331	1.00	0.00	H
ATOM	912	HB3	SER	58	35.165	32.424	50.688	1.00	0.00	H
ATOM	913	OG	SER	58	35.462	31.028	52.190	1.00	0.00	O
ATOM	914	HG	SER	58	35.863	30.158	52.125	1.00	0.00	H
ATOM	915	C	SER	58	32.879	31.307	50.488	1.00	0.00	C
ATOM	916	O	SER	58	32.341	32.083	49.657	1.00	0.00	O
ATOM	917	N	THR	59	32.317	30.941	51.632	1.00	0.00	N
ATOM	918	H	THR	59	32.764	30.348	52.316	1.00	0.00	H
ATOM	919	CA	THR	59	31.086	31.487	52.112	1.00	0.00	C
ATOM	920	HA	THR	59	30.764	32.132	51.294	1.00	0.00	H
ATOM	921	CB	THR	59	30.011	30.392	52.361	1.00	0.00	C
ATOM	922	HB	THR	59	29.916	29.827	51.433	1.00	0.00	H

```
ATOM    923  CG2 THR    59      30.247  29.394  53.523  1.00  0.00           C
ATOM    924 HG21 THR    59      29.485  28.614  53.507  1.00  0.00           H
ATOM    925 HG22 THR    59      31.241  28.953  53.452  1.00  0.00           H
ATOM    926 HG23 THR    59      30.291  29.926  54.474  1.00  0.00           H
ATOM    927  OG1 THR    59      28.743  30.902  52.693  1.00  0.00           O
ATOM    928  HG1 THR    59      28.581  31.590  52.042  1.00  0.00           H
ATOM    929  C   THR    59      31.208  32.431  53.381  1.00  0.00           C
ATOM    930  O   THR    59      32.221  32.311  54.113  1.00  0.00           O
ATOM    931  N   VAL    60      30.487  33.571  53.380  1.00  0.00           N
ATOM    932  H   VAL    60      29.718  33.625  52.728  1.00  0.00           H
ATOM    933  CA  VAL    60      30.921  34.870  54.111  1.00  0.00           C
ATOM    934  HA  VAL    60      31.549  34.547  54.940  1.00  0.00           H
ATOM    935  CB  VAL    60      31.865  35.693  53.228  1.00  0.00           C
ATOM    936  HB  VAL    60      32.700  35.025  53.017  1.00  0.00           H
ATOM    937  CG1 VAL    60      31.183  36.248  51.940  1.00  0.00           C
ATOM    938 HG11 VAL    60      30.716  35.434  51.385  1.00  0.00           H
ATOM    939 HG12 VAL    60      30.518  37.096  52.099  1.00  0.00           H
ATOM    940 HG13 VAL    60      32.010  36.595  51.319  1.00  0.00           H
ATOM    941  CG2 VAL    60      32.481  36.842  53.997  1.00  0.00           C
ATOM    942 HG21 VAL    60      33.345  37.253  53.475  1.00  0.00           H
ATOM    943 HG22 VAL    60      31.704  37.595  54.128  1.00  0.00           H
ATOM    944 HG23 VAL    60      32.879  36.527  54.962  1.00  0.00           H
ATOM    945  C   VAL    60      29.735  35.720  54.613  1.00  0.00           C
ATOM    946  O   VAL    60      28.737  35.832  53.919  1.00  0.00           O
ATOM    947  N   THR    61      29.814  36.334  55.788  1.00  0.00           N
ATOM    948  H   THR    61      30.634  36.273  56.375  1.00  0.00           H
ATOM    949  CA  THR    61      28.902  37.407  56.256  1.00  0.00           C
ATOM    950  HA  THR    61      28.100  37.548  55.531  1.00  0.00           H
ATOM    951  CB  THR    61      28.349  37.040  57.653  1.00  0.00           C
ATOM    952  HB  THR    61      27.845  37.899  58.096  1.00  0.00           H
ATOM    953  CG2 THR    61      27.406  35.853  57.676  1.00  0.00           C
ATOM    954 HG21 THR    61      27.829  35.222  56.894  1.00  0.00           H
ATOM    955 HG22 THR    61      27.434  35.250  58.583  1.00  0.00           H
ATOM    956 HG23 THR    61      26.380  36.037  57.358  1.00  0.00           H
ATOM    957  OG1 THR    61      29.312  36.612  58.645  1.00  0.00           O
ATOM    958  HG1 THR    61      29.730  37.469  58.766  1.00  0.00           H
ATOM    959  C   THR    61      29.644  38.757  56.267  1.00  0.00           C
ATOM    960  O   THR    61      30.868  38.757  56.269  1.00  0.00           O
ATOM    961  N   LEU    62      28.931  39.901  56.083  1.00  0.00           N
ATOM    962  H   LEU    62      27.924  39.833  56.130  1.00  0.00           H
ATOM    963  CA  LEU    62      29.553  41.228  55.790  1.00  0.00           C
ATOM    964  HA  LEU    62      30.095  41.102  54.852  1.00  0.00           H
ATOM    965  CB  LEU    62      28.364  42.285  55.558  1.00  0.00           C
ATOM    966  HB2 LEU    62      27.819  42.419  56.493  1.00  0.00           H
ATOM    967  HB3 LEU    62      28.712  43.300  55.369  1.00  0.00           H
ATOM    968  CG  LEU    62      27.387  41.864  54.421  1.00  0.00           C
ATOM    969  HG  LEU    62      27.056  40.852  54.657  1.00  0.00           H
ATOM    970  CD1 LEU    62      26.262  42.958  54.345  1.00  0.00           C
ATOM    971 HD11 LEU    62      25.377  42.515  53.888  1.00  0.00           H
ATOM    972 HD12 LEU    62      25.973  43.437  55.281  1.00  0.00           H
ATOM    973 HD13 LEU    62      26.618  43.847  53.827
  1.00  0.00           H
ATOM    974  CD2 LEU    62      28.069  41.872  53.069  1.00  0.00           C
ATOM    975 HD21 LEU    62      28.738  41.019  52.951  1.00  0.00           H
ATOM    976 HD22 LEU    62      27.260  41.498  52.442  1.00  0.00           H
ATOM    977 HD23 LEU    62      28.350  42.808  52.587  1.00  0.00           H
ATOM    978  C   LEU    62      30.490  41.670  56.853  1.00  0.00           C
ATOM    979  O   LEU    62      31.552  42.160  56.517  1.00  0.00           O
ATOM    980  N   ASP    63      30.188  41.383  58.137  1.00  0.00           N
ATOM    981  H   ASP    63      29.377  40.811  58.319  1.00  0.00           H
ATOM    982  CA  ASP    63      31.070  41.553  59.208  1.00  0.00           C
ATOM    983  HA  ASP    63      31.376  42.598  59.212  1.00  0.00           H
ATOM    984  CB  ASP    63      30.487  41.070  60.571  1.00  0.00           C
ATOM    985  HB2 ASP    63      31.186  41.218  61.395  1.00  0.00           H
```

ATOM	986	HB3	ASP	63	29.575	41.596	60.856	1.00	0.00	H
ATOM	987	CG	ASP	63	30.059	39.601	60.514	1.00	0.00	C
ATOM	988	OD1	ASP	63	30.242	38.887	61.555	1.00	0.00	O
ATOM	989	OD2	ASP	63	29.536	39.059	59.533	1.00	0.00	O
ATOM	990	C	ASP	63	32.418	40.750	58.966	1.00	0.00	C
ATOM	991	O	ASP	63	33.364	40.964	59.732	1.00	0.00	O
ATOM	992	N	THR	64	32.581	39.812	58.021	1.00	0.00	N
ATOM	993	H	THR	64	31.867	39.791	57.307	1.00	0.00	H
ATOM	994	CA	THR	64	33.770	38.994	57.712	1.00	0.00	C
ATOM	995	HA	THR	64	34.666	39.416	58.166	1.00	0.00	H
ATOM	996	CB	THR	64	33.509	37.492	58.061	1.00	0.00	C
ATOM	997	HB	THR	64	34.434	36.940	57.899	1.00	0.00	H
ATOM	998	CG2	THR	64	33.261	37.223	59.525	1.00	0.00	C
ATOM	999	HG21	THR	64	34.002	37.746	60.130	1.00	0.00	H
ATOM	1000	HG22	THR	64	32.312	37.680	59.809	1.00	0.00	H
ATOM	1001	HG23	THR	64	33.301	36.143	59.667	1.00	0.00	H
ATOM	1002	OG1	THR	64	32.402	36.943	57.388	1.00	0.00	O
ATOM	1003	HG1	THR	64	31.738	37.607	57.192	1.00	0.00	H
ATOM	1004	C	THR	64	34.162	39.005	56.205	1.00	0.00	C
ATOM	1005	O	THR	64	35.019	38.243	55.715	1.00	0.00	O
ATOM	1006	N	LEU	65	33.569	39.989	55.470	1.00	0.00	N
ATOM	1007	H	LEU	65	32.765	40.486	55.827	1.00	0.00	H
ATOM	1008	CA	LEU	65	33.912	40.261	54.012	1.00	0.00	C
ATOM	1009	HA	LEU	65	33.967	39.275	53.551	1.00	0.00	H
ATOM	1010	CB	LEU	65	32.713	41.105	53.491	1.00	0.00	C
ATOM	1011	HB2	LEU	65	31.804	40.546	53.712	1.00	0.00	H
ATOM	1012	HB3	LEU	65	32.589	42.062	53.997	1.00	0.00	H
ATOM	1013	CG	LEU	65	32.792	41.383	51.975	1.00	0.00	C
ATOM	1014	HG	LEU	65	33.704	41.934	51.745	1.00	0.00	H
ATOM	1015	CD1	LEU	65	32.615	40.171	50.990	1.00	0.00	C
ATOM	1016	HD11	LEU	65	32.482	40.582	49.990	1.00	0.00	H
ATOM	1017	HD12	LEU	65	33.501	39.536	51.007	1.00	0.00	H
ATOM	1018	HD13	LEU	65	31.825	39.560	51.428	1.00	0.00	H
ATOM	1019	CD2	LEU	65	31.766	42.453	51.683	1.00	0.00	C
ATOM	1020	HD21	LEU	65	31.994	43.267	52.370	1.00	0.00	H
ATOM	1021	HD22	LEU	65	31.774	42.854	50.670	1.00	0.00	H
ATOM	1022	HD23	LEU	65	30.719	42.185	51.830	1.00	0.00	H
ATOM	1023	C	LEU	65	35.271	41.048	53.840	1.00	0.00	C
ATOM	1024	O	LEU	65	35.582	41.936	54.571	1.00	0.00	O
ATOM	1025	N	THR	66	36.034	40.651	52.859	1.00	0.00	N
ATOM	1026	H	THR	66	35.746	39.891	52.258	1.00	0.00	H
ATOM	1027	CA	THR	66	37.361	41.170	52.641	1.00	0.00	C
ATOM	1028	HA	THR	66	37.753	41.563	53.579	1.00	0.00	H
ATOM	1029	CB	THR	66	38.357	40.001	52.324	1.00	0.00	C
ATOM	1030	HB	THR	66	38.022	39.634	51.353	1.00	0.00	H
ATOM	1031	CG2	THR	66	39.792	40.411	52.200	1.00	0.00	C
ATOM	1032	HG21	THR	66	40.445	39.540	52.247	1.00	0.00	H
ATOM	1033	HG22	THR	66	39.891	41.090	51.353	1.00	0.00	H
ATOM	1034	HG23	THR	66	40.034	40.961	53.109	1.00	0.00	H
ATOM	1035	OG1	THR	66	38.265	38.949	53.296	1.00	0.00	O
ATOM	1036	HG1	THR	66	37.390	38.594	53.125	1.00	0.00	H
ATOM	1037	C	THR	66	37.271	42.263	51.568	1.00	0.00	C
ATOM	1038	O	THR	66	36.336	42.316	50.741	1.00	0.00	O
ATOM	1039	N	GLU	67	38.290	43.148	51.380	1.00	0.00	N
ATOM	1040	H	GLU	67	39.034	43.180	52.062	1.00	0.00	H
ATOM	1041	CA	GLU	67	38.196	44.319	50.480	1.00	0.00	C
ATOM	1042	HA	GLU	67	37.215	44.793	50.441	1.00	0.00	H
ATOM	1043	CB	GLU	67	39.281	45.362	50.873	1.00	0.00	C
ATOM	1044	HB2	GLU	67	39.075	46.291	50.341	1.00	0.00	H
ATOM	1045	HB3	GLU	67	39.091	45.680	51.899	1.00	0.00	H
ATOM	1046	CG	GLU	67	40.820	45.095	50.732	1.00	0.00	C
ATOM	1047	HG2	GLU	67	41.039	45.015	49.667	1.00	0.00	H
ATOM	1048	HG3	GLU	67	41.363	45.955	51.123	1.00	0.00	H
ATOM	1049	CD	GLU	67	41.217	43.860	51.531	1.00	0.00	C

ATOM	1050	OE1	GLU	67	41.733	42.888	50.938	1.00	0.00	O
ATOM	1051	OE2	GLU	67	40.942	43.808	52.766	1.00	0.00	O
ATOM	1052	C	GLU	67	38.408	44.004	49.000	1.00	0.00	C
ATOM	1053	O	GLU	67	38.075	44.861	48.186	1.00	0.00	O
ATOM	1054	N	LYS	68	38.922	42.818	48.604	1.00	0.00	N
ATOM	1055	H	LYS	68	39.109	42.187	49.370	1.00	0.00	H
ATOM	1056	CA	LYS	68	39.424	42.600	47.233	1.00	0.00	C
ATOM	1057	HA	LYS	68	40.267	43.248	46.994	1.00	0.00	H
ATOM	1058	CB	LYS	68	39.669	41.062	47.091	1.00	0.00	C
ATOM	1059	HB2	LYS	68	40.019	40.653	48.039	1.00	0.00	H
ATOM	1060	HB3	LYS	68	38.794	40.531	46.714	1.00	0.00	H
ATOM	1061	CG	LYS	68	40.855	40.882	46.119	1.00	0.00	C
ATOM	1062	HG2	LYS	68	40.627	41.427	45.202	1.00	0.00	H
ATOM	1063	HG3	LYS	68	41.720	41.184	46.709	1.00	0.00	H
ATOM	1064	CD	LYS	68	40.993	39.381	45.796	1.00	0.00	C
ATOM	1065	HD2	LYS	68	40.281	38.992	45.069	1.00	0.00	H
ATOM	1066	HD3	LYS	68	41.881	39.117	45.220	1.00	0.00	H
ATOM	1067	CE	LYS	68	41.094	38.345	46.916	1.00	0.00	C
ATOM	1068	HE2	LYS	68	40.326	38.578	47.654	1.00	0.00	H
ATOM	1069	HE3	LYS	68	40.876	37.349	46.530	1.00	0.00	H
ATOM	1070	NZ	LYS	68	42.358	38.328	47.603	1.00	0.00	N
ATOM	1071	HZ1	LYS	68	42.340	38.846	48.470	1.00	0.00	H
ATOM	1072	HZ2	LYS	68	42.613	37.389	47.871	1.00	0.00	H
ATOM	1073	HZ3	LYS	68	43.076	38.769	47.045	1.00	0.00	H
ATOM	1074	C	LYS	68	38.412	42.929	46.110	1.00	0.00	C
ATOM	1075	O	LYS	68	38.804	43.464	45.067	1.00	0.00	O
ATOM	1076	N	HIE	69	37.145	42.492	46.189	1.00	0.00	N
ATOM	1077	H	HIE	69	36.832	42.070	47.051	1.00	0.00	H
ATOM	1078	CA	HIE	69	36.120	42.719	45.151	1.00	0.00	C
ATOM	1079	HA	HIE	69	36.447	42.366	44.173	1.00	0.00	H
ATOM	1080	CB	HIE	69	34.919	41.737	45.418	1.00	0.00	C
ATOM	1081	HB2	HIE	69	35.305	40.717	45.407	1.00	0.00	H
ATOM	1082	HB3	HIE	69	34.589	41.773	46.456	1.00	0.00	H
ATOM	1083	CG	HIE	69	33.827	41.819	44.430	1.00	0.00	C
ATOM	1084	ND1	HIE	69	33.800	41.127	43.222	1.00	0.00	N
ATOM	1085	CE1	HIE	69	32.521	41.315	42.735	1.00	0.00	C
ATOM	1086	HE1	HIE	69	32.178	40.946	41.779	1.00	0.00	H
ATOM	1087	NE2	HIE	69	31.901	42.176	43.533	1.00	0.00	N
ATOM	1088	HE2	HIE	69	31.053	42.637	43.235	1.00	0.00	H
ATOM	1089	CD2	HIE	69	32.703	42.472	44.541	1.00	0.00	C
ATOM	1090	HD2	HIE	69	32.533	43.187	45.332	1.00	0.00	H
ATOM	1091	C	HIE	69	35.678	44.152	45.032	1.00	0.00	C
ATOM	1092	O	HIE	69	35.553	44.639	43.897	1.00	0.00	O
ATOM	1093	N	ALA	70	35.691	44.886	46.110	1.00	0.00	N
ATOM	1094	H	ALA	70	36.204	44.490	46.884	1.00	0.00	H
ATOM	1095	CA	ALA	70	35.471	46.348	46.020	1.00	0.00	C
ATOM	1096	HA	ALA	70	34.511	46.617	45.579	1.00	0.00	H
ATOM	1097	CB	ALA	70	35.675	46.967	47.442	1.00	0.00	C
ATOM	1098	HB1	ALA	70	35.398	48.014	47.327	1.00	0.00	H
ATOM										
	1099	HB2	ALA	70	35.056	46.387	48.126	1.00	0.00	H
ATOM	1100	HB3	ALA	70	36.737	46.823	47.642	1.00	0.00	H
ATOM	1101	C	ALA	70	36.472	46.893	45.088	1.00	0.00	C
ATOM	1102	O	ALA	70	36.179	47.629	44.165	1.00	0.00	O
ATOM	1103	N	GLU	71	37.735	46.624	45.353	1.00	0.00	N
ATOM	1104	H	GLU	71	37.967	45.974	46.091	1.00	0.00	H
ATOM	1105	CA	GLU	71	38.932	47.139	44.644	1.00	0.00	C
ATOM	1106	HA	GLU	71	38.877	48.203	44.409	1.00	0.00	H
ATOM	1107	CB	GLU	71	40.203	46.715	45.453	1.00	0.00	C
ATOM	1108	HB2	GLU	71	40.423	45.657	45.309	1.00	0.00	H
ATOM	1109	HB3	GLU	71	41.081	47.118	44.949	1.00	0.00	H
ATOM	1110	CG	GLU	71	40.339	47.040	46.928	1.00	0.00	C
ATOM	1111	HG2	GLU	71	39.413	46.725	47.410	1.00	0.00	H
ATOM	1112	HG3	GLU	71	41.131	46.413	47.335	1.00	0.00	H

ATOM	1113	CD	GLU	71	40.612	48.583	47.207	1.00	0.00	C
ATOM	1114	OE1	GLU	71	40.642	48.925	48.399	1.00	0.00	O
ATOM	1115	OE2	GLU	71	40.974	49.340	46.299	1.00	0.00	O
ATOM	1116	C	GLU	71	39.085	46.455	43.200	1.00	0.00	C
ATOM	1117	O	GLU	71	39.892	46.909	42.383	1.00	0.00	O
ATOM	1118	N	GLN	72	38.340	45.427	42.871	1.00	0.00	N
ATOM	1119	H	GLN	72	37.698	45.042	43.550	1.00	0.00	H
ATOM	1120	CA	GLN	72	38.160	44.926	41.544	1.00	0.00	C
ATOM	1121	HA	GLN	72	39.041	45.036	40.913	1.00	0.00	H
ATOM	1122	CB	GLN	72	37.673	43.529	41.645	1.00	0.00	C
ATOM	1123	HB2	GLN	72	36.671	43.584	42.071	1.00	0.00	H
ATOM	1124	HB3	GLN	72	38.354	42.914	42.233	1.00	0.00	H
ATOM	1125	CG	GLN	72	37.586	42.746	40.283	1.00	0.00	C
ATOM	1126	HG2	GLN	72	37.371	43.351	39.403	1.00	0.00	H
ATOM	1127	HG3	GLN	72	38.583	42.362	40.067	1.00	0.00	H
ATOM	1128	CD	GLN	72	36.522	41.703	40.166	1.00	0.00	C
ATOM	1129	OE1	GLN	72	36.867	40.592	39.824	1.00	0.00	O
ATOM	1130	NE2	GLN	72	35.267	41.981	40.539	1.00	0.00	N
ATOM	1131	HE21	GLN	72	35.103	42.899	40.928	1.00	0.00	H
ATOM	1132	HE22	GLN	72	34.720	41.159	40.750	1.00	0.00	H
ATOM	1133	C	GLN	72	37.144	45.768	40.761	1.00	0.00	C
ATOM	1134	O	GLN	72	37.274	46.054	39.548	1.00	0.00	O
ATOM	1135	N	GLU	73	36.093	46.228	41.442	1.00	0.00	N
ATOM	1136	H	GLU	73	35.872	45.762	42.310	1.00	0.00	H
ATOM	1137	CA	GLU	73	35.159	47.231	40.920	1.00	0.00	C
ATOM	1138	HA	GLU	73	35.179	46.985	39.858	1.00	0.00	H
ATOM	1139	CB	GLU	73	33.710	46.939	41.491	1.00	0.00	C
ATOM	1140	HB2	GLU	73	33.551	47.187	42.540	1.00	0.00	H
ATOM	1141	HB3	GLU	73	33.039	47.675	41.050	1.00	0.00	H
ATOM	1142	CG	GLU	73	33.066	45.540	41.318	1.00	0.00	C
ATOM	1143	HG2	GLU	73	33.571	44.763	41.891	1.00	0.00	H
ATOM	1144	HG3	GLU	73	32.102	45.626	41.819	1.00	0.00	H
ATOM	1145	CD	GLU	73	32.767	45.142	39.861	1.00	0.00	C
ATOM	1146	OE1	GLU	73	33.705	45.205	38.986	1.00	0.00	O
ATOM	1147	OE2	GLU	73	31.582	45.018	39.579	1.00	0.00	O
ATOM	1148	C	GLU	73	35.490	48.715	41.052	1.00	0.00	C
ATOM	1149	O	GLU	73	34.952	49.606	40.415	1.00	0.00	O
ATOM	1150	N	ASN	74	36.549	48.991	41.836	1.00	0.00	N
ATOM	1151	H	ASN	74	36.912	48.288	42.463	1.00	0.00	H
ATOM	1152	CA	ASN	74	37.057	50.347	42.141	1.00	0.00	C
ATOM	1153	HA	ASN	74	37.956	50.090	42.702	1.00	0.00	H
ATOM	1154	CB	ASN	74	37.554	51.133	40.805	1.00	0.00	C
ATOM	1155	HB2	ASN	74	36.681	51.428	40.223	1.00	0.00	H
ATOM	1156	HB3	ASN	74	38.034	52.055	41.132	1.00	0.00	H
ATOM	1157	CG	ASN	74	38.465	50.386	39.864	1.00	0.00	C
ATOM	1158	OD1	ASN	74	39.701	50.655	39.688	1.00	0.00	O
ATOM	1159	ND2	ASN	74	37.948	49.397	39.201	1.00	0.00	N
ATOM	1160	HD21	ASN	74	38.477	48.995	38.441	1.00	0.00	H
ATOM	1161	HD22	ASN	74	36.972	49.143	39.231	1.00	0.00	H
ATOM	1162	C	ASN	74	36.191	51.077	43.243	1.00	0.00	C
ATOM	1163	O	ASN	74	36.173	52.315	43.322	1.00	0.00	O
ATOM	1164	N	MET	75	35.500	50.341	44.084	1.00	0.00	N
ATOM	1165	H	MET	75	35.598	49.341	43.979	1.00	0.00	H
ATOM	1166	CA	MET	75	34.901	50.906	45.341	1.00	0.00	C
ATOM	1167	HA	MET	75	34.709	51.958	45.129	1.00	0.00	H
ATOM	1168	CB	MET	75	33.575	50.195	45.653	1.00	0.00	C
ATOM	1169	HB2	MET	75	33.701	49.123	45.509	1.00	0.00	H
ATOM	1170	HB3	MET	75	33.247	50.344	46.683	1.00	0.00	H
ATOM	1171	CG	MET	75	32.462	50.801	44.802	1.00	0.00	C
ATOM	1172	HG2	MET	75	32.255	51.814	45.144	1.00	0.00	H
ATOM	1173	HG3	MET	75	32.760	50.741	43.755	1.00	0.00	H
ATOM	1174	SD	MET	75	30.868	49.887	44.803	1.00	0.00	S
ATOM	1175	CE	MET	75	31.413	48.504	43.722	1.00	0.00	C
ATOM	1176	HE1	MET	75	31.577	48.740	42.670	1.00	0.00	H

ATOM	1177	HE2	MET	75	32.234	47.983	44.215	1.00	0.00	H
ATOM	1178	HE3	MET	75	30.592	47.797	43.606	1.00	0.00	H
ATOM	1179	C	MET	75	35.898	50.835	46.535	1.00	0.00	C
ATOM	1180	O	MET	75	37.037	50.260	46.354	1.00	0.00	O
ATOM	1181	N	THR	76	35.540	51.299	47.750	1.00	0.00	N
ATOM	1182	H	THR	76	34.703	51.864	47.775	1.00	0.00	H
ATOM	1183	CA	THR	76	36.091	50.964	49.123	1.00	0.00	C
ATOM	1184	HA	THR	76	36.957	50.307	49.042	1.00	0.00	H
ATOM	1185	CB	THR	76	36.582	52.207	49.819	1.00	0.00	C
ATOM	1186	HB	THR	76	37.134	52.794	49.086	1.00	0.00	H
ATOM	1187	CG2	THR	76	35.475	53.154	50.164	1.00	0.00	C
ATOM	1188	HG21	THR	76	35.142	53.132	51.201	1.00	0.00	H
ATOM	1189	HG22	THR	76	35.812	54.187	50.079	1.00	0.00	H
ATOM	1190	HG23	THR	76	34.566	53.086	49.566	1.00	0.00	H
ATOM	1191	OG1	THR	76	37.454	51.980	50.889	1.00	0.00	O
ATOM	1192	HG1	THR	76	38.127	51.370	50.579	1.00	0.00	H
ATOM	1193	C	THR	76	35.105	50.100	49.964	1.00	0.00	C
ATOM	1194	O	THR	76	33.899	50.215	49.678	1.00	0.00	O
ATOM	1195	N	LEU	77	35.634	49.210	50.800	1.00	0.00	N
ATOM	1196	H	LEU	77	36.640	49.122	50.800	1.00	0.00	H
ATOM	1197	CA	LEU	77	34.903	48.138	51.450	1.00	0.00	C
ATOM	1198	HA	LEU	77	34.640	47.447	50.649	1.00	0.00	H
ATOM	1199	CB	LEU	77	35.952	47.437	52.373	1.00	0.00	C
ATOM	1200	HB2	LEU	77	36.827	47.150	51.790	1.00	0.00	H
ATOM	1201	HB3	LEU	77	36.232	48.286	52.997	1.00	0.00	H
ATOM	1202	CG	LEU	77	35.378	46.340	53.335	1.00	0.00	C
ATOM	1203	HG	LEU	77	34.538	46.752	53.894	1.00	0.00	H
ATOM	1204	CD1	LEU	77	34.815	45.188	52.510	1.00	0.00	C
ATOM	1205	HD11	LEU	77	33.887	45.410	51.982	1.00	0.00	H
ATOM	1206	HD12	LEU	77	35.602	44.860	51.832	1.00	0.00	H
ATOM	1207	HD13	LEU	77	34.532	44.379	53.184	1.00	0.00	H
ATOM	1208	CD2	LEU	77	36.575	45.731	54.034	1.00	0.00	C
ATOM	1209	HD21	LEU	77	37.048	46.422	54.731	1.00	0.00	H
ATOM	1210	HD22	LEU	77	36.136	44.950	54.654	1.00	0.00	H
ATOM	1211	HD23	LEU	77	37.393	45.350	53.422	1.00	0.00	H
ATOM	1212	C	LEU	77	33.585	48.704	52.137	1.00	0.00	C
ATOM	1213	O	LEU	77	32.572	48.031	52.008	1.00	0.00	O
ATOM	1214	N	THR	78	33.588	49.895	52.742	1.00	0.00	N
ATOM	1215	H	THR	78	34.470	50.374	52.852	1.00	0.00	H
ATOM	1216	CA	THR	78	32.401	50.485	53.331	1.00	0.00	C
ATOM	1217	HA	THR	78	31.978	49.719	53.982	1.00	0.00	H
ATOM	1218	CB	THR	78	32.762	51.696	54.136	1.00	0.00	C
ATOM	1219	HB	THR	78	31.900	52.294	54.431	1.00	0.00	H
ATOM	1220	CG2	THR	78	33.568	51.259	55.309	1.00	0.00	C
ATOM	1221	HG21	THR	78	33.275	50.347	55.828	1.00	0.00	H
ATOM	1222	HG22	THR	78	34.596	51.107	54.983	1.00	0.00	H
ATOM	1223	HG23	THR	78	33.417	52.018	56.077	1.00	0.00	H
ATOM	1224	OG1	THR	78	33.475	52.567	53.382	1.00	0.00	O
ATOM	1225	HG1	THR	78	32.977	53.387	53.412	1.00	0.00	H
ATOM	1226	C	THR	78	31.271	50.861	52.325	1.00	0.00	C
ATOM	1227	O	THR	78	30.092	50.979	52.658	1.00	0.00	O
ATOM	1228	N	GLU	79	31.584	51.046	50.999	1.00	0.00	N
ATOM	1229	H	GLU	79	32.554	50.813	50.841	1.00	0.00	H
ATOM	1230	CA	GLU	79	30.682	51.237	49.895	1.00	0.00	C
ATOM	1231	HA	GLU	79	29.748	51.628	50.300	1.00	0.00	H
ATOM	1232	CB	GLU	79	31.220	52.347	48.922	1.00	0.00	C
ATOM	1233	HB2	GLU	79	32.066	51.951	48.361	1.00	0.00	H
ATOM	1234	HB3	GLU	79	30.411	52.524	48.213	1.00	0.00	H
ATOM	1235	CG	GLU	79	31.601	53.620	49.772	1.00	0.00	C
ATOM	1236	HG2	GLU	79	32.233	53.517	50.654	1.00	0.00	H
ATOM	1237	HG3	GLU	79	32.057	54.299	49.051	1.00	0.00	H
ATOM	1238	CD	GLU	79	30.335	54.361	50.263	1.00	0.00	C
ATOM	1239	OE1	GLU	79	30.301	54.902	51.414	1.00	0.00	O

ATOM	1240	OE2	GLU	79	29.463	54.669	49.416	1.00	0.00	O
ATOM	1241	C	GLU	79	30.341	49.947	49.140	1.00	0.00	C
ATOM	1242	O	GLU	79	29.162	49.870	48.778	1.00	0.00	O
ATOM	1243	N	LEU	80	31.179	48.970	49.106	1.00	0.00	N
ATOM	1244	H	LEU	80	32.113	49.159	49.438	1.00	0.00	H
ATOM	1245	CA	LEU	80	30.742	47.599	48.649	1.00	0.00	C
ATOM	1246	HA	LEU	80	30.365	47.713	47.633	1.00	0.00	H
ATOM	1247	CB	LEU	80	31.903	46.591	48.475	1.00	0.00	C
ATOM	1248	HB2	LEU	80	32.721	47.113	47.978	1.00	0.00	H
ATOM	1249	HB3	LEU	80	32.220	46.391	49.499	1.00	0.00	H
ATOM	1250	CG	LEU	80	31.435	45.211	47.839	1.00	0.00	C
ATOM	1251	HG	LEU	80	30.554	44.819	48.348	1.00	0.00	H
ATOM	1252	CD1	LEU	80	31.176	45.368	46.324	1.00	0.00	C
ATOM	1253	HD11	LEU	80	31.987	45.741	45.698	1.00	0.00	H
ATOM	1254	HD12	LEU	80	30.851	44.413	45.913	1.00	0.00	H
ATOM	1255	HD13	LEU	80	30.334	46.053	46.222	1.00	0.00	H
ATOM	1256	CD2	LEU	80	32.631	44.246	47.953	1.00	0.00	C
ATOM	1257	HD21	LEU	80	32.363	43.256	47.584	1.00	0.00	H
ATOM	1258	HD22	LEU	80	33.502	44.706	47.487	1.00	0.00	H
ATOM	1259	HD23	LEU	80	32.953	44.208	48.994	1.00	0.00	H
ATOM	1260	C	LEU	80	29.673	46.995	49.607	1.00	0.00	C
ATOM	1261	O	LEU	80	28.647	46.716	49.062	1.00	0.00	O
ATOM	1262	N	LYS	81	29.878	47.032	50.970	1.00	0.00	N
ATOM	1263	H	LYS	81	30.754	47.330	51.374	1.00	0.00	H
ATOM	1264	CA	LYS	81	28.852	46.650	51.924	1.00	0.00	C
ATOM	1265	HA	LYS	81	28.661	45.605	51.679	1.00	0.00	H
ATOM	1266	CB	LYS	81	29.367	46.786	53.312	1.00	0.00	C
ATOM	1267	HB2	LYS	81	29.688	47.825	53.377	1.00	0.00	H
ATOM	1268	HB3	LYS	81	28.538	46.648	54.005	1.00	0.00	H
ATOM	1269	CG	LYS	81	30.484	45.814	53.676	1.00	0.00	C
ATOM	1270	HG2	LYS	81	30.029	44.825	53.740	1.00	0.00	H
ATOM	1271	HG3	LYS	81	31.235	45.868	52.887	1.00	0.00	H
ATOM	1272	CD	LYS	81	31.099	46.117	55.034	1.00	0.00	C
ATOM	1273	HD2	LYS	81	31.778	46.967	54.973	1.00	0.00	H
ATOM	1274	HD3	LYS	81	30.360	46.484	55.747	1.00	0.00	H
ATOM	1275	CE	LYS	81	31.966	44.965	55.553	1.00	0.00	C
ATOM	1276	HE2	LYS	81	31.463	44.014	55.377	1.00	0.00	H
ATOM	1277	HE3	LYS	81	32.874	44.897	54.952	1.00	0.00	H
ATOM	1278	NZ	LYS	81	32.345	45.132	56.999	1.00	0.00	N
ATOM	1279	HZ1	LYS	81	31.558	45.220	57.627	1.00	0.00	H
ATOM	1280	HZ2	LYS	81	32.863	44.324	57.310	1.00	0.00	H
ATOM	1281	HZ3	LYS	81	33.020	45.864	57.167	1.00	0.00	H
ATOM	1282	C	LYS	81	27.495	47.462	51.797	1.00	0.00	C
ATOM	1283	O	LYS	81	26.407	47.091	52.286	1.00	0.00	O
ATOM	1284	N	LYS	82	27.626	48.571	51.067	1.00	0.00	N
ATOM	1285	H	LYS	82	28.525	48.800	50.666	1.00	0.00	H
ATOM	1286	CA	LYS	82	26.513	49.560	50.911	1.00	0.00	C
ATOM	1287	HA	LYS	82	25.920	49.410	51.813	1.00	0.00	H
ATOM	1288	CB	LYS	82	27.138	51.014	50.984	1.00	0.00	C
ATOM	1289	HB2	LYS	82	28.046	51.144	51.573	1.00	0.00	H
ATOM	1290	HB3	LYS	82	27.484	51.168	49.962	1.00	0.00	H
ATOM	1291	CG	LYS	82	26.050	52.007	51.324	1.00	0.00	C
ATOM	1292	HG2	LYS	82	25.395	52.039	50.453	1.00	0.00	H
ATOM	1293	HG3	LYS	82	25.611	51.665	52.261	1.00	0.00	H
ATOM	1294	CD	LYS	82	26.758	53.340	51.386	1.00	0.00	C
ATOM	1295	HD2	LYS	82	27.211	53.391	50.395	1.00	0.00	H
ATOM	1296	HD3	LYS	82	25.988	54.110	51.439	1.00	0.00	H
ATOM	1297	CE	LYS	82	27.469	53.384	52.820	1.00	0.00	C
ATOM	1298	HE2	LYS	82	26.741	52.972	53.518	1.00	0.00	H
ATOM	1299	HE3	LYS	82	28.202	52.578	52.818	1.00	0.00	H
ATOM	1300	NZ	LYS	82	28.055	54.712	53.067	1.00	0.00	N
ATOM	1301	HZ1	LYS	82	28.225	54.763	54.062	1.00	0.00	H
ATOM	1302	HZ2	LYS	82	28.870	54.915	52.506	1.00	0.00	H
ATOM	1303	HZ3	LYS	82	27.454	55.504	52.896	1.00	0.00	H

ATOM	1304	C	LYS	82	25.735	49.232	49.630	1.00	0.00	C
ATOM	1305	O	LYS	82	24.539	49.449	49.619	1.00	0.00	O
ATOM	1306	N	VAL	83	26.433	48.942	48.523	1.00	0.00	N
ATOM	1307	H	VAL	83	27.436	48.851	48.592	1.00	0.00	H
ATOM	1308	CA	VAL	83	25.802	48.584	47.226	1.00	0.00	C
ATOM	1309	HA	VAL	83	24.919	49.218	47.149	1.00	0.00	H
ATOM	1310	CB	VAL	83	26.623	48.919	45.931	1.00	0.00	C
ATOM	1311	HB	VAL	83	25.912	48.682	45.139	1.00	0.00	H
ATOM	1312	CG1	VAL	83	26.987	50.425	45.778	1.00	0.00	C
ATOM	1313	HG11	VAL	83	27.318	50.572	44.750	1.00	0.00	H
ATOM	1314	HG12	VAL	83	26.004	50.875	45.920	1.00	0.00	H
ATOM	1315	HG13	VAL	83	27.656	50.734	46.580	1.00	0.00	H
ATOM	1316	CG2	VAL	83	27.817	48.000	45.743	1.00	0.00	C
ATOM	1317	HG21	VAL	83	28.179	47.740	46.739	1.00	0.00	H
ATOM	1318	HG22	VAL	83	27.617	47.030	45.291	1.00	0.00	H
ATOM	1319	HG23	VAL	83	28.691	48.462	45.284	1.00	0.00	H
ATOM	1320	C	VAL	83	25.342	47.174	47.326	1.00	0.00	C
ATOM	1321	O	VAL	83	24.291	46.931	46.789	1.00	0.00	O
ATOM	1322	N	ILE	84	26.028	46.272	48.044	1.00	0.00	N
ATOM	1323	H	ILE	84	26.885	46.598	48.467	1.00	0.00	H
ATOM	1324	CA	ILE	84	25.495	44.871	48.437	1.00	0.00	C
ATOM	1325	HA	ILE	84	25.225	44.468	47.462	1.00	0.00	H
ATOM	1326	CB	ILE	84	26.498	43.851	49.086	1.00	0.00	C
ATOM	1327	HB	ILE	84	26.954	44.266	49.985	1.00	0.00	H
ATOM	1328	CG2	ILE	84	25.692	42.584	49.546	1.00	0.00	C
ATOM	1329	HG21	ILE	84	24.879	42.833	50.229	1.00	0.00	H
ATOM	1330	HG22	ILE	84	25.193	42.193	48.660	1.00	0.00	H
ATOM	1331	HG23	ILE	84	26.262	41.742	49.942	1.00	0.00	H
ATOM	1332	CG1	ILE	84	27.583	43.539	48.061	1.00	0.00	C
ATOM	1333	HG12	ILE	84	27.037	43.187	47.186	1.00	0.00	H
ATOM	1334	HG13	ILE	84	28.186	44.416	47.825	1.00	0.00	H
ATOM	1335	CD1	ILE	84	28.595	42.500	48.517	1.00	0.00	C
ATOM	1336	HD11	ILE	84	29.058	42.673	49.489	1.00	0.00	H
ATOM	1337	HD12	ILE	84	28.069	41.553	48.633	1.00	0.00	H
ATOM	1338	HD13	ILE	84	29.303	42.346	47.702	1.00	0.00	H
ATOM	1339	C	ILE	84	24.131	45.111	49.115	1.00	0.00	C
ATOM	1340	O	ILE	84	23.097	44.615	48.643	1.00	0.00	O
ATOM	1341	N	ALA	85	24.107	45.723	50.333	1.00	0.00	N
ATOM	1342	H	ALA	85	25.006	45.998	50.702	1.00	0.00	H
ATOM	1343	CA	ALA	85	22.934	45.720	51.077	1.00	0.00	C
ATOM	1344	HA	ALA	85	22.610	44.681	51.032	1.00	0.00	H
ATOM	1345	CB	ALA	85	23.267	46.129	52.551	1.00	0.00	C
ATOM	1346	HB1	ALA	85	22.368	46.010	53.155	1.00	0.00	H
ATOM	1347	HB2	ALA	85	24.051	45.513	52.991	1.00	0.00	H
ATOM	1348	HB3	ALA	85	23.469	47.201	52.577	1.00	0.00	H
ATOM	1349	C	ALA	85	21.785	46.567	50.602	1.00	0.00	C
ATOM	1350	O	ALA	85	20.655	46.347	51.086	1.00	0.00	O
ATOM	1351	N	ASP	86	22.063	47.337	49.530	1.00	0.00	N
ATOM	1352	H	ASP	86	23.009	47.648	49.362	1.00	0.00	H
ATOM	1353	CA	ASP	86	20.948	47.848	48.709	1.00	0.00	C
ATOM	1354	HA	ASP	86	20.319	48.396	49.411	1.00	0.00	H
ATOM	1355	CB	ASP	86	21.549	48.790	47.670	1.00	0.00	C
ATOM	1356	HB2	ASP	86	22.430	49.274	48.092	1.00	0.00	H
ATOM	1357	HB3	ASP	86	21.764	48.218	46.767	1.00	0.00	H
ATOM	1358	CG	ASP	86	20.519	49.905	47.307	1.00	0.00	C
ATOM	1359	OD1	ASP	86	20.665	50.418	46.225	1.00	0.00	O
ATOM	1360	OD2	ASP	86	19.639	50.327	48.085	1.00	0.00	O
ATOM	1361	C	ASP	86	20.036	46.798	48.116	1.00	0.00	C
ATOM	1362	O	ASP	86	18.865	47.056	47.862	1.00	0.00	O
ATOM	1363	N	ILE	87	20.539	45.604	47.848	1.00	0.00	N
ATOM	1364	H	ILE	87	21.460	45.463	48.240	1.00	0.00	H
ATOM	1365	CA	ILE	87	19.894	44.537	47.027	1.00	0.00	C
ATOM	1366	HA	ILE	87	18.953	44.951	46.667	1.00	0.00	H

ATOM	1367	CB	ILE	87	20.850	44.050	45.845	1.00	0.00	C
ATOM	1368	HB	ILE	87	21.799	43.868	46.352	1.00	0.00	H
ATOM	1369	CG2	ILE	87	20.504	42.674	45.214	1.00	0.00	C
ATOM	1370	HG21	ILE	87	21.306	42.217	44.635	1.00	0.00	H
ATOM	1371	HG22	ILE	87	20.249	41.923	45.962	1.00	0.00	H
ATOM	1372	HG23	ILE	87	19.692	42.885	44.517	1.00	0.00	H
ATOM	1373	CG1	ILE	87	21.061	45.192	44.807	1.00	0.00	C
ATOM	1374	HG12	ILE	87	20.047	45.408	44.471	1.00	0.00	H
ATOM	1375	HG13	ILE	87	21.451	46.106	45.256	1.00	0.00	H
ATOM	1376	CD1	ILE	87	21.833	44.844	43.561	1.00	0.00	C
ATOM	1377	HD11	ILE	87	22.774	44.427	43.920	1.00	0.00	H
ATOM	1378	HD12	ILE	87	21.357	44.057	42.976	1.00	0.00	H
ATOM	1379	HD13	ILE	87	22.050	45.735	42.972	1.00	0.00	H
ATOM	1380	C	ILE	87	19.429	43.323	47.843	1.00	0.00	C
ATOM	1381	O	ILE	87	18.249	42.992	47.807	1.00	0.00	O
ATOM	1382	N	TYR	88	20.295	42.751	48.620	1.00	0.00	N
ATOM	1383	H	TYR	88	21.257	43.041	48.728	1.00	0.00	H
ATOM	1384	CA	TYR	88	19.946	41.742	49.598	1.00	0.00	C
ATOM	1385	HA	TYR	88	18.961	41.282	49.524	1.00	0.00	H
ATOM	1386	CB	TYR	88	21.015	40.701	49.758	1.00	0.00	C
ATOM	1387	HB2	TYR	88	22.036	41.083	49.713	1.00	0.00	H
ATOM	1388	HB3	TYR	88	21.009	40.103	50.669	1.00	0.00	H
ATOM	1389	CG	TYR	88	20.830	39.676	48.673	1.00	0.00	C
ATOM	1390	CD1	TYR	88	19.931	38.625	48.847	1.00	0.00	C
ATOM	1391	HD1	TYR	88	19.362	38.583	49.763	1.00	0.00	H
ATOM	1392	CE1	TYR	88	19.860	37.596	47.872	1.00	0.00	C
ATOM	1393	HE1	TYR	88	19.064	36.878	48.008	1.00	0.00	H
ATOM	1394	CZ	TYR	88	20.705	37.692	46.718	1.00	0.00	C
ATOM	1395	OH	TYR	88	20.545	36.805	45.699	1.00	0.00	O
ATOM	1396	HH	TYR	88	19.891	36.143	45.937	1.00	0.00	H
ATOM	1397	CE2	TYR	88	21.655	38.724	46.566	1.00	0.00	C
ATOM	1398	HE2	TYR	88	22.285	38.776	45.690	1.00	0.00	H
ATOM	1399	CD2	TYR	88	21.646	39.798	47.472	1.00	0.00	C
ATOM	1400	HD2	TYR	88	22.292	40.648	47.312	1.00	0.00	H
ATOM	1401	C	TYR	88	19.925	42.394	51.016	1.00	0.00	C
ATOM	1402	O	TYR	88	20.635	43.337	51.315	1.00	0.00	O
ATOM	1403	N	PRO	89	19.043	41.999	51.939	1.00	0.00	N
ATOM	1404	CD	PRO	89	17.752	41.371	51.665	1.00	0.00	C
ATOM	1405	HD2	PRO	89	18.000	40.310	51.625	1.00	0.00	H
ATOM	1406	HD3	PRO	89	17.277	41.711	50.745	1.00	0.00	H
ATOM	1407	CG	PRO	89	16.932	41.790	52.864	1.00	0.00	C
ATOM	1408	HG2	PRO	89	16.112	41.105	53.078	1.00	0.00	H
ATOM	1409	HG3	PRO	89	16.571	42.802	52.683	1.00	0.00	H
ATOM	1410	CB	PRO	89	17.920	41.765	54.028	1.00	0.00	C
ATOM	1411	HB2	PRO	89	18.103	40.727	54.302	1.00	0.00	H
ATOM	1412	HB3	PRO	89	17.619	42.348	54.898	1.00	0.00	H
ATOM	1413	CA	PRO	89	19.084	42.402	53.376	1.00	0.00	C
ATOM	1414	HA	PRO	89	19.014	43.487	53.435	1.00	0.00	H
ATOM	1415	C	PRO	89	20.315	41.855	54.139	1.00	0.00	C
ATOM	1416	O	PRO	89	20.658	40.709	53.895	1.00	0.00	O
ATOM	1417	N	GLY	90	20.860	42.653	55.114	1.00	0.00	N
ATOM	1418	H	GLY	90	20.402	43.548	55.198	1.00	0.00	H
ATOM	1419	CA	GLY	90	22.140	42.544	55.824	1.00	0.00	C
ATOM	1420	HA2	GLY	90	22.945	42.976	55.230	1.00	0.00	H
ATOM	1421	HA3	GLY	90	22.136	43.241	56.662	1.00	0.00	H
ATOM	1422	C	GLY	90	22.330	41.367	56.647	1.00	0.00	C
ATOM	1423	O	GLY	90	23.485	40.896	56.785	1.00	0.00	O
ATOM	1424	N	GLN	91	21.252	40.686	56.945	1.00	0.00	N
ATOM	1425	H	GLN	91	20.291	40.991	56.907	1.00	0.00	H
ATOM	1426	CA	GLN	91	21.370	39.372	57.596	1.00	0.00	C
ATOM	1427	HA	GLN	91	22.031	39.488	58.456	1.00	0.00	H
ATOM	1428	CB	GLN	91	19.941	38.959	58.054	1.00	0.00	C
ATOM	1429	HB2	GLN	91	19.279	38.855	57.195	1.00	0.00	H
ATOM	1430	HB3	GLN	91	19.991	38.041	58.639	1.00	0.00	H

ATOM	1431	CG	GLN	91	19.254	39.998	58.939	1.00	0.00	C
ATOM	1432	HG2	GLN	91	19.860	40.044	59.843	1.00	0.00	H
ATOM	1433	HG3	GLN	91	19.219	41.035	58.604	1.00	0.00	H
ATOM	1434	CD	GLN	91	17.811	39.585	59.366	1.00	0.00	C
ATOM	1435	OE1	GLN	91	16.984	39.354	58.575	1.00	0.00	O
ATOM	1436	NE2	GLN	91	17.576	39.370	60.636	1.00	0.00	N
ATOM	1437	HE21	GLN	91	18.258	39.475	61.373	1.00	0.00	H
ATOM	1438	HE22	GLN	91	16.591	39.309	60.849	1.00	0.00	H
ATOM	1439	C	GLN	91	21.942	38.264	56.698	1.00	0.00	C
ATOM	1440	O	GLN	91	22.503	37.261	57.209	1.00	0.00	O
ATOM	1441	N	THR	92	21.914	38.430	55.331	1.00	0.00	N
ATOM	1442	H	THR	92	21.562	39.324	55.020	1.00	0.00	H
ATOM	1443	CA	THR	92	22.233	37.468	54.280	1.00	0.00	C
ATOM	1444	HA	THR	92	21.612	36.575	54.359	1.00	0.00	H
ATOM	1445	CB	THR	92	22.038	38.131	52.953	1.00	0.00	C
ATOM	1446	HB	THR	92	22.662	39.023	52.980	1.00	0.00	H
ATOM	1447	CG2	THR	92	22.378	37.184	51.838	1.00	0.00	C
ATOM	1448	HG21	THR	92	21.723	36.313	51.794	1.00	0.00	H
ATOM	1449	HG22	THR	92	22.161	37.735	50.923	1.00	0.00	H
ATOM	1450	HG23	THR	92	23.418	36.868	51.905	1.00	0.00	H
ATOM	1451	OG1	THR	92	20.655	38.447	52.708	1.00	0.00	O
ATOM	1452	HG1	THR	92	20.550	39.351	53.012	1.00	0.00	H
ATOM	1453	C	THR	92	23.702	37.016	54.464	1.00	0.00	C
ATOM	1454	O	THR	92	24.627	37.812	54.768	1.00	0.00	O
ATOM	1455	N	GLN	93	23.908	35.696	54.426	1.00	0.00	N
ATOM	1456	H	GLN	93	23.112	35.075	54.421	1.00	0.00	H
ATOM	1457	CA	GLN	93	25.155	34.997	54.250	1.00	0.00	C
ATOM	1458	HA	GLN	93	25.912	35.754	54.455	1.00	0.00	H
ATOM	1459	CB	GLN	93	25.312	33.733	55.000	1.00	0.00	C
ATOM	1460	HB2	GLN	93	24.975	33.820	56.033	1.00	0.00	H
ATOM	1461	HB3	GLN	93	24.506	33.062	54.703	1.00	0.00	H
ATOM	1462	CG	GLN	93	26.620	32.921	54.765	1.00	0.00	C
ATOM	1463	HG2	GLN	93	26.855	32.693	53.726	1.00	0.00	H
ATOM	1464	HG3	GLN	93	27.432	33.511	55.189	1.00	0.00	H
ATOM	1465	CD	GLN	93	26.559	31.591	55.577	1.00	0.00	C
ATOM	1466	OE1	GLN	93	25.878	31.508	56.566	1.00	0.00	O
ATOM	1467	NE2	GLN	93	27.280	30.598	55.258	1.00	0.00	N
ATOM	1468	HE21	GLN	93	27.778	30.632	54.380	1.00	0.00	H
ATOM	1469	HE22	GLN	93	27.161	29.811	55.879	1.00	0.00	H
ATOM	1470	C	GLN	93	25.371	34.639	52.752	1.00	0.00	C
ATOM	1471	O	GLN	93	24.449	34.167	52.107	1.00	0.00	O
ATOM	1472	N	PHE	94	26.543	34.907	52.233	1.00	0.00	N
ATOM	1473	H	PHE	94	27.304	35.219	52.820	1.00	0.00	H
ATOM	1474	CA	PHE	94	26.861	34.924	50.811	1.00	0.00	C
ATOM										
1475	HA	PHE	94	25.909	34.757	50.306	1.00	0.00	H	
ATOM	1476	CB	PHE	94	27.390	36.327	50.485	1.00	0.00	C
ATOM	1477	HB2	PHE	94	28.266	36.556	51.092	1.00	0.00	H
ATOM	1478	HB3	PHE	94	27.632	36.412	49.426	1.00	0.00	H
ATOM	1479	CG	PHE	94	26.400	37.395	50.762	1.00	0.00	C
ATOM	1480	CD1	PHE	94	25.458	37.675	49.803	1.00	0.00	C
ATOM	1481	HD1	PHE	94	25.467	37.058	48.917	1.00	0.00	H
ATOM	1482	CE1	PHE	94	24.505	38.762	49.955	1.00	0.00	C
ATOM	1483	HE1	PHE	94	23.779	38.899	49.167	1.00	0.00	H
ATOM	1484	CZ	PHE	94	24.579	39.458	51.196	1.00	0.00	C
ATOM	1485	HZ	PHE	94	23.799	40.171	51.418	1.00	0.00	H
ATOM	1486	CE2	PHE	94	25.530	39.135	52.197	1.00	0.00	C
ATOM	1487	HE2	PHE	94	25.605	39.680	53.127	1.00	0.00	H
ATOM	1488	CD2	PHE	94	26.400	38.123	51.978	1.00	0.00	C
ATOM	1489	HD2	PHE	94	27.125	37.808	52.714	1.00	0.00	H
ATOM	1490	C	PHE	94	27.880	33.881	50.343	1.00	0.00	C
ATOM	1491	O	PHE	94	28.585	33.253	51.216	1.00	0.00	O
ATOM	1492	N	TYR	95	28.000	33.723	49.041	1.00	0.00	N
ATOM	1493	H	TYR	95	27.318	34.238	48.502	1.00	0.00	H

ATOM	1494	CA	TYR	95	29.028	32.924	48.394	1.00	0.00	C
ATOM	1495	HA	TYR	95	29.610	32.443	49.180	1.00	0.00	H
ATOM	1496	CB	TYR	95	28.325	31.732	47.707	1.00	0.00	C
ATOM	1497	HB2	TYR	95	27.578	31.285	48.363	1.00	0.00	H
ATOM	1498	HB3	TYR	95	27.684	32.110	46.910	1.00	0.00	H
ATOM	1499	CG	TYR	95	29.273	30.670	47.183	1.00	0.00	C
ATOM	1500	CD1	TYR	95	28.990	30.305	45.830	1.00	0.00	C
ATOM	1501	HD1	TYR	95	28.346	30.893	45.194	1.00	0.00	H
ATOM	1502	CE1	TYR	95	29.649	29.272	45.205	1.00	0.00	C
ATOM	1503	HE1	TYR	95	29.480	28.904	44.203	1.00	0.00	H
ATOM	1504	CZ	TYR	95	30.562	28.610	46.007	1.00	0.00	C
ATOM	1505	OH	TYR	95	31.227	27.538	45.562	1.00	0.00	O
ATOM	1506	HH	TYR	95	31.072	27.340	44.636	1.00	0.00	H
ATOM	1507	CE2	TYR	95	30.771	28.984	47.360	1.00	0.00	C
ATOM	1508	HE2	TYR	95	31.485	28.525	48.028	1.00	0.00	H
ATOM	1509	CD2	TYR	95	30.189	30.019	47.994	1.00	0.00	C
ATOM	1510	HD2	TYR	95	30.488	30.305	48.993	1.00	0.00	H
ATOM	1511	C	TYR	95	30.050	33.729	47.547	1.00	0.00	C
ATOM	1512	O	TYR	95	29.745	34.805	47.034	1.00	0.00	O
ATOM	1513	N	VAL	96	31.277	33.304	47.439	1.00	0.00	N
ATOM	1514	H	VAL	96	31.595	32.548	48.029	1.00	0.00	H
ATOM	1515	CA	VAL	96	32.324	34.009	46.698	1.00	0.00	C
ATOM	1516	HA	VAL	96	31.846	34.679	45.983	1.00	0.00	H
ATOM	1517	CB	VAL	96	33.362	34.820	47.530	1.00	0.00	C
ATOM	1518	HB	VAL	96	34.097	34.198	48.040	1.00	0.00	H
ATOM	1519	CG1	VAL	96	34.118	35.945	46.661	1.00	0.00	C
ATOM	1520	HG11	VAL	96	34.794	36.524	47.290	1.00	0.00	H
ATOM	1521	HG12	VAL	96	34.574	35.456	45.799	1.00	0.00	H
ATOM	1522	HG13	VAL	96	33.374	36.562	46.158	1.00	0.00	H
ATOM	1523	CG2	VAL	96	32.806	35.711	48.735	1.00	0.00	C
ATOM	1524	HG21	VAL	96	33.640	36.278	49.149	1.00	0.00	H
ATOM	1525	HG22	VAL	96	31.948	36.307	48.422	1.00	0.00	H
ATOM	1526	HG23	VAL	96	32.495	35.032	49.530	1.00	0.00	H
ATOM	1527	C	VAL	96	33.114	33.026	45.785	1.00	0.00	C
ATOM	1528	O	VAL	96	33.592	31.965	46.300	1.00	0.00	O
ATOM	1529	N	ILE	97	33.240	33.326	44.510	1.00	0.00	N
ATOM	1530	H	ILE	97	33.049	34.281	44.240	1.00	0.00	H
ATOM	1531	CA	ILE	97	34.028	32.507	43.561	1.00	0.00	C
ATOM	1532	HA	ILE	97	34.500	31.754	44.192	1.00	0.00	H
ATOM	1533	CB	ILE	97	33.058	31.906	42.480	1.00	0.00	C
ATOM	1534	HB	ILE	97	32.559	32.749	42.001	1.00	0.00	H
ATOM	1535	CG2	ILE	97	33.744	31.194	41.335	1.00	0.00	C
ATOM	1536	HG21	ILE	97	33.050	30.869	40.560	1.00	0.00	H
ATOM	1537	HG22	ILE	97	34.440	31.892	40.870	1.00	0.00	H
ATOM	1538	HG23	ILE	97	34.358	30.358	41.668	1.00	0.00	H
ATOM	1539	CG1	ILE	97	32.026	30.950	43.193	1.00	0.00	C
ATOM	1540	HG12	ILE	97	32.372	29.927	43.343	1.00	0.00	H
ATOM	1541	HG13	ILE	97	31.815	31.255	44.218	1.00	0.00	H
ATOM	1542	CD1	ILE	97	30.803	30.872	42.334	1.00	0.00	C
ATOM	1543	HD11	ILE	97	30.290	29.956	42.628	1.00	0.00	H
ATOM	1544	HD12	ILE	97	30.157	31.656	42.729	1.00	0.00	H
ATOM	1545	HD13	ILE	97	30.960	30.938	41.257	1.00	0.00	H
ATOM	1546	C	ILE	97	35.179	33.313	42.863	1.00	0.00	C
ATOM	1547	O	ILE	97	34.974	34.457	42.430	1.00	0.00	O
ATOM	1548	N	GLU	98	36.303	32.664	42.886	1.00	0.00	N
ATOM	1549	H	GLU	98	36.302	31.751	43.316	1.00	0.00	H
ATOM	1550	CA	GLU	98	37.594	33.068	42.330	1.00	0.00	C
ATOM	1551	HA	GLU	98	37.433	34.033	41.849	1.00	0.00	H
ATOM	1552	CB	GLU	98	38.582	33.353	43.525	1.00	0.00	C
ATOM	1553	HB2	GLU	98	38.868	32.381	43.927	1.00	0.00	H
ATOM	1554	HB3	GLU	98	39.471	33.805	43.085	1.00	0.00	H
ATOM	1555	CG	GLU	98	38.152	34.142	44.770	1.00	0.00	C
ATOM	1556	HG2	GLU	98	37.822	35.113	44.399	1.00	0.00	H
ATOM	1557	HG3	GLU	98	37.259	33.661	45.167	1.00	0.00	H

ATOM	1558	CD	GLU	98	39.235	34.167	45.804	1.00	0.00	C
ATOM	1559	OE1	GLU	98	38.903	34.172	47.034	1.00	0.00	O
ATOM	1560	OE2	GLU	98	40.395	34.438	45.411	1.00	0.00	O
ATOM	1561	C	GLU	98	38.236	32.097	41.316	1.00	0.00	C
ATOM	1562	O	GLU	98	38.075	30.912	41.309	1.00	0.00	O
ATOM	1563	N	PHE	99	38.875	32.613	40.254	1.00	0.00	N
ATOM	1564	H	PHE	99	39.228	33.557	40.186	1.00	0.00	H
ATOM	1565	CA	PHE	99	39.288	31.819	39.053	1.00	0.00	C
ATOM	1566	HA	PHE	99	39.657	30.889	39.485	1.00	0.00	H
ATOM	1567	CB	PHE	99	38.099	31.457	38.065	1.00	0.00	C
ATOM	1568	HB2	PHE	99	38.347	30.754	37.270	1.00	0.00	H
ATOM	1569	HB3	PHE	99	37.319	31.165	38.769	1.00	0.00	H
ATOM	1570	CG	PHE	99	37.466	32.630	37.375	1.00	0.00	C
ATOM	1571	CD1	PHE	99	38.209	33.587	36.654	1.00	0.00	C
ATOM	1572	HD1	PHE	99	39.280	33.580	36.513	1.00	0.00	H
ATOM	1573	CE1	PHE	99	37.566	34.620	35.978	1.00	0.00	C
ATOM	1574	HE1	PHE	99	38.084	35.475	35.570	1.00	0.00	H
ATOM	1575	CZ	PHE	99	36.148	34.717	35.988	1.00	0.00	C
ATOM	1576	HZ	PHE	99	35.665	35.623	35.655	1.00	0.00	H
ATOM	1577	CE2	PHE	99	35.415	33.719	36.592	1.00	0.00	C
ATOM	1578	HE2	PHE	99	34.336	33.728	36.565	1.00	0.00	H
ATOM	1579	CD2	PHE	99	36.060	32.695	37.264	1.00	0.00	C
ATOM	1580	HD2	PHE	99	35.463	31.930	37.736	1.00	0.00	H
ATOM	1581	C	PHE	99	40.508	32.465	38.378	1.00	0.00	C
ATOM	1582	O	PHE	99	40.972	33.543	38.764	1.00	0.00	O
ATOM	1583	N	LYS	100	41.036	31.753	37.386	1.00	0.00	N
ATOM	1584	H	LYS	100	40.845	30.761	37.375	1.00	0.00	H
ATOM	1585	CA	LYS	100	41.871	32.392	36.310	1.00	0.00	C
ATOM	1586	HA	LYS	100	41.417	33.376	36.192	1.00	0.00	H
ATOM	1587	CB	LYS	100	43.286	32.461	36.767	1.00	0.00	C
ATOM	1588	HB2	LYS	100	43.876	32.891	35.958	1.00	0.00	H
ATOM	1589	HB3	LYS	100	43.424	33.212	37.545	1.00	0.00	H
ATOM	1590	CG	LYS	100	43.995	31.231	37.275	1.00	0.00	C
ATOM	1591	HG2	LYS	100	43.394	30.835	38.093	1.00	0.00	H
ATOM	1592	HG3	LYS	100	44.195	30.445	36.548	1.00	0.00	H
ATOM	1593	CD	LYS	100	45.378	31.684	37.797	1.00	0.00	C
ATOM	1594	HD2	LYS	100	45.921	32.023	36.915	1.00	0.00	H
ATOM	1595	HD3	LYS	100	45.343	32.497	38.521	1.00	0.00	H
ATOM	1596	CE	LYS	100	46.204	30.553	38.371	1.00	0.00	C
ATOM	1597	HE2	LYS	100	45.641	30.120	39.197	1.00	0.00	H
ATOM	1598	HE3	LYS	100	46.304	29.794	37.594	1.00	0.00	H
ATOM	1599	NZ	LYS	100	47.512	30.987	38.961	1.00	0.00	N
ATOM	1600	HZ1	LYS	100						
47.984	30.255	39.472	1.00	0.00	H					
ATOM	1601	HZ2	LYS	100	47.972	31.357	38.142	1.00	0.00	H
ATOM	1602	HZ3	LYS	100	47.371	31.687	39.677	1.00	0.00	H
ATOM	1603	C	LYS	100	41.810	31.727	34.936	1.00	0.00	C
ATOM	1604	O	LYS	100	41.555	30.564	34.915	1.00	0.00	O
ATOM	1605	N	CYX	101	42.310	32.376	33.910	1.00	0.00	N
ATOM	1606	H	CYX	101	42.727	33.280	34.079	1.00	0.00	H
ATOM	1607	CA	CYX	101	42.567	31.752	32.636	1.00	0.00	C
ATOM	1608	HA	CYX	101	41.764	31.024	32.517	1.00	0.00	H
ATOM	1609	CB	CYX	101	42.464	32.772	31.496	1.00	0.00	C
ATOM	1610	HB2	CYX	101	41.419	33.062	31.378	1.00	0.00	H
ATOM	1611	HB3	CYX	101	43.205	33.565	31.592	1.00	0.00	H
ATOM	1612	SG	CYX	101	42.892	32.012	29.907	1.00	0.00	S
ATOM	1613	C	CYX	101	43.958	31.096	32.615	1.00	0.00	C
ATOM	1614	O	CYX	101	44.878	31.573	33.332	1.00	0.00	O
ATOM	1615	N	LEU	102	44.125	29.925	31.987	1.00	0.00	N
ATOM	1616	H	LEU	102	43.273	29.558	31.587	1.00	0.00	H
ATOM	1617	CA	LEU	102	45.491	29.261	31.980	1.00	0.00	C
ATOM	1618	HA	LEU	102	45.905	29.465	32.967	1.00	0.00	H
ATOM	1619	CB	LEU	102	45.324	27.731	31.793	1.00	0.00	C
ATOM	1620	HB2	LEU	102	44.861	27.484	30.838	1.00	0.00	H

ATOM	1621	HB3	LEU	102	46.378	27.462	31.866	1.00	0.00	H
ATOM	1622	CG	LEU	102	44.429	26.961	32.803	1.00	0.00	C
ATOM	1623	HG	LEU	102	43.392	27.248	32.627	1.00	0.00	H
ATOM	1624	CD1	LEU	102	44.669	25.478	32.339	1.00	0.00	C
ATOM	1625	HD11	LEU	102	44.279	24.627	32.895	1.00	0.00	H
ATOM	1626	HD12	LEU	102	44.292	25.359	31.323	1.00	0.00	H
ATOM	1627	HD13	LEU	102	45.725	25.245	32.198	1.00	0.00	H
ATOM	1628	CD2	LEU	102	44.765	27.059	34.272	1.00	0.00	C
ATOM	1629	HD21	LEU	102	44.551	28.071	34.613	1.00	0.00	H
ATOM	1630	HD22	LEU	102	44.026	26.390	34.715	1.00	0.00	H
ATOM	1631	HD23	LEU	102	45.794	26.838	34.559	1.00	0.00	H
ATOM	1632	C	LEU	102	46.409	29.936	31.026	1.00	0.00	C
ATOM	1633	O	LEU	102	46.184	29.916	29.800	1.00	0.00	O
ATOM	1634	OXT	LEU	102	47.466	30.486	31.437	1.00	0.00	O
HETATM	314	N	LYN	20	38.245	40.994	35.869	1.00	0.00	N
HETATM	315	H	LYN	20	38.960	40.666	35.236	1.00	0.00	H
HETATM	316	CA	LYN	20	37.380	39.950	36.483	1.00	0.00	C
HETATM	317	HA	LYN	20	36.972	40.360	37.407	1.00	0.00	H
HETATM	318	CB	LYN	20	36.091	39.742	35.588	1.00	0.00	C
HETATM	319	HB2	LYN	20	36.320	39.166	34.690	1.00	0.00	H
HETATM	320	HB3	LYN	20	35.738	40.713	35.242	1.00	0.00	H
HETATM	321	CG	LYN	20	35.017	38.964	36.416	1.00	0.00	C
HETATM	322	HG2	LYN	20	34.299	38.595	35.684	1.00	0.00	H
HETATM	323	HG3	LYN	20	35.419	38.076	36.901	1.00	0.00	H
HETATM	324	CD	LYN	20	34.315	39.856	37.390	1.00	0.00	C
HETATM	325	HD2	LYN	20	33.990	40.833	37.033	1.00	0.00	H
HETATM	326	HD3	LYN	20	34.951	40.034	38.257	1.00	0.00	H
HETATM	327	CE	LYN	20	33.049	39.122	37.863	1.00	0.00	C
HETATM	328	HE2	LYN	20	32.277	39.161	37.094	1.00	0.00	H
HETATM	329	HE3	LYN	20	33.311	38.084	38.069	1.00	0.00	H
HETATM	330	NZ	LYN	20	32.549	39.747	39.006	1.00	0.00	N
HETATM	331	HZ2	LYN	20	32.528	40.717	38.724	1.00	0.00	H
HETATM	332	HZ3	LYN	20	33.265	39.544	39.689	1.00	0.00	H
HETATM	333	C	LYN	20	38.217	38.677	36.883	1.00	0.00	C
HETATM	334	O	LYN	20	38.910	38.057	36.076	1.00	0.00	O
HETATM	1636	N	LIG	103	29.243	42.026	41.038	1.00	0.00	N
HETATM	1637	C	LIG	103	28.852	41.042	40.297	1.00	0.00	C
HETATM	1638	O	LIG	103	29.311	43.164	42.985	1.00	0.00	O
HETATM	1639	C5'	LIG	103	25.520	38.845	46.048	1.00	0.00	C
HETATM	1640	O5'	LIG	103	25.409	38.236	44.730	1.00	0.00	O
HETATM	1641	C4'	LIG	103	25.961	40.314	45.898	1.00	0.00	C
HETATM	1642	O4'	LIG	103	27.168	40.381	44.993	1.00	0.00	O
HETATM	1643	C3'	LIG	103	24.960	41.157	45.136	1.00	0.00	C
HETATM	1644	O3'	LIG	103	24.150	41.862	46.111	1.00	0.00	O
HETATM	1645	C2'	LIG	103	25.790	42.103	44.162	1.00	0.00	C
HETATM	1646	O2'	LIG	103	25.746	43.547	44.551	1.00	0.00	O
HETATM	1647	C1'	LIG	103	27.243	41.609	44.229	1.00	0.00	C
HETATM	1648	N1	LIG	103	27.872	41.388	42.889	1.00	0.00	N
HETATM	1649	O1	LIG	103	28.633	38.764	38.462	1.00	0.00	O
HETATM	1650	N2	LIG	103	29.440	40.852	39.107	1.00	0.00	N
HETATM	1651	C6	LIG	103	28.878	42.195	42.410	1.00	0.00	C
HETATM	1652	C7	LIG	103	27.353	40.383	42.131	1.00	0.00	C
HETATM	1653	C8	LIG	103	27.806	40.171	40.826	1.00	0.00	C
HETATM	1654	C9	LIG	103	29.171	39.854	38.186	1.00	0.00	C
HETATM	1655	C10	LIG	103	29.729	40.122	36.746	1.00	0.00	C
HETATM	1656	H	LIG	103	30.149	41.532	38.861	1.00	0.00	H
HETATM	1657	H1	LIG	103	27.292	39.488	40.159	1.00	0.00	H
HETATM	1658	H4	LIG	103	27.876	42.233	44.870	1.00	0.00	H
HETATM	1659	H6	LIG	103	26.095	40.762	46.890	1.00	0.00	H
HETATM	1660	H7	LIG	103	24.312	40.512	44.532	1.00	0.00	H
HETATM	1661	H8	LIG	103	25.367	41.990	43.156	1.00	0.00	H
HETATM	1662	H9	LIG	103	24.489	38.873	46.423	1.00	0.00	H
HETATM	1663	H10	LIG	103	26.202	38.292	46.707	1.00	0.00	H
HETATM	1664	H11	LIG	103	30.327	41.042	36.725	1.00	0.00	H

```
HETATM 1665  H12 LIG   103       28.897  40.297  36.052  1.00  0.00           H
HETATM 1666  H13 LIG   103       30.321  39.274  36.379  1.00  0.00           H
HETATM 1667  H14 LIG   103       26.446  40.013  42.591  1.00  0.00           H
HETATM 1668  H2' LIG   103       26.281  44.040  43.905  1.00  0.00           H
HETATM 1669  H3' LIG   103       24.551  42.747  46.071  1.00  0.00           H
HETATM 1670  H5' LIG   103       26.264  38.351  44.277  1.00  0.00           H
ENDMDL
MODEL     3
SHEET    1   1 1 ILE    22  ASP    26  0
SHEET    2   2 1 VAL    37  VAL    40  0
SHEET    3   3 1 CYX    50  VAL    60  0
SHEET    4   4 1 PHE    94  CYX   101  0
HELIX    1   1 GLN     9  PHE     11  1                                  3
HELIX    2   2 ASP    13  LEU     16  1                                  4
HELIX    3   3 LYS    68  GLN     72  1                                  5
HELIX    4   4 LEU    77  ALA     85  1                                  9
ATOM      1  N   GLN     1       33.312  17.406  34.807  1.00  0.00           N
ATOM      2  H1  GLN     1       33.073  16.429  34.905  1.00  0.00           H
ATOM      3  H2  GLN     1       34.316  17.436  34.921  1.00  0.00           H
ATOM      4  H3  GLN     1       32.858  18.031  35.458  1.00  0.00           H
ATOM      5  CA  GLN     1       32.835  17.880  33.442  1.00  0.00           C
ATOM      6  HA  GLN     1       33.542  17.566  32.674  1.00  0.00           H
ATOM      7  CB  GLN     1       31.410  17.416  33.219  1.00  0.00           C
ATOM      8  HB2 GLN     1       30.804  17.568  34.114  1.00  0.00           H
ATOM      9  HB3 GLN     1       31.012  17.908  32.332  1.00  0.00           H
ATOM     10  CG  GLN     1       31.204  15.909  32.887  1.00  0.00           C
ATOM     11  HG2 GLN     1       31.499  15.280  33.727  1.00  0.00           H
ATOM     12  HG3 GLN     1       30.144  15.711  32.727  1.00  0.00           H
ATOM     13  CD  GLN     1       31.947  15.488  31.627  1.00  0.00           C
ATOM     14  OE1 GLN     1       32.290  16.290  30.801  1.00  0.00           O
ATOM     15  NE2 GLN     1       31.959  14.183  31.364  1.00  0.00           N
ATOM     16 HE21 GLN     1       31.818  13.561  32.147  1.00  0.00           H
ATOM     17 HE22 GLN     1       32.479  13.770  30.602  1.00  0.00           H
ATOM     18  C   GLN     1       32.999  19.382  33.375  1.00  0.00           C
ATOM     19  O   GLN     1       32.701  20.030  34.346  1.00  0.00           O
ATOM     20  N   PRO     2       33.501  19.995  32.197  1.00  0.00           N
ATOM     21  CD  PRO     2       34.240  19.399  31.072  1.00  0.00           C
ATOM     22  HD2 PRO     2       33.480  19.053  30.372  1.00  0.00           H
ATOM     23  HD3 PRO     2       34.841  18.509  31.251  1.00  0.00           H
ATOM     24  CG  PRO     2       35.119  20.491  30.476  1.00  0.00           C
ATOM     25  HG2 PRO     2       35.257  20.244  29.423  1.00  0.00           H
ATOM     26  HG3 PRO     2       36.090  20.481  30.972  1.00  0.00           H
ATOM     27  CB  PRO     2       34.254  21.691  30.775  1.00  0.00           C
ATOM     28  HB2 PRO     2       33.414  21.790  30.087  1.00  0.00           H
ATOM     29  HB3 PRO     2       34.839
  22.608  30.842  1.00  0.00           H
ATOM     30  CA  PRO     2       33.610  21.444  32.081  1.00  0.00           C
ATOM     31  HA  PRO     2       34.280  21.904  32.807  1.00  0.00           H
ATOM     32  C   PRO     2       32.201  22.165  32.102  1.00  0.00           C
ATOM     33  O   PRO     2       31.146  21.505  31.966  1.00  0.00           O
ATOM     34  N   ASN     3       32.135  23.519  32.173  1.00  0.00           N
ATOM     35  H   ASN     3       32.996  24.043  32.215  1.00  0.00           H
ATOM     36  CA  ASN     3       30.911  24.236  32.097  1.00  0.00           C
ATOM     37  HA  ASN     3       30.117  23.561  32.419  1.00  0.00           H
ATOM     38  CB  ASN     3       30.979  25.340  33.200  1.00  0.00           C
ATOM     39  HB2 ASN     3       31.910  25.906  33.174  1.00  0.00           H
ATOM     40  HB3 ASN     3       30.089  25.968  33.177  1.00  0.00           H
ATOM     41  CG  ASN     3       30.840  24.762  34.570  1.00  0.00           C
ATOM     42  OD1 ASN     3       29.723  24.467  35.059  1.00  0.00           O
ATOM     43  ND2 ASN     3       31.876  24.545  35.284  1.00  0.00           N
ATOM     44 HD21 ASN     3       32.786  24.644  34.855  1.00  0.00           H
ATOM     45 HD22 ASN     3       31.702  24.160  36.201  1.00  0.00           H
ATOM     46  C   ASN     3       30.570  24.762  30.683  1.00  0.00           C
ATOM     47  O   ASN     3       31.486  25.242  29.948  1.00  0.00           O
```

ATOM	48	N	ASP	4	29.245	24.729	30.303	1.00	0.00	N
ATOM	49	H	ASP	4	28.567	24.737	31.051	1.00	0.00	H
ATOM	50	CA	ASP	4	28.716	25.253	29.071	1.00	0.00	C
ATOM	51	HA	ASP	4	29.339	24.985	28.218	1.00	0.00	H
ATOM	52	CB	ASP	4	27.366	24.502	28.797	1.00	0.00	C
ATOM	53	HB2	ASP	4	26.764	24.528	29.706	1.00	0.00	H
ATOM	54	HB3	ASP	4	26.763	25.010	28.046	1.00	0.00	H
ATOM	55	CG	ASP	4	27.367	22.980	28.314	1.00	0.00	C
ATOM	56	OD1	ASP	4	28.382	22.340	28.055	1.00	0.00	O
ATOM	57	OD2	ASP	4	26.225	22.481	28.065	1.00	0.00	O
ATOM	58	C	ASP	4	28.413	26.723	28.983	1.00	0.00	C
ATOM	59	O	ASP	4	28.564	27.258	27.910	1.00	0.00	O
ATOM	60	N	ILE	5	28.073	27.369	30.099	1.00	0.00	N
ATOM	61	H	ILE	5	27.973	26.940	31.008	1.00	0.00	H
ATOM	62	CA	ILE	5	27.869	28.837	30.060	1.00	0.00	C
ATOM	63	HA	ILE	5	27.181	28.939	29.221	1.00	0.00	H
ATOM	64	CB	ILE	5	27.141	29.198	31.283	1.00	0.00	C
ATOM	65	HB	ILE	5	27.547	28.639	32.125	1.00	0.00	H
ATOM	66	CG2	ILE	5	27.088	30.710	31.608	1.00	0.00	C
ATOM	67	HG21	ILE	5	26.671	31.253	30.761	1.00	0.00	H
ATOM	68	HG22	ILE	5	26.469	30.962	32.469	1.00	0.00	H
ATOM	69	HG23	ILE	5	28.122	31.037	31.716	1.00	0.00	H
ATOM	70	CG1	ILE	5	25.690	28.709	31.052	1.00	0.00	C
ATOM	71	HG12	ILE	5	25.290	29.360	30.273	1.00	0.00	H
ATOM	72	HG13	ILE	5	25.622	27.651	30.798	1.00	0.00	H
ATOM	73	CD1	ILE	5	24.754	28.766	32.247	1.00	0.00	C
ATOM	74	HD11	ILE	5	25.181	28.089	32.987	1.00	0.00	H
ATOM	75	HD12	ILE	5	24.555	29.770	32.623	1.00	0.00	H
ATOM	76	HD13	ILE	5	23.779	28.451	31.879	1.00	0.00	H
ATOM	77	C	ILE	5	29.185	29.589	29.810	1.00	0.00	C
ATOM	78	O	ILE	5	30.181	29.506	30.530	1.00	0.00	O
ATOM	79	N	THR	6	29.148	30.567	28.905	1.00	0.00	N
ATOM	80	H	THR	6	28.281	30.548	28.388	1.00	0.00	H
ATOM	81	CA	THR	6	30.311	31.395	28.601	1.00	0.00	C
ATOM	82	HA	THR	6	31.132	31.398	29.316	1.00	0.00	H
ATOM	83	CB	THR	6	30.928	30.847	27.294	1.00	0.00	C
ATOM	84	HB	THR	6	31.137	29.795	27.482	1.00	0.00	H
ATOM	85	CG2	THR	6	30.095	31.022	26.013	1.00	0.00	C
ATOM	86	HG21	THR	6	29.337	30.241	25.971	1.00	0.00	H
ATOM	87	HG22	THR	6	29.736	32.018	25.751	1.00	0.00	H
ATOM	88	HG23	THR	6	30.752	30.702	25.205	1.00	0.00	H
ATOM	89	OG1	THR	6	32.085	31.636	27.110	1.00	0.00	O
ATOM	90	HG1	THR	6	32.767	31.147	27.576	1.00	0.00	H
ATOM	91	C	THR	6	29.810	32.844	28.495	1.00	0.00	C
ATOM	92	O	THR	6	28.698	33.118	28.134	1.00	0.00	O
ATOM	93	N	PHE	7	30.633	33.771	28.956	1.00	0.00	N
ATOM	94	H	PHE	7	31.561	33.463	29.208	1.00	0.00	H
ATOM	95	CA	PHE	7	30.322	35.155	29.235	1.00	0.00	C
ATOM	96	HA	PHE	7	29.460	35.170	29.901	1.00	0.00	H
ATOM	97	CB	PHE	7	31.438	35.702	30.071	1.00	0.00	C
ATOM	98	HB2	PHE	7	32.275	35.643	29.377	1.00	0.00	H
ATOM	99	HB3	PHE	7	31.254	36.742	30.338	1.00	0.00	H
ATOM	100	CG	PHE	7	31.638	34.936	31.395	1.00	0.00	C
ATOM	101	CD1	PHE	7	32.639	33.927	31.669	1.00	0.00	C
ATOM	102	HD1	PHE	7	33.209	33.562	30.828	1.00	0.00	H
ATOM	103	CE1	PHE	7	32.699	33.260	32.909	1.00	0.00	C
ATOM	104	HE1	PHE	7	33.396	32.445	33.037	1.00	0.00	H
ATOM	105	CZ	PHE	7	31.816	33.572	33.885	1.00	0.00	C
ATOM	106	HZ	PHE	7	31.902	33.051	34.826	1.00	0.00	H
ATOM	107	CE2	PHE	7	30.856	34.606	33.761	1.00	0.00	C
ATOM	108	HE2	PHE	7	30.039	34.795	34.441	1.00	0.00	H
ATOM	109	CD2	PHE	7	30.805	35.204	32.474	1.00	0.00	C
ATOM	110	HD2	PHE	7	30.030	35.942	32.333	1.00	0.00	H
ATOM	111	C	PHE	7	30.058	35.921	27.946	1.00	0.00	C

ATOM	112	O	PHE	7	30.679	35.695	26.917	1.00	0.00	O
ATOM	113	N	PHE	8	29.194	36.922	28.060	1.00	0.00	N
ATOM	114	H	PHE	8	28.783	37.011	28.978	1.00	0.00	H
ATOM	115	CA	PHE	8	28.754	37.920	27.045	1.00	0.00	C
ATOM	116	HA	PHE	8	28.364	37.269	26.263	1.00	0.00	H
ATOM	117	CB	PHE	8	27.682	38.844	27.685	1.00	0.00	C
ATOM	118	HB2	PHE	8	27.965	39.128	28.699	1.00	0.00	H
ATOM	119	HB3	PHE	8	27.659	39.769	27.110	1.00	0.00	H
ATOM	120	CG	PHE	8	26.314	38.304	27.964	1.00	0.00	C
ATOM	121	CD1	PHE	8	25.648	37.581	27.006	1.00	0.00	C
ATOM	122	HD1	PHE	8	26.143	37.397	26.064	1.00	0.00	H
ATOM	123	CE1	PHE	8	24.377	37.035	27.171	1.00	0.00	C
ATOM	124	HE1	PHE	8	23.893	36.473	26.385	1.00	0.00	H
ATOM	125	CZ	PHE	8	23.674	37.217	28.391	1.00	0.00	C
ATOM	126	HZ	PHE	8	22.709	36.750	28.525	1.00	0.00	H
ATOM	127	CE2	PHE	8	24.330	37.940	29.363	1.00	0.00	C
ATOM	128	HE2	PHE	8	23.917	38.001	30.360	1.00	0.00	H
ATOM	129	CD2	PHE	8	25.646	38.428	29.176	1.00	0.00	C
ATOM	130	HD2	PHE	8	26.175	39.048	29.883	1.00	0.00	H
ATOM	131	C	PHE	8	30.027	38.660	26.515	1.00	0.00	C
ATOM	132	O	PHE	8	30.962	39.018	27.246	1.00	0.00	O
ATOM	133	N	GLN	9	30.007	39.076	25.200	1.00	0.00	N
ATOM	134	H	GLN	9	29.190	38.755	24.700	1.00	0.00	H
ATOM	135	CA	GLN	9	31.232	39.384	24.497	1.00	0.00	C
ATOM	136	HA	GLN	9	31.882	38.510	24.504	1.00	0.00	H
ATOM	137	CB	GLN	9	30.958	39.728	23.061	1.00	0.00	C
ATOM	138	HB2	GLN	9	30.241	40.549	23.034	1.00	0.00	H
ATOM	139	HB3	GLN	9	31.902	39.925	22.554	1.00	0.00	H
ATOM	140	CG	GLN	9	30.359	38.571	22.302	1.00	0.00	C
ATOM	141	HG2	GLN	9	30.954	37.673	22.467	1.00	0.00	H
ATOM	142	HG3	GLN	9	29.376	38.313	22.696	1.00	0.00	H
ATOM	143	CD	GLN	9	30.212	38.795	20.776	1.00	0.00	C
ATOM	144	OE1	GLN	9	31.017	39.475	20.159	1.00	0.00	O
ATOM	145	NE2	GLN	9	29.251	38.097	20.110	1.00	0.00	N
ATOM	146	HE21	GLN	9	29.271	38.302	19.121	1.00	0.00	H
ATOM	147	HE22	GLN	9	28.608	37.477	20.580	1.00	0.00	H
ATOM	148	C	GLN	9	32.176	40.510	25.092	1.00	0.00	C
ATOM	149	O	GLN	9	33.352	40.284	25.095	1.00	0.00	O
ATOM	150	N	ARG	10	31.574	41.650	25.476	1.00	0.00	N
ATOM	151	H	ARG	10	30.611	41.785	25.199	1.00	0.00	H
ATOM	152	CA	ARG	10	32.309	42.774	26.144	1.00	0.00	C
ATOM	153	HA	ARG	10	33.197	42.956	25.541	1.00	0.00	H
ATOM	154	CB	ARG	10	31.452	44.051	26.063	1.00	0.00	
		C								
ATOM	155	HB2	ARG	10	30.811	43.942	25.188	1.00	0.00	H
ATOM	156	HB3	ARG	10	30.822	44.007	26.952	1.00	0.00	H
ATOM	157	CG	ARG	10	32.139	45.447	25.932	1.00	0.00	C
ATOM	158	HG2	ARG	10	32.925	45.694	26.646	1.00	0.00	H
ATOM	159	HG3	ARG	10	32.457	45.617	24.904	1.00	0.00	H
ATOM	160	CD	ARG	10	31.125	46.565	26.193	1.00	0.00	C
ATOM	161	HD2	ARG	10	31.499	47.466	25.709	1.00	0.00	H
ATOM	162	HD3	ARG	10	30.232	46.283	25.634	1.00	0.00	H
ATOM	163	NE	ARG	10	30.880	46.858	27.598	1.00	0.00	N
ATOM	164	HE	ARG	10	31.697	47.122	28.129	1.00	0.00	H
ATOM	165	CZ	ARG	10	29.754	46.807	28.315	1.00	0.00	C
ATOM	166	NH1	ARG	10	28.551	46.776	27.762	1.00	0.00	N
ATOM	167	HH11	ARG	10	27.769	46.944	28.377	1.00	0.00	H
ATOM	168	HH12	ARG	10	28.584	47.098	26.805	1.00	0.00	H
ATOM	169	NH2	ARG	10	29.691	46.929	29.554	1.00	0.00	N
ATOM	170	HH21	ARG	10	30.569	46.851	30.048	1.00	0.00	H
ATOM	171	HH22	ARG	10	28.798	47.017	30.017	1.00	0.00	H
ATOM	172	C	ARG	10	32.741	42.530	27.563	1.00	0.00	C
ATOM	173	O	ARG	10	33.393	43.428	28.118	1.00	0.00	O
ATOM	174	N	PHE	11	32.545	41.295	28.121	1.00	0.00	N

ATOM	175	H	PHE	11	32.183	40.536	27.564	1.00	0.00	H
ATOM	176	CA	PHE	11	32.876	40.829	29.482	1.00	0.00	C
ATOM	177	HA	PHE	11	33.242	41.707	30.014	1.00	0.00	H
ATOM	178	CB	PHE	11	31.570	40.607	30.212	1.00	0.00	C
ATOM	179	HB2	PHE	11	31.702	40.160	31.197	1.00	0.00	H
ATOM	180	HB3	PHE	11	31.070	39.721	29.819	1.00	0.00	H
ATOM	181	CG	PHE	11	30.587	41.770	30.276	1.00	0.00	C
ATOM	182	CD1	PHE	11	30.823	42.875	31.136	1.00	0.00	C
ATOM	183	HD1	PHE	11	31.681	42.994	31.782	1.00	0.00	H
ATOM	184	CE1	PHE	11	29.902	43.931	31.065	1.00	0.00	C
ATOM	185	HE1	PHE	11	30.003	44.781	31.724	1.00	0.00	H
ATOM	186	CZ	PHE	11	28.792	43.827	30.234	1.00	0.00	C
ATOM	187	HZ	PHE	11	28.117	44.669	30.189	1.00	0.00	H
ATOM	188	CE2	PHE	11	28.527	42.738	29.407	1.00	0.00	C
ATOM	189	HE2	PHE	11	27.574	42.717	28.899	1.00	0.00	H
ATOM	190	CD2	PHE	11	29.461	41.654	29.476	1.00	0.00	C
ATOM	191	HD2	PHE	11	29.326	40.801	28.826	1.00	0.00	H
ATOM	192	C	PHE	11	33.752	39.578	29.544	1.00	0.00	C
ATOM	193	O	PHE	11	34.051	39.188	30.661	1.00	0.00	O
ATOM	194	N	GLN	12	34.181	39.083	28.389	1.00	0.00	N
ATOM	195	H	GLN	12	33.889	39.435	27.488	1.00	0.00	H
ATOM	196	CA	GLN	12	35.059	37.966	28.272	1.00	0.00	C
ATOM	197	HA	GLN	12	34.669	37.167	28.902	1.00	0.00	H
ATOM	198	CB	GLN	12	35.060	37.391	26.897	1.00	0.00	C
ATOM	199	HB2	GLN	12	35.236	38.181	26.166	1.00	0.00	H
ATOM	200	HB3	GLN	12	35.932	36.744	26.803	1.00	0.00	H
ATOM	201	CG	GLN	12	33.794	36.700	26.580	1.00	0.00	C
ATOM	202	HG2	GLN	12	33.693	35.859	27.266	1.00	0.00	H
ATOM	203	HG3	GLN	12	32.988	37.433	26.646	1.00	0.00	H
ATOM	204	CD	GLN	12	33.778	36.037	25.200	1.00	0.00	C
ATOM	205	OE1	GLN	12	34.661	36.197	24.358	1.00	0.00	O
ATOM	206	NE2	GLN	12	32.672	35.401	24.881	1.00	0.00	N
ATOM	207	HE21	GLN	12	31.937	35.257	25.559	1.00	0.00	H
ATOM	208	HE22	GLN	12	32.486	35.038	23.957	1.00	0.00	H
ATOM	209	C	GLN	12	36.509	38.338	28.695	1.00	0.00	C
ATOM	210	O	GLN	12	37.215	37.509	29.286	1.00	0.00	O
ATOM	211	N	ASP	13	37.062	39.504	28.203	1.00	0.00	N
ATOM	212	H	ASP	13	36.534	40.045	27.533	1.00	0.00	H
ATOM	213	CA	ASP	13	38.544	39.700	28.156	1.00	0.00	C
ATOM	214	HA	ASP	13	38.988	38.774	27.791	1.00	0.00	H
ATOM	215	CB	ASP	13	38.993	40.918	27.293	1.00	0.00	C
ATOM	216	HB2	ASP	13	38.698	41.741	27.945	1.00	0.00	H
ATOM	217	HB3	ASP	13	40.072	40.965	27.149	1.00	0.00	H
ATOM	218	CG	ASP	13	38.344	40.955	25.905	1.00	0.00	C
ATOM	219	OD1	ASP	13	38.056	42.002	25.314	1.00	0.00	O
ATOM	220	OD2	ASP	13	38.113	39.874	25.309	1.00	0.00	O
ATOM	221	C	ASP	13	39.233	39.929	29.500	1.00	0.00	C
ATOM	222	O	ASP	13	40.374	39.596	29.614	1.00	0.00	O
ATOM	223	N	ASP	14	38.382	40.356	30.414	1.00	0.00	N
ATOM	224	H	ASP	14	37.404	40.464	30.190	1.00	0.00	H
ATOM	225	CA	ASP	14	38.744	40.837	31.725	1.00	0.00	C
ATOM	226	HA	ASP	14	39.588	41.522	31.806	1.00	0.00	H
ATOM	227	CB	ASP	14	37.492	41.428	32.390	1.00	0.00	C
ATOM	228	HB2	ASP	14	36.772	40.617	32.500	1.00	0.00	H
ATOM	229	HB3	ASP	14	37.769	41.640	33.423	1.00	0.00	H
ATOM	230	CG	ASP	14	36.908	42.610	31.602	1.00	0.00	C
ATOM	231	OD1	ASP	14	37.641	43.142	30.740	1.00	0.00	O
ATOM	232	OD2	ASP	14	35.703	42.907	31.615	1.00	0.00	O
ATOM	233	C	ASP	14	39.312	39.745	32.594	1.00	0.00	C
ATOM	234	O	ASP	14	40.142	40.056	33.427	1.00	0.00	O
ATOM	235	N	ILE	15	39.023	38.475	32.322	1.00	0.00	N
ATOM	236	H	ILE	15	38.506	38.200	31.500	1.00	0.00	H
ATOM	237	CA	ILE	15	39.548	37.377	33.075	1.00	0.00	C
ATOM	238	HA	ILE	15	39.649	37.554	34.147	1.00	0.00	H

```
ATOM    239  CB  ILE    15      38.557  36.166  32.873  1.00  0.00            C
ATOM    240  HB  ILE    15      38.734  35.760  31.877  1.00  0.00            H
ATOM    241  CG2 ILE    15      39.015  35.037  33.916  1.00  0.00            C
ATOM    242 HG21 ILE    15      38.341  34.181  33.924  1.00  0.00            H
ATOM    243 HG22 ILE    15      40.049  34.793  33.670  1.00  0.00            H
ATOM    244 HG23 ILE    15      39.001  35.496  34.905  1.00  0.00            H
ATOM    245  CG1 ILE    15      37.072  36.577  33.053  1.00  0.00            C
ATOM    246 HG12 ILE    15      36.955  36.966  34.064  1.00  0.00            H
ATOM    247 HG13 ILE    15      36.839  37.390  32.364  1.00  0.00            H
ATOM    248  CD1 ILE    15      36.086  35.449  32.726  1.00  0.00            C
ATOM    249 HD11 ILE    15      35.075  35.846  32.632  1.00  0.00            H
ATOM    250 HD12 ILE    15      36.342  35.010  31.762  1.00  0.00            H
ATOM    251 HD13 ILE    15      36.101  34.782  33.588  1.00  0.00            H
ATOM    252  C   ILE    15      40.949  36.945  32.606  1.00  0.00            C
ATOM    253  O   ILE    15      41.847  36.705  33.411  1.00  0.00            O
ATOM    254  N   LEU    16      41.259  37.006  31.311  1.00  0.00            N
ATOM    255  H   LEU    16      40.529  37.145  30.626  1.00  0.00            H
ATOM    256  CA  LEU    16      42.613  36.970  30.742  1.00  0.00            C
ATOM    257  HA  LEU    16      42.968  36.064  31.233  1.00  0.00            H
ATOM    258  CB  LEU    16      42.664  36.793  29.193  1.00  0.00            C
ATOM    259  HB2 LEU    16      42.189  35.846  28.936  1.00  0.00            H
ATOM    260  HB3 LEU    16      42.037  37.642  28.921  1.00  0.00            H
ATOM    261  CG  LEU    16      43.993  36.751  28.380  1.00  0.00            C
ATOM    262  HG  LEU    16      44.557  37.673  28.517  1.00  0.00            H
ATOM    263  CD1 LEU    16      44.868  35.614  28.827  1.00  0.00            C
ATOM    264 HD11 LEU    16      45.387  35.842  29.758  1.00  0.00            H
ATOM    265 HD12 LEU    16      44.285  34.693  28.872  1.00  0.00            H
ATOM    266 HD13 LEU    16      45.568  35.452  28.008  1.00  0.00            H
ATOM    267  CD2 LEU    16      43.727  36.604  26.897  1.00  0.00            C
ATOM    268 HD21 LEU    16      44.664  36.378  26.387  1.00  0.00            H
ATOM    269 HD22 LEU    16      43.066  35.748  26.768  1.00  0.00            H
ATOM    270 HD23 LEU    16      43.310  37.511  26.461  1.00  0.00            H
ATOM    271  C   LEU    16      43.493  38.124  31.157  1.00  0.00            C
ATOM    272  O   LEU    16      44.657  37.936  31.611  1.00  0.00            O
ATOM    273  N   ALA    17      42.892  39.277  31.068  1.00  0.00            N
ATOM    274  H   ALA    17      41.959  39.355  30.689  1.00  0.00            H
ATOM    275  CA  ALA    17      43.484  40.542  31.547  1.00  0.00            C
ATOM    276  HA  ALA    17      44.515  40.577  31.198  1.00  0.00            H
ATOM    277  CB  ALA    17      42.713  41.707  30.960  1.00  0.00            C
ATOM    278  HB1 ALA    17      42.571  41.563  29.888  1.00  0.00            H
ATOM    279  HB2 ALA    17      41.722  41.680  31.412  1.00  0.00            H
ATOM    280
  HB3 ALA    17      43.255  42.621  31.206  1.00  0.00            H
ATOM    281  C   ALA    17      43.734  40.668  33.053  1.00  0.00            C
ATOM    282  O   ALA    17      44.183  41.701  33.519  1.00  0.00            O
ATOM    283  N   GLY    18      43.313  39.653  33.808  1.00  0.00            N
ATOM    284  H   GLY    18      42.916  38.853  33.336  1.00  0.00            H
ATOM    285  CA  GLY    18      43.384  39.649  35.291  1.00  0.00            C
ATOM    286  HA2 GLY    18      43.010  38.725  35.730  1.00  0.00            H
ATOM    287  HA3 GLY    18      44.368  39.966  35.638  1.00  0.00            H
ATOM    288  C   GLY    18      42.542  40.818  35.972  1.00  0.00            C
ATOM    289  O   GLY    18      42.779  41.017  37.172  1.00  0.00            O
ATOM    290  N   ARG    19      41.621  41.510  35.304  1.00  0.00            N
ATOM    291  H   ARG    19      41.487  41.276  34.331  1.00  0.00            H
ATOM    292  CA  ARG    19      40.677  42.514  35.846  1.00  0.00            C
ATOM    293  HA  ARG    19      41.249  43.121  36.549  1.00  0.00            H
ATOM    294  CB  ARG    19      40.074  43.328  34.682  1.00  0.00            C
ATOM    295  HB2 ARG    19      39.892  42.583  33.908  1.00  0.00            H
ATOM    296  HB3 ARG    19      39.244  43.864  35.143  1.00  0.00            H
ATOM    297  CG  ARG    19      41.048  44.331  34.070  1.00  0.00            C
ATOM    298  HG2 ARG    19      41.547  44.932  34.828  1.00  0.00            H
ATOM    299  HG3 ARG    19      41.768  43.861  33.400  1.00  0.00            H
ATOM    300  CD  ARG    19      40.265  45.404  33.224  1.00  0.00            C
ATOM    301  HD2 ARG    19      39.424  45.725  33.837  1.00  0.00            H
```

ATOM	302	HD3	ARG	19	40.976	46.203	33.013	1.00	0.00	H
ATOM	303	NE	ARG	19	39.734	44.892	31.986	1.00	0.00	N
ATOM	304	HE	ARG	19	38.783	44.553	32.013	1.00	0.00	H
ATOM	305	CZ	ARG	19	40.220	45.030	30.724	1.00	0.00	C
ATOM	306	NH1	ARG	19	41.294	45.664	30.379	1.00	0.00	N
ATOM	307	HH11	ARG	19	41.764	46.358	30.942	1.00	0.00	H
ATOM	308	HH12	ARG	19	41.776	45.339	29.554	1.00	0.00	H
ATOM	309	NH2	ARG	19	39.514	44.614	29.744	1.00	0.00	N
ATOM	310	HH21	ARG	19	39.763	44.791	28.780	1.00	0.00	H
ATOM	311	HH22	ARG	19	38.632	44.139	29.874	1.00	0.00	H
ATOM	312	C	ARG	19	39.516	41.888	36.647	1.00	0.00	C
ATOM	313	O	ARG	19	39.001	42.554	37.468	1.00	0.00	O
ATOM	335	N	THR	21	39.198	38.161	38.578	1.00	0.00	N
ATOM	336	H	THR	21	38.734	38.749	39.255	1.00	0.00	H
ATOM	337	CA	THR	21	39.809	36.979	39.113	1.00	0.00	C
ATOM	338	HA	THR	21	39.739	36.093	38.482	1.00	0.00	H
ATOM	339	CB	THR	21	41.352	37.240	39.281	1.00	0.00	C
ATOM	340	HB	THR	21	41.850	37.695	38.425	1.00	0.00	H
ATOM	341	CG2	THR	21	41.762	38.000	40.578	1.00	0.00	C
ATOM	342	HG21	THR	21	42.843	37.871	40.626	1.00	0.00	H
ATOM	343	HG22	THR	21	41.407	39.021	40.440	1.00	0.00	H
ATOM	344	HG23	THR	21	41.328	37.561	41.477	1.00	0.00	H
ATOM	345	OG1	THR	21	41.987	36.028	39.493	1.00	0.00	O
ATOM	346	HG1	THR	21	41.601	35.425	38.853	1.00	0.00	H
ATOM	347	C	THR	21	39.246	36.536	40.471	1.00	0.00	C
ATOM	348	O	THR	21	39.450	35.406	41.005	1.00	0.00	O
ATOM	349	N	ILE	22	38.327	37.362	41.004	1.00	0.00	N
ATOM	350	H	ILE	22	38.190	38.265	40.572	1.00	0.00	H
ATOM	351	CA	ILE	22	37.551	37.132	42.206	1.00	0.00	C
ATOM	352	HA	ILE	22	37.491	36.063	42.410	1.00	0.00	H
ATOM	353	CB	ILE	22	38.126	37.658	43.503	1.00	0.00	C
ATOM	354	HB	ILE	22	39.204	37.498	43.534	1.00	0.00	H
ATOM	355	CG2	ILE	22	38.064	39.222	43.549	1.00	0.00	C
ATOM	356	HG21	ILE	22	38.357	39.605	44.527	1.00	0.00	H
ATOM	357	HG22	ILE	22	38.771	39.649	42.838	1.00	0.00	H
ATOM	358	HG23	ILE	22	37.052	39.573	43.351	1.00	0.00	H
ATOM	359	CG1	ILE	22	37.447	37.081	44.765	1.00	0.00	C
ATOM	360	HG12	ILE	22	36.421	37.418	44.920	1.00	0.00	H
ATOM	361	HG13	ILE	22	37.470	36.004	44.599	1.00	0.00	H
ATOM	362	CD1	ILE	22	38.161	37.450	46.038	1.00	0.00	C
ATOM	363	HD11	ILE	22	39.246	37.359	45.984	1.00	0.00	H
ATOM	364	HD12	ILE	22	37.864	38.492	46.155	1.00	0.00	H
ATOM	365	HD13	ILE	22	37.898	36.866	46.920	1.00	0.00	H
ATOM	366	C	ILE	22	36.090	37.442	41.936	1.00	0.00	C
ATOM	367	O	ILE	22	35.870	38.406	41.150	1.00	0.00	O
ATOM	368	N	THR	23	35.117	36.709	42.453	1.00	0.00	N
ATOM	369	H	THR	23	35.296	35.883	43.006	1.00	0.00	H
ATOM	370	CA	THR	23	33.722	37.148	42.308	1.00	0.00	C
ATOM	371	HA	THR	23	33.658	38.237	42.296	1.00	0.00	H
ATOM	372	CB	THR	23	33.074	36.592	40.981	1.00	0.00	C
ATOM	373	HB	THR	23	33.823	36.647	40.192	1.00	0.00	H
ATOM	374	CG2	THR	23	32.654	35.048	41.175	1.00	0.00	C
ATOM	375	HG21	THR	23	32.468	34.582	40.207	1.00	0.00	H
ATOM	376	HG22	THR	23	33.498	34.542	41.643	1.00	0.00	H
ATOM	377	HG23	THR	23	31.763	34.951	41.794	1.00	0.00	H
ATOM	378	OG1	THR	23	32.093	37.477	40.602	1.00	0.00	O
ATOM	379	HG1	THR	23	32.523	38.280	40.295	1.00	0.00	H
ATOM	380	C	THR	23	32.759	36.895	43.471	1.00	0.00	C
ATOM	381	O	THR	23	32.912	35.918	44.173	1.00	0.00	O
ATOM	382	N	ILE	24	31.694	37.713	43.617	1.00	0.00	N
ATOM	383	H	ILE	24	31.501	38.431	42.934	1.00	0.00	H
ATOM	384	CA	ILE	24	30.761	37.602	44.792	1.00	0.00	C
ATOM	385	HA	ILE	24	31.183	36.932	45.540	1.00	0.00	H
ATOM	386	CB	ILE	24	30.670	39.017	45.476	1.00	0.00	C

ATOM	387	HB	ILE	24	30.371	39.727	44.705	1.00	0.00	H
ATOM	388	CG2	ILE	24	29.673	38.852	46.663	1.00	0.00	C
ATOM	389	HG21	ILE	24	30.025	38.158	47.427	1.00	0.00	H
ATOM	390	HG22	ILE	24	29.522	39.752	47.259	1.00	0.00	H
ATOM	391	HG23	ILE	24	28.691	38.548	46.300	1.00	0.00	H
ATOM	392	CG1	ILE	24	32.030	39.482	46.072	1.00	0.00	C
ATOM	393	HG12	ILE	24	32.450	38.806	46.816	1.00	0.00	H
ATOM	394	HG13	ILE	24	32.767	39.707	45.302	1.00	0.00	H
ATOM	395	CD1	ILE	24	31.990	40.767	46.905	1.00	0.00	C
ATOM	396	HD11	ILE	24	31.495	40.491	47.836	1.00	0.00	H
ATOM	397	HD12	ILE	24	33.028	41.047	47.084	1.00	0.00	H
ATOM	398	HD13	ILE	24	31.479	41.559	46.358	1.00	0.00	H
ATOM	399	C	ILE	24	29.468	37.045	44.309	1.00	0.00	C
ATOM	400	O	ILE	24	29.042	37.285	43.194	1.00	0.00	O
ATOM	401	N	ARG	25	28.779	36.199	45.129	1.00	0.00	N
ATOM	402	H	ARG	25	29.313	35.878	45.925	1.00	0.00	H
ATOM	403	CA	ARG	25	27.419	35.628	44.839	1.00	0.00	C
ATOM	404	HA	ARG	25	26.955	36.320	44.136	1.00	0.00	H
ATOM	405	CB	ARG	25	27.600	34.255	44.217	1.00	0.00	C
ATOM	406	HB2	ARG	25	28.224	33.626	44.851	1.00	0.00	H
ATOM	407	HB3	ARG	25	26.607	33.807	44.222	1.00	0.00	H
ATOM	408	CG	ARG	25	28.250	34.285	42.785	1.00	0.00	C
ATOM	409	HG2	ARG	25	29.291	34.605	42.754	1.00	0.00	H
ATOM	410	HG3	ARG	25	28.256	33.259	42.414	1.00	0.00	H
ATOM	411	CD	ARG	25	27.392	35.044	41.750	1.00	0.00	C
ATOM	412	HD2	ARG	25	26.384	34.626	41.752	1.00	0.00	H
ATOM	413	HD3	ARG	25	27.366	36.099	42.024	1.00	0.00	H
ATOM	414	NE	ARG	25	28.015	34.773	40.438	1.00	0.00	N
ATOM	415	HE	ARG	25	27.750	33.905	39.997	1.00	0.00	H
ATOM	416	CZ	ARG	25	29.024	35.411	39.931	1.00	0.00	C
ATOM	417	NH1	ARG	25	29.667	36.319	40.674	1.00	0.00	N
ATOM	418	HH11	ARG	25	30.404	36.887	40.281	1.00	0.00	H
ATOM	419	HH12	ARG	25	29.420	36.437	41.646	1.00	0.00	H
ATOM	420	NH2	ARG	25	29.430	35.317	38.665	1.00	0.00	N
ATOM	421	HH21	ARG	25	30.235	35.781	38.269	1.00	0.00	H
ATOM	422	HH22	ARG	25	28.988	34.534	38.204	1.00	0.00	H
ATOM	423	C	ARG	25	26.608	35.550	46.163	1.00	0.00	C
ATOM	424	O	ARG	25	27.128	35.730	47.261	1.00	0.00	O
ATOM	425	N	ASP	26	25.315	35.344	46.083	1.00	0.00	N
ATOM	426	H	ASP	26	25.000					
35.162	45.140	1.00		0.00	H					
ATOM	427	CA	ASP	26	24.468	34.771	47.112	1.00	0.00	C
ATOM	428	HA	ASP	26	24.789	35.218	48.053	1.00	0.00	H
ATOM	429	CB	ASP	26	22.962	34.901	46.880	1.00	0.00	C
ATOM	430	HB2	ASP	26	22.401	34.200	47.499	1.00	0.00	H
ATOM	431	HB3	ASP	26	22.686	35.898	47.224	1.00	0.00	H
ATOM	432	CG	ASP	26	22.377	34.569	45.492	1.00	0.00	C
ATOM	433	OD1	ASP	26	23.155	34.259	44.539	1.00	0.00	O
ATOM	434	OD2	ASP	26	21.095	34.507	45.366	1.00	0.00	O
ATOM	435	C	ASP	26	24.899	33.323	47.375	1.00	0.00	C
ATOM	436	O	ASP	26	25.536	32.674	46.519	1.00	0.00	O
ATOM	437	N	GLU	27	24.807	32.859	48.644	1.00	0.00	N
ATOM	438	H	GLU	27	24.289	33.408	49.317	1.00	0.00	H
ATOM	439	CA	GLU	27	25.067	31.463	49.055	1.00	0.00	C
ATOM	440	HA	GLU	27	26.050	31.244	48.639	1.00	0.00	H
ATOM	441	CB	GLU	27	25.044	31.417	50.657	1.00	0.00	C
ATOM	442	HB2	GLU	27	25.818	32.106	50.997	1.00	0.00	H
ATOM	443	HB3	GLU	27	24.169	31.947	51.036	1.00	0.00	H
ATOM	444	CG	GLU	27	25.283	30.033	51.277	1.00	0.00	C
ATOM	445	HG2	GLU	27	24.743	30.006	52.223	1.00	0.00	H
ATOM	446	HG3	GLU	27	24.864	29.272	50.618	1.00	0.00	H
ATOM	447	CD	GLU	27	26.768	29.668	51.534	1.00	0.00	C
ATOM	448	OE1	GLU	27	27.695	30.379	51.071	1.00	0.00	O
ATOM	449	OE2	GLU	27	26.963	28.705	52.324	1.00	0.00	O

ATOM	450	C	GLU	27	24.038	30.517	48.373	1.00	0.00	C
ATOM	451	O	GLU	27	22.856	30.555	48.768	1.00	0.00	O
ATOM	452	N	SER	28	24.507	29.588	47.437	1.00	0.00	N
ATOM	453	H	SER	28	25.508	29.462	47.388	1.00	0.00	H
ATOM	454	CA	SER	28	23.735	28.622	46.821	1.00	0.00	C
ATOM	455	HA	SER	28	23.174	28.172	47.639	1.00	0.00	H
ATOM	456	CB	SER	28	22.765	29.254	45.877	1.00	0.00	C
ATOM	457	HB2	SER	28	21.938	29.735	46.398	1.00	0.00	H
ATOM	458	HB3	SER	28	23.374	29.954	45.304	1.00	0.00	H
ATOM	459	OG	SER	28	22.265	28.293	45.040	1.00	0.00	O
ATOM	460	HG	SER	28	22.404	28.730	44.196	1.00	0.00	H
ATOM	461	C	SER	28	24.571	27.526	46.254	1.00	0.00	C
ATOM	462	O	SER	28	25.567	27.738	45.551	1.00	0.00	O
ATOM	463	N	GLU	29	24.080	26.313	46.490	1.00	0.00	N
ATOM	464	H	GLU	29	23.105	26.346	46.750	1.00	0.00	H
ATOM	465	CA	GLU	29	24.733	25.052	46.115	1.00	0.00	C
ATOM	466	HA	GLU	29	25.624	25.156	46.734	1.00	0.00	H
ATOM	467	CB	GLU	29	23.810	23.872	46.583	1.00	0.00	C
ATOM	468	HB2	GLU	29	23.763	24.024	47.661	1.00	0.00	H
ATOM	469	HB3	GLU	29	22.824	24.182	46.239	1.00	0.00	H
ATOM	470	CG	GLU	29	24.219	22.488	46.228	1.00	0.00	C
ATOM	471	HG2	GLU	29	23.580	21.753	46.717	1.00	0.00	H
ATOM	472	HG3	GLU	29	23.978	22.351	45.173	1.00	0.00	H
ATOM	473	CD	GLU	29	25.676	22.106	46.406	1.00	0.00	C
ATOM	474	OE1	GLU	29	26.274	21.405	45.557	1.00	0.00	O
ATOM	475	OE2	GLU	29	26.244	22.353	47.500	1.00	0.00	O
ATOM	476	C	GLU	29	25.011	24.927	44.603	1.00	0.00	C
ATOM	477	O	GLU	29	26.093	24.441	44.245	1.00	0.00	O
ATOM	478	N	SER	30	24.139	25.478	43.740	1.00	0.00	N
ATOM	479	H	SER	30	23.310	25.922	44.109	1.00	0.00	H
ATOM	480	CA	SER	30	24.193	25.601	42.271	1.00	0.00	C
ATOM	481	HA	SER	30	24.294	24.554	41.988	1.00	0.00	H
ATOM	482	CB	SER	30	22.952	26.288	41.712	1.00	0.00	C
ATOM	483	HB2	SER	30	23.062	27.372	41.693	1.00	0.00	H
ATOM	484	HB3	SER	30	22.845	25.913	40.694	1.00	0.00	H
ATOM	485	OG	SER	30	21.755	25.954	42.416	1.00	0.00	O
ATOM	486	HG	SER	30	21.252	26.680	42.043	1.00	0.00	H
ATOM	487	C	SER	30	25.347	26.328	41.633	1.00	0.00	C
ATOM	488	O	SER	30	25.803	25.963	40.534	1.00	0.00	O
ATOM	489	N	HIE	31	25.903	27.317	42.380	1.00	0.00	N
ATOM	490	H	HIE	31	25.555	27.492	43.313	1.00	0.00	H
ATOM	491	CA	HIE	31	27.096	28.062	41.943	1.00	0.00	C
ATOM	492	HA	HIE	31	26.868	28.595	41.020	1.00	0.00	H
ATOM	493	CB	HIE	31	27.334	29.171	42.908	1.00	0.00	C
ATOM	494	HB2	HIE	31	27.433	28.878	43.953	1.00	0.00	H
ATOM	495	HB3	HIE	31	28.216	29.742	42.621	1.00	0.00	H
ATOM	496	CG	HIE	31	26.165	30.229	43.090	1.00	0.00	C
ATOM	497	ND1	HIE	31	25.595	30.979	42.039	1.00	0.00	N
ATOM	498	CE1	HIE	31	24.881	31.971	42.646	1.00	0.00	C
ATOM	499	HE1	HIE	31	24.337	32.811	42.240	1.00	0.00	H
ATOM	500	NE2	HIE	31	24.901	31.802	43.949	1.00	0.00	N
ATOM	501	HE2	HIE	31	24.572	32.531	44.565	1.00	0.00	H
ATOM	502	CD2	HIE	31	25.796	30.807	44.301	1.00	0.00	C
ATOM	503	HD2	HIE	31	26.235	30.630	45.272	1.00	0.00	H
ATOM	504	C	HIE	31	28.357	27.231	41.747	1.00	0.00	C
ATOM	505	O	HIE	31	28.459	26.142	42.364	1.00	0.00	O
ATOM	506	N	PHE	32	29.413	27.645	41.090	1.00	0.00	N
ATOM	507	H	PHE	32	29.309	28.354	40.378	1.00	0.00	H
ATOM	508	CA	PHE	32	30.679	26.793	40.898	1.00	0.00	C
ATOM	509	HA	PHE	32	30.383	25.951	40.271	1.00	0.00	H
ATOM	510	CB	PHE	32	31.680	27.575	39.942	1.00	0.00	C
ATOM	511	HB2	PHE	32	32.208	28.371	40.466	1.00	0.00	H
ATOM	512	HB3	PHE	32	32.454	26.932	39.523	1.00	0.00	H
ATOM	513	CG	PHE	32	30.998	28.304	38.727	1.00	0.00	C

ATOM	514	CD1	PHE	32	30.972	29.670	38.709	1.00	0.00	C
ATOM	515	HD1	PHE	32	31.351	30.210	39.563	1.00	0.00	H
ATOM	516	CE1	PHE	32	30.369	30.405	37.650	1.00	0.00	C
ATOM	517	HE1	PHE	32	30.437	31.481	37.603	1.00	0.00	H
ATOM	518	CZ	PHE	32	29.825	29.703	36.538	1.00	0.00	C
ATOM	519	HZ	PHE	32	29.485	30.286	35.694	1.00	0.00	H
ATOM	520	CE2	PHE	32	29.838	28.250	36.503	1.00	0.00	C
ATOM	521	HE2	PHE	32	29.385	27.770	35.647	1.00	0.00	H
ATOM	522	CD2	PHE	32	30.462	27.575	37.629	1.00	0.00	C
ATOM	523	HD2	PHE	32	30.545	26.500	37.572	1.00	0.00	H
ATOM	524	C	PHE	32	31.313	26.257	42.145	1.00	0.00	C
ATOM	525	O	PHE	32	31.350	26.971	43.178	1.00	0.00	O
ATOM	526	N	LYS	33	31.943	25.096	42.084	1.00	0.00	N
ATOM	527	H	LYS	33	31.924	24.717	41.149	1.00	0.00	H
ATOM	528	CA	LYS	33	32.655	24.420	43.174	1.00	0.00	C
ATOM	529	HA	LYS	33	32.486	24.980	44.094	1.00	0.00	H
ATOM	530	CB	LYS	33	32.185	22.942	43.372	1.00	0.00	C
ATOM	531	HB2	LYS	33	32.501	22.498	42.428	1.00	0.00	H
ATOM	532	HB3	LYS	33	32.786	22.501	44.167	1.00	0.00	H
ATOM	533	CG	LYS	33	30.721	22.780	43.724	1.00	0.00	C
ATOM	534	HG2	LYS	33	30.096	22.869	42.835	1.00	0.00	H
ATOM	535	HG3	LYS	33	30.518	21.715	43.835	1.00	0.00	H
ATOM	536	CD	LYS	33	30.125	23.624	44.809	1.00	0.00	C
ATOM	537	HD2	LYS	33	30.832	23.550	45.635	1.00	0.00	H
ATOM	538	HD3	LYS	33	30.049	24.661	44.482	1.00	0.00	H
ATOM	539	CE	LYS	33	28.795	23.099	45.259	1.00	0.00	C
ATOM	540	HE2	LYS	33	28.051	23.230	44.474	1.00	0.00	H
ATOM	541	HE3	LYS	33	28.911	22.058	45.561	1.00	0.00	H
ATOM	542	NZ	LYS	33	28.308	23.866	46.388	1.00	0.00	N
ATOM	543	HZ1	LYS	33	27.939	24.751	46.072	1.00	0.00	H
ATOM	544	HZ2	LYS	33	27.525	23.349	46.763	1.00	0.00	H
ATOM	545	HZ3	LYS	33	29.018	24.003	47.092	1.00	0.00	H
ATOM	546	C	LYS	33	34.158	24.544	42.973	1.00	0.00	C
ATOM	547	O	LYS	33	34.612	24.889	41.833	1.00	0.00	O
ATOM	548	N	THR	34	35.018	24.166	44.000	1.00	0.00	N
ATOM	549	H	THR	34	34.730	23.902	44.930	1.00	0.00	H
ATOM	550	CA	THR	34	36.477	24.286	43.777	1.00	0.00	C
ATOM	551	HA	THR	34	36.701	25.302	43.453	1.00	0.00	
	H									
ATOM	552	CB	THR	34	37.313	24.068	45.048	1.00	0.00	C
ATOM	553	HB	THR	34	37.267	23.047	45.425	1.00	0.00	H
ATOM	554	CG2	THR	34	38.788	24.340	44.964	1.00	0.00	C
ATOM	555	HG21	THR	34	39.080	24.567	45.989	1.00	0.00	H
ATOM	556	HG22	THR	34	39.302	23.568	44.393	1.00	0.00	H
ATOM	557	HG23	THR	34	39.044	25.258	44.433	1.00	0.00	H
ATOM	558	OG1	THR	34	36.727	24.816	46.145	1.00	0.00	O
ATOM	559	HG1	THR	34	36.676	24.233	46.907	1.00	0.00	H
ATOM	560	C	THR	34	36.979	23.320	42.750	1.00	0.00	C
ATOM	561	O	THR	34	36.594	22.192	42.861	1.00	0.00	O
ATOM	562	N	GLY	35	37.765	23.702	41.798	1.00	0.00	N
ATOM	563	H	GLY	35	38.259	24.576	41.898	1.00	0.00	H
ATOM	564	CA	GLY	35	38.181	22.826	40.738	1.00	0.00	C
ATOM	565	HA2	GLY	35	39.046	23.357	40.340	1.00	0.00	H
ATOM	566	HA3	GLY	35	38.445	21.844	41.130	1.00	0.00	H
ATOM	567	C	GLY	35	37.193	22.651	39.535	1.00	0.00	C
ATOM	568	O	GLY	35	37.498	21.861	38.650	1.00	0.00	O
ATOM	569	N	ASP	36	36.119	23.387	39.502	1.00	0.00	N
ATOM	570	H	ASP	36	36.031	23.994	40.304	1.00	0.00	H
ATOM	571	CA	ASP	36	35.336	23.611	38.330	1.00	0.00	C
ATOM	572	HA	ASP	36	35.047	22.667	37.867	1.00	0.00	H
ATOM	573	CB	ASP	36	34.035	24.287	38.675	1.00	0.00	C
ATOM	574	HB2	ASP	36	34.134	25.267	39.143	1.00	0.00	H
ATOM	575	HB3	ASP	36	33.375	24.320	37.808	1.00	0.00	H
ATOM	576	CG	ASP	36	33.094	23.339	39.443	1.00	0.00	C

ATOM	577	OD1	ASP	36	31.920	23.775	39.724	1.00	0.00	O
ATOM	578	OD2	ASP	36	33.429	22.138	39.709	1.00	0.00	O
ATOM	579	C	ASP	36	36.076	24.339	37.280	1.00	0.00	C
ATOM	580	O	ASP	36	36.992	25.137	37.526	1.00	0.00	O
ATOM	581	N	VAL	37	35.643	24.168	36.002	1.00	0.00	N
ATOM	582	H	VAL	37	34.732	23.770	35.824	1.00	0.00	H
ATOM	583	CA	VAL	37	36.395	24.541	34.795	1.00	0.00	C
ATOM	584	HA	VAL	37	37.178	25.164	35.229	1.00	0.00	H
ATOM	585	CB	VAL	37	37.123	23.391	34.071	1.00	0.00	C
ATOM	586	HB	VAL	37	36.436	22.580	33.823	1.00	0.00	H
ATOM	587	CG1	VAL	37	37.906	23.729	32.783	1.00	0.00	C
ATOM	588	HG11	VAL	37	37.195	23.985	31.998	1.00	0.00	H
ATOM	589	HG12	VAL	37	38.464	24.637	33.014	1.00	0.00	H
ATOM	590	HG13	VAL	37	38.531	22.888	32.482	1.00	0.00	H
ATOM	591	CG2	VAL	37	38.064	22.672	35.016	1.00	0.00	C
ATOM	592	HG21	VAL	37	37.656	22.493	36.011	1.00	0.00	H
ATOM	593	HG22	VAL	37	38.419	21.727	34.606	1.00	0.00	H
ATOM	594	HG23	VAL	37	38.883	23.303	35.361	1.00	0.00	H
ATOM	595	C	VAL	37	35.510	25.281	33.798	1.00	0.00	C
ATOM	596	O	VAL	37	34.440	24.767	33.435	1.00	0.00	O
ATOM	597	N	LEU	38	35.920	26.442	33.229	1.00	0.00	N
ATOM	598	H	LEU	38	36.900	26.664	33.325	1.00	0.00	H
ATOM	599	CA	LEU	38	35.085	27.367	32.389	1.00	0.00	C
ATOM	600	HA	LEU	38	34.167	26.849	32.106	1.00	0.00	H
ATOM	601	CB	LEU	38	34.781	28.637	33.299	1.00	0.00	C
ATOM	602	HB2	LEU	38	35.657	29.065	33.788	1.00	0.00	H
ATOM	603	HB3	LEU	38	34.435	29.422	32.626	1.00	0.00	H
ATOM	604	CG	LEU	38	33.706	28.562	34.411	1.00	0.00	C
ATOM	605	HG	LEU	38	32.847	28.020	34.013	1.00	0.00	H
ATOM	606	CD1	LEU	38	34.086	27.804	35.670	1.00	0.00	C
ATOM	607	HD11	LEU	38	33.788	26.758	35.605	1.00	0.00	H
ATOM	608	HD12	LEU	38	35.153	27.782	35.890	1.00	0.00	H
ATOM	609	HD13	LEU	38	33.668	28.370	36.504	1.00	0.00	H
ATOM	610	CD2	LEU	38	33.145	29.961	34.782	1.00	0.00	C
ATOM	611	HD21	LEU	38	32.264	29.718	35.376	1.00	0.00	H
ATOM	612	HD22	LEU	38	33.823	30.570	35.381	1.00	0.00	H
ATOM	613	HD23	LEU	38	32.769	30.301	33.817	1.00	0.00	H
ATOM	614	C	LEU	38	35.765	27.725	31.068	1.00	0.00	C
ATOM	615	O	LEU	38	36.966	27.517	30.930	1.00	0.00	O
ATOM	616	N	ARG	39	35.048	28.316	30.090	1.00	0.00	N
ATOM	617	H	ARG	39	34.040	28.261	30.136	1.00	0.00	H
ATOM	618	CA	ARG	39	35.632	28.549	28.763	1.00	0.00	C
ATOM	619	HA	ARG	39	36.713	28.601	28.894	1.00	0.00	H
ATOM	620	CB	ARG	39	35.333	27.451	27.793	1.00	0.00	C
ATOM	621	HB2	ARG	39	34.320	27.575	27.408	1.00	0.00	H
ATOM	622	HB3	ARG	39	35.989	27.638	26.942	1.00	0.00	H
ATOM	623	CG	ARG	39	35.590	26.038	28.315	1.00	0.00	C
ATOM	624	HG2	ARG	39	36.610	25.882	28.667	1.00	0.00	H
ATOM	625	HG3	ARG	39	34.930	25.811	29.152	1.00	0.00	H
ATOM	626	CD	ARG	39	35.348	24.977	27.247	1.00	0.00	C
ATOM	627	HD2	ARG	39	36.026	25.212	26.426	1.00	0.00	H
ATOM	628	HD3	ARG	39	35.667	23.992	27.588	1.00	0.00	H
ATOM	629	NE	ARG	39	33.917	24.832	26.827	1.00	0.00	N
ATOM	630	HE	ARG	39	33.194	25.300	27.355	1.00	0.00	H
ATOM	631	CZ	ARG	39	33.478	24.281	25.683	1.00	0.00	C
ATOM	632	NH1	ARG	39	34.337	23.836	24.783	1.00	0.00	N
ATOM	633	HH11	ARG	39	35.305	23.805	25.069	1.00	0.00	H
ATOM	634	HH12	ARG	39	34.014	23.208	24.061	1.00	0.00	H
ATOM	635	NH2	ARG	39	32.205	24.184	25.301	1.00	0.00	N
ATOM	636	HH21	ARG	39	32.059	23.970	24.324	1.00	0.00	H
ATOM	637	HH22	ARG	39	31.524	24.770	25.763	1.00	0.00	H
ATOM	638	C	ARG	39	35.062	29.875	28.281	1.00	0.00	C
ATOM	639	O	ARG	39	33.927	30.333	28.540	1.00	0.00	O
ATOM	640	N	VAL	40	35.847	30.626	27.470	1.00	0.00	N

ATOM	641	H	VAL	40	36.747	30.199	27.302	1.00	0.00	H
ATOM	642	CA	VAL	40	35.460	31.863	26.743	1.00	0.00	C
ATOM	643	HA	VAL	40	34.385	31.792	26.582	1.00	0.00	H
ATOM	644	CB	VAL	40	35.803	33.115	27.492	1.00	0.00	C
ATOM	645	HB	VAL	40	35.633	33.994	26.869	1.00	0.00	H
ATOM	646	CG1	VAL	40	34.796	33.237	28.707	1.00	0.00	C
ATOM	647	HG11	VAL	40	34.970	32.420	29.408	1.00	0.00	H
ATOM	648	HG12	VAL	40	35.058	34.168	29.209	1.00	0.00	H
ATOM	649	HG13	VAL	40	33.744	33.301	28.425	1.00	0.00	H
ATOM	650	CG2	VAL	40	37.199	33.199	28.036	1.00	0.00	C
ATOM	651	HG21	VAL	40	37.936	33.154	27.234	1.00	0.00	H
ATOM	652	HG22	VAL	40	37.390	34.053	28.686	1.00	0.00	H
ATOM	653	HG23	VAL	40	37.330	32.319	28.664	1.00	0.00	H
ATOM	654	C	VAL	40	35.978	32.015	25.328	1.00	0.00	C
ATOM	655	O	VAL	40	37.162	31.746	25.064	1.00	0.00	O
ATOM	656	N	GLY	41	35.033	32.429	24.439	1.00	0.00	N
ATOM	657	H	GLY	41	34.066	32.559	24.698	1.00	0.00	H
ATOM	658	CA	GLY	41	35.468	32.858	23.112	1.00	0.00	C
ATOM	659	HA2	GLY	41	36.361	33.478	23.200	1.00	0.00	H
ATOM	660	HA3	GLY	41	35.868	31.952	22.658	1.00	0.00	H
ATOM	661	C	GLY	41	34.442	33.630	22.267	1.00	0.00	C
ATOM	662	O	GLY	41	33.252	33.604	22.631	1.00	0.00	O
ATOM	663	N	ARG	42	34.930	34.236	21.222	1.00	0.00	N
ATOM	664	H	ARG	42	35.931	34.219	21.094	1.00	0.00	H
ATOM	665	CA	ARG	42	34.165	34.901	20.243	1.00	0.00	C
ATOM	666	HA	ARG	42	33.261	34.325	20.051	1.00	0.00	H
ATOM	667	CB	ARG	42	33.610	36.202	20.837	1.00	0.00	C
ATOM	668	HB2	ARG	42	32.730	36.600	20.329	1.00	0.00	H
ATOM	669	HB3	ARG	42	33.289	36.001	21.859	1.00	0.00	H
ATOM	670	CG	ARG	42	34.675	37.351	21.126	1.00	0.00	C
ATOM	671	HG2	ARG	42	35.550	36.984	21.662	1.00	0.00	H
ATOM	672	HG3	ARG	42	34.976	37.850	20.205	1.00	0.00	H
ATOM	673	CD	ARG	42	33.887	38.290	22.009	1.00	0.00	C
ATOM	674	HD2	ARG	42	32.963	38.546	21.491	1.00	0.00	H
ATOM	675	HD3	ARG	42	33.609	37.707	22.887	1.00	0.00	H
ATOM	676	NE	ARG	42	34.602	39.500	22.226	1.00	0.00	N
ATOM	677	HE	ARG	42	34.269	40.285	21.686	1.00	0.00	H
ATOM	678	CZ	ARG	42	35.588	39.636	23.091	1.00	0.00	C
ATOM	679	NH1	ARG	42	36.082	38.653	23.842	1.00	0.00	N
ATOM	680	HH11	ARG	42	36.784	38.841	24.543	1.00	0.00	H
ATOM	681	HH12	ARG	42	35.557	37.792	23.887	1.00	0.00	H
ATOM	682	NH2	ARG	42	36.174	40.738	23.332	1.00	0.00	N
ATOM	683	HH21	ARG	42	35.963	41.568	22.797	1.00	0.00	H
ATOM	684	HH22	ARG	42	37.013	40.583	23.873	1.00	0.00	H
ATOM	685	C	ARG	42	34.808	35.003	18.930	1.00	0.00	C
ATOM	686	O	ARG	42	34.412	35.805	18.120	1.00	0.00	O
ATOM	687	N	PHE	43	35.814	34.237	18.557	1.00	0.00	N
ATOM	688	H	PHE	43	35.928	33.410	19.127	1.00	0.00	H
ATOM	689	CA	PHE	43	36.787	34.419	17.436	1.00	0.00	C
ATOM	690	HA	PHE	43	36.447	35.195	16.750	1.00	0.00	H
ATOM	691	CB	PHE	43	38.127	35.060	17.932	1.00	0.00	C
ATOM	692	HB2	PHE	43	38.634	34.253	18.461	1.00	0.00	H
ATOM	693	HB3	PHE	43	38.768	35.350	17.099	1.00	0.00	H
ATOM	694	CG	PHE	43	38.119	36.163	18.975	1.00	0.00	C
ATOM	695	CD1	PHE	43	37.417	37.340	18.819	1.00	0.00	C
ATOM	696	HD1	PHE	43	36.682	37.322	18.028	1.00	0.00	H
ATOM	697	CE1	PHE	43	37.435	38.282	19.814	1.00	0.00	C
ATOM	698	HE1	PHE	43	36.906	39.217	19.706	1.00	0.00	H
ATOM	699	CZ	PHE	43	38.149	38.072	21.009	1.00	0.00	C
ATOM	700	HZ	PHE	43	38.133	38.863	21.745	1.00	0.00	H
ATOM	701	CE2	PHE	43	38.908	36.914	21.182	1.00	0.00	C
ATOM	702	HE2	PHE	43	39.496	36.705	22.063	1.00	0.00	H
ATOM	703	CD2	PHE	43	38.860	35.970	20.168	1.00	0.00	C

ATOM	704	HD2	PHE	43	39.289	35.001	20.374	1.00	0.00	H
ATOM	705	C	PHE	43	37.103	33.137	16.702	1.00	0.00	C
ATOM	706	O	PHE	43	37.114	32.042	17.149	1.00	0.00	O
ATOM	707	N	GLU	44	37.416	33.242	15.404	1.00	0.00	N
ATOM	708	H	GLU	44	37.583	34.134	14.962	1.00	0.00	H
ATOM	709	CA	GLU	44	37.719	32.114	14.564	1.00	0.00	C
ATOM	710	HA	GLU	44	36.857	31.454	14.473	1.00	0.00	H
ATOM	711	CB	GLU	44	37.898	32.504	13.081	1.00	0.00	C
ATOM	712	HB2	GLU	44	36.915	32.875	12.792	1.00	0.00	H
ATOM	713	HB3	GLU	44	38.621	33.320	13.110	1.00	0.00	H
ATOM	714	CG	GLU	44	38.300	31.361	12.129	1.00	0.00	C
ATOM	715	HG2	GLU	44	39.376	31.235	12.016	1.00	0.00	H
ATOM	716	HG3	GLU	44	37.918	30.410	12.501	1.00	0.00	H
ATOM	717	CD	GLU	44	37.637	31.409	10.677	1.00	0.00	C
ATOM	718	OE1	GLU	44	36.457	31.361	10.471	1.00	0.00	O
ATOM	719	OE2	GLU	44	38.386	31.649	9.699	1.00	0.00	O
ATOM	720	C	GLU	44	38.902	31.225	15.052	1.00	0.00	C
ATOM	721	O	GLU	44	38.789	29.947	15.010	1.00	0.00	O
ATOM	722	N	ASP	45	39.962	31.750	15.591	1.00	0.00	N
ATOM	723	H	ASP	45	40.083	32.749	15.676	1.00	0.00	H
ATOM	724	CA	ASP	45	41.016	30.915	16.153	1.00	0.00	C
ATOM	725	HA	ASP	45	41.505	30.336	15.369	1.00	0.00	H
ATOM	726	CB	ASP	45	42.160	31.850	16.715	1.00	0.00	C
ATOM	727	HB2	ASP	45	42.407	32.608	15.971	1.00	0.00	H
ATOM	728	HB3	ASP	45	41.839	32.382	17.611	1.00	0.00	H
ATOM	729	CG	ASP	45	43.483	31.168	16.959	1.00	0.00	C
ATOM	730	OD1	ASP	45	43.504	29.905	16.910	1.00	0.00	O
ATOM	731	OD2	ASP	45	44.492	31.827	17.310	1.00	0.00	O
ATOM	732	C	ASP	45	40.518	29.954	17.217	1.00	0.00	C
ATOM	733	O	ASP	45	40.082	30.391	18.241	1.00	0.00	O
ATOM	734	N	ASP	46	40.547	28.614	16.965	1.00	0.00	N
ATOM	735	H	ASP	46	40.854	28.400	16.027	1.00	0.00	H
ATOM	736	CA	ASP	46	40.042	27.547	17.814	1.00	0.00	C
ATOM	737	HA	ASP	46	40.001	26.658	17.184	1.00	0.00	H
ATOM	738	CB	ASP	46	40.998	27.215	19.009	1.00	0.00	C
ATOM	739	HB2	ASP	46	41.080	28.190	19.489	1.00	0.00	H
ATOM	740	HB3	ASP	46	40.719	26.506	19.790	1.00	0.00	H
ATOM	741	CG	ASP	46	42.370	26.899	18.498	1.00	0.00	C
ATOM	742	OD1	ASP	46	42.530	26.288	17.422	1.00	0.00	O
ATOM	743	OD2	ASP	46	43.388	27.218	19.128	1.00	0.00	O
ATOM	744	C	ASP	46	38.589	27.637	18.317	1.00	0.00	C
ATOM	745	O	ASP	46	38.077	26.674	18.837	1.00	0.00	O
ATOM	746	N	GLY	47	37.858	28.743	18.148	1.00	0.00	N
ATOM	747	H	GLY	47	38.395	29.521	17.792	1.00	0.00	H
ATOM	748	CA	GLY	47	36.656	28.983	18.897	1.00	0.00	C
ATOM	749	HA2	GLY	47	36.045	29.756	18.430	1.00	0.00	H
ATOM	750	HA3	GLY	47	35.964	28.141	18.891	1.00	0.00	H
ATOM	751	C	GLY	47	36.792	29.329	20.369	1.00	0.00	C
ATOM	752	O	GLY	47	35.947	30.051	20.836	1.00	0.00	O
ATOM	753	N	TYR	48	37.801	28.858	21.027	1.00	0.00	N
ATOM	754	H	TYR	48	38.434	28.260	20.518	1.00	0.00	H
ATOM	755	CA	TYR	48	38.104	29.407	22.423	1.00	0.00	C
ATOM	756	HA	TYR	48	37.389	30.198	22.647	1.00	0.00	H
ATOM	757	CB	TYR	48	37.785	28.314	23.401	1.00	0.00	C
ATOM	758	HB2	TYR	48	38.523	27.527	23.252	1.00	0.00	H
ATOM	759	HB3	TYR	48	38.034	28.613	24.419	1.00	0.00	H
ATOM	760	CG	TYR	48	36.373	27.798	23.429	1.00	0.00	C
ATOM	761	CD1	TYR	48	35.366	28.578	23.973	1.00	0.00	C
ATOM	762	HD1	TYR	48	35.737	29.388	24.585	1.00	0.00	H
ATOM	763	CE1	TYR	48	34.016	28.301	23.705	1.00	0.00	C
ATOM	764	HE1	TYR	48	33.216	28.886	24.132	1.00	0.00	H
ATOM	765	CZ	TYR	48	33.682	27.143	22.945	1.00	0.00	C
ATOM	766	OH	TYR	48	32.390	26.943	22.581	1.00	0.00	O
ATOM	767	HH	TYR	48	32.415	26.164	22.020	1.00	0.00	H

```
ATOM    768  CE2 TYR    48      34.762  26.343  22.419  1.00  0.00           C
ATOM    769  HE2 TYR    48      34.554  25.424  21.892  1.00  0.00           H
ATOM    770  CD2 TYR    48      36.110  26.680  22.706  1.00  0.00           C
ATOM    771  HD2 TYR    48      36.946  26.126  22.304  1.00  0.00           H
ATOM    772  C   TYR    48      39.479  30.026  22.536  1.00  0.00           C
ATOM    773  O   TYR    48      40.355  29.521  21.856  1.00  0.00           O
ATOM    774  N   PHE    49      39.641  31.122  23.316  1.00  0.00           N
ATOM    775  H   PHE    49      38.833  31.589  23.701  1.00  0.00           H
ATOM    776  CA  PHE    49      40.894  31.835  23.379  1.00  0.00           C
ATOM    777  HA  PHE    49      41.547  31.530  22.561  1.00  0.00           H
ATOM    778  CB  PHE    49      40.692  33.328  23.071  1.00  0.00           C
ATOM    779  HB2 PHE    49      41.704  33.663  22.843  1.00  0.00           H
ATOM    780  HB3 PHE    49      40.194  33.523  22.121  1.00  0.00           H
ATOM    781  CG  PHE    49      40.103  34.189  24.142  1.00  0.00           C
ATOM    782  CD1 PHE    49      38.826  34.766  23.920  1.00  0.00           C
ATOM    783  HD1 PHE    49      38.256  34.523  23.036  1.00  0.00           H
ATOM    784  CE1 PHE    49      38.202  35.584  24.857  1.00  0.00           C
ATOM    785  HE1 PHE    49      37.281  36.106  24.642  1.00  0.00           H
ATOM    786  CZ  PHE    49      38.882  35.903  26.005  1.00  0.00           C
ATOM    787  HZ  PHE    49      38.406  36.626  26.652  1.00  0.00           H
ATOM    788  CE2 PHE    49      40.186  35.338  26.291  1.00  0.00           C
ATOM    789  HE2 PHE    49      40.674  35.554  27.230  1.00  0.00           H
ATOM    790  CD2 PHE    49      40.769  34.431  25.366  1.00  0.00           C
ATOM    791  HD2 PHE    49      41.718  33.968  25.592  1.00  0.00           H
ATOM    792  C   PHE    49      41.540  31.570  24.771  1.00  0.00           C
ATOM    793  O   PHE    49      42.708  31.756  24.998  1.00  0.00           O
ATOM    794  N   CYX    50      40.711  31.215  25.748  1.00  0.00           N
ATOM    795  H   CYX    50      39.708  31.199  25.630  1.00  0.00           H
ATOM    796  CA  CYX    50      41.111  30.797  27.080  1.00  0.00           C
ATOM    797  HA  CYX    50      42.108  30.356  27.061  1.00  0.00           H
ATOM    798  CB  CYX    50      41.009  31.998  27.962  1.00  0.00           C
ATOM    799  HB2 CYX    50      40.515  32.798  27.409  1.00  0.00           H
ATOM    800  HB3 CYX    50      40.553  31.798  28.931  1.00  0.00           H
ATOM    801  SG  CYX    50      42.639  32.575  28.516  1.00  0.00           S
ATOM    802  C   CYX    50      40.095
  29.803  27.750  1.00  0.00           C
ATOM    803  O   CYX    50      38.857  29.952  27.691  1.00  0.00           O
ATOM    804  N   THR    51      40.703  28.744  28.319  1.00  0.00           N
ATOM    805  H   THR    51      41.709  28.831  28.327  1.00  0.00           H
ATOM    806  CA  THR    51      40.038  27.940  29.304  1.00  0.00           C
ATOM    807  HA  THR    51      38.986  28.206  29.399  1.00  0.00           H
ATOM    808  CB  THR    51      40.155  26.463  28.987  1.00  0.00           C
ATOM    809  HB  THR    51      41.198  26.158  28.890  1.00  0.00           H
ATOM    810  CG2 THR    51      39.638  25.506  30.085  1.00  0.00           C
ATOM    811 HG21 THR    51      38.600  25.742  30.323  1.00  0.00           H
ATOM    812 HG22 THR    51      39.652  24.522  29.614  1.00  0.00           H
ATOM    813 HG23 THR    51      40.201  25.585  31.014  1.00  0.00           H
ATOM    814  OG1 THR    51      39.611  26.037  27.708  1.00  0.00           O
ATOM    815  HG1 THR    51      40.086  26.675  27.171  1.00  0.00           H
ATOM    816  C   THR    51      40.467  28.400  30.743  1.00  0.00           C
ATOM    817  O   THR    51      41.637  28.771  30.953  1.00  0.00           O
ATOM    818  N   ILE    52      39.478  28.309  31.643  1.00  0.00           N
ATOM    819  H   ILE    52      38.633  27.879  31.296  1.00  0.00           H
ATOM    820  CA  ILE    52      39.486  28.901  32.951  1.00  0.00           C
ATOM    821  HA  ILE    52      40.469  29.332  33.145  1.00  0.00           H
ATOM    822  CB  ILE    52      38.431  30.078  32.987  1.00  0.00           C
ATOM    823  HB  ILE    52      37.535  29.500  32.765  1.00  0.00           H
ATOM    824  CG2 ILE    52      38.149  30.696  34.434  1.00  0.00           C
ATOM    825 HG21 ILE    52      37.755  29.937  35.109  1.00  0.00           H
ATOM    826 HG22 ILE    52      39.054  31.070  34.912  1.00  0.00           H
ATOM    827 HG23 ILE    52      37.395  31.484  34.422  1.00  0.00           H
ATOM    828  CG1 ILE    52      38.754  31.205  32.011  1.00  0.00           C
ATOM    829 HG12 ILE    52      39.443  31.827  32.582  1.00  0.00           H
ATOM    830 HG13 ILE    52      39.255  30.867  31.104  1.00  0.00           H
```

ATOM	831	CD1	ILE	52	37.384	31.837	31.557	1.00	0.00	C
ATOM	832	HD11	ILE	52	36.693	31.936	32.394	1.00	0.00	H
ATOM	833	HD12	ILE	52	37.501	32.805	31.070	1.00	0.00	H
ATOM	834	HD13	ILE	52	37.024	31.168	30.775	1.00	0.00	H
ATOM	835	C	ILE	52	39.226	27.876	34.083	1.00	0.00	C
ATOM	836	O	ILE	52	38.577	26.898	33.749	1.00	0.00	O
ATOM	837	N	GLU	53	39.724	27.981	35.263	1.00	0.00	N
ATOM	838	H	GLU	53	40.323	28.783	35.396	1.00	0.00	H
ATOM	839	CA	GLU	53	39.494	27.047	36.307	1.00	0.00	C
ATOM	840	HA	GLU	53	38.588	26.446	36.220	1.00	0.00	H
ATOM	841	CB	GLU	53	40.718	26.077	36.464	1.00	0.00	C
ATOM	842	HB2	GLU	53	40.788	25.503	35.541	1.00	0.00	H
ATOM	843	HB3	GLU	53	41.609	26.689	36.608	1.00	0.00	H
ATOM	844	CG	GLU	53	40.520	25.090	37.633	1.00	0.00	C
ATOM	845	HG2	GLU	53	40.562	25.579	38.606	1.00	0.00	H
ATOM	846	HG3	GLU	53	39.582	24.561	37.457	1.00	0.00	H
ATOM	847	CD	GLU	53	41.867	24.289	37.694	1.00	0.00	C
ATOM	848	OE1	GLU	53	42.079	23.701	38.804	1.00	0.00	O
ATOM	849	OE2	GLU	53	42.572	24.147	36.699	1.00	0.00	O
ATOM	850	C	GLU	53	39.267	27.861	37.679	1.00	0.00	C
ATOM	851	O	GLU	53	39.722	28.996	37.869	1.00	0.00	O
ATOM	852	N	VAL	54	38.617	27.295	38.685	1.00	0.00	N
ATOM	853	H	VAL	54	38.130	26.433	38.487	1.00	0.00	H
ATOM	854	CA	VAL	54	38.313	27.851	39.965	1.00	0.00	C
ATOM	855	HA	VAL	54	38.259	28.937	39.892	1.00	0.00	H
ATOM	856	CB	VAL	54	36.871	27.503	40.425	1.00	0.00	C
ATOM	857	HB	VAL	54	36.756	26.421	40.484	1.00	0.00	H
ATOM	858	CG1	VAL	54	36.499	28.139	41.769	1.00	0.00	C
ATOM	859	HG11	VAL	54	37.354	28.033	42.436	1.00	0.00	H
ATOM	860	HG12	VAL	54	36.419	29.212	41.595	1.00	0.00	H
ATOM	861	HG13	VAL	54	35.577	27.773	42.220	1.00	0.00	H
ATOM	862	CG2	VAL	54	35.752	27.966	39.449	1.00	0.00	C
ATOM	863	HG21	VAL	54	35.911	29.039	39.353	1.00	0.00	H
ATOM	864	HG22	VAL	54	35.993	27.507	38.489	1.00	0.00	H
ATOM	865	HG23	VAL	54	34.746	27.718	39.788	1.00	0.00	H
ATOM	866	C	VAL	54	39.385	27.509	40.921	1.00	0.00	C
ATOM	867	O	VAL	54	39.430	26.347	41.376	1.00	0.00	O
ATOM	868	N	THR	55	40.270	28.374	41.337	1.00	0.00	N
ATOM	869	H	THR	55	39.998	29.291	41.011	1.00	0.00	H
ATOM	870	CA	THR	55	41.270	28.191	42.370	1.00	0.00	C
ATOM	871	HA	THR	55	41.994	27.413	42.127	1.00	0.00	H
ATOM	872	CB	THR	55	42.307	29.415	42.276	1.00	0.00	C
ATOM	873	HB	THR	55	43.008	29.353	43.108	1.00	0.00	H
ATOM	874	CG2	THR	55	43.058	29.621	40.995	1.00	0.00	C
ATOM	875	HG21	THR	55	42.317	30.115	40.367	1.00	0.00	H
ATOM	876	HG22	THR	55	44.054	30.024	41.179	1.00	0.00	H
ATOM	877	HG23	THR	55	43.385	28.680	40.550	1.00	0.00	H
ATOM	878	OG1	THR	55	41.634	30.600	42.568	1.00	0.00	O
ATOM	879	HG1	THR	55	42.306	31.157	42.968	1.00	0.00	H
ATOM	880	C	THR	55	40.665	27.902	43.743	1.00	0.00	C
ATOM	881	O	THR	55	41.161	27.074	44.402	1.00	0.00	O
ATOM	882	N	ALA	56	39.692	28.745	44.133	1.00	0.00	N
ATOM	883	H	ALA	56	39.414	29.473	43.489	1.00	0.00	H
ATOM	884	CA	ALA	56	39.264	28.617	45.544	1.00	0.00	C
ATOM	885	HA	ALA	56	39.334	27.570	45.836	1.00	0.00	H
ATOM	886	CB	ALA	56	40.296	29.423	46.364	1.00	0.00	C
ATOM	887	HB1	ALA	56	40.029	29.357	47.419	1.00	0.00	H
ATOM	888	HB2	ALA	56	41.339	29.151	46.205	1.00	0.00	H
ATOM	889	HB3	ALA	56	40.179	30.474	46.095	1.00	0.00	H
ATOM	890	C	ALA	56	37.839	29.051	45.818	1.00	0.00	C
ATOM	891	O	ALA	56	37.244	29.739	44.990	1.00	0.00	O
ATOM	892	N	THR	57	37.207	28.642	46.920	1.00	0.00	N
ATOM	893	H	THR	57	37.727	28.090	47.588	1.00	0.00	H
ATOM	894	CA	THR	57	35.752	29.005	47.178	1.00	0.00	C

ATOM	895	HA	THR	57	35.455	29.953	46.727	1.00	0.00	H
ATOM	896	CB	THR	57	34.785	28.001	46.487	1.00	0.00	C
ATOM	897	HB	THR	57	33.832	28.527	46.549	1.00	0.00	H
ATOM	898	CG2	THR	57	34.997	27.733	45.024	1.00	0.00	C
ATOM	899	HG21	THR	57	34.832	28.604	44.390	1.00	0.00	H
ATOM	900	HG22	THR	57	35.952	27.271	44.772	1.00	0.00	H
ATOM	901	HG23	THR	57	34.207	27.069	44.671	1.00	0.00	H
ATOM	902	OG1	THR	57	34.749	26.829	47.146	1.00	0.00	O
ATOM	903	HG1	THR	57	35.456	26.267	46.819	1.00	0.00	H
ATOM	904	C	THR	57	35.507	29.188	48.673	1.00	0.00	C
ATOM	905	O	THR	57	36.143	28.535	49.482	1.00	0.00	O
ATOM	906	N	SER	58	34.464	30.014	49.038	1.00	0.00	N
ATOM	907	H	SER	58	34.016	30.645	48.388	1.00	0.00	H
ATOM	908	CA	SER	58	34.161	30.280	50.477	1.00	0.00	C
ATOM	909	HA	SER	58	33.965	29.287	50.882	1.00	0.00	H
ATOM	910	CB	SER	58	35.324	31.014	51.213	1.00	0.00	C
ATOM	911	HB2	SER	58	34.975	31.237	52.222	1.00	0.00	H
ATOM	912	HB3	SER	58	36.149	30.301	51.211	1.00	0.00	H
ATOM	913	OG	SER	58	35.679	32.180	50.568	1.00	0.00	O
ATOM	914	HG	SER	58	36.155	31.916	49.777	1.00	0.00	H
ATOM	915	C	SER	58	32.880	30.996	50.725	1.00	0.00	C
ATOM	916	O	SER	58	32.405	31.821	49.916	1.00	0.00	O
ATOM	917	N	THR	59	32.204	30.716	51.850	1.00	0.00	N
ATOM	918	H	THR	59	32.562	30.018	52.486	1.00	0.00	H
ATOM	919	CA	THR	59	31.054	31.433	52.420	1.00	0.00	C
ATOM	920	HA	THR	59	30.337	31.645	51.627	1.00	0.00	H
ATOM	921	CB	THR	59	30.399	30.565	53.478	1.00	0.00	C
ATOM	922	HB	THR	59	30.578	29.519	53.229	1.00	0.00	H
ATOM	923	CG2	THR	59	30.990	30.703	54.934	1.00	0.00	C
ATOM	924	HG21	THR	59	30.587	29.878	55.521	1.00	0.00	H
ATOM	925	HG22	THR	59	32.080	30.691	54.937	1.00	0.00	H
ATOM	926	HG23	THR	59	30.730	31.613	55.474	1.00	0.00	H
ATOM	927	OG1	THR	59	28.975	30.868	53.457	1.00	0.00	
	O									
ATOM	928	HG1	THR	59	28.686	30.669	52.563	1.00	0.00	H
ATOM	929	C	THR	59	31.542	32.836	52.959	1.00	0.00	C
ATOM	930	O	THR	59	32.695	32.948	53.271	1.00	0.00	O
ATOM	931	N	VAL	60	30.680	33.815	53.183	1.00	0.00	N
ATOM	932	H	VAL	60	29.744	33.675	52.828	1.00	0.00	H
ATOM	933	CA	VAL	60	31.020	35.113	53.832	1.00	0.00	C
ATOM	934	HA	VAL	60	31.588	34.924	54.743	1.00	0.00	H
ATOM	935	CB	VAL	60	31.801	36.013	52.815	1.00	0.00	C
ATOM	936	HB	VAL	60	32.447	35.373	52.214	1.00	0.00	H
ATOM	937	CG1	VAL	60	30.849	36.804	51.822	1.00	0.00	C
ATOM	938	HG11	VAL	60	30.130	36.105	51.393	1.00	0.00	H
ATOM	939	HG12	VAL	60	30.240	37.609	52.233	1.00	0.00	H
ATOM	940	HG13	VAL	60	31.458	37.301	51.067	1.00	0.00	H
ATOM	941	CG2	VAL	60	32.765	37.083	53.328	1.00	0.00	C
ATOM	942	HG21	VAL	60	33.318	37.554	52.515	1.00	0.00	H
ATOM	943	HG22	VAL	60	32.352	37.994	53.761	1.00	0.00	H
ATOM	944	HG23	VAL	60	33.439	36.655	54.070	1.00	0.00	H
ATOM	945	C	VAL	60	29.761	35.809	54.279	1.00	0.00	C
ATOM	946	O	VAL	60	28.683	35.502	53.774	1.00	0.00	O
ATOM	947	N	THR	61	29.939	36.738	55.203	1.00	0.00	N
ATOM	948	H	THR	61	30.898	37.002	55.380	1.00	0.00	H
ATOM	949	CA	THR	61	28.831	37.709	55.578	1.00	0.00	C
ATOM	950	HA	THR	61	27.998	37.750	54.876	1.00	0.00	H
ATOM	951	CB	THR	61	28.308	37.315	56.977	1.00	0.00	C
ATOM	952	HB	THR	61	27.683	38.090	57.421	1.00	0.00	H
ATOM	953	CG2	THR	61	27.397	36.052	57.014	1.00	0.00	C
ATOM	954	HG21	THR	61	26.583	36.220	56.309	1.00	0.00	H
ATOM	955	HG22	THR	61	27.951	35.170	56.693	1.00	0.00	H
ATOM	956	HG23	THR	61	27.078	35.936	58.050	1.00	0.00	H
ATOM	957	OG1	THR	61	29.395	36.997	57.824	1.00	0.00	O

ATOM	958	HG1	THR	61	29.642	37.700	58.429	1.00	0.00	H
ATOM	959	C	THR	61	29.365	39.095	55.710	1.00	0.00	C
ATOM	960	O	THR	61	30.538	39.407	55.748	1.00	0.00	O
ATOM	961	N	LEU	62	28.461	40.061	55.949	1.00	0.00	N
ATOM	962	H	LEU	62	27.500	39.784	55.803	1.00	0.00	H
ATOM	963	CA	LEU	62	28.701	41.513	55.999	1.00	0.00	C
ATOM	964	HA	LEU	62	29.094	41.764	55.014	1.00	0.00	H
ATOM	965	CB	LEU	62	27.392	42.293	56.111	1.00	0.00	C
ATOM	966	HB2	LEU	62	26.957	42.007	57.068	1.00	0.00	H
ATOM	967	HB3	LEU	62	27.654	43.350	56.150	1.00	0.00	H
ATOM	968	CG	LEU	62	26.394	42.089	54.943	1.00	0.00	C
ATOM	969	HG	LEU	62	26.266	41.050	54.639	1.00	0.00	H
ATOM	970	CD1	LEU	62	25.063	42.572	55.396	1.00	0.00	C
ATOM	971	HD11	LEU	62	24.583	42.756	54.434	1.00	0.00	H
ATOM	972	HD12	LEU	62	24.748	41.889	56.185	1.00	0.00	H
ATOM	973	HD13	LEU	62	25.057	43.592	55.780	1.00	0.00	H
ATOM	974	CD2	LEU	62	26.881	42.778	53.680	1.00	0.00	C
ATOM	975	HD21	LEU	62	27.777	42.270	53.324	1.00	0.00	H
ATOM	976	HD22	LEU	62	26.161	42.768	52.861	1.00	0.00	H
ATOM	977	HD23	LEU	62	27.098	43.824	53.900	1.00	0.00	H
ATOM	978	C	LEU	62	29.782	41.808	57.018	1.00	0.00	C
ATOM	979	O	LEU	62	30.567	42.781	56.844	1.00	0.00	O
ATOM	980	N	ASP	63	29.834	41.107	58.159	1.00	0.00	N
ATOM	981	H	ASP	63	29.020	40.529	58.314	1.00	0.00	H
ATOM	982	CA	ASP	63	30.813	41.380	59.304	1.00	0.00	C
ATOM	983	HA	ASP	63	30.832	42.455	59.486	1.00	0.00	H
ATOM	984	CB	ASP	63	30.342	40.630	60.573	1.00	0.00	C
ATOM	985	HB2	ASP	63	31.054	41.004	61.309	1.00	0.00	H
ATOM	986	HB3	ASP	63	29.357	41.032	60.811	1.00	0.00	H
ATOM	987	CG	ASP	63	30.265	39.117	60.524	1.00	0.00	C
ATOM	988	OD1	ASP	63	29.964	38.423	61.473	1.00	0.00	O
ATOM	989	OD2	ASP	63	30.710	38.510	59.564	1.00	0.00	O
ATOM	990	C	ASP	63	32.294	40.962	58.955	1.00	0.00	C
ATOM	991	O	ASP	63	33.202	41.367	59.583	1.00	0.00	O
ATOM	992	N	THR	64	32.547	40.222	57.864	1.00	0.00	N
ATOM	993	H	THR	64	31.753	39.929	57.312	1.00	0.00	H
ATOM	994	CA	THR	64	33.826	39.575	57.453	1.00	0.00	C
ATOM	995	HA	THR	64	34.698	39.912	58.015	1.00	0.00	H
ATOM	996	CB	THR	64	33.829	38.103	57.901	1.00	0.00	C
ATOM	997	HB	THR	64	34.652	37.598	57.396	1.00	0.00	H
ATOM	998	CG2	THR	64	34.051	37.953	59.408	1.00	0.00	C
ATOM	999	HG21	THR	64	33.654	38.815	59.943	1.00	0.00	H
ATOM	1000	HG22	THR	64	33.558	37.024	59.694	1.00	0.00	H
ATOM	1001	HG23	THR	64	35.126	37.964	59.592	1.00	0.00	H
ATOM	1002	OG1	THR	64	32.579	37.514	57.586	1.00	0.00	O
ATOM	1003	HG1	THR	64	31.990	37.841	58.269	1.00	0.00	H
ATOM	1004	C	THR	64	34.072	39.655	55.952	1.00	0.00	C
ATOM	1005	O	THR	64	35.049	39.121	55.519	1.00	0.00	O
ATOM	1006	N	LEU	65	33.267	40.421	55.249	1.00	0.00	N
ATOM	1007	H	LEU	65	32.359	40.691	55.602	1.00	0.00	H
ATOM	1008	CA	LEU	65	33.534	40.845	53.946	1.00	0.00	C
ATOM	1009	HA	LEU	65	33.487	39.932	53.352	1.00	0.00	H
ATOM	1010	CB	LEU	65	32.408	41.655	53.376	1.00	0.00	C
ATOM	1011	HB2	LEU	65	31.486	41.288	53.827	1.00	0.00	H
ATOM	1012	HB3	LEU	65	32.601	42.685	53.678	1.00	0.00	H
ATOM	1013	CG	LEU	65	32.172	41.693	51.890	1.00	0.00	C
ATOM	1014	HG	LEU	65	33.163	41.905	51.486	1.00	0.00	H
ATOM	1015	CD1	LEU	65	31.704	40.420	51.274	1.00	0.00	C
ATOM	1016	HD11	LEU	65	30.656	40.240	51.512	1.00	0.00	H
ATOM	1017	HD12	LEU	65	31.685	40.546	50.191	1.00	0.00	H
ATOM	1018	HD13	LEU	65	32.284	39.567	51.627	1.00	0.00	H
ATOM	1019	CD2	LEU	65	31.261	42.864	51.507	1.00	0.00	C
ATOM	1020	HD21	LEU	65	30.315	42.865	52.049	1.00	0.00	H
ATOM	1021	HD22	LEU	65	31.694	43.835	51.752	1.00	0.00	H

```
ATOM   1022 HD23 LEU    65      31.117  42.917  50.427  1.00  0.00           H
ATOM   1023  C   LEU    65      34.924  41.503  53.822  1.00  0.00           C
ATOM   1024  O   LEU    65      35.349  42.369  54.619  1.00  0.00           O
ATOM   1025  N   THR    66      35.645  41.043  52.781  1.00  0.00           N
ATOM   1026  H   THR    66      35.340  40.293  52.178  1.00  0.00           H
ATOM   1027  CA  THR    66      37.079  41.489  52.594  1.00  0.00           C
ATOM   1028  HA  THR    66      37.518  42.142  53.347  1.00  0.00           H
ATOM   1029  CB  THR    66      38.007  40.268  52.340  1.00  0.00           C
ATOM   1030  HB  THR    66      37.735  39.816  51.386  1.00  0.00           H
ATOM   1031  CG2 THR    66      39.517  40.441  52.181  1.00  0.00           C
ATOM   1032 HG21 THR    66      39.905  40.734  53.156  1.00  0.00           H
ATOM   1033 HG22 THR    66      40.026  39.525  51.880  1.00  0.00           H
ATOM   1034 HG23 THR    66      39.655  41.091  51.316  1.00  0.00           H
ATOM   1035  OG1 THR    66      37.945  39.267  53.370  1.00  0.00           O
ATOM   1036  HG1 THR    66      37.071  38.869  53.372  1.00  0.00           H
ATOM   1037  C   THR    66      37.017  42.408  51.369  1.00  0.00           C
ATOM   1038  O   THR    66      36.209  42.370  50.474  1.00  0.00           O
ATOM   1039  N   GLU    67      38.095  43.201  51.164  1.00  0.00           N
ATOM   1040  H   GLU    67      38.882  43.123  51.793  1.00  0.00           H
ATOM   1041  CA  GLU    67      38.165  44.304  50.164  1.00  0.00           C
ATOM   1042  HA  GLU    67      37.213  44.759  49.891  1.00  0.00           H
ATOM   1043  CB  GLU    67      38.979  45.489  50.762  1.00  0.00           C
ATOM   1044  HB2 GLU    67      38.836  46.383  50.154  1.00  0.00           H
ATOM   1045  HB3 GLU    67      38.533  45.724  51.728  1.00  0.00           H
ATOM   1046  CG  GLU    67      40.517  45.277  50.748  1.00  0.00           C
ATOM   1047  HG2 GLU    67      40.943  45.377  49.749  1.00  0.00           H
ATOM   1048  HG3 GLU    67      40.987  46.029  51.380  1.00  0.00           H
ATOM   1049  CD  GLU    67      40.948  43.979  51.403  1.00  0.00           C
ATOM   1050  OE1 GLU    67      41.727  43.204  50.865  1.00  0.00           O
ATOM   1051  OE2 GLU    67      40.472  43.683  52.531  1.00  0.00           O
ATOM   1052  C   GLU    67      38.825  43.947  48.823  1.00  0.00           C
ATOM   1053
 O   GLU    67      38.816  44.774  47.934  1.00  0.00           O
ATOM   1054  N   LYS    68      39.332  42.710  48.660  1.00  0.00           N
ATOM   1055  H   LYS    68      39.363  42.087  49.455  1.00  0.00           H
ATOM   1056  CA  LYS    68      39.987  42.332  47.380  1.00  0.00           C
ATOM   1057  HA  LYS    68      40.886  42.923  47.214  1.00  0.00           H
ATOM   1058  CB  LYS    68      40.513  40.897  47.598  1.00  0.00           C
ATOM   1059  HB2 LYS    68      40.966  40.773  48.582  1.00  0.00           H
ATOM   1060  HB3 LYS    68      39.665  40.212  47.579  1.00  0.00           H
ATOM   1061  CG  LYS    68      41.517  40.371  46.521  1.00  0.00           C
ATOM   1062  HG2 LYS    68      41.768  39.319  46.658  1.00  0.00           H
ATOM   1063  HG3 LYS    68      41.002  40.575  45.582  1.00  0.00           H
ATOM   1064  CD  LYS    68      42.912  41.068  46.497  1.00  0.00           C
ATOM   1065  HD2 LYS    68      43.464  40.578  45.695  1.00  0.00           H
ATOM   1066  HD3 LYS    68      42.809  42.144  46.351  1.00  0.00           H
ATOM   1067  CE  LYS    68      43.716  40.814  47.766  1.00  0.00           C
ATOM   1068  HE2 LYS    68      43.235  41.222  48.655  1.00  0.00           H
ATOM   1069  HE3 LYS    68      43.757  39.732  47.886  1.00  0.00           H
ATOM   1070  NZ  LYS    68      45.008  41.487  47.728  1.00  0.00           N
ATOM   1071  HZ1 LYS    68      44.984  42.496  47.696  1.00  0.00           H
ATOM   1072  HZ2 LYS    68      45.482  41.353  48.609  1.00  0.00           H
ATOM   1073  HZ3 LYS    68      45.651  41.264  46.981  1.00  0.00           H
ATOM   1074  C   LYS    68      39.051  42.426  46.176  1.00  0.00           C
ATOM   1075  O   LYS    68      39.488  42.678  45.056  1.00  0.00           O
ATOM   1076  N   HIE    69      37.699  42.212  46.360  1.00  0.00           N
ATOM   1077  H   HIE    69      37.408  41.898  47.275  1.00  0.00           H
ATOM   1078  CA  HIE    69      36.678  42.410  45.324  1.00  0.00           C
ATOM   1079  HA  HIE    69      37.064  42.076  44.361  1.00  0.00           H
ATOM   1080  CB  HIE    69      35.468  41.439  45.472  1.00  0.00           C
ATOM   1081  HB2 HIE    69      35.941  40.458  45.428  1.00  0.00           H
ATOM   1082  HB3 HIE    69      35.021  41.680  46.436  1.00  0.00           H
ATOM   1083  CG  HIE    69      34.491  41.634  44.391  1.00  0.00           C
ATOM   1084  ND1 HIE    69      34.478  40.962  43.198  1.00  0.00           N
```

ATOM	1085	CE1	HIE	69	33.428	41.348	42.602	1.00	0.00	C
ATOM	1086	HE1	HIE	69	33.298	41.131	41.552	1.00	0.00	H
ATOM	1087	NE2	HIE	69	32.656	42.134	43.337	1.00	0.00	N
ATOM	1088	HE2	HIE	69	31.804	42.538	42.975	1.00	0.00	H
ATOM	1089	CD2	HIE	69	33.330	42.349	44.501	1.00	0.00	C
ATOM	1090	HD2	HIE	69	32.907	42.966	45.281	1.00	0.00	H
ATOM	1091	C	HIE	69	36.293	43.854	45.120	1.00	0.00	C
ATOM	1092	O	HIE	69	36.012	44.272	43.952	1.00	0.00	O
ATOM	1093	N	ALA	70	36.278	44.688	46.180	1.00	0.00	N
ATOM	1094	H	ALA	70	36.622	44.290	47.042	1.00	0.00	H
ATOM	1095	CA	ALA	70	36.018	46.136	46.025	1.00	0.00	C
ATOM	1096	HA	ALA	70	35.080	46.277	45.489	1.00	0.00	H
ATOM	1097	CB	ALA	70	35.755	46.771	47.415	1.00	0.00	C
ATOM	1098	HB1	ALA	70	35.465	47.810	47.258	1.00	0.00	H
ATOM	1099	HB2	ALA	70	34.917	46.296	47.923	1.00	0.00	H
ATOM	1100	HB3	ALA	70	36.638	46.626	48.038	1.00	0.00	H
ATOM	1101	C	ALA	70	37.155	46.806	45.204	1.00	0.00	C
ATOM	1102	O	ALA	70	36.899	47.564	44.308	1.00	0.00	O
ATOM	1103	N	GLU	71	38.415	46.403	45.531	1.00	0.00	N
ATOM	1104	H	GLU	71	38.586	45.744	46.277	1.00	0.00	H
ATOM	1105	CA	GLU	71	39.623	46.838	44.788	1.00	0.00	C
ATOM	1106	HA	GLU	71	39.767	47.918	44.772	1.00	0.00	H
ATOM	1107	CB	GLU	71	40.943	46.296	45.516	1.00	0.00	C
ATOM	1108	HB2	GLU	71	40.899	45.220	45.682	1.00	0.00	H
ATOM	1109	HB3	GLU	71	41.751	46.490	44.811	1.00	0.00	H
ATOM	1110	CG	GLU	71	41.270	46.910	46.900	1.00	0.00	C
ATOM	1111	HG2	GLU	71	40.608	46.516	47.672	1.00	0.00	H
ATOM	1112	HG3	GLU	71	42.239	46.528	47.221	1.00	0.00	H
ATOM	1113	CD	GLU	71	41.185	48.433	46.871	1.00	0.00	C
ATOM	1114	OE1	GLU	71	41.754	48.988	45.913	1.00	0.00	O
ATOM	1115	OE2	GLU	71	40.729	49.091	47.853	1.00	0.00	O
ATOM	1116	C	GLU	71	39.621	46.446	43.269	1.00	0.00	C
ATOM	1117	O	GLU	71	39.906	47.311	42.410	1.00	0.00	O
ATOM	1118	N	GLN	72	39.056	45.235	42.902	1.00	0.00	N
ATOM	1119	H	GLN	72	38.843	44.616	43.671	1.00	0.00	H
ATOM	1120	CA	GLN	72	38.783	44.758	41.533	1.00	0.00	C
ATOM	1121	HA	GLN	72	39.735	44.677	41.006	1.00	0.00	H
ATOM	1122	CB	GLN	72	38.268	43.358	41.609	1.00	0.00	C
ATOM	1123	HB2	GLN	72	37.233	43.301	41.946	1.00	0.00	H
ATOM	1124	HB3	GLN	72	38.753	42.786	42.400	1.00	0.00	H
ATOM	1125	CG	GLN	72	38.413	42.719	40.171	1.00	0.00	C
ATOM	1126	HG2	GLN	72	37.826	43.278	39.441	1.00	0.00	H
ATOM	1127	HG3	GLN	72	39.475	42.671	39.934	1.00	0.00	H
ATOM	1128	CD	GLN	72	37.903	41.342	40.407	1.00	0.00	C
ATOM	1129	OE1	GLN	72	38.602	40.352	40.439	1.00	0.00	O
ATOM	1130	NE2	GLN	72	36.621	41.127	40.527	1.00	0.00	N
ATOM	1131	HE21	GLN	72	36.004	41.925	40.565	1.00	0.00	H
ATOM	1132	HE22	GLN	72	36.308	40.225	40.854	1.00	0.00	H
ATOM	1133	C	GLN	72	37.835	45.627	40.827	1.00	0.00	C
ATOM	1134	O	GLN	72	38.006	45.947	39.640	1.00	0.00	O
ATOM	1135	N	GLU	73	36.734	45.975	41.577	1.00	0.00	N
ATOM	1136	H	GLU	73	36.705	45.663	42.537	1.00	0.00	H
ATOM	1137	CA	GLU	73	35.706	46.865	41.011	1.00	0.00	C
ATOM	1138	HA	GLU	73	35.674	46.504	39.984	1.00	0.00	H
ATOM	1139	CB	GLU	73	34.306	46.645	41.698	1.00	0.00	C
ATOM	1140	HB2	GLU	73	34.200	47.045	42.706	1.00	0.00	H
ATOM	1141	HB3	GLU	73	33.705	47.255	41.023	1.00	0.00	H
ATOM	1142	CG	GLU	73	33.737	45.200	41.586	1.00	0.00	C
ATOM	1143	HG2	GLU	73	34.244	44.539	42.291	1.00	0.00	H
ATOM	1144	HG3	GLU	73	32.673	45.186	41.822	1.00	0.00	H
ATOM	1145	CD	GLU	73	34.086	44.601	40.165	1.00	0.00	C
ATOM	1146	OE1	GLU	73	34.706	43.546	40.058	1.00	0.00	O
ATOM	1147	OE2	GLU	73	33.755	45.199	39.122	1.00	0.00	O
ATOM	1148	C	GLU	73	36.146	48.354	41.087	1.00	0.00	C

ATOM	1149	O	GLU	73	35.286	49.256	40.799	1.00	0.00	O
ATOM	1150	N	ASN	74	37.423	48.682	41.435	1.00	0.00	N
ATOM	1151	H	ASN	74	38.037	47.916	41.671	1.00	0.00	H
ATOM	1152	CA	ASN	74	37.953	50.067	41.689	1.00	0.00	C
ATOM	1153	HA	ASN	74	38.940	49.868	42.104	1.00	0.00	H
ATOM	1154	CB	ASN	74	38.097	50.828	40.360	1.00	0.00	C
ATOM	1155	HB2	ASN	74	37.059	51.049	40.110	1.00	0.00	H
ATOM	1156	HB3	ASN	74	38.647	51.764	40.449	1.00	0.00	H
ATOM	1157	CG	ASN	74	38.575	49.938	39.245	1.00	0.00	C
ATOM	1158	OD1	ASN	74	39.804	49.689	39.085	1.00	0.00	O
ATOM	1159	ND2	ASN	74	37.718	49.415	38.443	1.00	0.00	N
ATOM	1160	HD21	ASN	74	37.980	48.600	37.907	1.00	0.00	H
ATOM	1161	HD22	ASN	74	36.730	49.597	38.549	1.00	0.00	H
ATOM	1162	C	ASN	74	37.225	50.907	42.838	1.00	0.00	C
ATOM	1163	O	ASN	74	37.345	52.106	42.893	1.00	0.00	O
ATOM	1164	N	MET	75	36.348	50.240	43.641	1.00	0.00	N
ATOM	1165	H	MET	75	36.241	49.244	43.513	1.00	0.00	H
ATOM	1166	CA	MET	75	35.481	50.790	44.687	1.00	0.00	C
ATOM	1167	HA	MET	75	35.151	51.792	44.415	1.00	0.00	H
ATOM	1168	CB	MET	75	34.185	49.916	44.749	1.00	0.00	C
ATOM	1169	HB2	MET	75	34.446	48.858	44.770	1.00	0.00	H
ATOM	1170	HB3	MET	75	33.597	50.153	45.636	1.00	0.00	H
ATOM	1171	CG	MET	75	33.247	49.969	43.511	1.00	0.00	C
ATOM	1172	HG2	MET	75	32.952	51.002	43.325	1.00	0.00	H
ATOM	1173	HG3	MET	75	33.799	49.662	42.624	1.00	0.00	H
ATOM	1174	SD	MET	75	31.748	48.907	43.755	1.00	0.00	S
ATOM	1175	CE	MET	75	30.632	49.870	44.968	1.00	0.00	C
ATOM	1176	HE1	MET	75	31.145	49.985	45.924	1.00	0.00	H
ATOM	1177	HE2	MET	75	30.318	50.847	44.601	1.00	0.00	H
ATOM	1178	HE3	MET	75	29.756					
49.231	45.071	1.00	0.00			H				
ATOM	1179	C	MET	75	36.075	50.824	46.071	1.00	0.00	C
ATOM	1180	O	MET	75	37.113	50.108	46.422	1.00	0.00	O
ATOM	1181	N	THR	76	35.308	51.407	46.991	1.00	0.00	N
ATOM	1182	H	THR	76	34.427	51.816	46.713	1.00	0.00	H
ATOM	1183	CA	THR	76	35.466	51.166	48.454	1.00	0.00	C
ATOM	1184	HA	THR	76	36.476	50.801	48.642	1.00	0.00	H
ATOM	1185	CB	THR	76	35.435	52.515	49.233	1.00	0.00	C
ATOM	1186	HB	THR	76	35.710	53.395	48.649	1.00	0.00	H
ATOM	1187	CG2	THR	76	34.108	52.903	49.926	1.00	0.00	C
ATOM	1188	HG21	THR	76	33.435	53.109	49.093	1.00	0.00	H
ATOM	1189	HG22	THR	76	33.709	52.187	50.646	1.00	0.00	H
ATOM	1190	HG23	THR	76	34.120	53.899	50.369	1.00	0.00	H
ATOM	1191	OG1	THR	76	36.438	52.353	50.257	1.00	0.00	O
ATOM	1192	HG1	THR	76	36.067	52.162	51.121	1.00	0.00	H
ATOM	1193	C	THR	76	34.536	50.165	49.080	1.00	0.00	C
ATOM	1194	O	THR	76	33.349	50.041	48.647	1.00	0.00	O
ATOM	1195	N	LEU	77	34.988	49.448	50.034	1.00	0.00	N
ATOM	1196	H	LEU	77	35.853	49.696	50.494	1.00	0.00	H
ATOM	1197	CA	LEU	77	34.298	48.285	50.555	1.00	0.00	C
ATOM	1198	HA	LEU	77	34.056	47.717	49.657	1.00	0.00	H
ATOM	1199	CB	LEU	77	35.361	47.611	51.479	1.00	0.00	C
ATOM	1200	HB2	LEU	77	36.193	47.312	50.842	1.00	0.00	H
ATOM	1201	HB3	LEU	77	35.629	48.431	52.144	1.00	0.00	H
ATOM	1202	CG	LEU	77	34.719	46.378	52.197	1.00	0.00	C
ATOM	1203	HG	LEU	77	33.838	46.663	52.773	1.00	0.00	H
ATOM	1204	CD1	LEU	77	34.308	45.298	51.304	1.00	0.00	C
ATOM	1205	HD11	LEU	77	34.016	44.486	51.970	1.00	0.00	H
ATOM	1206	HD12	LEU	77	33.482	45.561	50.643	1.00	0.00	H
ATOM	1207	HD13	LEU	77	35.131	44.978	50.665	1.00	0.00	H
ATOM	1208	CD2	LEU	77	35.654	45.700	53.234	1.00	0.00	C
ATOM	1209	HD21	LEU	77	35.119	45.128	53.993	1.00	0.00	H
ATOM	1210	HD22	LEU	77	36.460	45.108	52.801	1.00	0.00	H
ATOM	1211	HD23	LEU	77	36.211	46.377	53.881	1.00	0.00	H

ATOM	1212	C	LEU	77	32.956	48.648	51.258	1.00	0.00	C
ATOM	1213	O	LEU	77	32.123	47.771	51.294	1.00	0.00	O
ATOM	1214	N	THR	78	32.898	49.816	51.878	1.00	0.00	N
ATOM	1215	H	THR	78	33.719	50.403	51.837	1.00	0.00	H
ATOM	1216	CA	THR	78	31.683	50.295	52.632	1.00	0.00	C
ATOM	1217	HA	THR	78	31.284	49.401	53.112	1.00	0.00	H
ATOM	1218	CB	THR	78	32.096	51.274	53.695	1.00	0.00	C
ATOM	1219	HB	THR	78	31.197	51.765	54.070	1.00	0.00	H
ATOM	1220	CG2	THR	78	32.681	50.419	54.797	1.00	0.00	C
ATOM	1221	HG21	THR	78	33.541	49.883	54.398	1.00	0.00	H
ATOM	1222	HG22	THR	78	33.031	51.150	55.527	1.00	0.00	H
ATOM	1223	HG23	THR	78	31.857	49.858	55.239	1.00	0.00	H
ATOM	1224	OG1	THR	78	33.063	52.130	53.105	1.00	0.00	O
ATOM	1225	HG1	THR	78	33.229	52.759	53.812	1.00	0.00	H
ATOM	1226	C	THR	78	30.589	50.845	51.719	1.00	0.00	C
ATOM	1227	O	THR	78	29.374	50.816	52.106	1.00	0.00	O
ATOM	1228	N	GLU	79	30.977	51.124	50.453	1.00	0.00	N
ATOM	1229	H	GLU	79	31.947	51.024	50.192	1.00	0.00	H
ATOM	1230	CA	GLU	79	30.056	51.417	49.311	1.00	0.00	C
ATOM	1231	HA	GLU	79	29.121	51.775	49.740	1.00	0.00	H
ATOM	1232	CB	GLU	79	30.609	52.606	48.559	1.00	0.00	C
ATOM	1233	HB2	GLU	79	31.603	52.335	48.203	1.00	0.00	H
ATOM	1234	HB3	GLU	79	30.020	52.831	47.670	1.00	0.00	H
ATOM	1235	CG	GLU	79	30.719	53.892	49.392	1.00	0.00	C
ATOM	1236	HG2	GLU	79	31.313	53.652	50.274	1.00	0.00	H
ATOM	1237	HG3	GLU	79	31.234	54.524	48.667	1.00	0.00	H
ATOM	1238	CD	GLU	79	29.343	54.523	49.665	1.00	0.00	C
ATOM	1239	OE1	GLU	79	28.835	54.377	50.820	1.00	0.00	O
ATOM	1240	OE2	GLU	79	28.779	55.037	48.779	1.00	0.00	O
ATOM	1241	C	GLU	79	29.809	50.073	48.521	1.00	0.00	C
ATOM	1242	O	GLU	79	28.732	49.882	48.016	1.00	0.00	O
ATOM	1243	N	LEU	80	30.641	49.036	48.656	1.00	0.00	N
ATOM	1244	H	LEU	80	31.504	49.210	49.150	1.00	0.00	H
ATOM	1245	CA	LEU	80	30.261	47.606	48.260	1.00	0.00	C
ATOM	1246	HA	LEU	80	29.779	47.666	47.285	1.00	0.00	H
ATOM	1247	CB	LEU	80	31.566	46.750	48.129	1.00	0.00	C
ATOM	1248	HB2	LEU	80	32.177	47.332	47.440	1.00	0.00	H
ATOM	1249	HB3	LEU	80	32.026	46.727	49.117	1.00	0.00	H
ATOM	1250	CG	LEU	80	31.377	45.237	47.761	1.00	0.00	C
ATOM	1251	HG	LEU	80	30.892	44.765	48.616	1.00	0.00	H
ATOM	1252	CD1	LEU	80	30.553	44.787	46.533	1.00	0.00	C
ATOM	1253	HD11	LEU	80	31.225	45.110	45.737	1.00	0.00	H
ATOM	1254	HD12	LEU	80	30.175	43.766	46.565	1.00	0.00	H
ATOM	1255	HD13	LEU	80	29.608	45.328	46.487	1.00	0.00	H
ATOM	1256	CD2	LEU	80	32.695	44.561	47.574	1.00	0.00	C
ATOM	1257	HD21	LEU	80	32.609	43.479	47.679	1.00	0.00	H
ATOM	1258	HD22	LEU	80	33.162	44.917	46.656	1.00	0.00	H
ATOM	1259	HD23	LEU	80	33.331	44.952	48.366	1.00	0.00	H
ATOM	1260	C	LEU	80	29.277	47.082	49.288	1.00	0.00	C
ATOM	1261	O	LEU	80	28.248	46.460	48.887	1.00	0.00	O
ATOM	1262	N	LYS	81	29.384	47.234	50.666	1.00	0.00	N
ATOM	1263	H	LYS	81	30.289	47.545	50.987	1.00	0.00	H
ATOM	1264	CA	LYS	81	28.201	47.083	51.529	1.00	0.00	C
ATOM	1265	HA	LYS	81	27.941	46.029	51.434	1.00	0.00	H
ATOM	1266	CB	LYS	81	28.499	47.287	53.023	1.00	0.00	C
ATOM	1267	HB2	LYS	81	28.811	48.323	53.157	1.00	0.00	H
ATOM	1268	HB3	LYS	81	27.642	47.127	53.677	1.00	0.00	H
ATOM	1269	CG	LYS	81	29.611	46.380	53.539	1.00	0.00	C
ATOM	1270	HG2	LYS	81	29.288	45.349	53.394	1.00	0.00	H
ATOM	1271	HG3	LYS	81	30.475	46.538	52.892	1.00	0.00	H
ATOM	1272	CD	LYS	81	29.980	46.598	55.000	1.00	0.00	C
ATOM	1273	HD2	LYS	81	30.294	47.604	55.275	1.00	0.00	H
ATOM	1274	HD3	LYS	81	29.054	46.385	55.535	1.00	0.00	H
ATOM	1275	CE	LYS	81	31.094	45.613	55.457	1.00	0.00	C

ATOM	1276	HE2	LYS	81	30.849	44.572	55.246	1.00	0.00	H
ATOM	1277	HE3	LYS	81	31.996	45.874	54.904	1.00	0.00	H
ATOM	1278	NZ	LYS	81	31.212	45.757	56.910	1.00	0.00	N
ATOM	1279	HZ1	LYS	81	30.379	45.494	57.418	1.00	0.00	H
ATOM	1280	HZ2	LYS	81	31.963	45.134	57.170	1.00	0.00	H
ATOM	1281	HZ3	LYS	81	31.447	46.689	57.222	1.00	0.00	H
ATOM	1282	C	LYS	81	26.882	47.678	51.038	1.00	0.00	C
ATOM	1283	O	LYS	81	25.760	47.120	50.960	1.00	0.00	O
ATOM	1284	N	LYS	82	27.001	48.973	50.718	1.00	0.00	N
ATOM	1285	H	LYS	82	27.853	49.431	51.010	1.00	0.00	H
ATOM	1286	CA	LYS	82	25.911	49.866	50.285	1.00	0.00	C
ATOM	1287	HA	LYS	82	25.210	50.005	51.108	1.00	0.00	H
ATOM	1288	CB	LYS	82	26.403	51.339	49.939	1.00	0.00	C
ATOM	1289	HB2	LYS	82	26.852	51.805	50.817	1.00	0.00	H
ATOM	1290	HB3	LYS	82	27.279	51.406	49.293	1.00	0.00	H
ATOM	1291	CG	LYS	82	25.251	52.142	49.373	1.00	0.00	C
ATOM	1292	HG2	LYS	82	24.922	51.735	48.417	1.00	0.00	H
ATOM	1293	HG3	LYS	82	24.349	52.147	49.984	1.00	0.00	H
ATOM	1294	CD	LYS	82	25.719	53.591	49.131	1.00	0.00	C
ATOM	1295	HD2	LYS	82	26.565	53.682	48.450	1.00	0.00	H
ATOM	1296	HD3	LYS	82	24.932	54.097	48.570	1.00	0.00	H
ATOM	1297	CE	LYS	82	25.826	54.333	50.417	1.00	0.00	C
ATOM	1298	HE2	LYS	82	24.912	54.486	50.991	1.00	0.00	H
ATOM	1299	HE3	LYS	82	26.396	53.850	51.211	1.00	0.00	H
ATOM	1300	NZ	LYS	82	26.540	55.654	50.215	1.00	0.00	N
ATOM	1301	HZ1	LYS	82	27.507	55.438	50.022	1.00	0.00	H
ATOM	1302	HZ2	LYS	82	26.143	56.173	49.444	1.00	0.00	H
ATOM	1303	HZ3	LYS	82	26.398	56.297	50.981	1.00	0.00	H
ATOM	1304	C	LYS	82	25.098	49.406	49.068	1.00	0.00	C
ATOM	1305	O	LYS	82	23.902	49.295	49.150	1.00	0.00	O
ATOM	1306	N	VAL	83	25.736	48.944	48.009	1.00	0.00	N
ATOM	1307	H	VAL	83	26.702	49.207	47.887	1.00	0.00	H
ATOM	1308	CA	VAL	83	24.939	48.431	46.927	1.00	0.00	C
ATOM	1309	HA	VAL	83	24.092	49.111	46.834	1.00	0.00	H
ATOM	1310	CB	VAL	83	25.793	48.441	45.637	1.00	0.00	C
ATOM	1311	HB	VAL	83	25.194	48.127	44.781	1.00	0.00	H
ATOM	1312	CG1	VAL	83	26.143	49.885	45.213	1.00	0.00	C
ATOM	1313	HG11	VAL	83	26.699	50.331	46.038	1.00	0.00	H
ATOM	1314	HG12	VAL	83	26.706	49.933	44.280	1.00	0.00	H
ATOM	1315	HG13	VAL	83	25.235	50.485	45.167	1.00	0.00	H
ATOM	1316	CG2	VAL	83	27.032	47.550	45.641	1.00	0.00	C
ATOM	1317	HG21	VAL	83	26.841	46.533	45.981	1.00	0.00	H
ATOM	1318	HG22	VAL	83	27.319	47.391	44.601	1.00	0.00	H
ATOM	1319	HG23	VAL	83	27.874	48.088	46.075	1.00	0.00	H
ATOM	1320	C	VAL	83	24.310	47.086	47.134	1.00	0.00	C
ATOM	1321	O	VAL	83	23.126	46.990	46.785	1.00	0.00	O
ATOM	1322	N	ILE	84	25.084	46.147	47.636	1.00	0.00	N
ATOM	1323	H	ILE	84	26.050	46.319	47.878	1.00	0.00	H
ATOM	1324	CA	ILE	84	24.456	44.804	47.945	1.00	0.00	C
ATOM	1325	HA	ILE	84	24.117	44.339	47.019	1.00	0.00	H
ATOM	1326	CB	ILE	84	25.444	43.847	48.656	1.00	0.00	C
ATOM	1327	HB	ILE	84	25.784	44.374	49.548	1.00	0.00	H
ATOM	1328	CG2	ILE	84	24.799	42.542	49.125	1.00	0.00	C
ATOM	1329	HG21	ILE	84	23.873	42.588	49.698	1.00	0.00	H
ATOM	1330	HG22	ILE	84	24.596	41.938	48.241	1.00	0.00	H
ATOM	1331	HG23	ILE	84	25.440	42.008	49.827	1.00	0.00	H
ATOM	1332	CG1	ILE	84	26.632	43.533	47.745	1.00	0.00	C
ATOM	1333	HG12	ILE	84	26.348	42.775	47.014	1.00	0.00	H
ATOM	1334	HG13	ILE	84	26.957	44.448	47.250	1.00	0.00	H
ATOM	1335	CD1	ILE	84	27.900	43.018	48.474	1.00	0.00	C
ATOM	1336	HD11	ILE	84	28.586	42.778	47.661	1.00	0.00	H
ATOM	1337	HD12	ILE	84	28.393	43.726	49.141	1.00	0.00	H
ATOM	1338	HD13	ILE	84	27.730	42.029	48.901	1.00	0.00	H

ATOM	1339	C	ILE	84	23.190	44.975	48.902	1.00	0.00	C
ATOM	1340	O	ILE	84	22.111	44.368	48.690	1.00	0.00	O
ATOM	1341	N	ALA	85	23.353	45.824	49.971	1.00	0.00	N
ATOM	1342	H	ALA	85	24.227	46.321	50.083	1.00	0.00	H
ATOM	1343	CA	ALA	85	22.314	46.115	50.905	1.00	0.00	C
ATOM	1344	HA	ALA	85	21.997	45.235	51.467	1.00	0.00	H
ATOM	1345	CB	ALA	85	22.890	47.025	51.939	1.00	0.00	C
ATOM	1346	HB1	ALA	85	23.878	46.770	52.320	1.00	0.00	H
ATOM	1347	HB2	ALA	85	23.069	48.035	51.570	1.00	0.00	H
ATOM	1348	HB3	ALA	85	22.213	47.087	52.793	1.00	0.00	H
ATOM	1349	C	ALA	85	20.992	46.760	50.347	1.00	0.00	C
ATOM	1350	O	ALA	85	19.937	46.513	50.918	1.00	0.00	O
ATOM	1351	N	ASP	86	21.042	47.451	49.228	1.00	0.00	N
ATOM	1352	H	ASP	86	21.956	47.778	48.949	1.00	0.00	H
ATOM	1353	CA	ASP	86	19.822	47.979	48.502	1.00	0.00	C
ATOM	1354	HA	ASP	86	19.174	48.520	49.194	1.00	0.00	H
ATOM	1355	CB	ASP	86	20.141	49.011	47.394	1.00	0.00	C
ATOM	1356	HB2	ASP	86	21.055	49.473	47.765	1.00	0.00	H
ATOM	1357	HB3	ASP	86	20.244	48.491	46.442	1.00	0.00	H
ATOM	1358	CG	ASP	86	19.199	50.241	47.264	1.00	0.00	C
ATOM	1359	OD1	ASP	86	19.465	50.986	46.257	1.00	0.00	O
ATOM	1360	OD2	ASP	86	18.505	50.647	48.243	1.00	0.00	O
ATOM	1361	C	ASP	86	19.010	46.848	47.749	1.00	0.00	C
ATOM	1362	O	ASP	86	17.817	47.057	47.369	1.00	0.00	O
ATOM	1363	N	ILE	87	19.652	45.671	47.610	1.00	0.00	N
ATOM	1364	H	ILE	87	20.577	45.557	47.996	1.00	0.00	H
ATOM	1365	CA	ILE	87	19.139	44.548	46.944	1.00	0.00	C
ATOM	1366	HA	ILE	87	18.282	44.945	46.400	1.00	0.00	H
ATOM	1367	CB	ILE	87	20.174	43.997	45.997	1.00	0.00	C
ATOM	1368	HB	ILE	87	21.067	43.671	46.532	1.00	0.00	H
ATOM	1369	CG2	ILE	87	19.581	42.746	45.290	1.00	0.00	C
ATOM	1370	HG21	ILE	87	20.396	42.210	44.802	1.00	0.00	H
ATOM	1371	HG22	ILE	87	19.208	42.002	45.993	1.00	0.00	H
ATOM	1372	HG23	ILE	87	18.817	43.024	44.563	1.00	0.00	H
ATOM	1373	CG1	ILE	87	20.506	44.966	44.947	1.00	0.00	C
ATOM	1374	HG12	ILE	87	19.716	44.999	44.197	1.00	0.00	H
ATOM	1375	HG13	ILE	87	20.651	45.955	45.379	1.00	0.00	H
ATOM	1376	CD1	ILE	87	21.853	44.529	44.214	1.00	0.00	C
ATOM	1377	HD11	ILE	87	22.089	45.291	43.471	1.00	0.00	H
ATOM	1378	HD12	ILE	87	22.658	44.519	44.948	1.00	0.00	H
ATOM	1379	HD13	ILE	87	21.698	43.569	43.722	1.00	0.00	H
ATOM	1380	C	ILE	87	18.662	43.453	47.908	1.00	0.00	C
ATOM	1381	O	ILE	87	17.573	43.028	47.772	1.00	0.00	O
ATOM	1382	N	TYR	88	19.541	43.013	48.849	1.00	0.00	N
ATOM	1383	H	TYR	88	20.451	43.447	48.921	1.00	0.00	H
ATOM	1384	CA	TYR	88	19.221	41.931	49.863	1.00	0.00	C
ATOM	1385	HA	TYR	88	18.285	41.452	49.576	1.00	0.00	H
ATOM	1386	CB	TYR	88	20.362	40.894	49.859	1.00	0.00	C
ATOM	1387	HB2	TYR	88	21.270	41.376	50.219	1.00	0.00	H
ATOM	1388	HB3	TYR	88	20.151	40.090	50.564	1.00	0.00	H
ATOM	1389	CG	TYR	88	20.736	40.332	48.510	1.00	0.00	C
ATOM	1390	CD1	TYR	88	20.223	39.048	48.139	1.00	0.00	C
ATOM	1391	HD1	TYR	88	19.371	38.682	48.694	1.00	0.00	H
ATOM	1392	CE1	TYR	88	20.558	38.471	46.870	1.00	0.00	C
ATOM	1393	HE1	TYR	88	20.237	37.468	46.633	1.00	0.00	H
ATOM	1394	CZ	TYR	88	21.378	39.189	45.932	1.00	0.00	C
ATOM	1395	OH	TYR	88	21.764	38.738	44.697	1.00	0.00	O
ATOM	1396	HH	TYR	88	21.985	39.484	44.135	1.00	0.00	H
ATOM	1397	CE2	TYR	88	21.895	40.467	46.334	1.00	0.00	C
ATOM	1398	HE2	TYR	88	22.622	40.892	45.658	1.00	0.00	H
ATOM	1399	CD2	TYR	88	21.541	40.977	47.580	1.00	0.00	C
ATOM	1400	HD2	TYR	88	21.930	41.945	47.861	1.00	0.00	H
ATOM	1401	C	TYR	88	19.213	42.481	51.283	1.00	0.00	C
ATOM	1402	O	TYR	88	19.904	43.445	51.567	1.00	0.00	O

ATOM	1403	N	PRO	89	18.365	41.883	52.186	1.00	0.00	N
ATOM	1404	CD	PRO	89	17.546	40.635	51.866	1.00	0.00	C
ATOM	1405	HD2	PRO	89	18.235	39.814	51.670	1.00	0.00	H
ATOM	1406	HD3	PRO	89	16.869	40.952	51.073	1.00	0.00	H
ATOM	1407	CG	PRO	89	16.795	40.301	53.066	1.00	0.00	C
ATOM	1408	HG2	PRO	89	17.041	39.260	53.279	1.00	0.00	H
ATOM	1409	HG3	PRO	89	15.726	40.429	52.902	1.00	0.00	H
ATOM	1410	CB	PRO	89	17.343	41.184	54.237	1.00	0.00	C
ATOM	1411	HB2	PRO	89	17.613	40.476	55.020	1.00	0.00	H
ATOM	1412	HB3	PRO	89	16.596	41.912	54.555	1.00	0.00	H
ATOM	1413	CA	PRO	89	18.410	42.053	53.569	1.00	0.00	C
ATOM	1414	HA	PRO	89	18.149	43.090	53.779	1.00	0.00	H
ATOM	1415	C	PRO	89	19.799	41.663	54.109	1.00	0.00	C
ATOM	1416	O	PRO	89	20.392	40.715	53.647	1.00	0.00	O
ATOM	1417	N	GLY	90	20.205	42.303	55.179	1.00	0.00	N
ATOM	1418	H	GLY	90	19.566	43.049	55.413	1.00	0.00	H
ATOM	1419	CA	GLY	90	21.455	42.188	55.909	1.00	0.00	C
ATOM	1420	HA2	GLY	90	22.246	42.469	55.214	1.00	0.00	H
ATOM	1421	HA3	GLY	90	21.445	42.944	56.695	1.00	0.00	H
ATOM	1422	C	GLY	90	21.676	40.779	56.512	1.00	0.00	C
ATOM	1423	O	GLY	90	22.765	40.349	56.821	1.00	0.00	O
ATOM	1424	N	GLN	91	20.579	40.051	56.743	1.00	0.00	N
ATOM	1425	H	GLN	91	19.667	40.446	56.555	1.00	0.00	H
ATOM	1426	CA	GLN	91	20.632	38.659	57.279	1.00	0.00	C
ATOM	1427	HA	GLN	91	21.216	38.541	58.192	1.00	0.00	H
ATOM	1428	CB	GLN	91	19.185	38.370	57.636	1.00	0.00	C
ATOM	1429									
HB2	GLN	91	18.917	39.269	58.191	1.00	0.00	H		
ATOM	1430	HB3	GLN	91	18.603	38.307	56.717	1.00	0.00	H
ATOM	1431	CG	GLN	91	18.867	37.127	58.544	1.00	0.00	C
ATOM	1432	HG2	GLN	91	19.361	36.183	58.315	1.00	0.00	H
ATOM	1433	HG3	GLN	91	19.133	37.424	59.559	1.00	0.00	H
ATOM	1434	CD	GLN	91	17.481	36.698	58.573	1.00	0.00	C
ATOM	1435	OE1	GLN	91	17.062	35.675	58.055	1.00	0.00	O
ATOM	1436	NE2	GLN	91	16.604	37.558	59.040	1.00	0.00	N
ATOM	1437	HE21	GLN	91	16.808	38.484	59.386	1.00	0.00	H
ATOM	1438	HE22	GLN	91	15.676	37.183	58.901	1.00	0.00	H
ATOM	1439	C	GLN	91	21.213	37.563	56.338	1.00	0.00	C
ATOM	1440	O	GLN	91	21.382	36.453	56.750	1.00	0.00	O
ATOM	1441	N	THR	92	21.591	37.964	55.079	1.00	0.00	N
ATOM	1442	H	THR	92	21.665	38.961	54.940	1.00	0.00	H
ATOM	1443	CA	THR	92	21.918	37.038	53.961	1.00	0.00	C
ATOM	1444	HA	THR	92	21.247	36.196	54.125	1.00	0.00	H
ATOM	1445	CB	THR	92	21.503	37.684	52.585	1.00	0.00	C
ATOM	1446	HB	THR	92	22.155	38.528	52.362	1.00	0.00	H
ATOM	1447	CG2	THR	92	21.694	36.715	51.494	1.00	0.00	C
ATOM	1448	HG21	THR	92	22.763	36.634	51.291	1.00	0.00	H
ATOM	1449	HG22	THR	92	21.227	35.761	51.737	1.00	0.00	H
ATOM	1450	HG23	THR	92	21.251	37.108	50.578	1.00	0.00	H
ATOM	1451	OG1	THR	92	20.188	38.188	52.723	1.00	0.00	O
ATOM	1452	HG1	THR	92	20.178	39.042	53.163	1.00	0.00	H
ATOM	1453	C	THR	92	23.331	36.514	53.998	1.00	0.00	C
ATOM	1454	O	THR	92	24.309	37.147	54.352	1.00	0.00	O
ATOM	1455	N	GLN	93	23.405	35.172	53.709	1.00	0.00	N
ATOM	1456	H	GLN	93	22.584	34.826	53.231	1.00	0.00	H
ATOM	1457	CA	GLN	93	24.675	34.455	53.612	1.00	0.00	C
ATOM	1458	HA	GLN	93	25.414	34.855	54.306	1.00	0.00	H
ATOM	1459	CB	GLN	93	24.466	33.025	54.088	1.00	0.00	C
ATOM	1460	HB2	GLN	93	23.834	33.037	54.976	1.00	0.00	H
ATOM	1461	HB3	GLN	93	23.933	32.616	53.228	1.00	0.00	H
ATOM	1462	CG	GLN	93	25.753	32.219	54.262	1.00	0.00	C
ATOM	1463	HG2	GLN	93	25.416	31.183	54.282	1.00	0.00	H
ATOM	1464	HG3	GLN	93	26.446	32.430	53.448	1.00	0.00	H
ATOM	1465	CD	GLN	93	26.481	32.527	55.619	1.00	0.00	C

ATOM	1466	OE1	GLN	93	25.891	32.656	56.690	1.00	0.00	O
ATOM	1467	NE2	GLN	93	27.816	32.705	55.582	1.00	0.00	N
ATOM	1468	HE21	GLN	93	28.306	32.746	54.700	1.00	0.00	H
ATOM	1469	HE22	GLN	93	28.335	32.891	56.427	1.00	0.00	H
ATOM	1470	C	GLN	93	25.246	34.608	52.200	1.00	0.00	C
ATOM	1471	O	GLN	93	24.493	34.511	51.230	1.00	0.00	O
ATOM	1472	N	PHE	94	26.519	35.019	52.090	1.00	0.00	N
ATOM	1473	H	PHE	94	27.185	35.118	52.844	1.00	0.00	H
ATOM	1474	CA	PHE	94	27.097	35.315	50.681	1.00	0.00	C
ATOM	1475	HA	PHE	94	26.368	35.094	49.903	1.00	0.00	H
ATOM	1476	CB	PHE	94	27.465	36.817	50.532	1.00	0.00	C
ATOM	1477	HB2	PHE	94	28.107	37.190	51.329	1.00	0.00	H
ATOM	1478	HB3	PHE	94	27.929	36.970	49.557	1.00	0.00	H
ATOM	1479	CG	PHE	94	26.255	37.748	50.434	1.00	0.00	C
ATOM	1480	CD1	PHE	94	25.511	37.953	49.246	1.00	0.00	C
ATOM	1481	HD1	PHE	94	25.799	37.481	48.317	1.00	0.00	H
ATOM	1482	CE1	PHE	94	24.453	38.855	49.261	1.00	0.00	C
ATOM	1483	HE1	PHE	94	23.919	38.951	48.328	1.00	0.00	H
ATOM	1484	CZ	PHE	94	24.128	39.595	50.405	1.00	0.00	C
ATOM	1485	HZ	PHE	94	23.403	40.388	50.296	1.00	0.00	H
ATOM	1486	CE2	PHE	94	24.840	39.313	51.593	1.00	0.00	C
ATOM	1487	HE2	PHE	94	24.635	39.944	52.445	1.00	0.00	H
ATOM	1488	CD2	PHE	94	25.833	38.324	51.658	1.00	0.00	C
ATOM	1489	HD2	PHE	94	26.302	38.076	52.599	1.00	0.00	H
ATOM	1490	C	PHE	94	28.193	34.282	50.337	1.00	0.00	C
ATOM	1491	O	PHE	94	28.796	33.704	51.247	1.00	0.00	O
ATOM	1492	N	TYR	95	28.670	34.242	49.109	1.00	0.00	N
ATOM	1493	H	TYR	95	28.201	34.810	48.418	1.00	0.00	H
ATOM	1494	CA	TYR	95	29.651	33.292	48.530	1.00	0.00	C
ATOM	1495	HA	TYR	95	30.237	32.885	49.354	1.00	0.00	H
ATOM	1496	CB	TYR	95	28.801	32.197	47.919	1.00	0.00	C
ATOM	1497	HB2	TYR	95	27.983	31.897	48.573	1.00	0.00	H
ATOM	1498	HB3	TYR	95	28.167	32.673	47.170	1.00	0.00	H
ATOM	1499	CG	TYR	95	29.522	30.968	47.286	1.00	0.00	C
ATOM	1500	CD1	TYR	95	29.411	30.751	45.885	1.00	0.00	C
ATOM	1501	HD1	TYR	95	28.882	31.531	45.359	1.00	0.00	H
ATOM	1502	CE1	TYR	95	29.996	29.556	45.368	1.00	0.00	C
ATOM	1503	HE1	TYR	95	29.781	29.258	44.352	1.00	0.00	H
ATOM	1504	CZ	TYR	95	30.813	28.728	46.228	1.00	0.00	C
ATOM	1505	OH	TYR	95	31.422	27.682	45.681	1.00	0.00	O
ATOM	1506	HH	TYR	95	31.165	27.600	44.760	1.00	0.00	H
ATOM	1507	CE2	TYR	95	31.070	29.102	47.554	1.00	0.00	C
ATOM	1508	HE2	TYR	95	31.742	28.549	48.192	1.00	0.00	H
ATOM	1509	CD2	TYR	95	30.321	30.135	48.110	1.00	0.00	C
ATOM	1510	HD2	TYR	95	30.432	30.215	49.182	1.00	0.00	H
ATOM	1511	C	TYR	95	30.652	33.762	47.506	1.00	0.00	C
ATOM	1512	O	TYR	95	30.345	34.524	46.618	1.00	0.00	O
ATOM	1513	N	VAL	96	31.910	33.422	47.656	1.00	0.00	N
ATOM	1514	H	VAL	96	32.166	32.678	48.289	1.00	0.00	H
ATOM	1515	CA	VAL	96	33.072	34.069	46.997	1.00	0.00	C
ATOM	1516	HA	VAL	96	32.680	34.777	46.268	1.00	0.00	H
ATOM	1517	CB	VAL	96	33.934	34.874	47.974	1.00	0.00	C
ATOM	1518	HB	VAL	96	34.242	34.182	48.758	1.00	0.00	H
ATOM	1519	CG1	VAL	96	35.199	35.384	47.290	1.00	0.00	C
ATOM	1520	HG11	VAL	96	35.885	34.551	47.137	1.00	0.00	H
ATOM	1521	HG12	VAL	96	34.973	35.786	46.301	1.00	0.00	H
ATOM	1522	HG13	VAL	96	35.796	36.139	47.800	1.00	0.00	H
ATOM	1523	CG2	VAL	96	33.345	36.023	48.770	1.00	0.00	C
ATOM	1524	HG21	VAL	96	32.624	35.673	49.509	1.00	0.00	H
ATOM	1525	HG22	VAL	96	34.192	36.390	49.351	1.00	0.00	H
ATOM	1526	HG23	VAL	96	32.919	36.884	48.256	1.00	0.00	H
ATOM	1527	C	VAL	96	33.825	32.951	46.271	1.00	0.00	C
ATOM	1528	O	VAL	96	34.273	31.984	46.873	1.00	0.00	O
ATOM	1529	N	ILE	97	34.018	33.151	45.022	1.00	0.00	N

ATOM	1530	H	ILE	97	33.827	34.076	44.664	1.00	0.00	H
ATOM	1531	CA	ILE	97	34.666	32.178	44.120	1.00	0.00	C
ATOM	1532	HA	ILE	97	34.967	31.253	44.612	1.00	0.00	H
ATOM	1533	CB	ILE	97	33.660	31.812	42.931	1.00	0.00	C
ATOM	1534	HB	ILE	97	33.397	32.725	42.399	1.00	0.00	H
ATOM	1535	CG2	ILE	97	34.241	30.848	41.861	1.00	0.00	C
ATOM	1536	HG21	ILE	97	33.495	30.842	41.066	1.00	0.00	H
ATOM	1537	HG22	ILE	97	35.243	31.060	41.488	1.00	0.00	H
ATOM	1538	HG23	ILE	97	34.260	29.861	42.324	1.00	0.00	H
ATOM	1539	CG1	ILE	97	32.324	31.261	43.469	1.00	0.00	C
ATOM	1540	HG12	ILE	97	32.321	30.240	43.853	1.00	0.00	H
ATOM	1541	HG13	ILE	97	31.964	31.838	44.321	1.00	0.00	H
ATOM	1542	CD1	ILE	97	31.177	31.330	42.460	1.00	0.00	C
ATOM	1543	HD11	ILE	97	31.119	32.325	42.019	1.00	0.00	H
ATOM	1544	HD12	ILE	97	31.322	30.507	41.760	1.00	0.00	H
ATOM	1545	HD13	ILE	97	30.268	31.020	42.974	1.00	0.00	H
ATOM	1546	C	ILE	97	35.916	32.921	43.549	1.00	0.00	C
ATOM	1547	O	ILE	97	35.771	34.035	43.086	1.00	0.00	O
ATOM	1548	N	GLU	98	37.066	32.294	43.516	1.00	0.00	N
ATOM	1549	H	GLU	98	37.106	31.335	43.831	1.00	0.00	H
ATOM	1550	CA	GLU	98	38.375	32.741	42.941	1.00	0.00	C
ATOM	1551	HA	GLU	98	38.210	33.731	42.516	1.00	0.00	H
ATOM	1552	CB	GLU	98	39.517	32.644	43.916	1.00	0.00	C
ATOM	1553	HB2	GLU	98	39.656	31.607	44.223	1.00	0.00	H
ATOM	1554	HB3	GLU	98	40.438					
32.947	43.417	1.00	0.00			H				
ATOM	1555	CG	GLU	98	39.215	33.571	45.126	1.00	0.00	C
ATOM	1556	HG2	GLU	98	39.415	34.609	44.862	1.00	0.00	H
ATOM	1557	HG3	GLU	98	38.162	33.460	45.389	1.00	0.00	H
ATOM	1558	CD	GLU	98	39.977	33.268	46.404	1.00	0.00	C
ATOM	1559	OE1	GLU	98	39.362	33.351	47.497	1.00	0.00	O
ATOM	1560	OE2	GLU	98	41.228	33.067	46.295	1.00	0.00	O
ATOM	1561	C	GLU	98	38.707	31.844	41.723	1.00	0.00	C
ATOM	1562	O	GLU	98	38.578	30.618	41.753	1.00	0.00	O
ATOM	1563	N	PHE	99	39.216	32.449	40.603	1.00	0.00	N
ATOM	1564	H	PHE	99	39.120	33.453	40.664	1.00	0.00	H
ATOM	1565	CA	PHE	99	39.486	31.863	39.293	1.00	0.00	C
ATOM	1566	HA	PHE	99	39.808	30.850	39.538	1.00	0.00	H
ATOM	1567	CB	PHE	99	38.153	31.632	38.603	1.00	0.00	C
ATOM	1568	HB2	PHE	99	38.445	31.212	37.640	1.00	0.00	H
ATOM	1569	HB3	PHE	99	37.600	30.841	39.109	1.00	0.00	H
ATOM	1570	CG	PHE	99	37.370	32.870	38.288	1.00	0.00	C
ATOM	1571	CD1	PHE	99	36.424	33.243	39.277	1.00	0.00	C
ATOM	1572	HD1	PHE	99	36.459	32.701	40.211	1.00	0.00	H
ATOM	1573	CE1	PHE	99	35.534	34.359	38.988	1.00	0.00	C
ATOM	1574	HE1	PHE	99	34.886	34.652	39.801	1.00	0.00	H
ATOM	1575	CZ	PHE	99	35.583	35.052	37.736	1.00	0.00	C
ATOM	1576	HZ	PHE	99	34.785	35.769	37.609	1.00	0.00	H
ATOM	1577	CE2	PHE	99	36.563	34.624	36.778	1.00	0.00	C
ATOM	1578	HE2	PHE	99	36.698	35.101	35.819	1.00	0.00	H
ATOM	1579	CD2	PHE	99	37.396	33.513	37.009	1.00	0.00	C
ATOM	1580	HD2	PHE	99	38.193	33.121	36.395	1.00	0.00	H
ATOM	1581	C	PHE	99	40.439	32.572	38.288	1.00	0.00	C
ATOM	1582	O	PHE	99	40.563	33.817	38.294	1.00	0.00	O
ATOM	1583	N	LYS	100	41.026	31.782	37.396	1.00	0.00	N
ATOM	1584	H	LYS	100	40.643	30.849	37.409	1.00	0.00	H
ATOM	1585	CA	LYS	100	42.021	32.247	36.393	1.00	0.00	C
ATOM	1586	HA	LYS	100	41.651	33.237	36.128	1.00	0.00	H
ATOM	1587	CB	LYS	100	43.414	32.455	37.074	1.00	0.00	C
ATOM	1588	HB2	LYS	100	44.014	32.931	36.300	1.00	0.00	H
ATOM	1589	HB3	LYS	100	43.471	33.244	37.823	1.00	0.00	H
ATOM	1590	CG	LYS	100	44.189	31.155	37.573	1.00	0.00	C
ATOM	1591	HG2	LYS	100	43.521	30.617	38.247	1.00	0.00	H
ATOM	1592	HG3	LYS	100	44.538	30.546	36.739	1.00	0.00	H

ATOM	1593	CD	LYS	100	45.491	31.583	38.219	1.00	0.00	C
ATOM	1594	HD2	LYS	100	45.992	32.207	37.479	1.00	0.00	H
ATOM	1595	HD3	LYS	100	45.125	32.048	39.136	1.00	0.00	H
ATOM	1596	CE	LYS	100	46.380	30.390	38.746	1.00	0.00	C
ATOM	1597	HE2	LYS	100	45.684	29.770	39.312	1.00	0.00	H
ATOM	1598	HE3	LYS	100	46.635	29.885	37.814	1.00	0.00	H
ATOM	1599	NZ	LYS	100	47.647	30.823	39.416	1.00	0.00	N
ATOM	1600	HZ1	LYS	100	47.429	31.450	40.178	1.00	0.00	H
ATOM	1601	HZ2	LYS	100	48.192	30.051	39.772	1.00	0.00	H
ATOM	1602	HZ3	LYS	100	48.230	31.393	38.821	1.00	0.00	H
ATOM	1603	C	LYS	100	42.138	31.460	35.104	1.00	0.00	C
ATOM	1604	O	LYS	100	41.663	30.334	35.133	1.00	0.00	O
ATOM	1605	N	CYX	101	42.682	32.065	34.023	1.00	0.00	N
ATOM	1606	H	CYX	101	43.175	32.931	34.191	1.00	0.00	H
ATOM	1607	CA	CYX	101	42.899	31.215	32.853	1.00	0.00	C
ATOM	1608	HA	CYX	101	42.114	30.484	32.660	1.00	0.00	H
ATOM	1609	CB	CYX	101	42.796	32.133	31.548	1.00	0.00	C
ATOM	1610	HB2	CYX	101	41.816	32.590	31.414	1.00	0.00	H
ATOM	1611	HB3	CYX	101	43.504	32.947	31.706	1.00	0.00	H
ATOM	1612	SG	CYX	101	43.286	31.247	29.966	1.00	0.00	S
ATOM	1613	C	CYX	101	44.184	30.370	33.011	1.00	0.00	C
ATOM	1614	O	CYX	101	45.147	30.838	33.554	1.00	0.00	O
ATOM	1615	N	LEU	102	44.162	29.157	32.466	1.00	0.00	N
ATOM	1616	H	LEU	102	43.274	28.998	32.012	1.00	0.00	H
ATOM	1617	CA	LEU	102	45.334	28.285	32.313	1.00	0.00	C
ATOM	1618	HA	LEU	102	45.824	28.429	33.276	1.00	0.00	H
ATOM	1619	CB	LEU	102	45.011	26.749	32.233	1.00	0.00	C
ATOM	1620	HB2	LEU	102	44.974	26.520	31.168	1.00	0.00	H
ATOM	1621	HB3	LEU	102	45.927	26.254	32.557	1.00	0.00	H
ATOM	1622	CG	LEU	102	43.874	26.169	33.108	1.00	0.00	C
ATOM	1623	HG	LEU	102	42.909	26.488	32.717	1.00	0.00	H
ATOM	1624	CD1	LEU	102	43.745	24.680	32.966	1.00	0.00	C
ATOM	1625	HD11	LEU	102	42.866	24.286	33.476	1.00	0.00	H
ATOM	1626	HD12	LEU	102	43.593	24.457	31.911	1.00	0.00	H
ATOM	1627	HD13	LEU	102	44.668	24.239	33.344	1.00	0.00	H
ATOM	1628	CD2	LEU	102	43.968	26.630	34.604	1.00	0.00	C
ATOM	1629	HD21	LEU	102	43.560	27.632	34.731	1.00	0.00	H
ATOM	1630	HD22	LEU	102	43.330	26.027	35.248	1.00	0.00	H
ATOM	1631	HD23	LEU	102	45.024	26.597	34.876	1.00	0.00	H
ATOM	1632	C	LEU	102	46.264	28.830	31.125	1.00	0.00	C
ATOM	1633	O	LEU	102	45.833	28.819	29.945	1.00	0.00	O
ATOM	1634	OXT	LEU	102	47.474	28.954	31.361	1.00	0.00	O
HETATM	314	N	LYN	20	39.254	40.634	36.272	1.00	0.00	N
HETATM	315	H	LYN	20	39.837	40.391	35.485	1.00	0.00	H
HETATM	316	CA	LYN	20	38.298	39.672	36.822	1.00	0.00	C
HETATM	317	HA	LYN	20	37.845	40.045	37.740	1.00	0.00	H
HETATM	318	CB	LYN	20	37.109	39.384	35.913	1.00	0.00	C
HETATM	319	HB2	LYN	20	37.368	38.883	34.981	1.00	0.00	H
HETATM	320	HB3	LYN	20	36.680	40.344	35.627	1.00	0.00	H
HETATM	321	CG	LYN	20	35.973	38.549	36.519	1.00	0.00	C
HETATM	322	HG2	LYN	20	35.177	38.413	35.787	1.00	0.00	H
HETATM	323	HG3	LYN	20	36.343	37.551	36.756	1.00	0.00	H
HETATM	324	CD	LYN	20	35.444	39.298	37.731	1.00	0.00	C
HETATM	325	HD2	LYN	20	35.269	40.347	37.495	1.00	0.00	H
HETATM	326	HD3	LYN	20	36.163	39.209	38.546	1.00	0.00	H
HETATM	327	CE	LYN	20	34.184	38.687	38.334	1.00	0.00	C
HETATM	328	HE2	LYN	20	33.387	38.569	37.599	1.00	0.00	H
HETATM	329	HE3	LYN	20	34.477	37.678	38.626	1.00	0.00	H
HETATM	330	NZ	LYN	20	33.607	39.334	39.547	1.00	0.00	N
HETATM	331	HZ2	LYN	20	33.546	40.327	39.372	1.00	0.00	H
HETATM	332	HZ3	LYN	20	34.254	39.236	40.317	1.00	0.00	H
HETATM	333	C	LYN	20	39.086	38.426	37.256	1.00	0.00	C
HETATM	334	O	LYN	20	39.495	37.655	36.401	1.00	0.00	O
HETATM	1636	N	LIG	103	30.221	41.462	41.138	1.00	0.00	N

```
HETATM 1637  C   LIG   103       29.941  40.383  40.421  1.00  0.00           C
HETATM 1638  O   LIG   103       29.957  42.741  42.973  1.00  0.00           O
HETATM 1639  C5' LIG   103       25.433  38.837  44.822  1.00  0.00           C
HETATM 1640  O5' LIG   103       25.432  38.228  43.510  1.00  0.00           O
HETATM 1641  C4' LIG   103       25.984  40.239  44.867  1.00  0.00           C
HETATM 1642  O4' LIG   103       27.434  40.259  44.701  1.00  0.00           O
HETATM 1643  C3' LIG   103       25.524  41.235  43.812  1.00  0.00           C
HETATM 1644  O3' LIG   103       24.334  41.937  44.314  1.00  0.00           O
HETATM 1645  C2' LIG   103       26.674  42.208  43.586  1.00  0.00           C
HETATM 1646  O2' LIG   103       26.670  43.398  44.440  1.00  0.00           O
HETATM 1647  C1' LIG   103       27.922  41.348  43.944  1.00  0.00           C
HETATM 1648  N1  LIG   103       28.709  40.894  42.828  1.00  0.00           N
HETATM 1649  O1  LIG   103       29.716  38.308  38.330  1.00  0.00           O
HETATM 1650  N2  LIG   103       30.530  40.241  39.186  1.00  0.00           N
HETATM 1651  C6  LIG   103       29.607  41.723  42.352  1.00  0.00           C
HETATM 1652  C7  LIG   103       28.297  39.715  42.156  1.00  0.00           C
HETATM 1653  C8  LIG   103       28.909  39.513  40.903  1.00  0.00           C
HETATM 1654  C9  LIG   103       30.391  39.314  38.265  1.00  0.00           C
HETATM 1655  C10 LIG   103       31.062  39.705  36.915  1.00  0.00           C
HETATM 1656  H   LIG   103       31.247  40.928  38.986  1.00  0.00           H
HETATM 1657  H1  LIG   103       28.564  38.670  40.315  1.00  0.00           H
HETATM 1658  H4  LIG   103       28.574  41.870  44.653  1.00  0.00           H
HETATM 1659  H6  LIG   103       25.700  40.704  45.819  1.00  0.00
           H
HETATM 1660  H7  LIG   103       25.298  40.662  42.905  1.00  0.00           H
HETATM 1661  H8  LIG   103       26.871  42.421  42.528  1.00  0.00           H
HETATM 1662  H9  LIG   103       24.466  38.870  45.339  1.00  0.00           H
HETATM 1663  H10 LIG   103       26.159  38.307  45.451  1.00  0.00           H
HETATM 1664  H11 LIG   103       31.620  40.648  36.966  1.00  0.00           H
HETATM 1665  H12 LIG   103       30.282  39.803  36.150  1.00  0.00           H
HETATM 1666  H13 LIG   103       31.720  38.870  36.641  1.00  0.00           H
HETATM 1667  H14 LIG   103       27.542  39.135  42.669  1.00  0.00           H
HETATM 1668  H2' LIG   103       26.140  44.023  43.917  1.00  0.00           H
HETATM 1669  H3' LIG   103       23.609  41.630  43.743  1.00  0.00           H
HETATM 1670  H5' LIG   103       24.663  37.644  43.390  1.00  0.00           H
ENDMDL
MODEL     4
SHEET    1   1 1 ILE    22  ASP    26  0
SHEET    2   2 1 VAL    37  VAL    40  0
SHEET    3   3 1 CYX    50  VAL    60  0
SHEET    4   4 1 PHE    94  CYX   101  0
HELIX    1   1 GLN     9  PHE     11  1                                   3
HELIX    2   2 ASP    13  LEU     16  1                                   4
HELIX    3   3 LYS    68  GLN     72  1                                   5
HELIX    4   4 LEU    77  ILE     87  1                                  11
ATOM      1  N   GLN     1       31.413  17.549  35.011  1.00  0.00           N
ATOM      2  H1  GLN     1       30.434  17.794  35.067  1.00  0.00           H
ATOM      3  H2  GLN     1       31.519  16.559  35.181  1.00  0.00           H
ATOM      4  H3  GLN     1       31.968  18.034  35.703  1.00  0.00           H
ATOM      5  CA  GLN     1       31.916  17.890  33.627  1.00  0.00           C
ATOM      6  HA  GLN     1       32.848  17.366  33.413  1.00  0.00           H
ATOM      7  CB  GLN     1       30.907  17.430  32.545  1.00  0.00           C
ATOM      8  HB2 GLN     1       29.969  17.879  32.870  1.00  0.00           H
ATOM      9  HB3 GLN     1       31.155  17.885  31.586  1.00  0.00           H
ATOM     10  CG  GLN     1       30.840  15.900  32.434  1.00  0.00           C
ATOM     11  HG2 GLN     1       30.838  15.420  33.412  1.00  0.00           H
ATOM     12  HG3 GLN     1       29.935  15.571  31.925  1.00  0.00           H
ATOM     13  CD  GLN     1       31.982  15.349  31.594  1.00  0.00           C
ATOM     14  OE1 GLN     1       33.111  15.860  31.619  1.00  0.00           O
ATOM     15  NE2 GLN     1       31.695  14.419  30.712  1.00  0.00           N
ATOM     16 HE21 GLN     1       30.814  13.927  30.764  1.00  0.00           H
ATOM     17 HE22 GLN     1       32.492  13.999  30.255  1.00  0.00           H
ATOM     18  C   GLN     1       32.021  19.388  33.671  1.00  0.00           C
ATOM     19  O   GLN     1       31.273  20.050  34.456  1.00  0.00           O
```

ATOM	20	N	PRO	2	32.895	19.923	32.814	1.00	0.00	N
ATOM	21	CD	PRO	2	33.821	19.173	31.999	1.00	0.00	C
ATOM	22	HD2	PRO	2	33.315	18.784	31.115	1.00	0.00	H
ATOM	23	HD3	PRO	2	34.265	18.323	32.518	1.00	0.00	H
ATOM	24	CG	PRO	2	34.961	20.068	31.517	1.00	0.00	C
ATOM	25	HG2	PRO	2	35.350	19.724	30.559	1.00	0.00	H
ATOM	26	HG3	PRO	2	35.746	20.216	32.258	1.00	0.00	H
ATOM	27	CB	PRO	2	34.135	21.381	31.440	1.00	0.00	C
ATOM	28	HB2	PRO	2	33.607	21.398	30.487	1.00	0.00	H
ATOM	29	HB3	PRO	2	34.790	22.250	31.495	1.00	0.00	H
ATOM	30	CA	PRO	2	33.133	21.316	32.597	1.00	0.00	C
ATOM	31	HA	PRO	2	33.334	21.928	33.476	1.00	0.00	H
ATOM	32	C	PRO	2	31.863	21.886	32.042	1.00	0.00	C
ATOM	33	O	PRO	2	31.125	21.187	31.291	1.00	0.00	O
ATOM	34	N	ASN	3	31.597	23.118	32.388	1.00	0.00	N
ATOM	35	H	ASN	3	32.234	23.515	33.063	1.00	0.00	H
ATOM	36	CA	ASN	3	30.374	23.790	32.134	1.00	0.00	C
ATOM	37	HA	ASN	3	29.470	23.262	32.440	1.00	0.00	H
ATOM	38	CB	ASN	3	30.415	25.065	32.945	1.00	0.00	C
ATOM	39	HB2	ASN	3	31.251	25.708	32.671	1.00	0.00	H
ATOM	40	HB3	ASN	3	29.553	25.639	32.603	1.00	0.00	H
ATOM	41	CG	ASN	3	30.332	24.821	34.405	1.00	0.00	C
ATOM	42	OD1	ASN	3	29.253	24.631	34.927	1.00	0.00	O
ATOM	43	ND2	ASN	3	31.395	24.837	35.116	1.00	0.00	N
ATOM	44	HD21	ASN	3	32.294	25.098	34.735	1.00	0.00	H
ATOM	45	HD22	ASN	3	31.292	24.688	36.109	1.00	0.00	H
ATOM	46	C	ASN	3	29.998	24.023	30.661	1.00	0.00	C
ATOM	47	O	ASN	3	30.862	24.454	29.915	1.00	0.00	O
ATOM	48	N	ASP	4	28.764	23.771	30.229	1.00	0.00	N
ATOM	49	H	ASP	4	28.108	23.429	30.916	1.00	0.00	H
ATOM	50	CA	ASP	4	28.155	24.261	28.960	1.00	0.00	C
ATOM	51	HA	ASP	4	28.796	24.105	28.092	1.00	0.00	H
ATOM	52	CB	ASP	4	26.764	23.570	28.640	1.00	0.00	C
ATOM	53	HB2	ASP	4	26.100	23.821	29.466	1.00	0.00	H
ATOM	54	HB3	ASP	4	26.361	23.973	27.710	1.00	0.00	H
ATOM	55	CG	ASP	4	26.786	22.051	28.422	1.00	0.00	C
ATOM	56	OD1	ASP	4	27.887	21.464	28.304	1.00	0.00	O
ATOM	57	OD2	ASP	4	25.700	21.501	28.350	1.00	0.00	O
ATOM	58	C	ASP	4	27.895	25.776	29.044	1.00	0.00	C
ATOM	59	O	ASP	4	28.195	26.451	28.092	1.00	0.00	O
ATOM	60	N	ILE	5	27.559	26.304	30.226	1.00	0.00	N
ATOM	61	H	ILE	5	27.121	25.641	30.850	1.00	0.00	H
ATOM	62	CA	ILE	5	27.515	27.727	30.584	1.00	0.00	C
ATOM	63	HA	ILE	5	26.736	28.087	29.911	1.00	0.00	H
ATOM	64	CB	ILE	5	27.007	27.936	32.034	1.00	0.00	C
ATOM	65	HB	ILE	5	27.824	27.691	32.711	1.00	0.00	H
ATOM	66	CG2	ILE	5	26.671	29.459	32.231	1.00	0.00	C
ATOM	67	HG21	ILE	5	25.729	29.649	31.715	1.00	0.00	H
ATOM	68	HG22	ILE	5	26.671	29.821	33.259	1.00	0.00	H
ATOM	69	HG23	ILE	5	27.434	30.118	31.818	1.00	0.00	H
ATOM	70	CG1	ILE	5	25.811	27.128	32.486	1.00	0.00	C
ATOM	71	HG12	ILE	5	24.899	27.388	31.950	1.00	0.00	H
ATOM	72	HG13	ILE	5	26.068	26.122	32.154	1.00	0.00	H
ATOM	73	CD1	ILE	5	25.533	27.270	34.025	1.00	0.00	C
ATOM	74	HD11	ILE	5	25.300	28.285	34.346	1.00	0.00	H
ATOM	75	HD12	ILE	5	24.753	26.547	34.263	1.00	0.00	H
ATOM	76	HD13	ILE	5	26.490	27.016	34.482	1.00	0.00	H
ATOM	77	C	ILE	5	28.914	28.395	30.371	1.00	0.00	C
ATOM	78	O	ILE	5	29.918	27.865	30.799	1.00	0.00	O
ATOM	79	N	THR	6	28.885	29.592	29.694	1.00	0.00	N
ATOM	80	H	THR	6	27.948	29.971	29.678	1.00	0.00	H
ATOM	81	CA	THR	6	30.085	30.480	29.475	1.00	0.00	C
ATOM	82	HA	THR	6	30.885	30.075	30.096	1.00	0.00	H
ATOM	83	CB	THR	6	30.585	30.350	27.993	1.00	0.00	C

ATOM	84	HB	THR	6	30.751	29.319	27.680	1.00	0.00	H
ATOM	85	CG2	THR	6	29.643	31.039	26.985	1.00	0.00	C
ATOM	86	HG21	THR	6	28.682	30.536	27.087	1.00	0.00	H
ATOM	87	HG22	THR	6	29.230	32.041	27.099	1.00	0.00	H
ATOM	88	HG23	THR	6	30.046	30.952	25.976	1.00	0.00	H
ATOM	89	OG1	THR	6	31.815	31.048	28.024	1.00	0.00	O
ATOM	90	HG1	THR	6	32.450	30.332	27.939	1.00	0.00	H
ATOM	91	C	THR	6	29.771	31.944	29.697	1.00	0.00	C
ATOM	92	O	THR	6	28.606	32.208	30.057	1.00	0.00	O
ATOM	93	N	PHE	7	30.777	32.847	29.660	1.00	0.00	N
ATOM	94	H	PHE	7	31.559	32.351	29.257	1.00	0.00	H
ATOM	95	CA	PHE	7	30.680	34.294	29.631	1.00	0.00	C
ATOM	96	HA	PHE	7	29.914	34.576	30.354	1.00	0.00	H
ATOM	97	CB	PHE	7	32.055	34.845	30.192	1.00	0.00	C
ATOM	98	HB2	PHE	7	32.861	34.448	29.574	1.00	0.00	H
ATOM	99	HB3	PHE	7	32.013	35.934	30.215	1.00	0.00	H
ATOM	100	CG	PHE	7	32.251	34.228	31.588	1.00	0.00	C
ATOM	101	CD1	PHE	7	33.279	33.309	31.872	1.00	0.00	C
ATOM	102	HD1	PHE	7	33.960	33.024	31.084	1.00	0.00	H
ATOM	103	CE1	PHE	7	33.437	32.822	33.161	1.00	0.00	C
ATOM	104	HE1	PHE	7	34.063	31.958	33.325	1.00	0.00	H
ATOM	105	CZ	PHE	7	32.510	33.275	34.189	1.00	0.00	C
ATOM	106	HZ	PHE	7	32.563	32.779	35.147	1.00	0.00	H
ATOM	107	CE2	PHE	7	31.453	34.169	33.891	1.00	0.00	C
ATOM	108	HE2	PHE	7	30.694	34.281	34.651	1.00	0.00	H
ATOM	109	CD2	PHE	7	31.334	34.649	32.559	1.00	0.00	C
ATOM	110	HD2	PHE	7	30.543	35.297	32.211	1.00	0.00	H
ATOM	111	C	PHE	7	30.313	34.926	28.242	1.00	0.00	C
ATOM	112	O	PHE	7	30.920	34.556	27.229	1.00	0.00	O
ATOM	113	N	PHE	8	29.351	35.877	28.242	1.00	0.00	N
ATOM	114	H	PHE	8	28.825	36.141	29.064	1.00	0.00	H
ATOM	115	CA	PHE	8	28.936	36.649	27.044	1.00	0.00	C
ATOM	116	HA	PHE	8	28.561	35.896	26.350	1.00	0.00	H
ATOM	117	CB	PHE	8	27.661	37.431	27.487	1.00	0.00	C
ATOM	118	HB2	PHE	8	27.896	38.443	27.815	1.00	0.00	H
ATOM	119	HB3	PHE	8	27.033	37.482	26.597	1.00	0.00	H
ATOM	120	CG	PHE	8	26.703	36.779	28.564	1.00	0.00	C
ATOM	121	CD1	PHE	8	26.104	35.542	28.183	1.00	0.00	C
ATOM	122	HD1	PHE	8	26.503	35.062	27.302	1.00	0.00	H
ATOM	123	CE1	PHE	8	25.048	35.016	28.948	1.00	0.00	C
ATOM	124	HE1	PHE	8	24.425	34.178	28.672	1.00	0.00	H
ATOM	125	CZ	PHE	8	24.771	35.538	30.232	1.00	0.00	C
ATOM	126	HZ	PHE	8	24.038	35.106	30.897	1.00	0.00	H
ATOM	127	CE2	PHE	8	25.436	36.758	30.661	1.00	0.00	C
ATOM	128	HE2	PHE	8	25.277	37.218	31.626	1.00	0.00	H
ATOM	129	CD2	PHE	8	26.335	37.394	29.756	1.00	0.00	C
ATOM	130	HD2	PHE	8	26.777	38.355	29.979	1.00	0.00	H
ATOM	131	C	PHE	8	30.095	37.440	26.312	1.00	0.00	C
ATOM	132	O	PHE	8	31.049	38.010	26.863	1.00	0.00	O
ATOM	133	N	GLN	9	29.944	37.619	24.946	1.00	0.00	N
ATOM	134	H	GLN	9	29.106	37.341	24.458	1.00	0.00	H
ATOM	135	CA	GLN	9	31.134	37.843	24.063	1.00	0.00	C
ATOM	136	HA	GLN	9	31.771	36.978	24.250	1.00	0.00	H
ATOM	137	CB	GLN	9	30.614	37.692	22.585	1.00	0.00	C
ATOM	138	HB2	GLN	9	29.836	38.455	22.565	1.00	0.00	H
ATOM	139	HB3	GLN	9	31.407	37.972	21.892	1.00	0.00	H
ATOM	140	CG	GLN	9	30.010	36.419	22.101	1.00	0.00	C
ATOM	141	HG2	GLN	9	30.453	35.557	22.598	1.00	0.00	H
ATOM	142	HG3	GLN	9	28.977	36.237	22.398	1.00	0.00	H
ATOM	143	CD	GLN	9	30.024	36.341	20.602	1.00	0.00	C
ATOM	144	OE1	GLN	9	30.218	37.319	19.896	1.00	0.00	O
ATOM	145	NE2	GLN	9	29.749	35.148	19.973	1.00	0.00	N
ATOM	146	HE21	GLN	9	29.600	35.223	18.977	1.00	0.00	H

ATOM	147	HE22	GLN	9	29.316	34.450	20.561	1.00	0.00	H
ATOM	148	C	GLN	9	31.903	39.160	24.381	1.00	0.00	C
ATOM	149	O	GLN	9	33.133	39.209	24.175	1.00	0.00	O
ATOM	150	N	ARG	10	31.219	40.167	24.904	1.00	0.00	N
ATOM	151	H	ARG	10	30.219	40.028	24.957	1.00	0.00	H
ATOM	152	CA	ARG	10	31.756	41.440	25.406	1.00	0.00	C
ATOM	153	HA	ARG	10	32.689	41.638	24.878	1.00	0.00	H
ATOM	154	CB	ARG	10	30.712	42.532	25.059	1.00	0.00	C
ATOM	155	HB2	ARG	10	30.184	42.199	24.167	1.00	0.00	H
ATOM	156	HB3	ARG	10	29.945	42.593	25.831	1.00	0.00	H
ATOM	157	CG	ARG	10	31.241	43.917	24.891	1.00	0.00	C
ATOM	158	HG2	ARG	10	31.688	44.213	25.841	1.00	0.00	H
ATOM	159	HG3	ARG	10	32.048	43.915	24.159	1.00	0.00	H
ATOM	160	CD	ARG	10	30.176	44.901	24.413	1.00	0.00	C
ATOM	161	HD2	ARG	10	30.658	45.878	24.421	1.00	0.00	H
ATOM	162	HD3	ARG	10	29.861	44.753	23.380	1.00	0.00	H
ATOM	163	NE	ARG	10	28.940	45.021	25.207	1.00	0.00	N
ATOM	164	HE	ARG	10	29.037	45.435	26.124	1.00	0.00	H
ATOM	165	CZ	ARG	10	27.766	44.648	24.903	1.00	0.00	C
ATOM	166	NH1	ARG	10	27.438	43.827	23.920	1.00	0.00	N
ATOM	167	HH11	ARG	10	26.496	43.477	23.813	1.00	0.00	H
ATOM	168	HH12	ARG	10	28.089	43.646	23.169	1.00	0.00	H
ATOM	169	NH2	ARG	10	26.786	45.057	25.652	1.00	0.00	N
ATOM	170	HH21	ARG	10	27.054	45.707	26.379	1.00	0.00	H
ATOM	171	HH22	ARG	10	25.832	44.811	25.433	1.00	0.00	H
ATOM	172	C	ARG	10	32.215	41.475	26.896	1.00	0.00	C
ATOM	173	O	ARG	10	32.678	42.504	27.396	1.00	0.00	O
ATOM	174	N	PHE	11	32.429	40.307	27.570	1.00	0.00	N
ATOM	175	H	PHE	11	31.864	39.549	27.216	1.00	0.00	H
ATOM	176	CA	PHE	11	32.955	40.296	28.952	1.00	0.00	C
ATOM	177	HA	PHE	11	33.450	41.247	29.149	1.00	0.00	H
ATOM	178	CB	PHE	11	31.817	40.292	29.944	1.00	0.00	C
ATOM	179	HB2	PHE	11	32.340	40.547	30.866	1.00	0.00	H
ATOM	180	HB3	PHE	11	31.534	39.256	30.130	1.00	0.00	H
ATOM	181	CG	PHE	11	30.625	41.315	29.651	1.00	0.00	C
ATOM	182	CD1	PHE	11	30.665	42.648	30.083	1.00	0.00	C
ATOM	183	HD1	PHE	11	31.474	42.968	30.723	1.00	0.00	H
ATOM	184	CE1	PHE	11	29.545	43.478	29.855	1.00	0.00	C
ATOM	185	HE1	PHE	11	29.513	44.499	30.205	1.00	0.00	H
ATOM	186	CZ	PHE	11	28.529	43.027	28.937	1.00	0.00	C
ATOM	187	HZ	PHE	11	27.843	43.769	28.556	1.00	0.00	H
ATOM	188	CE2	PHE	11	28.636	41.706	28.427	1.00	0.00	C
ATOM	189	HE2	PHE	11	27.946	41.357	27.673	1.00	0.00	H
ATOM	190	CD2	PHE	11	29.625	40.856	28.845	1.00	0.00	C
ATOM	191	HD2	PHE	11	29.666	39.822	28.540	1.00	0.00	H
ATOM	192	C	PHE	11	33.935	39.168	29.225	1.00	0.00	C
ATOM	193	O	PHE	11	34.251	38.828	30.376	1.00	0.00	O
ATOM	194	N	GLN	12	34.391	38.415	28.199	1.00	0.00	N
ATOM	195	H	GLN	12	34.293	38.738	27.248	1.00	0.00	H
ATOM	196	CA	GLN	12	35.271	37.272	28.338	1.00	0.00	C
ATOM	197	HA	GLN	12	34.878	36.627	29.125	1.00	0.00	H
ATOM	198	CB	GLN	12	35.414	36.492	27.058	1.00	0.00	C
ATOM	199	HB2	GLN	12	35.765	37.221	26.327	1.00	0.00	H
ATOM	200	HB3	GLN	12	36.109	35.656	27.140	1.00	0.00	H
ATOM	201	CG	GLN	12	34.043	36.014	26.694	1.00	0.00	C
ATOM	202	HG2	GLN	12	33.538	35.486	27.504	1.00	0.00	H
ATOM	203	HG3	GLN	12	33.437	36.892	26.473	1.00	0.00	H
ATOM	204	CD	GLN	12	34.062	35.264	25.375	1.00	0.00	C
ATOM	205	OE1	GLN	12	34.820	35.545	24.431	1.00	0.00	O
ATOM	206	NE2	GLN	12	33.175	34.332	25.232	1.00	0.00	N
ATOM	207	HE21	GLN	12	32.550	34.094	25.990	1.00	0.00	H
ATOM	208	HE22	GLN	12	32.982	33.933	24.324	1.00	0.00	H
ATOM	209	C	GLN	12	36.697	37.732	28.676	1.00	0.00	C
ATOM	210	O	GLN	12	37.356	37.018	29.391	1.00	0.00	O

ATOM	211	N	ASP	13	37.143	38.786	28.064	1.00	0.00	N
ATOM	212	H	ASP	13	36.636	39.128	27.260	1.00	0.00	H
ATOM	213	CA	ASP	13	38.533	39.169	28.008	1.00	0.00	C
ATOM	214	HA	ASP	13	39.088	38.252	27.809	1.00	0.00	H
ATOM	215	CB	ASP	13	38.720	40.171	26.891	1.00	0.00	C
ATOM	216	HB2	ASP	13	38.001	40.988	26.941	1.00	0.00	H
ATOM	217	HB3	ASP	13	39.651	40.720	27.025	1.00	0.00	H
ATOM	218	CG	ASP	13	38.699	39.663	25.384	1.00	0.00	C
ATOM	219	OD1	ASP	13	38.751	40.474	24.456	1.00	0.00	O
ATOM	220	OD2	ASP	13	38.326	38.502	25.155	1.00	0.00	O
ATOM	221	C	ASP	13	39.102	39.555	29.400	1.00	0.00	C
ATOM	222	O	ASP	13	40.321	39.712	29.549	1.00	0.00	O
ATOM	223	N	ASP	14	38.276	40.052	30.313	1.00	0.00	N
ATOM	224	H	ASP	14	37.301	40.017	30.050	1.00	0.00	H
ATOM	225	CA	ASP	14	38.801	40.656	31.504	1.00	0.00	C
ATOM	226	HA	ASP	14	39.507	41.442	31.235	1.00	0.00	H
ATOM	227	CB	ASP	14	37.682	41.200	32.416	1.00	0.00	C
ATOM	228	HB2	ASP	14	37.074	40.343	32.711	1.00	0.00	H
ATOM	229	HB3	ASP	14	38.114	41.657	33.306	1.00	0.00	H
ATOM	230	CG	ASP	14	36.811	42.262	31.846	1.00	0.00	C
ATOM	231	OD1	ASP	14	37.141	42.949	30.858	1.00	0.00	O
ATOM	232	OD2	ASP	14	35.748	42.506	32.485	1.00	0.00	O
ATOM	233	C	ASP	14	39.630	39.614	32.365	1.00	0.00	C
ATOM	234	O	ASP	14	40.588	39.942	33.065	1.00	0.00	O
ATOM	235	N	ILE	15	39.317	38.305	32.268	1.00	0.00	N
ATOM	236	H	ILE	15	38.462	38.167	31.747	1.00	0.00	H
ATOM	237	CA	ILE	15	39.768	37.170	33.031	1.00	0.00	C
ATOM	238	HA	ILE	15	39.780	37.452	34.084	1.00	0.00	H
ATOM	239	CB	ILE	15	38.850	35.924	32.772	1.00	0.00	C
ATOM	240	HB	ILE	15	38.970	35.727	31.707	1.00	0.00	H
ATOM	241	CG2	ILE	15	39.474	34.670	33.449	1.00	0.00	C
ATOM	242	HG21	ILE	15	38.740	33.870	33.543	1.00	0.00	H
ATOM	243	HG22	ILE	15	40.307	34.270	32.871	1.00	0.00	H
ATOM	244	HG23	ILE	15	39.927	34.936	34.404	1.00	0.00	H
ATOM	245	CG1	ILE	15	37.361	36.186	33.176	1.00	0.00	C
ATOM	246	HG12	ILE	15	37.299	36.422	34.237	1.00	0.00	H
ATOM	247	HG13	ILE	15	36.969	37.057	32.652	1.00	0.00	H
ATOM	248	CD1	ILE	15	36.428	35.014	32.827	1.00	0.00	C
ATOM	249	HD11	ILE	15	36.282	35.001	31.747	1.00	0.00	H
ATOM	250	HD12	ILE	15	36.696	34.092	33.343	1.00	0.00	H
ATOM	251	HD13	ILE	15	35.484	35.263	33.310	1.00	0.00	H
ATOM	252	C	ILE	15	41.227	36.899	32.670	1.00	0.00	C
ATOM	253	O	ILE	15	42.082	36.854	33.552	1.00	0.00	O
ATOM	254	N	LEU	16	41.598	36.805	31.355	1.00	0.00	N
ATOM	255	H	LEU	16	40.844	36.850	30.685	1.00	0.00	H
ATOM	256	CA	LEU	16	42.881	36.945	30.807	1.00	0.00	C
ATOM	257	HA	LEU	16	43.510	36.183	31.267	1.00	0.00	H
ATOM	258	CB	LEU	16	42.862	36.800	29.255	1.00	0.00	C
ATOM	259	HB2	LEU	16	42.449	35.837	28.958	1.00	0.00	H
ATOM	260	HB3	LEU	16	42.172	37.505	28.791	1.00	0.00	H
ATOM	261	CG	LEU	16	44.249	36.951	28.630	1.00	0.00	C
ATOM	262	HG	LEU	16	44.613	37.964	28.796	1.00	0.00	H
ATOM	263	CD1	LEU	16	45.286	35.942	29.235	1.00	0.00	C
ATOM	264	HD11	LEU	16	46.070	35.721	28.511	1.00	0.00	H
ATOM	265	HD12	LEU	16	45.732	36.401	30.117	1.00	0.00	H
ATOM	266	HD13	LEU	16	44.789	34.988	29.412	1.00	0.00	H
ATOM	267	CD2	LEU	16	44.205	36.709	27.174	1.00	0.00	C
ATOM	268	HD21	LEU	16	45.226	36.867	26.824	1.00	0.00	H
ATOM	269	HD22	LEU	16	43.851	35.701	26.959	1.00	0.00	H
ATOM	270	HD23	LEU	16	43.541	37.441	26.715	1.00	0.00	H
ATOM	271	C	LEU	16	43.496	38.325	31.240	1.00	0.00	C
ATOM	272	O	LEU	16	44.628	38.262	31.801	1.00	0.00	O
ATOM	273	N	ALA	17	42.829	39.495	31.083	1.00	0.00	N

ATOM	274	H	ALA	17	41.976	39.517	30.543	1.00	0.00	H
ATOM	275	CA	ALA	17	43.521	40.761	31.372	1.00	0.00	C
ATOM	276	HA	ALA	17	44.457	40.716	30.817	1.00	0.00	H
ATOM	277	CB	ALA	17	42.795	41.984	30.805	1.00	0.00	C
ATOM	278	HB1	ALA	17	42.310	41.772	29.851	1.00	0.00	H
ATOM	279	HB2	ALA	17	41.944	42.340	31.385	1.00	0.00	H
ATOM	280	HB3	ALA	17	43.482	42.829	30.811	1.00	0.00	H
ATOM	281	C	ALA	17	43.847	41.085	32.881	1.00	0.00	C
ATOM	282	O	ALA	17	44.136	42.241	33.218	1.00	0.00	O
ATOM	283	N	GLY	18	43.670	40.164	33.824	1.00	0.00	N
ATOM	284	H	GLY	18	43.313	39.257	33.555	1.00	0.00	H
ATOM	285	CA	GLY	18	43.741	40.371	35.276	1.00	0.00	C
ATOM	286	HA2	GLY	18	43.622	39.394	35.746	1.00	0.00	H
ATOM	287	HA3	GLY	18	44.732	40.747	35.529	1.00	0.00	H
ATOM	288	C	GLY	18	42.674	41.260	35.893	1.00	0.00	C
ATOM	289	O	GLY	18	42.635	41.413	37.107	1.00	0.00	O
ATOM	290	N	ARG	19	41.878	41.890	35.078	1.00	0.00	N
ATOM	291	H	ARG	19	41.976	41.748	34.082	1.00	0.00	H
ATOM	292	CA	ARG	19	40.812	42.842	35.374	1.00	0.00	C
ATOM	293	HA	ARG	19	41.260	43.550	36.072	1.00	0.00	H
ATOM	294	CB	ARG	19	40.272	43.476	34.028	1.00	0.00	C
ATOM	295	HB2	ARG	19	39.989	42.744	33.272	1.00	0.00	H
ATOM	296	HB3	ARG	19	39.318	43.929	34.295	1.00	0.00	H
ATOM	297	CG	ARG	19	41.159	44.587	33.518	1.00	0.00	C
ATOM	298	HG2	ARG	19	41.535	45.328	34.224	1.00	0.00	H
ATOM	299	HG3	ARG	19	42.004	44.083	33.051	1.00	0.00	H
ATOM	300	CD	ARG	19	40.395	45.408	32.471	1.00	0.00	C
ATOM	301	HD2	ARG	19	39.730	46.010	33.091	1.00	0.00	H
ATOM	302	HD3	ARG	19	40.980	46.156	31.934	1.00	0.00	H
ATOM	303	NE	ARG	19	39.681	44.636	31.424	1.00	0.00	N
ATOM	304	HE	ARG	19	38.815	44.177	31.664	1.00	0.00	H
ATOM	305	CZ	ARG	19	40.093	44.553	30.219	1.00	0.00	C
ATOM	306	NH1	ARG	19	41.264	44.862	29.836	1.00	0.00	N
ATOM	307	HH11	ARG	19	41.845	45.429	30.437	1.00	0.00	H
ATOM	308	HH12	ARG	19	41.425	44.742	28.846	1.00	0.00	H
ATOM	309	NH2	ARG	19	39.336	44.034	29.333	1.00	0.00	N
ATOM	310	HH21	ARG	19	39.670	44.081	28.381	1.00	0.00	H
ATOM	311	HH22	ARG	19	38.438	43.649	29.589	1.00	0.00	H
ATOM	312	C	ARG	19	39.656	42.185	36.170	1.00	0.00	C
ATOM	313	O	ARG	19	39.007	42.905	36.893	1.00	0.00	O
ATOM	335	N	THR	21	38.852	38.588	38.462	1.00	0.00	N
ATOM	336	H	THR	21	38.463	39.346	39.003	1.00	0.00	H
ATOM	337	CA	THR	21	39.503	37.498	39.145	1.00	0.00	C
ATOM	338	HA	THR	21	39.472	36.656	38.452	1.00	0.00	H
ATOM	339	CB	THR	21	40.965	37.801	39.491	1.00	0.00	C
ATOM	340	HB	THR	21	41.571	38.079	38.628	1.00	0.00	H
ATOM	341	CG2	THR	21	41.189	38.831	40.642	1.00	0.00	C
ATOM	342	HG21	THR	21	42.267	38.905	40.782	1.00	0.00	H
ATOM	343	HG22	THR	21	40.724	39.795	40.438	1.00	0.00	H
ATOM	344	HG23	THR	21	40.763	38.452	41.571	1.00	0.00	H
ATOM	345	OG1	THR	21	41.519	36.560	40.033	1.00	0.00	O
ATOM	346	HG1	THR	21	41.231	35.811	39.508	1.00	0.00	H
ATOM	347	C	THR	21	38.673	36.973	40.282	1.00	0.00	C
ATOM	348	O	THR	21	38.493	35.782	40.409	1.00	0.00	O
ATOM	349	N	ILE	22	38.139	37.893	41.167	1.00	0.00	N
ATOM	350	H	ILE	22	38.268	38.868	40.934	1.00	0.00	H
ATOM	351	CA	ILE	22	37.213	37.565	42.311	1.00	0.00	C
ATOM	352	HA	ILE	22	37.126	36.482	42.405	1.00	0.00	H
ATOM	353	CB	ILE	22	37.811	38.077	43.646	1.00	0.00	C
ATOM	354	HB	ILE	22	38.737	37.543	43.864	1.00	0.00	H
ATOM	355	CG2	ILE	22	38.292	39.559	43.466	1.00	0.00	C
ATOM	356	HG21	ILE	22	38.858	39.823	44.360	1.00	0.00	H
ATOM	357	HG22	ILE	22	39.017	39.773	42.680	1.00	0.00	H
ATOM	358	HG23	ILE	22	37.500	40.307	43.440	1.00	0.00	H

```
ATOM    359  CG1 ILE    22      36.787  37.870  44.787  1.00  0.00            C
ATOM    360 HG12 ILE    22      35.904  38.486  44.617  1.00  0.00            H
ATOM    361 HG13 ILE    22      36.362  36.868  44.753  1.00  0.00            H
ATOM    362  CD1 ILE    22      37.322  38.088  46.169  1.00  0.00            C
ATOM    363 HD11 ILE    22      36.541  37.918  46.910  1.00  0.00            H
ATOM    364 HD12 ILE    22      38.057  37.283  46.207  1.00  0.00            H
ATOM    365 HD13 ILE    22      37.799  39.049  46.369  1.00  0.00            H
ATOM    366  C   ILE    22      35.753  38.069  42.033  1.00  0.00            C
ATOM    367  O   ILE    22      35.581  39.081  41.397  1.00  0.00            O
ATOM    368  N   THR    23      34.793  37.268  42.398  1.00  0.00            N
ATOM    369  H   THR    23      35.140  36.356  42.658  1.00  0.00            H
ATOM    370  CA  THR    23      33.360  37.597  42.272  1.00  0.00            C
ATOM    371  HA  THR    23      33.332  38.684  42.349  1.00  0.00            H
ATOM    372  CB  THR    23      32.736  37.078  40.912  1.00  0.00            C
ATOM    373  HB  THR    23      33.438  37.258  40.097  1.00  0.00            H
ATOM    374  CG2 THR    23      32.271  35.620  40.984  1.00  0.00            C
ATOM    375 HG21 THR    23      32.059  35.370  39.944  1.00  0.00            H
ATOM    376 HG22 THR    23      32.982  34.980  41.505  1.00  0.00            H
ATOM    377 HG23 THR    23      31.365  35.494  41.577  1.00  0.00            H
ATOM    378  OG1 THR    23      31.604  37.893  40.595  1.00  0.00            O
ATOM    379  HG1 THR    23      31.814  38.754  40.229  1.00  0.00            H
ATOM    380  C   THR    23      32.556  37.053
  43.421  1.00  0.00            C
ATOM    381  O   THR    23      33.027  36.088  44.055  1.00  0.00            O
ATOM    382  N   ILE    24      31.426  37.735  43.674  1.00  0.00            N
ATOM    383  H   ILE    24      31.397  38.621  43.191  1.00  0.00            H
ATOM    384  CA  ILE    24      30.498  37.511  44.813  1.00  0.00            C
ATOM    385  HA  ILE    24      30.710  36.573  45.327  1.00  0.00            H
ATOM    386  CB  ILE    24      30.535  38.612  45.830  1.00  0.00            C
ATOM    387  HB  ILE    24      30.396  39.549  45.291  1.00  0.00            H
ATOM    388  CG2 ILE    24      29.488  38.520  47.000  1.00  0.00            C
ATOM    389 HG21 ILE    24      29.508  37.525  47.444  1.00  0.00            H
ATOM    390 HG22 ILE    24      29.645  39.195  47.843  1.00  0.00            H
ATOM    391 HG23 ILE    24      28.475  38.764  46.681  1.00  0.00            H
ATOM    392  CG1 ILE    24      31.974  38.648  46.458  1.00  0.00            C
ATOM    393 HG12 ILE    24      32.107  37.989  47.315  1.00  0.00            H
ATOM    394 HG13 ILE    24      32.795  38.545  45.748  1.00  0.00            H
ATOM    395  CD1 ILE    24      32.333  40.012  47.098  1.00  0.00            C
ATOM    396 HD11 ILE    24      33.409  40.180  47.104  1.00  0.00            H
ATOM    397 HD12 ILE    24      32.001  40.831  46.460  1.00  0.00            H
ATOM    398 HD13 ILE    24      31.825  40.098  48.059  1.00  0.00            H
ATOM    399  C   ILE    24      29.102  37.095  44.310  1.00  0.00            C
ATOM    400  O   ILE    24      28.711  37.502  43.254  1.00  0.00            O
ATOM    401  N   ARG    25      28.409  36.349  45.160  1.00  0.00            N
ATOM    402  H   ARG    25      28.917  36.118  46.001  1.00  0.00            H
ATOM    403  CA  ARG    25      27.058  35.918  44.900  1.00  0.00            C
ATOM    404  HA  ARG    25      26.573  36.719  44.342  1.00  0.00            H
ATOM    405  CB  ARG    25      26.987  34.550  44.155  1.00  0.00            C
ATOM    406  HB2 ARG    25      27.692  33.851  44.604  1.00  0.00            H
ATOM    407  HB3 ARG    25      25.955  34.199  44.185  1.00  0.00            H
ATOM    408  CG  ARG    25      27.384  34.512  42.631  1.00  0.00            C
ATOM    409  HG2 ARG    25      28.377  34.960  42.697  1.00  0.00            H
ATOM    410  HG3 ARG    25      27.535  33.549  42.143  1.00  0.00            H
ATOM    411  CD  ARG    25      26.684  35.471  41.667  1.00  0.00            C
ATOM    412  HD2 ARG    25      25.633  35.256  41.476  1.00  0.00            H
ATOM    413  HD3 ARG    25      26.825  36.482  42.052  1.00  0.00            H
ATOM    414  NE  ARG    25      27.392  35.302  40.408  1.00  0.00            N
ATOM    415  HE  ARG    25      27.039  34.630  39.741  1.00  0.00            H
ATOM    416  CZ  ARG    25      28.346  36.036  39.873  1.00  0.00            C
ATOM    417  NH1 ARG    25      28.893  37.024  40.414  1.00  0.00            N
ATOM    418 HH11 ARG    25      29.743  37.322  39.956  1.00  0.00            H
ATOM    419 HH12 ARG    25      28.541  37.341  41.307  1.00  0.00            H
ATOM    420  NH2 ARG    25      28.832  35.705  38.719  1.00  0.00            N
ATOM    421 HH21 ARG    25      29.484  36.226  38.149  1.00  0.00            H
```

ATOM	422	HH22	ARG	25	28.593	34.788	38.371	1.00	0.00	H
ATOM	423	C	ARG	25	26.379	35.713	46.247	1.00	0.00	C
ATOM	424	O	ARG	25	26.974	35.618	47.276	1.00	0.00	O
ATOM	425	N	ASP	26	25.041	35.602	46.227	1.00	0.00	N
ATOM	426	H	ASP	26	24.557	35.700	45.346	1.00	0.00	H
ATOM	427	CA	ASP	26	24.287	35.119	47.419	1.00	0.00	C
ATOM	428	HA	ASP	26	24.714	35.569	48.316	1.00	0.00	H
ATOM	429	CB	ASP	26	22.834	35.569	47.292	1.00	0.00	C
ATOM	430	HB2	ASP	26	22.333	35.480	48.255	1.00	0.00	H
ATOM	431	HB3	ASP	26	22.796	36.656	47.225	1.00	0.00	H
ATOM	432	CG	ASP	26	22.048	34.984	46.166	1.00	0.00	C
ATOM	433	OD1	ASP	26	22.255	35.293	44.937	1.00	0.00	O
ATOM	434	OD2	ASP	26	21.104	34.265	46.626	1.00	0.00	O
ATOM	435	C	ASP	26	24.491	33.635	47.531	1.00	0.00	C
ATOM	436	O	ASP	26	24.526	32.919	46.529	1.00	0.00	O
ATOM	437	N	GLU	27	24.644	33.085	48.730	1.00	0.00	N
ATOM	438	H	GLU	27	24.472	33.641	49.556	1.00	0.00	H
ATOM	439	CA	GLU	27	24.896	31.651	48.937	1.00	0.00	C
ATOM	440	HA	GLU	27	25.912	31.462	48.587	1.00	0.00	H
ATOM	441	CB	GLU	27	24.970	31.436	50.443	1.00	0.00	C
ATOM	442	HB2	GLU	27	25.769	32.016	50.905	1.00	0.00	H
ATOM	443	HB3	GLU	27	23.993	31.777	50.787	1.00	0.00	H
ATOM	444	CG	GLU	27	25.073	30.008	51.080	1.00	0.00	C
ATOM	445	HG2	GLU	27	25.059	30.070	52.168	1.00	0.00	H
ATOM	446	HG3	GLU	27	24.215	29.457	50.695	1.00	0.00	H
ATOM	447	CD	GLU	27	26.351	29.332	50.653	1.00	0.00	C
ATOM	448	OE1	GLU	27	26.283	28.225	50.057	1.00	0.00	O
ATOM	449	OE2	GLU	27	27.460	29.688	51.136	1.00	0.00	O
ATOM	450	C	GLU	27	23.842	30.627	48.347	1.00	0.00	C
ATOM	451	O	GLU	27	22.599	30.658	48.570	1.00	0.00	O
ATOM	452	N	SER	28	24.345	29.686	47.543	1.00	0.00	N
ATOM	453	H	SER	28	25.336	29.669	47.344	1.00	0.00	H
ATOM	454	CA	SER	28	23.604	28.577	46.865	1.00	0.00	C
ATOM	455	HA	SER	28	22.804	28.255	47.532	1.00	0.00	H
ATOM	456	CB	SER	28	23.002	28.986	45.559	1.00	0.00	C
ATOM	457	HB2	SER	28	22.281	29.792	45.700	1.00	0.00	H
ATOM	458	HB3	SER	28	23.746	29.139	44.778	1.00	0.00	H
ATOM	459	OG	SER	28	22.199	27.984	44.970	1.00	0.00	O
ATOM	460	HG	SER	28	22.171	28.179	44.030	1.00	0.00	H
ATOM	461	C	SER	28	24.538	27.371	46.657	1.00	0.00	C
ATOM	462	O	SER	28	25.691	27.420	46.190	1.00	0.00	O
ATOM	463	N	GLU	29	23.870	26.236	46.751	1.00	0.00	N
ATOM	464	H	GLU	29	23.023	26.281	47.300	1.00	0.00	H
ATOM	465	CA	GLU	29	24.458	24.929	46.359	1.00	0.00	C
ATOM	466	HA	GLU	29	25.421	24.822	46.861	1.00	0.00	H
ATOM	467	CB	GLU	29	23.519	23.845	46.927	1.00	0.00	C
ATOM	468	HB2	GLU	29	23.469	24.108	47.984	1.00	0.00	H
ATOM	469	HB3	GLU	29	22.520	24.025	46.529	1.00	0.00	H
ATOM	470	CG	GLU	29	23.883	22.324	46.840	1.00	0.00	C
ATOM	471	HG2	GLU	29	23.252	21.838	47.585	1.00	0.00	H
ATOM	472	HG3	GLU	29	23.556	22.036	45.841	1.00	0.00	H
ATOM	473	CD	GLU	29	25.353	21.925	47.017	1.00	0.00	C
ATOM	474	OE1	GLU	29	25.698	20.905	46.411	1.00	0.00	O
ATOM	475	OE2	GLU	29	26.158	22.608	47.692	1.00	0.00	O
ATOM	476	C	GLU	29	24.532	24.746	44.790	1.00	0.00	C
ATOM	477	O	GLU	29	25.214	23.856	44.244	1.00	0.00	O
ATOM	478	N	SER	30	23.866	25.610	44.002	1.00	0.00	N
ATOM	479	H	SER	30	23.147	26.195	44.406	1.00	0.00	H
ATOM	480	CA	SER	30	23.821	25.553	42.529	1.00	0.00	C
ATOM	481	HA	SER	30	23.772	24.555	42.095	1.00	0.00	H
ATOM	482	CB	SER	30	22.525	26.214	42.030	1.00	0.00	C
ATOM	483	HB2	SER	30	22.377	26.010	40.969	1.00	0.00	H
ATOM	484	HB3	SER	30	21.652	25.892	42.597	1.00	0.00	H
ATOM	485	OG	SER	30	22.765	27.583	42.252	1.00	0.00	O

ATOM	486	HG	SER	30	23.140	27.966	41.456	1.00	0.00	H
ATOM	487	C	SER	30	25.107	26.146	41.912	1.00	0.00	C
ATOM	488	O	SER	30	25.397	25.928	40.779	1.00	0.00	O
ATOM	489	N	HIE	31	25.861	26.851	42.711	1.00	0.00	N
ATOM	490	H	HIE	31	25.712	26.900	43.708	1.00	0.00	H
ATOM	491	CA	HIE	31	26.983	27.639	42.064	1.00	0.00	C
ATOM	492	HA	HIE	31	26.680	28.095	41.122	1.00	0.00	H
ATOM	493	CB	HIE	31	27.415	28.779	42.862	1.00	0.00	C
ATOM	494	HB2	HIE	31	27.847	28.415	43.794	1.00	0.00	H
ATOM	495	HB3	HIE	31	28.128	29.391	42.312	1.00	0.00	H
ATOM	496	CG	HIE	31	26.329	29.765	43.349	1.00	0.00	C
ATOM	497	ND1	HIE	31	25.376	30.339	42.539	1.00	0.00	N
ATOM	498	CE1	HIE	31	24.773	31.214	43.375	1.00	0.00	C
ATOM	499	HE1	HIE	31	23.916	31.822	43.128	1.00	0.00	H
ATOM	500	NE2	HIE	31	25.440	31.295	44.561	1.00	0.00	N
ATOM	501	HE2	HIE	31	25.183	31.871	45.351	1.00	0.00	H
ATOM	502	CD2	HIE	31	26.387	30.313	44.608	1.00	0.00	C
ATOM	503	HD2	HIE	31	27.039	30.022	45.419	1.00	0.00	H
ATOM	504	C	HIE	31	28.288	26.796	41.832	1.00	0.00	C
ATOM	505	O	HIE	31	28.467	25.735	42.375	1.00	0.00	
O										
ATOM	506	N	PHE	32	29.263	27.368	41.079	1.00	0.00	N
ATOM	507	H	PHE	32	29.055	28.225	40.588	1.00	0.00	H
ATOM	508	CA	PHE	32	30.552	26.773	40.868	1.00	0.00	C
ATOM	509	HA	PHE	32	30.427	25.857	40.292	1.00	0.00	H
ATOM	510	CB	PHE	32	31.485	27.607	39.966	1.00	0.00	C
ATOM	511	HB2	PHE	32	31.906	28.472	40.480	1.00	0.00	H
ATOM	512	HB3	PHE	32	32.310	26.923	39.764	1.00	0.00	H
ATOM	513	CG	PHE	32	30.906	28.183	38.682	1.00	0.00	C
ATOM	514	CD1	PHE	32	30.842	29.560	38.419	1.00	0.00	C
ATOM	515	HD1	PHE	32	31.395	30.289	38.993	1.00	0.00	H
ATOM	516	CE1	PHE	32	30.139	30.086	37.285	1.00	0.00	C
ATOM	517	HE1	PHE	32	30.115	31.139	37.046	1.00	0.00	H
ATOM	518	CZ	PHE	32	29.482	29.189	36.424	1.00	0.00	C
ATOM	519	HZ	PHE	32	28.748	29.405	35.662	1.00	0.00	H
ATOM	520	CE2	PHE	32	29.575	27.872	36.643	1.00	0.00	C
ATOM	521	HE2	PHE	32	29.003	27.180	36.042	1.00	0.00	H
ATOM	522	CD2	PHE	32	30.250	27.359	37.743	1.00	0.00	C
ATOM	523	HD2	PHE	32	30.247	26.293	37.921	1.00	0.00	H
ATOM	524	C	PHE	32	31.313	26.313	42.139	1.00	0.00	C
ATOM	525	O	PHE	32	31.454	27.138	43.065	1.00	0.00	O
ATOM	526	N	LYS	33	31.963	25.102	42.099	1.00	0.00	N
ATOM	527	H	LYS	33	31.915	24.608	41.219	1.00	0.00	H
ATOM	528	CA	LYS	33	32.915	24.586	43.077	1.00	0.00	C
ATOM	529	HA	LYS	33	32.680	25.025	44.046	1.00	0.00	H
ATOM	530	CB	LYS	33	32.568	23.085	43.242	1.00	0.00	C
ATOM	531	HB2	LYS	33	32.910	22.696	42.283	1.00	0.00	H
ATOM	532	HB3	LYS	33	33.116	22.622	44.062	1.00	0.00	H
ATOM	533	CG	LYS	33	31.120	22.723	43.504	1.00	0.00	C
ATOM	534	HG2	LYS	33	30.587	22.936	42.578	1.00	0.00	H
ATOM	535	HG3	LYS	33	31.207	21.649	43.673	1.00	0.00	H
ATOM	536	CD	LYS	33	30.460	23.298	44.727	1.00	0.00	C
ATOM	537	HD2	LYS	33	31.149	23.130	45.554	1.00	0.00	H
ATOM	538	HD3	LYS	33	30.212	24.336	44.508	1.00	0.00	H
ATOM	539	CE	LYS	33	29.148	22.560	44.871	1.00	0.00	C
ATOM	540	HE2	LYS	33	28.530	22.653	43.979	1.00	0.00	H
ATOM	541	HE3	LYS	33	29.226	21.478	44.978	1.00	0.00	H
ATOM	542	NZ	LYS	33	28.322	23.017	46.035	1.00	0.00	N
ATOM	543	HZ1	LYS	33	28.925	22.980	46.844	1.00	0.00	H
ATOM	544	HZ2	LYS	33	28.139	24.010	45.986	1.00	0.00	H
ATOM	545	HZ3	LYS	33	27.498	22.458	46.199	1.00	0.00	H
ATOM	546	C	LYS	33	34.350	24.875	42.749	1.00	0.00	C
ATOM	547	O	LYS	33	34.681	25.120	41.624	1.00	0.00	O
ATOM	548	N	THR	34	35.277	24.894	43.763	1.00	0.00	N

ATOM	549	H	THR	34	34.989	24.746	44.720	1.00	0.00	H
ATOM	550	CA	THR	34	36.766	24.874	43.379	1.00	0.00	C
ATOM	551	HA	THR	34	37.079	25.785	42.868	1.00	0.00	H
ATOM	552	CB	THR	34	37.609	24.702	44.618	1.00	0.00	C
ATOM	553	HB	THR	34	37.398	23.765	45.132	1.00	0.00	H
ATOM	554	CG2	THR	34	39.105	24.707	44.499	1.00	0.00	C
ATOM	555	HG21	THR	34	39.598	24.640	45.469	1.00	0.00	H
ATOM	556	HG22	THR	34	39.454	23.893	43.863	1.00	0.00	H
ATOM	557	HG23	THR	34	39.464	25.592	43.975	1.00	0.00	H
ATOM	558	OG1	THR	34	37.292	25.809	45.520	1.00	0.00	O
ATOM	559	HG1	THR	34	37.417	25.380	46.371	1.00	0.00	H
ATOM	560	C	THR	34	36.978	23.667	42.421	1.00	0.00	C
ATOM	561	O	THR	34	36.420	22.599	42.688	1.00	0.00	O
ATOM	562	N	GLY	35	37.683	23.798	41.307	1.00	0.00	N
ATOM	563	H	GLY	35	38.213	24.637	41.115	1.00	0.00	H
ATOM	564	CA	GLY	35	37.927	22.784	40.340	1.00	0.00	C
ATOM	565	HA2	GLY	35	38.841	23.087	39.829	1.00	0.00	H
ATOM	566	HA3	GLY	35	38.051	21.802	40.796	1.00	0.00	H
ATOM	567	C	GLY	35	36.823	22.628	39.299	1.00	0.00	C
ATOM	568	O	GLY	35	36.890	21.700	38.466	1.00	0.00	O
ATOM	569	N	ASP	36	35.822	23.536	39.300	1.00	0.00	N
ATOM	570	H	ASP	36	35.987	24.321	39.915	1.00	0.00	H
ATOM	571	CA	ASP	36	34.889	23.707	38.191	1.00	0.00	C
ATOM	572	HA	ASP	36	34.700	22.717	37.774	1.00	0.00	H
ATOM	573	CB	ASP	36	33.504	24.309	38.630	1.00	0.00	C
ATOM	574	HB2	ASP	36	33.657	25.162	39.292	1.00	0.00	H
ATOM	575	HB3	ASP	36	32.963	24.574	37.722	1.00	0.00	H
ATOM	576	CG	ASP	36	32.550	23.300	39.273	1.00	0.00	C
ATOM	577	OD1	ASP	36	31.338	23.591	39.431	1.00	0.00	O
ATOM	578	OD2	ASP	36	32.983	22.164	39.468	1.00	0.00	O
ATOM	579	C	ASP	36	35.446	24.509	36.978	1.00	0.00	C
ATOM	580	O	ASP	36	36.322	25.338	37.183	1.00	0.00	O
ATOM	581	N	VAL	37	35.188	24.063	35.763	1.00	0.00	N
ATOM	582	H	VAL	37	34.513	23.314	35.798	1.00	0.00	H
ATOM	583	CA	VAL	37	35.880	24.449	34.469	1.00	0.00	C
ATOM	584	HA	VAL	37	36.641	25.175	34.756	1.00	0.00	H
ATOM	585	CB	VAL	37	36.680	23.272	33.878	1.00	0.00	C
ATOM	586	HB	VAL	37	36.002	22.437	33.699	1.00	0.00	H
ATOM	587	CG1	VAL	37	37.435	23.487	32.561	1.00	0.00	C
ATOM	588	HG11	VAL	37	36.598	23.749	31.914	1.00	0.00	H
ATOM	589	HG12	VAL	37	38.152	24.303	32.644	1.00	0.00	H
ATOM	590	HG13	VAL	37	37.927	22.581	32.209	1.00	0.00	H
ATOM	591	CG2	VAL	37	37.788	22.885	34.920	1.00	0.00	C
ATOM	592	HG21	VAL	37	37.420	22.990	35.941	1.00	0.00	H
ATOM	593	HG22	VAL	37	38.144	21.872	34.732	1.00	0.00	H
ATOM	594	HG23	VAL	37	38.579	23.629	34.831	1.00	0.00	H
ATOM	595	C	VAL	37	34.923	25.123	33.512	1.00	0.00	C
ATOM	596	O	VAL	37	33.753	24.725	33.375	1.00	0.00	O
ATOM	597	N	LEU	38	35.456	26.159	32.878	1.00	0.00	N
ATOM	598	H	LEU	38	36.430	26.329	33.086	1.00	0.00	H
ATOM	599	CA	LEU	38	34.823	27.048	31.913	1.00	0.00	C
ATOM	600	HA	LEU	38	33.866	26.648	31.576	1.00	0.00	H
ATOM	601	CB	LEU	38	34.348	28.378	32.610	1.00	0.00	C
ATOM	602	HB2	LEU	38	35.254	28.871	32.964	1.00	0.00	H
ATOM	603	HB3	LEU	38	34.014	29.051	31.819	1.00	0.00	H
ATOM	604	CG	LEU	38	33.192	28.264	33.580	1.00	0.00	C
ATOM	605	HG	LEU	38	32.724	27.299	33.385	1.00	0.00	H
ATOM	606	CD1	LEU	38	33.585	28.193	34.981	1.00	0.00	C
ATOM	607	HD11	LEU	38	33.797	27.188	35.343	1.00	0.00	H
ATOM	608	HD12	LEU	38	34.447	28.825	35.198	1.00	0.00	H
ATOM	609	HD13	LEU	38	32.738	28.477	35.606	1.00	0.00	H
ATOM	610	CD2	LEU	38	32.144	29.458	33.497	1.00	0.00	C
ATOM	611	HD21	LEU	38	31.158	29.054	33.728	1.00	0.00	H
ATOM	612	HD22	LEU	38	32.402	30.339	34.084	1.00	0.00	H

```
ATOM    613 HD23 LEU    38      32.102  29.740  32.445  1.00  0.00           H
ATOM    614  C   LEU    38      35.752  27.339  30.775  1.00  0.00           C
ATOM    615  O   LEU    38      36.958  27.164  30.911  1.00  0.00           O
ATOM    616  N   ARG    39      35.168  27.839  29.691  1.00  0.00           N
ATOM    617  H   ARG    39      34.169  27.687  29.719  1.00  0.00           H
ATOM    618  CA  ARG    39      35.820  28.278  28.454  1.00  0.00           C
ATOM    619  HA  ARG    39      36.763  28.707  28.794  1.00  0.00           H
ATOM    620  CB  ARG    39      36.058  27.239  27.338  1.00  0.00           C
ATOM    621  HB2 ARG    39      35.097  26.854  26.993  1.00  0.00           H
ATOM    622  HB3 ARG    39      36.712  27.705  26.601  1.00  0.00           H
ATOM    623  CG  ARG    39      36.869  26.002  27.822  1.00  0.00           C
ATOM    624  HG2 ARG    39      37.897  26.330  27.976  1.00  0.00           H
ATOM    625  HG3 ARG    39      36.419  25.641  28.747  1.00  0.00           H
ATOM    626  CD  ARG    39      36.868  24.772  26.874  1.00  0.00           C
ATOM    627  HD2 ARG    39      37.213  25.180  25.924  1.00  0.00           H
ATOM    628  HD3 ARG    39      37.460  23.955  27.285  1.00  0.00           H
ATOM    629  NE  ARG    39      35.533  24.124  26.698  1.00  0.00           N
ATOM    630  HE  ARG    39      34.801  24.404  27.334  1.00  0.00           H
ATOM    631  CZ  ARG
    39      35.018  23.389  25.699  1.00  0.00           C
ATOM    632  NH1 ARG    39      35.805  23.067  24.718  1.00  0.00           N
ATOM    633 HH11 ARG    39      36.749  23.418  24.799  1.00  0.00           H
ATOM    634 HH12 ARG    39      35.451  22.337  24.117  1.00  0.00           H
ATOM    635  NH2 ARG    39      33.823  22.916  25.775  1.00  0.00           N
ATOM    636 HH21 ARG    39      33.680  22.191  25.086  1.00  0.00           H
ATOM    637 HH22 ARG    39      33.186  23.250  26.484  1.00  0.00           H
ATOM    638  C   ARG    39      35.142  29.383  27.765  1.00  0.00           C
ATOM    639  O   ARG    39      33.920  29.435  27.783  1.00  0.00           O
ATOM    640  N   VAL    40      35.875  30.236  27.106  1.00  0.00           N
ATOM    641  H   VAL    40      36.858  30.003  27.074  1.00  0.00           H
ATOM    642  CA  VAL    40      35.453  31.448  26.421  1.00  0.00           C
ATOM    643  HA  VAL    40      34.377  31.354  26.283  1.00  0.00           H
ATOM    644  CB  VAL    40      35.857  32.683  27.200  1.00  0.00           C
ATOM    645  HB  VAL    40      35.723  33.564  26.571  1.00  0.00           H
ATOM    646  CG1 VAL    40      34.921  32.916  28.410  1.00  0.00           C
ATOM    647 HG11 VAL    40      34.974  32.193  29.224  1.00  0.00           H
ATOM    648 HG12 VAL    40      35.171  33.878  28.858  1.00  0.00           H
ATOM    649 HG13 VAL    40      33.914  32.885  27.996  1.00  0.00           H
ATOM    650  CG2 VAL    40      37.310  32.667  27.698  1.00  0.00           C
ATOM    651 HG21 VAL    40      37.998  32.715  26.855  1.00  0.00           H
ATOM    652 HG22 VAL    40      37.499  33.580  28.263  1.00  0.00           H
ATOM    653 HG23 VAL    40      37.425  31.774  28.313  1.00  0.00           H
ATOM    654  C   VAL    40      36.039  31.471  25.050  1.00  0.00           C
ATOM    655  O   VAL    40      37.216  31.183  24.811  1.00  0.00           O
ATOM    656  N   GLY    41      35.162  31.701  24.085  1.00  0.00           N
ATOM    657  H   GLY    41      34.186  31.665  24.341  1.00  0.00           H
ATOM    658  CA  GLY    41      35.505  31.866  22.679  1.00  0.00           C
ATOM    659  HA2 GLY    41      36.481  32.331  22.540  1.00  0.00           H
ATOM    660  HA3 GLY    41      35.550  30.852  22.279  1.00  0.00           H
ATOM    661  C   GLY    41      34.508  32.667  21.805  1.00  0.00           C
ATOM    662  O   GLY    41      33.322  32.746  22.182  1.00  0.00           O
ATOM    663  N   ARG    42      34.908  33.172  20.655  1.00  0.00           N
ATOM    664  H   ARG    42      35.845  32.978  20.332  1.00  0.00           H
ATOM    665  CA  ARG    42      34.122  33.902  19.631  1.00  0.00           C
ATOM    666  HA  ARG    42      33.295  33.251  19.352  1.00  0.00           H
ATOM    667  CB  ARG    42      33.728  35.279  20.202  1.00  0.00           C
ATOM    668  HB2 ARG    42      33.028  35.778  19.533  1.00  0.00           H
ATOM    669  HB3 ARG    42      33.246  35.227  21.178  1.00  0.00           H
ATOM    670  CG  ARG    42      34.766  36.350  20.449  1.00  0.00           C
ATOM    671  HG2 ARG    42      35.589  35.980  21.060  1.00  0.00           H
ATOM    672  HG3 ARG    42      35.177  36.747  19.521  1.00  0.00           H
ATOM    673  CD  ARG    42      34.281  37.490  21.272  1.00  0.00           C
ATOM    674  HD2 ARG    42      33.462  37.927  20.702  1.00  0.00           H
ATOM    675  HD3 ARG    42      33.955  37.025  22.203  1.00  0.00           H
```

ATOM	676	NE	ARG	42	35.314	38.525	21.430	1.00	0.00	N
ATOM	677	HE	ARG	42	35.322	39.335	20.828	1.00	0.00	H
ATOM	678	CZ	ARG	42	36.085	38.727	22.546	1.00	0.00	C
ATOM	679	NH1	ARG	42	36.208	37.887	23.585	1.00	0.00	N
ATOM	680	HH11	ARG	42	36.847	38.154	24.320	1.00	0.00	H
ATOM	681	HH12	ARG	42	35.738	36.994	23.551	1.00	0.00	H
ATOM	682	NH2	ARG	42	36.698	39.858	22.655	1.00	0.00	N
ATOM	683	HH21	ARG	42	36.766	40.517	21.892	1.00	0.00	H
ATOM	684	HH22	ARG	42	37.282	40.048	23.456	1.00	0.00	H
ATOM	685	C	ARG	42	34.858	34.046	18.343	1.00	0.00	C
ATOM	686	O	ARG	42	34.526	34.984	17.576	1.00	0.00	O
ATOM	687	N	PHE	43	35.901	33.215	18.062	1.00	0.00	N
ATOM	688	H	PHE	43	36.124	32.588	18.822	1.00	0.00	H
ATOM	689	CA	PHE	43	36.771	33.270	16.918	1.00	0.00	C
ATOM	690	HA	PHE	43	36.309	33.791	16.080	1.00	0.00	H
ATOM	691	CB	PHE	43	38.060	33.965	17.282	1.00	0.00	C
ATOM	692	HB2	PHE	43	38.614	33.296	17.942	1.00	0.00	H
ATOM	693	HB3	PHE	43	38.614	34.069	16.349	1.00	0.00	H
ATOM	694	CG	PHE	43	37.951	35.318	18.065	1.00	0.00	C
ATOM	695	CD1	PHE	43	37.464	36.465	17.378	1.00	0.00	C
ATOM	696	HD1	PHE	43	37.242	36.377	16.324	1.00	0.00	H
ATOM	697	CE1	PHE	43	37.252	37.661	18.019	1.00	0.00	C
ATOM	698	HE1	PHE	43	36.744	38.420	17.442	1.00	0.00	H
ATOM	699	CZ	PHE	43	37.582	37.764	19.366	1.00	0.00	C
ATOM	700	HZ	PHE	43	37.329	38.695	19.853	1.00	0.00	H
ATOM	701	CE2	PHE	43	38.125	36.723	20.076	1.00	0.00	C
ATOM	702	HE2	PHE	43	38.388	36.879	21.112	1.00	0.00	H
ATOM	703	CD2	PHE	43	38.320	35.445	19.446	1.00	0.00	C
ATOM	704	HD2	PHE	43	38.665	34.620	20.051	1.00	0.00	H
ATOM	705	C	PHE	43	37.158	31.872	16.400	1.00	0.00	C
ATOM	706	O	PHE	43	36.962	30.848	17.007	1.00	0.00	O
ATOM	707	N	GLU	44	37.744	31.786	15.182	1.00	0.00	N
ATOM	708	H	GLU	44	37.933	32.649	14.691	1.00	0.00	H
ATOM	709	CA	GLU	44	37.917	30.544	14.448	1.00	0.00	C
ATOM	710	HA	GLU	44	36.959	30.022	14.445	1.00	0.00	H
ATOM	711	CB	GLU	44	38.124	30.962	12.961	1.00	0.00	C
ATOM	712	HB2	GLU	44	37.153	31.328	12.628	1.00	0.00	H
ATOM	713	HB3	GLU	44	38.885	31.741	12.998	1.00	0.00	H
ATOM	714	CG	GLU	44	38.568	29.843	12.021	1.00	0.00	C
ATOM	715	HG2	GLU	44	39.588	29.577	12.300	1.00	0.00	H
ATOM	716	HG3	GLU	44	37.957	28.977	12.276	1.00	0.00	H
ATOM	717	CD	GLU	44	38.541	30.235	10.563	1.00	0.00	C
ATOM	718	OE1	GLU	44	38.765	29.311	9.693	1.00	0.00	O
ATOM	719	OE2	GLU	44	38.262	31.450	10.235	1.00	0.00	O
ATOM	720	C	GLU	44	39.146	29.685	14.943	1.00	0.00	C
ATOM	721	O	GLU	44	39.102	28.449	14.833	1.00	0.00	O
ATOM	722	N	ASP	45	40.106	30.290	15.644	1.00	0.00	N
ATOM	723	H	ASP	45	39.996	31.273	15.853	1.00	0.00	H
ATOM	724	CA	ASP	45	41.309	29.700	16.292	1.00	0.00	C
ATOM	725	HA	ASP	45	41.811	29.153	15.493	1.00	0.00	H
ATOM	726	CB	ASP	45	42.319	30.813	16.654	1.00	0.00	C
ATOM	727	HB2	ASP	45	42.620	31.312	15.733	1.00	0.00	H
ATOM	728	HB3	ASP	45	41.891	31.508	17.377	1.00	0.00	H
ATOM	729	CG	ASP	45	43.626	30.242	17.257	1.00	0.00	C
ATOM	730	OD1	ASP	45	44.034	29.116	16.981	1.00	0.00	O
ATOM	731	OD2	ASP	45	44.210	31.066	17.957	1.00	0.00	O
ATOM	732	C	ASP	45	40.956	28.746	17.410	1.00	0.00	C
ATOM	733	O	ASP	45	40.940	29.134	18.572	1.00	0.00	O
ATOM	734	N	ASP	46	40.823	27.430	17.040	1.00	0.00	N
ATOM	735	H	ASP	46	40.631	27.240	16.067	1.00	0.00	H
ATOM	736	CA	ASP	46	40.528	26.329	17.891	1.00	0.00	C
ATOM	737	HA	ASP	46	40.152	25.565	17.212	1.00	0.00	H
ATOM	738	CB	ASP	46	41.782	25.792	18.628	1.00	0.00	C
ATOM	739	HB2	ASP	46	41.918	26.434	19.498	1.00	0.00	H

```
ATOM    740  HB3 ASP    46      41.620  24.838  19.130  1.00  0.00           H
ATOM    741  CG  ASP    46      43.015  25.832  17.819  1.00  0.00           C
ATOM    742  OD1 ASP    46      42.969  25.460  16.630  1.00  0.00           O
ATOM    743  OD2 ASP    46      44.133  26.147  18.317  1.00  0.00           O
ATOM    744  C   ASP    46      39.289  26.550  18.822  1.00  0.00           C
ATOM    745  O   ASP    46      39.171  26.076  19.971  1.00  0.00           O
ATOM    746  N   GLY    47      38.382  27.417  18.356  1.00  0.00           N
ATOM    747  H   GLY    47      38.570  27.956  17.522  1.00  0.00           H
ATOM    748  CA  GLY    47      37.036  27.501  19.004  1.00  0.00           C
ATOM    749  HA2 GLY    47      36.443  27.852  18.159  1.00  0.00           H
ATOM    750  HA3 GLY    47      36.738  26.552  19.450  1.00  0.00           H
ATOM    751  C   GLY    47      37.037  28.469  20.167  1.00  0.00           C
ATOM    752  O   GLY    47      36.015  29.174  20.420  1.00  0.00           O
ATOM    753  N   TYR    48      38.119  28.701  20.904  1.00  0.00           N
ATOM    754  H   TYR    48      38.935  28.200  20.581  1.00  0.00           H
ATOM    755  CA  TYR    48      38.244  29.073  22.299  1.00  0.00           C
ATOM    756  HA  TYR    48      37.506  29.843
  22.522  1.00  0.00           H
ATOM    757  CB  TYR    48      38.070  27.846  23.231  1.00  0.00           C
ATOM    758  HB2 TYR    48      38.806  27.100  22.931  1.00  0.00           H
ATOM    759  HB3 TYR    48      38.390  28.169  24.223  1.00  0.00           H
ATOM    760  CG  TYR    48      36.679  27.233  23.302  1.00  0.00           C
ATOM    761  CD1 TYR    48      35.653  27.911  23.983  1.00  0.00           C
ATOM    762  HD1 TYR    48      35.912  28.854  24.441  1.00  0.00           H
ATOM    763  CE1 TYR    48      34.354  27.420  24.077  1.00  0.00           C
ATOM    764  HE1 TYR    48      33.593  28.012  24.564  1.00  0.00           H
ATOM    765  CZ  TYR    48      34.084  26.225  23.466  1.00  0.00           C
ATOM    766  OH  TYR    48      32.849  25.734  23.578  1.00  0.00           O
ATOM    767  HH  TYR    48      32.572  25.368  22.736  1.00  0.00           H
ATOM    768  CE2 TYR    48      35.090  25.494  22.759  1.00  0.00           C
ATOM    769  HE2 TYR    48      34.957  24.476  22.424  1.00  0.00           H
ATOM    770  CD2 TYR    48      36.362  26.083  22.667  1.00  0.00           C
ATOM    771  HD2 TYR    48      37.084  25.519  22.095  1.00  0.00           H
ATOM    772  C   TYR    48      39.631  29.663  22.477  1.00  0.00           C
ATOM    773  O   TYR    48      40.542  29.184  21.822  1.00  0.00           O
ATOM    774  N   PHE    49      39.813  30.687  23.316  1.00  0.00           N
ATOM    775  H   PHE    49      38.996  31.146  23.696  1.00  0.00           H
ATOM    776  CA  PHE    49      41.133  31.329  23.583  1.00  0.00           C
ATOM    777  HA  PHE    49      41.837  30.715  23.022  1.00  0.00           H
ATOM    778  CB  PHE    49      41.104  32.764  23.054  1.00  0.00           C
ATOM    779  HB2 PHE    49      42.150  33.067  22.989  1.00  0.00           H
ATOM    780  HB3 PHE    49      40.669  32.796  22.055  1.00  0.00           H
ATOM    781  CG  PHE    49      40.337  33.736  23.961  1.00  0.00           C
ATOM    782  CD1 PHE    49      39.004  33.868  23.593  1.00  0.00           C
ATOM    783  HD1 PHE    49      38.587  33.478  22.676  1.00  0.00           H
ATOM    784  CE1 PHE    49      38.183  34.661  24.375  1.00  0.00           C
ATOM    785  HE1 PHE    49      37.174  34.957  24.127  1.00  0.00           H
ATOM    786  CZ  PHE    49      38.678  35.145  25.619  1.00  0.00           C
ATOM    787  HZ  PHE    49      38.012  35.736  26.230  1.00  0.00           H
ATOM    788  CE2 PHE    49      40.054  35.048  25.919  1.00  0.00           C
ATOM    789  HE2 PHE    49      40.537  35.575  26.728  1.00  0.00           H
ATOM    790  CD2 PHE    49      40.896  34.364  25.078  1.00  0.00           C
ATOM    791  HD2 PHE    49      41.945  34.192  25.275  1.00  0.00           H
ATOM    792  C   PHE    49      41.535  31.207  25.000  1.00  0.00           C
ATOM    793  O   PHE    49      42.740  31.289  25.243  1.00  0.00           O
ATOM    794  N   CYX    50      40.607  30.999  25.959  1.00  0.00           N
ATOM    795  H   CYX    50      39.646  31.133  25.679  1.00  0.00           H
ATOM    796  CA  CYX    50      40.812  30.722  27.368  1.00  0.00           C
ATOM    797  HA  CYX    50      41.845  30.375  27.404  1.00  0.00           H
ATOM    798  CB  CYX    50      40.588  31.985  28.207  1.00  0.00           C
ATOM    799  HB2 CYX    50      39.850  32.667  27.783  1.00  0.00           H
ATOM    800  HB3 CYX    50      40.220  31.784  29.213  1.00  0.00           H
ATOM    801  SG  CYX    50      42.127  33.047  28.458  1.00  0.00           S
ATOM    802  C   CYX    50      40.093  29.519  28.015  1.00  0.00           C
```

ATOM	803	O	CYX	50	38.861	29.514	28.156	1.00	0.00	O
ATOM	804	N	THR	51	40.764	28.499	28.479	1.00	0.00	N
ATOM	805	H	THR	51	41.773	28.528	28.473	1.00	0.00	H
ATOM	806	CA	THR	51	40.203	27.544	29.503	1.00	0.00	C
ATOM	807	HA	THR	51	39.135	27.431	29.319	1.00	0.00	H
ATOM	808	CB	THR	51	40.727	26.052	29.409	1.00	0.00	C
ATOM	809	HB	THR	51	41.814	26.035	29.488	1.00	0.00	H
ATOM	810	CG2	THR	51	40.028	25.161	30.379	1.00	0.00	C
ATOM	811	HG21	THR	51	38.961	25.327	30.237	1.00	0.00	H
ATOM	812	HG22	THR	51	40.382	24.134	30.298	1.00	0.00	H
ATOM	813	HG23	THR	51	40.392	25.453	31.364	1.00	0.00	H
ATOM	814	OG1	THR	51	40.393	25.586	28.135	1.00	0.00	O
ATOM	815	HG1	THR	51	40.924	26.128	27.547	1.00	0.00	H
ATOM	816	C	THR	51	40.426	28.135	30.911	1.00	0.00	C
ATOM	817	O	THR	51	41.523	28.673	31.191	1.00	0.00	O
ATOM	818	N	ILE	52	39.438	28.136	31.813	1.00	0.00	N
ATOM	819	H	ILE	52	38.605	27.637	31.538	1.00	0.00	H
ATOM	820	CA	ILE	52	39.542	28.773	33.141	1.00	0.00	C
ATOM	821	HA	ILE	52	40.618	28.902	33.255	1.00	0.00	H
ATOM	822	CB	ILE	52	38.730	30.037	33.091	1.00	0.00	C
ATOM	823	HB	ILE	52	37.719	29.795	32.765	1.00	0.00	H
ATOM	824	CG2	ILE	52	38.349	30.614	34.460	1.00	0.00	C
ATOM	825	HG21	ILE	52	37.582	30.039	34.977	1.00	0.00	H
ATOM	826	HG22	ILE	52	39.143	30.543	35.204	1.00	0.00	H
ATOM	827	HG23	ILE	52	38.028	31.653	34.385	1.00	0.00	H
ATOM	828	CG1	ILE	52	39.212	31.120	32.268	1.00	0.00	C
ATOM	829	HG12	ILE	52	39.675	31.821	32.963	1.00	0.00	H
ATOM	830	HG13	ILE	52	40.031	30.786	31.631	1.00	0.00	H
ATOM	831	CD1	ILE	52	38.169	32.018	31.527	1.00	0.00	C
ATOM	832	HD11	ILE	52	37.277	32.206	32.123	1.00	0.00	H
ATOM	833	HD12	ILE	52	38.611	32.985	31.285	1.00	0.00	H
ATOM	834	HD13	ILE	52	37.877	31.521	30.601	1.00	0.00	H
ATOM	835	C	ILE	52	39.006	27.794	34.236	1.00	0.00	C
ATOM	836	O	ILE	52	37.910	27.225	34.078	1.00	0.00	O
ATOM	837	N	GLU	53	39.831	27.554	35.299	1.00	0.00	N
ATOM	838	H	GLU	53	40.766	27.924	35.197	1.00	0.00	H
ATOM	839	CA	GLU	53	39.427	26.763	36.393	1.00	0.00	C
ATOM	840	HA	GLU	53	38.534	26.166	36.203	1.00	0.00	H
ATOM	841	CB	GLU	53	40.578	25.698	36.681	1.00	0.00	C
ATOM	842	HB2	GLU	53	40.916	25.084	35.848	1.00	0.00	H
ATOM	843	HB3	GLU	53	41.387	26.392	36.908	1.00	0.00	H
ATOM	844	CG	GLU	53	40.201	24.793	37.882	1.00	0.00	C
ATOM	845	HG2	GLU	53	40.054	25.449	38.740	1.00	0.00	H
ATOM	846	HG3	GLU	53	39.238	24.318	37.697	1.00	0.00	H
ATOM	847	CD	GLU	53	41.098	23.590	38.229	1.00	0.00	C
ATOM	848	OE1	GLU	53	41.143	23.189	39.405	1.00	0.00	O
ATOM	849	OE2	GLU	53	41.794	23.001	37.408	1.00	0.00	O
ATOM	850	C	GLU	53	39.119	27.652	37.626	1.00	0.00	C
ATOM	851	O	GLU	53	39.855	28.580	37.935	1.00	0.00	O
ATOM	852	N	VAL	54	38.101	27.295	38.418	1.00	0.00	N
ATOM	853	H	VAL	54	37.596	26.478	38.105	1.00	0.00	H
ATOM	854	CA	VAL	54	37.786	28.083	39.680	1.00	0.00	C
ATOM	855	HA	VAL	54	37.953	29.139	39.470	1.00	0.00	H
ATOM	856	CB	VAL	54	36.263	27.854	39.952	1.00	0.00	C
ATOM	857	HB	VAL	54	36.033	26.800	40.100	1.00	0.00	H
ATOM	858	CG1	VAL	54	35.918	28.386	41.377	1.00	0.00	C
ATOM	859	HG11	VAL	54	36.345	27.783	42.178	1.00	0.00	H
ATOM	860	HG12	VAL	54	36.290	29.406	41.489	1.00	0.00	H
ATOM	861	HG13	VAL	54	34.851	28.542	41.533	1.00	0.00	H
ATOM	862	CG2	VAL	54	35.363	28.488	38.943	1.00	0.00	C
ATOM	863	HG21	VAL	54	35.689	29.523	38.851	1.00	0.00	H
ATOM	864	HG22	VAL	54	35.437	27.860	38.055	1.00	0.00	H
ATOM	865	HG23	VAL	54	34.330	28.524	39.291	1.00	0.00	H
ATOM	866	C	VAL	54	38.761	27.652	40.809	1.00	0.00	C

ATOM	867	O	VAL	54	38.935	26.480	41.106	1.00	0.00	O
ATOM	868	N	THR	55	39.598	28.646	41.230	1.00	0.00	N
ATOM	869	H	THR	55	39.211	29.579	41.187	1.00	0.00	H
ATOM	870	CA	THR	55	40.883	28.448	41.958	1.00	0.00	C
ATOM	871	HA	THR	55	41.366	27.508	41.696	1.00	0.00	H
ATOM	872	CB	THR	55	41.826	29.600	41.643	1.00	0.00	C
ATOM	873	HB	THR	55	42.726	29.375	42.217	1.00	0.00	H
ATOM	874	CG2	THR	55	42.216	29.725	40.194	1.00	0.00	C
ATOM	875	HG21	THR	55	41.414	29.937	39.486	1.00	0.00	H
ATOM	876	HG22	THR	55	43.020	30.459	40.151	1.00	0.00	H
ATOM	877	HG23	THR	55	42.700	28.810	39.853	1.00	0.00	H
ATOM	878	OG1	THR	55	41.422	30.849	42.120	1.00	0.00	O
ATOM	879	HG1	THR	55	41.503	30.824	43.077	1.00	0.00	H
ATOM	880	C	THR	55	40.706	28.328	43.488	1.00	0.00	C
ATOM	881	O	THR	55	41.554	27.721	44.182	1.00	0.00	O
ATOM	882	N	ALA	56	39.601	28.918	43.944	1.00	0.00	N
ATOM	883	H	ALA	56	38.966	29.314	43.265	1.00	0.00	H
ATOM	884	CA	ALA	56	39.069	28.654	45.278	1.00	0.00	C
ATOM	885	HA	ALA	56	39.060	27.590	45.517	1.00	0.00	H
ATOM	886	CB	ALA	56	40.010	29.428	46.204	1.00	0.00	C
ATOM	887	HB1	ALA	56	40.161	30.455	45.872	1.00	0.00	H
ATOM	888	HB2	ALA	56	39.513	29.463	47.174	1.00	0.00	H
ATOM	889	HB3	ALA	56	40.972	28.925	46.302	1.00	0.00	H
ATOM	890	C	ALA	56	37.646	29.227	45.474	1.00	0.00	C
ATOM	891	O	ALA	56	37.222	30.084	44.640	1.00	0.00	O
ATOM	892	N	THR	57	36.869	28.668	46.411	1.00	0.00	N
ATOM	893	H	THR	57	37.267	27.887	46.915	1.00	0.00	H
ATOM	894	CA	THR	57	35.503	29.040	46.760	1.00	0.00	C
ATOM	895	HA	THR	57	35.245	30.071	46.515	1.00	0.00	H
ATOM	896	CB	THR	57	34.419	28.146	46.130	1.00	0.00	C
ATOM	897	HB	THR	57	33.446	28.504	46.468	1.00	0.00	H
ATOM	898	CG2	THR	57	34.475	28.211	44.660	1.00	0.00	C
ATOM	899	HG21	THR	57	34.377	29.215	44.248	1.00	0.00	H
ATOM	900	HG22	THR	57	35.400	27.754	44.306	1.00	0.00	H
ATOM	901	HG23	THR	57	33.634	27.651	44.250	1.00	0.00	H
ATOM	902	OG1	THR	57	34.694	26.881	46.663	1.00	0.00	O
ATOM	903	HG1	THR	57	35.505	26.628	46.214	1.00	0.00	H
ATOM	904	C	THR	57	35.342	29.055	48.279	1.00	0.00	C
ATOM	905	O	THR	57	36.103	28.400	48.986	1.00	0.00	O
ATOM	906	N	SER	58	34.405	29.849	48.813	1.00	0.00	N
ATOM	907	H	SER	58	33.906	30.390	48.121	1.00	0.00	H
ATOM	908	CA	SER	58	34.247	30.095	50.232	1.00	0.00	C
ATOM	909	HA	SER	58	34.090	29.124	50.701	1.00	0.00	H
ATOM	910	CB	SER	58	35.400	30.710	50.877	1.00	0.00	C
ATOM	911	HB2	SER	58	35.284	30.869	51.948	1.00	0.00	H
ATOM	912	HB3	SER	58	36.206	29.998	50.699	1.00	0.00	H
ATOM	913	OG	SER	58	35.631	31.959	50.232	1.00	0.00	O
ATOM	914	HG	SER	58	35.806	31.822	49.298	1.00	0.00	H
ATOM	915	C	SER	58	32.972	30.853	50.589	1.00	0.00	C
ATOM	916	O	SER	58	32.520	31.762	49.867	1.00	0.00	O
ATOM	917	N	THR	59	32.283	30.613	51.756	1.00	0.00	N
ATOM	918	H	THR	59	32.691	29.902	52.346	1.00	0.00	H
ATOM	919	CA	THR	59	31.076	31.366	52.185	1.00	0.00	C
ATOM	920	HA	THR	59	30.608	31.587	51.225	1.00	0.00	H
ATOM	921	CB	THR	59	30.163	30.416	52.988	1.00	0.00	C
ATOM	922	HB	THR	59	30.088	29.445	52.500	1.00	0.00	H
ATOM	923	CG2	THR	59	30.631	30.115	54.342	1.00	0.00	C
ATOM	924	HG21	THR	59	29.951	29.314	54.630	1.00	0.00	H
ATOM	925	HG22	THR	59	31.600	29.627	54.242	1.00	0.00	H
ATOM	926	HG23	THR	59	30.720	31.010	54.958	1.00	0.00	H
ATOM	927	OG1	THR	59	28.862	30.928	53.221	1.00	0.00	O
ATOM	928	HG1	THR	59	28.312	30.812	52.443	1.00	0.00	H
ATOM	929	C	THR	59	31.536	32.657	52.902	1.00	0.00	C

ATOM	930	O	THR	59	32.546	32.705	53.512	1.00	0.00	O
ATOM	931	N	VAL	60	30.684	33.651	52.992	1.00	0.00	N
ATOM	932	H	VAL	60	29.741	33.539	52.646	1.00	0.00	H
ATOM	933	CA	VAL	60	30.950	34.958	53.699	1.00	0.00	C
ATOM	934	HA	VAL	60	31.521	34.716	54.595	1.00	0.00	H
ATOM	935	CB	VAL	60	31.832	35.884	52.811	1.00	0.00	C
ATOM	936	HB	VAL	60	32.425	35.196	52.209	1.00	0.00	H
ATOM	937	CG1	VAL	60	31.084	36.776	51.825	1.00	0.00	C
ATOM	938	HG11	VAL	60	30.329	36.235	51.253	1.00	0.00	H
ATOM	939	HG12	VAL	60	30.603	37.590	52.365	1.00	0.00	H
ATOM	940	HG13	VAL	60	31.708	37.277	51.086	1.00	0.00	H
ATOM	941	CG2	VAL	60	32.882	36.624	53.643	1.00	0.00	C
ATOM	942	HG21	VAL	60	33.496	37.382	53.157	1.00	0.00	H
ATOM	943	HG22	VAL	60	32.307	37.255	54.319	1.00	0.00	H
ATOM	944	HG23	VAL	60	33.471	35.878	54.176	1.00	0.00	H
ATOM	945	C	VAL	60	29.652	35.582	54.062	1.00	0.00	C
ATOM	946	O	VAL	60	28.635	35.312	53.445	1.00	0.00	O
ATOM	947	N	THR	61	29.744	36.537	54.957	1.00	0.00	N
ATOM	948	H	THR	61	30.627	36.621	55.440	1.00	0.00	H
ATOM	949	CA	THR	61	28.761	37.601	55.226	1.00	0.00	C
ATOM	950	HA	THR	61	27.848	37.444	54.652	1.00	0.00	H
ATOM	951	CB	THR	61	28.330	37.429	56.696	1.00	0.00	C
ATOM	952	HB	THR	61	27.762	38.279	57.075	1.00	0.00	H
ATOM	953	CG2	THR	61	27.449	36.187	56.897	1.00	0.00	C
ATOM	954	HG21	THR	61	26.636	36.324	56.183	1.00	0.00	H
ATOM	955	HG22	THR	61	28.059	35.292	56.769	1.00	0.00	H
ATOM	956	HG23	THR	61	26.923	36.160	57.851	1.00	0.00	H
ATOM	957	OG1	THR	61	29.355	37.167	57.648	1.00	0.00	O
ATOM	958	HG1	THR	61	29.336	37.971	58.171	1.00	0.00	H
ATOM	959	C	THR	61	29.337	39.025	55.026	1.00	0.00	C
ATOM	960	O	THR	61	30.531	39.228	54.892	1.00	0.00	O
ATOM	961	N	LEU	62	28.528	40.098	55.027	1.00	0.00	N
ATOM	962	H	LEU	62	27.520	40.115	55.079	1.00	0.00	H
ATOM	963	CA	LEU	62	29.037	41.493	54.857	1.00	0.00	C
ATOM	964	HA	LEU	62	29.533	41.567	53.889	1.00	0.00	H
ATOM	965	CB	LEU	62	27.778	42.416	54.959	1.00	0.00	C
ATOM	966	HB2	LEU	62	27.250	42.261	55.900	1.00	0.00	H
ATOM	967	HB3	LEU	62	28.208	43.415	55.029	1.00	0.00	H
ATOM	968	CG	LEU	62	26.831	42.298	53.782	1.00	0.00	C
ATOM	969	HG	LEU	62	26.580	41.245	53.654	1.00	0.00	H
ATOM	970	CD1	LEU	62	25.497	42.933	54.118	1.00	0.00	C
ATOM	971	HD11	LEU	62	25.188	42.577	55.101	1.00	0.00	H
ATOM	972	HD12	LEU	62	25.655	44.004	54.238	1.00	0.00	H
ATOM	973	HD13	LEU	62	24.803	42.626	53.335	1.00	0.00	H
ATOM	974	CD2	LEU	62	27.401	42.858	52.478	1.00	0.00	C
ATOM	975	HD21	LEU	62	26.562	42.733	51.792	1.00	0.00	H
ATOM	976	HD22	LEU	62	27.609	43.920	52.604	1.00	0.00	H
ATOM	977	HD23	LEU	62	28.221	42.299	52.029	1.00	0.00	H
ATOM	978	C	LEU	62	30.042	41.852	56.013	1.00	0.00	C
ATOM	979	O	LEU	62	31.056	42.440	55.728	1.00	0.00	O
ATOM	980	N	ASP	63	29.848	41.291	57.192	1.00	0.00	N
ATOM	981	H	ASP	63	29.125	40.590	57.263	1.00	0.00	H
ATOM	982	CA	ASP	63	30.682	41.467	58.382	1.00	0.00	C
ATOM	983	HA	ASP	63	31.254	42.393	58.339	1.00	0.00	H
ATOM	984	CB	ASP	63	29.802	41.423	59.624	1.00	0.00	C
ATOM	985	HB2	ASP	63	30.416	41.742	60.466	1.00	0.00	H
ATOM	986	HB3	ASP	63	29.056	42.185	59.393	1.00	0.00	H
ATOM	987	CG	ASP	63	29.006	40.177	59.956	1.00	0.00	C
ATOM	988	OD1	ASP	63	28.965	39.752	61.138	1.00	0.00	O
ATOM	989	OD2	ASP	63	28.664	39.425	59.008	1.00	0.00	O
ATOM	990	C	ASP	63	31.861	40.541	58.484	1.00	0.00	C
ATOM	991	O	ASP	63	32.620	40.694	59.425	1.00	0.00	O
ATOM	992	N	THR	64	32.105	39.673	57.470	1.00	0.00	N
ATOM	993	H	THR	64	31.305	39.445	56.898	1.00	0.00	H

ATOM	994	CA	THR	64	33.426	38.999	57.195	1.00	0.00	C
ATOM	995	HA	THR	64	34.141	39.493	57.851	1.00	0.00	H
ATOM	996	CB	THR	64	33.360	37.480	57.478	1.00	0.00	C
ATOM	997	HB	THR	64	34.226	36.946	57.086	1.00	0.00	H
ATOM	998	CG2	THR	64	33.321	37.196	58.980	1.00	0.00	C
ATOM	999	HG21	THR	64	32.309	37.396	59.332	1.00	0.00	H
ATOM	1000	HG22	THR	64	33.594	36.146	59.086	1.00	0.00	H
ATOM	1001	HG23	THR	64	34.075	37.864	59.393	1.00	0.00	H
ATOM	1002	OG1	THR	64	32.238	36.849	56.915	1.00	0.00	O
ATOM	1003	HG1	THR	64	31.461	37.165	57.382	1.00	0.00	H
ATOM	1004	C	THR	64	33.966	39.317	55.786	1.00	0.00	C
ATOM	1005	O	THR	64	35.032	38.842	55.350	1.00	0.00	O
ATOM	1006	N	LEU	65	33.292	40.190	55.037	1.00	0.00	N
ATOM	1007	H	LEU	65	32.356	40.387	55.360	1.00	0.00	H
ATOM	1008	CA	LEU	65	33.794	40.732	53.765	1.00	0.00	C
ATOM	1009	HA	LEU	65	34.053	39.848	53.183	1.00	0.00	H
ATOM	1010	CB	LEU	65	32.690	41.655	53.160	1.00	0.00	C
ATOM	1011	HB2	LEU	65	31.827	41.015	53.344	1.00	0.00	H
ATOM	1012	HB3	LEU	65	32.694	42.663	53.573	1.00	0.00	H
ATOM	1013	CG	LEU	65	32.915	41.793	51.657	1.00	0.00	C
ATOM	1014	HG	LEU	65	33.660	42.563	51.455	1.00	0.00	H
ATOM	1015	CD1	LEU	65	33.299	40.602	50.794	1.00	0.00	C
ATOM	1016	HD11	LEU	65	34.312	40.278	51.032	1.00	0.00	H
ATOM	1017	HD12	LEU	65	32.751	39.686	51.014	1.00	0.00	H
ATOM	1018	HD13	LEU	65	33.224	40.892	49.745	1.00	0.00	H
ATOM	1019	CD2	LEU	65	31.528	42.265	51.110	1.00	0.00	C
ATOM	1020	HD21	LEU	65	31.148	43.054	51.759	1.00	0.00	H
ATOM	1021	HD22	LEU	65	31.603	42.617	50.081	1.00	0.00	H
ATOM	1022	HD23	LEU	65	30.858	41.409	51.190	1.00	0.00	H
ATOM	1023	C	LEU	65	35.108	41.563	53.985	1.00	0.00	C
ATOM	1024	O	LEU	65	35.021	42.653	54.508	1.00	0.00	O
ATOM	1025	N	THR	66	36.257	41.093	53.400	1.00	0.00	N
ATOM	1026	H	THR	66	36.230	40.197	52.935	1.00	0.00	H
ATOM	1027	CA	THR	66	37.493	41.864	52.991	1.00	0.00	C
ATOM	1028	HA	THR	66	37.820	42.515	53.802	1.00	0.00	H
ATOM	1029	CB	THR	66	38.736	40.864	52.889	1.00	0.00	C
ATOM	1030	HB	THR	66	38.531	40.128	52.113	1.00	0.00	H
ATOM	1031	CG2	THR	66	40.046	41.613	52.597	1.00	0.00	C
ATOM	1032	HG21	THR	66	40.235	42.258	53.454	1.00	0.00	H
ATOM	1033	HG22	THR	66	40.892	40.929	52.527	1.00	0.00	H
ATOM	1034	HG23	THR	66	39.936	42.076	51.617	1.00	0.00	H
ATOM	1035	OG1	THR	66	38.951	40.159	54.137	1.00	0.00	O
ATOM	1036	HG1	THR	66	38.445	39.346	54.195	1.00	0.00	H
ATOM	1037	C	THR	66	37.174	42.781	51.798	1.00	0.00	C
ATOM	1038	O	THR	66	36.458	42.364	50.891	1.00	0.00	O
ATOM	1039	N	GLU	67	37.828	43.928	51.666	1.00	0.00	N
ATOM	1040	H	GLU	67	38.513	44.158	52.371	1.00	0.00	H
ATOM	1041	CA	GLU	67	37.715	44.866	50.562	1.00	0.00	C
ATOM	1042	HA	GLU	67	36.647	44.969	50.373	1.00	0.00	H
ATOM	1043	CB	GLU	67	38.180	46.236	50.937	1.00	0.00	C
ATOM	1044	HB2	GLU	67	37.644	46.944	50.306	1.00	0.00	H
ATOM	1045	HB3	GLU	67	37.733	46.308	51.928	1.00	0.00	H
ATOM	1046	CG	GLU	67	39.680	46.550	51.028	1.00	0.00	C
ATOM	1047	HG2	GLU	67	39.956	46.739	49.990	1.00	0.00	H
ATOM	1048	HG3	GLU	67	39.670	47.519	51.526	1.00	0.00	H
ATOM	1049	CD	GLU	67	40.513	45.585	51.836	1.00	0.00	C
ATOM	1050	OE1	GLU	67	41.530	45.144	51.313	1.00	0.00	O
ATOM	1051	OE2	GLU	67	40.172	45.194	52.977	1.00	0.00	O
ATOM	1052	C	GLU	67	38.422	44.387	49.305	1.00	0.00	C
ATOM	1053	O	GLU	67	38.274	45.117	48.334	1.00	0.00	O
ATOM	1054	N	LYS	68	39.178	43.264	49.218	1.00	0.00	N
ATOM	1055	H	LYS	68	39.327	42.671	50.022	1.00	0.00	H
ATOM	1056	CA	LYS	68	39.942	42.909	48.005	1.00	0.00	C

ATOM	1057	HA	LYS	68	40.680	43.710	47.956	1.00	0.00	H
ATOM	1058	CB	LYS	68	40.694	41.647	48.307	1.00	0.00	C
ATOM	1059	HB2	LYS	68	41.302	41.853	49.188	1.00	0.00	H
ATOM	1060	HB3	LYS	68	39.952	40.861	48.455	1.00	0.00	H
ATOM	1061	CG	LYS	68	41.635	41.013	47.220	1.00	0.00	C
ATOM	1062	HG2	LYS	68	42.124	40.135	47.643	1.00	0.00	H
ATOM	1063	HG3	LYS	68	40.986	40.691	46.405	1.00	0.00	H
ATOM	1064	CD	LYS	68	42.676	41.987	46.648	1.00	0.00	C
ATOM	1065	HD2	LYS	68	43.049	41.639	45.684	1.00	0.00	H
ATOM	1066	HD3	LYS	68	42.253	42.986	46.535	1.00	0.00	H
ATOM	1067	CE	LYS	68	43.753	42.129	47.681	1.00	0.00	C
ATOM	1068	HE2	LYS	68	43.302	42.446	48.621	1.00	0.00	H
ATOM	1069	HE3	LYS	68	44.194	41.149	47.861	1.00	0.00	H
ATOM	1070	NZ	LYS	68	44.762	43.150	47.458	1.00	0.00	N
ATOM	1071	HZ1	LYS	68	44.439	44.094	47.297	1.00	0.00	H
ATOM	1072	HZ2	LYS	68	45.335	43.152	48.290	1.00	0.00	H
ATOM	1073	HZ3	LYS	68	45.410	42.868	46.738	1.00	0.00	H
ATOM	1074	C	LYS	68	39.112	42.866	46.682	1.00	0.00	C
ATOM	1075	O	LYS	68	39.599	43.443	45.720	1.00	0.00	O
ATOM	1076	N	HIE	69	37.845	42.419	46.729	1.00	0.00	N
ATOM	1077	H	HIE	69	37.478	41.953	47.547	1.00	0.00	H
ATOM	1078	CA	HIE	69	36.844	42.627	45.624	1.00	0.00	C
ATOM	1079	HA	HIE	69	37.243	42.166	44.720	1.00	0.00	H
ATOM	1080	CB	HIE	69	35.562	41.826	45.993	1.00	0.00	C
ATOM	1081	HB2	HIE	69	35.841	40.776	46.077	1.00	0.00	H
ATOM	1082	HB3	HIE	69	35.145	42.091	46.965	1.00	0.00	H
ATOM	1083	CG	HIE	69	34.465	41.997	44.910	1.00	0.00	C
ATOM	1084	ND1	HIE	69	34.166	41.086	43.864	1.00	0.00	N
ATOM	1085	CE1	HIE	69	33.101	41.615	43.175	1.00	0.00	C
ATOM	1086	HE1	HIE	69	32.781	41.249	42.210	1.00	0.00	H
ATOM	1087	NE2	HIE	69	32.718	42.751	43.749	1.00	0.00	N
ATOM	1088	HE2	HIE	69	32.067	43.399	43.330	1.00	0.00	H
ATOM	1089	CD2	HIE	69	33.598	43.022	44.779	1.00	0.00	C
ATOM	1090	HD2	HIE	69	33.643	43.919	45.378	1.00	0.00	H
ATOM	1091	C	HIE	69	36.599	44.119	45.331	1.00	0.00	C
ATOM	1092	O	HIE	69	36.695	44.571	44.169	1.00	0.00	O
ATOM	1093	N	ALA	70	36.394	44.987	46.363	1.00	0.00	N
ATOM	1094	H	ALA	70	36.428	44.559	47.277	1.00	0.00	H
ATOM	1095	CA	ALA	70	36.174	46.401	46.210	1.00	0.00	C
ATOM	1096	HA	ALA	70	35.321	46.580	45.557	1.00	0.00	H
ATOM	1097	CB	ALA	70	35.706	46.920	47.662	1.00	0.00	C
ATOM	1098	HB1	ALA	70	34.754	46.472	47.949	1.00	0.00	H
ATOM	1099	HB2	ALA	70	36.551	46.808	48.340	1.00	0.00	H
ATOM	1100	HB3	ALA	70	35.528	47.993	47.606	1.00	0.00	H
ATOM	1101	C	ALA	70	37.375	47.181	45.596	1.00	0.00	C
ATOM	1102	O	ALA	70	37.209	48.158	44.947	1.00	0.00	O
ATOM	1103	N	GLU	71	38.572	46.707	45.729	1.00	0.00	N
ATOM	1104	H	GLU	71	38.665	45.848	46.251	1.00	0.00	H
ATOM	1105	CA	GLU	71	39.834	47.172	45.153	1.00	0.00	C
ATOM	1106	HA	GLU	71	39.729	48.257	45.154	1.00	0.00	H
ATOM	1107	CB	GLU	71	41.056	46.721	45.936	1.00	0.00	C
ATOM	1108	HB2	GLU	71	41.050	45.632	45.907	1.00	0.00	H
ATOM	1109	HB3	GLU	71	41.913	47.107	45.382	1.00	0.00	H
ATOM	1110	CG	GLU	71	41.164	47.335	47.319	1.00	0.00	C
ATOM	1111	HG2	GLU	71	40.251	46.995	47.809	1.00	0.00	H
ATOM	1112	HG3	GLU	71	42.057	46.923	47.788	1.00	0.00	H
ATOM	1113	CD	GLU	71	41.318	48.877	47.447	1.00	0.00	C
ATOM	1114	OE1	GLU	71	42.124	49.580	46.758	1.00	0.00	O
ATOM	1115	OE2	GLU	71	40.590	49.471	48.302	1.00	0.00	O
ATOM	1116	C	GLU	71	39.990	46.788	43.619	1.00	0.00	C
ATOM	1117	O	GLU	71	40.390	47.630	42.771	1.00	0.00	O
ATOM	1118	N	GLN	72	39.572	45.559	43.235	1.00	0.00	N
ATOM	1119	H	GLN	72	39.198	45.023	44.005	1.00	0.00	H
ATOM	1120	CA	GLN	72	39.361	45.159	41.829	1.00	0.00	C

```
ATOM   1121  HA  GLN    72      40.293  45.295  41.282  1.00  0.00           H
ATOM   1122  CB  GLN    72      38.928  43.684  41.761  1.00  0.00           C
ATOM   1123  HB2 GLN    72      38.050  43.638  42.406  1.00  0.00           H
ATOM   1124  HB3 GLN    72      39.684  43.092  42.275  1.00  0.00           H
ATOM   1125  CG  GLN    72      38.538  43.074  40.410  1.00  0.00           C
ATOM   1126  HG2 GLN    72      37.958  43.774  39.810  1.00  0.00           H
ATOM   1127  HG3 GLN    72      39.453  42.922  39.838  1.00  0.00           H
ATOM   1128  CD  GLN    72      37.732  41.734  40.587  1.00  0.00           C
ATOM   1129  OE1 GLN    72      38.227  40.643  40.212  1.00  0.00           O
ATOM   1130  NE2 GLN    72      36.473  41.727  41.011  1.00  0.00           N
ATOM   1131 HE21 GLN    72      35.905  42.563  41.000  1.00  0.00           H
ATOM   1132 HE22 GLN    72      36.052  40.818
  41.139  1.00  0.00           H
ATOM   1133  C   GLN    72      38.288  46.068  41.162  1.00  0.00           C
ATOM   1134  O   GLN    72      38.353  46.380  39.956  1.00  0.00           O
ATOM   1135  N   GLU    73      37.273  46.518  41.955  1.00  0.00           N
ATOM   1136  H   GLU    73      37.202  46.201  42.912  1.00  0.00           H
ATOM   1137  CA  GLU    73      36.045  47.192  41.446  1.00  0.00           C
ATOM   1138  HA  GLU    73      35.965  47.049  40.369  1.00  0.00           H
ATOM   1139  CB  GLU    73      34.761  46.554  41.940  1.00  0.00           C
ATOM   1140  HB2 GLU    73      34.792  46.552  43.029  1.00  0.00           H
ATOM   1141  HB3 GLU    73      33.869  47.097  41.628  1.00  0.00           H
ATOM   1142  CG  GLU    73      34.582  45.072  41.503  1.00  0.00           C
ATOM   1143  HG2 GLU    73      35.272  44.360  41.958  1.00  0.00           H
ATOM   1144  HG3 GLU    73      33.633  44.660  41.846  1.00  0.00           H
ATOM   1145  CD  GLU    73      34.647  44.908  39.935  1.00  0.00           C
ATOM   1146  OE1 GLU    73      35.148  43.792  39.624  1.00  0.00           O
ATOM   1147  OE2 GLU    73      34.196  45.728  39.145  1.00  0.00           O
ATOM   1148  C   GLU    73      35.949  48.718  41.575  1.00  0.00           C
ATOM   1149  O   GLU    73      35.086  49.410  41.051  1.00  0.00           O
ATOM   1150  N   ASN    74      37.064  49.287  42.111  1.00  0.00           N
ATOM   1151  H   ASN    74      37.714  48.581  42.424  1.00  0.00           H
ATOM   1152  CA  ASN    74      37.354  50.697  42.342  1.00  0.00           C
ATOM   1153  HA  ASN    74      38.291  50.654  42.897  1.00  0.00           H
ATOM   1154  CB  ASN    74      37.620  51.427  40.987  1.00  0.00           C
ATOM   1155  HB2 ASN    74      36.657  51.549  40.491  1.00  0.00           H
ATOM   1156  HB3 ASN    74      38.030  52.433  41.086  1.00  0.00           H
ATOM   1157  CG  ASN    74      38.580  50.663  40.141  1.00  0.00           C
ATOM   1158  OD1 ASN    74      39.629  50.229  40.637  1.00  0.00           O
ATOM   1159  ND2 ASN    74      38.251  50.370  38.938  1.00  0.00           N
ATOM   1160 HD21 ASN    74      38.784  49.688  38.416  1.00  0.00           H
ATOM   1161 HD22 ASN    74      37.324  50.565  38.588  1.00  0.00           H
ATOM   1162  C   ASN    74      36.413  51.381  43.325  1.00  0.00           C
ATOM   1163  O   ASN    74      35.980  52.507  43.073  1.00  0.00           O
ATOM   1164  N   MET    75      36.136  50.771  44.478  1.00  0.00           N
ATOM   1165  H   MET    75      36.665  49.928  44.650  1.00  0.00           H
ATOM   1166  CA  MET    75      35.201  51.346  45.523  1.00  0.00           C
ATOM   1167  HA  MET    75      34.986  52.381  45.261  1.00  0.00           H
ATOM   1168  CB  MET    75      33.853  50.653  45.508  1.00  0.00           C
ATOM   1169  HB2 MET    75      34.044  49.710  46.021  1.00  0.00           H
ATOM   1170  HB3 MET    75      33.127  51.274  46.029  1.00  0.00           H
ATOM   1171  CG  MET    75      33.233  50.395  44.124  1.00  0.00           C
ATOM   1172  HG2 MET    75      32.798  51.336  43.790  1.00  0.00           H
ATOM   1173  HG3 MET    75      33.973  50.064  43.394  1.00  0.00           H
ATOM   1174  SD  MET    75      31.867  49.070  44.166  1.00  0.00           S
ATOM   1175  CE  MET    75      30.807  49.711  45.483  1.00  0.00           C
ATOM   1176  HE1 MET    75      31.486  50.073  46.254  1.00  0.00           H
ATOM   1177  HE2 MET    75      30.249  50.577  45.126  1.00  0.00           H
ATOM   1178  HE3 MET    75      30.276  48.909  45.996  1.00  0.00           H
ATOM   1179  C   MET    75      35.787  51.273  46.941  1.00  0.00           C
ATOM   1180  O   MET    75      36.662  50.441  47.123  1.00  0.00           O
ATOM   1181  N   THR    76      35.403  52.058  47.943  1.00  0.00           N
ATOM   1182  H   THR    76      34.662  52.686  47.664  1.00  0.00           H
ATOM   1183  CA  THR    76      35.566  51.693  49.311  1.00  0.00           C
```

ATOM	1184	HA	THR	76	36.582	51.397	49.571	1.00	0.00	H
ATOM	1185	CB	THR	76	35.234	52.910	50.216	1.00	0.00	C
ATOM	1186	HB	THR	76	36.002	53.653	50.000	1.00	0.00	H
ATOM	1187	CG2	THR	76	33.852	53.612	50.057	1.00	0.00	C
ATOM	1188	HG21	THR	76	33.048	52.898	50.236	1.00	0.00	H
ATOM	1189	HG22	THR	76	33.812	54.355	50.853	1.00	0.00	H
ATOM	1190	HG23	THR	76	33.706	54.115	49.101	1.00	0.00	H
ATOM	1191	OG1	THR	76	35.324	52.455	51.464	1.00	0.00	O
ATOM	1192	HG1	THR	76	35.885	52.991	52.027	1.00	0.00	H
ATOM	1193	C	THR	76	34.572	50.549	49.694	1.00	0.00	C
ATOM	1194	O	THR	76	33.535	50.492	49.050	1.00	0.00	O
ATOM	1195	N	LEU	77	34.922	49.676	50.659	1.00	0.00	N
ATOM	1196	H	LEU	77	35.861	49.822	51.002	1.00	0.00	H
ATOM	1197	CA	LEU	77	33.992	48.628	51.212	1.00	0.00	C
ATOM	1198	HA	LEU	77	33.742	47.921	50.421	1.00	0.00	H
ATOM	1199	CB	LEU	77	34.774	47.779	52.232	1.00	0.00	C
ATOM	1200	HB2	LEU	77	35.594	47.410	51.616	1.00	0.00	H
ATOM	1201	HB3	LEU	77	35.161	48.457	52.992	1.00	0.00	H
ATOM	1202	CG	LEU	77	34.072	46.593	52.973	1.00	0.00	C
ATOM	1203	HG	LEU	77	33.230	46.991	53.538	1.00	0.00	H
ATOM	1204	CD1	LEU	77	33.522	45.519	52.101	1.00	0.00	C
ATOM	1205	HD11	LEU	77	33.035	44.791	52.750	1.00	0.00	H
ATOM	1206	HD12	LEU	77	32.669	46.046	51.674	1.00	0.00	H
ATOM	1207	HD13	LEU	77	34.278	45.221	51.375	1.00	0.00	H
ATOM	1208	CD2	LEU	77	35.092	45.968	53.978	1.00	0.00	C
ATOM	1209	HD21	LEU	77	34.642	45.035	54.316	1.00	0.00	H
ATOM	1210	HD22	LEU	77	36.066	45.803	53.517	1.00	0.00	H
ATOM	1211	HD23	LEU	77	35.078	46.661	54.819	1.00	0.00	H
ATOM	1212	C	LEU	77	32.715	49.222	51.814	1.00	0.00	C
ATOM	1213	O	LEU	77	31.758	48.470	51.760	1.00	0.00	O
ATOM	1214	N	THR	78	32.668	50.457	52.324	1.00	0.00	N
ATOM	1215	H	THR	78	33.514	51.006	52.391	1.00	0.00	H
ATOM	1216	CA	THR	78	31.448	51.056	52.965	1.00	0.00	C
ATOM	1217	HA	THR	78	31.089	50.443	53.792	1.00	0.00	H
ATOM	1218	CB	THR	78	31.791	52.335	53.679	1.00	0.00	C
ATOM	1219	HB	THR	78	30.855	52.582	54.179	1.00	0.00	H
ATOM	1220	CG2	THR	78	32.918	52.210	54.725	1.00	0.00	C
ATOM	1221	HG21	THR	78	33.872	52.032	54.231	1.00	0.00	H
ATOM	1222	HG22	THR	78	32.925	53.162	55.255	1.00	0.00	H
ATOM	1223	HG23	THR	78	32.739	51.390	55.420	1.00	0.00	H
ATOM	1224	OG1	THR	78	32.124	53.400	52.794	1.00	0.00	O
ATOM	1225	HG1	THR	78	31.395	53.545	52.188	1.00	0.00	H
ATOM	1226	C	THR	78	30.427	51.262	51.929	1.00	0.00	C
ATOM	1227	O	THR	78	29.265	50.998	52.160	1.00	0.00	O
ATOM	1228	N	GLU	79	30.776	51.641	50.664	1.00	0.00	N
ATOM	1229	H	GLU	79	31.757	51.860	50.575	1.00	0.00	H
ATOM	1230	CA	GLU	79	29.841	51.704	49.581	1.00	0.00	C
ATOM	1231	HA	GLU	79	28.916	52.226	49.832	1.00	0.00	H
ATOM	1232	CB	GLU	79	30.520	52.526	48.493	1.00	0.00	C
ATOM	1233	HB2	GLU	79	31.558	52.212	48.390	1.00	0.00	H
ATOM	1234	HB3	GLU	79	30.092	52.321	47.511	1.00	0.00	H
ATOM	1235	CG	GLU	79	30.404	54.094	48.642	1.00	0.00	C
ATOM	1236	HG2	GLU	79	31.078	54.350	49.460	1.00	0.00	H
ATOM	1237	HG3	GLU	79	30.846	54.535	47.749	1.00	0.00	H
ATOM	1238	CD	GLU	79	28.978	54.522	48.932	1.00	0.00	C
ATOM	1239	OE1	GLU	79	28.700	55.371	49.830	1.00	0.00	O
ATOM	1240	OE2	GLU	79	28.030	54.202	48.171	1.00	0.00	O
ATOM	1241	C	GLU	79	29.448	50.354	48.877	1.00	0.00	C
ATOM	1242	O	GLU	79	28.319	50.232	48.434	1.00	0.00	O
ATOM	1243	N	LEU	80	30.281	49.314	48.967	1.00	0.00	N
ATOM	1244	H	LEU	80	31.212	49.470	49.328	1.00	0.00	H
ATOM	1245	CA	LEU	80	30.026	47.955	48.578	1.00	0.00	C
ATOM	1246	HA	LEU	80	29.529	47.944	47.608	1.00	0.00	H
ATOM	1247	CB	LEU	80	31.266	47.110	48.229	1.00	0.00	C

ATOM	1248	HB2	LEU	80	31.711	47.495	47.311	1.00	0.00	H
ATOM	1249	HB3	LEU	80	32.016	47.084	49.019	1.00	0.00	H
ATOM	1250	CG	LEU	80	30.969	45.554	48.033	1.00	0.00	C
ATOM	1251	HG	LEU	80	30.128	45.236	48.648	1.00	0.00	H
ATOM	1252	CD1	LEU	80	30.625	45.326	46.518	1.00	0.00	C
ATOM	1253	HD11	LEU	80	31.443	45.650	45.873	1.00	0.00	H
ATOM	1254	HD12	LEU	80	30.342	44.284	46.363	1.00	0.00	H
ATOM	1255	HD13	LEU	80	29.722	45.913	46.346	1.00	0.00	H
ATOM	1256	CD2	LEU	80	32.298	44.845	48.270	1.00	0.00	C
ATOM	1257	HD21	LEU	80	32.214	43.766	48.134	1.00	0.00	H
ATOM	1258	HD22	LEU	80	33.138	45.179	47.661	1.00	0.00	H
ATOM	1259	HD23	LEU	80	32.590	45.034	49.303	1.00	0.00	H
ATOM	1260	C	LEU	80	29.107	47.306	49.531	1.00	0.00	C
ATOM	1261	O	LEU	80	28.156	46.713	49.157	1.00	0.00	O
ATOM	1262	N	LYS	81	29.308	47.533	50.834	1.00	0.00	N
ATOM	1263	H	LYS	81	30.165	47.922	51.202	1.00	0.00	H
ATOM	1264	CA	LYS	81	28.266	47.061	51.811	1.00	0.00	C
ATOM	1265	HA	LYS	81	28.251	45.972	51.752	1.00	0.00	H
ATOM	1266	CB	LYS	81	28.555	47.466	53.263	1.00	0.00	C
ATOM	1267	HB2	LYS	81	28.847	48.492	53.487	1.00	0.00	H
ATOM	1268	HB3	LYS	81	27.666	47.343	53.882	1.00	0.00	H
ATOM	1269	CG	LYS	81	29.475	46.339	53.898	1.00	0.00	C
ATOM	1270	HG2	LYS	81	29.012	45.354	53.860	1.00	0.00	H
ATOM	1271	HG3	LYS	81	30.368	46.373	53.271	1.00	0.00	H
ATOM	1272	CD	LYS	81	29.731	46.820	55.339	1.00	0.00	C
ATOM	1273	HD2	LYS	81	30.072	47.853	55.276	1.00	0.00	H
ATOM	1274	HD3	LYS	81	28.835	46.774	55.960	1.00	0.00	H
ATOM	1275	CE	LYS	81	30.718	45.935	56.156	1.00	0.00	C
ATOM	1276	HE2	LYS	81	30.393	44.898	56.237	1.00	0.00	H
ATOM	1277	HE3	LYS	81	31.652	45.916	55.593	1.00	0.00	H
ATOM	1278	NZ	LYS	81	30.865	46.381	57.554	1.00	0.00	N
ATOM	1279	HZ1	LYS	81	30.055	46.178	58.123	1.00	0.00	H
ATOM	1280	HZ2	LYS	81	31.649	45.889	57.957	1.00	0.00	H
ATOM	1281	HZ3	LYS	81	31.018	47.365	57.717	1.00	0.00	H
ATOM	1282	C	LYS	81	26.891	47.585	51.422	1.00	0.00	C
ATOM	1283	O	LYS	81	25.928	46.838	51.314	1.00	0.00	O
ATOM	1284	N	LYS	82	26.831	48.916	51.073	1.00	0.00	N
ATOM	1285	H	LYS	82	27.669	49.466	51.196	1.00	0.00	H
ATOM	1286	CA	LYS	82	25.571	49.620	50.661	1.00	0.00	C
ATOM	1287	HA	LYS	82	24.893	49.456	51.498	1.00	0.00	H
ATOM	1288	CB	LYS	82	25.852	51.138	50.561	1.00	0.00	C
ATOM	1289	HB2	LYS	82	26.270	51.331	51.549	1.00	0.00	H
ATOM	1290	HB3	LYS	82	26.482	51.338	49.696	1.00	0.00	H
ATOM	1291	CG	LYS	82	24.568	51.986	50.439	1.00	0.00	C
ATOM	1292	HG2	LYS	82	23.963	51.649	49.598	1.00	0.00	H
ATOM	1293	HG3	LYS	82	23.959	51.926	51.341	1.00	0.00	H
ATOM	1294	CD	LYS	82	24.835	53.449	50.056	1.00	0.00	C
ATOM	1295	HD2	LYS	82	25.206	53.447	49.031	1.00	0.00	H
ATOM	1296	HD3	LYS	82	23.892	53.982	50.171	1.00	0.00	H
ATOM	1297	CE	LYS	82	25.781	54.102	51.076	1.00	0.00	C
ATOM	1298	HE2	LYS	82	25.468	54.023	52.116	1.00	0.00	H
ATOM	1299	HE3	LYS	82	26.716	53.590	50.846	1.00	0.00	H
ATOM	1300	NZ	LYS	82	26.003	55.524	50.651	1.00	0.00	N
ATOM	1301	HZ1	LYS	82	26.553	55.442	49.809	1.00	0.00	H
ATOM	1302	HZ2	LYS	82	25.135	56.042	50.665	1.00	0.00	H
ATOM	1303	HZ3	LYS	82	26.608	55.887	51.372	1.00	0.00	H
ATOM	1304	C	LYS	82	24.856	49.098	49.428	1.00	0.00	C
ATOM	1305	O	LYS	82	23.652	48.821	49.555	1.00	0.00	O
ATOM	1306	N	VAL	83	25.539	48.859	48.298	1.00	0.00	N
ATOM	1307	H	VAL	83	26.529	49.062	48.302	1.00	0.00	H
ATOM	1308	CA	VAL	83	24.921	48.201	47.108	1.00	0.00	C
ATOM	1309	HA	VAL	83	24.065	48.822	46.844	1.00	0.00	H
ATOM	1310	CB	VAL	83	25.812	48.031	45.767	1.00	0.00	C

ATOM	1311	HB	VAL	83	25.289	47.322	45.125	1.00	0.00	H
ATOM	1312	CG1	VAL	83	25.948	49.421	45.089	1.00	0.00	C
ATOM	1313	HG11	VAL	83	26.843	49.964	45.394	1.00	0.00	H
ATOM	1314	HG12	VAL	83	26.173	49.188	44.048	1.00	0.00	H
ATOM	1315	HG13	VAL	83	25.005	49.962	45.161	1.00	0.00	H
ATOM	1316	CG2	VAL	83	27.291	47.574	46.035	1.00	0.00	C
ATOM	1317	HG21	VAL	83	27.402	46.554	46.401	1.00	0.00	H
ATOM	1318	HG22	VAL	83	27.986	47.600	45.195	1.00	0.00	H
ATOM	1319	HG23	VAL	83	27.715	48.231	46.795	1.00	0.00	H
ATOM	1320	C	VAL	83	24.270	46.897	47.484	1.00	0.00	C
ATOM	1321	O	VAL	83	23.148	46.652	46.971	1.00	0.00	O
ATOM	1322	N	ILE	84	24.903	46.140	48.376	1.00	0.00	N
ATOM	1323	H	ILE	84	25.800	46.336	48.795	1.00	0.00	H
ATOM	1324	CA	ILE	84	24.275	44.900	48.787	1.00	0.00	C
ATOM	1325	HA	ILE	84	23.822	44.278	48.015	1.00	0.00	H
ATOM	1326	CB	ILE	84	25.481	44.058	49.395	1.00	0.00	C
ATOM	1327	HB	ILE	84	25.759	44.383	50.399	1.00	0.00	H
ATOM	1328	CG2	ILE	84	24.969	42.594	49.671	1.00	0.00	C
ATOM	1329	HG21	ILE	84	25.749	41.845	49.807	1.00	0.00	H
ATOM	1330	HG22	ILE	84	24.276	42.584	50.513	1.00	0.00	H
ATOM	1331	HG23	ILE	84	24.418	42.225	48.805	1.00	0.00	H
ATOM	1332	CG1	ILE	84	26.617	43.966	48.354	1.00	0.00	C
ATOM	1333	HG12	ILE	84	26.124	43.545	47.478	1.00	0.00	H
ATOM	1334	HG13	ILE	84	26.882	44.990	48.090	1.00	0.00	H
ATOM	1335	CD1	ILE	84	27.953	43.314	48.789	1.00	0.00	C
ATOM	1336	HD11	ILE	84	28.710	43.592	48.056	1.00	0.00	H
ATOM	1337	HD12	ILE	84	28.245	43.722	49.758	1.00	0.00	H
ATOM	1338	HD13	ILE	84	27.830	42.233	48.862	1.00	0.00	H
ATOM	1339	C	ILE	84	23.087	45.124	49.772	1.00	0.00	C
ATOM	1340	O	ILE	84	22.017	44.478	49.646	1.00	0.00	O
ATOM	1341	N	ALA	85	23.263	46.020	50.698	1.00	0.00	N
ATOM	1342	H	ALA	85	24.215	46.312	50.865	1.00	0.00	H
ATOM	1343	CA	ALA	85	22.256	46.420	51.761	1.00	0.00	C
ATOM	1344	HA	ALA	85	22.061	45.416	52.139	1.00	0.00	H
ATOM	1345	CB	ALA	85	22.844	47.234	52.917	1.00	0.00	C
ATOM	1346	HB1	ALA	85	22.050	47.530	53.604	1.00	0.00	H
ATOM	1347	HB2	ALA	85	23.614	46.691	53.465	1.00	0.00	H
ATOM	1348	HB3	ALA	85	23.237	48.222	52.675	1.00	0.00	H
ATOM	1349	C	ALA	85	20.943	47.021	51.058	1.00	0.00	C
ATOM	1350	O	ALA	85	19.846	47.016	51.640	1.00	0.00	O
ATOM	1351	N	ASP	86	20.930	47.527	49.785	1.00	0.00	N
ATOM	1352	H	ASP	86	21.816	47.691	49.330	1.00	0.00	H
ATOM	1353	CA	ASP	86	19.601	47.822	49.166	1.00	0.00	C
ATOM	1354	HA	ASP	86	18.994	48.405	49.858	1.00	0.00	H
ATOM	1355	CB	ASP	86	19.764	48.752	47.923	1.00	0.00	C
ATOM	1356	HB2	ASP	86	20.319	49.651	48.193	1.00	0.00	H
ATOM	1357	HB3	ASP	86	20.370	48.209	47.198	1.00	0.00	H
ATOM	1358	CG	ASP	86	18.423	49.277	47.382	1.00	0.00	C
ATOM	1359	OD1	ASP	86	18.201	49.083	46.123	1.00	0.00	O
ATOM	1360	OD2	ASP	86	17.654	49.977	48.106	1.00	0.00	O
ATOM	1361	C	ASP	86	18.704	46.599	48.801	1.00	0.00	C
ATOM	1362	O	ASP	86	17.516	46.532	48.892	1.00	0.00	O
ATOM	1363	N	ILE	87	19.333	45.554	48.281	1.00	0.00	N
ATOM	1364	H	ILE	87	20.340	45.544	48.222	1.00	0.00	H
ATOM	1365	CA	ILE	87	18.797	44.332	47.678	1.00	0.00	C
ATOM	1366	HA	ILE	87	18.035	44.668	46.975	1.00	0.00	H
ATOM	1367	CB	ILE	87	19.795	43.721	46.750	1.00	0.00	C
ATOM	1368	HB	ILE	87	20.628	43.296	47.309	1.00	0.00	H
ATOM	1369	CG2	ILE	87	19.075	42.590	46.001	1.00	0.00	C
ATOM	1370	HG21	ILE	87	19.776	42.143	45.295	1.00	0.00	H
ATOM	1371	HG22	ILE	87	18.707	41.802	46.658	1.00	0.00	H
ATOM	1372	HG23	ILE	87	18.247	43.068	45.477	1.00	0.00	H
ATOM	1373	CG1	ILE	87	20.339	44.666	45.640	1.00	0.00	C
ATOM	1374	HG12	ILE	87	19.751	44.530	44.732	1.00	0.00	H

```
ATOM   1375 HG13 ILE    87      20.370  45.685  46.027  1.00  0.00           H
ATOM   1376  CD1 ILE    87      21.769  44.337  45.211  1.00  0.00           C
ATOM   1377 HD11 ILE    87      22.342  44.262  46.135  1.00  0.00           H
ATOM   1378 HD12 ILE    87      21.836  43.344  44.766  1.00  0.00           H
ATOM   1379 HD13 ILE    87      22.279  45.043  44.556  1.00  0.00           H
ATOM   1380  C   ILE    87      18.297  43.369  48.695  1.00  0.00           C
ATOM   1381  O   ILE    87      17.155  42.923  48.679  1.00  0.00           O
ATOM   1382  N   TYR    88      19.221  42.952  49.630  1.00  0.00           N
ATOM   1383  H   TYR
    88      20.165  43.304  49.573  1.00  0.00           H
ATOM   1384  CA  TYR    88      19.003  41.826  50.554  1.00  0.00           C
ATOM   1385  HA  TYR    88      17.982  41.538  50.308  1.00  0.00           H
ATOM   1386  CB  TYR    88      20.088  40.733  50.220  1.00  0.00           C
ATOM   1387  HB2 TYR    88      21.015  41.182  50.575  1.00  0.00           H
ATOM   1388  HB3 TYR    88      19.916  39.882  50.878  1.00  0.00           H
ATOM   1389  CG  TYR    88      20.305  40.285  48.786  1.00  0.00           C
ATOM   1390  CD1 TYR    88      19.316  39.480  48.125  1.00  0.00           C
ATOM   1391  HD1 TYR    88      18.411  39.316  48.692  1.00  0.00           H
ATOM   1392  CE1 TYR    88      19.536  39.043  46.798  1.00  0.00           C
ATOM   1393  HE1 TYR    88      18.821  38.433  46.267  1.00  0.00           H
ATOM   1394  CZ  TYR    88      20.713  39.495  46.129  1.00  0.00           C
ATOM   1395  OH  TYR    88      20.818  39.384  44.747  1.00  0.00           O
ATOM   1396  HH  TYR    88      21.511  39.906  44.334  1.00  0.00           H
ATOM   1397  CE2 TYR    88      21.670  40.308  46.776  1.00  0.00           C
ATOM   1398  HE2 TYR    88      22.618  40.494  46.294  1.00  0.00           H
ATOM   1399  CD2 TYR    88      21.394  40.778  48.073  1.00  0.00           C
ATOM   1400  HD2 TYR    88      21.901  41.578  48.591  1.00  0.00           H
ATOM   1401  C   TYR    88      19.134  42.374  52.007  1.00  0.00           C
ATOM   1402  O   TYR    88      19.960  43.260  52.250  1.00  0.00           O
ATOM   1403  N   PRO    89      18.434  41.797  52.991  1.00  0.00           N
ATOM   1404  CD  PRO    89      17.598  40.640  52.948  1.00  0.00           C
ATOM   1405  HD2 PRO    89      18.176  39.898  52.398  1.00  0.00           H
ATOM   1406  HD3 PRO    89      16.581  40.775  52.578  1.00  0.00           H
ATOM   1407  CG  PRO    89      17.418  40.170  54.413  1.00  0.00           C
ATOM   1408  HG2 PRO    89      18.172  39.402  54.587  1.00  0.00           H
ATOM   1409  HG3 PRO    89      16.408  39.808  54.607  1.00  0.00           H
ATOM   1410  CB  PRO    89      17.664  41.416  55.192  1.00  0.00           C
ATOM   1411  HB2 PRO    89      18.086  41.124  56.153  1.00  0.00           H
ATOM   1412  HB3 PRO    89      16.804  42.084  55.204  1.00  0.00           H
ATOM   1413  CA  PRO    89      18.754  42.064  54.402  1.00  0.00           C
ATOM   1414  HA  PRO    89      18.684  43.125  54.642  1.00  0.00           H
ATOM   1415  C   PRO    89      20.168  41.560  54.734  1.00  0.00           C
ATOM   1416  O   PRO    89      20.721  40.595  54.138  1.00  0.00           O
ATOM   1417  N   GLY    90      20.757  42.205  55.672  1.00  0.00           N
ATOM   1418  H   GLY    90      20.318  42.946  56.202  1.00  0.00           H
ATOM   1419  CA  GLY    90      22.089  41.768  56.153  1.00  0.00           C
ATOM   1420  HA2 GLY    90      22.790  41.969  55.342  1.00  0.00           H
ATOM   1421  HA3 GLY    90      22.363  42.328  57.046  1.00  0.00           H
ATOM   1422  C   GLY    90      22.286  40.294  56.622  1.00  0.00           C
ATOM   1423  O   GLY    90      23.410  39.765  56.535  1.00  0.00           O
ATOM   1424  N   GLN    91      21.261  39.692  57.267  1.00  0.00           N
ATOM   1425  H   GLN    91      20.411  40.210  57.434  1.00  0.00           H
ATOM   1426  CA  GLN    91      21.294  38.298  57.612  1.00  0.00           C
ATOM   1427  HA  GLN    91      22.163  38.189  58.262  1.00  0.00           H
ATOM   1428  CB  GLN    91      20.009  38.019  58.400  1.00  0.00           C
ATOM   1429  HB2 GLN    91      19.151  38.411  57.853  1.00  0.00           H
ATOM   1430  HB3 GLN    91      19.811  36.951  58.496  1.00  0.00           H
ATOM   1431  CG  GLN    91      20.242  38.679  59.817  1.00  0.00           C
ATOM   1432  HG2 GLN    91      20.571  39.712  59.703  1.00  0.00           H
ATOM   1433  HG3 GLN    91      19.290  38.819  60.330  1.00  0.00           H
ATOM   1434  CD  GLN    91      21.121  37.983  60.867  1.00  0.00           C
ATOM   1435  OE1 GLN    91      22.286  38.204  60.859  1.00  0.00           O
ATOM   1436  NE2 GLN    91      20.619  37.098  61.741  1.00  0.00           N
ATOM   1437 HE21 GLN    91      19.633  36.882  61.712  1.00  0.00           H
```

ATOM	1438	HE22	GLN	91	21.205	36.681	62.450	1.00	0.00	H
ATOM	1439	C	GLN	91	21.359	37.315	56.404	1.00	0.00	C
ATOM	1440	O	GLN	91	21.474	36.130	56.742	1.00	0.00	O
ATOM	1441	N	THR	92	21.359	37.697	55.165	1.00	0.00	N
ATOM	1442	H	THR	92	21.157	38.687	55.148	1.00	0.00	H
ATOM	1443	CA	THR	92	21.697	36.860	54.060	1.00	0.00	C
ATOM	1444	HA	THR	92	20.977	36.042	54.029	1.00	0.00	H
ATOM	1445	CB	THR	92	21.674	37.560	52.685	1.00	0.00	C
ATOM	1446	HB	THR	92	22.539	38.214	52.579	1.00	0.00	H
ATOM	1447	CG2	THR	92	21.573	36.628	51.435	1.00	0.00	C
ATOM	1448	HG21	THR	92	21.222	35.656	51.777	1.00	0.00	H
ATOM	1449	HG22	THR	92	21.028	37.200	50.684	1.00	0.00	H
ATOM	1450	HG23	THR	92	22.591	36.484	51.075	1.00	0.00	H
ATOM	1451	OG1	THR	92	20.556	38.362	52.701	1.00	0.00	O
ATOM	1452	HG1	THR	92	20.749	39.188	53.150	1.00	0.00	H
ATOM	1453	C	THR	92	23.136	36.224	54.194	1.00	0.00	C
ATOM	1454	O	THR	92	24.037	36.966	54.642	1.00	0.00	O
ATOM	1455	N	GLN	93	23.356	34.994	53.706	1.00	0.00	N
ATOM	1456	H	GLN	93	22.639	34.565	53.139	1.00	0.00	H
ATOM	1457	CA	GLN	93	24.717	34.438	53.519	1.00	0.00	C
ATOM	1458	HA	GLN	93	25.445	34.960	54.143	1.00	0.00	H
ATOM	1459	CB	GLN	93	24.651	32.971	53.946	1.00	0.00	C
ATOM	1460	HB2	GLN	93	24.166	33.003	54.922	1.00	0.00	H
ATOM	1461	HB3	GLN	93	24.148	32.376	53.185	1.00	0.00	H
ATOM	1462	CG	GLN	93	26.092	32.405	54.104	1.00	0.00	C
ATOM	1463	HG2	GLN	93	26.015	31.320	54.177	1.00	0.00	H
ATOM	1464	HG3	GLN	93	26.692	32.670	53.234	1.00	0.00	H
ATOM	1465	CD	GLN	93	26.857	32.789	55.381	1.00	0.00	C
ATOM	1466	OE1	GLN	93	26.286	33.306	56.375	1.00	0.00	O
ATOM	1467	NE2	GLN	93	28.107	32.443	55.478	1.00	0.00	N
ATOM	1468	HE21	GLN	93	28.725	32.051	54.782	1.00	0.00	H
ATOM	1469	HE22	GLN	93	28.412	32.535	56.437	1.00	0.00	H
ATOM	1470	C	GLN	93	25.154	34.711	52.066	1.00	0.00	C
ATOM	1471	O	GLN	93	24.382	34.557	51.114	1.00	0.00	O
ATOM	1472	N	PHE	94	26.376	35.149	51.853	1.00	0.00	N
ATOM	1473	H	PHE	94	26.985	35.207	52.656	1.00	0.00	H
ATOM	1474	CA	PHE	94	27.077	35.399	50.549	1.00	0.00	C
ATOM	1475	HA	PHE	94	26.287	35.167	49.836	1.00	0.00	H
ATOM	1476	CB	PHE	94	27.522	36.909	50.427	1.00	0.00	C
ATOM	1477	HB2	PHE	94	28.190	37.158	51.251	1.00	0.00	H
ATOM	1478	HB3	PHE	94	28.150	36.998	49.539	1.00	0.00	H
ATOM	1479	CG	PHE	94	26.379	37.849	50.460	1.00	0.00	C
ATOM	1480	CD1	PHE	94	25.575	37.919	49.334	1.00	0.00	C
ATOM	1481	HD1	PHE	94	25.899	37.451	48.417	1.00	0.00	H
ATOM	1482	CE1	PHE	94	24.329	38.631	49.306	1.00	0.00	C
ATOM	1483	HE1	PHE	94	23.688	38.609	48.437	1.00	0.00	H
ATOM	1484	CZ	PHE	94	23.922	39.367	50.490	1.00	0.00	C
ATOM	1485	HZ	PHE	94	23.064	40.021	50.530	1.00	0.00	H
ATOM	1486	CE2	PHE	94	24.820	39.344	51.580	1.00	0.00	C
ATOM	1487	HE2	PHE	94	24.638	39.913	52.480	1.00	0.00	H
ATOM	1488	CD2	PHE	94	25.990	38.524	51.630	1.00	0.00	C
ATOM	1489	HD2	PHE	94	26.606	38.415	52.510	1.00	0.00	H
ATOM	1490	C	PHE	94	28.123	34.359	50.222	1.00	0.00	C
ATOM	1491	O	PHE	94	28.564	33.589	51.153	1.00	0.00	O
ATOM	1492	N	TYR	95	28.584	34.208	48.987	1.00	0.00	N
ATOM	1493	H	TYR	95	28.308	34.892	48.298	1.00	0.00	H
ATOM	1494	CA	TYR	95	29.457	33.210	48.505	1.00	0.00	C
ATOM	1495	HA	TYR	95	29.944	32.731	49.353	1.00	0.00	H
ATOM	1496	CB	TYR	95	28.539	32.281	47.701	1.00	0.00	C
ATOM	1497	HB2	TYR	95	27.720	32.007	48.367	1.00	0.00	H
ATOM	1498	HB3	TYR	95	28.082	32.860	46.899	1.00	0.00	H
ATOM	1499	CG	TYR	95	29.089	31.045	47.131	1.00	0.00	C
ATOM	1500	CD1	TYR	95	29.522	31.018	45.813	1.00	0.00	C
ATOM	1501	HD1	TYR	95	29.541	31.968	45.301	1.00	0.00	H

ATOM	1502	CE1	TYR	95	29.925	29.785	45.274	1.00	0.00	C
ATOM	1503	HE1	TYR	95	30.209	29.591	44.250	1.00	0.00	H
ATOM	1504	CZ	TYR	95	30.224	28.674	46.127	1.00	0.00	C
ATOM	1505	OH	TYR	95	30.507	27.508	45.576	1.00	0.00	O
ATOM	1506	HH	TYR	95	30.803	27.469	44.663	1.00	0.00	H
ATOM	1507	CE2	TYR	95	29.999	28.764	47.477	1.00	0.00	C
ATOM	1508	HE2	TYR	95	30.161	27.900	48.103	1.00	0.00	H
ATOM	1509	CD2	TYR	95	29.400	29.922	47.951	1.00	0.00	C
ATOM	1510	HD2	TYR	95	28.997	29.913	48.953	1.00	0.00	H
ATOM	1511	C	TYR	95	30.526	33.796	47.558	1.00	0.00	C
ATOM	1512	O	TYR	95	30.284	34.586	46.665	1.00	0.00	O
ATOM	1513	N	VAL	96	31.854	33.508	47.821	1.00	0.00	N
ATOM	1514	H	VAL	96	32.032	32.835	48.553	1.00	0.00	H
ATOM	1515	CA	VAL	96	32.943	34.106	47.008	1.00	0.00	C
ATOM	1516	HA	VAL	96	32.538	34.829	46.300	1.00	0.00	H
ATOM	1517	CB	VAL	96	34.045	34.673	47.910	1.00	0.00	C
ATOM	1518	HB	VAL	96	34.714	33.883	48.251	1.00	0.00	H
ATOM	1519	CG1	VAL	96	34.900	35.719	47.203	1.00	0.00	C
ATOM	1520	HG11	VAL	96	35.171	35.342	46.217	1.00	0.00	H
ATOM	1521	HG12	VAL	96	34.492	36.722	47.079	1.00	0.00	H
ATOM	1522	HG13	VAL	96	35.782	35.822	47.836	1.00	0.00	H
ATOM	1523	CG2	VAL	96	33.519	35.346	49.228	1.00	0.00	C
ATOM	1524	HG21	VAL	96	32.994	34.567	49.780	1.00	0.00	H
ATOM	1525	HG22	VAL	96	34.374	35.555	49.871	1.00	0.00	H
ATOM	1526	HG23	VAL	96	32.749	36.113	49.136	1.00	0.00	H
ATOM	1527	C	VAL	96	33.562	32.990	46.159	1.00	0.00	C
ATOM	1528	O	VAL	96	33.681	31.817	46.578	1.00	0.00	O
ATOM	1529	N	ILE	97	33.883	33.355	44.894	1.00	0.00	N
ATOM	1530	H	ILE	97	33.816	34.311	44.575	1.00	0.00	H
ATOM	1531	CA	ILE	97	34.317	32.467	43.781	1.00	0.00	C
ATOM	1532	HA	ILE	97	34.645	31.546	44.262	1.00	0.00	H
ATOM	1533	CB	ILE	97	33.230	32.163	42.702	1.00	0.00	C
ATOM	1534	HB	ILE	97	33.310	32.939	41.942	1.00	0.00	H
ATOM	1535	CG2	ILE	97	33.447	30.802	42.024	1.00	0.00	C
ATOM	1536	HG21	ILE	97	34.498	30.726	41.745	1.00	0.00	H
ATOM	1537	HG22	ILE	97	33.238	29.961	42.685	1.00	0.00	H
ATOM	1538	HG23	ILE	97	32.900	30.668	41.091	1.00	0.00	H
ATOM	1539	CG1	ILE	97	31.740	32.223	43.163	1.00	0.00	C
ATOM	1540	HG12	ILE	97	31.597	31.504	43.970	1.00	0.00	H
ATOM	1541	HG13	ILE	97	31.535	33.176	43.651	1.00	0.00	H
ATOM	1542	CD1	ILE	97	30.624	31.956	42.143	1.00	0.00	C
ATOM	1543	HD11	ILE	97	29.676	31.849	42.671	1.00	0.00	H
ATOM	1544	HD12	ILE	97	30.573	32.820	41.479	1.00	0.00	H
ATOM	1545	HD13	ILE	97	30.692	31.018	41.592	1.00	0.00	H
ATOM	1546	C	ILE	97	35.515	33.131	43.204	1.00	0.00	C
ATOM	1547	O	ILE	97	35.474	34.342	42.911	1.00	0.00	O
ATOM	1548	N	GLU	98	36.521	32.402	42.867	1.00	0.00	N
ATOM	1549	H	GLU	98	36.458	31.412	43.057	1.00	0.00	H
ATOM	1550	CA	GLU	98	37.834	32.961	42.457	1.00	0.00	C
ATOM	1551	HA	GLU	98	37.848	34.020	42.202	1.00	0.00	H
ATOM	1552	CB	GLU	98	38.870	33.019	43.576	1.00	0.00	C
ATOM	1553	HB2	GLU	98	38.758	32.010	43.973	1.00	0.00	H
ATOM	1554	HB3	GLU	98	39.890	33.172	43.224	1.00	0.00	H
ATOM	1555	CG	GLU	98	38.659	33.811	44.791	1.00	0.00	C
ATOM	1556	HG2	GLU	98	38.792	34.851	44.490	1.00	0.00	H
ATOM	1557	HG3	GLU	98	37.663	33.800	45.234	1.00	0.00	H
ATOM	1558	CD	GLU	98	39.740	33.605	45.826	1.00	0.00	C
ATOM	1559	OE1	GLU	98	39.426	33.260	47.028	1.00	0.00	O
ATOM	1560	OE2	GLU	98	40.962	33.815	45.588	1.00	0.00	O
ATOM	1561	C	GLU	98	38.442	32.291	41.180	1.00	0.00	C
ATOM	1562	O	GLU	98	38.467	31.103	41.157	1.00	0.00	O
ATOM	1563	N	PHE	99	38.950	33.010	40.111	1.00	0.00	N
ATOM	1564	H	PHE	99	39.017	34.017	40.128	1.00	0.00	H

ATOM	1565	CA	PHE	99	39.376	32.337	38.841	1.00	0.00	C
ATOM	1566	HA	PHE	99	39.848	31.389	39.099	1.00	0.00	H
ATOM	1567	CB	PHE	99	38.080	31.986	38.036	1.00	0.00	C
ATOM	1568	HB2	PHE	99	38.291	31.975	36.967	1.00	0.00	H
ATOM	1569	HB3	PHE	99	37.684	30.994	38.252	1.00	0.00	H
ATOM	1570	CG	PHE	99	37.015	33.047	38.206	1.00	0.00	C
ATOM	1571	CD1	PHE	99	35.919	32.792	39.046	1.00	0.00	C
ATOM	1572	HD1	PHE	99	36.030	31.997	39.769	1.00	0.00	H
ATOM	1573	CE1	PHE	99	34.945	33.780	39.094	1.00	0.00	C
ATOM	1574	HE1	PHE	99	34.193	33.711	39.865	1.00	0.00	H
ATOM	1575	CZ	PHE	99	34.877	34.885	38.269	1.00	0.00	C
ATOM	1576	HZ	PHE	99	34.052	35.579	38.325	1.00	0.00	H
ATOM	1577	CE2	PHE	99	35.830	34.964	37.288	1.00	0.00	C
ATOM	1578	HE2	PHE	99	35.708	35.737	36.543	1.00	0.00	H
ATOM	1579	CD2	PHE	99	36.952	34.112	37.296	1.00	0.00	C
ATOM	1580	HD2	PHE	99	37.719	34.402	36.592	1.00	0.00	H
ATOM	1581	C	PHE	99	40.336	33.172	37.993	1.00	0.00	C
ATOM	1582	O	PHE	99	40.597	34.352	38.263	1.00	0.00	O
ATOM	1583	N	LYS	100	40.893	32.512	36.992	1.00	0.00	N
ATOM	1584	H	LYS	100	40.652	31.534	36.922	1.00	0.00	H
ATOM	1585	CA	LYS	100	42.006	32.952	36.097	1.00	0.00	C
ATOM	1586	HA	LYS	100	41.875	33.970	35.728	1.00	0.00	H
ATOM	1587	CB	LYS	100	43.361	33.076	36.806	1.00	0.00	C
ATOM	1588	HB2	LYS	100	44.163	33.540	36.233	1.00	0.00	H
ATOM	1589	HB3	LYS	100	43.145	33.833	37.560	1.00	0.00	H
ATOM	1590	CG	LYS	100	43.863	31.746	37.448	1.00	0.00	C
ATOM	1591	HG2	LYS	100	43.230	31.389	38.259	1.00	0.00	H
ATOM	1592	HG3	LYS	100	43.989	31.136	36.552	1.00	0.00	H
ATOM	1593	CD	LYS	100	45.295	31.869	38.065	1.00	0.00	C
ATOM	1594	HD2	LYS	100	45.935	32.303	37.297	1.00	0.00	H
ATOM	1595	HD3	LYS	100	45.288	32.681	38.792	1.00	0.00	H
ATOM	1596	CE	LYS	100	45.907	30.598	38.606	1.00	0.00	C
ATOM	1597	HE2	LYS	100	45.564	30.383	39.618	1.00	0.00	H
ATOM	1598	HE3	LYS	100	45.479	29.866	37.922	1.00	0.00	H
ATOM	1599	NZ	LYS	100	47.378	30.619	38.523	1.00	0.00	N
ATOM	1600	HZ1	LYS	100	47.758	30.669	37.589	1.00	0.00	H
ATOM	1601	HZ2	LYS	100	47.588	31.508	38.953	1.00	0.00	H
ATOM	1602	HZ3	LYS	100	47.834	29.862	39.011	1.00	0.00	H
ATOM	1603	C	LYS	100	42.111	31.993	34.902	1.00	0.00	C
ATOM	1604	O	LYS	100	41.726	30.828	34.975	1.00	0.00	O
ATOM	1605	N	CYX	101	42.760	32.442	33.751	1.00	0.00	N
ATOM	1606	H	CYX	101	43.204	33.347	33.825	1.00	0.00	H
ATOM	1607	CA	CYX	101	43.014	31.769	32.494	1.00	0.00	C
ATOM	1608	HA	CYX	101	42.317	30.951	32.314	1.00	0.00	H
ATOM	1609	CB	CYX	101	42.935	32.800	31.343	1.00	0.00	C
ATOM	1610	HB2	CYX	101	41.961	33.272	31.472	1.00	0.00	H
ATOM	1611	HB3	CYX	101	43.777	33.483	31.454	1.00	0.00	H
ATOM	1612	SG	CYX	101	43.172	31.839	29.770	1.00	0.00	S
ATOM	1613	C	CYX	101	44.278	30.848	32.571	1.00	0.00	C
ATOM	1614	O	CYX	101	45.297	31.213	33.086	1.00	0.00	O
ATOM	1615	N	LEU	102	44.179	29.614	32.012	1.00	0.00	N
ATOM	1616	H	LEU	102	43.265	29.463	31.609	1.00	0.00	H
ATOM	1617	CA	LEU	102	45.339	28.672	31.906	1.00	0.00	C
ATOM	1618	HA	LEU	102	45.849	28.643	32.869	1.00	0.00	H
ATOM	1619	CB	LEU	102	44.876	27.207	31.786	1.00	0.00	C
ATOM	1620	HB2	LEU	102	44.549	26.962	30.775	1.00	0.00	H
ATOM	1621	HB3	LEU	102	45.824	26.669	31.829	1.00	0.00	H
ATOM	1622	CG	LEU	102	43.915	26.784	32.871	1.00	0.00	C
ATOM	1623	HG	LEU	102	42.920	27.229	32.908	1.00	0.00	H
ATOM	1624	CD1	LEU	102	43.679	25.272	32.693	1.00	0.00	C
ATOM	1625	HD11	LEU	102	43.003	24.971	33.493	1.00	0.00	H
ATOM	1626	HD12	LEU	102	43.227	25.162	31.707	1.00	0.00	H
ATOM	1627	HD13	LEU	102	44.578	24.658	32.763	1.00	0.00	H
ATOM	1628	CD2	LEU	102	44.367	27.102	34.384	1.00	0.00	C

```
ATOM   1629 HD21 LEU   102       44.350  28.164  34.629  1.00  0.00           H
ATOM   1630 HD22 LEU   102       43.589  26.607  34.967  1.00  0.00           H
ATOM   1631 HD23 LEU   102       45.299  26.635  34.699  1.00  0.00           H
ATOM   1632  C   LEU   102       46.498  28.939  30.909  1.00  0.00           C
ATOM   1633  O   LEU   102       46.257  29.297  29.775  1.00  0.00
      O
ATOM   1634  OXT LEU   102       47.676  28.694  31.294  1.00  0.00           O
HETATM  314  N   LYN    20       39.346  40.878  35.912  1.00  0.00           N
HETATM  315  H   LYN    20       39.876  40.275  35.300  1.00  0.00           H
HETATM  316  CA  LYN    20       38.250  40.155  36.682  1.00  0.00           C
HETATM  317  HA  LYN    20       38.049  40.811  37.530  1.00  0.00           H
HETATM  318  CB  LYN    20       37.006  39.919  35.814  1.00  0.00           C
HETATM  319  HB2 LYN    20       37.318  39.438  34.887  1.00  0.00           H
HETATM  320  HB3 LYN    20       36.756  40.936  35.515  1.00  0.00           H
HETATM  321  CG  LYN    20       35.980  39.053  36.478  1.00  0.00           C
HETATM  322  HG2 LYN    20       35.134  39.003  35.793  1.00  0.00           H
HETATM  323  HG3 LYN    20       36.424  38.071  36.641  1.00  0.00           H
HETATM  324  CD  LYN    20       35.496  39.778  37.734  1.00  0.00           C
HETATM  325  HD2 LYN    20       35.454  40.868  37.705  1.00  0.00           H
HETATM  326  HD3 LYN    20       36.237  39.596  38.512  1.00  0.00           H
HETATM  327  CE  LYN    20       34.145  39.202  38.126  1.00  0.00           C
HETATM  328  HE2 LYN    20       33.460  39.407  37.303  1.00  0.00           H
HETATM  329  HE3 LYN    20       34.169  38.119  38.245  1.00  0.00           H
HETATM  330  NZ  LYN    20       33.536  39.790  39.396  1.00  0.00           N
HETATM  331  HZ2 LYN    20       33.534  40.776  39.180  1.00  0.00           H
HETATM  332  HZ3 LYN    20       34.108  39.561  40.195  1.00  0.00           H
HETATM  333  C   LYN    20       38.813  38.849  37.170  1.00  0.00           C
HETATM  334  O   LYN    20       39.300  38.020  36.362  1.00  0.00           O
HETATM 1636  N   LIG   103       30.467  42.232  41.203  1.00  0.00           N
HETATM 1637  C   LIG   103       30.030  41.164  40.517  1.00  0.00           C
HETATM 1638  O   LIG   103       30.151  43.661  42.933  1.00  0.00           O
HETATM 1639  C5' LIG   103       25.662  39.945  45.614  1.00  0.00           C
HETATM 1640  O5' LIG   103       25.389  39.457  44.298  1.00  0.00           O
HETATM 1641  C4' LIG   103       26.375  41.224  45.631  1.00  0.00           C
HETATM 1642  O4' LIG   103       27.710  41.004  45.063  1.00  0.00           O
HETATM 1643  C3' LIG   103       25.811  42.326  44.702  1.00  0.00           C
HETATM 1644  O3' LIG   103       24.836  43.075  45.427  1.00  0.00           O
HETATM 1645  C2' LIG   103       27.018  43.106  44.181  1.00  0.00           C
HETATM 1646  O2' LIG   103       27.288  44.290  44.980  1.00  0.00           O
HETATM 1647  C1' LIG   103       28.203  42.128  44.297  1.00  0.00           C
HETATM 1648  N1  LIG   103       28.841  41.797  42.977  1.00  0.00           N
HETATM 1649  O1  LIG   103       29.134  39.292  38.398  1.00  0.00           O
HETATM 1650  N2  LIG   103       30.592  40.878  39.317  1.00  0.00           N
HETATM 1651  C6  LIG   103       29.829  42.640  42.397  1.00  0.00           C
HETATM 1652  C7  LIG   103       28.523  40.621  42.345  1.00  0.00           C
HETATM 1653  C8  LIG   103       29.115  40.313  41.171  1.00  0.00           C
HETATM 1654  C9  LIG   103       30.073  40.113  38.304  1.00  0.00           C
HETATM 1655  C10 LIG   103       30.636  40.486  36.914  1.00  0.00           C
HETATM 1656  H   LIG   103       31.220  41.633  39.066  1.00  0.00           H
HETATM 1657  H1  LIG   103       28.859  39.419  40.616  1.00  0.00           H
HETATM 1658  H4  LIG   103       29.033  42.610  44.826  1.00  0.00           H
HETATM 1659  H6  LIG   103       26.414  41.607  46.658  1.00  0.00           H
HETATM 1660  H7  LIG   103       25.332  41.837  43.846  1.00  0.00           H
HETATM 1661  H8  LIG   103       26.917  43.446  43.143  1.00  0.00           H
HETATM 1662  H9  LIG   103       24.762  40.041  46.233  1.00  0.00           H
HETATM 1663  H10 LIG   103       26.322  39.226  46.116  1.00  0.00           H
HETATM 1664  H11 LIG   103       30.290  41.500  36.682  1.00  0.00           H
HETATM 1665  H12 LIG   103       30.316  39.800  36.119  1.00  0.00           H
HETATM 1666  H13 LIG   103       31.723  40.599  36.822  1.00  0.00           H
HETATM 1667  H14 LIG   103       27.810  40.004  42.875  1.00  0.00           H
HETATM 1668  H2' LIG   103       26.402  44.651  45.156  1.00  0.00           H
HETATM 1669  H3' LIG   103       23.936  42.947  45.080  1.00  0.00           H
HETATM 1670  H5' LIG   103       24.823  38.668  44.241  1.00  0.00           H
ENDMDL
```

```
MODEL        5
SHEET    1   1 1 ILE    22  ASP    26  0
SHEET    2   2 1 VAL    37  VAL    40  0
SHEET    3   3 1 CYX    50  VAL    54  0
SHEET    4   4 1 THR    57  VAL    60  0
SHEET    5   5 1 PHE    94  CYX   101  0
HELIX    1   1 GLN      9  PHE     11  1                                  3
HELIX    2   2 ASP     13  ALA     17  1                                  5
HELIX    3   3 LEU     62  THR     64  1                                  3
HELIX    4   4 LYS     68  GLN     72  1                                  5
HELIX    5   5 LEU     77  ALA     85  1                                  9
ATOM      1  N   GLN     1      34.716  16.780  31.958  1.00  0.00           N
ATOM      2  H1  GLN     1      33.801  16.786  32.387  1.00  0.00           H
ATOM      3  H2  GLN     1      34.820  15.809  31.702  1.00  0.00           H
ATOM      4  H3  GLN     1      35.385  17.034  32.671  1.00  0.00           H
ATOM      5  CA  GLN     1      34.758  17.687  30.750  1.00  0.00           C
ATOM      6  HA  GLN     1      35.757  17.693  30.314  1.00  0.00           H
ATOM      7  CB  GLN     1      33.768  17.254  29.668  1.00  0.00           C
ATOM      8  HB2 GLN     1      32.827  17.276  30.219  1.00  0.00           H
ATOM      9  HB3 GLN     1      33.768  17.921  28.806  1.00  0.00           H
ATOM     10  CG  GLN     1      34.051  15.815  29.039  1.00  0.00           C
ATOM     11  HG2 GLN     1      34.265  15.070  29.806  1.00  0.00           H
ATOM     12  HG3 GLN     1      33.246  15.456  28.400  1.00  0.00           H
ATOM     13  CD  GLN     1      35.274  15.857  28.170  1.00  0.00           C
ATOM     14  OE1 GLN     1      35.890  16.865  27.995  1.00  0.00           O
ATOM     15  NE2 GLN     1      35.727  14.786  27.612  1.00  0.00           N
ATOM     16 HE21 GLN     1      35.185  13.937  27.690  1.00  0.00           H
ATOM     17 HE22 GLN     1      36.593  14.914  27.106  1.00  0.00           H
ATOM     18  C   GLN     1      34.401  19.097  31.162  1.00  0.00           C
ATOM     19  O   GLN     1      33.464  19.158  31.955  1.00  0.00           O
ATOM     20  N   PRO     2      34.949  20.125  30.498  1.00  0.00           N
ATOM     21  CD  PRO     2      35.999  19.982  29.503  1.00  0.00           C
ATOM     22  HD2 PRO     2      35.607  19.527  28.594  1.00  0.00           H
ATOM     23  HD3 PRO     2      36.686  19.201  29.828  1.00  0.00           H
ATOM     24  CG  PRO     2      36.526  21.337  29.303  1.00  0.00           C
ATOM     25  HG2 PRO     2      36.882  21.522  28.291  1.00  0.00           H
ATOM     26  HG3 PRO     2      37.332  21.401  30.035  1.00  0.00           H
ATOM     27  CB  PRO     2      35.350  22.245  29.630  1.00  0.00           C
ATOM     28  HB2 PRO     2      34.704  22.251  28.753  1.00  0.00           H
ATOM     29  HB3 PRO     2      35.677  23.245  29.916  1.00  0.00           H
ATOM     30  CA  PRO     2      34.625  21.513  30.748  1.00  0.00           C
ATOM     31  HA  PRO     2      35.054  21.699  31.732  1.00  0.00           H
ATOM     32  C   PRO     2      33.097  21.732  30.782  1.00  0.00           C
ATOM     33  O   PRO     2      32.294  21.058  30.058  1.00  0.00           O
ATOM     34  N   ASN     3      32.703  22.844  31.383  1.00  0.00           N
ATOM     35  H   ASN     3      33.435  23.333  31.878  1.00  0.00           H
ATOM     36  CA  ASN     3      31.288  23.201  31.506  1.00  0.00           C
ATOM     37  HA  ASN     3      30.643  22.354  31.742  1.00  0.00           H
ATOM     38  CB  ASN     3      31.191  24.252  32.569  1.00  0.00           C
ATOM     39  HB2 ASN     3      31.850  25.108  32.426  1.00  0.00           H
ATOM     40  HB3 ASN     3      30.208  24.719  32.534  1.00  0.00           H
ATOM     41  CG  ASN     3      31.327  23.718  33.978  1.00  0.00           C
ATOM     42  OD1 ASN     3      30.323  23.438  34.605  1.00  0.00           O
ATOM     43  ND2 ASN     3      32.504  23.482  34.500  1.00  0.00           N
ATOM     44 HD21 ASN     3      33.363  23.777  34.059  1.00  0.00           H
ATOM     45 HD22 ASN     3      32.524  23.034  35.405  1.00  0.00           H
ATOM     46  C   ASN     3      30.841  23.764  30.125  1.00  0.00           C
ATOM     47  O   ASN     3      31.677  24.369  29.373  1.00  0.00           O
ATOM     48  N   ASP     4      29.517  23.765  29.873  1.00  0.00           N
ATOM     49  H   ASP     4      28.942  23.374  30.605  1.00  0.00           H
ATOM     50  CA  ASP     4      28.880  24.401  28.766  1.00  0.00           C
ATOM     51  HA  ASP     4      29.598  24.314  27.951  1.00  0.00           H
ATOM     52  CB  ASP     4      27.566  23.740  28.505  1.00  0.00           C
ATOM     53  HB2 ASP     4      26.910  23.877  29.364  1.00  0.00           H
```

ATOM	54	HB3	ASP	4	27.132	24.398	27.751	1.00	0.00	H
ATOM	55	CG	ASP	4	27.659	22.296	28.023	1.00	0.00	C
ATOM	56	OD1	ASP	4	28.603	21.908	27.281	1.00	0.00	O
ATOM	57	OD2	ASP	4	26.823	21.464	28.486	1.00	0.00	O
ATOM	58	C	ASP	4	28.663	25.862	29.059	1.00	0.00	C
ATOM	59	O	ASP	4	29.041	26.769	28.265	1.00	0.00	O
ATOM	60	N	ILE	5	27.917	26.083	30.188	1.00	0.00	N
ATOM	61	H	ILE	5	27.789	25.248				
30.742	1.00		0.00		H					
ATOM	62	CA	ILE	5	27.762	27.335	30.820	1.00	0.00	C
ATOM	63	HA	ILE	5	26.998	27.972	30.376	1.00	0.00	H
ATOM	64	CB	ILE	5	27.313	27.084	32.254	1.00	0.00	C
ATOM	65	HB	ILE	5	28.019	26.422	32.755	1.00	0.00	H
ATOM	66	CG2	ILE	5	27.271	28.385	33.051	1.00	0.00	C
ATOM	67	HG21	ILE	5	26.702	29.126	32.489	1.00	0.00	H
ATOM	68	HG22	ILE	5	26.734	28.295	33.996	1.00	0.00	H
ATOM	69	HG23	ILE	5	28.309	28.704	33.151	1.00	0.00	H
ATOM	70	CG1	ILE	5	25.912	26.453	32.345	1.00	0.00	C
ATOM	71	HG12	ILE	5	25.138	27.160	32.046	1.00	0.00	H
ATOM	72	HG13	ILE	5	25.736	25.784	31.502	1.00	0.00	H
ATOM	73	CD1	ILE	5	25.614	25.861	33.695	1.00	0.00	C
ATOM	74	HD11	ILE	5	25.288	26.685	34.329	1.00	0.00	H
ATOM	75	HD12	ILE	5	24.772	25.179	33.574	1.00	0.00	H
ATOM	76	HD13	ILE	5	26.438	25.383	34.223	1.00	0.00	H
ATOM	77	C	ILE	5	29.109	28.151	30.754	1.00	0.00	C
ATOM	78	O	ILE	5	30.221	27.713	30.933	1.00	0.00	O
ATOM	79	N	THR	6	29.002	29.445	30.412	1.00	0.00	N
ATOM	80	H	THR	6	28.041	29.726	30.278	1.00	0.00	H
ATOM	81	CA	THR	6	30.094	30.387	29.944	1.00	0.00	C
ATOM	82	HA	THR	6	30.932	30.124	30.589	1.00	0.00	H
ATOM	83	CB	THR	6	30.454	29.970	28.529	1.00	0.00	C
ATOM	84	HB	THR	6	30.577	28.898	28.373	1.00	0.00	H
ATOM	85	CG2	THR	6	29.683	30.447	27.340	1.00	0.00	C
ATOM	86	HG21	THR	6	30.089	30.056	26.408	1.00	0.00	H
ATOM	87	HG22	THR	6	28.637	30.176	27.476	1.00	0.00	H
ATOM	88	HG23	THR	6	29.649	31.534	27.253	1.00	0.00	H
ATOM	89	OG1	THR	6	31.673	30.459	28.303	1.00	0.00	O
ATOM	90	HG1	THR	6	32.259	29.698	28.285	1.00	0.00	H
ATOM	91	C	THR	6	29.739	31.877	30.086	1.00	0.00	C
ATOM	92	O	THR	6	28.593	32.308	30.333	1.00	0.00	O
ATOM	93	N	PHE	7	30.776	32.725	30.053	1.00	0.00	N
ATOM	94	H	PHE	7	31.698	32.346	29.892	1.00	0.00	H
ATOM	95	CA	PHE	7	30.559	34.181	29.977	1.00	0.00	C
ATOM	96	HA	PHE	7	29.818	34.521	30.699	1.00	0.00	H
ATOM	97	CB	PHE	7	31.862	34.941	30.406	1.00	0.00	C
ATOM	98	HB2	PHE	7	32.656	34.714	29.694	1.00	0.00	H
ATOM	99	HB3	PHE	7	31.764	36.025	30.350	1.00	0.00	H
ATOM	100	CG	PHE	7	32.396	34.537	31.820	1.00	0.00	C
ATOM	101	CD1	PHE	7	33.413	33.592	32.022	1.00	0.00	C
ATOM	102	HD1	PHE	7	33.935	33.208	31.158	1.00	0.00	H
ATOM	103	CE1	PHE	7	33.828	33.273	33.403	1.00	0.00	C
ATOM	104	HE1	PHE	7	34.630	32.574	33.587	1.00	0.00	H
ATOM	105	CZ	PHE	7	33.130	33.810	34.512	1.00	0.00	C
ATOM	106	HZ	PHE	7	33.425	33.669	35.541	1.00	0.00	H
ATOM	107	CE2	PHE	7	31.930	34.565	34.302	1.00	0.00	C
ATOM	108	HE2	PHE	7	31.341	34.968	35.114	1.00	0.00	H
ATOM	109	CD2	PHE	7	31.631	34.921	32.957	1.00	0.00	C
ATOM	110	HD2	PHE	7	30.820	35.623	32.836	1.00	0.00	H
ATOM	111	C	PHE	7	30.160	34.616	28.507	1.00	0.00	C
ATOM	112	O	PHE	7	30.759	34.149	27.581	1.00	0.00	O
ATOM	113	N	PHE	8	29.218	35.554	28.369	1.00	0.00	N
ATOM	114	H	PHE	8	28.978	35.932	29.274	1.00	0.00	H
ATOM	115	CA	PHE	8	28.786	36.070	27.016	1.00	0.00	C
ATOM	116	HA	PHE	8	28.531	35.283	26.307	1.00	0.00	H

ATOM	117	CB	PHE	8	27.517	36.921	27.148	1.00	0.00	C
ATOM	118	HB2	PHE	8	27.822	37.967	27.192	1.00	0.00	H
ATOM	119	HB3	PHE	8	26.992	36.710	26.217	1.00	0.00	H
ATOM	120	CG	PHE	8	26.493	36.500	28.229	1.00	0.00	C
ATOM	121	CD1	PHE	8	26.023	35.206	28.384	1.00	0.00	C
ATOM	122	HD1	PHE	8	26.311	34.342	27.803	1.00	0.00	H
ATOM	123	CE1	PHE	8	25.144	34.941	29.486	1.00	0.00	C
ATOM	124	HE1	PHE	8	24.698	33.968	29.630	1.00	0.00	H
ATOM	125	CZ	PHE	8	24.782	35.976	30.378	1.00	0.00	C
ATOM	126	HZ	PHE	8	24.004	35.823	31.112	1.00	0.00	H
ATOM	127	CE2	PHE	8	25.254	37.266	30.196	1.00	0.00	C
ATOM	128	HE2	PHE	8	24.970	38.069	30.861	1.00	0.00	H
ATOM	129	CD2	PHE	8	26.132	37.518	29.155	1.00	0.00	C
ATOM	130	HD2	PHE	8	26.489	38.527	29.013	1.00	0.00	H
ATOM	131	C	PHE	8	29.975	36.922	26.385	1.00	0.00	C
ATOM	132	O	PHE	8	30.795	37.457	27.047	1.00	0.00	O
ATOM	133	N	GLN	9	30.097	36.901	25.006	1.00	0.00	N
ATOM	134	H	GLN	9	29.379	36.309	24.613	1.00	0.00	H
ATOM	135	CA	GLN	9	31.145	37.486	24.189	1.00	0.00	C
ATOM	136	HA	GLN	9	32.055	36.941	24.437	1.00	0.00	H
ATOM	137	CB	GLN	9	30.799	37.326	22.680	1.00	0.00	C
ATOM	138	HB2	GLN	9	29.886	37.872	22.436	1.00	0.00	H
ATOM	139	HB3	GLN	9	31.652	37.642	22.080	1.00	0.00	H
ATOM	140	CG	GLN	9	30.507	35.853	22.271	1.00	0.00	C
ATOM	141	HG2	GLN	9	31.316	35.207	22.607	1.00	0.00	H
ATOM	142	HG3	GLN	9	29.621	35.538	22.821	1.00	0.00	H
ATOM	143	CD	GLN	9	30.326	35.592	20.817	1.00	0.00	C
ATOM	144	OE1	GLN	9	30.439	36.468	19.947	1.00	0.00	O
ATOM	145	NE2	GLN	9	29.969	34.413	20.381	1.00	0.00	N
ATOM	146	HE21	GLN	9	29.686	34.181	19.440	1.00	0.00	H
ATOM	147	HE22	GLN	9	29.754	33.680	21.042	1.00	0.00	H
ATOM	148	C	GLN	9	31.570	38.872	24.552	1.00	0.00	C
ATOM	149	O	GLN	9	32.724	39.254	24.328	1.00	0.00	O
ATOM	150	N	ARG	10	30.669	39.714	25.104	1.00	0.00	N
ATOM	151	H	ARG	10	29.759	39.329	25.314	1.00	0.00	H
ATOM	152	CA	ARG	10	31.003	41.115	25.465	1.00	0.00	C
ATOM	153	HA	ARG	10	31.716	41.518	24.746	1.00	0.00	H
ATOM	154	CB	ARG	10	29.754	41.917	25.279	1.00	0.00	C
ATOM	155	HB2	ARG	10	29.219	41.767	24.341	1.00	0.00	H
ATOM	156	HB3	ARG	10	29.139	41.830	26.175	1.00	0.00	H
ATOM	157	CG	ARG	10	30.023	43.391	25.091	1.00	0.00	C
ATOM	158	HG2	ARG	10	30.563	43.710	25.983	1.00	0.00	H
ATOM	159	HG3	ARG	10	30.763	43.509	24.299	1.00	0.00	H
ATOM	160	CD	ARG	10	28.814	44.309	24.856	1.00	0.00	C
ATOM	161	HD2	ARG	10	29.332	45.217	24.549	1.00	0.00	H
ATOM	162	HD3	ARG	10	28.237	43.849	24.054	1.00	0.00	H
ATOM	163	NE	ARG	10	28.074	44.577	26.022	1.00	0.00	N
ATOM	164	HE	ARG	10	28.533	45.097	26.756	1.00	0.00	H
ATOM	165	CZ	ARG	10	26.814	44.124	26.274	1.00	0.00	C
ATOM	166	NH1	ARG	10	26.105	43.428	25.457	1.00	0.00	N
ATOM	167	HH11	ARG	10	25.105	43.372	25.593	1.00	0.00	H
ATOM	168	HH12	ARG	10	26.471	43.187	24.548	1.00	0.00	H
ATOM	169	NH2	ARG	10	26.263	44.463	27.405	1.00	0.00	N
ATOM	170	HH21	ARG	10	26.543	45.129	28.112	1.00	0.00	H
ATOM	171	HH22	ARG	10	25.427	43.970	27.683	1.00	0.00	H
ATOM	172	C	ARG	10	31.679	41.168	26.933	1.00	0.00	C
ATOM	173	O	ARG	10	32.234	42.242	27.237	1.00	0.00	O
ATOM	174	N	PHE	11	31.756	40.109	27.693	1.00	0.00	N
ATOM	175	H	PHE	11	31.279	39.265	27.409	1.00	0.00	H
ATOM	176	CA	PHE	11	32.290	40.115	29.086	1.00	0.00	C
ATOM	177	HA	PHE	11	32.734	41.086	29.306	1.00	0.00	H
ATOM	178	CB	PHE	11	31.121	39.809	30.079	1.00	0.00	C
ATOM	179	HB2	PHE	11	31.467	39.725	31.110	1.00	0.00	H
ATOM	180	HB3	PHE	11	30.640	38.906	29.708	1.00	0.00	H

ATOM	181	CG	PHE	11	30.166	40.963	30.109	1.00	0.00	C
ATOM	182	CD1	PHE	11	30.526	42.156	30.726	1.00	0.00	C
ATOM	183	HD1	PHE	11	31.483	42.156	31.227	1.00	0.00	H
ATOM	184	CE1	PHE	11	29.809	43.343	30.493	1.00	0.00	C
ATOM	185	HE1	PHE	11	30.071	44.330	30.844	1.00	0.00	H
ATOM	186	CZ	PHE	11	28.671	43.314	29.712	1.00	0.00	C
ATOM	187	HZ	PHE	11	28.054	44.197	29.644	1.00	0.00	H
ATOM	188	CE2	PHE	11	28.310	42.096	29.107	1.00	0.00	C
ATOM	189	HE2	PHE	11	27.410	42.028	28.516	1.00	0.00	H
ATOM	190	CD2	PHE	11	29.011	40.908	29.312	1.00	0.00	C
ATOM	191	HD2	PHE	11	28.708	39.940	28.940	1.00	0.00	H
ATOM	192	C	PHE	11	33.439	39.119	29.324	1.00	0.00	C
ATOM	193	O	PHE	11	33.660	38.647	30.470	1.00	0.00	O
ATOM	194	N	GLN	12	34.088	38.712	28.250	1.00	0.00	N
ATOM	195	H	GLN	12	34.001	39.288	27.425	1.00	0.00	H
ATOM	196	CA	GLN	12	35.214	37.744	28.405	1.00	0.00	C
ATOM	197	HA	GLN	12	34.973	37.073	29.229	1.00	0.00	H
ATOM	198	CB	GLN	12	35.389	36.872	27.127	1.00	0.00	C
ATOM	199	HB2	GLN	12	35.720	37.479	26.285	1.00	0.00	H
ATOM	200	HB3	GLN	12	36.204	36.163	27.270	1.00	0.00	H
ATOM	201	CG	GLN	12	34.031	36.145	26.758	1.00	0.00	C
ATOM	202	HG2	GLN	12	34.008	35.298	27.444	1.00	0.00	H
ATOM	203	HG3	GLN	12	33.262	36.915	26.818	1.00	0.00	H
ATOM	204	CD	GLN	12	34.073	35.532	25.381	1.00	0.00	C
ATOM	205	OE1	GLN	12	34.743	35.978	24.492	1.00	0.00	O
ATOM	206	NE2	GLN	12	33.243	34.563	25.098	1.00	0.00	N
ATOM	207	HE21	GLN	12	32.892	34.003	25.862	1.00	0.00	H
ATOM	208	HE22	GLN	12	33.429	34.152	24.194	1.00	0.00	H
ATOM	209	C	GLN	12	36.609	38.380	28.653	1.00	0.00	C
ATOM	210	O	GLN	12	37.349	37.920	29.551	1.00	0.00	O
ATOM	211	N	ASP	13	36.784	39.500	27.918	1.00	0.00	N
ATOM	212	H	ASP	13	36.047	39.826	27.310	1.00	0.00	H
ATOM	213	CA	ASP	13	38.143	40.152	27.827	1.00	0.00	C
ATOM	214	HA	ASP	13	38.781	39.381	27.394	1.00	0.00	H
ATOM	215	CB	ASP	13	38.181	41.271	26.856	1.00	0.00	C
ATOM	216	HB2	ASP	13	37.408	41.998	27.104	1.00	0.00	H
ATOM	217	HB3	ASP	13	39.151	41.765	26.813	1.00	0.00	H
ATOM	218	CG	ASP	13	37.861	40.775	25.408	1.00	0.00	C
ATOM	219	OD1	ASP	13	37.428	41.554	24.624	1.00	0.00	O
ATOM	220	OD2	ASP	13	37.978	39.582	25.107	1.00	0.00	O
ATOM	221	C	ASP	13	38.724	40.544	29.153	1.00	0.00	C
ATOM	222	O	ASP	13	39.912	40.401	29.341	1.00	0.00	O
ATOM	223	N	ASP	14	37.862	41.111	30.008	1.00	0.00	N
ATOM	224	H	ASP	14	36.930	41.088	29.620	1.00	0.00	H
ATOM	225	CA	ASP	14	38.221	41.635	31.359	1.00	0.00	C
ATOM	226	HA	ASP	14	38.703	42.612	31.299	1.00	0.00	H
ATOM	227	CB	ASP	14	36.925	41.778	32.290	1.00	0.00	C
ATOM	228	HB2	ASP	14	36.352	40.851	32.295	1.00	0.00	H
ATOM	229	HB3	ASP	14	37.214	41.962	33.325	1.00	0.00	H
ATOM	230	CG	ASP	14	36.071	43.038	31.873	1.00	0.00	C
ATOM	231	OD1	ASP	14	36.634	44.082	31.536	1.00	0.00	O
ATOM	232	OD2	ASP	14	34.803	43.043	31.973	1.00	0.00	O
ATOM	233	C	ASP	14	39.069	40.578	32.179	1.00	0.00	C
ATOM	234	O	ASP	14	39.915	40.885	33.027	1.00	0.00	O
ATOM	235	N	ILE	15	38.835	39.278	31.979	1.00	0.00	N
ATOM	236	H	ILE	15	38.194	38.950	31.270	1.00	0.00	H
ATOM	237	CA	ILE	15	39.468	38.085	32.693	1.00	0.00	C
ATOM	238	HA	ILE	15	39.646	38.342	33.737	1.00	0.00	H
ATOM	239	CB	ILE	15	38.589	36.799	32.590	1.00	0.00	C
ATOM	240	HB	ILE	15	38.358	36.563	31.552	1.00	0.00	H
ATOM	241	CG2	ILE	15	39.382	35.632	33.226	1.00	0.00	C
ATOM	242	HG21	ILE	15	38.831	34.692	33.217	1.00	0.00	H
ATOM	243	HG22	ILE	15	40.194	35.533	32.505	1.00	0.00	H

ATOM	244	HG23	ILE	15	39.780	35.850	34.216	1.00	0.00	H
ATOM	245	CG1	ILE	15	37.221	36.946	33.235	1.00	0.00	C
ATOM	246	HG12	ILE	15	37.324	37.372	34.233	1.00	0.00	H
ATOM	247	HG13	ILE	15	36.650	37.699	32.692	1.00	0.00	H
ATOM	248	CD1	ILE	15	36.388	35.703	33.194	1.00	0.00	C
ATOM	249	HD11	ILE	15	36.471	35.217	32.222	1.00	0.00	H
ATOM	250	HD12	ILE	15	36.751	35.059	33.994	1.00	0.00	H
ATOM	251	HD13	ILE	15	35.315	35.873	33.271	1.00	0.00	H
ATOM	252	C	ILE	15	40.939	37.925	32.164	1.00	0.00	C
ATOM	253	O	ILE	15	41.815	37.591	32.957	1.00	0.00	O
ATOM	254	N	LEU	16	41.156	38.116	30.854	1.00	0.00	N
ATOM	255	H	LEU	16	40.371	38.199	30.224	1.00	0.00	H
ATOM	256	CA	LEU	16	42.539	38.065	30.244	1.00	0.00	C
ATOM	257	HA	LEU	16	43.098	37.312	30.799	1.00	0.00	H
ATOM	258	CB	LEU	16	42.403	37.704	28.776	1.00	0.00	C
ATOM	259	HB2	LEU	16	41.683	36.891	28.675	1.00	0.00	H
ATOM	260	HB3	LEU	16	42.021	38.632	28.350	1.00	0.00	H
ATOM	261	CG	LEU	16	43.743	37.336	28.094	1.00	0.00	C
ATOM	262	HG	LEU	16	44.393	38.205	28.189	1.00	0.00	H
ATOM	263	CD1	LEU	16	44.329	36.059	28.744	1.00	0.00	C
ATOM	264	HD11	LEU	16	44.900	35.528	27.982	1.00	0.00	H
ATOM	265	HD12	LEU	16	44.922	36.278	29.632	1.00	0.00	H
ATOM	266	HD13	LEU	16	43.578	35.349	29.093	1.00	0.00	H
ATOM	267	CD2	LEU	16	43.684	37.059	26.681	1.00	0.00	C
ATOM	268	HD21	LEU	16	44.669	36.883	26.248	1.00	0.00	H
ATOM	269	HD22	LEU	16	43.076	36.212	26.361	1.00	0.00	H
ATOM	270	HD23	LEU	16	43.361	37.959	26.157	1.00	0.00	H
ATOM	271	C	LEU	16	43.283	39.348	30.510	1.00	0.00	C
ATOM	272	O	LEU	16	44.524	39.252	30.605	1.00	0.00	O
ATOM	273	N	ALA	17	42.699	40.526	30.625	1.00	0.00	N
ATOM	274	H	ALA	17	41.744	40.545	30.299	1.00	0.00	H
ATOM	275	CA	ALA	17	43.305	41.756	31.028	1.00	0.00	C
ATOM	276	HA	ALA	17	44.207	41.939	30.445	1.00	0.00	H
ATOM	277	CB	ALA	17	42.213	42.823	30.806	1.00	0.00	C
ATOM	278	HB1	ALA	17	41.819	42.810	29.790	1.00	0.00	H
ATOM	279	HB2	ALA	17	41.351	42.747	31.470	1.00	0.00	H
ATOM	280	HB3	ALA	17	42.520	43.801	31.178	1.00	0.00	H
ATOM	281	C	ALA	17	43.766	41.700	32.498	1.00	0.00	C
ATOM	282	O	ALA	17	44.781	42.292	32.923	1.00	0.00	O
ATOM	283	N	GLY	18	42.982	41.043	33.313	1.00	0.00	N
ATOM	284	H	GLY	18	42.238	40.510	32.887	1.00	0.00	H
ATOM	285	CA	GLY	18	43.064	40.927	34.798	1.00	0.00	C
ATOM	286	HA2	GLY	18	42.893	39.894	35.101	1.00	0.00	H
ATOM	287	HA3	GLY	18	44.084	41.165	35.097	1.00	0.00	H
ATOM	288	C	GLY	18	42.142	41.816	35.590	1.00	0.00	C
ATOM	289	O	GLY	18	42.191	41.683	36.807	1.00	0.00	O
ATOM	290	N	ARG	19	41.219	42.588	34.937	1.00	0.00	N
ATOM	291	H	ARG	19	41.124	42.509	33.934	1.00	0.00	H
ATOM	292	CA	ARG	19	40.227	43.446	35.617	1.00	0.00	C
ATOM	293	HA	ARG	19	40.716	44.092	36.344	1.00	0.00	H
ATOM	294	CB	ARG	19	39.477	44.355	34.632	1.00	0.00	C
ATOM	295	HB2	ARG	19	39.143	43.728	33.807	1.00	0.00	H
ATOM	296	HB3	ARG	19	38.662	44.722	35.256	1.00	0.00	H
ATOM	297	CG	ARG	19	40.386	45.415	33.903	1.00	0.00	C
ATOM	298	HG2	ARG	19	40.763	46.111	34.652	1.00	0.00	H
ATOM	299	HG3	ARG	19	41.270	45.008	33.412	1.00	0.00	H
ATOM	300	CD	ARG	19	39.640	46.210	32.828	1.00	0.00	C
ATOM	301	HD2	ARG	19	38.696	46.610	33.195	1.00	0.00	H
ATOM	302	HD3	ARG	19	40.229	47.034	32.423	1.00	0.00	H
ATOM	303	NE	ARG	19	39.158	45.340	31.801	1.00	0.00	N
ATOM	304	HE	ARG	19	38.282	44.885	32.019	1.00	0.00	H
ATOM	305	CZ	ARG	19	39.394	45.264	30.500	1.00	0.00	C
ATOM	306	NH1	ARG	19	40.405	45.800	29.878	1.00	0.00	N
ATOM	307	HH11	ARG	19	41.205	46.223	30.326	1.00	0.00	H

```
ATOM    308 HH12 ARG    19      40.473  45.691  28.876  1.00  0.00           H
ATOM    309  NH2 ARG    19      38.596  44.615  29.745  1.00  0.00           N
ATOM    310 HH21 ARG    19      38.841  44.608  28.765  1.00  0.00           H
ATOM    311 HH22 ARG    19      37.685  44.370  30.107  1.00  0.00           H
ATOM    312  C   ARG
    19      39.156  42.644  36.358  1.00  0.00           C
ATOM    313  O   ARG    19      38.582  43.233  37.286  1.00  0.00           O
ATOM    335  N   THR    21      38.731  38.850  38.224  1.00  0.00           N
ATOM    336  H   THR    21      38.388  39.555  38.859  1.00  0.00           H
ATOM    337  CA  THR    21      39.496  37.688  38.791  1.00  0.00           C
ATOM    338  HA  THR    21      39.535  36.947  37.993  1.00  0.00           H
ATOM    339  CB  THR    21      40.946  38.075  39.160  1.00  0.00           C
ATOM    340  HB  THR    21      41.333  38.568  38.268  1.00  0.00           H
ATOM    341  CG2 THR    21      41.157  39.087  40.311  1.00  0.00           C
ATOM    342 HG21 THR    21      40.544  39.984  40.414  1.00  0.00           H
ATOM    343 HG22 THR    21      40.898  38.525  41.207  1.00  0.00           H
ATOM    344 HG23 THR    21      42.166  39.484  40.200  1.00  0.00           H
ATOM    345  OG1 THR    21      41.783  36.977  39.547  1.00  0.00           O
ATOM    346  HG1 THR    21      42.236  36.647  38.767  1.00  0.00           H
ATOM    347  C   THR    21      38.711  37.153  40.047  1.00  0.00           C
ATOM    348  O   THR    21      38.891  35.986  40.400  1.00  0.00           O
ATOM    349  N   ILE    22      37.886  37.957  40.716  1.00  0.00           N
ATOM    350  H   ILE    22      37.965  38.940  40.496  1.00  0.00           H
ATOM    351  CA  ILE    22      36.982  37.644  41.815  1.00  0.00           C
ATOM    352  HA  ILE    22      36.862  36.569  41.960  1.00  0.00           H
ATOM    353  CB  ILE    22      37.635  38.172  43.166  1.00  0.00           C
ATOM    354  HB  ILE    22      38.550  37.581  43.213  1.00  0.00           H
ATOM    355  CG2 ILE    22      38.055  39.679  43.088  1.00  0.00           C
ATOM    356 HG21 ILE    22      37.169  40.231  42.773  1.00  0.00           H
ATOM    357 HG22 ILE    22      38.445  40.004  44.053  1.00  0.00           H
ATOM    358 HG23 ILE    22      38.886  39.808  42.395  1.00  0.00           H
ATOM    359  CG1 ILE    22      36.703  38.025  44.424  1.00  0.00           C
ATOM    360 HG12 ILE    22      35.991  38.837  44.276  1.00  0.00           H
ATOM    361 HG13 ILE    22      36.163  37.089  44.282  1.00  0.00           H
ATOM    362  CD1 ILE    22      37.365  38.007  45.792  1.00  0.00           C
ATOM    363 HD11 ILE    22      38.100  38.812  45.819  1.00  0.00           H
ATOM    364 HD12 ILE    22      36.688  38.156  46.633  1.00  0.00           H
ATOM    365 HD13 ILE    22      37.830  37.034  45.948  1.00  0.00           H
ATOM    366  C   ILE    22      35.595  38.294  41.623  1.00  0.00           C
ATOM    367  O   ILE    22      35.377  39.246  40.874  1.00  0.00           O
ATOM    368  N   THR    23      34.606  37.755  42.385  1.00  0.00           N
ATOM    369  H   THR    23      34.727  36.907  42.919  1.00  0.00           H
ATOM    370  CA  THR    23      33.112  37.991  42.236  1.00  0.00           C
ATOM    371  HA  THR    23      32.975  39.072  42.255  1.00  0.00           H
ATOM    372  CB  THR    23      32.497  37.385  40.902  1.00  0.00           C
ATOM    373  HB  THR    23      33.294  37.559  40.180  1.00  0.00           H
ATOM    374  CG2 THR    23      32.050  35.938  40.966  1.00  0.00           C
ATOM    375 HG21 THR    23      32.020  35.561  39.944  1.00  0.00           H
ATOM    376 HG22 THR    23      32.792  35.325  41.480  1.00  0.00           H
ATOM    377 HG23 THR    23      31.099  35.841  41.491  1.00  0.00           H
ATOM    378  OG1 THR    23      31.394  38.131  40.501  1.00  0.00           O
ATOM    379  HG1 THR    23      31.770  38.830  39.963  1.00  0.00           H
ATOM    380  C   THR    23      32.387  37.396  43.513  1.00  0.00           C
ATOM    381  O   THR    23      32.960  36.645  44.277  1.00  0.00           O
ATOM    382  N   ILE    24      31.113  37.820  43.720  1.00  0.00           N
ATOM    383  H   ILE    24      30.677  38.230  42.906  1.00  0.00           H
ATOM    384  CA  ILE    24      30.239  37.485  44.830  1.00  0.00           C
ATOM    385  HA  ILE    24      30.666  36.658  45.398  1.00  0.00           H
ATOM    386  CB  ILE    24      30.097  38.721  45.728  1.00  0.00           C
ATOM    387  HB  ILE    24      29.602  39.546  45.216  1.00  0.00           H
ATOM    388  CG2 ILE    24      29.263  38.256  46.964  1.00  0.00           C
ATOM    389 HG21 ILE    24      29.709  37.380  47.435  1.00  0.00           H
ATOM    390 HG22 ILE    24      29.116  39.109  47.625  1.00  0.00           H
ATOM    391 HG23 ILE    24      28.227  38.023  46.715  1.00  0.00           H
```

ATOM	392	CG1	ILE	24	31.485	39.162	46.241	1.00	0.00	C
ATOM	393	HG12	ILE	24	32.028	38.277	46.574	1.00	0.00	H
ATOM	394	HG13	ILE	24	31.982	39.692	45.429	1.00	0.00	H
ATOM	395	CD1	ILE	24	31.556	40.333	47.164	1.00	0.00	C
ATOM	396	HD11	ILE	24	32.627	40.335	47.369	1.00	0.00	H
ATOM	397	HD12	ILE	24	31.266	41.265	46.680	1.00	0.00	H
ATOM	398	HD13	ILE	24	30.960	40.296	48.076	1.00	0.00	H
ATOM	399	C	ILE	24	28.824	37.023	44.320	1.00	0.00	C
ATOM	400	O	ILE	24	28.375	37.627	43.335	1.00	0.00	O
ATOM	401	N	ARG	25	28.211	36.013	44.899	1.00	0.00	N
ATOM	402	H	ARG	25	28.678	35.615	45.701	1.00	0.00	H
ATOM	403	CA	ARG	25	26.827	35.594	44.683	1.00	0.00	C
ATOM	404	HA	ARG	25	26.198	36.343	44.201	1.00	0.00	H
ATOM	405	CB	ARG	25	26.889	34.174	44.126	1.00	0.00	C
ATOM	406	HB2	ARG	25	27.367	33.587	44.909	1.00	0.00	H
ATOM	407	HB3	ARG	25	25.881	33.774	44.024	1.00	0.00	H
ATOM	408	CG	ARG	25	27.551	33.924	42.779	1.00	0.00	C
ATOM	409	HG2	ARG	25	28.609	34.185	42.817	1.00	0.00	H
ATOM	410	HG3	ARG	25	27.554	32.843	42.643	1.00	0.00	H
ATOM	411	CD	ARG	25	26.843	34.686	41.606	1.00	0.00	C
ATOM	412	HD2	ARG	25	25.919	34.136	41.424	1.00	0.00	H
ATOM	413	HD3	ARG	25	26.756	35.702	41.992	1.00	0.00	H
ATOM	414	NE	ARG	25	27.695	34.653	40.359	1.00	0.00	N
ATOM	415	HE	ARG	25	27.599	33.811	39.809	1.00	0.00	H
ATOM	416	CZ	ARG	25	28.546	35.593	39.895	1.00	0.00	C
ATOM	417	NH1	ARG	25	28.678	36.763	40.483	1.00	0.00	N
ATOM	418	HH11	ARG	25	29.376	37.372	40.081	1.00	0.00	H
ATOM	419	HH12	ARG	25	28.009	36.933	41.220	1.00	0.00	H
ATOM	420	NH2	ARG	25	29.149	35.241	38.823	1.00	0.00	N
ATOM	421	HH21	ARG	25	29.863	35.820	38.404	1.00	0.00	H
ATOM	422	HH22	ARG	25	29.017	34.317	38.440	1.00	0.00	H
ATOM	423	C	ARG	25	26.163	35.500	46.095	1.00	0.00	C
ATOM	424	O	ARG	25	26.849	35.750	47.080	1.00	0.00	O
ATOM	425	N	ASP	26	24.950	35.019	46.218	1.00	0.00	N
ATOM	426	H	ASP	26	24.381	34.913	45.391	1.00	0.00	H
ATOM	427	CA	ASP	26	24.327	34.527	47.493	1.00	0.00	C
ATOM	428	HA	ASP	26	24.892	34.898	48.349	1.00	0.00	H
ATOM	429	CB	ASP	26	22.922	35.023	47.688	1.00	0.00	C
ATOM	430	HB2	ASP	26	22.495	34.722	48.645	1.00	0.00	H
ATOM	431	HB3	ASP	26	22.907	36.113	47.667	1.00	0.00	H
ATOM	432	CG	ASP	26	22.007	34.485	46.617	1.00	0.00	C
ATOM	433	OD1	ASP	26	22.249	34.726	45.420	1.00	0.00	O
ATOM	434	OD2	ASP	26	21.009	33.813	46.970	1.00	0.00	O
ATOM	435	C	ASP	26	24.463	32.970	47.555	1.00	0.00	C
ATOM	436	O	ASP	26	24.530	32.335	46.526	1.00	0.00	O
ATOM	437	N	GLU	27	24.455	32.415	48.715	1.00	0.00	N
ATOM	438	H	GLU	27	24.353	33.064	49.483	1.00	0.00	H
ATOM	439	CA	GLU	27	24.703	30.992	49.070	1.00	0.00	C
ATOM	440	HA	GLU	27	25.655	30.786	48.582	1.00	0.00	H
ATOM	441	CB	GLU	27	24.680	30.976	50.605	1.00	0.00	C
ATOM	442	HB2	GLU	27	25.300	31.769	51.023	1.00	0.00	H
ATOM	443	HB3	GLU	27	23.699	31.283	50.967	1.00	0.00	H
ATOM	444	CG	GLU	27	25.114	29.580	51.233	1.00	0.00	C
ATOM	445	HG2	GLU	27	25.111	29.564	52.322	1.00	0.00	H
ATOM	446	HG3	GLU	27	24.235	29.003	50.943	1.00	0.00	H
ATOM	447	CD	GLU	27	26.367	29.037	50.655	1.00	0.00	C
ATOM	448	OE1	GLU	27	26.388	28.572	49.476	1.00	0.00	O
ATOM	449	OE2	GLU	27	27.323	28.874	51.474	1.00	0.00	O
ATOM	450	C	GLU	27	23.637	30.053	48.434	1.00	0.00	C
ATOM	451	O	GLU	27	22.428	30.226	48.304	1.00	0.00	O
ATOM	452	N	SER	28	24.092	28.863	47.940	1.00	0.00	N
ATOM	453	H	SER	28	25.071	28.654	48.076	1.00	0.00	H
ATOM	454	CA	SER	28	23.386	27.755	47.278	1.00	0.00	C
ATOM	455	HA	SER	28	22.695	27.274	47.971	1.00	0.00	H

ATOM	456	CB	SER	28	22.617	28.250	46.061	1.00	0.00	C
ATOM	457	HB2	SER	28	21.802	28.867	46.439	1.00	0.00	H
ATOM	458	HB3	SER	28	23.282	28.849	45.440	1.00	0.00	H
ATOM	459	OG	SER	28	22.125	27.152	45.337	1.00	0.00	O
ATOM	460	HG	SER	28	21.205	26.992	45.560	1.00	0.00	H
ATOM	461	C	SER	28	24.376	26.620	46.869	1.00	0.00	C
ATOM	462	O	SER	28	25.483	26.889	46.395	1.00	0.00	O
ATOM	463	N	GLU	29	23.990	25.333	47.101	1.00	0.00	N
ATOM	464	H	GLU	29	23.232	25.236	47.762	1.00	0.00	H
ATOM	465	CA	GLU	29	24.656	24.120	46.654	1.00	0.00	C
ATOM	466	HA	GLU	29	25.682	24.082	47.020	1.00	0.00	H
ATOM	467	CB	GLU	29	23.886	22.986	47.327	1.00	0.00	C
ATOM	468	HB2	GLU	29	23.905	23.044	48.416	1.00	0.00	H
ATOM	469	HB3	GLU	29	22.841	22.971	47.021	1.00	0.00	H
ATOM	470	CG	GLU	29	24.352	21.557	46.947	1.00	0.00	C
ATOM	471	HG2	GLU	29	23.796	20.855	47.568	1.00	0.00	H
ATOM	472	HG3	GLU	29	24.124	21.336	45.904	1.00	0.00	H
ATOM	473	CD	GLU	29	25.852	21.377	47.156	1.00	0.00	C
ATOM	474	OE1	GLU	29	26.588	20.554	46.458	1.00	0.00	O
ATOM	475	OE2	GLU	29	26.428	22.027	48.081	1.00	0.00	O
ATOM	476	C	GLU	29	24.731	24.018	45.116	1.00	0.00	C
ATOM	477	O	GLU	29	25.571	23.333	44.592	1.00	0.00	O
ATOM	478	N	SER	30	23.893	24.796	44.364	1.00	0.00	N
ATOM	479	H	SER	30	23.184	25.299	44.879	1.00	0.00	H
ATOM	480	CA	SER	30	23.868	24.849	42.888	1.00	0.00	C
ATOM	481	HA	SER	30	23.570	23.892	42.461	1.00	0.00	H
ATOM	482	CB	SER	30	22.688	25.794	42.408	1.00	0.00	C
ATOM	483	HB2	SER	30	22.652	25.805	41.319	1.00	0.00	H
ATOM	484	HB3	SER	30	21.819	25.287	42.828	1.00	0.00	H
ATOM	485	OG	SER	30	23.004	27.165	42.871	1.00	0.00	O
ATOM	486	HG	SER	30	22.709	27.226	43.783	1.00	0.00	H
ATOM	487	C	SER	30	25.232	25.300	42.338	1.00	0.00	C
ATOM	488	O	SER	30	25.540	24.899	41.199	1.00	0.00	O
ATOM	489	N	HIE	31	25.931	26.182	43.031	1.00	0.00	N
ATOM	490	H	HIE	31	25.704	26.353	44.000	1.00	0.00	H
ATOM	491	CA	HIE	31	27.131	26.935	42.558	1.00	0.00	C
ATOM	492	HA	HIE	31	26.960	27.373	41.575	1.00	0.00	H
ATOM	493	CB	HIE	31	27.363	28.121	43.523	1.00	0.00	C
ATOM	494	HB2	HIE	31	27.639	27.685	44.482	1.00	0.00	H
ATOM	495	HB3	HIE	31	28.111	28.703	42.984	1.00	0.00	H
ATOM	496	CG	HIE	31	26.241	29.107	43.583	1.00	0.00	C
ATOM	497	ND1	HIE	31	25.600	29.719	42.526	1.00	0.00	N
ATOM	498	CE1	HIE	31	24.825	30.704	43.004	1.00	0.00	C
ATOM	499	HE1	HIE	31	24.231	31.356	42.382	1.00	0.00	H
ATOM	500	NE2	HIE	31	24.960	30.700	44.314	1.00	0.00	N
ATOM	501	HE2	HIE	31	24.580	31.439	44.889	1.00	0.00	H
ATOM	502	CD2	HIE	31	25.824	29.737	44.706	1.00	0.00	C
ATOM	503	HD2	HIE	31	26.385	29.731	45.628	1.00	0.00	H
ATOM	504	C	HIE	31	28.351	26.074	42.493	1.00	0.00	C
ATOM	505	O	HIE	31	28.506	25.067	43.238	1.00	0.00	O
ATOM	506	N	PHE	32	29.311	26.493	41.645	1.00	0.00	N
ATOM	507	H	PHE	32	29.107	27.290	41.058	1.00	0.00	H
ATOM	508	CA	PHE	32	30.650	25.827	41.561	1.00	0.00	C
ATOM	509	HA	PHE	32	30.558	24.798	41.214	1.00	0.00	H
ATOM	510	CB	PHE	32	31.613	26.556	40.665	1.00	0.00	C
ATOM	511	HB2	PHE	32	32.000	27.473	41.111	1.00	0.00	H
ATOM	512	HB3	PHE	32	32.546	26.004	40.555	1.00	0.00	H
ATOM	513	CG	PHE	32	31.150	26.844	39.212	1.00	0.00	C
ATOM	514	CD1	PHE	32	31.108	28.156	38.665	1.00	0.00	C
ATOM	515	HD1	PHE	32	31.332	29.009	39.287	1.00	0.00	H
ATOM	516	CE1	PHE	32	30.622	28.380	37.422	1.00	0.00	C
ATOM	517	HE1	PHE	32	30.505	29.380	37.033	1.00	0.00	H
ATOM	518	CZ	PHE	32	30.363	27.256	36.533	1.00	0.00	C

ATOM	519	HZ	PHE	32	30.081	27.469	35.512	1.00	0.00	H
ATOM	520	CE2	PHE	32	30.608	25.941	36.966	1.00	0.00	C
ATOM	521	HE2	PHE	32	30.477	25.056	36.361	1.00	0.00	H
ATOM	522	CD2	PHE	32	30.950	25.710	38.327	1.00	0.00	C
ATOM	523	HD2	PHE	32	31.119	24.717	38.717	1.00	0.00	H
ATOM	524	C	PHE	32	31.390	25.711	42.890	1.00	0.00	C
ATOM	525	O	PHE	32	31.342	26.501	43.841	1.00	0.00	O
ATOM	526	N	LYS	33	32.179	24.613	43.017	1.00	0.00	N
ATOM	527	H	LYS	33	32.452	24.267	42.109	1.00	0.00	H
ATOM	528	CA	LYS	33	33.192	24.339	44.025	1.00	0.00	C
ATOM	529	HA	LYS	33	33.023	24.872	44.961	1.00	0.00	H
ATOM	530	CB	LYS	33	33.212	22.840	44.228	1.00	0.00	C
ATOM	531	HB2	LYS	33	33.616	22.287	43.380	1.00	0.00	H
ATOM	532	HB3	LYS	33	33.869	22.633	45.073	1.00	0.00	H
ATOM	533	CG	LYS	33	31.891	22.123	44.661	1.00	0.00	C
ATOM	534	HG2	LYS	33	31.339	21.943	43.739	1.00	0.00	H
ATOM	535	HG3	LYS	33	32.157	21.146	45.068	1.00	0.00	H
ATOM	536	CD	LYS	33	30.981	22.852	45.708	1.00	0.00	C
ATOM	537	HD2	LYS	33	31.502	22.951	46.660	1.00	0.00	H
ATOM	538	HD3	LYS	33	30.830	23.893	45.420	1.00	0.00	H
ATOM	539	CE	LYS	33	29.712	22.014	45.930	1.00	0.00	C
ATOM	540	HE2	LYS	33	29.232	21.979	44.953	1.00	0.00	H
ATOM	541	HE3	LYS	33	29.988	20.995	46.200	1.00	0.00	H
ATOM	542	NZ	LYS	33	28.898	22.545	47.036	1.00	0.00	N
ATOM	543	HZ1	LYS	33	29.435	22.569	47.891	1.00	0.00	H
ATOM	544	HZ2	LYS	33	28.626	23.498	46.839	1.00	0.00	H
ATOM	545	HZ3	LYS	33	28.131	21.903	47.176	1.00	0.00	H
ATOM	546	C	LYS	33	34.551	24.804	43.475	1.00	0.00	C
ATOM	547	O	LYS	33	34.677	25.284	42.371	1.00	0.00	O
ATOM	548	N	THR	34	35.575	24.660	44.275	1.00	0.00	N
ATOM	549	H	THR	34	35.507	24.465	45.265	1.00	0.00	H
ATOM	550	CA	THR	34	36.920	25.015	43.760	1.00	0.00	C
ATOM	551	HA	THR	34	36.788	25.995	43.301	1.00	0.00	H
ATOM	552	CB	THR	34	37.930	25.285	44.875	1.00	0.00	C
ATOM	553	HB	THR	34	38.097	24.509	45.621	1.00	0.00	H
ATOM	554	CG2	THR	34	39.275	25.640	44.269	1.00	0.00	C
ATOM	555	HG21	THR	34	39.917	25.988	45.080	1.00	0.00	H
ATOM	556	HG22	THR	34	39.794	24.729	43.971	1.00	0.00	H
ATOM	557	HG23	THR	34	39.161	26.343	43.445	1.00	0.00	H
ATOM	558	OG1	THR	34	37.361	26.367	45.527	1.00	0.00	O
ATOM	559	HG1	THR	34	37.885	26.747	46.236	1.00	0.00	H
ATOM	560	C	THR	34	37.484	24.024	42.676	1.00	0.00	C
ATOM	561	O	THR	34	37.211	22.831	42.809	1.00	0.00	O
ATOM	562	N	GLY	35	38.078	24.486	41.569	1.00	0.00	N
ATOM	563	H	GLY	35	38.382	25.448	41.518	1.00	0.00	H
ATOM	564	CA	GLY	35	38.706	23.556	40.601	1.00	0.00	C
ATOM	565	HA2	GLY	35	39.566	23.996	40.095	1.00	0.00	H
ATOM	566	HA3	GLY	35	39.163	22.691	41.081	1.00	0.00	H
ATOM	567	C	GLY	35	37.649	23.119	39.485	1.00	0.00	C
ATOM	568	O	GLY	35	37.898	22.152	38.769	1.00	0.00	O
ATOM	569	N	ASP	36	36.507	23.773	39.338	1.00	0.00	N
ATOM	570	H	ASP	36	36.384	24.609	39.891	1.00	0.00	H
ATOM	571	CA	ASP	36	35.693	23.606	38.064	1.00	0.00	C
ATOM	572	HA	ASP	36	35.665	22.573	37.718	1.00	0.00	H
ATOM	573	CB	ASP	36	34.249	24.004	38.412	1.00	0.00	C
ATOM	574	HB2	ASP	36	34.374	24.986	38.868	1.00	0.00	H
ATOM	575	HB3	ASP	36	33.567	23.978	37.562	1.00	0.00	H
ATOM	576	CG	ASP	36	33.660	23.124	39.456	1.00	0.00	C
ATOM	577	OD1	ASP	36	32.779	23.489	40.276	1.00	0.00	O
ATOM	578	OD2	ASP	36	34.036	21.920	39.383	1.00	0.00	O
ATOM	579	C	ASP	36	36.160	24.428	36.872	1.00	0.00	C
ATOM	580	O	ASP	36	36.452	25.633	36.927	1.00	0.00	O
ATOM	581	N	VAL	37	36.120	23.845	35.703	1.00	0.00	N
ATOM	582	H	VAL	37	35.817	22.884	35.655	1.00	0.00	H

ATOM	583	CA	VAL	37	36.741	24.345	34.528	1.00	0.00	C
ATOM	584	HA	VAL	37	37.468	25.149	34.643	1.00	0.00	H
ATOM	585	CB	VAL	37	37.642	23.251	33.880	1.00	0.00	C
ATOM	586	HB	VAL	37	37.011	22.378	33.709	1.00	0.00	H
ATOM	587	CG1	VAL	37	38.128	23.629	32.496	1.00	0.00	C
ATOM	588	HG11	VAL	37	37.314	23.855	31.807	1.00	0.00	H
ATOM	589	HG12	VAL	37	38.861	24.436	32.508	1.00	0.00	H
ATOM	590	HG13	VAL	37	38.625	22.802	31.989	1.00	0.00	H
ATOM	591	CG2	VAL	37	38.800	22.808	34.765	1.00	0.00	C
ATOM	592	HG21	VAL	37	38.431	22.337	35.677	1.00	0.00	H
ATOM	593	HG22	VAL	37	39.201	21.942	34.240	1.00	0.00	H
ATOM	594	HG23	VAL	37	39.533	23.592	34.950	1.00	0.00	H
ATOM	595	C	VAL	37	35.674	24.790	33.495	1.00	0.00	C
ATOM	596	O	VAL	37	34.871	23.977	33.065	1.00	0.00	O
ATOM	597	N	LEU	38	35.761	26.049	32.990	1.00	0.00	N
ATOM	598	H	LEU	38	36.504	26.653	33.311	1.00	0.00	H
ATOM	599	CA	LEU	38	34.990	26.669	31.996	1.00	0.00	C
ATOM	600	HA	LEU	38	34.105	26.070	31.779	1.00	0.00	H
ATOM	601	CB	LEU	38	34.430	28.016	32.580	1.00	0.00	C
ATOM	602	HB2	LEU	38	35.251	28.619	32.966	1.00	0.00	H
ATOM	603	HB3	LEU	38	34.051	28.607	31.746	1.00	0.00	H
ATOM	604	CG	LEU	38	33.315	28.100	33.607	1.00	0.00	C
ATOM	605	HG	LEU	38	32.434	27.633	33.166	1.00	0.00	H
ATOM	606	CD1	LEU	38	33.672	27.468	34.960	1.00	0.00	C
ATOM	607	HD11	LEU	38	33.719	26.386	34.840	1.00	0.00	H
ATOM	608	HD12	LEU	38	34.694	27.716	35.244	1.00	0.00	H
ATOM	609	HD13	LEU	38	32.949	27.816	35.697	1.00	0.00	H
ATOM	610	CD2	LEU	38	33.013	29.537	33.967	1.00	0.00	C
ATOM	611	HD21	LEU	38	32.151	29.579	34.632	1.00	0.00	H
ATOM	612	HD22	LEU	38	33.891	29.946	34.468	1.00	0.00	H
ATOM	613	HD23	LEU	38	32.834	30.194	33.116	1.00	0.00	H
ATOM	614	C	LEU	38	35.925	26.917	30.772	1.00	0.00	C
ATOM	615	O	LEU	38	37.133	26.908	30.966	1.00	0.00	O
ATOM	616	N	ARG	39	35.397	27.299	29.617	1.00	0.00	N
ATOM	617	H	ARG	39	34.388	27.279	29.606	1.00	0.00	H
ATOM	618	CA	ARG	39	36.022	27.969	28.524	1.00	0.00	C
ATOM	619	HA	ARG	39	37.011	28.316	28.827	1.00	0.00	H
ATOM	620	CB	ARG	39	36.139	26.976	27.315	1.00	0.00	C
ATOM	621	HB2	ARG	39	35.192	26.614	26.917	1.00	0.00	H
ATOM	622	HB3	ARG	39	36.500	27.591	26.490	1.00	0.00	H
ATOM	623	CG	ARG	39	37.045	25.776	27.595	1.00	0.00	C
ATOM	624	HG2	ARG	39	37.978	26.107	28.051	1.00	0.00	H
ATOM	625	HG3	ARG	39	36.431	25.265	28.337	1.00	0.00	H
ATOM	626	CD	ARG	39	37.331	24.881	26.402	1.00	0.00	C
ATOM	627	HD2	ARG	39	37.781	25.429	25.574	1.00	0.00	H
ATOM	628	HD3	ARG	39	38.080	24.130	26.655	1.00	0.00	H
ATOM	629	NE	ARG	39	36.087	24.260	25.966	1.00	0.00	N
ATOM	630	HE	ARG	39	35.201	24.640	26.269	1.00	0.00	H
ATOM	631	CZ	ARG	39	36.039	23.056	25.371	1.00	0.00	C
ATOM	632	NH1	ARG	39	37.057	22.347	25.052	1.00	0.00	N
ATOM	633	HH11	ARG	39	37.949	22.819	25.021	1.00	0.00	H
ATOM	634	HH12	ARG	39	36.896	21.587	24.405	1.00	0.00	H
ATOM	635	NH2	ARG	39	34.935	22.455	25.176	1.00	0.00	N
ATOM	636	HH21	ARG	39	34.898	21.538	24.753	1.00	0.00	H
ATOM	637	HH22	ARG	39	34.164	22.860	25.689	1.00	0.00	H
ATOM	638	C	ARG	39	35.281	29.251	28.232	1.00	0.00	C
ATOM	639	O	ARG	39	34.105	29.414	28.551	1.00	0.00	O
ATOM	640	N	VAL	40	35.884	30.009	27.345	1.00	0.00	N
ATOM	641	H	VAL	40	36.836	29.792	27.086	1.00	0.00	H
ATOM	642	CA	VAL	40	35.366	31.224	26.571	1.00	0.00	C
ATOM	643	HA	VAL	40	34.284	31.102	26.609	1.00	0.00	H
ATOM	644	CB	VAL	40	35.643	32.569	27.317	1.00	0.00	C
ATOM	645	HB	VAL	40	35.167	33.382	26.768	1.00	0.00	H

ATOM	646	CG1	VAL	40	35.112	32.558	28.668	1.00	0.00	C
ATOM	647	HG11	VAL	40	35.534	31.727	29.233	1.00	0.00	H
ATOM	648	HG12	VAL	40	35.392	33.518	29.101	1.00	0.00	H
ATOM	649	HG13	VAL	40	34.033	32.438	28.568	1.00	0.00	H
ATOM	650	CG2	VAL	40	37.163	33.029	27.315	1.00	0.00	C
ATOM	651	HG21	VAL	40	37.818	32.158	27.274	1.00	0.00	H
ATOM	652	HG22	VAL	40	37.454	33.616	26.444	1.00	0.00	H
ATOM	653	HG23	VAL	40	37.418	33.498	28.266	1.00	0.00	H
ATOM	654	C	VAL	40	35.952	31.286	25.154	1.00	0.00	C
ATOM	655	O	VAL	40	37.139	30.993	24.987	1.00	0.00	O
ATOM	656	N	GLY	41	35.194	31.652	24.222	1.00	0.00	N
ATOM	657	H	GLY	41	34.343	32.086	24.551	1.00	0.00	H
ATOM	658	CA	GLY	41	35.530	31.747	22.820	1.00	0.00	C
ATOM	659	HA2	GLY	41	36.574	32.015	22.662	1.00	0.00	H
ATOM	660	HA3	GLY	41	35.209	30.826	22.334	1.00	0.00	H
ATOM	661	C	GLY	41	34.557	32.720	22.147	1.00	0.00	C
ATOM	662	O	GLY	41	33.398	32.869	22.593	1.00	0.00	O
ATOM	663	N	ARG	42	35.013	33.210	21.013	1.00	0.00	N
ATOM	664	H	ARG	42	35.942	32.969	20.701	1.00	0.00	H
ATOM	665	CA	ARG	42	34.151	33.903	19.989	1.00	0.00	C
ATOM	666	HA	ARG	42	33.189	33.393	19.927	1.00	0.00	H
ATOM	667	CB	ARG	42	33.850	35.344	20.467	1.00	0.00	C
ATOM	668	HB2	ARG	42	32.975	35.744	19.957	1.00	0.00	H
ATOM	669	HB3	ARG	42	33.519	35.122	21.481	1.00	0.00	H
ATOM	670	CG	ARG	42	35.013	36.419	20.491	1.00	0.00	C
ATOM	671	HG2	ARG	42	35.841	36.079	21.114	1.00	0.00	H
ATOM	672	HG3	ARG	42	35.351	36.607	19.472	1.00	0.00	H
ATOM	673	CD	ARG	42	34.489	37.695	21.128	1.00	0.00	C
ATOM	674	HD2	ARG	42	33.740	38.157	20.484	1.00	0.00	H
ATOM	675	HD3	ARG	42	34.115	37.482	22.130	1.00	0.00	H
ATOM	676	NE	ARG	42	35.678	38.647	21.347	1.00	0.00	N
ATOM	677	HE	ARG	42	36.047	39.234	20.613	1.00	0.00	H
ATOM	678	CZ	ARG	42	36.110	39.093	22.488	1.00	0.00	C
ATOM	679	NH1	ARG	42	35.782	38.564	23.650	1.00	0.00	N
ATOM	680	HH11	ARG	42	36.235	39.052	24.409	1.00	0.00	H
ATOM	681	HH12	ARG	42	35.335	37.660	23.710	1.00	0.00	H
ATOM	682	NH2	ARG	42	36.902	40.082	22.424	1.00	0.00	N
ATOM	683	HH21	ARG	42	37.183	40.552	21.575	1.00	0.00	H
ATOM	684	HH22	ARG	42	37.141	40.502	23.312	1.00	0.00	H
ATOM	685	C	ARG	42	34.695	33.925	18.509	1.00	0.00	C
ATOM	686	O	ARG	42	34.265	34.840	17.779	1.00	0.00	O
ATOM	687	N	PHE	43	35.539	33.008	18.044	1.00	0.00	N
ATOM	688	H	PHE	43	35.941	32.359	18.704	1.00	0.00	H
ATOM	689	CA	PHE	43	36.141	32.995	16.727	1.00	0.00	C
ATOM	690	HA	PHE	43	35.639	33.521	15.914	1.00	0.00	H
ATOM	691	CB	PHE	43	37.539	33.693	16.830	1.00	0.00	C
ATOM	692	HB2	PHE	43	38.296	33.122	17.367	1.00	0.00	H
ATOM	693	HB3	PHE	43	37.907	33.929	15.832	1.00	0.00	H
ATOM	694	CG	PHE	43	37.547	35.029	17.510	1.00	0.00	C
ATOM	695	CD1	PHE	43	37.165	36.145	16.767	1.00	0.00	C
ATOM	696	HD1	PHE	43	36.688	36.092	15.799	1.00	0.00	H
ATOM	697	CE1	PHE	43	37.343	37.432	17.317	1.00	0.00	C
ATOM	698	HE1	PHE	43	37.042	38.343	16.823	1.00	0.00	H
ATOM	699	CZ	PHE	43	37.978	37.591	18.567	1.00	0.00	C
ATOM	700	HZ	PHE	43	38.070	38.594	18.956	1.00	0.00	H
ATOM	701	CE2	PHE	43	38.368	36.487	19.313	1.00	0.00	C
ATOM	702	HE2	PHE	43	38.865	36.583	20.267	1.00	0.00	H
ATOM	703	CD2	PHE	43	38.164	35.178	18.742	1.00	0.00	C
ATOM	704	HD2	PHE	43	38.495	34.291	19.264	1.00	0.00	H
ATOM	705	C	PHE	43	36.349	31.551	16.209	1.00	0.00	C
ATOM	706	O	PHE	43	36.338	30.621	16.952	1.00	0.00	O
ATOM	707	N	GLU	44	36.671	31.446	14.897	1.00	0.00	N
ATOM	708	H	GLU	44	36.585	32.215	14.250	1.00	0.00	H
ATOM	709	CA	GLU							

				44	36.794	30.064	14.362	1.00	0.00	C
ATOM	710	HA	GLU	44	35.927	29.488	14.682	1.00	0.00	H
ATOM	711	CB	GLU	44	36.860	30.315	12.872	1.00	0.00	C
ATOM	712	HB2	GLU	44	35.935	30.829	12.613	1.00	0.00	H
ATOM	713	HB3	GLU	44	37.786	30.862	12.696	1.00	0.00	H
ATOM	714	CG	GLU	44	37.040	29.134	11.883	1.00	0.00	C
ATOM	715	HG2	GLU	44	37.975	28.575	11.911	1.00	0.00	H
ATOM	716	HG3	GLU	44	36.278	28.400	12.140	1.00	0.00	H
ATOM	717	CD	GLU	44	36.688	29.614	10.469	1.00	0.00	C
ATOM	718	OE1	GLU	44	35.815	28.907	9.881	1.00	0.00	O
ATOM	719	OE2	GLU	44	37.070	30.680	9.889	1.00	0.00	O
ATOM	720	C	GLU	44	38.060	29.240	14.714	1.00	0.00	C
ATOM	721	O	GLU	44	38.025	28.028	14.550	1.00	0.00	O
ATOM	722	N	ASP	45	39.131	29.892	15.166	1.00	0.00	N
ATOM	723	H	ASP	45	38.993	30.883	15.306	1.00	0.00	H
ATOM	724	CA	ASP	45	40.262	29.231	15.769	1.00	0.00	C
ATOM	725	HA	ASP	45	40.604	28.550	14.989	1.00	0.00	H
ATOM	726	CB	ASP	45	41.325	30.313	16.002	1.00	0.00	C
ATOM	727	HB2	ASP	45	41.445	30.904	15.094	1.00	0.00	H
ATOM	728	HB3	ASP	45	40.983	31.027	16.751	1.00	0.00	H
ATOM	729	CG	ASP	45	42.626	29.709	16.464	1.00	0.00	C
ATOM	730	OD1	ASP	45	42.935	28.543	16.111	1.00	0.00	O
ATOM	731	OD2	ASP	45	43.468	30.445	17.004	1.00	0.00	O
ATOM	732	C	ASP	45	40.037	28.411	17.077	1.00	0.00	C
ATOM	733	O	ASP	45	39.771	28.997	18.134	1.00	0.00	O
ATOM	734	N	ASP	46	40.178	27.059	16.998	1.00	0.00	N
ATOM	735	H	ASP	46	40.521	26.660	16.136	1.00	0.00	H
ATOM	736	CA	ASP	46	39.905	25.998	18.030	1.00	0.00	C
ATOM	737	HA	ASP	46	39.837	25.011	17.571	1.00	0.00	H
ATOM	738	CB	ASP	46	41.112	25.944	19.040	1.00	0.00	C
ATOM	739	HB2	ASP	46	41.159	26.882	19.594	1.00	0.00	H
ATOM	740	HB3	ASP	46	40.941	25.061	19.655	1.00	0.00	H
ATOM	741	CG	ASP	46	42.451	25.748	18.385	1.00	0.00	C
ATOM	742	OD1	ASP	46	42.638	25.070	17.334	1.00	0.00	O
ATOM	743	OD2	ASP	46	43.455	26.350	18.809	1.00	0.00	O
ATOM	744	C	ASP	46	38.532	26.123	18.769	1.00	0.00	C
ATOM	745	O	ASP	46	38.180	25.395	19.689	1.00	0.00	O
ATOM	746	N	GLY	47	37.709	27.122	18.366	1.00	0.00	N
ATOM	747	H	GLY	47	38.047	27.784	17.681	1.00	0.00	H
ATOM	748	CA	GLY	47	36.560	27.576	19.154	1.00	0.00	C
ATOM	749	HA2	GLY	47	35.802	28.173	18.645	1.00	0.00	H
ATOM	750	HA3	GLY	47	35.995	26.684	19.423	1.00	0.00	H
ATOM	751	C	GLY	47	36.802	28.441	20.379	1.00	0.00	C
ATOM	752	O	GLY	47	35.965	29.269	20.706	1.00	0.00	O
ATOM	753	N	TYR	48	37.920	28.405	21.099	1.00	0.00	N
ATOM	754	H	TYR	48	38.671	27.802	20.793	1.00	0.00	H
ATOM	755	CA	TYR	48	38.164	29.105	22.443	1.00	0.00	C
ATOM	756	HA	TYR	48	37.464	29.940	22.477	1.00	0.00	H
ATOM	757	CB	TYR	48	37.928	28.053	23.589	1.00	0.00	C
ATOM	758	HB2	TYR	48	38.849	27.513	23.809	1.00	0.00	H
ATOM	759	HB3	TYR	48	37.801	28.696	24.460	1.00	0.00	H
ATOM	760	CG	TYR	48	36.704	27.100	23.493	1.00	0.00	C
ATOM	761	CD1	TYR	48	35.472	27.664	23.851	1.00	0.00	C
ATOM	762	HD1	TYR	48	35.391	28.680	24.206	1.00	0.00	H
ATOM	763	CE1	TYR	48	34.241	26.987	23.623	1.00	0.00	C
ATOM	764	HE1	TYR	48	33.371	27.516	23.982	1.00	0.00	H
ATOM	765	CZ	TYR	48	34.316	25.700	23.006	1.00	0.00	C
ATOM	766	OH	TYR	48	33.237	24.982	22.836	1.00	0.00	O
ATOM	767	HH	TYR	48	33.272	24.101	22.457	1.00	0.00	H
ATOM	768	CE2	TYR	48	35.543	25.199	22.520	1.00	0.00	C
ATOM	769	HE2	TYR	48	35.531	24.262	21.982	1.00	0.00	H
ATOM	770	CD2	TYR	48	36.752	25.822	22.802	1.00	0.00	C
ATOM	771	HD2	TYR	48	37.666	25.426	22.383	1.00	0.00	H
ATOM	772	C	TYR	48	39.517	29.711	22.456	1.00	0.00	C

ATOM	773	O	TYR	48	40.404	29.225	21.807	1.00	0.00	O
ATOM	774	N	PHE	49	39.642	30.889	23.063	1.00	0.00	N
ATOM	775	H	PHE	49	38.842	31.336	23.486	1.00	0.00	H
ATOM	776	CA	PHE	49	40.922	31.552	23.300	1.00	0.00	C
ATOM	777	HA	PHE	49	41.748	31.029	22.818	1.00	0.00	H
ATOM	778	CB	PHE	49	40.941	32.966	22.675	1.00	0.00	C
ATOM	779	HB2	PHE	49	41.948	33.371	22.572	1.00	0.00	H
ATOM	780	HB3	PHE	49	40.622	32.936	21.633	1.00	0.00	H
ATOM	781	CG	PHE	49	40.083	34.004	23.306	1.00	0.00	C
ATOM	782	CD1	PHE	49	38.751	34.208	22.896	1.00	0.00	C
ATOM	783	HD1	PHE	49	38.311	33.695	22.053	1.00	0.00	H
ATOM	784	CE1	PHE	49	37.856	34.917	23.719	1.00	0.00	C
ATOM	785	HE1	PHE	49	36.793	34.857	23.539	1.00	0.00	H
ATOM	786	CZ	PHE	49	38.349	35.481	24.887	1.00	0.00	C
ATOM	787	HZ	PHE	49	37.650	35.942	25.569	1.00	0.00	H
ATOM	788	CE2	PHE	49	39.711	35.426	25.284	1.00	0.00	C
ATOM	789	HE2	PHE	49	39.979	35.722	26.288	1.00	0.00	H
ATOM	790	CD2	PHE	49	40.582	34.664	24.456	1.00	0.00	C
ATOM	791	HD2	PHE	49	41.619	34.595	24.752	1.00	0.00	H
ATOM	792	C	PHE	49	41.372	31.491	24.756	1.00	0.00	C
ATOM	793	O	PHE	49	42.410	32.159	24.991	1.00	0.00	O
ATOM	794	N	CYX	50	40.594	30.922	25.726	1.00	0.00	N
ATOM	795	H	CYX	50	39.661	30.695	25.412	1.00	0.00	H
ATOM	796	CA	CYX	50	40.938	30.681	27.119	1.00	0.00	C
ATOM	797	HA	CYX	50	41.946	30.346	27.363	1.00	0.00	H
ATOM	798	CB	CYX	50	40.580	32.005	27.901	1.00	0.00	C
ATOM	799	HB2	CYX	50	39.894	32.619	27.316	1.00	0.00	H
ATOM	800	HB3	CYX	50	40.127	31.756	28.861	1.00	0.00	H
ATOM	801	SG	CYX	50	42.009	33.106	28.125	1.00	0.00	S
ATOM	802	C	CYX	50	40.120	29.535	27.630	1.00	0.00	C
ATOM	803	O	CYX	50	38.877	29.605	27.591	1.00	0.00	O
ATOM	804	N	THR	51	40.795	28.488	28.167	1.00	0.00	N
ATOM	805	H	THR	51	41.785	28.441	27.968	1.00	0.00	H
ATOM	806	CA	THR	51	40.400	27.598	29.291	1.00	0.00	C
ATOM	807	HA	THR	51	39.352	27.345	29.133	1.00	0.00	H
ATOM	808	CB	THR	51	41.230	26.234	29.232	1.00	0.00	C
ATOM	809	HB	THR	51	42.263	26.546	29.383	1.00	0.00	H
ATOM	810	CG2	THR	51	40.812	25.311	30.413	1.00	0.00	C
ATOM	811	HG21	THR	51	39.734	25.156	30.376	1.00	0.00	H
ATOM	812	HG22	THR	51	41.260	24.365	30.107	1.00	0.00	H
ATOM	813	HG23	THR	51	41.230	25.691	31.345	1.00	0.00	H
ATOM	814	OG1	THR	51	41.000	25.567	27.953	1.00	0.00	O
ATOM	815	HG1	THR	51	40.897	26.167	27.211	1.00	0.00	H
ATOM	816	C	THR	51	40.629	28.314	30.687	1.00	0.00	C
ATOM	817	O	THR	51	41.637	29.053	30.887	1.00	0.00	O
ATOM	818	N	ILE	52	39.696	28.133	31.573	1.00	0.00	N
ATOM	819	H	ILE	52	38.935	27.498	31.381	1.00	0.00	H
ATOM	820	CA	ILE	52	39.750	28.835	32.875	1.00	0.00	C
ATOM	821	HA	ILE	52	40.760	29.212	33.031	1.00	0.00	H
ATOM	822	CB	ILE	52	38.723	30.007	32.858	1.00	0.00	C
ATOM	823	HB	ILE	52	37.675	29.724	32.955	1.00	0.00	H
ATOM	824	CG2	ILE	52	38.801	30.982	34.030	1.00	0.00	C
ATOM	825	HG21	ILE	52	37.916	31.615	33.959	1.00	0.00	H
ATOM	826	HG22	ILE	52	38.995	30.404	34.935	1.00	0.00	H
ATOM	827	HG23	ILE	52	39.701	31.582	33.901	1.00	0.00	H
ATOM	828	CG1	ILE	52	38.736	30.855	31.500	1.00	0.00	C
ATOM	829	HG12	ILE	52	39.787	31.045	31.283	1.00	0.00	H
ATOM	830	HG13	ILE	52	38.369	30.196	30.713	1.00	0.00	H
ATOM	831	CD1	ILE	52	37.915	32.191	31.335	1.00	0.00	C
ATOM	832	HD11	ILE	52	38.362	33.016	31.890	1.00	0.00	H
ATOM	833	HD12	ILE	52	37.578	32.435	30.328	1.00	0.00	H
ATOM	834	HD13	ILE	52	36.964	32.033				
31.844	1.00	0.00			H					
ATOM	835	C	ILE	52	39.423	27.845	33.931	1.00	0.00	C

ATOM	836	O	ILE	52	38.465	27.061	33.745	1.00	0.00	O
ATOM	837	N	GLU	53	39.920	28.016	35.151	1.00	0.00	N
ATOM	838	H	GLU	53	40.559	28.748	35.427	1.00	0.00	H
ATOM	839	CA	GLU	53	39.489	27.188	36.281	1.00	0.00	C
ATOM	840	HA	GLU	53	38.544	26.702	36.038	1.00	0.00	H
ATOM	841	CB	GLU	53	40.445	25.995	36.394	1.00	0.00	C
ATOM	842	HB2	GLU	53	40.518	25.519	35.416	1.00	0.00	H
ATOM	843	HB3	GLU	53	41.470	26.351	36.494	1.00	0.00	H
ATOM	844	CG	GLU	53	39.995	24.902	37.376	1.00	0.00	C
ATOM	845	HG2	GLU	53	39.882	25.361	38.358	1.00	0.00	H
ATOM	846	HG3	GLU	53	39.057	24.388	37.164	1.00	0.00	H
ATOM	847	CD	GLU	53	41.132	23.902	37.516	1.00	0.00	C
ATOM	848	OE1	GLU	53	42.181	24.269	38.081	1.00	0.00	O
ATOM	849	OE2	GLU	53	41.094	22.736	37.131	1.00	0.00	O
ATOM	850	C	GLU	53	39.296	28.075	37.541	1.00	0.00	C
ATOM	851	O	GLU	53	39.990	29.114	37.745	1.00	0.00	O
ATOM	852	N	VAL	54	38.240	27.762	38.279	1.00	0.00	N
ATOM	853	H	VAL	54	37.608	27.062	37.915	1.00	0.00	H
ATOM	854	CA	VAL	54	37.898	28.416	39.614	1.00	0.00	C
ATOM	855	HA	VAL	54	37.881	29.487	39.406	1.00	0.00	H
ATOM	856	CB	VAL	54	36.508	27.988	40.023	1.00	0.00	C
ATOM	857	HB	VAL	54	36.492	26.897	40.017	1.00	0.00	H
ATOM	858	CG1	VAL	54	36.058	28.462	41.405	1.00	0.00	C
ATOM	859	HG11	VAL	54	36.686	28.035	42.187	1.00	0.00	H
ATOM	860	HG12	VAL	54	36.107	29.542	41.541	1.00	0.00	H
ATOM	861	HG13	VAL	54	35.034	28.177	41.651	1.00	0.00	H
ATOM	862	CG2	VAL	54	35.378	28.392	39.053	1.00	0.00	C
ATOM	863	HG21	VAL	54	35.505	27.823	38.132	1.00	0.00	H
ATOM	864	HG22	VAL	54	34.407	27.938	39.255	1.00	0.00	H
ATOM	865	HG23	VAL	54	35.289	29.467	38.901	1.00	0.00	H
ATOM	866	C	VAL	54	38.965	28.048	40.615	1.00	0.00	C
ATOM	867	O	VAL	54	39.212	26.898	40.860	1.00	0.00	O
ATOM	868	N	THR	55	39.527	29.106	41.230	1.00	0.00	N
ATOM	869	H	THR	55	39.157	30.004	40.953	1.00	0.00	H
ATOM	870	CA	THR	55	40.792	29.078	42.075	1.00	0.00	C
ATOM	871	HA	THR	55	41.287	28.137	41.831	1.00	0.00	H
ATOM	872	CB	THR	55	41.823	30.180	41.780	1.00	0.00	C
ATOM	873	HB	THR	55	42.675	30.118	42.457	1.00	0.00	H
ATOM	874	CG2	THR	55	42.343	30.163	40.334	1.00	0.00	C
ATOM	875	HG21	THR	55	41.493	30.113	39.653	1.00	0.00	H
ATOM	876	HG22	THR	55	43.073	30.951	40.148	1.00	0.00	H
ATOM	877	HG23	THR	55	42.888	29.229	40.203	1.00	0.00	H
ATOM	878	OG1	THR	55	41.231	31.479	41.935	1.00	0.00	O
ATOM	879	HG1	THR	55	41.379	31.709	42.855	1.00	0.00	H
ATOM	880	C	THR	55	40.457	28.928	43.584	1.00	0.00	C
ATOM	881	O	THR	55	41.135	28.214	44.258	1.00	0.00	O
ATOM	882	N	ALA	56	39.406	29.681	44.019	1.00	0.00	N
ATOM	883	H	ALA	56	38.970	30.390	43.446	1.00	0.00	H
ATOM	884	CA	ALA	56	38.860	29.604	45.331	1.00	0.00	C
ATOM	885	HA	ALA	56	38.953	28.632	45.819	1.00	0.00	H
ATOM	886	CB	ALA	56	39.726	30.581	46.149	1.00	0.00	C
ATOM	887	HB1	ALA	56	39.561	31.590	45.770	1.00	0.00	H
ATOM	888	HB2	ALA	56	39.358	30.429	47.163	1.00	0.00	H
ATOM	889	HB3	ALA	56	40.778	30.305	46.069	1.00	0.00	H
ATOM	890	C	ALA	56	37.349	29.910	45.473	1.00	0.00	C
ATOM	891	O	ALA	56	36.786	30.533	44.596	1.00	0.00	O
ATOM	892	N	THR	57	36.709	29.336	46.522	1.00	0.00	N
ATOM	893	H	THR	57	37.316	28.800	47.125	1.00	0.00	H
ATOM	894	CA	THR	57	35.293	29.567	46.777	1.00	0.00	C
ATOM	895	HA	THR	57	35.048	30.584	46.470	1.00	0.00	H
ATOM	896	CB	THR	57	34.286	28.567	46.166	1.00	0.00	C
ATOM	897	HB	THR	57	33.263	28.726	46.508	1.00	0.00	H
ATOM	898	CG2	THR	57	34.225	28.637	44.663	1.00	0.00	C
ATOM	899	HG21	THR	57	33.824	27.722	44.227	1.00	0.00	H

ATOM	900	HG22	THR	57	33.673	29.471	44.229	1.00	0.00	H
ATOM	901	HG23	THR	57	35.224	28.775	44.249	1.00	0.00	H
ATOM	902	OG1	THR	57	34.788	27.208	46.487	1.00	0.00	O
ATOM	903	HG1	THR	57	35.728	27.301	46.319	1.00	0.00	H
ATOM	904	C	THR	57	35.056	29.561	48.299	1.00	0.00	C
ATOM	905	O	THR	57	35.630	28.744	49.015	1.00	0.00	O
ATOM	906	N	SER	58	34.216	30.491	48.740	1.00	0.00	N
ATOM	907	H	SER	58	33.612	31.045	48.150	1.00	0.00	H
ATOM	908	CA	SER	58	34.044	30.695	50.217	1.00	0.00	C
ATOM	909	HA	SER	58	34.063	29.712	50.688	1.00	0.00	H
ATOM	910	CB	SER	58	35.236	31.483	50.667	1.00	0.00	C
ATOM	911	HB2	SER	58	35.093	31.776	51.708	1.00	0.00	H
ATOM	912	HB3	SER	58	36.082	30.797	50.625	1.00	0.00	H
ATOM	913	OG	SER	58	35.380	32.684	50.093	1.00	0.00	O
ATOM	914	HG	SER	58	35.855	32.521	49.273	1.00	0.00	H
ATOM	915	C	SER	58	32.772	31.354	50.659	1.00	0.00	C
ATOM	916	O	SER	58	32.133	32.105	49.909	1.00	0.00	O
ATOM	917	N	THR	59	32.229	30.948	51.843	1.00	0.00	N
ATOM	918	H	THR	59	32.829	30.230	52.223	1.00	0.00	H
ATOM	919	CA	THR	59	30.886	31.378	52.377	1.00	0.00	C
ATOM	920	HA	THR	59	30.367	31.942	51.602	1.00	0.00	H
ATOM	921	CB	THR	59	29.892	30.209	52.562	1.00	0.00	C
ATOM	922	HB	THR	59	29.832	29.679	51.612	1.00	0.00	H
ATOM	923	CG2	THR	59	30.255	29.144	53.687	1.00	0.00	C
ATOM	924	HG21	THR	59	29.426	28.540	54.054	1.00	0.00	H
ATOM	925	HG22	THR	59	31.046	28.521	53.270	1.00	0.00	H
ATOM	926	HG23	THR	59	30.499	29.690	54.599	1.00	0.00	H
ATOM	927	OG1	THR	59	28.584	30.668	52.846	1.00	0.00	O
ATOM	928	HG1	THR	59	27.969	30.170	52.303	1.00	0.00	H
ATOM	929	C	THR	59	30.960	32.293	53.618	1.00	0.00	C
ATOM	930	O	THR	59	31.858	32.052	54.437	1.00	0.00	O
ATOM	931	N	VAL	60	30.054	33.283	53.747	1.00	0.00	N
ATOM	932	H	VAL	60	29.293	33.298	53.084	1.00	0.00	H
ATOM	933	CA	VAL	60	30.393	34.559	54.288	1.00	0.00	C
ATOM	934	HA	VAL	60	31.196	34.551	55.025	1.00	0.00	H
ATOM	935	CB	VAL	60	31.123	35.397	53.155	1.00	0.00	C
ATOM	936	HB	VAL	60	30.592	35.224	52.218	1.00	0.00	H
ATOM	937	CG1	VAL	60	31.007	36.905	53.310	1.00	0.00	C
ATOM	938	HG11	VAL	60	31.631	37.439	52.592	1.00	0.00	H
ATOM	939	HG12	VAL	60	30.008	37.326	53.201	1.00	0.00	H
ATOM	940	HG13	VAL	60	31.384	37.128	54.308	1.00	0.00	H
ATOM	941	CG2	VAL	60	32.617	35.147	53.062	1.00	0.00	C
ATOM	942	HG21	VAL	60	32.787	35.688	52.131	1.00	0.00	H
ATOM	943	HG22	VAL	60	33.104	35.530	53.959	1.00	0.00	H
ATOM	944	HG23	VAL	60	32.875	34.108	52.858	1.00	0.00	H
ATOM	945	C	VAL	60	29.148	35.326	54.730	1.00	0.00	C
ATOM	946	O	VAL	60	28.162	35.495	53.974	1.00	0.00	O
ATOM	947	N	THR	61	29.262	35.822	55.988	1.00	0.00	N
ATOM	948	H	THR	61	30.178	35.671	56.388	1.00	0.00	H
ATOM	949	CA	THR	61	28.489	36.958	56.446	1.00	0.00	C
ATOM	950	HA	THR	61	27.559	37.118	55.902	1.00	0.00	H
ATOM	951	CB	THR	61	28.161	36.773	57.960	1.00	0.00	C
ATOM	952	HB	THR	61	27.667	37.719	58.180	1.00	0.00	H
ATOM	953	CG2	THR	61	27.256	35.576	58.226	1.00	0.00	C
ATOM	954	HG21	THR	61	26.409	35.564	57.542	1.00	0.00	H
ATOM	955	HG22	THR	61	27.833	34.658	58.116	1.00	0.00	H
ATOM	956	HG23	THR	61	26.818	35.670	59.220	1.00	0.00	H
ATOM	957	OG1	THR	61	29.351	36.739	58.682	1.00	0.00	O
ATOM	958	HG1	THR	61	29.185	37.173	59.523	1.00	0.00	H
ATOM	959	C	THR	61	29.290	38.287	56.200	1.00	0.00	C
ATOM	960	O	THR	61	30.489	38.262	56.177	1.00	0.00	O
ATOM	961	N	LEU	62	28.642	39.403	55.974	1.00	0.00	N
ATOM	962	H	LEU	62	27.637	39.459	55.894	1.00	0.00	H

ATOM	963	CA	LEU	62	29.301	40.588	55.406	1.00	0.00	C
ATOM	964	HA	LEU	62	29.727	40.352	54.430	1.00	0.00	H
ATOM	965	CB	LEU	62	28.222	41.702	55.274	1.00	0.00	C
ATOM	966	HB2	LEU	62	27.603	41.703	56.171	1.00	0.00	H
ATOM	967	HB3	LEU	62	28.590	42.718	55.125	1.00	0.00	H
ATOM	968	CG	LEU	62	27.251	41.411	54.168	1.00	0.00	C
ATOM	969	HG	LEU	62	26.656	40.550	54.476	1.00	0.00	H
ATOM	970	CD1	LEU	62	26.337	42.666	54.169	1.00	0.00	C
ATOM	971	HD11	LEU	62	25.898	42.903	55.138	1.00	0.00	H
ATOM	972	HD12	LEU	62	26.870	43.556	53.835	1.00	0.00	H
ATOM	973	HD13	LEU	62	25.525	42.447	53.475	1.00	0.00	H
ATOM	974	CD2	LEU	62	27.769	41.140	52.717	1.00	0.00	C
ATOM	975	HD21	LEU	62	27.014	41.233	51.937	1.00	0.00	H
ATOM	976	HD22	LEU	62	28.550	41.839	52.420	1.00	0.00	H
ATOM	977	HD23	LEU	62	28.130	40.114	52.645	1.00	0.00	H
ATOM	978	C	LEU	62	30.481	41.143	56.215	1.00	0.00	C
ATOM	979	O	LEU	62	31.476	41.683	55.686	1.00	0.00	O
ATOM	980	N	ASP	63	30.372	40.943	57.550	1.00	0.00	N
ATOM	981	H	ASP	63	29.513	40.458	57.766	1.00	0.00	H
ATOM	982	CA	ASP	63	31.297	41.394	58.596	1.00	0.00	C
ATOM	983	HA	ASP	63	31.433	42.464	58.437	1.00	0.00	H
ATOM	984	CB	ASP	63	30.606	41.159	60.029	1.00	0.00	C
ATOM	985	HB2	ASP	63	31.278	41.547	60.795	1.00	0.00	H
ATOM	986	HB3	ASP	63	29.630	41.639	60.098	1.00	0.00	H
ATOM	987	CG	ASP	63	30.301	39.709	60.352	1.00	0.00	C
ATOM	988	OD1	ASP	63	31.073	39.101	61.130	1.00	0.00	O
ATOM	989	OD2	ASP	63	29.204	39.250	59.984	1.00	0.00	O
ATOM	990	C	ASP	63	32.621	40.538	58.583	1.00	0.00	C
ATOM	991	O	ASP	63	33.621	40.892	59.181	1.00	0.00	O
ATOM	992	N	THR	64	32.545	39.482	57.734	1.00	0.00	N
ATOM	993	H	THR	64	31.626	39.323	57.346	1.00	0.00	H
ATOM	994	CA	THR	64	33.630	38.544	57.513	1.00	0.00	C
ATOM	995	HA	THR	64	34.514	38.875	58.059	1.00	0.00	H
ATOM	996	CB	THR	64	33.253	37.091	57.897	1.00	0.00	C
ATOM	997	HB	THR	64	34.129	36.468	57.720	1.00	0.00	H
ATOM	998	CG2	THR	64	33.012	37.009	59.404	1.00	0.00	C
ATOM	999	HG21	THR	64	31.966	37.223	59.625	1.00	0.00	H
ATOM	1000	HG22	THR	64	33.354	36.027	59.730	1.00	0.00	H
ATOM	1001	HG23	THR	64	33.694	37.777	59.772	1.00	0.00	H
ATOM	1002	OG1	THR	64	32.180	36.598	57.241	1.00	0.00	O
ATOM	1003	HG1	THR	64	31.668	37.344	56.920	1.00	0.00	H
ATOM	1004	C	THR	64	34.020	38.510	56.031	1.00	0.00	C
ATOM	1005	O	THR	64	34.816	37.654	55.693	1.00	0.00	O
ATOM	1006	N	LEU	65	33.468	39.380	55.142	1.00	0.00	N
ATOM	1007	H	LEU	65	32.708	39.990	55.406	1.00	0.00	H
ATOM	1008	CA	LEU	65	33.903	39.512	53.760	1.00	0.00	C
ATOM	1009	HA	LEU	65	34.167	38.519	53.395	1.00	0.00	H
ATOM	1010	CB	LEU	65	32.754	40.204	52.956	1.00	0.00	C
ATOM	1011	HB2	LEU	65	31.828	39.636	53.043	1.00	0.00	H
ATOM	1012	HB3	LEU	65	32.534	41.140	53.470	1.00	0.00	H
ATOM	1013	CG	LEU	65	33.175	40.376	51.436	1.00	0.00	C
ATOM	1014	HG	LEU	65	34.157	40.825	51.286	1.00	0.00	H
ATOM	1015	CD1	LEU	65	33.076	39.170	50.467	1.00	0.00	C
ATOM	1016	HD11	LEU	65	33.747	38.405	50.857	1.00	0.00	H
ATOM	1017	HD12	LEU	65	32.071	38.750	50.413	1.00	0.00	H
ATOM	1018	HD13	LEU	65	33.491	39.515	49.520	1.00	0.00	H
ATOM	1019	CD2	LEU	65	32.168	41.406	50.819	1.00	0.00	C
ATOM	1020	HD21	LEU	65	32.055	42.365	51.324	1.00	0.00	H
ATOM	1021	HD22	LEU	65	32.425	41.478	49.762	1.00	0.00	H
ATOM	1022	HD23	LEU	65	31.184	40.937	50.832	1.00	0.00	H
ATOM	1023	C	LEU	65	35.123	40.452	53.747	1.00	0.00	C
ATOM	1024	O	LEU	65	35.039	41.550	54.269	1.00	0.00	O
ATOM	1025	N	THR	66	36.295	40.110	53.124	1.00	0.00	N
ATOM	1026	H	THR	66	36.263	39.283	52.545	1.00	0.00	H

ATOM	1027	CA	THR	66	37.590	40.883	53.035	1.00	0.00	C
ATOM	1028	HA	THR	66	37.790	41.356	53.996	1.00	0.00	H
ATOM	1029	CB	THR	66	38.846	40.003	52.660	1.00	0.00	C
ATOM	1030	HB	THR	66	38.638	39.575	51.679	1.00	0.00	H
ATOM	1031	CG2	THR	66	40.231	40.576	52.446	1.00	0.00	C
ATOM	1032	HG21	THR	66	40.982	39.788	52.388	1.00	0.00	H
ATOM	1033	HG22	THR	66	40.276	41.232	51.577	1.00	0.00	H
ATOM	1034	HG23	THR	66	40.511	41.130	53.342	1.00	0.00	H
ATOM	1035	OG1	THR	66	38.945	38.929	53.632	1.00	0.00	O
ATOM	1036	HG1	THR	66	38.296	38.302	53.304	1.00	0.00	H
ATOM	1037	C	THR	66	37.540	41.986	52.022	1.00	0.00	C
ATOM	1038	O	THR	66	36.616	41.900	51.148	1.00	0.00	O
ATOM	1039	N	GLU	67	38.324	43.111	51.976	1.00	0.00	N
ATOM	1040	H	GLU	67	39.173	43.040	52.518	1.00	0.00	H
ATOM	1041	CA	GLU	67	38.255	44.250	51.067	1.00	0.00	C
ATOM	1042	HA	GLU	67	37.176	44.386	51.130	1.00	0.00	H
ATOM	1043	CB	GLU	67	38.967	45.546	51.582	1.00	0.00	C
ATOM	1044	HB2	GLU	67	38.619	46.268	50.844	1.00	0.00	H
ATOM	1045	HB3	GLU	67	38.690	45.893	52.577	1.00	0.00	H
ATOM	1046	CG	GLU	67	40.507	45.464	51.570	1.00	0.00	C
ATOM	1047	HG2	GLU	67	40.697	44.842	50.694	1.00	0.00	H
ATOM	1048	HG3	GLU	67	40.874	46.468	51.358	1.00	0.00	H
ATOM	1049	CD	GLU	67	41.134	44.854	52.879	1.00	0.00	C
ATOM	1050	OE1	GLU	67	41.851	45.524	53.667	1.00	0.00	O
ATOM	1051	OE2	GLU	67	40.834	43.690	53.111	1.00	0.00	O
ATOM	1052	C	GLU	67	38.471	43.867	49.584	1.00	0.00	C
ATOM	1053	O	GLU	67	38.169	44.676	48.722	1.00	0.00	O
ATOM	1054	N	LYS	68	39.016	42.674	49.316	1.00	0.00	N
ATOM	1055	H	LYS	68	39.118	42.054	50.108	1.00	0.00	H
ATOM	1056	CA	LYS	68	39.574	42.266	48.027	1.00	0.00	C
ATOM	1057	HA	LYS	68	40.581	42.666	47.909	1.00	0.00	H
ATOM	1058	CB	LYS	68	39.843	40.771	48.073	1.00	0.00	C
ATOM	1059	HB2	LYS	68	40.297	40.467	49.017	1.00	0.00	H
ATOM	1060	HB3	LYS	68	38.908	40.212	48.051	1.00	0.00	H
ATOM	1061	CG	LYS	68	40.757	40.365	46.858	1.00	0.00	C
ATOM	1062	HG2	LYS	68	40.581	39.297	46.735	1.00	0.00	H
ATOM	1063	HG3	LYS	68	40.341	40.942	46.033	1.00	0.00	H
ATOM	1064	CD	LYS	68	42.245	40.618	47.097	1.00	0.00	C
ATOM	1065	HD2	LYS	68	42.814	40.012	46.393	1.00	0.00	H
ATOM	1066	HD3	LYS	68	42.502	41.652	46.868	1.00	0.00	H
ATOM	1067	CE	LYS	68	42.863	40.266	48.428	1.00	0.00	C
ATOM	1068	HE2	LYS	68	42.666	41.099	49.102	1.00	0.00	H
ATOM	1069	HE3	LYS	68	42.556	39.310	48.855	1.00	0.00	H
ATOM	1070	NZ	LYS	68	44.342	40.232	48.354	1.00	0.00	N
ATOM	1071	HZ1	LYS	68	44.762	40.918	47.742	1.00	0.00	H
ATOM	1072	HZ2	LYS	68	44.763	40.222	49.272	1.00	0.00	H
ATOM	1073	HZ3	LYS	68	44.602	39.304	48.050	1.00	0.00	H
ATOM	1074	C	LYS	68	38.719	42.649	46.774	1.00	0.00	C
ATOM	1075	O	LYS	68	39.288	43.180	45.802	1.00	0.00	O
ATOM	1076	N	HIE	69	37.471	42.181	46.700	1.00	0.00	N
ATOM	1077	H	HIE	69	37.121	41.660	47.491	1.00	0.00	H
ATOM	1078	CA	HIE	69	36.494	42.425	45.612	1.00	0.00	C
ATOM	1079	HA	HIE	69	37.041	42.067	44.740	1.00	0.00	H
ATOM	1080	CB	HIE	69	35.272	41.541	45.738	1.00	0.00	C
ATOM	1081	HB2	HIE	69	35.541	40.487	45.668	1.00	0.00	H
ATOM	1082	HB3	HIE	69	34.898	41.611	46.759	1.00	0.00	H
ATOM	1083	CG	HIE	69	34.134	41.836	44.746	1.00	0.00	C
ATOM	1084	ND1	HIE	69	34.003	41.345	43.492	1.00	0.00	N
ATOM	1085	CE1	HIE	69	32.908	41.885	42.878	1.00	0.00	C
ATOM	1086	HE1	HIE	69	32.629	41.637	41.865	1.00	0.00	H
ATOM	1087	NE2	HIE	69	32.415	42.801	43.691	1.00	0.00	N
ATOM	1088	HE2	HIE	69	31.575	43.324	43.490	1.00	0.00	H
ATOM	1089	CD2	HIE	69	33.128	42.750	44.901	1.00	0.00	C

ATOM	1090	HD2	HIE	69	33.231	43.449	45.717	1.00	0.00	H
ATOM	1091	C	HIE	69	36.164	43.932	45.417	1.00	0.00	C
ATOM	1092	O	HIE	69	36.166	44.459	44.326	1.00	0.00	O
ATOM	1093	N	ALA	70	35.963	44.665	46.534	1.00	0.00	N
ATOM	1094	H	ALA	70	35.908	44.133	47.392	1.00	0.00	H
ATOM	1095	CA	ALA	70	35.794	46.071	46.539	1.00	0.00	C
ATOM	1096	HA	ALA	70	35.012	46.313	45.821	1.00	0.00	H
ATOM	1097	CB	ALA	70	35.515	46.618	47.959	1.00	0.00	C
ATOM	1098	HB1	ALA	70	34.801	46.005	48.508	1.00	0.00	H
ATOM	1099	HB2	ALA	70	36.449	46.686	48.517	1.00	0.00	H
ATOM	1100	HB3	ALA	70	35.102	47.612	47.788	1.00	0.00	H
ATOM	1101	C	ALA	70	37.115	46.752	46.015	1.00	0.00	C
ATOM	1102	O	ALA	70	37.058	47.699	45.213	1.00	0.00	O
ATOM	1103	N	GLU	71	38.297	46.230	46.198	1.00	0.00	N
ATOM	1104	H	GLU	71	38.286	45.520	46.917	1.00	0.00	H
ATOM	1105	CA	GLU	71	39.587	46.731	45.654	1.00	0.00	C
ATOM	1106	HA	GLU	71	39.690	47.799	45.846	1.00	0.00	H
ATOM	1107	CB	GLU	71	40.793	46.158	46.462	1.00	0.00	C
ATOM	1108	HB2	GLU	71	40.688	45.096	46.682	1.00	0.00	H
ATOM	1109	HB3	GLU	71	41.658	46.436	45.861	1.00	0.00	H
ATOM	1110	CG	GLU	71	40.774	46.934	47.758	1.00	0.00	C
ATOM	1111	HG2	GLU	71	39.822	47.338	48.100	1.00	0.00	H
ATOM	1112	HG3	GLU	71	40.815	46.218	48.578	1.00	0.00	H
ATOM	1113	CD	GLU	71	41.864	47.938	47.957	1.00	0.00	C
ATOM	1114	OE1	GLU	71	43.079	47.585	48.069	1.00	0.00	O
ATOM	1115	OE2	GLU	71	41.553	49.162	48.043	1.00	0.00	O
ATOM	1116	C	GLU	71	39.773	46.475	44.169	1.00	0.00	C
ATOM	1117	O	GLU	71	40.368	47.292	43.564	1.00	0.00	O
ATOM	1118	N	GLN	72	39.115	45.420	43.597	1.00	0.00	N
ATOM	1119	H	GLN	72	38.552	44.846	44.209	1.00	0.00	H
ATOM	1120	CA	GLN	72	39.141	45.069	42.199	1.00	0.00	C
ATOM	1121	HA	GLN	72	40.172	45.254	41.898	1.00	0.00	H
ATOM	1122	CB	GLN	72	38.651	43.655	41.991	1.00	0.00	C
ATOM	1123	HB2	GLN	72	37.674	43.555	42.465	1.00	0.00	H
ATOM	1124	HB3	GLN	72	39.333	43.075	42.613	1.00	0.00	H
ATOM	1125	CG	GLN	72	38.572	43.112	40.492	1.00	0.00	C
ATOM	1126	HG2	GLN	72	38.266	43.837	39.738	1.00	0.00	H
ATOM	1127	HG3	GLN	72	39.562	42.774	40.189	1.00	0.00	H
ATOM	1128	CD	GLN	72	37.677	41.937	40.265	1.00	0.00	C
ATOM	1129	OE1	GLN	72	38.124	40.836	40.016	1.00	0.00	O
ATOM	1130	NE2	GLN	72	36.379	42.067	40.538	1.00	0.00	N
ATOM	1131	HE21	GLN	72	35.996	42.976	40.749	1.00	0.00	H
ATOM	1132	HE22	GLN	72	35.865	41.199	40.605	1.00	0.00	H
ATOM	1133	C	GLN	72	38.204	46.056	41.403	1.00	0.00	C
ATOM	1134	O	GLN	72	38.558	46.401	40.342	1.00	0.00	O
ATOM	1135	N	GLU	73	37.126	46.432	42.012	1.00	0.00	N
ATOM	1136	H	GLU	73	37.016	45.985	42.911	1.00	0.00	H
ATOM	1137	CA	GLU	73	36.067	47.410	41.685	1.00	0.00	C
ATOM	1138	HA	GLU	73	35.747	47.474	40.645	1.00	0.00	H
ATOM	1139	CB	GLU	73	34.749	46.895	42.293	1.00	0.00	C
ATOM	1140	HB2	GLU	73	34.917	46.856	43.369	1.00	0.00	H
ATOM	1141	HB3	GLU	73	33.987	47.657	42.136	1.00	0.00	H
ATOM	1142	CG	GLU	73	34.309	45.475	41.860	1.00	0.00	C
ATOM	1143	HG2	GLU	73	35.072	44.710	42.009	1.00	0.00	H
ATOM	1144	HG3	GLU	73	33.468	45.157	42.476	1.00	0.00	H
ATOM	1145	CD	GLU	73	34.067	45.395	40.322	1.00	0.00	C
ATOM	1146	OE1	GLU	73	34.365	44.384	39.696	1.00	0.00	O
ATOM	1147	OE2	GLU	73	33.657	46.461	39.692	1.00	0.00	O
ATOM	1148	C	GLU	73	36.470	48.861	41.970	1.00	0.00	C
ATOM	1149	O	GLU	73	35.668	49.704	41.492	1.00	0.00	O
ATOM	1150	N	ASN	74	37.690	49.108	42.503	1.00	0.00	N
ATOM	1151	H	ASN	74	38.309	48.325	42.662	1.00	0.00	H
ATOM	1152	CA	ASN	74	38.196	50.346	42.968	1.00	0.00	C
ATOM	1153	HA	ASN	74	39.070	50.040	43.542	1.00	0.00	H

ATOM	1154	CB	ASN	74	38.700	51.179	41.683	1.00	0.00	C
ATOM	1155	HB2	ASN	74	37.867	51.288	40.989	1.00	0.00	H
ATOM	1156	HB3	ASN	74	38.983	52.157	42.074	1.00	0.00	H
ATOM	1157	CG	ASN	74	39.992	50.600	41.074	1.00	0.00	C
ATOM	1158	OD1	ASN	74	41.114	51.005	41.452	1.00	0.00	O
ATOM	1159	ND2	ASN	74	39.962	49.629	40.216	1.00	0.00	N
ATOM	1160	HD21	ASN	74	40.817	49.108	40.092	1.00	0.00	H
ATOM	1161	HD22	ASN	74	39.078	49.211	39.961	1.00	0.00	H
ATOM	1162	C	ASN	74	37.175	51.012	43.844	1.00	0.00	C
ATOM	1163	O	ASN	74	37.062	52.242	43.796	1.00	0.00	O
ATOM	1164	N	MET	75	36.379	50.236	44.566	1.00	0.00	N
ATOM	1165	H	MET	75	36.474	49.230	44.537	1.00	0.00	H
ATOM	1166	CA	MET	75	35.469	50.742	45.594	1.00	0.00	C
ATOM	1167	HA	MET	75	35.225	51.784	45.379	1.00	0.00	H
ATOM	1168	CB	MET	75	34.125	50.044	45.588	1.00	0.00	C
ATOM	1169	HB2	MET	75	34.206	48.997	45.880	1.00	0.00	H
ATOM	1170	HB3	MET	75	33.520	50.630	46.280	1.00	0.00	H
ATOM	1171	CG	MET	75	33.371	50.076	44.300	1.00	0.00	C
ATOM	1172	HG2	MET	75	33.173	51.131	44.110	1.00	0.00	H
ATOM	1173	HG3	MET	75	34.036	49.783	43.489	1.00	0.00	H
ATOM	1174	SD	MET	75	31.905	49.044	44.205	1.00	0.00	S
ATOM	1175	CE	MET	75	30.852	50.202	44.968	1.00	0.00	C
ATOM	1176	HE1	MET	75	29.964	49.699	45.349	1.00	0.00	H
ATOM	1177	HE2	MET	75	31.273	50.831	45.752	1.00	0.00	H
ATOM	1178	HE3	MET	75	30.417	50.862	44.217	1.00	0.00	H
ATOM	1179	C	MET	75	36.173	50.654	47.010	1.00	0.00	C
ATOM	1180	O	MET	75	37.131	49.852	47.309	1.00	0.00	O
ATOM	1181	N	THR	76	35.824	51.509	47.979	1.00	0.00	N
ATOM	1182	H	THR	76	35.140	52.207	47.723	1.00	0.00	H
ATOM	1183	CA	THR	76	35.985	51.238	49.409	1.00	0.00	C
ATOM	1184	HA	THR	76	36.965	50.781	49.543	1.00	0.00	H
ATOM	1185	CB	THR	76	35.918	52.525	50.232	1.00	0.00	C
ATOM	1186	HB	THR	76	36.559	53.305	49.820	1.00	0.00	H
ATOM	1187	CG2	THR	76	34.458	53.160	50.303	1.00	0.00	C
ATOM	1188	HG21	THR	76	33.814	52.530	50.916	1.00	0.00	H
ATOM	1189	HG22	THR	76	34.532	54.149	50.755	1.00	0.00	H
ATOM	1190	HG23	THR	76	34.131	53.359	49.283	1.00	0.00	H
ATOM	1191	OG1	THR	76	36.335	52.398	51.607	1.00	0.00	O
ATOM	1192	HG1	THR	76	37.281	52.493	51.734	1.00	0.00	H
ATOM	1193	C	THR	76	34.910	50.189	49.856	1.00	0.00	C
ATOM	1194	O	THR	76	33.760	50.124	49.368	1.00	0.00	O
ATOM	1195	N	LEU	77	35.288	49.218	50.744	1.00	0.00	N
ATOM	1196	H	LEU	77	36.182	49.392	51.182	1.00	0.00	H
ATOM	1197	CA	LEU	77	34.528	48.041	51.147	1.00	0.00	C
ATOM	1198	HA	LEU	77	34.186	47.619	50.202	1.00	0.00	H
ATOM	1199	CB	LEU	77	35.322	46.993	52.006	1.00	0.00	C
ATOM	1200	HB2	LEU	77	35.915	46.441	51.278	1.00	0.00	H
ATOM	1201	HB3	LEU	77	36.005	47.530	52.664	1.00	0.00	H
ATOM	1202	CG	LEU	77	34.589	45.760	52.605	1.00	0.00	C
ATOM	1203	HG	LEU	77	33.682	46.143	53.075	1.00	0.00	H
ATOM	1204	CD1	LEU	77	34.230	44.813	51.555	1.00	0.00	C
ATOM	1205	HD11	LEU	77	33.201	44.994	51.240	1.00	0.00	H
ATOM	1206	HD12	LEU	77	34.891	44.987	50.708	1.00	0.00	H
ATOM	1207	HD13	LEU	77	34.201	43.772	51.876	1.00	0.00	H
ATOM	1208	CD2	LEU	77	35.428	45.083	53.604	1.00	0.00	C
ATOM	1209	HD21	LEU	77	34.919	44.226	54.046	1.00	0.00	H
ATOM	1210	HD22	LEU	77	36.355	44.697				
53.181	1.00	0.00			H					
ATOM	1211	HD23	LEU	77	35.551	45.752	54.456	1.00	0.00	H
ATOM	1212	C	LEU	77	33.192	48.572	51.770	1.00	0.00	C
ATOM	1213	O	LEU	77	32.199	47.847	51.599	1.00	0.00	O
ATOM	1214	N	THR	78	33.090	49.709	52.446	1.00	0.00	N
ATOM	1215	H	THR	78	33.962	50.130	52.733	1.00	0.00	H
ATOM	1216	CA	THR	78	31.816	50.303	52.823	1.00	0.00	C

ATOM	1217	HA	THR	78	31.212	49.555	53.335	1.00	0.00	H
ATOM	1218	CB	THR	78	32.023	51.424	53.859	1.00	0.00	C
ATOM	1219	HB	THR	78	31.052	51.543	54.340	1.00	0.00	H
ATOM	1220	CG2	THR	78	33.132	51.252	54.903	1.00	0.00	C
ATOM	1221	HG21	THR	78	34.101	51.191	54.408	1.00	0.00	H
ATOM	1222	HG22	THR	78	33.138	52.076	55.618	1.00	0.00	H
ATOM	1223	HG23	THR	78	32.910	50.333	55.446	1.00	0.00	H
ATOM	1224	OG1	THR	78	32.391	52.637	53.254	1.00	0.00	O
ATOM	1225	HG1	THR	78	31.601	53.095	52.958	1.00	0.00	H
ATOM	1226	C	THR	78	30.911	50.805	51.692	1.00	0.00	C
ATOM	1227	O	THR	78	29.719	50.913	52.010	1.00	0.00	O
ATOM	1228	N	GLU	79	31.372	51.108	50.481	1.00	0.00	N
ATOM	1229	H	GLU	79	32.354	50.969	50.289	1.00	0.00	H
ATOM	1230	CA	GLU	79	30.510	51.428	49.283	1.00	0.00	C
ATOM	1231	HA	GLU	79	29.544	51.857	49.553	1.00	0.00	H
ATOM	1232	CB	GLU	79	31.177	52.215	48.212	1.00	0.00	C
ATOM	1233	HB2	GLU	79	32.168	51.842	47.953	1.00	0.00	H
ATOM	1234	HB3	GLU	79	30.539	52.038	47.345	1.00	0.00	H
ATOM	1235	CG	GLU	79	31.240	53.726	48.463	1.00	0.00	C
ATOM	1236	HG2	GLU	79	31.873	53.992	49.310	1.00	0.00	H
ATOM	1237	HG3	GLU	79	31.673	54.215	47.590	1.00	0.00	H
ATOM	1238	CD	GLU	79	29.838	54.312	48.825	1.00	0.00	C
ATOM	1239	OE1	GLU	79	29.588	54.653	49.994	1.00	0.00	O
ATOM	1240	OE2	GLU	79	28.958	54.278	47.946	1.00	0.00	O
ATOM	1241	C	GLU	79	30.131	50.129	48.552	1.00	0.00	C
ATOM	1242	O	GLU	79	29.005	50.056	48.152	1.00	0.00	O
ATOM	1243	N	LEU	80	30.998	49.117	48.634	1.00	0.00	N
ATOM	1244	H	LEU	80	31.833	49.245	49.186	1.00	0.00	H
ATOM	1245	CA	LEU	80	30.562	47.786	48.180	1.00	0.00	C
ATOM	1246	HA	LEU	80	30.155	47.906	47.176	1.00	0.00	H
ATOM	1247	CB	LEU	80	31.752	46.822	48.013	1.00	0.00	C
ATOM	1248	HB2	LEU	80	32.420	47.246	47.264	1.00	0.00	H
ATOM	1249	HB3	LEU	80	32.283	46.670	48.951	1.00	0.00	H
ATOM	1250	CG	LEU	80	31.289	45.384	47.617	1.00	0.00	C
ATOM	1251	HG	LEU	80	30.334	45.195	48.109	1.00	0.00	H
ATOM	1252	CD1	LEU	80	31.031	45.357	46.062	1.00	0.00	C
ATOM	1253	HD11	LEU	80	32.011	45.272	45.592	1.00	0.00	H
ATOM	1254	HD12	LEU	80	30.358	44.566	45.732	1.00	0.00	H
ATOM	1255	HD13	LEU	80	30.446	46.215	45.728	1.00	0.00	H
ATOM	1256	CD2	LEU	80	32.379	44.289	47.975	1.00	0.00	C
ATOM	1257	HD21	LEU	80	32.180	43.372	47.420	1.00	0.00	H
ATOM	1258	HD22	LEU	80	33.332	44.694	47.632	1.00	0.00	H
ATOM	1259	HD23	LEU	80	32.447	44.090	49.044	1.00	0.00	H
ATOM	1260	C	LEU	80	29.410	47.198	49.081	1.00	0.00	C
ATOM	1261	O	LEU	80	28.402	46.682	48.577	1.00	0.00	O
ATOM	1262	N	LYS	81	29.455	47.375	50.418	1.00	0.00	N
ATOM	1263	H	LYS	81	30.333	47.583	50.871	1.00	0.00	H
ATOM	1264	CA	LYS	81	28.359	47.026	51.383	1.00	0.00	C
ATOM	1265	HA	LYS	81	27.951	46.095	50.991	1.00	0.00	H
ATOM	1266	CB	LYS	81	28.907	46.898	52.846	1.00	0.00	C
ATOM	1267	HB2	LYS	81	29.360	47.852	53.117	1.00	0.00	H
ATOM	1268	HB3	LYS	81	28.035	46.871	53.499	1.00	0.00	H
ATOM	1269	CG	LYS	81	29.812	45.658	53.145	1.00	0.00	C
ATOM	1270	HG2	LYS	81	29.239	44.732	53.203	1.00	0.00	H
ATOM	1271	HG3	LYS	81	30.550	45.599	52.344	1.00	0.00	H
ATOM	1272	CD	LYS	81	30.711	45.722	54.311	1.00	0.00	C
ATOM	1273	HD2	LYS	81	31.415	46.551	54.243	1.00	0.00	H
ATOM	1274	HD3	LYS	81	30.174	45.837	55.253	1.00	0.00	H
ATOM	1275	CE	LYS	81	31.592	44.541	54.465	1.00	0.00	C
ATOM	1276	HE2	LYS	81	30.948	43.676	54.305	1.00	0.00	H
ATOM	1277	HE3	LYS	81	32.263	44.690	53.618	1.00	0.00	H
ATOM	1278	NZ	LYS	81	32.270	44.447	55.748	1.00	0.00	N
ATOM	1279	HZ1	LYS	81	31.637	44.210	56.500	1.00	0.00	H
ATOM	1280	HZ2	LYS	81	32.882	43.645	55.802	1.00	0.00	H

ATOM	1281	HZ3	LYS	81	32.772	45.270	56.047	1.00	0.00	H
ATOM	1282	C	LYS	81	27.211	47.974	51.294	1.00	0.00	C
ATOM	1283	O	LYS	81	26.125	47.546	51.672	1.00	0.00	O
ATOM	1284	N	LYS	82	27.261	49.206	50.662	1.00	0.00	N
ATOM	1285	H	LYS	82	28.191	49.475	50.371	1.00	0.00	H
ATOM	1286	CA	LYS	82	26.161	50.088	50.524	1.00	0.00	C
ATOM	1287	HA	LYS	82	25.458	50.178	51.353	1.00	0.00	H
ATOM	1288	CB	LYS	82	26.694	51.523	50.222	1.00	0.00	C
ATOM	1289	HB2	LYS	82	27.444	51.747	50.980	1.00	0.00	H
ATOM	1290	HB3	LYS	82	27.124	51.584	49.222	1.00	0.00	H
ATOM	1291	CG	LYS	82	25.633	52.653	50.251	1.00	0.00	C
ATOM	1292	HG2	LYS	82	24.855	52.507	49.503	1.00	0.00	H
ATOM	1293	HG3	LYS	82	25.182	52.636	51.244	1.00	0.00	H
ATOM	1294	CD	LYS	82	26.122	54.078	50.214	1.00	0.00	C
ATOM	1295	HD2	LYS	82	26.770	54.226	49.350	1.00	0.00	H
ATOM	1296	HD3	LYS	82	25.197	54.654	50.184	1.00	0.00	H
ATOM	1297	CE	LYS	82	27.027	54.432	51.434	1.00	0.00	C
ATOM	1298	HE2	LYS	82	26.334	54.291	52.265	1.00	0.00	H
ATOM	1299	HE3	LYS	82	27.850	53.727	51.552	1.00	0.00	H
ATOM	1300	NZ	LYS	82	27.548	55.801	51.234	1.00	0.00	N
ATOM	1301	HZ1	LYS	82	28.116	55.878	50.403	1.00	0.00	H
ATOM	1302	HZ2	LYS	82	26.770	56.443	51.174	1.00	0.00	H
ATOM	1303	HZ3	LYS	82	28.219	55.997	51.964	1.00	0.00	H
ATOM	1304	C	LYS	82	25.218	49.556	49.396	1.00	0.00	C
ATOM	1305	O	LYS	82	24.028	49.335	49.600	1.00	0.00	O
ATOM	1306	N	VAL	83	25.837	49.159	48.271	1.00	0.00	N
ATOM	1307	H	VAL	83	26.817	49.379	48.168	1.00	0.00	H
ATOM	1308	CA	VAL	83	25.123	48.579	47.072	1.00	0.00	C
ATOM	1309	HA	VAL	83	24.250	49.200	46.868	1.00	0.00	H
ATOM	1310	CB	VAL	83	25.979	48.643	45.786	1.00	0.00	C
ATOM	1311	HB	VAL	83	25.402	48.180	44.985	1.00	0.00	H
ATOM	1312	CG1	VAL	83	26.445	50.051	45.378	1.00	0.00	C
ATOM	1313	HG11	VAL	83	27.066	50.490	46.159	1.00	0.00	H
ATOM	1314	HG12	VAL	83	26.998	50.179	44.448	1.00	0.00	H
ATOM	1315	HG13	VAL	83	25.628	50.734	45.145	1.00	0.00	H
ATOM	1316	CG2	VAL	83	27.243	47.875	45.997	1.00	0.00	C
ATOM	1317	HG21	VAL	83	27.072	46.852	46.328	1.00	0.00	H
ATOM	1318	HG22	VAL	83	27.704	47.768	45.014	1.00	0.00	H
ATOM	1319	HG23	VAL	83	27.954	48.379	46.652	1.00	0.00	H
ATOM	1320	C	VAL	83	24.478	47.215	47.346	1.00	0.00	C
ATOM	1321	O	VAL	83	23.289	47.024	46.947	1.00	0.00	O
ATOM	1322	N	ILE	84	25.051	46.365	48.211	1.00	0.00	N
ATOM	1323	H	ILE	84	25.989	46.575	48.519	1.00	0.00	H
ATOM	1324	CA	ILE	84	24.356	45.134	48.624	1.00	0.00	C
ATOM	1325	HA	ILE	84	23.902	44.682	47.743	1.00	0.00	H
ATOM	1326	CB	ILE	84	25.391	44.079	49.252	1.00	0.00	C
ATOM	1327	HB	ILE	84	25.823	44.536	50.142	1.00	0.00	H
ATOM	1328	CG2	ILE	84	24.590	42.769	49.503	1.00	0.00	C
ATOM	1329	HG21	ILE	84	25.148	41.843	49.365	1.00	0.00	H
ATOM	1330	HG22	ILE	84	24.100	42.717	50.475	1.00	0.00	H
ATOM	1331	HG23	ILE	84	23.866	42.763	48.689	1.00	0.00	H
ATOM	1332	CG1	ILE	84	26.657	43.838	48.401	1.00	0.00	C
ATOM	1333	HG12	ILE	84	26.388	43.175	47.578	1.00	0.00	H
ATOM	1334	HG13	ILE	84	26.992	44.828	48.096	1.00	0.00	H
ATOM	1335	CD1	ILE	84	27.717	43.119	49.264	1.00	0.00	C
ATOM	1336	HD11	ILE	84	28.657	43.294	48.739	1.00	0.00	H
ATOM	1337	HD12	ILE	84	27.757	43.452	50.300	1.00	0.00	H
ATOM	1338	HD13	ILE	84	27.434	42.067	49.236	1.00	0.00	H
ATOM	1339	C	ILE	84	23.116	45.433	49.608	1.00	0.00	C
ATOM	1340	O	ILE	84	22.012	44.768	49.619	1.00	0.00	O
ATOM	1341	N	ALA	85	23.342	46.383	50.506	1.00	0.00	N
ATOM	1342	H	ALA	85	24.287	46.723	50.608	1.00	0.00	H
ATOM	1343	CA	ALA	85	22.298	46.823	51.376	1.00	0.00	C

ATOM	1344	HA	ALA	85	21.781	45.981	51.837	1.00	0.00	H
ATOM	1345	CB	ALA	85	22.713	47.628	52.539	1.00	0.00	C
ATOM	1346	HB1	ALA	85	21.804	47.853	53.097	1.00	0.00	H
ATOM	1347	HB2	ALA	85	23.569	47.262	53.106	1.00	0.00	H
ATOM	1348	HB3	ALA	85	23.063	48.570	52.114	1.00	0.00	H
ATOM	1349	C	ALA	85	21.141	47.577	50.733	1.00	0.00	C
ATOM	1350	O	ALA	85	20.049	47.673	51.284	1.00	0.00	O
ATOM	1351	N	ASP	86	21.351	48.148	49.529	1.00	0.00	N
ATOM	1352	H	ASP	86	22.230	48.055	49.041	1.00	0.00	H
ATOM	1353	CA	ASP	86	20.276	48.779	48.728	1.00	0.00	C
ATOM	1354	HA	ASP	86	19.779	49.520	49.354	1.00	0.00	H
ATOM	1355	CB	ASP	86	20.865	49.600	47.554	1.00	0.00	C
ATOM	1356	HB2	ASP	86	21.795	50.069	47.876	1.00	0.00	H
ATOM	1357	HB3	ASP	86	21.106	48.968	46.698	1.00	0.00	H
ATOM	1358	CG	ASP	86	20.021	50.753	47.005	1.00	0.00	C
ATOM	1359	OD1	ASP	86	20.020	50.954	45.798	1.00	0.00	O
ATOM	1360	OD2	ASP	86	19.290	51.378	47.837	1.00	0.00	O
ATOM	1361	C	ASP	86	19.109	47.827	48.316	1.00	0.00	C
ATOM	1362	O	ASP	86	18.013	48.285	47.941	1.00	0.00	O
ATOM	1363	N	ILE	87	19.342	46.493	48.435	1.00	0.00	N
ATOM	1364	H	ILE	87	20.195	46.218	48.900	1.00	0.00	H
ATOM	1365	CA	ILE	87	18.480	45.332	48.007	1.00	0.00	C
ATOM	1366	HA	ILE	87	17.470	45.697	47.818	1.00	0.00	H
ATOM	1367	CB	ILE	87	18.908	44.623	46.639	1.00	0.00	C
ATOM	1368	HB	ILE	87	19.941	44.277	46.696	1.00	0.00	H
ATOM	1369	CG2	ILE	87	17.986	43.451	46.383	1.00	0.00	C
ATOM	1370	HG21	ILE	87	18.230	42.977	45.432	1.00	0.00	H
ATOM	1371	HG22	ILE	87	18.082	42.675	47.142	1.00	0.00	H
ATOM	1372	HG23	ILE	87	16.949	43.771	46.272	1.00	0.00	H
ATOM	1373	CG1	ILE	87	18.863	45.637	45.422	1.00	0.00	C
ATOM	1374	HG12	ILE	87	17.857	45.855	45.066	1.00	0.00	H
ATOM	1375	HG13	ILE	87	19.462	46.471	45.791	1.00	0.00	H
ATOM	1376	CD1	ILE	87	19.428	44.910	44.237	1.00	0.00	C
ATOM	1377	HD11	ILE	87	20.383	44.390	44.327	1.00	0.00	H
ATOM	1378	HD12	ILE	87	18.676	44.168	43.974	1.00	0.00	H
ATOM	1379	HD13	ILE	87	19.545	45.702	43.496	1.00	0.00	H
ATOM	1380	C	ILE	87	18.256	44.453	49.287	1.00	0.00	C
ATOM	1381	O	ILE	87	17.107	44.378	49.672	1.00	0.00	O
ATOM	1382	N	TYR	88	19.246	43.683	49.655	1.00	0.00	N
ATOM	1383	H	TYR	88	20.085	43.738	49.094	1.00	0.00	H
ATOM	1384	CA	TYR	88	19.261	42.602	50.637	1.00	0.00	C
ATOM	1385	HA	TYR	88	18.350	42.002	50.643	1.00	0.00	H
ATOM	1386	CB	TYR	88	20.410	41.672	50.279	1.00	0.00	C
ATOM	1387	HB2	TYR	88	21.329	42.259	50.298	1.00	0.00	H
ATOM	1388	HB3	TYR	88	20.529	40.919	51.059	1.00	0.00	H
ATOM	1389	CG	TYR	88	20.358	41.055	48.872	1.00	0.00	C
ATOM	1390	CD1	TYR	88	19.552	39.879	48.643	1.00	0.00	C
ATOM	1391	HD1	TYR	88	18.911	39.544	49.445	1.00	0.00	H
ATOM	1392	CE1	TYR	88	19.623	39.338	47.381	1.00	0.00	C
ATOM	1393	HE1	TYR	88	19.042	38.459	47.146	1.00	0.00	H
ATOM	1394	CZ	TYR	88	20.423	39.848	46.384	1.00	0.00	C
ATOM	1395	OH	TYR	88	20.446	39.346	45.116	1.00	0.00	O
ATOM	1396	HH	TYR	88	20.699	40.058	44.523	1.00	0.00	H
ATOM	1397	CE2	TYR	88	21.226	40.992	46.605	1.00	0.00	C
ATOM	1398	HE2	TYR	88	21.897	41.337	45.831	1.00	0.00	H
ATOM	1399	CD2	TYR	88	21.119	41.606	47.868	1.00	0.00	C
ATOM	1400	HD2	TYR	88	21.824	42.357	48.191	1.00	0.00	H
ATOM	1401	C	TYR	88	19.275	43.195	52.030	1.00	0.00	C
ATOM	1402	O	TYR	88	19.854	44.276	52.266	1.00	0.00	O
ATOM	1403	N	PRO	89	18.817	42.462	53.063	1.00	0.00	N
ATOM	1404	CD	PRO	89	17.997	41.259	53.036	1.00	0.00	C
ATOM	1405	HD2	PRO	89	18.586	40.531	52.478	1.00	0.00	H
ATOM	1406	HD3	PRO	89	17.075	41.538	52.526	1.00	0.00	H
ATOM	1407	CG	PRO	89	17.923	40.722	54.475	1.00	0.00	C

ATOM	1408	HG2	PRO	89	18.743	40.029	54.666	1.00	0.00	H
ATOM	1409	HG3	PRO	89	16.960	40.236	54.630	1.00	0.00	H
ATOM	1410	CB	PRO	89	18.294	41.901	55.286	1.00	0.00	C
ATOM	1411	HB2	PRO	89	18.588	41.748	56.324	1.00	0.00	H
ATOM	1412	HB3	PRO	89	17.391	42.506	55.355	1.00	0.00	H
ATOM	1413	CA	PRO	89	19.249	42.657	54.415	1.00	0.00	C
ATOM	1414	HA	PRO	89	19.289	43.717	54.667	1.00	0.00	H
ATOM	1415	C	PRO	89	20.707	42.146	54.668	1.00	0.00	C
ATOM	1416	O	PRO	89	21.058	41.139	54.060	1.00	0.00	O
ATOM	1417	N	GLY	90	21.527	42.906	55.367	1.00	0.00	N
ATOM	1418	H	GLY	90	21.050	43.726	55.713	1.00	0.00	H
ATOM	1419	CA	GLY	90	22.846	42.569	55.825	1.00	0.00	C
ATOM	1420	HA2	GLY	90	23.539	42.474	54.989	1.00	0.00	H
ATOM	1421	HA3	GLY	90	23.243	43.331	56.495	1.00	0.00	H
ATOM	1422	C	GLY	90	22.974	41.210	56.481	1.00	0.00	C
ATOM	1423	O	GLY	90	23.952	40.534	56.210	1.00	0.00	O
ATOM	1424	N	GLN	91	21.923	40.638	57.127	1.00	0.00	N
ATOM	1425	H	GLN	91	21.167	41.269	57.353	1.00	0.00	H
ATOM	1426	CA	GLN	91	21.955	39.228	57.634	1.00	0.00	C
ATOM	1427	HA	GLN	91	22.934	39.115	58.099	1.00	0.00	H
ATOM	1428	CB	GLN	91	20.726	39.068	58.518	1.00	0.00	C
ATOM	1429	HB2	GLN	91	19.873	39.607	58.107	1.00	0.00	H
ATOM	1430	HB3	GLN	91	20.440	38.018	58.599	1.00	0.00	H
ATOM	1431	CG	GLN	91	20.899	39.586	59.931	1.00	0.00	C
ATOM	1432	HG2	GLN	91	21.417	40.542	60.017	1.00	0.00	H
ATOM	1433	HG3	GLN	91	19.914	39.652	60.393	1.00	0.00	H
ATOM	1434	CD	GLN	91	21.711	38.677	60.865	1.00	0.00	C
ATOM	1435	OE1	GLN	91	22.497	37.890	60.370	1.00	0.00	O
ATOM	1436	NE2	GLN	91	21.656	38.872	62.154	1.00	0.00	N
ATOM	1437	HE21	GLN	91	20.954	39.516	62.491	1.00	0.00	H
ATOM	1438	HE22	GLN	91	22.085	38.316	62.878	1.00	0.00	H
ATOM	1439	C	GLN	91	22.033	38.080	56.581	1.00	0.00	C
ATOM	1440	O	GLN	91	22.461	36.995	56.958	1.00	0.00	O
ATOM	1441	N	THR	92	21.949	38.441	55.328	1.00	0.00	N
ATOM	1442	H	THR	92	21.879	39.443	55.215	1.00	0.00	H
ATOM	1443	CA	THR	92	22.092	37.506	54.231	1.00	0.00	C
ATOM	1444	HA	THR	92	21.310	36.751	54.321	1.00	0.00	H
ATOM	1445	CB	THR	92	21.833	38.157	52.830	1.00	0.00	C
ATOM	1446	HB	THR	92	22.609	38.876	52.569	1.00	0.00	H
ATOM	1447	CG2	THR	92	21.734	37.212	51.605	1.00	0.00	C
ATOM	1448	HG21	THR	92	21.096	36.332	51.680	1.00	0.00	H
ATOM	1449	HG22	THR	92	21.424	37.734	50.699	1.00	0.00	H
ATOM	1450	HG23	THR	92	22.732	36.866	51.333	1.00	0.00	H
ATOM	1451	OG1	THR	92	20.672	38.834	52.837	1.00	0.00	O
ATOM	1452	HG1	THR	92	20.869	39.662	53.282	1.00	0.00	H
ATOM	1453	C	THR	92	23.478	36.804	54.106	1.00	0.00	C
ATOM	1454	O	THR	92	24.537	37.432	54.275	1.00	0.00	O
ATOM	1455	N	GLN	93	23.428	35.525	53.683	1.00	0.00	N
ATOM	1456	H	GLN	93	22.537	35.185	53.350	1.00	0.00	H
ATOM	1457	CA	GLN	93	24.543	34.660	53.351	1.00	0.00	C
ATOM	1458	HA	GLN	93	25.369	34.910	54.015	1.00	0.00	H
ATOM	1459	CB	GLN	93	24.091	33.223	53.775	1.00	0.00	C
ATOM	1460	HB2	GLN	93	23.564	33.167	54.728	1.00	0.00	H
ATOM	1461	HB3	GLN							
	93	23.409	32.817	53.028	1.00	0.00	H			
ATOM	1462	CG	GLN	93	25.348	32.295	53.825	1.00	0.00	C
ATOM	1463	HG2	GLN	93	25.130	31.232	53.726	1.00	0.00	H
ATOM	1464	HG3	GLN	93	26.060	32.550	53.039	1.00	0.00	H
ATOM	1465	CD	GLN	93	26.118	32.432	55.155	1.00	0.00	C
ATOM	1466	OE1	GLN	93	25.776	33.228	56.064	1.00	0.00	O
ATOM	1467	NE2	GLN	93	27.223	31.796	55.424	1.00	0.00	N
ATOM	1468	HE21	GLN	93	27.784	31.463	54.653	1.00	0.00	H
ATOM	1469	HE22	GLN	93	27.650	32.146	56.270	1.00	0.00	H
ATOM	1470	C	GLN	93	25.024	34.748	51.887	1.00	0.00	C

ATOM	1471	O	GLN	93	24.331	34.431	50.939	1.00	0.00	O
ATOM	1472	N	PHE	94	26.297	35.077	51.728	1.00	0.00	N
ATOM	1473	H	PHE	94	26.898	35.298	52.510	1.00	0.00	H
ATOM	1474	CA	PHE	94	27.048	35.338	50.469	1.00	0.00	C
ATOM	1475	HA	PHE	94	26.374	35.176	49.629	1.00	0.00	H
ATOM	1476	CB	PHE	94	27.603	36.742	50.290	1.00	0.00	C
ATOM	1477	HB2	PHE	94	28.378	36.876	51.045	1.00	0.00	H
ATOM	1478	HB3	PHE	94	28.141	36.815	49.345	1.00	0.00	H
ATOM	1479	CG	PHE	94	26.528	37.823	50.545	1.00	0.00	C
ATOM	1480	CD1	PHE	94	25.696	38.205	49.467	1.00	0.00	C
ATOM	1481	HD1	PHE	94	25.820	37.718	48.510	1.00	0.00	H
ATOM	1482	CE1	PHE	94	24.752	39.187	49.610	1.00	0.00	C
ATOM	1483	HE1	PHE	94	24.105	39.523	48.812	1.00	0.00	H
ATOM	1484	CZ	PHE	94	24.574	39.799	50.879	1.00	0.00	C
ATOM	1485	HZ	PHE	94	23.688	40.386	51.074	1.00	0.00	H
ATOM	1486	CE2	PHE	94	25.361	39.380	51.973	1.00	0.00	C
ATOM	1487	HE2	PHE	94	25.091	39.713	52.964	1.00	0.00	H
ATOM	1488	CD2	PHE	94	26.320	38.416	51.826	1.00	0.00	C
ATOM	1489	HD2	PHE	94	26.942	38.127	52.660	1.00	0.00	H
ATOM	1490	C	PHE	94	28.140	34.330	50.266	1.00	0.00	C
ATOM	1491	O	PHE	94	28.615	33.700	51.185	1.00	0.00	O
ATOM	1492	N	TYR	95	28.455	34.155	48.988	1.00	0.00	N
ATOM	1493	H	TYR	95	27.849	34.653	48.352	1.00	0.00	H
ATOM	1494	CA	TYR	95	29.323	33.108	48.485	1.00	0.00	C
ATOM	1495	HA	TYR	95	29.989	32.840	49.304	1.00	0.00	H
ATOM	1496	CB	TYR	95	28.451	31.878	47.962	1.00	0.00	C
ATOM	1497	HB2	TYR	95	27.743	31.630	48.752	1.00	0.00	H
ATOM	1498	HB3	TYR	95	27.746	32.194	47.195	1.00	0.00	H
ATOM	1499	CG	TYR	95	29.095	30.595	47.519	1.00	0.00	C
ATOM	1500	CD1	TYR	95	29.405	30.338	46.206	1.00	0.00	C
ATOM	1501	HD1	TYR	95	29.168	31.005	45.389	1.00	0.00	H
ATOM	1502	CE1	TYR	95	30.084	29.171	45.816	1.00	0.00	C
ATOM	1503	HE1	TYR	95	30.222	28.996	44.760	1.00	0.00	H
ATOM	1504	CZ	TYR	95	30.469	28.239	46.820	1.00	0.00	C
ATOM	1505	OH	TYR	95	31.138	27.109	46.519	1.00	0.00	O
ATOM	1506	HH	TYR	95	31.138	27.026	45.562	1.00	0.00	H
ATOM	1507	CE2	TYR	95	30.218	28.541	48.207	1.00	0.00	C
ATOM	1508	HE2	TYR	95	30.573	27.919	49.015	1.00	0.00	H
ATOM	1509	CD2	TYR	95	29.474	29.628	48.497	1.00	0.00	C
ATOM	1510	HD2	TYR	95	29.252	29.891	49.521	1.00	0.00	H
ATOM	1511	C	TYR	95	30.136	33.649	47.331	1.00	0.00	C
ATOM	1512	O	TYR	95	29.649	34.061	46.222	1.00	0.00	O
ATOM	1513	N	VAL	96	31.408	33.723	47.638	1.00	0.00	N
ATOM	1514	H	VAL	96	31.621	33.223	48.490	1.00	0.00	H
ATOM	1515	CA	VAL	96	32.538	34.290	46.865	1.00	0.00	C
ATOM	1516	HA	VAL	96	32.136	35.057	46.203	1.00	0.00	H
ATOM	1517	CB	VAL	96	33.576	34.866	47.855	1.00	0.00	C
ATOM	1518	HB	VAL	96	33.957	34.091	48.521	1.00	0.00	H
ATOM	1519	CG1	VAL	96	34.864	35.347	47.126	1.00	0.00	C
ATOM	1520	HG11	VAL	96	35.514	34.536	46.796	1.00	0.00	H
ATOM	1521	HG12	VAL	96	34.652	35.944	46.240	1.00	0.00	H
ATOM	1522	HG13	VAL	96	35.390	36.024	47.798	1.00	0.00	H
ATOM	1523	CG2	VAL	96	32.995	36.024	48.638	1.00	0.00	C
ATOM	1524	HG21	VAL	96	32.152	35.765	49.280	1.00	0.00	H
ATOM	1525	HG22	VAL	96	33.719	36.609	49.206	1.00	0.00	H
ATOM	1526	HG23	VAL	96	32.599	36.676	47.861	1.00	0.00	H
ATOM	1527	C	VAL	96	33.020	33.288	45.903	1.00	0.00	C
ATOM	1528	O	VAL	96	33.239	32.132	46.308	1.00	0.00	O
ATOM	1529	N	ILE	97	33.302	33.651	44.613	1.00	0.00	N
ATOM	1530	H	ILE	97	33.334	34.659	44.553	1.00	0.00	H
ATOM	1531	CA	ILE	97	33.914	32.787	43.614	1.00	0.00	C
ATOM	1532	HA	ILE	97	34.311	31.934	44.162	1.00	0.00	H
ATOM	1533	CB	ILE	97	32.834	32.322	42.597	1.00	0.00	C
ATOM	1534	HB	ILE	97	32.478	33.178	42.026	1.00	0.00	H

```
ATOM   1535  CG2 ILE    97      33.456  31.258  41.697  1.00  0.00           C
ATOM   1536 HG21 ILE    97      33.919  30.505  42.336  1.00  0.00           H
ATOM   1537 HG22 ILE    97      32.759  30.882  40.948  1.00  0.00           H
ATOM   1538 HG23 ILE    97      34.241  31.756  41.129  1.00  0.00           H
ATOM   1539  CG1 ILE    97      31.628  31.722  43.370  1.00  0.00           C
ATOM   1540 HG12 ILE    97      32.085  31.194  44.206  1.00  0.00           H
ATOM   1541 HG13 ILE    97      31.158  32.568  43.870  1.00  0.00           H
ATOM   1542  CD1 ILE    97      30.559  30.823  42.571  1.00  0.00           C
ATOM   1543 HD11 ILE    97      30.456  31.076  41.516  1.00  0.00           H
ATOM   1544 HD12 ILE    97      30.968  29.814  42.526  1.00  0.00           H
ATOM   1545 HD13 ILE    97      29.581  30.946  43.036  1.00  0.00           H
ATOM   1546  C   ILE    97      35.049  33.584  42.860  1.00  0.00           C
ATOM   1547  O   ILE    97      34.911  34.770  42.676  1.00  0.00           O
ATOM   1548  N   GLU    98      36.208  32.956  42.565  1.00  0.00           N
ATOM   1549  H   GLU    98      36.122  31.954  42.478  1.00  0.00           H
ATOM   1550  CA  GLU    98      37.415  33.550  41.916  1.00  0.00           C
ATOM   1551  HA  GLU    98      37.148  34.535  41.535  1.00  0.00           H
ATOM   1552  CB  GLU    98      38.512  33.579  42.956  1.00  0.00           C
ATOM   1553  HB2 GLU    98      38.919  32.587  43.151  1.00  0.00           H
ATOM   1554  HB3 GLU    98      39.349  34.199  42.638  1.00  0.00           H
ATOM   1555  CG  GLU    98      38.062  34.079  44.366  1.00  0.00           C
ATOM   1556  HG2 GLU    98      37.582  35.030  44.135  1.00  0.00           H
ATOM   1557  HG3 GLU    98      37.381  33.359  44.820  1.00  0.00           H
ATOM   1558  CD  GLU    98      39.204  34.150  45.394  1.00  0.00           C
ATOM   1559  OE1 GLU    98      38.969  33.863  46.594  1.00  0.00           O
ATOM   1560  OE2 GLU    98      40.355  34.501  44.991  1.00  0.00           O
ATOM   1561  C   GLU    98      37.942  32.673  40.757  1.00  0.00           C
ATOM   1562  O   GLU    98      37.614  31.510  40.735  1.00  0.00           O
ATOM   1563  N   PHE    99      38.644  33.286  39.720  1.00  0.00           N
ATOM   1564  H   PHE    99      38.864  34.270  39.778  1.00  0.00           H
ATOM   1565  CA  PHE    99      39.105  32.589  38.471  1.00  0.00           C
ATOM   1566  HA  PHE    99      39.468  31.610  38.784  1.00  0.00           H
ATOM   1567  CB  PHE    99      37.926  32.310  37.579  1.00  0.00           C
ATOM   1568  HB2 PHE    99      38.203  32.426  36.532  1.00  0.00           H
ATOM   1569  HB3 PHE    99      37.500  31.314  37.696  1.00  0.00           H
ATOM   1570  CG  PHE    99      36.718  33.223  37.675  1.00  0.00           C
ATOM   1571  CD1 PHE    99      35.624  32.922  38.563  1.00  0.00           C
ATOM   1572  HD1 PHE    99      35.559  32.029  39.167  1.00  0.00           H
ATOM   1573  CE1 PHE    99      34.514  33.775  38.687  1.00  0.00           C
ATOM   1574  HE1 PHE    99      33.605  33.470  39.185  1.00  0.00           H
ATOM   1575  CZ  PHE    99      34.420  34.948  37.888  1.00  0.00           C
ATOM   1576  HZ  PHE    99      33.523  35.543  37.805  1.00  0.00           H
ATOM   1577  CE2 PHE    99      35.523  35.308  37.121  1.00  0.00           C
ATOM   1578  HE2 PHE    99      35.516  36.264  36.618  1.00  0.00           H
ATOM   1579  CD2 PHE    99      36.625  34.464  36.937  1.00  0.00           C
ATOM   1580  HD2 PHE    99      37.510  34.675  36.356  1.00  0.00           H
ATOM   1581  C   PHE    99      40.244  33.220  37.595  1.00  0.00           C
ATOM   1582  O   PHE    99      40.411  34.421  37.607  1.00  0.00           O
ATOM   1583  N   LYS   100      41.002  32.386  36.920  1.00  0.00           N
ATOM   1584  H   LYS   100      40.870  31.394  37.051  1.00  0.00           H
ATOM   1585  CA  LYS   100      41.948  32.855  35.859  1.00  0.00           C
ATOM   1586  HA  LYS   100      41.510  33.803
  35.548  1.00  0.00          H
ATOM   1587  CB  LYS   100      43.363  33.174  36.469  1.00  0.00           C
ATOM   1588  HB2 LYS   100      43.721  33.956  35.800  1.00  0.00           H
ATOM   1589  HB3 LYS   100      43.252  33.761  37.381  1.00  0.00           H
ATOM   1590  CG  LYS   100      44.156  31.843  36.699  1.00  0.00           C
ATOM   1591  HG2 LYS   100      43.553  31.276  37.410  1.00  0.00           H
ATOM   1592  HG3 LYS   100      44.251  31.378  35.718  1.00  0.00           H
ATOM   1593  CD  LYS   100      45.573  32.076  37.200  1.00  0.00           C
ATOM   1594  HD2 LYS   100      46.243  32.538  36.473  1.00  0.00           H
ATOM   1595  HD3 LYS   100      45.503  32.716  38.080  1.00  0.00           H
ATOM   1596  CE  LYS   100      46.203  30.761  37.727  1.00  0.00           C
ATOM   1597  HE2 LYS   100      45.760  30.477  38.681  1.00  0.00           H
```

ATOM	1598	HE3	LYS	100	45.904	29.918	37.105	1.00	0.00	H
ATOM	1599	NZ	LYS	100	47.666	30.792	38.017	1.00	0.00	N
ATOM	1600	HZ1	LYS	100	48.225	31.006	37.203	1.00	0.00	H
ATOM	1601	HZ2	LYS	100	47.865	31.577	38.619	1.00	0.00	H
ATOM	1602	HZ3	LYS	100	47.957	29.938	38.473	1.00	0.00	H
ATOM	1603	C	LYS	100	42.062	31.955	34.632	1.00	0.00	C
ATOM	1604	O	LYS	100	42.048	30.740	34.704	1.00	0.00	O
ATOM	1605	N	CYX	101	42.338	32.541	33.479	1.00	0.00	N
ATOM	1606	H	CYX	101	42.209	33.531	33.328	1.00	0.00	H
ATOM	1607	CA	CYX	101	42.788	31.807	32.309	1.00	0.00	C
ATOM	1608	HA	CYX	101	41.988	31.086	32.140	1.00	0.00	H
ATOM	1609	CB	CYX	101	42.769	32.799	31.120	1.00	0.00	C
ATOM	1610	HB2	CYX	101	41.836	33.360	31.073	1.00	0.00	H
ATOM	1611	HB3	CYX	101	43.514	33.580	31.270	1.00	0.00	H
ATOM	1612	SG	CYX	101	43.191	32.115	29.512	1.00	0.00	S
ATOM	1613	C	CYX	101	44.200	31.147	32.506	1.00	0.00	C
ATOM	1614	O	CYX	101	45.127	31.757	33.062	1.00	0.00	O
ATOM	1615	N	LEU	102	44.370	29.884	32.023	1.00	0.00	N
ATOM	1616	H	LEU	102	43.544	29.461	31.623	1.00	0.00	H
ATOM	1617	CA	LEU	102	45.594	29.054	32.199	1.00	0.00	C
ATOM	1618	HA	LEU	102	46.011	29.266	33.183	1.00	0.00	H
ATOM	1619	CB	LEU	102	45.314	27.519	32.055	1.00	0.00	C
ATOM	1620	HB2	LEU	102	44.969	27.507	31.021	1.00	0.00	H
ATOM	1621	HB3	LEU	102	46.260	26.982	32.123	1.00	0.00	H
ATOM	1622	CG	LEU	102	44.356	26.794	32.974	1.00	0.00	C
ATOM	1623	HG	LEU	102	43.380	27.180	32.679	1.00	0.00	H
ATOM	1624	CD1	LEU	102	44.504	25.285	32.716	1.00	0.00	C
ATOM	1625	HD11	LEU	102	45.526	24.916	32.790	1.00	0.00	H
ATOM	1626	HD12	LEU	102	43.859	24.625	33.295	1.00	0.00	H
ATOM	1627	HD13	LEU	102	44.228	25.165	31.669	1.00	0.00	H
ATOM	1628	CD2	LEU	102	44.668	27.093	34.449	1.00	0.00	C
ATOM	1629	HD21	LEU	102	45.706	26.763	34.493	1.00	0.00	H
ATOM	1630	HD22	LEU	102	44.730	28.178	34.536	1.00	0.00	H
ATOM	1631	HD23	LEU	102	43.873	26.639	35.040	1.00	0.00	H
ATOM	1632	C	LEU	102	46.694	29.507	31.169	1.00	0.00	C
ATOM	1633	O	LEU	102	46.324	29.875	30.004	1.00	0.00	O
ATOM	1634	OXT	LEU	102	47.907	29.413	31.480	1.00	0.00	O
HETATM	314	N	LYN	20	39.023	41.371	36.007	1.00	0.00	N
HETATM	315	H	LYN	20	39.565	40.971	35.254	1.00	0.00	H
HETATM	316	CA	LYN	20	38.036	40.448	36.592	1.00	0.00	C
HETATM	317	HA	LYN	20	37.823	40.971	37.524	1.00	0.00	H
HETATM	318	CB	LYN	20	36.730	40.369	35.739	1.00	0.00	C
HETATM	319	HB2	LYN	20	36.909	39.729	34.875	1.00	0.00	H
HETATM	320	HB3	LYN	20	36.435	41.338	35.339	1.00	0.00	H
HETATM	321	CG	LYN	20	35.500	39.733	36.374	1.00	0.00	C
HETATM	322	HG2	LYN	20	34.735	39.566	35.615	1.00	0.00	H
HETATM	323	HG3	LYN	20	35.793	38.730	36.683	1.00	0.00	H
HETATM	324	CD	LYN	20	34.857	40.334	37.685	1.00	0.00	C
HETATM	325	HD2	LYN	20	34.652	41.401	37.588	1.00	0.00	H
HETATM	326	HD3	LYN	20	35.634	40.190	38.436	1.00	0.00	H
HETATM	327	CE	LYN	20	33.597	39.612	38.128	1.00	0.00	C
HETATM	328	HE2	LYN	20	32.849	39.701	37.341	1.00	0.00	H
HETATM	329	HE3	LYN	20	33.926	38.602	38.370	1.00	0.00	H
HETATM	330	NZ	LYN	20	33.012	40.223	39.367	1.00	0.00	N
HETATM	331	HZ2	LYN	20	33.025	41.207	39.144	1.00	0.00	H
HETATM	332	HZ3	LYN	20	33.811	40.184	39.984	1.00	0.00	H
HETATM	333	C	LYN	20	38.777	39.170	36.938	1.00	0.00	C
HETATM	334	O	LYN	20	39.395	38.459	36.119	1.00	0.00	O
HETATM	1636	N	LIG	103	29.809	41.840	41.328	1.00	0.00	N
HETATM	1637	C	LIG	103	29.317	40.932	40.496	1.00	0.00	C
HETATM	1638	O	LIG	103	29.722	43.050	43.251	1.00	0.00	O
HETATM	1639	C5'	LIG	103	24.744	39.885	45.649	1.00	0.00	C
HETATM	1640	O5'	LIG	103	24.347	39.284	44.379	1.00	0.00	O
HETATM	1641	C4'	LIG	103	25.641	41.080	45.506	1.00	0.00	C

```
HETATM 1642  O4' LIG   103      26.851  40.729  44.862  1.00  0.00           O
HETATM 1643  C3' LIG   103      25.127  42.266  44.633  1.00  0.00           C
HETATM 1644  O3' LIG   103      24.269  43.188  45.324  1.00  0.00           O
HETATM 1645  C2' LIG   103      26.456  42.947  44.142  1.00  0.00           C
HETATM 1646  O2' LIG   103      26.756  44.101  44.969  1.00  0.00           O
HETATM 1647  C1' LIG   103      27.582  41.853  44.355  1.00  0.00           C
HETATM 1648  N1  LIG   103      28.247  41.376  43.076  1.00  0.00           N
HETATM 1649  O1  LIG   103      29.355  39.024  38.464  1.00  0.00           O
HETATM 1650  N2  LIG   103      29.591  41.122  39.188  1.00  0.00           N
HETATM 1651  C6  LIG   103      29.321  42.111  42.592  1.00  0.00           C
HETATM 1652  C7  LIG   103      27.700  40.398  42.266  1.00  0.00           C
HETATM 1653  C8  LIG   103      28.255  40.149  41.000  1.00  0.00           C
HETATM 1654  C9  LIG   103      29.527  40.209  38.212  1.00  0.00           C
HETATM 1655  C10 LIG   103      29.803  40.803  36.838  1.00  0.00           C
HETATM 1656  H   LIG   103      30.005  42.012  38.940  1.00  0.00           H
HETATM 1657  H1  LIG   103      27.849  39.484  40.247  1.00  0.00           H
HETATM 1658  H4  LIG   103      28.346  42.198  45.063  1.00  0.00           H
HETATM 1659  H6  LIG   103      25.892  41.458  46.504  1.00  0.00           H
HETATM 1660  H7  LIG   103      24.631  41.897  43.726  1.00  0.00           H
HETATM 1661  H8  LIG   103      26.435  43.318  43.110  1.00  0.00           H
HETATM 1662  H9  LIG   103      23.836  40.158  46.202  1.00  0.00           H
HETATM 1663  H10 LIG   103      25.321  39.177  46.257  1.00  0.00           H
HETATM 1664  H11 LIG   103      29.989  41.878  36.723  1.00  0.00           H
HETATM 1665  H12 LIG   103      29.041  40.538  36.096  1.00  0.00           H
HETATM 1666  H13 LIG   103      30.666  40.276  36.414  1.00  0.00           H
HETATM 1667  H14 LIG   103      26.861  39.867  42.697  1.00  0.00           H
HETATM 1668  H2' LIG   103      26.002  44.713  44.927  1.00  0.00           H
HETATM 1669  H3' LIG   103      23.927  43.775  44.629  1.00  0.00           H
HETATM 1670  H5' LIG   103      23.568  38.745  44.603  1.00  0.00           H
ENDMDL
MODEL      6
SHEET    1   1 1 ILE    22  ASP    26  0
SHEET    2   2 1 VAL    37  VAL    40  0
SHEET    3   3 1 CYX    50  VAL    60  0
SHEET    4   4 1 PHE    94  CYX   101  0
HELIX    1   1 GLN      9  LEU     16  1                                   8
HELIX    2   2 LYS     68  GLN     72  1                                   5
HELIX    3   3 LEU     77  ILE     87  1                                  11
ATOM      1  N   GLN     1      35.199  17.633  32.590  1.00  0.00           N
ATOM      2  H1  GLN     1      35.197  16.624  32.543  1.00  0.00           H
ATOM      3  H2  GLN     1      36.186  17.831  32.518  1.00  0.00           H
ATOM      4  H3  GLN     1      34.974  17.942  33.525  1.00  0.00           H
ATOM      5  CA  GLN     1      34.236  18.236  31.612  1.00  0.00           C
ATOM      6  HA  GLN     1      34.630  18.276  30.598  1.00  0.00           H
ATOM      7  CB  GLN     1      32.754  17.624  31.523  1.00  0.00           C
ATOM      8  HB2 GLN     1      32.410  17.657  32.556  1.00  0.00           H
ATOM      9  HB3 GLN     1      32.156  18.254  30.864  1.00  0.00           H
ATOM     10  CG  GLN     1      32.575  16.186  30.989  1.00  0.00           C
ATOM     11  HG2 GLN     1      33.319  15.576  31.501  1.00  0.00           H
ATOM     12  HG3 GLN     1      31.620  15.730  31.250  1.00  0.00           H
ATOM     13  CD  GLN     1      32.895  16.089  29.514  1.00  0.00           C
ATOM     14  OE1 GLN     1      33.580  16.979  28.901  1.00  0.00           O
ATOM     15  NE2 GLN     1      32.553  14.956  28.991  1.00  0.00           N
ATOM     16 HE21 GLN     1      32.050  14.300  29.574
  1.00  0.00           H
ATOM     17 HE22 GLN     1      32.785  14.783  28.023  1.00  0.00           H
ATOM     18  C   GLN     1      34.080  19.631  32.110  1.00  0.00           C
ATOM     19  O   GLN     1      33.905  19.852  33.322  1.00  0.00           O
ATOM     20  N   PRO     2      34.085  20.672  31.263  1.00  0.00           N
ATOM     21  CD  PRO     2      34.210  20.469  29.800  1.00  0.00           C
ATOM     22  HD2 PRO     2      33.483  19.711  29.508  1.00  0.00           H
ATOM     23  HD3 PRO     2      35.244  20.135  29.717  1.00  0.00           H
ATOM     24  CG  PRO     2      33.963  21.836  29.128  1.00  0.00           C
ATOM     25  HG2 PRO     2      32.886  21.922  28.986  1.00  0.00           H
```

ATOM	26	HG3	PRO	2	34.528	21.830	28.196	1.00	0.00	H
ATOM	27	CB	PRO	2	34.384	22.783	30.223	1.00	0.00	C
ATOM	28	HB2	PRO	2	33.959	23.780	30.107	1.00	0.00	H
ATOM	29	HB3	PRO	2	35.470	22.872	30.259	1.00	0.00	H
ATOM	30	CA	PRO	2	33.848	22.071	31.445	1.00	0.00	C
ATOM	31	HA	PRO	2	34.295	22.405	32.381	1.00	0.00	H
ATOM	32	C	PRO	2	32.313	22.299	31.707	1.00	0.00	C
ATOM	33	O	PRO	2	31.458	21.422	31.362	1.00	0.00	O
ATOM	34	N	ASN	3	31.966	23.488	32.162	1.00	0.00	N
ATOM	35	H	ASN	3	32.653	24.092	32.588	1.00	0.00	H
ATOM	36	CA	ASN	3	30.561	23.878	32.473	1.00	0.00	C
ATOM	37	HA	ASN	3	29.948	22.993	32.640	1.00	0.00	H
ATOM	38	CB	ASN	3	30.618	24.875	33.766	1.00	0.00	C
ATOM	39	HB2	ASN	3	31.518	25.470	33.606	1.00	0.00	H
ATOM	40	HB3	ASN	3	29.711	25.476	33.838	1.00	0.00	H
ATOM	41	CG	ASN	3	30.737	24.105	35.029	1.00	0.00	C
ATOM	42	OD1	ASN	3	29.876	23.452	35.524	1.00	0.00	O
ATOM	43	ND2	ASN	3	31.868	24.015	35.653	1.00	0.00	N
ATOM	44	HD21	ASN	3	32.535	24.664	35.261	1.00	0.00	H
ATOM	45	HD22	ASN	3	31.904	23.510	36.527	1.00	0.00	H
ATOM	46	C	ASN	3	29.978	24.528	31.201	1.00	0.00	C
ATOM	47	O	ASN	3	30.690	25.245	30.448	1.00	0.00	O
ATOM	48	N	ASP	4	28.698	24.280	30.924	1.00	0.00	N
ATOM	49	H	ASP	4	28.162	23.835	31.657	1.00	0.00	H
ATOM	50	CA	ASP	4	27.925	24.743	29.730	1.00	0.00	C
ATOM	51	HA	ASP	4	28.500	24.801	28.805	1.00	0.00	H
ATOM	52	CB	ASP	4	26.725	23.894	29.334	1.00	0.00	C
ATOM	53	HB2	ASP	4	26.008	23.982	30.151	1.00	0.00	H
ATOM	54	HB3	ASP	4	26.293	24.265	28.405	1.00	0.00	H
ATOM	55	CG	ASP	4	26.918	22.405	29.149	1.00	0.00	C
ATOM	56	OD1	ASP	4	27.950	21.917	28.671	1.00	0.00	O
ATOM	57	OD2	ASP	4	26.005	21.587	29.545	1.00	0.00	O
ATOM	58	C	ASP	4	27.479	26.134	30.096	1.00	0.00	C
ATOM	59	O	ASP	4	26.670	26.706	29.352	1.00	0.00	O
ATOM	60	N	ILE	5	27.862	26.799	31.162	1.00	0.00	N
ATOM	61	H	ILE	5	28.589	26.322	31.675	1.00	0.00	H
ATOM	62	CA	ILE	5	27.529	28.109	31.699	1.00	0.00	C
ATOM	63	HA	ILE	5	26.784	28.678	31.142	1.00	0.00	H
ATOM	64	CB	ILE	5	26.977	28.081	33.143	1.00	0.00	C
ATOM	65	HB	ILE	5	27.860	27.738	33.682	1.00	0.00	H
ATOM	66	CG2	ILE	5	26.617	29.479	33.730	1.00	0.00	C
ATOM	67	HG21	ILE	5	25.833	30.029	33.208	1.00	0.00	H
ATOM	68	HG22	ILE	5	26.392	29.379	34.792	1.00	0.00	H
ATOM	69	HG23	ILE	5	27.459	30.148	33.552	1.00	0.00	H
ATOM	70	CG1	ILE	5	25.749	27.108	33.264	1.00	0.00	C
ATOM	71	HG12	ILE	5	26.172	26.123	33.066	1.00	0.00	H
ATOM	72	HG13	ILE	5	25.239	27.253	34.215	1.00	0.00	H
ATOM	73	CD1	ILE	5	24.653	27.322	32.203	1.00	0.00	C
ATOM	74	HD11	ILE	5	23.780	26.802	32.596	1.00	0.00	H
ATOM	75	HD12	ILE	5	24.474	28.397	32.182	1.00	0.00	H
ATOM	76	HD13	ILE	5	24.986	26.886	31.260	1.00	0.00	H
ATOM	77	C	ILE	5	28.804	28.981	31.648	1.00	0.00	C
ATOM	78	O	ILE	5	29.852	28.430	31.919	1.00	0.00	O
ATOM	79	N	THR	6	28.668	30.220	31.176	1.00	0.00	N
ATOM	80	H	THR	6	27.769	30.582	30.889	1.00	0.00	H
ATOM	81	CA	THR	6	29.881	31.100	30.969	1.00	0.00	C
ATOM	82	HA	THR	6	30.419	31.070	31.916	1.00	0.00	H
ATOM	83	CB	THR	6	30.644	30.635	29.726	1.00	0.00	C
ATOM	84	HB	THR	6	30.761	29.553	29.677	1.00	0.00	H
ATOM	85	CG2	THR	6	29.908	31.004	28.457	1.00	0.00	C
ATOM	86	HG21	THR	6	28.893	30.608	28.455	1.00	0.00	H
ATOM	87	HG22	THR	6	29.720	32.074	28.363	1.00	0.00	H
ATOM	88	HG23	THR	6	30.495	30.730	27.580	1.00	0.00	H
ATOM	89	OG1	THR	6	31.959	31.259	29.684	1.00	0.00	O

ATOM	90	HG1	THR	6	32.565	30.646	29.261	1.00	0.00	H
ATOM	91	C	THR	6	29.512	32.582	30.827	1.00	0.00	C
ATOM	92	O	THR	6	28.313	32.986	30.841	1.00	0.00	O
ATOM	93	N	PHE	7	30.554	33.452	30.771	1.00	0.00	N
ATOM	94	H	PHE	7	31.474	33.035	30.769	1.00	0.00	H
ATOM	95	CA	PHE	7	30.565	34.865	30.678	1.00	0.00	C
ATOM	96	HA	PHE	7	30.062	35.250	31.566	1.00	0.00	H
ATOM	97	CB	PHE	7	32.094	35.220	30.829	1.00	0.00	C
ATOM	98	HB2	PHE	7	32.559	34.809	29.933	1.00	0.00	H
ATOM	99	HB3	PHE	7	32.133	36.309	30.816	1.00	0.00	H
ATOM	100	CG	PHE	7	32.728	34.579	32.139	1.00	0.00	C
ATOM	101	CD1	PHE	7	33.535	33.406	32.071	1.00	0.00	C
ATOM	102	HD1	PHE	7	33.737	32.844	31.172	1.00	0.00	H
ATOM	103	CE1	PHE	7	34.089	32.907	33.230	1.00	0.00	C
ATOM	104	HE1	PHE	7	34.701	32.020	33.159	1.00	0.00	H
ATOM	105	CZ	PHE	7	33.845	33.370	34.477	1.00	0.00	C
ATOM	106	HZ	PHE	7	34.192	32.829	35.345	1.00	0.00	H
ATOM	107	CE2	PHE	7	33.058	34.561	34.619	1.00	0.00	C
ATOM	108	HE2	PHE	7	32.960	35.039	35.583	1.00	0.00	H
ATOM	109	CD2	PHE	7	32.517	35.104	33.406	1.00	0.00	C
ATOM	110	HD2	PHE	7	31.941	35.997	33.596	1.00	0.00	H
ATOM	111	C	PHE	7	30.003	35.368	29.374	1.00	0.00	C
ATOM	112	O	PHE	7	30.205	34.804	28.315	1.00	0.00	O
ATOM	113	N	PHE	8	29.445	36.622	29.348	1.00	0.00	N
ATOM	114	H	PHE	8	29.156	37.023	30.229	1.00	0.00	H
ATOM	115	CA	PHE	8	28.979	37.285	28.117	1.00	0.00	C
ATOM	116	HA	PHE	8	28.554	36.530	27.456	1.00	0.00	H
ATOM	117	CB	PHE	8	27.836	38.231	28.499	1.00	0.00	C
ATOM	118	HB2	PHE	8	28.232	39.204	28.791	1.00	0.00	H
ATOM	119	HB3	PHE	8	27.228	38.390	27.608	1.00	0.00	H
ATOM	120	CG	PHE	8	26.920	37.713	29.528	1.00	0.00	C
ATOM	121	CD1	PHE	8	26.796	38.367	30.768	1.00	0.00	C
ATOM	122	HD1	PHE	8	27.376	39.248	30.998	1.00	0.00	H
ATOM	123	CE1	PHE	8	25.872	37.867	31.691	1.00	0.00	C
ATOM	124	HE1	PHE	8	25.726	38.339	32.651	1.00	0.00	H
ATOM	125	CZ	PHE	8	25.117	36.668	31.373	1.00	0.00	C
ATOM	126	HZ	PHE	8	24.361	36.408	32.099	1.00	0.00	H
ATOM	127	CE2	PHE	8	25.235	36.133	30.108	1.00	0.00	C
ATOM	128	HE2	PHE	8	24.567	35.305	29.920	1.00	0.00	H
ATOM	129	CD2	PHE	8	26.182	36.592	29.191	1.00	0.00	C
ATOM	130	HD2	PHE	8	26.241	36.102	28.230	1.00	0.00	H
ATOM	131	C	PHE	8	30.106	37.975	27.333	1.00	0.00	C
ATOM	132	O	PHE	8	31.235	38.208	27.814	1.00	0.00	O
ATOM	133	N	GLN	9	29.939	38.204	26.091	1.00	0.00	N
ATOM	134	H	GLN	9	29.093	37.869	25.652	1.00	0.00	H
ATOM	135	CA	GLN	9	30.929	38.811	25.197	1.00	0.00	C
ATOM	136	HA	GLN	9	31.774	38.125	25.268	1.00	0.00	H
ATOM	137	CB	GLN	9	30.398	38.781	23.703	1.00	0.00	C
ATOM	138	HB2	GLN	9	29.407	39.221	23.589	1.00	0.00	H
ATOM	139	HB3	GLN	9	31.113	39.211	23.001	1.00	0.00	H
ATOM	140	CG	GLN	9	30.347	37.355	23.169	1.00	0.00	C
ATOM	141	HG2	GLN	9	31.332	36.926	23.350	1.00	0.00	H
ATOM	142	HG3	GLN	9	29.598	36.798	23.733	1.00	0.00	H
ATOM	143	CD	GLN	9	30.173	37.190	21.719	1.00	0.00	C
ATOM	144	OE1	GLN	9	30.019	38.162	20.991	1.00	0.00	O
ATOM	145	NE2	GLN	9	29.940	36.020	21.214	1.00	0.00	N
ATOM	146	HE21	GLN	9	29.591	35.919	20.271	1.00	0.00	H
ATOM	147	HE22	GLN	9	29.948	35.168	21.755	1.00	0.00	H
ATOM	148	C	GLN	9	31.575	40.117	25.571	1.00	0.00	C
ATOM	149	O	GLN	9	32.738	40.197	25.352	1.00	0.00	O
ATOM	150	N	ARG	10	30.961	41.037	26.298	1.00	0.00	N
ATOM	151	H	ARG	10	30.006	40.803	26.527	1.00	0.00	H
ATOM	152	CA	ARG	10	31.432	42.257	26.874	1.00	0.00	C

ATOM	153	HA	ARG	10	32.020	42.827	26.154	1.00	0.00	H
ATOM	154	CB	ARG	10	30.247	43.046	27.468	1.00	0.00	C
ATOM	155	HB2	ARG	10	29.520	43.085	26.656	1.00	0.00	H
ATOM	156	HB3	ARG	10	29.847	42.494	28.317	1.00	0.00	H
ATOM	157	CG	ARG	10	30.598	44.513	27.910	1.00	0.00	C
ATOM	158	HG2	ARG	10	31.352	44.566	28.695	1.00	0.00	H
ATOM	159	HG3	ARG	10	31.030	44.998	27.034	1.00	0.00	H
ATOM	160	CD	ARG	10	29.487	45.309	28.383	1.00	0.00	C
ATOM	161	HD2	ARG	10	28.731	44.724	28.906	1.00	0.00	H
ATOM	162	HD3	ARG	10	29.943	45.953	29.135	1.00	0.00	H
ATOM	163	NE	ARG	10	28.887	46.193	27.317	1.00	0.00	N
ATOM	164	HE	ARG	10	29.180	45.967	26.377	1.00	0.00	H
ATOM	165	CZ	ARG	10	27.980	47.084	27.356	1.00	0.00	C
ATOM	166	NH1	ARG	10	27.359	47.386	28.437	1.00	0.00	N
ATOM	167	HH11	ARG	10	26.684	48.131	28.341	1.00	0.00	H
ATOM	168	HH12	ARG	10	27.724	47.025	29.306	1.00	0.00	H
ATOM	169	NH2	ARG	10	27.757	47.708	26.227	1.00	0.00	N
ATOM	170	HH21	ARG	10	28.225	47.373	25.397	1.00	0.00	H
ATOM	171	HH22	ARG	10	26.999	48.367	26.125	1.00	0.00	H
ATOM	172	C	ARG	10	32.492	41.974	27.980	1.00	0.00	C
ATOM	173	O	ARG	10	33.206	42.931	28.338	1.00	0.00	O
ATOM	174	N	PHE	11	32.699	40.771	28.515	1.00	0.00	N
ATOM	175	H	PHE	11	31.965	40.118	28.283	1.00	0.00	H
ATOM	176	CA	PHE	11	33.609	40.504	29.641	1.00	0.00	C
ATOM	177	HA	PHE	11	34.082	41.411	30.016	1.00	0.00	H
ATOM	178	CB	PHE	11	32.754	40.027	30.877	1.00	0.00	C
ATOM	179	HB2	PHE	11	33.384	40.039	31.766	1.00	0.00	H
ATOM	180	HB3	PHE	11	32.343	39.027	30.741	1.00	0.00	H
ATOM	181	CG	PHE	11	31.602	40.964	31.231	1.00	0.00	C
ATOM	182	CD1	PHE	11	31.850	42.237	31.763	1.00	0.00	C
ATOM	183	HD1	PHE	11	32.788	42.558	32.192	1.00	0.00	H
ATOM	184	CE1	PHE	11	30.824	43.216	31.799	1.00	0.00	C
ATOM	185	HE1	PHE	11	30.972	44.201	32.220	1.00	0.00	H
ATOM	186	CZ	PHE	11	29.461	42.802	31.505	1.00	0.00	C
ATOM	187	HZ	PHE	11	28.654	43.504	31.654	1.00	0.00	H
ATOM	188	CE2	PHE	11	29.280	41.515	30.951	1.00	0.00	C
ATOM	189	HE2	PHE	11	28.301	41.387	30.513	1.00	0.00	H
ATOM	190	CD2	PHE	11	30.332	40.639	30.765	1.00	0.00	C
ATOM	191	HD2	PHE	11	30.209	39.706	30.234	1.00	0.00	H
ATOM	192	C	PHE	11	34.772	39.514	29.403	1.00	0.00	C
ATOM	193	O	PHE	11	35.583	39.290	30.287	1.00	0.00	O
ATOM	194	N	GLN	12	34.891	38.959	28.209	1.00	0.00	N
ATOM	195	H	GLN	12	34.192	39.172	27.510	1.00	0.00	H
ATOM	196	CA	GLN	12	35.742	37.777	28.060	1.00	0.00	C
ATOM	197	HA	GLN	12	35.635	37.126	28.929	1.00	0.00	H
ATOM	198	CB	GLN	12	35.462	37.034	26.763	1.00	0.00	C
ATOM	199	HB2	GLN	12	35.505	37.726	25.921	1.00	0.00	H
ATOM	200	HB3	GLN	12	36.211	36.243	26.720	1.00	0.00	H
ATOM	201	CG	GLN	12	34.128	36.265	26.586	1.00	0.00	C
ATOM	202	HG2	GLN	12	34.133	35.423	27.279	1.00	0.00	H
ATOM	203	HG3	GLN	12	33.335	36.923	26.940	1.00	0.00	H
ATOM	204	CD	GLN	12	33.895	35.681	25.175	1.00	0.00	C
ATOM	205	OE1	GLN	12	34.504	36.076	24.223	1.00	0.00	O
ATOM	206	NE2	GLN	12	33.065	34.748	24.915	1.00	0.00	N
ATOM	207	HE21	GLN	12	32.375	34.365	25.546	1.00	0.00	H
ATOM	208	HE22	GLN	12	32.846	34.432	23.980	1.00	0.00	H
ATOM	209	C	GLN	12	37.221	38.196	27.991	1.00	0.00	C
ATOM	210	O	GLN	12	38.118	37.426	28.342	1.00	0.00	O
ATOM	211	N	ASP	13	37.443	39.345	27.364	1.00	0.00	N
ATOM	212	H	ASP	13	36.688	39.865	26.941	1.00	0.00	H
ATOM	213	CA	ASP	13	38.822	39.839	27.110	1.00	0.00	C
ATOM	214	HA	ASP	13	39.454	39.027	26.749	1.00	0.00	H
ATOM	215	CB	ASP	13	38.774	40.882	25.935	1.00	0.00	C
ATOM	216	HB2	ASP	13	38.218	41.779	26.207	1.00	0.00	H

```
ATOM    217  HB3 ASP    13      39.807  41.226  25.886  1.00  0.00           H
ATOM    218  CG  ASP    13      38.284  40.428  24.539  1.00  0.00           C
ATOM    219  OD1 ASP    13      38.134  41.298  23.653  1.00  0.00           O
ATOM    220  OD2 ASP    13      38.050  39.202  24.323  1.00  0.00           O
ATOM    221  C   ASP    13      39.478  40.422  28.363  1.00  0.00           C
ATOM    222  O   ASP    13      40.696  40.237  28.568  1.00  0.00           O
ATOM    223  N   ASP    14      38.698  40.843  29.365  1.00  0.00           N
ATOM    224  H   ASP    14      37.705  40.776  29.188  1.00  0.00           H
ATOM    225  CA  ASP    14      39.136  41.134  30.682  1.00  0.00           C
ATOM    226  HA  ASP    14      39.953  41.856  30.706  1.00  0.00           H
ATOM    227  CB  ASP    14      38.078  41.866  31.515  1.00  0.00           C
ATOM    228  HB2 ASP    14      37.172  41.262  31.567  1.00  0.00           H
ATOM    229  HB3 ASP    14      38.338  41.832  32.573  1.00  0.00           H
ATOM    230  CG  ASP    14      37.877  43.330  31.023  1.00  0.00           C
ATOM    231  OD1 ASP    14      38.871  43.998  30.745  1.00  0.00           O
ATOM    232  OD2 ASP    14      36.729  43.779  31.192  1.00  0.00           O
ATOM    233  C   ASP    14      39.687  39.832  31.380  1.00  0.00           C
ATOM    234  O   ASP    14      40.713  39.815  32.070  1.00  0.00           O
ATOM    235  N   ILE    15      39.015  38.670  31.099  1.00  0.00           N
ATOM    236  H   ILE    15      38.213  38.804  30.501  1.00  0.00           H
ATOM    237  CA  ILE    15      39.285  37.378  31.700  1.00  0.00           C
ATOM    238  HA  ILE    15      39.628  37.487  32.729  1.00  0.00           H
ATOM    239  CB  ILE    15      38.074  36.436  31.818  1.00  0.00           C
ATOM    240  HB  ILE    15      37.683  36.284  30.812  1.00  0.00           H
ATOM    241  CG2 ILE    15      38.580  35.099  32.430  1.00  0.00           C
ATOM    242 HG21 ILE    15      39.309  35.192  33.236  1.00  0.00           H
ATOM    243 HG22 ILE    15      37.699  34.511  32.690  1.00  0.00           H
ATOM    244 HG23 ILE    15      39.178  34.672  31.626  1.00  0.00           H
ATOM    245  CG1 ILE    15      37.047  36.987  32.751  1.00  0.00           C
ATOM    246 HG12 ILE    15      37.480  36.969  33.752  1.00  0.00           H
ATOM    247 HG13 ILE    15      36.928  38.062  32.623  1.00  0.00           H
ATOM    248  CD1 ILE    15      35.609  36.415  32.679  1.00  0.00           C
ATOM    249 HD11 ILE    15      35.060  36.810  33.533  1.00  0.00           H
ATOM    250 HD12 ILE    15      35.039  36.880  31.875  1.00  0.00           H
ATOM    251 HD13 ILE    15      35.518  35.335  32.567  1.00  0.00           H
ATOM    252  C   ILE    15      40.563  36.750  31.070  1.00  0.00           C
ATOM    253  O   ILE    15      41.376  36.177  31.806  1.00  0.00           O
ATOM    254  N   LEU    16      40.689  36.898  29.730  1.00  0.00           N
ATOM    255  H   LEU    16      39.922  37.158  29.127  1.00  0.00           H
ATOM    256  CA  LEU    16      41.975  36.676  29.028  1.00  0.00           C
ATOM    257  HA  LEU    16      42.185  35.623  29.218  1.00  0.00           H
ATOM    258  CB  LEU    16      41.838  36.781  27.461  1.00  0.00           C
ATOM    259  HB2 LEU    16      41.137  36.003  27.157  1.00  0.00           H
ATOM    260  HB3 LEU    16      41.320  37.733  27.335  1.00  0.00           H
ATOM    261  CG  LEU    16      43.150  36.957  26.558  1.00  0.00           C
ATOM    262  HG  LEU    16      43.719  37.834  26.867  1.00  0.00           H
ATOM    263  CD1 LEU    16      44.108  35.743  26.558  1.00  0.00           C
ATOM    264 HD11 LEU    16      43.702  34.927  25.962  1.00  0.00           H
ATOM    265 HD12 LEU    16      45.107  36.032  26.233  1.00  0.00           H
ATOM    266 HD13 LEU    16      44.180  35.286  27.545  1.00  0.00           H
ATOM    267  CD2 LEU    16
   42.743  37.313  25.128  1.00  0.00           C
ATOM    268 HD21 LEU    16      43.597  37.794  24.651  1.00  0.00           H
ATOM    269 HD22 LEU    16      42.464  36.439  24.541  1.00  0.00           H
ATOM    270 HD23 LEU    16      41.994  38.105  25.069  1.00  0.00           H
ATOM    271  C   LEU    16      43.224  37.449  29.486  1.00  0.00           C
ATOM    272  O   LEU    16      44.327  36.913  29.651  1.00  0.00           O
ATOM    273  N   ALA    17      42.964  38.695  29.958  1.00  0.00           N
ATOM    274  H   ALA    17      42.019  39.047  29.912  1.00  0.00           H
ATOM    275  CA  ALA    17      43.908  39.515  30.665  1.00  0.00           C
ATOM    276  HA  ALA    17      44.924  39.288  30.343  1.00  0.00           H
ATOM    277  CB  ALA    17      43.545  40.915  30.371  1.00  0.00           C
ATOM    278  HB1 ALA    17      43.462  41.004  29.288  1.00  0.00           H
ATOM    279  HB2 ALA    17      42.517  41.079  30.694  1.00  0.00           H
```

ATOM	280	HB3	ALA	17	44.264	41.594	30.828	1.00	0.00	H
ATOM	281	C	ALA	17	44.056	39.302	32.171	1.00	0.00	C
ATOM	282	O	ALA	17	45.025	39.699	32.731	1.00	0.00	O
ATOM	283	N	GLY	18	43.195	38.479	32.842	1.00	0.00	N
ATOM	284	H	GLY	18	42.447	38.002	32.359	1.00	0.00	H
ATOM	285	CA	GLY	18	43.154	38.398	34.308	1.00	0.00	C
ATOM	286	HA2	GLY	18	42.552	37.562	34.663	1.00	0.00	H
ATOM	287	HA3	GLY	18	44.140	38.127	34.685	1.00	0.00	H
ATOM	288	C	GLY	18	42.651	39.717	35.042	1.00	0.00	C
ATOM	289	O	GLY	18	42.813	39.773	36.255	1.00	0.00	O
ATOM	290	N	ARG	19	42.048	40.691	34.307	1.00	0.00	N
ATOM	291	H	ARG	19	42.071	40.631	33.299	1.00	0.00	H
ATOM	292	CA	ARG	19	41.443	41.915	34.931	1.00	0.00	C
ATOM	293	HA	ARG	19	42.124	42.285	35.697	1.00	0.00	H
ATOM	294	CB	ARG	19	41.337	43.017	33.812	1.00	0.00	C
ATOM	295	HB2	ARG	19	40.716	42.639	32.999	1.00	0.00	H
ATOM	296	HB3	ARG	19	40.883	43.886	34.289	1.00	0.00	H
ATOM	297	CG	ARG	19	42.713	43.374	33.098	1.00	0.00	C
ATOM	298	HG2	ARG	19	43.449	43.871	33.731	1.00	0.00	H
ATOM	299	HG3	ARG	19	43.150	42.438	32.753	1.00	0.00	H
ATOM	300	CD	ARG	19	42.459	44.209	31.859	1.00	0.00	C
ATOM	301	HD2	ARG	19	43.322	44.060	31.209	1.00	0.00	H
ATOM	302	HD3	ARG	19	41.508	43.912	31.416	1.00	0.00	H
ATOM	303	NE	ARG	19	42.465	45.586	32.329	1.00	0.00	N
ATOM	304	HE	ARG	19	43.290	45.948	32.786	1.00	0.00	H
ATOM	305	CZ	ARG	19	41.503	46.498	32.053	1.00	0.00	C
ATOM	306	NH1	ARG	19	40.236	46.229	31.780	1.00	0.00	N
ATOM	307	HH11	ARG	19	40.025	45.276	31.526	1.00	0.00	H
ATOM	308	HH12	ARG	19	39.537	46.920	31.546	1.00	0.00	H
ATOM	309	NH2	ARG	19	41.772	47.744	32.100	1.00	0.00	N
ATOM	310	HH21	ARG	19	41.104	48.391	31.708	1.00	0.00	H
ATOM	311	HH22	ARG	19	42.661	47.979	32.518	1.00	0.00	H
ATOM	312	C	ARG	19	40.176	41.703	35.666	1.00	0.00	C
ATOM	313	O	ARG	19	39.892	42.401	36.608	1.00	0.00	O
ATOM	335	N	THR	21	38.637	38.589	37.783	1.00	0.00	N
ATOM	336	H	THR	21	38.246	39.385	38.266	1.00	0.00	H
ATOM	337	CA	THR	21	39.275	37.498	38.492	1.00	0.00	C
ATOM	338	HA	THR	21	39.155	36.606	37.877	1.00	0.00	H
ATOM	339	CB	THR	21	40.748	37.821	38.715	1.00	0.00	C
ATOM	340	HB	THR	21	41.283	38.017	37.786	1.00	0.00	H
ATOM	341	CG2	THR	21	40.941	39.045	39.640	1.00	0.00	C
ATOM	342	HG21	THR	21	41.976	39.378	39.713	1.00	0.00	H
ATOM	343	HG22	THR	21	40.402	39.901	39.233	1.00	0.00	H
ATOM	344	HG23	THR	21	40.550	38.769	40.619	1.00	0.00	H
ATOM	345	OG1	THR	21	41.345	36.698	39.355	1.00	0.00	O
ATOM	346	HG1	THR	21	40.641	36.098	39.614	1.00	0.00	H
ATOM	347	C	THR	21	38.573	37.008	39.880	1.00	0.00	C
ATOM	348	O	THR	21	38.813	35.882	40.371	1.00	0.00	O
ATOM	349	N	ILE	22	37.680	37.795	40.441	1.00	0.00	N
ATOM	350	H	ILE	22	37.453	38.568	39.831	1.00	0.00	H
ATOM	351	CA	ILE	22	36.892	37.569	41.646	1.00	0.00	C
ATOM	352	HA	ILE	22	36.844	36.491	41.804	1.00	0.00	H
ATOM	353	CB	ILE	22	37.597	38.108	42.854	1.00	0.00	C
ATOM	354	HB	ILE	22	38.563	37.637	43.030	1.00	0.00	H
ATOM	355	CG2	ILE	22	37.653	39.615	42.745	1.00	0.00	C
ATOM	356	HG21	ILE	22	36.619	39.947	42.653	1.00	0.00	H
ATOM	357	HG22	ILE	22	38.099	39.897	43.698	1.00	0.00	H
ATOM	358	HG23	ILE	22	38.272	40.029	41.948	1.00	0.00	H
ATOM	359	CG1	ILE	22	36.746	37.649	44.133	1.00	0.00	C
ATOM	360	HG12	ILE	22	35.848	38.246	44.290	1.00	0.00	H
ATOM	361	HG13	ILE	22	36.489	36.599	43.992	1.00	0.00	H
ATOM	362	CD1	ILE	22	37.649	37.728	45.382	1.00	0.00	C
ATOM	363	HD11	ILE	22	37.664	38.785	45.645	1.00	0.00	H
ATOM	364	HD12	ILE	22	37.275	37.101	46.191	1.00	0.00	H

ATOM	365	HD13	ILE	22	38.663	37.415	45.132	1.00	0.00	H
ATOM	366	C	ILE	22	35.423	38.148	41.419	1.00	0.00	C
ATOM	367	O	ILE	22	35.137	39.099	40.688	1.00	0.00	O
ATOM	368	N	THR	23	34.462	37.358	41.838	1.00	0.00	N
ATOM	369	H	THR	23	34.662	36.474	42.284	1.00	0.00	H
ATOM	370	CA	THR	23	33.004	37.637	41.770	1.00	0.00	C
ATOM	371	HA	THR	23	32.992	38.725	41.847	1.00	0.00	H
ATOM	372	CB	THR	23	32.503	37.282	40.386	1.00	0.00	C
ATOM	373	HB	THR	23	33.280	37.427	39.637	1.00	0.00	H
ATOM	374	CG2	THR	23	31.860	35.840	40.276	1.00	0.00	C
ATOM	375	HG21	THR	23	31.221	35.640	41.137	1.00	0.00	H
ATOM	376	HG22	THR	23	31.182	35.885	39.423	1.00	0.00	H
ATOM	377	HG23	THR	23	32.620	35.084	40.079	1.00	0.00	H
ATOM	378	OG1	THR	23	31.595	38.330	40.196	1.00	0.00	O
ATOM	379	HG1	THR	23	32.035	39.032	39.709	1.00	0.00	H
ATOM	380	C	THR	23	32.115	37.032	42.934	1.00	0.00	C
ATOM	381	O	THR	23	32.569	36.032	43.511	1.00	0.00	O
ATOM	382	N	ILE	24	31.020	37.745	43.285	1.00	0.00	N
ATOM	383	H	ILE	24	30.868	38.622	42.808	1.00	0.00	H
ATOM	384	CA	ILE	24	30.144	37.482	44.470	1.00	0.00	C
ATOM	385	HA	ILE	24	30.654	36.662	44.977	1.00	0.00	H
ATOM	386	CB	ILE	24	30.250	38.600	45.503	1.00	0.00	C
ATOM	387	HB	ILE	24	30.025	39.497	44.926	1.00	0.00	H
ATOM	388	CG2	ILE	24	29.309	38.555	46.669	1.00	0.00	C
ATOM	389	HG21	ILE	24	29.506	37.612	47.179	1.00	0.00	H
ATOM	390	HG22	ILE	24	29.251	39.395	47.360	1.00	0.00	H
ATOM	391	HG23	ILE	24	28.328	38.487	46.199	1.00	0.00	H
ATOM	392	CG1	ILE	24	31.580	38.961	45.988	1.00	0.00	C
ATOM	393	HG12	ILE	24	31.911	38.094	46.560	1.00	0.00	H
ATOM	394	HG13	ILE	24	32.258	39.140	45.153	1.00	0.00	H
ATOM	395	CD1	ILE	24	31.710	40.208	46.945	1.00	0.00	C
ATOM	396	HD11	ILE	24	32.746	40.451	47.179	1.00	0.00	H
ATOM	397	HD12	ILE	24	31.300	41.057	46.397	1.00	0.00	H
ATOM	398	HD13	ILE	24	31.134	40.091	47.863	1.00	0.00	H
ATOM	399	C	ILE	24	28.631	37.163	44.118	1.00	0.00	C
ATOM	400	O	ILE	24	28.083	37.748	43.180	1.00	0.00	O
ATOM	401	N	ARG	25	28.113	36.100	44.717	1.00	0.00	N
ATOM	402	H	ARG	25	28.588	35.744	45.534	1.00	0.00	H
ATOM	403	CA	ARG	25	26.680	35.637	44.576	1.00	0.00	C
ATOM	404	HA	ARG	25	26.092	36.361	44.012	1.00	0.00	H
ATOM	405	CB	ARG	25	26.672	34.364	43.785	1.00	0.00	C
ATOM	406	HB2	ARG	25	27.185	33.583	44.347	1.00	0.00	H
ATOM	407	HB3	ARG	25	25.636	34.026	43.762	1.00	0.00	H
ATOM	408	CG	ARG	25	27.198	34.532	42.337	1.00	0.00	C
ATOM	409	HG2	ARG	25	28.198	34.964	42.300	1.00	0.00	H
ATOM	410	HG3	ARG	25	27.358	33.537	41.920	1.00	0.00	H
ATOM	411	CD	ARG	25	26.367	35.413	41.466	1.00	0.00	C
ATOM	412	HD2	ARG	25	25.366	34.999	41.576	1.00	0.00	H
ATOM	413	HD3	ARG	25	26.321	36.455	41.785			
1.00	0.00			H						
ATOM	414	NE	ARG	25	26.880	35.450	40.055	1.00	0.00	N
ATOM	415	HE	ARG	25	26.595	34.762	39.372	1.00	0.00	H
ATOM	416	CZ	ARG	25	27.755	36.390	39.658	1.00	0.00	C
ATOM	417	NH1	ARG	25	28.352	37.277	40.323	1.00	0.00	N
ATOM	418	HH11	ARG	25	28.830	38.027	39.844	1.00	0.00	H
ATOM	419	HH12	ARG	25	28.156	37.422	41.303	1.00	0.00	H
ATOM	420	NH2	ARG	25	28.056	36.393	38.380	1.00	0.00	N
ATOM	421	HH21	ARG	25	28.561	37.160	37.962	1.00	0.00	H
ATOM	422	HH22	ARG	25	27.410	35.999	37.712	1.00	0.00	H
ATOM	423	C	ARG	25	26.032	35.487	45.888	1.00	0.00	C
ATOM	424	O	ARG	25	26.700	35.166	46.882	1.00	0.00	O
ATOM	425	N	ASP	26	24.675	35.448	45.934	1.00	0.00	N
ATOM	426	H	ASP	26	24.083	35.629	45.135	1.00	0.00	H
ATOM	427	CA	ASP	26	24.042	34.728	47.022	1.00	0.00	C

ATOM	428	HA	ASP	26	24.446	35.046	47.983	1.00	0.00	H
ATOM	429	CB	ASP	26	22.536	35.149	47.087	1.00	0.00	C
ATOM	430	HB2	ASP	26	22.223	34.691	48.026	1.00	0.00	H
ATOM	431	HB3	ASP	26	22.479	36.224	47.258	1.00	0.00	H
ATOM	432	CG	ASP	26	21.712	34.686	45.811	1.00	0.00	C
ATOM	433	OD1	ASP	26	21.973	35.082	44.659	1.00	0.00	O
ATOM	434	OD2	ASP	26	20.770	33.846	46.035	1.00	0.00	O
ATOM	435	C	ASP	26	24.210	33.201	46.996	1.00	0.00	C
ATOM	436	O	ASP	26	24.506	32.618	45.924	1.00	0.00	O
ATOM	437	N	GLU	27	24.205	32.651	48.209	1.00	0.00	N
ATOM	438	H	GLU	27	24.030	33.354	48.914	1.00	0.00	H
ATOM	439	CA	GLU	27	24.609	31.265	48.541	1.00	0.00	C
ATOM	440	HA	GLU	27	25.584	31.011	48.125	1.00	0.00	H
ATOM	441	CB	GLU	27	24.892	31.141	49.976	1.00	0.00	C
ATOM	442	HB2	GLU	27	25.657	31.871	50.244	1.00	0.00	H
ATOM	443	HB3	GLU	27	23.993	31.475	50.495	1.00	0.00	H
ATOM	444	CG	GLU	27	25.339	29.821	50.559	1.00	0.00	C
ATOM	445	HG2	GLU	27	25.316	30.061	51.622	1.00	0.00	H
ATOM	446	HG3	GLU	27	24.745	28.934	50.332	1.00	0.00	H
ATOM	447	CD	GLU	27	26.785	29.502	50.262	1.00	0.00	C
ATOM	448	OE1	GLU	27	27.099	28.951	49.185	1.00	0.00	O
ATOM	449	OE2	GLU	27	27.641	29.680	51.202	1.00	0.00	O
ATOM	450	C	GLU	27	23.643	30.207	47.987	1.00	0.00	C
ATOM	451	O	GLU	27	22.400	30.326	48.146	1.00	0.00	O
ATOM	452	N	SER	28	24.275	29.143	47.462	1.00	0.00	N
ATOM	453	H	SER	28	25.282	29.097	47.521	1.00	0.00	H
ATOM	454	CA	SER	28	23.520	27.919	47.088	1.00	0.00	C
ATOM	455	HA	SER	28	22.863	27.753	47.942	1.00	0.00	H
ATOM	456	CB	SER	28	22.582	28.129	45.919	1.00	0.00	C
ATOM	457	HB2	SER	28	21.765	28.713	46.344	1.00	0.00	H
ATOM	458	HB3	SER	28	23.193	28.587	45.141	1.00	0.00	H
ATOM	459	OG	SER	28	22.099	26.874	45.544	1.00	0.00	O
ATOM	460	HG	SER	28	21.158	27.002	45.404	1.00	0.00	H
ATOM	461	C	SER	28	24.547	26.773	46.939	1.00	0.00	C
ATOM	462	O	SER	28	25.656	26.953	46.341	1.00	0.00	O
ATOM	463	N	GLU	29	24.182	25.533	47.382	1.00	0.00	N
ATOM	464	H	GLU	29	23.220	25.298	47.583	1.00	0.00	H
ATOM	465	CA	GLU	29	25.103	24.434	47.242	1.00	0.00	C
ATOM	466	HA	GLU	29	26.005	24.865	47.676	1.00	0.00	H
ATOM	467	CB	GLU	29	24.552	23.221	48.031	1.00	0.00	C
ATOM	468	HB2	GLU	29	23.976	23.553	48.894	1.00	0.00	H
ATOM	469	HB3	GLU	29	23.965	22.615	47.340	1.00	0.00	H
ATOM	470	CG	GLU	29	25.702	22.340	48.511	1.00	0.00	C
ATOM	471	HG2	GLU	29	25.364	21.327	48.731	1.00	0.00	H
ATOM	472	HG3	GLU	29	26.534	22.275	47.810	1.00	0.00	H
ATOM	473	CD	GLU	29	26.316	22.833	49.754	1.00	0.00	C
ATOM	474	OE1	GLU	29	26.233	22.163	50.815	1.00	0.00	O
ATOM	475	OE2	GLU	29	27.025	23.832	49.706	1.00	0.00	O
ATOM	476	C	GLU	29	25.392	24.136	45.760	1.00	0.00	C
ATOM	477	O	GLU	29	26.308	23.407	45.449	1.00	0.00	O
ATOM	478	N	SER	30	24.623	24.660	44.857	1.00	0.00	N
ATOM	479	H	SER	30	23.893	25.294	45.148	1.00	0.00	H
ATOM	480	CA	SER	30	24.602	24.268	43.470	1.00	0.00	C
ATOM	481	HA	SER	30	24.833	23.206	43.403	1.00	0.00	H
ATOM	482	CB	SER	30	23.168	24.299	42.903	1.00	0.00	C
ATOM	483	HB2	SER	30	23.154	23.899	41.889	1.00	0.00	H
ATOM	484	HB3	SER	30	22.503	23.778	43.592	1.00	0.00	H
ATOM	485	OG	SER	30	22.815	25.646	42.731	1.00	0.00	O
ATOM	486	HG	SER	30	22.544	25.983	43.588	1.00	0.00	H
ATOM	487	C	SER	30	25.626	24.872	42.521	1.00	0.00	C
ATOM	488	O	SER	30	25.776	24.424	41.429	1.00	0.00	O
ATOM	489	N	HIE	31	26.370	25.961	42.965	1.00	0.00	N
ATOM	490	H	HIE	31	26.075	26.233	43.892	1.00	0.00	H
ATOM	491	CA	HIE	31	27.345	26.669	42.155	1.00	0.00	C

ATOM	492	HA	HIE	31	26.845	26.934	41.221	1.00	0.00	H
ATOM	493	CB	HIE	31	27.750	27.946	42.925	1.00	0.00	C
ATOM	494	HB2	HIE	31	28.267	27.696	43.852	1.00	0.00	H
ATOM	495	HB3	HIE	31	28.430	28.552	42.327	1.00	0.00	H
ATOM	496	CG	HIE	31	26.627	28.975	43.295	1.00	0.00	C
ATOM	497	ND1	HIE	31	25.686	29.584	42.528	1.00	0.00	N
ATOM	498	CE1	HIE	31	25.248	30.699	43.226	1.00	0.00	C
ATOM	499	HE1	HIE	31	24.580	31.452	42.835	1.00	0.00	H
ATOM	500	NE2	HIE	31	25.742	30.639	44.499	1.00	0.00	N
ATOM	501	HE2	HIE	31	25.371	31.264	45.200	1.00	0.00	H
ATOM	502	CD2	HIE	31	26.592	29.572	44.567	1.00	0.00	C
ATOM	503	HD2	HIE	31	27.117	29.218	45.443	1.00	0.00	H
ATOM	504	C	HIE	31	28.570	25.850	41.779	1.00	0.00	C
ATOM	505	O	HIE	31	28.707	24.714	42.152	1.00	0.00	O
ATOM	506	N	PHE	32	29.446	26.506	40.951	1.00	0.00	N
ATOM	507	H	PHE	32	29.231	27.442	40.636	1.00	0.00	H
ATOM	508	CA	PHE	32	30.799	26.027	40.655	1.00	0.00	C
ATOM	509	HA	PHE	32	30.725	25.041	40.198	1.00	0.00	H
ATOM	510	CB	PHE	32	31.504	26.875	39.672	1.00	0.00	C
ATOM	511	HB2	PHE	32	31.800	27.744	40.259	1.00	0.00	H
ATOM	512	HB3	PHE	32	32.382	26.357	39.284	1.00	0.00	H
ATOM	513	CG	PHE	32	30.638	27.378	38.507	1.00	0.00	C
ATOM	514	CD1	PHE	32	30.732	28.716	38.210	1.00	0.00	C
ATOM	515	HD1	PHE	32	31.412	29.302	38.812	1.00	0.00	H
ATOM	516	CE1	PHE	32	30.074	29.253	37.137	1.00	0.00	C
ATOM	517	HE1	PHE	32	30.207	30.264	36.780	1.00	0.00	H
ATOM	518	CZ	PHE	32	29.373	28.438	36.263	1.00	0.00	C
ATOM	519	HZ	PHE	32	29.004	28.749	35.297	1.00	0.00	H
ATOM	520	CE2	PHE	32	29.401	27.054	36.494	1.00	0.00	C
ATOM	521	HE2	PHE	32	28.794	26.409	35.877	1.00	0.00	H
ATOM	522	CD2	PHE	32	30.004	26.510	37.649	1.00	0.00	C
ATOM	523	HD2	PHE	32	29.935	25.486	37.983	1.00	0.00	H
ATOM	524	C	PHE	32	31.639	25.960	41.974	1.00	0.00	C
ATOM	525	O	PHE	32	31.498	26.859	42.762	1.00	0.00	O
ATOM	526	N	LYS	33	32.340	24.832	42.165	1.00	0.00	N
ATOM	527	H	LYS	33	32.370	24.170	41.403	1.00	0.00	H
ATOM	528	CA	LYS	33	33.143	24.469	43.316	1.00	0.00	C
ATOM	529	HA	LYS	33	32.834	25.034	44.194	1.00	0.00	H
ATOM	530	CB	LYS	33	33.022	23.011	43.623	1.00	0.00	C
ATOM	531	HB2	LYS	33	33.562	22.474	42.844	1.00	0.00	H
ATOM	532	HB3	LYS	33	33.467	22.754	44.585	1.00	0.00	H
ATOM	533	CG	LYS	33	31.587	22.492	43.800	1.00	0.00	C
ATOM	534	HG2	LYS	33	31.013	22.499	42.874	1.00	0.00	H
ATOM	535	HG3	LYS	33	31.582	21.533	44.319	1.00	0.00	H
ATOM	536	CD	LYS	33	30.750	23.466	44.659	1.00	0.00	C
ATOM	537	HD2	LYS	33	31.350	23.566	45.564	1.00	0.00	H
ATOM	538	HD3	LYS	33	30.598	24.428	44.170	1.00	0.00	H
ATOM	539	CE	LYS	33	29.403	22.937	45.110	1.00	0.00	C
ATOM	540	HE2	LYS	33	29.599	22.047	45.708	1.00	0.00	H
ATOM	541	HE3	LYS	33	28.856	23.709	45.652	1.00	0.00	H
ATOM	542	NZ	LYS	33	28.544	22.572	43.979	1.00	0.00	N
ATOM	543	HZ1	LYS	33	28.850	21.720	43.531	1.00	0.00	H
ATOM	544	HZ2	LYS	33	27.638	22.371	44.377	1.00	0.00	H
ATOM	545	HZ3	LYS	33	28.478	23.339	43.324	1.00	0.00	H
ATOM	546	C	LYS	33	34.577	24.766	43.093	1.00	0.00	C
ATOM	547	O	LYS	33	35.027	24.853	41.906	1.00	0.00	O
ATOM	548	N	THR	34	35.372	24.804	44.126	1.00	0.00	N
ATOM	549	H	THR	34	34.980	24.542	45.019	1.00	0.00	H
ATOM	550	CA	THR	34	36.861	24.807	43.981	1.00	0.00	C
ATOM	551	HA	THR	34	37.059	25.764	43.499	1.00	0.00	H
ATOM	552	CB	THR	34	37.640	24.866	45.361	1.00	0.00	C
ATOM	553	HB	THR	34	37.238	24.147	46.074	1.00	0.00	H
ATOM	554	CG2	THR	34	39.208	24.758	45.270	1.00	0.00	C

ATOM	555	HG21	THR	34	39.650	25.626	44.782	1.00	0.00	H
ATOM	556	HG22	THR	34	39.468	24.678	46.326	1.00	0.00	H
ATOM	557	HG23	THR	34	39.595	23.826	44.860	1.00	0.00	H
ATOM	558	OG1	THR	34	37.402	26.053	45.992	1.00	0.00	O
ATOM	559	HG1	THR	34	36.471	26.119	46.220	1.00	0.00	H
ATOM	560	C	THR	34	37.346	23.544	43.123	1.00	0.00	C
ATOM	561	O	THR	34	36.924	22.409	43.322	1.00	0.00	O
ATOM	562	N	GLY	35	38.069	23.893	42.059	1.00	0.00	N
ATOM	563	H	GLY	35	38.251	24.879	41.935	1.00	0.00	H
ATOM	564	CA	GLY	35	38.586	22.989	41.001	1.00	0.00	C
ATOM	565	HA2	GLY	35	39.582	23.374	40.782	1.00	0.00	H
ATOM	566	HA3	GLY	35	38.714	21.963	41.349	1.00	0.00	H
ATOM	567	C	GLY	35	37.723	22.939	39.650	1.00	0.00	C
ATOM	568	O	GLY	35	38.170	22.416	38.646	1.00	0.00	O
ATOM	569	N	ASP	36	36.488	23.456	39.623	1.00	0.00	N
ATOM	570	H	ASP	36	36.126	23.823	40.491	1.00	0.00	H
ATOM	571	CA	ASP	36	35.615	23.573	38.340	1.00	0.00	C
ATOM	572	HA	ASP	36	35.478	22.584	37.901	1.00	0.00	H
ATOM	573	CB	ASP	36	34.244	24.108	38.765	1.00	0.00	C
ATOM	574	HB2	ASP	36	34.359	25.008	39.371	1.00	0.00	H
ATOM	575	HB3	ASP	36	33.846	24.404	37.794	1.00	0.00	H
ATOM	576	CG	ASP	36	33.372	23.037	39.439	1.00	0.00	C
ATOM	577	OD1	ASP	36	32.210	23.390	39.853	1.00	0.00	O
ATOM	578	OD2	ASP	36	33.880	21.881	39.683	1.00	0.00	O
ATOM	579	C	ASP	36	36.255	24.362	37.203	1.00	0.00	C
ATOM	580	O	ASP	36	37.049	25.241	37.395	1.00	0.00	O
ATOM	581	N	VAL	37	35.806	23.948	36.006	1.00	0.00	N
ATOM	582	H	VAL	37	35.089	23.245	35.892	1.00	0.00	H
ATOM	583	CA	VAL	37	36.408	24.404	34.724	1.00	0.00	C
ATOM	584	HA	VAL	37	37.214	25.072	35.029	1.00	0.00	H
ATOM	585	CB	VAL	37	37.013	23.241	33.891	1.00	0.00	C
ATOM	586	HB	VAL	37	36.254	22.634	33.398	1.00	0.00	H
ATOM	587	CG1	VAL	37	37.874	23.627	32.646	1.00	0.00	C
ATOM	588	HG11	VAL	37	37.159	24.242	32.100	1.00	0.00	H
ATOM	589	HG12	VAL	37	38.710	24.215	33.026	1.00	0.00	H
ATOM	590	HG13	VAL	37	38.151	22.797	31.997	1.00	0.00	H
ATOM	591	CG2	VAL	37	37.871	22.337	34.727	1.00	0.00	C
ATOM	592	HG21	VAL	37	38.475	22.806	35.504	1.00	0.00	H
ATOM	593	HG22	VAL	37	37.214	21.675	35.290	1.00	0.00	H
ATOM	594	HG23	VAL	37	38.512	21.666	34.154	1.00	0.00	H
ATOM	595	C	VAL	37	35.357	25.143	33.881	1.00	0.00	C
ATOM	596	O	VAL	37	34.193	24.720	33.837	1.00	0.00	O
ATOM	597	N	LEU	38	35.722	26.229	33.223	1.00	0.00	N
ATOM	598	H	LEU	38	36.650	26.585	33.408	1.00	0.00	H
ATOM	599	CA	LEU	38	34.856	27.096	32.311	1.00	0.00	C
ATOM	600	HA	LEU	38	33.925	26.569	32.101	1.00	0.00	H
ATOM	601	CB	LEU	38	34.507	28.368	33.101	1.00	0.00	C
ATOM	602	HB2	LEU	38	35.385	29.003	33.214	1.00	0.00	H
ATOM	603	HB3	LEU	38	33.863	28.927	32.424	1.00	0.00	H
ATOM	604	CG	LEU	38	33.746	28.198	34.417	1.00	0.00	C
ATOM	605	HG	LEU	38	33.042	27.370	34.335	1.00	0.00	H
ATOM	606	CD1	LEU	38	34.501	28.192	35.714	1.00	0.00	C
ATOM	607	HD11	LEU	38	35.004	29.156	35.633	1.00	0.00	H
ATOM	608	HD12	LEU	38	33.746	28.199	36.501	1.00	0.00	H
ATOM	609	HD13	LEU	38	35.217	27.372	35.686	1.00	0.00	H
ATOM	610	CD2	LEU	38	32.927	29.514	34.662	1.00	0.00	C
ATOM	611	HD21	LEU	38	32.178	29.652	33.883	1.00	0.00	H
ATOM	612	HD22	LEU	38	32.332	29.403	35.569	1.00	0.00	H
ATOM	613	HD23	LEU	38	33.572	30.393	34.670	1.00	0.00	H
ATOM	614	C	LEU	38	35.648	27.359	31.041	1.00	0.00	C
ATOM	615	O	LEU	38	36.839	27.084	31.019	1.00	0.00	O
ATOM	616	N	ARG	39	35.013	28.024	30.030	1.00	0.00	N
ATOM	617	H	ARG	39	34.086	28.359	30.249	1.00	0.00	H
ATOM	618	CA	ARG	39	35.579	28.429	28.675	1.00	0.00	C

ATOM	619	HA	ARG	39	36.664	28.482	28.768	1.00	0.00	H
ATOM	620	CB	ARG	39	35.213	27.412	27.627	1.00	0.00	C
ATOM	621	HB2	ARG	39	34.128	27.314	27.636	1.00	0.00	H
ATOM	622	HB3	ARG	39	35.568	27.726	26.645	1.00	0.00	H
ATOM	623	CG	ARG	39	35.901	26.063	27.940	1.00	0.00	C
ATOM	624	HG2	ARG	39	36.947	26.371	27.934	1.00	0.00	H
ATOM	625	HG3	ARG	39	35.646	25.759	28.955	1.00	0.00	H
ATOM	626	CD	ARG	39	35.663	24.901	27.051	1.00	0.00	C
ATOM	627	HD2	ARG	39	36.103	25.234	26.112	1.00	0.00	H
ATOM	628	HD3	ARG	39	36.171	24.054	27.510	1.00	0.00	H
ATOM	629	NE	ARG	39	34.250	24.629	26.876	1.00	0.00	N
ATOM	630	HE	ARG	39	33.564	25.241	27.294	1.00	0.00	H
ATOM	631	CZ	ARG	39	33.697	23.595	26.237	1.00	0.00	C
ATOM	632	NH1	ARG	39	34.409	22.800	25.493	1.00	0.00	N
ATOM	633	HH11	ARG	39	35.412	22.905	25.529	1.00	0.00	H
ATOM	634	HH12	ARG	39	34.028	22.055	24.928	1.00	0.00	H
ATOM	635	NH2	ARG	39	32.407	23.301	26.367	1.00	0.00	N
ATOM	636	HH21	ARG	39	32.139	22.387	26.029	1.00	0.00	H
ATOM	637	HH22	ARG	39	31.838	23.843	27.002	1.00	0.00	H
ATOM	638	C	ARG	39	35.129	29.831	28.177	1.00	0.00	C
ATOM	639	O	ARG	39	33.969	30.166	28.348	1.00	0.00	O
ATOM	640	N	VAL	40	35.978	30.594	27.422	1.00	0.00	N
ATOM	641	H	VAL	40	36.836	30.179	27.092	1.00	0.00	H
ATOM	642	CA	VAL	40	35.585	31.844	26.681	1.00	0.00	C
ATOM	643	HA	VAL	40	34.503	31.950	26.608	1.00	0.00	H
ATOM	644	CB	VAL	40	36.150	33.133	27.355	1.00	0.00	C
ATOM	645	HB	VAL	40	35.943	33.993	26.717	1.00	0.00	H
ATOM	646	CG1	VAL	40	35.547	33.393	28.741	1.00	0.00	C
ATOM	647	HG11	VAL	40	35.971	32.751	29.513	1.00	0.00	H
ATOM	648	HG12	VAL	40	35.699	34.432	29.035	1.00	0.00	H
ATOM	649	HG13	VAL	40	34.483	33.195	28.617	1.00	0.00	H
ATOM	650	CG2	VAL	40	37.671	33.181	27.522	1.00	0.00	C
ATOM	651	HG21	VAL	40	38.070	34.058	28.034	1.00	0.00	H
ATOM	652	HG22	VAL	40	38.042	32.282	28.013	1.00	0.00	H
ATOM	653	HG23	VAL	40	38.164	33.236	26.552	1.00	0.00	H
ATOM	654	C	VAL	40	36.062	31.741	25.232	1.00	0.00	C
ATOM	655	O	VAL	40	37.081	31.111	24.899	1.00	0.00	O
ATOM	656	N	GLY	41	35.232	32.369	24.346	1.00	0.00	N
ATOM	657	H	GLY	41	34.437	32.907	24.660	1.00	0.00	H
ATOM	658	CA	GLY	41	35.333	32.308	22.927	1.00	0.00	C
ATOM	659	HA2	GLY	41	36.342	32.666	22.719	1.00	0.00	H
ATOM	660	HA3	GLY	41	35.411	31.243	22.711	1.00	0.00	H
ATOM	661	C	GLY	41	34.158	32.940	22.165	1.00	0.00	C
ATOM	662	O	GLY	41	33.080	33.058	22.606	1.00	0.00	O
ATOM	663	N	ARG	42	34.426	33.362	20.924	1.00	0.00	N
ATOM	664	H	ARG	42						
35.374	33.346	20.573	1.00	0.00	H					
ATOM	665	CA	ARG	42	33.406	33.893	19.965	1.00	0.00	C
ATOM	666	HA	ARG	42	32.578	33.186	19.905	1.00	0.00	H
ATOM	667	CB	ARG	42	33.063	35.355	20.377	1.00	0.00	C
ATOM	668	HB2	ARG	42	32.379	35.733	19.616	1.00	0.00	H
ATOM	669	HB3	ARG	42	32.435	35.270	21.263	1.00	0.00	H
ATOM	670	CG	ARG	42	34.223	36.387	20.623	1.00	0.00	C
ATOM	671	HG2	ARG	42	34.923	36.088	21.403	1.00	0.00	H
ATOM	672	HG3	ARG	42	34.741	36.562	19.680	1.00	0.00	H
ATOM	673	CD	ARG	42	33.606	37.695	21.059	1.00	0.00	C
ATOM	674	HD2	ARG	42	32.822	38.039	20.383	1.00	0.00	H
ATOM	675	HD3	ARG	42	33.192	37.530	22.054	1.00	0.00	H
ATOM	676	NE	ARG	42	34.675	38.739	21.055	1.00	0.00	N
ATOM	677	HE	ARG	42	34.581	39.538	20.445	1.00	0.00	H
ATOM	678	CZ	ARG	42	35.683	38.895	21.893	1.00	0.00	C
ATOM	679	NH1	ARG	42	35.918	38.188	22.971	1.00	0.00	N
ATOM	680	HH11	ARG	42	36.726	38.382	23.544	1.00	0.00	H
ATOM	681	HH12	ARG	42	35.245	37.495	23.267	1.00	0.00	H

ATOM	682	NH2	ARG	42	36.591	39.764	21.682	1.00	0.00	N
ATOM	683	HH21	ARG	42	36.414	40.433	20.947	1.00	0.00	H
ATOM	684	HH22	ARG	42	37.247	39.951	22.426	1.00	0.00	H
ATOM	685	C	ARG	42	33.903	33.836	18.516	1.00	0.00	C
ATOM	686	O	ARG	42	33.129	34.038	17.575	1.00	0.00	O
ATOM	687	N	PHE	43	35.197	33.482	18.296	1.00	0.00	N
ATOM	688	H	PHE	43	35.674	33.011	19.051	1.00	0.00	H
ATOM	689	CA	PHE	43	35.878	33.429	16.995	1.00	0.00	C
ATOM	690	HA	PHE	43	35.433	33.944	16.144	1.00	0.00	H
ATOM	691	CB	PHE	43	37.326	33.951	17.263	1.00	0.00	C
ATOM	692	HB2	PHE	43	37.806	33.261	17.956	1.00	0.00	H
ATOM	693	HB3	PHE	43	37.765	33.814	16.275	1.00	0.00	H
ATOM	694	CG	PHE	43	37.452	35.355	17.769	1.00	0.00	C
ATOM	695	CD1	PHE	43	37.386	36.394	16.767	1.00	0.00	C
ATOM	696	HD1	PHE	43	37.290	36.187	15.712	1.00	0.00	H
ATOM	697	CE1	PHE	43	37.452	37.751	17.179	1.00	0.00	C
ATOM	698	HE1	PHE	43	37.253	38.562	16.494	1.00	0.00	H
ATOM	699	CZ	PHE	43	37.713	38.027	18.497	1.00	0.00	C
ATOM	700	HZ	PHE	43	37.937	39.009	18.890	1.00	0.00	H
ATOM	701	CE2	PHE	43	37.863	37.000	19.486	1.00	0.00	C
ATOM	702	HE2	PHE	43	37.977	37.288	20.521	1.00	0.00	H
ATOM	703	CD2	PHE	43	37.670	35.690	19.129	1.00	0.00	C
ATOM	704	HD2	PHE	43	37.781	34.956	19.914	1.00	0.00	H
ATOM	705	C	PHE	43	36.000	31.977	16.595	1.00	0.00	C
ATOM	706	O	PHE	43	36.112	31.150	17.541	1.00	0.00	O
ATOM	707	N	GLU	44	35.884	31.712	15.303	1.00	0.00	N
ATOM	708	H	GLU	44	35.632	32.461	14.672	1.00	0.00	H
ATOM	709	CA	GLU	44	35.947	30.341	14.774	1.00	0.00	C
ATOM	710	HA	GLU	44	35.251	29.715	15.334	1.00	0.00	H
ATOM	711	CB	GLU	44	35.450	30.414	13.271	1.00	0.00	C
ATOM	712	HB2	GLU	44	34.390	30.665	13.296	1.00	0.00	H
ATOM	713	HB3	GLU	44	35.915	31.260	12.763	1.00	0.00	H
ATOM	714	CG	GLU	44	35.353	29.082	12.430	1.00	0.00	C
ATOM	715	HG2	GLU	44	35.204	28.380	13.251	1.00	0.00	H
ATOM	716	HG3	GLU	44	34.475	29.127	11.787	1.00	0.00	H
ATOM	717	CD	GLU	44	36.630	28.814	11.736	1.00	0.00	C
ATOM	718	OE1	GLU	44	37.021	27.604	11.696	1.00	0.00	O
ATOM	719	OE2	GLU	44	37.362	29.717	11.306	1.00	0.00	O
ATOM	720	C	GLU	44	37.348	29.686	14.950	1.00	0.00	C
ATOM	721	O	GLU	44	37.383	28.523	15.205	1.00	0.00	O
ATOM	722	N	ASP	45	38.440	30.360	14.873	1.00	0.00	N
ATOM	723	H	ASP	45	38.414	31.364	14.775	1.00	0.00	H
ATOM	724	CA	ASP	45	39.874	29.961	15.024	1.00	0.00	C
ATOM	725	HA	ASP	45	40.009	29.287	14.179	1.00	0.00	H
ATOM	726	CB	ASP	45	40.852	31.126	14.862	1.00	0.00	C
ATOM	727	HB2	ASP	45	40.749	31.502	13.845	1.00	0.00	H
ATOM	728	HB3	ASP	45	40.602	31.915	15.572	1.00	0.00	H
ATOM	729	CG	ASP	45	42.315	30.747	15.059	1.00	0.00	C
ATOM	730	OD1	ASP	45	42.782	29.600	15.053	1.00	0.00	O
ATOM	731	OD2	ASP	45	43.146	31.688	15.185	1.00	0.00	O
ATOM	732	C	ASP	45	40.083	29.230	16.349	1.00	0.00	C
ATOM	733	O	ASP	45	40.176	29.868	17.411	1.00	0.00	O
ATOM	734	N	ASP	46	40.160	27.928	16.325	1.00	0.00	N
ATOM	735	H	ASP	46	40.218	27.461	15.430	1.00	0.00	H
ATOM	736	CA	ASP	46	40.174	27.054	17.550	1.00	0.00	C
ATOM	737	HA	ASP	46	40.151	26.036	17.160	1.00	0.00	H
ATOM	738	CB	ASP	46	41.534	27.211	18.372	1.00	0.00	C
ATOM	739	HB2	ASP	46	41.732	28.277	18.489	1.00	0.00	H
ATOM	740	HB3	ASP	46	41.457	26.675	19.318	1.00	0.00	H
ATOM	741	CG	ASP	46	42.654	26.503	17.669	1.00	0.00	C
ATOM	742	OD1	ASP	46	42.410	25.612	16.798	1.00	0.00	O
ATOM	743	OD2	ASP	46	43.835	26.845	17.856	1.00	0.00	O
ATOM	744	C	ASP	46	38.968	27.093	18.477	1.00	0.00	C
ATOM	745	O	ASP	46	38.971	26.495	19.526	1.00	0.00	O

ATOM	746	N	GLY	47	37.846	27.710	18.157	1.00	0.00	N
ATOM	747	H	GLY	47	38.050	28.216	17.306	1.00	0.00	H
ATOM	748	CA	GLY	47	36.564	27.958	18.811	1.00	0.00	C
ATOM	749	HA2	GLY	47	36.010	28.624	18.148	1.00	0.00	H
ATOM	750	HA3	GLY	47	36.078	27.006	19.022	1.00	0.00	H
ATOM	751	C	GLY	47	36.554	28.790	20.155	1.00	0.00	C
ATOM	752	O	GLY	47	35.569	29.307	20.537	1.00	0.00	O
ATOM	753	N	TYR	48	37.691	28.935	20.850	1.00	0.00	N
ATOM	754	H	TYR	48	38.417	28.404	20.389	1.00	0.00	H
ATOM	755	CA	TYR	48	37.879	29.311	22.268	1.00	0.00	C
ATOM	756	HA	TYR	48	37.172	30.067	22.608	1.00	0.00	H
ATOM	757	CB	TYR	48	37.771	28.078	23.149	1.00	0.00	C
ATOM	758	HB2	TYR	48	38.507	27.332	22.848	1.00	0.00	H
ATOM	759	HB3	TYR	48	37.979	28.391	24.172	1.00	0.00	H
ATOM	760	CG	TYR	48	36.369	27.489	23.190	1.00	0.00	C
ATOM	761	CD1	TYR	48	35.251	28.161	23.753	1.00	0.00	C
ATOM	762	HD1	TYR	48	35.399	29.175	24.095	1.00	0.00	H
ATOM	763	CE1	TYR	48	34.021	27.411	24.039	1.00	0.00	C
ATOM	764	HE1	TYR	48	33.211	27.980	24.472	1.00	0.00	H
ATOM	765	CZ	TYR	48	33.879	26.070	23.628	1.00	0.00	C
ATOM	766	OH	TYR	48	32.700	25.432	23.880	1.00	0.00	O
ATOM	767	HH	TYR	48	32.588	24.571	23.471	1.00	0.00	H
ATOM	768	CE2	TYR	48	34.938	25.465	22.907	1.00	0.00	C
ATOM	769	HE2	TYR	48	34.793	24.452	22.560	1.00	0.00	H
ATOM	770	CD2	TYR	48	36.143	26.177	22.776	1.00	0.00	C
ATOM	771	HD2	TYR	48	36.966	25.598	22.385	1.00	0.00	H
ATOM	772	C	TYR	48	39.286	29.916	22.402	1.00	0.00	C
ATOM	773	O	TYR	48	40.187	29.451	21.707	1.00	0.00	O
ATOM	774	N	PHE	49	39.472	30.867	23.304	1.00	0.00	N
ATOM	775	H	PHE	49	38.714	31.217	23.872	1.00	0.00	H
ATOM	776	CA	PHE	49	40.714	31.526	23.559	1.00	0.00	C
ATOM	777	HA	PHE	49	41.580	31.020	23.132	1.00	0.00	H
ATOM	778	CB	PHE	49	40.673	32.950	22.896	1.00	0.00	C
ATOM	779	HB2	PHE	49	41.702	33.275	23.046	1.00	0.00	H
ATOM	780	HB3	PHE	49	40.421	32.943	21.836	1.00	0.00	H
ATOM	781	CG	PHE	49	39.708	33.860	23.577	1.00	0.00	C
ATOM	782	CD1	PHE	49	38.523	34.190	22.898	1.00	0.00	C
ATOM	783	HD1	PHE	49	38.208	33.662	22.011	1.00	0.00	H
ATOM	784	CE1	PHE	49	37.573	35.065	23.386	1.00	0.00	C
ATOM	785	HE1	PHE	49	36.665	35.310	22.853	1.00	0.00	H
ATOM	786	CZ	PHE	49	37.927	35.754	24.528	1.00	0.00	C
ATOM	787	HZ	PHE	49	37.286	36.553	24.869	1.00	0.00	H
ATOM	788	CE2	PHE	49	39.136	35.495	25.239	1.00	0.00	C
ATOM	789	HE2	PHE	49	39.396	35.984	26.167			
1.00	0.00			H						
ATOM	790	CD2	PHE	49	40.051	34.482	24.762	1.00	0.00	C
ATOM	791	HD2	PHE	49	40.908	34.099	25.295	1.00	0.00	H
ATOM	792	C	PHE	49	41.163	31.620	25.044	1.00	0.00	C
ATOM	793	O	PHE	49	42.209	32.204	25.259	1.00	0.00	O
ATOM	794	N	CYX	50	40.469	31.010	25.970	1.00	0.00	N
ATOM	795	H	CYX	50	39.569	30.669	25.665	1.00	0.00	H
ATOM	796	CA	CYX	50	40.851	30.743	27.297	1.00	0.00	C
ATOM	797	HA	CYX	50	41.868	30.350	27.278	1.00	0.00	H
ATOM	798	CB	CYX	50	40.761	32.005	28.128	1.00	0.00	C
ATOM	799	HB2	CYX	50	40.245	32.783	27.564	1.00	0.00	H
ATOM	800	HB3	CYX	50	40.120	31.744	28.970	1.00	0.00	H
ATOM	801	SG	CYX	50	42.392	32.603	28.761	1.00	0.00	S
ATOM	802	C	CYX	50	39.915	29.687	27.832	1.00	0.00	C
ATOM	803	O	CYX	50	38.726	29.682	27.581	1.00	0.00	O
ATOM	804	N	THR	51	40.508	28.878	28.687	1.00	0.00	N
ATOM	805	H	THR	51	41.492	29.033	28.853	1.00	0.00	H
ATOM	806	CA	THR	51	39.966	27.874	29.557	1.00	0.00	C
ATOM	807	HA	THR	51	38.880	27.829	29.470	1.00	0.00	H
ATOM	808	CB	THR	51	40.488	26.442	29.100	1.00	0.00	C

ATOM	809	HB	THR	51	41.546	26.388	29.361	1.00	0.00	H
ATOM	810	CG2	THR	51	39.572	25.320	29.731	1.00	0.00	C
ATOM	811	HG21	THR	51	39.660	25.262	30.816	1.00	0.00	H
ATOM	812	HG22	THR	51	38.523	25.570	29.563	1.00	0.00	H
ATOM	813	HG23	THR	51	39.796	24.317	29.368	1.00	0.00	H
ATOM	814	OG1	THR	51	40.223	26.253	27.757	1.00	0.00	O
ATOM	815	HG1	THR	51	40.939	26.743	27.345	1.00	0.00	H
ATOM	816	C	THR	51	40.364	28.283	31.036	1.00	0.00	C
ATOM	817	O	THR	51	41.492	28.685	31.373	1.00	0.00	O
ATOM	818	N	ILE	52	39.422	28.177	31.972	1.00	0.00	N
ATOM	819	H	ILE	52	38.603	27.752	31.561	1.00	0.00	H
ATOM	820	CA	ILE	52	39.456	28.875	33.285	1.00	0.00	C
ATOM	821	HA	ILE	52	40.401	29.355	33.541	1.00	0.00	H
ATOM	822	CB	ILE	52	38.379	29.987	33.249	1.00	0.00	C
ATOM	823	HB	ILE	52	37.398	29.586	32.991	1.00	0.00	H
ATOM	824	CG2	ILE	52	38.180	30.728	34.597	1.00	0.00	C
ATOM	825	HG21	ILE	52	37.477	31.547	34.448	1.00	0.00	H
ATOM	826	HG22	ILE	52	37.694	30.162	35.391	1.00	0.00	H
ATOM	827	HG23	ILE	52	39.087	31.237	34.926	1.00	0.00	H
ATOM	828	CG1	ILE	52	38.729	31.093	32.235	1.00	0.00	C
ATOM	829	HG12	ILE	52	39.215	31.921	32.748	1.00	0.00	H
ATOM	830	HG13	ILE	52	39.467	30.738	31.516	1.00	0.00	H
ATOM	831	CD1	ILE	52	37.517	31.604	31.412	1.00	0.00	C
ATOM	832	HD11	ILE	52	36.751	31.831	32.154	1.00	0.00	H
ATOM	833	HD12	ILE	52	37.912	32.582	31.136	1.00	0.00	H
ATOM	834	HD13	ILE	52	37.242	30.876	30.649	1.00	0.00	H
ATOM	835	C	ILE	52	39.139	27.914	34.424	1.00	0.00	C
ATOM	836	O	ILE	52	38.169	27.140	34.391	1.00	0.00	O
ATOM	837	N	GLU	53	39.937	27.911	35.501	1.00	0.00	N
ATOM	838	H	GLU	53	40.600	28.668	35.573	1.00	0.00	H
ATOM	839	CA	GLU	53	39.648	27.144	36.712	1.00	0.00	C
ATOM	840	HA	GLU	53	38.783	26.497	36.564	1.00	0.00	H
ATOM	841	CB	GLU	53	40.861	26.276	37.120	1.00	0.00	C
ATOM	842	HB2	GLU	53	41.823	26.787	37.135	1.00	0.00	H
ATOM	843	HB3	GLU	53	40.685	25.824	38.096	1.00	0.00	H
ATOM	844	CG	GLU	53	40.865	25.071	36.260	1.00	0.00	C
ATOM	845	HG2	GLU	53	40.027	24.515	36.679	1.00	0.00	H
ATOM	846	HG3	GLU	53	40.728	25.273	35.199	1.00	0.00	H
ATOM	847	CD	GLU	53	41.972	24.090	36.503	1.00	0.00	C
ATOM	848	OE1	GLU	53	42.991	24.455	37.124	1.00	0.00	O
ATOM	849	OE2	GLU	53	41.882	22.906	36.116	1.00	0.00	O
ATOM	850	C	GLU	53	39.212	28.038	37.928	1.00	0.00	C
ATOM	851	O	GLU	53	39.718	29.201	38.054	1.00	0.00	O
ATOM	852	N	VAL	54	38.249	27.514	38.713	1.00	0.00	N
ATOM	853	H	VAL	54	38.040	26.555	38.472	1.00	0.00	H
ATOM	854	CA	VAL	54	37.818	28.096	40.000	1.00	0.00	C
ATOM	855	HA	VAL	54	37.786	29.183	39.927	1.00	0.00	H
ATOM	856	CB	VAL	54	36.406	27.649	40.431	1.00	0.00	C
ATOM	857	HB	VAL	54	36.250	26.571	40.471	1.00	0.00	H
ATOM	858	CG1	VAL	54	36.048	28.108	41.869	1.00	0.00	C
ATOM	859	HG11	VAL	54	35.038	27.857	42.194	1.00	0.00	H
ATOM	860	HG12	VAL	54	36.733	27.654	42.585	1.00	0.00	H
ATOM	861	HG13	VAL	54	36.196	29.182	41.986	1.00	0.00	H
ATOM	862	CG2	VAL	54	35.312	28.430	39.616	1.00	0.00	C
ATOM	863	HG21	VAL	54	35.576	29.481	39.729	1.00	0.00	H
ATOM	864	HG22	VAL	54	35.423	28.101	38.583	1.00	0.00	H
ATOM	865	HG23	VAL	54	34.304	28.325	40.018	1.00	0.00	H
ATOM	866	C	VAL	54	38.786	27.679	41.046	1.00	0.00	C
ATOM	867	O	VAL	54	38.833	26.548	41.453	1.00	0.00	O
ATOM	868	N	THR	55	39.664	28.622	41.425	1.00	0.00	N
ATOM	869	H	THR	55	39.664	29.539	40.999	1.00	0.00	H
ATOM	870	CA	THR	55	40.864	28.288	42.205	1.00	0.00	C
ATOM	871	HA	THR	55	41.018	27.210	42.228	1.00	0.00	H
ATOM	872	CB	THR	55	42.204	28.976	41.658	1.00	0.00	C

ATOM	873	HB	THR	55	42.983	28.347	42.091	1.00	0.00	H
ATOM	874	CG2	THR	55	42.352	28.854	40.166	1.00	0.00	C
ATOM	875	HG21	THR	55	42.171	27.818	39.876	1.00	0.00	H
ATOM	876	HG22	THR	55	41.625	29.498	39.670	1.00	0.00	H
ATOM	877	HG23	THR	55	43.387	29.020	39.870	1.00	0.00	H
ATOM	878	OG1	THR	55	42.444	30.277	42.144	1.00	0.00	O
ATOM	879	HG1	THR	55	42.872	30.087	42.983	1.00	0.00	H
ATOM	880	C	THR	55	40.776	28.555	43.690	1.00	0.00	C
ATOM	881	O	THR	55	41.520	27.988	44.489	1.00	0.00	O
ATOM	882	N	ALA	56	39.709	29.267	44.106	1.00	0.00	N
ATOM	883	H	ALA	56	39.045	29.603	43.422	1.00	0.00	H
ATOM	884	CA	ALA	56	39.393	29.440	45.527	1.00	0.00	C
ATOM	885	HA	ALA	56	39.663	28.524	46.052	1.00	0.00	H
ATOM	886	CB	ALA	56	40.384	30.474	46.108	1.00	0.00	C
ATOM	887	HB1	ALA	56	40.140	30.472	47.170	1.00	0.00	H
ATOM	888	HB2	ALA	56	41.411	30.142	45.950	1.00	0.00	H
ATOM	889	HB3	ALA	56	40.130	31.454	45.704	1.00	0.00	H
ATOM	890	C	ALA	56	37.914	29.914	45.718	1.00	0.00	C
ATOM	891	O	ALA	56	37.366	30.528	44.789	1.00	0.00	O
ATOM	892	N	THR	57	37.316	29.718	46.903	1.00	0.00	N
ATOM	893	H	THR	57	37.708	29.230	47.696	1.00	0.00	H
ATOM	894	CA	THR	57	35.902	30.081	47.190	1.00	0.00	C
ATOM	895	HA	THR	57	35.716	31.059	46.745	1.00	0.00	H
ATOM	896	CB	THR	57	34.893	28.954	46.821	1.00	0.00	C
ATOM	897	HB	THR	57	33.888	29.091	47.219	1.00	0.00	H
ATOM	898	CG2	THR	57	34.837	28.700	45.326	1.00	0.00	C
ATOM	899	HG21	THR	57	35.804	28.402	44.922	1.00	0.00	H
ATOM	900	HG22	THR	57	34.038	28.026	45.020	1.00	0.00	H
ATOM	901	HG23	THR	57	34.490	29.656	44.933	1.00	0.00	H
ATOM	902	OG1	THR	57	35.295	27.722	47.420	1.00	0.00	O
ATOM	903	HG1	THR	57	35.121	27.674	48.363	1.00	0.00	H
ATOM	904	C	THR	57	35.746	30.326	48.679	1.00	0.00	C
ATOM	905	O	THR	57	36.610	29.921	49.492	1.00	0.00	O
ATOM	906	N	SER	58	34.645	30.963	49.074	1.00	0.00	N
ATOM	907	H	SER	58	34.047	31.380	48.374	1.00	0.00	H
ATOM	908	CA	SER	58	34.280	31.112	50.460	1.00	0.00	C
ATOM	909	HA	SER	58	34.454	30.125	50.890	1.00	0.00	H
ATOM	910	CB	SER	58	35.106	32.172	51.273	1.00	0.00	C
ATOM	911	HB2	SER	58	34.659	32.253	52.264	1.00	0.00	H
ATOM	912	HB3	SER	58	36.114	31.770	51.364	1.00	0.00	H
ATOM	913	OG	SER	58	35.129	33.455	50.648	1.00	0.00	O
ATOM	914	HG	SER	58	35.905	33.455	50.082	1.00	0.00	H
ATOM	915	C	SER	58	32.790	31.554	50.536	1.00	0.00	C
ATOM	916	O	SER	58	32.269	32.167	49.616	1.00	0.00	O
ATOM	917	N	THR	59	32.188	31.156	51.697	1.00	0.00	N
ATOM	918	H	THR	59	32.667	30.547	52.344	1.00	0.00	H
ATOM	919	CA	THR	59	30.915	31.751	52.108	1.00	0.00	C
ATOM	920	HA	THR	59	30.318	32.079	51.258	1.00	0.00	H
ATOM	921	CB	THR	59	30.070	30.742	52.885	1.00	0.00	C
ATOM	922	HB	THR	59	30.006	29.812	52.319	1.00	0.00	H
ATOM	923	CG2	THR	59	30.681	30.303	54.200	1.00	0.00	C
ATOM	924	HG21	THR	59	30.844	31.097	54.929	1.00	0.00	H
ATOM	925	HG22	THR	59	30.007	29.525	54.560	1.00	0.00	H
ATOM	926	HG23	THR	59	31.667	29.865	54.045	1.00	0.00	H
ATOM	927	OG1	THR	59	28.761	31.134	53.074	1.00	0.00	O
ATOM	928	HG1	THR	59	28.283	30.997	52.253	1.00	0.00	H
ATOM	929	C	THR	59	31.198	33.016	52.969	1.00	0.00	C
ATOM	930	O	THR	59	31.878	33.011	53.948	1.00	0.00	O
ATOM	931	N	VAL	60	30.588	34.161	52.711	1.00	0.00	N
ATOM	932	H	VAL	60	29.856	34.094	52.018	1.00	0.00	H
ATOM	933	CA	VAL	60	30.754	35.435	53.375	1.00	0.00	C
ATOM	934	HA	VAL	60	31.257	35.263	54.326	1.00	0.00	H
ATOM	935	CB	VAL	60	31.559	36.437	52.599	1.00	0.00	C

ATOM	936	HB	VAL	60	31.643	37.292	53.269	1.00	0.00	H
ATOM	937	CG1	VAL	60	32.978	36.017	52.353	1.00	0.00	C
ATOM	938	HG11	VAL	60	33.578	36.745	51.807	1.00	0.00	H
ATOM	939	HG12	VAL	60	33.545	35.912	53.278	1.00	0.00	H
ATOM	940	HG13	VAL	60	33.207	35.139	51.748	1.00	0.00	H
ATOM	941	CG2	VAL	60	30.854	36.900	51.280	1.00	0.00	C
ATOM	942	HG21	VAL	60	31.408	37.537	50.590	1.00	0.00	H
ATOM	943	HG22	VAL	60	30.537	36.005	50.746	1.00	0.00	H
ATOM	944	HG23	VAL	60	29.933	37.446	51.481	1.00	0.00	H
ATOM	945	C	VAL	60	29.392	36.106	53.619	1.00	0.00	C
ATOM	946	O	VAL	60	28.409	35.787	52.949	1.00	0.00	O
ATOM	947	N	THR	61	29.366	37.147	54.494	1.00	0.00	N
ATOM	948	H	THR	61	30.249	37.361	54.935	1.00	0.00	H
ATOM	949	CA	THR	61	28.298	38.069	54.929	1.00	0.00	C
ATOM	950	HA	THR	61	27.495	38.127	54.195	1.00	0.00	H
ATOM	951	CB	THR	61	27.612	37.715	56.257	1.00	0.00	C
ATOM	952	HB	THR	61	26.775	38.342	56.565	1.00	0.00	H
ATOM	953	CG2	THR	61	26.842	36.402	56.077	1.00	0.00	C
ATOM	954	HG21	THR	61	26.095	36.436	56.869	1.00	0.00	H
ATOM	955	HG22	THR	61	26.352	36.299	55.109	1.00	0.00	H
ATOM	956	HG23	THR	61	27.538	35.582	56.252	1.00	0.00	H
ATOM	957	OG1	THR	61	28.587	37.462	57.255	1.00	0.00	O
ATOM	958	HG1	THR	61	28.632	38.327	57.669	1.00	0.00	H
ATOM	959	C	THR	61	28.840	39.576	55.019	1.00	0.00	C
ATOM	960	O	THR	61	30.071	39.749	54.907	1.00	0.00	O
ATOM	961	N	LEU	62	27.957	40.583	55.106	1.00	0.00	N
ATOM	962	H	LEU	62	27.004	40.298	54.934	1.00	0.00	H
ATOM	963	CA	LEU	62	28.323	42.022	55.312	1.00	0.00	C
ATOM	964	HA	LEU	62	28.712	42.450	54.388	1.00	0.00	H
ATOM	965	CB	LEU	62	27.026	42.774	55.676	1.00	0.00	C
ATOM	966	HB2	LEU	62	26.634	42.299	56.576	1.00	0.00	H
ATOM	967	HB3	LEU	62	27.195	43.805	55.986	1.00	0.00	H
ATOM	968	CG	LEU	62	26.031	42.861	54.446	1.00	0.00	C
ATOM	969	HG	LEU	62	25.767	41.890	54.029	1.00	0.00	H
ATOM	970	CD1	LEU	62	24.652	43.378	54.876	1.00	0.00	C
ATOM	971	HD11	LEU	62	24.258	42.758	55.683	1.00	0.00	H
ATOM	972	HD12	LEU	62	24.722	44.444	55.094	1.00	0.00	H
ATOM	973	HD13	LEU	62	23.969	43.366	54.027	1.00	0.00	H
ATOM	974	CD2	LEU	62	26.525	43.920	53.400	1.00	0.00	C
ATOM	975	HD21	LEU	62	26.591	44.894	53.885	1.00	0.00	H
ATOM	976	HD22	LEU	62	27.472	43.623	52.949	1.00	0.00	H
ATOM	977	HD23	LEU	62	25.847	43.914	52.546	1.00	0.00	H
ATOM	978	C	LEU	62	29.407	42.323	56.352	1.00	0.00	C
ATOM	979	O	LEU	62	30.064	43.352	56.269	1.00	0.00	O
ATOM	980	N	ASP	63	29.516	41.568	57.392	1.00	0.00	N
ATOM	981	H	ASP	63	28.883	40.780	57.425	1.00	0.00	H
ATOM	982	CA	ASP	63	30.371	41.689	58.610	1.00	0.00	C
ATOM	983	HA	ASP	63	30.699	42.727	58.566	1.00	0.00	H
ATOM	984	CB	ASP	63	29.625	41.468	59.950	1.00	0.00	C
ATOM	985	HB2	ASP	63	30.241	41.192	60.806	1.00	0.00	H
ATOM	986	HB3	ASP	63	29.221	42.399	60.347	1.00	0.00	H
ATOM	987	CG	ASP	63	28.523	40.395	59.952	1.00	0.00	C
ATOM	988	OD1	ASP	63	28.422	39.694	58.933	1.00	0.00	O
ATOM	989	OD2	ASP	63	27.643	40.351	60.844	1.00	0.00	O
ATOM	990	C	ASP	63	31.548	40.784	58.465	1.00	0.00	C
ATOM	991	O	ASP	63	32.346	40.711	59.409	1.00	0.00	O
ATOM	992	N	THR	64	31.752	40.166	57.275	1.00	0.00	N
ATOM	993	H	THR	64	31.074	40.272	56.534	1.00	0.00	H
ATOM	994	CA	THR	64	32.921	39.233	57.117	1.00	0.00	C
ATOM	995	HA	THR	64	33.702	39.539	57.814	1.00	0.00	H
ATOM	996	CB	THR	64	32.577	37.791	57.456	1.00	0.00	C
ATOM	997	HB	THR	64	33.540	37.284	57.493	1.00	0.00	H
ATOM	998	CG2	THR	64	31.789	37.577	58.692	1.00	0.00	C
ATOM	999	HG21	THR	64	32.425	38.069	59.427	1.00	0.00	H

ATOM	1000	HG22	THR	64	30.823	38.072	58.595	1.00	0.00	H
ATOM	1001	HG23	THR	64	31.720	36.526	58.976	1.00	0.00	H
ATOM	1002	OG1	THR	64	31.892	37.151	56.366	1.00	0.00	O
ATOM	1003	HG1	THR	64	32.472	36.500	55.963	1.00	0.00	H
ATOM	1004	C	THR	64	33.640	39.205	55.713	1.00	0.00	C
ATOM	1005	O	THR	64	34.862	39.001	55.623	1.00	0.00	O
ATOM	1006	N	LEU	65	32.964	39.653	54.677	1.00	0.00	N
ATOM	1007	H	LEU	65	31.975	39.763	54.848	1.00	0.00	H
ATOM	1008	CA	LEU	65	33.515	40.137	53.411	1.00	0.00	C
ATOM	1009	HA	LEU	65	33.716	39.232	52.838	1.00	0.00	H
ATOM	1010	CB	LEU	65	32.382	40.756	52.591	1.00	0.00	C
ATOM	1011	HB2	LEU	65	31.518	40.093	52.567	1.00	0.00	H
ATOM	1012	HB3	LEU	65	31.998	41.617	53.138	1.00	0.00	H
ATOM	1013	CG	LEU	65	32.817	41.183	51.195	1.00	0.00	C
ATOM	1014	HG	LEU	65	33.549	41.988	51.266	1.00	0.00	H
ATOM	1015	CD1	LEU	65	33.477	40.118	50.256	1.00	0.00	C
ATOM	1016	HD11	LEU	65	32.884	39.216	50.109	1.00	0.00	H
ATOM	1017	HD12	LEU	65	33.639	40.623	49.303	1.00	0.00	H
ATOM	1018	HD13	LEU	65	34.454	39.956	50.712	1.00	0.00	H
ATOM	1019	CD2	LEU	65	31.666	41.889	50.507	1.00	0.00	C
ATOM	1020	HD21	LEU	65	31.568	42.892	50.919	1.00	0.00	H
ATOM	1021	HD22	LEU	65	31.907	42.056	49.458	1.00	0.00	H
ATOM	1022	HD23	LEU	65	30.714	41.359	50.458	1.00	0.00	H
ATOM	1023	C	LEU	65	34.656	41.163	53.575	1.00	0.00	C
ATOM	1024	O	LEU	65	34.521	42.295	54.119	1.00	0.00	O
ATOM	1025	N	THR	66	35.829	40.814	52.951	1.00	0.00	N
ATOM	1026	H	THR	66	35.859	39.909	52.504	1.00	0.00	H
ATOM	1027	CA	THR	66	37.100	41.523	52.848	1.00	0.00	C
ATOM	1028	HA	THR	66	37.230	42.223	53.673	1.00	0.00	H
ATOM	1029	CB	THR	66	38.279	40.536	52.929	1.00	0.00	C
ATOM	1030	HB	THR	66	38.169	39.842	52.095	1.00	0.00	H
ATOM	1031	CG2	THR	66	39.643	41.212	52.854	1.00	0.00	C
ATOM	1032	HG21	THR	66	39.788	42.089	53.486	1.00	0.00	H
ATOM	1033	HG22	THR	66	40.346	40.485	53.261	1.00	0.00	H
ATOM	1034	HG23	THR	66	39.906	41.493	51.834	1.00	0.00	H
ATOM	1035	OG1	THR	66	38.215	39.808	54.103	1.00	0.00	O
ATOM	1036	HG1	THR	66	37.475	39.196	54.078	1.00	0.00	H
ATOM	1037	C	THR	66	37.141	42.344	51.562	1.00	0.00	C
ATOM	1038	O	THR	66	36.413	42.094	50.614	1.00	0.00	O
ATOM	1039	N	GLU	67	37.950	43.368	51.481	1.00	0.00	N
ATOM	1040	H	GLU	67						
38.481	43.577	52.314	1.00	0.00	H					
ATOM	1041	CA	GLU	67	37.845	44.367	50.427	1.00	0.00	C
ATOM	1042	HA	GLU	67	36.769	44.503	50.319	1.00	0.00	H
ATOM	1043	CB	GLU	67	38.579	45.714	50.823	1.00	0.00	C
ATOM	1044	HB2	GLU	67	38.310	46.528	50.149	1.00	0.00	H
ATOM	1045	HB3	GLU	67	38.252	46.090	51.791	1.00	0.00	H
ATOM	1046	CG	GLU	67	40.102	45.611	50.889	1.00	0.00	C
ATOM	1047	HG2	GLU	67	40.582	45.351	49.946	1.00	0.00	H
ATOM	1048	HG3	GLU	67	40.358	46.639	51.147	1.00	0.00	H
ATOM	1049	CD	GLU	67	40.644	44.814	52.057	1.00	0.00	C
ATOM	1050	OE1	GLU	67	41.830	44.445	51.916	1.00	0.00	O
ATOM	1051	OE2	GLU	67	40.017	44.580	53.052	1.00	0.00	O
ATOM	1052	C	GLU	67	38.390	43.922	49.013	1.00	0.00	C
ATOM	1053	O	GLU	67	38.475	44.816	48.179	1.00	0.00	O
ATOM	1054	N	LYS	68	38.759	42.669	48.712	1.00	0.00	N
ATOM	1055	H	LYS	68	38.705	41.980	49.449	1.00	0.00	H
ATOM	1056	CA	LYS	68	39.551	42.334	47.492	1.00	0.00	C
ATOM	1057	HA	LYS	68	40.385	43.035	47.466	1.00	0.00	H
ATOM	1058	CB	LYS	68	40.114	40.900	47.619	1.00	0.00	C
ATOM	1059	HB2	LYS	68	40.548	40.887	48.618	1.00	0.00	H
ATOM	1060	HB3	LYS	68	39.204	40.300	47.601	1.00	0.00	H
ATOM	1061	CG	LYS	68	41.070	40.459	46.560	1.00	0.00	C
ATOM	1062	HG2	LYS	68	41.562	39.516	46.796	1.00	0.00	H

ATOM	1063	HG3	LYS	68	40.361	40.349	45.740	1.00	0.00	H
ATOM	1064	CD	LYS	68	42.269	41.469	46.417	1.00	0.00	C
ATOM	1065	HD2	LYS	68	42.791	41.397	45.463	1.00	0.00	H
ATOM	1066	HD3	LYS	68	41.969	42.500	46.228	1.00	0.00	H
ATOM	1067	CE	LYS	68	43.249	41.336	47.628	1.00	0.00	C
ATOM	1068	HE2	LYS	68	42.694	41.213	48.559	1.00	0.00	H
ATOM	1069	HE3	LYS	68	43.738	40.373	47.479	1.00	0.00	H
ATOM	1070	NZ	LYS	68	44.233	42.497	47.615	1.00	0.00	N
ATOM	1071	HZ1	LYS	68	43.860	43.369	47.964	1.00	0.00	H
ATOM	1072	HZ2	LYS	68	45.163	42.331	47.971	1.00	0.00	H
ATOM	1073	HZ3	LYS	68	44.437	42.631	46.635	1.00	0.00	H
ATOM	1074	C	LYS	68	38.717	42.385	46.219	1.00	0.00	C
ATOM	1075	O	LYS	68	39.246	42.718	45.165	1.00	0.00	O
ATOM	1076	N	HIE	69	37.464	41.964	46.254	1.00	0.00	N
ATOM	1077	H	HIE	69	37.075	41.602	47.112	1.00	0.00	H
ATOM	1078	CA	HIE	69	36.563	42.318	45.193	1.00	0.00	C
ATOM	1079	HA	HIE	69	36.902	41.897	44.246	1.00	0.00	H
ATOM	1080	CB	HIE	69	35.197	41.641	45.481	1.00	0.00	C
ATOM	1081	HB2	HIE	69	35.402	40.579	45.615	1.00	0.00	H
ATOM	1082	HB3	HIE	69	34.681	42.048	46.351	1.00	0.00	H
ATOM	1083	CG	HIE	69	34.140	41.800	44.422	1.00	0.00	C
ATOM	1084	ND1	HIE	69	34.149	41.096	43.189	1.00	0.00	N
ATOM	1085	CE1	HIE	69	33.010	41.440	42.550	1.00	0.00	C
ATOM	1086	HE1	HIE	69	32.768	41.075	41.563	1.00	0.00	H
ATOM	1087	NE2	HIE	69	32.423	42.432	43.224	1.00	0.00	N
ATOM	1088	HE2	HIE	69	31.645	42.966	42.863	1.00	0.00	H
ATOM	1089	CD2	HIE	69	33.055	42.683	44.468	1.00	0.00	C
ATOM	1090	HD2	HIE	69	32.750	43.319	45.286	1.00	0.00	H
ATOM	1091	C	HIE	69	36.314	43.809	44.923	1.00	0.00	C
ATOM	1092	O	HIE	69	36.370	44.273	43.745	1.00	0.00	O
ATOM	1093	N	ALA	70	36.151	44.628	45.960	1.00	0.00	N
ATOM	1094	H	ALA	70	36.144	44.191	46.871	1.00	0.00	H
ATOM	1095	CA	ALA	70	35.991	46.062	45.788	1.00	0.00	C
ATOM	1096	HA	ALA	70	35.219	46.261	45.045	1.00	0.00	H
ATOM	1097	CB	ALA	70	35.503	46.745	47.076	1.00	0.00	C
ATOM	1098	HB1	ALA	70	35.631	47.820	46.946	1.00	0.00	H
ATOM	1099	HB2	ALA	70	34.490	46.561	47.432	1.00	0.00	H
ATOM	1100	HB3	ALA	70	36.215	46.587	47.886	1.00	0.00	H
ATOM	1101	C	ALA	70	37.273	46.748	45.086	1.00	0.00	C
ATOM	1102	O	ALA	70	37.209	47.827	44.528	1.00	0.00	O
ATOM	1103	N	GLU	71	38.446	46.178	45.346	1.00	0.00	N
ATOM	1104	H	GLU	71	38.435	45.544	46.132	1.00	0.00	H
ATOM	1105	CA	GLU	71	39.692	46.583	44.630	1.00	0.00	C
ATOM	1106	HA	GLU	71	39.824	47.665	44.642	1.00	0.00	H
ATOM	1107	CB	GLU	71	40.905	45.940	45.350	1.00	0.00	C
ATOM	1108	HB2	GLU	71	40.903	44.850	45.385	1.00	0.00	H
ATOM	1109	HB3	GLU	71	41.763	46.108	44.699	1.00	0.00	H
ATOM	1110	CG	GLU	71	41.227	46.546	46.703	1.00	0.00	C
ATOM	1111	HG2	GLU	71	40.328	46.543	47.320	1.00	0.00	H
ATOM	1112	HG3	GLU	71	41.924	45.884	47.217	1.00	0.00	H
ATOM	1113	CD	GLU	71	41.842	47.957	46.605	1.00	0.00	C
ATOM	1114	OE1	GLU	71	43.017	48.113	46.228	1.00	0.00	O
ATOM	1115	OE2	GLU	71	41.143	48.924	46.975	1.00	0.00	O
ATOM	1116	C	GLU	71	39.500	46.227	43.155	1.00	0.00	C
ATOM	1117	O	GLU	71	39.946	46.946	42.321	1.00	0.00	O
ATOM	1118	N	GLN	72	38.855	45.145	42.715	1.00	0.00	N
ATOM	1119	H	GLN	72	38.398	44.528	43.371	1.00	0.00	H
ATOM	1120	CA	GLN	72	38.618	44.943	41.318	1.00	0.00	C
ATOM	1121	HA	GLN	72	39.535	45.002	40.732	1.00	0.00	H
ATOM	1122	CB	GLN	72	38.097	43.544	41.096	1.00	0.00	C
ATOM	1123	HB2	GLN	72	37.178	43.407	41.665	1.00	0.00	H
ATOM	1124	HB3	GLN	72	38.874	42.908	41.519	1.00	0.00	H
ATOM	1125	CG	GLN	72	37.810	43.177	39.635	1.00	0.00	C
ATOM	1126	HG2	GLN	72	37.200	43.864	39.047	1.00	0.00	H

ATOM	1127	HG3	GLN	72	38.784	43.094	39.155	1.00	0.00	H
ATOM	1128	CD	GLN	72	37.112	41.807	39.666	1.00	0.00	C
ATOM	1129	OE1	GLN	72	37.637	40.778	39.426	1.00	0.00	O
ATOM	1130	NE2	GLN	72	35.879	41.732	39.896	1.00	0.00	N
ATOM	1131	HE21	GLN	72	35.323	42.570	39.990	1.00	0.00	H
ATOM	1132	HE22	GLN	72	35.589	40.854	40.302	1.00	0.00	H
ATOM	1133	C	GLN	72	37.558	46.022	40.862	1.00	0.00	C
ATOM	1134	O	GLN	72	37.679	46.559	39.751	1.00	0.00	O
ATOM	1135	N	GLU	73	36.537	46.339	41.671	1.00	0.00	N
ATOM	1136	H	GLU	73	36.503	45.828	42.542	1.00	0.00	H
ATOM	1137	CA	GLU	73	35.395	47.201	41.353	1.00	0.00	C
ATOM	1138	HA	GLU	73	35.262	47.224	40.272	1.00	0.00	H
ATOM	1139	CB	GLU	73	34.172	46.592	42.146	1.00	0.00	C
ATOM	1140	HB2	GLU	73	34.461	46.557	43.197	1.00	0.00	H
ATOM	1141	HB3	GLU	73	33.331	47.271	42.011	1.00	0.00	H
ATOM	1142	CG	GLU	73	33.726	45.170	41.709	1.00	0.00	C
ATOM	1143	HG2	GLU	73	34.445	44.441	42.085	1.00	0.00	H
ATOM	1144	HG3	GLU	73	32.783	44.904	42.186	1.00	0.00	H
ATOM	1145	CD	GLU	73	33.619	44.969	40.192	1.00	0.00	C
ATOM	1146	OE1	GLU	73	34.378	44.201	39.555	1.00	0.00	O
ATOM	1147	OE2	GLU	73	32.740	45.591	39.554	1.00	0.00	O
ATOM	1148	C	GLU	73	35.698	48.661	41.675	1.00	0.00	C
ATOM	1149	O	GLU	73	34.790	49.490	41.574	1.00	0.00	O
ATOM	1150	N	ASN	74	36.956	49.004	41.857	1.00	0.00	N
ATOM	1151	H	ASN	74	37.638	48.265	41.766	1.00	0.00	H
ATOM	1152	CA	ASN	74	37.430	50.387	42.017	1.00	0.00	C
ATOM	1153	HA	ASN	74	38.461	50.343	42.367	1.00	0.00	H
ATOM	1154	CB	ASN	74	37.632	51.083	40.671	1.00	0.00	C
ATOM	1155	HB2	ASN	74	36.622	51.223	40.284	1.00	0.00	H
ATOM	1156	HB3	ASN	74	38.078	52.030	40.973	1.00	0.00	H
ATOM	1157	CG	ASN	74	38.468	50.387	39.592	1.00	0.00	C
ATOM	1158	OD1	ASN	74	38.326	50.609	38.402	1.00	0.00	O
ATOM	1159	ND2	ASN	74	39.484	49.536	39.967	1.00	0.00	N
ATOM	1160	HD21	ASN	74	39.812	48.911	39.246	1.00	0.00	H
ATOM	1161	HD22	ASN	74	39.736	49.505	40.944	1.00	0.00	H
ATOM	1162	C	ASN	74	36.689	51.171	43.110	1.00	0.00	C
ATOM	1163	O	ASN	74	36.382	52.325	42.954	1.00	0.00	O
ATOM	1164	N	MET	75	36.267	50.569	44.211	1.00	0.00	N
ATOM	1165	H	MET	75	36.569	49.648	44.499			
1.00	0.00			H						
ATOM	1166	CA	MET	75	35.418	51.187	45.243	1.00	0.00	C
ATOM	1167	HA	MET	75	35.491	52.272	45.324	1.00	0.00	H
ATOM	1168	CB	MET	75	33.942	50.817	45.087	1.00	0.00	C
ATOM	1169	HB2	MET	75	33.432	51.348	45.890	1.00	0.00	H
ATOM	1170	HB3	MET	75	33.530	51.088	44.114	1.00	0.00	H
ATOM	1171	CG	MET	75	33.624	49.292	45.205	1.00	0.00	C
ATOM	1172	HG2	MET	75	34.138	48.882	44.337	1.00	0.00	H
ATOM	1173	HG3	MET	75	34.110	48.826	46.062	1.00	0.00	H
ATOM	1174	SD	MET	75	31.949	48.725	45.241	1.00	0.00	S
ATOM	1175	CE	MET	75	31.287	49.435	43.716	1.00	0.00	C
ATOM	1176	HE1	MET	75	30.207	49.547	43.793	1.00	0.00	H
ATOM	1177	HE2	MET	75	31.720	50.427	43.589	1.00	0.00	H
ATOM	1178	HE3	MET	75	31.606	48.838	42.861	1.00	0.00	H
ATOM	1179	C	MET	75	35.941	50.754	46.621	1.00	0.00	C
ATOM	1180	O	MET	75	36.822	49.875	46.722	1.00	0.00	O
ATOM	1181	N	THR	76	35.363	51.246	47.704	1.00	0.00	N
ATOM	1182	H	THR	76	34.468	51.702	47.612	1.00	0.00	H
ATOM	1183	CA	THR	76	35.860	51.027	49.074	1.00	0.00	C
ATOM	1184	HA	THR	76	36.873	50.643	48.947	1.00	0.00	H
ATOM	1185	CB	THR	76	35.929	52.341	49.908	1.00	0.00	C
ATOM	1186	HB	THR	76	36.584	52.996	49.334	1.00	0.00	H
ATOM	1187	CG2	THR	76	34.572	52.879	50.276	1.00	0.00	C
ATOM	1188	HG21	THR	76	33.939	53.099	49.416	1.00	0.00	H
ATOM	1189	HG22	THR	76	34.096	52.100	50.870	1.00	0.00	H

ATOM	1190	HG23	THR	76	34.747	53.797	50.835	1.00	0.00	H
ATOM	1191	OG1	THR	76	36.596	52.032	51.135	1.00	0.00	O
ATOM	1192	HG1	THR	76	37.516	51.906	50.890	1.00	0.00	H
ATOM	1193	C	THR	76	35.020	50.014	49.769	1.00	0.00	C
ATOM	1194	O	THR	76	33.840	49.913	49.447	1.00	0.00	O
ATOM	1195	N	LEU	77	35.472	49.151	50.701	1.00	0.00	N
ATOM	1196	H	LEU	77	36.465	49.102	50.874	1.00	0.00	H
ATOM	1197	CA	LEU	77	34.593	48.168	51.342	1.00	0.00	C
ATOM	1198	HA	LEU	77	34.212	47.542	50.535	1.00	0.00	H
ATOM	1199	CB	LEU	77	35.469	47.369	52.353	1.00	0.00	C
ATOM	1200	HB2	LEU	77	36.441	47.059	51.968	1.00	0.00	H
ATOM	1201	HB3	LEU	77	35.754	48.088	53.120	1.00	0.00	H
ATOM	1202	CG	LEU	77	34.753	46.205	53.090	1.00	0.00	C
ATOM	1203	HG	LEU	77	33.828	46.650	53.458	1.00	0.00	H
ATOM	1204	CD1	LEU	77	34.371	44.989	52.256	1.00	0.00	C
ATOM	1205	HD11	LEU	77	33.690	45.215	51.437	1.00	0.00	H
ATOM	1206	HD12	LEU	77	35.293	44.612	51.815	1.00	0.00	H
ATOM	1207	HD13	LEU	77	33.858	44.296	52.923	1.00	0.00	H
ATOM	1208	CD2	LEU	77	35.659	45.666	54.176	1.00	0.00	C
ATOM	1209	HD21	LEU	77	36.673	45.491	53.813	1.00	0.00	H
ATOM	1210	HD22	LEU	77	35.667	46.345	55.028	1.00	0.00	H
ATOM	1211	HD23	LEU	77	35.289	44.758	54.651	1.00	0.00	H
ATOM	1212	C	LEU	77	33.285	48.678	51.908	1.00	0.00	C
ATOM	1213	O	LEU	77	32.301	47.985	51.761	1.00	0.00	O
ATOM	1214	N	THR	78	33.258	49.832	52.627	1.00	0.00	N
ATOM	1215	H	THR	78	34.082	50.410	52.723	1.00	0.00	H
ATOM	1216	CA	THR	78	32.055	50.376	53.253	1.00	0.00	C
ATOM	1217	HA	THR	78	31.647	49.493	53.745	1.00	0.00	H
ATOM	1218	CB	THR	78	32.424	51.429	54.310	1.00	0.00	C
ATOM	1219	HB	THR	78	31.472	51.843	54.642	1.00	0.00	H
ATOM	1220	CG2	THR	78	33.200	50.969	55.529	1.00	0.00	C
ATOM	1221	HG21	THR	78	32.562	50.208	55.978	1.00	0.00	H
ATOM	1222	HG22	THR	78	34.169	50.630	55.163	1.00	0.00	H
ATOM	1223	HG23	THR	78	33.285	51.841	56.175	1.00	0.00	H
ATOM	1224	OG1	THR	78	33.288	52.386	53.753	1.00	0.00	O
ATOM	1225	HG1	THR	78	32.851	53.234	53.855	1.00	0.00	H
ATOM	1226	C	THR	78	30.967	50.878	52.217	1.00	0.00	C
ATOM	1227	O	THR	78	29.811	50.887	52.498	1.00	0.00	O
ATOM	1228	N	GLU	79	31.396	51.325	50.992	1.00	0.00	N
ATOM	1229	H	GLU	79	32.399	51.430	50.938	1.00	0.00	H
ATOM	1230	CA	GLU	79	30.586	51.455	49.785	1.00	0.00	C
ATOM	1231	HA	GLU	79	29.705	52.018	50.093	1.00	0.00	H
ATOM	1232	CB	GLU	79	31.266	52.271	48.639	1.00	0.00	C
ATOM	1233	HB2	GLU	79	32.264	51.900	48.405	1.00	0.00	H
ATOM	1234	HB3	GLU	79	30.783	52.135	47.673	1.00	0.00	H
ATOM	1235	CG	GLU	79	31.342	53.743	48.932	1.00	0.00	C
ATOM	1236	HG2	GLU	79	31.892	53.808	49.871	1.00	0.00	H
ATOM	1237	HG3	GLU	79	31.831	54.318	48.146	1.00	0.00	H
ATOM	1238	CD	GLU	79	30.010	54.308	49.013	1.00	0.00	C
ATOM	1239	OE1	GLU	79	29.833	55.230	49.827	1.00	0.00	O
ATOM	1240	OE2	GLU	79	29.087	53.960	48.247	1.00	0.00	O
ATOM	1241	C	GLU	79	30.080	50.150	49.246	1.00	0.00	C
ATOM	1242	O	GLU	79	28.907	50.050	48.982	1.00	0.00	O
ATOM	1243	N	LEU	80	30.873	49.112	49.095	1.00	0.00	N
ATOM	1244	H	LEU	80	31.828	49.257	49.394	1.00	0.00	H
ATOM	1245	CA	LEU	80	30.459	47.780	48.614	1.00	0.00	C
ATOM	1246	HA	LEU	80	30.087	47.982	47.609	1.00	0.00	H
ATOM	1247	CB	LEU	80	31.732	46.823	48.535	1.00	0.00	C
ATOM	1248	HB2	LEU	80	32.448	47.182	47.796	1.00	0.00	H
ATOM	1249	HB3	LEU	80	32.209	46.834	49.515	1.00	0.00	H
ATOM	1250	CG	LEU	80	31.313	45.372	48.189	1.00	0.00	C
ATOM	1251	HG	LEU	80	30.761	44.944	49.026	1.00	0.00	H
ATOM	1252	CD1	LEU	80	30.544	45.271	46.823	1.00	0.00	C
ATOM	1253	HD11	LEU	80	30.885	45.980	46.068	1.00	0.00	H

```
ATOM   1254 HD12 LEU    80      30.633  44.214  46.571  1.00  0.00           H
ATOM   1255 HD13 LEU    80      29.500  45.551  46.964  1.00  0.00           H
ATOM   1256  CD2 LEU    80      32.530  44.489  48.055  1.00  0.00           C
ATOM   1257 HD21 LEU    80      32.146  43.471  47.997  1.00  0.00           H
ATOM   1258 HD22 LEU    80      32.996  44.799  47.120  1.00  0.00           H
ATOM   1259 HD23 LEU    80      33.117  44.652  48.959  1.00  0.00           H
ATOM   1260  C   LEU    80      29.311  47.152  49.474  1.00  0.00           C
ATOM   1261  O   LEU    80      28.195  46.910  49.039  1.00  0.00           O
ATOM   1262  N   LYS    81      29.569  47.202  50.821  1.00  0.00           N
ATOM   1263  H   LYS    81      30.485  47.499  51.126  1.00  0.00           H
ATOM   1264  CA  LYS    81      28.536  46.780  51.746  1.00  0.00           C
ATOM   1265  HA  LYS    81      28.281  45.729  51.604  1.00  0.00           H
ATOM   1266  CB  LYS    81      29.151  46.907  53.168  1.00  0.00           C
ATOM   1267  HB2 LYS    81      29.522  47.925  53.291  1.00  0.00           H
ATOM   1268  HB3 LYS    81      28.291  46.758  53.822  1.00  0.00           H
ATOM   1269  CG  LYS    81      30.248  45.799  53.332  1.00  0.00           C
ATOM   1270  HG2 LYS    81      29.735  44.837  53.326  1.00  0.00           H
ATOM   1271  HG3 LYS    81      31.113  45.894  52.675  1.00  0.00           H
ATOM   1272  CD  LYS    81      30.942  46.186  54.693  1.00  0.00           C
ATOM   1273  HD2 LYS    81      31.469  47.128  54.537  1.00  0.00           H
ATOM   1274  HD3 LYS    81      30.197  46.187  55.489  1.00  0.00           H
ATOM   1275  CE  LYS    81      32.042  45.192  55.212  1.00  0.00           C
ATOM   1276  HE2 LYS    81      31.715  44.173  55.004  1.00  0.00           H
ATOM   1277  HE3 LYS    81      32.894  45.432  54.577  1.00  0.00           H
ATOM   1278  NZ  LYS    81      32.408  45.198  56.648  1.00  0.00           N
ATOM   1279  HZ1 LYS    81      31.558  45.202  57.194  1.00  0.00           H
ATOM   1280  HZ2 LYS    81      32.898  44.374  56.969  1.00  0.00           H
ATOM   1281  HZ3 LYS    81      32.957  46.017  56.866  1.00  0.00           H
ATOM   1282  C   LYS    81      27.240  47.557  51.624  1.00  0.00           C
ATOM   1283  O   LYS    81      26.228  46.923  51.744  1.00  0.00           O
ATOM   1284  N   LYS    82      27.336  48.879  51.424  1.00  0.00           N
ATOM   1285  H   LYS    82      28.227  49.351  51.370  1.00  0.00           H
ATOM   1286  CA  LYS    82      26.031  49.586  51.373  1.00  0.00           C
ATOM   1287  HA  LYS    82      25.353  49.314  52.182  1.00  0.00           H
ATOM   1288  CB  LYS    82      26.396  51.076  51.406  1.00  0.00           C
ATOM   1289  HB2 LYS    82      27.146  51.258  52.175  1.00  0.00           H
ATOM   1290  HB3 LYS    82      26.778  51.336  50.419  1.00  0.00           H
ATOM
   1291  CG  LYS    82      25.255  52.022  51.832  1.00  0.00           C
ATOM   1292  HG2 LYS    82      24.486  51.886  51.072  1.00  0.00           H
ATOM   1293  HG3 LYS    82      24.991  51.676  52.831  1.00  0.00           H
ATOM   1294  CD  LYS    82      25.646  53.510  51.794  1.00  0.00           C
ATOM   1295  HD2 LYS    82      24.706  54.051  51.688  1.00  0.00           H
ATOM   1296  HD3 LYS    82      26.123  53.683  52.760  1.00  0.00           H
ATOM   1297  CE  LYS    82      26.599  53.765  50.595  1.00  0.00           C
ATOM   1298  HE2 LYS    82      27.548  53.241  50.717  1.00  0.00           H
ATOM   1299  HE3 LYS    82      26.208  53.444  49.631  1.00  0.00           H
ATOM   1300  NZ  LYS    82      26.920  55.189  50.463  1.00  0.00           N
ATOM   1301  HZ1 LYS    82      27.882  55.290  50.173  1.00  0.00           H
ATOM   1302  HZ2 LYS    82      26.449  55.608  49.674  1.00  0.00           H
ATOM   1303  HZ3 LYS    82      26.732  55.728  51.296  1.00  0.00           H
ATOM   1304  C   LYS    82      25.264  49.192  50.066  1.00  0.00           C
ATOM   1305  O   LYS    82      24.015  49.155  50.145  1.00  0.00           O
ATOM   1306  N   VAL    83      25.865  48.988  48.882  1.00  0.00           N
ATOM   1307  H   VAL    83      26.810  49.316  48.744  1.00  0.00           H
ATOM   1308  CA  VAL    83      25.090  48.528  47.661  1.00  0.00           C
ATOM   1309  HA  VAL    83      24.216  49.173  47.574  1.00  0.00           H
ATOM   1310  CB  VAL    83      25.902  48.774  46.342  1.00  0.00           C
ATOM   1311  HB  VAL    83      25.177  48.568  45.554  1.00  0.00           H
ATOM   1312  CG1 VAL    83      26.244  50.253  46.124  1.00  0.00           C
ATOM   1313 HG11 VAL    83      26.856  50.710  46.901  1.00  0.00           H
ATOM   1314 HG12 VAL    83      26.802  50.315  45.189  1.00  0.00           H
ATOM   1315 HG13 VAL    83      25.299  50.795  46.102  1.00  0.00           H
ATOM   1316  CG2 VAL    83      27.191  47.883  46.149  1.00  0.00           C
```

ATOM	1317	HG21	VAL	83	27.083	46.819	46.357	1.00	0.00	H
ATOM	1318	HG22	VAL	83	27.572	48.009	45.137	1.00	0.00	H
ATOM	1319	HG23	VAL	83	28.031	48.163	46.787	1.00	0.00	H
ATOM	1320	C	VAL	83	24.457	47.164	47.768	1.00	0.00	C
ATOM	1321	O	VAL	83	23.329	47.033	47.274	1.00	0.00	O
ATOM	1322	N	ILE	84	25.174	46.216	48.431	1.00	0.00	N
ATOM	1323	H	ILE	84	26.117	46.442	48.714	1.00	0.00	H
ATOM	1324	CA	ILE	84	24.695	44.893	48.722	1.00	0.00	C
ATOM	1325	HA	ILE	84	24.260	44.435	47.834	1.00	0.00	H
ATOM	1326	CB	ILE	84	25.768	43.972	49.313	1.00	0.00	C
ATOM	1327	HB	ILE	84	26.231	44.553	50.111	1.00	0.00	H
ATOM	1328	CG2	ILE	84	25.197	42.677	49.839	1.00	0.00	C
ATOM	1329	HG21	ILE	84	26.000	42.177	50.383	1.00	0.00	H
ATOM	1330	HG22	ILE	84	24.412	42.886	50.566	1.00	0.00	H
ATOM	1331	HG23	ILE	84	24.739	42.000	49.119	1.00	0.00	H
ATOM	1332	CG1	ILE	84	26.950	43.576	48.343	1.00	0.00	C
ATOM	1333	HG12	ILE	84	26.486	42.906	47.618	1.00	0.00	H
ATOM	1334	HG13	ILE	84	27.360	44.462	47.860	1.00	0.00	H
ATOM	1335	CD1	ILE	84	28.159	42.888	49.032	1.00	0.00	C
ATOM	1336	HD11	ILE	84	28.519	43.577	49.796	1.00	0.00	H
ATOM	1337	HD12	ILE	84	27.896	42.001	49.607	1.00	0.00	H
ATOM	1338	HD13	ILE	84	28.857	42.568	48.259	1.00	0.00	H
ATOM	1339	C	ILE	84	23.588	45.071	49.763	1.00	0.00	C
ATOM	1340	O	ILE	84	22.539	44.416	49.591	1.00	0.00	O
ATOM	1341	N	ALA	85	23.576	45.938	50.749	1.00	0.00	N
ATOM	1342	H	ALA	85	24.358	46.578	50.782	1.00	0.00	H
ATOM	1343	CA	ALA	85	22.518	46.189	51.671	1.00	0.00	C
ATOM	1344	HA	ALA	85	22.094	45.244	52.014	1.00	0.00	H
ATOM	1345	CB	ALA	85	23.037	46.986	52.963	1.00	0.00	C
ATOM	1346	HB1	ALA	85	22.287	46.900	53.749	1.00	0.00	H
ATOM	1347	HB2	ALA	85	23.896	46.456	53.377	1.00	0.00	H
ATOM	1348	HB3	ALA	85	23.296	48.019	52.729	1.00	0.00	H
ATOM	1349	C	ALA	85	21.354	46.925	50.948	1.00	0.00	C
ATOM	1350	O	ALA	85	20.211	46.782	51.405	1.00	0.00	O
ATOM	1351	N	ASP	86	21.635	47.721	49.897	1.00	0.00	N
ATOM	1352	H	ASP	86	22.553	47.843	49.493	1.00	0.00	H
ATOM	1353	CA	ASP	86	20.624	48.284	49.040	1.00	0.00	C
ATOM	1354	HA	ASP	86	20.005	48.912	49.681	1.00	0.00	H
ATOM	1355	CB	ASP	86	21.128	49.229	47.920	1.00	0.00	C
ATOM	1356	HB2	ASP	86	21.884	49.848	48.404	1.00	0.00	H
ATOM	1357	HB3	ASP	86	21.726	48.705	47.175	1.00	0.00	H
ATOM	1358	CG	ASP	86	20.129	50.185	47.277	1.00	0.00	C
ATOM	1359	OD1	ASP	86	20.407	50.672	46.175	1.00	0.00	O
ATOM	1360	OD2	ASP	86	18.998	50.315	47.863	1.00	0.00	O
ATOM	1361	C	ASP	86	19.726	47.148	48.408	1.00	0.00	C
ATOM	1362	O	ASP	86	18.496	47.276	48.332	1.00	0.00	O
ATOM	1363	N	ILE	87	20.330	46.048	47.900	1.00	0.00	N
ATOM	1364	H	ILE	87	21.294	45.944	48.180	1.00	0.00	H
ATOM	1365	CA	ILE	87	19.654	45.012	47.097	1.00	0.00	C
ATOM	1366	HA	ILE	87	18.753	45.385	46.609	1.00	0.00	H
ATOM	1367	CB	ILE	87	20.686	44.512	46.021	1.00	0.00	C
ATOM	1368	HB	ILE	87	21.590	44.201	46.544	1.00	0.00	H
ATOM	1369	CG2	ILE	87	20.245	43.294	45.235	1.00	0.00	C
ATOM	1370	HG21	ILE	87	21.068	42.866	44.662	1.00	0.00	H
ATOM	1371	HG22	ILE	87	19.860	42.597	45.982	1.00	0.00	H
ATOM	1372	HG23	ILE	87	19.469	43.607	44.537	1.00	0.00	H
ATOM	1373	CG1	ILE	87	21.039	45.615	44.968	1.00	0.00	C
ATOM	1374	HG12	ILE	87	20.267	45.757	44.213	1.00	0.00	H
ATOM	1375	HG13	ILE	87	21.163	46.574	45.473	1.00	0.00	H
ATOM	1376	CD1	ILE	87	22.435	45.453	44.208	1.00	0.00	C
ATOM	1377	HD11	ILE	87	22.455	44.566	43.574	1.00	0.00	H
ATOM	1378	HD12	ILE	87	22.553	46.427	43.733	1.00	0.00	H
ATOM	1379	HD13	ILE	87	23.143	45.326	45.027	1.00	0.00	H
ATOM	1380	C	ILE	87	19.003	43.954	48.048	1.00	0.00	C

ATOM 1381 O ILE 87 17.871 43.500 47.786 1.00 0.00 O
ATOM 1382 N TYR 88 19.753 43.572 49.079 1.00 0.00 N
ATOM 1383 H TYR 88 20.636 44.034 49.248 1.00 0.00 H
ATOM 1384 CA TYR 88 19.437 42.368 49.846 1.00 0.00 C
ATOM 1385 HA TYR 88 18.630 41.816 49.363 1.00 0.00 H
ATOM 1386 CB TYR 88 20.684 41.477 49.681 1.00 0.00 C
ATOM 1387 HB2 TYR 88 21.565 42.112 49.775 1.00 0.00 H
ATOM 1388 HB3 TYR 88 20.767 40.734 50.474 1.00 0.00 H
ATOM 1389 CG TYR 88 20.727 40.617 48.395 1.00 0.00 C
ATOM 1390 CD1 TYR 88 20.001 39.393 48.314 1.00 0.00 C
ATOM 1391 HD1 TYR 88 19.445 38.939 49.122 1.00 0.00 H
ATOM 1392 CE1 TYR 88 20.144 38.602 47.130 1.00 0.00 C
ATOM 1393 HE1 TYR 88 19.532 37.719 47.023 1.00 0.00 H
ATOM 1394 CZ TYR 88 20.935 38.994 46.020 1.00 0.00 C
ATOM 1395 OH TYR 88 21.022 38.253 44.928 1.00 0.00 O
ATOM 1396 HH TYR 88 21.834 38.580 44.533 1.00 0.00 H
ATOM 1397 CE2 TYR 88 21.720 40.170 46.177 1.00 0.00 C
ATOM 1398 HE2 TYR 88 22.450 40.439 45.428 1.00 0.00 H
ATOM 1399 CD2 TYR 88 21.678 41.010 47.332 1.00 0.00 C
ATOM 1400 HD2 TYR 88 22.168 41.962 47.470 1.00 0.00 H
ATOM 1401 C TYR 88 19.131 42.545 51.328 1.00 0.00 C
ATOM 1402 O TYR 88 19.537 43.526 51.957 1.00 0.00 O
ATOM 1403 N PRO 89 18.398 41.547 51.914 1.00 0.00 N
ATOM 1404 CD PRO 89 17.535 40.535 51.355 1.00 0.00 C
ATOM 1405 HD2 PRO 89 18.079 39.749 50.831 1.00 0.00 H
ATOM 1406 HD3 PRO 89 16.973 40.980 50.533 1.00 0.00 H
ATOM 1407 CG PRO 89 16.490 40.114 52.394 1.00 0.00 C
ATOM 1408 HG2 PRO 89 16.123 39.117 52.155 1.00 0.00 H
ATOM 1409 HG3 PRO 89 15.736 40.898 52.460 1.00 0.00 H
ATOM 1410 CB PRO 89 17.415 40.178 53.600 1.00 0.00 C
ATOM 1411 HB2 PRO 89 17.924 39.218 53.680 1.00 0.00 H
ATOM 1412 HB3 PRO 89 16.643 40.217 54.368 1.00 0.00 H
ATOM 1413 CA PRO 89 18.330 41.410 53.360 1.00 0.00 C
ATOM 1414 HA PRO 89 17.855 42.339 53.677 1.00 0.00 H
ATOM 1415 C PRO 89 19.717 41.132 53.968 1.00 0.00 C
ATOM 1416 O PRO 89
20.551 40.417 53.441 1.00 0.00 O
ATOM 1417 N GLY 90 19.934 41.775 55.118 1.00 0.00 N
ATOM 1418 H GLY 90 19.180 42.345 55.473 1.00 0.00 H
ATOM 1419 CA GLY 90 21.188 41.619 55.899 1.00 0.00 C
ATOM 1420 HA2 GLY 90 22.028 42.016 55.327 1.00 0.00 H
ATOM 1421 HA3 GLY 90 21.102 42.189 56.825 1.00 0.00 H
ATOM 1422 C GLY 90 21.543 40.167 56.227 1.00 0.00 C
ATOM 1423 O GLY 90 22.689 39.769 56.192 1.00 0.00 O
ATOM 1424 N GLN 91 20.527 39.407 56.619 1.00 0.00 N
ATOM 1425 H GLN 91 19.594 39.797 56.625 1.00 0.00 H
ATOM 1426 CA GLN 91 20.571 37.977 56.923 1.00 0.00 C
ATOM 1427 HA GLN 91 21.289 37.865 57.735 1.00 0.00 H
ATOM 1428 CB GLN 91 19.181 37.497 57.474 1.00 0.00 C
ATOM 1429 HB2 GLN 91 18.415 37.718 56.731 1.00 0.00 H
ATOM 1430 HB3 GLN 91 19.242 36.431 57.692 1.00 0.00 H
ATOM 1431 CG GLN 91 18.916 38.192 58.906 1.00 0.00 C
ATOM 1432 HG2 GLN 91 19.822 38.103 59.507 1.00 0.00 H
ATOM 1433 HG3 GLN 91 18.744 39.266 58.820 1.00 0.00 H
ATOM 1434 CD GLN 91 17.743 37.510 59.631 1.00 0.00 C
ATOM 1435 OE1 GLN 91 16.853 36.886 59.002 1.00 0.00 O
ATOM 1436 NE2 GLN 91 17.593 37.620 60.932 1.00 0.00 N
ATOM 1437 HE21 GLN 91 18.070 38.377 61.401 1.00 0.00 H
ATOM 1438 HE22 GLN 91 16.867 37.112 61.417 1.00 0.00 H
ATOM 1439 C GLN 91 21.050 37.090 55.713 1.00 0.00 C
ATOM 1440 O GLN 91 21.256 35.902 55.863 1.00 0.00 O
ATOM 1441 N THR 92 21.280 37.600 54.495 1.00 0.00 N
ATOM 1442 H THR 92 21.599 38.558 54.487 1.00 0.00 H
ATOM 1443 CA THR 92 21.623 36.810 53.307 1.00 0.00 C

ATOM	1444	HA	THR	92	20.980	35.939	53.176	1.00	0.00	H
ATOM	1445	CB	THR	92	21.514	37.737	52.032	1.00	0.00	C
ATOM	1446	HB	THR	92	22.268	38.500	52.225	1.00	0.00	H
ATOM	1447	CG2	THR	92	21.809	36.952	50.665	1.00	0.00	C
ATOM	1448	HG21	THR	92	21.407	37.544	49.843	1.00	0.00	H
ATOM	1449	HG22	THR	92	22.884	36.814	50.550	1.00	0.00	H
ATOM	1450	HG23	THR	92	21.219	36.036	50.642	1.00	0.00	H
ATOM	1451	OG1	THR	92	20.231	38.323	51.792	1.00	0.00	O
ATOM	1452	HG1	THR	92	20.202	39.192	52.201	1.00	0.00	H
ATOM	1453	C	THR	92	23.020	36.363	53.387	1.00	0.00	C
ATOM	1454	O	THR	92	23.935	37.197	53.586	1.00	0.00	O
ATOM	1455	N	GLN	93	23.193	35.068	53.338	1.00	0.00	N
ATOM	1456	H	GLN	93	22.377	34.484	53.228	1.00	0.00	H
ATOM	1457	CA	GLN	93	24.500	34.433	53.205	1.00	0.00	C
ATOM	1458	HA	GLN	93	25.226	35.033	53.753	1.00	0.00	H
ATOM	1459	CB	GLN	93	24.454	32.903	53.604	1.00	0.00	C
ATOM	1460	HB2	GLN	93	23.838	32.753	54.490	1.00	0.00	H
ATOM	1461	HB3	GLN	93	23.875	32.390	52.837	1.00	0.00	H
ATOM	1462	CG	GLN	93	25.812	32.266	53.825	1.00	0.00	C
ATOM	1463	HG2	GLN	93	25.792	31.178	53.771	1.00	0.00	H
ATOM	1464	HG3	GLN	93	26.378	32.611	52.960	1.00	0.00	H
ATOM	1465	CD	GLN	93	26.404	32.695	55.207	1.00	0.00	C
ATOM	1466	OE1	GLN	93	25.738	33.019	56.177	1.00	0.00	O
ATOM	1467	NE2	GLN	93	27.710	32.572	55.295	1.00	0.00	N
ATOM	1468	HE21	GLN	93	28.169	32.141	54.505	1.00	0.00	H
ATOM	1469	HE22	GLN	93	28.153	32.654	56.198	1.00	0.00	H
ATOM	1470	C	GLN	93	24.847	34.523	51.721	1.00	0.00	C
ATOM	1471	O	GLN	93	24.109	34.165	50.851	1.00	0.00	O
ATOM	1472	N	PHE	94	26.157	34.925	51.444	1.00	0.00	N
ATOM	1473	H	PHE	94	26.654	35.210	52.276	1.00	0.00	H
ATOM	1474	CA	PHE	94	26.760	35.041	50.122	1.00	0.00	C
ATOM	1475	HA	PHE	94	26.052	34.628	49.404	1.00	0.00	H
ATOM	1476	CB	PHE	94	27.206	36.495	49.883	1.00	0.00	C
ATOM	1477	HB2	PHE	94	28.062	36.701	50.525	1.00	0.00	H
ATOM	1478	HB3	PHE	94	27.531	36.575	48.845	1.00	0.00	H
ATOM	1479	CG	PHE	94	26.083	37.507	49.964	1.00	0.00	C
ATOM	1480	CD1	PHE	94	25.366	37.839	48.801	1.00	0.00	C
ATOM	1481	HD1	PHE	94	25.575	37.283	47.899	1.00	0.00	H
ATOM	1482	CE1	PHE	94	24.437	38.928	48.781	1.00	0.00	C
ATOM	1483	HE1	PHE	94	23.785	39.112	47.940	1.00	0.00	H
ATOM	1484	CZ	PHE	94	24.070	39.537	49.994	1.00	0.00	C
ATOM	1485	HZ	PHE	94	23.217	40.189	50.111	1.00	0.00	H
ATOM	1486	CE2	PHE	94	24.864	39.249	51.114	1.00	0.00	C
ATOM	1487	HE2	PHE	94	24.742	39.800	52.034	1.00	0.00	H
ATOM	1488	CD2	PHE	94	25.832	38.219	51.178	1.00	0.00	C
ATOM	1489	HD2	PHE	94	26.427	38.086	52.069	1.00	0.00	H
ATOM	1490	C	PHE	94	27.919	34.132	49.981	1.00	0.00	C
ATOM	1491	O	PHE	94	28.527	33.531	50.906	1.00	0.00	O
ATOM	1492	N	TYR	95	28.352	33.914	48.774	1.00	0.00	N
ATOM	1493	H	TYR	95	27.819	34.392	48.061	1.00	0.00	H
ATOM	1494	CA	TYR	95	29.432	33.069	48.254	1.00	0.00	C
ATOM	1495	HA	TYR	95	29.995	32.660	49.094	1.00	0.00	H
ATOM	1496	CB	TYR	95	28.727	31.872	47.528	1.00	0.00	C
ATOM	1497	HB2	TYR	95	28.202	31.253	48.256	1.00	0.00	H
ATOM	1498	HB3	TYR	95	28.077	32.249	46.739	1.00	0.00	H
ATOM	1499	CG	TYR	95	29.623	30.726	46.906	1.00	0.00	C
ATOM	1500	CD1	TYR	95	29.630	30.440	45.560	1.00	0.00	C
ATOM	1501	HD1	TYR	95	29.060	31.112	44.936	1.00	0.00	H
ATOM	1502	CE1	TYR	95	30.236	29.259	45.052	1.00	0.00	C
ATOM	1503	HE1	TYR	95	30.157	29.023	44.001	1.00	0.00	H
ATOM	1504	CZ	TYR	95	31.085	28.503	45.900	1.00	0.00	C
ATOM	1505	OH	TYR	95	31.684	27.343	45.561	1.00	0.00	O
ATOM	1506	HH	TYR	95	31.696	27.173	44.616	1.00	0.00	H
ATOM	1507	CE2	TYR	95	31.199	28.888	47.237	1.00	0.00	C

```
ATOM   1508  HE2 TYR    95      31.815  28.276  47.878  1.00  0.00           H
ATOM   1509  CD2 TYR    95      30.404  29.920  47.761  1.00  0.00           C
ATOM   1510  HD2 TYR    95      30.601  30.278  48.761  1.00  0.00           H
ATOM   1511  C   TYR    95      30.447  33.852  47.347  1.00  0.00           C
ATOM   1512  O   TYR    95      30.007  34.392  46.342  1.00  0.00           O
ATOM   1513  N   VAL    96      31.749  33.809  47.581  1.00  0.00           N
ATOM   1514  H   VAL    96      32.120  33.108  48.206  1.00  0.00           H
ATOM   1515  CA  VAL    96      32.847  34.376  46.671  1.00  0.00           C
ATOM   1516  HA  VAL    96      32.459  35.080  45.935  1.00  0.00           H
ATOM   1517  CB  VAL    96      33.917  34.946  47.638  1.00  0.00           C
ATOM   1518  HB  VAL    96      34.220  34.130  48.293  1.00  0.00           H
ATOM   1519  CG1 VAL    96      35.205  35.417  46.887  1.00  0.00           C
ATOM   1520 HG11 VAL    96      35.789  34.556  46.559  1.00  0.00           H
ATOM   1521 HG12 VAL    96      34.962  35.900  45.941  1.00  0.00           H
ATOM   1522 HG13 VAL    96      35.786  36.072  47.536  1.00  0.00           H
ATOM   1523  CG2 VAL    96      33.522  36.090  48.587  1.00  0.00           C
ATOM   1524 HG21 VAL    96      33.468  37.043  48.060  1.00  0.00           H
ATOM   1525 HG22 VAL    96      32.625  35.789  49.130  1.00  0.00           H
ATOM   1526 HG23 VAL    96      34.241  36.287  49.382  1.00  0.00           H
ATOM   1527  C   VAL    96      33.479  33.299  45.827  1.00  0.00           C
ATOM   1528  O   VAL    96      33.747  32.239  46.320  1.00  0.00           O
ATOM   1529  N   ILE    97      33.818  33.601  44.560  1.00  0.00           N
ATOM   1530  H   ILE    97      33.664  34.522  44.176  1.00  0.00           H
ATOM   1531  CA  ILE    97      34.410  32.758  43.619  1.00  0.00           C
ATOM   1532  HA  ILE    97      34.887  31.934  44.152  1.00  0.00           H
ATOM   1533  CB  ILE    97      33.352  32.294  42.622  1.00  0.00           C
ATOM   1534  HB  ILE    97      33.151  33.093  41.907  1.00  0.00           H
ATOM   1535  CG2 ILE    97      33.848  31.045  41.826  1.00  0.00           C
ATOM   1536 HG21 ILE    97      34.067  30.223  42.507  1.00  0.00           H
ATOM   1537 HG22 ILE    97      33.144  30.648  41.095  1.00  0.00           H
ATOM   1538 HG23 ILE    97      34.777  31.308  41.319  1.00  0.00           H
ATOM   1539  CG1 ILE    97      31.977  31.958  43.276  1.00  0.00           C
ATOM   1540 HG12 ILE    97      32.174  31.184  44.017  1.00  0.00           H
ATOM   1541 HG13 ILE    97      31.624  32.852  43.791
  1.00  0.00           H
ATOM   1542  CD1 ILE    97      30.825  31.575  42.304  1.00  0.00           C
ATOM   1543 HD11 ILE    97      30.068  31.188  42.986  1.00  0.00           H
ATOM   1544 HD12 ILE    97      30.465  32.389  41.675  1.00  0.00           H
ATOM   1545 HD13 ILE    97      31.153  30.714  41.720  1.00  0.00           H
ATOM   1546  C   ILE    97      35.577  33.500  42.976  1.00  0.00           C
ATOM   1547  O   ILE    97      35.435  34.610  42.449  1.00  0.00           O
ATOM   1548  N   GLU    98      36.711  32.848  42.988  1.00  0.00           N
ATOM   1549  H   GLU    98      36.686  31.939  43.427  1.00  0.00           H
ATOM   1550  CA  GLU    98      37.977  33.369  42.448  1.00  0.00           C
ATOM   1551  HA  GLU    98      37.867  34.366  42.020  1.00  0.00           H
ATOM   1552  CB  GLU    98      39.132  33.448  43.556  1.00  0.00           C
ATOM   1553  HB2 GLU    98      39.652  32.498  43.679  1.00  0.00           H
ATOM   1554  HB3 GLU    98      39.832  34.224  43.246  1.00  0.00           H
ATOM   1555  CG  GLU    98      38.497  33.874  44.877  1.00  0.00           C
ATOM   1556  HG2 GLU    98      37.886  34.767  44.746  1.00  0.00           H
ATOM   1557  HG3 GLU    98      37.892  33.079  45.314  1.00  0.00           H
ATOM   1558  CD  GLU    98      39.558  34.237  45.946  1.00  0.00           C
ATOM   1559  OE1 GLU    98      39.206  34.124  47.143  1.00  0.00           O
ATOM   1560  OE2 GLU    98      40.647  34.728  45.675  1.00  0.00           O
ATOM   1561  C   GLU    98      38.490  32.468  41.329  1.00  0.00           C
ATOM   1562  O   GLU    98      38.238  31.236  41.402  1.00  0.00           O
ATOM   1563  N   PHE    99      38.994  33.041  40.249  1.00  0.00           N
ATOM   1564  H   PHE    99      39.181  34.033  40.227  1.00  0.00           H
ATOM   1565  CA  PHE    99      39.268  32.293  39.024  1.00  0.00           C
ATOM   1566  HA  PHE    99      39.589  31.306  39.356  1.00  0.00           H
ATOM   1567  CB  PHE    99      37.903  32.099  38.390  1.00  0.00           C
ATOM   1568  HB2 PHE    99      38.026  31.442  37.529  1.00  0.00           H
ATOM   1569  HB3 PHE    99      37.284  31.505  39.062  1.00  0.00           H
ATOM   1570  CG  PHE    99      37.115  33.377  37.960  1.00  0.00           C
```

ATOM	1571	CD1	PHE	99	36.385	34.129	38.949	1.00	0.00	C
ATOM	1572	HD1	PHE	99	36.319	33.718	39.945	1.00	0.00	H
ATOM	1573	CE1	PHE	99	35.722	35.353	38.652	1.00	0.00	C
ATOM	1574	HE1	PHE	99	35.209	35.929	39.408	1.00	0.00	H
ATOM	1575	CZ	PHE	99	35.905	35.916	37.385	1.00	0.00	C
ATOM	1576	HZ	PHE	99	35.616	36.949	37.265	1.00	0.00	H
ATOM	1577	CE2	PHE	99	36.673	35.198	36.399	1.00	0.00	C
ATOM	1578	HE2	PHE	99	36.786	35.632	35.416	1.00	0.00	H
ATOM	1579	CD2	PHE	99	37.198	33.875	36.694	1.00	0.00	C
ATOM	1580	HD2	PHE	99	37.916	33.405	36.036	1.00	0.00	H
ATOM	1581	C	PHE	99	40.201	32.911	38.032	1.00	0.00	C
ATOM	1582	O	PHE	99	40.261	34.151	37.848	1.00	0.00	O
ATOM	1583	N	LYS	100	41.056	32.021	37.441	1.00	0.00	N
ATOM	1584	H	LYS	100	40.899	31.062	37.715	1.00	0.00	H
ATOM	1585	CA	LYS	100	42.131	32.392	36.502	1.00	0.00	C
ATOM	1586	HA	LYS	100	41.905	33.322	35.981	1.00	0.00	H
ATOM	1587	CB	LYS	100	43.406	32.617	37.252	1.00	0.00	C
ATOM	1588	HB2	LYS	100	44.163	33.102	36.634	1.00	0.00	H
ATOM	1589	HB3	LYS	100	43.266	33.497	37.880	1.00	0.00	H
ATOM	1590	CG	LYS	100	44.145	31.406	37.915	1.00	0.00	C
ATOM	1591	HG2	LYS	100	44.942	31.710	38.595	1.00	0.00	H
ATOM	1592	HG3	LYS	100	43.505	30.865	38.613	1.00	0.00	H
ATOM	1593	CD	LYS	100	44.709	30.383	36.918	1.00	0.00	C
ATOM	1594	HD2	LYS	100	43.897	29.777	36.518	1.00	0.00	H
ATOM	1595	HD3	LYS	100	45.203	30.929	36.114	1.00	0.00	H
ATOM	1596	CE	LYS	100	45.855	29.474	37.554	1.00	0.00	C
ATOM	1597	HE2	LYS	100	46.659	30.159	37.824	1.00	0.00	H
ATOM	1598	HE3	LYS	100	45.360	28.951	38.372	1.00	0.00	H
ATOM	1599	NZ	LYS	100	46.313	28.515	36.555	1.00	0.00	N
ATOM	1600	HZ1	LYS	100	45.578	27.841	36.400	1.00	0.00	H
ATOM	1601	HZ2	LYS	100	46.740	28.984	35.769	1.00	0.00	H
ATOM	1602	HZ3	LYS	100	47.054	27.999	37.010	1.00	0.00	H
ATOM	1603	C	LYS	100	42.207	31.435	35.342	1.00	0.00	C
ATOM	1604	O	LYS	100	41.790	30.286	35.400	1.00	0.00	O
ATOM	1605	N	CYX	101	42.734	31.899	34.208	1.00	0.00	N
ATOM	1606	H	CYX	101	43.054	32.856	34.263	1.00	0.00	H
ATOM	1607	CA	CYX	101	42.865	31.121	32.966	1.00	0.00	C
ATOM	1608	HA	CYX	101	42.096	30.348	32.948	1.00	0.00	H
ATOM	1609	CB	CYX	101	42.503	32.048	31.803	1.00	0.00	C
ATOM	1610	HB2	CYX	101	41.427	32.174	31.688	1.00	0.00	H
ATOM	1611	HB3	CYX	101	43.023	33.005	31.861	1.00	0.00	H
ATOM	1612	SG	CYX	101	42.931	31.273	30.193	1.00	0.00	S
ATOM	1613	C	CYX	101	44.163	30.308	32.945	1.00	0.00	C
ATOM	1614	O	CYX	101	45.191	30.897	33.157	1.00	0.00	O
ATOM	1615	N	LEU	102	44.065	29.049	32.478	1.00	0.00	N
ATOM	1616	H	LEU	102	43.134	28.863	32.132	1.00	0.00	H
ATOM	1617	CA	LEU	102	45.170	28.055	32.236	1.00	0.00	C
ATOM	1618	HA	LEU	102	45.481	27.833	33.256	1.00	0.00	H
ATOM	1619	CB	LEU	102	44.591	26.778	31.423	1.00	0.00	C
ATOM	1620	HB2	LEU	102	43.863	27.150	30.701	1.00	0.00	H
ATOM	1621	HB3	LEU	102	45.384	26.306	30.842	1.00	0.00	H
ATOM	1622	CG	LEU	102	43.996	25.723	32.392	1.00	0.00	C
ATOM	1623	HG	LEU	102	44.797	25.183	32.897	1.00	0.00	H
ATOM	1624	CD1	LEU	102	42.979	26.141	33.497	1.00	0.00	C
ATOM	1625	HD11	LEU	102	42.150	26.648	33.003	1.00	0.00	H
ATOM	1626	HD12	LEU	102	42.546	25.348	34.106	1.00	0.00	H
ATOM	1627	HD13	LEU	102	43.374	26.891	34.184	1.00	0.00	H
ATOM	1628	CD2	LEU	102	43.342	24.651	31.549	1.00	0.00	C
ATOM	1629	HD21	LEU	102	42.565	25.097	30.928	1.00	0.00	H
ATOM	1630	HD22	LEU	102	44.087	24.075	30.998	1.00	0.00	H
ATOM	1631	HD23	LEU	102	42.875	23.951	32.242	1.00	0.00	H
ATOM	1632	C	LEU	102	46.404	28.615	31.535	1.00	0.00	C
ATOM	1633	O	LEU	102	46.438	28.611	30.250	1.00	0.00	O
ATOM	1634	OXT	LEU	102	47.346	29.014	32.244	1.00	0.00	O

```
HETATM  314  N   LYN    20      39.380  40.705  35.232  1.00  0.00           N
HETATM  315  H   LYN    20      39.800  40.135  34.513  1.00  0.00           H
HETATM  316  CA  LYN    20      38.235  40.122  35.981  1.00  0.00           C
HETATM  317  HA  LYN    20      37.830  40.714  36.802  1.00  0.00           H
HETATM  318  CB  LYN    20      36.987  40.087  34.965  1.00  0.00           C
HETATM  319  HB2 LYN    20      37.378  39.610  34.067  1.00  0.00           H
HETATM  320  HB3 LYN    20      36.736  41.120  34.728  1.00  0.00           H
HETATM  321  CG  LYN    20      35.608  39.422  35.393  1.00  0.00           C
HETATM  322  HG2 LYN    20      34.809  39.548  34.663  1.00  0.00           H
HETATM  323  HG3 LYN    20      35.692  38.339  35.487  1.00  0.00           H
HETATM  324  CD  LYN    20      34.996  40.030  36.693  1.00  0.00           C
HETATM  325  HD2 LYN    20      34.685  41.045  36.450  1.00  0.00           H
HETATM  326  HD3 LYN    20      35.838  40.085  37.383  1.00  0.00           H
HETATM  327  CE  LYN    20      33.842  39.250  37.343  1.00  0.00           C
HETATM  328  HE2 LYN    20      33.035  39.283  36.610  1.00  0.00           H
HETATM  329  HE3 LYN    20      34.121  38.242  37.652  1.00  0.00           H
HETATM  330  NZ  LYN    20      33.372  39.873  38.599  1.00  0.00           N
HETATM  331  HZ2 LYN    20      33.457  40.869  38.464  1.00  0.00           H
HETATM  332  HZ3 LYN    20      34.109  39.605  39.234  1.00  0.00           H
HETATM  333  C   LYN    20      38.737  38.750  36.474  1.00  0.00           C
HETATM  334  O   LYN    20      39.256  37.869  35.776  1.00  0.00           O
HETATM 1636  N   LIG   103      29.847  42.653  41.030  1.00  0.00           N
HETATM 1637  C   LIG   103      29.485  41.641  40.231  1.00  0.00           C
HETATM 1638  O   LIG   103      29.825  43.724  42.915  1.00  0.00           O
HETATM 1639  C5' LIG   103      25.569  39.666  45.286  1.00  0.00           C
HETATM 1640  O5' LIG   103      25.456  39.198  43.895  1.00  0.00           O
HETATM 1641  C4' LIG   103      26.180  41.028  45.391  1.00  0.00           C
HETATM 1642  O4' LIG   103      27.471  40.981  44.741  1.00  0.00           O
HETATM 1643  C3' LIG   103      25.378  42.175  44.776  1.00  0.00           C
HETATM 1644  O3' LIG   103      24.676  42.958  45.725  1.00  0.00           O
HETATM 1645  C2' LIG   103      26.397  43.098  44.125  1.00  0.00           C
HETATM 1646  O2' LIG   103      26.555  44.368  44.775  1.00  0.00           O
HETATM
 1647  C1' LIG   103      27.716  42.251  44.102  1.00  0.00           C
HETATM 1648  N1  LIG   103      28.373  41.921  42.803  1.00  0.00           N
HETATM 1649  O1  LIG   103      29.445  39.288  38.393  1.00  0.00           O
HETATM 1650  N2  LIG   103      30.038  41.477  39.001  1.00  0.00           N
HETATM 1651  C6  LIG   103      29.331  42.818  42.280  1.00  0.00           C
HETATM 1652  C7  LIG   103      27.910  40.892  42.047  1.00  0.00           C
HETATM 1653  C8  LIG   103      28.417  40.783  40.724  1.00  0.00           C
HETATM 1654  C9  LIG   103      29.980  40.347  38.176  1.00  0.00           C
HETATM 1655  C10 LIG   103      30.476  40.562  36.794  1.00  0.00           C
HETATM 1656  H   LIG   103      30.626  42.212  38.626  1.00  0.00           H
HETATM 1657  H1  LIG   103      27.874  40.097  40.084  1.00  0.00           H
HETATM 1658  H4  LIG   103      28.495  42.817  44.627  1.00  0.00           H
HETATM 1659  H6  LIG   103      26.366  41.403  46.406  1.00  0.00           H
HETATM 1660  H7  LIG   103      24.672  41.774  44.038  1.00  0.00           H
HETATM 1661  H8  LIG   103      26.004  43.421  43.153  1.00  0.00           H
HETATM 1662  H9  LIG   103      24.554  39.661  45.702  1.00  0.00           H
HETATM 1663  H10 LIG   103      26.240  38.971  45.804  1.00  0.00           H
HETATM 1664  H11 LIG   103      29.646  40.752  36.103  1.00  0.00           H
HETATM 1665  H12 LIG   103      30.965  39.686  36.349  1.00  0.00           H
HETATM 1666  H13 LIG   103      31.071  41.474  36.664  1.00  0.00           H
HETATM 1667  H14 LIG   103      27.095  40.318  42.468  1.00  0.00           H
HETATM 1668  H2' LIG   103      27.137  44.846  44.159  1.00  0.00           H
HETATM 1669  H3' LIG   103      25.198  43.759  45.904  1.00  0.00           H
HETATM 1670  H5' LIG   103      25.036  38.325  43.985  1.00  0.00           H
ENDMDL
MODEL      7
SHEET    1   1 1 ILE    22  ASP    26  0
SHEET    2   2 1 VAL    37  VAL    40  0
SHEET    3   3 1 CYX    50  VAL    60  0
SHEET    4   4 1 PHE    94  CYX   101  0
HELIX    1   1 GLN     9  ALA     17  1                                  9
```

```
HELIX    2   2 GLU     67  GLU     73  1                                   7
HELIX    3   3 LEU     77  ILE     87  1                                  11
ATOM      1  N   GLN     1      34.850  17.516  32.595  1.00  0.00           N
ATOM      2  H1  GLN     1      35.749  17.815  32.944  1.00  0.00           H
ATOM      3  H2  GLN     1      34.153  17.413  33.319  1.00  0.00           H
ATOM      4  H3  GLN     1      35.161  16.627  32.228  1.00  0.00           H
ATOM      5  CA  GLN     1      34.354  18.381  31.526  1.00  0.00           C
ATOM      6  HA  GLN     1      35.148  18.464  30.784  1.00  0.00           H
ATOM      7  CB  GLN     1      33.067  17.734  31.015  1.00  0.00           C
ATOM      8  HB2 GLN     1      32.381  17.603  31.853  1.00  0.00           H
ATOM      9  HB3 GLN     1      32.541  18.384  30.315  1.00  0.00           H
ATOM     10  CG  GLN     1      33.227  16.292  30.334  1.00  0.00           C
ATOM     11  HG2 GLN     1      33.719  15.528  30.936  1.00  0.00           H
ATOM     12  HG3 GLN     1      32.229  15.895  30.147  1.00  0.00           H
ATOM     13  CD  GLN     1      33.997  16.314  28.981  1.00  0.00           C
ATOM     14  OE1 GLN     1      34.536  17.355  28.619  1.00  0.00           O
ATOM     15  NE2 GLN     1      34.220  15.250  28.242  1.00  0.00           N
ATOM     16 HE21 GLN     1      33.846  14.346  28.496  1.00  0.00           H
ATOM     17 HE22 GLN     1      35.011  15.254  27.614  1.00  0.00           H
ATOM     18  C   GLN     1      34.108  19.774  32.136  1.00  0.00           C
ATOM     19  O   GLN     1      33.587  19.832  33.194  1.00  0.00           O
ATOM     20  N   PRO     2      34.334  20.819  31.359  1.00  0.00           N
ATOM     21  CD  PRO     2      35.153  20.774  30.146  1.00  0.00           C
ATOM     22  HD2 PRO     2      34.563  20.314  29.353  1.00  0.00           H
ATOM     23  HD3 PRO     2      36.076  20.213  30.290  1.00  0.00           H
ATOM     24  CG  PRO     2      35.452  22.245  29.740  1.00  0.00           C
ATOM     25  HG2 PRO     2      35.419  22.265  28.651  1.00  0.00           H
ATOM     26  HG3 PRO     2      36.391  22.594  30.171  1.00  0.00           H
ATOM     27  CB  PRO     2      34.232  22.908  30.235  1.00  0.00           C
ATOM     28  HB2 PRO     2      33.406  22.834  29.529  1.00  0.00           H
ATOM     29  HB3 PRO     2      34.436  23.916  30.597  1.00  0.00           H
ATOM     30  CA  PRO     2      33.921  22.167  31.566  1.00  0.00           C
ATOM     31  HA  PRO     2      34.527  22.628  32.347  1.00  0.00           H
ATOM     32  C   PRO     2      32.469  22.310  31.954  1.00  0.00           C
ATOM     33  O   PRO     2      31.570  21.684  31.376  1.00  0.00           O
ATOM     34  N   ASN     3      32.244  23.191  32.932  1.00  0.00           N
ATOM     35  H   ASN     3      33.060  23.524  33.426  1.00  0.00           H
ATOM     36  CA  ASN     3      30.971  23.745  33.321  1.00  0.00           C
ATOM     37  HA  ASN     3      30.345  22.994  33.802  1.00  0.00           H
ATOM     38  CB  ASN     3      31.274  24.868  34.363  1.00  0.00           C
ATOM     39  HB2 ASN     3      32.090  25.493  34.000  1.00  0.00           H
ATOM     40  HB3 ASN     3      30.380  25.475  34.505  1.00  0.00           H
ATOM     41  CG  ASN     3      31.591  24.177  35.719  1.00  0.00           C
ATOM     42  OD1 ASN     3      30.726  23.685  36.444  1.00  0.00           O
ATOM     43  ND2 ASN     3      32.821  23.838  36.009  1.00  0.00           N
ATOM     44 HD21 ASN     3      33.523  24.086  35.327  1.00  0.00           H
ATOM     45 HD22 ASN     3      33.055  23.472  36.921  1.00  0.00           H
ATOM     46  C   ASN     3      30.163  24.213  32.079  1.00  0.00           C
ATOM     47  O   ASN     3      30.719  24.831  31.213  1.00  0.00           O
ATOM     48  N   ASP     4      28.802  24.020  31.996  1.00  0.00           N
ATOM     49  H   ASP     4      28.285  23.483  32.677  1.00  0.00           H
ATOM     50  CA  ASP     4      27.994  24.631  30.916  1.00  0.00           C
ATOM     51  HA  ASP     4      28.552  24.539  29.984  1.00  0.00           H
ATOM     52  CB  ASP     4      26.612  23.877  30.669  1.00  0.00           C
ATOM     53  HB2 ASP     4      26.056  23.867  31.607  1.00  0.00           H
ATOM     54  HB3 ASP     4      26.027  24.472  29.967  1.00  0.00           H
ATOM     55  CG  ASP     4      26.749  22.435  30.153  1.00  0.00           C
ATOM     56  OD1 ASP     4      27.767  21.897  29.646  1.00  0.00           O
ATOM     57  OD2 ASP     4      25.695  21.792  30.089  1.00  0.00           O
ATOM     58  C   ASP     4      27.805  26.096  31.102  1.00  0.00           C
ATOM     59  O   ASP     4      27.827  26.747  30.060  1.00  0.00           O
ATOM     60  N   ILE     5      27.682  26.552  32.372  1.00  0.00           N
ATOM     61  H   ILE     5      27.887  25.869  33.087  1.00  0.00           H
ATOM     62  CA  ILE     5      27.443  27.956  32.691  1.00  0.00           C
```

ATOM	63	HA	ILE	5	26.669	28.243	31.979	1.00	0.00	H
ATOM	64	CB	ILE	5	26.880	28.125	34.121	1.00	0.00	C
ATOM	65	HB	ILE	5	27.495	27.583	34.839	1.00	0.00	H
ATOM	66	CG2	ILE	5	26.751	29.592	34.587	1.00	0.00	C
ATOM	67	HG21	ILE	5	26.362	30.180	33.755	1.00	0.00	H
ATOM	68	HG22	ILE	5	26.090	29.678	35.449	1.00	0.00	H
ATOM	69	HG23	ILE	5	27.733	29.990	34.844	1.00	0.00	H
ATOM	70	CG1	ILE	5	25.457	27.569	34.264	1.00	0.00	C
ATOM	71	HG12	ILE	5	25.501	26.496	34.078	1.00	0.00	H
ATOM	72	HG13	ILE	5	25.124	27.788	35.278	1.00	0.00	H
ATOM	73	CD1	ILE	5	24.378	28.221	33.320	1.00	0.00	C
ATOM	74	HD11	ILE	5	23.408	27.728	33.384	1.00	0.00	H
ATOM	75	HD12	ILE	5	24.277	29.298	33.457	1.00	0.00	H
ATOM	76	HD13	ILE	5	24.615	28.027	32.274	1.00	0.00	H
ATOM	77	C	ILE	5	28.765	28.734	32.475	1.00	0.00	C
ATOM	78	O	ILE	5	29.814	28.089	32.684	1.00	0.00	O
ATOM	79	N	THR	6	28.804	30.023	32.136	1.00	0.00	N
ATOM	80	H	THR	6	27.901	30.431	31.939	1.00	0.00	H
ATOM	81	CA	THR	6	30.024	30.864	32.141	1.00	0.00	C
ATOM	82	HA	THR	6	30.508	30.593	33.079	1.00	0.00	H
ATOM	83	CB	THR	6	31.002	30.539	30.963	1.00	0.00	C
ATOM	84	HB	THR	6	31.318	29.501	31.065	1.00	0.00	H
ATOM	85	CG2	THR	6	30.338	30.790	29.660	1.00	0.00	C
ATOM	86	HG21	THR	6	31.072	30.652	28.866	1.00	0.00	H
ATOM	87	HG22	THR	6	29.525	30.087	29.483	1.00	0.00	H
ATOM	88	HG23	THR	6	29.960	31.813	29.659	1.00	0.00	H
ATOM	89	OG1	THR	6	32.150	31.374	30.907	1.00	0.00	O
ATOM	90	HG1	THR	6	32.626	31.075	30.129	1.00	0.00	H
ATOM	91	C	THR	6	29.531	32.293	32.163	1.00	0.00	C
ATOM	92	O	THR	6	28.352	32.674	31.954	1.00	0.00	O
ATOM	93	N	PHE	7	30.519	33.226	32.331	1.00	0.00	N
ATOM	94	H	PHE	7	31.472	32.894	32.387	1.00	0.00	H
ATOM	95	CA	PHE	7	30.161	34.666	32.618	1.00	0.00	C
ATOM	96	HA	PHE	7	29.327	34.586	33.315	1.00	0.00	H
ATOM	97									
CB	PHE	7	31.342	35.335	33.238	1.00	0.00	C		
ATOM	98	HB2	PHE	7	32.126	35.390	32.483	1.00	0.00	H
ATOM	99	HB3	PHE	7	30.989	36.327	33.520	1.00	0.00	H
ATOM	100	CG	PHE	7	31.947	34.685	34.445	1.00	0.00	C
ATOM	101	CD1	PHE	7	33.203	34.135	34.345	1.00	0.00	C
ATOM	102	HD1	PHE	7	33.645	34.182	33.361	1.00	0.00	H
ATOM	103	CE1	PHE	7	33.744	33.407	35.430	1.00	0.00	C
ATOM	104	HE1	PHE	7	34.722	32.952	35.370	1.00	0.00	H
ATOM	105	CZ	PHE	7	33.084	33.405	36.681	1.00	0.00	C
ATOM	106	HZ	PHE	7	33.446	32.915	37.573	1.00	0.00	H
ATOM	107	CE2	PHE	7	31.850	34.129	36.876	1.00	0.00	C
ATOM	108	HE2	PHE	7	31.537	34.198	37.907	1.00	0.00	H
ATOM	109	CD2	PHE	7	31.340	34.817	35.745	1.00	0.00	C
ATOM	110	HD2	PHE	7	30.408	35.351	35.850	1.00	0.00	H
ATOM	111	C	PHE	7	29.640	35.366	31.370	1.00	0.00	C
ATOM	112	O	PHE	7	29.557	34.764	30.272	1.00	0.00	O
ATOM	113	N	PHE	8	29.255	36.656	31.485	1.00	0.00	N
ATOM	114	H	PHE	8	28.994	36.883	32.434	1.00	0.00	H
ATOM	115	CA	PHE	8	28.712	37.344	30.336	1.00	0.00	C
ATOM	116	HA	PHE	8	28.109	36.706	29.689	1.00	0.00	H
ATOM	117	CB	PHE	8	27.738	38.439	30.818	1.00	0.00	C
ATOM	118	HB2	PHE	8	28.296	39.191	31.375	1.00	0.00	H
ATOM	119	HB3	PHE	8	27.359	39.018	29.977	1.00	0.00	H
ATOM	120	CG	PHE	8	26.537	37.920	31.629	1.00	0.00	C
ATOM	121	CD1	PHE	8	25.918	38.891	32.470	1.00	0.00	C
ATOM	122	HD1	PHE	8	26.320	39.871	32.675	1.00	0.00	H
ATOM	123	CE1	PHE	8	24.711	38.616	33.123	1.00	0.00	C
ATOM	124	HE1	PHE	8	24.363	39.377	33.806	1.00	0.00	H
ATOM	125	CZ	PHE	8	24.165	37.274	32.998	1.00	0.00	C

ATOM	126	HZ	PHE	8	23.304	36.975	33.575	1.00	0.00	H
ATOM	127	CE2	PHE	8	24.750	36.372	32.160	1.00	0.00	C
ATOM	128	HE2	PHE	8	24.346	35.377	32.042	1.00	0.00	H
ATOM	129	CD2	PHE	8	25.953	36.656	31.438	1.00	0.00	C
ATOM	130	HD2	PHE	8	26.278	35.985	30.657	1.00	0.00	H
ATOM	131	C	PHE	8	29.833	37.784	29.402	1.00	0.00	C
ATOM	132	O	PHE	8	30.942	38.098	29.813	1.00	0.00	O
ATOM	133	N	GLN	9	29.459	37.850	28.147	1.00	0.00	N
ATOM	134	H	GLN	9	28.471	37.900	27.943	1.00	0.00	H
ATOM	135	CA	GLN	9	30.355	38.087	27.011	1.00	0.00	C
ATOM	136	HA	GLN	9	31.158	37.351	27.027	1.00	0.00	H
ATOM	137	CB	GLN	9	29.520	37.836	25.621	1.00	0.00	C
ATOM	138	HB2	GLN	9	28.672	38.514	25.533	1.00	0.00	H
ATOM	139	HB3	GLN	9	30.229	38.081	24.831	1.00	0.00	H
ATOM	140	CG	GLN	9	29.053	36.389	25.570	1.00	0.00	C
ATOM	141	HG2	GLN	9	29.735	35.748	26.131	1.00	0.00	H
ATOM	142	HG3	GLN	9	28.044	36.359	25.979	1.00	0.00	H
ATOM	143	CD	GLN	9	29.093	35.795	24.134	1.00	0.00	C
ATOM	144	OE1	GLN	9	29.083	36.430	23.101	1.00	0.00	O
ATOM	145	NE2	GLN	9	29.331	34.515	24.019	1.00	0.00	N
ATOM	146	HE21	GLN	9	29.306	34.114	23.093	1.00	0.00	H
ATOM	147	HE22	GLN	9	29.372	33.963	24.865	1.00	0.00	H
ATOM	148	C	GLN	9	31.074	39.438	27.075	1.00	0.00	C
ATOM	149	O	GLN	9	32.252	39.528	26.682	1.00	0.00	O
ATOM	150	N	ARG	10	30.435	40.513	27.603	1.00	0.00	N
ATOM	151	H	ARG	10	29.436	40.536	27.752	1.00	0.00	H
ATOM	152	CA	ARG	10	31.125	41.816	27.936	1.00	0.00	C
ATOM	153	HA	ARG	10	31.178	42.343	26.984	1.00	0.00	H
ATOM	154	CB	ARG	10	30.093	42.639	28.736	1.00	0.00	C
ATOM	155	HB2	ARG	10	29.277	42.955	28.086	1.00	0.00	H
ATOM	156	HB3	ARG	10	29.653	42.004	29.506	1.00	0.00	H
ATOM	157	CG	ARG	10	30.677	43.821	29.514	1.00	0.00	C
ATOM	158	HG2	ARG	10	31.153	43.309	30.351	1.00	0.00	H
ATOM	159	HG3	ARG	10	31.375	44.313	28.837	1.00	0.00	H
ATOM	160	CD	ARG	10	29.574	44.762	30.038	1.00	0.00	C
ATOM	161	HD2	ARG	10	28.854	44.309	30.719	1.00	0.00	H
ATOM	162	HD3	ARG	10	29.973	45.557	30.669	1.00	0.00	H
ATOM	163	NE	ARG	10	28.870	45.415	28.914	1.00	0.00	N
ATOM	164	HE	ARG	10	28.045	44.916	28.615	1.00	0.00	H
ATOM	165	CZ	ARG	10	29.216	46.414	28.121	1.00	0.00	C
ATOM	166	NH1	ARG	10	30.340	47.077	28.245	1.00	0.00	N
ATOM	167	HH11	ARG	10	30.528	47.852	27.625	1.00	0.00	H
ATOM	168	HH12	ARG	10	31.120	46.726	28.781	1.00	0.00	H
ATOM	169	NH2	ARG	10	28.365	46.783	27.173	1.00	0.00	N
ATOM	170	HH21	ARG	10	27.462	46.338	27.095	1.00	0.00	H
ATOM	171	HH22	ARG	10	28.672	47.538	26.577	1.00	0.00	H
ATOM	172	C	ARG	10	32.477	41.685	28.673	1.00	0.00	C
ATOM	173	O	ARG	10	33.296	42.546	28.429	1.00	0.00	O
ATOM	174	N	PHE	11	32.691	40.651	29.472	1.00	0.00	N
ATOM	175	H	PHE	11	31.910	40.035	29.645	1.00	0.00	H
ATOM	176	CA	PHE	11	33.828	40.437	30.421	1.00	0.00	C
ATOM	177	HA	PHE	11	34.403	41.363	30.439	1.00	0.00	H
ATOM	178	CB	PHE	11	33.166	40.274	31.758	1.00	0.00	C
ATOM	179	HB2	PHE	11	33.965	40.015	32.452	1.00	0.00	H
ATOM	180	HB3	PHE	11	32.537	39.391	31.649	1.00	0.00	H
ATOM	181	CG	PHE	11	32.203	41.331	32.262	1.00	0.00	C
ATOM	182	CD1	PHE	11	32.830	42.465	32.850	1.00	0.00	C
ATOM	183	HD1	PHE	11	33.910	42.471	32.823	1.00	0.00	H
ATOM	184	CE1	PHE	11	32.047	43.440	33.478	1.00	0.00	C
ATOM	185	HE1	PHE	11	32.536	44.351	33.789	1.00	0.00	H
ATOM	186	CZ	PHE	11	30.639	43.440	33.354	1.00	0.00	C
ATOM	187	HZ	PHE	11	29.987	44.248	33.651	1.00	0.00	H
ATOM	188	CE2	PHE	11	30.049	42.286	32.807	1.00	0.00	C
ATOM	189	HE2	PHE	11	28.988	42.300	32.605	1.00	0.00	H

```
ATOM    190  CD2 PHE    11      30.782  41.232  32.272  1.00  0.00           C
ATOM    191  HD2 PHE    11      30.337  40.313  31.919  1.00  0.00           H
ATOM    192  C   PHE    11      34.865  39.281  30.218  1.00  0.00           C
ATOM    193  O   PHE    11      35.793  39.043  30.971  1.00  0.00           O
ATOM    194  N   GLN    12      34.758  38.570  29.063  1.00  0.00           N
ATOM    195  H   GLN    12      34.000  38.821  28.443  1.00  0.00           H
ATOM    196  CA  GLN    12      35.716  37.482  28.648  1.00  0.00           C
ATOM    197  HA  GLN    12      35.798  36.626  29.317  1.00  0.00           H
ATOM    198  CB  GLN    12      35.366  36.822  27.288  1.00  0.00           C
ATOM    199  HB2 GLN    12      35.629  37.435  26.426  1.00  0.00           H
ATOM    200  HB3 GLN    12      35.948  35.934  27.038  1.00  0.00           H
ATOM    201  CG  GLN    12      33.886  36.506  27.167  1.00  0.00           C
ATOM    202  HG2 GLN    12      33.681  35.776  27.951  1.00  0.00           H
ATOM    203  HG3 GLN    12      33.279  37.388  27.373  1.00  0.00           H
ATOM    204  CD  GLN    12      33.446  35.932  25.815  1.00  0.00           C
ATOM    205  OE1 GLN    12      34.083  36.068  24.815  1.00  0.00           O
ATOM    206  NE2 GLN    12      32.472  35.114  25.777  1.00  0.00           N
ATOM    207 HE21 GLN    12      31.962  34.873  26.615  1.00  0.00           H
ATOM    208 HE22 GLN    12      32.241  34.788  24.850  1.00  0.00           H
ATOM    209  C   GLN    12      37.210  37.952  28.563  1.00  0.00           C
ATOM    210  O   GLN    12      38.129  37.416  29.132  1.00  0.00           O
ATOM    211  N   ASP    13      37.348  39.144  27.986  1.00  0.00           N
ATOM    212  H   ASP    13      36.532  39.723  27.848  1.00  0.00           H
ATOM    213  CA  ASP    13      38.645  39.833  27.814  1.00  0.00           C
ATOM    214  HA  ASP    13      39.339  39.099  27.402  1.00  0.00           H
ATOM    215  CB  ASP    13      38.562  40.868  26.708  1.00  0.00           C
ATOM    216  HB2 ASP    13      38.092  41.773  27.093  1.00  0.00           H
ATOM    217  HB3 ASP    13      39.573  41.086  26.365  1.00  0.00           H
ATOM    218  CG  ASP    13      37.708  40.552  25.456  1.00  0.00           C
ATOM    219  OD1 ASP    13      37.148  41.482  24.824  1.00  0.00           O
ATOM    220  OD2 ASP    13      37.724  39.361  25.038  1.00  0.00           O
ATOM    221  C   ASP    13      39.353  40.337  29.104  1.00  0.00           C
ATOM    222  O   ASP    13      40.575
  40.539  29.184  1.00  0.00           O
ATOM    223  N   ASP    14      38.527  40.626  30.161  1.00  0.00           N
ATOM    224  H   ASP    14      37.525  40.522  30.081  1.00  0.00           H
ATOM    225  CA  ASP    14      39.118  41.011  31.509  1.00  0.00           C
ATOM    226  HA  ASP    14      39.929  41.715  31.329  1.00  0.00           H
ATOM    227  CB  ASP    14      38.154  41.774  32.406  1.00  0.00           C
ATOM    228  HB2 ASP    14      37.319  41.110  32.635  1.00  0.00           H
ATOM    229  HB3 ASP    14      38.746  42.054  33.277  1.00  0.00           H
ATOM    230  CG  ASP    14      37.812  43.068  31.715  1.00  0.00           C
ATOM    231  OD1 ASP    14      38.628  44.020  31.999  1.00  0.00           O
ATOM    232  OD2 ASP    14      36.773  43.230  31.028  1.00  0.00           O
ATOM    233  C   ASP    14      39.571  39.728  32.239  1.00  0.00           C
ATOM    234  O   ASP    14      40.453  39.856  33.082  1.00  0.00           O
ATOM    235  N   ILE    15      39.151  38.477  31.930  1.00  0.00           N
ATOM    236  H   ILE    15      38.378  38.477  31.279  1.00  0.00           H
ATOM    237  CA  ILE    15      39.785  37.321  32.525  1.00  0.00           C
ATOM    238  HA  ILE    15      39.943  37.492  33.590  1.00  0.00           H
ATOM    239  CB  ILE    15      38.855  36.123  32.412  1.00  0.00           C
ATOM    240  HB  ILE    15      38.798  35.791  31.376  1.00  0.00           H
ATOM    241  CG2 ILE    15      39.341  35.096  33.350  1.00  0.00           C
ATOM    242 HG21 ILE    15      39.093  35.428  34.359  1.00  0.00           H
ATOM    243 HG22 ILE    15      38.666  34.249  33.223  1.00  0.00           H
ATOM    244 HG23 ILE    15      40.392  34.806  33.345  1.00  0.00           H
ATOM    245  CG1 ILE    15      37.390  36.538  32.683  1.00  0.00           C
ATOM    246 HG12 ILE    15      37.275  37.094  33.615  1.00  0.00           H
ATOM    247 HG13 ILE    15      36.943  37.204  31.945  1.00  0.00           H
ATOM    248  CD1 ILE    15      36.410  35.375  32.750  1.00  0.00           C
ATOM    249 HD11 ILE    15      36.510  34.771  31.848  1.00  0.00           H
ATOM    250 HD12 ILE    15      36.562  34.694  33.587  1.00  0.00           H
ATOM    251 HD13 ILE    15      35.412  35.805  32.845  1.00  0.00           H
ATOM    252  C   ILE    15      41.142  36.968  31.940  1.00  0.00           C
```

ATOM	253	O	ILE	15	42.082	36.737	32.712	1.00	0.00	O
ATOM	254	N	LEU	16	41.283	37.198	30.623	1.00	0.00	N
ATOM	255	H	LEU	16	40.499	37.704	30.235	1.00	0.00	H
ATOM	256	CA	LEU	16	42.584	37.021	29.923	1.00	0.00	C
ATOM	257	HA	LEU	16	42.964	36.034	30.184	1.00	0.00	H
ATOM	258	CB	LEU	16	42.319	37.043	28.440	1.00	0.00	C
ATOM	259	HB2	LEU	16	41.576	36.267	28.257	1.00	0.00	H
ATOM	260	HB3	LEU	16	41.907	38.022	28.195	1.00	0.00	H
ATOM	261	CG	LEU	16	43.523	36.906	27.403	1.00	0.00	C
ATOM	262	HG	LEU	16	44.138	37.800	27.513	1.00	0.00	H
ATOM	263	CD1	LEU	16	44.327	35.694	27.620	1.00	0.00	C
ATOM	264	HD11	LEU	16	43.785	34.755	27.519	1.00	0.00	H
ATOM	265	HD12	LEU	16	45.157	35.840	26.929	1.00	0.00	H
ATOM	266	HD13	LEU	16	44.776	35.778	28.611	1.00	0.00	H
ATOM	267	CD2	LEU	16	42.886	36.983	26.028	1.00	0.00	C
ATOM	268	HD21	LEU	16	43.688	36.800	25.313	1.00	0.00	H
ATOM	269	HD22	LEU	16	42.183	36.150	26.021	1.00	0.00	H
ATOM	270	HD23	LEU	16	42.359	37.929	25.908	1.00	0.00	H
ATOM	271	C	LEU	16	43.610	38.187	30.259	1.00	0.00	C
ATOM	272	O	LEU	16	44.796	37.867	30.327	1.00	0.00	O
ATOM	273	N	ALA	17	43.067	39.366	30.524	1.00	0.00	N
ATOM	274	H	ALA	17	42.068	39.385	30.378	1.00	0.00	H
ATOM	275	CA	ALA	17	43.862	40.489	30.890	1.00	0.00	C
ATOM	276	HA	ALA	17	44.791	40.479	30.321	1.00	0.00	H
ATOM	277	CB	ALA	17	43.274	41.809	30.353	1.00	0.00	C
ATOM	278	HB1	ALA	17	43.054	41.680	29.294	1.00	0.00	H
ATOM	279	HB2	ALA	17	42.380	42.114	30.898	1.00	0.00	H
ATOM	280	HB3	ALA	17	44.005	42.610	30.471	1.00	0.00	H
ATOM	281	C	ALA	17	44.183	40.546	32.364	1.00	0.00	C
ATOM	282	O	ALA	17	44.989	41.427	32.696	1.00	0.00	O
ATOM	283	N	GLY	18	43.453	39.796	33.276	1.00	0.00	N
ATOM	284	H	GLY	18	42.642	39.267	32.991	1.00	0.00	H
ATOM	285	CA	GLY	18	43.741	39.774	34.736	1.00	0.00	C
ATOM	286	HA2	GLY	18	43.237	38.920	35.188	1.00	0.00	H
ATOM	287	HA3	GLY	18	44.809	39.810	34.951	1.00	0.00	H
ATOM	288	C	GLY	18	43.189	40.981	35.423	1.00	0.00	C
ATOM	289	O	GLY	18	43.543	41.215	36.530	1.00	0.00	O
ATOM	290	N	ARG	19	42.222	41.691	34.849	1.00	0.00	N
ATOM	291	H	ARG	19	42.042	41.433	33.889	1.00	0.00	H
ATOM	292	CA	ARG	19	41.335	42.693	35.525	1.00	0.00	C
ATOM	293	HA	ARG	19	41.935	43.019	36.374	1.00	0.00	H
ATOM	294	CB	ARG	19	41.026	43.918	34.550	1.00	0.00	C
ATOM	295	HB2	ARG	19	40.564	43.477	33.667	1.00	0.00	H
ATOM	296	HB3	ARG	19	40.265	44.514	35.052	1.00	0.00	H
ATOM	297	CG	ARG	19	42.286	44.613	34.012	1.00	0.00	C
ATOM	298	HG2	ARG	19	42.965	44.946	34.797	1.00	0.00	H
ATOM	299	HG3	ARG	19	42.891	43.948	33.395	1.00	0.00	H
ATOM	300	CD	ARG	19	41.773	45.737	33.167	1.00	0.00	C
ATOM	301	HD2	ARG	19	40.969	45.464	32.483	1.00	0.00	H
ATOM	302	HD3	ARG	19	41.468	46.575	33.792	1.00	0.00	H
ATOM	303	NE	ARG	19	42.962	46.175	32.414	1.00	0.00	N
ATOM	304	HE	ARG	19	43.854	46.022	32.862	1.00	0.00	H
ATOM	305	CZ	ARG	19	42.962	46.725	31.172	1.00	0.00	C
ATOM	306	NH1	ARG	19	41.938	46.909	30.425	1.00	0.00	N
ATOM	307	HH11	ARG	19	41.037	46.500	30.624	1.00	0.00	H
ATOM	308	HH12	ARG	19	41.817	47.476	29.598	1.00	0.00	H
ATOM	309	NH2	ARG	19	44.158	47.128	30.699	1.00	0.00	N
ATOM	310	HH21	ARG	19	44.138	47.543	29.778	1.00	0.00	H
ATOM	311	HH22	ARG	19	44.971	46.970	31.276	1.00	0.00	H
ATOM	312	C	ARG	19	40.041	42.131	36.138	1.00	0.00	C
ATOM	313	O	ARG	19	39.411	42.813	36.926	1.00	0.00	O
ATOM	335	N	THR	21	39.120	38.505	38.393	1.00	0.00	N
ATOM	336	H	THR	21	38.525	39.136	38.911	1.00	0.00	H
ATOM	337	CA	THR	21	39.673	37.359	39.176	1.00	0.00	C

ATOM	338	HA	THR	21	39.642	36.485	38.525	1.00	0.00	H
ATOM	339	CB	THR	21	41.193	37.566	39.405	1.00	0.00	C
ATOM	340	HB	THR	21	41.638	37.539	38.410	1.00	0.00	H
ATOM	341	CG2	THR	21	41.425	38.894	40.147	1.00	0.00	C
ATOM	342	HG21	THR	21	41.401	39.682	39.395	1.00	0.00	H
ATOM	343	HG22	THR	21	40.704	39.167	40.917	1.00	0.00	H
ATOM	344	HG23	THR	21	42.473	38.917	40.448	1.00	0.00	H
ATOM	345	OG1	THR	21	41.911	36.598	40.164	1.00	0.00	O
ATOM	346	HG1	THR	21	41.573	35.759	39.842	1.00	0.00	H
ATOM	347	C	THR	21	38.994	36.937	40.461	1.00	0.00	C
ATOM	348	O	THR	21	39.154	35.769	40.909	1.00	0.00	O
ATOM	349	N	ILE	22	38.073	37.828	40.992	1.00	0.00	N
ATOM	350	H	ILE	22	38.105	38.793	40.696	1.00	0.00	H
ATOM	351	CA	ILE	22	37.057	37.484	42.017	1.00	0.00	C
ATOM	352	HA	ILE	22	37.084	36.408	42.185	1.00	0.00	H
ATOM	353	CB	ILE	22	37.501	38.087	43.386	1.00	0.00	C
ATOM	354	HB	ILE	22	38.469	37.662	43.647	1.00	0.00	H
ATOM	355	CG2	ILE	22	37.609	39.655	43.256	1.00	0.00	C
ATOM	356	HG21	ILE	22	36.591	40.044	43.223	1.00	0.00	H
ATOM	357	HG22	ILE	22	38.058	40.061	44.163	1.00	0.00	H
ATOM	358	HG23	ILE	22	38.125	40.008	42.363	1.00	0.00	H
ATOM	359	CG1	ILE	22	36.577	37.619	44.610	1.00	0.00	C
ATOM	360	HG12	ILE	22	35.543	37.945	44.491	1.00	0.00	H
ATOM	361	HG13	ILE	22	36.479	36.533	44.636	1.00	0.00	H
ATOM	362	CD1	ILE	22	37.148	37.835	45.957	1.00	0.00	C
ATOM	363	HD11	ILE	22	36.558	37.317	46.714	1.00	0.00	H
ATOM	364	HD12	ILE	22	38.165	37.441	45.936	1.00	0.00	H
ATOM	365	HD13	ILE	22	37.121	38.916	46.096	1.00	0.00	H
ATOM	366	C	ILE	22	35.707	38.031	41.607	1.00	0.00	C
ATOM	367	O	ILE	22	35.657	39.090	41.013	1.00	0.00	O
ATOM	368	N	THR	23	34.614	37.338	42.003	1.00	0.00	
	N									
ATOM	369	H	THR	23	34.706	36.505	42.567	1.00	0.00	H
ATOM	370	CA	THR	23	33.233	37.845	41.902	1.00	0.00	C
ATOM	371	HA	THR	23	33.187	38.934	41.889	1.00	0.00	H
ATOM	372	CB	THR	23	32.738	37.365	40.516	1.00	0.00	C
ATOM	373	HB	THR	23	33.505	37.182	39.764	1.00	0.00	H
ATOM	374	CG2	THR	23	31.979	36.008	40.438	1.00	0.00	C
ATOM	375	HG21	THR	23	31.281	35.824	41.254	1.00	0.00	H
ATOM	376	HG22	THR	23	31.526	35.865	39.456	1.00	0.00	H
ATOM	377	HG23	THR	23	32.756	35.252	40.550	1.00	0.00	H
ATOM	378	OG1	THR	23	31.910	38.344	40.001	1.00	0.00	O
ATOM	379	HG1	THR	23	32.427	39.053	39.613	1.00	0.00	H
ATOM	380	C	THR	23	32.405	37.329	43.089	1.00	0.00	C
ATOM	381	O	THR	23	32.664	36.282	43.680	1.00	0.00	O
ATOM	382	N	ILE	24	31.232	38.007	43.373	1.00	0.00	N
ATOM	383	H	ILE	24	30.909	38.647	42.662	1.00	0.00	H
ATOM	384	CA	ILE	24	30.390	37.706	44.488	1.00	0.00	C
ATOM	385	HA	ILE	24	30.744	36.923	45.161	1.00	0.00	H
ATOM	386	CB	ILE	24	30.338	38.974	45.413	1.00	0.00	C
ATOM	387	HB	ILE	24	30.142	39.868	44.820	1.00	0.00	H
ATOM	388	CG2	ILE	24	29.115	38.687	46.405	1.00	0.00	C
ATOM	389	HG21	ILE	24	28.215	38.777	45.795	1.00	0.00	H
ATOM	390	HG22	ILE	24	29.218	37.649	46.723	1.00	0.00	H
ATOM	391	HG23	ILE	24	28.964	39.394	47.221	1.00	0.00	H
ATOM	392	CG1	ILE	24	31.680	39.217	46.158	1.00	0.00	C
ATOM	393	HG12	ILE	24	31.897	38.309	46.721	1.00	0.00	H
ATOM	394	HG13	ILE	24	32.489	39.416	45.456	1.00	0.00	H
ATOM	395	CD1	ILE	24	31.547	40.370	47.104	1.00	0.00	C
ATOM	396	HD11	ILE	24	30.774	41.088	46.831	1.00	0.00	H
ATOM	397	HD12	ILE	24	31.256	39.889	48.038	1.00	0.00	H
ATOM	398	HD13	ILE	24	32.562	40.750	47.215	1.00	0.00	H
ATOM	399	C	ILE	24	29.013	37.195	43.913	1.00	0.00	C
ATOM	400	O	ILE	24	28.480	37.746	42.990	1.00	0.00	O

ATOM	401	N	ARG	25	28.348	36.155	44.446	1.00	0.00	N
ATOM	402	H	ARG	25	28.817	35.733	45.235	1.00	0.00	H
ATOM	403	CA	ARG	25	26.942	35.675	44.209	1.00	0.00	C
ATOM	404	HA	ARG	25	26.419	36.493	43.712	1.00	0.00	H
ATOM	405	CB	ARG	25	26.939	34.373	43.405	1.00	0.00	C
ATOM	406	HB2	ARG	25	27.580	33.694	43.967	1.00	0.00	H
ATOM	407	HB3	ARG	25	25.897	34.056	43.387	1.00	0.00	H
ATOM	408	CG	ARG	25	27.471	34.447	41.905	1.00	0.00	C
ATOM	409	HG2	ARG	25	28.546	34.583	42.031	1.00	0.00	H
ATOM	410	HG3	ARG	25	27.297	33.491	41.412	1.00	0.00	H
ATOM	411	CD	ARG	25	26.881	35.540	41.001	1.00	0.00	C
ATOM	412	HD2	ARG	25	25.911	35.249	40.599	1.00	0.00	H
ATOM	413	HD3	ARG	25	26.677	36.500	41.473	1.00	0.00	H
ATOM	414	NE	ARG	25	27.668	35.676	39.687	1.00	0.00	N
ATOM	415	HE	ARG	25	27.209	35.369	38.841	1.00	0.00	H
ATOM	416	CZ	ARG	25	28.692	36.465	39.446	1.00	0.00	C
ATOM	417	NH1	ARG	25	29.189	37.204	40.323	1.00	0.00	N
ATOM	418	HH11	ARG	25	30.055	37.711	40.209	1.00	0.00	H
ATOM	419	HH12	ARG	25	28.656	37.245	41.181	1.00	0.00	H
ATOM	420	NH2	ARG	25	29.159	36.608	38.226	1.00	0.00	N
ATOM	421	HH21	ARG	25	29.755	37.384	37.977	1.00	0.00	H
ATOM	422	HH22	ARG	25	28.770	36.019	37.502	1.00	0.00	H
ATOM	423	C	ARG	25	26.289	35.498	45.570	1.00	0.00	C
ATOM	424	O	ARG	25	26.896	35.640	46.648	1.00	0.00	O
ATOM	425	N	ASP	26	25.000	35.073	45.564	1.00	0.00	N
ATOM	426	H	ASP	26	24.580	34.965	44.651	1.00	0.00	H
ATOM	427	CA	ASP	26	24.295	34.509	46.746	1.00	0.00	C
ATOM	428	HA	ASP	26	24.734	34.947	47.642	1.00	0.00	H
ATOM	429	CB	ASP	26	22.825	34.982	46.789	1.00	0.00	C
ATOM	430	HB2	ASP	26	22.350	34.526	47.658	1.00	0.00	H
ATOM	431	HB3	ASP	26	22.894	36.070	46.796	1.00	0.00	H
ATOM	432	CG	ASP	26	22.142	34.710	45.461	1.00	0.00	C
ATOM	433	OD1	ASP	26	22.274	35.446	44.472	1.00	0.00	O
ATOM	434	OD2	ASP	26	21.392	33.694	45.422	1.00	0.00	O
ATOM	435	C	ASP	26	24.393	32.977	46.914	1.00	0.00	C
ATOM	436	O	ASP	26	24.501	32.229	45.914	1.00	0.00	O
ATOM	437	N	GLU	27	24.315	32.472	48.146	1.00	0.00	N
ATOM	438	H	GLU	27	24.099	33.066	48.934	1.00	0.00	H
ATOM	439	CA	GLU	27	24.672	31.098	48.547	1.00	0.00	C
ATOM	440	HA	GLU	27	25.680	30.825	48.235	1.00	0.00	H
ATOM	441	CB	GLU	27	24.745	30.998	50.095	1.00	0.00	C
ATOM	442	HB2	GLU	27	25.383	31.816	50.432	1.00	0.00	H
ATOM	443	HB3	GLU	27	23.809	31.429	50.448	1.00	0.00	H
ATOM	444	CG	GLU	27	25.078	29.636	50.749	1.00	0.00	C
ATOM	445	HG2	GLU	27	24.940	29.795	51.819	1.00	0.00	H
ATOM	446	HG3	GLU	27	24.291	28.938	50.465	1.00	0.00	H
ATOM	447	CD	GLU	27	26.420	29.078	50.401	1.00	0.00	C
ATOM	448	OE1	GLU	27	26.580	28.363	49.429	1.00	0.00	O
ATOM	449	OE2	GLU	27	27.365	29.315	51.243	1.00	0.00	O
ATOM	450	C	GLU	27	23.761	30.063	47.875	1.00	0.00	C
ATOM	451	O	GLU	27	22.541	30.222	47.823	1.00	0.00	O
ATOM	452	N	SER	28	24.338	28.968	47.443	1.00	0.00	N
ATOM	453	H	SER	28	25.334	28.846	47.555	1.00	0.00	H
ATOM	454	CA	SER	28	23.704	27.912	46.617	1.00	0.00	C
ATOM	455	HA	SER	28	22.799	27.499	47.062	1.00	0.00	H
ATOM	456	CB	SER	28	23.440	28.410	45.163	1.00	0.00	C
ATOM	457	HB2	SER	28	22.708	29.206	45.288	1.00	0.00	H
ATOM	458	HB3	SER	28	24.395	28.674	44.711	1.00	0.00	H
ATOM	459	OG	SER	28	22.768	27.417	44.405	1.00	0.00	O
ATOM	460	HG	SER	28	22.687	27.682	43.487	1.00	0.00	H
ATOM	461	C	SER	28	24.787	26.788	46.511	1.00	0.00	C
ATOM	462	O	SER	28	25.908	26.978	45.969	1.00	0.00	O
ATOM	463	N	GLU	29	24.456	25.531	46.868	1.00	0.00	N
ATOM	464	H	GLU	29	23.581	25.399	47.355	1.00	0.00	H

ATOM	465	CA	GLU	29	25.334	24.353	46.662	1.00	0.00	C
ATOM	466	HA	GLU	29	26.301	24.630	47.080	1.00	0.00	H
ATOM	467	CB	GLU	29	24.751	23.108	47.383	1.00	0.00	C
ATOM	468	HB2	GLU	29	24.552	23.324	48.432	1.00	0.00	H
ATOM	469	HB3	GLU	29	23.771	22.892	46.958	1.00	0.00	H
ATOM	470	CG	GLU	29	25.560	21.736	47.237	1.00	0.00	C
ATOM	471	HG2	GLU	29	25.007	21.020	47.844	1.00	0.00	H
ATOM	472	HG3	GLU	29	25.544	21.455	46.183	1.00	0.00	H
ATOM	473	CD	GLU	29	27.014	21.895	47.666	1.00	0.00	C
ATOM	474	OE1	GLU	29	27.440	22.679	48.559	1.00	0.00	O
ATOM	475	OE2	GLU	29	27.802	20.941	47.306	1.00	0.00	O
ATOM	476	C	GLU	29	25.592	24.081	45.147	1.00	0.00	C
ATOM	477	O	GLU	29	26.639	23.511	44.765	1.00	0.00	O
ATOM	478	N	SER	30	24.735	24.550	44.233	1.00	0.00	N
ATOM	479	H	SER	30	23.840	24.926	44.511	1.00	0.00	H
ATOM	480	CA	SER	30	24.935	24.292	42.831	1.00	0.00	C
ATOM	481	HA	SER	30	25.319	23.273	42.850	1.00	0.00	H
ATOM	482	CB	SER	30	23.696	24.231	41.989	1.00	0.00	C
ATOM	483	HB2	SER	30	23.921	23.732	41.047	1.00	0.00	H
ATOM	484	HB3	SER	30	23.115	23.468	42.508	1.00	0.00	H
ATOM	485	OG	SER	30	22.975	25.442	41.730	1.00	0.00	O
ATOM	486	HG	SER	30	22.026	25.306	41.688	1.00	0.00	H
ATOM	487	C	SER	30	26.081	25.176	42.145	1.00	0.00	C
ATOM	488	O	SER	30	26.344	24.966	40.943	1.00	0.00	O
ATOM	489	N	HIE	31	26.658	26.211	42.777	1.00	0.00	N
ATOM	490	H	HIE	31	26.474	26.336	43.762	1.00	0.00	H
ATOM	491	CA	HIE	31	27.749	26.927	42.126	1.00	0.00	C
ATOM	492	HA	HIE	31	27.513	27.236	41.108	1.00	0.00	H
ATOM	493	CB	HIE	31	28.138	28.311	42.873	1.00	0.00	C
ATOM	494									
HB2	HIE	31	28.580	28.035	43.830	1.00	0.00	H		
ATOM	495	HB3	HIE	31	28.978	28.807	42.387	1.00	0.00	H
ATOM	496	CG	HIE	31	27.046	29.251	43.140	1.00	0.00	C
ATOM	497	ND1	HIE	31	26.171	29.779	42.217	1.00	0.00	N
ATOM	498	CE1	HIE	31	25.594	30.842	42.845	1.00	0.00	C
ATOM	499	HE1	HIE	31	24.704	31.317	42.460	1.00	0.00	H
ATOM	500	NE2	HIE	31	26.183	31.004	44.053	1.00	0.00	N
ATOM	501	HE2	HIE	31	25.758	31.564	44.778	1.00	0.00	H
ATOM	502	CD2	HIE	31	27.053	29.929	44.348	1.00	0.00	C
ATOM	503	HD2	HIE	31	27.660	29.760	45.224	1.00	0.00	H
ATOM	504	C	HIE	31	28.958	26.054	41.956	1.00	0.00	C
ATOM	505	O	HIE	31	29.100	24.933	42.439	1.00	0.00	O
ATOM	506	N	PHE	32	29.978	26.739	41.342	1.00	0.00	N
ATOM	507	H	PHE	32	29.785	27.667	40.994	1.00	0.00	H
ATOM	508	CA	PHE	32	31.212	26.216	41.034	1.00	0.00	C
ATOM	509	HA	PHE	32	31.086	25.290	40.474	1.00	0.00	H
ATOM	510	CB	PHE	32	31.978	27.224	40.161	1.00	0.00	C
ATOM	511	HB2	PHE	32	32.244	28.090	40.766	1.00	0.00	H
ATOM	512	HB3	PHE	32	32.886	26.786	39.747	1.00	0.00	H
ATOM	513	CG	PHE	32	31.366	27.712	38.872	1.00	0.00	C
ATOM	514	CD1	PHE	32	31.426	29.104	38.548	1.00	0.00	C
ATOM	515	HD1	PHE	32	31.895	29.764	39.263	1.00	0.00	H
ATOM	516	CE1	PHE	32	30.836	29.631	37.372	1.00	0.00	C
ATOM	517	HE1	PHE	32	30.849	30.678	37.107	1.00	0.00	H
ATOM	518	CZ	PHE	32	30.290	28.747	36.510	1.00	0.00	C
ATOM	519	HZ	PHE	32	29.795	29.034	35.594	1.00	0.00	H
ATOM	520	CE2	PHE	32	30.150	27.391	36.811	1.00	0.00	C
ATOM	521	HE2	PHE	32	29.697	26.724	36.093	1.00	0.00	H
ATOM	522	CD2	PHE	32	30.675	26.875	37.950	1.00	0.00	C
ATOM	523	HD2	PHE	32	30.643	25.810	38.122	1.00	0.00	H
ATOM	524	C	PHE	32	32.025	26.032	42.315	1.00	0.00	C
ATOM	525	O	PHE	32	31.873	26.717	43.305	1.00	0.00	O
ATOM	526	N	LYS	33	32.775	24.911	42.327	1.00	0.00	N
ATOM	527	H	LYS	33	32.661	24.312	41.522	1.00	0.00	H

ATOM	528	CA	LYS	33	33.657	24.551	43.489	1.00	0.00	C
ATOM	529	HA	LYS	33	33.333	25.130	44.353	1.00	0.00	H
ATOM	530	CB	LYS	33	33.619	23.004	43.686	1.00	0.00	C
ATOM	531	HB2	LYS	33	34.147	22.496	42.880	1.00	0.00	H
ATOM	532	HB3	LYS	33	34.174	22.726	44.582	1.00	0.00	H
ATOM	533	CG	LYS	33	32.178	22.517	43.855	1.00	0.00	C
ATOM	534	HG2	LYS	33	31.625	22.834	42.971	1.00	0.00	H
ATOM	535	HG3	LYS	33	32.341	21.439	43.827	1.00	0.00	H
ATOM	536	CD	LYS	33	31.520	23.103	45.083	1.00	0.00	C
ATOM	537	HD2	LYS	33	32.117	23.033	45.991	1.00	0.00	H
ATOM	538	HD3	LYS	33	31.415	24.186	45.027	1.00	0.00	H
ATOM	539	CE	LYS	33	30.226	22.477	45.458	1.00	0.00	C
ATOM	540	HE2	LYS	33	30.453	21.480	45.837	1.00	0.00	H
ATOM	541	HE3	LYS	33	29.799	23.016	46.304	1.00	0.00	H
ATOM	542	NZ	LYS	33	29.192	22.491	44.352	1.00	0.00	N
ATOM	543	HZ1	LYS	33	29.468	21.905	43.577	1.00	0.00	H
ATOM	544	HZ2	LYS	33	28.229	22.357	44.628	1.00	0.00	H
ATOM	545	HZ3	LYS	33	29.283	23.395	43.912	1.00	0.00	H
ATOM	546	C	LYS	33	35.057	24.966	43.215	1.00	0.00	C
ATOM	547	O	LYS	33	35.378	25.098	42.011	1.00	0.00	O
ATOM	548	N	THR	34	35.867	24.896	44.212	1.00	0.00	N
ATOM	549	H	THR	34	35.587	24.650	45.150	1.00	0.00	H
ATOM	550	CA	THR	34	37.377	24.931	44.003	1.00	0.00	C
ATOM	551	HA	THR	34	37.674	25.951	43.763	1.00	0.00	H
ATOM	552	CB	THR	34	38.070	24.747	45.353	1.00	0.00	C
ATOM	553	HB	THR	34	37.850	23.787	45.820	1.00	0.00	H
ATOM	554	CG2	THR	34	39.573	24.998	45.242	1.00	0.00	C
ATOM	555	HG21	THR	34	39.984	25.952	44.910	1.00	0.00	H
ATOM	556	HG22	THR	34	40.011	24.724	46.202	1.00	0.00	H
ATOM	557	HG23	THR	34	39.969	24.283	44.521	1.00	0.00	H
ATOM	558	OG1	THR	34	37.697	25.807	46.190	1.00	0.00	O
ATOM	559	HG1	THR	34	38.094	25.540	47.022	1.00	0.00	H
ATOM	560	C	THR	34	37.971	24.032	42.945	1.00	0.00	C
ATOM	561	O	THR	34	37.784	22.859	42.934	1.00	0.00	O
ATOM	562	N	GLY	35	38.746	24.630	42.001	1.00	0.00	N
ATOM	563	H	GLY	35	38.886	25.626	42.096	1.00	0.00	H
ATOM	564	CA	GLY	35	39.369	23.861	40.877	1.00	0.00	C
ATOM	565	HA2	GLY	35	40.091	24.611	40.555	1.00	0.00	H
ATOM	566	HA3	GLY	35	39.994	23.043	41.236	1.00	0.00	H
ATOM	567	C	GLY	35	38.369	23.413	39.781	1.00	0.00	C
ATOM	568	O	GLY	35	38.836	22.787	38.836	1.00	0.00	O
ATOM	569	N	ASP	36	37.087	23.734	39.921	1.00	0.00	N
ATOM	570	H	ASP	36	36.903	24.394	40.662	1.00	0.00	H
ATOM	571	CA	ASP	36	36.203	23.738	38.692	1.00	0.00	C
ATOM	572	HA	ASP	36	36.110	22.731	38.289	1.00	0.00	H
ATOM	573	CB	ASP	36	34.757	24.315	39.003	1.00	0.00	C
ATOM	574	HB2	ASP	36	34.821	25.175	39.671	1.00	0.00	H
ATOM	575	HB3	ASP	36	34.253	24.629	38.089	1.00	0.00	H
ATOM	576	CG	ASP	36	33.754	23.359	39.608	1.00	0.00	C
ATOM	577	OD1	ASP	36	32.584	23.768	39.633	1.00	0.00	O
ATOM	578	OD2	ASP	36	33.984	22.171	39.867	1.00	0.00	O
ATOM	579	C	ASP	36	36.757	24.478	37.449	1.00	0.00	C
ATOM	580	O	ASP	36	37.458	25.482	37.587	1.00	0.00	O
ATOM	581	N	VAL	37	36.419	24.005	36.216	1.00	0.00	N
ATOM	582	H	VAL	37	35.737	23.270	36.096	1.00	0.00	H
ATOM	583	CA	VAL	37	36.887	24.632	34.935	1.00	0.00	C
ATOM	584	HA	VAL	37	37.722	25.285	35.189	1.00	0.00	H
ATOM	585	CB	VAL	37	37.598	23.541	34.048	1.00	0.00	C
ATOM	586	HB	VAL	37	37.038	22.606	34.080	1.00	0.00	H
ATOM	587	CG1	VAL	37	37.912	23.961	32.637	1.00	0.00	C
ATOM	588	HG11	VAL	37	37.029	24.070	32.008	1.00	0.00	H
ATOM	589	HG12	VAL	37	38.271	24.982	32.759	1.00	0.00	H
ATOM	590	HG13	VAL	37	38.595	23.311	32.089	1.00	0.00	H
ATOM	591	CG2	VAL	37	38.939	23.148	34.714	1.00	0.00	C

ATOM	592	HG21	VAL	37	39.600	24.013	34.770	1.00	0.00	H
ATOM	593	HG22	VAL	37	38.822	22.760	35.726	1.00	0.00	H
ATOM	594	HG23	VAL	37	39.457	22.377	34.143	1.00	0.00	H
ATOM	595	C	VAL	37	35.769	25.287	34.060	1.00	0.00	C
ATOM	596	O	VAL	37	34.689	24.657	33.826	1.00	0.00	O
ATOM	597	N	LEU	38	35.937	26.531	33.601	1.00	0.00	N
ATOM	598	H	LEU	38	36.727	27.101	33.870	1.00	0.00	H
ATOM	599	CA	LEU	38	35.112	27.169	32.566	1.00	0.00	C
ATOM	600	HA	LEU	38	34.233	26.564	32.350	1.00	0.00	H
ATOM	601	CB	LEU	38	34.541	28.523	33.031	1.00	0.00	C
ATOM	602	HB2	LEU	38	35.451	29.006	33.386	1.00	0.00	H
ATOM	603	HB3	LEU	38	34.245	29.060	32.130	1.00	0.00	H
ATOM	604	CG	LEU	38	33.387	28.637	34.122	1.00	0.00	C
ATOM	605	HG	LEU	38	32.456	28.227	33.731	1.00	0.00	H
ATOM	606	CD1	LEU	38	33.817	27.884	35.378	1.00	0.00	C
ATOM	607	HD11	LEU	38	33.113	28.185	36.153	1.00	0.00	H
ATOM	608	HD12	LEU	38	33.763	26.803	35.243	1.00	0.00	H
ATOM	609	HD13	LEU	38	34.724	28.370	35.740	1.00	0.00	H
ATOM	610	CD2	LEU	38	33.256	30.175	34.339	1.00	0.00	C
ATOM	611	HD21	LEU	38	33.023	30.592	33.358	1.00	0.00	H
ATOM	612	HD22	LEU	38	32.446	30.302	35.057	1.00	0.00	H
ATOM	613	HD23	LEU	38	34.197	30.530	34.761	1.00	0.00	H
ATOM	614	C	LEU	38	35.858	27.297	31.245	1.00	0.00	C
ATOM	615	O	LEU	38	37.038	27.158	31.159	1.00	0.00	O
ATOM	616	N	ARG	39	35.073	27.711	30.240	1.00	0.00	N
ATOM	617	H	ARG	39	34.097	27.679	30.500	1.00	0.00	H
ATOM	618	CA	ARG	39	35.670	28.294	29.043	1.00	0.00	C
ATOM	619	HA	ARG	39	36.682					
28.619		29.287	1.00	0.00		H				
ATOM	620	CB	ARG	39	35.541	27.304	27.886	1.00	0.00	C
ATOM	621	HB2	ARG	39	34.499	27.004	27.767	1.00	0.00	H
ATOM	622	HB3	ARG	39	35.992	27.748	26.999	1.00	0.00	H
ATOM	623	CG	ARG	39	36.257	25.985	28.228	1.00	0.00	C
ATOM	624	HG2	ARG	39	37.258	26.242	28.573	1.00	0.00	H
ATOM	625	HG3	ARG	39	35.740	25.531	29.073	1.00	0.00	H
ATOM	626	CD	ARG	39	36.159	24.966	27.112	1.00	0.00	C
ATOM	627	HD2	ARG	39	36.396	25.483	26.182	1.00	0.00	H
ATOM	628	HD3	ARG	39	36.847	24.127	27.225	1.00	0.00	H
ATOM	629	NE	ARG	39	34.710	24.492	27.000	1.00	0.00	N
ATOM	630	HE	ARG	39	34.008	24.963	27.555	1.00	0.00	H
ATOM	631	CZ	ARG	39	34.266	23.525	26.258	1.00	0.00	C
ATOM	632	NH1	ARG	39	34.973	23.060	25.304	1.00	0.00	N
ATOM	633	HH11	ARG	39	35.921	23.378	25.165	1.00	0.00	H
ATOM	634	HH12	ARG	39	34.422	22.711	24.533	1.00	0.00	H
ATOM	635	NH2	ARG	39	32.989	23.313	26.415	1.00	0.00	N
ATOM	636	HH21	ARG	39	32.665	22.499	25.913	1.00	0.00	H
ATOM	637	HH22	ARG	39	32.359	23.807	27.031	1.00	0.00	H
ATOM	638	C	ARG	39	34.964	29.577	28.690	1.00	0.00	C
ATOM	639	O	ARG	39	33.836	29.790	29.053	1.00	0.00	O
ATOM	640	N	VAL	40	35.641	30.365	27.723	1.00	0.00	N
ATOM	641	H	VAL	40	36.539	29.991	27.449	1.00	0.00	H
ATOM	642	CA	VAL	40	35.226	31.670	27.090	1.00	0.00	C
ATOM	643	HA	VAL	40	34.136	31.671	27.075	1.00	0.00	H
ATOM	644	CB	VAL	40	35.525	32.922	27.975	1.00	0.00	C
ATOM	645	HB	VAL	40	35.017	33.670	27.366	1.00	0.00	H
ATOM	646	CG1	VAL	40	34.818	32.835	29.268	1.00	0.00	C
ATOM	647	HG11	VAL	40	35.278	32.057	29.878	1.00	0.00	H
ATOM	648	HG12	VAL	40	34.801	33.772	29.823	1.00	0.00	H
ATOM	649	HG13	VAL	40	33.810	32.458	29.098	1.00	0.00	H
ATOM	650	CG2	VAL	40	36.997	33.083	28.044	1.00	0.00	C
ATOM	651	HG21	VAL	40	37.371	33.721	28.845	1.00	0.00	H
ATOM	652	HG22	VAL	40	37.479	32.117	28.194	1.00	0.00	H
ATOM	653	HG23	VAL	40	37.366	33.535	27.125	1.00	0.00	H
ATOM	654	C	VAL	40	35.723	31.875	25.636	1.00	0.00	C

ATOM	655	O	VAL	40	36.615	31.206	25.157	1.00	0.00	O
ATOM	656	N	GLY	41	35.107	32.882	25.015	1.00	0.00	N
ATOM	657	H	GLY	41	34.312	33.361	25.413	1.00	0.00	H
ATOM	658	CA	GLY	41	35.205	33.069	23.546	1.00	0.00	C
ATOM	659	HA2	GLY	41	35.862	33.929	23.422	1.00	0.00	H
ATOM	660	HA3	GLY	41	35.622	32.235	22.981	1.00	0.00	H
ATOM	661	C	GLY	41	33.850	33.387	22.901	1.00	0.00	C
ATOM	662	O	GLY	41	32.746	33.276	23.471	1.00	0.00	O
ATOM	663	N	ARG	42	33.936	33.877	21.721	1.00	0.00	N
ATOM	664	H	ARG	42	34.851	33.883	21.294	1.00	0.00	H
ATOM	665	CA	ARG	42	32.794	34.419	20.931	1.00	0.00	C
ATOM	666	HA	ARG	42	32.052	33.627	21.026	1.00	0.00	H
ATOM	667	CB	ARG	42	32.396	35.794	21.540	1.00	0.00	C
ATOM	668	HB2	ARG	42	31.453	36.119	21.098	1.00	0.00	H
ATOM	669	HB3	ARG	42	32.031	35.724	22.565	1.00	0.00	H
ATOM	670	CG	ARG	42	33.370	36.958	21.428	1.00	0.00	C
ATOM	671	HG2	ARG	42	34.344	36.756	21.873	1.00	0.00	H
ATOM	672	HG3	ARG	42	33.432	37.297	20.393	1.00	0.00	H
ATOM	673	CD	ARG	42	32.882	38.155	22.302	1.00	0.00	C
ATOM	674	HD2	ARG	42	31.857	38.468	22.097	1.00	0.00	H
ATOM	675	HD3	ARG	42	32.702	37.857	23.335	1.00	0.00	H
ATOM	676	NE	ARG	42	33.799	39.285	22.187	1.00	0.00	N
ATOM	677	HE	ARG	42	33.651	39.951	21.443	1.00	0.00	H
ATOM	678	CZ	ARG	42	34.869	39.477	22.920	1.00	0.00	C
ATOM	679	NH1	ARG	42	35.357	38.600	23.785	1.00	0.00	N
ATOM	680	HH11	ARG	42	36.135	38.883	24.364	1.00	0.00	H
ATOM	681	HH12	ARG	42	34.884	37.718	23.915	1.00	0.00	H
ATOM	682	NH2	ARG	42	35.453	40.559	22.730	1.00	0.00	N
ATOM	683	HH21	ARG	42	35.103	41.131	21.975	1.00	0.00	H
ATOM	684	HH22	ARG	42	36.150	40.918	23.366	1.00	0.00	H
ATOM	685	C	ARG	42	33.118	34.408	19.419	1.00	0.00	C
ATOM	686	O	ARG	42	32.491	35.123	18.603	1.00	0.00	O
ATOM	687	N	PHE	43	34.231	33.732	19.030	1.00	0.00	N
ATOM	688	H	PHE	43	34.703	33.264	19.790	1.00	0.00	H
ATOM	689	CA	PHE	43	34.860	33.864	17.624	1.00	0.00	C
ATOM	690	HA	PHE	43	34.214	34.510	17.028	1.00	0.00	H
ATOM	691	CB	PHE	43	36.255	34.420	17.946	1.00	0.00	C
ATOM	692	HB2	PHE	43	36.717	33.556	18.424	1.00	0.00	H
ATOM	693	HB3	PHE	43	36.833	34.633	17.046	1.00	0.00	H
ATOM	694	CG	PHE	43	36.395	35.727	18.748	1.00	0.00	C
ATOM	695	CD1	PHE	43	35.672	36.889	18.277	1.00	0.00	C
ATOM	696	HD1	PHE	43	34.956	36.805	17.474	1.00	0.00	H
ATOM	697	CE1	PHE	43	35.974	38.141	18.821	1.00	0.00	C
ATOM	698	HE1	PHE	43	35.507	39.054	18.483	1.00	0.00	H
ATOM	699	CZ	PHE	43	36.923	38.225	19.843	1.00	0.00	C
ATOM	700	HZ	PHE	43	37.179	39.223	20.164	1.00	0.00	H
ATOM	701	CE2	PHE	43	37.508	37.111	20.384	1.00	0.00	C
ATOM	702	HE2	PHE	43	38.149	37.171	21.251	1.00	0.00	H
ATOM	703	CD2	PHE	43	37.346	35.836	19.728	1.00	0.00	C
ATOM	704	HD2	PHE	43	37.868	34.959	20.082	1.00	0.00	H
ATOM	705	C	PHE	43	34.903	32.541	16.897	1.00	0.00	C
ATOM	706	O	PHE	43	34.600	31.452	17.420	1.00	0.00	O
ATOM	707	N	GLU	44	35.297	32.604	15.636	1.00	0.00	N
ATOM	708	H	GLU	44	35.416	33.513	15.212	1.00	0.00	H
ATOM	709	CA	GLU	44	35.447	31.447	14.684	1.00	0.00	C
ATOM	710	HA	GLU	44	34.686	30.695	14.895	1.00	0.00	H
ATOM	711	CB	GLU	44	35.310	31.938	13.257	1.00	0.00	C
ATOM	712	HB2	GLU	44	34.288	32.215	13.000	1.00	0.00	H
ATOM	713	HB3	GLU	44	35.854	32.873	13.125	1.00	0.00	H
ATOM	714	CG	GLU	44	35.750	30.948	12.100	1.00	0.00	C
ATOM	715	HG2	GLU	44	35.298	29.961	12.199	1.00	0.00	H
ATOM	716	HG3	GLU	44	35.321	31.271	11.151	1.00	0.00	H
ATOM	717	CD	GLU	44	37.200	30.900	11.768	1.00	0.00	C
ATOM	718	OE1	GLU	44	37.652	29.769	11.588	1.00	0.00	O

ATOM	719	OE2	GLU	44	37.849	31.954	11.638	1.00	0.00	O
ATOM	720	C	GLU	44	36.633	30.521	14.987	1.00	0.00	C
ATOM	721	O	GLU	44	36.550	29.328	14.723	1.00	0.00	O
ATOM	722	N	ASP	45	37.626	31.063	15.705	1.00	0.00	N
ATOM	723	H	ASP	45	37.653	32.039	15.964	1.00	0.00	H
ATOM	724	CA	ASP	45	38.937	30.380	15.839	1.00	0.00	C
ATOM	725	HA	ASP	45	39.193	29.873	14.907	1.00	0.00	H
ATOM	726	CB	ASP	45	40.040	31.402	16.131	1.00	0.00	C
ATOM	727	HB2	ASP	45	40.166	32.138	15.337	1.00	0.00	H
ATOM	728	HB3	ASP	45	39.886	31.846	17.115	1.00	0.00	H
ATOM	729	CG	ASP	45	41.392	30.771	16.239	1.00	0.00	C
ATOM	730	OD1	ASP	45	41.576	29.603	15.880	1.00	0.00	O
ATOM	731	OD2	ASP	45	42.364	31.465	16.597	1.00	0.00	O
ATOM	732	C	ASP	45	38.926	29.256	16.936	1.00	0.00	C
ATOM	733	O	ASP	45	39.266	29.589	18.055	1.00	0.00	O
ATOM	734	N	ASP	46	38.538	28.014	16.595	1.00	0.00	N
ATOM	735	H	ASP	46	38.409	27.846	15.608	1.00	0.00	H
ATOM	736	CA	ASP	46	38.328	26.884	17.560	1.00	0.00	C
ATOM	737	HA	ASP	46	37.834	26.072	17.025	1.00	0.00	H
ATOM	738	CB	ASP	46	39.649	26.287	18.105	1.00	0.00	C
ATOM	739	HB2	ASP	46	40.298	26.970	18.653	1.00	0.00	H
ATOM	740	HB3	ASP	46	39.332	25.545	18.839	1.00	0.00	H
ATOM	741	CG	ASP	46	40.436	25.489	17.066	1.00	0.00	C
ATOM	742	OD1	ASP	46	39.870	25.268	15.983	1.00	0.00	O
ATOM	743	OD2	ASP	46	41.612	25.010	17.241	1.00	0.00	O
ATOM	744	C	ASP	46	37.251	27.177	18.584	1.00	0.00	
	C									
ATOM	745	O	ASP	46	36.990	26.480	19.557	1.00	0.00	O
ATOM	746	N	GLY	47	36.512	28.262	18.491	1.00	0.00	N
ATOM	747	H	GLY	47	36.647	28.881	17.705	1.00	0.00	H
ATOM	748	CA	GLY	47	35.325	28.531	19.346	1.00	0.00	C
ATOM	749	HA2	GLY	47	34.619	29.190	18.841	1.00	0.00	H
ATOM	750	HA3	GLY	47	34.753	27.662	19.670	1.00	0.00	H
ATOM	751	C	GLY	47	35.630	29.333	20.617	1.00	0.00	C
ATOM	752	O	GLY	47	34.897	30.297	20.959	1.00	0.00	O
ATOM	753	N	TYR	48	36.669	28.894	21.370	1.00	0.00	N
ATOM	754	H	TYR	48	37.071	28.052	20.982	1.00	0.00	H
ATOM	755	CA	TYR	48	37.104	29.260	22.708	1.00	0.00	C
ATOM	756	HA	TYR	48	36.502	30.111	23.029	1.00	0.00	H
ATOM	757	CB	TYR	48	36.820	28.099	23.703	1.00	0.00	C
ATOM	758	HB2	TYR	48	37.387	27.220	23.402	1.00	0.00	H
ATOM	759	HB3	TYR	48	37.203	28.322	24.699	1.00	0.00	H
ATOM	760	CG	TYR	48	35.365	27.779	23.871	1.00	0.00	C
ATOM	761	CD1	TYR	48	34.565	28.644	24.674	1.00	0.00	C
ATOM	762	HD1	TYR	48	34.951	29.636	24.860	1.00	0.00	H
ATOM	763	CE1	TYR	48	33.247	28.315	25.058	1.00	0.00	C
ATOM	764	HE1	TYR	48	32.714	28.964	25.738	1.00	0.00	H
ATOM	765	CZ	TYR	48	32.763	27.078	24.691	1.00	0.00	C
ATOM	766	OH	TYR	48	31.537	26.694	25.223	1.00	0.00	O
ATOM	767	HH	TYR	48	31.244	25.823	24.945	1.00	0.00	H
ATOM	768	CE2	TYR	48	33.504	26.283	23.813	1.00	0.00	C
ATOM	769	HE2	TYR	48	33.092	25.373	23.404	1.00	0.00	H
ATOM	770	CD2	TYR	48	34.835	26.634	23.361	1.00	0.00	C
ATOM	771	HD2	TYR	48	35.444	26.032	22.703	1.00	0.00	H
ATOM	772	C	TYR	48	38.604	29.711	22.839	1.00	0.00	C
ATOM	773	O	TYR	48	39.393	29.131	22.160	1.00	0.00	O
ATOM	774	N	PHE	49	38.829	30.772	23.590	1.00	0.00	N
ATOM	775	H	PHE	49	38.009	31.175	24.021	1.00	0.00	H
ATOM	776	CA	PHE	49	40.120	31.445	23.701	1.00	0.00	C
ATOM	777	HA	PHE	49	40.792	30.870	23.063	1.00	0.00	H
ATOM	778	CB	PHE	49	40.192	32.903	23.141	1.00	0.00	C
ATOM	779	HB2	PHE	49	41.237	33.171	22.983	1.00	0.00	H
ATOM	780	HB3	PHE	49	39.823	32.918	22.115	1.00	0.00	H
ATOM	781	CG	PHE	49	39.498	33.981	23.959	1.00	0.00	C

ATOM	782	CD1	PHE	49	38.424	34.684	23.410	1.00	0.00	C
ATOM	783	HD1	PHE	49	37.906	34.453	22.492	1.00	0.00	H
ATOM	784	CE1	PHE	49	37.834	35.714	24.163	1.00	0.00	C
ATOM	785	HE1	PHE	49	36.973	36.275	23.833	1.00	0.00	H
ATOM	786	CZ	PHE	49	38.434	36.252	25.304	1.00	0.00	C
ATOM	787	HZ	PHE	49	37.990	37.128	25.753	1.00	0.00	H
ATOM	788	CE2	PHE	49	39.499	35.545	25.863	1.00	0.00	C
ATOM	789	HE2	PHE	49	39.895	35.915	26.797	1.00	0.00	H
ATOM	790	CD2	PHE	49	40.058	34.409	25.184	1.00	0.00	C
ATOM	791	HD2	PHE	49	40.976	33.938	25.506	1.00	0.00	H
ATOM	792	C	PHE	49	40.801	31.322	25.077	1.00	0.00	C
ATOM	793	O	PHE	49	42.080	31.427	25.193	1.00	0.00	O
ATOM	794	N	CYX	50	40.006	30.870	26.083	1.00	0.00	N
ATOM	795	H	CYX	50	39.031	30.665	25.916	1.00	0.00	H
ATOM	796	CA	CYX	50	40.458	30.926	27.463	1.00	0.00	C
ATOM	797	HA	CYX	50	41.502	30.642	27.589	1.00	0.00	H
ATOM	798	CB	CYX	50	40.274	32.326	28.160	1.00	0.00	C
ATOM	799	HB2	CYX	50	39.670	32.905	27.461	1.00	0.00	H
ATOM	800	HB3	CYX	50	39.861	32.152	29.153	1.00	0.00	H
ATOM	801	SG	CYX	50	41.735	33.290	28.347	1.00	0.00	S
ATOM	802	C	CYX	50	39.685	29.791	28.262	1.00	0.00	C
ATOM	803	O	CYX	50	38.506	29.895	28.498	1.00	0.00	O
ATOM	804	N	THR	51	40.415	28.752	28.642	1.00	0.00	N
ATOM	805	H	THR	51	41.368	28.616	28.338	1.00	0.00	H
ATOM	806	CA	THR	51	40.034	27.792	29.722	1.00	0.00	C
ATOM	807	HA	THR	51	39.002	27.447	29.662	1.00	0.00	H
ATOM	808	CB	THR	51	40.854	26.498	29.650	1.00	0.00	C
ATOM	809	HB	THR	51	41.899	26.807	29.646	1.00	0.00	H
ATOM	810	CG2	THR	51	40.673	25.486	30.697	1.00	0.00	C
ATOM	811	HG21	THR	51	40.893	25.942	31.662	1.00	0.00	H
ATOM	812	HG22	THR	51	39.605	25.264	30.734	1.00	0.00	H
ATOM	813	HG23	THR	51	41.156	24.538	30.460	1.00	0.00	H
ATOM	814	OG1	THR	51	40.505	25.767	28.461	1.00	0.00	O
ATOM	815	HG1	THR	51	41.118	25.055	28.268	1.00	0.00	H
ATOM	816	C	THR	51	40.316	28.476	31.062	1.00	0.00	C
ATOM	817	O	THR	51	41.425	29.051	31.282	1.00	0.00	O
ATOM	818	N	ILE	52	39.407	28.392	32.049	1.00	0.00	N
ATOM	819	H	ILE	52	38.564	27.908	31.772	1.00	0.00	H
ATOM	820	CA	ILE	52	39.574	29.115	33.299	1.00	0.00	C
ATOM	821	HA	ILE	52	40.510	29.666	33.393	1.00	0.00	H
ATOM	822	CB	ILE	52	38.586	30.342	33.290	1.00	0.00	C
ATOM	823	HB	ILE	52	37.562	30.013	33.114	1.00	0.00	H
ATOM	824	CG2	ILE	52	38.527	31.078	34.638	1.00	0.00	C
ATOM	825	HG21	ILE	52	38.025	32.036	34.501	1.00	0.00	H
ATOM	826	HG22	ILE	52	38.050	30.380	35.326	1.00	0.00	H
ATOM	827	HG23	ILE	52	39.548	31.317	34.936	1.00	0.00	H
ATOM	828	CG1	ILE	52	39.012	31.383	32.198	1.00	0.00	C
ATOM	829	HG12	ILE	52	39.576	32.215	32.619	1.00	0.00	H
ATOM	830	HG13	ILE	52	39.816	30.933	31.615	1.00	0.00	H
ATOM	831	CD1	ILE	52	37.794	31.869	31.368	1.00	0.00	C
ATOM	832	HD11	ILE	52	38.127	32.800	30.908	1.00	0.00	H
ATOM	833	HD12	ILE	52	37.460	31.140	30.629	1.00	0.00	H
ATOM	834	HD13	ILE	52	36.881	32.129	31.904	1.00	0.00	H
ATOM	835	C	ILE	52	39.384	28.124	34.469	1.00	0.00	C
ATOM	836	O	ILE	52	38.251	27.612	34.660	1.00	0.00	O
ATOM	837	N	GLU	53	40.428	28.013	35.311	1.00	0.00	N
ATOM	838	H	GLU	53	41.249	28.585	35.170	1.00	0.00	H
ATOM	839	CA	GLU	53	40.302	27.405	36.611	1.00	0.00	C
ATOM	840	HA	GLU	53	39.573	26.595	36.637	1.00	0.00	H
ATOM	841	CB	GLU	53	41.548	26.740	37.053	1.00	0.00	C
ATOM	842	HB2	GLU	53	41.924	26.030	36.315	1.00	0.00	H
ATOM	843	HB3	GLU	53	42.357	27.471	37.071	1.00	0.00	H
ATOM	844	CG	GLU	53	41.570	26.105	38.476	1.00	0.00	C
ATOM	845	HG2	GLU	53	41.431	26.917	39.190	1.00	0.00	H

ATOM	846	HG3	GLU	53	40.741	25.400	38.414	1.00	0.00	H
ATOM	847	CD	GLU	53	42.848	25.293	38.822	1.00	0.00	C
ATOM	848	OE1	GLU	53	42.799	24.114	39.265	1.00	0.00	O
ATOM	849	OE2	GLU	53	43.908	25.900	38.555	1.00	0.00	O
ATOM	850	C	GLU	53	39.621	28.389	37.673	1.00	0.00	C
ATOM	851	O	GLU	53	39.969	29.545	37.693	1.00	0.00	O
ATOM	852	N	VAL	54	38.848	27.795	38.562	1.00	0.00	N
ATOM	853	H	VAL	54	38.463	26.865	38.463	1.00	0.00	H
ATOM	854	CA	VAL	54	38.432	28.470	39.806	1.00	0.00	C
ATOM	855	HA	VAL	54	38.278	29.533	39.619	1.00	0.00	H
ATOM	856	CB	VAL	54	37.186	27.888	40.393	1.00	0.00	C
ATOM	857	HB	VAL	54	37.230	26.811	40.555	1.00	0.00	H
ATOM	858	CG1	VAL	54	36.878	28.493	41.776	1.00	0.00	C
ATOM	859	HG11	VAL	54	35.977	28.021	42.168	1.00	0.00	H
ATOM	860	HG12	VAL	54	37.632	28.337	42.548	1.00	0.00	H
ATOM	861	HG13	VAL	54	36.597	29.542	41.690	1.00	0.00	H
ATOM	862	CG2	VAL	54	35.987	28.236	39.481	1.00	0.00	C
ATOM	863	HG21	VAL	54	35.127	27.621	39.747	1.00	0.00	H
ATOM	864	HG22	VAL	54	35.719	29.280	39.644	1.00	0.00	H
ATOM	865	HG23	VAL	54	36.210	28.158	38.418	1.00	0.00	H
ATOM	866	C	VAL	54	39.554	28.235	40.822	1.00	0.00	C
ATOM	867	O	VAL	54	39.788	27.065	41.221	1.00	0.00	O
ATOM	868	N	THR	55	40.302	29.273	41.174	1.00	0.00	N
ATOM	869	H	THR	55	39.987	30.154	40.793	1.00	0.00	H
ATOM	870									
CA	THR	55	41.397	29.204	42.150	1.00	0.00	C		
ATOM	871	HA	THR	55	42.032	28.344	41.940	1.00	0.00	H
ATOM	872	CB	THR	55	42.376	30.403	41.988	1.00	0.00	C
ATOM	873	HB	THR	55	43.206	30.193	42.663	1.00	0.00	H
ATOM	874	CG2	THR	55	43.008	30.525	40.628	1.00	0.00	C
ATOM	875	HG21	THR	55	43.345	29.611	40.140	1.00	0.00	H
ATOM	876	HG22	THR	55	42.237	31.005	40.026	1.00	0.00	H
ATOM	877	HG23	THR	55	43.909	31.138	40.663	1.00	0.00	H
ATOM	878	OG1	THR	55	41.850	31.672	42.240	1.00	0.00	O
ATOM	879	HG1	THR	55	41.744	31.899	43.167	1.00	0.00	H
ATOM	880	C	THR	55	40.999	29.032	43.598	1.00	0.00	C
ATOM	881	O	THR	55	41.828	28.503	44.329	1.00	0.00	O
ATOM	882	N	ALA	56	39.809	29.573	44.000	1.00	0.00	N
ATOM	883	H	ALA	56	39.402	30.230	43.350	1.00	0.00	H
ATOM	884	CA	ALA	56	39.342	29.488	45.411	1.00	0.00	C
ATOM	885	HA	ALA	56	39.472	28.492	45.834	1.00	0.00	H
ATOM	886	CB	ALA	56	40.006	30.497	46.266	1.00	0.00	C
ATOM	887	HB1	ALA	56	39.634	30.363	47.282	1.00	0.00	H
ATOM	888	HB2	ALA	56	41.091	30.493	46.163	1.00	0.00	H
ATOM	889	HB3	ALA	56	39.751	31.505	45.937	1.00	0.00	H
ATOM	890	C	ALA	56	37.863	29.743	45.406	1.00	0.00	C
ATOM	891	O	ALA	56	37.311	30.410	44.549	1.00	0.00	O
ATOM	892	N	THR	57	37.168	29.188	46.386	1.00	0.00	N
ATOM	893	H	THR	57	37.648	28.535	46.988	1.00	0.00	H
ATOM	894	CA	THR	57	35.771	29.471	46.743	1.00	0.00	C
ATOM	895	HA	THR	57	35.680	30.469	46.314	1.00	0.00	H
ATOM	896	CB	THR	57	34.762	28.483	46.126	1.00	0.00	C
ATOM	897	HB	THR	57	33.754	28.609	46.523	1.00	0.00	H
ATOM	898	CG2	THR	57	34.674	28.581	44.628	1.00	0.00	C
ATOM	899	HG21	THR	57	35.660	28.265	44.287	1.00	0.00	H
ATOM	900	HG22	THR	57	33.910	27.864	44.327	1.00	0.00	H
ATOM	901	HG23	THR	57	34.343	29.589	44.385	1.00	0.00	H
ATOM	902	OG1	THR	57	35.140	27.128	46.356	1.00	0.00	O
ATOM	903	HG1	THR	57	36.084	26.957	46.398	1.00	0.00	H
ATOM	904	C	THR	57	35.653	29.600	48.284	1.00	0.00	C
ATOM	905	O	THR	57	36.437	28.992	49.043	1.00	0.00	O
ATOM	906	N	SER	58	34.706	30.360	48.758	1.00	0.00	N
ATOM	907	H	SER	58	34.081	30.644	48.017	1.00	0.00	H
ATOM	908	CA	SER	58	34.376	30.522	50.165	1.00	0.00	C

ATOM	909	HA	SER	58	34.459	29.515	50.573	1.00	0.00	H
ATOM	910	CB	SER	58	35.508	31.413	50.834	1.00	0.00	C
ATOM	911	HB2	SER	58	35.400	31.362	51.918	1.00	0.00	H
ATOM	912	HB3	SER	58	36.486	30.980	50.623	1.00	0.00	H
ATOM	913	OG	SER	58	35.432	32.744	50.340	1.00	0.00	O
ATOM	914	HG	SER	58	35.918	32.802	49.514	1.00	0.00	H
ATOM	915	C	SER	58	32.927	31.086	50.339	1.00	0.00	C
ATOM	916	O	SER	58	32.247	31.403	49.384	1.00	0.00	O
ATOM	917	N	THR	59	32.500	31.387	51.560	1.00	0.00	N
ATOM	918	H	THR	59	33.175	31.108	52.256	1.00	0.00	H
ATOM	919	CA	THR	59	31.290	32.003	51.964	1.00	0.00	C
ATOM	920	HA	THR	59	31.025	32.702	51.169	1.00	0.00	H
ATOM	921	CB	THR	59	30.159	30.911	52.069	1.00	0.00	C
ATOM	922	HB	THR	59	29.930	30.651	51.036	1.00	0.00	H
ATOM	923	CG2	THR	59	30.726	29.668	52.825	1.00	0.00	C
ATOM	924	HG21	THR	59	31.267	30.005	53.708	1.00	0.00	H
ATOM	925	HG22	THR	59	29.906	29.011	53.116	1.00	0.00	H
ATOM	926	HG23	THR	59	31.350	29.008	52.223	1.00	0.00	H
ATOM	927	OG1	THR	59	28.961	31.332	52.647	1.00	0.00	O
ATOM	928	HG1	THR	59	28.348	30.686	52.289	1.00	0.00	H
ATOM	929	C	THR	59	31.384	32.851	53.264	1.00	0.00	C
ATOM	930	O	THR	59	32.077	32.570	54.261	1.00	0.00	O
ATOM	931	N	VAL	60	30.559	33.931	53.296	1.00	0.00	N
ATOM	932	H	VAL	60	30.051	34.083	52.436	1.00	0.00	H
ATOM	933	CA	VAL	60	30.515	35.018	54.258	1.00	0.00	C
ATOM	934	HA	VAL	60	30.905	34.617	55.192	1.00	0.00	H
ATOM	935	CB	VAL	60	31.582	36.115	53.921	1.00	0.00	C
ATOM	936	HB	VAL	60	31.563	36.968	54.599	1.00	0.00	H
ATOM	937	CG1	VAL	60	33.012	35.665	54.008	1.00	0.00	C
ATOM	938	HG11	VAL	60	33.566	36.595	53.886	1.00	0.00	H
ATOM	939	HG12	VAL	60	33.181	35.018	54.870	1.00	0.00	H
ATOM	940	HG13	VAL	60	33.297	35.014	53.182	1.00	0.00	H
ATOM	941	CG2	VAL	60	31.298	36.671	52.505	1.00	0.00	C
ATOM	942	HG21	VAL	60	31.919	37.561	52.393	1.00	0.00	H
ATOM	943	HG22	VAL	60	31.669	35.997	51.733	1.00	0.00	H
ATOM	944	HG23	VAL	60	30.240	36.826	52.297	1.00	0.00	H
ATOM	945	C	VAL	60	29.167	35.661	54.310	1.00	0.00	C
ATOM	946	O	VAL	60	28.273	35.467	53.462	1.00	0.00	O
ATOM	947	N	THR	61	29.016	36.476	55.299	1.00	0.00	N
ATOM	948	H	THR	61	29.762	36.641	55.961	1.00	0.00	H
ATOM	949	CA	THR	61	27.995	37.510	55.417	1.00	0.00	C
ATOM	950	HA	THR	61	27.333	37.356	54.565	1.00	0.00	H
ATOM	951	CB	THR	61	27.274	37.529	56.745	1.00	0.00	C
ATOM	952	HB	THR	61	26.495	38.280	56.876	1.00	0.00	H
ATOM	953	CG2	THR	61	26.534	36.301	57.107	1.00	0.00	C
ATOM	954	HG21	THR	61	25.763	36.420	57.869	1.00	0.00	H
ATOM	955	HG22	THR	61	26.035	35.973	56.195	1.00	0.00	H
ATOM	956	HG23	THR	61	27.202	35.515	57.460	1.00	0.00	H
ATOM	957	OG1	THR	61	28.290	37.588	57.710	1.00	0.00	O
ATOM	958	HG1	THR	61	27.954	37.874	58.563	1.00	0.00	H
ATOM	959	C	THR	61	28.510	38.968	55.222	1.00	0.00	C
ATOM	960	O	THR	61	29.688	39.163	55.060	1.00	0.00	O
ATOM	961	N	LEU	62	27.613	39.964	55.245	1.00	0.00	N
ATOM	962	H	LEU	62	26.652	39.720	55.434	1.00	0.00	H
ATOM	963	CA	LEU	62	28.044	41.372	55.070	1.00	0.00	C
ATOM	964	HA	LEU	62	28.663	41.431	54.176	1.00	0.00	H
ATOM	965	CB	LEU	62	26.900	42.404	54.855	1.00	0.00	C
ATOM	966	HB2	LEU	62	26.235	42.391	55.718	1.00	0.00	H
ATOM	967	HB3	LEU	62	27.259	43.431	54.923	1.00	0.00	H
ATOM	968	CG	LEU	62	26.007	42.286	53.653	1.00	0.00	C
ATOM	969	HG	LEU	62	25.786	41.222	53.584	1.00	0.00	H
ATOM	970	CD1	LEU	62	24.705	43.127	53.871	1.00	0.00	C
ATOM	971	HD11	LEU	62	24.299	42.796	54.826	1.00	0.00	H
ATOM	972	HD12	LEU	62	24.938	44.188	53.949	1.00	0.00	H

```
ATOM    973 HD13 LEU    62      24.060  42.799  53.055  1.00  0.00           H
ATOM    974  CD2 LEU    62      26.809  42.730  52.436  1.00  0.00           C
ATOM    975 HD21 LEU    62      27.086  43.779  52.541  1.00  0.00           H
ATOM    976 HD22 LEU    62      27.697  42.106  52.337  1.00  0.00           H
ATOM    977 HD23 LEU    62      26.125  42.780  51.590  1.00  0.00           H
ATOM    978  C   LEU    62      28.978  41.890  56.168  1.00  0.00           C
ATOM    979  O   LEU    62      29.937  42.577  55.860  1.00  0.00           O
ATOM    980  N   ASP    63      28.763  41.421  57.428  1.00  0.00           N
ATOM    981  H   ASP    63      27.990  40.792  57.585  1.00  0.00           H
ATOM    982  CA  ASP    63      29.558  41.772  58.657  1.00  0.00           C
ATOM    983  HA  ASP    63      29.954  42.736  58.335  1.00  0.00           H
ATOM    984  CB  ASP    63      28.743  41.947  59.915  1.00  0.00           C
ATOM    985  HB2 ASP    63      29.383  42.399  60.672  1.00  0.00           H
ATOM    986  HB3 ASP    63      27.808  42.486  59.759  1.00  0.00           H
ATOM    987  CG  ASP    63      28.265  40.573  60.337  1.00  0.00           C
ATOM    988  OD1 ASP    63      28.093  39.661  59.569  1.00  0.00           O
ATOM    989  OD2 ASP    63      27.906  40.366  61.496  1.00  0.00           O
ATOM    990  C   ASP    63      30.787  40.811  58.842  1.00  0.00           C
ATOM    991  O   ASP    63      31.522  41.070  59.803  1.00  0.00           O
ATOM    992  N   THR    64      30.978  39.832  57.955  1.00  0.00           N
ATOM    993  H   THR    64      30.304  39.768  57.207  1.00  0.00           H
ATOM    994  CA  THR    64      32.158  39.044  57.839  1.00  0.00           C
ATOM    995  HA  THR    64      32.967
  39.485  58.421  1.00  0.00           H
ATOM    996  CB  THR    64      31.904  37.572  58.260  1.00  0.00           C
ATOM    997  HB  THR    64      32.844  37.026  58.180  1.00  0.00           H
ATOM    998  CG2 THR    64      31.473  37.404  59.740  1.00  0.00           C
ATOM    999 HG21 THR    64      30.558  37.971  59.913  1.00  0.00           H
ATOM   1000 HG22 THR    64      31.376  36.393  60.138  1.00  0.00           H
ATOM   1001 HG23 THR    64      32.288  37.831  60.324  1.00  0.00           H
ATOM   1002  OG1 THR    64      30.956  36.906  57.439  1.00  0.00           O
ATOM   1003  HG1 THR    64      30.134  37.157  57.868  1.00  0.00           H
ATOM   1004  C   THR    64      32.814  39.241  56.454  1.00  0.00           C
ATOM   1005  O   THR    64      33.774  38.596  56.163  1.00  0.00           O
ATOM   1006  N   LEU    65      32.409  40.235  55.693  1.00  0.00           N
ATOM   1007  H   LEU    65      31.589  40.694  56.061  1.00  0.00           H
ATOM   1008  CA  LEU    65      32.798  40.386  54.287  1.00  0.00           C
ATOM   1009  HA  LEU    65      32.719  39.422  53.784  1.00  0.00           H
ATOM   1010  CB  LEU    65      31.884  41.481  53.667  1.00  0.00           C
ATOM   1011  HB2 LEU    65      30.830  41.373  53.924  1.00  0.00           H
ATOM   1012  HB3 LEU    65      32.299  42.412  54.052  1.00  0.00           H
ATOM   1013  CG  LEU    65      31.903  41.696  52.072  1.00  0.00           C
ATOM   1014  HG  LEU    65      32.961  41.862  51.872  1.00  0.00           H
ATOM   1015  CD1 LEU    65      31.521  40.355  51.447  1.00  0.00           C
ATOM   1016 HD11 LEU    65      32.220  39.590  51.787  1.00  0.00           H
ATOM   1017 HD12 LEU    65      30.510  40.073  51.740  1.00  0.00           H
ATOM   1018 HD13 LEU    65      31.650  40.505  50.375  1.00  0.00           H
ATOM   1019  CD2 LEU    65      31.099  42.847  51.530  1.00  0.00           C
ATOM   1020 HD21 LEU    65      30.089  42.528  51.784  1.00  0.00           H
ATOM   1021 HD22 LEU    65      31.342  43.759  52.075  1.00  0.00           H
ATOM   1022 HD23 LEU    65      31.165  43.100  50.472  1.00  0.00           H
ATOM   1023  C   LEU    65      34.306  40.778  54.062  1.00  0.00           C
ATOM   1024  O   LEU    65      34.792  41.538  54.840  1.00  0.00           O
ATOM   1025  N   THR    66      34.967  40.313  53.026  1.00  0.00           N
ATOM   1026  H   THR    66      34.625  39.513  52.512  1.00  0.00           H
ATOM   1027  CA  THR    66      36.379  40.618  52.786  1.00  0.00           C
ATOM   1028  HA  THR    66      36.809  41.087  53.671  1.00  0.00           H
ATOM   1029  CB  THR    66      37.218  39.477  52.277  1.00  0.00           C
ATOM   1030  HB  THR    66      36.800  39.203  51.309  1.00  0.00           H
ATOM   1031  CG2 THR    66      38.767  39.742  52.157  1.00  0.00           C
ATOM   1032 HG21 THR    66      39.026  40.719  51.749  1.00  0.00           H
ATOM   1033 HG22 THR    66      39.079  39.779  53.200  1.00  0.00           H
ATOM   1034 HG23 THR    66      39.305  38.999  51.569  1.00  0.00           H
ATOM   1035  OG1 THR    66      37.077  38.370  53.131  1.00  0.00           O
```

ATOM	1036	HG1	THR	66	37.294	38.593	54.039	1.00	0.00	H
ATOM	1037	C	THR	66	36.398	41.710	51.767	1.00	0.00	C
ATOM	1038	O	THR	66	35.659	41.636	50.834	1.00	0.00	O
ATOM	1039	N	GLU	67	37.253	42.726	51.816	1.00	0.00	N
ATOM	1040	H	GLU	67	37.829	42.771	52.644	1.00	0.00	H
ATOM	1041	CA	GLU	67	37.649	43.645	50.761	1.00	0.00	C
ATOM	1042	HA	GLU	67	36.746	44.180	50.470	1.00	0.00	H
ATOM	1043	CB	GLU	67	38.574	44.739	51.240	1.00	0.00	C
ATOM	1044	HB2	GLU	67	38.509	45.578	50.548	1.00	0.00	H
ATOM	1045	HB3	GLU	67	38.368	45.241	52.185	1.00	0.00	H
ATOM	1046	CG	GLU	67	40.037	44.215	51.318	1.00	0.00	C
ATOM	1047	HG2	GLU	67	40.238	43.623	50.426	1.00	0.00	H
ATOM	1048	HG3	GLU	67	40.707	45.071	51.244	1.00	0.00	H
ATOM	1049	CD	GLU	67	40.390	43.552	52.666	1.00	0.00	C
ATOM	1050	OE1	GLU	67	39.535	43.010	53.324	1.00	0.00	O
ATOM	1051	OE2	GLU	67	41.570	43.533	53.059	1.00	0.00	O
ATOM	1052	C	GLU	67	38.106	43.081	49.399	1.00	0.00	C
ATOM	1053	O	GLU	67	38.236	43.818	48.436	1.00	0.00	O
ATOM	1054	N	LYS	68	38.307	41.748	49.264	1.00	0.00	N
ATOM	1055	H	LYS	68	37.868	41.173	49.970	1.00	0.00	H
ATOM	1056	CA	LYS	68	39.045	41.199	48.128	1.00	0.00	C
ATOM	1057	HA	LYS	68	40.009	41.707	48.133	1.00	0.00	H
ATOM	1058	CB	LYS	68	39.197	39.642	48.322	1.00	0.00	C
ATOM	1059	HB2	LYS	68	39.457	39.388	49.350	1.00	0.00	H
ATOM	1060	HB3	LYS	68	38.257	39.147	48.076	1.00	0.00	H
ATOM	1061	CG	LYS	68	40.232	38.930	47.497	1.00	0.00	C
ATOM	1062	HG2	LYS	68	40.216	37.849	47.632	1.00	0.00	H
ATOM	1063	HG3	LYS	68	39.896	39.054	46.467	1.00	0.00	H
ATOM	1064	CD	LYS	68	41.699	39.356	47.643	1.00	0.00	C
ATOM	1065	HD2	LYS	68	42.267	38.870	46.849	1.00	0.00	H
ATOM	1066	HD3	LYS	68	41.723	40.445	47.584	1.00	0.00	H
ATOM	1067	CE	LYS	68	42.254	38.965	48.999	1.00	0.00	C
ATOM	1068	HE2	LYS	68	41.605	39.448	49.730	1.00	0.00	H
ATOM	1069	HE3	LYS	68	42.190	37.905	49.244	1.00	0.00	H
ATOM	1070	NZ	LYS	68	43.654	39.488	49.122	1.00	0.00	N
ATOM	1071	HZ1	LYS	68	44.042	39.313	50.038	1.00	0.00	H
ATOM	1072	HZ2	LYS	68	44.232	39.023	48.436	1.00	0.00	H
ATOM	1073	HZ3	LYS	68	43.859	40.475	49.041	1.00	0.00	H
ATOM	1074	C	LYS	68	38.497	41.663	46.757	1.00	0.00	C
ATOM	1075	O	LYS	68	39.270	42.155	45.934	1.00	0.00	O
ATOM	1076	N	HIE	69	37.152	41.752	46.647	1.00	0.00	N
ATOM	1077	H	HIE	69	36.535	41.356	47.341	1.00	0.00	H
ATOM	1078	CA	HIE	69	36.436	42.238	45.467	1.00	0.00	C
ATOM	1079	HA	HIE	69	36.888	41.750	44.604	1.00	0.00	H
ATOM	1080	CB	HIE	69	34.999	41.776	45.611	1.00	0.00	C
ATOM	1081	HB2	HIE	69	34.943	40.699	45.772	1.00	0.00	H
ATOM	1082	HB3	HIE	69	34.584	42.298	46.473	1.00	0.00	H
ATOM	1083	CG	HIE	69	34.149	42.025	44.398	1.00	0.00	C
ATOM	1084	ND1	HIE	69	34.276	41.378	43.146	1.00	0.00	N
ATOM	1085	CE1	HIE	69	33.302	41.889	42.394	1.00	0.00	C
ATOM	1086	HE1	HIE	69	33.176	41.578	41.367	1.00	0.00	H
ATOM	1087	NE2	HIE	69	32.589	42.789	43.072	1.00	0.00	N
ATOM	1088	HE2	HIE	69	31.861	43.334	42.633	1.00	0.00	H
ATOM	1089	CD2	HIE	69	33.172	42.927	44.321	1.00	0.00	C
ATOM	1090	HD2	HIE	69	32.720	43.458	45.147	1.00	0.00	H
ATOM	1091	C	HIE	69	36.470	43.746	45.326	1.00	0.00	C
ATOM	1092	O	HIE	69	36.498	44.299	44.234	1.00	0.00	O
ATOM	1093	N	ALA	70	36.418	44.422	46.467	1.00	0.00	N
ATOM	1094	H	ALA	70	36.532	43.930	47.341	1.00	0.00	H
ATOM	1095	CA	ALA	70	36.473	45.909	46.527	1.00	0.00	C
ATOM	1096	HA	ALA	70	35.560	46.263	46.048	1.00	0.00	H
ATOM	1097	CB	ALA	70	36.318	46.378	47.982	1.00	0.00	C
ATOM	1098	HB1	ALA	70	37.259	46.398	48.532	1.00	0.00	H
ATOM	1099	HB2	ALA	70	35.869	47.370	47.927	1.00	0.00	H

ATOM	1100	HB3	ALA	70	35.525	45.849	48.510	1.00	0.00	H
ATOM	1101	C	ALA	70	37.711	46.497	45.855	1.00	0.00	C
ATOM	1102	O	ALA	70	37.705	47.425	45.075	1.00	0.00	O
ATOM	1103	N	GLU	71	38.834	45.849	46.124	1.00	0.00	N
ATOM	1104	H	GLU	71	38.788	45.059	46.753	1.00	0.00	H
ATOM	1105	CA	GLU	71	40.065	46.189	45.481	1.00	0.00	C
ATOM	1106	HA	GLU	71	40.228	47.249	45.675	1.00	0.00	H
ATOM	1107	CB	GLU	71	41.186	45.398	46.166	1.00	0.00	C
ATOM	1108	HB2	GLU	71	40.944	44.335	46.197	1.00	0.00	H
ATOM	1109	HB3	GLU	71	42.119	45.585	45.634	1.00	0.00	H
ATOM	1110	CG	GLU	71	41.443	45.914	47.628	1.00	0.00	C
ATOM	1111	HG2	GLU	71	40.501	45.993	48.172	1.00	0.00	H
ATOM	1112	HG3	GLU	71	42.112	45.268	48.197	1.00	0.00	H
ATOM	1113	CD	GLU	71	41.974	47.351	47.676	1.00	0.00	C
ATOM	1114	OE1	GLU	71	43.181	47.546	47.968	1.00	0.00	O
ATOM	1115	OE2	GLU	71	41.233	48.330	47.454	1.00	0.00	O
ATOM	1116	C	GLU	71	40.036	45.977	43.970	1.00	0.00	C
ATOM	1117	O	GLU	71	40.694	46.572	43.185	1.00	0.00	O
ATOM	1118	N	GLN	72	39.339	44.894	43.494	1.00	0.00	N
ATOM	1119	H	GLN	72	38.628	44.460	44.065	1.00	0.00	H
ATOM	1120	CA	GLN	72	39.287	44.616	42.059	1.00	0.00	C
ATOM	1121	HA	GLN	72	40.303	44.774	41.697	1.00	0.00	H
ATOM	1122	CB	GLN	72	38.875	43.209	41.944	1.00	0.00	C
ATOM	1123	HB2	GLN	72	37.858	43.270	42.333	1.00	0.00	H
ATOM	1124	HB3	GLN	72	39.527	42.543	42.509	1.00	0.00	H
ATOM	1125	CG	GLN	72	38.913	42.701	40.477	1.00	0.00	C
ATOM	1126	HG2	GLN	72	38.853	43.564	39.815	1.00	0.00	H
ATOM	1127	HG3	GLN	72	39.836	42.210	40.169	1.00	0.00	H
ATOM	1128	CD	GLN	72	37.823	41.670	40.273	1.00	0.00	C
ATOM	1129	OE1	GLN	72	38.140	40.501	40.002	1.00	0.00	O
ATOM	1130	NE2	GLN	72	36.575	41.870	40.483	1.00	0.00	N
ATOM	1131	HE21	GLN	72	36.156	42.788	40.447	1.00	0.00	H
ATOM	1132	HE22	GLN	72	36.018	41.039	40.624	1.00	0.00	H
ATOM	1133	C	GLN	72	38.323	45.525	41.349	1.00	0.00	C
ATOM	1134	O	GLN	72	38.665	46.054	40.308	1.00	0.00	O
ATOM	1135	N	GLU	73	37.179	45.877	41.948	1.00	0.00	N
ATOM	1136	H	GLU	73	37.079	45.460	42.862	1.00	0.00	H
ATOM	1137	CA	GLU	73	36.245	46.871	41.378	1.00	0.00	C
ATOM	1138	HA	GLU	73	36.211	46.749	40.296	1.00	0.00	H
ATOM	1139	CB	GLU	73	34.815	46.515	41.856	1.00	0.00	C
ATOM	1140	HB2	GLU	73	34.744	46.747	42.919	1.00	0.00	H
ATOM	1141	HB3	GLU	73	34.014	47.041	41.338	1.00	0.00	H
ATOM	1142	CG	GLU	73	34.363	45.101	41.674	1.00	0.00	C
ATOM	1143	HG2	GLU	73	34.964	44.445	42.303	1.00	0.00	H
ATOM	1144	HG3	GLU	73	33.309	45.042	41.944	1.00	0.00	H
ATOM	1145	CD	GLU	73	34.373	44.673	40.172	1.00	0.00	C
ATOM	1146	OE1	GLU	73	35.456	44.209	39.663	1.00	0.00	O
ATOM	1147	OE2	GLU	73	33.369	44.953	39.455	1.00	0.00	O
ATOM	1148	C	GLU	73	36.711	48.409	41.600	1.00	0.00	C
ATOM	1149	O	GLU	73	35.988	49.217	41.140	1.00	0.00	O
ATOM	1150	N	ASN	74	37.873	48.672	42.269	1.00	0.00	N
ATOM	1151	H	ASN	74	38.247	47.856	42.732	1.00	0.00	H
ATOM	1152	CA	ASN	74	38.440	49.993	42.684	1.00	0.00	C
ATOM	1153	HA	ASN	74	39.316	49.756	43.287	1.00	0.00	H
ATOM	1154	CB	ASN	74	38.983	50.690	41.445	1.00	0.00	C
ATOM	1155	HB2	ASN	74	38.186	50.941	40.746	1.00	0.00	H
ATOM	1156	HB3	ASN	74	39.304	51.698	41.709	1.00	0.00	H
ATOM	1157	CG	ASN	74	40.154	49.979	40.691	1.00	0.00	C
ATOM	1158	OD1	ASN	74	39.932	49.089	39.862	1.00	0.00	O
ATOM	1159	ND2	ASN	74	41.415	50.261	41.027	1.00	0.00	N
ATOM	1160	HD21	ASN	74	42.069	49.756	40.445	1.00	0.00	H
ATOM	1161	HD22	ASN	74	41.668	51.029	41.629	1.00	0.00	H
ATOM	1162	C	ASN	74	37.498	50.775	43.557	1.00	0.00	C

ATOM	1163	O	ASN	74	37.448	51.987	43.441	1.00	0.00	O
ATOM	1164	N	MET	75	36.759	50.122	44.432	1.00	0.00	N
ATOM	1165	H	MET	75	36.996	49.151	44.581	1.00	0.00	H
ATOM	1166	CA	MET	75	35.939	50.699	45.467	1.00	0.00	C
ATOM	1167	HA	MET	75	35.926	51.789	45.448	1.00	0.00	H
ATOM	1168	CB	MET	75	34.460	50.297	45.414	1.00	0.00	C
ATOM	1169	HB2	MET	75	33.812	50.798	46.134	1.00	0.00	H
ATOM	1170	HB3	MET	75	34.009	50.636	44.482	1.00	0.00	H
ATOM	1171	CG	MET	75	34.323	48.778	45.358	1.00	0.00	C
ATOM	1172	HG2	MET	75	35.008	48.351	44.626	1.00	0.00	H
ATOM	1173	HG3	MET	75	34.544	48.459	46.377	1.00	0.00	H
ATOM	1174	SD	MET	75	32.574	48.485	45.040	1.00	0.00	S
ATOM	1175	CE	MET	75	32.163	49.289	43.440	1.00	0.00	C
ATOM	1176	HE1	MET	75	31.175	48.934	43.148	1.00	0.00	H
ATOM	1177	HE2	MET	75	31.990	50.355	43.583	1.00	0.00	H
ATOM	1178	HE3	MET	75	32.921	49.103	42.679	1.00	0.00	H
ATOM	1179	C	MET	75	36.561	50.358	46.815	1.00	0.00	C
ATOM	1180	O	MET	75	37.556	49.600	46.951	1.00	0.00	O
ATOM	1181	N	THR	76	36.056	51.000	47.878	1.00	0.00	N
ATOM	1182	H	THR	76	35.254	51.566	47.640	1.00	0.00	H
ATOM	1183	CA	THR	76	36.399	50.662	49.295	1.00	0.00	C
ATOM	1184	HA	THR	76	37.114	49.842	49.359	1.00	0.00	H
ATOM	1185	CB	THR	76	37.083	51.883	49.987	1.00	0.00	C
ATOM	1186	HB	THR	76	37.819	52.381	49.356	1.00	0.00	H
ATOM	1187	CG2	THR	76	36.045	52.873	50.418	1.00	0.00	C
ATOM	1188	HG21	THR	76	35.421	52.458	51.209	1.00	0.00	H
ATOM	1189	HG22	THR	76	36.576	53.728	50.839	1.00	0.00	H
ATOM	1190	HG23	THR	76	35.553	53.299	49.544	1.00	0.00	H
ATOM	1191	OG1	THR	76	37.681	51.303	51.226	1.00	0.00	O
ATOM	1192	HG1	THR	76	38.374	50.685	50.984	1.00	0.00	H
ATOM	1193	C	THR	76	35.199	50.043	49.985	1.00	0.00	C
ATOM	1194	O	THR	76	34.059	50.255	49.571	1.00	0.00	O
ATOM	1195	N	LEU	77	35.412	49.094	50.905	1.00	0.00	N
ATOM	1196	H	LEU	77	36.385	48.988	51.156	1.00	0.00	H
ATOM	1197	CA	LEU	77	34.472	48.099	51.293	1.00	0.00	C
ATOM	1198	HA	LEU	77	34.153	47.695	50.332	1.00	0.00	H
ATOM	1199	CB	LEU	77	35.249	47.007	52.156	1.00	0.00	C
ATOM	1200	HB2	LEU	77	36.016	46.671	51.458	1.00	0.00	H
ATOM	1201	HB3	LEU	77	35.745	47.591	52.932	1.00	0.00	H
ATOM	1202	CG	LEU	77	34.523	45.877	52.780	1.00	0.00	C
ATOM	1203	HG	LEU	77	33.772	46.388	53.383	1.00	0.00	H
ATOM	1204	CD1	LEU	77	33.962	44.968	51.790	1.00	0.00	C
ATOM	1205	HD11	LEU	77	34.610	44.926	50.914	1.00	0.00	H
ATOM	1206	HD12	LEU	77	33.760	43.994	52.235	1.00	0.00	H
ATOM	1207	HD13	LEU	77	33.032	45.448	51.486	1.00	0.00	H
ATOM	1208	CD2	LEU	77	35.325	45.133	53.806	1.00	0.00	C
ATOM	1209	HD21	LEU	77	35.811	45.780	54.536	1.00	0.00	H
ATOM	1210	HD22	LEU	77	34.669	44.454	54.351	1.00	0.00	H
ATOM	1211	HD23	LEU	77	36.150	44.558	53.384	1.00	0.00	H
ATOM	1212	C	LEU	77	33.153	48.608	51.922	1.00	0.00	C
ATOM	1213	O	LEU	77	32.124	48.035	51.639	1.00	0.00	O
ATOM	1214	N	THR	78	33.283	49.774	52.613	1.00	0.00	N
ATOM	1215	H	THR	78	34.212	50.153	52.734	1.00	0.00	H
ATOM	1216	CA	THR	78	32.166	50.481	53.207	1.00	0.00	C
ATOM	1217	HA	THR	78	31.698	50.029	54.081	1.00	0.00	H
ATOM	1218	CB	THR	78	32.722	51.788	53.695	1.00	0.00	C
ATOM	1219	HB	THR	78	31.894	52.374	54.094	1.00	0.00	H
ATOM	1220	CG2	THR	78	33.730	51.655	54.820	1.00	0.00	C
ATOM	1221	HG21	THR	78	34.663	51.187	54.506	1.00	0.00	H
ATOM	1222	HG22	THR	78	33.926	52.587	55.351	1.00	0.00	H
ATOM	1223	HG23	THR	78	33.229	50.987	55.521	1.00	0.00	H
ATOM	1224	OG1	THR	78	33.475	52.480	52.740	1.00	0.00	O
ATOM	1225	HG1	THR	78	33.875	53.240	53.170	1.00	0.00	H
ATOM	1226	C	THR	78	31.070	50.711	52.166	1.00	0.00	C

ATOM	1227	O	THR	78	29.826	50.566	52.448	1.00	0.00	O
ATOM	1228	N	GLU	79	31.422	51.138	50.944	1.00	0.00	N
ATOM	1229	H	GLU	79	32.404	51.379	50.928	1.00	0.00	H
ATOM	1230	CA	GLU	79	30.538	51.425	49.766	1.00	0.00	C
ATOM	1231	HA	GLU	79	29.578	51.774	50.147	1.00	0.00	H
ATOM	1232	CB	GLU	79	31.040	52.511	48.856	1.00	0.00	C
ATOM	1233	HB2	GLU	79	31.911	52.159	48.302	1.00	0.00	H
ATOM	1234	HB3	GLU	79	30.259	52.845	48.173	1.00	0.00	H
ATOM	1235	CG	GLU	79	31.595	53.808	49.598	1.00	0.00	C
ATOM	1236	HG2	GLU	79	32.553	53.602	50.076	1.00	0.00	H
ATOM	1237	HG3	GLU	79	31.768	54.601	48.870	1.00	0.00	H
ATOM	1238	CD	GLU	79	30.467	54.342	50.514	1.00	0.00	C
ATOM	1239	OE1	GLU	79	30.817	54.798	51.634	1.00	0.00	O
ATOM	1240	OE2	GLU	79	29.282	54.194	50.227	1.00	0.00	O
ATOM	1241	C	GLU	79	30.161	50.243	48.882	1.00	0.00	C
ATOM	1242	O	GLU	79	29.044	50.145	48.450	1.00	0.00	O
ATOM	1243	N	LEU	80	31.048	49.269	48.807	1.00	0.00	N
ATOM	1244	H	LEU	80	31.963	49.354	49.224	1.00	0.00	H
ATOM	1245	CA	LEU	80	30.697	47.898	48.284	1.00	0.00	C
ATOM	1246									
HA	LEU	80	30.374	47.952	47.244	1.00	0.00	H		
ATOM	1247	CB	LEU	80	31.890	46.883	48.296	1.00	0.00	C
ATOM	1248	HB2	LEU	80	32.748	47.294	47.762	1.00	0.00	H
ATOM	1249	HB3	LEU	80	32.016	46.749	49.369	1.00	0.00	H
ATOM	1250	CG	LEU	80	31.588	45.443	47.696	1.00	0.00	C
ATOM	1251	HG	LEU	80	30.839	45.023	48.367	1.00	0.00	H
ATOM	1252	CD1	LEU	80	31.095	45.413	46.222	1.00	0.00	C
ATOM	1253	HD11	LEU	80	31.883	45.861	45.615	1.00	0.00	H
ATOM	1254	HD12	LEU	80	30.847	44.421	45.847	1.00	0.00	H
ATOM	1255	HD13	LEU	80	30.156	45.963	46.274	1.00	0.00	H
ATOM	1256	CD2	LEU	80	32.878	44.600	47.867	1.00	0.00	C
ATOM	1257	HD21	LEU	80	32.638	43.572	47.598	1.00	0.00	H
ATOM	1258	HD22	LEU	80	33.663	45.063	47.268	1.00	0.00	H
ATOM	1259	HD23	LEU	80	33.235	44.484	48.891	1.00	0.00	H
ATOM	1260	C	LEU	80	29.538	47.240	49.119	1.00	0.00	C
ATOM	1261	O	LEU	80	28.524	46.862	48.530	1.00	0.00	O
ATOM	1262	N	LYS	81	29.582	47.279	50.459	1.00	0.00	N
ATOM	1263	H	LYS	81	30.380	47.669	50.939	1.00	0.00	H
ATOM	1264	CA	LYS	81	28.443	46.846	51.292	1.00	0.00	C
ATOM	1265	HA	LYS	81	28.287	45.812	50.984	1.00	0.00	H
ATOM	1266	CB	LYS	81	28.846	46.927	52.797	1.00	0.00	C
ATOM	1267	HB2	LYS	81	29.082	47.967	53.020	1.00	0.00	H
ATOM	1268	HB3	LYS	81	27.955	46.683	53.375	1.00	0.00	H
ATOM	1269	CG	LYS	81	29.907	45.935	53.119	1.00	0.00	C
ATOM	1270	HG2	LYS	81	29.508	44.946	52.892	1.00	0.00	H
ATOM	1271	HG3	LYS	81	30.734	46.189	52.457	1.00	0.00	H
ATOM	1272	CD	LYS	81	30.350	45.951	54.565	1.00	0.00	C
ATOM	1273	HD2	LYS	81	30.807	46.934	54.682	1.00	0.00	H
ATOM	1274	HD3	LYS	81	29.506	45.756	55.227	1.00	0.00	H
ATOM	1275	CE	LYS	81	31.501	45.005	54.821	1.00	0.00	C
ATOM	1276	HE2	LYS	81	31.143	43.995	54.622	1.00	0.00	H
ATOM	1277	HE3	LYS	81	32.369	45.204	54.194	1.00	0.00	H
ATOM	1278	NZ	LYS	81	32.091	45.254	56.097	1.00	0.00	N
ATOM	1279	HZ1	LYS	81	31.425	45.118	56.844	1.00	0.00	H
ATOM	1280	HZ2	LYS	81	32.908	44.690	56.285	1.00	0.00	H
ATOM	1281	HZ3	LYS	81	32.242	46.237	56.281	1.00	0.00	H
ATOM	1282	C	LYS	81	27.111	47.500	51.070	1.00	0.00	C
ATOM	1283	O	LYS	81	26.097	46.798	51.081	1.00	0.00	O
ATOM	1284	N	LYS	82	27.153	48.778	50.816	1.00	0.00	N
ATOM	1285	H	LYS	82	28.086	49.167	50.806	1.00	0.00	H
ATOM	1286	CA	LYS	82	26.064	49.723	50.560	1.00	0.00	C
ATOM	1287	HA	LYS	82	25.258	49.620	51.286	1.00	0.00	H
ATOM	1288	CB	LYS	82	26.598	51.147	50.763	1.00	0.00	C
ATOM	1289	HB2	LYS	82	27.367	51.155	51.536	1.00	0.00	H

ATOM	1290	HB3	LYS	82	27.054	51.598	49.881	1.00	0.00	H
ATOM	1291	CG	LYS	82	25.497	52.025	51.322	1.00	0.00	C
ATOM	1292	HG2	LYS	82	24.711	52.103	50.570	1.00	0.00	H
ATOM	1293	HG3	LYS	82	25.013	51.608	52.205	1.00	0.00	H
ATOM	1294	CD	LYS	82	25.929	53.517	51.665	1.00	0.00	C
ATOM	1295	HD2	LYS	82	25.027	53.931	52.115	1.00	0.00	H
ATOM	1296	HD3	LYS	82	26.863	53.524	52.228	1.00	0.00	H
ATOM	1297	CE	LYS	82	26.196	54.336	50.447	1.00	0.00	C
ATOM	1298	HE2	LYS	82	27.015	53.945	49.843	1.00	0.00	H
ATOM	1299	HE3	LYS	82	25.276	54.295	49.865	1.00	0.00	H
ATOM	1300	NZ	LYS	82	26.288	55.796	50.603	1.00	0.00	N
ATOM	1301	HZ1	LYS	82	26.503	56.250	49.726	1.00	0.00	H
ATOM	1302	HZ2	LYS	82	25.511	56.359	50.915	1.00	0.00	H
ATOM	1303	HZ3	LYS	82	27.068	56.087	51.175	1.00	0.00	H
ATOM	1304	C	LYS	82	25.420	49.478	49.158	1.00	0.00	C
ATOM	1305	O	LYS	82	24.201	49.778	49.070	1.00	0.00	O
ATOM	1306	N	VAL	83	26.151	49.093	48.093	1.00	0.00	N
ATOM	1307	H	VAL	83	27.157	49.036	48.165	1.00	0.00	H
ATOM	1308	CA	VAL	83	25.528	48.732	46.842	1.00	0.00	C
ATOM	1309	HA	VAL	83	24.604	49.306	46.752	1.00	0.00	H
ATOM	1310	CB	VAL	83	26.424	48.786	45.570	1.00	0.00	C
ATOM	1311	HB	VAL	83	25.854	48.288	44.785	1.00	0.00	H
ATOM	1312	CG1	VAL	83	26.558	50.271	45.146	1.00	0.00	C
ATOM	1313	HG11	VAL	83	26.817	50.189	44.091	1.00	0.00	H
ATOM	1314	HG12	VAL	83	25.645	50.841	45.322	1.00	0.00	H
ATOM	1315	HG13	VAL	83	27.332	50.787	45.714	1.00	0.00	H
ATOM	1316	CG2	VAL	83	27.657	47.962	45.625	1.00	0.00	C
ATOM	1317	HG21	VAL	83	28.308	48.474	46.333	1.00	0.00	H
ATOM	1318	HG22	VAL	83	27.439	46.931	45.904	1.00	0.00	H
ATOM	1319	HG23	VAL	83	28.166	48.089	44.670	1.00	0.00	H
ATOM	1320	C	VAL	83	24.955	47.295	46.948	1.00	0.00	C
ATOM	1321	O	VAL	83	23.910	47.045	46.360	1.00	0.00	O
ATOM	1322	N	ILE	84	25.538	46.433	47.821	1.00	0.00	N
ATOM	1323	H	ILE	84	26.372	46.691	48.330	1.00	0.00	H
ATOM	1324	CA	ILE	84	25.115	44.988	47.953	1.00	0.00	C
ATOM	1325	HA	ILE	84	24.776	44.733	46.948	1.00	0.00	H
ATOM	1326	CB	ILE	84	26.226	44.065	48.437	1.00	0.00	C
ATOM	1327	HB	ILE	84	26.760	44.469	49.297	1.00	0.00	H
ATOM	1328	CG2	ILE	84	25.606	42.689	48.787	1.00	0.00	C
ATOM	1329	HG21	ILE	84	24.902	42.884	49.597	1.00	0.00	H
ATOM	1330	HG22	ILE	84	25.278	42.144	47.901	1.00	0.00	H
ATOM	1331	HG23	ILE	84	26.417	42.124	49.245	1.00	0.00	H
ATOM	1332	CG1	ILE	84	27.397	43.885	47.364	1.00	0.00	C
ATOM	1333	HG12	ILE	84	27.007	43.358	46.494	1.00	0.00	H
ATOM	1334	HG13	ILE	84	27.656	44.898	47.054	1.00	0.00	H
ATOM	1335	CD1	ILE	84	28.566	43.052	47.926	1.00	0.00	C
ATOM	1336	HD11	ILE	84	28.204	42.051	48.164	1.00	0.00	H
ATOM	1337	HD12	ILE	84	29.376	42.931	47.207	1.00	0.00	H
ATOM	1338	HD13	ILE	84	29.088	43.565	48.734	1.00	0.00	H
ATOM	1339	C	ILE	84	23.832	44.931	48.770	1.00	0.00	C
ATOM	1340	O	ILE	84	22.813	44.362	48.389	1.00	0.00	O
ATOM	1341	N	ALA	85	23.850	45.571	49.957	1.00	0.00	N
ATOM	1342	H	ALA	85	24.687	46.031	50.283	1.00	0.00	H
ATOM	1343	CA	ALA	85	22.645	45.726	50.825	1.00	0.00	C
ATOM	1344	HA	ALA	85	22.219	44.725	50.895	1.00	0.00	H
ATOM	1345	CB	ALA	85	23.091	46.191	52.209	1.00	0.00	C
ATOM	1346	HB1	ALA	85	22.283	45.855	52.860	1.00	0.00	H
ATOM	1347	HB2	ALA	85	23.884	45.554	52.599	1.00	0.00	H
ATOM	1348	HB3	ALA	85	23.516	47.193	52.271	1.00	0.00	H
ATOM	1349	C	ALA	85	21.540	46.633	50.211	1.00	0.00	C
ATOM	1350	O	ALA	85	20.441	46.707	50.732	1.00	0.00	O
ATOM	1351	N	ASP	86	21.856	47.427	49.178	1.00	0.00	N
ATOM	1352	H	ASP	86	22.730	47.354	48.678	1.00	0.00	H
ATOM	1353	CA	ASP	86	20.766	48.126	48.535	1.00	0.00	C

ATOM	1354	HA	ASP	86	20.034	48.369	49.306	1.00	0.00	H
ATOM	1355	CB	ASP	86	21.241	49.411	47.861	1.00	0.00	C
ATOM	1356	HB2	ASP	86	22.038	49.833	48.474	1.00	0.00	H
ATOM	1357	HB3	ASP	86	21.724	49.138	46.923	1.00	0.00	H
ATOM	1358	CG	ASP	86	20.123	50.448	47.755	1.00	0.00	C
ATOM	1359	OD1	ASP	86	20.129	51.092	46.662	1.00	0.00	O
ATOM	1360	OD2	ASP	86	19.338	50.762	48.711	1.00	0.00	O
ATOM	1361	C	ASP	86	20.134	47.130	47.526	1.00	0.00	C
ATOM	1362	O	ASP	86	18.937	47.108	47.344	1.00	0.00	O
ATOM	1363	N	ILE	87	20.945	46.382	46.791	1.00	0.00	N
ATOM	1364	H	ILE	87	21.916	46.286	47.049	1.00	0.00	H
ATOM	1365	CA	ILE	87	20.491	45.430	45.679	1.00	0.00	C
ATOM	1366	HA	ILE	87	19.659	45.779	45.066	1.00	0.00	H
ATOM	1367	CB	ILE	87	21.672	45.126	44.671	1.00	0.00	C
ATOM	1368	HB	ILE	87	22.550	44.730	45.181	1.00	0.00	H
ATOM	1369	CG2	ILE	87	21.239	43.975	43.686	1.00	0.00	C
ATOM	1370	HG21	ILE	87	20.324	44.300	43.192	1.00	0.00	H
ATOM	1371	HG22	ILE	87	21.990					
43.828	42.910	1.00	0.00			H				
ATOM	1372	HG23	ILE	87	21.102	43.017	44.189	1.00	0.00	H
ATOM	1373	CG1	ILE	87	22.018	46.369	43.852	1.00	0.00	C
ATOM	1374	HG12	ILE	87	21.297	46.618	43.073	1.00	0.00	H
ATOM	1375	HG13	ILE	87	22.009	47.101	44.660	1.00	0.00	H
ATOM	1376	CD1	ILE	87	23.332	46.207	43.091	1.00	0.00	C
ATOM	1377	HD11	ILE	87	23.660	47.223	42.873	1.00	0.00	H
ATOM	1378	HD12	ILE	87	24.107	45.786	43.732	1.00	0.00	H
ATOM	1379	HD13	ILE	87	23.283	45.720	42.117	1.00	0.00	H
ATOM	1380	C	ILE	87	19.859	44.164	46.355	1.00	0.00	C
ATOM	1381	O	ILE	87	18.859	43.673	45.857	1.00	0.00	O
ATOM	1382	N	TYR	88	20.375	43.700	47.451	1.00	0.00	N
ATOM	1383	H	TYR	88	21.213	44.055	47.889	1.00	0.00	H
ATOM	1384	CA	TYR	88	19.863	42.490	48.228	1.00	0.00	C
ATOM	1385	HA	TYR	88	19.015	42.063	47.692	1.00	0.00	H
ATOM	1386	CB	TYR	88	21.039	41.492	48.338	1.00	0.00	C
ATOM	1387	HB2	TYR	88	21.948	42.070	48.497	1.00	0.00	H
ATOM	1388	HB3	TYR	88	20.900	40.925	49.259	1.00	0.00	H
ATOM	1389	CG	TYR	88	21.239	40.593	47.107	1.00	0.00	C
ATOM	1390	CD1	TYR	88	20.617	39.354	47.040	1.00	0.00	C
ATOM	1391	HD1	TYR	88	19.983	39.067	47.866	1.00	0.00	H
ATOM	1392	CE1	TYR	88	20.784	38.557	45.922	1.00	0.00	C
ATOM	1393	HE1	TYR	88	20.332	37.578	45.860	1.00	0.00	H
ATOM	1394	CZ	TYR	88	21.396	39.045	44.807	1.00	0.00	C
ATOM	1395	OH	TYR	88	21.501	38.295	43.656	1.00	0.00	O
ATOM	1396	HH	TYR	88	21.942	38.728	42.921	1.00	0.00	H
ATOM	1397	CE2	TYR	88	21.952	40.317	44.795	1.00	0.00	C
ATOM	1398	HE2	TYR	88	22.599	40.615	43.983	1.00	0.00	H
ATOM	1399	CD2	TYR	88	21.822	41.120	45.962	1.00	0.00	C
ATOM	1400	HD2	TYR	88	22.272	42.098	46.056	1.00	0.00	H
ATOM	1401	C	TYR	88	19.500	42.843	49.679	1.00	0.00	C
ATOM	1402	O	TYR	88	19.928	43.856	50.221	1.00	0.00	O
ATOM	1403	N	PRO	89	18.673	41.996	50.354	1.00	0.00	N
ATOM	1404	CD	PRO	89	18.046	40.772	49.858	1.00	0.00	C
ATOM	1405	HD2	PRO	89	18.750	40.056	49.432	1.00	0.00	H
ATOM	1406	HD3	PRO	89	17.264	41.017	49.139	1.00	0.00	H
ATOM	1407	CG	PRO	89	17.323	40.150	51.026	1.00	0.00	C
ATOM	1408	HG2	PRO	89	17.925	39.413	51.556	1.00	0.00	H
ATOM	1409	HG3	PRO	89	16.336	39.742	50.804	1.00	0.00	H
ATOM	1410	CB	PRO	89	17.146	41.270	52.039	1.00	0.00	C
ATOM	1411	HB2	PRO	89	17.081	40.875	53.052	1.00	0.00	H
ATOM	1412	HB3	PRO	89	16.329	41.947	51.791	1.00	0.00	H
ATOM	1413	CA	PRO	89	18.389	42.111	51.836	1.00	0.00	C
ATOM	1414	HA	PRO	89	18.238	43.152	52.125	1.00	0.00	H
ATOM	1415	C	PRO	89	19.621	41.639	52.676	1.00	0.00	C
ATOM	1416	O	PRO	89	20.367	40.750	52.254	1.00	0.00	O

ATOM	1417	N	GLY	90	19.827	42.390	53.727	1.00	0.00	N
ATOM	1418	H	GLY	90	19.120	43.107	53.797	1.00	0.00	H
ATOM	1419	CA	GLY	90	21.026	42.320	54.568	1.00	0.00	C
ATOM	1420	HA2	GLY	90	21.909	42.699	54.051	1.00	0.00	H
ATOM	1421	HA3	GLY	90	20.835	42.892	55.475	1.00	0.00	H
ATOM	1422	C	GLY	90	21.379	41.011	55.172	1.00	0.00	C
ATOM	1423	O	GLY	90	22.572	40.598	55.310	1.00	0.00	O
ATOM	1424	N	GLN	91	20.347	40.163	55.391	1.00	0.00	N
ATOM	1425	H	GLN	91	19.445	40.428	55.019	1.00	0.00	H
ATOM	1426	CA	GLN	91	20.544	38.694	55.789	1.00	0.00	C
ATOM	1427	HA	GLN	91	21.071	38.707	56.743	1.00	0.00	H
ATOM	1428	CB	GLN	91	19.126	38.139	56.080	1.00	0.00	C
ATOM	1429	HB2	GLN	91	18.687	38.155	55.082	1.00	0.00	H
ATOM	1430	HB3	GLN	91	19.103	37.086	56.361	1.00	0.00	H
ATOM	1431	CG	GLN	91	18.232	38.919	57.020	1.00	0.00	C
ATOM	1432	HG2	GLN	91	17.582	38.194	57.511	1.00	0.00	H
ATOM	1433	HG3	GLN	91	18.761	39.312	57.889	1.00	0.00	H
ATOM	1434	CD	GLN	91	17.339	39.969	56.325	1.00	0.00	C
ATOM	1435	OE1	GLN	91	17.705	41.015	55.946	1.00	0.00	O
ATOM	1436	NE2	GLN	91	16.122	39.550	55.910	1.00	0.00	N
ATOM	1437	HE21	GLN	91	15.894	38.569	55.839	1.00	0.00	H
ATOM	1438	HE22	GLN	91	15.602	40.086	55.230	1.00	0.00	H
ATOM	1439	C	GLN	91	21.329	37.886	54.769	1.00	0.00	C
ATOM	1440	O	GLN	91	22.030	37.032	55.287	1.00	0.00	O
ATOM	1441	N	THR	92	21.208	38.112	53.461	1.00	0.00	N
ATOM	1442	H	THR	92	20.672	38.934	53.221	1.00	0.00	H
ATOM	1443	CA	THR	92	21.765	37.233	52.384	1.00	0.00	C
ATOM	1444	HA	THR	92	21.154	36.332	52.342	1.00	0.00	H
ATOM	1445	CB	THR	92	21.836	37.931	51.024	1.00	0.00	C
ATOM	1446	HB	THR	92	22.575	38.728	50.936	1.00	0.00	H
ATOM	1447	CG2	THR	92	21.954	36.875	49.912	1.00	0.00	C
ATOM	1448	HG21	THR	92	22.283	37.484	49.070	1.00	0.00	H
ATOM	1449	HG22	THR	92	22.515	35.956	50.078	1.00	0.00	H
ATOM	1450	HG23	THR	92	20.966	36.538	49.600	1.00	0.00	H
ATOM	1451	OG1	THR	92	20.516	38.539	50.731	1.00	0.00	O
ATOM	1452	HG1	THR	92	20.618	39.380	51.183	1.00	0.00	H
ATOM	1453	C	THR	92	23.185	36.645	52.728	1.00	0.00	C
ATOM	1454	O	THR	92	24.087	37.411	52.992	1.00	0.00	O
ATOM	1455	N	GLN	93	23.302	35.289	52.811	1.00	0.00	N
ATOM	1456	H	GLN	93	22.467	34.735	52.685	1.00	0.00	H
ATOM	1457	CA	GLN	93	24.606	34.597	52.796	1.00	0.00	C
ATOM	1458	HA	GLN	93	25.234	35.219	53.435	1.00	0.00	H
ATOM	1459	CB	GLN	93	24.474	33.192	53.337	1.00	0.00	C
ATOM	1460	HB2	GLN	93	23.978	33.132	54.306	1.00	0.00	H
ATOM	1461	HB3	GLN	93	23.919	32.680	52.551	1.00	0.00	H
ATOM	1462	CG	GLN	93	25.822	32.586	53.629	1.00	0.00	C
ATOM	1463	HG2	GLN	93	25.655	31.511	53.583	1.00	0.00	H
ATOM	1464	HG3	GLN	93	26.407	32.732	52.722	1.00	0.00	H
ATOM	1465	CD	GLN	93	26.534	32.864	55.002	1.00	0.00	C
ATOM	1466	OE1	GLN	93	25.876	33.003	56.063	1.00	0.00	O
ATOM	1467	NE2	GLN	93	27.858	32.661	55.048	1.00	0.00	N
ATOM	1468	HE21	GLN	93	28.331	32.484	54.173	1.00	0.00	H
ATOM	1469	HE22	GLN	93	28.321	32.793	55.936	1.00	0.00	H
ATOM	1470	C	GLN	93	25.198	34.680	51.345	1.00	0.00	C
ATOM	1471	O	GLN	93	24.493	34.346	50.362	1.00	0.00	O
ATOM	1472	N	PHE	94	26.419	35.173	51.185	1.00	0.00	N
ATOM	1473	H	PHE	94	26.944	35.267	52.042	1.00	0.00	H
ATOM	1474	CA	PHE	94	27.044	35.429	49.956	1.00	0.00	C
ATOM	1475	HA	PHE	94	26.327	35.231	49.159	1.00	0.00	H
ATOM	1476	CB	PHE	94	27.445	36.910	49.800	1.00	0.00	C
ATOM	1477	HB2	PHE	94	28.136	37.048	50.632	1.00	0.00	H
ATOM	1478	HB3	PHE	94	27.967	36.978	48.845	1.00	0.00	H
ATOM	1479	CG	PHE	94	26.335	37.980	49.781	1.00	0.00	C
ATOM	1480	CD1	PHE	94	25.760	38.360	48.534	1.00	0.00	C

ATOM	1481	HD1	PHE	94	26.087	37.920	47.604	1.00	0.00	H
ATOM	1482	CE1	PHE	94	24.725	39.350	48.566	1.00	0.00	C
ATOM	1483	HE1	PHE	94	24.370	39.695	47.606	1.00	0.00	H
ATOM	1484	CZ	PHE	94	24.335	39.961	49.750	1.00	0.00	C
ATOM	1485	HZ	PHE	94	23.520	40.664	49.653	1.00	0.00	H
ATOM	1486	CE2	PHE	94	24.880	39.553	51.008	1.00	0.00	C
ATOM	1487	HE2	PHE	94	24.505	39.923	51.951	1.00	0.00	H
ATOM	1488	CD2	PHE	94	25.867	38.546	50.995	1.00	0.00	C
ATOM	1489	HD2	PHE	94	26.259	38.297	51.970	1.00	0.00	H
ATOM	1490	C	PHE	94	28.300	34.528	49.733	1.00	0.00	C
ATOM	1491	O	PHE	94	29.214	34.443	50.573	1.00	0.00	O
ATOM	1492	N	TYR	95	28.388	33.980	48.480	1.00	0.00	N
ATOM	1493	H	TYR	95	27.807	34.411	47.776	1.00	0.00	H
ATOM	1494	CA	TYR	95	29.345	32.965	48.056	1.00	0.00	C
ATOM	1495	HA	TYR	95	29.991	32.785	48.916	1.00	0.00	H
ATOM	1496	CB	TYR	95	28.571	31.782	47.556	1.00	0.00	C
ATOM	1497	HB2	TYR	95	27.905	31.436	48.346	1.00	0.00	H
ATOM	1498	HB3	TYR	95	27.914	32.078	46.738	1.00	0.00	H
ATOM	1499	CG	TYR	95	29.384	30.579	47.111	1.00	0.00	C
ATOM	1500	CD1	TYR	95	30.122	30.552	45.925	1.00	0.00	C
ATOM	1501	HD1	TYR	95	30.248	31.462	45.358	1.00	0.00	H
ATOM	1502	CE1	TYR	95	30.753	29.347	45.476	1.00	0.00	C
ATOM	1503	HE1	TYR	95	31.257	29.299	44.521	1.00	0.00	H
ATOM	1504	CZ	TYR	95	30.775	28.181	46.329	1.00	0.00	C
ATOM	1505	OH	TYR	95	31.327	27.042	45.866	1.00	0.00	O
ATOM	1506	HH	TYR	95	31.499	27.168	44.929	1.00	0.00	H
ATOM	1507	CE2	TYR	95	30.070	28.274	47.568	1.00	0.00	C
ATOM	1508	HE2	TYR	95	29.889	27.338	48.076	1.00	0.00	H
ATOM	1509	CD2	TYR	95	29.378	29.430	47.912	1.00	0.00	C
ATOM	1510	HD2	TYR	95	28.780	29.310	48.803	1.00	0.00	H
ATOM	1511	C	TYR	95	30.310	33.605	47.026	1.00	0.00	C
ATOM	1512	O	TYR	95	30.022	34.436	46.190	1.00	0.00	O
ATOM	1513	N	VAL	96	31.569	33.252	47.224	1.00	0.00	N
ATOM	1514	H	VAL	96	31.781	32.558	47.926	1.00	0.00	H
ATOM	1515	CA	VAL	96	32.771	34.018	46.749	1.00	0.00	C
ATOM	1516	HA	VAL	96	32.446	34.880	46.164	1.00	0.00	H
ATOM	1517	CB	VAL	96	33.544	34.612	47.878	1.00	0.00	C
ATOM	1518	HB	VAL	96	33.888	33.831	48.557	1.00	0.00	H
ATOM	1519	CG1	VAL	96	34.833	35.444	47.538	1.00	0.00	C
ATOM	1520	HG11	VAL	96	35.149	35.897	48.478	1.00	0.00	H
ATOM	1521	HG12	VAL	96	35.553	34.716	47.164	1.00	0.00	H
ATOM	1522	HG13	VAL	96	34.763	36.228	46.786	1.00	0.00	H
ATOM	1523	CG2	VAL	96	32.593	35.579	48.625	1.00	0.00	C
ATOM	1524	HG21	VAL	96	32.122	36.258	47.916	1.00	0.00	H
ATOM	1525	HG22	VAL	96	31.778	35.033	49.099	1.00	0.00	H
ATOM	1526	HG23	VAL	96	33.087	36.226	49.351	1.00	0.00	H
ATOM	1527	C	VAL	96	33.688	33.177	45.857	1.00	0.00	C
ATOM	1528	O	VAL	96	34.295	32.186	46.243	1.00	0.00	O
ATOM	1529	N	ILE	97	33.751	33.576	44.560	1.00	0.00	N
ATOM	1530	H	ILE	97	33.506	34.523	44.310	1.00	0.00	H
ATOM	1531	CA	ILE	97	34.349	32.720	43.475	1.00	0.00	C
ATOM	1532	HA	ILE	97	34.658	31.848	44.051	1.00	0.00	H
ATOM	1533	CB	ILE	97	33.270	32.343	42.370	1.00	0.00	C
ATOM	1534	HB	ILE	97	32.926	33.214	41.813	1.00	0.00	H
ATOM	1535	CG2	ILE	97	33.949	31.318	41.380	1.00	0.00	C
ATOM	1536	HG21	ILE	97	33.409	31.150	40.448	1.00	0.00	H
ATOM	1537	HG22	ILE	97	34.875	31.779	41.033	1.00	0.00	H
ATOM	1538	HG23	ILE	97	34.241	30.362	41.816	1.00	0.00	H
ATOM	1539	CG1	ILE	97	31.993	31.707	43.022	1.00	0.00	C
ATOM	1540	HG12	ILE	97	32.470	30.980	43.680	1.00	0.00	H
ATOM	1541	HG13	ILE	97	31.573	32.498	43.644	1.00	0.00	H
ATOM	1542	CD1	ILE	97	30.960	31.131	42.048	1.00	0.00	C
ATOM	1543	HD11	ILE	97	29.942	31.147	42.439	1.00	0.00	H

ATOM	1544	HD12	ILE	97	30.906	31.722	41.134	1.00	0.00	H
ATOM	1545	HD13	ILE	97	31.187	30.093	41.808	1.00	0.00	H
ATOM	1546	C	ILE	97	35.522	33.435	42.842	1.00	0.00	C
ATOM	1547	O	ILE	97	35.381	34.582	42.448	1.00	0.00	O
ATOM	1548	N	GLU	98	36.716	32.830	42.927	1.00	0.00	N
ATOM	1549	H	GLU	98	36.694	31.919	43.364	1.00	0.00	H
ATOM	1550	CA	GLU	98	38.022	33.336	42.548	1.00	0.00	C
ATOM	1551	HA	GLU	98	37.730	34.245	42.023	1.00	0.00	H
ATOM	1552	CB	GLU	98	39.044	33.465	43.714	1.00	0.00	C
ATOM	1553	HB2	GLU	98	39.485	32.498	43.957	1.00	0.00	H
ATOM	1554	HB3	GLU	98	39.839	34.046	43.249	1.00	0.00	H
ATOM	1555	CG	GLU	98	38.405	34.251	44.792	1.00	0.00	C
ATOM	1556	HG2	GLU	98	38.217	35.265	44.439	1.00	0.00	H
ATOM	1557	HG3	GLU	98	37.526	33.732	45.176	1.00	0.00	H
ATOM	1558	CD	GLU	98	39.351	34.329	45.920	1.00	0.00	C
ATOM	1559	OE1	GLU	98	39.038	33.955	47.088	1.00	0.00	O
ATOM	1560	OE2	GLU	98	40.536	34.695	45.664	1.00	0.00	O
ATOM	1561	C	GLU	98	38.678	32.477	41.411	1.00	0.00	C
ATOM	1562	O	GLU	98	38.640	31.272	41.461	1.00	0.00	O
ATOM	1563	N	PHE	99	39.212	33.054	40.268	1.00	0.00	N
ATOM	1564	H	PHE	99	39.221	34.057	40.155	1.00	0.00	H
ATOM	1565	CA	PHE	99	39.564	32.486	39.052	1.00	0.00	C
ATOM	1566	HA	PHE	99	40.050	31.532	39.251	1.00	0.00	H
ATOM	1567	CB	PHE	99	38.288	32.193	38.263	1.00	0.00	C
ATOM	1568	HB2	PHE	99	38.488	31.777	37.275	1.00	0.00	H
ATOM	1569	HB3	PHE	99	37.728	31.423	38.793	1.00	0.00	H
ATOM	1570	CG	PHE	99	37.446	33.409	38.063	1.00	0.00	C
ATOM	1571	CD1	PHE	99	36.492	33.742	39.012	1.00	0.00	C
ATOM	1572	HD1	PHE	99	36.221	33.105	39.841	1.00	0.00	H
ATOM	1573	CE1	PHE	99	35.888	34.960	38.991	1.00	0.00	C
ATOM	1574	HE1	PHE	99	35.317	35.179	39.882	1.00	0.00	H
ATOM	1575	CZ	PHE	99	36.043	35.807	37.921	1.00	0.00	C
ATOM	1576	HZ	PHE	99	35.580	36.780	37.851	1.00	0.00	H
ATOM	1577	CE2	PHE	99	36.927	35.404	36.846	1.00	0.00	C
ATOM	1578	HE2	PHE	99	37.110	35.950	35.932	1.00	0.00	H
ATOM	1579	CD2	PHE	99	37.701	34.283	37.010	1.00	0.00	C
ATOM	1580	HD2	PHE	99	38.450	33.975	36.294	1.00	0.00	H
ATOM	1581	C	PHE	99	40.623	33.173	38.219	1.00	0.00	C
ATOM	1582	O	PHE	99	41.030	34.326	38.396	1.00	0.00	O
ATOM	1583	N	LYS	100	41.149	32.370	37.280	1.00	0.00	N
ATOM	1584	H	LYS	100	40.859	31.403	37.275	1.00	0.00	H
ATOM	1585	CA	LYS	100	42.105	32.834	36.257	1.00	0.00	C
ATOM	1586	HA	LYS	100	41.801	33.828	35.926	1.00	0.00	H
ATOM	1587	CB	LYS	100	43.543	33.009	36.851	1.00	0.00	C
ATOM	1588	HB2	LYS	100	44.182	33.560	36.161	1.00	0.00	H
ATOM	1589	HB3	LYS	100	43.503	33.739	37.658	1.00	0.00	H
ATOM	1590	CG	LYS	100	44.132	31.688	37.331	1.00	0.00	C
ATOM	1591	HG2	LYS	100	43.377	31.112	37.867	1.00	0.00	H
ATOM	1592	HG3	LYS	100	44.345	31.039	36.481	1.00	0.00	H
ATOM	1593	CD	LYS	100	45.454	31.856	38.016	1.00	0.00	C
ATOM	1594	HD2	LYS	100	46.152	32.373	37.357	1.00	0.00	H
ATOM	1595	HD3	LYS	100	45.340	32.554	38.844	1.00	0.00	H
ATOM	1596	CE	LYS	100	46.081	30.492	38.444	1.00	0.00	C
ATOM	1597	HE2	LYS	100	45.552	30.186	39.348	1.00	0.00	H
ATOM	1598	HE3	LYS	100	45.931	29.776	37.636	1.00	0.00	H
ATOM	1599	NZ	LYS	100	47.492	30.620	38.674	1.00	0.00	N
ATOM	1600	HZ1	LYS	100	47.968	29.854	39.132	1.00	0.00	H
ATOM	1601	HZ2	LYS	100	48.023	30.742	37.824	1.00	0.00	H
ATOM	1602	HZ3	LYS	100	47.649	31.455	39.220	1.00	0.00	H
ATOM	1603	C	LYS	100	42.172	31.855	35.065	1.00	0.00	C
ATOM	1604	O	LYS	100	42.100	30.601	35.222	1.00	0.00	O
ATOM	1605	N	CYX	101	42.352	32.541	33.915	1.00	0.00	N
ATOM	1606	H	CYX	101	42.591	33.520	33.976	1.00	0.00	H
ATOM	1607	CA	CYX	101	42.692	31.952	32.640	1.00	0.00	C

```
ATOM   1608  HA  CYX   101      42.034  31.090  32.520  1.00  0.00           H
ATOM   1609  CB  CYX   101      42.342  32.924  31.491  1.00  0.00           C
ATOM   1610  HB2 CYX   101      41.298  33.211  31.617  1.00  0.00           H
ATOM   1611  HB3 CYX   101      42.808  33.891  31.685  1.00  0.00           H
ATOM   1612  SG  CYX   101      42.766  32.313  29.804  1.00  0.00           S
ATOM   1613  C   CYX   101      44.143  31.513  32.645  1.00  0.00           C
ATOM   1614  O   CYX   101      45.024  32.150  33.155  1.00  0.00           O
ATOM   1615  N   LEU   102      44.359  30.256  32.173  1.00  0.00           N
ATOM   1616  H   LEU   102      43.640  29.788  31.640  1.00  0.00           H
ATOM   1617  CA  LEU   102      45.729  29.565  32.125  1.00  0.00           C
ATOM   1618  HA  LEU   102      46.137  29.826  33.103  1.00  0.00           H
ATOM   1619  CB  LEU   102      45.449  28.077  32.027  1.00  0.00           C
ATOM   1620  HB2 LEU   102      44.675  28.036  31.260  1.00  0.00           H
ATOM   1621  HB3 LEU   102      46.253  27.574  31.489  1.00  0.00           H
ATOM   1622
 CG  LEU   102      44.973  27.247  33.233  1.00  0.00           C
ATOM   1623  HG  LEU   102      45.693  27.399  34.038  1.00  0.00           H
ATOM   1624  CD1 LEU   102      43.517  27.659  33.632  1.00  0.00           C
ATOM   1625 HD11 LEU   102      43.578  28.689  33.982  1.00  0.00           H
ATOM   1626 HD12 LEU   102      42.807  27.545  32.813  1.00  0.00           H
ATOM   1627 HD13 LEU   102      43.134  27.102  34.487  1.00  0.00           H
ATOM   1628  CD2 LEU   102      45.024  25.751  32.847  1.00  0.00           C
ATOM   1629 HD21 LEU   102      44.277  25.508  32.090  1.00  0.00           H
ATOM   1630 HD22 LEU   102      46.011  25.485  32.467  1.00  0.00           H
ATOM   1631 HD23 LEU   102      44.769  25.163  33.728  1.00  0.00           H
ATOM   1632  C   LEU   102      46.719  30.092  31.126  1.00  0.00           C
ATOM   1633  O   LEU   102      46.460  29.996  29.902  1.00  0.00           O
ATOM   1634  OXT LEU   102      47.841  30.432  31.571  1.00  0.00           O
HETATM  314  N   LYN    20      39.739  40.841  35.868  1.00  0.00           N
HETATM  315  H   LYN    20      40.029  40.464  34.976  1.00  0.00           H
HETATM  316  CA  LYN    20      38.675  40.060  36.590  1.00  0.00           C
HETATM  317  HA  LYN    20      38.340  40.607  37.472  1.00  0.00           H
HETATM  318  CB  LYN    20      37.441  39.837  35.682  1.00  0.00           C
HETATM  319  HB2 LYN    20      37.660  39.259  34.784  1.00  0.00           H
HETATM  320  HB3 LYN    20      37.196  40.832  35.313  1.00  0.00           H
HETATM  321  CG  LYN    20      36.272  39.277  36.472  1.00  0.00           C
HETATM  322  HG2 LYN    20      35.553  38.797  35.807  1.00  0.00           H
HETATM  323  HG3 LYN    20      36.679  38.445  37.045  1.00  0.00           H
HETATM  324  CD  LYN    20      35.611  40.366  37.242  1.00  0.00           C
HETATM  325  HD2 LYN    20      35.229  41.086  36.518  1.00  0.00           H
HETATM  326  HD3 LYN    20      36.297  40.716  38.014  1.00  0.00           H
HETATM  327  CE  LYN    20      34.463  39.566  37.901  1.00  0.00           C
HETATM  328  HE2 LYN    20      33.710  39.199  37.205  1.00  0.00           H
HETATM  329  HE3 LYN    20      34.843  38.747  38.513  1.00  0.00           H
HETATM  330  NZ  LYN    20      33.813  40.469  38.849  1.00  0.00           N
HETATM  331  HZ2 LYN    20      33.555  41.350  38.426  1.00  0.00           H
HETATM  332  HZ3 LYN    20      34.557  40.729  39.480  1.00  0.00           H
HETATM  333  C   LYN    20      39.308  38.764  37.069  1.00  0.00           C
HETATM  334  O   LYN    20      39.772  37.995  36.264  1.00  0.00           O
HETATM 1636  N   LIG   103      30.170  42.445  40.212  1.00  0.00           N
HETATM 1637  C   LIG   103      29.874  41.416  39.516  1.00  0.00           C
HETATM 1638  O   LIG   103      30.034  43.568  42.118  1.00  0.00           O
HETATM 1639  C5' LIG   103      25.672  39.280  44.063  1.00  0.00           C
HETATM 1640  O5' LIG   103      25.573  39.028  42.659  1.00  0.00           O
HETATM 1641  C4' LIG   103      26.141  40.763  44.234  1.00  0.00           C
HETATM 1642  O4' LIG   103      27.599  40.780  43.889  1.00  0.00           O
HETATM 1643  C3' LIG   103      25.553  41.825  43.275  1.00  0.00           C
HETATM 1644  O3' LIG   103      24.497  42.495  43.843  1.00  0.00           O
HETATM 1645  C2' LIG   103      26.713  42.802  43.021  1.00  0.00           C
HETATM 1646  O2' LIG   103      26.526  43.959  43.881  1.00  0.00           O
HETATM 1647  C1' LIG   103      28.080  42.021  43.264  1.00  0.00           C
HETATM 1648  N1  LIG   103      28.778  41.668  41.999  1.00  0.00           N
HETATM 1649  O1  LIG   103      30.054  39.151  37.617  1.00  0.00           O
HETATM 1650  N2  LIG   103      30.580  41.271  38.435  1.00  0.00           N
```

```
HETATM 1651  C6  LIG   103       29.644  42.631  41.514  1.00  0.00           C
HETATM 1652  C7  LIG   103       28.337  40.639  41.183  1.00  0.00           C
HETATM 1653  C8  LIG   103       28.962  40.443  40.018  1.00  0.00           C
HETATM 1654  C9  LIG   103       30.618  40.244  37.524  1.00  0.00           C
HETATM 1655  C10 LIG   103       31.467  40.454  36.270  1.00  0.00           C
HETATM 1656  H   LIG   103       31.278  41.986  38.268  1.00  0.00           H
HETATM 1657  H1  LIG   103       28.619  39.638  39.377  1.00  0.00           H
HETATM 1658  H4  LIG   103       28.696  42.543  44.005  1.00  0.00           H
HETATM 1659  H6  LIG   103       26.248  41.163  45.250  1.00  0.00           H
HETATM 1660  H7  LIG   103       25.138  41.270  42.424  1.00  0.00           H
HETATM 1661  H8  LIG   103       26.777  43.068  41.959  1.00  0.00           H
HETATM 1662  H9  LIG   103       24.674  39.067  44.465  1.00  0.00           H
HETATM 1663  H10 LIG   103       26.402  38.629  44.562  1.00  0.00           H
HETATM 1664  H11 LIG   103       30.851  40.701  35.396  1.00  0.00           H
HETATM 1665  H12 LIG   103       32.068  39.561  36.058  1.00  0.00           H
HETATM 1666  H13 LIG   103       32.194  41.276  36.263  1.00  0.00           H
HETATM 1667  H14 LIG   103       27.559  40.015  41.601  1.00  0.00           H
HETATM 1668  H2' LIG   103       27.280  44.544  43.688  1.00  0.00           H
HETATM 1669  H3' LIG   103       24.896  43.344  44.099  1.00  0.00           H
HETATM 1670  H5' LIG   103       24.913  38.316  42.586  1.00  0.00           H
ENDMDL
MODEL      8
SHEET    1   1 1 ILE    22  ASP    26  0
SHEET    2   2 1 VAL    37  VAL    40  0
SHEET    3   3 1 CYX    50  VAL    60  0
SHEET    4   4 1 PHE    94  CYX   101  0
HELIX    1   1 GLN      9  LEU     16  1                                   8
HELIX    2   2 LEU     62  THR     64  1                                   3
HELIX    3   3 LEU     77  ILE     87  1                                  11
ATOM      1  N   GLN     1       35.495  17.376  33.292  1.00  0.00           N
ATOM      2  H1  GLN     1       36.066  17.916  33.926  1.00  0.00           H
ATOM      3  H2  GLN     1       34.585  17.242  33.709  1.00  0.00           H
ATOM      4  H3  GLN     1       35.914  16.475  33.110  1.00  0.00           H
ATOM      5  CA  GLN     1       35.322  18.128  31.969  1.00  0.00           C
ATOM      6  HA  GLN     1       36.257  18.181  31.410  1.00  0.00           H
ATOM      7  CB  GLN     1       34.300  17.449  31.039  1.00  0.00           C
ATOM      8  HB2 GLN     1       33.410  17.228  31.629  1.00  0.00           H
ATOM      9  HB3 GLN     1       34.055  18.190  30.277  1.00  0.00           H
ATOM     10  CG  GLN     1       34.827  16.253  30.298  1.00  0.00           C
ATOM     11  HG2 GLN     1       35.065  15.450  30.997  1.00  0.00           H
ATOM     12  HG3 GLN     1       33.924  15.974  29.756  1.00  0.00           H
ATOM     13  CD  GLN     1       35.964  16.495  29.299  1.00  0.00           C
ATOM     14  OE1 GLN     1       36.541  17.536  29.273  1.00  0.00           O
ATOM     15  NE2 GLN     1       36.435  15.470  28.533  1.00  0.00           N
ATOM     16 HE21 GLN     1       36.109  14.536  28.733  1.00  0.00           H
ATOM     17 HE22 GLN     1       37.280  15.650  28.012  1.00  0.00           H
ATOM     18  C   GLN     1       34.936  19.575  32.325  1.00  0.00           C
ATOM     19  O   GLN     1       34.401  19.787  33.415  1.00  0.00           O
ATOM     20  N   PRO     2       35.130  20.508  31.391  1.00  0.00           N
ATOM     21  CD  PRO     2       35.912  20.382  30.222  1.00  0.00           C
ATOM     22  HD2 PRO     2       35.417  19.742  29.493  1.00  0.00           H
ATOM     23  HD3 PRO     2       36.867  19.903  30.437  1.00  0.00           H
ATOM     24  CG  PRO     2       36.265  21.808  29.783  1.00  0.00           C
ATOM     25  HG2 PRO     2       36.431  21.816  28.706  1.00  0.00           H
ATOM     26  HG3 PRO     2       37.189  22.146  30.251  1.00  0.00           H
ATOM     27  CB  PRO     2       34.964  22.589  30.186  1.00  0.00           C
ATOM     28  HB2 PRO     2       34.280  22.404  29.358  1.00  0.00           H
ATOM     29  HB3 PRO     2       35.116  23.658  30.337  1.00  0.00           H
ATOM     30  CA  PRO     2       34.611  21.851  31.509  1.00  0.00           C
ATOM     31  HA  PRO     2       35.074  22.424  32.311  1.00  0.00           H
ATOM     32  C   PRO     2       33.078  21.970  31.705  1.00  0.00           C
ATOM     33  O   PRO     2       32.289  21.106  31.310  1.00  0.00           O
ATOM     34  N   ASN     3       32.662  23.124  32.248  1.00  0.00           N
ATOM     35  H   ASN     3       33.320  23.870  32.418  1.00  0.00           H
```

ATOM	36	CA	ASN	3	31.264	23.520	32.439	1.00	0.00	C
ATOM	37	HA	ASN	3	30.743	22.575	32.593	1.00	0.00	H
ATOM	38	CB	ASN	3	31.051	24.530	33.622	1.00	0.00	C
ATOM	39	HB2	ASN	3	31.463	25.512	33.390	1.00	0.00	H
ATOM	40	HB3	ASN	3	29.977	24.656	33.758	1.00	0.00	H
ATOM	41	CG	ASN	3	31.458	24.023	35.047	1.00	0.00	C
ATOM	42	OD1	ASN	3	30.613	23.728	35.878	1.00	0.00	O
ATOM	43	ND2	ASN	3	32.723	23.844	35.377	1.00	0.00	N
ATOM	44	HD21	ASN	3	33.472	24.092	34.747	1.00	0.00	H
ATOM	45	HD22	ASN	3	32.928	23.433	36.277	1.00	0.00	H
ATOM	46	C	ASN	3	30.713	24.220	31.147	1.00	0.00	C
ATOM	47	O	ASN	3	31.455	24.892	30.462	1.00	0.00	O
ATOM	48	N	ASP	4	29.443	24.098	30.838	1.00	0.00	N
ATOM	49	H	ASP	4	28.806	23.558	31.408	1.00	0.00	H
ATOM	50	CA	ASP	4	28.709	24.796	29.771	1.00	0.00	C
ATOM	51	HA	ASP	4	29.459	24.903	28.988	1.00	0.00	H
ATOM	52	CB	ASP							
				4	27.578	23.949	29.192	1.00	0.00	C
ATOM	53	HB2	ASP	4	26.875	23.798	30.010	1.00	0.00	H
ATOM	54	HB3	ASP	4	27.143	24.449	28.328	1.00	0.00	H
ATOM	55	CG	ASP	4	28.123	22.583	28.800	1.00	0.00	C
ATOM	56	OD1	ASP	4	28.935	22.505	27.891	1.00	0.00	O
ATOM	57	OD2	ASP	4	27.762	21.530	29.415	1.00	0.00	O
ATOM	58	C	ASP	4	28.324	26.207	30.202	1.00	0.00	C
ATOM	59	O	ASP	4	27.921	27.025	29.378	1.00	0.00	O
ATOM	60	N	ILE	5	28.478	26.583	31.497	1.00	0.00	N
ATOM	61	H	ILE	5	28.820	25.861	32.116	1.00	0.00	H
ATOM	62	CA	ILE	5	28.260	27.955	32.040	1.00	0.00	C
ATOM	63	HA	ILE	5	27.608	28.476	31.339	1.00	0.00	H
ATOM	64	CB	ILE	5	27.658	27.980	33.445	1.00	0.00	C
ATOM	65	HB	ILE	5	28.448	27.651	34.122	1.00	0.00	H
ATOM	66	CG2	ILE	5	27.239	29.412	33.866	1.00	0.00	C
ATOM	67	HG21	ILE	5	26.447	29.673	33.165	1.00	0.00	H
ATOM	68	HG22	ILE	5	26.893	29.471	34.899	1.00	0.00	H
ATOM	69	HG23	ILE	5	28.175	29.969	33.827	1.00	0.00	H
ATOM	70	CG1	ILE	5	26.513	27.028	33.635	1.00	0.00	C
ATOM	71	HG12	ILE	5	26.861	25.997	33.579	1.00	0.00	H
ATOM	72	HG13	ILE	5	26.181	27.183	34.661	1.00	0.00	H
ATOM	73	CD1	ILE	5	25.409	27.188	32.589	1.00	0.00	C
ATOM	74	HD11	ILE	5	24.601	26.462	32.676	1.00	0.00	H
ATOM	75	HD12	ILE	5	24.941	28.173	32.581	1.00	0.00	H
ATOM	76	HD13	ILE	5	25.666	27.197	31.529	1.00	0.00	H
ATOM	77	C	ILE	5	29.597	28.777	31.968	1.00	0.00	C
ATOM	78	O	ILE	5	30.684	28.372	32.259	1.00	0.00	O
ATOM	79	N	THR	6	29.495	30.021	31.460	1.00	0.00	N
ATOM	80	H	THR	6	28.578	30.399	31.271	1.00	0.00	H
ATOM	81	CA	THR	6	30.597	30.880	31.114	1.00	0.00	C
ATOM	82	HA	THR	6	31.429	30.924	31.817	1.00	0.00	H
ATOM	83	CB	THR	6	31.162	30.413	29.725	1.00	0.00	C
ATOM	84	HB	THR	6	31.724	29.482	29.802	1.00	0.00	H
ATOM	85	CG2	THR	6	30.097	30.155	28.608	1.00	0.00	C
ATOM	86	HG21	THR	6	30.163	30.959	27.874	1.00	0.00	H
ATOM	87	HG22	THR	6	30.195	29.181	28.127	1.00	0.00	H
ATOM	88	HG23	THR	6	29.087	30.277	28.998	1.00	0.00	H
ATOM	89	OG1	THR	6	32.048	31.423	29.194	1.00	0.00	O
ATOM	90	HG1	THR	6	32.777	31.042	28.698	1.00	0.00	H
ATOM	91	C	THR	6	30.191	32.380	31.099	1.00	0.00	C
ATOM	92	O	THR	6	29.002	32.717	30.872	1.00	0.00	O
ATOM	93	N	PHE	7	31.051	33.220	31.577	1.00	0.00	N
ATOM	94	H	PHE	7	31.946	32.804	31.792	1.00	0.00	H
ATOM	95	CA	PHE	7	30.945	34.652	31.763	1.00	0.00	C
ATOM	96	HA	PHE	7	30.098	34.785	32.437	1.00	0.00	H
ATOM	97	CB	PHE	7	32.240	35.233	32.419	1.00	0.00	C
ATOM	98	HB2	PHE	7	33.081	35.048	31.750	1.00	0.00	H

ATOM	99	HB3	PHE	7	32.184	36.318	32.500	1.00	0.00	H
ATOM	100	CG	PHE	7	32.541	34.741	33.783	1.00	0.00	C
ATOM	101	CD1	PHE	7	33.635	33.953	34.176	1.00	0.00	C
ATOM	102	HD1	PHE	7	34.380	33.753	33.420	1.00	0.00	H
ATOM	103	CE1	PHE	7	33.753	33.463	35.476	1.00	0.00	C
ATOM	104	HE1	PHE	7	34.577	32.820	35.750	1.00	0.00	H
ATOM	105	CZ	PHE	7	32.888	33.782	36.434	1.00	0.00	C
ATOM	106	HZ	PHE	7	32.984	33.492	37.469	1.00	0.00	H
ATOM	107	CE2	PHE	7	31.818	34.611	36.054	1.00	0.00	C
ATOM	108	HE2	PHE	7	31.040	34.757	36.788	1.00	0.00	H
ATOM	109	CD2	PHE	7	31.550	34.950	34.742	1.00	0.00	C
ATOM	110	HD2	PHE	7	30.612	35.447	34.539	1.00	0.00	H
ATOM	111	C	PHE	7	30.735	35.357	30.391	1.00	0.00	C
ATOM	112	O	PHE	7	31.357	35.053	29.334	1.00	0.00	O
ATOM	113	N	PHE	8	29.762	36.277	30.400	1.00	0.00	N
ATOM	114	H	PHE	8	29.320	36.515	31.276	1.00	0.00	H
ATOM	115	CA	PHE	8	29.194	36.774	29.158	1.00	0.00	C
ATOM	116	HA	PHE	8	29.081	35.910	28.504	1.00	0.00	H
ATOM	117	CB	PHE	8	27.883	37.498	29.410	1.00	0.00	C
ATOM	118	HB2	PHE	8	28.095	38.396	29.989	1.00	0.00	H
ATOM	119	HB3	PHE	8	27.458	37.730	28.434	1.00	0.00	H
ATOM	120	CG	PHE	8	26.699	36.712	30.032	1.00	0.00	C
ATOM	121	CD1	PHE	8	25.710	37.378	30.694	1.00	0.00	C
ATOM	122	HD1	PHE	8	25.746	38.452	30.805	1.00	0.00	H
ATOM	123	CE1	PHE	8	24.594	36.679	31.189	1.00	0.00	C
ATOM	124	HE1	PHE	8	23.751	37.219	31.596	1.00	0.00	H
ATOM	125	CZ	PHE	8	24.481	35.277	31.045	1.00	0.00	C
ATOM	126	HZ	PHE	8	23.598	34.802	31.449	1.00	0.00	H
ATOM	127	CE2	PHE	8	25.526	34.612	30.441	1.00	0.00	C
ATOM	128	HE2	PHE	8	25.469	33.538	30.341	1.00	0.00	H
ATOM	129	CD2	PHE	8	26.660	35.322	30.005	1.00	0.00	C
ATOM	130	HD2	PHE	8	27.495	34.740	29.644	1.00	0.00	H
ATOM	131	C	PHE	8	30.239	37.676	28.364	1.00	0.00	C
ATOM	132	O	PHE	8	31.236	38.117	28.929	1.00	0.00	O
ATOM	133	N	GLN	9	29.936	38.032	27.092	1.00	0.00	N
ATOM	134	H	GLN	9	29.049	37.686	26.756	1.00	0.00	H
ATOM	135	CA	GLN	9	30.848	38.761	26.186	1.00	0.00	C
ATOM	136	HA	GLN	9	31.653	38.054	25.990	1.00	0.00	H
ATOM	137	CB	GLN	9	30.249	39.083	24.792	1.00	0.00	C
ATOM	138	HB2	GLN	9	29.388	39.744	24.886	1.00	0.00	H
ATOM	139	HB3	GLN	9	31.088	39.595	24.320	1.00	0.00	H
ATOM	140	CG	GLN	9	29.894	37.759	24.040	1.00	0.00	C
ATOM	141	HG2	GLN	9	30.048	37.925	22.974	1.00	0.00	H
ATOM	142	HG3	GLN	9	30.451	36.939	24.493	1.00	0.00	H
ATOM	143	CD	GLN	9	28.410	37.394	24.265	1.00	0.00	C
ATOM	144	OE1	GLN	9	27.776	37.869	25.167	1.00	0.00	O
ATOM	145	NE2	GLN	9	27.897	36.479	23.482	1.00	0.00	N
ATOM	146	HE21	GLN	9	26.982	36.110	23.706	1.00	0.00	H
ATOM	147	HE22	GLN	9	28.325	36.201	22.610	1.00	0.00	H
ATOM	148	C	GLN	9	31.471	39.998	26.792	1.00	0.00	C
ATOM	149	O	GLN	9	32.647	40.260	26.569	1.00	0.00	O
ATOM	150	N	ARG	10	30.715	40.686	27.599	1.00	0.00	N
ATOM	151	H	ARG	10	29.738	40.445	27.689	1.00	0.00	H
ATOM	152	CA	ARG	10	31.225	41.883	28.289	1.00	0.00	C
ATOM	153	HA	ARG	10	31.462	42.617	27.519	1.00	0.00	H
ATOM	154	CB	ARG	10	30.064	42.557	29.084	1.00	0.00	C
ATOM	155	HB2	ARG	10	29.140	42.654	28.515	1.00	0.00	H
ATOM	156	HB3	ARG	10	29.768	41.980	29.961	1.00	0.00	H
ATOM	157	CG	ARG	10	30.417	43.998	29.616	1.00	0.00	C
ATOM	158	HG2	ARG	10	31.249	43.991	30.320	1.00	0.00	H
ATOM	159	HG3	ARG	10	30.688	44.690	28.817	1.00	0.00	H
ATOM	160	CD	ARG	10	29.288	44.650	30.329	1.00	0.00	C
ATOM	161	HD2	ARG	10	28.979	44.012	31.157	1.00	0.00	H
ATOM	162	HD3	ARG	10	29.664	45.571	30.776	1.00	0.00	H

ATOM	163	NE	ARG	10	28.086	45.057	29.575	1.00	0.00	N
ATOM	164	HE	ARG	10	28.298	45.468	28.677	1.00	0.00	H
ATOM	165	CZ	ARG	10	26.855	45.005	29.850	1.00	0.00	C
ATOM	166	NH1	ARG	10	26.448	44.782	31.087	1.00	0.00	N
ATOM	167	HH11	ARG	10	25.453	44.688	31.235	1.00	0.00	H
ATOM	168	HH12	ARG	10	27.044	44.584	31.878	1.00	0.00	H
ATOM	169	NH2	ARG	10	25.857	45.307	29.033	1.00	0.00	N
ATOM	170	HH21	ARG	10	25.986	45.221	28.035	1.00	0.00	H
ATOM	171	HH22	ARG	10	24.921	45.215	29.402	1.00	0.00	H
ATOM	172	C	ARG	10	32.456	41.628	29.217	1.00	0.00	C
ATOM	173	O	ARG	10	33.123	42.611	29.528	1.00	0.00	O
ATOM	174	N	PHE	11	32.719	40.369	29.620	1.00	0.00	N
ATOM	175	H	PHE	11	31.968	39.699	29.534	1.00	0.00	H
ATOM	176	CA	PHE	11	33.691	40.046	30.619	1.00	0.00	C
ATOM	177	HA	PHE	11	34.159	40.969				
30.963	1.00	0.00		H						
ATOM	178	CB	PHE	11	32.992	39.516	31.910	1.00	0.00	C
ATOM	179	HB2	PHE	11	33.710	39.209	32.670	1.00	0.00	H
ATOM	180	HB3	PHE	11	32.504	38.550	31.779	1.00	0.00	H
ATOM	181	CG	PHE	11	31.832	40.361	32.489	1.00	0.00	C
ATOM	182	CD1	PHE	11	32.244	41.485	33.206	1.00	0.00	C
ATOM	183	HD1	PHE	11	33.307	41.667	33.249	1.00	0.00	H
ATOM	184	CE1	PHE	11	31.349	42.393	33.701	1.00	0.00	C
ATOM	185	HE1	PHE	11	31.730	43.299	34.149	1.00	0.00	H
ATOM	186	CZ	PHE	11	29.921	42.251	33.479	1.00	0.00	C
ATOM	187	HZ	PHE	11	29.230	42.945	33.936	1.00	0.00	H
ATOM	188	CE2	PHE	11	29.554	41.161	32.637	1.00	0.00	C
ATOM	189	HE2	PHE	11	28.501	40.957	32.513	1.00	0.00	H
ATOM	190	CD2	PHE	11	30.470	40.247	32.082	1.00	0.00	C
ATOM	191	HD2	PHE	11	30.115	39.354	31.590	1.00	0.00	H
ATOM	192	C	PHE	11	34.850	39.286	30.122	1.00	0.00	C
ATOM	193	O	PHE	11	35.779	39.000	30.789	1.00	0.00	O
ATOM	194	N	GLN	12	34.789	38.824	28.885	1.00	0.00	N
ATOM	195	H	GLN	12	34.046	39.155	28.287	1.00	0.00	H
ATOM	196	CA	GLN	12	35.701	37.716	28.387	1.00	0.00	C
ATOM	197	HA	GLN	12	35.725	36.943	29.155	1.00	0.00	H
ATOM	198	CB	GLN	12	35.260	37.110	27.101	1.00	0.00	C
ATOM	199	HB2	GLN	12	35.204	37.912	26.365	1.00	0.00	H
ATOM	200	HB3	GLN	12	35.954	36.313	26.831	1.00	0.00	H
ATOM	201	CG	GLN	12	33.825	36.569	27.043	1.00	0.00	C
ATOM	202	HG2	GLN	12	33.787	35.675	27.665	1.00	0.00	H
ATOM	203	HG3	GLN	12	33.112	37.291	27.440	1.00	0.00	H
ATOM	204	CD	GLN	12	33.485	36.075	25.627	1.00	0.00	C
ATOM	205	OE1	GLN	12	34.093	36.412	24.613	1.00	0.00	O
ATOM	206	NE2	GLN	12	32.526	35.216	25.436	1.00	0.00	N
ATOM	207	HE21	GLN	12	32.165	34.864	26.311	1.00	0.00	H
ATOM	208	HE22	GLN	12	32.287	34.812	24.542	1.00	0.00	H
ATOM	209	C	GLN	12	37.136	38.248	28.208	1.00	0.00	C
ATOM	210	O	GLN	12	38.152	37.680	28.546	1.00	0.00	O
ATOM	211	N	ASP	13	37.169	39.474	27.667	1.00	0.00	N
ATOM	212	H	ASP	13	36.324	39.995	27.487	1.00	0.00	H
ATOM	213	CA	ASP	13	38.424	40.287	27.510	1.00	0.00	C
ATOM	214	HA	ASP	13	39.130	39.607	27.030	1.00	0.00	H
ATOM	215	CB	ASP	13	38.195	41.474	26.543	1.00	0.00	C
ATOM	216	HB2	ASP	13	37.500	42.199	26.966	1.00	0.00	H
ATOM	217	HB3	ASP	13	39.174	41.897	26.321	1.00	0.00	H
ATOM	218	CG	ASP	13	37.556	41.108	25.218	1.00	0.00	C
ATOM	219	OD1	ASP	13	36.853	42.002	24.672	1.00	0.00	O
ATOM	220	OD2	ASP	13	37.534	39.927	24.743	1.00	0.00	O
ATOM	221	C	ASP	13	39.030	40.681	28.798	1.00	0.00	C
ATOM	222	O	ASP	13	40.222	40.874	28.915	1.00	0.00	O
ATOM	223	N	ASP	14	38.225	40.820	29.853	1.00	0.00	N
ATOM	224	H	ASP	14	37.240	40.652	29.705	1.00	0.00	H
ATOM	225	CA	ASP	14	38.724	41.143	31.206	1.00	0.00	C

ATOM	226	HA	ASP	14	39.376	42.006	31.074	1.00	0.00	H
ATOM	227	CB	ASP	14	37.604	41.654	32.217	1.00	0.00	C
ATOM	228	HB2	ASP	14	36.803	40.922	32.318	1.00	0.00	H
ATOM	229	HB3	ASP	14	38.102	41.734	33.183	1.00	0.00	H
ATOM	230	CG	ASP	14	36.917	43.063	31.912	1.00	0.00	C
ATOM	231	OD1	ASP	14	36.000	43.424	32.671	1.00	0.00	O
ATOM	232	OD2	ASP	14	37.381	43.708	30.932	1.00	0.00	O
ATOM	233	C	ASP	14	39.536	40.055	31.850	1.00	0.00	C
ATOM	234	O	ASP	14	40.538	40.372	32.495	1.00	0.00	O
ATOM	235	N	ILE	15	39.343	38.806	31.370	1.00	0.00	N
ATOM	236	H	ILE	15	38.677	38.700	30.618	1.00	0.00	H
ATOM	237	CA	ILE	15	40.043	37.663	31.773	1.00	0.00	C
ATOM	238	HA	ILE	15	40.178	37.711	32.854	1.00	0.00	H
ATOM	239	CB	ILE	15	39.082	36.412	31.661	1.00	0.00	C
ATOM	240	HB	ILE	15	38.512	36.406	30.732	1.00	0.00	H
ATOM	241	CG2	ILE	15	39.901	35.128	31.908	1.00	0.00	C
ATOM	242	HG21	ILE	15	39.182	34.320	32.045	1.00	0.00	H
ATOM	243	HG22	ILE	15	40.471	34.919	31.002	1.00	0.00	H
ATOM	244	HG23	ILE	15	40.592	35.227	32.744	1.00	0.00	H
ATOM	245	CG1	ILE	15	37.911	36.452	32.738	1.00	0.00	C
ATOM	246	HG12	ILE	15	38.411	36.411	33.706	1.00	0.00	H
ATOM	247	HG13	ILE	15	37.478	37.444	32.611	1.00	0.00	H
ATOM	248	CD1	ILE	15	36.832	35.440	32.498	1.00	0.00	C
ATOM	249	HD11	ILE	15	35.837	35.866	32.370	1.00	0.00	H
ATOM	250	HD12	ILE	15	36.893	34.762	31.647	1.00	0.00	H
ATOM	251	HD13	ILE	15	36.811	34.826	33.399	1.00	0.00	H
ATOM	252	C	ILE	15	41.397	37.418	31.008	1.00	0.00	C
ATOM	253	O	ILE	15	42.373	37.248	31.665	1.00	0.00	O
ATOM	254	N	LEU	16	41.446	37.508	29.694	1.00	0.00	N
ATOM	255	H	LEU	16	40.606	37.441	29.138	1.00	0.00	H
ATOM	256	CA	LEU	16	42.760	37.487	28.883	1.00	0.00	C
ATOM	257	HA	LEU	16	43.270	36.602	29.264	1.00	0.00	H
ATOM	258	CB	LEU	16	42.319	37.458	27.358	1.00	0.00	C
ATOM	259	HB2	LEU	16	41.574	36.666	27.299	1.00	0.00	H
ATOM	260	HB3	LEU	16	41.884	38.435	27.147	1.00	0.00	H
ATOM	261	CG	LEU	16	43.603	37.329	26.463	1.00	0.00	C
ATOM	262	HG	LEU	16	44.524	37.092	26.996	1.00	0.00	H
ATOM	263	CD1	LEU	16	43.386	36.169	25.490	1.00	0.00	C
ATOM	264	HD11	LEU	16	44.304	35.881	24.977	1.00	0.00	H
ATOM	265	HD12	LEU	16	42.891	35.321	25.964	1.00	0.00	H
ATOM	266	HD13	LEU	16	42.640	36.480	24.758	1.00	0.00	H
ATOM	267	CD2	LEU	16	43.680	38.660	25.577	1.00	0.00	C
ATOM	268	HD21	LEU	16	42.813	38.723	24.918	1.00	0.00	H
ATOM	269	HD22	LEU	16	43.698	39.488	26.285	1.00	0.00	H
ATOM	270	HD23	LEU	16	44.592	38.628	24.981	1.00	0.00	H
ATOM	271	C	LEU	16	43.647	38.718	29.260	1.00	0.00	C
ATOM	272	O	LEU	16	44.879	38.609	29.222	1.00	0.00	O
ATOM	273	N	ALA	17	43.033	39.889	29.604	1.00	0.00	N
ATOM	274	H	ALA	17	42.027	39.861	29.514	1.00	0.00	H
ATOM	275	CA	ALA	17	43.643	41.021	30.269	1.00	0.00	C
ATOM	276	HA	ALA	17	44.604	41.206	29.791	1.00	0.00	H
ATOM	277	CB	ALA	17	42.879	42.240	29.986	1.00	0.00	C
ATOM	278	HB1	ALA	17	42.528	42.348	28.959	1.00	0.00	H
ATOM	279	HB2	ALA	17	42.022	42.333	30.653	1.00	0.00	H
ATOM	280	HB3	ALA	17	43.443	43.115	30.313	1.00	0.00	H
ATOM	281	C	ALA	17	43.927	40.872	31.777	1.00	0.00	C
ATOM	282	O	ALA	17	44.591	41.703	32.356	1.00	0.00	O
ATOM	283	N	GLY	18	43.512	39.762	32.345	1.00	0.00	N
ATOM	284	H	GLY	18	43.137	39.024	31.766	1.00	0.00	H
ATOM	285	CA	GLY	18	43.638	39.485	33.754	1.00	0.00	C
ATOM	286	HA2	GLY	18	43.290	38.453	33.703	1.00	0.00	H
ATOM	287	HA3	GLY	18	44.693	39.488	34.029	1.00	0.00	H
ATOM	288	C	GLY	18	42.914	40.411	34.797	1.00	0.00	C
ATOM	289	O	GLY	18	43.014	40.046	35.971	1.00	0.00	O

ATOM	290	N	ARG	19	42.233	41.504	34.397	1.00	0.00	N
ATOM	291	H	ARG	19	42.025	41.636	33.418	1.00	0.00	H
ATOM	292	CA	ARG	19	41.493	42.346	35.399	1.00	0.00	C
ATOM	293	HA	ARG	19	42.193	42.509	36.218	1.00	0.00	H
ATOM	294	CB	ARG	19	41.075	43.752	34.888	1.00	0.00	C
ATOM	295	HB2	ARG	19	40.350	43.619	34.085	1.00	0.00	H
ATOM	296	HB3	ARG	19	40.656	44.328	35.714	1.00	0.00	H
ATOM	297	CG	ARG	19	42.258	44.580	34.379	1.00	0.00	C
ATOM	298	HG2	ARG	19	42.994	44.606	35.182	1.00	0.00	H
ATOM	299	HG3	ARG	19	42.631	44.018	33.523	1.00	0.00	H
ATOM	300	CD	ARG	19	41.778	45.957	34.087	1.00	0.00	C
ATOM	301	HD2	ARG	19	41.124	45.962	33.215	1.00	0.00	H
ATOM	302	HD3	ARG	19	41.125	46.242	34.912	1.00	0.00	
H										
ATOM	303	NE	ARG	19	42.834	46.875	33.956	1.00	0.00	N
ATOM	304	HE	ARG	19	43.665	46.461	33.558	1.00	0.00	H
ATOM	305	CZ	ARG	19	42.748	48.148	34.100	1.00	0.00	C
ATOM	306	NH1	ARG	19	41.713	48.828	34.553	1.00	0.00	N
ATOM	307	HH11	ARG	19	40.771	48.467	34.585	1.00	0.00	H
ATOM	308	HH12	ARG	19	41.824	49.821	34.705	1.00	0.00	H
ATOM	309	NH2	ARG	19	43.796	48.873	33.869	1.00	0.00	N
ATOM	310	HH21	ARG	19	43.594	49.861	33.817	1.00	0.00	H
ATOM	311	HH22	ARG	19	44.601	48.569	33.339	1.00	0.00	H
ATOM	312	C	ARG	19	40.325	41.679	36.040	1.00	0.00	C
ATOM	313	O	ARG	19	39.994	42.183	37.104	1.00	0.00	O
ATOM	335	N	THR	21	39.197	38.145	37.856	1.00	0.00	N
ATOM	336	H	THR	21	38.798	38.919	38.368	1.00	0.00	H
ATOM	337	CA	THR	21	39.729	36.979	38.609	1.00	0.00	C
ATOM	338	HA	THR	21	39.643	36.138	37.921	1.00	0.00	H
ATOM	339	CB	THR	21	41.264	37.147	38.744	1.00	0.00	C
ATOM	340	HB	THR	21	41.716	37.193	37.754	1.00	0.00	H
ATOM	341	CG2	THR	21	41.686	38.296	39.629	1.00	0.00	C
ATOM	342	HG21	THR	21	41.418	38.254	40.686	1.00	0.00	H
ATOM	343	HG22	THR	21	42.771	38.373	39.553	1.00	0.00	H
ATOM	344	HG23	THR	21	41.199	39.214	39.301	1.00	0.00	H
ATOM	345	OG1	THR	21	41.895	36.036	39.349	1.00	0.00	O
ATOM	346	HG1	THR	21	41.639	35.179	39.001	1.00	0.00	H
ATOM	347	C	THR	21	39.008	36.495	39.899	1.00	0.00	C
ATOM	348	O	THR	21	39.362	35.595	40.589	1.00	0.00	O
ATOM	349	N	ILE	22	38.028	37.237	40.367	1.00	0.00	N
ATOM	350	H	ILE	22	37.787	38.065	39.841	1.00	0.00	H
ATOM	351	CA	ILE	22	37.168	36.979	41.533	1.00	0.00	C
ATOM	352	HA	ILE	22	37.077	35.894	41.587	1.00	0.00	H
ATOM	353	CB	ILE	22	37.789	37.509	42.849	1.00	0.00	C
ATOM	354	HB	ILE	22	38.781	37.057	42.862	1.00	0.00	H
ATOM	355	CG2	ILE	22	37.936	38.992	42.869	1.00	0.00	C
ATOM	356	HG21	ILE	22	36.932	39.403	42.978	1.00	0.00	H
ATOM	357	HG22	ILE	22	38.471	39.336	43.754	1.00	0.00	H
ATOM	358	HG23	ILE	22	38.479	39.261	41.963	1.00	0.00	H
ATOM	359	CG1	ILE	22	37.034	36.865	44.027	1.00	0.00	C
ATOM	360	HG12	ILE	22	36.013	37.245	44.032	1.00	0.00	H
ATOM	361	HG13	ILE	22	37.064	35.779	43.940	1.00	0.00	H
ATOM	362	CD1	ILE	22	37.723	37.123	45.401	1.00	0.00	C
ATOM	363	HD11	ILE	22	37.254	36.506	46.169	1.00	0.00	H
ATOM	364	HD12	ILE	22	38.767	36.816	45.339	1.00	0.00	H
ATOM	365	HD13	ILE	22	37.627	38.172	45.680	1.00	0.00	H
ATOM	366	C	ILE	22	35.756	37.535	41.223	1.00	0.00	C
ATOM	367	O	ILE	22	35.586	38.385	40.354	1.00	0.00	O
ATOM	368	N	THR	23	34.703	36.986	41.844	1.00	0.00	N
ATOM	369	H	THR	23	34.927	36.224	42.468	1.00	0.00	H
ATOM	370	CA	THR	23	33.318	37.442	41.683	1.00	0.00	C
ATOM	371	HA	THR	23	33.272	38.520	41.840	1.00	0.00	H
ATOM	372	CB	THR	23	32.695	37.085	40.327	1.00	0.00	C
ATOM	373	HB	THR	23	33.401	37.342	39.537	1.00	0.00	H

ATOM	374	CG2	THR	23	32.238	35.647	40.215	1.00	0.00	C
ATOM	375	HG21	THR	23	31.849	35.435	39.218	1.00	0.00	H
ATOM	376	HG22	THR	23	33.083	34.974	40.359	1.00	0.00	H
ATOM	377	HG23	THR	23	31.440	35.441	40.929	1.00	0.00	H
ATOM	378	OG1	THR	23	31.667	37.949	40.066	1.00	0.00	O
ATOM	379	HG1	THR	23	31.998	38.723	39.604	1.00	0.00	H
ATOM	380	C	THR	23	32.436	36.983	42.833	1.00	0.00	C
ATOM	381	O	THR	23	32.768	35.967	43.535	1.00	0.00	O
ATOM	382	N	ILE	24	31.362	37.660	43.134	1.00	0.00	N
ATOM	383	H	ILE	24	31.041	38.338	42.460	1.00	0.00	H
ATOM	384	CA	ILE	24	30.520	37.496	44.333	1.00	0.00	C
ATOM	385	HA	ILE	24	30.825	36.655	44.957	1.00	0.00	H
ATOM	386	CB	ILE	24	30.565	38.720	45.262	1.00	0.00	C
ATOM	387	HB	ILE	24	30.506	39.599	44.621	1.00	0.00	H
ATOM	388	CG2	ILE	24	29.428	38.658	46.379	1.00	0.00	C
ATOM	389	HG21	ILE	24	29.673	37.912	47.135	1.00	0.00	H
ATOM	390	HG22	ILE	24	29.495	39.633	46.861	1.00	0.00	H
ATOM	391	HG23	ILE	24	28.432	38.668	45.935	1.00	0.00	H
ATOM	392	CG1	ILE	24	31.905	38.757	46.068	1.00	0.00	C
ATOM	393	HG12	ILE	24	32.135	37.926	46.735	1.00	0.00	H
ATOM	394	HG13	ILE	24	32.698	38.958	45.347	1.00	0.00	H
ATOM	395	CD1	ILE	24	32.032	39.867	47.071	1.00	0.00	C
ATOM	396	HD11	ILE	24	31.433	39.664	47.958	1.00	0.00	H
ATOM	397	HD12	ILE	24	33.049	39.858	47.466	1.00	0.00	H
ATOM	398	HD13	ILE	24	31.827	40.848	46.642	1.00	0.00	H
ATOM	399	C	ILE	24	29.048	37.167	43.868	1.00	0.00	C
ATOM	400	O	ILE	24	28.509	37.846	42.960	1.00	0.00	O
ATOM	401	N	ARG	25	28.419	36.263	44.633	1.00	0.00	N
ATOM	402	H	ARG	25	28.859	35.956	45.489	1.00	0.00	H
ATOM	403	CA	ARG	25	27.030	35.775	44.287	1.00	0.00	C
ATOM	404	HA	ARG	25	26.519	36.552	43.718	1.00	0.00	H
ATOM	405	CB	ARG	25	27.111	34.444	43.506	1.00	0.00	C
ATOM	406	HB2	ARG	25	27.652	33.793	44.193	1.00	0.00	H
ATOM	407	HB3	ARG	25	26.083	34.107	43.375	1.00	0.00	H
ATOM	408	CG	ARG	25	27.728	34.569	42.152	1.00	0.00	C
ATOM	409	HG2	ARG	25	28.639	35.158	42.253	1.00	0.00	H
ATOM	410	HG3	ARG	25	27.941	33.554	41.816	1.00	0.00	H
ATOM	411	CD	ARG	25	26.761	35.187	41.068	1.00	0.00	C
ATOM	412	HD2	ARG	25	25.947	34.470	40.957	1.00	0.00	H
ATOM	413	HD3	ARG	25	26.604	36.220	41.378	1.00	0.00	H
ATOM	414	NE	ARG	25	27.406	35.374	39.814	1.00	0.00	N
ATOM	415	HE	ARG	25	27.039	34.770	39.092	1.00	0.00	H
ATOM	416	CZ	ARG	25	28.271	36.314	39.430	1.00	0.00	C
ATOM	417	NH1	ARG	25	28.797	37.206	40.247	1.00	0.00	N
ATOM	418	HH11	ARG	25	29.391	37.956	39.924	1.00	0.00	H
ATOM	419	HH12	ARG	25	28.642	37.011	41.226	1.00	0.00	H
ATOM	420	NH2	ARG	25	28.491	36.550	38.159	1.00	0.00	N
ATOM	421	HH21	ARG	25	29.191	37.266	38.025	1.00	0.00	H
ATOM	422	HH22	ARG	25	28.294	35.813	37.498	1.00	0.00	H
ATOM	423	C	ARG	25	26.232	35.487	45.579	1.00	0.00	C
ATOM	424	O	ARG	25	26.930	35.214	46.539	1.00	0.00	O
ATOM	425	N	ASP	26	24.899	35.491	45.633	1.00	0.00	N
ATOM	426	H	ASP	26	24.375	35.647	44.784	1.00	0.00	H
ATOM	427	CA	ASP	26	24.210	35.063	46.882	1.00	0.00	C
ATOM	428	HA	ASP	26	24.712	35.456	47.766	1.00	0.00	H
ATOM	429	CB	ASP	26	22.797	35.556	47.066	1.00	0.00	C
ATOM	430	HB2	ASP	26	22.275	35.226	47.965	1.00	0.00	H
ATOM	431	HB3	ASP	26	22.846	36.644	47.029	1.00	0.00	H
ATOM	432	CG	ASP	26	21.951	35.123	45.881	1.00	0.00	C
ATOM	433	OD1	ASP	26	22.156	35.720	44.807	1.00	0.00	O
ATOM	434	OD2	ASP	26	20.976	34.338	46.073	1.00	0.00	O
ATOM	435	C	ASP	26	24.256	33.553	46.997	1.00	0.00	C
ATOM	436	O	ASP	26	24.226	32.878	45.987	1.00	0.00	O
ATOM	437	N	GLU	27	24.228	33.081	48.259	1.00	0.00	N

```
ATOM    438  H   GLU    27      24.327  33.767  48.992  1.00  0.00           H
ATOM    439  CA  GLU    27      24.461  31.697  48.753  1.00  0.00           C
ATOM    440  HA  GLU    27      25.409  31.416  48.295  1.00  0.00           H
ATOM    441  CB  GLU    27      24.544  31.667  50.271  1.00  0.00           C
ATOM    442  HB2 GLU    27      25.299  32.333  50.688  1.00  0.00           H
ATOM    443  HB3 GLU    27      23.628  32.062  50.709  1.00  0.00           H
ATOM    444  CG  GLU    27      24.748  30.273  50.763  1.00  0.00           C
ATOM    445  HG2 GLU    27      24.727  30.190  51.849  1.00  0.00           H
ATOM    446  HG3 GLU    27      24.021  29.548  50.398  1.00  0.00           H
ATOM    447  CD  GLU    27      26.104  29.680  50.364  1.00  0.00           C
ATOM    448  OE1 GLU    27      26.125  29.143  49.211  1.00  0.00           O
ATOM    449  OE2 GLU
    27      26.994  29.677  51.152  1.00  0.00           O
ATOM    450  C   GLU    27      23.436  30.793  48.086  1.00  0.00           C
ATOM    451  O   GLU    27      22.244  31.004  48.199  1.00  0.00           O
ATOM    452  N   SER    28      23.947  29.775  47.371  1.00  0.00           N
ATOM    453  H   SER    28      24.956  29.812  47.362  1.00  0.00           H
ATOM    454  CA  SER    28      23.277  28.716  46.666  1.00  0.00           C
ATOM    455  HA  SER    28      22.363  28.370  47.150  1.00  0.00           H
ATOM    456  CB  SER    28      22.874  29.184  45.262  1.00  0.00           C
ATOM    457  HB2 SER    28      22.220  30.054  45.327  1.00  0.00           H
ATOM    458  HB3 SER    28      23.786  29.469  44.739  1.00  0.00           H
ATOM    459  OG  SER    28      22.130  28.178  44.680  1.00  0.00           O
ATOM    460  HG  SER    28      21.210  28.451  44.701  1.00  0.00           H
ATOM    461  C   SER    28      24.175  27.471  46.420  1.00  0.00           C
ATOM    462  O   SER    28      25.293  27.671  45.956  1.00  0.00           O
ATOM    463  N   GLU    29      23.586  26.283  46.456  1.00  0.00           N
ATOM    464  H   GLU    29      22.591  26.303  46.624  1.00  0.00           H
ATOM    465  CA  GLU    29      24.304  25.075  46.181  1.00  0.00           C
ATOM    466  HA  GLU    29      25.300  25.230  46.600  1.00  0.00           H
ATOM    467  CB  GLU    29      23.653  23.964  46.919  1.00  0.00           C
ATOM    468  HB2 GLU    29      23.679  24.191  47.985  1.00  0.00           H
ATOM    469  HB3 GLU    29      22.635  23.956  46.532  1.00  0.00           H
ATOM    470  CG  GLU    29      24.253  22.476  46.827  1.00  0.00           C
ATOM    471  HG2 GLU    29      23.691  21.916  47.573  1.00  0.00           H
ATOM    472  HG3 GLU    29      23.974  22.061  45.859  1.00  0.00           H
ATOM    473  CD  GLU    29      25.775  22.349  47.092  1.00  0.00           C
ATOM    474  OE1 GLU    29      26.341  23.306  47.661  1.00  0.00           O
ATOM    475  OE2 GLU    29      26.319  21.254  46.866  1.00  0.00           O
ATOM    476  C   GLU    29      24.574  24.766  44.692  1.00  0.00           C
ATOM    477  O   GLU    29      25.389  23.955  44.310  1.00  0.00           O
ATOM    478  N   SER    30      23.799  25.460  43.877  1.00  0.00           N
ATOM    479  H   SER    30      23.170  26.163  44.239  1.00  0.00           H
ATOM    480  CA  SER    30      23.965  25.418  42.473  1.00  0.00           C
ATOM    481  HA  SER    30      23.898  24.390  42.115  1.00  0.00           H
ATOM    482  CB  SER    30      22.827  26.129  41.718  1.00  0.00           C
ATOM    483  HB2 SER    30      23.021  26.124  40.646  1.00  0.00           H
ATOM    484  HB3 SER    30      21.878  25.643  41.942  1.00  0.00           H
ATOM    485  OG  SER    30      22.803  27.552  42.032  1.00  0.00           O
ATOM    486  HG  SER    30      22.278  27.686  42.825  1.00  0.00           H
ATOM    487  C   SER    30      25.230  26.045  41.969  1.00  0.00           C
ATOM    488  O   SER    30      25.674  25.845  40.860  1.00  0.00           O
ATOM    489  N   HIE    31      25.953  26.831  42.746  1.00  0.00           N
ATOM    490  H   HIE    31      25.612  27.088  43.662  1.00  0.00           H
ATOM    491  CA  HIE    31      27.199  27.438  42.362  1.00  0.00           C
ATOM    492  HA  HIE    31      27.089  28.040  41.461  1.00  0.00           H
ATOM    493  CB  HIE    31      27.675  28.450  43.358  1.00  0.00           C
ATOM    494  HB2 HIE    31      27.704  27.964  44.333  1.00  0.00           H
ATOM    495  HB3 HIE    31      28.702  28.671  43.065  1.00  0.00           H
ATOM    496  CG  HIE    31      26.802  29.659  43.363  1.00  0.00           C
ATOM    497  ND1 HIE    31      26.219  30.330  42.345  1.00  0.00           N
ATOM    498  CE1 HIE    31      25.580  31.380  42.930  1.00  0.00           C
ATOM    499  HE1 HIE    31      25.129  32.150  42.321  1.00  0.00           H
ATOM    500  NE2 HIE    31      25.817  31.468  44.224  1.00  0.00           N
```

ATOM	501	HE2	HIE	31	25.513	32.226	44.818	1.00	0.00	H
ATOM	502	CD2	HIE	31	26.674	30.447	44.456	1.00	0.00	C
ATOM	503	HD2	HIE	31	27.178	30.301	45.400	1.00	0.00	H
ATOM	504	C	HIE	31	28.283	26.436	42.160	1.00	0.00	C
ATOM	505	O	HIE	31	28.423	25.465	42.924	1.00	0.00	O
ATOM	506	N	PHE	32	29.197	26.751	41.251	1.00	0.00	N
ATOM	507	H	PHE	32	29.048	27.624	40.767	1.00	0.00	H
ATOM	508	CA	PHE	32	30.554	26.164	41.037	1.00	0.00	C
ATOM	509	HA	PHE	32	30.399	25.299	40.393	1.00	0.00	H
ATOM	510	CB	PHE	32	31.452	27.037	40.183	1.00	0.00	C
ATOM	511	HB2	PHE	32	31.640	27.982	40.689	1.00	0.00	H
ATOM	512	HB3	PHE	32	32.420	26.557	40.030	1.00	0.00	H
ATOM	513	CG	PHE	32	30.997	27.419	38.833	1.00	0.00	C
ATOM	514	CD1	PHE	32	31.144	28.728	38.327	1.00	0.00	C
ATOM	515	HD1	PHE	32	31.520	29.476	39.010	1.00	0.00	H
ATOM	516	CE1	PHE	32	30.792	29.015	37.019	1.00	0.00	C
ATOM	517	HE1	PHE	32	30.906	29.978	36.543	1.00	0.00	H
ATOM	518	CZ	PHE	32	30.295	27.981	36.201	1.00	0.00	C
ATOM	519	HZ	PHE	32	30.167	28.254	35.164	1.00	0.00	H
ATOM	520	CE2	PHE	32	30.083	26.643	36.654	1.00	0.00	C
ATOM	521	HE2	PHE	32	29.775	25.882	35.952	1.00	0.00	H
ATOM	522	CD2	PHE	32	30.490	26.363	37.982	1.00	0.00	C
ATOM	523	HD2	PHE	32	30.249	25.362	38.309	1.00	0.00	H
ATOM	524	C	PHE	32	31.423	26.013	42.302	1.00	0.00	C
ATOM	525	O	PHE	32	31.132	26.783	43.279	1.00	0.00	O
ATOM	526	N	LYS	33	32.384	25.084	42.336	1.00	0.00	N
ATOM	527	H	LYS	33	32.635	24.588	41.493	1.00	0.00	H
ATOM	528	CA	LYS	33	33.260	24.734	43.495	1.00	0.00	C
ATOM	529	HA	LYS	33	33.083	25.387	44.348	1.00	0.00	H
ATOM	530	CB	LYS	33	32.974	23.252	43.883	1.00	0.00	C
ATOM	531	HB2	LYS	33	33.469	22.625	43.140	1.00	0.00	H
ATOM	532	HB3	LYS	33	33.499	23.108	44.826	1.00	0.00	H
ATOM	533	CG	LYS	33	31.546	22.779	43.931	1.00	0.00	C
ATOM	534	HG2	LYS	33	30.983	22.810	42.998	1.00	0.00	H
ATOM	535	HG3	LYS	33	31.559	21.785	44.376	1.00	0.00	H
ATOM	536	CD	LYS	33	30.752	23.529	44.950	1.00	0.00	C
ATOM	537	HD2	LYS	33	31.371	23.543	45.847	1.00	0.00	H
ATOM	538	HD3	LYS	33	30.581	24.470	44.426	1.00	0.00	H
ATOM	539	CE	LYS	33	29.517	22.815	45.377	1.00	0.00	C
ATOM	540	HE2	LYS	33	29.021	22.422	44.490	1.00	0.00	H
ATOM	541	HE3	LYS	33	29.795	21.929	45.949	1.00	0.00	H
ATOM	542	NZ	LYS	33	28.572	23.662	45.987	1.00	0.00	N
ATOM	543	HZ1	LYS	33	28.904	24.148	46.807	1.00	0.00	H
ATOM	544	HZ2	LYS	33	28.171	24.294	45.309	1.00	0.00	H
ATOM	545	HZ3	LYS	33	27.740	23.186	46.307	1.00	0.00	H
ATOM	546	C	LYS	33	34.756	24.877	43.172	1.00	0.00	C
ATOM	547	O	LYS	33	35.068	24.983	41.970	1.00	0.00	O
ATOM	548	N	THR	34	35.672	24.806	44.135	1.00	0.00	N
ATOM	549	H	THR	34	35.429	24.635	45.101	1.00	0.00	H
ATOM	550	CA	THR	34	37.107	24.895	43.843	1.00	0.00	C
ATOM	551	HA	THR	34	37.397	25.798	43.304	1.00	0.00	H
ATOM	552	CB	THR	34	37.954	24.973	45.153	1.00	0.00	C
ATOM	553	HB	THR	34	37.929	23.995	45.636	1.00	0.00	H
ATOM	554	CG2	THR	34	39.458	25.291	44.847	1.00	0.00	C
ATOM	555	HG21	THR	34	40.052	25.276	45.761	1.00	0.00	H
ATOM	556	HG22	THR	34	39.763	24.599	44.062	1.00	0.00	H
ATOM	557	HG23	THR	34	39.383	26.274	44.381	1.00	0.00	H
ATOM	558	OG1	THR	34	37.312	25.898	45.951	1.00	0.00	O
ATOM	559	HG1	THR	34	37.776	25.861	46.791	1.00	0.00	H
ATOM	560	C	THR	34	37.527	23.624	43.165	1.00	0.00	C
ATOM	561	O	THR	34	37.302	22.520	43.746	1.00	0.00	O
ATOM	562	N	GLY	35	38.299	23.732	42.084	1.00	0.00	N
ATOM	563	H	GLY	35	38.748	24.595	41.813	1.00	0.00	H
ATOM	564	CA	GLY	35	38.591	22.566	41.261	1.00	0.00	C

ATOM	565	HA2	GLY	35	39.654	22.497	41.026	1.00	0.00	H
ATOM	566	HA3	GLY	35	38.363	21.686	41.862	1.00	0.00	H
ATOM	567	C	GLY	35	37.882	22.580	39.907	1.00	0.00	C
ATOM	568	O	GLY	35	38.372	21.957	38.950	1.00	0.00	O
ATOM	569	N	ASP	36	36.754	23.312	39.757	1.00	0.00	N
ATOM	570	H	ASP	36	36.438	23.864	40.542	1.00	0.00	H
ATOM	571	CA	ASP	36	36.066	23.499	38.472	1.00	0.00	C
ATOM	572	HA	ASP	36	35.656	22.521	38.223	1.00	0.00	H
ATOM	573	CB	ASP	36	34.834	24.349	38.660	1.00	0.00	C
ATOM	574	HB2	ASP	36	34.851	25.151				
39.398	1.00	0.00			H					
ATOM	575	HB3	ASP	36	34.689	24.917	37.741	1.00	0.00	H
ATOM	576	CG	ASP	36	33.540	23.526	38.899	1.00	0.00	C
ATOM	577	OD1	ASP	36	32.811	23.573	39.935	1.00	0.00	O
ATOM	578	OD2	ASP	36	33.176	22.830	37.933	1.00	0.00	O
ATOM	579	C	ASP	36	36.902	24.051	37.271	1.00	0.00	C
ATOM	580	O	ASP	36	37.851	24.842	37.475	1.00	0.00	O
ATOM	581	N	VAL	37	36.474	23.764	36.036	1.00	0.00	N
ATOM	582	H	VAL	37	35.638	23.205	35.932	1.00	0.00	H
ATOM	583	CA	VAL	37	37.124	24.341	34.791	1.00	0.00	C
ATOM	584	HA	VAL	37	37.821	25.081	35.186	1.00	0.00	H
ATOM	585	CB	VAL	37	37.978	23.307	33.938	1.00	0.00	C
ATOM	586	HB	VAL	37	37.257	22.495	33.836	1.00	0.00	H
ATOM	587	CG1	VAL	37	38.634	23.946	32.678	1.00	0.00	C
ATOM	588	HG11	VAL	37	39.061	23.165	32.048	1.00	0.00	H
ATOM	589	HG12	VAL	37	37.830	24.477	32.169	1.00	0.00	H
ATOM	590	HG13	VAL	37	39.467	24.583	32.973	1.00	0.00	H
ATOM	591	CG2	VAL	37	39.193	22.863	34.723	1.00	0.00	C
ATOM	592	HG21	VAL	37	39.861	23.688	34.972	1.00	0.00	H
ATOM	593	HG22	VAL	37	38.843	22.616	35.724	1.00	0.00	H
ATOM	594	HG23	VAL	37	39.691	21.990	34.300	1.00	0.00	H
ATOM	595	C	VAL	37	36.050	25.013	33.815	1.00	0.00	C
ATOM	596	O	VAL	37	34.920	24.444	33.605	1.00	0.00	O
ATOM	597	N	LEU	38	36.385	26.219	33.220	1.00	0.00	N
ATOM	598	H	LEU	38	37.302	26.599	33.408	1.00	0.00	H
ATOM	599	CA	LEU	38	35.536	27.036	32.370	1.00	0.00	C
ATOM	600	HA	LEU	38	34.754	26.395	31.963	1.00	0.00	H
ATOM	601	CB	LEU	38	34.983	28.304	33.082	1.00	0.00	C
ATOM	602	HB2	LEU	38	35.887	28.855	33.343	1.00	0.00	H
ATOM	603	HB3	LEU	38	34.296	28.737	32.354	1.00	0.00	H
ATOM	604	CG	LEU	38	34.134	28.063	34.373	1.00	0.00	C
ATOM	605	HG	LEU	38	33.506	27.186	34.216	1.00	0.00	H
ATOM	606	CD1	LEU	38	34.963	27.872	35.613	1.00	0.00	C
ATOM	607	HD11	LEU	38	35.325	26.845	35.572	1.00	0.00	H
ATOM	608	HD12	LEU	38	35.761	28.567	35.878	1.00	0.00	H
ATOM	609	HD13	LEU	38	34.307	27.969	36.479	1.00	0.00	H
ATOM	610	CD2	LEU	38	33.208	29.348	34.590	1.00	0.00	C
ATOM	611	HD21	LEU	38	32.544	29.485	33.736	1.00	0.00	H
ATOM	612	HD22	LEU	38	32.743	29.258	35.572	1.00	0.00	H
ATOM	613	HD23	LEU	38	33.925	30.169	34.607	1.00	0.00	H
ATOM	614	C	LEU	38	36.305	27.500	31.108	1.00	0.00	C
ATOM	615	O	LEU	38	37.568	27.615	31.204	1.00	0.00	O
ATOM	616	N	ARG	39	35.540	27.929	30.090	1.00	0.00	N
ATOM	617	H	ARG	39	34.541	27.791	30.025	1.00	0.00	H
ATOM	618	CA	ARG	39	36.130	28.433	28.880	1.00	0.00	C
ATOM	619	HA	ARG	39	37.176	28.648	29.100	1.00	0.00	H
ATOM	620	CB	ARG	39	36.209	27.366	27.745	1.00	0.00	C
ATOM	621	HB2	ARG	39	35.197	26.963	27.704	1.00	0.00	H
ATOM	622	HB3	ARG	39	36.529	27.805	26.801	1.00	0.00	H
ATOM	623	CG	ARG	39	37.188	26.292	28.128	1.00	0.00	C
ATOM	624	HG2	ARG	39	38.223	26.626	28.053	1.00	0.00	H
ATOM	625	HG3	ARG	39	36.923	25.930	29.121	1.00	0.00	H
ATOM	626	CD	ARG	39	36.906	25.265	27.028	1.00	0.00	C
ATOM	627	HD2	ARG	39	36.878	25.697	26.027	1.00	0.00	H

ATOM	628	HD3	ARG	39	37.712	24.546	27.169	1.00	0.00	H
ATOM	629	NE	ARG	39	35.611	24.486	27.132	1.00	0.00	N
ATOM	630	HE	ARG	39	34.837	24.935	27.601	1.00	0.00	H
ATOM	631	CZ	ARG	39	35.301	23.327	26.535	1.00	0.00	C
ATOM	632	NH1	ARG	39	36.059	22.715	25.720	1.00	0.00	N
ATOM	633	HH11	ARG	39	37.002	22.964	25.454	1.00	0.00	H
ATOM	634	HH12	ARG	39	35.673	21.930	25.217	1.00	0.00	H
ATOM	635	NH2	ARG	39	34.169	22.769	26.669	1.00	0.00	N
ATOM	636	HH21	ARG	39	33.970	21.933	26.138	1.00	0.00	H
ATOM	637	HH22	ARG	39	33.428	23.276	27.133	1.00	0.00	H
ATOM	638	C	ARG	39	35.446	29.636	28.309	1.00	0.00	C
ATOM	639	O	ARG	39	34.223	29.708	28.447	1.00	0.00	O
ATOM	640	N	VAL	40	36.128	30.621	27.790	1.00	0.00	N
ATOM	641	H	VAL	40	37.135	30.543	27.805	1.00	0.00	H
ATOM	642	CA	VAL	40	35.557	31.929	27.406	1.00	0.00	C
ATOM	643	HA	VAL	40	34.469	31.942	27.350	1.00	0.00	H
ATOM	644	CB	VAL	40	35.946	33.079	28.379	1.00	0.00	C
ATOM	645	HB	VAL	40	35.754	34.012	27.849	1.00	0.00	H
ATOM	646	CG1	VAL	40	35.129	33.104	29.622	1.00	0.00	C
ATOM	647	HG11	VAL	40	35.375	34.017	30.162	1.00	0.00	H
ATOM	648	HG12	VAL	40	34.068	32.993	29.399	1.00	0.00	H
ATOM	649	HG13	VAL	40	35.514	32.422	30.380	1.00	0.00	H
ATOM	650	CG2	VAL	40	37.428	33.308	28.589	1.00	0.00	C
ATOM	651	HG21	VAL	40	37.578	34.250	29.117	1.00	0.00	H
ATOM	652	HG22	VAL	40	37.891	32.577	29.253	1.00	0.00	H
ATOM	653	HG23	VAL	40	38.050	33.298	27.693	1.00	0.00	H
ATOM	654	C	VAL	40	35.975	32.291	25.984	1.00	0.00	C
ATOM	655	O	VAL	40	37.216	32.317	25.636	1.00	0.00	O
ATOM	656	N	GLY	41	35.005	32.676	25.227	1.00	0.00	N
ATOM	657	H	GLY	41	34.014	32.551	25.373	1.00	0.00	H
ATOM	658	CA	GLY	41	35.240	33.361	23.934	1.00	0.00	C
ATOM	659	HA2	GLY	41	35.596	34.356	24.202	1.00	0.00	H
ATOM	660	HA3	GLY	41	36.042	32.826	23.426	1.00	0.00	H
ATOM	661	C	GLY	41	33.981	33.539	23.057	1.00	0.00	C
ATOM	662	O	GLY	41	32.865	33.306	23.531	1.00	0.00	O
ATOM	663	N	ARG	42	34.151	34.074	21.832	1.00	0.00	N
ATOM	664	H	ARG	42	35.128	34.213	21.622	1.00	0.00	H
ATOM	665	CA	ARG	42	33.071	34.422	20.861	1.00	0.00	C
ATOM	666	HA	ARG	42	32.261	33.709	21.018	1.00	0.00	H
ATOM	667	CB	ARG	42	32.568	35.825	21.190	1.00	0.00	C
ATOM	668	HB2	ARG	42	31.808	36.151	20.481	1.00	0.00	H
ATOM	669	HB3	ARG	42	32.152	35.724	22.193	1.00	0.00	H
ATOM	670	CG	ARG	42	33.602	36.945	21.168	1.00	0.00	C
ATOM	671	HG2	ARG	42	34.575	36.769	21.628	1.00	0.00	H
ATOM	672	HG3	ARG	42	33.722	37.265	20.133	1.00	0.00	H
ATOM	673	CD	ARG	42	33.128	38.260	21.826	1.00	0.00	C
ATOM	674	HD2	ARG	42	32.178	38.548	21.372	1.00	0.00	H
ATOM	675	HD3	ARG	42	33.075	38.024	22.888	1.00	0.00	H
ATOM	676	NE	ARG	42	34.101	39.366	21.670	1.00	0.00	N
ATOM	677	HE	ARG	42	33.935	40.050	20.946	1.00	0.00	H
ATOM	678	CZ	ARG	42	35.005	39.759	22.637	1.00	0.00	C
ATOM	679	NH1	ARG	42	35.103	39.377	23.886	1.00	0.00	N
ATOM	680	HH11	ARG	42	35.809	39.860	24.422	1.00	0.00	H
ATOM	681	HH12	ARG	42	34.664	38.541	24.244	1.00	0.00	H
ATOM	682	NH2	ARG	42	35.974	40.617	22.321	1.00	0.00	N
ATOM	683	HH21	ARG	42	35.946	41.050	21.409	1.00	0.00	H
ATOM	684	HH22	ARG	42	36.723	40.811	22.970	1.00	0.00	H
ATOM	685	C	ARG	42	33.354	34.350	19.307	1.00	0.00	C
ATOM	686	O	ARG	42	32.331	34.282	18.577	1.00	0.00	O
ATOM	687	N	PHE	43	34.608	34.197	18.921	1.00	0.00	N
ATOM	688	H	PHE	43	35.252	34.312	19.690	1.00	0.00	H
ATOM	689	CA	PHE	43	35.043	34.193	17.486	1.00	0.00	C
ATOM	690	HA	PHE	43	34.285	34.760	16.947	1.00	0.00	H
ATOM	691	CB	PHE	43	36.410	34.961	17.408	1.00	0.00	C

ATOM	692	HB2	PHE	43	37.226	34.452	17.921	1.00	0.00	H
ATOM	693	HB3	PHE	43	36.764	35.104	16.387	1.00	0.00	H
ATOM	694	CG	PHE	43	36.345	36.385	17.960	1.00	0.00	C
ATOM	695	CD1	PHE	43	35.302	37.352	17.668	1.00	0.00	C
ATOM	696	HD1	PHE	43	34.486	36.999	17.055	1.00	0.00	H
ATOM	697	CE1	PHE	43	35.326	38.642	18.212	1.00	0.00	C
ATOM	698	HE1	PHE	43	34.513	39.279	17.896	1.00	0.00	H
ATOM	699	CZ	PHE	43	36.261	38.931	19.187	1.00	0.00	C
ATOM	700	HZ	PHE	43	36.220	39.951	19.537	1.00	0.00	H
ATOM	701	CE2	PHE	43	37.331	38.069	19.428	1.00	0.00	C
ATOM	702	HE2	PHE	43	38.092	38.299	20.159	1.00	0.00	H
ATOM	703	CD2	PHE	43	37.331	36.702	18.911	1.00	0.00	C
ATOM	704	HD2	PHE	43	38.074	35.976	19.206	1.00	0.00	H
ATOM	705	C	PHE	43	35.124	32.797	16.801	1.00	0.00	C
ATOM	706	O	PHE	43	34.909	31.724	17.380	1.00	0.00	O
ATOM	707	N	GLU	44	35.352	32.817	15.494	1.00	0.00	N
ATOM	708	H	GLU	44	35.560	33.703	15.054	1.00	0.00	H
ATOM	709	CA	GLU	44	35.302	31.649	14.558	1.00	0.00	C
ATOM	710	HA	GLU	44	34.324	31.170	14.622	1.00	0.00	H
ATOM	711	CB	GLU	44	35.396	32.337	13.150	1.00	0.00	C
ATOM	712	HB2	GLU	44	34.829	33.261	13.261	1.00	0.00	H
ATOM	713	HB3	GLU	44	36.365	32.778	12.917	1.00	0.00	H
ATOM	714	CG	GLU	44	34.776	31.536	12.008	1.00	0.00	C
ATOM	715	HG2	GLU	44	33.742	31.225	12.155	1.00	0.00	H
ATOM	716	HG3	GLU	44	34.718	32.243	11.181	1.00	0.00	H
ATOM	717	CD	GLU	44	35.578	30.428	11.498	1.00	0.00	C
ATOM	718	OE1	GLU	44	35.052	29.321	11.211	1.00	0.00	O
ATOM	719	OE2	GLU	44	36.838	30.580	11.382	1.00	0.00	O
ATOM	720	C	GLU	44	36.368	30.560	14.753	1.00	0.00	C
ATOM	721	O	GLU	44	36.084	29.367	14.563	1.00	0.00	O
ATOM	722	N	ASP	45	37.579	30.984	15.177	1.00	0.00	N
ATOM	723	H	ASP	45	37.678	31.984	15.290	1.00	0.00	H
ATOM	724	CA	ASP	45	38.648	30.176	15.825	1.00	0.00	C
ATOM	725	HA	ASP	45	39.101	29.524	15.077	1.00	0.00	H
ATOM	726	CB	ASP	45	39.740	31.182	16.225	1.00	0.00	C
ATOM	727	HB2	ASP	45	39.984	31.603	15.249	1.00	0.00	H
ATOM	728	HB3	ASP	45	39.497	31.866	17.039	1.00	0.00	H
ATOM	729	CG	ASP	45	41.047	30.541	16.592	1.00	0.00	C
ATOM	730	OD1	ASP	45	41.460	29.526	15.973	1.00	0.00	O
ATOM	731	OD2	ASP	45	41.683	30.967	17.585	1.00	0.00	O
ATOM	732	C	ASP	45	38.208	29.266	17.010	1.00	0.00	C
ATOM	733	O	ASP	45	38.496	29.603	18.165	1.00	0.00	O
ATOM	734	N	ASP	46	37.519	28.195	16.733	1.00	0.00	N
ATOM	735	H	ASP	46	37.243	28.087	15.767	1.00	0.00	H
ATOM	736	CA	ASP	46	37.205	27.124	17.688	1.00	0.00	C
ATOM	737	HA	ASP	46	36.373	26.578	17.244	1.00	0.00	H
ATOM	738	CB	ASP	46	38.420	26.135	17.813	1.00	0.00	C
ATOM	739	HB2	ASP	46	39.143	26.767	18.329	1.00	0.00	H
ATOM	740	HB3	ASP	46	38.216	25.319	18.505	1.00	0.00	H
ATOM	741	CG	ASP	46	38.926	25.557	16.516	1.00	0.00	C
ATOM	742	OD1	ASP	46	38.117	25.193	15.627	1.00	0.00	O
ATOM	743	OD2	ASP	46	40.156	25.553	16.276	1.00	0.00	O
ATOM	744	C	ASP	46	36.647	27.677	18.989	1.00	0.00	C
ATOM	745	O	ASP	46	37.037	27.214	20.046	1.00	0.00	O
ATOM	746	N	GLY	47	35.660	28.656	18.916	1.00	0.00	N
ATOM	747	H	GLY	47	35.407	28.846	17.956	1.00	0.00	H
ATOM	748	CA	GLY	47	34.799	29.103	20.003	1.00	0.00	C
ATOM	749	HA2	GLY	47	33.953	29.625	19.555	1.00	0.00	H
ATOM	750	HA3	GLY	47	34.459	28.214	20.534	1.00	0.00	H
ATOM	751	C	GLY	47	35.515	29.972	21.050	1.00	0.00	C
ATOM	752	O	GLY	47	34.911	31.024	21.397	1.00	0.00	O
ATOM	753	N	TYR	48	36.781	29.718	21.418	1.00	0.00	N
ATOM	754	H	TYR	48	37.233	29.016	20.848	1.00	0.00	H

ATOM	755	CA	TYR	48	37.416	30.260	22.676	1.00	0.00	C
ATOM	756	HA	TYR	48	36.711	30.994	23.067	1.00	0.00	H
ATOM	757	CB	TYR	48	37.498	29.060	23.644	1.00	0.00	C
ATOM	758	HB2	TYR	48	38.207	28.321	23.269	1.00	0.00	H
ATOM	759	HB3	TYR	48	37.872	29.352	24.625	1.00	0.00	H
ATOM	760	CG	TYR	48	36.241	28.264	23.861	1.00	0.00	C
ATOM	761	CD1	TYR	48	35.164	28.791	24.636	1.00	0.00	C
ATOM	762	HD1	TYR	48	35.271	29.735	25.150	1.00	0.00	H
ATOM	763	CE1	TYR	48	33.985	28.009	24.815	1.00	0.00	C
ATOM	764	HE1	TYR	48	33.139	28.396	25.364	1.00	0.00	H
ATOM	765	CZ	TYR	48	33.911	26.762	24.199	1.00	0.00	C
ATOM	766	OH	TYR	48	32.733	26.127	24.269	1.00	0.00	O
ATOM	767	HH	TYR	48	32.039	26.501	24.817	1.00	0.00	H
ATOM	768	CE2	TYR	48	35.016	26.214	23.565	1.00	0.00	C
ATOM	769	HE2	TYR	48	34.983	25.216	23.157	1.00	0.00	H
ATOM	770	CD2	TYR	48	36.111	27.014	23.243	1.00	0.00	C
ATOM	771	HD2	TYR	48	36.836	26.596	22.562	1.00	0.00	H
ATOM	772	C	TYR	48	38.773	30.988	22.520	1.00	0.00	C
ATOM	773	O	TYR	48	39.473	30.871	21.479	1.00	0.00	O
ATOM	774	N	PHE	49	39.227	31.746	23.594	1.00	0.00	N
ATOM	775	H	PHE	49	38.536	31.853	24.322	1.00	0.00	H
ATOM	776	CA	PHE	49	40.591	32.288	23.733	1.00	0.00	C
ATOM	777	HA	PHE	49	41.257	31.688	23.113	1.00	0.00	H
ATOM	778	CB	PHE	49	40.603	33.728	23.188	1.00	0.00	C
ATOM	779	HB2	PHE	49	41.594	34.066	23.491	1.00	0.00	H
ATOM	780	HB3	PHE	49	40.732	33.738	22.105	1.00	0.00	H
ATOM	781	CG	PHE	49	39.561	34.678	23.682	1.00	0.00	C
ATOM	782	CD1	PHE	49	38.756	35.333	22.755	1.00	0.00	C
ATOM	783	HD1	PHE	49	38.941	35.138	21.709	1.00	0.00	H
ATOM	784	CE1	PHE	49	38.016	36.457	23.096	1.00	0.00	C
ATOM	785	HE1	PHE	49	37.458	37.072	22.407	1.00	0.00	H
ATOM	786	CZ	PHE	49	37.815	36.649	24.466	1.00	0.00	C
ATOM	787	HZ	PHE	49	37.289	37.550	24.752	1.00	0.00	H
ATOM	788	CE2	PHE	49	38.532	35.981	25.458	1.00	0.00	C
ATOM	789	HE2	PHE	49	38.495	36.193	26.517	1.00	0.00	H
ATOM	790	CD2	PHE	49	39.470	34.976	25.054	1.00	0.00	C
ATOM	791	HD2	PHE	49	40.115	34.506	25.781	1.00	0.00	H
ATOM	792	C	PHE	49	41.191	32.166	25.130	1.00	0.00	C
ATOM	793	O	PHE	49	42.336	32.501	25.317	1.00	0.00	O
ATOM	794	N	CYX	50	40.385	31.881	26.180	1.00	0.00	N
ATOM	795	H	CYX	50	39.399	31.808	25.973	1.00	0.00	H
ATOM	796	CA	CYX	50	40.829	31.631	27.598	1.00	0.00	C
ATOM	797	HA	CYX	50	41.895	31.402	27.604	1.00	0.00	H
ATOM	798	CB	CYX	50	40.654	32.838	28.478	1.00	0.00	C
ATOM	799	HB2	CYX	50	40.080	33.630	27.997	1.00	0.00	H
ATOM	800	HB3	CYX	50	40.077	32.510	29.344	1.00	0.00	H
ATOM	801	SG	CYX	50	42.113	33.696	29.015	1.00	0.00	S
ATOM	802	C	CYX	50	40.178	30.377	28.214	1.00	0.00	C
ATOM	803	O	CYX	50	38.932	30.307	28.410	1.00	0.00	O
ATOM	804	N	THR	51	40.988	29.441	28.671	1.00	0.00	N
ATOM	805	H	THR	51	41.920	29.457	28.283	1.00	0.00	H
ATOM	806	CA	THR	51	40.674	28.240	29.518	1.00	0.00	C
ATOM	807	HA	THR	51	39.634	27.914	29.494	1.00	0.00	H
ATOM	808	CB	THR	51	41.597	27.114	29.082	1.00	0.00	C
ATOM	809	HB	THR	51	42.543	27.483	29.477	1.00	0.00	H
ATOM	810	CG2	THR	51	41.365	25.820	29.718	1.00	0.00	C
ATOM	811	HG21	THR	51	42.142	25.111	29.433	1.00	0.00	H
ATOM	812	HG22	THR	51	41.288	25.925	30.801	1.00	0.00	H
ATOM	813	HG23	THR	51	40.428	25.426	29.321	1.00	0.00	H
ATOM	814	OG1	THR	51	41.470	26.892	27.700	1.00	0.00	O
ATOM	815	HG1	THR	51	41.827	27.676	27.278	1.00	0.00	H
ATOM	816	C	THR	51	40.954	28.551	30.966	1.00	0.00	C
ATOM	817	O	THR	51	41.910	29.257	31.270	1.00	0.00	O
ATOM	818	N	ILE	52	40.119	28.125	31.966	1.00	0.00	N

ATOM	819	H	ILE	52	39.350	27.524	31.704	1.00	0.00	H
ATOM	820	CA	ILE	52	40.120	28.666	33.365	1.00	0.00	C
ATOM	821	HA	ILE	52	41.018	29.259	33.540	1.00	0.00	H
ATOM	822	CB	ILE	52	38.990	29.718	33.455	1.00	0.00	C
ATOM	823	HB	ILE	52	38.058	29.258	33.126	1.00	0.00	H
ATOM	824	CG2	ILE	52	38.776	30.275	34.840	1.00	0.00	C
ATOM	825	HG21	ILE	52	38.141	31.161	34.845	1.00	0.00	H
ATOM	826	HG22	ILE	52	38.230	29.534	35.423	1.00	0.00	H
ATOM	827	HG23	ILE	52	39.735	30.537	35.286	1.00	0.00	H
ATOM	828	CG1	ILE	52	39.127	30.942	32.484	1.00	0.00	C
ATOM	829	HG12	ILE	52	39.765	31.668	32.988	1.00	0.00	H
ATOM	830	HG13	ILE	52	39.749	30.705	31.621	1.00	0.00	H
ATOM	831	CD1	ILE	52	37.785	31.615	32.096	1.00	0.00	C
ATOM	832	HD11	ILE	52	37.938	32.630	31.728	1.00	0.00	H
ATOM	833	HD12	ILE	52	37.371	30.954	31.336	1.00	0.00	H
ATOM	834	HD13	ILE	52	37.141	31.770	32.960	1.00	0.00	H
ATOM	835	C	ILE	52	40.025	27.575	34.479	1.00	0.00	C
ATOM	836	O	ILE	52	39.227	26.684	34.214	1.00	0.00	O
ATOM	837	N	GLU	53	40.689	27.730	35.591	1.00	0.00	N
ATOM	838	H	GLU	53	41.310	28.525	35.533	1.00	0.00	H
ATOM	839	CA	GLU	53	40.563	26.938	36.812	1.00	0.00	C
ATOM	840	HA	GLU	53	39.761	26.225	36.622	1.00	0.00	H
ATOM	841	CB	GLU	53	41.765	26.111	37.115	1.00	0.00	C
ATOM	842	HB2	GLU	53	41.975	25.576	36.189	1.00	0.00	H
ATOM	843	HB3	GLU	53	42.669	26.695	37.292	1.00	0.00	H
ATOM	844	CG	GLU	53	41.586	25.011	38.169	1.00	0.00	C
ATOM	845	HG2	GLU	53	41.254	25.539	39.063	1.00	0.00	H
ATOM	846	HG3	GLU	53	40.882	24.313	37.714	1.00	0.00	H
ATOM	847	CD	GLU	53	42.793	24.185	38.533	1.00	0.00	C
ATOM	848	OE1	GLU	53	43.624	23.863	37.691	1.00	0.00	O
ATOM	849	OE2	GLU	53	42.926	23.896	39.786	1.00	0.00	O
ATOM	850	C	GLU	53	40.163	27.798	38.018	1.00	0.00	C
ATOM	851	O	GLU	53	40.769	28.836	38.298	1.00	0.00	O
ATOM	852	N	VAL	54	39.281	27.329	38.815	1.00	0.00	N
ATOM	853	H	VAL	54	38.821	26.508	38.448	1.00	0.00	H
ATOM	854	CA	VAL	54	38.850	27.962	40.068	1.00	0.00	C
ATOM	855	HA	VAL	54	38.896	29.046	39.957	1.00	0.00	H
ATOM	856	CB	VAL	54	37.389	27.586	40.414	1.00	0.00	C
ATOM	857	HB	VAL	54	37.426	26.545	40.735	1.00	0.00	H
ATOM	858	CG1	VAL	54	36.835	28.459	41.509	1.00	0.00	C
ATOM	859	HG11	VAL	54	35.929	28.076	41.977	1.00	0.00	H
ATOM	860	HG12	VAL	54	37.546	28.394	42.332	1.00	0.00	H
ATOM	861	HG13	VAL	54	36.744	29.458	41.081	1.00	0.00	H
ATOM	862	CG2	VAL	54	36.467	27.768	39.273	1.00	0.00	C
ATOM	863	HG21	VAL	54	36.498	28.802	38.931	1.00	0.00	H
ATOM	864	HG22	VAL	54	36.714	27.114	38.436	1.00	0.00	H
ATOM	865	HG23	VAL	54	35.436	27.500	39.502	1.00	0.00	H
ATOM	866	C	VAL	54	39.778	27.590	41.235	1.00	0.00	C
ATOM	867	O	VAL	54	39.863	26.410	41.595	1.00	0.00	O
ATOM	868	N	THR	55	40.487	28.631	41.703	1.00	0.00	N
ATOM	869	H	THR	55	40.149	29.531	41.394	1.00	0.00	H
ATOM	870	CA	THR	55	41.624	28.436	42.645	1.00	0.00	C
ATOM	871	HA	THR	55	41.882	27.389	42.481	1.00	0.00	H
ATOM	872	CB	THR	55	42.805	29.349	42.189	1.00	0.00	C
ATOM	873	HB	THR	55	43.691	29.157	42.796	1.00	0.00	H
ATOM	874	CG2	THR	55	43.204	29.192	40.720	1.00	0.00	C
ATOM	875	HG21	THR	55	43.144	28.159	40.374	1.00	0.00	H
ATOM	876	HG22	THR	55	42.553	29.816	40.107	1.00	0.00	H
ATOM	877	HG23	THR	55	44.193	29.634	40.610	1.00	0.00	H
ATOM	878	OG1	THR	55	42.606	30.748	42.329	1.00	0.00	O
ATOM	879	HG1	THR	55	42.472	31.072	43.223	1.00	0.00	H
ATOM	880	C	THR	55	41.207	28.580	44.159	1.00	0.00	C
ATOM	881	O	THR	55	41.932	27.951	44.954	1.00	0.00	O

ATOM	882	N	ALA	56	39.991	29.107	44.411	1.00	0.00	N
ATOM	883	H	ALA	56	39.372	29.437	43.685	1.00	0.00	H
ATOM	884	CA	ALA	56	39.392	29.022	45.770	1.00	0.00	C
ATOM	885	HA	ALA	56	39.507	28.047	46.242	1.00	0.00	H
ATOM	886	CB	ALA	56	40.095	30.042	46.694	1.00	0.00	C
ATOM	887	HB1	ALA	56	39.597	30.159	47.656	1.00	0.00	H
ATOM	888	HB2	ALA	56	41.106	29.673	46.868	1.00	0.00	H
ATOM	889	HB3	ALA	56	40.056	31.057	46.298	1.00	0.00	H
ATOM	890	C	ALA	56	37.823	29.357	45.750	1.00	0.00	C
ATOM	891	O	ALA	56	37.419	30.171	44.945	1.00	0.00	O
ATOM	892	N	THR	57	37.094	28.864	46.690	1.00	0.00	N
ATOM	893	H	THR	57	37.552	28.286	47.379	1.00	0.00	H
ATOM	894	CA	THR	57	35.684	29.292	47.020	1.00	0.00	C
ATOM	895	HA	THR	57	35.538	30.308	46.652	1.00	0.00	H
ATOM	896	CB	THR	57	34.643	28.362	46.343	1.00	0.00	C
ATOM	897	HB	THR	57	33.650	28.790	46.481	1.00	0.00	H
ATOM	898	CG2	THR	57	34.858	28.333	44.794	1.00	0.00	C
ATOM	899	HG21	THR	57	35.119	29.346	44.484	1.00	0.00	H
ATOM	900	HG22	THR	57	35.610	27.571	44.588	1.00	0.00	H
ATOM	901	HG23	THR	57	33.957	27.951	44.314	1.00	0.00	H
ATOM	902	OG1	THR	57	34.678	27.031	46.861	1.00	0.00	O
ATOM	903	HG1	THR	57	35.534	26.625	46.705	1.00	0.00	H
ATOM	904	C	THR	57	35.447	29.251	48.513	1.00	0.00	C
ATOM	905	O	THR	57	36.088	28.486	49.278	1.00	0.00	O
ATOM	906	N	SER	58	34.742	30.224	48.986	1.00	0.00	N
ATOM	907	H	SER	58	34.428	30.881	48.286	1.00	0.00	H
ATOM	908	CA	SER	58	34.314	30.334	50.337	1.00	0.00	C
ATOM	909	HA	SER	58	34.236	29.284	50.619	1.00	0.00	H
ATOM	910	CB	SER	58	35.352	31.132	51.200	1.00	0.00	C
ATOM	911	HB2	SER	58	34.941	31.323	52.192	1.00	0.00	H
ATOM	912	HB3	SER	58	36.310	30.615	51.221	1.00	0.00	H
ATOM	913	OG	SER	58	35.559	32.368	50.557	1.00	0.00	O
ATOM	914	HG	SER	58	36.080	32.199	49.769	1.00	0.00	H
ATOM	915	C	SER	58	32.880	30.968	50.498	1.00	0.00	C
ATOM	916	O	SER	58	32.302	31.433	49.509	1.00	0.00	O
ATOM	917	N	THR	59	32.358	30.921	51.716	1.00	0.00	N
ATOM	918	H	THR	59	32.773	30.489	52.528	1.00	0.00	H
ATOM	919	CA	THR	59	30.968	31.480	51.938	1.00	0.00	C
ATOM	920	HA	THR	59	30.516	31.949	51.064	1.00	0.00	H
ATOM	921	CB	THR	59	30.062	30.314	52.235	1.00	0.00	C
ATOM	922	HB	THR	59	29.958	29.692	51.345	1.00	0.00	H
ATOM	923	CG2	THR	59	30.267	29.539	53.512	1.00	0.00	C
ATOM	924	HG21	THR	59	30.372	30.216	54.358	1.00	0.00	H
ATOM	925	HG22	THR	59	29.411	28.925	53.792	1.00	0.00	H
ATOM	926	HG23	THR	59	31.082	28.851	53.287	1.00	0.00	H
ATOM	927	OG1	THR	59	28.809	30.902	52.525	1.00	0.00	O
ATOM	928	HG1	THR	59	28.149	30.553	51.923	1.00	0.00	H
ATOM	929	C	THR	59	31.042	32.528	53.051	1.00	0.00	C
ATOM	930	O	THR	59	31.880	32.276	53.918	1.00	0.00	O
ATOM	931	N	VAL	60	30.360	33.685	52.949	1.00	0.00	N
ATOM	932	H	VAL	60	29.686	33.732	52.199	1.00	0.00	H
ATOM	933	CA	VAL	60	30.652	34.975	53.688	1.00	0.00	C
ATOM	934	HA	VAL	60	31.093	34.630	54.624	1.00	0.00	H
ATOM	935	CB	VAL	60	31.625	35.802	52.767	1.00	0.00	C
ATOM	936	HB	VAL	60	31.712	36.786	53.228	1.00	0.00	H
ATOM	937	CG1	VAL	60	33.056	35.167	52.682	1.00	0.00	C
ATOM	938	HG11	VAL	60	33.614	35.825	52.017	1.00	0.00	H
ATOM	939	HG12	VAL	60	33.486	35.186	53.683	1.00	0.00	H
ATOM	940	HG13	VAL	60	33.079	34.242	52.105	1.00	0.00	H
ATOM	941	CG2	VAL	60	31.056	35.972	51.342	1.00	0.00	C
ATOM	942	HG21	VAL	60	31.684	36.573	50.684	1.00	0.00	H
ATOM	943	HG22	VAL	60	30.888	35.042	50.798	1.00	0.00	H
ATOM	944	HG23	VAL	60	30.108	36.508	51.364	1.00	0.00	H
ATOM	945	C	VAL	60	29.328	35.714	54.036	1.00	0.00	C

```
ATOM    946  O   VAL    60      28.352  35.464  53.362  1.00  0.00           O
ATOM    947  N   THR    61      29.365  36.746  54.924  1.00  0.00           N
ATOM    948  H   THR    61      30.219  36.825  55.457  1.00  0.00           H
ATOM    949  CA  THR    61      28.335  37.797  55.045  1.00  0.00           C
ATOM    950  HA  THR    61      27.583  37.731
  54.258  1.00  0.00           H
ATOM    951  CB  THR    61      27.735  37.837  56.405  1.00  0.00           C
ATOM    952  HB  THR    61      27.104  38.719  56.522  1.00  0.00           H
ATOM    953  CG2 THR    61      26.834  36.643  56.707  1.00  0.00           C
ATOM    954 HG21 THR    61      27.413  35.733  56.866  1.00  0.00           H
ATOM    955 HG22 THR    61      26.396  36.686  57.705  1.00  0.00           H
ATOM    956 HG23 THR    61      26.049  36.488  55.968  1.00  0.00           H
ATOM    957  OG1 THR    61      28.639  37.897  57.547  1.00  0.00           O
ATOM    958  HG1 THR    61      28.183  38.430  58.202  1.00  0.00           H
ATOM    959  C   THR    61      29.064  39.117  54.879  1.00  0.00           C
ATOM    960  O   THR    61      30.288  39.210  54.938  1.00  0.00           O
ATOM    961  N   LEU    62      28.303  40.204  54.708  1.00  0.00           N
ATOM    962  H   LEU    62      27.307  40.045  54.751  1.00  0.00           H
ATOM    963  CA  LEU    62      28.802  41.568  54.570  1.00  0.00           C
ATOM    964  HA  LEU    62      29.295  41.690  53.606  1.00  0.00           H
ATOM    965  CB  LEU    62      27.690  42.546  54.601  1.00  0.00           C
ATOM    966  HB2 LEU    62      27.137  42.491  55.539  1.00  0.00           H
ATOM    967  HB3 LEU    62      28.226  43.491  54.516  1.00  0.00           H
ATOM    968  CG  LEU    62      26.696  42.555  53.376  1.00  0.00           C
ATOM    969  HG  LEU    62      26.150  41.611  53.367  1.00  0.00           H
ATOM    970  CD1 LEU    62      25.648  43.637  53.514  1.00  0.00           C
ATOM    971 HD11 LEU    62      24.933  43.628  52.691  1.00  0.00           H
ATOM    972 HD12 LEU    62      25.122  43.472  54.454  1.00  0.00           H
ATOM    973 HD13 LEU    62      26.124  44.616  53.579  1.00  0.00           H
ATOM    974  CD2 LEU    62      27.502  42.792  52.134  1.00  0.00           C
ATOM    975 HD21 LEU    62      27.163  42.035  51.427  1.00  0.00           H
ATOM    976 HD22 LEU    62      27.117  43.668  51.613  1.00  0.00           H
ATOM    977 HD23 LEU    62      28.584  42.895  52.206  1.00  0.00           H
ATOM    978  C   LEU    62      29.690  42.005  55.768  1.00  0.00           C
ATOM    979  O   LEU    62      30.834  42.390  55.463  1.00  0.00           O
ATOM    980  N   ASP    63      29.221  41.709  57.007  1.00  0.00           N
ATOM    981  H   ASP    63      28.239  41.476  56.967  1.00  0.00           H
ATOM    982  CA  ASP    63      29.906  41.959  58.243  1.00  0.00           C
ATOM    983  HA  ASP    63      30.300  42.975  58.199  1.00  0.00           H
ATOM    984  CB  ASP    63      28.973  41.917  59.436  1.00  0.00           C
ATOM    985  HB2 ASP    63      29.525  42.258  60.312  1.00  0.00           H
ATOM    986  HB3 ASP    63      28.084  42.543  59.363  1.00  0.00           H
ATOM    987  CG  ASP    63      28.547  40.469  59.798  1.00  0.00           C
ATOM    988  OD1 ASP    63      27.751  39.778  59.093  1.00  0.00           O
ATOM    989  OD2 ASP    63      29.023  39.930  60.815  1.00  0.00           O
ATOM    990  C   ASP    63      31.230  41.177  58.434  1.00  0.00           C
ATOM    991  O   ASP    63      32.154  41.507  59.254  1.00  0.00           O
ATOM    992  N   THR    64      31.377  40.105  57.643  1.00  0.00           N
ATOM    993  H   THR    64      30.715  39.782  56.952  1.00  0.00           H
ATOM    994  CA  THR    64      32.578  39.234  57.591  1.00  0.00           C
ATOM    995  HA  THR    64      33.282  39.487  58.383  1.00  0.00           H
ATOM    996  CB  THR    64      32.189  37.725  57.909  1.00  0.00           C
ATOM    997  HB  THR    64      33.111  37.144  57.901  1.00  0.00           H
ATOM    998  CG2 THR    64      31.672  37.517  59.363  1.00  0.00           C
ATOM    999 HG21 THR    64      32.391  37.943  60.064  1.00  0.00           H
ATOM   1000 HG22 THR    64      30.765  38.075  59.597  1.00  0.00           H
ATOM   1001 HG23 THR    64      31.665  36.432  59.463  1.00  0.00           H
ATOM   1002  OG1 THR    64      31.272  37.239  57.059  1.00  0.00           O
ATOM   1003  HG1 THR    64      30.417  37.633  57.246  1.00  0.00           H
ATOM   1004  C   THR    64      33.482  39.348  56.433  1.00  0.00           C
ATOM   1005  O   THR    64      34.396  38.576  56.321  1.00  0.00           O
ATOM   1006  N   LEU    65      33.260  40.237  55.403  1.00  0.00           N
ATOM   1007  H   LEU    65      32.466  40.853  55.495  1.00  0.00           H
ATOM   1008  CA  LEU    65      33.876  40.153  54.105  1.00  0.00           C
```

ATOM	1009	HA	LEU	65	34.036	39.089	53.929	1.00	0.00	H
ATOM	1010	CB	LEU	65	32.853	40.717	53.120	1.00	0.00	C
ATOM	1011	HB2	LEU	65	31.856	40.317	53.312	1.00	0.00	H
ATOM	1012	HB3	LEU	65	32.768	41.788	53.303	1.00	0.00	H
ATOM	1013	CG	LEU	65	33.046	40.411	51.620	1.00	0.00	C
ATOM	1014	HG	LEU	65	34.053	40.636	51.268	1.00	0.00	H
ATOM	1015	CD1	LEU	65	32.609	39.068	51.133	1.00	0.00	C
ATOM	1016	HD11	LEU	65	32.710	39.016	50.049	1.00	0.00	H
ATOM	1017	HD12	LEU	65	33.069	38.177	51.561	1.00	0.00	H
ATOM	1018	HD13	LEU	65	31.571	38.928	51.433	1.00	0.00	H
ATOM	1019	CD2	LEU	65	32.112	41.386	50.843	1.00	0.00	C
ATOM	1020	HD21	LEU	65	31.076	41.064	50.952	1.00	0.00	H
ATOM	1021	HD22	LEU	65	32.336	42.344	51.312	1.00	0.00	H
ATOM	1022	HD23	LEU	65	32.385	41.311	49.790	1.00	0.00	H
ATOM	1023	C	LEU	65	35.224	40.886	53.989	1.00	0.00	C
ATOM	1024	O	LEU	65	35.503	41.768	54.794	1.00	0.00	O
ATOM	1025	N	THR	66	36.021	40.446	52.975	1.00	0.00	N
ATOM	1026	H	THR	66	35.636	39.649	52.488	1.00	0.00	H
ATOM	1027	CA	THR	66	37.382	40.936	52.778	1.00	0.00	C
ATOM	1028	HA	THR	66	37.762	41.465	53.652	1.00	0.00	H
ATOM	1029	CB	THR	66	38.386	39.719	52.735	1.00	0.00	C
ATOM	1030	HB	THR	66	38.299	39.332	51.720	1.00	0.00	H
ATOM	1031	CG2	THR	66	39.781	40.087	52.896	1.00	0.00	C
ATOM	1032	HG21	THR	66	39.902	40.597	53.853	1.00	0.00	H
ATOM	1033	HG22	THR	66	40.434	39.216	52.846	1.00	0.00	H
ATOM	1034	HG23	THR	66	40.095	40.692	52.046	1.00	0.00	H
ATOM	1035	OG1	THR	66	38.072	38.727	53.705	1.00	0.00	O
ATOM	1036	HG1	THR	66	37.568	38.034	53.273	1.00	0.00	H
ATOM	1037	C	THR	66	37.425	41.894	51.497	1.00	0.00	C
ATOM	1038	O	THR	66	36.548	41.788	50.623	1.00	0.00	O
ATOM	1039	N	GLU	67	38.401	42.776	51.448	1.00	0.00	N
ATOM	1040	H	GLU	67	39.052	42.848	52.215	1.00	0.00	H
ATOM	1041	CA	GLU	67	38.504	43.679	50.310	1.00	0.00	C
ATOM	1042	HA	GLU	67	37.530	44.112	50.083	1.00	0.00	H
ATOM	1043	CB	GLU	67	39.419	44.950	50.528	1.00	0.00	C
ATOM	1044	HB2	GLU	67	39.281	45.618	49.677	1.00	0.00	H
ATOM	1045	HB3	GLU	67	39.045	45.524	51.375	1.00	0.00	H
ATOM	1046	CG	GLU	67	40.936	44.723	50.772	1.00	0.00	C
ATOM	1047	HG2	GLU	67	41.010	44.090	51.656	1.00	0.00	H
ATOM	1048	HG3	GLU	67	41.225	44.245	49.836	1.00	0.00	H
ATOM	1049	CD	GLU	67	41.816	45.972	51.071	1.00	0.00	C
ATOM	1050	OE1	GLU	67	41.392	47.053	50.622	1.00	0.00	O
ATOM	1051	OE2	GLU	67	42.837	45.796	51.679	1.00	0.00	O
ATOM	1052	C	GLU	67	38.890	43.089	48.968	1.00	0.00	C
ATOM	1053	O	GLU	67	38.739	43.834	47.949	1.00	0.00	O
ATOM	1054	N	LYS	68	39.233	41.799	48.717	1.00	0.00	N
ATOM	1055	H	LYS	68	39.260	41.128	49.472	1.00	0.00	H
ATOM	1056	CA	LYS	68	39.810	41.356	47.364	1.00	0.00	C
ATOM	1057	HA	LYS	68	40.729	41.861	47.070	1.00	0.00	H
ATOM	1058	CB	LYS	68	40.239	39.834	47.530	1.00	0.00	C
ATOM	1059	HB2	LYS	68	40.730	39.645	48.484	1.00	0.00	H
ATOM	1060	HB3	LYS	68	39.379	39.164	47.543	1.00	0.00	H
ATOM	1061	CG	LYS	68	41.123	39.174	46.374	1.00	0.00	C
ATOM	1062	HG2	LYS	68	40.858	38.123	46.484	1.00	0.00	H
ATOM	1063	HG3	LYS	68	40.762	39.447	45.382	1.00	0.00	H
ATOM	1064	CD	LYS	68	42.601	39.279	46.472	1.00	0.00	C
ATOM	1065	HD2	LYS	68	42.921	40.318	46.548	1.00	0.00	H
ATOM	1066	HD3	LYS	68	43.006	38.522	47.143	1.00	0.00	H
ATOM	1067	CE	LYS	68	43.149	38.745	45.178	1.00	0.00	C
ATOM	1068	HE2	LYS	68	42.877	37.690	45.120	1.00	0.00	H
ATOM	1069	HE3	LYS	68	42.762	39.256	44.297	1.00	0.00	H
ATOM	1070	NZ	LYS	68	44.609	38.763	44.994	1.00	0.00	N
ATOM	1071	HZ1	LYS	68	44.989	38.433	45.871	1.00	0.00	H
ATOM	1072	HZ2	LYS	68	44.842	38.103	44.266	1.00	0.00	H

ATOM	1073	HZ3	LYS	68	45.070	39.653	44.873	1.00	0.00	H
ATOM	1074	C	LYS	68	38.830	41.574	46.165	1.00	0.00	C
ATOM	1075	O	LYS	68	39.310	41.772	45.006	1.00	0.00	
	O									
ATOM	1076	N	HIE	69	37.477	41.477	46.328	1.00	0.00	N
ATOM	1077	H	HIE	69	37.179	40.995	47.164	1.00	0.00	H
ATOM	1078	CA	HIE	69	36.514	41.843	45.329	1.00	0.00	C
ATOM	1079	HA	HIE	69	36.867	41.402	44.396	1.00	0.00	H
ATOM	1080	CB	HIE	69	35.223	41.057	45.524	1.00	0.00	C
ATOM	1081	HB2	HIE	69	35.396	39.992	45.680	1.00	0.00	H
ATOM	1082	HB3	HIE	69	34.830	41.393	46.483	1.00	0.00	H
ATOM	1083	CG	HIE	69	34.228	41.320	44.497	1.00	0.00	C
ATOM	1084	ND1	HIE	69	34.325	40.984	43.167	1.00	0.00	N
ATOM	1085	CE1	HIE	69	33.253	41.485	42.569	1.00	0.00	C
ATOM	1086	HE1	HIE	69	33.140	41.482	41.495	1.00	0.00	H
ATOM	1087	NE2	HIE	69	32.584	42.162	43.492	1.00	0.00	N
ATOM	1088	HE2	HIE	69	31.874	42.823	43.212	1.00	0.00	H
ATOM	1089	CD2	HIE	69	33.123	42.128	44.688	1.00	0.00	C
ATOM	1090	HD2	HIE	69	32.898	42.749	45.543	1.00	0.00	H
ATOM	1091	C	HIE	69	36.304	43.391	45.102	1.00	0.00	C
ATOM	1092	O	HIE	69	36.211	43.820	43.969	1.00	0.00	O
ATOM	1093	N	ALA	70	36.088	44.136	46.168	1.00	0.00	N
ATOM	1094	H	ALA	70	36.287	43.682	47.048	1.00	0.00	H
ATOM	1095	CA	ALA	70	35.827	45.602	46.122	1.00	0.00	C
ATOM	1096	HA	ALA	70	34.878	45.692	45.592	1.00	0.00	H
ATOM	1097	CB	ALA	70	35.587	46.030	47.569	1.00	0.00	C
ATOM	1098	HB1	ALA	70	36.414	45.852	48.257	1.00	0.00	H
ATOM	1099	HB2	ALA	70	35.173	47.019	47.762	1.00	0.00	H
ATOM	1100	HB3	ALA	70	34.867	45.303	47.945	1.00	0.00	H
ATOM	1101	C	ALA	70	36.972	46.401	45.524	1.00	0.00	C
ATOM	1102	O	ALA	70	36.829	47.408	44.807	1.00	0.00	O
ATOM	1103	N	GLU	71	38.220	45.886	45.704	1.00	0.00	N
ATOM	1104	H	GLU	71	38.390	45.194	46.421	1.00	0.00	H
ATOM	1105	CA	GLU	71	39.390	46.346	44.967	1.00	0.00	C
ATOM	1106	HA	GLU	71	39.439	47.429	45.068	1.00	0.00	H
ATOM	1107	CB	GLU	71	40.590	45.693	45.606	1.00	0.00	C
ATOM	1108	HB2	GLU	71	40.529	44.624	45.810	1.00	0.00	H
ATOM	1109	HB3	GLU	71	41.421	45.822	44.912	1.00	0.00	H
ATOM	1110	CG	GLU	71	40.831	46.332	47.039	1.00	0.00	C
ATOM	1111	HG2	GLU	71	39.949	46.378	47.679	1.00	0.00	H
ATOM	1112	HG3	GLU	71	41.614	45.830	47.607	1.00	0.00	H
ATOM	1113	CD	GLU	71	41.158	47.840	46.883	1.00	0.00	C
ATOM	1114	OE1	GLU	71	40.333	48.670	47.311	1.00	0.00	O
ATOM	1115	OE2	GLU	71	42.184	48.128	46.237	1.00	0.00	O
ATOM	1116	C	GLU	71	39.420	46.029	43.447	1.00	0.00	C
ATOM	1117	O	GLU	71	39.979	46.767	42.688	1.00	0.00	O
ATOM	1118	N	GLN	72	38.839	44.895	43.045	1.00	0.00	N
ATOM	1119	H	GLN	72	38.253	44.358	43.667	1.00	0.00	H
ATOM	1120	CA	GLN	72	38.635	44.656	41.621	1.00	0.00	C
ATOM	1121	HA	GLN	72	39.607	44.789	41.146	1.00	0.00	H
ATOM	1122	CB	GLN	72	38.252	43.175	41.470	1.00	0.00	C
ATOM	1123	HB2	GLN	72	37.233	43.075	41.842	1.00	0.00	H
ATOM	1124	HB3	GLN	72	38.863	42.451	42.010	1.00	0.00	H
ATOM	1125	CG	GLN	72	38.296	42.723	39.979	1.00	0.00	C
ATOM	1126	HG2	GLN	72	37.850	43.396	39.246	1.00	0.00	H
ATOM	1127	HG3	GLN	72	39.367	42.665	39.792	1.00	0.00	H
ATOM	1128	CD	GLN	72	37.698	41.355	39.696	1.00	0.00	C
ATOM	1129	OE1	GLN	72	38.415	40.376	39.489	1.00	0.00	O
ATOM	1130	NE2	GLN	72	36.416	41.245	39.720	1.00	0.00	N
ATOM	1131	HE21	GLN	72	35.720	41.970	39.829	1.00	0.00	H
ATOM	1132	HE22	GLN	72	36.050	40.310	39.615	1.00	0.00	H
ATOM	1133	C	GLN	72	37.640	45.599	41.048	1.00	0.00	C
ATOM	1134	O	GLN	72	37.885	46.213	40.014	1.00	0.00	O
ATOM	1135	N	GLU	73	36.398	45.846	41.623	1.00	0.00	N

ATOM	1136	H	GLU	73	36.225	45.493	42.553	1.00	0.00	H
ATOM	1137	CA	GLU	73	35.341	46.863	41.160	1.00	0.00	C
ATOM	1138	HA	GLU	73	35.427	46.877	40.073	1.00	0.00	H
ATOM	1139	CB	GLU	73	33.934	46.432	41.583	1.00	0.00	C
ATOM	1140	HB2	GLU	73	33.781	46.740	42.617	1.00	0.00	H
ATOM	1141	HB3	GLU	73	33.213	47.077	41.081	1.00	0.00	H
ATOM	1142	CG	GLU	73	33.631	44.942	41.464	1.00	0.00	C
ATOM	1143	HG2	GLU	73	34.345	44.283	41.958	1.00	0.00	H
ATOM	1144	HG3	GLU	73	32.651	44.798	41.918	1.00	0.00	H
ATOM	1145	CD	GLU	73	33.745	44.587	39.938	1.00	0.00	C
ATOM	1146	OE1	GLU	73	33.079	45.268	39.142	1.00	0.00	O
ATOM	1147	OE2	GLU	73	34.407	43.584	39.496	1.00	0.00	O
ATOM	1148	C	GLU	73	35.577	48.302	41.568	1.00	0.00	C
ATOM	1149	O	GLU	73	34.705	49.159	41.514	1.00	0.00	O
ATOM	1150	N	ASN	74	36.757	48.655	41.996	1.00	0.00	N
ATOM	1151	H	ASN	74	37.411	47.889	42.064	1.00	0.00	H
ATOM	1152	CA	ASN	74	37.340	49.949	42.182	1.00	0.00	C
ATOM	1153	HA	ASN	74	38.225	49.896	42.815	1.00	0.00	H
ATOM	1154	CB	ASN	74	37.628	50.587	40.819	1.00	0.00	C
ATOM	1155	HB2	ASN	74	36.679	50.621	40.285	1.00	0.00	H
ATOM	1156	HB3	ASN	74	37.994	51.602	40.978	1.00	0.00	H
ATOM	1157	CG	ASN	74	38.601	49.871	39.933	1.00	0.00	C
ATOM	1158	OD1	ASN	74	39.751	49.669	40.225	1.00	0.00	O
ATOM	1159	ND2	ASN	74	38.305	49.507	38.723	1.00	0.00	N
ATOM	1160	HD21	ASN	74	39.008	49.047	38.164	1.00	0.00	H
ATOM	1161	HD22	ASN	74	37.377	49.782	38.435	1.00	0.00	H
ATOM	1162	C	ASN	74	36.498	50.863	43.069	1.00	0.00	C
ATOM	1163	O	ASN	74	36.273	52.101	42.879	1.00	0.00	O
ATOM	1164	N	MET	75	36.149	50.321	44.220	1.00	0.00	N
ATOM	1165	H	MET	75	36.363	49.351	44.407	1.00	0.00	H
ATOM	1166	CA	MET	75	35.412	51.047	45.310	1.00	0.00	C
ATOM	1167	HA	MET	75	35.722	52.089	45.236	1.00	0.00	H
ATOM	1168	CB	MET	75	33.913	50.952	45.132	1.00	0.00	C
ATOM	1169	HB2	MET	75	33.556	51.954	45.368	1.00	0.00	H
ATOM	1170	HB3	MET	75	33.726	50.933	44.059	1.00	0.00	H
ATOM	1171	CG	MET	75	33.151	49.892	45.941	1.00	0.00	C
ATOM	1172	HG2	MET	75	33.604	48.915	45.776	1.00	0.00	H
ATOM	1173	HG3	MET	75	33.257	50.174	46.989	1.00	0.00	H
ATOM	1174	SD	MET	75	31.336	49.706	45.529	1.00	0.00	S
ATOM	1175	CE	MET	75	31.520	48.869	43.976	1.00	0.00	C
ATOM	1176	HE1	MET	75	30.501	48.745	43.611	1.00	0.00	H
ATOM	1177	HE2	MET	75	32.075	49.518	43.298	1.00	0.00	H
ATOM	1178	HE3	MET	75	32.042	47.915	44.046	1.00	0.00	H
ATOM	1179	C	MET	75	35.885	50.582	46.750	1.00	0.00	C
ATOM	1180	O	MET	75	36.371	49.440	46.960	1.00	0.00	O
ATOM	1181	N	THR	76	35.791	51.448	47.785	1.00	0.00	N
ATOM	1182	H	THR	76	35.334	52.346	47.738	1.00	0.00	H
ATOM	1183	CA	THR	76	35.957	51.019	49.189	1.00	0.00	C
ATOM	1184	HA	THR	76	36.904	50.481	49.150	1.00	0.00	H
ATOM	1185	CB	THR	76	36.148	52.173	50.154	1.00	0.00	C
ATOM	1186	HB	THR	76	37.040	52.783	50.013	1.00	0.00	H
ATOM	1187	CG2	THR	76	34.936	53.124	50.220	1.00	0.00	C
ATOM	1188	HG21	THR	76	34.779	53.415	49.182	1.00	0.00	H
ATOM	1189	HG22	THR	76	34.027	52.657	50.598	1.00	0.00	H
ATOM	1190	HG23	THR	76	35.264	54.026	50.737	1.00	0.00	H
ATOM	1191	OG1	THR	76	36.342	51.593	51.407	1.00	0.00	O
ATOM	1192	HG1	THR	76	36.333	52.410	51.911	1.00	0.00	H
ATOM	1193	C	THR	76	34.891	49.955	49.608	1.00	0.00	C
ATOM	1194	O	THR	76	33.744	50.004	49.311	1.00	0.00	O
ATOM	1195	N	LEU	77	35.254	48.964	50.501	1.00	0.00	N
ATOM	1196	H	LEU	77	36.160	48.998	50.947	1.00	0.00	H
ATOM	1197	CA	LEU	77	34.361	47.883	51.028	1.00	0.00	C
ATOM	1198	HA	LEU	77	33.917	47.321	50.206	1.00	0.00	H
ATOM	1199	CB	LEU	77	35.307	46.917	51.818	1.00	0.00	C

```
ATOM   1200  HB2 LEU    77      36.090  46.545  51.157  1.00  0.00           H
ATOM   1201  HB3 LEU
    77      35.831  47.515  52.564  1.00  0.00           H
ATOM   1202  CG  LEU    77      34.666  45.634  52.318  1.00  0.00           C
ATOM   1203  HG  LEU    77      33.647  45.869  52.626  1.00  0.00           H
ATOM   1204  CD1 LEU    77      34.558  44.677  51.125  1.00  0.00           C
ATOM   1205 HD11 LEU    77      35.564  44.539  50.730  1.00  0.00           H
ATOM   1206 HD12 LEU    77      34.149  43.717  51.438  1.00  0.00           H
ATOM   1207 HD13 LEU    77      33.872  45.099  50.391  1.00  0.00           H
ATOM   1208  CD2 LEU    77      35.427  45.029  53.462  1.00  0.00           C
ATOM   1209 HD21 LEU    77      35.640  45.779  54.224  1.00  0.00           H
ATOM   1210 HD22 LEU    77      34.950  44.182  53.956  1.00  0.00           H
ATOM   1211 HD23 LEU    77      36.397  44.647  53.141  1.00  0.00           H
ATOM   1212  C   LEU    77      33.271  48.423  51.908  1.00  0.00           C
ATOM   1213  O   LEU    77      32.264  47.689  51.993  1.00  0.00           O
ATOM   1214  N   THR    78      33.267  49.636  52.437  1.00  0.00           N
ATOM   1215  H   THR    78      34.125  50.165  52.501  1.00  0.00           H
ATOM   1216  CA  THR    78      31.985  50.237  53.050  1.00  0.00           C
ATOM   1217  HA  THR    78      31.577  49.576  53.815  1.00  0.00           H
ATOM   1218  CB  THR    78      32.267  51.525  53.826  1.00  0.00           C
ATOM   1219  HB  THR    78      31.352  52.006  54.169  1.00  0.00           H
ATOM   1220  CG2 THR    78      33.030  51.199  55.117  1.00  0.00           C
ATOM   1221 HG21 THR    78      33.956  50.707  54.822  1.00  0.00           H
ATOM   1222 HG22 THR    78      33.033  52.190  55.571  1.00  0.00           H
ATOM   1223 HG23 THR    78      32.421  50.701  55.871  1.00  0.00           H
ATOM   1224  OG1 THR    78      32.918  52.513  53.063  1.00  0.00           O
ATOM   1225  HG1 THR    78      32.310  53.252  52.988  1.00  0.00           H
ATOM   1226  C   THR    78      30.988  50.636  51.948  1.00  0.00           C
ATOM   1227  O   THR    78      29.775  50.505  52.035  1.00  0.00           O
ATOM   1228  N   GLU    79      31.436  51.110  50.759  1.00  0.00           N
ATOM   1229  H   GLU    79      32.415  51.046  50.516  1.00  0.00           H
ATOM   1230  CA  GLU    79      30.552  51.421  49.620  1.00  0.00           C
ATOM   1231  HA  GLU    79      29.649  51.908  49.989  1.00  0.00           H
ATOM   1232  CB  GLU    79      31.298  52.325  48.622  1.00  0.00           C
ATOM   1233  HB2 GLU    79      32.244  51.816  48.441  1.00  0.00           H
ATOM   1234  HB3 GLU    79      30.859  52.334  47.624  1.00  0.00           H
ATOM   1235  CG  GLU    79      31.420  53.766  49.156  1.00  0.00           C
ATOM   1236  HG2 GLU    79      32.108  53.784  50.002  1.00  0.00           H
ATOM   1237  HG3 GLU    79      31.892  54.279  48.317  1.00  0.00           H
ATOM   1238  CD  GLU    79      30.061  54.513  49.380  1.00  0.00           C
ATOM   1239  OE1 GLU    79      29.780  54.916  50.521  1.00  0.00           O
ATOM   1240  OE2 GLU    79      29.169  54.514  48.514  1.00  0.00           O
ATOM   1241  C   GLU    79      30.037  50.088  49.149  1.00  0.00           C
ATOM   1242  O   GLU    79      28.815  49.918  48.892  1.00  0.00           O
ATOM   1243  N   LEU    80      30.893  49.058  48.945  1.00  0.00           N
ATOM   1244  H   LEU    80      31.889  49.220  49.003  1.00  0.00           H
ATOM   1245  CA  LEU    80      30.433  47.680  48.496  1.00  0.00           C
ATOM   1246  HA  LEU    80      29.976  47.717  47.507  1.00  0.00           H
ATOM   1247  CB  LEU    80      31.735  46.796  48.336  1.00  0.00           C
ATOM   1248  HB2 LEU    80      32.557  47.224  47.764  1.00  0.00           H
ATOM   1249  HB3 LEU    80      32.058  46.481  49.328  1.00  0.00           H
ATOM   1250  CG  LEU    80      31.250  45.489  47.569  1.00  0.00           C
ATOM   1251  HG  LEU    80      30.221  45.254  47.845  1.00  0.00           H
ATOM   1252  CD1 LEU    80      31.136  45.611  46.089  1.00  0.00           C
ATOM   1253 HD11 LEU    80      32.113  45.661  45.609  1.00  0.00           H
ATOM   1254 HD12 LEU    80      30.594  44.727  45.754  1.00  0.00           H
ATOM   1255 HD13 LEU    80      30.647  46.562  45.878  1.00  0.00           H
ATOM   1256  CD2 LEU    80      32.058  44.249  48.066  1.00  0.00           C
ATOM   1257 HD21 LEU    80      31.727  43.356  47.537  1.00  0.00           H
ATOM   1258 HD22 LEU    80      33.100  44.446  47.812  1.00  0.00           H
ATOM   1259 HD23 LEU    80      31.880  44.120  49.133  1.00  0.00           H
ATOM   1260  C   LEU    80      29.342  47.010  49.435  1.00  0.00           C
ATOM   1261  O   LEU    80      28.320  46.444  49.031  1.00  0.00           O
ATOM   1262  N   LYS    81      29.495  47.126  50.742  1.00  0.00           N
```

ATOM	1263	H	LYS	81	30.233	47.745	51.046	1.00	0.00	H
ATOM	1264	CA	LYS	81	28.415	46.699	51.678	1.00	0.00	C
ATOM	1265	HA	LYS	81	28.192	45.672	51.387	1.00	0.00	H
ATOM	1266	CB	LYS	81	28.946	46.827	53.084	1.00	0.00	C
ATOM	1267	HB2	LYS	81	29.264	47.843	53.320	1.00	0.00	H
ATOM	1268	HB3	LYS	81	28.086	46.568	53.700	1.00	0.00	H
ATOM	1269	CG	LYS	81	30.060	45.753	53.438	1.00	0.00	C
ATOM	1270	HG2	LYS	81	29.692	44.742	53.263	1.00	0.00	H
ATOM	1271	HG3	LYS	81	30.936	45.934	52.815	1.00	0.00	H
ATOM	1272	CD	LYS	81	30.445	45.810	54.894	1.00	0.00	C
ATOM	1273	HD2	LYS	81	29.910	46.594	55.430	1.00	0.00	H
ATOM	1274	HD3	LYS	81	30.159	44.849	55.320	1.00	0.00	H
ATOM	1275	CE	LYS	81	31.979	46.033	55.036	1.00	0.00	C
ATOM	1276	HE2	LYS	81	32.580	45.175	54.739	1.00	0.00	H
ATOM	1277	HE3	LYS	81	32.374	46.963	54.627	1.00	0.00	H
ATOM	1278	NZ	LYS	81	32.190	46.147	56.492	1.00	0.00	N
ATOM	1279	HZ1	LYS	81	33.048	46.626	56.726	1.00	0.00	H
ATOM	1280	HZ2	LYS	81	31.372	46.644	56.814	1.00	0.00	H
ATOM	1281	HZ3	LYS	81	32.179	45.211	56.873	1.00	0.00	H
ATOM	1282	C	LYS	81	27.076	47.445	51.456	1.00	0.00	C
ATOM	1283	O	LYS	81	25.973	46.900	51.466	1.00	0.00	O
ATOM	1284	N	LYS	82	27.173	48.725	51.032	1.00	0.00	N
ATOM	1285	H	LYS	82	28.059	49.178	50.850	1.00	0.00	H
ATOM	1286	CA	LYS	82	25.919	49.575	50.823	1.00	0.00	C
ATOM	1287	HA	LYS	82	25.266	49.465	51.689	1.00	0.00	H
ATOM	1288	CB	LYS	82	26.365	51.057	50.814	1.00	0.00	C
ATOM	1289	HB2	LYS	82	27.453	51.125	50.794	1.00	0.00	H
ATOM	1290	HB3	LYS	82	25.954	51.430	49.876	1.00	0.00	H
ATOM	1291	CG	LYS	82	25.840	51.880	51.984	1.00	0.00	C
ATOM	1292	HG2	LYS	82	24.773	52.096	51.932	1.00	0.00	H
ATOM	1293	HG3	LYS	82	25.982	51.334	52.917	1.00	0.00	H
ATOM	1294	CD	LYS	82	26.521	53.212	51.988	1.00	0.00	C
ATOM	1295	HD2	LYS	82	26.339	53.856	52.848	1.00	0.00	H
ATOM	1296	HD3	LYS	82	27.598	53.046	51.978	1.00	0.00	H
ATOM	1297	CE	LYS	82	26.287	54.176	50.885	1.00	0.00	C
ATOM	1298	HE2	LYS	82	26.441	53.720	49.906	1.00	0.00	H
ATOM	1299	HE3	LYS	82	25.254	54.509	50.992	1.00	0.00	H
ATOM	1300	NZ	LYS	82	27.120	55.398	50.857	1.00	0.00	N
ATOM	1301	HZ1	LYS	82	26.934	55.944	51.686	1.00	0.00	H
ATOM	1302	HZ2	LYS	82	28.091	55.232	50.636	1.00	0.00	H
ATOM	1303	HZ3	LYS	82	26.800	56.101	50.206	1.00	0.00	H
ATOM	1304	C	LYS	82	25.067	49.154	49.653	1.00	0.00	C
ATOM	1305	O	LYS	82	23.843	49.143	49.751	1.00	0.00	O
ATOM	1306	N	VAL	83	25.752	48.966	48.546	1.00	0.00	N
ATOM	1307	H	VAL	83	26.690	49.336	48.579	1.00	0.00	H
ATOM	1308	CA	VAL	83	25.174	48.595	47.252	1.00	0.00	C
ATOM	1309	HA	VAL	83	24.254	49.139	47.038	1.00	0.00	H
ATOM	1310	CB	VAL	83	26.160	48.984	46.088	1.00	0.00	C
ATOM	1311	HB	VAL	83	25.654	48.788	45.142	1.00	0.00	H
ATOM	1312	CG1	VAL	83	26.691	50.389	46.256	1.00	0.00	C
ATOM	1313	HG11	VAL	83	25.909	51.149	46.255	1.00	0.00	H
ATOM	1314	HG12	VAL	83	27.258	50.446	47.185	1.00	0.00	H
ATOM	1315	HG13	VAL	83	27.378	50.672	45.459	1.00	0.00	H
ATOM	1316	CG2	VAL	83	27.429	48.122	46.103	1.00	0.00	C
ATOM	1317	HG21	VAL	83	28.057	48.116	46.994	1.00	0.00	H
ATOM	1318	HG22	VAL	83	27.240	47.100	45.773	1.00	0.00	H
ATOM	1319	HG23	VAL	83	28.116	48.568	45.384	1.00	0.00	H
ATOM	1320	C	VAL	83	24.712	47.148	47.192	1.00	0.00	C
ATOM	1321	O	VAL	83	23.740	46.869	46.514	1.00	0.00	O
ATOM	1322	N	ILE	84	25.274	46.243	47.948	1.00	0.00	N
ATOM	1323	H	ILE	84	26.162	46.395	48.401	1.00	0.00	H
ATOM	1324	CA	ILE	84	24.730	44.844	48.235	1.00	0.00	C
ATOM	1325	HA	ILE	84	24.504	44.427	47.254	1.00	0.00	H
ATOM	1326	CB	ILE	84	25.850	43.939				

48.874 1.00 0.00 C

ATOM	1327	HB	ILE	84	26.238	44.427	49.768	1.00	0.00	H
ATOM	1328	CG2	ILE	84	25.158	42.622	49.150	1.00	0.00	C
ATOM	1329	HG21	ILE	84	24.280	42.872	49.746	1.00	0.00	H
ATOM	1330	HG22	ILE	84	24.906	42.132	48.209	1.00	0.00	H
ATOM	1331	HG23	ILE	84	25.684	41.916	49.792	1.00	0.00	H
ATOM	1332	CG1	ILE	84	26.928	43.704	47.821	1.00	0.00	C
ATOM	1333	HG12	ILE	84	26.509	43.287	46.905	1.00	0.00	H
ATOM	1334	HG13	ILE	84	27.460	44.635	47.629	1.00	0.00	H
ATOM	1335	CD1	ILE	84	28.024	42.767	48.393	1.00	0.00	C
ATOM	1336	HD11	ILE	84	27.702	41.744	48.585	1.00	0.00	H
ATOM	1337	HD12	ILE	84	28.779	42.768	47.607	1.00	0.00	H
ATOM	1338	HD13	ILE	84	28.517	43.240	49.242	1.00	0.00	H
ATOM	1339	C	ILE	84	23.431	45.028	49.053	1.00	0.00	C
ATOM	1340	O	ILE	84	22.446	44.522	48.578	1.00	0.00	O
ATOM	1341	N	ALA	85	23.449	45.702	50.228	1.00	0.00	N
ATOM	1342	H	ALA	85	24.314	46.075	50.595	1.00	0.00	H
ATOM	1343	CA	ALA	85	22.249	45.879	50.972	1.00	0.00	C
ATOM	1344	HA	ALA	85	21.917	44.909	51.340	1.00	0.00	H
ATOM	1345	CB	ALA	85	22.697	46.569	52.245	1.00	0.00	C
ATOM	1346	HB1	ALA	85	21.863	46.628	52.945	1.00	0.00	H
ATOM	1347	HB2	ALA	85	23.329	45.893	52.822	1.00	0.00	H
ATOM	1348	HB3	ALA	85	23.050	47.576	52.023	1.00	0.00	H
ATOM	1349	C	ALA	85	21.087	46.620	50.324	1.00	0.00	C
ATOM	1350	O	ALA	85	19.944	46.488	50.755	1.00	0.00	O
ATOM	1351	N	ASP	86	21.288	47.316	49.235	1.00	0.00	N
ATOM	1352	H	ASP	86	22.261	47.333	48.966	1.00	0.00	H
ATOM	1353	CA	ASP	86	20.178	47.891	48.410	1.00	0.00	C
ATOM	1354	HA	ASP	86	19.392	48.348	49.011	1.00	0.00	H
ATOM	1355	CB	ASP	86	20.942	48.878	47.521	1.00	0.00	C
ATOM	1356	HB2	ASP	86	21.275	49.654	48.210	1.00	0.00	H
ATOM	1357	HB3	ASP	86	21.739	48.397	46.953	1.00	0.00	H
ATOM	1358	CG	ASP	86	19.974	49.547	46.465	1.00	0.00	C
ATOM	1359	OD1	ASP	86	20.353	49.712	45.280	1.00	0.00	O
ATOM	1360	OD2	ASP	86	18.987	50.106	47.005	1.00	0.00	O
ATOM	1361	C	ASP	86	19.387	46.740	47.599	1.00	0.00	C
ATOM	1362	O	ASP	86	18.147	46.716	47.365	1.00	0.00	O
ATOM	1363	N	ILE	87	20.168	45.776	47.154	1.00	0.00	N
ATOM	1364	H	ILE	87	21.113	45.784	47.511	1.00	0.00	H
ATOM	1365	CA	ILE	87	19.802	44.693	46.209	1.00	0.00	C
ATOM	1366	HA	ILE	87	18.944	45.004	45.612	1.00	0.00	H
ATOM	1367	CB	ILE	87	20.990	44.469	45.168	1.00	0.00	C
ATOM	1368	HB	ILE	87	21.929	44.101	45.582	1.00	0.00	H
ATOM	1369	CG2	ILE	87	20.637	43.379	44.056	1.00	0.00	C
ATOM	1370	HG21	ILE	87	21.478	43.180	43.392	1.00	0.00	H
ATOM	1371	HG22	ILE	87	20.272	42.448	44.490	1.00	0.00	H
ATOM	1372	HG23	ILE	87	19.808	43.644	43.400	1.00	0.00	H
ATOM	1373	CG1	ILE	87	21.402	45.769	44.391	1.00	0.00	C
ATOM	1374	HG12	ILE	87	20.613	45.889	43.649	1.00	0.00	H
ATOM	1375	HG13	ILE	87	21.268	46.701	44.941	1.00	0.00	H
ATOM	1376	CD1	ILE	87	22.814	45.768	43.660	1.00	0.00	C
ATOM	1377	HD11	ILE	87	23.067	46.812	43.472	1.00	0.00	H
ATOM	1378	HD12	ILE	87	23.538	45.397	44.385	1.00	0.00	H
ATOM	1379	HD13	ILE	87	22.792	45.133	42.775	1.00	0.00	H
ATOM	1380	C	ILE	87	19.294	43.409	46.900	1.00	0.00	C
ATOM	1381	O	ILE	87	18.477	42.656	46.328	1.00	0.00	O
ATOM	1382	N	TYR	88	19.783	43.211	48.154	1.00	0.00	N
ATOM	1383	H	TYR	88	20.426	43.909	48.500	1.00	0.00	H
ATOM	1384	CA	TYR	88	19.437	42.133	48.996	1.00	0.00	C
ATOM	1385	HA	TYR	88	18.584	41.648	48.521	1.00	0.00	H
ATOM	1386	CB	TYR	88	20.583	41.104	49.014	1.00	0.00	C
ATOM	1387	HB2	TYR	88	21.486	41.676	49.235	1.00	0.00	H
ATOM	1388	HB3	TYR	88	20.304	40.368	49.767	1.00	0.00	H
ATOM	1389	CG	TYR	88	20.977	40.394	47.756	1.00	0.00	C

ATOM	1390	CD1	TYR	88	20.429	39.083	47.623	1.00	0.00	C
ATOM	1391	HD1	TYR	88	19.845	38.625	48.407	1.00	0.00	H
ATOM	1392	CE1	TYR	88	20.648	38.372	46.423	1.00	0.00	C
ATOM	1393	HE1	TYR	88	20.204	37.396	46.296	1.00	0.00	H
ATOM	1394	CZ	TYR	88	21.512	38.913	45.452	1.00	0.00	C
ATOM	1395	OH	TYR	88	21.742	38.240	44.339	1.00	0.00	O
ATOM	1396	HH	TYR	88	21.809	37.324	44.619	1.00	0.00	H
ATOM	1397	CE2	TYR	88	22.001	40.223	45.500	1.00	0.00	C
ATOM	1398	HE2	TYR	88	22.391	40.667	44.595	1.00	0.00	H
ATOM	1399	CD2	TYR	88	21.728	41.018	46.694	1.00	0.00	C
ATOM	1400	HD2	TYR	88	22.029	42.055	46.663	1.00	0.00	H
ATOM	1401	C	TYR	88	19.041	42.575	50.394	1.00	0.00	C
ATOM	1402	O	TYR	88	19.349	43.637	50.839	1.00	0.00	O
ATOM	1403	N	PRO	89	18.419	41.670	51.240	1.00	0.00	N
ATOM	1404	CD	PRO	89	17.646	40.470	50.920	1.00	0.00	C
ATOM	1405	HD2	PRO	89	18.207	39.541	50.816	1.00	0.00	H
ATOM	1406	HD3	PRO	89	17.195	40.668	49.948	1.00	0.00	H
ATOM	1407	CG	PRO	89	16.693	40.258	52.037	1.00	0.00	C
ATOM	1408	HG2	PRO	89	16.413	39.212	51.919	1.00	0.00	H
ATOM	1409	HG3	PRO	89	15.897	41.003	52.042	1.00	0.00	H
ATOM	1410	CB	PRO	89	17.583	40.511	53.233	1.00	0.00	C
ATOM	1411	HB2	PRO	89	18.267	39.672	53.365	1.00	0.00	H
ATOM	1412	HB3	PRO	89	17.006	40.728	54.132	1.00	0.00	H
ATOM	1413	CA	PRO	89	18.402	41.757	52.692	1.00	0.00	C
ATOM	1414	HA	PRO	89	17.883	42.681	52.943	1.00	0.00	H
ATOM	1415	C	PRO	89	19.824	41.691	53.269	1.00	0.00	C
ATOM	1416	O	PRO	89	20.477	40.777	52.794	1.00	0.00	O
ATOM	1417	N	GLY	90	20.327	42.571	54.125	1.00	0.00	N
ATOM	1418	H	GLY	90	19.896	43.482	54.079	1.00	0.00	H
ATOM	1419	CA	GLY	90	21.672	42.457	54.660	1.00	0.00	C
ATOM	1420	HA2	GLY	90	22.506	42.625	53.978	1.00	0.00	H
ATOM	1421	HA3	GLY	90	21.695	43.138	55.510	1.00	0.00	H
ATOM	1422	C	GLY	90	21.894	41.113	55.348	1.00	0.00	C
ATOM	1423	O	GLY	90	23.042	40.627	55.326	1.00	0.00	O
ATOM	1424	N	GLN	91	20.865	40.393	55.788	1.00	0.00	N
ATOM	1425	H	GLN	91	19.971	40.857	55.709	1.00	0.00	H
ATOM	1426	CA	GLN	91	20.814	39.086	56.383	1.00	0.00	C
ATOM	1427	HA	GLN	91	21.476	39.124	57.248	1.00	0.00	H
ATOM	1428	CB	GLN	91	19.410	38.743	56.890	1.00	0.00	C
ATOM	1429	HB2	GLN	91	18.685	38.600	56.090	1.00	0.00	H
ATOM	1430	HB3	GLN	91	19.610	37.773	57.346	1.00	0.00	H
ATOM	1431	CG	GLN	91	18.964	39.493	58.223	1.00	0.00	C
ATOM	1432	HG2	GLN	91	17.982	39.132	58.533	1.00	0.00	H
ATOM	1433	HG3	GLN	91	19.699	39.307	59.006	1.00	0.00	H
ATOM	1434	CD	GLN	91	18.879	40.991	58.012	1.00	0.00	C
ATOM	1435	OE1	GLN	91	18.358	41.535	56.977	1.00	0.00	O
ATOM	1436	NE2	GLN	91	19.480	41.771	58.881	1.00	0.00	N
ATOM	1437	HE21	GLN	91	19.824	41.259	59.681	1.00	0.00	H
ATOM	1438	HE22	GLN	91	19.245	42.748	58.981	1.00	0.00	H
ATOM	1439	C	GLN	91	21.304	38.020	55.322	1.00	0.00	C
ATOM	1440	O	GLN	91	21.543	36.866	55.685	1.00	0.00	O
ATOM	1441	N	THR	92	21.441	38.408	54.045	1.00	0.00	N
ATOM	1442	H	THR	92	21.395	39.373	53.748	1.00	0.00	H
ATOM	1443	CA	THR	92	21.819	37.341	53.087	1.00	0.00	C
ATOM	1444	HA	THR	92	21.214	36.434	53.109	1.00	0.00	H
ATOM	1445	CB	THR	92	21.707	37.885	51.601	1.00	0.00	C
ATOM	1446	HB	THR	92	22.557	38.540	51.410	1.00	0.00	H
ATOM	1447	CG2	THR	92	21.701	36.782	50.548	1.00	0.00	C
ATOM	1448	HG21	THR	92	21.199	37.050	49.619	1.00	0.00	H
ATOM	1449	HG22	THR	92	22.706	36.437	50.298	1.00	0.00	H
ATOM	1450	HG23	THR	92	21.204	35.872	50.883	1.00	0.00	H
ATOM	1451	OG1	THR	92	20.454	38.469	51.394	1.00	0.00	O
ATOM	1452	HG1	THR	92	20.351	39.308	51.847	1.00	0.00	H

ATOM	1453	C	THR	92	23.260	36.864	53.356	1.00	0.00	C
ATOM	1454	O	THR	92	24.139	37.728	53.620	1.00	0.00	O
ATOM	1455	N	GLN	93	23.531	35.544	53.226	1.00	0.00	N
ATOM	1456	H	GLN	93	22.743	34.937	53.048	1.00	0.00	H
ATOM	1457	CA	GLN	93	24.860	34.945	53.122	1.00	0.00	C
ATOM	1458	HA	GLN	93	25.654	35.492	53.629	1.00	0.00	H
ATOM	1459	CB	GLN	93	24.844	33.481	53.738	1.00	0.00	C
ATOM	1460	HB2	GLN	93	24.533	33.574	54.779	1.00	0.00	H
ATOM	1461	HB3	GLN	93	23.993	33.044	53.213	1.00	0.00	H
ATOM	1462	CG	GLN	93	26.056	32.623	53.687	1.00	0.00	C
ATOM	1463	HG2	GLN	93	25.657	31.610	53.719	1.00	0.00	H
ATOM	1464	HG3	GLN	93	26.641	32.791	52.783	1.00	0.00	H
ATOM	1465	CD	GLN	93	27.020	32.946	54.905	1.00	0.00	C
ATOM	1466	OE1	GLN	93	26.625	33.536	55.910	1.00	0.00	O
ATOM	1467	NE2	GLN	93	28.143	32.304	54.931	1.00	0.00	N
ATOM	1468	HE21	GLN	93	28.366	31.753	54.116	1.00	0.00	H
ATOM	1469	HE22	GLN	93	28.697	32.221	55.772	1.00	0.00	H
ATOM	1470	C	GLN	93	25.246	34.963	51.579	1.00	0.00	C
ATOM	1471	O	GLN	93	24.481	34.717	50.655	1.00	0.00	O
ATOM	1472	N	PHE	94	26.492	35.304	51.279	1.00	0.00	N
ATOM	1473	H	PHE	94	27.131	35.442	52.049	1.00	0.00	H
ATOM	1474	CA	PHE	94	27.095	35.248	49.929	1.00	0.00	C
ATOM	1475	HA	PHE	94	26.282	35.022	49.239	1.00	0.00	H
ATOM	1476	CB	PHE	94	27.632	36.638	49.581	1.00	0.00	C
ATOM	1477	HB2	PHE	94	28.449	36.884	50.259	1.00	0.00	H
ATOM	1478	HB3	PHE	94	28.060	36.548	48.582	1.00	0.00	H
ATOM	1479	CG	PHE	94	26.540	37.698	49.639	1.00	0.00	C
ATOM	1480	CD1	PHE	94	25.645	37.837	48.542	1.00	0.00	C
ATOM	1481	HD1	PHE	94	25.747	37.242	47.646	1.00	0.00	H
ATOM	1482	CE1	PHE	94	24.582	38.675	48.596	1.00	0.00	C
ATOM	1483	HE1	PHE	94	23.827	38.792	47.831	1.00	0.00	H
ATOM	1484	CZ	PHE	94	24.270	39.332	49.808	1.00	0.00	C
ATOM	1485	HZ	PHE	94	23.365	39.905	49.942	1.00	0.00	H
ATOM	1486	CE2	PHE	94	25.144	39.191	50.936	1.00	0.00	C
ATOM	1487	HE2	PHE	94	24.993	39.874	51.758	1.00	0.00	H
ATOM	1488	CD2	PHE	94	26.273	38.403	50.833	1.00	0.00	C
ATOM	1489	HD2	PHE	94	26.919	38.207	51.676	1.00	0.00	H
ATOM	1490	C	PHE	94	28.161	34.196	49.749	1.00	0.00	C
ATOM	1491	O	PHE	94	28.455	33.406	50.643	1.00	0.00	O
ATOM	1492	N	TYR	95	28.699	34.053	48.535	1.00	0.00	N
ATOM	1493	H	TYR	95	28.277	34.613	47.809	1.00	0.00	H
ATOM	1494	CA	TYR	95	29.650	33.051	48.119	1.00	0.00	C
ATOM	1495	HA	TYR	95	30.185	32.735	49.014	1.00	0.00	H
ATOM	1496	CB	TYR	95	28.818	31.851	47.525	1.00	0.00	C
ATOM	1497	HB2	TYR	95	28.106	31.551	48.295	1.00	0.00	H
ATOM	1498	HB3	TYR	95	28.242	32.293	46.713	1.00	0.00	H
ATOM	1499	CG	TYR	95	29.579	30.651	47.102	1.00	0.00	C
ATOM	1500	CD1	TYR	95	29.882	30.499	45.672	1.00	0.00	C
ATOM	1501	HD1	TYR	95	29.673	31.290	44.967	1.00	0.00	H
ATOM	1502	CE1	TYR	95	30.528	29.333	45.234	1.00	0.00	C
ATOM	1503	HE1	TYR	95	30.703	29.158	44.182	1.00	0.00	H
ATOM	1504	CZ	TYR	95	30.755	28.240	46.145	1.00	0.00	C
ATOM	1505	OH	TYR	95	31.306	27.015	45.762	1.00	0.00	O
ATOM	1506	HH	TYR	95	31.268	26.895	44.809	1.00	0.00	H
ATOM	1507	CE2	TYR	95	30.426	28.370	47.516	1.00	0.00	C
ATOM	1508	HE2	TYR	95	30.715	27.598	48.213	1.00	0.00	H
ATOM	1509	CD2	TYR	95	29.822	29.604	47.984	1.00	0.00	C
ATOM	1510	HD2	TYR	95	29.663	29.615	49.052	1.00	0.00	H
ATOM	1511	C	TYR	95	30.713	33.602	47.133	1.00	0.00	C
ATOM	1512	O	TYR	95	30.425	34.494	46.363	1.00	0.00	O
ATOM	1513	N	VAL	96	31.919	32.993	47.088	1.00	0.00	N
ATOM	1514	H	VAL	96	32.127	32.207	47.687	1.00	0.00	H
ATOM	1515	CA	VAL	96	33.052	33.626	46.393	1.00	0.00	C
ATOM	1516	HA	VAL	96	32.697	34.533	45.905	1.00	0.00	H

ATOM	1517	CB	VAL	96	34.098	33.897	47.505	1.00	0.00	C
ATOM	1518	HB	VAL	96	34.470	32.925	47.826	1.00	0.00	H
ATOM	1519	CG1	VAL	96	35.312	34.605	46.920	1.00	0.00	C
ATOM	1520	HG11	VAL	96	35.980	34.771	47.765	1.00	0.00	H
ATOM	1521	HG12	VAL	96	35.754	33.871	46.246	1.00	0.00	H
ATOM	1522	HG13	VAL	96	34.969	35.490	46.383	1.00	0.00	H
ATOM	1523	CG2	VAL	96	33.429	34.738	48.644	1.00	0.00	C
ATOM	1524	HG21	VAL	96	32.574	34.344	49.194	1.00	0.00	H
ATOM	1525	HG22	VAL	96	34.243	35.047	49.300	1.00	0.00	H
ATOM	1526	HG23	VAL	96	33.060	35.665	48.206	1.00	0.00	H
ATOM	1527	C	VAL	96	33.537	32.644	45.385	1.00	0.00	C
ATOM	1528	O	VAL	96	33.602	31.450	45.703	1.00	0.00	O
ATOM	1529	N	ILE	97	33.848	33.176	44.216	1.00	0.00	N
ATOM	1530	H	ILE	97	33.556	34.100	43.931	1.00	0.00	H
ATOM	1531	CA	ILE	97	34.352	32.331	43.084	1.00	0.00	C
ATOM	1532	HA	ILE	97	34.530	31.288	43.345	1.00	0.00	H
ATOM	1533	CB	ILE	97	33.375	32.232	41.890	1.00	0.00	C
ATOM	1534	HB	ILE	97	32.985	33.185	41.532	1.00	0.00	H
ATOM	1535	CG2	ILE	97	33.996	31.524	40.656	1.00	0.00	C
ATOM	1536	HG21	ILE	97	34.388	32.336	40.044	1.00	0.00	H
ATOM	1537	HG22	ILE	97	34.832	30.885	40.943	1.00	0.00	H
ATOM	1538	HG23	ILE	97	33.293	30.883	40.126	1.00	0.00	H
ATOM	1539	CG1	ILE	97	32.122	31.484	42.303	1.00	0.00	C
ATOM	1540	HG12	ILE	97	32.088	30.428	42.035	1.00	0.00	H
ATOM	1541	HG13	ILE	97	32.035	31.567	43.386	1.00	0.00	H
ATOM	1542	CD1	ILE	97	30.734	32.116	41.860	1.00	0.00	C
ATOM	1543	HD11	ILE	97	30.553	33.074	42.346	1.00	0.00	H
ATOM	1544	HD12	ILE	97	30.500	32.138	40.796	1.00	0.00	H
ATOM	1545	HD13	ILE	97	30.072	31.490	42.459	1.00	0.00	H
ATOM	1546	C	ILE	97	35.758	32.996	42.738	1.00	0.00	C
ATOM	1547	O	ILE	97	35.694	33.978	41.967	1.00	0.00	O
ATOM	1548	N	GLU	98	36.896	32.504	43.241	1.00	0.00	N
ATOM	1549	H	GLU	98	36.798	31.634	43.745	1.00	0.00	H
ATOM	1550	CA	GLU	98	38.230	32.916	42.879	1.00	0.00	C
ATOM	1551	HA	GLU	98	38.259	33.947	42.527	1.00	0.00	H
ATOM	1552	CB	GLU	98	39.237	32.825	44.047	1.00	0.00	C
ATOM	1553	HB2	GLU	98	39.325	31.794	44.389	1.00	0.00	H
ATOM	1554	HB3	GLU	98	40.239	33.164	43.787	1.00	0.00	H
ATOM	1555	CG	GLU	98	38.759	33.576	45.339	1.00	0.00	C
ATOM	1556	HG2	GLU	98	38.485	34.603	45.097	1.00	0.00	H
ATOM	1557	HG3	GLU	98	37.893	32.982	45.632	1.00	0.00	H
ATOM	1558	CD	GLU	98	39.760	33.607	46.494	1.00	0.00	C
ATOM	1559	OE1	GLU	98	39.264	33.665	47.643	1.00	0.00	O
ATOM	1560	OE2	GLU	98	40.976	33.602	46.209	1.00	0.00	O
ATOM	1561	C	GLU	98	38.884	32.054	41.703	1.00	0.00	C
ATOM	1562	O	GLU	98	38.899	30.822	41.800	1.00	0.00	O
ATOM	1563	N	PHE	99	39.400	32.631	40.630	1.00	0.00	N
ATOM	1564	H	PHE	99	39.453	33.634	40.524	1.00	0.00	H
ATOM	1565	CA	PHE	99	39.723	31.771	39.411	1.00	0.00	C
ATOM	1566	HA	PHE	99	40.117	30.812	39.746	1.00	0.00	H
ATOM	1567	CB	PHE	99	38.451	31.504	38.527	1.00	0.00	C
ATOM	1568	HB2	PHE	99	38.772	30.807	37.752	1.00	0.00	H
ATOM	1569	HB3	PHE	99	37.710	30.986	39.136	1.00	0.00	H
ATOM	1570	CG	PHE	99	37.810	32.800	37.994	1.00	0.00	C
ATOM	1571	CD1	PHE	99	36.823	33.368	38.854	1.00	0.00	C
ATOM	1572	HD1	PHE	99	36.714	32.964	39.850	1.00	0.00	H
ATOM	1573	CE1	PHE	99	36.080	34.452	38.467	1.00	0.00	C
ATOM	1574	HE1	PHE	99	35.314	34.794	39.147	1.00	0.00	H
ATOM	1575	CZ	PHE	99	36.274	34.998	37.212	1.00	0.00	C
ATOM	1576	HZ	PHE	99	35.795	35.932	36.957	1.00	0.00	H
ATOM	1577	CE2	PHE	99	37.347	34.649	36.457	1.00	0.00	C
ATOM	1578	HE2	PHE	99	37.636	35.281	35.632	1.00	0.00	H
ATOM	1579	CD2	PHE	99	38.142	33.538	36.808	1.00	0.00	C

ATOM	1580	HD2	PHE	99	38.870	33.283	36.054	1.00	0.00	H
ATOM	1581	C	PHE	99	40.800	32.429	38.536	1.00	0.00	C
ATOM	1582	O	PHE	99	41.284	33.580	38.735	1.00	0.00	O
ATOM	1583	N	LYS	100	41.231	31.657	37.583	1.00	0.00	N
ATOM	1584	H	LYS	100	41.058	30.662	37.598	1.00	0.00	H
ATOM	1585	CA	LYS	100	42.250	32.175	36.607	1.00	0.00	C
ATOM	1586	HA	LYS	100	42.002	33.223	36.436	1.00	0.00	H
ATOM	1587	CB	LYS	100	43.667	32.186	37.260	1.00	0.00	C
ATOM	1588	HB2	LYS	100	44.350	32.828	36.700	1.00	0.00	H
ATOM	1589	HB3	LYS	100	43.641	32.681	38.231	1.00	0.00	H
ATOM	1590	CG	LYS	100	44.262	30.836	37.532	1.00	0.00	C
ATOM	1591	HG2	LYS	100	43.605	30.293	38.212	1.00	0.00	H
ATOM	1592	HG3	LYS	100	44.333	30.207	36.645	1.00	0.00	H
ATOM	1593	CD	LYS	100	45.691	30.842	38.023	1.00	0.00	C
ATOM	1594	HD2	LYS	100	45.776	31.650	38.748	1.00	0.00	H
ATOM	1595	HD3	LYS	100	45.891	29.902	38.539	1.00	0.00	H
ATOM	1596	CE	LYS	100	46.621	30.975	36.839	1.00	0.00	C
ATOM	1597	HE2	LYS	100	46.603	30.026	36.303	1.00	0.00	H
ATOM	1598	HE3	LYS	100	46.267	31.738	36.146	1.00	0.00	H
ATOM	1599	NZ	LYS	100	47.931	31.325	37.225	1.00	0.00	N
ATOM	1600	HZ1	LYS	100	47.990	32.261	37.599	1.00	0.00	H
ATOM	1601	HZ2	LYS	100	48.188	30.568	37.844	1.00	0.00	H
ATOM	1602	HZ3	LYS	100	48.592	31.235	36.466	1.00	0.00	H
ATOM	1603	C	LYS	100	42.465	31.551	35.225	1.00	0.00	C
ATOM	1604	O	LYS	100	42.362	30.351	35.114	1.00	0.00	O
ATOM	1605	N	CYX	101	42.646	32.330	34.165	1.00	0.00	N
ATOM	1606	H	CYX	101	42.590	33.318	34.366	1.00	0.00	H
ATOM	1607	CA	CYX	101	42.933	31.741	32.884	1.00	0.00	C
ATOM	1608	HA	CYX	101	42.265	30.893	32.736	1.00	0.00	H
ATOM	1609	CB	CYX	101	42.806	32.853	31.823	1.00	0.00	C
ATOM	1610	HB2	CYX	101	41.752	33.133	31.847	1.00	0.00	H
ATOM	1611	HB3	CYX	101	43.524	33.643	32.047	1.00	0.00	H
ATOM	1612	SG	CYX	101	43.202	32.410	30.111	1.00	0.00	S
ATOM	1613	C	CYX	101	44.314	31.155	32.909	1.00	0.00	C
ATOM	1614	O	CYX	101	45.221	31.812	33.441	1.00	0.00	O
ATOM	1615	N	LEU	102	44.575	29.849	32.429	1.00	0.00	N
ATOM	1616	H	LEU	102	43.828	29.302	32.027	1.00	0.00	H
ATOM	1617	CA	LEU	102	45.871	29.225	32.521	1.00	0.00	C
ATOM	1618	HA	LEU	102	46.364	29.331	33.487	1.00	0.00	H
ATOM	1619	CB	LEU	102	45.553	27.693	32.363	1.00	0.00	C
ATOM	1620	HB2	LEU	102	45.157	27.633	31.349	1.00	0.00	H
ATOM	1621	HB3	LEU	102	46.516	27.193	32.466	1.00	0.00	H
ATOM	1622	CG	LEU	102	44.535	27.135	33.368	1.00	0.00	C
ATOM	1623	HG	LEU	102	43.502	27.397	33.138	1.00	0.00	H
ATOM	1624	CD1	LEU	102	44.474	25.620	33.351	1.00	0.00	C
ATOM	1625	HD11	LEU	102	43.854	25.503	32.461	1.00	0.00	H
ATOM	1626	HD12	LEU	102	45.435	25.157	33.129	1.00	0.00	H
ATOM	1627	HD13	LEU	102	43.891	25.213	34.178	1.00	0.00	H
ATOM	1628	CD2	LEU	102	44.813	27.654	34.775	1.00	0.00	C
ATOM	1629	HD21	LEU	102	45.862	27.404	34.934	1.00	0.00	H
ATOM	1630	HD22	LEU	102	44.767	28.740	34.858	1.00	0.00	H
ATOM	1631	HD23	LEU	102	44.245	27.124	35.539	1.00	0.00	H
ATOM	1632	C	LEU	102	46.796	29.656	31.406	1.00	0.00	C
ATOM	1633	O	LEU	102	46.338	29.633	30.185	1.00	0.00	O
ATOM	1634	OXT	LEU	102	48.002	30.025	31.657	1.00	0.00	O
HETATM	314	N	LYN	20	39.711	40.649	35.454	1.00	0.00	N
HETATM	315	H	LYN	20	40.150	40.377	34.586	1.00	0.00	H
HETATM	316	CA	LYN	20	38.747	39.768	36.091	1.00	0.00	C
HETATM	317	HA	LYN	20	38.377	40.108	37.058	1.00	0.00	H
HETATM	318	CB	LYN	20	37.451	39.600	35.172	1.00	0.00	C
HETATM	319	HB2	LYN	20	37.808	39.117	34.262	1.00	0.00	H
HETATM	320	HB3	LYN	20	36.997	40.547	34.880	1.00	0.00	H
HETATM	321	CG	LYN	20	36.251	38.812	35.814	1.00	0.00	C
HETATM	322	HG2	LYN	20	35.488	38.639	35.056	1.00	0.00	H

```
HETATM  323  HG3 LYN    20       36.552  37.828  36.174  1.00  0.00            H
HETATM  324  CD  LYN    20       35.531  39.498  36.977  1.00  0.00            C
HETATM  325  HD2 LYN    20       35.464  40.562  36.752  1.00  0.00            H
HETATM  326  HD3 LYN    20       36.197  39.441  37.838  1.00  0.00            H
HETATM  327  CE  LYN    20       34.149  38.992  37.370  1.00  0.00            C
HETATM  328  HE2 LYN    20       33.570  39.106  36.454  1.00  0.00            H
HETATM  329  HE3 LYN    20       34.085  37.915  37.518  1.00  0.00            H
HETATM  330  NZ  LYN    20       33.494  39.702  38.505  1.00  0.00            N
HETATM  331  HZ2 LYN    20       33.370  40.644  38.162  1.00  0.00            H
HETATM  332  HZ3 LYN    20       34.151  39.689  39.271  1.00  0.00            H
HETATM  333  C   LYN    20       39.377  38.479  36.585  1.00  0.00            C
HETATM  334  O   LYN    20       40.049  37.709  35.830  1.00  0.00            O
HETATM 1636  N   LIG   103       30.173  42.245  40.227  1.00  0.00            N
HETATM 1637  C   LIG   103       29.664  41.363  39.439  1.00  0.00            C
HETATM 1638  O   LIG   103       30.260  43.527  42.038  1.00  0.00            O
HETATM 1639  C5' LIG   103       25.622  39.950  44.617  1.00  0.00            C
HETATM 1640  O5' LIG   103       25.679  39.040  43.505  1.00  0.00            O
HETATM 1641  C4' LIG   103       26.225  41.380  44.487  1.00  0.00            C
HETATM 1642  O4' LIG   103       27.445  41.222  43.821  1.00  0.00            O
HETATM 1643  C3' LIG   103       25.453  42.286  43.582  1.00  0.00            C
HETATM 1644  O3' LIG   103       24.471  42.923  44.352  1.00  0.00            O
HETATM 1645  C2' LIG   103       26.541  43.261  43.091  1.00  0.00            C
HETATM 1646  O2' LIG   103       26.564  44.392  43.910  1.00  0.00            O
HETATM 1647  C1' LIG   103       27.900  42.432  43.146  1.00  0.00            C
HETATM 1648  N1  LIG   103       28.523  42.003  41.769  1.00  0.00            N
HETATM 1649  O1  LIG   103       29.434  39.047  37.757  1.00  0.00            O
HETATM 1650  N2  LIG   103       30.328  41.060  38.339  1.00  0.00            N
HETATM 1651  C6  LIG   103       29.700  42.598  41.445  1.00  0.00            C
HETATM 1652  C7  LIG   103       27.834  41.106  41.009  1.00  0.00            C
HETATM 1653  C8  LIG   103       28.418  40.744  39.812  1.00  0.00            C
HETATM 1654  C9  LIG   103       30.038  40.052  37.453  1.00  0.00            C
HETATM 1655  C10 LIG   103       30.834  40.121  36.123  1.00  0.00            C
HETATM 1656  H   LIG   103       31.125  41.673  38.223  1.00  0.00            H
HETATM 1657  H1  LIG   103       27.910  40.055  39.149  1.00  0.00            H
HETATM 1658  H4  LIG   103       28.619  42.890  43.835  1.00  0.00            H
HETATM 1659  H6  LIG   103       26.429  41.812  45.475  1.00  0.00            H
HETATM 1660  H7  LIG   103       25.076  41.787  42.681  1.00  0.00            H
HETATM 1661  H8  LIG   103       26.334  43.590  42.065  1.00  0.00            H
HETATM 1662  H9  LIG   103       24.555  40.126  44.800  1.00  0.00            H
HETATM 1663  H10 LIG   103       25.936  39.380  45.500  1.00  0.00            H
HETATM 1664  H11 LIG   103       30.226  39.786  35.274  1.00  0.00            H
HETATM 1665  H12 LIG   103       31.747  39.514  36.180  1.00  0.00            H
HETATM 1666  H13 LIG   103       31.117  41.176  36.013  1.00  0.00            H
HETATM 1667  H14 LIG   103       26.869  40.751  41.345  1.00  0.00            H
HETATM 1668  H2' LIG   103       27.273  44.968  43.573  1.00  0.00            H
HETATM 1669  H3' LIG   103       24.684  43.820  44.043  1.00  0.00            H
HETATM 1670  H5' LIG   103       26.613  38.961  43.242  1.00  0.00            H
ENDMDL
MODEL      9
SHEET    1   1 1 ILE    22  ASP    26  0
SHEET    2   2 1 VAL    37  VAL    40  0
SHEET    3   3 1 CYX    50  VAL    60  0
SHEET    4   4 1 PHE    94  CYX   101  0
HELIX    1   1 GLN      9  ALA     17  1                                   9
HELIX    2   2 LYS     68  GLU     73  1                                   6
HELIX    3   3 LEU     77  ILE     87  1                                  11
ATOM      1  N   GLN     1       35.740  17.450  32.265  1.00  0.00            N
ATOM      2  H1  GLN     1       35.739  16.512  31.894  1.00  0.00            H
ATOM      3  H2  GLN     1       36.620  17.714  32.687  1.00  0.00            H
ATOM      4  H3  GLN     1       35.092  17.516  33.038  1.00  0.00            H
ATOM      5  CA  GLN     1       35.291  18.447  31.258  1.00  0.00            C
ATOM      6  HA  GLN     1       36.079  18.406  30.507  1.00  0.00            H
ATOM      7  CB  GLN     1
      33.974  18.012  30.498  1.00  0.00            C
```

ATOM	8	HB2	GLN	1	33.273	17.659	31.253	1.00	0.00	H
ATOM	9	HB3	GLN	1	33.618	18.822	29.862	1.00	0.00	H
ATOM	10	CG	GLN	1	34.171	16.770	29.650	1.00	0.00	C
ATOM	11	HG2	GLN	1	34.732	16.060	30.256	1.00	0.00	H
ATOM	12	HG3	GLN	1	33.219	16.330	29.353	1.00	0.00	H
ATOM	13	CD	GLN	1	35.024	16.934	28.328	1.00	0.00	C
ATOM	14	OE1	GLN	1	35.330	18.000	27.793	1.00	0.00	O
ATOM	15	NE2	GLN	1	35.252	15.854	27.700	1.00	0.00	N
ATOM	16	HE21	GLN	1	34.838	14.980	27.990	1.00	0.00	H
ATOM	17	HE22	GLN	1	35.602	16.028	26.769	1.00	0.00	H
ATOM	18	C	GLN	1	35.059	19.748	31.908	1.00	0.00	C
ATOM	19	O	GLN	1	34.672	19.756	33.081	1.00	0.00	O
ATOM	20	N	PRO	2	35.266	20.837	31.192	1.00	0.00	N
ATOM	21	CD	PRO	2	35.985	20.875	29.918	1.00	0.00	C
ATOM	22	HD2	PRO	2	35.653	20.091	29.238	1.00	0.00	H
ATOM	23	HD3	PRO	2	37.043	20.941	30.173	1.00	0.00	H
ATOM	24	CG	PRO	2	35.671	22.302	29.313	1.00	0.00	C
ATOM	25	HG2	PRO	2	34.694	22.324	28.829	1.00	0.00	H
ATOM	26	HG3	PRO	2	36.367	22.746	28.601	1.00	0.00	H
ATOM	27	CB	PRO	2	35.582	23.074	30.628	1.00	0.00	C
ATOM	28	HB2	PRO	2	34.978	23.953	30.397	1.00	0.00	H
ATOM	29	HB3	PRO	2	36.583	23.360	30.952	1.00	0.00	H
ATOM	30	CA	PRO	2	34.881	22.222	31.629	1.00	0.00	C
ATOM	31	HA	PRO	2	35.262	22.363	32.639	1.00	0.00	H
ATOM	32	C	PRO	2	33.357	22.410	31.528	1.00	0.00	C
ATOM	33	O	PRO	2	32.654	21.879	30.680	1.00	0.00	O
ATOM	34	N	ASN	3	32.786	23.209	32.500	1.00	0.00	N
ATOM	35	H	ASN	3	33.412	23.704	33.119	1.00	0.00	H
ATOM	36	CA	ASN	3	31.350	23.584	32.445	1.00	0.00	C
ATOM	37	HA	ASN	3	30.692	22.721	32.349	1.00	0.00	H
ATOM	38	CB	ASN	3	30.941	24.424	33.704	1.00	0.00	C
ATOM	39	HB2	ASN	3	31.569	25.313	33.643	1.00	0.00	H
ATOM	40	HB3	ASN	3	29.898	24.741	33.693	1.00	0.00	H
ATOM	41	CG	ASN	3	31.178	23.710	35.012	1.00	0.00	C
ATOM	42	OD1	ASN	3	30.156	23.384	35.645	1.00	0.00	O
ATOM	43	ND2	ASN	3	32.363	23.571	35.537	1.00	0.00	N
ATOM	44	HD21	ASN	3	33.068	23.937	34.915	1.00	0.00	H
ATOM	45	HD22	ASN	3	32.487	23.096	36.419	1.00	0.00	H
ATOM	46	C	ASN	3	30.951	24.490	31.257	1.00	0.00	C
ATOM	47	O	ASN	3	31.783	25.198	30.693	1.00	0.00	O
ATOM	48	N	ASP	4	29.617	24.458	30.925	1.00	0.00	N
ATOM	49	H	ASP	4	28.983	23.862	31.437	1.00	0.00	H
ATOM	50	CA	ASP	4	28.968	25.308	29.873	1.00	0.00	C
ATOM	51	HA	ASP	4	29.796	25.648	29.250	1.00	0.00	H
ATOM	52	CB	ASP	4	28.005	24.464	29.054	1.00	0.00	C
ATOM	53	HB2	ASP	4	27.159	24.193	29.685	1.00	0.00	H
ATOM	54	HB3	ASP	4	27.624	25.065	28.228	1.00	0.00	H
ATOM	55	CG	ASP	4	28.691	23.214	28.428	1.00	0.00	C
ATOM	56	OD1	ASP	4	29.813	23.286	27.868	1.00	0.00	O
ATOM	57	OD2	ASP	4	28.148	22.059	28.571	1.00	0.00	O
ATOM	58	C	ASP	4	28.334	26.635	30.256	1.00	0.00	C
ATOM	59	O	ASP	4	27.916	27.405	29.384	1.00	0.00	O
ATOM	60	N	ILE	5	28.182	26.936	31.549	1.00	0.00	N
ATOM	61	H	ILE	5	28.457	26.198	32.180	1.00	0.00	H
ATOM	62	CA	ILE	5	27.877	28.335	32.053	1.00	0.00	C
ATOM	63	HA	ILE	5	27.121	28.798	31.420	1.00	0.00	H
ATOM	64	CB	ILE	5	27.235	28.289	33.486	1.00	0.00	C
ATOM	65	HB	ILE	5	28.003	27.842	34.117	1.00	0.00	H
ATOM	66	CG2	ILE	5	27.195	29.653	34.231	1.00	0.00	C
ATOM	67	HG21	ILE	5	26.612	30.364	33.646	1.00	0.00	H
ATOM	68	HG22	ILE	5	26.621	29.482	35.142	1.00	0.00	H
ATOM	69	HG23	ILE	5	28.175	30.106	34.380	1.00	0.00	H
ATOM	70	CG1	ILE	5	25.954	27.478	33.565	1.00	0.00	C
ATOM	71	HG12	ILE	5	26.184	26.423	33.415	1.00	0.00	H

ATOM	72	HG13	ILE	5	25.546	27.544	34.574	1.00	0.00	H
ATOM	73	CD1	ILE	5	24.848	28.045	32.685	1.00	0.00	C
ATOM	74	HD11	ILE	5	23.946	27.495	32.950	1.00	0.00	H
ATOM	75	HD12	ILE	5	24.731	29.114	32.861	1.00	0.00	H
ATOM	76	HD13	ILE	5	24.992	27.967	31.607	1.00	0.00	H
ATOM	77	C	ILE	5	29.200	29.157	31.993	1.00	0.00	C
ATOM	78	O	ILE	5	30.242	28.673	32.398	1.00	0.00	O
ATOM	79	N	THR	6	29.109	30.426	31.613	1.00	0.00	N
ATOM	80	H	THR	6	28.154	30.647	31.371	1.00	0.00	H
ATOM	81	CA	THR	6	30.262	31.297	31.300	1.00	0.00	C
ATOM	82	HA	THR	6	31.082	30.975	31.943	1.00	0.00	H
ATOM	83	CB	THR	6	30.691	31.158	29.852	1.00	0.00	C
ATOM	84	HB	THR	6	30.749	30.115	29.540	1.00	0.00	H
ATOM	85	CG2	THR	6	29.815	31.832	28.754	1.00	0.00	C
ATOM	86	HG21	THR	6	30.135	32.856	28.562	1.00	0.00	H
ATOM	87	HG22	THR	6	29.914	31.290	27.814	1.00	0.00	H
ATOM	88	HG23	THR	6	28.800	31.713	29.134	1.00	0.00	H
ATOM	89	OG1	THR	6	32.009	31.673	29.677	1.00	0.00	O
ATOM	90	HG1	THR	6	32.564	30.917	29.473	1.00	0.00	H
ATOM	91	C	THR	6	30.030	32.813	31.544	1.00	0.00	C
ATOM	92	O	THR	6	28.892	33.153	31.867	1.00	0.00	O
ATOM	93	N	PHE	7	31.021	33.693	31.508	1.00	0.00	N
ATOM	94	H	PHE	7	31.863	33.302	31.113	1.00	0.00	H
ATOM	95	CA	PHE	7	30.932	35.137	31.568	1.00	0.00	C
ATOM	96	HA	PHE	7	30.131	35.358	32.274	1.00	0.00	H
ATOM	97	CB	PHE	7	32.294	35.677	32.190	1.00	0.00	C
ATOM	98	HB2	PHE	7	33.039	35.556	31.403	1.00	0.00	H
ATOM	99	HB3	PHE	7	32.138	36.740	32.368	1.00	0.00	H
ATOM	100	CG	PHE	7	32.679	35.047	33.542	1.00	0.00	C
ATOM	101	CD1	PHE	7	33.594	33.955	33.631	1.00	0.00	C
ATOM	102	HD1	PHE	7	34.203	33.579	32.822	1.00	0.00	H
ATOM	103	CE1	PHE	7	33.742	33.394	34.892	1.00	0.00	C
ATOM	104	HE1	PHE	7	34.395	32.534	34.911	1.00	0.00	H
ATOM	105	CZ	PHE	7	33.135	33.849	36.039	1.00	0.00	C
ATOM	106	HZ	PHE	7	33.262	33.324	36.975	1.00	0.00	H
ATOM	107	CE2	PHE	7	32.263	34.881	35.929	1.00	0.00	C
ATOM	108	HE2	PHE	7	31.729	35.216	36.806	1.00	0.00	H
ATOM	109	CD2	PHE	7	32.049	35.521	34.688	1.00	0.00	C
ATOM	110	HD2	PHE	7	31.463	36.427	34.746	1.00	0.00	H
ATOM	111	C	PHE	7	30.654	35.667	30.176	1.00	0.00	C
ATOM	112	O	PHE	7	31.067	35.064	29.191	1.00	0.00	O
ATOM	113	N	PHE	8	29.952	36.805	30.166	1.00	0.00	N
ATOM	114	H	PHE	8	29.630	37.214	31.031	1.00	0.00	H
ATOM	115	CA	PHE	8	29.595	37.564	28.992	1.00	0.00	C
ATOM	116	HA	PHE	8	29.441	36.827	28.203	1.00	0.00	H
ATOM	117	CB	PHE	8	28.353	38.422	29.208	1.00	0.00	C
ATOM	118	HB2	PHE	8	28.755	39.274	29.755	1.00	0.00	H
ATOM	119	HB3	PHE	8	28.117	38.608	28.160	1.00	0.00	H
ATOM	120	CG	PHE	8	27.207	37.884	29.987	1.00	0.00	C
ATOM	121	CD1	PHE	8	26.564	38.676	30.966	1.00	0.00	C
ATOM	122	HD1	PHE	8	27.021	39.608	31.263	1.00	0.00	H
ATOM	123	CE1	PHE	8	25.578	38.088	31.789	1.00	0.00	C
ATOM	124	HE1	PHE	8	25.040	38.699	32.500	1.00	0.00	H
ATOM	125	CZ	PHE	8	25.125	36.800	31.555	1.00	0.00	C
ATOM	126	HZ	PHE	8	24.362	36.427	32.221	1.00	0.00	H
ATOM	127	CE2	PHE	8	25.755	35.988	30.550	1.00	0.00	C
ATOM	128	HE2	PHE	8	25.454	34.972	30.345	1.00	0.00	H
ATOM	129	CD2	PHE	8	26.687	36.575	29.648	1.00	0.00	C
ATOM	130	HD2	PHE	8	27.140	36.008	28.848	1.00	0.00	H
ATOM	131	C	PHE	8	30.731	38.392	28.348	1.00	0.00	C
ATOM	132	O	PHE	8	31.861	38.468	28.772			
1.00	0.00			O						
ATOM	133	N	GLN	9	30.455	38.800	27.158	1.00	0.00	N
ATOM	134	H	GLN	9	29.516	38.711	26.798	1.00	0.00	H

ATOM	135	CA	GLN	9	31.492	39.554	26.352	1.00	0.00	C
ATOM	136	HA	GLN	9	32.273	38.814	26.174	1.00	0.00	H
ATOM	137	CB	GLN	9	30.900	40.166	24.984	1.00	0.00	C
ATOM	138	HB2	GLN	9	29.849	40.428	25.112	1.00	0.00	H
ATOM	139	HB3	GLN	9	31.496	41.029	24.686	1.00	0.00	H
ATOM	140	CG	GLN	9	31.049	39.203	23.790	1.00	0.00	C
ATOM	141	HG2	GLN	9	30.549	39.666	22.939	1.00	0.00	H
ATOM	142	HG3	GLN	9	32.101	38.979	23.618	1.00	0.00	H
ATOM	143	CD	GLN	9	30.327	37.857	23.987	1.00	0.00	C
ATOM	144	OE1	GLN	9	29.195	37.827	24.334	1.00	0.00	O
ATOM	145	NE2	GLN	9	30.941	36.711	23.725	1.00	0.00	N
ATOM	146	HE21	GLN	9	30.556	35.798	23.919	1.00	0.00	H
ATOM	147	HE22	GLN	9	31.787	36.692	23.175	1.00	0.00	H
ATOM	148	C	GLN	9	31.967	40.842	27.042	1.00	0.00	C
ATOM	149	O	GLN	9	33.134	41.158	26.744	1.00	0.00	O
ATOM	150	N	ARG	10	31.180	41.518	27.939	1.00	0.00	N
ATOM	151	H	ARG	10	30.232	41.173	27.994	1.00	0.00	H
ATOM	152	CA	ARG	10	31.604	42.701	28.743	1.00	0.00	C
ATOM	153	HA	ARG	10	31.915	43.428	27.993	1.00	0.00	H
ATOM	154	CB	ARG	10	30.352	43.177	29.550	1.00	0.00	C
ATOM	155	HB2	ARG	10	29.487	43.253	28.890	1.00	0.00	H
ATOM	156	HB3	ARG	10	30.166	42.440	30.332	1.00	0.00	H
ATOM	157	CG	ARG	10	30.616	44.570	30.252	1.00	0.00	C
ATOM	158	HG2	ARG	10	31.562	44.448	30.781	1.00	0.00	H
ATOM	159	HG3	ARG	10	30.651	45.258	29.407	1.00	0.00	H
ATOM	160	CD	ARG	10	29.527	44.927	31.231	1.00	0.00	C
ATOM	161	HD2	ARG	10	29.405	44.082	31.908	1.00	0.00	H
ATOM	162	HD3	ARG	10	29.807	45.783	31.845	1.00	0.00	H
ATOM	163	NE	ARG	10	28.180	45.063	30.572	1.00	0.00	N
ATOM	164	HE	ARG	10	28.135	45.502	29.665	1.00	0.00	H
ATOM	165	CZ	ARG	10	26.987	44.707	31.029	1.00	0.00	C
ATOM	166	NH1	ARG	10	26.854	44.427	32.301	1.00	0.00	N
ATOM	167	HH11	ARG	10	25.954	44.071	32.592	1.00	0.00	H
ATOM	168	HH12	ARG	10	27.658	44.592	32.890	1.00	0.00	H
ATOM	169	NH2	ARG	10	25.992	44.650	30.184	1.00	0.00	N
ATOM	170	HH21	ARG	10	26.092	44.905	29.213	1.00	0.00	H
ATOM	171	HH22	ARG	10	25.068	44.505	30.565	1.00	0.00	H
ATOM	172	C	ARG	10	32.882	42.519	29.554	1.00	0.00	C
ATOM	173	O	ARG	10	33.651	43.459	29.656	1.00	0.00	O
ATOM	174	N	PHE	11	33.035	41.272	30.131	1.00	0.00	N
ATOM	175	H	PHE	11	32.360	40.563	29.881	1.00	0.00	H
ATOM	176	CA	PHE	11	34.123	40.924	31.033	1.00	0.00	C
ATOM	177	HA	PHE	11	34.403	41.862	31.513	1.00	0.00	H
ATOM	178	CB	PHE	11	33.575	40.068	32.214	1.00	0.00	C
ATOM	179	HB2	PHE	11	34.291	40.133	33.032	1.00	0.00	H
ATOM	180	HB3	PHE	11	33.486	39.002	32.005	1.00	0.00	H
ATOM	181	CG	PHE	11	32.220	40.526	32.693	1.00	0.00	C
ATOM	182	CD1	PHE	11	32.126	41.694	33.521	1.00	0.00	C
ATOM	183	HD1	PHE	11	33.084	42.040	33.879	1.00	0.00	H
ATOM	184	CE1	PHE	11	30.887	42.239	33.868	1.00	0.00	C
ATOM	185	HE1	PHE	11	30.878	43.087	34.536	1.00	0.00	H
ATOM	186	CZ	PHE	11	29.710	41.612	33.357	1.00	0.00	C
ATOM	187	HZ	PHE	11	28.730	42.038	33.510	1.00	0.00	H
ATOM	188	CE2	PHE	11	29.851	40.494	32.535	1.00	0.00	C
ATOM	189	HE2	PHE	11	28.915	40.032	32.258	1.00	0.00	H
ATOM	190	CD2	PHE	11	31.120	39.860	32.193	1.00	0.00	C
ATOM	191	HD2	PHE	11	31.209	38.948	31.622	1.00	0.00	H
ATOM	192	C	PHE	11	35.349	40.133	30.386	1.00	0.00	C
ATOM	193	O	PHE	11	36.336	39.891	31.023	1.00	0.00	O
ATOM	194	N	GLN	12	35.225	39.711	29.167	1.00	0.00	N
ATOM	195	H	GLN	12	34.346	39.967	28.742	1.00	0.00	H
ATOM	196	CA	GLN	12	36.079	38.727	28.474	1.00	0.00	C
ATOM	197	HA	GLN	12	36.263	37.769	28.961	1.00	0.00	H
ATOM	198	CB	GLN	12	35.540	38.409	27.087	1.00	0.00	C

ATOM	199	HB2	GLN	12	35.129	39.286	26.589	1.00	0.00	H
ATOM	200	HB3	GLN	12	36.365	38.131	26.429	1.00	0.00	H
ATOM	201	CG	GLN	12	34.629	37.176	27.135	1.00	0.00	C
ATOM	202	HG2	GLN	12	35.077	36.348	27.684	1.00	0.00	H
ATOM	203	HG3	GLN	12	33.809	37.541	27.754	1.00	0.00	H
ATOM	204	CD	GLN	12	34.299	36.589	25.702	1.00	0.00	C
ATOM	205	OE1	GLN	12	34.831	36.906	24.698	1.00	0.00	O
ATOM	206	NE2	GLN	12	33.471	35.554	25.761	1.00	0.00	N
ATOM	207	HE21	GLN	12	33.025	35.263	26.620	1.00	0.00	H
ATOM	208	HE22	GLN	12	33.272	35.086	24.889	1.00	0.00	H
ATOM	209	C	GLN	12	37.552	39.183	28.238	1.00	0.00	C
ATOM	210	O	GLN	12	38.456	38.391	28.440	1.00	0.00	O
ATOM	211	N	ASP	13	37.704	40.440	27.709	1.00	0.00	N
ATOM	212	H	ASP	13	36.903	40.817	27.223	1.00	0.00	H
ATOM	213	CA	ASP	13	39.056	41.061	27.466	1.00	0.00	C
ATOM	214	HA	ASP	13	39.689	40.217	27.191	1.00	0.00	H
ATOM	215	CB	ASP	13	38.940	42.068	26.323	1.00	0.00	C
ATOM	216	HB2	ASP	13	38.370	42.899	26.738	1.00	0.00	H
ATOM	217	HB3	ASP	13	39.991	42.340	26.234	1.00	0.00	H
ATOM	218	CG	ASP	13	38.571	41.569	24.945	1.00	0.00	C
ATOM	219	OD1	ASP	13	38.333	42.409	24.043	1.00	0.00	O
ATOM	220	OD2	ASP	13	38.463	40.320	24.752	1.00	0.00	O
ATOM	221	C	ASP	13	39.661	41.542	28.834	1.00	0.00	C
ATOM	222	O	ASP	13	40.865	41.675	28.952	1.00	0.00	O
ATOM	223	N	ASP	14	38.854	41.641	29.921	1.00	0.00	N
ATOM	224	H	ASP	14	37.853	41.632	29.783	1.00	0.00	H
ATOM	225	CA	ASP	14	39.293	41.820	31.294	1.00	0.00	C
ATOM	226	HA	ASP	14	40.204	42.417	31.231	1.00	0.00	H
ATOM	227	CB	ASP	14	38.145	42.363	32.111	1.00	0.00	C
ATOM	228	HB2	ASP	14	37.239	42.126	31.553	1.00	0.00	H
ATOM	229	HB3	ASP	14	38.009	41.686	32.954	1.00	0.00	H
ATOM	230	CG	ASP	14	38.283	43.799	32.526	1.00	0.00	C
ATOM	231	OD1	ASP	14	37.338	44.259	33.201	1.00	0.00	O
ATOM	232	OD2	ASP	14	39.150	44.566	32.050	1.00	0.00	O
ATOM	233	C	ASP	14	39.857	40.462	31.770	1.00	0.00	C
ATOM	234	O	ASP	14	40.999	40.404	32.253	1.00	0.00	O
ATOM	235	N	ILE	15	39.066	39.386	31.652	1.00	0.00	N
ATOM	236	H	ILE	15	38.117	39.512	31.330	1.00	0.00	H
ATOM	237	CA	ILE	15	39.618	38.031	32.045	1.00	0.00	C
ATOM	238	HA	ILE	15	39.839	38.075	33.111	1.00	0.00	H
ATOM	239	CB	ILE	15	38.583	36.922	31.839	1.00	0.00	C
ATOM	240	HB	ILE	15	38.163	36.901	30.833	1.00	0.00	H
ATOM	241	CG2	ILE	15	39.263	35.558	32.241	1.00	0.00	C
ATOM	242	HG21	ILE	15	39.817	35.605	33.179	1.00	0.00	H
ATOM	243	HG22	ILE	15	38.638	34.705	32.504	1.00	0.00	H
ATOM	244	HG23	ILE	15	40.000	35.257	31.497	1.00	0.00	H
ATOM	245	CG1	ILE	15	37.357	37.160	32.803	1.00	0.00	C
ATOM	246	HG12	ILE	15	37.657	37.120	33.850	1.00	0.00	H
ATOM	247	HG13	ILE	15	37.010	38.177	32.619	1.00	0.00	H
ATOM	248	CD1	ILE	15	36.216	36.173	32.597	1.00	0.00	C
ATOM	249	HD11	ILE	15	35.490	36.448	33.362	1.00	0.00	H
ATOM	250	HD12	ILE	15	35.943	36.165	31.541	1.00	0.00	H
ATOM	251	HD13	ILE	15	36.388	35.117	32.807	1.00	0.00	H
ATOM	252	C	ILE	15	40.970	37.709	31.333	1.00	0.00	C
ATOM	253	O	ILE	15	41.854	37.316	32.029	1.00	0.00	O
ATOM	254	N	LEU	16	41.063	37.921	30.085	1.00	0.00	N
ATOM	255	H	LEU	16	40.155	38.108	29.686	1.00	0.00	H
ATOM	256	CA	LEU	16	42.253	37.797	29.231	1.00	0.00	C
ATOM	257	HA	LEU	16	42.378	36.722	29.093	1.00	0.00	H
ATOM	258	CB	LEU	16	42.000	38.299	27.771	1.00	0.00	C
ATOM	259	HB2	LEU	16	41.129	37.785	27.368	1.00	0.00	H
ATOM	260	HB3	LEU	16	41.742	39.357	27.803	1.00	0.00	H
ATOM	261	CG	LEU	16	43.260	38.099	26.942	1.00	0.00	C

ATOM	262	HG	LEU	16	44.106	38.495	27.504	1.00	0.00	H
ATOM	263	CD1	LEU	16	43.507	36.647	26.537	1.00	0.00	C
ATOM	264	HD11	LEU	16	44.500	36.519	26.105	1.00	0.00	H
ATOM	265	HD12	LEU	16	43.488	36.059	27.454	1.00	0.00	H
ATOM	266	HD13	LEU	16	42.721	36.404	25.821	1.00	0.00	H
ATOM	267	CD2	LEU	16	43.151	38.993	25.674	1.00	0.00	C
ATOM	268	HD21	LEU	16	43.536	39.999	25.841	1.00	0.00	H
ATOM	269	HD22	LEU	16	43.912	38.546	25.033	1.00	0.00	H
ATOM	270	HD23	LEU	16	42.143	38.972	25.262	1.00	0.00	H
ATOM	271	C	LEU	16	43.397	38.500	29.843	1.00	0.00	C
ATOM	272	O	LEU	16	44.450	37.903	30.003	1.00	0.00	O
ATOM	273	N	ALA	17	43.228	39.700	30.417	1.00	0.00	N
ATOM	274	H	ALA	17	42.275	40.033	30.448	1.00	0.00	H
ATOM	275	CA	ALA	17	44.251	40.497	31.046	1.00	0.00	C
ATOM	276	HA	ALA	17	45.240	40.290	30.638	1.00	0.00	H
ATOM	277	CB	ALA	17	43.973	41.940	30.636	1.00	0.00	C
ATOM	278	HB1	ALA	17	44.022	42.096	29.557	1.00	0.00	H
ATOM	279	HB2	ALA	17	43.031	42.318	31.032	1.00	0.00	H
ATOM	280	HB3	ALA	17	44.675	42.624	31.113	1.00	0.00	H
ATOM	281	C	ALA	17	44.450	40.383	32.527	1.00	0.00	C
ATOM	282	O	ALA	17	45.289	41.147	33.112	1.00	0.00	O
ATOM	283	N	GLY	18	43.616	39.532	33.174	1.00	0.00	N
ATOM	284	H	GLY	18	42.982	38.978	32.616	1.00	0.00	H
ATOM	285	CA	GLY	18	43.588	39.225	34.614	1.00	0.00	C
ATOM	286	HA2	GLY	18	43.237	38.197	34.704	1.00	0.00	H
ATOM	287	HA3	GLY	18	44.638	39.262	34.906	1.00	0.00	H
ATOM	288	C	GLY	18	42.812	40.150	35.456	1.00	0.00	C
ATOM	289	O	GLY	18	42.968	40.277	36.666	1.00	0.00	O
ATOM	290	N	ARG	19	42.001	40.953	34.854	1.00	0.00	N
ATOM	291	H	ARG	19	41.908	40.751	33.868	1.00	0.00	H
ATOM	292	CA	ARG	19	41.372	42.199	35.477	1.00	0.00	C
ATOM	293	HA	ARG	19	42.024	42.519	36.289	1.00	0.00	H
ATOM	294	CB	ARG	19	41.235	43.325	34.467	1.00	0.00	C
ATOM	295	HB2	ARG	19	41.121	42.919	33.462	1.00	0.00	H
ATOM	296	HB3	ARG	19	40.430	44.031	34.672	1.00	0.00	H
ATOM	297	CG	ARG	19	42.554	44.075	34.347	1.00	0.00	C
ATOM	298	HG2	ARG	19	42.888	44.626	35.226	1.00	0.00	H
ATOM	299	HG3	ARG	19	43.404	43.405	34.222	1.00	0.00	H
ATOM	300	CD	ARG	19	42.647	45.128	33.174	1.00	0.00	C
ATOM	301	HD2	ARG	19	42.844	44.575	32.256	1.00	0.00	H
ATOM	302	HD3	ARG	19	41.732	45.716	33.100	1.00	0.00	H
ATOM	303	NE	ARG	19	43.699	46.127	33.469	1.00	0.00	N
ATOM	304	HE	ARG	19	44.649	45.917	33.194	1.00	0.00	H
ATOM	305	CZ	ARG	19	43.578	47.285	34.088	1.00	0.00	C
ATOM	306	NH1	ARG	19	42.425	47.792	34.497	1.00	0.00	N
ATOM	307	HH11	ARG	19	41.562	47.268	34.441	1.00	0.00	H
ATOM	308	HH12	ARG	19	42.323	48.770	34.728	1.00	0.00	H
ATOM	309	NH2	ARG	19	44.537	48.076	34.329	1.00	0.00	N
ATOM	310	HH21	ARG	19	44.328	49.005	34.665	1.00	0.00	H
ATOM	311	HH22	ARG	19	45.480	47.716	34.287	1.00	0.00	H
ATOM	312	C	ARG	19	39.945	41.925	36.074	1.00	0.00	C
ATOM	313	O	ARG	19	39.542	42.647	36.911	1.00	0.00	O
ATOM	335	N	THR	21	38.577	38.491	38.075	1.00	0.00	N
ATOM	336	H	THR	21	38.280	39.245	38.679	1.00	0.00	H
ATOM	337	CA	THR	21	39.345	37.342	38.701	1.00	0.00	C
ATOM	338	HA	THR	21	39.384	36.509	37.999	1.00	0.00	H
ATOM	339	CB	THR	21	40.827	37.613	38.930	1.00	0.00	C
ATOM	340	HB	THR	21	41.405	37.744	38.015	1.00	0.00	H
ATOM	341	CG2	THR	21	41.048	38.776	39.850	1.00	0.00	C
ATOM	342	HG21	THR	21	40.461	38.562	40.745	1.00	0.00	H
ATOM	343	HG22	THR	21	42.090	38.949	40.117	1.00	0.00	H
ATOM	344	HG23	THR	21	40.614	39.711	39.494	1.00	0.00	H
ATOM	345	OG1	THR	21	41.349	36.468	39.480	1.00	0.00	O
ATOM	346	HG1	THR	21	40.980	35.665	39.106	1.00	0.00	H

ATOM	347	C	THR	21	38.671	36.878	39.990	1.00	0.00	C
ATOM	348	O	THR	21	38.943	35.778	40.418	1.00	0.00	O
ATOM	349	N	ILE	22	37.747	37.670	40.521	1.00	0.00	N
ATOM	350	H	ILE	22	37.551	38.547	40.062	1.00	0.00	H
ATOM	351	CA	ILE	22	36.935	37.363	41.693	1.00	0.00	C
ATOM	352	HA	ILE	22	36.930	36.290	41.885	1.00	0.00	H
ATOM	353	CB	ILE	22	37.430	38.059	42.921	1.00	0.00	C
ATOM	354	HB	ILE	22	38.388	37.640	43.227	1.00	0.00	H
ATOM	355	CG2	ILE	22	37.417	39.604	42.856	1.00	0.00	C
ATOM	356	HG21	ILE	22	36.453	39.977	42.510	1.00	0.00	H
ATOM	357	HG22	ILE	22	37.633	40.023	43.839	1.00	0.00	H
ATOM	358	HG23	ILE	22	38.320	39.879	42.311	1.00	0.00	H
ATOM	359	CG1	ILE	22	36.551	37.635	44.106	1.00	0.00	C
ATOM	360	HG12	ILE	22	35.588	38.144	44.104	1.00	0.00	H
ATOM	361	HG13	ILE	22	36.138	36.630	44.020	1.00	0.00	H
ATOM	362	CD1	ILE	22	37.269	37.722	45.414	1.00	0.00	C
ATOM	363	HD11	ILE	22	38.133	37.066	45.309	1.00	0.00	H
ATOM	364	HD12	ILE	22	37.546	38.755	45.623	1.00	0.00	H
ATOM	365	HD13	ILE	22	36.641	37.314	46.206	1.00	0.00	H
ATOM	366	C	ILE	22	35.480	37.701	41.352	1.00	0.00	C
ATOM	367	O	ILE	22	35.236	38.643	40.612	1.00	0.00	O
ATOM	368	N	THR	23	34.541	36.901	41.792	1.00	0.00	N
ATOM	369	H	THR	23	34.781	36.092	42.348	1.00	0.00	H
ATOM	370	CA	THR	23	33.130	37.403	41.870	1.00	0.00	C
ATOM	371	HA	THR	23	33.223	38.467	42.089	1.00	0.00	H
ATOM	372	CB	THR	23	32.456	37.171	40.499	1.00	0.00	C
ATOM	373	HB	THR	23	33.238	37.234	39.742	1.00	0.00	H
ATOM	374	CG2	THR	23	31.905	35.731	40.360	1.00	0.00	C
ATOM	375	HG21	THR	23	31.234	35.789	39.503	1.00	0.00	H
ATOM	376	HG22	THR	23	32.658	34.952	40.244	1.00	0.00	H
ATOM	377	HG23	THR	23	31.301	35.466	41.228	1.00	0.00	H
ATOM	378	OG1	THR	23	31.415	38.090	40.237	1.00	0.00	O
ATOM	379	HG1	THR	23	31.892	38.665	39.635	1.00	0.00	H
ATOM	380	C	THR	23	32.419	36.901	43.017	1.00	0.00	C
ATOM	381	O	THR	23	32.964	36.115	43.786	1.00	0.00	O
ATOM	382	N	ILE	24	31.148	37.269	43.201	1.00	0.00	N
ATOM	383	H	ILE	24	30.725	37.827	42.472	1.00	0.00	H
ATOM	384	CA	ILE	24	30.372	37.021	44.391	1.00	0.00	C
ATOM	385	HA	ILE	24	30.681	36.143	44.958	1.00	0.00	H
ATOM	386	CB	ILE	24	30.314	38.263	45.329	1.00	0.00	C
ATOM	387	HB	ILE	24	29.653	38.977	44.838	1.00	0.00	H
ATOM	388	CG2	ILE	24	29.639	38.096	46.693	1.00	0.00	C
ATOM	389	HG21	ILE	24	30.229	37.486	47.376	1.00	0.00	H
ATOM	390	HG22	ILE	24	29.417	39.065	47.139	1.00	0.00	H
ATOM	391	HG23	ILE	24	28.718	37.549	46.490	1.00	0.00	H
ATOM	392	CG1	ILE	24	31.661	39.075	45.419	1.00	0.00	C
ATOM	393	HG12	ILE	24	32.469	38.345	45.471	1.00	0.00	H
ATOM	394	HG13	ILE	24	31.954	39.541	44.478	1.00	0.00	H
ATOM	395	CD1	ILE	24	31.809	40.137	46.530	1.00	0.00	C
ATOM	396	HD11	ILE	24	31.100	40.949	46.367	1.00	0.00	H
ATOM	397	HD12	ILE	24	31.709	39.719	47.532	1.00	0.00	H
ATOM	398	HD13	ILE	24	32.829	40.504	46.417	1.00	0.00	H
ATOM	399	C	ILE	24	28.972	36.556	43.932	1.00	0.00	C
ATOM	400	O	ILE	24	28.561	37.051	42.840	1.00	0.00	O
ATOM	401	N	ARG	25	28.252	35.806	44.752	1.00	0.00	N
ATOM	402	H	ARG	25	28.666	35.423	45.591	1.00	0.00	H
ATOM	403	CA	ARG	25	26.902	35.410	44.451	1.00	0.00	C
ATOM	404	HA	ARG	25	26.435	36.131	43.782	1.00	0.00	H
ATOM	405	CB	ARG	25	26.931	34.013	43.799	1.00	0.00	C
ATOM	406	HB2	ARG	25	27.541	33.401	44.464	1.00	0.00	H
ATOM	407	HB3	ARG	25	25.899	33.660	43.822	1.00	0.00	H
ATOM	408	CG	ARG	25	27.521	33.850	42.411	1.00	0.00	C
ATOM	409	HG2	ARG	25	28.575	34.126	42.455	1.00	0.00	H

ATOM	410	HG3	ARG	25	27.576	32.803	42.112	1.00	0.00	H
ATOM	411	CD	ARG	25	26.717	34.590	41.360	1.00	0.00	C
ATOM	412	HD2	ARG	25	25.734	34.126	41.283	1.00	0.00	H
ATOM	413	HD3	ARG	25	26.676	35.665	41.532	1.00	0.00	H
ATOM	414	NE	ARG	25	27.389	34.402	40.035	1.00	0.00	N
ATOM	415	HE	ARG	25	27.142	33.607	39.461	1.00	0.00	H
ATOM	416	CZ	ARG	25	28.179	35.263	39.439	1.00	0.00	C
ATOM	417	NH1	ARG	25	28.679	36.256	40.119	1.00	0.00	N
ATOM	418	HH11	ARG	25	29.213	36.975	39.651	1.00	0.00	H
ATOM	419	HH12	ARG	25	28.645	36.305	41.127	1.00	0.00	H
ATOM	420	NH2	ARG	25	28.477	35.100	38.165	1.00	0.00	N
ATOM	421	HH21	ARG	25	28.976	35.763	37.590	1.00	0.00	H
ATOM	422	HH22	ARG	25	28.007	34.392	37.620	1.00	0.00	H
ATOM	423	C	ARG	25	26.121	35.348	45.772	1.00	0.00	C
ATOM	424	O	ARG	25	26.714	35.337	46.875	1.00	0.00	O
ATOM	425	N	ASP	26	24.808	35.227	45.825	1.00	0.00	N
ATOM	426	H	ASP	26	24.335	34.985	44.967	1.00	0.00	H
ATOM	427	CA	ASP	26	24.059	34.888	47.114	1.00	0.00	C
ATOM	428	HA	ASP	26	24.596	35.256	47.988	1.00	0.00	H
ATOM	429	CB	ASP	26	22.691	35.579	47.159	1.00	0.00	C
ATOM	430	HB2	ASP	26	22.316	35.643	48.181	1.00	0.00	H
ATOM	431	HB3	ASP	26	22.744	36.644	46.932	1.00	0.00	H
ATOM	432	CG	ASP	26	21.580	34.914	46.316	1.00	0.00	C
ATOM	433	OD1	ASP	26	21.684	34.920	45.062	1.00	0.00	O
ATOM	434	OD2	ASP	26	20.590	34.345	46.889	1.00	0.00	O
ATOM	435	C	ASP	26	23.929	33.347	47.166	1.00	0.00	C
ATOM	436	O	ASP	26	23.658	32.739	46.122	1.00	0.00	O
ATOM	437	N	GLU	27	24.065	32.793	48.336	1.00	0.00	N
ATOM	438	H	GLU	27	24.244	33.443	49.088	1.00	0.00	H
ATOM	439	CA	GLU	27	24.453	31.414	48.591	1.00	0.00	C
ATOM	440	HA	GLU	27	25.443	31.245	48.166	1.00	0.00	H
ATOM	441	CB	GLU	27	24.666	31.323	50.075	1.00	0.00	C
ATOM	442	HB2	GLU	27	25.469	32.033	50.273	1.00	0.00	H
ATOM	443	HB3	GLU	27	23.745	31.583	50.594	1.00	0.00	H
ATOM	444	CG	GLU	27	25.172	29.899	50.404	1.00	0.00	C
ATOM	445	HG2	GLU	27	25.418	29.897	51.465	1.00	0.00	H
ATOM	446	HG3	GLU	27	24.379	29.216	50.100	1.00	0.00	H
ATOM	447	CD	GLU	27	26.452	29.650	49.676	1.00	0.00	C
ATOM	448	OE1	GLU	27	26.373	29.179	48.524	1.00	0.00	O
ATOM	449	OE2	GLU	27	27.518	29.796	50.262	1.00	0.00	O
ATOM	450	C	GLU	27	23.433	30.374	48.007	1.00	0.00	C
ATOM	451	O	GLU	27	22.214	30.404	48.238	1.00	0.00	O
ATOM	452	N	SER	28	23.935	29.494	47.149	1.00	0.00	N
ATOM	453	H	SER	28	24.944	29.457	47.130	1.00	0.00	H
ATOM	454	CA	SER	28	23.175	28.421	46.490	1.00	0.00	C
ATOM	455	HA	SER	28	22.573	27.964	47.276	1.00	0.00	H
ATOM	456	CB	SER	28	22.458	28.982	45.247	1.00	0.00	C
ATOM	457	HB2	SER	28	22.114	29.983	45.507	1.00	0.00	H
ATOM	458	HB3	SER	28	23.067	28.846	44.353	1.00	0.00	H
ATOM	459	OG	SER	28	21.285	28.259	44.956	1.00	0.00	O
ATOM	460	HG	SER	28	20.793	28.094	45.763	1.00	0.00	H
ATOM	461	C	SER	28	23.964	27.246	45.947	1.00	0.00	C
ATOM	462	O	SER	28	25.100	27.431	45.530	1.00	0.00	O
ATOM	463	N	GLU	29	23.447	26.029	45.862	1.00	0.00	N
ATOM	464	H	GLU	29	22.484	25.858	46.116	1.00	0.00	H
ATOM	465	CA	GLU	29	24.207	24.806	45.457	1.00	0.00	C
ATOM	466	HA	GLU	29	24.964	24.756	46.241	1.00	0.00	H
ATOM	467	CB	GLU	29	23.192	23.649	45.539	1.00	0.00	C
ATOM	468	HB2	GLU	29	22.693	23.638	46.509	1.00	0.00	H
ATOM	469	HB3	GLU	29	22.437	23.809	44.769	1.00	0.00	H
ATOM	470	CG	GLU	29	23.878	22.282	45.214	1.00	0.00	C
ATOM	471	HG2	GLU	29	23.184	21.496	45.513	1.00	0.00	H
ATOM	472	HG3	GLU	29	24.012	22.281	44.132	1.00	0.00	H
ATOM	473	CD	GLU	29	25.178	21.999	45.996	1.00	0.00	C

ATOM	474	OE1	GLU	29	25.242	22.203	47.221	1.00	0.00	O
ATOM	475	OE2	GLU	29	26.169	21.550	45.336	1.00	0.00	O
ATOM	476	C	GLU	29	24.936	24.868	44.098	1.00	0.00	C
ATOM	477	O	GLU	29	25.934	24.165	43.799	1.00	0.00	O
ATOM	478	N	SER	30	24.380	25.643	43.174	1.00	0.00	N
ATOM	479	H	SER	30	23.490	26.016	43.468	1.00	0.00	H
ATOM	480	CA	SER	30	24.647	25.698	41.708	1.00	0.00	C
ATOM	481	HA	SER	30	24.927	24.665	41.502	1.00	0.00	H
ATOM	482	CB	SER	30	23.404	26.061	40.887	1.00	0.00	C
ATOM	483	HB2	SER	30	23.644	26.053	39.825	1.00	0.00	H
ATOM	484	HB3	SER	30	22.615	25.330	41.062	1.00	0.00	H
ATOM	485	OG	SER	30	22.991	27.362	41.336	1.00	0.00	O
ATOM	486	HG	SER	30	22.119	27.139	41.668	1.00	0.00	H
ATOM	487	C	SER	30	25.907	26.524	41.295	1.00	0.00	C
ATOM	488	O	SER	30	26.360	26.385	40.110	1.00	0.00	O
ATOM	489	N	HIE	31	26.434	27.378	42.143	1.00	0.00	N
ATOM	490	H	HIE	31	25.974	27.441	43.039	1.00	0.00	H
ATOM	491	CA	HIE	31	27.532	28.213	41.862	1.00	0.00	C
ATOM	492	HA	HIE	31	27.416	28.664	40.876	1.00	0.00	H
ATOM	493	CB	HIE	31	27.681	29.406	42.776	1.00	0.00	C
ATOM	494	HB2	HIE	31	28.142	29.076	43.706	1.00	0.00	H
ATOM	495	HB3	HIE	31	28.299	30.170	42.304	1.00	0.00	H
ATOM	496	CG	HIE	31	26.343	30.094	43.080	1.00	0.00	C
ATOM	497	ND1	HIE	31	25.417	30.569	42.148	1.00	0.00	N
ATOM	498	CE1	HIE	31	24.455	31.201	42.848	1.00	0.00	C
ATOM	499	HE1	HIE	31	23.547	31.640	42.462	1.00	0.00	H
ATOM	500	NE2	HIE	31	24.835	31.232	44.129	1.00	0.00	N
ATOM	501	HE2	HIE	31	24.304	31.727	44.832	1.00	0.00	H
ATOM	502	CD2	HIE	31	25.967	30.547	44.311	1.00	0.00	C
ATOM	503	HD2	HIE	31	26.506	30.379	45.232	1.00	0.00	H
ATOM	504	C	HIE	31	28.880	27.455	41.903	1.00	0.00	C
ATOM	505	O	HIE	31	29.017	26.399	42.542	1.00	0.00	O
ATOM	506	N	PHE	32	29.970	27.839	41.134	1.00	0.00	N
ATOM	507	H	PHE	32	29.888	28.731	40.669	1.00	0.00	H
ATOM	508	CA	PHE	32	31.086	26.966	40.807	1.00	0.00	C
ATOM	509	HA	PHE	32	30.774	26.047	40.313	1.00	0.00	H
ATOM	510	CB	PHE	32	31.970	27.721	39.798	1.00	0.00	C
ATOM	511	HB2	PHE	32	32.661	28.319	40.392	1.00	0.00	H
ATOM	512	HB3	PHE	32	32.644	27.004	39.329	1.00	0.00	H
ATOM	513	CG	PHE	32	31.329	28.601	38.799	1.00	0.00	C
ATOM	514	CD1	PHE	32	31.711	29.945	38.649	1.00	0.00	C
ATOM	515	HD1	PHE	32	32.481	30.366	39.276	1.00	0.00	H
ATOM	516	CE1	PHE	32	31.184	30.686	37.604	1.00	0.00	C
ATOM	517	HE1	PHE	32	31.553	31.689	37.445	1.00	0.00	H
ATOM	518	CZ	PHE	32	30.163	30.147	36.739	1.00	0.00	C
ATOM	519	HZ	PHE	32	29.870	30.758	35.898	1.00	0.00	H
ATOM	520	CE2	PHE	32	29.799	28.786	36.932	1.00	0.00	C
ATOM	521	HE2	PHE	32	28.949	28.396	36.392	1.00	0.00	H
ATOM	522	CD2	PHE	32	30.408	28.022	37.946	1.00	0.00	C
ATOM	523	HD2	PHE	32	30.149	26.974	37.933	1.00	0.00	H
ATOM	524	C	PHE	32	31.936	26.627	42.016	1.00	0.00	C
ATOM	525	O	PHE	32	32.280	27.447	42.874	1.00	0.00	O
ATOM	526	N	LYS	33	32.366	25.363	42.067	1.00	0.00	N
ATOM	527	H	LYS	33	32.057	24.709	41.363	1.00	0.00	H
ATOM	528	CA	LYS	33	33.231	24.700	43.090	1.00	0.00	C
ATOM	529	HA	LYS	33	32.978	25.151	44.049			
1.00	0.00			H						
ATOM	530	CB	LYS	33	33.098	23.138	43.091	1.00	0.00	C
ATOM	531	HB2	LYS	33	33.413	22.750	42.122	1.00	0.00	H
ATOM	532	HB3	LYS	33	33.717	22.696	43.872	1.00	0.00	H
ATOM	533	CG	LYS	33	31.622	22.579	43.218	1.00	0.00	C
ATOM	534	HG2	LYS	33	31.086	22.729	42.282	1.00	0.00	H
ATOM	535	HG3	LYS	33	31.824	21.525	43.405	1.00	0.00	H
ATOM	536	CD	LYS	33	30.842	23.227	44.348	1.00	0.00	C

ATOM	537	HD2	LYS	33	31.499	23.131	45.213	1.00	0.00	H
ATOM	538	HD3	LYS	33	30.568	24.249	44.082	1.00	0.00	H
ATOM	539	CE	LYS	33	29.580	22.325	44.512	1.00	0.00	C
ATOM	540	HE2	LYS	33	29.123	22.233	43.527	1.00	0.00	H
ATOM	541	HE3	LYS	33	29.869	21.340	44.880	1.00	0.00	H
ATOM	542	NZ	LYS	33	28.636	22.944	45.471	1.00	0.00	N
ATOM	543	HZ1	LYS	33	28.447	23.834	45.030	1.00	0.00	H
ATOM	544	HZ2	LYS	33	27.791	22.392	45.494	1.00	0.00	H
ATOM	545	HZ3	LYS	33	29.158	22.967	46.334	1.00	0.00	H
ATOM	546	C	LYS	33	34.728	25.117	42.850	1.00	0.00	C
ATOM	547	O	LYS	33	35.143	25.262	41.728	1.00	0.00	O
ATOM	548	N	THR	34	35.529	24.936	43.924	1.00	0.00	N
ATOM	549	H	THR	34	35.156	24.786	44.850	1.00	0.00	H
ATOM	550	CA	THR	34	36.986	24.807	43.895	1.00	0.00	C
ATOM	551	HA	THR	34	37.396	25.712	43.444	1.00	0.00	H
ATOM	552	CB	THR	34	37.659	24.661	45.271	1.00	0.00	C
ATOM	553	HB	THR	34	37.332	23.764	45.799	1.00	0.00	H
ATOM	554	CG2	THR	34	39.178	24.863	45.136	1.00	0.00	C
ATOM	555	HG21	THR	34	39.638	24.736	46.115	1.00	0.00	H
ATOM	556	HG22	THR	34	39.691	24.099	44.551	1.00	0.00	H
ATOM	557	HG23	THR	34	39.342	25.843	44.689	1.00	0.00	H
ATOM	558	OG1	THR	34	37.395	25.781	46.060	1.00	0.00	O
ATOM	559	HG1	THR	34	37.104	25.470	46.921	1.00	0.00	H
ATOM	560	C	THR	34	37.172	23.638	42.970	1.00	0.00	C
ATOM	561	O	THR	34	36.642	22.520	43.243	1.00	0.00	O
ATOM	562	N	GLY	35	37.916	23.809	41.833	1.00	0.00	N
ATOM	563	H	GLY	35	38.320	24.722	41.676	1.00	0.00	H
ATOM	564	CA	GLY	35	38.183	22.715	40.887	1.00	0.00	C
ATOM	565	HA2	GLY	35	39.212	22.868	40.562	1.00	0.00	H
ATOM	566	HA3	GLY	35	38.280	21.783	41.442	1.00	0.00	H
ATOM	567	C	GLY	35	37.286	22.521	39.714	1.00	0.00	C
ATOM	568	O	GLY	35	37.471	21.636	38.878	1.00	0.00	O
ATOM	569	N	ASP	36	36.241	23.370	39.635	1.00	0.00	N
ATOM	570	H	ASP	36	36.112	23.934	40.463	1.00	0.00	H
ATOM	571	CA	ASP	36	35.496	23.536	38.396	1.00	0.00	C
ATOM	572	HA	ASP	36	35.195	22.585	37.958	1.00	0.00	H
ATOM	573	CB	ASP	36	34.194	24.361	38.516	1.00	0.00	C
ATOM	574	HB2	ASP	36	34.280	25.128	39.286	1.00	0.00	H
ATOM	575	HB3	ASP	36	33.996	24.897	37.588	1.00	0.00	H
ATOM	576	CG	ASP	36	33.045	23.496	38.740	1.00	0.00	C
ATOM	577	OD1	ASP	36	32.211	23.806	39.614	1.00	0.00	O
ATOM	578	OD2	ASP	36	32.808	22.494	38.005	1.00	0.00	O
ATOM	579	C	ASP	36	36.329	24.286	37.283	1.00	0.00	C
ATOM	580	O	ASP	36	36.943	25.354	37.479	1.00	0.00	O
ATOM	581	N	VAL	37	36.427	23.681	36.096	1.00	0.00	N
ATOM	582	H	VAL	37	35.818	22.882	35.992	1.00	0.00	H
ATOM	583	CA	VAL	37	37.045	24.294	34.902	1.00	0.00	C
ATOM	584	HA	VAL	37	37.791	24.979	35.304	1.00	0.00	H
ATOM	585	CB	VAL	37	37.708	23.264	34.043	1.00	0.00	C
ATOM	586	HB	VAL	37	37.021	22.530	33.619	1.00	0.00	H
ATOM	587	CG1	VAL	37	38.500	23.856	32.874	1.00	0.00	C
ATOM	588	HG11	VAL	37	38.657	23.036	32.172	1.00	0.00	H
ATOM	589	HG12	VAL	37	37.985	24.595	32.260	1.00	0.00	H
ATOM	590	HG13	VAL	37	39.526	24.117	33.132	1.00	0.00	H
ATOM	591	CG2	VAL	37	38.824	22.696	34.867	1.00	0.00	C
ATOM	592	HG21	VAL	37	39.533	22.061	34.335	1.00	0.00	H
ATOM	593	HG22	VAL	37	39.555	23.376	35.303	1.00	0.00	H
ATOM	594	HG23	VAL	37	38.487	22.053	35.680	1.00	0.00	H
ATOM	595	C	VAL	37	35.954	25.059	34.184	1.00	0.00	C
ATOM	596	O	VAL	37	34.871	24.554	33.926	1.00	0.00	O
ATOM	597	N	LEU	38	36.256	26.265	33.779	1.00	0.00	N
ATOM	598	H	LEU	38	37.159	26.709	33.862	1.00	0.00	H
ATOM	599	CA	LEU	38	35.415	27.065	32.914	1.00	0.00	C
ATOM	600	HA	LEU	38	34.544	26.493	32.594	1.00	0.00	H

ATOM	601	CB	LEU	38	34.961	28.307	33.706	1.00	0.00	C
ATOM	602	HB2	LEU	38	35.898	28.808	33.946	1.00	0.00	H
ATOM	603	HB3	LEU	38	34.289	28.969	33.160	1.00	0.00	H
ATOM	604	CG	LEU	38	34.238	28.086	35.037	1.00	0.00	C
ATOM	605	HG	LEU	38	34.850	27.499	35.722	1.00	0.00	H
ATOM	606	CD1	LEU	38	34.148	29.503	35.630	1.00	0.00	C
ATOM	607	HD11	LEU	38	33.453	30.096	35.035	1.00	0.00	H
ATOM	608	HD12	LEU	38	33.667	29.546	36.607	1.00	0.00	H
ATOM	609	HD13	LEU	38	35.106	30.012	35.739	1.00	0.00	H
ATOM	610	CD2	LEU	38	32.860	27.396	34.870	1.00	0.00	C
ATOM	611	HD21	LEU	38	32.481	26.958	35.793	1.00	0.00	H
ATOM	612	HD22	LEU	38	32.117	28.138	34.581	1.00	0.00	H
ATOM	613	HD23	LEU	38	32.960	26.624	34.107	1.00	0.00	H
ATOM	614	C	LEU	38	36.139	27.508	31.590	1.00	0.00	C
ATOM	615	O	LEU	38	37.406	27.405	31.555	1.00	0.00	O
ATOM	616	N	ARG	39	35.394	28.079	30.617	1.00	0.00	N
ATOM	617	H	ARG	39	34.394	28.166	30.730	1.00	0.00	H
ATOM	618	CA	ARG	39	35.949	28.656	29.338	1.00	0.00	C
ATOM	619	HA	ARG	39	37.008	28.914	29.361	1.00	0.00	H
ATOM	620	CB	ARG	39	35.982	27.620	28.211	1.00	0.00	C
ATOM	621	HB2	ARG	39	34.956	27.375	27.934	1.00	0.00	H
ATOM	622	HB3	ARG	39	36.335	28.146	27.324	1.00	0.00	H
ATOM	623	CG	ARG	39	36.859	26.379	28.479	1.00	0.00	C
ATOM	624	HG2	ARG	39	37.910	26.650	28.585	1.00	0.00	H
ATOM	625	HG3	ARG	39	36.752	25.868	29.435	1.00	0.00	H
ATOM	626	CD	ARG	39	36.843	25.407	27.333	1.00	0.00	C
ATOM	627	HD2	ARG	39	37.125	26.022	26.479	1.00	0.00	H
ATOM	628	HD3	ARG	39	37.531	24.561	27.339	1.00	0.00	H
ATOM	629	NE	ARG	39	35.499	24.820	27.042	1.00	0.00	N
ATOM	630	HE	ARG	39	34.766	25.024	27.706	1.00	0.00	H
ATOM	631	CZ	ARG	39	35.216	24.274	25.915	1.00	0.00	C
ATOM	632	NH1	ARG	39	36.108	24.078	25.021	1.00	0.00	N
ATOM	633	HH11	ARG	39	37.054	24.371	25.216	1.00	0.00	H
ATOM	634	HH12	ARG	39	35.890	23.696	24.112	1.00	0.00	H
ATOM	635	NH2	ARG	39	33.986	23.659	25.679	1.00	0.00	N
ATOM	636	HH21	ARG	39	33.899	23.070	24.863	1.00	0.00	H
ATOM	637	HH22	ARG	39	33.343	23.435	26.424	1.00	0.00	H
ATOM	638	C	ARG	39	35.296	29.957	28.911	1.00	0.00	C
ATOM	639	O	ARG	39	34.142	30.129	29.167	1.00	0.00	O
ATOM	640	N	VAL	40	36.023	30.814	28.191	1.00	0.00	N
ATOM	641	H	VAL	40	37.018	30.675	28.086	1.00	0.00	H
ATOM	642	CA	VAL	40	35.500	31.936	27.373	1.00	0.00	C
ATOM	643	HA	VAL	40	34.438	31.715	27.262	1.00	0.00	H
ATOM	644	CB	VAL	40	35.777	33.305	28.096	1.00	0.00	C
ATOM	645	HB	VAL	40	35.224	33.987	27.449	1.00	0.00	H
ATOM	646	CG1	VAL	40	35.263	33.436	29.498	1.00	0.00	C
ATOM	647	HG11	VAL	40	34.240	33.061	29.526	1.00	0.00	H
ATOM	648	HG12	VAL	40	35.877	32.914	30.230	1.00	0.00	H
ATOM	649	HG13	VAL	40	35.186	34.485	29.785	1.00	0.00	H
ATOM	650	CG2	VAL	40	37.197	33.787	28.099	1.00	0.00	C
ATOM	651	HG21	VAL	40	37.290	34.862	28.255	1.00	0.00	H
ATOM	652	HG22	VAL	40	37.822	33.268	28.824	1.00	0.00	H
ATOM	653	HG23	VAL	40	37.605	33.570	27.112	1.00	0.00	H
ATOM	654	C	VAL	40	36.049	31.967	25.940	1.00	0.00	C
ATOM										
655	O	VAL	40	37.119	31.379	25.649	1.00	0.00	O	
ATOM	656	N	GLY	41	35.227	32.566	25.117	1.00	0.00	N
ATOM	657	H	GLY	41	34.381	32.920	25.540	1.00	0.00	H
ATOM	658	CA	GLY	41	35.432	32.734	23.705	1.00	0.00	C
ATOM	659	HA2	GLY	41	36.400	33.202	23.530	1.00	0.00	H
ATOM	660	HA3	GLY	41	35.449	31.751	23.234	1.00	0.00	H
ATOM	661	C	GLY	41	34.446	33.587	22.923	1.00	0.00	C
ATOM	662	O	GLY	41	33.368	33.862	23.468	1.00	0.00	O
ATOM	663	N	ARG	42	34.730	34.075	21.708	1.00	0.00	N

ATOM	664	H	ARG	42	35.652	33.894	21.338	1.00	0.00	H
ATOM	665	CA	ARG	42	33.713	34.697	20.832	1.00	0.00	C
ATOM	666	HA	ARG	42	32.711	34.287	20.962	1.00	0.00	H
ATOM	667	CB	ARG	42	33.580	36.261	21.232	1.00	0.00	C
ATOM	668	HB2	ARG	42	32.723	36.704	20.724	1.00	0.00	H
ATOM	669	HB3	ARG	42	33.331	36.478	22.271	1.00	0.00	H
ATOM	670	CG	ARG	42	34.809	37.083	20.865	1.00	0.00	C
ATOM	671	HG2	ARG	42	35.695	36.749	21.405	1.00	0.00	H
ATOM	672	HG3	ARG	42	34.979	37.189	19.794	1.00	0.00	H
ATOM	673	CD	ARG	42	34.443	38.475	21.230	1.00	0.00	C
ATOM	674	HD2	ARG	42	33.628	38.789	20.578	1.00	0.00	H
ATOM	675	HD3	ARG	42	33.983	38.452	22.218	1.00	0.00	H
ATOM	676	NE	ARG	42	35.602	39.426	21.189	1.00	0.00	N
ATOM	677	HE	ARG	42	35.804	39.697	20.237	1.00	0.00	H
ATOM	678	CZ	ARG	42	36.375	39.730	22.213	1.00	0.00	C
ATOM	679	NH1	ARG	42	36.219	39.246	23.424	1.00	0.00	N
ATOM	680	HH11	ARG	42	36.808	39.600	24.164	1.00	0.00	H
ATOM	681	HH12	ARG	42	35.519	38.553	23.648	1.00	0.00	H
ATOM	682	NH2	ARG	42	37.398	40.584	22.137	1.00	0.00	N
ATOM	683	HH21	ARG	42	37.554	41.189	21.343	1.00	0.00	H
ATOM	684	HH22	ARG	42	38.041	40.677	22.910	1.00	0.00	H
ATOM	685	C	ARG	42	33.876	34.532	19.344	1.00	0.00	C
ATOM	686	O	ARG	42	32.982	34.938	18.594	1.00	0.00	O
ATOM	687	N	PHE	43	35.122	34.096	18.950	1.00	0.00	N
ATOM	688	H	PHE	43	35.540	33.554	19.694	1.00	0.00	H
ATOM	689	CA	PHE	43	35.629	33.822	17.625	1.00	0.00	C
ATOM	690	HA	PHE	43	35.081	34.458	16.931	1.00	0.00	H
ATOM	691	CB	PHE	43	37.102	34.205	17.660	1.00	0.00	C
ATOM	692	HB2	PHE	43	37.664	33.343	18.022	1.00	0.00	H
ATOM	693	HB3	PHE	43	37.481	34.248	16.640	1.00	0.00	H
ATOM	694	CG	PHE	43	37.424	35.558	18.317	1.00	0.00	C
ATOM	695	CD1	PHE	43	36.990	36.752	17.806	1.00	0.00	C
ATOM	696	HD1	PHE	43	36.542	36.629	16.832	1.00	0.00	H
ATOM	697	CE1	PHE	43	37.380	37.967	18.375	1.00	0.00	C
ATOM	698	HE1	PHE	43	37.043	38.897	17.943	1.00	0.00	H
ATOM	699	CZ	PHE	43	38.109	37.957	19.591	1.00	0.00	C
ATOM	700	HZ	PHE	43	38.300	38.913	20.053	1.00	0.00	H
ATOM	701	CE2	PHE	43	38.645	36.766	20.102	1.00	0.00	C
ATOM	702	HE2	PHE	43	39.245	36.839	20.998	1.00	0.00	H
ATOM	703	CD2	PHE	43	38.272	35.554	19.464	1.00	0.00	C
ATOM	704	HD2	PHE	43	38.566	34.615	19.910	1.00	0.00	H
ATOM	705	C	PHE	43	35.505	32.304	17.233	1.00	0.00	C
ATOM	706	O	PHE	43	35.396	31.463	18.145	1.00	0.00	O
ATOM	707	N	GLU	44	35.557	31.999	15.930	1.00	0.00	N
ATOM	708	H	GLU	44	35.693	32.708	15.223	1.00	0.00	H
ATOM	709	CA	GLU	44	35.342	30.630	15.431	1.00	0.00	C
ATOM	710	HA	GLU	44	34.487	30.143	15.900	1.00	0.00	H
ATOM	711	CB	GLU	44	35.107	30.725	13.887	1.00	0.00	C
ATOM	712	HB2	GLU	44	34.544	31.617	13.611	1.00	0.00	H
ATOM	713	HB3	GLU	44	36.060	31.013	13.444	1.00	0.00	H
ATOM	714	CG	GLU	44	34.537	29.439	13.313	1.00	0.00	C
ATOM	715	HG2	GLU	44	35.219	28.638	13.594	1.00	0.00	H
ATOM	716	HG3	GLU	44	33.590	29.373	13.848	1.00	0.00	H
ATOM	717	CD	GLU	44	34.544	29.490	11.800	1.00	0.00	C
ATOM	718	OE1	GLU	44	34.145	30.575	11.259	1.00	0.00	O
ATOM	719	OE2	GLU	44	34.850	28.486	11.106	1.00	0.00	O
ATOM	720	C	GLU	44	36.519	29.640	15.716	1.00	0.00	C
ATOM	721	O	GLU	44	36.247	28.465	15.991	1.00	0.00	O
ATOM	722	N	ASP	45	37.730	30.184	15.862	1.00	0.00	N
ATOM	723	H	ASP	45	37.747	31.139	15.532	1.00	0.00	H
ATOM	724	CA	ASP	45	39.048	29.530	16.089	1.00	0.00	C
ATOM	725	HA	ASP	45	39.210	28.871	15.235	1.00	0.00	H
ATOM	726	CB	ASP	45	40.227	30.586	16.179	1.00	0.00	C
ATOM	727	HB2	ASP	45	40.243	31.099	15.217	1.00	0.00	H

ATOM	728	HB3	ASP	45	39.989	31.329	16.940	1.00	0.00	H
ATOM	729	CG	ASP	45	41.598	29.978	16.387	1.00	0.00	C
ATOM	730	OD1	ASP	45	41.984	29.220	15.469	1.00	0.00	O
ATOM	731	OD2	ASP	45	42.413	30.419	17.240	1.00	0.00	O
ATOM	732	C	ASP	45	38.982	28.632	17.318	1.00	0.00	C
ATOM	733	O	ASP	45	39.092	29.097	18.406	1.00	0.00	O
ATOM	734	N	ASP	46	38.834	27.304	17.142	1.00	0.00	N
ATOM	735	H	ASP	46	38.817	27.055	16.164	1.00	0.00	H
ATOM	736	CA	ASP	46	38.544	26.282	18.196	1.00	0.00	C
ATOM	737	HA	ASP	46	38.048	25.426	17.739	1.00	0.00	H
ATOM	738	CB	ASP	46	39.928	25.684	18.698	1.00	0.00	C
ATOM	739	HB2	ASP	46	40.496	26.489	19.166	1.00	0.00	H
ATOM	740	HB3	ASP	46	39.880	24.822	19.363	1.00	0.00	H
ATOM	741	CG	ASP	46	40.766	25.161	17.581	1.00	0.00	C
ATOM	742	OD1	ASP	46	40.340	24.334	16.738	1.00	0.00	O
ATOM	743	OD2	ASP	46	42.010	25.286	17.674	1.00	0.00	O
ATOM	744	C	ASP	46	37.585	26.654	19.401	1.00	0.00	C
ATOM	745	O	ASP	46	37.773	26.252	20.568	1.00	0.00	O
ATOM	746	N	GLY	47	36.685	27.627	19.042	1.00	0.00	N
ATOM	747	H	GLY	47	36.808	28.052	18.134	1.00	0.00	H
ATOM	748	CA	GLY	47	35.656	28.337	19.832	1.00	0.00	C
ATOM	749	HA2	GLY	47	35.142	29.025	19.161	1.00	0.00	H
ATOM	750	HA3	GLY	47	34.908	27.636	20.205	1.00	0.00	H
ATOM	751	C	GLY	47	36.108	29.224	20.964	1.00	0.00	C
ATOM	752	O	GLY	47	35.567	30.318	21.164	1.00	0.00	O
ATOM	753	N	TYR	48	37.076	28.815	21.772	1.00	0.00	N
ATOM	754	H	TYR	48	37.489	27.923	21.540	1.00	0.00	H
ATOM	755	CA	TYR	48	37.419	29.316	23.098	1.00	0.00	C
ATOM	756	HA	TYR	48	36.804	30.171	23.379	1.00	0.00	H
ATOM	757	CB	TYR	48	37.239	28.294	24.187	1.00	0.00	C
ATOM	758	HB2	TYR	48	37.866	27.414	24.047	1.00	0.00	H
ATOM	759	HB3	TYR	48	37.488	28.799	25.122	1.00	0.00	H
ATOM	760	CG	TYR	48	35.733	27.905	24.266	1.00	0.00	C
ATOM	761	CD1	TYR	48	34.854	28.678	25.065	1.00	0.00	C
ATOM	762	HD1	TYR	48	35.267	29.368	25.786	1.00	0.00	H
ATOM	763	CE1	TYR	48	33.453	28.489	24.951	1.00	0.00	C
ATOM	764	HE1	TYR	48	32.708	29.042	25.503	1.00	0.00	H
ATOM	765	CZ	TYR	48	32.979	27.451	24.127	1.00	0.00	C
ATOM	766	OH	TYR	48	31.642	27.246	23.963	1.00	0.00	O
ATOM	767	HH	TYR	48	31.418	26.402	23.564	1.00	0.00	H
ATOM	768	CE2	TYR	48	33.834	26.551	23.458	1.00	0.00	C
ATOM	769	HE2	TYR	48	33.416	25.676	22.985	1.00	0.00	H
ATOM	770	CD2	TYR	48	35.229	26.824	23.443	1.00	0.00	C
ATOM	771	HD2	TYR	48	35.893	26.224	22.838	1.00	0.00	H
ATOM	772	C	TYR	48	38.908	29.787	23.063	1.00	0.00	C
ATOM	773	O	TYR	48	39.688	29.137	22.350	1.00	0.00	O
ATOM	774	N	PHE	49	39.237	30.772	23.917	1.00	0.00	N
ATOM	775	H	PHE	49	38.580	31.199	24.552	1.00	0.00	H
ATOM	776	CA	PHE	49	40.574	31.310	23.911	1.00	0.00	C
ATOM	777	HA	PHE	49	41.268	30.675	23.359	1.00	0.00	H
ATOM	778	CB	PHE	49	40.618	32.668	23.220	1.00	0.00	C
ATOM	779	HB2	PHE	49	41.643	33.027	23.312	1.00	0.00	H
ATOM	780	HB3	PHE	49						
40.364	32.626	22.161	1.00	0.00	H					
ATOM	781	CG	PHE	49	39.877	33.772	23.930	1.00	0.00	C
ATOM	782	CD1	PHE	49	38.771	34.453	23.431	1.00	0.00	C
ATOM	783	HD1	PHE	49	38.422	34.051	22.491	1.00	0.00	H
ATOM	784	CE1	PHE	49	38.070	35.477	24.116	1.00	0.00	C
ATOM	785	HE1	PHE	49	37.186	35.875	23.640	1.00	0.00	H
ATOM	786	CZ	PHE	49	38.620	35.930	25.314	1.00	0.00	C
ATOM	787	HZ	PHE	49	38.081	36.706	25.837	1.00	0.00	H
ATOM	788	CE2	PHE	49	39.787	35.302	25.852	1.00	0.00	C
ATOM	789	HE2	PHE	49	40.236	35.565	26.798	1.00	0.00	H
ATOM	790	CD2	PHE	49	40.394	34.304	25.136	1.00	0.00	C

ATOM	791	HD2	PHE	49	41.298	33.893	25.561	1.00	0.00	H
ATOM	792	C	PHE	49	41.192	31.404	25.383	1.00	0.00	C
ATOM	793	O	PHE	49	42.386	31.448	25.566	1.00	0.00	O
ATOM	794	N	CYX	50	40.352	31.357	26.418	1.00	0.00	N
ATOM	795	H	CYX	50	39.417	31.059	26.176	1.00	0.00	H
ATOM	796	CA	CYX	50	40.795	31.138	27.758	1.00	0.00	C
ATOM	797	HA	CYX	50	41.861	30.908	27.768	1.00	0.00	H
ATOM	798	CB	CYX	50	40.581	32.472	28.522	1.00	0.00	C
ATOM	799	HB2	CYX	50	39.865	33.055	27.942	1.00	0.00	H
ATOM	800	HB3	CYX	50	40.083	32.291	29.474	1.00	0.00	H
ATOM	801	SG	CYX	50	42.109	33.347	29.013	1.00	0.00	S
ATOM	802	C	CYX	50	39.998	29.957	28.323	1.00	0.00	C
ATOM	803	O	CYX	50	38.777	30.074	28.472	1.00	0.00	O
ATOM	804	N	THR	51	40.669	28.905	28.778	1.00	0.00	N
ATOM	805	H	THR	51	41.655	28.881	28.561	1.00	0.00	H
ATOM	806	CA	THR	51	40.185	27.984	29.832	1.00	0.00	C
ATOM	807	HA	THR	51	39.130	27.712	29.845	1.00	0.00	H
ATOM	808	CB	THR	51	40.904	26.622	29.726	1.00	0.00	C
ATOM	809	HB	THR	51	41.975	26.781	29.597	1.00	0.00	H
ATOM	810	CG2	THR	51	40.613	25.665	30.873	1.00	0.00	C
ATOM	811	HG21	THR	51	40.405	24.693	30.425	1.00	0.00	H
ATOM	812	HG22	THR	51	41.490	25.516	31.502	1.00	0.00	H
ATOM	813	HG23	THR	51	39.772	26.057	31.446	1.00	0.00	H
ATOM	814	OG1	THR	51	40.604	25.885	28.546	1.00	0.00	O
ATOM	815	HG1	THR	51	40.888	26.549	27.913	1.00	0.00	H
ATOM	816	C	THR	51	40.657	28.552	31.243	1.00	0.00	C
ATOM	817	O	THR	51	41.794	29.042	31.285	1.00	0.00	O
ATOM	818	N	ILE	52	39.815	28.320	32.288	1.00	0.00	N
ATOM	819	H	ILE	52	38.948	27.831	32.120	1.00	0.00	H
ATOM	820	CA	ILE	52	39.862	29.039	33.589	1.00	0.00	C
ATOM	821	HA	ILE	52	40.800	29.588	33.674	1.00	0.00	H
ATOM	822	CB	ILE	52	38.819	30.137	33.635	1.00	0.00	C
ATOM	823	HB	ILE	52	37.865	29.765	33.260	1.00	0.00	H
ATOM	824	CG2	ILE	52	38.665	30.658	35.053	1.00	0.00	C
ATOM	825	HG21	ILE	52	37.730	31.217	35.047	1.00	0.00	H
ATOM	826	HG22	ILE	52	38.589	29.858	35.789	1.00	0.00	H
ATOM	827	HG23	ILE	52	39.505	31.261	35.398	1.00	0.00	H
ATOM	828	CG1	ILE	52	38.973	31.336	32.711	1.00	0.00	C
ATOM	829	HG12	ILE	52	39.623	32.055	33.211	1.00	0.00	H
ATOM	830	HG13	ILE	52	39.386	31.081	31.736	1.00	0.00	H
ATOM	831	CD1	ILE	52	37.600	32.005	32.456	1.00	0.00	C
ATOM	832	HD11	ILE	52	36.969	31.136	32.268	1.00	0.00	H
ATOM	833	HD12	ILE	52	37.075	32.420	33.316	1.00	0.00	H
ATOM	834	HD13	ILE	52	37.615	32.692	31.611	1.00	0.00	H
ATOM	835	C	ILE	52	39.667	28.003	34.691	1.00	0.00	C
ATOM	836	O	ILE	52	38.617	27.410	34.751	1.00	0.00	O
ATOM	837	N	GLU	53	40.608	27.856	35.645	1.00	0.00	N
ATOM	838	H	GLU	53	41.391	28.491	35.615	1.00	0.00	H
ATOM	839	CA	GLU	53	40.347	27.103	36.863	1.00	0.00	C
ATOM	840	HA	GLU	53	39.649	26.305	36.612	1.00	0.00	H
ATOM	841	CB	GLU	53	41.681	26.600	37.383	1.00	0.00	C
ATOM	842	HB2	GLU	53	41.964	25.652	36.926	1.00	0.00	H
ATOM	843	HB3	GLU	53	42.445	27.311	37.068	1.00	0.00	H
ATOM	844	CG	GLU	53	41.838	26.286	38.843	1.00	0.00	C
ATOM	845	HG2	GLU	53	41.817	27.241	39.367	1.00	0.00	H
ATOM	846	HG3	GLU	53	41.040	25.647	39.220	1.00	0.00	H
ATOM	847	CD	GLU	53	43.235	25.476	39.230	1.00	0.00	C
ATOM	848	OE1	GLU	53	43.501	25.333	40.480	1.00	0.00	O
ATOM	849	OE2	GLU	53	44.098	25.253	38.308	1.00	0.00	O
ATOM	850	C	GLU	53	39.626	28.015	37.841	1.00	0.00	C
ATOM	851	O	GLU	53	40.001	29.210	38.055	1.00	0.00	O
ATOM	852	N	VAL	54	38.760	27.438	38.665	1.00	0.00	N
ATOM	853	H	VAL	54	38.446	26.497	38.474	1.00	0.00	H
ATOM	854	CA	VAL	54	38.384	27.997	40.003	1.00	0.00	C

```
ATOM    855  HA  VAL    54      38.431  29.085  39.989  1.00  0.00           H
ATOM    856  CB  VAL    54      36.889  27.696  40.282  1.00  0.00           C
ATOM    857  HB  VAL    54      36.712  26.629  40.150  1.00  0.00           H
ATOM    858  CG1 VAL    54      36.414  28.186  41.673  1.00  0.00           C
ATOM    859 HG11 VAL    54      35.406  27.828  41.879  1.00  0.00           H
ATOM    860 HG12 VAL    54      37.128  27.813  42.408  1.00  0.00           H
ATOM    861 HG13 VAL    54      36.442  29.271  41.768  1.00  0.00           H
ATOM    862  CG2 VAL    54      36.019  28.444  39.236  1.00  0.00           C
ATOM    863 HG21 VAL    54      34.968  28.302  39.487  1.00  0.00           H
ATOM    864 HG22 VAL    54      36.129  29.516  39.394  1.00  0.00           H
ATOM    865 HG23 VAL    54      36.272  28.065  38.247  1.00  0.00           H
ATOM    866  C   VAL    54      39.335  27.480  41.102  1.00  0.00           C
ATOM    867  O   VAL    54      39.409  26.292  41.424  1.00  0.00           O
ATOM    868  N   THR    55      40.094  28.390  41.628  1.00  0.00           N
ATOM    869  H   THR    55      39.946  29.324  41.272  1.00  0.00           H
ATOM    870  CA  THR    55      41.287  28.195  42.454  1.00  0.00           C
ATOM    871  HA  THR    55      41.742  27.246  42.171  1.00  0.00           H
ATOM    872  CB  THR    55      42.344  29.365  42.233  1.00  0.00           C
ATOM    873  HB  THR    55      43.065  29.313  43.049  1.00  0.00           H
ATOM    874  CG2 THR    55      42.957  29.410  40.835  1.00  0.00           C
ATOM    875 HG21 THR    55      42.207  29.488  40.047  1.00  0.00           H
ATOM    876 HG22 THR    55      43.755  30.147  40.734  1.00  0.00           H
ATOM    877 HG23 THR    55      43.308  28.387  40.700  1.00  0.00           H
ATOM    878  OG1 THR    55      41.782  30.641  42.431  1.00  0.00           O
ATOM    879  HG1 THR    55      41.981  31.045  43.279  1.00  0.00           H
ATOM    880  C   THR    55      40.945  28.222  43.944  1.00  0.00           C
ATOM    881  O   THR    55      41.542  27.437  44.705  1.00  0.00           O
ATOM    882  N   ALA    56      40.000  29.015  44.345  1.00  0.00           N
ATOM    883  H   ALA    56      39.582  29.632  43.662  1.00  0.00           H
ATOM    884  CA  ALA    56      39.315  28.975  45.657  1.00  0.00           C
ATOM    885  HA  ALA    56      39.333  27.987  46.116  1.00  0.00           H
ATOM    886  CB  ALA    56      40.150  29.733  46.711  1.00  0.00           C
ATOM    887  HB1 ALA    56      39.809  29.668  47.744  1.00  0.00           H
ATOM    888  HB2 ALA    56      41.118  29.236  46.771  1.00  0.00           H
ATOM    889  HB3 ALA    56      40.329  30.782  46.479  1.00  0.00           H
ATOM    890  C   ALA    56      37.817  29.334  45.729  1.00  0.00           C
ATOM    891  O   ALA    56      37.311  30.078  44.873  1.00  0.00           O
ATOM    892  N   THR    57      37.111  28.827  46.705  1.00  0.00           N
ATOM    893  H   THR    57      37.428  28.164  47.397  1.00  0.00           H
ATOM    894  CA  THR    57      35.798  29.358  46.971  1.00  0.00           C
ATOM    895  HA  THR    57      35.670  30.333  46.499  1.00  0.00           H
ATOM    896  CB  THR    57      34.734  28.418  46.457  1.00  0.00           C
ATOM    897  HB  THR    57      33.761  28.852  46.688  1.00  0.00           H
ATOM    898  CG2 THR    57      34.745  28.365  44.903  1.00  0.00           C
ATOM    899 HG21 THR    57      34.065  27.548  44.663  1.00  0.00           H
ATOM    900 HG22 THR    57      34.248  29.196  44.402  1.00  0.00           H
ATOM    901 HG23 THR    57      35.703  28.192  44.411  1.00  0.00           H
ATOM    902  OG1 THR    57      34.893  27.105  46.944  1.00  0.00           O
ATOM    903  HG1 THR    57      35.585  26.726  46.397  1.00  0.00           H
ATOM    904  C   THR    57      35.525  29.544  48.447  1.00  0.00           C
ATOM    905  O   THR    57      36.123  28.878  49.297
  1.00  0.00           O
ATOM    906  N   SER    58      34.499  30.324  48.827  1.00  0.00           N
ATOM    907  H   SER    58      33.963  30.957  48.251  1.00  0.00           H
ATOM    908  CA  SER    58      34.161  30.561  50.284  1.00  0.00           C
ATOM    909  HA  SER    58      34.228  29.597  50.789  1.00  0.00           H
ATOM    910  CB  SER    58      35.217  31.472  50.989  1.00  0.00           C
ATOM    911  HB2 SER    58      35.059  31.496  52.069  1.00  0.00           H
ATOM    912  HB3 SER    58      36.195  30.993  50.950  1.00  0.00           H
ATOM    913  OG  SER    58      35.451  32.783  50.468  1.00  0.00           O
ATOM    914  HG  SER    58      35.932  32.637  49.648  1.00  0.00           H
ATOM    915  C   SER    58      32.734  31.042  50.571  1.00  0.00           C
ATOM    916  O   SER    58      32.019  31.304  49.575  1.00  0.00           O
ATOM    917  N   THR    59      32.257  31.154  51.767  1.00  0.00           N
```

ATOM	918	H	THR	59	32.946	30.948	52.476	1.00	0.00	H
ATOM	919	CA	THR	59	30.898	31.455	52.230	1.00	0.00	C
ATOM	920	HA	THR	59	30.422	32.149	51.537	1.00	0.00	H
ATOM	921	CB	THR	59	30.116	30.220	52.294	1.00	0.00	C
ATOM	922	HB	THR	59	30.228	29.719	51.332	1.00	0.00	H
ATOM	923	CG2	THR	59	30.468	29.245	53.402	1.00	0.00	C
ATOM	924	HG21	THR	59	29.929	28.338	53.131	1.00	0.00	H
ATOM	925	HG22	THR	59	31.555	29.178	53.447	1.00	0.00	H
ATOM	926	HG23	THR	59	30.074	29.704	54.309	1.00	0.00	H
ATOM	927	OG1	THR	59	28.770	30.509	52.493	1.00	0.00	O
ATOM	928	HG1	THR	59	28.380	30.340	51.631	1.00	0.00	H
ATOM	929	C	THR	59	30.845	32.258	53.554	1.00	0.00	C
ATOM	930	O	THR	59	31.633	31.888	54.445	1.00	0.00	O
ATOM	931	N	VAL	60	30.169	33.362	53.598	1.00	0.00	N
ATOM	932	H	VAL	60	29.613	33.589	52.785	1.00	0.00	H
ATOM	933	CA	VAL	60	30.390	34.426	54.615	1.00	0.00	C
ATOM	934	HA	VAL	60	30.312	34.084	55.647	1.00	0.00	H
ATOM	935	CB	VAL	60	31.725	35.130	54.335	1.00	0.00	C
ATOM	936	HB	VAL	60	31.854	35.960	55.030	1.00	0.00	H
ATOM	937	CG1	VAL	60	33.020	34.368	54.691	1.00	0.00	C
ATOM	938	HG11	VAL	60	33.931	34.960	54.769	1.00	0.00	H
ATOM	939	HG12	VAL	60	33.002	33.942	55.694	1.00	0.00	H
ATOM	940	HG13	VAL	60	33.180	33.611	53.924	1.00	0.00	H
ATOM	941	CG2	VAL	60	31.909	35.790	52.933	1.00	0.00	C
ATOM	942	HG21	VAL	60	32.906	36.223	52.845	1.00	0.00	H
ATOM	943	HG22	VAL	60	32.002	35.110	52.086	1.00	0.00	H
ATOM	944	HG23	VAL	60	31.121	36.533	52.812	1.00	0.00	H
ATOM	945	C	VAL	60	29.244	35.467	54.449	1.00	0.00	C
ATOM	946	O	VAL	60	28.356	35.306	53.613	1.00	0.00	O
ATOM	947	N	THR	61	29.387	36.505	55.242	1.00	0.00	N
ATOM	948	H	THR	61	30.200	36.575	55.836	1.00	0.00	H
ATOM	949	CA	THR	61	28.435	37.720	55.242	1.00	0.00	C
ATOM	950	HA	THR	61	27.777	37.673	54.374	1.00	0.00	H
ATOM	951	CB	THR	61	27.567	37.671	56.516	1.00	0.00	C
ATOM	952	HB	THR	61	26.873	38.509	56.575	1.00	0.00	H
ATOM	953	CG2	THR	61	26.675	36.476	56.521	1.00	0.00	C
ATOM	954	HG21	THR	61	26.044	36.496	57.410	1.00	0.00	H
ATOM	955	HG22	THR	61	26.036	36.547	55.641	1.00	0.00	H
ATOM	956	HG23	THR	61	27.207	35.526	56.467	1.00	0.00	H
ATOM	957	OG1	THR	61	28.384	37.512	57.677	1.00	0.00	O
ATOM	958	HG1	THR	61	28.884	38.309	57.868	1.00	0.00	H
ATOM	959	C	THR	61	29.159	39.036	54.964	1.00	0.00	C
ATOM	960	O	THR	61	30.392	39.020	54.910	1.00	0.00	O
ATOM	961	N	LEU	62	28.464	40.158	54.722	1.00	0.00	N
ATOM	962	H	LEU	62	27.459	40.163	54.828	1.00	0.00	H
ATOM	963	CA	LEU	62	29.008	41.493	54.436	1.00	0.00	C
ATOM	964	HA	LEU	62	29.684	41.391	53.587	1.00	0.00	H
ATOM	965	CB	LEU	62	27.846	42.465	54.276	1.00	0.00	C
ATOM	966	HB2	LEU	62	27.251	42.541	55.185	1.00	0.00	H
ATOM	967	HB3	LEU	62	28.208	43.482	54.129	1.00	0.00	H
ATOM	968	CG	LEU	62	26.870	42.059	53.048	1.00	0.00	C
ATOM	969	HG	LEU	62	26.231	41.215	53.307	1.00	0.00	H
ATOM	970	CD1	LEU	62	25.893	43.192	52.903	1.00	0.00	C
ATOM	971	HD11	LEU	62	24.991	42.934	52.347	1.00	0.00	H
ATOM	972	HD12	LEU	62	25.625	43.492	53.917	1.00	0.00	H
ATOM	973	HD13	LEU	62	26.314	44.116	52.509	1.00	0.00	H
ATOM	974	CD2	LEU	62	27.722	41.743	51.805	1.00	0.00	C
ATOM	975	HD21	LEU	62	27.100	41.421	50.969	1.00	0.00	H
ATOM	976	HD22	LEU	62	28.324	42.612	51.542	1.00	0.00	H
ATOM	977	HD23	LEU	62	28.403	40.904	51.952	1.00	0.00	H
ATOM	978	C	LEU	62	30.001	41.967	55.429	1.00	0.00	C
ATOM	979	O	LEU	62	31.129	42.366	55.040	1.00	0.00	O
ATOM	980	N	ASP	63	29.747	41.717	56.693	1.00	0.00	N
ATOM	981	H	ASP	63	28.863	41.285	56.927	1.00	0.00	H

```
ATOM    982  CA  ASP    63      30.720  41.947  57.720  1.00  0.00           C
ATOM    983  HA  ASP    63      30.951  43.008  57.638  1.00  0.00           H
ATOM    984  CB  ASP    63      30.008  41.694  59.120  1.00  0.00           C
ATOM    985  HB2 ASP    63      30.691  41.983  59.919  1.00  0.00           H
ATOM    986  HB3 ASP    63      29.112  42.311  59.067  1.00  0.00           H
ATOM    987  CG  ASP    63      29.527  40.241  59.447  1.00  0.00           C
ATOM    988  OD1 ASP    63      29.981  39.258  58.790  1.00  0.00           O
ATOM    989  OD2 ASP    63      28.780  40.088  60.438  1.00  0.00           O
ATOM    990  C   ASP    63      32.074  41.160  57.739  1.00  0.00           C
ATOM    991  O   ASP    63      33.114  41.606  58.258  1.00  0.00           O
ATOM    992  N   THR    64      32.114  40.025  56.977  1.00  0.00           N
ATOM    993  H   THR    64      31.278  39.742  56.486  1.00  0.00           H
ATOM    994  CA  THR    64      33.323  39.186  56.910  1.00  0.00           C
ATOM    995  HA  THR    64      34.062  39.584  57.604  1.00  0.00           H
ATOM    996  CB  THR    64      32.896  37.825  57.653  1.00  0.00           C
ATOM    997  HB  THR    64      33.601  37.071  57.306  1.00  0.00           H
ATOM    998  CG2 THR    64      33.056  37.976  59.187  1.00  0.00           C
ATOM    999 HG21 THR    64      32.513  38.856  59.530  1.00  0.00           H
ATOM   1000 HG22 THR    64      32.709  37.109  59.750  1.00  0.00           H
ATOM   1001 HG23 THR    64      34.091  38.168  59.470  1.00  0.00           H
ATOM   1002  OG1 THR    64      31.522  37.370  57.335  1.00  0.00           O
ATOM   1003  HG1 THR    64      30.998  37.993  57.845  1.00  0.00           H
ATOM   1004  C   THR    64      33.959  39.074  55.552  1.00  0.00           C
ATOM   1005  O   THR    64      34.868  38.319  55.402  1.00  0.00           O
ATOM   1006  N   LEU    65      33.455  39.818  54.601  1.00  0.00           N
ATOM   1007  H   LEU    65      32.467  40.012  54.669  1.00  0.00           H
ATOM   1008  CA  LEU    65      33.955  40.040  53.256  1.00  0.00           C
ATOM   1009  HA  LEU    65      34.024  39.026  52.861  1.00  0.00           H
ATOM   1010  CB  LEU    65      32.896  40.812  52.422  1.00  0.00           C
ATOM   1011  HB2 LEU    65      31.961  40.276  52.579  1.00  0.00           H
ATOM   1012  HB3 LEU    65      32.784  41.835  52.781  1.00  0.00           H
ATOM   1013  CG  LEU    65      33.101  40.955  50.867  1.00  0.00           C
ATOM   1014  HG  LEU    65      33.909  41.657  50.660  1.00  0.00           H
ATOM   1015  CD1 LEU    65      33.380  39.748  50.025  1.00  0.00           C
ATOM   1016 HD11 LEU    65      33.473  40.062  48.985  1.00  0.00           H
ATOM   1017 HD12 LEU    65      34.284  39.244  50.367  1.00  0.00           H
ATOM   1018 HD13 LEU    65      32.579  39.011  50.097  1.00  0.00           H
ATOM   1019  CD2 LEU    65      31.830  41.594  50.278  1.00  0.00           C
ATOM   1020 HD21 LEU    65      31.079  40.860  49.986  1.00  0.00           H
ATOM   1021 HD22 LEU    65      31.347  42.333  50.917  1.00  0.00           H
ATOM   1022 HD23 LEU    65      32.166  42.084  49.364  1.00  0.00           H
ATOM   1023  C   LEU    65      35.316  40.713  53.294  1.00  0.00           C
ATOM   1024  O   LEU    65      35.469  41.656  54.029  1.00  0.00           O
ATOM   1025  N   THR    66      36.198  40.326  52.351  1.00  0.00           N
ATOM   1026  H   THR    66      36.016  39.549  51.733  1.00  0.00           H
ATOM   1027  CA  THR    66      37.528  40.859  52.183  1.00  0.00           C
ATOM   1028  HA  THR    66      37.916  41.241  53.127  1.00  0.00           H
ATOM   1029  CB  THR    66      38.542  39.729  51.859  1.00  0.00           C
ATOM   1030  HB  THR    66      38.361  39.489  50.811  1.00  0.00           H
ATOM
   1031  CG2 THR    66      40.060  40.177  51.936  1.00  0.00           C
ATOM   1032 HG21 THR    66      40.142  40.907  52.742  1.00  0.00           H
ATOM   1033 HG22 THR    66      40.829  39.447  52.186  1.00  0.00           H
ATOM   1034 HG23 THR    66      40.415  40.599  50.996  1.00  0.00           H
ATOM   1035  OG1 THR    66      38.445  38.574  52.664  1.00  0.00           O
ATOM   1036  HG1 THR    66      37.580  38.162  52.593  1.00  0.00           H
ATOM   1037  C   THR    66      37.484  41.860  51.066  1.00  0.00           C
ATOM   1038  O   THR    66      36.712  41.661  50.148  1.00  0.00           O
ATOM   1039  N   GLU    67      38.451  42.812  51.000  1.00  0.00           N
ATOM   1040  H   GLU    67      39.159  42.796  51.721  1.00  0.00           H
ATOM   1041  CA  GLU    67      38.335  43.899  50.054  1.00  0.00           C
ATOM   1042  HA  GLU    67      37.286  44.171  50.166  1.00  0.00           H
ATOM   1043  CB  GLU    67      39.156  45.116  50.611  1.00  0.00           C
ATOM   1044  HB2 GLU    67      38.823  45.976  50.030  1.00  0.00           H
```

ATOM	1045	HB3	GLU	67	38.921	45.296	51.660	1.00	0.00	H
ATOM	1046	CG	GLU	67	40.724	45.050	50.433	1.00	0.00	C
ATOM	1047	HG2	GLU	67	41.023	44.017	50.609	1.00	0.00	H
ATOM	1048	HG3	GLU	67	40.948	45.313	49.399	1.00	0.00	H
ATOM	1049	CD	GLU	67	41.459	45.931	51.429	1.00	0.00	C
ATOM	1050	OE1	GLU	67	42.191	46.857	50.905	1.00	0.00	O
ATOM	1051	OE2	GLU	67	41.255	45.674	52.646	1.00	0.00	O
ATOM	1052	C	GLU	67	38.447	43.616	48.523	1.00	0.00	C
ATOM	1053	O	GLU	67	38.174	44.493	47.735	1.00	0.00	O
ATOM	1054	N	LYS	68	38.755	42.363	48.199	1.00	0.00	N
ATOM	1055	H	LYS	68	38.984	41.876	49.053	1.00	0.00	H
ATOM	1056	CA	LYS	68	39.277	41.953	46.873	1.00	0.00	C
ATOM	1057	HA	LYS	68	40.197	42.506	46.684	1.00	0.00	H
ATOM	1058	CB	LYS	68	39.669	40.455	46.897	1.00	0.00	C
ATOM	1059	HB2	LYS	68	40.382	40.414	47.720	1.00	0.00	H
ATOM	1060	HB3	LYS	68	38.841	39.814	47.199	1.00	0.00	H
ATOM	1061	CG	LYS	68	40.391	39.794	45.710	1.00	0.00	C
ATOM	1062	HG2	LYS	68	40.355	38.712	45.834	1.00	0.00	H
ATOM	1063	HG3	LYS	68	39.867	40.047	44.788	1.00	0.00	H
ATOM	1064	CD	LYS	68	41.893	40.054	45.598	1.00	0.00	C
ATOM	1065	HD2	LYS	68	42.230	41.074	45.415	1.00	0.00	H
ATOM	1066	HD3	LYS	68	42.448	39.720	46.475	1.00	0.00	H
ATOM	1067	CE	LYS	68	42.314	39.175	44.333	1.00	0.00	C
ATOM	1068	HE2	LYS	68	42.037	38.131	44.478	1.00	0.00	H
ATOM	1069	HE3	LYS	68	41.650	39.597	43.578	1.00	0.00	H
ATOM	1070	NZ	LYS	68	43.757	39.256	43.931	1.00	0.00	N
ATOM	1071	HZ1	LYS	68	44.055	38.562	43.261	1.00	0.00	H
ATOM	1072	HZ2	LYS	68	43.949	40.176	43.561	1.00	0.00	H
ATOM	1073	HZ3	LYS	68	44.292	39.013	44.752	1.00	0.00	H
ATOM	1074	C	LYS	68	38.405	42.423	45.713	1.00	0.00	C
ATOM	1075	O	LYS	68	38.923	42.937	44.712	1.00	0.00	O
ATOM	1076	N	HIE	69	37.060	42.360	45.874	1.00	0.00	N
ATOM	1077	H	HIE	69	36.740	41.856	46.689	1.00	0.00	H
ATOM	1078	CA	HIE	69	36.094	42.647	44.810	1.00	0.00	C
ATOM	1079	HA	HIE	69	36.489	42.384	43.829	1.00	0.00	H
ATOM	1080	CB	HIE	69	34.760	42.000	45.112	1.00	0.00	C
ATOM	1081	HB2	HIE	69	34.794	40.979	45.492	1.00	0.00	H
ATOM	1082	HB3	HIE	69	34.242	42.453	45.956	1.00	0.00	H
ATOM	1083	CG	HIE	69	33.803	42.050	43.951	1.00	0.00	C
ATOM	1084	ND1	HIE	69	33.812	41.217	42.814	1.00	0.00	N
ATOM	1085	CE1	HIE	69	32.712	41.468	42.063	1.00	0.00	C
ATOM	1086	HE1	HIE	69	32.386	40.881	41.217	1.00	0.00	H
ATOM	1087	NE2	HIE	69	32.073	42.508	42.714	1.00	0.00	N
ATOM	1088	HE2	HIE	69	31.161	42.859	42.456	1.00	0.00	H
ATOM	1089	CD2	HIE	69	32.673	42.778	43.886	1.00	0.00	C
ATOM	1090	HD2	HIE	69	32.371	43.499	44.632	1.00	0.00	H
ATOM	1091	C	HIE	69	35.897	44.114	44.750	1.00	0.00	C
ATOM	1092	O	HIE	69	35.913	44.632	43.643	1.00	0.00	O
ATOM	1093	N	ALA	70	35.845	44.807	45.889	1.00	0.00	N
ATOM	1094	H	ALA	70	35.991	44.266	46.729	1.00	0.00	H
ATOM	1095	CA	ALA	70	35.791	46.288	46.055	1.00	0.00	C
ATOM	1096	HA	ALA	70	34.871	46.590	45.554	1.00	0.00	H
ATOM	1097	CB	ALA	70	35.663	46.735	47.500	1.00	0.00	C
ATOM	1098	HB1	ALA	70	35.659	47.818	47.617	1.00	0.00	H
ATOM	1099	HB2	ALA	70	34.734	46.310	47.880	1.00	0.00	H
ATOM	1100	HB3	ALA	70	36.516	46.402	48.091	1.00	0.00	H
ATOM	1101	C	ALA	70	37.045	46.879	45.307	1.00	0.00	C
ATOM	1102	O	ALA	70	36.851	47.505	44.222	1.00	0.00	O
ATOM	1103	N	GLU	71	38.224	46.334	45.592	1.00	0.00	N
ATOM	1104	H	GLU	71	38.293	45.736	46.404	1.00	0.00	H
ATOM	1105	CA	GLU	71	39.493	46.804	44.922	1.00	0.00	C
ATOM	1106	HA	GLU	71	39.663	47.864	45.107	1.00	0.00	H
ATOM	1107	CB	GLU	71	40.609	46.018	45.502	1.00	0.00	C
ATOM	1108	HB2	GLU	71	40.344	44.968	45.615	1.00	0.00	H

ATOM	1109	HB3	GLU	71	41.545	46.080	44.945	1.00	0.00	H
ATOM	1110	CG	GLU	71	40.978	46.396	46.915	1.00	0.00	C
ATOM	1111	HG2	GLU	71	40.204	46.053	47.601	1.00	0.00	H
ATOM	1112	HG3	GLU	71	41.809	45.765	47.232	1.00	0.00	H
ATOM	1113	CD	GLU	71	41.387	47.873	47.172	1.00	0.00	C
ATOM	1114	OE1	GLU	71	40.535	48.689	47.601	1.00	0.00	O
ATOM	1115	OE2	GLU	71	42.549	48.217	46.790	1.00	0.00	O
ATOM	1116	C	GLU	71	39.560	46.575	43.382	1.00	0.00	C
ATOM	1117	O	GLU	71	40.232	47.285	42.642	1.00	0.00	O
ATOM	1118	N	GLN	72	38.874	45.531	42.935	1.00	0.00	N
ATOM	1119	H	GLN	72	38.295	45.097	43.641	1.00	0.00	H
ATOM	1120	CA	GLN	72	38.681	45.182	41.517	1.00	0.00	C
ATOM	1121	HA	GLN	72	39.612	45.311	40.966	1.00	0.00	H
ATOM	1122	CB	GLN	72	38.355	43.668	41.427	1.00	0.00	C
ATOM	1123	HB2	GLN	72	37.431	43.511	41.985	1.00	0.00	H
ATOM	1124	HB3	GLN	72	39.073	43.078	41.996	1.00	0.00	H
ATOM	1125	CG	GLN	72	38.151	42.995	40.043	1.00	0.00	C
ATOM	1126	HG2	GLN	72	37.758	43.661	39.275	1.00	0.00	H
ATOM	1127	HG3	GLN	72	39.156	42.751	39.698	1.00	0.00	H
ATOM	1128	CD	GLN	72	37.352	41.695	40.069	1.00	0.00	C
ATOM	1129	OE1	GLN	72	37.839	40.623	39.767	1.00	0.00	O
ATOM	1130	NE2	GLN	72	36.099	41.717	40.421	1.00	0.00	N
ATOM	1131	HE21	GLN	72	35.499	42.527	40.361	1.00	0.00	H
ATOM	1132	HE22	GLN	72	35.698	40.814	40.631	1.00	0.00	H
ATOM	1133	C	GLN	72	37.584	46.059	40.813	1.00	0.00	C
ATOM	1134	O	GLN	72	37.720	46.298	39.629	1.00	0.00	O
ATOM	1135	N	GLU	73	36.510	46.464	41.540	1.00	0.00	N
ATOM	1136	H	GLU	73	36.272	45.884	42.331	1.00	0.00	H
ATOM	1137	CA	GLU	73	35.407	47.250	41.043	1.00	0.00	C
ATOM	1138	HA	GLU	73	35.308	47.198	39.959	1.00	0.00	H
ATOM	1139	CB	GLU	73	34.045	46.648	41.464	1.00	0.00	C
ATOM	1140	HB2	GLU	73	33.912	46.757	42.540	1.00	0.00	H
ATOM	1141	HB3	GLU	73	33.201	47.206	41.056	1.00	0.00	H
ATOM	1142	CG	GLU	73	33.759	45.165	41.024	1.00	0.00	C
ATOM	1143	HG2	GLU	73	34.321	44.461	41.637	1.00	0.00	H
ATOM	1144	HG3	GLU	73	32.724	44.934	41.279	1.00	0.00	H
ATOM	1145	CD	GLU	73	33.973	44.730	39.588	1.00	0.00	C
ATOM	1146	OE1	GLU	73	33.635	45.533	38.624	1.00	0.00	O
ATOM	1147	OE2	GLU	73	34.356	43.561	39.375	1.00	0.00	O
ATOM	1148	C	GLU	73	35.516	48.810	41.396	1.00	0.00	C
ATOM	1149	O	GLU	73	34.555	49.545	41.420	1.00	0.00	O
ATOM	1150	N	ASN	74	36.753	49.231	41.730	1.00	0.00	N
ATOM	1151	H	ASN	74	37.395	48.480	41.941	1.00	0.00	H
ATOM	1152	CA	ASN	74	37.272	50.550	42.082	1.00	0.00	C
ATOM	1153	HA	ASN	74	38.325	50.329	42.259	1.00	0.00	H
ATOM	1154	CB	ASN	74	37.254	51.498	40.869	1.00	0.00	C
ATOM	1155	HB2	ASN	74	36.265	51.439	40.415	1.00	0.00	H
ATOM	1156	HB3	ASN	74	37.543	52.521	41.112	1.00	0.00	H
ATOM	1157	CG	ASN	74	38.231	50.974	39.828	1.00	0.00	C
ATOM	1158	OD1	ASN	74	39.428	50.826	39.998	1.00	0.00	O
ATOM	1159	ND2	ASN	74	37.830	50.931	38.587	1.00	0.00	N
ATOM	1160	HD21	ASN	74	38.416	50.573	37.847	1.00	0.00	H
ATOM	1161	HD22	ASN	74	36.906	51.215	38.295	1.00	0.00	H
ATOM	1162	C	ASN	74	36.778	51.286	43.365	1.00	0.00	C
ATOM	1163	O	ASN	74	37.003	52.493	43.530	1.00	0.00	O
ATOM	1164	N	MET	75	36.108	50.640	44.332	1.00	0.00	N
ATOM	1165	H	MET	75	35.960	49.647	44.221	1.00	0.00	H
ATOM	1166	CA	MET	75	35.443	51.211	45.497	1.00	0.00	C
ATOM	1167	HA	MET	75	35.663	52.279	45.497	1.00	0.00	H
ATOM	1168	CB	MET	75	33.945	50.913	45.373	1.00	0.00	C
ATOM	1169	HB2	MET	75	33.441	51.476	46.157	1.00	0.00	H
ATOM	1170	HB3	MET	75	33.679	51.338	44.405	1.00	0.00	H
ATOM	1171	CG	MET	75	33.632	49.366	45.541	1.00	0.00	C

ATOM	1172	HG2	MET	75	34.143	48.791	44.767	1.00	0.00	H
ATOM	1173	HG3	MET	75	33.948	49.087	46.547	1.00	0.00	H
ATOM	1174	SD	MET	75	31.948	48.837	45.457	1.00	0.00	S
ATOM	1175	CE	MET	75	31.653	49.089	43.682	1.00	0.00	C
ATOM	1176	HE1	MET	75	32.302	48.414	43.124	1.00	0.00	H
ATOM	1177	HE2	MET	75	30.590	48.902	43.524	1.00	0.00	H
ATOM	1178	HE3	MET	75	31.895	50.136	43.501	1.00	0.00	H
ATOM	1179	C	MET	75	36.048	50.754	46.852	1.00	0.00	C
ATOM	1180	O	MET	75	36.875	49.846	46.874	1.00	0.00	O
ATOM	1181	N	THR	76	35.705	51.421	47.956	1.00	0.00	N
ATOM	1182	H	THR	76	35.033	52.151	47.775	1.00	0.00	H
ATOM	1183	CA	THR	76	36.055	51.070	49.358	1.00	0.00	C
ATOM	1184	HA	THR	76	37.064	50.674	49.461	1.00	0.00	H
ATOM	1185	CB	THR	76	36.150	52.316	50.209	1.00	0.00	C
ATOM	1186	HB	THR	76	36.841	53.020	49.747	1.00	0.00	H
ATOM	1187	CG2	THR	76	34.832	53.127	50.257	1.00	0.00	C
ATOM	1188	HG21	THR	76	34.595	53.570	49.290	1.00	0.00	H
ATOM	1189	HG22	THR	76	34.055	52.404	50.509	1.00	0.00	H
ATOM	1190	HG23	THR	76	34.883	53.856	51.064	1.00	0.00	H
ATOM	1191	OG1	THR	76	36.601	52.088	51.545	1.00	0.00	O
ATOM	1192	HG1	THR	76	37.547	51.928	51.523	1.00	0.00	H
ATOM	1193	C	THR	76	35.083	50.061	49.867	1.00	0.00	C
ATOM	1194	O	THR	76	33.918	50.162	49.491	1.00	0.00	O
ATOM	1195	N	LEU	77	35.507	49.181	50.764	1.00	0.00	N
ATOM	1196	H	LEU	77	36.446	49.219	51.136	1.00	0.00	H
ATOM	1197	CA	LEU	77	34.734	47.936	51.155	1.00	0.00	C
ATOM	1198	HA	LEU	77	34.508	47.376	50.248	1.00	0.00	H
ATOM	1199	CB	LEU	77	35.619	47.044	51.970	1.00	0.00	C
ATOM	1200	HB2	LEU	77	36.448	46.700	51.351	1.00	0.00	H
ATOM	1201	HB3	LEU	77	36.055	47.624	52.784	1.00	0.00	H
ATOM	1202	CG	LEU	77	35.006	45.835	52.577	1.00	0.00	C
ATOM	1203	HG	LEU	77	34.312	46.105	53.373	1.00	0.00	H
ATOM	1204	CD1	LEU	77	34.206	44.904	51.536	1.00	0.00	C
ATOM	1205	HD11	LEU	77	33.643	44.113	52.031	1.00	0.00	H
ATOM	1206	HD12	LEU	77	33.392	45.444	51.054	1.00	0.00	H
ATOM	1207	HD13	LEU	77	34.859	44.438	50.797	1.00	0.00	H
ATOM	1208	CD2	LEU	77	36.074	44.981	53.189	1.00	0.00	C
ATOM	1209	HD21	LEU	77	35.491	44.216	53.704	1.00	0.00	H
ATOM	1210	HD22	LEU	77	36.920	44.763	52.537	1.00	0.00	H
ATOM	1211	HD23	LEU	77	36.457	45.685	53.928	1.00	0.00	H
ATOM	1212	C	LEU	77	33.362	48.310	51.755	1.00	0.00	C
ATOM	1213	O	LEU	77	32.418	47.531	51.680	1.00	0.00	O
ATOM	1214	N	THR	78	33.170	49.427	52.472	1.00	0.00	N
ATOM	1215	H	THR	78	34.004	49.984	52.581	1.00	0.00	H
ATOM	1216	CA	THR	78	31.845	49.785	53.059	1.00	0.00	C
ATOM	1217	HA	THR	78	31.388	48.933	53.563	1.00	0.00	H
ATOM	1218	CB	THR	78	31.894	50.849	54.176	1.00	0.00	C
ATOM	1219	HB	THR	78	30.915	51.075	54.600	1.00	0.00	H
ATOM	1220	CG2	THR	78	32.794	50.616	55.359	1.00	0.00	C
ATOM	1221	HG21	THR	78	33.779	50.314	55.004	1.00	0.00	H
ATOM	1222	HG22	THR	78	32.892	51.479	56.017	1.00	0.00	H
ATOM	1223	HG23	THR	78	32.501	49.736	55.929	1.00	0.00	H
ATOM	1224	OG1	THR	78	32.512	51.979	53.554	1.00	0.00	O
ATOM	1225	HG1	THR	78	31.856	52.610	53.245	1.00	0.00	H
ATOM	1226	C	THR	78	30.806	50.264	51.993	1.00	0.00	C
ATOM	1227	O	THR	78	29.605	49.974	52.136	1.00	0.00	O
ATOM	1228	N	GLU	79	31.318	50.855	50.879	1.00	0.00	N
ATOM	1229	H	GLU	79	32.302	51.077	50.862	1.00	0.00	H
ATOM	1230	CA	GLU	79	30.490	51.016	49.637	1.00	0.00	C
ATOM	1231	HA	GLU	79	29.538	51.438	49.956	1.00	0.00	H
ATOM	1232	CB	GLU	79	31.171	51.997	48.623	1.00	0.00	C
ATOM	1233	HB2	GLU	79	32.162	51.607	48.388	1.00	0.00	H
ATOM	1234	HB3	GLU	79	30.442	52.142	47.825	1.00	0.00	H
ATOM	1235	CG	GLU	79	31.265	53.400	49.289	1.00	0.00	C

ATOM	1236	HG2	GLU	79	32.015	53.294	50.073	1.00	0.00	H
ATOM	1237	HG3	GLU	79	31.771	53.998	48.531	1.00	0.00	H
ATOM	1238	CD	GLU	79	29.931	54.056	49.892	1.00	0.00	C
ATOM	1239	OE1	GLU	79	29.994	54.836	50.893	1.00	0.00	O
ATOM	1240	OE2	GLU	79	28.845	53.903	49.282	1.00	0.00	O
ATOM	1241	C	GLU	79	30.080	49.695	48.952	1.00	0.00	C
ATOM	1242	O	GLU	79	28.991	49.669	48.390	1.00	0.00	O
ATOM	1243	N	LEU	80	30.894	48.665	48.995	1.00	0.00	N
ATOM	1244	H	LEU	80	31.775	48.830	49.459	1.00	0.00	H
ATOM	1245	CA	LEU	80	30.609	47.346	48.441	1.00	0.00	C
ATOM	1246	HA	LEU	80	30.280	47.547	47.421	1.00	0.00	H
ATOM	1247	CB	LEU	80	31.890	46.515	48.224	1.00	0.00	C
ATOM	1248	HB2	LEU	80	32.651	47.049	47.655	1.00	0.00	H
ATOM	1249	HB3	LEU	80	32.254	46.292	49.227	1.00	0.00	H
ATOM	1250	CG	LEU	80	31.445	45.150	47.618	1.00	0.00	C
ATOM	1251	HG	LEU	80	30.793	44.611	48.306	1.00	0.00	H
ATOM	1252	CD1	LEU	80	30.916	45.204	46.186	1.00	0.00	C
ATOM	1253	HD11	LEU	80	31.800	45.332	45.561	1.00	0.00	H
ATOM	1254	HD12	LEU	80	30.452	44.223	46.087	1.00	0.00	H
ATOM	1255	HD13	LEU	80	30.199	46.008	46.021	1.00	0.00	H
ATOM	1256	CD2	LEU	80	32.655	44.174	47.772	1.00	0.00	C
ATOM	1257	HD21	LEU	80	32.370	43.193	47.389	1.00	0.00	H
ATOM	1258	HD22	LEU	80	33.439	44.591	47.139	1.00	0.00	H
ATOM	1259	HD23	LEU	80	33.107	44.122	48.761	1.00	0.00	H
ATOM	1260	C	LEU	80	29.569	46.638	49.248	1.00	0.00	C
ATOM	1261	O	LEU	80	28.648	46.115	48.709	1.00	0.00	O
ATOM	1262	N	LYS	81	29.531	46.839	50.576	1.00	0.00	N
ATOM	1263	H	LYS	81	30.239	47.463	50.937	1.00	0.00	H
ATOM	1264	CA	LYS	81	28.432	46.425	51.493	1.00	0.00	C
ATOM	1265	HA	LYS	81	28.228	45.363	51.357	1.00	0.00	H
ATOM	1266	CB	LYS	81	28.772	46.641	52.918	1.00	0.00	C
ATOM	1267	HB2	LYS	81	28.834	47.699	53.169	1.00	0.00	H
ATOM	1268	HB3	LYS	81	27.869	46.330	53.445	1.00	0.00	H
ATOM	1269	CG	LYS	81	30.010	45.864	53.355	1.00	0.00	C
ATOM	1270	HG2	LYS	81	29.716	44.815	53.397	1.00	0.00	H
ATOM	1271	HG3	LYS	81	30.852	45.918	52.666	1.00	0.00	H
ATOM	1272	CD	LYS	81	30.410	46.265	54.757	1.00	0.00	C
ATOM	1273	HD2	LYS	81	30.535	47.346	54.810	1.00	0.00	H
ATOM	1274	HD3	LYS	81	29.668	45.940	55.486	1.00	0.00	H
ATOM	1275	CE	LYS	81	31.750	45.671	55.086	1.00	0.00	C
ATOM	1276	HE2	LYS	81	31.744	44.582	55.049	1.00	0.00	H
ATOM	1277	HE3	LYS	81	32.437	45.929	54.281	1.00	0.00	H
ATOM	1278	NZ	LYS	81	32.231	46.191	56.337	1.00	0.00	N
ATOM	1279	HZ1	LYS	81	32.967	45.555	56.610	1.00	0.00	H
ATOM	1280	HZ2	LYS	81	32.560	47.122	56.124	1.00	0.00	H
ATOM	1281	HZ3	LYS	81	31.453	46.197	56.981			
1.00	0.00			H						
ATOM	1282	C	LYS	81	27.135	47.131	51.098	1.00	0.00	C
ATOM	1283	O	LYS	81	26.160	46.557	50.740	1.00	0.00	O
ATOM	1284	N	LYS	82	27.196	48.489	51.104	1.00	0.00	N
ATOM	1285	H	LYS	82	28.047	48.885	51.475	1.00	0.00	H
ATOM	1286	CA	LYS	82	26.063	49.320	50.628	1.00	0.00	C
ATOM	1287	HA	LYS	82	25.261	49.272	51.365	1.00	0.00	H
ATOM	1288	CB	LYS	82	26.363	50.851	50.598	1.00	0.00	C
ATOM	1289	HB2	LYS	82	27.274	51.059	50.036	1.00	0.00	H
ATOM	1290	HB3	LYS	82	25.544	51.340	50.071	1.00	0.00	H
ATOM	1291	CG	LYS	82	26.483	51.335	52.075	1.00	0.00	C
ATOM	1292	HG2	LYS	82	25.477	51.322	52.496	1.00	0.00	H
ATOM	1293	HG3	LYS	82	27.010	50.527	52.582	1.00	0.00	H
ATOM	1294	CD	LYS	82	27.120	52.724	52.220	1.00	0.00	C
ATOM	1295	HD2	LYS	82	27.072	52.948	53.286	1.00	0.00	H
ATOM	1296	HD3	LYS	82	28.144	52.689	51.848	1.00	0.00	H
ATOM	1297	CE	LYS	82	26.347	53.790	51.392	1.00	0.00	C
ATOM	1298	HE2	LYS	82	26.228	53.332	50.410	1.00	0.00	H

ATOM	1299	HE3	LYS	82	25.389	54.018	51.857	1.00	0.00	H
ATOM	1300	NZ	LYS	82	27.201	55.081	51.274	1.00	0.00	N
ATOM	1301	HZ1	LYS	82	26.824	55.889	50.799	1.00	0.00	H
ATOM	1302	HZ2	LYS	82	27.345	55.507	52.179	1.00	0.00	H
ATOM	1303	HZ3	LYS	82	28.095	54.945	50.825	1.00	0.00	H
ATOM	1304	C	LYS	82	25.423	48.886	49.305	1.00	0.00	C
ATOM	1305	O	LYS	82	24.222	48.871	49.343	1.00	0.00	O
ATOM	1306	N	VAL	83	26.207	48.585	48.186	1.00	0.00	N
ATOM	1307	H	VAL	83	27.214	48.517	48.221	1.00	0.00	H
ATOM	1308	CA	VAL	83	25.420	48.212	46.974	1.00	0.00	C
ATOM	1309	HA	VAL	83	24.480	48.763	46.951	1.00	0.00	H
ATOM	1310	CB	VAL	83	26.271	48.418	45.675	1.00	0.00	C
ATOM	1311	HB	VAL	83	25.665	47.975	44.884	1.00	0.00	H
ATOM	1312	CG1	VAL	83	26.393	49.850	45.237	1.00	0.00	C
ATOM	1313	HG11	VAL	83	27.019	50.218	46.051	1.00	0.00	H
ATOM	1314	HG12	VAL	83	26.879	49.943	44.266	1.00	0.00	H
ATOM	1315	HG13	VAL	83	25.382	50.251	45.321	1.00	0.00	H
ATOM	1316	CG2	VAL	83	27.674	47.755	45.544	1.00	0.00	C
ATOM	1317	HG21	VAL	83	27.551	46.681	45.683	1.00	0.00	H
ATOM	1318	HG22	VAL	83	28.054	48.016	44.555	1.00	0.00	H
ATOM	1319	HG23	VAL	83	28.407	48.166	46.237	1.00	0.00	H
ATOM	1320	C	VAL	83	24.891	46.824	47.084	1.00	0.00	C
ATOM	1321	O	VAL	83	23.848	46.561	46.493	1.00	0.00	O
ATOM	1322	N	ILE	84	25.505	45.882	47.752	1.00	0.00	N
ATOM	1323	H	ILE	84	26.414	46.092	48.138	1.00	0.00	H
ATOM	1324	CA	ILE	84	25.005	44.436	47.818	1.00	0.00	C
ATOM	1325	HA	ILE	84	24.646	44.201	46.817	1.00	0.00	H
ATOM	1326	CB	ILE	84	26.025	43.420	48.299	1.00	0.00	C
ATOM	1327	HB	ILE	84	26.367	43.566	49.323	1.00	0.00	H
ATOM	1328	CG2	ILE	84	25.340	42.070	48.279	1.00	0.00	C
ATOM	1329	HG21	ILE	84	24.602	41.969	49.074	1.00	0.00	H
ATOM	1330	HG22	ILE	84	24.953	41.806	47.295	1.00	0.00	H
ATOM	1331	HG23	ILE	84	26.123	41.426	48.681	1.00	0.00	H
ATOM	1332	CG1	ILE	84	27.197	43.406	47.247	1.00	0.00	C
ATOM	1333	HG12	ILE	84	26.774	42.959	46.347	1.00	0.00	H
ATOM	1334	HG13	ILE	84	27.520	44.413	46.985	1.00	0.00	H
ATOM	1335	CD1	ILE	84	28.413	42.549	47.633	1.00	0.00	C
ATOM	1336	HD11	ILE	84	28.997	42.910	48.480	1.00	0.00	H
ATOM	1337	HD12	ILE	84	28.071	41.541	47.869	1.00	0.00	H
ATOM	1338	HD13	ILE	84	29.092	42.536	46.780	1.00	0.00	H
ATOM	1339	C	ILE	84	23.696	44.451	48.659	1.00	0.00	C
ATOM	1340	O	ILE	84	22.660	44.037	48.109	1.00	0.00	O
ATOM	1341	N	ALA	85	23.721	45.134	49.740	1.00	0.00	N
ATOM	1342	H	ALA	85	24.562	45.622	50.010	1.00	0.00	H
ATOM	1343	CA	ALA	85	22.606	45.126	50.673	1.00	0.00	C
ATOM	1344	HA	ALA	85	22.087	44.168	50.670	1.00	0.00	H
ATOM	1345	CB	ALA	85	23.173	45.409	52.023	1.00	0.00	C
ATOM	1346	HB1	ALA	85	22.319	45.474	52.697	1.00	0.00	H
ATOM	1347	HB2	ALA	85	23.851	44.616	52.340	1.00	0.00	H
ATOM	1348	HB3	ALA	85	23.708	46.349	51.886	1.00	0.00	H
ATOM	1349	C	ALA	85	21.556	46.234	50.285	1.00	0.00	C
ATOM	1350	O	ALA	85	20.425	46.207	50.825	1.00	0.00	O
ATOM	1351	N	ASP	86	21.864	47.146	49.353	1.00	0.00	N
ATOM	1352	H	ASP	86	22.762	47.038	48.903	1.00	0.00	H
ATOM	1353	CA	ASP	86	20.832	47.974	48.672	1.00	0.00	C
ATOM	1354	HA	ASP	86	20.321	48.468	49.499	1.00	0.00	H
ATOM	1355	CB	ASP	86	21.411	49.142	47.821	1.00	0.00	C
ATOM	1356	HB2	ASP	86	21.978	49.890	48.374	1.00	0.00	H
ATOM	1357	HB3	ASP	86	22.052	48.714	47.050	1.00	0.00	H
ATOM	1358	CG	ASP	86	20.231	49.844	47.225	1.00	0.00	C
ATOM	1359	OD1	ASP	86	20.021	49.549	46.041	1.00	0.00	O
ATOM	1360	OD2	ASP	86	19.526	50.597	48.012	1.00	0.00	O
ATOM	1361	C	ASP	86	19.816	47.095	47.972	1.00	0.00	C
ATOM	1362	O	ASP	86	18.670	47.463	47.823	1.00	0.00	O

ATOM	1363	N	ILE	87	20.158	45.935	47.496	1.00	0.00	N
ATOM	1364	H	ILE	87	21.130	45.678	47.401	1.00	0.00	H
ATOM	1365	CA	ILE	87	19.203	45.001	46.758	1.00	0.00	C
ATOM	1366	HA	ILE	87	18.236	45.426	46.491	1.00	0.00	H
ATOM	1367	CB	ILE	87	19.818	44.605	45.384	1.00	0.00	C
ATOM	1368	HB	ILE	87	20.851	44.263	45.432	1.00	0.00	H
ATOM	1369	CG2	ILE	87	19.007	43.508	44.719	1.00	0.00	C
ATOM	1370	HG21	ILE	87	19.446	43.285	43.747	1.00	0.00	H
ATOM	1371	HG22	ILE	87	19.142	42.586	45.284	1.00	0.00	H
ATOM	1372	HG23	ILE	87	17.948	43.765	44.705	1.00	0.00	H
ATOM	1373	CG1	ILE	87	19.856	45.927	44.533	1.00	0.00	C
ATOM	1374	HG12	ILE	87	18.847	46.340	44.559	1.00	0.00	H
ATOM	1375	HG13	ILE	87	20.572	46.648	44.926	1.00	0.00	H
ATOM	1376	CD1	ILE	87	20.368	45.726	43.044	1.00	0.00	C
ATOM	1377	HD11	ILE	87	20.971	46.575	42.723	1.00	0.00	H
ATOM	1378	HD12	ILE	87	20.870	44.763	42.948	1.00	0.00	H
ATOM	1379	HD13	ILE	87	19.467	45.736	42.431	1.00	0.00	H
ATOM	1380	C	ILE	87	18.820	43.723	47.603	1.00	0.00	C
ATOM	1381	O	ILE	87	17.615	43.382	47.822	1.00	0.00	O
ATOM	1382	N	TYR	88	19.819	43.007	48.044	1.00	0.00	N
ATOM	1383	H	TYR	88	20.789	43.210	47.845	1.00	0.00	H
ATOM	1384	CA	TYR	88	19.643	41.810	48.871	1.00	0.00	C
ATOM	1385	HA	TYR	88	18.795	41.235	48.501	1.00	0.00	H
ATOM	1386	CB	TYR	88	20.923	40.936	48.712	1.00	0.00	C
ATOM	1387	HB2	TYR	88	21.788	41.590	48.833	1.00	0.00	H
ATOM	1388	HB3	TYR	88	20.818	40.281	49.577	1.00	0.00	H
ATOM	1389	CG	TYR	88	20.895	40.114	47.454	1.00	0.00	C
ATOM	1390	CD1	TYR	88	20.407	38.761	47.468	1.00	0.00	C
ATOM	1391	HD1	TYR	88	20.124	38.322	48.415	1.00	0.00	H
ATOM	1392	CE1	TYR	88	20.375	38.029	46.257	1.00	0.00	C
ATOM	1393	HE1	TYR	88	20.077	36.992	46.187	1.00	0.00	H
ATOM	1394	CZ	TYR	88	20.958	38.598	45.107	1.00	0.00	C
ATOM	1395	OH	TYR	88	21.196	37.845	44.042	1.00	0.00	O
ATOM	1396	HH	TYR	88	20.964	36.913	44.040	1.00	0.00	H
ATOM	1397	CE2	TYR	88	21.428	39.950	45.099	1.00	0.00	C
ATOM	1398	HE2	TYR	88	21.923	40.386	44.243	1.00	0.00	H
ATOM	1399	CD2	TYR	88	21.365	40.728	46.251	1.00	0.00	C
ATOM	1400	HD2	TYR	88	21.795	41.717	46.321	1.00	0.00	H
ATOM	1401	C	TYR	88	19.273	42.013	50.341	1.00	0.00	C
ATOM	1402	O	TYR	88	19.791	42.981	50.889	1.00	0.00	O
ATOM	1403	N	PRO	89	18.460	41.217	51.034	1.00	0.00	N
ATOM	1404	CD	PRO	89	17.649	40.148	50.501	1.00	0.00	C
ATOM	1405	HD2	PRO	89	18.281	39.417	49.996	1.00	0.00	H
ATOM	1406	HD3	PRO	89	16.847	40.562	49.890	1.00	0.00	H
ATOM	1407	CG	PRO	89	16.920	39.520	51.704	1.00	0.00	C
ATOM	1408	HG2	PRO	89	17.033	38.443	51.830	1.00	0.00	H
ATOM	1409	HG3	PRO	89	15.888	39.853	51.595	1.00	0.00	H
ATOM	1410	CB	PRO	89	17.378	40.261	53.004	1.00	0.00	C
ATOM	1411	HB2	PRO	89	17.748	39.517	53.709	1.00	0.00	H
ATOM	1412	HB3	PRO	89	16.562	40.811	53.473	1.00	0.00	H
ATOM	1413	CA	PRO	89	18.337	41.345	52.528	1.00	0.00	C
ATOM	1414	HA	PRO	89	17.880	42.317	52.718	1.00	0.00	H
ATOM	1415	C	PRO	89	19.707	41.144	53.237	1.00	0.00	C
ATOM	1416	O	PRO	89	20.423	40.157	52.921	1.00	0.00	O
ATOM	1417	N	GLY	90	20.194	42.031	54.087	1.00	0.00	N
ATOM	1418	H	GLY	90	19.712	42.865	54.389	1.00	0.00	H
ATOM	1419	CA	GLY	90	21.517	42.019	54.595	1.00	0.00	C
ATOM	1420	HA2	GLY	90	22.171	42.120	53.729	1.00	0.00	H
ATOM	1421	HA3	GLY	90	21.750	42.909	55.180	1.00	0.00	H
ATOM	1422	C	GLY	90	21.931	40.761	55.288	1.00	0.00	C
ATOM	1423	O	GLY	90	23.103	40.421	55.204	1.00	0.00	O
ATOM	1424	N	GLN	91	20.995	40.008	55.892	1.00	0.00	N
ATOM	1425	H	GLN	91	20.044	40.340	55.830	1.00	0.00	H

ATOM	1426	CA	GLN	91	21.243	38.687	56.447	1.00	0.00	C
ATOM	1427	HA	GLN	91	22.200	38.732	56.968	1.00	0.00	H
ATOM	1428	CB	GLN	91	20.137	38.293	57.523	1.00	0.00	C
ATOM	1429	HB2	GLN	91	19.275	37.762	57.118	1.00	0.00	H
ATOM	1430	HB3	GLN	91	20.653	37.586	58.172	1.00	0.00	H
ATOM	1431	CG	GLN	91	19.586	39.474	58.373	1.00	0.00	C
ATOM	1432	HG2	GLN	91	19.112	38.968	59.213	1.00	0.00	H
ATOM	1433	HG3	GLN	91	20.365	40.090	58.825	1.00	0.00	H
ATOM	1434	CD	GLN	91	18.538	40.383	57.666	1.00	0.00	C
ATOM	1435	OE1	GLN	91	17.478	39.924	57.309	1.00	0.00	O
ATOM	1436	NE2	GLN	91	18.785	41.625	57.317	1.00	0.00	N
ATOM	1437	HE21	GLN	91	19.575	42.164	57.643	1.00	0.00	H
ATOM	1438	HE22	GLN	91	18.067	42.149	56.839	1.00	0.00	H
ATOM	1439	C	GLN	91	21.416	37.525	55.481	1.00	0.00	C
ATOM	1440	O	GLN	91	21.660	36.397	55.862	1.00	0.00	O
ATOM	1441	N	THR	92	21.279	37.710	54.141	1.00	0.00	N
ATOM	1442	H	THR	92	21.107	38.653	53.824	1.00	0.00	H
ATOM	1443	CA	THR	92	21.589	36.710	53.167	1.00	0.00	C
ATOM	1444	HA	THR	92	20.928	35.845	53.225	1.00	0.00	H
ATOM	1445	CB	THR	92	21.318	37.342	51.842	1.00	0.00	C
ATOM	1446	HB	THR	92	21.873	38.279	51.788	1.00	0.00	H
ATOM	1447	CG2	THR	92	21.707	36.548	50.609	1.00	0.00	C
ATOM	1448	HG21	THR	92	20.863	35.882	50.426	1.00	0.00	H
ATOM	1449	HG22	THR	92	21.769	37.177	49.721	1.00	0.00	H
ATOM	1450	HG23	THR	92	22.601	35.934	50.719	1.00	0.00	H
ATOM	1451	OG1	THR	92	19.924	37.543	51.774	1.00	0.00	O
ATOM	1452	HG1	THR	92	19.780	38.182	52.475	1.00	0.00	H
ATOM	1453	C	THR	92	23.065	36.404	53.239	1.00	0.00	C
ATOM	1454	O	THR	92	23.907	37.280	53.245	1.00	0.00	O
ATOM	1455	N	GLN	93	23.411	35.097	53.266	1.00	0.00	N
ATOM	1456	H	GLN	93	22.700	34.382	53.208	1.00	0.00	H
ATOM	1457	CA	GLN	93	24.844	34.586	53.221	1.00	0.00	C
ATOM	1458	HA	GLN	93	25.414	35.279	53.840	1.00	0.00	H
ATOM	1459	CB	GLN	93	24.874	33.114	53.657	1.00	0.00	C
ATOM	1460	HB2	GLN	93	24.315	33.143	54.593	1.00	0.00	H
ATOM	1461	HB3	GLN	93	24.349	32.495	52.930	1.00	0.00	H
ATOM	1462	CG	GLN	93	26.331	32.579	53.857	1.00	0.00	C
ATOM	1463	HG2	GLN	93	26.903	32.485	52.935	1.00	0.00	H
ATOM	1464	HG3	GLN	93	26.713	33.305	54.575	1.00	0.00	H
ATOM	1465	CD	GLN	93	26.129	31.233	54.663	1.00	0.00	C
ATOM	1466	OE1	GLN	93	25.426	30.309	54.207	1.00	0.00	O
ATOM	1467	NE2	GLN	93	26.900	31.143	55.765	1.00	0.00	N
ATOM	1468	HE21	GLN	93	27.443	31.917	56.121	1.00	0.00	H
ATOM	1469	HE22	GLN	93	26.881	30.293	56.309	1.00	0.00	H
ATOM	1470	C	GLN	93	25.340	34.629	51.755	1.00	0.00	C
ATOM	1471	O	GLN	93	24.543	34.488	50.806	1.00	0.00	O
ATOM	1472	N	PHE	94	26.544	34.976	51.478	1.00	0.00	N
ATOM	1473	H	PHE	94	27.167	35.008	52.272	1.00	0.00	H
ATOM	1474	CA	PHE	94	26.996	35.238	50.142	1.00	0.00	C
ATOM	1475	HA	PHE	94	26.189	35.097	49.424	1.00	0.00	H
ATOM	1476	CB	PHE	94	27.528	36.725	49.904	1.00	0.00	C
ATOM	1477	HB2	PHE	94	28.273	36.999	50.650	1.00	0.00	H
ATOM	1478	HB3	PHE	94	27.874	36.730	48.870	1.00	0.00	H
ATOM	1479	CG	PHE	94	26.303	37.691	49.958	1.00	0.00	C
ATOM	1480	CD1	PHE	94	25.412	37.738	48.851	1.00	0.00	C
ATOM	1481	HD1	PHE	94	25.611	37.226	47.922	1.00	0.00	H
ATOM	1482	CE1	PHE	94	24.241	38.537	48.867	1.00	0.00	C
ATOM	1483	HE1	PHE	94	23.547	38.500	48.040	1.00	0.00	H
ATOM	1484	CZ	PHE	94	23.983	39.358	49.994	1.00	0.00	C
ATOM	1485	HZ	PHE	94	23.100	39.979	50.012	1.00	0.00	H
ATOM	1486	CE2	PHE	94	24.811	39.292	51.116	1.00	0.00	C
ATOM	1487	HE2	PHE	94	24.501	39.883	51.965	1.00	0.00	H
ATOM	1488	CD2	PHE	94	25.932	38.405	51.175	1.00	0.00	C
ATOM	1489	HD2	PHE	94	26.649	38.442	51.981	1.00	0.00	H

ATOM	1490	C	PHE	94	28.110	34.262	49.878	1.00	0.00	C
ATOM	1491	O	PHE	94	28.755	33.773	50.822	1.00	0.00	O
ATOM	1492	N	TYR	95	28.299	33.914	48.619	1.00	0.00	N
ATOM	1493	H	TYR	95	27.646	34.266	47.934	1.00	0.00	H
ATOM	1494	CA	TYR	95	29.353	32.927	48.144	1.00	0.00	C
ATOM	1495	HA	TYR	95	29.929	32.622	49.019	1.00	0.00	H
ATOM	1496	CB	TYR	95	28.701	31.846	47.321	1.00	0.00	C
ATOM	1497	HB2	TYR	95	27.901	31.424	47.931	1.00	0.00	H
ATOM	1498	HB3	TYR	95	28.271	32.346	46.453	1.00	0.00	H
ATOM	1499	CG	TYR	95	29.567	30.752	46.713	1.00	0.00	C
ATOM	1500	CD1	TYR	95	29.827	30.526	45.332	1.00	0.00	C
ATOM	1501	HD1	TYR	95	29.409	31.157	44.562	1.00	0.00	H
ATOM	1502	CE1	TYR	95	30.584	29.422	45.001	1.00	0.00	C
ATOM	1503	HE1	TYR	95	30.863	29.349	43.960	1.00	0.00	H
ATOM	1504	CZ	TYR	95	30.990	28.469	45.900	1.00	0.00	C
ATOM	1505	OH	TYR	95	31.602	27.334	45.550	1.00	0.00	O
ATOM	1506	HH	TYR	95	32.064	27.496	44.724	1.00	0.00	H
ATOM	1507	CE2	TYR	95	30.712	28.673	47.239	1.00	0.00	C
ATOM	1508	HE2	TYR	95	31.032	27.996	48.018	1.00	0.00	H
ATOM	1509	CD2	TYR	95	30.040	29.833	47.649	1.00	0.00	C
ATOM	1510	HD2	TYR	95	29.863	30.009	48.699	1.00	0.00	H
ATOM	1511	C	TYR	95	30.412	33.703	47.282	1.00	0.00	C
ATOM	1512	O	TYR	95	29.942	34.602	46.564	1.00	0.00	O
ATOM	1513	N	VAL	96	31.666	33.407	47.434	1.00	0.00	N
ATOM	1514	H	VAL	96	31.853	32.528	47.896	1.00	0.00	H
ATOM	1515	CA	VAL	96	32.734	34.104	46.804	1.00	0.00	C
ATOM	1516	HA	VAL	96	32.349	34.789	46.048	1.00	0.00	H
ATOM	1517	CB	VAL	96	33.695	34.858	47.800	1.00	0.00	C
ATOM	1518	HB	VAL	96	33.908	34.235	48.669	1.00	0.00	H
ATOM	1519	CG1	VAL	96	35.089	35.270	47.205	1.00	0.00	C
ATOM	1520	HG11	VAL	96	35.739	35.405	48.069	1.00	0.00	H
ATOM	1521	HG12	VAL	96	35.649	34.544	46.615	1.00	0.00	H
ATOM	1522	HG13	VAL	96	34.879	36.105	46.537	1.00	0.00	H
ATOM	1523	CG2	VAL	96	33.047	36.130	48.419	1.00	0.00	C
ATOM	1524	HG21	VAL	96	32.759	36.784	47.595	1.00	0.00	H
ATOM	1525	HG22	VAL	96	32.100	35.914	48.913	1.00	0.00	H
ATOM	1526	HG23	VAL	96	33.649	36.599	49.197	1.00	0.00	H
ATOM	1527	C	VAL	96	33.537	33.111	45.990	1.00	0.00	C
ATOM	1528	O	VAL	96	33.742	31.986	46.455	1.00	0.00	O
ATOM	1529	N	ILE	97	33.883	33.460	44.738	1.00	0.00	N
ATOM	1530	H	ILE	97	33.687	34.387	44.387	1.00	0.00	H
ATOM	1531	CA	ILE	97	34.605	32.562	43.828	1.00	0.00	C
ATOM	1532	HA	ILE	97						
34.797	31.600	44.304	1.00	0.00	H					
ATOM	1533	CB	ILE	97	33.734	32.181	42.636	1.00	0.00	C
ATOM	1534	HB	ILE	97	33.737	32.960	41.873	1.00	0.00	H
ATOM	1535	CG2	ILE	97	34.323	30.962	41.860	1.00	0.00	C
ATOM	1536	HG21	ILE	97	35.319	31.210	41.495	1.00	0.00	H
ATOM	1537	HG22	ILE	97	34.301	30.010	42.391	1.00	0.00	H
ATOM	1538	HG23	ILE	97	33.779	30.983	40.917	1.00	0.00	H
ATOM	1539	CG1	ILE	97	32.259	31.958	43.039	1.00	0.00	C
ATOM	1540	HG12	ILE	97	32.252	31.241	43.861	1.00	0.00	H
ATOM	1541	HG13	ILE	97	31.909	32.896	43.470	1.00	0.00	H
ATOM	1542	CD1	ILE	97	31.337	31.635	41.933	1.00	0.00	C
ATOM	1543	HD11	ILE	97	30.302	31.915	42.128	1.00	0.00	H
ATOM	1544	HD12	ILE	97	31.765	32.026	41.009	1.00	0.00	H
ATOM	1545	HD13	ILE	97	31.290	30.554	41.795	1.00	0.00	H
ATOM	1546	C	ILE	97	35.908	33.183	43.329	1.00	0.00	C
ATOM	1547	O	ILE	97	35.838	34.351	42.906	1.00	0.00	O
ATOM	1548	N	GLU	98	37.096	32.497	43.359	1.00	0.00	N
ATOM	1549	H	GLU	98	37.038	31.584	43.788	1.00	0.00	H
ATOM	1550	CA	GLU	98	38.362	33.060	42.878	1.00	0.00	C
ATOM	1551	HA	GLU	98	38.253	34.114	42.621	1.00	0.00	H
ATOM	1552	CB	GLU	98	39.440	32.966	43.964	1.00	0.00	C

ATOM	1553	HB2	GLU	98	39.554	31.892	44.115	1.00	0.00	H
ATOM	1554	HB3	GLU	98	40.382	33.436	43.683	1.00	0.00	H
ATOM	1555	CG	GLU	98	38.849	33.488	45.287	1.00	0.00	C
ATOM	1556	HG2	GLU	98	38.431	34.488	45.173	1.00	0.00	H
ATOM	1557	HG3	GLU	98	38.096	32.797	45.664	1.00	0.00	H
ATOM	1558	CD	GLU	98	39.799	33.536	46.500	1.00	0.00	C
ATOM	1559	OE1	GLU	98	39.238	33.855	47.586	1.00	0.00	O
ATOM	1560	OE2	GLU	98	41.059	33.510	46.332	1.00	0.00	O
ATOM	1561	C	GLU	98	38.842	32.233	41.652	1.00	0.00	C
ATOM	1562	O	GLU	98	38.971	31.014	41.752	1.00	0.00	O
ATOM	1563	N	PHE	99	38.990	32.908	40.487	1.00	0.00	N
ATOM	1564	H	PHE	99	39.054	33.911	40.384	1.00	0.00	H
ATOM	1565	CA	PHE	99	39.326	32.217	39.257	1.00	0.00	C
ATOM	1566	HA	PHE	99	39.730	31.212	39.380	1.00	0.00	H
ATOM	1567	CB	PHE	99	37.978	32.022	38.441	1.00	0.00	C
ATOM	1568	HB2	PHE	99	38.077	31.289	37.641	1.00	0.00	H
ATOM	1569	HB3	PHE	99	37.285	31.506	39.106	1.00	0.00	H
ATOM	1570	CG	PHE	99	37.318	33.287	37.934	1.00	0.00	C
ATOM	1571	CD1	PHE	99	36.578	34.053	38.811	1.00	0.00	C
ATOM	1572	HD1	PHE	99	36.367	33.782	39.835	1.00	0.00	H
ATOM	1573	CE1	PHE	99	35.880	35.197	38.327	1.00	0.00	C
ATOM	1574	HE1	PHE	99	35.354	35.797	39.055	1.00	0.00	H
ATOM	1575	CZ	PHE	99	35.898	35.573	36.945	1.00	0.00	C
ATOM	1576	HZ	PHE	99	35.295	36.375	36.544	1.00	0.00	H
ATOM	1577	CE2	PHE	99	36.741	34.808	36.131	1.00	0.00	C
ATOM	1578	HE2	PHE	99	36.803	35.150	35.109	1.00	0.00	H
ATOM	1579	CD2	PHE	99	37.369	33.635	36.596	1.00	0.00	C
ATOM	1580	HD2	PHE	99	37.960	33.155	35.831	1.00	0.00	H
ATOM	1581	C	PHE	99	40.364	32.906	38.370	1.00	0.00	C
ATOM	1582	O	PHE	99	40.424	34.151	38.361	1.00	0.00	O
ATOM	1583	N	LYS	100	41.153	32.114	37.661	1.00	0.00	N
ATOM	1584	H	LYS	100	41.087	31.114	37.795	1.00	0.00	H
ATOM	1585	CA	LYS	100	42.284	32.598	36.802	1.00	0.00	C
ATOM	1586	HA	LYS	100	42.129	33.652	36.570	1.00	0.00	H
ATOM	1587	CB	LYS	100	43.660	32.443	37.459	1.00	0.00	C
ATOM	1588	HB2	LYS	100	44.351	32.841	36.716	1.00	0.00	H
ATOM	1589	HB3	LYS	100	43.756	33.106	38.320	1.00	0.00	H
ATOM	1590	CG	LYS	100	44.073	31.001	37.774	1.00	0.00	C
ATOM	1591	HG2	LYS	100	43.367	30.635	38.519	1.00	0.00	H
ATOM	1592	HG3	LYS	100	43.957	30.354	36.904	1.00	0.00	H
ATOM	1593	CD	LYS	100	45.574	30.866	38.217	1.00	0.00	C
ATOM	1594	HD2	LYS	100	46.235	31.544	37.676	1.00	0.00	H
ATOM	1595	HD3	LYS	100	45.596	31.255	39.236	1.00	0.00	H
ATOM	1596	CE	LYS	100	46.036	29.515	37.861	1.00	0.00	C
ATOM	1597	HE2	LYS	100	45.389	28.756	38.301	1.00	0.00	H
ATOM	1598	HE3	LYS	100	46.036	29.489	36.771	1.00	0.00	H
ATOM	1599	NZ	LYS	100	47.446	29.227	38.288	1.00	0.00	N
ATOM	1600	HZ1	LYS	100	47.856	28.442	37.801	1.00	0.00	H
ATOM	1601	HZ2	LYS	100	48.029	30.051	38.274	1.00	0.00	H
ATOM	1602	HZ3	LYS	100	47.419	29.116	39.292	1.00	0.00	H
ATOM	1603	C	LYS	100	42.298	31.749	35.520	1.00	0.00	C
ATOM	1604	O	LYS	100	41.920	30.628	35.581	1.00	0.00	O
ATOM	1605	N	CYX	101	42.849	32.325	34.449	1.00	0.00	N
ATOM	1606	H	CYX	101	43.178	33.276	34.376	1.00	0.00	H
ATOM	1607	CA	CYX	101	43.083	31.750	33.220	1.00	0.00	C
ATOM	1608	HA	CYX	101	42.404	30.933	32.977	1.00	0.00	H
ATOM	1609	CB	CYX	101	42.669	32.709	31.988	1.00	0.00	C
ATOM	1610	HB2	CYX	101	41.640	33.012	32.189	1.00	0.00	H
ATOM	1611	HB3	CYX	101	43.227	33.644	31.981	1.00	0.00	H
ATOM	1612	SG	CYX	101	42.986	32.012	30.324	1.00	0.00	S
ATOM	1613	C	CYX	101	44.562	31.188	33.098	1.00	0.00	C
ATOM	1614	O	CYX	101	45.477	31.804	33.706	1.00	0.00	O
ATOM	1615	N	LEU	102	44.657	29.990	32.404	1.00	0.00	N
ATOM	1616	H	LEU	102	43.792	29.706	31.967	1.00	0.00	H

```
ATOM   1617  CA  LEU   102      45.810  29.194  32.147  1.00  0.00            C
ATOM   1618  HA  LEU   102      46.547  29.282  32.945  1.00  0.00            H
ATOM   1619  CB  LEU   102      45.378  27.722  32.262  1.00  0.00            C
ATOM   1620  HB2 LEU   102      44.755  27.512  31.392  1.00  0.00            H
ATOM   1621  HB3 LEU   102      46.296  27.134  32.270  1.00  0.00            H
ATOM   1622  CG  LEU   102      44.632  27.343  33.688  1.00  0.00            C
ATOM   1623  HG  LEU   102      43.751  27.981  33.771  1.00  0.00            H
ATOM   1624  CD1 LEU   102      44.305  25.815  33.589  1.00  0.00            C
ATOM   1625 HD11 LEU   102      43.809  25.619  32.639  1.00  0.00            H
ATOM   1626 HD12 LEU   102      45.212  25.213  33.610  1.00  0.00            H
ATOM   1627 HD13 LEU   102      43.671  25.780  34.475  1.00  0.00            H
ATOM   1628  CD2 LEU   102      45.591  27.624  34.824  1.00  0.00            C
ATOM   1629 HD21 LEU   102      46.484  27.055  34.566  1.00  0.00            H
ATOM   1630 HD22 LEU   102      45.734  28.682  35.043  1.00  0.00            H
ATOM   1631 HD23 LEU   102      45.147  27.031  35.623  1.00  0.00            H
ATOM   1632  C   LEU   102      46.638  29.610  30.905  1.00  0.00            C
ATOM   1633  O   LEU   102      46.125  29.318  29.825  1.00  0.00            O
ATOM   1634  OXT LEU   102      47.694  30.158  31.143  1.00  0.00            O
HETATM  314  N   LYN    20      39.242  40.907  35.654  1.00  0.00            N
HETATM  315  H   LYN    20      39.767  40.431  34.935  1.00  0.00            H
HETATM  316  CA  LYN    20      38.128  40.192  36.340  1.00  0.00            C
HETATM  317  HA  LYN    20      37.852  40.764  37.226  1.00  0.00            H
HETATM  318  CB  LYN    20      36.914  40.168  35.430  1.00  0.00            C
HETATM  319  HB2 LYN    20      37.325  39.725  34.521  1.00  0.00            H
HETATM  320  HB3 LYN    20      36.616  41.199  35.234  1.00  0.00            H
HETATM  321  CG  LYN    20      35.713  39.284  35.852  1.00  0.00            C
HETATM  322  HG2 LYN    20      35.005  39.182  35.030  1.00  0.00            H
HETATM  323  HG3 LYN    20      36.076  38.285  36.093  1.00  0.00            H
HETATM  324  CD  LYN    20      34.993  39.860  37.046  1.00  0.00            C
HETATM  325  HD2 LYN    20      34.507  40.779  36.719  1.00  0.00            H
HETATM  326  HD3 LYN    20      35.672  39.989  37.889  1.00  0.00            H
HETATM  327  CE  LYN    20      34.013  38.849  37.513  1.00  0.00            C
HETATM  328  HE2 LYN    20      33.269  38.676  36.736  1.00  0.00            H
HETATM  329  HE3 LYN    20      34.486  37.907  37.790  1.00  0.00            H
HETATM  330  NZ  LYN    20      33.360  39.445  38.709  1.00  0.00            N
HETATM  331  HZ2 LYN    20      33.197  40.428  38.544  1.00  0.00            H
HETATM  332  HZ3 LYN    20      34.081  39.321  39.405  1.00  0.00            H
HETATM  333  C   LYN    20      38.633  38.793  36.761  1.00  0.00            C
HETATM  334  O   LYN    20      39.087  37.902  36.028  1.00  0.00            O
HETATM 1636  N   LIG   103      29.519  41.702  40.479  1.00  0.00            N
HETATM 1637  C   LIG   103      29.184  40.677  39.755
  1.00  0.00           C
HETATM 1638  O   LIG   103      29.299  43.051  42.262  1.00  0.00            O
HETATM 1639  C5' LIG   103      24.893  38.989  44.893  1.00  0.00            C
HETATM 1640  O5' LIG   103      25.264  38.250  43.739  1.00  0.00            O
HETATM 1641  C4' LIG   103      25.559  40.378  44.812  1.00  0.00            C
HETATM 1642  O4' LIG   103      26.776  40.203  44.062  1.00  0.00            O
HETATM 1643  C3' LIG   103      24.782  41.407  43.950  1.00  0.00            C
HETATM 1644  O3' LIG   103      23.765  42.235  44.587  1.00  0.00            O
HETATM 1645  C2' LIG   103      25.909  42.343  43.271  1.00  0.00            C
HETATM 1646  O2' LIG   103      25.973  43.522  44.027  1.00  0.00            O
HETATM 1647  C1' LIG   103      27.171  41.460  43.329  1.00  0.00            C
HETATM 1648  N1  LIG   103      27.852  41.248  42.049  1.00  0.00            N
HETATM 1649  O1  LIG   103      29.608  38.379  37.967  1.00  0.00            O
HETATM 1650  N2  LIG   103      29.797  40.570  38.538  1.00  0.00            N
HETATM 1651  C6  LIG   103      28.955  41.989  41.713  1.00  0.00            C
HETATM 1652  C7  LIG   103      27.377  40.160  41.287  1.00  0.00            C
HETATM 1653  C8  LIG   103      28.057  39.885  40.096  1.00  0.00            C
HETATM 1654  C9  LIG   103      29.877  39.547  37.649  1.00  0.00            C
HETATM 1655  C10 LIG   103      30.548  39.914  36.383  1.00  0.00            C
HETATM 1656  H   LIG   103      30.367  41.336  38.200  1.00  0.00            H
HETATM 1657  H1  LIG   103      27.683  39.144  39.399  1.00  0.00            H
HETATM 1658  H4  LIG   103      27.894  42.053  43.904  1.00  0.00            H
HETATM 1659  H6  LIG   103      25.661  40.809  45.817  1.00  0.00            H
```

```
HETATM 1660  H7  LIG   103      24.320  40.872  43.111  1.00  0.00           H
HETATM 1661  H8  LIG   103      25.662  42.590  42.231  1.00  0.00           H
HETATM 1662  H9  LIG   103      23.823  39.127  45.098  1.00  0.00           H
HETATM 1663  H10 LIG   103      25.283  38.475  45.779  1.00  0.00           H
HETATM 1664  H11 LIG   103      29.830  39.979  35.557  1.00  0.00           H
HETATM 1665  H12 LIG   103      31.208  39.067  36.158  1.00  0.00           H
HETATM 1666  H13 LIG   103      31.176  40.808  36.488  1.00  0.00           H
HETATM 1667  H14 LIG   103      26.571  39.602  41.744  1.00  0.00           H
HETATM 1668  H2' LIG   103      25.036  43.768  43.934  1.00  0.00           H
HETATM 1669  H3' LIG   103      22.944  42.151  44.073  1.00  0.00           H
HETATM 1670  H5' LIG   103      26.174  38.521  43.526  1.00  0.00           H
ENDMDL
MODEL     10
SHEET    1   1 1 ILE    22  ASP    26  0
SHEET    2   2 1 VAL    37  VAL    40  0
SHEET    3   3 1 CYX    50  VAL    60  0
SHEET    4   4 1 PHE    94  CYX   101  0
HELIX    1   1 GLN     9  LEU     16  1                                   8
HELIX    2   2 GLU    67  HIE     69  1                                   3
HELIX    3   3 LEU    77  ALA     85  1                                   9
ATOM      1  N   GLN     1      34.324  16.871  32.480  1.00  0.00           N
ATOM      2  H1  GLN     1      35.219  17.231  32.780  1.00  0.00           H
ATOM      3  H2  GLN     1      33.864  16.922  33.378  1.00  0.00           H
ATOM      4  H3  GLN     1      34.314  15.894  32.224  1.00  0.00           H
ATOM      5  CA  GLN     1      33.699  17.804  31.480  1.00  0.00           C
ATOM      6  HA  GLN     1      34.256  17.612  30.563  1.00  0.00           H
ATOM      7  CB  GLN     1      32.251  17.290  31.196  1.00  0.00           C
ATOM      8  HB2 GLN     1      31.798  16.828  32.074  1.00  0.00           H
ATOM      9  HB3 GLN     1      31.632  18.151  30.943  1.00  0.00           H
ATOM     10  CG  GLN     1      32.201  16.236  30.071  1.00  0.00           C
ATOM     11  HG2 GLN     1      32.675  15.281  30.295  1.00  0.00           H
ATOM     12  HG3 GLN     1      31.145  16.026  29.903  1.00  0.00           H
ATOM     13  CD  GLN     1      32.803  16.797  28.804  1.00  0.00           C
ATOM     14  OE1 GLN     1      32.807  17.992  28.503  1.00  0.00           O
ATOM     15  NE2 GLN     1      33.249  15.984  27.886  1.00  0.00           N
ATOM     16 HE21 GLN     1      33.155  14.979  27.938  1.00  0.00           H
ATOM     17 HE22 GLN     1      33.520  16.346  26.983  1.00  0.00           H
ATOM     18  C   GLN     1      33.657  19.246  31.904  1.00  0.00           C
ATOM     19  O   GLN     1      33.211  19.422  33.033  1.00  0.00           O
ATOM     20  N   PRO     2      34.305  20.239  31.215  1.00  0.00           N
ATOM     21  CD  PRO     2      35.262  20.022  30.101  1.00  0.00           C
ATOM     22  HD2 PRO     2      34.626  20.108  29.219  1.00  0.00           H
ATOM     23  HD3 PRO     2      35.848  19.104  30.143  1.00  0.00           H
ATOM     24  CG  PRO     2      36.281  21.206  30.138  1.00  0.00           C
ATOM     25  HG2 PRO     2      36.646  21.487  29.149  1.00  0.00           H
ATOM     26  HG3 PRO     2      37.034  21.101  30.918  1.00  0.00           H
ATOM     27  CB  PRO     2      35.328  22.327  30.738  1.00  0.00           C
ATOM     28  HB2 PRO     2      34.768  22.785  29.923  1.00  0.00           H
ATOM     29  HB3 PRO     2      35.852  23.100  31.300  1.00  0.00           H
ATOM     30  CA  PRO     2      34.308  21.617  31.681  1.00  0.00           C
ATOM     31  HA  PRO     2      34.688  21.567  32.701  1.00  0.00           H
ATOM     32  C   PRO     2      32.826  22.207  31.674  1.00  0.00           C
ATOM     33  O   PRO     2      31.988  21.700  30.919  1.00  0.00           O
ATOM     34  N   ASN     3      32.541  23.283  32.421  1.00  0.00           N
ATOM     35  H   ASN     3      33.363  23.776  32.740  1.00  0.00           H
ATOM     36  CA  ASN     3      31.230  23.972  32.609  1.00  0.00           C
ATOM     37  HA  ASN     3      30.642  23.147  33.012  1.00  0.00           H
ATOM     38  CB  ASN     3      31.388  25.118  33.598  1.00  0.00           C
ATOM     39  HB2 ASN     3      32.234  25.770  33.379  1.00  0.00           H
ATOM     40  HB3 ASN     3      30.560  25.822  33.528  1.00  0.00           H
ATOM     41  CG  ASN     3      31.581  24.529  34.988  1.00  0.00           C
ATOM     42  OD1 ASN     3      30.645  23.912  35.579  1.00  0.00           O
ATOM     43  ND2 ASN     3      32.730  24.658  35.520  1.00  0.00           N
ATOM     44 HD21 ASN     3      33.484  24.878  34.886  1.00  0.00           H
```

ATOM	45	HD22	ASN	3	32.905	24.204	36.406	1.00	0.00	H
ATOM	46	C	ASN	3	30.527	24.443	31.325	1.00	0.00	C
ATOM	47	O	ASN	3	31.268	24.942	30.527	1.00	0.00	O
ATOM	48	N	ASP	4	29.241	24.278	31.139	1.00	0.00	N
ATOM	49	H	ASP	4	28.704	23.913	31.912	1.00	0.00	H
ATOM	50	CA	ASP	4	28.549	24.895	29.981	1.00	0.00	C
ATOM	51	HA	ASP	4	29.297	24.762	29.200	1.00	0.00	H
ATOM	52	CB	ASP	4	27.264	24.146	29.647	1.00	0.00	C
ATOM	53	HB2	ASP	4	26.632	23.943	30.512	1.00	0.00	H
ATOM	54	HB3	ASP	4	26.662	24.696	28.925	1.00	0.00	H
ATOM	55	CG	ASP	4	27.544	22.720	29.216	1.00	0.00	C
ATOM	56	OD1	ASP	4	28.640	22.371	28.775	1.00	0.00	O
ATOM	57	OD2	ASP	4	26.724	21.824	29.466	1.00	0.00	O
ATOM	58	C	ASP	4	28.248	26.378	30.265	1.00	0.00	C
ATOM	59	O	ASP	4	28.129	27.197	29.357	1.00	0.00	O
ATOM	60	N	ILE	5	28.069	26.723	31.568	1.00	0.00	N
ATOM	61	H	ILE	5	28.214	26.006	32.264	1.00	0.00	H
ATOM	62	CA	ILE	5	27.756	28.126	32.022	1.00	0.00	C
ATOM	63	HA	ILE	5	27.050	28.647	31.373	1.00	0.00	H
ATOM	64	CB	ILE	5	27.096	28.036	33.477	1.00	0.00	C
ATOM	65	HB	ILE	5	27.851	27.491	34.041	1.00	0.00	H
ATOM	66	CG2	ILE	5	26.982	29.397	34.319	1.00	0.00	C
ATOM	67	HG21	ILE	5	27.879	30.008	34.215	1.00	0.00	H
ATOM	68	HG22	ILE	5	26.174	29.945	33.835	1.00	0.00	H
ATOM	69	HG23	ILE	5	26.720	29.111	35.338	1.00	0.00	H
ATOM	70	CG1	ILE	5	25.650	27.364	33.472	1.00	0.00	C
ATOM	71	HG12	ILE	5	25.808	26.303	33.282	1.00	0.00	H
ATOM	72	HG13	ILE	5	25.234	27.532	34.465	1.00	0.00	H
ATOM	73	CD1	ILE	5	24.630	27.868	32.442	1.00	0.00	C
ATOM	74	HD11	ILE	5	23.780	27.202	32.298	1.00	0.00	H
ATOM	75	HD12	ILE	5	24.407	28.903	32.702	1.00	0.00	H
ATOM	76	HD13	ILE	5	25.097	27.927	31.458	1.00	0.00	H
ATOM	77	C	ILE	5	29.088	28.935	32.053	1.00	0.00	C
ATOM	78	O	ILE	5	30.198	28.475	32.347	1.00	0.00	O
ATOM	79	N	THR	6	28.996	30.193	31.700	1.00	0.00	N
ATOM	80	H	THR	6	28.070	30.441	31.381	1.00	0.00	H
ATOM	81	CA	THR	6	30.099	31.126	31.647	1.00	0.00	C
ATOM	82	HA	THR	6	30.778	31.025	32.494	1.00	0.00	H
ATOM	83	CB	THR	6	30.948	30.957	30.392	1.00	0.00	C
ATOM	84	HB	THR	6	31.168	29.893	30.320	1.00	0.00	H
ATOM	85	CG2	THR	6	30.302	31.332	29.045	1.00	0.00	C
ATOM	86	HG21	THR	6	29.724	32.253	29.129	1.00	0.00	H
ATOM	87	HG22	THR	6	31.044	31.346	28.248	1.00	0.00	H
ATOM	88	HG23	THR	6	29.631	30.539	28.715	1.00	0.00	H
ATOM	89	OG1	THR	6	32.114	31.736	30.549	1.00	0.00	O
ATOM	90	HG1	THR	6	32.747	31.278	29.991	1.00	0.00	H
ATOM	91	C	THR	6	29.601	32.581	31.693	1.00	0.00	C
ATOM	92	O	THR	6	28.446	32.934	31.537	1.00	0.00	O
ATOM	93	N	PHE	7	30.496	33.555	31.901	1.00	0.00	N
ATOM	94	H	PHE	7	31.444	33.257	31.718	1.00	0.00	H
ATOM	95	CA	PHE	7	30.327	35.048	31.923	1.00	0.00	C
ATOM	96	HA	PHE	7	29.616	35.202	32.735	1.00	0.00	H
ATOM	97	CB	PHE	7	31.627	35.760	32.282	1.00	0.00	C
ATOM	98	HB2	PHE	7	32.310	35.486	31.478	1.00	0.00	H
ATOM	99	HB3	PHE	7	31.534	36.845	32.303	1.00	0.00	H
ATOM	100	CG	PHE	7	32.300	35.258	33.552	1.00	0.00	C
ATOM	101	CD1	PHE	7	33.076	34.130	33.655	1.00	0.00	C
ATOM	102	HD1	PHE	7	33.252	33.494	32.800	1.00	0.00	H
ATOM	103	CE1	PHE	7	33.550	33.674	34.931	1.00	0.00	C
ATOM	104	HE1	PHE	7	34.219	32.827	34.955	1.00	0.00	H
ATOM	105	CZ	PHE	7	33.354	34.531	36.077	1.00	0.00	C
ATOM	106	HZ	PHE	7	33.738	34.185	37.026	1.00	0.00	H
ATOM	107	CE2	PHE	7	32.596	35.662	35.940	1.00	0.00	C

ATOM	108	HE2	PHE	7	32.376	36.263	36.810	1.00	0.00	H
ATOM	109	CD2	PHE	7	32.016	35.953	34.668	1.00	0.00	C
ATOM	110	HD2	PHE	7	31.288	36.749	34.603	1.00	0.00	H
ATOM	111	C	PHE	7	29.763	35.641	30.584	1.00	0.00	C
ATOM	112	O	PHE	7	30.201	35.180	29.514	1.00	0.00	O
ATOM	113	N	PHE	8	28.949	36.733	30.686	1.00	0.00	N
ATOM	114	H	PHE	8	28.925	37.005	31.658	1.00	0.00	H
ATOM	115	CA	PHE	8	28.542	37.718	29.625	1.00	0.00	C
ATOM	116	HA	PHE	8	27.861	37.265	28.905	1.00	0.00	H
ATOM	117	CB	PHE	8	27.680	38.833	30.235	1.00	0.00	C
ATOM	118	HB2	PHE	8	28.076	39.163	31.195	1.00	0.00	H
ATOM	119	HB3	PHE	8	27.568	39.727	29.623	1.00	0.00	H
ATOM	120	CG	PHE	8	26.237	38.455	30.519	1.00	0.00	C
ATOM	121	CD1	PHE	8	25.295	39.464	30.740	1.00	0.00	C
ATOM	122	HD1	PHE	8	25.685	40.470	30.693	1.00	0.00	H
ATOM	123	CE1	PHE	8	23.944	39.146	31.025	1.00	0.00	C
ATOM	124	HE1	PHE	8	23.234	39.959	30.963	1.00	0.00	H
ATOM	125	CZ	PHE	8	23.517	37.816	31.123	1.00	0.00	C
ATOM	126	HZ	PHE	8	22.481	37.511	31.085	1.00	0.00	H
ATOM	127	CE2	PHE	8	24.441	36.846	30.890	1.00	0.00	C
ATOM	128	HE2	PHE	8	24.115	35.824	30.769	1.00	0.00	H
ATOM	129	CD2	PHE	8	25.801	37.140	30.641	1.00	0.00	C
ATOM	130	HD2	PHE	8	26.407	36.312	30.303	1.00	0.00	H
ATOM	131	C	PHE	8	29.763	38.296	28.947	1.00	0.00	C
ATOM	132	O	PHE	8	30.890	38.248	29.396	1.00	0.00	O
ATOM	133	N	GLN	9	29.531	38.716	27.721	1.00	0.00	N
ATOM	134	H	GLN	9	28.576	38.887	27.439	1.00	0.00	H
ATOM	135	CA	GLN	9	30.517	39.062	26.739	1.00	0.00	C
ATOM	136	HA	GLN	9	31.257	38.262	26.725	1.00	0.00	H
ATOM	137	CB	GLN	9	29.906	39.091	25.287	1.00	0.00	C
ATOM	138	HB2	GLN	9	29.300	39.988	25.164	1.00	0.00	H
ATOM	139	HB3	GLN	9	30.738	39.147	24.583	1.00	0.00	H
ATOM	140	CG	GLN	9	29.090	37.839	24.872	1.00	0.00	C
ATOM	141	HG2	GLN	9	29.110	37.814	23.783	1.00	0.00	H
ATOM	142	HG3	GLN	9	29.588	36.935	25.224	1.00	0.00	H
ATOM	143	CD	GLN	9	27.671	37.778	25.155	1.00	0.00	C
ATOM	144	OE1	GLN	9	27.162	38.486	26.047	1.00	0.00	O
ATOM	145	NE2	GLN	9	26.941	36.986	24.440	1.00	0.00	N
ATOM	146	HE21	GLN	9	26.006	36.755	24.743	1.00	0.00	H
ATOM	147	HE22	GLN	9	27.413	36.566	23.652	1.00	0.00	H
ATOM	148	C	GLN	9	31.397	40.261	27.102	1.00	0.00	C
ATOM	149	O	GLN	9	32.484	40.411	26.586	1.00	0.00	O
ATOM	150	N	ARG	10	30.953	41.228	27.929	1.00	0.00	N
ATOM	151	H	ARG	10	30.090	41.020	28.411	1.00	0.00	H
ATOM	152	CA	ARG	10	31.711	42.422	28.358	1.00	0.00	C
ATOM	153	HA	ARG	10	31.973	43.108	27.552	1.00	0.00	H
ATOM	154	CB	ARG	10	30.760	43.138	29.322	1.00	0.00	C
ATOM	155	HB2	ARG	10	29.977	43.509	28.660	1.00	0.00	H
ATOM	156	HB3	ARG	10	30.470	42.460	30.125	1.00	0.00	H
ATOM	157	CG	ARG	10	31.068	44.533	29.918	1.00	0.00	C
ATOM	158	HG2	ARG	10	31.810	44.388	30.702	1.00	0.00	H
ATOM	159	HG3	ARG	10	31.546	45.173	29.175	1.00	0.00	H
ATOM	160	CD	ARG	10	29.783	45.164	30.476	1.00	0.00	C
ATOM	161	HD2	ARG	10	29.182	44.471	31.065	1.00	0.00	H
ATOM	162	HD3	ARG	10	30.044	46.058	31.043	1.00	0.00	H
ATOM	163	NE	ARG	10	28.981	45.673	29.396	1.00	0.00	N
ATOM	164	HE	ARG	10	29.485	45.741	28.522	1.00	0.00	H
ATOM	165	CZ	ARG	10	27.669	45.917	29.296	1.00	0.00	C
ATOM	166	NH1	ARG	10	26.774	45.828	30.275	1.00	0.00	N
ATOM	167	HH11	ARG	10	25.819	46.094	30.081	1.00	0.00	H
ATOM	168	HH12	ARG	10	27.093	45.907	31.230	1.00	0.00	H
ATOM	169	NH2	ARG	10	27.170	46.135	28.153	1.00	0.00	N
ATOM	170	HH21	ARG	10	27.776	46.026	27.353	1.00	0.00	H
ATOM	171	HH22	ARG	10	26.165	46.034	28.144	1.00	0.00	H

ATOM	172	C	ARG	10	33.066	42.019	29.066	1.00	0.00	C
ATOM	173	O	ARG	10	33.915	42.835	29.147	1.00	0.00	O
ATOM	174	N	PHE	11	33.117	40.873	29.771	1.00	0.00	N
ATOM	175	H	PHE	11	32.353	40.248	29.561	1.00	0.00	H
ATOM	176	CA	PHE	11	34.073	40.501	30.806	1.00	0.00	C
ATOM	177	HA	PHE	11	34.498	41.423	31.205	1.00	0.00	H
ATOM	178	CB	PHE	11	33.277	39.908	31.984	1.00	0.00	C
ATOM	179	HB2	PHE	11	33.977	39.831	32.818	1.00	0.00	H
ATOM	180	HB3	PHE	11	32.952	38.889	31.778	1.00	0.00	H
ATOM	181	CG	PHE	11	32.052	40.772	32.350	1.00	0.00	C
ATOM	182	CD1	PHE	11	32.319	42.074	32.803	1.00	0.00	C
ATOM	183	HD1	PHE	11	33.337	42.342	33.051	1.00	0.00	H
ATOM	184	CE1	PHE	11	31.261	42.951	33.203	1.00	0.00	C
ATOM	185	HE1	PHE	11	31.580	43.908	33.591	1.00	0.00	H
ATOM	186	CZ	PHE	11	29.953	42.485	33.181	1.00	0.00	C
ATOM	187	HZ	PHE	11	29.146	43.189	33.326	1.00	0.00	H
ATOM	188	CE2	PHE	11	29.685	41.250	32.551	1.00	0.00	C
ATOM	189	HE2	PHE	11	28.675	40.884	32.436	1.00	0.00	H
ATOM	190	CD2	PHE	11	30.717	40.379	32.072	1.00	0.00	C
ATOM	191	HD2	PHE	11	30.484	39.433	31.605	1.00	0.00	H
ATOM	192	C	PHE	11	35.229	39.532	30.353	1.00	0.00	C
ATOM	193	O	PHE	11	36.154	39.246	31.094	1.00	0.00	O
ATOM	194	N	GLN	12	35.229	39.058	29.131	1.00	0.00	N
ATOM	195	H	GLN	12	34.623	39.525	28.471	1.00	0.00	H
ATOM	196	CA	GLN	12	36.064	37.991	28.615	1.00	0.00	C
ATOM	197	HA	GLN	12	36.035	37.169	29.331	1.00	0.00	H
ATOM	198	CB	GLN	12	35.455	37.558	27.253	1.00	0.00	C
ATOM	199	HB2	GLN	12	35.435	38.400	26.561	1.00	0.00	H
ATOM	200	HB3	GLN	12	36.045	36.819	26.710	1.00	0.00	H
ATOM	201	CG	GLN	12	33.988	37.116	27.295	1.00	0.00	C
ATOM	202	HG2	GLN	12	33.782	36.302	27.991	1.00	0.00	H
ATOM	203	HG3	GLN	12	33.433	37.940	27.744	1.00	0.00	H
ATOM	204	CD	GLN	12	33.457	36.775	25.924	1.00	0.00	C
ATOM	205	OE1	GLN	12	33.903	37.252	24.924	1.00	0.00	O
ATOM	206	NE2	GLN	12	32.427	35.969	25.835	1.00	0.00	N
ATOM	207	HE21	GLN	12	32.139	35.432	26.640	1.00	0.00	H
ATOM	208	HE22	GLN	12	32.209	35.719	24.881	1.00	0.00	H
ATOM	209	C	GLN	12	37.520	38.495	28.474	1.00	0.00	C
ATOM	210	O	GLN	12	38.404	37.777	28.861	1.00	0.00	O
ATOM	211	N	ASP	13	37.732	39.697	28.030	1.00	0.00	N
ATOM	212	H	ASP	13	36.915	40.263	27.844	1.00	0.00	H
ATOM	213	CA	ASP	13	39.015	40.313	27.839	1.00	0.00	C
ATOM	214	HA	ASP	13	39.638	39.494	27.482	1.00	0.00	H
ATOM	215	CB	ASP	13	38.795	41.465	26.792	1.00	0.00	C
ATOM	216	HB2	ASP	13	38.157	42.259	27.182	1.00	0.00	H
ATOM	217	HB3	ASP	13	39.785	41.893	26.636	1.00	0.00	H
ATOM	218	CG	ASP	13	38.075	41.089	25.490	1.00	0.00	C
ATOM	219	OD1	ASP	13	37.704	41.996	24.752	1.00	0.00	O
ATOM	220	OD2	ASP	13	38.037	39.887	25.162	1.00	0.00	O
ATOM	221	C	ASP	13	39.568	40.888	29.178	1.00	0.00	C
ATOM	222	O	ASP	13	40.770	41.068	29.343	1.00	0.00	O
ATOM	223	N	ASP	14	38.738	41.142	30.214	1.00	0.00	N
ATOM	224	H	ASP	14	37.749	40.956	30.128	1.00	0.00	H
ATOM	225	CA	ASP	14	39.223	41.497	31.619	1.00	0.00	C
ATOM	226	HA	ASP	14	40.084	42.159	31.522	1.00	0.00	H
ATOM	227	CB	ASP	14	38.082	42.103	32.400	1.00	0.00	C
ATOM	228	HB2	ASP	14	37.223	41.435	32.337	1.00	0.00	H
ATOM	229	HB3	ASP	14	38.331	42.136	33.461	1.00	0.00	H
ATOM	230	CG	ASP	14	37.806	43.563	32.071	1.00	0.00	C
ATOM	231	OD1	ASP	14	37.956	44.445	32.899	1.00	0.00	O
ATOM	232	OD2	ASP	14	37.373	43.813	30.875	1.00	0.00	O
ATOM	233	C	ASP	14	39.814	40.235	32.283	1.00	0.00	C
ATOM	234	O	ASP	14	40.847	40.280	32.884	1.00	0.00	O

ATOM	235	N	ILE	15	39.003	39.163	32.210	1.00	0.00	N
ATOM	236	H	ILE	15	38.081	39.376	31.859	1.00	0.00	H
ATOM	237	CA	ILE	15	39.378	37.785	32.607	1.00	0.00	C
ATOM	238	HA	ILE	15	39.417	37.714	33.695	1.00	0.00	H
ATOM	239	CB	ILE	15	38.263	36.721	32.222	1.00	0.00	C
ATOM	240	HB	ILE	15	38.104	36.980	31.175	1.00	0.00	H
ATOM	241	CG2	ILE	15	38.752	35.240	32.203	1.00	0.00	C
ATOM	242	HG21	ILE	15	38.925	35.001	33.252	1.00	0.00	H
ATOM	243	HG22	ILE	15	37.950	34.773	31.631	1.00	0.00	H
ATOM	244	HG23	ILE	15	39.734	35.086	31.758	1.00	0.00	H
ATOM	245	CG1	ILE	15	37.023	36.921	33.120	1.00	0.00	C
ATOM	246	HG12	ILE	15	37.209	36.590	34.142	1.00	0.00	H
ATOM	247	HG13	ILE	15	36.756	37.976	33.072	1.00	0.00	H
ATOM	248	CD1	ILE	15	35.804	36.204	32.651	1.00	0.00	C
ATOM	249	HD11	ILE	15	35.812	35.203	33.080	1.00	0.00	H
ATOM	250	HD12	ILE	15	34.936	36.730	33.050	1.00	0.00	H
ATOM	251	HD13	ILE	15	35.723	36.073	31.571	1.00	0.00	H
ATOM	252	C	ILE	15	40.726	37.336	31.970	1.00	0.00	C
ATOM	253	O	ILE	15	41.610	36.768	32.596	1.00	0.00	O
ATOM	254	N	LEU	16	40.885	37.505	30.711	1.00	0.00	N
ATOM	255	H	LEU	16	40.141	37.941	30.186	1.00	0.00	H
ATOM	256	CA	LEU	16	42.166	37.287	29.944	1.00	0.00	C
ATOM	257	HA	LEU	16	42.445	36.233	29.974	1.00	0.00	H
ATOM	258	CB	LEU	16	41.815	37.422	28.484	1.00	0.00	C
ATOM	259	HB2	LEU	16	41.107	36.597	28.389	1.00	0.00	H
ATOM	260	HB3	LEU	16	41.359	38.412	28.445	1.00	0.00	H
ATOM	261	CG	LEU	16	43.033	37.367	27.589	1.00	0.00	C
ATOM	262	HG	LEU	16	43.705	38.149	27.945	1.00	0.00	H
ATOM	263	CD1	LEU	16	43.617	35.949	27.585	1.00	0.00	C
ATOM	264	HD11	LEU	16	44.501	35.970	26.949	1.00	0.00	H
ATOM	265	HD12	LEU	16	43.976	35.741	28.593	1.00	0.00	H
ATOM	266	HD13	LEU	16	42.867	35.233	27.246	1.00	0.00	H
ATOM	267	CD2	LEU	16	42.703	37.772	26.183	1.00	0.00	C
ATOM	268	HD21	LEU	16	42.551	38.849	26.115	1.00	0.00	H
ATOM	269	HD22	LEU	16	43.596	37.695	25.562	1.00	0.00	H
ATOM	270	HD23	LEU	16	41.838	37.231	25.800	1.00	0.00	H
ATOM	271	C	LEU	16	43.359	38.078	30.503	1.00	0.00	C
ATOM	272	O	LEU	16	44.482	37.552	30.648	1.00	0.00	O
ATOM	273	N	ALA	17	43.105	39.277	31.013	1.00	0.00	N
ATOM	274	H	ALA	17	42.143	39.582	30.978	1.00	0.00	H
ATOM	275	CA	ALA	17	44.081	40.211	31.645	1.00	0.00	C
ATOM	276	HA	ALA	17	45.020	39.904	31.185	1.00	0.00	H
ATOM	277	CB	ALA	17	43.828	41.614	31.092	1.00	0.00	C
ATOM	278	HB1	ALA	17	43.354	41.501	30.117	1.00	0.00	H
ATOM	279	HB2	ALA	17	43.125	42.144	31.736	1.00	0.00	H
ATOM	280	HB3	ALA	17	44.744	42.203	31.097	1.00	0.00	H
ATOM	281	C	ALA	17	44.237	40.048	33.174	1.00	0.00	C
ATOM	282	O	ALA	17	44.928	40.920	33.672	1.00	0.00	O
ATOM	283	N	GLY	18	43.608	39.117	33.829	1.00	0.00	N
ATOM	284	H	GLY	18	42.922	38.564	33.337	1.00	0.00	H
ATOM	285	CA	GLY	18	43.639	38.964	35.300	1.00	0.00	C
ATOM	286	HA2	GLY	18	43.181	38.010	35.562	1.00	0.00	H
ATOM	287	HA3	GLY	18	44.682	38.915	35.611	1.00	0.00	H
ATOM	288	C	GLY	18	42.902	40.114	36.116	1.00	0.00	C
ATOM	289	O	GLY	18	43.000	40.102	37.389	1.00	0.00	O
ATOM	290	N	ARG	19	42.299	41.100	35.436	1.00	0.00	N
ATOM	291	H	ARG	19	42.370	41.154	34.430	1.00	0.00	H
ATOM	292	CA	ARG	19	41.442	42.198	36.037	1.00	0.00	C
ATOM	293	HA	ARG	19	42.054	42.593	36.848	1.00	0.00	H
ATOM	294	CB	ARG	19	41.118	43.338	35.029	1.00	0.00	C
ATOM	295	HB2	ARG	19	40.499	43.003	34.195	1.00	0.00	H
ATOM	296	HB3	ARG	19	40.591	44.155	35.523	1.00	0.00	H
ATOM	297	CG	ARG	19	42.448	43.899	34.486	1.00	0.00	C
ATOM	298	HG2	ARG	19	43.191	43.910	35.283	1.00	0.00	H

```
ATOM    299  HG3 ARG    19      42.828  43.212  33.730  1.00  0.00            H
ATOM    300  CD  ARG    19      42.275  45.305  33.930  1.00  0.00            C
ATOM    301  HD2 ARG    19      41.349  45.345  33.356  1.00  0.00            H
ATOM    302  HD3 ARG    19      42.175  46.095  34.675  1.00  0.00            H
ATOM    303  NE  ARG    19      43.434  45.788  33.061  1.00  0.00            N
ATOM    304  HE  ARG    19      44.141  45.153  32.719  1.00  0.00            H
ATOM    305  CZ  ARG    19      43.538  47.034  32.620  1.00  0.00            C
ATOM    306  NH1 ARG    19      42.813  48.064  32.894  1.00  0.00            N
ATOM    307 HH11 ARG    19      42.073  47.951  33.572  1.00  0.00            H
ATOM    308 HH12 ARG    19      43.073  48.916  32.419  1.00  0.00            H
ATOM    309  NH2 ARG    19      44.526  47.333  31.803  1.00  0.00            N
ATOM    310 HH21 ARG    19      44.485  48.137  31.193  1.00  0.00            H
ATOM    311 HH22 ARG    19      45.169  46.613  31.506  1.00  0.00            H
ATOM    312  C   ARG    19      40.199  41.680  36.574  1.00  0.00            C
ATOM    313  O   ARG    19      39.592  42.414  37.359  1.00  0.00            O
ATOM    335  N   THR    21      39.046  38.035  38.449  1.00  0.00            N
ATOM    336  H   THR    21      38.891  38.841  39.035  1.00  0.00            H
ATOM    337  CA  THR    21      39.613  36.816  39.103  1.00  0.00            C
ATOM    338  HA  THR    21      39.383  35.954  38.477  1.00  0.00            H
ATOM    339  CB  THR    21      41.082  37.094  39.182  1.00  0.00            C
ATOM    340  HB  THR    21      41.391  37.393  38.181  1.00  0.00            H
ATOM    341  CG2 THR    21      41.446  38.236  40.122  1.00  0.00            C
ATOM    342 HG21 THR    21      41.158  38.015  41.149  1.00  0.00            H
ATOM    343 HG22 THR    21      42.516  38.437  40.057  1.00  0.00            H
ATOM    344 HG23 THR    21      41.075  39.169  39.698  1.00  0.00            H
ATOM    345  OG1 THR    21      41.730  35.967  39.633  1.00  0.00            O
ATOM    346  HG1 THR    21      41.297  35.193  39.265  1.00  0.00            H
ATOM    347  C   THR    21      39.020  36.380  40.459  1.00  0.00            C
ATOM    348  O   THR    21      39.361  35.302  40.986  1.00  0.00            O
ATOM    349  N   ILE    22      38.227  37.198  41.068  1.00  0.00            N
ATOM    350  H   ILE    22      38.123  38.137  40.710  1.00  0.00            H
ATOM    351  CA  ILE    22      37.352  36.871  42.170  1.00  0.00            C
ATOM    352  HA  ILE    22      37.295  35.787  42.265  1.00  0.00            H
ATOM    353  CB  ILE    22      37.916  37.543  43.474  1.00  0.00            C
ATOM    354  HB  ILE    22      38.849  37.044  43.741  1.00  0.00            H
ATOM    355  CG2 ILE    22      38.058  39.154  43.377  1.00  0.00            C
ATOM    356 HG21 ILE    22      37.028  39.491  43.274  1.00  0.00            H
ATOM    357 HG22 ILE    22      38.384  39.715  44.253  1.00  0.00            H
ATOM    358 HG23 ILE    22      38.702  39.426  42.540  1.00  0.00            H
ATOM    359  CG1 ILE    22
 36.896  37.217  44.604  1.00  0.00            C
ATOM    360 HG12 ILE    22      36.102  37.960  44.533  1.00  0.00            H
ATOM    361 HG13 ILE    22      36.471  36.217  44.523  1.00  0.00            H
ATOM    362  CD1 ILE    22      37.564  37.356  46.021  1.00  0.00            C
ATOM    363 HD11 ILE    22      36.752  37.393  46.746  1.00  0.00            H
ATOM    364 HD12 ILE    22      38.264  36.555  46.260  1.00  0.00            H
ATOM    365 HD13 ILE    22      38.085  38.292  46.222  1.00  0.00            H
ATOM    366  C   ILE    22      35.932  37.388  41.781  1.00  0.00            C
ATOM    367  O   ILE    22      35.801  38.291  40.964  1.00  0.00            O
ATOM    368  N   THR    23      34.867  36.775  42.275  1.00  0.00            N
ATOM    369  H   THR    23      35.119  36.024  42.901  1.00  0.00            H
ATOM    370  CA  THR    23      33.475  37.246  42.179  1.00  0.00            C
ATOM    371  HA  THR    23      33.598  38.326  42.274  1.00  0.00            H
ATOM    372  CB  THR    23      32.883  36.949  40.716  1.00  0.00            C
ATOM    373  HB  THR    23      33.720  36.895  40.021  1.00  0.00            H
ATOM    374  CG2 THR    23      32.327  35.554  40.714  1.00  0.00            C
ATOM    375 HG21 THR    23      32.110  35.323  39.671  1.00  0.00            H
ATOM    376 HG22 THR    23      33.083  34.924  41.184  1.00  0.00            H
ATOM    377 HG23 THR    23      31.411  35.451  41.296  1.00  0.00            H
ATOM    378  OG1 THR    23      31.901  37.955  40.312  1.00  0.00            O
ATOM    379  HG1 THR    23      32.411  38.719  40.034  1.00  0.00            H
ATOM    380  C   THR    23      32.611  36.772  43.338  1.00  0.00            C
ATOM    381  O   THR    23      32.956  35.887  44.055  1.00  0.00            O
ATOM    382  N   ILE    24      31.443  37.422  43.502  1.00  0.00            N
```

ATOM	383	H	ILE	24	31.090	37.932	42.705	1.00	0.00	H
ATOM	384	CA	ILE	24	30.440	37.336	44.588	1.00	0.00	C
ATOM	385	HA	ILE	24	30.714	36.618	45.360	1.00	0.00	H
ATOM	386	CB	ILE	24	30.267	38.621	45.383	1.00	0.00	C
ATOM	387	HB	ILE	24	29.785	39.364	44.748	1.00	0.00	H
ATOM	388	CG2	ILE	24	29.394	38.266	46.654	1.00	0.00	C
ATOM	389	HG21	ILE	24	29.877	37.509	47.271	1.00	0.00	H
ATOM	390	HG22	ILE	24	29.273	39.126	47.315	1.00	0.00	H
ATOM	391	HG23	ILE	24	28.363	38.145	46.319	1.00	0.00	H
ATOM	392	CG1	ILE	24	31.632	39.282	45.693	1.00	0.00	C
ATOM	393	HG12	ILE	24	32.257	38.728	46.393	1.00	0.00	H
ATOM	394	HG13	ILE	24	32.254	39.451	44.814	1.00	0.00	H
ATOM	395	CD1	ILE	24	31.388	40.705	46.287	1.00	0.00	C
ATOM	396	HD11	ILE	24	30.811	41.253	45.542	1.00	0.00	H
ATOM	397	HD12	ILE	24	30.873	40.642	47.246	1.00	0.00	H
ATOM	398	HD13	ILE	24	32.358	41.175	46.450	1.00	0.00	H
ATOM	399	C	ILE	24	29.087	36.890	43.933	1.00	0.00	C
ATOM	400	O	ILE	24	28.711	37.435	42.886	1.00	0.00	O
ATOM	401	N	ARG	25	28.379	35.920	44.526	1.00	0.00	N
ATOM	402	H	ARG	25	28.818	35.587	45.372	1.00	0.00	H
ATOM	403	CA	ARG	25	27.060	35.305	44.174	1.00	0.00	C
ATOM	404	HA	ARG	25	26.532	35.897	43.426	1.00	0.00	H
ATOM	405	CB	ARG	25	27.311	33.906	43.507	1.00	0.00	C
ATOM	406	HB2	ARG	25	27.812	33.144	44.105	1.00	0.00	H
ATOM	407	HB3	ARG	25	26.276	33.609	43.339	1.00	0.00	H
ATOM	408	CG	ARG	25	28.060	33.913	42.160	1.00	0.00	C
ATOM	409	HG2	ARG	25	29.108	34.184	42.298	1.00	0.00	H
ATOM	410	HG3	ARG	25	28.107	32.946	41.661	1.00	0.00	H
ATOM	411	CD	ARG	25	27.469	34.798	41.017	1.00	0.00	C
ATOM	412	HD2	ARG	25	26.526	34.337	40.724	1.00	0.00	H
ATOM	413	HD3	ARG	25	27.304	35.839	41.296	1.00	0.00	H
ATOM	414	NE	ARG	25	28.285	34.828	39.876	1.00	0.00	N
ATOM	415	HE	ARG	25	28.079	34.128	39.177	1.00	0.00	H
ATOM	416	CZ	ARG	25	29.122	35.769	39.578	1.00	0.00	C
ATOM	417	NH1	ARG	25	29.396	36.793	40.284	1.00	0.00	N
ATOM	418	HH11	ARG	25	30.115	37.435	39.985	1.00	0.00	H
ATOM	419	HH12	ARG	25	28.918	37.027	41.143	1.00	0.00	H
ATOM	420	NH2	ARG	25	29.830	35.667	38.476	1.00	0.00	N
ATOM	421	HH21	ARG	25	30.275	36.509	38.138	1.00	0.00	H
ATOM	422	HH22	ARG	25	29.740	34.956	37.765	1.00	0.00	H
ATOM	423	C	ARG	25	26.329	35.101	45.488	1.00	0.00	C
ATOM	424	O	ARG	25	26.981	34.779	46.460	1.00	0.00	O
ATOM	425	N	ASP	26	25.048	35.156	45.503	1.00	0.00	N
ATOM	426	H	ASP	26	24.628	35.159	44.583	1.00	0.00	H
ATOM	427	CA	ASP	26	24.180	34.816	46.641	1.00	0.00	C
ATOM	428	HA	ASP	26	24.712	35.199	47.512	1.00	0.00	H
ATOM	429	CB	ASP	26	22.803	35.460	46.528	1.00	0.00	C
ATOM	430	HB2	ASP	26	22.232	35.296	47.441	1.00	0.00	H
ATOM	431	HB3	ASP	26	22.897	36.528	46.335	1.00	0.00	H
ATOM	432	CG	ASP	26	21.873	35.001	45.343	1.00	0.00	C
ATOM	433	OD1	ASP	26	22.013	35.519	44.221	1.00	0.00	O
ATOM	434	OD2	ASP	26	21.016	34.082	45.437	1.00	0.00	O
ATOM	435	C	ASP	26	24.158	33.274	46.801	1.00	0.00	C
ATOM	436	O	ASP	26	24.036	32.495	45.838	1.00	0.00	O
ATOM	437	N	GLU	27	24.191	32.779	48.059	1.00	0.00	N
ATOM	438	H	GLU	27	24.133	33.397	48.856	1.00	0.00	H
ATOM	439	CA	GLU	27	24.486	31.389	48.450	1.00	0.00	C
ATOM	440	HA	GLU	27	25.547	31.150	48.383	1.00	0.00	H
ATOM	441	CB	GLU	27	24.312	31.373	49.988	1.00	0.00	C
ATOM	442	HB2	GLU	27	25.066	31.950	50.524	1.00	0.00	H
ATOM	443	HB3	GLU	27	23.397	31.891	50.276	1.00	0.00	H
ATOM	444	CG	GLU	27	24.311	29.971	50.575	1.00	0.00	C
ATOM	445	HG2	GLU	27	23.943	30.047	51.598	1.00	0.00	H
ATOM	446	HG3	GLU	27	23.612	29.291	50.089	1.00	0.00	H

ATOM	447	CD	GLU	27	25.718	29.236	50.556	1.00	0.00	C
ATOM	448	OE1	GLU	27	25.902	28.308	49.715	1.00	0.00	O
ATOM	449	OE2	GLU	27	26.605	29.584	51.407	1.00	0.00	O
ATOM	450	C	GLU	27	23.715	30.286	47.704	1.00	0.00	C
ATOM	451	O	GLU	27	22.480	30.251	47.719	1.00	0.00	O
ATOM	452	N	SER	28	24.343	29.333	47.077	1.00	0.00	N
ATOM	453	H	SER	28	25.338	29.189	47.161	1.00	0.00	H
ATOM	454	CA	SER	28	23.728	28.205	46.352	1.00	0.00	C
ATOM	455	HA	SER	28	22.797	27.821	46.772	1.00	0.00	H
ATOM	456	CB	SER	28	23.313	28.564	44.934	1.00	0.00	C
ATOM	457	HB2	SER	28	22.640	29.403	45.107	1.00	0.00	H
ATOM	458	HB3	SER	28	24.160	28.772	44.279	1.00	0.00	H
ATOM	459	OG	SER	28	22.639	27.529	44.231	1.00	0.00	O
ATOM	460	HG	SER	28	21.766	27.784	43.922	1.00	0.00	H
ATOM	461	C	SER	28	24.701	27.055	46.259	1.00	0.00	C
ATOM	462	O	SER	28	25.880	27.264	45.912	1.00	0.00	O
ATOM	463	N	GLU	29	24.314	25.809	46.523	1.00	0.00	N
ATOM	464	H	GLU	29	23.334	25.696	46.742	1.00	0.00	H
ATOM	465	CA	GLU	29	25.239	24.637	46.329	1.00	0.00	C
ATOM	466	HA	GLU	29	26.130	24.807	46.932	1.00	0.00	H
ATOM	467	CB	GLU	29	24.629	23.325	46.894	1.00	0.00	C
ATOM	468	HB2	GLU	29	24.136	23.506	47.850	1.00	0.00	H
ATOM	469	HB3	GLU	29	23.797	23.159	46.212	1.00	0.00	H
ATOM	470	CG	GLU	29	25.455	22.025	46.936	1.00	0.00	C
ATOM	471	HG2	GLU	29	24.770	21.266	47.312	1.00	0.00	H
ATOM	472	HG3	GLU	29	25.726	21.716	45.926	1.00	0.00	H
ATOM	473	CD	GLU	29	26.764	21.973	47.766	1.00	0.00	C
ATOM	474	OE1	GLU	29	27.487	23.034	47.864	1.00	0.00	O
ATOM	475	OE2	GLU	29	27.240	20.888	48.162	1.00	0.00	O
ATOM	476	C	GLU	29	25.624	24.335	44.912	1.00	0.00	C
ATOM	477	O	GLU	29	26.546	23.597	44.676	1.00	0.00	O
ATOM	478	N	SER	30	25.021	25.030	43.931	1.00	0.00	N
ATOM	479	H	SER	30	24.402	25.755	44.264	1.00	0.00	H
ATOM	480	CA	SER	30	25.195	24.805	42.515	1.00	0.00	C
ATOM	481	HA	SER	30	25.517	23.767	42.452	1.00	0.00	H
ATOM	482	CB	SER	30	23.933	24.971	41.702	1.00	0.00	C
ATOM	483	HB2	SER	30	24.121	24.759	40.650	1.00	0.00	H
ATOM	484	HB3	SER	30	23.159	24.305	42.082	1.00	0.00	H
ATOM	485	OG	SER	30	23.506	26.334	41.776	1.00	0.00	O
ATOM	486	HG	SER	30	22.965	26.414	42.566	1.00	0.00	H
ATOM	487	C	SER	30	26.319	25.640	41.871	1.00	0.00	C
ATOM	488	O	SER	30	26.486	25.560	40.616	1.00	0.00	O
ATOM	489	N	HIE	31	26.984	26.500	42.628	1.00	0.00	N
ATOM	490	H	HIE	31	26.794	26.483	43.620	1.00	0.00	H
ATOM	491	CA	HIE	31	27.974	27.418	42.124	1.00	0.00	C
ATOM	492	HA	HIE	31	27.574	27.791	41.182	1.00	0.00	H
ATOM	493	CB	HIE	31	28.303	28.515	43.147	1.00	0.00	C
ATOM	494	HB2	HIE	31	28.562	27.984	44.064	1.00	0.00	H
ATOM	495	HB3	HIE	31	29.209	29.007	42.791	1.00	0.00	H
ATOM	496	CG	HIE	31	27.222	29.521	43.328	1.00	0.00	C
ATOM	497	ND1	HIE	31	26.602	30.179	42.331	1.00	0.00	N
ATOM	498	CE1	HIE	31	25.627	30.931	42.871	1.00	0.00	C
ATOM	499	HE1	HIE	31	25.015	31.613	42.300	1.00	0.00	H
ATOM	500	NE2	HIE	31	25.745	30.941	44.211	1.00	0.00	N
ATOM	501	HE2	HIE	31	25.138	31.441	44.846	1.00	0.00	H
ATOM	502	CD2	HIE	31	26.795	30.131	44.508	1.00	0.00	C
ATOM	503	HD2	HIE	31	27.122	29.898	45.511	1.00	0.00	H
ATOM	504	C	HIE	31	29.324	26.648	41.887	1.00	0.00	C
ATOM	505	O	HIE	31	29.465	25.547	42.361	1.00	0.00	O
ATOM	506	N	PHE	32	30.270	27.316	41.218	1.00	0.00	N
ATOM	507	H	PHE	32	30.099	28.244	40.859	1.00	0.00	H
ATOM	508	CA	PHE	32	31.527	26.704	40.928	1.00	0.00	C
ATOM	509	HA	PHE	32	31.199	25.829	40.367	1.00	0.00	H

ATOM	510	CB	PHE	32	32.443	27.568	40.064	1.00	0.00	C
ATOM	511	HB2	PHE	32	32.946	28.275	40.723	1.00	0.00	H
ATOM	512	HB3	PHE	32	33.226	26.900	39.705	1.00	0.00	H
ATOM	513	CG	PHE	32	31.854	28.251	38.873	1.00	0.00	C
ATOM	514	CD1	PHE	32	31.323	29.565	38.893	1.00	0.00	C
ATOM	515	HD1	PHE	32	31.413	30.156	39.792	1.00	0.00	H
ATOM	516	CE1	PHE	32	30.585	30.124	37.818	1.00	0.00	C
ATOM	517	HE1	PHE	32	30.167	31.112	37.937	1.00	0.00	H
ATOM	518	CZ	PHE	32	30.444	29.394	36.615	1.00	0.00	C
ATOM	519	HZ	PHE	32	29.941	29.960	35.846	1.00	0.00	H
ATOM	520	CE2	PHE	32	30.876	28.026	36.570	1.00	0.00	C
ATOM	521	HE2	PHE	32	30.886	27.411	35.682	1.00	0.00	H
ATOM	522	CD2	PHE	32	31.660	27.498	37.665	1.00	0.00	C
ATOM	523	HD2	PHE	32	32.035	26.489	37.579	1.00	0.00	H
ATOM	524	C	PHE	32	32.287	26.107	42.173	1.00	0.00	C
ATOM	525	O	PHE	32	32.611	26.816	43.152	1.00	0.00	O
ATOM	526	N	LYS	33	32.574	24.808	42.104	1.00	0.00	N
ATOM	527	H	LYS	33	32.070	24.279	41.406	1.00	0.00	H
ATOM	528	CA	LYS	33	33.383	24.034	42.988	1.00	0.00	C
ATOM	529	HA	LYS	33	33.104	24.275	44.014	1.00	0.00	H
ATOM	530	CB	LYS	33	33.211	22.531	42.647	1.00	0.00	C
ATOM	531	HB2	LYS	33	33.496	22.320	41.616	1.00	0.00	H
ATOM	532	HB3	LYS	33	33.821	21.911	43.304	1.00	0.00	H
ATOM	533	CG	LYS	33	31.774	22.149	42.805	1.00	0.00	C
ATOM	534	HG2	LYS	33	31.139	22.960	42.448	1.00	0.00	H
ATOM	535	HG3	LYS	33	31.612	21.313	42.124	1.00	0.00	H
ATOM	536	CD	LYS	33	31.367	21.841	44.253	1.00	0.00	C
ATOM	537	HD2	LYS	33	31.781	20.845	44.419	1.00	0.00	H
ATOM	538	HD3	LYS	33	31.901	22.605	44.816	1.00	0.00	H
ATOM	539	CE	LYS	33	29.768	21.951	44.476	1.00	0.00	C
ATOM	540	HE2	LYS	33	29.284	21.136	43.937	1.00	0.00	H
ATOM	541	HE3	LYS	33	29.642	21.709	45.532	1.00	0.00	H
ATOM	542	NZ	LYS	33	29.247	23.287	44.284	1.00	0.00	N
ATOM	543	HZ1	LYS	33	29.484	23.539	43.334	1.00	0.00	H
ATOM	544	HZ2	LYS	33	28.253	23.374	44.438	1.00	0.00	H
ATOM	545	HZ3	LYS	33	29.558	23.942	44.987	1.00	0.00	H
ATOM	546	C	LYS	33	34.839	24.559	42.813	1.00	0.00	C
ATOM	547	O	LYS	33	35.205	24.957	41.731	1.00	0.00	O
ATOM	548	N	THR	34	35.685	24.340	43.808	1.00	0.00	N
ATOM	549	H	THR	34	35.240	24.052	44.667	1.00	0.00	H
ATOM	550	CA	THR	34	37.192	24.503	43.661	1.00	0.00	C
ATOM	551	HA	THR	34	37.320	25.494	43.224	1.00	0.00	H
ATOM	552	CB	THR	34	37.843	24.439	45.050	1.00	0.00	C
ATOM	553	HB	THR	34	37.458	23.544	45.535	1.00	0.00	H
ATOM	554	CG2	THR	34	39.356	24.471	45.080	1.00	0.00	C
ATOM	555	HG21	THR	34	39.752	23.558	44.636	1.00	0.00	H
ATOM	556	HG22	THR	34	39.668	25.367	44.543	1.00	0.00	H
ATOM	557	HG23	THR	34	39.722	24.495	46.107	1.00	0.00	H
ATOM	558	OG1	THR	34	37.397	25.580	45.776	1.00	0.00	O
ATOM	559	HG1	THR	34	37.785	25.571	46.653	1.00	0.00	H
ATOM	560	C	THR	34	37.854	23.467	42.706	1.00	0.00	C
ATOM	561	O	THR	34	37.413	22.317	42.647	1.00	0.00	O
ATOM	562	N	GLY	35	38.877	23.898	41.926	1.00	0.00	N
ATOM	563	H	GLY	35	39.206	24.845	42.051	1.00	0.00	H
ATOM	564	CA	GLY	35	39.539	23.081	40.938	1.00	0.00	C
ATOM	565	HA2	GLY	35	40.334	23.657	40.465	1.00	0.00	H
ATOM	566	HA3	GLY	35	40.008	22.175	41.324	1.00	0.00	H
ATOM	567	C	GLY	35	38.659	22.680	39.786	1.00	0.00	C
ATOM	568	O	GLY	35	39.117	21.942	38.993	1.00	0.00	O
ATOM	569	N	ASP	36	37.489	23.254	39.649	1.00	0.00	N
ATOM	570	H	ASP	36	37.144	23.846	40.392	1.00	0.00	H
ATOM	571	CA	ASP	36	36.627	23.072	38.509	1.00	0.00	C
ATOM	572	HA	ASP	36	36.567	22.012	38.260	1.00	0.00	H
ATOM	573	CB	ASP	36	35.140	23.336	38.833	1.00	0.00	C

ATOM	574	HB2	ASP	36	34.896	22.739	39.711	1.00	0.00	H
ATOM	575	HB3	ASP	36	34.963	24.366	39.141	1.00	0.00	H
ATOM	576	CG	ASP	36	34.136	22.856	37.729	1.00	0.00	C
ATOM	577	OD1	ASP	36	32.939	23.116	37.807	1.00	0.00	O
ATOM	578	OD2	ASP	36	34.530	21.984	36.957	1.00	0.00	O
ATOM	579	C	ASP	36	37.092	23.982	37.395	1.00	0.00	C
ATOM	580	O	ASP	36	37.890	24.932	37.552	1.00	0.00	O
ATOM	581	N	VAL	37	36.651	23.686	36.167	1.00	0.00	N
ATOM	582	H	VAL	37	35.992	22.921	36.146	1.00	0.00	H
ATOM	583	CA	VAL	37	37.208	24.148	34.901	1.00	0.00	C
ATOM	584	HA	VAL	37	37.927	24.933	35.141	1.00	0.00	H
ATOM	585	CB	VAL	37	37.951	23.010	34.184	1.00	0.00	C
ATOM	586	HB	VAL	37	37.253	22.384	33.630	1.00	0.00	H
ATOM	587	CG1	VAL	37	38.813	23.708	33.106	1.00	0.00	C
ATOM	588	HG11	VAL	37	39.414	22.973	32.571	1.00	0.00	H
ATOM	589	HG12	VAL	37	38.187	24.287	32.427	1.00	0.00	H
ATOM	590	HG13	VAL	37	39.525	24.334	33.644	1.00	0.00	H
ATOM	591	CG2	VAL	37	38.807	22.168	35.130	1.00	0.00	C
ATOM	592	HG21	VAL	37	39.324	22.779	35.871	1.00	0.00	H
ATOM	593	HG22	VAL	37	38.133	21.474	35.633	1.00	0.00	H
ATOM	594	HG23	VAL	37	39.548	21.523	34.658	1.00	0.00	H
ATOM	595	C	VAL	37	36.130	24.854	34.039	1.00	0.00	C
ATOM	596	O	VAL	37	35.148	24.169	33.700	1.00	0.00	O
ATOM	597	N	LEU	38	36.360	26.115	33.541	1.00	0.00	N
ATOM	598	H	LEU	38	37.111	26.659	33.943	1.00	0.00	H
ATOM	599	CA	LEU	38	35.451	26.914	32.716	1.00	0.00	C
ATOM	600	HA	LEU	38	34.554	26.321	32.534	1.00	0.00	H
ATOM	601	CB	LEU	38	35.072	28.225	33.570	1.00	0.00	C
ATOM	602	HB2	LEU	38	35.969	28.841	33.628	1.00	0.00	H
ATOM	603	HB3	LEU	38	34.397	28.825	32.959	1.00	0.00	H
ATOM	604	CG	LEU	38	34.457	28.010	34.986	1.00	0.00	C
ATOM	605	HG	LEU	38	33.646	27.299	34.831	1.00	0.00	H
ATOM	606	CD1	LEU	38	35.492	27.659	36.122	1.00	0.00	C
ATOM	607	HD11	LEU	38	34.994	27.933	37.051	1.00	0.00	H
ATOM	608	HD12	LEU	38	35.734	26.595	36.123	1.00	0.00	H
ATOM	609	HD13	LEU	38	36.439	28.196	36.078	1.00	0.00	H
ATOM										
610	CD2	LEU	38	33.947	29.428	35.298	1.00	0.00	C	
ATOM	611	HD21	LEU	38	33.633	29.578	36.331	1.00	0.00	H
ATOM	612	HD22	LEU	38	34.655	30.235	35.115	1.00	0.00	H
ATOM	613	HD23	LEU	38	33.158	29.599	34.564	1.00	0.00	H
ATOM	614	C	LEU	38	36.158	27.360	31.434	1.00	0.00	C
ATOM	615	O	LEU	38	37.362	27.086	31.305	1.00	0.00	O
ATOM	616	N	ARG	39	35.435	28.154	30.655	1.00	0.00	N
ATOM	617	H	ARG	39	34.444	28.304	30.776	1.00	0.00	H
ATOM	618	CA	ARG	39	35.962	28.618	29.290	1.00	0.00	C
ATOM	619	HA	ARG	39	36.972	29.005	29.418	1.00	0.00	H
ATOM	620	CB	ARG	39	35.898	27.520	28.222	1.00	0.00	C
ATOM	621	HB2	ARG	39	34.898	27.092	28.143	1.00	0.00	H
ATOM	622	HB3	ARG	39	35.922	28.008	27.247	1.00	0.00	H
ATOM	623	CG	ARG	39	37.074	26.518	28.306	1.00	0.00	C
ATOM	624	HG2	ARG	39	38.018	27.063	28.310	1.00	0.00	H
ATOM	625	HG3	ARG	39	37.095	25.939	29.230	1.00	0.00	H
ATOM	626	CD	ARG	39	37.182	25.594	27.049	1.00	0.00	C
ATOM	627	HD2	ARG	39	36.982	26.141	26.127	1.00	0.00	H
ATOM	628	HD3	ARG	39	38.241	25.341	27.009	1.00	0.00	H
ATOM	629	NE	ARG	39	36.322	24.402	26.985	1.00	0.00	N
ATOM	630	HE	ARG	39	35.373	24.631	27.245	1.00	0.00	H
ATOM	631	CZ	ARG	39	36.598	23.137	26.812	1.00	0.00	C
ATOM	632	NH1	ARG	39	37.790	22.616	26.773	1.00	0.00	N
ATOM	633	HH11	ARG	39	38.715	23.019	26.823	1.00	0.00	H
ATOM	634	HH12	ARG	39	37.897	21.622	26.631	1.00	0.00	H
ATOM	635	NH2	ARG	39	35.648	22.296	26.442	1.00	0.00	N
ATOM	636	HH21	ARG	39	35.862	21.321	26.288	1.00	0.00	H

ATOM	637	HH22	ARG	39	34.675	22.568	26.484	1.00	0.00	H
ATOM	638	C	ARG	39	35.137	29.890	28.931	1.00	0.00	C
ATOM	639	O	ARG	39	33.942	29.961	29.077	1.00	0.00	O
ATOM	640	N	VAL	40	35.804	30.853	28.309	1.00	0.00	N
ATOM	641	H	VAL	40	36.784	30.692	28.125	1.00	0.00	H
ATOM	642	CA	VAL	40	35.283	32.004	27.570	1.00	0.00	C
ATOM	643	HA	VAL	40	34.200	31.944	27.679	1.00	0.00	H
ATOM	644	CB	VAL	40	35.664	33.392	28.288	1.00	0.00	C
ATOM	645	HB	VAL	40	35.121	34.242	27.876	1.00	0.00	H
ATOM	646	CG1	VAL	40	35.261	33.331	29.754	1.00	0.00	C
ATOM	647	HG11	VAL	40	35.452	34.308	30.197	1.00	0.00	H
ATOM	648	HG12	VAL	40	34.214	33.090	29.936	1.00	0.00	H
ATOM	649	HG13	VAL	40	35.924	32.590	30.199	1.00	0.00	H
ATOM	650	CG2	VAL	40	37.197	33.623	28.074	1.00	0.00	C
ATOM	651	HG21	VAL	40	37.465	34.504	28.655	1.00	0.00	H
ATOM	652	HG22	VAL	40	37.808	32.752	28.313	1.00	0.00	H
ATOM	653	HG23	VAL	40	37.324	33.849	27.015	1.00	0.00	H
ATOM	654	C	VAL	40	35.614	32.073	26.071	1.00	0.00	C
ATOM	655	O	VAL	40	36.738	31.856	25.700	1.00	0.00	O
ATOM	656	N	GLY	41	34.568	32.600	25.378	1.00	0.00	N
ATOM	657	H	GLY	41	33.671	32.656	25.837	1.00	0.00	H
ATOM	658	CA	GLY	41	34.767	33.127	23.992	1.00	0.00	C
ATOM	659	HA2	GLY	41	35.579	33.855	24.015	1.00	0.00	H
ATOM	660	HA3	GLY	41	35.174	32.226	23.533	1.00	0.00	H
ATOM	661	C	GLY	41	33.626	33.916	23.311	1.00	0.00	C
ATOM	662	O	GLY	41	32.458	33.818	23.781	1.00	0.00	O
ATOM	663	N	ARG	42	33.950	34.481	22.152	1.00	0.00	N
ATOM	664	H	ARG	42	34.915	34.332	21.892	1.00	0.00	H
ATOM	665	CA	ARG	42	32.976	35.124	21.271	1.00	0.00	C
ATOM	666	HA	ARG	42	32.071	34.533	21.408	1.00	0.00	H
ATOM	667	CB	ARG	42	32.641	36.520	21.802	1.00	0.00	C
ATOM	668	HB2	ARG	42	31.657	36.840	21.458	1.00	0.00	H
ATOM	669	HB3	ARG	42	32.427	36.555	22.871	1.00	0.00	H
ATOM	670	CG	ARG	42	33.730	37.551	21.525	1.00	0.00	C
ATOM	671	HG2	ARG	42	34.692	37.147	21.840	1.00	0.00	H
ATOM	672	HG3	ARG	42	33.632	37.731	20.455	1.00	0.00	H
ATOM	673	CD	ARG	42	33.302	38.940	22.173	1.00	0.00	C
ATOM	674	HD2	ARG	42	32.343	39.211	21.732	1.00	0.00	H
ATOM	675	HD3	ARG	42	33.139	38.833	23.245	1.00	0.00	H
ATOM	676	NE	ARG	42	34.372	39.945	21.950	1.00	0.00	N
ATOM	677	HE	ARG	42	34.248	40.602	21.195	1.00	0.00	H
ATOM	678	CZ	ARG	42	35.417	40.113	22.731	1.00	0.00	C
ATOM	679	NH1	ARG	42	35.571	39.469	23.857	1.00	0.00	N
ATOM	680	HH11	ARG	42	36.385	39.700	24.407	1.00	0.00	H
ATOM	681	HH12	ARG	42	34.838	38.935	24.303	1.00	0.00	H
ATOM	682	NH2	ARG	42	36.327	40.973	22.464	1.00	0.00	N
ATOM	683	HH21	ARG	42	35.949	41.569	21.742	1.00	0.00	H
ATOM	684	HH22	ARG	42	36.924	41.315	23.204	1.00	0.00	H
ATOM	685	C	ARG	42	33.204	35.127	19.778	1.00	0.00	C
ATOM	686	O	ARG	42	32.341	35.606	19.043	1.00	0.00	O
ATOM	687	N	PHE	43	34.290	34.535	19.243	1.00	0.00	N
ATOM	688	H	PHE	43	34.876	34.025	19.889	1.00	0.00	H
ATOM	689	CA	PHE	43	34.673	34.483	17.839	1.00	0.00	C
ATOM	690	HA	PHE	43	33.973	35.028	17.206	1.00	0.00	H
ATOM	691	CB	PHE	43	36.060	35.178	17.734	1.00	0.00	C
ATOM	692	HB2	PHE	43	36.804	34.471	18.101	1.00	0.00	H
ATOM	693	HB3	PHE	43	36.255	35.269	16.665	1.00	0.00	H
ATOM	694	CG	PHE	43	36.130	36.577	18.269	1.00	0.00	C
ATOM	695	CD1	PHE	43	35.373	37.623	17.661	1.00	0.00	C
ATOM	696	HD1	PHE	43	34.719	37.411	16.828	1.00	0.00	H
ATOM	697	CE1	PHE	43	35.424	38.906	18.213	1.00	0.00	C
ATOM	698	HE1	PHE	43	34.780	39.646	17.761	1.00	0.00	H
ATOM	699	CZ	PHE	43	36.175	39.171	19.315	1.00	0.00	C
ATOM	700	HZ	PHE	43	36.281	40.165	19.724	1.00	0.00	H

ATOM	701	CE2	PHE	43	36.870	38.135	19.981	1.00	0.00	C
ATOM	702	HE2	PHE	43	37.517	38.321	20.825	1.00	0.00	H
ATOM	703	CD2	PHE	43	36.915	36.888	19.402	1.00	0.00	C
ATOM	704	HD2	PHE	43	37.474	36.136	19.940	1.00	0.00	H
ATOM	705	C	PHE	43	34.552	33.006	17.489	1.00	0.00	C
ATOM	706	O	PHE	43	34.700	32.058	18.386	1.00	0.00	O
ATOM	707	N	GLU	44	34.341	32.795	16.198	1.00	0.00	N
ATOM	708	H	GLU	44	34.155	33.610	15.634	1.00	0.00	H
ATOM	709	CA	GLU	44	34.105	31.436	15.578	1.00	0.00	C
ATOM	710	HA	GLU	44	33.368	30.903	16.178	1.00	0.00	H
ATOM	711	CB	GLU	44	33.481	31.649	14.116	1.00	0.00	C
ATOM	712	HB2	GLU	44	32.629	32.301	14.310	1.00	0.00	H
ATOM	713	HB3	GLU	44	34.201	32.125	13.450	1.00	0.00	H
ATOM	714	CG	GLU	44	32.993	30.480	13.362	1.00	0.00	C
ATOM	715	HG2	GLU	44	33.857	29.890	13.057	1.00	0.00	H
ATOM	716	HG3	GLU	44	32.263	30.030	14.036	1.00	0.00	H
ATOM	717	CD	GLU	44	32.048	30.803	12.215	1.00	0.00	C
ATOM	718	OE1	GLU	44	31.370	29.843	11.730	1.00	0.00	O
ATOM	719	OE2	GLU	44	32.047	31.968	11.703	1.00	0.00	O
ATOM	720	C	GLU	44	35.406	30.666	15.379	1.00	0.00	C
ATOM	721	O	GLU	44	35.439	29.386	15.398	1.00	0.00	O
ATOM	722	N	ASP	45	36.418	31.410	15.040	1.00	0.00	N
ATOM	723	H	ASP	45	36.227	32.402	15.037	1.00	0.00	H
ATOM	724	CA	ASP	45	37.791	30.920	14.904	1.00	0.00	C
ATOM	725	HA	ASP	45	37.857	30.059	14.240	1.00	0.00	H
ATOM	726	CB	ASP	45	38.572	32.074	14.378	1.00	0.00	C
ATOM	727	HB2	ASP	45	38.165	32.518	13.469	1.00	0.00	H
ATOM	728	HB3	ASP	45	38.583	32.833	15.161	1.00	0.00	H
ATOM	729	CG	ASP	45	39.964	31.724	13.982	1.00	0.00	C
ATOM	730	OD1	ASP	45	40.132	30.639	13.365	1.00	0.00	O
ATOM	731	OD2	ASP	45	40.942	32.436	14.392	1.00	0.00	O
ATOM	732	C	ASP	45	38.178	30.403	16.316	1.00	0.00	C
ATOM	733	O	ASP	45	38.344	31.181	17.229	1.00	0.00	O
ATOM	734	N	ASP	46	38.422	29.085	16.457	1.00	0.00	N
ATOM	735	H	ASP	46	38.292	28.520	15.629	1.00	0.00	H
ATOM	736	CA	ASP	46	38.819	28.350	17.754	1.00	0.00	C
ATOM	737	HA	ASP	46	38.892	27.299	17.478	1.00	0.00	H
ATOM	738	CB	ASP	46	40.197	28.804	18.302	1.00	0.00	C
ATOM	739	HB2	ASP	46	40.007	29.853	18.533	1.00	0.00	H
ATOM	740	HB3	ASP	46	40.456	28.268	19.214	1.00	0.00	H
ATOM	741	CG	ASP	46	41.302	28.678	17.306	1.00	0.00	C
ATOM	742	OD1	ASP	46	41.414	27.605	16.703	1.00	0.00	O
ATOM	743	OD2	ASP	46	42.178	29.563	17.168	1.00	0.00	O
ATOM	744	C	ASP	46	37.835	28.298	18.936	1.00	0.00	C
ATOM	745	O	ASP	46	37.891	27.343	19.686	1.00	0.00	O
ATOM	746	N	GLY	47	36.854	29.195	18.972	1.00	0.00	N
ATOM	747	H	GLY	47	36.807	29.895	18.246	1.00	0.00	H
ATOM	748	CA	GLY	47	35.633	28.932	19.771	1.00	0.00	C
ATOM	749	HA2	GLY	47	34.945	29.607	19.262	1.00	0.00	H
ATOM	750	HA3	GLY	47	35.280	27.903	19.710	1.00	0.00	H
ATOM	751	C	GLY	47	35.845	29.301	21.280	1.00	0.00	C
ATOM	752	O	GLY	47	34.813	29.595	21.964	1.00	0.00	O
ATOM	753	N	TYR	48	37.081	29.500	21.734	1.00	0.00	N
ATOM	754	H	TYR	48	37.786	29.236	21.061	1.00	0.00	H
ATOM	755	CA	TYR	48	37.470	29.853	23.092	1.00	0.00	C
ATOM	756	HA	TYR	48	36.802	30.605	23.514	1.00	0.00	H
ATOM	757	CB	TYR	48	37.591	28.662	24.032	1.00	0.00	C
ATOM	758	HB2	TYR	48	38.527	28.137	23.840	1.00	0.00	H
ATOM	759	HB3	TYR	48	37.713	28.995	25.063	1.00	0.00	H
ATOM	760	CG	TYR	48	36.461	27.640	23.982	1.00	0.00	C
ATOM	761	CD1	TYR	48	35.196	27.998	24.381	1.00	0.00	C
ATOM	762	HD1	TYR	48	34.895	28.944	24.805	1.00	0.00	H
ATOM	763	CE1	TYR	48	34.131	27.113	24.390	1.00	0.00	C

ATOM	764	HE1	TYR	48	33.190	27.392	24.841	1.00	0.00	H
ATOM	765	CZ	TYR	48	34.380	25.836	23.934	1.00	0.00	C
ATOM	766	OH	TYR	48	33.475	24.849	24.177	1.00	0.00	O
ATOM	767	HH	TYR	48	33.717	24.003	23.794	1.00	0.00	H
ATOM	768	CE2	TYR	48	35.608	25.460	23.323	1.00	0.00	C
ATOM	769	HE2	TYR	48	35.689	24.458	22.928	1.00	0.00	H
ATOM	770	CD2	TYR	48	36.692	26.350	23.485	1.00	0.00	C
ATOM	771	HD2	TYR	48	37.578	26.148	22.901	1.00	0.00	H
ATOM	772	C	TYR	48	38.825	30.596	22.977	1.00	0.00	C
ATOM	773	O	TYR	48	39.693	30.324	22.119	1.00	0.00	O
ATOM	774	N	PHE	49	39.137	31.494	23.949	1.00	0.00	N
ATOM	775	H	PHE	49	38.452	31.629	24.677	1.00	0.00	H
ATOM	776	CA	PHE	49	40.429	32.267	24.083	1.00	0.00	C
ATOM	777	HA	PHE	49	41.145	31.679	23.509	1.00	0.00	H
ATOM	778	CB	PHE	49	40.357	33.640	23.374	1.00	0.00	C
ATOM	779	HB2	PHE	49	41.289	34.196	23.479	1.00	0.00	H
ATOM	780	HB3	PHE	49	40.189	33.361	22.334	1.00	0.00	H
ATOM	781	CG	PHE	49	39.249	34.669	23.687	1.00	0.00	C
ATOM	782	CD1	PHE	49	38.339	35.133	22.702	1.00	0.00	C
ATOM	783	HD1	PHE	49	38.347	34.631	21.745	1.00	0.00	H
ATOM	784	CE1	PHE	49	37.312	35.963	23.085	1.00	0.00	C
ATOM	785	HE1	PHE	49	36.513	36.180	22.390	1.00	0.00	H
ATOM	786	CZ	PHE	49	37.318	36.626	24.326	1.00	0.00	C
ATOM	787	HZ	PHE	49	36.629	37.421	24.568	1.00	0.00	H
ATOM	788	CE2	PHE	49	38.320	36.242	25.255	1.00	0.00	C
ATOM	789	HE2	PHE	49	38.315	36.767	26.199	1.00	0.00	H
ATOM	790	CD2	PHE	49	39.208	35.207	24.965	1.00	0.00	C
ATOM	791	HD2	PHE	49	39.994	34.986	25.672	1.00	0.00	H
ATOM	792	C	PHE	49	40.974	32.325	25.443	1.00	0.00	C
ATOM	793	O	PHE	49	42.044	32.815	25.625	1.00	0.00	O
ATOM	794	N	CYX	50	40.223	31.701	26.379	1.00	0.00	N
ATOM	795	H	CYX	50	39.304	31.338	26.174	1.00	0.00	H
ATOM	796	CA	CYX	50	40.756	31.353	27.670	1.00	0.00	C
ATOM	797	HA	CYX	50	41.798	31.086	27.502	1.00	0.00	H
ATOM	798	CB	CYX	50	40.631	32.517	28.550	1.00	0.00	C
ATOM	799	HB2	CYX	50	39.855	33.157	28.132	1.00	0.00	H
ATOM	800	HB3	CYX	50	40.026	32.167	29.386	1.00	0.00	H
ATOM	801	SG	CYX	50	42.012	33.517	29.115	1.00	0.00	S
ATOM	802	C	CYX	50	40.128	30.060	28.217	1.00	0.00	C
ATOM	803	O	CYX	50	38.943	30.107	28.423	1.00	0.00	O
ATOM	804	N	THR	51	40.949	29.061	28.508	1.00	0.00	N
ATOM	805	H	THR	51	41.854	28.955	28.076	1.00	0.00	H
ATOM	806	CA	THR	51	40.564	28.014	29.553	1.00	0.00	C
ATOM	807	HA	THR	51	39.490	27.836	29.581	1.00	0.00	H
ATOM	808	CB	THR	51	41.292	26.686	29.237	1.00	0.00	C
ATOM	809	HB	THR	51	42.361	26.895	29.252	1.00	0.00	H
ATOM	810	CG2	THR	51	40.898	25.564	30.194	1.00	0.00	C
ATOM	811	HG21	THR	51	41.532	24.695	30.016	1.00	0.00	H
ATOM	812	HG22	THR	51	40.952	25.837	31.247	1.00	0.00	H
ATOM	813	HG23	THR	51	39.885	25.255	29.938	1.00	0.00	H
ATOM	814	OG1	THR	51	40.921	26.357	27.924	1.00	0.00	O
ATOM	815	HG1	THR	51	40.964	27.153	27.388	1.00	0.00	H
ATOM	816	C	THR	51	40.935	28.493	31.013	1.00	0.00	C
ATOM	817	O	THR	51	41.947	29.186	31.199	1.00	0.00	O
ATOM	818	N	ILE	52	40.127	28.138	32.005	1.00	0.00	N
ATOM	819	H	ILE	52	39.336	27.616	31.653	1.00	0.00	H
ATOM	820	CA	ILE	52	40.085	28.885	33.245	1.00	0.00	C
ATOM	821	HA	ILE	52	41.022	29.421	33.392	1.00	0.00	H
ATOM	822	CB	ILE	52	38.986	29.972	33.394	1.00	0.00	C
ATOM	823	HB	ILE	52	38.003	29.618	33.085	1.00	0.00	H
ATOM	824	CG2	ILE	52	38.595	30.580	34.756	1.00	0.00	C
ATOM	825	HG21	ILE	52	37.790	31.296	34.594	1.00	0.00	H
ATOM	826	HG22	ILE	52	38.394	29.719	35.393	1.00	0.00	H
ATOM	827	HG23	ILE	52	39.463	31.161	35.065	1.00	0.00	H

ATOM	828	CG1	ILE	52	39.274	31.109	32.358	1.00	0.00	C
ATOM	829	HG12	ILE	52	39.848	31.901	32.841	1.00	0.00	H
ATOM	830	HG13	ILE	52	39.633	30.676	31.425	1.00	0.00	H
ATOM	831	CD1	ILE	52	37.980	31.783	31.932	1.00	0.00	C
ATOM	832	HD11	ILE	52	37.274	30.999	31.661	1.00	0.00	H
ATOM	833	HD12	ILE	52	37.463	32.380	32.684	1.00	0.00	H
ATOM	834	HD13	ILE	52	38.207	32.447	31.097	1.00	0.00	H
ATOM	835	C	ILE	52	39.839	27.950	34.459	1.00	0.00	C
ATOM	836	O	ILE	52	38.991	27.086	34.316	1.00	0.00	O
ATOM	837	N	GLU	53	40.435	28.076	35.648	1.00	0.00	N
ATOM	838	H	GLU	53	41.124	28.804	35.780	1.00	0.00	H
ATOM	839	CA	GLU	53	40.298	27.149	36.795	1.00	0.00	C
ATOM	840	HA	GLU	53	39.486	26.454	36.578	1.00	0.00	H
ATOM	841	CB	GLU	53	41.634	26.339	36.851	1.00	0.00	C
ATOM	842	HB2	GLU	53	41.664	25.837	35.885	1.00	0.00	H
ATOM	843	HB3	GLU	53	42.460	27.042	36.959	1.00	0.00	H
ATOM	844	CG	GLU	53	41.616	25.318	38.009	1.00	0.00	C
ATOM	845	HG2	GLU	53	41.669	25.865	38.951	1.00	0.00	H
ATOM	846	HG3	GLU	53	40.714	24.709	37.959	1.00	0.00	H
ATOM	847	CD	GLU	53	42.874	24.447	37.822	1.00	0.00	C
ATOM	848	OE1	GLU	53	43.790	24.645	38.652	1.00	0.00	O
ATOM	849	OE2	GLU	53	42.957	23.571	36.853	1.00	0.00	O
ATOM	850	C	GLU	53	39.972	27.836	38.112	1.00	0.00	C
ATOM	851	O	GLU	53	40.570	28.859	38.445	1.00	0.00	O
ATOM	852	N	VAL	54	39.057	27.290	38.900	1.00	0.00	N
ATOM	853	H	VAL	54	38.681	26.416	38.560	1.00	0.00	H
ATOM	854	CA	VAL	54	38.769	27.742	40.243	1.00	0.00	C
ATOM	855	HA	VAL	54	38.649	28.826	40.213	1.00	0.00	H
ATOM	856	CB	VAL	54	37.449	27.215	40.827	1.00	0.00	C
ATOM	857	HB	VAL	54	37.460	26.139	41.008	1.00	0.00	H
ATOM	858	CG1	VAL	54	37.121	27.964	42.051	1.00	0.00	C
ATOM	859	HG11	VAL	54	36.140	27.693	42.444	1.00	0.00	H
ATOM	860	HG12	VAL	54	37.760	27.833	42.925	1.00		
0.00			H							
ATOM	861	HG13	VAL	54	36.953	28.978	41.691	1.00	0.00	H
ATOM	862	CG2	VAL	54	36.350	27.635	39.879	1.00	0.00	C
ATOM	863	HG21	VAL	54	35.409	27.382	40.368	1.00	0.00	H
ATOM	864	HG22	VAL	54	36.437	28.691	39.624	1.00	0.00	H
ATOM	865	HG23	VAL	54	36.472	27.091	38.943	1.00	0.00	H
ATOM	866	C	VAL	54	39.915	27.381	41.191	1.00	0.00	C
ATOM	867	O	VAL	54	40.369	26.253	41.247	1.00	0.00	O
ATOM	868	N	THR	55	40.395	28.387	41.940	1.00	0.00	N
ATOM	869	H	THR	55	39.836	29.227	41.961	1.00	0.00	H
ATOM	870	CA	THR	55	41.554	28.338	42.814	1.00	0.00	C
ATOM	871	HA	THR	55	42.046	27.375	42.675	1.00	0.00	H
ATOM	872	CB	THR	55	42.555	29.525	42.530	1.00	0.00	C
ATOM	873	HB	THR	55	43.347	29.392	43.267	1.00	0.00	H
ATOM	874	CG2	THR	55	43.271	29.499	41.159	1.00	0.00	C
ATOM	875	HG21	THR	55	42.691	29.781	40.280	1.00	0.00	H
ATOM	876	HG22	THR	55	44.063	30.248	41.169	1.00	0.00	H
ATOM	877	HG23	THR	55	43.786	28.572	40.904	1.00	0.00	H
ATOM	878	OG1	THR	55	42.045	30.823	42.488	1.00	0.00	O
ATOM	879	HG1	THR	55	42.068	31.133	43.395	1.00	0.00	H
ATOM	880	C	THR	55	41.199	28.385	44.222	1.00	0.00	C
ATOM	881	O	THR	55	41.867	27.727	45.071	1.00	0.00	O
ATOM	882	N	ALA	56	40.166	29.152	44.616	1.00	0.00	N
ATOM	883	H	ALA	56	39.683	29.740	43.952	1.00	0.00	H
ATOM	884	CA	ALA	56	39.585	29.137	45.946	1.00	0.00	C
ATOM	885	HA	ALA	56	39.727	28.163	46.414	1.00	0.00	H
ATOM	886	CB	ALA	56	40.263	29.975	47.063	1.00	0.00	C
ATOM	887	HB1	ALA	56	39.837	29.754	48.041	1.00	0.00	H
ATOM	888	HB2	ALA	56	41.346	29.871	47.116	1.00	0.00	H
ATOM	889	HB3	ALA	56	40.218	31.029	46.785	1.00	0.00	H
ATOM	890	C	ALA	56	38.123	29.366	46.019	1.00	0.00	C

ATOM	891	O	ALA	56	37.597	30.064	45.150	1.00	0.00	O
ATOM	892	N	THR	57	37.389	28.805	47.027	1.00	0.00	N
ATOM	893	H	THR	57	37.841	28.225	47.720	1.00	0.00	H
ATOM	894	CA	THR	57	35.883	29.115	47.252	1.00	0.00	C
ATOM	895	HA	THR	57	35.645	30.027	46.705	1.00	0.00	H
ATOM	896	CB	THR	57	34.950	27.951	46.669	1.00	0.00	C
ATOM	897	HB	THR	57	33.921	28.186	46.940	1.00	0.00	H
ATOM	898	CG2	THR	57	35.006	27.881	45.192	1.00	0.00	C
ATOM	899	HG21	THR	57	35.990	27.601	44.820	1.00	0.00	H
ATOM	900	HG22	THR	57	34.297	27.085	44.966	1.00	0.00	H
ATOM	901	HG23	THR	57	34.683	28.810	44.722	1.00	0.00	H
ATOM	902	OG1	THR	57	35.322	26.696	47.185	1.00	0.00	O
ATOM	903	HG1	THR	57	36.200	26.443	46.890	1.00	0.00	H
ATOM	904	C	THR	57	35.486	29.301	48.703	1.00	0.00	C
ATOM	905	O	THR	57	35.993	28.609	49.564	1.00	0.00	O
ATOM	906	N	SER	58	34.525	30.187	49.005	1.00	0.00	N
ATOM	907	H	SER	58	33.969	30.518	48.229	1.00	0.00	H
ATOM	908	CA	SER	58	34.104	30.515	50.393	1.00	0.00	C
ATOM	909	HA	SER	58	34.068	29.630	51.029	1.00	0.00	H
ATOM	910	CB	SER	58	35.168	31.449	51.019	1.00	0.00	C
ATOM	911	HB2	SER	58	34.847	31.672	52.036	1.00	0.00	H
ATOM	912	HB3	SER	58	36.084	30.864	50.949	1.00	0.00	H
ATOM	913	OG	SER	58	35.192	32.653	50.293	1.00	0.00	O
ATOM	914	HG	SER	58	35.672	32.524	49.472	1.00	0.00	H
ATOM	915	C	SER	58	32.663	31.157	50.477	1.00	0.00	C
ATOM	916	O	SER	58	32.075	31.560	49.457	1.00	0.00	O
ATOM	917	N	THR	59	32.049	31.272	51.694	1.00	0.00	N
ATOM	918	H	THR	59	32.691	31.064	52.446	1.00	0.00	H
ATOM	919	CA	THR	59	30.700	31.672	52.052	1.00	0.00	C
ATOM	920	HA	THR	59	30.319	32.206	51.183	1.00	0.00	H
ATOM	921	CB	THR	59	29.807	30.436	52.304	1.00	0.00	C
ATOM	922	HB	THR	59	29.787	29.886	51.362	1.00	0.00	H
ATOM	923	CG2	THR	59	30.193	29.462	53.477	1.00	0.00	C
ATOM	924	HG21	THR	59	29.472	28.646	53.465	1.00	0.00	H
ATOM	925	HG22	THR	59	31.157	29.001	53.261	1.00	0.00	H
ATOM	926	HG23	THR	59	30.260	29.972	54.437	1.00	0.00	H
ATOM	927	OG1	THR	59	28.509	30.978	52.599	1.00	0.00	O
ATOM	928	HG1	THR	59	27.806	30.451	52.213	1.00	0.00	H
ATOM	929	C	THR	59	30.771	32.703	53.231	1.00	0.00	C
ATOM	930	O	THR	59	31.474	32.465	54.196	1.00	0.00	O
ATOM	931	N	VAL	60	30.172	33.897	53.077	1.00	0.00	N
ATOM	932	H	VAL	60	29.748	34.086	52.181	1.00	0.00	H
ATOM	933	CA	VAL	60	30.323	35.134	53.932	1.00	0.00	C
ATOM	934	HA	VAL	60	30.522	34.753	54.932	1.00	0.00	H
ATOM	935	CB	VAL	60	31.502	36.004	53.436	1.00	0.00	C
ATOM	936	HB	VAL	60	31.435	36.911	54.038	1.00	0.00	H
ATOM	937	CG1	VAL	60	32.865	35.339	53.662	1.00	0.00	C
ATOM	938	HG11	VAL	60	33.661	36.078	53.578	1.00	0.00	H
ATOM	939	HG12	VAL	60	32.933	34.946	54.676	1.00	0.00	H
ATOM	940	HG13	VAL	60	32.938	34.536	52.928	1.00	0.00	H
ATOM	941	CG2	VAL	60	31.348	36.408	51.944	1.00	0.00	C
ATOM	942	HG21	VAL	60	32.082	37.100	51.532	1.00	0.00	H
ATOM	943	HG22	VAL	60	31.310	35.509	51.330	1.00	0.00	H
ATOM	944	HG23	VAL	60	30.336	36.805	51.854	1.00	0.00	H
ATOM	945	C	VAL	60	29.032	35.945	53.901	1.00	0.00	C
ATOM	946	O	VAL	60	28.394	36.271	52.901	1.00	0.00	O
ATOM	947	N	THR	61	28.753	36.494	55.055	1.00	0.00	N
ATOM	948	H	THR	61	29.364	36.120	55.767	1.00	0.00	H
ATOM	949	CA	THR	61	27.672	37.455	55.411	1.00	0.00	C
ATOM	950	HA	THR	61	26.962	37.442	54.583	1.00	0.00	H
ATOM	951	CB	THR	61	26.949	37.021	56.779	1.00	0.00	C
ATOM	952	HB	THR	61	26.650	37.963	57.239	1.00	0.00	H
ATOM	953	CG2	THR	61	25.659	36.222	56.442	1.00	0.00	C
ATOM	954	HG21	THR	61	24.976	36.759	55.783	1.00	0.00	H

ATOM	955	HG22	THR	61	25.953	35.315	55.914	1.00	0.00	H
ATOM	956	HG23	THR	61	25.167	35.970	57.382	1.00	0.00	H
ATOM	957	OG1	THR	61	27.673	36.206	57.725	1.00	0.00	O
ATOM	958	HG1	THR	61	28.307	36.821	58.103	1.00	0.00	H
ATOM	959	C	THR	61	28.466	38.799	55.437	1.00	0.00	C
ATOM	960	O	THR	61	29.724	38.918	55.345	1.00	0.00	O
ATOM	961	N	LEU	62	27.752	39.888	55.517	1.00	0.00	N
ATOM	962	H	LEU	62	26.744	39.851	55.543	1.00	0.00	H
ATOM	963	CA	LEU	62	28.219	41.326	55.455	1.00	0.00	C
ATOM	964	HA	LEU	62	28.972	41.439	54.674	1.00	0.00	H
ATOM	965	CB	LEU	62	27.066	42.312	55.312	1.00	0.00	C
ATOM	966	HB2	LEU	62	26.382	42.102	56.134	1.00	0.00	H
ATOM	967	HB3	LEU	62	27.494	43.289	55.534	1.00	0.00	H
ATOM	968	CG	LEU	62	26.160	42.293	54.053	1.00	0.00	C
ATOM	969	HG	LEU	62	25.708	41.306	53.959	1.00	0.00	H
ATOM	970	CD1	LEU	62	25.119	43.413	54.183	1.00	0.00	C
ATOM	971	HD11	LEU	62	24.511	43.530	53.286	1.00	0.00	H
ATOM	972	HD12	LEU	62	24.482	43.256	55.054	1.00	0.00	H
ATOM	973	HD13	LEU	62	25.757	44.291	54.269	1.00	0.00	H
ATOM	974	CD2	LEU	62	27.035	42.677	52.890	1.00	0.00	C
ATOM	975	HD21	LEU	62	27.428	43.676	53.080	1.00	0.00	H
ATOM	976	HD22	LEU	62	27.613	41.784	52.651	1.00	0.00	H
ATOM	977	HD23	LEU	62	26.398	42.802	52.015	1.00	0.00	H
ATOM	978	C	LEU	62	29.064	41.774	56.584	1.00	0.00	C
ATOM	979	O	LEU	62	29.744	42.770	56.563	1.00	0.00	O
ATOM	980	N	ASP	63	28.996	40.995	57.625	1.00	0.00	N
ATOM	981	H	ASP	63	28.445	40.160	57.488	1.00	0.00	H
ATOM	982	CA	ASP	63	29.940	41.110	58.736	1.00	0.00	C
ATOM	983	HA	ASP	63	30.023	42.137	59.088	1.00	0.00	H
ATOM	984	CB	ASP	63	29.276	40.287	59.894	1.00	0.00	C
ATOM	985	HB2	ASP	63	29.907	40.227	60.781	1.00	0.00	H
ATOM	986	HB3	ASP	63	28.399	40.827	60.250	1.00	0.00	H
ATOM	987	CG	ASP	63	28.873	38.886	59.565	1.00	0.00	C
ATOM	988	OD1	ASP	63	29.387	38.180	58.645	1.00	0.00	O
ATOM	989	OD2	ASP	63	27.833	38.501	60.038	1.00	0.00	O
ATOM	990	C	ASP	63	31.431	40.592	58.475	1.00	0.00	C
ATOM	991	O	ASP	63	32.283	40.897	59.240	1.00	0.00	O
ATOM	992	N	THR	64	31.649	39.768	57.408	1.00	0.00	N
ATOM	993	H	THR	64	30.847	39.603	56.815	1.00	0.00	H
ATOM	994	CA	THR	64	32.805	38.890	57.169	1.00	0.00	C
ATOM	995	HA	THR	64	33.662	39.249	57.738	1.00	0.00	H
ATOM	996	CB	THR	64	32.433	37.415	57.466	1.00	0.00	C
ATOM	997	HB	THR	64	33.092	36.697	56.978	1.00	0.00	H
ATOM	998	CG2	THR	64	32.663	37.076	58.907	1.00	0.00	C
ATOM	999	HG21	THR	64	32.018	37.705	59.521	1.00	0.00	H
ATOM	1000	HG22	THR	64	32.302	36.049	58.946	1.00	0.00	H
ATOM	1001	HG23	THR	64	33.733	37.165	59.098	1.00	0.00	H
ATOM	1002	OG1	THR	64	31.118	36.906	57.083	1.00	0.00	O
ATOM	1003	HG1	THR	64	30.535	37.321	57.722	1.00	0.00	H
ATOM	1004	C	THR	64	33.356	38.954	55.756	1.00	0.00	C
ATOM	1005	O	THR	64	34.375	38.376	55.476	1.00	0.00	O
ATOM	1006	N	LEU	65	32.647	39.717	54.872	1.00	0.00	N
ATOM	1007	H	LEU	65	31.753	40.081	55.171	1.00	0.00	H
ATOM	1008	CA	LEU	65	33.007	39.996	53.476	1.00	0.00	C
ATOM	1009	HA	LEU	65	33.159	38.987	53.094	1.00	0.00	H
ATOM	1010	CB	LEU	65	31.818	40.647	52.643	1.00	0.00	C
ATOM	1011	HB2	LEU	65	30.979	39.962	52.517	1.00	0.00	H
ATOM	1012	HB3	LEU	65	31.600	41.578	53.167	1.00	0.00	H
ATOM	1013	CG	LEU	65	32.290	40.997	51.204	1.00	0.00	C
ATOM	1014	HG	LEU	65	33.084	41.744	51.181	1.00	0.00	H
ATOM	1015	CD1	LEU	65	32.676	39.844	50.306	1.00	0.00	C
ATOM	1016	HD11	LEU	65	33.454	39.195	50.709	1.00	0.00	H
ATOM	1017	HD12	LEU	65	31.860	39.166	50.058	1.00	0.00	H

ATOM	1018	HD13	LEU	65	32.902	40.275	49.331	1.00	0.00	H
ATOM	1019	CD2	LEU	65	31.155	41.770	50.543	1.00	0.00	C
ATOM	1020	HD21	LEU	65	30.204	41.270	50.724	1.00	0.00	H
ATOM	1021	HD22	LEU	65	31.107	42.782	50.948	1.00	0.00	H
ATOM	1022	HD23	LEU	65	31.224	41.842	49.458	1.00	0.00	H
ATOM	1023	C	LEU	65	34.375	40.728	53.303	1.00	0.00	C
ATOM	1024	O	LEU	65	34.571	41.829	53.805	1.00	0.00	O
ATOM	1025	N	THR	66	35.366	40.014	52.819	1.00	0.00	N
ATOM	1026	H	THR	66	35.265	39.093	52.417	1.00	0.00	H
ATOM	1027	CA	THR	66	36.659	40.632	52.697	1.00	0.00	C
ATOM	1028	HA	THR	66	36.906	41.214	53.585	1.00	0.00	H
ATOM	1029	CB	THR	66	37.716	39.438	52.555	1.00	0.00	C
ATOM	1030	HB	THR	66	37.473	38.993	51.590	1.00	0.00	H
ATOM	1031	CG2	THR	66	39.246	39.824	52.595	1.00	0.00	C
ATOM	1032	HG21	THR	66	39.283	40.416	53.510	1.00	0.00	H
ATOM	1033	HG22	THR	66	39.959	39.000	52.576	1.00	0.00	H
ATOM	1034	HG23	THR	66	39.390	40.483	51.739	1.00	0.00	H
ATOM	1035	OG1	THR	66	37.563	38.515	53.657	1.00	0.00	O
ATOM	1036	HG1	THR	66	36.671	38.168	53.581	1.00	0.00	H
ATOM	1037	C	THR	66	36.820	41.716	51.536	1.00	0.00	C
ATOM	1038	O	THR	66	36.213	41.569	50.525	1.00	0.00	O
ATOM	1039	N	GLU	67	37.710	42.669	51.644	1.00	0.00	N
ATOM	1040	H	GLU	67	38.214	42.831	52.503	1.00	0.00	H
ATOM	1041	CA	GLU	67	37.904	43.735	50.589	1.00	0.00	C
ATOM	1042	HA	GLU	67	36.896	44.110	50.408	1.00	0.00	H
ATOM	1043	CB	GLU	67	38.693	44.906	51.171	1.00	0.00	C
ATOM	1044	HB2	GLU	67	38.303	45.745	50.596	1.00	0.00	H
ATOM	1045	HB3	GLU	67	38.330	45.051	52.188	1.00	0.00	H
ATOM	1046	CG	GLU	67	40.207	44.993	51.066	1.00	0.00	C
ATOM	1047	HG2	GLU	67	40.536	44.004	51.379	1.00	0.00	H
ATOM	1048	HG3	GLU	67	40.593	45.120	50.055	1.00	0.00	H
ATOM	1049	CD	GLU	67	40.927	45.922	52.082	1.00	0.00	C
ATOM	1050	OE1	GLU	67	41.147	45.401	53.229	1.00	0.00	O
ATOM	1051	OE2	GLU	67	41.209	47.028	51.735	1.00	0.00	O
ATOM	1052	C	GLU	67	38.431	43.308	49.210	1.00	0.00	C
ATOM	1053	O	GLU	67	38.393	44.129	48.283	1.00	0.00	O
ATOM	1054	N	LYS	68	38.845	42.062	48.987	1.00	0.00	N
ATOM	1055	H	LYS	68	38.612	41.389	49.702	1.00	0.00	H
ATOM	1056	CA	LYS	68	39.443	41.587	47.752	1.00	0.00	C
ATOM	1057	HA	LYS	68	40.344	42.170	47.563	1.00	0.00	H
ATOM	1058	CB	LYS	68	39.809	40.055	47.955	1.00	0.00	C
ATOM	1059	HB2	LYS	68	40.097	40.033	49.006	1.00	0.00	H
ATOM	1060	HB3	LYS	68	38.969	39.377	47.801	1.00	0.00	H
ATOM	1061	CG	LYS	68	40.958	39.455	47.113	1.00	0.00	C
ATOM	1062	HG2	LYS	68	41.131	38.394	47.291	1.00	0.00	H
ATOM	1063	HG3	LYS	68	40.671	39.487	46.062	1.00	0.00	H
ATOM	1064	CD	LYS	68	42.304	40.254	47.395	1.00	0.00	C
ATOM	1065	HD2	LYS	68	42.152	41.280	47.059	1.00	0.00	H
ATOM	1066	HD3	LYS	68	42.572	40.416	48.440	1.00	0.00	H
ATOM	1067	CE	LYS	68	43.381	39.600	46.567	1.00	0.00	C
ATOM	1068	HE2	LYS	68	43.446	38.559	46.882	1.00	0.00	H
ATOM	1069	HE3	LYS	68	43.063	39.583	45.525	1.00	0.00	H
ATOM	1070	NZ	LYS	68	44.705	40.192	46.603	1.00	0.00	N
ATOM	1071	HZ1	LYS	68	45.100	40.299	45.680	1.00	0.00	H
ATOM	1072	HZ2	LYS	68	44.721	41.126	46.990	1.00	0.00	H
ATOM	1073	HZ3	LYS	68	45.365	39.734	47.215	1.00	0.00	H
ATOM	1074	C	LYS	68	38.758	41.847	46.409	1.00	0.00	C
ATOM	1075	O	LYS	68	39.456	42.049	45.429	1.00	0.00	O
ATOM	1076	N	HIE	69	37.420	41.871	46.392	1.00	0.00	N
ATOM	1077	H	HIE	69	36.941	41.580	47.232	1.00	0.00	H
ATOM	1078	CA	HIE	69	36.552	42.201	45.291	1.00	0.00	C
ATOM	1079	HA	HIE	69	36.934	41.662	44.424	1.00	0.00	H
ATOM	1080	CB	HIE	69	35.070	41.782	45.463	1.00	0.00	C
ATOM	1081	HB2	HIE	69	35.191	40.794	45.905	1.00	0.00	H

ATOM	1082	HB3	HIE	69	34.560	42.328	46.257	1.00	0.00	H
ATOM	1083	CG	HIE	69	34.182	41.775	44.229	1.00	0.00	C
ATOM	1084	ND1	HIE	69	34.011	40.714	43.342	1.00	0.00	N
ATOM	1085	CE1	HIE	69	33.163	41.151	42.392	1.00	0.00	C
ATOM	1086	HE1	HIE	69	32.848	40.590	41.525	1.00	0.00	H
ATOM	1087	NE2	HIE	69	32.714	42.389	42.701	1.00	0.00	N
ATOM	1088	HE2	HIE	69	31.937	42.832	42.233	1.00	0.00	H
ATOM	1089	CD2	HIE	69	33.308	42.774	43.877	1.00	0.00	C
ATOM	1090	HD2	HIE	69	33.224	43.702	44.421	1.00	0.00	H
ATOM	1091	C	HIE	69	36.620	43.702	44.956	1.00	0.00	C
ATOM	1092	O	HIE	69	36.678	44.207	43.803	1.00	0.00	O
ATOM	1093	N	ALA	70	36.420	44.570	46.003	1.00	0.00	N
ATOM	1094	H	ALA	70	36.324	44.293	46.970	1.00	0.00	H
ATOM	1095	CA	ALA	70	36.123	46.011	45.860	1.00	0.00	C
ATOM	1096	HA	ALA	70	35.230	46.105	45.243	1.00	0.00	H
ATOM	1097	CB	ALA	70	35.771	46.610	47.268	1.00	0.00	C
ATOM	1098	HB1	ALA	70	36.624	46.567	47.947	1.00	0.00	H
ATOM	1099	HB2	ALA	70	35.405	47.633	47.186	1.00	0.00	H
ATOM	1100	HB3	ALA	70	34.941	46.008	47.638	1.00	0.00	H
ATOM	1101	C	ALA	70	37.250	46.758	45.113	1.00	0.00	C
ATOM	1102	O	ALA	70	36.961	47.507	44.228	1.00	0.00	O
ATOM	1103	N	GLU	71	38.528	46.386	45.378	1.00	0.00	N
ATOM	1104	H	GLU	71	38.691	45.927	46.263	1.00	0.00	H
ATOM	1105	CA	GLU	71	39.726	46.909	44.763	1.00	0.00	C
ATOM	1106	HA	GLU	71	39.784	47.997	44.791	1.00	0.00	H
ATOM	1107	CB	GLU	71	40.934	46.428	45.466	1.00	0.00	C
ATOM	1108	HB2	GLU	71	40.896	45.340	45.506	1.00	0.00	H
ATOM	1109	HB3	GLU	71	41.859	46.763	44.998	1.00	0.00	H
ATOM	1110	CG	GLU	71	41.087	46.809	46.921	1.00	0.00	C
ATOM	1111	HG2	GLU	71						
40.142	46.909	47.454	1.00	0.00	H					
ATOM	1112	HG3	GLU	71	41.533	45.932	47.391	1.00	0.00	H
ATOM	1113	CD	GLU	71	41.879	48.063	47.103	1.00	0.00	C
ATOM	1114	OE1	GLU	71	41.551	49.108	46.504	1.00	0.00	O
ATOM	1115	OE2	GLU	71	42.889	48.160	47.791	1.00	0.00	O
ATOM	1116	C	GLU	71	39.819	46.427	43.253	1.00	0.00	C
ATOM	1117	O	GLU	71	40.304	47.150	42.361	1.00	0.00	O
ATOM	1118	N	GLN	72	39.345	45.195	42.862	1.00	0.00	N
ATOM	1119	H	GLN	72	38.994	44.646	43.633	1.00	0.00	H
ATOM	1120	CA	GLN	72	39.125	44.774	41.531	1.00	0.00	C
ATOM	1121	HA	GLN	72	40.073	44.691	40.998	1.00	0.00	H
ATOM	1122	CB	GLN	72	38.531	43.365	41.625	1.00	0.00	C
ATOM	1123	HB2	GLN	72	37.471	43.544	41.805	1.00	0.00	H
ATOM	1124	HB3	GLN	72	39.112	42.788	42.345	1.00	0.00	H
ATOM	1125	CG	GLN	72	38.625	42.632	40.334	1.00	0.00	C
ATOM	1126	HG2	GLN	72	38.286	43.184	39.457	1.00	0.00	H
ATOM	1127	HG3	GLN	72	39.652	42.350	40.099	1.00	0.00	H
ATOM	1128	CD	GLN	72	37.842	41.338	40.359	1.00	0.00	C
ATOM	1129	OE1	GLN	72	38.291	40.197	40.202	1.00	0.00	O
ATOM	1130	NE2	GLN	72	36.544	41.436	40.397	1.00	0.00	N
ATOM	1131	HE21	GLN	72	36.050	42.302	40.240	1.00	0.00	H
ATOM	1132	HE22	GLN	72	36.023	40.595	40.602	1.00	0.00	H
ATOM	1133	C	GLN	72	38.137	45.705	40.798	1.00	0.00	C
ATOM	1134	O	GLN	72	38.479	46.225	39.710	1.00	0.00	O
ATOM	1135	N	GLU	73	37.030	46.076	41.440	1.00	0.00	N
ATOM	1136	H	GLU	73	36.937	45.801	42.408	1.00	0.00	H
ATOM	1137	CA	GLU	73	35.992	46.944	40.929	1.00	0.00	C
ATOM	1138	HA	GLU	73	35.893	46.812	39.851	1.00	0.00	H
ATOM	1139	CB	GLU	73	34.668	46.548	41.578	1.00	0.00	C
ATOM	1140	HB2	GLU	73	34.835	46.675	42.648	1.00	0.00	H
ATOM	1141	HB3	GLU	73	33.811	47.148	41.270	1.00	0.00	H
ATOM	1142	CG	GLU	73	34.315	45.084	41.216	1.00	0.00	C
ATOM	1143	HG2	GLU	73	34.820	44.339	41.830	1.00	0.00	H
ATOM	1144	HG3	GLU	73	33.271	44.955	41.500	1.00	0.00	H

ATOM	1145	CD	GLU	73	34.403	44.637	39.731	1.00	0.00	C
ATOM	1146	OE1	GLU	73	33.826	45.323	38.838	1.00	0.00	O
ATOM	1147	OE2	GLU	73	35.069	43.601	39.434	1.00	0.00	O
ATOM	1148	C	GLU	73	36.358	48.456	41.122	1.00	0.00	C
ATOM	1149	O	GLU	73	35.574	49.322	40.769	1.00	0.00	O
ATOM	1150	N	ASN	74	37.633	48.718	41.471	1.00	0.00	N
ATOM	1151	H	ASN	74	38.292	47.955	41.520	1.00	0.00	H
ATOM	1152	CA	ASN	74	38.112	50.099	41.829	1.00	0.00	C
ATOM	1153	HA	ASN	74	39.097	49.895	42.248	1.00	0.00	H
ATOM	1154	CB	ASN	74	38.570	50.781	40.506	1.00	0.00	C
ATOM	1155	HB2	ASN	74	37.710	50.747	39.837	1.00	0.00	H
ATOM	1156	HB3	ASN	74	38.837	51.791	40.816	1.00	0.00	H
ATOM	1157	CG	ASN	74	39.736	50.123	39.801	1.00	0.00	C
ATOM	1158	OD1	ASN	74	40.744	49.883	40.401	1.00	0.00	O
ATOM	1159	ND2	ASN	74	39.567	49.816	38.512	1.00	0.00	N
ATOM	1160	HD21	ASN	74	40.349	49.356	38.068	1.00	0.00	H
ATOM	1161	HD22	ASN	74	38.738	49.962	37.953	1.00	0.00	H
ATOM	1162	C	ASN	74	37.245	50.924	42.803	1.00	0.00	C
ATOM	1163	O	ASN	74	36.921	52.088	42.579	1.00	0.00	O
ATOM	1164	N	MET	75	36.761	50.340	43.895	1.00	0.00	N
ATOM	1165	H	MET	75	36.930	49.350	43.996	1.00	0.00	H
ATOM	1166	CA	MET	75	35.882	51.003	44.888	1.00	0.00	C
ATOM	1167	HA	MET	75	36.041	52.082	44.894	1.00	0.00	H
ATOM	1168	CB	MET	75	34.400	50.855	44.682	1.00	0.00	C
ATOM	1169	HB2	MET	75	33.958	51.363	45.539	1.00	0.00	H
ATOM	1170	HB3	MET	75	34.197	51.405	43.763	1.00	0.00	H
ATOM	1171	CG	MET	75	33.999	49.424	44.508	1.00	0.00	C
ATOM	1172	HG2	MET	75	34.132	49.032	43.499	1.00	0.00	H
ATOM	1173	HG3	MET	75	34.584	48.722	45.104	1.00	0.00	H
ATOM	1174	SD	MET	75	32.269	49.004	44.899	1.00	0.00	S
ATOM	1175	CE	MET	75	31.622	49.857	43.372	1.00	0.00	C
ATOM	1176	HE1	MET	75	31.829	49.290	42.464	1.00	0.00	H
ATOM	1177	HE2	MET	75	30.556	50.075	43.419	1.00	0.00	H
ATOM	1178	HE3	MET	75	32.141	50.815	43.378	1.00	0.00	H
ATOM	1179	C	MET	75	36.242	50.526	46.276	1.00	0.00	C
ATOM	1180	O	MET	75	37.005	49.561	46.511	1.00	0.00	O
ATOM	1181	N	THR	76	35.673	51.228	47.233	1.00	0.00	N
ATOM	1182	H	THR	76	35.294	52.102	46.899	1.00	0.00	H
ATOM	1183	CA	THR	76	35.829	50.907	48.663	1.00	0.00	C
ATOM	1184	HA	THR	76	36.782	50.400	48.815	1.00	0.00	H
ATOM	1185	CB	THR	76	35.949	52.149	49.591	1.00	0.00	C
ATOM	1186	HB	THR	76	36.605	52.860	49.090	1.00	0.00	H
ATOM	1187	CG2	THR	76	34.568	52.756	49.920	1.00	0.00	C
ATOM	1188	HG21	THR	76	33.992	52.997	49.027	1.00	0.00	H
ATOM	1189	HG22	THR	76	34.033	52.217	50.702	1.00	0.00	H
ATOM	1190	HG23	THR	76	34.727	53.652	50.519	1.00	0.00	H
ATOM	1191	OG1	THR	76	36.507	51.743	50.812	1.00	0.00	O
ATOM	1192	HG1	THR	76	36.550	52.534	51.353	1.00	0.00	H
ATOM	1193	C	THR	76	34.805	49.881	49.133	1.00	0.00	C
ATOM	1194	O	THR	76	33.684	50.007	48.643	1.00	0.00	O
ATOM	1195	N	LEU	77	35.231	48.884	50.013	1.00	0.00	N
ATOM	1196	H	LEU	77	36.179	48.843	50.360	1.00	0.00	H
ATOM	1197	CA	LEU	77	34.376	47.814	50.525	1.00	0.00	C
ATOM	1198	HA	LEU	77	34.106	47.081	49.765	1.00	0.00	H
ATOM	1199	CB	LEU	77	35.071	47.085	51.617	1.00	0.00	C
ATOM	1200	HB2	LEU	77	35.972	46.628	51.208	1.00	0.00	H
ATOM	1201	HB3	LEU	77	35.370	47.688	52.476	1.00	0.00	H
ATOM	1202	CG	LEU	77	34.354	45.872	52.311	1.00	0.00	C
ATOM	1203	HG	LEU	77	33.517	46.379	52.792	1.00	0.00	H
ATOM	1204	CD1	LEU	77	33.840	44.771	51.393	1.00	0.00	C
ATOM	1205	HD11	LEU	77	33.144	45.184	50.663	1.00	0.00	H
ATOM	1206	HD12	LEU	77	34.705	44.306	50.918	1.00	0.00	H
ATOM	1207	HD13	LEU	77	33.276	43.989	51.901	1.00	0.00	H
ATOM	1208	CD2	LEU	77	35.212	45.175	53.313	1.00	0.00	C

ATOM	1209	HD21	LEU	77	34.832	44.288	53.820	1.00	0.00	H
ATOM	1210	HD22	LEU	77	36.078	44.741	52.814	1.00	0.00	H
ATOM	1211	HD23	LEU	77	35.530	45.898	54.066	1.00	0.00	H
ATOM	1212	C	LEU	77	33.047	48.308	51.129	1.00	0.00	C
ATOM	1213	O	LEU	77	31.961	47.748	50.951	1.00	0.00	O
ATOM	1214	N	THR	78	33.088	49.452	51.902	1.00	0.00	N
ATOM	1215	H	THR	78	33.933	49.873	52.262	1.00	0.00	H
ATOM	1216	CA	THR	78	31.852	50.075	52.394	1.00	0.00	C
ATOM	1217	HA	THR	78	31.348	49.225	52.854	1.00	0.00	H
ATOM	1218	CB	THR	78	31.954	51.164	53.426	1.00	0.00	C
ATOM	1219	HB	THR	78	30.988	51.616	53.651	1.00	0.00	H
ATOM	1220	CG2	THR	78	32.639	50.579	54.688	1.00	0.00	C
ATOM	1221	HG21	THR	78	33.721	50.590	54.548	1.00	0.00	H
ATOM	1222	HG22	THR	78	32.415	51.173	55.575	1.00	0.00	H
ATOM	1223	HG23	THR	78	32.394	49.530	54.848	1.00	0.00	H
ATOM	1224	OG1	THR	78	32.793	52.158	52.949	1.00	0.00	O
ATOM	1225	HG1	THR	78	32.182	52.750	52.504	1.00	0.00	H
ATOM	1226	C	THR	78	30.965	50.475	51.217	1.00	0.00	C
ATOM	1227	O	THR	78	29.732	50.260	51.380	1.00	0.00	O
ATOM	1228	N	GLU	79	31.388	51.017	50.068	1.00	0.00	N
ATOM	1229	H	GLU	79	32.378	51.217	50.039	1.00	0.00	H
ATOM	1230	CA	GLU	79	30.612	51.357	48.889	1.00	0.00	C
ATOM	1231	HA	GLU	79	29.712	51.899	49.180	1.00	0.00	H
ATOM	1232	CB	GLU	79	31.443	52.224	47.895	1.00	0.00	C
ATOM	1233	HB2	GLU	79	32.505	51.987	47.955	1.00	0.00	H
ATOM	1234	HB3	GLU	79	31.101	52.068	46.871	1.00	0.00	H
ATOM	1235	CG	GLU	79	31.317	53.756	48.125	1.00	0.00	C
ATOM	1236	HG2	GLU	79	32.031	54.098	48.873	1.00	0.00	H
ATOM	1237	HG3	GLU	79	31.592	54.208	47.171	1.00	0.00	H
ATOM	1238	CD	GLU	79	29.914	54.304	48.416	1.00	0.00	C
ATOM	1239	OE1	GLU	79	29.857	54.914	49.502	1.00	0.00	O
ATOM	1240	OE2	GLU	79	28.924	54.078	47.681	1.00	0.00	O
ATOM	1241	C	GLU	79	30.169	50.026	48.171	1.00	0.00	C
ATOM	1242	O	GLU	79	29.138	49.950	47.496	1.00	0.00	O
ATOM	1243	N	LEU	80	30.881	48.861	48.332	1.00	0.00	N
ATOM	1244	H	LEU	80	31.795	48.828	48.758	1.00	0.00	H
ATOM	1245	CA	LEU	80	30.537	47.583	47.715	1.00	0.00	C
ATOM	1246	HA	LEU	80	30.131	47.773	46.721	1.00	0.00	H
ATOM	1247	CB	LEU	80	31.894	46.702	47.667	1.00	0.00	C
ATOM	1248	HB2	LEU	80	32.503	47.222	46.928	1.00	0.00	H
ATOM	1249	HB3	LEU	80	32.435	46.688	48.613	1.00	0.00	H
ATOM	1250	CG	LEU	80	31.608	45.255	47.251	1.00	0.00	C
ATOM	1251	HG	LEU	80	30.856	44.969	47.986	1.00	0.00	H
ATOM	1252	CD1	LEU	80	31.012	45.068	45.847	1.00	0.00	C
ATOM	1253	HD11	LEU	80	31.690	45.463	45.089	1.00	0.00	H
ATOM	1254	HD12	LEU	80	30.707	44.056	45.582	1.00	0.00	H
ATOM	1255	HD13	LEU	80	30.106	45.671	45.897	1.00	0.00	H
ATOM	1256	CD2	LEU	80	32.791	44.313	47.413	1.00	0.00	C
ATOM	1257	HD21	LEU	80	32.600	43.265	47.177	1.00	0.00	H
ATOM	1258	HD22	LEU	80	33.555	44.618	46.698	1.00	0.00	H
ATOM	1259	HD23	LEU	80	33.219	44.498	48.398	1.00	0.00	H
ATOM	1260	C	LEU	80	29.385	46.956	48.472	1.00	0.00	C
ATOM	1261	O	LEU	80	28.386	46.575	47.950	1.00	0.00	O
ATOM	1262	N	LYS	81	29.515	46.967	49.823	1.00	0.00	N
ATOM	1263	H	LYS	81	30.303	47.434	50.248	1.00	0.00	H
ATOM	1264	CA	LYS	81	28.453	46.638	50.782	1.00	0.00	C
ATOM	1265	HA	LYS	81	28.140	45.595	50.791	1.00	0.00	H
ATOM	1266	CB	LYS	81	28.916	46.967	52.241	1.00	0.00	C
ATOM	1267	HB2	LYS	81	29.626	47.787	52.339	1.00	0.00	H
ATOM	1268	HB3	LYS	81	28.002	46.953	52.836	1.00	0.00	H
ATOM	1269	CG	LYS	81	29.752	45.753	52.657	1.00	0.00	C
ATOM	1270	HG2	LYS	81	29.225	44.799	52.656	1.00	0.00	H
ATOM	1271	HG3	LYS	81	30.626	45.597	52.024	1.00	0.00	H

ATOM	1272	CD	LYS	81	30.262	46.060	54.038	1.00	0.00	C
ATOM	1273	HD2	LYS	81	30.853	46.974	54.097	1.00	0.00	H
ATOM	1274	HD3	LYS	81	29.446	46.199	54.747	1.00	0.00	H
ATOM	1275	CE	LYS	81	31.168	44.865	54.453	1.00	0.00	C
ATOM	1276	HE2	LYS	81	30.651	43.941	54.192	1.00	0.00	H
ATOM	1277	HE3	LYS	81	32.067	45.007	53.854	1.00	0.00	H
ATOM	1278	NZ	LYS	81	31.716	44.823	55.854	1.00	0.00	N
ATOM	1279	HZ1	LYS	81	32.443	45.517	55.761	1.00	0.00	H
ATOM	1280	HZ2	LYS	81	30.988	44.995	56.532	1.00	0.00	H
ATOM	1281	HZ3	LYS	81	32.204	43.946	55.968	1.00	0.00	H
ATOM	1282	C	LYS	81	27.158	47.423	50.583	1.00	0.00	C
ATOM	1283	O	LYS	81	26.141	46.798	50.464	1.00	0.00	O
ATOM	1284	N	LYS	82	27.253	48.743	50.367	1.00	0.00	N
ATOM	1285	H	LYS	82	28.147	49.151	50.603	1.00	0.00	H
ATOM	1286	CA	LYS	82	26.077	49.563	49.981	1.00	0.00	C
ATOM	1287	HA	LYS	82	25.384	49.446	50.814	1.00	0.00	H
ATOM	1288	CB	LYS	82	26.527	51.085	49.839	1.00	0.00	C
ATOM	1289	HB2	LYS	82	27.508	51.148	49.368	1.00	0.00	H
ATOM	1290	HB3	LYS	82	25.757	51.592	49.258	1.00	0.00	H
ATOM	1291	CG	LYS	82	26.709	51.742	51.216	1.00	0.00	C
ATOM	1292	HG2	LYS	82	25.767	51.753	51.763	1.00	0.00	H
ATOM	1293	HG3	LYS	82	27.449	51.137	51.741	1.00	0.00	H
ATOM	1294	CD	LYS	82	27.391	53.087	51.269	1.00	0.00	C
ATOM	1295	HD2	LYS	82	27.486	53.358	52.321	1.00	0.00	H
ATOM	1296	HD3	LYS	82	28.349	52.962	50.765	1.00	0.00	H
ATOM	1297	CE	LYS	82	26.672	54.198	50.510	1.00	0.00	C
ATOM	1298	HE2	LYS	82	26.602	53.993	49.441	1.00	0.00	H
ATOM	1299	HE3	LYS	82	25.630	54.278	50.818	1.00	0.00	H
ATOM	1300	NZ	LYS	82	27.436	55.375	50.655	1.00	0.00	N
ATOM	1301	HZ1	LYS	82	27.168	56.058	49.960	1.00	0.00	H
ATOM	1302	HZ2	LYS	82	27.207	55.880	51.499	1.00	0.00	H
ATOM	1303	HZ3	LYS	82	28.415	55.214	50.459	1.00	0.00	H
ATOM	1304	C	LYS	82	25.482	48.998	48.638	1.00	0.00	C
ATOM	1305	O	LYS	82	24.340	48.583	48.654	1.00	0.00	O
ATOM	1306	N	VAL	83	26.219	48.838	47.547	1.00	0.00	N
ATOM	1307	H	VAL	83	27.171	49.181	47.539	1.00	0.00	H
ATOM	1308	CA	VAL	83	25.541	48.474	46.264	1.00	0.00	C
ATOM	1309	HA	VAL	83	24.666	49.103	46.103	1.00	0.00	H
ATOM	1310	CB	VAL	83	26.492	48.685	45.050	1.00	0.00	C
ATOM	1311	HB	VAL	83	25.974	48.432	44.125	1.00	0.00	H
ATOM	1312	CG1	VAL	83	26.844	50.152	44.844	1.00	0.00	C
ATOM	1313	HG11	VAL	83	27.693	50.333	45.505	1.00	0.00	H
ATOM	1314	HG12	VAL	83	27.109	50.358	43.806	1.00	0.00	H
ATOM	1315	HG13	VAL	83	25.987	50.789	45.059	1.00	0.00	H
ATOM	1316	CG2	VAL	83	27.772	47.805	45.014	1.00	0.00	C
ATOM	1317	HG21	VAL	83	27.533	46.742	45.048	1.00	0.00	H
ATOM	1318	HG22	VAL	83	28.393	48.030	44.147	1.00	0.00	H
ATOM	1319	HG23	VAL	83	28.308	48.100	45.916	1.00	0.00	H
ATOM	1320	C	VAL	83	24.939	47.093	46.278	1.00	0.00	C
ATOM	1321	O	VAL	83	24.052	46.832	45.489	1.00	0.00	O
ATOM	1322	N	ILE	84	25.511	46.224	47.152	1.00	0.00	N
ATOM	1323	H	ILE	84	26.229	46.536	47.791	1.00	0.00	H
ATOM	1324	CA	ILE	84	24.877	44.891	47.248	1.00	0.00	C
ATOM	1325	HA	ILE	84	24.588	44.554	46.253	1.00	0.00	H
ATOM	1326	CB	ILE	84	26.005	43.901	47.704	1.00	0.00	C
ATOM	1327	HB	ILE	84	26.444	44.326	48.608	1.00	0.00	H
ATOM	1328	CG2	ILE	84	25.480	42.506	47.966	1.00	0.00	C
ATOM	1329	HG21	ILE	84	24.685	42.611	48.705	1.00	0.00	H
ATOM	1330	HG22	ILE	84	25.195	42.063	47.012	1.00	0.00	H
ATOM	1331	HG23	ILE	84	26.214	41.854	48.439	1.00	0.00	H
ATOM	1332	CG1	ILE	84	27.048	43.877	46.523	1.00	0.00	C
ATOM	1333	HG12	ILE	84	26.687	43.328	45.652	1.00	0.00	H
ATOM	1334	HG13	ILE	84	27.287	44.832	46.056	1.00	0.00	H
ATOM	1335	CD1	ILE	84	28.276	43.098	46.893	1.00	0.00	C

ATOM	1336	HD11	ILE	84	28.939	43.669	47.542	1.00	0.00	H
ATOM	1337	HD12	ILE	84	28.010	42.193	47.439	1.00	0.00	H
ATOM	1338	HD13	ILE	84	28.887	42.892	46.014	1.00	0.00	H
ATOM	1339	C	ILE	84	23.597	44.928	48.137	1.00	0.00	C
ATOM	1340	O	ILE	84	22.609	44.305	47.773	1.00	0.00	O
ATOM	1341	N	ALA	85	23.586	45.603	49.256	1.00	0.00	N
ATOM	1342	H	ALA	85	24.540	45.724	49.565	1.00	0.00	H
ATOM	1343	CA	ALA	85	22.493	45.789	50.267	1.00	0.00	C
ATOM	1344	HA	ALA	85	21.969	44.854	50.466	1.00	0.00	H
ATOM	1345	CB	ALA	85	23.155	46.213	51.612	1.00	0.00	C
ATOM	1346	HB1	ALA	85	22.428	46.409	52.401	1.00	0.00	H
ATOM	1347	HB2	ALA	85	23.825	45.378	51.815	1.00	0.00	H
ATOM	1348	HB3	ALA	85	23.675	47.146	51.393	1.00	0.00	H
ATOM	1349	C	ALA	85	21.308	46.672	49.675	1.00	0.00	C
ATOM	1350	O	ALA	85	20.245	46.857	50.310	1.00	0.00	O
ATOM	1351	N	ASP	86	21.590	47.282	48.554	1.00	0.00	N
ATOM	1352	H	ASP	86	22.569	47.287	48.304	1.00	0.00	H
ATOM	1353	CA	ASP	86	20.729	48.101	47.636	1.00	0.00	C
ATOM	1354	HA	ASP	86	20.087	48.655	48.321	1.00	0.00	H
ATOM	1355	CB	ASP	86	21.653	49.137	46.950	1.00	0.00	C
ATOM	1356	HB2	ASP	86	22.272	49.570	47.737	1.00	0.00	H
ATOM	1357	HB3	ASP	86	22.336	48.710	46.217	1.00	0.00	H
ATOM	1358	CG	ASP	86	20.791	50.304	46.398	1.00	0.00	C
ATOM	1359	OD1	ASP	86	21.487	51.293	46.103	1.00	0.00	O
ATOM	1360	OD2	ASP	86	19.580	50.361	46.396	1.00	0.00	O
ATOM	1361	C	ASP	86	19.928	47.149	46.728	1.00	0.00	C
ATOM										
1362	O	ASP	86	18.986	47.578	46.018	1.00	0.00	O	
ATOM	1363	N	ILE	87	20.277	45.860	46.656	1.00	0.00	N
ATOM	1364	H	ILE	87	21.132	45.556	47.097	1.00	0.00	H
ATOM	1365	CA	ILE	87	19.375	44.821	46.082	1.00	0.00	C
ATOM	1366	HA	ILE	87	18.453	45.276	45.717	1.00	0.00	H
ATOM	1367	CB	ILE	87	20.159	44.283	44.871	1.00	0.00	C
ATOM	1368	HB	ILE	87	21.122	44.003	45.297	1.00	0.00	H
ATOM	1369	CG2	ILE	87	19.442	43.040	44.278	1.00	0.00	C
ATOM	1370	HG21	ILE	87	19.378	42.236	45.012	1.00	0.00	H
ATOM	1371	HG22	ILE	87	18.395	43.287	44.100	1.00	0.00	H
ATOM	1372	HG23	ILE	87	20.049	42.665	43.453	1.00	0.00	H
ATOM	1373	CG1	ILE	87	20.423	45.335	43.696	1.00	0.00	C
ATOM	1374	HG12	ILE	87	19.442	45.434	43.233	1.00	0.00	H
ATOM	1375	HG13	ILE	87	20.746	46.315	44.048	1.00	0.00	H
ATOM	1376	CD1	ILE	87	21.393	44.948	42.621	1.00	0.00	C
ATOM	1377	HD11	ILE	87	22.262	44.449	43.050	1.00	0.00	H
ATOM	1378	HD12	ILE	87	20.823	44.292	41.962	1.00	0.00	H
ATOM	1379	HD13	ILE	87	21.651	45.822	42.023	1.00	0.00	H
ATOM	1380	C	ILE	87	19.010	43.734	47.144	1.00	0.00	C
ATOM	1381	O	ILE	87	17.861	43.314	47.234	1.00	0.00	O
ATOM	1382	N	TYR	88	19.929	43.206	47.917	1.00	0.00	N
ATOM	1383	H	TYR	88	20.869	43.577	47.910	1.00	0.00	H
ATOM	1384	CA	TYR	88	19.654	42.043	48.832	1.00	0.00	C
ATOM	1385	HA	TYR	88	18.853	41.392	48.481	1.00	0.00	H
ATOM	1386	CB	TYR	88	20.892	41.104	48.729	1.00	0.00	C
ATOM	1387	HB2	TYR	88	21.817	41.661	48.878	1.00	0.00	H
ATOM	1388	HB3	TYR	88	20.805	40.361	49.521	1.00	0.00	H
ATOM	1389	CG	TYR	88	21.132	40.371	47.393	1.00	0.00	C
ATOM	1390	CD1	TYR	88	20.543	39.066	47.236	1.00	0.00	C
ATOM	1391	HD1	TYR	88	19.949	38.582	47.998	1.00	0.00	H
ATOM	1392	CE1	TYR	88	20.742	38.403	46.006	1.00	0.00	C
ATOM	1393	HE1	TYR	88	20.406	37.385	45.880	1.00	0.00	H
ATOM	1394	CZ	TYR	88	21.559	38.954	45.021	1.00	0.00	C
ATOM	1395	OH	TYR	88	21.929	38.218	43.958	1.00	0.00	O
ATOM	1396	HH	TYR	88	21.716	37.289	44.071	1.00	0.00	H
ATOM	1397	CE2	TYR	88	22.221	40.148	45.235	1.00	0.00	C
ATOM	1398	HE2	TYR	88	22.887	40.572	44.499	1.00	0.00	H

ATOM	1399	CD2	TYR	88	22.126	40.775	46.434	1.00	0.00	C
ATOM	1400	HD2	TYR	88	22.784	41.623	46.552	1.00	0.00	H
ATOM	1401	C	TYR	88	19.379	42.548	50.231	1.00	0.00	C
ATOM	1402	O	TYR	88	19.799	43.642	50.603	1.00	0.00	O
ATOM	1403	N	PRO	89	18.552	41.849	51.061	1.00	0.00	N
ATOM	1404	CD	PRO	89	17.533	40.870	50.680	1.00	0.00	C
ATOM	1405	HD2	PRO	89	17.927	39.891	50.407	1.00	0.00	H
ATOM	1406	HD3	PRO	89	17.023	41.238	49.790	1.00	0.00	H
ATOM	1407	CG	PRO	89	16.534	40.815	51.833	1.00	0.00	C
ATOM	1408	HG2	PRO	89	16.196	39.796	52.022	1.00	0.00	H
ATOM	1409	HG3	PRO	89	15.715	41.514	51.662	1.00	0.00	H
ATOM	1410	CB	PRO	89	17.392	41.346	53.023	1.00	0.00	C
ATOM	1411	HB2	PRO	89	17.918	40.521	53.506	1.00	0.00	H
ATOM	1412	HB3	PRO	89	16.774	41.798	53.799	1.00	0.00	H
ATOM	1413	CA	PRO	89	18.385	42.242	52.464	1.00	0.00	C
ATOM	1414	HA	PRO	89	17.968	43.243	52.569	1.00	0.00	H
ATOM	1415	C	PRO	89	19.715	42.082	53.242	1.00	0.00	C
ATOM	1416	O	PRO	89	20.506	41.225	52.961	1.00	0.00	O
ATOM	1417	N	GLY	90	19.771	42.816	54.335	1.00	0.00	N
ATOM	1418	H	GLY	90	19.035	43.486	54.509	1.00	0.00	H
ATOM	1419	CA	GLY	90	20.948	42.825	55.210	1.00	0.00	C
ATOM	1420	HA2	GLY	90	21.787	43.341	54.744	1.00	0.00	H
ATOM	1421	HA3	GLY	90	20.592	43.312	56.118	1.00	0.00	H
ATOM	1422	C	GLY	90	21.523	41.508	55.660	1.00	0.00	C
ATOM	1423	O	GLY	90	22.712	41.253	55.564	1.00	0.00	O
ATOM	1424	N	GLN	91	20.669	40.622	56.068	1.00	0.00	N
ATOM	1425	H	GLN	91	19.753	40.856	55.714	1.00	0.00	H
ATOM	1426	CA	GLN	91	20.976	39.256	56.526	1.00	0.00	C
ATOM	1427	HA	GLN	91	21.778	39.313	57.260	1.00	0.00	H
ATOM	1428	CB	GLN	91	19.694	38.756	57.201	1.00	0.00	C
ATOM	1429	HB2	GLN	91	18.837	38.620	56.541	1.00	0.00	H
ATOM	1430	HB3	GLN	91	19.861	37.699	57.410	1.00	0.00	H
ATOM	1431	CG	GLN	91	19.248	39.345	58.560	1.00	0.00	C
ATOM	1432	HG2	GLN	91	18.670	38.604	59.112	1.00	0.00	H
ATOM	1433	HG3	GLN	91	20.085	39.680	59.172	1.00	0.00	H
ATOM	1434	CD	GLN	91	18.318	40.482	58.470	1.00	0.00	C
ATOM	1435	OE1	GLN	91	18.038	40.960	57.363	1.00	0.00	O
ATOM	1436	NE2	GLN	91	17.816	40.938	59.561	1.00	0.00	N
ATOM	1437	HE21	GLN	91	18.100	40.549	60.449	1.00	0.00	H
ATOM	1438	HE22	GLN	91	17.320	41.817	59.582	1.00	0.00	H
ATOM	1439	C	GLN	91	21.397	38.245	55.443	1.00	0.00	C
ATOM	1440	O	GLN	91	21.803	37.161	55.764	1.00	0.00	O
ATOM	1441	N	THR	92	21.541	38.686	54.242	1.00	0.00	N
ATOM	1442	H	THR	92	21.366	39.665	54.065	1.00	0.00	H
ATOM	1443	CA	THR	92	21.887	37.778	53.193	1.00	0.00	C
ATOM	1444	HA	THR	92	21.186	36.947	53.261	1.00	0.00	H
ATOM	1445	CB	THR	92	21.474	38.434	51.862	1.00	0.00	C
ATOM	1446	HB	THR	92	22.164	39.258	51.681	1.00	0.00	H
ATOM	1447	CG2	THR	92	21.523	37.389	50.688	1.00	0.00	C
ATOM	1448	HG21	THR	92	21.010	37.716	49.784	1.00	0.00	H
ATOM	1449	HG22	THR	92	22.517	37.202	50.283	1.00	0.00	H
ATOM	1450	HG23	THR	92	20.986	36.500	51.022	1.00	0.00	H
ATOM	1451	OG1	THR	92	20.113	38.886	51.882	1.00	0.00	O
ATOM	1452	HG1	THR	92	20.126	39.781	52.229	1.00	0.00	H
ATOM	1453	C	THR	92	23.321	37.314	53.268	1.00	0.00	C
ATOM	1454	O	THR	92	24.271	38.097	53.265	1.00	0.00	O
ATOM	1455	N	GLN	93	23.533	36.045	53.104	1.00	0.00	N
ATOM	1456	H	GLN	93	22.722	35.454	52.982	1.00	0.00	H
ATOM	1457	CA	GLN	93	24.784	35.258	52.874	1.00	0.00	C
ATOM	1458	HA	GLN	93	25.652	35.752	53.310	1.00	0.00	H
ATOM	1459	CB	GLN	93	24.769	33.952	53.642	1.00	0.00	C
ATOM	1460	HB2	GLN	93	24.300	34.232	54.584	1.00	0.00	H
ATOM	1461	HB3	GLN	93	24.152	33.187	53.169	1.00	0.00	H
ATOM	1462	CG	GLN	93	26.167	33.345	53.770	1.00	0.00	C

ATOM	1463	HG2	GLN	93	26.442	32.742	52.905	1.00	0.00	H
ATOM	1464	HG3	GLN	93	26.946	34.034	54.096	1.00	0.00	H
ATOM	1465	CD	GLN	93	26.130	32.365	54.943	1.00	0.00	C
ATOM	1466	OE1	GLN	93	25.142	32.182	55.628	1.00	0.00	O
ATOM	1467	NE2	GLN	93	27.142	31.470	54.977	1.00	0.00	N
ATOM	1468	HE21	GLN	93	27.733	31.383	54.164	1.00	0.00	H
ATOM	1469	HE22	GLN	93	27.012	30.698	55.616	1.00	0.00	H
ATOM	1470	C	GLN	93	25.118	35.014	51.358	1.00	0.00	C
ATOM	1471	O	GLN	93	24.316	34.507	50.515	1.00	0.00	O
ATOM	1472	N	PHE	94	26.357	35.400	51.015	1.00	0.00	N
ATOM	1473	H	PHE	94	26.881	35.895	51.722	1.00	0.00	H
ATOM	1474	CA	PHE	94	26.897	35.266	49.665	1.00	0.00	C
ATOM	1475	HA	PHE	94	26.079	34.948	49.019	1.00	0.00	H
ATOM	1476	CB	PHE	94	27.343	36.658	49.178	1.00	0.00	C
ATOM	1477	HB2	PHE	94	28.116	37.067	49.828	1.00	0.00	H
ATOM	1478	HB3	PHE	94	27.669	36.560	48.143	1.00	0.00	H
ATOM	1479	CG	PHE	94	26.238	37.658	49.171	1.00	0.00	C
ATOM	1480	CD1	PHE	94	25.666	37.956	47.935	1.00	0.00	C
ATOM	1481	HD1	PHE	94	25.979	37.326	47.116	1.00	0.00	H
ATOM	1482	CE1	PHE	94	24.629	38.921	47.796	1.00	0.00	C
ATOM	1483	HE1	PHE	94	24.185	39.204	46.853	1.00	0.00	H
ATOM	1484	CZ	PHE	94	24.281	39.662	48.991	1.00	0.00	C
ATOM	1485	HZ	PHE	94	23.649	40.527	48.858	1.00	0.00	H
ATOM	1486	CE2	PHE	94	25.026	39.510	50.230	1.00	0.00	C
ATOM	1487	HE2	PHE	94						
24.812	40.130	51.088	1.00	0.00	H					
ATOM	1488	CD2	PHE	94	25.964	38.495	50.295	1.00	0.00	C
ATOM	1489	HD2	PHE	94	26.596	38.404	51.165	1.00	0.00	H
ATOM	1490	C	PHE	94	28.132	34.280	49.603	1.00	0.00	C
ATOM	1491	O	PHE	94	29.050	34.290	50.421	1.00	0.00	O
ATOM	1492	N	TYR	95	28.177	33.501	48.578	1.00	0.00	N
ATOM	1493	H	TYR	95	27.498	33.689	47.853	1.00	0.00	H
ATOM	1494	CA	TYR	95	29.388	32.749	48.099	1.00	0.00	C
ATOM	1495	HA	TYR	95	29.878	32.383	49.000	1.00	0.00	H
ATOM	1496	CB	TYR	95	28.742	31.490	47.483	1.00	0.00	C
ATOM	1497	HB2	TYR	95	27.912	31.188	48.122	1.00	0.00	H
ATOM	1498	HB3	TYR	95	28.261	31.857	46.576	1.00	0.00	H
ATOM	1499	CG	TYR	95	29.664	30.317	47.117	1.00	0.00	C
ATOM	1500	CD1	TYR	95	30.322	30.273	45.865	1.00	0.00	C
ATOM	1501	HD1	TYR	95	30.288	31.207	45.326	1.00	0.00	H
ATOM	1502	CE1	TYR	95	30.980	29.158	45.405	1.00	0.00	C
ATOM	1503	HE1	TYR	95	31.435	29.218	44.428	1.00	0.00	H
ATOM	1504	CZ	TYR	95	31.070	27.992	46.200	1.00	0.00	C
ATOM	1505	OH	TYR	95	31.730	26.883	45.721	1.00	0.00	O
ATOM	1506	HH	TYR	95	32.069	27.088	44.846	1.00	0.00	H
ATOM	1507	CE2	TYR	95	30.540	28.078	47.508	1.00	0.00	C
ATOM	1508	HE2	TYR	95	30.578	27.175	48.101	1.00	0.00	H
ATOM	1509	CD2	TYR	95	29.733	29.130	47.924	1.00	0.00	C
ATOM	1510	HD2	TYR	95	29.213	29.128	48.870	1.00	0.00	H
ATOM	1511	C	TYR	95	30.234	33.583	47.164	1.00	0.00	C
ATOM	1512	O	TYR	95	29.865	34.580	46.519	1.00	0.00	O
ATOM	1513	N	VAL	96	31.548	33.203	47.162	1.00	0.00	N
ATOM	1514	H	VAL	96	31.916	32.524	47.813	1.00	0.00	H
ATOM	1515	CA	VAL	96	32.652	33.929	46.558	1.00	0.00	C
ATOM	1516	HA	VAL	96	32.139	34.559	45.833	1.00	0.00	H
ATOM	1517	CB	VAL	96	33.439	34.685	47.624	1.00	0.00	C
ATOM	1518	HB	VAL	96	33.866	33.928	48.282	1.00	0.00	H
ATOM	1519	CG1	VAL	96	34.557	35.531	46.990	1.00	0.00	C
ATOM	1520	HG11	VAL	96	35.259	34.798	46.591	1.00	0.00	H
ATOM	1521	HG12	VAL	96	34.109	36.181	46.239	1.00	0.00	H
ATOM	1522	HG13	VAL	96	35.023	36.177	47.735	1.00	0.00	H
ATOM	1523	CG2	VAL	96	32.456	35.610	48.357	1.00	0.00	C
ATOM	1524	HG21	VAL	96	31.642	35.029	48.791	1.00	0.00	H
ATOM	1525	HG22	VAL	96	32.951	36.208	49.122	1.00	0.00	H

ATOM	1526	HG23	VAL	96	32.032	36.236	47.571	1.00	0.00	H
ATOM	1527	C	VAL	96	33.472	32.906	45.824	1.00	0.00	C
ATOM	1528	O	VAL	96	33.923	31.911	46.423	1.00	0.00	O
ATOM	1529	N	ILE	97	33.857	33.184	44.583	1.00	0.00	N
ATOM	1530	H	ILE	97	33.763	34.132	44.247	1.00	0.00	H
ATOM	1531	CA	ILE	97	34.614	32.232	43.719	1.00	0.00	C
ATOM	1532	HA	ILE	97	34.962	31.313	44.190	1.00	0.00	H
ATOM	1533	CB	ILE	97	33.789	31.762	42.473	1.00	0.00	C
ATOM	1534	HB	ILE	97	33.525	32.622	41.860	1.00	0.00	H
ATOM	1535	CG2	ILE	97	34.505	30.604	41.709	1.00	0.00	C
ATOM	1536	HG21	ILE	97	34.133	30.501	40.690	1.00	0.00	H
ATOM	1537	HG22	ILE	97	35.539	30.915	41.552	1.00	0.00	H
ATOM	1538	HG23	ILE	97	34.560	29.738	42.369	1.00	0.00	H
ATOM	1539	CG1	ILE	97	32.330	31.286	42.810	1.00	0.00	C
ATOM	1540	HG12	ILE	97	32.038	30.372	42.293	1.00	0.00	H
ATOM	1541	HG13	ILE	97	32.375	30.996	43.860	1.00	0.00	H
ATOM	1542	CD1	ILE	97	31.268	32.371	42.622	1.00	0.00	C
ATOM	1543	HD11	ILE	97	30.309	32.070	43.043	1.00	0.00	H
ATOM	1544	HD12	ILE	97	31.627	33.241	43.172	1.00	0.00	H
ATOM	1545	HD13	ILE	97	31.052	32.493	41.561	1.00	0.00	H
ATOM	1546	C	ILE	97	35.870	32.957	43.235	1.00	0.00	C
ATOM	1547	O	ILE	97	35.817	33.966	42.556	1.00	0.00	O
ATOM	1548	N	GLU	98	37.004	32.458	43.672	1.00	0.00	N
ATOM	1549	H	GLU	98	36.969	31.583	44.172	1.00	0.00	H
ATOM	1550	CA	GLU	98	38.280	32.834	43.080	1.00	0.00	C
ATOM	1551	HA	GLU	98	38.174	33.839	42.670	1.00	0.00	H
ATOM	1552	CB	GLU	98	39.376	33.021	44.160	1.00	0.00	C
ATOM	1553	HB2	GLU	98	39.226	32.052	44.633	1.00	0.00	H
ATOM	1554	HB3	GLU	98	40.371	33.005	43.714	1.00	0.00	H
ATOM	1555	CG	GLU	98	39.131	34.154	45.142	1.00	0.00	C
ATOM	1556	HG2	GLU	98	39.733	34.978	44.760	1.00	0.00	H
ATOM	1557	HG3	GLU	98	38.077	34.425	45.213	1.00	0.00	H
ATOM	1558	CD	GLU	98	39.725	33.745	46.499	1.00	0.00	C
ATOM	1559	OE1	GLU	98	38.994	33.206	47.352	1.00	0.00	O
ATOM	1560	OE2	GLU	98	40.969	33.872	46.672	1.00	0.00	O
ATOM	1561	C	GLU	98	38.803	31.850	41.989	1.00	0.00	C
ATOM	1562	O	GLU	98	38.944	30.655	42.227	1.00	0.00	O
ATOM	1563	N	PHE	99	39.158	32.442	40.850	1.00	0.00	N
ATOM	1564	H	PHE	99	38.968	33.430	40.760	1.00	0.00	H
ATOM	1565	CA	PHE	99	39.464	31.756	39.535	1.00	0.00	C
ATOM	1566	HA	PHE	99	39.923	30.801	39.787	1.00	0.00	H
ATOM	1567	CB	PHE	99	38.198	31.478	38.767	1.00	0.00	C
ATOM	1568	HB2	PHE	99	38.425	30.861	37.897	1.00	0.00	H
ATOM	1569	HB3	PHE	99	37.458	30.901	39.321	1.00	0.00	H
ATOM	1570	CG	PHE	99	37.501	32.780	38.353	1.00	0.00	C
ATOM	1571	CD1	PHE	99	36.647	33.372	39.308	1.00	0.00	C
ATOM	1572	HD1	PHE	99	36.505	32.913	40.275	1.00	0.00	H
ATOM	1573	CE1	PHE	99	36.070	34.640	39.054	1.00	0.00	C
ATOM	1574	HE1	PHE	99	35.478	35.144	39.804	1.00	0.00	H
ATOM	1575	CZ	PHE	99	36.329	35.261	37.840	1.00	0.00	C
ATOM	1576	HZ	PHE	99	36.019	36.290	37.732	1.00	0.00	H
ATOM	1577	CE2	PHE	99	37.083	34.617	36.819	1.00	0.00	C
ATOM	1578	HE2	PHE	99	37.223	35.119	35.872	1.00	0.00	H
ATOM	1579	CD2	PHE	99	37.735	33.390	37.089	1.00	0.00	C
ATOM	1580	HD2	PHE	99	38.342	32.936	36.320	1.00	0.00	H
ATOM	1581	C	PHE	99	40.556	32.569	38.712	1.00	0.00	C
ATOM	1582	O	PHE	99	40.700	33.764	38.783	1.00	0.00	O
ATOM	1583	N	LYS	100	41.218	31.816	37.815	1.00	0.00	N
ATOM	1584	H	LYS	100	41.170	30.809	37.756	1.00	0.00	H
ATOM	1585	CA	LYS	100	42.165	32.500	36.827	1.00	0.00	C
ATOM	1586	HA	LYS	100	41.768	33.480	36.560	1.00	0.00	H
ATOM	1587	CB	LYS	100	43.577	32.787	37.397	1.00	0.00	C
ATOM	1588	HB2	LYS	100	44.234	33.346	36.732	1.00	0.00	H
ATOM	1589	HB3	LYS	100	43.368	33.491	38.202	1.00	0.00	H

ATOM	1590	CG	LYS	100	44.355	31.674	38.067	1.00	0.00	C
ATOM	1591	HG2	LYS	100	45.171	32.243	38.511	1.00	0.00	H
ATOM	1592	HG3	LYS	100	43.595	31.343	38.774	1.00	0.00	H
ATOM	1593	CD	LYS	100	44.855	30.510	37.283	1.00	0.00	C
ATOM	1594	HD2	LYS	100	44.101	29.921	36.762	1.00	0.00	H
ATOM	1595	HD3	LYS	100	45.474	30.815	36.439	1.00	0.00	H
ATOM	1596	CE	LYS	100	45.611	29.536	38.208	1.00	0.00	C
ATOM	1597	HE2	LYS	100	44.934	29.244	39.011	1.00	0.00	H
ATOM	1598	HE3	LYS	100	45.879	28.663	37.614	1.00	0.00	H
ATOM	1599	NZ	LYS	100	46.888	30.035	38.702	1.00	0.00	N
ATOM	1600	HZ1	LYS	100	47.488	30.316	37.939	1.00	0.00	H
ATOM	1601	HZ2	LYS	100	46.731	30.861	39.262	1.00	0.00	H
ATOM	1602	HZ3	LYS	100	47.468	29.390	39.219	1.00	0.00	H
ATOM	1603	C	LYS	100	42.280	31.760	35.485	1.00	0.00	C
ATOM	1604	O	LYS	100	41.929	30.561	35.367	1.00	0.00	O
ATOM	1605	N	CYX	101	42.793	32.426	34.443	1.00	0.00	N
ATOM	1606	H	CYX	101	43.092	33.360	34.681	1.00	0.00	H
ATOM	1607	CA	CYX	101	43.097	31.825	33.111	1.00	0.00	C
ATOM	1608	HA	CYX	101	42.353	31.108	32.762	1.00	0.00	H
ATOM	1609	CB	CYX	101	42.957	32.949	32.098	1.00	0.00	C
ATOM	1610	HB2	CYX	101	41.916	33.265	32.152	1.00	0.00	H
ATOM	1611	HB3	CYX	101	43.654	33.772	32.257	1.00	0.00	H
ATOM	1612	SG	CYX	101	43.235	32.421	30.358	1.00		
0.00			S							
ATOM	1613	C	CYX	101	44.536	31.134	33.185	1.00	0.00	C
ATOM	1614	O	CYX	101	45.445	31.731	33.730	1.00	0.00	O
ATOM	1615	N	LEU	102	44.538	29.889	32.638	1.00	0.00	N
ATOM	1616	H	LEU	102	43.685	29.634	32.161	1.00	0.00	H
ATOM	1617	CA	LEU	102	45.740	29.024	32.522	1.00	0.00	C
ATOM	1618	HA	LEU	102	46.428	29.152	33.357	1.00	0.00	H
ATOM	1619	CB	LEU	102	45.155	27.581	32.440	1.00	0.00	C
ATOM	1620	HB2	LEU	102	44.394	27.609	31.660	1.00	0.00	H
ATOM	1621	HB3	LEU	102	46.025	26.970	32.198	1.00	0.00	H
ATOM	1622	CG	LEU	102	44.499	26.930	33.631	1.00	0.00	C
ATOM	1623	HG	LEU	102	43.606	27.507	33.873	1.00	0.00	H
ATOM	1624	CD1	LEU	102	43.980	25.609	33.156	1.00	0.00	C
ATOM	1625	HD11	LEU	102	43.700	25.076	34.065	1.00	0.00	H
ATOM	1626	HD12	LEU	102	43.114	25.695	32.499	1.00	0.00	H
ATOM	1627	HD13	LEU	102	44.732	25.054	32.595	1.00	0.00	H
ATOM	1628	CD2	LEU	102	45.506	26.847	34.766	1.00	0.00	C
ATOM	1629	HD21	LEU	102	46.398	26.246	34.580	1.00	0.00	H
ATOM	1630	HD22	LEU	102	45.924	27.819	35.028	1.00	0.00	H
ATOM	1631	HD23	LEU	102	44.988	26.420	35.624	1.00	0.00	H
ATOM	1632	C	LEU	102	46.691	29.442	31.390	1.00	0.00	C
ATOM	1633	O	LEU	102	46.298	30.082	30.397	1.00	0.00	O
ATOM	1634	OXT	LEU	102	47.831	29.019	31.448	1.00	0.00	O
HETATM	314	N	LYN	20	39.731	40.498	36.155	1.00	0.00	N
HETATM	315	H	LYN	20	40.137	40.076	35.332	1.00	0.00	H
HETATM	316	CA	LYN	20	38.603	39.687	36.716	1.00	0.00	C
HETATM	317	HA	LYN	20	38.357	40.167	37.664	1.00	0.00	H
HETATM	318	CB	LYN	20	37.364	39.725	35.740	1.00	0.00	C
HETATM	319	HB2	LYN	20	37.649	39.281	34.786	1.00	0.00	H
HETATM	320	HB3	LYN	20	37.054	40.743	35.504	1.00	0.00	H
HETATM	321	CG	LYN	20	36.106	39.087	36.331	1.00	0.00	C
HETATM	322	HG2	LYN	20	35.240	39.328	35.717	1.00	0.00	H
HETATM	323	HG3	LYN	20	36.222	38.003	36.288	1.00	0.00	H
HETATM	324	CD	LYN	20	35.675	39.578	37.718	1.00	0.00	C
HETATM	325	HD2	LYN	20	35.817	40.658	37.753	1.00	0.00	H
HETATM	326	HD3	LYN	20	36.400	39.154	38.413	1.00	0.00	H
HETATM	327	CE	LYN	20	34.216	39.201	37.876	1.00	0.00	C
HETATM	328	HE2	LYN	20	33.647	39.667	37.071	1.00	0.00	H
HETATM	329	HE3	LYN	20	34.164	38.112	37.876	1.00	0.00	H
HETATM	330	NZ	LYN	20	33.695	39.653	39.148	1.00	0.00	N
HETATM	331	HZ2	LYN	20	33.311	40.565	38.948	1.00	0.00	H

```
HETATM  332  HZ3 LYN    20      34.508  39.735  39.742  1.00  0.00           H
HETATM  333  C   LYN    20      39.070  38.321  37.134  1.00  0.00           C
HETATM  334  O   LYN    20      39.420  37.435  36.338  1.00  0.00           O
HETATM 1636  N   LIG   103      30.307  41.854  39.940  1.00  0.00           N
HETATM 1637  C   LIG   103      30.100  40.873  39.144  1.00  0.00           C
HETATM 1638  O   LIG   103      30.094  43.060  41.768  1.00  0.00           O
HETATM 1639  C5' LIG   103      25.576  39.063  44.349  1.00  0.00           C
HETATM 1640  O5' LIG   103      25.739  38.252  43.170  1.00  0.00           O
HETATM 1641  C4' LIG   103      26.241  40.450  44.118  1.00  0.00           C
HETATM 1642  O4' LIG   103      27.570  40.259  43.522  1.00  0.00           O
HETATM 1643  C3' LIG   103      25.537  41.237  42.959  1.00  0.00           C
HETATM 1644  O3' LIG   103      24.438  42.002  43.513  1.00  0.00           O
HETATM 1645  C2' LIG   103      26.701  42.171  42.425  1.00  0.00           C
HETATM 1646  O2' LIG   103      26.521  43.348  43.119  1.00  0.00           O
HETATM 1647  C1' LIG   103      28.023  41.484  42.822  1.00  0.00           C
HETATM 1648  N1  LIG   103      28.796  41.155  41.569  1.00  0.00           N
HETATM 1649  O1  LIG   103      30.570  38.453  37.499  1.00  0.00           O
HETATM 1650  N2  LIG   103      30.746  40.770  37.977  1.00  0.00           N
HETATM 1651  C6  LIG   103      29.683  42.084  41.115  1.00  0.00           C
HETATM 1652  C7  LIG   103      28.408  40.083  40.787  1.00  0.00           C
HETATM 1653  C8  LIG   103      28.970  40.038  39.481  1.00  0.00           C
HETATM 1654  C9  LIG   103      30.717  39.637  37.126  1.00  0.00           C
HETATM 1655  C10 LIG   103      31.225  39.912  35.704  1.00  0.00           C
HETATM 1656  H   LIG   103      31.221  41.615  37.684  1.00  0.00           H
HETATM 1657  H1  LIG   103      28.686  39.232  38.815  1.00  0.00           H
HETATM 1658  H4  LIG   103      28.622  42.021  43.567  1.00  0.00           H
HETATM 1659  H6  LIG   103      26.081  41.163  44.937  1.00  0.00           H
HETATM 1660  H7  LIG   103      25.167  40.618  42.132  1.00  0.00           H
HETATM 1661  H8  LIG   103      26.538  42.291  41.346  1.00  0.00           H
HETATM 1662  H9  LIG   103      24.521  39.096  44.648  1.00  0.00           H
HETATM 1663  H10 LIG   103      26.105  38.606  45.195  1.00  0.00           H
HETATM 1664  H11 LIG   103      31.995  40.678  35.553  1.00  0.00           H
HETATM 1665  H12 LIG   103      30.450  40.088  34.948  1.00  0.00           H
HETATM 1666  H13 LIG   103      31.642  38.982  35.297  1.00  0.00           H
HETATM 1667  H14 LIG   103      27.517  39.534  41.062  1.00  0.00           H
HETATM 1668  H2' LIG   103      25.700  43.244  43.630  1.00  0.00           H
HETATM 1669  H3' LIG   103      23.808  42.142  42.785  1.00  0.00           H
HETATM 1670  H5' LIG   103      26.665  38.133  42.895  1.00  0.00           H
ENDMDL
MODEL     11
SHEET    1   1 1 ILE    22  ASP    26  0
SHEET    2   2 1 VAL    37  VAL    40  0
SHEET    3   3 1 CYX    50  VAL    60  0
SHEET    4   4 1 PHE    94  CYX   101  0
HELIX    1   1 GLN     12  LEU     16  1                                   5
HELIX    2   2 LYS     68  GLN     72  1                                   5
HELIX    3   3 LEU     77  ILE     87  1                                  11
ATOM      1  N   GLN     1      35.085  18.242  33.839  1.00  0.00           N
ATOM      2  H1  GLN     1      35.359  17.270  33.860  1.00  0.00           H
ATOM      3  H2  GLN     1      35.910  18.757  34.112  1.00  0.00           H
ATOM      4  H3  GLN     1      34.433  18.529  34.555  1.00  0.00           H
ATOM      5  CA  GLN     1      34.580  18.592  32.496  1.00  0.00           C
ATOM      6  HA  GLN     1      35.356  18.292  31.791  1.00  0.00           H
ATOM      7  CB  GLN     1      33.400  17.696  32.011  1.00  0.00           C
ATOM      8  HB2 GLN     1      32.500  17.929  32.581  1.00  0.00           H
ATOM      9  HB3 GLN     1      33.209  18.002  30.982  1.00  0.00           H
ATOM     10  CG  GLN     1      33.689  16.174  32.074  1.00  0.00           C
ATOM     11  HG2 GLN     1      34.062  15.835  33.041  1.00  0.00           H
ATOM     12  HG3 GLN     1      32.844  15.600  31.694  1.00  0.00           H
ATOM     13  CD  GLN     1      34.707  15.860  30.973  1.00  0.00           C
ATOM     14  OE1 GLN     1      35.233  16.751  30.317  1.00  0.00           O
ATOM     15  NE2 GLN     1      35.020  14.596  30.812  1.00  0.00           N
ATOM     16 HE21 GLN     1      34.549  13.911  31.385  1.00  0.00           H
ATOM     17 HE22 GLN     1      35.965  14.403  30.513  1.00  0.00           H
```

ATOM	18	C	GLN	1	34.288	20.080	32.262	1.00	0.00	C
ATOM	19	O	GLN	1	33.923	20.735	33.276	1.00	0.00	O
ATOM	20	N	PRO	2	34.381	20.764	31.093	1.00	0.00	N
ATOM	21	CD	PRO	2	34.790	20.240	29.797	1.00	0.00	C
ATOM	22	HD2	PRO	2	33.972	19.935	29.145	1.00	0.00	H
ATOM	23	HD3	PRO	2	35.502	19.417	29.832	1.00	0.00	H
ATOM	24	CG	PRO	2	35.510	21.432	29.116	1.00	0.00	C
ATOM	25	HG2	PRO	2	35.553	21.343	28.030	1.00	0.00	H
ATOM	26	HG3	PRO	2	36.534	21.455	29.489	1.00	0.00	H
ATOM	27	CB	PRO	2	34.746	22.523	29.560	1.00	0.00	C
ATOM	28	HB2	PRO	2	33.826	22.570	28.975	1.00	0.00	H
ATOM	29	HB3	PRO	2	35.322	23.439	29.428	1.00	0.00	H
ATOM	30	CA	PRO	2	34.233	22.205	30.972	1.00	0.00	C
ATOM	31	HA	PRO	2	34.863	22.719	31.699	1.00	0.00	H
ATOM	32	C	PRO	2	32.755	22.648	31.190	1.00	0.00	C
ATOM	33	O	PRO	2	31.868	22.166	30.412	1.00	0.00	O
ATOM	34	N	ASN	3	32.519	23.716	31.988	1.00	0.00	N
ATOM	35	H	ASN	3	33.358	24.068	32.425	1.00	0.00	H
ATOM	36	CA	ASN	3	31.222	24.225	32.388	1.00	0.00	C
ATOM	37	HA	ASN	3	30.559	23.419	32.702	1.00	0.00	H
ATOM	38	CB	ASN	3	31.358	25.155	33.623	1.00	0.00	C
ATOM	39	HB2	ASN	3	32.277	25.742	33.600	1.00	0.00	H
ATOM	40	HB3	ASN	3	30.545	25.881	33.653	1.00	0.00	H
ATOM	41	CG	ASN	3	31.200	24.391	34.916	1.00	0.00	C
ATOM	42	OD1	ASN	3	30.206	23.822	35.267	1.00	0.00	O
ATOM	43	ND2	ASN	3	32.160	24.407	35.788	1.00	0.00	N
ATOM	44	HD21	ASN	3	33.141	24.588	35.637	1.00	0.00	H
ATOM	45	HD22	ASN	3	31.957	24.008	36.693	1.00	0.00	H
ATOM	46	C	ASN	3	30.467	24.915	31.259	1.00	0.00	C
ATOM	47	O	ASN	3	31.116	25.476	30.357	1.00	0.00	O
ATOM	48	N	ASP	4	29.086	24.818	31.231	1.00	0.00	N
ATOM	49	H	ASP	4	28.608	24.345	31.985	1.00	0.00	H
ATOM	50	CA	ASP	4	28.201	25.433	30.259	1.00	0.00	C
ATOM	51	HA	ASP	4	28.686	25.509	29.286	1.00	0.00	H
ATOM	52	CB	ASP	4	26.988	24.502	29.983	1.00	0.00	C
ATOM	53	HB2	ASP	4	26.209	24.477	30.745	1.00	0.00	H
ATOM	54	HB3	ASP	4	26.481	24.840	29.079	1.00	0.00	H
ATOM	55	CG	ASP	4	27.377	23.122	29.617	1.00	0.00	C
ATOM	56	OD1	ASP	4	28.114	22.875	28.646	1.00	0.00	O
ATOM	57	OD2	ASP	4	26.883	22.249	30.352	1.00	0.00	O
ATOM	58	C	ASP	4	27.799	26.818	30.589	1.00	0.00	C
ATOM	59	O	ASP	4	27.588	27.599	29.659	1.00	0.00	O
ATOM	60	N	ILE	5	27.725	27.251	31.840	1.00	0.00	N
ATOM	61	H	ILE	5	27.928	26.583	32.569	1.00	0.00	H
ATOM	62	CA	ILE	5	27.578	28.607	32.272	1.00	0.00	C
ATOM	63	HA	ILE	5	27.081	29.189	31.496	1.00	0.00	H
ATOM	64	CB	ILE	5	26.749	28.741	33.611	1.00	0.00	C
ATOM	65	HB	ILE	5	27.243	28.282	34.468	1.00	0.00	H
ATOM	66	CG2	ILE	5	26.512	30.142	34.150	1.00	0.00	C
ATOM	67	HG21	ILE	5	27.448	30.648	34.387	1.00	0.00	H
ATOM	68	HG22	ILE	5	25.870	30.681	33.452	1.00	0.00	H
ATOM	69	HG23	ILE	5	25.919	30.038	35.060	1.00	0.00	H
ATOM	70	CG1	ILE	5	25.371	28.076	33.583	1.00	0.00	C
ATOM	71	HG12	ILE	5	25.397	27.030	33.277	1.00	0.00	H
ATOM	72	HG13	ILE	5	24.858	28.252	34.528	1.00	0.00	H
ATOM	73	CD1	ILE	5	24.373	28.359	32.494	1.00	0.00	C
ATOM	74	HD11	ILE	5	23.354	28.011	32.659	1.00	0.00	H
ATOM	75	HD12	ILE	5	24.424	29.433	32.312	1.00	0.00	H
ATOM	76	HD13	ILE	5	24.674	27.940	31.533	1.00	0.00	H
ATOM	77	C	ILE	5	28.967	29.268	32.365	1.00	0.00	C
ATOM	78	O	ILE	5	29.939	28.654	32.801	1.00	0.00	O
ATOM	79	N	THR	6	29.059	30.539	31.954	1.00	0.00	N
ATOM	80	H	THR	6	28.267	31.000	31.529	1.00	0.00	H

ATOM	81	CA	THR	6	30.296	31.318	31.930	1.00	0.00	C
ATOM	82	HA	THR	6	30.911	30.844	32.694	1.00	0.00	H
ATOM	83	CB	THR	6	30.982	31.084	30.600	1.00	0.00	C
ATOM	84	HB	THR	6	31.281	30.039	30.525	1.00	0.00	H
ATOM	85	CG2	THR	6	30.207	31.446	29.353	1.00	0.00	C
ATOM	86	HG21	THR	6	29.329	30.833	29.155	1.00	0.00	H
ATOM	87	HG22	THR	6	29.921	32.495	29.435	1.00	0.00	H
ATOM	88	HG23	THR	6	30.813	31.185	28.486	1.00	0.00	H
ATOM	89	OG1	THR	6	32.182	31.812	30.577	1.00	0.00	O
ATOM	90	HG1	THR	6	32.901	31.390	30.100	1.00	0.00	H
ATOM	91	C	THR	6	30.039	32.887	32.132	1.00	0.00	C
ATOM	92	O	THR	6	28.924	33.373	32.007	1.00	0.00	O
ATOM	93	N	PHE	7	31.064	33.654	32.386	1.00	0.00	N
ATOM	94	H	PHE	7	31.923	33.139	32.257	1.00	0.00	H
ATOM	95	CA	PHE	7	31.052	35.131	32.487	1.00	0.00	C
ATOM	96	HA	PHE	7	30.384	35.440	33.292	1.00	0.00	H
ATOM	97	CB	PHE	7	32.489	35.597	32.832	1.00	0.00	C
ATOM	98	HB2	PHE	7	33.102	35.143	32.051	1.00	0.00	H
ATOM	99	HB3	PHE	7	32.722	36.651	32.686	1.00	0.00	H
ATOM	100	CG	PHE	7	32.937	35.191	34.256	1.00	0.00	C
ATOM	101	CD1	PHE	7	33.413	33.879	34.569	1.00	0.00	C
ATOM	102	HD1	PHE	7	33.645	33.235	33.734	1.00	0.00	H
ATOM	103	CE1	PHE	7	33.543	33.447	35.879	1.00	0.00	C
ATOM	104	HE1	PHE	7	33.790	32.422	36.109	1.00	0.00	H
ATOM	105	CZ	PHE	7	33.367	34.392	36.879	1.00	0.00	C
ATOM	106	HZ	PHE	7	33.535	34.051	37.889	1.00	0.00	H
ATOM	107	CE2	PHE	7	32.943	35.702	36.602	1.00	0.00	C
ATOM	108	HE2	PHE	7	32.824	36.433	37.389	1.00	0.00	H
ATOM	109	CD2	PHE	7	32.751	36.112	35.284	1.00	0.00	C
ATOM	110	HD2	PHE	7	32.461	37.116	35.013	1.00	0.00	H
ATOM	111	C	PHE	7	30.610	35.689	31.099	1.00	0.00	C
ATOM	112	O	PHE	7	30.772	35.076	29.994	1.00	0.00	O
ATOM	113	N	PHE	8	30.070	36.848	31.077	1.00	0.00	N
ATOM	114	H	PHE	8	29.943	37.273	31.985	1.00	0.00	H
ATOM	115	CA	PHE	8	29.584	37.667	29.923	1.00	0.00	C
ATOM	116	HA	PHE	8	29.234	36.999	29.137	1.00	0.00	H
ATOM	117	CB	PHE	8	28.358	38.579	30.284	1.00	0.00	C
ATOM	118	HB2	PHE	8	28.632	39.214	31.126	1.00	0.00	H
ATOM	119	HB3	PHE	8	27.967	39.157	29.446	1.00	0.00	H
ATOM	120	CG	PHE	8	27.090	37.851	30.884	1.00	0.00	C
ATOM	121	CD1	PHE	8	26.675	38.111	32.182	1.00	0.00	C
ATOM	122	HD1	PHE	8	27.144	38.882	32.774	1.00	0.00	H
ATOM	123	CE1	PHE	8	25.531	37.548	32.779	1.00	0.00	C
ATOM	124	HE1	PHE	8	25.357	37.663	33.839	1.00	0.00	H
ATOM	125	CZ	PHE	8	24.823	36.612	32.014	1.00	0.00	C
ATOM	126	HZ	PHE	8	23.961	36.096	32.411	1.00	0.00	H
ATOM	127	CE2	PHE	8	25.082	36.419	30.616	1.00	0.00	C
ATOM	128	HE2	PHE	8	24.431	35.837	29.980	1.00	0.00	H
ATOM	129	CD2	PHE	8	26.306	37.004	30.103	1.00	0.00	C
ATOM	130	HD2	PHE	8	26.603	36.702	29.110	1.00	0.00	H
ATOM	131	C	PHE	8	30.682	38.479	29.255	1.00	0.00	C
ATOM	132	O	PHE	8	31.806	38.708	29.737	1.00	0.00	O
ATOM	133	N	GLN	9	30.244	39.065	28.089	1.00	0.00	N
ATOM	134	H	GLN	9	29.242	39.089	27.966	1.00	0.00	H
ATOM	135	CA	GLN	9	31.138	39.427	27.055	1.00	0.00	C
ATOM	136	HA	GLN	9	31.793	38.559	27.144	1.00	0.00	H
ATOM	137	CB	GLN	9	30.438	39.419	25.725	1.00	0.00	C
ATOM	138	HB2	GLN	9	29.664	40.187	25.710	1.00	0.00	H
ATOM	139	HB3	GLN	9	31.254	39.599	25.027	1.00	0.00	H
ATOM	140	CG	GLN	9	29.920	38.015	25.241	1.00	0.00	C
ATOM	141	HG2	GLN	9	29.646	38.092	24.190	1.00	0.00	H
ATOM	142	HG3	GLN	9	30.746	37.305	25.294	1.00	0.00	H
ATOM	143	CD	GLN	9	28.713	37.446	25.999	1.00	0.00	C
ATOM	144	OE1	GLN	9	27.964	38.083	26.749	1.00	0.00	O

ATOM	145	NE2	GLN	9	28.269	36.313	25.675	1.00	0.00	N
ATOM	146	HE21	GLN	9	27.407	36.047	26.129	1.00	0.00	H
ATOM	147	HE22	GLN	9	28.769	35.658	25.091	1.00	0.00	H
ATOM	148	C	GLN	9	32.033	40.568	27.375	1.00	0.00	C
ATOM	149	O	GLN	9	33.110	40.650	26.809	1.00	0.00	O
ATOM	150	N	ARG	10	31.614	41.524	28.209	1.00	0.00	N
ATOM	151	H	ARG	10	30.633	41.542	28.451	1.00	0.00	H
ATOM	152	CA	ARG	10	32.395	42.767	28.555	1.00	0.00	C
ATOM	153	HA	ARG	10	32.834	43.212	27.663	1.00	0.00	H
ATOM	154	CB	ARG	10	31.446	43.878	29.201	1.00	0.00	C
ATOM	155	HB2	ARG	10	30.587	43.324	29.579	1.00	0.00	H
ATOM	156	HB3	ARG	10	32.007	44.405	29.973	1.00	0.00	H
ATOM	157	CG	ARG	10	31.026	44.960	28.153	1.00	0.00	C
ATOM	158	HG2	ARG	10	32.016	45.373	27.962	1.00	0.00	H
ATOM	159	HG3	ARG	10	30.661	44.391	27.297	1.00	0.00	H
ATOM	160	CD	ARG	10	29.992	46.019	28.599	1.00	0.00	C
ATOM	161	HD2	ARG	10	29.078	45.443	28.745	1.00	0.00	H
ATOM	162	HD3	ARG	10	30.219	46.427	29.584	1.00	0.00	H
ATOM	163	NE	ARG	10	29.805	47.149	27.653	1.00	0.00	N
ATOM	164	HE	ARG	10	30.372	47.212	26.820	1.00	0.00	H
ATOM	165	CZ	ARG	10	29.136	48.246	27.898	1.00	0.00	C
ATOM	166	NH1	ARG	10	28.282	48.338	28.791	1.00	0.00	N
ATOM	167	HH11	ARG	10	27.864	49.256	28.763	1.00	0.00	H
ATOM	168	HH12	ARG	10	28.211	47.637	29.515	1.00	0.00	H
ATOM	169	NH2	ARG	10	29.279	49.228	27.063	1.00	0.00	N
ATOM	170	HH21	ARG	10	29.985	49.188	26.342	1.00	0.00	H
ATOM	171	HH22	ARG	10	28.799	50.104	27.211	1.00	0.00	H
ATOM	172	C	ARG	10	33.611	42.619	29.470	1.00	0.00	C
ATOM	173	O	ARG	10	34.286	43.626	29.740	1.00	0.00	O
ATOM	174	N	PHE	11	33.770	41.379	29.926	1.00	0.00	N
ATOM	175	H	PHE	11	32.969	40.776	29.804	1.00	0.00	H
ATOM	176	CA	PHE	11	34.664	40.929	31.020	1.00	0.00	C
ATOM	177	HA	PHE	11	35.473	41.634	31.207	1.00	0.00	H
ATOM	178	CB	PHE	11	33.862	40.782	32.316	1.00	0.00	C
ATOM	179	HB2	PHE	11	34.569	40.787	33.147	1.00	0.00	H
ATOM	180	HB3	PHE	11	33.524	39.750	32.401	1.00	0.00	H
ATOM	181	CG	PHE	11	32.670	41.708	32.485	1.00	0.00	C
ATOM	182	CD1	PHE	11	32.847	43.095	32.845	1.00	0.00	C
ATOM	183	HD1	PHE	11	33.843	43.513	32.874	1.00	0.00	H
ATOM	184	CE1	PHE	11	31.731	43.844	33.085	1.00	0.00	C
ATOM	185	HE1	PHE	11	31.981	44.853	33.374	1.00	0.00	H
ATOM	186	CZ	PHE	11	30.427	43.423	32.881	1.00	0.00	C
ATOM	187	HZ	PHE	11	29.620	44.088	33.151	1.00	0.00	H
ATOM	188	CE2	PHE	11	30.217	42.132	32.487	1.00	0.00	C
ATOM	189	HE2	PHE	11	29.191	41.824	32.352	1.00	0.00	H
ATOM	190	CD2	PHE	11	31.352	41.266	32.284	1.00	0.00	C
ATOM	191	HD2	PHE	11	31.205	40.222	32.052	1.00	0.00	H
ATOM	192	C	PHE	11	35.410	39.663	30.638	1.00	0.00	C
ATOM	193	O	PHE	11	36.309	39.200	31.333	1.00	0.00	O
ATOM	194	N	GLN	12	35.172	39.025	29.509	1.00	0.00	N
ATOM	195	H	GLN	12	34.390	39.368	28.969	1.00	0.00	H
ATOM	196	CA	GLN	12	35.963	37.957	28.975	1.00	0.00	C
ATOM	197	HA	GLN	12	36.008	37.091	29.637	1.00	0.00	H
ATOM	198	CB	GLN	12	35.391	37.583	27.604	1.00	0.00	C
ATOM	199	HB2	GLN	12	35.134	38.488	27.052	1.00	0.00	H
ATOM	200	HB3	GLN	12	36.127	36.997	27.053	1.00	0.00	H
ATOM	201	CG	GLN	12	34.009	36.756	27.673	1.00	0.00	C
ATOM	202	HG2	GLN	12	34.211	35.806	28.167	1.00	0.00	H
ATOM	203	HG3	GLN	12	33.362	37.425	28.239	1.00	0.00	H
ATOM	204	CD	GLN	12	33.573	36.407	26.227	1.00	0.00	C
ATOM	205	OE1	GLN	12	34.063	36.845	25.171	1.00	0.00	O
ATOM	206	NE2	GLN	12	32.665	35.421	26.082	1.00	0.00	N
ATOM	207	HE21	GLN	12	32.364	34.934	26.915	1.00	0.00	H

ATOM	208	HE22	GLN	12	32.715	35.009	25.162	1.00	0.00	H
ATOM	209	C	GLN	12	37.413	38.481	28.721	1.00	0.00	C
ATOM	210	O	GLN	12	38.368	37.890	29.157	1.00	0.00	O
ATOM	211	N	ASP	13	37.669	39.618	28.091	1.00	0.00	N
ATOM	212	H	ASP	13	36.956	40.239	27.739	1.00	0.00	H
ATOM	213	CA	ASP	13	39.023	40.123	27.965	1.00	0.00	C
ATOM	214	HA	ASP	13	39.686	39.364	27.550	1.00	0.00	H
ATOM	215	CB	ASP	13	39.154	41.255	26.892	1.00	0.00	C
ATOM	216	HB2	ASP	13	38.887	42.198	27.368	1.00	0.00	H
ATOM	217	HB3	ASP	13	40.227	41.380	26.745	1.00	0.00	H
ATOM	218	CG	ASP	13	38.522	41.035	25.468	1.00	0.00	C
ATOM	219	OD1	ASP	13	38.195	41.968	24.733	1.00	0.00	O
ATOM	220	OD2	ASP	13	38.190	39.887	25.105	1.00	0.00	O
ATOM	221	C	ASP	13	39.626	40.552	29.286	1.00	0.00	C
ATOM	222	O	ASP	13	40.877	40.492	29.466	1.00	0.00	O
ATOM	223	N	ASP	14	38.934	41.016	30.286	1.00	0.00	N
ATOM	224	H	ASP	14	37.946	41.175	30.156	1.00	0.00	H
ATOM	225	CA	ASP	14	39.295	41.166	31.722	1.00	0.00	C
ATOM	226	HA	ASP	14	40.053	41.949	31.676	1.00	0.00	H
ATOM	227	CB	ASP	14	38.186	41.756	32.619	1.00	0.00	C
ATOM	228	HB2	ASP	14	37.376	41.028	32.620	1.00	0.00	H
ATOM	229	HB3	ASP	14	38.705	41.767	33.577	1.00	0.00	H
ATOM	230	CG	ASP	14	37.683	43.093	32.254	1.00	0.00	C
ATOM	231	OD1	ASP	14	36.589	43.626	32.576	1.00	0.00	O
ATOM	232	OD2	ASP	14	38.439	43.806	31.569	1.00	0.00	O
ATOM	233	C	ASP	14	39.817	39.878	32.350	1.00	0.00	C
ATOM	234	O	ASP	14	40.865	40.021	33.013	1.00	0.00	O
ATOM	235	N	ILE	15	39.153	38.727	32.096	1.00	0.00	N
ATOM	236	H	ILE	15	38.290	38.762	31.573	1.00	0.00	H
ATOM	237	CA	ILE	15	39.731	37.477	32.641	1.00	0.00	C
ATOM	238	HA	ILE	15	40.071	37.640	33.664	1.00	0.00	H
ATOM	239	CB	ILE	15	38.695	36.372	32.481	1.00	0.00	C
ATOM	240	HB	ILE	15	38.439	36.481	31.427	1.00	0.00	H
ATOM	241	CG2	ILE	15	39.157	34.934	32.741	1.00	0.00	C
ATOM	242	HG21	ILE	15	38.381	34.185	32.587	1.00	0.00	H
ATOM	243	HG22	ILE	15	39.918	34.698	31.996	1.00	0.00	H
ATOM	244	HG23	ILE	15	39.631	34.809	33.715	1.00	0.00	H
ATOM	245	CG1	ILE	15	37.420	36.735	33.202	1.00	0.00	C
ATOM	246	HG12	ILE	15	37.732	36.756	34.247	1.00	0.00	H
ATOM	247	HG13	ILE	15	36.973	37.690	32.928	1.00	0.00	H
ATOM	248	CD1	ILE	15	36.269	35.775	33.167	1.00	0.00	C
ATOM	249	HD11	ILE	15	36.510	34.801	33.595	1.00	0.00	H
ATOM	250	HD12	ILE	15	35.398	36.046	33.765	1.00	0.00	H
ATOM	251	HD13	ILE	15	35.930	35.585	32.149	1.00	0.00	H
ATOM	252	C	ILE	15	41.083	37.128	31.892	1.00	0.00	C
ATOM	253	O	ILE	15	41.919	36.519	32.567	1.00	0.00	O
ATOM	254	N	LEU	16	41.329	37.442	30.644	1.00	0.00	N
ATOM	255	H	LEU	16	40.657	38.029	30.171	1.00	0.00	H
ATOM	256	CA	LEU	16	42.567	37.183	29.909	1.00	0.00	C
ATOM	257	HA	LEU	16	42.770	36.133	30.123	1.00	0.00	H
ATOM	258	CB	LEU	16	42.206	37.537	28.419	1.00	0.00	C
ATOM	259	HB2	LEU	16	41.382	36.920	28.059	1.00	0.00	H
ATOM	260	HB3	LEU	16	42.018	38.611	28.418	1.00	0.00	H
ATOM	261	CG	LEU	16	43.409	37.332	27.439	1.00	0.00	C
ATOM	262	HG	LEU	16	44.188	37.997	27.812	1.00	0.00	H
ATOM	263	CD1	LEU	16	43.920	35.874	27.270	1.00	0.00	C
ATOM	264	HD11	LEU	16	44.311	35.644	28.261	1.00	0.00	H
ATOM	265	HD12	LEU	16	43.133	35.143	27.086	1.00	0.00	H
ATOM	266	HD13	LEU	16	44.684	35.810	26.496	1.00	0.00	H
ATOM	267	CD2	LEU	16	42.978	37.772	26.034	1.00	0.00	C
ATOM	268	HD21	LEU	16	42.561	38.778	26.001	1.00	0.00	H
ATOM	269	HD22	LEU	16	43.899	37.702	25.455	1.00	0.00	H
ATOM	270	HD23	LEU	16	42.220	37.083	25.663	1.00	0.00	H
ATOM	271	C	LEU	16	43.746	38.081	30.372	1.00	0.00	C

ATOM	272	O	LEU	16	44.875	37.587	30.475	1.00	0.00	O
ATOM	273	N	ALA	17	43.561	39.403	30.555	1.00	0.00	N
ATOM	274	H	ALA	17	42.696	39.763	30.179	1.00	0.00	H
ATOM	275	CA	ALA	17	44.423	40.310	31.305	1.00	0.00	C
ATOM	276	HA	ALA	17	45.414	40.189	30.867	1.00	0.00	H
ATOM	277	CB	ALA	17	43.927	41.756	31.093	1.00	0.00	C
ATOM	278	HB1	ALA	17	44.060	42.045	30.050	1.00	0.00	H
ATOM	279	HB2	ALA	17	42.870	41.816	31.354	1.00	0.00	H
ATOM	280	HB3	ALA	17	44.495	42.422	31.742	1.00	0.00	H
ATOM	281	C	ALA	17	44.420	40.102	32.859	1.00	0.00	C
ATOM	282	O	ALA	17	45.024	40.865	33.639	1.00	0.00	O
ATOM	283	N	GLY	18	43.531	39.166	33.263	1.00	0.00	N
ATOM	284	H	GLY	18	43.110	38.489	32.643	1.00	0.00	H
ATOM	285	CA	GLY	18	43.460	38.771	34.664	1.00	0.00	C
ATOM	286	HA2	GLY	18	42.738	37.967	34.805	1.00	0.00	H
ATOM	287	HA3	GLY	18	44.500	38.557	34.911	1.00	0.00	H
ATOM	288	C	GLY	18	43.044	39.934	35.621	1.00	0.00	C
ATOM	289	O	GLY	18	43.226	39.860	36.810	1.00	0.00	O
ATOM	290	N	ARG	19	42.305	40.971	35.086	1.00	0.00	N
ATOM	291	H	ARG	19	42.129	40.886	34.095	1.00	0.00	H
ATOM	292	CA	ARG	19	41.670	42.115	35.833	1.00	0.00	C
ATOM	293	HA	ARG	19	42.390	42.472				
36.569	1.00	0.00			H					
ATOM	294	CB	ARG	19	41.393	43.274	34.826	1.00	0.00	C
ATOM	295	HB2	ARG	19	40.755	42.976	33.994	1.00	0.00	H
ATOM	296	HB3	ARG	19	40.860	44.093	35.310	1.00	0.00	H
ATOM	297	CG	ARG	19	42.704	43.870	34.453	1.00	0.00	C
ATOM	298	HG2	ARG	19	43.023	44.682	35.105	1.00	0.00	H
ATOM	299	HG3	ARG	19	43.484	43.112	34.528	1.00	0.00	H
ATOM	300	CD	ARG	19	42.625	44.423	33.062	1.00	0.00	C
ATOM	301	HD2	ARG	19	42.418	43.591	32.390	1.00	0.00	H
ATOM	302	HD3	ARG	19	41.840	45.170	32.933	1.00	0.00	H
ATOM	303	NE	ARG	19	43.959	44.972	32.675	1.00	0.00	N
ATOM	304	HE	ARG	19	44.638	44.841	33.411	1.00	0.00	H
ATOM	305	CZ	ARG	19	44.260	45.896	31.756	1.00	0.00	C
ATOM	306	NH1	ARG	19	43.506	45.916	30.688	1.00	0.00	N
ATOM	307	HH11	ARG	19	42.854	45.156	30.554	1.00	0.00	H
ATOM	308	HH12	ARG	19	43.665	46.640	30.002	1.00	0.00	H
ATOM	309	NH2	ARG	19	45.290	46.630	31.897	1.00	0.00	N
ATOM	310	HH21	ARG	19	45.577	47.164	31.088	1.00	0.00	H
ATOM	311	HH22	ARG	19	45.915	46.351	32.638	1.00	0.00	H
ATOM	312	C	ARG	19	40.377	41.640	36.500	1.00	0.00	C
ATOM	313	O	ARG	19	39.820	42.393	37.292	1.00	0.00	O
ATOM	335	N	THR	21	39.288	38.313	38.455	1.00	0.00	N
ATOM	336	H	THR	21	39.112	39.177	38.948	1.00	0.00	H
ATOM	337	CA	THR	21	39.808	37.105	39.146	1.00	0.00	C
ATOM	338	HA	THR	21	39.437	36.276	38.544	1.00	0.00	H
ATOM	339	CB	THR	21	41.358	37.087	39.057	1.00	0.00	C
ATOM	340	HB	THR	21	41.811	37.302	38.088	1.00	0.00	H
ATOM	341	CG2	THR	21	41.897	38.325	39.789	1.00	0.00	C
ATOM	342	HG21	THR	21	42.985	38.326	39.848	1.00	0.00	H
ATOM	343	HG22	THR	21	41.583	39.223	39.256	1.00	0.00	H
ATOM	344	HG23	THR	21	41.496	38.338	40.803	1.00	0.00	H
ATOM	345	OG1	THR	21	41.940	35.940	39.580	1.00	0.00	O
ATOM	346	HG1	THR	21	41.408	35.223	39.229	1.00	0.00	H
ATOM	347	C	THR	21	39.164	36.767	40.528	1.00	0.00	C
ATOM	348	O	THR	21	39.535	35.705	41.140	1.00	0.00	O
ATOM	349	N	ILE	22	38.144	37.546	40.995	1.00	0.00	N
ATOM	350	H	ILE	22	38.135	38.532	40.779	1.00	0.00	H
ATOM	351	CA	ILE	22	37.230	37.154	42.073	1.00	0.00	C
ATOM	352	HA	ILE	22	37.179	36.083	42.274	1.00	0.00	H
ATOM	353	CB	ILE	22	37.692	37.735	43.390	1.00	0.00	C
ATOM	354	HB	ILE	22	38.571	37.175	43.709	1.00	0.00	H
ATOM	355	CG2	ILE	22	38.045	39.213	43.388	1.00	0.00	C

ATOM	356	HG21	ILE	22	38.478	39.389	44.373	1.00	0.00	H
ATOM	357	HG22	ILE	22	38.817	39.362	42.634	1.00	0.00	H
ATOM	358	HG23	ILE	22	37.194	39.879	43.247	1.00	0.00	H
ATOM	359	CG1	ILE	22	36.625	37.403	44.496	1.00	0.00	C
ATOM	360	HG12	ILE	22	35.801	38.116	44.476	1.00	0.00	H
ATOM	361	HG13	ILE	22	36.152	36.443	44.290	1.00	0.00	H
ATOM	362	CD1	ILE	22	37.113	37.473	45.950	1.00	0.00	C
ATOM	363	HD11	ILE	22	37.652	38.382	46.218	1.00	0.00	H
ATOM	364	HD12	ILE	22	36.252	37.392	46.612	1.00	0.00	H
ATOM	365	HD13	ILE	22	37.765	36.629	46.172	1.00	0.00	H
ATOM	366	C	ILE	22	35.756	37.659	41.737	1.00	0.00	C
ATOM	367	O	ILE	22	35.611	38.707	41.008	1.00	0.00	O
ATOM	368	N	THR	23	34.696	36.900	42.043	1.00	0.00	N
ATOM	369	H	THR	23	34.891	36.000	42.460	1.00	0.00	H
ATOM	370	CA	THR	23	33.303	37.388	41.924	1.00	0.00	C
ATOM	371	HA	THR	23	33.256	38.476	41.959	1.00	0.00	H
ATOM	372	CB	THR	23	32.694	36.978	40.541	1.00	0.00	C
ATOM	373	HB	THR	23	33.461	36.891	39.771	1.00	0.00	H
ATOM	374	CG2	THR	23	31.937	35.633	40.534	1.00	0.00	C
ATOM	375	HG21	THR	23	30.947	35.608	40.990	1.00	0.00	H
ATOM	376	HG22	THR	23	31.715	35.332	39.510	1.00	0.00	H
ATOM	377	HG23	THR	23	32.548	34.826	40.939	1.00	0.00	H
ATOM	378	OG1	THR	23	31.736	37.944	40.148	1.00	0.00	O
ATOM	379	HG1	THR	23	32.108	38.500	39.458	1.00	0.00	H
ATOM	380	C	THR	23	32.422	36.990	43.071	1.00	0.00	C
ATOM	381	O	THR	23	32.716	36.028	43.784	1.00	0.00	O
ATOM	382	N	ILE	24	31.265	37.704	43.259	1.00	0.00	N
ATOM	383	H	ILE	24	31.102	38.485	42.640	1.00	0.00	H
ATOM	384	CA	ILE	24	30.322	37.479	44.366	1.00	0.00	C
ATOM	385	HA	ILE	24	30.501	36.531	44.873	1.00	0.00	H
ATOM	386	CB	ILE	24	30.344	38.633	45.369	1.00	0.00	C
ATOM	387	HB	ILE	24	30.439	39.509	44.727	1.00	0.00	H
ATOM	388	CG2	ILE	24	29.214	38.704	46.397	1.00	0.00	C
ATOM	389	HG21	ILE	24	28.224	38.670	45.942	1.00	0.00	H
ATOM	390	HG22	ILE	24	29.223	37.854	47.080	1.00	0.00	H
ATOM	391	HG23	ILE	24	29.396	39.539	47.073	1.00	0.00	H
ATOM	392	CG1	ILE	24	31.698	38.709	46.160	1.00	0.00	C
ATOM	393	HG12	ILE	24	31.617	38.081	47.047	1.00	0.00	H
ATOM	394	HG13	ILE	24	32.474	38.346	45.486	1.00	0.00	H
ATOM	395	CD1	ILE	24	31.959	40.084	46.774	1.00	0.00	C
ATOM	396	HD11	ILE	24	31.498	40.144	47.760	1.00	0.00	H
ATOM	397	HD12	ILE	24	33.041	40.136	46.889	1.00	0.00	H
ATOM	398	HD13	ILE	24	31.527	40.821	46.098	1.00	0.00	H
ATOM	399	C	ILE	24	28.935	37.133	43.807	1.00	0.00	C
ATOM	400	O	ILE	24	28.485	37.807	42.858	1.00	0.00	O
ATOM	401	N	ARG	25	28.285	36.106	44.283	1.00	0.00	N
ATOM	402	H	ARG	25	28.797	35.434	44.836	1.00	0.00	H
ATOM	403	CA	ARG	25	26.935	35.647	44.066	1.00	0.00	C
ATOM	404	HA	ARG	25	26.450	36.382	43.424	1.00	0.00	H
ATOM	405	CB	ARG	25	27.041	34.270	43.356	1.00	0.00	C
ATOM	406	HB2	ARG	25	27.494	33.562	44.049	1.00	0.00	H
ATOM	407	HB3	ARG	25	26.009	33.974	43.162	1.00	0.00	H
ATOM	408	CG	ARG	25	27.817	34.258	42.050	1.00	0.00	C
ATOM	409	HG2	ARG	25	28.835	34.624	42.186	1.00	0.00	H
ATOM	410	HG3	ARG	25	27.842	33.220	41.717	1.00	0.00	H
ATOM	411	CD	ARG	25	27.139	35.172	41.016	1.00	0.00	C
ATOM	412	HD2	ARG	25	26.214	34.619	40.856	1.00	0.00	H
ATOM	413	HD3	ARG	25	26.763	36.147	41.329	1.00	0.00	H
ATOM	414	NE	ARG	25	27.901	35.302	39.727	1.00	0.00	N
ATOM	415	HE	ARG	25	27.628	34.682	38.978	1.00	0.00	H
ATOM	416	CZ	ARG	25	28.547	36.376	39.327	1.00	0.00	C
ATOM	417	NH1	ARG	25	28.862	37.366	40.143	1.00	0.00	N
ATOM	418	HH11	ARG	25	29.548	38.071	39.917	1.00	0.00	H
ATOM	419	HH12	ARG	25	28.638	37.171	41.108	1.00	0.00	H

ATOM	420	NH2	ARG	25	28.965	36.452	38.139	1.00	0.00	N
ATOM	421	HH21	ARG	25	29.315	37.246	37.623	1.00	0.00	H
ATOM	422	HH22	ARG	25	28.807	35.628	37.576	1.00	0.00	H
ATOM	423	C	ARG	25	26.146	35.494	45.445	1.00	0.00	C
ATOM	424	O	ARG	25	26.784	35.484	46.512	1.00	0.00	O
ATOM	425	N	ASP	26	24.867	35.495	45.444	1.00	0.00	N
ATOM	426	H	ASP	26	24.325	35.615	44.600	1.00	0.00	H
ATOM	427	CA	ASP	26	24.173	35.212	46.703	1.00	0.00	C
ATOM	428	HA	ASP	26	24.631	35.788	47.506	1.00	0.00	H
ATOM	429	CB	ASP	26	22.636	35.684	46.682	1.00	0.00	C
ATOM	430	HB2	ASP	26	22.362	35.627	47.735	1.00	0.00	H
ATOM	431	HB3	ASP	26	22.603	36.720	46.345	1.00	0.00	H
ATOM	432	CG	ASP	26	21.552	34.863	45.993	1.00	0.00	C
ATOM	433	OD1	ASP	26	21.709	34.440	44.841	1.00	0.00	O
ATOM	434	OD2	ASP	26	20.514	34.575	46.619	1.00	0.00	O
ATOM	435	C	ASP	26	24.213	33.679	46.993	1.00	0.00	C
ATOM	436	O	ASP	26	24.381	32.839	46.067	1.00	0.00	O
ATOM	437	N	GLU	27	24.040	33.189	48.239	1.00	0.00	N
ATOM	438	H	GLU	27	23.987	33.798	49.043	1.00	0.00	H
ATOM	439	CA	GLU	27	24.133	31.768	48.487	1.00	0.00	C
ATOM	440	HA	GLU	27	25.139	31.507	48.160	1.00	0.00	H
ATOM	441	CB	GLU	27	24.185	31.495	49.993	1.00	0.00	C
ATOM	442	HB2	GLU	27	24.971	32.160	50.350	1.00	0.00	H
ATOM	443	HB3	GLU	27	23.175	31.634	50.377	1.00	0.00	H
ATOM	444	CG	GLU	27	24.666	30.114	50.396	1.00	0.00	C
ATOM	445	HG2	GLU	27	24.583	29.969	51.473	1.00	0.00	H
ATOM	446	HG3	GLU	27	24.027	29.344	49.964	1.00	0.00	H
ATOM	447	CD	GLU	27	26.044	29.722	50.066	1.00	0.00	C
ATOM	448	OE1	GLU	27	26.344	29.475	48.910	1.00	0.00	O
ATOM	449	OE2	GLU	27	26.892	29.670	50.975	1.00	0.00	O
ATOM	450	C	GLU	27	23.158	30.824	47.636	1.00	0.00	C
ATOM	451	O	GLU	27	21.949	31.009	47.550	1.00	0.00	O
ATOM	452	N	SER	28	23.760	29.687	47.121	1.00	0.00	N
ATOM	453	H	SER	28	24.717	29.536	47.409	1.00	0.00	H
ATOM	454	CA	SER	28	23.140	28.552	46.393	1.00	0.00	C
ATOM	455	HA	SER	28	22.248	28.294	46.964	1.00	0.00	H
ATOM	456	CB	SER	28	22.776	28.995	45.027	1.00	0.00	C
ATOM	457	HB2	SER	28	22.071	29.827	45.043	1.00	0.00	H
ATOM	458	HB3	SER	28	23.673	29.410	44.565	1.00	0.00	H
ATOM	459	OG	SER	28	22.374	28.073	44.057	1.00	0.00	O
ATOM	460	HG	SER	28	21.417	27.996	44.041	1.00	0.00	H
ATOM	461	C	SER	28	24.219	27.477	46.302	1.00	0.00	C
ATOM	462	O	SER	28	25.388	27.803	46.204	1.00	0.00	O
ATOM	463	N	GLU	29	23.837	26.212	46.205	1.00	0.00	N
ATOM	464	H	GLU	29	22.873	26.025	46.445	1.00	0.00	H
ATOM	465	CA	GLU	29	24.764	25.110	45.943	1.00	0.00	C
ATOM	466	HA	GLU	29	25.626	25.284	46.587	1.00	0.00	H
ATOM	467	CB	GLU	29	24.098	23.787	46.366	1.00	0.00	C
ATOM	468	HB2	GLU	29	23.989	23.761	47.450	1.00	0.00	H
ATOM	469	HB3	GLU	29	23.084	23.770	45.967	1.00	0.00	H
ATOM	470	CG	GLU	29	24.624	22.379	45.900	1.00	0.00	C
ATOM	471	HG2	GLU	29	23.888	21.609	46.131	1.00	0.00	H
ATOM	472	HG3	GLU	29	24.586	22.381	44.811	1.00	0.00	H
ATOM	473	CD	GLU	29	26.004	22.047	46.409	1.00	0.00	C
ATOM	474	OE1	GLU	29	26.616	22.793	47.167	1.00	0.00	O
ATOM	475	OE2	GLU	29	26.555	20.983	46.143	1.00	0.00	O
ATOM	476	C	GLU	29	25.130	24.969	44.424	1.00	0.00	C
ATOM	477	O	GLU	29	26.082	24.249	44.143	1.00	0.00	O
ATOM	478	N	SER	30	24.376	25.643	43.545	1.00	0.00	N
ATOM	479	H	SER	30	23.514	26.080	43.842	1.00	0.00	H
ATOM	480	CA	SER	30	24.585	25.636	42.084	1.00	0.00	C
ATOM	481	HA	SER	30	24.569	24.609	41.719	1.00	0.00	H
ATOM	482	CB	SER	30	23.327	26.186	41.424	1.00	0.00	C

ATOM	483	HB2	SER	30	23.387	25.936	40.365	1.00	0.00	H
ATOM	484	HB3	SER	30	22.404	25.736	41.788	1.00	0.00	H
ATOM	485	OG	SER	30	23.242	27.591	41.476	1.00	0.00	O
ATOM	486	HG	SER	30	23.055	27.804	42.392	1.00	0.00	H
ATOM	487	C	SER	30	25.747	26.428	41.505	1.00	0.00	C
ATOM	488	O	SER	30	25.885	26.535	40.281	1.00	0.00	O
ATOM	489	N	HIE	31	26.507	27.025	42.374	1.00	0.00	N
ATOM	490	H	HIE	31	26.412	26.754	43.343	1.00	0.00	H
ATOM	491	CA	HIE	31	27.712	27.720	41.981	1.00	0.00	C
ATOM	492	HA	HIE	31	27.493	28.388	41.148	1.00	0.00	H
ATOM	493	CB	HIE	31	28.166	28.668	43.070	1.00	0.00	C
ATOM	494	HB2	HIE	31	28.480	28.234	44.020	1.00	0.00	H
ATOM	495	HB3	HIE	31	29.035	29.243	42.749	1.00	0.00	H
ATOM	496	CG	HIE	31	27.069	29.692	43.417	1.00	0.00	C
ATOM	497	ND1	HIE	31	26.318	30.422	42.491	1.00	0.00	N
ATOM	498	CE1	HIE	31	25.478	31.140	43.265	1.00	0.00	C
ATOM	499	HE1	HIE	31	24.652	31.697	42.850	1.00	0.00	H
ATOM	500	NE2	HIE	31	25.733	30.997	44.561	1.00	0.00	N
ATOM	501	HE2	HIE	31	25.309	31.482	45.339	1.00	0.00	H
ATOM	502	CD2	HIE	31	26.729	30.066	44.680	1.00	0.00	C
ATOM	503	HD2	HIE	31	27.017	29.601	45.611	1.00	0.00	H
ATOM	504	C	HIE	31	28.848	26.819	41.499	1.00	0.00	C
ATOM	505	O	HIE	31	28.911	25.569	41.700	1.00	0.00	O
ATOM	506	N	PHE	32	29.840	27.457	40.916	1.00	0.00	N
ATOM	507	H	PHE	32	29.749	28.412	40.596	1.00	0.00	H
ATOM	508	CA	PHE	32	31.103	26.682	40.746	1.00	0.00	C
ATOM	509	HA	PHE	32	30.995	25.661	40.382	1.00	0.00	H
ATOM	510	CB	PHE	32	31.944	27.390	39.763	1.00	0.00	C
ATOM	511	HB2	PHE	32	32.491	28.147	40.325	1.00	0.00	H
ATOM	512	HB3	PHE	32	32.619	26.733	39.214	1.00	0.00	H
ATOM	513	CG	PHE	32	31.107	28.012	38.652	1.00	0.00	C
ATOM	514	CD1	PHE	32	31.035	29.414	38.604	1.00	0.00	C
ATOM	515	HD1	PHE	32	31.482	29.987	39.403	1.00	0.00	H
ATOM	516	CE1	PHE	32	30.256	30.028	37.615	1.00	0.00	C
ATOM	517	HE1	PHE	32	30.090	31.095	37.607	1.00	0.00	H
ATOM	518	CZ	PHE	32	29.804	29.212	36.518	1.00	0.00	C
ATOM	519	HZ	PHE	32	29.243	29.736	35.758	1.00	0.00	H
ATOM	520	CE2	PHE	32	29.983	27.840	36.551	1.00	0.00	C
ATOM	521	HE2	PHE	32	29.597	27.214	35.759	1.00	0.00	H
ATOM	522	CD2	PHE	32	30.595	27.229	37.638	1.00	0.00	C
ATOM	523	HD2	PHE	32	30.791	26.167	37.638	1.00	0.00	H
ATOM	524	C	PHE	32	31.839	26.600	42.123	1.00	0.00	C
ATOM	525	O	PHE	32	31.731	27.426	42.973	1.00	0.00	O
ATOM	526	N	LYS	33	32.634	25.497	42.238	1.00	0.00	N
ATOM	527	H	LYS	33	32.654	24.838	41.473	1.00	0.00	H
ATOM	528	CA	LYS	33	33.422	25.085	43.395	1.00	0.00	C
ATOM	529	HA	LYS	33	33.317	25.827	44.186	1.00	0.00	H
ATOM	530	CB	LYS	33	32.910	23.736	43.932	1.00	0.00	C
ATOM	531	HB2	LYS	33	32.991	22.956	43.174	1.00	0.00	H
ATOM	532	HB3	LYS	33	33.543	23.551	44.799	1.00	0.00	H
ATOM	533	CG	LYS	33	31.402	23.806	44.253	1.00	0.00	C
ATOM	534	HG2	LYS	33	31.248	24.703	44.853	1.00	0.00	H
ATOM	535	HG3	LYS	33	30.887	23.880	43.296	1.00	0.00	H
ATOM	536	CD	LYS	33	30.878	22.647	45.067	1.00	0.00	C
ATOM	537	HD2	LYS	33	31.196	21.676	44.688	1.00	0.00	H
ATOM	538	HD3	LYS	33	31.310	22.689	46.067	1.00	0.00	H
ATOM	539	CE	LYS	33	29.340	22.626	45.295	1.00	0.00	C
ATOM	540	HE2	LYS	33	28.731	22.577	44.392	1.00	0.00	H
ATOM	541	HE3	LYS	33	29.055	21.721	45.831	1.00	0.00	H
ATOM	542	NZ	LYS	33	28.906	23.772	46.058	1.00	0.00	N
ATOM	543	HZ1	LYS	33	27.901	23.772	46.156	1.00	0.00	H
ATOM	544	HZ2	LYS	33	29.259	23.930	46.991	1.00	0.00	H
ATOM	545	HZ3	LYS	33	29.218	24.571	45.524	1.00	0.00	H
ATOM	546	C	LYS	33	34.947	25.058	43.155	1.00	0.00	C

ATOM	547	O	LYS	33	35.366	24.859	41.999	1.00	0.00	O
ATOM	548	N	THR	34	35.797	25.130	44.178	1.00	0.00	N
ATOM	549	H	THR	34	35.381	25.301	45.081	1.00	0.00	H
ATOM	550	CA	THR	34	37.267	24.996	44.184	1.00	0.00	C
ATOM	551	HA	THR	34	37.730	25.811	43.627	1.00	0.00	H
ATOM	552	CB	THR	34	37.835	25.004	45.634	1.00	0.00	C
ATOM	553	HB	THR	34	37.460	24.103	46.120	1.00	0.00	H
ATOM	554	CG2	THR	34	39.364	25.089	45.634	1.00	0.00	C
ATOM	555	HG21	THR	34	39.740	24.067	45.600	1.00	0.00	H
ATOM	556	HG22	THR	34	39.773	25.636	44.785	1.00	0.00	H
ATOM	557	HG23	THR	34	39.758	25.491	46.567	1.00	0.00	H
ATOM	558	OG1	THR	34	37.544	26.173	46.291	1.00	0.00	O
ATOM	559	HG1	THR	34	36.602	26.359	46.307	1.00	0.00	H
ATOM	560	C	THR	34	37.732	23.858	43.343	1.00	0.00	C
ATOM	561	O	THR	34	37.513	22.735	43.793	1.00	0.00	O
ATOM	562	N	GLY	35	38.355	24.093	42.170	1.00	0.00	N
ATOM	563	H	GLY	35	38.843	24.977	42.140	1.00	0.00	H
ATOM	564	CA	GLY	35	38.866	23.077	41.201	1.00	0.00	C
ATOM	565	HA2	GLY							
	35	39.793	23.429	40.750	1.00	0.00	H			
ATOM	566	HA3	GLY	35	39.023	22.194	41.820	1.00	0.00	H
ATOM	567	C	GLY	35	37.895	22.864	40.040	1.00	0.00	C
ATOM	568	O	GLY	35	38.444	22.231	39.123	1.00	0.00	O
ATOM	569	N	ASP	36	36.668	23.347	39.879	1.00	0.00	N
ATOM	570	H	ASP	36	36.343	23.875	40.677	1.00	0.00	H
ATOM	571	CA	ASP	36	35.830	23.337	38.677	1.00	0.00	C
ATOM	572	HA	ASP	36	35.713	22.327	38.288	1.00	0.00	H
ATOM	573	CB	ASP	36	34.462	23.882	38.997	1.00	0.00	C
ATOM	574	HB2	ASP	36	34.542	24.943	39.230	1.00	0.00	H
ATOM	575	HB3	ASP	36	33.809	23.958	38.127	1.00	0.00	H
ATOM	576	CG	ASP	36	33.729	23.053	40.049	1.00	0.00	C
ATOM	577	OD1	ASP	36	32.485	23.270	40.204	1.00	0.00	O
ATOM	578	OD2	ASP	36	34.267	22.015	40.647	1.00	0.00	O
ATOM	579	C	ASP	36	36.419	24.108	37.486	1.00	0.00	C
ATOM	580	O	ASP	36	37.155	25.063	37.737	1.00	0.00	O
ATOM	581	N	VAL	37	35.983	23.848	36.234	1.00	0.00	N
ATOM	582	H	VAL	37	35.440	23.031	35.992	1.00	0.00	H
ATOM	583	CA	VAL	37	36.700	24.497	35.079	1.00	0.00	C
ATOM	584	HA	VAL	37	37.307	25.325	35.441	1.00	0.00	H
ATOM	585	CB	VAL	37	37.630	23.522	34.273	1.00	0.00	C
ATOM	586	HB	VAL	37	37.051	22.635	34.018	1.00	0.00	H
ATOM	587	CG1	VAL	37	38.252	24.088	32.937	1.00	0.00	C
ATOM	588	HG11	VAL	37	37.436	24.165	32.219	1.00	0.00	H
ATOM	589	HG12	VAL	37	38.697	25.066	33.125	1.00	0.00	H
ATOM	590	HG13	VAL	37	38.974	23.385	32.526	1.00	0.00	H
ATOM	591	CG2	VAL	37	38.810	23.144	35.215	1.00	0.00	C
ATOM	592	HG21	VAL	37	39.287	24.049	35.593	1.00	0.00	H
ATOM	593	HG22	VAL	37	38.463	22.585	36.083	1.00	0.00	H
ATOM	594	HG23	VAL	37	39.579	22.577	34.690	1.00	0.00	H
ATOM	595	C	VAL	37	35.712	25.151	34.083	1.00	0.00	C
ATOM	596	O	VAL	37	34.619	24.611	33.904	1.00	0.00	O
ATOM	597	N	LEU	38	36.008	26.389	33.572	1.00	0.00	N
ATOM	598	H	LEU	38	36.907	26.785	33.808	1.00	0.00	H
ATOM	599	CA	LEU	38	35.174	27.298	32.762	1.00	0.00	C
ATOM	600	HA	LEU	38	34.201	26.888	32.494	1.00	0.00	H
ATOM	601	CB	LEU	38	34.939	28.653	33.558	1.00	0.00	C
ATOM	602	HB2	LEU	38	35.904	29.028	33.896	1.00	0.00	H
ATOM	603	HB3	LEU	38	34.420	29.336	32.884	1.00	0.00	H
ATOM	604	CG	LEU	38	33.974	28.430	34.703	1.00	0.00	C
ATOM	605	HG	LEU	38	33.368	27.526	34.655	1.00	0.00	H
ATOM	606	CD1	LEU	38	34.775	28.283	36.029	1.00	0.00	C
ATOM	607	HD11	LEU	38	35.199	29.238	36.340	1.00	0.00	H
ATOM	608	HD12	LEU	38	34.161	28.025	36.892	1.00	0.00	H
ATOM	609	HD13	LEU	38	35.495	27.467	35.973	1.00	0.00	H

ATOM	610	CD2	LEU	38	33.045	29.592	34.908	1.00	0.00	C
ATOM	611	HD21	LEU	38	32.411	29.464	35.784	1.00	0.00	H
ATOM	612	HD22	LEU	38	33.622	30.508	35.033	1.00	0.00	H
ATOM	613	HD23	LEU	38	32.463	29.709	33.994	1.00	0.00	H
ATOM	614	C	LEU	38	35.920	27.598	31.420	1.00	0.00	C
ATOM	615	O	LEU	38	37.070	27.193	31.221	1.00	0.00	O
ATOM	616	N	ARG	39	35.251	28.350	30.540	1.00	0.00	N
ATOM	617	H	ARG	39	34.308	28.630	30.773	1.00	0.00	H
ATOM	618	CA	ARG	39	35.867	28.779	29.316	1.00	0.00	C
ATOM	619	HA	ARG	39	36.935	28.969	29.434	1.00	0.00	H
ATOM	620	CB	ARG	39	35.596	27.807	28.077	1.00	0.00	C
ATOM	621	HB2	ARG	39	34.521	27.759	27.910	1.00	0.00	H
ATOM	622	HB3	ARG	39	35.949	28.246	27.143	1.00	0.00	H
ATOM	623	CG	ARG	39	36.252	26.462	28.258	1.00	0.00	C
ATOM	624	HG2	ARG	39	37.282	26.596	28.588	1.00	0.00	H
ATOM	625	HG3	ARG	39	35.840	25.915	29.107	1.00	0.00	H
ATOM	626	CD	ARG	39	36.124	25.484	27.088	1.00	0.00	C
ATOM	627	HD2	ARG	39	36.454	25.982	26.177	1.00	0.00	H
ATOM	628	HD3	ARG	39	36.778	24.650	27.344	1.00	0.00	H
ATOM	629	NE	ARG	39	34.667	25.083	26.897	1.00	0.00	N
ATOM	630	HE	ARG	39	34.013	25.611	27.458	1.00	0.00	H
ATOM	631	CZ	ARG	39	34.183	23.980	26.398	1.00	0.00	C
ATOM	632	NH1	ARG	39	34.955	23.128	25.830	1.00	0.00	N
ATOM	633	HH11	ARG	39	35.925	23.365	25.674	1.00	0.00	H
ATOM	634	HH12	ARG	39	34.459	22.266	25.656	1.00	0.00	H
ATOM	635	NH2	ARG	39	32.917	23.694	26.496	1.00	0.00	N
ATOM	636	HH21	ARG	39	32.503	22.838	26.156	1.00	0.00	H
ATOM	637	HH22	ARG	39	32.352	24.425	26.904	1.00	0.00	H
ATOM	638	C	ARG	39	35.405	30.165	28.926	1.00	0.00	C
ATOM	639	O	ARG	39	34.300	30.490	29.283	1.00	0.00	O
ATOM	640	N	VAL	40	36.254	30.941	28.159	1.00	0.00	N
ATOM	641	H	VAL	40	37.220	30.653	28.110	1.00	0.00	H
ATOM	642	CA	VAL	40	35.913	32.217	27.474	1.00	0.00	C
ATOM	643	HA	VAL	40	34.826	32.185	27.554	1.00	0.00	H
ATOM	644	CB	VAL	40	36.321	33.511	28.157	1.00	0.00	C
ATOM	645	HB	VAL	40	35.961	34.340	27.548	1.00	0.00	H
ATOM	646	CG1	VAL	40	35.628	33.792	29.481	1.00	0.00	C
ATOM	647	HG11	VAL	40	35.910	33.098	30.275	1.00	0.00	H
ATOM	648	HG12	VAL	40	35.962	34.785	29.782	1.00	0.00	H
ATOM	649	HG13	VAL	40	34.545	33.715	29.391	1.00	0.00	H
ATOM	650	CG2	VAL	40	37.810	33.790	28.297	1.00	0.00	C
ATOM	651	HG21	VAL	40	37.924	34.752	28.798	1.00	0.00	H
ATOM	652	HG22	VAL	40	38.131	33.011	28.988	1.00	0.00	H
ATOM	653	HG23	VAL	40	38.388	33.601	27.392	1.00	0.00	H
ATOM	654	C	VAL	40	36.237	32.189	26.035	1.00	0.00	C
ATOM	655	O	VAL	40	37.349	31.752	25.626	1.00	0.00	O
ATOM	656	N	GLY	41	35.279	32.801	25.290	1.00	0.00	N
ATOM	657	H	GLY	41	34.486	33.127	25.823	1.00	0.00	H
ATOM	658	CA	GLY	41	35.292	32.976	23.907	1.00	0.00	C
ATOM	659	HA2	GLY	41	36.068	33.708	23.684	1.00	0.00	H
ATOM	660	HA3	GLY	41	35.519	32.008	23.460	1.00	0.00	H
ATOM	661	C	GLY	41	33.993	33.470	23.301	1.00	0.00	C
ATOM	662	O	GLY	41	32.934	33.489	23.967	1.00	0.00	O
ATOM	663	N	ARG	42	34.137	34.095	22.150	1.00	0.00	N
ATOM	664	H	ARG	42	34.999	34.068	21.626	1.00	0.00	H
ATOM	665	CA	ARG	42	32.958	34.686	21.386	1.00	0.00	C
ATOM	666	HA	ARG	42	32.010	34.197	21.609	1.00	0.00	H
ATOM	667	CB	ARG	42	32.717	36.135	21.890	1.00	0.00	C
ATOM	668	HB2	ARG	42	31.863	36.467	21.300	1.00	0.00	H
ATOM	669	HB3	ARG	42	32.484	36.088	22.953	1.00	0.00	H
ATOM	670	CG	ARG	42	33.778	37.163	21.665	1.00	0.00	C
ATOM	671	HG2	ARG	42	34.699	36.748	22.074	1.00	0.00	H
ATOM	672	HG3	ARG	42	33.812	37.381	20.597	1.00	0.00	H
ATOM	673	CD	ARG	42	33.423	38.503	22.318	1.00	0.00	C

ATOM	674	HD2	ARG	42	32.539	38.889	21.811	1.00	0.00	H
ATOM	675	HD3	ARG	42	33.131	38.351	23.357	1.00	0.00	H
ATOM	676	NE	ARG	42	34.496	39.516	22.043	1.00	0.00	N
ATOM	677	HE	ARG	42	34.335	40.227	21.344	1.00	0.00	H
ATOM	678	CZ	ARG	42	35.496	39.662	22.900	1.00	0.00	C
ATOM	679	NH1	ARG	42	35.863	38.857	23.761	1.00	0.00	N
ATOM	680	HH11	ARG	42	36.701	39.041	24.294	1.00	0.00	H
ATOM	681	HH12	ARG	42	35.444	37.939	23.792	1.00	0.00	H
ATOM	682	NH2	ARG	42	36.276	40.647	22.799	1.00	0.00	N
ATOM	683	HH21	ARG	42	36.267	41.336	22.061	1.00	0.00	H
ATOM	684	HH22	ARG	42	37.069	40.742	23.418	1.00	0.00	H
ATOM	685	C	ARG	42	33.032	34.606	19.844	1.00	0.00	C
ATOM	686	O	ARG	42	32.002	34.831	19.231	1.00	0.00	O
ATOM	687	N	PHE	43	34.234	34.346	19.369	1.00	0.00	N
ATOM	688	H	PHE	43	34.965	34.189	20.048	1.00	0.00	H
ATOM	689	CA	PHE	43	34.558	34.144	17.930	1.00	0.00	C
ATOM	690	HA	PHE	43	33.838	34.687				
17.318	1.00	0.00			H					
ATOM	691	CB	PHE	43	35.984	34.692	17.699	1.00	0.00	C
ATOM	692	HB2	PHE	43	36.679	34.037	18.226	1.00	0.00	H
ATOM	693	HB3	PHE	43	36.272	34.563	16.656	1.00	0.00	H
ATOM	694	CG	PHE	43	36.210	36.040	18.200	1.00	0.00	C
ATOM	695	CD1	PHE	43	35.486	37.067	17.675	1.00	0.00	C
ATOM	696	HD1	PHE	43	34.836	37.020	16.814	1.00	0.00	H
ATOM	697	CE1	PHE	43	35.613	38.323	18.300	1.00	0.00	C
ATOM	698	HE1	PHE	43	35.090	39.161	17.866	1.00	0.00	H
ATOM	699	CZ	PHE	43	36.381	38.505	19.411	1.00	0.00	C
ATOM	700	HZ	PHE	43	36.518	39.480	19.856	1.00	0.00	H
ATOM	701	CE2	PHE	43	37.125	37.432	19.919	1.00	0.00	C
ATOM	702	HE2	PHE	43	37.879	37.689	20.647	1.00	0.00	H
ATOM	703	CD2	PHE	43	37.104	36.238	19.292	1.00	0.00	C
ATOM	704	HD2	PHE	43	37.506	35.329	19.713	1.00	0.00	H
ATOM	705	C	PHE	43	34.477	32.597	17.591	1.00	0.00	C
ATOM	706	O	PHE	43	34.783	31.710	18.353	1.00	0.00	O
ATOM	707	N	GLU	44	34.109	32.351	16.358	1.00	0.00	N
ATOM	708	H	GLU	44	33.852	33.130	15.768	1.00	0.00	H
ATOM	709	CA	GLU	44	34.087	30.958	15.848	1.00	0.00	C
ATOM	710	HA	GLU	44	33.622	30.388	16.653	1.00	0.00	H
ATOM	711	CB	GLU	44	33.102	30.806	14.578	1.00	0.00	C
ATOM	712	HB2	GLU	44	32.121	31.136	14.919	1.00	0.00	H
ATOM	713	HB3	GLU	44	33.465	31.416	13.751	1.00	0.00	H
ATOM	714	CG	GLU	44	32.948	29.419	13.953	1.00	0.00	C
ATOM	715	HG2	GLU	44	32.426	29.466	12.998	1.00	0.00	H
ATOM	716	HG3	GLU	44	33.957	29.116	13.669	1.00	0.00	H
ATOM	717	CD	GLU	44	32.252	28.404	14.939	1.00	0.00	C
ATOM	718	OE1	GLU	44	32.594	28.392	16.141	1.00	0.00	O
ATOM	719	OE2	GLU	44	31.389	27.733	14.431	1.00	0.00	O
ATOM	720	C	GLU	44	35.491	30.400	15.460	1.00	0.00	C
ATOM	721	O	GLU	44	35.698	29.174	15.254	1.00	0.00	O
ATOM	722	N	ASP	45	36.410	31.286	15.140	1.00	0.00	N
ATOM	723	H	ASP	45	36.137	32.256	15.216	1.00	0.00	H
ATOM	724	CA	ASP	45	37.850	30.972	14.807	1.00	0.00	C
ATOM	725	HA	ASP	45	37.754	30.119	14.135	1.00	0.00	H
ATOM	726	CB	ASP	45	38.502	32.223	14.051	1.00	0.00	C
ATOM	727	HB2	ASP	45	37.914	32.431	13.158	1.00	0.00	H
ATOM	728	HB3	ASP	45	38.611	33.115	14.669	1.00	0.00	H
ATOM	729	CG	ASP	45	39.857	32.014	13.440	1.00	0.00	C
ATOM	730	OD1	ASP	45	40.461	30.874	13.429	1.00	0.00	O
ATOM	731	OD2	ASP	45	40.406	33.011	12.967	1.00	0.00	O
ATOM	732	C	ASP	45	38.622	30.659	16.011	1.00	0.00	C
ATOM	733	O	ASP	45	38.682	31.455	16.916	1.00	0.00	O
ATOM	734	N	ASP	46	38.955	29.378	16.130	1.00	0.00	N
ATOM	735	H	ASP	46	38.737	28.705	15.408	1.00	0.00	H
ATOM	736	CA	ASP	46	39.598	28.755	17.299	1.00	0.00	C

ATOM	737	HA	ASP	46	39.616	27.680	17.114	1.00	0.00	H
ATOM	738	CB	ASP	46	40.977	29.397	17.648	1.00	0.00	C
ATOM	739	HB2	ASP	46	40.762	30.408	17.991	1.00	0.00	H
ATOM	740	HB3	ASP	46	41.491	28.766	18.374	1.00	0.00	H
ATOM	741	CG	ASP	46	41.916	29.527	16.384	1.00	0.00	C
ATOM	742	OD1	ASP	46	42.424	28.486	15.864	1.00	0.00	O
ATOM	743	OD2	ASP	46	42.146	30.656	15.819	1.00	0.00	O
ATOM	744	C	ASP	46	38.739	28.687	18.627	1.00	0.00	C
ATOM	745	O	ASP	46	39.164	28.128	19.605	1.00	0.00	O
ATOM	746	N	GLY	47	37.562	29.337	18.696	1.00	0.00	N
ATOM	747	H	GLY	47	37.452	29.901	17.865	1.00	0.00	H
ATOM	748	CA	GLY	47	36.409	29.242	19.531	1.00	0.00	C
ATOM	749	HA2	GLY	47	35.502	29.680	19.113	1.00	0.00	H
ATOM	750	HA3	GLY	47	36.119	28.201	19.672	1.00	0.00	H
ATOM	751	C	GLY	47	36.515	29.776	20.967	1.00	0.00	C
ATOM	752	O	GLY	47	35.694	30.480	21.462	1.00	0.00	O
ATOM	753	N	TYR	48	37.620	29.533	21.629	1.00	0.00	N
ATOM	754	H	TYR	48	38.381	29.030	21.196	1.00	0.00	H
ATOM	755	CA	TYR	48	37.928	29.943	23.019	1.00	0.00	C
ATOM	756	HA	TYR	48	37.225	30.759	23.180	1.00	0.00	H
ATOM	757	CB	TYR	48	37.594	28.678	23.943	1.00	0.00	C
ATOM	758	HB2	TYR	48	38.335	27.907	23.733	1.00	0.00	H
ATOM	759	HB3	TYR	48	37.831	28.826	24.996	1.00	0.00	H
ATOM	760	CG	TYR	48	36.241	28.071	23.832	1.00	0.00	C
ATOM	761	CD1	TYR	48	35.085	28.731	24.161	1.00	0.00	C
ATOM	762	HD1	TYR	48	35.181	29.765	24.459	1.00	0.00	H
ATOM	763	CE1	TYR	48	33.860	28.039	24.388	1.00	0.00	C
ATOM	764	HE1	TYR	48	33.000	28.464	24.885	1.00	0.00	H
ATOM	765	CZ	TYR	48	33.772	26.716	24.089	1.00	0.00	C
ATOM	766	OH	TYR	48	32.531	26.176	24.134	1.00	0.00	O
ATOM	767	HH	TYR	48	32.489	25.295	23.753	1.00	0.00	H
ATOM	768	CE2	TYR	48	34.864	26.057	23.435	1.00	0.00	C
ATOM	769	HE2	TYR	48	34.757	25.085	22.976	1.00	0.00	H
ATOM	770	CD2	TYR	48	36.028	26.759	23.387	1.00	0.00	C
ATOM	771	HD2	TYR	48	36.791	26.256	22.810	1.00	0.00	H
ATOM	772	C	TYR	48	39.359	30.528	23.110	1.00	0.00	C
ATOM	773	O	TYR	48	40.222	30.244	22.310	1.00	0.00	O
ATOM	774	N	PHE	49	39.655	31.402	24.148	1.00	0.00	N
ATOM	775	H	PHE	49	38.898	31.561	24.797	1.00	0.00	H
ATOM	776	CA	PHE	49	40.873	32.098	24.390	1.00	0.00	C
ATOM	777	HA	PHE	49	41.729	31.674	23.865	1.00	0.00	H
ATOM	778	CB	PHE	49	40.830	33.494	23.863	1.00	0.00	C
ATOM	779	HB2	PHE	49	41.868	33.822	23.920	1.00	0.00	H
ATOM	780	HB3	PHE	49	40.602	33.483	22.798	1.00	0.00	H
ATOM	781	CG	PHE	49	39.864	34.430	24.484	1.00	0.00	C
ATOM	782	CD1	PHE	49	38.677	34.858	23.764	1.00	0.00	C
ATOM	783	HD1	PHE	49	38.398	34.402	22.826	1.00	0.00	H
ATOM	784	CE1	PHE	49	37.847	35.894	24.239	1.00	0.00	C
ATOM	785	HE1	PHE	49	36.976	36.095	23.634	1.00	0.00	H
ATOM	786	CZ	PHE	49	38.080	36.530	25.443	1.00	0.00	C
ATOM	787	HZ	PHE	49	37.577	37.452	25.697	1.00	0.00	H
ATOM	788	CE2	PHE	49	39.172	36.097	26.137	1.00	0.00	C
ATOM	789	HE2	PHE	49	39.391	36.488	27.119	1.00	0.00	H
ATOM	790	CD2	PHE	49	40.077	35.088	25.695	1.00	0.00	C
ATOM	791	HD2	PHE	49	40.947	34.849	26.288	1.00	0.00	H
ATOM	792	C	PHE	49	41.439	32.042	25.860	1.00	0.00	C
ATOM	793	O	PHE	49	42.614	32.203	26.129	1.00	0.00	O
ATOM	794	N	CYX	50	40.587	31.667	26.825	1.00	0.00	N
ATOM	795	H	CYX	50	39.611	31.528	26.605	1.00	0.00	H
ATOM	796	CA	CYX	50	41.037	31.107	28.093	1.00	0.00	C
ATOM	797	HA	CYX	50	42.029	30.686	27.928	1.00	0.00	H
ATOM	798	CB	CYX	50	41.040	32.213	29.249	1.00	0.00	C
ATOM	799	HB2	CYX	50	40.190	32.873	29.076	1.00	0.00	H
ATOM	800	HB3	CYX	50	41.087	31.660	30.187	1.00	0.00	H

ATOM	801	SG	CYX	50	42.593	33.187	29.308	1.00	0.00	S
ATOM	802	C	CYX	50	40.190	29.943	28.507	1.00	0.00	C
ATOM	803	O	CYX	50	38.979	30.116	28.475	1.00	0.00	O
ATOM	804	N	THR	51	40.733	28.783	28.779	1.00	0.00	N
ATOM	805	H	THR	51	41.728	28.665	28.659	1.00	0.00	H
ATOM	806	CA	THR	51	40.121	27.804	29.758	1.00	0.00	C
ATOM	807	HA	THR	51	39.041	27.883	29.633	1.00	0.00	H
ATOM	808	CB	THR	51	40.681	26.399	29.456	1.00	0.00	C
ATOM	809	HB	THR	51	41.751	26.335	29.655	1.00	0.00	H
ATOM	810	CG2	THR	51	39.971	25.336	30.362	1.00	0.00	C
ATOM	811	HG21	THR	51	40.222	24.371	29.923	1.00	0.00	H
ATOM	812	HG22	THR	51	40.306	25.388	31.398	1.00	0.00	H
ATOM	813	HG23	THR	51	38.901	25.545	30.342	1.00	0.00	H
ATOM	814	OG1	THR	51	40.401	26.026	28.140	1.00	0.00	O
ATOM	815	HG1	THR	51	40.809	26.660	27.546	1.00	0.00	
	H									
ATOM	816	C	THR	51	40.463	28.307	31.158	1.00	0.00	C
ATOM	817	O	THR	51	41.464	29.040	31.360	1.00	0.00	O
ATOM	818	N	ILE	52	39.594	28.155	32.178	1.00	0.00	N
ATOM	819	H	ILE	52	38.873	27.462	32.031	1.00	0.00	H
ATOM	820	CA	ILE	52	39.693	28.796	33.499	1.00	0.00	C
ATOM	821	HA	ILE	52	40.717	29.118	33.690	1.00	0.00	H
ATOM	822	CB	ILE	52	38.731	30.013	33.627	1.00	0.00	C
ATOM	823	HB	ILE	52	37.752	29.619	33.354	1.00	0.00	H
ATOM	824	CG2	ILE	52	38.551	30.592	35.094	1.00	0.00	C
ATOM	825	HG21	ILE	52	37.824	30.072	35.717	1.00	0.00	H
ATOM	826	HG22	ILE	52	39.540	30.583	35.555	1.00	0.00	H
ATOM	827	HG23	ILE	52	38.169	31.612	35.055	1.00	0.00	H
ATOM	828	CG1	ILE	52	38.996	31.167	32.631	1.00	0.00	C
ATOM	829	HG12	ILE	52	39.630	31.868	33.172	1.00	0.00	H
ATOM	830	HG13	ILE	52	39.511	30.778	31.752	1.00	0.00	H
ATOM	831	CD1	ILE	52	37.685	31.804	32.153	1.00	0.00	C
ATOM	832	HD11	ILE	52	37.176	32.326	32.964	1.00	0.00	H
ATOM	833	HD12	ILE	52	37.968	32.475	31.342	1.00	0.00	H
ATOM	834	HD13	ILE	52	37.126	30.985	31.701	1.00	0.00	H
ATOM	835	C	ILE	52	39.419	27.820	34.599	1.00	0.00	C
ATOM	836	O	ILE	52	38.522	27.039	34.474	1.00	0.00	O
ATOM	837	N	GLU	53	40.169	27.805	35.715	1.00	0.00	N
ATOM	838	H	GLU	53	40.941	28.455	35.741	1.00	0.00	H
ATOM	839	CA	GLU	53	40.007	26.937	36.882	1.00	0.00	C
ATOM	840	HA	GLU	53	39.197	26.226	36.712	1.00	0.00	H
ATOM	841	CB	GLU	53	41.287	26.160	37.086	1.00	0.00	C
ATOM	842	HB2	GLU	53	41.411	25.485	36.238	1.00	0.00	H
ATOM	843	HB3	GLU	53	42.217	26.727	37.096	1.00	0.00	H
ATOM	844	CG	GLU	53	41.300	25.459	38.489	1.00	0.00	C
ATOM	845	HG2	GLU	53	41.190	26.207	39.275	1.00	0.00	H
ATOM	846	HG3	GLU	53	40.394	24.853	38.527	1.00	0.00	H
ATOM	847	CD	GLU	53	42.647	24.712	38.828	1.00	0.00	C
ATOM	848	OE1	GLU	53	42.943	23.676	38.191	1.00	0.00	O
ATOM	849	OE2	GLU	53	43.376	25.087	39.768	1.00	0.00	O
ATOM	850	C	GLU	53	39.658	27.872	38.016	1.00	0.00	C
ATOM	851	O	GLU	53	40.199	28.943	38.101	1.00	0.00	O
ATOM	852	N	VAL	54	38.843	27.432	39.000	1.00	0.00	N
ATOM	853	H	VAL	54	38.321	26.574	38.911	1.00	0.00	H
ATOM	854	CA	VAL	54	38.405	28.035	40.246	1.00	0.00	C
ATOM	855	HA	VAL	54	38.332	29.109	40.074	1.00	0.00	H
ATOM	856	CB	VAL	54	37.018	27.507	40.701	1.00	0.00	C
ATOM	857	HB	VAL	54	36.969	26.419	40.686	1.00	0.00	H
ATOM	858	CG1	VAL	54	36.640	28.045	42.017	1.00	0.00	C
ATOM	859	HG11	VAL	54	37.415	27.767	42.732	1.00	0.00	H
ATOM	860	HG12	VAL	54	36.557	29.129	41.939	1.00	0.00	H
ATOM	861	HG13	VAL	54	35.648	27.661	42.247	1.00	0.00	H
ATOM	862	CG2	VAL	54	35.929	28.007	39.693	1.00	0.00	C
ATOM	863	HG21	VAL	54	36.139	27.712	38.665	1.00	0.00	H

ATOM	864	HG22	VAL	54	34.972	27.654	40.077	1.00	0.00	H
ATOM	865	HG23	VAL	54	35.930	29.092	39.579	1.00	0.00	H
ATOM	866	C	VAL	54	39.482	27.717	41.265	1.00	0.00	C
ATOM	867	O	VAL	54	39.765	26.569	41.623	1.00	0.00	O
ATOM	868	N	THR	55	40.178	28.750	41.828	1.00	0.00	N
ATOM	869	H	THR	55	39.954	29.723	41.677	1.00	0.00	H
ATOM	870	CA	THR	55	41.327	28.623	42.755	1.00	0.00	C
ATOM	871	HA	THR	55	41.885	27.761	42.389	1.00	0.00	H
ATOM	872	CB	THR	55	42.266	29.865	42.566	1.00	0.00	C
ATOM	873	HB	THR	55	43.033	29.732	43.330	1.00	0.00	H
ATOM	874	CG2	THR	55	42.921	29.961	41.197	1.00	0.00	C
ATOM	875	HG21	THR	55	42.159	29.864	40.424	1.00	0.00	H
ATOM	876	HG22	THR	55	43.428	30.926	41.165	1.00	0.00	H
ATOM	877	HG23	THR	55	43.641	29.148	41.097	1.00	0.00	H
ATOM	878	OG1	THR	55	41.527	31.131	42.761	1.00	0.00	O
ATOM	879	HG1	THR	55	42.114	31.867	42.570	1.00	0.00	H
ATOM	880	C	THR	55	40.869	28.481	44.276	1.00	0.00	C
ATOM	881	O	THR	55	41.529	27.812	45.059	1.00	0.00	O
ATOM	882	N	ALA	56	39.849	29.259	44.706	1.00	0.00	N
ATOM	883	H	ALA	56	39.387	29.842	44.023	1.00	0.00	H
ATOM	884	CA	ALA	56	39.277	29.436	46.037	1.00	0.00	C
ATOM	885	HA	ALA	56	39.314	28.433	46.461	1.00	0.00	H
ATOM	886	CB	ALA	56	40.083	30.403	46.968	1.00	0.00	C
ATOM	887	HB1	ALA	56	39.745	30.328	48.002	1.00	0.00	H
ATOM	888	HB2	ALA	56	41.131	30.120	47.067	1.00	0.00	H
ATOM	889	HB3	ALA	56	40.014	31.410	46.556	1.00	0.00	H
ATOM	890	C	ALA	56	37.786	29.715	45.989	1.00	0.00	C
ATOM	891	O	ALA	56	37.272	30.441	45.027	1.00	0.00	O
ATOM	892	N	THR	57	37.013	29.255	46.915	1.00	0.00	N
ATOM	893	H	THR	57	37.515	28.812	47.671	1.00	0.00	H
ATOM	894	CA	THR	57	35.535	29.447	47.010	1.00	0.00	C
ATOM	895	HA	THR	57	35.194	30.406	46.619	1.00	0.00	H
ATOM	896	CB	THR	57	34.677	28.458	46.227	1.00	0.00	C
ATOM	897	HB	THR	57	33.670	28.435	46.644	1.00	0.00	H
ATOM	898	CG2	THR	57	34.571	28.698	44.769	1.00	0.00	C
ATOM	899	HG21	THR	57	34.094	29.663	44.595	1.00	0.00	H
ATOM	900	HG22	THR	57	35.581	28.731	44.359	1.00	0.00	H
ATOM	901	HG23	THR	57	34.034	27.917	44.230	1.00	0.00	H
ATOM	902	OG1	THR	57	35.115	27.080	46.383	1.00	0.00	O
ATOM	903	HG1	THR	57	34.609	26.671	47.089	1.00	0.00	H
ATOM	904	C	THR	57	35.162	29.348	48.459	1.00	0.00	C
ATOM	905	O	THR	57	35.821	28.663	49.209	1.00	0.00	O
ATOM	906	N	SER	58	34.085	30.073	48.830	1.00	0.00	N
ATOM	907	H	SER	58	33.635	30.689	48.170	1.00	0.00	H
ATOM	908	CA	SER	58	33.608	30.034	50.200	1.00	0.00	C
ATOM	909	HA	SER	58	33.343	29.033	50.537	1.00	0.00	H
ATOM	910	CB	SER	58	34.673	30.656	51.086	1.00	0.00	C
ATOM	911	HB2	SER	58	34.276	30.702	52.101	1.00	0.00	H
ATOM	912	HB3	SER	58	35.593	30.072	51.092	1.00	0.00	H
ATOM	913	OG	SER	58	35.052	31.943	50.624	1.00	0.00	O
ATOM	914	HG	SER	58	34.275	32.363	50.246	1.00	0.00	H
ATOM	915	C	SER	58	32.412	30.949	50.430	1.00	0.00	C
ATOM	916	O	SER	58	32.324	32.078	49.924	1.00	0.00	O
ATOM	917	N	THR	59	31.578	30.510	51.346	1.00	0.00	N
ATOM	918	H	THR	59	31.654	29.643	51.861	1.00	0.00	H
ATOM	919	CA	THR	59	30.469	31.242	51.924	1.00	0.00	C
ATOM	920	HA	THR	59	29.919	31.566	51.040	1.00	0.00	H
ATOM	921	CB	THR	59	29.594	30.284	52.726	1.00	0.00	C
ATOM	922	HB	THR	59	29.483	29.445	52.040	1.00	0.00	H
ATOM	923	CG2	THR	59	30.221	29.834	53.962	1.00	0.00	C
ATOM	924	HG21	THR	59	31.249	29.614	53.672	1.00	0.00	H
ATOM	925	HG22	THR	59	30.230	30.600	54.738	1.00	0.00	H
ATOM	926	HG23	THR	59	29.736	28.899	54.238	1.00	0.00	H
ATOM	927	OG1	THR	59	28.305	30.813	53.026	1.00	0.00	O

ATOM	928	HG1	THR	59	27.790	30.778	52.216	1.00	0.00	H
ATOM	929	C	THR	59	30.832	32.532	52.720	1.00	0.00	C
ATOM	930	O	THR	59	31.719	32.517	53.590	1.00	0.00	O
ATOM	931	N	VAL	60	30.030	33.639	52.724	1.00	0.00	N
ATOM	932	H	VAL	60	29.163	33.628	52.207	1.00	0.00	H
ATOM	933	CA	VAL	60	30.403	34.965	53.243	1.00	0.00	C
ATOM	934	HA	VAL	60	31.251	34.827	53.914	1.00	0.00	H
ATOM	935	CB	VAL	60	30.830	35.980	52.162	1.00	0.00	C
ATOM	936	HB	VAL	60	30.118	35.869	51.343	1.00	0.00	H
ATOM	937	CG1	VAL	60	30.856	37.401	52.728	1.00	0.00	C
ATOM	938	HG11	VAL	60	31.545	37.570	53.554	1.00	0.00	H
ATOM	939	HG12	VAL	60	31.057	38.116	51.928	1.00	0.00	H
ATOM	940	HG13	VAL	60	29.797	37.630	52.848	1.00	0.00	H
ATOM	941	CG2	VAL							
60	32.246	35.615	51.701	1.00	0.00	C				
ATOM	942	HG21	VAL	60	32.798	36.466	51.303	1.00	0.00	H
ATOM	943	HG22	VAL	60	32.864	35.323	52.551	1.00	0.00	H
ATOM	944	HG23	VAL	60	32.111	34.733	51.075	1.00	0.00	H
ATOM	945	C	VAL	60	29.166	35.534	53.996	1.00	0.00	C
ATOM	946	O	VAL	60	28.101	35.550	53.420	1.00	0.00	O
ATOM	947	N	THR	61	29.307	36.171	55.171	1.00	0.00	N
ATOM	948	H	THR	61	30.174	36.111	55.685	1.00	0.00	H
ATOM	949	CA	THR	61	28.443	37.240	55.635	1.00	0.00	C
ATOM	950	HA	THR	61	27.674	37.423	54.885	1.00	0.00	H
ATOM	951	CB	THR	61	27.925	37.033	57.057	1.00	0.00	C
ATOM	952	HB	THR	61	27.381	37.910	57.410	1.00	0.00	H
ATOM	953	CG2	THR	61	27.006	35.772	57.131	1.00	0.00	C
ATOM	954	HG21	THR	61	26.506	35.796	58.098	1.00	0.00	H
ATOM	955	HG22	THR	61	26.250	35.751	56.347	1.00	0.00	H
ATOM	956	HG23	THR	61	27.578	34.845	57.165	1.00	0.00	H
ATOM	957	OG1	THR	61	28.977	36.862	57.975	1.00	0.00	O
ATOM	958	HG1	THR	61	29.197	37.722	58.340	1.00	0.00	H
ATOM	959	C	THR	61	29.233	38.603	55.535	1.00	0.00	C
ATOM	960	O	THR	61	30.460	38.684	55.539	1.00	0.00	O
ATOM	961	N	LEU	62	28.500	39.779	55.416	1.00	0.00	N
ATOM	962	H	LEU	62	27.505	39.656	55.292	1.00	0.00	H
ATOM	963	CA	LEU	62	29.130	41.037	55.010	1.00	0.00	C
ATOM	964	HA	LEU	62	29.742	40.869	54.125	1.00	0.00	H
ATOM	965	CB	LEU	62	28.027	42.086	54.709	1.00	0.00	C
ATOM	966	HB2	LEU	62	27.357	42.084	55.569	1.00	0.00	H
ATOM	967	HB3	LEU	62	28.595	43.015	54.654	1.00	0.00	H
ATOM	968	CG	LEU	62	27.372	41.798	53.330	1.00	0.00	C
ATOM	969	HG	LEU	62	27.140	40.733	53.285	1.00	0.00	H
ATOM	970	CD1	LEU	62	26.080	42.600	53.124	1.00	0.00	C
ATOM	971	HD11	LEU	62	25.529	42.373	52.211	1.00	0.00	H
ATOM	972	HD12	LEU	62	25.271	42.429	53.835	1.00	0.00	H
ATOM	973	HD13	LEU	62	26.355	43.653	53.173	1.00	0.00	H
ATOM	974	CD2	LEU	62	28.189	42.052	52.136	1.00	0.00	C
ATOM	975	HD21	LEU	62	29.071	41.413	52.174	1.00	0.00	H
ATOM	976	HD22	LEU	62	27.673	41.667	51.256	1.00	0.00	H
ATOM	977	HD23	LEU	62	28.469	43.098	52.010	1.00	0.00	H
ATOM	978	C	LEU	62	30.175	41.563	56.040	1.00	0.00	C
ATOM	979	O	LEU	62	30.984	42.468	55.616	1.00	0.00	O
ATOM	980	N	ASP	63	30.176	41.115	57.245	1.00	0.00	N
ATOM	981	H	ASP	63	29.407	40.587	57.633	1.00	0.00	H
ATOM	982	CA	ASP	63	31.254	41.429	58.148	1.00	0.00	C
ATOM	983	HA	ASP	63	31.450	42.475	57.915	1.00	0.00	H
ATOM	984	CB	ASP	63	30.755	41.319	59.612	1.00	0.00	C
ATOM	985	HB2	ASP	63	31.639	41.677	60.140	1.00	0.00	H
ATOM	986	HB3	ASP	63	29.876	41.948	59.742	1.00	0.00	H
ATOM	987	CG	ASP	63	30.331	39.867	59.993	1.00	0.00	C
ATOM	988	OD1	ASP	63	29.839	39.086	59.169	1.00	0.00	O
ATOM	989	OD2	ASP	63	30.573	39.518	61.147	1.00	0.00	O
ATOM	990	C	ASP	63	32.589	40.757	57.798	1.00	0.00	C

ATOM	991	O	ASP	63	33.607	41.382	57.927	1.00	0.00	O
ATOM	992	N	THR	64	32.508	39.459	57.462	1.00	0.00	N
ATOM	993	H	THR	64	31.544	39.175	57.554	1.00	0.00	H
ATOM	994	CA	THR	64	33.633	38.538	57.083	1.00	0.00	C
ATOM	995	HA	THR	64	34.480	38.583	57.767	1.00	0.00	H
ATOM	996	CB	THR	64	33.326	37.019	57.149	1.00	0.00	C
ATOM	997	HB	THR	64	34.267	36.616	56.776	1.00	0.00	H
ATOM	998	CG2	THR	64	33.171	36.541	58.588	1.00	0.00	C
ATOM	999	HG21	THR	64	33.897	36.977	59.274	1.00	0.00	H
ATOM	1000	HG22	THR	64	32.134	36.735	58.864	1.00	0.00	H
ATOM	1001	HG23	THR	64	33.191	35.452	58.635	1.00	0.00	H
ATOM	1002	OG1	THR	64	32.320	36.687	56.230	1.00	0.00	O
ATOM	1003	HG1	THR	64	32.039	37.515	55.836	1.00	0.00	H
ATOM	1004	C	THR	64	34.279	38.808	55.674	1.00	0.00	C
ATOM	1005	O	THR	64	35.248	38.153	55.270	1.00	0.00	O
ATOM	1006	N	LEU	65	33.713	39.752	54.897	1.00	0.00	N
ATOM	1007	H	LEU	65	32.773	40.093	55.043	1.00	0.00	H
ATOM	1008	CA	LEU	65	34.175	40.054	53.518	1.00	0.00	C
ATOM	1009	HA	LEU	65	34.389	39.062	53.120	1.00	0.00	H
ATOM	1010	CB	LEU	65	32.942	40.653	52.743	1.00	0.00	C
ATOM	1011	HB2	LEU	65	32.096	39.974	52.845	1.00	0.00	H
ATOM	1012	HB3	LEU	65	32.680	41.613	53.187	1.00	0.00	H
ATOM	1013	CG	LEU	65	33.249	40.914	51.307	1.00	0.00	C
ATOM	1014	HG	LEU	65	34.089	41.594	51.167	1.00	0.00	H
ATOM	1015	CD1	LEU	65	33.544	39.595	50.498	1.00	0.00	C
ATOM	1016	HD11	LEU	65	34.446	39.123	50.889	1.00	0.00	H
ATOM	1017	HD12	LEU	65	32.703	38.921	50.660	1.00	0.00	H
ATOM	1018	HD13	LEU	65	33.727	39.795	49.442	1.00	0.00	H
ATOM	1019	CD2	LEU	65	32.040	41.590	50.741	1.00	0.00	C
ATOM	1020	HD21	LEU	65	31.762	42.465	51.328	1.00	0.00	H
ATOM	1021	HD22	LEU	65	32.261	41.787	49.693	1.00	0.00	H
ATOM	1022	HD23	LEU	65	31.179	40.922	50.765	1.00	0.00	H
ATOM	1023	C	LEU	65	35.360	40.983	53.411	1.00	0.00	C
ATOM	1024	O	LEU	65	35.176	42.140	53.834	1.00	0.00	O
ATOM	1025	N	THR	66	36.527	40.492	52.822	1.00	0.00	N
ATOM	1026	H	THR	66	36.513	39.495	52.657	1.00	0.00	H
ATOM	1027	CA	THR	66	37.741	41.293	52.554	1.00	0.00	C
ATOM	1028	HA	THR	66	37.981	41.965	53.378	1.00	0.00	H
ATOM	1029	CB	THR	66	38.983	40.385	52.251	1.00	0.00	C
ATOM	1030	HB	THR	66	38.676	39.702	51.459	1.00	0.00	H
ATOM	1031	CG2	THR	66	40.318	41.022	51.772	1.00	0.00	C
ATOM	1032	HG21	THR	66	40.444	41.896	52.411	1.00	0.00	H
ATOM	1033	HG22	THR	66	41.129	40.315	51.947	1.00	0.00	H
ATOM	1034	HG23	THR	66	40.276	41.442	50.767	1.00	0.00	H
ATOM	1035	OG1	THR	66	39.333	39.671	53.345	1.00	0.00	O
ATOM	1036	HG1	THR	66	40.099	39.149	53.093	1.00	0.00	H
ATOM	1037	C	THR	66	37.535	42.090	51.237	1.00	0.00	C
ATOM	1038	O	THR	66	36.709	41.779	50.331	1.00	0.00	O
ATOM	1039	N	GLU	67	38.285	43.136	51.029	1.00	0.00	N
ATOM	1040	H	GLU	67	38.941	43.186	51.795	1.00	0.00	H
ATOM	1041	CA	GLU	67	38.335	44.161	49.912	1.00	0.00	C
ATOM	1042	HA	GLU	67	37.326	44.427	49.599	1.00	0.00	H
ATOM	1043	CB	GLU	67	38.910	45.513	50.344	1.00	0.00	C
ATOM	1044	HB2	GLU	67	38.467	46.322	49.764	1.00	0.00	H
ATOM	1045	HB3	GLU	67	38.514	45.734	51.335	1.00	0.00	H
ATOM	1046	CG	GLU	67	40.426	45.644	50.246	1.00	0.00	C
ATOM	1047	HG2	GLU	67	40.690	45.534	49.195	1.00	0.00	H
ATOM	1048	HG3	GLU	67	40.743	46.675	50.399	1.00	0.00	H
ATOM	1049	CD	GLU	67	41.253	44.725	51.159	1.00	0.00	C
ATOM	1050	OE1	GLU	67	40.693	44.136	52.116	1.00	0.00	O
ATOM	1051	OE2	GLU	67	42.465	44.595	50.856	1.00	0.00	O
ATOM	1052	C	GLU	67	38.746	43.683	48.520	1.00	0.00	C
ATOM	1053	O	GLU	67	38.701	44.513	47.559	1.00	0.00	O
ATOM	1054	N	LYS	68	39.191	42.456	48.315	1.00	0.00	N

```
ATOM   1055  H   LYS    68      38.962  41.803  49.051  1.00  0.00           H
ATOM   1056  CA  LYS    68      39.810  41.949  47.099  1.00  0.00           C
ATOM   1057  HA  LYS    68      40.848  42.267  47.008  1.00  0.00           H
ATOM   1058  CB  LYS    68      39.849  40.382  47.183  1.00  0.00           C
ATOM   1059  HB2 LYS    68      40.281  40.040  48.123  1.00  0.00           H
ATOM   1060  HB3 LYS    68      38.815  40.050  47.092  1.00  0.00           H
ATOM   1061  CG  LYS    68      40.591  39.663  46.083  1.00  0.00           C
ATOM   1062  HG2 LYS    68      40.368  38.603  46.205  1.00  0.00           H
ATOM   1063  HG3 LYS    68      40.197  39.981  45.118  1.00  0.00           H
ATOM   1064  CD  LYS    68      42.122  39.917  46.079  1.00  0.00           C
ATOM   1065  HD2 LYS    68      42.302  40.895  45.634  1.00  0.00           H
ATOM   1066  HD3 LYS    68      42.488  39.909
  47.105  1.00  0.00           H
ATOM   1067  CE  LYS    68      42.684  38.908  45.033  1.00  0.00           C
ATOM   1068  HE2 LYS    68      42.284  37.937  45.325  1.00  0.00           H
ATOM   1069  HE3 LYS    68      42.338  39.011  44.005  1.00  0.00           H
ATOM   1070  NZ  LYS    68      44.152  38.864  45.082  1.00  0.00           N
ATOM   1071  HZ1 LYS    68      44.507  38.404  44.256  1.00  0.00           H
ATOM   1072  HZ2 LYS    68      44.520  39.804  45.033  1.00  0.00           H
ATOM   1073  HZ3 LYS    68      44.472  38.427  45.934  1.00  0.00           H
ATOM   1074  C   LYS    68      39.076  42.352  45.842  1.00  0.00           C
ATOM   1075  O   LYS    68      39.654  42.821  44.893  1.00  0.00           O
ATOM   1076  N   HIE    69      37.790  42.004  45.848  1.00  0.00           N
ATOM   1077  H   HIE    69      37.414  41.551  46.669  1.00  0.00           H
ATOM   1078  CA  HIE    69      36.786  42.335  44.830  1.00  0.00           C
ATOM   1079  HA  HIE    69      37.234  42.109  43.862  1.00  0.00           H
ATOM   1080  CB  HIE    69      35.495  41.430  45.015  1.00  0.00           C
ATOM   1081  HB2 HIE    69      35.887  40.415  45.084  1.00  0.00           H
ATOM   1082  HB3 HIE    69      35.005  41.737  45.940  1.00  0.00           H
ATOM   1083  CG  HIE    69      34.399  41.513  43.960  1.00  0.00           C
ATOM   1084  ND1 HIE    69      34.194  40.664  42.817  1.00  0.00           N
ATOM   1085  CE1 HIE    69      33.212  41.223  42.138  1.00  0.00           C
ATOM   1086  HE1 HIE    69      32.874  40.821  41.195  1.00  0.00           H
ATOM   1087  NE2 HIE    69      32.648  42.218  42.793  1.00  0.00           N
ATOM   1088  HE2 HIE    69      31.977  42.821  42.338  1.00  0.00           H
ATOM   1089  CD2 HIE    69      33.478  42.555  43.834  1.00  0.00           C
ATOM   1090  HD2 HIE    69      33.356  43.355  44.548  1.00  0.00           H
ATOM   1091  C   HIE    69      36.413  43.814  44.673  1.00  0.00           C
ATOM   1092  O   HIE    69      36.269  44.245  43.554  1.00  0.00           O
ATOM   1093  N   ALA    70      36.442  44.616  45.740  1.00  0.00           N
ATOM   1094  H   ALA    70      36.346  44.192  46.651  1.00  0.00           H
ATOM   1095  CA  ALA    70      36.061  46.077  45.706  1.00  0.00           C
ATOM   1096  HA  ALA    70      35.234  46.152  45.000  1.00  0.00           H
ATOM   1097  CB  ALA    70      35.570  46.551  47.046  1.00  0.00           C
ATOM   1098  HB1 ALA    70      36.429  46.778  47.680  1.00  0.00           H
ATOM   1099  HB2 ALA    70      34.932  47.423  46.899  1.00  0.00           H
ATOM   1100  HB3 ALA    70      35.030  45.750  47.550  1.00  0.00           H
ATOM   1101  C   ALA    70      37.196  46.830  45.074  1.00  0.00           C
ATOM   1102  O   ALA    70      37.024  47.471  44.051  1.00  0.00           O
ATOM   1103  N   GLU    71      38.430  46.645  45.526  1.00  0.00           N
ATOM   1104  H   GLU    71      38.479  46.016  46.316  1.00  0.00           H
ATOM   1105  CA  GLU    71      39.676  46.996  44.795  1.00  0.00           C
ATOM   1106  HA  GLU    71      39.712  48.082  44.704  1.00  0.00           H
ATOM   1107  CB  GLU    71      40.867  46.572  45.642  1.00  0.00           C
ATOM   1108  HB2 GLU    71      40.780  45.524  45.928  1.00  0.00           H
ATOM   1109  HB3 GLU    71      41.766  46.721  45.043  1.00  0.00           H
ATOM   1110  CG  GLU    71      40.987  47.446  46.837  1.00  0.00           C
ATOM   1111  HG2 GLU    71      40.135  47.218  47.477  1.00  0.00           H
ATOM   1112  HG3 GLU    71      41.950  47.118  47.228  1.00  0.00           H
ATOM   1113  CD  GLU    71      41.101  48.935  46.553  1.00  0.00           C
ATOM   1114  OE1 GLU    71      41.959  49.324  45.733  1.00  0.00           O
ATOM   1115  OE2 GLU    71      40.567  49.808  47.235  1.00  0.00           O
ATOM   1116  C   GLU    71      39.757  46.466  43.306  1.00  0.00           C
ATOM   1117  O   GLU    71      40.097  47.264  42.433  1.00  0.00           O
```

ATOM	1118	N	GLN	72	39.280	45.276	42.946	1.00	0.00	N
ATOM	1119	H	GLN	72	39.143	44.604	43.688	1.00	0.00	H
ATOM	1120	CA	GLN	72	39.233	44.830	41.524	1.00	0.00	C
ATOM	1121	HA	GLN	72	40.217	44.756	41.060	1.00	0.00	H
ATOM	1122	CB	GLN	72	38.852	43.327	41.614	1.00	0.00	C
ATOM	1123	HB2	GLN	72	37.962	43.252	42.238	1.00	0.00	H
ATOM	1124	HB3	GLN	72	39.619	42.773	42.154	1.00	0.00	H
ATOM	1125	CG	GLN	72	38.640	42.674	40.215	1.00	0.00	C
ATOM	1126	HG2	GLN	72	38.019	43.329	39.604	1.00	0.00	H
ATOM	1127	HG3	GLN	72	39.614	42.564	39.740	1.00	0.00	H
ATOM	1128	CD	GLN	72	37.934	41.349	40.293	1.00	0.00	C
ATOM	1129	OE1	GLN	72	38.524	40.292	40.271	1.00	0.00	O
ATOM	1130	NE2	GLN	72	36.682	41.385	40.560	1.00	0.00	N
ATOM	1131	HE21	GLN	72	36.302	42.307	40.403	1.00	0.00	H
ATOM	1132	HE22	GLN	72	36.146	40.563	40.804	1.00	0.00	H
ATOM	1133	C	GLN	72	38.257	45.762	40.673	1.00	0.00	C
ATOM	1134	O	GLN	72	38.696	46.285	39.641	1.00	0.00	O
ATOM	1135	N	GLU	73	37.066	46.062	41.225	1.00	0.00	N
ATOM	1136	H	GLU	73	36.848	45.644	42.118	1.00	0.00	H
ATOM	1137	CA	GLU	73	36.045	46.850	40.526	1.00	0.00	C
ATOM	1138	HA	GLU	73	36.239	46.419	39.543	1.00	0.00	H
ATOM	1139	CB	GLU	73	34.609	46.431	40.905	1.00	0.00	C
ATOM	1140	HB2	GLU	73	34.385	46.798	41.907	1.00	0.00	H
ATOM	1141	HB3	GLU	73	34.014	47.103	40.287	1.00	0.00	H
ATOM	1142	CG	GLU	73	34.202	44.931	40.738	1.00	0.00	C
ATOM	1143	HG2	GLU	73	34.669	44.293	41.489	1.00	0.00	H
ATOM	1144	HG3	GLU	73	33.126	44.823	40.878	1.00	0.00	H
ATOM	1145	CD	GLU	73	34.521	44.436	39.375	1.00	0.00	C
ATOM	1146	OE1	GLU	73	35.473	43.646	39.272	1.00	0.00	O
ATOM	1147	OE2	GLU	73	33.922	44.833	38.331	1.00	0.00	O
ATOM	1148	C	GLU	73	36.354	48.364	40.716	1.00	0.00	C
ATOM	1149	O	GLU	73	35.468	49.154	40.317	1.00	0.00	O
ATOM	1150	N	ASN	74	37.572	48.722	41.176	1.00	0.00	N
ATOM	1151	H	ASN	74	38.252	48.032	41.459	1.00	0.00	H
ATOM	1152	CA	ASN	74	38.054	50.088	41.325	1.00	0.00	C
ATOM	1153	HA	ASN	74	39.000	49.926	41.840	1.00	0.00	H
ATOM	1154	CB	ASN	74	38.346	50.904	39.992	1.00	0.00	C
ATOM	1155	HB2	ASN	74	37.413	51.273	39.567	1.00	0.00	H
ATOM	1156	HB3	ASN	74	38.926	51.729	40.405	1.00	0.00	H
ATOM	1157	CG	ASN	74	38.964	49.983	38.943	1.00	0.00	C
ATOM	1158	OD1	ASN	74	38.447	49.687	37.827	1.00	0.00	O
ATOM	1159	ND2	ASN	74	40.177	49.648	39.050	1.00	0.00	N
ATOM	1160	HD21	ASN	74	40.593	49.048	38.351	1.00	0.00	H
ATOM	1161	HD22	ASN	74	40.825	50.115	39.668	1.00	0.00	H
ATOM	1162	C	ASN	74	37.191	50.883	42.384	1.00	0.00	C
ATOM	1163	O	ASN	74	36.765	52.018	42.093	1.00	0.00	O
ATOM	1164	N	MET	75	36.934	50.302	43.576	1.00	0.00	N
ATOM	1165	H	MET	75	37.285	49.370	43.740	1.00	0.00	H
ATOM	1166	CA	MET	75	35.956	50.866	44.551	1.00	0.00	C
ATOM	1167	HA	MET	75	35.959	51.954	44.473	1.00	0.00	H
ATOM	1168	CB	MET	75	34.463	50.570	44.208	1.00	0.00	C
ATOM	1169	HB2	MET	75	33.842	51.145	44.895	1.00	0.00	H
ATOM	1170	HB3	MET	75	34.331	50.881	43.172	1.00	0.00	H
ATOM	1171	CG	MET	75	34.093	49.162	44.347	1.00	0.00	C
ATOM	1172	HG2	MET	75	34.810	48.673	43.688	1.00	0.00	H
ATOM	1173	HG3	MET	75	34.255	48.852	45.380	1.00	0.00	H
ATOM	1174	SD	MET	75	32.388	48.768	43.979	1.00	0.00	S
ATOM	1175	CE	MET	75	32.392	48.606	42.112	1.00	0.00	C
ATOM	1176	HE1	MET	75	33.395	48.614	41.684	1.00	0.00	H
ATOM	1177	HE2	MET	75	31.834	47.722	41.807	1.00	0.00	H
ATOM	1178	HE3	MET	75	31.970	49.535	41.726	1.00	0.00	H
ATOM	1179	C	MET	75	36.339	50.606	45.979	1.00	0.00	C
ATOM	1180	O	MET	75	36.846	49.529	46.261	1.00	0.00	O
ATOM	1181	N	THR	76	35.881	51.532	46.848	1.00	0.00	N

ATOM	1182	H	THR	76	35.473	52.396	46.519	1.00	0.00	H
ATOM	1183	CA	THR	76	35.909	51.334	48.318	1.00	0.00	C
ATOM	1184	HA	THR	76	36.878	50.950	48.638	1.00	0.00	H
ATOM	1185	CB	THR	76	35.699	52.637	49.153	1.00	0.00	C
ATOM	1186	HB	THR	76	35.930	53.513	48.547	1.00	0.00	H
ATOM	1187	CG2	THR	76	34.376	52.691	49.843	1.00	0.00	C
ATOM	1188	HG21	THR	76	34.144	53.664	50.275	1.00	0.00	H
ATOM	1189	HG22	THR	76	33.666	52.277	49.127	1.00	0.00	H
ATOM	1190	HG23	THR	76	34.336	52.037	50.714	1.00	0.00	H
ATOM	1191	OG1	THR	76	36.559	52.781	50.287	1.00	0.00	O
ATOM	1192	HG1	THR	76	37.452	52.877	49.948	1.00	0.00	H
ATOM	1193	C	THR	76	35.076	50.076	48.831	1.00	0.00	C
ATOM	1194	O	THR	76	34.001	49.858	48.256	1.00	0.00	O
ATOM	1195	N	LEU	77	35.361	49.431	49.938	1.00	0.00	N
ATOM	1196	H	LEU	77	36.251	49.638	50.370	1.00	0.00	H
ATOM	1197	CA	LEU	77	34.578	48.372	50.522	1.00	0.00	C
ATOM	1198	HA	LEU	77	34.323	47.688	49.714	1.00	0.00	H
ATOM	1199	CB	LEU	77	35.523	47.589	51.428	1.00	0.00	C
ATOM	1200	HB2	LEU	77	36.325	47.225	50.786	1.00	0.00	H
ATOM	1201	HB3	LEU	77	35.957	48.187	52.230	1.00	0.00	H
ATOM	1202	CG	LEU	77	34.918	46.379	52.232	1.00	0.00	C
ATOM	1203	HG	LEU	77	34.120	46.807	52.837	1.00	0.00	H
ATOM	1204	CD1	LEU	77	34.356	45.312	51.391	1.00	0.00	C
ATOM	1205	HD11	LEU	77	33.954	44.479	51.967	1.00	0.00	H
ATOM	1206	HD12	LEU	77	33.558	45.747	50.790	1.00	0.00	H
ATOM	1207	HD13	LEU	77	35.171	44.853	50.832	1.00	0.00	H
ATOM	1208	CD2	LEU	77	35.921	45.720	53.151	1.00	0.00	C
ATOM	1209	HD21	LEU	77	36.737	45.210	52.638	1.00	0.00	H
ATOM	1210	HD22	LEU	77	36.348	46.477	53.809	1.00	0.00	H
ATOM	1211	HD23	LEU	77	35.351	45.019	53.760	1.00	0.00	H
ATOM	1212	C	LEU	77	33.331	48.770	51.296	1.00	0.00	C
ATOM	1213	O	LEU	77	32.297	48.193	51.000	1.00	0.00	O
ATOM	1214	N	THR	78	33.357	49.906	52.030	1.00	0.00	N
ATOM	1215	H	THR	78	34.235	50.292	52.347	1.00	0.00	H
ATOM	1216	CA	THR	78	32.148	50.435	52.697	1.00	0.00	C
ATOM	1217	HA	THR	78	31.791	49.691	53.411	1.00	0.00	H
ATOM	1218	CB	THR	78	32.328	51.711	53.530	1.00	0.00	C
ATOM	1219	HB	THR	78	31.397	52.025	54.001	1.00	0.00	H
ATOM	1220	CG2	THR	78	33.351	51.631	54.612	1.00	0.00	C
ATOM	1221	HG21	THR	78	33.408	52.519	55.242	1.00	0.00	H
ATOM	1222	HG22	THR	78	33.239	50.814	55.325	1.00	0.00	H
ATOM	1223	HG23	THR	78	34.343	51.400	54.225	1.00	0.00	H
ATOM	1224	OG1	THR	78	32.793	52.710	52.645	1.00	0.00	O
ATOM	1225	HG1	THR	78	32.151	53.414	52.531	1.00	0.00	H
ATOM	1226	C	THR	78	31.050	50.762	51.744	1.00	0.00	C
ATOM	1227	O	THR	78	29.885	50.607	52.034	1.00	0.00	O
ATOM	1228	N	GLU	79	31.322	51.240	50.531	1.00	0.00	N
ATOM	1229	H	GLU	79	32.304	51.391	50.350	1.00	0.00	H
ATOM	1230	CA	GLU	79	30.280	51.457	49.455	1.00	0.00	C
ATOM	1231	HA	GLU	79	29.382	51.786	49.976	1.00	0.00	H
ATOM	1232	CB	GLU	79	30.731	52.474	48.525	1.00	0.00	C
ATOM	1233	HB2	GLU	79	31.673	52.115	48.112	1.00	0.00	H
ATOM	1234	HB3	GLU	79	30.140	52.466	47.609	1.00	0.00	H
ATOM	1235	CG	GLU	79	30.914	53.965	49.028	1.00	0.00	C
ATOM	1236	HG2	GLU	79	31.614	53.955	49.863	1.00	0.00	H
ATOM	1237	HG3	GLU	79	31.194	54.538	48.144	1.00	0.00	H
ATOM	1238	CD	GLU	79	29.460	54.592	49.357	1.00	0.00	C
ATOM	1239	OE1	GLU	79	29.122	54.797	50.550	1.00	0.00	O
ATOM	1240	OE2	GLU	79	28.765	54.969	48.361	1.00	0.00	O
ATOM	1241	C	GLU	79	29.752	50.148	48.833	1.00	0.00	C
ATOM	1242	O	GLU	79	28.568	50.072	48.598	1.00	0.00	O
ATOM	1243	N	LEU	80	30.638	49.168	48.477	1.00	0.00	N
ATOM	1244	H	LEU	80	31.594	49.420	48.681	1.00	0.00	H

ATOM	1245	CA	LEU	80	30.194	47.916	47.790	1.00	0.00	C
ATOM	1246	HA	LEU	80	29.664	48.120	46.858	1.00	0.00	H
ATOM	1247	CB	LEU	80	31.467	47.080	47.469	1.00	0.00	C
ATOM	1248	HB2	LEU	80	31.984	47.498	46.604	1.00	0.00	H
ATOM	1249	HB3	LEU	80	32.149	47.115	48.319	1.00	0.00	H
ATOM	1250	CG	LEU	80	31.188	45.623	46.999	1.00	0.00	C
ATOM	1251	HG	LEU	80	30.461	45.170	47.673	1.00	0.00	H
ATOM	1252	CD1	LEU	80	30.412	45.562	45.613	1.00	0.00	C
ATOM	1253	HD11	LEU	80	31.168	45.898	44.905	1.00	0.00	H
ATOM	1254	HD12	LEU	80	30.110	44.523	45.484	1.00	0.00	H
ATOM	1255	HD13	LEU	80	29.535	46.210	45.657	1.00	0.00	H
ATOM	1256	CD2	LEU	80	32.359	44.667	47.075	1.00	0.00	C
ATOM	1257	HD21	LEU	80	32.059	43.631	46.916	1.00	0.00	H
ATOM	1258	HD22	LEU	80	33.157	45.023	46.423	1.00	0.00	H
ATOM	1259	HD23	LEU	80	32.626	44.685	48.132	1.00	0.00	H
ATOM	1260	C	LEU	80	29.264	47.070	48.718	1.00	0.00	C
ATOM	1261	O	LEU	80	28.324	46.498	48.218	1.00	0.00	O
ATOM	1262	N	LYS	81	29.535	47.064	50.023	1.00	0.00	N
ATOM	1263	H	LYS	81	30.434	47.458	50.262	1.00	0.00	H
ATOM	1264	CA	LYS	81	28.530	46.583	51.018	1.00	0.00	C
ATOM	1265	HA	LYS	81	28.208	45.569	50.781	1.00	0.00	H
ATOM	1266	CB	LYS	81	29.034	46.701	52.428	1.00	0.00	C
ATOM	1267	HB2	LYS	81	29.339	47.732	52.609	1.00	0.00	H
ATOM	1268	HB3	LYS	81	28.206	46.482	53.103	1.00	0.00	H
ATOM	1269	CG	LYS	81	30.232	45.852	52.682	1.00	0.00	C
ATOM	1270	HG2	LYS	81	30.027	44.786	52.572	1.00	0.00	H
ATOM	1271	HG3	LYS	81	31.076	46.032	52.017	1.00	0.00	H
ATOM	1272	CD	LYS	81	30.706	45.967	54.127	1.00	0.00	C
ATOM	1273	HD2	LYS	81	30.849	47.035	54.293	1.00	0.00	H
ATOM	1274	HD3	LYS	81	29.965	45.521	54.791	1.00	0.00	H
ATOM	1275	CE	LYS	81	31.960	45.180	54.363	1.00	0.00	C
ATOM	1276	HE2	LYS	81	31.862	44.100	54.249	1.00	0.00	H
ATOM	1277	HE3	LYS	81	32.615	45.571	53.584	1.00	0.00	H
ATOM	1278	NZ	LYS	81	32.550	45.452	55.713	1.00	0.00	N
ATOM	1279	HZ1	LYS	81	32.710	46.421	55.948	1.00	0.00	H
ATOM	1280	HZ2	LYS	81	31.954	45.037	56.415	1.00	0.00	H
ATOM	1281	HZ3	LYS	81	33.464	45.054	55.876	1.00	0.00	H
ATOM	1282	C	LYS	81	27.204	47.296	50.927	1.00	0.00	C
ATOM	1283	O	LYS	81	26.180	46.665	50.815	1.00	0.00	O
ATOM	1284	N	LYS	82	27.267	48.619	50.871	1.00	0.00	N
ATOM	1285	H	LYS	82	28.176	49.052	50.799	1.00	0.00	H
ATOM	1286	CA	LYS	82	26.140	49.510	50.925	1.00	0.00	C
ATOM	1287	HA	LYS	82	25.518	49.172	51.754	1.00	0.00	H
ATOM	1288	CB	LYS	82	26.507	50.974	51.297	1.00	0.00	C
ATOM	1289	HB2	LYS	82	27.214	50.968	52.126	1.00	0.00	H
ATOM	1290	HB3	LYS	82	27.081	51.376	50.462	1.00	0.00	H
ATOM	1291	CG	LYS	82	25.271	51.801	51.672	1.00	0.00	C
ATOM	1292	HG2	LYS	82	24.522	52.006	50.906	1.00	0.00	H
ATOM	1293	HG3	LYS	82	24.758	51.345	52.519	1.00	0.00	H
ATOM	1294	CD	LYS	82	25.791	53.136	52.271	1.00	0.00	C
ATOM	1295	HD2	LYS	82	25.044	53.550	52.948	1.00	0.00	H
ATOM	1296	HD3	LYS	82	26.705	52.897	52.813	1.00	0.00	H
ATOM	1297	CE	LYS	82	26.112	54.144	51.183	1.00	0.00	C
ATOM	1298	HE2	LYS	82	26.328	55.112	51.635	1.00	0.00	H
ATOM	1299	HE3	LYS	82	27.023	53.791	50.700	1.00	0.00	H
ATOM	1300	NZ	LYS	82	25.007	54.398	50.230	1.00	0.00	N
ATOM	1301	HZ1	LYS	82	24.150	54.603	50.723	1.00	0.00	H
ATOM	1302	HZ2	LYS	82	25.272	55.222	49.711	1.00	0.00	H
ATOM	1303	HZ3	LYS	82	24.749	53.637	49.618	1.00	0.00	H
ATOM	1304	C	LYS	82	25.177	49.446	49.721	1.00	0.00	C
ATOM	1305	O	LYS	82	23.931	49.412	49.907	1.00	0.00	O
ATOM	1306	N	VAL	83	25.692	49.365	48.528	1.00	0.00	N
ATOM	1307	H	VAL	83	26.696	49.265	48.538	1.00	0.00	H
ATOM	1308	CA	VAL	83	24.873	49.092	47.339	1.00	0.00	C

```
ATOM   1309  HA  VAL    83      23.997  49.740  47.305  1.00  0.00           H
ATOM   1310  CB  VAL    83      25.623  49.347  46.009  1.00  0.00           C
ATOM   1311  HB  VAL    83      25.064  48.873  45.201  1.00  0.00           H
ATOM   1312  CG1 VAL    83      26.018  50.749  45.784  1.00  0.00           C
ATOM   1313 HG11 VAL    83      25.154  51.412  45.830  1.00  0.00           H
ATOM   1314 HG12 VAL    83      26.657  51.028  46.621  1.00  0.00           H
ATOM   1315 HG13 VAL    83      26.623  50.843  44.882  1.00  0.00           H
ATOM   1316  CG2 VAL    83      26.888  48.438  46.063  1.00  0.00           C
ATOM   1317 HG21 VAL
    83      26.614  47.407  45.842  1.00  0.00           H
ATOM   1318 HG22 VAL    83      27.589  48.810  45.316  1.00  0.00           H
ATOM   1319 HG23 VAL    83      27.428  48.560  47.001  1.00  0.00           H
ATOM   1320  C   VAL    83      24.308  47.626  47.365  1.00  0.00           C
ATOM   1321  O   VAL    83      23.182  47.387  46.955  1.00  0.00           O
ATOM   1322  N   ILE    84      25.052  46.625  47.925  1.00  0.00           N
ATOM   1323  H   ILE    84      25.970  46.924  48.225  1.00  0.00           H
ATOM   1324  CA  ILE    84      24.536  45.252  48.051  1.00  0.00           C
ATOM   1325  HA  ILE    84      24.103  44.971  47.091  1.00  0.00           H
ATOM   1326  CB  ILE    84      25.748  44.299  48.418  1.00  0.00           C
ATOM   1327  HB  ILE    84      26.465  44.935  48.937  1.00  0.00           H
ATOM   1328  CG2 ILE    84      25.474  43.110  49.363  1.00  0.00           C
ATOM   1329 HG21 ILE    84      25.135  43.597  50.278  1.00  0.00           H
ATOM   1330 HG22 ILE    84      24.674  42.498  48.946  1.00  0.00           H
ATOM   1331 HG23 ILE    84      26.362  42.496  49.508  1.00  0.00           H
ATOM   1332  CG1 ILE    84      26.349  43.801  47.051  1.00  0.00           C
ATOM   1333 HG12 ILE    84      25.542  43.182  46.658  1.00  0.00           H
ATOM   1334 HG13 ILE    84      26.436  44.655  46.378  1.00  0.00           H
ATOM   1335  CD1 ILE    84      27.751  43.220  47.085  1.00  0.00           C
ATOM   1336 HD11 ILE    84      27.694  42.246  47.573  1.00  0.00           H
ATOM   1337 HD12 ILE    84      28.208  43.006  46.119  1.00  0.00           H
ATOM   1338 HD13 ILE    84      28.439  43.956  47.502  1.00  0.00           H
ATOM   1339  C   ILE    84      23.376  45.187  49.024  1.00  0.00           C
ATOM   1340  O   ILE    84      22.381  44.485  48.794  1.00  0.00           O
ATOM   1341  N   ALA    85      23.470  45.844  50.215  1.00  0.00           N
ATOM   1342  H   ALA    85      24.248  46.446  50.441  1.00  0.00           H
ATOM   1343  CA  ALA    85      22.403  45.888  51.147  1.00  0.00           C
ATOM   1344  HA  ALA    85      22.126  44.893  51.496  1.00  0.00           H
ATOM   1345  CB  ALA    85      22.857  46.662  52.383  1.00  0.00           C
ATOM   1346  HB1 ALA    85      23.819  46.280  52.727  1.00  0.00           H
ATOM   1347  HB2 ALA    85      23.061  47.692  52.088  1.00  0.00           H
ATOM   1348  HB3 ALA    85      22.089  46.601  53.154  1.00  0.00           H
ATOM   1349  C   ALA    85      21.070  46.402  50.704  1.00  0.00           C
ATOM   1350  O   ALA    85      20.059  46.240  51.329  1.00  0.00           O
ATOM   1351  N   ASP    86      20.970  47.143  49.632  1.00  0.00           N
ATOM   1352  H   ASP    86      21.890  47.465  49.370  1.00  0.00           H
ATOM   1353  CA  ASP    86      19.776  47.714  48.987  1.00  0.00           C
ATOM   1354  HA  ASP    86      19.211  48.047  49.859  1.00  0.00           H
ATOM   1355  CB  ASP    86      20.016  48.994  48.136  1.00  0.00           C
ATOM   1356  HB2 ASP    86      20.658  48.608  47.345  1.00  0.00           H
ATOM   1357  HB3 ASP    86      19.109  49.296  47.614  1.00  0.00           H
ATOM   1358  CG  ASP    86      20.747  50.154  48.859  1.00  0.00           C
ATOM   1359  OD1 ASP    86      21.333  51.030  48.261  1.00  0.00           O
ATOM   1360  OD2 ASP    86      20.442  50.319  50.053  1.00  0.00           O
ATOM   1361  C   ASP    86      19.015  46.662  48.100  1.00  0.00           C
ATOM   1362  O   ASP    86      17.730  46.664  48.039  1.00  0.00           O
ATOM   1363  N   ILE    87      19.791  45.862  47.398  1.00  0.00           N
ATOM   1364  H   ILE    87      20.756  46.156  47.443  1.00  0.00           H
ATOM   1365  CA  ILE    87      19.295  44.704  46.603  1.00  0.00           C
ATOM   1366  HA  ILE    87      18.506  44.972  45.900  1.00  0.00           H
ATOM   1367  CB  ILE    87      20.420  44.375  45.594  1.00  0.00           C
ATOM   1368  HB  ILE    87      21.365  44.240  46.121  1.00  0.00           H
ATOM   1369  CG2 ILE    87      20.131  43.074  44.883  1.00  0.00           C
ATOM   1370 HG21 ILE    87      19.120  43.240  44.512  1.00  0.00           H
ATOM   1371 HG22 ILE    87      20.810  42.932  44.041  1.00  0.00           H
```

ATOM	1372	HG23	ILE	87	20.104	42.213	45.550	1.00	0.00	H
ATOM	1373	CG1	ILE	87	20.654	45.472	44.568	1.00	0.00	C
ATOM	1374	HG12	ILE	87	19.903	45.350	43.788	1.00	0.00	H
ATOM	1375	HG13	ILE	87	20.499	46.417	45.089	1.00	0.00	H
ATOM	1376	CD1	ILE	87	22.022	45.363	43.839	1.00	0.00	C
ATOM	1377	HD11	ILE	87	22.310	46.265	43.299	1.00	0.00	H
ATOM	1378	HD12	ILE	87	22.799	45.213	44.589	1.00	0.00	H
ATOM	1379	HD13	ILE	87	22.087	44.657	43.011	1.00	0.00	H
ATOM	1380	C	ILE	87	18.830	43.573	47.475	1.00	0.00	C
ATOM	1381	O	ILE	87	17.758	42.946	47.241	1.00	0.00	O
ATOM	1382	N	TYR	88	19.688	43.291	48.510	1.00	0.00	N
ATOM	1383	H	TYR	88	20.477	43.899	48.675	1.00	0.00	H
ATOM	1384	CA	TYR	88	19.529	42.078	49.308	1.00	0.00	C
ATOM	1385	HA	TYR	88	18.719	41.509	48.853	1.00	0.00	H
ATOM	1386	CB	TYR	88	20.887	41.363	49.235	1.00	0.00	C
ATOM	1387	HB2	TYR	88	21.616	42.047	49.669	1.00	0.00	H
ATOM	1388	HB3	TYR	88	20.764	40.519	49.915	1.00	0.00	H
ATOM	1389	CG	TYR	88	21.274	40.800	47.849	1.00	0.00	C
ATOM	1390	CD1	TYR	88	20.641	39.637	47.415	1.00	0.00	C
ATOM	1391	HD1	TYR	88	19.790	39.325	48.002	1.00	0.00	H
ATOM	1392	CE1	TYR	88	20.985	39.070	46.131	1.00	0.00	C
ATOM	1393	HE1	TYR	88	20.703	38.140	45.662	1.00	0.00	H
ATOM	1394	CZ	TYR	88	22.078	39.661	45.449	1.00	0.00	C
ATOM	1395	OH	TYR	88	22.640	39.022	44.350	1.00	0.00	O
ATOM	1396	HH	TYR	88	22.060	38.460	43.831	1.00	0.00	H
ATOM	1397	CE2	TYR	88	22.729	40.868	45.900	1.00	0.00	C
ATOM	1398	HE2	TYR	88	23.491	41.315	45.278	1.00	0.00	H
ATOM	1399	CD2	TYR	88	22.278	41.397	47.079	1.00	0.00	C
ATOM	1400	HD2	TYR	88	22.784	42.271	47.464	1.00	0.00	H
ATOM	1401	C	TYR	88	19.031	42.300	50.806	1.00	0.00	C
ATOM	1402	O	TYR	88	19.360	43.349	51.470	1.00	0.00	O
ATOM	1403	N	PRO	89	18.143	41.437	51.379	1.00	0.00	N
ATOM	1404	CD	PRO	89	17.265	40.467	50.694	1.00	0.00	C
ATOM	1405	HD2	PRO	89	17.689	39.833	49.916	1.00	0.00	H
ATOM	1406	HD3	PRO	89	16.416	41.039	50.319	1.00	0.00	H
ATOM	1407	CG	PRO	89	16.791	39.516	51.811	1.00	0.00	C
ATOM	1408	HG2	PRO	89	17.549	38.746	51.953	1.00	0.00	H
ATOM	1409	HG3	PRO	89	15.779	39.150	51.637	1.00	0.00	H
ATOM	1410	CB	PRO	89	16.641	40.461	52.988	1.00	0.00	C
ATOM	1411	HB2	PRO	89	16.679	39.874	53.907	1.00	0.00	H
ATOM	1412	HB3	PRO	89	15.671	40.948	52.891	1.00	0.00	H
ATOM	1413	CA	PRO	89	17.789	41.489	52.819	1.00	0.00	C
ATOM	1414	HA	PRO	89	17.420	42.484	53.069	1.00	0.00	H
ATOM	1415	C	PRO	89	19.014	41.097	53.699	1.00	0.00	C
ATOM	1416	O	PRO	89	19.883	40.314	53.345	1.00	0.00	O
ATOM	1417	N	GLY	90	19.189	41.725	54.900	1.00	0.00	N
ATOM	1418	H	GLY	90	18.420	42.316	55.182	1.00	0.00	H
ATOM	1419	CA	GLY	90	20.425	41.713	55.711	1.00	0.00	C
ATOM	1420	HA2	GLY	90	21.308	42.052	55.170	1.00	0.00	H
ATOM	1421	HA3	GLY	90	20.261	42.329	56.596	1.00	0.00	H
ATOM	1422	C	GLY	90	20.832	40.366	56.251	1.00	0.00	C
ATOM	1423	O	GLY	90	22.007	40.108	56.539	1.00	0.00	O
ATOM	1424	N	GLN	91	19.772	39.532	56.355	1.00	0.00	N
ATOM	1425	H	GLN	91	18.916	40.011	56.117	1.00	0.00	H
ATOM	1426	CA	GLN	91	19.722	38.089	56.627	1.00	0.00	C
ATOM	1427	HA	GLN	91	20.384	37.937	57.479	1.00	0.00	H
ATOM	1428	CB	GLN	91	18.334	37.693	56.950	1.00	0.00	C
ATOM	1429	HB2	GLN	91	17.702	37.664	56.062	1.00	0.00	H
ATOM	1430	HB3	GLN	91	18.257	36.673	57.324	1.00	0.00	H
ATOM	1431	CG	GLN	91	17.626	38.604	57.934	1.00	0.00	C
ATOM	1432	HG2	GLN	91	16.797	37.974	58.255	1.00	0.00	H
ATOM	1433	HG3	GLN	91	18.294	38.817	58.769	1.00	0.00	H
ATOM	1434	CD	GLN	91	17.046	39.921	57.379	1.00	0.00	C
ATOM	1435	OE1	GLN	91	16.992	40.146	56.251	1.00	0.00	O

```
ATOM   1436  NE2 GLN    91      16.360  40.724  58.197  1.00  0.00           N
ATOM   1437 HE21 GLN    91      16.151  40.340  59.108  1.00  0.00           H
ATOM   1438 HE22 GLN    91      16.051  41.583  57.764  1.00  0.00           H
ATOM   1439  C   GLN    91      20.232  37.250  55.409  1.00  0.00           C
ATOM   1440  O   GLN    91      20.174  36.025  55.536  1.00  0.00           O
ATOM   1441  N   THR    92      20.793  37.809  54.350  1.00  0.00           N
ATOM   1442  H   THR    92      20.944  38.804
  54.441  1.00  0.00           H
ATOM   1443  CA  THR    92      21.361  37.081  53.223  1.00  0.00           C
ATOM   1444  HA  THR    92      20.831  36.194  52.876  1.00  0.00           H
ATOM   1445  CB  THR    92      21.392  37.986  52.003  1.00  0.00           C
ATOM   1446  HB  THR    92      22.028  38.841  52.228  1.00  0.00           H
ATOM   1447  CG2 THR    92      22.041  37.308  50.822  1.00  0.00           C
ATOM   1448 HG21 THR    92      23.118  37.395  50.961  1.00  0.00           H
ATOM   1449 HG22 THR    92      21.716  36.278  50.670  1.00  0.00           H
ATOM   1450 HG23 THR    92      21.837  37.847  49.897  1.00  0.00           H
ATOM   1451  OG1 THR    92      20.142  38.497  51.540  1.00  0.00           O
ATOM   1452  HG1 THR    92      19.721  38.873  52.316  1.00  0.00           H
ATOM   1453  C   THR    92      22.745  36.532  53.476  1.00  0.00           C
ATOM   1454  O   THR    92      23.528  37.261  54.153  1.00  0.00           O
ATOM   1455  N   GLN    93      22.986  35.276  53.171  1.00  0.00           N
ATOM   1456  H   GLN    93      22.188  34.734  52.874  1.00  0.00           H
ATOM   1457  CA  GLN    93      24.316  34.676  52.941  1.00  0.00           C
ATOM   1458  HA  GLN    93      25.011  35.250  53.554  1.00  0.00           H
ATOM   1459  CB  GLN    93      24.123  33.214  53.270  1.00  0.00           C
ATOM   1460  HB2 GLN    93      23.650  33.051  54.237  1.00  0.00           H
ATOM   1461  HB3 GLN    93      23.436  32.677  52.616  1.00  0.00           H
ATOM   1462  CG  GLN    93      25.530  32.584  53.452  1.00  0.00           C
ATOM   1463  HG2 GLN    93      25.436  31.501  53.368  1.00  0.00           H
ATOM   1464  HG3 GLN    93      26.303  32.971  52.789  1.00  0.00           H
ATOM   1465  CD  GLN    93      26.080  32.803  54.889  1.00  0.00           C
ATOM   1466  OE1 GLN    93      25.482  33.004  55.899  1.00  0.00           O
ATOM   1467  NE2 GLN    93      27.360  32.762  55.086  1.00  0.00           N
ATOM   1468 HE21 GLN    93      27.926  32.360  54.353  1.00  0.00           H
ATOM   1469 HE22 GLN    93      27.662  32.737  56.050  1.00  0.00           H
ATOM   1470  C   GLN    93      24.770  34.821  51.483  1.00  0.00           C
ATOM   1471  O   GLN    93      23.978  34.817  50.523  1.00  0.00           O
ATOM   1472  N   PHE    94      26.051  34.919  51.330  1.00  0.00           N
ATOM   1473  H   PHE    94      26.646  34.823  52.140  1.00  0.00           H
ATOM   1474  CA  PHE    94      26.661  35.114  50.024  1.00  0.00           C
ATOM   1475  HA  PHE    94      25.957  35.016  49.198  1.00  0.00           H
ATOM   1476  CB  PHE    94      27.215  36.544  49.954  1.00  0.00           C
ATOM   1477  HB2 PHE    94      28.007  36.656  50.694  1.00  0.00           H
ATOM   1478  HB3 PHE    94      27.767  36.640  49.018  1.00  0.00           H
ATOM   1479  CG  PHE    94      26.268  37.796  49.967  1.00  0.00           C
ATOM   1480  CD1 PHE    94      25.662  38.320  48.768  1.00  0.00           C
ATOM   1481  HD1 PHE    94      25.852  37.822  47.830  1.00  0.00           H
ATOM   1482  CE1 PHE    94      24.777  39.396  48.836  1.00  0.00           C
ATOM   1483  HE1 PHE    94      24.312  39.749  47.926  1.00  0.00           H
ATOM   1484  CZ  PHE    94      24.567  40.012  50.099  1.00  0.00           C
ATOM   1485  HZ  PHE    94      23.883  40.829  50.268  1.00  0.00           H
ATOM   1486  CE2 PHE    94      25.278  39.525  51.212  1.00  0.00           C
ATOM   1487  HE2 PHE    94      25.078  39.959  52.182  1.00  0.00           H
ATOM   1488  CD2 PHE    94      26.199  38.551  51.105  1.00  0.00           C
ATOM   1489  HD2 PHE    94      26.742  38.214  51.975  1.00  0.00           H
ATOM   1490  C   PHE    94      27.730  34.096  49.773  1.00  0.00           C
ATOM   1491  O   PHE    94      28.189  33.384  50.682  1.00  0.00           O
ATOM   1492  N   TYR    95      28.297  34.021  48.563  1.00  0.00           N
ATOM   1493  H   TYR    95      27.972  34.705  47.894  1.00  0.00           H
ATOM   1494  CA  TYR    95      29.194  32.947  47.959  1.00  0.00           C
ATOM   1495  HA  TYR    95      29.642  32.345  48.749  1.00  0.00           H
ATOM   1496  CB  TYR    95      28.310  31.903  47.155  1.00  0.00           C
ATOM   1497  HB2 TYR    95      27.524  31.504  47.798  1.00  0.00           H
ATOM   1498  HB3 TYR    95      27.903  32.350  46.249  1.00  0.00           H
```

ATOM	1499	CG	TYR	95	29.181	30.734	46.731	1.00	0.00	C
ATOM	1500	CD1	TYR	95	29.724	30.609	45.467	1.00	0.00	C
ATOM	1501	HD1	TYR	95	29.488	31.408	44.780	1.00	0.00	H
ATOM	1502	CE1	TYR	95	30.570	29.533	45.130	1.00	0.00	C
ATOM	1503	HE1	TYR	95	30.880	29.453	44.099	1.00	0.00	H
ATOM	1504	CZ	TYR	95	30.684	28.410	46.038	1.00	0.00	C
ATOM	1505	OH	TYR	95	31.319	27.283	45.640	1.00	0.00	O
ATOM	1506	HH	TYR	95	31.439	27.166	44.695	1.00	0.00	H
ATOM	1507	CE2	TYR	95	30.112	28.531	47.278	1.00	0.00	C
ATOM	1508	HE2	TYR	95	30.226	27.663	47.910	1.00	0.00	H
ATOM	1509	CD2	TYR	95	29.324	29.674	47.672	1.00	0.00	C
ATOM	1510	HD2	TYR	95	28.934	29.756	48.676	1.00	0.00	H
ATOM	1511	C	TYR	95	30.275	33.635	47.088	1.00	0.00	C
ATOM	1512	O	TYR	95	29.869	34.440	46.229	1.00	0.00	O
ATOM	1513	N	VAL	96	31.539	33.457	47.481	1.00	0.00	N
ATOM	1514	H	VAL	96	31.738	32.744	48.168	1.00	0.00	H
ATOM	1515	CA	VAL	96	32.741	33.946	46.717	1.00	0.00	C
ATOM	1516	HA	VAL	96	32.327	34.699	46.045	1.00	0.00	H
ATOM	1517	CB	VAL	96	33.726	34.666	47.717	1.00	0.00	C
ATOM	1518	HB	VAL	96	33.828	33.909	48.495	1.00	0.00	H
ATOM	1519	CG1	VAL	96	35.194	34.789	47.034	1.00	0.00	C
ATOM	1520	HG11	VAL	96	35.275	35.535	46.243	1.00	0.00	H
ATOM	1521	HG12	VAL	96	35.772	35.174	47.874	1.00	0.00	H
ATOM	1522	HG13	VAL	96	35.586	33.829	46.702	1.00	0.00	H
ATOM	1523	CG2	VAL	96	33.344	35.981	48.356	1.00	0.00	C
ATOM	1524	HG21	VAL	96	33.542	36.677	47.541	1.00	0.00	H
ATOM	1525	HG22	VAL	96	32.259	36.026	48.460	1.00	0.00	H
ATOM	1526	HG23	VAL	96	33.823	36.121	49.324	1.00	0.00	H
ATOM	1527	C	VAL	96	33.311	32.851	45.787	1.00	0.00	C
ATOM	1528	O	VAL	96	33.557	31.685	46.137	1.00	0.00	O
ATOM	1529	N	ILE	97	33.716	33.204	44.562	1.00	0.00	N
ATOM	1530	H	ILE	97	33.508	34.174	44.370	1.00	0.00	H
ATOM	1531	CA	ILE	97	34.412	32.440	43.545	1.00	0.00	C
ATOM	1532	HA	ILE	97	34.757	31.507	43.990	1.00	0.00	H
ATOM	1533	CB	ILE	97	33.468	32.094	42.339	1.00	0.00	C
ATOM	1534	HB	ILE	97	33.310	33.038	41.818	1.00	0.00	H
ATOM	1535	CG2	ILE	97	34.148	31.079	41.424	1.00	0.00	C
ATOM	1536	HG21	ILE	97	35.151	31.455	41.226	1.00	0.00	H
ATOM	1537	HG22	ILE	97	34.211	30.124	41.948	1.00	0.00	H
ATOM	1538	HG23	ILE	97	33.574	30.908	40.514	1.00	0.00	H
ATOM	1539	CG1	ILE	97	32.153	31.428	42.707	1.00	0.00	C
ATOM	1540	HG12	ILE	97	32.010	30.514	42.131	1.00	0.00	H
ATOM	1541	HG13	ILE	97	32.264	31.040	43.720	1.00	0.00	H
ATOM	1542	CD1	ILE	97	30.907	32.312	42.612	1.00	0.00	C
ATOM	1543	HD11	ILE	97	31.020	32.728	41.611	1.00	0.00	H
ATOM	1544	HD12	ILE	97	29.981	31.738	42.658	1.00	0.00	H
ATOM	1545	HD13	ILE	97	30.898	33.055	43.408	1.00	0.00	H
ATOM	1546	C	ILE	97	35.650	33.197	43.034	1.00	0.00	C
ATOM	1547	O	ILE	97	35.487	34.290	42.521	1.00	0.00	O
ATOM	1548	N	GLU	98	36.840	32.619	43.190	1.00	0.00	N
ATOM	1549	H	GLU	98	36.949	31.705	43.607	1.00	0.00	H
ATOM	1550	CA	GLU	98	38.110	33.185	42.802	1.00	0.00	C
ATOM	1551	HA	GLU	98	38.028	34.197	42.406	1.00	0.00	H
ATOM	1552	CB	GLU	98	39.091	33.131	43.977	1.00	0.00	C
ATOM	1553	HB2	GLU	98	39.012	32.133	44.408	1.00	0.00	H
ATOM	1554	HB3	GLU	98	40.027	33.453	43.522	1.00	0.00	H
ATOM	1555	CG	GLU	98	38.698	34.143	44.988	1.00	0.00	C
ATOM	1556	HG2	GLU	98	38.352	35.064	44.518	1.00	0.00	H
ATOM	1557	HG3	GLU	98	37.787	33.860	45.517	1.00	0.00	H
ATOM	1558	CD	GLU	98	39.803	34.453	46.068	1.00	0.00	C
ATOM	1559	OE1	GLU	98	39.698	34.027	47.226	1.00	0.00	O
ATOM	1560	OE2	GLU	98	40.689	35.242	45.758	1.00	0.00	O
ATOM	1561	C	GLU	98	38.753	32.262	41.714	1.00	0.00	C
ATOM	1562	O	GLU	98	38.535	31.047	41.772	1.00	0.00	O

ATOM	1563	N	PHE	99	39.438	32.728	40.662	1.00	0.00	N
ATOM	1564	H	PHE	99	39.746	33.685	40.565	1.00	0.00	H
ATOM	1565	CA	PHE	99	39.638	31.925	39.433	1.00	0.00	C
ATOM	1566	HA	PHE	99	39.961	30.936	39.758	1.00	0.00	H
ATOM	1567	CB	PHE	99	38.261	31.822	38.611	1.00	0.00	C
ATOM	1568	HB2	PHE	99	38.468	31.267	37.696	1.00	0.00	H
ATOM	1569	HB3	PHE	99	37.661	31.279	39.341	1.00	0.00	H
ATOM	1570	CG	PHE	99	37.537	33.160	38.341	1.00	0.00	C
ATOM	1571	CD1	PHE	99	36.680	33.621	39.333	1.00	0.00	C
ATOM	1572	HD1	PHE	99	36.472	32.971	40.171	1.00	0.00	H
ATOM	1573	CE1	PHE	99	35.987	34.805	39.153	1.00	0.00	C
ATOM	1574	HE1	PHE	99	35.165	35.034	39.815	1.00	0.00	H
ATOM	1575	CZ	PHE	99	36.218	35.550	38.038	1.00	0.00	C
ATOM	1576	HZ	PHE	99	35.598	36.432	37.997	1.00	0.00	H
ATOM	1577	CE2	PHE	99	37.253	35.262	37.063	1.00	0.00	C
ATOM	1578	HE2	PHE	99	37.454	35.954	36.259	1.00	0.00	H
ATOM	1579	CD2	PHE	99	37.909	33.997	37.232	1.00	0.00	C
ATOM	1580	HD2	PHE	99	38.694	33.724	36.542	1.00	0.00	H
ATOM	1581	C	PHE	99	40.728	32.496	38.572	1.00	0.00	C
ATOM	1582	O	PHE	99	40.945	33.749	38.564	1.00	0.00	O
ATOM	1583	N	LYS	100	41.350	31.697	37.719	1.00	0.00	N
ATOM	1584	H	LYS	100	41.151	30.713	37.827	1.00	0.00	H
ATOM	1585	CA	LYS	100	42.487	32.122	36.919	1.00	0.00	C
ATOM	1586	HA	LYS	100	42.481	33.201	36.766	1.00	0.00	H
ATOM	1587	CB	LYS	100	43.822	31.916	37.653	1.00	0.00	C
ATOM	1588	HB2	LYS	100	44.476	32.488	36.996	1.00	0.00	H
ATOM	1589	HB3	LYS	100	43.782	32.543	38.543	1.00	0.00	H
ATOM	1590	CG	LYS	100	44.296	30.524	38.004	1.00	0.00	C
ATOM	1591	HG2	LYS	100	43.502	30.136	38.642	1.00	0.00	H
ATOM	1592	HG3	LYS	100	44.382	29.973	37.067	1.00	0.00	H
ATOM	1593	CD	LYS	100	45.668	30.725	38.658	1.00	0.00	C
ATOM	1594	HD2	LYS	100	46.305	31.319	38.002	1.00	0.00	H
ATOM	1595	HD3	LYS	100	45.620	31.428	39.489	1.00	0.00	H
ATOM	1596	CE	LYS	100	46.330	29.385	39.171	1.00	0.00	C
ATOM	1597	HE2	LYS	100	45.518	28.901	39.714	1.00	0.00	H
ATOM	1598	HE3	LYS	100	46.641	28.730	38.357	1.00	0.00	H
ATOM	1599	NZ	LYS	100	47.452	29.639	40.009	1.00	0.00	N
ATOM	1600	HZ1	LYS	100	47.282	30.292	40.760	1.00	0.00	H
ATOM	1601	HZ2	LYS	100	47.719	28.812	40.525	1.00	0.00	H
ATOM	1602	HZ3	LYS	100	48.244	29.919	39.448	1.00	0.00	H
ATOM	1603	C	LYS	100	42.449	31.517	35.531	1.00	0.00	C
ATOM	1604	O	LYS	100	41.963	30.384	35.391	1.00	0.00	O
ATOM	1605	N	CYX	101	42.964	32.138	34.529	1.00	0.00	N
ATOM	1606	H	CYX	101	43.349	33.068	34.610	1.00	0.00	H
ATOM	1607	CA	CYX	101	43.173	31.451	33.254	1.00	0.00	C
ATOM	1608	HA	CYX	101	42.308	30.845	32.984	1.00	0.00	H
ATOM	1609	CB	CYX	101	43.324	32.575	32.219	1.00	0.00	C
ATOM	1610	HB2	CYX	101	42.374	33.100	32.115	1.00	0.00	H
ATOM	1611	HB3	CYX	101	43.984	33.319	32.662	1.00	0.00	H
ATOM	1612	SG	CYX	101	43.735	32.163	30.509	1.00	0.00	S
ATOM	1613	C	CYX	101	44.464	30.497	33.223	1.00	0.00	C
ATOM	1614	O	CYX	101	45.384	30.652	34.061	1.00	0.00	O
ATOM	1615	N	LEU	102	44.554	29.533	32.329	1.00	0.00	N
ATOM	1616	H	LEU	102	43.757	29.162	31.831	1.00	0.00	H
ATOM	1617	CA	LEU	102	45.657	28.558	32.360	1.00	0.00	C
ATOM	1618	HA	LEU	102	46.201	28.534	33.303	1.00	0.00	H
ATOM	1619	CB	LEU	102	44.947	27.217	32.111	1.00	0.00	C
ATOM	1620	HB2	LEU	102	44.486	27.181	31.124	1.00	0.00	H
ATOM	1621	HB3	LEU	102	45.765	26.500	32.037	1.00	0.00	H
ATOM	1622	CG	LEU	102	43.890	26.712	33.125	1.00	0.00	C
ATOM	1623	HG	LEU	102	43.005	27.342	33.042	1.00	0.00	H
ATOM	1624	CD1	LEU	102	43.548	25.355	32.542	1.00	0.00	C
ATOM	1625	HD11	LEU	102	44.487	24.826	32.375	1.00	0.00	H

ATOM	1626	HD12	LEU	102	43.120	24.721	33.318	1.00	0.00	H
ATOM	1627	HD13	LEU	102	42.780	25.352	31.768	1.00	0.00	H
ATOM	1628	CD2	LEU	102	44.348	26.636	34.621	1.00	0.00	C
ATOM	1629	HD21	LEU	102	45.301	27.161	34.687	1.00	0.00	H
ATOM	1630	HD22	LEU	102	43.608	26.955	35.355	1.00	0.00	H
ATOM	1631	HD23	LEU	102	44.593	25.601	34.856	1.00	0.00	H
ATOM	1632	C	LEU	102	46.664	28.775	31.298	1.00	0.00	C
ATOM	1633	O	LEU	102	46.384	28.669	30.085	1.00	0.00	O
ATOM	1634	OXT	LEU	102	47.807	29.027	31.743	1.00	0.00	O
HETATM	314	N	LYN	20	39.901	40.455	36.184	1.00	0.00	N
HETATM	315	H	LYN	20	40.428	39.938	35.494	1.00	0.00	H
HETATM	316	CA	LYN	20	38.688	39.854	36.663	1.00	0.00	C
HETATM	317	HA	LYN	20	38.252	40.413	37.491	1.00	0.00	H
HETATM	318	CB	LYN	20	37.626	39.813	35.595	1.00	0.00	C
HETATM	319	HB2	LYN	20	38.103	39.341	34.737	1.00	0.00	H
HETATM	320	HB3	LYN	20	37.454	40.820	35.215	1.00	0.00	H
HETATM	321	CG	LYN	20	36.434	39.018	36.139	1.00	0.00	C
HETATM	322	HG2	LYN	20	35.702	39.101	35.335	1.00	0.00	H
HETATM	323	HG3	LYN	20	36.679	37.972	36.316	1.00	0.00	H
HETATM	324	CD	LYN	20	35.683	39.605	37.342	1.00	0.00	C
HETATM	325	HD2	LYN	20	35.573	40.666	37.113	1.00	0.00	H
HETATM	326	HD3	LYN	20	36.211	39.399	38.272	1.00	0.00	H
HETATM	327	CE	LYN	20	34.330	39.062	37.610	1.00	0.00	C
HETATM	328	HE2	LYN	20	33.719	39.263	36.730	1.00	0.00	H
HETATM	329	HE3	LYN	20	34.276	37.980	37.716	1.00	0.00	H
HETATM	330	NZ	LYN	20	33.730	39.708	38.755	1.00	0.00	N
HETATM	331	HZ2	LYN	20	33.459	40.589	38.342	1.00	0.00	H
HETATM	332	HZ3	LYN	20	34.526	39.900	39.346	1.00	0.00	H
HETATM	333	C	LYN	20	39.173	38.463	37.159	1.00	0.00	C
HETATM	334	O	LYN	20	39.472	37.502	36.373	1.00	0.00	O
HETATM	1636	N	LIG	103	30.703	41.892	39.706	1.00	0.00	N
HETATM	1637	C	LIG	103	30.373	40.901	38.898	1.00	0.00	C
HETATM	1638	O	LIG	103	30.448	43.267	41.410	1.00	0.00	O
HETATM	1639	C5'	LIG	103	25.781	39.418	43.869	1.00	0.00	C
HETATM	1640	O5'	LIG	103	25.900	38.712	42.709	1.00	0.00	O
HETATM	1641	C4'	LIG	103	26.466	40.843	43.736	1.00	0.00	C
HETATM	1642	O4'	LIG	103	27.741	40.668	43.063	1.00	0.00	O
HETATM	1643	C3'	LIG	103	25.763	41.936	42.924	1.00	0.00	C
HETATM	1644	O3'	LIG	103	24.874	42.781	43.735	1.00	0.00	O
HETATM	1645	C2'	LIG	103	26.941	42.702	42.199	1.00	0.00	C
HETATM	1646	O2'	LIG	103	27.049	43.998	42.864	1.00	0.00	O
HETATM	1647	C1'	LIG	103	28.257	41.864	42.395	1.00	0.00	C
HETATM	1648	N1	LIG	103	28.946	41.560	41.171	1.00	0.00	N
HETATM	1649	O1	LIG	103	30.394	38.666	37.011	1.00	0.00	O
HETATM	1650	N2	LIG	103	31.022	40.742	37.698	1.00	0.00	N
HETATM	1651	C6	LIG	103	30.039	42.238	40.837	1.00	0.00	C
HETATM	1652	C7	LIG	103	28.465	40.531	40.352	1.00	0.00	C
HETATM	1653	C8	LIG	103	29.183	40.126	39.268	1.00	0.00	C
HETATM	1654	C9	LIG	103	30.848	39.782	36.756	1.00	0.00	C
HETATM	1655	C10	LIG	103	31.468	40.105	35.448	1.00	0.00	C
HETATM	1656	H	LIG	103	31.799	41.371	37.535	1.00	0.00	H
HETATM	1657	H1	LIG	103	28.843	39.392	38.547	1.00	0.00	H
HETATM	1658	H4	LIG	103	29.002	42.349	43.036	1.00	0.00	H
HETATM	1659	H6	LIG	103	26.695	41.193	44.750	1.00	0.00	H
HETATM	1660	H7	LIG	103	25.165	41.530	42.099	1.00	0.00	H
HETATM	1661	H8	LIG	103	26.735	42.807	41.127	1.00	0.00	H
HETATM	1662	H9	LIG	103	24.722	39.684	43.975	1.00	0.00	H
HETATM	1663	H10	LIG	103	25.993	38.971	44.848	1.00	0.00	H
HETATM	1664	H11	LIG	103	31.304	41.146	35.142	1.00	0.00	H
HETATM	1665	H12	LIG	103	31.203	39.327	34.721	1.00	0.00	H
HETATM	1666	H13	LIG	103	32.560	40.001	35.458	1.00	0.00	H
HETATM	1667	H14	LIG	103	27.565	40.029	40.680	1.00	0.00	H
HETATM	1668	H2'	LIG	103	27.631	44.649	42.435	1.00	0.00	H
HETATM	1669	H3'	LIG	103	25.464	43.552	43.811	1.00	0.00	H

```
HETATM 1670  H5' LIG   103       26.867  38.603  42.741  1.00  0.00           H
ENDMDL
MODEL     12
SHEET     1   1 1 ILE    22  ASP    26  0
SHEET     2   2 1 VAL    37  VAL    40  0
SHEET     3   3 1 CYX    50  VAL    60  0
SHEET     4   4 1 PHE    94  CYX
 101  0
HELIX     1   1 GLN      9  LEU     16  1                                   8
HELIX     2   2 LEU     62  THR     64  1                                   3
HELIX     3   3 LEU     77  ILE     87  1                                  11
ATOM      1  N   GLN     1       33.236  17.891  32.455  1.00  0.00           N
ATOM      2  H1  GLN     1       32.360  17.826  32.953  1.00  0.00           H
ATOM      3  H2  GLN     1       33.530  16.954  32.219  1.00  0.00           H
ATOM      4  H3  GLN     1       33.938  18.313  33.047  1.00  0.00           H
ATOM      5  CA  GLN     1       33.115  18.771  31.276  1.00  0.00           C
ATOM      6  HA  GLN     1       34.089  18.840  30.790  1.00  0.00           H
ATOM      7  CB  GLN     1       32.095  18.249  30.211  1.00  0.00           C
ATOM      8  HB2 GLN     1       31.142  18.004  30.681  1.00  0.00           H
ATOM      9  HB3 GLN     1       31.912  18.982  29.425  1.00  0.00           H
ATOM     10  CG  GLN     1       32.582  17.020  29.477  1.00  0.00           C
ATOM     11  HG2 GLN     1       32.651  16.159  30.142  1.00  0.00           H
ATOM     12  HG3 GLN     1       31.848  16.785  28.706  1.00  0.00           H
ATOM     13  CD  GLN     1       33.854  17.240  28.683  1.00  0.00           C
ATOM     14  OE1 GLN     1       34.594  18.241  28.773  1.00  0.00           O
ATOM     15  NE2 GLN     1       34.198  16.353  27.791  1.00  0.00           N
ATOM     16 HE21 GLN     1       33.641  15.519  27.673  1.00  0.00           H
ATOM     17 HE22 GLN     1       34.932  16.593  27.140  1.00  0.00           H
ATOM     18  C   GLN     1       32.938  20.240  31.713  1.00  0.00           C
ATOM     19  O   GLN     1       32.212  20.452  32.729  1.00  0.00           O
ATOM     20  N   PRO     2       33.482  21.273  30.942  1.00  0.00           N
ATOM     21  CD  PRO     2       34.436  21.253  29.823  1.00  0.00           C
ATOM     22  HD2 PRO     2       33.831  21.282  28.917  1.00  0.00           H
ATOM     23  HD3 PRO     2       35.057  20.357  29.848  1.00  0.00           H
ATOM     24  CG  PRO     2       35.285  22.524  29.990  1.00  0.00           C
ATOM     25  HG2 PRO     2       35.781  22.744  29.044  1.00  0.00           H
ATOM     26  HG3 PRO     2       35.911  22.318  30.858  1.00  0.00           H
ATOM     27  CB  PRO     2       34.185  23.526  30.288  1.00  0.00           C
ATOM     28  HB2 PRO     2       33.653  23.824  29.385  1.00  0.00           H
ATOM     29  HB3 PRO     2       34.621  24.425  30.725  1.00  0.00           H
ATOM     30  CA  PRO     2       33.257  22.760  31.241  1.00  0.00           C
ATOM     31  HA  PRO     2       33.530  22.925  32.283  1.00  0.00           H
ATOM     32  C   PRO     2       31.787  23.072  31.077  1.00  0.00           C
ATOM     33  O   PRO     2       31.144  22.593  30.145  1.00  0.00           O
ATOM     34  N   ASN     3       31.243  24.006  31.827  1.00  0.00           N
ATOM     35  H   ASN     3       31.852  24.326  32.566  1.00  0.00           H
ATOM     36  CA  ASN     3       29.913  24.652  31.647  1.00  0.00           C
ATOM     37  HA  ASN     3       29.099  23.935  31.747  1.00  0.00           H
ATOM     38  CB  ASN     3       29.670  25.732  32.705  1.00  0.00           C
ATOM     39  HB2 ASN     3       30.530  26.400  32.746  1.00  0.00           H
ATOM     40  HB3 ASN     3       28.761  26.281  32.459  1.00  0.00           H
ATOM     41  CG  ASN     3       29.368  25.117  34.096  1.00  0.00           C
ATOM     42  OD1 ASN     3       28.306  24.532  34.336  1.00  0.00           O
ATOM     43  ND2 ASN     3       30.365  25.053  34.976  1.00  0.00           N
ATOM     44 HD21 ASN     3       31.308  25.330  34.743  1.00  0.00           H
ATOM     45 HD22 ASN     3       30.186  24.296  35.621  1.00  0.00           H
ATOM     46  C   ASN     3       29.845  25.183  30.194  1.00  0.00           C
ATOM     47  O   ASN     3       30.908  25.517  29.694  1.00  0.00           O
ATOM     48  N   ASP     4       28.638  25.272  29.562  1.00  0.00           N
ATOM     49  H   ASP     4       27.796  24.962  30.026  1.00  0.00           H
ATOM     50  CA  ASP     4       28.500  25.970  28.306  1.00  0.00           C
ATOM     51  HA  ASP     4       29.435  25.938  27.749  1.00  0.00           H
ATOM     52  CB  ASP     4       27.448  25.205  27.446  1.00  0.00           C
ATOM     53  HB2 ASP     4       26.512  25.214  28.005  1.00  0.00           H
```

ATOM	54	HB3	ASP	4	27.377	25.727	26.492	1.00	0.00	H
ATOM	55	CG	ASP	4	27.920	23.751	27.213	1.00	0.00	C
ATOM	56	OD1	ASP	4	27.290	22.718	27.389	1.00	0.00	O
ATOM	57	OD2	ASP	4	29.050	23.571	26.704	1.00	0.00	O
ATOM	58	C	ASP	4	28.020	27.372	28.556	1.00	0.00	C
ATOM	59	O	ASP	4	28.398	28.296	27.848	1.00	0.00	O
ATOM	60	N	ILE	5	27.222	27.590	29.622	1.00	0.00	N
ATOM	61	H	ILE	5	27.103	26.770	30.200	1.00	0.00	H
ATOM	62	CA	ILE	5	26.859	28.810	30.362	1.00	0.00	C
ATOM	63	HA	ILE	5	26.511	29.538	29.630	1.00	0.00	H
ATOM	64	CB	ILE	5	25.702	28.526	31.356	1.00	0.00	C
ATOM	65	HB	ILE	5	25.996	27.605	31.860	1.00	0.00	H
ATOM	66	CG2	ILE	5	25.569	29.671	32.431	1.00	0.00	C
ATOM	67	HG21	ILE	5	24.832	29.326	33.154	1.00	0.00	H
ATOM	68	HG22	ILE	5	26.498	29.967	32.919	1.00	0.00	H
ATOM	69	HG23	ILE	5	25.196	30.573	31.946	1.00	0.00	H
ATOM	70	CG1	ILE	5	24.326	28.227	30.734	1.00	0.00	C
ATOM	71	HG12	ILE	5	24.330	27.323	30.127	1.00	0.00	H
ATOM	72	HG13	ILE	5	23.531	28.058	31.460	1.00	0.00	H
ATOM	73	CD1	ILE	5	23.872	29.152	29.623	1.00	0.00	C
ATOM	74	HD11	ILE	5	24.527	29.125	28.751	1.00	0.00	H
ATOM	75	HD12	ILE	5	22.871	28.832	29.336	1.00	0.00	H
ATOM	76	HD13	ILE	5	23.844	30.118	30.127	1.00	0.00	H
ATOM	77	C	ILE	5	28.100	29.445	31.009	1.00	0.00	C
ATOM	78	O	ILE	5	29.034	28.728	31.275	1.00	0.00	O
ATOM	79	N	THR	6	28.150	30.822	31.161	1.00	0.00	N
ATOM	80	H	THR	6	27.321	31.334	30.893	1.00	0.00	H
ATOM	81	CA	THR	6	29.418	31.597	31.308	1.00	0.00	C
ATOM	82	HA	THR	6	30.031	31.128	32.079	1.00	0.00	H
ATOM	83	CB	THR	6	30.094	31.442	29.950	1.00	0.00	C
ATOM	84	HB	THR	6	30.131	30.378	29.721	1.00	0.00	H
ATOM	85	CG2	THR	6	29.413	32.179	28.858	1.00	0.00	C
ATOM	86	HG21	THR	6	29.436	33.266	28.951	1.00	0.00	H
ATOM	87	HG22	THR	6	29.951	31.888	27.956	1.00	0.00	H
ATOM	88	HG23	THR	6	28.434	31.740	28.669	1.00	0.00	H
ATOM	89	OG1	THR	6	31.392	32.020	29.997	1.00	0.00	O
ATOM	90	HG1	THR	6	32.051	31.429	29.626	1.00	0.00	H
ATOM	91	C	THR	6	29.114	33.119	31.720	1.00	0.00	C
ATOM	92	O	THR	6	27.954	33.480	32.064	1.00	0.00	O
ATOM	93	N	PHE	7	30.204	33.945	31.785	1.00	0.00	N
ATOM	94	H	PHE	7	31.059	33.574	31.395	1.00	0.00	H
ATOM	95	CA	PHE	7	30.177	35.433	32.042	1.00	0.00	C
ATOM	96	HA	PHE	7	29.546	35.469	32.931	1.00	0.00	H
ATOM	97	CB	PHE	7	31.610	35.864	32.284	1.00	0.00	C
ATOM	98	HB2	PHE	7	32.231	35.786	31.392	1.00	0.00	H
ATOM	99	HB3	PHE	7	31.652	36.927	32.521	1.00	0.00	H
ATOM	100	CG	PHE	7	32.267	35.269	33.589	1.00	0.00	C
ATOM	101	CD1	PHE	7	33.219	34.228	33.513	1.00	0.00	C
ATOM	102	HD1	PHE	7	33.555	33.819	32.572	1.00	0.00	H
ATOM	103	CE1	PHE	7	33.766	33.661	34.668	1.00	0.00	C
ATOM	104	HE1	PHE	7	34.619	32.999	34.683	1.00	0.00	H
ATOM	105	CZ	PHE	7	33.303	34.124	35.939	1.00	0.00	C
ATOM	106	HZ	PHE	7	33.798	33.647	36.772	1.00	0.00	H
ATOM	107	CE2	PHE	7	32.363	35.147	35.986	1.00	0.00	C
ATOM	108	HE2	PHE	7	32.016	35.462	36.958	1.00	0.00	H
ATOM	109	CD2	PHE	7	31.814	35.763	34.808	1.00	0.00	C
ATOM	110	HD2	PHE	7	31.067	36.536	34.916	1.00	0.00	H
ATOM	111	C	PHE	7	29.512	36.318	30.959	1.00	0.00	C
ATOM	112	O	PHE	7	29.474	35.922	29.754	1.00	0.00	O
ATOM	113	N	PHE	8	29.010	37.446	31.338	1.00	0.00	N
ATOM	114	H	PHE	8	29.084	37.662	32.322	1.00	0.00	H
ATOM	115	CA	PHE	8	28.548	38.349	30.267	1.00	0.00	C
ATOM	116	HA	PHE	8	27.886	37.854	29.556	1.00	0.00	H
ATOM	117	CB	PHE	8	27.654	39.442	30.890	1.00	0.00	C

```
ATOM    118  HB2 PHE     8      28.263  39.956  31.635  1.00  0.00           H
ATOM    119  HB3 PHE     8      27.384  40.113  30.076  1.00  0.00           H
ATOM    120  CG  PHE     8      26.389  39.026  31.601  1.00  0.00           C
ATOM    121  CD1 PHE     8      25.437  38.227  30.858  1.00  0.00           C
ATOM    122  HD1 PHE     8      25.570  38.028  29.805  1.00  0.00           H
ATOM    123  CE1 PHE
  8      24.263  37.766  31.458  1.00  0.00           C
ATOM    124  HE1 PHE     8      23.564  37.148  30.916  1.00  0.00           H
ATOM    125  CZ  PHE     8      24.086  37.993  32.815  1.00  0.00           C
ATOM    126  HZ  PHE     8      23.187  37.592  33.258  1.00  0.00           H
ATOM    127  CE2 PHE     8      24.908  38.889  33.533  1.00  0.00           C
ATOM    128  HE2 PHE     8      24.637  39.242  34.518  1.00  0.00           H
ATOM    129  CD2 PHE     8      26.131  39.200  32.942  1.00  0.00           C
ATOM    130  HD2 PHE     8      26.818  39.743  33.574  1.00  0.00           H
ATOM    131  C   PHE     8      29.733  39.062  29.452  1.00  0.00           C
ATOM    132  O   PHE     8      30.921  38.934  29.755  1.00  0.00           O
ATOM    133  N   GLN     9      29.363  39.606  28.308  1.00  0.00           N
ATOM    134  H   GLN     9      28.391  39.748  28.075  1.00  0.00           H
ATOM    135  CA  GLN     9      30.292  39.973  27.258  1.00  0.00           C
ATOM    136  HA  GLN     9      30.867  39.093  26.969  1.00  0.00           H
ATOM    137  CB  GLN     9      29.629  40.531  25.965  1.00  0.00           C
ATOM    138  HB2 GLN     9      28.727  41.111  26.159  1.00  0.00           H
ATOM    139  HB3 GLN     9      30.430  41.138  25.545  1.00  0.00           H
ATOM    140  CG  GLN     9      29.240  39.464  24.994  1.00  0.00           C
ATOM    141  HG2 GLN     9      28.877  39.967  24.098  1.00  0.00           H
ATOM    142  HG3 GLN     9      30.109  38.819  24.871  1.00  0.00           H
ATOM    143  CD  GLN     9      28.069  38.659  25.453  1.00  0.00           C
ATOM    144  OE1 GLN     9      27.317  38.922  26.373  1.00  0.00           O
ATOM    145  NE2 GLN     9      28.001  37.447  24.929  1.00  0.00           N
ATOM    146 HE21 GLN     9      27.362  36.783  25.342  1.00  0.00           H
ATOM    147 HE22 GLN     9      28.639  37.142  24.208  1.00  0.00           H
ATOM    148  C   GLN     9      31.293  41.037  27.677  1.00  0.00           C
ATOM    149  O   GLN     9      32.464  40.917  27.289  1.00  0.00           O
ATOM    150  N   ARG    10      30.932  42.128  28.337  1.00  0.00           N
ATOM    151  H   ARG    10      29.951  42.155  28.575  1.00  0.00           H
ATOM    152  CA  ARG    10      31.770  43.221  28.858  1.00  0.00           C
ATOM    153  HA  ARG    10      32.266  43.694  28.009  1.00  0.00           H
ATOM    154  CB  ARG    10      30.949  44.313  29.422  1.00  0.00           C
ATOM    155  HB2 ARG    10      30.292  43.790  30.117  1.00  0.00           H
ATOM    156  HB3 ARG    10      31.565  44.962  30.047  1.00  0.00           H
ATOM    157  CG  ARG    10      30.070  45.115  28.456  1.00  0.00           C
ATOM    158  HG2 ARG    10      30.749  45.879  28.075  1.00  0.00           H
ATOM    159  HG3 ARG    10      29.776  44.472  27.627  1.00  0.00           H
ATOM    160  CD  ARG    10      28.800  45.781  29.087  1.00  0.00           C
ATOM    161  HD2 ARG    10      28.027  45.043  29.303  1.00  0.00           H
ATOM    162  HD3 ARG    10      29.086  46.290  30.008  1.00  0.00           H
ATOM    163  NE  ARG    10      28.268  46.872  28.173  1.00  0.00           N
ATOM    164  HE  ARG    10      27.920  46.602  27.264  1.00  0.00           H
ATOM    165  CZ  ARG    10      28.214  48.180  28.468  1.00  0.00           C
ATOM    166  NH1 ARG    10      28.566  48.759  29.590  1.00  0.00           N
ATOM    167 HH11 ARG    10      28.803  49.737  29.673  1.00  0.00           H
ATOM    168 HH12 ARG    10      28.864  48.223  30.393  1.00  0.00           H
ATOM    169  NH2 ARG    10      27.787  48.966  27.545  1.00  0.00           N
ATOM    170 HH21 ARG    10      27.736  48.631  26.594  1.00  0.00           H
ATOM    171 HH22 ARG    10      27.722  49.950  27.767  1.00  0.00           H
ATOM    172  C   ARG    10      32.921  42.783  29.697  1.00  0.00           C
ATOM    173  O   ARG    10      33.837  43.533  29.895  1.00  0.00           O
ATOM    174  N   PHE    11      32.972  41.518  30.192  1.00  0.00           N
ATOM    175  H   PHE    11      32.154  40.936  30.081  1.00  0.00           H
ATOM    176  CA  PHE    11      34.124  41.056  30.872  1.00  0.00           C
ATOM    177  HA  PHE    11      34.712  41.864  31.308  1.00  0.00           H
ATOM    178  CB  PHE    11      33.514  40.320  32.125  1.00  0.00           C
ATOM    179  HB2 PHE    11      34.257  39.747  32.680  1.00  0.00           H
ATOM    180  HB3 PHE    11      32.781  39.559  31.857  1.00  0.00           H
```

ATOM	181	CG	PHE	11	33.031	41.321	33.108	1.00	0.00	C
ATOM	182	CD1	PHE	11	33.926	42.179	33.769	1.00	0.00	C
ATOM	183	HD1	PHE	11	34.997	42.084	33.673	1.00	0.00	H
ATOM	184	CE1	PHE	11	33.443	43.197	34.486	1.00	0.00	C
ATOM	185	HE1	PHE	11	34.072	43.956	34.927	1.00	0.00	H
ATOM	186	CZ	PHE	11	32.059	43.422	34.525	1.00	0.00	C
ATOM	187	HZ	PHE	11	31.652	44.296	35.010	1.00	0.00	H
ATOM	188	CE2	PHE	11	31.115	42.482	33.994	1.00	0.00	C
ATOM	189	HE2	PHE	11	30.080	42.633	34.260	1.00	0.00	H
ATOM	190	CD2	PHE	11	31.651	41.487	33.199	1.00	0.00	C
ATOM	191	HD2	PHE	11	30.954	40.807	32.735	1.00	0.00	H
ATOM	192	C	PHE	11	35.104	40.256	30.025	1.00	0.00	C
ATOM	193	O	PHE	11	36.032	39.693	30.618	1.00	0.00	O
ATOM	194	N	GLN	12	34.954	40.013	28.723	1.00	0.00	N
ATOM	195	H	GLN	12	34.163	40.429	28.252	1.00	0.00	H
ATOM	196	CA	GLN	12	35.724	38.944	27.985	1.00	0.00	C
ATOM	197	HA	GLN	12	35.640	38.028	28.569	1.00	0.00	H
ATOM	198	CB	GLN	12	35.163	38.648	26.609	1.00	0.00	C
ATOM	199	HB2	GLN	12	35.002	39.613	26.128	1.00	0.00	H
ATOM	200	HB3	GLN	12	35.926	38.230	25.952	1.00	0.00	H
ATOM	201	CG	GLN	12	33.956	37.795	26.617	1.00	0.00	C
ATOM	202	HG2	GLN	12	34.178	36.833	27.080	1.00	0.00	H
ATOM	203	HG3	GLN	12	33.160	38.205	27.238	1.00	0.00	H
ATOM	204	CD	GLN	12	33.350	37.472	25.221	1.00	0.00	C
ATOM	205	OE1	GLN	12	34.077	37.482	24.192	1.00	0.00	O
ATOM	206	NE2	GLN	12	32.061	37.100	25.176	1.00	0.00	N
ATOM	207	HE21	GLN	12	31.470	37.089	25.994	1.00	0.00	H
ATOM	208	HE22	GLN	12	31.668	36.803	24.293	1.00	0.00	H
ATOM	209	C	GLN	12	37.192	39.264	27.825	1.00	0.00	C
ATOM	210	O	GLN	12	37.948	38.400	28.313	1.00	0.00	O
ATOM	211	N	ASP	13	37.621	40.446	27.329	1.00	0.00	N
ATOM	212	H	ASP	13	36.964	41.158	27.041	1.00	0.00	H
ATOM	213	CA	ASP	13	39.080	40.690	27.196	1.00	0.00	C
ATOM	214	HA	ASP	13	39.525	39.740	26.899	1.00	0.00	H
ATOM	215	CB	ASP	13	39.344	41.667	26.013	1.00	0.00	C
ATOM	216	HB2	ASP	13	39.064	42.658	26.369	1.00	0.00	H
ATOM	217	HB3	ASP	13	40.420	41.724	25.850	1.00	0.00	H
ATOM	218	CG	ASP	13	38.631	41.282	24.714	1.00	0.00	C
ATOM	219	OD1	ASP	13	38.297	42.250	24.021	1.00	0.00	O
ATOM	220	OD2	ASP	13	38.418	40.100	24.410	1.00	0.00	O
ATOM	221	C	ASP	13	39.723	41.055	28.516	1.00	0.00	C
ATOM	222	O	ASP	13	40.987	40.951	28.544	1.00	0.00	O
ATOM	223	N	ASP	14	38.941	41.309	29.554	1.00	0.00	N
ATOM	224	H	ASP	14	38.062	41.747	29.316	1.00	0.00	H
ATOM	225	CA	ASP	14	39.592	41.637	30.833	1.00	0.00	C
ATOM	226	HA	ASP	14	40.594	42.032	30.664	1.00	0.00	H
ATOM	227	CB	ASP	14	38.861	42.828	31.487	1.00	0.00	C
ATOM	228	HB2	ASP	14	37.837	42.527	31.709	1.00	0.00	H
ATOM	229	HB3	ASP	14	39.320	43.036	32.454	1.00	0.00	H
ATOM	230	CG	ASP	14	38.931	44.207	30.728	1.00	0.00	C
ATOM	231	OD1	ASP	14	37.833	44.794	30.508	1.00	0.00	O
ATOM	232	OD2	ASP	14	39.999	44.635	30.320	1.00	0.00	O
ATOM	233	C	ASP	14	39.794	40.402	31.673	1.00	0.00	C
ATOM	234	O	ASP	14	40.702	40.298	32.535	1.00	0.00	O
ATOM	235	N	ILE	15	39.008	39.377	31.408	1.00	0.00	N
ATOM	236	H	ILE	15	38.179	39.481	30.841	1.00	0.00	H
ATOM	237	CA	ILE	15	39.291	38.049	31.867	1.00	0.00	C
ATOM	238	HA	ILE	15	39.557	38.027	32.924	1.00	0.00	H
ATOM	239	CB	ILE	15	38.101	37.127	31.614	1.00	0.00	C
ATOM	240	HB	ILE	15	37.811	37.230	30.568	1.00	0.00	H
ATOM	241	CG2	ILE	15	38.505	35.649	31.871	1.00	0.00	C
ATOM	242	HG21	ILE	15	39.313	35.301	31.227	1.00	0.00	H
ATOM	243	HG22	ILE	15	38.790	35.570	32.920	1.00	0.00	H
ATOM	244	HG23	ILE	15	37.688	35.008	31.539	1.00	0.00	H

```
ATOM    245  CG1 ILE    15      36.862  37.446  32.465  1.00  0.00           C
ATOM    246 HG12 ILE    15      37.127  37.083  33.459  1.00  0.00           H
ATOM    247 HG13 ILE    15      36.679  38.506  32.640  1.00  0.00           H
ATOM    248  CD1 ILE    15      35.536  36.762  32.074
  1.00  0.00           C
ATOM    249 HD11 ILE    15      35.656  35.685  31.943  1.00  0.00           H
ATOM    250 HD12 ILE    15      34.785  36.987  32.831  1.00  0.00           H
ATOM    251 HD13 ILE    15      35.259  37.144  31.092  1.00  0.00           H
ATOM    252  C   ILE    15      40.516  37.517  31.166  1.00  0.00           C
ATOM    253  O   ILE    15      41.378  36.885  31.721  1.00  0.00           O
ATOM    254  N   LEU    16      40.684  37.781  29.841  1.00  0.00           N
ATOM    255  H   LEU    16      39.955  38.340  29.423  1.00  0.00           H
ATOM    256  CA  LEU    16      41.937  37.458  29.142  1.00  0.00           C
ATOM    257  HA  LEU    16      42.132  36.397  29.295  1.00  0.00           H
ATOM    258  CB  LEU    16      41.761  37.753  27.644  1.00  0.00           C
ATOM    259  HB2 LEU    16      40.911  37.186  27.263  1.00  0.00           H
ATOM    260  HB3 LEU    16      41.627  38.828  27.527  1.00  0.00           H
ATOM    261  CG  LEU    16      42.997  37.498  26.672  1.00  0.00           C
ATOM    262  HG  LEU    16      43.838  38.145  26.922  1.00  0.00           H
ATOM    263  CD1 LEU    16      43.481  35.990  26.807  1.00  0.00           C
ATOM    264 HD11 LEU    16      43.560  35.601  27.822  1.00  0.00           H
ATOM    265 HD12 LEU    16      42.783  35.408  26.207  1.00  0.00           H
ATOM    266 HD13 LEU    16      44.381  35.948  26.193  1.00  0.00           H
ATOM    267  CD2 LEU    16      42.646  37.906  25.263  1.00  0.00           C
ATOM    268 HD21 LEU    16      42.156  38.878  25.332  1.00  0.00           H
ATOM    269 HD22 LEU    16      43.520  38.092  24.640  1.00  0.00           H
ATOM    270 HD23 LEU    16      42.100  37.114  24.751  1.00  0.00           H
ATOM    271  C   LEU    16      43.218  38.184  29.614  1.00  0.00           C
ATOM    272  O   LEU    16      44.276  37.599  29.887  1.00  0.00           O
ATOM    273  N   ALA    17      43.142  39.473  29.873  1.00  0.00           N
ATOM    274  H   ALA    17      42.315  39.973  29.580  1.00  0.00           H
ATOM    275  CA  ALA    17      44.268  40.258  30.424  1.00  0.00           C
ATOM    276  HA  ALA    17      45.125  40.185  29.754  1.00  0.00           H
ATOM    277  CB  ALA    17      43.911  41.718  30.334  1.00  0.00           C
ATOM    278  HB1 ALA    17      44.733  42.328  30.710  1.00  0.00           H
ATOM    279  HB2 ALA    17      43.838  41.825  29.253  1.00  0.00           H
ATOM    280  HB3 ALA    17      42.979  42.028  30.805  1.00  0.00           H
ATOM    281  C   ALA    17      44.716  39.880  31.818  1.00  0.00           C
ATOM    282  O   ALA    17      45.662  40.486  32.382  1.00  0.00           O
ATOM    283  N   GLY    18      44.016  38.909  32.406  1.00  0.00           N
ATOM    284  H   GLY    18      43.378  38.402  31.809  1.00  0.00           H
ATOM    285  CA  GLY    18      44.187  38.545  33.825  1.00  0.00           C
ATOM    286  HA2 GLY    18      43.734  37.581  34.056  1.00  0.00           H
ATOM    287  HA3 GLY    18      45.271  38.517  33.939  1.00  0.00           H
ATOM    288  C   GLY    18      43.724  39.647  34.860  1.00  0.00           C
ATOM    289  O   GLY    18      44.204  39.722  35.994  1.00  0.00           O
ATOM    290  N   ARG    19      42.815  40.542  34.422  1.00  0.00           N
ATOM    291  H   ARG    19      42.327  40.332  33.562  1.00  0.00           H
ATOM    292  CA  ARG    19      42.271  41.640  35.268  1.00  0.00           C
ATOM    293  HA  ARG    19      43.061  41.922  35.962  1.00  0.00           H
ATOM    294  CB  ARG    19      41.789  42.813  34.482  1.00  0.00           C
ATOM    295  HB2 ARG    19      41.068  42.463  33.744  1.00  0.00           H
ATOM    296  HB3 ARG    19      41.315  43.475  35.206  1.00  0.00           H
ATOM    297  CG  ARG    19      42.903  43.636  33.754  1.00  0.00           C
ATOM    298  HG2 ARG    19      43.339  44.294  34.506  1.00  0.00           H
ATOM    299  HG3 ARG    19      43.656  43.007  33.280  1.00  0.00           H
ATOM    300  CD  ARG    19      42.265  44.595  32.646  1.00  0.00           C
ATOM    301  HD2 ARG    19      41.986  44.036  31.753  1.00  0.00           H
ATOM    302  HD3 ARG    19      41.307  44.946  33.028  1.00  0.00           H
ATOM    303  NE  ARG    19      43.166  45.682  32.276  1.00  0.00           N
ATOM    304  HE  ARG    19      44.024  45.752  32.802  1.00  0.00           H
ATOM    305  CZ  ARG    19      42.908  46.629  31.444  1.00  0.00           C
ATOM    306  NH1 ARG    19      41.921  46.568  30.585  1.00  0.00           N
ATOM    307 HH11 ARG    19      41.150  45.929  30.716  1.00  0.00           H
```

ATOM	308	HH12	ARG	19	41.872	47.331	29.925	1.00	0.00	H
ATOM	309	NH2	ARG	19	43.638	47.690	31.436	1.00	0.00	N
ATOM	310	HH21	ARG	19	43.274	48.482	30.928	1.00	0.00	H
ATOM	311	HH22	ARG	19	44.439	47.752	32.048	1.00	0.00	H
ATOM	312	C	ARG	19	41.099	41.114	36.159	1.00	0.00	C
ATOM	313	O	ARG	19	41.128	41.180	37.448	1.00	0.00	O
ATOM	335	N	THR	21	39.316	38.095	37.945	1.00	0.00	N
ATOM	336	H	THR	21	39.317	38.864	38.600	1.00	0.00	H
ATOM	337	CA	THR	21	39.832	36.804	38.417	1.00	0.00	C
ATOM	338	HA	THR	21	39.501	36.013	37.744	1.00	0.00	H
ATOM	339	CB	THR	21	41.444	36.713	38.517	1.00	0.00	C
ATOM	340	HB	THR	21	41.702	36.708	37.458	1.00	0.00	H
ATOM	341	CG2	THR	21	41.987	37.989	39.230	1.00	0.00	C
ATOM	342	HG21	THR	21	41.709	38.895	38.692	1.00	0.00	H
ATOM	343	HG22	THR	21	41.599	38.060	40.246	1.00	0.00	H
ATOM	344	HG23	THR	21	43.076	37.956	39.216	1.00	0.00	H
ATOM	345	OG1	THR	21	42.115	35.611	39.127	1.00	0.00	O
ATOM	346	HG1	THR	21	41.585	34.834	38.936	1.00	0.00	H
ATOM	347	C	THR	21	39.249	36.308	39.766	1.00	0.00	C
ATOM	348	O	THR	21	39.411	35.171	40.132	1.00	0.00	O
ATOM	349	N	ILE	22	38.407	37.182	40.344	1.00	0.00	N
ATOM	350	H	ILE	22	38.305	38.090	39.914	1.00	0.00	H
ATOM	351	CA	ILE	22	37.563	36.920	41.514	1.00	0.00	C
ATOM	352	HA	ILE	22	37.502	35.868	41.791	1.00	0.00	H
ATOM	353	CB	ILE	22	38.178	37.560	42.740	1.00	0.00	C
ATOM	354	HB	ILE	22	39.242	37.328	42.781	1.00	0.00	H
ATOM	355	CG2	ILE	22	37.911	39.103	42.919	1.00	0.00	C
ATOM	356	HG21	ILE	22	38.376	39.518	43.814	1.00	0.00	H
ATOM	357	HG22	ILE	22	38.284	39.602	42.025	1.00	0.00	H
ATOM	358	HG23	ILE	22	36.893	39.452	43.088	1.00	0.00	H
ATOM	359	CG1	ILE	22	37.579	36.882	44.005	1.00	0.00	C
ATOM	360	HG12	ILE	22	36.540	37.168	44.172	1.00	0.00	H
ATOM	361	HG13	ILE	22	37.329	35.848	43.772	1.00	0.00	H
ATOM	362	CD1	ILE	22	38.567	36.855	45.234	1.00	0.00	C
ATOM	363	HD11	ILE	22	38.725	37.861	45.621	1.00	0.00	H
ATOM	364	HD12	ILE	22	38.021	36.394	46.058	1.00	0.00	H
ATOM	365	HD13	ILE	22	39.445	36.250	45.011	1.00	0.00	H
ATOM	366	C	ILE	22	36.121	37.450	41.399	1.00	0.00	C
ATOM	367	O	ILE	22	35.981	38.449	40.661	1.00	0.00	O
ATOM	368	N	THR	23	35.062	36.808	41.940	1.00	0.00	N
ATOM	369	H	THR	23	35.167	35.984	42.515	1.00	0.00	H
ATOM	370	CA	THR	23	33.683	37.306	41.872	1.00	0.00	C
ATOM	371	HA	THR	23	33.725	38.395	41.838	1.00	0.00	H
ATOM	372	CB	THR	23	33.073	36.801	40.547	1.00	0.00	C
ATOM	373	HB	THR	23	33.885	36.899	39.826	1.00	0.00	H
ATOM	374	CG2	THR	23	32.519	35.335	40.439	1.00	0.00	C
ATOM	375	HG21	THR	23	31.787	35.131	41.220	1.00	0.00	H
ATOM	376	HG22	THR	23	32.068	35.154	39.463	1.00	0.00	H
ATOM	377	HG23	THR	23	33.408	34.706	40.486	1.00	0.00	H
ATOM	378	OG1	THR	23	32.095	37.729	40.221	1.00	0.00	O
ATOM	379	HG1	THR	23	32.680	38.390	39.842	1.00	0.00	H
ATOM	380	C	THR	23	32.793	36.871	42.982	1.00	0.00	C
ATOM	381	O	THR	23	33.156	35.909	43.667	1.00	0.00	O
ATOM	382	N	ILE	24	31.639	37.542	43.294	1.00	0.00	N
ATOM	383	H	ILE	24	31.259	38.272	42.709	1.00	0.00	H
ATOM	384	CA	ILE	24	30.789	37.232	44.458	1.00	0.00	C
ATOM	385	HA	ILE	24	31.210	36.441	45.077	1.00	0.00	H
ATOM	386	CB	ILE	24	30.714	38.470	45.372	1.00	0.00	C
ATOM	387	HB	ILE	24	30.375	39.368	44.857	1.00	0.00	H
ATOM	388	CG2	ILE	24	29.647	38.291	46.584	1.00	0.00	C
ATOM	389	HG21	ILE	24	29.474	39.234	47.101	1.00	0.00	H
ATOM	390	HG22	ILE	24	28.668	38.000	46.203	1.00	0.00	H
ATOM	391	HG23	ILE	24	29.957	37.525	47.295	1.00	0.00	H
ATOM	392	CG1	ILE	24	32.138	38.810	45.878	1.00	0.00	C

```
ATOM    393 HG12 ILE    24      32.735  37.931  46.119  1.00  0.00           H
ATOM    394 HG13 ILE    24      32.715  39.308  45.099  1.00  0.00           H
ATOM
    395  CD1 ILE    24      32.157  39.793  47.053  1.00  0.00           C
ATOM    396 HD11 ILE    24      31.714  39.198  47.850  1.00  0.00           H
ATOM    397 HD12 ILE    24      33.176  39.996  47.384  1.00  0.00           H
ATOM    398 HD13 ILE    24      31.531  40.675  46.919  1.00  0.00           H
ATOM    399  C   ILE    24      29.431  36.683  43.985  1.00  0.00           C
ATOM    400  O   ILE    24      28.926  37.003  42.895  1.00  0.00           O
ATOM    401  N   ARG    25      28.760  35.855  44.803  1.00  0.00           N
ATOM    402  H   ARG    25      29.170  35.537  45.670  1.00  0.00           H
ATOM    403  CA  ARG    25      27.462  35.256  44.541  1.00  0.00           C
ATOM    404  HA  ARG    25      26.824  35.854  43.890  1.00  0.00           H
ATOM    405  CB  ARG    25      27.771  33.861  43.829  1.00  0.00           C
ATOM    406  HB2 ARG    25      28.509  33.306  44.408  1.00  0.00           H
ATOM    407  HB3 ARG    25      26.844  33.287  43.820  1.00  0.00           H
ATOM    408  CG  ARG    25      28.238  33.909  42.351  1.00  0.00           C
ATOM    409  HG2 ARG    25      29.245  34.323  42.386  1.00  0.00           H
ATOM    410  HG3 ARG    25      28.330  32.906  41.933  1.00  0.00           H
ATOM    411  CD  ARG    25      27.321  34.750  41.363  1.00  0.00           C
ATOM    412  HD2 ARG    25      26.346  34.264  41.339  1.00  0.00           H
ATOM    413  HD3 ARG    25      27.294  35.786  41.702  1.00  0.00           H
ATOM    414  NE  ARG    25      27.853  34.777  40.008  1.00  0.00           N
ATOM    415  HE  ARG    25      27.303  34.320  39.295  1.00  0.00           H
ATOM    416  CZ  ARG    25      28.724  35.562  39.473  1.00  0.00           C
ATOM    417  NH1 ARG    25      29.400  36.348  40.263  1.00  0.00           N
ATOM    418 HH11 ARG    25      30.178  36.912  39.951  1.00  0.00           H
ATOM    419 HH12 ARG    25      29.182  36.440  41.245  1.00  0.00           H
ATOM    420  NH2 ARG    25      28.903  35.727  38.206  1.00  0.00           N
ATOM    421 HH21 ARG    25      29.259  36.629  37.924  1.00  0.00           H
ATOM    422 HH22 ARG    25      28.330  35.196  37.566  1.00  0.00           H
ATOM    423  C   ARG    25      26.623  35.180  45.809  1.00  0.00           C
ATOM    424  O   ARG    25      27.175  35.324  46.899  1.00  0.00           O
ATOM    425  N   ASP    26      25.272  35.009  45.764  1.00  0.00           N
ATOM    426  H   ASP    26      24.887  34.772  44.861  1.00  0.00           H
ATOM    427  CA  ASP    26      24.436  34.730  46.981  1.00  0.00           C
ATOM    428  HA  ASP    26      24.841  35.408  47.732  1.00  0.00           H
ATOM    429  CB  ASP    26      22.988  35.215  46.759  1.00  0.00           C
ATOM    430  HB2 ASP    26      22.456  35.190  47.710  1.00  0.00           H
ATOM    431  HB3 ASP    26      23.028  36.274  46.500  1.00  0.00           H
ATOM    432  CG  ASP    26      22.241  34.426  45.668  1.00  0.00           C
ATOM    433  OD1 ASP    26      22.900  33.780  44.815  1.00  0.00           O
ATOM    434  OD2 ASP    26      20.994  34.428  45.568  1.00  0.00           O
ATOM    435  C   ASP    26      24.515  33.220  47.448  1.00  0.00           C
ATOM    436  O   ASP    26      24.671  32.381  46.613  1.00  0.00           O
ATOM    437  N   GLU    27      24.444  32.941  48.703  1.00  0.00           N
ATOM    438  H   GLU    27      24.283  33.702  49.348  1.00  0.00           H
ATOM    439  CA  GLU    27      24.799  31.629  49.245  1.00  0.00           C
ATOM    440  HA  GLU    27      25.752  31.229  48.898  1.00  0.00           H
ATOM    441  CB  GLU    27      24.805  31.777  50.836  1.00  0.00           C
ATOM    442  HB2 GLU    27      25.599  32.439  51.182  1.00  0.00           H
ATOM    443  HB3 GLU    27      23.888  32.272  51.154  1.00  0.00           H
ATOM    444  CG  GLU    27      24.950  30.481  51.558  1.00  0.00           C
ATOM    445  HG2 GLU    27      25.049  30.743  52.611  1.00  0.00           H
ATOM    446  HG3 GLU    27      24.021  29.926  51.435  1.00  0.00           H
ATOM    447  CD  GLU    27      26.190  29.685  51.221  1.00  0.00           C
ATOM    448  OE1 GLU    27      26.962  29.450  52.167  1.00  0.00           O
ATOM    449  OE2 GLU    27      26.244  29.201  50.088  1.00  0.00           O
ATOM    450  C   GLU    27      23.746  30.604  48.875  1.00  0.00           C
ATOM    451  O   GLU    27      22.570  30.638  49.335  1.00  0.00           O
ATOM    452  N   SER    28      24.144  29.754  47.878  1.00  0.00           N
ATOM    453  H   SER    28      24.979  30.005  47.366  1.00  0.00           H
ATOM    454  CA  SER    28      23.307  28.710  47.211  1.00  0.00           C
ATOM    455  HA  SER    28      22.540  28.515  47.961  1.00  0.00           H
```

ATOM	456	CB	SER	28	22.523	29.315	46.016	1.00	0.00	C
ATOM	457	HB2	SER	28	21.843	30.137	46.239	1.00	0.00	H
ATOM	458	HB3	SER	28	23.149	29.664	45.195	1.00	0.00	H
ATOM	459	OG	SER	28	21.779	28.265	45.425	1.00	0.00	O
ATOM	460	HG	SER	28	20.963	28.237	45.929	1.00	0.00	H
ATOM	461	C	SER	28	24.265	27.541	46.733	1.00	0.00	C
ATOM	462	O	SER	28	25.383	27.790	46.276	1.00	0.00	O
ATOM	463	N	GLU	29	23.870	26.322	46.948	1.00	0.00	N
ATOM	464	H	GLU	29	23.003	26.318	47.467	1.00	0.00	H
ATOM	465	CA	GLU	29	24.603	25.108	46.612	1.00	0.00	C
ATOM	466	HA	GLU	29	25.523	25.079	47.197	1.00	0.00	H
ATOM	467	CB	GLU	29	23.659	23.990	47.095	1.00	0.00	C
ATOM	468	HB2	GLU	29	23.634	23.891	48.181	1.00	0.00	H
ATOM	469	HB3	GLU	29	22.642	24.290	46.844	1.00	0.00	H
ATOM	470	CG	GLU	29	23.991	22.537	46.700	1.00	0.00	C
ATOM	471	HG2	GLU	29	23.151	21.905	46.989	1.00	0.00	H
ATOM	472	HG3	GLU	29	24.190	22.379	45.640	1.00	0.00	H
ATOM	473	CD	GLU	29	25.260	21.978	47.468	1.00	0.00	C
ATOM	474	OE1	GLU	29	26.208	22.803	47.710	1.00	0.00	O
ATOM	475	OE2	GLU	29	25.482	20.751	47.633	1.00	0.00	O
ATOM	476	C	GLU	29	24.846	25.084	45.092	1.00	0.00	C
ATOM	477	O	GLU	29	25.753	24.382	44.676	1.00	0.00	O
ATOM	478	N	SER	30	24.048	25.876	44.382	1.00	0.00	N
ATOM	479	H	SER	30	23.235	26.222	44.869	1.00	0.00	H
ATOM	480	CA	SER	30	23.940	25.842	42.929	1.00	0.00	C
ATOM	481	HA	SER	30	23.884	24.779	42.696	1.00	0.00	H
ATOM	482	CB	SER	30	22.660	26.494	42.444	1.00	0.00	C
ATOM	483	HB2	SER	30	22.552	26.322	41.373	1.00	0.00	H
ATOM	484	HB3	SER	30	21.778	26.023	42.878	1.00	0.00	H
ATOM	485	OG	SER	30	22.575	27.824	42.828	1.00	0.00	O
ATOM	486	HG	SER	30	22.353	27.886	43.760	1.00	0.00	H
ATOM	487	C	SER	30	25.126	26.373	42.183	1.00	0.00	C
ATOM	488	O	SER	30	25.443	25.867	41.144	1.00	0.00	O
ATOM	489	N	HIE	31	25.852	27.322	42.770	1.00	0.00	N
ATOM	490	H	HIE	31	25.729	27.536	43.750	1.00	0.00	H
ATOM	491	CA	HIE	31	26.995	28.000	42.130	1.00	0.00	C
ATOM	492	HA	HIE	31	26.719	28.404	41.156	1.00	0.00	H
ATOM	493	CB	HIE	31	27.349	29.297	42.987	1.00	0.00	C
ATOM	494	HB2	HIE	31	27.647	28.937	43.971	1.00	0.00	H
ATOM	495	HB3	HIE	31	28.268	29.812	42.707	1.00	0.00	H
ATOM	496	CG	HIE	31	26.196	30.330	43.129	1.00	0.00	C
ATOM	497	ND1	HIE	31	25.425	30.833	42.120	1.00	0.00	N
ATOM	498	CE1	HIE	31	24.594	31.661	42.713	1.00	0.00	C
ATOM	499	HE1	HIE	31	23.793	32.180	42.208	1.00	0.00	H
ATOM	500	NE2	HIE	31	24.781	31.674	44.042	1.00	0.00	N
ATOM	501	HE2	HIE	31	24.134	32.065	44.712	1.00	0.00	H
ATOM	502	CD2	HIE	31	25.793	30.819	44.273	1.00	0.00	C
ATOM	503	HD2	HIE	31	26.228	30.614	45.241	1.00	0.00	H
ATOM	504	C	HIE	31	28.254	27.086	41.933	1.00	0.00	C
ATOM	505	O	HIE	31	28.294	25.912	42.365	1.00	0.00	O
ATOM	506	N	PHE	32	29.258	27.721	41.293	1.00	0.00	N
ATOM	507	H	PHE	32	28.976	28.569	40.821	1.00	0.00	H
ATOM	508	CA	PHE	32	30.525	27.122	40.997	1.00	0.00	C
ATOM	509	HA	PHE	32	30.232	26.224	40.455	1.00	0.00	H
ATOM	510	CB	PHE	32	31.351	28.081	40.199	1.00	0.00	C
ATOM	511	HB2	PHE	32	31.522	28.969	40.807	1.00	0.00	H
ATOM	512	HB3	PHE	32	32.275	27.588	39.899	1.00	0.00	H
ATOM	513	CG	PHE	32	30.598	28.666	39.004	1.00	0.00	C
ATOM	514	CD1	PHE	32	30.878	29.954	38.564	1.00	0.00	C
ATOM	515	HD1	PHE	32	31.539	30.618	39.101	1.00	0.00	H
ATOM	516	CE1	PHE	32	30.194	30.470	37.483	1.00	0.00	C
ATOM	517	HE1	PHE	32	30.459	31.447	37.105	1.00	0.00	H
ATOM	518	CZ	PHE	32	29.180	29.764	36.834	1.00	0.00	C
ATOM	519	HZ	PHE	32	28.719	30.293	36.013	1.00	0.00	H

```
ATOM    520  CE2 PHE    32
   28.841  28.494  37.227  1.00  0.00           C
ATOM    521  HE2 PHE    32      28.077  27.978  36.666  1.00  0.00           H
ATOM    522  CD2 PHE    32      29.661  27.907  38.201  1.00  0.00           C
ATOM    523  HD2 PHE    32      29.427  26.887  38.464  1.00  0.00           H
ATOM    524  C   PHE    32      31.276  26.635  42.229  1.00  0.00           C
ATOM    525  O   PHE    32      31.471  27.432  43.131  1.00  0.00           O
ATOM    526  N   LYS    33      31.882  25.467  42.295  1.00  0.00           N
ATOM    527  H   LYS    33      31.708  24.849  41.514  1.00  0.00           H
ATOM    528  CA  LYS    33      32.777  25.056  43.410  1.00  0.00           C
ATOM    529  HA  LYS    33      32.707  25.842  44.161  1.00  0.00           H
ATOM    530  CB  LYS    33      32.235  23.696  43.994  1.00  0.00           C
ATOM    531  HB2 LYS    33      32.029  23.089  43.112  1.00  0.00           H
ATOM    532  HB3 LYS    33      32.978  23.353  44.714  1.00  0.00           H
ATOM    533  CG  LYS    33      30.850  23.757  44.703  1.00  0.00           C
ATOM    534  HG2 LYS    33      30.769  24.585  45.407  1.00  0.00           H
ATOM    535  HG3 LYS    33      30.047  23.980  44.001  1.00  0.00           H
ATOM    536  CD  LYS    33      30.510  22.394  45.323  1.00  0.00           C
ATOM    537  HD2 LYS    33      30.866  21.625  44.636  1.00  0.00           H
ATOM    538  HD3 LYS    33      31.094  22.308  46.239  1.00  0.00           H
ATOM    539  CE  LYS    33      29.011  22.208  45.703  1.00  0.00           C
ATOM    540  HE2 LYS    33      28.432  21.990  44.805  1.00  0.00           H
ATOM    541  HE3 LYS    33      28.962  21.279  46.270  1.00  0.00           H
ATOM    542  NZ  LYS    33      28.556  23.377  46.571  1.00  0.00           N
ATOM    543  HZ1 LYS    33      28.352  24.193  46.012  1.00  0.00           H
ATOM    544  HZ2 LYS    33      27.703  23.096  47.033  1.00  0.00           H
ATOM    545  HZ3 LYS    33      29.172  23.780  47.262  1.00  0.00           H
ATOM    546  C   LYS    33      34.275  24.980  42.985  1.00  0.00           C
ATOM    547  O   LYS    33      34.465  24.859  41.788  1.00  0.00           O
ATOM    548  N   THR    34      35.230  25.078  43.891  1.00  0.00           N
ATOM    549  H   THR    34      34.878  25.209  44.828  1.00  0.00           H
ATOM    550  CA  THR    34      36.658  25.045  43.594  1.00  0.00           C
ATOM    551  HA  THR    34      37.015  25.957  43.115  1.00  0.00           H
ATOM    552  CB  THR    34      37.461  24.935  44.972  1.00  0.00           C
ATOM    553  HB  THR    34      37.021  24.062  45.453  1.00  0.00           H
ATOM    554  CG2 THR    34      39.001  24.686  44.720  1.00  0.00           C
ATOM    555 HG21 THR    34      39.063  23.646  44.403  1.00  0.00           H
ATOM    556 HG22 THR    34      39.277  25.367  43.913  1.00  0.00           H
ATOM    557 HG23 THR    34      39.590  24.989  45.586  1.00  0.00           H
ATOM    558  OG1 THR    34      37.296  26.104  45.659  1.00  0.00           O
ATOM    559  HG1 THR    34      37.385  25.902  46.593  1.00  0.00           H
ATOM    560  C   THR    34      37.018  23.808  42.775  1.00  0.00           C
ATOM    561  O   THR    34      36.710  22.627  43.125  1.00  0.00           O
ATOM    562  N   GLY    35      37.614  24.007  41.584  1.00  0.00           N
ATOM    563  H   GLY    35      37.730  24.947  41.230  1.00  0.00           H
ATOM    564  CA  GLY    35      37.927  22.974  40.635  1.00  0.00           C
ATOM    565  HA2 GLY    35      38.869  23.257  40.167  1.00  0.00           H
ATOM    566  HA3 GLY    35      38.037  22.048  41.198  1.00  0.00           H
ATOM    567  C   GLY    35      36.843  22.873  39.503  1.00  0.00           C
ATOM    568  O   GLY    35      37.112  22.254  38.434  1.00  0.00           O
ATOM    569  N   ASP    36      35.668  23.537  39.591  1.00  0.00           N
ATOM    570  H   ASP    36      35.370  24.079  40.390  1.00  0.00           H
ATOM    571  CA  ASP    36      34.689  23.659  38.468  1.00  0.00           C
ATOM    572  HA  ASP    36      34.570  22.704  37.956  1.00  0.00           H
ATOM    573  CB  ASP    36      33.247  24.128  38.974  1.00  0.00           C
ATOM    574  HB2 ASP    36      33.336  24.951  39.684  1.00  0.00           H
ATOM    575  HB3 ASP    36      32.640  24.220  38.073  1.00  0.00           H
ATOM    576  CG  ASP    36      32.369  23.152  39.757  1.00  0.00           C
ATOM    577  OD1 ASP    36      31.254  23.519  40.183  1.00  0.00           O
ATOM    578  OD2 ASP    36      32.601  21.908  39.749  1.00  0.00           O
ATOM    579  C   ASP    36      35.210  24.540  37.346  1.00  0.00           C
ATOM    580  O   ASP    36      35.999  25.483  37.415  1.00  0.00           O
ATOM    581  N   VAL    37      34.664  24.157  36.180  1.00  0.00           N
ATOM    582  H   VAL    37      34.052  23.354  36.155  1.00  0.00           H
```

ATOM	583	CA	VAL	37	35.347	24.542	34.906	1.00	0.00	C
ATOM	584	HA	VAL	37	36.136	25.260	35.131	1.00	0.00	H
ATOM	585	CB	VAL	37	35.948	23.371	34.174	1.00	0.00	C
ATOM	586	HB	VAL	37	35.104	22.879	33.691	1.00	0.00	H
ATOM	587	CG1	VAL	37	36.899	23.720	33.007	1.00	0.00	C
ATOM	588	HG11	VAL	37	36.548	24.255	32.124	1.00	0.00	H
ATOM	589	HG12	VAL	37	37.645	24.334	33.512	1.00	0.00	H
ATOM	590	HG13	VAL	37	37.294	22.762	32.672	1.00	0.00	H
ATOM	591	CG2	VAL	37	36.732	22.325	35.076	1.00	0.00	C
ATOM	592	HG21	VAL	37	37.643	22.726	35.522	1.00	0.00	H
ATOM	593	HG22	VAL	37	35.996	21.847	35.722	1.00	0.00	H
ATOM	594	HG23	VAL	37	37.005	21.506	34.409	1.00	0.00	H
ATOM	595	C	VAL	37	34.325	25.304	34.009	1.00	0.00	C
ATOM	596	O	VAL	37	33.161	24.905	33.800	1.00	0.00	O
ATOM	597	N	LEU	38	34.808	26.348	33.291	1.00	0.00	N
ATOM	598	H	LEU	38	35.785	26.580	33.402	1.00	0.00	H
ATOM	599	CA	LEU	38	34.082	27.184	32.336	1.00	0.00	C
ATOM	600	HA	LEU	38	33.174	26.655	32.046	1.00	0.00	H
ATOM	601	CB	LEU	38	33.651	28.498	33.113	1.00	0.00	C
ATOM	602	HB2	LEU	38	34.580	28.940	33.476	1.00	0.00	H
ATOM	603	HB3	LEU	38	33.162	29.175	32.411	1.00	0.00	H
ATOM	604	CG	LEU	38	32.724	28.313	34.329	1.00	0.00	C
ATOM	605	HG	LEU	38	32.323	27.311	34.176	1.00	0.00	H
ATOM	606	CD1	LEU	38	33.571	28.310	35.652	1.00	0.00	C
ATOM	607	HD11	LEU	38	32.804	28.160	36.412	1.00	0.00	H
ATOM	608	HD12	LEU	38	34.452	27.669	35.681	1.00	0.00	H
ATOM	609	HD13	LEU	38	34.054	29.277	35.787	1.00	0.00	H
ATOM	610	CD2	LEU	38	31.679	29.453	34.325	1.00	0.00	C
ATOM	611	HD21	LEU	38	30.909	29.086	35.004	1.00	0.00	H
ATOM	612	HD22	LEU	38	32.100	30.419	34.602	1.00	0.00	H
ATOM	613	HD23	LEU	38	31.126	29.510	33.388	1.00	0.00	H
ATOM	614	C	LEU	38	34.939	27.559	31.103	1.00	0.00	C
ATOM	615	O	LEU	38	36.109	27.140	31.036	1.00	0.00	O
ATOM	616	N	ARG	39	34.374	28.166	29.983	1.00	0.00	N
ATOM	617	H	ARG	39	33.388	28.365	30.068	1.00	0.00	H
ATOM	618	CA	ARG	39	35.105	28.748	28.893	1.00	0.00	C
ATOM	619	HA	ARG	39	36.072	29.053	29.293	1.00	0.00	H
ATOM	620	CB	ARG	39	35.139	27.691	27.765	1.00	0.00	C
ATOM	621	HB2	ARG	39	34.148	27.575	27.326	1.00	0.00	H
ATOM	622	HB3	ARG	39	35.902	27.940	27.028	1.00	0.00	H
ATOM	623	CG	ARG	39	35.665	26.349	28.197	1.00	0.00	C
ATOM	624	HG2	ARG	39	36.596	26.491	28.747	1.00	0.00	H
ATOM	625	HG3	ARG	39	35.003	25.812	28.877	1.00	0.00	H
ATOM	626	CD	ARG	39	36.081	25.480	26.974	1.00	0.00	C
ATOM	627	HD2	ARG	39	36.429	26.057	26.117	1.00	0.00	H
ATOM	628	HD3	ARG	39	36.929	24.862	27.268	1.00	0.00	H
ATOM	629	NE	ARG	39	34.942	24.637	26.718	1.00	0.00	N
ATOM	630	HE	ARG	39	34.045	25.095	26.790	1.00	0.00	H
ATOM	631	CZ	ARG	39	35.003	23.388	26.412	1.00	0.00	C
ATOM	632	NH1	ARG	39	36.069	22.784	26.125	1.00	0.00	N
ATOM	633	HH11	ARG	39	36.952	23.255	25.995	1.00	0.00	H
ATOM	634	HH12	ARG	39	36.067	21.776	26.201	1.00	0.00	H
ATOM	635	NH2	ARG	39	33.891	22.755	26.309	1.00	0.00	N
ATOM	636	HH21	ARG	39	33.877	21.905	25.763	1.00	0.00	H
ATOM	637	HH22	ARG	39	33.029	23.227	26.544	1.00	0.00	H
ATOM	638	C	ARG	39	34.479	30.080	28.395	1.00	0.00	C
ATOM	639	O	ARG	39	33.331	30.339	28.651	1.00	0.00	O
ATOM	640	N	VAL	40	35.269	30.868	27.657	1.00	0.00	N
ATOM	641	H	VAL	40	36.201	30.483	27.598	1.00	0.00	H
ATOM	642	CA	VAL	40	35.047	32.174	27.084	1.00	0.00	C
ATOM	643	HA	VAL	40	33.986	32.245	26.847	1.00	0.00	H
ATOM	644	CB	VAL	40	35.341	33.337	28.075	1.00	0.00	C
ATOM	645	HB	VAL	40	35.352	34.220	27.436			
1.00	0.00	H								

ATOM	646	CG1	VAL	40	34.311	33.580	29.202	1.00	0.00	C
ATOM	647	HG11	VAL	40	34.160	32.632	29.718	1.00	0.00	H
ATOM	648	HG12	VAL	40	34.581	34.449	29.804	1.00	0.00	H
ATOM	649	HG13	VAL	40	33.331	33.797	28.780	1.00	0.00	H
ATOM	650	CG2	VAL	40	36.761	33.214	28.658	1.00	0.00	C
ATOM	651	HG21	VAL	40	37.122	34.199	28.951	1.00	0.00	H
ATOM	652	HG22	VAL	40	36.741	32.511	29.490	1.00	0.00	H
ATOM	653	HG23	VAL	40	37.475	32.981	27.867	1.00	0.00	H
ATOM	654	C	VAL	40	35.672	32.366	25.702	1.00	0.00	C
ATOM	655	O	VAL	40	36.872	32.077	25.507	1.00	0.00	O
ATOM	656	N	GLY	41	34.873	32.836	24.754	1.00	0.00	N
ATOM	657	H	GLY	41	33.917	33.075	24.978	1.00	0.00	H
ATOM	658	CA	GLY	41	35.262	33.113	23.403	1.00	0.00	C
ATOM	659	HA2	GLY	41	36.194	33.679	23.406	1.00	0.00	H
ATOM	660	HA3	GLY	41	35.480	32.157	22.925	1.00	0.00	H
ATOM	661	C	GLY	41	34.172	33.729	22.522	1.00	0.00	C
ATOM	662	O	GLY	41	33.029	33.644	22.926	1.00	0.00	O
ATOM	663	N	ARG	42	34.461	34.360	21.384	1.00	0.00	N
ATOM	664	H	ARG	42	35.427	34.445	21.102	1.00	0.00	H
ATOM	665	CA	ARG	42	33.474	34.964	20.503	1.00	0.00	C
ATOM	666	HA	ARG	42	32.487	34.529	20.667	1.00	0.00	H
ATOM	667	CB	ARG	42	33.252	36.466	20.818	1.00	0.00	C
ATOM	668	HB2	ARG	42	32.460	36.872	20.189	1.00	0.00	H
ATOM	669	HB3	ARG	42	32.891	36.506	21.845	1.00	0.00	H
ATOM	670	CG	ARG	42	34.484	37.325	20.786	1.00	0.00	C
ATOM	671	HG2	ARG	42	35.157	36.872	21.515	1.00	0.00	H
ATOM	672	HG3	ARG	42	34.934	37.230	19.798	1.00	0.00	H
ATOM	673	CD	ARG	42	34.189	38.754	21.121	1.00	0.00	C
ATOM	674	HD2	ARG	42	33.469	39.123	20.389	1.00	0.00	H
ATOM	675	HD3	ARG	42	33.768	38.880	22.120	1.00	0.00	H
ATOM	676	NE	ARG	42	35.474	39.602	21.098	1.00	0.00	N
ATOM	677	HE	ARG	42	35.742	39.996	20.209	1.00	0.00	H
ATOM	678	CZ	ARG	42	36.241	39.706	22.079	1.00	0.00	C
ATOM	679	NH1	ARG	42	36.246	38.922	23.116	1.00	0.00	N
ATOM	680	HH11	ARG	42	36.787	39.233	23.911	1.00	0.00	H
ATOM	681	HH12	ARG	42	35.559	38.190	23.226	1.00	0.00	H
ATOM	682	NH2	ARG	42	37.229	40.512	21.972	1.00	0.00	N
ATOM	683	HH21	ARG	42	37.387	41.077	21.150	1.00	0.00	H
ATOM	684	HH22	ARG	42	37.781	40.682	22.801	1.00	0.00	H
ATOM	685	C	ARG	42	33.786	34.695	18.977	1.00	0.00	C
ATOM	686	O	ARG	42	33.150	35.226	18.051	1.00	0.00	O
ATOM	687	N	PHE	43	34.788	33.841	18.674	1.00	0.00	N
ATOM	688	H	PHE	43	35.151	33.322	19.460	1.00	0.00	H
ATOM	689	CA	PHE	43	35.352	33.591	17.376	1.00	0.00	C
ATOM	690	HA	PHE	43	34.780	34.083	16.588	1.00	0.00	H
ATOM	691	CB	PHE	43	36.817	34.095	17.311	1.00	0.00	C
ATOM	692	HB2	PHE	43	37.346	33.454	18.018	1.00	0.00	H
ATOM	693	HB3	PHE	43	37.293	33.994	16.337	1.00	0.00	H
ATOM	694	CG	PHE	43	36.965	35.579	17.681	1.00	0.00	C
ATOM	695	CD1	PHE	43	36.769	36.624	16.764	1.00	0.00	C
ATOM	696	HD1	PHE	43	36.479	36.418	15.745	1.00	0.00	H
ATOM	697	CE1	PHE	43	37.040	37.946	17.231	1.00	0.00	C
ATOM	698	HE1	PHE	43	36.746	38.736	16.557	1.00	0.00	H
ATOM	699	CZ	PHE	43	37.448	38.216	18.514	1.00	0.00	C
ATOM	700	HZ	PHE	43	37.576	39.254	18.781	1.00	0.00	H
ATOM	701	CE2	PHE	43	37.901	37.220	19.383	1.00	0.00	C
ATOM	702	HE2	PHE	43	38.210	37.434	20.395	1.00	0.00	H
ATOM	703	CD2	PHE	43	37.514	35.923	18.971	1.00	0.00	C
ATOM	704	HD2	PHE	43	37.716	35.112	19.654	1.00	0.00	H
ATOM	705	C	PHE	43	35.261	32.036	17.114	1.00	0.00	C
ATOM	706	O	PHE	43	35.447	31.228	18.027	1.00	0.00	O
ATOM	707	N	GLU	44	35.033	31.562	15.839	1.00	0.00	N
ATOM	708	H	GLU	44	34.730	32.136	15.066	1.00	0.00	H
ATOM	709	CA	GLU	44	35.178	30.192	15.499	1.00	0.00	C

ATOM	710	HA	GLU	44	34.585	29.649	16.237	1.00	0.00	H
ATOM	711	CB	GLU	44	34.757	29.971	14.021	1.00	0.00	C
ATOM	712	HB2	GLU	44	33.843	30.561	13.954	1.00	0.00	H
ATOM	713	HB3	GLU	44	35.546	30.415	13.415	1.00	0.00	H
ATOM	714	CG	GLU	44	34.395	28.569	13.520	1.00	0.00	C
ATOM	715	HG2	GLU	44	33.687	28.777	12.717	1.00	0.00	H
ATOM	716	HG3	GLU	44	35.314	28.284	13.009	1.00	0.00	H
ATOM	717	CD	GLU	44	33.768	27.606	14.478	1.00	0.00	C
ATOM	718	OE1	GLU	44	32.668	28.005	14.997	1.00	0.00	O
ATOM	719	OE2	GLU	44	34.394	26.559	14.826	1.00	0.00	O
ATOM	720	C	GLU	44	36.640	29.599	15.601	1.00	0.00	C
ATOM	721	O	GLU	44	36.911	28.436	15.911	1.00	0.00	O
ATOM	722	N	ASP	45	37.663	30.472	15.387	1.00	0.00	N
ATOM	723	H	ASP	45	37.400	31.432	15.221	1.00	0.00	H
ATOM	724	CA	ASP	45	39.124	30.141	15.333	1.00	0.00	C
ATOM	725	HA	ASP	45	39.170	29.350	14.586	1.00	0.00	H
ATOM	726	CB	ASP	45	40.023	31.271	14.829	1.00	0.00	C
ATOM	727	HB2	ASP	45	39.676	31.492	13.819	1.00	0.00	H
ATOM	728	HB3	ASP	45	39.939	32.229	15.342	1.00	0.00	H
ATOM	729	CG	ASP	45	41.499	30.869	14.842	1.00	0.00	C
ATOM	730	OD1	ASP	45	41.784	29.655	14.762	1.00	0.00	O
ATOM	731	OD2	ASP	45	42.433	31.742	14.981	1.00	0.00	O
ATOM	732	C	ASP	45	39.607	29.693	16.681	1.00	0.00	C
ATOM	733	O	ASP	45	39.636	30.434	17.626	1.00	0.00	O
ATOM	734	N	ASP	46	39.891	28.407	16.731	1.00	0.00	N
ATOM	735	H	ASP	46	39.865	27.872	15.875	1.00	0.00	H
ATOM	736	CA	ASP	46	40.234	27.644	17.961	1.00	0.00	C
ATOM	737	HA	ASP	46	40.179	26.607	17.631	1.00	0.00	H
ATOM	738	CB	ASP	46	41.647	27.942	18.547	1.00	0.00	C
ATOM	739	HB2	ASP	46	41.542	28.764	19.255	1.00	0.00	H
ATOM	740	HB3	ASP	46	41.787	27.072	19.188	1.00	0.00	H
ATOM	741	CG	ASP	46	42.901	28.073	17.677	1.00	0.00	C
ATOM	742	OD1	ASP	46	43.050	27.062	16.920	1.00	0.00	O
ATOM	743	OD2	ASP	46	43.563	29.171	17.450	1.00	0.00	O
ATOM	744	C	ASP	46	39.111	27.655	19.032	1.00	0.00	C
ATOM	745	O	ASP	46	39.214	27.054	20.089	1.00	0.00	O
ATOM	746	N	GLY	47	37.959	28.342	18.819	1.00	0.00	N
ATOM	747	H	GLY	47	37.898	28.744	17.895	1.00	0.00	H
ATOM	748	CA	GLY	47	36.866	28.378	19.762	1.00	0.00	C
ATOM	749	HA2	GLY	47	35.956	28.575	19.195	1.00	0.00	H
ATOM	750	HA3	GLY	47	36.774	27.368	20.160	1.00	0.00	H
ATOM	751	C	GLY	47	36.955	29.312	20.960	1.00	0.00	C
ATOM	752	O	GLY	47	35.976	30.039	21.211	1.00	0.00	O
ATOM	753	N	TYR	48	38.098	29.423	21.634	1.00	0.00	N
ATOM	754	H	TYR	48	38.814	28.748	21.406	1.00	0.00	H
ATOM	755	CA	TYR	48	38.214	30.031	22.914	1.00	0.00	C
ATOM	756	HA	TYR	48	37.344	30.658	23.108	1.00	0.00	H
ATOM	757	CB	TYR	48	38.283	28.906	23.990	1.00	0.00	C
ATOM	758	HB2	TYR	48	39.164	28.269	23.922	1.00	0.00	H
ATOM	759	HB3	TYR	48	38.371	29.380	24.967	1.00	0.00	H
ATOM	760	CG	TYR	48	37.085	27.999	23.907	1.00	0.00	C
ATOM	761	CD1	TYR	48	35.790	28.463	24.304	1.00	0.00	C
ATOM	762	HD1	TYR	48	35.495	29.479	24.523	1.00	0.00	H
ATOM	763	CE1	TYR	48	34.716	27.539	24.096	1.00	0.00	C
ATOM	764	HE1	TYR	48	33.743	27.924	24.360	1.00	0.00	H
ATOM	765	CZ	TYR	48	34.861	26.230	23.581	1.00	0.00	C
ATOM	766	OH	TYR	48	33.861	25.348	23.570	1.00	0.00	O
ATOM	767	HH	TYR	48	34.108	24.495	23.204	1.00	0.00	H
ATOM	768	CE2	TYR	48	36.250	25.854	23.308	1.00	0.00	C
ATOM	769	HE2	TYR	48	36.453	24.816	23.087	1.00	0.00	H
ATOM	770	CD2	TYR	48	37.278	26.721	23.472	1.00	0.00	C
ATOM	771	HD2	TYR	48	38.272	26.324	23.330	1.00	0.00	H
ATOM	772	C	TYR	48	39.485	30.863	23.121	1.00	0.00	C

ATOM	773	O	TYR	48	40.326	30.878	22.242	1.00	0.00	O
ATOM	774	N	PHE	49	39.570	31.650	24.212	1.00	0.00	N
ATOM	775	H	PHE	49	38.784	31.605	24.845	1.00	0.00	H
ATOM	776	CA	PHE	49	40.799	32.330	24.635	1.00	0.00	C
ATOM	777	HA	PHE	49	41.663	32.007	24.056	1.00	0.00	H
ATOM	778	CB	PHE	49	40.580	33.815	24.318	1.00	0.00	C
ATOM	779	HB2	PHE	49	41.545	34.286	24.503	1.00	0.00	H
ATOM	780	HB3	PHE	49	40.418	33.892	23.243	1.00	0.00	H
ATOM	781	CG	PHE	49	39.468	34.572	25.065	1.00	0.00	C
ATOM	782	CD1	PHE	49	38.240	34.875	24.475	1.00	0.00	C
ATOM	783	HD1	PHE	49	38.131	34.636	23.428	1.00	0.00	H
ATOM	784	CE1	PHE	49	37.217	35.512	25.179	1.00	0.00	C
ATOM	785	HE1	PHE	49	36.296	35.884	24.757	1.00	0.00	H
ATOM	786	CZ	PHE	49	37.443	35.767	26.494	1.00	0.00	C
ATOM	787	HZ	PHE	49	36.714	36.430	26.937	1.00	0.00	H
ATOM	788	CE2	PHE	49	38.676	35.460	27.138	1.00	0.00	C
ATOM	789	HE2	PHE	49	38.889	35.840	28.126	1.00	0.00	H
ATOM	790	CD2	PHE	49	39.727	34.989	26.424	1.00	0.00	C
ATOM	791	HD2	PHE	49	40.674	34.770	26.896	1.00	0.00	H
ATOM	792	C	PHE	49	41.218	32.028	26.067	1.00	0.00	C
ATOM	793	O	PHE	49	42.404	31.767	26.312	1.00	0.00	O
ATOM	794	N	CYX	50	40.188	31.725	26.885	1.00	0.00	N
ATOM	795	H	CYX	50	39.348	32.179	26.555	1.00	0.00	H
ATOM	796	CA	CYX	50	40.355	31.074	28.200	1.00	0.00	C
ATOM	797	HA	CYX	50	41.376	30.735	28.376	1.00	0.00	H
ATOM	798	CB	CYX	50	40.215	32.009	29.430	1.00	0.00	C
ATOM	799	HB2	CYX	50	39.201	32.403	29.477	1.00	0.00	H
ATOM	800	HB3	CYX	50	40.397	31.485	30.368	1.00	0.00	H
ATOM	801	SG	CYX	50	41.273	33.508	29.592	1.00	0.00	S
ATOM	802	C	CYX	50	39.401	29.909	28.322	1.00	0.00	C
ATOM	803	O	CYX	50	38.200	30.184	28.356	1.00	0.00	O
ATOM	804	N	THR	51	39.905	28.619	28.488	1.00	0.00	N
ATOM	805	H	THR	51	40.862	28.464	28.201	1.00	0.00	H
ATOM	806	CA	THR	51	39.338	27.706	29.537	1.00	0.00	C
ATOM	807	HA	THR	51	38.284	27.621	29.272	1.00	0.00	H
ATOM	808	CB	THR	51	40.012	26.333	29.496	1.00	0.00	C
ATOM	809	HB	THR	51	41.063	26.482	29.747	1.00	0.00	H
ATOM	810	CG2	THR	51	39.324	25.327	30.375	1.00	0.00	C
ATOM	811	HG21	THR	51	38.269	25.203	30.131	1.00	0.00	H
ATOM	812	HG22	THR	51	39.953	24.437	30.368	1.00	0.00	H
ATOM	813	HG23	THR	51	39.409	25.660	31.410	1.00	0.00	H
ATOM	814	OG1	THR	51	40.205	25.665	28.266	1.00	0.00	O
ATOM	815	HG1	THR	51	40.803	24.962	28.529	1.00	0.00	H
ATOM	816	C	THR	51	39.544	28.339	30.978	1.00	0.00	C
ATOM	817	O	THR	51	40.584	28.906	31.249	1.00	0.00	O
ATOM	818	N	ILE	52	38.653	28.121	31.880	1.00	0.00	N
ATOM	819	H	ILE	52	37.860	27.512	31.736	1.00	0.00	H
ATOM	820	CA	ILE	52	38.664	28.731	33.267	1.00	0.00	C
ATOM	821	HA	ILE	52	39.656	29.179	33.337	1.00	0.00	H
ATOM	822	CB	ILE	52	37.516	29.794	33.434	1.00	0.00	C
ATOM	823	HB	ILE	52	36.633	29.400	32.930	1.00	0.00	H
ATOM	824	CG2	ILE	52	37.155	30.134	34.941	1.00	0.00	C
ATOM	825	HG21	ILE	52	36.738	29.232	35.390	1.00	0.00	H
ATOM	826	HG22	ILE	52	38.086	30.375	35.451	1.00	0.00	H
ATOM	827	HG23	ILE	52	36.454	30.966	35.000	1.00	0.00	H
ATOM	828	CG1	ILE	52	37.838	31.067	32.606	1.00	0.00	C
ATOM	829	HG12	ILE	52	38.600	31.683	33.085	1.00	0.00	H
ATOM	830	HG13	ILE	52	38.277	30.775	31.652	1.00	0.00	H
ATOM	831	CD1	ILE	52	36.589	31.943	32.212	1.00	0.00	C
ATOM	832	HD11	ILE	52	36.213	32.553	33.033	1.00	0.00	H
ATOM	833	HD12	ILE	52	36.773	32.753	31.506	1.00	0.00	H
ATOM	834	HD13	ILE	52	35.823	31.307	31.769	1.00	0.00	H
ATOM	835	C	ILE	52	38.425	27.613	34.269	1.00	0.00	C
ATOM	836	O	ILE	52	37.567	26.720	34.038	1.00	0.00	O

ATOM	837	N	GLU	53	39.127	27.743	35.412	1.00	0.00	N
ATOM	838	H	GLU	53	39.843	28.445	35.535	1.00	0.00	H
ATOM	839	CA	GLU	53	38.962	26.817	36.523	1.00	0.00	C
ATOM	840	HA	GLU	53	38.061	26.209	36.445	1.00	0.00	H
ATOM	841	CB	GLU	53	40.130	25.785	36.529	1.00	0.00	C
ATOM	842	HB2	GLU	53	40.420	25.429	35.542	1.00	0.00	H
ATOM	843	HB3	GLU	53	41.004	26.250	36.985	1.00	0.00	H
ATOM	844	CG	GLU	53	39.726	24.600	37.374	1.00	0.00	C
ATOM	845	HG2	GLU	53	39.506	24.996	38.366	1.00	0.00	H
ATOM	846	HG3	GLU	53	38.849	24.155	36.907	1.00	0.00	H
ATOM	847	CD	GLU	53	40.688	23.453	37.361	1.00	0.00	C
ATOM	848	OE1	GLU	53	40.712	22.612	36.409	1.00	0.00	O
ATOM	849	OE2	GLU	53	41.380	23.188	38.385	1.00	0.00	O
ATOM	850	C	GLU	53	38.850	27.604	37.920	1.00	0.00	C
ATOM	851	O	GLU	53	39.853	28.280	38.215	1.00	0.00	O
ATOM	852	N	VAL	54	37.883	27.327	38.787	1.00	0.00	N
ATOM	853	H	VAL	54	37.189	26.657	38.488	1.00	0.00	H
ATOM	854	CA	VAL	54	37.829	27.970	40.112	1.00	0.00	C
ATOM	855	HA	VAL	54	37.840	29.052	39.979	1.00	0.00	H
ATOM	856	CB	VAL	54	36.453	27.640	40.749	1.00	0.00	C
ATOM	857	HB	VAL	54	36.357	26.555	40.727	1.00	0.00	H
ATOM	858	CG1	VAL	54	36.261	28.303	42.126	1.00	0.00	C
ATOM	859	HG11	VAL	54	37.122	28.185	42.786	1.00	0.00	H
ATOM	860	HG12	VAL	54	36.129	29.379	42.015	1.00	0.00	H
ATOM	861	HG13	VAL	54	35.409	27.833	42.616	1.00	0.00	H
ATOM	862	CG2	VAL	54	35.349	28.204	39.882	1.00	0.00	C
ATOM	863	HG21	VAL	54	34.405	28.088	40.414	1.00	0.00	H
ATOM	864	HG22	VAL	54	35.368	29.233	39.525	1.00	0.00	H
ATOM	865	HG23	VAL	54	35.285	27.709	38.914	1.00	0.00	H
ATOM	866	C	VAL	54	38.978	27.586	41.014	1.00	0.00	C
ATOM	867	O	VAL	54	39.161	26.351	41.143	1.00	0.00	O
ATOM	868	N	THR	55	39.754	28.430	41.644	1.00	0.00	N
ATOM	869	H	THR	55	39.418	29.380	41.577	1.00	0.00	H
ATOM	870	CA	THR	55	40.946	28.036	42.326	1.00	0.00	C
ATOM	871	HA	THR	55	41.356	27.058	42.076	1.00	0.00	H
ATOM	872	CB	THR	55	42.050	29.071	42.159	1.00	0.00	C
ATOM	873	HB	THR	55	42.804	28.804	42.899	1.00	0.00	H
ATOM	874	CG2	THR	55	42.638	29.070	40.752	1.00	0.00	C
ATOM	875	HG21	THR	55	43.592	29.599	40.780	1.00	0.00	H
ATOM	876	HG22	THR	55	42.782	28.022	40.492	1.00	0.00	H
ATOM	877	HG23	THR	55	42.012	29.563	40.009	1.00	0.00	H
ATOM	878	OG1	THR	55	41.747	30.407	42.480	1.00	0.00	O
ATOM	879	HG1	THR	55	42.296	30.639	43.234	1.00	0.00	H
ATOM	880	C	THR	55	40.735	27.902	43.841	1.00	0.00	C
ATOM	881	O	THR	55	41.319	26.994	44.509	1.00	0.00	O
ATOM	882	N	ALA	56	39.869	28.782	44.396	1.00	0.00	N
ATOM	883	H	ALA	56	39.436	29.502	43.837	1.00	0.00	H
ATOM	884	CA	ALA	56	39.453	28.891	45.775	1.00	0.00	C
ATOM	885	HA	ALA	56	39.321	27.906	46.223	1.00	0.00	H
ATOM	886	CB	ALA	56	40.470	29.720	46.557	1.00	0.00	C
ATOM	887	HB1	ALA	56	40.884	30.590	46.049	1.00	0.00	H
ATOM	888	HB2	ALA	56	39.890	30.135	47.381	1.00	0.00	H
ATOM	889	HB3	ALA	56	41.251	29.042	46.902	1.00	0.00	H
ATOM	890	C	ALA	56	38.043	29.568	45.807	1.00	0.00	C
ATOM	891	O	ALA	56	37.822	30.482	45.002	1.00	0.00	O
ATOM	892	N	THR	57	37.152	29.209	46.690	1.00	0.00	N
ATOM	893	H	THR	57	37.399	28.478	47.342	1.00	0.00	H
ATOM	894	CA	THR	57	35.975	29.965	47.122	1.00	0.00	C
ATOM	895	HA	THR	57	35.984	30.930	46.614	1.00	0.00	H
ATOM	896	CB	THR	57						
					34.720	29.245	46.607	1.00	0.00	C
ATOM	897	HB	THR	57	33.941	29.986	46.790	1.00	0.00	H
ATOM	898	CG2	THR	57	34.869	28.855	45.107	1.00	0.00	C
ATOM	899	HG21	THR	57	33.873	28.578	44.760	1.00	0.00	H

ATOM	900	HG22	THR	57	35.118	29.729	44.505	1.00	0.00	H
ATOM	901	HG23	THR	57	35.564	28.050	44.872	1.00	0.00	H
ATOM	902	OG1	THR	57	34.444	28.145	47.386	1.00	0.00	O
ATOM	903	HG1	THR	57	33.485	28.113	47.425	1.00	0.00	H
ATOM	904	C	THR	57	35.809	30.100	48.631	1.00	0.00	C
ATOM	905	O	THR	57	36.508	29.342	49.288	1.00	0.00	O
ATOM	906	N	SER	58	34.915	31.005	49.095	1.00	0.00	N
ATOM	907	H	SER	58	34.370	31.599	48.486	1.00	0.00	H
ATOM	908	CA	SER	58	34.631	30.983	50.538	1.00	0.00	C
ATOM	909	HA	SER	58	34.576	29.924	50.790	1.00	0.00	H
ATOM	910	CB	SER	58	35.822	31.551	51.369	1.00	0.00	C
ATOM	911	HB2	SER	58	35.714	31.401	52.444	1.00	0.00	H
ATOM	912	HB3	SER	58	36.763	31.053	51.135	1.00	0.00	H
ATOM	913	OG	SER	58	35.893	32.979	51.093	1.00	0.00	O
ATOM	914	HG	SER	58	36.171	33.394	51.913	1.00	0.00	H
ATOM	915	C	SER	58	33.254	31.599	50.839	1.00	0.00	C
ATOM	916	O	SER	58	32.809	32.537	50.185	1.00	0.00	O
ATOM	917	N	THR	59	32.429	30.968	51.705	1.00	0.00	N
ATOM	918	H	THR	59	32.721	30.017	51.883	1.00	0.00	H
ATOM	919	CA	THR	59	31.127	31.518	52.175	1.00	0.00	C
ATOM	920	HA	THR	59	30.734	32.120	51.355	1.00	0.00	H
ATOM	921	CB	THR	59	30.134	30.430	52.628	1.00	0.00	C
ATOM	922	HB	THR	59	30.093	29.561	51.971	1.00	0.00	H
ATOM	923	CG2	THR	59	30.261	29.877	54.056	1.00	0.00	C
ATOM	924	HG21	THR	59	29.434	29.182	54.199	1.00	0.00	H
ATOM	925	HG22	THR	59	31.138	29.249	54.207	1.00	0.00	H
ATOM	926	HG23	THR	59	30.256	30.601	54.871	1.00	0.00	H
ATOM	927	OG1	THR	59	28.816	31.029	52.617	1.00	0.00	O
ATOM	928	HG1	THR	59	28.093	30.410	52.496	1.00	0.00	H
ATOM	929	C	THR	59	31.387	32.644	53.187	1.00	0.00	C
ATOM	930	O	THR	59	32.201	32.477	54.063	1.00	0.00	O
ATOM	931	N	VAL	60	30.620	33.729	53.134	1.00	0.00	N
ATOM	932	H	VAL	60	29.987	33.688	52.348	1.00	0.00	H
ATOM	933	CA	VAL	60	30.818	35.058	53.770	1.00	0.00	C
ATOM	934	HA	VAL	60	31.325	35.025	54.735	1.00	0.00	H
ATOM	935	CB	VAL	60	31.752	35.873	52.819	1.00	0.00	C
ATOM	936	HB	VAL	60	31.298	35.849	51.829	1.00	0.00	H
ATOM	937	CG1	VAL	60	32.013	37.344	53.267	1.00	0.00	C
ATOM	938	HG11	VAL	60	32.583	37.244	54.191	1.00	0.00	H
ATOM	939	HG12	VAL	60	32.429	37.885	52.418	1.00	0.00	H
ATOM	940	HG13	VAL	60	31.034	37.777	53.472	1.00	0.00	H
ATOM	941	CG2	VAL	60	33.176	35.248	52.712	1.00	0.00	C
ATOM	942	HG21	VAL	60	33.816	35.816	52.037	1.00	0.00	H
ATOM	943	HG22	VAL	60	33.669	35.220	53.684	1.00	0.00	H
ATOM	944	HG23	VAL	60	33.242	34.227	52.334	1.00	0.00	H
ATOM	945	C	VAL	60	29.538	35.765	54.051	1.00	0.00	C
ATOM	946	O	VAL	60	28.832	36.135	53.148	1.00	0.00	O
ATOM	947	N	THR	61	29.293	36.081	55.360	1.00	0.00	N
ATOM	948	H	THR	61	29.968	35.640	55.967	1.00	0.00	H
ATOM	949	CA	THR	61	28.323	37.086	55.713	1.00	0.00	C
ATOM	950	HA	THR	61	27.507	37.095	54.990	1.00	0.00	H
ATOM	951	CB	THR	61	27.601	36.991	57.056	1.00	0.00	C
ATOM	952	HB	THR	61	27.088	37.916	57.317	1.00	0.00	H
ATOM	953	CG2	THR	61	26.522	35.883	57.193	1.00	0.00	C
ATOM	954	HG21	THR	61	25.745	36.248	56.521	1.00	0.00	H
ATOM	955	HG22	THR	61	26.952	34.899	57.000	1.00	0.00	H
ATOM	956	HG23	THR	61	26.026	35.795	58.160	1.00	0.00	H
ATOM	957	OG1	THR	61	28.531	36.725	58.089	1.00	0.00	O
ATOM	958	HG1	THR	61	28.993	37.531	58.334	1.00	0.00	H
ATOM	959	C	THR	61	28.991	38.488	55.608	1.00	0.00	C
ATOM	960	O	THR	61	30.209	38.582	55.808	1.00	0.00	O
ATOM	961	N	LEU	62	28.193	39.585	55.471	1.00	0.00	N
ATOM	962	H	LEU	62	27.187	39.495	55.486	1.00	0.00	H
ATOM	963	CA	LEU	62	28.794	40.890	55.070	1.00	0.00	C

ATOM	964	HA	LEU	62	29.570	40.783	54.313	1.00	0.00	H
ATOM	965	CB	LEU	62	27.643	41.917	54.634	1.00	0.00	C
ATOM	966	HB2	LEU	62	26.914	41.932	55.444	1.00	0.00	H
ATOM	967	HB3	LEU	62	27.993	42.946	54.563	1.00	0.00	H
ATOM	968	CG	LEU	62	26.915	41.575	53.387	1.00	0.00	C
ATOM	969	HG	LEU	62	26.419	40.612	53.512	1.00	0.00	H
ATOM	970	CD1	LEU	62	25.883	42.638	53.055	1.00	0.00	C
ATOM	971	HD11	LEU	62	25.293	42.227	52.236	1.00	0.00	H
ATOM	972	HD12	LEU	62	25.354	42.878	53.977	1.00	0.00	H
ATOM	973	HD13	LEU	62	26.373	43.576	52.792	1.00	0.00	H
ATOM	974	CD2	LEU	62	27.823	41.555	52.164	1.00	0.00	C
ATOM	975	HD21	LEU	62	28.636	42.266	52.304	1.00	0.00	H
ATOM	976	HD22	LEU	62	28.320	40.590	52.074	1.00	0.00	H
ATOM	977	HD23	LEU	62	27.263	41.804	51.262	1.00	0.00	H
ATOM	978	C	LEU	62	29.544	41.563	56.242	1.00	0.00	C
ATOM	979	O	LEU	62	30.338	42.439	55.939	1.00	0.00	O
ATOM	980	N	ASP	63	29.358	41.197	57.505	1.00	0.00	N
ATOM	981	H	ASP	63	28.756	40.403	57.672	1.00	0.00	H
ATOM	982	CA	ASP	63	30.146	41.536	58.658	1.00	0.00	C
ATOM	983	HA	ASP	63	30.223	42.617	58.768	1.00	0.00	H
ATOM	984	CB	ASP	63	29.535	41.027	60.014	1.00	0.00	C
ATOM	985	HB2	ASP	63	30.129	41.221	60.908	1.00	0.00	H
ATOM	986	HB3	ASP	63	28.611	41.580	60.184	1.00	0.00	H
ATOM	987	CG	ASP	63	29.234	39.527	59.935	1.00	0.00	C
ATOM	988	OD1	ASP	63	29.630	38.925	58.874	1.00	0.00	O
ATOM	989	OD2	ASP	63	28.491	38.971	60.765	1.00	0.00	O
ATOM	990	C	ASP	63	31.602	41.105	58.596	1.00	0.00	C
ATOM	991	O	ASP	63	32.457	41.550	59.391	1.00	0.00	O
ATOM	992	N	THR	64	32.023	40.208	57.734	1.00	0.00	N
ATOM	993	H	THR	64	31.293	39.738	57.216	1.00	0.00	H
ATOM	994	CA	THR	64	33.348	39.537	57.507	1.00	0.00	C
ATOM	995	HA	THR	64	34.156	40.148	57.909	1.00	0.00	H
ATOM	996	CB	THR	64	33.372	38.184	58.294	1.00	0.00	C
ATOM	997	HB	THR	64	34.014	37.467	57.783	1.00	0.00	H
ATOM	998	CG2	THR	64	33.747	38.394	59.701	1.00	0.00	C
ATOM	999	HG21	THR	64	34.462	39.214	59.777	1.00	0.00	H
ATOM	1000	HG22	THR	64	32.852	38.746	60.214	1.00	0.00	H
ATOM	1001	HG23	THR	64	34.007	37.532	60.314	1.00	0.00	H
ATOM	1002	OG1	THR	64	32.037	37.606	58.223	1.00	0.00	O
ATOM	1003	HG1	THR	64	31.402	38.239	58.565	1.00	0.00	H
ATOM	1004	C	THR	64	33.728	39.382	56.048	1.00	0.00	C
ATOM	1005	O	THR	64	34.449	38.482	55.627	1.00	0.00	O
ATOM	1006	N	LEU	65	33.303	40.352	55.236	1.00	0.00	N
ATOM	1007	H	LEU	65	32.550	40.944	55.556	1.00	0.00	H
ATOM	1008	CA	LEU	65	33.626	40.545	53.839	1.00	0.00	C
ATOM	1009	HA	LEU	65	33.786	39.536	53.458	1.00	0.00	H
ATOM	1010	CB	LEU	65	32.421	41.226	53.094	1.00	0.00	C
ATOM	1011	HB2	LEU	65	31.619	40.499	53.218	1.00	0.00	H
ATOM	1012	HB3	LEU	65	32.193	42.163	53.599	1.00	0.00	H
ATOM	1013	CG	LEU	65	32.642	41.624	51.565	1.00	0.00	C
ATOM	1014	HG	LEU	65	33.597	42.147	51.591	1.00	0.00	H
ATOM	1015	CD1	LEU	65	32.811	40.424	50.645	1.00	0.00	C
ATOM	1016	HD11	LEU	65	33.738	39.902	50.887	1.00	0.00	H
ATOM	1017	HD12	LEU	65	31.901	39.829	50.580	1.00	0.00	H
ATOM	1018	HD13	LEU	65	32.912	40.832	49.639	1.00	0.00	H
ATOM	1019	CD2	LEU	65	31.456	42.436	51.126	1.00	0.00	C
ATOM	1020	HD21	LEU	65	30.557	41.820	51.166	1.00	0.00	H
ATOM	1021	HD22	LEU	65	31.332	43.237	51.853			
1.00	0.00			H						
ATOM	1022	HD23	LEU	65	31.653	42.797	50.116	1.00	0.00	H
ATOM	1023	C	LEU	65	34.936	41.268	53.755	1.00	0.00	C
ATOM	1024	O	LEU	65	35.012	42.430	54.231	1.00	0.00	O
ATOM	1025	N	THR	66	35.992	40.651	53.147	1.00	0.00	N
ATOM	1026	H	THR	66	35.905	39.706	52.798	1.00	0.00	H

ATOM	1027	CA	THR	66	37.320	41.225	52.775	1.00	0.00	C
ATOM	1028	HA	THR	66	37.724	41.820	53.594	1.00	0.00	H
ATOM	1029	CB	THR	66	38.300	40.095	52.409	1.00	0.00	C
ATOM	1030	HB	THR	66	38.003	39.633	51.467	1.00	0.00	H
ATOM	1031	CG2	THR	66	39.749	40.595	52.276	1.00	0.00	C
ATOM	1032	HG21	THR	66	40.030	41.280	53.076	1.00	0.00	H
ATOM	1033	HG22	THR	66	40.403	39.725	52.224	1.00	0.00	H
ATOM	1034	HG23	THR	66	39.922	41.078	51.314	1.00	0.00	H
ATOM	1035	OG1	THR	66	38.253	39.163	53.435	1.00	0.00	O
ATOM	1036	HG1	THR	66	37.617	38.474	53.227	1.00	0.00	H
ATOM	1037	C	THR	66	37.245	42.122	51.582	1.00	0.00	C
ATOM	1038	O	THR	66	36.354	42.031	50.743	1.00	0.00	O
ATOM	1039	N	GLU	67	38.183	43.064	51.392	1.00	0.00	N
ATOM	1040	H	GLU	67	38.968	43.145	52.023	1.00	0.00	H
ATOM	1041	CA	GLU	67	38.154	44.118	50.346	1.00	0.00	C
ATOM	1042	HA	GLU	67	37.071	44.174	50.242	1.00	0.00	H
ATOM	1043	CB	GLU	67	38.805	45.425	50.805	1.00	0.00	C
ATOM	1044	HB2	GLU	67	38.399	46.060	50.017	1.00	0.00	H
ATOM	1045	HB3	GLU	67	38.284	45.739	51.708	1.00	0.00	H
ATOM	1046	CG	GLU	67	40.306	45.508	51.015	1.00	0.00	C
ATOM	1047	HG2	GLU	67	40.688	45.039	50.107	1.00	0.00	H
ATOM	1048	HG3	GLU	67	40.573	46.562	51.092	1.00	0.00	H
ATOM	1049	CD	GLU	67	40.901	44.765	52.194	1.00	0.00	C
ATOM	1050	OE1	GLU	67	40.225	44.219	53.074	1.00	0.00	O
ATOM	1051	OE2	GLU	67	42.171	44.753	52.216	1.00	0.00	O
ATOM	1052	C	GLU	67	38.629	43.650	48.986	1.00	0.00	C
ATOM	1053	O	GLU	67	38.648	44.408	48.010	1.00	0.00	O
ATOM	1054	N	LYS	68	38.875	42.371	48.792	1.00	0.00	N
ATOM	1055	H	LYS	68	38.763	41.870	49.662	1.00	0.00	H
ATOM	1056	CA	LYS	68	39.599	41.760	47.722	1.00	0.00	C
ATOM	1057	HA	LYS	68	40.564	42.251	47.592	1.00	0.00	H
ATOM	1058	CB	LYS	68	39.685	40.271	48.021	1.00	0.00	C
ATOM	1059	HB2	LYS	68	39.937	40.088	49.065	1.00	0.00	H
ATOM	1060	HB3	LYS	68	38.745	39.772	47.784	1.00	0.00	H
ATOM	1061	CG	LYS	68	40.727	39.552	47.143	1.00	0.00	C
ATOM	1062	HG2	LYS	68	40.790	38.538	47.538	1.00	0.00	H
ATOM	1063	HG3	LYS	68	40.343	39.392	46.136	1.00	0.00	H
ATOM	1064	CD	LYS	68	42.112	40.187	47.139	1.00	0.00	C
ATOM	1065	HD2	LYS	68	42.086	41.221	46.800	1.00	0.00	H
ATOM	1066	HD3	LYS	68	42.495	40.108	48.157	1.00	0.00	H
ATOM	1067	CE	LYS	68	42.952	39.333	46.190	1.00	0.00	C
ATOM	1068	HE2	LYS	68	43.650	38.659	46.687	1.00	0.00	H
ATOM	1069	HE3	LYS	68	42.295	38.692	45.603	1.00	0.00	H
ATOM	1070	NZ	LYS	68	43.773	40.162	45.269	1.00	0.00	N
ATOM	1071	HZ1	LYS	68	43.170	40.882	44.895	1.00	0.00	H
ATOM	1072	HZ2	LYS	68	44.586	40.627	45.647	1.00	0.00	H
ATOM	1073	HZ3	LYS	68	44.161	39.541	44.573	1.00	0.00	H
ATOM	1074	C	LYS	68	38.892	41.971	46.412	1.00	0.00	C
ATOM	1075	O	LYS	68	39.548	42.153	45.405	1.00	0.00	O
ATOM	1076	N	HIE	69	37.553	42.046	46.394	1.00	0.00	N
ATOM	1077	H	HIE	69	37.135	41.742	47.262	1.00	0.00	H
ATOM	1078	CA	HIE	69	36.695	42.280	45.245	1.00	0.00	C
ATOM	1079	HA	HIE	69	37.205	42.000	44.323	1.00	0.00	H
ATOM	1080	CB	HIE	69	35.403	41.529	45.269	1.00	0.00	C
ATOM	1081	HB2	HIE	69	35.580	40.460	45.388	1.00	0.00	H
ATOM	1082	HB3	HIE	69	34.790	41.812	46.125	1.00	0.00	H
ATOM	1083	CG	HIE	69	34.447	41.806	44.096	1.00	0.00	C
ATOM	1084	ND1	HIE	69	34.417	41.163	42.916	1.00	0.00	N
ATOM	1085	CE1	HIE	69	33.366	41.631	42.298	1.00	0.00	C
ATOM	1086	HE1	HIE	69	32.913	41.315	41.369	1.00	0.00	H
ATOM	1087	NE2	HIE	69	32.731	42.550	42.958	1.00	0.00	N
ATOM	1088	HE2	HIE	69	31.803	42.775	42.632	1.00	0.00	H
ATOM	1089	CD2	HIE	69	33.351	42.604	44.176	1.00	0.00	C
ATOM	1090	HD2	HIE	69	32.889	43.082	45.027	1.00	0.00	H

ATOM	1091	C	HIE	69	36.580	43.807	45.083	1.00	0.00	C
ATOM	1092	O	HIE	69	36.811	44.302	43.982	1.00	0.00	O
ATOM	1093	N	ALA	70	36.342	44.491	46.195	1.00	0.00	N
ATOM	1094	H	ALA	70	36.227	43.986	47.061	1.00	0.00	H
ATOM	1095	CA	ALA	70	36.255	45.925	46.113	1.00	0.00	C
ATOM	1096	HA	ALA	70	35.392	46.255	45.534	1.00	0.00	H
ATOM	1097	CB	ALA	70	36.088	46.448	47.552	1.00	0.00	C
ATOM	1098	HB1	ALA	70	36.060	47.534	47.452	1.00	0.00	H
ATOM	1099	HB2	ALA	70	35.197	46.006	47.998	1.00	0.00	H
ATOM	1100	HB3	ALA	70	36.923	46.222	48.214	1.00	0.00	H
ATOM	1101	C	ALA	70	37.425	46.645	45.381	1.00	0.00	C
ATOM	1102	O	ALA	70	37.124	47.532	44.578	1.00	0.00	O
ATOM	1103	N	GLU	71	38.680	46.302	45.685	1.00	0.00	N
ATOM	1104	H	GLU	71	38.755	45.514	46.312	1.00	0.00	H
ATOM	1105	CA	GLU	71	39.949	46.586	44.992	1.00	0.00	C
ATOM	1106	HA	GLU	71	40.198	47.644	45.080	1.00	0.00	H
ATOM	1107	CB	GLU	71	41.144	45.800	45.595	1.00	0.00	C
ATOM	1108	HB2	GLU	71	40.740	44.833	45.895	1.00	0.00	H
ATOM	1109	HB3	GLU	71	41.953	45.684	44.875	1.00	0.00	H
ATOM	1110	CG	GLU	71	41.669	46.357	46.939	1.00	0.00	C
ATOM	1111	HG2	GLU	71	40.855	46.380	47.663	1.00	0.00	H
ATOM	1112	HG3	GLU	71	42.414	45.647	47.298	1.00	0.00	H
ATOM	1113	CD	GLU	71	42.106	47.841	46.790	1.00	0.00	C
ATOM	1114	OE1	GLU	71	42.891	48.125	45.872	1.00	0.00	O
ATOM	1115	OE2	GLU	71	41.590	48.720	47.518	1.00	0.00	O
ATOM	1116	C	GLU	71	39.716	46.330	43.465	1.00	0.00	C
ATOM	1117	O	GLU	71	40.140	47.149	42.655	1.00	0.00	O
ATOM	1118	N	GLN	72	39.171	45.207	43.027	1.00	0.00	N
ATOM	1119	H	GLN	72	38.577	44.617	43.592	1.00	0.00	H
ATOM	1120	CA	GLN	72	39.189	44.807	41.555	1.00	0.00	C
ATOM	1121	HA	GLN	72	40.188	44.891	41.125	1.00	0.00	H
ATOM	1122	CB	GLN	72	38.765	43.281	41.507	1.00	0.00	C
ATOM	1123	HB2	GLN	72	37.757	43.229	41.916	1.00	0.00	H
ATOM	1124	HB3	GLN	72	39.574	42.753	42.012	1.00	0.00	H
ATOM	1125	CG	GLN	72	38.656	42.695	40.003	1.00	0.00	C
ATOM	1126	HG2	GLN	72	38.028	43.402	39.459	1.00	0.00	H
ATOM	1127	HG3	GLN	72	39.611	42.709	39.477	1.00	0.00	H
ATOM	1128	CD	GLN	72	37.980	41.290	40.001	1.00	0.00	C
ATOM	1129	OE1	GLN	72	38.447	40.266	39.620	1.00	0.00	O
ATOM	1130	NE2	GLN	72	36.790	41.240	40.482	1.00	0.00	N
ATOM	1131	HE21	GLN	72	36.348	42.032	40.928	1.00	0.00	H
ATOM	1132	HE22	GLN	72	36.274	40.378	40.588	1.00	0.00	H
ATOM	1133	C	GLN	72	38.215	45.785	40.793	1.00	0.00	C
ATOM	1134	O	GLN	72	38.470	46.214	39.698	1.00	0.00	O
ATOM	1135	N	GLU	73	37.104	46.108	41.466	1.00	0.00	N
ATOM	1136	H	GLU	73	36.919	45.706	42.374	1.00	0.00	H
ATOM	1137	CA	GLU	73	36.076	46.986	40.932	1.00	0.00	C
ATOM	1138	HA	GLU	73	36.041	46.837	39.853	1.00	0.00	H
ATOM	1139	CB	GLU	73	34.644	46.649	41.468	1.00	0.00	C
ATOM	1140	HB2	GLU	73	34.562	46.847	42.537	1.00	0.00	H
ATOM	1141	HB3	GLU	73	33.903	47.225	40.914	1.00	0.00	H
ATOM	1142	CG	GLU	73	34.318	45.140	41.190	1.00	0.00	C
ATOM	1143	HG2	GLU	73	34.987	44.540	41.807	1.00	0.00	H
ATOM	1144	HG3	GLU	73	33.369	44.884	41.661	1.00	0.00	H
ATOM	1145	CD	GLU	73	34.511	44.661	39.738	1.00	0.00	C
ATOM	1146	OE1	GLU	73	34.025	45.330	38.795	1.00	0.00	O
ATOM	1147	OE2	GLU	73	35.191	43.602	39.588	1.00	0.00	O
ATOM	1148	C	GLU	73	36.443	48.454	41.019	1.00	0.00	C
ATOM	1149	O	GLU	73	35.767	49.300	40.482	1.00	0.00	O
ATOM	1150	N	ASN	74	37.568	48.805	41.681	1.00	0.00	N
ATOM	1151	H	ASN	74	37.974	47.945	42.021	1.00	0.00	H
ATOM	1152	CA	ASN	74	38.165	50.109	41.933	1.00	0.00	C
ATOM	1153	HA	ASN	74	38.980	50.005	42.651	1.00	0.00	H

ATOM	1154	CB	ASN	74	38.610	50.742	40.580	1.00	0.00	C
ATOM	1155	HB2	ASN	74	37.811	50.631	39.847	1.00	0.00	H
ATOM	1156	HB3	ASN	74	38.814	51.799	40.751	1.00	0.00	H
ATOM	1157	CG	ASN	74	39.872	50.158	40.025	1.00	0.00	C
ATOM	1158	OD1	ASN	74	40.491	50.714	39.084	1.00	0.00	O
ATOM	1159	ND2	ASN	74	40.304	49.011	40.514	1.00	0.00	N
ATOM	1160	HD21	ASN	74	41.066	48.589	40.003	1.00	0.00	H
ATOM	1161	HD22	ASN	74	39.919	48.474	41.278	1.00	0.00	H
ATOM	1162	C	ASN	74	37.136	51.053	42.659	1.00	0.00	C
ATOM	1163	O	ASN	74	36.801	52.173	42.183	1.00	0.00	O
ATOM	1164	N	MET	75	36.734	50.534	43.797	1.00	0.00	N
ATOM	1165	H	MET	75	37.051	49.638	44.139	1.00	0.00	H
ATOM	1166	CA	MET	75	35.880	51.248	44.741	1.00	0.00	C
ATOM	1167	HA	MET	75	36.183	52.295	44.696	1.00	0.00	H
ATOM	1168	CB	MET	75	34.399	51.077	44.315	1.00	0.00	C
ATOM	1169	HB2	MET	75	33.836	51.676	45.031	1.00	0.00	H
ATOM	1170	HB3	MET	75	34.341	51.448	43.292	1.00	0.00	H
ATOM	1171	CG	MET	75	33.894	49.664	44.446	1.00	0.00	C
ATOM	1172	HG2	MET	75	34.249	49.093	43.588	1.00	0.00	H
ATOM	1173	HG3	MET	75	34.226	49.277	45.409	1.00	0.00	H
ATOM	1174	SD	MET	75	32.133	49.678	44.528	1.00	0.00	S
ATOM	1175	CE	MET	75	31.802	47.936	44.158	1.00	0.00	C
ATOM	1176	HE1	MET	75	31.748	47.329	45.062	1.00	0.00	H
ATOM	1177	HE2	MET	75	30.841	47.820	43.656	1.00	0.00	H
ATOM	1178	HE3	MET	75	32.530	47.615	43.413	1.00	0.00	H
ATOM	1179	C	MET	75	36.302	50.839	46.185	1.00	0.00	C
ATOM	1180	O	MET	75	37.286	50.043	46.312	1.00	0.00	O
ATOM	1181	N	THR	76	35.687	51.330	47.265	1.00	0.00	N
ATOM	1182	H	THR	76	34.831	51.856	47.168	1.00	0.00	H
ATOM	1183	CA	THR	76	35.980	50.961	48.654	1.00	0.00	C
ATOM	1184	HA	THR	76	36.979	50.537	48.754	1.00	0.00	H
ATOM	1185	CB	THR	76	36.066	52.394	49.450	1.00	0.00	C
ATOM	1186	HB	THR	76	36.865	52.890	48.899	1.00	0.00	H
ATOM	1187	CG2	THR	76	34.756	53.237	49.523	1.00	0.00	C
ATOM	1188	HG21	THR	76	34.032	52.516	49.904	1.00	0.00	H
ATOM	1189	HG22	THR	76	34.780	54.137	50.137	1.00	0.00	H
ATOM	1190	HG23	THR	76	34.508	53.443	48.482	1.00	0.00	H
ATOM	1191	OG1	THR	76	36.575	52.256	50.787	1.00	0.00	O
ATOM	1192	HG1	THR	76	37.471	52.597	50.739	1.00	0.00	H
ATOM	1193	C	THR	76	34.943	50.021	49.353	1.00	0.00	C
ATOM	1194	O	THR	76	33.862	49.867	48.742	1.00	0.00	O
ATOM	1195	N	LEU	77	35.282	49.157	50.328	1.00	0.00	N
ATOM	1196	H	LEU	77	36.203	49.261	50.731	1.00	0.00	H
ATOM	1197	CA	LEU	77	34.513	48.035	50.818	1.00	0.00	C
ATOM	1198	HA	LEU	77	34.320	47.353	49.988	1.00	0.00	H
ATOM	1199	CB	LEU	77	35.443	47.370	51.819	1.00	0.00	C
ATOM	1200	HB2	LEU	77	36.321	46.988	51.298	1.00	0.00	H
ATOM	1201	HB3	LEU	77	35.828	48.137	52.491	1.00	0.00	H
ATOM	1202	CG	LEU	77	34.802	46.405	52.774	1.00	0.00	C
ATOM	1203	HG	LEU	77	34.035	46.797	53.441	1.00	0.00	H
ATOM	1204	CD1	LEU	77	34.265	45.193	51.971	1.00	0.00	C
ATOM	1205	HD11	LEU	77	34.025	44.420	52.701	1.00	0.00	H
ATOM	1206	HD12	LEU	77	33.367	45.494	51.429	1.00	0.00	H
ATOM	1207	HD13	LEU	77	35.027	44.775	51.314	1.00	0.00	H
ATOM	1208	CD2	LEU	77	35.847	45.725	53.659	1.00	0.00	C
ATOM	1209	HD21	LEU	77	35.351	45.023	54.331	1.00	0.00	H
ATOM	1210	HD22	LEU	77	36.500	45.192	52.967	1.00	0.00	H
ATOM	1211	HD23	LEU	77	36.321	46.451	54.317	1.00	0.00	H
ATOM	1212	C	LEU	77	33.132	48.505	51.374	1.00	0.00	C
ATOM	1213	O	LEU	77	32.167	47.801	51.140	1.00	0.00	O
ATOM	1214	N	THR	78	33.042	49.649	52.096	1.00	0.00	N
ATOM	1215	H	THR	78	33.921	50.131	52.223	1.00	0.00	H
ATOM	1216	CA	THR	78	31.771	50.301	52.505	1.00	0.00	C
ATOM	1217	HA	THR	78	31.366	49.680	53.305	1.00	0.00	H

ATOM	1218	CB	THR	78	32.035	51.667	53.116	1.00	0.00	C
ATOM	1219	HB	THR	78	31.063	52.119	53.304	1.00	0.00	H
ATOM	1220	CG2	THR	78	32.748	51.620	54.479	1.00	0.00	C
ATOM	1221	HG21	THR	78	32.674	52.623	54.899	1.00	0.00	H
ATOM	1222	HG22	THR	78	32.199	50.897	55.083	1.00	0.00	H
ATOM	1223	HG23	THR	78	33.800	51.361	54.359	1.00	0.00	H
ATOM	1224	OG1	THR	78	32.804	52.388	52.225	1.00	0.00	O
ATOM	1225	HG1	THR	78	33.343	53.034	52.688	1.00	0.00	H
ATOM	1226	C	THR	78	30.786	50.335	51.346	1.00	0.00	C
ATOM	1227	O	THR	78	29.657	49.904	51.563	1.00	0.00	O
ATOM	1228	N	GLU	79	31.205	50.785	50.151	1.00	0.00	N
ATOM	1229	H	GLU	79	32.180	51.013	50.025	1.00	0.00	H
ATOM	1230	CA	GLU	79	30.311	50.815	48.958	1.00	0.00	C
ATOM	1231	HA	GLU	79	29.356	51.202	49.313	1.00	0.00	H
ATOM	1232	CB	GLU	79	31.001	51.652	47.905	1.00	0.00	C
ATOM	1233	HB2	GLU	79	32.036	51.359	47.725	1.00	0.00	H
ATOM	1234	HB3	GLU	79	30.517	51.467	46.947	1.00	0.00	H
ATOM	1235	CG	GLU	79	30.950	53.141	48.238	1.00	0.00	C
ATOM	1236	HG2	GLU	79	31.332	53.303	49.246	1.00	0.00	H
ATOM	1237	HG3	GLU	79	31.669	53.506	47.506	1.00	0.00	H
ATOM	1238	CD	GLU	79	29.573	53.818	48.132	1.00	0.00	C
ATOM	1239	OE1	GLU	79	29.305	54.739	48.926	1.00	0.00	O
ATOM	1240	OE2	GLU	79	28.690	53.414	47.331	1.00	0.00	O
ATOM	1241	C	GLU	79	29.888	49.433	48.510	1.00	0.00	C
ATOM	1242	O	GLU	79	28.700	49.202	48.412	1.00	0.00	O
ATOM	1243	N	LEU	80	30.754	48.452	48.341	1.00	0.00	N
ATOM	1244	H	LEU	80	31.708	48.702	48.559	1.00	0.00	H
ATOM	1245	CA	LEU	80	30.385	47.092	47.971	1.00	0.00	C
ATOM	1246	HA	LEU	80	29.904	47.152	46.995	1.00	0.00	H
ATOM	1247	CB	LEU	80	31.650	46.228	47.908	1.00	0.00	C
ATOM	1248	HB2	LEU	80	32.261	46.597	47.083	1.00	0.00	H
ATOM	1249	HB3	LEU	80	32.257	46.307	48.810	1.00	0.00	H
ATOM	1250	CG	LEU	80	31.592	44.758	47.622	1.00	0.00	C
ATOM	1251	HG	LEU	80	30.959	44.347	48.410	1.00	0.00	H
ATOM	1252	CD1	LEU	80	30.886	44.344	46.326	1.00	0.00	C
ATOM	1253	HD11	LEU	80	30.759	43.263	46.372	1.00	0.00	H
ATOM	1254	HD12	LEU	80	29.872	44.737	46.263	1.00	0.00	H
ATOM	1255	HD13	LEU	80	31.446	44.672	45.451	1.00	0.00	H
ATOM	1256	CD2	LEU	80	32.932	44.100	47.677	1.00	0.00	C
ATOM	1257	HD21	LEU	80	32.900	43.010	47.678	1.00	0.00	H
ATOM	1258	HD22	LEU	80	33.455	44.275	46.736	1.00	0.00	H
ATOM	1259	HD23	LEU	80	33.612	44.412	48.469	1.00	0.00	H
ATOM	1260	C	LEU	80	29.410	46.492	48.999	1.00	0.00	C
ATOM	1261	O	LEU	80	28.540	45.664	48.693	1.00	0.00	O
ATOM	1262	N	LYS	81	29.666	46.808	50.285	1.00	0.00	N
ATOM	1263	H	LYS	81	30.554	47.262	50.446	1.00	0.00	H
ATOM	1264	CA	LYS	81	28.729	46.481	51.385	1.00	0.00	C
ATOM	1265	HA	LYS	81	28.624	45.402	51.265	1.00	0.00	H
ATOM	1266	CB	LYS	81	29.356	46.745	52.786	1.00	0.00	C
ATOM	1267	HB2	LYS	81	29.714	47.770	52.881	1.00	0.00	H
ATOM	1268	HB3	LYS	81	28.512	46.774	53.475	1.00	0.00	H
ATOM	1269	CG	LYS	81	30.460	45.782	53.221	1.00	0.00	C
ATOM	1270	HG2	LYS	81	29.925	44.862	53.458	1.00	0.00	H
ATOM	1271	HG3	LYS	81	31.178	45.778	52.402	1.00	0.00	H
ATOM	1272	CD	LYS	81	31.258	46.269	54.462	1.00	0.00	C
ATOM	1273	HD2	LYS	81	31.932	47.098	54.251	1.00	0.00	H
ATOM	1274	HD3	LYS	81	30.533	46.599	55.207	1.00	0.00	H
ATOM	1275	CE	LYS	81	32.030	45.102	55.043	1.00	0.00	C
ATOM	1276	HE2	LYS	81	31.363	44.244	55.135	1.00	0.00	H
ATOM	1277	HE3	LYS	81	32.802	44.755	54.356	1.00	0.00	H
ATOM	1278	NZ	LYS	81	32.699	45.397	56.325	1.00	0.00	N
ATOM	1279	HZ1	LYS	81	33.394	46.124	56.231	1.00	0.00	H
ATOM	1280	HZ2	LYS	81	32.024	45.740	56.994	1.00	0.00	H

ATOM	1281	HZ3	LYS	81	33.135	44.573	56.716	1.00	0.00	H
ATOM	1282	C	LYS	81	27.350	47.095	51.269	1.00	0.00	C
ATOM	1283	O	LYS	81	26.345	46.419	51.419	1.00	0.00	O
ATOM	1284	N	LYS	82	27.221	48.357	50.870	1.00	0.00	N
ATOM	1285	H	LYS	82	28.111	48.806	50.705	1.00	0.00	H
ATOM	1286	CA	LYS	82	26.002	49.094	50.552	1.00	0.00	C
ATOM	1287	HA	LYS	82	25.300	48.992	51.379	1.00	0.00	H
ATOM	1288	CB	LYS	82	26.262	50.602	50.332	1.00	0.00	C
ATOM	1289	HB2	LYS	82	26.940	50.889	51.136	1.00	0.00	H
ATOM	1290	HB3	LYS	82	26.771	50.624	49.368	1.00	0.00	H
ATOM	1291	CG	LYS	82	24.980	51.402	50.358	1.00	0.00	C
ATOM	1292	HG2	LYS	82	24.368	51.177	49.484	1.00	0.00	H
ATOM	1293	HG3	LYS	82	24.451	51.007	51.224	1.00	0.00	H
ATOM	1294	CD	LYS	82	25.188	52.913	50.433	1.00	0.00	C
ATOM	1295	HD2	LYS	82	24.230	53.371	50.186	1.00	0.00	H
ATOM	1296	HD3	LYS	82	25.433	53.272	51.433	1.00	0.00	H
ATOM	1297	CE	LYS	82	26.270	53.541	49.495	1.00	0.00	C
ATOM	1298	HE2	LYS	82	26.355	54.598	49.747	1.00	0.00	H
ATOM	1299	HE3	LYS	82	27.245	53.121	49.746	1.00	0.00	H
ATOM	1300	NZ	LYS	82	26.148	53.362	48.080	1.00	0.00	N
ATOM	1301	HZ1	LYS	82	25.721	54.151	47.618	1.00	0.00	H
ATOM	1302	HZ2	LYS	82	27.064	53.282	47.663	1.00	0.00	H
ATOM	1303	HZ3	LYS	82	25.544	52.572	47.905	1.00	0.00	H
ATOM	1304	C	LYS	82	25.337	48.556	49.268	1.00	0.00	C
ATOM	1305	O	LYS	82	24.107	48.435	49.342	1.00	0.00	O
ATOM	1306	N	VAL	83	26.005	48.238	48.180	1.00	0.00	N
ATOM	1307	H	VAL	83	26.997	48.411	48.108	1.00	0.00	H
ATOM	1308	CA	VAL	83	25.285	47.798	46.909	1.00	0.00	C
ATOM	1309	HA	VAL	83	24.481	48.512	46.728	1.00	0.00	H
ATOM	1310	CB	VAL	83	26.052	47.875	45.658	1.00	0.00	C
ATOM	1311	HB	VAL	83	25.413	47.760	44.783	1.00	0.00	H
ATOM	1312	CG1	VAL	83	26.788	49.230	45.504	1.00	0.00	C
ATOM	1313	HG11	VAL	83	26.022	50.001	45.420	1.00	0.00	H
ATOM	1314	HG12	VAL	83	27.527	49.463	46.270	1.00	0.00	H
ATOM	1315	HG13	VAL	83	27.232	49.218	44.509	1.00	0.00	H
ATOM	1316	CG2	VAL	83	27.156	46.888	45.457	1.00	0.00	C
ATOM	1317	HG21	VAL	83	26.789	45.865	45.540	1.00	0.00	H
ATOM	1318	HG22	VAL	83	27.690	47.146	44.541	1.00	0.00	H
ATOM	1319	HG23	VAL	83	27.782	46.969	46.344	1.00	0.00	H
ATOM	1320	C	VAL	83	24.672	46.445	47.153	1.00	0.00	C
ATOM	1321	O	VAL	83	23.564	46.229	46.801	1.00	0.00	O
ATOM	1322	N	ILE	84	25.396	45.493	47.739	1.00	0.00	N
ATOM	1323	H	ILE	84	26.348	45.678	48.022	1.00	0.00	H
ATOM	1324	CA	ILE	84	24.930	44.109	47.954	1.00	0.00	C
ATOM	1325	HA	ILE	84	24.625	43.649	47.014	1.00	0.00	H
ATOM	1326	CB	ILE	84	26.042	43.242	48.640	1.00	0.00	C
ATOM	1327	HB	ILE	84	26.322	43.950	49.419	1.00	0.00	H
ATOM	1328	CG2	ILE	84	25.480	41.960	49.166	1.00	0.00	C
ATOM	1329	HG21	ILE	84	24.950	42.088	50.109	1.00	0.00	H
ATOM	1330	HG22	ILE	84	24.727	41.514	48.515	1.00	0.00	H
ATOM	1331	HG23	ILE	84	26.180	41.140	49.325	1.00	0.00	H
ATOM	1332	CG1	ILE	84	27.149	42.972	47.579	1.00	0.00	C
ATOM	1333	HG12	ILE	84	26.705	42.192	46.961	1.00	0.00	H
ATOM	1334	HG13	ILE	84	27.347	43.859	46.979	1.00	0.00	H
ATOM	1335	CD1	ILE	84	28.384	42.479	48.296	1.00	0.00	C
ATOM	1336	HD11	ILE	84	29.186	42.219	47.605	1.00	0.00	H
ATOM	1337	HD12	ILE	84	28.745	43.325	48.882	1.00	0.00	H
ATOM	1338	HD13	ILE	84	28.136	41.655	48.966	1.00	0.00	H
ATOM	1339	C	ILE	84	23.728	44.140	48.936	1.00	0.00	C
ATOM	1340	O	ILE	84	22.758	43.413	48.784	1.00	0.00	O
ATOM	1341	N	ALA	85	23.806	45.065	49.904	1.00	0.00	N
ATOM	1342	H	ALA	85	24.685	45.559	49.975	1.00	0.00	H
ATOM	1343	CA	ALA	85	22.759	45.561	50.788	1.00	0.00	C
ATOM	1344	HA	ALA	85	22.351	44.674	51.272	1.00	0.00	H

ATOM	1345	CB	ALA	85	23.392	46.369	51.937	1.00	0.00	C
ATOM	1346	HB1	ALA	85	24.264	45.794	52.246	1.00	0.00	H
ATOM	1347	HB2	ALA	85	23.855	47.292	51.587	1.00	0.00	H
ATOM	1348	HB3	ALA	85	22.659	46.447	52.739	1.00	0.00	H
ATOM	1349	C	ALA	85	21.540	46.249	50.137	1.00	0.00	C
ATOM	1350	O	ALA	85	20.404	46.051	50.590	1.00	0.00	O
ATOM	1351	N	ASP	86	21.735	47.002	49.022	1.00	0.00	N
ATOM	1352	H	ASP	86	22.696	47.185	48.772	1.00	0.00	H
ATOM	1353	CA	ASP	86	20.673	47.564	48.234	1.00	0.00	C
ATOM	1354	HA	ASP	86	19.868	47.947	48.861	1.00	0.00	H
ATOM	1355	CB	ASP	86	21.274	48.747	47.379	1.00	0.00	C
ATOM	1356	HB2	ASP	86	22.260	48.395	47.079	1.00	0.00	H
ATOM	1357	HB3	ASP	86	20.630	48.817	46.502	1.00	0.00	H
ATOM	1358	CG	ASP	86	21.258	50.032	48.176	1.00	0.00	C
ATOM	1359	OD1	ASP	86	21.970	50.933	47.686	1.00	0.00	O
ATOM	1360	OD2	ASP	86	20.498	50.225	49.179	1.00	0.00	O
ATOM	1361	C	ASP	86	20.021	46.538	47.284	1.00	0.00	C
ATOM	1362	O	ASP	86	18.978	46.834	46.709	1.00	0.00	O
ATOM	1363	N	ILE	87	20.600	45.313	47.122	1.00	0.00	N
ATOM	1364	H	ILE	87	21.460	45.182	47.635	1.00	0.00	H
ATOM	1365	CA	ILE	87	20.023	44.196	46.389	1.00	0.00	C
ATOM	1366	HA	ILE	87	19.179	44.503	45.772	1.00	0.00	H
ATOM	1367	CB	ILE	87	21.052	43.502	45.409	1.00	0.00	C
ATOM	1368	HB	ILE	87	21.838	43.068	46.027	1.00	0.00	H
ATOM	1369	CG2	ILE	87	20.476	42.353	44.644	1.00	0.00	C
ATOM	1370	HG21	ILE	87	19.562	42.651	44.130	1.00	0.00	H
ATOM	1371	HG22	ILE	87	21.156	42.020	43.860	1.00	0.00	H
ATOM	1372	HG23	ILE	87	20.234	41.542	45.332	1.00	0.00	H
ATOM	1373	CG1	ILE	87	21.541	44.592	44.359	1.00	0.00	C
ATOM	1374	HG12	ILE	87	20.631	45.026	43.944	1.00	0.00	H
ATOM	1375	HG13	ILE	87	21.996	45.409	44.919	1.00	0.00	H
ATOM	1376	CD1	ILE	87	22.497	44.141	43.288	1.00	0.00	C
ATOM	1377	HD11	ILE	87	23.403	44.742	43.219	1.00	0.00	H
ATOM	1378	HD12	ILE	87	22.875	43.149	43.535	1.00	0.00	H
ATOM	1379	HD13	ILE	87	21.975	44.128	42.331	1.00	0.00	H
ATOM	1380	C	ILE	87	19.307	43.209	47.304	1.00	0.00	C
ATOM	1381	O	ILE	87	18.281	42.608	46.903	1.00	0.00	O
ATOM	1382	N	TYR	88	19.889	42.889	48.481	1.00	0.00	N
ATOM	1383	H	TYR	88	20.831	43.218	48.638	1.00	0.00	H
ATOM	1384	CA	TYR	88	19.285	41.890	49.420	1.00	0.00	C
ATOM	1385	HA	TYR	88	18.312	41.596	49.025	1.00	0.00	H
ATOM	1386	CB	TYR	88	20.195	40.679	49.411	1.00	0.00	C
ATOM	1387	HB2	TYR	88	21.162	41.100	49.688	1.00	0.00	H
ATOM	1388	HB3	TYR	88	19.811	40.015	50.184	1.00	0.00	H
ATOM	1389	CG	TYR	88	20.367	39.870	48.132	1.00	0.00	C
ATOM	1390	CD1	TYR	88	19.396	38.941	47.725	1.00	0.00	C
ATOM	1391	HD1	TYR	88	18.475	38.829	48.278	1.00	0.00	H
ATOM	1392	CE1	TYR	88	19.571	38.138	46.568	1.00	0.00	C
ATOM	1393	HE1	TYR	88	18.870	37.320	46.482	1.00	0.00	H
ATOM	1394	CZ	TYR	88	20.761	38.161	45.829	1.00	0.00	C
ATOM	1395	OH	TYR	88	20.847	37.287	44.747	1.00	0.00	O
ATOM	1396	HH	TYR	88	20.885	36.384	45.069	1.00	0.00	H
ATOM	1397	CE2	TYR	88	21.766	39.060	46.222			
1.00	0.00		C							
ATOM	1398	HE2	TYR	88	22.672	39.256	45.669	1.00	0.00	H
ATOM	1399	CD2	TYR	88	21.547	39.943	47.313	1.00	0.00	C
ATOM	1400	HD2	TYR	88	22.329	40.650	47.548	1.00	0.00	H
ATOM	1401	C	TYR	88	19.063	42.341	50.852	1.00	0.00	C
ATOM	1402	O	TYR	88	19.768	43.224	51.358	1.00	0.00	O
ATOM	1403	N	PRO	89	18.021	41.753	51.459	1.00	0.00	N
ATOM	1404	CD	PRO	89	17.103	40.734	51.021	1.00	0.00	C
ATOM	1405	HD2	PRO	89	17.600	39.875	50.568	1.00	0.00	H
ATOM	1406	HD3	PRO	89	16.327	41.139	50.371	1.00	0.00	H
ATOM	1407	CG	PRO	89	16.334	40.198	52.163	1.00	0.00	C

ATOM	1408	HG2	PRO	89	16.819	39.412	52.743	1.00	0.00	H
ATOM	1409	HG3	PRO	89	15.303	39.969	51.892	1.00	0.00	H
ATOM	1410	CB	PRO	89	16.315	41.474	53.026	1.00	0.00	C
ATOM	1411	HB2	PRO	89	16.080	41.343	54.082	1.00	0.00	H
ATOM	1412	HB3	PRO	89	15.611	42.221	52.659	1.00	0.00	H
ATOM	1413	CA	PRO	89	17.704	42.074	52.850	1.00	0.00	C
ATOM	1414	HA	PRO	89	17.654	43.135	53.094	1.00	0.00	H
ATOM	1415	C	PRO	89	18.697	41.546	53.851	1.00	0.00	C
ATOM	1416	O	PRO	89	19.388	40.530	53.676	1.00	0.00	O
ATOM	1417	N	GLY	90	18.848	42.275	54.980	1.00	0.00	N
ATOM	1418	H	GLY	90	18.316	43.128	55.075	1.00	0.00	H
ATOM	1419	CA	GLY	90	19.752	41.990	56.070	1.00	0.00	C
ATOM	1420	HA2	GLY	90	20.767	42.317	55.848	1.00	0.00	H
ATOM	1421	HA3	GLY	90	19.505	42.580	56.954	1.00	0.00	H
ATOM	1422	C	GLY	90	19.941	40.523	56.521	1.00	0.00	C
ATOM	1423	O	GLY	90	21.069	40.148	56.929	1.00	0.00	O
ATOM	1424	N	GLN	91	18.979	39.599	56.362	1.00	0.00	N
ATOM	1425	H	GLN	91	18.175	39.840	55.802	1.00	0.00	H
ATOM	1426	CA	GLN	91	18.952	38.174	56.666	1.00	0.00	C
ATOM	1427	HA	GLN	91	19.471	38.124	57.623	1.00	0.00	H
ATOM	1428	CB	GLN	91	17.468	37.667	56.776	1.00	0.00	C
ATOM	1429	HB2	GLN	91	16.985	37.572	55.803	1.00	0.00	H
ATOM	1430	HB3	GLN	91	17.589	36.632	57.094	1.00	0.00	H
ATOM	1431	CG	GLN	91	16.606	38.395	57.849	1.00	0.00	C
ATOM	1432	HG2	GLN	91	15.679	37.836	57.981	1.00	0.00	H
ATOM	1433	HG3	GLN	91	16.883	38.320	58.901	1.00	0.00	H
ATOM	1434	CD	GLN	91	16.242	39.795	57.464	1.00	0.00	C
ATOM	1435	OE1	GLN	91	16.180	40.231	56.307	1.00	0.00	O
ATOM	1436	NE2	GLN	91	15.929	40.622	58.384	1.00	0.00	N
ATOM	1437	HE21	GLN	91	16.188	40.406	59.335	1.00	0.00	H
ATOM	1438	HE22	GLN	91	15.680	41.541	58.048	1.00	0.00	H
ATOM	1439	C	GLN	91	19.644	37.286	55.617	1.00	0.00	C
ATOM	1440	O	GLN	91	19.643	36.061	55.813	1.00	0.00	O
ATOM	1441	N	THR	92	20.326	37.818	54.635	1.00	0.00	N
ATOM	1442	H	THR	92	20.539	38.805	54.652	1.00	0.00	H
ATOM	1443	CA	THR	92	20.949	37.033	53.538	1.00	0.00	C
ATOM	1444	HA	THR	92	20.425	36.084	53.432	1.00	0.00	H
ATOM	1445	CB	THR	92	20.779	37.793	52.194	1.00	0.00	C
ATOM	1446	HB	THR	92	21.380	38.701	52.196	1.00	0.00	H
ATOM	1447	CG2	THR	92	21.156	37.111	50.855	1.00	0.00	C
ATOM	1448	HG21	THR	92	22.232	36.953	50.773	1.00	0.00	H
ATOM	1449	HG22	THR	92	20.757	36.098	50.895	1.00	0.00	H
ATOM	1450	HG23	THR	92	20.840	37.764	50.041	1.00	0.00	H
ATOM	1451	OG1	THR	92	19.504	38.257	51.956	1.00	0.00	O
ATOM	1452	HG1	THR	92	19.336	38.932	52.618	1.00	0.00	H
ATOM	1453	C	THR	92	22.402	36.723	53.651	1.00	0.00	C
ATOM	1454	O	THR	92	23.058	37.414	54.405	1.00	0.00	O
ATOM	1455	N	GLN	93	22.996	35.726	52.928	1.00	0.00	N
ATOM	1456	H	GLN	93	22.394	35.074	52.447	1.00	0.00	H
ATOM	1457	CA	GLN	93	24.429	35.392	53.091	1.00	0.00	C
ATOM	1458	HA	GLN	93	24.931	36.247	53.545	1.00	0.00	H
ATOM	1459	CB	GLN	93	24.558	34.173	54.021	1.00	0.00	C
ATOM	1460	HB2	GLN	93	24.162	34.413	55.008	1.00	0.00	H
ATOM	1461	HB3	GLN	93	23.918	33.399	53.599	1.00	0.00	H
ATOM	1462	CG	GLN	93	25.948	33.595	54.275	1.00	0.00	C
ATOM	1463	HG2	GLN	93	26.544	33.429	53.376	1.00	0.00	H
ATOM	1464	HG3	GLN	93	26.544	34.361	54.768	1.00	0.00	H
ATOM	1465	CD	GLN	93	26.005	32.355	55.173	1.00	0.00	C
ATOM	1466	OE1	GLN	93	25.101	32.042	55.929	1.00	0.00	O
ATOM	1467	NE2	GLN	93	27.112	31.659	55.139	1.00	0.00	N
ATOM	1468	HE21	GLN	93	27.781	31.766	54.389	1.00	0.00	H
ATOM	1469	HE22	GLN	93	27.111	30.728	55.534	1.00	0.00	H
ATOM	1470	C	GLN	93	25.089	35.206	51.739	1.00	0.00	C
ATOM	1471	O	GLN	93	24.396	34.867	50.843	1.00	0.00	O

ATOM	1472	N	PHE	94	26.369	35.531	51.626	1.00	0.00	N
ATOM	1473	H	PHE	94	26.894	35.736	52.464	1.00	0.00	H
ATOM	1474	CA	PHE	94	27.128	35.682	50.364	1.00	0.00	C
ATOM	1475	HA	PHE	94	26.484	35.417	49.526	1.00	0.00	H
ATOM	1476	CB	PHE	94	27.504	37.154	50.266	1.00	0.00	C
ATOM	1477	HB2	PHE	94	28.064	37.443	51.156	1.00	0.00	H
ATOM	1478	HB3	PHE	94	28.126	37.351	49.392	1.00	0.00	H
ATOM	1479	CG	PHE	94	26.272	38.015	50.132	1.00	0.00	C
ATOM	1480	CD1	PHE	94	25.587	38.090	48.889	1.00	0.00	C
ATOM	1481	HD1	PHE	94	25.946	37.424	48.118	1.00	0.00	H
ATOM	1482	CE1	PHE	94	24.374	38.689	48.672	1.00	0.00	C
ATOM	1483	HE1	PHE	94	23.823	38.671	47.744	1.00	0.00	H
ATOM	1484	CZ	PHE	94	23.687	39.134	49.756	1.00	0.00	C
ATOM	1485	HZ	PHE	94	22.735	39.636	49.659	1.00	0.00	H
ATOM	1486	CE2	PHE	94	24.332	39.134	51.009	1.00	0.00	C
ATOM	1487	HE2	PHE	94	23.823	39.550	51.865	1.00	0.00	H
ATOM	1488	CD2	PHE	94	25.612	38.594	51.200	1.00	0.00	C
ATOM	1489	HD2	PHE	94	25.942	38.505	52.225	1.00	0.00	H
ATOM	1490	C	PHE	94	28.308	34.685	50.225	1.00	0.00	C
ATOM	1491	O	PHE	94	28.642	34.013	51.191	1.00	0.00	O
ATOM	1492	N	TYR	95	28.874	34.628	48.994	1.00	0.00	N
ATOM	1493	H	TYR	95	28.400	35.168	48.284	1.00	0.00	H
ATOM	1494	CA	TYR	95	29.832	33.566	48.632	1.00	0.00	C
ATOM	1495	HA	TYR	95	30.425	33.254	49.493	1.00	0.00	H
ATOM	1496	CB	TYR	95	29.114	32.200	48.188	1.00	0.00	C
ATOM	1497	HB2	TYR	95	28.545	31.738	48.996	1.00	0.00	H
ATOM	1498	HB3	TYR	95	28.412	32.556	47.433	1.00	0.00	H
ATOM	1499	CG	TYR	95	29.933	31.113	47.645	1.00	0.00	C
ATOM	1500	CD1	TYR	95	29.932	30.731	46.277	1.00	0.00	C
ATOM	1501	HD1	TYR	95	29.594	31.527	45.629	1.00	0.00	H
ATOM	1502	CE1	TYR	95	30.496	29.555	45.782	1.00	0.00	C
ATOM	1503	HE1	TYR	95	30.403	29.241	44.753	1.00	0.00	H
ATOM	1504	CZ	TYR	95	31.187	28.687	46.661	1.00	0.00	C
ATOM	1505	OH	TYR	95	31.674	27.454	46.364	1.00	0.00	O
ATOM	1506	HH	TYR	95	31.635	27.175	45.446	1.00	0.00	H
ATOM	1507	CE2	TYR	95	31.301	29.132	47.986	1.00	0.00	C
ATOM	1508	HE2	TYR	95	31.747	28.470	48.713	1.00	0.00	H
ATOM	1509	CD2	TYR	95	30.677	30.256	48.524	1.00	0.00	C
ATOM	1510	HD2	TYR	95	30.701	30.411	49.592	1.00	0.00	H
ATOM	1511	C	TYR	95	30.859	34.084	47.584	1.00	0.00	C
ATOM	1512	O	TYR	95	30.597	34.373	46.395	1.00	0.00	O
ATOM	1513	N	VAL	96	32.113	34.118	47.982	1.00	0.00	N
ATOM	1514	H	VAL	96	32.336	33.763	48.901	1.00	0.00	H
ATOM	1515	CA	VAL	96	33.331	34.582	47.123	1.00	0.00	C
ATOM	1516	HA	VAL	96	33.024	35.323	46.385	1.00	0.00	H
ATOM	1517	CB	VAL	96	34.353	35.255	48.035	1.00	0.00	C
ATOM	1518	HB	VAL	96	34.937	34.651	48.731	1.00	0.00	H
ATOM	1519	CG1	VAL	96	35.468	35.853	47.202	1.00	0.00	C
ATOM	1520	HG11	VAL	96	35.172	36.638	46.507	1.00	0.00	H
ATOM	1521	HG12	VAL	96	36.323	36.223	47.766	1.00	0.00	H
ATOM	1522	HG13	VAL	96	36.035	35.125	46.621	1.00	0.00	H
ATOM	1523	CG2	VAL	96	33.741	36.420	48.879	1.00	0.00	C
ATOM	1524	HG21	VAL	96	33.198	37.158	48.290	1.00	0.00	H
ATOM	1525	HG22	VAL	96	33.014	36.068	49.612	1.00	0.00	H
ATOM	1526	HG23	VAL	96	34.565	36.951	49.357	1.00	0.00	H
ATOM	1527	C	VAL	96	33.927	33.375	46.339	1.00	0.00	C
ATOM	1528	O	VAL	96	34.052	32.324	46.882	1.00	0.00	O
ATOM	1529	N	ILE	97	34.335	33.620	45.092	1.00	0.00	N
ATOM	1530	H	ILE	97	34.024	34.506	44.719	1.00	0.00	H
ATOM	1531	CA	ILE	97	34.873	32.637	44.093	1.00	0.00	C
ATOM	1532	HA	ILE	97	35.211	31.727	44.590	1.00	0.00	H
ATOM	1533	CB	ILE	97	33.681	32.275	43.198	1.00	0.00	C
ATOM	1534	HB	ILE	97	33.370	33.150	42.627	1.00	0.00	H

ATOM	1535	CG2	ILE	97	34.083	31.263	42.141	1.00	0.00	C
ATOM	1536	HG21	ILE	97	35.149	31.321	41.921	1.00	0.00	H
ATOM	1537	HG22	ILE	97	33.888	30.261	42.525	1.00	0.00	H
ATOM	1538	HG23	ILE	97	33.373	31.237	41.315	1.00	0.00	H
ATOM	1539	CG1	ILE	97	32.403	31.776	43.777	1.00	0.00	C
ATOM	1540	HG12	ILE	97	32.726	30.924	44.374	1.00	0.00	H
ATOM	1541	HG13	ILE	97	32.116	32.601	44.428	1.00	0.00	H
ATOM	1542	CD1	ILE	97	31.171	31.405	42.919	1.00	0.00	C
ATOM	1543	HD11	ILE	97	31.289	30.443	42.420	1.00	0.00	H
ATOM	1544	HD12	ILE	97	30.230	31.486	43.463	1.00	0.00	H
ATOM	1545	HD13	ILE	97	31.190	32.158	42.131	1.00	0.00	H
ATOM	1546	C	ILE	97	36.005	33.362	43.322	1.00	0.00	C
ATOM	1547	O	ILE	97	35.753	34.246	42.508	1.00	0.00	O
ATOM	1548	N	GLU	98	37.284	32.967	43.565	1.00	0.00	N
ATOM	1549	H	GLU	98	37.351	32.092	44.067	1.00	0.00	H
ATOM	1550	CA	GLU	98	38.436	33.205	42.736	1.00	0.00	C
ATOM	1551	HA	GLU	98	38.466	34.217	42.330	1.00	0.00	H
ATOM	1552	CB	GLU	98	39.674	33.221	43.657	1.00	0.00	C
ATOM	1553	HB2	GLU	98	39.530	33.890	44.506	1.00	0.00	H
ATOM	1554	HB3	GLU	98	39.820	32.229	44.083	1.00	0.00	H
ATOM	1555	CG	GLU	98	40.940	33.747	42.944	1.00	0.00	C
ATOM	1556	HG2	GLU	98	41.133	33.165	42.043	1.00	0.00	H
ATOM	1557	HG3	GLU	98	40.866	34.818	42.753	1.00	0.00	H
ATOM	1558	CD	GLU	98	42.176	33.774	43.794	1.00	0.00	C
ATOM	1559	OE1	GLU	98	42.238	32.941	44.702	1.00	0.00	O
ATOM	1560	OE2	GLU	98	43.148	34.495	43.544	1.00	0.00	O
ATOM	1561	C	GLU	98	38.668	32.114	41.628	1.00	0.00	C
ATOM	1562	O	GLU	98	38.311	31.019	41.891	1.00	0.00	O
ATOM	1563	N	PHE	99	39.304	32.417	40.526	1.00	0.00	N
ATOM	1564	H	PHE	99	39.473	33.396	40.344	1.00	0.00	H
ATOM	1565	CA	PHE	99	39.397	31.541	39.284	1.00	0.00	C
ATOM	1566	HA	PHE	99	39.622	30.510	39.558	1.00	0.00	H
ATOM	1567	CB	PHE	99	38.043	31.619	38.514	1.00	0.00	C
ATOM	1568	HB2	PHE	99	38.090	31.095	37.560	1.00	0.00	H
ATOM	1569	HB3	PHE	99	37.362	31.217	39.264	1.00	0.00	H
ATOM	1570	CG	PHE	99	37.609	33.070	38.191	1.00	0.00	C
ATOM	1571	CD1	PHE	99	36.812	33.842	39.063	1.00	0.00	C
ATOM	1572	HD1	PHE	99	36.559	33.427	40.028	1.00	0.00	H
ATOM	1573	CE1	PHE	99	36.297	35.035	38.707	1.00	0.00	C
ATOM	1574	HE1	PHE	99	35.641	35.549	39.394	1.00	0.00	H
ATOM	1575	CZ	PHE	99	36.631	35.592	37.432	1.00	0.00	C
ATOM	1576	HZ	PHE	99	36.194	36.527	37.113	1.00	0.00	H
ATOM	1577	CE2	PHE	99	37.535	34.908	36.554	1.00	0.00	C
ATOM	1578	HE2	PHE	99	37.765	35.294	35.571	1.00	0.00	H
ATOM	1579	CD2	PHE	99	38.010	33.637	36.961	1.00	0.00	C
ATOM	1580	HD2	PHE	99	38.733	33.086	36.379	1.00	0.00	H
ATOM	1581	C	PHE	99	40.552	31.996	38.339	1.00	0.00	C
ATOM	1582	O	PHE	99	40.987	33.156	38.251	1.00	0.00	O
ATOM	1583	N	LYS	100	41.139	31.061	37.588	1.00	0.00	N
ATOM	1584	H	LYS	100	40.739	30.134	37.568	1.00	0.00	H
ATOM	1585	CA	LYS	100	42.226	31.326	36.653	1.00	0.00	C
ATOM	1586	HA	LYS	100	42.606	32.335	36.815	1.00	0.00	H
ATOM	1587	CB	LYS	100	43.511	30.517	37.120	1.00	0.00	C
ATOM	1588	HB2	LYS	100	44.276	30.688	36.363	1.00	0.00	H
ATOM	1589	HB3	LYS	100	43.903	30.900	38.061	1.00	0.00	H
ATOM	1590	CG	LYS	100	43.181	28.968	37.369	1.00	0.00	C
ATOM	1591	HG2	LYS	100	42.304	28.756	37.981	1.00	0.00	H
ATOM	1592	HG3	LYS	100	42.914	28.718	36.342	1.00	0.00	H
ATOM	1593	CD	LYS	100	44.384	28.230	37.930	1.00	0.00	C
ATOM	1594	HD2	LYS	100	45.317	28.273	37.368	1.00	0.00	H
ATOM	1595	HD3	LYS	100	44.548	28.710	38.894	1.00	0.00	H
ATOM	1596	CE	LYS	100	44.317	26.639	38.179	1.00	0.00	C
ATOM	1597	HE2	LYS	100	43.480	26.365	38.822	1.00	0.00	H
ATOM	1598	HE3	LYS	100	44.243	26.143	37.212	1.00	0.00	H

ATOM	1599	NZ	LYS	100	45.516	26.234	38.978	1.00	0.00	N
ATOM	1600	HZ1	LYS	100	45.554	25.255	39.220	1.00	0.00	H
ATOM	1601	HZ2	LYS	100	46.331	26.263	38.382	1.00	0.00	H
ATOM	1602	HZ3	LYS	100	45.643	26.885	39.741	1.00	0.00	H
ATOM	1603	C	LYS	100	41.832	30.999	35.304	1.00	0.00	C
ATOM	1604	O	LYS	100	41.011	30.132	35.021	1.00	0.00	O
ATOM	1605	N	CYX	101	42.387	31.845	34.402	1.00	0.00	N
ATOM	1606	H	CYX	101	42.991	32.608	34.673	1.00	0.00	H
ATOM	1607	CA	CYX	101	42.375	31.667	32.950	1.00	0.00	C
ATOM	1608	HA	CYX	101	41.505	31.219	32.470	1.00	0.00	H
ATOM	1609	CB	CYX	101	42.795	32.957	32.287	1.00	0.00	C
ATOM	1610	HB2	CYX	101	42.063	33.748	32.453	1.00	0.00	H
ATOM	1611	HB3	CYX	101	43.787	33.144	32.698	1.00	0.00	H
ATOM	1612	SG	CYX	101	43.052	32.834	30.414	1.00	0.00	S
ATOM	1613	C	CYX	101	43.554	30.677	32.686	1.00	0.00	C
ATOM	1614	O	CYX	101	44.750	30.879	32.955	1.00	0.00	O
ATOM	1615	N	LEU	102	43.300	29.496	32.179	1.00	0.00	N
ATOM	1616	H	LEU	102	42.343	29.219	32.011	1.00	0.00	H
ATOM	1617	CA	LEU	102	44.234	28.380	31.730	1.00	0.00	C
ATOM	1618	HA	LEU	102	45.123	28.341	32.358	1.00	0.00	H
ATOM	1619	CB	LEU	102	43.498	27.061	31.913	1.00	0.00	C
ATOM	1620	HB2	LEU	102	42.589	26.979	31.318	1.00	0.00	H
ATOM	1621	HB3	LEU	102	44.112	26.267	31.488	1.00	0.00	H
ATOM	1622	CG	LEU	102	43.144	26.669	33.385	1.00	0.00	C
ATOM	1623	HG	LEU	102	42.668	27.501	33.904	1.00	0.00	H
ATOM	1624	CD1	LEU	102	42.447	25.340	33.400	1.00	0.00	C
ATOM	1625	HD11	LEU	102	43.114	24.640	32.897	1.00	0.00	H
ATOM	1626	HD12	LEU	102	42.239	24.993	34.412	1.00	0.00	H
ATOM	1627	HD13	LEU	102	41.522	25.349	32.824	1.00	0.00	H
ATOM	1628	CD2	LEU	102	44.461	26.499	34.256	1.00	0.00	C
ATOM	1629	HD21	LEU	102	45.037	27.416	34.128	1.00	0.00	H
ATOM	1630	HD22	LEU	102	44.253	26.306	35.309	1.00	0.00	H
ATOM	1631	HD23	LEU	102	45.021	25.648	33.871	1.00	0.00	H
ATOM	1632	C	LEU	102	44.895	28.669	30.417	1.00	0.00	C
ATOM	1633	O	LEU	102	44.443	29.579	29.703	1.00	0.00	O
ATOM	1634	OXT	LEU	102	45.871	27.965	30.170	1.00	0.00	O
HETATM	314	N	LYN	20	40.021	40.586	35.547	1.00	0.00	N
HETATM	315	H	LYN	20	40.088	40.629	34.541	1.00	0.00	H
HETATM	316	CA	LYN	20	38.953	39.936	36.234	1.00	0.00	C
HETATM	317	HA	LYN	20	38.623	40.417	37.154	1.00	0.00	H
HETATM	318	CB	LYN	20	37.749	40.169	35.354	1.00	0.00	C
HETATM	319	HB2	LYN	20	37.984	39.765	34.369	1.00	0.00	H
HETATM	320	HB3	LYN	20	37.571	41.229	35.176	1.00	0.00	H
HETATM	321	CG	LYN	20	36.532	39.382	35.811	1.00	0.00	C
HETATM	322	HG2	LYN	20	35.783	39.493	35.026	1.00	0.00	H
HETATM	323	HG3	LYN	20	36.690	38.353	36.133	1.00	0.00	H
HETATM	324	CD	LYN	20	35.857	39.981	37.040	1.00	0.00	C
HETATM	325	HD2	LYN	20	35.356	40.926	36.830	1.00	0.00	H
HETATM	326	HD3	LYN	20	36.625	40.186	37.786	1.00	0.00	H
HETATM	327	CE	LYN	20						
					34.814	38.949	37.572	1.00	0.00	C
HETATM	328	HE2	LYN	20	34.001	38.726	36.880	1.00	0.00	H
HETATM	329	HE3	LYN	20	35.327	38.028	37.850	1.00	0.00	H
HETATM	330	NZ	LYN	20	34.118	39.375	38.796	1.00	0.00	N
HETATM	331	HZ2	LYN	20	33.897	40.352	38.668	1.00	0.00	H
HETATM	332	HZ3	LYN	20	34.871	39.343	39.468	1.00	0.00	H
HETATM	333	C	LYN	20	39.293	38.442	36.678	1.00	0.00	C
HETATM	334	O	LYN	20	39.521	37.575	35.801	1.00	0.00	O
HETATM	1636	N	LIG	103	30.296	41.554	40.778	1.00	0.00	N
HETATM	1637	C	LIG	103	30.017	40.549	39.997	1.00	0.00	C
HETATM	1638	O	LIG	103	29.951	42.849	42.586	1.00	0.00	O
HETATM	1639	C5'	LIG	103	25.605	38.380	44.510	1.00	0.00	C
HETATM	1640	O5'	LIG	103	25.185	38.061	43.167	1.00	0.00	O
HETATM	1641	C4'	LIG	103	26.072	39.847	44.653	1.00	0.00	C

```
HETATM 1642  O4' LIG   103      27.451  40.000  44.294  1.00  0.00           O
HETATM 1643  C3' LIG   103      25.326  40.922  43.803  1.00  0.00           C
HETATM 1644  O3' LIG   103      24.377  41.514  44.829  1.00  0.00           O
HETATM 1645  C2' LIG   103      26.444  41.908  43.320  1.00  0.00           C
HETATM 1646  O2' LIG   103      26.248  43.119  44.045  1.00  0.00           O
HETATM 1647  C1' LIG   103      27.790  41.232  43.582  1.00  0.00           C
HETATM 1648  N1  LIG   103      28.575  41.003  42.339  1.00  0.00           N
HETATM 1649  O1  LIG   103      30.132  38.417  37.866  1.00  0.00           O
HETATM 1650  N2  LIG   103      30.795  40.437  38.793  1.00  0.00           N
HETATM 1651  C6  LIG   103      29.621  41.849  41.974  1.00  0.00           C
HETATM 1652  C7  LIG   103      28.157  39.972  41.509  1.00  0.00           C
HETATM 1653  C8  LIG   103      28.933  39.661  40.396  1.00  0.00           C
HETATM 1654  C9  LIG   103      30.700  39.483  37.817  1.00  0.00           C
HETATM 1655  C10 LIG   103      31.374  39.758  36.489  1.00  0.00           C
HETATM 1656  H   LIG   103      31.382  41.253  38.671  1.00  0.00           H
HETATM 1657  H1  LIG   103      28.552  38.880  39.748  1.00  0.00           H
HETATM 1658  H4  LIG   103      28.392  41.840  44.268  1.00  0.00           H
HETATM 1659  H6  LIG   103      25.969  40.038  45.728  1.00  0.00           H
HETATM 1660  H7  LIG   103      24.804  40.505  42.934  1.00  0.00           H
HETATM 1661  H8  LIG   103      26.258  41.994  42.242  1.00  0.00           H
HETATM 1662  H9  LIG   103      24.763  38.210  45.192  1.00  0.00           H
HETATM 1663  H10 LIG   103      26.411  37.722  44.858  1.00  0.00           H
HETATM 1664  H11 LIG   103      31.334  38.933  35.768  1.00  0.00           H
HETATM 1665  H12 LIG   103      32.439  39.968  36.646  1.00  0.00           H
HETATM 1666  H13 LIG   103      30.934  40.635  35.998  1.00  0.00           H
HETATM 1667  H14 LIG   103      27.284  39.474  41.909  1.00  0.00           H
HETATM 1668  H2' LIG   103      26.971  43.735  43.832  1.00  0.00           H
HETATM 1669  H3' LIG   103      24.642  42.450  44.805  1.00  0.00           H
HETATM 1670  H5' LIG   103      24.811  37.179  43.331  1.00  0.00           H
ENDMDL
MODEL     13
SHEET     1   1 1 ILE    22  ASP    26  0
SHEET     2   2 1 VAL    37  VAL    40  0
SHEET     3   3 1 CYX    50  VAL    60  0
SHEET     4   4 1 PHE    94  CYX   101  0
HELIX     1   1 GLN     9  LEU     16  1                                   8
HELIX     2   2 LEU    62  THR     64  1                                   3
HELIX     3   3 LYS    68  GLN     72  1                                   5
HELIX     4   4 LEU    77  ILE     87  1                                  11
ATOM      1  N   GLN     1      35.927  16.460  31.774  1.00  0.00           N
ATOM      2  H1  GLN     1      35.986  15.538  31.368  1.00  0.00           H
ATOM      3  H2  GLN     1      36.911  16.624  31.929  1.00  0.00           H
ATOM      4  H3  GLN     1      35.372  16.418  32.617  1.00  0.00           H
ATOM      5  CA  GLN     1      35.508  17.637  30.878  1.00  0.00           C
ATOM      6  HA  GLN     1      36.256  17.789  30.099  1.00  0.00           H
ATOM      7  CB  GLN     1      34.172  17.310  30.102  1.00  0.00           C
ATOM      8  HB2 GLN     1      33.466  17.114  30.910  1.00  0.00           H
ATOM      9  HB3 GLN     1      33.817  18.204  29.588  1.00  0.00           H
ATOM     10  CG  GLN     1      34.098  16.097  29.184  1.00  0.00           C
ATOM     11  HG2 GLN     1      34.586  15.313  29.762  1.00  0.00           H
ATOM     12  HG3 GLN     1      33.092  15.845  28.846  1.00  0.00           H
ATOM     13  CD  GLN     1      34.807  16.342  27.903  1.00  0.00           C
ATOM     14  OE1 GLN     1      35.649  17.264  27.771  1.00  0.00           O
ATOM     15  NE2 GLN     1      34.444  15.555  26.864  1.00  0.00           N
ATOM     16 HE21 GLN     1      33.715  14.859  26.930  1.00  0.00           H
ATOM     17 HE22 GLN     1      35.214  15.523  26.211  1.00  0.00           H
ATOM     18  C   GLN     1      35.370  18.936  31.668  1.00  0.00           C
ATOM     19  O   GLN     1      34.965  18.865  32.832  1.00  0.00           O
ATOM     20  N   PRO     2      35.633  20.097  30.989  1.00  0.00           N
ATOM     21  CD  PRO     2      36.377  20.252  29.764  1.00  0.00           C
ATOM     22  HD2 PRO     2      35.791  19.859  28.934  1.00  0.00           H
ATOM     23  HD3 PRO     2      37.270  19.630  29.810  1.00  0.00           H
ATOM     24  CG  PRO     2      36.722  21.739  29.632  1.00  0.00           C
ATOM     25  HG2 PRO     2      36.838  22.021  28.586  1.00  0.00           H
```

ATOM	26	HG3	PRO	2	37.570	21.894	30.300	1.00	0.00	H
ATOM	27	CB	PRO	2	35.653	22.429	30.391	1.00	0.00	C
ATOM	28	HB2	PRO	2	34.809	22.631	29.732	1.00	0.00	H
ATOM	29	HB3	PRO	2	35.940	23.395	30.806	1.00	0.00	H
ATOM	30	CA	PRO	2	35.177	21.408	31.410	1.00	0.00	C
ATOM	31	HA	PRO	2	35.683	21.554	32.364	1.00	0.00	H
ATOM	32	C	PRO	2	33.644	21.542	31.514	1.00	0.00	C
ATOM	33	O	PRO	2	32.809	20.862	30.981	1.00	0.00	O
ATOM	34	N	ASN	3	33.229	22.457	32.410	1.00	0.00	N
ATOM	35	H	ASN	3	33.944	22.912	32.959	1.00	0.00	H
ATOM	36	CA	ASN	3	31.822	22.816	32.589	1.00	0.00	C
ATOM	37	HA	ASN	3	31.171	21.951	32.715	1.00	0.00	H
ATOM	38	CB	ASN	3	31.649	23.710	33.852	1.00	0.00	C
ATOM	39	HB2	ASN	3	32.170	24.662	33.748	1.00	0.00	H
ATOM	40	HB3	ASN	3	30.613	24.006	34.017	1.00	0.00	H
ATOM	41	CG	ASN	3	32.118	23.050	35.149	1.00	0.00	C
ATOM	42	OD1	ASN	3	31.272	22.499	35.865	1.00	0.00	O
ATOM	43	ND2	ASN	3	33.379	23.000	35.478	1.00	0.00	N
ATOM	44	HD21	ASN	3	34.057	23.481	34.903	1.00	0.00	H
ATOM	45	HD22	ASN	3	33.618	22.444	36.286	1.00	0.00	H
ATOM	46	C	ASN	3	31.204	23.536	31.357	1.00	0.00	C
ATOM	47	O	ASN	3	31.961	24.374	30.807	1.00	0.00	O
ATOM	48	N	ASP	4	29.941	23.251	30.892	1.00	0.00	N
ATOM	49	H	ASP	4	29.328	22.803	31.557	1.00	0.00	H
ATOM	50	CA	ASP	4	29.394	24.045	29.786	1.00	0.00	C
ATOM	51	HA	ASP	4	30.136	24.141	28.993	1.00	0.00	H
ATOM	52	CB	ASP	4	28.179	23.351	29.259	1.00	0.00	C
ATOM	53	HB2	ASP	4	27.375	23.362	29.995	1.00	0.00	H
ATOM	54	HB3	ASP	4	27.839	23.964	28.423	1.00	0.00	H
ATOM	55	CG	ASP	4	28.279	21.901	28.714	1.00	0.00	C
ATOM	56	OD1	ASP	4	28.981	21.063	29.272	1.00	0.00	O
ATOM	57	OD2	ASP	4	27.534	21.585	27.721	1.00	0.00	O
ATOM	58	C	ASP	4	29.048	25.444	30.240	1.00	0.00	C
ATOM	59	O	ASP	4	28.964	26.391	29.416	1.00	0.00	O
ATOM	60	N	ILE	5	28.673	25.655	31.565	1.00	0.00	N
ATOM	61	H	ILE	5	28.900	24.862	32.147	1.00	0.00	H
ATOM	62	CA	ILE	5	28.262	27.027	32.110	1.00	0.00	C
ATOM	63	HA	ILE	5	27.609	27.483	31.367	1.00	0.00	H
ATOM	64	CB	ILE	5	27.342	26.972	33.396	1.00	0.00	C
ATOM	65	HB	ILE	5	27.847	26.370	34.151	1.00	0.00	H
ATOM	66	CG2	ILE	5	27.108	28.376	33.982	1.00	0.00	C
ATOM	67	HG21	ILE	5	26.925	29.188	33.280	1.00	0.00	H
ATOM	68	HG22	ILE	5	26.275	28.412	34.684	1.00	0.00	H
ATOM	69	HG23	ILE	5	28.009	28.645	34.534	1.00	0.00	H
ATOM	70	CG1	ILE	5	26.028	26.286	33.167	1.00	0.00	C
ATOM	71	HG12	ILE	5	26.402	25.267	33.071	1.00	0.00	H
ATOM	72	HG13	ILE	5	25.496	26.349	34.117	1.00	0.00	H
ATOM	73	CD1	ILE	5	25.133	26.728	32.064	1.00	0.00	C
ATOM	74	HD11	ILE	5	25.476	26.332	31.108	1.00	0.00	H
ATOM	75	HD12	ILE	5	24.087	26.432	32.150	1.00	0.00	H
ATOM	76	HD13	ILE	5	25.173	27.813	31.963	1.00	0.00	H
ATOM	77	C	ILE	5	29.481					
27.956	32.320	1.00	0.00			C				
ATOM	78	O	ILE	5	30.520	27.419	32.605	1.00	0.00	O
ATOM	79	N	THR	6	29.373	29.260	32.146	1.00	0.00	N
ATOM	80	H	THR	6	28.487	29.693	31.926	1.00	0.00	H
ATOM	81	CA	THR	6	30.563	30.153	32.018	1.00	0.00	C
ATOM	82	HA	THR	6	31.275	29.865	32.792	1.00	0.00	H
ATOM	83	CB	THR	6	31.256	30.012	30.608	1.00	0.00	C
ATOM	84	HB	THR	6	31.517	28.957	30.528	1.00	0.00	H
ATOM	85	CG2	THR	6	30.497	30.470	29.444	1.00	0.00	C
ATOM	86	HG21	THR	6	30.221	31.524	29.462	1.00	0.00	H
ATOM	87	HG22	THR	6	31.037	30.191	28.539	1.00	0.00	H
ATOM	88	HG23	THR	6	29.537	29.957	29.489	1.00	0.00	H

ATOM	89	OG1	THR	6	32.467	30.718	30.631	1.00	0.00	O
ATOM	90	HG1	THR	6	32.989	30.292	29.948	1.00	0.00	H
ATOM	91	C	THR	6	30.226	31.625	32.249	1.00	0.00	C
ATOM	92	O	THR	6	29.066	31.884	32.632	1.00	0.00	O
ATOM	93	N	PHE	7	31.122	32.565	32.216	1.00	0.00	N
ATOM	94	H	PHE	7	32.074	32.305	32.005	1.00	0.00	H
ATOM	95	CA	PHE	7	30.802	33.989	32.202	1.00	0.00	C
ATOM	96	HA	PHE	7	30.051	34.154	32.974	1.00	0.00	H
ATOM	97	CB	PHE	7	32.039	34.812	32.602	1.00	0.00	C
ATOM	98	HB2	PHE	7	32.892	34.538	31.981	1.00	0.00	H
ATOM	99	HB3	PHE	7	31.805	35.857	32.397	1.00	0.00	H
ATOM	100	CG	PHE	7	32.371	34.532	34.028	1.00	0.00	C
ATOM	101	CD1	PHE	7	33.621	34.039	34.288	1.00	0.00	C
ATOM	102	HD1	PHE	7	34.268	33.716	33.486	1.00	0.00	H
ATOM	103	CE1	PHE	7	34.077	33.770	35.633	1.00	0.00	C
ATOM	104	HE1	PHE	7	34.965	33.216	35.896	1.00	0.00	H
ATOM	105	CZ	PHE	7	33.184	34.033	36.671	1.00	0.00	C
ATOM	106	HZ	PHE	7	33.546	33.859	37.673	1.00	0.00	H
ATOM	107	CE2	PHE	7	31.919	34.604	36.437	1.00	0.00	C
ATOM	108	HE2	PHE	7	31.288	34.755	37.301	1.00	0.00	H
ATOM	109	CD2	PHE	7	31.529	34.838	35.082	1.00	0.00	C
ATOM	110	HD2	PHE	7	30.495	35.136	34.988	1.00	0.00	H
ATOM	111	C	PHE	7	30.152	34.503	30.895	1.00	0.00	C
ATOM	112	O	PHE	7	30.590	34.181	29.795	1.00	0.00	O
ATOM	113	N	PHE	8	29.056	35.291	30.956	1.00	0.00	N
ATOM	114	H	PHE	8	28.554	35.405	31.825	1.00	0.00	H
ATOM	115	CA	PHE	8	28.403	35.841	29.790	1.00	0.00	C
ATOM	116	HA	PHE	8	28.106	34.967	29.210	1.00	0.00	H
ATOM	117	CB	PHE	8	27.057	36.540	30.264	1.00	0.00	C
ATOM	118	HB2	PHE	8	27.272	37.450	30.824	1.00	0.00	H
ATOM	119	HB3	PHE	8	26.558	36.914	29.370	1.00	0.00	H
ATOM	120	CG	PHE	8	26.110	35.574	30.940	1.00	0.00	C
ATOM	121	CD1	PHE	8	25.912	35.606	32.318	1.00	0.00	C
ATOM	122	HD1	PHE	8	26.393	36.379	32.899	1.00	0.00	H
ATOM	123	CE1	PHE	8	25.094	34.676	33.029	1.00	0.00	C
ATOM	124	HE1	PHE	8	24.921	34.726	34.095	1.00	0.00	H
ATOM	125	CZ	PHE	8	24.434	33.697	32.247	1.00	0.00	C
ATOM	126	HZ	PHE	8	23.826	32.918	32.682	1.00	0.00	H
ATOM	127	CE2	PHE	8	24.615	33.688	30.848	1.00	0.00	C
ATOM	128	HE2	PHE	8	23.972	33.022	30.291	1.00	0.00	H
ATOM	129	CD2	PHE	8	25.331	34.660	30.176	1.00	0.00	C
ATOM	130	HD2	PHE	8	25.310	34.678	29.097	1.00	0.00	H
ATOM	131	C	PHE	8	29.178	36.821	28.919	1.00	0.00	C
ATOM	132	O	PHE	8	30.123	37.384	29.429	1.00	0.00	O
ATOM	133	N	GLN	9	28.897	36.930	27.589	1.00	0.00	N
ATOM	134	H	GLN	9	28.081	36.501	27.174	1.00	0.00	H
ATOM	135	CA	GLN	9	29.922	37.367	26.565	1.00	0.00	C
ATOM	136	HA	GLN	9	30.785	36.720	26.728	1.00	0.00	H
ATOM	137	CB	GLN	9	29.318	37.354	25.140	1.00	0.00	C
ATOM	138	HB2	GLN	9	28.308	37.757	25.213	1.00	0.00	H
ATOM	139	HB3	GLN	9	29.837	38.095	24.531	1.00	0.00	H
ATOM	140	CG	GLN	9	29.297	35.949	24.498	1.00	0.00	C
ATOM	141	HG2	GLN	9	30.299	35.572	24.295	1.00	0.00	H
ATOM	142	HG3	GLN	9	28.831	35.278	25.221	1.00	0.00	H
ATOM	143	CD	GLN	9	28.497	35.962	23.192	1.00	0.00	C
ATOM	144	OE1	GLN	9	28.398	37.006	22.491	1.00	0.00	O
ATOM	145	NE2	GLN	9	27.936	34.879	22.672	1.00	0.00	N
ATOM	146	HE21	GLN	9	27.578	35.032	21.739	1.00	0.00	H
ATOM	147	HE22	GLN	9	28.031	34.011	23.179	1.00	0.00	H
ATOM	148	C	GLN	9	30.395	38.783	26.919	1.00	0.00	C
ATOM	149	O	GLN	9	31.540	39.215	26.681	1.00	0.00	O
ATOM	150	N	ARG	10	29.538	39.552	27.622	1.00	0.00	N
ATOM	151	H	ARG	10	28.599	39.233	27.817	1.00	0.00	H
ATOM	152	CA	ARG	10	29.770	40.974	28.021	1.00	0.00	C

ATOM	153	HA	ARG	10	29.907	41.533	27.095	1.00	0.00	H
ATOM	154	CB	ARG	10	28.572	41.487	28.859	1.00	0.00	C
ATOM	155	HB2	ARG	10	27.676	41.467	28.238	1.00	0.00	H
ATOM	156	HB3	ARG	10	28.502	40.862	29.749	1.00	0.00	H
ATOM	157	CG	ARG	10	28.675	42.970	29.385	1.00	0.00	C
ATOM	158	HG2	ARG	10	27.796	43.259	29.959	1.00	0.00	H
ATOM	159	HG3	ARG	10	29.522	42.944	30.071	1.00	0.00	H
ATOM	160	CD	ARG	10	28.983	43.946	28.259	1.00	0.00	C
ATOM	161	HD2	ARG	10	29.913	43.650	27.773	1.00	0.00	H
ATOM	162	HD3	ARG	10	28.118	43.907	27.597	1.00	0.00	H
ATOM	163	NE	ARG	10	29.125	45.272	28.787	1.00	0.00	N
ATOM	164	HE	ARG	10	28.414	45.968	28.616	1.00	0.00	H
ATOM	165	CZ	ARG	10	30.245	45.836	29.251	1.00	0.00	C
ATOM	166	NH1	ARG	10	31.353	45.168	29.705	1.00	0.00	N
ATOM	167	HH11	ARG	10	32.148	45.634	30.121	1.00	0.00	H
ATOM	168	HH12	ARG	10	31.330	44.159	29.704	1.00	0.00	H
ATOM	169	NH2	ARG	10	30.388	47.117	29.308	1.00	0.00	N
ATOM	170	HH21	ARG	10	29.600	47.736	29.190	1.00	0.00	H
ATOM	171	HH22	ARG	10	31.149	47.524	29.834	1.00	0.00	H
ATOM	172	C	ARG	10	31.045	41.289	28.861	1.00	0.00	C
ATOM	173	O	ARG	10	31.530	42.383	28.914	1.00	0.00	O
ATOM	174	N	PHE	11	31.517	40.307	29.569	1.00	0.00	N
ATOM	175	H	PHE	11	30.989	39.451	29.467	1.00	0.00	H
ATOM	176	CA	PHE	11	32.628	40.297	30.562	1.00	0.00	C
ATOM	177	HA	PHE	11	32.740	41.329	30.894	1.00	0.00	H
ATOM	178	CB	PHE	11	32.235	39.560	31.872	1.00	0.00	C
ATOM	179	HB2	PHE	11	32.927	39.831	32.670	1.00	0.00	H
ATOM	180	HB3	PHE	11	32.263	38.530	31.518	1.00	0.00	H
ATOM	181	CG	PHE	11	30.782	39.774	32.311	1.00	0.00	C
ATOM	182	CD1	PHE	11	30.410	41.065	32.790	1.00	0.00	C
ATOM	183	HD1	PHE	11	31.047	41.935	32.721	1.00	0.00	H
ATOM	184	CE1	PHE	11	29.135	41.237	33.263	1.00	0.00	C
ATOM	185	HE1	PHE	11	28.842	42.215	33.617	1.00	0.00	H
ATOM	186	CZ	PHE	11	28.259	40.125	33.374	1.00	0.00	C
ATOM	187	HZ	PHE	11	27.339	40.147	33.941	1.00	0.00	H
ATOM	188	CE2	PHE	11	28.597	38.872	32.838	1.00	0.00	C
ATOM	189	HE2	PHE	11	27.929	38.028	32.926	1.00	0.00	H
ATOM	190	CD2	PHE	11	29.845	38.721	32.309	1.00	0.00	C
ATOM	191	HD2	PHE	11	30.155	37.730	32.012	1.00	0.00	H
ATOM	192	C	PHE	11	33.917	39.806	29.922	1.00	0.00	C
ATOM	193	O	PHE	11	34.956	39.803	30.612	1.00	0.00	O
ATOM	194	N	GLN	12	33.896	39.392	28.601	1.00	0.00	N
ATOM	195	H	GLN	12	33.089	39.533	28.011	1.00	0.00	H
ATOM	196	CA	GLN	12	35.042	38.657	28.138	1.00	0.00	C
ATOM	197	HA	GLN	12	35.277	37.927	28.913	1.00	0.00	H
ATOM	198	CB	GLN	12	34.752	37.892	26.912	1.00	0.00	C
ATOM	199	HB2	GLN	12	34.248	38.560	26.213	1.00	0.00	H
ATOM	200	HB3	GLN	12	35.732	37.637	26.509	1.00	0.00	H
ATOM	201	CG	GLN	12	33.751	36.683	27.016	1.00	0.00	C
ATOM	202	HG2	GLN	12	34.315	35.865	27.465	1.00	0.00	H
ATOM	203	HG3	GLN	12	32.907	36.914	27.666	1.00	0.00	H
ATOM	204	CD	GLN	12	33.205	36.159	25.710	1.00	0.00	C
ATOM	205	OE1	GLN	12	33.603	36.527	24.602	1.00	0.00	O
ATOM	206	NE2	GLN	12	32.403	35.111	25.732	1.00	0.00	N
ATOM	207	HE21	GLN	12	32.120	34.709	26.614	1.00	0.00	H
ATOM	208	HE22	GLN	12	32.308	34.419	25.002	1.00	0.00	H
ATOM	209	C	GLN	12	36.285	39.522	28.013	1.00	0.00	C
ATOM	210	O	GLN	12	37.397	39.011	28.237	1.00	0.00	O
ATOM	211	N	ASP	13	36.196	40.776	27.532	1.00	0.00	N
ATOM	212	H	ASP	13	35.322	41.043	27.103	1.00	0.00	H
ATOM	213	CA	ASP	13	37.365	41.585	27.256	1.00	0.00	C
ATOM	214	HA	ASP	13	38.134	41.204	26.583	1.00	0.00	H
ATOM	215	CB	ASP	13	36.920	42.938	26.687	1.00	0.00	C

ATOM	216	HB2	ASP	13	36.288	43.474	27.394	1.00	0.00	H
ATOM	217	HB3	ASP	13	37.830	43.539	26.662	1.00	0.00	H
ATOM	218	CG	ASP	13	36.230	42.730	25.318	1.00	0.00	C
ATOM	219	OD1	ASP	13	35.233	43.442	25.102	1.00	0.00	O
ATOM	220	OD2	ASP	13	36.633	41.880	24.504	1.00	0.00	O
ATOM	221	C	ASP	13	38.154	41.839	28.536	1.00	0.00	C
ATOM	222	O	ASP	13	39.398	41.821	28.543	1.00	0.00	O
ATOM	223	N	ASP	14	37.407	41.958	29.675	1.00	0.00	N
ATOM	224	H	ASP	14	36.422	42.093	29.496	1.00	0.00	H
ATOM	225	CA	ASP	14	37.925	42.117	31.041	1.00	0.00	C
ATOM	226	HA	ASP	14	38.729	42.852	31.086	1.00	0.00	H
ATOM	227	CB	ASP	14	36.785	42.635	31.917	1.00	0.00	C
ATOM	228	HB2	ASP	14	35.891	42.030	31.767	1.00	0.00	H
ATOM	229	HB3	ASP	14	37.030	42.500	32.971	1.00	0.00	H
ATOM	230	CG	ASP	14	36.517	44.093	31.645	1.00	0.00	C
ATOM	231	OD1	ASP	14	37.073	44.946	32.340	1.00	0.00	O
ATOM	232	OD2	ASP	14	35.929	44.364	30.635	1.00	0.00	O
ATOM	233	C	ASP	14	38.535	40.820	31.627	1.00	0.00	C
ATOM	234	O	ASP	14	39.470	40.879	32.426	1.00	0.00	O
ATOM	235	N	ILE	15	38.072	39.681	31.070	1.00	0.00	N
ATOM	236	H	ILE	15	37.396	39.625	30.322	1.00	0.00	H
ATOM	237	CA	ILE	15	38.661	38.333	31.410	1.00	0.00	C
ATOM	238	HA	ILE	15	39.039	38.359	32.432	1.00	0.00	H
ATOM	239	CB	ILE	15	37.609	37.172	31.454	1.00	0.00	C
ATOM	240	HB	ILE	15	37.044	37.162	30.523	1.00	0.00	H
ATOM	241	CG2	ILE	15	38.307	35.869	31.679	1.00	0.00	C
ATOM	242	HG21	ILE	15	37.576	35.112	31.963	1.00	0.00	H
ATOM	243	HG22	ILE	15	38.856	35.458	30.831	1.00	0.00	H
ATOM	244	HG23	ILE	15	39.009	36.009	32.501	1.00	0.00	H
ATOM	245	CG1	ILE	15	36.533	37.371	32.563	1.00	0.00	C
ATOM	246	HG12	ILE	15	36.983	37.226	33.544	1.00	0.00	H
ATOM	247	HG13	ILE	15	36.130	38.373	32.409	1.00	0.00	H
ATOM	248	CD1	ILE	15	35.331	36.441	32.470	1.00	0.00	C
ATOM	249	HD11	ILE	15	35.630	35.417	32.696	1.00	0.00	H
ATOM	250	HD12	ILE	15	34.510	36.613	33.166	1.00	0.00	H
ATOM	251	HD13	ILE	15	35.055	36.362	31.418	1.00	0.00	H
ATOM	252	C	ILE	15	39.866	38.017	30.471	1.00	0.00	C
ATOM	253	O	ILE	15	40.825	37.490	30.954	1.00	0.00	O
ATOM	254	N	LEU	16	39.823	38.556	29.270	1.00	0.00	N
ATOM	255	H	LEU	16	39.063	39.188	29.062	1.00	0.00	H
ATOM	256	CA	LEU	16	40.975	38.529	28.341	1.00	0.00	C
ATOM	257	HA	LEU	16	41.187	37.460	28.326	1.00	0.00	H
ATOM	258	CB	LEU	16	40.532	38.892	26.948	1.00	0.00	C
ATOM	259	HB2	LEU	16	39.794	38.153	26.637	1.00	0.00	H
ATOM	260	HB3	LEU	16	39.941	39.807	26.916	1.00	0.00	H
ATOM	261	CG	LEU	16	41.447	38.879	25.750	1.00	0.00	C
ATOM	262	HG	LEU	16	42.061	39.771	25.878	1.00	0.00	H
ATOM	263	CD1	LEU	16	42.341	37.570	25.701	1.00	0.00	C
ATOM	264	HD11	LEU	16	42.953	37.583	24.798	1.00	0.00	H
ATOM	265	HD12	LEU	16	42.949	37.513	26.604	1.00	0.00	H
ATOM	266	HD13	LEU	16	41.761	36.649	25.749	1.00	0.00	H
ATOM	267	CD2	LEU	16	40.725	38.965	24.345	1.00	0.00	C
ATOM	268	HD21	LEU	16	40.002	39.779	24.395	1.00	0.00	H
ATOM	269	HD22	LEU	16	41.523	39.186	23.638	1.00	0.00	H
ATOM	270	HD23	LEU	16	40.207	38.016	24.201	1.00	0.00	H
ATOM	271	C	LEU	16	42.165	39.396	28.841	1.00	0.00	C
ATOM	272	O	LEU	16	43.244	38.880	28.765	1.00	0.00	O
ATOM	273	N	ALA	17	42.016	40.573	29.432	1.00	0.00	N
ATOM	274	H	ALA	17	41.078	40.946	29.401	1.00	0.00	H
ATOM	275	CA	ALA	17	43.056	41.282	30.065	1.00	0.00	C
ATOM	276	HA	ALA	17	43.785	41.349	29.257	1.00	0.00	H
ATOM	277	CB	ALA	17	42.627	42.711	30.448	1.00	0.00	C
ATOM	278	HB1	ALA	17	42.150	43.164	29.579	1.00	0.00	H
ATOM	279	HB2	ALA	17	41.918	42.778	31.274	1.00	0.00	H

```
ATOM    280  HB3 ALA    17      43.542  43.254  30.683  1.00  0.00           H
ATOM    281  C   ALA    17      43.670  40.595  31.283  1.00  0.00           C
ATOM    282  O   ALA    17      44.771  40.883  31.677  1.00  0.00           O
ATOM    283  N   GLY    18      42.964  39.708  31.853  1.00  0.00           N
ATOM    284  H   GLY    18      42.135  39.343  31.405  1.00  0.00           H
ATOM    285  CA  GLY    18      43.342  39.133  33.156  1.00  0.00           C
ATOM    286  HA2 GLY    18      42.827  38.176  33.246  1.00  0.00           H
ATOM    287  HA3 GLY    18      44.386  38.839  33.260  1.00  0.00           H
ATOM    288  C   GLY    18      43.086  40.002  34.389  1.00  0.00           C
ATOM    289  O   GLY    18      43.637  39.733  35.468  1.00  0.00           O
ATOM    290  N   ARG    19      42.294  41.082  34.309  1.00  0.00           N
ATOM    291  H   ARG    19      41.975  41.317  33.380  1.00  0.00           H
ATOM    292  CA  ARG    19      41.795  41.892  35.350  1.00  0.00           C
ATOM    293  HA  ARG    19      42.609  41.927  36.074  1.00  0.00           H
ATOM    294  CB  ARG    19      41.373  43.285  34.894  1.00  0.00           C
ATOM    295  HB2 ARG    19      42.097  43.677  34.178  1.00  0.00           H
ATOM    296  HB3 ARG    19      40.445  43.310  34.324  1.00  0.00           H
ATOM    297  CG  ARG    19      41.138  44.307  36.004  1.00  0.00           C
ATOM    298  HG2 ARG    19      40.275  44.022  36.606  1.00  0.00           H
ATOM    299  HG3 ARG    19      42.054  44.358  36.594  1.00  0.00           H
ATOM    300  CD  ARG    19      40.927  45.628  35.330  1.00  0.00           C
ATOM    301  HD2 ARG    19      41.811  45.917  34.762  1.00  0.00           H
ATOM    302  HD3 ARG    19      40.220  45.533  34.506  1.00  0.00           H
ATOM    303  NE  ARG    19      40.675  46.721  36.264  1.00  0.00           N
ATOM    304  HE  ARG    19      41.513  47.245  36.471  1.00  0.00           H
ATOM    305  CZ  ARG    19      39.492  47.034  36.824  1.00  0.00           C
ATOM    306  NH1 ARG    19      38.373  46.451  36.574  1.00  0.00           N
ATOM    307 HH11 ARG    19      38.428  45.652  35.959  1.00  0.00           H
ATOM    308 HH12 ARG    19      37.541  46.546  37.139  1.00  0.00           H
ATOM    309  NH2 ARG    19      39.549  47.885  37.765  1.00  0.00           N
ATOM    310 HH21 ARG    19      38.677  48.212  38.155  1.00  0.00           H
ATOM    311 HH22 ARG    19      40.467  48.222  38.018  1.00  0.00           H
ATOM    312  C   ARG    19      40.692  41.157  36.093  1.00  0.00           C
ATOM    313  O   ARG    19      40.796  40.940  37.281  1.00  0.00           O
ATOM    335  N   THR    21      38.996  38.298  37.871  1.00  0.00           N
ATOM    336  H   THR    21      38.676  39.024  38.496  1.00  0.00           H
ATOM    337  CA  THR    21      39.602  37.137  38.506  1.00  0.00           C
ATOM    338  HA  THR    21      39.458  36.255  37.883  1.00  0.00           H
ATOM    339  CB  THR    21      41.119  37.336  38.651  1.00  0.00           C
ATOM    340  HB  THR    21      41.398  37.667  37.650  1.00  0.00           H
ATOM    341  CG2 THR    21      41.343  38.291  39.802  1.00  0.00           C
ATOM    342 HG21 THR    21      40.667  39.148  39.811  1.00  0.00           H
ATOM    343 HG22 THR    21      41.217  37.727  40.726  1.00  0.00           H
ATOM    344 HG23 THR    21      42.415  38.487  39.795  1.00  0.00           H
ATOM    345  OG1 THR    21      41.887  36.183  39.044  1.00  0.00           O
ATOM    346  HG1 THR    21      41.559  35.494  38.461  1.00  0.00           H
ATOM    347  C   THR    21      38.940  36.666  39.840  1.00  0.00           C
ATOM    348  O   THR    21      39.203  35.668  40.461  1.00  0.00           O
ATOM    349
  N   ILE    22      37.987  37.470  40.343  1.00  0.00           N
ATOM    350  H   ILE    22      37.944  38.382  39.912  1.00  0.00           H
ATOM    351  CA  ILE    22      37.142  37.228  41.465  1.00  0.00           C
ATOM    352  HA  ILE    22      37.047  36.144  41.510  1.00  0.00           H
ATOM    353  CB  ILE    22      37.755  37.756  42.797  1.00  0.00           C
ATOM    354  HB  ILE    22      38.646  37.142  42.924  1.00  0.00           H
ATOM    355  CG2 ILE    22      38.081  39.265  42.688  1.00  0.00           C
ATOM    356 HG21 ILE    22      38.599  39.713  43.536  1.00  0.00           H
ATOM    357 HG22 ILE    22      38.809  39.296  41.878  1.00  0.00           H
ATOM    358 HG23 ILE    22      37.141  39.753  42.428  1.00  0.00           H
ATOM    359  CG1 ILE    22      36.822  37.528  44.103  1.00  0.00           C
ATOM    360 HG12 ILE    22      36.143  38.378  44.172  1.00  0.00           H
ATOM    361 HG13 ILE    22      36.321  36.565  44.011  1.00  0.00           H
ATOM    362  CD1 ILE    22      37.668  37.541  45.330  1.00  0.00           C
ATOM    363 HD11 ILE    22      38.040  38.544  45.539  1.00  0.00           H
```

ATOM	364	HD12	ILE	22	37.110	37.241	46.217	1.00	0.00	H
ATOM	365	HD13	ILE	22	38.505	36.847	45.249	1.00	0.00	H
ATOM	366	C	ILE	22	35.717	37.714	41.098	1.00	0.00	C
ATOM	367	O	ILE	22	35.623	38.602	40.227	1.00	0.00	O
ATOM	368	N	THR	23	34.670	37.141	41.652	1.00	0.00	N
ATOM	369	H	THR	23	34.891	36.353	42.242	1.00	0.00	H
ATOM	370	CA	THR	23	33.336	37.616	41.495	1.00	0.00	C
ATOM	371	HA	THR	23	33.435	38.673	41.248	1.00	0.00	H
ATOM	372	CB	THR	23	32.740	37.042	40.185	1.00	0.00	C
ATOM	373	HB	THR	23	33.408	37.133	39.329	1.00	0.00	H
ATOM	374	CG2	THR	23	32.396	35.561	40.247	1.00	0.00	C
ATOM	375	HG21	THR	23	31.734	35.416	39.393	1.00	0.00	H
ATOM	376	HG22	THR	23	33.231	34.864	40.314	1.00	0.00	H
ATOM	377	HG23	THR	23	31.617	35.410	40.995	1.00	0.00	H
ATOM	378	OG1	THR	23	31.638	37.747	39.790	1.00	0.00	O
ATOM	379	HG1	THR	23	31.960	38.488	39.272	1.00	0.00	H
ATOM	380	C	THR	23	32.468	37.355	42.771	1.00	0.00	C
ATOM	381	O	THR	23	32.911	36.554	43.629	1.00	0.00	O
ATOM	382	N	ILE	24	31.262	37.942	42.862	1.00	0.00	N
ATOM	383	H	ILE	24	30.955	38.719	42.295	1.00	0.00	H
ATOM	384	CA	ILE	24	30.339	37.600	44.001	1.00	0.00	C
ATOM	385	HA	ILE	24	30.797	36.821	44.610	1.00	0.00	H
ATOM	386	CB	ILE	24	30.327	38.775	45.063	1.00	0.00	C
ATOM	387	HB	ILE	24	29.794	39.614	44.617	1.00	0.00	H
ATOM	388	CG2	ILE	24	29.599	38.305	46.299	1.00	0.00	C
ATOM	389	HG21	ILE	24	29.927	38.843	47.189	1.00	0.00	H
ATOM	390	HG22	ILE	24	28.544	38.540	46.162	1.00	0.00	H
ATOM	391	HG23	ILE	24	29.738	37.246	46.519	1.00	0.00	H
ATOM	392	CG1	ILE	24	31.751	39.256	45.377	1.00	0.00	C
ATOM	393	HG12	ILE	24	32.304	38.372	45.690	1.00	0.00	H
ATOM	394	HG13	ILE	24	32.229	39.663	44.485	1.00	0.00	H
ATOM	395	CD1	ILE	24	31.911	40.399	46.432	1.00	0.00	C
ATOM	396	HD11	ILE	24	32.845	40.960	46.450	1.00	0.00	H
ATOM	397	HD12	ILE	24	31.122	41.147	46.360	1.00	0.00	H
ATOM	398	HD13	ILE	24	31.876	40.053	47.465	1.00	0.00	H
ATOM	399	C	ILE	24	28.933	37.064	43.556	1.00	0.00	C
ATOM	400	O	ILE	24	28.351	37.592	42.660	1.00	0.00	O
ATOM	401	N	ARG	25	28.286	36.266	44.391	1.00	0.00	N
ATOM	402	H	ARG	25	28.757	36.159	45.278	1.00	0.00	H
ATOM	403	CA	ARG	25	27.056	35.510	44.302	1.00	0.00	C
ATOM	404	HA	ARG	25	26.417	36.001	43.567	1.00	0.00	H
ATOM	405	CB	ARG	25	27.250	34.090	43.810	1.00	0.00	C
ATOM	406	HB2	ARG	25	27.792	33.529	44.570	1.00	0.00	H
ATOM	407	HB3	ARG	25	26.283	33.596	43.726	1.00	0.00	H
ATOM	408	CG	ARG	25	27.894	34.038	42.382	1.00	0.00	C
ATOM	409	HG2	ARG	25	28.879	34.494	42.483	1.00	0.00	H
ATOM	410	HG3	ARG	25	27.999	32.979	42.148	1.00	0.00	H
ATOM	411	CD	ARG	25	27.165	34.756	41.238	1.00	0.00	C
ATOM	412	HD2	ARG	25	26.255	34.180	41.075	1.00	0.00	H
ATOM	413	HD3	ARG	25	27.011	35.818	41.431	1.00	0.00	H
ATOM	414	NE	ARG	25	27.842	34.673	39.953	1.00	0.00	N
ATOM	415	HE	ARG	25	27.729	33.834	39.402	1.00	0.00	H
ATOM	416	CZ	ARG	25	28.466	35.714	39.396	1.00	0.00	C
ATOM	417	NH1	ARG	25	28.669	36.772	40.093	1.00	0.00	N
ATOM	418	HH11	ARG	25	29.173	37.468	39.563	1.00	0.00	H
ATOM	419	HH12	ARG	25	28.127	37.068	40.893	1.00	0.00	H
ATOM	420	NH2	ARG	25	29.058	35.595	38.247	1.00	0.00	N
ATOM	421	HH21	ARG	25	29.430	36.444	37.843	1.00	0.00	H
ATOM	422	HH22	ARG	25	28.871	34.805	37.646	1.00	0.00	H
ATOM	423	C	ARG	25	26.273	35.560	45.620	1.00	0.00	C
ATOM	424	O	ARG	25	26.865	35.886	46.672	1.00	0.00	O
ATOM	425	N	ASP	26	25.004	35.279	45.516	1.00	0.00	N
ATOM	426	H	ASP	26	24.677	35.024	44.595	1.00	0.00	H
ATOM	427	CA	ASP	26	24.240	34.750	46.632	1.00	0.00	C

ATOM	428	HA	ASP	26	24.648	35.248	47.511	1.00	0.00	H
ATOM	429	CB	ASP	26	22.713	35.042	46.561	1.00	0.00	C
ATOM	430	HB2	ASP	26	22.120	34.687	47.404	1.00	0.00	H
ATOM	431	HB3	ASP	26	22.645	36.130	46.558	1.00	0.00	H
ATOM	432	CG	ASP	26	22.060	34.457	45.332	1.00	0.00	C
ATOM	433	OD1	ASP	26	22.437	34.740	44.194	1.00	0.00	O
ATOM	434	OD2	ASP	26	21.007	33.781	45.479	1.00	0.00	O
ATOM	435	C	ASP	26	24.395	33.236	46.782	1.00	0.00	C
ATOM	436	O	ASP	26	24.591	32.523	45.814	1.00	0.00	O
ATOM	437	N	GLU	27	24.199	32.784	48.020	1.00	0.00	N
ATOM	438	H	GLU	27	24.000	33.386	48.806	1.00	0.00	H
ATOM	439	CA	GLU	27	24.583	31.349	48.331	1.00	0.00	C
ATOM	440	HA	GLU	27	25.576	31.278	47.891	1.00	0.00	H
ATOM	441	CB	GLU	27	24.792	31.038	49.885	1.00	0.00	C
ATOM	442	HB2	GLU	27	25.334	31.922	50.221	1.00	0.00	H
ATOM	443	HB3	GLU	27	23.856	30.917	50.431	1.00	0.00	H
ATOM	444	CG	GLU	27	25.717	29.780	50.089	1.00	0.00	C
ATOM	445	HG2	GLU	27	25.700	29.204	49.164	1.00	0.00	H
ATOM	446	HG3	GLU	27	26.709	30.208	50.230	1.00	0.00	H
ATOM	447	CD	GLU	27	25.411	28.920	51.403	1.00	0.00	C
ATOM	448	OE1	GLU	27	24.655	27.944	51.191	1.00	0.00	O
ATOM	449	OE2	GLU	27	25.966	29.201	52.507	1.00	0.00	O
ATOM	450	C	GLU	27	23.649	30.307	47.625	1.00	0.00	C
ATOM	451	O	GLU	27	22.454	30.234	47.957	1.00	0.00	O
ATOM	452	N	SER	28	24.280	29.428	46.783	1.00	0.00	N
ATOM	453	H	SER	28	25.277	29.526	46.658	1.00	0.00	H
ATOM	454	CA	SER	28	23.573	28.314	46.155	1.00	0.00	C
ATOM	455	HA	SER	28	22.853	27.885	46.851	1.00	0.00	H
ATOM	456	CB	SER	28	22.807	28.824	44.959	1.00	0.00	C
ATOM	457	HB2	SER	28	22.005	29.463	45.328	1.00	0.00	H
ATOM	458	HB3	SER	28	23.513	29.480	44.451	1.00	0.00	H
ATOM	459	OG	SER	28	22.312	27.843	44.058	1.00	0.00	O
ATOM	460	HG	SER	28	21.399	27.745	44.338	1.00	0.00	H
ATOM	461	C	SER	28	24.624	27.245	45.768	1.00	0.00	C
ATOM	462	O	SER	28	25.720	27.570	45.313	1.00	0.00	O
ATOM	463	N	GLU	29	24.255	25.998	45.899	1.00	0.00	N
ATOM	464	H	GLU	29	23.287	25.907	46.173	1.00	0.00	H
ATOM	465	CA	GLU	29	24.870	24.834	45.313	1.00	0.00	C
ATOM	466	HA	GLU	29	25.893	24.709	45.669	1.00	0.00	H
ATOM	467	CB	GLU	29	24.034	23.578	45.671	1.00	0.00	C
ATOM	468	HB2	GLU	29	24.229	23.160	46.657	1.00	0.00	H
ATOM	469	HB3	GLU	29	22.970	23.798	45.760	1.00	0.00	H
ATOM	470	CG	GLU	29	23.881	22.433	44.638	1.00	0.00	C
ATOM	471	HG2	GLU	29	23.041	21.791	44.904	1.00	0.00	H
ATOM	472	HG3	GLU	29	23.664	22.875	43.666	1.00	0.00	H
ATOM	473	CD	GLU	29	25.064	21.498	44.595	1.00	0.00	C
ATOM	474	OE1	GLU	29	25.528					
21.016	43.528		1.00	0.00		O				
ATOM	475	OE2	GLU	29	25.676	21.162	45.650	1.00	0.00	O
ATOM	476	C	GLU	29	24.983	25.019	43.838	1.00	0.00	C
ATOM	477	O	GLU	29	26.017	24.679	43.269	1.00	0.00	O
ATOM	478	N	SER	30	24.011	25.576	43.102	1.00	0.00	N
ATOM	479	H	SER	30	23.169	25.917	43.543	1.00	0.00	H
ATOM	480	CA	SER	30	24.093	25.577	41.659	1.00	0.00	C
ATOM	481	HA	SER	30	24.344	24.559	41.360	1.00	0.00	H
ATOM	482	CB	SER	30	22.650	25.941	41.226	1.00	0.00	C
ATOM	483	HB2	SER	30	22.559	25.665	40.175	1.00	0.00	H
ATOM	484	HB3	SER	30	21.944	25.331	41.788	1.00	0.00	H
ATOM	485	OG	SER	30	22.419	27.314	41.403	1.00	0.00	O
ATOM	486	HG	SER	30	22.127	27.508	42.296	1.00	0.00	H
ATOM	487	C	SER	30	25.212	26.373	41.003	1.00	0.00	C
ATOM	488	O	SER	30	25.448	26.265	39.821	1.00	0.00	O
ATOM	489	N	HIE	31	25.952	27.239	41.705	1.00	0.00	N
ATOM	490	H	HIE	31	25.713	27.247	42.687	1.00	0.00	H

ATOM	491	CA	HIE	31	27.171	27.968	41.251	1.00	0.00	C
ATOM	492	HA	HIE	31	27.062	28.318	40.224	1.00	0.00	H
ATOM	493	CB	HIE	31	27.465	29.091	42.240	1.00	0.00	C
ATOM	494	HB2	HIE	31	27.591	28.630	43.220	1.00	0.00	H
ATOM	495	HB3	HIE	31	28.360	29.638	41.944	1.00	0.00	H
ATOM	496	CG	HIE	31	26.220	30.038	42.368	1.00	0.00	C
ATOM	497	ND1	HIE	31	25.454	30.622	41.293	1.00	0.00	N
ATOM	498	CE1	HIE	31	24.511	31.406	41.925	1.00	0.00	C
ATOM	499	HE1	HIE	31	23.695	31.829	41.359	1.00	0.00	H
ATOM	500	NE2	HIE	31	24.635	31.410	43.256	1.00	0.00	N
ATOM	501	HE2	HIE	31	24.133	31.868	44.003	1.00	0.00	H
ATOM	502	CD2	HIE	31	25.732	30.582	43.515	1.00	0.00	C
ATOM	503	HD2	HIE	31	26.099	30.277	44.484	1.00	0.00	H
ATOM	504	C	HIE	31	28.432	27.077	41.113	1.00	0.00	C
ATOM	505	O	HIE	31	28.571	25.912	41.501	1.00	0.00	O
ATOM	506	N	PHE	32	29.465	27.668	40.495	1.00	0.00	N
ATOM	507	H	PHE	32	29.289	28.567	40.068	1.00	0.00	H
ATOM	508	CA	PHE	32	30.808	27.097	40.304	1.00	0.00	C
ATOM	509	HA	PHE	32	30.689	26.072	39.954	1.00	0.00	H
ATOM	510	CB	PHE	32	31.761	27.927	39.431	1.00	0.00	C
ATOM	511	HB2	PHE	32	32.061	28.657	40.183	1.00	0.00	H
ATOM	512	HB3	PHE	32	32.668	27.432	39.083	1.00	0.00	H
ATOM	513	CG	PHE	32	31.157	28.723	38.235	1.00	0.00	C
ATOM	514	CD1	PHE	32	31.126	30.098	38.318	1.00	0.00	C
ATOM	515	HD1	PHE	32	31.243	30.656	39.236	1.00	0.00	H
ATOM	516	CE1	PHE	32	30.878	30.854	37.150	1.00	0.00	C
ATOM	517	HE1	PHE	32	30.872	31.933	37.181	1.00	0.00	H
ATOM	518	CZ	PHE	32	30.551	30.181	35.966	1.00	0.00	C
ATOM	519	HZ	PHE	32	30.308	30.768	35.093	1.00	0.00	H
ATOM	520	CE2	PHE	32	30.673	28.792	35.854	1.00	0.00	C
ATOM	521	HE2	PHE	32	30.576	28.403	34.851	1.00	0.00	H
ATOM	522	CD2	PHE	32	30.985	28.039	37.030	1.00	0.00	C
ATOM	523	HD2	PHE	32	31.063	26.963	36.993	1.00	0.00	H
ATOM	524	C	PHE	32	31.438	26.660	41.686	1.00	0.00	C
ATOM	525	O	PHE	32	31.149	27.258	42.726	1.00	0.00	O
ATOM	526	N	LYS	33	32.121	25.556	41.657	1.00	0.00	N
ATOM	527	H	LYS	33	32.234	25.178	40.727	1.00	0.00	H
ATOM	528	CA	LYS	33	32.940	24.991	42.885	1.00	0.00	C
ATOM	529	HA	LYS	33	32.842	25.601	43.783	1.00	0.00	H
ATOM	530	CB	LYS	33	32.448	23.597	43.266	1.00	0.00	C
ATOM	531	HB2	LYS	33	32.434	23.088	42.303	1.00	0.00	H
ATOM	532	HB3	LYS	33	33.096	23.128	44.006	1.00	0.00	H
ATOM	533	CG	LYS	33	30.991	23.478	43.801	1.00	0.00	C
ATOM	534	HG2	LYS	33	30.916	24.212	44.603	1.00	0.00	H
ATOM	535	HG3	LYS	33	30.306	23.923	43.078	1.00	0.00	H
ATOM	536	CD	LYS	33	30.513	22.063	44.164	1.00	0.00	C
ATOM	537	HD2	LYS	33	30.629	21.395	43.311	1.00	0.00	H
ATOM	538	HD3	LYS	33	31.151	21.699	44.969	1.00	0.00	H
ATOM	539	CE	LYS	33	28.993	22.053	44.478	1.00	0.00	C
ATOM	540	HE2	LYS	33	28.827	22.509	45.455	1.00	0.00	H
ATOM	541	HE3	LYS	33	28.490	22.657	43.723	1.00	0.00	H
ATOM	542	NZ	LYS	33	28.352	20.691	44.449	1.00	0.00	N
ATOM	543	HZ1	LYS	33	28.682	20.044	45.150	1.00	0.00	H
ATOM	544	HZ2	LYS	33	27.369	20.845	44.624	1.00	0.00	H
ATOM	545	HZ3	LYS	33	28.473	20.182	43.584	1.00	0.00	H
ATOM	546	C	LYS	33	34.436	24.939	42.587	1.00	0.00	C
ATOM	547	O	LYS	33	34.912	24.936	41.445	1.00	0.00	O
ATOM	548	N	THR	34	35.207	24.944	43.663	1.00	0.00	N
ATOM	549	H	THR	34	34.772	24.799	44.562	1.00	0.00	H
ATOM	550	CA	THR	34	36.654	24.919	43.590	1.00	0.00	C
ATOM	551	HA	THR	34	36.959	25.879	43.170	1.00	0.00	H
ATOM	552	CB	THR	34	37.291	24.930	45.003	1.00	0.00	C
ATOM	553	HB	THR	34	36.852	24.192	45.673	1.00	0.00	H
ATOM	554	CG2	THR	34	38.866	24.771	45.101	1.00	0.00	C

ATOM	555	HG21	THR	34	39.452	25.242	44.312	1.00	0.00	H
ATOM	556	HG22	THR	34	39.230	25.236	46.016	1.00	0.00	H
ATOM	557	HG23	THR	34	39.204	23.736	45.059	1.00	0.00	H
ATOM	558	OG1	THR	34	36.954	26.212	45.636	1.00	0.00	O
ATOM	559	HG1	THR	34	35.994	26.188	45.636	1.00	0.00	H
ATOM	560	C	THR	34	37.290	23.780	42.780	1.00	0.00	C
ATOM	561	O	THR	34	36.665	22.723	42.770	1.00	0.00	O
ATOM	562	N	GLY	35	38.334	24.054	41.893	1.00	0.00	N
ATOM	563	H	GLY	35	38.694	24.997	41.933	1.00	0.00	H
ATOM	564	CA	GLY	35	38.825	23.133	40.809	1.00	0.00	C
ATOM	565	HA2	GLY	35	39.661	23.646	40.334	1.00	0.00	H
ATOM	566	HA3	GLY	35	39.217	22.232	41.281	1.00	0.00	H
ATOM	567	C	GLY	35	37.844	22.930	39.587	1.00	0.00	C
ATOM	568	O	GLY	35	38.281	22.212	38.614	1.00	0.00	O
ATOM	569	N	ASP	36	36.607	23.554	39.543	1.00	0.00	N
ATOM	570	H	ASP	36	36.428	24.276	40.227	1.00	0.00	H
ATOM	571	CA	ASP	36	35.767	23.676	38.261	1.00	0.00	C
ATOM	572	HA	ASP	36	35.486	22.688	37.894	1.00	0.00	H
ATOM	573	CB	ASP	36	34.494	24.502	38.337	1.00	0.00	C
ATOM	574	HB2	ASP	36	34.622	25.366	38.991	1.00	0.00	H
ATOM	575	HB3	ASP	36	34.184	24.945	37.390	1.00	0.00	H
ATOM	576	CG	ASP	36	33.212	23.806	38.835	1.00	0.00	C
ATOM	577	OD1	ASP	36	32.207	24.451	39.191	1.00	0.00	O
ATOM	578	OD2	ASP	36	33.006	22.561	38.699	1.00	0.00	O
ATOM	579	C	ASP	36	36.560	24.383	37.139	1.00	0.00	C
ATOM	580	O	ASP	36	37.060	25.514	37.286	1.00	0.00	O
ATOM	581	N	VAL	37	36.686	23.640	36.009	1.00	0.00	N
ATOM	582	H	VAL	37	36.159	22.779	36.023	1.00	0.00	H
ATOM	583	CA	VAL	37	37.279	24.170	34.811	1.00	0.00	C
ATOM	584	HA	VAL	37	38.021	24.883	35.173	1.00	0.00	H
ATOM	585	CB	VAL	37	38.022	23.014	34.115	1.00	0.00	C
ATOM	586	HB	VAL	37	37.382	22.255	33.665	1.00	0.00	H
ATOM	587	CG1	VAL	37	39.043	23.520	33.012	1.00	0.00	C
ATOM	588	HG11	VAL	37	39.599	22.695	32.567	1.00	0.00	H
ATOM	589	HG12	VAL	37	38.429	23.972	32.233	1.00	0.00	H
ATOM	590	HG13	VAL	37	39.727	24.247	33.450	1.00	0.00	H
ATOM	591	CG2	VAL	37	38.967	22.241	35.064	1.00	0.00	C
ATOM	592	HG21	VAL	37	39.649	22.892	35.611	1.00	0.00	H
ATOM	593	HG22	VAL	37	38.345	21.740	35.807	1.00	0.00	H
ATOM	594	HG23	VAL	37	39.462	21.460	34.486	1.00	0.00	H
ATOM	595	C	VAL	37	36.259	24.787	33.856	1.00	0.00	C
ATOM	596	O	VAL	37	35.352	24.073	33.443	1.00	0.00	O
ATOM	597	N	LEU	38	36.449	26.051	33.421	1.00	0.00	N
ATOM	598	H	LEU	38	37.272	26.449	33.852	1.00	0.00	H
ATOM	599	CA	LEU	38	35.568	26.872	32.619	1.00	0.00	C
ATOM	600	HA	LEU	38	34.687	26.271	32.393	1.00	0.00	H
ATOM	601	CB	LEU	38	35.059	28.152	33.369	1.00	0.00	C
ATOM	602	HB2	LEU	38	35.658	29.044	33.185	1.00	0.00	H
ATOM	603	HB3	LEU	38	34.111	28.481	32.947	1.00	0.00	H
ATOM	604	CG	LEU	38	34.855	28.055	34.890	1.00	0.00	C
ATOM	605	HG	LEU	38	35.814	27.856	35.366	1.00	0.00	H
ATOM	606	CD1	LEU	38	34.245	29.383	35.349	1.00	0.00	C
ATOM	607	HD11	LEU	38	34.704	30.277	34.928	1.00	0.00	H
ATOM	608	HD12	LEU	38	33.184	29.473	35.118	1.00	0.00	H
ATOM	609	HD13	LEU	38	34.361	29.477	36.429	1.00	0.00	H
ATOM	610	CD2	LEU	38	33.866	26.943	35.339	1.00	0.00	C
ATOM	611	HD21	LEU	38	33.816	26.910	36.427	1.00	0.00	H
ATOM	612	HD22	LEU	38	32.917	27.244	34.894	1.00	0.00	H
ATOM	613	HD23	LEU	38	34.054	25.979	34.865	1.00	0.00	H
ATOM	614	C	LEU	38	36.220	27.312	31.289	1.00	0.00	C
ATOM	615	O	LEU	38	37.470	27.332	31.234	1.00	0.00	O
ATOM	616	N	ARG	39	35.463	27.770	30.339	1.00	0.00	N
ATOM	617	H	ARG	39	34.459	27.739	30.454	1.00	0.00	H

ATOM	618	CA	ARG	39	35.896	28.222	29.069	1.00	0.00	C
ATOM	619	HA	ARG	39	36.964	28.391	29.199	1.00	0.00	H
ATOM	620	CB	ARG	39	35.857	27.076	28.107	1.00	0.00	C
ATOM	621	HB2	ARG	39	34.853	26.681	27.946	1.00	0.00	H
ATOM	622	HB3	ARG	39	36.291	27.399	27.160	1.00	0.00	H
ATOM	623	CG	ARG	39	36.805	25.912	28.524	1.00	0.00	C
ATOM	624	HG2	ARG	39	37.763	26.289	28.881	1.00	0.00	H
ATOM	625	HG3	ARG	39	36.237	25.456	29.334	1.00	0.00	H
ATOM	626	CD	ARG	39	37.028	24.941	27.348	1.00	0.00	C
ATOM	627	HD2	ARG	39	37.320	25.385	26.396	1.00	0.00	H
ATOM	628	HD3	ARG	39	37.782	24.217	27.658	1.00	0.00	H
ATOM	629	NE	ARG	39	35.731	24.193	27.077	1.00	0.00	N
ATOM	630	HE	ARG	39	34.870	24.494	27.513	1.00	0.00	H
ATOM	631	CZ	ARG	39	35.547	23.106	26.421	1.00	0.00	C
ATOM	632	NH1	ARG	39	36.339	22.499	25.623	1.00	0.00	N
ATOM	633	HH11	ARG	39	37.238	22.925	25.441	1.00	0.00	H
ATOM	634	HH12	ARG	39	36.074	21.547	25.413	1.00	0.00	H
ATOM	635	NH2	ARG	39	34.384	22.585	26.498	1.00	0.00	N
ATOM	636	HH21	ARG	39	34.093	21.860	25.858	1.00	0.00	H
ATOM	637	HH22	ARG	39	33.709	23.151	26.992	1.00	0.00	H
ATOM	638	C	ARG	39	35.191	29.525	28.575	1.00	0.00	C
ATOM	639	O	ARG	39	33.984	29.644	28.788	1.00	0.00	O
ATOM	640	N	VAL	40	35.874	30.521	28.055	1.00	0.00	N
ATOM	641	H	VAL	40	36.833	30.282	27.848	1.00	0.00	H
ATOM	642	CA	VAL	40	35.390	31.817	27.631	1.00	0.00	C
ATOM	643	HA	VAL	40	34.312	31.746	27.778	1.00	0.00	H
ATOM	644	CB	VAL	40	35.846	32.905	28.646	1.00	0.00	C
ATOM	645	HB	VAL	40	35.658	33.871	28.176	1.00	0.00	H
ATOM	646	CG1	VAL	40	35.028	32.763	29.934	1.00	0.00	C
ATOM	647	HG11	VAL	40	35.051	31.809	30.462	1.00	0.00	H
ATOM	648	HG12	VAL	40	35.300	33.564	30.622	1.00	0.00	H
ATOM	649	HG13	VAL	40	33.992	33.088	29.829	1.00	0.00	H
ATOM	650	CG2	VAL	40	37.369	32.840	28.903	1.00	0.00	C
ATOM	651	HG21	VAL	40	37.598	31.903	29.409	1.00	0.00	H
ATOM	652	HG22	VAL	40	38.048	32.987	28.063	1.00	0.00	H
ATOM	653	HG23	VAL	40	37.722	33.577	29.624	1.00	0.00	H
ATOM	654	C	VAL	40	35.711	32.229	26.186	1.00	0.00	C
ATOM	655	O	VAL	40	36.787	32.031	25.650	1.00	0.00	O
ATOM	656	N	GLY	41	34.694	32.747	25.477	1.00	0.00	N
ATOM	657	H	GLY	41	33.820	32.854	25.975	1.00	0.00	H
ATOM	658	CA	GLY	41	34.871	33.378	24.158	1.00	0.00	C
ATOM	659	HA2	GLY	41	35.184	34.412	24.303	1.00	0.00	H
ATOM	660	HA3	GLY	41	35.678	32.931	23.578	1.00	0.00	H
ATOM	661	C	GLY	41	33.585	33.525	23.313	1.00	0.00	C
ATOM	662	O	GLY	41	32.591	32.895	23.566	1.00	0.00	O
ATOM	663	N	ARG	42	33.692	34.332	22.241	1.00	0.00	N
ATOM	664	H	ARG	42	34.562	34.780	21.993	1.00	0.00	H
ATOM	665	CA	ARG	42	32.569	34.643	21.336	1.00	0.00	C
ATOM	666	HA	ARG	42	31.660	34.079	21.547	1.00	0.00	H
ATOM	667	CB	ARG	42	32.075	36.107	21.689	1.00	0.00	C
ATOM	668	HB2	ARG	42	31.157	36.292	21.132	1.00	0.00	H
ATOM	669	HB3	ARG	42	31.887	36.003	22.758	1.00	0.00	H
ATOM	670	CG	ARG	42	33.114	37.200	21.431	1.00	0.00	C
ATOM	671	HG2	ARG	42	33.976	36.957	22.052	1.00	0.00	H
ATOM	672	HG3	ARG	42	33.317	37.104	20.364	1.00	0.00	H
ATOM	673	CD	ARG	42	32.444	38.547	21.828	1.00	0.00	C
ATOM	674	HD2	ARG	42	31.613	38.776	21.159	1.00	0.00	H
ATOM	675	HD3	ARG	42	32.001	38.564	22.823	1.00	0.00	H
ATOM	676	NE	ARG	42	33.495	39.645	21.732	1.00	0.00	N
ATOM	677	HE	ARG	42	33.379	40.213	20.905	1.00	0.00	H
ATOM	678	CZ	ARG	42	34.335	40.059	22.633	1.00	0.00	C
ATOM	679	NH1	ARG	42	34.457	39.394	23.765	1.00	0.00	N
ATOM	680	HH11	ARG	42	35.206	39.554	24.424	1.00	0.00	H
ATOM	681	HH12	ARG	42	33.885	38.588	23.970	1.00	0.00	H

```
ATOM    682  NH2 ARG    42      35.153  41.032  22.384  1.00  0.00           N
ATOM    683 HH21 ARG    42      34.998  41.609  21.571  1.00  0.00           H
ATOM    684 HH22 ARG    42      35.807  41.358  23.083  1.00  0.00           H
ATOM    685  C   ARG    42      32.930  34.474  19.869  1.00  0.00           C
ATOM    686  O   ARG    42      32.063  34.509  19.007  1.00  0.00           O
ATOM    687  N   PHE    43      34.240  34.213  19.579  1.00  0.00           N
ATOM    688  H   PHE    43      34.899  33.990  20.311  1.00  0.00           H
ATOM    689  CA  PHE    43      34.698  34.083  18.180  1.00  0.00           C
ATOM    690  HA  PHE    43      33.937  34.286  17.428  1.00  0.00           H
ATOM    691  CB  PHE    43      35.906  34.956  17.793  1.00  0.00           C
ATOM    692  HB2 PHE    43      36.755  34.730  18.439  1.00  0.00           H
ATOM    693  HB3 PHE    43      36.237  34.626  16.808  1.00  0.00           H
ATOM    694  CG  PHE    43      35.695  36.391  18.032  1.00  0.00           C
ATOM    695  CD1 PHE    43      34.995  37.177  17.110  1.00  0.00           C
ATOM    696  HD1 PHE    43      34.509  36.676  16.286  1.00  0.00           H
ATOM    697  CE1 PHE    43      34.793  38.591  17.284  1.00  0.00           C
ATOM    698  HE1 PHE    43      34.275  39.224  16.578  1.00  0.00           H
ATOM    699  CZ  PHE    43      35.243  39.105  18.523  1.00  0.00           C
ATOM    700  HZ  PHE    43      35.047  40.102  18.888  1.00  0.00           H
ATOM    701  CE2 PHE    43      35.896  38.370  19.516  1.00  0.00           C
ATOM    702  HE2 PHE    43      36.204  38.846  20.435  1.00  0.00           H
ATOM    703  CD2 PHE    43      36.164  36.965  19.245  1.00  0.00           C
ATOM    704  HD2 PHE    43      36.820  36.378  19.870  1.00  0.00           H
ATOM    705  C   PHE    43      35.121  32.639  17.865  1.00  0.00           C
ATOM    706  O   PHE    43      35.386  31.902  18.814  1.00  0.00           O
ATOM    707  N   GLU    44      35.306  32.182  16.622  1.00  0.00           N
ATOM    708  H   GLU    44      34.939  32.802  15.914  1.00  0.00           H
ATOM    709  CA  GLU    44      35.618  30.848  16.152  1.00  0.00           C
ATOM    710  HA  GLU    44      34.832  30.219  16.570  1.00  0.00           H
ATOM    711  CB  GLU    44      35.462  30.805  14.565  1.00  0.00           C
ATOM    712  HB2 GLU    44      34.627  31.425  14.237  1.00  0.00           H
ATOM    713  HB3 GLU    44      36.410  31.062  14.094  1.00  0.00           H
ATOM    714  CG  GLU    44      35.097  29.411  14.150  1.00  0.00           C
ATOM    715  HG2 GLU    44      35.249  29.367  13.072  1.00  0.00           H
ATOM    716  HG3 GLU    44      35.762  28.686  14.617  1.00  0.00           H
ATOM    717  CD  GLU    44      33.683  28.966  14.481  1.00  0.00           C
ATOM    718  OE1 GLU    44      33.085  29.435  15.484  1.00  0.00           O
ATOM    719  OE2 GLU    44      33.071  28.210  13.696  1.00  0.00           O
ATOM    720  C   GLU    44      37.000  30.294  16.610  1.00  0.00           C
ATOM    721  O   GLU    44      37.215  29.103  16.793  1.00  0.00           O
ATOM    722  N   ASP    45      38.008  31.140  16.622  1.00  0.00           N
ATOM    723  H   ASP    45      37.795  32.103  16.406  1.00  0.00           H
ATOM    724  CA  ASP    45      39.447  30.663  16.755  1.00  0.00           C
ATOM    725
 HA  ASP    45      39.544  29.931  15.954  1.00  0.00           H
ATOM    726  CB  ASP    45      40.412  31.764  16.477  1.00  0.00           C
ATOM    727  HB2 ASP    45      40.326  32.014  15.419  1.00  0.00           H
ATOM    728  HB3 ASP    45      40.203  32.671  17.042  1.00  0.00           H
ATOM    729  CG  ASP    45      41.877  31.377  16.723  1.00  0.00           C
ATOM    730  OD1 ASP    45      42.276  30.219  16.429  1.00  0.00           O
ATOM    731  OD2 ASP    45      42.658  32.134  17.326  1.00  0.00           O
ATOM    732  C   ASP    45      39.699  29.926  18.095  1.00  0.00           C
ATOM    733  O   ASP    45      39.763  30.561  19.151  1.00  0.00           O
ATOM    734  N   ASP    46      39.991  28.576  18.106  1.00  0.00           N
ATOM    735  H   ASP    46      40.129  28.153  17.199  1.00  0.00           H
ATOM    736  CA  ASP    46      40.032  27.577  19.236  1.00  0.00           C
ATOM    737  HA  ASP    46      39.906  26.660  18.661  1.00  0.00           H
ATOM    738  CB  ASP    46      41.338  27.331  19.957  1.00  0.00           C
ATOM    739  HB2 ASP    46      41.537  28.206  20.576  1.00  0.00           H
ATOM    740  HB3 ASP    46      41.314  26.467  20.620  1.00  0.00           H
ATOM    741  CG  ASP    46      42.502  27.203  19.065  1.00  0.00           C
ATOM    742  OD1 ASP    46      42.418  26.489  18.018  1.00  0.00           O
ATOM    743  OD2 ASP    46      43.455  27.976  19.222  1.00  0.00           O
ATOM    744  C   ASP    46      38.778  27.614  20.165  1.00  0.00           C
```

ATOM	745	O	ASP	46	38.822	26.979	21.210	1.00	0.00	O
ATOM	746	N	GLY	47	37.680	28.163	19.742	1.00	0.00	N
ATOM	747	H	GLY	47	37.629	28.633	18.849	1.00	0.00	H
ATOM	748	CA	GLY	47	36.354	28.090	20.408	1.00	0.00	C
ATOM	749	HA2	GLY	47	35.555	28.226	19.679	1.00	0.00	H
ATOM	750	HA3	GLY	47	36.436	27.102	20.861	1.00	0.00	H
ATOM	751	C	GLY	47	36.175	29.029	21.607	1.00	0.00	C
ATOM	752	O	GLY	47	35.049	29.496	21.829	1.00	0.00	O
ATOM	753	N	TYR	48	37.289	29.172	22.358	1.00	0.00	N
ATOM	754	H	TYR	48	38.154	28.754	22.047	1.00	0.00	H
ATOM	755	CA	TYR	48	37.457	29.818	23.632	1.00	0.00	C
ATOM	756	HA	TYR	48	36.733	30.630	23.705	1.00	0.00	H
ATOM	757	CB	TYR	48	37.159	28.872	24.797	1.00	0.00	C
ATOM	758	HB2	TYR	48	37.969	28.146	24.720	1.00	0.00	H
ATOM	759	HB3	TYR	48	37.283	29.460	25.706	1.00	0.00	H
ATOM	760	CG	TYR	48	35.878	28.059	24.672	1.00	0.00	C
ATOM	761	CD1	TYR	48	34.654	28.542	25.249	1.00	0.00	C
ATOM	762	HD1	TYR	48	34.679	29.555	25.623	1.00	0.00	H
ATOM	763	CE1	TYR	48	33.510	27.700	25.174	1.00	0.00	C
ATOM	764	HE1	TYR	48	32.567	28.116	25.497	1.00	0.00	H
ATOM	765	CZ	TYR	48	33.584	26.450	24.654	1.00	0.00	C
ATOM	766	OH	TYR	48	32.569	25.568	24.855	1.00	0.00	O
ATOM	767	HH	TYR	48	31.689	25.943	24.935	1.00	0.00	H
ATOM	768	CE2	TYR	48	34.812	25.941	24.174	1.00	0.00	C
ATOM	769	HE2	TYR	48	34.876	24.879	23.990	1.00	0.00	H
ATOM	770	CD2	TYR	48	35.915	26.743	24.052	1.00	0.00	C
ATOM	771	HD2	TYR	48	36.848	26.457	23.591	1.00	0.00	H
ATOM	772	C	TYR	48	38.958	30.213	23.660	1.00	0.00	C
ATOM	773	O	TYR	48	39.884	29.419	23.347	1.00	0.00	O
ATOM	774	N	PHE	49	39.181	31.482	24.103	1.00	0.00	N
ATOM	775	H	PHE	49	38.424	32.099	24.357	1.00	0.00	H
ATOM	776	CA	PHE	49	40.555	32.034	24.279	1.00	0.00	C
ATOM	777	HA	PHE	49	41.146	31.753	23.406	1.00	0.00	H
ATOM	778	CB	PHE	49	40.351	33.601	24.258	1.00	0.00	C
ATOM	779	HB2	PHE	49	41.265	34.191	24.178	1.00	0.00	H
ATOM	780	HB3	PHE	49	39.903	33.954	23.330	1.00	0.00	H
ATOM	781	CG	PHE	49	39.514	34.236	25.379	1.00	0.00	C
ATOM	782	CD1	PHE	49	38.307	34.874	24.945	1.00	0.00	C
ATOM	783	HD1	PHE	49	37.858	34.576	24.008	1.00	0.00	H
ATOM	784	CE1	PHE	49	37.496	35.568	25.900	1.00	0.00	C
ATOM	785	HE1	PHE	49	36.518	35.895	25.576	1.00	0.00	H
ATOM	786	CZ	PHE	49	38.024	35.853	27.109	1.00	0.00	C
ATOM	787	HZ	PHE	49	37.343	36.397	27.748	1.00	0.00	H
ATOM	788	CE2	PHE	49	39.260	35.306	27.513	1.00	0.00	C
ATOM	789	HE2	PHE	49	39.519	35.502	28.543	1.00	0.00	H
ATOM	790	CD2	PHE	49	40.038	34.474	26.667	1.00	0.00	C
ATOM	791	HD2	PHE	49	40.991	34.037	26.928	1.00	0.00	H
ATOM	792	C	PHE	49	41.274	31.665	25.581	1.00	0.00	C
ATOM	793	O	PHE	49	42.423	32.046	25.611	1.00	0.00	O
ATOM	794	N	CYX	50	40.604	31.063	26.584	1.00	0.00	N
ATOM	795	H	CYX	50	39.631	30.866	26.395	1.00	0.00	H
ATOM	796	CA	CYX	50	41.230	30.543	27.773	1.00	0.00	C
ATOM	797	HA	CYX	50	42.250	30.230	27.551	1.00	0.00	H
ATOM	798	CB	CYX	50	41.331	31.685	28.805	1.00	0.00	C
ATOM	799	HB2	CYX	50	40.618	32.468	28.544	1.00	0.00	H
ATOM	800	HB3	CYX	50	41.171	31.277	29.804	1.00	0.00	H
ATOM	801	SG	CYX	50	42.914	32.584	28.943	1.00	0.00	S
ATOM	802	C	CYX	50	40.461	29.350	28.465	1.00	0.00	C
ATOM	803	O	CYX	50	39.264	29.332	28.462	1.00	0.00	O
ATOM	804	N	THR	51	41.226	28.416	29.049	1.00	0.00	N
ATOM	805	H	THR	51	42.206	28.660	29.056	1.00	0.00	H
ATOM	806	CA	THR	51	40.709	27.422	29.928	1.00	0.00	C
ATOM	807	HA	THR	51	39.663	27.242	29.678	1.00	0.00	H
ATOM	808	CB	THR	51	41.467	26.086	29.784	1.00	0.00	C

ATOM	809	HB	THR	51	42.526	26.141	30.034	1.00	0.00	H
ATOM	810	CG2	THR	51	40.878	25.148	30.806	1.00	0.00	C
ATOM	811	HG21	THR	51	41.500	24.252	30.800	1.00	0.00	H
ATOM	812	HG22	THR	51	40.870	25.452	31.852	1.00	0.00	H
ATOM	813	HG23	THR	51	39.892	24.889	30.422	1.00	0.00	H
ATOM	814	OG1	THR	51	41.287	25.440	28.543	1.00	0.00	O
ATOM	815	HG1	THR	51	42.027	25.738	28.010	1.00	0.00	H
ATOM	816	C	THR	51	40.956	28.016	31.356	1.00	0.00	C
ATOM	817	O	THR	51	42.117	28.226	31.622	1.00	0.00	O
ATOM	818	N	ILE	52	39.890	28.405	32.060	1.00	0.00	N
ATOM	819	H	ILE	52	39.025	28.320	31.545	1.00	0.00	H
ATOM	820	CA	ILE	52	39.896	29.007	33.433	1.00	0.00	C
ATOM	821	HA	ILE	52	40.838	29.529	33.602	1.00	0.00	H
ATOM	822	CB	ILE	52	38.637	29.865	33.760	1.00	0.00	C
ATOM	823	HB	ILE	52	37.688	29.333	33.709	1.00	0.00	H
ATOM	824	CG2	ILE	52	38.484	30.546	35.166	1.00	0.00	C
ATOM	825	HG21	ILE	52	38.590	29.800	35.954	1.00	0.00	H
ATOM	826	HG22	ILE	52	39.337	31.208	35.309	1.00	0.00	H
ATOM	827	HG23	ILE	52	37.474	30.950	35.226	1.00	0.00	H
ATOM	828	CG1	ILE	52	38.602	30.989	32.623	1.00	0.00	C
ATOM	829	HG12	ILE	52	39.602	31.424	32.642	1.00	0.00	H
ATOM	830	HG13	ILE	52	38.381	30.568	31.642	1.00	0.00	H
ATOM	831	CD1	ILE	52	37.667	32.199	32.861	1.00	0.00	C
ATOM	832	HD11	ILE	52	37.938	32.678	33.802	1.00	0.00	H
ATOM	833	HD12	ILE	52	37.900	32.911	32.069	1.00	0.00	H
ATOM	834	HD13	ILE	52	36.605	31.961	32.802	1.00	0.00	H
ATOM	835	C	ILE	52	39.822	27.870	34.441	1.00	0.00	C
ATOM	836	O	ILE	52	39.061	26.924	34.173	1.00	0.00	O
ATOM	837	N	GLU	53	40.329	28.031	35.598	1.00	0.00	N
ATOM	838	H	GLU	53	40.969	28.812	35.618	1.00	0.00	H
ATOM	839	CA	GLU	53	40.015	27.080	36.740	1.00	0.00	C
ATOM	840	HA	GLU	53	39.101	26.515	36.562	1.00	0.00	H
ATOM	841	CB	GLU	53	41.139	25.935	36.882	1.00	0.00	C
ATOM	842	HB2	GLU	53	41.224	25.335	35.975	1.00	0.00	H
ATOM	843	HB3	GLU	53	42.093	26.352	37.204	1.00	0.00	H
ATOM	844	CG	GLU	53	40.814	24.957	37.954	1.00	0.00	C
ATOM	845	HG2	GLU	53	40.628	25.487	38.887	1.00	0.00	H
ATOM	846	HG3	GLU	53	39.819	24.572	37.727	1.00	0.00	H
ATOM	847	CD	GLU	53	41.735	23.748	38.081	1.00	0.00	C
ATOM	848	OE1	GLU	53	42.423	23.644	39.050	1.00	0.00	O
ATOM	849	OE2	GLU	53	41.811	22.866	37.193	1.00	0.00	O
ATOM	850	C	GLU	53	39.762					
27.885	38.067	1.00	0.00		C					
ATOM	851	O	GLU	53	40.618	28.686	38.442	1.00	0.00	O
ATOM	852	N	VAL	54	38.660	27.672	38.791	1.00	0.00	N
ATOM	853	H	VAL	54	37.967	27.109	38.319	1.00	0.00	H
ATOM	854	CA	VAL	54	38.368	28.262	40.103	1.00	0.00	C
ATOM	855	HA	VAL	54	38.363	29.351	40.067	1.00	0.00	H
ATOM	856	CB	VAL	54	36.847	28.005	40.372	1.00	0.00	C
ATOM	857	HB	VAL	54	36.705	26.976	40.045	1.00	0.00	H
ATOM	858	CG1	VAL	54	36.330	28.186	41.792	1.00	0.00	C
ATOM	859	HG11	VAL	54	36.025	29.223	41.930	1.00	0.00	H
ATOM	860	HG12	VAL	54	35.418	27.604	41.929	1.00	0.00	H
ATOM	861	HG13	VAL	54	37.070	27.988	42.567	1.00	0.00	H
ATOM	862	CG2	VAL	54	36.010	28.855	39.395	1.00	0.00	C
ATOM	863	HG21	VAL	54	34.983	28.491	39.449	1.00	0.00	H
ATOM	864	HG22	VAL	54	35.963	29.909	39.665	1.00	0.00	H
ATOM	865	HG23	VAL	54	36.267	28.680	38.350	1.00	0.00	H
ATOM	866	C	VAL	54	39.339	27.807	41.205	1.00	0.00	C
ATOM	867	O	VAL	54	39.380	26.659	41.493	1.00	0.00	O
ATOM	868	N	THR	55	40.000	28.755	41.875	1.00	0.00	N
ATOM	869	H	THR	55	39.773	29.716	41.663	1.00	0.00	H
ATOM	870	CA	THR	55	41.122	28.519	42.871	1.00	0.00	C
ATOM	871	HA	THR	55	41.507	27.515	42.696	1.00	0.00	H

ATOM	872	CB	THR	55	42.328	29.448	42.535	1.00	0.00	C
ATOM	873	HB	THR	55	43.191	29.144	43.128	1.00	0.00	H
ATOM	874	CG2	THR	55	42.799	29.295	41.053	1.00	0.00	C
ATOM	875	HG21	THR	55	43.745	29.784	40.820	1.00	0.00	H
ATOM	876	HG22	THR	55	42.875	28.239	40.795	1.00	0.00	H
ATOM	877	HG23	THR	55	42.091	29.748	40.359	1.00	0.00	H
ATOM	878	OG1	THR	55	42.127	30.841	42.867	1.00	0.00	O
ATOM	879	HG1	THR	55	42.192	30.994	43.812	1.00	0.00	H
ATOM	880	C	THR	55	40.664	28.559	44.333	1.00	0.00	C
ATOM	881	O	THR	55	41.324	27.806	45.112	1.00	0.00	O
ATOM	882	N	ALA	56	39.644	29.300	44.626	1.00	0.00	N
ATOM	883	H	ALA	56	39.184	29.896	43.953	1.00	0.00	H
ATOM	884	CA	ALA	56	38.932	29.089	45.887	1.00	0.00	C
ATOM	885	HA	ALA	56	38.888	28.047	46.200	1.00	0.00	H
ATOM	886	CB	ALA	56	39.550	29.927	47.040	1.00	0.00	C
ATOM	887	HB1	ALA	56	40.609	29.691	47.135	1.00	0.00	H
ATOM	888	HB2	ALA	56	39.504	31.006	46.896	1.00	0.00	H
ATOM	889	HB3	ALA	56	39.094	29.745	48.014	1.00	0.00	H
ATOM	890	C	ALA	56	37.446	29.628	45.807	1.00	0.00	C
ATOM	891	O	ALA	56	37.027	30.446	45.062	1.00	0.00	O
ATOM	892	N	THR	57	36.731	29.036	46.710	1.00	0.00	N
ATOM	893	H	THR	57	37.184	28.299	47.232	1.00	0.00	H
ATOM	894	CA	THR	57	35.331	29.453	46.953	1.00	0.00	C
ATOM	895	HA	THR	57	35.225	30.506	46.690	1.00	0.00	H
ATOM	896	CB	THR	57	34.338	28.646	46.149	1.00	0.00	C
ATOM	897	HB	THR	57	33.354	29.008	46.447	1.00	0.00	H
ATOM	898	CG2	THR	57	34.293	28.781	44.624	1.00	0.00	C
ATOM	899	HG21	THR	57	33.359	28.364	44.251	1.00	0.00	H
ATOM	900	HG22	THR	57	34.342	29.821	44.301	1.00	0.00	H
ATOM	901	HG23	THR	57	35.011	28.190	44.055	1.00	0.00	H
ATOM	902	OG1	THR	57	34.372	27.239	46.482	1.00	0.00	O
ATOM	903	HG1	THR	57	33.507	27.118	46.879	1.00	0.00	H
ATOM	904	C	THR	57	34.980	29.347	48.423	1.00	0.00	C
ATOM	905	O	THR	57	35.531	28.478	49.137	1.00	0.00	O
ATOM	906	N	SER	58	34.155	30.236	48.963	1.00	0.00	N
ATOM	907	H	SER	58	33.912	30.995	48.344	1.00	0.00	H
ATOM	908	CA	SER	58	33.785	30.400	50.371	1.00	0.00	C
ATOM	909	HA	SER	58	33.782	29.488	50.969	1.00	0.00	H
ATOM	910	CB	SER	58	34.741	31.471	50.924	1.00	0.00	C
ATOM	911	HB2	SER	58	34.401	31.690	51.936	1.00	0.00	H
ATOM	912	HB3	SER	58	35.748	31.087	51.089	1.00	0.00	H
ATOM	913	OG	SER	58	34.794	32.633	50.139	1.00	0.00	O
ATOM	914	HG	SER	58	35.406	32.540	49.405	1.00	0.00	H
ATOM	915	C	SER	58	32.274	30.858	50.434	1.00	0.00	C
ATOM	916	O	SER	58	31.721	31.523	49.515	1.00	0.00	O
ATOM	917	N	THR	59	31.593	30.454	51.481	1.00	0.00	N
ATOM	918	H	THR	59	32.053	29.934	52.214	1.00	0.00	H
ATOM	919	CA	THR	59	30.261	31.103	51.830	1.00	0.00	C
ATOM	920	HA	THR	59	29.766	31.543	50.965	1.00	0.00	H
ATOM	921	CB	THR	59	29.213	30.070	52.300	1.00	0.00	C
ATOM	922	HB	THR	59	29.144	29.259	51.575	1.00	0.00	H
ATOM	923	CG2	THR	59	29.426	29.489	53.674	1.00	0.00	C
ATOM	924	HG21	THR	59	30.417	29.057	53.810	1.00	0.00	H
ATOM	925	HG22	THR	59	29.286	30.304	54.386	1.00	0.00	H
ATOM	926	HG23	THR	59	28.689	28.706	53.851	1.00	0.00	H
ATOM	927	OG1	THR	59	27.994	30.808	52.324	1.00	0.00	O
ATOM	928	HG1	THR	59	27.268	30.180	52.299	1.00	0.00	H
ATOM	929	C	THR	59	30.496	32.236	52.878	1.00	0.00	C
ATOM	930	O	THR	59	31.498	32.214	53.650	1.00	0.00	O
ATOM	931	N	VAL	60	29.654	33.305	52.828	1.00	0.00	N
ATOM	932	H	VAL	60	28.979	33.236	52.080	1.00	0.00	H
ATOM	933	CA	VAL	60	30.040	34.734	53.146	1.00	0.00	C
ATOM	934	HA	VAL	60	30.800	34.666	53.926	1.00	0.00	H
ATOM	935	CB	VAL	60	30.633	35.445	51.910	1.00	0.00	C

ATOM	936	HB	VAL	60	30.009	35.184	51.055	1.00	0.00	H
ATOM	937	CG1	VAL	60	30.574	36.956	51.999	1.00	0.00	C
ATOM	938	HG11	VAL	60	31.125	37.216	52.903	1.00	0.00	H
ATOM	939	HG12	VAL	60	30.929	37.380	51.060	1.00	0.00	H
ATOM	940	HG13	VAL	60	29.527	37.245	52.095	1.00	0.00	H
ATOM	941	CG2	VAL	60	32.051	34.954	51.585	1.00	0.00	C
ATOM	942	HG21	VAL	60	32.462	35.614	50.821	1.00	0.00	H
ATOM	943	HG22	VAL	60	32.723	35.121	52.426	1.00	0.00	H
ATOM	944	HG23	VAL	60	32.013	33.927	51.220	1.00	0.00	H
ATOM	945	C	VAL	60	28.824	35.480	53.759	1.00	0.00	C
ATOM	946	O	VAL	60	27.850	35.674	53.081	1.00	0.00	O
ATOM	947	N	THR	61	28.978	35.985	55.008	1.00	0.00	N
ATOM	948	H	THR	61	29.597	35.542	55.671	1.00	0.00	H
ATOM	949	CA	THR	61	28.226	37.167	55.449	1.00	0.00	C
ATOM	950	HA	THR	61	27.339	37.306	54.830	1.00	0.00	H
ATOM	951	CB	THR	61	27.748	36.982	56.850	1.00	0.00	C
ATOM	952	HB	THR	61	27.334	37.923	57.212	1.00	0.00	H
ATOM	953	CG2	THR	61	26.675	35.892	56.993	1.00	0.00	C
ATOM	954	HG21	THR	61	26.280	35.650	57.980	1.00	0.00	H
ATOM	955	HG22	THR	61	25.832	36.235	56.395	1.00	0.00	H
ATOM	956	HG23	THR	61	27.115	34.993	56.561	1.00	0.00	H
ATOM	957	OG1	THR	61	28.849	36.615	57.624	1.00	0.00	O
ATOM	958	HG1	THR	61	29.037	37.366	58.192	1.00	0.00	H
ATOM	959	C	THR	61	28.985	38.455	55.371	1.00	0.00	C
ATOM	960	O	THR	61	30.163	38.545	55.570	1.00	0.00	O
ATOM	961	N	LEU	62	28.251	39.585	55.276	1.00	0.00	N
ATOM	962	H	LEU	62	27.243	39.539	55.234	1.00	0.00	H
ATOM	963	CA	LEU	62	28.929	40.876	54.962	1.00	0.00	C
ATOM	964	HA	LEU	62	29.523	40.669	54.072	1.00	0.00	H
ATOM	965	CB	LEU	62	27.886	42.018	54.489	1.00	0.00	C
ATOM	966	HB2	LEU	62	27.191	42.298	55.280	1.00	0.00	H
ATOM	967	HB3	LEU	62	28.559	42.855	54.300	1.00	0.00	H
ATOM	968	CG	LEU	62	27.048	41.784	53.147	1.00	0.00	C
ATOM	969	HG	LEU	62	26.537	40.822	53.188	1.00	0.00	H
ATOM	970	CD1	LEU	62	26.029	42.969	52.980	1.00	0.00	C
ATOM	971	HD11	LEU	62	26.587	43.868	52.717	1.00	0.00	H
ATOM	972	HD12	LEU	62	25.378	42.641	52.169	1.00	0.00	H
ATOM	973	HD13	LEU	62	25.461	43.147	53.894	1.00	0.00	H
ATOM	974	CD2	LEU	62	27.901	41.730	51.869	1.00	0.00	C
ATOM	975	HD21	LEU	62	27.281	41.622	50.979	1.00	0.00	H
ATOM	976	HD22	LEU	62	28.428	42.662	51.665	1.00	0.00	H
ATOM	977	HD23	LEU	62	28.545	40.854	51.937	1.00	0.00	H
ATOM	978	C	LEU	62	29.825	41.405	56.153	1.00	0.00	C
ATOM	979	O	LEU	62	30.702	42.203	55.984	1.00	0.00	O
ATOM	980	N	ASP	63	29.641	40.946	57.424	1.00	0.00	N
ATOM	981	H	ASP	63	28.881	40.322	57.654	1.00	0.00	H
ATOM	982	CA	ASP	63	30.579	41.101	58.512	1.00	0.00	C
ATOM	983	HA	ASP	63	30.765	42.163	58.672	1.00	0.00	H
ATOM	984	CB	ASP	63	30.019	40.749	59.888	1.00	0.00	C
ATOM	985	HB2	ASP	63	30.768	41.102	60.598	1.00	0.00	H
ATOM	986	HB3	ASP	63	29.136	41.341	60.130	1.00	0.00	H
ATOM	987	CG	ASP	63	29.693	39.264	60.008	1.00	0.00	C
ATOM	988	OD1	ASP	63	29.082	38.705	59.086	1.00	0.00	O
ATOM	989	OD2	ASP	63	30.095	38.702	61.066	1.00	0.00	O
ATOM	990	C	ASP	63	32.007	40.373	58.234	1.00	0.00	C
ATOM	991	O	ASP	63	33.084	40.801	58.632	1.00	0.00	O
ATOM	992	N	THR	64	31.985	39.265	57.450	1.00	0.00	N
ATOM	993	H	THR	64	31.089	39.036	57.044	1.00	0.00	H
ATOM	994	CA	THR	64	33.172	38.537	57.047	1.00	0.00	C
ATOM	995	HA	THR	64	33.981	38.575	57.776	1.00	0.00	H
ATOM	996	CB	THR	64	32.974	37.038	56.795	1.00	0.00	C
ATOM	997	HB	THR	64	33.974	36.607	56.764	1.00	0.00	H
ATOM	998	CG2	THR	64	32.265	36.265	57.889	1.00	0.00	C

ATOM	999	HG21	THR	64	31.491	36.880	58.348	1.00	0.00	H
ATOM	1000	HG22	THR	64	31.786	35.411	57.410	1.00	0.00	H
ATOM	1001	HG23	THR	64	32.874	35.919	58.725	1.00	0.00	H
ATOM	1002	OG1	THR	64	32.236	36.740	55.611	1.00	0.00	O
ATOM	1003	HG1	THR	64	31.490	37.342	55.651	1.00	0.00	H
ATOM	1004	C	THR	64	33.782	39.141	55.730	1.00	0.00	C
ATOM	1005	O	THR	64	34.807	38.680	55.290	1.00	0.00	O
ATOM	1006	N	LEU	65	33.154	40.080	55.060	1.00	0.00	N
ATOM	1007	H	LEU	65	32.308	40.516	55.399	1.00	0.00	H
ATOM	1008	CA	LEU	65	33.547	40.493	53.729	1.00	0.00	C
ATOM	1009	HA	LEU	65	33.625	39.600	53.109	1.00	0.00	H
ATOM	1010	CB	LEU	65	32.456	41.419	53.164	1.00	0.00	C
ATOM	1011	HB2	LEU	65	31.488	40.918	53.215	1.00	0.00	H
ATOM	1012	HB3	LEU	65	32.382	42.317	53.778	1.00	0.00	H
ATOM	1013	CG	LEU	65	32.586	41.774	51.664	1.00	0.00	C
ATOM	1014	HG	LEU	65	33.521	42.280	51.424	1.00	0.00	H
ATOM	1015	CD1	LEU	65	32.508	40.551	50.743	1.00	0.00	C
ATOM	1016	HD11	LEU	65	33.254	39.784	50.951	1.00	0.00	H
ATOM	1017	HD12	LEU	65	31.558	40.084	51.002	1.00	0.00	H
ATOM	1018	HD13	LEU	65	32.607	40.900	49.715	1.00	0.00	H
ATOM	1019	CD2	LEU	65	31.425	42.661	51.331	1.00	0.00	C
ATOM	1020	HD21	LEU	65	30.406	42.358	51.570	1.00	0.00	H
ATOM	1021	HD22	LEU	65	31.571	43.621	51.828	1.00	0.00	H
ATOM	1022	HD23	LEU	65	31.376	42.847	50.258	1.00	0.00	H
ATOM	1023	C	LEU	65	34.956	41.108	53.685	1.00	0.00	C
ATOM	1024	O	LEU	65	35.414	41.713	54.617	1.00	0.00	O
ATOM	1025	N	THR	66	35.725	41.104	52.588	1.00	0.00	N
ATOM	1026	H	THR	66	35.279	40.844	51.720	1.00	0.00	H
ATOM	1027	CA	THR	66	37.138	41.542	52.464	1.00	0.00	C
ATOM	1028	HA	THR	66	37.468	42.181	53.284	1.00	0.00	H
ATOM	1029	CB	THR	66	37.947	40.260	52.337	1.00	0.00	C
ATOM	1030	HB	THR	66	37.521	39.623	51.560	1.00	0.00	H
ATOM	1031	CG2	THR	66	39.384	40.335	51.784	1.00	0.00	C
ATOM	1032	HG21	THR	66	39.926	41.024	52.432	1.00	0.00	H
ATOM	1033	HG22	THR	66	39.978	39.437	51.957	1.00	0.00	H
ATOM	1034	HG23	THR	66	39.445	40.619	50.735	1.00	0.00	H
ATOM	1035	OG1	THR	66	37.903	39.489	53.472	1.00	0.00	O
ATOM	1036	HG1	THR	66	37.061	39.028	53.511	1.00	0.00	H
ATOM	1037	C	THR	66	37.302	42.306	51.218	1.00	0.00	C
ATOM	1038	O	THR	66	36.436	42.214	50.329	1.00	0.00	O
ATOM	1039	N	GLU	67	38.271	43.219	51.080	1.00	0.00	N
ATOM	1040	H	GLU	67	38.848	43.425	51.885	1.00	0.00	H
ATOM	1041	CA	GLU	67	38.354	44.073	49.980	1.00	0.00	C
ATOM	1042	HA	GLU	67	37.316	44.403	49.919	1.00	0.00	H
ATOM	1043	CB	GLU	67	39.186	45.320	50.241	1.00	0.00	C
ATOM	1044	HB2	GLU	67	38.859	46.010	49.461	1.00	0.00	H
ATOM	1045	HB3	GLU	67	38.825	45.714	51.191	1.00	0.00	H
ATOM	1046	CG	GLU	67	40.681	45.146	50.241	1.00	0.00	C
ATOM	1047	HG2	GLU	67	40.975	44.986	49.204	1.00	0.00	H
ATOM	1048	HG3	GLU	67	41.177	46.028	50.643	1.00	0.00	H
ATOM	1049	CD	GLU	67	41.205	44.051	51.175	1.00	0.00	C
ATOM	1050	OE1	GLU	67	40.688	43.863	52.338	1.00	0.00	O
ATOM	1051	OE2	GLU	67	42.243	43.428	50.757	1.00	0.00	O
ATOM	1052	C	GLU	67	38.671	43.442	48.586	1.00	0.00	C
ATOM	1053	O	GLU	67	38.702	44.226	47.618	1.00	0.00	O
ATOM	1054	N	LYS	68	38.940	42.137	48.465	1.00	0.00	N
ATOM	1055	H	LYS	68	38.896	41.567	49.297	1.00	0.00	H
ATOM	1056	CA	LYS	68	39.319	41.498	47.181	1.00	0.00	C
ATOM	1057	HA	LYS	68	40.316	41.856	46.927	1.00	0.00	H
ATOM	1058	CB	LYS	68	39.387	40.047	47.480	1.00	0.00	C
ATOM	1059	HB2	LYS	68	38.435	39.818	47.958	1.00	0.00	H
ATOM	1060	HB3	LYS	68	39.575	39.480	46.568	1.00	0.00	H
ATOM	1061	CG	LYS	68	40.694	39.726	48.278	1.00	0.00	C
ATOM	1062	HG2	LYS	68	41.551	40.017	47.671	1.00	0.00	H

```
ATOM   1063  HG3 LYS    68      40.655  40.451  49.090  1.00  0.00           H
ATOM   1064  CD  LYS    68      40.818  38.332  48.870  1.00  0.00           C
ATOM   1065  HD2 LYS    68      41.725  38.346  49.474  1.00  0.00           H
ATOM   1066  HD3 LYS    68      39.966  38.194  49.538  1.00  0.00           H
ATOM   1067  CE  LYS    68      40.798  37.123  47.823  1.00  0.00           C
ATOM   1068  HE2 LYS    68      40.988  36.204  48.376  1.00  0.00           H
ATOM   1069  HE3 LYS    68      39.826  37.066  47.334  1.00  0.00           H
ATOM   1070  NZ  LYS    68      41.863  37.285  46.813  1.00  0.00           N
ATOM   1071  HZ1 LYS    68      41.653  37.879  46.022  1.00  0.00           H
ATOM   1072  HZ2 LYS    68      42.728  37.474  47.299  1.00  0.00           H
ATOM   1073  HZ3 LYS    68      41.981  36.405  46.332  1.00  0.00           H
ATOM   1074  C   LYS    68      38.524  41.950  45.888  1.00  0.00           C
ATOM   1075  O   LYS    68      39.152  42.464  45.024  1.00  0.00           O
ATOM   1076  N   HIE    69      37.267  41.800  45.874  1.00  0.00           N
ATOM   1077  H   HIE    69      36.824  41.310  46.638  1.00  0.00           H
ATOM   1078  CA  HIE    69      36.354  42.336  44.851  1.00  0.00           C
ATOM   1079  HA  HIE    69      36.798  42.019  43.907  1.00  0.00           H
ATOM   1080  CB  HIE    69      34.953  41.706  44.947  1.00  0.00           C
ATOM   1081  HB2 HIE    69      35.009  40.659  45.244  1.00  0.00           H
ATOM   1082  HB3 HIE    69      34.523  42.203  45.817  1.00  0.00           H
ATOM   1083  CG  HIE    69      34.089  41.748  43.693  1.00  0.00           C
ATOM   1084  ND1 HIE    69      34.356  41.181  42.453  1.00  0.00           N
ATOM   1085  CE1 HIE    69      33.297  41.483  41.731  1.00  0.00           C
ATOM   1086  HE1 HIE    69      33.163  41.333  40.670  1.00  0.00           H
ATOM   1087  NE2 HIE    69      32.304  42.030  42.453  1.00  0.00           N
ATOM   1088  HE2 HIE    69      31.374  42.309  42.174  1.00  0.00           H
ATOM   1089  CD2 HIE    69      32.834  42.288  43.695  1.00  0.00           C
ATOM   1090  HD2 HIE    69      32.473  42.846  44.547  1.00  0.00           H
ATOM   1091  C   HIE    69      36.290  43.833  44.854  1.00  0.00           C
ATOM   1092  O   HIE    69      35.942  44.302  43.766  1.00  0.00           O
ATOM   1093  N   ALA    70      36.513  44.465  46.009  1.00  0.00           N
ATOM   1094  H   ALA    70      36.758  43.969  46.854  1.00  0.00           H
ATOM   1095  CA  ALA    70      36.396  45.931  46.039  1.00  0.00           C
ATOM   1096  HA  ALA    70      35.471  46.092  45.485  1.00  0.00           H
ATOM   1097  CB  ALA    70      36.243  46.454  47.462  1.00  0.00           C
ATOM   1098  HB1 ALA    70      35.938  47.501  47.478  1.00  0.00           H
ATOM   1099  HB2 ALA    70      35.566  45.910  48.120  1.00  0.00           H
ATOM   1100  HB3 ALA    70      37.239  46.506  47.901  1.00  0.00           H
ATOM   1101
 C   ALA    70      37.592  46.718  45.283  1.00  0.00           C
ATOM   1102  O   ALA    70      37.419  47.783  44.693  1.00  0.00           O
ATOM   1103  N   GLU    71      38.849  46.187  45.292  1.00  0.00           N
ATOM   1104  H   GLU    71      39.079  45.349  45.806  1.00  0.00           H
ATOM   1105  CA  GLU    71      39.897  46.691  44.361  1.00  0.00           C
ATOM   1106  HA  GLU    71      40.116  47.759  44.370  1.00  0.00           H
ATOM   1107  CB  GLU    71      41.229  46.039  44.650  1.00  0.00           C
ATOM   1108  HB2 GLU    71      41.118  44.955  44.653  1.00  0.00           H
ATOM   1109  HB3 GLU    71      41.885  46.213  43.796  1.00  0.00           H
ATOM   1110  CG  GLU    71      41.911  46.502  45.991  1.00  0.00           C
ATOM   1111  HG2 GLU    71      41.240  46.347  46.836  1.00  0.00           H
ATOM   1112  HG3 GLU    71      42.796  45.874  46.092  1.00  0.00           H
ATOM   1113  CD  GLU    71      42.306  47.998  46.123  1.00  0.00           C
ATOM   1114  OE1 GLU    71      43.356  48.389  45.614  1.00  0.00           O
ATOM   1115  OE2 GLU    71      41.585  48.865  46.668  1.00  0.00           O
ATOM   1116  C   GLU    71      39.613  46.475  42.859  1.00  0.00           C
ATOM   1117  O   GLU    71      39.751  47.423  42.094  1.00  0.00           O
ATOM   1118  N   GLN    72      39.130  45.309  42.555  1.00  0.00           N
ATOM   1119  H   GLN    72      39.177  44.607  43.279  1.00  0.00           H
ATOM   1120  CA  GLN    72      38.732  44.973  41.143  1.00  0.00           C
ATOM   1121  HA  GLN    72      39.652  45.201  40.605  1.00  0.00           H
ATOM   1122  CB  GLN    72      38.527  43.456  41.093  1.00  0.00           C
ATOM   1123  HB2 GLN    72      37.879  43.126  41.906  1.00  0.00           H
ATOM   1124  HB3 GLN    72      39.398  42.972  41.537  1.00  0.00           H
ATOM   1125  CG  GLN    72      38.196  42.841  39.648  1.00  0.00           C
```

ATOM	1126	HG2	GLN	72	37.536	43.523	39.113	1.00	0.00	H
ATOM	1127	HG3	GLN	72	39.158	42.732	39.147	1.00	0.00	H
ATOM	1128	CD	GLN	72	37.550	41.478	39.753	1.00	0.00	C
ATOM	1129	OE1	GLN	72	38.306	40.443	39.695	1.00	0.00	O
ATOM	1130	NE2	GLN	72	36.277	41.388	40.130	1.00	0.00	N
ATOM	1131	HE21	GLN	72	35.704	42.207	39.977	1.00	0.00	H
ATOM	1132	HE22	GLN	72	35.875	40.476	40.291	1.00	0.00	H
ATOM	1133	C	GLN	72	37.533	45.795	40.497	1.00	0.00	C
ATOM	1134	O	GLN	72	37.588	46.285	39.388	1.00	0.00	O
ATOM	1135	N	GLU	73	36.540	46.151	41.337	1.00	0.00	N
ATOM	1136	H	GLU	73	36.406	45.653	42.205	1.00	0.00	H
ATOM	1137	CA	GLU	73	35.392	47.037	41.030	1.00	0.00	C
ATOM	1138	HA	GLU	73	35.329	46.994	39.942	1.00	0.00	H
ATOM	1139	CB	GLU	73	34.161	46.468	41.687	1.00	0.00	C
ATOM	1140	HB2	GLU	73	34.328	46.336	42.757	1.00	0.00	H
ATOM	1141	HB3	GLU	73	33.393	47.241	41.690	1.00	0.00	H
ATOM	1142	CG	GLU	73	33.721	45.098	41.199	1.00	0.00	C
ATOM	1143	HG2	GLU	73	34.346	44.439	41.801	1.00	0.00	H
ATOM	1144	HG3	GLU	73	32.647	45.057	41.380	1.00	0.00	H
ATOM	1145	CD	GLU	73	33.975	44.735	39.706	1.00	0.00	C
ATOM	1146	OE1	GLU	73	34.355	43.612	39.387	1.00	0.00	O
ATOM	1147	OE2	GLU	73	33.550	45.483	38.776	1.00	0.00	O
ATOM	1148	C	GLU	73	35.715	48.473	41.360	1.00	0.00	C
ATOM	1149	O	GLU	73	34.892	49.353	41.127	1.00	0.00	O
ATOM	1150	N	ASN	74	36.912	48.779	41.741	1.00	0.00	N
ATOM	1151	H	ASN	74	37.603	48.045	41.804	1.00	0.00	H
ATOM	1152	CA	ASN	74	37.413	50.075	42.205	1.00	0.00	C
ATOM	1153	HA	ASN	74	38.330	49.890	42.764	1.00	0.00	H
ATOM	1154	CB	ASN	74	37.781	50.930	40.982	1.00	0.00	C
ATOM	1155	HB2	ASN	74	37.001	50.899	40.222	1.00	0.00	H
ATOM	1156	HB3	ASN	74	37.891	51.948	41.356	1.00	0.00	H
ATOM	1157	CG	ASN	74	39.043	50.651	40.219	1.00	0.00	C
ATOM	1158	OD1	ASN	74	39.448	51.359	39.332	1.00	0.00	O
ATOM	1159	ND2	ASN	74	39.781	49.591	40.428	1.00	0.00	N
ATOM	1160	HD21	ASN	74	40.442	49.276	39.732	1.00	0.00	H
ATOM	1161	HD22	ASN	74	39.570	48.976	41.201	1.00	0.00	H
ATOM	1162	C	ASN	74	36.461	50.805	43.253	1.00	0.00	C
ATOM	1163	O	ASN	74	36.132	51.974	43.056	1.00	0.00	O
ATOM	1164	N	MET	75	36.177	50.196	44.396	1.00	0.00	N
ATOM	1165	H	MET	75	36.695	49.356	44.613	1.00	0.00	H
ATOM	1166	CA	MET	75	35.281	50.685	45.444	1.00	0.00	C
ATOM	1167	HA	MET	75	34.934	51.718	45.403	1.00	0.00	H
ATOM	1168	CB	MET	75	33.975	49.912	45.339	1.00	0.00	C
ATOM	1169	HB2	MET	75	33.692	50.206	44.329	1.00	0.00	H
ATOM	1170	HB3	MET	75	34.202	48.846	45.371	1.00	0.00	H
ATOM	1171	CG	MET	75	32.832	50.241	46.299	1.00	0.00	C
ATOM	1172	HG2	MET	75	33.001	49.488	47.068	1.00	0.00	H
ATOM	1173	HG3	MET	75	32.948	51.203	46.800	1.00	0.00	H
ATOM	1174	SD	MET	75	31.137	49.903	45.744	1.00	0.00	S
ATOM	1175	CE	MET	75	30.852	51.568	45.208	1.00	0.00	C
ATOM	1176	HE1	MET	75	31.301	52.298	45.882	1.00	0.00	H
ATOM	1177	HE2	MET	75	31.274	51.699	44.212	1.00	0.00	H
ATOM	1178	HE3	MET	75	29.767	51.628	45.124	1.00	0.00	H
ATOM	1179	C	MET	75	35.964	50.486	46.793	1.00	0.00	C
ATOM	1180	O	MET	75	36.855	49.620	46.908	1.00	0.00	O
ATOM	1181	N	THR	76	35.631	51.303	47.810	1.00	0.00	N
ATOM	1182	H	THR	76	35.068	52.087	47.514	1.00	0.00	H
ATOM	1183	CA	THR	76	36.028	50.967	49.195	1.00	0.00	C
ATOM	1184	HA	THR	76	36.979	50.435	49.233	1.00	0.00	H
ATOM	1185	CB	THR	76	36.316	52.238	50.036	1.00	0.00	C
ATOM	1186	HB	THR	76	36.923	52.961	49.491	1.00	0.00	H
ATOM	1187	CG2	THR	76	35.045	52.897	50.462	1.00	0.00	C
ATOM	1188	HG21	THR	76	34.388	52.245	51.036	1.00	0.00	H
ATOM	1189	HG22	THR	76	35.282	53.817	50.996	1.00	0.00	H

ATOM	1190	HG23	THR	76	34.503	53.096	49.538	1.00	0.00	H
ATOM	1191	OG1	THR	76	37.007	51.980	51.239	1.00	0.00	O
ATOM	1192	HG1	THR	76	37.949	52.122	51.118	1.00	0.00	H
ATOM	1193	C	THR	76	35.019	49.998	49.896	1.00	0.00	C
ATOM	1194	O	THR	76	33.842	50.068	49.575	1.00	0.00	O
ATOM	1195	N	LEU	77	35.546	49.061	50.686	1.00	0.00	N
ATOM	1196	H	LEU	77	36.534	49.097	50.888	1.00	0.00	H
ATOM	1197	CA	LEU	77	34.851	47.881	51.215	1.00	0.00	C
ATOM	1198	HA	LEU	77	34.707	47.238	50.347	1.00	0.00	H
ATOM	1199	CB	LEU	77	35.804	47.175	52.207	1.00	0.00	C
ATOM	1200	HB2	LEU	77	36.641	47.005	51.531	1.00	0.00	H
ATOM	1201	HB3	LEU	77	36.143	47.906	52.942	1.00	0.00	H
ATOM	1202	CG	LEU	77	35.417	45.900	53.004	1.00	0.00	C
ATOM	1203	HG	LEU	77	34.731	46.184	53.802	1.00	0.00	H
ATOM	1204	CD1	LEU	77	34.826	44.798	52.172	1.00	0.00	C
ATOM	1205	HD11	LEU	77	34.953	43.843	52.681	1.00	0.00	H
ATOM	1206	HD12	LEU	77	33.785	45.090	52.035	1.00	0.00	H
ATOM	1207	HD13	LEU	77	35.275	44.770	51.180	1.00	0.00	H
ATOM	1208	CD2	LEU	77	36.677	45.427	53.790	1.00	0.00	C
ATOM	1209	HD21	LEU	77	37.455	45.162	53.074	1.00	0.00	H
ATOM	1210	HD22	LEU	77	36.902	46.205	54.520	1.00	0.00	H
ATOM	1211	HD23	LEU	77	36.434	44.550	54.391	1.00	0.00	H
ATOM	1212	C	LEU	77	33.477	48.197	51.876	1.00	0.00	C
ATOM	1213	O	LEU	77	32.503	47.466	51.653	1.00	0.00	O
ATOM	1214	N	THR	78	33.308	49.279	52.624	1.00	0.00	N
ATOM	1215	H	THR	78	34.065	49.936	52.753	1.00	0.00	H
ATOM	1216	CA	THR	78	32.092	49.673	53.365	1.00	0.00	C
ATOM	1217	HA	THR	78	31.749	48.787	53.899	1.00	0.00	H
ATOM	1218	CB	THR	78	32.484	50.682	54.505	1.00	0.00	C
ATOM	1219	HB	THR	78	31.517	51.008	54.887	1.00	0.00	H
ATOM	1220	CG2	THR	78	33.211	49.906	55.672	1.00	0.00	C
ATOM	1221	HG21	THR	78	33.572	50.595	56.436	1.00	0.00	H
ATOM	1222	HG22	THR	78	32.469	49.187	56.021	1.00	0.00	H
ATOM	1223	HG23	THR	78	34.096	49.357	55.348	1.00	0.00	H
ATOM	1224	OG1	THR	78	33.179	51.901	54.159	1.00	0.00	O
ATOM	1225	HG1	THR	78	34.042	51.604	53.863	1.00	0.00	H
ATOM	1226	C	THR	78	31.090					
50.193	52.340	1.00	0.00	C						
ATOM	1227	O	THR	78	29.896	49.991	52.515	1.00	0.00	O
ATOM	1228	N	GLU	79	31.468	50.965	51.365	1.00	0.00	N
ATOM	1229	H	GLU	79	32.401	51.342	51.285	1.00	0.00	H
ATOM	1230	CA	GLU	79	30.511	51.274	50.293	1.00	0.00	C
ATOM	1231	HA	GLU	79	29.582	51.612	50.749	1.00	0.00	H
ATOM	1232	CB	GLU	79	31.025	52.379	49.299	1.00	0.00	C
ATOM	1233	HB2	GLU	79	31.899	52.054	48.733	1.00	0.00	H
ATOM	1234	HB3	GLU	79	30.305	52.451	48.484	1.00	0.00	H
ATOM	1235	CG	GLU	79	31.236	53.797	49.792	1.00	0.00	C
ATOM	1236	HG2	GLU	79	31.966	53.854	50.598	1.00	0.00	H
ATOM	1237	HG3	GLU	79	31.670	54.247	48.900	1.00	0.00	H
ATOM	1238	CD	GLU	79	29.955	54.542	50.200	1.00	0.00	C
ATOM	1239	OE1	GLU	79	29.006	54.701	49.399	1.00	0.00	O
ATOM	1240	OE2	GLU	79	29.900	54.899	51.383	1.00	0.00	O
ATOM	1241	C	GLU	79	30.076	50.029	49.386	1.00	0.00	C
ATOM	1242	O	GLU	79	28.931	49.871	49.020	1.00	0.00	O
ATOM	1243	N	LEU	80	31.019	49.127	49.259	1.00	0.00	N
ATOM	1244	H	LEU	80	31.925	49.318	49.664	1.00	0.00	H
ATOM	1245	CA	LEU	80	30.727	47.823	48.635	1.00	0.00	C
ATOM	1246	HA	LEU	80	30.295	48.054	47.662	1.00	0.00	H
ATOM	1247	CB	LEU	80	32.075	46.978	48.467	1.00	0.00	C
ATOM	1248	HB2	LEU	80	32.862	47.691	48.217	1.00	0.00	H
ATOM	1249	HB3	LEU	80	32.287	46.440	49.390	1.00	0.00	H
ATOM	1250	CG	LEU	80	31.900	45.892	47.447	1.00	0.00	C
ATOM	1251	HG	LEU	80	30.877	45.533	47.563	1.00	0.00	H
ATOM	1252	CD1	LEU	80	31.967	46.420	46.054	1.00	0.00	C

ATOM	1253	HD11	LEU	80	32.896	46.989	46.013	1.00	0.00	H
ATOM	1254	HD12	LEU	80	31.888	45.592	45.350	1.00	0.00	H
ATOM	1255	HD13	LEU	80	31.085	47.055	45.964	1.00	0.00	H
ATOM	1256	CD2	LEU	80	32.908	44.776	47.648	1.00	0.00	C
ATOM	1257	HD21	LEU	80	33.646	45.036	46.890	1.00	0.00	H
ATOM	1258	HD22	LEU	80	33.494	44.775	48.567	1.00	0.00	H
ATOM	1259	HD23	LEU	80	32.396	43.851	47.383	1.00	0.00	H
ATOM	1260	C	LEU	80	29.715	46.995	49.419	1.00	0.00	C
ATOM	1261	O	LEU	80	28.758	46.435	48.827	1.00	0.00	O
ATOM	1262	N	LYS	81	29.775	46.938	50.823	1.00	0.00	N
ATOM	1263	H	LYS	81	30.625	47.240	51.276	1.00	0.00	H
ATOM	1264	CA	LYS	81	28.734	46.369	51.656	1.00	0.00	C
ATOM	1265	HA	LYS	81	28.434	45.353	51.398	1.00	0.00	H
ATOM	1266	CB	LYS	81	29.162	46.412	53.211	1.00	0.00	C
ATOM	1267	HB2	LYS	81	29.490	47.406	53.514	1.00	0.00	H
ATOM	1268	HB3	LYS	81	28.270	46.192	53.798	1.00	0.00	H
ATOM	1269	CG	LYS	81	30.294	45.462	53.524	1.00	0.00	C
ATOM	1270	HG2	LYS	81	30.038	44.470	53.153	1.00	0.00	H
ATOM	1271	HG3	LYS	81	31.215	45.787	53.039	1.00	0.00	H
ATOM	1272	CD	LYS	81	30.693	45.426	54.972	1.00	0.00	C
ATOM	1273	HD2	LYS	81	30.603	46.425	55.399	1.00	0.00	H
ATOM	1274	HD3	LYS	81	29.981	44.858	55.572	1.00	0.00	H
ATOM	1275	CE	LYS	81	32.118	44.943	55.254	1.00	0.00	C
ATOM	1276	HE2	LYS	81	32.278	44.066	54.627	1.00	0.00	H
ATOM	1277	HE3	LYS	81	32.899	45.671	55.030	1.00	0.00	H
ATOM	1278	NZ	LYS	81	32.303	44.550	56.624	1.00	0.00	N
ATOM	1279	HZ1	LYS	81	31.825	43.668	56.734	1.00	0.00	H
ATOM	1280	HZ2	LYS	81	33.283	44.351	56.761	1.00	0.00	H
ATOM	1281	HZ3	LYS	81	32.035	45.200	57.350	1.00	0.00	H
ATOM	1282	C	LYS	81	27.418	47.050	51.396	1.00	0.00	C
ATOM	1283	O	LYS	81	26.406	46.371	51.201	1.00	0.00	O
ATOM	1284	N	LYS	82	27.391	48.360	51.288	1.00	0.00	N
ATOM	1285	H	LYS	82	28.259	48.767	51.604	1.00	0.00	H
ATOM	1286	CA	LYS	82	26.232	49.248	51.091	1.00	0.00	C
ATOM	1287	HA	LYS	82	25.533	48.888	51.847	1.00	0.00	H
ATOM	1288	CB	LYS	82	26.760	50.674	51.405	1.00	0.00	C
ATOM	1289	HB2	LYS	82	27.364	50.620	52.311	1.00	0.00	H
ATOM	1290	HB3	LYS	82	27.414	50.976	50.586	1.00	0.00	H
ATOM	1291	CG	LYS	82	25.546	51.578	51.600	1.00	0.00	C
ATOM	1292	HG2	LYS	82	25.117	51.842	50.633	1.00	0.00	H
ATOM	1293	HG3	LYS	82	24.811	51.063	52.218	1.00	0.00	H
ATOM	1294	CD	LYS	82	25.932	52.995	52.164	1.00	0.00	C
ATOM	1295	HD2	LYS	82	25.016	53.536	52.407	1.00	0.00	H
ATOM	1296	HD3	LYS	82	26.485	52.810	53.084	1.00	0.00	H
ATOM	1297	CE	LYS	82	26.766	53.757	51.161	1.00	0.00	C
ATOM	1298	HE2	LYS	82	27.693	53.207	51.002	1.00	0.00	H
ATOM	1299	HE3	LYS	82	26.278	53.842	50.189	1.00	0.00	H
ATOM	1300	NZ	LYS	82	27.145	55.071	51.685	1.00	0.00	N
ATOM	1301	HZ1	LYS	82	27.553	55.086	52.609	1.00	0.00	H
ATOM	1302	HZ2	LYS	82	27.911	55.362	51.095	1.00	0.00	H
ATOM	1303	HZ3	LYS	82	26.373	55.721	51.645	1.00	0.00	H
ATOM	1304	C	LYS	82	25.535	49.197	49.677	1.00	0.00	C
ATOM	1305	O	LYS	82	24.318	49.377	49.665	1.00	0.00	O
ATOM	1306	N	VAL	83	26.181	48.882	48.561	1.00	0.00	N
ATOM	1307	H	VAL	83	27.185	48.772	48.570	1.00	0.00	H
ATOM	1308	CA	VAL	83	25.537	48.567	47.283	1.00	0.00	C
ATOM	1309	HA	VAL	83	24.704	49.268	47.237	1.00	0.00	H
ATOM	1310	CB	VAL	83	26.494	48.754	46.125	1.00	0.00	C
ATOM	1311	HB	VAL	83	25.847	48.619	45.258	1.00	0.00	H
ATOM	1312	CG1	VAL	83	27.001	50.239	45.999	1.00	0.00	C
ATOM	1313	HG11	VAL	83	27.185	50.635	45.000	1.00	0.00	H
ATOM	1314	HG12	VAL	83	26.143	50.794	46.381	1.00	0.00	H
ATOM	1315	HG13	VAL	83	27.936	50.343	46.550	1.00	0.00	H
ATOM	1316	CG2	VAL	83	27.719	47.853	45.885	1.00	0.00	C

ATOM	1317	HG21	VAL	83	28.154	48.033	44.902	1.00	0.00	H
ATOM	1318	HG22	VAL	83	28.477	48.145	46.612	1.00	0.00	H
ATOM	1319	HG23	VAL	83	27.361	46.827	45.957	1.00	0.00	H
ATOM	1320	C	VAL	83	24.864	47.217	47.194	1.00	0.00	C
ATOM	1321	O	VAL	83	23.758	47.160	46.646	1.00	0.00	O
ATOM	1322	N	ILE	84	25.558	46.217	47.765	1.00	0.00	N
ATOM	1323	H	ILE	84	26.443	46.501	48.161	1.00	0.00	H
ATOM	1324	CA	ILE	84	25.046	44.915	47.956	1.00	0.00	C
ATOM	1325	HA	ILE	84	24.676	44.556	46.996	1.00	0.00	H
ATOM	1326	CB	ILE	84	26.086	43.925	48.550	1.00	0.00	C
ATOM	1327	HB	ILE	84	26.569	44.326	49.441	1.00	0.00	H
ATOM	1328	CG2	ILE	84	25.342	42.615	48.858	1.00	0.00	C
ATOM	1329	HG21	ILE	84	24.719	42.598	49.753	1.00	0.00	H
ATOM	1330	HG22	ILE	84	24.709	42.310	48.025	1.00	0.00	H
ATOM	1331	HG23	ILE	84	26.033	41.794	49.052	1.00	0.00	H
ATOM	1332	CG1	ILE	84	27.258	43.533	47.601	1.00	0.00	C
ATOM	1333	HG12	ILE	84	27.007	42.685	46.963	1.00	0.00	H
ATOM	1334	HG13	ILE	84	27.404	44.374	46.922	1.00	0.00	H
ATOM	1335	CD1	ILE	84	28.571	43.177	48.276	1.00	0.00	C
ATOM	1336	HD11	ILE	84	28.952	44.077	48.760	1.00	0.00	H
ATOM	1337	HD12	ILE	84	28.562	42.310	48.939	1.00	0.00	H
ATOM	1338	HD13	ILE	84	29.333	43.092	47.504	1.00	0.00	H
ATOM	1339	C	ILE	84	23.748	44.876	48.854	1.00	0.00	C
ATOM	1340	O	ILE	84	22.735	44.192	48.546	1.00	0.00	O
ATOM	1341	N	ALA	85	23.721	45.543	49.971	1.00	0.00	N
ATOM	1342	H	ALA	85	24.545	46.080	50.198	1.00	0.00	H
ATOM	1343	CA	ALA	85	22.665	45.660	50.944	1.00	0.00	C
ATOM	1344	HA	ALA	85	22.318	44.722	51.377	1.00	0.00	H
ATOM	1345	CB	ALA	85	23.232	46.417	52.131	1.00	0.00	C
ATOM	1346	HB1	ALA	85	23.546	47.429	51.875	1.00	0.00	H
ATOM	1347	HB2	ALA	85	22.487	46.693	52.878	1.00	0.00	H
ATOM	1348	HB3	ALA	85	24.063	45.830	52.522	1.00	0.00	H
ATOM	1349	C	ALA	85	21.442	46.426	50.358	1.00	0.00	C
ATOM	1350	O	ALA	85	20.344	46.279	50.976	1.00	0.00	O
ATOM	1351	N	ASP	86	21.466	47.033	49.177	1.00	0.00	
	N									
ATOM	1352	H	ASP	86	22.310	47.135	48.632	1.00	0.00	H
ATOM	1353	CA	ASP	86	20.297	47.479	48.417	1.00	0.00	C
ATOM	1354	HA	ASP	86	19.520	47.734	49.138	1.00	0.00	H
ATOM	1355	CB	ASP	86	20.670	48.656	47.550	1.00	0.00	C
ATOM	1356	HB2	ASP	86	21.413	49.256	48.076	1.00	0.00	H
ATOM	1357	HB3	ASP	86	21.164	48.294	46.648	1.00	0.00	H
ATOM	1358	CG	ASP	86	19.485	49.388	47.051	1.00	0.00	C
ATOM	1359	OD1	ASP	86	19.226	49.424	45.809	1.00	0.00	O
ATOM	1360	OD2	ASP	86	18.776	49.838	47.940	1.00	0.00	O
ATOM	1361	C	ASP	86	19.663	46.445	47.421	1.00	0.00	C
ATOM	1362	O	ASP	86	18.463	46.415	47.280	1.00	0.00	O
ATOM	1363	N	ILE	87	20.556	45.669	46.840	1.00	0.00	N
ATOM	1364	H	ILE	87	21.545	45.830	46.972	1.00	0.00	H
ATOM	1365	CA	ILE	87	20.239	44.633	45.857	1.00	0.00	C
ATOM	1366	HA	ILE	87	19.553	44.951	45.073	1.00	0.00	H
ATOM	1367	CB	ILE	87	21.621	44.284	45.104	1.00	0.00	C
ATOM	1368	HB	ILE	87	22.427	44.130	45.820	1.00	0.00	H
ATOM	1369	CG2	ILE	87	21.441	43.127	44.202	1.00	0.00	C
ATOM	1370	HG21	ILE	87	21.338	42.182	44.734	1.00	0.00	H
ATOM	1371	HG22	ILE	87	20.662	43.315	43.463	1.00	0.00	H
ATOM	1372	HG23	ILE	87	22.376	43.040	43.649	1.00	0.00	H
ATOM	1373	CG1	ILE	87	22.023	45.473	44.230	1.00	0.00	C
ATOM	1374	HG12	ILE	87	21.348	45.650	43.393	1.00	0.00	H
ATOM	1375	HG13	ILE	87	21.938	46.400	44.798	1.00	0.00	H
ATOM	1376	CD1	ILE	87	23.509	45.523	43.786	1.00	0.00	C
ATOM	1377	HD11	ILE	87	24.098	45.321	44.680	1.00	0.00	H
ATOM	1378	HD12	ILE	87	23.681	44.896	42.911	1.00	0.00	H
ATOM	1379	HD13	ILE	87	23.762	46.562	43.571	1.00	0.00	H

ATOM	1380	C	ILE	87	19.674	43.330	46.387	1.00	0.00	C
ATOM	1381	O	ILE	87	18.701	42.838	45.839	1.00	0.00	O
ATOM	1382	N	TYR	88	20.323	42.858	47.539	1.00	0.00	N
ATOM	1383	H	TYR	88	21.086	43.426	47.880	1.00	0.00	H
ATOM	1384	CA	TYR	88	19.862	41.784	48.456	1.00	0.00	C
ATOM	1385	HA	TYR	88	18.826	41.575	48.191	1.00	0.00	H
ATOM	1386	CB	TYR	88	20.717	40.553	48.309	1.00	0.00	C
ATOM	1387	HB2	TYR	88	21.743	40.774	48.600	1.00	0.00	H
ATOM	1388	HB3	TYR	88	20.277	39.886	49.052	1.00	0.00	H
ATOM	1389	CG	TYR	88	20.590	39.680	46.996	1.00	0.00	C
ATOM	1390	CD1	TYR	88	19.595	38.706	46.827	1.00	0.00	C
ATOM	1391	HD1	TYR	88	18.801	38.650	47.556	1.00	0.00	H
ATOM	1392	CE1	TYR	88	19.665	37.791	45.759	1.00	0.00	C
ATOM	1393	HE1	TYR	88	18.998	36.942	45.740	1.00	0.00	H
ATOM	1394	CZ	TYR	88	20.687	37.889	44.798	1.00	0.00	C
ATOM	1395	OH	TYR	88	20.736	36.947	43.777	1.00	0.00	O
ATOM	1396	HH	TYR	88	21.343	36.217	43.919	1.00	0.00	H
ATOM	1397	CE2	TYR	88	21.637	38.965	44.916	1.00	0.00	C
ATOM	1398	HE2	TYR	88	22.521	39.037	44.299	1.00	0.00	H
ATOM	1399	CD2	TYR	88	21.603	39.828	46.052	1.00	0.00	C
ATOM	1400	HD2	TYR	88	22.379	40.561	46.218	1.00	0.00	H
ATOM	1401	C	TYR	88	19.930	42.230	49.894	1.00	0.00	C
ATOM	1402	O	TYR	88	20.857	42.975	50.267	1.00	0.00	O
ATOM	1403	N	PRO	89	18.961	41.969	50.791	1.00	0.00	N
ATOM	1404	CD	PRO	89	17.866	40.998	50.618	1.00	0.00	C
ATOM	1405	HD2	PRO	89	18.214	39.975	50.758	1.00	0.00	H
ATOM	1406	HD3	PRO	89	17.419	40.971	49.624	1.00	0.00	H
ATOM	1407	CG	PRO	89	16.813	41.354	51.645	1.00	0.00	C
ATOM	1408	HG2	PRO	89	16.199	40.471	51.818	1.00	0.00	H
ATOM	1409	HG3	PRO	89	16.318	42.285	51.369	1.00	0.00	H
ATOM	1410	CB	PRO	89	17.782	41.703	52.863	1.00	0.00	C
ATOM	1411	HB2	PRO	89	18.033	40.823	53.455	1.00	0.00	H
ATOM	1412	HB3	PRO	89	17.406	42.519	53.481	1.00	0.00	H
ATOM	1413	CA	PRO	89	19.069	42.302	52.193	1.00	0.00	C
ATOM	1414	HA	PRO	89	18.888	43.375	52.244	1.00	0.00	H
ATOM	1415	C	PRO	89	20.494	42.071	52.822	1.00	0.00	C
ATOM	1416	O	PRO	89	21.056	41.047	52.542	1.00	0.00	O
ATOM	1417	N	GLY	90	20.939	42.997	53.695	1.00	0.00	N
ATOM	1418	H	GLY	90	20.222	43.670	53.927	1.00	0.00	H
ATOM	1419	CA	GLY	90	22.290	42.970	54.315	1.00	0.00	C
ATOM	1420	HA2	GLY	90	22.986	43.030	53.478	1.00	0.00	H
ATOM	1421	HA3	GLY	90	22.444	43.767	55.042	1.00	0.00	H
ATOM	1422	C	GLY	90	22.551	41.644	55.075	1.00	0.00	C
ATOM	1423	O	GLY	90	23.730	41.184	55.156	1.00	0.00	O
ATOM	1424	N	GLN	91	21.528	41.006	55.639	1.00	0.00	N
ATOM	1425	H	GLN	91	20.611	41.373	55.426	1.00	0.00	H
ATOM	1426	CA	GLN	91	21.551	39.695	56.400	1.00	0.00	C
ATOM	1427	HA	GLN	91	22.491	39.586	56.943	1.00	0.00	H
ATOM	1428	CB	GLN	91	20.476	39.739	57.538	1.00	0.00	C
ATOM	1429	HB2	GLN	91	19.486	39.623	57.098	1.00	0.00	H
ATOM	1430	HB3	GLN	91	20.680	38.959	58.271	1.00	0.00	H
ATOM	1431	CG	GLN	91	20.538	41.053	58.363	1.00	0.00	C
ATOM	1432	HG2	GLN	91	20.454	41.890	57.669	1.00	0.00	H
ATOM	1433	HG3	GLN	91	19.758	41.112	59.123	1.00	0.00	H
ATOM	1434	CD	GLN	91	21.750	41.326	59.235	1.00	0.00	C
ATOM	1435	OE1	GLN	91	22.332	40.412	59.747	1.00	0.00	O
ATOM	1436	NE2	GLN	91	22.310	42.517	59.315	1.00	0.00	N
ATOM	1437	HE21	GLN	91	21.938	43.231	58.706	1.00	0.00	H
ATOM	1438	HE22	GLN	91	23.132	42.732	59.862	1.00	0.00	H
ATOM	1439	C	GLN	91	21.442	38.430	55.434	1.00	0.00	C
ATOM	1440	O	GLN	91	21.090	37.379	55.875	1.00	0.00	O
ATOM	1441	N	THR	92	21.716	38.583	54.135	1.00	0.00	N
ATOM	1442	H	THR	92	21.837	39.565	53.932	1.00	0.00	H
ATOM	1443	CA	THR	92	21.834	37.458	53.250	1.00	0.00	C

```
ATOM   1444  HA  THR    92      20.964  36.851  53.500  1.00  0.00           H
ATOM   1445  CB  THR    92      21.486  37.890  51.841  1.00  0.00           C
ATOM   1446  HB  THR    92      22.260  38.568  51.482  1.00  0.00           H
ATOM   1447  CG2 THR    92      21.133  36.749  50.867  1.00  0.00           C
ATOM   1448 HG21 THR    92      20.247  36.204  51.195  1.00  0.00           H
ATOM   1449 HG22 THR    92      21.152  37.190  49.872  1.00  0.00           H
ATOM   1450 HG23 THR    92      21.952  36.032  50.913  1.00  0.00           H
ATOM   1451  OG1 THR    92      20.242  38.640  51.868  1.00  0.00           O
ATOM   1452  HG1 THR    92      20.409  39.499  52.262  1.00  0.00           H
ATOM   1453  C   THR    92      23.176  36.649  53.290  1.00  0.00           C
ATOM   1454  O   THR    92      24.211  37.153  53.564  1.00  0.00           O
ATOM   1455  N   GLN    93      23.043  35.401  52.963  1.00  0.00           N
ATOM   1456  H   GLN    93      22.158  34.914  52.955  1.00  0.00           H
ATOM   1457  CA  GLN    93      24.253  34.565  52.766  1.00  0.00           C
ATOM   1458  HA  GLN    93      25.091  34.862  53.397  1.00  0.00           H
ATOM   1459  CB  GLN    93      23.883  33.236  53.338  1.00  0.00           C
ATOM   1460  HB2 GLN    93      23.287  33.413  54.233  1.00  0.00           H
ATOM   1461  HB3 GLN    93      23.163  32.887  52.598  1.00  0.00           H
ATOM   1462  CG  GLN    93      25.053  32.169  53.523  1.00  0.00           C
ATOM   1463  HG2 GLN    93      24.712  31.138  53.613  1.00  0.00           H
ATOM   1464  HG3 GLN    93      25.708  32.253  52.656  1.00  0.00           H
ATOM   1465  CD  GLN    93      25.903  32.456  54.741  1.00  0.00           C
ATOM   1466  OE1 GLN    93      25.338  32.725  55.837  1.00  0.00           O
ATOM   1467  NE2 GLN    93      27.164  32.474  54.591  1.00  0.00           N
ATOM   1468 HE21 GLN    93      27.453  32.102  53.698  1.00  0.00           H
ATOM   1469 HE22 GLN    93      27.719  32.389  55.431  1.00  0.00           H
ATOM   1470  C   GLN    93      24.704  34.583  51.302  1.00  0.00           C
ATOM   1471  O   GLN    93      23.899  34.335  50.439  1.00  0.00           O
ATOM   1472  N   PHE    94      25.911  35.060  51.057  1.00  0.00           N
ATOM   1473  H   PHE    94      26.467  35.229  51.884  1.00  0.00           H
ATOM   1474  CA  PHE    94      26.606  35.289  49.800  1.00  0.00           C
ATOM   1475  HA  PHE    94      25.957  35.239  48.925  1.00  0.00           H
ATOM   1476  CB  PHE    94      27.097  36.723  49.881  1.00  0.00           C
ATOM   1477
 HB2 PHE    94      27.827  36.766  50.689  1.00  0.00           H
ATOM   1478  HB3 PHE    94      27.808  37.077  49.134  1.00  0.00           H
ATOM   1479  CG  PHE    94      26.003  37.780  50.145  1.00  0.00           C
ATOM   1480  CD1 PHE    94      25.417  38.321  48.964  1.00  0.00           C
ATOM   1481  HD1 PHE    94      25.694  37.876  48.019  1.00  0.00           H
ATOM   1482  CE1 PHE    94      24.473  39.353  49.008  1.00  0.00           C
ATOM   1483  HE1 PHE    94      24.036  39.809  48.132  1.00  0.00           H
ATOM   1484  CZ  PHE    94      24.156  39.929  50.292  1.00  0.00           C
ATOM   1485  HZ  PHE    94      23.402  40.701  50.347  1.00  0.00           H
ATOM   1486  CE2 PHE    94      24.877  39.484  51.377  1.00  0.00           C
ATOM   1487  HE2 PHE    94      24.628  39.893  52.345  1.00  0.00           H
ATOM   1488  CD2 PHE    94      25.811  38.412  51.339  1.00  0.00           C
ATOM   1489  HD2 PHE    94      26.331  38.062  52.217  1.00  0.00           H
ATOM   1490  C   PHE    94      27.658  34.220  49.695  1.00  0.00           C
ATOM   1491  O   PHE    94      27.982  33.461  50.583  1.00  0.00           O
ATOM   1492  N   TYR    95      28.221  34.214  48.544  1.00  0.00           N
ATOM   1493  H   TYR    95      27.824  34.825  47.846  1.00  0.00           H
ATOM   1494  CA  TYR    95      29.226  33.265  48.042  1.00  0.00           C
ATOM   1495  HA  TYR    95      29.681  32.763  48.896  1.00  0.00           H
ATOM   1496  CB  TYR    95      28.436  32.111  47.429  1.00  0.00           C
ATOM   1497  HB2 TYR    95      27.819  31.659  48.207  1.00  0.00           H
ATOM   1498  HB3 TYR    95      27.878  32.482  46.571  1.00  0.00           H
ATOM   1499  CG  TYR    95      29.205  30.903  46.821  1.00  0.00           C
ATOM   1500  CD1 TYR    95      29.572  30.867  45.491  1.00  0.00           C
ATOM   1501  HD1 TYR    95      29.406  31.759  44.904  1.00  0.00           H
ATOM   1502  CE1 TYR    95      30.174  29.651  45.008  1.00  0.00           C
ATOM   1503  HE1 TYR    95      30.514  29.636  43.983  1.00  0.00           H
ATOM   1504  CZ  TYR    95      30.416  28.545  45.808  1.00  0.00           C
ATOM   1505  OH  TYR    95      31.031  27.458  45.326  1.00  0.00           O
ATOM   1506  HH  TYR    95      31.044  27.489  44.366  1.00  0.00           H
```

ATOM	1507	CE2	TYR	95	30.058	28.654	47.214	1.00	0.00	C
ATOM	1508	HE2	TYR	95	30.130	27.825	47.903	1.00	0.00	H
ATOM	1509	CD2	TYR	95	29.419	29.821	47.666	1.00	0.00	C
ATOM	1510	HD2	TYR	95	29.165	29.817	48.716	1.00	0.00	H
ATOM	1511	C	TYR	95	30.298	33.903	47.170	1.00	0.00	C
ATOM	1512	O	TYR	95	30.049	34.432	46.110	1.00	0.00	O
ATOM	1513	N	VAL	96	31.579	33.922	47.603	1.00	0.00	N
ATOM	1514	H	VAL	96	31.788	33.409	48.447	1.00	0.00	H
ATOM	1515	CA	VAL	96	32.653	34.507	46.789	1.00	0.00	C
ATOM	1516	HA	VAL	96	32.258	35.280	46.131	1.00	0.00	H
ATOM	1517	CB	VAL	96	33.588	35.284	47.703	1.00	0.00	C
ATOM	1518	HB	VAL	96	33.949	34.680	48.534	1.00	0.00	H
ATOM	1519	CG1	VAL	96	34.866	35.830	47.036	1.00	0.00	C
ATOM	1520	HG11	VAL	96	34.734	36.376	46.102	1.00	0.00	H
ATOM	1521	HG12	VAL	96	35.357	36.559	47.680	1.00	0.00	H
ATOM	1522	HG13	VAL	96	35.515	34.989	46.790	1.00	0.00	H
ATOM	1523	CG2	VAL	96	32.887	36.518	48.198	1.00	0.00	C
ATOM	1524	HG21	VAL	96	32.470	37.018	47.323	1.00	0.00	H
ATOM	1525	HG22	VAL	96	32.059	36.294	48.870	1.00	0.00	H
ATOM	1526	HG23	VAL	96	33.526	37.183	48.780	1.00	0.00	H
ATOM	1527	C	VAL	96	33.365	33.481	45.941	1.00	0.00	C
ATOM	1528	O	VAL	96	33.502	32.363	46.450	1.00	0.00	O
ATOM	1529	N	ILE	97	33.804	33.820	44.728	1.00	0.00	N
ATOM	1530	H	ILE	97	33.692	34.789	44.464	1.00	0.00	H
ATOM	1531	CA	ILE	97	34.458	32.931	43.831	1.00	0.00	C
ATOM	1532	HA	ILE	97	34.733	32.043	44.400	1.00	0.00	H
ATOM	1533	CB	ILE	97	33.524	32.565	42.695	1.00	0.00	C
ATOM	1534	HB	ILE	97	33.356	33.476	42.121	1.00	0.00	H
ATOM	1535	CG2	ILE	97	34.049	31.553	41.590	1.00	0.00	C
ATOM	1536	HG21	ILE	97	33.306	31.437	40.799	1.00	0.00	H
ATOM	1537	HG22	ILE	97	34.896	31.978	41.053	1.00	0.00	H
ATOM	1538	HG23	ILE	97	34.265	30.561	41.986	1.00	0.00	H
ATOM	1539	CG1	ILE	97	32.141	32.082	43.154	1.00	0.00	C
ATOM	1540	HG12	ILE	97	32.157	31.052	43.508	1.00	0.00	H
ATOM	1541	HG13	ILE	97	31.748	32.742	43.926	1.00	0.00	H
ATOM	1542	CD1	ILE	97	30.995	32.163	42.160	1.00	0.00	C
ATOM	1543	HD11	ILE	97	30.088	32.129	42.764	1.00	0.00	H
ATOM	1544	HD12	ILE	97	31.025	33.008	41.474	1.00	0.00	H
ATOM	1545	HD13	ILE	97	31.029	31.268	41.538	1.00	0.00	H
ATOM	1546	C	ILE	97	35.772	33.491	43.256	1.00	0.00	C
ATOM	1547	O	ILE	97	35.721	34.591	42.696	1.00	0.00	O
ATOM	1548	N	GLU	98	36.953	32.778	43.217	1.00	0.00	N
ATOM	1549	H	GLU	98	37.016	31.904	43.719	1.00	0.00	H
ATOM	1550	CA	GLU	98	38.220	33.266	42.651	1.00	0.00	C
ATOM	1551	HA	GLU	98	38.191	34.232	42.147	1.00	0.00	H
ATOM	1552	CB	GLU	98	39.172	33.385	43.908	1.00	0.00	C
ATOM	1553	HB2	GLU	98	38.611	33.983	44.625	1.00	0.00	H
ATOM	1554	HB3	GLU	98	39.366	32.419	44.374	1.00	0.00	H
ATOM	1555	CG	GLU	98	40.545	34.093	43.594	1.00	0.00	C
ATOM	1556	HG2	GLU	98	41.151	33.393	43.019	1.00	0.00	H
ATOM	1557	HG3	GLU	98	40.381	34.891	42.871	1.00	0.00	H
ATOM	1558	CD	GLU	98	41.320	34.582	44.828	1.00	0.00	C
ATOM	1559	OE1	GLU	98	41.144	33.941	45.878	1.00	0.00	O
ATOM	1560	OE2	GLU	98	42.253	35.404	44.783	1.00	0.00	O
ATOM	1561	C	GLU	98	38.820	32.348	41.580	1.00	0.00	C
ATOM	1562	O	GLU	98	38.598	31.136	41.644	1.00	0.00	O
ATOM	1563	N	PHE	99	39.339	32.830	40.481	1.00	0.00	N
ATOM	1564	H	PHE	99	39.543	33.818	40.422	1.00	0.00	H
ATOM	1565	CA	PHE	99	39.621	32.059	39.265	1.00	0.00	C
ATOM	1566	HA	PHE	99	39.884	31.043	39.558	1.00	0.00	H
ATOM	1567	CB	PHE	99	38.270	31.981	38.484	1.00	0.00	C
ATOM	1568	HB2	PHE	99	38.497	31.468	37.549	1.00	0.00	H
ATOM	1569	HB3	PHE	99	37.667	31.324	39.110	1.00	0.00	H
ATOM	1570	CG	PHE	99	37.566	33.280	38.191	1.00	0.00	C

```
ATOM   1571  CD1 PHE    99      36.651  33.778  39.196  1.00  0.00           C
ATOM   1572  HD1 PHE    99      36.330  33.217  40.061  1.00  0.00           H
ATOM   1573  CE1 PHE    99      35.985  35.015  38.941  1.00  0.00           C
ATOM   1574  HE1 PHE    99      35.256  35.345  39.667  1.00  0.00           H
ATOM   1575  CZ  PHE    99      36.077  35.667  37.732  1.00  0.00           C
ATOM   1576  HZ  PHE    99      35.656  36.652  37.590  1.00  0.00           H
ATOM   1577  CE2 PHE    99      36.909  35.084  36.727  1.00  0.00           C
ATOM   1578  HE2 PHE    99      36.969  35.670  35.822  1.00  0.00           H
ATOM   1579  CD2 PHE    99      37.654  33.915  36.934  1.00  0.00           C
ATOM   1580  HD2 PHE    99      38.306  33.518  36.170  1.00  0.00           H
ATOM   1581  C   PHE    99      40.693  32.715  38.383  1.00  0.00           C
ATOM   1582  O   PHE    99      40.836  33.926  38.204  1.00  0.00           O
ATOM   1583  N   LYS   100      41.451  31.800  37.627  1.00  0.00           N
ATOM   1584  H   LYS   100      41.326  30.809  37.777  1.00  0.00           H
ATOM   1585  CA  LYS   100      42.553  32.151  36.729  1.00  0.00           C
ATOM   1586  HA  LYS   100      42.525  33.209  36.464  1.00  0.00           H
ATOM   1587  CB  LYS   100      43.929  32.140  37.403  1.00  0.00           C
ATOM   1588  HB2 LYS   100      44.666  32.698  36.826  1.00  0.00           H
ATOM   1589  HB3 LYS   100      43.798  32.508  38.420  1.00  0.00           H
ATOM   1590  CG  LYS   100      44.546  30.759  37.650  1.00  0.00           C
ATOM   1591  HG2 LYS   100      43.723  30.058  37.785  1.00  0.00           H
ATOM   1592  HG3 LYS   100      45.096  30.366  36.794  1.00  0.00           H
ATOM   1593  CD  LYS   100      45.497  30.766  38.865  1.00  0.00           C
ATOM   1594  HD2 LYS   100      46.248  31.550  38.776  1.00  0.00           H
ATOM   1595  HD3 LYS   100      44.973  31.115  39.755  1.00  0.00           H
ATOM   1596  CE  LYS   100      46.042  29.343  39.109  1.00  0.00           C
ATOM   1597  HE2 LYS   100      45.326  28.597  39.456  1.00  0.00           H
ATOM   1598  HE3 LYS   100      46.444  29.025  38.147  1.00  0.00           H
ATOM   1599  NZ  LYS   100      47.157  29.436  40.075  1.00  0.00           N
ATOM   1600  HZ1 LYS   100      46.844  29.388  41.034  1.00  0.00           H
ATOM   1601  HZ2 LYS   100      47.907  28.771  39.950  1.00  0.00           H
ATOM   1602  HZ3 LYS   100      47.586
  30.323  39.853  1.00  0.00           H
ATOM   1603  C   LYS   100      42.580  31.344  35.521  1.00  0.00           C
ATOM   1604  O   LYS   100      42.100  30.204  35.607  1.00  0.00           O
ATOM   1605  N   CYX   101      43.007  31.960  34.416  1.00  0.00           N
ATOM   1606  H   CYX   101      43.293  32.926  34.490  1.00  0.00           H
ATOM   1607  CA  CYX   101      43.365  31.165  33.205  1.00  0.00           C
ATOM   1608  HA  CYX   101      42.675  30.386  32.881  1.00  0.00           H
ATOM   1609  CB  CYX   101      43.390  32.212  32.012  1.00  0.00           C
ATOM   1610  HB2 CYX   101      42.368  32.557  31.853  1.00  0.00           H
ATOM   1611  HB3 CYX   101      44.034  33.060  32.242  1.00  0.00           H
ATOM   1612  SG  CYX   101      43.979  31.598  30.362  1.00  0.00           S
ATOM   1613  C   CYX   101      44.762  30.535  33.333  1.00  0.00           C
ATOM   1614  O   CYX   101      45.665  31.055  33.955  1.00  0.00           O
ATOM   1615  N   LEU   102      44.916  29.357  32.636  1.00  0.00           N
ATOM   1616  H   LEU   102      44.075  28.935  32.267  1.00  0.00           H
ATOM   1617  CA  LEU   102      46.141  28.659  32.416  1.00  0.00           C
ATOM   1618  HA  LEU   102      46.739  28.796  33.317  1.00  0.00           H
ATOM   1619  CB  LEU   102      45.849  27.080  32.418  1.00  0.00           C
ATOM   1620  HB2 LEU   102      45.146  26.832  31.623  1.00  0.00           H
ATOM   1621  HB3 LEU   102      46.779  26.547  32.218  1.00  0.00           H
ATOM   1622  CG  LEU   102      45.501  26.292  33.673  1.00  0.00           C
ATOM   1623  HG  LEU   102      45.423  25.247  33.375  1.00  0.00           H
ATOM   1624  CD1 LEU   102      46.633  26.325  34.730  1.00  0.00           C
ATOM   1625 HD11 LEU   102      46.677  27.257  35.295  1.00  0.00           H
ATOM   1626 HD12 LEU   102      46.515  25.494  35.425  1.00  0.00           H
ATOM   1627 HD13 LEU   102      47.544  26.067  34.193  1.00  0.00           H
ATOM   1628  CD2 LEU   102      44.217  26.722  34.276  1.00  0.00           C
ATOM   1629 HD21 LEU   102      44.421  27.593  34.899  1.00  0.00           H
ATOM   1630 HD22 LEU   102      43.424  26.832  33.536  1.00  0.00           H
ATOM   1631 HD23 LEU   102      43.816  26.005  34.991  1.00  0.00           H
ATOM   1632  C   LEU   102      46.949  29.036  31.158  1.00  0.00           C
ATOM   1633  O   LEU   102      48.172  29.245  31.269  1.00  0.00           O
```

```
ATOM   1634  OXT LEU   102       46.375  28.997  30.096  1.00  0.00           O
HETATM  314  N   LYN    20       39.598  40.662  35.457  1.00  0.00           N
HETATM  315  H   LYN    20       39.505  40.628  34.451  1.00  0.00           H
HETATM  316  CA  LYN    20       38.551  40.019  36.197  1.00  0.00           C
HETATM  317  HA  LYN    20       38.323  40.655  37.053  1.00  0.00           H
HETATM  318  CB  LYN    20       37.271  40.023  35.271  1.00  0.00           C
HETATM  319  HB2 LYN    20       37.423  39.529  34.311  1.00  0.00           H
HETATM  320  HB3 LYN    20       36.977  41.035  34.993  1.00  0.00           H
HETATM  321  CG  LYN    20       35.978  39.373  35.878  1.00  0.00           C
HETATM  322  HG2 LYN    20       35.123  39.559  35.227  1.00  0.00           H
HETATM  323  HG3 LYN    20       36.009  38.312  35.633  1.00  0.00           H
HETATM  324  CD  LYN    20       35.506  39.839  37.269  1.00  0.00           C
HETATM  325  HD2 LYN    20       35.397  40.923  37.281  1.00  0.00           H
HETATM  326  HD3 LYN    20       36.249  39.458  37.970  1.00  0.00           H
HETATM  327  CE  LYN    20       34.152  39.179  37.453  1.00  0.00           C
HETATM  328  HE2 LYN    20       33.543  39.485  36.601  1.00  0.00           H
HETATM  329  HE3 LYN    20       34.292  38.104  37.348  1.00  0.00           H
HETATM  330  NZ  LYN    20       33.513  39.594  38.718  1.00  0.00           N
HETATM  331  HZ2 LYN    20       33.357  40.583  38.583  1.00  0.00           H
HETATM  332  HZ3 LYN    20       34.240  39.433  39.401  1.00  0.00           H
HETATM  333  C   LYN    20       38.941  38.591  36.541  1.00  0.00           C
HETATM  334  O   LYN    20       39.267  37.731  35.698  1.00  0.00           O
HETATM 1636  N   LIG   103       29.522  41.697  40.635  1.00  0.00           N
HETATM 1637  C   LIG   103       29.061  40.811  39.782  1.00  0.00           C
HETATM 1638  O   LIG   103       29.453  42.952  42.452  1.00  0.00           O
HETATM 1639  C5' LIG   103       25.526  38.698  45.075  1.00  0.00           C
HETATM 1640  O5' LIG   103       25.545  38.237  43.740  1.00  0.00           O
HETATM 1641  C4' LIG   103       25.961  40.167  45.088  1.00  0.00           C
HETATM 1642  O4' LIG   103       27.257  40.164  44.438  1.00  0.00           O
HETATM 1643  C3' LIG   103       25.061  41.093  44.272  1.00  0.00           C
HETATM 1644  O3' LIG   103       24.148  41.795  45.016  1.00  0.00           O
HETATM 1645  C2' LIG   103       26.127  42.081  43.688  1.00  0.00           C
HETATM 1646  O2' LIG   103       26.355  43.269  44.569  1.00  0.00           O
HETATM 1647  C1' LIG   103       27.489  41.369  43.711  1.00  0.00           C
HETATM 1648  N1  LIG   103       28.100  41.080  42.344  1.00  0.00           N
HETATM 1649  O1  LIG   103       29.380  38.646  37.800  1.00  0.00           O
HETATM 1650  N2  LIG   103       29.801  40.706  38.672  1.00  0.00           N
HETATM 1651  C6  LIG   103       29.045  41.994  41.860  1.00  0.00           C
HETATM 1652  C7  LIG   103       27.490  40.156  41.477  1.00  0.00           C
HETATM 1653  C8  LIG   103       27.928  39.984  40.212  1.00  0.00           C
HETATM 1654  C9  LIG   103       29.876  39.733  37.690  1.00  0.00           C
HETATM 1655  C10 LIG   103       30.779  40.036  36.486  1.00  0.00           C
HETATM 1656  H   LIG   103       30.347  41.554  38.573  1.00  0.00           H
HETATM 1657  H1  LIG   103       27.593  39.152  39.602  1.00  0.00           H
HETATM 1658  H4  LIG   103       28.191  41.940  44.330  1.00  0.00           H
HETATM 1659  H6  LIG   103       25.983  40.543  46.119  1.00  0.00           H
HETATM 1660  H7  LIG   103       24.529  40.664  43.414  1.00  0.00           H
HETATM 1661  H8  LIG   103       25.857  42.394  42.672  1.00  0.00           H
HETATM 1662  H9  LIG   103       24.547  38.658  45.569  1.00  0.00           H
HETATM 1663  H10 LIG   103       26.233  38.235  45.774  1.00  0.00           H
HETATM 1664  H11 LIG   103       31.658  40.471  36.977  1.00  0.00           H
HETATM 1665  H12 LIG   103       30.307  40.674  35.730  1.00  0.00           H
HETATM 1666  H13 LIG   103       31.093  39.034  36.167  1.00  0.00           H
HETATM 1667  H14 LIG   103       26.557  39.736  41.827  1.00  0.00           H
HETATM 1668  H2' LIG   103       27.162  43.757  44.327  1.00  0.00           H
HETATM 1669  H3' LIG   103       24.632  42.615  45.212  1.00  0.00           H
HETATM 1670  H5' LIG   103       26.485  38.173  43.497  1.00  0.00           H
ENDMDL
MODEL     14
SHEET     1   1 1 ILE    22  ASP    26  0
SHEET     2   2 1 VAL    37  VAL    40  0
SHEET     3   3 1 CYX    50  VAL    60  0
SHEET     4   4 1 PHE    94  CYX   101  0
HELIX     1   1 GLN      9  LEU     16  1                                   8
```

```
HELIX    2   2 LYS     68  GLU     73  1                                   6
HELIX    3   3 LEU     77  ILE     87  1                                  11
ATOM      1  N   GLN     1      36.603  17.753  33.512  1.00  0.00           N
ATOM      2  H1  GLN     1      37.414  17.203  33.266  1.00  0.00           H
ATOM      3  H2  GLN     1      36.791  18.486  34.181  1.00  0.00           H
ATOM      4  H3  GLN     1      35.890  17.178  33.937  1.00  0.00           H
ATOM      5  CA  GLN     1      36.079  18.387  32.283  1.00  0.00           C
ATOM      6  HA  GLN     1      36.965  18.724  31.744  1.00  0.00           H
ATOM      7  CB  GLN     1      35.193  17.495  31.402  1.00  0.00           C
ATOM      8  HB2 GLN     1      34.317  17.144  31.948  1.00  0.00           H
ATOM      9  HB3 GLN     1      34.839  18.081  30.553  1.00  0.00           H
ATOM     10  CG  GLN     1      35.947  16.257  30.811  1.00  0.00           C
ATOM     11  HG2 GLN     1      35.276  15.771  30.104  1.00  0.00           H
ATOM     12  HG3 GLN     1      36.852  16.587  30.300  1.00  0.00           H
ATOM     13  CD  GLN     1      36.244  15.122  31.809  1.00  0.00           C
ATOM     14  OE1 GLN     1      35.454  14.797  32.672  1.00  0.00           O
ATOM     15  NE2 GLN     1      37.380  14.550  31.732  1.00  0.00           N
ATOM     16 HE21 GLN     1      38.120  14.759  31.077  1.00  0.00           H
ATOM     17 HE22 GLN     1      37.677  14.024  32.541  1.00  0.00           H
ATOM     18  C   GLN     1      35.356  19.759  32.465  1.00  0.00           C
ATOM     19  O   GLN     1      34.778  19.867  33.550  1.00  0.00           O
ATOM     20  N   PRO     2      35.483  20.732  31.523  1.00  0.00           N
ATOM     21  CD  PRO     2      36.274  20.872  30.326  1.00  0.00           C
ATOM     22  HD2 PRO     2      35.708  20.456  29.494  1.00  0.00           H
ATOM     23  HD3 PRO     2      37.243  20.394  30.470  1.00  0.00           H
ATOM     24  CG  PRO     2      36.562  22.379  30.064  1.00  0.00           C
ATOM     25  HG2 PRO     2      36.808  22.729  29.061  1.00  0.00           H
ATOM     26  HG3 PRO     2      37.204  22.791  30.842  1.00  0.00           H
ATOM     27  CB  PRO     2      35.279  22.883  30.586  1.00  0.00           C
ATOM     28  HB2 PRO     2      34.603  22.870  29.730  1.00  0.00           H
ATOM     29  HB3 PRO     2      35.476  23.897  30.932  1.00  0.00           H
ATOM     30  CA  PRO     2      34.794  22.032  31.724  1.00  0.00           C
ATOM     31  HA  PRO     2      35.230  22.520  32.596  1.00  0.00           H
ATOM     32  C   PRO     2      33.315  22.040
  31.890  1.00  0.00           C
ATOM     33  O   PRO     2      32.623  21.166  31.487  1.00  0.00           O
ATOM     34  N   ASN     3      32.920  22.976  32.731  1.00  0.00           N
ATOM     35  H   ASN     3      33.564  23.678  33.064  1.00  0.00           H
ATOM     36  CA  ASN     3      31.584  23.195  32.900  1.00  0.00           C
ATOM     37  HA  ASN     3      30.966  22.321  33.103  1.00  0.00           H
ATOM     38  CB  ASN     3      31.457  24.027  34.192  1.00  0.00           C
ATOM     39  HB2 ASN     3      32.056  23.696  35.040  1.00  0.00           H
ATOM     40  HB3 ASN     3      31.736  25.071  34.051  1.00  0.00           H
ATOM     41  CG  ASN     3      29.985  23.951  34.799  1.00  0.00           C
ATOM     42  OD1 ASN     3      29.012  23.700  34.119  1.00  0.00           O
ATOM     43  ND2 ASN     3      29.795  24.291  36.057  1.00  0.00           N
ATOM     44 HD21 ASN     3      30.551  24.476  36.700  1.00  0.00           H
ATOM     45 HD22 ASN     3      28.826  24.398  36.322  1.00  0.00           H
ATOM     46  C   ASN     3      31.009  23.829  31.664  1.00  0.00           C
ATOM     47  O   ASN     3      31.667  24.635  31.047  1.00  0.00           O
ATOM     48  N   ASP     4      29.710  23.633  31.358  1.00  0.00           N
ATOM     49  H   ASP     4      29.100  23.112  31.972  1.00  0.00           H
ATOM     50  CA  ASP     4      28.923  24.272  30.297  1.00  0.00           C
ATOM     51  HA  ASP     4      29.559  24.425  29.426  1.00  0.00           H
ATOM     52  CB  ASP     4      27.799  23.217  29.862  1.00  0.00           C
ATOM     53  HB2 ASP     4      27.029  23.142  30.631  1.00  0.00           H
ATOM     54  HB3 ASP     4      27.344  23.565  28.935  1.00  0.00           H
ATOM     55  CG  ASP     4      28.493  21.957  29.358  1.00  0.00           C
ATOM     56  OD1 ASP     4      29.411  22.020  28.488  1.00  0.00           O
ATOM     57  OD2 ASP     4      28.167  20.909  29.888  1.00  0.00           O
ATOM     58  C   ASP     4      28.361  25.679  30.663  1.00  0.00           C
ATOM     59  O   ASP     4      27.982  26.457  29.797  1.00  0.00           O
ATOM     60  N   ILE     5      28.305  25.889  32.011  1.00  0.00           N
ATOM     61  H   ILE     5      28.567  25.116  32.606  1.00  0.00           H
```

ATOM	62	CA	ILE	5	28.047	27.181	32.620	1.00	0.00	C
ATOM	63	HA	ILE	5	27.162	27.735	32.308	1.00	0.00	H
ATOM	64	CB	ILE	5	27.634	27.057	34.132	1.00	0.00	C
ATOM	65	HB	ILE	5	28.337	26.348	34.567	1.00	0.00	H
ATOM	66	CG2	ILE	5	27.869	28.174	35.115	1.00	0.00	C
ATOM	67	HG21	ILE	5	27.213	27.949	35.957	1.00	0.00	H
ATOM	68	HG22	ILE	5	28.939	28.269	35.303	1.00	0.00	H
ATOM	69	HG23	ILE	5	27.589	29.161	34.750	1.00	0.00	H
ATOM	70	CG1	ILE	5	26.176	26.590	34.186	1.00	0.00	C
ATOM	71	HG12	ILE	5	25.938	25.824	33.446	1.00	0.00	H
ATOM	72	HG13	ILE	5	26.016	26.174	35.181	1.00	0.00	H
ATOM	73	CD1	ILE	5	25.170	27.742	33.970	1.00	0.00	C
ATOM	74	HD11	ILE	5	24.236	27.182	34.011	1.00	0.00	H
ATOM	75	HD12	ILE	5	25.264	28.439	34.803	1.00	0.00	H
ATOM	76	HD13	ILE	5	25.251	28.289	33.030	1.00	0.00	H
ATOM	77	C	ILE	5	29.260	28.125	32.481	1.00	0.00	C
ATOM	78	O	ILE	5	30.381	27.712	32.697	1.00	0.00	O
ATOM	79	N	THR	6	28.984	29.392	32.143	1.00	0.00	N
ATOM	80	H	THR	6	28.019	29.691	32.126	1.00	0.00	H
ATOM	81	CA	THR	6	29.989	30.473	31.857	1.00	0.00	C
ATOM	82	HA	THR	6	30.817	30.222	32.519	1.00	0.00	H
ATOM	83	CB	THR	6	30.452	30.213	30.432	1.00	0.00	C
ATOM	84	HB	THR	6	30.796	29.185	30.311	1.00	0.00	H
ATOM	85	CG2	THR	6	29.442	30.610	29.373	1.00	0.00	C
ATOM	86	HG21	THR	6	29.173	31.653	29.538	1.00	0.00	H
ATOM	87	HG22	THR	6	29.736	30.385	28.348	1.00	0.00	H
ATOM	88	HG23	THR	6	28.557	29.995	29.539	1.00	0.00	H
ATOM	89	OG1	THR	6	31.528	31.064	30.310	1.00	0.00	O
ATOM	90	HG1	THR	6	32.322	30.526	30.257	1.00	0.00	H
ATOM	91	C	THR	6	29.517	31.890	32.259	1.00	0.00	C
ATOM	92	O	THR	6	28.305	32.149	32.366	1.00	0.00	O
ATOM	93	N	PHE	7	30.402	32.813	32.481	1.00	0.00	N
ATOM	94	H	PHE	7	31.395	32.754	32.300	1.00	0.00	H
ATOM	95	CA	PHE	7	30.019	34.182	32.776	1.00	0.00	C
ATOM	96	HA	PHE	7	29.229	34.162	33.527	1.00	0.00	H
ATOM	97	CB	PHE	7	31.222	34.932	33.164	1.00	0.00	C
ATOM	98	HB2	PHE	7	31.980	34.882	32.382	1.00	0.00	H
ATOM	99	HB3	PHE	7	31.033	36.004	33.196	1.00	0.00	H
ATOM	100	CG	PHE	7	31.913	34.612	34.511	1.00	0.00	C
ATOM	101	CD1	PHE	7	33.246	34.034	34.527	1.00	0.00	C
ATOM	102	HD1	PHE	7	33.723	33.698	33.617	1.00	0.00	H
ATOM	103	CE1	PHE	7	33.945	33.887	35.815	1.00	0.00	C
ATOM	104	HE1	PHE	7	34.988	33.615	35.897	1.00	0.00	H
ATOM	105	CZ	PHE	7	33.204	34.044	37.012	1.00	0.00	C
ATOM	106	HZ	PHE	7	33.717	33.843	37.941	1.00	0.00	H
ATOM	107	CE2	PHE	7	31.923	34.563	36.975	1.00	0.00	C
ATOM	108	HE2	PHE	7	31.488	34.691	37.955	1.00	0.00	H
ATOM	109	CD2	PHE	7	31.305	34.854	35.768	1.00	0.00	C
ATOM	110	HD2	PHE	7	30.382	35.407	35.668	1.00	0.00	H
ATOM	111	C	PHE	7	29.406	34.820	31.529	1.00	0.00	C
ATOM	112	O	PHE	7	29.676	34.464	30.330	1.00	0.00	O
ATOM	113	N	PHE	8	28.581	35.886	31.796	1.00	0.00	N
ATOM	114	H	PHE	8	28.458	36.271	32.721	1.00	0.00	H
ATOM	115	CA	PHE	8	27.880	36.658	30.730	1.00	0.00	C
ATOM	116	HA	PHE	8	27.459	35.889	30.082	1.00	0.00	H
ATOM	117	CB	PHE	8	26.816	37.571	31.314	1.00	0.00	C
ATOM	118	HB2	PHE	8	27.280	38.366	31.895	1.00	0.00	H
ATOM	119	HB3	PHE	8	26.328	38.133	30.518	1.00	0.00	H
ATOM	120	CG	PHE	8	25.796	37.019	32.306	1.00	0.00	C
ATOM	121	CD1	PHE	8	25.553	35.633	32.177	1.00	0.00	C
ATOM	122	HD1	PHE	8	25.959	34.968	31.429	1.00	0.00	H
ATOM	123	CE1	PHE	8	24.459	35.129	32.913	1.00	0.00	C
ATOM	124	HE1	PHE	8	24.236	34.074	32.847	1.00	0.00	H
ATOM	125	CZ	PHE	8	23.636	35.984	33.631	1.00	0.00	C

ATOM	126	HZ	PHE	8	22.767	35.637	34.171	1.00	0.00	H
ATOM	127	CE2	PHE	8	23.910	37.331	33.815	1.00	0.00	C
ATOM	128	HE2	PHE	8	23.301	38.072	34.312	1.00	0.00	H
ATOM	129	CD2	PHE	8	24.994	37.862	33.090	1.00	0.00	C
ATOM	130	HD2	PHE	8	24.995	38.933	32.958	1.00	0.00	H
ATOM	131	C	PHE	8	28.904	37.361	29.817	1.00	0.00	C
ATOM	132	O	PHE	8	30.099	37.532	30.061	1.00	0.00	O
ATOM	133	N	GLN	9	28.461	37.777	28.615	1.00	0.00	N
ATOM	134	H	GLN	9	27.455	37.713	28.559	1.00	0.00	H
ATOM	135	CA	GLN	9	29.282	38.120	27.468	1.00	0.00	C
ATOM	136	HA	GLN	9	30.107	37.410	27.408	1.00	0.00	H
ATOM	137	CB	GLN	9	28.340	38.067	26.170	1.00	0.00	C
ATOM	138	HB2	GLN	9	28.073	37.020	26.035	1.00	0.00	H
ATOM	139	HB3	GLN	9	27.490	38.685	26.461	1.00	0.00	H
ATOM	140	CG	GLN	9	28.928	38.599	24.916	1.00	0.00	C
ATOM	141	HG2	GLN	9	29.306	39.595	25.147	1.00	0.00	H
ATOM	142	HG3	GLN	9	29.863	38.078	24.713	1.00	0.00	H
ATOM	143	CD	GLN	9	28.121	38.558	23.641	1.00	0.00	C
ATOM	144	OE1	GLN	9	26.924	38.243	23.607	1.00	0.00	O
ATOM	145	NE2	GLN	9	28.743	38.940	22.552	1.00	0.00	N
ATOM	146	HE21	GLN	9	28.232	38.895	21.684	1.00	0.00	H
ATOM	147	HE22	GLN	9	29.609	39.456	22.607	1.00	0.00	H
ATOM	148	C	GLN	9	30.049	39.394	27.709	1.00	0.00	C
ATOM	149	O	GLN	9	31.124	39.540	27.120	1.00	0.00	O
ATOM	150	N	ARG	10	29.495	40.281	28.525	1.00	0.00	N
ATOM	151	H	ARG	10	28.571	40.065	28.871	1.00	0.00	H
ATOM	152	CA	ARG	10	30.016	41.591	28.859	1.00	0.00	C
ATOM	153	HA	ARG	10	30.184	42.153	27.939	1.00	0.00	H
ATOM	154	CB	ARG	10	28.927	42.313	29.642	1.00	0.00	C
ATOM	155	HB2	ARG	10	27.956	42.244	29.154	1.00	0.00	H
ATOM	156	HB3	ARG	10	28.755	41.765	30.568	1.00	0.00	H
ATOM	157	CG	ARG	10	29.272	43.716	30.019	1.00	0.00	
C										
ATOM	158	HG2	ARG	10	28.451	44.066	30.644	1.00	0.00	H
ATOM	159	HG3	ARG	10	30.213	43.691	30.569	1.00	0.00	H
ATOM	160	CD	ARG	10	29.269	44.723	28.871	1.00	0.00	C
ATOM	161	HD2	ARG	10	28.669	44.429	28.011	1.00	0.00	H
ATOM	162	HD3	ARG	10	28.893	45.690	29.205	1.00	0.00	H
ATOM	163	NE	ARG	10	30.687	44.872	28.237	1.00	0.00	N
ATOM	164	HE	ARG	10	31.469	44.821	28.873	1.00	0.00	H
ATOM	165	CZ	ARG	10	30.927	44.788	26.975	1.00	0.00	C
ATOM	166	NH1	ARG	10	30.049	44.518	26.020	1.00	0.00	N
ATOM	167	HH11	ARG	10	30.377	44.342	25.081	1.00	0.00	H
ATOM	168	HH12	ARG	10	29.064	44.422	26.221	1.00	0.00	H
ATOM	169	NH2	ARG	10	32.117	44.865	26.492	1.00	0.00	N
ATOM	170	HH21	ARG	10	32.887	45.045	27.120	1.00	0.00	H
ATOM	171	HH22	ARG	10	32.285	44.893	25.497	1.00	0.00	H
ATOM	172	C	ARG	10	31.457	41.614	29.433	1.00	0.00	C
ATOM	173	O	ARG	10	32.182	42.621	29.297	1.00	0.00	O
ATOM	174	N	PHE	11	31.855	40.547	30.166	1.00	0.00	N
ATOM	175	H	PHE	11	31.174	39.814	30.310	1.00	0.00	H
ATOM	176	CA	PHE	11	33.099	40.525	30.964	1.00	0.00	C
ATOM	177	HA	PHE	11	33.426	41.563	31.023	1.00	0.00	H
ATOM	178	CB	PHE	11	32.749	40.071	32.356	1.00	0.00	C
ATOM	179	HB2	PHE	11	33.649	40.128	32.968	1.00	0.00	H
ATOM	180	HB3	PHE	11	32.404	39.038	32.391	1.00	0.00	H
ATOM	181	CG	PHE	11	31.658	40.910	32.994	1.00	0.00	C
ATOM	182	CD1	PHE	11	31.996	42.017	33.804	1.00	0.00	C
ATOM	183	HD1	PHE	11	33.035	42.315	33.794	1.00	0.00	H
ATOM	184	CE1	PHE	11	31.023	42.894	34.329	1.00	0.00	C
ATOM	185	HE1	PHE	11	31.379	43.775	34.843	1.00	0.00	H
ATOM	186	CZ	PHE	11	29.686	42.565	34.170	1.00	0.00	C
ATOM	187	HZ	PHE	11	29.005	43.343	34.481	1.00	0.00	H
ATOM	188	CE2	PHE	11	29.288	41.368	33.520	1.00	0.00	C

ATOM	189	HE2	PHE	11	28.236	41.156	33.405	1.00	0.00	H
ATOM	190	CD2	PHE	11	30.321	40.487	33.019	1.00	0.00	C
ATOM	191	HD2	PHE	11	30.075	39.528	32.586	1.00	0.00	H
ATOM	192	C	PHE	11	34.283	39.718	30.290	1.00	0.00	C
ATOM	193	O	PHE	11	35.348	39.518	30.842	1.00	0.00	O
ATOM	194	N	GLN	12	34.071	39.126	29.090	1.00	0.00	N
ATOM	195	H	GLN	12	33.192	39.318	28.631	1.00	0.00	H
ATOM	196	CA	GLN	12	34.966	38.074	28.478	1.00	0.00	C
ATOM	197	HA	GLN	12	35.201	37.201	29.087	1.00	0.00	H
ATOM	198	CB	GLN	12	34.361	37.668	27.171	1.00	0.00	C
ATOM	199	HB2	GLN	12	33.992	38.524	26.605	1.00	0.00	H
ATOM	200	HB3	GLN	12	35.208	37.275	26.609	1.00	0.00	H
ATOM	201	CG	GLN	12	33.275	36.574	27.372	1.00	0.00	C
ATOM	202	HG2	GLN	12	33.579	35.851	28.128	1.00	0.00	H
ATOM	203	HG3	GLN	12	32.368	37.035	27.761	1.00	0.00	H
ATOM	204	CD	GLN	12	32.894	35.703	26.128	1.00	0.00	C
ATOM	205	OE1	GLN	12	33.002	36.241	25.023	1.00	0.00	O
ATOM	206	NE2	GLN	12	32.312	34.547	26.270	1.00	0.00	N
ATOM	207	HE21	GLN	12	32.097	34.111	27.154	1.00	0.00	H
ATOM	208	HE22	GLN	12	31.879	34.152	25.447	1.00	0.00	H
ATOM	209	C	GLN	12	36.363	38.591	28.269	1.00	0.00	C
ATOM	210	O	GLN	12	37.368	37.929	28.650	1.00	0.00	O
ATOM	211	N	ASP	13	36.532	39.741	27.602	1.00	0.00	N
ATOM	212	H	ASP	13	35.713	40.216	27.250	1.00	0.00	H
ATOM	213	CA	ASP	13	37.882	40.318	27.226	1.00	0.00	C
ATOM	214	HA	ASP	13	38.470	39.519	26.775	1.00	0.00	H
ATOM	215	CB	ASP	13	37.810	41.461	26.178	1.00	0.00	C
ATOM	216	HB2	ASP	13	37.383	42.355	26.630	1.00	0.00	H
ATOM	217	HB3	ASP	13	38.755	41.816	25.767	1.00	0.00	H
ATOM	218	CG	ASP	13	36.936	41.110	25.028	1.00	0.00	C
ATOM	219	OD1	ASP	13	36.848	39.835	24.719	1.00	0.00	O
ATOM	220	OD2	ASP	13	36.229	41.928	24.427	1.00	0.00	O
ATOM	221	C	ASP	13	38.676	40.809	28.417	1.00	0.00	C
ATOM	222	O	ASP	13	39.929	40.796	28.475	1.00	0.00	O
ATOM	223	N	ASP	14	37.950	41.146	29.499	1.00	0.00	N
ATOM	224	H	ASP	14	36.944	41.144	29.405	1.00	0.00	H
ATOM	225	CA	ASP	14	38.509	41.466	30.813	1.00	0.00	C
ATOM	226	HA	ASP	14	39.420	42.044	30.658	1.00	0.00	H
ATOM	227	CB	ASP	14	37.460	42.314	31.600	1.00	0.00	C
ATOM	228	HB2	ASP	14	36.563	41.707	31.731	1.00	0.00	H
ATOM	229	HB3	ASP	14	37.935	42.687	32.507	1.00	0.00	H
ATOM	230	CG	ASP	14	37.213	43.676	30.899	1.00	0.00	C
ATOM	231	OD1	ASP	14	36.106	44.229	31.129	1.00	0.00	O
ATOM	232	OD2	ASP	14	38.099	44.237	30.263	1.00	0.00	O
ATOM	233	C	ASP	14	38.874	40.276	31.653	1.00	0.00	C
ATOM	234	O	ASP	14	39.766	40.374	32.555	1.00	0.00	O
ATOM	235	N	ILE	15	38.320	39.060	31.351	1.00	0.00	N
ATOM	236	H	ILE	15	37.579	39.077	30.665	1.00	0.00	H
ATOM	237	CA	ILE	15	38.714	37.753	31.973	1.00	0.00	C
ATOM	238	HA	ILE	15	38.945	37.777	33.038	1.00	0.00	H
ATOM	239	CB	ILE	15	37.495	36.810	31.739	1.00	0.00	C
ATOM	240	HB	ILE	15	37.141	36.872	30.710	1.00	0.00	H
ATOM	241	CG2	ILE	15	37.926	35.366	31.961	1.00	0.00	C
ATOM	242	HG21	ILE	15	37.055	34.752	31.731	1.00	0.00	H
ATOM	243	HG22	ILE	15	38.788	35.115	31.342	1.00	0.00	H
ATOM	244	HG23	ILE	15	38.167	35.236	33.016	1.00	0.00	H
ATOM	245	CG1	ILE	15	36.246	37.119	32.644	1.00	0.00	C
ATOM	246	HG12	ILE	15	36.491	36.720	33.629	1.00	0.00	H
ATOM	247	HG13	ILE	15	36.182	38.196	32.806	1.00	0.00	H
ATOM	248	CD1	ILE	15	34.962	36.363	32.254	1.00	0.00	C
ATOM	249	HD11	ILE	15	34.124	36.771	32.821	1.00	0.00	H
ATOM	250	HD12	ILE	15	34.752	36.512	31.196	1.00	0.00	H
ATOM	251	HD13	ILE	15	35.059	35.285	32.384	1.00	0.00	H
ATOM	252	C	ILE	15	39.996	37.305	31.278	1.00	0.00	C

```
ATOM    253  O   ILE    15      41.052  36.984  31.889  1.00  0.00           O
ATOM    254  N   LEU    16      40.012  37.402  29.968  1.00  0.00           N
ATOM    255  H   LEU    16      39.247  37.890  29.525  1.00  0.00           H
ATOM    256  CA  LEU    16      41.193  37.252  29.052  1.00  0.00           C
ATOM    257  HA  LEU    16      41.508  36.208  29.047  1.00  0.00           H
ATOM    258  CB  LEU    16      40.810  37.590  27.591  1.00  0.00           C
ATOM    259  HB2 LEU    16      39.961  36.933  27.404  1.00  0.00           H
ATOM    260  HB3 LEU    16      40.449  38.616  27.667  1.00  0.00           H
ATOM    261  CG  LEU    16      42.002  37.485  26.612  1.00  0.00           C
ATOM    262  HG  LEU    16      42.766  38.133  27.045  1.00  0.00           H
ATOM    263  CD1 LEU    16      42.615  36.123  26.439  1.00  0.00           C
ATOM    264 HD11 LEU    16      41.911  35.379  26.066  1.00  0.00           H
ATOM    265 HD12 LEU    16      43.402  36.133  25.686  1.00  0.00           H
ATOM    266 HD13 LEU    16      42.872  35.774  27.439  1.00  0.00           H
ATOM    267  CD2 LEU    16      41.691  37.983  25.174  1.00  0.00           C
ATOM    268 HD21 LEU    16      40.973  37.343  24.664  1.00  0.00           H
ATOM    269 HD22 LEU    16      41.196  38.954  25.198  1.00  0.00           H
ATOM    270 HD23 LEU    16      42.617  37.989  24.598  1.00  0.00           H
ATOM    271  C   LEU    16      42.441  38.104  29.482  1.00  0.00           C
ATOM    272  O   LEU    16      43.571  37.621  29.649  1.00  0.00           O
ATOM    273  N   ALA    17      42.212  39.329  29.854  1.00  0.00           N
ATOM    274  H   ALA    17      41.258  39.653  29.791  1.00  0.00           H
ATOM    275  CA  ALA    17      43.200  40.280  30.431  1.00  0.00           C
ATOM    276  HA  ALA    17      44.158  40.112  29.939  1.00  0.00           H
ATOM    277  CB  ALA    17      42.690  41.742  30.266  1.00  0.00           C
ATOM    278  HB1 ALA    17      43.447  42.440  30.624  1.00  0.00           H
ATOM    279  HB2 ALA    17      42.591  41.923  29.195  1.00  0.00           H
ATOM    280  HB3 ALA    17      41.646  41.811  30.570  1.00  0.00           H
ATOM    281  C   ALA    17      43.586  40.017  31.935  1.00  0.00           C
ATOM    282  O   ALA    17      44.534  40.549  32.470  1.00  0.00           O
ATOM    283  N   GLY
    18      42.871  38.977  32.605  1.00  0.00           N
ATOM    284  H   GLY    18      42.082  38.656  32.063  1.00  0.00           H
ATOM    285  CA  GLY    18      42.836  38.833  34.085  1.00  0.00           C
ATOM    286  HA2 GLY    18      42.152  38.028  34.352  1.00  0.00           H
ATOM    287  HA3 GLY    18      43.851  38.567  34.382  1.00  0.00           H
ATOM    288  C   GLY    18      42.478  40.063  34.864  1.00  0.00           C
ATOM    289  O   GLY    18      42.727  40.102  36.103  1.00  0.00           O
ATOM    290  N   ARG    19      41.830  41.080  34.249  1.00  0.00           N
ATOM    291  H   ARG    19      41.497  40.905  33.311  1.00  0.00           H
ATOM    292  CA  ARG    19      41.163  42.187  35.064  1.00  0.00           C
ATOM    293  HA  ARG    19      41.904  42.594  35.750  1.00  0.00           H
ATOM    294  CB  ARG    19      40.810  43.214  34.013  1.00  0.00           C
ATOM    295  HB2 ARG    19      41.796  43.543  33.686  1.00  0.00           H
ATOM    296  HB3 ARG    19      40.267  42.739  33.196  1.00  0.00           H
ATOM    297  CG  ARG    19      40.026  44.429  34.421  1.00  0.00           C
ATOM    298  HG2 ARG    19      39.688  44.854  33.475  1.00  0.00           H
ATOM    299  HG3 ARG    19      39.090  44.094  34.868  1.00  0.00           H
ATOM    300  CD  ARG    19      41.036  45.285  35.276  1.00  0.00           C
ATOM    301  HD2 ARG    19      41.622  44.673  35.962  1.00  0.00           H
ATOM    302  HD3 ARG    19      41.766  45.714  34.589  1.00  0.00           H
ATOM    303  NE  ARG    19      40.285  46.434  35.956  1.00  0.00           N
ATOM    304  HE  ARG    19      39.765  47.005  35.307  1.00  0.00           H
ATOM    305  CZ  ARG    19      40.324  46.790  37.222  1.00  0.00           C
ATOM    306  NH1 ARG    19      41.167  46.215  37.983  1.00  0.00           N
ATOM    307 HH11 ARG    19      41.468  45.268  37.801  1.00  0.00           H
ATOM    308 HH12 ARG    19      41.428  46.678  38.842  1.00  0.00           H
ATOM    309  NH2 ARG    19      39.626  47.749  37.747  1.00  0.00           N
ATOM    310 HH21 ARG    19      39.587  47.883  38.747  1.00  0.00           H
ATOM    311 HH22 ARG    19      38.868  48.202  37.257  1.00  0.00           H
ATOM    312  C   ARG    19      39.940  41.830  35.857  1.00  0.00           C
ATOM    313  O   ARG    19      39.592  42.426  36.893  1.00  0.00           O
ATOM    335  N   THR    21      38.985  38.677  37.904  1.00  0.00           N
ATOM    336  H   THR    21      38.847  39.537  38.416  1.00  0.00           H
```

ATOM	337	CA	THR	21	39.734	37.521	38.487	1.00	0.00	C
ATOM	338	HA	THR	21	39.580	36.679	37.813	1.00	0.00	H
ATOM	339	CB	THR	21	41.207	37.947	38.434	1.00	0.00	C
ATOM	340	HB	THR	21	41.411	38.057	37.370	1.00	0.00	H
ATOM	341	CG2	THR	21	41.563	39.207	39.277	1.00	0.00	C
ATOM	342	HG21	THR	21	41.051	40.104	38.926	1.00	0.00	H
ATOM	343	HG22	THR	21	41.394	38.947	40.322	1.00	0.00	H
ATOM	344	HG23	THR	21	42.638	39.287	39.113	1.00	0.00	H
ATOM	345	OG1	THR	21	42.021	36.868	38.952	1.00	0.00	O
ATOM	346	HG1	THR	21	41.619	36.029	38.716	1.00	0.00	H
ATOM	347	C	THR	21	39.245	37.074	39.852	1.00	0.00	C
ATOM	348	O	THR	21	39.688	36.078	40.435	1.00	0.00	O
ATOM	349	N	ILE	22	38.266	37.780	40.447	1.00	0.00	N
ATOM	350	H	ILE	22	38.065	38.732	40.172	1.00	0.00	H
ATOM	351	CA	ILE	22	37.514	37.527	41.677	1.00	0.00	C
ATOM	352	HA	ILE	22	37.425	36.441	41.726	1.00	0.00	H
ATOM	353	CB	ILE	22	38.227	38.039	42.941	1.00	0.00	C
ATOM	354	HB	ILE	22	39.232	37.620	42.898	1.00	0.00	H
ATOM	355	CG2	ILE	22	38.315	39.516	42.964	1.00	0.00	C
ATOM	356	HG21	ILE	22	38.497	39.976	41.993	1.00	0.00	H
ATOM	357	HG22	ILE	22	37.387	40.036	43.199	1.00	0.00	H
ATOM	358	HG23	ILE	22	39.134	39.840	43.606	1.00	0.00	H
ATOM	359	CG1	ILE	22	37.550	37.614	44.223	1.00	0.00	C
ATOM	360	HG12	ILE	22	36.620	38.162	44.373	1.00	0.00	H
ATOM	361	HG13	ILE	22	37.244	36.568	44.231	1.00	0.00	H
ATOM	362	CD1	ILE	22	38.231	37.795	45.636	1.00	0.00	C
ATOM	363	HD11	ILE	22	38.356	38.829	45.956	1.00	0.00	H
ATOM	364	HD12	ILE	22	37.915	37.173	46.473	1.00	0.00	H
ATOM	365	HD13	ILE	22	39.176	37.271	45.496	1.00	0.00	H
ATOM	366	C	ILE	22	36.115	38.112	41.618	1.00	0.00	C
ATOM	367	O	ILE	22	35.893	39.110	40.958	1.00	0.00	O
ATOM	368	N	THR	23	35.114	37.418	42.207	1.00	0.00	N
ATOM	369	H	THR	23	35.445	36.547	42.596	1.00	0.00	H
ATOM	370	CA	THR	23	33.698	37.802	42.201	1.00	0.00	C
ATOM	371	HA	THR	23	33.741	38.890	42.253	1.00	0.00	H
ATOM	372	CB	THR	23	33.034	37.449	40.852	1.00	0.00	C
ATOM	373	HB	THR	23	33.817	37.572	40.104	1.00	0.00	H
ATOM	374	CG2	THR	23	32.472	36.025	40.814	1.00	0.00	C
ATOM	375	HG21	THR	23	33.252	35.327	41.120	1.00	0.00	H
ATOM	376	HG22	THR	23	31.652	35.986	41.530	1.00	0.00	H
ATOM	377	HG23	THR	23	32.232	35.776	39.780	1.00	0.00	H
ATOM	378	OG1	THR	23	32.002	38.407	40.630	1.00	0.00	O
ATOM	379	HG1	THR	23	32.392	39.145	40.157	1.00	0.00	H
ATOM	380	C	THR	23	32.806	37.297	43.406	1.00	0.00	C
ATOM	381	O	THR	23	33.002	36.194	43.927	1.00	0.00	O
ATOM	382	N	ILE	24	31.825	38.147	43.756	1.00	0.00	N
ATOM	383	H	ILE	24	31.755	39.045	43.300	1.00	0.00	H
ATOM	384	CA	ILE	24	30.784	37.823	44.761	1.00	0.00	C
ATOM	385	HA	ILE	24	31.210	37.041	45.390	1.00	0.00	H
ATOM	386	CB	ILE	24	30.515	39.006	45.630	1.00	0.00	C
ATOM	387	HB	ILE	24	30.186	39.796	44.956	1.00	0.00	H
ATOM	388	CG2	ILE	24	29.431	38.883	46.726	1.00	0.00	C
ATOM	389	HG21	ILE	24	28.510	38.763	46.157	1.00	0.00	H
ATOM	390	HG22	ILE	24	29.560	38.142	47.516	1.00	0.00	H
ATOM	391	HG23	ILE	24	29.218	39.785	47.299	1.00	0.00	H
ATOM	392	CG1	ILE	24	31.753	39.417	46.507	1.00	0.00	C
ATOM	393	HG12	ILE	24	31.795	38.949	47.490	1.00	0.00	H
ATOM	394	HG13	ILE	24	32.659	39.141	45.967	1.00	0.00	H
ATOM	395	CD1	ILE	24	31.924	40.920	46.647	1.00	0.00	C
ATOM	396	HD11	ILE	24	31.905	41.404	45.670	1.00	0.00	H
ATOM	397	HD12	ILE	24	31.094	41.307	47.237	1.00	0.00	H
ATOM	398	HD13	ILE	24	32.903	41.096	47.092	1.00	0.00	H
ATOM	399	C	ILE	24	29.502	37.371	44.057	1.00	0.00	C
ATOM	400	O	ILE	24	29.217	37.683	42.895	1.00	0.00	O

ATOM	401	N	ARG	25	28.725	36.533	44.751	1.00	0.00	N
ATOM	402	H	ARG	25	29.223	36.154	45.543	1.00	0.00	H
ATOM	403	CA	ARG	25	27.428	35.870	44.467	1.00	0.00	C
ATOM	404	HA	ARG	25	26.803	36.553	43.891	1.00	0.00	H
ATOM	405	CB	ARG	25	27.625	34.535	43.739	1.00	0.00	C
ATOM	406	HB2	ARG	25	28.323	33.934	44.323	1.00	0.00	H
ATOM	407	HB3	ARG	25	26.721	33.926	43.763	1.00	0.00	H
ATOM	408	CG	ARG	25	28.200	34.594	42.368	1.00	0.00	C
ATOM	409	HG2	ARG	25	29.179	35.069	42.430	1.00	0.00	H
ATOM	410	HG3	ARG	25	28.352	33.604	41.936	1.00	0.00	H
ATOM	411	CD	ARG	25	27.231	35.317	41.425	1.00	0.00	C
ATOM	412	HD2	ARG	25	26.264	34.814	41.453	1.00	0.00	H
ATOM	413	HD3	ARG	25	27.046	36.291	41.878	1.00	0.00	H
ATOM	414	NE	ARG	25	27.622	35.246	40.008	1.00	0.00	N
ATOM	415	HE	ARG	25	27.142	34.514	39.505	1.00	0.00	H
ATOM	416	CZ	ARG	25	28.411	36.160	39.440	1.00	0.00	C
ATOM	417	NH1	ARG	25	29.125	36.972	40.174	1.00	0.00	N
ATOM	418	HH11	ARG	25	29.830	37.507	39.689	1.00	0.00	H
ATOM	419	HH12	ARG	25	29.053	36.998	41.181	1.00	0.00	H
ATOM	420	NH2	ARG	25	28.536	36.414	38.183	1.00	0.00	N
ATOM	421	HH21	ARG	25	29.127	37.183	37.901	1.00	0.00	H
ATOM	422	HH22	ARG	25	27.765	36.077	37.625	1.00	0.00	H
ATOM	423	C	ARG	25	26.674	35.650	45.738	1.00	0.00	C
ATOM	424	O	ARG	25	27.286	35.666	46.833	1.00	0.00	O
ATOM	425	N	ASP	26	25.404	35.553	45.580	1.00	0.00	N
ATOM	426	H	ASP	26	25.005	35.283	44.692	1.00	0.00	H
ATOM	427	CA	ASP	26	24.531	35.190	46.795	1.00	0.00	C
ATOM	428	HA	ASP	26	25.021	35.385	47.750	1.00	0.00	H
ATOM	429	CB	ASP	26	23.247	35.937	46.702	1.00	0.00	C
ATOM	430	HB2	ASP	26	22.600	35.765	47.562	1.00	0.00	H
ATOM	431	HB3	ASP	26	23.441	37.008	46.650	1.00	0.00	H
ATOM	432	CG	ASP	26	22.398	35.642	45.488	1.00	0.00	C
ATOM	433	OD1	ASP	26	21.460	34.851	45.629	1.00	0.00	O
ATOM	434	OD2	ASP	26	22.613	36.260	44.430	1.00	0.00	O
ATOM	435	C	ASP	26	24.339	33.631	46.754	1.00	0.00	C
ATOM	436	O	ASP	26	24.383	33.122	45.626	1.00	0.00	O
ATOM	437	N	GLU	27	24.296	32.900	47.840	1.00	0.00	N
ATOM	438	H	GLU	27	24.515	33.303	48.740	1.00	0.00	H
ATOM	439	CA	GLU	27	24.241	31.463	47.858	1.00	0.00	C
ATOM	440	HA	GLU	27	25.233	31.087	47.611	1.00	0.00	H
ATOM	441	CB	GLU	27	23.821	30.932	49.259	1.00	0.00	C
ATOM	442	HB2	GLU	27	23.028	31.532	49.703	1.00	0.00	H
ATOM	443	HB3	GLU	27	23.245	30.012	49.159	1.00	0.00	H
ATOM	444	CG	GLU	27	25.009	30.830	50.224	1.00	0.00	C
ATOM	445	HG2	GLU	27	25.566	31.766	50.274	1.00	0.00	H
ATOM	446	HG3	GLU	27	24.648	30.749	51.250	1.00	0.00	H
ATOM	447	CD	GLU	27	25.909	29.636	49.875	1.00	0.00	C
ATOM	448	OE1	GLU	27	25.816	29.033	48.770	1.00	0.00	O
ATOM	449	OE2	GLU	27	26.509	29.098	50.815	1.00	0.00	O
ATOM	450	C	GLU	27	23.290	30.726	46.917	1.00	0.00	C
ATOM	451	O	GLU	27	22.191	31.169	46.686	1.00	0.00	O
ATOM	452	N	SER	28	23.779	29.567	46.467	1.00	0.00	N
ATOM	453	H	SER	28	24.516	29.107	46.981	1.00	0.00	H
ATOM	454	CA	SER	28	23.048	28.620	45.577	1.00	0.00	C
ATOM	455	HA	SER	28	22.071	28.420	46.018	1.00	0.00	H
ATOM	456	CB	SER	28	22.680	29.340	44.264	1.00	0.00	C
ATOM	457	HB2	SER	28	21.992	30.184	44.330	1.00	0.00	H
ATOM	458	HB3	SER	28	23.555	29.847	43.857	1.00	0.00	H
ATOM	459	OG	SER	28	22.102	28.466	43.323	1.00	0.00	O
ATOM	460	HG	SER	28	21.440	27.951	43.789	1.00	0.00	H
ATOM	461	C	SER	28	23.802	27.342	45.330	1.00	0.00	C
ATOM	462	O	SER	28	24.976	27.329	45.199	1.00	0.00	O
ATOM	463	N	GLU	29	23.117	26.161	45.281	1.00	0.00	N

ATOM	464	H	GLU	29	22.127	26.198	45.481	1.00	0.00	H
ATOM	465	CA	GLU	29	23.799	24.811	45.123	1.00	0.00	C
ATOM	466	HA	GLU	29	24.520	24.685	45.931	1.00	0.00	H
ATOM	467	CB	GLU	29	22.750	23.673	45.179	1.00	0.00	C
ATOM	468	HB2	GLU	29	21.986	23.847	45.936	1.00	0.00	H
ATOM	469	HB3	GLU	29	22.187	23.610	44.248	1.00	0.00	H
ATOM	470	CG	GLU	29	23.490	22.397	45.493	1.00	0.00	C
ATOM	471	HG2	GLU	29	22.821	21.598	45.176	1.00	0.00	H
ATOM	472	HG3	GLU	29	24.445	22.294	44.976	1.00	0.00	H
ATOM	473	CD	GLU	29	23.719	22.221	46.963	1.00	0.00	C
ATOM	474	OE1	GLU	29	24.826	22.454	47.412	1.00	0.00	O
ATOM	475	OE2	GLU	29	22.779	21.737	47.647	1.00	0.00	O
ATOM	476	C	GLU	29	24.553	24.780	43.796	1.00	0.00	C
ATOM	477	O	GLU	29	25.649	24.173	43.696	1.00	0.00	O
ATOM	478	N	SER	30	24.149	25.614	42.799	1.00	0.00	N
ATOM	479	H	SER	30	23.390	26.240	43.032	1.00	0.00	H
ATOM	480	CA	SER	30	24.639	25.518	41.420	1.00	0.00	C
ATOM	481	HA	SER	30	24.622	24.471	41.117	1.00	0.00	H
ATOM	482	CB	SER	30	23.803	26.352	40.485	1.00	0.00	C
ATOM	483	HB2	SER	30	24.321	26.357	39.526	1.00	0.00	H
ATOM	484	HB3	SER	30	22.781	25.978	40.441	1.00	0.00	H
ATOM	485	OG	SER	30	23.645	27.693	41.006	1.00	0.00	O
ATOM	486	HG	SER	30	22.960	27.773	41.672	1.00	0.00	H
ATOM	487	C	SER	30	26.153	25.905	41.325	1.00	0.00	C
ATOM	488	O	SER	30	26.854	25.565	40.375	1.00	0.00	O
ATOM	489	N	HIE	31	26.661	26.648	42.293	1.00	0.00	N
ATOM	490	H	HIE	31	25.959	26.925	42.964	1.00	0.00	H
ATOM	491	CA	HIE	31	27.922	27.400	42.300	1.00	0.00	C
ATOM	492	HA	HIE	31	27.794	28.068	41.448	1.00	0.00	H
ATOM	493	CB	HIE	31	28.006	28.182	43.594	1.00	0.00	C
ATOM	494	HB2	HIE	31	27.746	27.551	44.444	1.00	0.00	H
ATOM	495	HB3	HIE	31	29.036	28.520	43.703	1.00	0.00	H
ATOM	496	CG	HIE	31	27.120	29.432	43.564	1.00	0.00	C
ATOM	497	ND1	HIE	31	26.808	30.165	42.466	1.00	0.00	N
ATOM	498	CE1	HIE	31	25.969	31.202	42.759	1.00	0.00	C
ATOM	499	HE1	HIE	31	25.343	31.783	42.098	1.00	0.00	H
ATOM	500	NE2	HIE	31	25.780	31.109	44.115	1.00	0.00	N
ATOM	501	HE2	HIE	31	25.127	31.693	44.618	1.00	0.00	H
ATOM	502	CD2	HIE	31	26.487	30.040	44.642	1.00	0.00	C
ATOM	503	HD2	HIE	31	26.673	29.698	45.649	1.00	0.00	H
ATOM	504	C	HIE	31	29.159	26.595	42.082	1.00	0.00	C
ATOM	505	O	HIE	31	29.184	25.365	42.215	1.00	0.00	O
ATOM	506	N	PHE	32	30.210	27.251	41.512	1.00	0.00	N
ATOM	507	H	PHE	32	30.153	28.253	41.395	1.00	0.00	H
ATOM	508	CA	PHE	32	31.485	26.716	41.404	1.00	0.00	C
ATOM	509	HA	PHE	32	31.437	25.781	40.845	1.00	0.00	H
ATOM	510	CB	PHE	32	32.420	27.720	40.651	1.00	0.00	C
ATOM	511	HB2	PHE	32	32.637	28.561	41.311	1.00	0.00	H
ATOM	512	HB3	PHE	32	33.415	27.276	40.683	1.00	0.00	H
ATOM	513	CG	PHE	32	32.123	28.042	39.270	1.00	0.00	C
ATOM	514	CD1	PHE	32	32.091	29.334	38.856	1.00	0.00	C
ATOM	515	HD1	PHE	32	32.522	30.073	39.515	1.00	0.00	H
ATOM	516	CE1	PHE	32	31.757	29.675	37.556	1.00	0.00	C
ATOM	517	HE1	PHE	32	31.698	30.716	37.275	1.00	0.00	H
ATOM	518	CZ	PHE	32	31.254	28.672	36.674	1.00	0.00	C
ATOM	519	HZ	PHE	32	31.029	28.885	35.640	1.00	0.00	H
ATOM	520	CE2	PHE	32	31.347	27.330	37.028	1.00	0.00	C
ATOM	521	HE2	PHE	32	31.016	26.574	36.331	1.00	0.00	H
ATOM	522	CD2	PHE	32	31.835	27.035	38.329	1.00	0.00	C
ATOM	523	HD2	PHE	32	31.857	26.004	38.652	1.00	0.00	H
ATOM	524	C	PHE	32	32.137	26.393	42.793	1.00	0.00	C
ATOM	525	O	PHE	32	31.830	26.942	43.819	1.00	0.00	O
ATOM	526	N	LYS	33	33.035	25.425	42.729	1.00	0.00	N
ATOM	527	H	LYS	33	33.217	25.139	41.777	1.00	0.00	H

ATOM	528	CA	LYS	33	33.714	24.701	43.789	1.00	0.00	C
ATOM	529	HA	LYS	33	33.554	25.120	44.783	1.00	0.00	H
ATOM	530	CB	LYS	33	33.382	23.198	43.828	1.00	0.00	C
ATOM	531	HB2	LYS	33	33.628	22.663	42.912	1.00	0.00	H
ATOM	532	HB3	LYS	33	33.841	22.673	44.665	1.00	0.00	H
ATOM	533	CG	LYS	33	31.861	23.157	43.976	1.00	0.00	C
ATOM	534	HG2	LYS	33	31.510	23.891	44.700	1.00	0.00	H
ATOM	535	HG3	LYS	33	31.431	23.323	42.988	1.00	0.00	H
ATOM	536	CD	LYS	33	31.409	21.714	44.418	1.00	0.00	C
ATOM	537	HD2	LYS	33	31.905	20.947	43.821	1.00	0.00	H
ATOM	538	HD3	LYS	33	31.623	21.587	45.479	1.00	0.00	H
ATOM	539	CE	LYS	33	29.909	21.644	44.374	1.00	0.00	C
ATOM	540	HE2	LYS	33	29.554	22.636	44.658	1.00	0.00	H
ATOM	541	HE3	LYS	33	29.461	21.549	43.385	1.00	0.00	H
ATOM	542	NZ	LYS	33	29.364	20.694	45.321	1.00	0.00	N
ATOM	543	HZ1	LYS	33	28.379	20.573	45.130	1.00	0.00	H
ATOM	544	HZ2	LYS	33	29.794	19.789	45.191	1.00	0.00	H
ATOM	545	HZ3	LYS	33	29.597	21.101	46.215	1.00	0.00	H
ATOM	546	C	LYS	33	35.262	24.848	43.482	1.00	0.00	C
ATOM	547	O	LYS	33	35.750	25.178	42.361	1.00	0.00	O
ATOM	548	N	THR	34	36.100	24.619	44.459	1.00	0.00	N
ATOM	549	H	THR	34	35.712	24.144	45.261	1.00	0.00	H
ATOM	550	CA	THR	34	37.561	24.644	44.238	1.00	0.00	C
ATOM	551	HA	THR	34	37.832	25.603	43.796	1.00	0.00	H
ATOM	552	CB	THR	34	38.366	24.618	45.522	1.00	0.00	C
ATOM	553	HB	THR	34	37.822	24.022	46.255	1.00	0.00	H
ATOM	554	CG2	THR	34	39.835	24.261	45.477	1.00	0.00	
C										
ATOM	555	HG21	THR	34	40.169	24.189	46.511	1.00	0.00	H
ATOM	556	HG22	THR	34	40.027	23.292	45.018	1.00	0.00	H
ATOM	557	HG23	THR	34	40.362	25.010	44.883	1.00	0.00	H
ATOM	558	OG1	THR	34	38.255	25.889	46.039	1.00	0.00	O
ATOM	559	HG1	THR	34	37.322	26.040	45.871	1.00	0.00	H
ATOM	560	C	THR	34	38.050	23.562	43.243	1.00	0.00	C
ATOM	561	O	THR	34	37.711	22.422	43.452	1.00	0.00	O
ATOM	562	N	GLY	35	38.788	23.953	42.243	1.00	0.00	N
ATOM	563	H	GLY	35	39.152	24.894	42.205	1.00	0.00	H
ATOM	564	CA	GLY	35	39.289	23.148	41.110	1.00	0.00	C
ATOM	565	HA2	GLY	35	39.916	23.903	40.636	1.00	0.00	H
ATOM	566	HA3	GLY	35	39.885	22.260	41.324	1.00	0.00	H
ATOM	567	C	GLY	35	38.301	22.804	39.941	1.00	0.00	C
ATOM	568	O	GLY	35	38.687	21.961	39.121	1.00	0.00	O
ATOM	569	N	ASP	36	37.190	23.556	39.943	1.00	0.00	N
ATOM	570	H	ASP	36	36.930	24.232	40.647	1.00	0.00	H
ATOM	571	CA	ASP	36	36.320	23.519	38.817	1.00	0.00	C
ATOM	572	HA	ASP	36	36.045	22.516	38.490	1.00	0.00	H
ATOM	573	CB	ASP	36	35.051	24.284	39.094	1.00	0.00	C
ATOM	574	HB2	ASP	36	35.278	25.263	39.514	1.00	0.00	H
ATOM	575	HB3	ASP	36	34.483	24.370	38.168	1.00	0.00	H
ATOM	576	CG	ASP	36	34.052	23.581	39.973	1.00	0.00	C
ATOM	577	OD1	ASP	36	32.928	24.054	40.322	1.00	0.00	O
ATOM	578	OD2	ASP	36	34.318	22.426	40.375	1.00	0.00	O
ATOM	579	C	ASP	36	36.947	24.212	37.584	1.00	0.00	C
ATOM	580	O	ASP	36	37.789	25.008	37.805	1.00	0.00	O
ATOM	581	N	VAL	37	36.545	23.949	36.376	1.00	0.00	N
ATOM	582	H	VAL	37	35.835	23.231	36.347	1.00	0.00	H
ATOM	583	CA	VAL	37	36.965	24.669	35.171	1.00	0.00	C
ATOM	584	HA	VAL	37	37.729	25.422	35.368	1.00	0.00	H
ATOM	585	CB	VAL	37	37.719	23.647	34.197	1.00	0.00	C
ATOM	586	HB	VAL	37	37.040	22.803	34.078	1.00	0.00	H
ATOM	587	CG1	VAL	37	38.177	24.212	32.852	1.00	0.00	C
ATOM	588	HG11	VAL	37	38.598	23.388	32.276	1.00	0.00	H
ATOM	589	HG12	VAL	37	37.387	24.738	32.317	1.00	0.00	H
ATOM	590	HG13	VAL	37	38.893	25.031	32.925	1.00	0.00	H

ATOM	591	CG2	VAL	37	39.005	23.192	34.740	1.00	0.00	C
ATOM	592	HG21	VAL	37	39.743	23.992	34.804	1.00	0.00	H
ATOM	593	HG22	VAL	37	38.842	22.837	35.757	1.00	0.00	H
ATOM	594	HG23	VAL	37	39.326	22.248	34.299	1.00	0.00	H
ATOM	595	C	VAL	37	35.768	25.405	34.372	1.00	0.00	C
ATOM	596	O	VAL	37	34.746	24.726	34.202	1.00	0.00	O
ATOM	597	N	LEU	38	36.044	26.510	33.689	1.00	0.00	N
ATOM	598	H	LEU	38	37.021	26.763	33.736	1.00	0.00	H
ATOM	599	CA	LEU	38	35.131	27.139	32.769	1.00	0.00	C
ATOM	600	HA	LEU	38	34.548	26.304	32.381	1.00	0.00	H
ATOM	601	CB	LEU	38	34.167	28.139	33.435	1.00	0.00	C
ATOM	602	HB2	LEU	38	33.525	28.646	32.715	1.00	0.00	H
ATOM	603	HB3	LEU	38	33.450	27.498	33.949	1.00	0.00	H
ATOM	604	CG	LEU	38	34.814	29.159	34.435	1.00	0.00	C
ATOM	605	HG	LEU	38	35.819	29.477	34.155	1.00	0.00	H
ATOM	606	CD1	LEU	38	33.902	30.471	34.504	1.00	0.00	C
ATOM	607	HD11	LEU	38	33.838	30.932	33.518	1.00	0.00	H
ATOM	608	HD12	LEU	38	32.855	30.307	34.758	1.00	0.00	H
ATOM	609	HD13	LEU	38	34.289	31.108	35.300	1.00	0.00	H
ATOM	610	CD2	LEU	38	34.755	28.615	35.868	1.00	0.00	C
ATOM	611	HD21	LEU	38	35.367	29.220	36.536	1.00	0.00	H
ATOM	612	HD22	LEU	38	33.735	28.620	36.250	1.00	0.00	H
ATOM	613	HD23	LEU	38	35.195	27.630	36.023	1.00	0.00	H
ATOM	614	C	LEU	38	35.978	27.719	31.526	1.00	0.00	C
ATOM	615	O	LEU	38	37.189	27.562	31.391	1.00	0.00	O
ATOM	616	N	ARG	39	35.270	28.382	30.603	1.00	0.00	N
ATOM	617	H	ARG	39	34.263	28.429	30.653	1.00	0.00	H
ATOM	618	CA	ARG	39	35.825	28.868	29.298	1.00	0.00	C
ATOM	619	HA	ARG	39	36.901	29.031	29.340	1.00	0.00	H
ATOM	620	CB	ARG	39	35.613	27.848	28.201	1.00	0.00	C
ATOM	621	HB2	ARG	39	34.598	27.730	27.824	1.00	0.00	H
ATOM	622	HB3	ARG	39	36.160	28.346	27.400	1.00	0.00	H
ATOM	623	CG	ARG	39	36.412	26.521	28.548	1.00	0.00	C
ATOM	624	HG2	ARG	39	37.390	26.662	29.006	1.00	0.00	H
ATOM	625	HG3	ARG	39	35.924	25.938	29.329	1.00	0.00	H
ATOM	626	CD	ARG	39	36.609	25.625	27.361	1.00	0.00	C
ATOM	627	HD2	ARG	39	37.156	26.204	26.616	1.00	0.00	H
ATOM	628	HD3	ARG	39	37.053	24.698	27.725	1.00	0.00	H
ATOM	629	NE	ARG	39	35.390	25.085	26.737	1.00	0.00	N
ATOM	630	HE	ARG	39	34.512	25.255	27.205	1.00	0.00	H
ATOM	631	CZ	ARG	39	35.367	24.194	25.780	1.00	0.00	C
ATOM	632	NH1	ARG	39	36.458	23.829	25.162	1.00	0.00	N
ATOM	633	HH11	ARG	39	37.352	24.213	25.436	1.00	0.00	H
ATOM	634	HH12	ARG	39	36.393	23.099	24.467	1.00	0.00	H
ATOM	635	NH2	ARG	39	34.259	23.586	25.521	1.00	0.00	N
ATOM	636	HH21	ARG	39	34.160	22.900	24.785	1.00	0.00	H
ATOM	637	HH22	ARG	39	33.500	23.630	26.186	1.00	0.00	H
ATOM	638	C	ARG	39	35.099	30.184	28.938	1.00	0.00	C
ATOM	639	O	ARG	39	34.044	30.522	29.429	1.00	0.00	O
ATOM	640	N	VAL	40	35.653	30.996	28.057	1.00	0.00	N
ATOM	641	H	VAL	40	36.602	30.717	27.853	1.00	0.00	H
ATOM	642	CA	VAL	40	35.196	32.199	27.409	1.00	0.00	C
ATOM	643	HA	VAL	40	34.114	32.314	27.475	1.00	0.00	H
ATOM	644	CB	VAL	40	35.623	33.471	28.145	1.00	0.00	C
ATOM	645	HB	VAL	40	35.321	34.231	27.424	1.00	0.00	H
ATOM	646	CG1	VAL	40	35.024	33.787	29.550	1.00	0.00	C
ATOM	647	HG11	VAL	40	35.363	32.983	30.205	1.00	0.00	H
ATOM	648	HG12	VAL	40	35.253	34.807	29.862	1.00	0.00	H
ATOM	649	HG13	VAL	40	33.936	33.751	29.496	1.00	0.00	H
ATOM	650	CG2	VAL	40	37.135	33.616	28.190	1.00	0.00	C
ATOM	651	HG21	VAL	40	37.540	32.703	28.628	1.00	0.00	H
ATOM	652	HG22	VAL	40	37.579	33.759	27.205	1.00	0.00	H
ATOM	653	HG23	VAL	40	37.435	34.418	28.865	1.00	0.00	H
ATOM	654	C	VAL	40	35.543	32.280	25.930	1.00	0.00	C

ATOM	655	O	VAL	40	36.664	32.078	25.586	1.00	0.00	O
ATOM	656	N	GLY	41	34.488	32.452	25.106	1.00	0.00	N
ATOM	657	H	GLY	41	33.622	32.764	25.523	1.00	0.00	H
ATOM	658	CA	GLY	41	34.550	32.407	23.667	1.00	0.00	C
ATOM	659	HA2	GLY	41	35.490	32.864	23.358	1.00	0.00	H
ATOM	660	HA3	GLY	41	34.677	31.439	23.183	1.00	0.00	H
ATOM	661	C	GLY	41	33.365	33.186	23.029	1.00	0.00	C
ATOM	662	O	GLY	41	32.324	33.427	23.653	1.00	0.00	O
ATOM	663	N	ARG	42	33.527	33.627	21.753	1.00	0.00	N
ATOM	664	H	ARG	42	34.480	33.524	21.437	1.00	0.00	H
ATOM	665	CA	ARG	42	32.437	34.023	20.920	1.00	0.00	C
ATOM	666	HA	ARG	42	31.583	33.406	21.198	1.00	0.00	H
ATOM	667	CB	ARG	42	31.971	35.475	21.260	1.00	0.00	C
ATOM	668	HB2	ARG	42	31.219	35.682	20.499	1.00	0.00	H
ATOM	669	HB3	ARG	42	31.468	35.526	22.225	1.00	0.00	H
ATOM	670	CG	ARG	42	32.950	36.632	21.211	1.00	0.00	C
ATOM	671	HG2	ARG	42	33.914	36.410	21.668	1.00	0.00	H
ATOM	672	HG3	ARG	42	33.040	36.848	20.147	1.00	0.00	H
ATOM	673	CD	ARG	42	32.452	37.755	22.009	1.00	0.00	C
ATOM	674	HD2	ARG	42	31.445	37.963	21.648	1.00	0.00	H
ATOM	675	HD3	ARG	42	32.367	37.519	23.070	1.00	0.00	H
ATOM	676	NE	ARG	42	33.296	38.971	21.826	1.00	0.00	N
ATOM	677	HE	ARG	42	33.112	39.624	21.078	1.00	0.00	H
ATOM	678	CZ	ARG	42	34.271	39.264	22.668	1.00	0.00	C
ATOM	679	NH1	ARG	42	34.513	38.692	23.828	1.00	0.00	N
ATOM	680	HH11	ARG							
42	35.200	39.099	24.447	1.00	0.00	H				
ATOM	681	HH12	ARG	42	33.766	38.057	24.071	1.00	0.00	H
ATOM	682	NH2	ARG	42	35.181	40.144	22.426	1.00	0.00	N
ATOM	683	HH21	ARG	42	35.167	40.633	21.542	1.00	0.00	H
ATOM	684	HH22	ARG	42	35.868	40.376	23.130	1.00	0.00	H
ATOM	685	C	ARG	42	32.593	33.925	19.413	1.00	0.00	C
ATOM	686	O	ARG	42	31.798	34.389	18.624	1.00	0.00	O
ATOM	687	N	PHE	43	33.666	33.315	19.027	1.00	0.00	N
ATOM	688	H	PHE	43	34.203	32.795	19.706	1.00	0.00	H
ATOM	689	CA	PHE	43	34.286	33.313	17.676	1.00	0.00	C
ATOM	690	HA	PHE	43	33.601	33.770	16.962	1.00	0.00	H
ATOM	691	CB	PHE	43	35.683	33.944	17.844	1.00	0.00	C
ATOM	692	HB2	PHE	43	36.351	33.377	18.491	1.00	0.00	H
ATOM	693	HB3	PHE	43	36.191	33.884	16.881	1.00	0.00	H
ATOM	694	CG	PHE	43	35.672	35.427	18.196	1.00	0.00	C
ATOM	695	CD1	PHE	43	34.825	36.289	17.546	1.00	0.00	C
ATOM	696	HD1	PHE	43	34.202	35.862	16.774	1.00	0.00	H
ATOM	697	CE1	PHE	43	34.898	37.666	17.823	1.00	0.00	C
ATOM	698	HE1	PHE	43	34.317	38.425	17.321	1.00	0.00	H
ATOM	699	CZ	PHE	43	35.717	38.164	18.828	1.00	0.00	C
ATOM	700	HZ	PHE	43	35.724	39.227	19.019	1.00	0.00	H
ATOM	701	CE2	PHE	43	36.606	37.256	19.513	1.00	0.00	C
ATOM	702	HE2	PHE	43	37.307	37.642	20.237	1.00	0.00	H
ATOM	703	CD2	PHE	43	36.556	35.867	19.189	1.00	0.00	C
ATOM	704	HD2	PHE	43	37.148	35.150	19.738	1.00	0.00	H
ATOM	705	C	PHE	43	34.477	31.927	17.104	1.00	0.00	C
ATOM	706	O	PHE	43	34.256	30.906	17.752	1.00	0.00	O
ATOM	707	N	GLU	44	34.865	31.872	15.848	1.00	0.00	N
ATOM	708	H	GLU	44	35.051	32.742	15.370	1.00	0.00	H
ATOM	709	CA	GLU	44	35.076	30.627	15.100	1.00	0.00	C
ATOM	710	HA	GLU	44	34.423	29.849	15.493	1.00	0.00	H
ATOM	711	CB	GLU	44	34.559	30.833	13.607	1.00	0.00	C
ATOM	712	HB2	GLU	44	33.469	30.848	13.636	1.00	0.00	H
ATOM	713	HB3	GLU	44	34.898	31.814	13.272	1.00	0.00	H
ATOM	714	CG	GLU	44	35.002	29.835	12.538	1.00	0.00	C
ATOM	715	HG2	GLU	44	34.468	30.215	11.667	1.00	0.00	H
ATOM	716	HG3	GLU	44	36.081	29.934	12.428	1.00	0.00	H
ATOM	717	CD	GLU	44	34.551	28.336	12.787	1.00	0.00	C

ATOM	718	OE1	GLU	44	35.416	27.423	12.526	1.00	0.00	O
ATOM	719	OE2	GLU	44	33.344	28.172	13.137	1.00	0.00	O
ATOM	720	C	GLU	44	36.541	30.194	15.077	1.00	0.00	C
ATOM	721	O	GLU	44	36.803	29.001	15.026	1.00	0.00	O
ATOM	722	N	ASP	45	37.539	31.100	15.029	1.00	0.00	N
ATOM	723	H	ASP	45	37.300	32.080	15.061	1.00	0.00	H
ATOM	724	CA	ASP	45	38.941	30.772	15.126	1.00	0.00	C
ATOM	725	HA	ASP	45	39.135	29.891	14.514	1.00	0.00	H
ATOM	726	CB	ASP	45	39.864	31.888	14.624	1.00	0.00	C
ATOM	727	HB2	ASP	45	39.510	32.304	13.681	1.00	0.00	H
ATOM	728	HB3	ASP	45	39.860	32.672	15.381	1.00	0.00	H
ATOM	729	CG	ASP	45	41.289	31.382	14.442	1.00	0.00	C
ATOM	730	OD1	ASP	45	41.508	30.211	14.091	1.00	0.00	O
ATOM	731	OD2	ASP	45	42.261	32.160	14.605	1.00	0.00	O
ATOM	732	C	ASP	45	39.263	30.395	16.646	1.00	0.00	C
ATOM	733	O	ASP	45	38.898	31.119	17.582	1.00	0.00	O
ATOM	734	N	ASP	46	39.954	29.270	16.889	1.00	0.00	N
ATOM	735	H	ASP	46	40.218	28.781	16.046	1.00	0.00	H
ATOM	736	CA	ASP	46	40.238	28.652	18.222	1.00	0.00	C
ATOM	737	HA	ASP	46	40.829	27.752	18.055	1.00	0.00	H
ATOM	738	CB	ASP	46	41.242	29.574	18.928	1.00	0.00	C
ATOM	739	HB2	ASP	46	40.773	30.455	19.366	1.00	0.00	H
ATOM	740	HB3	ASP	46	41.804	29.125	19.746	1.00	0.00	H
ATOM	741	CG	ASP	46	42.382	30.002	17.979	1.00	0.00	C
ATOM	742	OD1	ASP	46	43.008	29.108	17.428	1.00	0.00	O
ATOM	743	OD2	ASP	46	42.744	31.210	17.937	1.00	0.00	O
ATOM	744	C	ASP	46	39.072	28.309	19.167	1.00	0.00	C
ATOM	745	O	ASP	46	39.294	27.425	20.070	1.00	0.00	O
ATOM	746	N	GLY	47	37.827	28.708	18.798	1.00	0.00	N
ATOM	747	H	GLY	47	37.756	29.411	18.077	1.00	0.00	H
ATOM	748	CA	GLY	47	36.662	28.322	19.632	1.00	0.00	C
ATOM	749	HA2	GLY	47	35.778	28.574	19.047	1.00	0.00	H
ATOM	750	HA3	GLY	47	36.622	27.246	19.801	1.00	0.00	H
ATOM	751	C	GLY	47	36.498	29.046	20.944	1.00	0.00	C
ATOM	752	O	GLY	47	35.430	29.617	21.200	1.00	0.00	O
ATOM	753	N	TYR	48	37.613	29.075	21.684	1.00	0.00	N
ATOM	754	H	TYR	48	38.416	28.511	21.447	1.00	0.00	H
ATOM	755	CA	TYR	48	37.703	29.686	23.054	1.00	0.00	C
ATOM	756	HA	TYR	48	36.931	30.449	23.140	1.00	0.00	H
ATOM	757	CB	TYR	48	37.496	28.710	24.178	1.00	0.00	C
ATOM	758	HB2	TYR	48	38.353	28.060	24.357	1.00	0.00	H
ATOM	759	HB3	TYR	48	37.432	29.267	25.112	1.00	0.00	H
ATOM	760	CG	TYR	48	36.273	27.874	23.941	1.00	0.00	C
ATOM	761	CD1	TYR	48	35.012	28.338	24.458	1.00	0.00	C
ATOM	762	HD1	TYR	48	34.958	29.161	25.155	1.00	0.00	H
ATOM	763	CE1	TYR	48	33.841	27.610	24.180	1.00	0.00	C
ATOM	764	HE1	TYR	48	32.873	27.951	24.518	1.00	0.00	H
ATOM	765	CZ	TYR	48	33.914	26.458	23.393	1.00	0.00	C
ATOM	766	OH	TYR	48	32.825	25.890	22.861	1.00	0.00	O
ATOM	767	HH	TYR	48	33.037	25.142	22.297	1.00	0.00	H
ATOM	768	CE2	TYR	48	35.171	25.974	22.999	1.00	0.00	C
ATOM	769	HE2	TYR	48	35.239	25.089	22.382	1.00	0.00	H
ATOM	770	CD2	TYR	48	36.328	26.660	23.293	1.00	0.00	C
ATOM	771	HD2	TYR	48	37.276	26.306	22.916	1.00	0.00	H
ATOM	772	C	TYR	48	39.127	30.331	23.128	1.00	0.00	C
ATOM	773	O	TYR	48	40.123	29.698	22.797	1.00	0.00	O
ATOM	774	N	PHE	49	39.167	31.526	23.658	1.00	0.00	N
ATOM	775	H	PHE	49	38.261	31.921	23.867	1.00	0.00	H
ATOM	776	CA	PHE	49	40.342	32.313	23.801	1.00	0.00	C
ATOM	777	HA	PHE	49	41.063	31.813	23.154	1.00	0.00	H
ATOM	778	CB	PHE	49	40.278	33.747	23.257	1.00	0.00	C
ATOM	779	HB2	PHE	49	41.094	34.357	23.647	1.00	0.00	H
ATOM	780	HB3	PHE	49	40.566	33.754	22.207	1.00	0.00	H
ATOM	781	CG	PHE	49	39.006	34.513	23.534	1.00	0.00	C

ATOM	782	CD1	PHE	49	37.931	34.554	22.586	1.00	0.00	C
ATOM	783	HD1	PHE	49	38.043	34.022	21.653	1.00	0.00	H
ATOM	784	CE1	PHE	49	36.714	35.259	22.837	1.00	0.00	C
ATOM	785	HE1	PHE	49	36.024	35.421	22.022	1.00	0.00	H
ATOM	786	CZ	PHE	49	36.545	35.896	24.121	1.00	0.00	C
ATOM	787	HZ	PHE	49	35.670	36.513	24.257	1.00	0.00	H
ATOM	788	CE2	PHE	49	37.497	35.776	25.102	1.00	0.00	C
ATOM	789	HE2	PHE	49	37.375	36.298	26.039	1.00	0.00	H
ATOM	790	CD2	PHE	49	38.696	35.106	24.796	1.00	0.00	C
ATOM	791	HD2	PHE	49	39.474	35.006	25.539	1.00	0.00	H
ATOM	792	C	PHE	49	40.834	32.302	25.295	1.00	0.00	C
ATOM	793	O	PHE	49	41.957	32.800	25.602	1.00	0.00	O
ATOM	794	N	CYX	50	40.021	31.710	26.227	1.00	0.00	N
ATOM	795	H	CYX	50	39.117	31.383	25.916	1.00	0.00	H
ATOM	796	CA	CYX	50	40.535	31.442	27.583	1.00	0.00	C
ATOM	797	HA	CYX	50	41.601	31.287	27.409	1.00	0.00	H
ATOM	798	CB	CYX	50	40.461	32.745	28.410	1.00	0.00	C
ATOM	799	HB2	CYX	50	40.010	33.593	27.894	1.00	0.00	H
ATOM	800	HB3	CYX	50	39.875	32.468	29.286	1.00	0.00	H
ATOM	801	SG	CYX	50	42.007	33.361	29.004	1.00	0.00	S
ATOM	802	C	CYX	50	39.864	30.286	28.338	1.00	0.00	C
ATOM	803	O	CYX	50	38.588	30.291	28.448	1.00	0.00	O
ATOM	804	N	THR	51	40.674	29.356	28.853	1.00	0.00	N
ATOM	805	H	THR	51	41.670	29.421				
28.702	1.00	0.00			H					
ATOM	806	CA	THR	51	40.156	28.255	29.750	1.00	0.00	C
ATOM	807	HA	THR	51	39.068	28.297	29.802	1.00	0.00	H
ATOM	808	CB	THR	51	40.651	26.901	29.215	1.00	0.00	C
ATOM	809	HB	THR	51	41.739	26.911	29.133	1.00	0.00	H
ATOM	810	CG2	THR	51	40.286	25.637	30.064	1.00	0.00	C
ATOM	811	HG21	THR	51	40.362	24.686	29.537	1.00	0.00	H
ATOM	812	HG22	THR	51	40.772	25.549	31.035	1.00	0.00	H
ATOM	813	HG23	THR	51	39.212	25.756	30.200	1.00	0.00	H
ATOM	814	OG1	THR	51	39.961	26.792	28.029	1.00	0.00	O
ATOM	815	HG1	THR	51	40.463	27.384	27.463	1.00	0.00	H
ATOM	816	C	THR	51	40.758	28.400	31.204	1.00	0.00	C
ATOM	817	O	THR	51	41.940	28.708	31.385	1.00	0.00	O
ATOM	818	N	ILE	52	39.853	28.308	32.136	1.00	0.00	N
ATOM	819	H	ILE	52	39.000	27.832	31.877	1.00	0.00	H
ATOM	820	CA	ILE	52	39.864	29.046	33.430	1.00	0.00	C
ATOM	821	HA	ILE	52	40.787	29.530	33.747	1.00	0.00	H
ATOM	822	CB	ILE	52	38.791	30.175	33.387	1.00	0.00	C
ATOM	823	HB	ILE	52	37.939	29.744	32.862	1.00	0.00	H
ATOM	824	CG2	ILE	52	38.319	30.653	34.794	1.00	0.00	C
ATOM	825	HG21	ILE	52	39.088	31.264	35.267	1.00	0.00	H
ATOM	826	HG22	ILE	52	37.432	31.287	34.798	1.00	0.00	H
ATOM	827	HG23	ILE	52	38.177	29.863	35.530	1.00	0.00	H
ATOM	828	CG1	ILE	52	39.182	31.351	32.428	1.00	0.00	C
ATOM	829	HG12	ILE	52	39.767	32.049	33.028	1.00	0.00	H
ATOM	830	HG13	ILE	52	39.874	31.026	31.650	1.00	0.00	H
ATOM	831	CD1	ILE	52	38.032	32.102	31.822	1.00	0.00	C
ATOM	832	HD11	ILE	52	37.361	31.429	31.289	1.00	0.00	H
ATOM	833	HD12	ILE	52	37.625	32.733	32.611	1.00	0.00	H
ATOM	834	HD13	ILE	52	38.288	32.824	31.046	1.00	0.00	H
ATOM	835	C	ILE	52	39.742	28.054	34.535	1.00	0.00	C
ATOM	836	O	ILE	52	38.761	27.349	34.504	1.00	0.00	O
ATOM	837	N	GLU	53	40.547	28.122	35.534	1.00	0.00	N
ATOM	838	H	GLU	53	41.317	28.775	35.493	1.00	0.00	H
ATOM	839	CA	GLU	53	40.390	27.221	36.714	1.00	0.00	C
ATOM	840	HA	GLU	53	39.618	26.468	36.556	1.00	0.00	H
ATOM	841	CB	GLU	53	41.706	26.521	36.980	1.00	0.00	C
ATOM	842	HB2	GLU	53	42.063	26.106	36.038	1.00	0.00	H
ATOM	843	HB3	GLU	53	42.401	27.260	37.376	1.00	0.00	H
ATOM	844	CG	GLU	53	41.670	25.454	38.004	1.00	0.00	C

ATOM	845	HG2	GLU	53	41.270	25.843	38.941	1.00	0.00	H
ATOM	846	HG3	GLU	53	40.993	24.648	37.724	1.00	0.00	H
ATOM	847	CD	GLU	53	42.992	24.803	38.283	1.00	0.00	C
ATOM	848	OE1	GLU	53	42.991	23.621	38.699	1.00	0.00	O
ATOM	849	OE2	GLU	53	44.000	25.556	38.342	1.00	0.00	O
ATOM	850	C	GLU	53	39.978	28.038	38.004	1.00	0.00	C
ATOM	851	O	GLU	53	40.472	29.136	38.282	1.00	0.00	O
ATOM	852	N	VAL	54	39.036	27.551	38.780	1.00	0.00	N
ATOM	853	H	VAL	54	38.716	26.616	38.575	1.00	0.00	H
ATOM	854	CA	VAL	54	38.539	28.121	40.000	1.00	0.00	C
ATOM	855	HA	VAL	54	38.588	29.209	39.943	1.00	0.00	H
ATOM	856	CB	VAL	54	37.104	27.790	40.267	1.00	0.00	C
ATOM	857	HB	VAL	54	37.018	26.711	40.389	1.00	0.00	H
ATOM	858	CG1	VAL	54	36.679	28.419	41.554	1.00	0.00	C
ATOM	859	HG11	VAL	54	37.293	28.143	42.411	1.00	0.00	H
ATOM	860	HG12	VAL	54	36.733	29.506	41.607	1.00	0.00	H
ATOM	861	HG13	VAL	54	35.627	28.179	41.704	1.00	0.00	H
ATOM	862	CG2	VAL	54	36.035	28.179	39.290	1.00	0.00	C
ATOM	863	HG21	VAL	54	35.037	27.791	39.487	1.00	0.00	H
ATOM	864	HG22	VAL	54	35.968	29.267	39.310	1.00	0.00	H
ATOM	865	HG23	VAL	54	36.390	27.775	38.343	1.00	0.00	H
ATOM	866	C	VAL	54	39.500	27.640	41.097	1.00	0.00	C
ATOM	867	O	VAL	54	39.430	26.535	41.603	1.00	0.00	O
ATOM	868	N	THR	55	40.420	28.541	41.462	1.00	0.00	N
ATOM	869	H	THR	55	40.403	29.427	40.976	1.00	0.00	H
ATOM	870	CA	THR	55	41.561	28.417	42.373	1.00	0.00	C
ATOM	871	HA	THR	55	41.977	27.410	42.327	1.00	0.00	H
ATOM	872	CB	THR	55	42.684	29.369	42.019	1.00	0.00	C
ATOM	873	HB	THR	55	43.350	29.471	42.875	1.00	0.00	H
ATOM	874	CG2	THR	55	43.485	28.817	40.899	1.00	0.00	C
ATOM	875	HG21	THR	55	42.717	28.791	40.126	1.00	0.00	H
ATOM	876	HG22	THR	55	44.328	29.451	40.622	1.00	0.00	H
ATOM	877	HG23	THR	55	43.789	27.793	41.117	1.00	0.00	H
ATOM	878	OG1	THR	55	42.215	30.734	41.634	1.00	0.00	O
ATOM	879	HG1	THR	55	41.902	31.193	42.417	1.00	0.00	H
ATOM	880	C	THR	55	41.084	28.653	43.812	1.00	0.00	C
ATOM	881	O	THR	55	41.588	27.985	44.675	1.00	0.00	O
ATOM	882	N	ALA	56	40.091	29.482	44.134	1.00	0.00	N
ATOM	883	H	ALA	56	39.678	30.167	43.517	1.00	0.00	H
ATOM	884	CA	ALA	56	39.490	29.575	45.512	1.00	0.00	C
ATOM	885	HA	ALA	56	39.716	28.616	45.978	1.00	0.00	H
ATOM	886	CB	ALA	56	40.253	30.658	46.368	1.00	0.00	C
ATOM	887	HB1	ALA	56	39.726	30.696	47.322	1.00	0.00	H
ATOM	888	HB2	ALA	56	41.276	30.372	46.610	1.00	0.00	H
ATOM	889	HB3	ALA	56	40.336	31.637	45.896	1.00	0.00	H
ATOM	890	C	ALA	56	37.947	29.834	45.565	1.00	0.00	C
ATOM	891	O	ALA	56	37.408	30.430	44.651	1.00	0.00	O
ATOM	892	N	THR	57	37.242	29.277	46.576	1.00	0.00	N
ATOM	893	H	THR	57	37.737	28.775	47.299	1.00	0.00	H
ATOM	894	CA	THR	57	35.760	29.294	46.765	1.00	0.00	C
ATOM	895	HA	THR	57	35.371	30.095	46.136	1.00	0.00	H
ATOM	896	CB	THR	57	35.100	28.041	46.191	1.00	0.00	C
ATOM	897	HB	THR	57	34.099	28.146	46.611	1.00	0.00	H
ATOM	898	CG2	THR	57	35.140	27.988	44.624	1.00	0.00	C
ATOM	899	HG21	THR	57	34.845	28.961	44.231	1.00	0.00	H
ATOM	900	HG22	THR	57	36.089	27.653	44.207	1.00	0.00	H
ATOM	901	HG23	THR	57	34.299	27.384	44.289	1.00	0.00	H
ATOM	902	OG1	THR	57	35.707	26.815	46.631	1.00	0.00	O
ATOM	903	HG1	THR	57	35.392	26.430	47.451	1.00	0.00	H
ATOM	904	C	THR	57	35.356	29.479	48.242	1.00	0.00	C
ATOM	905	O	THR	57	36.003	28.744	48.999	1.00	0.00	O
ATOM	906	N	SER	58	34.289	30.182	48.572	1.00	0.00	N
ATOM	907	H	SER	58	33.693	30.615	47.882	1.00	0.00	H
ATOM	908	CA	SER	58	33.796	30.121	49.972	1.00	0.00	C

ATOM	909	HA	SER	58	33.745	29.134	50.432	1.00	0.00	H
ATOM	910	CB	SER	58	34.739	30.918	50.919	1.00	0.00	C
ATOM	911	HB2	SER	58	35.718	30.472	50.747	1.00	0.00	H
ATOM	912	HB3	SER	58	34.966	31.928	50.578	1.00	0.00	H
ATOM	913	OG	SER	58	34.385	30.825	52.294	1.00	0.00	O
ATOM	914	HG	SER	58	34.369	29.938	52.661	1.00	0.00	H
ATOM	915	C	SER	58	32.310	30.704	50.184	1.00	0.00	C
ATOM	916	O	SER	58	31.843	31.601	49.438	1.00	0.00	O
ATOM	917	N	THR	59	31.565	30.159	51.128	1.00	0.00	N
ATOM	918	H	THR	59	32.043	29.403	51.595	1.00	0.00	H
ATOM	919	CA	THR	59	30.303	30.663	51.699	1.00	0.00	C
ATOM	920	HA	THR	59	29.653	31.012	50.898	1.00	0.00	H
ATOM	921	CB	THR	59	29.511	29.444	52.214	1.00	0.00	C
ATOM	922	HB	THR	59	29.282	28.800	51.365	1.00	0.00	H
ATOM	923	CG2	THR	59	30.138	28.530	53.219	1.00	0.00	C
ATOM	924	HG21	THR	59	29.551	27.616	53.318	1.00	0.00	H
ATOM	925	HG22	THR	59	31.113	28.257	52.816	1.00	0.00	H
ATOM	926	HG23	THR	59	30.325	28.946	54.209	1.00	0.00	H
ATOM	927	OG1	THR	59	28.224	29.801	52.744	1.00	0.00	O
ATOM	928	HG1	THR	59	27.555	29.614	52.081	1.00	0.00	H
ATOM	929	C	THR	59	30.590	31.687	52.732	1.00	0.00	C
ATOM	930	O	THR	59	31.472	31.498	53.551	1.00	0.00	O
ATOM	931	N	VAL	60	30.015	32.893	52.638	1.00	0.00	N
ATOM	932	H	VAL	60	29.278	33.040	51.963	1.00	0.00	H
ATOM	933	CA	VAL	60	30.502	34.077	53.362	1.00	0.00	C
ATOM	934	HA	VAL	60	31.071	33.769	54.239	1.00	0.00	H
ATOM	935	CB	VAL	60	31.636	34.794	52.531	1.00	0.00	C
ATOM	936	HB	VAL	60	32.313	33.947	52.415	1.00	0.00	H
ATOM	937	CG1	VAL	60	31.130	35.493	51.204	1.00	0.00	C
ATOM	938	HG11	VAL	60	30.696	34.664	50.646	1.00	0.00	H
ATOM	939	HG12	VAL	60	30.556	36.387	51.448	1.00	0.00	H
ATOM	940	HG13	VAL	60	31.977	35.797	50.588	1.00	0.00	H
ATOM	941	CG2	VAL	60	32.634	35.845	53.257	1.00	0.00	C
ATOM	942	HG21	VAL	60	33.483	35.380	53.758	1.00	0.00	H
ATOM	943	HG22	VAL	60	33.151	36.370	52.453	1.00	0.00	H
ATOM	944	HG23	VAL	60	32.029	36.504	53.880	1.00	0.00	H
ATOM	945	C	VAL	60	29.339	34.996	53.803	1.00	0.00	C
ATOM	946	O	VAL	60	28.403	35.224	52.996	1.00	0.00	O
ATOM	947	N	THR	61	29.620	35.778	54.857	1.00	0.00	N
ATOM	948	H	THR	61	30.499	35.626	55.331	1.00	0.00	H
ATOM	949	CA	THR	61	28.598	36.761	55.341	1.00	0.00	C
ATOM	950	HA	THR	61	27.698	36.581	54.754	1.00	0.00	H
ATOM	951	CB	THR	61	28.229	36.641	56.809	1.00	0.00	C
ATOM	952	HB	THR	61	27.431	37.362	56.985	1.00	0.00	H
ATOM	953	CG2	THR	61	27.729	35.287	57.155	1.00	0.00	C
ATOM	954	HG21	THR	61	27.051	34.881	56.405	1.00	0.00	H
ATOM	955	HG22	THR	61	28.454	34.506	57.384	1.00	0.00	H
ATOM	956	HG23	THR	61	27.211	35.427	58.104	1.00	0.00	H
ATOM	957	OG1	THR	61	29.260	36.905	57.721	1.00	0.00	O
ATOM	958	HG1	THR	61	28.956	37.739	58.086	1.00	0.00	H
ATOM	959	C	THR	61	29.168	38.148	54.909	1.00	0.00	C
ATOM	960	O	THR	61	30.405	38.299	54.856	1.00	0.00	O
ATOM	961	N	LEU	62	28.316	39.068	54.629	1.00	0.00	N
ATOM	962	H	LEU	62	27.351	38.771	54.636	1.00	0.00	H
ATOM	963	CA	LEU	62	28.704	40.358	54.054	1.00	0.00	C
ATOM	964	HA	LEU	62	29.157	40.048	53.112	1.00	0.00	H
ATOM	965	CB	LEU	62	27.334	41.009	53.859	1.00	0.00	C
ATOM	966	HB2	LEU	62	26.849	40.472	53.044	1.00	0.00	H
ATOM	967	HB3	LEU	62	26.761	40.956	54.785	1.00	0.00	H
ATOM	968	CG	LEU	62	27.419	42.497	53.610	1.00	0.00	C
ATOM	969	HG	LEU	62	28.185	42.969	54.223	1.00	0.00	H
ATOM	970	CD1	LEU	62	27.731	42.727	52.135	1.00	0.00	C
ATOM	971	HD11	LEU	62	27.534	43.748	51.808	1.00	0.00	H

ATOM	972	HD12	LEU	62	28.780	42.546	51.900	1.00	0.00	H
ATOM	973	HD13	LEU	62	27.066	42.095	51.546	1.00	0.00	H
ATOM	974	CD2	LEU	62	26.126	43.212	53.771	1.00	0.00	C
ATOM	975	HD21	LEU	62	25.610	43.084	54.723	1.00	0.00	H
ATOM	976	HD22	LEU	62	26.201	44.290	53.628	1.00	0.00	H
ATOM	977	HD23	LEU	62	25.446	42.883	52.985	1.00	0.00	H
ATOM	978	C	LEU	62	29.678	41.114	54.941	1.00	0.00	C
ATOM	979	O	LEU	62	30.576	41.698	54.393	1.00	0.00	O
ATOM	980	N	ASP	63	29.660	40.846	56.253	1.00	0.00	N
ATOM	981	H	ASP	63	28.948	40.183	56.523	1.00	0.00	H
ATOM	982	CA	ASP	63	30.570	41.275	57.324	1.00	0.00	C
ATOM	983	HA	ASP	63	30.983	42.265	57.127	1.00	0.00	H
ATOM	984	CB	ASP	63	29.758	41.355	58.607	1.00	0.00	C
ATOM	985	HB2	ASP	63	30.396	41.756	59.395	1.00	0.00	H
ATOM	986	HB3	ASP	63	28.983	42.091	58.393	1.00	0.00	H
ATOM	987	CG	ASP	63	29.131	40.069	59.084	1.00	0.00	C
ATOM	988	OD1	ASP	63	29.109	39.863	60.324	1.00	0.00	O
ATOM	989	OD2	ASP	63	28.753	39.259	58.229	1.00	0.00	O
ATOM	990	C	ASP	63	31.820	40.446	57.629	1.00	0.00	C
ATOM	991	O	ASP	63	32.673	41.048	58.268	1.00	0.00	O
ATOM	992	N	THR	64	31.973	39.199	57.152	1.00	0.00	N
ATOM	993	H	THR	64	31.255	38.894	56.512	1.00	0.00	H
ATOM	994	CA	THR	64	33.161	38.303	57.350	1.00	0.00	C
ATOM	995	HA	THR	64	33.861	38.667	58.101	1.00	0.00	H
ATOM	996	CB	THR	64	32.781	36.893	57.773	1.00	0.00	C
ATOM	997	HB	THR	64	33.687	36.289	57.776	1.00	0.00	H
ATOM	998	CG2	THR	64	32.185	37.001	59.235	1.00	0.00	C
ATOM	999	HG21	THR	64	31.911	36.025	59.637	1.00	0.00	H
ATOM	1000	HG22	THR	64	32.942	37.424	59.897	1.00	0.00	H
ATOM	1001	HG23	THR	64	31.370	37.723	59.269	1.00	0.00	H
ATOM	1002	OG1	THR	64	31.788	36.393	56.823	1.00	0.00	O
ATOM	1003	HG1	THR	64	30.950	36.804	57.048	1.00	0.00	H
ATOM	1004	C	THR	64	33.971	38.407	55.935	1.00	0.00	C
ATOM	1005	O	THR	64	35.144	37.994	55.924	1.00	0.00	O
ATOM	1006	N	LEU	65	33.408	38.975	54.869	1.00	0.00	N
ATOM	1007	H	LEU	65	32.457	39.289	55.005	1.00	0.00	H
ATOM	1008	CA	LEU	65	33.947	39.083	53.494	1.00	0.00	C
ATOM	1009	HA	LEU	65	34.130	38.076	53.118	1.00	0.00	H
ATOM	1010	CB	LEU	65	32.878	39.608	52.636	1.00	0.00	C
ATOM	1011	HB2	LEU	65	32.049	38.901	52.663	1.00	0.00	H
ATOM	1012	HB3	LEU	65	32.537	40.558	53.047	1.00	0.00	H
ATOM	1013	CG	LEU	65	33.218	39.747	51.133	1.00	0.00	C
ATOM	1014	HG	LEU	65	34.061	40.421	50.992	1.00	0.00	H
ATOM	1015	CD1	LEU	65	33.682	38.408	50.462	1.00	0.00	C
ATOM	1016	HD11	LEU	65	33.794	38.687	49.414	1.00	0.00	H
ATOM	1017	HD12	LEU	65	34.524	37.875	50.902	1.00	0.00	H
ATOM	1018	HD13	LEU	65	32.821	37.739	50.464	1.00	0.00	H
ATOM	1019	CD2	LEU	65	31.993	40.303	50.360	1.00	0.00	C
ATOM	1020	HD21	LEU	65	31.168	39.605	50.224	1.00	0.00	H
ATOM	1021	HD22	LEU	65	31.700	41.192	50.921	1.00	0.00	H
ATOM	1022	HD23	LEU	65	32.392	40.654	49.408	1.00	0.00	H
ATOM	1023	C	LEU	65	35.217	39.973	53.319	1.00	0.00	C
ATOM	1024	O	LEU	65	35.311	41.183	53.653	1.00	0.00	O
ATOM	1025	N	THR	66	36.206	39.459	52.627	1.00	0.00	N
ATOM	1026	H	THR	66	36.245	38.459	52.490	1.00	0.00	H
ATOM	1027	CA	THR	66	37.483	40.052	52.235	1.00	0.00	C
ATOM	1028	HA	THR	66	37.911	40.188	53.228	1.00	0.00	H
ATOM	1029	CB	THR	66	38.311	39.031	51.470	1.00	0.00	C
ATOM	1030	HB	THR	66	38.360	38.162	52.127	1.00	0.00	H
ATOM	1031	CG2	THR	66	37.776	38.572	50.156	1.00	0.00	C
ATOM	1032	HG21	THR	66	37.601	39.371	49.437	1.00	0.00	H
ATOM	1033	HG22	THR	66	38.518	38.000	49.600	1.00	0.00	H
ATOM	1034	HG23	THR	66	36.872	37.969	50.251	1.00	0.00	H
ATOM	1035	OG1	THR	66	39.553	39.604	51.145	1.00	0.00	O

ATOM	1036	HG1	THR	66	40.239	39.440	51.798	1.00	0.00	H
ATOM	1037	C	THR	66	37.321	41.294	51.427	1.00	0.00	C
ATOM	1038	O	THR	66	36.381	41.374	50.653	1.00	0.00	O
ATOM	1039	N	GLU	67	38.172	42.300	51.692	1.00	0.00	N
ATOM	1040	H	GLU	67	38.903	42.101	52.361	1.00	0.00	H
ATOM	1041	CA	GLU	67	38.288	43.534	50.900	1.00	0.00	C
ATOM	1042	HA	GLU	67	37.291	43.937	50.718	1.00	0.00	H
ATOM	1043	CB	GLU	67	39.102	44.614	51.596	1.00	0.00	C
ATOM	1044	HB2	GLU	67	38.834	45.588	51.186	1.00	0.00	H
ATOM	1045	HB3	GLU	67	38.814	44.642	52.647	1.00	0.00	H
ATOM	1046	CG	GLU	67	40.668	44.446	51.735	1.00	0.00	C
ATOM	1047	HG2	GLU	67	40.839	43.480	52.209	1.00	0.00	H
ATOM	1048	HG3	GLU	67	40.998	44.469	50.697	1.00	0.00	H
ATOM	1049	CD	GLU	67	41.437	45.555	52.447	1.00	0.00	C
ATOM	1050	OE1	GLU	67	40.949	46.573	52.979	1.00	0.00	O
ATOM	1051	OE2	GLU	67	42.674	45.457	52.393	1.00	0.00	O
ATOM	1052	C	GLU	67	38.904	43.355	49.499	1.00	0.00	C
ATOM	1053	O	GLU	67	38.900	44.285	48.649	1.00	0.00	O
ATOM	1054	N	LYS	68	39.371	42.181	49.148	1.00	0.00	N
ATOM	1055	H	LYS	68	39.408	41.528	49.916	1.00	0.00	H
ATOM	1056	CA	LYS	68	40.051	41.795	47.891	1.00	0.00	C
ATOM	1057	HA	LYS	68	40.993	42.333	47.778	1.00	0.00	H
ATOM	1058	CB	LYS	68	40.409	40.265	47.941	1.00	0.00	C
ATOM	1059	HB2	LYS	68	41.081	40.063	48.776	1.00	0.00	H
ATOM	1060	HB3	LYS	68	39.467	39.737	48.089	1.00	0.00	H
ATOM	1061	CG	LYS	68	41.126	39.894	46.661	1.00	0.00	C
ATOM	1062	HG2	LYS	68	40.364	39.978	45.885	1.00	0.00	H
ATOM	1063	HG3	LYS	68	41.975	40.548	46.465	1.00	0.00	H
ATOM	1064	CD	LYS	68	41.453	38.441	46.675	1.00	0.00	C
ATOM	1065	HD2	LYS	68	40.743	37.884	47.288	1.00	0.00	H
ATOM	1066	HD3	LYS	68	41.387	38.194	45.615	1.00	0.00	H
ATOM	1067	CE	LYS	68	42.938	38.027	47.168	1.00	0.00	C
ATOM	1068	HE2	LYS	68	43.619	38.534	46.485	1.00	0.00	H
ATOM	1069	HE3	LYS	68	43.108	38.407	48.176	1.00	0.00	H
ATOM	1070	NZ	LYS	68	43.072	36.553	47.059	1.00	0.00	N
ATOM	1071	HZ1	LYS	68	43.941	36.173	47.406	1.00	0.00	H
ATOM	1072	HZ2	LYS	68	42.374	36.074	47.608	1.00	0.00	H
ATOM	1073	HZ3	LYS	68	42.954	36.282	46.093	1.00	0.00	H
ATOM	1074	C	LYS	68	39.170	42.156	46.668	1.00	0.00	C
ATOM	1075	O	LYS	68	39.662	42.426	45.584	1.00	0.00	O
ATOM	1076	N	HIE	69	37.850	42.088	46.815	1.00	0.00	N
ATOM	1077	H	HIE	69	37.444	41.976	47.732	1.00	0.00	H
ATOM	1078	CA	HIE	69	36.931	42.321	45.701	1.00	0.00	C
ATOM	1079	HA	HIE	69	37.377	41.981	44.767	1.00	0.00	H
ATOM	1080	CB	HIE	69	35.711	41.413	45.786	1.00	0.00	C
ATOM	1081	HB2	HIE	69	36.157	40.420	45.840	1.00	0.00	H
ATOM	1082	HB3	HIE	69	35.194	41.665	46.712	1.00	0.00	H
ATOM	1083	CG	HIE	69	34.775	41.600	44.635	1.00	0.00	C
ATOM	1084	ND1	HIE	69	34.797	40.973	43.414	1.00	0.00	N
ATOM	1085	CE1	HIE	69	33.922	41.569	42.623	1.00	0.00	C
ATOM	1086	HE1	HIE	69	33.691	41.306	41.602	1.00	0.00	H
ATOM	1087	NE2	HIE	69	33.210	42.469	43.341	1.00	0.00	N
ATOM	1088	HE2	HIE	69	32.318	42.830	43.036	1.00	0.00	H
ATOM	1089	CD2	HIE	69	33.775	42.521	44.578	1.00	0.00	C
ATOM	1090	HD2	HIE	69	33.547	43.227	45.363	1.00	0.00	H
ATOM	1091	C	HIE	69	36.651	43.803	45.434	1.00	0.00	C
ATOM	1092	O	HIE	69	36.740	44.355	44.293	1.00	0.00	O
ATOM	1093	N	ALA	70	36.400	44.560	46.536	1.00	0.00	N
ATOM	1094	H	ALA	70	36.466	44.054	47.408	1.00	0.00	H
ATOM	1095	CA	ALA	70	36.401	46.068	46.602	1.00	0.00	C
ATOM	1096	HA	ALA	70	35.525	46.331	46.010	1.00	0.00	H
ATOM	1097	CB	ALA	70	36.183	46.505	48.010	1.00	0.00	C
ATOM	1098	HB1	ALA	70	35.983	47.575	47.947	1.00	0.00	H

ATOM	1099	HB2	ALA	70	35.412	45.914	48.504	1.00	0.00	H
ATOM	1100	HB3	ALA	70	37.068	46.355	48.629	1.00	0.00	H
ATOM	1101	C	ALA	70	37.667	46.697	45.870	1.00	0.00	C
ATOM	1102	O	ALA	70	37.504	47.627	45.078	1.00	0.00	O
ATOM	1103	N	GLU	71	38.787	46.078	46.053	1.00	0.00	N
ATOM	1104	H	GLU	71	38.903	45.391	46.785	1.00	0.00	H
ATOM	1105	CA	GLU	71	40.016	46.473	45.360	1.00	0.00	C
ATOM	1106	HA	GLU	71	40.104	47.520	45.647	1.00	0.00	H
ATOM	1107	CB	GLU	71	41.211	45.757	45.887	1.00	0.00	C
ATOM	1108	HB2	GLU	71	40.941	44.714	46.056	1.00	0.00	H
ATOM	1109	HB3	GLU	71	41.995	45.679	45.134	1.00	0.00	H
ATOM	1110	CG	GLU	71	41.717	46.359	47.242	1.00	0.00	C
ATOM	1111	HG2	GLU	71	40.814	46.376	47.853	1.00	0.00	H
ATOM	1112	HG3	GLU	71	42.256	45.592	47.798	1.00	0.00	H
ATOM	1113	CD	GLU	71	42.395	47.694	47.287	1.00	0.00	C
ATOM	1114	OE1	GLU	71	42.778	48.172	48.415	1.00	0.00	O
ATOM	1115	OE2	GLU	71	42.612	48.392	46.272	1.00	0.00	O
ATOM	1116	C	GLU	71	39.965	46.320	43.852	1.00	0.00	C
ATOM	1117	O	GLU	71	40.514	47.154	43.127	1.00	0.00	O
ATOM	1118	N	GLN	72	39.446	45.194	43.427	1.00	0.00	N
ATOM	1119	H	GLN	72	38.922	44.704	44.138	1.00	0.00	H
ATOM	1120	CA	GLN	72	39.338	44.894	41.979	1.00	0.00	C
ATOM	1121	HA	GLN	72	40.324	44.997	41.525	1.00	0.00	H
ATOM	1122	CB	GLN	72	38.724	43.465	41.786	1.00	0.00	C
ATOM	1123	HB2	GLN	72	37.812	43.322	42.366	1.00	0.00	H
ATOM	1124	HB3	GLN	72	39.387	42.721	42.228	1.00	0.00	H
ATOM	1125	CG	GLN	72	38.634	43.118	40.309	1.00	0.00	C
ATOM	1126	HG2	GLN	72	38.092	43.859	39.721	1.00	0.00	H
ATOM	1127	HG3	GLN	72	39.599	43.018	39.813	1.00	0.00	H
ATOM	1128	CD	GLN	72	37.861	41.808	40.174	1.00	0.00	C
ATOM	1129	OE1	GLN	72	38.466	40.798	39.747	1.00	0.00	O
ATOM	1130	NE2	GLN	72	36.553	41.721	40.482	1.00	0.00	N
ATOM	1131	HE21	GLN	72	35.972	42.507	40.737	1.00	0.00	H
ATOM	1132	HE22	GLN	72	36.254	40.818	40.823	1.00	0.00	H
ATOM	1133	C	GLN	72	38.398	45.968	41.286	1.00	0.00	C
ATOM	1134	O	GLN	72	38.570	46.327	40.122	1.00	0.00	O
ATOM	1135	N	GLU	73	37.292	46.301	42.067	1.00	0.00	N
ATOM	1136	H	GLU	73	37.180	45.850	42.963	1.00	0.00	H
ATOM	1137	CA	GLU	73	36.119	47.217	41.637	1.00	0.00	C
ATOM	1138	HA	GLU	73	36.029	47.262	40.552	1.00	0.00	H
ATOM	1139	CB	GLU	73	34.819	46.706	42.254	1.00	0.00	C
ATOM	1140	HB2	GLU	73	35.017	46.710	43.326	1.00	0.00	H
ATOM	1141	HB3	GLU	73	34.026	47.404	41.988	1.00	0.00	H
ATOM	1142	CG	GLU	73	34.517	45.248	41.805	1.00	0.00	C
ATOM	1143	HG2	GLU	73	35.290	44.529	42.078	1.00	0.00	H
ATOM	1144	HG3	GLU	73	33.728	44.968	42.502	1.00	0.00	H
ATOM	1145	CD	GLU	73	33.913	45.071	40.437	1.00	0.00	C
ATOM	1146	OE1	GLU	73	34.599	44.490	39.536	1.00	0.00	O
ATOM	1147	OE2	GLU	73	32.829	45.609	40.223	1.00	0.00	O
ATOM	1148	C	GLU	73	36.392	48.726	41.907	1.00	0.00	C
ATOM	1149	O	GLU	73	35.496	49.576	41.652	1.00	0.00	O
ATOM	1150	N	ASN	74	37.542	49.104	42.483	1.00	0.00	N
ATOM	1151	H	ASN	74	38.148	48.375	42.831	1.00	0.00	H
ATOM	1152	CA	ASN	74	37.974	50.475	42.713	1.00	0.00	C
ATOM	1153	HA	ASN	74	38.888	50.450	43.307	1.00	0.00	H
ATOM	1154	CB	ASN	74	38.334	51.151	41.393	1.00	0.00	C
ATOM	1155	HB2	ASN	74	37.482	51.360	40.747	1.00	0.00	H
ATOM	1156	HB3	ASN	74	38.765	52.131	41.602	1.00	0.00	H
ATOM	1157	CG	ASN	74	39.467	50.464	40.617	1.00	0.00	C
ATOM	1158	OD1	ASN	74	39.746	49.290	40.683	1.00	0.00	O
ATOM	1159	ND2	ASN	74	40.196	51.151	39.845	1.00	0.00	N
ATOM	1160	HD21	ASN	74	41.060	50.740	39.521	1.00	0.00	H
ATOM	1161	HD22	ASN	74	39.996	52.104	39.577	1.00	0.00	H
ATOM	1162	C	ASN	74	36.956	51.189	43.589	1.00	0.00	C

```
ATOM   1163  O   ASN    74      36.405  52.204  43.187  1.00  0.00           O
ATOM   1164  N   MET    75      36.715  50.578  44.774  1.00  0.00           N
ATOM   1165  H   MET    75      37.055  49.642  44.946  1.00  0.00           H
ATOM   1166  CA  MET    75      35.611  51.014  45.677  1.00  0.00           C
ATOM   1167  HA  MET    75      35.648  52.103  45.637  1.00  0.00           H
ATOM   1168  CB  MET    75      34.190  50.533  45.162  1.00  0.00           C
ATOM   1169  HB2 MET    75      33.354  51.071  45.606  1.00  0.00           H
ATOM   1170  HB3 MET    75      34.147  50.779  44.101  1.00  0.00           H
ATOM   1171  CG  MET    75      33.755  49.098  45.427  1.00  0.00           C
ATOM   1172  HG2 MET    75      34.641  48.491  45.240  1.00  0.00           H
ATOM   1173  HG3 MET    75      33.533  49.104  46.495  1.00  0.00           H
ATOM   1174  SD  MET    75      32.420  48.506  44.422  1.00  0.00           S
ATOM   1175  CE  MET    75      31.154  49.750  44.698  1.00  0.00           C
ATOM   1176  HE1 MET    75      31.479  50.767  44.475  1.00  0.00           H
ATOM   1177  HE2 MET    75      30.329  49.505  44.029  1.00  0.00           H
ATOM   1178  HE3 MET    75      30.686  49.630  45.674  1.00  0.00           H
ATOM   1179  C   MET    75      36.017  50.625  47.134  1.00  0.00           C
ATOM   1180  O   MET    75      36.613  49.577  47.280  1.00  0.00           O
ATOM   1181  N   THR    76      35.754  51.421
  48.155  1.00  0.00           N
ATOM   1182  H   THR    76      35.158  52.218  47.983  1.00  0.00           H
ATOM   1183  CA  THR    76      36.055  51.026  49.561  1.00  0.00           C
ATOM   1184  HA  THR    76      36.999  50.480  49.590  1.00  0.00           H
ATOM   1185  CB  THR    76      36.294  52.227  50.482  1.00  0.00           C
ATOM   1186  HB  THR    76      36.998  52.859  49.941  1.00  0.00           H
ATOM   1187  CG2 THR    76      34.996  53.110  50.667  1.00  0.00           C
ATOM   1188 HG21 THR    76      34.201  52.813  49.983  1.00  0.00           H
ATOM   1189 HG22 THR    76      34.609  53.020  51.682  1.00  0.00           H
ATOM   1190 HG23 THR    76      35.304  54.151  50.572  1.00  0.00           H
ATOM   1191  OG1 THR    76      36.725  51.885  51.767  1.00  0.00           O
ATOM   1192  HG1 THR    76      37.626  51.579  51.647  1.00  0.00           H
ATOM   1193  C   THR    76      34.975  50.004  50.088  1.00  0.00           C
ATOM   1194  O   THR    76      33.881  50.101  49.657  1.00  0.00           O
ATOM   1195  N   LEU    77      35.347  49.047  50.928  1.00  0.00           N
ATOM   1196  H   LEU    77      36.321  49.010  51.192  1.00  0.00           H
ATOM   1197  CA  LEU    77      34.437  48.001  51.500  1.00  0.00           C
ATOM   1198  HA  LEU    77      34.242  47.377  50.627  1.00  0.00           H
ATOM   1199  CB  LEU    77      35.184  47.176  52.645  1.00  0.00           C
ATOM   1200  HB2 LEU    77      36.086  46.788  52.173  1.00  0.00           H
ATOM   1201  HB3 LEU    77      35.475  47.907  53.399  1.00  0.00           H
ATOM   1202  CG  LEU    77      34.496  45.977  53.247  1.00  0.00           C
ATOM   1203  HG  LEU    77      33.654  46.309  53.855  1.00  0.00           H
ATOM   1204  CD1 LEU    77      33.934  45.082  52.212  1.00  0.00           C
ATOM   1205 HD11 LEU    77      32.882  45.310  52.045  1.00  0.00           H
ATOM   1206 HD12 LEU    77      34.568  44.999  51.330  1.00  0.00           H
ATOM   1207 HD13 LEU    77      34.059  44.062  52.575  1.00  0.00           H
ATOM   1208  CD2 LEU    77      35.565  45.233  54.122  1.00  0.00           C
ATOM   1209 HD21 LEU    77      35.091  44.692  54.941  1.00  0.00           H
ATOM   1210 HD22 LEU    77      36.171  44.589  53.485  1.00  0.00           H
ATOM   1211 HD23 LEU    77      36.318  45.952  54.446  1.00  0.00           H
ATOM   1212  C   LEU    77      33.006  48.542  51.868  1.00  0.00           C
ATOM   1213  O   LEU    77      31.982  47.972  51.524  1.00  0.00           O
ATOM   1214  N   THR    78      32.868  49.701  52.532  1.00  0.00           N
ATOM   1215  H   THR    78      33.689  50.026  53.023  1.00  0.00           H
ATOM   1216  CA  THR    78      31.621  50.336  52.904  1.00  0.00           C
ATOM   1217  HA  THR    78      31.024  49.670  53.527  1.00  0.00           H
ATOM   1218  CB  THR    78      32.020  51.453  53.928  1.00  0.00           C
ATOM   1219  HB  THR    78      32.601  52.255  53.474  1.00  0.00           H
ATOM   1220  CG2 THR    78      30.871  52.216  54.614  1.00  0.00           C
ATOM   1221 HG21 THR    78      30.198  51.480  55.054  1.00  0.00           H
ATOM   1222 HG22 THR    78      31.283  52.957  55.300  1.00  0.00           H
ATOM   1223 HG23 THR    78      30.332  52.856  53.915  1.00  0.00           H
ATOM   1224  OG1 THR    78      32.936  51.010  54.957  1.00  0.00           O
ATOM   1225  HG1 THR    78      33.817  51.086  54.583  1.00  0.00           H
```

ATOM	1226	C	THR	78	30.715	50.849	51.725	1.00	0.00	C
ATOM	1227	O	THR	78	29.504	50.801	51.807	1.00	0.00	O
ATOM	1228	N	GLU	79	31.381	51.226	50.687	1.00	0.00	N
ATOM	1229	H	GLU	79	32.381	51.086	50.712	1.00	0.00	H
ATOM	1230	CA	GLU	79	30.834	51.459	49.325	1.00	0.00	C
ATOM	1231	HA	GLU	79	29.890	52.000	49.392	1.00	0.00	H
ATOM	1232	CB	GLU	79	31.766	52.349	48.490	1.00	0.00	C
ATOM	1233	HB2	GLU	79	32.768	51.958	48.663	1.00	0.00	H
ATOM	1234	HB3	GLU	79	31.504	52.210	47.441	1.00	0.00	H
ATOM	1235	CG	GLU	79	31.833	53.794	48.925	1.00	0.00	C
ATOM	1236	HG2	GLU	79	31.819	53.822	50.016	1.00	0.00	H
ATOM	1237	HG3	GLU	79	32.798	54.208	48.632	1.00	0.00	H
ATOM	1238	CD	GLU	79	30.634	54.685	48.407	1.00	0.00	C
ATOM	1239	OE1	GLU	79	30.462	55.814	48.961	1.00	0.00	O
ATOM	1240	OE2	GLU	79	29.815	54.145	47.586	1.00	0.00	O
ATOM	1241	C	GLU	79	30.456	50.206	48.530	1.00	0.00	C
ATOM	1242	O	GLU	79	29.531	50.246	47.752	1.00	0.00	O
ATOM	1243	N	LEU	80	31.202	49.162	48.724	1.00	0.00	N
ATOM	1244	H	LEU	80	32.100	49.244	49.179	1.00	0.00	H
ATOM	1245	CA	LEU	80	30.888	47.821	48.244	1.00	0.00	C
ATOM	1246	HA	LEU	80	30.590	47.865	47.196	1.00	0.00	H
ATOM	1247	CB	LEU	80	32.054	46.855	48.359	1.00	0.00	C
ATOM	1248	HB2	LEU	80	32.950	47.316	47.943	1.00	0.00	H
ATOM	1249	HB3	LEU	80	32.217	46.743	49.432	1.00	0.00	H
ATOM	1250	CG	LEU	80	31.762	45.480	47.783	1.00	0.00	C
ATOM	1251	HG	LEU	80	30.893	44.994	48.223	1.00	0.00	H
ATOM	1252	CD1	LEU	80	31.534	45.362	46.320	1.00	0.00	C
ATOM	1253	HD11	LEU	80	32.440	45.581	45.753	1.00	0.00	H
ATOM	1254	HD12	LEU	80	31.353	44.305	46.123	1.00	0.00	H
ATOM	1255	HD13	LEU	80	30.821	46.103	45.959	1.00	0.00	H
ATOM	1256	CD2	LEU	80	32.944	44.573	48.290	1.00	0.00	C
ATOM	1257	HD21	LEU	80	33.836	44.732	47.683	1.00	0.00	H
ATOM	1258	HD22	LEU	80	33.151	44.813	49.333	1.00	0.00	H
ATOM	1259	HD23	LEU	80	32.705	43.511	48.275	1.00	0.00	H
ATOM	1260	C	LEU	80	29.643	47.303	48.980	1.00	0.00	C
ATOM	1261	O	LEU	80	28.639	47.119	48.277	1.00	0.00	O
ATOM	1262	N	LYS	81	29.617	47.204	50.279	1.00	0.00	N
ATOM	1263	H	LYS	81	30.471	47.432	50.768	1.00	0.00	H
ATOM	1264	CA	LYS	81	28.400	46.798	51.042	1.00	0.00	C
ATOM	1265	HA	LYS	81	28.010	45.872	50.617	1.00	0.00	H
ATOM	1266	CB	LYS	81	28.655	46.596	52.525	1.00	0.00	C
ATOM	1267	HB2	LYS	81	28.839	47.553	53.014	1.00	0.00	H
ATOM	1268	HB3	LYS	81	27.763	46.137	52.951	1.00	0.00	H
ATOM	1269	CG	LYS	81	29.893	45.749	52.975	1.00	0.00	C
ATOM	1270	HG2	LYS	81	29.727	44.737	52.608	1.00	0.00	H
ATOM	1271	HG3	LYS	81	30.769	46.143	52.461	1.00	0.00	H
ATOM	1272	CD	LYS	81	30.041	45.584	54.469	1.00	0.00	C
ATOM	1273	HD2	LYS	81	29.069	45.260	54.839	1.00	0.00	H
ATOM	1274	HD3	LYS	81	30.724	44.756	54.664	1.00	0.00	H
ATOM	1275	CE	LYS	81	30.465	46.803	55.311	1.00	0.00	C
ATOM	1276	HE2	LYS	81	31.365	47.221	54.859	1.00	0.00	H
ATOM	1277	HE3	LYS	81	29.693	47.573	55.284	1.00	0.00	H
ATOM	1278	NZ	LYS	81	30.729	46.542	56.772	1.00	0.00	N
ATOM	1279	HZ1	LYS	81	29.912	46.192	57.250	1.00	0.00	H
ATOM	1280	HZ2	LYS	81	31.463	45.853	56.851	1.00	0.00	H
ATOM	1281	HZ3	LYS	81	31.016	47.392	57.234	1.00	0.00	H
ATOM	1282	C	LYS	81	27.264	47.758	50.928	1.00	0.00	C
ATOM	1283	O	LYS	81	26.113	47.298	51.036	1.00	0.00	O
ATOM	1284	N	LYS	82	27.500	49.033	50.560	1.00	0.00	N
ATOM	1285	H	LYS	82	28.493	49.215	50.521	1.00	0.00	H
ATOM	1286	CA	LYS	82	26.483	49.963	50.171	1.00	0.00	C
ATOM	1287	HA	LYS	82	25.804	50.107	51.012	1.00	0.00	H
ATOM	1288	CB	LYS	82	27.067	51.311	49.689	1.00	0.00	C
ATOM	1289	HB2	LYS	82	28.044	51.485	50.140	1.00	0.00	H

ATOM	1290	HB3	LYS	82	27.243	51.284	48.615	1.00	0.00	H
ATOM	1291	CG	LYS	82	26.088	52.445	49.967	1.00	0.00	C
ATOM	1292	HG2	LYS	82	25.051	52.411	49.631	1.00	0.00	H
ATOM	1293	HG3	LYS	82	25.888	52.423	51.038	1.00	0.00	H
ATOM	1294	CD	LYS	82	26.664	53.805	49.658	1.00	0.00	C
ATOM	1295	HD2	LYS	82	26.066	54.519	50.224	1.00	0.00	H
ATOM	1296	HD3	LYS	82	27.678	53.904	50.045	1.00	0.00	H
ATOM	1297	CE	LYS	82	26.547	54.086	48.182	1.00	0.00	C
ATOM	1298	HE2	LYS	82	26.870	53.164	47.699	1.00	0.00	H
ATOM	1299	HE3	LYS	82	25.504	54.250	47.911	1.00	0.00	H
ATOM	1300	NZ	LYS	82	27.372	55.273	47.811	1.00	0.00	N
ATOM	1301	HZ1	LYS	82	27.248	55.424	46.820	1.00	0.00	H
ATOM	1302	HZ2	LYS	82	27.008	56.110	48.242	1.00	0.00	H
ATOM	1303	HZ3	LYS	82	28.343	55.035	47.956	1.00	0.00	H
ATOM	1304	C	LYS	82	25.519	49.389	49.084	1.00	0.00	C
ATOM	1305	O	LYS	82	24.315	49.232	49.301	1.00	0.00	O
ATOM	1306	N	VAL	83	26.065	49.120	47.853	1.00	0.00	N
ATOM	1307	H	VAL	83	27.068	49.182	47.754	1.00	0.00	H
ATOM	1308	CA	VAL	83	25.256	48.700	46.717	1.00	0.00	C
ATOM	1309	HA	VAL	83	24.370	49.310	46.542	1.00	0.00	H
ATOM	1310	CB	VAL	83	26.145	48.766	45.486	1.00	0.00	C
ATOM	1311	HB	VAL	83	25.684	48.217	44.666	1.00	0.00	H
ATOM	1312	CG1	VAL	83	26.322	50.268	45.091	1.00	0.00	C
ATOM	1313	HG11	VAL	83	26.766	50.327	44.097	1.00	0.00	H
ATOM	1314	HG12	VAL	83	25.422	50.863	45.250	1.00	0.00	H
ATOM	1315	HG13	VAL	83	27.105	50.683	45.726	1.00	0.00	H
ATOM	1316	CG2	VAL	83	27.583	48.239	45.561	1.00	0.00	C
ATOM	1317	HG21	VAL	83	28.215	48.515	46.406	1.00	0.00	H
ATOM	1318	HG22	VAL	83	27.612	47.149	45.591	1.00	0.00	H
ATOM	1319	HG23	VAL	83	28.107	48.580	44.669	1.00	0.00	H
ATOM	1320	C	VAL	83	24.720	47.346	47.046	1.00	0.00	C
ATOM	1321	O	VAL	83	23.545	47.126	46.757	1.00	0.00	O
ATOM	1322	N	ILE	84	25.429	46.411	47.703	1.00	0.00	N
ATOM	1323	H	ILE	84	26.321	46.618	48.131	1.00	0.00	H
ATOM	1324	CA	ILE	84	24.764	45.129	48.071	1.00	0.00	C
ATOM	1325	HA	ILE	84	24.372	44.694	47.150	1.00	0.00	H
ATOM	1326	CB	ILE	84	25.799	44.193	48.713	1.00	0.00	C
ATOM	1327	HB	ILE	84	26.354	44.600	49.558	1.00	0.00	H
ATOM	1328	CG2	ILE	84	25.232	42.780	49.167	1.00	0.00	C
ATOM	1329	HG21	ILE	84	24.412	42.952	49.862	1.00	0.00	H
ATOM	1330	HG22	ILE	84	24.840	42.259	48.292	1.00	0.00	H
ATOM	1331	HG23	ILE	84	25.975	42.181	49.693	1.00	0.00	H
ATOM	1332	CG1	ILE	84	26.977	43.766	47.782	1.00	0.00	C
ATOM	1333	HG12	ILE	84	26.657	43.095	46.984	1.00	0.00	H
ATOM	1334	HG13	ILE	84	27.461	44.608	47.286	1.00	0.00	H
ATOM	1335	CD1	ILE	84	28.216	43.117	48.583	1.00	0.00	C
ATOM	1336	HD11	ILE	84	28.636	43.829	49.293	1.00	0.00	H
ATOM	1337	HD12	ILE	84	27.910	42.296	49.231	1.00	0.00	H
ATOM	1338	HD13	ILE	84	28.962	42.801	47.854	1.00	0.00	H
ATOM	1339	C	ILE	84	23.525	45.379	48.968	1.00	0.00	C
ATOM	1340	O	ILE	84	22.506	44.756	48.813	1.00	0.00	O
ATOM	1341	N	ALA	85	23.633	46.260	49.984	1.00	0.00	N
ATOM	1342	H	ALA	85	24.495	46.779	50.068	1.00	0.00	H
ATOM	1343	CA	ALA	85	22.546	46.588	50.960	1.00	0.00	C
ATOM	1344	HA	ALA	85	22.185	45.639	51.355	1.00	0.00	H
ATOM	1345	CB	ALA	85	23.118	47.492	52.056	1.00	0.00	C
ATOM	1346	HB1	ALA	85	23.656	48.361	51.680	1.00	0.00	H
ATOM	1347	HB2	ALA	85	22.253	47.819	52.634	1.00	0.00	H
ATOM	1348	HB3	ALA	85	23.848	46.897	52.604	1.00	0.00	H
ATOM	1349	C	ALA	85	21.322	47.311	50.359	1.00	0.00	C
ATOM	1350	O	ALA	85	20.382	47.682	51.106	1.00	0.00	O
ATOM	1351	N	ASP	86	21.313	47.681	49.082	1.00	0.00	N
ATOM	1352	H	ASP	86	22.132	47.538	48.508	1.00	0.00	H

ATOM	1353	CA	ASP	86	20.086	48.053	48.340	1.00	0.00	C
ATOM	1354	HA	ASP	86	19.242	48.271	48.994	1.00	0.00	H
ATOM	1355	CB	ASP	86	20.384	49.225	47.391	1.00	0.00	C
ATOM	1356	HB2	ASP	86	21.173	48.823	46.757	1.00	0.00	H
ATOM	1357	HB3	ASP	86	19.629	49.479	46.647	1.00	0.00	H
ATOM	1358	CG	ASP	86	20.763	50.540	47.983	1.00	0.00	C
ATOM	1359	OD1	ASP	86	21.403	51.330	47.235	1.00	0.00	O
ATOM	1360	OD2	ASP	86	20.292	50.776	49.059	1.00	0.00	O
ATOM	1361	C	ASP	86	19.630	47.018	47.393	1.00	0.00	C
ATOM	1362	O	ASP	86	18.427	46.969	47.065	1.00	0.00	O
ATOM	1363	N	ILE	87	20.509	46.119	46.917	1.00	0.00	N
ATOM	1364	H	ILE	87	21.444	46.268	47.267	1.00	0.00	H
ATOM	1365	CA	ILE	87	20.120	45.048	45.997	1.00	0.00	C
ATOM	1366	HA	ILE	87	19.316	45.429	45.367	1.00	0.00	H
ATOM	1367	CB	ILE	87	21.323	44.605	45.148	1.00	0.00	C
ATOM	1368	HB	ILE	87	22.199	44.404	45.765	1.00	0.00	H
ATOM	1369	CG2	ILE	87	20.956	43.341	44.278	1.00	0.00	C
ATOM	1370	HG21	ILE	87	20.044	43.482	43.698	1.00	0.00	H
ATOM	1371	HG22	ILE	87	21.806	43.137	43.626	1.00	0.00	H
ATOM	1372	HG23	ILE	87	20.782	42.467	44.906	1.00	0.00	H
ATOM	1373	CG1	ILE	87	21.680	45.756	44.143	1.00	0.00	C
ATOM	1374	HG12	ILE	87	20.797	45.938	43.529	1.00	0.00	H
ATOM	1375	HG13	ILE	87	21.649	46.751	44.584	1.00	0.00	H
ATOM	1376	CD1	ILE	87	23.021	45.672	43.477	1.00	0.00	C
ATOM	1377	HD11	ILE	87	23.060	46.547	42.828	1.00	0.00	H
ATOM	1378	HD12	ILE	87	23.772	45.571	44.260	1.00	0.00	H
ATOM	1379	HD13	ILE	87	23.070	44.756	42.888	1.00	0.00	H
ATOM	1380	C	ILE	87	19.532	43.891	46.794	1.00	0.00	C
ATOM	1381	O	ILE	87	18.495	43.358	46.326	1.00	0.00	O
ATOM	1382	N	TYR	88	20.068	43.488	47.983	1.00	0.00	N
ATOM	1383	H	TYR	88	20.774	44.104	48.361	1.00	0.00	H
ATOM	1384	CA	TYR	88	19.585	42.362	48.781	1.00	0.00	C
ATOM	1385	HA	TYR	88	18.636	42.077	48.326	1.00	0.00	H
ATOM	1386	CB	TYR	88	20.604	41.221	48.756	1.00	0.00	C
ATOM	1387	HB2	TYR	88	21.497	41.589	49.261	1.00	0.00	H
ATOM	1388	HB3	TYR	88	20.323	40.387	49.398	1.00	0.00	H
ATOM	1389	CG	TYR	88	21.025	40.572	47.542	1.00	0.00	C
ATOM	1390	CD1	TYR	88	20.368	39.398	47.117	1.00	0.00	C
ATOM	1391	HD1	TYR	88	19.554	38.923	47.643	1.00	0.00	H
ATOM	1392	CE1	TYR	88	20.705	38.844	45.865	1.00	0.00	C
ATOM	1393	HE1	TYR	88	20.167	37.970	45.527	1.00	0.00	H
ATOM	1394	CZ	TYR	88	21.891	39.319	45.163	1.00	0.00	C
ATOM	1395	OH	TYR	88	22.294	38.753	44.034	1.00	0.00	O
ATOM	1396	HH	TYR	88	22.230	37.797	44.117	1.00	0.00	H
ATOM	1397	CE2	TYR	88	22.605	40.389	45.714	1.00	0.00	C
ATOM	1398	HE2	TYR	88	23.455	40.806	45.195	1.00	0.00	H
ATOM	1399	CD2	TYR	88	22.178	41.087	46.866	1.00	0.00	C
ATOM	1400	HD2	TYR	88	22.749	41.923	47.244	1.00	0.00	H
ATOM	1401	C	TYR	88	19.334	42.733	50.233	1.00	0.00	C
ATOM	1402	O	TYR	88	20.062	43.613	50.681	1.00	0.00	O
ATOM	1403	N	PRO	89	18.381	42.065	50.947	1.00	0.00	N
ATOM	1404	CD	PRO	89	17.199	41.447	50.332	1.00	0.00	C
ATOM	1405	HD2	PRO	89	17.289	40.447	49.907	1.00	0.00	H
ATOM	1406	HD3	PRO	89	16.787	41.961	49.462	1.00	0.00	H
ATOM	1407	CG	PRO	89	16.079	41.457	51.431	1.00	0.00	C
ATOM	1408	HG2	PRO	89	15.423	40.593	51.332	1.00	0.00	H
ATOM	1409	HG3	PRO	89	15.588	42.424	51.320	1.00	0.00	H
ATOM	1410	CB	PRO	89	16.826	41.548	52.742	1.00	0.00	C
ATOM	1411	HB2	PRO	89	17.130	40.605	53.199	1.00	0.00	H
ATOM	1412	HB3	PRO	89	16.361	42.204	53.480	1.00	0.00	H
ATOM	1413	CA	PRO	89	18.197	42.236	52.390	1.00	0.00	C
ATOM	1414	HA	PRO	89	18.112	43.320	52.453	1.00	0.00	H
ATOM	1415	C	PRO	89	19.400	41.819	53.244	1.00	0.00	C
ATOM	1416	O	PRO	89	19.990	40.787	52.913	1.00	0.00	O

ATOM	1417	N	GLY	90	19.787	42.505	54.326	1.00	0.00	N
ATOM	1418	H	GLY	90	19.100	43.109	54.755	1.00	0.00	H
ATOM	1419	CA	GLY	90	21.036	42.185	55.112	1.00	0.00	C
ATOM	1420	HA2	GLY	90	21.922	42.405	54.516	1.00	0.00	H
ATOM	1421	HA3	GLY	90	20.877	42.809	55.991	1.00	0.00	H
ATOM	1422	C	GLY	90	21.098	40.802	55.719	1.00	0.00	C
ATOM	1423	O	GLY	90	22.178	40.287	56.053	1.00	0.00	O
ATOM	1424	N	GLN	91	19.962	40.091	55.781	1.00	0.00	N
ATOM	1425	H	GLN	91	19.130	40.512	55.393	1.00	0.00	H
ATOM	1426	CA	GLN	91	19.893	38.727	56.268	1.00	0.00	C
ATOM	1427	HA	GLN	91	20.708	38.640	56.986	1.00	0.00	H
ATOM	1428	CB	GLN	91	18.466	38.365	56.765	1.00	0.00	C
ATOM	1429	HB2	GLN	91	17.695	38.367	55.995	1.00	0.00	H
ATOM	1430	HB3	GLN	91	18.537	37.289	56.926	1.00	0.00	H
ATOM	1431	CG	GLN	91	17.951	39.091	58.049	1.00	0.00	C
ATOM	1432	HG2	GLN							
				91	17.638	40.125	57.907	1.00	0.00	H
ATOM	1433	HG3	GLN	91	17.127	38.489	58.435	1.00	0.00	H
ATOM	1434	CD	GLN	91	18.995	39.132	59.214	1.00	0.00	C
ATOM	1435	OE1	GLN	91	19.702	38.175	59.490	1.00	0.00	O
ATOM	1436	NE2	GLN	91	19.048	40.159	59.938	1.00	0.00	N
ATOM	1437	HE21	GLN	91	18.395	40.924	59.856	1.00	0.00	H
ATOM	1438	HE22	GLN	91	19.646	40.020	60.740	1.00	0.00	H
ATOM	1439	C	GLN	91	20.295	37.719	55.125	1.00	0.00	C
ATOM	1440	O	GLN	91	20.380	36.509	55.288	1.00	0.00	O
ATOM	1441	N	THR	92	20.659	38.237	53.897	1.00	0.00	N
ATOM	1442	H	THR	92	20.677	39.234	53.732	1.00	0.00	H
ATOM	1443	CA	THR	92	21.189	37.421	52.742	1.00	0.00	C
ATOM	1444	HA	THR	92	20.357	36.731	52.603	1.00	0.00	H
ATOM	1445	CB	THR	92	21.227	38.277	51.434	1.00	0.00	C
ATOM	1446	HB	THR	92	21.874	39.141	51.588	1.00	0.00	H
ATOM	1447	CG2	THR	92	21.744	37.594	50.198	1.00	0.00	C
ATOM	1448	HG21	THR	92	21.169	36.681	50.044	1.00	0.00	H
ATOM	1449	HG22	THR	92	21.525	38.134	49.277	1.00	0.00	H
ATOM	1450	HG23	THR	92	22.787	37.278	50.143	1.00	0.00	H
ATOM	1451	OG1	THR	92	19.909	38.651	51.150	1.00	0.00	O
ATOM	1452	HG1	THR	92	19.607	39.251	51.837	1.00	0.00	H
ATOM	1453	C	THR	92	22.513	36.711	52.992	1.00	0.00	C
ATOM	1454	O	THR	92	23.479	37.354	53.442	1.00	0.00	O
ATOM	1455	N	GLN	93	22.636	35.471	52.632	1.00	0.00	N
ATOM	1456	H	GLN	93	21.891	34.986	52.152	1.00	0.00	H
ATOM	1457	CA	GLN	93	23.958	34.797	52.672	1.00	0.00	C
ATOM	1458	HA	GLN	93	24.625	35.213	53.428	1.00	0.00	H
ATOM	1459	CB	GLN	93	23.692	33.370	53.179	1.00	0.00	C
ATOM	1460	HB2	GLN	93	23.098	33.355	54.092	1.00	0.00	H
ATOM	1461	HB3	GLN	93	23.086	32.873	52.420	1.00	0.00	H
ATOM	1462	CG	GLN	93	24.982	32.488	53.243	1.00	0.00	C
ATOM	1463	HG2	GLN	93	24.606	31.467	53.306	1.00	0.00	H
ATOM	1464	HG3	GLN	93	25.525	32.493	52.297	1.00	0.00	H
ATOM	1465	CD	GLN	93	25.871	32.955	54.357	1.00	0.00	C
ATOM	1466	OE1	GLN	93	25.513	33.752	55.189	1.00	0.00	O
ATOM	1467	NE2	GLN	93	27.203	32.546	54.344	1.00	0.00	N
ATOM	1468	HE21	GLN	93	27.593	31.969	53.612	1.00	0.00	H
ATOM	1469	HE22	GLN	93	27.714	32.820	55.170	1.00	0.00	H
ATOM	1470	C	GLN	93	24.768	34.833	51.359	1.00	0.00	C
ATOM	1471	O	GLN	93	24.161	34.658	50.276	1.00	0.00	O
ATOM	1472	N	PHE	94	26.112	34.944	51.351	1.00	0.00	N
ATOM	1473	H	PHE	94	26.385	35.161	52.299	1.00	0.00	H
ATOM	1474	CA	PHE	94	26.876	35.139	50.137	1.00	0.00	C
ATOM	1475	HA	PHE	94	26.171	35.102	49.307	1.00	0.00	H
ATOM	1476	CB	PHE	94	27.460	36.537	50.149	1.00	0.00	C
ATOM	1477	HB2	PHE	94	28.231	36.679	50.905	1.00	0.00	H
ATOM	1478	HB3	PHE	94	28.060	36.604	49.240	1.00	0.00	H
ATOM	1479	CG	PHE	94	26.434	37.642	50.125	1.00	0.00	C

ATOM	1480	CD1	PHE	94	25.812	38.122	48.950	1.00	0.00	C
ATOM	1481	HD1	PHE	94	26.062	37.705	47.985	1.00	0.00	H
ATOM	1482	CE1	PHE	94	24.855	39.143	48.977	1.00	0.00	C
ATOM	1483	HE1	PHE	94	24.344	39.478	48.086	1.00	0.00	H
ATOM	1484	CZ	PHE	94	24.535	39.731	50.239	1.00	0.00	C
ATOM	1485	HZ	PHE	94	23.797	40.515	50.323	1.00	0.00	H
ATOM	1486	CE2	PHE	94	25.147	39.303	51.410	1.00	0.00	C
ATOM	1487	HE2	PHE	94	24.849	39.733	52.355	1.00	0.00	H
ATOM	1488	CD2	PHE	94	26.155	38.334	51.345	1.00	0.00	C
ATOM	1489	HD2	PHE	94	26.702	38.183	52.265	1.00	0.00	H
ATOM	1490	C	PHE	94	27.915	34.002	49.847	1.00	0.00	C
ATOM	1491	O	PHE	94	28.017	32.938	50.491	1.00	0.00	O
ATOM	1492	N	TYR	95	28.548	34.212	48.688	1.00	0.00	N
ATOM	1493	H	TYR	95	28.197	35.026	48.204	1.00	0.00	H
ATOM	1494	CA	TYR	95	29.438	33.323	48.035	1.00	0.00	C
ATOM	1495	HA	TYR	95	30.079	32.907	48.813	1.00	0.00	H
ATOM	1496	CB	TYR	95	28.737	32.162	47.292	1.00	0.00	C
ATOM	1497	HB2	TYR	95	27.852	31.908	47.877	1.00	0.00	H
ATOM	1498	HB3	TYR	95	28.300	32.499	46.352	1.00	0.00	H
ATOM	1499	CG	TYR	95	29.483	30.903	47.057	1.00	0.00	C
ATOM	1500	CD1	TYR	95	30.380	30.808	45.951	1.00	0.00	C
ATOM	1501	HD1	TYR	95	30.400	31.673	45.305	1.00	0.00	H
ATOM	1502	CE1	TYR	95	31.236	29.700	45.817	1.00	0.00	C
ATOM	1503	HE1	TYR	95	31.975	29.604	45.035	1.00	0.00	H
ATOM	1504	CZ	TYR	95	31.084	28.577	46.772	1.00	0.00	C
ATOM	1505	OH	TYR	95	31.784	27.459	46.599	1.00	0.00	O
ATOM	1506	HH	TYR	95	32.125	27.246	45.727	1.00	0.00	H
ATOM	1507	CE2	TYR	95	30.307	28.710	47.944	1.00	0.00	C
ATOM	1508	HE2	TYR	95	30.175	27.898	48.644	1.00	0.00	H
ATOM	1509	CD2	TYR	95	29.508	29.860	48.084	1.00	0.00	C
ATOM	1510	HD2	TYR	95	28.852	29.891	48.940	1.00	0.00	H
ATOM	1511	C	TYR	95	30.470	34.007	47.208	1.00	0.00	C
ATOM	1512	O	TYR	95	30.243	35.064	46.604	1.00	0.00	O
ATOM	1513	N	VAL	96	31.649	33.465	47.216	1.00	0.00	N
ATOM	1514	H	VAL	96	31.739	32.596	47.723	1.00	0.00	H
ATOM	1515	CA	VAL	96	32.871	34.085	46.681	1.00	0.00	C
ATOM	1516	HA	VAL	96	32.480	34.839	45.998	1.00	0.00	H
ATOM	1517	CB	VAL	96	33.779	34.886	47.653	1.00	0.00	C
ATOM	1518	HB	VAL	96	33.161	35.724	47.974	1.00	0.00	H
ATOM	1519	CG1	VAL	96	34.197	34.123	48.954	1.00	0.00	C
ATOM	1520	HG11	VAL	96	34.588	34.940	49.562	1.00	0.00	H
ATOM	1521	HG12	VAL	96	33.292	33.639	49.318	1.00	0.00	H
ATOM	1522	HG13	VAL	96	34.933	33.335	48.793	1.00	0.00	H
ATOM	1523	CG2	VAL	96	35.084	35.462	47.061	1.00	0.00	C
ATOM	1524	HG21	VAL	96	34.903	36.125	46.215	1.00	0.00	H
ATOM	1525	HG22	VAL	96	35.687	36.042	47.761	1.00	0.00	H
ATOM	1526	HG23	VAL	96	35.780	34.683	46.750	1.00	0.00	H
ATOM	1527	C	VAL	96	33.743	33.149	45.858	1.00	0.00	C
ATOM	1528	O	VAL	96	34.001	31.961	46.117	1.00	0.00	O
ATOM	1529	N	ILE	97	34.101	33.598	44.627	1.00	0.00	N
ATOM	1530	H	ILE	97	33.793	34.505	44.306	1.00	0.00	H
ATOM	1531	CA	ILE	97	34.807	32.807	43.634	1.00	0.00	C
ATOM	1532	HA	ILE	97	35.311	31.962	44.103	1.00	0.00	H
ATOM	1533	CB	ILE	97	33.833	32.413	42.498	1.00	0.00	C
ATOM	1534	HB	ILE	97	33.430	33.313	42.035	1.00	0.00	H
ATOM	1535	CG2	ILE	97	34.652	31.545	41.510	1.00	0.00	C
ATOM	1536	HG21	ILE	97	34.150	31.538	40.543	1.00	0.00	H
ATOM	1537	HG22	ILE	97	35.658	31.953	41.405	1.00	0.00	H
ATOM	1538	HG23	ILE	97	34.807	30.515	41.831	1.00	0.00	H
ATOM	1539	CG1	ILE	97	32.592	31.692	42.895	1.00	0.00	C
ATOM	1540	HG12	ILE	97	32.494	30.783	42.302	1.00	0.00	H
ATOM	1541	HG13	ILE	97	32.578	31.353	43.931	1.00	0.00	H
ATOM	1542	CD1	ILE	97	31.315	32.559	42.651	1.00	0.00	C
ATOM	1543	HD11	ILE	97	31.175	32.951	41.643	1.00	0.00	H

```
ATOM   1544 HD12 ILE    97      30.490  31.922  42.970  1.00  0.00           H
ATOM   1545 HD13 ILE    97      31.350  33.377  43.369  1.00  0.00           H
ATOM   1546  C   ILE    97      36.046  33.578  43.141  1.00  0.00           C
ATOM   1547  O   ILE    97      36.027  34.713  42.680  1.00  0.00           O
ATOM   1548  N   GLU    98      37.224  33.038  43.310  1.00  0.00           N
ATOM   1549  H   GLU    98      37.253  32.168  43.824  1.00  0.00           H
ATOM   1550  CA  GLU    98      38.464  33.437  42.694  1.00  0.00           C
ATOM   1551  HA  GLU    98      38.321  34.354  42.121  1.00  0.00           H
ATOM   1552  CB  GLU    98      39.433  33.804  43.872  1.00  0.00           C
ATOM   1553  HB2 GLU    98      38.813  34.359  44.575  1.00  0.00           H
ATOM   1554  HB3 GLU    98      39.755  32.853  44.297  1.00  0.00           H
ATOM   1555  CG  GLU    98      40.525  34.778  43.597  1.00  0.00           C
ATOM   1556  HG2 GLU    98      40.958  34.406  42.669  1.00  0.00           H
ATOM   1557  HG3 GLU    98      40.174  35.789
  43.387  1.00  0.00           H
ATOM   1558  CD  GLU    98      41.625  34.822  44.589  1.00  0.00           C
ATOM   1559  OE1 GLU    98      41.739  33.966  45.479  1.00  0.00           O
ATOM   1560  OE2 GLU    98      42.474  35.719  44.530  1.00  0.00           O
ATOM   1561  C   GLU    98      39.030  32.400  41.706  1.00  0.00           C
ATOM   1562  O   GLU    98      39.036  31.193  41.928  1.00  0.00           O
ATOM   1563  N   PHE    99      39.564  32.945  40.625  1.00  0.00           N
ATOM   1564  H   PHE    99      39.768  33.934  40.614  1.00  0.00           H
ATOM   1565  CA  PHE    99      39.763  32.203  39.417  1.00  0.00           C
ATOM   1566  HA  PHE    99      40.181  31.254  39.754  1.00  0.00           H
ATOM   1567  CB  PHE    99      38.408  31.983  38.721  1.00  0.00           C
ATOM   1568  HB2 PHE    99      38.718  31.477  37.808  1.00  0.00           H
ATOM   1569  HB3 PHE    99      37.681  31.379  39.265  1.00  0.00           H
ATOM   1570  CG  PHE    99      37.733  33.246  38.283  1.00  0.00           C
ATOM   1571  CD1 PHE    99      36.931  33.988  39.287  1.00  0.00           C
ATOM   1572  HD1 PHE    99      36.841  33.614  40.296  1.00  0.00           H
ATOM   1573  CE1 PHE    99      36.241  35.127  38.841  1.00  0.00           C
ATOM   1574  HE1 PHE    99      35.745  35.695  39.613  1.00  0.00           H
ATOM   1575  CZ  PHE    99      36.273  35.612  37.537  1.00  0.00           C
ATOM   1576  HZ  PHE    99      35.657  36.425  37.183  1.00  0.00           H
ATOM   1577  CE2 PHE    99      37.104  34.970  36.632  1.00  0.00           C
ATOM   1578  HE2 PHE    99      37.116  35.338  35.616  1.00  0.00           H
ATOM   1579  CD2 PHE    99      37.787  33.818  36.968  1.00  0.00           C
ATOM   1580  HD2 PHE    99      38.458  33.337  36.271  1.00  0.00           H
ATOM   1581  C   PHE    99      40.908  32.839  38.568  1.00  0.00           C
ATOM   1582  O   PHE    99      41.168  34.043  38.696  1.00  0.00           O
ATOM   1583  N   LYS   100      41.575  32.012  37.700  1.00  0.00           N
ATOM   1584  H   LYS   100      41.309  31.041  37.791  1.00  0.00           H
ATOM   1585  CA  LYS   100      42.556  32.440  36.678  1.00  0.00           C
ATOM   1586  HA  LYS   100      42.340  33.476  36.419  1.00  0.00           H
ATOM   1587  CB  LYS   100      44.012  32.255  37.095  1.00  0.00           C
ATOM   1588  HB2 LYS   100      44.659  32.679  36.327  1.00  0.00           H
ATOM   1589  HB3 LYS   100      44.232  32.863  37.972  1.00  0.00           H
ATOM   1590  CG  LYS   100      44.353  30.748  37.293  1.00  0.00           C
ATOM   1591  HG2 LYS   100      43.855  30.314  38.159  1.00  0.00           H
ATOM   1592  HG3 LYS   100      44.139  30.086  36.453  1.00  0.00           H
ATOM   1593  CD  LYS   100      45.870  30.569  37.579  1.00  0.00           C
ATOM   1594  HD2 LYS   100      46.365  31.049  36.735  1.00  0.00           H
ATOM   1595  HD3 LYS   100      46.129  30.947  38.568  1.00  0.00           H
ATOM   1596  CE  LYS   100      46.143  29.039  37.548  1.00  0.00           C
ATOM   1597  HE2 LYS   100      45.478  28.436  38.165  1.00  0.00           H
ATOM   1598  HE3 LYS   100      45.970  28.726  36.518  1.00  0.00           H
ATOM   1599  NZ  LYS   100      47.504  28.768  38.062  1.00  0.00           N
ATOM   1600  HZ1 LYS   100      48.154  29.250  37.458  1.00  0.00           H
ATOM   1601  HZ2 LYS   100      47.694  29.123  38.989  1.00  0.00           H
ATOM   1602  HZ3 LYS   100      47.726  27.795  38.220  1.00  0.00           H
ATOM   1603  C   LYS   100      42.406  31.805  35.315  1.00  0.00           C
ATOM   1604  O   LYS   100      42.112  30.627  35.314  1.00  0.00           O
ATOM   1605  N   CYX   101      42.566  32.506  34.166  1.00  0.00           N
ATOM   1606  H   CYX   101      42.889  33.457  34.273  1.00  0.00           H
```

ATOM	1607	CA	CYX	101	42.870	31.725	32.957	1.00	0.00	C
ATOM	1608	HA	CYX	101	42.099	30.983	32.754	1.00	0.00	H
ATOM	1609	CB	CYX	101	42.682	32.607	31.736	1.00	0.00	C
ATOM	1610	HB2	CYX	101	41.758	33.169	31.874	1.00	0.00	H
ATOM	1611	HB3	CYX	101	43.445	33.384	31.677	1.00	0.00	H
ATOM	1612	SG	CYX	101	42.809	31.873	30.118	1.00	0.00	S
ATOM	1613	C	CYX	101	44.187	30.940	33.007	1.00	0.00	C
ATOM	1614	O	CYX	101	45.227	31.395	33.424	1.00	0.00	O
ATOM	1615	N	LEU	102	44.209	29.695	32.587	1.00	0.00	N
ATOM	1616	H	LEU	102	43.339	29.270	32.299	1.00	0.00	H
ATOM	1617	CA	LEU	102	45.348	28.776	32.556	1.00	0.00	C
ATOM	1618	HA	LEU	102	45.770	28.727	33.561	1.00	0.00	H
ATOM	1619	CB	LEU	102	44.864	27.279	32.420	1.00	0.00	C
ATOM	1620	HB2	LEU	102	44.432	27.099	31.435	1.00	0.00	H
ATOM	1621	HB3	LEU	102	45.686	26.591	32.222	1.00	0.00	H
ATOM	1622	CG	LEU	102	43.922	26.683	33.489	1.00	0.00	C
ATOM	1623	HG	LEU	102	43.105	27.386	33.650	1.00	0.00	H
ATOM	1624	CD1	LEU	102	43.356	25.347	32.996	1.00	0.00	C
ATOM	1625	HD11	LEU	102	42.905	24.748	33.787	1.00	0.00	H
ATOM	1626	HD12	LEU	102	42.547	25.450	32.272	1.00	0.00	H
ATOM	1627	HD13	LEU	102	44.182	24.757	32.601	1.00	0.00	H
ATOM	1628	CD2	LEU	102	44.673	26.391	34.754	1.00	0.00	C
ATOM	1629	HD21	LEU	102	44.083	25.872	35.510	1.00	0.00	H
ATOM	1630	HD22	LEU	102	45.583	25.815	34.585	1.00	0.00	H
ATOM	1631	HD23	LEU	102	44.885	27.360	35.208	1.00	0.00	H
ATOM	1632	C	LEU	102	46.460	29.213	31.622	1.00	0.00	C
ATOM	1633	O	LEU	102	47.653	29.153	31.989	1.00	0.00	O
ATOM	1634	OXT	LEU	102	46.118	29.645	30.443	1.00	0.00	O
HETATM	314	N	LYN	20	39.231	40.796	35.350	1.00	0.00	N
HETATM	315	H	LYN	20	39.513	40.408	34.461	1.00	0.00	H
HETATM	316	CA	LYN	20	38.225	40.122	36.083	1.00	0.00	C
HETATM	317	HA	LYN	20	37.859	40.719	36.918	1.00	0.00	H
HETATM	318	CB	LYN	20	37.065	39.909	35.121	1.00	0.00	C
HETATM	319	HB2	LYN	20	37.417	39.458	34.194	1.00	0.00	H
HETATM	320	HB3	LYN	20	36.671	40.899	34.886	1.00	0.00	H
HETATM	321	CG	LYN	20	35.949	39.079	35.789	1.00	0.00	C
HETATM	322	HG2	LYN	20	35.142	38.928	35.073	1.00	0.00	H
HETATM	323	HG3	LYN	20	36.378	38.111	36.045	1.00	0.00	H
HETATM	324	CD	LYN	20	35.371	39.909	36.974	1.00	0.00	C
HETATM	325	HD2	LYN	20	34.889	40.854	36.728	1.00	0.00	H
HETATM	326	HD3	LYN	20	36.176	40.206	37.646	1.00	0.00	H
HETATM	327	CE	LYN	20	34.360	39.065	37.785	1.00	0.00	C
HETATM	328	HE2	LYN	20	33.579	38.745	37.095	1.00	0.00	H
HETATM	329	HE3	LYN	20	34.890	38.242	38.264	1.00	0.00	H
HETATM	330	NZ	LYN	20	33.854	39.900	38.912	1.00	0.00	N
HETATM	331	HZ2	LYN	20	33.808	40.815	38.488	1.00	0.00	H
HETATM	332	HZ3	LYN	20	34.566	39.775	39.618	1.00	0.00	H
HETATM	333	C	LYN	20	38.826	38.796	36.568	1.00	0.00	C
HETATM	334	O	LYN	20	39.110	37.901	35.784	1.00	0.00	O
HETATM	1636	N	LIG	103	30.426	41.784	41.046	1.00	0.00	N
HETATM	1637	C	LIG	103	29.972	40.809	40.221	1.00	0.00	C
HETATM	1638	O	LIG	103	30.488	42.823	43.004	1.00	0.00	O
HETATM	1639	C5'	LIG	103	25.763	39.281	44.485	1.00	0.00	C
HETATM	1640	O5'	LIG	103	25.449	39.184	43.097	1.00	0.00	O
HETATM	1641	C4'	LIG	103	26.250	40.674	44.913	1.00	0.00	C
HETATM	1642	O4'	LIG	103	27.694	40.616	44.682	1.00	0.00	O
HETATM	1643	C3'	LIG	103	25.813	41.889	44.079	1.00	0.00	C
HETATM	1644	O3'	LIG	103	24.968	42.722	44.849	1.00	0.00	O
HETATM	1645	C2'	LIG	103	27.126	42.638	43.662	1.00	0.00	C
HETATM	1646	O2'	LIG	103	27.319	43.890	44.289	1.00	0.00	O
HETATM	1647	C1'	LIG	103	28.237	41.657	43.959	1.00	0.00	C
HETATM	1648	N1	LIG	103	28.877	41.238	42.710	1.00	0.00	N
HETATM	1649	O1	LIG	103	30.073	38.774	38.196	1.00	0.00	O
HETATM	1650	N2	LIG	103	30.557	40.824	38.972	1.00	0.00	N

```
HETATM 1651  C6  LIG   103      29.975  41.948  42.358  1.00  0.00           C
HETATM 1652  C7  LIG   103      28.315  40.284  41.890  1.00  0.00           C
HETATM 1653  C8  LIG   103      28.875  40.051  40.680  1.00  0.00           C
HETATM 1654  C9  LIG   103      30.566  39.856  37.998  1.00  0.00           C
HETATM 1655  C10 LIG   103      31.223  40.286  36.691  1.00  0.00           C
HETATM 1656  H   LIG   103      31.129  41.621  38.725  1.00  0.00           H
HETATM 1657  H1  LIG   103      28.388  39.292  40.078  1.00  0.00           H
HETATM 1658  H4  LIG   103      28.946  42.116  44.657  1.00  0.00           H
HETATM 1659  H6  LIG   103      26.084  40.808  45.989  1.00  0.00           H
HETATM 1660  H7  LIG   103      25.203  41.568  43.227  1.00  0.00           H
HETATM 1661  H8  LIG   103      27.048  42.757  42.574  1.00  0.00           H
HETATM 1662  H9  LIG   103      24.852  39.043  45.049  1.00  0.00
      H
HETATM 1663  H10 LIG   103      26.547  38.604  44.844  1.00  0.00           H
HETATM 1664  H11 LIG   103      30.732  41.097  36.140  1.00  0.00           H
HETATM 1665  H12 LIG   103      31.312  39.450  35.986  1.00  0.00           H
HETATM 1666  H13 LIG   103      32.274  40.509  36.911  1.00  0.00           H
HETATM 1667  H14 LIG   103      27.560  39.689  42.385  1.00  0.00           H
HETATM 1668  H2' LIG   103      27.971  44.396  43.774  1.00  0.00           H
HETATM 1669  H3' LIG   103      25.561  43.463  45.064  1.00  0.00           H
HETATM 1670  H5' LIG   103      25.335  38.223  42.987  1.00  0.00           H
ENDMDL
MODEL    15
SHEET    1   1 1 ILE    22  ASP    26  0
SHEET    2   2 1 VAL    37  VAL    40  0
SHEET    3   3 1 CYX    50  THR    61  0
SHEET    4   4 1 GLN    93  CYX   101  0
HELIX    1   1 GLN     12  LEU     16  1                                  5
HELIX    2   2 LYS     68  GLN     72  1                                  5
HELIX    3   3 LEU     77  ALA     85  1                                  9
ATOM      1  N   GLN     1      34.978  17.782  34.202  1.00  0.00           N
ATOM      2  H1  GLN     1      35.481  18.410  34.814  1.00  0.00           H
ATOM      3  H2  GLN     1      33.995  17.779  34.432  1.00  0.00           H
ATOM      4  H3  GLN     1      35.334  16.839  34.252  1.00  0.00           H
ATOM      5  CA  GLN     1      35.094  18.290  32.831  1.00  0.00           C
ATOM      6  HA  GLN     1      36.133  18.138  32.541  1.00  0.00           H
ATOM      7  CB  GLN     1      34.216  17.542  31.840  1.00  0.00           C
ATOM      8  HB2 GLN     1      33.257  18.035  31.995  1.00  0.00           H
ATOM      9  HB3 GLN     1      34.459  17.864  30.827  1.00  0.00           H
ATOM     10  CG  GLN     1      34.087  16.083  31.938  1.00  0.00           C
ATOM     11  HG2 GLN     1      33.581  15.865  32.879  1.00  0.00           H
ATOM     12  HG3 GLN     1      33.442  15.723  31.136  1.00  0.00           H
ATOM     13  CD  GLN     1      35.480  15.354  31.883  1.00  0.00           C
ATOM     14  OE1 GLN     1      36.515  15.826  32.457  1.00  0.00           O
ATOM     15  NE2 GLN     1      35.575  14.193  31.308  1.00  0.00           N
ATOM     16 HE21 GLN     1      34.817  13.812  30.760  1.00  0.00           H
ATOM     17 HE22 GLN     1      36.484  13.758  31.249  1.00  0.00           H
ATOM     18  C   GLN     1      34.766  19.811  32.923  1.00  0.00           C
ATOM     19  O   GLN     1      34.165  20.282  33.868  1.00  0.00           O
ATOM     20  N   PRO     2      35.118  20.501  31.875  1.00  0.00           N
ATOM     21  CD  PRO     2      36.046  20.170  30.808  1.00  0.00           C
ATOM     22  HD2 PRO     2      35.666  19.545  29.999  1.00  0.00           H
ATOM     23  HD3 PRO     2      36.927  19.589  31.083  1.00  0.00           H
ATOM     24  CG  PRO     2      36.565  21.571  30.361  1.00  0.00           C
ATOM     25  HG2 PRO     2      36.905  21.561  29.326  1.00  0.00           H
ATOM     26  HG3 PRO     2      37.348  21.764  31.095  1.00  0.00           H
ATOM     27  CB  PRO     2      35.368  22.548  30.605  1.00  0.00           C
ATOM     28  HB2 PRO     2      34.706  22.570  29.739  1.00  0.00           H
ATOM     29  HB3 PRO     2      35.714  23.569  30.762  1.00  0.00           H
ATOM     30  CA  PRO     2      34.732  21.930  31.802  1.00  0.00           C
ATOM     31  HA  PRO     2      35.205  22.504  32.599  1.00  0.00           H
ATOM     32  C   PRO     2      33.255  22.198  31.792  1.00  0.00           C
ATOM     33  O   PRO     2      32.573  21.703  30.878  1.00  0.00           O
ATOM     34  N   ASN     3      32.783  23.105  32.636  1.00  0.00           N
```

ATOM	35	H	ASN	3	33.434	23.605	33.224	1.00	0.00	H
ATOM	36	CA	ASN	3	31.366	23.485	32.732	1.00	0.00	C
ATOM	37	HA	ASN	3	30.745	22.590	32.723	1.00	0.00	H
ATOM	38	CB	ASN	3	31.144	24.330	33.993	1.00	0.00	C
ATOM	39	HB2	ASN	3	31.903	24.005	34.705	1.00	0.00	H
ATOM	40	HB3	ASN	3	31.370	25.386	33.845	1.00	0.00	H
ATOM	41	CG	ASN	3	29.780	24.201	34.581	1.00	0.00	C
ATOM	42	OD1	ASN	3	28.800	24.636	33.916	1.00	0.00	O
ATOM	43	ND2	ASN	3	29.630	23.537	35.729	1.00	0.00	N
ATOM	44	HD21	ASN	3	30.378	23.556	36.408	1.00	0.00	H
ATOM	45	HD22	ASN	3	28.676	23.654	36.041	1.00	0.00	H
ATOM	46	C	ASN	3	30.925	24.260	31.478	1.00	0.00	C
ATOM	47	O	ASN	3	31.608	25.121	30.916	1.00	0.00	O
ATOM	48	N	ASP	4	29.701	23.926	30.951	1.00	0.00	N
ATOM	49	H	ASP	4	29.218	23.224	31.492	1.00	0.00	H
ATOM	50	CA	ASP	4	29.021	24.744	29.952	1.00	0.00	C
ATOM	51	HA	ASP	4	29.888	24.938	29.320	1.00	0.00	H
ATOM	52	CB	ASP	4	28.011	23.857	29.191	1.00	0.00	C
ATOM	53	HB2	ASP	4	27.421	23.248	29.878	1.00	0.00	H
ATOM	54	HB3	ASP	4	27.288	24.550	28.760	1.00	0.00	H
ATOM	55	CG	ASP	4	28.651	23.057	28.061	1.00	0.00	C
ATOM	56	OD1	ASP	4	29.862	22.671	28.086	1.00	0.00	O
ATOM	57	OD2	ASP	4	27.868	23.040	27.075	1.00	0.00	O
ATOM	58	C	ASP	4	28.442	26.127	30.447	1.00	0.00	C
ATOM	59	O	ASP	4	28.066	26.967	29.674	1.00	0.00	O
ATOM	60	N	ILE	5	28.460	26.411	31.768	1.00	0.00	N
ATOM	61	H	ILE	5	28.643	25.683	32.443	1.00	0.00	H
ATOM	62	CA	ILE	5	28.271	27.752	32.335	1.00	0.00	C
ATOM	63	HA	ILE	5	27.436	28.238	31.829	1.00	0.00	H
ATOM	64	CB	ILE	5	27.846	27.698	33.905	1.00	0.00	C
ATOM	65	HB	ILE	5	28.717	27.295	34.421	1.00	0.00	H
ATOM	66	CG2	ILE	5	27.723	29.123	34.417	1.00	0.00	C
ATOM	67	HG21	ILE	5	27.381	29.053	35.450	1.00	0.00	H
ATOM	68	HG22	ILE	5	28.659	29.679	34.378	1.00	0.00	H
ATOM	69	HG23	ILE	5	27.037	29.594	33.714	1.00	0.00	H
ATOM	70	CG1	ILE	5	26.654	26.710	34.244	1.00	0.00	C
ATOM	71	HG12	ILE	5	26.681	25.818	33.617	1.00	0.00	H
ATOM	72	HG13	ILE	5	26.844	26.324	35.245	1.00	0.00	H
ATOM	73	CD1	ILE	5	25.204	27.320	34.222	1.00	0.00	C
ATOM	74	HD11	ILE	5	25.217	28.005	35.070	1.00	0.00	H
ATOM	75	HD12	ILE	5	25.046	27.816	33.265	1.00	0.00	H
ATOM	76	HD13	ILE	5	24.608	26.416	34.351	1.00	0.00	H
ATOM	77	C	ILE	5	29.498	28.645	32.071	1.00	0.00	C
ATOM	78	O	ILE	5	30.679	28.326	32.385	1.00	0.00	O
ATOM	79	N	THR	6	29.206	29.864	31.599	1.00	0.00	N
ATOM	80	H	THR	6	28.245	30.174	31.637	1.00	0.00	H
ATOM	81	CA	THR	6	30.205	30.896	31.128	1.00	0.00	C
ATOM	82	HA	THR	6	31.140	30.658	31.635	1.00	0.00	H
ATOM	83	CB	THR	6	30.455	30.655	29.677	1.00	0.00	C
ATOM	84	HB	THR	6	30.767	29.628	29.495	1.00	0.00	H
ATOM	85	CG2	THR	6	29.291	31.114	28.792	1.00	0.00	C
ATOM	86	HG21	THR	6	29.502	30.830	27.762	1.00	0.00	H
ATOM	87	HG22	THR	6	28.328	30.756	29.159	1.00	0.00	H
ATOM	88	HG23	THR	6	29.319	32.203	28.770	1.00	0.00	H
ATOM	89	OG1	THR	6	31.480	31.535	29.162	1.00	0.00	O
ATOM	90	HG1	THR	6	32.332	31.191	29.440	1.00	0.00	H
ATOM	91	C	THR	6	29.821	32.349	31.453	1.00	0.00	C
ATOM	92	O	THR	6	28.790	32.549	32.178	1.00	0.00	O
ATOM	93	N	PHE	7	30.642	33.357	31.129	1.00	0.00	N
ATOM	94	H	PHE	7	31.290	33.205	30.369	1.00	0.00	H
ATOM	95	CA	PHE	7	30.422	34.806	31.398	1.00	0.00	C
ATOM	96	HA	PHE	7	29.651	34.920	32.160	1.00	0.00	H
ATOM	97	CB	PHE	7	31.626	35.528	32.080	1.00	0.00	C
ATOM	98	HB2	PHE	7	32.599	35.442	31.597	1.00	0.00	H

ATOM	99	HB3	PHE	7	31.414	36.596	32.063	1.00	0.00	H
ATOM	100	CG	PHE	7	32.003	35.113	33.453	1.00	0.00	C
ATOM	101	CD1	PHE	7	32.902	34.082	33.744	1.00	0.00	C
ATOM	102	HD1	PHE	7	33.218	33.352	33.014	1.00	0.00	H
ATOM	103	CE1	PHE	7	33.353	33.925	35.067	1.00	0.00	C
ATOM	104	HE1	PHE	7	34.005	33.108	35.335	1.00	0.00	H
ATOM	105	CZ	PHE	7	32.979	34.866	36.095	1.00	0.00	C
ATOM	106	HZ	PHE	7	33.538	34.784	37.015	1.00	0.00	H
ATOM	107	CE2	PHE	7	32.060	35.869	35.749	1.00	0.00	C
ATOM	108	HE2	PHE	7	31.715	36.533	36.529	1.00	0.00	H
ATOM	109	CD2	PHE	7	31.545	36.006	34.457	1.00	0.00	C
ATOM	110	HD2	PHE	7	30.784	36.764	34.341	1.00	0.00	H
ATOM	111	C	PHE	7	30.050	35.537	30.072	1.00	0.00	C
ATOM	112	O	PHE	7	30.364	35.096	28.971	1.00	0.00	O
ATOM	113	N	PHE	8	29.400	36.664	30.244	1.00	0.00	N
ATOM	114	H	PHE	8	28.946	36.895	31.117	1.00	0.00	H
ATOM	115	CA	PHE	8	28.931	37.436	29.086	1.00	0.00	C
ATOM	116	HA	PHE	8	28.383	36.751	28.439	1.00	0.00	H
ATOM	117	CB	PHE	8	28.061	38.603	29.511	1.00	0.00	C
ATOM	118	HB2	PHE	8	28.650	39.247	30.165	1.00	0.00	H
ATOM	119	HB3	PHE	8	27.683	38.930	28.543	1.00	0.00	H
ATOM	120	CG	PHE	8	26.825	38.166	30.291	1.00	0.00	C
ATOM	121	CD1	PHE	8	25.863	37.351	29.663	1.00	0.00	C
ATOM	122	HD1	PHE	8	26.040	36.973	28.667	1.00	0.00	H
ATOM	123	CE1	PHE	8	24.724	36.978	30.298	1.00	0.00	C
ATOM	124	HE1	PHE	8	24.023	36.336	29.786	1.00	0.00	H
ATOM	125	CZ	PHE	8	24.554	37.423	31.649	1.00	0.00	C
ATOM	126	HZ	PHE	8	23.795	36.967	32.268	1.00	0.00	H
ATOM	127	CE2	PHE	8	25.552	38.180	32.317	1.00	0.00	C
ATOM	128	HE2	PHE	8	25.513	38.382	33.377	1.00	0.00	H
ATOM	129	CD2	PHE	8	26.625	38.687	31.583	1.00	0.00	C
ATOM	130	HD2	PHE	8	27.210	39.470	32.043	1.00	0.00	H
ATOM	131	C	PHE	8	30.062	38.081	28.253	1.00	0.00	C
ATOM	132	O	PHE	8	31.236	38.260	28.637	1.00	0.00	O
ATOM	133	N	GLN	9	29.749	38.334	26.994	1.00	0.00	N
ATOM	134	H	GLN	9	28.839	38.011	26.697	1.00	0.00	H
ATOM	135	CA	GLN	9	30.664	38.957	25.938	1.00	0.00	C
ATOM	136	HA	GLN	9	31.498	38.275	25.774	1.00	0.00	H
ATOM	137	CB	GLN	9	29.936	39.193	24.612	1.00	0.00	C
ATOM	138	HB2	GLN	9	29.757	38.196	24.211	1.00	0.00	H
ATOM	139	HB3	GLN	9	28.977	39.697	24.734	1.00	0.00	H
ATOM	140	CG	GLN	9	30.835	39.933	23.561	1.00	0.00	C
ATOM	141	HG2	GLN	9	30.885	40.952	23.947	1.00	0.00	H
ATOM	142	HG3	GLN	9	31.827	39.503	23.428	1.00	0.00	H
ATOM	143	CD	GLN	9	30.253	39.965	22.213	1.00	0.00	C
ATOM	144	OE1	GLN	9	29.011	39.795	22.092	1.00	0.00	O
ATOM	145	NE2	GLN	9	30.950	40.292	21.130	1.00	0.00	N
ATOM	146	HE21	GLN	9	30.429	40.417	20.274	1.00	0.00	H
ATOM	147	HE22	GLN	9	31.922	40.557	21.200	1.00	0.00	H
ATOM	148	C	GLN	9	31.281	40.243	26.499	1.00	0.00	C
ATOM	149	O	GLN	9	32.409	40.508	26.091	1.00	0.00	O
ATOM	150	N	ARG	10	30.600	41.122	27.390	1.00	0.00	N
ATOM	151	H	ARG	10	29.632	40.914	27.592	1.00	0.00	H
ATOM	152	CA	ARG	10	31.200	42.290	28.085	1.00	0.00	C
ATOM	153	HA	ARG	10	31.502	42.996	27.312	1.00	0.00	H
ATOM	154	CB	ARG	10	30.089	42.947	29.008	1.00	0.00	C
ATOM	155	HB2	ARG	10	29.253	43.256	28.382	1.00	0.00	H
ATOM	156	HB3	ARG	10	29.745	42.190	29.713	1.00	0.00	H
ATOM	157	CG	ARG	10	30.553	44.216	29.780	1.00	0.00	C
ATOM	158	HG2	ARG	10	29.757	44.617	30.408	1.00	0.00	H
ATOM	159	HG3	ARG	10	31.435	43.967	30.369	1.00	0.00	H
ATOM	160	CD	ARG	10	30.768	45.292	28.761	1.00	0.00	C
ATOM	161	HD2	ARG	10	30.338	45.056	27.787	1.00	0.00	H

ATOM	162	HD3	ARG	10	30.270	46.223	29.032	1.00	0.00	H
ATOM	163	NE	ARG	10	32.236	45.599	28.555	1.00	0.00	N
ATOM	164	HE	ARG	10	32.978	45.027	28.931	1.00	0.00	H
ATOM	165	CZ	ARG	10	32.828	46.483	27.863	1.00	0.00	C
ATOM	166	NH1	ARG	10	32.182	47.430	27.316	1.00	0.00	N
ATOM	167	HH11	ARG	10	32.712	48.123	26.805	1.00	0.00	H
ATOM	168	HH12	ARG	10	31.185	47.448	27.480	1.00	0.00	H
ATOM	169	NH2	ARG	10	34.104	46.459	27.802	1.00	0.00	N
ATOM	170	HH21	ARG	10	34.712	45.755	28.199	1.00	0.00	H
ATOM	171	HH22	ARG	10	34.538	47.048	27.107	1.00	0.00	H
ATOM	172	C	ARG	10	32.440	41.981	28.927	1.00	0.00	C
ATOM	173	O	ARG	10	33.331	42.832	29.082	1.00	0.00	O
ATOM	174	N	PHE	11	32.530	40.821	29.605	1.00	0.00	N
ATOM	175	H	PHE	11	31.837	40.118	29.392	1.00	0.00	H
ATOM	176	CA	PHE	11	33.551	40.427	30.566	1.00	0.00	C
ATOM	177	HA	PHE	11	34.013	41.368	30.868	1.00	0.00	H
ATOM	178	CB	PHE	11	32.766	39.824	31.813	1.00	0.00	C
ATOM	179	HB2	PHE	11	33.513	39.646	32.588	1.00	0.00	H
ATOM	180	HB3	PHE	11	32.216	38.941	31.487	1.00	0.00	H
ATOM	181	CG	PHE	11	31.869	40.883	32.379	1.00	0.00	C
ATOM	182	CD1	PHE	11	32.421	42.007	33.072	1.00	0.00	C
ATOM	183	HD1	PHE	11	33.487	42.043	33.244	1.00	0.00	H
ATOM	184	CE1	PHE	11	31.497	43.007	33.443	1.00	0.00	C
ATOM	185	HE1	PHE	11	31.881	43.890	33.933	1.00	0.00	H
ATOM	186	CZ	PHE	11	30.145	42.833	33.500	1.00	0.00	C
ATOM	187	HZ	PHE	11	29.456	43.505	33.991	1.00	0.00	H
ATOM	188	CE2	PHE	11	29.634	41.657	32.960	1.00	0.00	C
ATOM	189	HE2	PHE	11	28.568	41.487	32.984	1.00	0.00	H
ATOM	190	CD2	PHE	11	30.461	40.788	32.258	1.00	0.00	C
ATOM	191	HD2	PHE	11	30.137	39.826	31.889	1.00	0.00	H
ATOM	192	C	PHE	11	34.729	39.540	30.124	1.00	0.00	C
ATOM	193	O	PHE	11	35.787	39.518	30.750	1.00	0.00	O
ATOM	194	N	GLN	12	34.650	38.900	28.961	1.00	0.00	N
ATOM	195	H	GLN	12	33.774	38.787	28.472	1.00	0.00	H
ATOM	196	CA	GLN	12	35.640	37.992	28.431	1.00	0.00	C
ATOM	197	HA	GLN	12	35.713	37.167	29.139	1.00	0.00	H
ATOM	198	CB	GLN	12	35.163	37.408	27.089	1.00	0.00	C
ATOM	199	HB2	GLN	12	34.806	38.166	26.393	1.00	0.00	H
ATOM	200	HB3	GLN	12	35.984	36.743	26.819	1.00	0.00	H
ATOM	201	CG	GLN	12	33.880	36.491	27.207	1.00	0.00	C
ATOM	202	HG2	GLN	12	33.966	35.700	27.952	1.00	0.00	H
ATOM	203	HG3	GLN	12	33.017	37.051	27.565	1.00	0.00	H
ATOM	204	CD	GLN	12	33.525	35.967	25.793	1.00	0.00	C
ATOM	205	OE1	GLN	12	33.967	36.272	24.734	1.00	0.00	O
ATOM	206	NE2	GLN	12	32.470	35.150	25.794	1.00	0.00	N
ATOM	207	HE21	GLN	12	31.892	34.830	26.559	1.00	0.00	H
ATOM	208	HE22	GLN	12	32.214	34.726	24.913	1.00	0.00	H
ATOM	209	C	GLN	12	36.990	38.566	28.237	1.00	0.00	C
ATOM	210	O	GLN	12	37.949	38.074	28.854	1.00	0.00	O
ATOM	211	N	ASP	13	37.082	39.661	27.516	1.00	0.00	N
ATOM	212	H	ASP	13	36.254	40.084	27.122	1.00	0.00	H
ATOM	213	CA	ASP	13	38.339	40.305	27.268	1.00	0.00	C
ATOM	214	HA	ASP	13	38.916	39.514	26.787	1.00	0.00	H
ATOM	215	CB	ASP	13	38.206	41.456	26.261	1.00	0.00	C
ATOM	216	HB2	ASP	13	37.587	42.288	26.596	1.00	0.00	H
ATOM	217	HB3	ASP	13	39.234	41.798	26.139	1.00	0.00	H
ATOM	218	CG	ASP	13	37.752	41.003	24.864	1.00	0.00	C
ATOM	219	OD1	ASP	13	37.645	39.811	24.582	1.00	0.00	O
ATOM	220	OD2	ASP	13	37.434	41.881	24.047	1.00	0.00	O
ATOM	221	C	ASP	13	38.961	40.878	28.618	1.00	0.00	C
ATOM	222	O	ASP	13	40.152	41.117	28.662	1.00	0.00	O
ATOM	223	N	ASP	14	38.215	41.036	29.691	1.00	0.00	N
ATOM	224	H	ASP	14	37.238	40.779	29.720	1.00	0.00	H
ATOM	225	CA	ASP	14	38.735	41.422	31.013	1.00	0.00	C

ATOM	226	HA	ASP	14	39.538	42.146	30.873	1.00	0.00	H
ATOM	227	CB	ASP	14	37.754	41.990	32.009	1.00	0.00	C
ATOM	228	HB2	ASP	14	37.060	41.211	32.325	1.00	0.00	H
ATOM	229	HB3	ASP	14	38.338	42.277	32.883	1.00	0.00	H
ATOM	230	CG	ASP	14	36.953	43.265	31.506	1.00	0.00	C
ATOM	231	OD1	ASP	14	35.938	43.733	32.115	1.00	0.00	O
ATOM	232	OD2	ASP	14	37.410	43.926	30.528	1.00	0.00	O
ATOM	233	C	ASP	14	39.376	40.181	31.668	1.00	0.00	C
ATOM	234	O	ASP	14	40.435	40.270	32.305	1.00	0.00	O
ATOM	235	N	ILE	15	38.683	38.984	31.516	1.00	0.00	N
ATOM	236	H	ILE	15	37.882	38.968	30.900	1.00	0.00	H
ATOM	237	CA	ILE	15	39.106	37.743	32.133	1.00	0.00	C
ATOM	238	HA	ILE	15	39.296	37.935	33.189	1.00	0.00	H
ATOM	239	CB	ILE	15	37.970	36.747	32.111	1.00	0.00	C
ATOM	240	HB	ILE	15	37.715	36.576	31.066	1.00	0.00	H
ATOM	241	CG2	ILE	15	38.357	35.345	32.729	1.00	0.00	C
ATOM	242	HG21	ILE	15	37.489	34.695	32.620	1.00	0.00	H
ATOM	243	HG22	ILE	15	39.212	34.919	32.205	1.00	0.00	H
ATOM	244	HG23	ILE	15	38.530	35.288	33.804	1.00	0.00	H
ATOM	245	CG1	ILE	15	36.693	37.303	32.818	1.00	0.00	C
ATOM	246	HG12	ILE	15	36.734	37.278	33.907	1.00	0.00	H
ATOM	247	HG13	ILE	15	36.578	38.368	32.614	1.00	0.00	H
ATOM	248	CD1	ILE	15	35.398	36.585	32.347	1.00	0.00	C
ATOM	249	HD11	ILE	15	34.500	36.996	32.809	1.00	0.00	H
ATOM	250	HD12	ILE	15	35.299	36.736	31.272	1.00	0.00	H
ATOM	251	HD13	ILE	15	35.511	35.507	32.462	1.00	0.00	H
ATOM	252	C	ILE	15	40.396	37.211	31.500	1.00	0.00	C
ATOM	253	O	ILE	15	41.314	36.824	32.183	1.00	0.00	O
ATOM	254	N	LEU	16	40.475	37.304	30.146	1.00	0.00	N
ATOM	255	H	LEU	16	39.612	37.527	29.672	1.00	0.00	H
ATOM	256	CA	LEU	16	41.669	37.128	29.333	1.00	0.00	C
ATOM	257	HA	LEU	16	42.116	36.151	29.515	1.00	0.00	H
ATOM	258	CB	LEU	16	41.250	37.252	27.846	1.00	0.00	C
ATOM	259	HB2	LEU	16	40.665	36.382	27.550	1.00	0.00	H
ATOM	260	HB3	LEU	16	40.606	38.131	27.796	1.00	0.00	H
ATOM	261	CG	LEU	16	42.405	37.351	26.868	1.00	0.00	C
ATOM	262	HG	LEU	16	43.197	38.010	27.222	1.00	0.00	H
ATOM	263	CD1	LEU	16	43.092	35.998	26.675	1.00	0.00	C
ATOM	264	HD11	LEU	16	42.365	35.285	26.285	1.00	0.00	H
ATOM	265	HD12	LEU	16	43.973	36.003	26.033	1.00	0.00	H
ATOM	266	HD13	LEU	16	43.423	35.768	27.687	1.00	0.00	H
ATOM	267	CD2	LEU	16	41.981	37.728	25.414	1.00	0.00	C
ATOM	268	HD21	LEU	16	41.299	36.977	25.015	1.00	0.00	H
ATOM	269	HD22	LEU	16	41.450	38.679	25.361	1.00	0.00	H
ATOM	270	HD23	LEU	16	42.817	37.694	24.714	1.00	0.00	H
ATOM	271	C	LEU	16	42.857	38.065	29.688	1.00	0.00	C
ATOM	272	O	LEU	16	43.984	37.596	29.804	1.00	0.00	O
ATOM	273	N	ALA	17	42.628	39.338	29.918	1.00	0.00	N
ATOM	274	H	ALA	17	41.665	39.636	29.986	1.00	0.00	H
ATOM	275	CA	ALA	17	43.597	40.347	30.324	1.00	0.00	C
ATOM	276	HA	ALA	17	44.575	40.236	29.856	1.00	0.00	H
ATOM	277	CB	ALA	17	43.005	41.755	30.029	1.00	0.00	C
ATOM	278	HB1	ALA	17	42.757	41.852	28.972	1.00	0.00	H
ATOM	279	HB2	ALA	17	42.107	41.862	30.637	1.00	0.00	H
ATOM	280	HB3	ALA	17	43.666	42.540	30.397	1.00	0.00	H
ATOM	281	C	ALA	17	43.881	40.209	31.820	1.00	0.00	C
ATOM	282	O	ALA	17	44.594	41.089	32.301	1.00	0.00	O
ATOM	283	N	GLY	18	43.284	39.237	32.572	1.00	0.00	N
ATOM	284	H	GLY	18	42.579	38.646	32.154	1.00	0.00	H
ATOM	285	CA	GLY	18	43.356	39.057	34.043	1.00	0.00	C
ATOM	286	HA2	GLY	18	42.755	38.166	34.222	1.00	0.00	H
ATOM	287	HA3	GLY	18	44.283	38.686	34.478	1.00	0.00	H
ATOM	288	C	GLY	18	42.759	40.194	34.823	1.00	0.00	C

ATOM	289	O	GLY	18	42.996	40.117	36.009	1.00	0.00	O
ATOM	290	N	ARG	19	42.145	41.101	34.117	1.00	0.00	N
ATOM	291	H	ARG	19	42.058	40.865	33.138	1.00	0.00	H
ATOM	292	CA	ARG	19	41.497	42.342	34.752	1.00	0.00	C
ATOM	293	HA	ARG	19	42.273	42.789	35.374	1.00	0.00	H
ATOM	294	CB	ARG	19	41.240	43.341	33.687	1.00	0.00	C
ATOM	295	HB2	ARG	19	42.183	43.426	33.148	1.00	0.00	H
ATOM	296	HB3	ARG	19	40.418	42.974	33.073	1.00	0.00	H
ATOM	297	CG	ARG	19	40.730	44.688	34.150	1.00	0.00	C
ATOM	298	HG2	ARG	19	40.646	45.294	33.247	1.00	0.00	H
ATOM	299	HG3	ARG	19	39.748	44.541	34.600	1.00	0.00	H
ATOM	300	CD	ARG	19	41.721	45.368	35.125	1.00	0.00	C
ATOM	301	HD2	ARG	19	41.885	44.661	35.939	1.00	0.00	H
ATOM	302	HD3	ARG	19	42.652	45.611	34.611	1.00	0.00	H
ATOM	303	NE	ARG	19	41.155	46.636	35.629	1.00	0.00	N
ATOM	304	HE	ARG	19	41.357	47.532	35.211	1.00	0.00	H
ATOM	305	CZ	ARG	19	40.466	46.764	36.823	1.00	0.00	C
ATOM	306	NH1	ARG	19	40.042	45.795	37.548	1.00	0.00	N
ATOM	307	HH11	ARG	19	39.963	44.834	37.249	1.00	0.00	H
ATOM	308	HH12	ARG	19	39.587	46.026	38.420	1.00	0.00	H
ATOM	309	NH2	ARG	19	40.335	48.002	37.265	1.00	0.00	N
ATOM	310	HH21	ARG	19	39.679	48.122	38.023	1.00	0.00	H
ATOM	311	HH22	ARG	19	40.515	48.746	36.606	1.00	0.00	H
ATOM	312	C	ARG	19	40.290	42.057	35.596	1.00	0.00	C
ATOM	313	O	ARG	19	40.029	42.842	36.482	1.00	0.00	O
ATOM	335	N	THR	21	38.898	38.718	37.799	1.00	0.00	N
ATOM	336	H	THR	21	38.534	39.469	38.369	1.00	0.00	H
ATOM	337	CA	THR	21	39.431	37.564	38.535	1.00	0.00	C
ATOM	338	HA	THR	21	39.204	36.666	37.960	1.00	0.00	H
ATOM	339	CB	THR	21	40.962	37.751	38.569	1.00	0.00	C
ATOM	340	HB	THR	21	41.334	37.721	37.545	1.00	0.00	H
ATOM	341	CG2	THR	21	41.535	39.089	39.208	1.00	0.00	C
ATOM	342	HG21	THR	21	41.180	39.963	38.662	1.00	0.00	H
ATOM	343	HG22	THR	21	41.018	39.118	40.167	1.00	0.00	H
ATOM	344	HG23	THR	21	42.622	39.049	39.274	1.00	0.00	H
ATOM	345	OG1	THR	21	41.664	36.653	39.257	1.00	0.00	O
ATOM	346	HG1	THR	21	41.266	35.798	39.075	1.00	0.00	H
ATOM	347	C	THR	21	38.847	37.224	39.952	1.00	0.00	C
ATOM	348	O	THR	21	39.267	36.253	40.549	1.00	0.00	O
ATOM	349	N	ILE	22	37.857	37.928	40.460	1.00	0.00	N
ATOM	350	H	ILE	22	37.745	38.893	40.184	1.00	0.00	H
ATOM	351	CA	ILE	22	36.986	37.556	41.587	1.00	0.00	C
ATOM	352	HA	ILE	22	36.980	36.468	41.654	1.00	0.00	H
ATOM	353	CB	ILE	22	37.627	38.005	42.914	1.00	0.00	C
ATOM	354	HB	ILE	22	38.306	37.179	43.130	1.00	0.00	H
ATOM	355	CG2	ILE	22	38.175	39.439	42.793	1.00	0.00	C
ATOM	356	HG21	ILE	22	38.808	39.674	41.937	1.00	0.00	H
ATOM	357	HG22	ILE	22	37.323	40.104	42.655	1.00	0.00	H
ATOM	358	HG23	ILE	22	38.674	39.807	43.690	1.00	0.00	H
ATOM	359	CG1	ILE	22	36.615	38.003	44.157	1.00	0.00	C
ATOM	360	HG12	ILE	22	35.770	38.691	44.131	1.00	0.00	H
ATOM	361	HG13	ILE	22	36.203	36.998	44.071	1.00	0.00	H
ATOM	362	CD1	ILE	22	37.359	38.027	45.497	1.00	0.00	C
ATOM	363	HD11	ILE	22	38.249	37.398	45.498	1.00	0.00	H
ATOM	364	HD12	ILE	22	37.623	39.050	45.763	1.00	0.00	H
ATOM	365	HD13	ILE	22	36.854	37.642	46.383	1.00	0.00	H
ATOM	366	C	ILE	22	35.611	38.087	41.350	1.00	0.00	C
ATOM	367	O	ILE	22	35.491	39.119	40.711	1.00	0.00	O
ATOM	368	N	THR	23	34.598	37.393	41.853	1.00	0.00	N
ATOM	369	H	THR	23	34.783	36.553	42.381	1.00	0.00	H
ATOM	370	CA	THR	23	33.200	37.815	41.822	1.00	0.00	C
ATOM	371	HA	THR	23	33.184	38.896	41.960	1.00	0.00	H
ATOM	372	CB	THR	23	32.686	37.456	40.413	1.00	0.00	C
ATOM	373	HB	THR	23	33.498	37.555	39.693	1.00	0.00	H

ATOM	374	CG2	THR	23	32.143	36.054	40.268	1.00	0.00	C
ATOM	375	HG21	THR	23	32.796	35.342	40.772	1.00	0.00	H
ATOM	376	HG22	THR	23	31.171	35.947	40.749	1.00	0.00	H
ATOM	377	HG23	THR	23	31.909	35.759	39.245	1.00	0.00	H
ATOM	378	OG1	THR	23	31.764	38.441	40.127	1.00	0.00	O
ATOM	379	HG1	THR	23	32.232	39.117	39.634	1.00	0.00	H
ATOM	380	C	THR	23	32.330	37.373	43.039	1.00	0.00	C
ATOM	381	O	THR	23	32.789	36.511	43.874	1.00	0.00	O
ATOM	382	N	ILE	24	31.144	37.896	43.152	1.00	0.00	N
ATOM	383	H	ILE	24	30.786	38.524	42.447	1.00	0.00	H
ATOM	384	CA	ILE	24	30.300	37.810	44.336			
1.00	0.00			C						
ATOM	385	HA	ILE	24	30.610	36.951	44.931	1.00	0.00	H
ATOM	386	CB	ILE	24	30.307	39.164	45.181	1.00	0.00	C
ATOM	387	HB	ILE	24	29.960	39.888	44.444	1.00	0.00	H
ATOM	388	CG2	ILE	24	29.310	39.135	46.321	1.00	0.00	C
ATOM	389	HG21	ILE	24	28.345	38.781	45.958	1.00	0.00	H
ATOM	390	HG22	ILE	24	29.764	38.504	47.085	1.00	0.00	H
ATOM	391	HG23	ILE	24	29.070	40.122	46.719	1.00	0.00	H
ATOM	392	CG1	ILE	24	31.687	39.602	45.672	1.00	0.00	C
ATOM	393	HG12	ILE	24	32.092	38.808	46.300	1.00	0.00	H
ATOM	394	HG13	ILE	24	32.348	39.695	44.811	1.00	0.00	H
ATOM	395	CD1	ILE	24	31.768	40.817	46.482	1.00	0.00	C
ATOM	396	HD11	ILE	24	31.121	41.492	45.923	1.00	0.00	H
ATOM	397	HD12	ILE	24	31.352	40.624	47.472	1.00	0.00	H
ATOM	398	HD13	ILE	24	32.807	41.102	46.648	1.00	0.00	H
ATOM	399	C	ILE	24	28.886	37.470	43.897	1.00	0.00	C
ATOM	400	O	ILE	24	28.354	37.945	42.915	1.00	0.00	O
ATOM	401	N	ARG	25	28.254	36.475	44.569	1.00	0.00	N
ATOM	402	H	ARG	25	28.936	36.056	45.185	1.00	0.00	H
ATOM	403	CA	ARG	25	26.854	36.025	44.375	1.00	0.00	C
ATOM	404	HA	ARG	25	26.269	36.739	43.796	1.00	0.00	H
ATOM	405	CB	ARG	25	26.827	34.818	43.598	1.00	0.00	C
ATOM	406	HB2	ARG	25	27.192	33.958	44.159	1.00	0.00	H
ATOM	407	HB3	ARG	25	25.772	34.672	43.364	1.00	0.00	H
ATOM	408	CG	ARG	25	27.451	34.896	42.178	1.00	0.00	C
ATOM	409	HG2	ARG	25	28.469	35.285	42.176	1.00	0.00	H
ATOM	410	HG3	ARG	25	27.532	33.904	41.734	1.00	0.00	H
ATOM	411	CD	ARG	25	26.641	35.811	41.230	1.00	0.00	C
ATOM	412	HD2	ARG	25	25.627	35.415	41.286	1.00	0.00	H
ATOM	413	HD3	ARG	25	26.572	36.797	41.691	1.00	0.00	H
ATOM	414	NE	ARG	25	27.202	35.852	39.887	1.00	0.00	N
ATOM	415	HE	ARG	25	26.691	35.266	39.242	1.00	0.00	H
ATOM	416	CZ	ARG	25	28.061	36.662	39.330	1.00	0.00	C
ATOM	417	NH1	ARG	25	28.813	37.403	40.062	1.00	0.00	N
ATOM	418	HH11	ARG	25	29.592	37.873	39.624	1.00	0.00	H
ATOM	419	HH12	ARG	25	28.786	37.269	41.064	1.00	0.00	H
ATOM	420	NH2	ARG	25	28.239	36.692	38.053	1.00	0.00	N
ATOM	421	HH21	ARG	25	29.084	37.182	37.797	1.00	0.00	H
ATOM	422	HH22	ARG	25	28.046	35.878	37.488	1.00	0.00	H
ATOM	423	C	ARG	25	26.135	35.949	45.747	1.00	0.00	C
ATOM	424	O	ARG	25	26.745	35.834	46.823	1.00	0.00	O
ATOM	425	N	ASP	26	24.785	36.070	45.805	1.00	0.00	N
ATOM	426	H	ASP	26	24.240	36.104	44.955	1.00	0.00	H
ATOM	427	CA	ASP	26	23.931	35.341	46.788	1.00	0.00	C
ATOM	428	HA	ASP	26	24.191	35.677	47.792	1.00	0.00	H
ATOM	429	CB	ASP	26	22.473	35.760	46.601	1.00	0.00	C
ATOM	430	HB2	ASP	26	21.913	35.161	47.318	1.00	0.00	H
ATOM	431	HB3	ASP	26	22.356	36.809	46.869	1.00	0.00	H
ATOM	432	CG	ASP	26	21.883	35.475	45.221	1.00	0.00	C
ATOM	433	OD1	ASP	26	20.649	35.429	45.130	1.00	0.00	O
ATOM	434	OD2	ASP	26	22.532	35.503	44.194	1.00	0.00	O
ATOM	435	C	ASP	26	24.184	33.815	46.761	1.00	0.00	C
ATOM	436	O	ASP	26	24.500	33.230	45.692	1.00	0.00	O

ATOM	437	N	GLU	27	23.824	33.105	47.802	1.00	0.00	N
ATOM	438	H	GLU	27	23.521	33.613	48.621	1.00	0.00	H
ATOM	439	CA	GLU	27	23.934	31.643	47.958	1.00	0.00	C
ATOM	440	HA	GLU	27	24.965	31.551	47.615	1.00	0.00	H
ATOM	441	CB	GLU	27	23.866	31.168	49.414	1.00	0.00	C
ATOM	442	HB2	GLU	27	23.303	31.918	49.970	1.00	0.00	H
ATOM	443	HB3	GLU	27	23.289	30.244	49.407	1.00	0.00	H
ATOM	444	CG	GLU	27	25.180	31.012	50.177	1.00	0.00	C
ATOM	445	HG2	GLU	27	25.697	31.969	50.104	1.00	0.00	H
ATOM	446	HG3	GLU	27	24.895	30.784	51.205	1.00	0.00	H
ATOM	447	CD	GLU	27	26.007	29.871	49.562	1.00	0.00	C
ATOM	448	OE1	GLU	27	25.812	29.506	48.364	1.00	0.00	O
ATOM	449	OE2	GLU	27	26.860	29.256	50.242	1.00	0.00	O
ATOM	450	C	GLU	27	23.063	30.747	47.073	1.00	0.00	C
ATOM	451	O	GLU	27	21.836	30.917	46.980	1.00	0.00	O
ATOM	452	N	SER	28	23.664	29.608	46.720	1.00	0.00	N
ATOM	453	H	SER	28	24.620	29.492	47.023	1.00	0.00	H
ATOM	454	CA	SER	28	23.114	28.494	45.894	1.00	0.00	C
ATOM	455	HA	SER	28	22.189	28.187	46.383	1.00	0.00	H
ATOM	456	CB	SER	28	22.723	29.136	44.525	1.00	0.00	C
ATOM	457	HB2	SER	28	22.155	30.054	44.673	1.00	0.00	H
ATOM	458	HB3	SER	28	23.551	29.520	43.930	1.00	0.00	H
ATOM	459	OG	SER	28	22.005	28.175	43.705	1.00	0.00	O
ATOM	460	HG	SER	28	21.112	28.084	44.045	1.00	0.00	H
ATOM	461	C	SER	28	24.140	27.386	45.665	1.00	0.00	C
ATOM	462	O	SER	28	25.337	27.660	45.501	1.00	0.00	O
ATOM	463	N	GLU	29	23.613	26.137	45.567	1.00	0.00	N
ATOM	464	H	GLU	29	22.613	26.038	45.676	1.00	0.00	H
ATOM	465	CA	GLU	29	24.283	24.825	45.275	1.00	0.00	C
ATOM	466	HA	GLU	29	25.282	25.041	45.654	1.00	0.00	H
ATOM	467	CB	GLU	29	23.771	23.679	46.173	1.00	0.00	C
ATOM	468	HB2	GLU	29	23.921	24.097	47.169	1.00	0.00	H
ATOM	469	HB3	GLU	29	22.696	23.648	45.997	1.00	0.00	H
ATOM	470	CG	GLU	29	24.353	22.318	46.176	1.00	0.00	C
ATOM	471	HG2	GLU	29	23.808	21.590	46.778	1.00	0.00	H
ATOM	472	HG3	GLU	29	24.294	21.918	45.163	1.00	0.00	H
ATOM	473	CD	GLU	29	25.816	22.182	46.692	1.00	0.00	C
ATOM	474	OE1	GLU	29	26.412	23.125	47.265	1.00	0.00	O
ATOM	475	OE2	GLU	29	26.437	21.096	46.551	1.00	0.00	O
ATOM	476	C	GLU	29	24.549	24.653	43.797	1.00	0.00	C
ATOM	477	O	GLU	29	25.256	23.830	43.298	1.00	0.00	O
ATOM	478	N	SER	30	23.966	25.490	42.968	1.00	0.00	N
ATOM	479	H	SER	30	23.588	26.270	43.487	1.00	0.00	H
ATOM	480	CA	SER	30	24.167	25.440	41.529	1.00	0.00	C
ATOM	481	HA	SER	30	24.170	24.412	41.167	1.00	0.00	H
ATOM	482	CB	SER	30	22.971	26.166	40.855	1.00	0.00	C
ATOM	483	HB2	SER	30	23.105	25.982	39.789	1.00	0.00	H
ATOM	484	HB3	SER	30	21.998	25.839	41.219	1.00	0.00	H
ATOM	485	OG	SER	30	23.069	27.506	41.174	1.00	0.00	O
ATOM	486	HG	SER	30	22.575	27.621	41.989	1.00	0.00	H
ATOM	487	C	SER	30	25.503	26.190	41.129	1.00	0.00	C
ATOM	488	O	SER	30	25.934	26.089	39.957	1.00	0.00	O
ATOM	489	N	HIE	31	26.103	26.971	41.995	1.00	0.00	N
ATOM	490	H	HIE	31	25.639	27.058	42.887	1.00	0.00	H
ATOM	491	CA	HIE	31	27.398	27.590	41.811	1.00	0.00	C
ATOM	492	HA	HIE	31	27.344	28.216	40.920	1.00	0.00	H
ATOM	493	CB	HIE	31	27.752	28.486	43.065	1.00	0.00	C
ATOM	494	HB2	HIE	31	27.654	28.009	44.039	1.00	0.00	H
ATOM	495	HB3	HIE	31	28.816	28.723	43.079	1.00	0.00	H
ATOM	496	CG	HIE	31	26.976	29.742	43.114	1.00	0.00	C
ATOM	497	ND1	HIE	31	26.691	30.565	41.995	1.00	0.00	N
ATOM	498	CE1	HIE	31	26.024	31.597	42.584	1.00	0.00	C
ATOM	499	HE1	HIE	31	25.649	32.439	42.021	1.00	0.00	H
ATOM	500	NE2	HIE	31	26.013	31.513	43.935	1.00	0.00	N

ATOM	501	HE2	HIE	31	25.488	32.042	44.617	1.00	0.00	H
ATOM	502	CD2	HIE	31	26.450	30.302	44.240	1.00	0.00	C
ATOM	503	HD2	HIE	31	26.560	29.777	45.178	1.00	0.00	H
ATOM	504	C	HIE	31	28.546	26.557	41.651	1.00	0.00	C
ATOM	505	O	HIE	31	28.482	25.388	41.866	1.00	0.00	O
ATOM	506	N	PHE	32	29.724	27.142	41.312	1.00	0.00	N
ATOM	507	H	PHE	32	29.757	28.152	41.305	1.00	0.00	H
ATOM	508	CA	PHE	32	31.044	26.454	41.201	1.00	0.00	C
ATOM	509	HA	PHE	32	30.935	25.481	40.721	1.00	0.00	H
ATOM										
510	CB	PHE	32	32.028	27.271	40.361	1.00	0.00	C	
ATOM	511	HB2	PHE	32	32.545	28.044	40.929	1.00	0.00	H
ATOM	512	HB3	PHE	32	32.920	26.671	40.182	1.00	0.00	H
ATOM	513	CG	PHE	32	31.460	27.822	39.059	1.00	0.00	C
ATOM	514	CD1	PHE	32	31.479	29.201	38.780	1.00	0.00	C
ATOM	515	HD1	PHE	32	31.818	29.889	39.541	1.00	0.00	H
ATOM	516	CE1	PHE	32	31.129	29.711	37.547	1.00	0.00	C
ATOM	517	HE1	PHE	32	31.286	30.738	37.251	1.00	0.00	H
ATOM	518	CZ	PHE	32	30.790	28.732	36.533	1.00	0.00	C
ATOM	519	HZ	PHE	32	30.558	29.030	35.520	1.00	0.00	H
ATOM	520	CE2	PHE	32	30.731	27.360	36.800	1.00	0.00	C
ATOM	521	HE2	PHE	32	30.453	26.691	36.000	1.00	0.00	H
ATOM	522	CD2	PHE	32	31.039	26.885	38.071	1.00	0.00	C
ATOM	523	HD2	PHE	32	30.999	25.836	38.326	1.00	0.00	H
ATOM	524	C	PHE	32	31.730	26.173	42.545	1.00	0.00	C
ATOM	525	O	PHE	32	31.623	26.994	43.470	1.00	0.00	O
ATOM	526	N	LYS	33	32.520	25.141	42.597	1.00	0.00	N
ATOM	527	H	LYS	33	32.644	24.594	41.757	1.00	0.00	H
ATOM	528	CA	LYS	33	33.382	24.828	43.713	1.00	0.00	C
ATOM	529	HA	LYS	33	33.234	25.562	44.503	1.00	0.00	H
ATOM	530	CB	LYS	33	33.045	23.402	44.246	1.00	0.00	C
ATOM	531	HB2	LYS	33	32.862	22.717	43.418	1.00	0.00	H
ATOM	532	HB3	LYS	33	33.894	23.198	44.898	1.00	0.00	H
ATOM	533	CG	LYS	33	31.737	23.423	45.121	1.00	0.00	C
ATOM	534	HG2	LYS	33	31.953	24.088	45.958	1.00	0.00	H
ATOM	535	HG3	LYS	33	30.910	23.860	44.561	1.00	0.00	H
ATOM	536	CD	LYS	33	31.256	22.054	45.651	1.00	0.00	C
ATOM	537	HD2	LYS	33	31.062	21.457	44.760	1.00	0.00	H
ATOM	538	HD3	LYS	33	32.016	21.566	46.262	1.00	0.00	H
ATOM	539	CE	LYS	33	29.992	22.116	46.554	1.00	0.00	C
ATOM	540	HE2	LYS	33	29.539	21.160	46.814	1.00	0.00	H
ATOM	541	HE3	LYS	33	30.341	22.633	47.447	1.00	0.00	H
ATOM	542	NZ	LYS	33	28.871	22.874	45.945	1.00	0.00	N
ATOM	543	HZ1	LYS	33	28.058	22.798	46.539	1.00	0.00	H
ATOM	544	HZ2	LYS	33	29.069	23.859	45.837	1.00	0.00	H
ATOM	545	HZ3	LYS	33	28.618	22.476	45.052	1.00	0.00	H
ATOM	546	C	LYS	33	34.849	24.955	43.225	1.00	0.00	C
ATOM	547	O	LYS	33	35.148	24.836	42.047	1.00	0.00	O
ATOM	548	N	THR	34	35.830	25.212	44.202	1.00	0.00	N
ATOM	549	H	THR	34	35.556	25.309	45.169	1.00	0.00	H
ATOM	550	CA	THR	34	37.290	25.274	43.845	1.00	0.00	C
ATOM	551	HA	THR	34	37.528	26.170	43.272	1.00	0.00	H
ATOM	552	CB	THR	34	38.112	25.351	45.124	1.00	0.00	C
ATOM	553	HB	THR	34	38.003	24.522	45.824	1.00	0.00	H
ATOM	554	CG2	THR	34	39.611	25.408	44.790	1.00	0.00	C
ATOM	555	HG21	THR	34	40.128	25.547	45.738	1.00	0.00	H
ATOM	556	HG22	THR	34	40.032	24.553	44.262	1.00	0.00	H
ATOM	557	HG23	THR	34	39.797	26.302	44.193	1.00	0.00	H
ATOM	558	OG1	THR	34	37.730	26.521	45.742	1.00	0.00	O
ATOM	559	HG1	THR	34	36.963	26.329	46.284	1.00	0.00	H
ATOM	560	C	THR	34	37.709	23.996	43.036	1.00	0.00	C
ATOM	561	O	THR	34	37.406	22.887	43.476	1.00	0.00	O
ATOM	562	N	GLY	35	38.373	24.286	41.997	1.00	0.00	N
ATOM	563	H	GLY	35	38.530	25.245	41.723	1.00	0.00	H

ATOM	564	CA	GLY	35	38.877	23.192	41.154	1.00	0.00	C
ATOM	565	HA2	GLY	35	39.829	23.469	40.701	1.00	0.00	H
ATOM	566	HA3	GLY	35	39.066	22.311	41.766	1.00	0.00	H
ATOM	567	C	GLY	35	38.007	22.851	39.985	1.00	0.00	C
ATOM	568	O	GLY	35	38.452	22.032	39.174	1.00	0.00	O
ATOM	569	N	ASP	36	36.818	23.450	39.825	1.00	0.00	N
ATOM	570	H	ASP	36	36.553	24.141	40.513	1.00	0.00	H
ATOM	571	CA	ASP	36	36.045	23.388	38.640	1.00	0.00	C
ATOM	572	HA	ASP	36	35.930	22.330	38.406	1.00	0.00	H
ATOM	573	CB	ASP	36	34.598	23.879	38.972	1.00	0.00	C
ATOM	574	HB2	ASP	36	34.710	24.892	39.361	1.00	0.00	H
ATOM	575	HB3	ASP	36	34.068	23.895	38.020	1.00	0.00	H
ATOM	576	CG	ASP	36	33.786	22.968	39.859	1.00	0.00	C
ATOM	577	OD1	ASP	36	32.631	23.448	40.242	1.00	0.00	O
ATOM	578	OD2	ASP	36	34.053	21.780	40.193	1.00	0.00	O
ATOM	579	C	ASP	36	36.720	24.097	37.462	1.00	0.00	C
ATOM	580	O	ASP	36	37.357	25.116	37.659	1.00	0.00	O
ATOM	581	N	VAL	37	36.642	23.545	36.240	1.00	0.00	N
ATOM	582	H	VAL	37	35.948	22.834	36.058	1.00	0.00	H
ATOM	583	CA	VAL	37	37.105	24.227	35.069	1.00	0.00	C
ATOM	584	HA	VAL	37	37.752	25.043	35.390	1.00	0.00	H
ATOM	585	CB	VAL	37	38.001	23.315	34.241	1.00	0.00	C
ATOM	586	HB	VAL	37	37.364	22.512	33.871	1.00	0.00	H
ATOM	587	CG1	VAL	37	38.526	23.968	32.996	1.00	0.00	C
ATOM	588	HG11	VAL	37	39.114	23.202	32.491	1.00	0.00	H
ATOM	589	HG12	VAL	37	37.728	24.277	32.322	1.00	0.00	H
ATOM	590	HG13	VAL	37	39.160	24.842	33.146	1.00	0.00	H
ATOM	591	CG2	VAL	37	39.108	22.686	35.052	1.00	0.00	C
ATOM	592	HG21	VAL	37	39.476	23.473	35.712	1.00	0.00	H
ATOM	593	HG22	VAL	37	38.783	21.864	35.691	1.00	0.00	H
ATOM	594	HG23	VAL	37	39.891	22.291	34.406	1.00	0.00	H
ATOM	595	C	VAL	37	35.878	24.693	34.236	1.00	0.00	C
ATOM	596	O	VAL	37	34.868	24.038	34.401	1.00	0.00	O
ATOM	597	N	LEU	38	35.974	25.797	33.527	1.00	0.00	N
ATOM	598	H	LEU	38	36.717	26.353	33.926	1.00	0.00	H
ATOM	599	CA	LEU	38	34.970	26.462	32.634	1.00	0.00	C
ATOM	600	HA	LEU	38	34.542	25.657	32.034	1.00	0.00	H
ATOM	601	CB	LEU	38	33.913	27.290	33.426	1.00	0.00	C
ATOM	602	HB2	LEU	38	33.221	27.585	32.638	1.00	0.00	H
ATOM	603	HB3	LEU	38	33.361	26.743	34.192	1.00	0.00	H
ATOM	604	CG	LEU	38	34.518	28.515	34.128	1.00	0.00	C
ATOM	605	HG	LEU	38	35.349	28.937	33.561	1.00	0.00	H
ATOM	606	CD1	LEU	38	33.455	29.572	34.158	1.00	0.00	C
ATOM	607	HD11	LEU	38	33.349	29.909	33.127	1.00	0.00	H
ATOM	608	HD12	LEU	38	32.553	29.152	34.602	1.00	0.00	H
ATOM	609	HD13	LEU	38	33.799	30.405	34.772	1.00	0.00	H
ATOM	610	CD2	LEU	38	34.981	28.099	35.550	1.00	0.00	C
ATOM	611	HD21	LEU	38	35.239	29.007	36.093	1.00	0.00	H
ATOM	612	HD22	LEU	38	34.193	27.610	36.122	1.00	0.00	H
ATOM	613	HD23	LEU	38	35.829	27.422	35.447	1.00	0.00	H
ATOM	614	C	LEU	38	35.662	27.411	31.569	1.00	0.00	C
ATOM	615	O	LEU	38	36.905	27.550	31.571	1.00	0.00	O
ATOM	616	N	ARG	39	34.988	27.930	30.580	1.00	0.00	N
ATOM	617	H	ARG	39	34.024	27.639	30.504	1.00	0.00	H
ATOM	618	CA	ARG	39	35.589	28.520	29.318	1.00	0.00	C
ATOM	619	HA	ARG	39	36.636	28.799	29.440	1.00	0.00	H
ATOM	620	CB	ARG	39	35.619	27.396	28.281	1.00	0.00	C
ATOM	621	HB2	ARG	39	34.625	26.956	28.204	1.00	0.00	H
ATOM	622	HB3	ARG	39	35.747	27.985	27.373	1.00	0.00	H
ATOM	623	CG	ARG	39	36.763	26.417	28.492	1.00	0.00	C
ATOM	624	HG2	ARG	39	37.728	26.904	28.628	1.00	0.00	H
ATOM	625	HG3	ARG	39	36.610	25.873	29.425	1.00	0.00	H
ATOM	626	CD	ARG	39	36.984	25.500	27.298	1.00	0.00	C
ATOM	627	HD2	ARG	39	37.170	26.173	26.461	1.00	0.00	H

ATOM	628	HD3	ARG	39	37.852	24.872	27.498	1.00	0.00	H
ATOM	629	NE	ARG	39	35.760	24.745	27.031	1.00	0.00	N
ATOM	630	HE	ARG	39	34.960	24.993	27.595	1.00	0.00	H
ATOM	631	CZ	ARG	39	35.606	23.732	26.201	1.00	0.00	C
ATOM	632	NH1	ARG	39	36.586	23.143	25.519	1.00	0.00	N
ATOM	633	HH11	ARG	39	37.520	23.529	25.544	1.00	0.00	H
ATOM	634	HH12	ARG	39	36.390	22.274	25.046	1.00	0.00	H
ATOM	635	NH2	ARG	39						
34.469	23.134	26.283	1.00	0.00	N					
ATOM	636	HH21	ARG	39	34.338	22.245	25.821	1.00	0.00	H
ATOM	637	HH22	ARG	39	33.733	23.489	26.878	1.00	0.00	H
ATOM	638	C	ARG	39	34.923	29.850	29.010	1.00	0.00	C
ATOM	639	O	ARG	39	33.792	30.061	29.384	1.00	0.00	O
ATOM	640	N	VAL	40	35.694	30.671	28.251	1.00	0.00	N
ATOM	641	H	VAL	40	36.642	30.374	28.068	1.00	0.00	H
ATOM	642	CA	VAL	40	35.179	31.815	27.448	1.00	0.00	C
ATOM	643	HA	VAL	40	34.101	31.684	27.363	1.00	0.00	H
ATOM	644	CB	VAL	40	35.534	33.158	28.152	1.00	0.00	C
ATOM	645	HB	VAL	40	35.338	33.962	27.443	1.00	0.00	H
ATOM	646	CG1	VAL	40	34.609	33.301	29.365	1.00	0.00	C
ATOM	647	HG11	VAL	40	35.063	32.803	30.221	1.00	0.00	H
ATOM	648	HG12	VAL	40	34.604	34.344	29.682	1.00	0.00	H
ATOM	649	HG13	VAL	40	33.618	33.066	28.977	1.00	0.00	H
ATOM	650	CG2	VAL	40	37.018	33.381	28.532	1.00	0.00	C
ATOM	651	HG21	VAL	40	37.690	33.487	27.680	1.00	0.00	H
ATOM	652	HG22	VAL	40	37.102	34.253	29.180	1.00	0.00	H
ATOM	653	HG23	VAL	40	37.342	32.501	29.089	1.00	0.00	H
ATOM	654	C	VAL	40	35.670	31.775	25.995	1.00	0.00	C
ATOM	655	O	VAL	40	36.874	31.383	25.766	1.00	0.00	O
ATOM	656	N	GLY	41	34.805	32.138	25.048	1.00	0.00	N
ATOM	657	H	GLY	41	33.838	31.983	25.295	1.00	0.00	H
ATOM	658	CA	GLY	41	35.037	32.019	23.618	1.00	0.00	C
ATOM	659	HA2	GLY	41	35.925	32.611	23.394	1.00	0.00	H
ATOM	660	HA3	GLY	41	35.078	30.976	23.304	1.00	0.00	H
ATOM	661	C	GLY	41	33.880	32.813	22.918	1.00	0.00	C
ATOM	662	O	GLY	41	32.853	32.924	23.459	1.00	0.00	O
ATOM	663	N	ARG	42	34.062	33.399	21.693	1.00	0.00	N
ATOM	664	H	ARG	42	34.903	33.061	21.248	1.00	0.00	H
ATOM	665	CA	ARG	42	33.037	33.927	20.795	1.00	0.00	C
ATOM	666	HA	ARG	42	32.185	33.257	20.914	1.00	0.00	H
ATOM	667	CB	ARG	42	32.561	35.323	21.270	1.00	0.00	C
ATOM	668	HB2	ARG	42	31.657	35.475	20.681	1.00	0.00	H
ATOM	669	HB3	ARG	42	32.231	35.185	22.300	1.00	0.00	H
ATOM	670	CG	ARG	42	33.641	36.437	21.224	1.00	0.00	C
ATOM	671	HG2	ARG	42	34.577	36.123	21.685	1.00	0.00	H
ATOM	672	HG3	ARG	42	33.844	36.610	20.167	1.00	0.00	H
ATOM	673	CD	ARG	42	33.153	37.799	21.816	1.00	0.00	C
ATOM	674	HD2	ARG	42	32.311	38.171	21.232	1.00	0.00	H
ATOM	675	HD3	ARG	42	32.757	37.698	22.827	1.00	0.00	H
ATOM	676	NE	ARG	42	34.197	38.814	21.607	1.00	0.00	N
ATOM	677	HE	ARG	42	33.999	39.482	20.876	1.00	0.00	H
ATOM	678	CZ	ARG	42	35.209	39.141	22.393	1.00	0.00	C
ATOM	679	NH1	ARG	42	35.509	38.471	23.459	1.00	0.00	N
ATOM	680	HH11	ARG	42	36.337	38.777	23.950	1.00	0.00	H
ATOM	681	HH12	ARG	42	34.880	37.717	23.695	1.00	0.00	H
ATOM	682	NH2	ARG	42	36.075	40.094	22.208	1.00	0.00	N
ATOM	683	HH21	ARG	42	36.148	40.521	21.296	1.00	0.00	H
ATOM	684	HH22	ARG	42	36.778	40.326	22.894	1.00	0.00	H
ATOM	685	C	ARG	42	33.488	33.937	19.303	1.00	0.00	C
ATOM	686	O	ARG	42	32.651	34.273	18.488	1.00	0.00	O
ATOM	687	N	PHE	43	34.681	33.582	19.043	1.00	0.00	N
ATOM	688	H	PHE	43	35.171	33.031	19.734	1.00	0.00	H
ATOM	689	CA	PHE	43	35.238	33.458	17.695	1.00	0.00	C
ATOM	690	HA	PHE	43	34.605	33.921	16.939	1.00	0.00	H

ATOM	691	CB	PHE	43	36.649	34.078	17.591	1.00	0.00	C
ATOM	692	HB2	PHE	43	37.304	33.304	17.994	1.00	0.00	H
ATOM	693	HB3	PHE	43	36.818	34.179	16.518	1.00	0.00	H
ATOM	694	CG	PHE	43	36.910	35.394	18.238	1.00	0.00	C
ATOM	695	CD1	PHE	43	36.457	36.589	17.757	1.00	0.00	C
ATOM	696	HD1	PHE	43	35.771	36.490	16.930	1.00	0.00	H
ATOM	697	CE1	PHE	43	36.762	37.749	18.501	1.00	0.00	C
ATOM	698	HE1	PHE	43	36.374	38.712	18.202	1.00	0.00	H
ATOM	699	CZ	PHE	43	37.460	37.678	19.733	1.00	0.00	C
ATOM	700	HZ	PHE	43	37.513	38.573	20.336	1.00	0.00	H
ATOM	701	CE2	PHE	43	37.982	36.444	20.142	1.00	0.00	C
ATOM	702	HE2	PHE	43	38.735	36.190	20.873	1.00	0.00	H
ATOM	703	CD2	PHE	43	37.847	35.352	19.302	1.00	0.00	C
ATOM	704	HD2	PHE	43	38.444	34.460	19.423	1.00	0.00	H
ATOM	705	C	PHE	43	35.356	32.006	17.268	1.00	0.00	C
ATOM	706	O	PHE	43	35.539	31.060	18.026	1.00	0.00	O
ATOM	707	N	GLU	44	35.255	31.781	15.981	1.00	0.00	N
ATOM	708	H	GLU	44	35.214	32.659	15.483	1.00	0.00	H
ATOM	709	CA	GLU	44	35.402	30.493	15.418	1.00	0.00	C
ATOM	710	HA	GLU	44	34.757	29.855	16.022	1.00	0.00	H
ATOM	711	CB	GLU	44	34.936	30.597	13.943	1.00	0.00	C
ATOM	712	HB2	GLU	44	33.946	31.049	13.859	1.00	0.00	H
ATOM	713	HB3	GLU	44	35.677	31.268	13.510	1.00	0.00	H
ATOM	714	CG	GLU	44	34.975	29.246	13.180	1.00	0.00	C
ATOM	715	HG2	GLU	44	34.741	29.544	12.159	1.00	0.00	H
ATOM	716	HG3	GLU	44	35.982	28.830	13.126	1.00	0.00	H
ATOM	717	CD	GLU	44	33.823	28.259	13.553	1.00	0.00	C
ATOM	718	OE1	GLU	44	33.403	27.499	12.598	1.00	0.00	O
ATOM	719	OE2	GLU	44	33.237	28.310	14.639	1.00	0.00	O
ATOM	720	C	GLU	44	36.800	29.946	15.515	1.00	0.00	C
ATOM	721	O	GLU	44	36.942	28.752	15.814	1.00	0.00	O
ATOM	722	N	ASP	45	37.820	30.819	15.399	1.00	0.00	N
ATOM	723	H	ASP	45	37.615	31.801	15.280	1.00	0.00	H
ATOM	724	CA	ASP	45	39.243	30.359	15.306	1.00	0.00	C
ATOM	725	HA	ASP	45	39.360	29.394	14.811	1.00	0.00	H
ATOM	726	CB	ASP	45	40.132	31.433	14.614	1.00	0.00	C
ATOM	727	HB2	ASP	45	39.600	31.540	13.668	1.00	0.00	H
ATOM	728	HB3	ASP	45	40.046	32.393	15.123	1.00	0.00	H
ATOM	729	CG	ASP	45	41.565	30.984	14.600	1.00	0.00	C
ATOM	730	OD1	ASP	45	41.901	29.756	14.492	1.00	0.00	O
ATOM	731	OD2	ASP	45	42.445	31.799	14.953	1.00	0.00	O
ATOM	732	C	ASP	45	39.780	29.923	16.669	1.00	0.00	C
ATOM	733	O	ASP	45	39.630	30.637	17.692	1.00	0.00	O
ATOM	734	N	ASP	46	40.335	28.713	16.783	1.00	0.00	N
ATOM	735	H	ASP	46	40.533	28.196	15.939	1.00	0.00	H
ATOM	736	CA	ASP	46	40.602	27.986	18.023	1.00	0.00	C
ATOM	737	HA	ASP	46	40.804	26.981	17.650	1.00	0.00	H
ATOM	738	CB	ASP	46	41.825	28.612	18.851	1.00	0.00	C
ATOM	739	HB2	ASP	46	41.483	29.588	19.196	1.00	0.00	H
ATOM	740	HB3	ASP	46	41.883	27.975	19.734	1.00	0.00	H
ATOM	741	CG	ASP	46	43.137	28.652	18.171	1.00	0.00	C
ATOM	742	OD1	ASP	46	43.802	27.633	18.046	1.00	0.00	O
ATOM	743	OD2	ASP	46	43.531	29.789	17.783	1.00	0.00	O
ATOM	744	C	ASP	46	39.478	27.720	18.955	1.00	0.00	C
ATOM	745	O	ASP	46	39.701	27.093	19.964	1.00	0.00	O
ATOM	746	N	GLY	47	38.223	28.139	18.657	1.00	0.00	N
ATOM	747	H	GLY	47	38.104	28.449	17.704	1.00	0.00	H
ATOM	748	CA	GLY	47	36.963	27.924	19.373	1.00	0.00	C
ATOM	749	HA2	GLY	47	36.105	28.158	18.743	1.00	0.00	H
ATOM	750	HA3	GLY	47	36.808	26.845	19.404	1.00	0.00	H
ATOM	751	C	GLY	47	36.891	28.568	20.809	1.00	0.00	C
ATOM	752	O	GLY	47	35.902	29.252	21.194	1.00	0.00	O
ATOM	753	N	TYR	48	37.938	28.388	21.586	1.00	0.00	N
ATOM	754	H	TYR	48	38.736	27.857	21.269	1.00	0.00	H

ATOM	755	CA	TYR	48	38.012	28.984	22.936	1.00	0.00	C
ATOM	756	HA	TYR	48	37.218	29.730	22.973	1.00	0.00	H
ATOM	757	CB	TYR	48	37.724	27.938	23.957	1.00	0.00	C
ATOM	758	HB2	TYR	48	38.376	27.067	23.885	1.00	0.00	H
ATOM	759	HB3	TYR	48	37.895	28.330	24.960	1.00	0.00	H
ATOM	760	CG	TYR	48	36.268	27.440	23.921			
1.00	0.00			C						
ATOM	761	CD1	TYR	48	35.232	28.179	24.561	1.00	0.00	C
ATOM	762	HD1	TYR	48	35.472	29.168	24.921	1.00	0.00	H
ATOM	763	CE1	TYR	48	33.937	27.593	24.701	1.00	0.00	C
ATOM	764	HE1	TYR	48	33.195	28.082	25.315	1.00	0.00	H
ATOM	765	CZ	TYR	48	33.685	26.312	24.251	1.00	0.00	C
ATOM	766	OH	TYR	48	32.456	25.746	24.453	1.00	0.00	O
ATOM	767	HH	TYR	48	32.152	25.203	23.723	1.00	0.00	H
ATOM	768	CE2	TYR	48	34.685	25.569	23.656	1.00	0.00	C
ATOM	769	HE2	TYR	48	34.549	24.546	23.338	1.00	0.00	H
ATOM	770	CD2	TYR	48	35.988	26.169	23.491	1.00	0.00	C
ATOM	771	HD2	TYR	48	36.808	25.642	23.026	1.00	0.00	H
ATOM	772	C	TYR	48	39.310	29.780	23.093	1.00	0.00	C
ATOM	773	O	TYR	48	40.365	29.465	22.421	1.00	0.00	O
ATOM	774	N	PHE	49	39.352	30.755	23.986	1.00	0.00	N
ATOM	775	H	PHE	49	38.643	30.792	24.706	1.00	0.00	H
ATOM	776	CA	PHE	49	40.543	31.612	24.184	1.00	0.00	C
ATOM	777	HA	PHE	49	41.310	31.033	23.669	1.00	0.00	H
ATOM	778	CB	PHE	49	40.524	32.986	23.541	1.00	0.00	C
ATOM	779	HB2	PHE	49	41.462	33.535	23.625	1.00	0.00	H
ATOM	780	HB3	PHE	49	40.381	32.820	22.474	1.00	0.00	H
ATOM	781	CG	PHE	49	39.503	33.984	23.983	1.00	0.00	C
ATOM	782	CD1	PHE	49	38.372	34.402	23.162	1.00	0.00	C
ATOM	783	HD1	PHE	49	38.315	33.942	22.187	1.00	0.00	H
ATOM	784	CE1	PHE	49	37.504	35.453	23.450	1.00	0.00	C
ATOM	785	HE1	PHE	49	36.785	35.823	22.733	1.00	0.00	H
ATOM	786	CZ	PHE	49	37.724	36.104	24.668	1.00	0.00	C
ATOM	787	HZ	PHE	49	37.083	36.941	24.905	1.00	0.00	H
ATOM	788	CE2	PHE	49	38.814	35.759	25.499	1.00	0.00	C
ATOM	789	HE2	PHE	49	38.957	36.353	26.390	1.00	0.00	H
ATOM	790	CD2	PHE	49	39.657	34.675	25.227	1.00	0.00	C
ATOM	791	HD2	PHE	49	40.507	34.514	25.873	1.00	0.00	H
ATOM	792	C	PHE	49	40.962	31.633	25.668	1.00	0.00	C
ATOM	793	O	PHE	49	42.077	32.093	25.848	1.00	0.00	O
ATOM	794	N	CYX	50	40.128	31.207	26.614	1.00	0.00	N
ATOM	795	H	CYX	50	39.370	30.592	26.351	1.00	0.00	H
ATOM	796	CA	CYX	50	40.544	31.219	28.012	1.00	0.00	C
ATOM	797	HA	CYX	50	41.609	30.992	28.062	1.00	0.00	H
ATOM	798	CB	CYX	50	40.349	32.550	28.728	1.00	0.00	C
ATOM	799	HB2	CYX	50	39.669	33.086	28.067	1.00	0.00	H
ATOM	800	HB3	CYX	50	39.969	32.394	29.738	1.00	0.00	H
ATOM	801	SG	CYX	50	41.962	33.415	28.939	1.00	0.00	S
ATOM	802	C	CYX	50	39.757	30.089	28.601	1.00	0.00	C
ATOM	803	O	CYX	50	38.505	30.086	28.752	1.00	0.00	O
ATOM	804	N	THR	51	40.508	29.091	29.099	1.00	0.00	N
ATOM	805	H	THR	51	41.502	29.147	28.931	1.00	0.00	H
ATOM	806	CA	THR	51	40.091	27.973	29.956	1.00	0.00	C
ATOM	807	HA	THR	51	39.010	27.922	29.821	1.00	0.00	H
ATOM	808	CB	THR	51	40.786	26.705	29.587	1.00	0.00	C
ATOM	809	HB	THR	51	41.860	26.891	29.547	1.00	0.00	H
ATOM	810	CG2	THR	51	40.559	25.494	30.519	1.00	0.00	C
ATOM	811	HG21	THR	51	41.310	24.723	30.345	1.00	0.00	H
ATOM	812	HG22	THR	51	40.635	25.799	31.562	1.00	0.00	H
ATOM	813	HG23	THR	51	39.612	24.994	30.318	1.00	0.00	H
ATOM	814	OG1	THR	51	40.374	26.351	28.316	1.00	0.00	O
ATOM	815	HG1	THR	51	40.793	26.880	27.631	1.00	0.00	H
ATOM	816	C	THR	51	40.452	28.341	31.410	1.00	0.00	C
ATOM	817	O	THR	51	41.477	28.947	31.665	1.00	0.00	O

ATOM	818	N	ILE	52	39.450	28.154	32.314	1.00	0.00	N
ATOM	819	H	ILE	52	38.563	27.807	31.978	1.00	0.00	H
ATOM	820	CA	ILE	52	39.512	28.837	33.633	1.00	0.00	C
ATOM	821	HA	ILE	52	40.476	29.334	33.743	1.00	0.00	H
ATOM	822	CB	ILE	52	38.550	30.092	33.625	1.00	0.00	C
ATOM	823	HB	ILE	52	37.585	29.734	33.268	1.00	0.00	H
ATOM	824	CG2	ILE	52	38.248	30.669	35.044	1.00	0.00	C
ATOM	825	HG21	ILE	52	39.190	30.867	35.556	1.00	0.00	H
ATOM	826	HG22	ILE	52	37.610	31.541	34.894	1.00	0.00	H
ATOM	827	HG23	ILE	52	37.648	29.987	35.645	1.00	0.00	H
ATOM	828	CG1	ILE	52	39.086	31.205	32.661	1.00	0.00	C
ATOM	829	HG12	ILE	52	39.388	32.090	33.221	1.00	0.00	H
ATOM	830	HG13	ILE	52	39.999	30.950	32.125	1.00	0.00	H
ATOM	831	CD1	ILE	52	37.873	31.648	31.764	1.00	0.00	C
ATOM	832	HD11	ILE	52	37.355	30.822	31.275	1.00	0.00	H
ATOM	833	HD12	ILE	52	37.179	32.204	32.396	1.00	0.00	H
ATOM	834	HD13	ILE	52	38.190	32.370	31.011	1.00	0.00	H
ATOM	835	C	ILE	52	39.288	27.962	34.808	1.00	0.00	C
ATOM	836	O	ILE	52	38.261	27.274	34.884	1.00	0.00	O
ATOM	837	N	GLU	53	40.218	27.987	35.752	1.00	0.00	N
ATOM	838	H	GLU	53	40.841	28.777	35.657	1.00	0.00	H
ATOM	839	CA	GLU	53	40.101	27.146	37.018	1.00	0.00	C
ATOM	840	HA	GLU	53	39.452	26.277	36.911	1.00	0.00	H
ATOM	841	CB	GLU	53	41.437	26.492	37.455	1.00	0.00	C
ATOM	842	HB2	GLU	53	41.744	25.785	36.685	1.00	0.00	H
ATOM	843	HB3	GLU	53	42.263	27.200	37.515	1.00	0.00	H
ATOM	844	CG	GLU	53	41.441	25.839	38.827	1.00	0.00	C
ATOM	845	HG2	GLU	53	41.145	26.591	39.557	1.00	0.00	H
ATOM	846	HG3	GLU	53	40.651	25.092	38.907	1.00	0.00	H
ATOM	847	CD	GLU	53	42.826	25.249	39.122	1.00	0.00	C
ATOM	848	OE1	GLU	53	43.221	24.229	38.491	1.00	0.00	O
ATOM	849	OE2	GLU	53	43.487	25.752	40.033	1.00	0.00	O
ATOM	850	C	GLU	53	39.521	28.133	38.042	1.00	0.00	C
ATOM	851	O	GLU	53	39.970	29.228	38.176	1.00	0.00	O
ATOM	852	N	VAL	54	38.563	27.717	38.808	1.00	0.00	N
ATOM	853	H	VAL	54	38.248	26.773	38.629	1.00	0.00	H
ATOM	854	CA	VAL	54	38.072	28.387	40.037	1.00	0.00	C
ATOM	855	HA	VAL	54	37.952	29.458	39.879	1.00	0.00	H
ATOM	856	CB	VAL	54	36.659	27.850	40.284	1.00	0.00	C
ATOM	857	HB	VAL	54	36.724	26.803	40.577	1.00	0.00	H
ATOM	858	CG1	VAL	54	36.031	28.606	41.444	1.00	0.00	C
ATOM	859	HG11	VAL	54	36.666	28.530	42.327	1.00	0.00	H
ATOM	860	HG12	VAL	54	35.998	29.664	41.188	1.00	0.00	H
ATOM	861	HG13	VAL	54	35.012	28.222	41.481	1.00	0.00	H
ATOM	862	CG2	VAL	54	35.751	28.009	39.064	1.00	0.00	C
ATOM	863	HG21	VAL	54	34.717	27.732	39.277	1.00	0.00	H
ATOM	864	HG22	VAL	54	35.650	29.016	38.663	1.00	0.00	H
ATOM	865	HG23	VAL	54	36.118	27.425	38.221	1.00	0.00	H
ATOM	866	C	VAL	54	39.140	28.203	41.120	1.00	0.00	C
ATOM	867	O	VAL	54	39.254	27.087	41.594	1.00	0.00	O
ATOM	868	N	THR	55	39.904	29.278	41.498	1.00	0.00	N
ATOM	869	H	THR	55	39.605	30.190	41.186	1.00	0.00	H
ATOM	870	CA	THR	55	41.178	29.058	42.146	1.00	0.00	C
ATOM	871	HA	THR	55	41.566	28.062	41.934	1.00	0.00	H
ATOM	872	CB	THR	55	42.194	30.140	41.821	1.00	0.00	C
ATOM	873	HB	THR	55	43.016	30.025	42.528	1.00	0.00	H
ATOM	874	CG2	THR	55	42.723	29.885	40.398	1.00	0.00	C
ATOM	875	HG21	THR	55	43.810	29.841	40.460	1.00	0.00	H
ATOM	876	HG22	THR	55	42.429	28.878	40.101	1.00	0.00	H
ATOM	877	HG23	THR	55	42.362	30.672	39.736	1.00	0.00	H
ATOM	878	OG1	THR	55	41.710	31.439	41.944	1.00	0.00	O
ATOM	879	HG1	THR	55	42.349	32.050	41.570	1.00	0.00	H
ATOM	880	C	THR	55	40.935	29.073	43.670	1.00	0.00	C
ATOM	881	O	THR	55	41.680	28.528	44.539	1.00	0.00	O

```
ATOM    882  N   ALA    56      39.813  29.721  44.131  1.00  0.00           N
ATOM    883  H   ALA    56      39.268  30.145  43.395  1.00  0.00           H
ATOM    884  CA  ALA    56      39.274  29.717  45.497  1.00  0.00           C
ATOM    885  HA  ALA    56      39.516  28.734  45.901  1.00  0.00           H
ATOM
    886  CB  ALA    56      39.954  30.863  46.293  1.00  0.00           C
ATOM    887  HB1 ALA    56      39.661  30.795  47.340  1.00  0.00           H
ATOM    888  HB2 ALA    56      41.040  30.765  46.305  1.00  0.00           H
ATOM    889  HB3 ALA    56      39.710  31.862  45.932  1.00  0.00           H
ATOM    890  C   ALA    56      37.681  29.934  45.407  1.00  0.00           C
ATOM    891  O   ALA    56      37.106  30.801  44.684  1.00  0.00           O
ATOM    892  N   THR    57      36.904  29.431  46.430  1.00  0.00           N
ATOM    893  H   THR    57      37.401  28.874  47.111  1.00  0.00           H
ATOM    894  CA  THR    57      35.541  29.834  46.707  1.00  0.00           C
ATOM    895  HA  THR    57      35.414  30.830  46.282  1.00  0.00           H
ATOM    896  CB  THR    57      34.539  28.793  46.122  1.00  0.00           C
ATOM    897  HB  THR    57      33.517  29.123  46.316  1.00  0.00           H
ATOM    898  CG2 THR    57      34.643  28.554  44.602  1.00  0.00           C
ATOM    899 HG21 THR    57      34.466  29.543  44.182  1.00  0.00           H
ATOM    900 HG22 THR    57      35.608  28.083  44.413  1.00  0.00           H
ATOM    901 HG23 THR    57      33.849  27.925  44.199  1.00  0.00           H
ATOM    902  OG1 THR    57      34.738  27.546  46.613  1.00  0.00           O
ATOM    903  HG1 THR    57      34.246  27.420  47.428  1.00  0.00           H
ATOM    904  C   THR    57      35.329  29.860  48.223  1.00  0.00           C
ATOM    905  O   THR    57      36.045  29.214  48.979  1.00  0.00           O
ATOM    906  N   SER    58      34.347  30.641  48.757  1.00  0.00           N
ATOM    907  H   SER    58      33.788  31.233  48.160  1.00  0.00           H
ATOM    908  CA  SER    58      33.890  30.645  50.206  1.00  0.00           C
ATOM    909  HA  SER    58      34.090  29.635  50.564  1.00  0.00           H
ATOM    910  CB  SER    58      34.663  31.726  50.972  1.00  0.00           C
ATOM    911  HB2 SER    58      35.725  31.631  50.746  1.00  0.00           H
ATOM    912  HB3 SER    58      34.465  32.698  50.521  1.00  0.00           H
ATOM    913  OG  SER    58      34.371  31.724  52.316  1.00  0.00           O
ATOM    914  HG  SER    58      33.437  31.917  52.429  1.00  0.00           H
ATOM    915  C   SER    58      32.370  30.980  50.173  1.00  0.00           C
ATOM    916  O   SER    58      31.957  31.836  49.384  1.00  0.00           O
ATOM    917  N   THR    59      31.540  30.254  50.942  1.00  0.00           N
ATOM    918  H   THR    59      32.017  29.523  51.449  1.00  0.00           H
ATOM    919  CA  THR    59      30.386  30.938  51.544  1.00  0.00           C
ATOM    920  HA  THR    59      29.931  31.313  50.627  1.00  0.00           H
ATOM    921  CB  THR    59      29.285  29.955  52.136  1.00  0.00           C
ATOM    922  HB  THR    59      29.106  29.137  51.438  1.00  0.00           H
ATOM    923  CG2 THR    59      29.656  29.362  53.473  1.00  0.00           C
ATOM    924 HG21 THR    59      29.061  28.481  53.712  1.00  0.00           H
ATOM    925 HG22 THR    59      30.705  29.068  53.521  1.00  0.00           H
ATOM    926 HG23 THR    59      29.429  30.052  54.286  1.00  0.00           H
ATOM    927  OG1 THR    59      28.064  30.632  52.307  1.00  0.00           O
ATOM    928  HG1 THR    59      27.382  30.343  51.697  1.00  0.00           H
ATOM    929  C   THR    59      30.858  31.975  52.590  1.00  0.00           C
ATOM    930  O   THR    59      31.861  31.773  53.247  1.00  0.00           O
ATOM    931  N   VAL    60      30.217  33.119  52.683  1.00  0.00           N
ATOM    932  H   VAL    60      29.380  33.264  52.136  1.00  0.00           H
ATOM    933  CA  VAL    60      30.626  34.193  53.634  1.00  0.00           C
ATOM    934  HA  VAL    60      30.846  33.747  54.604  1.00  0.00           H
ATOM    935  CB  VAL    60      31.812  35.053  52.982  1.00  0.00           C
ATOM    936  HB  VAL    60      32.619  34.354  52.762  1.00  0.00           H
ATOM    937  CG1 VAL    60      31.379  35.746  51.691  1.00  0.00           C
ATOM    938 HG11 VAL    60      30.984  35.067  50.936  1.00  0.00           H
ATOM    939 HG12 VAL    60      30.653  36.478  52.043  1.00  0.00           H
ATOM    940 HG13 VAL    60      32.214  36.307  51.270  1.00  0.00           H
ATOM    941  CG2 VAL    60      32.527  35.979  53.973  1.00  0.00           C
ATOM    942 HG21 VAL    60      32.502  35.525  54.964  1.00  0.00           H
ATOM    943 HG22 VAL    60      33.529  36.280  53.665  1.00  0.00           H
ATOM    944 HG23 VAL    60      32.027  36.947  53.992  1.00  0.00           H
```

ATOM	945	C	VAL	60	29.496	35.172	53.934	1.00	0.00	C
ATOM	946	O	VAL	60	28.624	35.457	53.088	1.00	0.00	O
ATOM	947	N	THR	61	29.515	35.737	55.143	1.00	0.00	N
ATOM	948	H	THR	61	30.298	35.559	55.757	1.00	0.00	H
ATOM	949	CA	THR	61	28.598	36.783	55.607	1.00	0.00	C
ATOM	950	HA	THR	61	27.649	36.473	55.171	1.00	0.00	H
ATOM	951	CB	THR	61	28.343	36.817	57.132	1.00	0.00	C
ATOM	952	HB	THR	61	27.641	37.625	57.335	1.00	0.00	H
ATOM	953	CG2	THR	61	27.493	35.669	57.644	1.00	0.00	C
ATOM	954	HG21	THR	61	28.037	34.729	57.544	1.00	0.00	H
ATOM	955	HG22	THR	61	27.312	35.948	58.682	1.00	0.00	H
ATOM	956	HG23	THR	61	26.563	35.683	57.076	1.00	0.00	H
ATOM	957	OG1	THR	61	29.447	37.017	57.984	1.00	0.00	O
ATOM	958	HG1	THR	61	29.607	37.896	58.337	1.00	0.00	H
ATOM	959	C	THR	61	29.085	38.154	55.241	1.00	0.00	C
ATOM	960	O	THR	61	30.262	38.442	55.175	1.00	0.00	O
ATOM	961	N	LEU	62	28.087	39.102	55.091	1.00	0.00	N
ATOM	962	H	LEU	62	27.116	38.827	55.149	1.00	0.00	H
ATOM	963	CA	LEU	62	28.461	40.469	54.639	1.00	0.00	C
ATOM	964	HA	LEU	62	28.979	40.324	53.691	1.00	0.00	H
ATOM	965	CB	LEU	62	27.208	41.256	54.161	1.00	0.00	C
ATOM	966	HB2	LEU	62	26.592	40.548	53.605	1.00	0.00	H
ATOM	967	HB3	LEU	62	26.631	41.530	55.044	1.00	0.00	H
ATOM	968	CG	LEU	62	27.543	42.487	53.243	1.00	0.00	C
ATOM	969	HG	LEU	62	28.115	43.279	53.725	1.00	0.00	H
ATOM	970	CD1	LEU	62	28.383	42.101	51.951	1.00	0.00	C
ATOM	971	HD11	LEU	62	28.640	43.012	51.411	1.00	0.00	H
ATOM	972	HD12	LEU	62	29.371	41.744	52.240	1.00	0.00	H
ATOM	973	HD13	LEU	62	27.860	41.342	51.368	1.00	0.00	H
ATOM	974	CD2	LEU	62	26.206	43.271	52.936	1.00	0.00	C
ATOM	975	HD21	LEU	62	25.777	43.493	53.912	1.00	0.00	H
ATOM	976	HD22	LEU	62	26.410	44.120	52.284	1.00	0.00	H
ATOM	977	HD23	LEU	62	25.537	42.590	52.408	1.00	0.00	H
ATOM	978	C	LEU	62	29.426	41.144	55.514	1.00	0.00	C
ATOM	979	O	LEU	62	30.275	41.937	55.077	1.00	0.00	O
ATOM	980	N	ASP	63	29.284	40.892	56.807	1.00	0.00	N
ATOM	981	H	ASP	63	28.529	40.253	57.014	1.00	0.00	H
ATOM	982	CA	ASP	63	30.202	41.423	57.817	1.00	0.00	C
ATOM	983	HA	ASP	63	30.519	42.438	57.582	1.00	0.00	H
ATOM	984	CB	ASP	63	29.612	41.544	59.168	1.00	0.00	C
ATOM	985	HB2	ASP	63	30.315	42.069	59.815	1.00	0.00	H
ATOM	986	HB3	ASP	63	28.716	42.164	59.120	1.00	0.00	H
ATOM	987	CG	ASP	63	29.127	40.230	59.897	1.00	0.00	C
ATOM	988	OD1	ASP	63	28.181	40.242	60.710	1.00	0.00	O
ATOM	989	OD2	ASP	63	29.574	39.137	59.624	1.00	0.00	O
ATOM	990	C	ASP	63	31.622	40.755	57.947	1.00	0.00	C
ATOM	991	O	ASP	63	32.463	41.249	58.696	1.00	0.00	O
ATOM	992	N	THR	64	31.959	39.692	57.163	1.00	0.00	N
ATOM	993	H	THR	64	31.307	39.380	56.457	1.00	0.00	H
ATOM	994	CA	THR	64	33.306	39.123	57.070	1.00	0.00	C
ATOM	995	HA	THR	64	34.009	39.844	57.488	1.00	0.00	H
ATOM	996	CB	THR	64	33.438	37.820	57.889	1.00	0.00	C
ATOM	997	HB	THR	64	34.463	37.449	57.849	1.00	0.00	H
ATOM	998	CG2	THR	64	32.994	37.904	59.378	1.00	0.00	C
ATOM	999	HG21	THR	64	33.637	37.234	59.949	1.00	0.00	H
ATOM	1000	HG22	THR	64	33.110	38.930	59.731	1.00	0.00	H
ATOM	1001	HG23	THR	64	31.959	37.620	59.564	1.00	0.00	H
ATOM	1002	OG1	THR	64	32.654	36.862	57.343	1.00	0.00	O
ATOM	1003	HG1	THR	64	31.743	37.101	57.531	1.00	0.00	H
ATOM	1004	C	THR	64	33.829	39.028	55.608	1.00	0.00	C
ATOM	1005	O	THR	64	34.895	38.529	55.387	1.00	0.00	O
ATOM	1006	N	LEU	65	33.083	39.528	54.623	1.00	0.00	N
ATOM	1007	H	LEU	65	32.100	39.669	54.808	1.00	0.00	H
ATOM	1008	CA	LEU	65	33.606	39.719	53.218	1.00	0.00	C

ATOM	1009	HA	LEU	65	33.933	38.712	52.959	1.00	0.00	H
ATOM	1010	CB	LEU	65	32.489	40.236	52.358	1.00	0.00	C
ATOM	1011	HB2	LEU	65						
31.655	39.536	52.401	1.00	0.00	H					
ATOM	1012	HB3	LEU	65	31.963	41.105	52.757	1.00	0.00	H
ATOM	1013	CG	LEU	65	32.785	40.606	50.897	1.00	0.00	C
ATOM	1014	HG	LEU	65	33.652	41.265	50.847	1.00	0.00	H
ATOM	1015	CD1	LEU	65	33.118	39.392	50.060	1.00	0.00	C
ATOM	1016	HD11	LEU	65	32.291	38.693	50.185	1.00	0.00	H
ATOM	1017	HD12	LEU	65	33.171	39.580	48.988	1.00	0.00	H
ATOM	1018	HD13	LEU	65	33.971	38.882	50.508	1.00	0.00	H
ATOM	1019	CD2	LEU	65	31.591	41.218	50.160	1.00	0.00	C
ATOM	1020	HD21	LEU	65	30.817	40.464	50.015	1.00	0.00	H
ATOM	1021	HD22	LEU	65	31.155	41.955	50.836	1.00	0.00	H
ATOM	1022	HD23	LEU	65	31.932	41.776	49.288	1.00	0.00	H
ATOM	1023	C	LEU	65	34.842	40.583	53.216	1.00	0.00	C
ATOM	1024	O	LEU	65	34.827	41.602	53.943	1.00	0.00	O
ATOM	1025	N	THR	66	35.904	40.014	52.572	1.00	0.00	N
ATOM	1026	H	THR	66	35.727	39.120	52.136	1.00	0.00	H
ATOM	1027	CA	THR	66	37.187	40.622	52.305	1.00	0.00	C
ATOM	1028	HA	THR	66	37.626	40.996	53.231	1.00	0.00	H
ATOM	1029	CB	THR	66	38.130	39.536	51.711	1.00	0.00	C
ATOM	1030	HB	THR	66	38.198	38.691	52.396	1.00	0.00	H
ATOM	1031	CG2	THR	66	37.789	39.101	50.311	1.00	0.00	C
ATOM	1032	HG21	THR	66	37.893	39.910	49.588	1.00	0.00	H
ATOM	1033	HG22	THR	66	38.395	38.223	50.084	1.00	0.00	H
ATOM	1034	HG23	THR	66	36.755	38.790	50.163	1.00	0.00	H
ATOM	1035	OG1	THR	66	39.490	40.046	51.637	1.00	0.00	O
ATOM	1036	HG1	THR	66	39.824	40.017	52.537	1.00	0.00	H
ATOM	1037	C	THR	66	37.123	41.759	51.364	1.00	0.00	C
ATOM	1038	O	THR	66	36.262	41.838	50.498	1.00	0.00	O
ATOM	1039	N	GLU	67	38.118	42.669	51.258	1.00	0.00	N
ATOM	1040	H	GLU	67	38.769	42.544	52.020	1.00	0.00	H
ATOM	1041	CA	GLU	67	38.172	43.871	50.422	1.00	0.00	C
ATOM	1042	HA	GLU	67	37.222	44.402	50.484	1.00	0.00	H
ATOM	1043	CB	GLU	67	39.196	44.903	50.860	1.00	0.00	C
ATOM	1044	HB2	GLU	67	39.153	45.743	50.164	1.00	0.00	H
ATOM	1045	HB3	GLU	67	38.793	45.193	51.830	1.00	0.00	H
ATOM	1046	CG	GLU	67	40.673	44.530	50.947	1.00	0.00	C
ATOM	1047	HG2	GLU	67	40.731	43.597	51.508	1.00	0.00	H
ATOM	1048	HG3	GLU	67	41.058	44.315	49.950	1.00	0.00	H
ATOM	1049	CD	GLU	67	41.584	45.518	51.638	1.00	0.00	C
ATOM	1050	OE1	GLU	67	41.122	46.601	51.853	1.00	0.00	O
ATOM	1051	OE2	GLU	67	42.692	45.198	52.184	1.00	0.00	O
ATOM	1052	C	GLU	67	38.385	43.611	48.938	1.00	0.00	C
ATOM	1053	O	GLU	67	38.330	44.458	48.053	1.00	0.00	O
ATOM	1054	N	LYS	68	38.666	42.370	48.603	1.00	0.00	N
ATOM	1055	H	LYS	68	38.654	41.685	49.345	1.00	0.00	H
ATOM	1056	CA	LYS	68	39.330	41.978	47.370	1.00	0.00	C
ATOM	1057	HA	LYS	68	40.202	42.569	47.092	1.00	0.00	H
ATOM	1058	CB	LYS	68	39.735	40.493	47.467	1.00	0.00	C
ATOM	1059	HB2	LYS	68	40.342	40.298	48.352	1.00	0.00	H
ATOM	1060	HB3	LYS	68	38.803	39.928	47.496	1.00	0.00	H
ATOM	1061	CG	LYS	68	40.634	40.018	46.342	1.00	0.00	C
ATOM	1062	HG2	LYS	68	40.134	39.786	45.402	1.00	0.00	H
ATOM	1063	HG3	LYS	68	41.433	40.727	46.132	1.00	0.00	H
ATOM	1064	CD	LYS	68	41.322	38.785	46.892	1.00	0.00	C
ATOM	1065	HD2	LYS	68	42.084	39.034	47.631	1.00	0.00	H
ATOM	1066	HD3	LYS	68	40.659	37.968	47.179	1.00	0.00	H
ATOM	1067	CE	LYS	68	42.193	38.226	45.728	1.00	0.00	C
ATOM	1068	HE2	LYS	68	41.576	38.070	44.843	1.00	0.00	H
ATOM	1069	HE3	LYS	68	42.985	38.888	45.378	1.00	0.00	H
ATOM	1070	NZ	LYS	68	42.846	36.990	46.089	1.00	0.00	N
ATOM	1071	HZ1	LYS	68	43.671	36.832	45.526	1.00	0.00	H

ATOM	1072	HZ2	LYS	68	43.024	36.985	47.083	1.00	0.00	H
ATOM	1073	HZ3	LYS	68	42.144	36.294	45.884	1.00	0.00	H
ATOM	1074	C	LYS	68	38.472	42.197	46.087	1.00	0.00	C
ATOM	1075	O	LYS	68	39.029	42.547	45.048	1.00	0.00	O
ATOM	1076	N	HIE	69	37.143	42.138	46.087	1.00	0.00	N
ATOM	1077	H	HIE	69	36.668	41.913	46.951	1.00	0.00	H
ATOM	1078	CA	HIE	69	36.315	42.527	44.970	1.00	0.00	C
ATOM	1079	HA	HIE	69	36.701	42.274	43.983	1.00	0.00	H
ATOM	1080	CB	HIE	69	34.948	41.902	45.099	1.00	0.00	C
ATOM	1081	HB2	HIE	69	34.828	40.890	45.485	1.00	0.00	H
ATOM	1082	HB3	HIE	69	34.356	42.574	45.720	1.00	0.00	H
ATOM	1083	CG	HIE	69	34.049	41.928	43.841	1.00	0.00	C
ATOM	1084	ND1	HIE	69	34.061	40.979	42.768	1.00	0.00	N
ATOM	1085	CE1	HIE	69	33.043	41.358	42.002	1.00	0.00	C
ATOM	1086	HE1	HIE	69	32.664	40.817	41.148	1.00	0.00	H
ATOM	1087	NE2	HIE	69	32.534	42.541	42.477	1.00	0.00	N
ATOM	1088	HE2	HIE	69	31.828	43.115	42.040	1.00	0.00	H
ATOM	1089	CD2	HIE	69	33.165	42.920	43.617	1.00	0.00	C
ATOM	1090	HD2	HIE	69	33.124	43.848	44.171	1.00	0.00	H
ATOM	1091	C	HIE	69	36.181	44.065	44.826	1.00	0.00	C
ATOM	1092	O	HIE	69	36.168	44.495	43.682	1.00	0.00	O
ATOM	1093	N	ALA	70	36.150	44.820	45.923	1.00	0.00	N
ATOM	1094	H	ALA	70	36.086	44.347	46.813	1.00	0.00	H
ATOM	1095	CA	ALA	70	36.155	46.223	45.996	1.00	0.00	C
ATOM	1096	HA	ALA	70	35.310	46.484	45.357	1.00	0.00	H
ATOM	1097	CB	ALA	70	35.902	46.603	47.455	1.00	0.00	C
ATOM	1098	HB1	ALA	70	35.655	47.664	47.485	1.00	0.00	H
ATOM	1099	HB2	ALA	70	35.114	46.057	47.974	1.00	0.00	H
ATOM	1100	HB3	ALA	70	36.763	46.343	48.070	1.00	0.00	H
ATOM	1101	C	ALA	70	37.554	46.770	45.492	1.00	0.00	C
ATOM	1102	O	ALA	70	37.540	47.733	44.691	1.00	0.00	O
ATOM	1103	N	GLU	71	38.731	46.054	45.715	1.00	0.00	N
ATOM	1104	H	GLU	71	38.690	45.289	46.371	1.00	0.00	H
ATOM	1105	CA	GLU	71	40.003	46.458	45.051	1.00	0.00	C
ATOM	1106	HA	GLU	71	40.076	47.538	45.186	1.00	0.00	H
ATOM	1107	CB	GLU	71	41.217	45.801	45.687	1.00	0.00	C
ATOM	1108	HB2	GLU	71	41.116	44.717	45.736	1.00	0.00	H
ATOM	1109	HB3	GLU	71	42.217	46.054	45.336	1.00	0.00	H
ATOM	1110	CG	GLU	71	41.315	46.211	47.207	1.00	0.00	C
ATOM	1111	HG2	GLU	71	40.374	46.073	47.739	1.00	0.00	H
ATOM	1112	HG3	GLU	71	42.072	45.579	47.671	1.00	0.00	H
ATOM	1113	CD	GLU	71	41.768	47.633	47.527	1.00	0.00	C
ATOM	1114	OE1	GLU	71	42.644	47.829	48.427	1.00	0.00	O
ATOM	1115	OE2	GLU	71	41.235	48.615	46.931	1.00	0.00	O
ATOM	1116	C	GLU	71	40.070	46.342	43.535	1.00	0.00	C
ATOM	1117	O	GLU	71	40.450	47.268	42.809	1.00	0.00	O
ATOM	1118	N	GLN	72	39.556	45.272	42.930	1.00	0.00	N
ATOM	1119	H	GLN	72	39.301	44.503	43.533	1.00	0.00	H
ATOM	1120	CA	GLN	72	39.332	44.986	41.541	1.00	0.00	C
ATOM	1121	HA	GLN	72	40.298	45.059	41.041	1.00	0.00	H
ATOM	1122	CB	GLN	72	38.778	43.604	41.312	1.00	0.00	C
ATOM	1123	HB2	GLN	72	37.789	43.666	41.765	1.00	0.00	H
ATOM	1124	HB3	GLN	72	39.402	42.912	41.876	1.00	0.00	H
ATOM	1125	CG	GLN	72	38.585	43.152	39.793	1.00	0.00	C
ATOM	1126	HG2	GLN	72	38.109	43.989	39.281	1.00	0.00	H
ATOM	1127	HG3	GLN	72	39.522	42.884	39.304	1.00	0.00	H
ATOM	1128	CD	GLN	72	37.713	41.885	39.757	1.00	0.00	C
ATOM	1129	OE1	GLN	72	38.265	40.814	39.646	1.00	0.00	O
ATOM	1130	NE2	GLN	72	36.396	41.908	39.823	1.00	0.00	N
ATOM	1131	HE21	GLN	72	35.895	42.778	39.700	1.00	0.00	H
ATOM	1132	HE22	GLN	72	35.940	41.037	40.055	1.00	0.00	H
ATOM	1133	C	GLN	72	38.464	46.054	40.857	1.00	0.00	C
ATOM	1134	O	GLN	72	38.805	46.678	39.896	1.00	0.00	O
ATOM	1135	N	GLU	73	37.324	46.296	41.457	1.00	0.00	N

```
ATOM   1136  H   GLU    73      37.180  45.905  42.376
  1.00  0.00           H
ATOM   1137  CA  GLU    73      36.265  47.187  40.936  1.00  0.00           C
ATOM   1138  HA  GLU    73      36.340  47.089  39.853  1.00  0.00           H
ATOM   1139  CB  GLU    73      34.908  46.613  41.439  1.00  0.00           C
ATOM   1140  HB2 GLU    73      34.885  46.317  42.488  1.00  0.00           H
ATOM   1141  HB3 GLU    73      34.167  47.376  41.201  1.00  0.00           H
ATOM   1142  CG  GLU    73      34.450  45.352  40.801  1.00  0.00           C
ATOM   1143  HG2 GLU    73      34.906  44.504  41.313  1.00  0.00           H
ATOM   1144  HG3 GLU    73      33.367  45.321  40.922  1.00  0.00           H
ATOM   1145  CD  GLU    73      34.714  45.227  39.299  1.00  0.00           C
ATOM   1146  OE1 GLU    73      35.298  44.245  38.766  1.00  0.00           O
ATOM   1147  OE2 GLU    73      34.277  46.162  38.533  1.00  0.00           O
ATOM   1148  C   GLU    73      36.442  48.653  41.280  1.00  0.00           C
ATOM   1149  O   GLU    73      35.588  49.427  40.941  1.00  0.00           O
ATOM   1150  N   ASN    74      37.618  48.978  41.851  1.00  0.00           N
ATOM   1151  H   ASN    74      38.277  48.223  41.976  1.00  0.00           H
ATOM   1152  CA  ASN    74      38.059  50.344  42.144  1.00  0.00           C
ATOM   1153  HA  ASN    74      39.007  50.210  42.665  1.00  0.00           H
ATOM   1154  CB  ASN    74      38.338  51.143  40.826  1.00  0.00           C
ATOM   1155  HB2 ASN    74      37.389  51.471  40.403  1.00  0.00           H
ATOM   1156  HB3 ASN    74      38.914  51.973  41.235  1.00  0.00           H
ATOM   1157  CG  ASN    74      39.229  50.418  39.753  1.00  0.00           C
ATOM   1158  OD1 ASN    74      38.822  50.190  38.601  1.00  0.00           O
ATOM   1159  ND2 ASN    74      40.435  50.068  40.167  1.00  0.00           N
ATOM   1160 HD21 ASN    74      40.900  49.531  39.450  1.00  0.00           H
ATOM   1161 HD22 ASN    74      40.763  50.311  41.091  1.00  0.00           H
ATOM   1162  C   ASN    74      37.151  51.080  43.095  1.00  0.00           C
ATOM   1163  O   ASN    74      36.831  52.270  43.008  1.00  0.00           O
ATOM   1164  N   MET    75      36.782  50.514  44.223  1.00  0.00           N
ATOM   1165  H   MET    75      37.022  49.538  44.318  1.00  0.00           H
ATOM   1166  CA  MET    75      35.993  51.141  45.285  1.00  0.00           C
ATOM   1167  HA  MET    75      36.069  52.228  45.275  1.00  0.00           H
ATOM   1168  CB  MET    75      34.489  50.772  45.237  1.00  0.00           C
ATOM   1169  HB2 MET    75      33.997  51.329  46.035  1.00  0.00           H
ATOM   1170  HB3 MET    75      34.104  51.080  44.265  1.00  0.00           H
ATOM   1171  CG  MET    75      34.101  49.274  45.415  1.00  0.00           C
ATOM   1172  HG2 MET    75      34.734  48.631  44.803  1.00  0.00           H
ATOM   1173  HG3 MET    75      34.324  49.132  46.473  1.00  0.00           H
ATOM   1174  SD  MET    75      32.294  49.032  45.113  1.00  0.00           S
ATOM   1175  CE  MET    75      32.406  49.051  43.333  1.00  0.00           C
ATOM   1176  HE1 MET    75      31.510  48.701  42.820  1.00  0.00           H
ATOM   1177  HE2 MET    75      32.727  50.010  42.925  1.00  0.00           H
ATOM   1178  HE3 MET    75      33.103  48.229  43.175  1.00  0.00           H
ATOM   1179  C   MET    75      36.514  50.610  46.603  1.00  0.00           C
ATOM   1180  O   MET    75      37.128  49.575  46.638  1.00  0.00           O
ATOM   1181  N   THR    76      36.339  51.399  47.645  1.00  0.00           N
ATOM   1182  H   THR    76      35.838  52.270  47.541  1.00  0.00           H
ATOM   1183  CA  THR    76      36.491  50.934  49.068  1.00  0.00           C
ATOM   1184  HA  THR    76      37.417  50.364  49.137  1.00  0.00           H
ATOM   1185  CB  THR    76      36.654  52.035  50.090  1.00  0.00           C
ATOM   1186  HB  THR    76      37.259  52.816  49.629  1.00  0.00           H
ATOM   1187  CG2 THR    76      35.436  52.797  50.339  1.00  0.00           C
ATOM   1188 HG21 THR    76      34.592  52.132  50.526  1.00  0.00           H
ATOM   1189 HG22 THR    76      35.583  53.302  51.294  1.00  0.00           H
ATOM   1190 HG23 THR    76      35.221  53.481  49.518  1.00  0.00           H
ATOM   1191  OG1 THR    76      37.216  51.510  51.392  1.00  0.00           O
ATOM   1192  HG1 THR    76      38.115  51.849  51.367  1.00  0.00           H
ATOM   1193  C   THR    76      35.433  49.929  49.502  1.00  0.00           C
ATOM   1194  O   THR    76      34.328  49.920  48.972  1.00  0.00           O
ATOM   1195  N   LEU    77      35.835  48.962  50.357  1.00  0.00           N
ATOM   1196  H   LEU    77      36.839  48.974  50.461  1.00  0.00           H
ATOM   1197  CA  LEU    77      34.978  47.890  50.878  1.00  0.00           C
ATOM   1198  HA  LEU    77      34.721  47.252  50.033  1.00  0.00           H
```

ATOM	1199	CB	LEU	77	35.723	47.082	51.969	1.00	0.00	C
ATOM	1200	HB2	LEU	77	36.596	46.573	51.562	1.00	0.00	H
ATOM	1201	HB3	LEU	77	36.141	47.744	52.728	1.00	0.00	H
ATOM	1202	CG	LEU	77	34.894	46.080	52.695	1.00	0.00	C
ATOM	1203	HG	LEU	77	34.187	46.619	53.325	1.00	0.00	H
ATOM	1204	CD1	LEU	77	34.193	44.993	51.854	1.00	0.00	C
ATOM	1205	HD11	LEU	77	33.416	45.439	51.234	1.00	0.00	H
ATOM	1206	HD12	LEU	77	34.859	44.476	51.164	1.00	0.00	H
ATOM	1207	HD13	LEU	77	33.683	44.343	52.565	1.00	0.00	H
ATOM	1208	CD2	LEU	77	35.809	45.335	53.691	1.00	0.00	C
ATOM	1209	HD21	LEU	77	36.343	45.990	54.379	1.00	0.00	H
ATOM	1210	HD22	LEU	77	35.097	44.804	54.324	1.00	0.00	H
ATOM	1211	HD23	LEU	77	36.452	44.600	53.209	1.00	0.00	H
ATOM	1212	C	LEU	77	33.595	48.460	51.333	1.00	0.00	C
ATOM	1213	O	LEU	77	32.576	47.838	51.060	1.00	0.00	O
ATOM	1214	N	THR	78	33.700	49.593	52.087	1.00	0.00	N
ATOM	1215	H	THR	78	34.634	49.970	52.167	1.00	0.00	H
ATOM	1216	CA	THR	78	32.502	50.293	52.576	1.00	0.00	C
ATOM	1217	HA	THR	78	31.988	49.802	53.403	1.00	0.00	H
ATOM	1218	CB	THR	78	32.976	51.700	53.110	1.00	0.00	C
ATOM	1219	HB	THR	78	33.684	52.098	52.383	1.00	0.00	H
ATOM	1220	CG2	THR	78	31.942	52.815	53.109	1.00	0.00	C
ATOM	1221	HG21	THR	78	31.073	52.527	53.699	1.00	0.00	H
ATOM	1222	HG22	THR	78	32.367	53.597	53.738	1.00	0.00	H
ATOM	1223	HG23	THR	78	31.726	53.170	52.101	1.00	0.00	H
ATOM	1224	OG1	THR	78	33.577	51.526	54.373	1.00	0.00	O
ATOM	1225	HG1	THR	78	34.426	51.123	54.178	1.00	0.00	H
ATOM	1226	C	THR	78	31.465	50.558	51.441	1.00	0.00	C
ATOM	1227	O	THR	78	30.225	50.471	51.674	1.00	0.00	O
ATOM	1228	N	GLU	79	31.922	50.923	50.287	1.00	0.00	N
ATOM	1229	H	GLU	79	32.796	50.501	50.009	1.00	0.00	H
ATOM	1230	CA	GLU	79	31.085	51.166	49.135	1.00	0.00	C
ATOM	1231	HA	GLU	79	30.249	51.753	49.515	1.00	0.00	H
ATOM	1232	CB	GLU	79	31.780	52.100	48.112	1.00	0.00	C
ATOM	1233	HB2	GLU	79	32.718	51.625	47.824	1.00	0.00	H
ATOM	1234	HB3	GLU	79	31.164	52.119	47.212	1.00	0.00	H
ATOM	1235	CG	GLU	79	31.998	53.509	48.629	1.00	0.00	C
ATOM	1236	HG2	GLU	79	32.763	53.490	49.406	1.00	0.00	H
ATOM	1237	HG3	GLU	79	32.382	53.995	47.733	1.00	0.00	H
ATOM	1238	CD	GLU	79	30.786	54.330	49.106	1.00	0.00	C
ATOM	1239	OE1	GLU	79	30.844	54.943	50.224	1.00	0.00	O
ATOM	1240	OE2	GLU	79	29.785	54.231	48.369	1.00	0.00	O
ATOM	1241	C	GLU	79	30.388	49.898	48.647	1.00	0.00	C
ATOM	1242	O	GLU	79	29.214	49.967	48.213	1.00	0.00	O
ATOM	1243	N	LEU	80	31.110	48.780	48.622	1.00	0.00	N
ATOM	1244	H	LEU	80	32.006	48.794	49.089	1.00	0.00	H
ATOM	1245	CA	LEU	80	30.668	47.524	48.142	1.00	0.00	C
ATOM	1246	HA	LEU	80	30.184	47.706	47.182	1.00	0.00	H
ATOM	1247	CB	LEU	80	31.938	46.614	47.997	1.00	0.00	C
ATOM	1248	HB2	LEU	80	32.734	47.131	47.462	1.00	0.00	H
ATOM	1249	HB3	LEU	80	32.113	46.371	49.044	1.00	0.00	H
ATOM	1250	CG	LEU	80	31.690	45.186	47.487	1.00	0.00	C
ATOM	1251	HG	LEU	80	31.030	44.632	48.154	1.00	0.00	H
ATOM	1252	CD1	LEU	80	31.052	45.183	46.089	1.00	0.00	C
ATOM	1253	HD11	LEU	80	31.674	45.721	45.374	1.00	0.00	H
ATOM	1254	HD12	LEU	80	30.990	44.163	45.708	1.00	0.00	H
ATOM	1255	HD13	LEU	80	30.103	45.708	46.196	1.00	0.00	H
ATOM	1256	CD2	LEU	80	32.947	44.379	47.497	1.00	0.00	C
ATOM	1257	HD21	LEU	80	33.680	44.673	46.747	1.00	0.00	H
ATOM	1258	HD22	LEU	80	33.400	44.336	48.489	1.00	0.00	H
ATOM	1259	HD23	LEU	80	32.671	43.361	47.225	1.00	0.00	H
ATOM	1260	C	LEU	80	29.636	46.880	49.089	1.00	0.00	C
ATOM	1261	O	LEU	80	28.675	46.321	48.637	1.00	0.00	O
ATOM										

	1262	N	LYS	81	29.796	46.938	50.455	1.00	0.00	N
ATOM	1263	H	LYS	81	30.596	47.475	50.758	1.00	0.00	H
ATOM	1264	CA	LYS	81	28.699	46.537	51.386	1.00	0.00	C
ATOM	1265	HA	LYS	81	28.492	45.467	51.326	1.00	0.00	H
ATOM	1266	CB	LYS	81	29.276	46.702	52.812	1.00	0.00	C
ATOM	1267	HB2	LYS	81	29.667	47.719	52.825	1.00	0.00	H
ATOM	1268	HB3	LYS	81	28.500	46.648	53.576	1.00	0.00	H
ATOM	1269	CG	LYS	81	30.410	45.727	53.135	1.00	0.00	C
ATOM	1270	HG2	LYS	81	30.009	44.715	53.193	1.00	0.00	H
ATOM	1271	HG3	LYS	81	31.109	45.573	52.313	1.00	0.00	H
ATOM	1272	CD	LYS	81	31.298	46.000	54.383	1.00	0.00	C
ATOM	1273	HD2	LYS	81	32.160	45.362	54.184	1.00	0.00	H
ATOM	1274	HD3	LYS	81	31.467	47.069	54.259	1.00	0.00	H
ATOM	1275	CE	LYS	81	30.614	45.646	55.717	1.00	0.00	C
ATOM	1276	HE2	LYS	81	29.865	46.365	56.047	1.00	0.00	H
ATOM	1277	HE3	LYS	81	30.182	44.676	55.470	1.00	0.00	H
ATOM	1278	NZ	LYS	81	31.601	45.352	56.788	1.00	0.00	N
ATOM	1279	HZ1	LYS	81	32.117	46.188	57.022	1.00	0.00	H
ATOM	1280	HZ2	LYS	81	31.068	45.181	57.629	1.00	0.00	H
ATOM	1281	HZ3	LYS	81	32.218	44.581	56.580	1.00	0.00	H
ATOM	1282	C	LYS	81	27.361	47.290	51.154	1.00	0.00	C
ATOM	1283	O	LYS	81	26.226	46.729	51.201	1.00	0.00	O
ATOM	1284	N	LYS	82	27.494	48.569	50.898	1.00	0.00	N
ATOM	1285	H	LYS	82	28.360	49.024	51.154	1.00	0.00	H
ATOM	1286	CA	LYS	82	26.362	49.464	50.588	1.00	0.00	C
ATOM	1287	HA	LYS	82	25.648	49.497	51.411	1.00	0.00	H
ATOM	1288	CB	LYS	82	26.890	50.962	50.503	1.00	0.00	C
ATOM	1289	HB2	LYS	82	27.859	51.003	50.005	1.00	0.00	H
ATOM	1290	HB3	LYS	82	26.209	51.571	49.909	1.00	0.00	H
ATOM	1291	CG	LYS	82	26.958	51.702	51.866	1.00	0.00	C
ATOM	1292	HG2	LYS	82	25.949	51.916	52.220	1.00	0.00	H
ATOM	1293	HG3	LYS	82	27.448	51.119	52.646	1.00	0.00	H
ATOM	1294	CD	LYS	82	27.734	53.019	51.738	1.00	0.00	C
ATOM	1295	HD2	LYS	82	27.694	53.584	52.670	1.00	0.00	H
ATOM	1296	HD3	LYS	82	28.795	52.777	51.675	1.00	0.00	H
ATOM	1297	CE	LYS	82	27.279	53.933	50.558	1.00	0.00	C
ATOM	1298	HE2	LYS	82	27.345	53.467	49.576	1.00	0.00	H
ATOM	1299	HE3	LYS	82	26.241	54.240	50.691	1.00	0.00	H
ATOM	1300	NZ	LYS	82	28.019	55.172	50.601	1.00	0.00	N
ATOM	1301	HZ1	LYS	82	27.814	55.591	51.497	1.00	0.00	H
ATOM	1302	HZ2	LYS	82	29.026	55.140	50.526	1.00	0.00	H
ATOM	1303	HZ3	LYS	82	27.660	55.813	49.908	1.00	0.00	H
ATOM	1304	C	LYS	82	25.578	49.074	49.361	1.00	0.00	C
ATOM	1305	O	LYS	82	24.352	49.005	49.408	1.00	0.00	O
ATOM	1306	N	VAL	83	26.211	48.850	48.199	1.00	0.00	N
ATOM	1307	H	VAL	83	27.217	48.895	48.277	1.00	0.00	H
ATOM	1308	CA	VAL	83	25.385	48.350	47.112	1.00	0.00	C
ATOM	1309	HA	VAL	83	24.532	49.028	47.075	1.00	0.00	H
ATOM	1310	CB	VAL	83	25.951	48.323	45.715	1.00	0.00	C
ATOM	1311	HB	VAL	83	25.482	47.727	44.931	1.00	0.00	H
ATOM	1312	CG1	VAL	83	25.929	49.748	45.140	1.00	0.00	C
ATOM	1313	HG11	VAL	83	26.423	49.822	44.171	1.00	0.00	H
ATOM	1314	HG12	VAL	83	24.902	50.086	44.999	1.00	0.00	H
ATOM	1315	HG13	VAL	83	26.406	50.344	45.919	1.00	0.00	H
ATOM	1316	CG2	VAL	83	27.429	47.830	45.658	1.00	0.00	C
ATOM	1317	HG21	VAL	83	28.094	48.314	46.373	1.00	0.00	H
ATOM	1318	HG22	VAL	83	27.454	46.743	45.741	1.00	0.00	H
ATOM	1319	HG23	VAL	83	27.772	48.113	44.663	1.00	0.00	H
ATOM	1320	C	VAL	83	24.768	46.939	47.259	1.00	0.00	C
ATOM	1321	O	VAL	83	23.698	46.650	46.735	1.00	0.00	O
ATOM	1322	N	ILE	84	25.478	46.062	47.981	1.00	0.00	N
ATOM	1323	H	ILE	84	26.389	46.373	48.291	1.00	0.00	H
ATOM	1324	CA	ILE	84	24.932	44.695	48.122	1.00	0.00	C
ATOM	1325	HA	ILE	84	24.660	44.380	47.115	1.00	0.00	H

ATOM	1326	CB	ILE	84	26.022	43.713	48.639	1.00	0.00	C
ATOM	1327	HB	ILE	84	26.465	44.223	49.494	1.00	0.00	H
ATOM	1328	CG2	ILE	84	25.477	42.321	49.015	1.00	0.00	C
ATOM	1329	HG21	ILE	84	24.831	42.299	49.892	1.00	0.00	H
ATOM	1330	HG22	ILE	84	24.766	42.077	48.226	1.00	0.00	H
ATOM	1331	HG23	ILE	84	26.327	41.643	49.098	1.00	0.00	H
ATOM	1332	CG1	ILE	84	27.170	43.624	47.661	1.00	0.00	C
ATOM	1333	HG12	ILE	84	26.870	43.015	46.808	1.00	0.00	H
ATOM	1334	HG13	ILE	84	27.467	44.614	47.317	1.00	0.00	H
ATOM	1335	CD1	ILE	84	28.476	42.991	48.233	1.00	0.00	C
ATOM	1336	HD11	ILE	84	28.817	43.743	48.945	1.00	0.00	H
ATOM	1337	HD12	ILE	84	28.314	42.017	48.693	1.00	0.00	H
ATOM	1338	HD13	ILE	84	29.214	42.867	47.441	1.00	0.00	H
ATOM	1339	C	ILE	84	23.736	44.735	49.047	1.00	0.00	C
ATOM	1340	O	ILE	84	22.674	44.238	48.615	1.00	0.00	O
ATOM	1341	N	ALA	85	23.838	45.467	50.188	1.00	0.00	N
ATOM	1342	H	ALA	85	24.771	45.776	50.424	1.00	0.00	H
ATOM	1343	CA	ALA	85	22.687	45.643	51.195	1.00	0.00	C
ATOM	1344	HA	ALA	85	22.253	44.681	51.469	1.00	0.00	H
ATOM	1345	CB	ALA	85	23.335	46.323	52.390	1.00	0.00	C
ATOM	1346	HB1	ALA	85	23.869	47.193	52.011	1.00	0.00	H
ATOM	1347	HB2	ALA	85	22.547	46.754	53.010	1.00	0.00	H
ATOM	1348	HB3	ALA	85	24.001	45.796	53.073	1.00	0.00	H
ATOM	1349	C	ALA	85	21.499	46.528	50.659	1.00	0.00	C
ATOM	1350	O	ALA	85	20.392	46.437	51.170	1.00	0.00	O
ATOM	1351	N	ASP	86	21.746	47.319	49.617	1.00	0.00	N
ATOM	1352	H	ASP	86	22.671	47.358	49.214	1.00	0.00	H
ATOM	1353	CA	ASP	86	20.705	48.090	48.911	1.00	0.00	C
ATOM	1354	HA	ASP	86	20.215	48.547	49.771	1.00	0.00	H
ATOM	1355	CB	ASP	86	21.343	49.180	48.001	1.00	0.00	C
ATOM	1356	HB2	ASP	86	22.159	48.748	47.422	1.00	0.00	H
ATOM	1357	HB3	ASP	86	20.646	49.406	47.194	1.00	0.00	H
ATOM	1358	CG	ASP	86	21.645	50.431	48.629	1.00	0.00	C
ATOM	1359	OD1	ASP	86	22.236	51.273	47.916	1.00	0.00	O
ATOM	1360	OD2	ASP	86	21.229	50.806	49.742	1.00	0.00	O
ATOM	1361	C	ASP	86	19.607	47.164	48.263	1.00	0.00	C
ATOM	1362	O	ASP	86	18.499	47.604	48.025	1.00	0.00	O
ATOM	1363	N	ILE	87	20.015	45.924	47.964	1.00	0.00	N
ATOM	1364	H	ILE	87	20.954	45.637	48.203	1.00	0.00	H
ATOM	1365	CA	ILE	87	19.031	44.958	47.372	1.00	0.00	C
ATOM	1366	HA	ILE	87	18.034	45.365	47.205	1.00	0.00	H
ATOM	1367	CB	ILE	87	19.714	44.410	46.050	1.00	0.00	C
ATOM	1368	HB	ILE	87	20.681	43.958	46.272	1.00	0.00	H
ATOM	1369	CG2	ILE	87	18.899	43.223	45.565	1.00	0.00	C
ATOM	1370	HG21	ILE	87	17.862	43.452	45.320	1.00	0.00	H
ATOM	1371	HG22	ILE	87	19.404	42.825	44.684	1.00	0.00	H
ATOM	1372	HG23	ILE	87	18.971	42.407	46.284	1.00	0.00	H
ATOM	1373	CG1	ILE	87	19.794	45.398	44.861	1.00	0.00	C
ATOM	1374	HG12	ILE	87	18.846	45.473	44.330	1.00	0.00	H
ATOM	1375	HG13	ILE	87	20.044	46.384	45.253	1.00	0.00	H
ATOM	1376	CD1	ILE	87	20.839	45.011	43.822	1.00	0.00	C
ATOM	1377	HD11	ILE	87	20.801	45.709	42.986	1.00	0.00	H
ATOM	1378	HD12	ILE	87	21.739	45.106	44.429	1.00	0.00	H
ATOM	1379	HD13	ILE	87	20.690	43.972	43.526	1.00	0.00	H
ATOM	1380	C	ILE	87	18.706	43.807	48.357	1.00	0.00	C
ATOM	1381	O	ILE	87	17.593	43.274	48.523	1.00	0.00	O
ATOM	1382	N	TYR	88	19.776	43.367	49.012	1.00	0.00	N
ATOM	1383	H	TYR	88	20.678	43.738	48.752	1.00	0.00	H
ATOM	1384	CA	TYR	88	19.772	42.103	49.750	1.00	0.00	C
ATOM	1385	HA	TYR	88	19.034	41.442	49.294	1.00	0.00	H
ATOM	1386	CB	TYR	88	21.043	41.381	49.515	1.00	0.00	C
ATOM	1387	HB2	TYR	88						
	21.866	42.027	49.820	1.00	0.00		H			
ATOM	1388	HB3	TYR	88	21.053	40.500	50.157	1.00	0.00	H

ATOM	1389	CG	TYR	88	21.186	40.861	48.061	1.00	0.00	C
ATOM	1390	CD1	TYR	88	20.453	39.708	47.742	1.00	0.00	C
ATOM	1391	HD1	TYR	88	19.727	39.266	48.409	1.00	0.00	H
ATOM	1392	CE1	TYR	88	20.593	39.155	46.492	1.00	0.00	C
ATOM	1393	HE1	TYR	88	19.941	38.358	46.168	1.00	0.00	H
ATOM	1394	CZ	TYR	88	21.592	39.672	45.577	1.00	0.00	C
ATOM	1395	OH	TYR	88	21.690	39.124	44.303	1.00	0.00	O
ATOM	1396	HH	TYR	88	20.825	38.847	43.987	1.00	0.00	H
ATOM	1397	CE2	TYR	88	22.310	40.791	45.902	1.00	0.00	C
ATOM	1398	HE2	TYR	88	22.918	41.286	45.160	1.00	0.00	H
ATOM	1399	CD2	TYR	88	22.089	41.444	47.115	1.00	0.00	C
ATOM	1400	HD2	TYR	88	22.589	42.359	47.400	1.00	0.00	H
ATOM	1401	C	TYR	88	19.566	42.300	51.248	1.00	0.00	C
ATOM	1402	O	TYR	88	20.293	43.046	51.888	1.00	0.00	O
ATOM	1403	N	PRO	89	18.480	41.852	51.898	1.00	0.00	N
ATOM	1404	CD	PRO	89	17.154	41.470	51.377	1.00	0.00	C
ATOM	1405	HD2	PRO	89	17.168	40.409	51.132	1.00	0.00	H
ATOM	1406	HD3	PRO	89	16.935	42.058	50.484	1.00	0.00	H
ATOM	1407	CG	PRO	89	16.150	41.786	52.536	1.00	0.00	C
ATOM	1408	HG2	PRO	89	15.301	41.102	52.560	1.00	0.00	H
ATOM	1409	HG3	PRO	89	15.845	42.830	52.596	1.00	0.00	H
ATOM	1410	CB	PRO	89	16.969	41.361	53.765	1.00	0.00	C
ATOM	1411	HB2	PRO	89	16.892	40.276	53.816	1.00	0.00	H
ATOM	1412	HB3	PRO	89	16.695	41.794	54.727	1.00	0.00	H
ATOM	1413	CA	PRO	89	18.352	41.948	53.392	1.00	0.00	C
ATOM	1414	HA	PRO	89	18.266	43.018	53.583	1.00	0.00	H
ATOM	1415	C	PRO	89	19.488	41.337	54.257	1.00	0.00	C
ATOM	1416	O	PRO	89	20.082	40.377	53.860	1.00	0.00	O
ATOM	1417	N	GLY	90	19.774	41.935	55.437	1.00	0.00	N
ATOM	1418	H	GLY	90	19.110	42.625	55.755	1.00	0.00	H
ATOM	1419	CA	GLY	90	21.082	41.780	56.035	1.00	0.00	C
ATOM	1420	HA2	GLY	90	21.875	42.291	55.489	1.00	0.00	H
ATOM	1421	HA3	GLY	90	20.772	42.060	57.042	1.00	0.00	H
ATOM	1422	C	GLY	90	21.425	40.339	56.483	1.00	0.00	C
ATOM	1423	O	GLY	90	22.635	40.110	56.655	1.00	0.00	O
ATOM	1424	N	GLN	91	20.433	39.308	56.575	1.00	0.00	N
ATOM	1425	H	GLN	91	19.482	39.603	56.406	1.00	0.00	H
ATOM	1426	CA	GLN	91	20.802	37.950	56.997	1.00	0.00	C
ATOM	1427	HA	GLN	91	21.632	38.033	57.699	1.00	0.00	H
ATOM	1428	CB	GLN	91	19.491	37.349	57.575	1.00	0.00	C
ATOM	1429	HB2	GLN	91	18.791	37.148	56.764	1.00	0.00	H
ATOM	1430	HB3	GLN	91	19.849	36.459	58.093	1.00	0.00	H
ATOM	1431	CG	GLN	91	18.772	38.256	58.516	1.00	0.00	C
ATOM	1432	HG2	GLN	91	18.267	39.078	58.010	1.00	0.00	H
ATOM	1433	HG3	GLN	91	17.882	37.671	58.746	1.00	0.00	H
ATOM	1434	CD	GLN	91	19.578	38.694	59.722	1.00	0.00	C
ATOM	1435	OE1	GLN	91	20.370	37.892	60.235	1.00	0.00	O
ATOM	1436	NE2	GLN	91	19.550	39.983	60.065	1.00	0.00	N
ATOM	1437	HE21	GLN	91	19.018	40.713	59.614	1.00	0.00	H
ATOM	1438	HE22	GLN	91	20.053	40.148	60.925	1.00	0.00	H
ATOM	1439	C	GLN	91	21.340	37.042	55.867	1.00	0.00	C
ATOM	1440	O	GLN	91	22.076	36.181	56.230	1.00	0.00	O
ATOM	1441	N	THR	92	20.918	37.300	54.611	1.00	0.00	N
ATOM	1442	H	THR	92	20.156	37.947	54.464	1.00	0.00	H
ATOM	1443	CA	THR	92	21.397	36.721	53.329	1.00	0.00	C
ATOM	1444	HA	THR	92	20.790	35.927	52.894	1.00	0.00	H
ATOM	1445	CB	THR	92	21.348	37.844	52.251	1.00	0.00	C
ATOM	1446	HB	THR	92	21.963	38.690	52.555	1.00	0.00	H
ATOM	1447	CG2	THR	92	21.696	37.368	50.883	1.00	0.00	C
ATOM	1448	HG21	THR	92	21.021	36.593	50.519	1.00	0.00	H
ATOM	1449	HG22	THR	92	21.717	38.199	50.177	1.00	0.00	H
ATOM	1450	HG23	THR	92	22.671	36.887	50.948	1.00	0.00	H
ATOM	1451	OG1	THR	92	19.982	38.317	52.209	1.00	0.00	O
ATOM	1452	HG1	THR	92	20.029	38.997	52.885	1.00	0.00	H

ATOM	1453	C	THR	92	22.851	36.232	53.439	1.00	0.00	C
ATOM	1454	O	THR	92	23.711	36.975	53.929	1.00	0.00	O
ATOM	1455	N	GLN	93	23.105	35.043	52.853	1.00	0.00	N
ATOM	1456	H	GLN	93	22.313	34.485	52.571	1.00	0.00	H
ATOM	1457	CA	GLN	93	24.431	34.476	52.858	1.00	0.00	C
ATOM	1458	HA	GLN	93	25.069	34.892	53.638	1.00	0.00	H
ATOM	1459	CB	GLN	93	24.336	32.955	53.268	1.00	0.00	C
ATOM	1460	HB2	GLN	93	23.909	32.957	54.271	1.00	0.00	H
ATOM	1461	HB3	GLN	93	23.733	32.454	52.511	1.00	0.00	H
ATOM	1462	CG	GLN	93	25.762	32.302	53.430	1.00	0.00	C
ATOM	1463	HG2	GLN	93	25.634	31.222	53.507	1.00	0.00	H
ATOM	1464	HG3	GLN	93	26.434	32.470	52.589	1.00	0.00	H
ATOM	1465	CD	GLN	93	26.581	32.709	54.629	1.00	0.00	C
ATOM	1466	OE1	GLN	93	26.232	33.497	55.474	1.00	0.00	O
ATOM	1467	NE2	GLN	93	27.687	32.022	54.789	1.00	0.00	N
ATOM	1468	HE21	GLN	93	27.847	31.354	54.047	1.00	0.00	H
ATOM	1469	HE22	GLN	93	28.344	32.278	55.512	1.00	0.00	H
ATOM	1470	C	GLN	93	25.066	34.677	51.498	1.00	0.00	C
ATOM	1471	O	GLN	93	24.511	34.259	50.503	1.00	0.00	O
ATOM	1472	N	PHE	94	26.289	35.177	51.346	1.00	0.00	N
ATOM	1473	H	PHE	94	26.811	35.382	52.186	1.00	0.00	H
ATOM	1474	CA	PHE	94	26.901	35.350	50.019	1.00	0.00	C
ATOM	1475	HA	PHE	94	26.133	35.399	49.246	1.00	0.00	H
ATOM	1476	CB	PHE	94	27.646	36.723	50.042	1.00	0.00	C
ATOM	1477	HB2	PHE	94	28.339	36.808	50.878	1.00	0.00	H
ATOM	1478	HB3	PHE	94	28.224	36.910	49.136	1.00	0.00	H
ATOM	1479	CG	PHE	94	26.672	37.761	50.287	1.00	0.00	C
ATOM	1480	CD1	PHE	94	25.926	38.225	49.180	1.00	0.00	C
ATOM	1481	HD1	PHE	94	26.158	37.979	48.154	1.00	0.00	H
ATOM	1482	CE1	PHE	94	24.824	39.110	49.337	1.00	0.00	C
ATOM	1483	HE1	PHE	94	24.246	39.520	48.522	1.00	0.00	H
ATOM	1484	CZ	PHE	94	24.356	39.409	50.639	1.00	0.00	C
ATOM	1485	HZ	PHE	94	23.610	40.184	50.738	1.00	0.00	H
ATOM	1486	CE2	PHE	94	25.103	39.009	51.766	1.00	0.00	C
ATOM	1487	HE2	PHE	94	24.783	39.300	52.756	1.00	0.00	H
ATOM	1488	CD2	PHE	94	26.272	38.197	51.602	1.00	0.00	C
ATOM	1489	HD2	PHE	94	26.799	37.914	52.502	1.00	0.00	H
ATOM	1490	C	PHE	94	27.909	34.234	49.733	1.00	0.00	C
ATOM	1491	O	PHE	94	28.306	33.431	50.583	1.00	0.00	O
ATOM	1492	N	TYR	95	28.184	34.108	48.430	1.00	0.00	N
ATOM	1493	H	TYR	95	27.866	34.742	47.711	1.00	0.00	H
ATOM	1494	CA	TYR	95	29.158	33.153	47.964	1.00	0.00	C
ATOM	1495	HA	TYR	95	29.583	32.688	48.853	1.00	0.00	H
ATOM	1496	CB	TYR	95	28.427	32.035	47.160	1.00	0.00	C
ATOM	1497	HB2	TYR	95	27.543	31.775	47.740	1.00	0.00	H
ATOM	1498	HB3	TYR	95	28.097	32.617	46.300	1.00	0.00	H
ATOM	1499	CG	TYR	95	29.169	30.770	46.803	1.00	0.00	C
ATOM	1500	CD1	TYR	95	29.575	30.467	45.511	1.00	0.00	C
ATOM	1501	HD1	TYR	95	29.283	31.210	44.783	1.00	0.00	H
ATOM	1502	CE1	TYR	95	30.389	29.356	45.292	1.00	0.00	C
ATOM	1503	HE1	TYR	95	30.882	29.118	44.363	1.00	0.00	H
ATOM	1504	CZ	TYR	95	30.717	28.477	46.369	1.00	0.00	C
ATOM	1505	OH	TYR	95	31.442	27.352	46.070	1.00	0.00	O
ATOM	1506	HH	TYR	95	31.605	27.292	45.126	1.00	0.00	H
ATOM	1507	CE2	TYR	95	30.352	28.805	47.707	1.00	0.00	C
ATOM	1508	HE2	TYR	95	30.675	28.222	48.556	1.00	0.00	H
ATOM	1509	CD2	TYR	95	29.525	29.971	47.897	1.00	0.00	C
ATOM	1510	HD2	TYR	95	29.105	30.267	48.846	1.00	0.00	H
ATOM	1511	C	TYR	95	30.180	33.944	47.161	1.00	0.00	C
ATOM	1512	O	TYR	95	29.811	34.753	46.335			
1.00	0.00	O								
ATOM	1513	N	VAL	96	31.442	33.845	47.470	1.00	0.00	N
ATOM	1514	H	VAL	96	31.784	33.142	48.108	1.00	0.00	H
ATOM	1515	CA	VAL	96	32.497	34.565	46.790	1.00	0.00	C

ATOM	1516	HA	VAL	96	32.011	35.267	46.112	1.00	0.00	H
ATOM	1517	CB	VAL	96	33.242	35.506	47.764	1.00	0.00	C
ATOM	1518	HB	VAL	96	32.569	36.211	48.254	1.00	0.00	H
ATOM	1519	CG1	VAL	96	33.804	34.855	49.030	1.00	0.00	C
ATOM	1520	HG11	VAL	96	34.326	35.653	49.558	1.00	0.00	H
ATOM	1521	HG12	VAL	96	33.011	34.418	49.637	1.00	0.00	H
ATOM	1522	HG13	VAL	96	34.634	34.179	48.819	1.00	0.00	H
ATOM	1523	CG2	VAL	96	34.348	36.297	47.040	1.00	0.00	C
ATOM	1524	HG21	VAL	96	33.879	36.862	46.234	1.00	0.00	H
ATOM	1525	HG22	VAL	96	34.764	37.030	47.732	1.00	0.00	H
ATOM	1526	HG23	VAL	96	35.156	35.654	46.691	1.00	0.00	H
ATOM	1527	C	VAL	96	33.337	33.606	45.994	1.00	0.00	C
ATOM	1528	O	VAL	96	33.719	32.543	46.488	1.00	0.00	O
ATOM	1529	N	ILE	97	33.518	33.875	44.741	1.00	0.00	N
ATOM	1530	H	ILE	97	33.503	34.837	44.430	1.00	0.00	H
ATOM	1531	CA	ILE	97	34.077	32.936	43.724	1.00	0.00	C
ATOM	1532	HA	ILE	97	34.489	31.996	44.093	1.00	0.00	H
ATOM	1533	CB	ILE	97	33.094	32.629	42.545	1.00	0.00	C
ATOM	1534	HB	ILE	97	32.841	33.546	42.012	1.00	0.00	H
ATOM	1535	CG2	ILE	97	33.495	31.601	41.453	1.00	0.00	C
ATOM	1536	HG21	ILE	97	33.679	30.613	41.873	1.00	0.00	H
ATOM	1537	HG22	ILE	97	32.686	31.518	40.728	1.00	0.00	H
ATOM	1538	HG23	ILE	97	34.438	31.897	40.992	1.00	0.00	H
ATOM	1539	CG1	ILE	97	31.630	32.262	43.074	1.00	0.00	C
ATOM	1540	HG12	ILE	97	31.288	31.392	42.512	1.00	0.00	H
ATOM	1541	HG13	ILE	97	31.779	31.954	44.109	1.00	0.00	H
ATOM	1542	CD1	ILE	97	30.582	33.335	42.968	1.00	0.00	C
ATOM	1543	HD11	ILE	97	29.694	32.886	43.412	1.00	0.00	H
ATOM	1544	HD12	ILE	97	30.885	34.257	43.464	1.00	0.00	H
ATOM	1545	HD13	ILE	97	30.377	33.431	41.902	1.00	0.00	H
ATOM	1546	C	ILE	97	35.316	33.640	43.128	1.00	0.00	C
ATOM	1547	O	ILE	97	35.284	34.879	42.901	1.00	0.00	O
ATOM	1548	N	GLU	98	36.390	32.969	42.963	1.00	0.00	N
ATOM	1549	H	GLU	98	36.310	31.966	43.036	1.00	0.00	H
ATOM	1550	CA	GLU	98	37.662	33.585	42.460	1.00	0.00	C
ATOM	1551	HA	GLU	98	37.474	34.508	41.911	1.00	0.00	H
ATOM	1552	CB	GLU	98	38.647	33.907	43.633	1.00	0.00	C
ATOM	1553	HB2	GLU	98	38.267	34.686	44.294	1.00	0.00	H
ATOM	1554	HB3	GLU	98	38.700	32.986	44.214	1.00	0.00	H
ATOM	1555	CG	GLU	98	40.058	34.431	43.201	1.00	0.00	C
ATOM	1556	HG2	GLU	98	40.394	33.606	42.572	1.00	0.00	H
ATOM	1557	HG3	GLU	98	39.902	35.304	42.568	1.00	0.00	H
ATOM	1558	CD	GLU	98	41.148	34.749	44.331	1.00	0.00	C
ATOM	1559	OE1	GLU	98	40.826	35.310	45.396	1.00	0.00	O
ATOM	1560	OE2	GLU	98	42.345	34.539	44.042	1.00	0.00	O
ATOM	1561	C	GLU	98	38.291	32.570	41.504	1.00	0.00	C
ATOM	1562	O	GLU	98	38.115	31.347	41.567	1.00	0.00	O
ATOM	1563	N	PHE	99	38.861	33.029	40.447	1.00	0.00	N
ATOM	1564	H	PHE	99	39.257	33.958	40.449	1.00	0.00	H
ATOM	1565	CA	PHE	99	39.126	32.262	39.226	1.00	0.00	C
ATOM	1566	HA	PHE	99	39.517	31.278	39.483	1.00	0.00	H
ATOM	1567	CB	PHE	99	37.887	32.058	38.400	1.00	0.00	C
ATOM	1568	HB2	PHE	99	38.150	31.321	37.641	1.00	0.00	H
ATOM	1569	HB3	PHE	99	37.125	31.556	38.995	1.00	0.00	H
ATOM	1570	CG	PHE	99	37.254	33.382	37.992	1.00	0.00	C
ATOM	1571	CD1	PHE	99	36.254	33.947	38.830	1.00	0.00	C
ATOM	1572	HD1	PHE	99	35.812	33.318	39.589	1.00	0.00	H
ATOM	1573	CE1	PHE	99	35.792	35.281	38.590	1.00	0.00	C
ATOM	1574	HE1	PHE	99	35.060	35.782	39.207	1.00	0.00	H
ATOM	1575	CZ	PHE	99	36.201	35.985	37.420	1.00	0.00	C
ATOM	1576	HZ	PHE	99	35.723	36.916	37.152	1.00	0.00	H
ATOM	1577	CE2	PHE	99	37.189	35.392	36.597	1.00	0.00	C
ATOM	1578	HE2	PHE	99	37.570	35.840	35.692	1.00	0.00	H
ATOM	1579	CD2	PHE	99	37.687	34.143	36.868	1.00	0.00	C

ATOM	1580	HD2	PHE	99	38.422	33.692	36.217	1.00	0.00	H
ATOM	1581	C	PHE	99	40.227	32.926	38.442	1.00	0.00	C
ATOM	1582	O	PHE	99	40.353	34.165	38.477	1.00	0.00	O
ATOM	1583	N	LYS	100	40.959	32.151	37.606	1.00	0.00	N
ATOM	1584	H	LYS	100	40.766	31.174	37.434	1.00	0.00	H
ATOM	1585	CA	LYS	100	41.815	32.819	36.621	1.00	0.00	C
ATOM	1586	HA	LYS	100	41.197	33.677	36.358	1.00	0.00	H
ATOM	1587	CB	LYS	100	43.129	33.287	37.355	1.00	0.00	C
ATOM	1588	HB2	LYS	100	43.621	33.939	36.634	1.00	0.00	H
ATOM	1589	HB3	LYS	100	42.813	33.943	38.166	1.00	0.00	H
ATOM	1590	CG	LYS	100	44.209	32.382	37.794	1.00	0.00	C
ATOM	1591	HG2	LYS	100	43.852	31.567	38.424	1.00	0.00	H
ATOM	1592	HG3	LYS	100	44.719	32.037	36.895	1.00	0.00	H
ATOM	1593	CD	LYS	100	45.278	33.214	38.670	1.00	0.00	C
ATOM	1594	HD2	LYS	100	45.285	34.268	38.390	1.00	0.00	H
ATOM	1595	HD3	LYS	100	44.972	33.179	39.716	1.00	0.00	H
ATOM	1596	CE	LYS	100	46.667	32.619	38.595	1.00	0.00	C
ATOM	1597	HE2	LYS	100	46.862	32.146	39.558	1.00	0.00	H
ATOM	1598	HE3	LYS	100	46.671	31.862	37.810	1.00	0.00	H
ATOM	1599	NZ	LYS	100	47.714	33.543	38.201	1.00	0.00	N
ATOM	1600	HZ1	LYS	100	47.544	33.843	37.251	1.00	0.00	H
ATOM	1601	HZ2	LYS	100	47.916	34.277	38.865	1.00	0.00	H
ATOM	1602	HZ3	LYS	100	48.572	33.012	38.223	1.00	0.00	H
ATOM	1603	C	LYS	100	42.096	32.006	35.389	1.00	0.00	C
ATOM	1604	O	LYS	100	41.717	30.829	35.376	1.00	0.00	O
ATOM	1605	N	CYX	101	42.633	32.509	34.276	1.00	0.00	N
ATOM	1606	H	CYX	101	42.834	33.498	34.288	1.00	0.00	H
ATOM	1607	CA	CYX	101	42.768	31.756	33.036	1.00	0.00	C
ATOM	1608	HA	CYX	101	41.936	31.054	32.978	1.00	0.00	H
ATOM	1609	CB	CYX	101	42.646	32.770	31.878	1.00	0.00	C
ATOM	1610	HB2	CYX	101	41.626	33.156	31.879	1.00	0.00	H
ATOM	1611	HB3	CYX	101	43.357	33.586	32.009	1.00	0.00	H
ATOM	1612	SG	CYX	101	42.786	32.033	30.186	1.00	0.00	S
ATOM	1613	C	CYX	101	44.101	30.896	33.015	1.00	0.00	C
ATOM	1614	O	CYX	101	45.096	31.340	33.525	1.00	0.00	O
ATOM	1615	N	LEU	102	44.057	29.705	32.464	1.00	0.00	N
ATOM	1616	H	LEU	102	43.191	29.530	31.975	1.00	0.00	H
ATOM	1617	CA	LEU	102	45.233	28.810	32.318	1.00	0.00	C
ATOM	1618	HA	LEU	102	45.909	29.036	33.143	1.00	0.00	H
ATOM	1619	CB	LEU	102	44.636	27.398	32.539	1.00	0.00	C
ATOM	1620	HB2	LEU	102	43.987	27.112	31.712	1.00	0.00	H
ATOM	1621	HB3	LEU	102	45.572	26.850	32.426	1.00	0.00	H
ATOM	1622	CG	LEU	102	43.977	27.106	33.843	1.00	0.00	C
ATOM	1623	HG	LEU	102	43.040	27.664	33.815	1.00	0.00	H
ATOM	1624	CD1	LEU	102	43.477	25.702	33.787	1.00	0.00	C
ATOM	1625	HD11	LEU	102	42.756	25.493	34.577	1.00	0.00	H
ATOM	1626	HD12	LEU	102	42.916	25.525	32.870	1.00	0.00	H
ATOM	1627	HD13	LEU	102	44.301	24.990	33.826	1.00	0.00	H
ATOM	1628	CD2	LEU	102	44.859	27.474	35.095	1.00	0.00	C
ATOM	1629	HD21	LEU	102	44.431	27.113	36.030	1.00	0.00	H
ATOM	1630	HD22	LEU	102	45.743	26.903	34.811	1.00	0.00	H
ATOM	1631	HD23	LEU	102	44.882	28.563	35.078	1.00	0.00	H
ATOM	1632	C	LEU	102	45.952	29.001	30.959	1.00	0.00	C
ATOM	1633	O	LEU	102	47.035	29.636	30.975	1.00	0.00	O
ATOM	1634	OXT	LEU	102	45.407	28.663	29.895	1.00	0.00	O
HETATM	314	N	LYN	20	39.505	41.033	35.268	1.00	0.00	N
HETATM	315	H	LYN	20	39.805	40.507	34.460	1.00	0.00	H
HETATM	316	CA	LYN	20	38.512	40.280	36.003	1.00	0.00	C
HETATM	317	HA	LYN	20	38.345	40.925	36.866	1.00	0.00	H
HETATM	318	CB	LYN	20	37.273	40.201	35.108	1.00	0.00	C
HETATM	319	HB2	LYN	20	37.480	39.627	34.205	1.00	0.00	H
HETATM	320	HB3	LYN	20	37.002	41.168	34.688	1.00	0.00	H
HETATM	321	CG	LYN	20	36.085	39.559	35.801	1.00	0.00	C

```
HETATM  322  HG2 LYN    20      35.260  39.679  35.098  1.00  0.00           H
HETATM  323  HG3 LYN    20      36.364  38.517  35.960  1.00  0.00           H
HETATM  324  CD  LYN    20      35.679  40.048  37.166  1.00  0.00           C
HETATM  325  HD2 LYN    20      35.630  41.135  37.095  1.00  0.00           H
HETATM  326  HD3 LYN    20      36.336  39.676  37.952  1.00  0.00           H
HETATM  327  CE  LYN    20      34.231  39.509  37.544  1.00  0.00           C
HETATM  328  HE2 LYN    20      33.603  39.731  36.681  1.00  0.00           H
HETATM  329  HE3 LYN    20      34.302  38.444  37.763  1.00  0.00           H
HETATM  330  NZ  LYN    20      33.622  40.176  38.602  1.00  0.00           N
HETATM  331  HZ2 LYN    20      33.463  41.141  38.350  1.00  0.00           H
HETATM  332  HZ3 LYN    20      34.193  40.101  39.432  1.00  0.00           H
HETATM  333  C   LYN    20      39.026  38.949  36.497  1.00  0.00           C
HETATM  334  O   LYN    20      39.634  38.250  35.713  1.00  0.00           O
HETATM 1636  N   LIG   103      30.163  42.360  39.436  1.00  0.00           N
HETATM 1637  C   LIG   103      29.698  41.337  38.774  1.00  0.00           C
HETATM 1638  O   LIG   103      30.235  43.615  41.327  1.00  0.00           O
HETATM 1639  C5' LIG   103      25.928  39.761  43.980  1.00  0.00           C
HETATM 1640  O5' LIG   103      25.455  39.414  42.633  1.00  0.00           O
HETATM 1641  C4' LIG   103      26.759  41.123  43.953  1.00  0.00           C
HETATM 1642  O4' LIG   103      28.014  41.041  43.351  1.00  0.00           O
HETATM 1643  C3' LIG   103      25.998  42.275  43.250  1.00  0.00           C
HETATM 1644  O3' LIG   103      25.088  43.033  44.113  1.00  0.00           O
HETATM 1645  C2' LIG   103      27.127  43.201  42.696  1.00  0.00           C
HETATM 1646  O2' LIG   103      27.305  44.248  43.667  1.00  0.00           O
HETATM 1647  C1' LIG   103      28.326  42.258  42.626  1.00  0.00           C
HETATM 1648  N1  LIG   103      28.742  41.895  41.294  1.00  0.00           N
HETATM 1649  O1  LIG   103      30.175  38.740  37.575  1.00  0.00           O
HETATM 1650  N2  LIG   103      30.307  41.025  37.620  1.00  0.00           N
HETATM 1651  C6  LIG   103      29.768  42.664  40.750  1.00  0.00           C
HETATM 1652  C7  LIG   103      28.112  40.924  40.582  1.00  0.00           C
HETATM 1653  C8  LIG   103      28.586  40.595  39.317  1.00  0.00           C
HETATM 1654  C9  LIG   103      30.457  39.799  37.090  1.00  0.00           C
HETATM 1655  C10 LIG   103      31.285  39.884  35.830  1.00  0.00           C
HETATM 1656  H   LIG   103      30.662  41.740  36.996  1.00  0.00           H
HETATM 1657  H1  LIG   103      28.131  39.794  38.747  1.00  0.00           H
HETATM 1658  H4  LIG   103      29.123  42.846  43.094  1.00  0.00           H
HETATM 1659  H6  LIG   103      26.928  41.380  45.006  1.00  0.00           H
HETATM 1660  H7  LIG   103      25.396  41.926  42.402  1.00  0.00           H
HETATM 1661  H8  LIG   103      26.927  43.581  41.687  1.00  0.00           H
HETATM 1662  H9  LIG   103      25.029  39.887  44.597  1.00  0.00           H
HETATM 1663  H10 LIG   103      26.445  38.940  44.494  1.00  0.00           H
HETATM 1664  H11 LIG   103      30.633  40.056  34.966  1.00  0.00           H
HETATM 1665  H12 LIG   103      31.803  38.935  35.644  1.00  0.00           H
HETATM 1666  H13 LIG   103      32.114  40.601  35.888  1.00  0.00           H
HETATM 1667  H14 LIG   103      27.270  40.428  41.046  1.00  0.00           H
HETATM 1668  H2' LIG   103      27.874  44.882  43.197  1.00  0.00           H
HETATM 1669  H3' LIG   103      25.581  43.869  44.054  1.00  0.00           H
HETATM 1670  H5' LIG   103      24.881  38.644  42.784  1.00  0.00           H
ENDMDL
MODEL     16
SHEET    1   1 1 ILE    22  ASP    26  0
SHEET    2   2 1 VAL    37  VAL    40  0
SHEET    3   3 1 CYX    50  THR    61  0
SHEET    4   4 1 GLN    93  CYX   101  0
HELIX    1   1 GLN     12  ALA     17  1                                   6
HELIX    2   2 LEU     77  ILE     87  1                                  11
ATOM      1  N   GLN     1      35.456  17.461  33.535  1.00  0.00           N
ATOM      2  H1  GLN     1      34.564  17.638  33.973  1.00  0.00           H
ATOM      3  H2  GLN     1      35.674  16.475  33.516  1.00  0.00           H
ATOM      4  H3  GLN     1      36.134  17.966  34.088  1.00  0.00           H
ATOM      5  CA  GLN     1      35.265  18.095  32.248  1.00  0.00           C
ATOM      6  HA  GLN     1      36.083  18.003  31.534  1.00  0.00           H
ATOM      7  CB  GLN     1      33.986  17.471  31.646  1.00  0.00           C
ATOM      8  HB2 GLN     1      33.189  17.364  32.382  1.00  0.00           H
```

```
ATOM      9  HB3 GLN     1      33.590  18.159  30.899  1.00  0.00           H
ATOM     10  CG  GLN     1      34.119  16.104  30.978  1.00  0.00           C
ATOM     11  HG2 GLN     1      33.238  15.945  30.356  1.00  0.00           H
ATOM     12  HG3 GLN     1      35.030  16.129  30.381  1.00  0.00           H
ATOM     13  CD  GLN     1      34.356  14.951  31.936  1.00  0.00           C
ATOM     14  OE1 GLN     1      35.410  14.836  32.565  1.00  0.00           O
ATOM     15  NE2 GLN     1      33.367  14.072  32.048  1.00  0.00           N
ATOM     16 HE21 GLN     1      32.443  14.215  31.667  1.00  0.00           H
ATOM     17 HE22 GLN     1      33.548  13.277  32.643  1.00  0.00           H
ATOM     18  C   GLN     1      35.157  19.599  32.467  1.00  0.00           C
ATOM     19  O   GLN     1      34.870  20.038  33.587  1.00  0.00           O
ATOM     20  N   PRO     2      35.356  20.352  31.368  1.00  0.00           N
ATOM     21  CD  PRO     2      35.694  19.895  29.995  1.00  0.00           C
ATOM     22  HD2 PRO     2      35.084  19.089  29.588  1.00  0.00           H
ATOM     23  HD3 PRO     2      36.749  19.626  29.937  1.00  0.00           H
ATOM     24  CG  PRO     2      35.720  21.164  29.093  1.00  0.00           C
ATOM     25  HG2 PRO     2      34.703  21.370  28.757  1.00  0.00           H
ATOM     26  HG3 PRO     2      36.457  21.059  28.298  1.00  0.00           H
ATOM     27  CB  PRO     2      35.963  22.361  30.126  1.00  0.00           C
ATOM     28  HB2 PRO     2      35.558  23.313  29.781  1.00  0.00           H
ATOM     29  HB3 PRO     2      37.022  22.413  30.376  1.00  0.00           H
ATOM     30  CA  PRO     2      35.188  21.791  31.348  1.00  0.00           C
ATOM     31  HA  PRO     2      35.690  22.230  32.210  1.00  0.00           H
ATOM     32  C   PRO     2      33.691  22.023  31.334  1.00  0.00           C
ATOM     33  O   PRO     2      32.953  21.425  30.540  1.00  0.00           O
ATOM     34  N   ASN     3      33.165  22.996  32.127  1.00  0.00           N
ATOM     35  H   ASN     3      33.719  23.444  32.842  1.00  0.00           H
ATOM     36  CA  ASN     3      31.734  23.253  32.209  1.00  0.00           C
ATOM     37  HA  ASN     3      31.159  22.327  32.193  1.00  0.00           H
ATOM     38  CB  ASN     3      31.381  23.911  33.572  1.00  0.00           C
ATOM     39  HB2 ASN     3      31.854  24.879  33.731  1.00  0.00           H
ATOM     40  HB3 ASN     3      30.328  24.192  33.546  1.00  0.00           H
ATOM     41  CG  ASN     3      31.611  23.202  34.907  1.00  0.00           C
ATOM     42  OD1 ASN     3      30.780  22.541  35.422  1.00  0.00           O
ATOM     43  ND2 ASN     3      32.844  23.267  35.408  1.00  0.00           N
ATOM     44 HD21 ASN     3      33.592  23.688  34.877  1.00  0.00           H
ATOM     45 HD22 ASN     3      33.087  22.652  36.172  1.00  0.00           H
ATOM     46  C   ASN     3      31.281  24.187  31.035  1.00  0.00           C
ATOM     47  O   ASN     3      32.095  24.995  30.616  1.00  0.00           O
ATOM     48  N   ASP     4      30.108  24.034  30.402  1.00  0.00           N
ATOM     49  H   ASP     4      29.576  23.189  30.560  1.00  0.00           H
ATOM     50  CA  ASP     4      29.616  24.950  29.378  1.00  0.00           C
ATOM     51  HA  ASP     4      30.322  24.986  28.549  1.00  0.00           H
ATOM     52  CB  ASP     4      28.408  24.268  28.753  1.00  0.00           C
ATOM     53  HB2 ASP     4      28.660  23.212  28.665  1.00  0.00           H
ATOM     54  HB3 ASP     4      27.547  24.239  29.421  1.00  0.00           H
ATOM     55  CG  ASP     4      27.983  24.864  27.415  1.00  0.00           C
ATOM     56  OD1 ASP     4      28.875  25.059  26.547  1.00  0.00           O
ATOM     57  OD2 ASP     4      26.810  25.212  27.154  1.00  0.00           O
ATOM     58  C   ASP     4      29.192  26.354  29.847  1.00  0.00           C
ATOM     59  O   ASP     4      29.468  27.304  29.125  1.00  0.00           O
ATOM     60  N   ILE     5      28.745  26.598  31.130  1.00  0.00           N
ATOM     61  H   ILE     5      28.523  25.765  31.659  1.00  0.00           H
ATOM     62  CA  ILE     5      28.387  27.858  31.678  1.00  0.00           C
ATOM     63  HA  ILE     5      27.658  28.317  31.010  1.00  0.00           H
ATOM     64  CB  ILE     5      27.704  27.587  33.050  1.00  0.00           C
ATOM     65  HB  ILE     5      28.281  26.854  33.613  1.00  0.00           H
ATOM     66  CG2 ILE     5      27.337  28.851  33.777  1.00  0.00           C
ATOM     67 HG21 ILE     5      26.702  28.718  34.652  1.00  0.00           H
ATOM     68 HG22 ILE     5      28.311  29.157  34.160  1.00  0.00           H
ATOM     69
 HG23 ILE     5      26.957  29.620  33.106  1.00  0.00           H
ATOM     70  CG1 ILE     5      26.397  26.886  32.880  1.00  0.00           C
ATOM     71 HG12 ILE     5      26.602  25.977  32.314  1.00  0.00           H
```

```
ATOM     72 HG13 ILE     5       26.156  26.569  33.894  1.00  0.00           H
ATOM     73  CD1 ILE     5       25.207  27.575  32.262  1.00  0.00           C
ATOM     74 HD11 ILE     5       24.413  27.654  33.005  1.00  0.00           H
ATOM     75 HD12 ILE     5       25.550  28.549  31.914  1.00  0.00           H
ATOM     76 HD13 ILE     5       24.849  27.026  31.391  1.00  0.00           H
ATOM     77  C   ILE     5       29.619  28.773  31.705  1.00  0.00           C
ATOM     78  O   ILE     5       30.793  28.294  31.880  1.00  0.00           O
ATOM     79  N   THR     6       29.389  30.096  31.631  1.00  0.00           N
ATOM     80  H   THR     6       28.409  30.309  31.516  1.00  0.00           H
ATOM     81  CA  THR     6       30.392  31.101  31.358  1.00  0.00           C
ATOM     82  HA  THR     6       31.193  30.754  32.010  1.00  0.00           H
ATOM     83  CB  THR     6       30.805  30.931  29.899  1.00  0.00           C
ATOM     84  HB  THR     6       30.994  29.859  29.847  1.00  0.00           H
ATOM     85  CG2 THR     6       29.751  31.214  28.864  1.00  0.00           C
ATOM     86 HG21 THR     6       29.802  32.294  28.730  1.00  0.00           H
ATOM     87 HG22 THR     6       29.961  30.793  27.881  1.00  0.00           H
ATOM     88 HG23 THR     6       28.759  30.874  29.162  1.00  0.00           H
ATOM     89  OG1 THR     6       31.978  31.677  29.601  1.00  0.00           O
ATOM     90  HG1 THR     6       32.704  31.050  29.561  1.00  0.00           H
ATOM     91  C   THR     6       29.943  32.512  31.701  1.00  0.00           C
ATOM     92  O   THR     6       28.762  32.832  31.846  1.00  0.00           O
ATOM     93  N   PHE     7       30.908  33.497  31.779  1.00  0.00           N
ATOM     94  H   PHE     7       31.860  33.165  31.833  1.00  0.00           H
ATOM     95  CA  PHE     7       30.554  34.968  31.971  1.00  0.00           C
ATOM     96  HA  PHE     7       29.730  35.038  32.681  1.00  0.00           H
ATOM     97  CB  PHE     7       31.734  35.706  32.668  1.00  0.00           C
ATOM     98  HB2 PHE     7       32.580  35.416  32.045  1.00  0.00           H
ATOM     99  HB3 PHE     7       31.549  36.768  32.513  1.00  0.00           H
ATOM    100  CG  PHE     7       32.121  35.439  34.120  1.00  0.00           C
ATOM    101  CD1 PHE     7       32.661  34.172  34.481  1.00  0.00           C
ATOM    102  HD1 PHE     7       32.690  33.334  33.800  1.00  0.00           H
ATOM    103  CE1 PHE     7       33.226  33.944  35.778  1.00  0.00           C
ATOM    104  HE1 PHE     7       33.692  33.008  36.048  1.00  0.00           H
ATOM    105  CZ  PHE     7       33.091  34.948  36.771  1.00  0.00           C
ATOM    106  HZ  PHE     7       33.513  34.835  37.759  1.00  0.00           H
ATOM    107  CE2 PHE     7       32.429  36.158  36.452  1.00  0.00           C
ATOM    108  HE2 PHE     7       32.276  36.915  37.207  1.00  0.00           H
ATOM    109  CD2 PHE     7       31.954  36.420  35.118  1.00  0.00           C
ATOM    110  HD2 PHE     7       31.488  37.343  34.803  1.00  0.00           H
ATOM    111  C   PHE     7       30.132  35.583  30.631  1.00  0.00           C
ATOM    112  O   PHE     7       30.456  34.975  29.592  1.00  0.00           O
ATOM    113  N   PHE     8       29.324  36.676  30.629  1.00  0.00           N
ATOM    114  H   PHE     8       28.946  36.922  31.532  1.00  0.00           H
ATOM    115  CA  PHE     8       28.699  37.184  29.428  1.00  0.00           C
ATOM    116  HA  PHE     8       28.357  36.273  28.935  1.00  0.00           H
ATOM    117  CB  PHE     8       27.532  38.083  29.761  1.00  0.00           C
ATOM    118  HB2 PHE     8       27.914  39.035  30.130  1.00  0.00           H
ATOM    119  HB3 PHE     8       27.022  38.178  28.803  1.00  0.00           H
ATOM    120  CG  PHE     8       26.558  37.568  30.754  1.00  0.00           C
ATOM    121  CD1 PHE     8       25.819  36.351  30.492  1.00  0.00           C
ATOM    122  HD1 PHE     8       26.106  35.941  29.535  1.00  0.00           H
ATOM    123  CE1 PHE     8       24.707  35.985  31.202  1.00  0.00           C
ATOM    124  HE1 PHE     8       24.109  35.154  30.857  1.00  0.00           H
ATOM    125  CZ  PHE     8       24.471  36.692  32.442  1.00  0.00           C
ATOM    126  HZ  PHE     8       23.663  36.382  33.089  1.00  0.00           H
ATOM    127  CE2 PHE     8       25.202  37.799  32.793  1.00  0.00           C
ATOM    128  HE2 PHE     8       24.948  38.267  33.733  1.00  0.00           H
ATOM    129  CD2 PHE     8       26.270  38.251  31.970  1.00  0.00           C
ATOM    130  HD2 PHE     8       26.808  39.151  32.228  1.00  0.00           H
ATOM    131  C   PHE     8       29.720  37.822  28.399  1.00  0.00           C
ATOM    132  O   PHE     8       30.833  38.157  28.768  1.00  0.00           O
ATOM    133  N   GLN     9       29.338  38.038  27.179  1.00  0.00           N
ATOM    134  H   GLN     9       28.367  37.868  26.959  1.00  0.00           H
ATOM    135  CA  GLN     9       30.196  38.616  26.057  1.00  0.00           C
```

ATOM	136	HA	GLN	9	31.010	37.904	25.915	1.00	0.00	H
ATOM	137	CB	GLN	9	29.230	38.764	24.860	1.00	0.00	C
ATOM	138	HB2	GLN	9	28.792	37.780	24.690	1.00	0.00	H
ATOM	139	HB3	GLN	9	28.442	39.502	25.008	1.00	0.00	H
ATOM	140	CG	GLN	9	29.968	39.093	23.591	1.00	0.00	C
ATOM	141	HG2	GLN	9	30.138	40.169	23.545	1.00	0.00	H
ATOM	142	HG3	GLN	9	30.965	38.659	23.523	1.00	0.00	H
ATOM	143	CD	GLN	9	29.265	38.758	22.251	1.00	0.00	C
ATOM	144	OE1	GLN	9	28.952	39.642	21.417	1.00	0.00	O
ATOM	145	NE2	GLN	9	28.967	37.548	22.044	1.00	0.00	N
ATOM	146	HE21	GLN	9	28.443	37.359	21.202	1.00	0.00	H
ATOM	147	HE22	GLN	9	28.992	36.910	22.827	1.00	0.00	H
ATOM	148	C	GLN	9	30.857	39.949	26.355	1.00	0.00	C
ATOM	149	O	GLN	9	31.813	40.238	25.646	1.00	0.00	O
ATOM	150	N	ARG	10	30.286	40.806	27.191	1.00	0.00	N
ATOM	151	H	ARG	10	29.425	40.491	27.614	1.00	0.00	H
ATOM	152	CA	ARG	10	30.823	42.126	27.645	1.00	0.00	C
ATOM	153	HA	ARG	10	30.952	42.729	26.747	1.00	0.00	H
ATOM	154	CB	ARG	10	29.752	42.748	28.652	1.00	0.00	C
ATOM	155	HB2	ARG	10	28.815	42.904	28.119	1.00	0.00	H
ATOM	156	HB3	ARG	10	29.594	41.965	29.394	1.00	0.00	H
ATOM	157	CG	ARG	10	30.083	44.186	29.214	1.00	0.00	C
ATOM	158	HG2	ARG	10	29.411	44.439	30.034	1.00	0.00	H
ATOM	159	HG3	ARG	10	31.092	44.160	29.625	1.00	0.00	H
ATOM	160	CD	ARG	10	29.928	45.392	28.153	1.00	0.00	C
ATOM	161	HD2	ARG	10	28.966	45.294	27.650	1.00	0.00	H
ATOM	162	HD3	ARG	10	29.848	46.333	28.697	1.00	0.00	H
ATOM	163	NE	ARG	10	30.970	45.427	27.078	1.00	0.00	N
ATOM	164	HE	ARG	10	30.814	44.838	26.273	1.00	0.00	H
ATOM	165	CZ	ARG	10	32.152	46.057	27.105	1.00	0.00	C
ATOM	166	NH1	ARG	10	32.421	47.013	27.882	1.00	0.00	N
ATOM	167	HH11	ARG	10	33.234	47.567	27.653	1.00	0.00	H
ATOM	168	HH12	ARG	10	31.617	47.431	28.325	1.00	0.00	H
ATOM	169	NH2	ARG	10	33.135	45.791	26.289	1.00	0.00	N
ATOM	170	HH21	ARG	10	33.080	45.040	25.615	1.00	0.00	H
ATOM	171	HH22	ARG	10	33.954	46.379	26.342	1.00	0.00	H
ATOM	172	C	ARG	10	32.219	42.045	28.351	1.00	0.00	C
ATOM	173	O	ARG	10	32.906	43.038	28.385	1.00	0.00	O
ATOM	174	N	PHE	11	32.461	40.942	29.034	1.00	0.00	N
ATOM	175	H	PHE	11	31.848	40.140	29.074	1.00	0.00	H
ATOM	176	CA	PHE	11	33.651	40.785	29.916	1.00	0.00	C
ATOM	177	HA	PHE	11	34.216	41.658	30.240	1.00	0.00	H
ATOM	178	CB	PHE	11	33.000	40.104	31.220	1.00	0.00	C
ATOM	179	HB2	PHE	11	33.853	40.094	31.899	1.00	0.00	H
ATOM	180	HB3	PHE	11	32.750	39.069	30.986	1.00	0.00	H
ATOM	181	CG	PHE	11	31.823	40.809	31.872	1.00	0.00	C
ATOM	182	CD1	PHE	11	31.733	42.219	31.904	1.00	0.00	C
ATOM	183	HD1	PHE	11	32.477	42.885	31.491	1.00	0.00	H
ATOM	184	CE1	PHE	11	30.678	42.871	32.442	1.00	0.00	C
ATOM	185	HE1	PHE	11	30.704	43.950	32.420	1.00	0.00	H
ATOM	186	CZ	PHE	11	29.679	42.157	33.072	1.00	0.00	C
ATOM	187	HZ	PHE	11	28.878	42.698	33.554	1.00	0.00	H
ATOM	188	CE2	PHE	11	29.690	40.740	32.965	1.00	0.00	C
ATOM	189	HE2	PHE	11	28.837	40.180	33.318	1.00	0.00	H
ATOM	190	CD2	PHE	11	30.798	40.038	32.465	1.00	0.00	C
ATOM	191	HD2	PHE	11	30.723	38.967	32.346	1.00	0.00	H
ATOM	192	C	PHE	11	34.699	39.742	29.461	1.00	0.00	C
ATOM	193	O	PHE	11	35.660	39.557	30.218	1.00	0.00	O
ATOM	194	N	GLN	12	34.533	39.030	28.372	1.00	0.00	N
ATOM	195	H	GLN	12	33.686	39.110	27.827	1.00	0.00	H
ATOM	196	CA	GLN	12	35.504	37.919	28.015	1.00	0.00	C
ATOM	197	HA	GLN	12	35.648	37.219	28.837	1.00	0.00	H
ATOM	198	CB	GLN	12	35.016	37.386	26.679	1.00	0.00	C

ATOM	199	HB2	GLN	12	34.943	38.106	25.863	1.00	0.00	H
ATOM	200	HB3	GLN	12	35.731	36.696	26.232	1.00	0.00	H
ATOM	201	CG	GLN	12	33.704	36.548	26.903	1.00	0.00	C
ATOM	202	HG2	GLN	12	34.046	35.733	27.541	1.00	0.00	H
ATOM	203	HG3	GLN	12	32.929	37.122	27.410	1.00	0.00	H
ATOM	204	CD	GLN	12	33.024	36.310	25.588	1.00	0.00	C
ATOM	205	OE1	GLN	12	33.198	37.055	24.635	1.00	0.00	O
ATOM	206	NE2	GLN	12	32.277	35.283	25.453	1.00	0.00	N
ATOM	207	HE21	GLN	12	32.097	34.682	26.245	1.00	0.00	H
ATOM	208	HE22	GLN	12	31.925	35.006	24.549	1.00	0.00	H
ATOM	209	C	GLN	12	36.849	38.485	27.750	1.00	0.00	C
ATOM	210	O	GLN	12	37.845	37.852	28.161	1.00	0.00	O
ATOM	211	N	ASP	13	36.976	39.672	27.285	1.00	0.00	N
ATOM	212	H	ASP	13	36.117	40.012	26.876	1.00	0.00	H
ATOM	213	CA	ASP	13	38.211	40.346	27.038	1.00	0.00	C
ATOM	214	HA	ASP	13	38.823	39.564	26.590	1.00	0.00	H
ATOM	215	CB	ASP	13	38.027	41.480	26.001	1.00	0.00	C
ATOM	216	HB2	ASP	13	37.375	42.272	26.370	1.00	0.00	H
ATOM	217	HB3	ASP	13	39.023	41.839	25.739	1.00	0.00	H
ATOM	218	CG	ASP	13	37.298	41.074	24.737	1.00	0.00	C
ATOM	219	OD1	ASP	13	37.295	39.876	24.367	1.00	0.00	O
ATOM	220	OD2	ASP	13	36.854	42.050	24.057	1.00	0.00	O
ATOM	221	C	ASP	13	38.890	40.909	28.307	1.00	0.00	C
ATOM	222	O	ASP	13	40.128	40.950	28.443	1.00	0.00	O
ATOM	223	N	ASP	14	38.128	41.081	29.372	1.00	0.00	N
ATOM	224	H	ASP	14	37.121	41.087	29.301	1.00	0.00	H
ATOM	225	CA	ASP	14	38.670	41.431	30.737	1.00	0.00	C
ATOM	226	HA	ASP	14	39.572	42.029	30.608	1.00	0.00	H
ATOM	227	CB	ASP	14	37.632	42.218	31.572	1.00	0.00	C
ATOM	228	HB2	ASP	14	36.911	41.509	31.978	1.00	0.00	H
ATOM	229	HB3	ASP	14	38.057	42.544	32.522	1.00	0.00	H
ATOM	230	CG	ASP	14	36.873	43.319	30.777	1.00	0.00	C
ATOM	231	OD1	ASP	14	35.639	43.392	30.939	1.00	0.00	O
ATOM	232	OD2	ASP	14	37.550	44.105	30.100	1.00	0.00	O
ATOM	233	C	ASP	14	39.119	40.228	31.603	1.00	0.00	C
ATOM	234	O	ASP	14	40.180	40.297	32.212	1.00	0.00	O
ATOM	235	N	ILE	15	38.409	39.059	31.447	1.00	0.00	N
ATOM	236	H	ILE	15	37.628	39.102	30.809	1.00	0.00	H
ATOM	237	CA	ILE	15	38.797	37.727	31.897	1.00	0.00	C
ATOM	238	HA	ILE	15	39.097	37.983	32.913	1.00	0.00	H
ATOM	239	CB	ILE	15	37.693	36.694	31.891	1.00	0.00	C
ATOM	240	HB	ILE	15	37.446	36.474	30.852	1.00	0.00	H
ATOM	241	CG2	ILE	15	38.247	35.429	32.559	1.00	0.00	C
ATOM	242	HG21	ILE	15	38.722	35.617	33.523	1.00	0.00	H
ATOM	243	HG22	ILE	15	37.414	34.726	32.557	1.00	0.00	H
ATOM	244	HG23	ILE	15	39.037	34.898	32.027	1.00	0.00	H
ATOM	245	CG1	ILE	15	36.566	37.259	32.857	1.00	0.00	C
ATOM	246	HG12	ILE	15	36.772	37.189	33.924	1.00	0.00	H
ATOM	247	HG13	ILE	15	36.345	38.313	32.688	1.00	0.00	H
ATOM	248	CD1	ILE	15	35.203	36.639	32.421	1.00	0.00	C
ATOM	249	HD11	ILE	15	35.054	36.877	31.368	1.00	0.00	H
ATOM	250	HD12	ILE	15	35.006	35.593	32.657	1.00	0.00	H
ATOM	251	HD13	ILE	15	34.383	37.133	32.944	1.00	0.00	H
ATOM	252	C	ILE	15	40.159	37.246	31.231	1.00	0.00	C
ATOM	253	O	ILE	15	41.087	36.979	31.920	1.00	0.00	O
ATOM	254	N	LEU	16	40.228	37.416	29.891	1.00	0.00	N
ATOM	255	H	LEU	16	39.385	37.787	29.477	1.00	0.00	H
ATOM	256	CA	LEU	16	41.433	37.162	29.054	1.00	0.00	C
ATOM	257	HA	LEU	16	41.786	36.134	29.143	1.00	0.00	H
ATOM	258	CB	LEU	16	41.127	37.584	27.555	1.00	0.00	C
ATOM	259	HB2	LEU	16	40.332	37.059	27.024	1.00	0.00	H
ATOM	260	HB3	LEU	16	40.919	38.653	27.577	1.00	0.00	H
ATOM	261	CG	LEU	16	42.273	37.355	26.545	1.00	0.00	C
ATOM	262	HG	LEU	16	43.075	38.054	26.776	1.00	0.00	H

ATOM	263	CD1	LEU	16	42.800	35.961	26.612	1.00	0.00	C
ATOM	264	HD11	LEU	16	43.171	35.736	27.611	1.00	0.00	H
ATOM	265	HD12	LEU	16	42.009	35.246	26.388	1.00	0.00	H
ATOM	266	HD13	LEU	16	43.549	35.831	25.832	1.00	0.00	H
ATOM	267	CD2	LEU	16	41.732	37.636	25.125	1.00	0.00	C
ATOM	268	HD21	LEU	16	41.306	38.632	25.243	1.00	0.00	H
ATOM	269	HD22	LEU	16	42.588	37.607	24.450	1.00	0.00	H
ATOM	270	HD23	LEU	16	40.970	36.921	24.815	1.00	0.00	H
ATOM	271	C	LEU	16	42.627	37.979	29.588	1.00	0.00	C
ATOM	272	O	LEU	16	43.703	37.404	29.695	1.00	0.00	O
ATOM	273	N	ALA	17	42.504	39.257	29.987	1.00	0.00	N
ATOM	274	H	ALA	17	41.636	39.731	29.780	1.00	0.00	H
ATOM	275	CA	ALA	17	43.525	40.094	30.626	1.00	0.00	C
ATOM	276	HA	ALA	17	44.491	39.839	30.188	1.00	0.00	H
ATOM	277	CB	ALA	17	43.100	41.562	30.305	1.00	0.00	C
ATOM	278	HB1	ALA	17	42.110	41.700	30.738	1.00	0.00	H
ATOM	279	HB2	ALA	17	43.807	42.245	30.778	1.00	0.00	H
ATOM	280	HB3	ALA	17	42.997	41.639	29.222	1.00	0.00	H
ATOM	281	C	ALA	17	43.772	39.839	32.152	1.00	0.00	C
ATOM	282	O	ALA	17	44.797	40.230	32.706	1.00	0.00	O
ATOM	283	N	GLY	18	42.934	39.023	32.803	1.00	0.00	N
ATOM	284	H	GLY	18	42.108	38.613	32.391	1.00	0.00	H
ATOM	285	CA	GLY	18	42.964	38.947	34.309	1.00	0.00	C
ATOM	286	HA2	GLY	18	42.444	38.053	34.651	1.00	0.00	H
ATOM	287	HA3	GLY	18	44.020	38.881	34.575	1.00	0.00	H
ATOM	288	C	GLY	18	42.403	40.108	35.085	1.00	0.00	C
ATOM	289	O	GLY	18	42.554	40.166	36.248	1.00	0.00	O
ATOM	290	N	ARG	19	41.704	41.062	34.408	1.00	0.00	N
ATOM	291	H	ARG	19	41.427	40.952	33.442	1.00	0.00	H
ATOM	292	CA	ARG	19	41.146	42.258	35.049	1.00	0.00	C
ATOM	293	HA	ARG	19	41.795	42.495	35.892	1.00	0.00	H
ATOM	294	CB	ARG	19	41.156	43.439	34.150	1.00	0.00	C
ATOM	295	HB2	ARG	19	42.082	43.382	33.579	1.00	0.00	H
ATOM	296	HB3	ARG	19	40.276	43.334	33.515	1.00	0.00	H
ATOM	297	CG	ARG	19	40.948	44.804	34.870	1.00	0.00	C
ATOM	298	HG2	ARG	19	41.041	45.559	34.089	1.00	0.00	H
ATOM	299	HG3	ARG	19	39.962	44.901	35.323	1.00	0.00	H
ATOM	300	CD	ARG	19	41.959	45.150	35.893	1.00	0.00	C
ATOM	301	HD2	ARG	19	42.246	44.231	36.404	1.00	0.00	H
ATOM	302	HD3	ARG	19	42.862	45.493	35.387	1.00	0.00	H
ATOM	303	NE	ARG	19	41.669	46.330	36.809	1.00	0.00	N
ATOM	304	HE	ARG	19	42.242	47.161	36.836	1.00	0.00	H
ATOM	305	CZ	ARG	19	40.758	46.331	37.676	1.00	0.00	C
ATOM	306	NH1	ARG	19	39.844	45.443	37.870	1.00	0.00	N
ATOM	307	HH11	ARG	19	39.816	44.546	37.406	1.00	0.00	H
ATOM	308	HH12	ARG	19	39.213	45.738	38.601	1.00	0.00	H
ATOM	309	NH2	ARG	19	40.673	47.355	38.411	1.00	0.00	N
ATOM	310	HH21	ARG	19	39.974	47.410	39.137	1.00	0.00	H
ATOM	311	HH22	ARG	19	41.034	48.184	37.960	1.00	0.00	H
ATOM	312	C	ARG	19	39.830	41.980	35.883	1.00	0.00	C
ATOM	313	O	ARG	19	39.393	42.855	36.684	1.00	0.00	O
ATOM	335	N	THR	21	38.391	38.503	37.928	1.00	0.00	N
ATOM	336	H	THR	21	38.080	39.255	38.526	1.00	0.00	H
ATOM	337	CA	THR	21	39.226	37.436	38.493	1.00	0.00	C
ATOM	338	HA	THR	21	39.084	36.581	37.832	1.00	0.00	H
ATOM	339	CB	THR	21	40.768	37.795	38.414	1.00	0.00	C
ATOM	340	HB	THR	21	41.115	37.826	37.381	1.00	0.00	H
ATOM	341	CG2	THR	21	41.077	39.091	39.132	1.00	0.00	C
ATOM	342	HG21	THR	21	42.166	39.113	39.093	1.00	0.00	H
ATOM	343	HG22	THR	21	40.635	39.917	38.573	1.00	0.00	H
ATOM	344	HG23	THR	21	40.669	39.008	40.140	1.00	0.00	H
ATOM	345	OG1	THR	21	41.496	36.721	39.024	1.00	0.00	O
ATOM	346	HG1	THR	21	41.023	35.903	38.855	1.00	0.00	H

```
ATOM    347  C   THR    21      38.787  37.040  39.945  1.00  0.00           C
ATOM    348  O   THR    21      39.103  35.922  40.412  1.00  0.00           O
ATOM    349  N   ILE    22      37.980  37.881  40.591  1.00  0.00           N
ATOM    350  H   ILE    22      37.842  38.793  40.179  1.00  0.00           H
ATOM    351  CA  ILE    22      37.234  37.550  41.792  1.00  0.00           C
ATOM    352  HA  ILE    22      37.162  36.483  42.007  1.00  0.00           H
ATOM    353  CB  ILE    22      37.785  38.145  43.088  1.00  0.00           C
ATOM    354  HB  ILE    22      38.851  37.918  43.091  1.00  0.00           H
ATOM    355  CG2 ILE    22      37.629  39.612  42.991  1.00  0.00           C
ATOM    356 HG21 ILE    22      37.917  39.954  41.997  1.00  0.00           H
ATOM    357 HG22 ILE    22      36.612  39.873  43.281  1.00  0.00           H
ATOM    358 HG23 ILE    22      38.281  40.051  43.748  1.00  0.00           H
ATOM    359  CG1 ILE    22      37.092  37.683  44.425  1.00  0.00           C
ATOM    360 HG12 ILE    22      36.110  38.122  44.600  1.00  0.00           H
ATOM    361 HG13 ILE    22      36.943  36.613  44.280  1.00  0.00           H
ATOM    362  CD1 ILE    22      37.964  37.887  45.673  1.00  0.00           C
ATOM    363 HD11 ILE    22      38.005  38.950  45.913  1.00  0.00           H
ATOM    364 HD12 ILE    22      37.430  37.469  46.525  1.00  0.00           H
ATOM    365 HD13 ILE    22      38.918  37.426  45.418  1.00  0.00           H
ATOM    366  C   ILE    22      35.746  37.998  41.526  1.00  0.00           C
ATOM    367  O   ILE    22      35.546  38.995  40.843  1.00  0.00           O
ATOM    368  N   THR    23      34.758  37.272  42.028  1.00  0.00           N
ATOM    369  H   THR    23      35.024  36.404  42.471  1.00  0.00           H
ATOM    370  CA  THR    23      33.341  37.603  41.984  1.00  0.00           C
ATOM    371  HA  THR    23      33.287  38.692  42.015  1.00  0.00           H
ATOM    372  CB  THR    23      32.688  37.179  40.602  1.00  0.00           C
ATOM    373  HB  THR    23      33.432  37.210  39.805  1.00  0.00           H
ATOM    374  CG2 THR    23      31.952  35.737  40.519  1.00  0.00           C
ATOM    375 HG21 THR    23      32.708  35.084  40.954  1.00  0.00           H
ATOM    376 HG22 THR    23      31.011  35.751  41.066  1.00  0.00           H
ATOM    377 HG23 THR    23      31.704  35.471  39.490  1.00  0.00           H
ATOM    378  OG1 THR    23      31.860  38.246  40.151  1.00  0.00           O
ATOM    379  HG1 THR    23      32.384  38.950  39.764  1.00  0.00           H
ATOM    380  C   THR    23      32.449  37.105  43.065  1.00  0.00           C
ATOM    381  O   THR    23      32.577  35.928  43.480  1.00  0.00           O
ATOM    382  N   ILE    24      31.524  37.883  43.532  1.00  0.00           N
ATOM    383  H   ILE    24      31.323  38.813  43.192  1.00  0.00           H
ATOM    384  CA  ILE    24      30.609  37.497  44.658  1.00  0.00           C
ATOM    385  HA  ILE    24      31.036  36.608  45.119  1.00  0.00           H
ATOM    386  CB  ILE    24      30.383  38.671  45.690  1.00  0.00           C
ATOM    387  HB  ILE    24      29.983  39.517  45.131  1.00  0.00           H
ATOM    388  CG2 ILE    24      29.439  38.309  46.827  1.00  0.00           C
ATOM    389 HG21 ILE    24      29.159  39.211  47.370  1.00  0.00           H
ATOM    390 HG22 ILE    24      28.540  37.809  46.465  1.00  0.00           H
ATOM    391 HG23 ILE    24      29.822  37.502  47.451  1.00  0.00           H
ATOM    392  CG1 ILE    24      31.704  39.257  46.250  1.00  0.00           C
ATOM    393 HG12 ILE    24      32.100  38.457  46.876  1.00  0.00           H
ATOM    394 HG13 ILE    24      32.416  39.444  45.447  1.00  0.00           H
ATOM    395  CD1 ILE    24      31.687  40.562  47.092  1.00  0.00           C
ATOM    396 HD11 ILE    24      32.621  40.715  47.633  1.00  0.00           H
ATOM    397 HD12 ILE    24      31.610  41.339  46.332  1.00  0.00           H
ATOM    398 HD13 ILE    24      30.821  40.722  47.733  1.00  0.00           H
ATOM    399  C   ILE    24      29.234  37.160  44.010  1.00  0.00           C
ATOM    400  O   ILE    24      28.695  37.898  43.162  1.00  0.00           O
ATOM    401  N   ARG    25      28.671  36.070  44.435  1.00  0.00           N
ATOM    402  H   ARG    25      29.149  35.610  45.197  1.00  0.00           H
ATOM    403  CA  ARG    25      27.308  35.620  44.180  1.00  0.00           C
ATOM    404  HA  ARG    25      26.785  36.379  43.599  1.00  0.00           H
ATOM    405  CB  ARG    25      27.342  34.256  43.508  1.00  0.00           C
ATOM    406  HB2 ARG    25      27.783  33.540  44.201  1.00  0.00           H
ATOM    407  HB3 ARG    25      26.272  34.051  43.468  1.00  0.00           H
ATOM    408  CG  ARG    25      28.069  34.241  42.158  1.00  0.00           C
ATOM    409  HG2 ARG    25      28.972  34.824  42.338  1.00  0.00           H
ATOM    410  HG3 ARG    25      28.310  33.248  41.780  1.00  0.00           H
```

```
ATOM    411  CD  ARG    25      27.304  35.065  41.084  1.00  0.00            C
ATOM    412  HD2 ARG    25      26.336  34.677  40.766  1.00  0.00            H
ATOM    413  HD3 ARG    25      27.051  36.033  41.519  1.00  0.00            H
ATOM    414  NE  ARG    25      28.160  35.301  39.902  1.00  0.00            N
ATOM    415  HE  ARG    25      28.056  34.735  39.071  1.00  0.00            H
ATOM    416  CZ  ARG    25      28.755  36.437  39.540  1.00  0.00            C
ATOM    417  NH1 ARG    25      28.981  37.376  40.375  1.00  0.00            N
ATOM    418 HH11 ARG    25      29.717  38.009  40.094  1.00  0.00            H
ATOM    419 HH12 ARG    25      28.476  37.486  41.242  1.00  0.00            H
ATOM    420  NH2 ARG    25      29.361  36.437  38.407  1.00  0.00            N
ATOM    421 HH21 ARG    25      29.777  37.281  38.038  1.00  0.00            H
ATOM    422 HH22 ARG    25      29.500  35.566  37.914  1.00  0.00            H
ATOM    423  C   ARG    25      26.523  35.524  45.490  1.00  0.00            C
ATOM    424  O   ARG    25      27.066  35.416  46.591  1.00  0.00            O
ATOM    425  N   ASP    26      25.161  35.388  45.430  1.00  0.00            N
ATOM    426  H   ASP    26      24.675  35.269  44.553  1.00  0.00            H
ATOM    427  CA  ASP    26      24.306  34.954  46.569  1.00  0.00            C
ATOM    428  HA  ASP    26      24.822  35.229  47.490  1.00  0.00            H
ATOM    429  CB  ASP    26      22.887  35.544  46.609  1.00  0.00            C
ATOM    430  HB2 ASP    26      22.440  35.484  47.602  1.00  0.00            H
ATOM    431  HB3 ASP    26      22.990  36.627  46.554  1.00  0.00            H
ATOM    432  CG  ASP    26      21.885  35.113  45.576  1.00  0.00            C
ATOM    433  OD1 ASP    26      21.332  33.987  45.721  1.00  0.00            O
ATOM    434  OD2 ASP    26      21.564  35.872  44.591  1.00  0.00            O
ATOM    435  C   ASP    26      24.311  33.411  46.596  1.00  0.00            C
ATOM    436  O   ASP    26      24.613  32.780  45.555  1.00  0.00            O
ATOM    437  N   GLU    27      24.173  32.741  47.747  1.00  0.00            N
ATOM    438  H   GLU    27      24.168  33.296  48.590  1.00  0.00            H
ATOM    439  CA  GLU    27      24.472  31.282  48.037  1.00  0.00            C
ATOM    440  HA  GLU    27      25.551  31.165  47.930  1.00  0.00            H
ATOM    441  CB  GLU    27      24.228  30.974  49.527  1.00  0.00            C
ATOM    442  HB2 GLU    27      23.541  31.696  49.968  1.00  0.00            H
ATOM    443  HB3 GLU    27      23.676  30.034  49.549  1.00  0.00            H
ATOM    444  CG  GLU    27      25.428  30.938  50.514  1.00  0.00            C
ATOM    445  HG2 GLU    27      26.074  31.809  50.405  1.00  0.00            H
ATOM    446  HG3 GLU    27      24.988  30.969  51.511  1.00  0.00            H
ATOM    447  CD  GLU    27      26.282  29.657  50.257  1.00  0.00            C
ATOM    448  OE1 GLU    27      26.148  29.125  49.116  1.00  0.00            O
ATOM    449  OE2 GLU    27      26.895  29.258  51.227  1.00  0.00            O
ATOM    450  C   GLU    27      23.830  30.229  47.073  1.00  0.00            C
ATOM    451  O   GLU    27      22.642  30.325  46.895  1.00  0.00            O
ATOM    452  N   SER    28      24.485  29.284  46.491  1.00  0.00            N
ATOM    453  H   SER    28      25.482  29.169  46.603  1.00  0.00            H
ATOM    454  CA  SER    28      23.807  28.103  45.931  1.00  0.00            C
ATOM    455  HA  SER    28      22.934  27.804  46.511  1.00  0.00            H
ATOM    456  CB  SER    28      23.242  28.415  44.485  1.00  0.00            C
ATOM    457  HB2 SER    28      22.500  29.214  44.507  1.00  0.00            H
ATOM    458  HB3 SER    28      24.118  28.763  43.936  1.00  0.00            H
ATOM    459  OG  SER    28      22.704  27.254  43.912  1.00  0.00            O
ATOM    460  HG  SER    28      22.070  26.838  44.501  1.00  0.00            H
ATOM    461  C   SER    28      24.911  26.999  45.782  1.00  0.00            C
ATOM    462  O   SER    28      26.120  27.245  45.688  1.00  0.00            O
ATOM    463  N   GLU    29      24.426  25.739  45.703  1.00  0.00            N
ATOM    464  H   GLU    29      23.441  25.620  45.886  1.00  0.00            H
ATOM    465  CA  GLU    29      25.277  24.592  45.359  1.00  0.00            C
ATOM    466
 HA  GLU    29      26.204  24.787  45.897  1.00  0.00            H
ATOM    467  CB  GLU    29      24.626  23.351  45.983  1.00  0.00            C
ATOM    468  HB2 GLU    29      24.752  23.311  47.065  1.00  0.00            H
ATOM    469  HB3 GLU    29      23.552  23.372  45.799  1.00  0.00            H
ATOM    470  CG  GLU    29      24.997  21.875  45.526  1.00  0.00            C
ATOM    471  HG2 GLU    29      24.419  21.210  46.168  1.00  0.00            H
ATOM    472  HG3 GLU    29      24.784  21.637  44.484  1.00  0.00            H
ATOM    473  CD  GLU    29      26.469  21.596  45.837  1.00  0.00            C
```

ATOM	474	OE1	GLU	29	27.146	20.708	45.264	1.00	0.00	O
ATOM	475	OE2	GLU	29	26.929	22.166	46.844	1.00	0.00	O
ATOM	476	C	GLU	29	25.553	24.554	43.800	1.00	0.00	C
ATOM	477	O	GLU	29	26.448	23.882	43.303	1.00	0.00	O
ATOM	478	N	SER	30	24.695	25.357	43.079	1.00	0.00	N
ATOM	479	H	SER	30	24.101	26.008	43.573	1.00	0.00	H
ATOM	480	CA	SER	30	24.748	25.385	41.616	1.00	0.00	C
ATOM	481	HA	SER	30	24.770	24.367	41.226	1.00	0.00	H
ATOM	482	CB	SER	30	23.633	26.223	40.991	1.00	0.00	C
ATOM	483	HB2	SER	30	23.738	26.166	39.907	1.00	0.00	H
ATOM	484	HB3	SER	30	22.618	25.889	41.212	1.00	0.00	H
ATOM	485	OG	SER	30	23.569	27.578	41.307	1.00	0.00	O
ATOM	486	HG	SER	30	22.979	27.576	42.065	1.00	0.00	H
ATOM	487	C	SER	30	26.026	25.998	41.043	1.00	0.00	C
ATOM	488	O	SER	30	26.426	25.673	39.870	1.00	0.00	O
ATOM	489	N	HIE	31	26.653	26.905	41.717	1.00	0.00	N
ATOM	490	H	HIE	31	26.352	27.069	42.667	1.00	0.00	H
ATOM	491	CA	HIE	31	27.866	27.608	41.210	1.00	0.00	C
ATOM	492	HA	HIE	31	27.569	28.037	40.252	1.00	0.00	H
ATOM	493	CB	HIE	31	28.195	28.807	42.117	1.00	0.00	C
ATOM	494	HB2	HIE	31	28.660	28.328	42.979	1.00	0.00	H
ATOM	495	HB3	HIE	31	28.918	29.429	41.591	1.00	0.00	H
ATOM	496	CG	HIE	31	27.064	29.717	42.505	1.00	0.00	C
ATOM	497	ND1	HIE	31	26.337	30.454	41.546	1.00	0.00	N
ATOM	498	CE1	HIE	31	25.524	31.225	42.332	1.00	0.00	C
ATOM	499	HE1	HIE	31	24.799	31.856	41.839	1.00	0.00	H
ATOM	500	NE2	HIE	31	25.725	31.062	43.638	1.00	0.00	N
ATOM	501	HE2	HIE	31	25.298	31.661	44.329	1.00	0.00	H
ATOM	502	CD2	HIE	31	26.770	30.177	43.780	1.00	0.00	C
ATOM	503	HD2	HIE	31	27.213	29.867	44.715	1.00	0.00	H
ATOM	504	C	HIE	31	29.087	26.708	41.014	1.00	0.00	C
ATOM	505	O	HIE	31	29.184	25.620	41.612	1.00	0.00	O
[truncated: 352,914 more chars]
