# Supplementary material for: YqfB protein from Escherichia coli: an atypical amidohydrolase active towards N4-acylcytosine derivatives
Source: Sci Rep. 2020 Jan 21;10:788. doi: 10.1038/s41598-020-57664-w (PMC6972931; doi:10.1038/s41598-020-57664-w)
Supplement: Supplementary file 3 — YqfB_model_Lys21_prot. [file 41598_2020_57664_MOESM3_ESM.pdf]

YqfB protein from Escherichia coli: an atypical amidohydrolase active towards N4-acylcytosine derivatives

Rūta Stanislauskienėa,#,*, Audrius Laurynėnasb,#, Rasa Rutkienėa,#, Agota Aučynaitėa, Daiva Tauraitėa, Rita Meškienėa, Nina Urbelienėa, Algirdas Kaupinisc, Mindaugas Valiusc, Laura Kalinienea, Rolandas Meškysa

a Department of Molecular Microbiology and Biotechnology, b Department of Bioanalysis, and c Proteomics Centre, Institute of Biochemistry, Life Sciences Center, Vilnius University, Sauletekio al. 7, Vilnius LT-10257, Lithuania
# Those authors contributed equally to this work
* corresponding author ruta.stanislauskiene@bchi.vu.lt

The coordinates regarding protein YqfB Lys21_prot structure are provided below.

```
MODEL      1
SHEET    1   1 1 ILE    22  ASP    26  0
SHEET    2   2 1 VAL    37  VAL    40  0
SHEET    3   3 1 CYX    50  VAL    60  0
SHEET    4   4 1 PHE    94  CYX   101  0
HELIX    1   1 GLN     9  PHE     11  1                                  3
HELIX    2   2 ASP    13  LEU     16  1                                  4
HELIX    3   3 GLU    67  GLN     72  1                                  6
HELIX    4   4 LEU    77  ALA     85  1                                  9
ATOM      1  N   GLN     1      36.770  17.469  30.222  1.00  0.00           N
ATOM      2  H1  GLN     1      37.461  17.975  30.758  1.00  0.00           H
ATOM      3  H2  GLN     1      36.339  16.800  30.844  1.00  0.00           H
ATOM      4  H3  GLN     1      37.303  16.960  29.533  1.00  0.00           H
ATOM      5  CA  GLN     1      35.898  18.390  29.440  1.00  0.00           C
ATOM      6  HA  GLN     1      36.579  18.846  28.720  1.00  0.00           H
ATOM      7  CB  GLN     1      34.731  17.703  28.660  1.00  0.00           C
ATOM      8  HB2 GLN     1      34.087  17.128  29.326  1.00  0.00           H
ATOM      9  HB3 GLN     1      34.004  18.395  28.236  1.00  0.00           H
ATOM     10  CG  GLN     1      35.125  16.745  27.481  1.00  0.00           C
ATOM     11  HG2 GLN     1      35.822  15.950  27.745  1.00  0.00           H
ATOM     12  HG3 GLN     1      34.214  16.295  27.087  1.00  0.00           H
ATOM     13  CD  GLN     1      35.719  17.494  26.321  1.00  0.00           C
ATOM     14  OE1 GLN     1      36.256  18.601  26.463  1.00  0.00           O
ATOM     15  NE2 GLN     1      35.929  16.723  25.291  1.00  0.00           N
ATOM     16 HE21 GLN     1      35.738  15.739  25.414  1.00  0.00           H
ATOM     17 HE22 GLN     1      36.472  17.069  24.513  1.00  0.00           H
ATOM     18  C   GLN     1      35.395  19.544  30.343  1.00  0.00           C
ATOM     19  O   GLN     1      34.952  19.216  31.438  1.00  0.00           O
ATOM     20  N   PRO     2      35.439  20.814  29.852  1.00  0.00           N
ATOM     21  CD  PRO     2      36.047  21.276  28.589  1.00  0.00           C
ATOM     22  HD2 PRO     2      35.662  20.641  27.791  1.00  0.00           H
ATOM     23  HD3 PRO     2      37.121  21.155  28.732  1.00  0.00           H
ATOM     24  CG  PRO     2      35.856  22.786  28.384  1.00  0.00           C
ATOM     25  HG2 PRO     2      34.944  22.847  27.791  1.00  0.00           H
ATOM     26  HG3 PRO     2      36.726  23.294  27.966  1.00  0.00           H
ATOM     27  CB  PRO     2      35.543  23.153  29.797  1.00  0.00           C
ATOM     28  HB2 PRO     2      34.810  23.960  29.760  1.00  0.00           H
ATOM     29  HB3 PRO     2      36.523  23.412  30.194  1.00  0.00           H
ATOM     30  CA  PRO     2      34.950  21.985  30.569  1.00  0.00           C
ATOM     31  HA  PRO     2      35.382  22.036  31.569  1.00  0.00           H
ATOM     32  C   PRO     2      33.436  22.043  30.578  1.00  0.00           C
ATOM     33  O   PRO     2      32.761  21.148  30.066  1.00  0.00           O
ATOM     34  N   ASN     3      32.913  23.072  31.257  1.00  0.00           N
ATOM     35  H   ASN     3      33.502  23.610  31.874  1.00  0.00           H
ATOM     36  CA  ASN     3      31.512  23.493  31.003  1.00  0.00           C
ATOM     37  HA  ASN     3      30.902  22.609  31.191  1.00  0.00           H
ATOM     38  CB  ASN     3      31.218  24.653  31.996  1.00  0.00           C
ATOM     39  HB2 ASN     3      31.960  25.421  31.777  1.00  0.00           H
ATOM     40  HB3 ASN     3      30.277  25.132  31.728  1.00  0.00           H
```

ATOM	41	CG	ASN	3	31.219	24.194	33.469	1.00	0.00	C
ATOM	42	OD1	ASN	3	30.126	24.140	34.104	1.00	0.00	O
ATOM	43	ND2	ASN	3	32.275	23.991	34.129	1.00	0.00	N
ATOM	44	HD21	ASN	3	33.172	24.096	33.676	1.00	0.00	H
ATOM	45	HD22	ASN	3	32.290	23.511	35.018	1.00	0.00	H
ATOM	46	C	ASN	3	31.328	24.008	29.565	1.00	0.00	C
ATOM	47	O	ASN	3	32.146	24.792	29.092	1.00	0.00	O
ATOM	48	N	ASP	4	30.164	23.720	28.988	1.00	0.00	N
ATOM	49	H	ASP	4	29.487	23.117	29.432	1.00	0.00	H
ATOM	50	CA	ASP	4	29.535	24.480	27.924	1.00	0.00	C
ATOM	51	HA	ASP	4	30.301	24.829	27.232	1.00	0.00	H
ATOM	52	CB	ASP	4	28.642	23.520	27.118	1.00	0.00	C
ATOM	53	HB2	ASP	4	27.732	23.290	27.672	1.00	0.00	H
ATOM	54	HB3	ASP	4	28.335	24.052	26.217	1.00	0.00	H
ATOM	55	CG	ASP	4	29.288	22.188	26.623	1.00	0.00	C
ATOM	56	OD1	ASP	4	28.509	21.361	26.078	1.00	0.00	O
ATOM	57	OD2	ASP	4	30.525	22.042	26.801	1.00	0.00	O
ATOM	58	C	ASP	4	28.817	25.759	28.464	1.00	0.00	C
ATOM	59	O	ASP	4	28.976	26.803	27.832	1.00	0.00	O
ATOM	60	N	ILE	5	28.380	25.726	29.742	1.00	0.00	N
ATOM	61	H	ILE	5	28.272	24.862	30.253	1.00	0.00	H
ATOM	62	CA	ILE	5	28.015	26.970	30.548	1.00	0.00	C
ATOM	63	HA	ILE	5	27.277	27.619	30.076	1.00	0.00	H
ATOM	64	CB	ILE	5	27.555	26.564	31.972	1.00	0.00	C
ATOM	65	HB	ILE	5	28.287	25.836	32.322	1.00	0.00	H
ATOM	66	CG2	ILE	5	27.530	27.670	33.035	1.00	0.00	C
ATOM	67	HG21	ILE	5	27.059	27.294	33.944	1.00	0.00	H
ATOM	68	HG22	ILE	5	28.577	27.883	33.243	1.00	0.00	H
ATOM	69	HG23	ILE	5	27.050	28.579	32.673	1.00	0.00	H
ATOM	70	CG1	ILE	5	26.138	25.943	31.876	1.00	0.00	C
ATOM	71	HG12	ILE	5	26.210	25.119	31.166	1.00	0.00	H
ATOM	72	HG13	ILE	5	25.756	25.503	32.797	1.00	0.00	H
ATOM	73	CD1	ILE	5	25.062	26.824	31.268	1.00	0.00	C
ATOM	74	HD11	ILE	5	24.863	27.706	31.876	1.00	0.00	H
ATOM	75	HD12	ILE	5	25.272	27.211	30.271	1.00	0.00	H
ATOM	76	HD13	ILE	5	24.080	26.360	31.179	1.00	0.00	H
ATOM	77	C	ILE	5	29.270	27.859	30.699	1.00	0.00	C
ATOM	78	O	ILE	5	30.371	27.371	30.745	1.00	0.00	O
ATOM	79	N	THR	6	29.124	29.143	30.590	1.00	0.00	N
ATOM	80	H	THR	6	28.195	29.492	30.405	1.00	0.00	H
ATOM	81	CA	THR	6	30.173	30.156	30.482	1.00	0.00	C
ATOM	82	HA	THR	6	31.017	29.835	31.093	1.00	0.00	H
ATOM	83	CB	THR	6	30.703	30.247	29.015	1.00	0.00	C
ATOM	84	HB	THR	6	31.016	29.253	28.695	1.00	0.00	H
ATOM	85	CG2	THR	6	29.634	30.793	27.956	1.00	0.00	C
ATOM	86	HG21	THR	6	29.418	31.861	27.999	1.00	0.00	H
ATOM	87	HG22	THR	6	30.036	30.546	26.974	1.00	0.00	H
ATOM	88	HG23	THR	6	28.742	30.205	28.171	1.00	0.00	H
ATOM	89	OG1	THR	6	31.918	30.979	28.952	1.00	0.00	O
ATOM	90	HG1	THR	6	32.547	30.472	29.472	1.00	0.00	H
ATOM	91	C	THR	6	29.664	31.549	30.883	1.00	0.00	C
ATOM	92	O	THR	6	28.459	31.789	30.928	1.00	0.00	O
ATOM	93	N	PHE	7	30.596	32.493	31.157	1.00	0.00	N
ATOM	94	H	PHE	7	31.568	32.221	31.181	1.00	0.00	H
ATOM	95	CA	PHE	7	30.165	33.859	31.294	1.00	0.00	C
ATOM	96	HA	PHE	7	29.288	33.842	31.941	1.00	0.00	H
ATOM	97	CB	PHE	7	31.367	34.639	31.965	1.00	0.00	C
ATOM	98	HB2	PHE	7	32.064	34.775	31.138	1.00	0.00	H
ATOM	99	HB3	PHE	7	30.977	35.561	32.398	1.00	0.00	H
ATOM	100	CG	PHE	7	32.169	34.025	33.115	1.00	0.00	C
ATOM	101	CD1	PHE	7	31.527	33.516	34.312	1.00	0.00	C
ATOM	102	HD1	PHE	7	30.511	33.786	34.560	1.00	0.00	H
ATOM	103	CE1	PHE	7	32.321	32.977	35.374	1.00	0.00	C
ATOM	104	HE1	PHE	7	31.934	32.737	36.354	1.00	0.00	H

ATOM	105	CZ	PHE	7	33.740	32.957	35.236	1.00	0.00	C
ATOM	106	HZ	PHE	7	34.370	32.550	36.013	1.00	0.00	H
ATOM	107	CE2	PHE	7	34.323	33.172	33.979	1.00	0.00	C
ATOM	108	HE2	PHE	7	35.362	32.987	33.750	1.00	0.00	H
ATOM	109	CD2	PHE	7	33.501	33.741	32.988	1.00	0.00	C
ATOM	110	HD2	PHE	7	33.861	33.890	31.981	1.00	0.00	H
ATOM	111	C	PHE	7	29.827	34.426	29.945	1.00	0.00	C
ATOM	112	O	PHE	7	30.637	34.267	28.977	1.00	0.00	O
ATOM	113	N	PHE	8	28.722	35.131	29.795	1.00	0.00	N
ATOM	114	H	PHE	8	28.092	35.187	30.583	1.00	0.00	H
ATOM	115	CA	PHE	8	28.160	35.794	28.557	1.00	0.00	C
ATOM	116	HA	PHE	8	27.954	35.052	27.787	1.00	0.00	H
ATOM	117	CB	PHE	8	26.844	36.536	28.800	1.00	0.00	C
ATOM	118	HB2	PHE	8	26.870	37.271	29.603	1.00	0.00	H
ATOM	119	HB3	PHE	8	26.655	37.157	27.924	1.00	0.00	H
ATOM	120	CG								
			PHE	8	25.719	35.617	29.058	1.00	0.00	C
ATOM	121	CD1	PHE	8	25.471	35.167	30.347	1.00	0.00	C
ATOM	122	HD1	PHE	8	26.024	35.560	31.187	1.00	0.00	H
ATOM	123	CE1	PHE	8	24.453	34.218	30.578	1.00	0.00	C
ATOM	124	HE1	PHE	8	24.266	33.941	31.605	1.00	0.00	H
ATOM	125	CZ	PHE	8	23.655	33.813	29.503	1.00	0.00	C
ATOM	126	HZ	PHE	8	22.838	33.124	29.658	1.00	0.00	H
ATOM	127	CE2	PHE	8	23.947	34.259	28.220	1.00	0.00	C
ATOM	128	HE2	PHE	8	23.239	33.994	27.448	1.00	0.00	H
ATOM	129	CD2	PHE	8	24.996	35.154	28.022	1.00	0.00	C
ATOM	130	HD2	PHE	8	25.223	35.471	27.013	1.00	0.00	H
ATOM	131	C	PHE	8	29.133	36.862	27.955	1.00	0.00	C
ATOM	132	O	PHE	8	29.868	37.591	28.665	1.00	0.00	O
ATOM	133	N	GLN	9	29.204	36.816	26.626	1.00	0.00	N
ATOM	134	H	GLN	9	28.586	36.187	26.135	1.00	0.00	H
ATOM	135	CA	GLN	9	30.306	37.341	25.845	1.00	0.00	C
ATOM	136	HA	GLN	9	31.163	36.678	25.964	1.00	0.00	H
ATOM	137	CB	GLN	9	29.981	37.260	24.275	1.00	0.00	C
ATOM	138	HB2	GLN	9	30.735	37.879	23.790	1.00	0.00	H
ATOM	139	HB3	GLN	9	30.162	36.242	23.931	1.00	0.00	H
ATOM	140	CG	GLN	9	28.534	37.544	23.743	1.00	0.00	C
ATOM	141	HG2	GLN	9	28.238	38.539	24.070	1.00	0.00	H
ATOM	142	HG3	GLN	9	28.754	37.523	22.675	1.00	0.00	H
ATOM	143	CD	GLN	9	27.562	36.455	24.120	1.00	0.00	C
ATOM	144	OE1	GLN	9	27.838	35.460	24.816	1.00	0.00	O
ATOM	145	NE2	GLN	9	26.379	36.459	23.595	1.00	0.00	N
ATOM	146	HE21	GLN	9	25.752	35.781	24.002	1.00	0.00	H
ATOM	147	HE22	GLN	9	26.075	37.199	22.978	1.00	0.00	H
ATOM	148	C	GLN	9	30.778	38.774	26.187	1.00	0.00	C
ATOM	149	O	GLN	9	31.927	38.874	26.150	1.00	0.00	O
ATOM	150	N	ARG	10	29.879	39.653	26.641	1.00	0.00	N
ATOM	151	H	ARG	10	28.930	39.325	26.752	1.00	0.00	H
ATOM	152	CA	ARG	10	30.119	41.084	27.063	1.00	0.00	C
ATOM	153	HA	ARG	10	30.558	41.611	26.216	1.00	0.00	H
ATOM	154	CB	ARG	10	28.815	41.745	27.450	1.00	0.00	C
ATOM	155	HB2	ARG	10	28.046	41.738	26.677	1.00	0.00	H
ATOM	156	HB3	ARG	10	28.333	41.166	28.237	1.00	0.00	H
ATOM	157	CG	ARG	10	28.872	43.178	28.033	1.00	0.00	C
ATOM	158	HG2	ARG	10	29.414	43.227	28.977	1.00	0.00	H
ATOM	159	HG3	ARG	10	29.404	43.805	27.319	1.00	0.00	H
ATOM	160	CD	ARG	10	27.540	43.668	28.499	1.00	0.00	C
ATOM	161	HD2	ARG	10	26.845	43.702	27.660	1.00	0.00	H
ATOM	162	HD3	ARG	10	27.214	42.891	29.189	1.00	0.00	H
ATOM	163	NE	ARG	10	27.547	45.019	29.263	1.00	0.00	N
ATOM	164	HE	ARG	10	28.366	45.609	29.263	1.00	0.00	H
ATOM	165	CZ	ARG	10	26.722	45.252	30.235	1.00	0.00	C
ATOM	166	NH1	ARG	10	25.617	44.470	30.280	1.00	0.00	N
ATOM	167	HH11	ARG	10	24.908	44.642	30.978	1.00	0.00	H

ATOM	168	HH12	ARG	10	25.470	43.809	29.530	1.00	0.00	H
ATOM	169	NH2	ARG	10	26.966	46.104	31.163	1.00	0.00	N
ATOM	170	HH21	ARG	10	27.780	46.679	31.002	1.00	0.00	H
ATOM	171	HH22	ARG	10	26.233	46.431	31.777	1.00	0.00	H
ATOM	172	C	ARG	10	31.207	41.146	28.159	1.00	0.00	C
ATOM	173	O	ARG	10	31.742	42.292	28.336	1.00	0.00	O
ATOM	174	N	PHE	11	31.452	40.118	28.971	1.00	0.00	N
ATOM	175	H	PHE	11	30.970	39.258	28.754	1.00	0.00	H
ATOM	176	CA	PHE	11	32.256	40.064	30.200	1.00	0.00	C
ATOM	177	HA	PHE	11	32.395	41.014	30.719	1.00	0.00	H
ATOM	178	CB	PHE	11	31.286	39.372	31.227	1.00	0.00	C
ATOM	179	HB2	PHE	11	31.774	39.230	32.192	1.00	0.00	H
ATOM	180	HB3	PHE	11	31.143	38.380	30.799	1.00	0.00	H
ATOM	181	CG	PHE	11	29.901	40.012	31.513	1.00	0.00	C
ATOM	182	CD1	PHE	11	29.797	41.335	31.989	1.00	0.00	C
ATOM	183	HD1	PHE	11	30.729	41.842	32.195	1.00	0.00	H
ATOM	184	CE1	PHE	11	28.640	42.107	31.821	1.00	0.00	C
ATOM	185	HE1	PHE	11	28.698	43.177	31.954	1.00	0.00	H
ATOM	186	CZ	PHE	11	27.469	41.410	31.404	1.00	0.00	C
ATOM	187	HZ	PHE	11	26.502	41.884	31.328	1.00	0.00	H
ATOM	188	CE2	PHE	11	27.501	40.043	31.025	1.00	0.00	C
ATOM	189	HE2	PHE	11	26.654	39.459	30.696	1.00	0.00	H
ATOM	190	CD2	PHE	11	28.719	39.331	31.059	1.00	0.00	C
ATOM	191	HD2	PHE	11	28.754	38.327	30.662	1.00	0.00	H
ATOM	192	C	PHE	11	33.574	39.226	30.138	1.00	0.00	C
ATOM	193	O	PHE	11	34.225	38.994	31.116	1.00	0.00	O
ATOM	194	N	GLN	12	33.976	38.696	28.951	1.00	0.00	N
ATOM	195	H	GLN	12	33.337	38.887	28.192	1.00	0.00	H
ATOM	196	CA	GLN	12	35.290	38.154	28.764	1.00	0.00	C
ATOM	197	HA	GLN	12	35.515	37.418	29.536	1.00	0.00	H
ATOM	198	CB	GLN	12	35.416	37.463	27.371	1.00	0.00	C
ATOM	199	HB2	GLN	12	35.316	38.286	26.664	1.00	0.00	H
ATOM	200	HB3	GLN	12	36.409	37.069	27.158	1.00	0.00	H
ATOM	201	CG	GLN	12	34.357	36.433	27.030	1.00	0.00	C
ATOM	202	HG2	GLN	12	34.427	35.642	27.776	1.00	0.00	H
ATOM	203	HG3	GLN	12	33.399	36.947	27.110	1.00	0.00	H
ATOM	204	CD	GLN	12	34.349	35.893	25.595	1.00	0.00	C
ATOM	205	OE1	GLN	12	34.883	36.469	24.663	1.00	0.00	O
ATOM	206	NE2	GLN	12	33.593	34.876	25.302	1.00	0.00	N
ATOM	207	HE21	GLN	12	33.024	34.441	26.013	1.00	0.00	H
ATOM	208	HE22	GLN	12	33.531	34.648	24.320	1.00	0.00	H
ATOM	209	C	GLN	12	36.435	39.170	28.805	1.00	0.00	C
ATOM	210	O	GLN	12	37.627	38.792	28.936	1.00	0.00	O
ATOM	211	N	ASP	13	36.211	40.393	28.355	1.00	0.00	N
ATOM	212	H	ASP	13	35.246	40.686	28.403	1.00	0.00	H
ATOM	213	CA	ASP	13	37.271	41.380	27.996	1.00	0.00	C
ATOM	214	HA	ASP	13	37.891	41.007	27.180	1.00	0.00	H
ATOM	215	CB	ASP	13	36.574	42.661	27.471	1.00	0.00	C
ATOM	216	HB2	ASP	13	36.172	43.232	28.309	1.00	0.00	H
ATOM	217	HB3	ASP	13	37.250	43.360	26.978	1.00	0.00	H
ATOM	218	CG	ASP	13	35.449	42.475	26.417	1.00	0.00	C
ATOM	219	OD1	ASP	13	34.591	43.380	26.280	1.00	0.00	O
ATOM	220	OD2	ASP	13	35.487	41.515	25.610	1.00	0.00	O
ATOM	221	C	ASP	13	38.234	41.711	29.145	1.00	0.00	C
ATOM	222	O	ASP	13	39.473	41.799	29.058	1.00	0.00	O
ATOM	223	N	ASP	14	37.604	41.781	30.290	1.00	0.00	N
ATOM	224	H	ASP	14	36.600	41.676	30.340	1.00	0.00	H
ATOM	225	CA	ASP	14	38.305	42.037	31.540	1.00	0.00	C
ATOM	226	HA	ASP	14	39.005	42.872	31.498	1.00	0.00	H
ATOM	227	CB	ASP	14	37.242	42.345	32.627	1.00	0.00	C
ATOM	228	HB2	ASP	14	36.401	41.664	32.499	1.00	0.00	H
ATOM	229	HB3	ASP	14	37.668	42.229	33.624	1.00	0.00	H
ATOM	230	CG	ASP	14	36.722	43.721	32.656	1.00	0.00	C
ATOM	231	OD1	ASP	14	35.786	43.926	33.489	1.00	0.00	O

```
ATOM    232  OD2 ASP    14      37.273  44.692  32.078  1.00  0.00           O
ATOM    233  C   ASP    14      39.144  40.843  31.975  1.00  0.00           C
ATOM    234  O   ASP    14      40.053  41.018  32.762  1.00  0.00           O
ATOM    235  N   ILE    15      38.891  39.646  31.424  1.00  0.00           N
ATOM    236  H   ILE    15      38.268  39.546  30.635  1.00  0.00           H
ATOM    237  CA  ILE    15      39.724  38.468  31.493  1.00  0.00           C
ATOM    238  HA  ILE    15      40.277  38.491  32.432  1.00  0.00           H
ATOM    239  CB  ILE    15      38.893  37.183  31.538  1.00  0.00           C
ATOM    240  HB  ILE    15      38.308  36.994  30.637  1.00  0.00           H
ATOM    241  CG2 ILE    15      39.981  36.074  31.637  1.00  0.00           C
ATOM    242 HG21 ILE    15      39.507  35.111  31.824  1.00  0.00           H
ATOM    243 HG22 ILE    15      40.536  36.048  30.699  1.00  0.00           H
ATOM    244 HG23 ILE    15      40.675  36.341  32.434  1.00  0.00           H
ATOM    245  CG1 ILE    15      37.935  37.180
  32.768  1.00  0.00           C
ATOM    246 HG12 ILE    15      38.375  37.333  33.753  1.00  0.00           H
ATOM    247 HG13 ILE    15      37.356  38.103  32.751  1.00  0.00           H
ATOM    248  CD1 ILE    15      37.010  35.973  32.883  1.00  0.00           C
ATOM    249 HD11 ILE    15      37.432  35.131  33.432  1.00  0.00           H
ATOM    250 HD12 ILE    15      36.028  36.231  33.279  1.00  0.00           H
ATOM    251 HD13 ILE    15      36.858  35.521  31.903  1.00  0.00           H
ATOM    252  C   ILE    15      40.881  38.497  30.480  1.00  0.00           C
ATOM    253  O   ILE    15      41.991  38.107  30.715  1.00  0.00           O
ATOM    254  N   LEU    16      40.668  39.094  29.327  1.00  0.00           N
ATOM    255  H   LEU    16      39.827  39.653  29.316  1.00  0.00           H
ATOM    256  CA  LEU    16      41.600  39.193  28.297  1.00  0.00           C
ATOM    257  HA  LEU    16      41.801  38.146  28.073  1.00  0.00           H
ATOM    258  CB  LEU    16      40.918  39.715  26.978  1.00  0.00           C
ATOM    259  HB2 LEU    16      39.919  39.354  26.733  1.00  0.00           H
ATOM    260  HB3 LEU    16      40.764  40.791  27.055  1.00  0.00           H
ATOM    261  CG  LEU    16      41.873  39.670  25.771  1.00  0.00           C
ATOM    262  HG  LEU    16      42.800  40.201  25.990  1.00  0.00           H
ATOM    263  CD1 LEU    16      42.185  38.235  25.235  1.00  0.00           C
ATOM    264 HD11 LEU    16      42.586  37.557  25.987  1.00  0.00           H
ATOM    265 HD12 LEU    16      41.218  37.820  24.949  1.00  0.00           H
ATOM    266 HD13 LEU    16      42.894  38.269  24.408  1.00  0.00           H
ATOM    267  CD2 LEU    16      41.112  40.393  24.691  1.00  0.00           C
ATOM    268 HD21 LEU    16      40.240  39.802  24.406  1.00  0.00           H
ATOM    269 HD22 LEU    16      40.762  41.370  25.025  1.00  0.00           H
ATOM    270 HD23 LEU    16      41.728  40.613  23.820  1.00  0.00           H
ATOM    271  C   LEU    16      42.884  39.954  28.751  1.00  0.00           C
ATOM    272  O   LEU    16      43.994  39.564  28.380  1.00  0.00           O
ATOM    273  N   ALA    17      42.777  40.986  29.651  1.00  0.00           N
ATOM    274  H   ALA    17      41.842  41.297  29.878  1.00  0.00           H
ATOM    275  CA  ALA    17      43.881  41.662  30.315  1.00  0.00           C
ATOM    276  HA  ALA    17      44.821  41.647  29.764  1.00  0.00           H
ATOM    277  CB  ALA    17      43.322  43.102  30.261  1.00  0.00           C
ATOM    278  HB1 ALA    17      43.889  43.779  30.901  1.00  0.00           H
ATOM    279  HB2 ALA    17      43.449  43.493  29.251  1.00  0.00           H
ATOM    280  HB3 ALA    17      42.261  43.142  30.505  1.00  0.00           H
ATOM    281  C   ALA    17      44.088  41.234  31.796  1.00  0.00           C
ATOM    282  O   ALA    17      45.044  41.692  32.423  1.00  0.00           O
ATOM    283  N   GLY    18      43.289  40.336  32.349  1.00  0.00           N
ATOM    284  H   GLY    18      42.544  39.998  31.757  1.00  0.00           H
ATOM    285  CA  GLY    18      43.343  39.853  33.747  1.00  0.00           C
ATOM    286  HA2 GLY    18      42.773  38.942  33.928  1.00  0.00           H
ATOM    287  HA3 GLY    18      44.352  39.573  34.050  1.00  0.00           H
ATOM    288  C   GLY    18      42.809  40.889  34.752  1.00  0.00           C
ATOM    289  O   GLY    18      43.188  40.742  35.931  1.00  0.00           O
ATOM    290  N   ARG    19      41.915  41.826  34.384  1.00  0.00           N
ATOM    291  H   ARG    19      41.520  41.747  33.458  1.00  0.00           H
ATOM    292  CA  ARG    19      41.386  42.863  35.332  1.00  0.00           C
ATOM    293  HA  ARG    19      42.238  43.254  35.888  1.00  0.00           H
ATOM    294  CB  ARG    19      40.776  44.009  34.533  1.00  0.00           C
```

ATOM	295	HB2	ARG	19	40.047	43.611	33.827	1.00	0.00	H
ATOM	296	HB3	ARG	19	40.152	44.622	35.185	1.00	0.00	H
ATOM	297	CG	ARG	19	41.831	44.829	33.758	1.00	0.00	C
ATOM	298	HG2	ARG	19	42.522	45.353	34.418	1.00	0.00	H
ATOM	299	HG3	ARG	19	42.463	44.263	33.074	1.00	0.00	H
ATOM	300	CD	ARG	19	41.110	45.938	33.001	1.00	0.00	C
ATOM	301	HD2	ARG	19	40.768	46.512	33.863	1.00	0.00	H
ATOM	302	HD3	ARG	19	41.857	46.572	32.524	1.00	0.00	H
ATOM	303	NE	ARG	19	40.112	45.564	32.051	1.00	0.00	N
ATOM	304	HE	ARG	19	39.147	45.567	32.351	1.00	0.00	H
ATOM	305	CZ	ARG	19	40.174	45.598	30.711	1.00	0.00	C
ATOM	306	NH1	ARG	19	41.178	45.977	30.049	1.00	0.00	N
ATOM	307	HH11	ARG	19	42.094	46.155	30.435	1.00	0.00	H
ATOM	308	HH12	ARG	19	41.146	45.861	29.046	1.00	0.00	H
ATOM	309	NH2	ARG	19	39.043	45.545	30.026	1.00	0.00	N
ATOM	310	HH21	ARG	19	39.007	45.673	29.025	1.00	0.00	H
ATOM	311	HH22	ARG	19	38.202	45.565	30.585	1.00	0.00	H
ATOM	312	C	ARG	19	40.335	42.266	36.306	1.00	0.00	C
ATOM	313	O	ARG	19	40.218	42.798	37.372	1.00	0.00	O
ATOM	314	N	LYS	20	39.541	41.215	35.954	1.00	0.00	N
ATOM	315	H	LYS	20	39.695	40.904	35.005	1.00	0.00	H
ATOM	316	CA	LYS	20	38.642	40.486	36.826	1.00	0.00	C
ATOM	317	HA	LYS	20	38.310	41.147	37.626	1.00	0.00	H
ATOM	318	CB	LYS	20	37.372	40.184	35.942	1.00	0.00	C
ATOM	319	HB2	LYS	20	37.668	39.474	35.170	1.00	0.00	H
ATOM	320	HB3	LYS	20	37.058	41.086	35.417	1.00	0.00	H
ATOM	321	CG	LYS	20	36.119	39.770	36.819	1.00	0.00	C
ATOM	322	HG2	LYS	20	35.484	39.100	36.239	1.00	0.00	H
ATOM	323	HG3	LYS	20	36.334	39.133	37.676	1.00	0.00	H
ATOM	324	CD	LYS	20	35.238	40.977	37.299	1.00	0.00	C
ATOM	325	HD2	LYS	20	34.875	41.530	36.433	1.00	0.00	H
ATOM	326	HD3	LYS	20	35.934	41.600	37.863	1.00	0.00	H
ATOM	327	CE	LYS	20	34.054	40.375	38.066	1.00	0.00	C
ATOM	328	HE2	LYS	20	33.537	39.638	37.452	1.00	0.00	H
ATOM	329	HE3	LYS	20	34.527	39.892	38.921	1.00	0.00	H
ATOM	330	NZ	LYS	20	33.059	41.398	38.529	1.00	0.00	N
ATOM	331	HZ1	LYS	20	32.495	41.727	37.758	1.00	0.00	H
ATOM	332	HZ2	LYS	20	33.542	42.153	38.993	1.00	0.00	H
ATOM	333	HZ3	LYS	20	32.403	40.932	39.139	1.00	0.00	H
ATOM	334	C	LYS	20	39.198	39.106	37.163	1.00	0.00	C
ATOM	335	O	LYS	20	39.703	38.420	36.298	1.00	0.00	O
ATOM	336	N	THR	21	39.090	38.668	38.416	1.00	0.00	N
ATOM	337	H	THR	21	38.680	39.361	39.025	1.00	0.00	H
ATOM	338	CA	THR	21	39.372	37.318	38.979	1.00	0.00	C
ATOM	339	HA	THR	21	39.319	36.516	38.242	1.00	0.00	H
ATOM	340	CB	THR	21	40.768	37.155	39.515	1.00	0.00	C
ATOM	341	HB	THR	21	40.933	36.121	39.815	1.00	0.00	H
ATOM	342	CG2	THR	21	41.791	37.322	38.459	1.00	0.00	C
ATOM	343	HG21	THR	21	41.363	37.050	37.493	1.00	0.00	H
ATOM	344	HG22	THR	21	42.149	38.351	38.433	1.00	0.00	H
ATOM	345	HG23	THR	21	42.634	36.661	38.661	1.00	0.00	H
ATOM	346	OG1	THR	21	41.026	38.006	40.574	1.00	0.00	O
ATOM	347	HG1	THR	21	40.910	37.487	41.374	1.00	0.00	H
ATOM	348	C	THR	21	38.447	36.810	40.027	1.00	0.00	C
ATOM	349	O	THR	21	38.512	35.653	40.310	1.00	0.00	O
ATOM	350	N	ILE	22	37.668	37.750	40.616	1.00	0.00	N
ATOM	351	H	ILE	22	37.701	38.693	40.257	1.00	0.00	H
ATOM	352	CA	ILE	22	36.721	37.403	41.649	1.00	0.00	C
ATOM	353	HA	ILE	22	36.702	36.315	41.704	1.00	0.00	H
ATOM	354	CB	ILE	22	37.324	37.905	43.016	1.00	0.00	C
ATOM	355	HB	ILE	22	38.329	37.484	43.016	1.00	0.00	H
ATOM	356	CG2	ILE	22	37.495	39.420	43.139	1.00	0.00	C
ATOM	357	HG21	ILE	22	38.181	39.754	42.360	1.00	0.00	H
ATOM	358	HG22	ILE	22	36.524	39.905	43.238	1.00	0.00	H

ATOM	359	HG23	ILE	22	38.008	39.562	44.090	1.00	0.00	H
ATOM	360	CG1	ILE	22	36.663	37.368	44.334	1.00	0.00	C
ATOM	361	HG12	ILE	22	35.617	37.634	44.486	1.00	0.00	H
ATOM	362	HG13	ILE	22	36.530	36.305	44.129	1.00	0.00	H
ATOM	363	CD1	ILE	22	37.459	37.504	45.587	1.00	0.00	C
ATOM	364	HD11	ILE	22	37.244	36.713	46.305	1.00	0.00	H
ATOM	365	HD12	ILE	22	38.521	37.277	45.497	1.00	0.00	H
ATOM	366	HD13	ILE	22	37.276	38.475	46.046	1.00	0.00	H
ATOM	367	C	ILE	22	35.251	37.833	41.487	1.00	0.00	C
ATOM	368	O	ILE	22	34.868	38.532	40.543	1.00	0.00	O
ATOM	369	N	THR	23	34.344	37.088	42.173	1.00	0.00	N
ATOM	370	H	THR	23	34.651	36.318	42.749	1.00	0.00	H
ATOM	371	CA	THR	23	32.900	37.336	42.225	1.00	0.00	C
ATOM	372	HA	THR	23	32.631	38.354	41.946	1.00	0.00	H
ATOM	373	CB	THR	23	32.229	36.374	41.266	1.00	0.00	C
ATOM	374	HB	THR	23	32.604	35.354	41.336	1.00	0.00	H
ATOM	375	CG2	THR	23	30.759	36.287	41.429	1.00	0.00	C
ATOM	376	HG21	THR	23	30.417	35.717	40.565	1.00	0.00	H
ATOM	377	HG22	THR	23	30.621	35.698	42.334	1.00	0.00	H
ATOM	378	HG23	THR	23	30.305	37.278	41.416	1.00	0.00	H
ATOM	379	OG1	THR	23	32.456	36.756	39.936	1.00	0.00	O
ATOM	380	HG1	THR	23	33.272	37.223	39.738	1.00	0.00	H
ATOM	381	C	THR	23	32.273	37.102	43.602	1.00	0.00	C
ATOM	382	O	THR	23	32.760	36.168	44.229	1.00	0.00	O
ATOM	383	N	ILE	24	31.274	37.937	43.906	1.00	0.00	N
ATOM	384	H	ILE	24	31.032	38.636	43.218	1.00	0.00	H
ATOM	385	CA	ILE	24	30.377	37.674	45.080	1.00	0.00	C
ATOM	386	HA	ILE	24	30.812	36.833	45.622	1.00	0.00	H
ATOM	387	CB	ILE	24	30.386	38.794	46.184	1.00	0.00	C
ATOM	388	HB	ILE	24	29.825	39.636	45.776	1.00	0.00	H
ATOM	389	CG2	ILE	24	29.616	38.314	47.437	1.00	0.00	C
ATOM	390	HG21	ILE	24	29.978	37.318	47.691	1.00	0.00	H
ATOM	391	HG22	ILE	24	29.833	38.931	48.310	1.00	0.00	H
ATOM	392	HG23	ILE	24	28.559	38.218	47.186	1.00	0.00	H
ATOM	393	CG1	ILE	24	31.759	39.276	46.511	1.00	0.00	C
ATOM	394	HG12	ILE	24	32.300	38.526	47.086	1.00	0.00	H
ATOM	395	HG13	ILE	24	32.370	39.380	45.614	1.00	0.00	H
ATOM	396	CD1	ILE	24	31.893	40.617	47.217	1.00	0.00	C
ATOM	397	HD11	ILE	24	32.905	40.958	47.436	1.00	0.00	H
ATOM	398	HD12	ILE	24	31.470	41.373	46.555	1.00	0.00	H
ATOM	399	HD13	ILE	24	31.327	40.639	48.149	1.00	0.00	H
ATOM	400	C	ILE	24	28.889	37.332	44.749	1.00	0.00	C
ATOM	401	O	ILE	24	28.338	37.849	43.775	1.00	0.00	O
ATOM	402	N	ARG	25	28.303	36.367	45.482	1.00	0.00	N
ATOM	403	H	ARG	25	28.852	35.921	46.202	1.00	0.00	H
ATOM	404	CA	ARG	25	26.956	35.897	45.324	1.00	0.00	C
ATOM	405	HA	ARG	25	26.286	36.653	44.915	1.00	0.00	H
ATOM	406	CB	ARG	25	26.900	34.684	44.352	1.00	0.00	C
ATOM	407	HB2	ARG	25	27.756	34.069	44.625	1.00	0.00	H
ATOM	408	HB3	ARG	25	25.998	34.101	44.538	1.00	0.00	H
ATOM	409	CG	ARG	25	27.029	34.900	42.783	1.00	0.00	C
ATOM	410	HG2	ARG	25	26.304	35.653	42.474	1.00	0.00	H
ATOM	411	HG3	ARG	25	28.068	35.173	42.600	1.00	0.00	H
ATOM	412	CD	ARG	25	26.799	33.612	41.981	1.00	0.00	C
ATOM	413	HD2	ARG	25	27.654	32.954	42.135	1.00	0.00	H
ATOM	414	HD3	ARG	25	25.886	33.122	42.322	1.00	0.00	H
ATOM	415	NE	ARG	25	26.623	33.895	40.549	1.00	0.00	N
ATOM	416	HE	ARG	25	27.502	33.882	40.049	1.00	0.00	H
ATOM	417	CZ	ARG	25	25.451	34.041	39.952	1.00	0.00	C
ATOM	418	NH1	ARG	25	24.285	34.193	40.502	1.00	0.00	N
ATOM	419	HH11	ARG	25	23.492	34.335	39.892	1.00	0.00	H
ATOM	420	HH12	ARG	25	24.169	34.231	41.505	1.00	0.00	H
ATOM	421	NH2	ARG	25	25.416	34.086	38.646	1.00	0.00	N

ATOM	422	HH21	ARG	25	24.564	33.953	38.120	1.00	0.00	H
ATOM	423	HH22	ARG	25	26.277	34.159	38.125	1.00	0.00	H
ATOM	424	C	ARG	25	26.441	35.604	46.712	1.00	0.00	C
ATOM	425	O	ARG	25	27.075	35.529	47.753	1.00	0.00	O
ATOM	426	N	ASP	26	25.087	35.520	46.684	1.00	0.00	N
ATOM	427	H	ASP	26	24.526	35.729	45.870	1.00	0.00	H
ATOM	428	CA	ASP	26	24.347	35.059	47.820	1.00	0.00	C
ATOM	429	HA	ASP	26	24.767	35.555	48.695	1.00	0.00	H
ATOM	430	CB	ASP	26	22.898	35.626	47.694	1.00	0.00	C
ATOM	431	HB2	ASP	26	22.378	35.495	48.643	1.00	0.00	H
ATOM	432	HB3	ASP	26	22.926	36.712	47.591	1.00	0.00	H
ATOM	433	CG	ASP	26	22.120	35.039	46.524	1.00	0.00	C
ATOM	434	OD1	ASP	26	22.100	35.703	45.463	1.00	0.00	O
ATOM	435	OD2	ASP	26	21.349	34.051	46.662	1.00	0.00	O
ATOM	436	C	ASP	26	24.454	33.542	47.860	1.00	0.00	C
ATOM	437	O	ASP	26	24.559	32.894	46.850	1.00	0.00	O
ATOM	438	N	GLU	27	24.358	32.933	49.071	1.00	0.00	N
ATOM	439	H	GLU	27	24.089	33.515	49.852	1.00	0.00	H
ATOM	440	CA	GLU	27	24.655	31.483	49.325	1.00	0.00	C
ATOM	441	HA	GLU	27	25.649	31.302	48.915	1.00	0.00	H
ATOM	442	CB	GLU	27	24.754	31.257	50.880	1.00	0.00	C
ATOM	443	HB2	GLU	27	25.564	31.865	51.282	1.00	0.00	H
ATOM	444	HB3	GLU	27	23.852	31.609	51.380	1.00	0.00	H
ATOM	445	CG	GLU	27	25.071	29.804	51.278	1.00	0.00	C
ATOM	446	HG2	GLU	27	25.194	29.785	52.361	1.00	0.00	H
ATOM	447	HG3	GLU	27	24.163	29.286	50.971	1.00	0.00	H
ATOM	448	CD	GLU	27	26.332	29.357	50.588	1.00	0.00	C
ATOM	449	OE1	GLU	27	26.355	28.584	49.637	1.00	0.00	O
ATOM	450	OE2	GLU	27	27.437	29.673	51.069	1.00	0.00	O
ATOM	451	C	GLU	27	23.594	30.610	48.664	1.00	0.00	C
ATOM	452	O	GLU	27	22.439	30.727	49.054	1.00	0.00	O
ATOM	453	N	SER	28	24.036	29.643	47.829	1.00	0.00	N
ATOM	454	H	SER	28	25.036	29.592	47.697	1.00	0.00	H
ATOM	455	CA	SER	28	23.142	28.718	47.117	1.00	0.00	C
ATOM	456	HA	SER	28	22.361	28.380	47.799	1.00	0.00	H
ATOM	457	CB	SER	28	22.462	29.425	45.943	1.00	0.00	C
ATOM	458	HB2	SER	28	21.962	30.296	46.366	1.00	0.00	H
ATOM	459	HB3	SER	28	23.183	29.842	45.240	1.00	0.00	H
ATOM	460	OG	SER	28	21.523	28.552	45.349	1.00	0.00	O
ATOM	461	HG	SER	28	21.849	28.317	44.478	1.00	0.00	H
ATOM	462	C	SER	28	23.866	27.534	46.619	1.00	0.00	C
ATOM	463	O	SER	28	25.017	27.678	46.238	1.00	0.00	O
ATOM	464	N	GLU	29	23.246	26.345	46.536	1.00	0.00	N
ATOM	465	H	GLU	29	22.411	26.189	47.082	1.00	0.00	H
ATOM	466	CA	GLU	29	23.942	25.163	46.067	1.00	0.00	C
ATOM	467	HA	GLU	29	24.910	25.193	46.570	1.00	0.00	H
ATOM	468	CB	GLU	29	23.273	23.852	46.668	1.00	0.00	C
ATOM	469	HB2	GLU	29	23.309	23.868	47.757	1.00	0.00	H
ATOM	470	HB3	GLU	29	22.259	23.956	46.285	1.00	0.00	H
ATOM	471	CG	GLU	29	23.836	22.414	46.356	1.00	0.00	C
ATOM	472	HG2	GLU	29	23.085	21.644	46.532	1.00	0.00	H
ATOM	473	HG3	GLU	29	23.882	22.217	45.285	1.00	0.00	H
ATOM	474	CD	GLU	29	25.146	22.208	47.044	1.00	0.00	C
ATOM	475	OE1	GLU	29	25.123	22.178	48.298	1.00	0.00	O
ATOM	476	OE2	GLU	29	26.171	21.834	46.406	1.00	0.00	O
ATOM	477	C	GLU	29	24.244	25.117	44.561	1.00	0.00	C
ATOM	478	O	GLU	29	25.235	24.467	44.079	1.00	0.00	O
ATOM	479	N	SER	30	23.451	25.842	43.833	1.00	0.00	N
ATOM	480	H	SER	30	22.743	26.297	44.391	1.00	0.00	H
ATOM	481	CA	SER	30	23.462	25.928	42.360	1.00	0.00	C
ATOM	482	HA	SER	30	23.339	24.920	41.964	1.00	0.00	H
ATOM	483	CB	SER	30	22.199	26.623	41.938	1.00	0.00	C
ATOM	484	HB2	SER	30	22.252	26.927	40.893	1.00	0.00	H
ATOM	485	HB3	SER	30	21.358	25.965	42.156	1.00	0.00	H

ATOM	486	OG	SER	30	22.074	27.794	42.635	1.00	0.00	O
ATOM	487	HG	SER	30	21.683	28.409	42.009	1.00	0.00	H
ATOM	488	C	SER	30	24.706	26.614	41.614	1.00	0.00	C
ATOM	489	O	SER	30	24.896	26.415	40.421	1.00	0.00	O
ATOM	490	N	HIE	31	25.616	27.154	42.377	1.00	0.00	N
ATOM	491	H	HIE	31	25.438	26.998	43.359	1.00	0.00	H
ATOM	492	CA	HIE	31	26.890	27.804	41.890	1.00	0.00	C
ATOM	493	HA	HIE	31	26.680	28.551	41.124	1.00	0.00	H
ATOM	494	CB	HIE	31	27.580	28.667	43.011	1.00	0.00	C
ATOM	495	HB2	HIE	31	27.951	28.097	43.862	1.00	0.00	H
ATOM	496	HB3	HIE	31	28.474	29.186	42.668	1.00	0.00	H
ATOM	497	CG	HIE	31	26.688	29.771	43.510	1.00	0.00	C
ATOM	498	ND1	HIE	31	25.760	30.427	42.758	1.00	0.00	N
ATOM	499	CE1	HIE	31	25.005	31.094	43.659	1.00	0.00	C
ATOM	500	HE1	HIE	31	24.224	31.783	43.373	1.00	0.00	H
ATOM	501	NE2	HIE	31	25.442	30.945	44.895	1.00	0.00	N
ATOM	502	HE2	HIE	31	25.079	31.463	45.682	1.00	0.00	H
ATOM	503	CD2	HIE	31	26.488	30.092	44.834	1.00	0.00	C
ATOM	504	HD2	HIE	31	27.047	29.722	45.681	1.00	0.00	H
ATOM	505	C	HIE	31	27.973	26.860	41.365	1.00	0.00	C
ATOM	506	O	HIE	31	27.964	25.590	41.469	1.00	0.00	O
ATOM	507	N	PHE	32	29.021	27.474	40.732	1.00	0.00	N
ATOM	508	H	PHE	32	29.068	28.481	40.661	1.00	0.00	H
ATOM	509	CA	PHE	32	30.342	26.692	40.464	1.00	0.00	C
ATOM	510	HA	PHE	32	30.019	25.788	39.947	1.00	0.00	H
ATOM	511	CB	PHE	32	31.289	27.570	39.615	1.00	0.00	C
ATOM	512	HB2	PHE	32	31.543	28.409	40.261	1.00	0.00	H
ATOM	513	HB3	PHE	32	32.220	27.015	39.500	1.00	0.00	H
ATOM	514	CG	PHE	32	30.677	28.010	38.302	1.00	0.00	C
ATOM	515	CD1	PHE	32	30.624	29.420	38.033	1.00	0.00	C
ATOM	516	HD1	PHE	32	30.927	30.104	38.812	1.00	0.00	H
ATOM	517	CE1	PHE	32	30.141	29.932	36.832	1.00	0.00	C
ATOM	518	HE1	PHE	32	29.993	30.994	36.709	1.00	0.00	H
ATOM	519	CZ	PHE	32	29.722	29.035	35.862	1.00	0.00	C
ATOM	520	HZ	PHE	32	29.473	29.403	34.877	1.00	0.00	H
ATOM	521	CE2	PHE	32	29.951	27.676	36.038	1.00	0.00	C
ATOM	522	HE2	PHE	32	29.723	27.054	35.185	1.00	0.00	H
ATOM	523	CD2	PHE	32	30.348	27.114	37.291	1.00	0.00	C
ATOM	524	HD2	PHE	32	30.604	26.077	37.444	1.00	0.00	H
ATOM	525	C	PHE	32	30.938	26.238	41.810	1.00	0.00	C
ATOM	526	O	PHE	32	30.765	26.951	42.816	1.00	0.00	O
ATOM	527	N	LYS	33	31.620	25.085	41.899	1.00	0.00	N
ATOM	528	H	LYS	33	31.657	24.565	41.034	1.00	0.00	H
ATOM	529	CA	LYS	33	32.387	24.626	43.030	1.00	0.00	C
ATOM	530	HA	LYS	33	32.029	25.112	43.937	1.00	0.00	H
ATOM	531	CB	LYS	33	31.996	23.096	43.060	1.00	0.00	C
ATOM	532	HB2	LYS	33	32.111	22.642	42.075	1.00	0.00	H
ATOM	533	HB3	LYS	33	32.788	22.568	43.591	1.00	0.00	H
ATOM	534	CG	LYS	33	30.612	22.832	43.642	1.00	0.00	C
ATOM	535	HG2	LYS	33	29.812	23.491	43.302	1.00	0.00	H
ATOM	536	HG3	LYS	33	30.304	21.887	43.192	1.00	0.00	H
ATOM	537	CD	LYS	33	30.595	22.667	45.201	1.00	0.00	C
ATOM	538	HD2	LYS	33	31.286	21.850	45.415	1.00	0.00	H
ATOM	539	HD3	LYS	33	31.100	23.527	45.640	1.00	0.00	H
ATOM	540	CE	LYS	33	29.309	22.372	45.939	1.00	0.00	C
ATOM	541	HE2	LYS	33	29.100	21.336	45.672	1.00	0.00	H
ATOM	542	HE3	LYS	33	29.422	22.458	47.019	1.00	0.00	H
ATOM	543	NZ	LYS	33	28.159	23.267	45.415	1.00	0.00	N
ATOM	544	HZ1	LYS	33	27.422	23.049	46.070	1.00	0.00	H
ATOM	545	HZ2	LYS	33	28.457	24.232	45.392	1.00	0.00	H
ATOM	546	HZ3	LYS	33	27.814	23.040	44.494	1.00	0.00	H
ATOM	547	C	LYS	33	33.869	24.785	42.903	1.00	0.00	C
ATOM	548	O	LYS	33	34.375	25.018	41.795	1.00	0.00	O

ATOM	549	N	THR	34	34.722	24.582	43.946	1.00	0.00	N
ATOM	550	H	THR	34	34.319	24.464	44.865	1.00	0.00	H
ATOM	551	CA	THR	34	36.218	24.613	43.906	1.00	0.00	C
ATOM	552	HA	THR	34	36.457	25.623	43.572	1.00	0.00	H
ATOM	553	CB	THR	34	36.929	24.483	45.239	1.00	0.00	C
ATOM	554	HB	THR	34	36.473	23.601	45.690	1.00	0.00	H
ATOM	555	CG2	THR	34	38.459	24.325	45.204	1.00	0.00	C
ATOM	556	HG21	THR	34	38.641	23.465	44.560	1.00	0.00	H
ATOM	557	HG22	THR	34	38.911	25.176	44.697	1.00	0.00	H
ATOM	558	HG23	THR	34	38.872	24.127	46.193	1.00	0.00	H
ATOM	559	OG1	THR	34	36.767	25.576	46.092	1.00	0.00	O
ATOM	560	HG1	THR	34	37.019	25.297	46.975	1.00	0.00	H
ATOM	561	C	THR	34	36.680	23.482	42.906	1.00	0.00	C
ATOM	562	O	THR	34	36.241	22.309	43.001	1.00	0.00	O
ATOM	563	N	GLY	35	37.586	23.777	41.962	1.00	0.00	N
ATOM	564	H	GLY	35	38.086	24.655	41.973	1.00	0.00	H
ATOM	565	CA	GLY	35	37.894	22.764	40.900	1.00	0.00	C
ATOM	566	HA2	GLY	35	38.925	23.007	40.639	1.00	0.00	H
ATOM	567	HA3	GLY	35	37.950	21.766	41.334	1.00	0.00	H
ATOM	568	C	GLY	35	36.970	22.691	39.636	1.00	0.00	C
ATOM	569	O	GLY	35	37.098	21.756	38.849	1.00	0.00	O
ATOM	570	N	ASP	36	36.083	23.721	39.405	1.00	0.00	N
ATOM	571	H	ASP	36	36.042	24.523	40.016	1.00	0.00	H
ATOM	572	CA	ASP	36	35.272	23.894	38.240	1.00	0.00	C
ATOM	573	HA	ASP	36	35.044	22.893	37.873	1.00	0.00	H
ATOM	574	CB	ASP	36	33.955	24.391	38.736	1.00	0.00	C
ATOM	575	HB2	ASP	36	34.109	25.249	39.392	1.00	0.00	H
ATOM	576	HB3	ASP	36	33.453	24.801	37.860	1.00	0.00	H
ATOM	577	CG	ASP	36	32.958	23.302	39.197	1.00	0.00	C
ATOM	578	OD1	ASP	36	33.350	22.103	39.320	1.00	0.00	O
ATOM	579	OD2	ASP	36	31.789	23.693	39.470	1.00	0.00	O
ATOM	580	C	ASP	36	36.053	24.638	37.110	1.00	0.00	C
ATOM	581	O	ASP	36	36.983	25.458	37.344	1.00	0.00	O
ATOM	582	N	VAL	37	35.696	24.223	35.888	1.00	0.00	N
ATOM	583	H	VAL	37	34.977	23.516	35.845	1.00	0.00	H
ATOM	584	CA	VAL	37	36.422	24.582	34.646	1.00	0.00	C
ATOM	585	HA	VAL	37	37.200	25.312	34.867	1.00	0.00	H
ATOM	586	CB	VAL	37	37.160	23.372	34.112	1.00	0.00	C
ATOM	587	HB	VAL	37	36.444	22.714	33.621	1.00	0.00	H
ATOM	588	CG1	VAL	37	38.177	23.679	33.047	1.00	0.00	C
ATOM	589	HG11	VAL	37	38.808	24.460	33.473	1.00	0.00	H
ATOM	590	HG12	VAL	37	38.770	22.855	32.651	1.00	0.00	H
ATOM	591	HG13	VAL	37	37.602	24.171	32.261	1.00	0.00	H
ATOM	592	CG2	VAL	37	37.897	22.503	35.204	1.00	0.00	C
ATOM	593	HG21	VAL	37	38.453	21.728	34.675	1.00	0.00	H
ATOM	594	HG22	VAL	37	38.632	23.157	35.674	1.00	0.00	H
ATOM	595	HG23	VAL	37	37.187	22.176	35.964	1.00	0.00	H
ATOM	596	C	VAL	37	35.606	25.195	33.523	1.00	0.00	C
ATOM	597	O	VAL	37	34.666	24.611	33.024	1.00	0.00	O
ATOM	598	N	LEU	38	36.111	26.364	32.989	1.00	0.00	N
ATOM	599	H	LEU	38	36.943	26.732	33.429	1.00	0.00	H
ATOM	600	CA	LEU	38	35.391	27.164	31.996	1.00	0.00	C
ATOM	601	HA	LEU	38	34.523	26.673	31.555	1.00	0.00	H
ATOM	602	CB	LEU	38	34.771	28.437	32.648	1.00	0.00	C
ATOM	603	HB2	LEU	38	35.546	29.186	32.811	1.00	0.00	H
ATOM	604	HB3	LEU	38	34.050	28.790	31.911	1.00	0.00	H
ATOM	605	CG	LEU	38	34.015	28.221	33.986	1.00	0.00	C
ATOM	606	HG	LEU	38	33.442	27.307	33.826	1.00	0.00	H
ATOM	607	CD1	LEU	38	34.839	28.123	35.273	1.00	0.00	C
ATOM	608	HD11	LEU	38	34.242	27.874	36.150	1.00	0.00	H
ATOM	609	HD12	LEU	38	35.663	27.425	35.121	1.00	0.00	H
ATOM	610	HD13	LEU	38	35.321	29.072	35.507	1.00	0.00	H
ATOM	611	CD2	LEU	38	33.030	29.419	34.124	1.00	0.00	C
ATOM	612	HD21	LEU	38	32.448	29.273	35.033	1.00	0.00	H

```
ATOM    613 HD22 LEU    38      33.431  30.431  34.190  1.00  0.00           H
ATOM    614 HD23 LEU    38      32.371  29.449  33.256  1.00  0.00           H
ATOM    615  C   LEU    38      36.350  27.470  30.836  1.00  0.00           C
ATOM    616  O   LEU    38      37.497  27.042  30.884  1.00  0.00           O
ATOM    617  N   ARG    39      35.817  28.140  29.768  1.00  0.00           N
ATOM    618  H   ARG    39      34.810  28.193  29.824  1.00  0.00           H
ATOM    619  CA  ARG    39      36.630  28.727  28.656  1.00  0.00           C
ATOM    620  HA  ARG    39      37.605  28.847  29.129  1.00  0.00           H
ATOM    621  CB  ARG    39      36.725  27.792
  27.492  1.00  0.00           C
ATOM    622  HB2 ARG    39      35.748  27.573  27.061  1.00  0.00           H
ATOM    623  HB3 ARG    39      37.382  28.380  26.853  1.00  0.00           H
ATOM    624  CG  ARG    39      37.269  26.455  27.886  1.00  0.00           C
ATOM    625  HG2 ARG    39      38.101  26.373  28.586  1.00  0.00           H
ATOM    626  HG3 ARG    39      36.677  25.734  28.449  1.00  0.00           H
ATOM    627  CD  ARG    39      37.751  25.813  26.653  1.00  0.00           C
ATOM    628  HD2 ARG    39      38.180  26.580  26.008  1.00  0.00           H
ATOM    629  HD3 ARG    39      38.514  25.099  26.964  1.00  0.00           H
ATOM    630  NE  ARG    39      36.564  25.086  26.028  1.00  0.00           N
ATOM    631  HE  ARG    39      35.653  25.448  26.269  1.00  0.00           H
ATOM    632  CZ  ARG    39      36.677  23.951  25.320  1.00  0.00           C
ATOM    633  NH1 ARG    39      37.875  23.516  25.030  1.00  0.00           N
ATOM    634 HH11 ARG    39      38.718  24.057  25.158  1.00  0.00           H
ATOM    635 HH12 ARG    39      37.837  22.844  24.277  1.00  0.00           H
ATOM    636  NH2 ARG    39      35.598  23.197  24.965  1.00  0.00           N
ATOM    637 HH21 ARG    39      35.774  22.316  24.505  1.00  0.00           H
ATOM    638 HH22 ARG    39      34.706  23.292  25.430  1.00  0.00           H
ATOM    639  C   ARG    39      36.041  30.056  28.231  1.00  0.00           C
ATOM    640  O   ARG    39      34.932  30.428  28.629  1.00  0.00           O
ATOM    641  N   VAL    40      36.880  30.854  27.448  1.00  0.00           N
ATOM    642  H   VAL    40      37.754  30.461  27.129  1.00  0.00           H
ATOM    643  CA  VAL    40      36.534  32.228  27.018  1.00  0.00           C
ATOM    644  HA  VAL    40      35.453  32.307  26.907  1.00  0.00           H
ATOM    645  CB  VAL    40      36.754  33.389  28.125  1.00  0.00           C
ATOM    646  HB  VAL    40      36.635  34.336  27.599  1.00  0.00           H
ATOM    647  CG1 VAL    40      35.666  33.423  29.181  1.00  0.00           C
ATOM    648 HG11 VAL    40      35.804  32.702  29.986  1.00  0.00           H
ATOM    649 HG12 VAL    40      35.486  34.441  29.524  1.00  0.00           H
ATOM    650 HG13 VAL    40      34.747  33.105  28.690  1.00  0.00           H
ATOM    651  CG2 VAL    40      38.212  33.490  28.660  1.00  0.00           C
ATOM    652 HG21 VAL    40      38.335  34.363  29.300  1.00  0.00           H
ATOM    653 HG22 VAL    40      38.512  32.602  29.217  1.00  0.00           H
ATOM    654 HG23 VAL    40      38.969  33.483  27.877  1.00  0.00           H
ATOM    655  C   VAL    40      37.250  32.649  25.753  1.00  0.00           C
ATOM    656  O   VAL    40      38.460  32.468  25.670  1.00  0.00           O
ATOM    657  N   GLY    41      36.385  33.131  24.797  1.00  0.00           N
ATOM    658  H   GLY    41      35.388  33.177  24.955  1.00  0.00           H
ATOM    659  CA  GLY    41      36.853  33.693  23.612  1.00  0.00           C
ATOM    660  HA2 GLY    41      37.227  34.697  23.806  1.00  0.00           H
ATOM    661  HA3 GLY    41      37.754  33.195  23.254  1.00  0.00           H
ATOM    662  C   GLY    41      35.753  33.802  22.508  1.00  0.00           C
ATOM    663  O   GLY    41      34.565  33.535  22.708  1.00  0.00           O
ATOM    664  N   ARG    42      36.113  34.254  21.373  1.00  0.00           N
ATOM    665  H   ARG    42      37.116  34.268  21.252  1.00  0.00           H
ATOM    666  CA  ARG    42      35.205  34.568  20.257  1.00  0.00           C
ATOM    667  HA  ARG    42      34.588  33.721  19.959  1.00  0.00           H
ATOM    668  CB  ARG    42      34.255  35.784  20.626  1.00  0.00           C
ATOM    669  HB2 ARG    42      33.640  35.967  19.744  1.00  0.00           H
ATOM    670  HB3 ARG    42      33.622  35.667  21.505  1.00  0.00           H
ATOM    671  CG  ARG    42      34.972  37.155  20.988  1.00  0.00           C
ATOM    672  HG2 ARG    42      35.519  37.004  21.919  1.00  0.00           H
ATOM    673  HG3 ARG    42      35.673  37.473  20.216  1.00  0.00           H
ATOM    674  CD  ARG    42      33.956  38.229  21.234  1.00  0.00           C
ATOM    675  HD2 ARG    42      33.177  38.186  20.474  1.00  0.00           H
```

ATOM	676	HD3	ARG	42	33.407	38.005	22.150	1.00	0.00	H
ATOM	677	NE	ARG	42	34.553	39.522	21.557	1.00	0.00	N
ATOM	678	HE	ARG	42	34.279	40.226	20.887	1.00	0.00	H
ATOM	679	CZ	ARG	42	35.121	39.981	22.599	1.00	0.00	C
ATOM	680	NH1	ARG	42	35.450	39.294	23.698	1.00	0.00	N
ATOM	681	HH11	ARG	42	35.719	39.899	24.462	1.00	0.00	H
ATOM	682	HH12	ARG	42	35.198	38.334	23.885	1.00	0.00	H
ATOM	683	NH2	ARG	42	35.360	41.236	22.748	1.00	0.00	N
ATOM	684	HH21	ARG	42	35.025	41.805	21.983	1.00	0.00	H
ATOM	685	HH22	ARG	42	35.533	41.555	23.690	1.00	0.00	H
ATOM	686	C	ARG	42	35.893	34.950	18.945	1.00	0.00	C
ATOM	687	O	ARG	42	35.231	35.336	18.002	1.00	0.00	O
ATOM	688	N	PHE	43	37.166	34.885	18.924	1.00	0.00	N
ATOM	689	H	PHE	43	37.624	34.660	19.796	1.00	0.00	H
ATOM	690	CA	PHE	43	37.980	35.213	17.764	1.00	0.00	C
ATOM	691	HA	PHE	43	37.353	35.713	17.026	1.00	0.00	H
ATOM	692	CB	PHE	43	39.077	36.228	18.227	1.00	0.00	C
ATOM	693	HB2	PHE	43	39.763	35.800	18.957	1.00	0.00	H
ATOM	694	HB3	PHE	43	39.671	36.413	17.333	1.00	0.00	H
ATOM	695	CG	PHE	43	38.631	37.560	18.713	1.00	0.00	C
ATOM	696	CD1	PHE	43	38.283	38.612	17.882	1.00	0.00	C
ATOM	697	HD1	PHE	43	38.296	38.379	16.828	1.00	0.00	H
ATOM	698	CE1	PHE	43	37.855	39.875	18.285	1.00	0.00	C
ATOM	699	HE1	PHE	43	37.659	40.614	17.522	1.00	0.00	H
ATOM	700	CZ	PHE	43	37.804	40.099	19.696	1.00	0.00	C
ATOM	701	HZ	PHE	43	37.375	40.963	20.182	1.00	0.00	H
ATOM	702	CE2	PHE	43	38.128	39.011	20.562	1.00	0.00	C
ATOM	703	HE2	PHE	43	37.993	39.087	21.631	1.00	0.00	H
ATOM	704	CD2	PHE	43	38.508	37.783	20.051	1.00	0.00	C
ATOM	705	HD2	PHE	43	38.842	37.032	20.751	1.00	0.00	H
ATOM	706	C	PHE	43	38.663	33.957	17.209	1.00	0.00	C
ATOM	707	O	PHE	43	39.024	33.025	17.976	1.00	0.00	O
ATOM	708	N	GLU	44	39.015	33.941	15.925	1.00	0.00	N
ATOM	709	H	GLU	44	38.737	34.805	15.483	1.00	0.00	H
ATOM	710	CA	GLU	44	39.375	32.730	15.174	1.00	0.00	C
ATOM	711	HA	GLU	44	38.632	32.049	15.590	1.00	0.00	H
ATOM	712	CB	GLU	44	39.071	32.902	13.685	1.00	0.00	C
ATOM	713	HB2	GLU	44	38.183	33.514	13.529	1.00	0.00	H
ATOM	714	HB3	GLU	44	39.901	33.314	13.110	1.00	0.00	H
ATOM	715	CG	GLU	44	38.942	31.533	13.013	1.00	0.00	C
ATOM	716	HG2	GLU	44	39.923	31.076	12.883	1.00	0.00	H
ATOM	717	HG3	GLU	44	38.391	30.873	13.682	1.00	0.00	H
ATOM	718	CD	GLU	44	38.165	31.760	11.756	1.00	0.00	C
ATOM	719	OE1	GLU	44	38.581	32.721	11.021	1.00	0.00	O
ATOM	720	OE2	GLU	44	37.277	31.013	11.306	1.00	0.00	O
ATOM	721	C	GLU	44	40.696	32.099	15.544	1.00	0.00	C
ATOM	722	O	GLU	44	41.220	31.146	14.954	1.00	0.00	O
ATOM	723	N	ASP	45	41.317	32.614	16.605	1.00	0.00	N
ATOM	724	H	ASP	45	40.690	33.038	17.273	1.00	0.00	H
ATOM	725	CA	ASP	45	42.509	32.088	17.168	1.00	0.00	C
ATOM	726	HA	ASP	45	43.183	31.731	16.390	1.00	0.00	H
ATOM	727	CB	ASP	45	43.294	33.305	17.841	1.00	0.00	C
ATOM	728	HB2	ASP	45	42.545	33.753	18.494	1.00	0.00	H
ATOM	729	HB3	ASP	45	44.103	32.987	18.498	1.00	0.00	H
ATOM	730	CG	ASP	45	43.851	34.200	16.738	1.00	0.00	C
ATOM	731	OD1	ASP	45	43.662	35.491	16.868	1.00	0.00	O
ATOM	732	OD2	ASP	45	44.583	33.664	15.873	1.00	0.00	O
ATOM	733	C	ASP	45	42.260	30.940	18.146	1.00	0.00	C
ATOM	734	O	ASP	45	42.302	31.079	19.390	1.00	0.00	O
ATOM	735	N	ASP	46	42.062	29.717	17.695	1.00	0.00	N
ATOM	736	H	ASP	46	42.201	29.576	16.705	1.00	0.00	H
ATOM	737	CA	ASP	46	41.729	28.509	18.447	1.00	0.00	C
ATOM	738	HA	ASP	46	41.630	27.744	17.677	1.00	0.00	H
ATOM	739	CB	ASP	46	42.959	27.972	19.364	1.00	0.00	C

```
ATOM    740  HB2 ASP    46      43.075  28.537  20.288  1.00  0.00           H
ATOM    741  HB3 ASP    46      42.871  26.936  19.690  1.00  0.00           H
ATOM    742  CG  ASP    46      44.286  28.186  18.665  1.00  0.00           C
ATOM    743  OD1 ASP    46      45.249  28.717  19.249  1.00  0.00           O
ATOM    744  OD2 ASP    46      44.308  27.732  17.542  1.00  0.00           O
ATOM    745  C   ASP    46      40.357  28.594  19.295  1.00  0.00           C
ATOM    746  O   ASP    46      40.102  27.667  20.064  1.00  0.00
      O
ATOM    747  N   GLY    47      39.554  29.662  19.125  1.00  0.00           N
ATOM    748  H   GLY    47      40.090  30.434  18.753  1.00  0.00           H
ATOM    749  CA  GLY    47      38.144  29.872  19.550  1.00  0.00           C
ATOM    750  HA2 GLY    47      37.698  30.636  18.913  1.00  0.00           H
ATOM    751  HA3 GLY    47      37.605  28.954  19.315  1.00  0.00           H
ATOM    752  C   GLY    47      37.989  30.284  21.037  1.00  0.00           C
ATOM    753  O   GLY    47      37.094  31.032  21.440  1.00  0.00           O
ATOM    754  N   TYR    48      38.982  29.791  21.783  1.00  0.00           N
ATOM    755  H   TYR    48      39.664  29.144  21.415  1.00  0.00           H
ATOM    756  CA  TYR    48      39.098  30.173  23.202  1.00  0.00           C
ATOM    757  HA  TYR    48      38.321  30.898  23.443  1.00  0.00           H
ATOM    758  CB  TYR    48      38.863  28.899  23.975  1.00  0.00           C
ATOM    759  HB2 TYR    48      39.751  28.267  23.970  1.00  0.00           H
ATOM    760  HB3 TYR    48      38.792  29.242  25.008  1.00  0.00           H
ATOM    761  CG  TYR    48      37.671  28.076  23.682  1.00  0.00           C
ATOM    762  CD1 TYR    48      36.393  28.491  23.973  1.00  0.00           C
ATOM    763  HD1 TYR    48      36.273  29.504  24.329  1.00  0.00           H
ATOM    764  CE1 TYR    48      35.305  27.629  23.836  1.00  0.00           C
ATOM    765  HE1 TYR    48      34.352  27.972  24.212  1.00  0.00           H
ATOM    766  CZ  TYR    48      35.463  26.377  23.167  1.00  0.00           C
ATOM    767  OH  TYR    48      34.419  25.514  23.013  1.00  0.00           O
ATOM    768  HH  TYR    48      34.741  24.643  22.767  1.00  0.00           H
ATOM    769  CE2 TYR    48      36.714  26.039  22.734  1.00  0.00           C
ATOM    770  HE2 TYR    48      36.849  25.078  22.261  1.00  0.00           H
ATOM    771  CD2 TYR    48      37.830  26.855  23.022  1.00  0.00           C
ATOM    772  HD2 TYR    48      38.786  26.437  22.742  1.00  0.00           H
ATOM    773  C   TYR    48      40.587  30.675  23.476  1.00  0.00           C
ATOM    774  O   TYR    48      41.539  30.044  23.135  1.00  0.00           O
ATOM    775  N   PHE    49      40.676  31.858  24.077  1.00  0.00           N
ATOM    776  H   PHE    49      39.801  32.318  24.285  1.00  0.00           H
ATOM    777  CA  PHE    49      41.960  32.354  24.489  1.00  0.00           C
ATOM    778  HA  PHE    49      42.717  32.027  23.776  1.00  0.00           H
ATOM    779  CB  PHE    49      41.995  33.901  24.301  1.00  0.00           C
ATOM    780  HB2 PHE    49      43.033  34.201  24.446  1.00  0.00           H
ATOM    781  HB3 PHE    49      41.627  34.262  23.341  1.00  0.00           H
ATOM    782  CG  PHE    49      41.249  34.679  25.358  1.00  0.00           C
ATOM    783  CD1 PHE    49      41.739  34.772  26.636  1.00  0.00           C
ATOM    784  HD1 PHE    49      42.711  34.339  26.828  1.00  0.00           H
ATOM    785  CE1 PHE    49      41.052  35.510  27.578  1.00  0.00           C
ATOM    786  HE1 PHE    49      41.454  35.613  28.576  1.00  0.00           H
ATOM    787  CZ  PHE    49      39.817  36.086  27.233  1.00  0.00           C
ATOM    788  HZ  PHE    49      39.332  36.741  27.940  1.00  0.00           H
ATOM    789  CE2 PHE    49      39.272  35.928  25.918  1.00  0.00           C
ATOM    790  HE2 PHE    49      38.395  36.468  25.595  1.00  0.00           H
ATOM    791  CD2 PHE    49      40.017  35.206  24.944  1.00  0.00           C
ATOM    792  HD2 PHE    49      39.701  35.144  23.913  1.00  0.00           H
ATOM    793  C   PHE    49      42.403  31.754  25.798  1.00  0.00           C
ATOM    794  O   PHE    49      43.591  31.719  26.102  1.00  0.00           O
ATOM    795  N   CYX    50      41.507  31.377  26.620  1.00  0.00           N
ATOM    796  H   CYX    50      40.564  31.661  26.396  1.00  0.00           H
ATOM    797  CA  CYX    50      41.851  30.775  27.893  1.00  0.00           C
ATOM    798  HA  CYX    50      42.852  30.342  27.922  1.00  0.00           H
ATOM    799  CB  CYX    50      41.899  31.898  28.940  1.00  0.00           C
ATOM    800  HB2 CYX    50      41.127  32.649  28.771  1.00  0.00           H
ATOM    801  HB3 CYX    50      41.786  31.343  29.870  1.00  0.00           H
ATOM    802  SG  CYX    50      43.451  32.751  29.202  1.00  0.00           S
```

ATOM	803	C	CYX	50	40.831	29.667	28.288	1.00	0.00	C
ATOM	804	O	CYX	50	39.651	29.960	28.346	1.00	0.00	O
ATOM	805	N	THR	51	41.271	28.447	28.596	1.00	0.00	N
ATOM	806	H	THR	51	42.265	28.350	28.445	1.00	0.00	H
ATOM	807	CA	THR	51	40.657	27.668	29.712	1.00	0.00	C
ATOM	808	HA	THR	51	39.587	27.682	29.505	1.00	0.00	H
ATOM	809	CB	THR	51	41.259	26.278	29.688	1.00	0.00	C
ATOM	810	HB	THR	51	42.347	26.321	29.659	1.00	0.00	H
ATOM	811	CG2	THR	51	40.864	25.365	30.901	1.00	0.00	C
ATOM	812	HG21	THR	51	39.859	24.979	30.740	1.00	0.00	H
ATOM	813	HG22	THR	51	41.575	24.540	30.860	1.00	0.00	H
ATOM	814	HG23	THR	51	41.012	25.948	31.811	1.00	0.00	H
ATOM	815	OG1	THR	51	40.710	25.723	28.582	1.00	0.00	O
ATOM	816	HG1	THR	51	40.872	26.267	27.808	1.00	0.00	H
ATOM	817	C	THR	51	40.914	28.317	31.030	1.00	0.00	C
ATOM	818	O	THR	51	41.920	28.934	31.159	1.00	0.00	O
ATOM	819	N	ILE	52	39.914	28.236	31.961	1.00	0.00	N
ATOM	820	H	ILE	52	39.063	27.718	31.794	1.00	0.00	H
ATOM	821	CA	ILE	52	39.936	28.831	33.284	1.00	0.00	C
ATOM	822	HA	ILE	52	40.938	29.249	33.382	1.00	0.00	H
ATOM	823	CB	ILE	52	39.089	30.068	33.308	1.00	0.00	C
ATOM	824	HB	ILE	52	38.153	29.794	32.820	1.00	0.00	H
ATOM	825	CG2	ILE	52	38.742	30.581	34.757	1.00	0.00	C
ATOM	826	HG21	ILE	52	38.172	29.823	35.294	1.00	0.00	H
ATOM	827	HG22	ILE	52	39.693	30.869	35.206	1.00	0.00	H
ATOM	828	HG23	ILE	52	38.072	31.438	34.711	1.00	0.00	H
ATOM	829	CG1	ILE	52	39.608	31.181	32.410	1.00	0.00	C
ATOM	830	HG12	ILE	52	40.500	31.590	32.885	1.00	0.00	H
ATOM	831	HG13	ILE	52	40.103	30.711	31.560	1.00	0.00	H
ATOM	832	CD1	ILE	52	38.527	32.235	31.959	1.00	0.00	C
ATOM	833	HD11	ILE	52	37.794	31.757	31.308	1.00	0.00	H
ATOM	834	HD12	ILE	52	37.909	32.533	32.807	1.00	0.00	H
ATOM	835	HD13	ILE	52	38.886	33.113	31.422	1.00	0.00	H
ATOM	836	C	ILE	52	39.551	27.797	34.375	1.00	0.00	C
ATOM	837	O	ILE	52	38.694	27.044	34.082	1.00	0.00	O
ATOM	838	N	GLU	53	40.059	27.911	35.615	1.00	0.00	N
ATOM	839	H	GLU	53	40.756	28.630	35.748	1.00	0.00	H
ATOM	840	CA	GLU	53	39.783	27.086	36.769	1.00	0.00	C
ATOM	841	HA	GLU	53	38.950	26.437	36.497	1.00	0.00	H
ATOM	842	CB	GLU	53	41.010	26.230	37.089	1.00	0.00	C
ATOM	843	HB2	GLU	53	41.515	25.781	36.233	1.00	0.00	H
ATOM	844	HB3	GLU	53	41.841	26.826	37.462	1.00	0.00	H
ATOM	845	CG	GLU	53	40.783	25.156	38.172	1.00	0.00	C
ATOM	846	HG2	GLU	53	40.441	25.650	39.081	1.00	0.00	H
ATOM	847	HG3	GLU	53	39.972	24.627	37.670	1.00	0.00	H
ATOM	848	CD	GLU	53	41.925	24.199	38.474	1.00	0.00	C
ATOM	849	OE1	GLU	53	42.777	23.955	37.587	1.00	0.00	O
ATOM	850	OE2	GLU	53	41.846	23.563	39.562	1.00	0.00	O
ATOM	851	C	GLU	53	39.257	27.936	37.932	1.00	0.00	C
ATOM	852	O	GLU	53	39.847	28.997	38.213	1.00	0.00	O
ATOM	853	N	VAL	54	38.364	27.505	38.734	1.00	0.00	N
ATOM	854	H	VAL	54	37.970	26.587	38.580	1.00	0.00	H
ATOM	855	CA	VAL	54	38.058	27.952	40.101	1.00	0.00	C
ATOM	856	HA	VAL	54	38.158	29.039	40.105	1.00	0.00	H
ATOM	857	CB	VAL	54	36.548	27.670	40.542	1.00	0.00	C
ATOM	858	HB	VAL	54	36.306	26.610	40.472	1.00	0.00	H
ATOM	859	CG1	VAL	54	36.301	28.213	41.943	1.00	0.00	C
ATOM	860	HG11	VAL	54	35.281	27.985	42.252	1.00	0.00	H
ATOM	861	HG12	VAL	54	36.951	27.758	42.691	1.00	0.00	H
ATOM	862	HG13	VAL	54	36.494	29.285	41.991	1.00	0.00	H
ATOM	863	CG2	VAL	54	35.590	28.447	39.600	1.00	0.00	C
ATOM	864	HG21	VAL	54	34.576	28.337	39.982	1.00	0.00	H
ATOM	865	HG22	VAL	54	35.893	29.492	39.528	1.00	0.00	H
ATOM	866	HG23	VAL	54	35.644	27.990	38.611	1.00	0.00	H

ATOM	867	C	VAL	54	39.090	27.557	41.140	1.00	0.00	C
ATOM	868	O	VAL	54	39.148	26.419	41.619	1.00	0.00	O
ATOM	869	N	THR	55	39.902	28.522	41.512	1.00	0.00	N
ATOM	870	H	THR	55	39.702	29.452	41.173	1.00	0.00	H
ATOM	871	CA	THR	55	41.078	28.355	42.410	1.00	0.00	C
ATOM	872	HA	THR							
55	41.584	27.436	42.118	1.00	0.00	H				
ATOM	873	CB	THR	55	42.109	29.430	42.240	1.00	0.00	C
ATOM	874	HB	THR	55	42.958	29.066	42.818	1.00	0.00	H
ATOM	875	CG2	THR	55	42.609	29.601	40.799	1.00	0.00	C
ATOM	876	HG21	THR	55	43.431	30.317	40.797	1.00	0.00	H
ATOM	877	HG22	THR	55	42.756	28.636	40.313	1.00	0.00	H
ATOM	878	HG23	THR	55	41.839	30.045	40.167	1.00	0.00	H
ATOM	879	OG1	THR	55	41.659	30.723	42.581	1.00	0.00	O
ATOM	880	HG1	THR	55	41.692	30.834	43.534	1.00	0.00	H
ATOM	881	C	THR	55	40.546	28.177	43.820	1.00	0.00	C
ATOM	882	O	THR	55	41.001	27.234	44.484	1.00	0.00	O
ATOM	883	N	ALA	56	39.591	28.996	44.279	1.00	0.00	N
ATOM	884	H	ALA	56	39.237	29.740	43.694	1.00	0.00	H
ATOM	885	CA	ALA	56	38.981	28.847	45.593	1.00	0.00	C
ATOM	886	HA	ALA	56	39.056	27.810	45.922	1.00	0.00	H
ATOM	887	CB	ALA	56	39.777	29.789	46.543	1.00	0.00	C
ATOM	888	HB1	ALA	56	40.829	29.562	46.373	1.00	0.00	H
ATOM	889	HB2	ALA	56	39.510	30.820	46.313	1.00	0.00	H
ATOM	890	HB3	ALA	56	39.538	29.502	47.567	1.00	0.00	H
ATOM	891	C	ALA	56	37.554	29.303	45.633	1.00	0.00	C
ATOM	892	O	ALA	56	37.058	30.086	44.806	1.00	0.00	O
ATOM	893	N	THR	57	36.954	28.941	46.772	1.00	0.00	N
ATOM	894	H	THR	57	37.429	28.360	47.448	1.00	0.00	H
ATOM	895	CA	THR	57	35.583	29.398	47.134	1.00	0.00	C
ATOM	896	HA	THR	57	35.291	30.324	46.640	1.00	0.00	H
ATOM	897	CB	THR	57	34.474	28.368	46.711	1.00	0.00	C
ATOM	898	HB	THR	57	33.488	28.736	46.993	1.00	0.00	H
ATOM	899	CG2	THR	57	34.602	28.152	45.159	1.00	0.00	C
ATOM	900	HG21	THR	57	33.762	27.507	44.902	1.00	0.00	H
ATOM	901	HG22	THR	57	34.589	29.147	44.714	1.00	0.00	H
ATOM	902	HG23	THR	57	35.585	27.809	44.834	1.00	0.00	H
ATOM	903	OG1	THR	57	34.712	27.120	47.382	1.00	0.00	O
ATOM	904	HG1	THR	57	35.487	26.786	46.927	1.00	0.00	H
ATOM	905	C	THR	57	35.467	29.695	48.638	1.00	0.00	C
ATOM	906	O	THR	57	36.223	29.155	49.466	1.00	0.00	O
ATOM	907	N	SER	58	34.502	30.539	49.061	1.00	0.00	N
ATOM	908	H	SER	58	33.990	31.106	48.399	1.00	0.00	H
ATOM	909	CA	SER	58	34.236	30.823	50.479	1.00	0.00	C
ATOM	910	HA	SER	58	34.400	29.956	51.119	1.00	0.00	H
ATOM	911	CB	SER	58	35.119	31.948	51.028	1.00	0.00	C
ATOM	912	HB2	SER	58	36.168	31.887	50.736	1.00	0.00	H
ATOM	913	HB3	SER	58	34.722	32.901	50.680	1.00	0.00	H
ATOM	914	OG	SER	58	35.108	32.066	52.434	1.00	0.00	O
ATOM	915	HG	SER	58	35.300	32.981	52.650	1.00	0.00	H
ATOM	916	C	SER	58	32.891	31.312	50.873	1.00	0.00	C
ATOM	917	O	SER	58	32.315	32.225	50.263	1.00	0.00	O
ATOM	918	N	THR	59	32.235	30.769	51.950	1.00	0.00	N
ATOM	919	H	THR	59	32.712	29.965	52.331	1.00	0.00	H
ATOM	920	CA	THR	59	30.981	31.313	52.463	1.00	0.00	C
ATOM	921	HA	THR	59	30.467	31.727	51.595	1.00	0.00	H
ATOM	922	CB	THR	59	30.129	30.207	52.975	1.00	0.00	C
ATOM	923	HB	THR	59	30.116	29.356	52.292	1.00	0.00	H
ATOM	924	CG2	THR	59	30.577	29.703	54.344	1.00	0.00	C
ATOM	925	HG21	THR	59	29.846	28.986	54.718	1.00	0.00	H
ATOM	926	HG22	THR	59	31.614	29.366	54.397	1.00	0.00	H
ATOM	927	HG23	THR	59	30.573	30.570	55.004	1.00	0.00	H
ATOM	928	OG1	THR	59	28.850	30.709	53.158	1.00	0.00	O
ATOM	929	HG1	THR	59	28.459	30.689	52.281	1.00	0.00	H

ATOM	930	C	THR	59	31.292	32.423	53.515	1.00	0.00	C
ATOM	931	O	THR	59	32.313	32.207	54.261	1.00	0.00	O
ATOM	932	N	VAL	60	30.583	33.542	53.492	1.00	0.00	N
ATOM	933	H	VAL	60	29.685	33.599	53.035	1.00	0.00	H
ATOM	934	CA	VAL	60	30.937	34.795	54.128	1.00	0.00	C
ATOM	935	HA	VAL	60	31.518	34.660	55.040	1.00	0.00	H
ATOM	936	CB	VAL	60	31.854	35.664	53.138	1.00	0.00	C
ATOM	937	HB	VAL	60	32.588	34.958	52.749	1.00	0.00	H
ATOM	938	CG1	VAL	60	31.136	36.393	52.035	1.00	0.00	C
ATOM	939	HG11	VAL	60	30.460	37.156	52.423	1.00	0.00	H
ATOM	940	HG12	VAL	60	31.875	36.697	51.294	1.00	0.00	H
ATOM	941	HG13	VAL	60	30.494	35.698	51.495	1.00	0.00	H
ATOM	942	CG2	VAL	60	32.708	36.770	53.765	1.00	0.00	C
ATOM	943	HG21	VAL	60	33.139	36.344	54.672	1.00	0.00	H
ATOM	944	HG22	VAL	60	33.383	37.146	52.996	1.00	0.00	H
ATOM	945	HG23	VAL	60	32.093	37.588	54.141	1.00	0.00	H
ATOM	946	C	VAL	60	29.705	35.507	54.688	1.00	0.00	C
ATOM	947	O	VAL	60	28.560	35.062	54.526	1.00	0.00	O
ATOM	948	N	THR	61	29.852	36.607	55.443	1.00	0.00	N
ATOM	949	H	THR	61	30.766	36.966	55.679	1.00	0.00	H
ATOM	950	CA	THR	61	28.764	37.407	56.002	1.00	0.00	C
ATOM	951	HA	THR	61	27.928	37.241	55.323	1.00	0.00	H
ATOM	952	CB	THR	61	28.372	36.997	57.385	1.00	0.00	C
ATOM	953	HB	THR	61	27.565	37.673	57.671	1.00	0.00	H
ATOM	954	CG2	THR	61	27.805	35.590	57.437	1.00	0.00	C
ATOM	955	HG21	THR	61	28.504	34.848	57.052	1.00	0.00	H
ATOM	956	HG22	THR	61	27.480	35.227	58.413	1.00	0.00	H
ATOM	957	HG23	THR	61	26.848	35.660	56.921	1.00	0.00	H
ATOM	958	OG1	THR	61	29.442	36.925	58.296	1.00	0.00	O
ATOM	959	HG1	THR	61	29.806	37.803	58.437	1.00	0.00	H
ATOM	960	C	THR	61	28.957	38.900	55.685	1.00	0.00	C
ATOM	961	O	THR	61	30.020	39.328	55.218	1.00	0.00	O
ATOM	962	N	LEU	62	27.924	39.746	55.654	1.00	0.00	N
ATOM	963	H	LEU	62	26.993	39.408	55.852	1.00	0.00	H
ATOM	964	CA	LEU	62	28.114	41.146	55.107	1.00	0.00	C
ATOM	965	HA	LEU	62	28.487	40.970	54.098	1.00	0.00	H
ATOM	966	CB	LEU	62	26.772	41.855	55.107	1.00	0.00	C
ATOM	967	HB2	LEU	62	26.005	41.092	54.979	1.00	0.00	H
ATOM	968	HB3	LEU	62	26.653	42.368	56.061	1.00	0.00	H
ATOM	969	CG	LEU	62	26.635	42.961	53.971	1.00	0.00	C
ATOM	970	HG	LEU	62	27.376	43.721	54.220	1.00	0.00	H
ATOM	971	CD1	LEU	62	26.965	42.511	52.538	1.00	0.00	C
ATOM	972	HD11	LEU	62	26.873	43.421	51.946	1.00	0.00	H
ATOM	973	HD12	LEU	62	27.973	42.123	52.399	1.00	0.00	H
ATOM	974	HD13	LEU	62	26.321	41.687	52.230	1.00	0.00	H
ATOM	975	CD2	LEU	62	25.233	43.656	53.873	1.00	0.00	C
ATOM	976	HD21	LEU	62	24.812	43.687	54.879	1.00	0.00	H
ATOM	977	HD22	LEU	62	25.434	44.699	53.633	1.00	0.00	H
ATOM	978	HD23	LEU	62	24.611	43.339	53.036	1.00	0.00	H
ATOM	979	C	LEU	62	29.092	42.060	55.840	1.00	0.00	C
ATOM	980	O	LEU	62	29.606	43.048	55.267	1.00	0.00	O
ATOM	981	N	ASP	63	29.323	41.742	57.117	1.00	0.00	N
ATOM	982	H	ASP	63	28.737	41.013	57.497	1.00	0.00	H
ATOM	983	CA	ASP	63	30.382	42.331	57.916	1.00	0.00	C
ATOM	984	HA	ASP	63	30.268	43.414	57.910	1.00	0.00	H
ATOM	985	CB	ASP	63	30.288	41.852	59.382	1.00	0.00	C
ATOM	986	HB2	ASP	63	31.132	42.259	59.938	1.00	0.00	H
ATOM	987	HB3	ASP	63	29.395	42.272	59.845	1.00	0.00	H
ATOM	988	CG	ASP	63	30.183	40.336	59.575	1.00	0.00	C
ATOM	989	OD1	ASP	63	30.534	39.581	58.642	1.00	0.00	O
ATOM	990	OD2	ASP	63	29.852	39.845	60.650	1.00	0.00	O
ATOM	991	C	ASP	63	31.798	41.969	57.387	1.00	0.00	C
ATOM	992	O	ASP	63	32.857	42.472	57.810	1.00	0.00	O
ATOM	993	N	THR	64	31.934	40.845	56.679	1.00	0.00	N

```
ATOM    994  H   THR    64      31.146  40.213  56.679  1.00  0.00           H
ATOM    995  CA  THR    64      33.251  40.269  56.295  1.00  0.00           C
ATOM    996  HA  THR    64      34.054  40.947  56.587  1.00  0.00           H
ATOM    997  CB  THR    64      33.584  39.027
  57.193  1.00  0.00          C
ATOM    998  HB  THR    64      34.311  38.354  56.740  1.00  0.00           H
ATOM    999  CG2 THR    64      33.982  39.372  58.593  1.00  0.00           C
ATOM   1000 HG21 THR    64      35.034  39.617  58.740  1.00  0.00           H
ATOM   1001 HG22 THR    64      33.372  40.238  58.853  1.00  0.00           H
ATOM   1002 HG23 THR    64      33.710  38.555  59.261  1.00  0.00           H
ATOM   1003  OG1 THR    64      32.340  38.260  57.277  1.00  0.00           O
ATOM   1004  HG1 THR    64      31.682  38.613  57.881  1.00  0.00           H
ATOM   1005  C   THR    64      33.444  40.014  54.807  1.00  0.00           C
ATOM   1006  O   THR    64      34.292  39.170  54.456  1.00  0.00           O
ATOM   1007  N   LEU    65      32.920  40.845  53.937  1.00  0.00           N
ATOM   1008  H   LEU    65      32.196  41.486  54.230  1.00  0.00           H
ATOM   1009  CA  LEU    65      33.288  40.730  52.469  1.00  0.00           C
ATOM   1010  HA  LEU    65      32.779  39.836  52.110  1.00  0.00           H
ATOM   1011  CB  LEU    65      32.483  41.800  51.669  1.00  0.00           C
ATOM   1012  HB2 LEU    65      32.986  42.754  51.831  1.00  0.00           H
ATOM   1013  HB3 LEU    65      32.793  41.557  50.653  1.00  0.00           H
ATOM   1014  CG  LEU    65      30.942  41.894  51.792  1.00  0.00           C
ATOM   1015  HG  LEU    65      30.675  42.255  52.786  1.00  0.00           H
ATOM   1016  CD1 LEU    65      30.506  43.017  50.825  1.00  0.00           C
ATOM   1017 HD11 LEU    65      31.037  43.968  50.871  1.00  0.00           H
ATOM   1018 HD12 LEU    65      30.669  42.775  49.774  1.00  0.00           H
ATOM   1019 HD13 LEU    65      29.457  43.227  51.029  1.00  0.00           H
ATOM   1020  CD2 LEU    65      30.247  40.563  51.438  1.00  0.00           C
ATOM   1021 HD21 LEU    65      30.250  39.899  52.303  1.00  0.00           H
ATOM   1022 HD22 LEU    65      29.196  40.701  51.188  1.00  0.00           H
ATOM   1023 HD23 LEU    65      30.664  39.998  50.604  1.00  0.00           H
ATOM   1024  C   LEU    65      34.736  40.911  52.031  1.00  0.00           C
ATOM   1025  O   LEU    65      35.407  41.736  52.604  1.00  0.00           O
ATOM   1026  N   THR    66      35.192  40.109  51.039  1.00  0.00           N
ATOM   1027  H   THR    66      34.609  39.352  50.712  1.00  0.00           H
ATOM   1028  CA  THR    66      36.626  40.294  50.671  1.00  0.00           C
ATOM   1029  HA  THR    66      37.275  40.143  51.533  1.00  0.00           H
ATOM   1030  CB  THR    66      37.043  39.092  49.778  1.00  0.00           C
ATOM   1031  HB  THR    66      36.845  38.098  50.177  1.00  0.00           H
ATOM   1032  CG2 THR    66      36.325  39.113  48.463  1.00  0.00           C
ATOM   1033 HG21 THR    66      35.247  39.198  48.601  1.00  0.00           H
ATOM   1034 HG22 THR    66      36.570  40.003  47.885  1.00  0.00           H
ATOM   1035 HG23 THR    66      36.456  38.159  47.952  1.00  0.00           H
ATOM   1036  OG1 THR    66      38.419  39.153  49.438  1.00  0.00           O
ATOM   1037  HG1 THR    66      38.956  39.077  50.230  1.00  0.00           H
ATOM   1038  C   THR    66      36.982  41.653  50.067  1.00  0.00           C
ATOM   1039  O   THR    66      36.303  42.124  49.138  1.00  0.00           O
ATOM   1040  N   GLU    67      38.166  42.219  50.435  1.00  0.00           N
ATOM   1041  H   GLU    67      38.759  41.803  51.140  1.00  0.00           H
ATOM   1042  CA  GLU    67      38.633  43.508  49.875  1.00  0.00           C
ATOM   1043  HA  GLU    67      37.844  44.260  49.856  1.00  0.00           H
ATOM   1044  CB  GLU    67      39.837  44.062  50.755  1.00  0.00           C
ATOM   1045  HB2 GLU    67      40.059  45.049  50.348  1.00  0.00           H
ATOM   1046  HB3 GLU    67      39.492  44.149  51.785  1.00  0.00           H
ATOM   1047  CG  GLU    67      41.145  43.134  50.789  1.00  0.00           C
ATOM   1048  HG2 GLU    67      41.290  42.700  49.799  1.00  0.00           H
ATOM   1049  HG3 GLU    67      42.044  43.665  51.100  1.00  0.00           H
ATOM   1050  CD  GLU    67      41.109  41.972  51.740  1.00  0.00           C
ATOM   1051  OE1 GLU    67      42.216  41.444  51.867  1.00  0.00           O
ATOM   1052  OE2 GLU    67      40.119  41.583  52.398  1.00  0.00           O
ATOM   1053  C   GLU    67      39.099  43.407  48.372  1.00  0.00           C
ATOM   1054  O   GLU    67      39.050  44.335  47.611  1.00  0.00           O
ATOM   1055  N   LYS    68      39.457  42.120  47.980  1.00  0.00           N
ATOM   1056  H   LYS    68      39.235  41.439  48.693  1.00  0.00           H
```

ATOM	1057	CA	LYS	68	39.903	41.836	46.602	1.00	0.00	C
ATOM	1058	HA	LYS	68	40.836	42.366	46.406	1.00	0.00	H
ATOM	1059	CB	LYS	68	40.201	40.277	46.416	1.00	0.00	C
ATOM	1060	HB2	LYS	68	39.353	39.715	46.805	1.00	0.00	H
ATOM	1061	HB3	LYS	68	40.266	40.066	45.349	1.00	0.00	H
ATOM	1062	CG	LYS	68	41.530	39.925	47.163	1.00	0.00	C
ATOM	1063	HG2	LYS	68	42.314	40.347	46.536	1.00	0.00	H
ATOM	1064	HG3	LYS	68	41.506	40.337	48.171	1.00	0.00	H
ATOM	1065	CD	LYS	68	41.788	38.390	47.124	1.00	0.00	C
ATOM	1066	HD2	LYS	68	41.435	38.052	46.149	1.00	0.00	H
ATOM	1067	HD3	LYS	68	42.851	38.181	47.239	1.00	0.00	H
ATOM	1068	CE	LYS	68	41.045	37.730	48.264	1.00	0.00	C
ATOM	1069	HE2	LYS	68	41.753	37.892	49.078	1.00	0.00	H
ATOM	1070	HE3	LYS	68	40.093	38.259	48.292	1.00	0.00	H
ATOM	1071	NZ	LYS	68	40.833	36.298	48.022	1.00	0.00	N
ATOM	1072	HZ1	LYS	68	40.481	36.119	47.093	1.00	0.00	H
ATOM	1073	HZ2	LYS	68	41.624	35.692	48.181	1.00	0.00	H
ATOM	1074	HZ3	LYS	68	40.113	36.019	48.673	1.00	0.00	H
ATOM	1075	C	LYS	68	38.901	42.372	45.590	1.00	0.00	C
ATOM	1076	O	LYS	68	39.281	43.009	44.617	1.00	0.00	O
ATOM	1077	N	HIE	69	37.629	42.175	45.848	1.00	0.00	N
ATOM	1078	H	HIE	69	37.385	41.673	46.689	1.00	0.00	H
ATOM	1079	CA	HIE	69	36.435	42.528	44.993	1.00	0.00	C
ATOM	1080	HA	HIE	69	36.615	42.346	43.934	1.00	0.00	H
ATOM	1081	CB	HIE	69	35.161	41.682	45.397	1.00	0.00	C
ATOM	1082	HB2	HIE	69	35.521	40.663	45.536	1.00	0.00	H
ATOM	1083	HB3	HIE	69	34.785	42.110	46.327	1.00	0.00	H
ATOM	1084	CG	HIE	69	34.097	41.640	44.401	1.00	0.00	C
ATOM	1085	ND1	HIE	69	33.866	40.603	43.458	1.00	0.00	N
ATOM	1086	CE1	HIE	69	32.776	40.941	42.875	1.00	0.00	C
ATOM	1087	HE1	HIE	69	32.241	40.392	42.114	1.00	0.00	H
ATOM	1088	NE2	HIE	69	32.234	42.075	43.384	1.00	0.00	N
ATOM	1089	HE2	HIE	69	31.425	42.544	43.002	1.00	0.00	H
ATOM	1090	CD2	HIE	69	33.154	42.573	44.282	1.00	0.00	C
ATOM	1091	HD2	HIE	69	33.133	43.540	44.760	1.00	0.00	H
ATOM	1092	C	HIE	69	36.188	44.042	45.113	1.00	0.00	C
ATOM	1093	O	HIE	69	35.858	44.679	44.117	1.00	0.00	O
ATOM	1094	N	ALA	70	36.340	44.652	46.316	1.00	0.00	N
ATOM	1095	H	ALA	70	36.615	44.105	47.119	1.00	0.00	H
ATOM	1096	CA	ALA	70	36.245	46.082	46.461	1.00	0.00	C
ATOM	1097	HA	ALA	70	35.243	46.431	46.206	1.00	0.00	H
ATOM	1098	CB	ALA	70	36.454	46.596	47.903	1.00	0.00	C
ATOM	1099	HB1	ALA	70	37.494	46.340	48.107	1.00	0.00	H
ATOM	1100	HB2	ALA	70	36.345	47.681	47.899	1.00	0.00	H
ATOM	1101	HB3	ALA	70	35.833	46.172	48.693	1.00	0.00	H
ATOM	1102	C	ALA	70	37.261	46.829	45.554	1.00	0.00	C
ATOM	1103	O	ALA	70	36.842	47.595	44.726	1.00	0.00	O
ATOM	1104	N	GLU	71	38.487	46.405	45.525	1.00	0.00	N
ATOM	1105	H	GLU	71	38.711	45.713	46.226	1.00	0.00	H
ATOM	1106	CA	GLU	71	39.461	46.829	44.520	1.00	0.00	C
ATOM	1107	HA	GLU	71	39.613	47.896	44.680	1.00	0.00	H
ATOM	1108	CB	GLU	71	40.844	46.187	44.915	1.00	0.00	C
ATOM	1109	HB2	GLU	71	40.723	45.104	44.938	1.00	0.00	H
ATOM	1110	HB3	GLU	71	41.641	46.550	44.267	1.00	0.00	H
ATOM	1111	CG	GLU	71	41.219	46.706	46.284	1.00	0.00	C
ATOM	1112	HG2	GLU	71	40.360	46.694	46.956	1.00	0.00	H
ATOM	1113	HG3	GLU	71	41.913	46.020	46.771	1.00	0.00	H
ATOM	1114	CD	GLU	71	41.855	48.079	46.324	1.00	0.00	C
ATOM	1115	OE1	GLU	71	41.081	49.080	46.073	1.00	0.00	O
ATOM	1116	OE2	GLU	71	43.114	48.103	46.326	1.00	0.00	O
ATOM	1117	C	GLU	71	39.038	46.527	43.088	1.00	0.00	C
ATOM	1118	O	GLU	71	39.248	47.426	42.309	1.00	0.00	O
ATOM	1119	N	GLN	72	38.592	45.295	42.753	1.00	0.00	N
ATOM	1120	H	GLN	72	38.559	44.633	43.514	1.00	0.00	H

ATOM	1121	CA	GLN	72	38.141	44.956	41.362	1.00	0.00	C
ATOM	1122	HA	GLN	72	39.002	45.150	40.724	1.00	0.00	H
ATOM	1123	CB	GLN	72	37.834	43.469	41.335	1.00	0.00	C
ATOM	1124	HB2	GLN	72	37.190	43.212	42.177	1.00	0.00	H
ATOM	1125	HB3	GLN	72	38.730	42.851	41.392	1.00	0.00	H
ATOM	1126	CG	GLN	72	37.075	42.987	40.148	1.00	0.00	C
ATOM	1127	HG2	GLN	72	36.119	43.504	40.075	1.00	0.00	H
ATOM	1128	HG3	GLN	72	37.665	43.243	39.268	1.00	0.00	H
ATOM	1129	CD	GLN	72	36.796	41.514	40.239	1.00	0.00	C
ATOM	1130	OE1	GLN	72	37.579	40.698	39.837	1.00	0.00	O
ATOM	1131	NE2	GLN	72	35.603	41.161	40.697	1.00	0.00	N
ATOM	1132	HE21	GLN	72	34.948	41.897	40.923	1.00	0.00	H
ATOM	1133	HE22	GLN	72	35.394	40.173	40.674	1.00	0.00	H
ATOM	1134	C	GLN	72	37.034	45.868	40.864	1.00	0.00	C
ATOM	1135	O	GLN	72	37.003	46.213	39.671	1.00	0.00	O
ATOM	1136	N	GLU	73	35.964	46.148	41.603	1.00	0.00	N
ATOM	1137	H	GLU	73	35.866	45.688	42.496	1.00	0.00	H
ATOM	1138	CA	GLU	73	34.862	47.028	41.179	1.00	0.00	C
ATOM	1139	HA	GLU	73	34.593	46.779	40.152	1.00	0.00	H
ATOM	1140	CB	GLU	73	33.534	46.646	41.884	1.00	0.00	C
ATOM	1141	HB2	GLU	73	33.605	46.978	42.920	1.00	0.00	H
ATOM	1142	HB3	GLU	73	32.657	47.212	41.572	1.00	0.00	H
ATOM	1143	CG	GLU	73	33.136	45.125	41.744	1.00	0.00	C
ATOM	1144	HG2	GLU	73	33.835	44.539	42.339	1.00	0.00	H
ATOM	1145	HG3	GLU	73	32.208	44.998	42.302	1.00	0.00	H
ATOM	1146	CD	GLU	73	32.983	44.716	40.300	1.00	0.00	C
ATOM	1147	OE1	GLU	73	33.528	43.611	39.954	1.00	0.00	O
ATOM	1148	OE2	GLU	73	32.323	45.452	39.560	1.00	0.00	O
ATOM	1149	C	GLU	73	35.162	48.566	41.334	1.00	0.00	C
ATOM	1150	O	GLU	73	34.304	49.369	41.381	1.00	0.00	O
ATOM	1151	N	ASN	74	36.459	48.906	41.488	1.00	0.00	N
ATOM	1152	H	ASN	74	37.036	48.096	41.657	1.00	0.00	H
ATOM	1153	CA	ASN	74	37.029	50.237	41.673	1.00	0.00	C
ATOM	1154	HA	ASN	74	38.086	50.101	41.901	1.00	0.00	H
ATOM	1155	CB	ASN	74	37.049	51.104	40.403	1.00	0.00	C
ATOM	1156	HB2	ASN	74	36.043	51.465	40.192	1.00	0.00	H
ATOM	1157	HB3	ASN	74	37.577	52.033	40.615	1.00	0.00	H
ATOM	1158	CG	ASN	74	37.584	50.392	39.129	1.00	0.00	C
ATOM	1159	OD1	ASN	74	36.886	49.899	38.249	1.00	0.00	O
ATOM	1160	ND2	ASN	74	38.855	50.327	38.892	1.00	0.00	N
ATOM	1161	HD21	ASN	74	39.033	49.715	38.109	1.00	0.00	H
ATOM	1162	HD22	ASN	74	39.516	50.472	39.641	1.00	0.00	H
ATOM	1163	C	ASN	74	36.386	50.977	42.794	1.00	0.00	C
ATOM	1164	O	ASN	74	36.085	52.182	42.714	1.00	0.00	O
ATOM	1165	N	MET	75	36.225	50.304	43.958	1.00	0.00	N
ATOM	1166	H	MET	75	36.489	49.330	43.942	1.00	0.00	H
ATOM	1167	CA	MET	75	35.288	50.661	44.997	1.00	0.00	C
ATOM	1168	HA	MET	75	34.858	51.621	44.710	1.00	0.00	H
ATOM	1169	CB	MET	75	34.261	49.536	45.036	1.00	0.00	C
ATOM	1170	HB2	MET	75	34.343	48.788	44.247	1.00	0.00	H
ATOM	1171	HB3	MET	75	34.341	49.068	46.017	1.00	0.00	H
ATOM	1172	CG	MET	75	32.796	50.063	44.930	1.00	0.00	C
ATOM	1173	HG2	MET	75	32.432	50.714	45.724	1.00	0.00	H
ATOM	1174	HG3	MET	75	32.834	50.697	44.045	1.00	0.00	H
ATOM	1175	SD	MET	75	31.505	48.894	44.452	1.00	0.00	S
ATOM	1176	CE	MET	75	30.142	50.024	44.256	1.00	0.00	C
ATOM	1177	HE1	MET	75	30.243	50.578	43.322	1.00	0.00	H
ATOM	1178	HE2	MET	75	29.177	49.519	44.233	1.00	0.00	H
ATOM	1179	HE3	MET	75	29.991	50.708	45.091	1.00	0.00	H
ATOM	1180	C	MET	75	36.028	50.786	46.328	1.00	0.00	C
ATOM	1181	O	MET	75	37.133	50.262	46.546	1.00	0.00	O
ATOM	1182	N	THR	76	35.351	51.352	47.345	1.00	0.00	N
ATOM	1183	H	THR	76	34.569	51.951	47.122	1.00	0.00	H

ATOM	1184	CA	THR	76	35.699	51.194	48.786	1.00	0.00	C
ATOM	1185	HA	THR	76	36.719	50.825	48.883	1.00	0.00	H
ATOM	1186	CB	THR	76	35.672	52.516	49.587	1.00	0.00	C
ATOM	1187	HB	THR	76	35.932	53.345	48.929	1.00	0.00	H
ATOM	1188	CG2	THR	76	34.206	52.728	50.087	1.00	0.00	C
ATOM	1189	HG21	THR	76	34.084	53.744	50.461	1.00	0.00	H
ATOM	1190	HG22	THR	76	33.477	52.527	49.302	1.00	0.00	H
ATOM	1191	HG23	THR	76	33.927	52.023	50.870	1.00	0.00	H
ATOM	1192	OG1	THR	76	36.456	52.242	50.604	1.00	0.00	O
ATOM	1193	HG1	THR	76	37.043	52.965	50.839	1.00	0.00	H
ATOM	1194	C	THR	76	34.823	50.051	49.363	1.00	0.00	C
ATOM	1195	O	THR	76	33.673	49.859	48.949	1.00	0.00	O
ATOM	1196	N	LEU	77	35.406	49.266	50.259	1.00	0.00	N
ATOM	1197	H	LEU	77	36.366	49.368	50.555	1.00	0.00	H
ATOM	1198	CA	LEU	77	34.786	48.040	50.720	1.00	0.00	C
ATOM	1199	HA	LEU	77	34.665	47.384	49.857	1.00	0.00	H
ATOM	1200	CB	LEU	77	35.799	47.414	51.668	1.00	0.00	C
ATOM	1201	HB2	LEU	77	36.749	47.307	51.143	1.00	0.00	H
ATOM	1202	HB3	LEU	77	35.966	48.044	52.542	1.00	0.00	H
ATOM	1203	CG	LEU	77	35.351	46.130	52.314	1.00	0.00	C
ATOM	1204	HG	LEU	77	34.474	46.336	52.927	1.00	0.00	H
ATOM	1205	CD1	LEU	77	34.983	45.057	51.262	1.00	0.00	C
ATOM	1206	HD11	LEU	77	35.675	44.988	50.422	1.00	0.00	H
ATOM	1207	HD12	LEU	77	34.969	44.076	51.738	1.00	0.00	H
ATOM	1208	HD13	LEU	77	33.946	45.277	51.006	1.00	0.00	H
ATOM	1209	CD2	LEU	77	36.490	45.571	53.160	1.00	0.00	C
ATOM	1210	HD21	LEU	77	36.172	44.635	53.617	1.00	0.00	H
ATOM	1211	HD22	LEU	77	37.406	45.599	52.568	1.00	0.00	H
ATOM	1212	HD23	LEU	77	36.635	46.142	54.078	1.00	0.00	H
ATOM	1213	C	LEU	77	33.369	48.224	51.342	1.00	0.00	C
ATOM	1214	O	LEU	77	32.526	47.422	51.013	1.00	0.00	O
ATOM	1215	N	THR	78	33.104	49.407	52.017	1.00	0.00	N
ATOM	1216	H	THR	78	33.924	49.975	52.178	1.00	0.00	H
ATOM	1217	CA	THR	78	31.720	49.740	52.416	1.00	0.00	C
ATOM	1218	HA	THR	78	31.326	48.826	52.858	1.00	0.00	H
ATOM	1219	CB	THR	78	31.678	50.812	53.500	1.00	0.00	C
ATOM	1220	HB	THR	78	30.639	51.081	53.689	1.00	0.00	H
ATOM	1221	CG2	THR	78	32.322	50.503	54.846	1.00	0.00	C
ATOM	1222	HG21	THR	78	32.146	51.348	55.512	1.00	0.00	H
ATOM	1223	HG22	THR	78	31.875	49.602	55.266	1.00	0.00	H
ATOM	1224	HG23	THR	78	33.395	50.462	54.660	1.00	0.00	H
ATOM	1225	OG1	THR	78	32.260	52.027	53.071	1.00	0.00	O
ATOM	1226	HG1	THR	78	32.002	52.608	53.790	1.00	0.00	H
ATOM	1227	C	THR	78	30.791	50.164	51.281	1.00	0.00	C
ATOM	1228	O	THR	78	29.599	49.922	51.450	1.00	0.00	O
ATOM	1229	N	GLU	79	31.100	50.864	50.196	1.00	0.00	N
ATOM	1230	H	GLU	79	32.086	51.049	50.085	1.00	0.00	H
ATOM	1231	CA	GLU	79	30.120	51.172	49.118	1.00	0.00	C
ATOM	1232	HA	GLU	79	29.175	51.605	49.446	1.00	0.00	H
ATOM	1233	CB	GLU	79	30.701	52.286	48.266	1.00	0.00	C
ATOM	1234	HB2	GLU	79	31.710	51.964	48.012	1.00	0.00	H
ATOM	1235	HB3	GLU	79	30.151	52.511	47.352	1.00	0.00	H
ATOM	1236	CG	GLU	79	30.893	53.642	48.926	1.00	0.00	C
ATOM	1237	HG2	GLU	79	30.949	53.468	50.000	1.00	0.00	H
ATOM	1238	HG3	GLU	79	31.869	53.977	48.574	1.00	0.00	H
ATOM	1239	CD	GLU	79	29.789	54.710	48.734	1.00	0.00	C
ATOM	1240	OE1	GLU	79	29.924	55.798	49.412	1.00	0.00	O
ATOM	1241	OE2	GLU	79	28.752	54.523	48.071	1.00	0.00	O
ATOM	1242	C	GLU	79	29.750	49.896	48.308	1.00	0.00	C
ATOM	1243	O	GLU	79	28.649	49.821	47.853	1.00	0.00	O
ATOM	1244	N	LEU	80	30.625	48.922	48.237	1.00	0.00	N
ATOM	1245	H	LEU	80	31.601	49.112	48.415	1.00	0.00	H
ATOM	1246	CA	LEU	80	30.270	47.565	47.850	1.00	0.00	C
ATOM	1247	HA	LEU	80	29.729	47.589	46.904	1.00	0.00	H

```
ATOM   1248  CB  LEU
    80      31.538  46.718  47.655  1.00  0.00           C
ATOM   1249  HB2 LEU    80      32.333  47.178  47.069  1.00  0.00            H
ATOM   1250  HB3 LEU    80      31.903  46.508  48.661  1.00  0.00            H
ATOM   1251  CG  LEU    80      31.333  45.252  47.070  1.00  0.00            C
ATOM   1252  HG  LEU    80      30.610  44.761  47.719  1.00  0.00            H
ATOM   1253  CD1 LEU    80      30.887  45.182  45.668  1.00  0.00            C
ATOM   1254 HD11 LEU    80      31.362  45.904  45.004  1.00  0.00            H
ATOM   1255 HD12 LEU    80      30.963  44.163  45.289  1.00  0.00            H
ATOM   1256 HD13 LEU    80      29.866  45.559  45.717  1.00  0.00            H
ATOM   1257  CD2 LEU    80      32.633  44.548  47.164  1.00  0.00            C
ATOM   1258 HD21 LEU    80      33.075  44.706  48.148  1.00  0.00            H
ATOM   1259 HD22 LEU    80      32.463  43.485  46.992  1.00  0.00            H
ATOM   1260 HD23 LEU    80      33.351  44.938  46.443  1.00  0.00            H
ATOM   1261  C   LEU    80      29.197  47.007  48.785  1.00  0.00            C
ATOM   1262  O   LEU    80      28.171  46.667  48.240  1.00  0.00            O
ATOM   1263  N   LYS    81      29.373  47.155  50.103  1.00  0.00            N
ATOM   1264  H   LYS    81      30.268  47.468  50.451  1.00  0.00            H
ATOM   1265  CA  LYS    81      28.341  46.585  50.999  1.00  0.00            C
ATOM   1266  HA  LYS    81      28.144  45.547  50.731  1.00  0.00            H
ATOM   1267  CB  LYS    81      28.820  46.733  52.500  1.00  0.00            C
ATOM   1268  HB2 LYS    81      28.907  47.809  52.651  1.00  0.00            H
ATOM   1269  HB3 LYS    81      27.999  46.457  53.160  1.00  0.00            H
ATOM   1270  CG  LYS    81      30.058  45.937  52.836  1.00  0.00            C
ATOM   1271  HG2 LYS    81      29.686  44.912  52.823  1.00  0.00            H
ATOM   1272  HG3 LYS    81      30.836  46.056  52.083  1.00  0.00            H
ATOM   1273  CD  LYS    81      30.621  45.933  54.276  1.00  0.00            C
ATOM   1274  HD2 LYS    81      30.767  46.975  54.559  1.00  0.00            H
ATOM   1275  HD3 LYS    81      29.824  45.551  54.915  1.00  0.00            H
ATOM   1276  CE  LYS    81      31.899  45.085  54.556  1.00  0.00            C
ATOM   1277  HE2 LYS    81      31.588  44.044  54.461  1.00  0.00            H
ATOM   1278  HE3 LYS    81      32.708  45.199  53.835  1.00  0.00            H
ATOM   1279  NZ  LYS    81      32.470  45.438  55.875  1.00  0.00            N
ATOM   1280  HZ1 LYS    81      31.768  45.254  56.578  1.00  0.00            H
ATOM   1281  HZ2 LYS    81      33.244  44.849  56.147  1.00  0.00            H
ATOM   1282  HZ3 LYS    81      32.887  46.357  55.892  1.00  0.00            H
ATOM   1283  C   LYS    81      27.049  47.324  50.886  1.00  0.00            C
ATOM   1284  O   LYS    81      26.017  46.695  50.922  1.00  0.00            O
ATOM   1285  N   LYS    82      27.123  48.614  50.608  1.00  0.00            N
ATOM   1286  H   LYS    82      27.993  49.122  50.535  1.00  0.00            H
ATOM   1287  CA  LYS    82      25.987  49.482  50.362  1.00  0.00            C
ATOM   1288  HA  LYS    82      25.303  49.328  51.195  1.00  0.00            H
ATOM   1289  CB  LYS    82      26.492  50.992  50.295  1.00  0.00            C
ATOM   1290  HB2 LYS    82      27.268  51.073  51.055  1.00  0.00            H
ATOM   1291  HB3 LYS    82      26.943  51.108  49.309  1.00  0.00            H
ATOM   1292  CG  LYS    82      25.387  52.040  50.443  1.00  0.00            C
ATOM   1293  HG2 LYS    82      24.427  51.664  50.089  1.00  0.00            H
ATOM   1294  HG3 LYS    82      25.183  52.258  51.491  1.00  0.00            H
ATOM   1295  CD  LYS    82      25.630  53.380  49.701  1.00  0.00            C
ATOM   1296  HD2 LYS    82      26.381  53.281  48.918  1.00  0.00            H
ATOM   1297  HD3 LYS    82      24.694  53.740  49.273  1.00  0.00            H
ATOM   1298  CE  LYS    82      26.150  54.532  50.559  1.00  0.00            C
ATOM   1299  HE2 LYS    82      25.279  54.976  51.043  1.00  0.00            H
ATOM   1300  HE3 LYS    82      26.833  54.116  51.298  1.00  0.00            H
ATOM   1301  NZ  LYS    82      26.944  55.591  49.799  1.00  0.00            N
ATOM   1302  HZ1 LYS    82      27.309  56.241  50.480  1.00  0.00            H
ATOM   1303  HZ2 LYS    82      27.691  55.163  49.270  1.00  0.00            H
ATOM   1304  HZ3 LYS    82      26.362  56.206  49.247  1.00  0.00            H
ATOM   1305  C   LYS    82      25.109  49.070  49.137  1.00  0.00            C
ATOM   1306  O   LYS    82      23.905  49.089  49.316  1.00  0.00            O
ATOM   1307  N   VAL    83      25.751  48.623  48.032  1.00  0.00            N
ATOM   1308  H   VAL    83      26.760  48.632  48.050  1.00  0.00            H
ATOM   1309  CA  VAL    83      25.177  48.209  46.723  1.00  0.00            C
ATOM   1310  HA  VAL    83      24.196  48.684  46.744  1.00  0.00            H
```

```
ATOM   1311  CB  VAL    83      25.952  48.671  45.509  1.00  0.00           C
ATOM   1312  HB  VAL    83      25.331  48.452  44.639  1.00  0.00           H
ATOM   1313  CG1 VAL    83      26.441  50.193  45.350  1.00  0.00           C
ATOM   1314 HG11 VAL    83      27.118  50.456  46.164  1.00  0.00           H
ATOM   1315 HG12 VAL    83      27.079  50.233  44.466  1.00  0.00           H
ATOM   1316 HG13 VAL    83      25.524  50.782  45.330  1.00  0.00           H
ATOM   1317  CG2 VAL    83      27.277  47.839  45.418  1.00  0.00           C
ATOM   1318 HG21 VAL    83      27.094  46.797  45.681  1.00  0.00           H
ATOM   1319 HG22 VAL    83      27.567  47.870  44.368  1.00  0.00           H
ATOM   1320 HG23 VAL    83      28.000  48.352  46.052  1.00  0.00           H
ATOM   1321  C   VAL    83      24.778  46.738  46.698  1.00  0.00           C
ATOM   1322  O   VAL    83      23.932  46.332  45.885  1.00  0.00           O
ATOM   1323  N   ILE    84      25.357  46.008  47.598  1.00  0.00           N
ATOM   1324  H   ILE    84      26.140  46.456  48.050  1.00  0.00           H
ATOM   1325  CA  ILE    84      24.838  44.688  47.986  1.00  0.00           C
ATOM   1326  HA  ILE    84      24.508  44.203  47.067  1.00  0.00           H
ATOM   1327  CB  ILE    84      25.962  43.781  48.482  1.00  0.00           C
ATOM   1328  HB  ILE    84      26.556  44.328  49.213  1.00  0.00           H
ATOM   1329  CG2 ILE    84      25.490  42.492  49.120  1.00  0.00           C
ATOM   1330 HG21 ILE    84      25.041  41.763  48.447  1.00  0.00           H
ATOM   1331 HG22 ILE    84      26.395  42.152  49.624  1.00  0.00           H
ATOM   1332 HG23 ILE    84      24.860  42.745  49.973  1.00  0.00           H
ATOM   1333  CG1 ILE    84      27.038  43.460  47.380  1.00  0.00           C
ATOM   1334 HG12 ILE    84      26.602  42.919  46.540  1.00  0.00           H
ATOM   1335 HG13 ILE    84      27.256  44.438  46.950  1.00  0.00           H
ATOM   1336  CD1 ILE    84      28.423  42.771  47.773  1.00  0.00           C
ATOM   1337 HD11 ILE    84      29.010  43.533  48.285  1.00  0.00           H
ATOM   1338 HD12 ILE    84      28.253  41.896  48.400  1.00  0.00           H
ATOM   1339 HD13 ILE    84      28.919  42.437  46.862  1.00  0.00           H
ATOM   1340  C   ILE    84      23.650  44.819  48.897  1.00  0.00           C
ATOM   1341  O   ILE    84      22.612  44.216  48.695  1.00  0.00           O
ATOM   1342  N   ALA    85      23.744  45.655  49.895  1.00  0.00           N
ATOM   1343  H   ALA    85      24.658  45.899  50.248  1.00  0.00           H
ATOM   1344  CA  ALA    85      22.616  45.930  50.864  1.00  0.00           C
ATOM   1345  HA  ALA    85      22.413  44.989  51.375  1.00  0.00           H
ATOM   1346  CB  ALA    85      23.113  46.816  51.981  1.00  0.00           C
ATOM   1347  HB1 ALA    85      22.267  47.033  52.633  1.00  0.00           H
ATOM   1348  HB2 ALA    85      23.733  46.212  52.645  1.00  0.00           H
ATOM   1349  HB3 ALA    85      23.655  47.677  51.591  1.00  0.00           H
ATOM   1350  C   ALA    85      21.348  46.502  50.265  1.00  0.00           C
ATOM   1351  O   ALA    85      20.261  46.064  50.625  1.00  0.00           O
ATOM   1352  N   ASP    86      21.429  47.474  49.370  1.00  0.00           N
ATOM   1353  H   ASP    86      22.328  47.894  49.179  1.00  0.00           H
ATOM   1354  CA  ASP    86      20.389  48.058  48.535  1.00  0.00           C
ATOM   1355  HA  ASP    86      19.710  48.600  49.194  1.00  0.00           H
ATOM   1356  CB  ASP    86      21.029  48.903  47.462  1.00  0.00           C
ATOM   1357  HB2 ASP    86      21.960  48.470  47.099  1.00  0.00           H
ATOM   1358  HB3 ASP    86      20.367  48.975  46.599  1.00  0.00           H
ATOM   1359  CG  ASP    86      21.247  50.407  47.901  1.00  0.00           C
ATOM   1360  OD1 ASP    86      20.739  50.796  48.943  1.00  0.00           O
ATOM   1361  OD2 ASP    86      21.954  51.187  47.230  1.00  0.00           O
ATOM   1362  C   ASP    86      19.520  46.912  47.936  1.00  0.00           C
ATOM   1363  O   ASP    86      18.361  47.144  47.583  1.00  0.00           O
ATOM   1364  N   ILE    87      20.081  45.760  47.600  1.00  0.00           N
ATOM   1365  H   ILE    87      21.046  45.574  47.835  1.00  0.00           H
ATOM   1366  CA  ILE    87      19.479  44.657  46.924  1.00  0.00           C
ATOM   1367  HA  ILE    87      18.622  45.063  46.387  1.00  0.00           H
ATOM   1368  CB  ILE    87      20.488  44.245  45.793  1.00  0.00           C
ATOM   1369  HB  ILE    87      21.451  44.061  46.272  1.00  0.00           H
ATOM   1370  CG2 ILE    87      20.078  42.915  45.149  1.00  0.00           C
ATOM   1371 HG21 ILE    87      20.953  42.612  44.575  1.00  0.00           H
ATOM   1372 HG22 ILE    87      19.865  42.168  45.914  1.00  0.00           H
ATOM   1373 HG23 ILE    87      19.217  43.076
  44.500  1.00  0.00           H
```

ATOM	1374	CG1	ILE	87	20.510	45.268	44.687	1.00	0.00	C
ATOM	1375	HG12	ILE	87	19.546	45.660	44.363	1.00	0.00	H
ATOM	1376	HG13	ILE	87	20.963	46.129	45.177	1.00	0.00	H
ATOM	1377	CD1	ILE	87	21.350	44.995	43.430	1.00	0.00	C
ATOM	1378	HD11	ILE	87	20.680	44.387	42.822	1.00	0.00	H
ATOM	1379	HD12	ILE	87	21.447	45.873	42.791	1.00	0.00	H
ATOM	1380	HD13	ILE	87	22.326	44.545	43.608	1.00	0.00	H
ATOM	1381	C	ILE	87	18.974	43.592	47.876	1.00	0.00	C
ATOM	1382	O	ILE	87	17.777	43.245	47.844	1.00	0.00	O
ATOM	1383	N	TYR	88	19.808	43.164	48.807	1.00	0.00	N
ATOM	1384	H	TYR	88	20.751	43.512	48.698	1.00	0.00	H
ATOM	1385	CA	TYR	88	19.594	42.081	49.803	1.00	0.00	C
ATOM	1386	HA	TYR	88	18.704	41.522	49.516	1.00	0.00	H
ATOM	1387	CB	TYR	88	20.844	41.202	49.812	1.00	0.00	C
ATOM	1388	HB2	TYR	88	21.684	41.858	50.039	1.00	0.00	H
ATOM	1389	HB3	TYR	88	20.847	40.501	50.648	1.00	0.00	H
ATOM	1390	CG	TYR	88	21.165	40.440	48.548	1.00	0.00	C
ATOM	1391	CD1	TYR	88	20.748	39.089	48.417	1.00	0.00	C
ATOM	1392	HD1	TYR	88	20.377	38.504	49.245	1.00	0.00	H
ATOM	1393	CE1	TYR	88	20.943	38.438	47.194	1.00	0.00	C
ATOM	1394	HE1	TYR	88	20.525	37.447	47.107	1.00	0.00	H
ATOM	1395	CZ	TYR	88	21.767	38.982	46.206	1.00	0.00	C
ATOM	1396	OH	TYR	88	21.921	38.219	45.052	1.00	0.00	O
ATOM	1397	HH	TYR	88	22.063	37.308	45.317	1.00	0.00	H
ATOM	1398	CE2	TYR	88	22.233	40.318	46.369	1.00	0.00	C
ATOM	1399	HE2	TYR	88	22.629	40.785	45.480	1.00	0.00	H
ATOM	1400	CD2	TYR	88	21.918	41.035	47.522	1.00	0.00	C
ATOM	1401	HD2	TYR	88	22.294	42.038	47.661	1.00	0.00	H
ATOM	1402	C	TYR	88	19.377	42.487	51.268	1.00	0.00	C
ATOM	1403	O	TYR	88	19.920	43.496	51.686	1.00	0.00	O
ATOM	1404	N	PRO	89	18.679	41.655	52.093	1.00	0.00	N
ATOM	1405	CD	PRO	89	18.036	40.437	51.686	1.00	0.00	C
ATOM	1406	HD2	PRO	89	18.616	39.833	50.988	1.00	0.00	H
ATOM	1407	HD3	PRO	89	17.130	40.711	51.146	1.00	0.00	H
ATOM	1408	CG	PRO	89	17.703	39.665	52.915	1.00	0.00	C
ATOM	1409	HG2	PRO	89	18.383	38.820	53.028	1.00	0.00	H
ATOM	1410	HG3	PRO	89	16.671	39.351	52.761	1.00	0.00	H
ATOM	1411	CB	PRO	89	17.671	40.740	54.064	1.00	0.00	C
ATOM	1412	HB2	PRO	89	18.041	40.209	54.941	1.00	0.00	H
ATOM	1413	HB3	PRO	89	16.634	41.071	54.133	1.00	0.00	H
ATOM	1414	CA	PRO	89	18.655	41.808	53.587	1.00	0.00	C
ATOM	1415	HA	PRO	89	18.398	42.805	53.943	1.00	0.00	H
ATOM	1416	C	PRO	89	19.986	41.473	54.038	1.00	0.00	C
ATOM	1417	O	PRO	89	20.458	40.322	53.874	1.00	0.00	O
ATOM	1418	N	GLY	90	20.571	42.347	54.881	1.00	0.00	N
ATOM	1419	H	GLY	90	20.033	43.154	55.169	1.00	0.00	H
ATOM	1420	CA	GLY	90	21.965	42.217	55.403	1.00	0.00	C
ATOM	1421	HA2	GLY	90	22.612	42.188	54.525	1.00	0.00	H
ATOM	1422	HA3	GLY	90	22.241	43.104	55.974	1.00	0.00	H
ATOM	1423	C	GLY	90	22.201	40.948	56.304	1.00	0.00	C
ATOM	1424	O	GLY	90	23.323	40.555	56.618	1.00	0.00	O
ATOM	1425	N	GLN	91	21.144	40.213	56.679	1.00	0.00	N
ATOM	1426	H	GLN	91	20.246	40.559	56.372	1.00	0.00	H
ATOM	1427	CA	GLN	91	21.190	38.879	57.405	1.00	0.00	C
ATOM	1428	HA	GLN	91	22.069	38.912	58.049	1.00	0.00	H
ATOM	1429	CB	GLN	91	19.859	38.666	58.232	1.00	0.00	C
ATOM	1430	HB2	GLN	91	19.025	38.765	57.537	1.00	0.00	H
ATOM	1431	HB3	GLN	91	19.866	37.642	58.607	1.00	0.00	H
ATOM	1432	CG	GLN	91	19.786	39.718	59.345	1.00	0.00	C
ATOM	1433	HG2	GLN	91	19.791	40.747	58.982	1.00	0.00	H
ATOM	1434	HG3	GLN	91	18.903	39.605	59.974	1.00	0.00	H
ATOM	1435	CD	GLN	91	20.937	39.667	60.303	1.00	0.00	C
ATOM	1436	OE1	GLN	91	21.674	40.661	60.380	1.00	0.00	O
ATOM	1437	NE2	GLN	91	21.239	38.625	61.031	1.00	0.00	N

ATOM	1438	HE21	GLN	91	20.603	37.841	61.064	1.00	0.00	H
ATOM	1439	HE22	GLN	91	21.836	38.758	61.835	1.00	0.00	H
ATOM	1440	C	GLN	91	21.453	37.713	56.401	1.00	0.00	C
ATOM	1441	O	GLN	91	21.414	36.530	56.701	1.00	0.00	O
ATOM	1442	N	THR	92	21.527	38.002	55.092	1.00	0.00	N
ATOM	1443	H	THR	92	21.566	38.985	54.861	1.00	0.00	H
ATOM	1444	CA	THR	92	21.708	36.947	54.085	1.00	0.00	C
ATOM	1445	HA	THR	92	20.981	36.151	54.244	1.00	0.00	H
ATOM	1446	CB	THR	92	21.710	37.614	52.706	1.00	0.00	C
ATOM	1447	HB	THR	92	22.125	38.610	52.861	1.00	0.00	H
ATOM	1448	CG2	THR	92	22.412	36.897	51.532	1.00	0.00	C
ATOM	1449	HG21	THR	92	21.956	35.912	51.441	1.00	0.00	H
ATOM	1450	HG22	THR	92	22.243	37.484	50.629	1.00	0.00	H
ATOM	1451	HG23	THR	92	23.483	36.778	51.694	1.00	0.00	H
ATOM	1452	OG1	THR	92	20.374	37.845	52.277	1.00	0.00	O
ATOM	1453	HG1	THR	92	20.139	38.624	52.787	1.00	0.00	H
ATOM	1454	C	THR	92	23.100	36.258	54.295	1.00	0.00	C
ATOM	1455	O	THR	92	24.052	36.942	54.729	1.00	0.00	O
ATOM	1456	N	GLN	93	23.219	34.927	54.127	1.00	0.00	N
ATOM	1457	H	GLN	93	22.432	34.442	53.723	1.00	0.00	H
ATOM	1458	CA	GLN	93	24.514	34.267	54.031	1.00	0.00	C
ATOM	1459	HA	GLN	93	25.215	34.743	54.718	1.00	0.00	H
ATOM	1460	CB	GLN	93	24.309	32.794	54.460	1.00	0.00	C
ATOM	1461	HB2	GLN	93	23.865	32.735	55.454	1.00	0.00	H
ATOM	1462	HB3	GLN	93	23.744	32.351	53.640	1.00	0.00	H
ATOM	1463	CG	GLN	93	25.716	32.073	54.446	1.00	0.00	C
ATOM	1464	HG2	GLN	93	25.450	31.026	54.594	1.00	0.00	H
ATOM	1465	HG3	GLN	93	26.286	32.200	53.525	1.00	0.00	H
ATOM	1466	CD	GLN	93	26.638	32.306	55.670	1.00	0.00	C
ATOM	1467	OE1	GLN	93	26.272	32.589	56.796	1.00	0.00	O
ATOM	1468	NE2	GLN	93	27.874	32.062	55.491	1.00	0.00	N
ATOM	1469	HE21	GLN	93	28.359	31.998	54.607	1.00	0.00	H
ATOM	1470	HE22	GLN	93	28.408	32.224	56.332	1.00	0.00	H
ATOM	1471	C	GLN	93	25.094	34.437	52.613	1.00	0.00	C
ATOM	1472	O	GLN	93	24.406	34.222	51.609	1.00	0.00	O
ATOM	1473	N	PHE	94	26.361	34.854	52.510	1.00	0.00	N
ATOM	1474	H	PHE	94	26.889	34.747	53.364	1.00	0.00	H
ATOM	1475	CA	PHE	94	27.026	35.160	51.200	1.00	0.00	C
ATOM	1476	HA	PHE	94	26.256	35.232	50.432	1.00	0.00	H
ATOM	1477	CB	PHE	94	27.603	36.640	51.156	1.00	0.00	C
ATOM	1478	HB2	PHE	94	28.328	36.777	51.960	1.00	0.00	H
ATOM	1479	HB3	PHE	94	28.178	36.776	50.241	1.00	0.00	H
ATOM	1480	CG	PHE	94	26.626	37.821	51.205	1.00	0.00	C
ATOM	1481	CD1	PHE	94	25.884	38.132	50.056	1.00	0.00	C
ATOM	1482	HD1	PHE	94	25.972	37.518	49.173	1.00	0.00	H
ATOM	1483	CE1	PHE	94	24.952	39.183	49.988	1.00	0.00	C
ATOM	1484	HE1	PHE	94	24.480	39.445	49.052	1.00	0.00	H
ATOM	1485	CZ	PHE	94	24.627	39.774	51.237	1.00	0.00	C
ATOM	1486	HZ	PHE	94	23.728	40.370	51.295	1.00	0.00	H
ATOM	1487	CE2	PHE	94	25.154	39.294	52.437	1.00	0.00	C
ATOM	1488	HE2	PHE	94	24.858	39.633	53.418	1.00	0.00	H
ATOM	1489	CD2	PHE	94	26.177	38.306	52.433	1.00	0.00	C
ATOM	1490	HD2	PHE	94	26.518	37.962	53.398	1.00	0.00	H
ATOM	1491	C	PHE	94	28.052	34.085	50.849	1.00	0.00	C
ATOM	1492	O	PHE	94	28.427	33.240	51.683	1.00	0.00	O
ATOM	1493	N	TYR	95	28.437	34.072	49.563	1.00	0.00	N
ATOM	1494	H	TYR	95	28.108	34.798	48.941	1.00	0.00	H
ATOM	1495	CA	TYR	95	29.307	33.118	48.913	1.00	0.00	C
ATOM	1496	HA	TYR	95	29.811	32.622	49.742	1.00	0.00	H
ATOM	1497	CB	TYR	95	28.369	32.043	48.200	1.00	0.00	C
ATOM	1498	HB2	TYR	95	27.756	31.467	48.893	1.00	0.00	
H										
ATOM	1499	HB3	TYR	95	27.647	32.564	47.571	1.00	0.00	H
ATOM	1500	CG	TYR	95	29.200	31.040	47.407	1.00	0.00	C

ATOM	1501	CD1	TYR	95	29.264	31.092	46.037	1.00	0.00	C
ATOM	1502	HD1	TYR	95	28.678	31.832	45.510	1.00	0.00	H
ATOM	1503	CE1	TYR	95	29.997	30.123	45.285	1.00	0.00	C
ATOM	1504	HE1	TYR	95	30.038	30.247	44.213	1.00	0.00	H
ATOM	1505	CZ	TYR	95	30.526	28.973	45.968	1.00	0.00	C
ATOM	1506	OH	TYR	95	31.195	27.975	45.284	1.00	0.00	O
ATOM	1507	HH	TYR	95	31.282	28.039	44.330	1.00	0.00	H
ATOM	1508	CE2	TYR	95	30.392	28.902	47.305	1.00	0.00	C
ATOM	1509	HE2	TYR	95	30.733	28.017	47.821	1.00	0.00	H
ATOM	1510	CD2	TYR	95	29.779	29.966	48.036	1.00	0.00	C
ATOM	1511	HD2	TYR	95	29.623	29.917	49.104	1.00	0.00	H
ATOM	1512	C	TYR	95	30.280	33.847	47.936	1.00	0.00	C
ATOM	1513	O	TYR	95	29.908	34.696	47.137	1.00	0.00	O
ATOM	1514	N	VAL	96	31.526	33.418	47.881	1.00	0.00	N
ATOM	1515	H	VAL	96	31.642	32.574	48.422	1.00	0.00	H
ATOM	1516	CA	VAL	96	32.638	33.925	47.023	1.00	0.00	C
ATOM	1517	HA	VAL	96	32.231	34.791	46.500	1.00	0.00	H
ATOM	1518	CB	VAL	96	33.779	34.461	47.924	1.00	0.00	C
ATOM	1519	HB	VAL	96	33.948	33.847	48.809	1.00	0.00	H
ATOM	1520	CG1	VAL	96	35.052	34.735	47.161	1.00	0.00	C
ATOM	1521	HG11	VAL	96	35.286	33.866	46.545	1.00	0.00	H
ATOM	1522	HG12	VAL	96	35.041	35.580	46.473	1.00	0.00	H
ATOM	1523	HG13	VAL	96	35.772	35.010	47.932	1.00	0.00	H
ATOM	1524	CG2	VAL	96	33.351	35.823	48.584	1.00	0.00	C
ATOM	1525	HG21	VAL	96	32.509	35.685	49.264	1.00	0.00	H
ATOM	1526	HG22	VAL	96	34.251	36.262	49.013	1.00	0.00	H
ATOM	1527	HG23	VAL	96	32.953	36.542	47.868	1.00	0.00	H
ATOM	1528	C	VAL	96	33.198	32.939	45.961	1.00	0.00	C
ATOM	1529	O	VAL	96	33.292	31.729	46.209	1.00	0.00	O
ATOM	1530	N	ILE	97	33.473	33.452	44.774	1.00	0.00	N
ATOM	1531	H	ILE	97	32.973	34.308	44.582	1.00	0.00	H
ATOM	1532	CA	ILE	97	34.203	32.655	43.719	1.00	0.00	C
ATOM	1533	HA	ILE	97	34.536	31.688	44.094	1.00	0.00	H
ATOM	1534	CB	ILE	97	33.197	32.390	42.515	1.00	0.00	C
ATOM	1535	HB	ILE	97	32.967	33.336	42.025	1.00	0.00	H
ATOM	1536	CG2	ILE	97	33.856	31.565	41.415	1.00	0.00	C
ATOM	1537	HG21	ILE	97	33.397	30.584	41.288	1.00	0.00	H
ATOM	1538	HG22	ILE	97	33.621	32.154	40.529	1.00	0.00	H
ATOM	1539	HG23	ILE	97	34.926	31.494	41.606	1.00	0.00	H
ATOM	1540	CG1	ILE	97	31.802	31.930	42.958	1.00	0.00	C
ATOM	1541	HG12	ILE	97	31.520	31.082	42.333	1.00	0.00	H
ATOM	1542	HG13	ILE	97	31.758	31.554	43.980	1.00	0.00	H
ATOM	1543	CD1	ILE	97	30.646	32.978	42.777	1.00	0.00	C
ATOM	1544	HD11	ILE	97	30.617	33.736	43.560	1.00	0.00	H
ATOM	1545	HD12	ILE	97	30.601	33.333	41.747	1.00	0.00	H
ATOM	1546	HD13	ILE	97	29.710	32.419	42.781	1.00	0.00	H
ATOM	1547	C	ILE	97	35.467	33.358	43.180	1.00	0.00	C
ATOM	1548	O	ILE	97	35.518	34.552	42.858	1.00	0.00	O
ATOM	1549	N	GLU	98	36.554	32.634	43.045	1.00	0.00	N
ATOM	1550	H	GLU	98	36.504	31.652	43.278	1.00	0.00	H
ATOM	1551	CA	GLU	98	37.841	33.190	42.740	1.00	0.00	C
ATOM	1552	HA	GLU	98	37.784	34.138	42.205	1.00	0.00	H
ATOM	1553	CB	GLU	98	38.642	33.416	44.033	1.00	0.00	C
ATOM	1554	HB2	GLU	98	38.023	33.847	44.820	1.00	0.00	H
ATOM	1555	HB3	GLU	98	38.920	32.461	44.478	1.00	0.00	H
ATOM	1556	CG	GLU	98	39.877	34.296	43.874	1.00	0.00	C
ATOM	1557	HG2	GLU	98	40.615	33.670	43.372	1.00	0.00	H
ATOM	1558	HG3	GLU	98	39.761	35.173	43.238	1.00	0.00	H
ATOM	1559	CD	GLU	98	40.524	34.666	45.190	1.00	0.00	C
ATOM	1560	OE1	GLU	98	40.749	35.887	45.396	1.00	0.00	O
ATOM	1561	OE2	GLU	98	40.844	33.790	45.959	1.00	0.00	O
ATOM	1562	C	GLU	98	38.544	32.190	41.862	1.00	0.00	C
ATOM	1563	O	GLU	98	38.349	31.000	41.906	1.00	0.00	O
ATOM	1564	N	PHE	99	39.272	32.681	40.841	1.00	0.00	N

ATOM	1565	H	PHE	99	39.374	33.671	40.671	1.00	0.00	H
ATOM	1566	CA	PHE	99	39.655	31.916	39.668	1.00	0.00	C
ATOM	1567	HA	PHE	99	39.906	30.903	39.981	1.00	0.00	H
ATOM	1568	CB	PHE	99	38.463	31.795	38.680	1.00	0.00	C
ATOM	1569	HB2	PHE	99	38.790	31.188	37.835	1.00	0.00	H
ATOM	1570	HB3	PHE	99	37.716	31.268	39.273	1.00	0.00	H
ATOM	1571	CG	PHE	99	37.846	33.144	38.190	1.00	0.00	C
ATOM	1572	CD1	PHE	99	38.498	33.968	37.228	1.00	0.00	C
ATOM	1573	HD1	PHE	99	39.348	33.566	36.696	1.00	0.00	H
ATOM	1574	CE1	PHE	99	37.941	35.183	36.752	1.00	0.00	C
ATOM	1575	HE1	PHE	99	38.489	35.711	35.986	1.00	0.00	H
ATOM	1576	CZ	PHE	99	36.745	35.655	37.313	1.00	0.00	C
ATOM	1577	HZ	PHE	99	36.301	36.518	36.841	1.00	0.00	H
ATOM	1578	CE2	PHE	99	36.093	34.891	38.270	1.00	0.00	C
ATOM	1579	HE2	PHE	99	35.100	35.179	38.580	1.00	0.00	H
ATOM	1580	CD2	PHE	99	36.602	33.631	38.671	1.00	0.00	C
ATOM	1581	HD2	PHE	99	36.129	33.020	39.426	1.00	0.00	H
ATOM	1582	C	PHE	99	40.812	32.599	38.899	1.00	0.00	C
ATOM	1583	O	PHE	99	41.192	33.756	39.202	1.00	0.00	O
ATOM	1584	N	LYS	100	41.444	31.837	37.927	1.00	0.00	N
ATOM	1585	H	LYS	100	41.100	30.888	37.876	1.00	0.00	H
ATOM	1586	CA	LYS	100	42.569	32.174	36.948	1.00	0.00	C
ATOM	1587	HA	LYS	100	42.596	33.218	36.631	1.00	0.00	H
ATOM	1588	CB	LYS	100	43.847	32.010	37.799	1.00	0.00	C
ATOM	1589	HB2	LYS	100	43.965	32.937	38.361	1.00	0.00	H
ATOM	1590	HB3	LYS	100	43.609	31.173	38.456	1.00	0.00	H
ATOM	1591	CG	LYS	100	45.151	31.700	37.027	1.00	0.00	C
ATOM	1592	HG2	LYS	100	45.037	30.690	36.631	1.00	0.00	H
ATOM	1593	HG3	LYS	100	45.279	32.428	36.226	1.00	0.00	H
ATOM	1594	CD	LYS	100	46.452	31.803	37.896	1.00	0.00	C
ATOM	1595	HD2	LYS	100	46.155	31.582	38.921	1.00	0.00	H
ATOM	1596	HD3	LYS	100	47.248	31.117	37.604	1.00	0.00	H
ATOM	1597	CE	LYS	100	47.031	33.227	37.903	1.00	0.00	C
ATOM	1598	HE2	LYS	100	46.231	33.933	37.680	1.00	0.00	H
ATOM	1599	HE3	LYS	100	47.431	33.531	38.870	1.00	0.00	H
ATOM	1600	NZ	LYS	100	48.065	33.460	36.806	1.00	0.00	N
ATOM	1601	HZ1	LYS	100	47.922	32.865	36.003	1.00	0.00	H
ATOM	1602	HZ2	LYS	100	47.944	34.436	36.572	1.00	0.00	H
ATOM	1603	HZ3	LYS	100	48.988	33.362	37.204	1.00	0.00	H
ATOM	1604	C	LYS	100	42.677	31.322	35.634	1.00	0.00	C
ATOM	1605	O	LYS	100	42.178	30.175	35.565	1.00	0.00	O
ATOM	1606	N	CYX	101	43.225	31.931	34.593	1.00	0.00	N
ATOM	1607	H	CYX	101	43.636	32.830	34.802	1.00	0.00	H
ATOM	1608	CA	CYX	101	43.489	31.248	33.290	1.00	0.00	C
ATOM	1609	HA	CYX	101	42.657	30.568	33.111	1.00	0.00	H
ATOM	1610	CB	CYX	101	43.413	32.211	32.136	1.00	0.00	C
ATOM	1611	HB2	CYX	101	42.361	32.308	31.874	1.00	0.00	H
ATOM	1612	HB3	CYX	101	43.908	33.155	32.364	1.00	0.00	H
ATOM	1613	SG	CYX	101	44.171	31.527	30.614	1.00	0.00	S
ATOM	1614	C	CYX	101	44.635	30.210	33.367	1.00	0.00	C
ATOM	1615	O	CYX	101	45.555	30.393	34.142	1.00	0.00	O
ATOM	1616	N	LEU	102	44.449	29.051	32.684	1.00	0.00	N
ATOM	1617	H	LEU	102	43.650	28.884	32.091	1.00	0.00	H
ATOM	1618	CA	LEU	102	45.492	27.993	32.579	1.00	0.00	C
ATOM	1619	HA	LEU	102	46.010	28.099	33.531	1.00	0.00	H
ATOM	1620	CB	LEU	102	44.909	26.567	32.541	1.00	0.00	C
ATOM	1621	HB2	LEU	102	44.499	26.415	31.544	1.00	0.00	H
ATOM	1622	HB3	LEU	102	45.768	25.908	32.666	1.00	0.00	H
ATOM	1623	CG	LEU	102	43.782	26.268	33.597	1.00	0.00	C
ATOM	1624	HG	LEU	102	42.833	26.719	33.307	1.00	0.00	H
ATOM	1625	CD1	LEU	102	43.542	24.778	33.683	1.00	0.00	C
ATOM	1626	HD11	LEU	102	42.681	24.668	34.342	1.00	0.00	H
ATOM	1627	HD12	LEU	102	43.347	24.364	32.694	1.00	0.00	H

```
ATOM   1628 HD13 LEU   102       44.452  24.299  34.040  1.00  0.00           H
ATOM   1629  CD2 LEU   102       44.218  26.614  35.007  1.00  0.00           C
ATOM   1630 HD21 LEU   102       44.180  27.702  35.066  1.00  0.00           H
ATOM   1631 HD22 LEU   102       43.608  26.306  35.856  1.00  0.00           H
ATOM   1632 HD23 LEU   102       45.252  26.303  35.160  1.00  0.00           H
ATOM   1633  C   LEU   102       46.618  28.189  31.530  1.00  0.00           C
ATOM   1634  O   LEU   102       46.226  28.400  30.364  1.00  0.00           O
ATOM   1635  OXT LEU   102       47.838  28.118  31.852  1.00  0.00           O
HETATM 1637  N   LIG   103       28.385  39.243  38.699  1.00  0.00           N
HETATM 1638  C   LIG   103       28.334  37.937  38.889  1.00  0.00           C
HETATM 1639  O   LIG   103       28.046  41.330  39.421  1.00  0.00           O
HETATM 1640  C5' LIG   103       26.122  40.477  45.058  1.00  0.00           C
HETATM 1641  O5' LIG   103       26.032  39.062  44.977  1.00  0.00           O
HETATM 1642  C4' LIG   103       26.037  41.115  43.685  1.00  0.00           C
HETATM 1643  O4' LIG   103       26.844  40.329  42.797  1.00  0.00           O
HETATM 1644  C3' LIG   103       24.636  41.215  43.054  1.00  0.00           C
HETATM 1645  O3' LIG   103       23.969  42.433  43.237  1.00  0.00           O
HETATM 1646  C2' LIG   103       24.987  40.892  41.563  1.00  0.00           C
HETATM 1647  O2' LIG   103       24.529  41.918  40.629  1.00  0.00           O
HETATM 1648  C1' LIG   103       26.490  40.693  41.509  1.00  0.00           C
HETATM 1649  N1  LIG   103       27.001  39.728  40.602  1.00  0.00           N
HETATM 1650  O1  LIG   103       28.356  35.003  38.707  1.00  0.00           O
HETATM 1651  N2  LIG   103       29.021  37.124  38.079  1.00  0.00           N
HETATM 1652  C6  LIG   103       27.795  40.128  39.587  1.00  0.00           C
HETATM 1653  C7  LIG   103       26.801  38.359  40.810  1.00  0.00           C
HETATM 1654  C8  LIG   103       27.486  37.449  39.989  1.00  0.00           C
HETATM 1655  C9  LIG   103       29.072  35.769  38.030  1.00  0.00           C
HETATM 1656  C10 LIG   103       30.207  35.254  37.090  1.00  0.00           C
HETATM 1657  H   LIG   103       29.501  37.657  37.365  1.00  0.00           H
HETATM 1658  H1  LIG   103       27.408  36.425  40.333  1.00  0.00           H
HETATM 1659  H4  LIG   103       26.875  41.711  41.381  1.00  0.00           H
HETATM 1660  H6  LIG   103       26.455  42.122  43.811  1.00  0.00           H
HETATM 1661  H7  LIG   103       24.002  40.459  43.533  1.00  0.00           H
HETATM 1662  H8  LIG   103       24.638  39.900  41.252  1.00  0.00           H
HETATM 1663  H9  LIG   103       25.311  40.934  45.639  1.00  0.00           H
HETATM 1664  H10 LIG   103       27.012  40.811  45.606  1.00  0.00           H
HETATM 1665  H11 LIG   103       31.161  35.567  37.534  1.00  0.00           H
HETATM 1666  H12 LIG   103       30.055  35.710  36.104  1.00  0.00           H
HETATM 1667  H13 LIG   103       30.104  34.163  37.113  1.00  0.00           H
HETATM 1668  H14 LIG   103       26.153  38.128  41.645  1.00  0.00           H
HETATM 1669  H2' LIG   103       25.171  42.649  40.623  1.00  0.00           H
HETATM 1670  H3' LIG   103       23.174  42.373  42.680  1.00  0.00           H
HETATM 1671  H5' LIG   103       26.639  38.843  44.249  1.00  0.00           H
ENDMDL
MODEL        2
SHEET    1   1 1 ILE    22  ASP    26  0
SHEET    2   2 1 VAL    37  VAL    40  0
SHEET    3   3 1 CYX    50  GLU    53  0
SHEET    4   4 1 ALA    56  VAL    60  0
SHEET    5   5 1 PHE    94  GLU    98  0
HELIX    1   1 GLN     9  PHE     11  1                                  3
HELIX    2   2 ASP    13  LEU     16  1                                  4
HELIX    3   3 LEU    62  THR     64  1                                  3
HELIX    4   4 GLU    67  GLU     73  1                                  7
HELIX    5   5 LEU    77  ALA     85  1                                  9
ATOM      1  N   GLN     1       38.140  17.636  33.331  1.00  0.00           N
ATOM      2  H1  GLN     1       38.573  16.752  33.103  1.00  0.00           H
ATOM      3  H2  GLN     1       38.792  18.341  33.644  1.00  0.00           H
ATOM      4  H3  GLN     1       37.513  17.471  34.107  1.00  0.00           H
ATOM      5  CA  GLN     1       37.362  18.012  32.189  1.00  0.00           C
ATOM      6  HA  GLN     1       38.084  18.309  31.428  1.00  0.00           H
ATOM      7  CB  GLN     1       36.511  16.813  31.638  1.00  0.00           C
ATOM      8  HB2 GLN     1       35.770  16.530  32.386  1.00  0.00           H
ATOM      9  HB3 GLN     1       35.909  17.210  30.822  1.00  0.00           H
```

ATOM	10	CG	GLN	1	37.235	15.564	31.178	1.00	0.00	C
ATOM	11	HG2	GLN	1	37.718	15.129	32.052	1.00	0.00	H
ATOM	12	HG3	GLN	1	36.583	14.838	30.691	1.00	0.00	H
ATOM	13	CD	GLN	1	38.331	15.871	30.148	1.00	0.00	C
ATOM	14	OE1	GLN	1	38.457	16.966	29.638	1.00	0.00	O
ATOM	15	NE2	GLN	1	39.223	14.933	29.788	1.00	0.00	N
ATOM	16	HE21	GLN	1	38.991	13.969	29.980	1.00	0.00	H
ATOM	17	HE22	GLN	1	39.946	15.125	29.109	1.00	0.00	H
ATOM	18	C	GLN	1	36.564	19.276	32.423	1.00	0.00	C
ATOM	19	O	GLN	1	36.138	19.491	33.570	1.00	0.00	O
ATOM	20	N	PRO	2	36.401	20.138	31.441	1.00	0.00	N
ATOM	21	CD	PRO	2	37.041	20.068	30.152	1.00	0.00	C
ATOM	22	HD2	PRO	2	36.835	19.146	29.610	1.00	0.00	H
ATOM	23	HD3	PRO	2	38.076	20.326	30.376	1.00	0.00	H
ATOM	24	CG	PRO	2	36.412	21.153	29.298	1.00	0.00	C
ATOM	25	HG2	PRO	2	35.584	20.777	28.698	1.00	0.00	H
ATOM	26	HG3	PRO	2	37.238	21.497	28.676	1.00	0.00	H
ATOM	27	CB	PRO	2	36.140	22.250	30.321	1.00	0.00	C
ATOM	28	HB2	PRO	2	35.341	22.923	30.007	1.00	0.00	H
ATOM	29	HB3	PRO	2	37.095	22.765	30.424	1.00	0.00	H
ATOM	30	CA	PRO	2	35.709	21.458	31.608	1.00	0.00	C
ATOM	31	HA	PRO	2	35.993	21.999	32.510	1.00	0.00	H
ATOM	32	C	PRO	2	34.179	21.297	31.700	1.00	0.00	C
ATOM	33	O	PRO	2	33.675	20.177	31.507	1.00	0.00	O
ATOM	34	N	ASN	3	33.477	22.405	31.996	1.00	0.00	N
ATOM	35	H	ASN	3	34.009	23.256	32.107	1.00	0.00	H
ATOM	36	CA	ASN	3	32.009	22.445	32.229	1.00	0.00	C
ATOM	37	HA	ASN	3	31.655	21.418	32.138	1.00	0.00	H
ATOM	38	CB	ASN	3	31.749	23.110	33.645	1.00	0.00	C
ATOM	39	HB2	ASN	3	32.209	24.098	33.684	1.00	0.00	H
ATOM	40	HB3	ASN	3	30.698	23.338	33.826	1.00	0.00	H
ATOM	41	CG	ASN	3	32.319	22.289	34.852	1.00	0.00	C
ATOM	42	OD1	ASN	3	31.682	21.398	35.427	1.00	0.00	O
ATOM	43	ND2	ASN	3	33.506	22.512	35.226	1.00	0.00	N
ATOM	44	HD21	ASN	3	34.124	23.130	34.721	1.00	0.00	H
ATOM	45	HD22	ASN	3	33.900	22.130	36.075	1.00	0.00	H
ATOM	46	C	ASN	3	31.282	23.227	31.167	1.00	0.00	C
ATOM	47	O	ASN	3	31.898	24.015	30.388	1.00	0.00	O
ATOM	48	N	ASP	4	29.968	23.102	31.092	1.00	0.00	N
ATOM	49	H	ASP	4	29.559	22.489	31.782	1.00	0.00	H
ATOM	50	CA	ASP	4	29.099	23.834	30.178	1.00	0.00	C
ATOM	51	HA	ASP	4	29.494	23.888	29.164	1.00	0.00	H
ATOM	52	CB	ASP	4	27.782	23.146	29.931	1.00	0.00	C
ATOM	53	HB2	ASP	4	27.283	23.024	30.893	1.00	0.00	H
ATOM	54	HB3	ASP	4	27.186	23.749	29.245	1.00	0.00	H
ATOM	55	CG	ASP	4	27.852	21.729	29.340	1.00	0.00	C
ATOM	56	OD1	ASP	4	26.833	21.008	29.495	1.00	0.00	O
ATOM	57	OD2	ASP	4	28.761	21.432	28.504	1.00	0.00	O
ATOM	58	C	ASP	4	28.851	25.263	30.677	1.00	0.00	C
ATOM	59	O	ASP	4	28.672	26.210	29.911	1.00	0.00	O
ATOM	60	N	ILE	5	28.562	25.369	31.992	1.00	0.00	N
ATOM	61	H	ILE	5	28.668	24.689	32.730	1.00	0.00	H
ATOM	62	CA	ILE	5	28.125	26.665	32.538	1.00	0.00	C
ATOM	63	HA	ILE	5	27.312	27.120	31.973	1.00	0.00	H
ATOM	64	CB	ILE	5	27.549	26.470	33.992	1.00	0.00	C
ATOM	65	HB	ILE	5	28.322	25.972	34.577	1.00	0.00	H
ATOM	66	CG2	ILE	5	27.072	27.833	34.596	1.00	0.00	C
ATOM	67	HG21	ILE	5	26.398	27.710	35.444	1.00	0.00	H
ATOM	68	HG22	ILE	5	27.974	28.424	34.753	1.00	0.00	H
ATOM	69	HG23	ILE	5	26.490	28.321	33.814	1.00	0.00	H
ATOM	70	CG1	ILE	5	26.357	25.471	33.971	1.00	0.00	C
ATOM	71 H	HG12	ILE	5	26.731	24.507	33.627	1.00	0.00	
ATOM	72	HG13	ILE	5	26.015	25.450	35.006	1.00	0.00	H

ATOM	73	CD1	ILE	5	25.192	25.838	33.036	1.00	0.00	C
ATOM	74	HD11	ILE	5	25.551	25.764	32.010	1.00	0.00	H
ATOM	75	HD12	ILE	5	24.317	25.188	33.083	1.00	0.00	H
ATOM	76	HD13	ILE	5	24.846	26.869	33.118	1.00	0.00	H
ATOM	77	C	ILE	5	29.309	27.657	32.516	1.00	0.00	C
ATOM	78	O	ILE	5	30.502	27.289	32.744	1.00	0.00	O
ATOM	79	N	THR	6	29.025	28.911	32.167	1.00	0.00	N
ATOM	80	H	THR	6	28.099	29.113	31.818	1.00	0.00	H
ATOM	81	CA	THR	6	30.146	29.959	32.115	1.00	0.00	C
ATOM	82	HA	THR	6	30.873	29.752	32.901	1.00	0.00	H
ATOM	83	CB	THR	6	30.860	29.712	30.770	1.00	0.00	C
ATOM	84	HB	THR	6	31.118	28.654	30.718	1.00	0.00	H
ATOM	85	CG2	THR	6	30.033	29.972	29.522	1.00	0.00	C
ATOM	86	HG21	THR	6	30.106	31.036	29.297	1.00	0.00	H
ATOM	87	HG22	THR	6	30.341	29.340	28.688	1.00	0.00	H
ATOM	88	HG23	THR	6	29.059	29.522	29.719	1.00	0.00	H
ATOM	89	OG1	THR	6	32.137	30.282	30.591	1.00	0.00	O
ATOM	90	HG1	THR	6	32.586	29.675	29.998	1.00	0.00	H
ATOM	91	C	THR	6	29.783	31.411	32.306	1.00	0.00	C
ATOM	92	O	THR	6	28.593	31.760	32.475	1.00	0.00	O
ATOM	93	N	PHE	7	30.786	32.331	32.271	1.00	0.00	N
ATOM	94	H	PHE	7	31.667	32.033	31.876	1.00	0.00	H
ATOM	95	CA	PHE	7	30.555	33.834	32.265	1.00	0.00	C
ATOM	96	HA	PHE	7	29.951	34.209	33.092	1.00	0.00	H
ATOM	97	CB	PHE	7	31.938	34.495	32.371	1.00	0.00	C
ATOM	98	HB2	PHE	7	32.516	34.177	31.503	1.00	0.00	H
ATOM	99	HB3	PHE	7	31.791	35.575	32.338	1.00	0.00	H
ATOM	100	CG	PHE	7	32.685	34.243	33.689	1.00	0.00	C
ATOM	101	CD1	PHE	7	32.132	34.399	34.959	1.00	0.00	C
ATOM	102	HD1	PHE	7	31.107	34.734	35.016	1.00	0.00	H
ATOM	103	CE1	PHE	7	32.813	34.113	36.152	1.00	0.00	C
ATOM	104	HE1	PHE	7	32.252	34.170	37.073	1.00	0.00	H
ATOM	105	CZ	PHE	7	34.087	33.660	36.107	1.00	0.00	C
ATOM	106	HZ	PHE	7	34.615	33.433	37.020	1.00	0.00	H
ATOM	107	CE2	PHE	7	34.729	33.523	34.845	1.00	0.00	C
ATOM	108	HE2	PHE	7	35.687	33.030	34.778	1.00	0.00	H
ATOM	109	CD2	PHE	7	34.007	33.731	33.628	1.00	0.00	C
ATOM	110	HD2	PHE	7	34.394	33.405	32.674	1.00	0.00	H
ATOM	111	C	PHE	7	29.879	34.408	31.026	1.00	0.00	C
ATOM	112	O	PHE	7	30.274	34.138	29.934	1.00	0.00	O
ATOM	113	N	PHE	8	28.812	35.227	31.215	1.00	0.00	N
ATOM	114	H	PHE	8	28.490	35.330	32.167	1.00	0.00	H
ATOM	115	CA	PHE	8	28.021	35.792	30.124	1.00	0.00	C
ATOM	116	HA	PHE	8	27.758	34.981	29.444	1.00	0.00	H
ATOM	117	CB	PHE	8	26.725	36.458	30.578	1.00	0.00	C
ATOM	118	HB2	PHE	8	26.849	37.014	31.508	1.00	0.00	H
ATOM	119	HB3	PHE	8	26.395	37.165	29.817	1.00	0.00	H
ATOM	120	CG	PHE	8	25.456	35.615	30.949	1.00	0.00	C
ATOM	121	CD1	PHE	8	25.568	34.282	31.394	1.00	0.00	C
ATOM	122	HD1	PHE	8	26.545	33.824	31.433	1.00	0.00	H
ATOM	123	CE1	PHE	8	24.522	33.453	31.712	1.00	0.00	C
ATOM	124	HE1	PHE	8	24.696	32.460	32.098	1.00	0.00	H
ATOM	125	CZ	PHE	8	23.230	34.014	31.546	1.00	0.00	C
ATOM	126	HZ	PHE	8	22.337	33.464	31.806	1.00	0.00	H
ATOM	127	CE2	PHE	8	23.031	35.328	31.079	1.00	0.00	C
ATOM	128	HE2	PHE	8	22.022	35.712	31.047	1.00	0.00	H
ATOM	129	CD2	PHE	8	24.164	36.104	30.766	1.00	0.00	C
ATOM	130	HD2	PHE	8	23.963	37.109	30.425	1.00	0.00	H
ATOM	131	C	PHE	8	28.823	36.763	29.333	1.00	0.00	C
ATOM	132	O	PHE	8	29.619	37.548	29.826	1.00	0.00	O
ATOM	133	N	GLN	9	28.566	36.659	28.061	1.00	0.00	N
ATOM	134	H	GLN	9	28.060	35.901	27.628	1.00	0.00	H
ATOM	135	CA	GLN	9	29.354	37.337	27.045	1.00	0.00	C
ATOM	136	HA	GLN	9	30.357	36.935	27.188	1.00	0.00	H

ATOM	137	CB	GLN	9	28.875	36.905	25.600	1.00	0.00	C
ATOM	138	HB2	GLN	9	29.691	37.222	24.949	1.00	0.00	H
ATOM	139	HB3	GLN	9	28.959	35.823	25.486	1.00	0.00	H
ATOM	140	CG	GLN	9	27.540	37.504	25.105	1.00	0.00	C
ATOM	141	HG2	GLN	9	27.770	38.511	24.757	1.00	0.00	H
ATOM	142	HG3	GLN	9	27.218	37.013	24.186	1.00	0.00	H
ATOM	143	CD	GLN	9	26.407	37.360	26.076	1.00	0.00	C
ATOM	144	OE1	GLN	9	26.152	36.341	26.736	1.00	0.00	O
ATOM	145	NE2	GLN	9	25.738	38.471	26.387	1.00	0.00	N
ATOM	146	HE21	GLN	9	24.852	38.283	26.833	1.00	0.00	H
ATOM	147	HE22	GLN	9	25.909	39.311	25.853	1.00	0.00	H
ATOM	148	C	GLN	9	29.398	38.867	27.234	1.00	0.00	C
ATOM	149	O	GLN	9	30.387	39.507	26.924	1.00	0.00	O
ATOM	150	N	ARG	10	28.374	39.366	27.912	1.00	0.00	N
ATOM	151	H	ARG	10	27.710	38.714	28.306	1.00	0.00	H
ATOM	152	CA	ARG	10	28.220	40.672	28.496	1.00	0.00	C
ATOM	153	HA	ARG	10	28.047	41.402	27.705	1.00	0.00	H
ATOM	154	CB	ARG	10	26.869	40.748	29.258	1.00	0.00	C
ATOM	155	HB2	ARG	10	26.128	40.116	28.768	1.00	0.00	H
ATOM	156	HB3	ARG	10	27.042	40.443	30.290	1.00	0.00	H
ATOM	157	CG	ARG	10	26.239	42.151	29.083	1.00	0.00	C
ATOM	158	HG2	ARG	10	26.843	42.959	29.496	1.00	0.00	H
ATOM	159	HG3	ARG	10	26.043	42.401	28.040	1.00	0.00	H
ATOM	160	CD	ARG	10	24.860	42.091	29.880	1.00	0.00	C
ATOM	161	HD2	ARG	10	24.275	41.275	29.453	1.00	0.00	H
ATOM	162	HD3	ARG	10	25.028	41.786	30.912	1.00	0.00	H
ATOM	163	NE	ARG	10	24.201	43.418	29.740	1.00	0.00	N
ATOM	164	HE	ARG	10	24.759	44.198	29.423	1.00	0.00	H
ATOM	165	CZ	ARG	10	23.130	43.717	30.323	1.00	0.00	C
ATOM	166	NH1	ARG	10	22.512	42.824	31.032	1.00	0.00	N
ATOM	167	HH11	ARG	10	21.712	43.154	31.552	1.00	0.00	H
ATOM	168	HH12	ARG	10	22.928	41.905	31.092	1.00	0.00	H
ATOM	169	NH2	ARG	10	22.577	44.895	30.248	1.00	0.00	N
ATOM	170	HH21	ARG	10	22.925	45.611	29.627	1.00	0.00	H
ATOM	171	HH22	ARG	10	21.935	45.127	30.992	1.00	0.00	H
ATOM	172	C	ARG	10	29.418	41.168	29.277	1.00	0.00	C
ATOM	173	O	ARG	10	29.736	42.386	29.254	1.00	0.00	O
ATOM	174	N	PHE	11	30.103	40.329	29.922	1.00	0.00	N
ATOM	175	H	PHE	11	29.855	39.356	29.817	1.00	0.00	H
ATOM	176	CA	PHE	11	31.256	40.539	30.823	1.00	0.00	C
ATOM	177	HA	PHE	11	31.375	41.614	30.962	1.00	0.00	H
ATOM	178	CB	PHE	11	30.849	40.080	32.250	1.00	0.00	C
ATOM	179	HB2	PHE	11	31.468	40.511	33.037	1.00	0.00	H
ATOM	180	HB3	PHE	11	30.986	39.000	32.301	1.00	0.00	H
ATOM	181	CG	PHE	11	29.343	40.364	32.603	1.00	0.00	C
ATOM	182	CD1	PHE	11	29.035	41.639	32.916	1.00	0.00	C
ATOM	183	HD1	PHE	11	29.737	42.457	32.850	1.00	0.00	H
ATOM	184	CE1	PHE	11	27.697	41.966	33.271	1.00	0.00	C
ATOM	185	HE1	PHE	11	27.440	42.993	33.480	1.00	0.00	H
ATOM	186	CZ	PHE	11	26.713	40.931	33.347	1.00	0.00	C
ATOM	187	HZ	PHE	11	25.722	41.180	33.699	1.00	0.00	H
ATOM	188	CE2	PHE	11	27.107	39.636	33.047	1.00	0.00	C
ATOM	189	HE2	PHE	11	26.314	38.906	33.114	1.00	0.00	H
ATOM	190	CD2	PHE	11	28.447	39.308	32.780	1.00	0.00	C
ATOM	191	HD2	PHE	11	28.669	38.257	32.672	1.00	0.00	H
ATOM	192	C	PHE	11	32.615	39.911	30.458	1.00	0.00	C
ATOM	193	O	PHE	11	33.571	39.801	31.274	1.00	0.00	O
ATOM	194	N	GLN	12	32.791	39.457	29.232	1.00	0.00	N
ATOM	195	H	GLN	12	31.986	39.558	28.632	1.00	0.00	H
ATOM	196	CA	GLN	12	34.001	38.691	28.900	1.00	0.00	C
ATOM	197	HA	GLN	12	34.435	38.218	29.782	1.00	0.00	H
ATOM	198	CB	GLN	12	33.675	37.647	27.841	1.00	0.00	C
ATOM	199	HB2	GLN	12	33.217	38.140	26.983	1.00	0.00	H

ATOM	200	HB3	GLN	12	34.604	37.277	27.408	1.00	0.00	H
ATOM	201	CG	GLN	12	32.994	36.462	28.322	1.00	0.00	C
ATOM	202	HG2	GLN	12	33.603	35.972	29.083	1.00	0.00	H
ATOM	203	HG3	GLN	12	32.065	36.834	28.753	1.00	0.00	H
ATOM	204	CD	GLN	12	32.699	35.430	27.191	1.00	0.00	C
ATOM	205	OE1	GLN	12	33.387	35.415	26.163	1.00	0.00	O
ATOM	206	NE2	GLN	12	31.685	34.596	27.342	1.00	0.00	N
ATOM	207	HE21	GLN	12	31.115	34.606	28.176	1.00	0.00	H
ATOM	208	HE22	GLN	12	31.405	34.220	26.448	1.00	0.00	H
ATOM	209	C	GLN	12	35.161	39.660	28.472	1.00	0.00	C
ATOM	210	O	GLN	12	36.299	39.201	28.599	1.00	0.00	O
ATOM	211	N	ASP	13	35.001	40.901	28.079	1.00	0.00	N
ATOM	212	H	ASP	13	34.083	41.302	27.953	1.00	0.00	H
ATOM	213	CA	ASP	13	36.111	41.790	27.689	1.00	0.00	C
ATOM	214	HA	ASP	13	36.720	41.118	27.085	1.00	0.00	H
ATOM	215	CB	ASP	13	35.598	43.008	26.964	1.00	0.00	C
ATOM	216	HB2	ASP	13	34.892	43.410	27.691	1.00	0.00	H
ATOM	217	HB3	ASP	13	36.340	43.716	26.600	1.00	0.00	H
ATOM	218	CG	ASP	13	34.911	42.516	25.663	1.00	0.00	C
ATOM	219	OD1	ASP	13	33.885	43.203	25.316	1.00	0.00	O
ATOM	220	OD2	ASP	13	35.263	41.510	25.124	1.00	0.00	O
ATOM	221	C	ASP	13	37.125	42.123	28.794	1.00	0.00	C
ATOM	222	O	ASP	13	38.296	42.434	28.490	1.00	0.00	O
ATOM	223	N	ASP	14	36.607	42.026	30.010	1.00	0.00	N
ATOM	224	H	ASP	14	35.604	42.142	30.047	1.00	0.00	H
ATOM	225	CA	ASP	14	37.414	42.265	31.172	1.00	0.00	C
ATOM	226	HA	ASP	14	38.266	42.826	30.788	1.00	0.00	H
ATOM	227	CB	ASP	14	36.611	43.008	32.291	1.00	0.00	C
ATOM	228	HB2	ASP	14	35.622	42.562	32.390	1.00	0.00	H
ATOM	229	HB3	ASP	14	37.060	42.921	33.281	1.00	0.00	H
ATOM	230	CG	ASP	14	36.352	44.488	31.912	1.00	0.00	C
ATOM	231	OD1	ASP	14	35.160	44.845	31.939	1.00	0.00	O
ATOM	232	OD2	ASP	14	37.207	45.227	31.439	1.00	0.00	O
ATOM	233	C	ASP	14	37.960	40.943	31.719	1.00	0.00	C
ATOM	234	O	ASP	14	38.860	40.908	32.536	1.00	0.00	O
ATOM	235	N	ILE	15	37.518	39.784	31.275	1.00	0.00	N
ATOM	236	H	ILE	15	36.855	39.794	30.514	1.00	0.00	H
ATOM	237	CA	ILE	15	38.203	38.510	31.629	1.00	0.00	C
ATOM	238	HA	ILE	15	38.703	38.579	32.594	1.00	0.00	H
ATOM	239	CB	ILE	15	37.212	37.343	31.643	1.00	0.00	C
ATOM	240	HB	ILE	15	36.716	37.447	30.679	1.00	0.00	H
ATOM	241	CG2	ILE	15	37.931	35.965	31.706	1.00	0.00	C
ATOM	242	HG21	ILE	15	37.218	35.141	31.700	1.00	0.00	H
ATOM	243	HG22	ILE	15	38.454	35.934	30.750	1.00	0.00	H
ATOM	244	HG23	ILE	15	38.681	36.051	32.491	1.00	0.00	H
ATOM	245	CG1	ILE	15	36.165	37.459	32.768	1.00	0.00	C
ATOM	246	HG12	ILE	15	36.559	37.114	33.723	1.00	0.00	H
ATOM	247	HG13	ILE	15	35.924	38.498	32.990	1.00	0.00	H
ATOM	248	CD1	ILE	15	34.885	36.637	32.376	1.00	0.00	C
ATOM	249	HD11	ILE	15	34.217	36.700	33.233	1.00	0.00	H
ATOM	250	HD12	ILE	15	34.499	37.000	31.424	1.00	0.00	H
ATOM	251	HD13	ILE	15	35.179	35.618	32.124	1.00	0.00	H
ATOM	252	C	ILE	15	39.313	38.351	30.603	1.00	0.00	C
ATOM	253	O	ILE	15	40.456	38.012	30.993	1.00	0.00	O
ATOM	254	N	LEU	16	39.082	38.661	29.318	1.00	0.00	N
ATOM	255	H	LEU	16	38.119	38.890	29.117	1.00	0.00	H
ATOM	256	CA	LEU	16	39.969	38.819	28.178	1.00	0.00	C
ATOM	257	HA	LEU	16	40.555	37.928	27.954	1.00	0.00	H
ATOM	258	CB	LEU	16	39.156	39.205	26.977	1.00	0.00	C
ATOM	259	HB2	LEU	16	38.312	38.540	26.790	1.00	0.00	H
ATOM	260	HB3	LEU	16	38.737	40.179	27.233	1.00	0.00	H
ATOM	261	CG	LEU	16	39.878	39.452	25.729	1.00	0.00	C
ATOM	262	HG	LEU	16	40.627	40.242	25.779	1.00	0.00	H
ATOM	263	CD1	LEU	16	40.597	38.181	25.213	1.00	0.00	C

```
ATOM    264 HD11 LEU    16      41.238  38.359  24.350  1.00  0.00              H
ATOM    265 HD12 LEU    16      41.299  37.657  25.860  1.00  0.00              H
ATOM    266 HD13 LEU    16      39.845  37.433  24.957  1.00  0.00              H
ATOM    267  CD2 LEU    16      38.795  39.830  24.696  1.00  0.00              C
ATOM    268 HD21 LEU    16      39.320  40.156  23.799  1.00  0.00              H
ATOM    269 HD22 LEU    16      38.230  38.935  24.434  1.00  0.00              H
ATOM    270 HD23 LEU    16      38.016  40.448  25.144  1.00  0.00              H
ATOM    271  C   LEU    16      41.119  39.820  28.439  1.00  0.00              C
ATOM    272  O   LEU    16      42.211  39.440  28.133  1.00  0.00              O
ATOM    273  N   ALA    17      40.790  41.032  28.934  1.00  0.00              N
ATOM    274  H   ALA    17      39.828  41.311  29.054  1.00  0.00              H
ATOM    275  CA  ALA    17      41.853  42.006  29.394  1.00  0.00              C
ATOM    276  HA  ALA    17      42.661  41.873  28.674  1.00  0.00              H
ATOM    277  CB  ALA    17      41.350  43.452  29.407  1.00  0.00              C
ATOM    278  HB1 ALA    17      42.081  44.182  29.755  1.00  0.00              H
ATOM    279  HB2 ALA    17      41.141  43.700  28.366  1.00  0.00              H
ATOM    280  HB3 ALA    17      40.439  43.499  30.005  1.00  0.00              H
ATOM    281  C   ALA    17      42.311  41.708  30.868  1.00  0.00              C
ATOM    282  O   ALA    17      43.185  42.478  31.299  1.00  0.00              O
ATOM    283  N   GLY    18      41.738  40.730  31.608  1.00  0.00              N
ATOM    284  H   GLY    18      41.026  40.205  31.120  1.00  0.00              H
ATOM    285  CA  GLY    18      42.177  40.352  32.921  1.00  0.00              C
ATOM    286  HA2 GLY    18      41.470  39.561  33.173  1.00  0.00              H
ATOM    287  HA3 GLY    18      43.225  40.054  32.940  1.00  0.00              H
ATOM    288  C   GLY    18      41.929  41.272  34.104  1.00  0.00              C
ATOM    289  O   GLY    18      42.429  40.992  35.189  1.00  0.00              O
ATOM    290  N   ARG    19      41.098  42.321  33.856  1.00  0.00              N
ATOM    291  H   ARG    19      40.781  42.456  32.907  1.00  0.00              H
ATOM    292  CA  ARG    19      40.699  43.206  34.903  1.00  0.00              C
ATOM    293  HA  ARG    19      41.551  43.281  35.578  1.00  0.00              H
ATOM    294  CB  ARG    19      40.376  44.604  34.274  1.00  0.00              C
ATOM    295  HB2 ARG    19      39.559  44.533  33.555  1.00  0.00              H
ATOM    296  HB3 ARG    19      40.048  45.241  35.095  1.00  0.00              H
ATOM    297  CG  ARG    19      41.602  45.339  33.534  1.00  0.00              C
ATOM    298  HG2 ARG    19      42.331  45.641  34.286  1.00  0.00              H
ATOM    299  HG3 ARG    19      42.014  44.815  32.671  1.00  0.00              H
ATOM    300  CD  ARG    19      41.092  46.680  32.893  1.00  0.00              C
ATOM    301  HD2 ARG    19      40.738  47.352  33.673  1.00  0.00              H
ATOM    302  HD3 ARG    19      41.951  47.216  32.490  1.00  0.00              H
ATOM    303  NE  ARG    19      40.134  46.417  31.743  1.00  0.00              N
ATOM    304  HE  ARG    19      39.210  46.152  32.055  1.00  0.00              H
ATOM    305  CZ  ARG    19      40.337  46.379  30.456  1.00  0.00              C
ATOM    306  NH1 ARG    19      41.474  46.751  29.954  1.00  0.00              N
ATOM    307 HH11 ARG    19      42.202  47.131  30.542  1.00  0.00              H
ATOM    308 HH12 ARG    19      41.590  46.720  28.950  1.00  0.00              H
ATOM    309  NH2 ARG    19      39.355  46.129  29.646  1.00  0.00              N
ATOM    310 HH21 ARG    19      39.429  46.339  28.661  1.00  0.00              H
ATOM    311 HH22 ARG    19      38.468  46.085  30.127  1.00  0.00              H
ATOM    312  C   ARG    19      39.493  42.649  35.725  1.00  0.00              C
ATOM    313  O   ARG    19      39.070  43.289  36.676  1.00  0.00              O
ATOM    314  N   LYS    20      39.059  41.395  35.467  1.00  0.00              N
ATOM    315  H   LYS    20      39.302  41.092  34.535  1.00  0.00              H
ATOM    316  CA  LYS    20      38.240  40.519  36.349  1.00  0.00              C
ATOM    317  HA  LYS    20      38.050  41.039  37.288  1.00  0.00              H
ATOM    318  CB  LYS    20      36.917  40.286  35.543  1.00  0.00              C
ATOM    319  HB2 LYS    20      36.931  39.578  34.716  1.00  0.00              H
ATOM    320  HB3 LYS    20      36.609  41.189  35.015  1.00  0.00              H
ATOM    321  CG  LYS    20      35.837  39.909  36.658  1.00  0.00              C
ATOM    322  HG2 LYS    20      35.082  39.353
  36.099  1.00  0.00              H
ATOM    323  HG3 LYS    20      36.187  39.309  37.499  1.00  0.00              H
ATOM    324  CD  LYS    20      35.134  41.184  37.104  1.00  0.00              C
ATOM    325  HD2 LYS    20      34.668  41.699  36.263  1.00  0.00              H
ATOM    326  HD3 LYS    20      35.897  41.843  37.518  1.00  0.00              H
```

ATOM	327	CE	LYS	20	34.115	40.805	38.164	1.00	0.00	C
ATOM	328	HE2	LYS	20	33.470	39.976	37.876	1.00	0.00	H
ATOM	329	HE3	LYS	20	34.650	40.490	39.061	1.00	0.00	H
ATOM	330	NZ	LYS	20	33.257	41.996	38.496	1.00	0.00	N
ATOM	331	HZ1	LYS	20	32.771	41.810	39.362	1.00	0.00	H
ATOM	332	HZ2	LYS	20	32.715	42.278	37.691	1.00	0.00	H
ATOM	333	HZ3	LYS	20	33.804	42.780	38.820	1.00	0.00	H
ATOM	334	C	LYS	20	38.949	39.170	36.657	1.00	0.00	C
ATOM	335	O	LYS	20	39.325	38.350	35.801	1.00	0.00	O
ATOM	336	N	THR	21	39.193	38.942	37.939	1.00	0.00	N
ATOM	337	H	THR	21	38.963	39.661	38.610	1.00	0.00	H
ATOM	338	CA	THR	21	39.784	37.711	38.478	1.00	0.00	C
ATOM	339	HA	THR	21	39.719	36.932	37.721	1.00	0.00	H
ATOM	340	CB	THR	21	41.303	37.856	38.826	1.00	0.00	C
ATOM	341	HB	THR	21	41.606	36.857	39.141	1.00	0.00	H
ATOM	342	CG2	THR	21	42.225	38.484	37.735	1.00	0.00	C
ATOM	343	HG21	THR	21	41.997	39.542	37.604	1.00	0.00	H
ATOM	344	HG22	THR	21	43.266	38.479	38.060	1.00	0.00	H
ATOM	345	HG23	THR	21	42.150	37.984	36.769	1.00	0.00	H
ATOM	346	OG1	THR	21	41.446	38.775	39.885	1.00	0.00	O
ATOM	347	HG1	THR	21	41.227	38.265	40.670	1.00	0.00	H
ATOM	348	C	THR	21	39.079	37.176	39.738	1.00	0.00	C
ATOM	349	O	THR	21	39.346	36.107	40.382	1.00	0.00	O
ATOM	350	N	ILE	22	38.028	37.942	40.170	1.00	0.00	N
ATOM	351	H	ILE	22	37.824	38.822	39.718	1.00	0.00	H
ATOM	352	CA	ILE	22	37.212	37.508	41.324	1.00	0.00	C
ATOM	353	HA	ILE	22	37.233	36.418	41.314	1.00	0.00	H
ATOM	354	CB	ILE	22	37.777	37.966	42.645	1.00	0.00	C
ATOM	355	HB	ILE	22	38.831	37.751	42.470	1.00	0.00	H
ATOM	356	CG2	ILE	22	37.623	39.468	42.903	1.00	0.00	C
ATOM	357	HG21	ILE	22	38.385	39.888	43.559	1.00	0.00	H
ATOM	358	HG22	ILE	22	37.762	39.905	41.914	1.00	0.00	H
ATOM	359	HG23	ILE	22	36.587	39.672	43.172	1.00	0.00	H
ATOM	360	CG1	ILE	22	37.360	37.255	43.898	1.00	0.00	C
ATOM	361	HG12	ILE	22	36.370	37.638	44.146	1.00	0.00	H
ATOM	362	HG13	ILE	22	37.186	36.200	43.685	1.00	0.00	H
ATOM	363	CD1	ILE	22	38.261	37.331	45.069	1.00	0.00	C
ATOM	364	HD11	ILE	22	38.967	36.529	44.853	1.00	0.00	H
ATOM	365	HD12	ILE	22	38.711	38.304	45.268	1.00	0.00	H
ATOM	366	HD13	ILE	22	37.783	36.922	45.959	1.00	0.00	H
ATOM	367	C	ILE	22	35.740	37.964	41.173	1.00	0.00	C
ATOM	368	O	ILE	22	35.450	38.883	40.485	1.00	0.00	O
ATOM	369	N	THR	23	34.807	37.223	41.791	1.00	0.00	N
ATOM	370	H	THR	23	35.071	36.359	42.243	1.00	0.00	H
ATOM	371	CA	THR	23	33.343	37.548	41.722	1.00	0.00	C
ATOM	372	HA	THR	23	33.350	38.638	41.721	1.00	0.00	H
ATOM	373	CB	THR	23	32.734	37.043	40.427	1.00	0.00	C
ATOM	374	HB	THR	23	33.157	36.083	40.127	1.00	0.00	H
ATOM	375	CG2	THR	23	31.214	36.892	40.321	1.00	0.00	C
ATOM	376	HG21	THR	23	30.849	37.874	40.620	1.00	0.00	H
ATOM	377	HG22	THR	23	30.938	36.491	39.345	1.00	0.00	H
ATOM	378	HG23	THR	23	30.913	36.134	41.043	1.00	0.00	H
ATOM	379	OG1	THR	23	33.073	37.877	39.310	1.00	0.00	O
ATOM	380	HG1	THR	23	33.761	38.471	39.618	1.00	0.00	H
ATOM	381	C	THR	23	32.425	37.102	42.896	1.00	0.00	C
ATOM	382	O	THR	23	32.698	36.070	43.494	1.00	0.00	O
ATOM	383	N	ILE	24	31.370	37.940	43.248	1.00	0.00	N
ATOM	384	H	ILE	24	31.161	38.718	42.639	1.00	0.00	H
ATOM	385	CA	ILE	24	30.422	37.490	44.245	1.00	0.00	C
ATOM	386	HA	ILE	24	30.732	36.531	44.660	1.00	0.00	H
ATOM	387	CB	ILE	24	30.356	38.503	45.348	1.00	0.00	C
ATOM	388	HB	ILE	24	30.055	39.432	44.865	1.00	0.00	H
ATOM	389	CG2	ILE	24	29.194	38.346	46.369	1.00	0.00	C
ATOM	390	HG21	ILE	24	29.328	37.585	47.138	1.00	0.00	H

ATOM	391	HG22	ILE	24	29.046	39.222	47.001	1.00	0.00	H
ATOM	392	HG23	ILE	24	28.245	38.232	45.846	1.00	0.00	H
ATOM	393	CG1	ILE	24	31.670	38.722	46.069	1.00	0.00	C
ATOM	394	HG12	ILE	24	31.954	37.973	46.809	1.00	0.00	H
ATOM	395	HG13	ILE	24	32.464	38.772	45.324	1.00	0.00	H
ATOM	396	CD1	ILE	24	31.812	40.124	46.702	1.00	0.00	C
ATOM	397	HD11	ILE	24	32.754	40.202	47.246	1.00	0.00	H
ATOM	398	HD12	ILE	24	31.817	40.817	45.861	1.00	0.00	H
ATOM	399	HD13	ILE	24	31.050	40.270	47.466	1.00	0.00	H
ATOM	400	C	ILE	24	29.070	37.226	43.661	1.00	0.00	C
ATOM	401	O	ILE	24	28.635	37.794	42.660	1.00	0.00	O
ATOM	402	N	ARG	25	28.399	36.164	44.244	1.00	0.00	N
ATOM	403	H	ARG	25	28.902	35.708	44.991	1.00	0.00	H
ATOM	404	CA	ARG	25	27.163	35.646	43.677	1.00	0.00	C
ATOM	405	HA	ARG	25	26.605	36.371	43.085	1.00	0.00	H
ATOM	406	CB	ARG	25	27.414	34.398	42.930	1.00	0.00	C
ATOM	407	HB2	ARG	25	28.317	33.972	43.367	1.00	0.00	H
ATOM	408	HB3	ARG	25	26.633	33.673	43.156	1.00	0.00	H
ATOM	409	CG	ARG	25	27.429	34.471	41.394	1.00	0.00	C
ATOM	410	HG2	ARG	25	27.973	35.378	41.130	1.00	0.00	H
ATOM	411	HG3	ARG	25	27.955	33.584	41.042	1.00	0.00	H
ATOM	412	CD	ARG	25	26.058	34.512	40.744	1.00	0.00	C
ATOM	413	HD2	ARG	25	25.722	33.492	40.930	1.00	0.00	H
ATOM	414	HD3	ARG	25	25.501	35.272	41.292	1.00	0.00	H
ATOM	415	NE	ARG	25	26.056	34.884	39.332	1.00	0.00	N
ATOM	416	HE	ARG	25	26.733	34.477	38.700	1.00	0.00	H
ATOM	417	CZ	ARG	25	25.166	35.591	38.619	1.00	0.00	C
ATOM	418	NH1	ARG	25	24.135	36.105	39.096	1.00	0.00	N
ATOM	419	HH11	ARG	25	23.558	36.511	38.372	1.00	0.00	H
ATOM	420	HH12	ARG	25	23.719	35.850	39.980	1.00	0.00	H
ATOM	421	NH2	ARG	25	25.379	35.835	37.402	1.00	0.00	N
ATOM	422	HH21	ARG	25	24.769	36.348	36.784	1.00	0.00	H
ATOM	423	HH22	ARG	25	26.000	35.243	36.869	1.00	0.00	H
ATOM	424	C	ARG	25	26.272	35.412	44.878	1.00	0.00	C
ATOM	425	O	ARG	25	26.661	34.903	45.934	1.00	0.00	O
ATOM	426	N	ASP	26	24.914	35.594	44.802	1.00	0.00	N
ATOM	427	H	ASP	26	24.535	35.963	43.943	1.00	0.00	H
ATOM	428	CA	ASP	26	23.985	35.198	45.855	1.00	0.00	C
ATOM	429	HA	ASP	26	24.417	35.539	46.796	1.00	0.00	H
ATOM	430	CB	ASP	26	22.677	35.942	45.903	1.00	0.00	C
ATOM	431	HB2	ASP	26	22.037	35.381	46.583	1.00	0.00	H
ATOM	432	HB3	ASP	26	22.831	36.947	46.298	1.00	0.00	H
ATOM	433	CG	ASP	26	22.028	36.128	44.521	1.00	0.00	C
ATOM	434	OD1	ASP	26	22.733	36.190	43.461	1.00	0.00	O
ATOM	435	OD2	ASP	26	20.789	36.269	44.510	1.00	0.00	O
ATOM	436	C	ASP	26	23.886	33.649	46.044	1.00	0.00	C
ATOM	437	O	ASP	26	23.902	32.959	44.995	1.00	0.00	O
ATOM	438	N	GLU	27	23.948	33.050	47.236	1.00	0.00	N
ATOM	439	H	GLU	27	23.972	33.680	48.026	1.00	0.00	H
ATOM	440	CA	GLU	27	24.152	31.620	47.602	1.00	0.00	C
ATOM	441	HA	GLU	27	25.177	31.383	47.319	1.00	0.00	H
ATOM	442	CB	GLU	27	24.135	31.553	49.169	1.00	0.00	C
ATOM	443	HB2	GLU	27	24.775	32.330	49.586	1.00	0.00	H
ATOM	444	HB3	GLU	27	23.108	31.701	49.502	1.00	0.00	H
ATOM	445	CG	GLU	27	24.467	30.095	49.657	1.00	0.00	C
ATOM	446	HG2	GLU	27	24.222	30.095	50.719	1.00	0.00	H
ATOM	447	HG3	GLU	27	23.792	29.363	49.211	1.00	0.00	H
ATOM	448	CD	GLU	27	25.827	29.579	49.389	1.00	0.00	C
ATOM	449	OE1	GLU	27	26.573	29.220	50.354	1.00	0.00	O
ATOM	450	OE2	GLU	27	26.174	29.561	48.194	1.00	0.00	O
ATOM	451	C	GLU	27	23.197	30.598	46.922	1.00	0.00	C
ATOM	452	O	GLU	27	21.977	30.777	46.905	1.00	0.00	O
ATOM	453	N	SER	28	23.715	29.490	46.440	1.00	0.00	N

ATOM	454	H	SER	28	24.657	29.288	46.741	1.00	0.00	H
ATOM	455	CA	SER	28	22.993	28.270	46.033	1.00	0.00	C
ATOM	456	HA	SER	28	22.169	28.002	46.696	1.00	0.00	H
ATOM	457	CB	SER	28	22.329	28.492	44.652	1.00	0.00	C
ATOM	458	HB2	SER	28	21.569	29.274	44.667	1.00	0.00	H
ATOM	459	HB3	SER	28	23.251	28.598	44.081	1.00	0.00	H
ATOM	460	OG	SER	28	21.671	27.273	44.250	1.00	0.00	O
ATOM	461	HG	SER	28	20.973	27.170	44.901	1.00	0.00	H
ATOM	462	C	SER	28	23.945	27.013	45.922	1.00	0.00	C
ATOM	463	O	SER	28	25.133	27.087	45.579	1.00	0.00	O
ATOM	464	N	GLU	29	23.352	25.821	46.086	1.00	0.00	N
ATOM	465	H	GLU	29	22.370	25.673	46.273	1.00	0.00	H
ATOM	466	CA	GLU	29	24.095	24.538	45.884	1.00	0.00	C
ATOM	467	HA	GLU	29	25.118	24.605	46.252	1.00	0.00	H
ATOM	468	CB	GLU	29	23.307	23.350	46.529	1.00	0.00	C
ATOM	469	HB2	GLU	29	23.068	23.619	47.558	1.00	0.00	H
ATOM	470	HB3	GLU	29	22.374	23.191	45.987	1.00	0.00	H
ATOM	471	CG	GLU	29	24.024	22.010	46.607	1.00	0.00	C
ATOM	472	HG2	GLU	29	23.472	21.430	47.347	1.00	0.00	H
ATOM	473	HG3	GLU	29	23.888	21.509	45.649	1.00	0.00	H
ATOM	474	CD	GLU	29	25.513	21.971	47.036	1.00	0.00	C
ATOM	475	OE1	GLU	29	26.247	21.102	46.562	1.00	0.00	O
ATOM	476	OE2	GLU	29	26.076	22.856	47.750	1.00	0.00	O
ATOM	477	C	GLU	29	24.362	24.258	44.389	1.00	0.00	C
ATOM	478	O	GLU	29	25.092	23.378	43.990	1.00	0.00	O
ATOM	479	N	SER	30	23.745	25.095	43.550	1.00	0.00	N
ATOM	480	H	SER	30	23.311	25.937	43.900	1.00	0.00	H
ATOM	481	CA	SER	30	23.877	25.069	42.098	1.00	0.00	C
ATOM	482	HA	SER	30	23.845	24.056	41.696	1.00	0.00	H
ATOM	483	CB	SER	30	22.729	25.715	41.380	1.00	0.00	C
ATOM	484	HB2	SER	30	22.839	25.760	40.297	1.00	0.00	H
ATOM	485	HB3	SER	30	21.793	25.182	41.550	1.00	0.00	H
ATOM	486	OG	SER	30	22.620	27.046	41.824	1.00	0.00	O
ATOM	487	HG	SER	30	22.051	27.094	42.596	1.00	0.00	H
ATOM	488	C	SER	30	25.166	25.729	41.518	1.00	0.00	C
ATOM	489	O	SER	30	25.512	25.469	40.383	1.00	0.00	O
ATOM	490	N	HIE	31	25.924	26.478	42.358	1.00	0.00	N
ATOM	491	H	HIE	31	25.606	26.496	43.316	1.00	0.00	H
ATOM	492	CA	HIE	31	27.243	27.102	42.080	1.00	0.00	C
ATOM	493	HA	HIE	31	27.129	27.682	41.164	1.00	0.00	H
ATOM	494	CB	HIE	31	27.528	28.040	43.200	1.00	0.00	C
ATOM	495	HB2	HIE	31	27.375	27.495	44.132	1.00	0.00	H
ATOM	496	HB3	HIE	31	28.598	28.245	43.220	1.00	0.00	H
ATOM	497	CG	HIE	31	26.704	29.332	43.197	1.00	0.00	C
ATOM	498	ND1	HIE	31	26.224	29.977	42.050	1.00	0.00	N
ATOM	499	CE1	HIE	31	25.543	31.086	42.477	1.00	0.00	C
ATOM	500	HE1	HIE	31	24.991	31.751	41.830	1.00	0.00	H
ATOM	501	NE2	HIE	31	25.581	31.098	43.817	1.00	0.00	N
ATOM	502	HE2	HIE	31	25.090	31.775	44.384	1.00	0.00	H
ATOM	503	CD2	HIE	31	26.366	30.087	44.319	1.00	0.00	C
ATOM	504	HD2	HIE	31	26.567	29.944	45.370	1.00	0.00	H
ATOM	505	C	HIE	31	28.404	26.133	41.780	1.00	0.00	C
ATOM	506	O	HIE	31	28.576	25.056	42.358	1.00	0.00	O
ATOM	507	N	PHE	32	29.431	26.579	41.005	1.00	0.00	N
ATOM	508	H	PHE	32	29.261	27.492	40.611	1.00	0.00	H
ATOM	509	CA	PHE	32	30.773	26.004	40.856	1.00	0.00	C
ATOM	510	HA	PHE	32	30.550	25.021	40.441	1.00	0.00	H
ATOM	511	CB	PHE	32	31.751	26.851	39.934	1.00	0.00	C
ATOM	512	HB2	PHE	32	32.160	27.706	40.473	1.00	0.00	H
ATOM	513	HB3	PHE	32	32.609	26.203	39.758	1.00	0.00	H
ATOM	514	CG	PHE	32	31.230	27.306	38.602	1.00	0.00	C
ATOM	515	CD1	PHE	32	31.106	28.653	38.196	1.00	0.00	C
ATOM	516	HD1	PHE	32	31.353	29.460	38.872	1.00	0.00	H
ATOM	517	CE1	PHE	32	30.581	28.961	36.960	1.00	0.00	C

ATOM	518	HE1	PHE	32	30.408	29.971	36.617	1.00	0.00	H
ATOM	519	CZ	PHE	32	30.311	27.896	36.045	1.00	0.00	C
ATOM	520	HZ	PHE	32	30.129	28.179	35.019	1.00	0.00	H
ATOM	521	CE2	PHE	32	30.474	26.586	36.357	1.00	0.00	C
ATOM	522	HE2	PHE	32	30.301	25.830	35.606	1.00	0.00	H
ATOM	523	CD2	PHE	32	30.894	26.275	37.691	1.00	0.00	C
ATOM	524	HD2	PHE	32	31.054	25.231	37.917	1.00	0.00	H
ATOM	525	C	PHE	32	31.510	25.723	42.172	1.00	0.00	C
ATOM	526	O	PHE	32	31.493	26.629	42.992	1.00	0.00	O
ATOM	527	N	LYS	33	32.212	24.569	42.325	1.00	0.00	N
ATOM	528	H	LYS	33	32.018	24.020	41.499	1.00	0.00	H
ATOM	529	CA	LYS	33	33.140	24.144	43.414	1.00	0.00	C
ATOM	530	HA	LYS	33	32.901	24.673	44.336	1.00	0.00	H
ATOM	531	CB	LYS	33	32.934	22.643	43.626	1.00	0.00	C
ATOM	532	HB2	LYS	33	33.198	22.132	42.699	1.00	0.00	H
ATOM	533	HB3	LYS	33	33.696	22.306	44.328	1.00	0.00	H
ATOM	534	CG	LYS	33	31.480	22.187	43.844	1.00	0.00	C
ATOM	535	HG2	LYS	33	30.966	22.487	42.931	1.00	0.00	H
ATOM	536	HG3	LYS	33	31.535	21.100	43.896	1.00	0.00	H
ATOM	537	CD	LYS	33	30.804	22.715	45.086	1.00	0.00	C
ATOM	538	HD2	LYS	33	31.496	22.578	45.917	1.00	0.00	H
ATOM	539	HD3	LYS	33	30.547	23.769	44.979	1.00	0.00	H
ATOM	540	CE	LYS	33	29.488	21.959	45.336	1.00	0.00	C
ATOM	541	HE2	LYS	33	28.968	22.007	44.379	1.00	0.00	H
ATOM	542	HE3	LYS	33	29.740	20.926	45.572	1.00	0.00	H
ATOM	543	NZ	LYS	33	28.746	22.558	46.413	1.00	0.00	N
ATOM	544	HZ1	LYS	33	27.978	21.950	46.660	1.00	0.00	H
ATOM	545	HZ2	LYS	33	29.200	22.712	47.302	1.00	0.00	H
ATOM	546	HZ3	LYS	33	28.321	23.441	46.169	1.00	0.00	H
ATOM	547	C	LYS	33	34.602	24.583	43.080	1.00	0.00	C
ATOM	548	O	LYS	33	34.885	25.114	42.012	1.00	0.00	O
ATOM	549	N	THR	34	35.507	24.420	43.961	1.00	0.00	N
ATOM	550	H	THR	34	35.331	23.875	44.793	1.00	0.00	H
ATOM	551	CA	THR	34	36.909	24.756	43.629	1.00	0.00	C
ATOM	552	HA	THR	34	36.884	25.719	43.120	1.00	0.00	H
ATOM	553	CB	THR	34	37.700	24.852	44.959	1.00	0.00	C
ATOM	554	HB	THR	34	37.732	23.944	45.560	1.00	0.00	H
ATOM	555	CG2	THR	34	39.078	25.429	44.821	1.00	0.00	C
ATOM	556	HG21	THR	34	39.022	26.237	44.091	1.00	0.00	H
ATOM	557	HG22	THR	34	39.476	25.751	45.783	1.00	0.00	H
ATOM	558	HG23	THR	34	39.807	24.677	44.516	1.00	0.00	H
ATOM	559	OG1	THR	34	37.089	25.821	45.707	1.00	0.00	O
ATOM	560	HG1	THR	34	37.479	25.968	46.572	1.00	0.00	H
ATOM	561	C	THR	34	37.603	23.706	42.689	1.00	0.00	C
ATOM	562	O	THR	34	37.364	22.517	42.776	1.00	0.00	O
ATOM	563	N	GLY	35	38.468	24.170	41.838	1.00	0.00	N
ATOM	564	H	GLY	35	38.665	25.153	41.952	1.00	0.00	H
ATOM	565	CA	GLY	35	39.027	23.328	40.788	1.00	0.00	C
ATOM	566	HA2	GLY	35	39.897	23.894	40.455	1.00	0.00	H
ATOM	567	HA3	GLY	35	39.357	22.381	41.213	1.00	0.00	H
ATOM	568	C	GLY	35	38.056	23.067	39.596	1.00	0.00	C
ATOM	569	O	GLY	35	38.437	22.248	38.747	1.00	0.00	O
ATOM	570	N	ASP	36	36.833	23.608	39.650	1.00	0.00	N
ATOM	571	H	ASP	36	36.560	24.237	40.391	1.00	0.00	H
ATOM	572	CA	ASP	36	35.972	23.388	38.473	1.00	0.00	C
ATOM	573	HA	ASP	36	35.805	22.346	38.204	1.00	0.00	H
ATOM	574	CB	ASP	36	34.492	23.903	38.750	1.00	0.00	C
ATOM	575	HB2	ASP	36	34.577	24.817	39.337	1.00	0.00	H
ATOM	576	HB3	ASP	36	34.039	24.208	37.807	1.00	0.00	H
ATOM	577	CG	ASP	36	33.504	22.928	39.533	1.00	0.00	C
ATOM	578	OD1	ASP	36	33.887	21.749	39.850	1.00	0.00	O
ATOM	579	OD2	ASP	36	32.372	23.380	39.843	1.00	0.00	O
ATOM	580	C	ASP	36	36.581	24.236	37.323	1.00	0.00	C

ATOM	581	O	ASP	36	36.937	25.443	37.487	1.00	0.00	O
ATOM	582	N	VAL	37	36.658	23.659	36.089	1.00	0.00	N
ATOM	583	H	VAL	37	36.411	22.687	35.979	1.00	0.00	H
ATOM	584	CA	VAL	37	37.303	24.402	35.002	1.00	0.00	C
ATOM	585	HA	VAL	37	37.907	25.244	35.338	1.00	0.00	H
ATOM	586	CB	VAL	37	38.445	23.500	34.395	1.00	0.00	C
ATOM	587	HB	VAL	37	38.081	22.489	34.209	1.00	0.00	H
ATOM	588	CG1	VAL	37	38.865	24.075	33.002	1.00	0.00	C
ATOM	589	HG11	VAL	37	38.712	25.150	33.099	1.00	0.00	H
ATOM	590	HG12	VAL	37	39.922	23.921	32.784	1.00	0.00	H
ATOM	591	HG13	VAL	37	38.204	23.554	32.310	1.00	0.00	H
ATOM	592	CG2	VAL	37	39.774	23.457	35.259	1.00	0.00	C
ATOM	593	HG21	VAL	37	39.599	23.335	36.327	1.00	0.00	H
ATOM	594	HG22	VAL	37	40.463	22.670	34.951	1.00	0.00	H
ATOM	595	HG23	VAL	37	40.274	24.421	35.155	1.00	0.00	H
ATOM	596	C	VAL	37	36.268	24.820	34.052	1.00	0.00	C
ATOM	597	O	VAL	37	35.248	24.146	33.789	1.00	0.00	O
ATOM	598	N	LEU	38	36.418	26.065	33.511	1.00	0.00	N
ATOM	599	H	LEU	38	37.313	26.517	33.629	1.00	0.00	H
ATOM	600	CA	LEU	38	35.515	26.783	32.612	1.00	0.00	C
ATOM	601	HA	LEU	38	34.697	26.136	32.296	1.00	0.00	H
ATOM	602	CB	LEU	38	34.984	28.104	33.241	1.00	0.00	C
ATOM	603	HB2	LEU	38	35.818	28.796	33.124	1.00	0.00	H
ATOM	604	HB3	LEU	38	34.210	28.564	32.627	1.00	0.00	H
ATOM	605	CG	LEU	38	34.635	28.097	34.720	1.00	0.00	C
ATOM	606	HG	LEU	38	35.546	27.819	35.249	1.00	0.00	H
ATOM	607	CD1	LEU	38	34.154	29.493	35.075	1.00	0.00	C
ATOM	608	HD11	LEU	38	33.386	29.748	34.345	1.00	0.00	H
ATOM	609	HD12	LEU	38	33.644	29.584	36.032	1.00	0.00	H
ATOM	610	HD13	LEU	38	35.005	30.175	35.095	1.00	0.00	H
ATOM	611	CD2	LEU	38	33.528	27.090	34.977	1.00	0.00	C
ATOM	612	HD21	LEU	38	32.746	27.346	34.262	1.00	0.00	H
ATOM	613	HD22	LEU	38	33.863	26.069	34.793	1.00	0.00	H
ATOM	614	HD23	LEU	38	33.130	27.172	35.987	1.00	0.00	H
ATOM	615	C	LEU	38	36.315	27.095	31.304	1.00	0.00	C
ATOM	616	O	LEU	38	37.525	27.190	31.355	1.00	0.00	O
ATOM	617	N	ARG	39	35.549	27.406	30.226	1.00	0.00	N
ATOM	618	H	ARG	39	34.546	27.428	30.344	1.00	0.00	H
ATOM	619	CA	ARG	39	36.075	27.998	29.010	1.00	0.00	C
ATOM	620	HA	ARG	39	37.114	28.288	29.163	1.00	0.00	H
ATOM	621	CB	ARG	39	36.043	26.924	27.816	1.00	0.00	C
ATOM	622	HB2	ARG	39	34.997	26.696	27.614	1.00	0.00	H
ATOM	623	HB3	ARG	39	36.407	27.375	26.893	1.00	0.00	H
ATOM	624	CG	ARG	39	36.921	25.758	28.132	1.00	0.00	C
ATOM	625	HG2	ARG	39	37.957	26.094	28.167	1.00	0.00	H
ATOM	626	HG3	ARG	39	36.574	25.382	29.095	1.00	0.00	H
ATOM	627	CD	ARG	39	36.796	24.676	27.044	1.00	0.00	C
ATOM	628	HD2	ARG	39	37.286	24.995	26.124	1.00	0.00	H
ATOM	629	HD3	ARG	39	37.407	23.854	27.415	1.00	0.00	H
ATOM	630	NE	ARG	39	35.461	24.007	26.941	1.00	0.00	N
ATOM	631	HE	ARG	39	34.639	24.454	27.321	1.00	0.00	H
ATOM	632	CZ	ARG	39	35.248	22.887	26.333	1.00	0.00	C
ATOM	633	NH1	ARG	39	36.134	22.067	25.844	1.00	0.00	N
ATOM	634	HH11	ARG	39	37.117	22.294	25.804	1.00	0.00	H
ATOM	635	HH12	ARG	39	35.798	21.138	25.638	1.00	0.00	H
ATOM	636	NH2	ARG	39	34.076	22.488	26.208	1.00	0.00	N
ATOM	637	HH21	ARG	39	33.996	21.635	25.672	1.00	0.00	H
ATOM	638	HH22	ARG	39	33.265	23.029	26.471	1.00	0.00	H
ATOM	639	C	ARG	39	35.311	29.242	28.510	1.00	0.00	C
ATOM	640	O	ARG	39	34.052	29.270	28.631	1.00	0.00	O
ATOM	641	N	VAL	40	36.099	30.229	28.018	1.00	0.00	N
ATOM	642	H	VAL	40	37.093	30.092	27.909	1.00	0.00	H
ATOM	643	CA	VAL	40	35.474	31.550	27.645	1.00	0.00	C
ATOM	644	HA	VAL	40	34.398	31.428	27.766	1.00	0.00	H

ATOM	645	CB	VAL	40	35.677	32.696	28.689	1.00	0.00	C
ATOM	646	HB	VAL	40	35.722	33.650	28.166	1.00	0.00	H
ATOM	647	CG1	VAL	40	34.510	32.950	29.704	1.00	0.00	C
ATOM	648	HG11	VAL	40	33.587	32.899	29.127	1.00	0.00	H
ATOM	649	HG12	VAL	40	34.650	32.139	30.420	1.00	0.00	H
ATOM	650	HG13	VAL	40	34.638	33.917	30.190	1.00	0.00	H
ATOM	651	CG2	VAL	40	37.020	32.563	29.360	1.00	0.00	C
ATOM	652	HG21	VAL	40	37.166	33.464	29.957	1.00	0.00	H
ATOM	653	HG22	VAL	40	36.913	31.820	30.150	1.00	0.00	H
ATOM	654	HG23	VAL	40	37.779	32.502	28.580	1.00	0.00	H
ATOM	655	C	VAL	40	35.703	32.010	26.174	1.00	0.00	C
ATOM	656	O	VAL	40	36.820	31.846	25.686	1.00	0.00	O
ATOM	657	N	GLY	41	34.658	32.574	25.527	1.00	0.00	N
ATOM	658	H	GLY	41	33.784	32.707	26.016	1.00	0.00	H
ATOM	659	CA	GLY	41	34.699	33.224	24.197	1.00	0.00	C
ATOM	660	HA2	GLY	41	35.316	34.087	24.444	1.00	0.00	H
ATOM	661	HA3	GLY	41	35.219	32.505	23.563	1.00	0.00	H
ATOM	662	C	GLY	41	33.387	33.572	23.557	1.00	0.00	C
ATOM	663	O	GLY	41	32.309	33.009	23.860	1.00	0.00	O
ATOM	664	N	ARG	42	33.480	34.284	22.432	1.00	0.00	N
ATOM	665	H	ARG	42	34.426	34.545	22.194	1.00	0.00	H
ATOM	666	CA	ARG	42	32.458	34.359	21.362	1.00	0.00	C
ATOM	667	HA	ARG	42	32.178	33.332	21.126	1.00	0.00	H
ATOM	668	CB	ARG	42	31.247	35.180	21.802	1.00	0.00	C
ATOM	669	HB2	ARG	42	30.552	35.108	20.966	1.00	0.00	H
ATOM	670	HB3	ARG	42	30.857	34.635	22.662	1.00	0.00	H
ATOM	671	CG	ARG	42	31.376	36.687	22.167	1.00	0.00	C
ATOM	672	HG2	ARG	42	31.777	37.277	21.343	1.00	0.00	H
ATOM	673	HG3	ARG	42	30.364	37.069	22.307	1.00	0.00	H
ATOM	674	CD	ARG	42	32.300	37.032	23.378	1.00	0.00	C
ATOM	675	HD2	ARG	42	32.051	36.485	24.287	1.00	0.00	H
ATOM	676	HD3	ARG	42	33.343	36.849	23.117	1.00	0.00	H
ATOM	677	NE	ARG	42	32.147	38.487	23.676	1.00	0.00	N
ATOM	678	HE	ARG	42	31.200	38.825	23.580	1.00	0.00	H
ATOM	679	CZ	ARG	42	33.053	39.293	24.172	1.00	0.00	C
ATOM	680	NH1	ARG	42	34.276	39.046	24.198	1.00	0.00	N
ATOM	681	HH11	ARG	42	34.867	39.678	24.720	1.00	0.00	H
ATOM	682	HH12	ARG	42	34.590	38.159	23.832	1.00	0.00	H
ATOM	683	NH2	ARG	42	32.648	40.367	24.702	1.00	0.00	N
ATOM	684	HH21	ARG	42	31.685	40.660	24.613	1.00	0.00	H
ATOM	685	HH22	ARG	42	33.303	41.093	24.952	1.00	0.00	H
ATOM	686	C	ARG	42	33.063	34.891	20.035	1.00	0.00	C
ATOM	687	O	ARG	42	32.271	35.419	19.271	1.00	0.00	O
ATOM	688	N	PHE	43	34.400	34.660	19.777	1.00	0.00	N
ATOM	689	H	PHE	43	34.963	34.420	20.581	1.00	0.00	H
ATOM	690	CA	PHE	43	35.093	34.893	18.525	1.00	0.00	C
ATOM	691	HA	PHE	43	34.309	35.020	17.779	1.00	0.00	H
ATOM	692	CB	PHE	43	35.897	36.180	18.630	1.00	0.00	C
ATOM	693	HB2	PHE	43	36.659	36.040	19.396	1.00	0.00	H
ATOM	694	HB3	PHE	43	36.497	36.293	17.728	1.00	0.00	H
ATOM	695	CG	PHE	43	35.216	37.543	19.001	1.00	0.00	C
ATOM	696	CD1	PHE	43	34.944	38.474	18.008	1.00	0.00	C
ATOM	697	HD1	PHE	43	35.201	38.252	16.982	1.00	0.00	H
ATOM	698	CE1	PHE	43	34.520	39.762	18.366	1.00	0.00	C
ATOM	699	HE1	PHE	43	34.257	40.393	17.530	1.00	0.00	H
ATOM	700	CZ	PHE	43	34.235	40.048	19.699	1.00	0.00	C
ATOM	701	HZ	PHE	43	33.897	41.026	20.008	1.00	0.00	H
ATOM	702	CE2	PHE	43	34.391	39.076	20.684	1.00	0.00	C
ATOM	703	HE2	PHE	43	34.054	39.248	21.696	1.00	0.00	H
ATOM	704	CD2	PHE	43	34.979	37.868	20.392	1.00	0.00	C
ATOM	705	HD2	PHE	43	35.270	37.103	21.098	1.00	0.00	H
ATOM	706	C	PHE	43	35.866	33.661	18.076	1.00	0.00	C
ATOM	707	O	PHE	43	36.446	32.956	18.883	1.00	0.00	O

ATOM	708	N	GLU	44	35.967	33.373	16.751	1.00	0.00	N
ATOM	709	H	GLU	44	35.394	33.886	16.097	1.00	0.00	H
ATOM	710	CA	GLU	44	36.473	32.095	16.285	1.00	0.00	C
ATOM	711	HA	GLU	44	35.878	31.403	16.882	1.00	0.00	H
ATOM	712	CB	GLU	44	36.034	31.807	14.812	1.00	0.00	C
ATOM	713	HB2	GLU	44	36.303	30.761	14.660	1.00	0.00	H
ATOM	714	HB3	GLU	44	34.966	32.012	14.731	1.00	0.00	H
ATOM	715	CG	GLU	44	36.780	32.654	13.724	1.00	0.00	C
ATOM	716	HG2	GLU	44	37.852	32.596	13.911	1.00	0.00	H
ATOM	717	HG3	GLU	44	36.606	32.321	12.701	1.00	0.00	H
ATOM	718	CD	GLU	44	36.373	34.141	13.725	1.00	0.00	C
ATOM	719	OE1	GLU	44	37.092	34.873	13.036	1.00	0.00	O
ATOM	720	OE2	GLU	44	35.303	34.475	14.262	1.00	0.00	O
ATOM	721	C	GLU	44	37.992	31.837	16.480	1.00	0.00	C
ATOM	722	O	GLU	44	38.360	30.687	16.401	1.00	0.00	O
ATOM	723	N	ASP	45	38.848	32.795	16.921	1.00	0.00	N
ATOM	724	H	ASP	45	38.488	33.736	16.974	1.00	0.00	H
ATOM	725	CA	ASP	45	40.299	32.823	17.177	1.00	0.00	C
ATOM	726	HA	ASP	45	40.824	32.408	16.316	1.00	0.00	H
ATOM	727	CB	ASP	45	40.790	34.218	17.570	1.00	0.00	C
ATOM	728	HB2	ASP	45	40.381	34.538	18.528	1.00	0.00	H
ATOM	729	HB3	ASP	45	41.874	34.108	17.576	1.00	0.00	H
ATOM	730	CG	ASP	45	40.385	35.277	16.492	1.00	0.00	C
ATOM	731	OD1	ASP	45	41.255	35.849	15.823	1.00	0.00	O
ATOM	732	OD2	ASP	45	39.225	35.553	16.096	1.00	0.00	O
ATOM	733	C	ASP	45	40.713	31.890	18.324	1.00	0.00	C
ATOM	734	O	ASP	45	40.490	32.178	19.527	1.00	0.00	O
ATOM	735	N	ASP	46	41.173	30.693	17.949	1.00	0.00	N
ATOM	736	H	ASP	46	41.296	30.441	16.979	1.00	0.00	H
ATOM	737	CA	ASP	46	41.378	29.488	18.746	1.00	0.00	C
ATOM	738	HA	ASP	46	41.587	28.694	18.028	1.00	0.00	H
ATOM	739	CB	ASP	46	42.568	29.583	19.719	1.00	0.00	C
ATOM	740	HB2	ASP	46	42.115	30.181	20.508	1.00	0.00	H
ATOM	741	HB3	ASP	46	42.917	28.636	20.131	1.00	0.00	H
ATOM	742	CG	ASP	46	43.882	30.219	19.194	1.00	0.00	C
ATOM	743	OD1	ASP	46	44.518	30.908	19.992	1.00	0.00	O
ATOM	744	OD2	ASP	46	44.346	29.787	18.120	1.00	0.00	O
ATOM	745	C	ASP	46	40.160	28.915	19.468	1.00	0.00	C
ATOM	746	O	ASP	46	40.344	28.027	20.361	1.00	0.00	O
ATOM	747	N	GLY	47	38.987	29.458	19.107	1.00	0.00	N
ATOM	748	H	GLY	47	38.950	30.186	18.409	1.00	0.00	H
ATOM	749	CA	GLY	47	37.724	28.953	19.579	1.00	0.00	C
ATOM	750	HA2	GLY	47	36.856	29.294	19.016	1.00	0.00	H
ATOM	751	HA3	GLY	47	37.693	27.891	19.339	1.00	0.00	H
ATOM	752	C	GLY	47	37.378	29.267	21.090	1.00	0.00	C
ATOM	753	O	GLY	47	36.181	29.316	21.466	1.00	0.00	O
ATOM	754	N	TYR	48	38.459	29.505	21.931	1.00	0.00	N
ATOM	755	H	TYR	48	39.361	29.497	21.478	1.00	0.00	H
ATOM	756	CA	TYR	48	38.456	30.081	23.275	1.00	0.00	C
ATOM	757	HA	TYR	48	37.530	30.629	23.446	1.00	0.00	H
ATOM	758	CB	TYR	48	38.555	28.887	24.269	1.00	0.00	C
ATOM	759	HB2	TYR	48	39.474	28.344	24.047	1.00	0.00	H
ATOM	760	HB3	TYR	48	38.729	29.173	25.306	1.00	0.00	H
ATOM	761	CG	TYR	48	37.319	27.958	24.184	1.00	0.00	C
ATOM	762	CD1	TYR	48	36.032	28.369	24.705	1.00	0.00	C
ATOM	763	HD1	TYR	48	35.983	29.329	25.197	1.00	0.00	H
ATOM	764	CE1	TYR	48	35.044	27.380	24.772	1.00	0.00	C
ATOM	765	HE1	TYR	48	34.028	27.386	25.140	1.00	0.00	H
ATOM	766	CZ	TYR	48	35.310	26.044	24.369	1.00	0.00	C
ATOM	767	OH	TYR	48	34.334	25.135	24.384	1.00	0.00	O
ATOM	768	HH	TYR	48	34.389	24.655	23.554	1.00	0.00	H
ATOM	769	CE2	TYR	48	36.475	25.740	23.689	1.00	0.00	C
ATOM	770	HE2	TYR	48	36.564	24.773	23.215	1.00	0.00	H
ATOM	771	CD2	TYR	48	37.585	26.684	23.689	1.00	0.00	C

ATOM	772	HD2	TYR	48	38.594	26.410	23.419	1.00	0.00	H
ATOM	773	C	TYR	48	39.605	31.016	23.415	1.00	0.00	C
ATOM	774	O	TYR	48	40.609	30.833	22.710	1.00	0.00	O
ATOM	775	N	PHE	49	39.595	32.095	24.232	1.00	0.00	N
ATOM	776	H	PHE	49	38.657	32.311	24.541	1.00	0.00	H
ATOM	777	CA	PHE	49	40.810	32.986	24.515	1.00	0.00	C
ATOM	778	HA	PHE	49	41.568	32.789	23.757	1.00	0.00	H
ATOM	779	CB	PHE	49	40.411	34.434	24.385	1.00	0.00	C
ATOM	780	HB2	PHE	49	41.239	35.140	24.439	1.00	0.00	H
ATOM	781	HB3	PHE	49	39.919	34.540	23.417	1.00	0.00	H
ATOM	782	CG	PHE	49	39.416	34.983	25.463	1.00	0.00	C
ATOM	783	CD1	PHE	49	39.786	35.418	26.776	1.00	0.00	C
ATOM	784	HD1	PHE	49	40.825	35.299	27.044	1.00	0.00	H
ATOM	785	CE1	PHE	49	38.833	35.829	27.701	1.00	0.00	C
ATOM	786	HE1	PHE	49	39.160	36.074	28.701	1.00	0.00	H
ATOM	787	CZ	PHE	49	37.463	35.821	27.410	1.00	0.00	C
ATOM	788	HZ	PHE	49	36.773	36.304	28.086	1.00	0.00	H
ATOM	789	CE2	PHE	49	37.087	35.341	26.172	1.00	0.00	C
ATOM	790	HE2	PHE	49	36.031	35.234	25.971	1.00	0.00	H
ATOM	791	CD2	PHE	49	38.036	34.945	25.206	1.00	0.00	C
ATOM	792	HD2	PHE	49	37.606	34.573	24.286	1.00	0.00	H
ATOM	793	C	PHE	49	41.513	32.791	25.892	1.00	0.00	C
ATOM	794	O	PHE	49	42.637	33.132	26.163	1.00	0.00	O
ATOM	795	N	CYX	50	40.790	32.210	26.864	1.00	0.00	N
ATOM	796	H	CYX	50	39.811	32.035	26.681	1.00	0.00	H
ATOM	797	CA	CYX	50	41.236	31.568	28.104	1.00	0.00	C
ATOM	798	HA	CYX	50	42.287	31.328	27.942	1.00	0.00	H
ATOM	799	CB	CYX	50	41.164	32.543	29.312	1.00	0.00	C
ATOM	800	HB2	CYX	50	40.358	33.263	29.168	1.00	0.00	H
ATOM	801	HB3	CYX	50	41.085	31.965	30.233	1.00	0.00	H
ATOM	802	SG	CYX	50	42.721	33.512	29.437	1.00	0.00	S
ATOM	803	C	CYX	50	40.349	30.299	28.352	1.00	0.00	C
ATOM	804	O	CYX	50	39.166	30.290	28.203	1.00	0.00	O
ATOM	805	N	THR	51	41.053	29.478	29.151	1.00	0.00	N
ATOM	806	H	THR	51	42.057	29.471	29.036	1.00	0.00	H
ATOM	807	CA	THR	51	40.481	28.384	29.971	1.00	0.00	C
ATOM	808	HA	THR	51	39.400	28.239	29.968	1.00	0.00	H
ATOM	809	CB	THR	51	41.161	27.063	29.608	1.00	0.00	C
ATOM	810	HB	THR	51	42.236	27.116	29.780	1.00	0.00	H
ATOM	811	CG2	THR	51	40.604	25.873	30.401	1.00	0.00	C
ATOM	812	HG21	THR	51	41.252	25.010	30.246	1.00	0.00	H
ATOM	813	HG22	THR	51	40.585	26.148	31.456	1.00	0.00	H
ATOM	814	HG23	THR	51	39.613	25.625	30.022	1.00	0.00	H
ATOM	815	OG1	THR	51	40.989	26.712	28.232	1.00	0.00	O
ATOM	816	HG1	THR	51	41.325	27.445	27.711	1.00	0.00	H
ATOM	817	C	THR	51	40.866	28.813	31.369	1.00	0.00	C
ATOM	818	O	THR	51	41.920	29.395	31.551	1.00	0.00	O
ATOM	819	N	ILE	52	40.121	28.498	32.377	1.00	0.00	N
ATOM	820	H	ILE	52	39.250	28.016	32.206	1.00	0.00	H
ATOM	821	CA	ILE	52	40.078	29.079	33.707	1.00	0.00	C
ATOM	822	HA	ILE	52	41.046	29.540	33.900	1.00	0.00	H
ATOM	823	CB	ILE	52	38.925	30.144	33.643	1.00	0.00	C
ATOM	824	HB	ILE	52	37.997	29.715	33.262	1.00	0.00	H
ATOM	825	CG2	ILE	52	38.500	30.495	35.054	1.00	0.00	C
ATOM	826	HG21	ILE	52	37.746	31.269	34.913	1.00	0.00	H
ATOM	827	HG22	ILE	52	37.934	29.788	35.662	1.00	0.00	H
ATOM	828	HG23	ILE	52	39.280	30.855	35.726	1.00	0.00	H
ATOM	829	CG1	ILE	52	39.341	31.413	32.814	1.00	0.00	C
ATOM	830	HG12	ILE	52	40.344	31.659	33.162	1.00	0.00	H
ATOM	831	HG13	ILE	52	39.480	31.115	31.774	1.00	0.00	H
ATOM	832	CD1	ILE	52	38.524	32.755	32.851	1.00	0.00	C
ATOM	833	HD11	ILE	52	39.065	33.492	32.257	1.00	0.00	H
ATOM	834	HD12	ILE	52	37.504	32.774	32.468	1.00	0.00	H

ATOM	835	HD13	ILE	52	38.608	33.187	33.848	1.00	0.00	H
ATOM	836	C	ILE	52	39.757	27.961	34.736	1.00	0.00	C
ATOM	837	O	ILE	52	38.937	27.066	34.407	1.00	0.00	O
ATOM	838	N	GLU	53	40.434	28.040	35.877	1.00	0.00	N
ATOM	839	H	GLU	53	41.104	28.781	36.027	1.00	0.00	H
ATOM	840	CA	GLU	53	40.139	27.269	37.096	1.00	0.00	C
ATOM	841	HA	GLU	53	39.482	26.408	36.969	1.00	0.00	H
ATOM	842	CB	GLU	53	41.394	26.696	37.814	1.00	0.00	C
ATOM	843	HB2	GLU	53	42.120	26.220	37.153	1.00	0.00	H
ATOM	844	HB3	GLU	53	41.758	27.589	38.323	1.00	0.00	H
ATOM	845	CG	GLU	53	41.066	25.633	38.932	1.00	0.00	C
ATOM	846	HG2	GLU	53	40.361	26.029	39.663	1.00	0.00	H
ATOM	847	HG3	GLU	53	40.678	24.693	38.540	1.00	0.00	H
ATOM	848	CD	GLU	53	42.337	25.279	39.710	1.00	0.00	C
ATOM	849	OE1	GLU	53	43.313	26.012	39.953	1.00	0.00	O
ATOM	850	OE2	GLU	53	42.500	24.119	40.196	1.00	0.00	O
ATOM	851	C	GLU	53	39.456	28.278	38.065	1.00	0.00	C
ATOM	852	O	GLU	53	39.839	29.468	38.199	1.00	0.00	O
ATOM	853	N	VAL	54	38.432	27.768	38.718	1.00	0.00	N
ATOM	854	H	VAL	54	38.018	26.903	38.402	1.00	0.00	H
ATOM	855	CA	VAL	54	37.793	28.387	39.906	1.00	0.00	C
ATOM	856	HA	VAL	54	37.683	29.467	39.810	1.00	0.00	H
ATOM	857	CB	VAL	54	36.334	27.929	40.014	1.00	0.00	C
ATOM	858	HB	VAL	54	36.256	26.862	40.228	1.00	0.00	H
ATOM	859	CG1	VAL	54	35.659	28.489	41.317	1.00	0.00	C
ATOM	860	HG11	VAL	54	34.631	28.126	41.312	1.00	0.00	H
ATOM	861	HG12	VAL	54	36.255	28.050	42.117	1.00	0.00	H
ATOM	862	HG13	VAL	54	35.709	29.574	41.420	1.00	0.00	H
ATOM	863	CG2	VAL	54	35.453	28.394	38.794	1.00	0.00	C
ATOM	864	HG21	VAL	54	35.431	29.477	38.919	1.00	0.00	H
ATOM	865	HG22	VAL	54	35.978	28.168	37.867	1.00	0.00	H
ATOM	866	HG23	VAL	54	34.464	27.937	38.743	1.00	0.00	H
ATOM	867	C	VAL	54	38.673	27.961	41.087	1.00	0.00	C
ATOM	868	O	VAL	54	38.619	26.914	41.706	1.00	0.00	O
ATOM	869	N	THR	55	39.733	28.814	41.210	1.00	0.00	N
ATOM	870	H	THR	55	39.692	29.677	40.686	1.00	0.00	H
ATOM	871	CA	THR	55	40.942	28.589	42.005	1.00	0.00	C
ATOM	872	HA	THR	55	41.417	27.632	41.789	1.00	0.00	H
ATOM	873	CB	THR	55	41.943	29.646	41.529	1.00	0.00	C
ATOM	874	HB	THR	55	42.796	29.583	42.205	1.00	0.00	H
ATOM	875	CG2	THR	55	42.589	29.462	40.180	1.00	0.00	C
ATOM	876	HG21	THR	55	43.138	28.520	40.193	1.00	0.00	H
ATOM	877	HG22	THR	55	41.882	29.537	39.354	1.00	0.00	H
ATOM	878	HG23	THR	55	43.397	30.184	40.061	1.00	0.00	H
ATOM	879	OG1	THR	55	41.452	30.985	41.639	1.00	0.00	O
ATOM	880	HG1	THR	55	41.701	31.181	42.546	1.00	0.00	H
ATOM	881	C	THR	55	40.609	28.415	43.506	1.00	0.00	C
ATOM	882	O	THR	55	41.232	27.651	44.227	1.00	0.00	O
ATOM	883	N	ALA	56	39.586	29.154	43.991	1.00	0.00	N
ATOM	884	H	ALA	56	39.092	29.772	43.364	1.00	0.00	H
ATOM	885	CA	ALA	56	39.013	28.932	45.353	1.00	0.00	C
ATOM	886	HA	ALA	56	39.077	27.892	45.671	1.00	0.00	H
ATOM	887	CB	ALA	56	39.666	29.763	46.383	1.00	0.00	C
ATOM	888	HB1	ALA	56	39.695	30.828	46.154	1.00	0.00	H
ATOM	889	HB2	ALA	56	39.062	29.594	47.274	1.00	0.00	H
ATOM	890	HB3	ALA	56	40.637	29.292	46.533	1.00	0.00	H
ATOM	891	C	ALA	56	37.529	29.329	45.353	1.00	0.00	C
ATOM	892	O	ALA	56	37.021	30.049	44.487	1.00	0.00	O
ATOM	893	N	THR	57	36.826	28.812	46.385	1.00	0.00	N
ATOM	894	H	THR	57	37.416	28.235	46.968	1.00	0.00	H
ATOM	895	CA	THR	57	35.425	29.106	46.714	1.00	0.00	C
ATOM	896	HA	THR	57	35.171	30.141	46.491	1.00	0.00	H
ATOM	897	CB	THR	57	34.491	28.121	46.019	1.00	0.00	C
ATOM	898	HB	THR	57	33.532	28.538	46.326	1.00	0.00	H

ATOM	899	CG2	THR	57	34.643	28.172	44.505	1.00	0.00	C
ATOM	900	HG21	THR	57	34.014	27.389	44.082	1.00	0.00	H
ATOM	901	HG22	THR	57	34.400	29.182	44.173	1.00	0.00	H
ATOM	902	HG23	THR	57	35.663	27.967	44.177	1.00	0.00	H
ATOM	903	OG1	THR	57	34.637	26.721	46.382	1.00	0.00	O
ATOM	904	HG1	THR	57	35.502	26.352	46.189	1.00	0.00	H
ATOM	905	C	THR	57	35.059	28.989	48.201	1.00	0.00	C
ATOM	906	O	THR	57	35.537	28.108	48.943	1.00	0.00	O
ATOM	907	N	SER	58	34.274	29.955	48.569	1.00	0.00	N
ATOM	908	H	SER	58	33.922	30.577	47.855	1.00	0.00	H
ATOM	909	CA	SER	58	33.877	30.154	49.961	1.00	0.00	C
ATOM	910	HA	SER	58	33.721	29.145	50.342	1.00	0.00	H
ATOM	911	CB	SER	58	34.990	30.962	50.793	1.00	0.00	C
ATOM	912	HB2	SER	58	35.919	30.392	50.791	1.00	0.00	H
ATOM	913	HB3	SER	58	35.154	31.894	50.250	1.00	0.00	H
ATOM	914	OG	SER	58	34.502	31.074	52.113	1.00	0.00	O
ATOM	915	HG	SER	58	34.625	30.175	52.426	1.00	0.00	H
ATOM	916	C	SER	58	32.490	30.885	50.119	1.00	0.00	C
ATOM	917	O	SER	58	32.325	31.954	49.585	1.00	0.00	O
ATOM	918	N	THR	59	31.557	30.422	51.045	1.00	0.00	N
ATOM	919	H	THR	59	31.763	29.525	51.458	1.00	0.00	H
ATOM	920	CA	THR	59	30.377	31.116	51.437	1.00	0.00	C
ATOM	921	HA	THR	59	29.934	31.572	50.552	1.00	0.00	H
ATOM	922	CB	THR	59	29.414	30.050	52.014	1.00	0.00	C
ATOM	923	HB	THR	59	29.425	29.351	51.178	1.00	0.00	H
ATOM	924	CG2	THR	59	29.676	29.284	53.273	1.00	0.00	C
ATOM	925	HG21	THR	59	30.625	28.790	53.066	1.00	0.00	H
ATOM	926	HG22	THR	59	29.812	29.883	54.173	1.00	0.00	H
ATOM	927	HG23	THR	59	28.925	28.525	53.491	1.00	0.00	H
ATOM	928	OG1	THR	59	28.061	30.608	52.022	1.00	0.00	O
ATOM	929	HG1	THR	59	27.498	30.216	51.351	1.00	0.00	H
ATOM	930	C	THR	59	30.808	32.171	52.455	1.00	0.00	C
ATOM	931	O	THR	59	31.734	31.970	53.246	1.00	0.00	O
ATOM	932	N	VAL	60	30.070	33.295	52.534	1.00	0.00	N
ATOM	933	H	VAL	60	29.235	33.289	51.967	1.00	0.00	H
ATOM	934	CA	VAL	60	30.389	34.511	53.285	1.00	0.00	C
ATOM	935	HA	VAL	60	30.819	34.276	54.258	1.00	0.00	H
ATOM	936	CB	VAL	60	31.520	35.248	52.469	1.00	0.00	C
ATOM	937	HB	VAL	60	32.355	34.587	52.237	1.00	0.00	H
ATOM	938	CG1	VAL	60	31.075	35.911	51.139	1.00	0.00	C
ATOM	939	HG11	VAL	60	30.695	35.051	50.587	1.00	0.00	H
ATOM	940	HG12	VAL	60	30.238	36.601	51.248	1.00	0.00	H
ATOM	941	HG13	VAL	60	31.893	36.447	50.661	1.00	0.00	H
ATOM	942	CG2	VAL	60	32.162	36.444	53.170	1.00	0.00	C
ATOM	943	HG21	VAL	60	32.392	36.062	54.164	1.00	0.00	H
ATOM	944	HG22	VAL	60	33.050	36.812	52.655	1.00	0.00	H
ATOM	945	HG23	VAL	60	31.448	37.264	53.114	1.00	0.00	H
ATOM	946	C	VAL	60	29.156	35.389	53.566	1.00	0.00	C
ATOM	947	O	VAL	60	28.112	35.285	52.928	1.00	0.00	O
ATOM	948	N	THR	61	29.243	36.212	54.559	1.00	0.00	N
ATOM	949	H	THR	61	30.113	36.207	55.074	1.00	0.00	H
ATOM	950	CA	THR	61	28.169	37.073	55.062	1.00	0.00	C
ATOM	951	HA	THR	61	27.188	36.975	54.596	1.00	0.00	H
ATOM	952	CB	THR	61	27.994	36.860	56.562	1.00	0.00	C
ATOM	953	HB	THR	61	27.318	37.633	56.926	1.00	0.00	H
ATOM	954	CG2	THR	61	27.390	35.423	56.643	1.00	0.00	C
ATOM	955	HG21	THR	61	28.111	34.684	56.294	1.00	0.00	H
ATOM	956	HG22	THR	61	27.438	35.166	57.701	1.00	0.00	H
ATOM	957	HG23	THR	61	26.404	35.342	56.187	1.00	0.00	H
ATOM	958	OG1	THR	61	29.204	36.960	57.323	1.00	0.00	O
ATOM	959	HG1	THR	61	29.134	37.823	57.740	1.00	0.00	H
ATOM	960	C	THR	61	28.725	38.522	54.855	1.00	0.00	C
ATOM	961	O	THR	61	29.948	38.726	54.756	1.00	0.00	O

ATOM	962	N	LEU	62	27.774	39.487	54.846	1.00	0.00	N
ATOM	963	H	LEU	62	26.834	39.245	55.126	1.00	0.00	H
ATOM	964	CA	LEU	62	28.008	40.787	54.268	1.00	0.00	C
ATOM	965	HA	LEU	62	28.519	40.618	53.320	1.00	0.00	H
ATOM	966	CB	LEU	62	26.652	41.496	54.159	1.00	0.00	C
ATOM	967	HB2	LEU	62	26.067	40.771	53.594	1.00	0.00	H
ATOM	968	HB3	LEU	62	26.287	41.691	55.167	1.00	0.00	H
ATOM	969	CG	LEU	62	26.524	42.758	53.321	1.00	0.00	C
ATOM	970	HG	LEU	62	26.820	43.652	53.869	1.00	0.00	H
ATOM	971	CD1	LEU	62	27.416	42.789	52.064	1.00	0.00	C
ATOM	972	HD11	LEU	62	28.452	42.774	52.404	1.00	0.00	H
ATOM	973	HD12	LEU	62	27.208	41.958	51.391	1.00	0.00	H
ATOM	974	HD13	LEU	62	27.344	43.760	51.574	1.00	0.00	H
ATOM	975	CD2	LEU	62	25.130	43.118	52.873	1.00	0.00	C
ATOM	976	HD21	LEU	62	24.651	42.293	52.346	1.00	0.00	H
ATOM	977	HD22	LEU	62	24.480	43.357	53.714	1.00	0.00	H
ATOM	978	HD23	LEU	62	25.116	44.074	52.348	1.00	0.00	H
ATOM	979	C	LEU	62	29.128	41.558	55.025	1.00	0.00	C
ATOM	980	O	LEU	62	29.989	42.130	54.394	1.00	0.00	O
ATOM	981	N	ASP	63	29.212	41.378	56.298	1.00	0.00	N
ATOM	982	H	ASP	63	28.506	40.734	56.622	1.00	0.00	H
ATOM	983	CA	ASP	63	30.176	41.958	57.233	1.00	0.00	C
ATOM	984	HA	ASP	63	30.363	42.993	56.947	1.00	0.00	H
ATOM	985	CB	ASP	63	29.529	42.009	58.658	1.00	0.00	C
ATOM	986	HB2	ASP	63	30.366	42.444	59.204	1.00	0.00	H
ATOM	987	HB3	ASP	63	28.567	42.520	58.609	1.00	0.00	H
ATOM	988	CG	ASP	63	29.349	40.624	59.282	1.00	0.00	C
ATOM	989	OD1	ASP	63	28.876	39.753	58.527	1.00	0.00	O
ATOM	990	OD2	ASP	63	29.538	40.329	60.476	1.00	0.00	O
ATOM	991	C	ASP	63	31.599	41.320	57.116	1.00	0.00	C
ATOM	992	O	ASP	63	32.494	41.881	57.715	1.00	0.00	O
ATOM	993	N	THR	64	31.722	40.222	56.403	1.00	0.00	N
ATOM	994	H	THR	64	30.878	39.941	55.924	1.00	0.00	H
ATOM	995	CA	THR	64	33.029	39.514	56.271	1.00	0.00	C
ATOM	996	HA	THR	64	33.851	40.164	56.573	1.00	0.00	H
ATOM	997	CB	THR	64	33.083	38.222	57.144	1.00	0.00	C
ATOM	998	HB	THR	64	34.007	37.666	56.982	1.00	0.00	H
ATOM	999	CG2	THR	64	32.666	38.507	58.564	1.00	0.00	C
ATOM	1000	HG21	THR	64	33.070	39.455	58.920	1.00	0.00	H
ATOM	1001	HG22	THR	64	31.589	38.554	58.725	1.00	0.00	H
ATOM	1002	HG23	THR	64	33.106	37.802	59.269	1.00	0.00	H
ATOM	1003	OG1	THR	64	32.144	37.301	56.595	1.00	0.00	O
ATOM	1004	HG1	THR	64	31.317	37.764	56.446	1.00	0.00	H
ATOM	1005	C	THR	64	33.449	39.170	54.849	1.00	0.00	C
ATOM	1006	O	THR	64	34.386	38.398	54.639	1.00	0.00	O
ATOM	1007	N	LEU	65	32.889	39.872	53.854	1.00	0.00	N
ATOM	1008	H	LEU	65	32.140	40.478	54.156	1.00	0.00	H
ATOM	1009	CA	LEU	65	33.463	39.850	52.475	1.00	0.00	C
ATOM	1010	HA	LEU	65	33.351	38.831	52.105	1.00	0.00	H
ATOM	1011	CB	LEU	65	32.855	40.957	51.704	1.00	0.00	C
ATOM	1012	HB2	LEU	65	32.974	41.976	52.071	1.00	0.00	H
ATOM	1013	HB3	LEU	65	33.390	41.036	50.757	1.00	0.00	H
ATOM	1014	CG	LEU	65	31.310	40.777	51.346	1.00	0.00	C
ATOM	1015	HG	LEU	65	30.707	40.555	52.227	1.00	0.00	H
ATOM	1016	CD1	LEU	65	30.870	42.108	50.781	1.00	0.00	C
ATOM	1017	HD11	LEU	65	29.821	42.056	50.489	1.00	0.00	H
ATOM	1018	HD12	LEU	65	30.992	42.873	51.548	1.00	0.00	H
ATOM	1019	HD13	LEU	65	31.469	42.428	49.929	1.00	0.00	H
ATOM	1020	CD2	LEU	65	31.164	39.715	50.294	1.00	0.00	C
ATOM	1021	HD21	LEU	65	31.761	39.928	49.407	1.00	0.00	H
ATOM	1022	HD22	LEU	65	31.376	38.730	50.709	1.00	0.00	H
ATOM	1023	HD23	LEU	65	30.113	39.725	50.008	1.00	0.00	H
ATOM	1024	C	LEU	65	34.967	40.194	52.441	1.00	0.00	C
ATOM	1025	O	LEU	65	35.424	41.178	53.044	1.00	0.00	O

ATOM	1026	N	THR	66	35.704	39.463	51.590	1.00	0.00	N
ATOM	1027	H	THR	66	35.384	38.614	51.149	1.00	0.00	H
ATOM	1028	CA	THR	66	37.104	39.804	51.197	1.00	0.00	C
ATOM	1029	HA	THR	66	37.709	39.660	52.093	1.00	0.00	H
ATOM	1030	CB	THR	66	37.590	38.884	50.112	1.00	0.00	C
ATOM	1031	HB	THR	66	37.694	37.929	50.626	1.00	0.00	H
ATOM	1032	CG2	THR	66	36.720	38.890	48.908	1.00	0.00	C
ATOM	1033	HG21	THR	66	36.934	39.739	48.258	1.00	0.00	H
ATOM	1034	HG22	THR	66	37.076	37.958	48.469	1.00	0.00	H
ATOM	1035	HG23	THR	66	35.695	38.827	49.272	1.00	0.00	H
ATOM	1036	OG1	THR	66	38.834	39.266	49.610	1.00	0.00	O
ATOM	1037	HG1	THR	66	39.446	39.069	50.322	1.00	0.00	H
ATOM	1038	C	THR	66	37.279	41.254	50.686	1.00	0.00	C
ATOM	1039	O	THR	66	36.473	41.747	49.900	1.00	0.00	O
ATOM	1040	N	GLU	67	38.367	41.890	51.112	1.00	0.00	N
ATOM	1041	H	GLU	67	39.064	41.445	51.692	1.00	0.00	H
ATOM	1042	CA	GLU	67	38.739	43.206	50.639	1.00	0.00	C
ATOM	1043	HA	GLU	67	37.831	43.803	50.736	1.00	0.00	H
ATOM	1044	CB	GLU	67	39.806	43.866	51.581	1.00	0.00	C
ATOM	1045	HB2	GLU	67	39.717	44.937	51.404	1.00	0.00	H
ATOM	1046	HB3	GLU	67	39.568	43.674	52.628	1.00	0.00	H
ATOM	1047	CG	GLU	67	41.218	43.504	51.335	1.00	0.00	C
ATOM	1048	HG2	GLU	67	41.411	43.667	50.274	1.00	0.00	H
ATOM	1049	HG3	GLU	67	41.764	44.234	51.933	1.00	0.00	H
ATOM	1050	CD	GLU	67	41.658	42.121	51.891	1.00	0.00	C
ATOM	1051	OE1	GLU	67	42.853	41.817	51.951	1.00	0.00	O
ATOM	1052	OE2	GLU	67	40.829	41.217	52.270	1.00	0.00	O
ATOM	1053	C	GLU	67	39.043	43.196	49.145	1.00	0.00	C
ATOM	1054	O	GLU	67	39.056	44.282	48.580	1.00	0.00	O
ATOM	1055	N	LYS	68	39.312	42.033	48.496	1.00	0.00	N
ATOM	1056	H	LYS	68	39.177	41.216	49.075	1.00	0.00	H
ATOM	1057	CA	LYS	68	39.875	41.854	47.163	1.00	0.00	C
ATOM	1058	HA	LYS	68	40.828	42.362	47.015	1.00	0.00	H
ATOM	1059	CB	LYS	68	40.190	40.349	46.900	1.00	0.00	C
ATOM	1060	HB2	LYS	68	39.442	39.649	47.271	1.00	0.00	H
ATOM	1061	HB3	LYS	68	40.351	40.243	45.827	1.00	0.00	H
ATOM	1062	CG	LYS	68	41.430	39.835	47.611	1.00	0.00	C
ATOM	1063	HG2	LYS	68	42.293	40.384	47.232	1.00	0.00	H
ATOM	1064	HG3	LYS	68	41.210	39.908	48.675	1.00	0.00	H
ATOM	1065	CD	LYS	68	41.640	38.354	47.339	1.00	0.00	C
ATOM	1066	HD2	LYS	68	41.730	38.146	46.273	1.00	0.00	H
ATOM	1067	HD3	LYS	68	42.519	38.071	47.918	1.00	0.00	H
ATOM	1068	CE	LYS	68	40.528	37.484	47.912	1.00	0.00	C
ATOM	1069	HE2	LYS	68	40.589	37.627	48.991	1.00	0.00	H
ATOM	1070	HE3	LYS	68	39.574	37.957	47.682	1.00	0.00	H
ATOM	1071	NZ	LYS	68	40.667	36.052	47.502	1.00	0.00	N
ATOM	1072	HZ1	LYS	68	39.831	35.494	47.602	1.00	0.00	H
ATOM	1073	HZ2	LYS	68	40.750	35.994	46.497	1.00	0.00	H
ATOM	1074	HZ3	LYS	68	41.509	35.623	47.858	1.00	0.00	H
ATOM	1075	C	LYS	68	38.831	42.355	46.136	1.00	0.00	C
ATOM	1076	O	LYS	68	39.255	43.072	45.192	1.00	0.00	O
ATOM	1077	N	HIE	69	37.536	42.039	46.274	1.00	0.00	N
ATOM	1078	H	HIE	69	37.371	41.497	47.110	1.00	0.00	H
ATOM	1079	CA	HIE	69	36.519	42.521	45.290	1.00	0.00	C
ATOM	1080	HA	HIE	69	36.689	42.181	44.268	1.00	0.00	H
ATOM	1081	CB	HIE	69	35.209	41.981	45.788	1.00	0.00	C
ATOM	1082	HB2	HIE	69	35.353	40.963	46.153	1.00	0.00	H
ATOM	1083	HB3	HIE	69	34.810	42.554	46.625	1.00	0.00	H
ATOM	1084	CG	HIE	69	34.208	42.020	44.671	1.00	0.00	C
ATOM	1085	ND1	HIE	69	33.963	40.963	43.801	1.00	0.00	N
ATOM	1086	CE1	HIE	69	32.936	41.341	42.963	1.00	0.00	C
ATOM	1087	HE1	HIE	69	32.440	40.706	42.243	1.00	0.00	H
ATOM	1088	NE2	HIE	69	32.535	42.577	43.353	1.00	0.00	N

ATOM	1089	HE2	HIE	69	31.784	43.108	42.936	1.00	0.00	H
ATOM	1090	CD2	HIE	69	33.368	43.019	44.369	1.00	0.00	C
ATOM	1091	HD2	HIE	69	33.187	43.944	44.896	1.00	0.00	H
ATOM	1092	C	HIE	69	36.456	43.994	45.251	1.00	0.00	C
ATOM	1093	O	HIE	69	36.206	44.559	44.164	1.00	0.00	O
ATOM	1094	N	ALA	70	36.826	44.784	46.302	1.00	0.00	N
ATOM	1095	H	ALA	70	37.221	44.298	47.095	1.00	0.00	H
ATOM	1096	CA	ALA	70	36.898	46.220	46.373	1.00	0.00	C
ATOM	1097	HA	ALA	70	35.986	46.619	45.930	1.00	0.00	H
ATOM	1098	CB	ALA	70	37.015	46.695	47.826	1.00	0.00	C
ATOM	1099	HB1	ALA	70	36.233	46.179	48.382	1.00	0.00	H
ATOM	1100	HB2	ALA	70	38.071	46.545	48.048	1.00	0.00	H
ATOM	1101	HB3	ALA	70	36.786	47.753	47.954	1.00	0.00	H
ATOM	1102	C	ALA	70	38.055	46.805	45.521	1.00	0.00	C
ATOM	1103	O	ALA	70	37.882	47.836	44.878	1.00	0.00	O
ATOM	1104	N	GLU	71	39.216	46.220	45.770	1.00	0.00	N
ATOM	1105	H	GLU	71	39.272	45.522	46.497	1.00	0.00	H
ATOM	1106	CA	GLU	71	40.409	46.482	44.956	1.00	0.00	C
ATOM	1107	HA	GLU	71	40.634	47.548	44.940	1.00	0.00	H
ATOM	1108	CB	GLU	71	41.573	45.730	45.531	1.00	0.00	C
ATOM	1109	HB2	GLU	71	41.358	44.662	45.559	1.00	0.00	H
ATOM	1110	HB3	GLU	71	42.408	45.952	44.868	1.00	0.00	H
ATOM	1111	CG	GLU	71	42.144	46.056	46.909	1.00	0.00	C
ATOM	1112	HG2	GLU	71	41.359	46.033	47.666	1.00	0.00	H
ATOM	1113	HG3	GLU	71	42.848	45.244	47.091	1.00	0.00	H
ATOM	1114	CD	GLU	71	42.795	47.461	46.980	1.00	0.00	C
ATOM	1115	OE1	GLU	71	43.213	47.883	45.890	1.00	0.00	O
ATOM	1116	OE2	GLU	71	42.840	48.122	48.025	1.00	0.00	O
ATOM	1117	C	GLU	71	40.277	46.111	43.495	1.00	0.00	C
ATOM	1118	O	GLU	71	40.977	46.615	42.668	1.00	0.00	O
ATOM	1119	N	GLN	72	39.272	45.341	43.057	1.00	0.00	N
ATOM	1120	H	GLN	72	38.719	44.806	43.710	1.00	0.00	H
ATOM	1121	CA	GLN	72	38.967	45.104	41.602	1.00	0.00	C
ATOM	1122	HA	GLN	72	39.884	44.946	41.035	1.00	0.00	H
ATOM	1123	CB	GLN	72	38.225	43.766	41.463	1.00	0.00	C
ATOM	1124	HB2	GLN	72	37.270	43.866	41.980	1.00	0.00	H
ATOM	1125	HB3	GLN	72	38.806	42.973	41.934	1.00	0.00	H
ATOM	1126	CG	GLN	72	38.035	43.198	40.033	1.00	0.00	C
ATOM	1127	HG2	GLN	72	37.484	43.882	39.389	1.00	0.00	H
ATOM	1128	HG3	GLN	72	38.949	43.196	39.437	1.00	0.00	H
ATOM	1129	CD	GLN	72	37.342	41.810	40.026	1.00	0.00	C
ATOM	1130	OE1	GLN	72	37.873	40.857	39.481	1.00	0.00	O
ATOM	1131	NE2	GLN	72	36.163	41.767	40.511	1.00	0.00	N
ATOM	1132	HE21	GLN	72	35.723	42.640	40.768	1.00	0.00	H
ATOM	1133	HE22	GLN	72	35.768	40.847	40.636	1.00	0.00	H
ATOM	1134	C	GLN	72	38.097	46.249	41.076	1.00	0.00	C
ATOM	1135	O	GLN	72	38.332	46.826	40.017	1.00	0.00	O
ATOM	1136	N	GLU	73	36.923	46.354	41.680	1.00	0.00	N
ATOM	1137	H	GLU	73	36.746	45.808	42.511	1.00	0.00	H
ATOM	1138	CA	GLU	73	35.744	47.111	41.253	1.00	0.00	C
ATOM	1139	HA	GLU	73	35.482	46.993	40.201	1.00	0.00	H
ATOM	1140	CB	GLU	73	34.596	46.752	42.206	1.00	0.00	C
ATOM	1141	HB2	GLU	73	34.902	46.907	43.240	1.00	0.00	H
ATOM	1142	HB3	GLU	73	33.778	47.448	42.022	1.00	0.00	H
ATOM	1143	CG	GLU	73	34.066	45.326	41.902	1.00	0.00	C
ATOM	1144	HG2	GLU	73	34.934	44.688	42.067	1.00	0.00	H
ATOM	1145	HG3	GLU	73	33.348	45.121	42.697	1.00	0.00	H
ATOM	1146	CD	GLU	73	33.571	45.189	40.452	1.00	0.00	C
ATOM	1147	OE1	GLU	73	32.599	45.930	40.048	1.00	0.00	O
ATOM	1148	OE2	GLU	73	34.036	44.205	39.779	1.00	0.00	O
ATOM	1149	C	GLU	73	36.009	48.563	41.328	1.00	0.00	C
ATOM	1150	O	GLU	73	35.090	49.378	40.972	1.00	0.00	O
ATOM	1151	N	ASN	74	37.114	48.988	42.024	1.00	0.00	N
ATOM	1152	H	ASN	74	37.647	48.272	42.497	1.00	0.00	H

ATOM	1153	CA	ASN	74	37.546	50.357	42.328	1.00	0.00	C
ATOM	1154	HA	ASN	74	38.508	50.257	42.832	1.00	0.00	H
ATOM	1155	CB	ASN	74	37.932	51.085	40.967	1.00	0.00	C
ATOM	1156	HB2	ASN	74	37.031	51.215	40.368	1.00	0.00	H
ATOM	1157	HB3	ASN	74	38.324	52.094	41.088	1.00	0.00	H
ATOM	1158	CG	ASN	74	38.978	50.349	40.213	1.00	0.00	C
ATOM	1159	OD1	ASN	74	39.995	49.982	40.793	1.00	0.00	O
ATOM	1160	ND2	ASN	74	38.732	50.156	38.924	1.00	0.00	N
ATOM	1161	HD21	ASN	74	39.391	49.589	38.412	1.00	0.00	H
ATOM	1162	HD22	ASN	74	37.834	50.478	38.591	1.00	0.00	H
ATOM	1163	C	ASN	74	36.646	51.113	43.314	1.00	0.00	C
ATOM	1164	O	ASN	74	36.155	52.253	43.158	1.00	0.00	O
ATOM	1165	N	MET	75	36.517	50.499	44.473	1.00	0.00	N
ATOM	1166	H	MET	75	36.905	49.568	44.454	1.00	0.00	H
ATOM	1167	CA	MET	75	35.815	50.981	45.660	1.00	0.00	C
ATOM	1168	HA	MET	75	35.573	52.037	45.534	1.00	0.00	H
ATOM	1169	CB	MET	75	34.442	50.228	45.741	1.00	0.00	C
ATOM	1170	HB2	MET	75	34.532	49.192	46.066	1.00	0.00	H
ATOM	1171	HB3	MET	75	33.880	50.706	46.543	1.00	0.00	H
ATOM	1172	CG	MET	75	33.563	50.256	44.518	1.00	0.00	C
ATOM	1173	HG2	MET	75	33.510	51.285	44.162	1.00	0.00	H
ATOM	1174	HG3	MET	75	34.035	49.639	43.753	1.00	0.00	H
ATOM	1175	SD	MET	75	31.827	49.619	44.769	1.00	0.00	S
ATOM	1176	CE	MET	75	31.170	51.242	45.376	1.00	0.00	C
ATOM	1177	HE1	MET	75	31.695	51.466	46.304	1.00	0.00	H
ATOM	1178	HE2	MET	75	31.262	52.075	44.678	1.00	0.00	H
ATOM	1179	HE3	MET	75	30.092	51.149	45.506	1.00	0.00	H
ATOM	1180	C	MET	75	36.584	50.791	47.019	1.00	0.00	C
ATOM	1181	O	MET	75	37.489	49.958	47.180	1.00	0.00	O
ATOM	1182	N	THR	76	36.061	51.409	48.054	1.00	0.00	N
ATOM	1183	H	THR	76	35.284	52.029	47.876	1.00	0.00	H
ATOM	1184	CA	THR	76	36.348	51.094	49.472	1.00	0.00	C
ATOM	1185	HA	THR	76	37.366	50.714	49.380	1.00	0.00	H
ATOM	1186	CB	THR	76	36.449	52.294	50.450	1.00	0.00	C
ATOM	1187	HB	THR	76	37.136	52.938	49.902	1.00	0.00	H
ATOM	1188	CG2	THR	76	35.068	52.971	50.841	1.00	0.00	C
ATOM	1189	HG21	THR	76	35.332	53.879	51.383	1.00	0.00	H
ATOM	1190	HG22	THR	76	34.533	53.170	49.913	1.00	0.00	H
ATOM	1191	HG23	THR	76	34.324	52.333	51.321	1.00	0.00	H
ATOM	1192	OG1	THR	76	37.195	51.813	51.537	1.00	0.00	O
ATOM	1193	HG1	THR	76	38.128	51.782	51.314	1.00	0.00	H
ATOM	1194	C	THR	76	35.425	49.991	49.918	1.00	0.00	C
ATOM	1195	O	THR	76	34.197	50.071	49.660	1.00	0.00	O
ATOM	1196	N	LEU	77	35.883	49.000	50.660	1.00	0.00	N
ATOM	1197	H	LEU	77	36.878	49.060	50.824	1.00	0.00	H
ATOM	1198	CA	LEU	77	35.116	47.917	51.170	1.00	0.00	C
ATOM	1199	HA	LEU	77	34.655	47.447	50.302	1.00	0.00	H
ATOM	1200	CB	LEU	77	36.031	46.910	51.979	1.00	0.00	C
ATOM	1201	HB2	LEU	77	36.851	46.562	51.350	1.00	0.00	H
ATOM	1202	HB3	LEU	77	36.430	47.450	52.838	1.00	0.00	H
ATOM	1203	CG	LEU	77	35.323	45.669	52.522	1.00	0.00	C
ATOM	1204	HG	LEU	77	34.660	45.997	53.323	1.00	0.00	H
ATOM	1205	CD1	LEU	77	34.645	44.914	51.409	1.00	0.00	C
ATOM	1206	HD11	LEU	77	34.265	43.960	51.770	1.00	0.00	H
ATOM	1207	HD12	LEU	77	33.778	45.397	50.960	1.00	0.00	H
ATOM	1208	HD13	LEU	77	35.434	44.629	50.714	1.00	0.00	H
ATOM	1209	CD2	LEU	77	36.108	44.708	53.433	1.00	0.00	C
ATOM	1210	HD21	LEU	77	36.892	44.254	52.827	1.00	0.00	H
ATOM	1211	HD22	LEU	77	36.601	45.341	54.170	1.00	0.00	H
ATOM	1212	HD23	LEU	77	35.458	43.990	53.932	1.00	0.00	H
ATOM	1213	C	LEU	77	33.924	48.430	52.087	1.00	0.00	C
ATOM	1214	O	LEU	77	32.873	47.827	51.922	1.00	0.00	O
ATOM	1215	N	THR	78	33.956	49.661	52.668	1.00	0.00	N

ATOM	1216	H	THR	78	34.754	50.276	52.584	1.00	0.00	H
ATOM	1217	CA	THR	78	32.767	50.077	53.402	1.00	0.00	C
ATOM	1218	HA	THR	78	32.379	49.169	53.866	1.00	0.00	H
ATOM	1219	CB	THR	78	33.270	51.085	54.459	1.00	0.00	C
ATOM	1220	HB	THR	78	32.363	51.555	54.841	1.00	0.00	H
ATOM	1221	CG2	THR	78	34.055	50.484	55.582	1.00	0.00	C
ATOM	1222	HG21	THR	78	33.361	49.740	55.975	1.00	0.00	H
ATOM	1223	HG22	THR	78	34.904	49.866	55.293	1.00	0.00	H
ATOM	1224	HG23	THR	78	34.333	51.268	56.287	1.00	0.00	H
ATOM	1225	OG1	THR	78	34.143	52.035	54.034	1.00	0.00	O
ATOM	1226	HG1	THR	78	34.200	52.640	54.777	1.00	0.00	H
ATOM	1227	C	THR	78	31.666	50.643	52.572	1.00	0.00	C
ATOM	1228	O	THR	78	30.549	50.535	52.929	1.00	0.00	O
ATOM	1229	N	GLU	79	32.043	51.208	51.383	1.00	0.00	N
ATOM	1230	H	GLU	79	32.987	51.083	51.043	1.00	0.00	H
ATOM	1231	CA	GLU	79	31.068	51.665	50.360	1.00	0.00	C
ATOM	1232	HA	GLU	79	30.250	52.169	50.874	1.00	0.00	H
ATOM	1233	CB	GLU	79	31.553	52.633	49.293	1.00	0.00	C
ATOM	1234	HB2	GLU	79	32.026	53.429	49.869	1.00	0.00	H
ATOM	1235	HB3	GLU	79	32.302	52.139	48.673	1.00	0.00	H
ATOM	1236	CG	GLU	79	30.477	53.282	48.392	1.00	0.00	C
ATOM	1237	HG2	GLU	79	31.030	53.915	47.697	1.00	0.00	H
ATOM	1238	HG3	GLU	79	29.874	52.515	47.905	1.00	0.00	H
ATOM	1239	CD	GLU	79	29.485	54.187	49.137	1.00	0.00	C
ATOM	1240	OE1	GLU	79	29.857	54.708	50.205	1.00	0.00	O
ATOM	1241	OE2	GLU	79	28.439	54.568	48.645	1.00	0.00	O
ATOM	1242	C	GLU	79	30.440	50.417	49.594	1.00	0.00	C
ATOM	1243	O	GLU	79	29.241	50.207	49.478	1.00	0.00	O
ATOM	1244	N	LEU	80	31.341	49.475	49.307	1.00	0.00	N
ATOM	1245	H	LEU	80	32.315	49.676	49.481	1.00	0.00	H
ATOM	1246	CA	LEU	80	31.002	48.164	48.692	1.00	0.00	C
ATOM	1247	HA	LEU	80	30.631	48.429	47.701	1.00	0.00	H
ATOM	1248	CB	LEU	80	32.298	47.312	48.535	1.00	0.00	C
ATOM	1249	HB2	LEU	80	33.061	47.983	48.141	1.00	0.00	H
ATOM	1250	HB3	LEU	80	32.608	46.982	49.527	1.00	0.00	H
ATOM	1251	CG	LEU	80	32.028	46.031	47.740	1.00	0.00	C
ATOM	1252	HG	LEU	80	31.263	45.549	48.349	1.00	0.00	H
ATOM	1253	CD1	LEU	80	31.580	46.296	46.308	1.00	0.00	C
ATOM	1254	HD11	LEU	80	31.537	45.282	45.911	1.00	0.00	H
ATOM	1255	HD12	LEU	80	30.638	46.844	46.334	1.00	0.00	H
ATOM	1256	HD13	LEU	80	32.353	46.829	45.755	1.00	0.00	H
ATOM	1257	CD2	LEU	80	33.182	45.032	47.525	1.00	0.00	C
ATOM	1258	HD21	LEU	80	33.921	45.592	46.951	1.00	0.00	H
ATOM	1259	HD22	LEU	80	33.543	44.881	48.542	1.00	0.00	H
ATOM	1260	HD23	LEU	80	32.962	44.079	47.042	1.00	0.00	H
ATOM	1261	C	LEU	80	29.893	47.436	49.452	1.00	0.00	C
ATOM	1262	O	LEU	80	28.918	47.024	48.808	1.00	0.00	O
ATOM	1263	N	LYS	81	29.982	47.380	50.812	1.00	0.00	N
ATOM	1264	H	LYS	81	30.833	47.729	51.229	1.00	0.00	H
ATOM	1265	CA	LYS	81	29.009	46.667	51.677	1.00	0.00	C
ATOM	1266	HA	LYS	81	28.865	45.625	51.392	1.00	0.00	H
ATOM	1267	CB	LYS	81	29.553	46.745	53.078	1.00	0.00	C
ATOM	1268	HB2	LYS	81	29.996	47.725	53.251	1.00	0.00	H
ATOM	1269	HB3	LYS	81	28.809	46.622	53.865	1.00	0.00	H
ATOM	1270	CG	LYS	81	30.610	45.661	53.236	1.00	0.00	C
ATOM	1271	HG2	LYS	81	30.059	44.721	53.222	1.00	0.00	H
ATOM	1272	HG3	LYS	81	31.311	45.630	52.403	1.00	0.00	H
ATOM	1273	CD	LYS	81	31.240	45.850	54.648	1.00	0.00	C
ATOM	1274	HD2	LYS	81	31.829	46.766	54.676	1.00	0.00	H
ATOM	1275	HD3	LYS	81	30.398	45.978	55.329	1.00	0.00	H
ATOM	1276	CE	LYS	81	32.219	44.767	55.084	1.00	0.00	C
ATOM	1277	HE2	LYS	81	31.820	43.787	54.820	1.00	0.00	H
ATOM	1278	HE3	LYS	81	33.092	44.918	54.447	1.00	0.00	H
ATOM	1279	NZ	LYS	81	32.650	45.015	56.540	1.00	0.00	N

```
ATOM   1280  HZ1 LYS    81      33.182  45.872  56.594  1.00  0.00           H
ATOM   1281  HZ2 LYS    81      31.809  45.203  57.066  1.00  0.00           H
ATOM   1282  HZ3 LYS    81      33.108  44.205  56.934  1.00  0.00           H
ATOM   1283  C   LYS    81      27.709  47.470  51.582  1.00  0.00           C
ATOM   1284  O   LYS    81      26.650  46.822  51.428  1.00  0.00           O
ATOM   1285  N   LYS    82      27.708  48.786  51.601  1.00  0.00           N
ATOM   1286  H   LYS    82      28.610  49.226  51.489  1.00  0.00           H
ATOM   1287  CA  LYS    82      26.496  49.589  51.449  1.00  0.00           C
ATOM   1288  HA  LYS    82      25.853  49.365  52.301  1.00  0.00           H
ATOM   1289  CB  LYS    82      26.976  51.029  51.565  1.00  0.00           C
ATOM   1290  HB2 LYS    82      27.609  51.233  52.430  1.00  0.00           H
ATOM   1291  HB3 LYS    82      27.505  51.216  50.630  1.00  0.00           H
ATOM   1292  CG  LYS    82      25.846  52.065  51.572  1.00  0.00           C
ATOM   1293  HG2 LYS    82      25.112  51.881  50.788  1.00  0.00           H
ATOM   1294  HG3 LYS    82      25.334  52.090  52.534  1.00  0.00           H
ATOM   1295  CD  LYS    82      26.303  53.558  51.333  1.00  0.00           C
ATOM   1296  HD2 LYS    82      26.620  53.578  50.291  1.00  0.00           H
ATOM   1297  HD3 LYS    82      25.462  54.245  51.427  1.00  0.00           H
ATOM   1298  CE  LYS    82      27.398  54.063  52.301  1.00  0.00           C
ATOM   1299  HE2 LYS    82      27.102  53.764  53.307  1.00  0.00           H
ATOM   1300  HE3 LYS    82      28.230  53.431  51.991  1.00  0.00           H
ATOM   1301  NZ  LYS    82      27.638  55.455  52.002  1.00  0.00           N
ATOM   1302  HZ1 LYS    82      28.205  55.886  52.719  1.00  0.00           H
ATOM   1303  HZ2 LYS    82      28.145  55.664  51.154  1.00  0.00           H
ATOM   1304  HZ3 LYS    82      26.805  56.026  52.001  1.00  0.00           H
ATOM   1305  C   LYS    82      25.757  49.320  50.162  1.00  0.00           C
ATOM   1306  O   LYS    82      24.594  48.953  50.127  1.00  0.00           O
ATOM   1307  N   VAL    83      26.456  49.401  48.994  1.00  0.00           N
ATOM   1308  H   VAL    83      27.446  49.599  49.033  1.00  0.00           H
ATOM   1309  CA  VAL    83      25.879  49.115  47.624  1.00  0.00           C
ATOM   1310  HA  VAL    83      24.914  49.622  47.579  1.00  0.00           H
ATOM   1311  CB  VAL    83      26.787  49.666  46.529  1.00  0.00           C
ATOM   1312  HB  VAL    83      26.316  49.609  45.548  1.00  0.00           H
ATOM   1313  CG1 VAL    83      26.829  51.119  46.753  1.00  0.00           C
ATOM   1314 HG11 VAL    83      27.326  51.444  47.666  1.00  0.00           H
ATOM   1315 HG12 VAL    83      27.408  51.568  45.947  1.00  0.00           H
ATOM   1316 HG13 VAL    83      25.831  51.557  46.763  1.00  0.00           H
ATOM   1317  CG2 VAL    83      28.064  48.962  46.396  1.00  0.00           C
ATOM   1318 HG21 VAL    83      28.029  47.916  46.091  1.00  0.00           H
ATOM   1319 HG22 VAL    83      28.551  49.462  45.558  1.00  0.00           H
ATOM   1320 HG23 VAL    83      28.678  49.145  47.278  1.00  0.00           H
ATOM   1321  C   VAL    83      25.489  47.713  47.306  1.00  0.00           C
ATOM   1322  O   VAL    83      24.818  47.553  46.294  1.00  0.00           O
ATOM   1323  N   ILE    84      25.833  46.773  48.142  1.00  0.00           N
ATOM   1324  H   ILE    84      26.255  47.171  48.969  1.00  0.00           H
ATOM   1325  CA  ILE
    84      25.264  45.383  48.146  1.00  0.00           C
ATOM   1326  HA  ILE    84      24.871  45.110  47.167  1.00  0.00           H
ATOM   1327  CB  ILE    84      26.354  44.378  48.480  1.00  0.00           C
ATOM   1328  HB  ILE    84      26.965  44.756  49.299  1.00  0.00           H
ATOM   1329  CG2 ILE    84      25.879  42.977  48.928  1.00  0.00           C
ATOM   1330 HG21 ILE    84      25.338  42.479  48.125  1.00  0.00           H
ATOM   1331 HG22 ILE    84      26.754  42.350  49.105  1.00  0.00           H
ATOM   1332 HG23 ILE    84      25.310  42.955  49.858  1.00  0.00           H
ATOM   1333  CG1 ILE    84      27.209  44.260  47.257  1.00  0.00           C
ATOM   1334 HG12 ILE    84      26.737  43.963  46.321  1.00  0.00           H
ATOM   1335 HG13 ILE    84      27.563  45.265  47.026  1.00  0.00           H
ATOM   1336  CD1 ILE    84      28.488  43.513  47.445  1.00  0.00           C
ATOM   1337 HD11 ILE    84      28.919  43.804  48.403  1.00  0.00           H
ATOM   1338 HD12 ILE    84      28.345  42.432  47.474  1.00  0.00           H
ATOM   1339 HD13 ILE    84      29.313  43.762  46.777  1.00  0.00           H
ATOM   1340  C   ILE    84      24.032  45.274  49.043  1.00  0.00           C
ATOM   1341  O   ILE    84      23.046  44.661  48.630  1.00  0.00           O
ATOM   1342  N   ALA    85      24.024  45.981  50.160  1.00  0.00           N
```

ATOM	1343	H	ALA	85	24.837	46.539	50.379	1.00	0.00	H
ATOM	1344	CA	ALA	85	22.826	46.251	50.986	1.00	0.00	C
ATOM	1345	HA	ALA	85	22.397	45.306	51.316	1.00	0.00	H
ATOM	1346	CB	ALA	85	23.276	46.929	52.242	1.00	0.00	C
ATOM	1347	HB1	ALA	85	23.986	47.714	51.987	1.00	0.00	H
ATOM	1348	HB2	ALA	85	22.480	47.573	52.617	1.00	0.00	H
ATOM	1349	HB3	ALA	85	23.619	46.125	52.894	1.00	0.00	H
ATOM	1350	C	ALA	85	21.718	46.955	50.212	1.00	0.00	C
ATOM	1351	O	ALA	85	20.517	46.667	50.476	1.00	0.00	O
ATOM	1352	N	ASP	86	21.966	47.796	49.158	1.00	0.00	N
ATOM	1353	H	ASP	86	22.876	48.122	48.866	1.00	0.00	H
ATOM	1354	CA	ASP	86	20.894	48.321	48.378	1.00	0.00	C
ATOM	1355	HA	ASP	86	20.105	48.762	48.987	1.00	0.00	H
ATOM	1356	CB	ASP	86	21.468	49.319	47.290	1.00	0.00	C
ATOM	1357	HB2	ASP	86	22.341	48.805	46.888	1.00	0.00	H
ATOM	1358	HB3	ASP	86	20.712	49.542	46.537	1.00	0.00	H
ATOM	1359	CG	ASP	86	21.910	50.702	47.858	1.00	0.00	C
ATOM	1360	OD1	ASP	86	21.262	51.370	48.670	1.00	0.00	O
ATOM	1361	OD2	ASP	86	23.033	51.165	47.404	1.00	0.00	O
ATOM	1362	C	ASP	86	20.147	47.214	47.556	1.00	0.00	C
ATOM	1363	O	ASP	86	18.932	47.407	47.323	1.00	0.00	O
ATOM	1364	N	ILE	87	20.765	46.070	47.308	1.00	0.00	N
ATOM	1365	H	ILE	87	21.745	45.972	47.527	1.00	0.00	H
ATOM	1366	CA	ILE	87	20.158	45.030	46.443	1.00	0.00	C
ATOM	1367	HA	ILE	87	19.408	45.588	45.884	1.00	0.00	H
ATOM	1368	CB	ILE	87	21.199	44.384	45.551	1.00	0.00	C
ATOM	1369	HB	ILE	87	21.881	43.732	46.095	1.00	0.00	H
ATOM	1370	CG2	ILE	87	20.586	43.449	44.521	1.00	0.00	C
ATOM	1371	HG21	ILE	87	21.280	42.968	43.833	1.00	0.00	H
ATOM	1372	HG22	ILE	87	19.919	42.769	45.053	1.00	0.00	H
ATOM	1373	HG23	ILE	87	20.118	44.080	43.766	1.00	0.00	H
ATOM	1374	CG1	ILE	87	22.142	45.326	44.755	1.00	0.00	C
ATOM	1375	HG12	ILE	87	21.573	45.703	43.905	1.00	0.00	H
ATOM	1376	HG13	ILE	87	22.394	46.201	45.354	1.00	0.00	H
ATOM	1377	CD1	ILE	87	23.462	44.758	44.256	1.00	0.00	C
ATOM	1378	HD11	ILE	87	24.004	45.635	43.903	1.00	0.00	H
ATOM	1379	HD12	ILE	87	24.087	44.394	45.072	1.00	0.00	H
ATOM	1380	HD13	ILE	87	23.283	44.008	43.486	1.00	0.00	H
ATOM	1381	C	ILE	87	19.442	43.995	47.259	1.00	0.00	C
ATOM	1382	O	ILE	87	18.427	43.423	46.894	1.00	0.00	O
ATOM	1383	N	TYR	88	20.100	43.691	48.349	1.00	0.00	N
ATOM	1384	H	TYR	88	20.881	44.282	48.597	1.00	0.00	H
ATOM	1385	CA	TYR	88	19.751	42.497	49.101	1.00	0.00	C
ATOM	1386	HA	TYR	88	18.810	42.026	48.819	1.00	0.00	H
ATOM	1387	CB	TYR	88	20.805	41.391	49.068	1.00	0.00	C
ATOM	1388	HB2	TYR	88	21.704	41.787	49.542	1.00	0.00	H
ATOM	1389	HB3	TYR	88	20.430	40.602	49.720	1.00	0.00	H
ATOM	1390	CG	TYR	88	21.050	40.801	47.655	1.00	0.00	C
ATOM	1391	CD1	TYR	88	20.157	39.916	47.153	1.00	0.00	C
ATOM	1392	HD1	TYR	88	19.321	39.511	47.705	1.00	0.00	H
ATOM	1393	CE1	TYR	88	20.403	39.279	45.951	1.00	0.00	C
ATOM	1394	HE1	TYR	88	19.646	38.699	45.442	1.00	0.00	H
ATOM	1395	CZ	TYR	88	21.530	39.681	45.148	1.00	0.00	C
ATOM	1396	OH	TYR	88	21.665	39.135	43.930	1.00	0.00	O
ATOM	1397	HH	TYR	88	21.380	38.219	43.911	1.00	0.00	H
ATOM	1398	CE2	TYR	88	22.431	40.645	45.627	1.00	0.00	C
ATOM	1399	HE2	TYR	88	23.151	40.991	44.900	1.00	0.00	H
ATOM	1400	CD2	TYR	88	22.194	41.271	46.890	1.00	0.00	C
ATOM	1401	HD2	TYR	88	22.866	42.034	47.253	1.00	0.00	H
ATOM	1402	C	TYR	88	19.497	42.818	50.551	1.00	0.00	C
ATOM	1403	O	TYR	88	20.187	43.576	51.211	1.00	0.00	O
ATOM	1404	N	PRO	89	18.536	42.235	51.302	1.00	0.00	N
ATOM	1405	CD	PRO	89	17.529	41.356	50.803	1.00	0.00	C
ATOM	1406	HD2	PRO	89	17.990	40.690	50.074	1.00	0.00	H

```
ATOM   1407  HD3 PRO    89      16.674  41.860  50.355  1.00  0.00           H
ATOM   1408  CG  PRO    89      17.120  40.542  51.969  1.00  0.00           C
ATOM   1409  HG2 PRO    89      17.879  39.776  52.139  1.00  0.00           H
ATOM   1410  HG3 PRO    89      16.156  40.048  51.857  1.00  0.00           H
ATOM   1411  CB  PRO    89      17.255  41.519  53.111  1.00  0.00           C
ATOM   1412  HB2 PRO    89      17.152  41.150  54.132  1.00  0.00           H
ATOM   1413  HB3 PRO    89      16.458  42.249  52.976  1.00  0.00           H
ATOM   1414  CA  PRO    89      18.502  42.262  52.771  1.00  0.00           C
ATOM   1415  HA  PRO    89      18.377  43.291  53.106  1.00  0.00           H
ATOM   1416  C   PRO    89      19.760  41.652  53.469  1.00  0.00           C
ATOM   1417  O   PRO    89      20.325  40.662  53.035  1.00  0.00           O
ATOM   1418  N   GLY    90      20.155  42.216  54.602  1.00  0.00           N
ATOM   1419  H   GLY    90      19.654  43.026  54.941  1.00  0.00           H
ATOM   1420  CA  GLY    90      21.499  41.977  55.305  1.00  0.00           C
ATOM   1421  HA2 GLY    90      22.317  42.139  54.603  1.00  0.00           H
ATOM   1422  HA3 GLY    90      21.606  42.647  56.159  1.00  0.00           H
ATOM   1423  C   GLY    90      21.711  40.548  55.795  1.00  0.00           C
ATOM   1424  O   GLY    90      22.814  40.095  56.115  1.00  0.00           O
ATOM   1425  N   GLN    91      20.592  39.801  55.785  1.00  0.00           N
ATOM   1426  H   GLN    91      19.748  40.115  55.328  1.00  0.00           H
ATOM   1427  CA  GLN    91      20.622  38.379  56.292  1.00  0.00           C
ATOM   1428  HA  GLN    91      21.459  38.224  56.973  1.00  0.00           H
ATOM   1429  CB  GLN    91      19.260  38.005  57.002  1.00  0.00           C
ATOM   1430  HB2 GLN    91      18.464  38.380  56.359  1.00  0.00           H
ATOM   1431  HB3 GLN    91      19.138  36.947  57.230  1.00  0.00           H
ATOM   1432  CG  GLN    91      19.336  38.729  58.432  1.00  0.00           C
ATOM   1433  HG2 GLN    91      20.112  38.222  59.007  1.00  0.00           H
ATOM   1434  HG3 GLN    91      19.661  39.762  58.308  1.00  0.00           H
ATOM   1435  CD  GLN    91      17.950  38.716  59.164  1.00  0.00           C
ATOM   1436  OE1 GLN    91      17.117  39.637  59.078  1.00  0.00           O
ATOM   1437  NE2 GLN    91      17.643  37.645  59.753  1.00  0.00           N
ATOM   1438 HE21 GLN    91      18.349  36.923  59.739  1.00  0.00           H
ATOM   1439 HE22 GLN    91      16.706  37.473  60.089  1.00  0.00           H
ATOM   1440  C   GLN    91      20.949  37.351  55.227  1.00  0.00           C
ATOM   1441  O   GLN    91      21.028  36.168  55.482  1.00  0.00           O
ATOM   1442  N   THR    92      21.093  37.820  54.022  1.00  0.00           N
ATOM   1443  H   THR    92      20.858  38.781  53.819  1.00  0.00           H
ATOM   1444  CA  THR    92      21.450  37.022  52.798  1.00  0.00           C
ATOM   1445  HA  THR    92      20.813  36.139  52.779  1.00  0.00           H
ATOM   1446  CB  THR    92      21.263  37.702  51.523  1.00  0.00           C
ATOM   1447  HB  THR    92      22.023  38.482  51.496  1.00  0.00           H
ATOM   1448  CG2 THR    92      21.290  36.782  50.310  1.00  0.00           C
ATOM   1449 HG21 THR    92      20.948  37.372  49.459  1.00  0.00           H
ATOM   1450 HG22 THR    92      22.294  36.443
  50.056  1.00  0.00           H
ATOM   1451 HG23 THR    92      20.553  36.015  50.550  1.00  0.00           H
ATOM   1452  OG1 THR    92      19.976  38.291  51.542  1.00  0.00           O
ATOM   1453  HG1 THR    92      19.955  39.092  52.072  1.00  0.00           H
ATOM   1454  C   THR    92      22.890  36.452  52.919  1.00  0.00           C
ATOM   1455  O   THR    92      23.867  37.130  53.242  1.00  0.00           O
ATOM   1456  N   GLN    93      22.997  35.152  52.570  1.00  0.00           N
ATOM   1457  H   GLN    93      22.141  34.630  52.456  1.00  0.00           H
ATOM   1458  CA  GLN    93      24.270  34.454  52.434  1.00  0.00           C
ATOM   1459  HA  GLN    93      24.946  34.826  53.205  1.00  0.00           H
ATOM   1460  CB  GLN    93      23.985  32.950  52.570  1.00  0.00           C
ATOM   1461  HB2 GLN    93      23.363  32.861  53.460  1.00  0.00           H
ATOM   1462  HB3 GLN    93      23.362  32.625  51.736  1.00  0.00           H
ATOM   1463  CG  GLN    93      25.276  32.127  52.738  1.00  0.00           C
ATOM   1464  HG2 GLN    93      24.961  31.089  52.849  1.00  0.00           H
ATOM   1465  HG3 GLN    93      25.889  32.166  51.837  1.00  0.00           H
ATOM   1466  CD  GLN    93      25.949  32.451  54.018  1.00  0.00           C
ATOM   1467  OE1 GLN    93      25.362  32.833  55.018  1.00  0.00           O
ATOM   1468  NE2 GLN    93      27.241  32.264  54.085  1.00  0.00           N
ATOM   1469 HE21 GLN    93      27.782  31.890  53.319  1.00  0.00           H
```

ATOM	1470	HE22	GLN	93	27.691	32.282	54.989	1.00	0.00	H
ATOM	1471	C	GLN	93	24.833	34.779	51.012	1.00	0.00	C
ATOM	1472	O	GLN	93	24.078	34.556	50.036	1.00	0.00	O
ATOM	1473	N	PHE	94	26.123	35.115	50.923	1.00	0.00	N
ATOM	1474	H	PHE	94	26.739	34.975	51.712	1.00	0.00	H
ATOM	1475	CA	PHE	94	26.842	35.347	49.709	1.00	0.00	C
ATOM	1476	HA	PHE	94	26.039	35.146	48.999	1.00	0.00	H
ATOM	1477	CB	PHE	94	27.425	36.724	49.643	1.00	0.00	C
ATOM	1478	HB2	PHE	94	28.123	36.892	50.464	1.00	0.00	H
ATOM	1479	HB3	PHE	94	28.014	36.882	48.740	1.00	0.00	H
ATOM	1480	CG	PHE	94	26.354	37.852	49.644	1.00	0.00	C
ATOM	1481	CD1	PHE	94	25.515	37.954	48.505	1.00	0.00	C
ATOM	1482	HD1	PHE	94	25.672	37.353	47.622	1.00	0.00	H
ATOM	1483	CE1	PHE	94	24.620	39.015	48.509	1.00	0.00	C
ATOM	1484	HE1	PHE	94	23.960	39.194	47.673	1.00	0.00	H
ATOM	1485	CZ	PHE	94	24.450	39.829	49.614	1.00	0.00	C
ATOM	1486	HZ	PHE	94	23.685	40.587	49.680	1.00	0.00	H
ATOM	1487	CE2	PHE	94	25.286	39.719	50.729	1.00	0.00	C
ATOM	1488	HE2	PHE	94	25.188	40.356	51.596	1.00	0.00	H
ATOM	1489	CD2	PHE	94	26.310	38.726	50.742	1.00	0.00	C
ATOM	1490	HD2	PHE	94	27.010	38.667	51.563	1.00	0.00	H
ATOM	1491	C	PHE	94	27.928	34.247	49.435	1.00	0.00	C
ATOM	1492	O	PHE	94	28.289	33.587	50.360	1.00	0.00	O
ATOM	1493	N	TYR	95	28.360	34.062	48.160	1.00	0.00	N
ATOM	1494	H	TYR	95	27.973	34.732	47.510	1.00	0.00	H
ATOM	1495	CA	TYR	95	29.368	33.074	47.632	1.00	0.00	C
ATOM	1496	HA	TYR	95	29.955	32.656	48.450	1.00	0.00	H
ATOM	1497	CB	TYR	95	28.544	31.926	46.968	1.00	0.00	C
ATOM	1498	HB2	TYR	95	27.721	31.628	47.616	1.00	0.00	H
ATOM	1499	HB3	TYR	95	28.208	32.327	46.011	1.00	0.00	H
ATOM	1500	CG	TYR	95	29.346	30.644	46.632	1.00	0.00	C
ATOM	1501	CD1	TYR	95	30.241	30.679	45.540	1.00	0.00	C
ATOM	1502	HD1	TYR	95	30.214	31.569	44.927	1.00	0.00	H
ATOM	1503	CE1	TYR	95	30.907	29.459	45.252	1.00	0.00	C
ATOM	1504	HE1	TYR	95	31.475	29.396	44.336	1.00	0.00	H
ATOM	1505	CZ	TYR	95	30.761	28.294	46.074	1.00	0.00	C
ATOM	1506	OH	TYR	95	31.337	27.038	45.763	1.00	0.00	O
ATOM	1507	HH	TYR	95	31.702	27.117	44.879	1.00	0.00	H
ATOM	1508	CE2	TYR	95	29.868	28.360	47.179	1.00	0.00	C
ATOM	1509	HE2	TYR	95	29.550	27.478	47.713	1.00	0.00	H
ATOM	1510	CD2	TYR	95	29.237	29.545	47.477	1.00	0.00	C
ATOM	1511	HD2	TYR	95	28.627	29.566	48.367	1.00	0.00	H
ATOM	1512	C	TYR	95	30.430	33.786	46.750	1.00	0.00	C
ATOM	1513	O	TYR	95	30.132	34.317	45.684	1.00	0.00	O
ATOM	1514	N	VAL	96	31.718	33.690	47.160	1.00	0.00	N
ATOM	1515	H	VAL	96	31.916	33.217	48.031	1.00	0.00	H
ATOM	1516	CA	VAL	96	32.884	34.160	46.371	1.00	0.00	C
ATOM	1517	HA	VAL	96	32.565	35.040	45.813	1.00	0.00	H
ATOM	1518	CB	VAL	96	34.064	34.554	47.166	1.00	0.00	C
ATOM	1519	HB	VAL	96	34.430	33.620	47.594	1.00	0.00	H
ATOM	1520	CG1	VAL	96	35.134	35.290	46.406	1.00	0.00	C
ATOM	1521	HG11	VAL	96	35.440	34.650	45.579	1.00	0.00	H
ATOM	1522	HG12	VAL	96	34.641	36.218	46.118	1.00	0.00	H
ATOM	1523	HG13	VAL	96	35.971	35.377	47.099	1.00	0.00	H
ATOM	1524	CG2	VAL	96	33.562	35.517	48.264	1.00	0.00	C
ATOM	1525	HG21	VAL	96	32.844	34.922	48.830	1.00	0.00	H
ATOM	1526	HG22	VAL	96	34.339	35.775	48.984	1.00	0.00	H
ATOM	1527	HG23	VAL	96	33.174	36.479	47.929	1.00	0.00	H
ATOM	1528	C	VAL	96	33.211	33.084	45.409	1.00	0.00	C
ATOM	1529	O	VAL	96	33.593	31.979	45.784	1.00	0.00	O
ATOM	1530	N	ILE	97	33.210	33.380	44.114	1.00	0.00	N
ATOM	1531	H	ILE	97	32.714	34.226	43.873	1.00	0.00	H
ATOM	1532	CA	ILE	97	34.008	32.689	43.049	1.00	0.00	C
ATOM	1533	HA	ILE	97	34.349	31.692	43.326	1.00	0.00	H

ATOM	1534	CB	ILE	97	33.109	32.664	41.738	1.00	0.00	C
ATOM	1535	HB	ILE	97	32.707	33.612	41.380	1.00	0.00	H
ATOM	1536	CG2	ILE	97	33.888	32.188	40.484	1.00	0.00	C
ATOM	1537	HG21	ILE	97	34.367	31.227	40.668	1.00	0.00	H
ATOM	1538	HG22	ILE	97	33.315	32.283	39.561	1.00	0.00	H
ATOM	1539	HG23	ILE	97	34.764	32.796	40.259	1.00	0.00	H
ATOM	1540	CG1	ILE	97	31.929	31.676	41.982	1.00	0.00	C
ATOM	1541	HG12	ILE	97	31.983	30.822	41.306	1.00	0.00	H
ATOM	1542	HG13	ILE	97	32.036	31.308	43.003	1.00	0.00	H
ATOM	1543	CD1	ILE	97	30.530	32.232	41.749	1.00	0.00	C
ATOM	1544	HD11	ILE	97	29.837	31.520	42.199	1.00	0.00	H
ATOM	1545	HD12	ILE	97	30.378	33.172	42.280	1.00	0.00	H
ATOM	1546	HD13	ILE	97	30.245	32.446	40.718	1.00	0.00	H
ATOM	1547	C	ILE	97	35.320	33.472	42.855	1.00	0.00	C
ATOM	1548	O	ILE	97	35.361	34.575	42.317	1.00	0.00	O
ATOM	1549	N	GLU	98	36.445	32.847	43.174	1.00	0.00	N
ATOM	1550	H	GLU	98	36.385	31.923	43.578	1.00	0.00	H
ATOM	1551	CA	GLU	98	37.773	33.337	42.704	1.00	0.00	C
ATOM	1552	HA	GLU	98	37.698	34.386	42.418	1.00	0.00	H
ATOM	1553	CB	GLU	98	38.725	33.265	43.893	1.00	0.00	C
ATOM	1554	HB2	GLU	98	38.350	33.882	44.710	1.00	0.00	H
ATOM	1555	HB3	GLU	98	38.834	32.295	44.378	1.00	0.00	H
ATOM	1556	CG	GLU	98	40.082	33.935	43.643	1.00	0.00	C
ATOM	1557	HG2	GLU	98	40.718	33.289	43.039	1.00	0.00	H
ATOM	1558	HG3	GLU	98	39.940	34.791	42.984	1.00	0.00	H
ATOM	1559	CD	GLU	98	40.918	34.352	44.900	1.00	0.00	C
ATOM	1560	OE1	GLU	98	41.303	35.527	44.959	1.00	0.00	O
ATOM	1561	OE2	GLU	98	41.114	33.535	45.861	1.00	0.00	O
ATOM	1562	C	GLU	98	38.282	32.453	41.565	1.00	0.00	C
ATOM	1563	O	GLU	98	37.993	31.211	41.592	1.00	0.00	O
ATOM	1564	N	PHE	99	38.860	33.087	40.495	1.00	0.00	N
ATOM	1565	H	PHE	99	38.981	34.082	40.367	1.00	0.00	H
ATOM	1566	CA	PHE	99	39.313	32.304	39.349	1.00	0.00	C
ATOM	1567	HA	PHE	99	39.474	31.333	39.819	1.00	0.00	H
ATOM	1568	CB	PHE	99	38.099	32.365	38.402	1.00	0.00	C
ATOM	1569	HB2	PHE	99	38.249	31.772	37.500	1.00	0.00	H
ATOM	1570	HB3	PHE	99	37.278	31.902	38.949	1.00	0.00	H
ATOM	1571	CG	PHE	99	37.713	33.629	37.812	1.00	0.00	C
ATOM	1572	CD1	PHE	99	38.257	33.987	36.588	1.00	0.00	C
ATOM	1573	HD1	PHE	99	38.874	33.372	35.950	1.00	0.00	H
ATOM	1574	CE1	PHE	99	37.896	35.192	35.984	1.00	0.00	C
ATOM	1575	HE1	PHE	99	38.312	35.389	35.006	1.00	0.00	H
ATOM	1576	CZ	PHE	99	37.046	36.104	36.678	1.00	0.00	C
ATOM	1577	HZ	PHE	99	36.737	37.051	36.263	1.00	0.00	H
ATOM	1578	CE2	PHE	99	36.424	35.684	37.884	1.00	0.00	C
ATOM	1579	HE2	PHE	99	35.595	36.272	38.247	1.00	0.00	H
ATOM	1580	CD2	PHE	99	36.812	34.447	38.503	1.00	0.00	C
ATOM	1581	HD2	PHE	99	36.349	34.163	39.437	1.00	0.00	H
ATOM	1582	C	PHE	99	40.595	32.793	38.792	1.00	0.00	C
ATOM	1583	O	PHE	99	40.946	33.961	38.939	1.00	0.00	O
ATOM	1584	N	LYS	100	41.319	32.040	37.923	1.00	0.00	N
ATOM	1585	H	LYS	100	40.940	31.109	37.819	1.00	0.00	H
ATOM	1586	CA	LYS	100	42.588	32.365	37.137	1.00	0.00	C
ATOM	1587	HA	LYS	100	42.462	33.419	36.887	1.00	0.00	H
ATOM	1588	CB	LYS	100	43.865	32.194	37.962	1.00	0.00	C
ATOM	1589	HB2	LYS	100	43.607	32.431	38.994	1.00	0.00	H
ATOM	1590	HB3	LYS	100	44.216	31.162	37.941	1.00	0.00	H
ATOM	1591	CG	LYS	100	44.937	33.153	37.435	1.00	0.00	C
ATOM	1592	HG2	LYS	100	45.184	32.937	36.396	1.00	0.00	H
ATOM	1593	HG3	LYS	100	44.651	34.201	37.531	1.00	0.00	H
ATOM	1594	CD	LYS	100	46.257	32.818	38.210	1.00	0.00	C
ATOM	1595	HD2	LYS	100	46.042	32.803	39.279	1.00	0.00	H
ATOM	1596	HD3	LYS	100	46.662	31.878	37.835	1.00	0.00	H

ATOM	1597	CE	LYS	100	47.357	33.853	37.848	1.00	0.00	C
ATOM	1598	HE2	LYS	100	47.617	33.687	36.803	1.00	0.00	H
ATOM	1599	HE3	LYS	100	46.954	34.865	37.874	1.00	0.00	H
ATOM	1600	NZ	LYS	100	48.553	33.729	38.683	1.00	0.00	N
ATOM	1601	HZ1	LYS	100	48.316	33.996	39.629	1.00	0.00	H
ATOM	1602	HZ2	LYS	100	48.826	32.760	38.772	1.00	0.00	H
ATOM	1603	HZ3	LYS	100	49.408	34.181	38.389	1.00	0.00	H
ATOM	1604	C	LYS	100	42.645	31.617	35.862	1.00	0.00	C
ATOM	1605	O	LYS	100	42.131	30.486	35.747	1.00	0.00	O
ATOM	1606	N	CYX	101	42.943	32.296	34.721	1.00	0.00	N
ATOM	1607	H	CYX	101	43.393	33.185	34.892	1.00	0.00	H
ATOM	1608	CA	CYX	101	43.188	31.702	33.356	1.00	0.00	C
ATOM	1609	HA	CYX	101	42.483	30.891	33.170	1.00	0.00	H
ATOM	1610	CB	CYX	101	43.095	32.822	32.312	1.00	0.00	C
ATOM	1611	HB2	CYX	101	42.103	33.269	32.244	1.00	0.00	H
ATOM	1612	HB3	CYX	101	43.754	33.663	32.526	1.00	0.00	H
ATOM	1613	SG	CYX	101	43.654	32.202	30.695	1.00	0.00	S
ATOM	1614	C	CYX	101	44.467	30.854	33.429	1.00	0.00	C
ATOM	1615	O	CYX	101	45.404	31.127	34.259	1.00	0.00	O
ATOM	1616	N	LEU	102	44.641	29.902	32.468	1.00	0.00	N
ATOM	1617	H	LEU	102	43.883	29.697	31.834	1.00	0.00	H
ATOM	1618	CA	LEU	102	45.690	28.888	32.470	1.00	0.00	C
ATOM	1619	HA	LEU	102	46.307	29.013	33.360	1.00	0.00	H
ATOM	1620	CB	LEU	102	44.974	27.599	32.490	1.00	0.00	C
ATOM	1621	HB2	LEU	102	44.430	27.479	31.553	1.00	0.00	H
ATOM	1622	HB3	LEU	102	45.766	26.850	32.431	1.00	0.00	H
ATOM	1623	CG	LEU	102	43.981	27.373	33.602	1.00	0.00	C
ATOM	1624	HG	LEU	102	43.209	28.127	33.442	1.00	0.00	H
ATOM	1625	CD1	LEU	102	43.393	25.984	33.678	1.00	0.00	C
ATOM	1626	HD11	LEU	102	44.154	25.210	33.777	1.00	0.00	H
ATOM	1627	HD12	LEU	102	42.516	25.989	34.325	1.00	0.00	H
ATOM	1628	HD13	LEU	102	43.014	25.794	32.674	1.00	0.00	H
ATOM	1629	CD2	LEU	102	44.508	27.753	34.930	1.00	0.00	C
ATOM	1630	HD21	LEU	102	45.446	27.206	35.027	1.00	0.00	H
ATOM	1631	HD22	LEU	102	44.822	28.795	34.999	1.00	0.00	H
ATOM	1632	HD23	LEU	102	43.744	27.538	35.676	1.00	0.00	H
ATOM	1633	C	LEU	102	46.658	29.026	31.313	1.00	0.00	C
ATOM	1634	O	LEU	102	47.843	29.058	31.644	1.00	0.00	O
ATOM	1635	OXT	LEU	102	46.245	29.335	30.207	1.00	0.00	O
HETATM	1637	N	LIG	103	28.693	40.603	37.978	1.00	0.00	N
HETATM	1638	C	LIG	103	29.076	39.333	38.063	1.00	0.00	C
HETATM	1639	O	LIG	103	27.395	42.194	38.768	1.00	0.00	O
HETATM	1640	C5'	LIG	103	24.968	38.694	43.217	1.00	0.00	C
HETATM	1641	O5'	LIG	103	24.727	37.686	42.248	1.00	0.00	O
HETATM	1642	C4'	LIG	103	24.883	40.195	42.587	1.00	0.00	C
HETATM	1643	O4'	LIG	103	26.168	40.358	41.888	1.00	0.00	O
HETATM	1644	C3'	LIG	103	23.791	40.516	41.617	1.00	0.00	C
HETATM	1645	O3'	LIG	103	23.257	41.873	41.796	1.00	0.00	O
HETATM	1646	C2'	LIG	103	24.474	40.334	40.254	1.00	0.00	C
HETATM	1647	O2'	LIG	103	23.808	41.158	39.233	1.00	0.00	O
HETATM	1648	C1'	LIG	103	25.982	40.793	40.556	1.00	0.00	C
HETATM	1649	N1	LIG	103	26.968	40.137	39.607	1.00	0.00	N
HETATM	1650	O1	LIG	103	29.788	36.959	36.698	1.00	0.00	O
HETATM	1651	N2	LIG	103	30.138	39.063	37.285	1.00	0.00	N
HETATM	1652	C6	LIG	103	27.666	40.998	38.809	1.00	0.00	C
HETATM	1653	C7	LIG	103	27.446	38.793	39.718	1.00	0.00	C
HETATM	1654	C8	LIG	103	28.501	38.391	38.946	1.00	0.00	C
HETATM	1655	C9	LIG	103	30.473	37.958	36.571	1.00	0.00	C
HETATM	1656	C10	LIG	103	31.636	38.033	35.576	1.00	0.00	C
HETATM	1657	H	LIG	103	30.622	39.892	36.961	1.00	0.00	H
HETATM	1658	H1	LIG	103	28.754	37.337	38.938	1.00	0.00	H
HETATM	1659	H4	LIG	103	25.925	41.884	40.468	1.00	0.00	H
HETATM	1660	H6	LIG	103	24.813	40.909	43.417	1.00	0.00	H
HETATM	1661	H7	LIG	103	23.002	39.762	41.727	1.00	0.00	H

```
HETATM 1662  H8  LIG   103       24.569  39.281  39.960  1.00  0.00           H
HETATM 1663  H9  LIG   103       24.417  38.578  44.158  1.00  0.00           H
HETATM 1664  H10 LIG   103       26.007  38.518  43.518  1.00  0.00           H
HETATM 1665  H11 LIG   103       31.645  36.992  35.232  1.00  0.00           H
HETATM 1666  H12 LIG   103       32.577  38.250  36.096  1.00  0.00           H
HETATM 1667  H13 LIG   103       31.413  38.603  34.666  1.00  0.00           H
HETATM 1668  H14 LIG   103       26.810  38.176  40.340  1.00  0.00           H
HETATM 1669  H2' LIG   103       23.649  42.049  39.588  1.00  0.00           H
HETATM 1670  H3' LIG   103       22.291  41.751  41.762  1.00  0.00           H
HETATM 1671  H5' LIG   103       23.888  37.274  42.519  1.00  0.00           H
ENDMDL
MODEL      3
SHEET    1   1 1 ILE    22  ASP    26  0
SHEET    2   2 1 VAL    37  VAL    40  0
SHEET    3   3 1 CYX    50  VAL    60  0
SHEET    4   4 1 PHE    94  CYX   101  0
HELIX    1   1 PHE     11  LEU     16  1                                   6
HELIX    2   2 GLU     67  GLN     72  1                                   6
HELIX    3   3 LEU     77  ILE     84  1                                   8
ATOM      1  N   GLN     1       36.575  17.163  34.498  1.00  0.00           N
ATOM      2  H1  GLN     1       37.213  17.824  34.920  1.00  0.00           H
ATOM      3  H2  GLN     1       35.650  17.118  34.901  1.00  0.00           H
ATOM      4  H3  GLN     1       37.136  16.331  34.617  1.00  0.00           H
ATOM      5  CA  GLN     1       36.339  17.590  33.016  1.00  0.00           C
ATOM      6  HA  GLN     1       37.320  17.939  32.695  1.00  0.00           H
ATOM      7  CB  GLN     1       35.818  16.392  32.152  1.00  0.00           C
ATOM      8  HB2 GLN     1       34.783  16.208  32.440  1.00  0.00           H
ATOM      9  HB3 GLN     1       35.941  16.544  31.080  1.00  0.00           H
ATOM     10  CG  GLN     1       36.489  14.971  32.407  1.00  0.00           C
ATOM     11  HG2 GLN     1       36.520  14.712  33.466  1.00  0.00           H
ATOM     12  HG3 GLN     1       36.032  14.206  31.779  1.00  0.00           H
ATOM     13  CD  GLN     1       37.900  14.984  31.932  1.00  0.00           C
ATOM     14  OE1 GLN     1       38.519  16.020  31.819  1.00  0.00           O
ATOM     15  NE2 GLN     1       38.557  13.933  31.621  1.00  0.00           N
ATOM     16 HE21 GLN     1       38.145  13.073  31.955  1.00  0.00           H
ATOM     17 HE22 GLN     1       39.478  14.087  31.238  1.00  0.00           H
ATOM     18  C   GLN     1       35.558  18.949  32.995  1.00  0.00           C
ATOM     19  O   GLN     1       35.009  19.241  34.122  1.00  0.00           O
ATOM     20  N   PRO     2       35.555  19.757  31.908  1.00  0.00           N
ATOM     21  CD  PRO     2       36.262  19.495  30.681  1.00  0.00           C
ATOM     22  HD2 PRO     2       35.592  19.154  29.891  1.00  0.00           H
ATOM     23  HD3 PRO     2       37.177  18.905  30.689  1.00  0.00           H
ATOM     24  CG  PRO     2       36.785  20.861  30.259  1.00  0.00           C
ATOM     25  HG2 PRO     2       36.962  20.832  29.183  1.00  0.00           H
ATOM
     26  HG3 PRO     2       37.712  21.027  30.808  1.00  0.00           H
ATOM     27  CB  PRO     2       35.621  21.736  30.584  1.00  0.00           C
ATOM     28  HB2 PRO     2       34.877  21.642  29.794  1.00  0.00           H
ATOM     29  HB3 PRO     2       35.933  22.764  30.765  1.00  0.00           H
ATOM     30  CA  PRO     2       35.102  21.132  31.895  1.00  0.00           C
ATOM     31  HA  PRO     2       35.591  21.699  32.687  1.00  0.00           H
ATOM     32  C   PRO     2       33.605  21.278  32.122  1.00  0.00           C
ATOM     33  O   PRO     2       32.860  20.279  31.981  1.00  0.00           O
ATOM     34  N   ASN     3       33.104  22.426  32.503  1.00  0.00           N
ATOM     35  H   ASN     3       33.729  23.207  32.646  1.00  0.00           H
ATOM     36  CA  ASN     3       31.658  22.777  32.530  1.00  0.00           C
ATOM     37  HA  ASN     3       31.130  21.884  32.865  1.00  0.00           H
ATOM     38  CB  ASN     3       31.392  23.825  33.626  1.00  0.00           C
ATOM     39  HB2 ASN     3       31.881  24.749  33.317  1.00  0.00           H
ATOM     40  HB3 ASN     3       30.322  23.997  33.523  1.00  0.00           H
ATOM     41  CG  ASN     3       31.864  23.362  35.005  1.00  0.00           C
ATOM     42  OD1 ASN     3       31.148  22.795  35.786  1.00  0.00           O
ATOM     43  ND2 ASN     3       33.124  23.255  35.240  1.00  0.00           N
ATOM     44 HD21 ASN     3       33.868  23.503  34.602  1.00  0.00           H
```

ATOM	45	HD22	ASN	3	33.135	22.542	35.956	1.00	0.00	H
ATOM	46	C	ASN	3	31.103	23.185	31.168	1.00	0.00	C
ATOM	47	O	ASN	3	31.858	23.824	30.471	1.00	0.00	O
ATOM	48	N	ASP	4	29.805	23.089	30.974	1.00	0.00	N
ATOM	49	H	ASP	4	29.273	22.531	31.626	1.00	0.00	H
ATOM	50	CA	ASP	4	29.058	23.784	29.946	1.00	0.00	C
ATOM	51	HA	ASP	4	29.742	23.806	29.098	1.00	0.00	H
ATOM	52	CB	ASP	4	27.805	22.924	29.619	1.00	0.00	C
ATOM	53	HB2	ASP	4	27.428	22.501	30.550	1.00	0.00	H
ATOM	54	HB3	ASP	4	27.043	23.503	29.099	1.00	0.00	H
ATOM	55	CG	ASP	4	28.139	21.841	28.529	1.00	0.00	C
ATOM	56	OD1	ASP	4	27.362	20.870	28.521	1.00	0.00	O
ATOM	57	OD2	ASP	4	29.226	21.825	27.876	1.00	0.00	O
ATOM	58	C	ASP	4	28.587	25.205	30.279	1.00	0.00	C
ATOM	59	O	ASP	4	28.157	26.004	29.386	1.00	0.00	O
ATOM	60	N	ILE	5	28.685	25.567	31.548	1.00	0.00	N
ATOM	61	H	ILE	5	29.010	24.833	32.162	1.00	0.00	H
ATOM	62	CA	ILE	5	28.404	26.875	32.158	1.00	0.00	C
ATOM	63	HA	ILE	5	27.734	27.358	31.446	1.00	0.00	H
ATOM	64	CB	ILE	5	27.629	26.737	33.473	1.00	0.00	C
ATOM	65	HB	ILE	5	28.370	26.230	34.090	1.00	0.00	H
ATOM	66	CG2	ILE	5	27.125	28.063	34.123	1.00	0.00	C
ATOM	67	HG21	ILE	5	26.724	27.766	35.092	1.00	0.00	H
ATOM	68	HG22	ILE	5	27.899	28.832	34.130	1.00	0.00	H
ATOM	69	HG23	ILE	5	26.375	28.596	33.538	1.00	0.00	H
ATOM	70	CG1	ILE	5	26.412	25.757	33.307	1.00	0.00	C
ATOM	71	HG12	ILE	5	26.678	24.741	33.015	1.00	0.00	H
ATOM	72	HG13	ILE	5	25.921	25.561	34.261	1.00	0.00	H
ATOM	73	CD1	ILE	5	25.342	26.242	32.304	1.00	0.00	C
ATOM	74	HD11	ILE	5	24.651	25.422	32.106	1.00	0.00	H
ATOM	75	HD12	ILE	5	24.772	27.129	32.580	1.00	0.00	H
ATOM	76	HD13	ILE	5	25.737	26.392	31.299	1.00	0.00	H
ATOM	77	C	ILE	5	29.668	27.746	32.181	1.00	0.00	C
ATOM	78	O	ILE	5	30.760	27.297	32.489	1.00	0.00	O
ATOM	79	N	THR	6	29.501	28.978	31.746	1.00	0.00	N
ATOM	80	H	THR	6	28.561	29.254	31.502	1.00	0.00	H
ATOM	81	CA	THR	6	30.613	29.859	31.944	1.00	0.00	C
ATOM	82	HA	THR	6	31.298	29.542	32.731	1.00	0.00	H
ATOM	83	CB	THR	6	31.451	29.963	30.647	1.00	0.00	C
ATOM	84	HB	THR	6	31.780	28.947	30.427	1.00	0.00	H
ATOM	85	CG2	THR	6	30.699	30.321	29.285	1.00	0.00	C
ATOM	86	HG21	THR	6	31.321	30.064	28.428	1.00	0.00	H
ATOM	87	HG22	THR	6	29.755	29.814	29.086	1.00	0.00	H
ATOM	88	HG23	THR	6	30.652	31.407	29.198	1.00	0.00	H
ATOM	89	OG1	THR	6	32.674	30.679	30.846	1.00	0.00	O
ATOM	90	HG1	THR	6	33.160	30.644	30.018	1.00	0.00	H
ATOM	91	C	THR	6	29.988	31.278	32.344	1.00	0.00	C
ATOM	92	O	THR	6	28.755	31.374	32.523	1.00	0.00	O
ATOM	93	N	PHE	7	30.833	32.287	32.567	1.00	0.00	N
ATOM	94	H	PHE	7	31.824	32.109	32.482	1.00	0.00	H
ATOM	95	CA	PHE	7	30.401	33.651	32.763	1.00	0.00	C
ATOM	96	HA	PHE	7	29.591	33.584	33.490	1.00	0.00	H
ATOM	97	CB	PHE	7	31.543	34.538	33.240	1.00	0.00	C
ATOM	98	HB2	PHE	7	32.232	34.603	32.398	1.00	0.00	H
ATOM	99	HB3	PHE	7	31.165	35.535	33.468	1.00	0.00	H
ATOM	100	CG	PHE	7	32.267	33.885	34.398	1.00	0.00	C
ATOM	101	CD1	PHE	7	31.577	33.840	35.660	1.00	0.00	C
ATOM	102	HD1	PHE	7	30.564	34.213	35.707	1.00	0.00	H
ATOM	103	CE1	PHE	7	32.229	33.474	36.843	1.00	0.00	C
ATOM	104	HE1	PHE	7	31.697	33.503	37.783	1.00	0.00	H
ATOM	105	CZ	PHE	7	33.517	33.050	36.827	1.00	0.00	C
ATOM	106	HZ	PHE	7	33.766	32.511	37.728	1.00	0.00	H
ATOM	107	CE2	PHE	7	34.255	33.070	35.578	1.00	0.00	C
ATOM	108	HE2	PHE	7	35.280	32.740	35.502	1.00	0.00	H

ATOM	109	CD2	PHE	7	33.647	33.611	34.340	1.00	0.00	C
ATOM	110	HD2	PHE	7	34.217	33.634	33.422	1.00	0.00	H
ATOM	111	C	PHE	7	29.639	34.170	31.572	1.00	0.00	C
ATOM	112	O	PHE	7	30.025	33.930	30.416	1.00	0.00	O
ATOM	113	N	PHE	8	28.537	34.901	31.786	1.00	0.00	N
ATOM	114	H	PHE	8	28.324	35.174	32.735	1.00	0.00	H
ATOM	115	CA	PHE	8	27.705	35.456	30.739	1.00	0.00	C
ATOM	116	HA	PHE	8	27.322	34.623	30.149	1.00	0.00	H
ATOM	117	CB	PHE	8	26.491	36.153	31.326	1.00	0.00	C
ATOM	118	HB2	PHE	8	26.871	36.948	31.967	1.00	0.00	H
ATOM	119	HB3	PHE	8	26.119	36.517	30.369	1.00	0.00	H
ATOM	120	CG	PHE	8	25.456	35.315	32.122	1.00	0.00	C
ATOM	121	CD1	PHE	8	25.181	33.979	31.732	1.00	0.00	C
ATOM	122	HD1	PHE	8	25.728	33.486	30.942	1.00	0.00	H
ATOM	123	CE1	PHE	8	24.269	33.182	32.403	1.00	0.00	C
ATOM	124	HE1	PHE	8	24.065	32.138	32.217	1.00	0.00	H
ATOM	125	CZ	PHE	8	23.363	33.826	33.275	1.00	0.00	C
ATOM	126	HZ	PHE	8	22.514	33.248	33.608	1.00	0.00	H
ATOM	127	CE2	PHE	8	23.610	35.114	33.721	1.00	0.00	C
ATOM	128	HE2	PHE	8	22.898	35.647	34.336	1.00	0.00	H
ATOM	129	CD2	PHE	8	24.687	35.792	33.154	1.00	0.00	C
ATOM	130	HD2	PHE	8	24.858	36.797	33.511	1.00	0.00	H
ATOM	131	C	PHE	8	28.479	36.390	29.798	1.00	0.00	C
ATOM	132	O	PHE	8	29.385	37.087	30.177	1.00	0.00	O
ATOM	133	N	GLN	9	28.012	36.448	28.608	1.00	0.00	N
ATOM	134	H	GLN	9	27.279	35.811	28.329	1.00	0.00	H
ATOM	135	CA	GLN	9	28.841	36.916	27.436	1.00	0.00	C
ATOM	136	HA	GLN	9	29.794	36.387	27.456	1.00	0.00	H
ATOM	137	CB	GLN	9	28.073	36.498	26.128	1.00	0.00	C
ATOM	138	HB2	GLN	9	28.812	36.518	25.327	1.00	0.00	H
ATOM	139	HB3	GLN	9	27.745	35.461	26.180	1.00	0.00	H
ATOM	140	CG	GLN	9	26.755	37.227	25.782	1.00	0.00	C
ATOM	141	HG2	GLN	9	26.961	38.297	25.792	1.00	0.00	H
ATOM	142	HG3	GLN	9	26.456	37.008	24.757	1.00	0.00	H
ATOM	143	CD	GLN	9	25.676	36.903	26.789	1.00	0.00	C
ATOM	144	OE1	GLN	9	25.667	35.872	27.492	1.00	0.00	O
ATOM	145	NE2	GLN	9	24.626	37.666	26.909	1.00	0.00	N
ATOM	146	HE21	GLN	9	24.067	37.611	27.748	1.00	0.00	H
ATOM	147	HE22	GLN	9	24.610	38.472	26.299	1.00	0.00	H
ATOM	148	C	GLN	9	29.216	38.444	27.438	1.00	0.00	C
ATOM	149	O	GLN	9	30.214	38.843	26.801	1.00	0.00	O
ATOM	150	N	ARG	10	28.536	39.324	28.184	1.00	0.00	N
ATOM	151	H	ARG	10						
					27.735	39.009	28.712	1.00	0.00	H
ATOM	152	CA	ARG	10	28.770	40.746	28.288	1.00	0.00	C
ATOM	153	HA	ARG	10	29.113	41.259	27.390	1.00	0.00	H
ATOM	154	CB	ARG	10	27.490	41.527	28.732	1.00	0.00	C
ATOM	155	HB2	ARG	10	26.694	41.225	28.049	1.00	0.00	H
ATOM	156	HB3	ARG	10	27.197	41.201	29.730	1.00	0.00	H
ATOM	157	CG	ARG	10	27.774	43.066	28.811	1.00	0.00	C
ATOM	158	HG2	ARG	10	28.612	43.211	29.492	1.00	0.00	H
ATOM	159	HG3	ARG	10	27.997	43.479	27.827	1.00	0.00	H
ATOM	160	CD	ARG	10	26.528	43.667	29.429	1.00	0.00	C
ATOM	161	HD2	ARG	10	26.441	43.242	30.428	1.00	0.00	H
ATOM	162	HD3	ARG	10	26.620	44.737	29.622	1.00	0.00	H
ATOM	163	NE	ARG	10	25.245	43.315	28.717	1.00	0.00	N
ATOM	164	HE	ARG	10	25.058	43.444	27.733	1.00	0.00	H
ATOM	165	CZ	ARG	10	24.254	42.544	29.221	1.00	0.00	C
ATOM	166	NH1	ARG	10	24.341	42.108	30.470	1.00	0.00	N
ATOM	167	HH11	ARG	10	23.488	41.928	30.980	1.00	0.00	H
ATOM	168	HH12	ARG	10	25.132	42.518	30.946	1.00	0.00	H
ATOM	169	NH2	ARG	10	23.208	42.200	28.533	1.00	0.00	N
ATOM	170	HH21	ARG	10	23.212	42.458	27.557	1.00	0.00	H
ATOM	171	HH22	ARG	10	22.435	41.721	28.972	1.00	0.00	H

ATOM	172	C	ARG	10	29.945	41.021	29.314	1.00	0.00	C
ATOM	173	O	ARG	10	30.773	41.924	29.030	1.00	0.00	O
ATOM	174	N	PHE	11	30.210	40.192	30.331	1.00	0.00	N
ATOM	175	H	PHE	11	29.655	39.347	30.346	1.00	0.00	H
ATOM	176	CA	PHE	11	31.280	40.321	31.275	1.00	0.00	C
ATOM	177	HA	PHE	11	31.255	41.275	31.800	1.00	0.00	H
ATOM	178	CB	PHE	11	31.018	39.270	32.404	1.00	0.00	C
ATOM	179	HB2	PHE	11	31.931	39.212	32.997	1.00	0.00	H
ATOM	180	HB3	PHE	11	30.841	38.346	31.853	1.00	0.00	H
ATOM	181	CG	PHE	11	29.845	39.610	33.298	1.00	0.00	C
ATOM	182	CD1	PHE	11	29.733	40.868	33.913	1.00	0.00	C
ATOM	183	HD1	PHE	11	30.480	41.628	33.744	1.00	0.00	H
ATOM	184	CE1	PHE	11	28.669	41.051	34.737	1.00	0.00	C
ATOM	185	HE1	PHE	11	28.577	41.997	35.247	1.00	0.00	H
ATOM	186	CZ	PHE	11	27.646	40.108	34.989	1.00	0.00	C
ATOM	187	HZ	PHE	11	26.792	40.266	35.631	1.00	0.00	H
ATOM	188	CE2	PHE	11	27.687	38.895	34.321	1.00	0.00	C
ATOM	189	HE2	PHE	11	26.885	38.175	34.360	1.00	0.00	H
ATOM	190	CD2	PHE	11	28.809	38.703	33.422	1.00	0.00	C
ATOM	191	HD2	PHE	11	28.798	37.776	32.867	1.00	0.00	H
ATOM	192	C	PHE	11	32.693	40.201	30.580	1.00	0.00	C
ATOM	193	O	PHE	11	33.701	40.643	31.046	1.00	0.00	O
ATOM	194	N	GLN	12	32.670	39.383	29.575	1.00	0.00	N
ATOM	195	H	GLN	12	31.826	39.213	29.047	1.00	0.00	H
ATOM	196	CA	GLN	12	33.846	38.724	28.983	1.00	0.00	C
ATOM	197	HA	GLN	12	34.397	38.124	29.708	1.00	0.00	H
ATOM	198	CB	GLN	12	33.512	37.663	27.952	1.00	0.00	C
ATOM	199	HB2	GLN	12	32.822	37.961	27.162	1.00	0.00	H
ATOM	200	HB3	GLN	12	34.452	37.310	27.526	1.00	0.00	H
ATOM	201	CG	GLN	12	32.863	36.391	28.578	1.00	0.00	C
ATOM	202	HG2	GLN	12	33.478	35.959	29.368	1.00	0.00	H
ATOM	203	HG3	GLN	12	31.924	36.505	29.118	1.00	0.00	H
ATOM	204	CD	GLN	12	32.540	35.303	27.584	1.00	0.00	C
ATOM	205	OE1	GLN	12	33.086	35.147	26.536	1.00	0.00	O
ATOM	206	NE2	GLN	12	31.567	34.482	27.847	1.00	0.00	N
ATOM	207	HE21	GLN	12	31.043	34.492	28.711	1.00	0.00	H
ATOM	208	HE22	GLN	12	31.491	33.744	27.163	1.00	0.00	H
ATOM	209	C	GLN	12	34.884	39.714	28.408	1.00	0.00	C
ATOM	210	O	GLN	12	36.073	39.432	28.433	1.00	0.00	O
ATOM	211	N	ASP	13	34.462	40.905	27.919	1.00	0.00	N
ATOM	212	H	ASP	13	33.495	41.190	27.981	1.00	0.00	H
ATOM	213	CA	ASP	13	35.432	41.994	27.715	1.00	0.00	C
ATOM	214	HA	ASP	13	35.966	41.702	26.811	1.00	0.00	H
ATOM	215	CB	ASP	13	34.636	43.260	27.363	1.00	0.00	C
ATOM	216	HB2	ASP	13	33.971	43.529	28.184	1.00	0.00	H
ATOM	217	HB3	ASP	13	35.309	44.106	27.211	1.00	0.00	H
ATOM	218	CG	ASP	13	33.675	43.173	26.189	1.00	0.00	C
ATOM	219	OD1	ASP	13	32.676	43.924	26.090	1.00	0.00	O
ATOM	220	OD2	ASP	13	33.897	42.297	25.328	1.00	0.00	O
ATOM	221	C	ASP	13	36.332	42.283	28.890	1.00	0.00	C
ATOM	222	O	ASP	13	37.552	42.689	28.715	1.00	0.00	O
ATOM	223	N	ASP	14	35.896	42.194	30.115	1.00	0.00	N
ATOM	224	H	ASP	14	34.996	41.786	30.324	1.00	0.00	H
ATOM	225	CA	ASP	14	36.809	42.584	31.230	1.00	0.00	C
ATOM	226	HA	ASP	14	37.403	43.437	30.899	1.00	0.00	H
ATOM	227	CB	ASP	14	35.964	43.193	32.331	1.00	0.00	C
ATOM	228	HB2	ASP	14	35.190	42.541	32.735	1.00	0.00	H
ATOM	229	HB3	ASP	14	36.601	43.388	33.194	1.00	0.00	H
ATOM	230	CG	ASP	14	35.270	44.451	31.853	1.00	0.00	C
ATOM	231	OD1	ASP	14	34.057	44.588	32.255	1.00	0.00	O
ATOM	232	OD2	ASP	14	35.981	45.386	31.326	1.00	0.00	O
ATOM	233	C	ASP	14	37.723	41.385	31.727	1.00	0.00	C
ATOM	234	O	ASP	14	38.870	41.599	32.200	1.00	0.00	O
ATOM	235	N	ILE	15	37.244	40.148	31.632	1.00	0.00	N

ATOM	236	H	ILE	15	36.394	40.144	31.086	1.00	0.00	H
ATOM	237	CA	ILE	15	38.027	38.930	31.737	1.00	0.00	C
ATOM	238	HA	ILE	15	38.605	38.957	32.661	1.00	0.00	H
ATOM	239	CB	ILE	15	37.111	37.696	31.730	1.00	0.00	C
ATOM	240	HB	ILE	15	36.662	37.885	30.755	1.00	0.00	H
ATOM	241	CG2	ILE	15	37.875	36.362	31.569	1.00	0.00	C
ATOM	242	HG21	ILE	15	37.171	35.536	31.470	1.00	0.00	H
ATOM	243	HG22	ILE	15	38.580	36.375	30.739	1.00	0.00	H
ATOM	244	HG23	ILE	15	38.466	36.162	32.464	1.00	0.00	H
ATOM	245	CG1	ILE	15	35.969	37.678	32.835	1.00	0.00	C
ATOM	246	HG12	ILE	15	36.500	37.312	33.714	1.00	0.00	H
ATOM	247	HG13	ILE	15	35.424	38.617	32.943	1.00	0.00	H
ATOM	248	CD1	ILE	15	34.880	36.615	32.707	1.00	0.00	C
ATOM	249	HD11	ILE	15	34.420	36.796	31.737	1.00	0.00	H
ATOM	250	HD12	ILE	15	35.239	35.589	32.803	1.00	0.00	H
ATOM	251	HD13	ILE	15	34.155	36.685	33.517	1.00	0.00	H
ATOM	252	C	ILE	15	39.013	38.902	30.522	1.00	0.00	C
ATOM	253	O	ILE	15	40.181	38.524	30.731	1.00	0.00	O
ATOM	254	N	LEU	16	38.641	39.263	29.297	1.00	0.00	N
ATOM	255	H	LEU	16	37.646	39.415	29.215	1.00	0.00	H
ATOM	256	CA	LEU	16	39.520	39.605	28.198	1.00	0.00	C
ATOM	257	HA	LEU	16	40.003	38.642	28.031	1.00	0.00	H
ATOM	258	CB	LEU	16	38.729	39.989	26.929	1.00	0.00	C
ATOM	259	HB2	LEU	16	37.973	39.209	26.823	1.00	0.00	H
ATOM	260	HB3	LEU	16	38.207	40.912	27.180	1.00	0.00	H
ATOM	261	CG	LEU	16	39.579	40.106	25.641	1.00	0.00	C
ATOM	262	HG	LEU	16	40.219	40.987	25.659	1.00	0.00	H
ATOM	263	CD1	LEU	16	40.365	38.848	25.368	1.00	0.00	C
ATOM	264	HD11	LEU	16	40.890	38.988	24.424	1.00	0.00	H
ATOM	265	HD12	LEU	16	41.146	38.859	26.128	1.00	0.00	H
ATOM	266	HD13	LEU	16	39.725	37.974	25.252	1.00	0.00	H
ATOM	267	CD2	LEU	16	38.648	40.364	24.511	1.00	0.00	C
ATOM	268	HD21	LEU	16	39.150	40.707	23.607	1.00	0.00	H
ATOM	269	HD22	LEU	16	38.132	39.444	24.235	1.00	0.00	H
ATOM	270	HD23	LEU	16	37.780	40.993	24.708	1.00	0.00	H
ATOM	271	C	LEU	16	40.627	40.599	28.580	1.00	0.00	C
ATOM	272	O	LEU	16	41.787	40.287	28.356	1.00	0.00	O
ATOM	273	N	ALA	17	40.290	41.727	29.174	1.00	0.00	N
ATOM	274	H	ALA	17	39.325	42.024	29.150	1.00	0.00	H
ATOM	275	CA	ALA	17	41.262	42.645	29.706	1.00	0.00	C
ATOM	276	HA	ALA	17	41.904	42.859	28.851			
1.00	0.00			H						
ATOM	277	CB	ALA	17	40.549	43.922	30.209	1.00	0.00	C
ATOM	278	HB1	ALA	17	41.236	44.620	30.689	1.00	0.00	H
ATOM	279	HB2	ALA	17	40.088	44.402	29.345	1.00	0.00	H
ATOM	280	HB3	ALA	17	39.807	43.546	30.913	1.00	0.00	H
ATOM	281	C	ALA	17	42.299	42.137	30.732	1.00	0.00	C
ATOM	282	O	ALA	17	43.309	42.789	30.997	1.00	0.00	O
ATOM	283	N	GLY	18	42.073	40.931	31.287	1.00	0.00	N
ATOM	284	H	GLY	18	41.292	40.352	31.014	1.00	0.00	H
ATOM	285	CA	GLY	18	42.816	40.362	32.434	1.00	0.00	C
ATOM	286	HA2	GLY	18	42.832	39.274	32.502	1.00	0.00	H
ATOM	287	HA3	GLY	18	43.829	40.755	32.356	1.00	0.00	H
ATOM	288	C	GLY	18	42.387	41.055	33.751	1.00	0.00	C
ATOM	289	O	GLY	18	43.034	40.848	34.739	1.00	0.00	O
ATOM	290	N	ARG	19	41.190	41.706	33.754	1.00	0.00	N
ATOM	291	H	ARG	19	40.729	41.793	32.859	1.00	0.00	H
ATOM	292	CA	ARG	19	40.758	42.635	34.871	1.00	0.00	C
ATOM	293	HA	ARG	19	41.691	42.862	35.387	1.00	0.00	H
ATOM	294	CB	ARG	19	40.183	43.942	34.261	1.00	0.00	C
ATOM	295	HB2	ARG	19	39.511	43.608	33.471	1.00	0.00	H
ATOM	296	HB3	ARG	19	39.556	44.405	35.023	1.00	0.00	H
ATOM	297	CG	ARG	19	41.181	45.051	33.786	1.00	0.00	C
ATOM	298	HG2	ARG	19	41.951	45.116	34.554	1.00	0.00	H

ATOM	299	HG3	ARG	19	41.615	44.694	32.851	1.00	0.00	H
ATOM	300	CD	ARG	19	40.510	46.413	33.474	1.00	0.00	C
ATOM	301	HD2	ARG	19	40.038	46.753	34.397	1.00	0.00	H
ATOM	302	HD3	ARG	19	41.213	47.231	33.320	1.00	0.00	H
ATOM	303	NE	ARG	19	39.464	46.305	32.444	1.00	0.00	N
ATOM	304	HE	ARG	19	38.651	45.803	32.770	1.00	0.00	H
ATOM	305	CZ	ARG	19	39.327	46.965	31.361	1.00	0.00	C
ATOM	306	NH1	ARG	19	40.260	47.717	30.834	1.00	0.00	N
ATOM	307	HH11	ARG	19	41.099	48.038	31.295	1.00	0.00	H
ATOM	308	HH12	ARG	19	40.243	48.089	29.895	1.00	0.00	H
ATOM	309	NH2	ARG	19	38.203	46.831	30.671	1.00	0.00	N
ATOM	310	HH21	ARG	19	38.136	47.372	29.821	1.00	0.00	H
ATOM	311	HH22	ARG	19	37.512	46.224	31.089	1.00	0.00	H
ATOM	312	C	ARG	19	39.848	42.083	35.903	1.00	0.00	C
ATOM	313	O	ARG	19	40.079	42.229	37.127	1.00	0.00	O
ATOM	314	N	LYS	20	38.846	41.316	35.565	1.00	0.00	N
ATOM	315	H	LYS	20	38.577	41.243	34.595	1.00	0.00	H
ATOM	316	CA	LYS	20	37.981	40.527	36.514	1.00	0.00	C
ATOM	317	HA	LYS	20	37.780	41.218	37.333	1.00	0.00	H
ATOM	318	CB	LYS	20	36.539	40.241	35.971	1.00	0.00	C
ATOM	319	HB2	LYS	20	36.480	39.421	35.255	1.00	0.00	H
ATOM	320	HB3	LYS	20	36.311	41.100	35.340	1.00	0.00	H
ATOM	321	CG	LYS	20	35.601	40.120	37.148	1.00	0.00	C
ATOM	322	HG2	LYS	20	34.858	39.351	36.934	1.00	0.00	H
ATOM	323	HG3	LYS	20	36.070	39.671	38.023	1.00	0.00	H
ATOM	324	CD	LYS	20	35.093	41.520	37.605	1.00	0.00	C
ATOM	325	HD2	LYS	20	34.746	42.121	36.765	1.00	0.00	H
ATOM	326	HD3	LYS	20	35.811	42.087	38.198	1.00	0.00	H
ATOM	327	CE	LYS	20	33.815	41.281	38.496	1.00	0.00	C
ATOM	328	HE2	LYS	20	33.117	40.654	37.941	1.00	0.00	H
ATOM	329	HE3	LYS	20	34.009	40.856	39.480	1.00	0.00	H
ATOM	330	NZ	LYS	20	32.960	42.463	38.769	1.00	0.00	N
ATOM	331	HZ1	LYS	20	32.205	42.263	39.410	1.00	0.00	H
ATOM	332	HZ2	LYS	20	32.661	42.900	37.910	1.00	0.00	H
ATOM	333	HZ3	LYS	20	33.487	43.263	39.089	1.00	0.00	H
ATOM	334	C	LYS	20	38.555	39.222	36.930	1.00	0.00	C
ATOM	335	O	LYS	20	38.722	38.329	36.050	1.00	0.00	O
ATOM	336	N	THR	21	38.876	39.017	38.203	1.00	0.00	N
ATOM	337	H	THR	21	38.613	39.791	38.795	1.00	0.00	H
ATOM	338	CA	THR	21	39.595	37.788	38.731	1.00	0.00	C
ATOM	339	HA	THR	21	39.606	37.123	37.867	1.00	0.00	H
ATOM	340	CB	THR	21	41.085	38.083	39.129	1.00	0.00	C
ATOM	341	HB	THR	21	41.540	37.098	39.229	1.00	0.00	H
ATOM	342	CG2	THR	21	41.800	38.896	38.090	1.00	0.00	C
ATOM	343	HG21	THR	21	41.312	39.847	37.876	1.00	0.00	H
ATOM	344	HG22	THR	21	42.832	39.033	38.415	1.00	0.00	H
ATOM	345	HG23	THR	21	41.871	38.331	37.161	1.00	0.00	H
ATOM	346	OG1	THR	21	41.106	38.829	40.302	1.00	0.00	O
ATOM	347	HG1	THR	21	41.761	38.475	40.908	1.00	0.00	H
ATOM	348	C	THR	21	38.883	37.030	39.886	1.00	0.00	C
ATOM	349	O	THR	21	39.248	35.888	40.271	1.00	0.00	O
ATOM	350	N	ILE	22	37.815	37.664	40.431	1.00	0.00	N
ATOM	351	H	ILE	22	37.517	38.451	39.870	1.00	0.00	H
ATOM	352	CA	ILE	22	36.996	37.324	41.528	1.00	0.00	C
ATOM	353	HA	ILE	22	36.938	36.235	41.548	1.00	0.00	H
ATOM	354	CB	ILE	22	37.719	37.749	42.885	1.00	0.00	C
ATOM	355	HB	ILE	22	38.760	37.447	42.996	1.00	0.00	H
ATOM	356	CG2	ILE	22	37.609	39.214	43.051	1.00	0.00	C
ATOM	357	HG21	ILE	22	38.330	39.630	43.755	1.00	0.00	H
ATOM	358	HG22	ILE	22	37.698	39.762	42.113	1.00	0.00	H
ATOM	359	HG23	ILE	22	36.609	39.442	43.420	1.00	0.00	H
ATOM	360	CG1	ILE	22	36.994	37.171	44.157	1.00	0.00	C
ATOM	361	HG12	ILE	22	35.944	37.452	44.231	1.00	0.00	H
ATOM	362	HG13	ILE	22	37.015	36.092	44.001	1.00	0.00	H

```
ATOM    363  CD1 ILE    22      37.614  37.567  45.470  1.00  0.00           C
ATOM    364 HD11 ILE    22      37.305  36.888  46.266  1.00  0.00           H
ATOM    365 HD12 ILE    22      38.690  37.530  45.298  1.00  0.00           H
ATOM    366 HD13 ILE    22      37.222  38.571  45.631  1.00  0.00           H
ATOM    367  C   ILE    22      35.600  37.850  41.399  1.00  0.00           C
ATOM    368  O   ILE    22      35.403  38.905  40.776  1.00  0.00           O
ATOM    369  N   THR    23      34.549  37.074  41.665  1.00  0.00           N
ATOM    370  H   THR    23      34.689  36.196  42.145  1.00  0.00           H
ATOM    371  CA  THR    23      33.079  37.461  41.584  1.00  0.00           C
ATOM    372  HA  THR    23      32.931  38.540  41.588  1.00  0.00           H
ATOM    373  CB  THR    23      32.495  37.024  40.259  1.00  0.00           C
ATOM    374  HB  THR    23      32.823  36.010  40.032  1.00  0.00           H
ATOM    375  CG2 THR    23      30.937  36.960  40.248  1.00  0.00           C
ATOM    376 HG21 THR    23      30.655  38.009  40.344  1.00  0.00           H
ATOM    377 HG22 THR    23      30.680  36.559  39.267  1.00  0.00           H
ATOM    378 HG23 THR    23      30.587  36.290  41.033  1.00  0.00           H
ATOM    379  OG1 THR    23      32.950  37.890  39.218  1.00  0.00           O
ATOM    380  HG1 THR    23      33.893  37.946  39.383  1.00  0.00           H
ATOM    381  C   THR    23      32.282  36.888  42.835  1.00  0.00           C
ATOM    382  O   THR    23      32.817  35.995  43.530  1.00  0.00           O
ATOM    383  N   ILE    24      31.240  37.520  43.222  1.00  0.00           N
ATOM    384  H   ILE    24      30.836  38.183  42.577  1.00  0.00           H
ATOM    385  CA  ILE    24      30.368  37.208  44.357  1.00  0.00           C
ATOM    386  HA  ILE    24      30.731  36.324  44.883  1.00  0.00           H
ATOM    387  CB  ILE    24      30.324  38.502  45.328  1.00  0.00           C
ATOM    388  HB  ILE    24      29.691  39.278  44.898  1.00  0.00           H
ATOM    389  CG2 ILE    24      29.722  37.935  46.606  1.00  0.00           C
ATOM    390 HG21 ILE    24      30.439  37.284  47.106  1.00  0.00           H
ATOM    391 HG22 ILE    24      29.537  38.794  47.252  1.00  0.00           H
ATOM    392 HG23 ILE    24      28.767  37.490  46.326  1.00  0.00           H
ATOM    393  CG1 ILE    24      31.742  39.077  45.662  1.00  0.00           C
ATOM    394 HG12 ILE    24      32.539  38.334  45.675  1.00  0.00           H
ATOM    395 HG13 ILE    24      31.945  39.696  44.788  1.00  0.00           H
ATOM    396  CD1 ILE    24      31.866  39.939  46.887  1.00  0.00           C
ATOM    397 HD11 ILE    24      32.850  40.396  46.993  1.00  0.00           H
ATOM    398 HD12 ILE    24      31.097  40.697  46.739  1.00  0.00           H
ATOM    399 HD13 ILE    24      31.660  39.431  47.829  1.00  0.00           H
ATOM    400  C   ILE    24      28.943  37.008  43.805  1.00  0.00           C
ATOM    401  O   ILE    24      28.513  37.822  43.017  1.00  0.00           O
ATOM
    402  N   ARG    25      28.278  36.001  44.274  1.00  0.00           N
ATOM    403  H   ARG    25      28.770  35.475  44.983  1.00  0.00           H
ATOM    404  CA  ARG    25      26.789  35.778  44.150  1.00  0.00           C
ATOM    405  HA  ARG    25      26.285  36.613  43.662  1.00  0.00           H
ATOM    406  CB  ARG    25      26.581  34.435  43.341  1.00  0.00           C
ATOM    407  HB2 ARG    25      27.171  33.586  43.689  1.00  0.00           H
ATOM    408  HB3 ARG    25      25.550  34.118  43.495  1.00  0.00           H
ATOM    409  CG  ARG    25      26.925  34.501  41.831  1.00  0.00           C
ATOM    410  HG2 ARG    25      27.881  35.003  41.675  1.00  0.00           H
ATOM    411  HG3 ARG    25      26.900  33.480  41.453  1.00  0.00           H
ATOM    412  CD  ARG    25      25.784  35.282  41.087  1.00  0.00           C
ATOM    413  HD2 ARG    25      24.893  34.660  41.186  1.00  0.00           H
ATOM    414  HD3 ARG    25      25.706  36.259  41.565  1.00  0.00           H
ATOM    415  NE  ARG    25      25.973  35.564  39.670  1.00  0.00           N
ATOM    416  HE  ARG    25      26.481  34.873  39.137  1.00  0.00           H
ATOM    417  CZ  ARG    25      25.322  36.373  38.878  1.00  0.00           C
ATOM    418  NH1 ARG    25      24.459  37.243  39.341  1.00  0.00           N
ATOM    419 HH11 ARG    25      23.758  37.639  38.733  1.00  0.00           H
ATOM    420 HH12 ARG    25      24.296  37.083  40.325  1.00  0.00           H
ATOM    421  NH2 ARG    25      25.494  36.245  37.591  1.00  0.00           N
ATOM    422 HH21 ARG    25      25.070  36.949  37.004  1.00  0.00           H
ATOM    423 HH22 ARG    25      25.716  35.374  37.129  1.00  0.00           H
ATOM    424  C   ARG    25      26.048  35.783  45.502  1.00  0.00           C
ATOM    425  O   ARG    25      26.664  35.710  46.562  1.00  0.00           O
```

ATOM	426	N	ASP	26	24.745	35.760	45.505	1.00	0.00	N
ATOM	427	H	ASP	26	24.273	35.808	44.614	1.00	0.00	H
ATOM	428	CA	ASP	26	23.955	35.242	46.571	1.00	0.00	C
ATOM	429	HA	ASP	26	24.349	35.635	47.508	1.00	0.00	H
ATOM	430	CB	ASP	26	22.490	35.684	46.425	1.00	0.00	C
ATOM	431	HB2	ASP	26	21.984	34.779	46.761	1.00	0.00	H
ATOM	432	HB3	ASP	26	22.318	36.484	47.145	1.00	0.00	H
ATOM	433	CG	ASP	26	21.864	35.918	45.036	1.00	0.00	C
ATOM	434	OD1	ASP	26	22.597	36.002	44.001	1.00	0.00	O
ATOM	435	OD2	ASP	26	20.606	36.044	44.973	1.00	0.00	O
ATOM	436	C	ASP	26	24.249	33.702	46.535	1.00	0.00	C
ATOM	437	O	ASP	26	24.536	33.117	45.482	1.00	0.00	O
ATOM	438	N	GLU	27	24.170	33.060	47.752	1.00	0.00	N
ATOM	439	H	GLU	27	23.914	33.533	48.607	1.00	0.00	H
ATOM	440	CA	GLU	27	24.546	31.677	48.019	1.00	0.00	C
ATOM	441	HA	GLU	27	25.590	31.475	47.776	1.00	0.00	H
ATOM	442	CB	GLU	27	24.467	31.452	49.539	1.00	0.00	C
ATOM	443	HB2	GLU	27	25.209	32.084	50.024	1.00	0.00	H
ATOM	444	HB3	GLU	27	23.439	31.733	49.768	1.00	0.00	H
ATOM	445	CG	GLU	27	24.652	29.997	50.164	1.00	0.00	C
ATOM	446	HG2	GLU	27	24.638	29.938	51.251	1.00	0.00	H
ATOM	447	HG3	GLU	27	23.794	29.402	49.847	1.00	0.00	H
ATOM	448	CD	GLU	27	26.029	29.331	49.842	1.00	0.00	C
ATOM	449	OE1	GLU	27	26.815	28.997	50.764	1.00	0.00	O
ATOM	450	OE2	GLU	27	26.332	28.961	48.696	1.00	0.00	O
ATOM	451	C	GLU	27	23.724	30.544	47.295	1.00	0.00	C
ATOM	452	O	GLU	27	22.501	30.589	47.346	1.00	0.00	O
ATOM	453	N	SER	28	24.435	29.544	46.751	1.00	0.00	N
ATOM	454	H	SER	28	25.442	29.577	46.814	1.00	0.00	H
ATOM	455	CA	SER	28	23.848	28.418	46.007	1.00	0.00	C
ATOM	456	HA	SER	28	23.040	28.078	46.654	1.00	0.00	H
ATOM	457	CB	SER	28	23.312	28.841	44.717	1.00	0.00	C
ATOM	458	HB2	SER	28	22.684	29.732	44.690	1.00	0.00	H
ATOM	459	HB3	SER	28	24.118	28.980	43.997	1.00	0.00	H
ATOM	460	OG	SER	28	22.553	27.697	44.312	1.00	0.00	O
ATOM	461	HG	SER	28	21.745	27.844	44.810	1.00	0.00	H
ATOM	462	C	SER	28	24.819	27.219	45.814	1.00	0.00	C
ATOM	463	O	SER	28	25.953	27.390	45.340	1.00	0.00	O
ATOM	464	N	GLU	29	24.283	26.013	46.055	1.00	0.00	N
ATOM	465	H	GLU	29	23.277	26.014	46.137	1.00	0.00	H
ATOM	466	CA	GLU	29	25.057	24.734	45.846	1.00	0.00	C
ATOM	467	HA	GLU	29	26.061	24.880	46.244	1.00	0.00	H
ATOM	468	CB	GLU	29	24.513	23.627	46.723	1.00	0.00	C
ATOM	469	HB2	GLU	29	24.475	24.005	47.744	1.00	0.00	H
ATOM	470	HB3	GLU	29	23.489	23.398	46.429	1.00	0.00	H
ATOM	471	CG	GLU	29	25.156	22.275	46.574	1.00	0.00	C
ATOM	472	HG2	GLU	29	24.683	21.523	47.207	1.00	0.00	H
ATOM	473	HG3	GLU	29	24.887	21.882	45.593	1.00	0.00	H
ATOM	474	CD	GLU	29	26.643	22.131	46.836	1.00	0.00	C
ATOM	475	OE1	GLU	29	27.312	21.249	46.245	1.00	0.00	O
ATOM	476	OE2	GLU	29	27.199	22.824	47.727	1.00	0.00	O
ATOM	477	C	GLU	29	25.138	24.334	44.333	1.00	0.00	C
ATOM	478	O	GLU	29	25.990	23.535	43.913	1.00	0.00	O
ATOM	479	N	SER	30	24.370	24.961	43.454	1.00	0.00	N
ATOM	480	H	SER	30	23.661	25.586	43.810	1.00	0.00	H
ATOM	481	CA	SER	30	24.466	24.902	41.913	1.00	0.00	C
ATOM	482	HA	SER	30	24.497	23.844	41.656	1.00	0.00	H
ATOM	483	CB	SER	30	23.131	25.444	41.250	1.00	0.00	C
ATOM	484	HB2	SER	30	23.151	25.302	40.169	1.00	0.00	H
ATOM	485	HB3	SER	30	22.261	24.888	41.601	1.00	0.00	H
ATOM	486	OG	SER	30	22.847	26.772	41.590	1.00	0.00	O
ATOM	487	HG	SER	30	22.324	26.697	42.393	1.00	0.00	H
ATOM	488	C	SER	30	25.708	25.672	41.414	1.00	0.00	C
ATOM	489	O	SER	30	26.153	25.356	40.325	1.00	0.00	O

ATOM	490	N	HIE	31	26.253	26.603	42.196	1.00	0.00	N
ATOM	491	H	HIE	31	25.759	26.883	43.031	1.00	0.00	H
ATOM	492	CA	HIE	31	27.462	27.287	41.765	1.00	0.00	C
ATOM	493	HA	HIE	31	27.160	27.778	40.840	1.00	0.00	H
ATOM	494	CB	HIE	31	27.774	28.413	42.710	1.00	0.00	C
ATOM	495	HB2	HIE	31	27.928	27.902	43.659	1.00	0.00	H
ATOM	496	HB3	HIE	31	28.618	29.012	42.365	1.00	0.00	H
ATOM	497	CG	HIE	31	26.682	29.468	42.958	1.00	0.00	C
ATOM	498	ND1	HIE	31	25.939	29.977	41.861	1.00	0.00	N
ATOM	499	CE1	HIE	31	25.235	30.969	42.399	1.00	0.00	C
ATOM	500	HE1	HIE	31	24.542	31.586	41.847	1.00	0.00	H
ATOM	501	NE2	HIE	31	25.558	31.177	43.752	1.00	0.00	N
ATOM	502	HE2	HIE	31	25.056	31.759	44.409	1.00	0.00	H
ATOM	503	CD2	HIE	31	26.469	30.171	44.113	1.00	0.00	C
ATOM	504	HD2	HIE	31	26.894	30.076	45.101	1.00	0.00	H
ATOM	505	C	HIE	31	28.717	26.480	41.420	1.00	0.00	C
ATOM	506	O	HIE	31	28.802	25.344	41.798	1.00	0.00	O
ATOM	507	N	PHE	32	29.676	27.094	40.744	1.00	0.00	N
ATOM	508	H	PHE	32	29.442	28.067	40.599	1.00	0.00	H
ATOM	509	CA	PHE	32	31.110	26.644	40.576	1.00	0.00	C
ATOM	510	HA	PHE	32	31.198	25.705	40.031	1.00	0.00	H
ATOM	511	CB	PHE	32	31.844	27.752	39.745	1.00	0.00	C
ATOM	512	HB2	PHE	32	32.014	28.582	40.430	1.00	0.00	H
ATOM	513	HB3	PHE	32	32.851	27.436	39.473	1.00	0.00	H
ATOM	514	CG	PHE	32	31.136	28.374	38.514	1.00	0.00	C
ATOM	515	CD1	PHE	32	30.879	29.747	38.338	1.00	0.00	C
ATOM	516	HD1	PHE	32	31.043	30.440	39.151	1.00	0.00	H
ATOM	517	CE1	PHE	32	30.538	30.233	37.077	1.00	0.00	C
ATOM	518	HE1	PHE	32	30.425	31.292	36.902	1.00	0.00	H
ATOM	519	CZ	PHE	32	30.120	29.309	36.071	1.00	0.00	C
ATOM	520	HZ	PHE	32	29.925	29.728	35.096	1.00	0.00	H
ATOM	521	CE2	PHE	32	30.310	27.919	36.207	1.00	0.00	C
ATOM	522	HE2	PHE	32	29.975	27.281	35.403	1.00	0.00	H
ATOM	523	CD2	PHE	32	30.761	27.465	37.474	1.00	0.00	C
ATOM	524	HD2	PHE	32	30.886	26.399	37.597	1.00	0.00	H
ATOM	525	C	PHE	32	31.726	26.425	41.997	1.00	0.00	C
ATOM	526	O	PHE	32	31.369	26.947	43.045	1.00	0.00	O
ATOM	527	N	LYS	33						
32.574	25.429	42.001	1.00	0.00			N			
ATOM	528	H	LYS	33	32.551	24.826	41.192	1.00	0.00	H
ATOM	529	CA	LYS	33	33.328	24.880	43.137	1.00	0.00	C
ATOM	530	HA	LYS	33	33.085	25.434	44.043	1.00	0.00	H
ATOM	531	CB	LYS	33	33.014	23.393	43.287	1.00	0.00	C
ATOM	532	HB2	LYS	33	33.437	22.837	42.450	1.00	0.00	H
ATOM	533	HB3	LYS	33	33.497	23.010	44.187	1.00	0.00	H
ATOM	534	CG	LYS	33	31.589	22.857	43.412	1.00	0.00	C
ATOM	535	HG2	LYS	33	31.140	22.976	42.426	1.00	0.00	H
ATOM	536	HG3	LYS	33	31.712	21.803	43.664	1.00	0.00	H
ATOM	537	CD	LYS	33	30.760	23.378	44.585	1.00	0.00	C
ATOM	538	HD2	LYS	33	31.267	23.082	45.504	1.00	0.00	H
ATOM	539	HD3	LYS	33	30.739	24.467	44.601	1.00	0.00	H
ATOM	540	CE	LYS	33	29.310	22.792	44.528	1.00	0.00	C
ATOM	541	HE2	LYS	33	29.410	21.710	44.618	1.00	0.00	H
ATOM	542	HE3	LYS	33	28.788	23.025	45.456	1.00	0.00	H
ATOM	543	NZ	LYS	33	28.642	23.137	43.282	1.00	0.00	N
ATOM	544	HZ1	LYS	33	28.522	22.261	42.797	1.00	0.00	H
ATOM	545	HZ2	LYS	33	27.789	23.660	43.427	1.00	0.00	H
ATOM	546	HZ3	LYS	33	29.147	23.803	42.715	1.00	0.00	H
ATOM	547	C	LYS	33	34.826	24.992	42.820	1.00	0.00	C
ATOM	548	O	LYS	33	35.214	25.214	41.673	1.00	0.00	O
ATOM	549	N	THR	34	35.609	24.899	43.853	1.00	0.00	N
ATOM	550	H	THR	34	35.199	24.813	44.772	1.00	0.00	H
ATOM	551	CA	THR	34	37.088	24.906	43.809	1.00	0.00	C
ATOM	552	HA	THR	34	37.486	25.842	43.416	1.00	0.00	H

ATOM	553	CB	THR	34	37.845	24.755	45.194	1.00	0.00	C
ATOM	554	HB	THR	34	37.419	23.875	45.675	1.00	0.00	H
ATOM	555	CG2	THR	34	39.346	24.664	45.123	1.00	0.00	C
ATOM	556	HG21	THR	34	39.625	25.658	44.773	1.00	0.00	H
ATOM	557	HG22	THR	34	39.807	24.519	46.100	1.00	0.00	H
ATOM	558	HG23	THR	34	39.783	23.937	44.438	1.00	0.00	H
ATOM	559	OG1	THR	34	37.524	25.840	45.907	1.00	0.00	O
ATOM	560	HG1	THR	34	36.753	25.511	46.376	1.00	0.00	H
ATOM	561	C	THR	34	37.545	23.829	42.807	1.00	0.00	C
ATOM	562	O	THR	34	37.177	22.691	42.865	1.00	0.00	O
ATOM	563	N	GLY	35	38.460	24.260	41.885	1.00	0.00	N
ATOM	564	H	GLY	35	38.802	25.210	41.901	1.00	0.00	H
ATOM	565	CA	GLY	35	39.074	23.356	40.838	1.00	0.00	C
ATOM	566	HA2	GLY	35	40.068	23.689	40.538	1.00	0.00	H
ATOM	567	HA3	GLY	35	39.242	22.391	41.315	1.00	0.00	H
ATOM	568	C	GLY	35	38.154	23.055	39.656	1.00	0.00	C
ATOM	569	O	GLY	35	38.624	22.293	38.819	1.00	0.00	O
ATOM	570	N	ASP	36	36.923	23.564	39.581	1.00	0.00	N
ATOM	571	H	ASP	36	36.492	24.247	40.187	1.00	0.00	H
ATOM	572	CA	ASP	36	36.126	23.410	38.330	1.00	0.00	C
ATOM	573	HA	ASP	36	36.163	22.338	38.135	1.00	0.00	H
ATOM	574	CB	ASP	36	34.733	23.995	38.583	1.00	0.00	C
ATOM	575	HB2	ASP	36	34.828	25.020	38.946	1.00	0.00	H
ATOM	576	HB3	ASP	36	34.270	23.985	37.596	1.00	0.00	H
ATOM	577	CG	ASP	36	33.899	23.263	39.552	1.00	0.00	C
ATOM	578	OD1	ASP	36	34.189	22.119	40.017	1.00	0.00	O
ATOM	579	OD2	ASP	36	32.759	23.798	39.810	1.00	0.00	O
ATOM	580	C	ASP	36	36.827	24.167	37.169	1.00	0.00	C
ATOM	581	O	ASP	36	37.480	25.149	37.421	1.00	0.00	O
ATOM	582	N	VAL	37	36.722	23.705	35.903	1.00	0.00	N
ATOM	583	H	VAL	37	36.111	22.919	35.741	1.00	0.00	H
ATOM	584	CA	VAL	37	37.260	24.350	34.653	1.00	0.00	C
ATOM	585	HA	VAL	37	37.943	25.148	34.943	1.00	0.00	H
ATOM	586	CB	VAL	37	38.237	23.439	33.862	1.00	0.00	C
ATOM	587	HB	VAL	37	37.591	22.597	33.617	1.00	0.00	H
ATOM	588	CG1	VAL	37	38.830	24.056	32.500	1.00	0.00	C
ATOM	589	HG11	VAL	37	39.352	24.999	32.662	1.00	0.00	H
ATOM	590	HG12	VAL	37	39.604	23.472	32.003	1.00	0.00	H
ATOM	591	HG13	VAL	37	37.990	24.269	31.840	1.00	0.00	H
ATOM	592	CG2	VAL	37	39.423	22.994	34.682	1.00	0.00	C
ATOM	593	HG21	VAL	37	39.127	22.637	35.669	1.00	0.00	H
ATOM	594	HG22	VAL	37	40.039	22.256	34.168	1.00	0.00	H
ATOM	595	HG23	VAL	37	40.137	23.810	34.787	1.00	0.00	H
ATOM	596	C	VAL	37	36.099	24.914	33.822	1.00	0.00	C
ATOM	597	O	VAL	37	35.078	24.311	33.618	1.00	0.00	O
ATOM	598	N	LEU	38	36.278	26.193	33.442	1.00	0.00	N
ATOM	599	H	LEU	38	37.209	26.541	33.627	1.00	0.00	H
ATOM	600	CA	LEU	38	35.447	27.222	32.769	1.00	0.00	C
ATOM	601	HA	LEU	38	34.496	26.809	32.430	1.00	0.00	H
ATOM	602	CB	LEU	38	35.268	28.401	33.736	1.00	0.00	C
ATOM	603	HB2	LEU	38	36.186	28.988	33.709	1.00	0.00	H
ATOM	604	HB3	LEU	38	34.574	29.044	33.195	1.00	0.00	H
ATOM	605	CG	LEU	38	34.684	28.085	35.129	1.00	0.00	C
ATOM	606	HG	LEU	38	35.331	27.426	35.708	1.00	0.00	H
ATOM	607	CD1	LEU	38	34.451	29.274	36.070	1.00	0.00	C
ATOM	608	HD11	LEU	38	34.150	29.012	37.084	1.00	0.00	H
ATOM	609	HD12	LEU	38	35.366	29.848	36.206	1.00	0.00	H
ATOM	610	HD13	LEU	38	33.624	29.867	35.680	1.00	0.00	H
ATOM	611	CD2	LEU	38	33.300	27.539	34.961	1.00	0.00	C
ATOM	612	HD21	LEU	38	32.886	27.254	35.928	1.00	0.00	H
ATOM	613	HD22	LEU	38	32.598	28.206	34.463	1.00	0.00	H
ATOM	614	HD23	LEU	38	33.280	26.617	34.380	1.00	0.00	H
ATOM	615	C	LEU	38	36.137	27.620	31.522	1.00	0.00	C
ATOM	616	O	LEU	38	37.366	27.560	31.366	1.00	0.00	O

ATOM	617	N	ARG	39	35.389	27.995	30.460	1.00	0.00	N
ATOM	618	H	ARG	39	34.412	27.760	30.546	1.00	0.00	H
ATOM	619	CA	ARG	39	35.946	28.532	29.220	1.00	0.00	C
ATOM	620	HA	ARG	39	36.974	28.857	29.385	1.00	0.00	H
ATOM	621	CB	ARG	39	35.993	27.450	28.196	1.00	0.00	C
ATOM	622	HB2	ARG	39	35.027	27.140	27.800	1.00	0.00	H
ATOM	623	HB3	ARG	39	36.554	27.832	27.342	1.00	0.00	H
ATOM	624	CG	ARG	39	36.762	26.145	28.622	1.00	0.00	C
ATOM	625	HG2	ARG	39	37.669	26.424	29.156	1.00	0.00	H
ATOM	626	HG3	ARG	39	36.138	25.751	29.424	1.00	0.00	H
ATOM	627	CD	ARG	39	36.920	25.121	27.489	1.00	0.00	C
ATOM	628	HD2	ARG	39	37.194	25.721	26.620	1.00	0.00	H
ATOM	629	HD3	ARG	39	37.705	24.435	27.805	1.00	0.00	H
ATOM	630	NE	ARG	39	35.665	24.413	27.106	1.00	0.00	N
ATOM	631	HE	ARG	39	34.744	24.703	27.402	1.00	0.00	H
ATOM	632	CZ	ARG	39	35.571	23.472	26.175	1.00	0.00	C
ATOM	633	NH1	ARG	39	36.587	23.101	25.439	1.00	0.00	N
ATOM	634	HH11	ARG	39	37.492	23.549	25.478	1.00	0.00	H
ATOM	635	HH12	ARG	39	36.472	22.314	24.816	1.00	0.00	H
ATOM	636	NH2	ARG	39	34.463	22.828	26.035	1.00	0.00	N
ATOM	637	HH21	ARG	39	34.539	21.984	25.487	1.00	0.00	H
ATOM	638	HH22	ARG	39	33.625	23.217	26.443	1.00	0.00	H
ATOM	639	C	ARG	39	35.282	29.756	28.769	1.00	0.00	C
ATOM	640	O	ARG	39	34.037	29.844	28.800	1.00	0.00	O
ATOM	641	N	VAL	40	36.051	30.754	28.346	1.00	0.00	N
ATOM	642	H	VAL	40	37.050	30.605	28.339	1.00	0.00	H
ATOM	643	CA	VAL	40	35.514	32.083	27.831	1.00	0.00	C
ATOM	644	HA	VAL	40	34.429	31.995	27.776	1.00	0.00	H
ATOM	645	CB	VAL	40	35.777	33.295	28.780	1.00	0.00	C
ATOM	646	HB	VAL	40	35.433	34.234	28.344	1.00	0.00	H
ATOM	647	CG1	VAL	40	34.998	32.993	30.097	1.00	0.00	C
ATOM	648	HG11	VAL	40	33.972	32.783	29.794	1.00	0.00	H
ATOM	649	HG12	VAL	40	35.452	32.142	30.604	1.00	0.00	H
ATOM	650	HG13	VAL	40	35.095	33.869	30.738	1.00	0.00	H
ATOM	651	CG2	VAL	40	37.270	33.393	29.059	1.00	0.00	C
ATOM	652	HG21	VAL	40	37.669	34.250	29.602	1.00	0.00	H
ATOM	653	HG22	VAL	40	37.576	32.503	29.609	1.00	0.00	H
ATOM	654	HG23	VAL	40	37.811	33.293	28.118	1.00	0.00	H
ATOM	655	C	VAL	40	35.999	32.249	26.366	1.00	0.00	C
ATOM	656	O	VAL	40	37.079	31.931	25.867	1.00	0.00	O
ATOM	657	N	GLY	41	35.064	32.912	25.638	1.00	0.00	N
ATOM	658	H	GLY	41	34.288	33.310	26.146	1.00	0.00	H
ATOM	659	CA	GLY	41	35.212	33.403	24.252	1.00	0.00	C
ATOM	660	HA2	GLY	41	35.419	34.471	24.303	1.00	0.00	H
ATOM	661	HA3	GLY	41	35.937	32.833	23.671	1.00	0.00	H
ATOM	662	C	GLY	41	33.947	33.225	23.430	1.00	0.00	C
ATOM	663	O	GLY	41	32.989	32.462	23.809	1.00	0.00	O
ATOM	664	N	ARG	42	33.934	34.085	22.419	1.00	0.00	N
ATOM	665	H	ARG	42	34.728	34.690	22.263	1.00	0.00	H
ATOM	666	CA	ARG	42	32.799	34.125	21.482	1.00	0.00	C
ATOM	667	HA	ARG	42	32.577	33.089	21.225	1.00	0.00	H
ATOM	668	CB	ARG	42	31.581	34.874	22.130	1.00	0.00	C
ATOM	669	HB2	ARG	42	30.726	34.627	21.500	1.00	0.00	H
ATOM	670	HB3	ARG	42	31.297	34.363	23.051	1.00	0.00	H
ATOM	671	CG	ARG	42	31.685	36.423	22.252	1.00	0.00	C
ATOM	672	HG2	ARG	42	32.112	36.973	21.414	1.00	0.00	H
ATOM	673	HG3	ARG	42	30.717	36.841	22.529	1.00	0.00	H
ATOM	674	CD	ARG	42	32.575	36.919	23.390	1.00	0.00	C
ATOM	675	HD2	ARG	42	32.565	36.120	24.131	1.00	0.00	H
ATOM	676	HD3	ARG	42	33.601	37.080	23.058	1.00	0.00	H
ATOM	677	NE	ARG	42	32.113	38.111	24.061	1.00	0.00	N
ATOM	678	HE	ARG	42	31.145	38.244	24.317	1.00	0.00	H
ATOM	679	CZ	ARG	42	32.796	39.222	24.423	1.00	0.00	C

ATOM	680	NH1	ARG	42	34.029	39.541	24.118	1.00	0.00	N
ATOM	681	HH11	ARG	42	34.428	40.402	24.464	1.00	0.00	H
ATOM	682	HH12	ARG	42	34.520	38.902	23.510	1.00	0.00	H
ATOM	683	NH2	ARG	42	32.191	40.188	25.098	1.00	0.00	N
ATOM	684	HH21	ARG	42	31.359	40.050	25.654	1.00	0.00	H
ATOM	685	HH22	ARG	42	32.798	40.984	25.243	1.00	0.00	H
ATOM	686	C	ARG	42	33.098	34.566	20.038	1.00	0.00	C
ATOM	687	O	ARG	42	32.181	34.938	19.286	1.00	0.00	O
ATOM	688	N	PHE	43	34.349	34.463	19.635	1.00	0.00	N
ATOM	689	H	PHE	43	35.031	34.165	20.317	1.00	0.00	H
ATOM	690	CA	PHE	43	34.938	34.777	18.220	1.00	0.00	C
ATOM	691	HA	PHE	43	34.118	34.725	17.505	1.00	0.00	H
ATOM	692	CB	PHE	43	35.568	36.159	18.127	1.00	0.00	C
ATOM	693	HB2	PHE	43	36.397	36.080	18.829	1.00	0.00	H
ATOM	694	HB3	PHE	43	35.976	36.350	17.135	1.00	0.00	H
ATOM	695	CG	PHE	43	34.682	37.308	18.570	1.00	0.00	C
ATOM	696	CD1	PHE	43	33.417	37.485	18.072	1.00	0.00	C
ATOM	697	HD1	PHE	43	33.201	36.806	17.260	1.00	0.00	H
ATOM	698	CE1	PHE	43	32.501	38.423	18.596	1.00	0.00	C
ATOM	699	HE1	PHE	43	31.541	38.649	18.156	1.00	0.00	H
ATOM	700	CZ	PHE	43	32.913	39.172	19.708	1.00	0.00	C
ATOM	701	HZ	PHE	43	32.254	39.931	20.101	1.00	0.00	H
ATOM	702	CE2	PHE	43	34.186	38.883	20.269	1.00	0.00	C
ATOM	703	HE2	PHE	43	34.477	39.380	21.183	1.00	0.00	H
ATOM	704	CD2	PHE	43	35.153	38.138	19.611	1.00	0.00	C
ATOM	705	HD2	PHE	43	36.110	38.039	20.103	1.00	0.00	H
ATOM	706	C	PHE	43	35.806	33.613	17.777	1.00	0.00	C
ATOM	707	O	PHE	43	36.180	32.705	18.559	1.00	0.00	O
ATOM	708	N	GLU	44	36.131	33.592	16.504	1.00	0.00	N
ATOM	709	H	GLU	44	35.761	34.248	15.830	1.00	0.00	H
ATOM	710	CA	GLU	44	36.924	32.519	15.917	1.00	0.00	C
ATOM	711	HA	GLU	44	36.408	31.619	16.248	1.00	0.00	H
ATOM	712	CB	GLU	44	36.704	32.640	14.367	1.00	0.00	C
ATOM	713	HB2	GLU	44	36.976	31.710	13.870	1.00	0.00	H
ATOM	714	HB3	GLU	44	35.621	32.641	14.238	1.00	0.00	H
ATOM	715	CG	GLU	44	37.411	33.769	13.585	1.00	0.00	C
ATOM	716	HG2	GLU	44	38.401	33.837	14.035	1.00	0.00	H
ATOM	717	HG3	GLU	44	37.602	33.382	12.584	1.00	0.00	H
ATOM	718	CD	GLU	44	36.768	35.124	13.809	1.00	0.00	C
ATOM	719	OE1	GLU	44	37.530	36.052	13.799	1.00	0.00	O
ATOM	720	OE2	GLU	44	35.524	35.121	13.975	1.00	0.00	O
ATOM	721	C	GLU	44	38.466	32.344	16.355	1.00	0.00	C
ATOM	722	O	GLU	44	39.126	31.444	15.854	1.00	0.00	O
ATOM	723	N	ASP	45	39.062	33.146	17.287	1.00	0.00	N
ATOM	724	H	ASP	45	38.548	33.768	17.894	1.00	0.00	H
ATOM	725	CA	ASP	45	40.512	32.925	17.748	1.00	0.00	C
ATOM	726	HA	ASP	45	41.089	32.681	16.857	1.00	0.00	H
ATOM	727	CB	ASP	45	40.993	34.255	18.329	1.00	0.00	C
ATOM	728	HB2	ASP	45	40.423	34.383	19.249	1.00	0.00	H
ATOM	729	HB3	ASP	45	42.054	34.112	18.538	1.00	0.00	H
ATOM	730	CG	ASP	45	40.855	35.461	17.479	1.00	0.00	C
ATOM	731	OD1	ASP	45	40.734	35.365	16.234	1.00	0.00	O
ATOM	732	OD2	ASP	45	40.996	36.598	18.017	1.00	0.00	O
ATOM	733	C	ASP	45	40.669	31.789	18.733	1.00	0.00	C
ATOM	734	O	ASP	45	40.471	32.015	19.909	1.00	0.00	O
ATOM	735	N	ASP	46	40.981	30.596	18.306	1.00	0.00	N
ATOM	736	H	ASP	46	41.281	30.409	17.360	1.00	0.00	H
ATOM	737	CA	ASP	46	40.888	29.312	19.094	1.00	0.00	C
ATOM	738	HA	ASP	46	41.089	28.553	18.337	1.00	0.00	H
ATOM	739	CB	ASP	46	42.095	29.115	20.115	1.00	0.00	C
ATOM	740	HB2	ASP	46	41.779	29.612	21.033	1.00	0.00	H
ATOM	741	HB3	ASP	46	42.178	28.053	20.348	1.00	0.00	H
ATOM	742	CG	ASP	46	43.392	29.589	19.587	1.00	0.00	C
ATOM	743	OD1	ASP	46	43.983	30.543	20.104	1.00	0.00	O

ATOM	744	OD2	ASP	46	43.920	28.935	18.682	1.00	0.00	O
ATOM	745	C	ASP	46	39.463	28.968	19.580	1.00	0.00	C
ATOM	746	O	ASP	46	39.217	27.938	20.201	1.00	0.00	O
ATOM	747	N	GLY	47	38.450	29.800	19.290	1.00	0.00	N
ATOM	748	H	GLY	47	38.730	30.629	18.785	1.00	0.00	H
ATOM	749	CA	GLY	47	37.045	29.727	19.607	1.00	0.00	C
ATOM	750	HA2	GLY	47	36.414	30.302	18.928	1.00	0.00	H
ATOM	751	HA3	GLY	47	36.678	28.705	19.528	1.00	0.00	H
ATOM	752	C	GLY	47	36.770	30.102	21.068	1.00	0.00	C
ATOM	753	O	GLY	47	35.990	31.030	21.305	1.00	0.00	O
ATOM	754	N	TYR	48	37.513	29.458	22.016	1.00	0.00	N
ATOM	755	H	TYR	48	37.970	28.609	21.714	1.00	0.00	H
ATOM	756	CA	TYR	48	37.773	29.909	23.428	1.00	0.00	C
ATOM	757	HA	TYR	48	37.094	30.715	23.704	1.00	0.00	H
ATOM	758	CB	TYR	48	37.602	28.779	24.502	1.00	0.00	C
ATOM	759	HB2	TYR	48	38.317	27.999	24.240	1.00	0.00	H
ATOM	760	HB3	TYR	48	37.761	29.197	25.496	1.00	0.00	H
ATOM	761	CG	TYR	48	36.216	28.085	24.499	1.00	0.00	C
ATOM	762	CD1	TYR	48	35.076	28.811	24.606	1.00	0.00	C
ATOM	763	HD1	TYR	48	35.085	29.864	24.843	1.00	0.00	H
ATOM	764	CE1	TYR	48	33.769	28.173	24.462	1.00	0.00	C
ATOM	765	HE1	TYR	48	32.881	28.783	24.543	1.00	0.00	H
ATOM	766	CZ	TYR	48	33.729	26.768	24.279	1.00	0.00	C
ATOM	767	OH	TYR	48	32.509	26.318	24.035	1.00	0.00	O
ATOM	768	HH	TYR	48	32.504	25.401	23.748	1.00	0.00	H
ATOM	769	CE2	TYR	48	34.891	25.961	24.121	1.00	0.00	C
ATOM	770	HE2	TYR	48	34.930	24.932	23.793	1.00	0.00	H
ATOM	771	CD2	TYR	48	36.169	26.668	24.335	1.00	0.00	C
ATOM	772	HD2	TYR	48	37.111	26.184	24.130	1.00	0.00	H
ATOM	773	C	TYR	48	39.159	30.611	23.541	1.00	0.00	C
ATOM	774	O	TYR	48	40.206	29.985	23.418	1.00	0.00	O
ATOM	775	N	PHE	49	39.159	31.897	23.857	1.00	0.00	N
ATOM	776	H	PHE	49	38.318	32.395	24.111	1.00	0.00	H
ATOM	777	CA	PHE	49	40.404	32.662	24.181	1.00	0.00	C
ATOM										
	778	HA	PHE	49	41.149	32.379	23.437	1.00	0.00	H
ATOM	779	CB	PHE	49	40.177	34.198	24.221	1.00	0.00	C
ATOM	780	HB2	PHE	49	41.154	34.648	24.398	1.00	0.00	H
ATOM	781	HB3	PHE	49	39.814	34.538	23.250	1.00	0.00	H
ATOM	782	CG	PHE	49	39.258	34.836	25.328	1.00	0.00	C
ATOM	783	CD1	PHE	49	39.876	35.213	26.496	1.00	0.00	C
ATOM	784	HD1	PHE	49	40.932	35.051	26.660	1.00	0.00	H
ATOM	785	CE1	PHE	49	39.153	35.843	27.535	1.00	0.00	C
ATOM	786	HE1	PHE	49	39.679	36.105	28.441	1.00	0.00	H
ATOM	787	CZ	PHE	49	37.764	35.951	27.320	1.00	0.00	C
ATOM	788	HZ	PHE	49	37.140	36.302	28.129	1.00	0.00	H
ATOM	789	CE2	PHE	49	37.135	35.741	26.049	1.00	0.00	C
ATOM	790	HE2	PHE	49	36.079	35.913	25.905	1.00	0.00	H
ATOM	791	CD2	PHE	49	37.879	35.083	25.088	1.00	0.00	C
ATOM	792	HD2	PHE	49	37.486	34.900	24.098	1.00	0.00	H
ATOM	793	C	PHE	49	41.003	32.326	25.583	1.00	0.00	C
ATOM	794	O	PHE	49	42.162	32.569	25.833	1.00	0.00	O
ATOM	795	N	CYX	50	40.239	31.661	26.450	1.00	0.00	N
ATOM	796	H	CYX	50	39.239	31.654	26.312	1.00	0.00	H
ATOM	797	CA	CYX	50	40.753	31.130	27.732	1.00	0.00	C
ATOM	798	HA	CYX	50	41.773	30.817	27.512	1.00	0.00	H
ATOM	799	CB	CYX	50	40.788	32.102	28.889	1.00	0.00	C
ATOM	800	HB2	CYX	50	39.913	32.749	28.950	1.00	0.00	H
ATOM	801	HB3	CYX	50	40.723	31.469	29.774	1.00	0.00	H
ATOM	802	SG	CYX	50	42.256	33.160	28.962	1.00	0.00	S
ATOM	803	C	CYX	50	39.987	29.900	28.154	1.00	0.00	C
ATOM	804	O	CYX	50	38.812	29.968	28.302	1.00	0.00	O
ATOM	805	N	THR	51	40.735	28.829	28.468	1.00	0.00	N
ATOM	806	H	THR	51	41.736	28.891	28.355	1.00	0.00	H

ATOM	807	CA	THR	51	40.317	27.849	29.507	1.00	0.00	C
ATOM	808	HA	THR	51	39.241	27.672	29.528	1.00	0.00	H
ATOM	809	CB	THR	51	41.033	26.565	29.241	1.00	0.00	C
ATOM	810	HB	THR	51	42.122	26.588	29.283	1.00	0.00	H
ATOM	811	CG2	THR	51	40.565	25.357	30.039	1.00	0.00	C
ATOM	812	HG21	THR	51	41.230	24.541	29.757	1.00	0.00	H
ATOM	813	HG22	THR	51	40.744	25.471	31.109	1.00	0.00	H
ATOM	814	HG23	THR	51	39.521	25.115	29.843	1.00	0.00	H
ATOM	815	OG1	THR	51	40.739	26.103	27.866	1.00	0.00	O
ATOM	816	HG1	THR	51	40.847	26.864	27.291	1.00	0.00	H
ATOM	817	C	THR	51	40.749	28.349	30.902	1.00	0.00	C
ATOM	818	O	THR	51	41.835	28.976	31.029	1.00	0.00	O
ATOM	819	N	ILE	52	39.889	28.308	31.826	1.00	0.00	N
ATOM	820	H	ILE	52	38.999	27.850	31.688	1.00	0.00	H
ATOM	821	CA	ILE	52	39.996	28.951	33.124	1.00	0.00	C
ATOM	822	HA	ILE	52	40.986	29.374	33.298	1.00	0.00	H
ATOM	823	CB	ILE	52	38.935	30.018	33.279	1.00	0.00	C
ATOM	824	HB	ILE	52	38.039	29.605	32.816	1.00	0.00	H
ATOM	825	CG2	ILE	52	38.682	30.299	34.783	1.00	0.00	C
ATOM	826	HG21	ILE	52	37.906	31.061	34.859	1.00	0.00	H
ATOM	827	HG22	ILE	52	38.260	29.425	35.279	1.00	0.00	H
ATOM	828	HG23	ILE	52	39.611	30.710	35.177	1.00	0.00	H
ATOM	829	CG1	ILE	52	39.339	31.304	32.504	1.00	0.00	C
ATOM	830	HG12	ILE	52	40.219	31.760	32.958	1.00	0.00	H
ATOM	831	HG13	ILE	52	39.608	30.967	31.502	1.00	0.00	H
ATOM	832	CD1	ILE	52	38.156	32.268	32.365	1.00	0.00	C
ATOM	833	HD11	ILE	52	38.408	33.098	31.705	1.00	0.00	H
ATOM	834	HD12	ILE	52	37.277	31.764	31.963	1.00	0.00	H
ATOM	835	HD13	ILE	52	37.760	32.672	33.296	1.00	0.00	H
ATOM	836	C	ILE	52	39.738	27.868	34.169	1.00	0.00	C
ATOM	837	O	ILE	52	38.758	27.186	34.054	1.00	0.00	O
ATOM	838	N	GLU	53	40.521	27.900	35.239	1.00	0.00	N
ATOM	839	H	GLU	53	41.118	28.687	35.447	1.00	0.00	H
ATOM	840	CA	GLU	53	40.334	27.043	36.384	1.00	0.00	C
ATOM	841	HA	GLU	53	39.608	26.282	36.099	1.00	0.00	H
ATOM	842	CB	GLU	53	41.732	26.428	36.584	1.00	0.00	C
ATOM	843	HB2	GLU	53	42.089	25.851	35.731	1.00	0.00	H
ATOM	844	HB3	GLU	53	42.478	27.221	36.523	1.00	0.00	H
ATOM	845	CG	GLU	53	42.015	25.582	37.815	1.00	0.00	C
ATOM	846	HG2	GLU	53	41.544	25.942	38.729	1.00	0.00	H
ATOM	847	HG3	GLU	53	41.585	24.608	37.582	1.00	0.00	H
ATOM	848	CD	GLU	53	43.526	25.518	38.165	1.00	0.00	C
ATOM	849	OE1	GLU	53	44.304	24.841	37.459	1.00	0.00	O
ATOM	850	OE2	GLU	53	43.929	26.065	39.241	1.00	0.00	O
ATOM	851	C	GLU	53	39.923	27.949	37.572	1.00	0.00	C
ATOM	852	O	GLU	53	40.305	29.126	37.659	1.00	0.00	O
ATOM	853	N	VAL	54	39.078	27.426	38.462	1.00	0.00	N
ATOM	854	H	VAL	54	38.740	26.495	38.263	1.00	0.00	H
ATOM	855	CA	VAL	54	38.569	28.187	39.615	1.00	0.00	C
ATOM	856	HA	VAL	54	38.571	29.271	39.512	1.00	0.00	H
ATOM	857	CB	VAL	54	37.087	27.842	39.874	1.00	0.00	C
ATOM	858	HB	VAL	54	36.866	26.777	39.797	1.00	0.00	H
ATOM	859	CG1	VAL	54	36.574	28.361	41.219	1.00	0.00	C
ATOM	860	HG11	VAL	54	35.517	28.121	41.338	1.00	0.00	H
ATOM	861	HG12	VAL	54	37.094	27.971	42.093	1.00	0.00	H
ATOM	862	HG13	VAL	54	36.644	29.448	41.258	1.00	0.00	H
ATOM	863	CG2	VAL	54	36.220	28.505	38.846	1.00	0.00	C
ATOM	864	HG21	VAL	54	36.356	29.581	38.738	1.00	0.00	H
ATOM	865	HG22	VAL	54	36.441	27.911	37.959	1.00	0.00	H
ATOM	866	HG23	VAL	54	35.183	28.406	39.164	1.00	0.00	H
ATOM	867	C	VAL	54	39.553	27.890	40.701	1.00	0.00	C
ATOM	868	O	VAL	54	39.753	26.759	41.131	1.00	0.00	O
ATOM	869	N	THR	55	40.286	28.886	41.234	1.00	0.00	N
ATOM	870	H	THR	55	40.015	29.822	40.968	1.00	0.00	H

ATOM	871	CA	THR	55	41.405	28.841	42.213	1.00	0.00	C
ATOM	872	HA	THR	55	41.859	27.851	42.175	1.00	0.00	H
ATOM	873	CB	THR	55	42.585	29.851	41.845	1.00	0.00	C
ATOM	874	HB	THR	55	43.395	29.738	42.566	1.00	0.00	H
ATOM	875	CG2	THR	55	43.169	29.767	40.416	1.00	0.00	C
ATOM	876	HG21	THR	55	42.449	30.190	39.715	1.00	0.00	H
ATOM	877	HG22	THR	55	44.111	30.312	40.369	1.00	0.00	H
ATOM	878	HG23	THR	55	43.185	28.686	40.276	1.00	0.00	H
ATOM	879	OG1	THR	55	42.150	31.211	42.088	1.00	0.00	O
ATOM	880	HG1	THR	55	42.409	31.407	42.992	1.00	0.00	H
ATOM	881	C	THR	55	41.044	28.928	43.710	1.00	0.00	C
ATOM	882	O	THR	55	41.823	28.404	44.540	1.00	0.00	O
ATOM	883	N	ALA	56	39.860	29.420	44.047	1.00	0.00	N
ATOM	884	H	ALA	56	39.304	29.659	43.238	1.00	0.00	H
ATOM	885	CA	ALA	56	39.286	29.461	45.436	1.00	0.00	C
ATOM	886	HA	ALA	56	39.447	28.459	45.837	1.00	0.00	H
ATOM	887	CB	ALA	56	40.003	30.474	46.346	1.00	0.00	C
ATOM	888	HB1	ALA	56	39.767	31.500	46.062	1.00	0.00	H
ATOM	889	HB2	ALA	56	39.567	30.472	47.345	1.00	0.00	H
ATOM	890	HB3	ALA	56	41.060	30.214	46.296	1.00	0.00	H
ATOM	891	C	ALA	56	37.807	29.615	45.465	1.00	0.00	C
ATOM	892	O	ALA	56	37.244	30.323	44.633	1.00	0.00	O
ATOM	893	N	THR	57	37.071	29.077	46.401	1.00	0.00	N
ATOM	894	H	THR	57	37.580	28.336	46.860	1.00	0.00	H
ATOM	895	CA	THR	57	35.659	29.397	46.750	1.00	0.00	C
ATOM	896	HA	THR	57	35.408	30.411	46.441	1.00	0.00	H
ATOM	897	CB	THR	57	34.689	28.510	45.923	1.00	0.00	C
ATOM	898	HB	THR	57	33.662	28.739	46.212	1.00	0.00	H
ATOM	899	CG2	THR	57	34.670	28.601	44.362	1.00	0.00	C
ATOM	900	HG21	THR	57	33.902	27.912	44.011	1.00	0.00	H
ATOM	901	HG22	THR	57	34.511	29.600	43.958	1.00	0.00	H
ATOM	902	HG23	THR	57	35.635	28.284	43.968	1.00	0.00	H
ATOM	903	OG1	THR	57	35.024	27.130	46.097	1.00	0.00	O
ATOM	904	HG1	THR	57	35.975	27.092	46.224	1.00	0.00	H
ATOM	905	C	THR	57	35.281	29.266	48.226	1.00	0.00	C
ATOM	906	O	THR	57	35.834	28.361	48.869	1.00	0.00	O
ATOM	907	N	SER	58	34.344	30.035	48.652	1.00	0.00	N
ATOM	908	H	SER	58	33.999	30.671	47.948	1.00	0.00	H
ATOM	909	CA	SER	58	33.605	29.847	49.946	1.00	0.00	C
ATOM	910	HA	SER	58	33.363	28.786	49.998	1.00	0.00	H
ATOM	911	CB	SER	58	34.600	30.068	51.051	1.00	0.00	C
ATOM	912	HB2	SER	58	35.426	29.359	51.115	1.00	0.00	H
ATOM	913	HB3	SER	58	35.019	31.058	50.873	1.00	0.00	H
ATOM	914	OG	SER	58	33.991	30.044	52.351	1.00	0.00	O
ATOM	915	HG	SER	58	33.984	29.117	52.600	1.00	0.00	H
ATOM	916	C	SER	58	32.352	30.694	50.149	1.00	0.00	C
ATOM	917	O	SER	58	32.075	31.586	49.319	1.00	0.00	O
ATOM	918	N	THR	59	31.501	30.388	51.135	1.00	0.00	N
ATOM	919	H	THR	59	31.847	29.718	51.807	1.00	0.00	H
ATOM	920	CA	THR	59	30.305	31.112	51.590	1.00	0.00	C
ATOM	921	HA	THR	59	29.875	31.611	50.722	1.00	0.00	H
ATOM	922	CB	THR	59	29.372	30.196	52.244	1.00	0.00	C
ATOM	923	HB	THR	59	29.475	29.272	51.674	1.00	0.00	H
ATOM	924	CG2	THR	59	29.696	29.886	53.736	1.00	0.00	C
ATOM	925	HG21	THR	59	30.589	29.261	53.746	1.00	0.00	H
ATOM	926	HG22	THR	59	29.825	30.766	54.366	1.00	0.00	H
ATOM	927	HG23	THR	59	28.926	29.221	54.127	1.00	0.00	H
ATOM	928	OG1	THR	59	28.043	30.725	52.126	1.00	0.00	O
ATOM	929	HG1	THR	59	27.509	30.125	51.600	1.00	0.00	H
ATOM	930	C	THR	59	30.683	32.285	52.460	1.00	0.00	C
ATOM	931	O	THR	59	31.865	32.382	52.929	1.00	0.00	O
ATOM	932	N	VAL	60	29.851	33.223	52.656	1.00	0.00	N
ATOM	933	H	VAL	60	28.959	33.148	52.189	1.00	0.00	H

ATOM	934	CA	VAL	60	30.238	34.471	53.298	1.00	0.00	C
ATOM	935	HA	VAL	60	30.792	34.329	54.227	1.00	0.00	H
ATOM	936	CB	VAL	60	31.272	35.194	52.383	1.00	0.00	C
ATOM	937	HB	VAL	60	31.874	34.377	51.985	1.00	0.00	H
ATOM	938	CG1	VAL	60	30.702	35.864	51.136	1.00	0.00	C
ATOM	939	HG11	VAL	60	30.154	35.066	50.635	1.00	0.00	H
ATOM	940	HG12	VAL	60	30.082	36.732	51.361	1.00	0.00	H
ATOM	941	HG13	VAL	60	31.514	36.249	50.519	1.00	0.00	H
ATOM	942	CG2	VAL	60	32.167	36.283	52.940	1.00	0.00	C
ATOM	943	HG21	VAL	60	32.574	35.936	53.890	1.00	0.00	H
ATOM	944	HG22	VAL	60	32.887	36.589	52.181	1.00	0.00	H
ATOM	945	HG23	VAL	60	31.598	37.163	53.234	1.00	0.00	H
ATOM	946	C	VAL	60	28.967	35.226	53.554	1.00	0.00	C
ATOM	947	O	VAL	60	27.951	35.039	52.900	1.00	0.00	O
ATOM	948	N	THR	61	29.088	36.242	54.391	1.00	0.00	N
ATOM	949	H	THR	61	30.042	36.561	54.488	1.00	0.00	H
ATOM	950	CA	THR	61	28.055	37.224	54.677	1.00	0.00	C
ATOM	951	HA	THR	61	27.166	36.976	54.097	1.00	0.00	H
ATOM	952	CB	THR	61	27.773	37.278	56.203	1.00	0.00	C
ATOM	953	HB	THR	61	26.950	37.988	56.293	1.00	0.00	H
ATOM	954	CG2	THR	61	27.314	35.976	56.796	1.00	0.00	C
ATOM	955	HG21	THR	61	26.420	35.568	56.323	1.00	0.00	H
ATOM	956	HG22	THR	61	28.112	35.234	56.819	1.00	0.00	H
ATOM	957	HG23	THR	61	26.956	36.209	57.799	1.00	0.00	H
ATOM	958	OG1	THR	61	28.869	37.776	56.939	1.00	0.00	O
ATOM	959	HG1	THR	61	28.475	38.343	57.606	1.00	0.00	H
ATOM	960	C	THR	61	28.448	38.615	54.149	1.00	0.00	C
ATOM	961	O	THR	61	29.471	38.836	53.537	1.00	0.00	O
ATOM	962	N	LEU	62	27.587	39.608	54.365	1.00	0.00	N
ATOM	963	H	LEU	62	26.729	39.491	54.885	1.00	0.00	H
ATOM	964	CA	LEU	62	27.866	41.012	53.939	1.00	0.00	C
ATOM	965	HA	LEU	62	28.125	41.045	52.880	1.00	0.00	H
ATOM	966	CB	LEU	62	26.624	41.908	54.115	1.00	0.00	C
ATOM	967	HB2	LEU	62	25.806	41.354	53.652	1.00	0.00	H
ATOM	968	HB3	LEU	62	26.319	41.928	55.161	1.00	0.00	H
ATOM	969	CG	LEU	62	26.672	43.325	53.588	1.00	0.00	C
ATOM	970	HG	LEU	62	27.706	43.651	53.701	1.00	0.00	H
ATOM	971	CD1	LEU	62	26.169	43.475	52.203	1.00	0.00	C
ATOM	972	HD11	LEU	62	26.248	44.494	51.823	1.00	0.00	H
ATOM	973	HD12	LEU	62	26.587	42.737	51.517	1.00	0.00	H
ATOM	974	HD13	LEU	62	25.095	43.331	52.321	1.00	0.00	H
ATOM	975	CD2	LEU	62	26.013	44.317	54.506	1.00	0.00	C
ATOM	976	HD21	LEU	62	24.932	44.184	54.541	1.00	0.00	H
ATOM	977	HD22	LEU	62	26.358	44.171	55.530	1.00	0.00	H
ATOM	978	HD23	LEU	62	26.136	45.340	54.148	1.00	0.00	H
ATOM	979	C	LEU	62	29.055	41.601	54.817	1.00	0.00	C
ATOM	980	O	LEU	62	30.023	42.181	54.312	1.00	0.00	O
ATOM	981	N	ASP	63	28.883	41.512	56.118	1.00	0.00	N
ATOM	982	H	ASP	63	28.205	40.867	56.496	1.00	0.00	H
ATOM	983	CA	ASP	63	29.783	42.048	57.173	1.00	0.00	C
ATOM	984	HA	ASP	63	30.063	42.996	56.714	1.00	0.00	H
ATOM	985	CB	ASP	63	29.117	42.110	58.541	1.00	0.00	C
ATOM	986	HB2	ASP	63	29.839	42.393	59.308	1.00	0.00	H
ATOM	987	HB3	ASP	63	28.370	42.894	58.668	1.00	0.00	H
ATOM	988	CG	ASP	63	28.520	40.814	59.131	1.00	0.00	C
ATOM	989	OD1	ASP	63	28.038	39.921	58.414	1.00	0.00	O
ATOM	990	OD2	ASP	63	28.457	40.637	60.369	1.00	0.00	O
ATOM	991	C	ASP	63	31.174	41.383	57.240	1.00	0.00	C
ATOM	992	O	ASP	63	32.056	41.883	57.879	1.00	0.00	O
ATOM	993	N	THR	64	31.312	40.226	56.522	1.00	0.00	N
ATOM	994	H	THR	64	30.468	39.814	56.150	1.00	0.00	H
ATOM	995	CA	THR	64	32.594	39.483	56.337	1.00	0.00	C
ATOM	996	HA	THR	64	33.343	39.943	56.981	1.00	0.00	H
ATOM	997	CB	THR	64	32.527	38.026	56.833	1.00	0.00	C

```
ATOM    998  HB  THR    64      33.521  37.582  56.897  1.00  0.00           H
ATOM    999  CG2 THR    64      32.064  37.946  58.246  1.00  0.00           C
ATOM   1000 HG21 THR    64      32.647  38.653  58.835  1.00  0.00           H
ATOM   1001 HG22 THR    64      30.999  38.149  58.358  1.00  0.00           H
ATOM   1002 HG23 THR    64      32.263  36.965  58.677  1.00  0.00           H
ATOM   1003  OG1 THR    64      31.662  37.217  56.173  1.00  0.00           O
ATOM   1004  HG1 THR    64      30.881  37.138  56.726  1.00  0.00           H
ATOM   1005  C   THR    64      33.113  39.462  54.869  1.00  0.00           C
ATOM   1006  O   THR    64      33.804  38.566  54.324  1.00  0.00           O
ATOM   1007  N   LEU    65      32.552  40.352  54.041  1.00  0.00           N
ATOM   1008  H   LEU    65      31.772  40.885  54.401  1.00  0.00           H
ATOM   1009  CA  LEU    65      32.759  40.418  52.553  1.00  0.00           C
ATOM   1010  HA  LEU    65      32.323  39.551  52.056  1.00  0.00           H
ATOM   1011  CB  LEU    65      32.032  41.688  51.873  1.00  0.00           C
ATOM   1012  HB2 LEU    65      32.034  42.492  52.610  1.00  0.00           H
ATOM   1013  HB3 LEU    65      32.614  42.040  51.021  1.00  0.00           H
ATOM   1014  CG  LEU    65      30.582  41.381  51.380  1.00  0.00           C
ATOM   1015  HG  LEU    65      30.106  40.836  52.195  1.00  0.00           H
ATOM   1016  CD1 LEU    65      29.863  42.688  51.094  1.00  0.00           C
ATOM   1017 HD11 LEU    65      28.788  42.521  51.022  1.00  0.00           H
ATOM   1018 HD12 LEU    65      29.987  43.394  51.915  1.00  0.00           H
ATOM   1019 HD13 LEU    65      30.255  43.123  50.174  1.00  0.00           H
ATOM   1020  CD2 LEU    65      30.614  40.543  50.059  1.00  0.00           C
ATOM   1021 HD21 LEU    65      31.393  39.787  50.158  1.00  0.00           H
ATOM   1022 HD22 LEU    65      29.655  40.179  49.691  1.00  0.00           H
ATOM   1023 HD23 LEU    65      31.034  41.110  49.229  1.00  0.00           H
ATOM   1024  C   LEU    65      34.210  40.542  52.110  1.00  0.00           C
ATOM   1025  O   LEU    65      34.948  41.257  52.800  1.00  0.00           O
ATOM   1026  N   THR    66      34.708  39.938  51.059  1.00  0.00           N
ATOM   1027  H   THR    66      34.129  39.333  50.494  1.00  0.00           H
ATOM   1028  CA  THR    66      36.164  40.032  50.737
  1.00  0.00           C
ATOM   1029  HA  THR    66      36.689  39.821  51.669  1.00  0.00           H
ATOM   1030  CB  THR    66      36.693  38.892  49.870  1.00  0.00           C
ATOM   1031  HB  THR    66      36.683  38.091  50.610  1.00  0.00           H
ATOM   1032  CG2 THR    66      35.782  38.692  48.621  1.00  0.00           C
ATOM   1033 HG21 THR    66      36.156  37.819  48.085  1.00  0.00           H
ATOM   1034 HG22 THR    66      34.757  38.464  48.913  1.00  0.00           H
ATOM   1035 HG23 THR    66      35.789  39.577  47.984  1.00  0.00           H
ATOM   1036  OG1 THR    66      37.993  39.149  49.439  1.00  0.00           O
ATOM   1037  HG1 THR    66      38.544  38.843  50.163  1.00  0.00           H
ATOM   1038  C   THR    66      36.578  41.398  50.216  1.00  0.00           C
ATOM   1039  O   THR    66      36.113  41.933  49.200  1.00  0.00           O
ATOM   1040  N   GLU    67      37.596  41.931  50.904  1.00  0.00           N
ATOM   1041  H   GLU    67      37.889  41.519  51.778  1.00  0.00           H
ATOM   1042  CA  GLU    67      38.357  43.203  50.605  1.00  0.00           C
ATOM   1043  HA  GLU    67      37.684  44.031  50.385  1.00  0.00           H
ATOM   1044  CB  GLU    67      39.260  43.494  51.798  1.00  0.00           C
ATOM   1045  HB2 GLU    67      39.699  44.466  51.579  1.00  0.00           H
ATOM   1046  HB3 GLU    67      38.677  43.513  52.719  1.00  0.00           H
ATOM   1047  CG  GLU    67      40.461  42.640  52.214  1.00  0.00           C
ATOM   1048  HG2 GLU    67      40.993  42.242  51.350  1.00  0.00           H
ATOM   1049  HG3 GLU    67      41.148  43.111  52.917  1.00  0.00           H
ATOM   1050  CD  GLU    67      40.017  41.299  52.851  1.00  0.00           C
ATOM   1051  OE1 GLU    67      40.595  40.239  52.537  1.00  0.00           O
ATOM   1052  OE2 GLU    67      39.045  41.229  53.604  1.00  0.00           O
ATOM   1053  C   GLU    67      39.265  43.100  49.319  1.00  0.00           C
ATOM   1054  O   GLU    67      39.669  44.142  48.802  1.00  0.00           O
ATOM   1055  N   LYS    68      39.297  41.883  48.667  1.00  0.00           N
ATOM   1056  H   LYS    68      38.771  41.138  49.102  1.00  0.00           H
ATOM   1057  CA  LYS    68      39.995  41.702  47.343  1.00  0.00           C
ATOM   1058  HA  LYS    68      40.846  42.381  47.324  1.00  0.00           H
ATOM   1059  CB  LYS    68      40.382  40.264  47.050  1.00  0.00           C
ATOM   1060  HB2 LYS    68      39.461  39.681  47.086  1.00  0.00           H
```

ATOM	1061	HB3	LYS	68	40.736	40.047	46.041	1.00	0.00	H
ATOM	1062	CG	LYS	68	41.446	39.729	48.052	1.00	0.00	C
ATOM	1063	HG2	LYS	68	42.326	40.369	48.017	1.00	0.00	H
ATOM	1064	HG3	LYS	68	41.105	39.745	49.088	1.00	0.00	H
ATOM	1065	CD	LYS	68	41.901	38.282	47.709	1.00	0.00	C
ATOM	1066	HD2	LYS	68	42.192	38.223	46.660	1.00	0.00	H
ATOM	1067	HD3	LYS	68	42.751	38.060	48.354	1.00	0.00	H
ATOM	1068	CE	LYS	68	40.860	37.242	48.059	1.00	0.00	C
ATOM	1069	HE2	LYS	68	40.524	37.323	49.094	1.00	0.00	H
ATOM	1070	HE3	LYS	68	39.928	37.375	47.509	1.00	0.00	H
ATOM	1071	NZ	LYS	68	41.392	35.872	47.876	1.00	0.00	N
ATOM	1072	HZ1	LYS	68	40.767	35.147	48.197	1.00	0.00	H
ATOM	1073	HZ2	LYS	68	41.688	35.714	46.922	1.00	0.00	H
ATOM	1074	HZ3	LYS	68	42.252	35.783	48.397	1.00	0.00	H
ATOM	1075	C	LYS	68	39.136	42.202	46.158	1.00	0.00	C
ATOM	1076	O	LYS	68	39.590	43.050	45.406	1.00	0.00	O
ATOM	1077	N	HIE	69	37.877	41.825	46.123	1.00	0.00	N
ATOM	1078	H	HIE	69	37.526	41.115	46.751	1.00	0.00	H
ATOM	1079	CA	HIE	69	36.856	42.390	45.259	1.00	0.00	C
ATOM	1080	HA	HIE	69	37.031	42.171	44.206	1.00	0.00	H
ATOM	1081	CB	HIE	69	35.579	41.673	45.626	1.00	0.00	C
ATOM	1082	HB2	HIE	69	35.910	40.642	45.738	1.00	0.00	H
ATOM	1083	HB3	HIE	69	35.232	42.049	46.589	1.00	0.00	H
ATOM	1084	CG	HIE	69	34.529	41.826	44.570	1.00	0.00	C
ATOM	1085	ND1	HIE	69	34.478	41.088	43.419	1.00	0.00	N
ATOM	1086	CE1	HIE	69	33.493	41.546	42.736	1.00	0.00	C
ATOM	1087	HE1	HIE	69	33.136	41.094	41.823	1.00	0.00	H
ATOM	1088	NE2	HIE	69	32.958	42.605	43.245	1.00	0.00	N
ATOM	1089	HE2	HIE	69	32.128	43.131	43.011	1.00	0.00	H
ATOM	1090	CD2	HIE	69	33.574	42.794	44.467	1.00	0.00	C
ATOM	1091	HD2	HIE	69	33.377	43.586	45.174	1.00	0.00	H
ATOM	1092	C	HIE	69	36.667	43.920	45.296	1.00	0.00	C
ATOM	1093	O	HIE	69	36.638	44.546	44.215	1.00	0.00	O
ATOM	1094	N	ALA	70	36.690	44.500	46.460	1.00	0.00	N
ATOM	1095	H	ALA	70	36.500	43.878	47.232	1.00	0.00	H
ATOM	1096	CA	ALA	70	36.736	45.962	46.714	1.00	0.00	C
ATOM	1097	HA	ALA	70	35.726	46.280	46.454	1.00	0.00	H
ATOM	1098	CB	ALA	70	37.067	46.286	48.226	1.00	0.00	C
ATOM	1099	HB1	ALA	70	36.157	46.086	48.794	1.00	0.00	H
ATOM	1100	HB2	ALA	70	37.887	45.669	48.593	1.00	0.00	H
ATOM	1101	HB3	ALA	70	37.275	47.348	48.352	1.00	0.00	H
ATOM	1102	C	ALA	70	37.655	46.718	45.746	1.00	0.00	C
ATOM	1103	O	ALA	70	37.302	47.536	44.896	1.00	0.00	O
ATOM	1104	N	GLU	71	38.924	46.418	45.793	1.00	0.00	N
ATOM	1105	H	GLU	71	39.225	45.648	46.373	1.00	0.00	H
ATOM	1106	CA	GLU	71	39.980	47.077	45.038	1.00	0.00	C
ATOM	1107	HA	GLU	71	39.942	48.165	45.109	1.00	0.00	H
ATOM	1108	CB	GLU	71	41.357	46.607	45.607	1.00	0.00	C
ATOM	1109	HB2	GLU	71	41.416	45.522	45.527	1.00	0.00	H
ATOM	1110	HB3	GLU	71	42.074	47.136	44.979	1.00	0.00	H
ATOM	1111	CG	GLU	71	41.637	47.033	47.064	1.00	0.00	C
ATOM	1112	HG2	GLU	71	41.144	46.359	47.765	1.00	0.00	H
ATOM	1113	HG3	GLU	71	42.676	46.964	47.386	1.00	0.00	H
ATOM	1114	CD	GLU	71	41.203	48.460	47.528	1.00	0.00	C
ATOM	1115	OE1	GLU	71	41.578	49.448	46.872	1.00	0.00	O
ATOM	1116	OE2	GLU	71	40.474	48.641	48.548	1.00	0.00	O
ATOM	1117	C	GLU	71	39.858	46.671	43.531	1.00	0.00	C
ATOM	1118	O	GLU	71	40.459	47.405	42.703	1.00	0.00	O
ATOM	1119	N	GLN	72	39.308	45.511	43.210	1.00	0.00	N
ATOM	1120	H	GLN	72	38.929	44.982	43.982	1.00	0.00	H
ATOM	1121	CA	GLN	72	39.060	45.111	41.795	1.00	0.00	C
ATOM	1122	HA	GLN	72	39.967	45.315	41.225	1.00	0.00	H
ATOM	1123	CB	GLN	72	38.730	43.646	41.668	1.00	0.00	C
ATOM	1124	HB2	GLN	72	37.961	43.377	42.394	1.00	0.00	H

```
ATOM   1125  HB3 GLN    72      39.584  43.001  41.865  1.00  0.00           H
ATOM   1126  CG  GLN    72      38.183  43.234  40.289  1.00  0.00           C
ATOM   1127  HG2 GLN    72      37.560  44.029  39.880  1.00  0.00           H
ATOM   1128  HG3 GLN    72      39.035  43.055  39.633  1.00  0.00           H
ATOM   1129  CD  GLN    72      37.384  41.945  40.325  1.00  0.00           C
ATOM   1130  OE1 GLN    72      37.834  40.893  39.984  1.00  0.00           O
ATOM   1131  NE2 GLN    72      36.200  41.937  40.928  1.00  0.00           N
ATOM   1132 HE21 GLN    72      35.830  42.706  41.468  1.00  0.00           H
ATOM   1133 HE22 GLN    72      35.902  40.992  41.124  1.00  0.00           H
ATOM   1134  C   GLN    72      37.948  46.047  41.248  1.00  0.00           C
ATOM   1135  O   GLN    72      38.182  46.724  40.236  1.00  0.00           O
ATOM   1136  N   GLU    73      36.858  46.198  41.977  1.00  0.00           N
ATOM   1137  H   GLU    73      36.775  45.727  42.867  1.00  0.00           H
ATOM   1138  CA  GLU    73      35.818  47.177  41.647  1.00  0.00           C
ATOM   1139  HA  GLU    73      35.466  46.987  40.633  1.00  0.00           H
ATOM   1140  CB  GLU    73      34.549  46.922  42.468  1.00  0.00           C
ATOM   1141  HB2 GLU    73      34.739  46.772  43.530  1.00  0.00           H
ATOM   1142  HB3 GLU    73      33.913  47.803  42.549  1.00  0.00           H
ATOM   1143  CG  GLU    73      33.674  45.671  42.068  1.00  0.00           C
ATOM   1144  HG2 GLU    73      34.402  44.859  42.075  1.00  0.00           H
ATOM   1145  HG3 GLU    73      32.836  45.476  42.737  1.00  0.00           H
ATOM   1146  CD  GLU    73      33.152  45.898  40.672  1.00  0.00           C
ATOM   1147  OE1 GLU    73      32.285  46.708  40.431  1.00  0.00           O
ATOM   1148  OE2 GLU    73      33.495  45.189  39.710  1.00  0.00           O
ATOM   1149  C   GLU    73      36.284  48.671  41.837  1.00  0.00           C
ATOM   1150  O   GLU    73      35.517  49.554  41.473  1.00  0.00           O
ATOM   1151  N   ASN    74      37.474  48.896  42.348  1.00  0.00           N
ATOM   1152  H   ASN    74      37.998  48.060  42.562  1.00  0.00           H
ATOM   1153  CA  ASN    74      38.010  50.260  42.785  1.00  0.00           C
ATOM
   1154  HA  ASN    74      38.994  50.169  43.245  1.00  0.00           H
ATOM   1155  CB  ASN    74      38.353  51.132  41.528  1.00  0.00           C
ATOM   1156  HB2 ASN    74      37.462  51.360  40.943  1.00  0.00           H
ATOM   1157  HB3 ASN    74      38.817  52.036  41.922  1.00  0.00           H
ATOM   1158  CG  ASN    74      39.287  50.548  40.595  1.00  0.00           C
ATOM   1159  OD1 ASN    74      39.329  50.995  39.446  1.00  0.00           O
ATOM   1160  ND2 ASN    74      40.112  49.584  40.940  1.00  0.00           N
ATOM   1161 HD21 ASN    74      40.739  49.304  40.201  1.00  0.00           H
ATOM   1162 HD22 ASN    74      39.883  49.040  41.761  1.00  0.00           H
ATOM   1163  C   ASN    74      37.162  50.939  43.885  1.00  0.00           C
ATOM   1164  O   ASN    74      37.268  52.129  44.180  1.00  0.00           O
ATOM   1165  N   MET    75      36.482  50.136  44.715  1.00  0.00           N
ATOM   1166  H   MET    75      36.653  49.140  44.710  1.00  0.00           H
ATOM   1167  CA  MET    75      35.809  50.696  45.938  1.00  0.00           C
ATOM   1168  HA  MET    75      35.618  51.758  45.781  1.00  0.00           H
ATOM   1169  CB  MET    75      34.435  49.942  46.041  1.00  0.00           C
ATOM   1170  HB2 MET    75      34.536  48.856  46.025  1.00  0.00           H
ATOM   1171  HB3 MET    75      33.865  50.241  46.920  1.00  0.00           H
ATOM   1172  CG  MET    75      33.425  50.322  45.024  1.00  0.00           C
ATOM   1173  HG2 MET    75      33.106  51.322  45.318  1.00  0.00           H
ATOM   1174  HG3 MET    75      33.888  50.254  44.041  1.00  0.00           H
ATOM   1175  SD  MET    75      31.975  49.229  45.095  1.00  0.00           S
ATOM   1176  CE  MET    75      30.969  49.903  43.707  1.00  0.00           C
ATOM   1177  HE1 MET    75      30.041  49.369  43.502  1.00  0.00           H
ATOM   1178  HE2 MET    75      30.733  50.939  43.951  1.00  0.00           H
ATOM   1179  HE3 MET    75      31.583  49.823  42.809  1.00  0.00           H
ATOM   1180  C   MET    75      36.561  50.546  47.187  1.00  0.00           C
ATOM   1181  O   MET    75      37.315  49.592  47.344  1.00  0.00           O
ATOM   1182  N   THR    76      36.202  51.395  48.123  1.00  0.00           N
ATOM   1183  H   THR    76      35.792  52.274  47.838  1.00  0.00           H
ATOM   1184  CA  THR    76      36.321  51.059  49.528  1.00  0.00           C
ATOM   1185  HA  THR    76      37.264  50.536  49.676  1.00  0.00           H
ATOM   1186  CB  THR    76      36.439  52.344  50.334  1.00  0.00           C
ATOM   1187  HB  THR    76      36.961  53.075  49.718  1.00  0.00           H
```

ATOM	1188	CG2	THR	76	35.084	52.813	50.879	1.00	0.00	C
ATOM	1189	HG21	THR	76	34.433	53.030	50.032	1.00	0.00	H
ATOM	1190	HG22	THR	76	34.655	52.081	51.563	1.00	0.00	H
ATOM	1191	HG23	THR	76	35.301	53.754	51.383	1.00	0.00	H
ATOM	1192	OG1	THR	76	37.206	52.040	51.483	1.00	0.00	O
ATOM	1193	HG1	THR	76	38.083	51.919	51.115	1.00	0.00	H
ATOM	1194	C	THR	76	35.323	50.080	50.075	1.00	0.00	C
ATOM	1195	O	THR	76	34.163	49.970	49.599	1.00	0.00	O
ATOM	1196	N	LEU	77	35.761	49.296	51.100	1.00	0.00	N
ATOM	1197	H	LEU	77	36.725	49.326	51.399	1.00	0.00	H
ATOM	1198	CA	LEU	77	34.979	48.178	51.636	1.00	0.00	C
ATOM	1199	HA	LEU	77	34.751	47.438	50.868	1.00	0.00	H
ATOM	1200	CB	LEU	77	35.832	47.431	52.660	1.00	0.00	C
ATOM	1201	HB2	LEU	77	36.859	47.270	52.334	1.00	0.00	H
ATOM	1202	HB3	LEU	77	36.021	48.066	53.525	1.00	0.00	H
ATOM	1203	CG	LEU	77	35.156	46.179	53.186	1.00	0.00	C
ATOM	1204	HG	LEU	77	34.230	46.533	53.641	1.00	0.00	H
ATOM	1205	CD1	LEU	77	34.853	45.039	52.242	1.00	0.00	C
ATOM	1206	HD11	LEU	77	34.398	44.219	52.798	1.00	0.00	H
ATOM	1207	HD12	LEU	77	34.195	45.214	51.391	1.00	0.00	H
ATOM	1208	HD13	LEU	77	35.820	44.664	51.906	1.00	0.00	H
ATOM	1209	CD2	LEU	77	35.912	45.565	54.405	1.00	0.00	C
ATOM	1210	HD21	LEU	77	35.292	44.761	54.804	1.00	0.00	H
ATOM	1211	HD22	LEU	77	36.850	45.136	54.052	1.00	0.00	H
ATOM	1212	HD23	LEU	77	36.144	46.216	55.248	1.00	0.00	H
ATOM	1213	C	LEU	77	33.563	48.512	52.260	1.00	0.00	C
ATOM	1214	O	LEU	77	32.646	47.816	51.861	1.00	0.00	O
ATOM	1215	N	THR	78	33.368	49.717	52.864	1.00	0.00	N
ATOM	1216	H	THR	78	34.187	50.238	53.145	1.00	0.00	H
ATOM	1217	CA	THR	78	32.027	50.197	53.207	1.00	0.00	C
ATOM	1218	HA	THR	78	31.568	49.425	53.825	1.00	0.00	H
ATOM	1219	CB	THR	78	32.073	51.394	54.016	1.00	0.00	C
ATOM	1220	HB	THR	78	31.026	51.671	54.139	1.00	0.00	H
ATOM	1221	CG2	THR	78	32.808	51.120	55.393	1.00	0.00	C
ATOM	1222	HG21	THR	78	33.860	50.870	55.258	1.00	0.00	H
ATOM	1223	HG22	THR	78	32.735	51.983	56.055	1.00	0.00	H
ATOM	1224	HG23	THR	78	32.390	50.337	56.025	1.00	0.00	H
ATOM	1225	OG1	THR	78	32.717	52.451	53.336	1.00	0.00	O
ATOM	1226	HG1	THR	78	32.449	53.311	53.671	1.00	0.00	H
ATOM	1227	C	THR	78	31.075	50.548	52.007	1.00	0.00	C
ATOM	1228	O	THR	78	29.877	50.273	52.135	1.00	0.00	O
ATOM	1229	N	GLU	79	31.619	51.069	50.865	1.00	0.00	N
ATOM	1230	H	GLU	79	32.600	51.299	50.941	1.00	0.00	H
ATOM	1231	CA	GLU	79	30.948	51.404	49.593	1.00	0.00	C
ATOM	1232	HA	GLU	79	30.048	51.991	49.774	1.00	0.00	H
ATOM	1233	CB	GLU	79	31.843	52.233	48.597	1.00	0.00	C
ATOM	1234	HB2	GLU	79	32.321	53.007	49.197	1.00	0.00	H
ATOM	1235	HB3	GLU	79	32.620	51.608	48.157	1.00	0.00	H
ATOM	1236	CG	GLU	79	31.101	52.782	47.433	1.00	0.00	C
ATOM	1237	HG2	GLU	79	31.868	53.102	46.727	1.00	0.00	H
ATOM	1238	HG3	GLU	79	30.447	52.005	47.038	1.00	0.00	H
ATOM	1239	CD	GLU	79	30.295	54.030	47.805	1.00	0.00	C
ATOM	1240	OE1	GLU	79	30.162	54.464	49.005	1.00	0.00	O
ATOM	1241	OE2	GLU	79	29.730	54.654	46.827	1.00	0.00	O
ATOM	1242	C	GLU	79	30.391	50.035	48.961	1.00	0.00	C
ATOM	1243	O	GLU	79	29.296	49.938	48.389	1.00	0.00	O
ATOM	1244	N	LEU	80	31.219	48.936	49.027	1.00	0.00	N
ATOM	1245	H	LEU	80	32.144	49.129	49.383	1.00	0.00	H
ATOM	1246	CA	LEU	80	30.821	47.611	48.465	1.00	0.00	C
ATOM	1247	HA	LEU	80	30.386	47.637	47.466	1.00	0.00	H
ATOM	1248	CB	LEU	80	32.112	46.646	48.480	1.00	0.00	C
ATOM	1249	HB2	LEU	80	32.882	47.272	48.030	1.00	0.00	H
ATOM	1250	HB3	LEU	80	32.306	46.499	49.542	1.00	0.00	H
ATOM	1251	CG	LEU	80	31.935	45.314	47.721	1.00	0.00	C

ATOM 1252 HG LEU 80 31.249 44.644 48.241 1.00 0.00 H
ATOM 1253 CD1 LEU 80 31.479 45.347 46.273 1.00 0.00 C
ATOM 1254 HD11 LEU 80 31.233 44.336 45.948 1.00 0.00 H
ATOM 1255 HD12 LEU 80 30.572 45.930 46.116 1.00 0.00 H
ATOM 1256 HD13 LEU 80 32.206 45.828 45.619 1.00 0.00 H
ATOM 1257 CD2 LEU 80 33.339 44.546 47.810 1.00 0.00 C
ATOM 1258 HD21 LEU 80 34.070 45.054 47.181 1.00 0.00 H
ATOM 1259 HD22 LEU 80 33.820 44.744 48.768 1.00 0.00 H
ATOM 1260 HD23 LEU 80 33.272 43.494 47.530 1.00 0.00 H
ATOM 1261 C LEU 80 29.781 46.991 49.402 1.00 0.00 C
ATOM 1262 O LEU 80 28.788 46.502 48.982 1.00 0.00 O
ATOM 1263 N LYS 81 29.922 47.178 50.707 1.00 0.00 N
ATOM 1264 H LYS 81 30.803 47.531 51.055 1.00 0.00 H
ATOM 1265 CA LYS 81 28.822 46.860 51.680 1.00 0.00 C
ATOM 1266 HA LYS 81 28.577 45.808 51.528 1.00 0.00 H
ATOM 1267 CB LYS 81 29.338 46.990 53.129 1.00 0.00 C
ATOM 1268 HB2 LYS 81 29.808 47.965 53.244 1.00 0.00 H
ATOM 1269 HB3 LYS 81 28.448 46.865 53.746 1.00 0.00 H
ATOM 1270 CG LYS 81 30.244 45.711 53.397 1.00 0.00 C
ATOM 1271 HG2 LYS 81 29.662 44.788 53.373 1.00 0.00 H
ATOM 1272 HG3 LYS 81 31.111 45.667 52.739 1.00 0.00 H
ATOM 1273 CD LYS 81 30.740 45.648 54.861 1.00 0.00 C
ATOM 1274 HD2 LYS 81 31.239 46.588 55.098 1.00 0.00 H
ATOM 1275 HD3 LYS 81 29.898 45.573 55.549 1.00 0.00 H
ATOM 1276 CE LYS 81 31.747 44.553 55.085 1.00 0.00 C
ATOM 1277 HE2 LYS 81 31.311 43.560 54.979 1.00 0.00 H
ATOM 1278 HE3 LYS 81 32.482 44.693 54.292 1.00 0.00 H
ATOM 1279 NZ LYS 81
32.357 44.764 56.410 1.00 0.00 N
ATOM 1280 HZ1 LYS 81 32.603 45.733 56.554 1.00 0.00 H
ATOM 1281 HZ2 LYS 81 31.725 44.451 57.134 1.00 0.00 H
ATOM 1282 HZ3 LYS 81 33.168 44.180 56.555 1.00 0.00 H
ATOM 1283 C LYS 81 27.558 47.661 51.319 1.00 0.00 C
ATOM 1284 O LYS 81 26.481 47.078 51.328 1.00 0.00 O
ATOM 1285 N LYS 82 27.695 48.967 50.999 1.00 0.00 N
ATOM 1286 H LYS 82 28.614 49.385 50.965 1.00 0.00 H
ATOM 1287 CA LYS 82 26.558 49.885 50.744 1.00 0.00 C
ATOM 1288 HA LYS 82 25.929 49.893 51.634 1.00 0.00 H
ATOM 1289 CB LYS 82 27.059 51.347 50.635 1.00 0.00 C
ATOM 1290 HB2 LYS 82 27.637 51.586 51.528 1.00 0.00 H
ATOM 1291 HB3 LYS 82 27.702 51.471 49.763 1.00 0.00 H
ATOM 1292 CG LYS 82 25.984 52.449 50.507 1.00 0.00 C
ATOM 1293 HG2 LYS 82 25.315 52.194 49.685 1.00 0.00 H
ATOM 1294 HG3 LYS 82 25.350 52.515 51.390 1.00 0.00 H
ATOM 1295 CD LYS 82 26.558 53.837 50.114 1.00 0.00 C
ATOM 1296 HD2 LYS 82 27.169 53.642 49.232 1.00 0.00 H
ATOM 1297 HD3 LYS 82 25.712 54.479 49.864 1.00 0.00 H
ATOM 1298 CE LYS 82 27.383 54.413 51.244 1.00 0.00 C
ATOM 1299 HE2 LYS 82 26.759 54.798 52.049 1.00 0.00 H
ATOM 1300 HE3 LYS 82 28.003 53.710 51.801 1.00 0.00 H
ATOM 1301 NZ LYS 82 28.182 55.536 50.685 1.00 0.00 N
ATOM 1302 HZ1 LYS 82 28.582 56.140 51.389 1.00 0.00 H
ATOM 1303 HZ2 LYS 82 29.018 55.154 50.267 1.00 0.00 H
ATOM 1304 HZ3 LYS 82 27.535 56.046 50.099 1.00 0.00 H
ATOM 1305 C LYS 82 25.741 49.534 49.542 1.00 0.00 C
ATOM 1306 O LYS 82 24.551 49.295 49.743 1.00 0.00 O
ATOM 1307 N VAL 83 26.337 49.552 48.322 1.00 0.00 N
ATOM 1308 H VAL 83 27.303 49.839 48.369 1.00 0.00 H
ATOM 1309 CA VAL 83 25.512 49.247 47.130 1.00 0.00 C
ATOM 1310 HA VAL 83 24.615 49.857 47.238 1.00 0.00 H
ATOM 1311 CB VAL 83 26.289 49.552 45.834 1.00 0.00 C
ATOM 1312 HB VAL 83 25.518 49.536 45.063 1.00 0.00 H
ATOM 1313 CG1 VAL 83 27.098 50.875 45.757 1.00 0.00 C
ATOM 1314 HG11 VAL 83 28.128 50.883 46.112 1.00 0.00 H

ATOM	1315	HG12	VAL	83	27.088	51.223	44.723	1.00	0.00	H
ATOM	1316	HG13	VAL	83	26.592	51.528	46.468	1.00	0.00	H
ATOM	1317	CG2	VAL	83	27.262	48.516	45.389	1.00	0.00	C
ATOM	1318	HG21	VAL	83	26.761	47.590	45.107	1.00	0.00	H
ATOM	1319	HG22	VAL	83	27.753	48.872	44.483	1.00	0.00	H
ATOM	1320	HG23	VAL	83	28.100	48.420	46.079	1.00	0.00	H
ATOM	1321	C	VAL	83	25.005	47.814	46.977	1.00	0.00	C
ATOM	1322	O	VAL	83	23.961	47.629	46.298	1.00	0.00	O
ATOM	1323	N	ILE	84	25.570	46.826	47.646	1.00	0.00	N
ATOM	1324	H	ILE	84	26.499	47.026	47.991	1.00	0.00	H
ATOM	1325	CA	ILE	84	25.108	45.423	47.782	1.00	0.00	C
ATOM	1326	HA	ILE	84	24.682	45.169	46.811	1.00	0.00	H
ATOM	1327	CB	ILE	84	26.196	44.378	48.132	1.00	0.00	C
ATOM	1328	HB	ILE	84	26.808	44.865	48.891	1.00	0.00	H
ATOM	1329	CG2	ILE	84	25.550	43.067	48.612	1.00	0.00	C
ATOM	1330	HG21	ILE	84	24.985	42.616	47.797	1.00	0.00	H
ATOM	1331	HG22	ILE	84	26.374	42.397	48.858	1.00	0.00	H
ATOM	1332	HG23	ILE	84	25.012	43.288	49.533	1.00	0.00	H
ATOM	1333	CG1	ILE	84	27.091	44.151	46.944	1.00	0.00	C
ATOM	1334	HG12	ILE	84	26.615	43.691	46.079	1.00	0.00	H
ATOM	1335	HG13	ILE	84	27.422	45.163	46.709	1.00	0.00	H
ATOM	1336	CD1	ILE	84	28.340	43.336	47.253	1.00	0.00	C
ATOM	1337	HD11	ILE	84	28.782	43.577	48.220	1.00	0.00	H
ATOM	1338	HD12	ILE	84	28.172	42.264	47.351	1.00	0.00	H
ATOM	1339	HD13	ILE	84	29.131	43.509	46.524	1.00	0.00	H
ATOM	1340	C	ILE	84	23.900	45.419	48.763	1.00	0.00	C
ATOM	1341	O	ILE	84	22.792	44.918	48.495	1.00	0.00	O
ATOM	1342	N	ALA	85	24.056	46.018	49.938	1.00	0.00	N
ATOM	1343	H	ALA	85	24.934	46.411	50.244	1.00	0.00	H
ATOM	1344	CA	ALA	85	22.928	46.325	50.858	1.00	0.00	C
ATOM	1345	HA	ALA	85	22.443	45.373	51.074	1.00	0.00	H
ATOM	1346	CB	ALA	85	23.523	46.898	52.172	1.00	0.00	C
ATOM	1347	HB1	ALA	85	23.955	47.888	52.036	1.00	0.00	H
ATOM	1348	HB2	ALA	85	22.761	46.817	52.949	1.00	0.00	H
ATOM	1349	HB3	ALA	85	24.395	46.295	52.425	1.00	0.00	H
ATOM	1350	C	ALA	85	21.819	47.288	50.267	1.00	0.00	C
ATOM	1351	O	ALA	85	20.730	47.301	50.839	1.00	0.00	O
ATOM	1352	N	ASP	86	22.053	47.861	49.134	1.00	0.00	N
ATOM	1353	H	ASP	86	22.956	47.670	48.724	1.00	0.00	H
ATOM	1354	CA	ASP	86	21.038	48.526	48.355	1.00	0.00	C
ATOM	1355	HA	ASP	86	20.296	48.934	49.042	1.00	0.00	H
ATOM	1356	CB	ASP	86	21.580	49.782	47.542	1.00	0.00	C
ATOM	1357	HB2	ASP	86	22.422	49.374	46.983	1.00	0.00	H
ATOM	1358	HB3	ASP	86	20.967	50.232	46.760	1.00	0.00	H
ATOM	1359	CG	ASP	86	22.026	50.959	48.336	1.00	0.00	C
ATOM	1360	OD1	ASP	86	21.696	51.171	49.519	1.00	0.00	O
ATOM	1361	OD2	ASP	86	22.718	51.806	47.739	1.00	0.00	O
ATOM	1362	C	ASP	86	20.242	47.539	47.461	1.00	0.00	C
ATOM	1363	O	ASP	86	19.275	47.975	46.767	1.00	0.00	O
ATOM	1364	N	ILE	87	20.579	46.238	47.409	1.00	0.00	N
ATOM	1365	H	ILE	87	21.267	45.817	48.018	1.00	0.00	H
ATOM	1366	CA	ILE	87	19.699	45.238	46.828	1.00	0.00	C
ATOM	1367	HA	ILE	87	18.838	45.804	46.471	1.00	0.00	H
ATOM	1368	CB	ILE	87	20.446	44.619	45.553	1.00	0.00	C
ATOM	1369	HB	ILE	87	21.488	44.437	45.818	1.00	0.00	H
ATOM	1370	CG2	ILE	87	19.731	43.351	45.022	1.00	0.00	C
ATOM	1371	HG21	ILE	87	20.313	42.902	44.218	1.00	0.00	H
ATOM	1372	HG22	ILE	87	19.677	42.585	45.795	1.00	0.00	H
ATOM	1373	HG23	ILE	87	18.737	43.545	44.619	1.00	0.00	H
ATOM	1374	CG1	ILE	87	20.479	45.686	44.413	1.00	0.00	C
ATOM	1375	HG12	ILE	87	19.458	45.973	44.164	1.00	0.00	H
ATOM	1376	HG13	ILE	87	20.880	46.636	44.767	1.00	0.00	H
ATOM	1377	CD1	ILE	87	21.102	45.216	43.112	1.00	0.00	C
ATOM	1378	HD11	ILE	87	21.288	46.129	42.546	1.00	0.00	H

ATOM	1379	HD12	ILE	87	22.067	44.720	43.218	1.00	0.00	H
ATOM	1380	HD13	ILE	87	20.423	44.659	42.466	1.00	0.00	H
ATOM	1381	C	ILE	87	19.152	44.193	47.858	1.00	0.00	C
ATOM	1382	O	ILE	87	17.955	43.839	47.705	1.00	0.00	O
ATOM	1383	N	TYR	88	20.029	43.706	48.729	1.00	0.00	N
ATOM	1384	H	TYR	88	20.984	44.032	48.786	1.00	0.00	H
ATOM	1385	CA	TYR	88	19.812	42.483	49.617	1.00	0.00	C
ATOM	1386	HA	TYR	88	18.846	42.026	49.406	1.00	0.00	H
ATOM	1387	CB	TYR	88	20.946	41.434	49.336	1.00	0.00	C
ATOM	1388	HB2	TYR	88	21.909	41.942	49.272	1.00	0.00	H
ATOM	1389	HB3	TYR	88	20.972	40.725	50.163	1.00	0.00	H
ATOM	1390	CG	TYR	88	20.829	40.680	48.011	1.00	0.00	C
ATOM	1391	CD1	TYR	88	19.679	39.887	47.727	1.00	0.00	C
ATOM	1392	HD1	TYR	88	18.988	39.805	48.553	1.00	0.00	H
ATOM	1393	CE1	TYR	88	19.583	39.246	46.502	1.00	0.00	C
ATOM	1394	HE1	TYR	88	18.759	38.550	46.450	1.00	0.00	H
ATOM	1395	CZ	TYR	88	20.503	39.368	45.485	1.00	0.00	C
ATOM	1396	OH	TYR	88	20.462	38.620	44.365	1.00	0.00	O
ATOM	1397	HH	TYR	88	20.577	37.689	44.567	1.00	0.00	H
ATOM	1398	CE2	TYR	88	21.544	40.316	45.611	1.00	0.00	C
ATOM	1399	HE2	TYR	88	22.276	40.488	44.835	1.00	0.00	H
ATOM	1400	CD2	TYR	88	21.699	40.925	46.935	1.00	0.00	C
ATOM	1401	HD2	TYR	88	22.505	41.615	47.137	1.00	0.00	H
ATOM	1402	C	TYR	88	19.770	42.870	51.090	1.00	0.00	C
ATOM	1403	O	TYR	88	20.698	43.618	51.512	1.00	0.00	O
ATOM	1404	N	PRO	89	18.879	42.269	51.954			
1.00	0.00	N								
ATOM	1405	CD	PRO	89	17.650	41.648	51.529	1.00	0.00	C
ATOM	1406	HD2	PRO	89	17.839	40.576	51.478	1.00	0.00	H
ATOM	1407	HD3	PRO	89	17.174	42.088	50.653	1.00	0.00	H
ATOM	1408	CG	PRO	89	16.688	41.781	52.715	1.00	0.00	C
ATOM	1409	HG2	PRO	89	15.920	41.008	52.747	1.00	0.00	H
ATOM	1410	HG3	PRO	89	16.111	42.699	52.600	1.00	0.00	H
ATOM	1411	CB	PRO	89	17.509	41.926	53.959	1.00	0.00	C
ATOM	1412	HB2	PRO	89	17.725	40.943	54.375	1.00	0.00	H
ATOM	1413	HB3	PRO	89	17.145	42.655	54.683	1.00	0.00	H
ATOM	1414	CA	PRO	89	18.871	42.404	53.405	1.00	0.00	C
ATOM	1415	HA	PRO	89	19.006	43.449	53.682	1.00	0.00	H
ATOM	1416	C	PRO	89	20.114	41.585	53.939	1.00	0.00	C
ATOM	1417	O	PRO	89	20.530	40.545	53.417	1.00	0.00	O
ATOM	1418	N	GLY	90	20.765	42.098	54.999	1.00	0.00	N
ATOM	1419	H	GLY	90	20.272	42.819	55.506	1.00	0.00	H
ATOM	1420	CA	GLY	90	22.021	41.631	55.530	1.00	0.00	C
ATOM	1421	HA2	GLY	90	22.790	41.554	54.761	1.00	0.00	H
ATOM	1422	HA3	GLY	90	22.427	42.332	56.259	1.00	0.00	H
ATOM	1423	C	GLY	90	22.005	40.169	56.060	1.00	0.00	C
ATOM	1424	O	GLY	90	23.052	39.563	56.023	1.00	0.00	O
ATOM	1425	N	GLN	91	20.848	39.627	56.453	1.00	0.00	N
ATOM	1426	H	GLN	91	20.018	40.202	56.465	1.00	0.00	H
ATOM	1427	CA	GLN	91	20.631	38.187	56.550	1.00	0.00	C
ATOM	1428	HA	GLN	91	21.257	37.820	57.363	1.00	0.00	H
ATOM	1429	CB	GLN	91	19.233	38.101	57.193	1.00	0.00	C
ATOM	1430	HB2	GLN	91	19.124	38.756	58.057	1.00	0.00	H
ATOM	1431	HB3	GLN	91	18.571	38.478	56.413	1.00	0.00	H
ATOM	1432	CG	GLN	91	18.689	36.713	57.517	1.00	0.00	C
ATOM	1433	HG2	GLN	91	18.920	36.054	56.680	1.00	0.00	H
ATOM	1434	HG3	GLN	91	19.207	36.260	58.363	1.00	0.00	H
ATOM	1435	CD	GLN	91	17.183	36.677	57.780	1.00	0.00	C
ATOM	1436	OE1	GLN	91	16.414	37.583	57.548	1.00	0.00	O
ATOM	1437	NE2	GLN	91	16.770	35.580	58.369	1.00	0.00	N
ATOM	1438	HE21	GLN	91	17.564	35.029	58.664	1.00	0.00	H
ATOM	1439	HE22	GLN	91	15.883	35.622	58.850	1.00	0.00	H
ATOM	1440	C	GLN	91	20.916	37.332	55.260	1.00	0.00	C
ATOM	1441	O	GLN	91	21.089	36.124	55.448	1.00	0.00	O

ATOM	1442	N	THR	92	21.024	37.826	54.054	1.00	0.00	N
ATOM	1443	H	THR	92	20.786	38.799	53.924	1.00	0.00	H
ATOM	1444	CA	THR	92	21.218	37.012	52.826	1.00	0.00	C
ATOM	1445	HA	THR	92	20.353	36.376	52.643	1.00	0.00	H
ATOM	1446	CB	THR	92	21.126	38.005	51.604	1.00	0.00	C
ATOM	1447	HB	THR	92	21.860	38.808	51.658	1.00	0.00	H
ATOM	1448	CG2	THR	92	21.390	37.357	50.229	1.00	0.00	C
ATOM	1449	HG21	THR	92	21.050	36.336	50.053	1.00	0.00	H
ATOM	1450	HG22	THR	92	20.751	37.881	49.519	1.00	0.00	H
ATOM	1451	HG23	THR	92	22.471	37.450	50.117	1.00	0.00	H
ATOM	1452	OG1	THR	92	19.794	38.603	51.571	1.00	0.00	O
ATOM	1453	HG1	THR	92	19.780	39.180	52.339	1.00	0.00	H
ATOM	1454	C	THR	92	22.591	36.410	52.858	1.00	0.00	C
ATOM	1455	O	THR	92	23.574	37.183	52.753	1.00	0.00	O
ATOM	1456	N	GLN	93	22.725	35.093	52.908	1.00	0.00	N
ATOM	1457	H	GLN	93	21.880	34.549	52.805	1.00	0.00	H
ATOM	1458	CA	GLN	93	23.999	34.407	52.665	1.00	0.00	C
ATOM	1459	HA	GLN	93	24.808	34.920	53.186	1.00	0.00	H
ATOM	1460	CB	GLN	93	23.912	33.012	53.112	1.00	0.00	C
ATOM	1461	HB2	GLN	93	23.366	32.962	54.055	1.00	0.00	H
ATOM	1462	HB3	GLN	93	23.384	32.500	52.307	1.00	0.00	H
ATOM	1463	CG	GLN	93	25.236	32.281	53.238	1.00	0.00	C
ATOM	1464	HG2	GLN	93	24.969	31.225	53.293	1.00	0.00	H
ATOM	1465	HG3	GLN	93	25.846	32.411	52.344	1.00	0.00	H
ATOM	1466	CD	GLN	93	26.021	32.706	54.498	1.00	0.00	C
ATOM	1467	OE1	GLN	93	25.416	33.249	55.456	1.00	0.00	O
ATOM	1468	NE2	GLN	93	27.238	32.378	54.578	1.00	0.00	N
ATOM	1469	HE21	GLN	93	27.664	32.042	53.726	1.00	0.00	H
ATOM	1470	HE22	GLN	93	27.775	32.552	55.415	1.00	0.00	H
ATOM	1471	C	GLN	93	24.498	34.616	51.227	1.00	0.00	C
ATOM	1472	O	GLN	93	23.737	34.578	50.246	1.00	0.00	O
ATOM	1473	N	PHE	94	25.815	34.805	51.002	1.00	0.00	N
ATOM	1474	H	PHE	94	26.472	34.794	51.769	1.00	0.00	H
ATOM	1475	CA	PHE	94	26.513	35.053	49.777	1.00	0.00	C
ATOM	1476	HA	PHE	94	25.855	35.148	48.913	1.00	0.00	H
ATOM	1477	CB	PHE	94	27.119	36.468	49.853	1.00	0.00	C
ATOM	1478	HB2	PHE	94	27.671	36.456	50.793	1.00	0.00	H
ATOM	1479	HB3	PHE	94	27.901	36.516	49.095	1.00	0.00	H
ATOM	1480	CG	PHE	94	26.194	37.602	49.772	1.00	0.00	C
ATOM	1481	CD1	PHE	94	25.588	38.147	48.575	1.00	0.00	C
ATOM	1482	HD1	PHE	94	25.874	37.796	47.595	1.00	0.00	H
ATOM	1483	CE1	PHE	94	24.832	39.338	48.594	1.00	0.00	C
ATOM	1484	HE1	PHE	94	24.360	39.643	47.672	1.00	0.00	H
ATOM	1485	CZ	PHE	94	24.686	40.016	49.829	1.00	0.00	C
ATOM	1486	HZ	PHE	94	24.138	40.944	49.891	1.00	0.00	H
ATOM	1487	CE2	PHE	94	25.250	39.601	51.008	1.00	0.00	C
ATOM	1488	HE2	PHE	94	25.217	40.102	51.965	1.00	0.00	H
ATOM	1489	CD2	PHE	94	25.910	38.277	51.003	1.00	0.00	C
ATOM	1490	HD2	PHE	94	26.287	37.880	51.934	1.00	0.00	H
ATOM	1491	C	PHE	94	27.687	34.034	49.534	1.00	0.00	C
ATOM	1492	O	PHE	94	28.287	33.377	50.403	1.00	0.00	O
ATOM	1493	N	TYR	95	28.234	34.075	48.285	1.00	0.00	N
ATOM	1494	H	TYR	95	27.918	34.821	47.681	1.00	0.00	H
ATOM	1495	CA	TYR	95	29.190	33.091	47.827	1.00	0.00	C
ATOM	1496	HA	TYR	95	29.791	32.789	48.685	1.00	0.00	H
ATOM	1497	CB	TYR	95	28.429	31.922	47.325	1.00	0.00	C
ATOM	1498	HB2	TYR	95	27.722	31.545	48.065	1.00	0.00	H
ATOM	1499	HB3	TYR	95	27.850	32.245	46.459	1.00	0.00	H
ATOM	1500	CG	TYR	95	29.302	30.789	47.018	1.00	0.00	C
ATOM	1501	CD1	TYR	95	29.573	30.611	45.657	1.00	0.00	C
ATOM	1502	HD1	TYR	95	29.207	31.347	44.957	1.00	0.00	H
ATOM	1503	CE1	TYR	95	30.265	29.394	45.281	1.00	0.00	C
ATOM	1504	HE1	TYR	95	30.477	29.190	44.242	1.00	0.00	H
ATOM	1505	CZ	TYR	95	30.678	28.519	46.250	1.00	0.00	C

ATOM	1506	OH	TYR	95	31.248	27.342	45.858	1.00	0.00	O
ATOM	1507	HH	TYR	95	31.171	27.253	44.905	1.00	0.00	H
ATOM	1508	CE2	TYR	95	30.418	28.790	47.618	1.00	0.00	C
ATOM	1509	HE2	TYR	95	30.890	28.140	48.340	1.00	0.00	H
ATOM	1510	CD2	TYR	95	29.682	29.917	48.032	1.00	0.00	C
ATOM	1511	HD2	TYR	95	29.329	29.963	49.051	1.00	0.00	H
ATOM	1512	C	TYR	95	30.164	33.657	46.796	1.00	0.00	C
ATOM	1513	O	TYR	95	29.726	34.440	45.949	1.00	0.00	O
ATOM	1514	N	VAL	96	31.403	33.345	46.940	1.00	0.00	N
ATOM	1515	H	VAL	96	31.724	32.838	47.752	1.00	0.00	H
ATOM	1516	CA	VAL	96	32.607	33.911	46.229	1.00	0.00	C
ATOM	1517	HA	VAL	96	32.271	34.752	45.624	1.00	0.00	H
ATOM	1518	CB	VAL	96	33.649	34.401	47.233	1.00	0.00	C
ATOM	1519	HB	VAL	96	34.018	33.502	47.726	1.00	0.00	H
ATOM	1520	CG1	VAL	96	34.855	35.109	46.547	1.00	0.00	C
ATOM	1521	HG11	VAL	96	35.369	34.367	45.937	1.00	0.00	H
ATOM	1522	HG12	VAL	96	34.419	35.944	45.999	1.00	0.00	H
ATOM	1523	HG13	VAL	96	35.556	35.490	47.292	1.00	0.00	H
ATOM	1524	CG2	VAL	96	33.057	35.373	48.282	1.00	0.00	C
ATOM	1525	HG21	VAL	96	32.167	35.052	48.823	1.00	0.00	H
ATOM	1526	HG22	VAL	96	33.811	35.485	49.060	1.00	0.00	H
ATOM	1527	HG23	VAL	96	32.901	36.326	47.778	1.00	0.00	H
ATOM	1528	C	VAL	96	33.230	32.831	45.191	1.00	0.00	C
ATOM	1529	O	VAL	96	33.442	31.771	45.626	1.00	0.00	O
ATOM	1530	N	ILE	97	33.511	33.310	43.929	1.00	0.00	N
ATOM	1531	H	ILE	97	33.343	34.274	43.678	1.00	0.00	H
ATOM	1532	CA	ILE	97	34.284	32.504	42.976	1.00	0.00	C
ATOM	1533	HA	ILE	97	34.569	31.574	43.467	1.00	0.00	H
ATOM	1534	CB	ILE	97	33.341	32.051	41.813	1.00	0.00	C
ATOM	1535	HB	ILE	97	33.158	32.882	41.132	1.00	0.00	H
ATOM	1536	CG2	ILE	97	33.976	31.051	40.856	1.00	0.00	C
ATOM	1537	HG21	ILE	97	33.396	30.875	39.950	1.00	0.00	H
ATOM	1538	HG22	ILE	97	34.982	31.378	40.593	1.00	0.00	H
ATOM	1539	HG23	ILE	97	34.152	30.068	41.290	1.00	0.00	H
ATOM	1540	CG1	ILE	97	31.973	31.349	42.249	1.00	0.00	C
ATOM	1541	HG12	ILE	97	31.611	30.531	41.625	1.00	0.00	H
ATOM	1542	HG13	ILE	97	32.104	31.003	43.274	1.00	0.00	H
ATOM	1543	CD1	ILE	97	30.930	32.533	42.294	1.00	0.00	C
ATOM	1544	HD11	ILE	97	29.985	32.191	42.717	1.00	0.00	H
ATOM	1545	HD12	ILE	97	31.231	33.473	42.759	1.00	0.00	H
ATOM	1546	HD13	ILE	97	30.670	32.785	41.266	1.00	0.00	H
ATOM	1547	C	ILE	97	35.504	33.166	42.491	1.00	0.00	C
ATOM	1548	O	ILE	97	35.422	34.164	41.726	1.00	0.00	O
ATOM	1549	N	GLU	98	36.695	32.703	42.855	1.00	0.00	N
ATOM	1550	H	GLU	98	36.849	31.800	43.281	1.00	0.00	H
ATOM	1551	CA	GLU	98	37.983	33.282	42.405	1.00	0.00	C
ATOM	1552	HA	GLU	98	37.799	34.290	42.032	1.00	0.00	H
ATOM	1553	CB	GLU	98	38.865	33.578	43.582	1.00	0.00	C
ATOM	1554	HB2	GLU	98	38.266	34.008	44.384	1.00	0.00	H
ATOM	1555	HB3	GLU	98	39.112	32.648	44.094	1.00	0.00	H
ATOM	1556	CG	GLU	98	40.084	34.426	43.385	1.00	0.00	C
ATOM	1557	HG2	GLU	98	40.735	33.985	42.632	1.00	0.00	H
ATOM	1558	HG3	GLU	98	39.582	35.349	43.091	1.00	0.00	H
ATOM	1559	CD	GLU	98	40.961	34.643	44.639	1.00	0.00	C
ATOM	1560	OE1	GLU	98	41.461	35.760	44.910	1.00	0.00	O
ATOM	1561	OE2	GLU	98	41.195	33.626	45.405	1.00	0.00	O
ATOM	1562	C	GLU	98	38.580	32.316	41.374	1.00	0.00	C
ATOM	1563	O	GLU	98	38.610	31.116	41.623	1.00	0.00	O
ATOM	1564	N	PHE	99	39.117	32.860	40.287	1.00	0.00	N
ATOM	1565	H	PHE	99	39.025	33.863	40.210	1.00	0.00	H
ATOM	1566	CA	PHE	99	39.445	32.177	39.118	1.00	0.00	C
ATOM	1567	HA	PHE	99	39.876	31.229	39.439	1.00	0.00	H
ATOM	1568	CB	PHE	99	38.172	32.061	38.303	1.00	0.00	C

ATOM	1569	HB2	PHE	99	38.477	31.443	37.458	1.00	0.00	H
ATOM	1570	HB3	PHE	99	37.409	31.469	38.807	1.00	0.00	H
ATOM	1571	CG	PHE	99	37.563	33.347	37.800	1.00	0.00	C
ATOM	1572	CD1	PHE	99	38.037	34.013	36.673	1.00	0.00	C
ATOM	1573	HD1	PHE	99	38.726	33.451	36.059	1.00	0.00	H
ATOM	1574	CE1	PHE	99	37.472	35.209	36.323	1.00	0.00	C
ATOM	1575	HE1	PHE	99	37.827	35.669	35.414	1.00	0.00	H
ATOM	1576	CZ	PHE	99	36.400	35.776	37.078	1.00	0.00	C
ATOM	1577	HZ	PHE	99	36.041	36.740	36.751	1.00	0.00	H
ATOM	1578	CE2	PHE	99	35.934	35.103	38.234	1.00	0.00	C
ATOM	1579	HE2	PHE	99	35.136	35.491	38.850	1.00	0.00	H
ATOM	1580	CD2	PHE	99	36.506	33.899	38.581	1.00	0.00	C
ATOM	1581	HD2	PHE	99	36.188	33.385	39.477	1.00	0.00	H
ATOM	1582	C	PHE	99	40.549	32.762	38.257	1.00	0.00	C
ATOM	1583	O	PHE	99	40.820	33.921	38.372	1.00	0.00	O
ATOM	1584	N	LYS	100	41.232	31.956	37.445	1.00	0.00	N
ATOM	1585	H	LYS	100	40.968	30.983	37.385	1.00	0.00	H
ATOM	1586	CA	LYS	100	42.366	32.411	36.560	1.00	0.00	C
ATOM	1587	HA	LYS	100	42.297	33.431	36.184	1.00	0.00	H
ATOM	1588	CB	LYS	100	43.682	32.336	37.364	1.00	0.00	C
ATOM	1589	HB2	LYS	100	43.991	31.293	37.425	1.00	0.00	H
ATOM	1590	HB3	LYS	100	44.448	32.759	36.713	1.00	0.00	H
ATOM	1591	CG	LYS	100	43.744	33.073	38.698	1.00	0.00	C
ATOM	1592	HG2	LYS	100	43.377	34.087	38.550	1.00	0.00	H
ATOM	1593	HG3	LYS	100	43.256	32.473	39.466	1.00	0.00	H
ATOM	1594	CD	LYS	100	45.276	33.202	39.199	1.00	0.00	C
ATOM	1595	HD2	LYS	100	45.282	33.677	40.180	1.00	0.00	H
ATOM	1596	HD3	LYS	100	45.743	32.218	39.190	1.00	0.00	H
ATOM	1597	CE	LYS	100	46.017	34.167	38.293	1.00	0.00	C
ATOM	1598	HE2	LYS	100	46.231	33.752	37.307	1.00	0.00	H
ATOM	1599	HE3	LYS	100	45.370	35.031	38.140	1.00	0.00	H
ATOM	1600	NZ	LYS	100	47.332	34.515	38.831	1.00	0.00	N
ATOM	1601	HZ1	LYS	100	47.801	35.252	38.322	1.00	0.00	H
ATOM	1602	HZ2	LYS	100	47.353	34.734	39.816	1.00	0.00	H
ATOM	1603	HZ3	LYS	100	47.905	33.692	38.709	1.00	0.00	H
ATOM	1604	C	LYS	100	42.472	31.560	35.325	1.00	0.00	C
ATOM	1605	O	LYS	100	42.180	30.360	35.363	1.00	0.00	O
ATOM	1606	N	CYX	101	42.922	32.155	34.299	1.00	0.00	N
ATOM	1607	H	CYX	101	43.144	33.131	34.429	1.00	0.00	H
ATOM	1608	CA	CYX	101	43.049	31.519	33.009	1.00	0.00	C
ATOM	1609	HA	CYX	101	42.190	30.854	32.920	1.00	0.00	H
ATOM	1610	CB	CYX	101	42.810	32.536	31.892	1.00	0.00	C
ATOM	1611	HB2	CYX	101	41.731	32.655	31.804	1.00	0.00	H
ATOM	1612	HB3	CYX	101	43.357	33.468	32.035	1.00	0.00	H
ATOM	1613	SG	CYX	101	43.338	32.075	30.241	1.00	0.00	S
ATOM	1614	C	CYX	101	44.402	30.864	32.864	1.00	0.00	C
ATOM	1615	O	CYX	101	45.394	31.230	33.511	1.00	0.00	O
ATOM	1616	N	LEU	102	44.419	29.853	32.030	1.00	0.00	N
ATOM	1617	H	LEU	102	43.517	29.634	31.632	1.00	0.00	H
ATOM	1618	CA	LEU	102	45.508	28.977	31.758	1.00	0.00	C
ATOM	1619	HA	LEU	102	46.214	29.166	32.567	1.00	0.00	H
ATOM	1620	CB	LEU	102	44.927	27.577	31.765	1.00	0.00	C
ATOM	1621	HB2	LEU	102	44.303	27.412	30.887	1.00	0.00	H
ATOM	1622	HB3	LEU	102	45.765	26.898	31.603	1.00	0.00	H
ATOM	1623	CG	LEU	102	44.148	27.034	33.002	1.00	0.00	C
ATOM	1624	HG	LEU	102	43.458	27.857	33.186	1.00	0.00	H
ATOM	1625	CD1	LEU	102	43.330	25.805	32.796	1.00	0.00	C
ATOM	1626	HD11	LEU	102	43.786	24.824	32.659	1.00	0.00	H
ATOM	1627	HD12	LEU	102	42.619	25.721	33.618	1.00	0.00	H
ATOM	1628	HD13	LEU	102	42.642	25.966	31.967	1.00	0.00	H
ATOM	1629	CD2	LEU	102	45.041	26.828	34.245	1.00	0.00	C
ATOM	1630	HD21	LEU	102	45.899	26.204	33.990	1.00	0.00	H
ATOM	1631	HD22	LEU	102	45.488	27.796	34.468	1.00	0.00	H
ATOM	1632	HD23	LEU	102	44.508	26.467	35.125	1.00	0.00	H

```
ATOM   1633  C   LEU   102       46.286  29.302  30.447  1.00  0.00           C
ATOM   1634  O   LEU   102       47.499  28.889  30.299  1.00  0.00           O
ATOM   1635  OXT LEU   102       45.786  30.075  29.594  1.00  0.00           O
HETATM 1637  N   LIG   103       28.624  40.675  38.077  1.00  0.00           N
HETATM 1638  C   LIG   103       28.933  39.404  37.965  1.00  0.00           C
HETATM 1639  O   LIG   103       27.469  42.316  39.126  1.00  0.00           O
HETATM 1640  C5' LIG   103       24.313  38.836  43.429  1.00  0.00           C
HETATM 1641  O5' LIG   103       23.922  37.798  42.575  1.00  0.00           O
HETATM 1642  C4' LIG   103       24.790  40.025  42.583  1.00  0.00           C
HETATM 1643  O4' LIG   103       25.996  39.699  41.899  1.00  0.00           O
HETATM 1644  C3' LIG   103       23.802  40.577  41.533  1.00  0.00           C
HETATM 1645  O3' LIG   103       22.886  41.529  42.108  1.00  0.00           O
HETATM 1646  C2' LIG   103       24.646  41.122  40.373  1.00  0.00           C
HETATM 1647  O2' LIG   103       24.522  42.522  40.233  1.00  0.00           O
HETATM 1648  C1' LIG   103       26.105  40.777  40.854  1.00  0.00           C
HETATM 1649  N1  LIG   103       26.962  40.215  39.751  1.00  0.00           N
HETATM 1650  O1  LIG   103       29.725  36.876  36.727  1.00  0.00           O
HETATM 1651  N2  LIG   103       29.954  39.155  37.137  1.00  0.00           N
HETATM 1652  C6  LIG   103       27.677  41.099  38.987  1.00  0.00           C
HETATM 1653  C7  LIG   103       27.269  38.812  39.630  1.00  0.00           C
HETATM 1654  C8  LIG   103       28.241  38.395  38.743  1.00  0.00           C
HETATM 1655  C9  LIG   103       30.420  37.942  36.652  1.00  0.00           C
HETATM 1656  C10 LIG   103
   31.798  37.807  36.071  1.00  0.00           C
HETATM 1657  H   LIG   103       30.454  40.006  36.908  1.00  0.00           H
HETATM 1658  H1  LIG   103       28.464  37.358  38.524  1.00  0.00           H
HETATM 1659  H4  LIG   103       26.534  41.732  41.182  1.00  0.00           H
HETATM 1660  H6  LIG   103       25.097  40.795  43.300  1.00  0.00           H
HETATM 1661  H7  LIG   103       23.216  39.757  41.098  1.00  0.00           H
HETATM 1662  H8  LIG   103       24.513  40.707  39.366  1.00  0.00           H
HETATM 1663  H9  LIG   103       23.594  39.043  44.231  1.00  0.00           H
HETATM 1664  H10 LIG   103       25.159  38.502  44.040  1.00  0.00           H
HETATM 1665  H11 LIG   103       32.560  38.317  36.673  1.00  0.00           H
HETATM 1666  H12 LIG   103       31.784  38.329  35.106  1.00  0.00           H
HETATM 1667  H13 LIG   103       32.062  36.749  35.958  1.00  0.00           H
HETATM 1668  H14 LIG   103       26.749  38.223  40.373  1.00  0.00           H
HETATM 1669  H2' LIG   103       24.166  42.771  41.105  1.00  0.00           H
HETATM 1670  H3' LIG   103       22.034  41.527  41.638  1.00  0.00           H
HETATM 1671  H5' LIG   103       23.368  37.150  43.044  1.00  0.00           H
ENDMDL
MODEL     4
SHEET    1   1 1 ILE    22  ASP    26  0
SHEET    2   2 1 VAL    37  VAL    40  0
SHEET    3   3 1 CYX    50  VAL    60  0
SHEET    4   4 1 PHE    94  CYX   101  0
HELIX    1   1 GLN     12  LEU     16  1                                  5
HELIX    2   2 LEU     62  THR     64  1                                  3
HELIX    3   3 GLU     67  GLU     73  1                                  7
HELIX    4   4 LEU     77  ALA     85  1                                  9
ATOM      1  N   GLN     1       36.698  17.459  31.686  1.00  0.00           N
ATOM      2  H1  GLN     1       36.153  17.528  32.534  1.00  0.00           H
ATOM      3  H2  GLN     1       36.879  16.474  31.560  1.00  0.00           H
ATOM      4  H3  GLN     1       37.630  17.791  31.894  1.00  0.00           H
ATOM      5  CA  GLN     1       36.012  18.187  30.575  1.00  0.00           C
ATOM      6  HA  GLN     1       36.820  18.367  29.866  1.00  0.00           H
ATOM      7  CB  GLN     1       34.970  17.343  29.862  1.00  0.00           C
ATOM      8  HB2 GLN     1       34.350  16.871  30.625  1.00  0.00           H
ATOM      9  HB3 GLN     1       34.351  18.006  29.258  1.00  0.00           H
ATOM     10  CG  GLN     1       35.652  16.248  28.994  1.00  0.00           C
ATOM     11  HG2 GLN     1       36.255  15.709  29.724  1.00  0.00           H
ATOM     12  HG3 GLN     1       34.905  15.604  28.528  1.00  0.00           H
ATOM     13  CD  GLN     1       36.566  16.803  27.911  1.00  0.00           C
ATOM     14  OE1 GLN     1       36.650  17.934  27.594  1.00  0.00           O
ATOM     15  NE2 GLN     1       37.219  15.977  27.172  1.00  0.00           N
```

ATOM	16	HE21	GLN	1	37.236	15.036	27.537	1.00	0.00	H
ATOM	17	HE22	GLN	1	37.622	16.212	26.276	1.00	0.00	H
ATOM	18	C	GLN	1	35.440	19.468	31.142	1.00	0.00	C
ATOM	19	O	GLN	1	34.794	19.373	32.174	1.00	0.00	O
ATOM	20	N	PRO	2	35.683	20.651	30.457	1.00	0.00	N
ATOM	21	CD	PRO	2	36.614	20.934	29.303	1.00	0.00	C
ATOM	22	HD2	PRO	2	36.106	20.820	28.346	1.00	0.00	H
ATOM	23	HD3	PRO	2	37.498	20.298	29.330	1.00	0.00	H
ATOM	24	CG	PRO	2	36.969	22.349	29.477	1.00	0.00	C
ATOM	25	HG2	PRO	2	37.282	22.729	28.505	1.00	0.00	H
ATOM	26	HG3	PRO	2	37.815	22.486	30.151	1.00	0.00	H
ATOM	27	CB	PRO	2	35.726	23.061	30.070	1.00	0.00	C
ATOM	28	HB2	PRO	2	34.976	23.281	29.312	1.00	0.00	H
ATOM	29	HB3	PRO	2	35.854	23.955	30.679	1.00	0.00	H
ATOM	30	CA	PRO	2	35.197	21.916	30.985	1.00	0.00	C
ATOM	31	HA	PRO	2	35.615	21.988	31.988	1.00	0.00	H
ATOM	32	C	PRO	2	33.700	22.114	31.113	1.00	0.00	C
ATOM	33	O	PRO	2	33.071	21.580	30.224	1.00	0.00	O
ATOM	34	N	ASN	3	33.156	22.888	32.039	1.00	0.00	N
ATOM	35	H	ASN	3	33.794	23.322	32.692	1.00	0.00	H
ATOM	36	CA	ASN	3	31.706	23.245	32.283	1.00	0.00	C
ATOM	37	HA	ASN	3	31.264	22.326	32.668	1.00	0.00	H
ATOM	38	CB	ASN	3	31.664	24.201	33.541	1.00	0.00	C
ATOM	39	HB2	ASN	3	32.391	25.009	33.456	1.00	0.00	H
ATOM	40	HB3	ASN	3	30.664	24.634	33.559	1.00	0.00	H
ATOM	41	CG	ASN	3	31.840	23.484	34.782	1.00	0.00	C
ATOM	42	OD1	ASN	3	30.951	22.925	35.381	1.00	0.00	O
ATOM	43	ND2	ASN	3	33.036	23.451	35.272	1.00	0.00	N
ATOM	44	HD21	ASN	3	33.710	23.815	34.614	1.00	0.00	H
ATOM	45	HD22	ASN	3	33.254	22.868	36.067	1.00	0.00	H
ATOM	46	C	ASN	3	30.977	23.699	31.010	1.00	0.00	C
ATOM	47	O	ASN	3	31.457	24.600	30.354	1.00	0.00	O
ATOM	48	N	ASP	4	29.824	23.194	30.678	1.00	0.00	N
ATOM	49	H	ASP	4	29.503	22.375	31.176	1.00	0.00	H
ATOM	50	CA	ASP	4	29.047	23.743	29.570	1.00	0.00	C
ATOM	51	HA	ASP	4	29.746	23.849	28.740	1.00	0.00	H
ATOM	52	CB	ASP	4	27.813	22.844	29.293	1.00	0.00	C
ATOM	53	HB2	ASP	4	27.101	22.662	30.098	1.00	0.00	H
ATOM	54	HB3	ASP	4	27.255	23.262	28.454	1.00	0.00	H
ATOM	55	CG	ASP	4	28.212	21.515	28.656	1.00	0.00	C
ATOM	56	OD1	ASP	4	27.322	20.699	28.375	1.00	0.00	O
ATOM	57	OD2	ASP	4	29.422	21.309	28.353	1.00	0.00	O
ATOM	58	C	ASP	4	28.409	25.091	29.927	1.00	0.00	C
ATOM	59	O	ASP	4	27.838	25.735	29.036	1.00	0.00	O
ATOM	60	N	ILE	5	28.417	25.581	31.123	1.00	0.00	N
ATOM	61	H	ILE	5	28.715	24.999	31.893	1.00	0.00	H
ATOM	62	CA	ILE	5	27.920	26.944	31.518	1.00	0.00	C
ATOM	63	HA	ILE	5	27.274	27.224	30.687	1.00	0.00	H
ATOM	64	CB	ILE	5	27.029	26.887	32.807	1.00	0.00	C
ATOM	65	HB	ILE	5	27.674	26.482	33.587	1.00	0.00	H
ATOM	66	CG2	ILE	5	26.704	28.322	33.333	1.00	0.00	C
ATOM	67	HG21	ILE	5	26.027	28.330	34.189	1.00	0.00	H
ATOM	68	HG22	ILE	5	27.567	28.955	33.539	1.00	0.00	H
ATOM	69	HG23	ILE	5	26.187	28.835	32.522	1.00	0.00	H
ATOM	70	CG1	ILE	5	25.843	25.982	32.609	1.00	0.00	C
ATOM	71	HG12	ILE	5	26.280	25.083	32.173	1.00	0.00	H
ATOM	72	HG13	ILE	5	25.511	25.705	33.610	1.00	0.00	H
ATOM	73	CD1	ILE	5	24.657	26.281	31.594	1.00	0.00	C
ATOM	74	HD11	ILE	5	24.143	25.321	31.550	1.00	0.00	H
ATOM	75	HD12	ILE	5	24.047	27.115	31.940	1.00	0.00	H
ATOM	76	HD13	ILE	5	25.024	26.490	30.589	1.00	0.00	H
ATOM	77	C	ILE	5	29.081	27.878	31.718	1.00	0.00	C
ATOM	78	O	ILE	5	30.031	27.448	32.383	1.00	0.00	O
ATOM	79	N	THR	6	28.970	29.125	31.201	1.00	0.00	N

```
ATOM     80  H   THR     6       28.126  29.388  30.714  1.00  0.00            H
ATOM     81  CA  THR     6       29.988  30.234  31.462  1.00  0.00            C
ATOM     82  HA  THR     6       30.472  30.043  32.420  1.00  0.00            H
ATOM     83  CB  THR     6       31.071  30.058  30.418  1.00  0.00            C
ATOM     84  HB  THR     6       31.355  29.006  30.392  1.00  0.00            H
ATOM     85  CG2 THR     6       30.576  30.539  29.047  1.00  0.00            C
ATOM     86 HG21 THR     6       31.321  30.440  28.259  1.00  0.00            H
ATOM     87 HG22 THR     6       29.664  29.960  28.906  1.00  0.00            H
ATOM     88 HG23 THR     6       30.256  31.581  29.029  1.00  0.00            H
ATOM     89  OG1 THR     6       32.154  30.869  30.681  1.00  0.00            O
ATOM     90  HG1 THR     6       32.914  30.487  30.237  1.00  0.00            H
ATOM     91  C   THR     6       29.368  31.601  31.489  1.00  0.00            C
ATOM     92  O   THR     6       28.163  31.731  31.338  1.00  0.00            O
ATOM     93  N   PHE     7       30.183  32.616  31.819  1.00  0.00            N
ATOM     94  H   PHE     7       31.175  32.422  31.819  1.00  0.00            H
ATOM     95  CA  PHE     7       29.697  34.011  32.035  1.00  0.00            C
ATOM     96  HA  PHE     7       28.824  34.040  32.689  1.00  0.00            H
ATOM     97  CB  PHE     7       31.066  34.709  32.485  1.00  0.00            C
ATOM     98  HB2 PHE     7       31.864  34.746  31.744  1.00  0.00            H
ATOM     99  HB3 PHE     7       30.849  35.772  32.605  1.00  0.00            H
ATOM    100  CG  PHE     7       31.749  34.228  33.699  1.00  0.00            C
ATOM    101  CD1 PHE     7       31.022  33.963  34.841  1.00  0.00            C
ATOM    102  HD1 PHE     7       29.962  34.130  34.952  1.00  0.00            H
ATOM    103  CE1 PHE     7       31.657  33.425  35.947  1.00  0.00            C
ATOM    104  HE1 PHE     7       31.074
  33.174  36.822  1.00  0.00            H
ATOM    105  CZ  PHE     7       33.000  33.071  35.930  1.00  0.00            C
ATOM    106  HZ  PHE     7       33.445  32.519  36.745  1.00  0.00            H
ATOM    107  CE2 PHE     7       33.746  33.356  34.763  1.00  0.00            C
ATOM    108  HE2 PHE     7       34.774  33.026  34.778  1.00  0.00            H
ATOM    109  CD2 PHE     7       33.116  33.903  33.676  1.00  0.00            C
ATOM    110  HD2 PHE     7       33.638  34.046  32.740  1.00  0.00            H
ATOM    111  C   PHE     7       29.118  34.651  30.765  1.00  0.00            C
ATOM    112  O   PHE     7       29.500  34.359  29.614  1.00  0.00            O
ATOM    113  N   PHE     8       28.035  35.470  30.948  1.00  0.00            N
ATOM    114  H   PHE     8       27.710  35.791  31.849  1.00  0.00            H
ATOM    115  CA  PHE     8       27.551  36.218  29.769  1.00  0.00            C
ATOM    116  HA  PHE     8       27.300  35.425  29.065  1.00  0.00            H
ATOM    117  CB  PHE     8       26.333  37.087  30.106  1.00  0.00            C
ATOM    118  HB2 PHE     8       26.702  37.860  30.780  1.00  0.00            H
ATOM    119  HB3 PHE     8       25.983  37.540  29.179  1.00  0.00            H
ATOM    120  CG  PHE     8       25.228  36.319  30.708  1.00  0.00            C
ATOM    121  CD1 PHE     8       24.657  36.701  31.990  1.00  0.00            C
ATOM    122  HD1 PHE     8       25.062  37.526  32.559  1.00  0.00            H
ATOM    123  CE1 PHE     8       23.547  35.915  32.377  1.00  0.00            C
ATOM    124  HE1 PHE     8       22.940  36.210  33.221  1.00  0.00            H
ATOM    125  CZ  PHE     8       23.063  34.878  31.579  1.00  0.00            C
ATOM    126  HZ  PHE     8       22.158  34.382  31.897  1.00  0.00            H
ATOM    127  CE2 PHE     8       23.664  34.466  30.368  1.00  0.00            C
ATOM    128  HE2 PHE     8       23.247  33.730  29.696  1.00  0.00            H
ATOM    129  CD2 PHE     8       24.842  35.165  30.015  1.00  0.00            C
ATOM    130  HD2 PHE     8       25.363  34.820  29.133  1.00  0.00            H
ATOM    131  C   PHE     8       28.574  37.062  29.024  1.00  0.00            C
ATOM    132  O   PHE     8       29.509  37.625  29.610  1.00  0.00            O
ATOM    133  N   GLN     9       28.430  37.196  27.734  1.00  0.00            N
ATOM    134  H   GLN     9       27.635  36.759  27.290  1.00  0.00            H
ATOM    135  CA  GLN     9       29.238  38.134  26.884  1.00  0.00            C
ATOM    136  HA  GLN     9       30.273  37.921  27.151  1.00  0.00            H
ATOM    137  CB  GLN     9       28.994  37.781  25.464  1.00  0.00            C
ATOM    138  HB2 GLN     9       30.027  37.827  25.120  1.00  0.00            H
ATOM    139  HB3 GLN     9       28.716  36.730  25.387  1.00  0.00            H
ATOM    140  CG  GLN     9       28.221  38.778  24.500  1.00  0.00            C
ATOM    141  HG2 GLN     9       28.597  39.790  24.643  1.00  0.00            H
ATOM    142  HG3 GLN     9       28.394  38.501  23.461  1.00  0.00            H
```

ATOM	143	CD	GLN	9	26.701	38.770	24.726	1.00	0.00	C
ATOM	144	OE1	GLN	9	26.186	39.206	25.800	1.00	0.00	O
ATOM	145	NE2	GLN	9	25.998	38.328	23.706	1.00	0.00	N
ATOM	146	HE21	GLN	9	25.001	38.459	23.799	1.00	0.00	H
ATOM	147	HE22	GLN	9	26.390	38.113	22.801	1.00	0.00	H
ATOM	148	C	GLN	9	29.142	39.636	27.284	1.00	0.00	C
ATOM	149	O	GLN	9	29.788	40.456	26.688	1.00	0.00	O
ATOM	150	N	ARG	10	28.221	40.056	28.178	1.00	0.00	N
ATOM	151	H	ARG	10	27.602	39.301	28.436	1.00	0.00	H
ATOM	152	CA	ARG	10	28.244	41.264	28.996	1.00	0.00	C
ATOM	153	HA	ARG	10	28.236	42.145	28.355	1.00	0.00	H
ATOM	154	CB	ARG	10	27.052	41.216	29.905	1.00	0.00	C
ATOM	155	HB2	ARG	10	27.162	40.352	30.560	1.00	0.00	H
ATOM	156	HB3	ARG	10	27.042	42.111	30.526	1.00	0.00	H
ATOM	157	CG	ARG	10	25.613	41.159	29.190	1.00	0.00	C
ATOM	158	HG2	ARG	10	25.451	40.199	28.701	1.00	0.00	H
ATOM	159	HG3	ARG	10	24.926	41.425	29.993	1.00	0.00	H
ATOM	160	CD	ARG	10	25.386	42.079	27.977	1.00	0.00	C
ATOM	161	HD2	ARG	10	25.855	43.048	28.148	1.00	0.00	H
ATOM	162	HD3	ARG	10	25.933	41.585	27.174	1.00	0.00	H
ATOM	163	NE	ARG	10	24.031	42.279	27.586	1.00	0.00	N
ATOM	164	HE	ARG	10	23.536	42.944	28.163	1.00	0.00	H
ATOM	165	CZ	ARG	10	23.386	41.772	26.540	1.00	0.00	C
ATOM	166	NH1	ARG	10	23.828	40.892	25.728	1.00	0.00	N
ATOM	167	HH11	ARG	10	23.303	40.558	24.930	1.00	0.00	H
ATOM	168	HH12	ARG	10	24.700	40.410	25.891	1.00	0.00	H
ATOM	169	NH2	ARG	10	22.251	42.347	26.169	1.00	0.00	N
ATOM	170	HH21	ARG	10	22.093	43.280	26.524	1.00	0.00	H
ATOM	171	HH22	ARG	10	21.728	41.944	25.406	1.00	0.00	H
ATOM	172	C	ARG	10	29.538	41.338	29.881	1.00	0.00	C
ATOM	173	O	ARG	10	29.901	42.424	30.339	1.00	0.00	O
ATOM	174	N	PHE	11	30.102	40.167	30.219	1.00	0.00	N
ATOM	175	H	PHE	11	29.617	39.351	29.876	1.00	0.00	H
ATOM	176	CA	PHE	11	31.239	40.051	31.104	1.00	0.00	C
ATOM	177	HA	PHE	11	31.623	41.030	31.389	1.00	0.00	H
ATOM	178	CB	PHE	11	30.767	39.392	32.379	1.00	0.00	C
ATOM	179	HB2	PHE	11	31.631	39.347	33.042	1.00	0.00	H
ATOM	180	HB3	PHE	11	30.488	38.360	32.166	1.00	0.00	H
ATOM	181	CG	PHE	11	29.580	40.132	33.111	1.00	0.00	C
ATOM	182	CD1	PHE	11	29.710	41.595	33.282	1.00	0.00	C
ATOM	183	HD1	PHE	11	30.514	42.148	32.820	1.00	0.00	H
ATOM	184	CE1	PHE	11	28.784	42.266	34.151	1.00	0.00	C
ATOM	185	HE1	PHE	11	28.755	43.345	34.194	1.00	0.00	H
ATOM	186	CZ	PHE	11	27.857	41.559	34.949	1.00	0.00	C
ATOM	187	HZ	PHE	11	27.195	42.145	35.569	1.00	0.00	H
ATOM	188	CE2	PHE	11	27.952	40.161	34.903	1.00	0.00	C
ATOM	189	HE2	PHE	11	27.328	39.585	35.570	1.00	0.00	H
ATOM	190	CD2	PHE	11	28.847	39.477	34.042	1.00	0.00	C
ATOM	191	HD2	PHE	11	28.896	38.409	34.195	1.00	0.00	H
ATOM	192	C	PHE	11	32.492	39.383	30.563	1.00	0.00	C
ATOM	193	O	PHE	11	33.603	39.849	30.781	1.00	0.00	O
ATOM	194	N	GLN	12	32.268	38.410	29.706	1.00	0.00	N
ATOM	195	H	GLN	12	31.363	37.992	29.541	1.00	0.00	H
ATOM	196	CA	GLN	12	33.421	37.811	28.949	1.00	0.00	C
ATOM	197	HA	GLN	12	33.952	37.160	29.643	1.00	0.00	H
ATOM	198	CB	GLN	12	32.973	36.944	27.764	1.00	0.00	C
ATOM	199	HB2	GLN	12	32.437	37.708	27.200	1.00	0.00	H
ATOM	200	HB3	GLN	12	33.824	36.530	27.224	1.00	0.00	H
ATOM	201	CG	GLN	12	31.984	35.769	28.111	1.00	0.00	C
ATOM	202	HG2	GLN	12	32.350	35.264	29.005	1.00	0.00	H
ATOM	203	HG3	GLN	12	31.018	36.239	28.286	1.00	0.00	H
ATOM	204	CD	GLN	12	31.922	34.713	27.036	1.00	0.00	C
ATOM	205	OE1	GLN	12	32.759	34.549	26.193	1.00	0.00	O
ATOM	206	NE2	GLN	12	30.985	33.784	27.258	1.00	0.00	N

ATOM	207	HE21	GLN	12	30.196	33.995	27.851	1.00	0.00	H
ATOM	208	HE22	GLN	12	31.100	32.818	26.986	1.00	0.00	H
ATOM	209	C	GLN	12	34.499	38.678	28.458	1.00	0.00	C
ATOM	210	O	GLN	12	35.624	38.326	28.693	1.00	0.00	O
ATOM	211	N	ASP	13	34.228	39.846	27.769	1.00	0.00	N
ATOM	212	H	ASP	13	33.253	40.079	27.644	1.00	0.00	H
ATOM	213	CA	ASP	13	35.176	40.765	27.320	1.00	0.00	C
ATOM	214	HA	ASP	13	35.844	40.104	26.768	1.00	0.00	H
ATOM	215	CB	ASP	13	34.519	41.719	26.310	1.00	0.00	C
ATOM	216	HB2	ASP	13	33.813	42.322	26.881	1.00	0.00	H
ATOM	217	HB3	ASP	13	35.200	42.450	25.871	1.00	0.00	H
ATOM	218	CG	ASP	13	33.800	40.997	25.148	1.00	0.00	C
ATOM	219	OD1	ASP	13	32.617	41.183	24.941	1.00	0.00	O
ATOM	220	OD2	ASP	13	34.454	40.148	24.442	1.00	0.00	O
ATOM	221	C	ASP	13	36.002	41.557	28.363	1.00	0.00	C
ATOM	222	O	ASP	13	37.032	42.177	28.016	1.00	0.00	O
ATOM	223	N	ASP	14	35.633	41.562	29.676	1.00	0.00	N
ATOM	224	H	ASP	14	34.995	40.836	29.970	1.00	0.00	H
ATOM	225	CA	ASP	14	36.558	42.007	30.779	1.00	0.00	C
ATOM	226	HA	ASP	14	36.965	42.978	30.494	1.00	0.00	H
ATOM	227	CB	ASP	14	35.690	42.222	32.039	1.00	0.00	C
ATOM	228	HB2	ASP	14	35.083	41.367	32.337	1.00	0.00	H
ATOM	229	HB3	ASP	14	36.317	42.574	32.858	1.00	0.00	H
ATOM	230	CG	ASP	14	34.699	43.385	31.796	1.00	0.00	C
ATOM	231	OD1	ASP	14	33.621	43.375	32.407	1.00	0.00	O
ATOM	232	OD2	ASP	14	35.003	44.380	31.100	1.00	0.00	O
ATOM	233	C	ASP	14	37.628	40.953	30.959	1.00	0.00	C
ATOM	234	O	ASP	14	38.769	41.327	31.182	1.00	0.00	O
ATOM	235	N	ILE	15	37.357	39.647	30.784	1.00	0.00	N
ATOM	236	H	ILE	15	36.417	39.337	30.580	1.00	0.00	H
ATOM	237	CA	ILE	15	38.377	38.603	30.879	1.00	0.00	C
ATOM	238	HA	ILE	15	39.016	38.912	31.707	1.00	0.00	H
ATOM	239	CB	ILE	15	37.627	37.203	31.202	1.00	0.00	C
ATOM	240	HB	ILE	15	37.064	36.976	30.298	1.00	0.00	H
ATOM	241	CG2	ILE	15	38.664	36.033	31.318	1.00	0.00	C
ATOM	242	HG21	ILE	15	38.113	35.179	31.710	1.00	0.00	H
ATOM	243	HG22	ILE	15	39.063	35.742	30.346	1.00	0.00	H
ATOM	244	HG23	ILE	15	39.369	36.348	32.087	1.00	0.00	H
ATOM	245	CG1	ILE	15	36.697	37.188	32.431	1.00	0.00	C
ATOM	246	HG12	ILE	15	37.428	37.135	33.239	1.00	0.00	H
ATOM	247	HG13	ILE	15	36.352	38.216	32.548	1.00	0.00	H
ATOM	248	CD1	ILE	15	35.592	36.162	32.485	1.00	0.00	C
ATOM	249	HD11	ILE	15	35.926	35.124	32.468	1.00	0.00	H
ATOM	250	HD12	ILE	15	35.075	36.294	33.435	1.00	0.00	H
ATOM	251	HD13	ILE	15	34.924	36.350	31.645	1.00	0.00	H
ATOM	252	C	ILE	15	39.288	38.454	29.663	1.00	0.00	C
ATOM	253	O	ILE	15	40.407	38.078	29.765	1.00	0.00	O
ATOM	254	N	LEU	16	38.813	38.917	28.507	1.00	0.00	N
ATOM	255	H	LEU	16	37.828	39.134	28.463	1.00	0.00	H
ATOM	256	CA	LEU	16	39.699	39.308	27.426	1.00	0.00	C
ATOM	257	HA	LEU	16	40.351	38.450	27.261	1.00	0.00	H
ATOM	258	CB	LEU	16	38.756	39.404	26.217	1.00	0.00	C
ATOM	259	HB2	LEU	16	38.245	38.442	26.170	1.00	0.00	H
ATOM	260	HB3	LEU	16	37.960	40.124	26.407	1.00	0.00	H
ATOM	261	CG	LEU	16	39.502	39.883	24.931	1.00	0.00	C
ATOM	262	HG	LEU	16	40.025	40.821	25.119	1.00	0.00	H
ATOM	263	CD1	LEU	16	40.480	38.873	24.453	1.00	0.00	C
ATOM	264	HD11	LEU	16	39.886	37.976	24.281	1.00	0.00	H
ATOM	265	HD12	LEU	16	40.984	39.266	23.569	1.00	0.00	H
ATOM	266	HD13	LEU	16	41.292	38.688	25.156	1.00	0.00	H
ATOM	267	CD2	LEU	16	38.482	40.194	23.862	1.00	0.00	C
ATOM	268	HD21	LEU	16	39.046	40.549	22.999	1.00	0.00	H
ATOM	269	HD22	LEU	16	37.923	39.285	23.637	1.00	0.00	H

ATOM	270	HD23	LEU	16	37.822	40.961	24.264	1.00	0.00	H
ATOM	271	C	LEU	16	40.583	40.551	27.696	1.00	0.00	C
ATOM	272	O	LEU	16	41.807	40.601	27.371	1.00	0.00	O
ATOM	273	N	ALA	17	40.028	41.596	28.283	1.00	0.00	N
ATOM	274	H	ALA	17	39.038	41.576	28.476	1.00	0.00	H
ATOM	275	CA	ALA	17	40.733	42.774	28.768	1.00	0.00	C
ATOM	276	HA	ALA	17	41.339	43.189	27.962	1.00	0.00	H
ATOM	277	CB	ALA	17	39.614	43.884	28.936	1.00	0.00	C
ATOM	278	HB1	ALA	17	40.063	44.807	29.303	1.00	0.00	H
ATOM	279	HB2	ALA	17	39.283	44.057	27.913	1.00	0.00	H
ATOM	280	HB3	ALA	17	38.763	43.617	29.563	1.00	0.00	H
ATOM	281	C	ALA	17	41.635	42.654	30.011	1.00	0.00	C
ATOM	282	O	ALA	17	42.255	43.598	30.370	1.00	0.00	O
ATOM	283	N	GLY	18	41.695	41.485	30.638	1.00	0.00	N
ATOM	284	H	GLY	18	41.086	40.744	30.318	1.00	0.00	H
ATOM	285	CA	GLY	18	42.370	41.149	31.900	1.00	0.00	C
ATOM	286	HA2	GLY	18	42.199	40.091	32.099	1.00	0.00	H
ATOM	287	HA3	GLY	18	43.451	41.234	31.786	1.00	0.00	H
ATOM	288	C	GLY	18	41.932	41.872	33.187	1.00	0.00	C
ATOM	289	O	GLY	18	42.657	41.908	34.220	1.00	0.00	O
ATOM	290	N	ARG	19	40.703	42.441	33.256	1.00	0.00	N
ATOM	291	H	ARG	19	40.085	42.263	32.477	1.00	0.00	H
ATOM	292	CA	ARG	19	40.165	43.242	34.434	1.00	0.00	C
ATOM	293	HA	ARG	19	40.964	43.843	34.868	1.00	0.00	H
ATOM	294	CB	ARG	19	39.084	44.220	33.897	1.00	0.00	C
ATOM	295	HB2	ARG	19	38.344	43.591	33.403	1.00	0.00	H
ATOM	296	HB3	ARG	19	38.630	44.693	34.768	1.00	0.00	H
ATOM	297	CG	ARG	19	39.702	45.337	33.087	1.00	0.00	C
ATOM	298	HG2	ARG	19	40.575	45.644	33.662	1.00	0.00	H
ATOM	299	HG3	ARG	19	39.993	44.966	32.104	1.00	0.00	H
ATOM	300	CD	ARG	19	38.765	46.492	32.801	1.00	0.00	C
ATOM	301	HD2	ARG	19	38.438	46.926	33.745	1.00	0.00	H
ATOM	302	HD3	ARG	19	39.419	47.305	32.484	1.00	0.00	H
ATOM	303	NE	ARG	19	37.644	46.180	31.821	1.00	0.00	N
ATOM	304	HE	ARG	19	36.906	45.573	32.148	1.00	0.00	H
ATOM	305	CZ	ARG	19	37.489	46.784	30.648	1.00	0.00	C
ATOM	306	NH1	ARG	19	38.350	47.546	30.071	1.00	0.00	N
ATOM	307	HH11	ARG	19	39.061	47.871	30.712	1.00	0.00	H
ATOM	308	HH12	ARG	19	38.087	47.949	29.183	1.00	0.00	H
ATOM	309	NH2	ARG	19	36.515	46.515	29.922	1.00	0.00	N
ATOM	310	HH21	ARG	19	36.435	47.000	29.040	1.00	0.00	H
ATOM	311	HH22	ARG	19	35.860	45.870	30.338	1.00	0.00	H
ATOM	312	C	ARG	19	39.645	42.301	35.561	1.00	0.00	C
ATOM	313	O	ARG	19	39.340	42.798	36.631	1.00	0.00	O
ATOM	314	N	LYS	20	39.513	41.001	35.352	1.00	0.00	N
ATOM	315	H	LYS	20	39.856	40.552	34.515	1.00	0.00	H
ATOM	316	CA	LYS	20	38.631	40.182	36.222	1.00	0.00	C
ATOM	317	HA	LYS	20	38.335	40.690	37.141	1.00	0.00	H
ATOM	318	CB	LYS	20	37.391	40.011	35.361	1.00	0.00	C
ATOM	319	HB2	LYS	20	37.531	39.240	34.604	1.00	0.00	H
ATOM	320	HB3	LYS	20	37.182	40.902	34.768	1.00	0.00	H
ATOM	321	CG	LYS	20	36.135	39.647	36.201	1.00	0.00	C
ATOM	322	HG2	LYS	20	35.448	39.057	35.595	1.00	0.00	H
ATOM	323	HG3	LYS	20	36.371	38.944	37.000	1.00	0.00	H
ATOM	324	CD	LYS	20	35.382	40.940	36.641	1.00	0.00	C
ATOM	325	HD2	LYS	20	34.886	41.341	35.757	1.00	0.00	H
ATOM	326	HD3	LYS	20	36.127	41.654	36.991	1.00	0.00	H
ATOM	327	CE	LYS	20	34.383	40.766	37.778	1.00	0.00	C
ATOM	328	HE2	LYS	20	33.603	40.142	37.342	1.00	0.00	H
ATOM	329	HE3	LYS	20	34.872	40.247	38.601	1.00	0.00	H
ATOM	330	NZ	LYS	20	33.844	42.014	38.380	1.00	0.00	N
ATOM	331	HZ1	LYS	20	34.553	42.492	38.918	1.00	0.00	H
ATOM	332	HZ2	LYS	20	32.972	41.828	38.856	1.00	0.00	H
ATOM	333	HZ3	LYS	20	33.495	42.533	37.588	1.00	0.00	H

```
ATOM    334  C   LYS    20      39.202  38.845  36.524  1.00  0.00           C
ATOM    335  O   LYS    20      39.464  38.002  35.644  1.00  0.00           O
ATOM    336  N   THR    21      39.300  38.516  37.838  1.00  0.00           N
ATOM    337  H   THR    21      39.207  39.224  38.551  1.00  0.00           H
ATOM    338  CA  THR    21      39.758  37.237  38.377  1.00  0.00           C
ATOM    339  HA  THR    21      39.523  36.482  37.625  1.00  0.00           H
ATOM    340  CB  THR    21      41.299  37.336  38.538  1.00  0.00           C
ATOM    341  HB  THR    21      41.627  36.328  38.792  1.00  0.00           H
ATOM    342  CG2 THR    21      42.158  37.875  37.364  1.00  0.00           C
ATOM    343 HG21 THR    21      43.185  37.965  37.715  1.00  0.00           H
ATOM    344 HG22 THR    21      42.090  37.268  36.461  1.00  0.00           H
ATOM    345 HG23 THR    21      41.816  38.888  37.150  1.00  0.00           H
ATOM    346  OG1 THR    21      41.630  38.239  39.614  1.00  0.00           O
ATOM    347  HG1 THR    21      41.786  37.636  40.344  1.00  0.00           H
ATOM    348  C   THR    21      39.023  36.754  39.663  1.00  0.00           C
ATOM    349  O   THR    21      39.230  35.621  40.084  1.00  0.00           O
ATOM    350  N   ILE    22      38.060  37.509  40.135  1.00  0.00           N
ATOM    351  H   ILE    22      38.029  38.407  39.675  1.00  0.00           H
ATOM    352  CA  ILE    22      37.208  37.245  41.297  1.00  0.00           C
ATOM    353  HA  ILE    22      37.140  36.160  41.383  1.00  0.00           H
ATOM    354  CB  ILE    22      37.747  37.809  42.602  1.00  0.00           C
ATOM    355
  HB  ILE    22      38.772  37.439  42.631  1.00  0.00           H
ATOM    356  CG2 ILE    22      37.932  39.285  42.803  1.00  0.00           C
ATOM    357 HG21 ILE    22      38.421  39.485  43.758  1.00  0.00           H
ATOM    358 HG22 ILE    22      38.652  39.810  42.175  1.00  0.00           H
ATOM    359 HG23 ILE    22      37.043  39.829  42.484  1.00  0.00           H
ATOM    360  CG1 ILE    22      37.040  37.235  43.861  1.00  0.00           C
ATOM    361 HG12 ILE    22      36.056  37.701  43.916  1.00  0.00           H
ATOM    362 HG13 ILE    22      36.857  36.168  43.738  1.00  0.00           H
ATOM    363  CD1 ILE    22      37.727  37.376  45.159  1.00  0.00           C
ATOM    364 HD11 ILE    22      37.448  38.314  45.639  1.00  0.00           H
ATOM    365 HD12 ILE    22      37.403  36.674  45.926  1.00  0.00           H
ATOM    366 HD13 ILE    22      38.803  37.320  44.996  1.00  0.00           H
ATOM    367  C   ILE    22      35.795  37.748  40.906  1.00  0.00           C
ATOM    368  O   ILE    22      35.627  38.642  40.117  1.00  0.00           O
ATOM    369  N   THR    23      34.806  37.108  41.445  1.00  0.00           N
ATOM    370  H   THR    23      35.000  36.296  42.013  1.00  0.00           H
ATOM    371  CA  THR    23      33.418  37.450  41.337  1.00  0.00           C
ATOM    372  HA  THR    23      33.290  38.524  41.198  1.00  0.00           H
ATOM    373  CB  THR    23      32.878  36.694  40.072  1.00  0.00           C
ATOM    374  HB  THR    23      33.487  35.854  39.739  1.00  0.00           H
ATOM    375  CG2 THR    23      31.426  36.320  40.125  1.00  0.00           C
ATOM    376 HG21 THR    23      30.780  37.180  40.299  1.00  0.00           H
ATOM    377 HG22 THR    23      31.145  35.842  39.186  1.00  0.00           H
ATOM    378 HG23 THR    23      31.200  35.589  40.901  1.00  0.00           H
ATOM    379  OG1 THR    23      33.010  37.738  39.162  1.00  0.00           O
ATOM    380  HG1 THR    23      33.906  38.079  39.216  1.00  0.00           H
ATOM    381  C   THR    23      32.648  37.053  42.626  1.00  0.00           C
ATOM    382  O   THR    23      32.720  35.908  43.091  1.00  0.00           O
ATOM    383  N   ILE    24      31.720  37.880  43.210  1.00  0.00           N
ATOM    384  H   ILE    24      31.682  38.847  42.920  1.00  0.00           H
ATOM    385  CA  ILE    24      30.808  37.583  44.293  1.00  0.00           C
ATOM    386  HA  ILE    24      31.239  36.852  44.976  1.00  0.00           H
ATOM    387  CB  ILE    24      30.595  38.879  45.167  1.00  0.00           C
ATOM    388  HB  ILE    24      30.685  39.779  44.559  1.00  0.00           H
ATOM    389  CG2 ILE    24      29.133  38.950  45.721  1.00  0.00           C
ATOM    390 HG21 ILE    24      28.410  38.742  44.931  1.00  0.00           H
ATOM    391 HG22 ILE    24      28.959  38.297  46.575  1.00  0.00           H
ATOM    392 HG23 ILE    24      28.801  39.925  46.080  1.00  0.00           H
ATOM    393  CG1 ILE    24      31.703  38.937  46.142  1.00  0.00           C
ATOM    394 HG12 ILE    24      31.562  38.059  46.770  1.00  0.00           H
ATOM    395 HG13 ILE    24      32.644  38.710  45.641  1.00  0.00           H
ATOM    396  CD1 ILE    24      31.857  40.225  46.969  1.00  0.00           C
```

ATOM	397	HD11	ILE	24	30.915	40.556	47.407	1.00	0.00	H
ATOM	398	HD12	ILE	24	32.690	40.154	47.668	1.00	0.00	H
ATOM	399	HD13	ILE	24	32.063	40.949	46.179	1.00	0.00	H
ATOM	400	C	ILE	24	29.595	36.882	43.684	1.00	0.00	C
ATOM	401	O	ILE	24	29.177	37.242	42.551	1.00	0.00	O
ATOM	402	N	ARG	25	28.933	36.044	44.480	1.00	0.00	N
ATOM	403	H	ARG	25	29.282	35.816	45.400	1.00	0.00	H
ATOM	404	CA	ARG	25	27.622	35.625	44.241	1.00	0.00	C
ATOM	405	HA	ARG	25	27.146	36.273	43.505	1.00	0.00	H
ATOM	406	CB	ARG	25	27.641	34.174	43.755	1.00	0.00	C
ATOM	407	HB2	ARG	25	28.461	33.694	44.287	1.00	0.00	H
ATOM	408	HB3	ARG	25	26.728	33.652	44.042	1.00	0.00	H
ATOM	409	CG	ARG	25	27.846	33.957	42.210	1.00	0.00	C
ATOM	410	HG2	ARG	25	28.714	34.525	41.876	1.00	0.00	H
ATOM	411	HG3	ARG	25	27.952	32.884	42.049	1.00	0.00	H
ATOM	412	CD	ARG	25	26.621	34.404	41.424	1.00	0.00	C
ATOM	413	HD2	ARG	25	25.777	33.751	41.647	1.00	0.00	H
ATOM	414	HD3	ARG	25	26.382	35.417	41.751	1.00	0.00	H
ATOM	415	NE	ARG	25	26.708	34.335	39.910	1.00	0.00	N
ATOM	416	HE	ARG	25	27.452	33.815	39.469	1.00	0.00	H
ATOM	417	CZ	ARG	25	25.808	34.813	39.115	1.00	0.00	C
ATOM	418	NH1	ARG	25	24.701	35.315	39.532	1.00	0.00	N
ATOM	419	HH11	ARG	25	24.153	35.856	38.877	1.00	0.00	H
ATOM	420	HH12	ARG	25	24.348	35.235	40.475	1.00	0.00	H
ATOM	421	NH2	ARG	25	26.016	34.639	37.881	1.00	0.00	N
ATOM	422	HH21	ARG	25	25.220	34.870	37.304	1.00	0.00	H
ATOM	423	HH22	ARG	25	26.925	34.449	37.483	1.00	0.00	H
ATOM	424	C	ARG	25	26.812	35.824	45.505	1.00	0.00	C
ATOM	425	O	ARG	25	27.387	35.972	46.633	1.00	0.00	O
ATOM	426	N	ASP	26	25.442	35.799	45.391	1.00	0.00	N
ATOM	427	H	ASP	26	25.009	35.552	44.513	1.00	0.00	H
ATOM	428	CA	ASP	26	24.622	35.462	46.567	1.00	0.00	C
ATOM	429	HA	ASP	26	25.032	35.827	47.508	1.00	0.00	H
ATOM	430	CB	ASP	26	23.239	36.086	46.549	1.00	0.00	C
ATOM	431	HB2	ASP	26	22.670	35.811	47.437	1.00	0.00	H
ATOM	432	HB3	ASP	26	23.289	37.172	46.623	1.00	0.00	H
ATOM	433	CG	ASP	26	22.324	35.818	45.328	1.00	0.00	C
ATOM	434	OD1	ASP	26	22.826	35.739	44.199	1.00	0.00	O
ATOM	435	OD2	ASP	26	21.052	35.742	45.549	1.00	0.00	O
ATOM	436	C	ASP	26	24.534	33.903	46.632	1.00	0.00	C
ATOM	437	O	ASP	26	24.634	33.238	45.595	1.00	0.00	O
ATOM	438	N	GLU	27	24.490	33.388	47.802	1.00	0.00	N
ATOM	439	H	GLU	27	24.295	33.987	48.591	1.00	0.00	H
ATOM	440	CA	GLU	27	24.728	31.952	47.996	1.00	0.00	C
ATOM	441	HA	GLU	27	25.656	31.637	47.518	1.00	0.00	H
ATOM	442	CB	GLU	27	24.772	31.746	49.541	1.00	0.00	C
ATOM	443	HB2	GLU	27	25.470	32.463	49.975	1.00	0.00	H
ATOM	444	HB3	GLU	27	23.773	31.973	49.912	1.00	0.00	H
ATOM	445	CG	GLU	27	25.175	30.428	50.110	1.00	0.00	C
ATOM	446	HG2	GLU	27	25.424	30.555	51.163	1.00	0.00	H
ATOM	447	HG3	GLU	27	24.382	29.686	50.013	1.00	0.00	H
ATOM	448	CD	GLU	27	26.485	29.809	49.477	1.00	0.00	C
ATOM	449	OE1	GLU	27	27.490	29.523	50.182	1.00	0.00	O
ATOM	450	OE2	GLU	27	26.333	29.396	48.308	1.00	0.00	O
ATOM	451	C	GLU	27	23.621	31.078	47.313	1.00	0.00	C
ATOM	452	O	GLU	27	22.452	31.462	47.193	1.00	0.00	O
ATOM	453	N	SER	28	24.019	29.999	46.804	1.00	0.00	N
ATOM	454	H	SER	28	25.017	29.916	46.940	1.00	0.00	H
ATOM	455	CA	SER	28	23.387	28.769	46.222	1.00	0.00	C
ATOM	456	HA	SER	28	22.666	28.385	46.943	1.00	0.00	H
ATOM	457	CB	SER	28	22.699	29.118	44.913	1.00	0.00	C
ATOM	458	HB2	SER	28	21.927	29.841	45.174	1.00	0.00	H
ATOM	459	HB3	SER	28	23.433	29.497	44.202	1.00	0.00	H
ATOM	460	OG	SER	28	22.035	27.898	44.445	1.00	0.00	O

```
ATOM    461  HG  SER    28      21.115  28.158  44.525  1.00  0.00           H
ATOM    462  C   SER    28      24.405  27.648  46.017  1.00  0.00           C
ATOM    463  O   SER    28      25.468  27.914  45.503  1.00  0.00           O
ATOM    464  N   GLU    29      24.060  26.423  46.448  1.00  0.00           N
ATOM    465  H   GLU    29      23.218  26.351  47.003  1.00  0.00           H
ATOM    466  CA  GLU    29      24.848  25.237  46.271  1.00  0.00           C
ATOM    467  HA  GLU    29      25.803  25.420  46.765  1.00  0.00           H
ATOM    468  CB  GLU    29      24.268  24.129  47.144  1.00  0.00           C
ATOM    469  HB2 GLU    29      24.323  24.476  48.176  1.00  0.00           H
ATOM    470  HB3 GLU    29      23.219  24.133  46.847  1.00  0.00           H
ATOM    471  CG  GLU    29      24.872  22.716  46.967  1.00  0.00           C
ATOM    472  HG2 GLU    29      24.102  22.037  47.334  1.00  0.00           H
ATOM    473  HG3 GLU    29      24.972  22.366  45.939  1.00  0.00           H
ATOM    474  CD  GLU    29      26.144  22.682  47.820  1.00  0.00           C
ATOM    475  OE1 GLU    29      27.265  22.451  47.209  1.00  0.00           O
ATOM    476  OE2 GLU    29      26.029  22.723  49.036  1.00  0.00           O
ATOM    477  C   GLU    29      25.019  24.836  44.838  1.00  0.00           C
ATOM    478  O   GLU    29      26.061  24.141  44.577  1.00  0.00           O
ATOM    479  N   SER    30      24.155  25.261  43.852  1.00  0.00           N
ATOM    480  H   SER    30      23.320
  25.745  44.149  1.00  0.00           H
ATOM    481  CA  SER    30      24.444  25.181  42.443  1.00  0.00           C
ATOM    482  HA  SER    30      24.380  24.116  42.220  1.00  0.00           H
ATOM    483  CB  SER    30      23.321  25.840  41.578  1.00  0.00           C
ATOM    484  HB2 SER    30      23.521  25.644  40.525  1.00  0.00           H
ATOM    485  HB3 SER    30      22.342  25.507  41.922  1.00  0.00           H
ATOM    486  OG  SER    30      23.326  27.218  41.826  1.00  0.00           O
ATOM    487  HG  SER    30      22.794  27.383  42.607  1.00  0.00           H
ATOM    488  C   SER    30      25.710  25.760  41.885  1.00  0.00           C
ATOM    489  O   SER    30      26.155  25.355  40.780  1.00  0.00           O
ATOM    490  N   HIE    31      26.320  26.770  42.544  1.00  0.00           N
ATOM    491  H   HIE    31      25.887  27.008  43.424  1.00  0.00           H
ATOM    492  CA  HIE    31      27.427  27.540  42.004  1.00  0.00           C
ATOM    493  HA  HIE    31      27.177  27.908  41.008  1.00  0.00           H
ATOM    494  CB  HIE    31      27.664  28.857  42.851  1.00  0.00           C
ATOM    495  HB2 HIE    31      27.841  28.571  43.889  1.00  0.00           H
ATOM    496  HB3 HIE    31      28.588  29.268  42.447  1.00  0.00           H
ATOM    497  CG  HIE    31      26.558  29.920  42.791  1.00  0.00           C
ATOM    498  ND1 HIE    31      26.068  30.470  41.577  1.00  0.00           N
ATOM    499  CE1 HIE    31      25.177  31.356  42.038  1.00  0.00           C
ATOM    500  HE1 HIE    31      24.428  31.828  41.419  1.00  0.00           H
ATOM    501  NE2 HIE    31      25.173  31.435  43.389  1.00  0.00           N
ATOM    502  HE2 HIE    31      24.502  31.976  43.915  1.00  0.00           H
ATOM    503  CD2 HIE    31      26.053  30.589  43.904  1.00  0.00           C
ATOM    504  HD2 HIE    31      26.240  30.436  44.956  1.00  0.00           H
ATOM    505  C   HIE    31      28.748  26.763  41.841  1.00  0.00           C
ATOM    506  O   HIE    31      28.926  25.706  42.449  1.00  0.00           O
ATOM    507  N   PHE    32      29.661  27.256  41.029  1.00  0.00           N
ATOM    508  H   PHE    32      29.501  28.235  40.837  1.00  0.00           H
ATOM    509  CA  PHE    32      30.949  26.597  40.867  1.00  0.00           C
ATOM    510  HA  PHE    32      30.651  25.621  40.485  1.00  0.00           H
ATOM    511  CB  PHE    32      31.792  27.332  39.822  1.00  0.00           C
ATOM    512  HB2 PHE    32      32.090  28.297  40.231  1.00  0.00           H
ATOM    513  HB3 PHE    32      32.641  26.663  39.683  1.00  0.00           H
ATOM    514  CG  PHE    32      31.184  27.490  38.483  1.00  0.00           C
ATOM    515  CD1 PHE    32      30.960  28.750  37.948  1.00  0.00           C
ATOM    516  HD1 PHE    32      31.229  29.649  38.482  1.00  0.00           H
ATOM    517  CE1 PHE    32      30.351  28.876  36.658  1.00  0.00           C
ATOM    518  HE1 PHE    32      30.134  29.834  36.210  1.00  0.00           H
ATOM    519  CZ  PHE    32      30.007  27.741  35.916  1.00  0.00           C
ATOM    520  HZ  PHE    32      29.601  27.876  34.925  1.00  0.00           H
ATOM    521  CE2 PHE    32      30.306  26.466  36.500  1.00  0.00           C
ATOM    522  HE2 PHE    32      30.026  25.565  35.973  1.00  0.00           H
ATOM    523  CD2 PHE    32      30.902  26.368  37.760  1.00  0.00           C
```

ATOM	524	HD2	PHE	32	31.062	25.415	38.243	1.00	0.00	H
ATOM	525	C	PHE	32	31.631	26.454	42.236	1.00	0.00	C
ATOM	526	O	PHE	32	31.400	27.282	43.160	1.00	0.00	O
ATOM	527	N	LYS	33	32.415	25.418	42.403	1.00	0.00	N
ATOM	528	H	LYS	33	32.278	24.715	41.690	1.00	0.00	H
ATOM	529	CA	LYS	33	33.213	25.038	43.608	1.00	0.00	C
ATOM	530	HA	LYS	33	33.064	25.669	44.485	1.00	0.00	H
ATOM	531	CB	LYS	33	32.940	23.544	44.105	1.00	0.00	C
ATOM	532	HB2	LYS	33	33.088	22.857	43.272	1.00	0.00	H
ATOM	533	HB3	LYS	33	33.670	23.204	44.839	1.00	0.00	H
ATOM	534	CG	LYS	33	31.528	23.340	44.694	1.00	0.00	C
ATOM	535	HG2	LYS	33	30.724	23.476	43.970	1.00	0.00	H
ATOM	536	HG3	LYS	33	31.378	22.288	44.936	1.00	0.00	H
ATOM	537	CD	LYS	33	31.220	24.140	45.965	1.00	0.00	C
ATOM	538	HD2	LYS	33	31.830	23.571	46.666	1.00	0.00	H
ATOM	539	HD3	LYS	33	31.368	25.209	45.808	1.00	0.00	H
ATOM	540	CE	LYS	33	29.746	24.027	46.439	1.00	0.00	C
ATOM	541	HE2	LYS	33	29.423	22.997	46.593	1.00	0.00	H
ATOM	542	HE3	LYS	33	29.524	24.634	47.316	1.00	0.00	H
ATOM	543	NZ	LYS	33	28.752	24.396	45.392	1.00	0.00	N
ATOM	544	HZ1	LYS	33	28.838	23.844	44.550	1.00	0.00	H
ATOM	545	HZ2	LYS	33	27.793	24.164	45.611	1.00	0.00	H
ATOM	546	HZ3	LYS	33	28.833	25.380	45.178	1.00	0.00	H
ATOM	547	C	LYS	33	34.736	25.283	43.313	1.00	0.00	C
ATOM	548	O	LYS	33	35.116	25.413	42.170	1.00	0.00	O
ATOM	549	N	THR	34	35.637	25.150	44.273	1.00	0.00	N
ATOM	550	H	THR	34	35.384	24.699	45.141	1.00	0.00	H
ATOM	551	CA	THR	34	37.134	25.145	43.964	1.00	0.00	C
ATOM	552	HA	THR	34	37.422	25.930	43.264	1.00	0.00	H
ATOM	553	CB	THR	34	37.987	25.147	45.263	1.00	0.00	C
ATOM	554	HB	THR	34	37.776	24.271	45.875	1.00	0.00	H
ATOM	555	CG2	THR	34	39.443	25.433	45.027	1.00	0.00	C
ATOM	556	HG21	THR	34	39.911	25.706	45.973	1.00	0.00	H
ATOM	557	HG22	THR	34	39.874	24.471	44.752	1.00	0.00	H
ATOM	558	HG23	THR	34	39.537	26.158	44.219	1.00	0.00	H
ATOM	559	OG1	THR	34	37.463	26.177	46.060	1.00	0.00	O
ATOM	560	HG1	THR	34	37.996	26.248	46.856	1.00	0.00	H
ATOM	561	C	THR	34	37.580	23.902	43.262	1.00	0.00	C
ATOM	562	O	THR	34	36.960	22.854	43.464	1.00	0.00	O
ATOM	563	N	GLY	35	38.508	23.956	42.288	1.00	0.00	N
ATOM	564	H	GLY	35	38.911	24.867	42.118	1.00	0.00	H
ATOM	565	CA	GLY	35	38.843	22.961	41.270	1.00	0.00	C
ATOM	566	HA2	GLY	35	39.843	23.081	40.855	1.00	0.00	H
ATOM	567	HA3	GLY	35	38.722	21.948	41.657	1.00	0.00	H
ATOM	568	C	GLY	35	38.065	22.871	39.990	1.00	0.00	C
ATOM	569	O	GLY	35	38.549	22.229	39.080	1.00	0.00	O
ATOM	570	N	ASP	36	36.853	23.465	39.873	1.00	0.00	N
ATOM	571	H	ASP	36	36.548	24.148	40.552	1.00	0.00	H
ATOM	572	CA	ASP	36	36.029	23.423	38.618	1.00	0.00	C
ATOM	573	HA	ASP	36	36.006	22.389	38.274	1.00	0.00	H
ATOM	574	CB	ASP	36	34.564	23.948	38.886	1.00	0.00	C
ATOM	575	HB2	ASP	36	34.682	24.974	39.235	1.00	0.00	H
ATOM	576	HB3	ASP	36	33.961	23.939	37.979	1.00	0.00	H
ATOM	577	CG	ASP	36	33.810	23.173	39.901	1.00	0.00	C
ATOM	578	OD1	ASP	36	34.216	22.001	40.278	1.00	0.00	O
ATOM	579	OD2	ASP	36	32.714	23.629	40.237	1.00	0.00	O
ATOM	580	C	ASP	36	36.808	24.140	37.493	1.00	0.00	C
ATOM	581	O	ASP	36	37.664	25.041	37.851	1.00	0.00	O
ATOM	582	N	VAL	37	36.630	23.705	36.198	1.00	0.00	N
ATOM	583	H	VAL	37	36.004	22.924	36.062	1.00	0.00	H
ATOM	584	CA	VAL	37	37.317	24.288	35.009	1.00	0.00	C
ATOM	585	HA	VAL	37	38.043	25.033	35.336	1.00	0.00	H
ATOM	586	CB	VAL	37	38.374	23.301	34.469	1.00	0.00	C
ATOM	587	HB	VAL	37	37.739	22.480	34.134	1.00	0.00	H

ATOM	588	CG1	VAL	37	39.148	23.847	33.280	1.00	0.00	C
ATOM	589	HG11	VAL	37	39.847	24.575	33.691	1.00	0.00	H
ATOM	590	HG12	VAL	37	39.705	23.066	32.763	1.00	0.00	H
ATOM	591	HG13	VAL	37	38.438	24.299	32.590	1.00	0.00	H
ATOM	592	CG2	VAL	37	39.403	22.715	35.415	1.00	0.00	C
ATOM	593	HG21	VAL	37	40.063	23.463	35.853	1.00	0.00	H
ATOM	594	HG22	VAL	37	38.939	22.017	36.114	1.00	0.00	H
ATOM	595	HG23	VAL	37	40.093	22.131	34.806	1.00	0.00	H
ATOM	596	C	VAL	37	36.288	24.740	33.997	1.00	0.00	C
ATOM	597	O	VAL	37	35.341	24.048	33.555	1.00	0.00	O
ATOM	598	N	LEU	38	36.576	25.968	33.521	1.00	0.00	N
ATOM	599	H	LEU	38	37.451	26.398	33.786	1.00	0.00	H
ATOM	600	CA	LEU	38	35.675	26.778	32.726	1.00	0.00	C
ATOM	601	HA	LEU	38	34.751	26.207	32.650	1.00	0.00	H
ATOM	602	CB	LEU	38	35.319	28.089	33.507	1.00	0.00	C
ATOM	603	HB2	LEU	38	36.116	28.820	33.374	1.00	0.00	H
ATOM	604	HB3	LEU	38	34.569	28.636	32.937	1.00	0.00	H
ATOM	605	CG	LEU	38	35.042	28.007	34.948	1.00	0.00	C
ATOM	606	HG	LEU	38	35.794	27.502	35.555	1.00	0.00	H
ATOM	607	CD1	LEU	38	34.859	29.426	35.440	1.00	0.00	C
ATOM	608	HD11	LEU	38	33.897	29.732	35.026	1.00	0.00	H
ATOM	609	HD12	LEU	38	34.840	29.348	36.527	1.00	0.00	H
ATOM	610	HD13	LEU	38	35.592	30.128	35.043	1.00	0.00	H
ATOM	611	CD2	LEU	38	33.714	27.342	35.189	1.00	0.00	C
ATOM	612	HD21	LEU	38	32.951	27.781	34.546	1.00	0.00	H
ATOM	613	HD22	LEU	38	33.816	26.288	34.932	1.00	0.00	H
ATOM	614	HD23	LEU	38	33.344	27.426	36.211	1.00	0.00	H
ATOM	615	C	LEU	38	36.319	27.179	31.330	1.00	0.00	C
ATOM	616	O	LEU	38	37.564	27.154	31.229	1.00	0.00	O
ATOM	617	N	ARG	39	35.548	27.722	30.388	1.00	0.00	N
ATOM	618	H	ARG	39	34.549	27.778	30.526	1.00	0.00	H
ATOM	619	CA	ARG	39	36.125	28.429	29.224	1.00	0.00	C
ATOM	620	HA	ARG	39	37.120	28.776	29.508	1.00	0.00	H
ATOM	621	CB	ARG	39	36.186	27.491	27.905	1.00	0.00	C
ATOM	622	HB2	ARG	39	35.161	27.147	27.760	1.00	0.00	H
ATOM	623	HB3	ARG	39	36.447	28.198	27.118	1.00	0.00	H
ATOM	624	CG	ARG	39	37.215	26.394	28.014	1.00	0.00	C
ATOM	625	HG2	ARG	39	38.199	26.851	28.115	1.00	0.00	H
ATOM	626	HG3	ARG	39	37.079	25.804	28.921	1.00	0.00	H
ATOM	627	CD	ARG	39	37.164	25.466	26.735	1.00	0.00	C
ATOM	628	HD2	ARG	39	37.251	26.062	25.828	1.00	0.00	H
ATOM	629	HD3	ARG	39	38.011	24.782	26.792	1.00	0.00	H
ATOM	630	NE	ARG	39	35.878	24.751	26.675	1.00	0.00	N
ATOM	631	HE	ARG	39	35.129	25.239	27.147	1.00	0.00	H
ATOM	632	CZ	ARG	39	35.560	23.632	26.131	1.00	0.00	C
ATOM	633	NH1	ARG	39	36.413	22.854	25.432	1.00	0.00	N
ATOM	634	HH11	ARG	39	37.339	23.204	25.229	1.00	0.00	H
ATOM	635	HH12	ARG	39	36.089	21.929	25.186	1.00	0.00	H
ATOM	636	NH2	ARG	39	34.350	23.219	26.353	1.00	0.00	N
ATOM	637	HH21	ARG	39	33.935	22.523	25.750	1.00	0.00	H
ATOM	638	HH22	ARG	39	33.768	23.764	26.973	1.00	0.00	H
ATOM	639	C	ARG	39	35.317	29.659	28.846	1.00	0.00	C
ATOM	640	O	ARG	39	34.139	29.770	29.196	1.00	0.00	O
ATOM	641	N	VAL	40	36.044	30.678	28.405	1.00	0.00	N
ATOM	642	H	VAL	40	37.051	30.686	28.316	1.00	0.00	H
ATOM	643	CA	VAL	40	35.425	31.917	27.827	1.00	0.00	C
ATOM	644	HA	VAL	40	34.345	31.795	27.890	1.00	0.00	H
ATOM	645	CB	VAL	40	35.740	33.013	28.884	1.00	0.00	C
ATOM	646	HB	VAL	40	35.479	34.003	28.512	1.00	0.00	H
ATOM	647	CG1	VAL	40	35.014	32.937	30.162	1.00	0.00	C
ATOM	648	HG11	VAL	40	34.000	32.767	29.797	1.00	0.00	H
ATOM	649	HG12	VAL	40	35.395	32.200	30.868	1.00	0.00	H
ATOM	650	HG13	VAL	40	35.134	33.899	30.661	1.00	0.00	H

ATOM	651	CG2	VAL	40	37.258	33.089	29.182	1.00	0.00	C
ATOM	652	HG21	VAL	40	37.604	33.961	29.739	1.00	0.00	H
ATOM	653	HG22	VAL	40	37.613	32.295	29.839	1.00	0.00	H
ATOM	654	HG23	VAL	40	37.849	33.081	28.266	1.00	0.00	H
ATOM	655	C	VAL	40	35.854	32.210	26.362	1.00	0.00	C
ATOM	656	O	VAL	40	36.931	31.761	25.910	1.00	0.00	O
ATOM	657	N	GLY	41	34.934	32.775	25.584	1.00	0.00	N
ATOM	658	H	GLY	41	34.012	33.017	25.920	1.00	0.00	H
ATOM	659	CA	GLY	41	35.116	33.018	24.144	1.00	0.00	C
ATOM	660	HA2	GLY	41	35.444	34.041	23.961	1.00	0.00	H
ATOM	661	HA3	GLY	41	35.877	32.341	23.757	1.00	0.00	H
ATOM	662	C	GLY	41	33.824	32.676	23.299	1.00	0.00	C
ATOM	663	O	GLY	41	33.182	31.645	23.542	1.00	0.00	O
ATOM	664	N	ARG	42	33.414	33.609	22.396	1.00	0.00	N
ATOM	665	H	ARG	42	33.983	34.433	22.269	1.00	0.00	H
ATOM	666	CA	ARG	42	32.228	33.640	21.626	1.00	0.00	C
ATOM	667	HA	ARG	42	31.684	32.700	21.710	1.00	0.00	H
ATOM	668	CB	ARG	42	31.199	34.618	22.094	1.00	0.00	C
ATOM	669	HB2	ARG	42	30.172	34.305	21.905	1.00	0.00	H
ATOM	670	HB3	ARG	42	31.245	34.500	23.176	1.00	0.00	H
ATOM	671	CG	ARG	42	31.424	36.049	21.640	1.00	0.00	C
ATOM	672	HG2	ARG	42	32.402	36.405	21.962	1.00	0.00	H
ATOM	673	HG3	ARG	42	31.342	36.002	20.554	1.00	0.00	H
ATOM	674	CD	ARG	42	30.290	37.026	22.105	1.00	0.00	C
ATOM	675	HD2	ARG	42	29.323	36.943	21.610	1.00	0.00	H
ATOM	676	HD3	ARG	42	30.070	36.760	23.140	1.00	0.00	H
ATOM	677	NE	ARG	42	30.701	38.431	21.925	1.00	0.00	N
ATOM	678	HE	ARG	42	30.137	39.018	21.328	1.00	0.00	H
ATOM	679	CZ	ARG	42	31.491	39.170	22.700	1.00	0.00	C
ATOM	680	NH1	ARG	42	32.211	38.654	23.599	1.00	0.00	N
ATOM	681	HH11	ARG	42	32.738	39.245	24.225	1.00	0.00	H
ATOM	682	HH12	ARG	42	32.580	37.717	23.512	1.00	0.00	H
ATOM	683	NH2	ARG	42	31.510	40.458	22.609	1.00	0.00	N
ATOM	684	HH21	ARG	42	30.767	40.988	22.175	1.00	0.00	H
ATOM	685	HH22	ARG	42	32.136	40.979	23.207	1.00	0.00	H
ATOM	686	C	ARG	42	32.461	33.731	20.107	1.00	0.00	C
ATOM	687	O	ARG	42	31.544	33.316	19.388	1.00	0.00	O
ATOM	688	N	PHE	43	33.646	34.155	19.687	1.00	0.00	N
ATOM	689	H	PHE	43	34.347	34.384	20.377	1.00	0.00	H
ATOM	690	CA	PHE	43	34.078	34.058	18.289	1.00	0.00	C
ATOM	691	HA	PHE	43	33.212	33.971	17.634	1.00	0.00	H
ATOM	692	CB	PHE	43	34.822	35.349	17.738	1.00	0.00	C
ATOM	693	HB2	PHE	43	35.788	35.439	18.234	1.00	0.00	H
ATOM	694	HB3	PHE	43	34.953	35.308	16.658	1.00	0.00	H
ATOM	695	CG	PHE	43	33.957	36.577	18.089	1.00	0.00	C
ATOM	696	CD1	PHE	43	32.669	36.745	17.590	1.00	0.00	C
ATOM	697	HD1	PHE	43	32.376	36.052	16.815	1.00	0.00	H
ATOM	698	CE1	PHE	43	31.807	37.771	17.988	1.00	0.00	C
ATOM	699	HE1	PHE	43	30.805	37.692	17.593	1.00	0.00	H
ATOM	700	CZ	PHE	43	32.247	38.662	19.003	1.00	0.00	C
ATOM	701	HZ	PHE	43	31.620	39.484	19.315	1.00	0.00	H
ATOM	702	CE2	PHE	43	33.558	38.568	19.526	1.00	0.00	C
ATOM	703	HE2	PHE	43	33.793	39.331	20.252	1.00	0.00	H
ATOM	704	CD2	PHE	43	34.366	37.540	19.128	1.00	0.00	C
ATOM	705	HD2	PHE	43	35.397	37.474	19.439	1.00	0.00	H
ATOM	706	C	PHE	43	34.941	32.791	17.920	1.00	0.00	C
ATOM	707	O	PHE	43	35.804	32.387	18.723	1.00	0.00	O
ATOM	708	N	GLU	44	34.754	32.376	16.692	1.00	0.00	N
ATOM	709	H	GLU	44	34.081	32.810	16.076	1.00	0.00	H
ATOM	710	CA	GLU	44	35.271	31.087	16.246	1.00	0.00	C
ATOM	711	HA	GLU	44	35.038	30.378	17.040	1.00	0.00	H
ATOM	712	CB	GLU	44	34.626	30.526	14.967	1.00	0.00	C
ATOM	713	HB2	GLU	44	34.986	29.510	14.807	1.00	0.00	H
ATOM	714	HB3	GLU	44	33.542	30.571	15.071	1.00	0.00	H

```
ATOM    715  CG  GLU    44      34.955  31.247  13.692  1.00  0.00           C
ATOM    716  HG2 GLU    44      35.474  32.178  13.919  1.00  0.00           H
ATOM    717  HG3 GLU    44      35.659  30.736  13.034  1.00  0.00           H
ATOM    718  CD  GLU    44      33.707  31.499  12.812  1.00  0.00           C
ATOM    719  OE1 GLU    44      33.444  32.679  12.610  1.00  0.00           O
ATOM    720  OE2 GLU    44      33.058  30.583  12.342  1.00  0.00           O
ATOM    721  C   GLU    44      36.803  30.920  16.169  1.00  0.00           C
ATOM    722  O   GLU    44      37.275  29.836  15.993  1.00  0.00           O
ATOM    723  N   ASP    45      37.647  31.938  16.335  1.00  0.00           N
ATOM    724  H   ASP    45      37.248  32.819  16.627  1.00  0.00           H
ATOM    725  CA  ASP    45      39.121  31.716  16.448  1.00  0.00           C
ATOM    726  HA  ASP    45      39.604  31.407  15.521  1.00  0.00           H
ATOM    727  CB  ASP    45      39.891  33.034  16.730  1.00  0.00           C
ATOM    728  HB2 ASP    45      39.596  33.450  17.693  1.00  0.00           H
ATOM    729  HB3 ASP    45      40.912  32.656  16.787  1.00  0.00           H
ATOM    730  CG  ASP    45      39.767  34.108  15.643  1.00  0.00           C
ATOM    731
 OD1 ASP    45      40.275  35.223  15.964  1.00  0.00           O
ATOM    732  OD2 ASP    45      39.311  33.983  14.504  1.00  0.00           O
ATOM    733  C   ASP    45      39.375  30.744  17.647  1.00  0.00           C
ATOM    734  O   ASP    45      39.086  31.037  18.792  1.00  0.00           O
ATOM    735  N   ASP    46      39.890  29.523  17.406  1.00  0.00           N
ATOM    736  H   ASP    46      40.270  29.343  16.488  1.00  0.00           H
ATOM    737  CA  ASP    46      39.944  28.388  18.374  1.00  0.00           C
ATOM    738  HA  ASP    46      40.151  27.569  17.685  1.00  0.00           H
ATOM    739  CB  ASP    46      41.122  28.579  19.361  1.00  0.00           C
ATOM    740  HB2 ASP    46      40.835  29.340  20.088  1.00  0.00           H
ATOM    741  HB3 ASP    46      41.268  27.632  19.881  1.00  0.00           H
ATOM    742  CG  ASP    46      42.464  29.001  18.776  1.00  0.00           C
ATOM    743  OD1 ASP    46      43.290  29.346  19.596  1.00  0.00           O
ATOM    744  OD2 ASP    46      42.741  28.981  17.539  1.00  0.00           O
ATOM    745  C   ASP    46      38.582  28.045  19.033  1.00  0.00           C
ATOM    746  O   ASP    46      38.575  27.162  19.908  1.00  0.00           O
ATOM    747  N   GLY    47      37.505  28.728  18.748  1.00  0.00           N
ATOM    748  H   GLY    47      37.652  29.535  18.160  1.00  0.00           H
ATOM    749  CA  GLY    47      36.244  28.749  19.500  1.00  0.00           C
ATOM    750  HA2 GLY    47      35.528  29.257  18.853  1.00  0.00           H
ATOM    751  HA3 GLY    47      35.918  27.718  19.635  1.00  0.00           H
ATOM    752  C   GLY    47      36.288  29.503  20.771  1.00  0.00           C
ATOM    753  O   GLY    47      35.308  29.942  21.297  1.00  0.00           O
ATOM    754  N   TYR    48      37.516  29.752  21.308  1.00  0.00           N
ATOM    755  H   TYR    48      38.239  29.642  20.612  1.00  0.00           H
ATOM    756  CA  TYR    48      37.777  30.192  22.658  1.00  0.00           C
ATOM    757  HA  TYR    48      36.902  30.763  22.969  1.00  0.00           H
ATOM    758  CB  TYR    48      37.887  28.946  23.566  1.00  0.00           C
ATOM    759  HB2 TYR    48      38.823  28.437  23.334  1.00  0.00           H
ATOM    760  HB3 TYR    48      37.934  29.328  24.586  1.00  0.00           H
ATOM    761  CG  TYR    48      36.691  27.983  23.580  1.00  0.00           C
ATOM    762  CD1 TYR    48      35.516  28.473  24.199  1.00  0.00           C
ATOM    763  HD1 TYR    48      35.485  29.489  24.564  1.00  0.00           H
ATOM    764  CE1 TYR    48      34.394  27.721  24.323  1.00  0.00           C
ATOM    765  HE1 TYR    48      33.525  28.254  24.681  1.00  0.00           H
ATOM    766  CZ  TYR    48      34.375  26.389  23.852  1.00  0.00           C
ATOM    767  OH  TYR    48      33.264  25.629  23.937  1.00  0.00           O
ATOM    768  HH  TYR    48      32.455  26.128  24.073  1.00  0.00           H
ATOM    769  CE2 TYR    48      35.587  25.879  23.254  1.00  0.00           C
ATOM    770  HE2 TYR    48      35.560  24.863  22.888  1.00  0.00           H
ATOM    771  CD2 TYR    48      36.723  26.707  23.078  1.00  0.00           C
ATOM    772  HD2 TYR    48      37.589  26.280  22.594  1.00  0.00           H
ATOM    773  C   TYR    48      39.049  31.055  22.724  1.00  0.00           C
ATOM    774  O   TYR    48      39.880  31.038  21.832  1.00  0.00           O
ATOM    775  N   PHE    49      39.185  31.836  23.826  1.00  0.00           N
ATOM    776  H   PHE    49      38.357  32.065  24.357  1.00  0.00           H
ATOM    777  CA  PHE    49      40.410  32.556  24.182  1.00  0.00           C
```

ATOM	778	HA	PHE	49	41.112	32.241	23.409	1.00	0.00	H
ATOM	779	CB	PHE	49	40.299	34.097	23.974	1.00	0.00	C
ATOM	780	HB2	PHE	49	41.308	34.482	24.118	1.00	0.00	H
ATOM	781	HB3	PHE	49	39.808	34.234	23.011	1.00	0.00	H
ATOM	782	CG	PHE	49	39.604	34.655	25.170	1.00	0.00	C
ATOM	783	CD1	PHE	49	40.275	35.068	26.310	1.00	0.00	C
ATOM	784	HD1	PHE	49	41.344	34.942	26.395	1.00	0.00	H
ATOM	785	CE1	PHE	49	39.600	35.545	27.439	1.00	0.00	C
ATOM	786	HE1	PHE	49	40.149	35.995	28.253	1.00	0.00	H
ATOM	787	CZ	PHE	49	38.201	35.762	27.320	1.00	0.00	C
ATOM	788	HZ	PHE	49	37.623	36.242	28.096	1.00	0.00	H
ATOM	789	CE2	PHE	49	37.541	35.408	26.125	1.00	0.00	C
ATOM	790	HE2	PHE	49	36.475	35.554	26.030	1.00	0.00	H
ATOM	791	CD2	PHE	49	38.233	34.831	25.039	1.00	0.00	C
ATOM	792	HD2	PHE	49	37.656	34.435	24.217	1.00	0.00	H
ATOM	793	C	PHE	49	41.131	32.156	25.508	1.00	0.00	C
ATOM	794	O	PHE	49	42.341	32.082	25.606	1.00	0.00	O
ATOM	795	N	CYX	50	40.293	31.724	26.455	1.00	0.00	N
ATOM	796	H	CYX	50	39.292	31.811	26.356	1.00	0.00	H
ATOM	797	CA	CYX	50	40.862	31.339	27.773	1.00	0.00	C
ATOM	798	HA	CYX	50	41.935	31.176	27.677	1.00	0.00	H
ATOM	799	CB	CYX	50	40.767	32.519	28.755	1.00	0.00	C
ATOM	800	HB2	CYX	50	40.036	33.191	28.306	1.00	0.00	H
ATOM	801	HB3	CYX	50	40.524	32.028	29.698	1.00	0.00	H
ATOM	802	SG	CYX	50	42.233	33.496	28.952	1.00	0.00	S
ATOM	803	C	CYX	50	40.159	30.150	28.350	1.00	0.00	C
ATOM	804	O	CYX	50	38.960	30.156	28.662	1.00	0.00	O
ATOM	805	N	THR	51	40.889	29.133	28.653	1.00	0.00	N
ATOM	806	H	THR	51	41.854	29.367	28.471	1.00	0.00	H
ATOM	807	CA	THR	51	40.488	28.080	29.644	1.00	0.00	C
ATOM	808	HA	THR	51	39.416	27.898	29.561	1.00	0.00	H
ATOM	809	CB	THR	51	41.196	26.762	29.167	1.00	0.00	C
ATOM	810	HB	THR	51	42.279	26.840	29.077	1.00	0.00	H
ATOM	811	CG2	THR	51	40.823	25.692	30.184	1.00	0.00	C
ATOM	812	HG21	THR	51	39.736	25.650	30.102	1.00	0.00	H
ATOM	813	HG22	THR	51	41.228	24.704	29.968	1.00	0.00	H
ATOM	814	HG23	THR	51	41.086	25.938	31.212	1.00	0.00	H
ATOM	815	OG1	THR	51	40.653	26.299	27.907	1.00	0.00	O
ATOM	816	HG1	THR	51	40.787	26.978	27.242	1.00	0.00	H
ATOM	817	C	THR	51	40.852	28.640	31.046	1.00	0.00	C
ATOM	818	O	THR	51	41.928	29.220	31.199	1.00	0.00	O
ATOM	819	N	ILE	52	39.906	28.431	32.013	1.00	0.00	N
ATOM	820	H	ILE	52	39.143	27.793	31.837	1.00	0.00	H
ATOM	821	CA	ILE	52	39.883	29.117	33.239	1.00	0.00	C
ATOM	822	HA	ILE	52	40.892	29.488	33.426	1.00	0.00	H
ATOM	823	CB	ILE	52	38.867	30.281	33.285	1.00	0.00	C
ATOM	824	HB	ILE	52	37.958	29.985	32.760	1.00	0.00	H
ATOM	825	CG2	ILE	52	38.575	30.739	34.750	1.00	0.00	C
ATOM	826	HG21	ILE	52	37.963	30.008	35.277	1.00	0.00	H
ATOM	827	HG22	ILE	52	39.498	30.945	35.292	1.00	0.00	H
ATOM	828	HG23	ILE	52	37.833	31.537	34.760	1.00	0.00	H
ATOM	829	CG1	ILE	52	39.479	31.378	32.388	1.00	0.00	C
ATOM	830	HG12	ILE	52	40.407	31.754	32.820	1.00	0.00	H
ATOM	831	HG13	ILE	52	39.602	31.142	31.332	1.00	0.00	H
ATOM	832	CD1	ILE	52	38.602	32.656	32.531	1.00	0.00	C
ATOM	833	HD11	ILE	52	38.700	33.184	33.480	1.00	0.00	H
ATOM	834	HD12	ILE	52	38.973	33.347	31.775	1.00	0.00	H
ATOM	835	HD13	ILE	52	37.588	32.310	32.331	1.00	0.00	H
ATOM	836	C	ILE	52	39.656	28.117	34.409	1.00	0.00	C
ATOM	837	O	ILE	52	38.597	27.459	34.499	1.00	0.00	O
ATOM	838	N	GLU	53	40.663	27.965	35.304	1.00	0.00	N
ATOM	839	H	GLU	53	41.493	28.540	35.307	1.00	0.00	H
ATOM	840	CA	GLU	53	40.427	27.191	36.521	1.00	0.00	C
ATOM	841	HA	GLU	53	39.848	26.277	36.390	1.00	0.00	H

ATOM	842	CB	GLU	53	41.827	26.530	36.975	1.00	0.00	C
ATOM	843	HB2	GLU	53	42.262	26.036	36.106	1.00	0.00	H
ATOM	844	HB3	GLU	53	42.516	27.324	37.262	1.00	0.00	H
ATOM	845	CG	GLU	53	41.565	25.463	38.040	1.00	0.00	C
ATOM	846	HG2	GLU	53	41.013	25.832	38.905	1.00	0.00	H
ATOM	847	HG3	GLU	53	40.911	24.656	37.709	1.00	0.00	H
ATOM	848	CD	GLU	53	42.886	24.923	38.533	1.00	0.00	C
ATOM	849	OE1	GLU	53	43.841	25.681	38.608	1.00	0.00	O
ATOM	850	OE2	GLU	53	43.010	23.716	38.869	1.00	0.00	O
ATOM	851	C	GLU	53	39.812	28.064	37.672	1.00	0.00	C
ATOM	852	O	GLU	53	40.269	29.174	37.861	1.00	0.00	O
ATOM	853	N	VAL	54	38.761	27.535	38.284	1.00	0.00	N
ATOM	854	H	VAL	54	38.290	26.686	38.007	1.00	0.00	H
ATOM	855	CA	VAL	54	38.281	28.021	39.529	1.00	0.00	C
ATOM	856	HA	VAL	54	38.138					
29.100	39.471	1.00	0.00			H				
ATOM	857	CB	VAL	54	36.863	27.521	39.929	1.00	0.00	C
ATOM	858	HB	VAL	54	37.033	26.448	40.023	1.00	0.00	H
ATOM	859	CG1	VAL	54	36.337	28.117	41.245	1.00	0.00	C
ATOM	860	HG11	VAL	54	35.277	27.917	41.400	1.00	0.00	H
ATOM	861	HG12	VAL	54	36.948	27.639	42.010	1.00	0.00	H
ATOM	862	HG13	VAL	54	36.474	29.195	41.327	1.00	0.00	H
ATOM	863	CG2	VAL	54	35.790	27.879	38.893	1.00	0.00	C
ATOM	864	HG21	VAL	54	36.292	27.762	37.933	1.00	0.00	H
ATOM	865	HG22	VAL	54	34.956	27.208	39.097	1.00	0.00	H
ATOM	866	HG23	VAL	54	35.471	28.922	38.917	1.00	0.00	H
ATOM	867	C	VAL	54	39.282	27.688	40.681	1.00	0.00	C
ATOM	868	O	VAL	54	39.326	26.520	41.127	1.00	0.00	O
ATOM	869	N	THR	55	40.103	28.661	41.094	1.00	0.00	N
ATOM	870	H	THR	55	39.951	29.632	40.858	1.00	0.00	H
ATOM	871	CA	THR	55	41.180	28.377	42.052	1.00	0.00	C
ATOM	872	HA	THR	55	41.453	27.340	41.857	1.00	0.00	H
ATOM	873	CB	THR	55	42.390	29.267	41.740	1.00	0.00	C
ATOM	874	HB	THR	55	43.254	28.914	42.303	1.00	0.00	H
ATOM	875	CG2	THR	55	42.876	29.259	40.263	1.00	0.00	C
ATOM	876	HG21	THR	55	42.815	28.208	39.981	1.00	0.00	H
ATOM	877	HG22	THR	55	42.334	29.919	39.585	1.00	0.00	H
ATOM	878	HG23	THR	55	43.934	29.511	40.349	1.00	0.00	H
ATOM	879	OG1	THR	55	42.026	30.623	42.019	1.00	0.00	O
ATOM	880	HG1	THR	55	42.551	30.890	42.776	1.00	0.00	H
ATOM	881	C	THR	55	40.815	28.658	43.538	1.00	0.00	C
ATOM	882	O	THR	55	41.479	28.032	44.368	1.00	0.00	O
ATOM	883	N	ALA	56	39.762	29.393	43.916	1.00	0.00	N
ATOM	884	H	ALA	56	39.100	29.856	43.310	1.00	0.00	H
ATOM	885	CA	ALA	56	39.160	29.384	45.262	1.00	0.00	C
ATOM	886	HA	ALA	56	39.236	28.341	45.569	1.00	0.00	H
ATOM	887	CB	ALA	56	40.090	30.330	46.076	1.00	0.00	C
ATOM	888	HB1	ALA	56	39.838	30.305	47.136	1.00	0.00	H
ATOM	889	HB2	ALA	56	41.157	30.125	45.992	1.00	0.00	H
ATOM	890	HB3	ALA	56	40.083	31.388	45.812	1.00	0.00	H
ATOM	891	C	ALA	56	37.705	29.703	45.346	1.00	0.00	C
ATOM	892	O	ALA	56	37.177	30.355	44.441	1.00	0.00	O
ATOM	893	N	THR	57	37.119	29.225	46.474	1.00	0.00	N
ATOM	894	H	THR	57	37.687	28.635	47.066	1.00	0.00	H
ATOM	895	CA	THR	57	35.810	29.631	46.904	1.00	0.00	C
ATOM	896	HA	THR	57	35.687	30.612	46.445	1.00	0.00	H
ATOM	897	CB	THR	57	34.672	28.714	46.502	1.00	0.00	C
ATOM	898	HB	THR	57	33.695	29.062	46.836	1.00	0.00	H
ATOM	899	CG2	THR	57	34.725	28.399	44.978	1.00	0.00	C
ATOM	900	HG21	THR	57	35.650	27.890	44.706	1.00	0.00	H
ATOM	901	HG22	THR	57	33.811	27.830	44.810	1.00	0.00	H
ATOM	902	HG23	THR	57	34.719	29.366	44.477	1.00	0.00	H
ATOM	903	OG1	THR	57	34.831	27.423	47.118	1.00	0.00	O
ATOM	904	HG1	THR	57	35.731	27.176	46.890	1.00	0.00	H

ATOM	905	C	THR	57	35.666	29.805	48.457	1.00	0.00	C
ATOM	906	O	THR	57	36.255	29.092	49.240	1.00	0.00	O
ATOM	907	N	SER	58	34.799	30.767	48.840	1.00	0.00	N
ATOM	908	H	SER	58	34.383	31.409	48.181	1.00	0.00	H
ATOM	909	CA	SER	58	34.353	30.889	50.280	1.00	0.00	C
ATOM	910	HA	SER	58	34.453	29.924	50.777	1.00	0.00	H
ATOM	911	CB	SER	58	35.226	31.821	50.983	1.00	0.00	C
ATOM	912	HB2	SER	58	35.161	32.860	50.657	1.00	0.00	H
ATOM	913	HB3	SER	58	34.899	31.738	52.020	1.00	0.00	H
ATOM	914	OG	SER	58	36.547	31.328	51.150	1.00	0.00	O
ATOM	915	HG	SER	58	36.493	30.631	51.809	1.00	0.00	H
ATOM	916	C	SER	58	32.930	31.444	50.370	1.00	0.00	C
ATOM	917	O	SER	58	32.499	32.341	49.665	1.00	0.00	O
ATOM	918	N	THR	59	32.210	30.878	51.332	1.00	0.00	N
ATOM	919	H	THR	59	32.517	30.147	51.958	1.00	0.00	H
ATOM	920	CA	THR	59	30.862	31.403	51.719	1.00	0.00	C
ATOM	921	HA	THR	59	30.464	31.927	50.850	1.00	0.00	H
ATOM	922	CB	THR	59	29.833	30.378	52.094	1.00	0.00	C
ATOM	923	HB	THR	59	29.966	29.637	51.305	1.00	0.00	H
ATOM	924	CG2	THR	59	29.985	29.652	53.460	1.00	0.00	C
ATOM	925	HG21	THR	59	29.294	28.820	53.590	1.00	0.00	H
ATOM	926	HG22	THR	59	31.003	29.268	53.515	1.00	0.00	H
ATOM	927	HG23	THR	59	29.809	30.256	54.351	1.00	0.00	H
ATOM	928	OG1	THR	59	28.544	30.918	52.028	1.00	0.00	O
ATOM	929	HG1	THR	59	28.244	30.514	51.210	1.00	0.00	H
ATOM	930	C	THR	59	30.944	32.520	52.783	1.00	0.00	C
ATOM	931	O	THR	59	31.787	32.526	53.674	1.00	0.00	O
ATOM	932	N	VAL	60	30.068	33.529	52.775	1.00	0.00	N
ATOM	933	H	VAL	60	29.329	33.465	52.090	1.00	0.00	H
ATOM	934	CA	VAL	60	30.315	34.790	53.537	1.00	0.00	C
ATOM	935	HA	VAL	60	30.718	34.362	54.455	1.00	0.00	H
ATOM	936	CB	VAL	60	31.363	35.638	52.832	1.00	0.00	C
ATOM	937	HB	VAL	60	32.224	34.976	52.735	1.00	0.00	H
ATOM	938	CG1	VAL	60	30.840	36.066	51.399	1.00	0.00	C
ATOM	939	HG11	VAL	60	30.547	35.193	50.816	1.00	0.00	H
ATOM	940	HG12	VAL	60	29.989	36.747	51.392	1.00	0.00	H
ATOM	941	HG13	VAL	60	31.557	36.425	50.660	1.00	0.00	H
ATOM	942	CG2	VAL	60	31.911	36.897	53.436	1.00	0.00	C
ATOM	943	HG21	VAL	60	32.300	36.594	54.408	1.00	0.00	H
ATOM	944	HG22	VAL	60	32.705	37.408	52.890	1.00	0.00	H
ATOM	945	HG23	VAL	60	31.130	37.647	53.555	1.00	0.00	H
ATOM	946	C	VAL	60	29.053	35.534	53.922	1.00	0.00	C
ATOM	947	O	VAL	60	28.038	35.450	53.236	1.00	0.00	O
ATOM	948	N	THR	61	29.185	36.280	55.033	1.00	0.00	N
ATOM	949	H	THR	61	30.129	36.330	55.384	1.00	0.00	H
ATOM	950	CA	THR	61	28.173	37.264	55.630	1.00	0.00	C
ATOM	951	HA	THR	61	27.179	36.981	55.284	1.00	0.00	H
ATOM	952	CB	THR	61	28.147	37.057	57.141	1.00	0.00	C
ATOM	953	HB	THR	61	27.463	37.780	57.582	1.00	0.00	H
ATOM	954	CG2	THR	61	27.831	35.616	57.562	1.00	0.00	C
ATOM	955	HG21	THR	61	28.163	34.861	56.850	1.00	0.00	H
ATOM	956	HG22	THR	61	28.281	35.522	58.551	1.00	0.00	H
ATOM	957	HG23	THR	61	26.770	35.686	57.799	1.00	0.00	H
ATOM	958	OG1	THR	61	29.435	37.382	57.654	1.00	0.00	O
ATOM	959	HG1	THR	61	29.351	38.262	58.027	1.00	0.00	H
ATOM	960	C	THR	61	28.454	38.698	55.270	1.00	0.00	C
ATOM	961	O	THR	61	29.621	38.985	54.945	1.00	0.00	O
ATOM	962	N	LEU	62	27.434	39.567	55.243	1.00	0.00	N
ATOM	963	H	LEU	62	26.586	39.329	55.738	1.00	0.00	H
ATOM	964	CA	LEU	62	27.643	40.939	54.770	1.00	0.00	C
ATOM	965	HA	LEU	62	28.119	40.907	53.790	1.00	0.00	H
ATOM	966	CB	LEU	62	26.260	41.491	54.500	1.00	0.00	C
ATOM	967	HB2	LEU	62	25.727	40.654	54.049	1.00	0.00	H
ATOM	968	HB3	LEU	62	25.714	41.661	55.427	1.00	0.00	H

ATOM	969	CG	LEU	62	25.973	42.649	53.543	1.00	0.00	C
ATOM	970	HG	LEU	62	26.308	43.614	53.923	1.00	0.00	H
ATOM	971	CD1	LEU	62	26.543	42.357	52.153	1.00	0.00	C
ATOM	972	HD11	LEU	62	26.324	43.206	51.507	1.00	0.00	H
ATOM	973	HD12	LEU	62	27.614	42.152	52.150	1.00	0.00	H
ATOM	974	HD13	LEU	62	26.018	41.518	51.699	1.00	0.00	H
ATOM	975	CD2	LEU	62	24.478	42.815	53.360	1.00	0.00	C
ATOM	976	HD21	LEU	62	24.076	41.947	52.838	1.00	0.00	H
ATOM	977	HD22	LEU	62	23.981	42.868	54.328	1.00	0.00	H
ATOM	978	HD23	LEU	62	24.181	43.612	52.679	1.00	0.00	H
ATOM	979	C	LEU	62	28.492	41.795	55.667	1.00	0.00	C
ATOM	980	O	LEU	62	29.075	42.786	55.223	1.00	0.00	O
ATOM	981	N	ASP	63	28.808	41.391	56.912	1.00	0.00	N
ATOM	982	H	ASP	63	28.318	40.620	57.345	1.00	0.00	H
ATOM	983	CA	ASP	63	29.830	42.055	57.732	1.00	0.00	C
ATOM	984	HA	ASP	63	29.775	43.091	57.398	1.00	0.00	H
ATOM	985	CB	ASP	63	29.639	41.823	59.302	1.00	0.00	C
ATOM	986	HB2	ASP	63	30.579	42.062	59.800	1.00	0.00	H
ATOM	987	HB3	ASP	63	28.946	42.517	59.777	1.00	0.00	H
ATOM	988	CG	ASP	63	29.302	40.410	59.704	1.00	0.00	C
ATOM	989	OD1	ASP	63	29.347	39.559	58.774	1.00	0.00	O
ATOM	990	OD2	ASP	63	28.776	40.183	60.842	1.00	0.00	O
ATOM	991	C	ASP	63	31.252	41.690	57.402	1.00	0.00	C
ATOM	992	O	ASP	63	32.216	42.365	57.819	1.00	0.00	O
ATOM	993	N	THR	64	31.464	40.688	56.491	1.00	0.00	N
ATOM	994	H	THR	64	30.650	40.225	56.112	1.00	0.00	H
ATOM	995	CA	THR	64	32.768	40.133	56.262	1.00	0.00	C
ATOM	996	HA	THR	64	33.528	40.876	56.506	1.00	0.00	H
ATOM	997	CB	THR	64	32.994	38.861	57.103	1.00	0.00	C
ATOM	998	HB	THR	64	34.038	38.607	56.917	1.00	0.00	H
ATOM	999	CG2	THR	64	32.880	39.095	58.589	1.00	0.00	C
ATOM	1000	HG21	THR	64	31.981	39.643	58.875	1.00	0.00	H
ATOM	1001	HG22	THR	64	32.733	38.111	59.035	1.00	0.00	H
ATOM	1002	HG23	THR	64	33.734	39.656	58.970	1.00	0.00	H
ATOM	1003	OG1	THR	64	32.198	37.741	56.734	1.00	0.00	O
ATOM	1004	HG1	THR	64	31.296	37.885	57.031	1.00	0.00	H
ATOM	1005	C	THR	64	33.078	39.734	54.804	1.00	0.00	C
ATOM	1006	O	THR	64	33.917	38.860	54.504	1.00	0.00	O
ATOM	1007	N	LEU	65	32.440	40.445	53.852	1.00	0.00	N
ATOM	1008	H	LEU	65	31.937	41.297	54.057	1.00	0.00	H
ATOM	1009	CA	LEU	65	32.747	40.267	52.402	1.00	0.00	C
ATOM	1010	HA	LEU	65	32.470	39.265	52.076	1.00	0.00	H
ATOM	1011	CB	LEU	65	32.042	41.316	51.517	1.00	0.00	C
ATOM	1012	HB2	LEU	65	32.425	42.307	51.754	1.00	0.00	H
ATOM	1013	HB3	LEU	65	32.397	41.123	50.504	1.00	0.00	H
ATOM	1014	CG	LEU	65	30.544	41.321	51.386	1.00	0.00	C
ATOM	1015	HG	LEU	65	30.114	41.471	52.377	1.00	0.00	H
ATOM	1016	CD1	LEU	65	29.984	42.378	50.453	1.00	0.00	C
ATOM	1017	HD11	LEU	65	28.911	42.407	50.262	1.00	0.00	H
ATOM	1018	HD12	LEU	65	30.194	43.301	50.996	1.00	0.00	H
ATOM	1019	HD13	LEU	65	30.561	42.465	49.533	1.00	0.00	H
ATOM	1020	CD2	LEU	65	29.980	40.084	50.873	1.00	0.00	C
ATOM	1021	HD21	LEU	65	30.225	39.247	51.528	1.00	0.00	H
ATOM	1022	HD22	LEU	65	28.892	40.077	50.944	1.00	0.00	H
ATOM	1023	HD23	LEU	65	30.248	39.949	49.825	1.00	0.00	H
ATOM	1024	C	LEU	65	34.223	40.395	52.104	1.00	0.00	C
ATOM	1025	O	LEU	65	34.951	41.112	52.873	1.00	0.00	O
ATOM	1026	N	THR	66	34.737	39.647	51.122	1.00	0.00	N
ATOM	1027	H	THR	66	34.115	39.041	50.606	1.00	0.00	H
ATOM	1028	CA	THR	66	36.129	39.624	50.661	1.00	0.00	C
ATOM	1029	HA	THR	66	36.767	39.203	51.439	1.00	0.00	H
ATOM	1030	CB	THR	66	36.380	38.673	49.482	1.00	0.00	C
ATOM	1031	HB	THR	66	36.285	37.648	49.839	1.00	0.00	H

ATOM	1032	CG2	THR	66	35.272	38.748	48.339	1.00	0.00	C
ATOM	1033	HG21	THR	66	35.657	38.210	47.473	1.00	0.00	H
ATOM	1034	HG22	THR	66	34.353	38.230	48.613	1.00	0.00	H
ATOM	1035	HG23	THR	66	34.986	39.794	48.226	1.00	0.00	H
ATOM	1036	OG1	THR	66	37.608	38.911	48.869	1.00	0.00	O
ATOM	1037	HG1	THR	66	38.218	38.780	49.598	1.00	0.00	H
ATOM	1038	C	THR	66	36.675	41.006	50.306	1.00	0.00	C
ATOM	1039	O	THR	66	36.286	41.626	49.361	1.00	0.00	O
ATOM	1040	N	GLU	67	37.772	41.466	50.968	1.00	0.00	N
ATOM	1041	H	GLU	67	38.143	40.843	51.669	1.00	0.00	H
ATOM	1042	CA	GLU	67	38.483	42.728	50.604	1.00	0.00	C
ATOM	1043	HA	GLU	67	37.796	43.575	50.610	1.00	0.00	H
ATOM	1044	CB	GLU	67	39.527	43.166	51.687	1.00	0.00	C
ATOM	1045	HB2	GLU	67	39.637	44.250	51.654	1.00	0.00	H
ATOM	1046	HB3	GLU	67	39.102	42.966	52.671	1.00	0.00	H
ATOM	1047	CG	GLU	67	41.011	42.635	51.739	1.00	0.00	C
ATOM	1048	HG2	GLU	67	41.340	42.679	50.702	1.00	0.00	H
ATOM	1049	HG3	GLU	67	41.685	43.236	52.351	1.00	0.00	H
ATOM	1050	CD	GLU	67	41.153	41.194	52.137	1.00	0.00	C
ATOM	1051	OE1	GLU	67	42.315	40.805	52.252	1.00	0.00	O
ATOM	1052	OE2	GLU	67	40.100	40.469	52.367	1.00	0.00	O
ATOM	1053	C	GLU	67	39.106	42.746	49.212	1.00	0.00	C
ATOM	1054	O	GLU	67	39.329	43.762	48.595	1.00	0.00	O
ATOM	1055	N	LYS	68	39.396	41.571	48.569	1.00	0.00	N
ATOM	1056	H	LYS	68	39.107	40.747	49.076	1.00	0.00	H
ATOM	1057	CA	LYS	68	39.917	41.413	47.245	1.00	0.00	C
ATOM	1058	HA	LYS	68	40.739	42.128	47.204	1.00	0.00	H
ATOM	1059	CB	LYS	68	40.568	40.054	47.012	1.00	0.00	C
ATOM	1060	HB2	LYS	68	39.786	39.294	47.003	1.00	0.00	H
ATOM	1061	HB3	LYS	68	41.112	40.139	46.072	1.00	0.00	H
ATOM	1062	CG	LYS	68	41.713	39.708	47.966	1.00	0.00	C
ATOM	1063	HG2	LYS	68	42.562	40.355	47.749	1.00	0.00	H
ATOM	1064	HG3	LYS	68	41.319	39.849	48.973	1.00	0.00	H
ATOM	1065	CD	LYS	68	42.145	38.261	47.743	1.00	0.00	C
ATOM	1066	HD2	LYS	68	42.132	38.051	46.674	1.00	0.00	H
ATOM	1067	HD3	LYS	68	43.123	37.996	48.142	1.00	0.00	H
ATOM	1068	CE	LYS	68	41.126	37.283	48.476	1.00	0.00	C
ATOM	1069	HE2	LYS	68	41.215	37.413	49.555	1.00	0.00	H
ATOM	1070	HE3	LYS	68	40.142	37.566	48.101	1.00	0.00	H
ATOM	1071	NZ	LYS	68	41.488	35.843	48.217	1.00	0.00	N
ATOM	1072	HZ1	LYS	68	41.423	35.559	47.250	1.00	0.00	H
ATOM	1073	HZ2	LYS	68	42.396	35.572	48.567	1.00	0.00	H
ATOM	1074	HZ3	LYS	68	40.824	35.271	48.719	1.00	0.00	H
ATOM	1075	C	LYS	68	38.895	41.809	46.154	1.00	0.00	C
ATOM	1076	O	LYS	68	39.355	42.393	45.117	1.00	0.00	O
ATOM	1077	N	HIE	69	37.652	41.465	46.262	1.00	0.00	N
ATOM	1078	H	HIE	69	37.346	40.965	47.084	1.00	0.00	H
ATOM	1079	CA	HIE	69	36.591	41.946	45.319	1.00	0.00	C
ATOM	1080	HA	HIE	69	36.830	41.647	44.298	1.00	0.00	H
ATOM	1081	CB	HIE	69	35.219	41.212	45.595	1.00	0.00	C
ATOM	1082	HB2	HIE	69	35.566	40.213	45.858	1.00	0.00	H
ATOM	1083	HB3	HIE	69	34.719	41.623	46.472	1.00	0.00	H
ATOM	1084	CG	HIE	69	34.300	41.219	44.396	1.00	0.00	C
ATOM	1085	ND1	HIE	69	34.316	40.448	43.191	1.00	0.00	N
ATOM	1086	CE1	HIE	69	33.337	40.978	42.364	1.00	0.00	C
ATOM	1087	HE1	HIE	69	33.195	40.639	41.348	1.00	0.00	H
ATOM	1088	NE2	HIE	69	32.702	41.959	43.046	1.00	0.00	N
ATOM	1089	HE2	HIE	69	31.920	42.479	42.675	1.00	0.00	H
ATOM	1090	CD2	HIE	69	33.342	42.148	44.291	1.00	0.00	C
ATOM	1091	HD2	HIE	69	32.979	42.797	45.075	1.00	0.00	H
ATOM	1092	C	HIE	69	36.494	43.437	45.300	1.00	0.00	C
ATOM	1093	O	HIE	69	36.254	44.040	44.284	1.00	0.00	O
ATOM	1094	N	ALA	70	36.605	44.152	46.435	1.00	0.00	N
ATOM	1095	H	ALA	70	36.895	43.634	47.253	1.00	0.00	H

ATOM	1096	CA	ALA	70	36.633	45.607	46.417	1.00	0.00	C
ATOM	1097	HA	ALA	70	35.757	45.983	45.888	1.00	0.00	H
ATOM	1098	CB	ALA	70	36.475	46.072	47.855	1.00	0.00	C
ATOM	1099	HB1	ALA	70	36.597	47.155	47.877	1.00	0.00	H
ATOM	1100	HB2	ALA	70	35.521	45.764	48.285	1.00	0.00	H
ATOM	1101	HB3	ALA	70	37.298	45.641	48.423	1.00	0.00	H
ATOM	1102	C	ALA	70	37.820	46.304	45.730	1.00	0.00	C
ATOM	1103	O	ALA	70	37.635	47.255	44.915	1.00	0.00	O
ATOM	1104	N	GLU	71	39.017	45.749	45.975	1.00	0.00	N
ATOM	1105	H	GLU	71	39.103	45.154	46.786	1.00	0.00	H
ATOM	1106	CA	GLU	71	40.257	46.120	45.330	1.00	0.00	C
ATOM	1107									
		HA	GLU	71	40.466	47.168	45.547	1.00	0.00	H
ATOM	1108	CB	GLU	71	41.435	45.359	45.978	1.00	0.00	C
ATOM	1109	HB2	GLU	71	41.240	45.395	47.049	1.00	0.00	H
ATOM	1110	HB3	GLU	71	41.432	44.304	45.705	1.00	0.00	H
ATOM	1111	CG	GLU	71	42.833	45.875	45.550	1.00	0.00	C
ATOM	1112	HG2	GLU	71	43.108	45.482	44.571	1.00	0.00	H
ATOM	1113	HG3	GLU	71	42.685	46.944	45.396	1.00	0.00	H
ATOM	1114	CD	GLU	71	43.979	45.538	46.518	1.00	0.00	C
ATOM	1115	OE1	GLU	71	44.124	44.397	46.974	1.00	0.00	O
ATOM	1116	OE2	GLU	71	44.800	46.385	46.812	1.00	0.00	O
ATOM	1117	C	GLU	71	40.165	45.928	43.871	1.00	0.00	C
ATOM	1118	O	GLU	71	40.681	46.822	43.170	1.00	0.00	O
ATOM	1119	N	GLN	72	39.515	44.844	43.441	1.00	0.00	N
ATOM	1120	H	GLN	72	39.061	44.294	44.157	1.00	0.00	H
ATOM	1121	CA	GLN	72	39.398	44.541	41.952	1.00	0.00	C
ATOM	1122	HA	GLN	72	40.390	44.510	41.501	1.00	0.00	H
ATOM	1123	CB	GLN	72	38.831	43.113	41.728	1.00	0.00	C
ATOM	1124	HB2	GLN	72	37.781	43.007	42.005	1.00	0.00	H
ATOM	1125	HB3	GLN	72	39.343	42.494	42.466	1.00	0.00	H
ATOM	1126	CG	GLN	72	38.798	42.546	40.305	1.00	0.00	C
ATOM	1127	HG2	GLN	72	38.511	43.278	39.550	1.00	0.00	H
ATOM	1128	HG3	GLN	72	39.810	42.245	40.039	1.00	0.00	H
ATOM	1129	CD	GLN	72	37.892	41.323	40.047	1.00	0.00	C
ATOM	1130	OE1	GLN	72	38.343	40.261	39.729	1.00	0.00	O
ATOM	1131	NE2	GLN	72	36.633	41.451	40.282	1.00	0.00	N
ATOM	1132	HE21	GLN	72	36.198	42.329	40.527	1.00	0.00	H
ATOM	1133	HE22	GLN	72	36.108	40.675	40.661	1.00	0.00	H
ATOM	1134	C	GLN	72	38.442	45.510	41.284	1.00	0.00	C
ATOM	1135	O	GLN	72	38.692	45.989	40.163	1.00	0.00	O
ATOM	1136	N	GLU	73	37.333	45.905	41.974	1.00	0.00	N
ATOM	1137	H	GLU	73	37.008	45.426	42.801	1.00	0.00	H
ATOM	1138	CA	GLU	73	36.368	46.850	41.449	1.00	0.00	C
ATOM	1139	HA	GLU	73	36.404	46.700	40.370	1.00	0.00	H
ATOM	1140	CB	GLU	73	34.944	46.579	41.886	1.00	0.00	C
ATOM	1141	HB2	GLU	73	34.909	46.747	42.963	1.00	0.00	H
ATOM	1142	HB3	GLU	73	34.265	47.293	41.420	1.00	0.00	H
ATOM	1143	CG	GLU	73	34.523	45.169	41.579	1.00	0.00	C
ATOM	1144	HG2	GLU	73	35.060	44.437	42.183	1.00	0.00	H
ATOM	1145	HG3	GLU	73	33.490	44.999	41.885	1.00	0.00	H
ATOM	1146	CD	GLU	73	34.580	44.845	40.053	1.00	0.00	C
ATOM	1147	OE1	GLU	73	33.764	45.303	39.307	1.00	0.00	O
ATOM	1148	OE2	GLU	73	35.290	43.910	39.673	1.00	0.00	O
ATOM	1149	C	GLU	73	36.841	48.323	41.502	1.00	0.00	C
ATOM	1150	O	GLU	73	36.006	49.212	41.205	1.00	0.00	O
ATOM	1151	N	ASN	74	37.912	48.604	42.193	1.00	0.00	N
ATOM	1152	H	ASN	74	38.377	47.806	42.602	1.00	0.00	H
ATOM	1153	CA	ASN	74	38.388	49.936	42.607	1.00	0.00	C
ATOM	1154	HA	ASN	74	39.344	49.772	43.104	1.00	0.00	H
ATOM	1155	CB	ASN	74	38.884	50.801	41.405	1.00	0.00	C
ATOM	1156	HB2	ASN	74	38.143	51.063	40.650	1.00	0.00	H
ATOM	1157	HB3	ASN	74	39.205	51.767	41.794	1.00	0.00	H
ATOM	1158	CG	ASN	74	40.076	50.172	40.737	1.00	0.00	C

ATOM	1159	OD1	ASN	74	40.914	49.521	41.319	1.00	0.00	O
ATOM	1160	ND2	ASN	74	40.261	50.302	39.474	1.00	0.00	N
ATOM	1161	HD21	ASN	74	41.047	49.869	39.011	1.00	0.00	H
ATOM	1162	HD22	ASN	74	39.489	50.630	38.911	1.00	0.00	H
ATOM	1163	C	ASN	74	37.406	50.650	43.616	1.00	0.00	C
ATOM	1164	O	ASN	74	37.264	51.864	43.661	1.00	0.00	O
ATOM	1165	N	MET	75	36.768	49.811	44.390	1.00	0.00	N
ATOM	1166	H	MET	75	37.077	48.850	44.404	1.00	0.00	H
ATOM	1167	CA	MET	75	35.762	50.135	45.366	1.00	0.00	C
ATOM	1168	HA	MET	75	35.493	51.166	45.135	1.00	0.00	H
ATOM	1169	CB	MET	75	34.487	49.242	45.329	1.00	0.00	C
ATOM	1170	HB2	MET	75	34.725	48.188	45.471	1.00	0.00	H
ATOM	1171	HB3	MET	75	33.889	49.593	46.172	1.00	0.00	H
ATOM	1172	CG	MET	75	33.769	49.384	43.996	1.00	0.00	C
ATOM	1173	HG2	MET	75	33.733	50.389	43.572	1.00	0.00	H
ATOM	1174	HG3	MET	75	34.207	48.715	43.255	1.00	0.00	H
ATOM	1175	SD	MET	75	32.072	48.899	43.933	1.00	0.00	S
ATOM	1176	CE	MET	75	31.415	50.212	44.931	1.00	0.00	C
ATOM	1177	HE1	MET	75	31.737	51.179	44.546	1.00	0.00	H
ATOM	1178	HE2	MET	75	30.351	50.105	44.716	1.00	0.00	H
ATOM	1179	HE3	MET	75	31.613	50.287	46.000	1.00	0.00	H
ATOM	1180	C	MET	75	36.419	50.118	46.711	1.00	0.00	C
ATOM	1181	O	MET	75	37.281	49.334	46.959	1.00	0.00	O
ATOM	1182	N	THR	76	35.890	50.837	47.708	1.00	0.00	N
ATOM	1183	H	THR	76	35.044	51.328	47.454	1.00	0.00	H
ATOM	1184	CA	THR	76	36.111	50.473	49.176	1.00	0.00	C
ATOM	1185	HA	THR	76	37.101	50.035	49.304	1.00	0.00	H
ATOM	1186	CB	THR	76	36.058	51.717	50.089	1.00	0.00	C
ATOM	1187	HB	THR	76	36.902	52.377	49.884	1.00	0.00	H
ATOM	1188	CG2	THR	76	34.801	52.558	50.015	1.00	0.00	C
ATOM	1189	HG21	THR	76	34.893	53.412	50.687	1.00	0.00	H
ATOM	1190	HG22	THR	76	34.549	52.880	49.005	1.00	0.00	H
ATOM	1191	HG23	THR	76	33.962	51.924	50.306	1.00	0.00	H
ATOM	1192	OG1	THR	76	36.316	51.415	51.439	1.00	0.00	O
ATOM	1193	HG1	THR	76	37.261	51.366	51.601	1.00	0.00	H
ATOM	1194	C	THR	76	35.086	49.504	49.681	1.00	0.00	C
ATOM	1195	O	THR	76	33.968	49.459	49.222	1.00	0.00	O
ATOM	1196	N	LEU	77	35.483	48.698	50.630	1.00	0.00	N
ATOM	1197	H	LEU	77	36.426	48.601	50.977	1.00	0.00	H
ATOM	1198	CA	LEU	77	34.603	47.538	51.113	1.00	0.00	C
ATOM	1199	HA	LEU	77	34.436	46.910	50.238	1.00	0.00	H
ATOM	1200	CB	LEU	77	35.417	46.740	52.113	1.00	0.00	C
ATOM	1201	HB2	LEU	77	36.349	46.510	51.599	1.00	0.00	H
ATOM	1202	HB3	LEU	77	35.729	47.242	53.029	1.00	0.00	H
ATOM	1203	CG	LEU	77	34.805	45.339	52.551	1.00	0.00	C
ATOM	1204	HG	LEU	77	33.831	45.420	53.033	1.00	0.00	H
ATOM	1205	CD1	LEU	77	34.667	44.404	51.400	1.00	0.00	C
ATOM	1206	HD11	LEU	77	33.666	44.412	50.968	1.00	0.00	H
ATOM	1207	HD12	LEU	77	35.495	44.356	50.692	1.00	0.00	H
ATOM	1208	HD13	LEU	77	34.746	43.454	51.926	1.00	0.00	H
ATOM	1209	CD2	LEU	77	35.765	44.721	53.561	1.00	0.00	C
ATOM	1210	HD21	LEU	77	36.797	44.823	53.225	1.00	0.00	H
ATOM	1211	HD22	LEU	77	35.601	45.132	54.556	1.00	0.00	H
ATOM	1212	HD23	LEU	77	35.614	43.655	53.729	1.00	0.00	H
ATOM	1213	C	LEU	77	33.252	47.980	51.685	1.00	0.00	C
ATOM	1214	O	LEU	77	32.267	47.236	51.492	1.00	0.00	O
ATOM	1215	N	THR	78	33.152	49.153	52.292	1.00	0.00	N
ATOM	1216	H	THR	78	33.986	49.621	52.617	1.00	0.00	H
ATOM	1217	CA	THR	78	31.931	49.761	52.865	1.00	0.00	C
ATOM	1218	HA	THR	78	31.467	49.030	53.528	1.00	0.00	H
ATOM	1219	CB	THR	78	32.338	50.992	53.689	1.00	0.00	C
ATOM	1220	HB	THR	78	31.392	51.393	54.052	1.00	0.00	H
ATOM	1221	CG2	THR	78	33.265	50.557	54.869	1.00	0.00	C
ATOM	1222	HG21	THR	78	33.322	51.403	55.554	1.00	0.00	H

ATOM	1223	HG22	THR	78	32.820	49.753	55.453	1.00	0.00	H
ATOM	1224	HG23	THR	78	34.290	50.440	54.516	1.00	0.00	H
ATOM	1225	OG1	THR	78	33.025	52.038	53.094	1.00	0.00	O
ATOM	1226	HG1	THR	78	32.363	52.577	52.656	1.00	0.00	H
ATOM	1227	C	THR	78	31.010	50.226	51.683	1.00	0.00	C
ATOM	1228	O	THR	78	29.784	50.258	51.847	1.00	0.00	O
ATOM	1229	N	GLU	79	31.552	50.637	50.534	1.00	0.00	N
ATOM	1230	H	GLU	79	32.557	50.686	50.449	1.00	0.00	H
ATOM	1231	CA	GLU	79	30.689	51.049	49.395	1.00	0.00	C
ATOM	1232	HA	GLU	79	29.778					
51.436	49.851	1.00	0.00	H						
ATOM	1233	CB	GLU	79	31.422	52.071	48.605	1.00	0.00	C
ATOM	1234	HB2	GLU	79	31.954	52.817	49.196	1.00	0.00	H
ATOM	1235	HB3	GLU	79	32.195	51.542	48.048	1.00	0.00	H
ATOM	1236	CG	GLU	79	30.588	52.930	47.608	1.00	0.00	C
ATOM	1237	HG2	GLU	79	31.156	53.647	47.015	1.00	0.00	H
ATOM	1238	HG3	GLU	79	30.045	52.354	46.859	1.00	0.00	H
ATOM	1239	CD	GLU	79	29.568	53.719	48.385	1.00	0.00	C
ATOM	1240	OE1	GLU	79	28.403	53.828	48.001	1.00	0.00	O
ATOM	1241	OE2	GLU	79	29.991	54.221	49.503	1.00	0.00	O
ATOM	1242	C	GLU	79	30.274	49.766	48.604	1.00	0.00	C
ATOM	1243	O	GLU	79	29.081	49.627	48.287	1.00	0.00	O
ATOM	1244	N	LEU	80	31.130	48.725	48.499	1.00	0.00	N
ATOM	1245	H	LEU	80	32.049	48.857	48.897	1.00	0.00	H
ATOM	1246	CA	LEU	80	30.716	47.372	48.043	1.00	0.00	C
ATOM	1247	HA	LEU	80	30.203	47.563	47.101	1.00	0.00	H
ATOM	1248	CB	LEU	80	31.989	46.514	48.040	1.00	0.00	C
ATOM	1249	HB2	LEU	80	32.779	47.054	47.518	1.00	0.00	H
ATOM	1250	HB3	LEU	80	32.344	46.321	49.053	1.00	0.00	H
ATOM	1251	CG	LEU	80	31.768	45.121	47.313	1.00	0.00	C
ATOM	1252	HG	LEU	80	31.113	44.402	47.804	1.00	0.00	H
ATOM	1253	CD1	LEU	80	31.316	45.452	45.885	1.00	0.00	C
ATOM	1254	HD11	LEU	80	31.512	44.528	45.342	1.00	0.00	H
ATOM	1255	HD12	LEU	80	30.277	45.725	45.699	1.00	0.00	H
ATOM	1256	HD13	LEU	80	32.001	46.228	45.545	1.00	0.00	H
ATOM	1257	CD2	LEU	80	33.158	44.560	47.351	1.00	0.00	C
ATOM	1258	HD21	LEU	80	33.799	45.085	46.643	1.00	0.00	H
ATOM	1259	HD22	LEU	80	33.657	44.543	48.321	1.00	0.00	H
ATOM	1260	HD23	LEU	80	33.049	43.535	46.996	1.00	0.00	H
ATOM	1261	C	LEU	80	29.567	46.773	48.851	1.00	0.00	C
ATOM	1262	O	LEU	80	28.445	46.430	48.352	1.00	0.00	O
ATOM	1263	N	LYS	81	29.673	46.881	50.166	1.00	0.00	N
ATOM	1264	H	LYS	81	30.523	47.204	50.605	1.00	0.00	H
ATOM	1265	CA	LYS	81	28.588	46.424	51.056	1.00	0.00	C
ATOM	1266	HA	LYS	81	28.271	45.414	50.791	1.00	0.00	H
ATOM	1267	CB	LYS	81	28.989	46.557	52.517	1.00	0.00	C
ATOM	1268	HB2	LYS	81	29.501	47.518	52.513	1.00	0.00	H
ATOM	1269	HB3	LYS	81	28.144	46.740	53.180	1.00	0.00	H
ATOM	1270	CG	LYS	81	29.834	45.319	52.958	1.00	0.00	C
ATOM	1271	HG2	LYS	81	29.141	44.516	53.208	1.00	0.00	H
ATOM	1272	HG3	LYS	81	30.593	45.056	52.222	1.00	0.00	H
ATOM	1273	CD	LYS	81	30.528	45.780	54.254	1.00	0.00	C
ATOM	1274	HD2	LYS	81	30.999	46.741	54.046	1.00	0.00	H
ATOM	1275	HD3	LYS	81	29.791	45.734	55.055	1.00	0.00	H
ATOM	1276	CE	LYS	81	31.555	44.704	54.541	1.00	0.00	C
ATOM	1277	HE2	LYS	81	31.001	43.768	54.624	1.00	0.00	H
ATOM	1278	HE3	LYS	81	32.334	44.586	53.788	1.00	0.00	H
ATOM	1279	NZ	LYS	81	32.256	45.057	55.779	1.00	0.00	N
ATOM	1280	HZ1	LYS	81	32.628	45.994	55.735	1.00	0.00	H
ATOM	1281	HZ2	LYS	81	31.723	44.978	56.634	1.00	0.00	H
ATOM	1282	HZ3	LYS	81	33.070	44.471	55.898	1.00	0.00	H
ATOM	1283	C	LYS	81	27.341	47.268	50.926	1.00	0.00	C
ATOM	1284	O	LYS	81	26.336	46.693	50.970	1.00	0.00	O
ATOM	1285	N	LYS	82	27.416	48.537	50.574	1.00	0.00	N

ATOM	1286	H	LYS	82	28.355	48.905	50.521	1.00	0.00	H
ATOM	1287	CA	LYS	82	26.222	49.395	50.325	1.00	0.00	C
ATOM	1288	HA	LYS	82	25.357	49.143	50.940	1.00	0.00	H
ATOM	1289	CB	LYS	82	26.471	50.933	50.457	1.00	0.00	C
ATOM	1290	HB2	LYS	82	27.279	51.137	49.753	1.00	0.00	H
ATOM	1291	HB3	LYS	82	25.575	51.458	50.124	1.00	0.00	H
ATOM	1292	CG	LYS	82	26.784	51.447	51.855	1.00	0.00	C
ATOM	1293	HG2	LYS	82	25.852	51.568	52.408	1.00	0.00	H
ATOM	1294	HG3	LYS	82	27.379	50.735	52.429	1.00	0.00	H
ATOM	1295	CD	LYS	82	27.597	52.692	51.711	1.00	0.00	C
ATOM	1296	HD2	LYS	82	28.542	52.624	51.172	1.00	0.00	H
ATOM	1297	HD3	LYS	82	27.067	53.434	51.114	1.00	0.00	H
ATOM	1298	CE	LYS	82	27.797	53.317	53.129	1.00	0.00	C
ATOM	1299	HE2	LYS	82	26.847	53.618	53.569	1.00	0.00	H
ATOM	1300	HE3	LYS	82	28.307	52.609	53.783	1.00	0.00	H
ATOM	1301	NZ	LYS	82	28.577	54.579	53.058	1.00	0.00	N
ATOM	1302	HZ1	LYS	82	28.698	54.773	54.041	1.00	0.00	H
ATOM	1303	HZ2	LYS	82	29.487	54.491	52.626	1.00	0.00	H
ATOM	1304	HZ3	LYS	82	28.003	55.306	52.656	1.00	0.00	H
ATOM	1305	C	LYS	82	25.506	49.098	49.022	1.00	0.00	C
ATOM	1306	O	LYS	82	24.295	48.897	48.986	1.00	0.00	O
ATOM	1307	N	VAL	83	26.263	48.864	47.932	1.00	0.00	N
ATOM	1308	H	VAL	83	27.267	48.834	48.026	1.00	0.00	H
ATOM	1309	CA	VAL	83	25.706	48.718	46.563	1.00	0.00	C
ATOM	1310	HA	VAL	83	24.905	49.425	46.353	1.00	0.00	H
ATOM	1311	CB	VAL	83	26.743	48.835	45.378	1.00	0.00	C
ATOM	1312	HB	VAL	83	26.182	48.661	44.460	1.00	0.00	H
ATOM	1313	CG1	VAL	83	27.356	50.251	45.351	1.00	0.00	C
ATOM	1314	HG11	VAL	83	27.979	50.430	46.226	1.00	0.00	H
ATOM	1315	HG12	VAL	83	27.916	50.453	44.439	1.00	0.00	H
ATOM	1316	HG13	VAL	83	26.613	51.035	45.496	1.00	0.00	H
ATOM	1317	CG2	VAL	83	27.888	47.820	45.515	1.00	0.00	C
ATOM	1318	HG21	VAL	83	27.344	46.903	45.290	1.00	0.00	H
ATOM	1319	HG22	VAL	83	28.694	48.014	44.808	1.00	0.00	H
ATOM	1320	HG23	VAL	83	28.379	47.943	46.480	1.00	0.00	H
ATOM	1321	C	VAL	83	24.916	47.370	46.451	1.00	0.00	C
ATOM	1322	O	VAL	83	24.012	47.297	45.660	1.00	0.00	O
ATOM	1323	N	ILE	84	25.289	46.346	47.233	1.00	0.00	N
ATOM	1324	H	ILE	84	26.041	46.638	47.840	1.00	0.00	H
ATOM	1325	CA	ILE	84	24.596	45.119	47.571	1.00	0.00	C
ATOM	1326	HA	ILE	84	24.187	44.828	46.603	1.00	0.00	H
ATOM	1327	CB	ILE	84	25.745	44.088	47.921	1.00	0.00	C
ATOM	1328	HB	ILE	84	26.410	44.569	48.640	1.00	0.00	H
ATOM	1329	CG2	ILE	84	25.050	42.879	48.622	1.00	0.00	C
ATOM	1330	HG21	ILE	84	24.484	43.112	49.523	1.00	0.00	H
ATOM	1331	HG22	ILE	84	24.355	42.378	47.949	1.00	0.00	H
ATOM	1332	HG23	ILE	84	25.826	42.192	48.961	1.00	0.00	H
ATOM	1333	CG1	ILE	84	26.512	43.619	46.633	1.00	0.00	C
ATOM	1334	HG12	ILE	84	25.924	43.317	45.766	1.00	0.00	H
ATOM	1335	HG13	ILE	84	27.061	44.428	46.152	1.00	0.00	H
ATOM	1336	CD1	ILE	84	27.616	42.584	46.783	1.00	0.00	C
ATOM	1337	HD11	ILE	84	28.345	42.546	45.973	1.00	0.00	H
ATOM	1338	HD12	ILE	84	28.166	42.656	47.722	1.00	0.00	H
ATOM	1339	HD13	ILE	84	27.116	41.623	46.892	1.00	0.00	H
ATOM	1340	C	ILE	84	23.457	45.196	48.645	1.00	0.00	C
ATOM	1341	O	ILE	84	22.428	44.662	48.282	1.00	0.00	O
ATOM	1342	N	ALA	85	23.561	45.904	49.751	1.00	0.00	N
ATOM	1343	H	ALA	85	24.438	46.404	49.810	1.00	0.00	H
ATOM	1344	CA	ALA	85	22.369	46.099	50.654	1.00	0.00	C
ATOM	1345	HA	ALA	85	21.867	45.134	50.704	1.00	0.00	H
ATOM	1346	CB	ALA	85	23.013	46.610	51.953	1.00	0.00	C
ATOM	1347	HB1	ALA	85	23.423	47.611	51.814	1.00	0.00	H
ATOM	1348	HB2	ALA	85	22.202	46.720	52.673	1.00	0.00	H
ATOM	1349	HB3	ALA	85	23.778	45.920	52.308	1.00	0.00	H

ATOM	1350	C	ALA	85	21.341	46.992	50.042	1.00	0.00	C
ATOM	1351	O	ALA	85	20.161	46.960	50.454	1.00	0.00	O
ATOM	1352	N	ASP	86	21.787	47.633	48.933	1.00	0.00	N
ATOM	1353	H	ASP	86	22.764	47.520	48.701	1.00	0.00	H
ATOM	1354	CA	ASP	86	20.799	48.261	47.964	1.00	0.00	C
ATOM	1355	HA	ASP	86	20.193	48.954	48.546	1.00	0.00	H
ATOM	1356	CB	ASP	86	21.461	48.851	46.764	1.00	0.00	C
ATOM	1357	HB2	ASP	86	22.338	49.406	47.098	1.00	0.00	H
ATOM	1358	HB3	ASP	86	21.670	48.077	46.025	1.00	0.00	H
ATOM	1359	CG	ASP	86	20.589	49.909	46.087	1.00	0.00	C
ATOM	1360	OD1	ASP	86	19.632	50.430	46.696	1.00	0.00	O
ATOM	1361	OD2	ASP	86	21.009	50.369	45.049	1.00	0.00	O
ATOM	1362	C	ASP	86	19.758	47.289	47.444	1.00	0.00	C
ATOM	1363	O	ASP	86	18.559	47.596	47.454	1.00	0.00	O
ATOM	1364	N	ILE	87	20.119	46.116	46.998	1.00	0.00	N
ATOM	1365	H	ILE	87	21.095	45.865	46.927	1.00	0.00	H
ATOM	1366	CA	ILE	87	19.102	45.171	46.646	1.00	0.00	C
ATOM	1367	HA	ILE	87	18.298	45.806	46.272	1.00	0.00	H
ATOM	1368	CB	ILE	87	19.595	44.360	45.421	1.00	0.00	C
ATOM	1369	HB	ILE	87	20.569	43.954	45.697	1.00	0.00	H
ATOM	1370	CG2	ILE	87	18.623	43.189	45.182	1.00	0.00	C
ATOM	1371	HG21	ILE	87	18.849	42.679	44.245	1.00	0.00	H
ATOM	1372	HG22	ILE	87	18.919	42.405	45.880	1.00	0.00	H
ATOM	1373	HG23	ILE	87	17.579	43.484	45.282	1.00	0.00	H
ATOM	1374	CG1	ILE	87	19.573	45.232	44.189	1.00	0.00	C
ATOM	1375	HG12	ILE	87	18.540	45.206	43.841	1.00	0.00	H
ATOM	1376	HG13	ILE	87	19.883	46.255	44.403	1.00	0.00	H
ATOM	1377	CD1	ILE	87	20.546	44.759	43.143	1.00	0.00	C
ATOM	1378	HD11	ILE	87	20.335	43.711	42.931	1.00	0.00	H
ATOM	1379	HD12	ILE	87	20.488	45.377	42.247	1.00	0.00	H
ATOM	1380	HD13	ILE	87	21.551	44.836	43.557	1.00	0.00	H
ATOM	1381	C	ILE	87	18.611	44.291	47.872	1.00	0.00	C
ATOM	1382	O	ILE	87	17.443	44.179	48.137	1.00	0.00	O
ATOM	1383	N	TYR	88	19.618	43.718	48.432	1.00	0.00	N
ATOM	1384	H	TYR	88	20.572	43.970	48.219	1.00	0.00	H
ATOM	1385	CA	TYR	88	19.480	42.578	49.317	1.00	0.00	C
ATOM	1386	HA	TYR	88	18.641	42.002	48.929	1.00	0.00	H
ATOM	1387	CB	TYR	88	20.720	41.639	49.106	1.00	0.00	C
ATOM	1388	HB2	TYR	88	21.602	42.251	49.295	1.00	0.00	H
ATOM	1389	HB3	TYR	88	20.731	40.800	49.801	1.00	0.00	H
ATOM	1390	CG	TYR	88	20.897	41.099	47.699	1.00	0.00	C
ATOM	1391	CD1	TYR	88	20.017	40.092	47.263	1.00	0.00	C
ATOM	1392	HD1	TYR	88	19.321	39.714	47.997	1.00	0.00	H
ATOM	1393	CE1	TYR	88	19.975	39.619	45.962	1.00	0.00	C
ATOM	1394	HE1	TYR	88	19.422	38.712	45.764	1.00	0.00	H
ATOM	1395	CZ	TYR	88	20.799	40.191	45.002	1.00	0.00	C
ATOM	1396	OH	TYR	88	20.889	39.701	43.734	1.00	0.00	O
ATOM	1397	HH	TYR	88	20.578	38.798	43.632	1.00	0.00	H
ATOM	1398	CE2	TYR	88	21.599	41.355	45.356	1.00	0.00	C
ATOM	1399	HE2	TYR	88	22.141	41.864	44.572	1.00	0.00	H
ATOM	1400	CD2	TYR	88	21.738	41.718	46.708	1.00	0.00	C
ATOM	1401	HD2	TYR	88	22.395	42.512	47.034	1.00	0.00	H
ATOM	1402	C	TYR	88	19.113	42.957	50.778	1.00	0.00	C
ATOM	1403	O	TYR	88	19.576	43.952	51.308	1.00	0.00	O
ATOM	1404	N	PRO	89	18.314	42.048	51.458	1.00	0.00	N
ATOM	1405	CD	PRO	89	17.637	40.922	50.911	1.00	0.00	C
ATOM	1406	HD2	PRO	89	18.101	40.338	50.115	1.00	0.00	H
ATOM	1407	HD3	PRO	89	16.666	41.263	50.552	1.00	0.00	H
ATOM	1408	CG	PRO	89	17.181	40.065	52.156	1.00	0.00	C
ATOM	1409	HG2	PRO	89	18.039	39.434	52.387	1.00	0.00	H
ATOM	1410	HG3	PRO	89	16.332	39.406	51.972	1.00	0.00	H
ATOM	1411	CB	PRO	89	16.952	41.169	53.192	1.00	0.00	C
ATOM	1412	HB2	PRO	89	17.001	40.830	54.227	1.00	0.00	H

ATOM	1413	HB3	PRO	89	16.091	41.801	52.976	1.00	0.00	H
ATOM	1414	CA	PRO	89	18.034	42.171	52.898	1.00	0.00	C
ATOM	1415	HA	PRO	89	17.693	43.164	53.191	1.00	0.00	H
ATOM	1416	C	PRO	89	19.281	41.791	53.675	1.00	0.00	C
ATOM	1417	O	PRO	89	19.973	40.895	53.201	1.00	0.00	O
ATOM	1418	N	GLY	90	19.658	42.486	54.708	1.00	0.00	N
ATOM	1419	H	GLY	90	19.044	43.200	55.069	1.00	0.00	H
ATOM	1420	CA	GLY	90	20.944	42.257	55.341	1.00	0.00	C
ATOM	1421	HA2	GLY	90	21.742	42.533	54.651	1.00	0.00	H
ATOM	1422	HA3	GLY	90	21.002	42.972	56.162	1.00	0.00	H
ATOM	1423	C	GLY	90	21.250	40.831	55.827	1.00	0.00	C
ATOM	1424	O	GLY	90	22.401	40.572	56.125	1.00	0.00	O
ATOM	1425	N	GLN	91	20.229	39.956	55.854	1.00	0.00	N
ATOM	1426	H	GLN	91	19.291	40.184	55.555	1.00	0.00	H
ATOM	1427	CA	GLN	91	20.546	38.570	56.334	1.00	0.00	C
ATOM	1428	HA	GLN	91	21.514	38.520	56.833	1.00	0.00	H
ATOM	1429	CB	GLN	91	19.547	38.060	57.361	1.00	0.00	C
ATOM	1430	HB2	GLN	91	18.514	38.111	57.017	1.00	0.00	H
ATOM	1431	HB3	GLN	91	19.680	36.992	57.533	1.00	0.00	H
ATOM	1432	CG	GLN	91	19.770	38.773	58.733	1.00	0.00	C
ATOM	1433	HG2	GLN	91	20.706	38.516	59.229	1.00	0.00	H
ATOM	1434	HG3	GLN	91	19.732	39.857	58.633	1.00	0.00	H
ATOM	1435	CD	GLN	91	18.698	38.571	59.789	1.00	0.00	C
ATOM	1436	OE1	GLN	91	17.766	37.794	59.714	1.00	0.00	O
ATOM	1437	NE2	GLN	91	18.627	39.384	60.808	1.00	0.00	N
ATOM	1438	HE21	GLN	91	19.340	40.079	60.978	1.00	0.00	H
ATOM	1439	HE22	GLN	91	17.923	39.101	61.475	1.00	0.00	H
ATOM	1440	C	GLN	91	20.780	37.519	55.210	1.00	0.00	C
ATOM	1441	O	GLN	91	20.612	36.299	55.375	1.00	0.00	O
ATOM	1442	N	THR	92	21.006	38.026	54.029	1.00	0.00	N
ATOM	1443	H	THR	92	20.969	39.033	53.958	1.00	0.00	H
ATOM	1444	CA	THR	92	21.444	37.217	52.898	1.00	0.00	C
ATOM	1445	HA	THR	92	20.706	36.471	52.601	1.00	0.00	H
ATOM	1446	CB	THR	92	21.522	38.225	51.699	1.00	0.00	C
ATOM	1447	HB	THR	92	22.246	39.013	51.903	1.00	0.00	H
ATOM	1448	CG2	THR	92	21.858	37.508	50.447	1.00	0.00	C
ATOM	1449	HG21	THR	92	22.888	37.159	50.509	1.00	0.00	H
ATOM	1450	HG22	THR	92	21.263	36.605	50.303	1.00	0.00	H
ATOM	1451	HG23	THR	92	21.706	38.143	49.574	1.00	0.00	H
ATOM	1452	OG1	THR	92	20.231	38.720	51.532	1.00	0.00	O
ATOM	1453	HG1	THR	92	20.147	39.459	52.139	1.00	0.00	H
ATOM	1454	C	THR	92	22.803	36.643	53.117	1.00	0.00	C
ATOM	1455	O	THR	92	23.716	37.360	53.426	1.00	0.00	O
ATOM	1456	N	GLN	93	22.994	35.368	52.841	1.00	0.00	N
ATOM	1457	H	GLN	93	22.184	34.816	52.600	1.00	0.00	H
ATOM	1458	CA	GLN	93	24.252	34.845	52.612	1.00	0.00	C
ATOM	1459	HA	GLN	93	24.934	35.273	53.345	1.00	0.00	H
ATOM	1460	CB	GLN	93	24.229	33.309	52.944	1.00	0.00	C
ATOM	1461	HB2	GLN	93	23.722	33.124	53.891	1.00	0.00	H
ATOM	1462	HB3	GLN	93	23.644	32.779	52.192	1.00	0.00	H
ATOM	1463	CG	GLN	93	25.656	32.625	53.094	1.00	0.00	C
ATOM	1464	HG2	GLN	93	25.503	31.563	53.287	1.00	0.00	H
ATOM	1465	HG3	GLN	93	26.189	32.833	52.166	1.00	0.00	H
ATOM	1466	CD	GLN	93	26.502	33.077	54.299	1.00	0.00	C
ATOM	1467	OE1	GLN	93	26.148	33.868	55.183	1.00	0.00	O
ATOM	1468	NE2	GLN	93	27.708	32.599	54.366	1.00	0.00	N
ATOM	1469	HE21	GLN	93	28.147	32.101	53.605	1.00	0.00	H
ATOM	1470	HE22	GLN	93	28.324	32.744	55.152	1.00	0.00	H
ATOM	1471	C	GLN	93	24.730	35.129	51.164	1.00	0.00	C
ATOM	1472	O	GLN	93	24.060	34.864	50.146	1.00	0.00	O
ATOM	1473	N	PHE	94	26.024	35.472	51.142	1.00	0.00	N
ATOM	1474	H	PHE	94	26.527	35.626	52.004	1.00	0.00	H
ATOM	1475	CA	PHE	94	26.831	35.675	49.928	1.00	0.00	C
ATOM	1476	HA	PHE	94	26.213	35.514	49.044	1.00	0.00	H

```
ATOM   1477  CB  PHE    94      27.413  37.088  49.965  1.00  0.00           C
ATOM   1478  HB2 PHE    94      27.881  37.315  50.922  1.00  0.00           H
ATOM   1479  HB3 PHE    94      28.202  37.231  49.227  1.00  0.00           H
ATOM   1480  CG  PHE    94      26.290  38.083  49.579  1.00  0.00           C
ATOM   1481  CD1 PHE    94      25.956  38.394  48.258  1.00  0.00           C
ATOM   1482  HD1 PHE    94      26.333  37.897  47.377  1.00  0.00           H
ATOM   1483
 CE1 PHE    94      24.820  39.262  48.043  1.00  0.00           C
ATOM   1484  HE1 PHE    94      24.546  39.472  47.020  1.00  0.00           H
ATOM   1485  CZ  PHE    94      24.218  39.882  49.142  1.00  0.00           C
ATOM   1486  HZ  PHE    94      23.364  40.532  49.021  1.00  0.00           H
ATOM   1487  CE2 PHE    94      24.567  39.640  50.444  1.00  0.00           C
ATOM   1488  HE2 PHE    94      23.967  40.118  51.204  1.00  0.00           H
ATOM   1489  CD2 PHE    94      25.631  38.722  50.650  1.00  0.00           C
ATOM   1490  HD2 PHE    94      25.921  38.478  51.660  1.00  0.00           H
ATOM   1491  C   PHE    94      27.867  34.603  49.880  1.00  0.00           C
ATOM   1492  O   PHE    94      27.978  33.647  50.617  1.00  0.00           O
ATOM   1493  N   TYR    95      28.584  34.703  48.769  1.00  0.00           N
ATOM   1494  H   TYR    95      28.259  35.405  48.119  1.00  0.00           H
ATOM   1495  CA  TYR    95      29.588  33.745  48.290  1.00  0.00           C
ATOM   1496  HA  TYR    95      30.152  33.325  49.123  1.00  0.00           H
ATOM   1497  CB  TYR    95      28.841  32.652  47.568  1.00  0.00           C
ATOM   1498  HB2 TYR    95      28.049  32.224  48.182  1.00  0.00           H
ATOM   1499  HB3 TYR    95      28.394  33.039  46.652  1.00  0.00           H
ATOM   1500  CG  TYR    95      29.621  31.443  47.098  1.00  0.00           C
ATOM   1501  CD1 TYR    95      29.763  31.183  45.687  1.00  0.00           C
ATOM   1502  HD1 TYR    95      29.434  31.988  45.047  1.00  0.00           H
ATOM   1503  CE1 TYR    95      30.352  29.984  45.262  1.00  0.00           C
ATOM   1504  HE1 TYR    95      30.307  29.729  44.213  1.00  0.00           H
ATOM   1505  CZ  TYR    95      30.724  28.993  46.269  1.00  0.00           C
ATOM   1506  OH  TYR    95      31.279  27.810  45.844  1.00  0.00           O
ATOM   1507  HH  TYR    95      31.322  27.815  44.885  1.00  0.00           H
ATOM   1508  CE2 TYR    95      30.535  29.240  47.648  1.00  0.00           C
ATOM   1509  HE2 TYR    95      30.739  28.442  48.347  1.00  0.00           H
ATOM   1510  CD2 TYR    95      30.072  30.506  48.046  1.00  0.00           C
ATOM   1511  HD2 TYR    95      29.917  30.641  49.106  1.00  0.00           H
ATOM   1512  C   TYR    95      30.737  34.374  47.428  1.00  0.00           C
ATOM   1513  O   TYR    95      30.630  35.508  46.905  1.00  0.00           O
ATOM   1514  N   VAL    96      31.828  33.689  47.361  1.00  0.00           N
ATOM   1515  H   VAL    96      31.842  32.897  47.987  1.00  0.00           H
ATOM   1516  CA  VAL    96      33.019  34.026  46.648  1.00  0.00           C
ATOM   1517  HA  VAL    96      32.918  34.965  46.102  1.00  0.00           H
ATOM   1518  CB  VAL    96      34.212  34.327  47.585  1.00  0.00           C
ATOM   1519  HB  VAL    96      34.359  33.495  48.273  1.00  0.00           H
ATOM   1520  CG1 VAL    96      35.533  34.479  46.762  1.00  0.00           C
ATOM   1521 HG11 VAL    96      35.582  35.318  46.067  1.00  0.00           H
ATOM   1522 HG12 VAL    96      36.479  34.530  47.302  1.00  0.00           H
ATOM   1523 HG13 VAL    96      35.620  33.582  46.148  1.00  0.00           H
ATOM   1524  CG2 VAL    96      34.051  35.549  48.395  1.00  0.00           C
ATOM   1525 HG21 VAL    96      33.758  36.358  47.726  1.00  0.00           H
ATOM   1526 HG22 VAL    96      33.404  35.388  49.257  1.00  0.00           H
ATOM   1527 HG23 VAL    96      35.030  35.772  48.818  1.00  0.00           H
ATOM   1528  C   VAL    96      33.340  32.911  45.612  1.00  0.00           C
ATOM   1529  O   VAL    96      33.566  31.809  45.991  1.00  0.00           O
ATOM   1530  N   ILE    97      33.495  33.297  44.361  1.00  0.00           N
ATOM   1531  H   ILE    97      33.471  34.265  44.074  1.00  0.00           H
ATOM   1532  CA  ILE    97      34.147  32.443  43.311  1.00  0.00           C
ATOM   1533  HA  ILE    97      34.470  31.525  43.803  1.00  0.00           H
ATOM   1534  CB  ILE    97      33.278  32.200  42.065  1.00  0.00           C
ATOM   1535  HB  ILE    97      32.904  33.170  41.736  1.00  0.00           H
ATOM   1536  CG2 ILE    97      34.087  31.565  40.915  1.00  0.00           C
ATOM   1537 HG21 ILE    97      34.867  32.166  40.447  1.00  0.00           H
ATOM   1538 HG22 ILE    97      34.490  30.589  41.186  1.00  0.00           H
ATOM   1539 HG23 ILE    97      33.383  31.285  40.130  1.00  0.00           H
```

ATOM	1540	CG1	ILE	97	32.083	31.341	42.473	1.00	0.00	C
ATOM	1541	HG12	ILE	97	32.553	30.400	42.758	1.00	0.00	H
ATOM	1542	HG13	ILE	97	31.723	31.749	43.417	1.00	0.00	H
ATOM	1543	CD1	ILE	97	30.912	31.200	41.545	1.00	0.00	C
ATOM	1544	HD11	ILE	97	30.541	32.212	41.386	1.00	0.00	H
ATOM	1545	HD12	ILE	97	31.114	30.799	40.551	1.00	0.00	H
ATOM	1546	HD13	ILE	97	30.083	30.647	41.989	1.00	0.00	H
ATOM	1547	C	ILE	97	35.417	33.174	42.823	1.00	0.00	C
ATOM	1548	O	ILE	97	35.288	34.402	42.575	1.00	0.00	O
ATOM	1549	N	GLU	98	36.492	32.546	42.787	1.00	0.00	N
ATOM	1550	H	GLU	98	36.548	31.640	43.228	1.00	0.00	H
ATOM	1551	CA	GLU	98	37.780	33.026	42.337	1.00	0.00	C
ATOM	1552	HA	GLU	98	37.579	33.953	41.800	1.00	0.00	H
ATOM	1553	CB	GLU	98	38.577	33.592	43.555	1.00	0.00	C
ATOM	1554	HB2	GLU	98	37.910	34.053	44.282	1.00	0.00	H
ATOM	1555	HB3	GLU	98	38.938	32.764	44.167	1.00	0.00	H
ATOM	1556	CG	GLU	98	39.851	34.515	43.270	1.00	0.00	C
ATOM	1557	HG2	GLU	98	40.611	34.021	42.664	1.00	0.00	H
ATOM	1558	HG3	GLU	98	39.558	35.345	42.628	1.00	0.00	H
ATOM	1559	CD	GLU	98	40.486	35.193	44.569	1.00	0.00	C
ATOM	1560	OE1	GLU	98	41.356	36.079	44.416	1.00	0.00	O
ATOM	1561	OE2	GLU	98	40.220	34.767	45.719	1.00	0.00	O
ATOM	1562	C	GLU	98	38.587	32.100	41.389	1.00	0.00	C
ATOM	1563	O	GLU	98	38.355	30.901	41.528	1.00	0.00	O
ATOM	1564	N	PHE	99	39.473	32.599	40.527	1.00	0.00	N
ATOM	1565	H	PHE	99	39.654	33.593	40.509	1.00	0.00	H
ATOM	1566	CA	PHE	99	39.950	31.855	39.290	1.00	0.00	C
ATOM	1567	HA	PHE	99	40.322	30.897	39.654	1.00	0.00	H
ATOM	1568	CB	PHE	99	38.781	31.840	38.350	1.00	0.00	C
ATOM	1569	HB2	PHE	99	38.937	31.205	37.479	1.00	0.00	H
ATOM	1570	HB3	PHE	99	37.980	31.281	38.831	1.00	0.00	H
ATOM	1571	CG	PHE	99	38.230	33.159	37.833	1.00	0.00	C
ATOM	1572	CD1	PHE	99	38.805	33.853	36.716	1.00	0.00	C
ATOM	1573	HD1	PHE	99	39.718	33.469	36.284	1.00	0.00	H
ATOM	1574	CE1	PHE	99	38.111	34.939	36.162	1.00	0.00	C
ATOM	1575	HE1	PHE	99	38.473	35.463	35.289	1.00	0.00	H
ATOM	1576	CZ	PHE	99	36.963	35.407	36.798	1.00	0.00	C
ATOM	1577	HZ	PHE	99	36.439	36.257	36.388	1.00	0.00	H
ATOM	1578	CE2	PHE	99	36.467	34.825	37.948	1.00	0.00	C
ATOM	1579	HE2	PHE	99	35.640	35.200	38.533	1.00	0.00	H
ATOM	1580	CD2	PHE	99	37.144	33.703	38.478	1.00	0.00	C
ATOM	1581	HD2	PHE	99	36.658	33.211	39.307	1.00	0.00	H
ATOM	1582	C	PHE	99	41.191	32.462	38.607	1.00	0.00	C
ATOM	1583	O	PHE	99	41.637	33.511	39.036	1.00	0.00	O
ATOM	1584	N	LYS	100	41.643	31.826	37.491	1.00	0.00	N
ATOM	1585	H	LYS	100	41.143	30.997	37.206	1.00	0.00	H
ATOM	1586	CA	LYS	100	42.862	32.153	36.761	1.00	0.00	C
ATOM	1587	HA	LYS	100	43.014	33.232	36.781	1.00	0.00	H
ATOM	1588	CB	LYS	100	44.046	31.469	37.439	1.00	0.00	C
ATOM	1589	HB2	LYS	100	43.823	30.539	37.960	1.00	0.00	H
ATOM	1590	HB3	LYS	100	44.799	31.308	36.668	1.00	0.00	H
ATOM	1591	CG	LYS	100	44.671	32.488	38.400	1.00	0.00	C
ATOM	1592	HG2	LYS	100	44.873	33.468	37.969	1.00	0.00	H
ATOM	1593	HG3	LYS	100	43.950	32.627	39.206	1.00	0.00	H
ATOM	1594	CD	LYS	100	45.906	31.834	39.074	1.00	0.00	C
ATOM	1595	HD2	LYS	100	45.486	31.067	39.724	1.00	0.00	H
ATOM	1596	HD3	LYS	100	46.469	31.393	38.252	1.00	0.00	H
ATOM	1597	CE	LYS	100	46.715	32.964	39.712	1.00	0.00	C
ATOM	1598	HE2	LYS	100	47.031	33.628	38.907	1.00	0.00	H
ATOM	1599	HE3	LYS	100	46.029	33.483	40.382	1.00	0.00	H
ATOM	1600	NZ	LYS	100	47.821	32.347	40.545	1.00	0.00	N
ATOM	1601	HZ1	LYS	100	48.560	31.966	39.971	1.00	0.00	H
ATOM	1602	HZ2	LYS	100	48.235	33.052	41.137	1.00	0.00	H
ATOM	1603	HZ3	LYS	100	47.532	31.561	41.109	1.00	0.00	H

```
ATOM   1604  C   LYS   100      42.845  31.494  35.293  1.00  0.00            C
ATOM   1605  O   LYS   100      42.403  30.365  35.157  1.00  0.00            O
ATOM   1606  N   CYX   101      43.279  32.222  34.267  1.00  0.00            N
ATOM   1607  H   CYX   101      43.811  33.051  34.488  1.00  0.00            H
ATOM   1608  CA  CYX   101      43.470
  31.679  32.877  1.00  0.00            C
ATOM   1609  HA  CYX   101      42.648  30.975  32.752  1.00  0.00            H
ATOM   1610  CB  CYX   101      43.389  32.757  31.741  1.00  0.00            C
ATOM   1611  HB2 CYX   101      42.546  33.429  31.903  1.00  0.00            H
ATOM   1612  HB3 CYX   101      44.211  33.448  31.919  1.00  0.00            H
ATOM   1613  SG  CYX   101      43.427  32.215  30.019  1.00  0.00            S
ATOM   1614  C   CYX   101      44.805  30.900  32.615  1.00  0.00            C
ATOM   1615  O   CYX   101      45.909  31.276  33.087  1.00  0.00            O
ATOM   1616  N   LEU   102      44.687  29.790  31.953  1.00  0.00            N
ATOM   1617  H   LEU   102      43.847  29.619  31.418  1.00  0.00            H
ATOM   1618  CA  LEU   102      45.890  28.923  31.610  1.00  0.00            C
ATOM   1619  HA  LEU   102      46.696  29.099  32.321  1.00  0.00            H
ATOM   1620  CB  LEU   102      45.462  27.393  31.741  1.00  0.00            C
ATOM   1621  HB2 LEU   102      44.464  27.380  31.304  1.00  0.00            H
ATOM   1622  HB3 LEU   102      46.071  26.733  31.121  1.00  0.00            H
ATOM   1623  CG  LEU   102      45.342  26.766  33.123  1.00  0.00            C
ATOM   1624  HG  LEU   102      46.004  27.401  33.713  1.00  0.00            H
ATOM   1625  CD1 LEU   102      43.950  26.747  33.817  1.00  0.00            C
ATOM   1626 HD11 LEU   102      43.621  27.772  33.990  1.00  0.00            H
ATOM   1627 HD12 LEU   102      43.267  26.196  33.172  1.00  0.00            H
ATOM   1628 HD13 LEU   102      44.015  26.116  34.703  1.00  0.00            H
ATOM   1629  CD2 LEU   102      45.845  25.330  33.177  1.00  0.00            C
ATOM   1630 HD21 LEU   102      45.700  24.904  34.170  1.00  0.00            H
ATOM   1631 HD22 LEU   102      45.176  24.690  32.603  1.00  0.00            H
ATOM   1632 HD23 LEU   102      46.868  25.264  32.803  1.00  0.00            H
ATOM   1633  C   LEU   102      46.453  29.337  30.233  1.00  0.00            C
ATOM   1634  O   LEU   102      47.087  30.435  30.176  1.00  0.00            O
ATOM   1635  OXT LEU   102      46.456  28.555  29.246  1.00  0.00            O
HETATM 1637  N   LIG   103      28.678  40.409  38.813  1.00  0.00            N
HETATM 1638  C   LIG   103      28.833  39.115  38.650  1.00  0.00            C
HETATM 1639  O   LIG   103      27.366  42.016  39.519  1.00  0.00            O
HETATM 1640  C5' LIG   103      24.509  38.632  43.987  1.00  0.00            C
HETATM 1641  O5' LIG   103      24.663  37.534  43.075  1.00  0.00            O
HETATM 1642  C4' LIG   103      24.581  40.056  43.287  1.00  0.00            C
HETATM 1643  O4' LIG   103      25.964  40.132  42.740  1.00  0.00            O
HETATM 1644  C3' LIG   103      23.615  40.180  42.117  1.00  0.00            C
HETATM 1645  O3' LIG   103      22.904  41.352  42.118  1.00  0.00            O
HETATM 1646  C2' LIG   103      24.493  40.216  40.858  1.00  0.00            C
HETATM 1647  O2' LIG   103      23.999  41.091  39.824  1.00  0.00            O
HETATM 1648  C1' LIG   103      25.931  40.573  41.349  1.00  0.00            C
HETATM 1649  N1  LIG   103      27.073  40.027  40.516  1.00  0.00            N
HETATM 1650  O1  LIG   103      29.341  36.434  37.370  1.00  0.00            O
HETATM 1651  N2  LIG   103      29.768  38.709  37.750  1.00  0.00            N
HETATM 1652  C6  LIG   103      27.678  40.871  39.638  1.00  0.00            C
HETATM 1653  C7  LIG   103      27.256  38.645  40.453  1.00  0.00            C
HETATM 1654  C8  LIG   103      28.086  38.169  39.515  1.00  0.00            C
HETATM 1655  C9  LIG   103      29.988  37.471  37.182  1.00  0.00            C
HETATM 1656  C10 LIG   103      31.192  37.513  36.193  1.00  0.00            C
HETATM 1657  H   LIG   103      30.344  39.501  37.493  1.00  0.00            H
HETATM 1658  H1  LIG   103      28.441  37.146  39.559  1.00  0.00            H
HETATM 1659  H4  LIG   103      26.048  41.662  41.405  1.00  0.00            H
HETATM 1660  H6  LIG   103      24.343  40.810  44.047  1.00  0.00            H
HETATM 1661  H7  LIG   103      22.881  39.366  42.083  1.00  0.00            H
HETATM 1662  H8  LIG   103      24.626  39.172  40.548  1.00  0.00            H
HETATM 1663  H9  LIG   103      23.494  38.555  44.396  1.00  0.00            H
HETATM 1664  H10 LIG   103      25.294  38.547  44.748  1.00  0.00            H
HETATM 1665  H11 LIG   103      30.827  37.908  35.237  1.00  0.00            H
HETATM 1666  H12 LIG   103      31.594  36.502  36.054  1.00  0.00            H
HETATM 1667  H13 LIG   103      31.983  38.190  36.537  1.00  0.00            H
```

```
HETATM 1668  H14 LIG   103       26.760  38.132  41.265  1.00  0.00           H
HETATM 1669  H2' LIG   103       23.312  40.566  39.379  1.00  0.00           H
HETATM 1670  H3' LIG   103       23.020  41.703  41.218  1.00  0.00           H
HETATM 1671  H5' LIG   103       23.973  36.869  43.245  1.00  0.00           H
ENDMDL
MODEL      5
SHEET    1   1 1 ILE    22  ASP    26  0
SHEET    2   2 1 VAL    37  VAL    40  0
SHEET    3   3 1 CYX    50  VAL    60  0
SHEET    4   4 1 PHE    94  CYX   101  0
HELIX    1   1 GLN     9  LEU     16  1                                   8
HELIX    2   2 ASP    45  GLY     47  1                                   3
HELIX    3   3 GLU    67  GLN     72  1                                   6
HELIX    4   4 LEU    77  ALA     85  1                                   9
ATOM      1  N   GLN     1       37.020  17.808  33.432  1.00  0.00           N
ATOM      2  H1  GLN     1       36.486  17.557  34.252  1.00  0.00           H
ATOM      3  H2  GLN     1       37.637  17.035  33.228  1.00  0.00           H
ATOM      4  H3  GLN     1       37.632  18.587  33.632  1.00  0.00           H
ATOM      5  CA  GLN     1       36.204  18.231  32.287  1.00  0.00           C
ATOM      6  HA  GLN     1       36.952  18.328  31.499  1.00  0.00           H
ATOM      7  CB  GLN     1       35.207  17.157  31.835  1.00  0.00           C
ATOM      8  HB2 GLN     1       34.600  17.139  32.741  1.00  0.00           H
ATOM      9  HB3 GLN     1       34.671  17.554  30.973  1.00  0.00           H
ATOM     10  CG  GLN     1       35.572  15.756  31.427  1.00  0.00           C
ATOM     11  HG2 GLN     1       35.926  15.209  32.302  1.00  0.00           H
ATOM     12  HG3 GLN     1       34.661  15.317  31.021  1.00  0.00           H
ATOM     13  CD  GLN     1       36.708  15.674  30.379  1.00  0.00           C
ATOM     14  OE1 GLN     1       36.944  16.588  29.637  1.00  0.00           O
ATOM     15  NE2 GLN     1       37.170  14.504  30.151  1.00  0.00           N
ATOM     16 HE21 GLN     1       36.810  13.734  30.696  1.00  0.00           H
ATOM     17 HE22 GLN     1       37.868  14.413  29.426  1.00  0.00           H
ATOM     18  C   GLN     1       35.688  19.640  32.517  1.00  0.00           C
ATOM     19  O   GLN     1       35.392  19.990  33.640  1.00  0.00           O
ATOM     20  N   PRO     2       35.628  20.480  31.441  1.00  0.00           N
ATOM     21  CD  PRO     2       36.045  20.282  30.129  1.00  0.00           C
ATOM     22  HD2 PRO     2       35.677  19.321  29.771  1.00  0.00           H
ATOM     23  HD3 PRO     2       37.124  20.404  30.051  1.00  0.00           H
ATOM     24  CG  PRO     2       35.340  21.340  29.319  1.00  0.00           C
ATOM     25  HG2 PRO     2       34.326  20.982  29.139  1.00  0.00           H
ATOM     26  HG3 PRO     2       35.939  21.495  28.422  1.00  0.00           H
ATOM     27  CB  PRO     2       35.318  22.543  30.267  1.00  0.00           C
ATOM     28  HB2 PRO     2       34.560  23.298  30.058  1.00  0.00           H
ATOM     29  HB3 PRO     2       36.323  22.953  30.187  1.00  0.00           H
ATOM     30  CA  PRO     2       35.108  21.832  31.631  1.00  0.00           C
ATOM     31  HA  PRO     2       35.718  22.383  32.347  1.00  0.00           H
ATOM     32  C   PRO     2       33.593  21.886  31.975  1.00  0.00           C
ATOM     33  O   PRO     2       32.846  21.119  31.337  1.00  0.00           O
ATOM     34  N   ASN     3       33.156  22.778  32.876  1.00  0.00           N
ATOM     35  H   ASN     3       33.870  23.255  33.407  1.00  0.00           H
ATOM     36  CA  ASN     3       31.779  23.093  33.140  1.00  0.00           C
ATOM     37  HA  ASN     3       31.212  22.231  33.493  1.00  0.00           H
ATOM     38  CB  ASN     3       31.682  24.129  34.293  1.00  0.00           C
ATOM     39  HB2 ASN     3       32.267  25.046  34.229  1.00  0.00           H
ATOM     40  HB3 ASN     3       30.662  24.495  34.414  1.00  0.00           H
ATOM     41  CG  ASN     3       32.193  23.595  35.563  1.00  0.00           C
ATOM     42  OD1 ASN     3       33.175  22.902  35.700  1.00  0.00           O
ATOM     43  ND2 ASN     3       31.561  23.961  36.689  1.00  0.00           N
ATOM     44 HD21 ASN     3       30.752  24.563  36.627  1.00  0.00           H
ATOM     45 HD22 ASN     3       31.993  23.755  37.579  1.00  0.00           H
ATOM     46  C   ASN     3       31.023  23.647  31.911  1.00  0.00           C
ATOM     47  O   ASN     3       31.374  24.547  31.268  1.00  0.00           O
ATOM     48  N   ASP     4       29.806  23.079  31.694  1.00  0.00           N
ATOM     49  H   ASP     4       29.456  22.396  32.352  1.00  0.00           H
ATOM     50  CA  ASP     4       28.782  23.522  30.693  1.00  0.00           C
```

```
ATOM     51  HA  ASP     4      29.096  23.438  29.652  1.00  0.00           H
ATOM     52  CB  ASP     4      27.505  22.569  30.688  1.00  0.00           C
ATOM     53  HB2 ASP     4      26.855  22.892  31.500  1.00  0.00           H
ATOM     54  HB3 ASP     4      26.945  22.776  29.776  1.00  0.00           H
ATOM     55  CG  ASP     4      27.830  21.058  30.967  1.00  0.00           C
ATOM     56  OD1 ASP     4      28.978  20.575  30.814  1.00  0.00           O
ATOM     57  OD2 ASP     4      26.932  20.222
  31.278  1.00  0.00           O
ATOM     58  C   ASP     4      28.138  24.882  31.140  1.00  0.00           C
ATOM     59  O   ASP     4      27.591  25.606  30.281  1.00  0.00           O
ATOM     60  N   ILE     5      28.254  25.269  32.423  1.00  0.00           N
ATOM     61  H   ILE     5      28.784  24.685  33.053  1.00  0.00           H
ATOM     62  CA  ILE     5      27.871  26.635  32.923  1.00  0.00           C
ATOM     63  HA  ILE     5      27.205  27.066  32.175  1.00  0.00           H
ATOM     64  CB  ILE     5      27.078  26.570  34.275  1.00  0.00           C
ATOM     65  HB  ILE     5      27.794  26.413  35.082  1.00  0.00           H
ATOM     66  CG2 ILE     5      26.476  27.942  34.578  1.00  0.00           C
ATOM     67 HG21 ILE     5      25.789  28.072  35.415  1.00  0.00           H
ATOM     68 HG22 ILE     5      27.287  28.571  34.944  1.00  0.00           H
ATOM     69 HG23 ILE     5      26.059  28.330  33.649  1.00  0.00           H
ATOM     70  CG1 ILE     5      26.058  25.450  34.389  1.00  0.00           C
ATOM     71 HG12 ILE     5      26.696  24.567  34.350  1.00  0.00           H
ATOM     72 HG13 ILE     5      25.597  25.391  35.375  1.00  0.00           H
ATOM     73  CD1 ILE     5      24.954  25.364  33.307  1.00  0.00           C
ATOM     74 HD11 ILE     5      24.329  24.479  33.427  1.00  0.00           H
ATOM     75 HD12 ILE     5      24.417  26.307  33.420  1.00  0.00           H
ATOM     76 HD13 ILE     5      25.403  25.331  32.316  1.00  0.00           H
ATOM     77  C   ILE     5      29.133  27.551  33.013  1.00  0.00           C
ATOM     78  O   ILE     5      30.228  27.137  33.363  1.00  0.00           O
ATOM     79  N   THR     6      29.026  28.812  32.563  1.00  0.00           N
ATOM     80  H   THR     6      28.129  29.110  32.205  1.00  0.00           H
ATOM     81  CA  THR     6      30.148  29.800  32.503  1.00  0.00           C
ATOM     82  HA  THR     6      30.926  29.547  33.223  1.00  0.00           H
ATOM     83  CB  THR     6      30.741  29.768  30.997  1.00  0.00           C
ATOM     84  HB  THR     6      30.955  28.789  30.566  1.00  0.00           H
ATOM     85  CG2 THR     6      29.772  30.410  30.000  1.00  0.00           C
ATOM     86 HG21 THR     6      29.531  31.425  30.315  1.00  0.00           H
ATOM     87 HG22 THR     6      30.240  30.393  29.015  1.00  0.00           H
ATOM     88 HG23 THR     6      28.804  29.924  29.880  1.00  0.00           H
ATOM     89  OG1 THR     6      31.940  30.481  30.909  1.00  0.00           O
ATOM     90  HG1 THR     6      32.418  30.166  30.140  1.00  0.00           H
ATOM     91  C   THR     6      29.620  31.186  32.846  1.00  0.00           C
ATOM     92  O   THR     6      28.429  31.273  33.147  1.00  0.00           O
ATOM     93  N   PHE     7      30.444  32.177  32.762  1.00  0.00           N
ATOM     94  H   PHE     7      31.402  32.034  32.471  1.00  0.00           H
ATOM     95  CA  PHE     7      30.068  33.591  32.946  1.00  0.00           C
ATOM     96  HA  PHE     7      29.447  33.659  33.841  1.00  0.00           H
ATOM     97  CB  PHE     7      31.327  34.364  33.094  1.00  0.00           C
ATOM     98  HB2 PHE     7      31.878  34.346  32.153  1.00  0.00           H
ATOM     99  HB3 PHE     7      31.027  35.404  33.227  1.00  0.00           H
ATOM    100  CG  PHE     7      32.248  33.928  34.162  1.00  0.00           C
ATOM    101  CD1 PHE     7      31.862  33.934  35.519  1.00  0.00           C
ATOM    102  HD1 PHE     7      30.933  34.425  35.768  1.00  0.00           H
ATOM    103  CE1 PHE     7      32.619  33.415  36.507  1.00  0.00           C
ATOM    104  HE1 PHE     7      32.289  33.441  37.536  1.00  0.00           H
ATOM    105  CZ  PHE     7      33.872  32.943  36.280  1.00  0.00           C
ATOM    106  HZ  PHE     7      34.439  32.535  37.103  1.00  0.00           H
ATOM    107  CE2 PHE     7      34.443  32.999  34.992  1.00  0.00           C
ATOM    108  HE2 PHE     7      35.447  32.614  34.884  1.00  0.00           H
ATOM    109  CD2 PHE     7      33.577  33.408  33.902  1.00  0.00           C
ATOM    110  HD2 PHE     7      33.884  33.392  32.867  1.00  0.00           H
ATOM    111  C   PHE     7      29.243  34.151  31.757  1.00  0.00           C
ATOM    112  O   PHE     7      29.547  33.898  30.610  1.00  0.00           O
ATOM    113  N   PHE     8      28.312  35.075  32.007  1.00  0.00           N
```

ATOM	114	H	PHE	8	28.034	35.057	32.978	1.00	0.00	H
ATOM	115	CA	PHE	8	27.453	35.794	30.988	1.00	0.00	C
ATOM	116	HA	PHE	8	27.119	35.075	30.241	1.00	0.00	H
ATOM	117	CB	PHE	8	26.189	36.330	31.789	1.00	0.00	C
ATOM	118	HB2	PHE	8	26.484	37.021	32.579	1.00	0.00	H
ATOM	119	HB3	PHE	8	25.635	37.024	31.157	1.00	0.00	H
ATOM	120	CG	PHE	8	25.215	35.321	32.445	1.00	0.00	C
ATOM	121	CD1	PHE	8	24.626	35.446	33.751	1.00	0.00	C
ATOM	122	HD1	PHE	8	24.928	36.301	34.339	1.00	0.00	H
ATOM	123	CE1	PHE	8	23.563	34.675	34.162	1.00	0.00	C
ATOM	124	HE1	PHE	8	23.108	34.803	35.134	1.00	0.00	H
ATOM	125	CZ	PHE	8	23.073	33.684	33.360	1.00	0.00	C
ATOM	126	HZ	PHE	8	22.219	33.076	33.623	1.00	0.00	H
ATOM	127	CE2	PHE	8	23.671	33.405	32.088	1.00	0.00	C
ATOM	128	HE2	PHE	8	23.196	32.686	31.436	1.00	0.00	H
ATOM	129	CD2	PHE	8	24.769	34.193	31.679	1.00	0.00	C
ATOM	130	HD2	PHE	8	25.098	34.106	30.655	1.00	0.00	H
ATOM	131	C	PHE	8	28.279	36.833	30.190	1.00	0.00	C
ATOM	132	O	PHE	8	29.202	37.422	30.672	1.00	0.00	O
ATOM	133	N	GLN	9	27.817	37.048	28.906	1.00	0.00	N
ATOM	134	H	GLN	9	26.892	36.752	28.634	1.00	0.00	H
ATOM	135	CA	GLN	9	28.559	37.703	27.823	1.00	0.00	C
ATOM	136	HA	GLN	9	29.457	37.145	27.556	1.00	0.00	H
ATOM	137	CB	GLN	9	27.574	37.564	26.679	1.00	0.00	C
ATOM	138	HB2	GLN	9	27.323	36.514	26.532	1.00	0.00	H
ATOM	139	HB3	GLN	9	26.655	38.076	26.961	1.00	0.00	H
ATOM	140	CG	GLN	9	28.120	38.109	25.346	1.00	0.00	C
ATOM	141	HG2	GLN	9	29.187	37.903	25.268	1.00	0.00	H
ATOM	142	HG3	GLN	9	27.731	37.448	24.571	1.00	0.00	H
ATOM	143	CD	GLN	9	27.827	39.576	25.000	1.00	0.00	C
ATOM	144	OE1	GLN	9	27.736	40.517	25.741	1.00	0.00	O
ATOM	145	NE2	GLN	9	27.644	39.896	23.757	1.00	0.00	N
ATOM	146	HE21	GLN	9	27.664	40.850	23.426	1.00	0.00	H
ATOM	147	HE22	GLN	9	27.737	39.247	22.988	1.00	0.00	H
ATOM	148	C	GLN	9	29.132	39.094	28.041	1.00	0.00	C
ATOM	149	O	GLN	9	30.312	39.369	27.765	1.00	0.00	O
ATOM	150	N	ARG	10	28.455	39.880	28.863	1.00	0.00	N
ATOM	151	H	ARG	10	27.548	39.527	29.133	1.00	0.00	H
ATOM	152	CA	ARG	10	28.715	41.240	29.304	1.00	0.00	C
ATOM	153	HA	ARG	10	28.951	41.842	28.427	1.00	0.00	H
ATOM	154	CB	ARG	10	27.520	41.889	30.029	1.00	0.00	C
ATOM	155	HB2	ARG	10	27.197	41.261	30.859	1.00	0.00	H
ATOM	156	HB3	ARG	10	27.936	42.786	30.488	1.00	0.00	H
ATOM	157	CG	ARG	10	26.439	42.212	29.006	1.00	0.00	C
ATOM	158	HG2	ARG	10	26.272	41.417	28.280	1.00	0.00	H
ATOM	159	HG3	ARG	10	25.510	42.253	29.574	1.00	0.00	H
ATOM	160	CD	ARG	10	26.651	43.546	28.263	1.00	0.00	C
ATOM	161	HD2	ARG	10	26.902	44.350	28.955	1.00	0.00	H
ATOM	162	HD3	ARG	10	27.568	43.431	27.687	1.00	0.00	H
ATOM	163	NE	ARG	10	25.466	43.925	27.441	1.00	0.00	N
ATOM	164	HE	ARG	10	24.899	44.620	27.904	1.00	0.00	H
ATOM	165	CZ	ARG	10	25.017	43.326	26.395	1.00	0.00	C
ATOM	166	NH1	ARG	10	25.487	42.241	25.811	1.00	0.00	N
ATOM	167	HH11	ARG	10	24.904	41.757	25.145	1.00	0.00	H
ATOM	168	HH12	ARG	10	26.348	41.819	26.128	1.00	0.00	H
ATOM	169	NH2	ARG	10	23.944	43.797	25.817	1.00	0.00	N
ATOM	170	HH21	ARG	10	23.718	44.763	26.008	1.00	0.00	H
ATOM	171	HH22	ARG	10	23.578	43.348	24.989	1.00	0.00	H
ATOM	172	C	ARG	10	29.948	41.305	30.214	1.00	0.00	C
ATOM	173	O	ARG	10	30.567	42.364	30.405	1.00	0.00	O
ATOM	174	N	PHE	11	30.242	40.231	30.928	1.00	0.00	N
ATOM	175	H	PHE	11	29.664	39.409	30.826	1.00	0.00	H
ATOM	176	CA	PHE	11	31.443	40.139	31.782	1.00	0.00	C
ATOM	177	HA	PHE	11	31.640	41.107	32.244	1.00	0.00	H

ATOM	178	CB	PHE	11	31.216	39.244	32.983	1.00	0.00	C
ATOM	179	HB2	PHE	11	32.068	39.467	33.625	1.00	0.00	H
ATOM	180	HB3	PHE	11	31.166	38.172	32.791	1.00	0.00	H
ATOM	181	CG	PHE	11	29.902	39.640	33.691	1.00	0.00	C
ATOM	182	CD1	PHE	11	29.692	40.978	34.187	1.00	0.00	C
ATOM	183	HD1	PHE	11	30.374	41.769	33.912	1.00	0.00	H
ATOM	184	CE1	PHE	11	28.531	41.256	34.956	1.00	0.00	C
ATOM	185	HE1	PHE	11	28.367	42.261	35.316	1.00	0.00	H
ATOM	186	CZ	PHE	11	27.543	40.232	35.211	1.00	0.00	C
ATOM	187	HZ	PHE	11	26.583	40.435	35.663	1.00	0.00	H
ATOM	188	CE2	PHE	11	27.756	38.922	34.705	1.00	0.00	C
ATOM	189	HE2	PHE	11	27.036	38.167	34.984	1.00	0.00	H
ATOM	190	CD2	PHE	11	28.971	38.588	34.078	1.00	0.00	C
ATOM	191	HD2	PHE	11	29.032	37.579	33.696	1.00	0.00	H
ATOM	192	C	PHE	11	32.678	39.725	31.008	1.00	0.00	C
ATOM	193	O	PHE	11	33.810	40.068	31.375	1.00	0.00	O
ATOM	194	N	GLN	12	32.483	39.048	29.855	1.00	0.00	N
ATOM	195	H	GLN	12	31.589	38.836	29.438	1.00	0.00	H
ATOM	196	CA	GLN	12	33.615	38.286	29.259	1.00	0.00	C
ATOM	197	HA	GLN	12	34.061	37.730	30.083	1.00	0.00	H
ATOM	198	CB	GLN	12	33.087	37.220	28.263	1.00	0.00	C
ATOM	199	HB2	GLN	12	32.505	37.650	27.449	1.00	0.00	H
ATOM	200	HB3	GLN	12	33.917	36.749	27.735	1.00	0.00	H
ATOM	201	CG	GLN	12	32.349	35.984	28.932	1.00	0.00	C
ATOM	202	HG2	GLN	12	32.951	35.647	29.774	1.00	0.00	H
ATOM	203	HG3	GLN	12	31.402	36.360	29.317	1.00	0.00	H
ATOM	204	CD	GLN	12	32.208	34.807	27.964	1.00	0.00	C
ATOM	205	OE1	GLN	12	32.657	34.813	26.811	1.00	0.00	O
ATOM	206	NE2	GLN	12	31.543	33.773	28.293	1.00	0.00	N
ATOM	207	HE21	GLN	12	30.990	33.659	29.131	1.00	0.00	H
ATOM	208	HE22	GLN	12	31.525	33.031	27.608	1.00	0.00	H
ATOM	209	C	GLN	12	34.714	39.199	28.591	1.00	0.00	C
ATOM	210	O	GLN	12	35.822	38.727	28.470	1.00	0.00	O
ATOM	211	N	ASP	13	34.407	40.501	28.261	1.00	0.00	N
ATOM	212	H	ASP	13	33.463	40.855	28.307	1.00	0.00	H
ATOM	213	CA	ASP	13	35.439	41.453	27.881	1.00	0.00	C
ATOM	214	HA	ASP	13	36.130	40.863	27.279	1.00	0.00	H
ATOM	215	CB	ASP	13	34.961	42.481	26.842	1.00	0.00	C
ATOM	216	HB2	ASP	13	34.153	43.043	27.312	1.00	0.00	H
ATOM	217	HB3	ASP	13	35.827	43.081	26.564	1.00	0.00	H
ATOM	218	CG	ASP	13	34.389	41.993	25.533	1.00	0.00	C
ATOM	219	OD1	ASP	13	33.410	42.649	25.054	1.00	0.00	O
ATOM	220	OD2	ASP	13	34.795	40.929	25.025	1.00	0.00	O
ATOM	221	C	ASP	13	36.194	42.068	29.077	1.00	0.00	C
ATOM	222	O	ASP	13	37.394	42.300	29.023	1.00	0.00	O
ATOM	223	N	ASP	14	35.510	42.188	30.199	1.00	0.00	N
ATOM	224	H	ASP	14	34.529	41.953	30.238	1.00	0.00	H
ATOM	225	CA	ASP	14	36.158	42.460	31.474	1.00	0.00	C
ATOM	226	HA	ASP	14	36.778	43.319	31.217	1.00	0.00	H
ATOM	227	CB	ASP	14	35.204	42.873	32.593	1.00	0.00	C
ATOM	228	HB2	ASP	14	34.388	42.158	32.690	1.00	0.00	H
ATOM	229	HB3	ASP	14	35.664	42.831	33.581	1.00	0.00	H
ATOM	230	CG	ASP	14	34.647	44.297	32.477	1.00	0.00	C
ATOM	231	OD1	ASP	14	33.572	44.512	32.932	1.00	0.00	O
ATOM	232	OD2	ASP	14	35.259	45.168	31.886	1.00	0.00	O
ATOM	233	C	ASP	14	37.087	41.330	31.983	1.00	0.00	C
ATOM	234	O	ASP	14	38.193	41.520	32.503	1.00	0.00	O
ATOM	235	N	ILE	15	36.750	40.050	31.558	1.00	0.00	N
ATOM	236	H	ILE	15	35.801	39.925	31.235	1.00	0.00	H
ATOM	237	CA	ILE	15	37.589	38.787	31.821	1.00	0.00	C
ATOM	238	HA	ILE	15	38.096	38.883	32.781	1.00	0.00	H
ATOM	239	CB	ILE	15	36.668	37.544	31.804	1.00	0.00	C
ATOM	240	HB	ILE	15	36.221	37.384	30.823	1.00	0.00	H

ATOM	241	CG2	ILE	15	37.494	36.264	31.997	1.00	0.00	C
ATOM	242	HG21	ILE	15	36.891	35.356	32.032	1.00	0.00	H
ATOM	243	HG22	ILE	15	38.330	36.264	31.299	1.00	0.00	H
ATOM	244	HG23	ILE	15	38.031	36.332	32.944	1.00	0.00	H
ATOM	245	CG1	ILE	15	35.558	37.617	32.908	1.00	0.00	C
ATOM	246	HG12	ILE	15	35.995	37.413	33.885	1.00	0.00	H
ATOM	247	HG13	ILE	15	35.064	38.588	32.945	1.00	0.00	H
ATOM	248	CD1	ILE	15	34.373	36.612	32.798	1.00	0.00	C
ATOM	249	HD11	ILE	15	34.161	36.515	31.733	1.00	0.00	H
ATOM	250	HD12	ILE	15	34.656	35.624	33.158	1.00	0.00	H
ATOM	251	HD13	ILE	15	33.505	36.959	33.359	1.00	0.00	H
ATOM	252	C	ILE	15	38.674	38.748	30.752	1.00	0.00	C
ATOM	253	O	ILE	15	39.797	38.264	31.015	1.00	0.00	O
ATOM	254	N	LEU	16	38.408	39.165	29.471	1.00	0.00	N
ATOM	255	H	LEU	16	37.455	39.331	29.180	1.00	0.00	H
ATOM	256	CA	LEU	16	39.395	39.349	28.436	1.00	0.00	C
ATOM	257	HA	LEU	16	39.939	38.407	28.356	1.00	0.00	H
ATOM	258	CB	LEU	16	38.774	39.718	27.101	1.00	0.00	C
ATOM	259	HB2	LEU	16	38.009	39.031	26.737	1.00	0.00	H
ATOM	260	HB3	LEU	16	38.373	40.727	27.198	1.00	0.00	H
ATOM	261	CG	LEU	16	39.769	39.883	25.916	1.00	0.00	C
ATOM	262	HG	LEU	16	40.365	40.787	26.043	1.00	0.00	H
ATOM	263	CD1	LEU	16	40.644	38.671	25.675	1.00	0.00	C
ATOM	264	HD11	LEU	16	40.029	37.798	25.456	1.00	0.00	H
ATOM	265	HD12	LEU	16	41.404	38.955	24.946	1.00	0.00	H
ATOM	266	HD13	LEU	16	41.219	38.405	26.562	1.00	0.00	H
ATOM	267	CD2	LEU	16	39.007	40.172	24.598	1.00	0.00	C
ATOM	268	HD21	LEU	16	38.433	39.299	24.287	1.00	0.00	H
ATOM	269	HD22	LEU	16	38.342	41.009	24.812	1.00	0.00	H
ATOM	270	HD23	LEU	16	39.653	40.294	23.728	1.00	0.00	H
ATOM	271	C	LEU	16	40.455	40.356	28.870	1.00	0.00	C
ATOM	272	O	LEU	16	41.619	40.080	28.729	1.00	0.00	O
ATOM	273	N	ALA	17	40.156	41.550	29.430	1.00	0.00	N
ATOM	274	H	ALA	17	39.195	41.862	29.408	1.00	0.00	H
ATOM	275	CA	ALA	17	41.188	42.501	29.868	1.00	0.00	C
ATOM	276	HA	ALA	17	41.881	42.691	29.048	1.00	0.00	H
ATOM	277	CB	ALA	17	40.490	43.795	30.310	1.00	0.00	C
ATOM	278	HB1	ALA	17	41.118	44.493	30.864	1.00	0.00	H
ATOM	279	HB2	ALA	17	40.123	44.344	29.443	1.00	0.00	H
ATOM	280	HB3	ALA	17	39.615	43.398	30.823	1.00	0.00	H
ATOM	281	C	ALA	17	41.932	42.030	31.146	1.00	0.00	C
ATOM	282	O	ALA	17	42.913	42.656	31.542	1.00	0.00	O
ATOM	283	N	GLY	18	41.566	40.967	31.867	1.00	0.00	N
ATOM	284	H	GLY	18	40.836	40.394	31.468	1.00	0.00	H
ATOM	285	CA	GLY	18	42.083	40.600	33.180	1.00	0.00	C
ATOM	286	HA2	GLY	18	41.731	39.611	33.478	1.00	0.00	H
ATOM	287	HA3	GLY	18	43.173	40.608	33.164	1.00	0.00	H
ATOM	288	C	GLY	18	41.562	41.525	34.294	1.00	0.00	C
ATOM	289	O	GLY	18	42.156	41.573	35.398	1.00	0.00	O
ATOM	290	N	ARG	19	40.431	42.211	34.075	1.00	0.00	N
ATOM	291	H	ARG	19	39.942	42.173	33.193	1.00	0.00	H
ATOM	292	CA	ARG	19	39.781	42.982	35.120	1.00	0.00	C
ATOM	293	HA	ARG	19	40.477	43.539	35.746	1.00	0.00	H
ATOM	294	CB	ARG	19	38.936	44.072	34.582	1.00	0.00	C
ATOM	295	HB2	ARG	19	38.298	43.727	33.769	1.00	0.00	H
ATOM	296	HB3	ARG	19	38.269	44.404	35.379	1.00	0.00	H
ATOM	297	CG	ARG	19	39.760	45.276	34.066	1.00	0.00	C
ATOM	298	HG2	ARG	19	40.637	45.466	34.686	1.00	0.00	H
ATOM	299	HG3	ARG	19	40.149	44.944	33.104	1.00	0.00	H
ATOM	300	CD	ARG	19	38.892	46.547	33.821	1.00	0.00	C
ATOM	301	HD2	ARG	19	38.214	46.888	34.604	1.00	0.00	H
ATOM	302	HD3	ARG	19	39.565	47.391	33.680	1.00	0.00	H
ATOM	303	NE	ARG	19	37.983	46.290	32.735	1.00	0.00	N
ATOM	304	HE	ARG	19	37.122	45.818	32.973	1.00	0.00	H

ATOM	305	CZ	ARG	19	38.183	46.571	31.468	1.00	0.00	C
ATOM	306	NH1	ARG	19	39.223	47.088	30.981	1.00	0.00	N
ATOM	307	HH11	ARG	19	40.087	47.196	31.493	1.00	0.00	H
ATOM	308	HH12	ARG	19	39.174	47.686	30.167	1.00	0.00	H
ATOM	309	NH2	ARG	19	37.235	46.415	30.629	1.00	0.00	N
ATOM	310	HH21	ARG	19	37.591	46.304	29.690	1.00	0.00	H
ATOM	311	HH22	ARG	19	36.325	46.078	30.909	1.00	0.00	H
ATOM	312	C	ARG	19	39.030	42.106	36.085	1.00	0.00	C
ATOM	313	O	ARG	19	39.403	42.076	37.235	1.00	0.00	O
ATOM	314	N	LYS	20	38.056	41.360	35.595	1.00	0.00	N
ATOM	315	H	LYS	20	38.113	41.359	34.587	1.00	0.00	H
ATOM	316	CA	LYS	20	37.244	40.420	36.416	1.00	0.00	C
ATOM	317	HA	LYS	20	37.149	40.936	37.371	1.00	0.00	H
ATOM	318	CB	LYS	20	35.933	40.305	35.744	1.00	0.00	C
ATOM	319	HB2	LYS	20	36.137	39.869	34.767	1.00	0.00	H
ATOM	320	HB3	LYS	20	35.541	41.315	35.620	1.00	0.00	H
ATOM	321	CG	LYS	20	34.952	39.361	36.279	1.00	0.00	C
ATOM	322	HG2	LYS	20	34.086	39.477	35.628	1.00	0.00	H
ATOM	323	HG3	LYS	20	35.216	38.312	36.144	1.00	0.00	H
ATOM	324	CD	LYS	20	34.567	39.649	37.746	1.00	0.00	C
ATOM	325	HD2	LYS	20	35.441	39.343	38.321	1.00	0.00	H
ATOM	326	HD3	LYS	20	33.669	39.065	37.948	1.00	0.00	H
ATOM	327	CE	LYS	20	34.362	41.148	37.997	1.00	0.00	C
ATOM	328	HE2	LYS	20	35.259	41.695	37.709	1.00	0.00	H
ATOM	329	HE3	LYS	20	33.441	41.480	37.517	1.00	0.00	H
ATOM	330	NZ	LYS	20	34.194	41.258	39.444	1.00	0.00	N
ATOM	331	HZ1	LYS	20	34.973	40.814	39.909	1.00	0.00	H
ATOM	332	HZ2	LYS	20	33.381	40.825	39.860	1.00	0.00	H
ATOM	333	HZ3	LYS	20	34.350	42.235	39.646	1.00	0.00	H
ATOM	334	C	LYS	20	38.024	39.103	36.604	1.00	0.00	C
ATOM	335	O	LYS	20	38.291	38.347	35.647	1.00	0.00	O
ATOM	336	N	THR	21	38.223	38.826	37.915	1.00	0.00	N
ATOM	337	H	THR	21	38.045	39.542	38.604	1.00	0.00	H
ATOM	338	CA	THR	21	38.807	37.516	38.429	1.00	0.00	C
ATOM	339	HA	THR	21	38.678	36.703	37.713	1.00	0.00	H
ATOM	340	CB	THR	21	40.321	37.630	38.620	1.00	0.00	C
ATOM	341	HB	THR	21	40.647	36.675	39.031	1.00	0.00	H
ATOM	342	CG2	THR	21	40.984	37.810	37.203	1.00	0.00	C
ATOM	343	HG21	THR	21	42.053	37.636	37.321	1.00	0.00	H
ATOM	344	HG22	THR	21	40.657	37.051	36.492	1.00	0.00	H
ATOM	345	HG23	THR	21	40.841	38.838	36.870	1.00	0.00	H
ATOM	346	OG1	THR	21	40.661	38.724	39.447	1.00	0.00	O
ATOM	347	HG1	THR	21	40.562	38.301	40.303	1.00	0.00	H
ATOM	348	C	THR	21	38.210	37.005	39.708	1.00	0.00	C
ATOM	349	O	THR	21	38.569	35.899	40.118	1.00	0.00	O
ATOM	350	N	ILE	22	37.355	37.815	40.353	1.00	0.00	N
ATOM	351	H	ILE	22	36.969	38.641	39.920	1.00	0.00	H
ATOM	352	CA	ILE	22	36.703	37.487	41.546	1.00	0.00	C
ATOM	353	HA	ILE	22	36.622	36.403	41.626	1.00	0.00	H
ATOM	354	CB	ILE	22	37.371	37.836	42.936	1.00	0.00	C
ATOM	355	HB	ILE	22	38.234	37.169	42.947	1.00	0.00	H
ATOM	356	CG2	ILE	22	37.677	39.339	42.773	1.00	0.00	C
ATOM	357	HG21	ILE	22	36.833	39.986	42.531	1.00	0.00	H
ATOM	358	HG22	ILE	22	38.228	39.617	43.672	1.00	0.00	H
ATOM	359	HG23	ILE	22	38.492	39.376	42.051	1.00	0.00	H
ATOM	360	CG1	ILE	22	36.570	37.507	44.201	1.00	0.00	C
ATOM	361	HG12	ILE	22	35.745	38.219	44.191	1.00	0.00	H
ATOM	362	HG13	ILE	22	36.153	36.516	44.020	1.00	0.00	H
ATOM	363	CD1	ILE	22	37.484	37.630	45.492	1.00	0.00	C
ATOM	364	HD11	ILE	22	38.434	37.189	45.188	1.00	0.00	H
ATOM	365	HD12	ILE	22	37.752	38.672	45.666	1.00	0.00	H
ATOM	366	HD13	ILE	22	36.973	37.249	46.376	1.00	0.00	H
ATOM	367	C	ILE	22	35.215	37.938	41.328	1.00	0.00	C

ATOM	368	O	ILE	22	34.955	38.995	40.755	1.00	0.00	O
ATOM	369	N	THR	23	34.317	37.189	41.921	1.00	0.00	N
ATOM	370	H	THR	23	34.665	36.379	42.416	1.00	0.00	H
ATOM	371	CA	THR	23	32.884	37.368	41.907	1.00	0.00	C
ATOM	372	HA	THR	23	32.719	38.433	41.744	1.00	0.00	H
ATOM	373	CB	THR	23	32.150	36.749	40.691	1.00	0.00	C
ATOM	374	HB	THR	23	32.632	35.830	40.356	1.00	0.00	H
ATOM	375	CG2	THR	23	30.697	36.416	40.829	1.00	0.00	C
ATOM	376	HG21	THR	23	30.177	37.339	41.083	1.00	0.00	H
ATOM	377	HG22	THR	23	30.367	36.067	39.851	1.00	0.00	H
ATOM	378	HG23	THR	23	30.578	35.632	41.576	1.00	0.00	H
ATOM	379	OG1	THR	23	32.102	37.647	39.593	1.00	0.00	O
ATOM	380	HG1	THR	23	31.782	38.491	39.923	1.00	0.00	H
ATOM	381	C	THR	23	32.218	37.022	43.255	1.00	0.00	C
ATOM	382	O	THR	23	32.458	35.991	43.835	1.00	0.00	O
ATOM	383	N	ILE	24	31.240	37.859	43.562	1.00	0.00	N
ATOM	384	H	ILE	24	31.265	38.725	43.044	1.00	0.00	H
ATOM	385	CA	ILE	24	30.343	37.669	44.712	1.00	0.00	C
ATOM	386	HA	ILE	24	30.606	36.758	45.247	1.00	0.00	H
ATOM	387	CB	ILE	24	30.500	38.811	45.764	1.00	0.00	C
ATOM	388	HB	ILE	24	30.042	39.652	45.241	1.00	0.00	H
ATOM	389	CG2	ILE	24	29.679	38.378	46.938	1.00	0.00	C
ATOM	390	HG21	ILE	24	29.723	39.259	47.579	1.00	0.00	H
ATOM	391	HG22	ILE	24	28.622	38.203	46.732	1.00	0.00	H
ATOM	392	HG23	ILE	24	30.099	37.500	47.426	1.00	0.00	H
ATOM	393	CG1	ILE	24	31.979	39.143	46.104	1.00	0.00	C
ATOM	394	HG12	ILE	24	32.683	38.342	45.879	1.00	0.00	H
ATOM	395	HG13	ILE	24	32.181	39.980	45.435	1.00	0.00	H
ATOM	396	CD1	ILE	24	32.203	39.642	47.529	1.00	0.00	C
ATOM	397	HD11	ILE	24	33.207	40.063	47.580	1.00	0.00	H
ATOM	398	HD12	ILE	24	31.501	40.451	47.735	1.00	0.00	H
ATOM	399	HD13	ILE	24	31.948	38.842	48.223	1.00	0.00	H
ATOM	400	C	ILE	24	28.913	37.416	44.184	1.00	0.00	C
ATOM	401	O	ILE	24	28.392	38.185	43.304	1.00	0.00	O
ATOM	402	N	ARG	25	28.269	36.351	44.641	1.00	0.00	N
ATOM	403	H	ARG	25	28.759	35.698	45.237	1.00	0.00	H
ATOM	404	CA	ARG	25	26.895	36.031	44.314	1.00	0.00	C
ATOM	405	HA	ARG	25	26.385	36.812	43.749	1.00	0.00	H
ATOM	406	CB	ARG	25	26.814	34.733	43.474	1.00	0.00	C
ATOM	407	HB2	ARG	25	27.479	33.959	43.857	1.00	0.00	H
ATOM	408	HB3	ARG	25	25.790	34.357	43.471	1.00	0.00	H
ATOM	409	CG	ARG	25	27.194	34.955	42.081	1.00	0.00	C
ATOM	410	HG2	ARG	25	28.158	35.462	42.035	1.00	0.00	H
ATOM	411	HG3	ARG	25	27.315	34.005	41.560	1.00	0.00	H
ATOM	412	CD	ARG	25	26.174	35.850	41.361	1.00	0.00	C
ATOM	413	HD2	ARG	25	25.237	35.326	41.546	1.00	0.00	H
ATOM	414	HD3	ARG	25	26.118	36.856	41.776	1.00	0.00	H
ATOM	415	NE	ARG	25	26.526	35.979	39.959	1.00	0.00	N
ATOM	416	HE	ARG	25	27.185	35.394	39.465	1.00	0.00	H
ATOM	417	CZ	ARG	25	25.949	36.943	39.207	1.00	0.00	C
ATOM	418	NH1	ARG	25	24.866	37.624	39.440	1.00	0.00	N
ATOM	419	HH11	ARG	25	24.383	38.215	38.777	1.00	0.00	H
ATOM	420	HH12	ARG	25	24.374	37.474	40.309	1.00	0.00	H
ATOM	421	NH2	ARG	25	26.465	37.309	38.067	1.00	0.00	N
ATOM	422	HH21	ARG	25	25.883	37.935	37.531	1.00	0.00	H
ATOM	423	HH22	ARG	25	27.383	36.955	37.838	1.00	0.00	H
ATOM	424	C	ARG	25	26.186	35.817	45.661	1.00	0.00	C
ATOM	425	O	ARG	25	26.903	35.639	46.622	1.00	0.00	O
ATOM	426	N	ASP	26	24.859	35.828	45.741	1.00	0.00	N
ATOM	427	H	ASP	26	24.256	36.030	44.955	1.00	0.00	H
ATOM	428	CA	ASP	26	24.154	35.188	46.881	1.00	0.00	C
ATOM	429	HA	ASP	26	24.655	35.504	47.797	1.00	0.00	H
ATOM	430	CB	ASP	26	22.704	35.652	46.998	1.00	0.00	C
ATOM	431	HB2	ASP	26	22.239	35.021	47.755	1.00	0.00	H

ATOM	432	HB3	ASP	26	22.676	36.666	47.398	1.00	0.00	H
ATOM	433	CG	ASP	26	21.812	35.476	45.814	1.00	0.00	C
ATOM	434	OD1	ASP	26	20.808	34.757	45.882	1.00	0.00	O
ATOM	435	OD2	ASP	26	22.218	35.917	44.763	1.00	0.00	O
ATOM	436	C	ASP	26	24.199	33.638	46.709	1.00	0.00	C
ATOM	437	O	ASP	26	24.171	33.153	45.600	1.00	0.00	O
ATOM	438	N	GLU	27	24.123	32.935	47.838	1.00	0.00	N
ATOM	439	H	GLU	27	23.880	33.463	48.664	1.00	0.00	H
ATOM	440	CA	GLU	27	24.457	31.433	48.049	1.00	0.00	C
ATOM	441	HA	GLU	27	25.522	31.355	47.832	1.00	0.00	H
ATOM	442	CB	GLU	27	24.222	31.183	49.574	1.00	0.00	C
ATOM	443	HB2	GLU	27	24.848	31.956	50.021	1.00	0.00	H
ATOM	444	HB3	GLU	27	23.186	31.426	49.808	1.00	0.00	H
ATOM	445	CG	GLU	27	24.542	29.764	50.173	1.00	0.00	C
ATOM	446	HG2	GLU	27	24.452	29.795	51.259	1.00	0.00	H
ATOM	447	HG3	GLU	27	23.815	29.022	49.847	1.00	0.00	H
ATOM	448	CD	GLU	27	25.943	29.190	49.849	1.00	0.00	C
ATOM	449	OE1	GLU	27	26.675	28.769	50.761	1.00	0.00	O
ATOM	450	OE2	GLU	27	26.317	29.210	48.685	1.00	0.00	O
ATOM	451	C	GLU	27	23.617	30.502	47.269	1.00	0.00	C
ATOM	452	O	GLU	27	22.419	30.733	47.210	1.00	0.00	O
ATOM	453	N	SER	28	24.255	29.530	46.637	1.00	0.00	N
ATOM	454	H	SER	28	25.263	29.556	46.671	1.00	0.00	H
ATOM	455	CA	SER	28	23.522	28.326	46.268	1.00	0.00	C
ATOM	456	HA	SER	28	22.905	27.955	47.087	1.00	0.00	H
ATOM	457	CB	SER	28	22.689	28.764	45.039	1.00	0.00	C
ATOM	458	HB2	SER	28	21.881	29.387	45.423	1.00	0.00	H
ATOM	459	HB3	SER	28	23.255	29.440	44.399	1.00	0.00	H
ATOM	460	OG	SER	28	22.061	27.720	44.359	1.00	0.00	O
ATOM	461	HG	SER	28	21.315	27.373	44.852	1.00	0.00	H
ATOM	462	C	SER	28	24.469	27.187	45.860	1.00	0.00	C
ATOM	463	O	SER	28	25.579	27.429	45.457	1.00	0.00	O
ATOM	464	N	GLU	29	24.077	25.939	46.034	1.00	0.00	N
ATOM	465	H	GLU	29	23.134	25.781	46.360	1.00	0.00	H
ATOM	466	CA	GLU	29	24.737	24.693	45.632	1.00	0.00	C
ATOM	467	HA	GLU	29	25.686	24.693	46.168	1.00	0.00	H
ATOM	468	CB	GLU	29	23.858	23.494	46.060	1.00	0.00	C
ATOM	469	HB2	GLU	29	23.583	23.520	47.114	1.00	0.00	H
ATOM	470	HB3	GLU	29	22.903	23.604	45.545	1.00	0.00	H
ATOM	471	CG	GLU	29	24.353	22.133	45.615	1.00	0.00	C
ATOM	472	HG2	GLU	29	24.362	22.108	44.526	1.00	0.00	H
ATOM	473	HG3	GLU	29	25.366	22.060	46.009	1.00	0.00	H
ATOM	474	CD	GLU	29	23.552	20.985	46.102	1.00	0.00	C
ATOM	475	OE1	GLU	29	24.173	19.959	46.422	1.00	0.00	O
ATOM	476	OE2	GLU	29	22.343	21.147	46.347	1.00	0.00	O
ATOM	477	C	GLU	29	24.972	24.665	44.075	1.00	0.00	C
ATOM	478	O	GLU	29	25.930	23.997	43.630	1.00	0.00	O
ATOM	479	N	SER	30	24.164	25.286	43.279	1.00	0.00	N
ATOM	480	H	SER	30	23.536	25.957	43.697	1.00	0.00	H
ATOM	481	CA	SER	30	24.367	25.507	41.832	1.00	0.00	C
ATOM	482	HA	SER	30	24.344	24.535	41.341	1.00	0.00	H
ATOM	483	CB	SER	30	23.243	26.428	41.312	1.00	0.00	C
ATOM	484	HB2	SER	30	23.358	26.493	40.231	1.00	0.00	H
ATOM	485	HB3	SER	30	22.267	26.021	41.580	1.00	0.00	H
ATOM	486	OG	SER	30	23.337	27.768	41.842	1.00	0.00	O
ATOM	487	HG	SER	30	22.762	27.890	42.601	1.00	0.00	H
ATOM	488	C	SER	30	25.709	26.092	41.427	1.00	0.00	C
ATOM	489	O	SER	30	26.115	25.961	40.221	1.00	0.00	O
ATOM	490	N	HIE	31	26.360	26.875	42.291	1.00	0.00	N
ATOM	491	H	HIE	31	25.933	26.891	43.206	1.00	0.00	H
ATOM	492	CA	HIE	31	27.519	27.733	41.911	1.00	0.00	C
ATOM	493	HA	HIE	31	27.315	28.359	41.042	1.00	0.00	H
ATOM	494	CB	HIE	31	27.850	28.731	43.064	1.00	0.00	C

ATOM	495	HB2	HIE	31	27.953	28.195	44.007	1.00	0.00	H
ATOM	496	HB3	HIE	31	28.810	29.044	42.654	1.00	0.00	H
ATOM	497	CG	HIE	31	26.863	29.956	43.085	1.00	0.00	C
ATOM	498	ND1	HIE	31	26.398	30.776	42.070	1.00	0.00	N
ATOM	499	CE1	HIE	31	25.529	31.651	42.578	1.00	0.00	C
ATOM	500	HE1	HIE	31	25.035	32.447	42.042	1.00	0.00	H
ATOM	501	NE2	HIE	31	25.489	31.443	43.944	1.00	0.00	N
ATOM	502	HE2	HIE	31	24.910	31.914	44.624	1.00	0.00	H
ATOM	503	CD2	HIE	31	26.266	30.353	44.235	1.00	0.00	C
ATOM	504	HD2	HIE	31	26.229	29.913	45.221	1.00	0.00	H
ATOM	505	C	HIE	31	28.768	26.867	41.709	1.00	0.00	C
ATOM	506	O	HIE	31	28.813	25.681	42.040	1.00	0.00	O
ATOM	507	N	PHE	32	29.829	27.495	41.102	1.00	0.00	N
ATOM	508	H	PHE	32	29.741	28.481	40.900	1.00	0.00	H
ATOM	509	CA	PHE	32	31.099	26.791	40.934	1.00	0.00	C
ATOM	510	HA	PHE	32	30.816	25.922	40.339	1.00	0.00	H
ATOM	511	CB	PHE	32	32.025	27.684	40.000	1.00	0.00	C
ATOM	512	HB2	PHE	32	32.440	28.448	40.658	1.00	0.00	H
ATOM	513	HB3	PHE	32	32.895	27.073	39.758	1.00	0.00	H
ATOM	514	CG	PHE	32	31.457	28.234	38.692	1.00	0.00	C
ATOM	515	CD1	PHE	32	31.517	29.626	38.325	1.00	0.00	C
ATOM	516	HD1	PHE	32	31.971	30.324	39.013	1.00	0.00	H
ATOM	517	CE1	PHE	32	30.934	30.056	37.128	1.00	0.00	C
ATOM	518	HE1	PHE	32	30.927	31.094	36.831	1.00	0.00	H
ATOM	519	CZ	PHE	32	30.272	29.205	36.213	1.00	0.00	C
ATOM	520	HZ	PHE	32	29.733	29.623	35.376	1.00	0.00	H
ATOM	521	CE2	PHE	32	30.228	27.866	36.540	1.00	0.00	C
ATOM	522	HE2	PHE	32	29.807	27.143	35.858	1.00	0.00	H
ATOM	523	CD2	PHE	32	30.769	27.409	37.785	1.00	0.00	C
ATOM	524	HD2	PHE	32	30.731	26.376	38.096	1.00	0.00	H
ATOM	525	C	PHE	32	31.728	26.448	42.279	1.00	0.00	C
ATOM	526	O	PHE	32	31.417	27.079	43.349	1.00	0.00	O
ATOM	527	N	LYS	33	32.704	25.512	42.282	1.00	0.00	N
ATOM	528	H	LYS	33	33.026	25.107	41.414	1.00	0.00	H
ATOM	529	CA	LYS	33	33.467	25.165	43.460	1.00	0.00	C
ATOM	530	HA	LYS	33	33.267	25.881	44.257	1.00	0.00	H
ATOM	531	CB	LYS	33	33.107	23.762	44.040	1.00	0.00	C
ATOM	532	HB2	LYS	33	33.407	23.030	43.290	1.00	0.00	H
ATOM	533	HB3	LYS	33	33.694	23.630	44.948	1.00	0.00	H
ATOM	534	CG	LYS	33	31.597	23.585	44.485	1.00	0.00	C
ATOM	535	HG2	LYS	33	31.559	22.945	45.367	1.00	0.00	H
ATOM	536	HG3	LYS	33	31.215	24.544	44.836	1.00	0.00	H
ATOM	537	CD	LYS	33	30.852	23.063	43.307	1.00	0.00	C
ATOM	538	HD2	LYS	33	31.003	23.784	42.504	1.00	0.00	H
ATOM	539	HD3	LYS	33	31.179	22.081	42.961	1.00	0.00	H
ATOM	540	CE	LYS	33	29.367	23.071	43.689	1.00	0.00	C
ATOM	541	HE2	LYS	33	28.985	22.286	44.341	1.00	0.00	H
ATOM	542	HE3	LYS	33	29.175	24.002	44.221	1.00	0.00	H
ATOM	543	NZ	LYS	33	28.446	23.044	42.504	1.00	0.00	N
ATOM	544	HZ1	LYS	33	28.307	22.079	42.244	1.00	0.00	H
ATOM	545	HZ2	LYS	33	27.592	23.565	42.643	1.00	0.00	H
ATOM	546	HZ3	LYS	33	28.852	23.500	41.699	1.00	0.00	H
ATOM	547	C	LYS	33	34.922	25.178	43.128	1.00	0.00	C
ATOM	548	O	LYS	33	35.299	25.370	41.949	1.00	0.00	O
ATOM	549	N	THR	34	35.844	25.129	44.074	1.00	0.00	N
ATOM	550	H	THR	34	35.507	25.168	45.025	1.00	0.00	H
ATOM	551	CA	THR	34	37.340	25.169	43.783	1.00	0.00	C
ATOM	552	HA	THR	34	37.592	26.040	43.178	1.00	0.00	H
ATOM	553	CB	THR	34	37.973	25.127	45.259	1.00	0.00	C
ATOM	554	HB	THR	34	37.536	24.305	45.827	1.00	0.00	H
ATOM	555	CG2	THR	34	39.472	24.937	45.172	1.00	0.00	C
ATOM	556	HG21	THR	34	39.932	24.899	46.159	1.00	0.00	H
ATOM	557	HG22	THR	34	39.606	23.950	44.731	1.00	0.00	H
ATOM	558	HG23	THR	34	40.029	25.761	44.724	1.00	0.00	

H

ATOM	559	OG1	THR	34	37.766	26.343	45.876	1.00	0.00	O
ATOM	560	HG1	THR	34	36.840	26.392	46.126	1.00	0.00	H
ATOM	561	C	THR	34	37.730	24.022	42.929	1.00	0.00	C
ATOM	562	O	THR	34	37.244	22.935	43.111	1.00	0.00	O
ATOM	563	N	GLY	35	38.599	24.197	41.933	1.00	0.00	N
ATOM	564	H	GLY	35	38.754	25.146	41.621	1.00	0.00	H
ATOM	565	CA	GLY	35	39.065	23.082	41.076	1.00	0.00	C
ATOM	566	HA2	GLY	35	40.110	23.255	40.822	1.00	0.00	H
ATOM	567	HA3	GLY	35	38.917	22.099	41.525	1.00	0.00	H
ATOM	568	C	GLY	35	38.224	22.990	39.769	1.00	0.00	C
ATOM	569	O	GLY	35	38.553	22.163	38.926	1.00	0.00	O
ATOM	570	N	ASP	36	37.137	23.819	39.562	1.00	0.00	N
ATOM	571	H	ASP	36	36.822	24.518	40.219	1.00	0.00	H
ATOM	572	CA	ASP	36	36.372	23.745	38.335	1.00	0.00	C
ATOM	573	HA	ASP	36	36.327	22.696	38.042	1.00	0.00	H
ATOM	574	CB	ASP	36	34.913	24.223	38.609	1.00	0.00	C
ATOM	575	HB2	ASP	36	34.905	25.105	39.250	1.00	0.00	H
ATOM	576	HB3	ASP	36	34.552	24.568	37.640	1.00	0.00	H
ATOM	577	CG	ASP	36	33.940	23.138	39.142	1.00	0.00	C
ATOM	578	OD1	ASP	36	34.339	21.968	39.352	1.00	0.00	O
ATOM	579	OD2	ASP	36	32.768	23.490	39.460	1.00	0.00	O
ATOM	580	C	ASP	36	37.079	24.405	37.182	1.00	0.00	C
ATOM	581	O	ASP	36	37.759	25.419	37.323	1.00	0.00	O
ATOM	582	N	VAL	37	36.985	23.894	35.985	1.00	0.00	N
ATOM	583	H	VAL	37	36.382	23.106	35.801	1.00	0.00	H
ATOM	584	CA	VAL	37	37.489	24.557	34.718	1.00	0.00	C
ATOM	585	HA	VAL	37	38.149	25.400	34.922	1.00	0.00	H
ATOM	586	CB	VAL	37	38.268	23.539	33.873	1.00	0.00	C
ATOM	587	HB	VAL	37	37.665	22.666	33.620	1.00	0.00	H
ATOM	588	CG1	VAL	37	38.783	24.126	32.526	1.00	0.00	C
ATOM	589	HG11	VAL	37	39.157	25.129	32.732	1.00	0.00	H
ATOM	590	HG12	VAL	37	39.531	23.463	32.092	1.00	0.00	H
ATOM	591	HG13	VAL	37	37.981	24.227	31.795	1.00	0.00	H
ATOM	592	CG2	VAL	37	39.643	23.186	34.607	1.00	0.00	C
ATOM	593	HG21	VAL	37	40.052	22.290	34.142	1.00	0.00	H
ATOM	594	HG22	VAL	37	40.409	23.962	34.607	1.00	0.00	H
ATOM	595	HG23	VAL	37	39.521	22.903	35.653	1.00	0.00	H
ATOM	596	C	VAL	37	36.389	25.115	33.891	1.00	0.00	C
ATOM	597	O	VAL	37	35.327	24.507	33.681	1.00	0.00	O
ATOM	598	N	LEU	38	36.516	26.392	33.438	1.00	0.00	N
ATOM	599	H	LEU	38	37.309	26.906	33.796	1.00	0.00	H
ATOM	600	CA	LEU	38	35.532	27.262	32.731	1.00	0.00	C
ATOM	601	HA	LEU	38	34.695	26.617	32.467	1.00	0.00	H
ATOM	602	CB	LEU	38	35.118	28.412	33.600	1.00	0.00	C
ATOM	603	HB2	LEU	38	35.968	29.092	33.547	1.00	0.00	H
ATOM	604	HB3	LEU	38	34.241	28.904	33.180	1.00	0.00	H
ATOM	605	CG	LEU	38	34.721	28.084	35.102	1.00	0.00	C
ATOM	606	HG	LEU	38	35.614	27.551	35.429	1.00	0.00	H
ATOM	607	CD1	LEU	38	34.480	29.380	35.895	1.00	0.00	C
ATOM	608	HD11	LEU	38	33.475	29.730	35.661	1.00	0.00	H
ATOM	609	HD12	LEU	38	34.633	29.152	36.949	1.00	0.00	H
ATOM	610	HD13	LEU	38	35.283	30.065	35.619	1.00	0.00	H
ATOM	611	CD2	LEU	38	33.542	27.133	35.259	1.00	0.00	C
ATOM	612	HD21	LEU	38	33.996	26.168	35.038	1.00	0.00	H
ATOM	613	HD22	LEU	38	33.069	27.232	36.237	1.00	0.00	H
ATOM	614	HD23	LEU	38	32.819	27.450	34.507	1.00	0.00	H
ATOM	615	C	LEU	38	36.060	27.618	31.310	1.00	0.00	C
ATOM	616	O	LEU	38	37.230	27.735	31.135	1.00	0.00	O
ATOM	617	N	ARG	39	35.125	27.920	30.411	1.00	0.00	N
ATOM	618	H	ARG	39	34.149	27.779	30.632	1.00	0.00	H
ATOM	619	CA	ARG	39	35.463	28.376	29.093	1.00	0.00	C
ATOM	620	HA	ARG	39	36.531	28.555	28.970	1.00	0.00	H
ATOM	621	CB	ARG	39	35.233	27.271	28.113	1.00	0.00	C

ATOM	622	HB2	ARG	39	34.153	27.160	28.004	1.00	0.00	H
ATOM	623	HB3	ARG	39	35.738	27.549	27.189	1.00	0.00	H
ATOM	624	CG	ARG	39	35.789	25.929	28.529	1.00	0.00	C
ATOM	625	HG2	ARG	39	36.796	26.074	28.923	1.00	0.00	H
ATOM	626	HG3	ARG	39	35.188	25.454	29.304	1.00	0.00	H
ATOM	627	CD	ARG	39	35.946	24.957	27.402	1.00	0.00	C
ATOM	628	HD2	ARG	39	36.262	25.470	26.494	1.00	0.00	H
ATOM	629	HD3	ARG	39	36.687	24.196	27.647	1.00	0.00	H
ATOM	630	NE	ARG	39	34.708	24.327	27.074	1.00	0.00	N
ATOM	631	HE	ARG	39	33.843	24.528	27.555	1.00	0.00	H
ATOM	632	CZ	ARG	39	34.473	23.555	25.971	1.00	0.00	C
ATOM	633	NH1	ARG	39	35.275	23.290	24.990	1.00	0.00	N
ATOM	634	HH11	ARG	39	36.226	23.571	25.186	1.00	0.00	H
ATOM	635	HH12	ARG	39	35.059	22.575	24.311	1.00	0.00	H
ATOM	636	NH2	ARG	39	33.301	23.072	25.872	1.00	0.00	N
ATOM	637	HH21	ARG	39	33.178	22.567	25.005	1.00	0.00	H
ATOM	638	HH22	ARG	39	32.582	23.300	26.544	1.00	0.00	H
ATOM	639	C	ARG	39	34.794	29.714	28.743	1.00	0.00	C
ATOM	640	O	ARG	39	33.602	29.903	28.883	1.00	0.00	O
ATOM	641	N	VAL	40	35.582	30.643	28.187	1.00	0.00	N
ATOM	642	H	VAL	40	36.508	30.256	28.070	1.00	0.00	H
ATOM	643	CA	VAL	40	35.142	32.040	27.821	1.00	0.00	C
ATOM	644	HA	VAL	40	34.076	32.129	28.034	1.00	0.00	H
ATOM	645	CB	VAL	40	35.814	33.017	28.832	1.00	0.00	C
ATOM	646	HB	VAL	40	35.517	34.028	28.553	1.00	0.00	H
ATOM	647	CG1	VAL	40	35.292	32.760	30.276	1.00	0.00	C
ATOM	648	HG11	VAL	40	34.206	32.789	30.367	1.00	0.00	H
ATOM	649	HG12	VAL	40	35.705	31.789	30.547	1.00	0.00	H
ATOM	650	HG13	VAL	40	35.751	33.494	30.939	1.00	0.00	H
ATOM	651	CG2	VAL	40	37.325	32.825	28.974	1.00	0.00	C
ATOM	652	HG21	VAL	40	37.714	33.710	29.477	1.00	0.00	H
ATOM	653	HG22	VAL	40	37.578	31.891	29.477	1.00	0.00	H
ATOM	654	HG23	VAL	40	37.738	32.985	27.977	1.00	0.00	H
ATOM	655	C	VAL	40	35.417	32.421	26.416	1.00	0.00	C
ATOM	656	O	VAL	40	36.514	32.162	25.923	1.00	0.00	O
ATOM	657	N	GLY	41	34.512	33.079	25.777	1.00	0.00	N
ATOM	658	H	GLY	41	33.661	33.343	26.252	1.00	0.00	H
ATOM	659	CA	GLY	41	34.683	33.287	24.341	1.00	0.00	C
ATOM	660	HA2	GLY	41	35.483	33.958	24.027	1.00	0.00	H
ATOM	661	HA3	GLY	41	34.994	32.316	23.955	1.00	0.00	H
ATOM	662	C	GLY	41	33.385	33.711	23.707	1.00	0.00	C
ATOM	663	O	GLY	41	32.335	33.390	24.215	1.00	0.00	O
ATOM	664	N	ARG	42	33.416	34.446	22.572	1.00	0.00	N
ATOM	665	H	ARG	42	34.330	34.780	22.299	1.00	0.00	H
ATOM	666	CA	ARG	42	32.289	34.535	21.632	1.00	0.00	C
ATOM	667	HA	ARG	42	31.534	33.748	21.655	1.00	0.00	H
ATOM	668	CB	ARG	42	31.371	35.644	22.072	1.00	0.00	C
ATOM	669	HB2	ARG	42	30.407	35.564	21.571	1.00	0.00	H
ATOM	670	HB3	ARG	42	31.263	35.638	23.157	1.00	0.00	H
ATOM	671	CG	ARG	42	31.789	37.063	21.736	1.00	0.00	C
ATOM	672	HG2	ARG	42	32.659	37.316	22.341	1.00	0.00	H
ATOM	673	HG3	ARG	42	32.040	37.191	20.683	1.00	0.00	H
ATOM	674	CD	ARG	42	30.713	38.200	22.040	1.00	0.00	C
ATOM	675	HD2	ARG	42	30.077	38.274	21.159	1.00	0.00	H
ATOM	676	HD3	ARG	42	30.129	37.942	22.924	1.00	0.00	H
ATOM	677	NE	ARG	42	31.408	39.494	22.140	1.00	0.00	N
ATOM	678	HE	ARG	42	31.538	39.957	21.251	1.00	0.00	H
ATOM	679	CZ	ARG	42	32.245	40.057	23.015	1.00	0.00	C
ATOM	680	NH1	ARG	42	32.516	39.497	24.139	1.00	0.00	N
ATOM	681	HH11	ARG	42	33.305	39.783	24.702	1.00	0.00	H
ATOM	682	HH12	ARG	42	32.330	38.521	24.322	1.00	0.00	H
ATOM	683	NH2	ARG	42	32.947	41.088	22.730	1.00	0.00	N
ATOM	684	HH21	ARG	42	32.783	41.462	21.806	1.00	0.00	H

ATOM	685	HH22	ARG	42	33.286	41.535	23.569	1.00	0.00	H
ATOM	686	C	ARG	42	32.671	34.480	20.183	1.00	0.00	C
ATOM	687	O	ARG	42	31.817	34.698	19.288	1.00	0.00	O
ATOM	688	N	PHE	43	33.911	34.171	19.838	1.00	0.00	N
ATOM	689	H	PHE	43	34.484	34.019	20.655	1.00	0.00	H
ATOM	690	CA	PHE	43	34.413	33.917	18.469	1.00	0.00	C
ATOM	691	HA	PHE	43	33.589	33.885	17.756	1.00	0.00	H
ATOM	692	CB	PHE	43	35.081	35.198	17.943	1.00	0.00	C
ATOM	693	HB2	PHE	43	36.114	35.135	18.287	1.00	0.00	H
ATOM	694	HB3	PHE	43	34.994	35.205	16.856	1.00	0.00	H
ATOM	695	CG	PHE	43	34.495	36.602	18.293	1.00	0.00	C
ATOM	696	CD1	PHE	43	33.436	37.197	17.556	1.00	0.00	C
ATOM	697	HD1	PHE	43	32.898	36.636	16.807	1.00	0.00	H
ATOM	698	CE1	PHE	43	32.986	38.492	17.894	1.00	0.00	C
ATOM	699	HE1	PHE	43	32.160	39.025	17.445	1.00	0.00	H
ATOM	700	CZ	PHE	43	33.581	39.246	18.912	1.00	0.00	C
ATOM	701	HZ	PHE	43	33.372	40.298	19.040	1.00	0.00	H
ATOM	702	CE2	PHE	43	34.546	38.705	19.677	1.00	0.00	C
ATOM	703	HE2	PHE	43	35.146	39.243	20.395	1.00	0.00	H
ATOM	704	CD2	PHE	43	35.033	37.364	19.285	1.00	0.00	C
ATOM	705	HD2	PHE	43	35.934	36.985	19.745	1.00	0.00	H
ATOM	706	C	PHE	43	35.162	32.590	18.308	1.00	0.00	C
ATOM	707	O	PHE	43	35.645	32.066	19.342	1.00	0.00	O
ATOM	708	N	GLU	44	35.176	31.968	17.153	1.00	0.00	N
ATOM	709	H	GLU	44	34.658	32.380	16.389	1.00	0.00	H
ATOM	710	CA	GLU	44	35.705	30.561	16.852	1.00	0.00	C
ATOM	711	HA	GLU	44	35.417	29.974	17.724	1.00	0.00	H
ATOM	712	CB	GLU	44	34.987	29.856	15.640	1.00	0.00	C
ATOM	713	HB2	GLU	44	35.077	28.801	15.897	1.00	0.00	H
ATOM	714	HB3	GLU	44	33.936	30.120	15.752	1.00	0.00	H
ATOM	715	CG	GLU	44	35.583	30.185	14.214	1.00	0.00	C
ATOM	716	HG2	GLU	44	36.616	29.848	14.130	1.00	0.00	H
ATOM	717	HG3	GLU	44	35.011	29.782	13.378	1.00	0.00	H
ATOM	718	CD	GLU	44	35.636	31.691	14.143	1.00	0.00	C
ATOM	719	OE1	GLU	44	36.646	32.278	13.661	1.00	0.00	O
ATOM	720	OE2	GLU	44	34.569	32.378	14.399	1.00	0.00	O
ATOM	721	C	GLU	44	37.244	30.362	16.735	1.00	0.00	C
ATOM	722	O	GLU	44	37.600	29.272	16.420	1.00	0.00	O
ATOM	723	N	ASP	45	38.076	31.367	17.042	1.00	0.00	N
ATOM	724	H	ASP	45	37.614	32.173	17.440	1.00	0.00	H
ATOM	725	CA	ASP	45	39.538	31.381	17.140	1.00	0.00	C
ATOM	726	HA	ASP	45	39.962	31.059	16.189	1.00	0.00	H
ATOM	727	CB	ASP	45	40.068	32.869	17.298	1.00	0.00	C
ATOM	728	HB2	ASP	45	39.812	33.291	18.270	1.00	0.00	H
ATOM	729	HB3	ASP	45	41.153	32.805	17.206	1.00	0.00	H
ATOM	730	CG	ASP	45	39.643	33.764	16.205	1.00	0.00	C
ATOM	731	OD1	ASP	45	40.207	33.746	15.077	1.00	0.00	O
ATOM	732	OD2	ASP	45	38.717	34.566	16.567	1.00	0.00	O
ATOM	733	C	ASP	45	40.055	30.460	18.191	1.00	0.00	C
ATOM	734	O	ASP	45	39.968	30.812	19.346	1.00	0.00	O
ATOM	735	N	ASP	46	40.316	29.203	17.862	1.00	0.00	N
ATOM	736	H	ASP	46	40.128	28.923	16.910	1.00	0.00	H
ATOM	737	CA	ASP	46	40.559	28.053	18.773	1.00	0.00	C
ATOM	738	HA	ASP	46	40.535	27.251	18.036	1.00	0.00	H
ATOM	739	CB	ASP	46	41.946	28.107	19.409	1.00	0.00	C
ATOM	740	HB2	ASP	46	41.775	28.797	20.234	1.00	0.00	H
ATOM	741	HB3	ASP	46	42.096	27.123	19.853	1.00	0.00	H
ATOM	742	CG	ASP	46	43.147	28.604	18.630	1.00	0.00	C
ATOM	743	OD1	ASP	46	44.218	28.372	19.206	1.00	0.00	O
ATOM	744	OD2	ASP	46	43.101	29.170	17.472	1.00	0.00	O
ATOM	745	C	ASP	46	39.270	27.858	19.657	1.00	0.00	C
ATOM	746	O	ASP	46	39.388	27.223	20.693	1.00	0.00	O
ATOM	747	N	GLY	47	38.031	28.148	19.271	1.00	0.00	N
ATOM	748	H	GLY	47	37.927	28.626	18.388	1.00	0.00	H

ATOM	749	CA	GLY	47	36.751	27.838	19.884	1.00	0.00	C
ATOM	750	HA2	GLY	47	35.964	27.950	19.138	1.00	0.00	H
ATOM	751	HA3	GLY	47	36.657	26.796	20.189	1.00	0.00	H
ATOM	752	C	GLY	47	36.427	28.671	21.100	1.00	0.00	C
ATOM	753	O	GLY	47	35.269	28.781	21.439	1.00	0.00	O
ATOM	754	N	TYR	48	37.450	29.346	21.712	1.00	0.00	N
ATOM	755	H	TYR	48	38.369	29.198	21.319	1.00	0.00	H
ATOM	756	CA	TYR	48	37.504	30.191	22.957	1.00	0.00	C
ATOM	757	HA	TYR	48	36.584	30.775	22.976	1.00	0.00	H
ATOM	758	CB	TYR	48	37.353	29.250	24.205	1.00	0.00	C
ATOM	759	HB2	TYR	48	38.210	28.575	24.213	1.00	0.00	H
ATOM	760	HB3	TYR	48	37.458	29.892	25.079	1.00	0.00	H
ATOM	761	CG	TYR	48	36.016	28.513	24.434	1.00	0.00	C
ATOM	762	CD1	TYR	48	34.959	29.131	25.065	1.00	0.00	C
ATOM	763	HD1	TYR	48	35.103	30.188	25.237	1.00	0.00	H
ATOM	764	CE1	TYR	48	33.794	28.458	25.385	1.00	0.00	C
ATOM	765	HE1	TYR	48	32.928	28.940	25.814	1.00	0.00	H
ATOM	766	CZ	TYR	48	33.608	27.135	24.926	1.00	0.00	C
ATOM	767	OH	TYR	48	32.433	26.451	25.189	1.00	0.00	O
ATOM	768	HH	TYR	48	31.903	27.024	25.748	1.00	0.00	H
ATOM	769	CE2	TYR	48	34.660	26.532	24.200	1.00	0.00	C
ATOM	770	HE2	TYR	48	34.511	25.586	23.701	1.00	0.00	H
ATOM	771	CD2	TYR	48	35.848	27.201	23.963	1.00	0.00	C
ATOM	772	HD2	TYR	48	36.603	26.771	23.323	1.00	0.00	H
ATOM	773	C	TYR	48	38.840	31.014	23.099	1.00	0.00	C
ATOM	774	O	TYR	48	39.864	30.616	22.754	1.00	0.00	O
ATOM	775	N	PHE	49	38.745	32.074	23.898	1.00	0.00	N
ATOM	776	H	PHE	49	37.847	32.217	24.336	1.00	0.00	H
ATOM	777	CA	PHE	49	39.879	32.909	24.262	1.00	0.00	C
ATOM	778	HA	PHE	49	40.509	32.882	23.372	1.00	0.00	H
ATOM	779	CB	PHE	49	39.544	34.347	24.515	1.00	0.00	C
ATOM	780	HB2	PHE	49	40.374	35.020	24.299	1.00	0.00	H
ATOM	781	HB3	PHE	49	38.838	34.517	23.703	1.00	0.00	H
ATOM	782	CG	PHE	49	39.013	34.874	25.791	1.00	0.00	C
ATOM	783	CD1	PHE	49	39.876	35.173	26.861	1.00	0.00	C
ATOM	784	HD1	PHE	49	40.937	34.981	26.788	1.00	0.00	H
ATOM	785	CE1	PHE	49	39.336	35.900	27.921	1.00	0.00	C
ATOM	786	HE1	PHE	49	39.974	36.044	28.781	1.00	0.00	H
ATOM	787	CZ	PHE	49	37.943	36.097	28.101	1.00	0.00	C
ATOM	788	HZ	PHE	49	37.670	36.745	28.920	1.00	0.00	H
ATOM	789	CE2	PHE	49	37.093	35.818	27.031	1.00	0.00	C
ATOM	790	HE2	PHE	49	36.041	36.060	27.052	1.00	0.00	H
ATOM	791	CD2	PHE	49	37.659	35.204	25.907	1.00	0.00	C
ATOM	792	HD2	PHE	49	36.984	34.870	25.133	1.00	0.00	H
ATOM	793	C	PHE	49	40.749	32.346	25.438	1.00	0.00	C
ATOM	794	O	PHE	49	41.887	32.740	25.667	1.00	0.00	O
ATOM	795	N	CYX	50	40.162	31.554	26.313	1.00	0.00	N
ATOM	796	H	CYX	50	39.218	31.278	26.081	1.00	0.00	H
ATOM	797	CA	CYX	50	40.709	31.142	27.590	1.00	0.00	C
ATOM	798	HA	CYX	50	41.766	30.886	27.525	1.00	0.00	H
ATOM	799	CB	CYX	50	40.633	32.289	28.680	1.00	0.00	C
ATOM	800	HB2	CYX	50	39.822	32.979	28.450	1.00	0.00	H
ATOM	801	HB3	CYX	50	40.455	31.823	29.649	1.00	0.00	H
ATOM	802	SG	CYX	50	42.237	33.161	28.855	1.00	0.00	S
ATOM	803	C	CYX	50	40.047	29.945	28.206	1.00	0.00	C
ATOM	804	O	CYX	50	38.813	29.784	28.101	1.00	0.00	O
ATOM	805	N	THR	51	40.843	29.205	28.990	1.00	0.00	N
ATOM	806	H	THR	51	41.844	29.340	28.988	1.00	0.00	H
ATOM	807	CA	THR	51	40.425	28.052	29.789	1.00	0.00	C
ATOM	808	HA	THR	51	39.343	27.924	29.768	1.00	0.00	H
ATOM	809	CB	THR	51	41.075	26.703				
29.318	1.00	0.00	C							
ATOM	810	HB	THR	51	42.151	26.784	29.162	1.00	0.00	H
ATOM	811	CG2	THR	51	40.888	25.528	30.225	1.00	0.00	C

ATOM	812	HG21	THR	51	41.259	25.652	31.242	1.00	0.00	H
ATOM	813	HG22	THR	51	39.826	25.288	30.167	1.00	0.00	H
ATOM	814	HG23	THR	51	41.424	24.630	29.916	1.00	0.00	H
ATOM	815	OG1	THR	51	40.524	26.504	28.052	1.00	0.00	O
ATOM	816	HG1	THR	51	41.017	26.968	27.370	1.00	0.00	H
ATOM	817	C	THR	51	40.732	28.440	31.204	1.00	0.00	C
ATOM	818	O	THR	51	41.959	28.599	31.439	1.00	0.00	O
ATOM	819	N	ILE	52	39.786	28.558	32.140	1.00	0.00	N
ATOM	820	H	ILE	52	38.925	28.218	31.737	1.00	0.00	H
ATOM	821	CA	ILE	52	39.926	29.272	33.385	1.00	0.00	C
ATOM	822	HA	ILE	52	40.928	29.697	33.468	1.00	0.00	H
ATOM	823	CB	ILE	52	38.834	30.411	33.252	1.00	0.00	C
ATOM	824	HB	ILE	52	37.875	29.977	32.971	1.00	0.00	H
ATOM	825	CG2	ILE	52	38.559	31.028	34.654	1.00	0.00	C
ATOM	826	HG21	ILE	52	38.211	30.293	35.379	1.00	0.00	H
ATOM	827	HG22	ILE	52	39.489	31.487	34.989	1.00	0.00	H
ATOM	828	HG23	ILE	52	37.760	31.769	34.654	1.00	0.00	H
ATOM	829	CG1	ILE	52	39.486	31.481	32.389	1.00	0.00	C
ATOM	830	HG12	ILE	52	40.362	31.827	32.938	1.00	0.00	H
ATOM	831	HG13	ILE	52	39.814	31.089	31.426	1.00	0.00	H
ATOM	832	CD1	ILE	52	38.593	32.651	32.203	1.00	0.00	C
ATOM	833	HD11	ILE	52	38.694	33.224	33.123	1.00	0.00	H
ATOM	834	HD12	ILE	52	38.939	33.250	31.360	1.00	0.00	H
ATOM	835	HD13	ILE	52	37.584	32.333	31.940	1.00	0.00	H
ATOM	836	C	ILE	52	39.661	28.313	34.503	1.00	0.00	C
ATOM	837	O	ILE	52	38.560	27.841	34.652	1.00	0.00	O
ATOM	838	N	GLU	53	40.593	28.240	35.460	1.00	0.00	N
ATOM	839	H	GLU	53	41.466	28.747	35.427	1.00	0.00	H
ATOM	840	CA	GLU	53	40.448	27.403	36.667	1.00	0.00	C
ATOM	841	HA	GLU	53	39.713	26.604	36.569	1.00	0.00	H
ATOM	842	CB	GLU	53	41.842	26.641	36.796	1.00	0.00	C
ATOM	843	HB2	GLU	53	42.060	26.095	35.877	1.00	0.00	H
ATOM	844	HB3	GLU	53	42.590	27.405	37.008	1.00	0.00	H
ATOM	845	CG	GLU	53	41.717	25.594	37.954	1.00	0.00	C
ATOM	846	HG2	GLU	53	41.845	26.139	38.889	1.00	0.00	H
ATOM	847	HG3	GLU	53	40.699	25.206	37.949	1.00	0.00	H
ATOM	848	CD	GLU	53	42.844	24.501	37.930	1.00	0.00	C
ATOM	849	OE1	GLU	53	44.049	24.824	37.937	1.00	0.00	O
ATOM	850	OE2	GLU	53	42.516	23.296	38.015	1.00	0.00	O
ATOM	851	C	GLU	53	39.981	28.216	37.867	1.00	0.00	C
ATOM	852	O	GLU	53	40.424	29.323	38.086	1.00	0.00	O
ATOM	853	N	VAL	54	39.117	27.654	38.720	1.00	0.00	N
ATOM	854	H	VAL	54	38.716	26.830	38.297	1.00	0.00	H
ATOM	855	CA	VAL	54	38.611	28.275	39.978	1.00	0.00	C
ATOM	856	HA	VAL	54	38.499	29.357	39.907	1.00	0.00	H
ATOM	857	CB	VAL	54	37.308	27.615	40.477	1.00	0.00	C
ATOM	858	HB	VAL	54	37.430	26.536	40.581	1.00	0.00	H
ATOM	859	CG1	VAL	54	36.687	28.355	41.683	1.00	0.00	C
ATOM	860	HG11	VAL	54	36.748	29.433	41.535	1.00	0.00	H
ATOM	861	HG12	VAL	54	35.630	28.127	41.824	1.00	0.00	H
ATOM	862	HG13	VAL	54	37.253	27.989	42.540	1.00	0.00	H
ATOM	863	CG2	VAL	54	36.240	27.703	39.379	1.00	0.00	C
ATOM	864	HG21	VAL	54	36.545	27.257	38.432	1.00	0.00	H
ATOM	865	HG22	VAL	54	35.392	27.098	39.701	1.00	0.00	H
ATOM	866	HG23	VAL	54	35.864	28.722	39.282	1.00	0.00	H
ATOM	867	C	VAL	54	39.706	28.128	40.993	1.00	0.00	C
ATOM	868	O	VAL	54	39.923	27.021	41.439	1.00	0.00	O
ATOM	869	N	THR	55	40.234	29.134	41.601	1.00	0.00	N
ATOM	870	H	THR	55	39.956	30.033	41.231	1.00	0.00	H
ATOM	871	CA	THR	55	41.471	28.999	42.474	1.00	0.00	C
ATOM	872	HA	THR	55	41.846	27.976	42.427	1.00	0.00	H
ATOM	873	CB	THR	55	42.441	30.048	42.057	1.00	0.00	C
ATOM	874	HB	THR	55	43.341	29.929	42.660	1.00	0.00	H
ATOM	875	CG2	THR	55	42.844	29.735	40.576	1.00	0.00	C

ATOM	876	HG21	THR	55	43.139	28.698	40.415	1.00	0.00	H
ATOM	877	HG22	THR	55	42.069	30.022	39.865	1.00	0.00	H
ATOM	878	HG23	THR	55	43.675	30.420	40.406	1.00	0.00	H
ATOM	879	OG1	THR	55	41.956	31.377	42.073	1.00	0.00	O
ATOM	880	HG1	THR	55	41.886	31.573	43.010	1.00	0.00	H
ATOM	881	C	THR	55	41.124	29.089	43.967	1.00	0.00	C
ATOM	882	O	THR	55	41.988	28.654	44.769	1.00	0.00	O
ATOM	883	N	ALA	56	40.032	29.756	44.364	1.00	0.00	N
ATOM	884	H	ALA	56	39.429	30.320	43.783	1.00	0.00	H
ATOM	885	CA	ALA	56	39.595	29.766	45.765	1.00	0.00	C
ATOM	886	HA	ALA	56	39.666	28.746	46.142	1.00	0.00	H
ATOM	887	CB	ALA	56	40.466	30.729	46.638	1.00	0.00	C
ATOM	888	HB1	ALA	56	41.514	30.435	46.700	1.00	0.00	H
ATOM	889	HB2	ALA	56	40.551	31.705	46.161	1.00	0.00	H
ATOM	890	HB3	ALA	56	39.980	30.826	47.609	1.00	0.00	H
ATOM	891	C	ALA	56	38.167	30.217	45.892	1.00	0.00	C
ATOM	892	O	ALA	56	37.841	31.342	45.446	1.00	0.00	O
ATOM	893	N	THR	57	37.356	29.429	46.588	1.00	0.00	N
ATOM	894	H	THR	57	37.704	28.502	46.786	1.00	0.00	H
ATOM	895	CA	THR	57	35.943	29.701	46.917	1.00	0.00	C
ATOM	896	HA	THR	57	35.579	30.596	46.412	1.00	0.00	H
ATOM	897	CB	THR	57	34.965	28.589	46.473	1.00	0.00	C
ATOM	898	HB	THR	57	33.991	28.773	46.927	1.00	0.00	H
ATOM	899	CG2	THR	57	34.716	28.556	44.905	1.00	0.00	C
ATOM	900	HG21	THR	57	35.582	28.373	44.268	1.00	0.00	H
ATOM	901	HG22	THR	57	33.845	27.950	44.652	1.00	0.00	H
ATOM	902	HG23	THR	57	34.390	29.567	44.661	1.00	0.00	H
ATOM	903	OG1	THR	57	35.433	27.394	46.753	1.00	0.00	O
ATOM	904	HG1	THR	57	35.087	27.037	47.574	1.00	0.00	H
ATOM	905	C	THR	57	35.655	29.918	48.394	1.00	0.00	C
ATOM	906	O	THR	57	36.336	29.361	49.244	1.00	0.00	O
ATOM	907	N	SER	58	34.759	30.836	48.675	1.00	0.00	N
ATOM	908	H	SER	58	34.312	31.421	47.983	1.00	0.00	H
ATOM	909	CA	SER	58	34.327	30.979	50.090	1.00	0.00	C
ATOM	910	HA	SER	58	34.381	30.020	50.605	1.00	0.00	H
ATOM	911	CB	SER	58	35.118	32.147	50.699	1.00	0.00	C
ATOM	912	HB2	SER	58	35.272	32.864	49.893	1.00	0.00	H
ATOM	913	HB3	SER	58	34.490	32.778	51.328	1.00	0.00	H
ATOM	914	OG	SER	58	36.237	31.808	51.399	1.00	0.00	O
ATOM	915	HG	SER	58	36.916	31.578	50.760	1.00	0.00	H
ATOM	916	C	SER	58	32.822	31.335	50.208	1.00	0.00	C
ATOM	917	O	SER	58	32.399	32.255	49.527	1.00	0.00	O
ATOM	918	N	THR	59	32.090	30.692	51.124	1.00	0.00	N
ATOM	919	H	THR	59	32.576	29.986	51.659	1.00	0.00	H
ATOM	920	CA	THR	59	30.793	31.106	51.644	1.00	0.00	C
ATOM	921	HA	THR	59	30.299	31.727	50.895	1.00	0.00	H
ATOM	922	CB	THR	59	29.870	29.913	51.865	1.00	0.00	C
ATOM	923	HB	THR	59	29.859	29.204	51.037	1.00	0.00	H
ATOM	924	CG2	THR	59	30.172	29.045	53.078	1.00	0.00	C
ATOM	925	HG21	THR	59	30.023	29.619	53.992	1.00	0.00	H
ATOM	926	HG22	THR	59	29.654	28.091	52.977	1.00	0.00	H
ATOM	927	HG23	THR	59	31.209	28.711	53.043	1.00	0.00	H
ATOM	928	OG1	THR	59	28.547	30.256	52.183	1.00	0.00	O
ATOM	929	HG1	THR	59	27.907	29.826	51.612	1.00	0.00	H
ATOM	930	C	THR	59	30.992	32.034	52.911	1.00	0.00	C
ATOM	931	O	THR	59	32.004	32.005	53.578	1.00	0.00	O
ATOM	932	N	VAL	60	30.017	33.007	53.037	1.00	0.00	N
ATOM	933	H	VAL	60	29.235	32.987	52.398	1.00	0.00	H
ATOM	934	CA	VAL	60	30.270	34.266	53.813	1.00	0.00	C
ATOM	935	HA	VAL	60	30.532	33.894	54.803	1.00	0.00	H
ATOM	936	CB	VAL	60	31.436	35.051	53.064	1.00	0.00	C
ATOM	937	HB	VAL	60	32.359	34.505	52.867	1.00	0.00	H
ATOM	938	CG1	VAL	60	31.161	35.535	51.665	1.00	0.00	C

ATOM	939	HG11	VAL	60	31.051	34.681	50.997	1.00	0.00	H
ATOM	940	HG12	VAL	60	30.225	36.086	51.567	1.00	0.00	H
ATOM	941	HG13	VAL	60	31.968	36.141	51.254	1.00	0.00	H
ATOM	942	CG2	VAL	60	31.889	36.223	53.908	1.00	0.00	C
ATOM	943	HG21	VAL	60	31.841	35.971	54.967	1.00	0.00	H
ATOM	944	HG22	VAL	60	32.960	36.398	53.814	1.00	0.00	H
ATOM	945	HG23	VAL	60	31.257	37.105	53.796	1.00	0.00	H
ATOM	946	C	VAL	60	29.053	35.193	53.921	1.00	0.00	C
ATOM	947	O	VAL	60	28.022	34.929	53.225	1.00	0.00	O
ATOM	948	N	THR	61	29.106	36.149	54.814	1.00	0.00	N
ATOM	949	H	THR	61	29.895	36.232	55.438	1.00	0.00	H
ATOM	950	CA	THR	61	28.075	37.112	54.986	1.00	0.00	C
ATOM	951	HA	THR	61	27.214	37.091	54.317	1.00	0.00	H
ATOM	952	CB	THR	61	27.470	36.811	56.361	1.00	0.00	C
ATOM	953	HB	THR	61	26.975	37.673	56.809	1.00	0.00	H
ATOM	954	CG2	THR	61	26.507	35.683	56.422	1.00	0.00	C
ATOM	955	HG21	THR	61	27.007	34.858	56.929	1.00	0.00	H
ATOM	956	HG22	THR	61	25.751	36.080	57.098	1.00	0.00	H
ATOM	957	HG23	THR	61	26.117	35.456	55.430	1.00	0.00	H
ATOM	958	OG1	THR	61	28.416	36.493	57.356	1.00	0.00	O
ATOM	959	HG1	THR	61	28.985	37.259	57.467	1.00	0.00	H
ATOM	960	C	THR	61	28.556	38.588	54.872	1.00	0.00	C
ATOM	961	O	THR	61	29.722	38.915	54.734	1.00	0.00	O
ATOM	962	N	LEU	62	27.604	39.435	54.730	1.00	0.00	N
ATOM	963	H	LEU	62	26.648	39.151	54.894	1.00	0.00	H
ATOM	964	CA	LEU	62	27.799	40.888	54.323	1.00	0.00	C
ATOM	965	HA	LEU	62	28.201	40.858	53.310	1.00	0.00	H
ATOM	966	CB	LEU	62	26.387	41.486	54.338	1.00	0.00	C
ATOM	967	HB2	LEU	62	25.693	40.842	53.797	1.00	0.00	H
ATOM	968	HB3	LEU	62	26.008	41.610	55.352	1.00	0.00	H
ATOM	969	CG	LEU	62	26.456	42.868	53.664	1.00	0.00	C
ATOM	970	HG	LEU	62	27.065	43.599	54.196	1.00	0.00	H
ATOM	971	CD1	LEU	62	26.888	42.812	52.300	1.00	0.00	C
ATOM	972	HD11	LEU	62	26.776	43.817	51.893	1.00	0.00	H
ATOM	973	HD12	LEU	62	27.931	42.503	52.227	1.00	0.00	H
ATOM	974	HD13	LEU	62	26.264	42.092	51.771	1.00	0.00	H
ATOM	975	CD2	LEU	62	24.981	43.360	53.657	1.00	0.00	C
ATOM	976	HD21	LEU	62	24.287	42.823	53.010	1.00	0.00	H
ATOM	977	HD22	LEU	62	24.497	43.340	54.634	1.00	0.00	H
ATOM	978	HD23	LEU	62	24.956	44.419	53.396	1.00	0.00	H
ATOM	979	C	LEU	62	28.815	41.734	55.183	1.00	0.00	C
ATOM	980	O	LEU	62	29.465	42.618	54.677	1.00	0.00	O
ATOM	981	N	ASP	63	28.889	41.323	56.442	1.00	0.00	N
ATOM	982	H	ASP	63	28.184	40.628	56.644	1.00	0.00	H
ATOM	983	CA	ASP	63	29.914	41.672	57.414	1.00	0.00	C
ATOM	984	HA	ASP	63	29.942	42.761	57.388	1.00	0.00	H
ATOM	985	CB	ASP	63	29.510	41.176	58.798	1.00	0.00	C
ATOM	986	HB2	ASP	63	30.255	41.651	59.435	1.00	0.00	H
ATOM	987	HB3	ASP	63	28.600	41.672	59.138	1.00	0.00	H
ATOM	988	CG	ASP	63	29.437	39.633	59.080	1.00	0.00	C
ATOM	989	OD1	ASP	63	29.572	38.757	58.161	1.00	0.00	O
ATOM	990	OD2	ASP	63	29.249	39.254	60.270	1.00	0.00	O
ATOM	991	C	ASP	63	31.394	41.279	57.048	1.00	0.00	C
ATOM	992	O	ASP	63	32.288	41.946	57.575	1.00	0.00	O
ATOM	993	N	THR	64	31.665	40.196	56.338	1.00	0.00	N
ATOM	994	H	THR	64	30.874	39.753	55.894	1.00	0.00	H
ATOM	995	CA	THR	64	32.903	39.520	56.211	1.00	0.00	C
ATOM	996	HA	THR	64	33.652	40.227	56.568	1.00	0.00	H
ATOM	997	CB	THR	64	32.892	38.293	57.122	1.00	0.00	C
ATOM	998	HB	THR	64	33.704	37.626	56.829	1.00	0.00	H
ATOM	999	CG2	THR	64	33.334	38.658	58.591	1.00	0.00	C
ATOM	1000	HG21	THR	64	34.135	39.396	58.584	1.00	0.00	H
ATOM	1001	HG22	THR	64	32.540	39.179	59.126	1.00	0.00	H
ATOM	1002	HG23	THR	64	33.718	37.734	59.023	1.00	0.00	H

```
ATOM   1003  OG1 THR    64      31.711  37.507  57.145  1.00  0.00           O
ATOM   1004  HG1 THR    64      31.014  38.129  57.368  1.00  0.00           H
ATOM   1005  C   THR    64      33.240  39.086  54.752  1.00  0.00           C
ATOM   1006  O   THR    64      34.333  38.711  54.453  1.00  0.00           O
ATOM   1007  N   LEU    65      32.452  39.529  53.739  1.00  0.00           N
ATOM   1008  H   LEU    65      31.517  39.882  53.887  1.00  0.00           H
ATOM   1009  CA  LEU    65      33.042  39.734  52.361  1.00  0.00           C
ATOM   1010  HA  LEU    65      33.072  38.772  51.850  1.00  0.00           H
ATOM   1011  CB  LEU    65      32.117  40.556  51.493  1.00  0.00           C
ATOM   1012  HB2 LEU    65      32.335  41.610  51.670  1.00  0.00           H
ATOM   1013  HB3 LEU    65      32.301  40.302  50.449  1.00  0.00           H
ATOM   1014  CG  LEU    65      30.587  40.461  51.572  1.00  0.00           C
ATOM   1015  HG  LEU    65      30.268  40.648  52.597  1.00  0.00           H
ATOM   1016  CD1 LEU    65      29.899  41.424  50.630  1.00  0.00           C
ATOM   1017 HD11 LEU    65      28.830  41.212  50.647  1.00  0.00           H
ATOM   1018 HD12 LEU    65      30.094  42.434  50.988  1.00  0.00           H
ATOM   1019 HD13 LEU    65      30.191  41.261  49.592  1.00  0.00           H
ATOM   1020  CD2 LEU    65      30.173  39.030  51.484  1.00  0.00           C
ATOM   1021 HD21 LEU    65      30.605  38.447  52.298  1.00  0.00           H
ATOM   1022 HD22 LEU    65      29.095  38.938  51.615  1.00  0.00           H
ATOM   1023 HD23 LEU    65      30.353  38.653  50.476  1.00  0.00           H
ATOM   1024  C   LEU    65      34.465  40.295  52.231  1.00  0.00           C
ATOM   1025  O   LEU    65      34.775  41.346  52.813  1.00  0.00           O
ATOM   1026  N   THR    66      35.343  39.592  51.507  1.00  0.00           N
ATOM   1027  H   THR    66      35.067  38.670  51.201  1.00  0.00           H
ATOM   1028  CA  THR    66      36.682  39.945  51.238  1.00  0.00           C
ATOM   1029  HA  THR    66      37.176  39.774  52.194  1.00  0.00           H
ATOM   1030  CB  THR    66      37.351  38.899  50.254  1.00  0.00           C
ATOM   1031  HB  THR    66      37.428  37.963  50.808  1.00  0.00           H
ATOM   1032  CG2 THR    66      36.643  38.730  48.933  1.00  0.00           C
ATOM   1033 HG21 THR    66      37.032  37.802  48.514  1.00  0.00           H
ATOM   1034 HG22 THR    66      35.563  38.607  49.020  1.00  0.00           H
ATOM   1035 HG23 THR    66      36.852  39.602  48.313  1.00  0.00           H
ATOM   1036  OG1 THR    66      38.641  39.280  49.982  1.00  0.00           O
ATOM   1037  HG1 THR    66      39.109  39.408  50.810  1.00  0.00           H
ATOM   1038  C   THR    66      36.891  41.322  50.702  1.00  0.00           C
ATOM   1039  O   THR    66      36.320  41.655  49.674  1.00  0.00           O
ATOM   1040  N   GLU    67      37.810  42.061  51.307  1.00  0.00           N
ATOM   1041  H   GLU    67      38.110  41.783  52.231  1.00  0.00           H
ATOM   1042  CA  GLU    67      38.410  43.284  50.695  1.00  0.00           C
ATOM   1043  HA  GLU    67      37.643  44.057  50.643  1.00  0.00           H
ATOM   1044  CB  GLU    67      39.480  43.817  51.698  1.00  0.00           C
ATOM   1045  HB2 GLU    67      39.643  44.834  51.340  1.00  0.00           H
ATOM   1046  HB3 GLU    67      39.164  43.908  52.737  1.00  0.00           H
ATOM   1047  CG  GLU    67      40.854  43.141  51.613  1.00  0.00           C
ATOM   1048  HG2 GLU    67      41.337  43.349  50.658  1.00  0.00           H
ATOM   1049  HG3 GLU    67      41.506  43.571  52.372  1.00  0.00           H
ATOM   1050  CD  GLU    67      40.713  41.665  52.083  1.00  0.00           C
ATOM   1051  OE1 GLU    67      40.268  41.406  53.222  1.00  0.00           O
ATOM   1052  OE2 GLU    67      40.886  40.767  51.234  1.00  0.00           O
ATOM   1053  C   GLU    67      39.062  43.131  49.252  1.00  0.00           C
ATOM   1054  O   GLU    67      39.041  44.033  48.411  1.00  0.00           O
ATOM   1055  N   LYS    68      39.541  41.911  48.889  1.00  0.00           N
ATOM   1056  H   LYS    68      39.452  41.309  49.694  1.00  0.00           H
ATOM   1057  CA  LYS    68      40.157  41.467  47.642  1.00  0.00           C
ATOM   1058  HA  LYS    68      41.024  42.103  47.468  1.00  0.00           H
ATOM   1059  CB  LYS    68      40.527  40.006  47.720  1.00  0.00           C
ATOM   1060  HB2 LYS
    68      40.913  39.681  48.685  1.00  0.00           H
ATOM   1061  HB3 LYS    68      39.639  39.455  47.410  1.00  0.00           H
ATOM   1062  CG  LYS    68      41.619  39.621  46.654  1.00  0.00           C
ATOM   1063  HG2 LYS    68      41.289  39.753  45.623  1.00  0.00           H
ATOM   1064  HG3 LYS    68      42.499  40.205  46.921  1.00  0.00           H
ATOM   1065  CD  LYS    68      41.951  38.184  46.820  1.00  0.00           C
```

ATOM	1066	HD2	LYS	68	42.143	38.030	47.882	1.00	0.00	H
ATOM	1067	HD3	LYS	68	41.130	37.530	46.524	1.00	0.00	H
ATOM	1068	CE	LYS	68	43.158	37.622	46.017	1.00	0.00	C
ATOM	1069	HE2	LYS	68	42.836	37.793	44.990	1.00	0.00	H
ATOM	1070	HE3	LYS	68	44.088	38.187	46.062	1.00	0.00	H
ATOM	1071	NZ	LYS	68	43.487	36.225	46.356	1.00	0.00	N
ATOM	1072	HZ1	LYS	68	43.478	36.098	47.358	1.00	0.00	H
ATOM	1073	HZ2	LYS	68	42.753	35.604	46.044	1.00	0.00	H
ATOM	1074	HZ3	LYS	68	44.374	35.943	45.961	1.00	0.00	H
ATOM	1075	C	LYS	68	39.228	41.763	46.474	1.00	0.00	C
ATOM	1076	O	LYS	68	39.622	42.522	45.592	1.00	0.00	O
ATOM	1077	N	HIE	69	37.936	41.370	46.606	1.00	0.00	N
ATOM	1078	H	HIE	69	37.675	40.782	47.386	1.00	0.00	H
ATOM	1079	CA	HIE	69	36.958	41.887	45.657	1.00	0.00	C
ATOM	1080	HA	HIE	69	37.249	41.438	44.707	1.00	0.00	H
ATOM	1081	CB	HIE	69	35.584	41.198	46.009	1.00	0.00	C
ATOM	1082	HB2	HIE	69	35.779	40.145	46.213	1.00	0.00	H
ATOM	1083	HB3	HIE	69	35.278	41.690	46.932	1.00	0.00	H
ATOM	1084	CG	HIE	69	34.603	41.292	44.890	1.00	0.00	C
ATOM	1085	ND1	HIE	69	34.407	40.347	43.885	1.00	0.00	N
ATOM	1086	CE1	HIE	69	33.492	40.940	43.082	1.00	0.00	C
ATOM	1087	HE1	HIE	69	33.177	40.517	42.141	1.00	0.00	H
ATOM	1088	NE2	HIE	69	33.207	42.194	43.434	1.00	0.00	N
ATOM	1089	HE2	HIE	69	32.474	42.748	43.013	1.00	0.00	H
ATOM	1090	CD2	HIE	69	33.845	42.430	44.586	1.00	0.00	C
ATOM	1091	HD2	HIE	69	34.055	43.400	45.010	1.00	0.00	H
ATOM	1092	C	HIE	69	36.816	43.449	45.431	1.00	0.00	C
ATOM	1093	O	HIE	69	36.865	43.917	44.302	1.00	0.00	O
ATOM	1094	N	ALA	70	36.773	44.209	46.522	1.00	0.00	N
ATOM	1095	H	ALA	70	36.830	43.749	47.419	1.00	0.00	H
ATOM	1096	CA	ALA	70	36.604	45.635	46.515	1.00	0.00	C
ATOM	1097	HA	ALA	70	35.645	45.821	46.031	1.00	0.00	H
ATOM	1098	CB	ALA	70	36.454	46.136	47.928	1.00	0.00	C
ATOM	1099	HB1	ALA	70	35.717	45.497	48.413	1.00	0.00	H
ATOM	1100	HB2	ALA	70	37.400	46.149	48.469	1.00	0.00	H
ATOM	1101	HB3	ALA	70	35.946	47.100	47.887	1.00	0.00	H
ATOM	1102	C	ALA	70	37.800	46.315	45.862	1.00	0.00	C
ATOM	1103	O	ALA	70	37.567	47.184	44.995	1.00	0.00	O
ATOM	1104	N	GLU	71	39.057	45.898	46.160	1.00	0.00	N
ATOM	1105	H	GLU	71	39.207	45.184	46.859	1.00	0.00	H
ATOM	1106	CA	GLU	71	40.277	46.331	45.394	1.00	0.00	C
ATOM	1107	HA	GLU	71	40.381	47.399	45.583	1.00	0.00	H
ATOM	1108	CB	GLU	71	41.521	45.681	45.997	1.00	0.00	C
ATOM	1109	HB2	GLU	71	41.541	45.861	47.072	1.00	0.00	H
ATOM	1110	HB3	GLU	71	41.596	44.643	45.673	1.00	0.00	H
ATOM	1111	CG	GLU	71	42.868	46.382	45.558	1.00	0.00	C
ATOM	1112	HG2	GLU	71	42.801	46.496	44.477	1.00	0.00	H
ATOM	1113	HG3	GLU	71	42.832	47.408	45.924	1.00	0.00	H
ATOM	1114	CD	GLU	71	44.117	45.615	45.989	1.00	0.00	C
ATOM	1115	OE1	GLU	71	44.174	44.923	47.042	1.00	0.00	O
ATOM	1116	OE2	GLU	71	45.137	45.838	45.259	1.00	0.00	O
ATOM	1117	C	GLU	71	40.217	45.962	43.902	1.00	0.00	C
ATOM	1118	O	GLU	71	40.697	46.768	43.095	1.00	0.00	O
ATOM	1119	N	GLN	72	39.794	44.709	43.563	1.00	0.00	N
ATOM	1120	H	GLN	72	39.424	44.078	44.258	1.00	0.00	H
ATOM	1121	CA	GLN	72	39.761	44.411	42.143	1.00	0.00	C
ATOM	1122	HA	GLN	72	40.702	44.577	41.618	1.00	0.00	H
ATOM	1123	CB	GLN	72	39.469	42.908	42.071	1.00	0.00	C
ATOM	1124	HB2	GLN	72	38.703	42.753	42.831	1.00	0.00	H
ATOM	1125	HB3	GLN	72	40.398	42.407	42.341	1.00	0.00	H
ATOM	1126	CG	GLN	72	39.213	42.300	40.709	1.00	0.00	C
ATOM	1127	HG2	GLN	72	39.781	42.952	40.045	1.00	0.00	H
ATOM	1128	HG3	GLN	72	39.639	41.297	40.748	1.00	0.00	H
ATOM	1129	CD	GLN	72	37.817	42.253	40.222	1.00	0.00	C

```
ATOM   1130  OE1 GLN    72      37.102  41.252  40.186  1.00  0.00           O
ATOM   1131  NE2 GLN    72      37.184  43.276  39.739  1.00  0.00           N
ATOM   1132 HE21 GLN    72      37.546  44.197  39.539  1.00  0.00           H
ATOM   1133 HE22 GLN    72      36.183  43.231  39.617  1.00  0.00           H
ATOM   1134  C   GLN    72      38.689  45.259  41.389  1.00  0.00           C
ATOM   1135  O   GLN    72      38.795  45.485  40.172  1.00  0.00           O
ATOM   1136  N   GLU    73      37.582  45.545  42.061  1.00  0.00           N
ATOM   1137  H   GLU    73      37.582  45.059  42.946  1.00  0.00           H
ATOM   1138  CA  GLU    73      36.480  46.428  41.569  1.00  0.00           C
ATOM   1139  HA  GLU    73      36.394  46.290  40.491  1.00  0.00           H
ATOM   1140  CB  GLU    73      35.221  46.060  42.327  1.00  0.00           C
ATOM   1141  HB2 GLU    73      35.477  46.021  43.386  1.00  0.00           H
ATOM   1142  HB3 GLU    73      34.488  46.856  42.206  1.00  0.00           H
ATOM   1143  CG  GLU    73      34.503  44.768  41.732  1.00  0.00           C
ATOM   1144  HG2 GLU    73      35.074  43.876  41.987  1.00  0.00           H
ATOM   1145  HG3 GLU    73      33.554  44.733  42.267  1.00  0.00           H
ATOM   1146  CD  GLU    73      34.215  44.875  40.247  1.00  0.00           C
ATOM   1147  OE1 GLU    73      33.728  45.884  39.800  1.00  0.00           O
ATOM   1148  OE2 GLU    73      34.616  43.914  39.490  1.00  0.00           O
ATOM   1149  C   GLU    73      36.788  47.940  41.739  1.00  0.00           C
ATOM   1150  O   GLU    73      35.876  48.766  41.394  1.00  0.00           O
ATOM   1151  N   ASN    74      37.970  48.353  42.179  1.00  0.00           N
ATOM   1152  H   ASN    74      38.629  47.655  42.493  1.00  0.00           H
ATOM   1153  CA  ASN    74      38.473  49.682  42.595  1.00  0.00           C
ATOM   1154  HA  ASN    74      39.517  49.449  42.806  1.00  0.00           H
ATOM   1155  CB  ASN    74      38.519  50.556  41.308  1.00  0.00           C
ATOM   1156  HB2 ASN    74      37.536  50.957  41.065  1.00  0.00           H
ATOM   1157  HB3 ASN    74      39.217  51.378  41.465  1.00  0.00           H
ATOM   1158  CG  ASN    74      39.018  49.819  40.087  1.00  0.00           C
ATOM   1159  OD1 ASN    74      40.115  49.217  40.123  1.00  0.00           O
ATOM   1160  ND2 ASN    74      38.291  49.812  39.007  1.00  0.00           N
ATOM   1161 HD21 ASN    74      38.434  49.113  38.292  1.00  0.00           H
ATOM   1162 HD22 ASN    74      37.521  50.437  38.819  1.00  0.00           H
ATOM   1163  C   ASN    74      37.808  50.341  43.766  1.00  0.00           C
ATOM   1164  O   ASN    74      38.227  51.454  44.170  1.00  0.00           O
ATOM   1165  N   MET    75      36.784  49.717  44.338  1.00  0.00           N
ATOM   1166  H   MET    75      36.698  48.725  44.167  1.00  0.00           H
ATOM   1167  CA  MET    75      35.944  50.276  45.376  1.00  0.00           C
ATOM   1168  HA  MET    75      35.801  51.329  45.134  1.00  0.00           H
ATOM   1169  CB  MET    75      34.644  49.511  45.564  1.00  0.00           C
ATOM   1170  HB2 MET    75      34.861  48.453  45.715  1.00  0.00           H
ATOM   1171  HB3 MET    75      33.959  49.985  46.269  1.00  0.00           H
ATOM   1172  CG  MET    75      33.907  49.543  44.239  1.00  0.00           C
ATOM   1173  HG2 MET    75      34.036  50.509  43.752  1.00  0.00           H
ATOM   1174  HG3 MET    75      34.339  48.811  43.555  1.00  0.00           H
ATOM   1175  SD  MET    75      32.143  49.111  44.284  1.00  0.00           S
ATOM   1176  CE  MET    75      31.382  50.253  45.381  1.00  0.00           C
ATOM   1177  HE1 MET    75      31.903  50.304  46.337  1.00  0.00           H
ATOM   1178  HE2 MET    75      31.398  51.259  44.960  1.00  0.00           H
ATOM   1179  HE3 MET    75      30.381  50.053  45.762  1.00  0.00           H
ATOM   1180  C   MET    75      36.538  50.170  46.749  1.00  0.00           C
ATOM   1181  O   MET    75      37.420  49.376  47.025  1.00  0.00           O
ATOM   1182  N   THR    76      36.085  51.057  47.674  1.00  0.00           N
ATOM   1183  H   THR    76      35.271  51.602  47.422  1.00  0.00           H
ATOM   1184  CA  THR    76      36.393  50.905  49.091  1.00  0.00           C
ATOM   1185  HA  THR    76      37.425  50.579
  49.225  1.00  0.00           H
ATOM   1186  CB  THR    76      36.388  52.213  49.894  1.00  0.00           C
ATOM   1187  HB  THR    76      36.996  52.977  49.410  1.00  0.00           H
ATOM   1188  CG2 THR    76      35.034  52.856  50.063  1.00  0.00           C
ATOM   1189 HG21 THR    76      34.454  52.292  50.794  1.00  0.00           H
ATOM   1190 HG22 THR    76      35.199  53.880  50.398  1.00  0.00           H
ATOM   1191 HG23 THR    76      34.483  52.961  49.130  1.00  0.00           H
ATOM   1192  OG1 THR    76      36.976  52.076  51.161  1.00  0.00           O
```

ATOM	1193	HG1	THR	76	37.920	52.233	51.085	1.00	0.00	H
ATOM	1194	C	THR	76	35.485	49.887	49.661	1.00	0.00	C
ATOM	1195	O	THR	76	34.331	49.981	49.344	1.00	0.00	O
ATOM	1196	N	LEU	77	35.977	48.946	50.480	1.00	0.00	N
ATOM	1197	H	LEU	77	36.955	49.010	50.722	1.00	0.00	H
ATOM	1198	CA	LEU	77	35.213	47.762	50.998	1.00	0.00	C
ATOM	1199	HA	LEU	77	35.114	47.004	50.222	1.00	0.00	H
ATOM	1200	CB	LEU	77	36.073	47.101	52.065	1.00	0.00	C
ATOM	1201	HB2	LEU	77	37.029	46.862	51.600	1.00	0.00	H
ATOM	1202	HB3	LEU	77	36.340	47.927	52.725	1.00	0.00	H
ATOM	1203	CG	LEU	77	35.475	45.930	52.838	1.00	0.00	C
ATOM	1204	HG	LEU	77	34.568	46.309	53.309	1.00	0.00	H
ATOM	1205	CD1	LEU	77	35.140	44.758	51.841	1.00	0.00	C
ATOM	1206	HD11	LEU	77	35.340	43.862	52.427	1.00	0.00	H
ATOM	1207	HD12	LEU	77	34.134	44.774	51.420	1.00	0.00	H
ATOM	1208	HD13	LEU	77	35.831	44.754	50.998	1.00	0.00	H
ATOM	1209	CD2	LEU	77	36.335	45.441	53.965	1.00	0.00	C
ATOM	1210	HD21	LEU	77	37.207	45.121	53.395	1.00	0.00	H
ATOM	1211	HD22	LEU	77	36.700	46.245	54.604	1.00	0.00	H
ATOM	1212	HD23	LEU	77	35.776	44.748	54.595	1.00	0.00	H
ATOM	1213	C	LEU	77	33.793	48.076	51.599	1.00	0.00	C
ATOM	1214	O	LEU	77	32.929	47.293	51.334	1.00	0.00	O
ATOM	1215	N	THR	78	33.591	49.089	52.381	1.00	0.00	N
ATOM	1216	H	THR	78	34.272	49.832	52.434	1.00	0.00	H
ATOM	1217	CA	THR	78	32.276	49.421	52.896	1.00	0.00	C
ATOM	1218	HA	THR	78	31.845	48.565	53.415	1.00	0.00	H
ATOM	1219	CB	THR	78	32.215	50.524	53.936	1.00	0.00	C
ATOM	1220	HB	THR	78	31.191	50.895	53.967	1.00	0.00	H
ATOM	1221	CG2	THR	78	32.733	50.251	55.339	1.00	0.00	C
ATOM	1222	HG21	THR	78	33.589	49.607	55.134	1.00	0.00	H
ATOM	1223	HG22	THR	78	32.925	51.214	55.814	1.00	0.00	H
ATOM	1224	HG23	THR	78	31.980	49.677	55.879	1.00	0.00	H
ATOM	1225	OG1	THR	78	32.971	51.621	53.379	1.00	0.00	O
ATOM	1226	HG1	THR	78	33.890	51.368	53.499	1.00	0.00	H
ATOM	1227	C	THR	78	31.276	49.876	51.811	1.00	0.00	C
ATOM	1228	O	THR	78	30.088	49.748	52.019	1.00	0.00	O
ATOM	1229	N	GLU	79	31.716	50.645	50.832	1.00	0.00	N
ATOM	1230	H	GLU	79	32.713	50.802	50.788	1.00	0.00	H
ATOM	1231	CA	GLU	79	30.845	51.231	49.761	1.00	0.00	C
ATOM	1232	HA	GLU	79	29.912	51.634	50.152	1.00	0.00	H
ATOM	1233	CB	GLU	79	31.449	52.339	48.925	1.00	0.00	C
ATOM	1234	HB2	GLU	79	31.823	53.023	49.687	1.00	0.00	H
ATOM	1235	HB3	GLU	79	32.344	52.068	48.364	1.00	0.00	H
ATOM	1236	CG	GLU	79	30.516	53.053	47.945	1.00	0.00	C
ATOM	1237	HG2	GLU	79	30.549	52.456	47.034	1.00	0.00	H
ATOM	1238	HG3	GLU	79	29.559	53.207	48.443	1.00	0.00	H
ATOM	1239	CD	GLU	79	31.123	54.383	47.591	1.00	0.00	C
ATOM	1240	OE1	GLU	79	30.844	55.343	48.317	1.00	0.00	O
ATOM	1241	OE2	GLU	79	31.692	54.509	46.438	1.00	0.00	O
ATOM	1242	C	GLU	79	30.451	50.027	48.863	1.00	0.00	C
ATOM	1243	O	GLU	79	29.314	49.889	48.306	1.00	0.00	O
ATOM	1244	N	LEU	80	31.288	49.047	48.725	1.00	0.00	N
ATOM	1245	H	LEU	80	32.176	49.217	49.175	1.00	0.00	H
ATOM	1246	CA	LEU	80	30.959	47.755	48.122	1.00	0.00	C
ATOM	1247	HA	LEU	80	30.562	47.854	47.111	1.00	0.00	H
ATOM	1248	CB	LEU	80	32.221	46.859	47.923	1.00	0.00	C
ATOM	1249	HB2	LEU	80	32.969	47.318	47.276	1.00	0.00	H
ATOM	1250	HB3	LEU	80	32.786	46.741	48.847	1.00	0.00	H
ATOM	1251	CG	LEU	80	31.963	45.484	47.329	1.00	0.00	C
ATOM	1252	HG	LEU	80	31.154	44.934	47.811	1.00	0.00	H
ATOM	1253	CD1	LEU	80	31.481	45.526	45.883	1.00	0.00	C
ATOM	1254	HD11	LEU	80	31.270	44.505	45.564	1.00	0.00	H
ATOM	1255	HD12	LEU	80	30.615	46.184	45.809	1.00	0.00	H
ATOM	1256	HD13	LEU	80	32.276	45.905	45.240	1.00	0.00	H

```
ATOM   1257  CD2 LEU    80       33.102  44.531  47.441  1.00  0.00           C
ATOM   1258 HD21 LEU    80       33.887  44.800  46.734  1.00  0.00           H
ATOM   1259 HD22 LEU    80       33.516  44.505  48.449  1.00  0.00           H
ATOM   1260 HD23 LEU    80       32.779  43.511  47.234  1.00  0.00           H
ATOM   1261  C   LEU    80       29.904  47.051  48.956  1.00  0.00           C
ATOM   1262  O   LEU    80       28.903  46.598  48.369  1.00  0.00           O
ATOM   1263  N   LYS    81       30.074  46.942  50.246  1.00  0.00           N
ATOM   1264  H   LYS    81       30.907  47.331  50.666  1.00  0.00           H
ATOM   1265  CA  LYS    81       28.992  46.344  51.102  1.00  0.00           C
ATOM   1266  HA  LYS    81       28.890  45.313  50.763  1.00  0.00           H
ATOM   1267  CB  LYS    81       29.604  46.311  52.508  1.00  0.00           C
ATOM   1268  HB2 LYS    81       30.112  47.255  52.700  1.00  0.00           H
ATOM   1269  HB3 LYS    81       28.836  46.193  53.272  1.00  0.00           H
ATOM   1270  CG  LYS    81       30.567  45.180  52.554  1.00  0.00           C
ATOM   1271  HG2 LYS    81       29.959  44.280  52.479  1.00  0.00           H
ATOM   1272  HG3 LYS    81       31.250  45.136  51.705  1.00  0.00           H
ATOM   1273  CD  LYS    81       31.394  45.046  53.838  1.00  0.00           C
ATOM   1274  HD2 LYS    81       32.019  45.923  54.011  1.00  0.00           H
ATOM   1275  HD3 LYS    81       30.629  45.119  54.611  1.00  0.00           H
ATOM   1276  CE  LYS    81       32.167  43.807  54.123  1.00  0.00           C
ATOM   1277  HE2 LYS    81       31.476  42.965  54.087  1.00  0.00           H
ATOM   1278  HE3 LYS    81       32.874  43.655  53.308  1.00  0.00           H
ATOM   1279  NZ  LYS    81       32.725  43.864  55.409  1.00  0.00           N
ATOM   1280  HZ1 LYS    81       31.956  43.821  56.062  1.00  0.00           H
ATOM   1281  HZ2 LYS    81       33.368  43.101  55.557  1.00  0.00           H
ATOM   1282  HZ3 LYS    81       33.196  44.751  55.517  1.00  0.00           H
ATOM   1283  C   LYS    81       27.619  47.031  51.028  1.00  0.00           C
ATOM   1284  O   LYS    81       26.498  46.373  51.019  1.00  0.00           O
ATOM   1285  N   LYS    82       27.650  48.392  50.959  1.00  0.00           N
ATOM   1286  H   LYS    82       28.528  48.870  51.104  1.00  0.00           H
ATOM   1287  CA  LYS    82       26.456  49.271  50.912  1.00  0.00           C
ATOM   1288  HA  LYS    82       25.777  48.862  51.660  1.00  0.00           H
ATOM   1289  CB  LYS    82       26.815  50.747  51.201  1.00  0.00           C
ATOM   1290  HB2 LYS    82       27.574  51.095  50.500  1.00  0.00           H
ATOM   1291  HB3 LYS    82       25.984  51.357  50.846  1.00  0.00           H
ATOM   1292  CG  LYS    82       27.170  51.094  52.641  1.00  0.00           C
ATOM   1293  HG2 LYS    82       26.275  51.152  53.260  1.00  0.00           H
ATOM   1294  HG3 LYS    82       27.817  50.280  52.970  1.00  0.00           H
ATOM   1295  CD  LYS    82       27.864  52.429  52.744  1.00  0.00           C
ATOM   1296  HD2 LYS    82       28.873  52.415  52.332  1.00  0.00           H
ATOM   1297  HD3 LYS    82       27.246  53.077  52.121  1.00  0.00           H
ATOM   1298  CE  LYS    82       27.826  52.922  54.198  1.00  0.00           C
ATOM   1299  HE2 LYS    82       27.036  52.457  54.788  1.00  0.00           H
ATOM   1300  HE3 LYS    82       28.792  52.608  54.593  1.00  0.00           H
ATOM   1301  NZ  LYS    82       27.735  54.384  54.219  1.00  0.00           N
ATOM   1302  HZ1 LYS    82       27.468  54.687  55.145  1.00  0.00           H
ATOM   1303  HZ2 LYS    82       28.638  54.825  54.124  1.00  0.00           H
ATOM   1304  HZ3 LYS    82       27.101  54.619  53.468  1.00  0.00           H
ATOM   1305  C   LYS    82       25.742  49.249  49.569  1.00  0.00           C
ATOM   1306  O   LYS    82       24.504  49.254  49.577  1.00  0.00           O
ATOM   1307  N   VAL    83       26.442  49.078  48.462  1.00  0.00           N
ATOM   1308  H   VAL    83       27.436  49.253  48.477  1.00  0.00           H
ATOM   1309  CA  VAL    83       25.832  48.820  47.128  1.00  0.00           C
ATOM   1310  HA  VAL    83       24.908  49.392  47.040  1.00  0.00
      H
ATOM   1311  CB  VAL    83       26.723  49.268  45.946  1.00  0.00           C
ATOM   1312  HB  VAL    83       26.044  49.085  45.112  1.00  0.00           H
ATOM   1313  CG1 VAL    83       27.042  50.765  46.077  1.00  0.00           C
ATOM   1314 HG11 VAL    83       27.606  51.147  45.226  1.00  0.00           H
ATOM   1315 HG12 VAL    83       26.137  51.361  46.196  1.00  0.00           H
ATOM   1316 HG13 VAL    83       27.631  50.961  46.972  1.00  0.00           H
ATOM   1317  CG2 VAL    83       27.964  48.466  45.575  1.00  0.00           C
ATOM   1318 HG21 VAL    83       27.785  47.417  45.335  1.00  0.00           H
ATOM   1319 HG22 VAL    83       28.429  48.980  44.735  1.00  0.00           H
```

ATOM	1320	HG23	VAL	83	28.708	48.413	46.370	1.00	0.00	H
ATOM	1321	C	VAL	83	25.388	47.389	46.864	1.00	0.00	C
ATOM	1322	O	VAL	83	24.405	47.209	46.138	1.00	0.00	O
ATOM	1323	N	ILE	84	25.971	46.400	47.548	1.00	0.00	N
ATOM	1324	H	ILE	84	26.885	46.598	47.928	1.00	0.00	H
ATOM	1325	CA	ILE	84	25.365	45.092	47.621	1.00	0.00	C
ATOM	1326	HA	ILE	84	25.201	44.663	46.633	1.00	0.00	H
ATOM	1327	CB	ILE	84	26.301	44.064	48.327	1.00	0.00	C
ATOM	1328	HB	ILE	84	26.796	44.542	49.172	1.00	0.00	H
ATOM	1329	CG2	ILE	84	25.535	42.830	48.889	1.00	0.00	C
ATOM	1330	HG21	ILE	84	24.849	43.095	49.694	1.00	0.00	H
ATOM	1331	HG22	ILE	84	24.974	42.421	48.049	1.00	0.00	H
ATOM	1332	HG23	ILE	84	26.250	42.120	49.301	1.00	0.00	H
ATOM	1333	CG1	ILE	84	27.449	43.672	47.316	1.00	0.00	C
ATOM	1334	HG12	ILE	84	26.927	43.094	46.553	1.00	0.00	H
ATOM	1335	HG13	ILE	84	27.873	44.570	46.867	1.00	0.00	H
ATOM	1336	CD1	ILE	84	28.573	42.759	47.909	1.00	0.00	C
ATOM	1337	HD11	ILE	84	29.276	42.518	47.111	1.00	0.00	H
ATOM	1338	HD12	ILE	84	29.197	43.269	48.643	1.00	0.00	H
ATOM	1339	HD13	ILE	84	28.200	41.821	48.319	1.00	0.00	H
ATOM	1340	C	ILE	84	24.049	45.220	48.354	1.00	0.00	C
ATOM	1341	O	ILE	84	23.051	44.728	47.870	1.00	0.00	O
ATOM	1342	N	ALA	85	23.990	45.824	49.494	1.00	0.00	N
ATOM	1343	H	ALA	85	24.898	46.018	49.890	1.00	0.00	H
ATOM	1344	CA	ALA	85	22.867	46.086	50.330	1.00	0.00	C
ATOM	1345	HA	ALA	85	22.444	45.104	50.544	1.00	0.00	H
ATOM	1346	CB	ALA	85	23.340	46.596	51.717	1.00	0.00	C
ATOM	1347	HB1	ALA	85	24.028	47.429	51.577	1.00	0.00	H
ATOM	1348	HB2	ALA	85	22.495	46.701	52.399	1.00	0.00	H
ATOM	1349	HB3	ALA	85	23.890	45.803	52.224	1.00	0.00	H
ATOM	1350	C	ALA	85	21.687	47.036	49.756	1.00	0.00	C
ATOM	1351	O	ALA	85	20.523	46.920	50.199	1.00	0.00	O
ATOM	1352	N	ASP	86	21.955	47.760	48.704	1.00	0.00	N
ATOM	1353	H	ASP	86	22.921	47.871	48.431	1.00	0.00	H
ATOM	1354	CA	ASP	86	20.969	48.350	47.795	1.00	0.00	C
ATOM	1355	HA	ASP	86	20.118	48.753	48.344	1.00	0.00	H
ATOM	1356	CB	ASP	86	21.693	49.525	47.095	1.00	0.00	C
ATOM	1357	HB2	ASP	86	22.156	50.154	47.855	1.00	0.00	H
ATOM	1358	HB3	ASP	86	22.575	49.231	46.525	1.00	0.00	H
ATOM	1359	CG	ASP	86	20.769	50.509	46.401	1.00	0.00	C
ATOM	1360	OD1	ASP	86	19.587	50.740	46.707	1.00	0.00	O
ATOM	1361	OD2	ASP	86	21.299	50.981	45.399	1.00	0.00	O
ATOM	1362	C	ASP	86	20.347	47.423	46.746	1.00	0.00	C
ATOM	1363	O	ASP	86	19.466	47.764	45.935	1.00	0.00	O
ATOM	1364	N	ILE	87	20.631	46.117	46.898	1.00	0.00	N
ATOM	1365	H	ILE	87	21.241	45.873	47.664	1.00	0.00	H
ATOM	1366	CA	ILE	87	20.224	45.005	46.028	1.00	0.00	C
ATOM	1367	HA	ILE	87	19.432	45.336	45.357	1.00	0.00	H
ATOM	1368	CB	ILE	87	21.384	44.604	45.070	1.00	0.00	C
ATOM	1369	HB	ILE	87	22.229	44.250	45.662	1.00	0.00	H
ATOM	1370	CG2	ILE	87	20.836	43.448	44.168	1.00	0.00	C
ATOM	1371	HG21	ILE	87	21.639	43.049	43.549	1.00	0.00	H
ATOM	1372	HG22	ILE	87	20.427	42.608	44.731	1.00	0.00	H
ATOM	1373	HG23	ILE	87	20.020	43.896	43.602	1.00	0.00	H
ATOM	1374	CG1	ILE	87	22.003	45.816	44.272	1.00	0.00	C
ATOM	1375	HG12	ILE	87	21.223	46.249	43.646	1.00	0.00	H
ATOM	1376	HG13	ILE	87	22.419	46.485	45.025	1.00	0.00	H
ATOM	1377	CD1	ILE	87	23.270	45.486	43.500	1.00	0.00	C
ATOM	1378	HD11	ILE	87	23.953	44.952	44.161	1.00	0.00	H
ATOM	1379	HD12	ILE	87	23.065	44.819	42.664	1.00	0.00	H
ATOM	1380	HD13	ILE	87	23.691	46.435	43.164	1.00	0.00	H
ATOM	1381	C	ILE	87	19.534	43.818	46.860	1.00	0.00	C
ATOM	1382	O	ILE	87	18.514	43.245	46.492	1.00	0.00	O
ATOM	1383	N	TYR	88	20.058	43.458	47.984	1.00	0.00	N

ATOM	1384	H	TYR	88	20.955	43.860	48.212	1.00	0.00	H
ATOM	1385	CA	TYR	88	19.631	42.294	48.831	1.00	0.00	C
ATOM	1386	HA	TYR	88	18.800	41.768	48.361	1.00	0.00	H
ATOM	1387	CB	TYR	88	20.876	41.376	48.947	1.00	0.00	C
ATOM	1388	HB2	TYR	88	21.798	41.918	49.155	1.00	0.00	H
ATOM	1389	HB3	TYR	88	20.779	40.665	49.768	1.00	0.00	H
ATOM	1390	CG	TYR	88	21.175	40.619	47.620	1.00	0.00	C
ATOM	1391	CD1	TYR	88	20.537	39.428	47.210	1.00	0.00	C
ATOM	1392	HD1	TYR	88	19.686	39.100	47.787	1.00	0.00	H
ATOM	1393	CE1	TYR	88	20.972	38.723	46.101	1.00	0.00	C
ATOM	1394	HE1	TYR	88	20.500	37.796	45.812	1.00	0.00	H
ATOM	1395	CZ	TYR	88	21.927	39.324	45.228	1.00	0.00	C
ATOM	1396	OH	TYR	88	22.371	38.646	44.122	1.00	0.00	O
ATOM	1397	HH	TYR	88	22.122	37.737	44.301	1.00	0.00	H
ATOM	1398	CE2	TYR	88	22.452	40.583	45.473	1.00	0.00	C
ATOM	1399	HE2	TYR	88	23.187	41.034	44.823	1.00	0.00	H
ATOM	1400	CD2	TYR	88	22.118	41.198	46.742	1.00	0.00	C
ATOM	1401	HD2	TYR	88	22.618	42.099	47.070	1.00	0.00	H
ATOM	1402	C	TYR	88	19.230	42.717	50.262	1.00	0.00	C
ATOM	1403	O	TYR	88	19.721	43.687	50.778	1.00	0.00	O
ATOM	1404	N	PRO	89	18.406	41.892	50.982	1.00	0.00	N
ATOM	1405	CD	PRO	89	17.593	40.817	50.569	1.00	0.00	C
ATOM	1406	HD2	PRO	89	18.124	40.115	49.924	1.00	0.00	H
ATOM	1407	HD3	PRO	89	16.858	41.362	49.976	1.00	0.00	H
ATOM	1408	CG	PRO	89	17.111	40.174	51.821	1.00	0.00	C
ATOM	1409	HG2	PRO	89	17.714	39.353	52.208	1.00	0.00	H
ATOM	1410	HG3	PRO	89	16.121	39.744	51.659	1.00	0.00	H
ATOM	1411	CB	PRO	89	17.091	41.205	52.875	1.00	0.00	C
ATOM	1412	HB2	PRO	89	17.208	40.726	53.847	1.00	0.00	H
ATOM	1413	HB3	PRO	89	16.144	41.741	52.900	1.00	0.00	H
ATOM	1414	CA	PRO	89	18.298	42.010	52.427	1.00	0.00	C
ATOM	1415	HA	PRO	89	18.069	43.062	52.591	1.00	0.00	H
ATOM	1416	C	PRO	89	19.595	41.578	53.038	1.00	0.00	C
ATOM	1417	O	PRO	89	20.256	40.585	52.673	1.00	0.00	O
ATOM	1418	N	GLY	90	19.891	42.330	54.112	1.00	0.00	N
ATOM	1419	H	GLY	90	19.316	43.143	54.277	1.00	0.00	H
ATOM	1420	CA	GLY	90	21.201	42.243	54.828	1.00	0.00	C
ATOM	1421	HA2	GLY	90	22.066	42.459	54.201	1.00	0.00	H
ATOM	1422	HA3	GLY	90	21.170	43.042	55.568	1.00	0.00	H
ATOM	1423	C	GLY	90	21.530	40.833	55.461	1.00	0.00	C
ATOM	1424	O	GLY	90	22.619	40.240	55.446	1.00	0.00	O
ATOM	1425	N	GLN	91	20.432	40.151	55.851	1.00	0.00	N
ATOM	1426	H	GLN	91	19.490	40.488	55.704	1.00	0.00	H
ATOM	1427	CA	GLN	91	20.345	38.746	56.333	1.00	0.00	C
ATOM	1428	HA	GLN	91	20.983	38.700	57.215	1.00	0.00	H
ATOM	1429	CB	GLN	91	18.823	38.529	56.728	1.00	0.00	C
ATOM	1430	HB2	GLN	91	18.434	39.538	56.875	1.00	0.00	H
ATOM	1431	HB3	GLN	91	18.232	38.106	55.915	1.00	0.00	H
ATOM	1432	CG	GLN	91	18.705	37.605	57.906	1.00	0.00	C
ATOM	1433	HG2	GLN	91	18.748	36.561	57.597	1.00	0.00	H
ATOM	1434	HG3	GLN	91	19.521	37.790	58.604	1.00	0.00	H
ATOM	1435	CD	GLN	91	17.289	37.655	58.560	1.00	0.00	C
ATOM	1436	OE1	GLN							
				91	16.240	37.677	57.910	1.00	0.00	O
ATOM	1437	NE2	GLN	91	17.321	37.804	59.891	1.00	0.00	N
ATOM	1438	HE21	GLN	91	18.146	37.867	60.469	1.00	0.00	H
ATOM	1439	HE22	GLN	91	16.389	37.725	60.272	1.00	0.00	H
ATOM	1440	C	GLN	91	20.755	37.680	55.269	1.00	0.00	C
ATOM	1441	O	GLN	91	20.711	36.464	55.579	1.00	0.00	O
ATOM	1442	N	THR	92	21.002	38.046	54.015	1.00	0.00	N
ATOM	1443	H	THR	92	20.956	39.033	53.805	1.00	0.00	H
ATOM	1444	CA	THR	92	21.229	37.198	52.798	1.00	0.00	C
ATOM	1445	HA	THR	92	20.434	36.459	52.700	1.00	0.00	H
ATOM	1446	CB	THR	92	21.252	38.000	51.433	1.00	0.00	C

ATOM	1447	HB	THR	92	21.934	38.833	51.601	1.00	0.00	H
ATOM	1448	CG2	THR	92	21.765	37.331	50.200	1.00	0.00	C
ATOM	1449	HG21	THR	92	22.815	37.045	50.251	1.00	0.00	H
ATOM	1450	HG22	THR	92	21.212	36.391	50.172	1.00	0.00	H
ATOM	1451	HG23	THR	92	21.539	37.841	49.264	1.00	0.00	H
ATOM	1452	OG1	THR	92	20.029	38.541	51.121	1.00	0.00	O
ATOM	1453	HG1	THR	92	20.047	39.364	51.615	1.00	0.00	H
ATOM	1454	C	THR	92	22.518	36.424	52.948	1.00	0.00	C
ATOM	1455	O	THR	92	23.476	37.051	53.279	1.00	0.00	O
ATOM	1456	N	GLN	93	22.668	35.106	52.746	1.00	0.00	N
ATOM	1457	H	GLN	93	21.835	34.626	52.436	1.00	0.00	H
ATOM	1458	CA	GLN	93	23.971	34.435	52.766	1.00	0.00	C
ATOM	1459	HA	GLN	93	24.633	34.804	53.550	1.00	0.00	H
ATOM	1460	CB	GLN	93	23.781	32.940	53.064	1.00	0.00	C
ATOM	1461	HB2	GLN	93	23.380	32.834	54.072	1.00	0.00	H
ATOM	1462	HB3	GLN	93	23.060	32.526	52.360	1.00	0.00	H
ATOM	1463	CG	GLN	93	25.131	32.217	52.974	1.00	0.00	C
ATOM	1464	HG2	GLN	93	24.980	31.163	53.205	1.00	0.00	H
ATOM	1465	HG3	GLN	93	25.608	32.405	52.012	1.00	0.00	H
ATOM	1466	CD	GLN	93	25.970	32.588	54.181	1.00	0.00	C
ATOM	1467	OE1	GLN	93	25.469	33.086	55.210	1.00	0.00	O
ATOM	1468	NE2	GLN	93	27.210	32.330	54.146	1.00	0.00	N
ATOM	1469	HE21	GLN	93	27.585	32.048	53.251	1.00	0.00	H
ATOM	1470	HE22	GLN	93	27.821	32.580	54.910	1.00	0.00	H
ATOM	1471	C	GLN	93	24.543	34.695	51.369	1.00	0.00	C
ATOM	1472	O	GLN	93	23.791	34.727	50.397	1.00	0.00	O
ATOM	1473	N	PHE	94	25.866	34.954	51.366	1.00	0.00	N
ATOM	1474	H	PHE	94	26.457	34.848	52.177	1.00	0.00	H
ATOM	1475	CA	PHE	94	26.646	35.190	50.164	1.00	0.00	C
ATOM	1476	HA	PHE	94	25.936	35.156	49.338	1.00	0.00	H
ATOM	1477	CB	PHE	94	27.262	36.554	50.142	1.00	0.00	C
ATOM	1478	HB2	PHE	94	27.934	36.707	50.987	1.00	0.00	H
ATOM	1479	HB3	PHE	94	27.928	36.593	49.280	1.00	0.00	H
ATOM	1480	CG	PHE	94	26.240	37.687	50.090	1.00	0.00	C
ATOM	1481	CD1	PHE	94	25.618	38.151	48.912	1.00	0.00	C
ATOM	1482	HD1	PHE	94	25.794	37.599	48.001	1.00	0.00	H
ATOM	1483	CE1	PHE	94	24.702	39.257	48.930	1.00	0.00	C
ATOM	1484	HE1	PHE	94	24.301	39.712	48.037	1.00	0.00	H
ATOM	1485	CZ	PHE	94	24.338	39.836	50.166	1.00	0.00	C
ATOM	1486	HZ	PHE	94	23.773	40.755	50.115	1.00	0.00	H
ATOM	1487	CE2	PHE	94	24.890	39.347	51.340	1.00	0.00	C
ATOM	1488	HE2	PHE	94	24.497	39.681	52.289	1.00	0.00	H
ATOM	1489	CD2	PHE	94	25.795	38.261	51.303	1.00	0.00	C
ATOM	1490	HD2	PHE	94	26.140	37.873	52.249	1.00	0.00	H
ATOM	1491	C	PHE	94	27.662	34.136	49.856	1.00	0.00	C
ATOM	1492	O	PHE	94	27.960	33.268	50.699	1.00	0.00	O
ATOM	1493	N	TYR	95	28.203	34.159	48.641	1.00	0.00	N
ATOM	1494	H	TYR	95	27.830	34.740	47.904	1.00	0.00	H
ATOM	1495	CA	TYR	95	29.132	33.109	48.132	1.00	0.00	C
ATOM	1496	HA	TYR	95	29.778	32.783	48.947	1.00	0.00	H
ATOM	1497	CB	TYR	95	28.344	32.020	47.509	1.00	0.00	C
ATOM	1498	HB2	TYR	95	27.619	31.686	48.250	1.00	0.00	H
ATOM	1499	HB3	TYR	95	27.884	32.409	46.599	1.00	0.00	H
ATOM	1500	CG	TYR	95	29.269	30.831	47.108	1.00	0.00	C
ATOM	1501	CD1	TYR	95	29.448	30.532	45.773	1.00	0.00	C
ATOM	1502	HD1	TYR	95	29.111	31.194	44.988	1.00	0.00	H
ATOM	1503	CE1	TYR	95	30.280	29.459	45.419	1.00	0.00	C
ATOM	1504	HE1	TYR	95	30.507	29.235	44.387	1.00	0.00	H
ATOM	1505	CZ	TYR	95	30.860	28.684	46.395	1.00	0.00	C
ATOM	1506	OH	TYR	95	31.544	27.623	45.953	1.00	0.00	O
ATOM	1507	HH	TYR	95	31.533	27.615	44.993	1.00	0.00	H
ATOM	1508	CE2	TYR	95	30.750	29.019	47.738	1.00	0.00	C
ATOM	1509	HE2	TYR	95	31.191	28.399	48.506	1.00	0.00	H
ATOM	1510	CD2	TYR	95	29.893	30.082	48.133	1.00	0.00	C

```
ATOM   1511  HD2 TYR    95      29.797  30.418  49.156  1.00  0.00           H
ATOM   1512  C   TYR    95      30.094  33.742  47.154  1.00  0.00           C
ATOM   1513  O   TYR    95      29.664  34.539  46.266  1.00  0.00           O
ATOM   1514  N   VAL    96      31.414  33.485  47.199  1.00  0.00           N
ATOM   1515  H   VAL    96      31.812  32.796  47.821  1.00  0.00           H
ATOM   1516  CA  VAL    96      32.516  34.097  46.517  1.00  0.00           C
ATOM   1517  HA  VAL    96      32.040  34.823  45.857  1.00  0.00           H
ATOM   1518  CB  VAL    96      33.460  34.872  47.481  1.00  0.00           C
ATOM   1519  HB  VAL    96      33.821  34.092  48.152  1.00  0.00           H
ATOM   1520  CG1 VAL    96      34.595  35.654  46.855  1.00  0.00           C
ATOM   1521 HG11 VAL    96      34.096  36.345  46.176  1.00  0.00           H
ATOM   1522 HG12 VAL    96      35.165  36.140  47.647  1.00  0.00           H
ATOM   1523 HG13 VAL    96      35.272  34.987  46.321  1.00  0.00           H
ATOM   1524  CG2 VAL    96      32.726  35.910  48.362  1.00  0.00           C
ATOM   1525 HG21 VAL    96      33.360  36.510  49.016  1.00  0.00           H
ATOM   1526 HG22 VAL    96      32.232  36.583  47.662  1.00  0.00           H
ATOM   1527 HG23 VAL    96      31.979  35.376  48.950  1.00  0.00           H
ATOM   1528  C   VAL    96      33.345  33.054  45.724  1.00  0.00           C
ATOM   1529  O   VAL    96      33.582  31.963  46.286  1.00  0.00           O
ATOM   1530  N   ILE    97      33.815  33.507  44.561  1.00  0.00           N
ATOM   1531  H   ILE    97      33.533  34.403  44.190  1.00  0.00           H
ATOM   1532  CA  ILE    97      34.477  32.552  43.615  1.00  0.00           C
ATOM   1533  HA  ILE    97      34.917  31.683  44.104  1.00  0.00           H
ATOM   1534  CB  ILE    97      33.579  32.073  42.473  1.00  0.00           C
ATOM   1535  HB  ILE    97      33.357  32.927  41.832  1.00  0.00           H
ATOM   1536  CG2 ILE    97      34.313  31.071  41.568  1.00  0.00           C
ATOM   1537 HG21 ILE    97      35.303  31.443  41.307  1.00  0.00           H
ATOM   1538 HG22 ILE    97      34.273  30.144  42.140  1.00  0.00           H
ATOM   1539 HG23 ILE    97      33.714  30.805  40.696  1.00  0.00           H
ATOM   1540  CG1 ILE    97      32.292  31.486  42.936  1.00  0.00           C
ATOM   1541 HG12 ILE    97      32.494  30.464  43.259  1.00  0.00           H
ATOM   1542 HG13 ILE    97      31.895  31.940  43.843  1.00  0.00           H
ATOM   1543  CD1 ILE    97      31.095  31.535  41.886  1.00  0.00           C
ATOM   1544 HD11 ILE    97      31.335  30.996  40.969  1.00  0.00           H
ATOM   1545 HD12 ILE    97      30.129  31.186  42.250  1.00  0.00           H
ATOM   1546 HD13 ILE    97      30.898  32.576  41.633  1.00  0.00           H
ATOM   1547  C   ILE    97      35.654  33.352  42.968  1.00  0.00           C
ATOM   1548  O   ILE    97      35.423  34.420  42.435  1.00  0.00           O
ATOM   1549  N   GLU    98      36.923  32.931  43.090  1.00  0.00           N
ATOM   1550  H   GLU    98      37.054  32.025  43.517  1.00  0.00           H
ATOM   1551  CA  GLU    98      38.077  33.508  42.469  1.00  0.00           C
ATOM   1552  HA  GLU    98      37.781  34.479  42.072  1.00  0.00           H
ATOM   1553  CB  GLU    98      39.149  33.825  43.541  1.00  0.00           C
ATOM   1554  HB2 GLU    98      38.664  34.435  44.303  1.00  0.00           H
ATOM   1555  HB3 GLU    98      39.551  32.893  43.939  1.00  0.00           H
ATOM   1556  CG  GLU    98      40.260  34.683  43.079  1.00  0.00           C
ATOM   1557  HG2 GLU    98      40.793  34.185  42.270  1.00  0.00           H
ATOM   1558  HG3 GLU    98      39.957  35.636  42.643  1.00  0.00           H
ATOM   1559  CD  GLU    98      41.331  35.153  44.197  1.00  0.00           C
ATOM   1560  OE1 GLU    98      42.123  36.020  43.794  1.00  0.00           O
ATOM   1561  OE2 GLU    98      41.454  34.621
  45.364  1.00  0.00          O
ATOM   1562  C   GLU    98      38.681  32.619  41.455  1.00  0.00           C
ATOM   1563  O   GLU    98      38.698  31.393  41.632  1.00  0.00           O
ATOM   1564  N   PHE    99      39.256  33.176  40.372  1.00  0.00           N
ATOM   1565  H   PHE    99      39.167  34.182  40.370  1.00  0.00           H
ATOM   1566  CA  PHE    99      39.534  32.476  39.089  1.00  0.00           C
ATOM   1567  HA  PHE    99      39.766  31.430  39.291  1.00  0.00           H
ATOM   1568  CB  PHE    99      38.243  32.245  38.247  1.00  0.00           C
ATOM   1569  HB2 PHE    99      38.564  31.800  37.306  1.00  0.00           H
ATOM   1570  HB3 PHE    99      37.694  31.457  38.762  1.00  0.00           H
ATOM   1571  CG  PHE    99      37.313  33.377  37.967  1.00  0.00           C
ATOM   1572  CD1 PHE    99      37.198  34.086  36.734  1.00  0.00           C
ATOM   1573  HD1 PHE    99      37.865  33.793  35.935  1.00  0.00           H
```

ATOM	1574	CE1	PHE	99	36.294	35.104	36.478	1.00	0.00	C
ATOM	1575	HE1	PHE	99	36.348	35.675	35.562	1.00	0.00	H
ATOM	1576	CZ	PHE	99	35.389	35.444	37.528	1.00	0.00	C
ATOM	1577	HZ	PHE	99	34.686	36.259	37.432	1.00	0.00	H
ATOM	1578	CE2	PHE	99	35.482	34.817	38.782	1.00	0.00	C
ATOM	1579	HE2	PHE	99	34.779	35.087	39.556	1.00	0.00	H
ATOM	1580	CD2	PHE	99	36.465	33.809	38.999	1.00	0.00	C
ATOM	1581	HD2	PHE	99	36.542	33.344	39.971	1.00	0.00	H
ATOM	1582	C	PHE	99	40.697	33.176	38.288	1.00	0.00	C
ATOM	1583	O	PHE	99	41.055	34.333	38.428	1.00	0.00	O
ATOM	1584	N	LYS	100	41.326	32.334	37.421	1.00	0.00	N
ATOM	1585	H	LYS	100	40.966	31.390	37.399	1.00	0.00	H
ATOM	1586	CA	LYS	100	42.515	32.615	36.693	1.00	0.00	C
ATOM	1587	HA	LYS	100	42.456	33.681	36.478	1.00	0.00	H
ATOM	1588	CB	LYS	100	43.778	32.198	37.547	1.00	0.00	C
ATOM	1589	HB2	LYS	100	43.429	31.408	38.211	1.00	0.00	H
ATOM	1590	HB3	LYS	100	44.566	31.782	36.920	1.00	0.00	H
ATOM	1591	CG	LYS	100	44.441	33.334	38.334	1.00	0.00	C
ATOM	1592	HG2	LYS	100	44.875	34.006	37.593	1.00	0.00	H
ATOM	1593	HG3	LYS	100	43.735	33.820	39.009	1.00	0.00	H
ATOM	1594	CD	LYS	100	45.636	32.818	39.216	1.00	0.00	C
ATOM	1595	HD2	LYS	100	45.318	32.119	39.990	1.00	0.00	H
ATOM	1596	HD3	LYS	100	46.326	32.298	38.552	1.00	0.00	H
ATOM	1597	CE	LYS	100	46.452	33.942	39.842	1.00	0.00	C
ATOM	1598	HE2	LYS	100	47.463	33.687	40.164	1.00	0.00	H
ATOM	1599	HE3	LYS	100	46.622	34.635	39.019	1.00	0.00	H
ATOM	1600	NZ	LYS	100	45.765	34.622	40.954	1.00	0.00	N
ATOM	1601	HZ1	LYS	100	45.574	33.980	41.710	1.00	0.00	H
ATOM	1602	HZ2	LYS	100	46.468	35.251	41.313	1.00	0.00	H
ATOM	1603	HZ3	LYS	100	44.951	35.102	40.595	1.00	0.00	H
ATOM	1604	C	LYS	100	42.657	31.864	35.376	1.00	0.00	C
ATOM	1605	O	LYS	100	42.596	30.636	35.383	1.00	0.00	O
ATOM	1606	N	CYX	101	42.873	32.574	34.276	1.00	0.00	N
ATOM	1607	H	CYX	101	43.045	33.567	34.339	1.00	0.00	H
ATOM	1608	CA	CYX	101	43.214	31.927	32.979	1.00	0.00	C
ATOM	1609	HA	CYX	101	42.497	31.139	32.752	1.00	0.00	H
ATOM	1610	CB	CYX	101	43.131	32.985	31.854	1.00	0.00	C
ATOM	1611	HB2	CYX	101	42.141	33.441	31.866	1.00	0.00	H
ATOM	1612	HB3	CYX	101	43.851	33.795	31.964	1.00	0.00	H
ATOM	1613	SG	CYX	101	43.253	32.077	30.292	1.00	0.00	S
ATOM	1614	C	CYX	101	44.557	31.206	33.108	1.00	0.00	C
ATOM	1615	O	CYX	101	45.536	31.746	33.548	1.00	0.00	O
ATOM	1616	N	LEU	102	44.519	30.001	32.546	1.00	0.00	N
ATOM	1617	H	LEU	102	43.590	29.696	32.295	1.00	0.00	H
ATOM	1618	CA	LEU	102	45.633	29.094	32.342	1.00	0.00	C
ATOM	1619	HA	LEU	102	46.236	28.917	33.232	1.00	0.00	H
ATOM	1620	CB	LEU	102	45.103	27.666	32.032	1.00	0.00	C
ATOM	1621	HB2	LEU	102	44.431	27.794	31.183	1.00	0.00	H
ATOM	1622	HB3	LEU	102	45.923	27.057	31.649	1.00	0.00	H
ATOM	1623	CG	LEU	102	44.347	26.929	33.077	1.00	0.00	C
ATOM	1624	HG	LEU	102	43.472	27.518	33.353	1.00	0.00	H
ATOM	1625	CD1	LEU	102	43.979	25.605	32.415	1.00	0.00	C
ATOM	1626	HD11	LEU	102	43.257	25.131	33.079	1.00	0.00	H
ATOM	1627	HD12	LEU	102	43.529	25.736	31.431	1.00	0.00	H
ATOM	1628	HD13	LEU	102	44.851	24.979	32.224	1.00	0.00	H
ATOM	1629	CD2	LEU	102	45.143	26.534	34.328	1.00	0.00	C
ATOM	1630	HD21	LEU	102	44.639	25.775	34.928	1.00	0.00	H
ATOM	1631	HD22	LEU	102	46.053	26.081	33.937	1.00	0.00	H
ATOM	1632	HD23	LEU	102	45.394	27.434	34.889	1.00	0.00	H
ATOM	1633	C	LEU	102	46.581	29.655	31.253	1.00	0.00	C
ATOM	1634	O	LEU	102	47.731	29.385	31.361	1.00	0.00	O
ATOM	1635	OXT	LEU	102	46.247	30.470	30.372	1.00	0.00	O
HETATM	1637	N	LIG	103	29.013	41.190	38.489	1.00	0.00	N
HETATM	1638	C	LIG	103	29.162	39.835	38.402	1.00	0.00	C

```
HETATM 1639  O   LIG   103       28.113  43.032  39.366  1.00  0.00           O
HETATM 1640  C5' LIG   103       26.539  40.625  45.129  1.00  0.00           C
HETATM 1641  O5' LIG   103       26.566  39.299  44.706  1.00  0.00           O
HETATM 1642  C4' LIG   103       26.423  41.581  43.897  1.00  0.00           C
HETATM 1643  O4' LIG   103       27.466  41.363  42.896  1.00  0.00           O
HETATM 1644  C3' LIG   103       25.126  41.464  43.066  1.00  0.00           C
HETATM 1645  O3' LIG   103       24.313  42.591  43.429  1.00  0.00           O
HETATM 1646  C2' LIG   103       25.521  41.644  41.576  1.00  0.00           C
HETATM 1647  O2' LIG   103       24.900  42.774  41.031  1.00  0.00           O
HETATM 1648  C1' LIG   103       27.018  41.845  41.562  1.00  0.00           C
HETATM 1649  N1  LIG   103       27.756  41.128  40.482  1.00  0.00           N
HETATM 1650  O1  LIG   103       29.327  37.003  37.495  1.00  0.00           O
HETATM 1651  N2  LIG   103       30.007  39.207  37.455  1.00  0.00           N
HETATM 1652  C6  LIG   103       28.257  41.835  39.434  1.00  0.00           C
HETATM 1653  C7  LIG   103       27.914  39.774  40.432  1.00  0.00           C
HETATM 1654  C8  LIG   103       28.641  39.086  39.506  1.00  0.00           C
HETATM 1655  C9  LIG   103       30.092  37.859  37.052  1.00  0.00           C
HETATM 1656  C10 LIG   103       31.260  37.448  36.148  1.00  0.00           C
HETATM 1657  H   LIG   103       30.521  39.852  36.867  1.00  0.00           H
HETATM 1658  H1  LIG   103       28.679  38.011  39.638  1.00  0.00           H
HETATM 1659  H4  LIG   103       27.352  42.889  41.579  1.00  0.00           H
HETATM 1660  H6  LIG   103       26.523  42.594  44.306  1.00  0.00           H
HETATM 1661  H7  LIG   103       24.614  40.507  43.221  1.00  0.00           H
HETATM 1662  H8  LIG   103       25.169  40.710  41.121  1.00  0.00           H
HETATM 1663  H9  LIG   103       25.618  40.750  45.713  1.00  0.00           H
HETATM 1664  H10 LIG   103       27.325  40.912  45.838  1.00  0.00           H
HETATM 1665  H11 LIG   103       31.836  36.718  36.729  1.00  0.00           H
HETATM 1666  H12 LIG   103       31.918  38.287  35.890  1.00  0.00           H
HETATM 1667  H13 LIG   103       30.793  36.891  35.327  1.00  0.00           H
HETATM 1668  H14 LIG   103       27.329  39.291  41.204  1.00  0.00           H
HETATM 1669  H2' LIG   103       24.948  42.841  40.062  1.00  0.00           H
HETATM 1670  H3' LIG   103       24.287  43.105  42.604  1.00  0.00           H
HETATM 1671  H5' LIG   103       27.240  39.226  44.008  1.00  0.00           H
ENDMDL
MODEL      6
SHEET    1   1 1 ILE    22  ASP    26  0
SHEET    2   2 1 VAL    37  VAL    40  0
SHEET    3   3 1 CYX    50  THR    61  0
SHEET    4   4 1 GLN    93  CYX   101  0
HELIX    1   1 GLN      9  LEU     16  1                                   8
HELIX    2   2 GLU     67  GLU     73  1                                   7
HELIX    3   3 LEU     77  ASP     86  1                                  10
ATOM      1  N   GLN     1       37.545  17.343  33.512  1.00  0.00           N
ATOM      2  H1  GLN     1       38.070  16.541  33.195  1.00  0.00           H
ATOM      3  H2  GLN     1       38.158  17.970  34.015  1.00  0.00           H
ATOM      4  H3  GLN     1       36.812  16.917  34.061  1.00  0.00           H
ATOM      5  CA  GLN     1       37.054  18.016  32.312  1.00  0.00           C
ATOM      6  HA  GLN     1       37.809  18.114  31.531  1.00  0.00           H
ATOM      7  CB  GLN     1       36.033  17.135  31.638  1.00  0.00           C
ATOM      8  HB2 GLN     1       35.302  16.996  32.435  1.00  0.00           H
ATOM      9  HB3 GLN     1       35.535  17.656  30.820  1.00  0.00           H
ATOM     10  CG  GLN     1       36.322  15.779  31.116  1.00  0.00           C
ATOM     11  HG2 GLN     1       36.547  15.142  31.971
  1.00  0.00           H
ATOM     12  HG3 GLN     1       35.449  15.393  30.587  1.00  0.00           H
ATOM     13  CD  GLN     1       37.441  15.713  30.057  1.00  0.00           C
ATOM     14  OE1 GLN     1       38.082  16.671  29.645  1.00  0.00           O
ATOM     15  NE2 GLN     1       37.691  14.477  29.602  1.00  0.00           N
ATOM     16 HE21 GLN     1       37.083  13.720  29.880  1.00  0.00           H
ATOM     17 HE22 GLN     1       38.607  14.368  29.188  1.00  0.00           H
ATOM     18  C   GLN     1       36.560  19.422  32.565  1.00  0.00           C
ATOM     19  O   GLN     1       36.064  19.650  33.710  1.00  0.00           O
ATOM     20  N   PRO     2       36.554  20.364  31.596  1.00  0.00           N
ATOM     21  CD  PRO     2       37.165  20.288  30.337  1.00  0.00           C
```

ATOM	22	HD2	PRO	2	37.161	19.340	29.797	1.00	0.00	H
ATOM	23	HD3	PRO	2	38.161	20.705	30.481	1.00	0.00	H
ATOM	24	CG	PRO	2	36.487	21.332	29.425	1.00	0.00	C
ATOM	25	HG2	PRO	2	35.632	20.864	28.937	1.00	0.00	H
ATOM	26	HG3	PRO	2	37.264	21.687	28.748	1.00	0.00	H
ATOM	27	CB	PRO	2	36.020	22.419	30.445	1.00	0.00	C
ATOM	28	HB2	PRO	2	35.051	22.835	30.172	1.00	0.00	H
ATOM	29	HB3	PRO	2	36.835	23.133	30.559	1.00	0.00	H
ATOM	30	CA	PRO	2	35.815	21.659	31.771	1.00	0.00	C
ATOM	31	HA	PRO	2	36.279	22.244	32.565	1.00	0.00	H
ATOM	32	C	PRO	2	34.333	21.489	32.036	1.00	0.00	C
ATOM	33	O	PRO	2	33.687	20.474	31.663	1.00	0.00	O
ATOM	34	N	ASN	3	33.701	22.483	32.670	1.00	0.00	N
ATOM	35	H	ASN	3	34.221	23.303	32.947	1.00	0.00	H
ATOM	36	CA	ASN	3	32.264	22.586	32.935	1.00	0.00	C
ATOM	37	HA	ASN	3	31.849	21.596	33.122	1.00	0.00	H
ATOM	38	CB	ASN	3	31.841	23.534	34.133	1.00	0.00	C
ATOM	39	HB2	ASN	3	32.075	24.575	33.911	1.00	0.00	H
ATOM	40	HB3	ASN	3	30.784	23.347	34.324	1.00	0.00	H
ATOM	41	CG	ASN	3	32.591	23.159	35.409	1.00	0.00	C
ATOM	42	OD1	ASN	3	33.215	22.121	35.514	1.00	0.00	O
ATOM	43	ND2	ASN	3	32.402	23.944	36.407	1.00	0.00	N
ATOM	44	HD21	ASN	3	31.792	24.729	36.226	1.00	0.00	H
ATOM	45	HD22	ASN	3	32.843	23.688	37.278	1.00	0.00	H
ATOM	46	C	ASN	3	31.510	23.064	31.690	1.00	0.00	C
ATOM	47	O	ASN	3	32.110	23.781	30.876	1.00	0.00	O
ATOM	48	N	ASP	4	30.217	22.639	31.465	1.00	0.00	N
ATOM	49	H	ASP	4	29.783	21.949	32.062	1.00	0.00	H
ATOM	50	CA	ASP	4	29.361	23.128	30.361	1.00	0.00	C
ATOM	51	HA	ASP	4	29.959	23.192	29.451	1.00	0.00	H
ATOM	52	CB	ASP	4	28.054	22.275	30.084	1.00	0.00	C
ATOM	53	HB2	ASP	4	27.486	22.250	31.013	1.00	0.00	H
ATOM	54	HB3	ASP	4	27.382	22.713	29.345	1.00	0.00	H
ATOM	55	CG	ASP	4	28.235	20.854	29.593	1.00	0.00	C
ATOM	56	OD1	ASP	4	29.455	20.473	29.428	1.00	0.00	O
ATOM	57	OD2	ASP	4	27.234	20.152	29.381	1.00	0.00	O
ATOM	58	C	ASP	4	28.945	24.549	30.649	1.00	0.00	C
ATOM	59	O	ASP	4	28.908	25.307	29.698	1.00	0.00	O
ATOM	60	N	ILE	5	28.656	24.982	31.902	1.00	0.00	N
ATOM	61	H	ILE	5	28.682	24.219	32.563	1.00	0.00	H
ATOM	62	CA	ILE	5	28.303	26.296	32.380	1.00	0.00	C
ATOM	63	HA	ILE	5	27.663	26.788	31.647	1.00	0.00	H
ATOM	64	CB	ILE	5	27.497	26.189	33.655	1.00	0.00	C
ATOM	65	HB	ILE	5	27.925	25.407	34.282	1.00	0.00	H
ATOM	66	CG2	ILE	5	27.548	27.565	34.468	1.00	0.00	C
ATOM	67	HG21	ILE	5	26.937	27.473	35.366	1.00	0.00	H
ATOM	68	HG22	ILE	5	28.544	27.673	34.900	1.00	0.00	H
ATOM	69	HG23	ILE	5	27.291	28.413	33.834	1.00	0.00	H
ATOM	70	CG1	ILE	5	26.121	25.663	33.324	1.00	0.00	C
ATOM	71	HG12	ILE	5	26.194	24.759	32.722	1.00	0.00	H
ATOM	72	HG13	ILE	5	25.497	25.384	34.173	1.00	0.00	H
ATOM	73	CD1	ILE	5	25.192	26.550	32.579	1.00	0.00	C
ATOM	74	HD11	ILE	5	25.402	26.804	31.540	1.00	0.00	H
ATOM	75	HD12	ILE	5	24.250	26.002	32.560	1.00	0.00	H
ATOM	76	HD13	ILE	5	25.081	27.440	33.198	1.00	0.00	H
ATOM	77	C	ILE	5	29.583	27.094	32.498	1.00	0.00	C
ATOM	78	O	ILE	5	30.635	26.497	32.810	1.00	0.00	O
ATOM	79	N	THR	6	29.548	28.395	32.246	1.00	0.00	N
ATOM	80	H	THR	6	28.659	28.861	32.133	1.00	0.00	H
ATOM	81	CA	THR	6	30.584	29.430	32.352	1.00	0.00	C
ATOM	82	HA	THR	6	31.201	29.108	33.190	1.00	0.00	H
ATOM	83	CB	THR	6	31.438	29.406	31.058	1.00	0.00	C
ATOM	84	HB	THR	6	31.703	28.394	30.751	1.00	0.00	H
ATOM	85	CG2	THR	6	30.914	30.097	29.801	1.00	0.00	C

ATOM	86	HG21	THR	6	31.579	29.904	28.959	1.00	0.00	H
ATOM	87	HG22	THR	6	29.973	29.611	29.544	1.00	0.00	H
ATOM	88	HG23	THR	6	30.773	31.150	30.047	1.00	0.00	H
ATOM	89	OG1	THR	6	32.683	29.981	31.366	1.00	0.00	O
ATOM	90	HG1	THR	6	33.153	29.839	30.542	1.00	0.00	H
ATOM	91	C	THR	6	30.046	30.857	32.659	1.00	0.00	C
ATOM	92	O	THR	6	28.908	30.949	33.158	1.00	0.00	O
ATOM	93	N	PHE	7	30.833	31.937	32.508	1.00	0.00	N
ATOM	94	H	PHE	7	31.821	31.774	32.374	1.00	0.00	H
ATOM	95	CA	PHE	7	30.344	33.257	32.862	1.00	0.00	C
ATOM	96	HA	PHE	7	29.634	33.135	33.680	1.00	0.00	H
ATOM	97	CB	PHE	7	31.374	34.123	33.541	1.00	0.00	C
ATOM	98	HB2	PHE	7	32.146	34.231	32.778	1.00	0.00	H
ATOM	99	HB3	PHE	7	30.874	35.088	33.611	1.00	0.00	H
ATOM	100	CG	PHE	7	32.084	33.736	34.888	1.00	0.00	C
ATOM	101	CD1	PHE	7	31.249	33.580	36.052	1.00	0.00	C
ATOM	102	HD1	PHE	7	30.194	33.815	36.068	1.00	0.00	H
ATOM	103	CE1	PHE	7	31.870	33.182	37.190	1.00	0.00	C
ATOM	104	HE1	PHE	7	31.248	33.016	38.057	1.00	0.00	H
ATOM	105	CZ	PHE	7	33.255	32.944	37.271	1.00	0.00	C
ATOM	106	HZ	PHE	7	33.714	32.633	38.199	1.00	0.00	H
ATOM	107	CE2	PHE	7	34.012	33.044	36.127	1.00	0.00	C
ATOM	108	HE2	PHE	7	35.054	32.760	36.113	1.00	0.00	H
ATOM	109	CD2	PHE	7	33.428	33.538	34.965	1.00	0.00	C
ATOM	110	HD2	PHE	7	34.065	33.856	34.152	1.00	0.00	H
ATOM	111	C	PHE	7	29.521	33.861	31.679	1.00	0.00	C
ATOM	112	O	PHE	7	29.796	33.487	30.496	1.00	0.00	O
ATOM	113	N	PHE	8	28.437	34.625	31.934	1.00	0.00	N
ATOM	114	H	PHE	8	28.285	34.874	32.901	1.00	0.00	H
ATOM	115	CA	PHE	8	27.450	35.051	30.959	1.00	0.00	C
ATOM	116	HA	PHE	8	27.242	34.254	30.246	1.00	0.00	H
ATOM	117	CB	PHE	8	26.115	35.619	31.502	1.00	0.00	C
ATOM	118	HB2	PHE	8	26.366	36.119	32.438	1.00	0.00	H
ATOM	119	HB3	PHE	8	25.606	36.295	30.814	1.00	0.00	H
ATOM	120	CG	PHE	8	25.110	34.551	31.906	1.00	0.00	C
ATOM	121	CD1	PHE	8	24.485	34.615	33.120	1.00	0.00	C
ATOM	122	HD1	PHE	8	24.832	35.343	33.840	1.00	0.00	H
ATOM	123	CE1	PHE	8	23.465	33.680	33.443	1.00	0.00	C
ATOM	124	HE1	PHE	8	23.063	33.748	34.443	1.00	0.00	H
ATOM	125	CZ	PHE	8	23.054	32.726	32.568	1.00	0.00	C
ATOM	126	HZ	PHE	8	22.300	31.984	32.787	1.00	0.00	H
ATOM	127	CE2	PHE	8	23.706	32.650	31.318	1.00	0.00	C
ATOM	128	HE2	PHE	8	23.516	31.804	30.674	1.00	0.00	H
ATOM	129	CD2	PHE	8	24.772	33.531	31.003	1.00	0.00	C
ATOM	130	HD2	PHE	8	25.334	33.408	30.090	1.00	0.00	H
ATOM	131	C	PHE	8	28.029	36.238	30.201	1.00	0.00	C
ATOM	132	O	PHE	8	28.892	36.956	30.691	1.00	0.00	O
ATOM	133	N	GLN	9	27.677	36.377	28.907	1.00	0.00	N
ATOM	134	H	GLN	9	27.054	35.652	28.580	1.00	0.00	H
ATOM	135	CA	GLN	9	28.346	37.084	27.857	1.00	0.00	C
ATOM	136	HA	GLN	9	29.341	36.644	27.792	1.00	0.00	H
ATOM										
	137	CB	GLN	9	27.553	36.976	26.481	1.00	0.00	C
ATOM	138	HB2	GLN	9	27.345	35.973	26.111	1.00	0.00	H
ATOM	139	HB3	GLN	9	26.600	37.483	26.629	1.00	0.00	H
ATOM	140	CG	GLN	9	28.242	37.641	25.259	1.00	0.00	C
ATOM	141	HG2	GLN	9	29.309	37.610	25.479	1.00	0.00	H
ATOM	142	HG3	GLN	9	27.998	37.062	24.368	1.00	0.00	H
ATOM	143	CD	GLN	9	27.838	39.069	24.987	1.00	0.00	C
ATOM	144	OE1	GLN	9	27.557	39.908	25.887	1.00	0.00	O
ATOM	145	NE2	GLN	9	27.718	39.428	23.715	1.00	0.00	N
ATOM	146	HE21	GLN	9	27.369	40.335	23.444	1.00	0.00	H
ATOM	147	HE22	GLN	9	27.811	38.699	23.021	1.00	0.00	H
ATOM	148	C	GLN	9	28.778	38.521	28.169	1.00	0.00	C

ATOM	149	O	GLN	9	29.860	39.043	27.756	1.00	0.00	O
ATOM	150	N	ARG	10	27.994	39.142	28.994	1.00	0.00	N
ATOM	151	H	ARG	10	27.374	38.587	29.566	1.00	0.00	H
ATOM	152	CA	ARG	10	28.032	40.539	29.450	1.00	0.00	C
ATOM	153	HA	ARG	10	28.233	41.287	28.684	1.00	0.00	H
ATOM	154	CB	ARG	10	26.715	40.925	30.194	1.00	0.00	C
ATOM	155	HB2	ARG	10	26.511	40.227	31.005	1.00	0.00	H
ATOM	156	HB3	ARG	10	26.919	41.904	30.630	1.00	0.00	H
ATOM	157	CG	ARG	10	25.499	40.992	29.276	1.00	0.00	C
ATOM	158	HG2	ARG	10	25.159	39.978	29.065	1.00	0.00	H
ATOM	159	HG3	ARG	10	24.712	41.432	29.888	1.00	0.00	H
ATOM	160	CD	ARG	10	25.665	41.814	28.036	1.00	0.00	C
ATOM	161	HD2	ARG	10	26.040	42.755	28.437	1.00	0.00	H
ATOM	162	HD3	ARG	10	26.488	41.465	27.413	1.00	0.00	H
ATOM	163	NE	ARG	10	24.519	41.997	27.219	1.00	0.00	N
ATOM	164	HE	ARG	10	23.688	42.195	27.760	1.00	0.00	H
ATOM	165	CZ	ARG	10	24.438	42.279	25.929	1.00	0.00	C
ATOM	166	NH1	ARG	10	25.403	41.888	25.153	1.00	0.00	N
ATOM	167	HH11	ARG	10	25.275	42.133	24.181	1.00	0.00	H
ATOM	168	HH12	ARG	10	26.264	41.458	25.458	1.00	0.00	H
ATOM	169	NH2	ARG	10	23.383	42.840	25.347	1.00	0.00	N
ATOM	170	HH21	ARG	10	22.572	43.029	25.920	1.00	0.00	H
ATOM	171	HH22	ARG	10	23.257	42.675	24.358	1.00	0.00	H
ATOM	172	C	ARG	10	29.210	40.832	30.320	1.00	0.00	C
ATOM	173	O	ARG	10	29.607	41.961	30.566	1.00	0.00	O
ATOM	174	N	PHE	11	29.869	39.757	30.889	1.00	0.00	N
ATOM	175	H	PHE	11	29.505	38.823	30.762	1.00	0.00	H
ATOM	176	CA	PHE	11	31.032	39.869	31.806	1.00	0.00	C
ATOM	177	HA	PHE	11	31.040	40.898	32.164	1.00	0.00	H
ATOM	178	CB	PHE	11	30.789	38.996	33.065	1.00	0.00	C
ATOM	179	HB2	PHE	11	31.588	39.195	33.779	1.00	0.00	H
ATOM	180	HB3	PHE	11	30.896	37.946	32.791	1.00	0.00	H
ATOM	181	CG	PHE	11	29.498	39.199	33.820	1.00	0.00	C
ATOM	182	CD1	PHE	11	29.421	40.327	34.646	1.00	0.00	C
ATOM	183	HD1	PHE	11	30.256	41.011	34.612	1.00	0.00	H
ATOM	184	CE1	PHE	11	28.297	40.515	35.496	1.00	0.00	C
ATOM	185	HE1	PHE	11	28.198	41.381	36.134	1.00	0.00	H
ATOM	186	CZ	PHE	11	27.212	39.618	35.274	1.00	0.00	C
ATOM	187	HZ	PHE	11	26.344	39.746	35.902	1.00	0.00	H
ATOM	188	CE2	PHE	11	27.212	38.636	34.296	1.00	0.00	C
ATOM	189	HE2	PHE	11	26.300	38.101	34.076	1.00	0.00	H
ATOM	190	CD2	PHE	11	28.428	38.341	33.646	1.00	0.00	C
ATOM	191	HD2	PHE	11	28.541	37.532	32.939	1.00	0.00	H
ATOM	192	C	PHE	11	32.430	39.621	31.189	1.00	0.00	C
ATOM	193	O	PHE	11	33.484	39.958	31.763	1.00	0.00	O
ATOM	194	N	GLN	12	32.440	38.828	30.134	1.00	0.00	N
ATOM	195	H	GLN	12	31.536	38.603	29.744	1.00	0.00	H
ATOM	196	CA	GLN	12	33.608	38.187	29.554	1.00	0.00	C
ATOM	197	HA	GLN	12	33.971	37.496	30.316	1.00	0.00	H
ATOM	198	CB	GLN	12	33.239	37.298	28.333	1.00	0.00	C
ATOM	199	HB2	GLN	12	32.564	37.801	27.640	1.00	0.00	H
ATOM	200	HB3	GLN	12	34.073	36.811	27.826	1.00	0.00	H
ATOM	201	CG	GLN	12	32.504	36.081	28.906	1.00	0.00	C
ATOM	202	HG2	GLN	12	33.174	35.593	29.614	1.00	0.00	H
ATOM	203	HG3	GLN	12	31.527	36.363	29.301	1.00	0.00	H
ATOM	204	CD	GLN	12	32.159	34.983	27.914	1.00	0.00	C
ATOM	205	OE1	GLN	12	32.559	35.006	26.736	1.00	0.00	O
ATOM	206	NE2	GLN	12	31.603	33.900	28.465	1.00	0.00	N
ATOM	207	HE21	GLN	12	31.000	33.908	29.276	1.00	0.00	H
ATOM	208	HE22	GLN	12	31.415	33.118	27.854	1.00	0.00	H
ATOM	209	C	GLN	12	34.680	39.166	29.182	1.00	0.00	C
ATOM	210	O	GLN	12	35.852	38.949	29.455	1.00	0.00	O
ATOM	211	N	ASP	13	34.328	40.313	28.580	1.00	0.00	N
ATOM	212	H	ASP	13	33.384	40.508	28.277	1.00	0.00	H

ATOM	213	CA	ASP	13	35.363	41.212	28.016	1.00	0.00	C
ATOM	214	HA	ASP	13	36.074	40.691	27.374	1.00	0.00	H
ATOM	215	CB	ASP	13	34.777	42.330	27.159	1.00	0.00	C
ATOM	216	HB2	ASP	13	33.842	42.612	27.644	1.00	0.00	H
ATOM	217	HB3	ASP	13	35.529	43.117	27.111	1.00	0.00	H
ATOM	218	CG	ASP	13	34.515	41.945	25.667	1.00	0.00	C
ATOM	219	OD1	ASP	13	34.075	42.784	24.823	1.00	0.00	O
ATOM	220	OD2	ASP	13	34.997	40.858	25.261	1.00	0.00	O
ATOM	221	C	ASP	13	36.241	41.834	29.134	1.00	0.00	C
ATOM	222	O	ASP	13	37.394	42.258	28.946	1.00	0.00	O
ATOM	223	N	ASP	14	35.736	41.851	30.392	1.00	0.00	N
ATOM	224	H	ASP	14	34.755	41.626	30.473	1.00	0.00	H
ATOM	225	CA	ASP	14	36.524	42.244	31.539	1.00	0.00	C
ATOM	226	HA	ASP	14	37.153	43.114	31.356	1.00	0.00	H
ATOM	227	CB	ASP	14	35.546	42.525	32.732	1.00	0.00	C
ATOM	228	HB2	ASP	14	34.889	41.664	32.851	1.00	0.00	H
ATOM	229	HB3	ASP	14	36.169	42.748	33.599	1.00	0.00	H
ATOM	230	CG	ASP	14	34.633	43.767	32.583	1.00	0.00	C
ATOM	231	OD1	ASP	14	33.429	43.476	32.407	1.00	0.00	O
ATOM	232	OD2	ASP	14	35.128	44.923	32.538	1.00	0.00	O
ATOM	233	C	ASP	14	37.529	41.140	31.860	1.00	0.00	C
ATOM	234	O	ASP	14	38.604	41.440	32.401	1.00	0.00	O
ATOM	235	N	ILE	15	37.213	39.808	31.676	1.00	0.00	N
ATOM	236	H	ILE	15	36.371	39.601	31.159	1.00	0.00	H
ATOM	237	CA	ILE	15	38.202	38.756	31.779	1.00	0.00	C
ATOM	238	HA	ILE	15	38.711	38.908	32.731	1.00	0.00	H
ATOM	239	CB	ILE	15	37.519	37.427	31.885	1.00	0.00	C
ATOM	240	HB	ILE	15	37.067	37.195	30.921	1.00	0.00	H
ATOM	241	CG2	ILE	15	38.552	36.282	32.093	1.00	0.00	C
ATOM	242	HG21	ILE	15	38.015	35.371	32.359	1.00	0.00	H
ATOM	243	HG22	ILE	15	39.088	36.081	31.166	1.00	0.00	H
ATOM	244	HG23	ILE	15	39.255	36.577	32.872	1.00	0.00	H
ATOM	245	CG1	ILE	15	36.427	37.252	33.015	1.00	0.00	C
ATOM	246	HG12	ILE	15	36.887	37.486	33.975	1.00	0.00	H
ATOM	247	HG13	ILE	15	35.647	37.971	32.761	1.00	0.00	H
ATOM	248	CD1	ILE	15	35.669	35.963	33.242	1.00	0.00	C
ATOM	249	HD11	ILE	15	35.167	35.550	32.368	1.00	0.00	H
ATOM	250	HD12	ILE	15	36.367	35.192	33.568	1.00	0.00	H
ATOM	251	HD13	ILE	15	34.933	36.201	34.008	1.00	0.00	H
ATOM	252	C	ILE	15	39.252	38.823	30.648	1.00	0.00	C
ATOM	253	O	ILE	15	40.456	38.533	30.884	1.00	0.00	O
ATOM	254	N	LEU	16	38.826	39.165	29.437	1.00	0.00	N
ATOM	255	H	LEU	16	37.842	39.320	29.271	1.00	0.00	H
ATOM	256	CA	LEU	16	39.808	39.451	28.360	1.00	0.00	C
ATOM	257	HA	LEU	16	40.479	38.594	28.311	1.00	0.00	H
ATOM	258	CB	LEU	16	38.972	39.579	27.063	1.00	0.00	C
ATOM	259	HB2	LEU	16	38.301	38.720	27.103	1.00	0.00	H
ATOM	260	HB3	LEU	16	38.451	40.536	27.091	1.00	0.00	H
ATOM	261	CG	LEU	16	39.907	39.601	25.833	1.00	0.00	C
ATOM	262	HG	LEU	16						
					40.715	40.297	26.059	1.00	0.00	H
ATOM	263	CD1	LEU	16	40.555	38.235	25.553	1.00	0.00	C
ATOM	264	HD11	LEU	16	39.857	37.399	25.503	1.00	0.00	H
ATOM	265	HD12	LEU	16	41.007	38.221	24.561	1.00	0.00	H
ATOM	266	HD13	LEU	16	41.313	37.877	26.250	1.00	0.00	H
ATOM	267	CD2	LEU	16	39.196	40.144	24.553	1.00	0.00	C
ATOM	268	HD21	LEU	16	38.490	39.351	24.307	1.00	0.00	H
ATOM	269	HD22	LEU	16	38.663	41.077	24.731	1.00	0.00	H
ATOM	270	HD23	LEU	16	39.899	40.449	23.777	1.00	0.00	H
ATOM	271	C	LEU	16	40.728	40.715	28.669	1.00	0.00	C
ATOM	272	O	LEU	16	41.958	40.675	28.438	1.00	0.00	O
ATOM	273	N	ALA	17	40.190	41.774	29.264	1.00	0.00	N
ATOM	274	H	ALA	17	39.186	41.816	29.172	1.00	0.00	H
ATOM	275	CA	ALA	17	40.969	42.834	29.947	1.00	0.00	C

ATOM	276	HA	ALA	17	41.661	43.184	29.179	1.00	0.00	H
ATOM	277	CB	ALA	17	40.114	44.027	30.280	1.00	0.00	C
ATOM	278	HB1	ALA	17	39.501	44.380	29.450	1.00	0.00	H
ATOM	279	HB2	ALA	17	39.487	43.695	31.108	1.00	0.00	H
ATOM	280	HB3	ALA	17	40.743	44.808	30.709	1.00	0.00	H
ATOM	281	C	ALA	17	41.878	42.416	31.126	1.00	0.00	C
ATOM	282	O	ALA	17	42.864	43.073	31.400	1.00	0.00	O
ATOM	283	N	GLY	18	41.596	41.208	31.691	1.00	0.00	N
ATOM	284	H	GLY	18	40.893	40.626	31.260	1.00	0.00	H
ATOM	285	CA	GLY	18	42.214	40.643	32.867	1.00	0.00	C
ATOM	286	HA2	GLY	18	42.144	39.566	32.715	1.00	0.00	H
ATOM	287	HA3	GLY	18	43.301	40.715	32.851	1.00	0.00	H
ATOM	288	C	GLY	18	41.654	41.143	34.166	1.00	0.00	C
ATOM	289	O	GLY	18	42.194	40.734	35.206	1.00	0.00	O
ATOM	290	N	ARG	19	40.751	42.104	34.124	1.00	0.00	N
ATOM	291	H	ARG	19	40.297	42.142	33.222	1.00	0.00	H
ATOM	292	CA	ARG	19	40.231	42.842	35.271	1.00	0.00	C
ATOM	293	HA	ARG	19	41.072	43.308	35.786	1.00	0.00	H
ATOM	294	CB	ARG	19	39.238	44.028	34.972	1.00	0.00	C
ATOM	295	HB2	ARG	19	38.473	43.848	34.217	1.00	0.00	H
ATOM	296	HB3	ARG	19	38.664	44.332	35.847	1.00	0.00	H
ATOM	297	CG	ARG	19	39.851	45.433	34.586	1.00	0.00	C
ATOM	298	HG2	ARG	19	40.613	45.755	35.295	1.00	0.00	H
ATOM	299	HG3	ARG	19	40.427	45.357	33.663	1.00	0.00	H
ATOM	300	CD	ARG	19	38.795	46.532	34.427	1.00	0.00	C
ATOM	301	HD2	ARG	19	38.237	46.571	35.362	1.00	0.00	H
ATOM	302	HD3	ARG	19	39.317	47.482	34.306	1.00	0.00	H
ATOM	303	NE	ARG	19	37.825	46.334	33.343	1.00	0.00	N
ATOM	304	HE	ARG	19	37.007	45.758	33.488	1.00	0.00	H
ATOM	305	CZ	ARG	19	38.037	46.559	32.058	1.00	0.00	C
ATOM	306	NH1	ARG	19	39.030	47.222	31.605	1.00	0.00	N
ATOM	307	HH11	ARG	19	39.737	47.544	32.250	1.00	0.00	H
ATOM	308	HH12	ARG	19	39.044	47.429	30.615	1.00	0.00	H
ATOM	309	NH2	ARG	19	37.286	46.012	31.202	1.00	0.00	N
ATOM	310	HH21	ARG	19	37.303	46.255	30.222	1.00	0.00	H
ATOM	311	HH22	ARG	19	36.430	45.602	31.548	1.00	0.00	H
ATOM	312	C	ARG	19	39.598	41.816	36.297	1.00	0.00	C
ATOM	313	O	ARG	19	40.019	41.694	37.447	1.00	0.00	O
ATOM	314	N	LYS	20	38.662	41.116	35.747	1.00	0.00	N
ATOM	315	H	LYS	20	38.497	41.312	34.770	1.00	0.00	H
ATOM	316	CA	LYS	20	37.711	40.278	36.429	1.00	0.00	C
ATOM	317	HA	LYS	20	37.425	40.755	37.366	1.00	0.00	H
ATOM	318	CB	LYS	20	36.373	40.098	35.635	1.00	0.00	C
ATOM	319	HB2	LYS	20	36.656	39.709	34.656	1.00	0.00	H
ATOM	320	HB3	LYS	20	35.964	41.098	35.495	1.00	0.00	H
ATOM	321	CG	LYS	20	35.247	39.188	36.148	1.00	0.00	C
ATOM	322	HG2	LYS	20	34.384	39.540	35.582	1.00	0.00	H
ATOM	323	HG3	LYS	20	35.501	38.143	35.975	1.00	0.00	H
ATOM	324	CD	LYS	20	35.026	39.349	37.631	1.00	0.00	C
ATOM	325	HD2	LYS	20	35.808	38.907	38.249	1.00	0.00	H
ATOM	326	HD3	LYS	20	33.996	39.045	37.812	1.00	0.00	H
ATOM	327	CE	LYS	20	34.975	40.848	38.098	1.00	0.00	C
ATOM	328	HE2	LYS	20	35.956	41.274	37.886	1.00	0.00	H
ATOM	329	HE3	LYS	20	34.315	41.428	37.453	1.00	0.00	H
ATOM	330	NZ	LYS	20	34.512	41.028	39.466	1.00	0.00	N
ATOM	331	HZ1	LYS	20	35.069	40.532	40.147	1.00	0.00	H
ATOM	332	HZ2	LYS	20	33.522	40.863	39.584	1.00	0.00	H
ATOM	333	HZ3	LYS	20	34.666	41.986	39.750	1.00	0.00	H
ATOM	334	C	LYS	20	38.367	38.942	36.819	1.00	0.00	C
ATOM	335	O	LYS	20	38.538	38.093	36.023	1.00	0.00	O
ATOM	336	N	THR	21	38.704	38.818	38.103	1.00	0.00	N
ATOM	337	H	THR	21	38.565	39.654	38.652	1.00	0.00	H
ATOM	338	CA	THR	21	39.360	37.700	38.777	1.00	0.00	C
ATOM	339	HA	THR	21	39.409	36.826	38.129	1.00	0.00	H

ATOM	340	CB	THR	21	40.796	38.047	39.108	1.00	0.00	C
ATOM	341	HB	THR	21	41.195	37.127	39.534	1.00	0.00	H
ATOM	342	CG2	THR	21	41.727	38.612	38.033	1.00	0.00	C
ATOM	343	HG21	THR	21	41.578	39.678	37.865	1.00	0.00	H
ATOM	344	HG22	THR	21	42.754	38.450	38.361	1.00	0.00	H
ATOM	345	HG23	THR	21	41.555	38.163	37.055	1.00	0.00	H
ATOM	346	OG1	THR	21	40.951	38.926	40.193	1.00	0.00	O
ATOM	347	HG1	THR	21	40.992	38.480	41.041	1.00	0.00	H
ATOM	348	C	THR	21	38.582	37.243	40.019	1.00	0.00	C
ATOM	349	O	THR	21	38.814	36.128	40.489	1.00	0.00	O
ATOM	350	N	ILE	22	37.672	38.020	40.614	1.00	0.00	N
ATOM	351	H	ILE	22	37.657	38.999	40.368	1.00	0.00	H
ATOM	352	CA	ILE	22	36.929	37.583	41.840	1.00	0.00	C
ATOM	353	HA	ILE	22	36.830	36.498	41.843	1.00	0.00	H
ATOM	354	CB	ILE	22	37.758	37.907	43.118	1.00	0.00	C
ATOM	355	HB	ILE	22	38.674	37.320	43.181	1.00	0.00	H
ATOM	356	CG2	ILE	22	38.250	39.343	43.103	1.00	0.00	C
ATOM	357	HG21	ILE	22	37.525	40.154	43.057	1.00	0.00	H
ATOM	358	HG22	ILE	22	38.840	39.489	44.008	1.00	0.00	H
ATOM	359	HG23	ILE	22	38.958	39.461	42.282	1.00	0.00	H
ATOM	360	CG1	ILE	22	36.941	37.468	44.389	1.00	0.00	C
ATOM	361	HG12	ILE	22	36.216	38.279	44.451	1.00	0.00	H
ATOM	362	HG13	ILE	22	36.431	36.511	44.275	1.00	0.00	H
ATOM	363	CD1	ILE	22	37.776	37.336	45.674	1.00	0.00	C
ATOM	364	HD11	ILE	22	38.712	36.776	45.676	1.00	0.00	H
ATOM	365	HD12	ILE	22	38.097	38.371	45.786	1.00	0.00	H
ATOM	366	HD13	ILE	22	37.144	37.151	46.543	1.00	0.00	H
ATOM	367	C	ILE	22	35.590	38.179	41.734	1.00	0.00	C
ATOM	368	O	ILE	22	35.351	39.293	41.283	1.00	0.00	O
ATOM	369	N	THR	23	34.579	37.430	42.145	1.00	0.00	N
ATOM	370	H	THR	23	34.876	36.517	42.459	1.00	0.00	H
ATOM	371	CA	THR	23	33.112	37.782	42.060	1.00	0.00	C
ATOM	372	HA	THR	23	32.918	38.854	42.022	1.00	0.00	H
ATOM	373	CB	THR	23	32.604	37.132	40.780	1.00	0.00	C
ATOM	374	HB	THR	23	33.009	36.120	40.765	1.00	0.00	H
ATOM	375	CG2	THR	23	31.030	36.908	40.632	1.00	0.00	C
ATOM	376	HG21	THR	23	30.547	37.759	41.111	1.00	0.00	H
ATOM	377	HG22	THR	23	30.720	36.743	39.599	1.00	0.00	H
ATOM	378	HG23	THR	23	30.756	36.039	41.232	1.00	0.00	H
ATOM	379	OG1	THR	23	33.084	37.922	39.770	1.00	0.00	O
ATOM	380	HG1	THR	23	32.497	38.681	39.799	1.00	0.00	H
ATOM	381	C	THR	23	32.427	37.213	43.270	1.00	0.00	C
ATOM	382	O	THR	23	32.671	36.102	43.704	1.00	0.00	O
ATOM	383	N	ILE	24	31.501	37.969	43.829	1.00	0.00	N
ATOM	384	H	ILE	24	31.364	38.876	43.405	1.00	0.00	H
ATOM	385	CA	ILE	24	30.548	37.681	44.951	1.00	0.00	C
ATOM	386	HA	ILE	24	30.802	36.708	45.373	1.00	0.00	H
ATOM	387	CB	ILE	24	30.457	38.808	45.987			
1.00	0.00			C						
ATOM	388	HB	ILE	24	30.281	39.688	45.369	1.00	0.00	H
ATOM	389	CG2	ILE	24	29.488	38.479	47.127	1.00	0.00	C
ATOM	390	HG21	ILE	24	29.556	39.344	47.787	1.00	0.00	H
ATOM	391	HG22	ILE	24	28.423	38.362	46.928	1.00	0.00	H
ATOM	392	HG23	ILE	24	29.803	37.557	47.617	1.00	0.00	H
ATOM	393	CG1	ILE	24	31.838	38.978	46.621	1.00	0.00	C
ATOM	394	HG12	ILE	24	32.131	38.317	47.437	1.00	0.00	H
ATOM	395	HG13	ILE	24	32.647	38.779	45.918	1.00	0.00	H
ATOM	396	CD1	ILE	24	32.027	40.398	47.086	1.00	0.00	C
ATOM	397	HD11	ILE	24	31.383	40.672	47.921	1.00	0.00	H
ATOM	398	HD12	ILE	24	33.082	40.412	47.359	1.00	0.00	H
ATOM	399	HD13	ILE	24	31.752	41.198	46.399	1.00	0.00	H
ATOM	400	C	ILE	24	29.120	37.505	44.323	1.00	0.00	C
ATOM	401	O	ILE	24	28.711	38.460	43.662	1.00	0.00	O
ATOM	402	N	ARG	25	28.453	36.372	44.542	1.00	0.00	N

ATOM	403	H	ARG	25	28.796	35.743	45.254	1.00	0.00	H
ATOM	404	CA	ARG	25	27.028	36.028	44.138	1.00	0.00	C
ATOM	405	HA	ARG	25	26.519	36.847	43.629	1.00	0.00	H
ATOM	406	CB	ARG	25	27.018	34.871	43.173	1.00	0.00	C
ATOM	407	HB2	ARG	25	27.820	34.152	43.335	1.00	0.00	H
ATOM	408	HB3	ARG	25	26.057	34.366	43.267	1.00	0.00	H
ATOM	409	CG	ARG	25	27.262	35.414	41.729	1.00	0.00	C
ATOM	410	HG2	ARG	25	26.598	36.224	41.431	1.00	0.00	H
ATOM	411	HG3	ARG	25	28.265	35.841	41.730	1.00	0.00	H
ATOM	412	CD	ARG	25	27.294	34.305	40.643	1.00	0.00	C
ATOM	413	HD2	ARG	25	28.198	33.714	40.791	1.00	0.00	H
ATOM	414	HD3	ARG	25	26.415	33.662	40.615	1.00	0.00	H
ATOM	415	NE	ARG	25	27.291	34.881	39.229	1.00	0.00	N
ATOM	416	HE	ARG	25	28.203	35.172	38.905	1.00	0.00	H
ATOM	417	CZ	ARG	25	26.279	34.942	38.373	1.00	0.00	C
ATOM	418	NH1	ARG	25	25.172	34.538	38.758	1.00	0.00	N
ATOM	419	HH11	ARG	25	24.373	34.775	38.188	1.00	0.00	H
ATOM	420	HH12	ARG	25	25.020	34.212	39.702	1.00	0.00	H
ATOM	421	NH2	ARG	25	26.401	35.467	37.163	1.00	0.00	N
ATOM	422	HH21	ARG	25	25.571	35.735	36.656	1.00	0.00	H
ATOM	423	HH22	ARG	25	27.282	35.868	36.873	1.00	0.00	H
ATOM	424	C	ARG	25	26.259	35.700	45.395	1.00	0.00	C
ATOM	425	O	ARG	25	26.861	35.323	46.435	1.00	0.00	O
ATOM	426	N	ASP	26	24.925	35.748	45.387	1.00	0.00	N
ATOM	427	H	ASP	26	24.499	36.178	44.578	1.00	0.00	H
ATOM	428	CA	ASP	26	23.971	35.173	46.364	1.00	0.00	C
ATOM	429	HA	ASP	26	24.405	35.438	47.328	1.00	0.00	H
ATOM	430	CB	ASP	26	22.548	35.836	46.187	1.00	0.00	C
ATOM	431	HB2	ASP	26	21.800	35.261	46.733	1.00	0.00	H
ATOM	432	HB3	ASP	26	22.589	36.805	46.685	1.00	0.00	H
ATOM	433	CG	ASP	26	22.166	36.085	44.695	1.00	0.00	C
ATOM	434	OD1	ASP	26	22.568	35.245	43.831	1.00	0.00	O
ATOM	435	OD2	ASP	26	21.502	37.073	44.400	1.00	0.00	O
ATOM	436	C	ASP	26	23.946	33.596	46.435	1.00	0.00	C
ATOM	437	O	ASP	26	23.862	32.880	45.394	1.00	0.00	O
ATOM	438	N	GLU	27	24.062	33.132	47.674	1.00	0.00	N
ATOM	439	H	GLU	27	23.977	33.703	48.502	1.00	0.00	H
ATOM	440	CA	GLU	27	24.302	31.708	47.889	1.00	0.00	C
ATOM	441	HA	GLU	27	25.305	31.541	47.497	1.00	0.00	H
ATOM	442	CB	GLU	27	24.221	31.538	49.434	1.00	0.00	C
ATOM	443	HB2	GLU	27	24.914	32.245	49.890	1.00	0.00	H
ATOM	444	HB3	GLU	27	23.206	31.836	49.699	1.00	0.00	H
ATOM	445	CG	GLU	27	24.466	30.175	50.066	1.00	0.00	C
ATOM	446	HG2	GLU	27	24.509	30.325	51.145	1.00	0.00	H
ATOM	447	HG3	GLU	27	23.609	29.530	49.871	1.00	0.00	H
ATOM	448	CD	GLU	27	25.750	29.504	49.612	1.00	0.00	C
ATOM	449	OE1	GLU	27	26.707	29.350	50.424	1.00	0.00	O
ATOM	450	OE2	GLU	27	25.784	28.960	48.455	1.00	0.00	O
ATOM	451	C	GLU	27	23.190	30.785	47.272	1.00	0.00	C
ATOM	452	O	GLU	27	21.989	31.128	47.134	1.00	0.00	O
ATOM	453	N	SER	28	23.654	29.623	46.842	1.00	0.00	N
ATOM	454	H	SER	28	24.586	29.451	47.192	1.00	0.00	H
ATOM	455	CA	SER	28	23.087	28.636	45.852	1.00	0.00	C
ATOM	456	HA	SER	28	22.163	28.352	46.355	1.00	0.00	H
ATOM	457	CB	SER	28	22.972	29.305	44.470	1.00	0.00	C
ATOM	458	HB2	SER	28	22.433	30.249	44.560	1.00	0.00	H
ATOM	459	HB3	SER	28	23.975	29.527	44.108	1.00	0.00	H
ATOM	460	OG	SER	28	22.306	28.375	43.628	1.00	0.00	O
ATOM	461	HG	SER	28	21.746	28.985	43.142	1.00	0.00	H
ATOM	462	C	SER	28	23.949	27.400	45.643	1.00	0.00	C
ATOM	463	O	SER	28	25.170	27.501	45.404	1.00	0.00	O
ATOM	464	N	GLU	29	23.307	26.250	45.743	1.00	0.00	N
ATOM	465	H	GLU	29	22.302	26.290	45.837	1.00	0.00	H
ATOM	466	CA	GLU	29	24.037	24.961	45.776	1.00	0.00	C

ATOM	467	HA	GLU	29	24.839	24.959	46.514	1.00	0.00	H
ATOM	468	CB	GLU	29	23.097	23.930	46.395	1.00	0.00	C
ATOM	469	HB2	GLU	29	22.934	24.226	47.431	1.00	0.00	H
ATOM	470	HB3	GLU	29	22.143	24.068	45.887	1.00	0.00	H
ATOM	471	CG	GLU	29	23.658	22.534	46.276	1.00	0.00	C
ATOM	472	HG2	GLU	29	23.641	22.267	45.219	1.00	0.00	H
ATOM	473	HG3	GLU	29	24.660	22.468	46.699	1.00	0.00	H
ATOM	474	CD	GLU	29	22.846	21.454	46.986	1.00	0.00	C
ATOM	475	OE1	GLU	29	23.299	20.263	46.873	1.00	0.00	O
ATOM	476	OE2	GLU	29	21.838	21.764	47.701	1.00	0.00	O
ATOM	477	C	GLU	29	24.776	24.554	44.431	1.00	0.00	C
ATOM	478	O	GLU	29	25.865	24.042	44.449	1.00	0.00	O
ATOM	479	N	SER	30	24.183	24.988	43.275	1.00	0.00	N
ATOM	480	H	SER	30	23.275	25.429	43.266	1.00	0.00	H
ATOM	481	CA	SER	30	24.666	24.749	41.928	1.00	0.00	C
ATOM	482	HA	SER	30	24.974	23.706	41.856	1.00	0.00	H
ATOM	483	CB	SER	30	23.521	25.055	40.912	1.00	0.00	C
ATOM	484	HB2	SER	30	23.884	24.790	39.919	1.00	0.00	H
ATOM	485	HB3	SER	30	22.615	24.589	41.299	1.00	0.00	H
ATOM	486	OG	SER	30	23.333	26.486	40.887	1.00	0.00	O
ATOM	487	HG	SER	30	23.350	26.651	39.942	1.00	0.00	H
ATOM	488	C	SER	30	25.885	25.607	41.487	1.00	0.00	C
ATOM	489	O	SER	30	26.420	25.529	40.373	1.00	0.00	O
ATOM	490	N	HIE	31	26.280	26.519	42.393	1.00	0.00	N
ATOM	491	H	HIE	31	25.723	26.654	43.224	1.00	0.00	H
ATOM	492	CA	HIE	31	27.518	27.408	42.229	1.00	0.00	C
ATOM	493	HA	HIE	31	27.458	27.900	41.259	1.00	0.00	H
ATOM	494	CB	HIE	31	27.625	28.404	43.406	1.00	0.00	C
ATOM	495	HB2	HIE	31	27.514	27.791	44.300	1.00	0.00	H
ATOM	496	HB3	HIE	31	28.629	28.828	43.409	1.00	0.00	H
ATOM	497	CG	HIE	31	26.791	29.609	43.443	1.00	0.00	C
ATOM	498	ND1	HIE	31	26.307	30.245	42.330	1.00	0.00	N
ATOM	499	CE1	HIE	31	25.611	31.383	42.780	1.00	0.00	C
ATOM	500	HE1	HIE	31	25.137	32.118	42.147	1.00	0.00	H
ATOM	501	NE2	HIE	31	25.822	31.404	44.099	1.00	0.00	N
ATOM	502	HE2	HIE	31	25.371	32.086	44.692	1.00	0.00	H
ATOM	503	CD2	HIE	31	26.525	30.352	44.569	1.00	0.00	C
ATOM	504	HD2	HIE	31	26.877	30.277	45.587	1.00	0.00	H
ATOM	505	C	HIE	31	28.726	26.460	42.146	1.00	0.00	C
ATOM	506	O	HIE	31	28.709	25.286	42.579	1.00	0.00	O
ATOM	507	N	PHE	32	29.839	27.015	41.582	1.00	0.00	N
ATOM	508	H	PHE	32	29.727	27.901	41.111	1.00	0.00	H
ATOM	509	CA	PHE	32	31.133	26.339	41.235	1.00	0.00	C
ATOM	510	HA	PHE	32	30.824	25.504	40.606	1.00	0.00	H
ATOM	511	CB	PHE	32	32.104	27.151	40.386	1.00	0.00	C
ATOM	512	HB2	PHE	32	32.335	28.065	40.933	1.00	0.00	H
ATOM										
	513	HB3	PHE	32	33.069	26.651	40.304	1.00	0.00	H
ATOM	514	CG	PHE	32	31.561	27.574	39.012	1.00	0.00	C
ATOM	515	CD1	PHE	32	31.421	28.947	38.758	1.00	0.00	C
ATOM	516	HD1	PHE	32	31.617	29.640	39.564	1.00	0.00	H
ATOM	517	CE1	PHE	32	31.112	29.331	37.461	1.00	0.00	C
ATOM	518	HE1	PHE	32	31.124	30.398	37.295	1.00	0.00	H
ATOM	519	CZ	PHE	32	30.742	28.373	36.509	1.00	0.00	C
ATOM	520	HZ	PHE	32	30.396	28.628	35.517	1.00	0.00	H
ATOM	521	CE2	PHE	32	30.749	26.974	36.808	1.00	0.00	C
ATOM	522	HE2	PHE	32	30.469	26.249	36.059	1.00	0.00	H
ATOM	523	CD2	PHE	32	31.207	26.638	38.077	1.00	0.00	C
ATOM	524	HD2	PHE	32	31.190	25.578	38.284	1.00	0.00	H
ATOM	525	C	PHE	32	31.867	25.765	42.421	1.00	0.00	C
ATOM	526	O	PHE	32	31.797	26.369	43.505	1.00	0.00	O
ATOM	527	N	LYS	33	32.670	24.683	42.243	1.00	0.00	N
ATOM	528	H	LYS	33	32.744	24.316	41.304	1.00	0.00	H
ATOM	529	CA	LYS	33	33.599	24.165	43.263	1.00	0.00	C

ATOM	530	HA	LYS	33	33.378	24.610	44.233	1.00	0.00	H
ATOM	531	CB	LYS	33	33.388	22.696	43.452	1.00	0.00	C
ATOM	532	HB2	LYS	33	33.382	22.296	42.438	1.00	0.00	H
ATOM	533	HB3	LYS	33	34.240	22.198	43.913	1.00	0.00	H
ATOM	534	CG	LYS	33	32.136	22.110	44.122	1.00	0.00	C
ATOM	535	HG2	LYS	33	31.955	21.058	43.898	1.00	0.00	H
ATOM	536	HG3	LYS	33	32.203	22.260	45.200	1.00	0.00	H
ATOM	537	CD	LYS	33	30.820	22.834	43.733	1.00	0.00	C
ATOM	538	HD2	LYS	33	30.901	23.860	44.093	1.00	0.00	H
ATOM	539	HD3	LYS	33	30.653	22.763	42.657	1.00	0.00	H
ATOM	540	CE	LYS	33	29.586	22.261	44.556	1.00	0.00	C
ATOM	541	HE2	LYS	33	29.609	21.175	44.470	1.00	0.00	H
ATOM	542	HE3	LYS	33	29.661	22.500	45.618	1.00	0.00	H
ATOM	543	NZ	LYS	33	28.342	22.777	43.927	1.00	0.00	N
ATOM	544	HZ1	LYS	33	28.146	22.122	43.184	1.00	0.00	H
ATOM	545	HZ2	LYS	33	27.562	22.842	44.566	1.00	0.00	H
ATOM	546	HZ3	LYS	33	28.528	23.664	43.481	1.00	0.00	H
ATOM	547	C	LYS	33	35.069	24.521	42.965	1.00	0.00	C
ATOM	548	O	LYS	33	35.456	24.742	41.814	1.00	0.00	O
ATOM	549	N	THR	34	35.837	24.756	43.975	1.00	0.00	N
ATOM	550	H	THR	34	35.596	24.547	44.933	1.00	0.00	H
ATOM	551	CA	THR	34	37.273	25.002	43.750	1.00	0.00	C
ATOM	552	HA	THR	34	37.475	25.881	43.137	1.00	0.00	H
ATOM	553	CB	THR	34	38.090	25.145	45.093	1.00	0.00	C
ATOM	554	HB	THR	34	37.853	24.307	45.748	1.00	0.00	H
ATOM	555	CG2	THR	34	39.577	25.407	44.934	1.00	0.00	C
ATOM	556	HG21	THR	34	39.786	26.157	44.170	1.00	0.00	H
ATOM	557	HG22	THR	34	39.888	25.689	45.940	1.00	0.00	H
ATOM	558	HG23	THR	34	40.092	24.470	44.725	1.00	0.00	H
ATOM	559	OG1	THR	34	37.632	26.296	45.763	1.00	0.00	O
ATOM	560	HG1	THR	34	36.684	26.150	45.818	1.00	0.00	H
ATOM	561	C	THR	34	37.826	23.780	42.929	1.00	0.00	C
ATOM	562	O	THR	34	37.389	22.600	43.111	1.00	0.00	O
ATOM	563	N	GLY	35	38.739	24.123	41.982	1.00	0.00	N
ATOM	564	H	GLY	35	39.083	25.071	41.937	1.00	0.00	H
ATOM	565	CA	GLY	35	39.163	23.173	40.936	1.00	0.00	C
ATOM	566	HA2	GLY	35	40.169	23.451	40.627	1.00	0.00	H
ATOM	567	HA3	GLY	35	39.221	22.180	41.383	1.00	0.00	H
ATOM	568	C	GLY	35	38.257	23.168	39.699	1.00	0.00	C
ATOM	569	O	GLY	35	38.596	22.437	38.772	1.00	0.00	O
ATOM	570	N	ASP	36	37.197	24.027	39.555	1.00	0.00	N
ATOM	571	H	ASP	36	36.834	24.445	40.400	1.00	0.00	H
ATOM	572	CA	ASP	36	36.331	24.016	38.398	1.00	0.00	C
ATOM	573	HA	ASP	36	36.240	22.966	38.118	1.00	0.00	H
ATOM	574	CB	ASP	36	34.984	24.610	38.744	1.00	0.00	C
ATOM	575	HB2	ASP	36	35.108	25.495	39.369	1.00	0.00	H
ATOM	576	HB3	ASP	36	34.527	25.125	37.899	1.00	0.00	H
ATOM	577	CG	ASP	36	34.029	23.622	39.294	1.00	0.00	C
ATOM	578	OD1	ASP	36	34.476	22.533	39.714	1.00	0.00	O
ATOM	579	OD2	ASP	36	32.811	23.909	39.304	1.00	0.00	O
ATOM	580	C	ASP	36	37.049	24.705	37.166	1.00	0.00	C
ATOM	581	O	ASP	36	37.411	25.890	37.239	1.00	0.00	O
ATOM	582	N	VAL	37	37.390	24.001	36.119	1.00	0.00	N
ATOM	583	H	VAL	37	37.009	23.070	36.029	1.00	0.00	H
ATOM	584	CA	VAL	37	37.872	24.533	34.867	1.00	0.00	C
ATOM	585	HA	VAL	37	38.584	25.331	35.081	1.00	0.00	H
ATOM	586	CB	VAL	37	38.743	23.503	34.125	1.00	0.00	C
ATOM	587	HB	VAL	37	38.135	22.659	33.797	1.00	0.00	H
ATOM	588	CG1	VAL	37	39.408	24.007	32.891	1.00	0.00	C
ATOM	589	HG11	VAL	37	39.977	24.924	33.042	1.00	0.00	H
ATOM	590	HG12	VAL	37	40.126	23.280	32.512	1.00	0.00	H
ATOM	591	HG13	VAL	37	38.555	24.211	32.243	1.00	0.00	H
ATOM	592	CG2	VAL	37	39.820	22.942	35.042	1.00	0.00	C
ATOM	593	HG21	VAL	37	40.312	22.115	34.529	1.00	0.00	H

ATOM	594	HG22	VAL	37	40.577	23.677	35.316	1.00	0.00	H
ATOM	595	HG23	VAL	37	39.507	22.633	36.039	1.00	0.00	H
ATOM	596	C	VAL	37	36.754	25.050	33.949	1.00	0.00	C
ATOM	597	O	VAL	37	35.822	24.284	33.697	1.00	0.00	O
ATOM	598	N	LEU	38	36.754	26.301	33.547	1.00	0.00	N
ATOM	599	H	LEU	38	37.568	26.858	33.762	1.00	0.00	H
ATOM	600	CA	LEU	38	35.817	26.986	32.634	1.00	0.00	C
ATOM	601	HA	LEU	38	35.001	26.265	32.593	1.00	0.00	H
ATOM	602	CB	LEU	38	35.201	28.238	33.257	1.00	0.00	C
ATOM	603	HB2	LEU	38	35.963	29.007	33.381	1.00	0.00	H
ATOM	604	HB3	LEU	38	34.364	28.518	32.616	1.00	0.00	H
ATOM	605	CG	LEU	38	34.681	28.026	34.732	1.00	0.00	C
ATOM	606	HG	LEU	38	35.412	27.665	35.456	1.00	0.00	H
ATOM	607	CD1	LEU	38	34.135	29.383	35.184	1.00	0.00	C
ATOM	608	HD11	LEU	38	33.474	29.165	36.022	1.00	0.00	H
ATOM	609	HD12	LEU	38	34.987	30.008	35.450	1.00	0.00	H
ATOM	610	HD13	LEU	38	33.484	29.777	34.403	1.00	0.00	H
ATOM	611	CD2	LEU	38	33.646	26.978	34.943	1.00	0.00	C
ATOM	612	HD21	LEU	38	34.100	26.040	34.625	1.00	0.00	H
ATOM	613	HD22	LEU	38	33.555	26.856	36.022	1.00	0.00	H
ATOM	614	HD23	LEU	38	32.685	27.195	34.477	1.00	0.00	H
ATOM	615	C	LEU	38	36.433	27.126	31.251	1.00	0.00	C
ATOM	616	O	LEU	38	37.662	27.220	31.103	1.00	0.00	O
ATOM	617	N	ARG	39	35.637	27.352	30.247	1.00	0.00	N
ATOM	618	H	ARG	39	34.634	27.352	30.371	1.00	0.00	H
ATOM	619	CA	ARG	39	36.067	27.910	28.933	1.00	0.00	C
ATOM	620	HA	ARG	39	37.046	28.389	28.957	1.00	0.00	H
ATOM	621	CB	ARG	39	36.175	26.827	27.882	1.00	0.00	C
ATOM	622	HB2	ARG	39	35.220	26.339	27.690	1.00	0.00	H
ATOM	623	HB3	ARG	39	36.388	27.285	26.916	1.00	0.00	H
ATOM	624	CG	ARG	39	37.215	25.799	28.136	1.00	0.00	C
ATOM	625	HG2	ARG	39	38.227	26.201	28.079	1.00	0.00	H
ATOM	626	HG3	ARG	39	37.042	25.374	29.125	1.00	0.00	H
ATOM	627	CD	ARG	39	37.097	24.562	27.200	1.00	0.00	C
ATOM	628	HD2	ARG	39	37.452	24.994	26.264	1.00	0.00	H
ATOM	629	HD3	ARG	39	37.728	23.734	27.522	1.00	0.00	H
ATOM	630	NE	ARG	39	35.735	24.002	27.097	1.00	0.00	N
ATOM	631	HE	ARG	39	34.995	24.453	27.618	1.00	0.00	H
ATOM	632	CZ	ARG	39	35.346	22.891	26.492	1.00	0.00	C
ATOM	633	NH1	ARG	39	36.102	22.136	25.739	1.00	0.00	N
ATOM	634	HH11	ARG	39	37.051	22.464	25.621	1.00	0.00	H
ATOM	635	HH12	ARG	39	35.836	21.194	25.491	1.00	0.00	H
ATOM	636	NH2	ARG	39	34.179	22.451	26.687	1.00	0.00	N
ATOM	637	HH21	ARG	39	33.896	21.584	26.254	1.00	0.00	H
ATOM	638	HH22	ARG	39						
					33.497	23.094	27.064	1.00	0.00	H
ATOM	639	C	ARG	39	35.096	28.959	28.506	1.00	0.00	C
ATOM	640	O	ARG	39	33.889	28.857	28.840	1.00	0.00	O
ATOM	641	N	VAL	40	35.587	29.991	27.769	1.00	0.00	N
ATOM	642	H	VAL	40	36.386	29.739	27.206	1.00	0.00	H
ATOM	643	CA	VAL	40	34.815	31.225	27.394	1.00	0.00	C
ATOM	644	HA	VAL	40	33.765	30.954	27.293	1.00	0.00	H
ATOM	645	CB	VAL	40	35.156	32.272	28.489	1.00	0.00	C
ATOM	646	HB	VAL	40	34.782	33.265	28.238	1.00	0.00	H
ATOM	647	CG1	VAL	40	34.690	31.886	29.876	1.00	0.00	C
ATOM	648	HG11	VAL	40	33.609	31.772	29.795	1.00	0.00	H
ATOM	649	HG12	VAL	40	35.121	31.002	30.346	1.00	0.00	H
ATOM	650	HG13	VAL	40	34.762	32.848	30.384	1.00	0.00	H
ATOM	651	CG2	VAL	40	36.682	32.473	28.515	1.00	0.00	C
ATOM	652	HG21	VAL	40	36.954	33.076	29.382	1.00	0.00	H
ATOM	653	HG22	VAL	40	37.147	31.489	28.548	1.00	0.00	H
ATOM	654	HG23	VAL	40	37.025	32.954	27.599	1.00	0.00	H
ATOM	655	C	VAL	40	35.194	31.794	25.971	1.00	0.00	C
ATOM	656	O	VAL	40	36.377	31.789	25.686	1.00	0.00	O

ATOM	657	N	GLY	41	34.206	32.188	25.158	1.00	0.00	N
ATOM	658	H	GLY	41	33.275	32.053	25.528	1.00	0.00	H
ATOM	659	CA	GLY	41	34.270	32.734	23.832	1.00	0.00	C
ATOM	660	HA2	GLY	41	35.124	33.405	23.742	1.00	0.00	H
ATOM	661	HA3	GLY	41	34.530	31.983	23.087	1.00	0.00	H
ATOM	662	C	GLY	41	32.904	33.258	23.341	1.00	0.00	C
ATOM	663	O	GLY	41	31.870	32.605	23.629	1.00	0.00	O
ATOM	664	N	ARG	42	32.842	34.289	22.421	1.00	0.00	N
ATOM	665	H	ARG	42	33.670	34.739	22.055	1.00	0.00	H
ATOM	666	CA	ARG	42	31.678	34.575	21.629	1.00	0.00	C
ATOM	667	HA	ARG	42	31.003	33.720	21.594	1.00	0.00	H
ATOM	668	CB	ARG	42	31.013	35.842	22.280	1.00	0.00	C
ATOM	669	HB2	ARG	42	30.007	35.976	21.880	1.00	0.00	H
ATOM	670	HB3	ARG	42	30.859	35.849	23.358	1.00	0.00	H
ATOM	671	CG	ARG	42	31.743	37.155	21.899	1.00	0.00	C
ATOM	672	HG2	ARG	42	32.754	37.103	22.303	1.00	0.00	H
ATOM	673	HG3	ARG	42	31.832	37.327	20.827	1.00	0.00	H
ATOM	674	CD	ARG	42	30.818	38.343	22.496	1.00	0.00	C
ATOM	675	HD2	ARG	42	30.034	38.364	21.739	1.00	0.00	H
ATOM	676	HD3	ARG	42	30.419	38.218	23.502	1.00	0.00	H
ATOM	677	NE	ARG	42	31.372	39.663	22.328	1.00	0.00	N
ATOM	678	HE	ARG	42	30.801	40.205	21.695	1.00	0.00	H
ATOM	679	CZ	ARG	42	32.322	40.216	23.026	1.00	0.00	C
ATOM	680	NH1	ARG	42	32.832	39.567	24.017	1.00	0.00	N
ATOM	681	HH11	ARG	42	33.447	40.035	24.668	1.00	0.00	H
ATOM	682	HH12	ARG	42	32.611	38.593	24.162	1.00	0.00	H
ATOM	683	NH2	ARG	42	32.746	41.427	22.895	1.00	0.00	N
ATOM	684	HH21	ARG	42	32.400	42.014	22.151	1.00	0.00	H
ATOM	685	HH22	ARG	42	33.344	41.830	23.603	1.00	0.00	H
ATOM	686	C	ARG	42	31.957	34.739	20.092	1.00	0.00	C
ATOM	687	O	ARG	42	31.196	34.339	19.290	1.00	0.00	O
ATOM	688	N	PHE	43	33.086	35.283	19.657	1.00	0.00	N
ATOM	689	H	PHE	43	33.707	35.642	20.367	1.00	0.00	H
ATOM	690	CA	PHE	43	33.551	35.167	18.279	1.00	0.00	C
ATOM	691	HA	PHE	43	32.679	35.024	17.641	1.00	0.00	H
ATOM	692	CB	PHE	43	34.200	36.414	17.846	1.00	0.00	C
ATOM	693	HB2	PHE	43	35.257	36.393	18.108	1.00	0.00	H
ATOM	694	HB3	PHE	43	34.392	36.393	16.774	1.00	0.00	H
ATOM	695	CG	PHE	43	33.541	37.697	18.326	1.00	0.00	C
ATOM	696	CD1	PHE	43	32.323	38.149	17.750	1.00	0.00	C
ATOM	697	HD1	PHE	43	31.891	37.567	16.947	1.00	0.00	H
ATOM	698	CE1	PHE	43	31.729	39.343	18.122	1.00	0.00	C
ATOM	699	HE1	PHE	43	30.876	39.774	17.619	1.00	0.00	H
ATOM	700	CZ	PHE	43	32.363	40.059	19.166	1.00	0.00	C
ATOM	701	HZ	PHE	43	32.004	41.049	19.411	1.00	0.00	H
ATOM	702	CE2	PHE	43	33.517	39.572	19.849	1.00	0.00	C
ATOM	703	HE2	PHE	43	33.967	40.082	20.688	1.00	0.00	H
ATOM	704	CD2	PHE	43	34.103	38.327	19.499	1.00	0.00	C
ATOM	705	HD2	PHE	43	35.024	38.019	19.971	1.00	0.00	H
ATOM	706	C	PHE	43	34.419	33.942	18.013	1.00	0.00	C
ATOM	707	O	PHE	43	34.903	33.251	18.954	1.00	0.00	O
ATOM	708	N	GLU	44	34.612	33.682	16.682	1.00	0.00	N
ATOM	709	H	GLU	44	34.243	34.320	15.994	1.00	0.00	H
ATOM	710	CA	GLU	44	35.005	32.308	16.204	1.00	0.00	C
ATOM	711	HA	GLU	44	34.679	31.542	16.909	1.00	0.00	H
ATOM	712	CB	GLU	44	34.493	32.097	14.734	1.00	0.00	C
ATOM	713	HB2	GLU	44	34.755	31.106	14.363	1.00	0.00	H
ATOM	714	HB3	GLU	44	33.407	32.021	14.689	1.00	0.00	H
ATOM	715	CG	GLU	44	34.939	33.212	13.777	1.00	0.00	C
ATOM	716	HG2	GLU	44	35.722	33.813	14.240	1.00	0.00	H
ATOM	717	HG3	GLU	44	35.224	32.772	12.821	1.00	0.00	H
ATOM	718	CD	GLU	44	33.836	34.306	13.443	1.00	0.00	C
ATOM	719	OE1	GLU	44	33.569	34.469	12.232	1.00	0.00	O
ATOM	720	OE2	GLU	44	33.174	34.930	14.362	1.00	0.00	O

ATOM	721	C	GLU	44	36.492	32.016	16.236	1.00	0.00	C
ATOM	722	O	GLU	44	36.853	30.836	15.980	1.00	0.00	O
ATOM	723	N	ASP	45	37.316	32.928	16.654	1.00	0.00	N
ATOM	724	H	ASP	45	36.943	33.841	16.876	1.00	0.00	H
ATOM	725	CA	ASP	45	38.767	32.710	16.816	1.00	0.00	C
ATOM	726	HA	ASP	45	39.250	32.442	15.877	1.00	0.00	H
ATOM	727	CB	ASP	45	39.544	33.970	17.316	1.00	0.00	C
ATOM	728	HB2	ASP	45	39.203	34.264	18.309	1.00	0.00	H
ATOM	729	HB3	ASP	45	40.566	33.656	17.527	1.00	0.00	H
ATOM	730	CG	ASP	45	39.446	35.231	16.427	1.00	0.00	C
ATOM	731	OD1	ASP	45	40.209	36.174	16.791	1.00	0.00	O
ATOM	732	OD2	ASP	45	38.667	35.349	15.463	1.00	0.00	O
ATOM	733	C	ASP	45	39.078	31.656	17.809	1.00	0.00	C
ATOM	734	O	ASP	45	38.647	31.784	18.948	1.00	0.00	O
ATOM	735	N	ASP	46	39.870	30.694	17.367	1.00	0.00	N
ATOM	736	H	ASP	46	40.107	30.685	16.386	1.00	0.00	H
ATOM	737	CA	ASP	46	40.324	29.549	18.168	1.00	0.00	C
ATOM	738	HA	ASP	46	40.644	28.778	17.468	1.00	0.00	H
ATOM	739	CB	ASP	46	41.561	29.944	18.929	1.00	0.00	C
ATOM	740	HB2	ASP	46	41.355	30.654	19.729	1.00	0.00	H
ATOM	741	HB3	ASP	46	41.913	29.044	19.434	1.00	0.00	H
ATOM	742	CG	ASP	46	42.764	30.394	18.032	1.00	0.00	C
ATOM	743	OD1	ASP	46	43.552	31.264	18.475	1.00	0.00	O
ATOM	744	OD2	ASP	46	42.897	29.975	16.876	1.00	0.00	O
ATOM	745	C	ASP	46	39.210	28.886	19.017	1.00	0.00	C
ATOM	746	O	ASP	46	39.551	28.083	19.908	1.00	0.00	O
ATOM	747	N	GLY	47	37.933	29.126	18.699	1.00	0.00	N
ATOM	748	H	GLY	47	37.774	29.716	17.895	1.00	0.00	H
ATOM	749	CA	GLY	47	36.761	28.728	19.479	1.00	0.00	C
ATOM	750	HA2	GLY	47	35.905	28.951	18.843	1.00	0.00	H
ATOM	751	HA3	GLY	47	36.827	27.651	19.633	1.00	0.00	H
ATOM	752	C	GLY	47	36.504	29.328	20.834	1.00	0.00	C
ATOM	753	O	GLY	47	35.387	29.451	21.327	1.00	0.00	O
ATOM	754	N	TYR	48	37.574	29.641	21.560	1.00	0.00	N
ATOM	755	H	TYR	48	38.452	29.237	21.266	1.00	0.00	H
ATOM	756	CA	TYR	48	37.555	30.199	22.915	1.00	0.00	C
ATOM	757	HA	TYR	48	36.614	30.700	23.145	1.00	0.00	H
ATOM	758	CB	TYR	48	37.838	29.070	23.960	1.00	0.00	C
ATOM	759	HB2	TYR	48	38.838	28.689	23.753	1.00	0.00	H
ATOM	760	HB3	TYR	48	37.870	29.375	25.005	1.00	0.00	H
ATOM	761	CG	TYR	48	36.878	27.879	23.910	1.00	0.00	C
ATOM	762	CD1	TYR	48	35.561	27.917	24.352	1.00	0.00	C
ATOM	763	HD1	TYR	48	35.239	28.905	24.649	1.00	0.00	H
ATOM	764	CE1	TYR	48	34.711	26.817	24.336	1.00	0.00	C
ATOM	765	HE1	TYR	48	33.705	26.851	24.729	1.00	0.00	H
ATOM	766	CZ	TYR	48	35.223	25.563	23.789	1.00	0.00	C
ATOM	767	OH	TYR	48	34.424	24.496	23.713	1.00	0.00	O
ATOM	768	HH	TYR	48	34.716	23.727	23.218	1.00	0.00	H
ATOM	769	CE2	TYR	48	36.557	25.547	23.370	1.00	0.00	C
ATOM	770	HE2	TYR	48	36.930	24.641	22.916	1.00	0.00	H
ATOM	771	CD2	TYR	48	37.430	26.598	23.569	1.00	0.00	C
ATOM	772	HD2	TYR	48	38.448	26.392	23.275	1.00	0.00	H
ATOM	773	C	TYR	48	38.724	31.209	23.001	1.00	0.00	C
ATOM	774	O	TYR	48	39.693	31.097	22.235	1.00	0.00	O
ATOM	775	N	PHE	49	38.587	32.126	23.918	1.00	0.00	N
ATOM	776	H	PHE	49	37.763	32.225	24.493	1.00	0.00	H
ATOM	777	CA	PHE	49	39.632	33.075	24.270	1.00	0.00	C
ATOM	778	HA	PHE	49	40.303	33.139	23.412	1.00	0.00	H
ATOM	779	CB	PHE	49	39.115	34.539	24.394	1.00	0.00	C
ATOM	780	HB2	PHE	49	39.957	35.200	24.188	1.00	0.00	H
ATOM	781	HB3	PHE	49	38.431	34.710	23.562	1.00	0.00	H
ATOM	782	CG	PHE	49	38.498	34.918	25.678	1.00	0.00	C
ATOM	783	CD1	PHE	49	39.208	35.196	26.882	1.00	0.00	C

ATOM	784	HD1	PHE	49	40.277	35.042	26.931	1.00	0.00	H
ATOM	785	CE1	PHE	49	38.424	35.695	28.031	1.00	0.00	C
ATOM	786	HE1	PHE	49	39.005	35.975	28.898	1.00	0.00	H
ATOM	787	CZ	PHE	49	37.021	35.786	27.980	1.00	0.00	C
ATOM	788	HZ	PHE	49	36.425	35.959	28.862	1.00	0.00	H
ATOM	789	CE2	PHE	49	36.406	35.399	26.777	1.00	0.00	C
ATOM	790	HE2	PHE	49	35.327	35.427	26.799	1.00	0.00	H
ATOM	791	CD2	PHE	49	37.061	35.011	25.643	1.00	0.00	C
ATOM	792	HD2	PHE	49	36.472	34.811	24.760	1.00	0.00	H
ATOM	793	C	PHE	49	40.488	32.554	25.452	1.00	0.00	C
ATOM	794	O	PHE	49	41.689	32.727	25.534	1.00	0.00	O
ATOM	795	N	CYX	50	39.824	31.998	26.452	1.00	0.00	N
ATOM	796	H	CYX	50	38.833	31.836	26.343	1.00	0.00	H
ATOM	797	CA	CYX	50	40.392	31.381	27.633	1.00	0.00	C
ATOM	798	HA	CYX	50	41.477	31.289	27.576	1.00	0.00	H
ATOM	799	CB	CYX	50	40.344	32.334	28.880	1.00	0.00	C
ATOM	800	HB2	CYX	50	39.333	32.713	29.033	1.00	0.00	H
ATOM	801	HB3	CYX	50	40.391	31.657	29.734	1.00	0.00	H
ATOM	802	SG	CYX	50	41.644	33.641	29.126	1.00	0.00	S
ATOM	803	C	CYX	50	39.878	29.955	27.922	1.00	0.00	C
ATOM	804	O	CYX	50	38.653	29.638	27.865	1.00	0.00	O
ATOM	805	N	THR	51	40.814	29.161	28.337	1.00	0.00	N
ATOM	806	H	THR	51	41.749	29.542	28.349	1.00	0.00	H
ATOM	807	CA	THR	51	40.519	28.216	29.396	1.00	0.00	C
ATOM	808	HA	THR	51	39.533	27.770	29.278	1.00	0.00	H
ATOM	809	CB	THR	51	41.401	26.975	29.298	1.00	0.00	C
ATOM	810	HB	THR	51	42.400	27.256	29.635	1.00	0.00	H
ATOM	811	CG2	THR	51	40.871	25.744	30.015	1.00	0.00	C
ATOM	812	HG21	THR	51	40.641	26.021	31.043	1.00	0.00	H
ATOM	813	HG22	THR	51	39.995	25.327	29.518	1.00	0.00	H
ATOM	814	HG23	THR	51	41.640	24.973	30.051	1.00	0.00	H
ATOM	815	OG1	THR	51	41.349	26.525	27.885	1.00	0.00	O
ATOM	816	HG1	THR	51	42.254	26.683	27.607	1.00	0.00	H
ATOM	817	C	THR	51	40.788	28.770	30.807	1.00	0.00	C
ATOM	818	O	THR	51	41.744	29.500	30.949	1.00	0.00	O
ATOM	819	N	ILE	52	39.920	28.546	31.771	1.00	0.00	N
ATOM	820	H	ILE	52	39.117	27.965	31.580	1.00	0.00	H
ATOM	821	CA	ILE	52	40.019	29.229	33.068	1.00	0.00	C
ATOM	822	HA	ILE	52	41.014	29.672	33.125	1.00	0.00	H
ATOM	823	CB	ILE	52	38.884	30.291	33.169	1.00	0.00	C
ATOM	824	HB	ILE	52	37.987	29.773	32.827	1.00	0.00	H
ATOM	825	CG2	ILE	52	38.629	30.809	34.554	1.00	0.00	C
ATOM	826	HG21	ILE	52	38.436	29.926	35.163	1.00	0.00	H
ATOM	827	HG22	ILE	52	39.558	31.246	34.917	1.00	0.00	H
ATOM	828	HG23	ILE	52	37.767	31.473	34.622	1.00	0.00	H
ATOM	829	CG1	ILE	52	39.231	31.457	32.290	1.00	0.00	C
ATOM	830	HG12	ILE	52	40.198	31.908	32.518	1.00	0.00	H
ATOM	831	HG13	ILE	52	39.305	31.047	31.282	1.00	0.00	H
ATOM	832	CD1	ILE	52	38.172	32.600	32.381	1.00	0.00	C
ATOM	833	HD11	ILE	52	38.257	33.247	33.254	1.00	0.00	H
ATOM	834	HD12	ILE	52	38.288	33.198	31.478	1.00	0.00	H
ATOM	835	HD13	ILE	52	37.273	32.008	32.208	1.00	0.00	H
ATOM	836	C	ILE	52	39.979	28.168	34.133	1.00	0.00	C
ATOM	837	O	ILE	52	39.540	27.102	33.952	1.00	0.00	O
ATOM	838	N	GLU	53	40.470	28.462	35.344	1.00	0.00	N
ATOM	839	H	GLU	53	41.127	29.228	35.384	1.00	0.00	H
ATOM	840	CA	GLU	53	40.400	27.635	36.569	1.00	0.00	C
ATOM	841	HA	GLU	53	39.616	26.887	36.453	1.00	0.00	H
ATOM	842	CB	GLU	53	41.721	26.790	36.827	1.00	0.00	C
ATOM	843	HB2	GLU	53	41.706	26.164	35.935	1.00	0.00	H
ATOM	844	HB3	GLU	53	42.665	27.333	36.882	1.00	0.00	H
ATOM	845	CG	GLU	53	41.558	25.821	38.045	1.00	0.00	C
ATOM	846	HG2	GLU	53	41.795	26.384	38.949	1.00	0.00	H
ATOM	847	HG3	GLU	53	40.557	25.394	37.980	1.00	0.00	H

```
ATOM    848  CD  GLU    53      42.512  24.645  38.079  1.00  0.00           C
ATOM    849  OE1 GLU    53      43.280  24.488  37.137  1.00  0.00           O
ATOM    850  OE2 GLU    53      42.534  23.896  39.061  1.00  0.00           O
ATOM    851  C   GLU    53      40.107  28.477  37.813  1.00  0.00           C
ATOM    852  O   GLU    53      40.734  29.492  38.137  1.00  0.00           O
ATOM    853  N   VAL    54      38.995  28.109  38.460  1.00  0.00           N
ATOM    854  H   VAL    54      38.552  27.275  38.102  1.00  0.00           H
ATOM    855  CA  VAL    54      38.526  28.575  39.733  1.00  0.00           C
ATOM    856  HA  VAL    54      38.484  29.665  39.739  1.00  0.00           H
ATOM    857  CB  VAL    54      37.104  28.105  39.908  1.00  0.00           C
ATOM    858  HB  VAL    54      37.160  27.017  39.880  1.00  0.00           H
ATOM    859  CG1 VAL    54      36.548  28.495  41.302  1.00  0.00           C
ATOM    860 HG11 VAL    54      36.481  29.556  41.546  1.00  0.00           H
ATOM    861 HG12 VAL    54      35.572  28.043  41.479  1.00  0.00           H
ATOM    862 HG13 VAL    54      37.132  28.106  42.137  1.00  0.00           H
ATOM    863  CG2 VAL    54      36.155  28.622  38.781  1.00  0.00           C
ATOM    864 HG21 VAL    54      36.189  29.709  38.708  1.00  0.00           H
ATOM    865 HG22 VAL    54      36.341  28.042  37.877  1.00  0.00           H
ATOM    866 HG23 VAL    54      35.115  28.522  39.093  1.00  0.00           H
ATOM    867  C   VAL    54      39.510  28.059  40.755  1.00  0.00           C
ATOM    868  O   VAL    54      39.733  26.814  40.944  1.00  0.00           O
ATOM    869  N   THR    55      40.204  29.008  41.320  1.00  0.00           N
ATOM    870  H   THR    55      40.011  29.973  41.096  1.00  0.00           H
ATOM    871  CA  THR    55      41.199  28.791  42.385  1.00  0.00           C
ATOM    872  HA  THR    55      41.613  27.799  42.209  1.00  0.00           H
ATOM    873  CB  THR    55      42.422  29.755  42.073  1.00  0.00           C
ATOM    874  HB  THR    55      43.212  29.715  42.823  1.00  0.00           H
ATOM    875  CG2 THR    55      43.089  29.572  40.697  1.00  0.00           C
ATOM    876 HG21 THR    55      43.664  28.651  40.788  1.00  0.00           H
ATOM    877 HG22 THR    55      42.266  29.530  39.983  1.00  0.00           H
ATOM    878 HG23 THR    55      43.785  30.386  40.497  1.00  0.00           H
ATOM    879  OG1 THR    55      41.947  31.073  41.981  1.00  0.00           O
ATOM    880  HG1 THR    55      42.090  31.609  42.764  1.00  0.00           H
ATOM    881  C   THR    55      40.763  28.821  43.847  1.00  0.00           C
ATOM    882  O   THR    55      41.384  28.082  44.612  1.00  0.00           O
ATOM    883  N   ALA    56      39.700  29.529  44.262  1.00  0.00           N
ATOM    884  H   ALA    56      39.154  30.093  43.627  1.00  0.00           H
ATOM    885  CA  ALA    56      39.209  29.475  45.669  1.00  0.00           C
ATOM    886  HA  ALA    56      39.479  28.554  46.188  1.00  0.00           H
ATOM    887  CB  ALA    56      39.868  30.577  46.401  1.00  0.00           C
ATOM    888  HB1 ALA    56      39.860  31.560  45.930  1.00  0.00           H
ATOM
    889  HB2 ALA    56      39.434  30.696  47.394  1.00  0.00           H
ATOM    890  HB3 ALA    56      40.878  30.269  46.669  1.00  0.00           H
ATOM    891  C   ALA    56      37.694  29.685  45.609  1.00  0.00           C
ATOM    892  O   ALA    56      37.298  30.537  44.789  1.00  0.00           O
ATOM    893  N   THR    57      36.946  29.122  46.591  1.00  0.00           N
ATOM    894  H   THR    57      37.397  28.454  47.200  1.00  0.00           H
ATOM    895  CA  THR    57      35.450  29.395  46.690  1.00  0.00           C
ATOM    896  HA  THR    57      35.240  30.414  46.368  1.00  0.00           H
ATOM    897  CB  THR    57      34.523  28.367  46.003  1.00  0.00           C
ATOM    898  HB  THR    57      33.509  28.724  46.184  1.00  0.00           H
ATOM    899  CG2 THR    57      34.500  28.432  44.464  1.00  0.00           C
ATOM    900 HG21 THR    57      33.484  28.121  44.219  1.00  0.00           H
ATOM    901 HG22 THR    57      34.605  29.488  44.217  1.00  0.00           H
ATOM    902 HG23 THR    57      35.242  27.761  44.032  1.00  0.00           H
ATOM    903  OG1 THR    57      34.638  27.011  46.497  1.00  0.00           O
ATOM    904  HG1 THR    57      34.378  27.128  47.413  1.00  0.00           H
ATOM    905  C   THR    57      35.083  29.479  48.197  1.00  0.00           C
ATOM    906  O   THR    57      35.563  28.662  49.017  1.00  0.00           O
ATOM    907  N   SER    58      34.163  30.286  48.612  1.00  0.00           N
ATOM    908  H   SER    58      33.597  30.732  47.904  1.00  0.00           H
ATOM    909  CA  SER    58      33.793  30.516  49.984  1.00  0.00           C
ATOM    910  HA  SER    58      33.988  29.645  50.608  1.00  0.00           H
```

ATOM	911	CB	SER	58	34.718	31.661	50.446	1.00	0.00	C
ATOM	912	HB2	SER	58	34.464	32.570	49.901	1.00	0.00	H
ATOM	913	HB3	SER	58	34.545	31.832	51.508	1.00	0.00	H
ATOM	914	OG	SER	58	36.089	31.552	50.123	1.00	0.00	O
ATOM	915	HG	SER	58	36.171	31.634	49.170	1.00	0.00	H
ATOM	916	C	SER	58	32.310	30.926	50.220	1.00	0.00	C
ATOM	917	O	SER	58	31.674	31.470	49.319	1.00	0.00	O
ATOM	918	N	THR	59	31.721	30.543	51.341	1.00	0.00	N
ATOM	919	H	THR	59	32.344	30.033	51.952	1.00	0.00	H
ATOM	920	CA	THR	59	30.370	31.004	51.778	1.00	0.00	C
ATOM	921	HA	THR	59	29.842	31.326	50.881	1.00	0.00	H
ATOM	922	CB	THR	59	29.584	29.802	52.277	1.00	0.00	C
ATOM	923	HB	THR	59	29.581	29.045	51.493	1.00	0.00	H
ATOM	924	CG2	THR	59	30.065	29.347	53.710	1.00	0.00	C
ATOM	925	HG21	THR	59	29.620	30.000	54.461	1.00	0.00	H
ATOM	926	HG22	THR	59	29.751	28.326	53.926	1.00	0.00	H
ATOM	927	HG23	THR	59	31.139	29.498	53.819	1.00	0.00	H
ATOM	928	OG1	THR	59	28.312	30.291	52.503	1.00	0.00	O
ATOM	929	HG1	THR	59	27.724	30.121	51.763	1.00	0.00	H
ATOM	930	C	THR	59	30.601	32.123	52.793	1.00	0.00	C
ATOM	931	O	THR	59	31.520	32.054	53.616	1.00	0.00	O
ATOM	932	N	VAL	60	29.869	33.235	52.685	1.00	0.00	N
ATOM	933	H	VAL	60	29.071	33.240	52.067	1.00	0.00	H
ATOM	934	CA	VAL	60	30.234	34.523	53.339	1.00	0.00	C
ATOM	935	HA	VAL	60	30.719	34.331	54.296	1.00	0.00	H
ATOM	936	CB	VAL	60	31.315	35.272	52.491	1.00	0.00	C
ATOM	937	HB	VAL	60	32.088	34.535	52.264	1.00	0.00	H
ATOM	938	CG1	VAL	60	30.892	35.827	51.091	1.00	0.00	C
ATOM	939	HG11	VAL	60	30.690	34.928	50.509	1.00	0.00	H
ATOM	940	HG12	VAL	60	30.018	36.479	51.052	1.00	0.00	H
ATOM	941	HG13	VAL	60	31.721	36.396	50.670	1.00	0.00	H
ATOM	942	CG2	VAL	60	31.956	36.471	53.182	1.00	0.00	C
ATOM	943	HG21	VAL	60	32.614	36.089	53.962	1.00	0.00	H
ATOM	944	HG22	VAL	60	32.729	36.883	52.533	1.00	0.00	H
ATOM	945	HG23	VAL	60	31.205	37.208	53.469	1.00	0.00	H
ATOM	946	C	VAL	60	29.035	35.369	53.714	1.00	0.00	C
ATOM	947	O	VAL	60	27.991	35.308	53.041	1.00	0.00	O
ATOM	948	N	THR	61	29.097	36.274	54.707	1.00	0.00	N
ATOM	949	H	THR	61	30.014	36.272	55.128	1.00	0.00	H
ATOM	950	CA	THR	61	28.009	37.148	55.178	1.00	0.00	C
ATOM	951	HA	THR	61	27.069	36.772	54.774	1.00	0.00	H
ATOM	952	CB	THR	61	27.820	37.079	56.696	1.00	0.00	C
ATOM	953	HB	THR	61	27.171	37.923	56.930	1.00	0.00	H
ATOM	954	CG2	THR	61	27.227	35.696	57.003	1.00	0.00	C
ATOM	955	HG21	THR	61	26.917	35.572	58.040	1.00	0.00	H
ATOM	956	HG22	THR	61	26.311	35.544	56.432	1.00	0.00	H
ATOM	957	HG23	THR	61	27.887	34.847	56.827	1.00	0.00	H
ATOM	958	OG1	THR	61	29.015	37.180	57.438	1.00	0.00	O
ATOM	959	HG1	THR	61	28.888	37.981	57.952	1.00	0.00	H
ATOM	960	C	THR	61	28.304	38.672	54.802	1.00	0.00	C
ATOM	961	O	THR	61	29.444	39.029	54.515	1.00	0.00	O
ATOM	962	N	LEU	62	27.281	39.571	54.859	1.00	0.00	N
ATOM	963	H	LEU	62	26.402	39.114	55.054	1.00	0.00	H
ATOM	964	CA	LEU	62	27.302	41.030	54.524	1.00	0.00	C
ATOM	965	HA	LEU	62	27.749	41.053	53.532	1.00	0.00	H
ATOM	966	CB	LEU	62	25.931	41.710	54.610	1.00	0.00	C
ATOM	967	HB2	LEU	62	25.352	41.022	53.993	1.00	0.00	H
ATOM	968	HB3	LEU	62	25.554	41.686	55.632	1.00	0.00	H
ATOM	969	CG	LEU	62	25.702	43.159	54.128	1.00	0.00	C
ATOM	970	HG	LEU	62	26.248	43.842	54.779	1.00	0.00	H
ATOM	971	CD1	LEU	62	26.281	43.545	52.739	1.00	0.00	C
ATOM	972	HD11	LEU	62	26.102	44.608	52.578	1.00	0.00	H
ATOM	973	HD12	LEU	62	27.330	43.252	52.779	1.00	0.00	H
ATOM	974	HD13	LEU	62	25.733	42.900	52.052	1.00	0.00	H

ATOM	975	CD2	LEU	62	24.162	43.320	54.221	1.00	0.00	C
ATOM	976	HD21	LEU	62	23.645	42.581	53.609	1.00	0.00	H
ATOM	977	HD22	LEU	62	23.818	43.274	55.255	1.00	0.00	H
ATOM	978	HD23	LEU	62	23.888	44.320	53.888	1.00	0.00	H
ATOM	979	C	LEU	62	28.307	41.832	55.311	1.00	0.00	C
ATOM	980	O	LEU	62	28.774	42.944	54.901	1.00	0.00	O
ATOM	981	N	ASP	63	28.797	41.425	56.454	1.00	0.00	N
ATOM	982	H	ASP	63	28.343	40.564	56.724	1.00	0.00	H
ATOM	983	CA	ASP	63	29.687	42.058	57.447	1.00	0.00	C
ATOM	984	HA	ASP	63	29.957	43.086	57.205	1.00	0.00	H
ATOM	985	CB	ASP	63	29.076	42.076	58.825	1.00	0.00	C
ATOM	986	HB2	ASP	63	29.783	42.502	59.535	1.00	0.00	H
ATOM	987	HB3	ASP	63	28.198	42.709	58.692	1.00	0.00	H
ATOM	988	CG	ASP	63	28.651	40.687	59.335	1.00	0.00	C
ATOM	989	OD1	ASP	63	28.543	39.655	58.582	1.00	0.00	O
ATOM	990	OD2	ASP	63	28.269	40.625	60.542	1.00	0.00	O
ATOM	991	C	ASP	63	31.085	41.388	57.464	1.00	0.00	C
ATOM	992	O	ASP	63	32.045	41.806	58.149	1.00	0.00	O
ATOM	993	N	THR	64	31.343	40.443	56.624	1.00	0.00	N
ATOM	994	H	THR	64	30.532	40.113	56.120	1.00	0.00	H
ATOM	995	CA	THR	64	32.575	39.611	56.389	1.00	0.00	C
ATOM	996	HA	THR	64	33.391	40.104	56.918	1.00	0.00	H
ATOM	997	CB	THR	64	32.459	38.173	57.001	1.00	0.00	C
ATOM	998	HB	THR	64	33.436	37.720	56.835	1.00	0.00	H
ATOM	999	CG2	THR	64	32.137	38.230	58.489	1.00	0.00	C
ATOM	1000	HG21	THR	64	31.056	38.330	58.593	1.00	0.00	H
ATOM	1001	HG22	THR	64	32.605	37.364	58.957	1.00	0.00	H
ATOM	1002	HG23	THR	64	32.642	39.109	58.890	1.00	0.00	H
ATOM	1003	OG1	THR	64	31.436	37.354	56.453	1.00	0.00	O
ATOM	1004	HG1	THR	64	30.546	37.582	56.731	1.00	0.00	H
ATOM	1005	C	THR	64	33.052	39.366	54.983	1.00	0.00	C
ATOM	1006	O	THR	64	33.954	38.620	54.699	1.00	0.00	O
ATOM	1007	N	LEU	65	32.455	39.995	53.962	1.00	0.00	N
ATOM	1008	H	LEU	65	31.727	40.618	54.281	1.00	0.00	H
ATOM	1009	CA	LEU	65	32.956	40.108	52.573	1.00	0.00	C
ATOM	1010	HA	LEU	65	32.832	39.089	52.206	1.00	0.00	H
ATOM	1011	CB	LEU	65	32.162	41.171	51.752	1.00	0.00	C
ATOM	1012	HB2	LEU	65	32.318	42.167	52.166	1.00	0.00	H
ATOM	1013	HB3	LEU	65	32.444	41.092	50.702	1.00	0.00	H
ATOM	1014	CG	LEU	65	30.678	40.938	51.762	1.00	0.00	C
ATOM	1015	HG	LEU	65	30.280	40.936	52.778	1.00	0.00	H
ATOM	1016	CD1	LEU	65	30.019	42.087	51.010	1.00	0.00	C
ATOM	1017	HD11	LEU	65	30.307	43.060	51.411	1.00	0.00	H
ATOM	1018	HD12	LEU	65	30.194	41.902	49.950	1.00	0.00	H
ATOM	1019	HD13	LEU	65	28.936	41.978	51.035	1.00	0.00	H
ATOM	1020	CD2	LEU	65	30.221	39.588	51.206	1.00	0.00	C
ATOM	1021	HD21	LEU	65	30.451	38.763	51.882	1.00	0.00	H
ATOM	1022	HD22	LEU	65	29.154	39.603	50.984	1.00	0.00	H
ATOM	1023	HD23	LEU	65	30.627	39.452	50.204	1.00	0.00	H
ATOM	1024	C	LEU	65	34.430	40.496	52.537	1.00	0.00	C
ATOM	1025	O	LEU	65	34.786	41.275	53.410	1.00	0.00	O
ATOM	1026	N	THR	66	35.252	40.009	51.563	1.00	0.00	N
ATOM	1027	H	THR	66	34.885	39.285	50.963	1.00	0.00	H
ATOM	1028	CA	THR	66	36.645	40.287	51.441	1.00	0.00	C
ATOM	1029	HA	THR	66	37.085	40.301	52.438	1.00	0.00	H
ATOM	1030	CB	THR	66	37.278	39.045	50.698	1.00	0.00	C
ATOM	1031	HB	THR	66	37.059	38.146	51.273	1.00	0.00	H
ATOM	1032	CG2	THR	66	36.868	38.942	49.164	1.00	0.00	C
ATOM	1033	HG21	THR	66	37.302	37.998	48.836	1.00	0.00	H
ATOM	1034	HG22	THR	66	35.798	38.944	48.958	1.00	0.00	H
ATOM	1035	HG23	THR	66	37.377	39.701	48.572	1.00	0.00	H
ATOM	1036	OG1	THR	66	38.684	39.143	50.570	1.00	0.00	O
ATOM	1037	HG1	THR	66	38.912	38.668	51.373	1.00	0.00	H

ATOM	1038	C	THR	66	37.062	41.511	50.714	1.00	0.00	C
ATOM	1039	O	THR	66	36.533	42.014	49.666	1.00	0.00	O
ATOM	1040	N	GLU	67	38.068	42.070	51.358	1.00	0.00	N
ATOM	1041	H	GLU	67	38.465	41.561	52.135	1.00	0.00	H
ATOM	1042	CA	GLU	67	38.776	43.334	50.988	1.00	0.00	C
ATOM	1043	HA	GLU	67	37.967	44.059	50.887	1.00	0.00	H
ATOM	1044	CB	GLU	67	39.818	43.790	52.046	1.00	0.00	C
ATOM	1045	HB2	GLU	67	40.005	44.860	51.976	1.00	0.00	H
ATOM	1046	HB3	GLU	67	39.389	43.751	53.048	1.00	0.00	H
ATOM	1047	CG	GLU	67	41.138	43.042	52.153	1.00	0.00	C
ATOM	1048	HG2	GLU	67	41.760	43.276	51.289	1.00	0.00	H
ATOM	1049	HG3	GLU	67	41.665	43.366	53.051	1.00	0.00	H
ATOM	1050	CD	GLU	67	40.962	41.565	52.333	1.00	0.00	C
ATOM	1051	OE1	GLU	67	40.150	41.126	53.201	1.00	0.00	O
ATOM	1052	OE2	GLU	67	41.596	40.769	51.582	1.00	0.00	O
ATOM	1053	C	GLU	67	39.382	43.112	49.597	1.00	0.00	C
ATOM	1054	O	GLU	67	39.307	44.117	48.806	1.00	0.00	O
ATOM	1055	N	LYS	68	39.855	41.923	49.187	1.00	0.00	N
ATOM	1056	H	LYS	68	39.821	41.147	49.832	1.00	0.00	H
ATOM	1057	CA	LYS	68	40.469	41.568	47.918	1.00	0.00	C
ATOM	1058	HA	LYS	68	41.380	42.146	47.771	1.00	0.00	H
ATOM	1059	CB	LYS	68	40.892	40.075	48.005	1.00	0.00	C
ATOM	1060	HB2	LYS	68	41.500	39.918	48.896	1.00	0.00	H
ATOM	1061	HB3	LYS	68	40.008	39.441	48.073	1.00	0.00	H
ATOM	1062	CG	LYS	68	41.634	39.606	46.768	1.00	0.00	C
ATOM	1063	HG2	LYS	68	40.967	39.794	45.926	1.00	0.00	H
ATOM	1064	HG3	LYS	68	42.480	40.242	46.503	1.00	0.00	H
ATOM	1065	CD	LYS	68	41.997	38.137	46.556	1.00	0.00	C
ATOM	1066	HD2	LYS	68	40.984	37.738	46.524	1.00	0.00	H
ATOM	1067	HD3	LYS	68	42.643	37.978	45.693	1.00	0.00	H
ATOM	1068	CE	LYS	68	42.634	37.421	47.733	1.00	0.00	C
ATOM	1069	HE2	LYS	68	43.717	37.328	47.665	1.00	0.00	H
ATOM	1070	HE3	LYS	68	42.440	37.904	48.691	1.00	0.00	H
ATOM	1071	NZ	LYS	68	42.062	36.072	47.760	1.00	0.00	N
ATOM	1072	HZ1	LYS	68	41.205	36.045	48.296	1.00	0.00	H
ATOM	1073	HZ2	LYS	68	41.948	35.789	46.797	1.00	0.00	H
ATOM	1074	HZ3	LYS	68	42.768	35.427	48.084	1.00	0.00	H
ATOM	1075	C	LYS	68	39.560	41.862	46.733	1.00	0.00	C
ATOM	1076	O	LYS	68	39.979	42.239	45.666	1.00	0.00	O
ATOM	1077	N	HIE	69	38.242	41.627	46.895	1.00	0.00	N
ATOM	1078	H	HIE	69	37.857	41.527	47.823	1.00	0.00	H
ATOM	1079	CA	HIE	69	37.299	41.958	45.782	1.00	0.00	C
ATOM	1080	HA	HIE	69	37.717	41.616	44.835	1.00	0.00	H
ATOM	1081	CB	HIE	69	36.008	41.255	46.062	1.00	0.00	C
ATOM	1082	HB2	HIE	69	36.140	40.173	46.078	1.00	0.00	H
ATOM	1083	HB3	HIE	69	35.771	41.483	47.101	1.00	0.00	H
ATOM	1084	CG	HIE	69	34.860	41.431	45.039	1.00	0.00	C
ATOM	1085	ND1	HIE	69	34.739	40.645	43.906	1.00	0.00	N
ATOM	1086	CE1	HIE	69	33.687	41.204	43.211	1.00	0.00	C
ATOM	1087	HE1	HIE	69	33.398	40.819	42.244	1.00	0.00	H
ATOM	1088	NE2	HIE	69	33.160	42.257	43.936	1.00	0.00	N
ATOM	1089	HE2	HIE	69	32.308	42.732	43.676	1.00	0.00	H
ATOM	1090	CD2	HIE	69	33.966	42.441	45.008	1.00	0.00	C
ATOM	1091	HD2	HIE	69	33.850	43.192	45.776	1.00	0.00	H
ATOM	1092	C	HIE	69	37.126	43.450	45.583	1.00	0.00	C
ATOM	1093	O	HIE	69	37.117	43.972	44.394	1.00	0.00	O
ATOM	1094	N	ALA	70	36.923	44.160	46.720	1.00	0.00	N
ATOM	1095	H	ALA	70	36.914	43.776	47.654	1.00	0.00	H
ATOM	1096	CA	ALA	70	36.698	45.611	46.577	1.00	0.00	C
ATOM	1097	HA	ALA	70	35.858	45.699	45.889	1.00	0.00	H
ATOM	1098	CB	ALA	70	36.313	46.148	47.947	1.00	0.00	C
ATOM	1099	HB1	ALA	70	36.121	47.192	47.698	1.00	0.00	H
ATOM	1100	HB2	ALA	70	35.368	45.686	48.234	1.00	0.00	H
ATOM	1101	HB3	ALA	70	37.152	46.000	48.626	1.00	0.00	H

```
ATOM   1102  C   ALA    70      37.977  46.245  45.968  1.00  0.00           C
ATOM   1103  O   ALA    70      37.895  47.108  45.115  1.00  0.00           O
ATOM   1104  N   GLU    71      39.202  45.765  46.305  1.00  0.00           N
ATOM   1105  H   GLU    71      39.186  45.119  47.082  1.00  0.00           H
ATOM   1106  CA  GLU    71      40.446  46.134  45.705  1.00  0.00           C
ATOM   1107  HA  GLU    71      40.581  47.197  45.902  1.00  0.00           H
ATOM   1108  CB  GLU    71      41.618  45.468  46.499  1.00  0.00           C
ATOM   1109  HB2 GLU    71      41.409  45.659  47.552  1.00  0.00           H
ATOM   1110  HB3 GLU    71      41.603  44.406  46.251  1.00  0.00           H
ATOM   1111  CG  GLU    71      42.943  46.107  46.170  1.00  0.00           C
ATOM   1112  HG2 GLU    71      43.188  45.766  45.164  1.00  0.00           H
ATOM   1113  HG3 GLU    71      42.875  47.190  46.274  1.00  0.00           H
ATOM   1114  CD  GLU    71      44.145  45.606  46.876  1.00  0.00           C
ATOM   1115  OE1 GLU    71      45.090  45.375  46.076  1.00  0.00           O
ATOM   1116  OE2 GLU    71      44.286  45.495  48.106  1.00  0.00           O
ATOM   1117  C   GLU    71      40.542  45.875  44.161  1.00  0.00           C
ATOM   1118  O   GLU    71      40.945  46.773  43.385  1.00  0.00           O
ATOM   1119  N   GLN    72      40.217  44.685  43.652  1.00  0.00           N
ATOM   1120  H   GLN    72      39.954  43.972  44.319  1.00  0.00           H
ATOM   1121  CA  GLN    72      40.066  44.221  42.250  1.00  0.00           C
ATOM   1122  HA  GLN    72      41.011  44.416  41.742  1.00  0.00           H
ATOM   1123  CB  GLN    72      39.641  42.708  42.196  1.00  0.00           C
ATOM   1124  HB2 GLN    72      38.684  42.615  42.709  1.00  0.00           H
ATOM   1125  HB3 GLN    72      40.361  42.100  42.743  1.00  0.00           H
ATOM   1126  CG  GLN    72      39.610  42.065  40.772  1.00  0.00           C
ATOM   1127  HG2 GLN    72      40.248  42.704  40.161  1.00  0.00           H
ATOM   1128  HG3 GLN    72      40.103  41.096  40.847  1.00  0.00           H
ATOM   1129  CD  GLN    72      38.192  41.915  40.089  1.00  0.00           C
ATOM   1130  OE1 GLN    72      37.552  40.890  39.912  1.00  0.00           O
ATOM   1131  NE2 GLN    72      37.667  43.083  39.554  1.00  0.00           N
ATOM   1132 HE21 GLN    72      38.218  43.928  39.599  1.00  0.00           H
ATOM   1133 HE22 GLN    72      36.771  43.075  39.088  1.00  0.00           H
ATOM   1134  C   GLN    72      39.052  45.140  41.523  1.00  0.00           C
ATOM   1135  O   GLN    72      39.278  45.415  40.388  1.00  0.00           O
ATOM   1136  N   GLU    73      37.990  45.538  42.173  1.00  0.00           N
ATOM   1137  H   GLU    73      37.824  45.236  43.121  1.00  0.00           H
ATOM   1138  CA  GLU    73      36.883  46.366  41.553  1.00  0.00           C
ATOM   1139  HA  GLU    73      36.838  46.079  40.503
  1.00  0.00           H
ATOM   1140  CB  GLU    73      35.542  45.990  42.230  1.00  0.00           C
ATOM   1141  HB2 GLU    73      35.686  46.214  43.287  1.00  0.00           H
ATOM   1142  HB3 GLU    73      34.790  46.700  41.884  1.00  0.00           H
ATOM   1143  CG  GLU    73      35.003  44.514  42.038  1.00  0.00           C
ATOM   1144  HG2 GLU    73      35.840  43.821  41.952  1.00  0.00           H
ATOM   1145  HG3 GLU    73      34.317  44.326  42.864  1.00  0.00           H
ATOM   1146  CD  GLU    73      34.099  44.341  40.782  1.00  0.00           C
ATOM   1147  OE1 GLU    73      34.587  43.749  39.780  1.00  0.00           O
ATOM   1148  OE2 GLU    73      32.951  44.818  40.832  1.00  0.00           O
ATOM   1149  C   GLU    73      37.167  47.917  41.527  1.00  0.00           C
ATOM   1150  O   GLU    73      36.517  48.644  40.831  1.00  0.00           O
ATOM   1151  N   ASN    74      38.265  48.325  42.159  1.00  0.00           N
ATOM   1152  H   ASN    74      38.794  47.644  42.684  1.00  0.00           H
ATOM   1153  CA  ASN    74      38.676  49.742  42.347  1.00  0.00           C
ATOM   1154  HA  ASN    74      39.619  49.831  42.886  1.00  0.00           H
ATOM   1155  CB  ASN    74      39.076  50.429  40.975  1.00  0.00           C
ATOM   1156  HB2 ASN    74      38.256  50.579  40.273  1.00  0.00           H
ATOM   1157  HB3 ASN    74      39.326  51.463  41.211  1.00  0.00           H
ATOM   1158  CG  ASN    74      40.219  49.785  40.166  1.00  0.00           C
ATOM   1159  OD1 ASN    74      40.236  49.775  38.951  1.00  0.00           O
ATOM   1160  ND2 ASN    74      41.194  49.189  40.784  1.00  0.00           N
ATOM   1161 HD21 ASN    74      42.017  48.846  40.310  1.00  0.00           H
ATOM   1162 HD22 ASN    74      41.176  49.099  41.790  1.00  0.00           H
ATOM   1163  C   ASN    74      37.635  50.579  43.218  1.00  0.00           C
ATOM   1164  O   ASN    74      37.363  51.763  42.943  1.00  0.00           O
```

ATOM	1165	N	MET	75	37.055	49.950	44.259	1.00	0.00	N
ATOM	1166	H	MET	75	37.421	49.025	44.441	1.00	0.00	H
ATOM	1167	CA	MET	75	36.255	50.569	45.292	1.00	0.00	C
ATOM	1168	HA	MET	75	36.139	51.632	45.085	1.00	0.00	H
ATOM	1169	CB	MET	75	34.875	49.925	45.248	1.00	0.00	C
ATOM	1170	HB2	MET	75	35.050	48.874	45.477	1.00	0.00	H
ATOM	1171	HB3	MET	75	34.249	50.366	46.024	1.00	0.00	H
ATOM	1172	CG	MET	75	34.161	49.919	43.883	1.00	0.00	C
ATOM	1173	HG2	MET	75	33.920	50.960	43.664	1.00	0.00	H
ATOM	1174	HG3	MET	75	34.605	49.291	43.112	1.00	0.00	H
ATOM	1175	SD	MET	75	32.538	49.145	43.945	1.00	0.00	S
ATOM	1176	CE	MET	75	31.574	50.207	44.911	1.00	0.00	C
ATOM	1177	HE1	MET	75	32.118	50.384	45.839	1.00	0.00	H
ATOM	1178	HE2	MET	75	31.297	51.086	44.329	1.00	0.00	H
ATOM	1179	HE3	MET	75	30.649	49.708	45.200	1.00	0.00	H
ATOM	1180	C	MET	75	36.842	50.425	46.727	1.00	0.00	C
ATOM	1181	O	MET	75	37.745	49.612	47.000	1.00	0.00	O
ATOM	1182	N	THR	76	36.176	51.071	47.671	1.00	0.00	N
ATOM	1183	H	THR	76	35.425	51.654	47.329	1.00	0.00	H
ATOM	1184	CA	THR	76	36.415	50.919	49.071	1.00	0.00	C
ATOM	1185	HA	THR	76	37.350	50.376	49.201	1.00	0.00	H
ATOM	1186	CB	THR	76	36.656	52.242	49.845	1.00	0.00	C
ATOM	1187	HB	THR	76	37.155	52.979	49.216	1.00	0.00	H
ATOM	1188	CG2	THR	76	35.385	52.918	50.155	1.00	0.00	C
ATOM	1189	HG21	THR	76	35.409	53.947	50.515	1.00	0.00	H
ATOM	1190	HG22	THR	76	34.825	52.872	49.221	1.00	0.00	H
ATOM	1191	HG23	THR	76	34.778	52.269	50.787	1.00	0.00	H
ATOM	1192	OG1	THR	76	37.372	51.999	51.076	1.00	0.00	O
ATOM	1193	HG1	THR	76	38.251	51.649	50.914	1.00	0.00	H
ATOM	1194	C	THR	76	35.412	49.983	49.701	1.00	0.00	C
ATOM	1195	O	THR	76	34.248	49.994	49.430	1.00	0.00	O
ATOM	1196	N	LEU	77	35.853	49.078	50.606	1.00	0.00	N
ATOM	1197	H	LEU	77	36.850	49.017	50.754	1.00	0.00	H
ATOM	1198	CA	LEU	77	35.023	47.868	51.042	1.00	0.00	C
ATOM	1199	HA	LEU	77	34.753	47.294	50.156	1.00	0.00	H
ATOM	1200	CB	LEU	77	35.841	47.033	51.974	1.00	0.00	C
ATOM	1201	HB2	LEU	77	36.752	46.719	51.464	1.00	0.00	H
ATOM	1202	HB3	LEU	77	36.108	47.565	52.887	1.00	0.00	H
ATOM	1203	CG	LEU	77	35.216	45.651	52.333	1.00	0.00	C
ATOM	1204	HG	LEU	77	34.282	45.843	52.861	1.00	0.00	H
ATOM	1205	CD1	LEU	77	34.982	44.686	51.124	1.00	0.00	C
ATOM	1206	HD11	LEU	77	35.028	43.660	51.490	1.00	0.00	H
ATOM	1207	HD12	LEU	77	34.023	44.817	50.623	1.00	0.00	H
ATOM	1208	HD13	LEU	77	35.846	44.783	50.467	1.00	0.00	H
ATOM	1209	CD2	LEU	77	36.100	44.974	53.432	1.00	0.00	C
ATOM	1210	HD21	LEU	77	35.647	44.039	53.762	1.00	0.00	H
ATOM	1211	HD22	LEU	77	37.083	44.753	53.018	1.00	0.00	H
ATOM	1212	HD23	LEU	77	36.088	45.688	54.257	1.00	0.00	H
ATOM	1213	C	LEU	77	33.739	48.381	51.735	1.00	0.00	C
ATOM	1214	O	LEU	77	32.800	47.680	51.577	1.00	0.00	O
ATOM	1215	N	THR	78	33.620	49.527	52.473	1.00	0.00	N
ATOM	1216	H	THR	78	34.481	50.052	52.519	1.00	0.00	H
ATOM	1217	CA	THR	78	32.350	50.165	53.070	1.00	0.00	C
ATOM	1218	HA	THR	78	31.824	49.291	53.452	1.00	0.00	H
ATOM	1219	CB	THR	78	32.706	51.185	54.086	1.00	0.00	C
ATOM	1220	HB	THR	78	31.934	51.950	54.160	1.00	0.00	H
ATOM	1221	CG2	THR	78	32.970	50.507	55.360	1.00	0.00	C
ATOM	1222	HG21	THR	78	33.217	51.241	56.128	1.00	0.00	H
ATOM	1223	HG22	THR	78	32.197	49.763	55.549	1.00	0.00	H
ATOM	1224	HG23	THR	78	33.866	49.886	55.343	1.00	0.00	H
ATOM	1225	OG1	THR	78	33.863	51.955	53.732	1.00	0.00	O
ATOM	1226	HG1	THR	78	34.574	51.311	53.700	1.00	0.00	H
ATOM	1227	C	THR	78	31.385	50.695	51.998	1.00	0.00	C
ATOM	1228	O	THR	78	30.190	50.588	52.181	1.00	0.00	O

ATOM	1229	N	GLU	79	31.926	51.109	50.859	1.00	0.00	N
ATOM	1230	H	GLU	79	32.909	50.965	50.684	1.00	0.00	H
ATOM	1231	CA	GLU	79	31.096	51.583	49.775	1.00	0.00	C
ATOM	1232	HA	GLU	79	30.354	52.266	50.187	1.00	0.00	H
ATOM	1233	CB	GLU	79	31.927	52.464	48.796	1.00	0.00	C
ATOM	1234	HB2	GLU	79	32.343	53.328	49.315	1.00	0.00	H
ATOM	1235	HB3	GLU	79	32.658	51.797	48.341	1.00	0.00	H
ATOM	1236	CG	GLU	79	31.081	53.020	47.648	1.00	0.00	C
ATOM	1237	HG2	GLU	79	30.854	52.190	46.980	1.00	0.00	H
ATOM	1238	HG3	GLU	79	30.185	53.525	48.014	1.00	0.00	H
ATOM	1239	CD	GLU	79	31.911	53.967	46.778	1.00	0.00	C
ATOM	1240	OE1	GLU	79	31.772	53.974	45.539	1.00	0.00	O
ATOM	1241	OE2	GLU	79	32.632	54.792	47.366	1.00	0.00	O
ATOM	1242	C	GLU	79	30.430	50.438	48.979	1.00	0.00	C
ATOM	1243	O	GLU	79	29.209	50.408	48.737	1.00	0.00	O
ATOM	1244	N	LEU	80	31.207	49.359	48.758	1.00	0.00	N
ATOM	1245	H	LEU	80	32.165	49.590	48.979	1.00	0.00	H
ATOM	1246	CA	LEU	80	30.875	48.033	48.190	1.00	0.00	C
ATOM	1247	HA	LEU	80	30.318	48.204	47.269	1.00	0.00	H
ATOM	1248	CB	LEU	80	32.128	47.120	47.999	1.00	0.00	C
ATOM	1249	HB2	LEU	80	32.858	47.710	47.444	1.00	0.00	H
ATOM	1250	HB3	LEU	80	32.549	46.898	48.980	1.00	0.00	H
ATOM	1251	CG	LEU	80	31.726	45.859	47.087	1.00	0.00	C
ATOM	1252	HG	LEU	80	30.737	45.968	46.642	1.00	0.00	H
ATOM	1253	CD1	LEU	80	32.698	45.835	45.897	1.00	0.00	C
ATOM	1254	HD11	LEU	80	32.423	45.062	45.182	1.00	0.00	H
ATOM	1255	HD12	LEU	80	32.603	46.788	45.377	1.00	0.00	H
ATOM	1256	HD13	LEU	80	33.683	45.722	46.350	1.00	0.00	H
ATOM	1257	CD2	LEU	80	31.994	44.496	47.739	1.00	0.00	C
ATOM	1258	HD21	LEU	80	32.956	44.449	48.248	1.00	0.00	H
ATOM	1259	HD22	LEU	80	31.159	44.207	48.378	1.00	0.00	H
ATOM	1260	HD23	LEU	80	32.117	43.792	46.917	1.00	0.00	H
ATOM	1261	C	LEU	80	29.727	47.328	48.930	1.00	0.00	C
ATOM	1262	O	LEU	80	28.753	46.916	48.343	1.00	0.00	O
ATOM	1263	N	LYS	81	29.819	47.327	50.295	1.00	0.00	N
ATOM	1264	H	LYS	81	30.661	47.729	50.680	1.00	0.00	H
ATOM	1265	CA	LYS	81	28.914	46.726	51.271	1.00	0.00	C
ATOM	1266	HA	LYS	81	28.683	45.700	50.985	1.00	0.00	H
ATOM	1267	CB	LYS	81	29.627	46.814	52.598	1.00	0.00	C
ATOM	1268	HB2	LYS	81	30.041	47.816	52.716	1.00	0.00	H
ATOM	1269	HB3	LYS	81	28.887	46.718	53.392	1.00	0.00	H
ATOM	1270	CG	LYS	81	30.585	45.686	52.768	1.00	0.00	C
ATOM	1271	HG2	LYS	81	30.156	44.696	52.614	1.00	0.00	H
ATOM	1272	HG3	LYS	81	31.403	45.729	52.049	1.00	0.00	H
ATOM	1273	CD	LYS	81	31.394	45.791	54.097	1.00	0.00	C
ATOM	1274	HD2	LYS	81	32.132	46.593	54.045	1.00	0.00	H
ATOM	1275	HD3	LYS	81	30.668	46.032	54.873	1.00	0.00	H
ATOM	1276	CE	LYS	81	32.030	44.386	54.349	1.00	0.00	C
ATOM	1277	HE2	LYS	81	31.183	43.703	54.428	1.00	0.00	H
ATOM	1278	HE3	LYS	81	32.672	44.115	53.511	1.00	0.00	H
ATOM	1279	NZ	LYS	81	32.760	44.395	55.636	1.00	0.00	N
ATOM	1280	HZ1	LYS	81	32.068	44.662	56.321	1.00	0.00	H
ATOM	1281	HZ2	LYS	81	33.026	43.452	55.881	1.00	0.00	H
ATOM	1282	HZ3	LYS	81	33.464	45.116	55.710	1.00	0.00	H
ATOM	1283	C	LYS	81	27.638	47.585	51.279	1.00	0.00	C
ATOM	1284	O	LYS	81	26.580	46.933	51.149	1.00	0.00	O
ATOM	1285	N	LYS	82	27.667	48.932	51.262	1.00	0.00	N
ATOM	1286	H	LYS	82	28.593	49.313	51.132	1.00	0.00	H
ATOM	1287	CA	LYS	82	26.554	49.833	51.172	1.00	0.00	C
ATOM	1288	HA	LYS	82	25.978	49.724	52.091	1.00	0.00	H
ATOM	1289	CB	LYS	82	27.147	51.268	51.215	1.00	0.00	C
ATOM	1290	HB2	LYS	82	28.083	51.363	50.668	1.00	0.00	H
ATOM	1291	HB3	LYS	82	26.376	51.874	50.738	1.00	0.00	H

ATOM	1292	CG	LYS	82	27.350	51.760	52.600	1.00	0.00	C
ATOM	1293	HG2	LYS	82	26.414	52.012	53.098	1.00	0.00	H
ATOM	1294	HG3	LYS	82	27.846	50.985	53.184	1.00	0.00	H
ATOM	1295	CD	LYS	82	28.090	53.067	52.611	1.00	0.00	C
ATOM	1296	HD2	LYS	82	29.150	52.904	52.417	1.00	0.00	H
ATOM	1297	HD3	LYS	82	27.542	53.730	51.942	1.00	0.00	H
ATOM	1298	CE	LYS	82	28.143	53.751	53.997	1.00	0.00	C
ATOM	1299	HE2	LYS	82	27.132	53.919	54.367	1.00	0.00	H
ATOM	1300	HE3	LYS	82	28.697	53.083	54.659	1.00	0.00	H
ATOM	1301	NZ	LYS	82	28.869	55.028	53.855	1.00	0.00	N
ATOM	1302	HZ1	LYS	82	29.834	54.813	53.647	1.00	0.00	H
ATOM	1303	HZ2	LYS	82	28.435	55.559	53.114	1.00	0.00	H
ATOM	1304	HZ3	LYS	82	28.863	55.517	54.737	1.00	0.00	H
ATOM	1305	C	LYS	82	25.624	49.645	49.978	1.00	0.00	C
ATOM	1306	O	LYS	82	24.481	49.261	50.066	1.00	0.00	O
ATOM	1307	N	VAL	83	26.243	49.856	48.770	1.00	0.00	N
ATOM	1308	H	VAL	83	27.219	50.114	48.741	1.00	0.00	H
ATOM	1309	CA	VAL	83	25.531	49.552	47.493	1.00	0.00	C
ATOM	1310	HA	VAL	83	24.666	50.214	47.438	1.00	0.00	H
ATOM	1311	CB	VAL	83	26.370	49.894	46.262	1.00	0.00	C
ATOM	1312	HB	VAL	83	25.649	49.641	45.483	1.00	0.00	H
ATOM	1313	CG1	VAL	83	26.670	51.390	46.230	1.00	0.00	C
ATOM	1314	HG11	VAL	83	25.720	51.922	46.282	1.00	0.00	H
ATOM	1315	HG12	VAL	83	27.389	51.679	46.997	1.00	0.00	H
ATOM	1316	HG13	VAL	83	27.098	51.572	45.245	1.00	0.00	H
ATOM	1317	CG2	VAL	83	27.613	49.012	46.085	1.00	0.00	C
ATOM	1318	HG21	VAL	83	28.329	49.177	46.889	1.00	0.00	H
ATOM	1319	HG22	VAL	83	27.403	47.949	45.960	1.00	0.00	H
ATOM	1320	HG23	VAL	83	28.230	49.331	45.245	1.00	0.00	H
ATOM	1321	C	VAL	83	24.972	48.104	47.395	1.00	0.00	C
ATOM	1322	O	VAL	83	23.872	47.891	46.871	1.00	0.00	O
ATOM	1323	N	ILE	84	25.769	47.108	47.819	1.00	0.00	N
ATOM	1324	H	ILE	84	26.657	47.281	48.269	1.00	0.00	H
ATOM	1325	CA	ILE	84	25.264	45.776	47.984	1.00	0.00	C
ATOM	1326	HA	ILE	84	24.818	45.591	47.006	1.00	0.00	H
ATOM	1327	CB	ILE	84	26.329	44.629	48.115	1.00	0.00	C
ATOM	1328	HB	ILE	84	26.958	44.968	48.938	1.00	0.00	H
ATOM	1329	CG2	ILE	84	25.790	43.290	48.441	1.00	0.00	C
ATOM	1330	HG21	ILE	84	25.082	43.392	49.262	1.00	0.00	H
ATOM	1331	HG22	ILE	84	25.271	43.002	47.527	1.00	0.00	H
ATOM	1332	HG23	ILE	84	26.516	42.533	48.741	1.00	0.00	H
ATOM	1333	CG1	ILE	84	27.199	44.486	46.833	1.00	0.00	C
ATOM	1334	HG12	ILE	84	26.609	44.068	46.016	1.00	0.00	H
ATOM	1335	HG13	ILE	84	27.496	45.494	46.545	1.00	0.00	H
ATOM	1336	CD1	ILE	84	28.480	43.597	46.954	1.00	0.00	C
ATOM	1337	HD11	ILE	84	28.850	43.532	45.930	1.00	0.00	H
ATOM	1338	HD12	ILE	84	29.149	44.061	47.680	1.00	0.00	H
ATOM	1339	HD13	ILE	84	28.028	42.643	47.224	1.00	0.00	H
ATOM	1340	C	ILE	84	24.109	45.599	48.912	1.00	0.00	C
ATOM	1341	O	ILE	84	23.088	45.002	48.574	1.00	0.00	O
ATOM	1342	N	ALA	85	24.185	46.113	50.149	1.00	0.00	N
ATOM	1343	H	ALA	85	25.027	46.653	50.292	1.00	0.00	H
ATOM	1344	CA	ALA	85	23.017	46.232	51.030	1.00	0.00	C
ATOM	1345	HA	ALA	85	22.704	45.224	51.304	1.00	0.00	H
ATOM	1346	CB	ALA	85	23.574	46.949	52.257	1.00	0.00	C
ATOM	1347	HB1	ALA	85	24.425	46.345	52.576	1.00	0.00	H
ATOM	1348	HB2	ALA	85	23.805	47.971	51.957	1.00	0.00	H
ATOM	1349	HB3	ALA	85	22.784	46.850	53.000	1.00	0.00	H
ATOM	1350	C	ALA	85	21.784	47.034	50.523	1.00	0.00	C
ATOM	1351	O	ALA	85	20.714	46.952	51.129	1.00	0.00	O
ATOM	1352	N	ASP	86	21.885	47.751	49.368	1.00	0.00	N
ATOM	1353	H	ASP	86	22.777	47.802	48.895	1.00	0.00	H
ATOM	1354	CA	ASP	86	20.726	48.347	48.662	1.00	0.00	C
ATOM	1355	HA	ASP	86	20.131	48.853	49.421	1.00	0.00	H

ATOM	1356	CB	ASP	86	21.281	49.380	47.699	1.00	0.00	C
ATOM	1357	HB2	ASP	86	22.135	49.908	48.123	1.00	0.00	H
ATOM	1358	HB3	ASP	86	21.665	48.830	46.841	1.00	0.00	H
ATOM	1359	CG	ASP	86	20.199	50.418	47.345	1.00	0.00	C
ATOM	1360	OD1	ASP	86	19.547	50.872	48.335	1.00	0.00	O
ATOM	1361	OD2	ASP	86	20.162	50.888	46.227	1.00	0.00	O
ATOM	1362	C	ASP	86	19.889	47.279	47.948	1.00	0.00	C
ATOM	1363	O	ASP	86	18.719	47.485	47.659	1.00	0.00	O
ATOM	1364	N	ILE	87	20.501	46.201	47.586	1.00	0.00	N
ATOM	1365	H	ILE	87	21.437	46.083	47.948	1.00	0.00	H
ATOM	1366	CA	ILE	87	19.875	45.112	46.763	1.00	0.00	C
ATOM	1367	HA	ILE	87	18.969	45.549	46.343	1.00	0.00	H
ATOM	1368	CB	ILE	87	21.004	44.731	45.689	1.00	0.00	C
ATOM	1369	HB	ILE	87	21.876	44.225	46.103	1.00	0.00	H
ATOM	1370	CG2	ILE	87	20.583	43.557	44.740	1.00	0.00	C
ATOM	1371	HG21	ILE	87	21.334	43.296	43.995	1.00	0.00	H
ATOM	1372	HG22	ILE	87	20.440	42.675	45.365	1.00	0.00	H
ATOM	1373	HG23	ILE	87	19.660	43.854	44.244	1.00	0.00	H
ATOM	1374	CG1	ILE	87	21.263	45.969	44.798	1.00	0.00	C
ATOM	1375	HG12	ILE	87	20.371	46.174	44.206	1.00	0.00	H
ATOM	1376	HG13	ILE	87	21.470	46.819	45.448	1.00	0.00	H
ATOM	1377	CD1	ILE	87	22.498	45.821	43.915	1.00	0.00	C
ATOM	1378	HD11	ILE	87	22.465	44.902	43.330	1.00	0.00	H
ATOM	1379	HD12	ILE	87	22.615	46.588	43.149	1.00	0.00	H
ATOM	1380	HD13	ILE	87	23.408	45.823	44.515	1.00	0.00	H
ATOM	1381	C	ILE	87	19.413	43.952	47.639	1.00	0.00	C
ATOM	1382	O	ILE	87	18.574	43.158	47.238	1.00	0.00	O
ATOM	1383	N	TYR	88	20.120	43.756	48.756	1.00	0.00	N
ATOM	1384	H	TYR	88	20.760	44.495	49.008	1.00	0.00	H
ATOM	1385	CA	TYR	88	20.131	42.591	49.604	1.00	0.00	C
ATOM	1386	HA	TYR	88	19.383	41.922	49.181	1.00	0.00	H
ATOM	1387	CB	TYR	88	21.552	41.897	49.616	1.00	0.00	C
ATOM	1388	HB2	TYR	88	22.294	42.683	49.750	1.00	0.00	H
ATOM	1389	HB3	TYR	88	21.606	41.185	50.440	1.00	0.00	H
ATOM	1390	CG	TYR	88						
					21.724	41.238	48.280	1.00	0.00	C
ATOM	1391	CD1	TYR	88	21.032	40.036	47.934	1.00	0.00	C
ATOM	1392	HD1	TYR	88	20.285	39.542	48.537	1.00	0.00	H
ATOM	1393	CE1	TYR	88	21.351	39.359	46.747	1.00	0.00	C
ATOM	1394	HE1	TYR	88	20.781	38.540	46.331	1.00	0.00	H
ATOM	1395	CZ	TYR	88	22.311	39.927	45.898	1.00	0.00	C
ATOM	1396	OH	TYR	88	22.682	39.352	44.702	1.00	0.00	O
ATOM	1397	HH	TYR	88	22.074	38.621	44.571	1.00	0.00	H
ATOM	1398	CE2	TYR	88	22.946	41.172	46.187	1.00	0.00	C
ATOM	1399	HE2	TYR	88	23.613	41.712	45.531	1.00	0.00	H
ATOM	1400	CD2	TYR	88	22.678	41.810	47.410	1.00	0.00	C
ATOM	1401	HD2	TYR	88	23.282	42.649	47.724	1.00	0.00	H
ATOM	1402	C	TYR	88	19.654	42.913	51.041	1.00	0.00	C
ATOM	1403	O	TYR	88	20.226	43.792	51.697	1.00	0.00	O
ATOM	1404	N	PRO	89	18.603	42.253	51.559	1.00	0.00	N
ATOM	1405	CD	PRO	89	17.591	41.394	50.832	1.00	0.00	C
ATOM	1406	HD2	PRO	89	18.097	40.581	50.311	1.00	0.00	H
ATOM	1407	HD3	PRO	89	17.014	41.926	50.076	1.00	0.00	H
ATOM	1408	CG	PRO	89	16.732	40.827	51.945	1.00	0.00	C
ATOM	1409	HG2	PRO	89	17.098	39.846	52.247	1.00	0.00	H
ATOM	1410	HG3	PRO	89	15.701	40.764	51.597	1.00	0.00	H
ATOM	1411	CB	PRO	89	16.857	41.729	53.052	1.00	0.00	C
ATOM	1412	HB2	PRO	89	16.748	41.241	54.021	1.00	0.00	H
ATOM	1413	HB3	PRO	89	16.080	42.492	52.987	1.00	0.00	H
ATOM	1414	CA	PRO	89	18.266	42.383	52.972	1.00	0.00	C
ATOM	1415	HA	PRO	89	18.148	43.369	53.420	1.00	0.00	H
ATOM	1416	C	PRO	89	19.218	41.619	53.847	1.00	0.00	C
ATOM	1417	O	PRO	89	19.772	40.593	53.458	1.00	0.00	O
ATOM	1418	N	GLY	90	19.550	42.125	55.059	1.00	0.00	N

ATOM	1419	H	GLY	90	19.083	42.894	55.517	1.00	0.00	H
ATOM	1420	CA	GLY	90	20.841	41.748	55.697	1.00	0.00	C
ATOM	1421	HA2	GLY	90	21.668	42.091	55.076	1.00	0.00	H
ATOM	1422	HA3	GLY	90	20.828	42.201	56.689	1.00	0.00	H
ATOM	1423	C	GLY	90	21.159	40.260	56.070	1.00	0.00	C
ATOM	1424	O	GLY	90	22.306	39.811	56.026	1.00	0.00	O
ATOM	1425	N	GLN	91	20.094	39.529	56.455	1.00	0.00	N
ATOM	1426	H	GLN	91	19.192	39.971	56.344	1.00	0.00	H
ATOM	1427	CA	GLN	91	20.101	38.085	56.609	1.00	0.00	C
ATOM	1428	HA	GLN	91	20.890	37.854	57.325	1.00	0.00	H
ATOM	1429	CB	GLN	91	18.747	37.545	57.176	1.00	0.00	C
ATOM	1430	HB2	GLN	91	18.825	36.536	57.580	1.00	0.00	H
ATOM	1431	HB3	GLN	91	18.580	38.075	58.113	1.00	0.00	H
ATOM	1432	CG	GLN	91	17.497	37.719	56.251	1.00	0.00	C
ATOM	1433	HG2	GLN	91	17.058	38.709	56.131	1.00	0.00	H
ATOM	1434	HG3	GLN	91	17.844	37.402	55.267	1.00	0.00	H
ATOM	1435	CD	GLN	91	16.444	36.768	56.759	1.00	0.00	C
ATOM	1436	OE1	GLN	91	16.221	35.764	56.138	1.00	0.00	O
ATOM	1437	NE2	GLN	91	15.732	37.029	57.827	1.00	0.00	N
ATOM	1438	HE21	GLN	91	16.061	37.723	58.484	1.00	0.00	H
ATOM	1439	HE22	GLN	91	15.096	36.293	58.101	1.00	0.00	H
ATOM	1440	C	GLN	91	20.482	37.321	55.277	1.00	0.00	C
ATOM	1441	O	GLN	91	20.531	36.078	55.253	1.00	0.00	O
ATOM	1442	N	THR	92	20.820	37.946	54.147	1.00	0.00	N
ATOM	1443	H	THR	92	20.860	38.956	54.169	1.00	0.00	H
ATOM	1444	CA	THR	92	21.389	37.301	52.966	1.00	0.00	C
ATOM	1445	HA	THR	92	20.719	36.543	52.561	1.00	0.00	H
ATOM	1446	CB	THR	92	21.483	38.272	51.755	1.00	0.00	C
ATOM	1447	HB	THR	92	22.165	39.078	52.024	1.00	0.00	H
ATOM	1448	CG2	THR	92	22.011	37.509	50.521	1.00	0.00	C
ATOM	1449	HG21	THR	92	23.078	37.291	50.547	1.00	0.00	H
ATOM	1450	HG22	THR	92	21.467	36.567	50.446	1.00	0.00	H
ATOM	1451	HG23	THR	92	21.774	38.039	49.598	1.00	0.00	H
ATOM	1452	OG1	THR	92	20.239	38.859	51.414	1.00	0.00	O
ATOM	1453	HG1	THR	92	19.964	39.502	52.072	1.00	0.00	H
ATOM	1454	C	THR	92	22.832	36.726	53.271	1.00	0.00	C
ATOM	1455	O	THR	92	23.752	37.506	53.643	1.00	0.00	O
ATOM	1456	N	GLN	93	22.996	35.383	53.032	1.00	0.00	N
ATOM	1457	H	GLN	93	22.139	34.849	52.993	1.00	0.00	H
ATOM	1458	CA	GLN	93	24.200	34.624	52.864	1.00	0.00	C
ATOM	1459	HA	GLN	93	25.025	35.074	53.418	1.00	0.00	H
ATOM	1460	CB	GLN	93	23.969	33.156	53.279	1.00	0.00	C
ATOM	1461	HB2	GLN	93	23.492	33.153	54.259	1.00	0.00	H
ATOM	1462	HB3	GLN	93	23.201	32.768	52.610	1.00	0.00	H
ATOM	1463	CG	GLN	93	25.269	32.377	53.257	1.00	0.00	C
ATOM	1464	HG2	GLN	93	24.959	31.349	53.070	1.00	0.00	H
ATOM	1465	HG3	GLN	93	25.925	32.644	52.428	1.00	0.00	H
ATOM	1466	CD	GLN	93	26.133	32.468	54.558	1.00	0.00	C
ATOM	1467	OE1	GLN	93	25.675	33.058	55.567	1.00	0.00	O
ATOM	1468	NE2	GLN	93	27.313	31.997	54.649	1.00	0.00	N
ATOM	1469	HE21	GLN	93	27.692	31.527	53.839	1.00	0.00	H
ATOM	1470	HE22	GLN	93	27.820	31.951	55.521	1.00	0.00	H
ATOM	1471	C	GLN	93	24.641	34.681	51.411	1.00	0.00	C
ATOM	1472	O	GLN	93	23.785	34.499	50.516	1.00	0.00	O
ATOM	1473	N	PHE	94	25.919	35.048	51.150	1.00	0.00	N
ATOM	1474	H	PHE	94	26.572	35.238	51.896	1.00	0.00	H
ATOM	1475	CA	PHE	94	26.596	35.108	49.883	1.00	0.00	C
ATOM	1476	HA	PHE	94	25.863	35.098	49.077	1.00	0.00	H
ATOM	1477	CB	PHE	94	27.411	36.344	49.732	1.00	0.00	C
ATOM	1478	HB2	PHE	94	28.185	36.416	50.497	1.00	0.00	H
ATOM	1479	HB3	PHE	94	27.982	36.490	48.816	1.00	0.00	H
ATOM	1480	CG	PHE	94	26.508	37.544	49.849	1.00	0.00	C
ATOM	1481	CD1	PHE	94	25.963	38.170	48.709	1.00	0.00	C
ATOM	1482	HD1	PHE	94	26.195	37.869	47.697	1.00	0.00	H

```
ATOM   1483  CE1 PHE    94      25.017  39.233  48.845  1.00  0.00           C
ATOM   1484  HE1 PHE    94      24.531  39.628  47.965  1.00  0.00           H
ATOM   1485  CZ  PHE    94      24.758  39.780  50.152  1.00  0.00           C
ATOM   1486  HZ  PHE    94      23.988  40.537  50.209  1.00  0.00           H
ATOM   1487  CE2 PHE    94      25.411  39.227  51.293  1.00  0.00           C
ATOM   1488  HE2 PHE    94      25.086  39.580  52.261  1.00  0.00           H
ATOM   1489  CD2 PHE    94      26.303  38.088  51.155  1.00  0.00           C
ATOM   1490  HD2 PHE    94      26.781  37.679  52.032  1.00  0.00           H
ATOM   1491  C   PHE    94      27.573  33.909  49.640  1.00  0.00           C
ATOM   1492  O   PHE    94      27.957  33.212  50.586  1.00  0.00           O
ATOM   1493  N   TYR    95      27.978  33.754  48.341  1.00  0.00           N
ATOM   1494  H   TYR    95      27.572  34.406  47.685  1.00  0.00           H
ATOM   1495  CA  TYR    95      29.058  32.874  47.891  1.00  0.00           C
ATOM   1496  HA  TYR    95      29.539  32.352  48.718  1.00  0.00           H
ATOM   1497  CB  TYR    95      28.364  31.659  47.122  1.00  0.00           C
ATOM   1498  HB2 TYR    95      27.551  31.308  47.756  1.00  0.00           H
ATOM   1499  HB3 TYR    95      27.936  32.043  46.195  1.00  0.00           H
ATOM   1500  CG  TYR    95      29.282  30.489  46.863  1.00  0.00           C
ATOM   1501  CD1 TYR    95      29.694  30.190  45.577  1.00  0.00           C
ATOM   1502  HD1 TYR    95      29.454  30.809  44.725  1.00  0.00           H
ATOM   1503  CE1 TYR    95      30.381  29.036  45.269  1.00  0.00           C
ATOM   1504  HE1 TYR    95      30.781  28.851  44.284  1.00  0.00           H
ATOM   1505  CZ  TYR    95      30.776  28.178  46.320  1.00  0.00           C
ATOM   1506  OH  TYR    95      31.589  27.129  46.057  1.00  0.00           O
ATOM   1507  HH  TYR    95      31.608  26.905  45.124  1.00  0.00           H
ATOM   1508  CE2 TYR    95      30.361  28.470  47.620  1.00  0.00           C
ATOM   1509  HE2 TYR    95      30.533  27.757  48.412  1.00  0.00           H
ATOM   1510  CD2 TYR    95      29.618  29.599  47.914  1.00  0.00           C
ATOM   1511  HD2 TYR    95      29.470  29.708  48.979  1.00  0.00           H
ATOM   1512  C   TYR    95      30.105  33.598  47.033  1.00  0.00           C
ATOM   1513  O   TYR    95      29.762  34.495  46.313  1.00  0.00           O
ATOM   1514  N   VAL    96      31.368  33.263  47.219  1.00  0.00           N
ATOM   1515  H   VAL    96      31.545  32.566  47.929
  1.00  0.00           H
ATOM   1516  CA  VAL    96      32.505  33.834  46.433  1.00  0.00           C
ATOM   1517  HA  VAL    96      32.150  34.682  45.848  1.00  0.00           H
ATOM   1518  CB  VAL    96      33.636  34.460  47.349  1.00  0.00           C
ATOM   1519  HB  VAL    96      34.232  33.601  47.658  1.00  0.00           H
ATOM   1520  CG1 VAL    96      34.633  35.414  46.666  1.00  0.00           C
ATOM   1521 HG11 VAL    96      34.167  36.260  46.162  1.00  0.00           H
ATOM   1522 HG12 VAL    96      35.386  35.668  47.413  1.00  0.00           H
ATOM   1523 HG13 VAL    96      35.116  34.936  45.814  1.00  0.00           H
ATOM   1524  CG2 VAL    96      33.099  35.165  48.609  1.00  0.00           C
ATOM   1525 HG21 VAL    96      33.871  35.798  49.047  1.00  0.00           H
ATOM   1526 HG22 VAL    96      32.143  35.629  48.371  1.00  0.00           H
ATOM   1527 HG23 VAL    96      32.924  34.292  49.239  1.00  0.00           H
ATOM   1528  C   VAL    96      33.174  32.858  45.489  1.00  0.00           C
ATOM   1529  O   VAL    96      33.475  31.753  45.884  1.00  0.00           O
ATOM   1530  N   ILE    97      33.393  33.205  44.263  1.00  0.00           N
ATOM   1531  H   ILE    97      33.038  34.114  44.005  1.00  0.00           H
ATOM   1532  CA  ILE    97      34.165  32.474  43.222  1.00  0.00           C
ATOM   1533  HA  ILE    97      34.571  31.547  43.625  1.00  0.00           H
ATOM   1534  CB  ILE    97      33.199  32.249  42.032  1.00  0.00           C
ATOM   1535  HB  ILE    97      33.040  33.160  41.454  1.00  0.00           H
ATOM   1536  CG2 ILE    97      33.865  31.199  41.100  1.00  0.00           C
ATOM   1537 HG21 ILE    97      34.782  31.506  40.597  1.00  0.00           H
ATOM   1538 HG22 ILE    97      33.964  30.214  41.555  1.00  0.00           H
ATOM   1539 HG23 ILE    97      33.184  30.988  40.275  1.00  0.00           H
ATOM   1540  CG1 ILE    97      31.737  31.691  42.438  1.00  0.00           C
ATOM   1541 HG12 ILE    97      31.447  31.024  41.627  1.00  0.00           H
ATOM   1542 HG13 ILE    97      31.750  31.112  43.362  1.00  0.00           H
ATOM   1543  CD1 ILE    97      30.672  32.808  42.475  1.00  0.00           C
ATOM   1544 HD11 ILE    97      30.499  33.242  41.491  1.00  0.00           H
ATOM   1545 HD12 ILE    97      29.787  32.382  42.949  1.00  0.00           H
```

ATOM	1546	HD13	ILE	97	31.060	33.596	43.121	1.00	0.00	H
ATOM	1547	C	ILE	97	35.361	33.264	42.789	1.00	0.00	C
ATOM	1548	O	ILE	97	35.259	34.330	42.179	1.00	0.00	O
ATOM	1549	N	GLU	98	36.616	32.873	43.035	1.00	0.00	N
ATOM	1550	H	GLU	98	36.797	31.999	43.507	1.00	0.00	H
ATOM	1551	CA	GLU	98	37.874	33.525	42.593	1.00	0.00	C
ATOM	1552	HA	GLU	98	37.680	34.508	42.162	1.00	0.00	H
ATOM	1553	CB	GLU	98	38.693	33.693	43.946	1.00	0.00	C
ATOM	1554	HB2	GLU	98	38.187	34.400	44.603	1.00	0.00	H
ATOM	1555	HB3	GLU	98	38.864	32.741	44.447	1.00	0.00	H
ATOM	1556	CG	GLU	98	40.063	34.295	43.555	1.00	0.00	C
ATOM	1557	HG2	GLU	98	40.699	33.527	43.114	1.00	0.00	H
ATOM	1558	HG3	GLU	98	39.912	35.142	42.887	1.00	0.00	H
ATOM	1559	CD	GLU	98	40.757	34.896	44.793	1.00	0.00	C
ATOM	1560	OE1	GLU	98	41.402	35.906	44.634	1.00	0.00	O
ATOM	1561	OE2	GLU	98	40.564	34.544	45.938	1.00	0.00	O
ATOM	1562	C	GLU	98	38.625	32.654	41.559	1.00	0.00	C
ATOM	1563	O	GLU	98	38.696	31.429	41.701	1.00	0.00	O
ATOM	1564	N	PHE	99	39.043	33.207	40.391	1.00	0.00	N
ATOM	1565	H	PHE	99	38.973	34.214	40.371	1.00	0.00	H
ATOM	1566	CA	PHE	99	39.333	32.476	39.152	1.00	0.00	C
ATOM	1567	HA	PHE	99	39.814	31.566	39.512	1.00	0.00	H
ATOM	1568	CB	PHE	99	38.014	32.150	38.406	1.00	0.00	C
ATOM	1569	HB2	PHE	99	38.252	31.592	37.500	1.00	0.00	H
ATOM	1570	HB3	PHE	99	37.366	31.496	38.988	1.00	0.00	H
ATOM	1571	CG	PHE	99	37.178	33.354	38.025	1.00	0.00	C
ATOM	1572	CD1	PHE	99	37.530	34.206	36.970	1.00	0.00	C
ATOM	1573	HD1	PHE	99	38.285	33.918	36.253	1.00	0.00	H
ATOM	1574	CE1	PHE	99	36.722	35.304	36.664	1.00	0.00	C
ATOM	1575	HE1	PHE	99	37.001	35.953	35.847	1.00	0.00	H
ATOM	1576	CZ	PHE	99	35.647	35.630	37.537	1.00	0.00	C
ATOM	1577	HZ	PHE	99	34.979	36.464	37.380	1.00	0.00	H
ATOM	1578	CE2	PHE	99	35.545	34.927	38.706	1.00	0.00	C
ATOM	1579	HE2	PHE	99	34.877	35.285	39.476	1.00	0.00	H
ATOM	1580	CD2	PHE	99	36.227	33.780	38.958	1.00	0.00	C
ATOM	1581	HD2	PHE	99	35.977	33.289	39.887	1.00	0.00	H
ATOM	1582	C	PHE	99	40.403	33.146	38.218	1.00	0.00	C
ATOM	1583	O	PHE	99	40.504	34.382	38.244	1.00	0.00	O
ATOM	1584	N	LYS	100	41.181	32.345	37.455	1.00	0.00	N
ATOM	1585	H	LYS	100	40.996	31.353	37.502	1.00	0.00	H
ATOM	1586	CA	LYS	100	42.245	32.866	36.613	1.00	0.00	C
ATOM	1587	HA	LYS	100	42.032	33.907	36.371	1.00	0.00	H
ATOM	1588	CB	LYS	100	43.636	32.647	37.303	1.00	0.00	C
ATOM	1589	HB2	LYS	100	43.816	31.581	37.444	1.00	0.00	H
ATOM	1590	HB3	LYS	100	44.425	32.933	36.608	1.00	0.00	H
ATOM	1591	CG	LYS	100	43.686	33.564	38.554	1.00	0.00	C
ATOM	1592	HG2	LYS	100	43.318	34.567	38.340	1.00	0.00	H
ATOM	1593	HG3	LYS	100	43.122	33.088	39.356	1.00	0.00	H
ATOM	1594	CD	LYS	100	45.112	33.811	39.066	1.00	0.00	C
ATOM	1595	HD2	LYS	100	45.691	32.916	39.294	1.00	0.00	H
ATOM	1596	HD3	LYS	100	45.610	34.320	38.241	1.00	0.00	H
ATOM	1597	CE	LYS	100	45.184	34.865	40.163	1.00	0.00	C
ATOM	1598	HE2	LYS	100	46.231	35.105	40.346	1.00	0.00	H
ATOM	1599	HE3	LYS	100	44.610	35.729	39.828	1.00	0.00	H
ATOM	1600	NZ	LYS	100	44.604	34.275	41.425	1.00	0.00	N
ATOM	1601	HZ1	LYS	100	43.612	34.465	41.420	1.00	0.00	H
ATOM	1602	HZ2	LYS	100	44.807	33.287	41.477	1.00	0.00	H
ATOM	1603	HZ3	LYS	100	44.932	34.677	42.291	1.00	0.00	H
ATOM	1604	C	LYS	100	42.372	32.075	35.296	1.00	0.00	C
ATOM	1605	O	LYS	100	42.221	30.836	35.286	1.00	0.00	O
ATOM	1606	N	CYX	101	42.774	32.752	34.273	1.00	0.00	N
ATOM	1607	H	CYX	101	43.159	33.671	34.440	1.00	0.00	H
ATOM	1608	CA	CYX	101	42.944	32.224	32.934	1.00	0.00	C
ATOM	1609	HA	CYX	101	42.270	31.416	32.647	1.00	0.00	H

```
ATOM   1610  CB  CYX   101      42.739  33.347  31.960  1.00  0.00           C
ATOM   1611  HB2 CYX   101      41.709  33.680  32.089  1.00  0.00           H
ATOM   1612  HB3 CYX   101      43.359  34.232  32.104  1.00  0.00           H
ATOM   1613  SG  CYX   101      42.896  32.679  30.307  1.00  0.00           S
ATOM   1614  C   CYX   101      44.281  31.464  32.857  1.00  0.00           C
ATOM   1615  O   CYX   101      45.252  31.919  33.480  1.00  0.00           O
ATOM   1616  N   LEU   102      44.290  30.243  32.227  1.00  0.00           N
ATOM   1617  H   LEU   102      43.405  29.824  31.976  1.00  0.00           H
ATOM   1618  CA  LEU   102      45.438  29.355  32.023  1.00  0.00           C
ATOM   1619  HA  LEU   102      46.060  29.518  32.903  1.00  0.00           H
ATOM   1620  CB  LEU   102      44.880  27.957  31.868  1.00  0.00           C
ATOM   1621  HB2 LEU   102      44.368  27.819  30.916  1.00  0.00           H
ATOM   1622  HB3 LEU   102      45.686  27.225  31.920  1.00  0.00           H
ATOM   1623  CG  LEU   102      43.834  27.408  32.900  1.00  0.00           C
ATOM   1624  HG  LEU   102      42.936  28.024  32.879  1.00  0.00           H
ATOM   1625  CD1 LEU   102      43.507  25.940  32.620  1.00  0.00           C
ATOM   1626 HD11 LEU   102      42.931  25.959  31.695  1.00  0.00           H
ATOM   1627 HD12 LEU   102      44.344  25.305  32.332  1.00  0.00           H
ATOM   1628 HD13 LEU   102      42.864  25.508  33.387  1.00  0.00           H
ATOM   1629  CD2 LEU   102      44.356  27.446  34.359  1.00  0.00           C
ATOM   1630 HD21 LEU   102      43.559  27.019  34.968  1.00  0.00           H
ATOM   1631 HD22 LEU   102      45.256  26.836  34.434  1.00  0.00           H
ATOM   1632 HD23 LEU   102      44.536  28.503  34.556  1.00  0.00           H
ATOM   1633  C   LEU   102      46.394  29.733  30.901  1.00  0.00           C
ATOM   1634  O   LEU   102      47.608  29.470  31.084  1.00  0.00           O
ATOM   1635  OXT LEU   102      46.003  30.407  29.934  1.00  0.00           O
HETATM 1637  N   LIG   103      29.062  40.669  38.597  1.00  0.00           N
HETATM 1638  C   LIG   103      29.096  39.386  38.675  1.00  0.00           C
HETATM 1639  O   LIG   103      28.246  42.634  39.278  1.00  0.00           O
HETATM 1640  C5' LIG   103      26.632  40.552  45.069  1.00  0.00           C
HETATM 1641  O5' LIG   103      26.288  39.286  44.552  1.00  0.00           O
HETATM
 1642  C4' LIG   103      26.689  41.500  43.865  1.00  0.00           C
HETATM 1643  O4' LIG   103      27.650  41.125  42.890  1.00  0.00           O
HETATM 1644  C3' LIG   103      25.349  41.748  43.175  1.00  0.00           C
HETATM 1645  O3' LIG   103      24.798  43.046  43.435  1.00  0.00           O
HETATM 1646  C2' LIG   103      25.640  41.632  41.636  1.00  0.00           C
HETATM 1647  O2' LIG   103      25.150  42.775  40.908  1.00  0.00           O
HETATM 1648  C1' LIG   103      27.180  41.606  41.676  1.00  0.00           C
HETATM 1649  N1  LIG   103      27.708  40.792  40.570  1.00  0.00           N
HETATM 1650  O1  LIG   103      28.929  36.699  37.407  1.00  0.00           O
HETATM 1651  N2  LIG   103      29.900  38.761  37.771  1.00  0.00           N
HETATM 1652  C6  LIG   103      28.273  41.418  39.494  1.00  0.00           C
HETATM 1653  C7  LIG   103      27.716  39.451  40.714  1.00  0.00           C
HETATM 1654  C8  LIG   103      28.480  38.712  39.822  1.00  0.00           C
HETATM 1655  C9  LIG   103      29.800  37.540  37.143  1.00  0.00           C
HETATM 1656  C10 LIG   103      30.828  37.142  36.086  1.00  0.00           C
HETATM 1657  H   LIG   103      30.611  39.404  37.445  1.00  0.00           H
HETATM 1658  H1  LIG   103      28.460  37.637  39.952  1.00  0.00           H
HETATM 1659  H4  LIG   103      27.501  42.634  41.468  1.00  0.00           H
HETATM 1660  H6  LIG   103      26.901  42.526  44.192  1.00  0.00           H
HETATM 1661  H7  LIG   103      24.560  41.021  43.406  1.00  0.00           H
HETATM 1662  H8  LIG   103      25.310  40.660  41.250  1.00  0.00           H
HETATM 1663  H9  LIG   103      26.020  40.888  45.915  1.00  0.00           H
HETATM 1664  H10 LIG   103      27.641  40.342  45.445  1.00  0.00           H
HETATM 1665  H11 LIG   103      31.433  36.261  36.337  1.00  0.00           H
HETATM 1666  H12 LIG   103      31.559  37.958  36.040  1.00  0.00           H
HETATM 1667  H13 LIG   103      30.452  37.167  35.057  1.00  0.00           H
HETATM 1668  H14 LIG   103      27.103  39.020  41.494  1.00  0.00           H
HETATM 1669  H2' LIG   103      25.195  42.508  39.974  1.00  0.00           H
HETATM 1670  H3' LIG   103      24.769  43.445  42.548  1.00  0.00           H
HETATM 1671  H5' LIG   103      27.040  39.071  43.970  1.00  0.00           H
ENDMDL
MODEL     7
```

```
SHEET    1   1 1 ILE    22  ASP    26  0
SHEET    2   2 1 VAL    37  VAL    40  0
SHEET    3   3 1 CYX    50  VAL    60  0
SHEET    4   4 1 PHE    94  CYX   101  0
HELIX    1   1 GLN     9  PHE     11  1                                  3
HELIX    2   2 ASP    13  LEU     16  1                                  4
HELIX    3   3 GLU    67  GLU     73  1                                  7
HELIX    4   4 LEU    77  ALA     85  1                                  9
ATOM      1  N   GLN     1      37.088  17.132  32.893  1.00  0.00          N
ATOM      2  H1  GLN     1      36.468  17.061  33.687  1.00  0.00          H
ATOM      3  H2  GLN     1      37.318  16.196  32.592  1.00  0.00          H
ATOM      4  H3  GLN     1      37.939  17.628  33.118  1.00  0.00          H
ATOM      5  CA  GLN     1      36.368  17.870  31.852  1.00  0.00          C
ATOM      6  HA  GLN     1      37.086  17.940  31.035  1.00  0.00          H
ATOM      7  CB  GLN     1      35.084  17.193  31.320  1.00  0.00          C
ATOM      8  HB2 GLN     1      34.555  16.941  32.239  1.00  0.00          H
ATOM      9  HB3 GLN     1      34.468  17.854  30.710  1.00  0.00          H
ATOM     10  CG  GLN     1      35.446  15.958  30.477  1.00  0.00          C
ATOM     11  HG2 GLN     1      36.172  15.345  31.012  1.00  0.00          H
ATOM     12  HG3 GLN     1      34.539  15.381  30.297  1.00  0.00          H
ATOM     13  CD  GLN     1      36.098  16.211  29.152  1.00  0.00          C
ATOM     14  OE1 GLN     1      36.590  17.330  28.835  1.00  0.00          O
ATOM     15  NE2 GLN     1      36.238  15.336  28.219  1.00  0.00          N
ATOM     16 HE21 GLN     1      35.808  14.421  28.230  1.00  0.00          H
ATOM     17 HE22 GLN     1      36.474  15.787  27.347  1.00  0.00          H
ATOM     18  C   GLN     1      36.014  19.222  32.332  1.00  0.00          C
ATOM     19  O   GLN     1      35.440  19.248  33.404  1.00  0.00          O
ATOM     20  N   PRO     2      36.244  20.297  31.576  1.00  0.00          N
ATOM     21  CD  PRO     2      37.017  20.293  30.340  1.00  0.00          C
ATOM     22  HD2 PRO     2      36.344  20.002  29.535  1.00  0.00          H
ATOM     23  HD3 PRO     2      37.926  19.695  30.396  1.00  0.00          H
ATOM     24  CG  PRO     2      37.411  21.749  30.230  1.00  0.00          C
ATOM     25  HG2 PRO     2      37.668  21.988  29.198  1.00  0.00          H
ATOM     26  HG3 PRO     2      38.307  21.823  30.845  1.00  0.00          H
ATOM     27  CB  PRO     2      36.209  22.562  30.782  1.00  0.00          C
ATOM     28  HB2 PRO     2      35.663  22.732  29.854  1.00  0.00          H
ATOM     29  HB3 PRO     2      36.489  23.482  31.293  1.00  0.00          H
ATOM     30  CA  PRO     2      35.631  21.654  31.826  1.00  0.00          C
ATOM     31  HA  PRO     2      35.821  22.007  32.839  1.00  0.00          H
ATOM     32  C   PRO     2      34.156  21.676  31.747  1.00  0.00          C
ATOM     33  O   PRO     2      33.562  20.730  31.248  1.00  0.00          O
ATOM     34  N   ASN     3      33.577  22.730  32.310  1.00  0.00          N
ATOM     35  H   ASN     3      34.118  23.485  32.706  1.00  0.00          H
ATOM     36  CA  ASN     3      32.156  22.837  32.565  1.00  0.00          C
ATOM     37  HA  ASN     3      31.883  21.868  32.983  1.00  0.00          H
ATOM     38  CB  ASN     3      32.017  24.059  33.493  1.00  0.00          C
ATOM     39  HB2 ASN     3      32.644  24.903  33.208  1.00  0.00          H
ATOM     40  HB3 ASN     3      30.995  24.397  33.321  1.00  0.00          H
ATOM     41  CG  ASN     3      32.290  23.700  34.992  1.00  0.00          C
ATOM     42  OD1 ASN     3      32.534  22.551  35.274  1.00  0.00          O
ATOM     43  ND2 ASN     3      32.075  24.605  35.939  1.00  0.00          N
ATOM     44 HD21 ASN     3      31.996  25.583  35.701  1.00  0.00          H
ATOM     45 HD22 ASN     3      32.104  24.276  36.893  1.00  0.00          H
ATOM     46  C   ASN     3      31.352  23.026  31.210  1.00  0.00          C
ATOM     47  O   ASN     3      31.827  23.779  30.359  1.00  0.00          O
ATOM     48  N   ASP     4      30.106  22.642  31.040  1.00  0.00          N
ATOM     49  H   ASP     4      29.603  22.247  31.822  1.00  0.00          H
ATOM     50  CA  ASP     4      29.237  23.093  29.942  1.00  0.00          C
ATOM     51  HA  ASP     4      29.856  23.224  29.055  1.00  0.00          H
ATOM     52  CB  ASP     4      28.234  21.899  29.525  1.00  0.00          C
ATOM     53  HB2 ASP     4      27.511  21.809  30.335  1.00  0.00          H
ATOM     54  HB3 ASP     4      27.669  22.317  28.691  1.00  0.00          H
ATOM     55  CG  ASP     4      28.861  20.551  29.129  1.00  0.00          C
ATOM     56  OD1 ASP     4      30.055  20.478  28.667  1.00  0.00          O
```

ATOM	57	OD2	ASP	4	28.152	19.491	29.331	1.00	0.00	O
ATOM	58	C	ASP	4	28.502	24.440	30.222	1.00	0.00	C
ATOM	59	O	ASP	4	27.939	24.958	29.261	1.00	0.00	O
ATOM	60	N	ILE	5	28.398	24.898	31.425	1.00	0.00	N
ATOM	61	H	ILE	5	28.895	24.357	32.119	1.00	0.00	H
ATOM	62	CA	ILE	5	28.090	26.330	31.838	1.00	0.00	C
ATOM	63	HA	ILE	5	27.514	26.790	31.035	1.00	0.00	H
ATOM	64	CB	ILE	5	27.161	26.195	33.112	1.00	0.00	C
ATOM	65	HB	ILE	5	27.605	25.621	33.927	1.00	0.00	H
ATOM	66	CG2	ILE	5	26.818	27.492	33.809	1.00	0.00	C
ATOM	67	HG21	ILE	5	26.136	27.230	34.618	1.00	0.00	H
ATOM	68	HG22	ILE	5	27.704	27.922	34.277	1.00	0.00	H
ATOM	69	HG23	ILE	5	26.317	28.130	33.080	1.00	0.00	H
ATOM	70	CG1	ILE	5	25.894	25.325	32.872	1.00	0.00	C
ATOM	71	HG12	ILE	5	26.118	24.300	32.575	1.00	0.00	H
ATOM	72	HG13	ILE	5	25.294	25.193	33.771	1.00	0.00	H
ATOM	73	CD1	ILE	5	24.957	26.022	31.878	1.00	0.00	C
ATOM	74	HD11	ILE	5	23.950	25.610	31.947	1.00	0.00	H
ATOM	75	HD12	ILE	5	24.858	27.095	32.044	1.00	0.00	H
ATOM	76	HD13	ILE	5	25.359	25.920	30.870	1.00	0.00	H
ATOM	77	C	ILE	5	29.298	27.308	31.980	1.00	0.00	C
ATOM	78	O	ILE	5	30.379	26.868	32.354	1.00	0.00	O
ATOM	79	N	THR	6	29.186	28.648	31.759	1.00	0.00	N
ATOM	80	H	THR	6	28.300	29.058	31.502	1.00	0.00	H
ATOM	81	CA	THR	6	30.293	29.635	31.895	1.00	0.00	C
ATOM	82	HA	THR	6	30.876	29.230	32.722	1.00	0.00	H
ATOM	83	CB	THR	6	31.175	29.546	30.666	1.00	0.00	C
ATOM	84	HB	THR	6	31.332	28.479	30.507	1.00	0.00	H
ATOM	85	CG2	THR	6	30.595	30.202	29.380	1.00	0.00	C
ATOM	86	HG21	THR	6	29.527	30.010	29.280	1.00	0.00	H
ATOM	87	HG22	THR	6	30.814	31.251	29.579	1.00	0.00	H
ATOM	88	HG23	THR	6	31.251	29.892	28.566	1.00	0.00	H
ATOM	89	OG1	THR	6	32.419	30.192	30.884	1.00	0.00	O
ATOM	90	HG1								
THR	6		32.930	30.009	30.092	1.00	0.00		H	
ATOM	91	C	THR	6	29.763	30.988	32.241	1.00	0.00	C
ATOM	92	O	THR	6	28.520	31.211	32.295	1.00	0.00	O
ATOM	93	N	PHE	7	30.584	31.994	32.323	1.00	0.00	N
ATOM	94	H	PHE	7	31.555	31.909	32.058	1.00	0.00	H
ATOM	95	CA	PHE	7	30.163	33.418	32.406	1.00	0.00	C
ATOM	96	HA	PHE	7	29.553	33.557	33.298	1.00	0.00	H
ATOM	97	CB	PHE	7	31.439	34.344	32.673	1.00	0.00	C
ATOM	98	HB2	PHE	7	32.032	34.315	31.759	1.00	0.00	H
ATOM	99	HB3	PHE	7	31.069	35.327	32.967	1.00	0.00	H
ATOM	100	CG	PHE	7	32.326	33.906	33.867	1.00	0.00	C
ATOM	101	CD1	PHE	7	31.966	33.999	35.201	1.00	0.00	C
ATOM	102	HD1	PHE	7	31.002	34.312	35.576	1.00	0.00	H
ATOM	103	CE1	PHE	7	32.893	33.593	36.231	1.00	0.00	C
ATOM	104	HE1	PHE	7	32.551	33.507	37.252	1.00	0.00	H
ATOM	105	CZ	PHE	7	34.231	33.326	35.960	1.00	0.00	C
ATOM	106	HZ	PHE	7	34.808	32.946	36.790	1.00	0.00	H
ATOM	107	CE2	PHE	7	34.635	33.409	34.583	1.00	0.00	C
ATOM	108	HE2	PHE	7	35.634	33.181	34.244	1.00	0.00	H
ATOM	109	CD2	PHE	7	33.729	33.643	33.578	1.00	0.00	C
ATOM	110	HD2	PHE	7	34.103	33.749	32.570	1.00	0.00	H
ATOM	111	C	PHE	7	29.222	33.901	31.239	1.00	0.00	C
ATOM	112	O	PHE	7	29.496	33.518	30.108	1.00	0.00	O
ATOM	113	N	PHE	8	28.357	34.847	31.484	1.00	0.00	N
ATOM	114	H	PHE	8	28.215	35.158	32.434	1.00	0.00	H
ATOM	115	CA	PHE	8	27.701	35.646	30.502	1.00	0.00	C
ATOM	116	HA	PHE	8	27.168	35.073	29.744	1.00	0.00	H
ATOM	117	CB	PHE	8	26.444	36.361	31.074	1.00	0.00	C
ATOM	118	HB2	PHE	8	26.732	36.997	31.912	1.00	0.00	H
ATOM	119	HB3	PHE	8	26.079	36.915	30.209	1.00	0.00	H

ATOM	120	CG	PHE	8	25.266	35.549	31.594	1.00	0.00	C
ATOM	121	CD1	PHE	8	24.727	35.657	32.890	1.00	0.00	C
ATOM	122	HD1	PHE	8	25.032	36.365	33.646	1.00	0.00	H
ATOM	123	CE1	PHE	8	23.587	34.907	33.155	1.00	0.00	C
ATOM	124	HE1	PHE	8	23.208	35.018	34.160	1.00	0.00	H
ATOM	125	CZ	PHE	8	22.859	34.152	32.208	1.00	0.00	C
ATOM	126	HZ	PHE	8	21.861	33.850	32.492	1.00	0.00	H
ATOM	127	CE2	PHE	8	23.433	33.961	30.981	1.00	0.00	C
ATOM	128	HE2	PHE	8	22.870	33.526	30.169	1.00	0.00	H
ATOM	129	CD2	PHE	8	24.605	34.643	30.677	1.00	0.00	C
ATOM	130	HD2	PHE	8	25.020	34.583	29.682	1.00	0.00	H
ATOM	131	C	PHE	8	28.636	36.550	29.678	1.00	0.00	C
ATOM	132	O	PHE	8	29.702	36.985	30.137	1.00	0.00	O
ATOM	133	N	GLN	9	28.274	36.735	28.451	1.00	0.00	N
ATOM	134	H	GLN	9	27.324	36.461	28.240	1.00	0.00	H
ATOM	135	CA	GLN	9	28.913	37.399	27.312	1.00	0.00	C
ATOM	136	HA	GLN	9	29.845	36.912	27.022	1.00	0.00	H
ATOM	137	CB	GLN	9	28.085	37.296	25.995	1.00	0.00	C
ATOM	138	HB2	GLN	9	28.202	36.239	25.759	1.00	0.00	H
ATOM	139	HB3	GLN	9	27.025	37.540	26.073	1.00	0.00	H
ATOM	140	CG	GLN	9	28.604	38.163	24.814	1.00	0.00	C
ATOM	141	HG2	GLN	9	29.693	38.171	24.857	1.00	0.00	H
ATOM	142	HG3	GLN	9	28.413	37.554	23.931	1.00	0.00	H
ATOM	143	CD	GLN	9	28.005	39.523	24.712	1.00	0.00	C
ATOM	144	OE1	GLN	9	27.393	40.054	25.604	1.00	0.00	O
ATOM	145	NE2	GLN	9	28.311	40.158	23.626	1.00	0.00	N
ATOM	146	HE21	GLN	9	28.026	41.119	23.503	1.00	0.00	H
ATOM	147	HE22	GLN	9	28.700	39.616	22.868	1.00	0.00	H
ATOM	148	C	GLN	9	29.298	38.861	27.751	1.00	0.00	C
ATOM	149	O	GLN	9	30.293	39.416	27.337	1.00	0.00	O
ATOM	150	N	ARG	10	28.480	39.488	28.601	1.00	0.00	N
ATOM	151	H	ARG	10	27.647	38.937	28.756	1.00	0.00	H
ATOM	152	CA	ARG	10	28.729	40.789	29.264	1.00	0.00	C
ATOM	153	HA	ARG	10	28.816	41.472	28.418	1.00	0.00	H
ATOM	154	CB	ARG	10	27.490	41.185	30.008	1.00	0.00	C
ATOM	155	HB2	ARG	10	27.124	40.255	30.443	1.00	0.00	H
ATOM	156	HB3	ARG	10	27.705	41.942	30.762	1.00	0.00	H
ATOM	157	CG	ARG	10	26.420	41.766	29.034	1.00	0.00	C
ATOM	158	HG2	ARG	10	26.270	40.977	28.296	1.00	0.00	H
ATOM	159	HG3	ARG	10	25.517	41.938	29.620	1.00	0.00	H
ATOM	160	CD	ARG	10	26.810	43.065	28.342	1.00	0.00	C
ATOM	161	HD2	ARG	10	27.571	43.513	28.981	1.00	0.00	H
ATOM	162	HD3	ARG	10	27.282	42.884	27.377	1.00	0.00	H
ATOM	163	NE	ARG	10	25.639	44.004	28.341	1.00	0.00	N
ATOM	164	HE	ARG	10	25.772	44.921	28.744	1.00	0.00	H
ATOM	165	CZ	ARG	10	24.757	43.992	27.377	1.00	0.00	C
ATOM	166	NH1	ARG	10	24.528	42.997	26.592	1.00	0.00	N
ATOM	167	HH11	ARG	10	23.825	43.119	25.877	1.00	0.00	H
ATOM	168	HH12	ARG	10	24.992	42.119	26.776	1.00	0.00	H
ATOM	169	NH2	ARG	10	23.959	45.045	27.220	1.00	0.00	N
ATOM	170	HH21	ARG	10	23.837	45.663	28.009	1.00	0.00	H
ATOM	171	HH22	ARG	10	23.198	45.165	26.568	1.00	0.00	H
ATOM	172	C	ARG	10	29.991	41.007	30.212	1.00	0.00	C
ATOM	173	O	ARG	10	30.481	42.144	30.425	1.00	0.00	O
ATOM	174	N	PHE	11	30.578	39.950	30.701	1.00	0.00	N
ATOM	175	H	PHE	11	30.077	39.124	30.402	1.00	0.00	H
ATOM	176	CA	PHE	11	31.860	39.941	31.489	1.00	0.00	C
ATOM	177	HA	PHE	11	32.090	40.938	31.865	1.00	0.00	H
ATOM	178	CB	PHE	11	31.642	39.067	32.683	1.00	0.00	C
ATOM	179	HB2	PHE	11	32.431	39.339	33.385	1.00	0.00	H
ATOM	180	HB3	PHE	11	31.685	38.000	32.466	1.00	0.00	H
ATOM	181	CG	PHE	11	30.405	39.304	33.453	1.00	0.00	C
ATOM	182	CD1	PHE	11	30.147	40.591	33.947	1.00	0.00	C
ATOM	183	HD1	PHE	11	30.785	41.405	33.637	1.00	0.00	H

ATOM	184	CE1	PHE	11	28.998	40.836	34.720	1.00	0.00	C
ATOM	185	HE1	PHE	11	28.681	41.822	35.030	1.00	0.00	H
ATOM	186	CZ	PHE	11	28.037	39.784	34.839	1.00	0.00	C
ATOM	187	HZ	PHE	11	27.162	39.836	35.469	1.00	0.00	H
ATOM	188	CE2	PHE	11	28.216	38.513	34.181	1.00	0.00	C
ATOM	189	HE2	PHE	11	27.527	37.682	34.150	1.00	0.00	H
ATOM	190	CD2	PHE	11	29.398	38.320	33.503	1.00	0.00	C
ATOM	191	HD2	PHE	11	29.606	37.371	33.031	1.00	0.00	H
ATOM	192	C	PHE	11	33.032	39.399	30.686	1.00	0.00	C
ATOM	193	O	PHE	11	34.114	39.482	31.174	1.00	0.00	O
ATOM	194	N	GLN	12	32.871	38.885	29.424	1.00	0.00	N
ATOM	195	H	GLN	12	31.923	38.975	29.087	1.00	0.00	H
ATOM	196	CA	GLN	12	33.872	38.000	28.747	1.00	0.00	C
ATOM	197	HA	GLN	12	34.391	37.440	29.525	1.00	0.00	H
ATOM	198	CB	GLN	12	33.206	36.896	27.888	1.00	0.00	C
ATOM	199	HB2	GLN	12	32.329	37.278	27.365	1.00	0.00	H
ATOM	200	HB3	GLN	12	33.952	36.626	27.140	1.00	0.00	H
ATOM	201	CG	GLN	12	32.773	35.603	28.671	1.00	0.00	C
ATOM	202	HG2	GLN	12	33.524	35.244	29.376	1.00	0.00	H
ATOM	203	HG3	GLN	12	31.933	35.818	29.330	1.00	0.00	H
ATOM	204	CD	GLN	12	32.361	34.395	27.770	1.00	0.00	C
ATOM	205	OE1	GLN	12	33.087	34.016	26.884	1.00	0.00	O
ATOM	206	NE2	GLN	12	31.359	33.564	28.030	1.00	0.00	N
ATOM	207	HE21	GLN	12	30.674	33.888	28.699	1.00	0.00	H
ATOM	208	HE22	GLN	12	31.160	32.975	27.234	1.00	0.00	H
ATOM	209	C	GLN	12	34.900	38.890	28.003	1.00	0.00	C
ATOM	210	O	GLN	12	36.099	38.599	28.035	1.00	0.00	O
ATOM	211	N	ASP	13	34.548	40.041	27.429	1.00	0.00	N
ATOM	212	H	ASP	13	33.565	40.265	27.490	1.00	0.00	H
ATOM	213	CA	ASP	13	35.526	41.046	26.891	1.00	0.00	C
ATOM	214	HA	ASP	13	36.226	40.490	26.268	1.00	0.00	H
ATOM	215	CB	ASP	13	34.806	42.091				
25.971	1.00	0.00			C					
ATOM	216	HB2	ASP	13	34.015	42.573	26.546	1.00	0.00	H
ATOM	217	HB3	ASP	13	35.564	42.831	25.715	1.00	0.00	H
ATOM	218	CG	ASP	13	34.157	41.534	24.720	1.00	0.00	C
ATOM	219	OD1	ASP	13	33.382	42.254	24.049	1.00	0.00	O
ATOM	220	OD2	ASP	13	34.416	40.393	24.426	1.00	0.00	O
ATOM	221	C	ASP	13	36.349	41.689	28.025	1.00	0.00	C
ATOM	222	O	ASP	13	37.427	42.149	27.849	1.00	0.00	O
ATOM	223	N	ASP	14	35.773	41.677	29.258	1.00	0.00	N
ATOM	224	H	ASP	14	34.831	41.316	29.301	1.00	0.00	H
ATOM	225	CA	ASP	14	36.578	41.942	30.458	1.00	0.00	C
ATOM	226	HA	ASP	14	37.227	42.779	30.202	1.00	0.00	H
ATOM	227	CB	ASP	14	35.727	42.308	31.714	1.00	0.00	C
ATOM	228	HB2	ASP	14	35.139	41.476	32.101	1.00	0.00	H
ATOM	229	HB3	ASP	14	36.297	42.727	32.543	1.00	0.00	H
ATOM	230	CG	ASP	14	34.667	43.405	31.500	1.00	0.00	C
ATOM	231	OD1	ASP	14	34.026	43.879	32.480	1.00	0.00	O
ATOM	232	OD2	ASP	14	34.482	43.943	30.418	1.00	0.00	O
ATOM	233	C	ASP	14	37.526	40.804	30.784	1.00	0.00	C
ATOM	234	O	ASP	14	38.565	41.136	31.401	1.00	0.00	O
ATOM	235	N	ILE	15	37.294	39.516	30.472	1.00	0.00	N
ATOM	236	H	ILE	15	36.580	39.334	29.781	1.00	0.00	H
ATOM	237	CA	ILE	15	38.176	38.424	30.894	1.00	0.00	C
ATOM	238	HA	ILE	15	38.687	38.613	31.839	1.00	0.00	H
ATOM	239	CB	ILE	15	37.351	37.152	31.003	1.00	0.00	C
ATOM	240	HB	ILE	15	36.798	36.997	30.076	1.00	0.00	H
ATOM	241	CG2	ILE	15	38.305	36.030	31.314	1.00	0.00	C
ATOM	242	HG21	ILE	15	39.006	35.685	30.553	1.00	0.00	H
ATOM	243	HG22	ILE	15	38.894	36.258	32.203	1.00	0.00	H
ATOM	244	HG23	ILE	15	37.674	35.207	31.647	1.00	0.00	H
ATOM	245	CG1	ILE	15	36.437	37.195	32.239	1.00	0.00	C
ATOM	246	HG12	ILE	15	36.980	37.015	33.167	1.00	0.00	H

ATOM	247	HG13	ILE	15	36.110	38.230	32.333	1.00	0.00	H
ATOM	248	CD1	ILE	15	35.098	36.433	32.226	1.00	0.00	C
ATOM	249	HD11	ILE	15	34.497	36.776	33.067	1.00	0.00	H
ATOM	250	HD12	ILE	15	34.642	36.628	31.256	1.00	0.00	H
ATOM	251	HD13	ILE	15	35.404	35.397	32.376	1.00	0.00	H
ATOM	252	C	ILE	15	39.249	38.165	29.822	1.00	0.00	C
ATOM	253	O	ILE	15	40.387	37.882	30.209	1.00	0.00	O
ATOM	254	N	LEU	16	38.981	38.524	28.549	1.00	0.00	N
ATOM	255	H	LEU	16	38.045	38.815	28.303	1.00	0.00	H
ATOM	256	CA	LEU	16	40.008	38.603	27.560	1.00	0.00	C
ATOM	257	HA	LEU	16	40.672	37.754	27.725	1.00	0.00	H
ATOM	258	CB	LEU	16	39.310	38.435	26.221	1.00	0.00	C
ATOM	259	HB2	LEU	16	38.751	37.511	26.367	1.00	0.00	H
ATOM	260	HB3	LEU	16	38.563	39.204	26.027	1.00	0.00	H
ATOM	261	CG	LEU	16	40.130	38.348	24.963	1.00	0.00	C
ATOM	262	HG	LEU	16	40.456	39.356	24.709	1.00	0.00	H
ATOM	263	CD1	LEU	16	41.253	37.404	25.059	1.00	0.00	C
ATOM	264	HD11	LEU	16	40.973	36.393	25.353	1.00	0.00	H
ATOM	265	HD12	LEU	16	41.601	37.258	24.036	1.00	0.00	H
ATOM	266	HD13	LEU	16	42.001	37.871	25.700	1.00	0.00	H
ATOM	267	CD2	LEU	16	39.208	37.960	23.796	1.00	0.00	C
ATOM	268	HD21	LEU	16	38.999	36.891	23.764	1.00	0.00	H
ATOM	269	HD22	LEU	16	38.332	38.601	23.906	1.00	0.00	H
ATOM	270	HD23	LEU	16	39.691	38.329	22.892	1.00	0.00	H
ATOM	271	C	LEU	16	40.905	39.865	27.833	1.00	0.00	C
ATOM	272	O	LEU	16	42.109	39.826	27.588	1.00	0.00	O
ATOM	273	N	ALA	17	40.305	40.917	28.391	1.00	0.00	N
ATOM	274	H	ALA	17	39.308	40.902	28.544	1.00	0.00	H
ATOM	275	CA	ALA	17	41.107	42.054	28.902	1.00	0.00	C
ATOM	276	HA	ALA	17	41.865	42.223	28.137	1.00	0.00	H
ATOM	277	CB	ALA	17	40.303	43.292	29.076	1.00	0.00	C
ATOM	278	HB1	ALA	17	40.132	43.554	28.032	1.00	0.00	H
ATOM	279	HB2	ALA	17	39.432	43.193	29.723	1.00	0.00	H
ATOM	280	HB3	ALA	17	41.005	44.072	29.373	1.00	0.00	H
ATOM	281	C	ALA	17	41.859	41.720	30.209	1.00	0.00	C
ATOM	282	O	ALA	17	42.627	42.560	30.693	1.00	0.00	O
ATOM	283	N	GLY	18	41.694	40.512	30.821	1.00	0.00	N
ATOM	284	H	GLY	18	41.022	39.859	30.444	1.00	0.00	H
ATOM	285	CA	GLY	18	42.455	40.118	32.048	1.00	0.00	C
ATOM	286	HA2	GLY	18	42.296	39.061	32.257	1.00	0.00	H
ATOM	287	HA3	GLY	18	43.509	40.311	31.844	1.00	0.00	H
ATOM	288	C	GLY	18	42.070	40.958	33.259	1.00	0.00	C
ATOM	289	O	GLY	18	42.784	40.945	34.247	1.00	0.00	O
ATOM	290	N	ARG	19	40.943	41.806	33.245	1.00	0.00	N
ATOM	291	H	ARG	19	40.409	41.947	32.399	1.00	0.00	H
ATOM	292	CA	ARG	19	40.657	42.676	34.340	1.00	0.00	C
ATOM	293	HA	ARG	19	41.562	43.003	34.852	1.00	0.00	H
ATOM	294	CB	ARG	19	40.073	43.976	33.819	1.00	0.00	C
ATOM	295	HB2	ARG	19	39.072	43.736	33.456	1.00	0.00	H
ATOM	296	HB3	ARG	19	39.920	44.632	34.676	1.00	0.00	H
ATOM	297	CG	ARG	19	40.927	44.681	32.757	1.00	0.00	C
ATOM	298	HG2	ARG	19	41.969	44.706	33.076	1.00	0.00	H
ATOM	299	HG3	ARG	19	40.855	44.167	31.798	1.00	0.00	H
ATOM	300	CD	ARG	19	40.448	46.096	32.527	1.00	0.00	C
ATOM	301	HD2	ARG	19	40.781	46.772	33.315	1.00	0.00	H
ATOM	302	HD3	ARG	19	41.001	46.466	31.663	1.00	0.00	H
ATOM	303	NE	ARG	19	38.960	46.273	32.423	1.00	0.00	N
ATOM	304	HE	ARG	19	38.546	46.148	33.335	1.00	0.00	H
ATOM	305	CZ	ARG	19	38.327	46.209	31.276	1.00	0.00	C
ATOM	306	NH1	ARG	19	38.904	46.268	30.123	1.00	0.00	N
ATOM	307	HH11	ARG	19	39.899	46.436	30.072	1.00	0.00	H
ATOM	308	HH12	ARG	19	38.338	46.304	29.288	1.00	0.00	H
ATOM	309	NH2	ARG	19	37.026	46.010	31.226	1.00	0.00	N
ATOM	310	HH21	ARG	19	36.476	46.114	30.386	1.00	0.00	H

ATOM	311	HH22	ARG	19	36.520	45.999	32.100	1.00	0.00	H
ATOM	312	C	ARG	19	39.775	41.944	35.395	1.00	0.00	C
ATOM	313	O	ARG	19	39.640	42.492	36.493	1.00	0.00	O
ATOM	314	N	LYS	20	39.200	40.801	35.151	1.00	0.00	N
ATOM	315	H	LYS	20	39.407	40.451	34.226	1.00	0.00	H
ATOM	316	CA	LYS	20	38.141	40.208	35.988	1.00	0.00	C
ATOM	317	HA	LYS	20	37.942	40.857	36.841	1.00	0.00	H
ATOM	318	CB	LYS	20	36.791	40.195	35.177	1.00	0.00	C
ATOM	319	HB2	LYS	20	36.979	39.639	34.259	1.00	0.00	H
ATOM	320	HB3	LYS	20	36.456	41.215	34.988	1.00	0.00	H
ATOM	321	CG	LYS	20	35.592	39.456	35.879	1.00	0.00	C
ATOM	322	HG2	LYS	20	34.690	39.614	35.289	1.00	0.00	H
ATOM	323	HG3	LYS	20	35.788	38.384	35.912	1.00	0.00	H
ATOM	324	CD	LYS	20	35.277	40.058	37.335	1.00	0.00	C
ATOM	325	HD2	LYS	20	36.042	39.910	38.097	1.00	0.00	H
ATOM	326	HD3	LYS	20	34.388	39.492	37.616	1.00	0.00	H
ATOM	327	CE	LYS	20	35.076	41.598	37.370	1.00	0.00	C
ATOM	328	HE2	LYS	20	35.941	42.091	36.926	1.00	0.00	H
ATOM	329	HE3	LYS	20	34.226	41.844	36.733	1.00	0.00	H
ATOM	330	NZ	LYS	20	34.827	42.109	38.781	1.00	0.00	N
ATOM	331	HZ1	LYS	20	35.577	41.890	39.423	1.00	0.00	H
ATOM	332	HZ2	LYS	20	34.014	41.687	39.207	1.00	0.00	H
ATOM	333	HZ3	LYS	20	34.577	43.083	38.692	1.00	0.00	H
ATOM	334	C	LYS	20	38.623	38.835	36.515	1.00	0.00	C
ATOM	335	O	LYS	20	38.698	37.777	35.822	1.00	0.00	O
ATOM	336	N	THR	21	38.991	38.969	37.744	1.00	0.00	N
ATOM	337	H	THR	21	39.064	39.876	38.185	1.00	0.00	H
ATOM	338	CA	THR	21	39.687	37.928	38.556	1.00	0.00	C
ATOM	339	HA	THR	21	40.021	37.123	37.902	1.00	0.00	H
ATOM	340	CB	THR	21	40.963	38.582	39.153	1.00	0.00	
	C									
ATOM	341	HB	THR	21	41.441	37.939	39.891	1.00	0.00	H
ATOM	342	CG2	THR	21	42.063	38.946	38.126	1.00	0.00	C
ATOM	343	HG21	THR	21	41.825	39.700	37.375	1.00	0.00	H
ATOM	344	HG22	THR	21	42.900	39.246	38.757	1.00	0.00	H
ATOM	345	HG23	THR	21	42.387	38.042	37.611	1.00	0.00	H
ATOM	346	OG1	THR	21	40.629	39.803	39.788	1.00	0.00	O
ATOM	347	HG1	THR	21	41.461	40.280	39.816	1.00	0.00	H
ATOM	348	C	THR	21	38.924	37.353	39.758	1.00	0.00	C
ATOM	349	O	THR	21	39.176	36.252	40.154	1.00	0.00	O
ATOM	350	N	ILE	22	37.999	38.118	40.357	1.00	0.00	N
ATOM	351	H	ILE	22	38.099	39.093	40.117	1.00	0.00	H
ATOM	352	CA	ILE	22	37.163	37.687	41.445	1.00	0.00	C
ATOM	353	HA	ILE	22	37.148	36.601	41.336	1.00	0.00	H
ATOM	354	CB	ILE	22	37.730	38.066	42.831	1.00	0.00	C
ATOM	355	HB	ILE	22	38.579	37.383	42.852	1.00	0.00	H
ATOM	356	CG2	ILE	22	38.269	39.500	42.914	1.00	0.00	C
ATOM	357	HG21	ILE	22	38.794	39.651	43.857	1.00	0.00	H
ATOM	358	HG22	ILE	22	39.056	39.710	42.189	1.00	0.00	H
ATOM	359	HG23	ILE	22	37.431	40.193	42.841	1.00	0.00	H
ATOM	360	CG1	ILE	22	36.821	37.651	44.014	1.00	0.00	C
ATOM	361	HG12	ILE	22	35.858	38.152	44.109	1.00	0.00	H
ATOM	362	HG13	ILE	22	36.623	36.599	43.808	1.00	0.00	H
ATOM	363	CD1	ILE	22	37.490	37.717	45.415	1.00	0.00	C
ATOM	364	HD11	ILE	22	37.767	38.750	45.627	1.00	0.00	H
ATOM	365	HD12	ILE	22	36.818	37.301	46.167	1.00	0.00	H
ATOM	366	HD13	ILE	22	38.431	37.171	45.460	1.00	0.00	H
ATOM	367	C	ILE	22	35.642	38.159	41.121	1.00	0.00	C
ATOM	368	O	ILE	22	35.429	39.019	40.244	1.00	0.00	O
ATOM	369	N	THR	23	34.690	37.485	41.685	1.00	0.00	N
ATOM	370	H	THR	23	34.913	36.641	42.191	1.00	0.00	H
ATOM	371	CA	THR	23	33.228	37.779	41.578	1.00	0.00	C
ATOM	372	HA	THR	23	33.135	38.836	41.327	1.00	0.00	H
ATOM	373	CB	THR	23	32.571	37.035	40.386	1.00	0.00	C

ATOM	374	HB	THR	23	32.833	35.981	40.478	1.00	0.00	H
ATOM	375	CG2	THR	23	31.059	37.175	40.350	1.00	0.00	C
ATOM	376	HG21	THR	23	30.852	38.222	40.573	1.00	0.00	H
ATOM	377	HG22	THR	23	30.731	36.918	39.342	1.00	0.00	H
ATOM	378	HG23	THR	23	30.490	36.513	41.002	1.00	0.00	H
ATOM	379	OG1	THR	23	33.130	37.609	39.250	1.00	0.00	O
ATOM	380	HG1	THR	23	34.001	37.925	39.500	1.00	0.00	H
ATOM	381	C	THR	23	32.403	37.372	42.831	1.00	0.00	C
ATOM	382	O	THR	23	32.459	36.246	43.353	1.00	0.00	O
ATOM	383	N	ILE	24	31.478	38.205	43.361	1.00	0.00	N
ATOM	384	H	ILE	24	31.331	39.033	42.803	1.00	0.00	H
ATOM	385	CA	ILE	24	30.509	37.872	44.385	1.00	0.00	C
ATOM	386	HA	ILE	24	30.851	36.933	44.822	1.00	0.00	H
ATOM	387	CB	ILE	24	30.442	38.936	45.563	1.00	0.00	C
ATOM	388	HB	ILE	24	29.982	39.817	45.116	1.00	0.00	H
ATOM	389	CG2	ILE	24	29.418	38.518	46.611	1.00	0.00	C
ATOM	390	HG21	ILE	24	29.541	38.996	47.583	1.00	0.00	H
ATOM	391	HG22	ILE	24	28.404	38.645	46.231	1.00	0.00	H
ATOM	392	HG23	ILE	24	29.544	37.492	46.956	1.00	0.00	H
ATOM	393	CG1	ILE	24	31.748	39.376	46.257	1.00	0.00	C
ATOM	394	HG12	ILE	24	32.080	38.691	47.036	1.00	0.00	H
ATOM	395	HG13	ILE	24	32.459	39.432	45.432	1.00	0.00	H
ATOM	396	CD1	ILE	24	31.678	40.802	46.698	1.00	0.00	C
ATOM	397	HD11	ILE	24	31.132	40.935	47.631	1.00	0.00	H
ATOM	398	HD12	ILE	24	32.673	41.116	47.011	1.00	0.00	H
ATOM	399	HD13	ILE	24	31.362	41.502	45.924	1.00	0.00	H
ATOM	400	C	ILE	24	29.119	37.688	43.731	1.00	0.00	C
ATOM	401	O	ILE	24	28.688	38.526	42.899	1.00	0.00	O
ATOM	402	N	ARG	25	28.463	36.574	44.073	1.00	0.00	N
ATOM	403	H	ARG	25	28.848	35.913	44.731	1.00	0.00	H
ATOM	404	CA	ARG	25	27.014	36.244	43.760	1.00	0.00	C
ATOM	405	HA	ARG	25	26.476	37.029	43.229	1.00	0.00	H
ATOM	406	CB	ARG	25	27.024	34.992	42.833	1.00	0.00	C
ATOM	407	HB2	ARG	25	27.781	34.280	43.166	1.00	0.00	H
ATOM	408	HB3	ARG	25	26.043	34.519	42.860	1.00	0.00	H
ATOM	409	CG	ARG	25	27.274	35.482	41.418	1.00	0.00	C
ATOM	410	HG2	ARG	25	26.497	36.177	41.098	1.00	0.00	H
ATOM	411	HG3	ARG	25	28.196	36.060	41.484	1.00	0.00	H
ATOM	412	CD	ARG	25	27.335	34.440	40.285	1.00	0.00	C
ATOM	413	HD2	ARG	25	28.200	33.831	40.549	1.00	0.00	H
ATOM	414	HD3	ARG	25	26.372	33.963	40.463	1.00	0.00	H
ATOM	415	NE	ARG	25	27.556	35.034	38.952	1.00	0.00	N
ATOM	416	HE	ARG	25	28.484	35.155	38.572	1.00	0.00	H
ATOM	417	CZ	ARG	25	26.627	35.535	38.164	1.00	0.00	C
ATOM	418	NH1	ARG	25	25.329	35.595	38.450	1.00	0.00	N
ATOM	419	HH11	ARG	25	24.625	35.752	37.743	1.00	0.00	H
ATOM	420	HH12	ARG	25	24.970	35.457	39.385	1.00	0.00	H
ATOM	421	NH2	ARG	25	26.990	36.230	37.093	1.00	0.00	N
ATOM	422	HH21	ARG	25	26.215	36.327	36.452	1.00	0.00	H
ATOM	423	HH22	ARG	25	27.950	36.439	36.861	1.00	0.00	H
ATOM	424	C	ARG	25	26.313	36.145	45.079	1.00	0.00	C
ATOM	425	O	ARG	25	26.926	35.738	46.071	1.00	0.00	O
ATOM	426	N	ASP	26	24.955	36.303	45.146	1.00	0.00	N
ATOM	427	H	ASP	26	24.531	36.586	44.276	1.00	0.00	H
ATOM	428	CA	ASP	26	24.063	35.682	46.170	1.00	0.00	C
ATOM	429	HA	ASP	26	24.428	36.015	47.140	1.00	0.00	H
ATOM	430	CB	ASP	26	22.652	36.315	45.897	1.00	0.00	C
ATOM	431	HB2	ASP	26	21.985	35.835	46.613	1.00	0.00	H
ATOM	432	HB3	ASP	26	22.666	37.387	46.093	1.00	0.00	H
ATOM	433	CG	ASP	26	22.114	36.261	44.425	1.00	0.00	C
ATOM	434	OD1	ASP	26	22.762	35.755	43.462	1.00	0.00	O
ATOM	435	OD2	ASP	26	21.079	36.987	44.134	1.00	0.00	O
ATOM	436	C	ASP	26	24.038	34.167	46.157	1.00	0.00	C
ATOM	437	O	ASP	26	24.136	33.521	45.062	1.00	0.00	O

ATOM	438	N	GLU	27	24.029	33.507	47.307	1.00	0.00	N
ATOM	439	H	GLU	27	23.855	34.091	48.113	1.00	0.00	H
ATOM	440	CA	GLU	27	24.187	32.065	47.383	1.00	0.00	C
ATOM	441	HA	GLU	27	25.231	31.755	47.344	1.00	0.00	H
ATOM	442	CB	GLU	27	23.849	31.606	48.833	1.00	0.00	C
ATOM	443	HB2	GLU	27	24.369	32.305	49.488	1.00	0.00	H
ATOM	444	HB3	GLU	27	22.809	31.864	49.032	1.00	0.00	H
ATOM	445	CG	GLU	27	24.079	30.133	49.272	1.00	0.00	C
ATOM	446	HG2	GLU	27	23.669	30.042	50.279	1.00	0.00	H
ATOM	447	HG3	GLU	27	23.491	29.510	48.598	1.00	0.00	H
ATOM	448	CD	GLU	27	25.548	29.792	49.104	1.00	0.00	C
ATOM	449	OE1	GLU	27	26.333	29.827	50.098	1.00	0.00	O
ATOM	450	OE2	GLU	27	25.898	29.390	47.964	1.00	0.00	O
ATOM	451	C	GLU	27	23.382	31.252	46.381	1.00	0.00	C
ATOM	452	O	GLU	27	22.249	31.533	46.034	1.00	0.00	O
ATOM	453	N	SER	28	23.947	30.107	46.003	1.00	0.00	N
ATOM	454	H	SER	28	24.843	29.910	46.425	1.00	0.00	H
ATOM	455	CA	SER	28	23.245	29.014	45.317	1.00	0.00	C
ATOM	456	HA	SER	28	22.352	28.939	45.938	1.00	0.00	H
ATOM	457	CB	SER	28	22.800	29.373	43.970	1.00	0.00	C
ATOM	458	HB2	SER	28	22.361	30.363	43.843	1.00	0.00	H
ATOM	459	HB3	SER	28	23.662	29.174	43.334	1.00	0.00	H
ATOM	460	OG	SER	28	21.654	28.670	43.528	1.00	0.00	O
ATOM	461	HG	SER	28	21.038	28.896	44.229	1.00	0.00	H
ATOM	462	C	SER	28	24.131	27.793	45.321	1.00	0.00	C
ATOM	463	O	SER	28	25.321	27.876	45.113	1.00	0.00	O
ATOM	464	N	GLU	29	23.589	26.584	45.433	1.00	0.00	N
ATOM	465	H	GLU	29	22.685	26.536	45.881	1.00	0.00	H
ATOM	466	CA	GLU							
29	24.386	25.317	45.404	1.00	0.00	C				
ATOM	467	HA	GLU	29	25.277	25.400	46.025	1.00	0.00	H
ATOM	468	CB	GLU	29	23.452	24.211	46.054	1.00	0.00	C
ATOM	469	HB2	GLU	29	23.211	24.674	47.011	1.00	0.00	H
ATOM	470	HB3	GLU	29	22.531	24.033	45.500	1.00	0.00	H
ATOM	471	CG	GLU	29	24.129	22.866	46.187	1.00	0.00	C
ATOM	472	HG2	GLU	29	24.275	22.368	45.229	1.00	0.00	H
ATOM	473	HG3	GLU	29	25.126	22.992	46.610	1.00	0.00	H
ATOM	474	CD	GLU	29	23.469	21.891	47.099	1.00	0.00	C
ATOM	475	OE1	GLU	29	23.852	20.682	47.102	1.00	0.00	O
ATOM	476	OE2	GLU	29	22.453	22.203	47.756	1.00	0.00	O
ATOM	477	C	GLU	29	24.916	24.963	44.069	1.00	0.00	C
ATOM	478	O	GLU	29	25.999	24.374	44.059	1.00	0.00	O
ATOM	479	N	SER	30	24.216	25.364	42.970	1.00	0.00	N
ATOM	480	H	SER	30	23.331	25.766	43.242	1.00	0.00	H
ATOM	481	CA	SER	30	24.716	25.302	41.559	1.00	0.00	C
ATOM	482	HA	SER	30	24.918	24.255	41.333	1.00	0.00	H
ATOM	483	CB	SER	30	23.832	25.916	40.563	1.00	0.00	C
ATOM	484	HB2	SER	30	24.340	25.879	39.599	1.00	0.00	H
ATOM	485	HB3	SER	30	22.898	25.354	40.542	1.00	0.00	H
ATOM	486	OG	SER	30	23.590	27.322	40.939	1.00	0.00	O
ATOM	487	HG	SER	30	24.085	27.909	40.363	1.00	0.00	H
ATOM	488	C	SER	30	26.115	26.030	41.273	1.00	0.00	C
ATOM	489	O	SER	30	26.681	25.765	40.218	1.00	0.00	O
ATOM	490	N	HIE	31	26.601	26.939	42.130	1.00	0.00	N
ATOM	491	H	HIE	31	25.967	27.267	42.845	1.00	0.00	H
ATOM	492	CA	HIE	31	27.768	27.761	41.926	1.00	0.00	C
ATOM	493	HA	HIE	31	27.642	28.312	40.994	1.00	0.00	H
ATOM	494	CB	HIE	31	27.875	28.831	42.995	1.00	0.00	C
ATOM	495	HB2	HIE	31	27.697	28.410	43.984	1.00	0.00	H
ATOM	496	HB3	HIE	31	28.919	29.142	42.947	1.00	0.00	H
ATOM	497	CG	HIE	31	27.033	30.080	42.907	1.00	0.00	C
ATOM	498	ND1	HIE	31	26.690	30.742	41.772	1.00	0.00	N
ATOM	499	CE1	HIE	31	25.844	31.753	42.110	1.00	0.00	C
ATOM	500	HE1	HIE	31	25.464	32.451	41.379	1.00	0.00	H

ATOM	501	NE2	HIE	31	25.626	31.754	43.457	1.00	0.00	N
ATOM	502	HE2	HIE	31	25.135	32.469	43.975	1.00	0.00	H
ATOM	503	CD2	HIE	31	26.337	30.666	43.962	1.00	0.00	C
ATOM	504	HD2	HIE	31	26.280	30.283	44.970	1.00	0.00	H
ATOM	505	C	HIE	31	29.055	26.944	41.824	1.00	0.00	C
ATOM	506	O	HIE	31	29.096	25.802	42.348	1.00	0.00	O
ATOM	507	N	PHE	32	30.081	27.442	41.166	1.00	0.00	N
ATOM	508	H	PHE	32	30.029	28.290	40.618	1.00	0.00	H
ATOM	509	CA	PHE	32	31.381	26.740	40.922	1.00	0.00	C
ATOM	510	HA	PHE	32	31.118	25.767	40.508	1.00	0.00	H
ATOM	511	CB	PHE	32	32.280	27.532	39.956	1.00	0.00	C
ATOM	512	HB2	PHE	32	32.655	28.395	40.506	1.00	0.00	H
ATOM	513	HB3	PHE	32	33.130	26.929	39.637	1.00	0.00	H
ATOM	514	CG	PHE	32	31.592	28.019	38.649	1.00	0.00	C
ATOM	515	CD1	PHE	32	31.492	29.322	38.283	1.00	0.00	C
ATOM	516	HD1	PHE	32	31.990	30.010	38.950	1.00	0.00	H
ATOM	517	CE1	PHE	32	30.840	29.746	37.109	1.00	0.00	C
ATOM	518	HE1	PHE	32	30.649	30.788	36.901	1.00	0.00	H
ATOM	519	CZ	PHE	32	30.175	28.794	36.373	1.00	0.00	C
ATOM	520	HZ	PHE	32	29.560	29.179	35.573	1.00	0.00	H
ATOM	521	CE2	PHE	32	30.242	27.377	36.783	1.00	0.00	C
ATOM	522	HE2	PHE	32	29.724	26.590	36.254	1.00	0.00	H
ATOM	523	CD2	PHE	32	30.908	27.028	37.947	1.00	0.00	C
ATOM	524	HD2	PHE	32	30.902	26.060	38.426	1.00	0.00	H
ATOM	525	C	PHE	32	32.097	26.543	42.285	1.00	0.00	C
ATOM	526	O	PHE	32	31.937	27.285	43.301	1.00	0.00	O
ATOM	527	N	LYS	33	32.892	25.509	42.359	1.00	0.00	N
ATOM	528	H	LYS	33	32.912	24.872	41.576	1.00	0.00	H
ATOM	529	CA	LYS	33	33.782	25.046	43.447	1.00	0.00	C
ATOM	530	HA	LYS	33	33.713	25.807	44.223	1.00	0.00	H
ATOM	531	CB	LYS	33	33.327	23.722	44.002	1.00	0.00	C
ATOM	532	HB2	LYS	33	32.891	23.019	43.292	1.00	0.00	H
ATOM	533	HB3	LYS	33	34.212	23.338	44.512	1.00	0.00	H
ATOM	534	CG	LYS	33	32.278	23.938	45.090	1.00	0.00	C
ATOM	535	HG2	LYS	33	32.338	23.099	45.782	1.00	0.00	H
ATOM	536	HG3	LYS	33	32.556	24.764	45.746	1.00	0.00	H
ATOM	537	CD	LYS	33	30.763	24.166	44.665	1.00	0.00	C
ATOM	538	HD2	LYS	33	30.849	24.923	43.887	1.00	0.00	H
ATOM	539	HD3	LYS	33	30.271	23.280	44.261	1.00	0.00	H
ATOM	540	CE	LYS	33	29.787	24.755	45.645	1.00	0.00	C
ATOM	541	HE2	LYS	33	29.563	24.086	46.477	1.00	0.00	H
ATOM	542	HE3	LYS	33	30.149	25.668	46.116	1.00	0.00	H
ATOM	543	NZ	LYS	33	28.522	25.073	44.988	1.00	0.00	N
ATOM	544	HZ1	LYS	33	28.011	25.810	45.454	1.00	0.00	H
ATOM	545	HZ2	LYS	33	28.700	25.462	44.073	1.00	0.00	H
ATOM	546	HZ3	LYS	33	27.956	24.244	44.895	1.00	0.00	H
ATOM	547	C	LYS	33	35.281	25.073	43.023	1.00	0.00	C
ATOM	548	O	LYS	33	35.650	24.871	41.852	1.00	0.00	O
ATOM	549	N	THR	34	36.199	25.377	43.960	1.00	0.00	N
ATOM	550	H	THR	34	35.879	25.528	44.906	1.00	0.00	H
ATOM	551	CA	THR	34	37.633	25.350	43.719	1.00	0.00	C
ATOM	552	HA	THR	34	37.962	26.210	43.136	1.00	0.00	H
ATOM	553	CB	THR	34	38.386	25.651	45.056	1.00	0.00	C
ATOM	554	HB	THR	34	37.914	25.164	45.909	1.00	0.00	H
ATOM	555	CG2	THR	34	39.912	25.505	44.925	1.00	0.00	C
ATOM	556	HG21	THR	34	40.285	24.515	45.188	1.00	0.00	H
ATOM	557	HG22	THR	34	40.156	25.863	43.925	1.00	0.00	H
ATOM	558	HG23	THR	34	40.310	26.272	45.590	1.00	0.00	H
ATOM	559	OG1	THR	34	38.104	27.040	45.259	1.00	0.00	O
ATOM	560	HG1	THR	34	37.155	27.187	45.239	1.00	0.00	H
ATOM	561	C	THR	34	37.999	24.038	43.204	1.00	0.00	C
ATOM	562	O	THR	34	37.771	22.992	43.846	1.00	0.00	O
ATOM	563	N	GLY	35	38.759	24.004	42.057	1.00	0.00	N
ATOM	564	H	GLY	35	39.095	24.868	41.657	1.00	0.00	H

```
ATOM    565  CA  GLY    35      39.054  22.862  41.226  1.00  0.00           C
ATOM    566  HA2 GLY    35      40.062  22.839  40.810  1.00  0.00           H
ATOM    567  HA3 GLY    35      38.859  21.907  41.713  1.00  0.00           H
ATOM    568  C   GLY    35      38.102  22.644  40.061  1.00  0.00           C
ATOM    569  O   GLY    35      38.287  21.686  39.296  1.00  0.00           O
ATOM    570  N   ASP    36      37.028  23.432  39.791  1.00  0.00           N
ATOM    571  H   ASP    36      37.043  24.255  40.377  1.00  0.00           H
ATOM    572  CA  ASP    36      36.231  23.476  38.533  1.00  0.00           C
ATOM    573  HA  ASP    36      36.104  22.485  38.097  1.00  0.00           H
ATOM    574  CB  ASP    36      34.842  24.141  38.773  1.00  0.00           C
ATOM    575  HB2 ASP    36      34.972  25.124  39.224  1.00  0.00           H
ATOM    576  HB3 ASP    36      34.358  24.369  37.824  1.00  0.00           H
ATOM    577  CG  ASP    36      33.864  23.431  39.724  1.00  0.00           C
ATOM    578  OD1 ASP    36      34.002  22.267  40.160  1.00  0.00           O
ATOM    579  OD2 ASP    36      32.792  24.005  40.000  1.00  0.00           O
ATOM    580  C   ASP    36      37.065  24.240  37.472  1.00  0.00           C
ATOM    581  O   ASP    36      37.811  25.147  37.783  1.00  0.00           O
ATOM    582  N   VAL    37      36.811  23.931  36.202  1.00  0.00           N
ATOM    583  H   VAL    37      36.199  23.164  35.959  1.00  0.00           H
ATOM    584  CA  VAL    37      37.473  24.639  35.071  1.00  0.00           C
ATOM    585  HA  VAL    37      38.079  25.446  35.483  1.00  0.00           H
ATOM    586  CB  VAL    37      38.440  23.737  34.344  1.00  0.00           C
ATOM    587  HB  VAL    37      37.817  23.062  33.757  1.00  0.00           H
ATOM    588  CG1 VAL    37      39.376  24.479  33.478  1.00  0.00           C
ATOM    589 HG11 VAL    37      39.960  25.251  33.977  1.00  0.00           H
ATOM    590 HG12 VAL    37      39.956  23.848  32.804  1.00  0.00           H
ATOM    591 HG13 VAL    37      38.794  25.122
  32.818  1.00  0.00           H
ATOM    592  CG2 VAL    37      39.390  22.912  35.263  1.00  0.00           C
ATOM    593 HG21 VAL    37      39.938  22.205  34.639  1.00  0.00           H
ATOM    594 HG22 VAL    37      40.102  23.564  35.769  1.00  0.00           H
ATOM    595 HG23 VAL    37      38.871  22.259  35.964  1.00  0.00           H
ATOM    596  C   VAL    37      36.254  25.197  34.242  1.00  0.00           C
ATOM    597  O   VAL    37      35.316  24.504  33.830  1.00  0.00           O
ATOM    598  N   LEU    38      36.413  26.438  33.827  1.00  0.00           N
ATOM    599  H   LEU    38      37.241  26.915  34.157  1.00  0.00           H
ATOM    600  CA  LEU    38      35.545  27.055  32.815  1.00  0.00           C
ATOM    601  HA  LEU    38      34.604  26.517  32.703  1.00  0.00           H
ATOM    602  CB  LEU    38      35.077  28.459  33.277  1.00  0.00           C
ATOM    603  HB2 LEU    38      35.856  29.207  33.130  1.00  0.00           H
ATOM    604  HB3 LEU    38      34.388  28.789  32.499  1.00  0.00           H
ATOM    605  CG  LEU    38      34.516  28.627  34.737  1.00  0.00           C
ATOM    606  HG  LEU    38      35.288  28.329  35.447  1.00  0.00           H
ATOM    607  CD1 LEU    38      34.142  30.058  35.062  1.00  0.00           C
ATOM    608 HD11 LEU    38      35.070  30.629  35.092  1.00  0.00           H
ATOM    609 HD12 LEU    38      33.419  30.318  34.290  1.00  0.00           H
ATOM    610 HD13 LEU    38      33.640  30.148  36.026  1.00  0.00           H
ATOM    611  CD2 LEU    38      33.259  27.747  34.717  1.00  0.00           C
ATOM    612 HD21 LEU    38      33.488  26.689  34.591  1.00  0.00           H
ATOM    613 HD22 LEU    38      32.640  27.737  35.614  1.00  0.00           H
ATOM    614 HD23 LEU    38      32.704  27.981  33.809  1.00  0.00           H
ATOM    615  C   LEU    38      36.213  27.138  31.467  1.00  0.00           C
ATOM    616  O   LEU    38      37.433  26.908  31.363  1.00  0.00           O
ATOM    617  N   ARG    39      35.424  27.465  30.423  1.00  0.00           N
ATOM    618  H   ARG    39      34.435  27.449  30.622  1.00  0.00           H
ATOM    619  CA  ARG    39      35.854  27.859  29.059  1.00  0.00           C
ATOM    620  HA  ARG    39      36.912  28.106  29.138  1.00  0.00           H
ATOM    621  CB  ARG    39      35.802  26.735  28.044  1.00  0.00           C
ATOM    622  HB2 ARG    39      34.833  26.238  28.073  1.00  0.00           H
ATOM    623  HB3 ARG    39      36.050  27.035  27.025  1.00  0.00           H
ATOM    624  CG  ARG    39      36.818  25.610  28.337  1.00  0.00           C
ATOM    625  HG2 ARG    39      37.857  25.923  28.438  1.00  0.00           H
ATOM    626  HG3 ARG    39      36.511  25.026  29.205  1.00  0.00           H
ATOM    627  CD  ARG    39      36.914  24.448  27.305  1.00  0.00           C
```

ATOM	628	HD2	ARG	39	37.284	24.979	26.427	1.00	0.00	H
ATOM	629	HD3	ARG	39	37.775	23.873	27.644	1.00	0.00	H
ATOM	630	NE	ARG	39	35.621	23.723	27.148	1.00	0.00	N
ATOM	631	HE	ARG	39	34.733	24.191	27.265	1.00	0.00	H
ATOM	632	CZ	ARG	39	35.520	22.399	26.884	1.00	0.00	C
ATOM	633	NH1	ARG	39	36.525	21.662	26.616	1.00	0.00	N
ATOM	634	HH11	ARG	39	37.402	22.070	26.325	1.00	0.00	H
ATOM	635	HH12	ARG	39	36.311	20.685	26.483	1.00	0.00	H
ATOM	636	NH2	ARG	39	34.398	21.748	26.864	1.00	0.00	N
ATOM	637	HH21	ARG	39	34.338	20.740	26.866	1.00	0.00	H
ATOM	638	HH22	ARG	39	33.537	22.242	27.054	1.00	0.00	H
ATOM	639	C	ARG	39	35.129	29.173	28.633	1.00	0.00	C
ATOM	640	O	ARG	39	33.988	29.316	28.955	1.00	0.00	O
ATOM	641	N	VAL	40	35.806	30.043	27.847	1.00	0.00	N
ATOM	642	H	VAL	40	36.742	29.796	27.554	1.00	0.00	H
ATOM	643	CA	VAL	40	35.291	31.288	27.387	1.00	0.00	C
ATOM	644	HA	VAL	40	34.203	31.354	27.369	1.00	0.00	H
ATOM	645	CB	VAL	40	35.647	32.469	28.406	1.00	0.00	C
ATOM	646	HB	VAL	40	35.329	33.392	27.920	1.00	0.00	H
ATOM	647	CG1	VAL	40	34.928	32.355	29.775	1.00	0.00	C
ATOM	648	HG11	VAL	40	33.847	32.486	29.713	1.00	0.00	H
ATOM	649	HG12	VAL	40	35.259	31.444	30.275	1.00	0.00	H
ATOM	650	HG13	VAL	40	35.248	33.214	30.363	1.00	0.00	H
ATOM	651	CG2	VAL	40	37.115	32.450	28.758	1.00	0.00	C
ATOM	652	HG21	VAL	40	37.307	33.401	29.256	1.00	0.00	H
ATOM	653	HG22	VAL	40	37.442	31.568	29.307	1.00	0.00	H
ATOM	654	HG23	VAL	40	37.739	32.441	27.864	1.00	0.00	H
ATOM	655	C	VAL	40	35.756	31.625	25.923	1.00	0.00	C
ATOM	656	O	VAL	40	36.773	31.092	25.387	1.00	0.00	O
ATOM	657	N	GLY	41	34.950	32.442	25.172	1.00	0.00	N
ATOM	658	H	GLY	41	34.103	32.722	25.647	1.00	0.00	H
ATOM	659	CA	GLY	41	34.930	32.656	23.698	1.00	0.00	C
ATOM	660	HA2	GLY	41	35.465	33.586	23.510	1.00	0.00	H
ATOM	661	HA3	GLY	41	35.380	31.853	23.113	1.00	0.00	H
ATOM	662	C	GLY	41	33.452	32.768	23.195	1.00	0.00	C
ATOM	663	O	GLY	41	32.495	32.341	23.901	1.00	0.00	O
ATOM	664	N	ARG	42	33.289	33.214	21.939	1.00	0.00	N
ATOM	665	H	ARG	42	34.163	33.433	21.482	1.00	0.00	H
ATOM	666	CA	ARG	42	32.023	33.291	21.153	1.00	0.00	C
ATOM	667	HA	ARG	42	31.427	32.401	21.354	1.00	0.00	H
ATOM	668	CB	ARG	42	31.134	34.429	21.733	1.00	0.00	C
ATOM	669	HB2	ARG	42	30.143	34.348	21.286	1.00	0.00	H
ATOM	670	HB3	ARG	42	30.866	34.267	22.778	1.00	0.00	H
ATOM	671	CG	ARG	42	31.610	35.887	21.457	1.00	0.00	C
ATOM	672	HG2	ARG	42	32.624	36.053	21.820	1.00	0.00	H
ATOM	673	HG3	ARG	42	31.409	36.189	20.430	1.00	0.00	H
ATOM	674	CD	ARG	42	30.687	36.728	22.299	1.00	0.00	C
ATOM	675	HD2	ARG	42	29.640	36.439	22.205	1.00	0.00	H
ATOM	676	HD3	ARG	42	30.935	36.419	23.314	1.00	0.00	H
ATOM	677	NE	ARG	42	30.823	38.193	22.250	1.00	0.00	N
ATOM	678	HE	ARG	42	30.370	38.569	21.429	1.00	0.00	H
ATOM	679	CZ	ARG	42	31.768	38.962	22.867	1.00	0.00	C
ATOM	680	NH1	ARG	42	32.493	38.573	23.883	1.00	0.00	N
ATOM	681	HH11	ARG	42	33.231	39.106	24.320	1.00	0.00	H
ATOM	682	HH12	ARG	42	32.474	37.629	24.244	1.00	0.00	H
ATOM	683	NH2	ARG	42	31.969	40.202	22.458	1.00	0.00	N
ATOM	684	HH21	ARG	42	31.310	40.691	21.870	1.00	0.00	H
ATOM	685	HH22	ARG	42	32.658	40.721	22.986	1.00	0.00	H
ATOM	686	C	ARG	42	32.155	33.451	19.648	1.00	0.00	C
ATOM	687	O	ARG	42	31.135	33.323	18.945	1.00	0.00	O
ATOM	688	N	PHE	43	33.371	33.503	19.048	1.00	0.00	N
ATOM	689	H	PHE	43	34.192	33.515	19.635	1.00	0.00	H
ATOM	690	CA	PHE	43	33.703	33.527	17.673	1.00	0.00	C
ATOM	691	HA	PHE	43	32.785	33.608	17.092	1.00	0.00	H

ATOM	692	CB	PHE	43	34.494	34.830	17.423	1.00	0.00	C
ATOM	693	HB2	PHE	43	35.469	34.790	17.909	1.00	0.00	H
ATOM	694	HB3	PHE	43	34.663	34.922	16.351	1.00	0.00	H
ATOM	695	CG	PHE	43	33.846	36.102	18.022	1.00	0.00	C
ATOM	696	CD1	PHE	43	32.657	36.653	17.456	1.00	0.00	C
ATOM	697	HD1	PHE	43	32.335	36.175	16.542	1.00	0.00	H
ATOM	698	CE1	PHE	43	32.089	37.771	17.996	1.00	0.00	C
ATOM	699	HE1	PHE	43	31.202	38.154	17.511	1.00	0.00	H
ATOM	700	CZ	PHE	43	32.655	38.436	19.121	1.00	0.00	C
ATOM	701	HZ	PHE	43	32.295	39.406	19.432	1.00	0.00	H
ATOM	702	CE2	PHE	43	33.880	37.899	19.627	1.00	0.00	C
ATOM	703	HE2	PHE	43	34.463	38.503	20.306	1.00	0.00	H
ATOM	704	CD2	PHE	43	34.457	36.711	19.155	1.00	0.00	C
ATOM	705	HD2	PHE	43	35.344	36.291	19.607	1.00	0.00	H
ATOM	706	C	PHE	43	34.415	32.241	17.183	1.00	0.00	C
ATOM	707	O	PHE	43	34.883	31.398	17.973	1.00	0.00	O
ATOM	708	N	GLU	44	34.467	32.093	15.849	1.00	0.00	N
ATOM	709	H	GLU	44	34.211	32.862	15.247	1.00	0.00	H
ATOM	710	CA	GLU	44	34.723	30.886	15.029	1.00	0.00	C
ATOM	711	HA	GLU	44	34.191	30.113	15.581	1.00	0.00	H
ATOM	712	CB	GLU	44	34.097	31.186	13.654	1.00	0.00	C
ATOM	713	HB2	GLU	44	33.858	30.212	13.227	1.00	0.00	H
ATOM	714	HB3	GLU	44	33.221	31.835	13.669	1.00	0.00	H
ATOM	715	CG	GLU	44	35.054	31.881	12.628	1.00	0.00	C
ATOM	716	HG2	GLU	44	36.005	31.357	12.534	1.00	0.00	
H										
ATOM	717	HG3	GLU	44	34.592	31.848	11.641	1.00	0.00	H
ATOM	718	CD	GLU	44	35.336	33.374	12.927	1.00	0.00	C
ATOM	719	OE1	GLU	44	36.271	33.887	12.310	1.00	0.00	O
ATOM	720	OE2	GLU	44	34.557	34.010	13.671	1.00	0.00	O
ATOM	721	C	GLU	44	36.103	30.304	15.105	1.00	0.00	C
ATOM	722	O	GLU	44	36.335	29.178	14.571	1.00	0.00	O
ATOM	723	N	ASP	45	37.071	30.867	15.844	1.00	0.00	N
ATOM	724	H	ASP	45	36.797	31.673	16.388	1.00	0.00	H
ATOM	725	CA	ASP	45	38.466	30.556	16.040	1.00	0.00	C
ATOM	726	HA	ASP	45	38.788	30.130	15.090	1.00	0.00	H
ATOM	727	CB	ASP	45	39.125	32.010	16.068	1.00	0.00	C
ATOM	728	HB2	ASP	45	38.740	32.649	16.863	1.00	0.00	H
ATOM	729	HB3	ASP	45	40.177	31.914	16.338	1.00	0.00	H
ATOM	730	CG	ASP	45	39.169	32.833	14.807	1.00	0.00	C
ATOM	731	OD1	ASP	45	39.197	34.067	14.814	1.00	0.00	O
ATOM	732	OD2	ASP	45	39.082	32.180	13.748	1.00	0.00	O
ATOM	733	C	ASP	45	38.864	29.691	17.249	1.00	0.00	C
ATOM	734	O	ASP	45	38.668	30.075	18.392	1.00	0.00	O
ATOM	735	N	ASP	46	39.354	28.460	16.972	1.00	0.00	N
ATOM	736	H	ASP	46	39.481	28.130	16.026	1.00	0.00	H
ATOM	737	CA	ASP	46	39.713	27.392	17.943	1.00	0.00	C
ATOM	738	HA	ASP	46	39.641	26.430	17.435	1.00	0.00	H
ATOM	739	CB	ASP	46	41.177	27.650	18.452	1.00	0.00	C
ATOM	740	HB2	ASP	46	41.258	28.679	18.802	1.00	0.00	H
ATOM	741	HB3	ASP	46	41.343	26.928	19.251	1.00	0.00	H
ATOM	742	CG	ASP	46	42.357	27.442	17.421	1.00	0.00	C
ATOM	743	OD1	ASP	46	43.542	27.563	17.797	1.00	0.00	O
ATOM	744	OD2	ASP	46	42.076	27.038	16.280	1.00	0.00	O
ATOM	745	C	ASP	46	38.664	27.191	19.103	1.00	0.00	C
ATOM	746	O	ASP	46	39.020	26.622	20.195	1.00	0.00	O
ATOM	747	N	GLY	47	37.421	27.620	18.879	1.00	0.00	N
ATOM	748	H	GLY	47	37.291	28.242	18.094	1.00	0.00	H
ATOM	749	CA	GLY	47	36.238	27.379	19.699	1.00	0.00	C
ATOM	750	HA2	GLY	47	35.327	27.504	19.112	1.00	0.00	H
ATOM	751	HA3	GLY	47	36.218	26.318	19.950	1.00	0.00	H
ATOM	752	C	GLY	47	36.209	28.088	21.052	1.00	0.00	C
ATOM	753	O	GLY	47	35.131	28.529	21.524	1.00	0.00	O
ATOM	754	N	TYR	48	37.371	28.282	21.701	1.00	0.00	N

ATOM	755	H	TYR	48	38.231	28.058	21.219	1.00	0.00	H
ATOM	756	CA	TYR	48	37.628	28.996	22.944	1.00	0.00	C
ATOM	757	HA	TYR	48	36.814	29.697	23.133	1.00	0.00	H
ATOM	758	CB	TYR	48	37.633	28.031	24.204	1.00	0.00	C
ATOM	759	HB2	TYR	48	38.638	27.662	24.402	1.00	0.00	H
ATOM	760	HB3	TYR	48	37.312	28.591	25.082	1.00	0.00	H
ATOM	761	CG	TYR	48	36.605	26.905	24.146	1.00	0.00	C
ATOM	762	CD1	TYR	48	35.285	26.967	24.661	1.00	0.00	C
ATOM	763	HD1	TYR	48	35.033	27.756	25.354	1.00	0.00	H
ATOM	764	CE1	TYR	48	34.376	25.887	24.590	1.00	0.00	C
ATOM	765	HE1	TYR	48	33.381	25.931	25.009	1.00	0.00	H
ATOM	766	CZ	TYR	48	34.725	24.752	23.847	1.00	0.00	C
ATOM	767	OH	TYR	48	33.740	23.854	23.641	1.00	0.00	O
ATOM	768	HH	TYR	48	33.905	23.466	22.779	1.00	0.00	H
ATOM	769	CE2	TYR	48	35.998	24.674	23.219	1.00	0.00	C
ATOM	770	HE2	TYR	48	36.283	23.853	22.578	1.00	0.00	H
ATOM	771	CD2	TYR	48	36.948	25.718	23.383	1.00	0.00	C
ATOM	772	HD2	TYR	48	37.931	25.631	22.945	1.00	0.00	H
ATOM	773	C	TYR	48	38.901	29.802	22.937	1.00	0.00	C
ATOM	774	O	TYR	48	39.866	29.348	22.290	1.00	0.00	O
ATOM	775	N	PHE	49	38.996	30.964	23.589	1.00	0.00	N
ATOM	776	H	PHE	49	38.268	31.286	24.210	1.00	0.00	H
ATOM	777	CA	PHE	49	40.260	31.676	23.884	1.00	0.00	C
ATOM	778	HA	PHE	49	40.973	31.345	23.128	1.00	0.00	H
ATOM	779	CB	PHE	49	40.034	33.149	23.757	1.00	0.00	C
ATOM	780	HB2	PHE	49	41.025	33.597	23.683	1.00	0.00	H
ATOM	781	HB3	PHE	49	39.462	33.331	22.847	1.00	0.00	H
ATOM	782	CG	PHE	49	39.207	33.728	24.896	1.00	0.00	C
ATOM	783	CD1	PHE	49	39.800	34.147	26.104	1.00	0.00	C
ATOM	784	HD1	PHE	49	40.865	34.006	26.217	1.00	0.00	H
ATOM	785	CE1	PHE	49	39.033	34.827	27.113	1.00	0.00	C
ATOM	786	HE1	PHE	49	39.537	35.079	28.034	1.00	0.00	H
ATOM	787	CZ	PHE	49	37.713	35.146	26.821	1.00	0.00	C
ATOM	788	HZ	PHE	49	37.184	35.721	27.567	1.00	0.00	H
ATOM	789	CE2	PHE	49	37.157	34.820	25.584	1.00	0.00	C
ATOM	790	HE2	PHE	49	36.140	35.110	25.362	1.00	0.00	H
ATOM	791	CD2	PHE	49	37.903	34.092	24.636	1.00	0.00	C
ATOM	792	HD2	PHE	49	37.488	33.781	23.689	1.00	0.00	H
ATOM	793	C	PHE	49	40.939	31.330	25.217	1.00	0.00	C
ATOM	794	O	PHE	49	42.238	31.387	25.295	1.00	0.00	O
ATOM	795	N	CYX	50	40.237	30.925	26.244	1.00	0.00	N
ATOM	796	H	CYX	50	39.261	30.735	26.067	1.00	0.00	H
ATOM	797	CA	CYX	50	40.741	30.577	27.603	1.00	0.00	C
ATOM	798	HA	CYX	50	41.769	30.266	27.414	1.00	0.00	H
ATOM	799	CB	CYX	50	40.749	31.817	28.491	1.00	0.00	C
ATOM	800	HB2	CYX	50	39.853	32.429	28.397	1.00	0.00	H
ATOM	801	HB3	CYX	50	40.793	31.443	29.514	1.00	0.00	H
ATOM	802	SG	CYX	50	42.166	32.931	28.490	1.00	0.00	S
ATOM	803	C	CYX	50	39.992	29.363	28.202	1.00	0.00	C
ATOM	804	O	CYX	50	38.764	29.299	28.330	1.00	0.00	O
ATOM	805	N	THR	51	40.809	28.461	28.763	1.00	0.00	N
ATOM	806	H	THR	51	41.779	28.539	28.494	1.00	0.00	H
ATOM	807	CA	THR	51	40.519	27.603	29.838	1.00	0.00	C
ATOM	808	HA	THR	51	39.486	27.257	29.852	1.00	0.00	H
ATOM	809	CB	THR	51	41.340	26.343	29.779	1.00	0.00	C
ATOM	810	HB	THR	51	42.401	26.594	29.784	1.00	0.00	H
ATOM	811	CG2	THR	51	41.251	25.437	31.001	1.00	0.00	C
ATOM	812	HG21	THR	51	40.291	24.923	30.976	1.00	0.00	H
ATOM	813	HG22	THR	51	42.078	24.735	31.102	1.00	0.00	H
ATOM	814	HG23	THR	51	41.298	26.124	31.846	1.00	0.00	H
ATOM	815	OG1	THR	51	40.868	25.587	28.685	1.00	0.00	O
ATOM	816	HG1	THR	51	41.151	26.093	27.919	1.00	0.00	H
ATOM	817	C	THR	51	40.735	28.279	31.112	1.00	0.00	C
ATOM	818	O	THR	51	41.767	28.873	31.262	1.00	0.00	O

```
ATOM    819  N   ILE    52      39.816  28.309  32.033  1.00  0.00           N
ATOM    820  H   ILE    52      38.932  27.841  31.895  1.00  0.00           H
ATOM    821  CA  ILE    52      39.923  29.144  33.266  1.00  0.00           C
ATOM    822  HA  ILE    52      40.937  29.543  33.320  1.00  0.00           H
ATOM    823  CB  ILE    52      39.023  30.420  33.145  1.00  0.00           C
ATOM    824  HB  ILE    52      37.975  30.125  33.135  1.00  0.00           H
ATOM    825  CG2 ILE    52      39.119  31.233  34.475  1.00  0.00           C
ATOM    826 HG21 ILE    52      38.822  30.607  35.315  1.00  0.00           H
ATOM    827 HG22 ILE    52      40.160  31.544  34.555  1.00  0.00           H
ATOM    828 HG23 ILE    52      38.375  32.029  34.448  1.00  0.00           H
ATOM    829  CG1 ILE    52      39.308  31.268  31.913  1.00  0.00           C
ATOM    830 HG12 ILE    52      40.375  31.235  31.689  1.00  0.00           H
ATOM    831 HG13 ILE    52      38.748  30.838  31.082  1.00  0.00           H
ATOM    832  CD1 ILE    52      38.885  32.717  32.008  1.00  0.00           C
ATOM    833 HD11 ILE    52      39.406  33.275  32.785  1.00  0.00           H
ATOM    834 HD12 ILE    52      39.191  33.270  31.121  1.00  0.00           H
ATOM    835 HD13 ILE    52      37.796  32.761  32.038  1.00  0.00           H
ATOM    836  C   ILE    52      39.691  28.337  34.546  1.00  0.00           C
ATOM    837  O   ILE    52      38.615  27.876  34.881  1.00  0.00           O
ATOM    838  N   GLU    53      40.763  28.118  35.278  1.00  0.00           N
ATOM    839  H   GLU    53      41.667  28.506  35.047  1.00  0.00           H
ATOM    840  CA  GLU    53      40.651  27.475  36.575  1.00  0.00           C
ATOM    841  HA  GLU    53      39.884  26.703  36.513  1.00  0.00           H
ATOM    842  CB  GLU
    53      41.974  26.676  37.047  1.00  0.00           C
ATOM    843  HB2 GLU    53      42.171  25.967  36.242  1.00  0.00           H
ATOM    844  HB3 GLU    53      42.754  27.438  37.011  1.00  0.00           H
ATOM    845  CG  GLU    53      41.874  26.014  38.383  1.00  0.00           C
ATOM    846  HG2 GLU    53      41.946  26.827  39.104  1.00  0.00           H
ATOM    847  HG3 GLU    53      40.889  25.557  38.482  1.00  0.00           H
ATOM    848  CD  GLU    53      42.973  24.935  38.614  1.00  0.00           C
ATOM    849  OE1 GLU    53      43.900  24.729  37.769  1.00  0.00           O
ATOM    850  OE2 GLU    53      42.919  24.331  39.720  1.00  0.00           O
ATOM    851  C   GLU    53      40.174  28.396  37.659  1.00  0.00           C
ATOM    852  O   GLU    53      40.734  29.491  37.876  1.00  0.00           O
ATOM    853  N   VAL    54      39.193  27.896  38.415  1.00  0.00           N
ATOM    854  H   VAL    54      38.804  27.010  38.125  1.00  0.00           H
ATOM    855  CA  VAL    54      38.718  28.508  39.649  1.00  0.00           C
ATOM    856  HA  VAL    54      38.658  29.597  39.635  1.00  0.00           H
ATOM    857  CB  VAL    54      37.232  28.080  39.842  1.00  0.00           C
ATOM    858  HB  VAL    54      37.255  26.991  39.834  1.00  0.00           H
ATOM    859  CG1 VAL    54      36.660  28.622  41.212  1.00  0.00           C
ATOM    860 HG11 VAL    54      36.645  29.696  41.030  1.00  0.00           H
ATOM    861 HG12 VAL    54      35.667  28.255  41.475  1.00  0.00           H
ATOM    862 HG13 VAL    54      37.409  28.320  41.943  1.00  0.00           H
ATOM    863  CG2 VAL    54      36.311  28.555  38.730  1.00  0.00           C
ATOM    864 HG21 VAL    54      36.612  27.976  37.857  1.00  0.00           H
ATOM    865 HG22 VAL    54      35.259  28.330  38.908  1.00  0.00           H
ATOM    866 HG23 VAL    54      36.481  29.593  38.449  1.00  0.00           H
ATOM    867  C   VAL    54      39.649  28.020  40.776  1.00  0.00           C
ATOM    868  O   VAL    54      39.695  26.867  41.067  1.00  0.00           O
ATOM    869  N   THR    55      40.357  29.060  41.385  1.00  0.00           N
ATOM    870  H   THR    55      40.150  29.995  41.064  1.00  0.00           H
ATOM    871  CA  THR    55      41.490  28.862  42.225  1.00  0.00           C
ATOM    872  HA  THR    55      41.831  27.836  42.084  1.00  0.00           H
ATOM    873  CB  THR    55      42.593  29.771  41.705  1.00  0.00           C
ATOM    874  HB  THR    55      43.476  29.643  42.331  1.00  0.00           H
ATOM    875  CG2 THR    55      43.174  29.516  40.320  1.00  0.00           C
ATOM    876 HG21 THR    55      43.004  28.459  40.112  1.00  0.00           H
ATOM    877 HG22 THR    55      42.654  30.041  39.518  1.00  0.00           H
ATOM    878 HG23 THR    55      44.244  29.690  40.205  1.00  0.00           H
ATOM    879  OG1 THR    55      42.292  31.095  41.729  1.00  0.00           O
ATOM    880  HG1 THR    55      42.259  31.327  42.659  1.00  0.00           H
ATOM    881  C   THR    55      41.175  29.080  43.702  1.00  0.00           C
```

ATOM	882	O	THR	55	42.053	28.584	44.516	1.00	0.00	O
ATOM	883	N	ALA	56	40.117	29.750	44.060	1.00	0.00	N
ATOM	884	H	ALA	56	39.419	30.013	43.379	1.00	0.00	H
ATOM	885	CA	ALA	56	39.605	29.864	45.412	1.00	0.00	C
ATOM	886	HA	ALA	56	39.692	28.942	45.987	1.00	0.00	H
ATOM	887	CB	ALA	56	40.392	31.007	46.152	1.00	0.00	C
ATOM	888	HB1	ALA	56	40.430	31.890	45.514	1.00	0.00	H
ATOM	889	HB2	ALA	56	40.096	31.254	47.171	1.00	0.00	H
ATOM	890	HB3	ALA	56	41.458	30.787	46.227	1.00	0.00	H
ATOM	891	C	ALA	56	38.080	30.225	45.195	1.00	0.00	C
ATOM	892	O	ALA	56	37.618	30.757	44.171	1.00	0.00	O
ATOM	893	N	THR	57	37.274	29.783	46.137	1.00	0.00	N
ATOM	894	H	THR	57	37.712	29.360	46.943	1.00	0.00	H
ATOM	895	CA	THR	57	35.862	29.948	46.315	1.00	0.00	C
ATOM	896	HA	THR	57	35.516	30.795	45.722	1.00	0.00	H
ATOM	897	CB	THR	57	35.043	28.732	45.845	1.00	0.00	C
ATOM	898	HB	THR	57	34.106	28.802	46.397	1.00	0.00	H
ATOM	899	CG2	THR	57	34.828	28.875	44.386	1.00	0.00	C
ATOM	900	HG21	THR	57	34.373	29.825	44.103	1.00	0.00	H
ATOM	901	HG22	THR	57	35.811	28.751	43.934	1.00	0.00	H
ATOM	902	HG23	THR	57	34.187	28.049	44.080	1.00	0.00	H
ATOM	903	OG1	THR	57	35.649	27.460	46.030	1.00	0.00	O
ATOM	904	HG1	THR	57	35.262	27.060	46.812	1.00	0.00	H
ATOM	905	C	THR	57	35.527	30.207	47.839	1.00	0.00	C
ATOM	906	O	THR	57	36.161	29.667	48.752	1.00	0.00	O
ATOM	907	N	SER	58	34.391	30.897	48.130	1.00	0.00	N
ATOM	908	H	SER	58	33.872	31.320	47.375	1.00	0.00	H
ATOM	909	CA	SER	58	33.767	30.913	49.464	1.00	0.00	C
ATOM	910	HA	SER	58	33.956	29.980	49.994	1.00	0.00	H
ATOM	911	CB	SER	58	34.406	32.056	50.311	1.00	0.00	C
ATOM	912	HB2	SER	58	34.453	32.984	49.741	1.00	0.00	H
ATOM	913	HB3	SER	58	33.932	32.288	51.266	1.00	0.00	H
ATOM	914	OG	SER	58	35.674	31.549	50.667	1.00	0.00	O
ATOM	915	HG	SER	58	36.012	31.305	49.803	1.00	0.00	H
ATOM	916	C	SER	58	32.273	31.186	49.609	1.00	0.00	C
ATOM	917	O	SER	58	31.773	32.198	49.013	1.00	0.00	O
ATOM	918	N	THR	59	31.579	30.543	50.603	1.00	0.00	N
ATOM	919	H	THR	59	32.092	29.779	51.021	1.00	0.00	H
ATOM	920	CA	THR	59	30.329	31.076	51.171	1.00	0.00	C
ATOM	921	HA	THR	59	29.878	31.675	50.380	1.00	0.00	H
ATOM	922	CB	THR	59	29.583	29.857	51.680	1.00	0.00	C
ATOM	923	HB	THR	59	29.376	29.133	50.891	1.00	0.00	H
ATOM	924	CG2	THR	59	30.255	29.216	52.902	1.00	0.00	C
ATOM	925	HG21	THR	59	29.828	28.238	53.125	1.00	0.00	H
ATOM	926	HG22	THR	59	31.282	29.003	52.604	1.00	0.00	H
ATOM	927	HG23	THR	59	30.180	29.870	53.770	1.00	0.00	H
ATOM	928	OG1	THR	59	28.354	30.255	52.174	1.00	0.00	O
ATOM	929	HG1	THR	59	27.872	30.511	51.384	1.00	0.00	H
ATOM	930	C	THR	59	30.625	32.131	52.234	1.00	0.00	C
ATOM	931	O	THR	59	31.547	32.068	53.037	1.00	0.00	O
ATOM	932	N	VAL	60	29.796	33.183	52.346	1.00	0.00	N
ATOM	933	H	VAL	60	28.978	33.236	51.755	1.00	0.00	H
ATOM	934	CA	VAL	60	29.962	34.469	53.180	1.00	0.00	C
ATOM	935	HA	VAL	60	30.467	34.219	54.113	1.00	0.00	H
ATOM	936	CB	VAL	60	30.966	35.340	52.430	1.00	0.00	C
ATOM	937	HB	VAL	60	31.678	34.646	51.985	1.00	0.00	H
ATOM	938	CG1	VAL	60	30.470	36.259	51.262	1.00	0.00	C
ATOM	939	HG11	VAL	60	29.993	35.674	50.475	1.00	0.00	H
ATOM	940	HG12	VAL	60	29.838	37.035	51.693	1.00	0.00	H
ATOM	941	HG13	VAL	60	31.372	36.736	50.880	1.00	0.00	H
ATOM	942	CG2	VAL	60	31.816	36.223	53.428	1.00	0.00	C
ATOM	943	HG21	VAL	60	32.035	35.753	54.386	1.00	0.00	H
ATOM	944	HG22	VAL	60	32.799	36.428	53.004	1.00	0.00	H
ATOM	945	HG23	VAL	60	31.395	37.209	53.629	1.00	0.00	H

ATOM	946	C	VAL	60	28.655	35.152	53.515	1.00	0.00	C
ATOM	947	O	VAL	60	27.578	34.886	53.024	1.00	0.00	O
ATOM	948	N	THR	61	28.782	36.154	54.396	1.00	0.00	N
ATOM	949	H	THR	61	29.658	36.454	54.799	1.00	0.00	H
ATOM	950	CA	THR	61	27.723	37.083	54.880	1.00	0.00	C
ATOM	951	HA	THR	61	26.871	36.944	54.214	1.00	0.00	H
ATOM	952	CB	THR	61	27.211	36.721	56.318	1.00	0.00	C
ATOM	953	HB	THR	61	26.709	37.561	56.796	1.00	0.00	H
ATOM	954	CG2	THR	61	26.254	35.494	56.387	1.00	0.00	C
ATOM	955	HG21	THR	61	25.842	35.507	57.397	1.00	0.00	H
ATOM	956	HG22	THR	61	25.553	35.640	55.565	1.00	0.00	H
ATOM	957	HG23	THR	61	26.880	34.644	56.119	1.00	0.00	H
ATOM	958	OG1	THR	61	28.322	36.362	57.148	1.00	0.00	O
ATOM	959	HG1	THR	61	28.697	37.169	57.510	1.00	0.00	H
ATOM	960	C	THR	61	28.131	38.535	54.574	1.00	0.00	C
ATOM	961	O	THR	61	29.326	38.804	54.282	1.00	0.00	O
ATOM	962	N	LEU	62	27.256	39.510	54.639	1.00	0.00	N
ATOM	963	H	LEU	62	26.301	39.263	54.857	1.00	0.00	H
ATOM	964	CA	LEU	62	27.470	40.881	54.194	1.00	0.00	C
ATOM	965	HA	LEU	62	27.911	40.951	53.199	1.00	0.00	H
ATOM	966	CB	LEU	62	26.017	41.547	54.173	1.00	0.00	C
ATOM	967	HB2	LEU	62	25.222	40.801				
54.149	1.00	0.00		H						
ATOM	968	HB3	LEU	62	25.815	42.208	55.016	1.00	0.00	H
ATOM	969	CG	LEU	62	25.861	42.366	52.943	1.00	0.00	C
ATOM	970	HG	LEU	62	26.116	41.813	52.038	1.00	0.00	H
ATOM	971	CD1	LEU	62	24.386	42.741	52.882	1.00	0.00	C
ATOM	972	HD11	LEU	62	24.382	43.461	52.063	1.00	0.00	H
ATOM	973	HD12	LEU	62	23.706	41.907	52.708	1.00	0.00	H
ATOM	974	HD13	LEU	62	24.153	43.226	53.830	1.00	0.00	H
ATOM	975	CD2	LEU	62	26.696	43.620	52.721	1.00	0.00	C
ATOM	976	HD21	LEU	62	26.702	44.346	53.533	1.00	0.00	H
ATOM	977	HD22	LEU	62	27.760	43.449	52.555	1.00	0.00	H
ATOM	978	HD23	LEU	62	26.366	44.109	51.804	1.00	0.00	H
ATOM	979	C	LEU	62	28.301	41.640	55.245	1.00	0.00	C
ATOM	980	O	LEU	62	28.854	42.666	54.914	1.00	0.00	O
ATOM	981	N	ASP	63	28.487	41.185	56.487	1.00	0.00	N
ATOM	982	H	ASP	63	28.057	40.292	56.682	1.00	0.00	H
ATOM	983	CA	ASP	63	29.480	41.646	57.488	1.00	0.00	C
ATOM	984	HA	ASP	63	29.425	42.732	57.568	1.00	0.00	H
ATOM	985	CB	ASP	63	29.091	40.947	58.872	1.00	0.00	C
ATOM	986	HB2	ASP	63	29.849	41.242	59.598	1.00	0.00	H
ATOM	987	HB3	ASP	63	28.160	41.344	59.278	1.00	0.00	H
ATOM	988	CG	ASP	63	29.030	39.437	58.807	1.00	0.00	C
ATOM	989	OD1	ASP	63	29.435	38.832	57.772	1.00	0.00	O
ATOM	990	OD2	ASP	63	28.721	38.878	59.857	1.00	0.00	O
ATOM	991	C	ASP	63	30.920	41.335	57.043	1.00	0.00	C
ATOM	992	O	ASP	63	31.793	41.976	57.632	1.00	0.00	O
ATOM	993	N	THR	64	31.252	40.488	56.108	1.00	0.00	N
ATOM	994	H	THR	64	30.499	39.956	55.694	1.00	0.00	H
ATOM	995	CA	THR	64	32.622	39.913	55.978	1.00	0.00	C
ATOM	996	HA	THR	64	33.451	40.573	56.233	1.00	0.00	H
ATOM	997	CB	THR	64	32.860	38.634	56.831	1.00	0.00	C
ATOM	998	HB	THR	64	33.828	38.207	56.570	1.00	0.00	H
ATOM	999	CG2	THR	64	32.949	38.908	58.338	1.00	0.00	C
ATOM	1000	HG21	THR	64	32.946	37.904	58.761	1.00	0.00	H
ATOM	1001	HG22	THR	64	33.780	39.596	58.497	1.00	0.00	H
ATOM	1002	HG23	THR	64	32.123	39.443	58.806	1.00	0.00	H
ATOM	1003	OG1	THR	64	31.846	37.694	56.642	1.00	0.00	O
ATOM	1004	HG1	THR	64	31.011	38.096	56.891	1.00	0.00	H
ATOM	1005	C	THR	64	32.927	39.568	54.466	1.00	0.00	C
ATOM	1006	O	THR	64	33.775	38.763	54.122	1.00	0.00	O
ATOM	1007	N	LEU	65	32.272	40.296	53.564	1.00	0.00	N
ATOM	1008	H	LEU	65	31.554	40.949	53.844	1.00	0.00	H

ATOM	1009	CA	LEU	65	32.733	40.551	52.172	1.00	0.00	C
ATOM	1010	HA	LEU	65	32.439	39.679	51.588	1.00	0.00	H
ATOM	1011	CB	LEU	65	31.865	41.762	51.645	1.00	0.00	C
ATOM	1012	HB2	LEU	65	31.905	42.601	52.341	1.00	0.00	H
ATOM	1013	HB3	LEU	65	32.366	42.106	50.740	1.00	0.00	H
ATOM	1014	CG	LEU	65	30.406	41.590	51.345	1.00	0.00	C
ATOM	1015	HG	LEU	65	29.899	41.621	52.310	1.00	0.00	H
ATOM	1016	CD1	LEU	65	29.923	42.803	50.519	1.00	0.00	C
ATOM	1017	HD11	LEU	65	30.324	43.742	50.901	1.00	0.00	H
ATOM	1018	HD12	LEU	65	30.294	42.791	49.494	1.00	0.00	H
ATOM	1019	HD13	LEU	65	28.836	42.859	50.458	1.00	0.00	H
ATOM	1020	CD2	LEU	65	30.046	40.293	50.705	1.00	0.00	C
ATOM	1021	HD21	LEU	65	30.776	40.045	49.934	1.00	0.00	H
ATOM	1022	HD22	LEU	65	29.940	39.534	51.480	1.00	0.00	H
ATOM	1023	HD23	LEU	65	29.018	40.318	50.344	1.00	0.00	H
ATOM	1024	C	LEU	65	34.251	40.859	52.119	1.00	0.00	C
ATOM	1025	O	LEU	65	34.839	41.535	52.974	1.00	0.00	O
ATOM	1026	N	THR	66	34.881	40.234	51.158	1.00	0.00	N
ATOM	1027	H	THR	66	34.338	39.583	50.608	1.00	0.00	H
ATOM	1028	CA	THR	66	36.344	40.273	51.011	1.00	0.00	C
ATOM	1029	HA	THR	66	36.704	40.212	52.039	1.00	0.00	H
ATOM	1030	CB	THR	66	36.707	39.084	50.137	1.00	0.00	C
ATOM	1031	HB	THR	66	36.340	38.230	50.706	1.00	0.00	H
ATOM	1032	CG2	THR	66	36.211	39.106	48.716	1.00	0.00	C
ATOM	1033	HG21	THR	66	36.902	39.653	48.074	1.00	0.00	H
ATOM	1034	HG22	THR	66	36.296	38.076	48.373	1.00	0.00	H
ATOM	1035	HG23	THR	66	35.259	39.593	48.505	1.00	0.00	H
ATOM	1036	OG1	THR	66	38.138	39.007	50.079	1.00	0.00	O
ATOM	1037	HG1	THR	66	38.470	38.763	50.945	1.00	0.00	H
ATOM	1038	C	THR	66	36.700	41.682	50.571	1.00	0.00	C
ATOM	1039	O	THR	66	36.221	42.132	49.527	1.00	0.00	O
ATOM	1040	N	GLU	67	37.672	42.180	51.326	1.00	0.00	N
ATOM	1041	H	GLU	67	37.896	41.708	52.191	1.00	0.00	H
ATOM	1042	CA	GLU	67	38.347	43.369	50.831	1.00	0.00	C
ATOM	1043	HA	GLU	67	37.572	44.134	50.865	1.00	0.00	H
ATOM	1044	CB	GLU	67	39.409	43.760	51.868	1.00	0.00	C
ATOM	1045	HB2	GLU	67	39.981	44.626	51.532	1.00	0.00	H
ATOM	1046	HB3	GLU	67	39.058	44.012	52.869	1.00	0.00	H
ATOM	1047	CG	GLU	67	40.498	42.685	52.063	1.00	0.00	C
ATOM	1048	HG2	GLU	67	40.828	42.273	51.110	1.00	0.00	H
ATOM	1049	HG3	GLU	67	41.402	43.170	52.432	1.00	0.00	H
ATOM	1050	CD	GLU	67	40.206	41.586	53.052	1.00	0.00	C
ATOM	1051	OE1	GLU	67	39.088	41.313	53.453	1.00	0.00	O
ATOM	1052	OE2	GLU	67	41.218	40.970	53.527	1.00	0.00	O
ATOM	1053	C	GLU	67	39.040	43.199	49.444	1.00	0.00	C
ATOM	1054	O	GLU	67	39.291	44.133	48.721	1.00	0.00	O
ATOM	1055	N	LYS	68	39.486	41.957	49.085	1.00	0.00	N
ATOM	1056	H	LYS	68	39.399	41.141	49.674	1.00	0.00	H
ATOM	1057	CA	LYS	68	40.200	41.625	47.857	1.00	0.00	C
ATOM	1058	HA	LYS	68	41.005	42.340	47.690	1.00	0.00	H
ATOM	1059	CB	LYS	68	40.777	40.219	48.060	1.00	0.00	C
ATOM	1060	HB2	LYS	68	41.127	39.985	49.065	1.00	0.00	H
ATOM	1061	HB3	LYS	68	39.910	39.602	47.826	1.00	0.00	H
ATOM	1062	CG	LYS	68	41.962	39.905	47.148	1.00	0.00	C
ATOM	1063	HG2	LYS	68	41.746	40.272	46.145	1.00	0.00	H
ATOM	1064	HG3	LYS	68	42.835	40.415	47.557	1.00	0.00	H
ATOM	1065	CD	LYS	68	42.244	38.373	47.092	1.00	0.00	C
ATOM	1066	HD2	LYS	68	42.398	37.925	48.073	1.00	0.00	H
ATOM	1067	HD3	LYS	68	41.418	37.868	46.591	1.00	0.00	H
ATOM	1068	CE	LYS	68	43.537	38.233	46.262	1.00	0.00	C
ATOM	1069	HE2	LYS	68	43.169	38.435	45.256	1.00	0.00	H
ATOM	1070	HE3	LYS	68	44.230	39.026	46.545	1.00	0.00	H
ATOM	1071	NZ	LYS	68	44.024	36.841	46.304	1.00	0.00	N
ATOM	1072	HZ1	LYS	68	43.357	36.126	46.050	1.00	0.00	H

ATOM	1073	HZ2	LYS	68	44.839	36.716	45.723	1.00	0.00	H
ATOM	1074	HZ3	LYS	68	44.159	36.428	47.216	1.00	0.00	H
ATOM	1075	C	LYS	68	39.408	41.723	46.580	1.00	0.00	C
ATOM	1076	O	LYS	68	39.977	42.141	45.559	1.00	0.00	O
ATOM	1077	N	HIE	69	38.066	41.532	46.659	1.00	0.00	N
ATOM	1078	H	HIE	69	37.754	41.433	47.614	1.00	0.00	H
ATOM	1079	CA	HIE	69	37.054	41.868	45.663	1.00	0.00	C
ATOM	1080	HA	HIE	69	37.497	41.608	44.702	1.00	0.00	H
ATOM	1081	CB	HIE	69	35.804	41.060	45.807	1.00	0.00	C
ATOM	1082	HB2	HIE	69	36.015	39.993	45.869	1.00	0.00	H
ATOM	1083	HB3	HIE	69	35.310	41.426	46.708	1.00	0.00	H
ATOM	1084	CG	HIE	69	34.840	41.233	44.738	1.00	0.00	C
ATOM	1085	ND1	HIE	69	35.056	40.609	43.474	1.00	0.00	N
ATOM	1086	CE1	HIE	69	34.238	41.210	42.586	1.00	0.00	C
ATOM	1087	HE1	HIE	69	34.232	41.105	41.511	1.00	0.00	H
ATOM	1088	NE2	HIE	69	33.562	42.179	43.261	1.00	0.00	N
ATOM	1089	HE2	HIE	69	32.933	42.885	42.908	1.00	0.00	H
ATOM	1090	CD2	HIE	69	33.935	42.167	44.586	1.00	0.00	C
ATOM	1091	HD2	HIE	69	33.565	42.826	45.357	1.00	0.00	H
ATOM	1092	C	HIE	69	36.803	43.364	45.560	1.00	0.00	C
ATOM	1093	O	HIE	69	36.985	43.955	44.488	1.00	0.00	O
ATOM	1094	N	ALA	70	36.632	44.093	46.715	1.00	0.00	N
ATOM	1095	H	ALA	70	36.362	43.630	47.571	1.00	0.00	H
ATOM	1096	CA	ALA	70	36.482	45.543	46.732	1.00	0.00	C
ATOM	1097	HA	ALA	70	35.533	45.809	46.268	1.00	0.00	H
ATOM	1098	CB	ALA	70	36.449	45.977	48.160	1.00	0.00	C
ATOM	1099	HB1	ALA	70	36.517	47.064	48.180	1.00	0.00	H
ATOM	1100	HB2	ALA	70	35.469	45.711	48.556	1.00	0.00	H
ATOM	1101	HB3	ALA	70	37.288	45.619	48.757	1.00	0.00	H
ATOM	1102	C	ALA	70	37.728	46.245	46.109	1.00	0.00	C
ATOM	1103	O	ALA	70	37.505	47.179	45.284	1.00	0.00	O
ATOM	1104	N	GLU	71	38.909	45.844	46.432	1.00	0.00	N
ATOM	1105	H	GLU	71	39.030	45.326	47.289	1.00	0.00	H
ATOM	1106	CA	GLU	71	40.027	46.494	45.805	1.00	0.00	C
ATOM	1107	HA	GLU	71	39.925	47.564	45.997	1.00	0.00	H
ATOM	1108	CB	GLU	71	41.290	45.964	46.563	1.00	0.00	C
ATOM	1109	HB2	GLU	71	41.156	46.041	47.642	1.00	0.00	H
ATOM	1110	HB3	GLU	71	41.379	44.935	46.212	1.00	0.00	H
ATOM	1111	CG	GLU	71	42.568	46.758	46.148	1.00	0.00	C
ATOM	1112	HG2	GLU	71	42.976	46.249	45.275	1.00	0.00	H
ATOM	1113	HG3	GLU	71	42.277	47.796	45.987	1.00	0.00	H
ATOM	1114	CD	GLU	71	43.619	46.783	47.293	1.00	0.00	C
ATOM	1115	OE1	GLU	71	44.513	45.925	47.260	1.00	0.00	O
ATOM	1116	OE2	GLU	71	43.546	47.632	48.181	1.00	0.00	O
ATOM	1117	C	GLU	71	40.194	46.232	44.272	1.00	0.00	C
ATOM	1118	O	GLU	71	40.722	47.099	43.568	1.00	0.00	O
ATOM	1119	N	GLN	72	39.696	45.138	43.760	1.00	0.00	N
ATOM	1120	H	GLN	72	39.241	44.588	44.474	1.00	0.00	H
ATOM	1121	CA	GLN	72	39.673	44.872	42.333	1.00	0.00	C
ATOM	1122	HA	GLN	72	40.569	45.242	41.834	1.00	0.00	H
ATOM	1123	CB	GLN	72	39.709	43.268	42.186	1.00	0.00	C
ATOM	1124	HB2	GLN	72	38.990	42.832	42.880	1.00	0.00	H
ATOM	1125	HB3	GLN	72	40.706	43.015	42.544	1.00	0.00	H
ATOM	1126	CG	GLN	72	39.223	42.686	40.835	1.00	0.00	C
ATOM	1127	HG2	GLN	72	39.573	43.248	39.969	1.00	0.00	H
ATOM	1128	HG3	GLN	72	39.690	41.702	40.853	1.00	0.00	H
ATOM	1129	CD	GLN	72	37.751	42.289	40.799	1.00	0.00	C
ATOM	1130	OE1	GLN	72	37.380	41.602	39.869	1.00	0.00	O
ATOM	1131	NE2	GLN	72	36.850	42.848	41.620	1.00	0.00	N
ATOM	1132	HE21	GLN	72	37.226	43.557	42.233	1.00	0.00	H
ATOM	1133	HE22	GLN	72	35.852	42.736	41.513	1.00	0.00	H
ATOM	1134	C	GLN	72	38.373	45.388	41.645	1.00	0.00	C
ATOM	1135	O	GLN	72	38.224	45.425	40.440	1.00	0.00	O

ATOM	1136	N	GLU	73	37.441	45.911	42.419	1.00	0.00	N
ATOM	1137	H	GLU	73	37.461	45.659	43.397	1.00	0.00	H
ATOM	1138	CA	GLU	73	36.524	46.998	41.949	1.00	0.00	C
ATOM	1139	HA	GLU	73	36.348	46.671	40.925	1.00	0.00	H
ATOM	1140	CB	GLU	73	35.181	46.976	42.740	1.00	0.00	C
ATOM	1141	HB2	GLU	73	35.354	47.160	43.800	1.00	0.00	H
ATOM	1142	HB3	GLU	73	34.581	47.779	42.312	1.00	0.00	H
ATOM	1143	CG	GLU	73	34.385	45.678	42.564	1.00	0.00	C
ATOM	1144	HG2	GLU	73	34.743	44.875	43.210	1.00	0.00	H
ATOM	1145	HG3	GLU	73	33.404	45.897	42.985	1.00	0.00	H
ATOM	1146	CD	GLU	73	34.212	45.235	41.114	1.00	0.00	C
ATOM	1147	OE1	GLU	73	34.040	44.031	40.935	1.00	0.00	O
ATOM	1148	OE2	GLU	73	34.276	46.109	40.171	1.00	0.00	O
ATOM	1149	C	GLU	73	37.091	48.457	41.983	1.00	0.00	C
ATOM	1150	O	GLU	73	36.494	49.413	41.441	1.00	0.00	O
ATOM	1151	N	ASN	74	38.251	48.605	42.597	1.00	0.00	N
ATOM	1152	H	ASN	74	38.578	47.774	43.069	1.00	0.00	H
ATOM	1153	CA	ASN	74	38.788	49.921	43.039	1.00	0.00	C
ATOM	1154	HA	ASN	74	39.495	49.514	43.760	1.00	0.00	H
ATOM	1155	CB	ASN	74	39.445	50.687	41.938	1.00	0.00	C
ATOM	1156	HB2	ASN	74	38.705	51.272	41.391	1.00	0.00	H
ATOM	1157	HB3	ASN	74	40.116	51.417	42.388	1.00	0.00	H
ATOM	1158	CG	ASN	74	40.324	49.879	40.984	1.00	0.00	C
ATOM	1159	OD1	ASN	74	40.421	50.161	39.767	1.00	0.00	O
ATOM	1160	ND2	ASN	74	41.051	48.828	41.316	1.00	0.00	N
ATOM	1161	HD21	ASN	74	41.437	48.287	40.555	1.00	0.00	H
ATOM	1162	HD22	ASN	74	40.980	48.520	42.275	1.00	0.00	H
ATOM	1163	C	ASN	74	37.901	50.744	43.983	1.00	0.00	C
ATOM	1164	O	ASN	74	38.006	51.989	44.110	1.00	0.00	O
ATOM	1165	N	MET	75	36.987	50.066	44.659	1.00	0.00	N
ATOM	1166	H	MET	75	36.904	49.072	44.502	1.00	0.00	H
ATOM	1167	CA	MET	75	36.148	50.672	45.745	1.00	0.00	C
ATOM	1168	HA	MET	75	36.173	51.743	45.542	1.00	0.00	H
ATOM	1169	CB	MET	75	34.700	50.210	45.592	1.00	0.00	C
ATOM	1170	HB2	MET	75	34.648	49.134	45.757	1.00	0.00	H
ATOM	1171	HB3	MET	75	34.086	50.736	46.322	1.00	0.00	H
ATOM	1172	CG	MET	75	34.034	50.423	44.245	1.00	0.00	C
ATOM	1173	HG2	MET	75	33.896	51.474	43.990	1.00	0.00	H
ATOM	1174	HG3	MET	75	34.689	49.929	43.528	1.00	0.00	H
ATOM	1175	SD	MET	75	32.421	49.601	44.105	1.00	0.00	S
ATOM	1176	CE	MET	75	31.409	50.589	45.309	1.00	0.00	C
ATOM	1177	HE1	MET	75	31.642	50.216	46.307	1.00	0.00	H
ATOM	1178	HE2	MET	75	31.616	51.658	45.255	1.00	0.00	H
ATOM	1179	HE3	MET	75	30.343	50.433	45.150	1.00	0.00	H
ATOM	1180	C	MET	75	36.731	50.299	47.135	1.00	0.00	C
ATOM	1181	O	MET	75	37.496	49.340	47.393	1.00	0.00	O
ATOM	1182	N	THR	76	36.461	51.148	48.090	1.00	0.00	N
ATOM	1183	H	THR	76	36.143	52.058	47.787	1.00	0.00	H
ATOM	1184	CA	THR	76	36.609	50.908	49.542	1.00	0.00	C
ATOM	1185	HA	THR	76	37.559	50.390	49.680	1.00	0.00	H
ATOM	1186	CB	THR	76	36.649	52.245	50.324	1.00	0.00	C
ATOM	1187	HB	THR	76	37.370	52.853	49.779	1.00	0.00	H
ATOM	1188	CG2	THR	76	35.359	53.110	50.430	1.00	0.00	C
ATOM	1189	HG21	THR	76	34.651	52.615	51.095	1.00	0.00	H
ATOM	1190	HG22	THR	76	35.629	54.103	50.787	1.00	0.00	H
ATOM	1191	HG23	THR	76	34.844	53.051	49.471	1.00	0.00	H
ATOM	1192	OG1	THR	76	37.026	51.877	51.672	1.00	0.00	O
ATOM	1193	HG1	THR	76	37.737	52.465	51.936	1.00	0.00	H
ATOM	1194	C	THR	76	35.519	49.921	50.059	1.00	0.00	C
ATOM	1195	O	THR	76	34.402	49.926	49.592	1.00	0.00	O
ATOM	1196	N	LEU	77	35.782	49.017	51.037	1.00	0.00	N
ATOM	1197	H	LEU	77	36.722	48.909	51.393	1.00	0.00	H
ATOM	1198	CA	LEU	77	34.775	47.877	51.299	1.00	0.00	C
ATOM	1199	HA	LEU	77	34.567	47.388	50.347	1.00	0.00	H

```
ATOM   1200  CB  LEU    77      35.350  46.850  52.335  1.00  0.00           C
ATOM   1201  HB2 LEU    77      36.315  46.469  52.002  1.00  0.00           H
ATOM   1202  HB3 LEU    77      35.702  47.302  53.263  1.00  0.00           H
ATOM   1203  CG  LEU    77      34.384  45.703  52.665  1.00  0.00           C
ATOM   1204  HG  LEU    77      33.474  46.116  53.102  1.00  0.00           H
ATOM   1205  CD1 LEU    77      34.009  44.795  51.458  1.00  0.00           C
ATOM   1206 HD11 LEU    77      33.464  44.030  52.012  1.00  0.00           H
ATOM   1207 HD12 LEU    77      33.312  45.355  50.835  1.00  0.00           H
ATOM   1208 HD13 LEU    77      34.857  44.461  50.859  1.00  0.00           H
ATOM   1209  CD2 LEU    77      35.117  44.752  53.651  1.00  0.00           C
ATOM   1210 HD21 LEU    77      34.611  43.823  53.911  1.00  0.00           H
ATOM   1211 HD22 LEU    77      36.092  44.667  53.171  1.00  0.00           H
ATOM   1212 HD23 LEU    77      35.379  45.309  54.551  1.00  0.00           H
ATOM   1213  C   LEU    77      33.366  48.396  51.802  1.00  0.00           C
ATOM   1214  O   LEU    77      32.316  47.802  51.568  1.00  0.00           O
ATOM   1215  N   THR    78      33.389  49.499  52.628  1.00  0.00           N
ATOM   1216  H   THR    78      34.280  49.885  52.906  1.00  0.00           H
ATOM   1217  CA  THR    78      32.130  50.109  53.182  1.00  0.00           C
ATOM   1218  HA  THR
    78      31.552  49.353  53.715  1.00  0.00           H
ATOM   1219  CB  THR    78      32.434  51.291  54.150  1.00  0.00           C
ATOM   1220  HB  THR    78      31.488  51.834  54.138  1.00  0.00           H
ATOM   1221  CG2 THR    78      32.823  50.821  55.538  1.00  0.00           C
ATOM   1222 HG21 THR    78      33.023  49.749  55.516  1.00  0.00           H
ATOM   1223 HG22 THR    78      33.806  51.183  55.836  1.00  0.00           H
ATOM   1224 HG23 THR    78      32.034  51.139  56.219  1.00  0.00           H
ATOM   1225  OG1 THR    78      33.386  52.188  53.600  1.00  0.00           O
ATOM   1226  HG1 THR    78      34.184  51.896  54.048  1.00  0.00           H
ATOM   1227  C   THR    78      31.199  50.525  52.084  1.00  0.00           C
ATOM   1228  O   THR    78      30.024  50.307  52.163  1.00  0.00           O
ATOM   1229  N   GLU    79      31.809  51.119  51.109  1.00  0.00           N
ATOM   1230  H   GLU    79      32.805  51.088  50.950  1.00  0.00           H
ATOM   1231  CA  GLU    79      31.117  51.645  49.958  1.00  0.00           C
ATOM   1232  HA  GLU    79      30.272  52.287  50.207  1.00  0.00           H
ATOM   1233  CB  GLU    79      32.065  52.394  49.068  1.00  0.00           C
ATOM   1234  HB2 GLU    79      32.567  53.230  49.553  1.00  0.00           H
ATOM   1235  HB3 GLU    79      32.752  51.710  48.570  1.00  0.00           H
ATOM   1236  CG  GLU    79      31.082  53.069  48.089  1.00  0.00           C
ATOM   1237  HG2 GLU    79      30.611  52.347  47.422  1.00  0.00           H
ATOM   1238  HG3 GLU    79      30.227  53.509  48.603  1.00  0.00           H
ATOM   1239  CD  GLU    79      31.722  54.058  47.079  1.00  0.00           C
ATOM   1240  OE1 GLU    79      31.118  54.427  46.039  1.00  0.00           O
ATOM   1241  OE2 GLU    79      32.866  54.527  47.340  1.00  0.00           O
ATOM   1242  C   GLU    79      30.418  50.435  49.193  1.00  0.00           C
ATOM   1243  O   GLU    79      29.246  50.468  48.919  1.00  0.00           O
ATOM   1244  N   LEU    80      31.178  49.382  48.833  1.00  0.00           N
ATOM   1245  H   LEU    80      32.076  49.376  49.295  1.00  0.00           H
ATOM   1246  CA  LEU    80      30.661  48.186  48.219  1.00  0.00           C
ATOM   1247  HA  LEU    80      30.240  48.475  47.255  1.00  0.00           H
ATOM   1248  CB  LEU    80      31.784  47.240  47.775  1.00  0.00           C
ATOM   1249  HB2 LEU    80      32.426  47.847  47.136  1.00  0.00           H
ATOM   1250  HB3 LEU    80      32.293  46.874  48.666  1.00  0.00           H
ATOM   1251  CG  LEU    80      31.183  46.012  47.006  1.00  0.00           C
ATOM   1252  HG  LEU    80      30.461  45.501  47.643  1.00  0.00           H
ATOM   1253  CD1 LEU    80      30.549  46.263  45.625  1.00  0.00           C
ATOM   1254 HD11 LEU    80      30.345  45.296  45.162  1.00  0.00           H
ATOM   1255 HD12 LEU    80      29.709  46.946  45.750  1.00  0.00           H
ATOM   1256 HD13 LEU    80      31.354  46.723  45.051  1.00  0.00           H
ATOM   1257  CD2 LEU    80      32.291  44.945  46.852  1.00  0.00           C
ATOM   1258 HD21 LEU    80      33.127  45.405  46.324  1.00  0.00           H
ATOM   1259 HD22 LEU    80      32.637  44.511  47.790  1.00  0.00           H
ATOM   1260 HD23 LEU    80      31.953  44.087  46.269  1.00  0.00           H
ATOM   1261  C   LEU    80      29.573  47.498  49.121  1.00  0.00           C
ATOM   1262  O   LEU    80      28.580  46.991  48.548  1.00  0.00           O
```

ATOM	1263	N	LYS	81	29.828	47.448	50.457	1.00	0.00	N
ATOM	1264	H	LYS	81	30.718	47.752	50.825	1.00	0.00	H
ATOM	1265	CA	LYS	81	28.822	46.973	51.426	1.00	0.00	C
ATOM	1266	HA	LYS	81	28.549	45.937	51.230	1.00	0.00	H
ATOM	1267	CB	LYS	81	29.337	47.072	52.859	1.00	0.00	C
ATOM	1268	HB2	LYS	81	30.080	47.857	52.991	1.00	0.00	H
ATOM	1269	HB3	LYS	81	28.544	47.244	53.587	1.00	0.00	H
ATOM	1270	CG	LYS	81	30.085	45.766	53.153	1.00	0.00	C
ATOM	1271	HG2	LYS	81	29.411	44.914	53.058	1.00	0.00	H
ATOM	1272	HG3	LYS	81	30.879	45.634	52.420	1.00	0.00	H
ATOM	1273	CD	LYS	81	30.748	45.810	54.506	1.00	0.00	C
ATOM	1274	HD2	LYS	81	31.434	46.651	54.612	1.00	0.00	H
ATOM	1275	HD3	LYS	81	29.905	45.884	55.193	1.00	0.00	H
ATOM	1276	CE	LYS	81	31.478	44.516	54.758	1.00	0.00	C
ATOM	1277	HE2	LYS	81	30.707	43.764	54.589	1.00	0.00	H
ATOM	1278	HE3	LYS	81	32.308	44.474	54.052	1.00	0.00	H
ATOM	1279	NZ	LYS	81	32.159	44.502	56.084	1.00	0.00	N
ATOM	1280	HZ1	LYS	81	32.685	43.655	56.237	1.00	0.00	H
ATOM	1281	HZ2	LYS	81	32.937	45.139	56.178	1.00	0.00	H
ATOM	1282	HZ3	LYS	81	31.530	44.606	56.868	1.00	0.00	H
ATOM	1283	C	LYS	81	27.513	47.715	51.248	1.00	0.00	C
ATOM	1284	O	LYS	81	26.544	47.064	51.188	1.00	0.00	O
ATOM	1285	N	LYS	82	27.457	49.013	51.210	1.00	0.00	N
ATOM	1286	H	LYS	82	28.321	49.534	51.208	1.00	0.00	H
ATOM	1287	CA	LYS	82	26.259	49.815	51.161	1.00	0.00	C
ATOM	1288	HA	LYS	82	25.639	49.463	51.986	1.00	0.00	H
ATOM	1289	CB	LYS	82	26.522	51.313	51.261	1.00	0.00	C
ATOM	1290	HB2	LYS	82	27.407	51.564	50.675	1.00	0.00	H
ATOM	1291	HB3	LYS	82	25.740	51.950	50.845	1.00	0.00	H
ATOM	1292	CG	LYS	82	26.723	51.731	52.720	1.00	0.00	C
ATOM	1293	HG2	LYS	82	25.878	51.427	53.339	1.00	0.00	H
ATOM	1294	HG3	LYS	82	27.612	51.182	53.032	1.00	0.00	H
ATOM	1295	CD	LYS	82	26.930	53.230	53.013	1.00	0.00	C
ATOM	1296	HD2	LYS	82	27.765	53.521	52.375	1.00	0.00	H
ATOM	1297	HD3	LYS	82	26.063	53.877	52.877	1.00	0.00	H
ATOM	1298	CE	LYS	82	27.343	53.395	54.535	1.00	0.00	C
ATOM	1299	HE2	LYS	82	26.443	53.606	55.112	1.00	0.00	H
ATOM	1300	HE3	LYS	82	27.804	52.510	54.975	1.00	0.00	H
ATOM	1301	NZ	LYS	82	28.171	54.646	54.633	1.00	0.00	N
ATOM	1302	HZ1	LYS	82	29.080	54.667	54.194	1.00	0.00	H
ATOM	1303	HZ2	LYS	82	27.602	55.417	54.313	1.00	0.00	H
ATOM	1304	HZ3	LYS	82	28.512	54.929	55.541	1.00	0.00	H
ATOM	1305	C	LYS	82	25.488	49.509	49.919	1.00	0.00	C
ATOM	1306	O	LYS	82	24.362	49.024	50.013	1.00	0.00	O
ATOM	1307	N	VAL	83	26.087	49.677	48.722	1.00	0.00	N
ATOM	1308	H	VAL	83	27.060	49.941	48.655	1.00	0.00	H
ATOM	1309	CA	VAL	83	25.479	49.304	47.482	1.00	0.00	C
ATOM	1310	HA	VAL	83	24.561	49.878	47.367	1.00	0.00	H
ATOM	1311	CB	VAL	83	26.233	49.736	46.200	1.00	0.00	C
ATOM	1312	HB	VAL	83	25.488	49.799	45.408	1.00	0.00	H
ATOM	1313	CG1	VAL	83	26.900	51.148	46.268	1.00	0.00	C
ATOM	1314	HG11	VAL	83	26.173	51.958	46.316	1.00	0.00	H
ATOM	1315	HG12	VAL	83	27.679	51.182	47.030	1.00	0.00	H
ATOM	1316	HG13	VAL	83	27.431	51.235	45.319	1.00	0.00	H
ATOM	1317	CG2	VAL	83	27.296	48.796	45.601	1.00	0.00	C
ATOM	1318	HG21	VAL	83	28.170	48.810	46.254	1.00	0.00	H
ATOM	1319	HG22	VAL	83	26.952	47.764	45.664	1.00	0.00	H
ATOM	1320	HG23	VAL	83	27.518	49.020	44.558	1.00	0.00	H
ATOM	1321	C	VAL	83	25.036	47.854	47.391	1.00	0.00	C
ATOM	1322	O	VAL	83	24.180	47.537	46.598	1.00	0.00	O
ATOM	1323	N	ILE	84	25.488	46.884	48.200	1.00	0.00	N
ATOM	1324	H	ILE	84	26.299	47.020	48.785	1.00	0.00	H
ATOM	1325	CA	ILE	84	24.982	45.521	48.229	1.00	0.00	C
ATOM	1326	HA	ILE	84	24.463	45.362	47.285	1.00	0.00	H

```
ATOM   1327  CB  ILE    84      26.142  44.471  48.299  1.00  0.00           C
ATOM   1328  HB  ILE    84      26.647  44.792  49.209  1.00  0.00           H
ATOM   1329  CG2 ILE    84      25.536  43.105  48.587  1.00  0.00           C
ATOM   1330 HG21 ILE    84      26.306  42.348  48.733  1.00  0.00           H
ATOM   1331 HG22 ILE    84      24.868  42.973  49.439  1.00  0.00           H
ATOM   1332 HG23 ILE    84      24.945  42.889  47.697  1.00  0.00           H
ATOM   1333  CG1 ILE    84      26.882  44.438  47.025  1.00  0.00           C
ATOM   1334 HG12 ILE    84      26.241  44.058  46.228  1.00  0.00           H
ATOM   1335 HG13 ILE    84      27.296  45.427  46.828  1.00  0.00           H
ATOM   1336  CD1 ILE    84      28.142  43.554  47.193  1.00  0.00           C
ATOM   1337 HD11 ILE    84      28.642  43.436  46.232  1.00  0.00           H
ATOM   1338 HD12 ILE    84      28.945  43.965  47.805  1.00  0.00           H
ATOM   1339 HD13 ILE    84      27.923  42.532  47.506  1.00  0.00           H
ATOM   1340  C   ILE    84      23.883  45.435  49.316  1.00  0.00           C
ATOM   1341  O   ILE    84      22.834  44.776  49.045  1.00  0.00           O
ATOM   1342  N   ALA    85      23.959  46.145  50.498  1.00  0.00           N
ATOM   1343  H   ALA    85      24.670  46.850
  50.627  1.00  0.00           H
ATOM   1344  CA  ALA    85      22.970  46.040  51.554  1.00  0.00           C
ATOM   1345  HA  ALA    85      22.632  45.004  51.541  1.00  0.00           H
ATOM   1346  CB  ALA    85      23.600  46.477  52.912  1.00  0.00           C
ATOM   1347  HB1 ALA    85      23.994  47.483  52.763  1.00  0.00           H
ATOM   1348  HB2 ALA    85      22.903  46.365  53.742  1.00  0.00           H
ATOM   1349  HB3 ALA    85      24.435  45.804  53.107  1.00  0.00           H
ATOM   1350  C   ALA    85      21.715  46.854  51.233  1.00  0.00           C
ATOM   1351  O   ALA    85      20.711  46.743  51.952  1.00  0.00           O
ATOM   1352  N   ASP    86      21.666  47.647  50.187  1.00  0.00           N
ATOM   1353  H   ASP    86      22.540  47.809  49.706  1.00  0.00           H
ATOM   1354  CA  ASP    86      20.395  48.268  49.641  1.00  0.00           C
ATOM   1355  HA  ASP    86      19.672  48.340  50.453  1.00  0.00           H
ATOM   1356  CB  ASP    86      20.609  49.756  49.285  1.00  0.00           C
ATOM   1357  HB2 ASP    86      21.010  50.169  50.211  1.00  0.00           H
ATOM   1358  HB3 ASP    86      21.373  49.948  48.531  1.00  0.00           H
ATOM   1359  CG  ASP    86      19.296  50.474  48.897  1.00  0.00           C
ATOM   1360  OD1 ASP    86      18.263  50.494  49.622  1.00  0.00           O
ATOM   1361  OD2 ASP    86      19.269  51.225  47.898  1.00  0.00           O
ATOM   1362  C   ASP    86      19.718  47.422  48.516  1.00  0.00           C
ATOM   1363  O   ASP    86      18.498  47.646  48.305  1.00  0.00           O
ATOM   1364  N   ILE    87      20.395  46.414  47.941  1.00  0.00           N
ATOM   1365  H   ILE    87      21.365  46.279  48.186  1.00  0.00           H
ATOM   1366  CA  ILE    87      19.849  45.416  47.054  1.00  0.00           C
ATOM   1367  HA  ILE    87      19.047  45.807  46.429  1.00  0.00           H
ATOM   1368  CB  ILE    87      20.986  44.964  45.996  1.00  0.00           C
ATOM   1369  HB  ILE    87      21.878  44.577  46.488  1.00  0.00           H
ATOM   1370  CG2 ILE    87      20.436  43.822  45.217  1.00  0.00           C
ATOM   1371 HG21 ILE    87      20.130  43.065  45.939  1.00  0.00           H
ATOM   1372 HG22 ILE    87      19.647  44.097  44.516  1.00  0.00           H
ATOM   1373 HG23 ILE    87      21.199  43.316  44.625  1.00  0.00           H
ATOM   1374  CG1 ILE    87      21.526  46.134  45.083  1.00  0.00           C
ATOM   1375 HG12 ILE    87      20.655  46.586  44.609  1.00  0.00           H
ATOM   1376 HG13 ILE    87      22.030  46.906  45.665  1.00  0.00           H
ATOM   1377  CD1 ILE    87      22.327  45.606  43.893  1.00  0.00           C
ATOM   1378 HD11 ILE    87      23.079  44.857  44.144  1.00  0.00           H
ATOM   1379 HD12 ILE    87      21.714  45.215  43.081  1.00  0.00           H
ATOM   1380 HD13 ILE    87      22.838  46.452  43.433  1.00  0.00           H
ATOM   1381  C   ILE    87      19.215  44.252  47.871  1.00  0.00           C
ATOM   1382  O   ILE    87      18.137  43.711  47.599  1.00  0.00           O
ATOM   1383  N   TYR    88      20.013  43.752  48.860  1.00  0.00           N
ATOM   1384  H   TYR    88      20.874  44.239  49.065  1.00  0.00           H
ATOM   1385  CA  TYR    88      19.672  42.464  49.530  1.00  0.00           C
ATOM   1386  HA  TYR    88      18.823  42.038  48.995  1.00  0.00           H
ATOM   1387  CB  TYR    88      20.815  41.464  49.450  1.00  0.00           C
ATOM   1388  HB2 TYR    88      21.715  41.858  49.920  1.00  0.00           H
ATOM   1389  HB3 TYR    88      20.466  40.566  49.959  1.00  0.00           H
```

ATOM	1390	CG	TYR	88	21.275	40.972	48.132	1.00	0.00	C
ATOM	1391	CD1	TYR	88	20.415	40.118	47.428	1.00	0.00	C
ATOM	1392	HD1	TYR	88	19.610	39.576	47.904	1.00	0.00	H
ATOM	1393	CE1	TYR	88	20.774	39.625	46.198	1.00	0.00	C
ATOM	1394	HE1	TYR	88	20.184	38.851	45.729	1.00	0.00	H
ATOM	1395	CZ	TYR	88	21.945	40.074	45.607	1.00	0.00	C
ATOM	1396	OH	TYR	88	22.259	39.619	44.360	1.00	0.00	O
ATOM	1397	HH	TYR	88	21.801	38.811	44.117	1.00	0.00	H
ATOM	1398	CE2	TYR	88	22.843	40.909	46.301	1.00	0.00	C
ATOM	1399	HE2	TYR	88	23.684	41.365	45.802	1.00	0.00	H
ATOM	1400	CD2	TYR	88	22.443	41.394	47.541	1.00	0.00	C
ATOM	1401	HD2	TYR	88	23.000	42.124	48.109	1.00	0.00	H
ATOM	1402	C	TYR	88	19.391	42.697	51.008	1.00	0.00	C
ATOM	1403	O	TYR	88	20.115	43.531	51.593	1.00	0.00	O
ATOM	1404	N	PRO	89	18.414	42.062	51.688	1.00	0.00	N
ATOM	1405	CD	PRO	89	17.485	41.149	51.091	1.00	0.00	C
ATOM	1406	HD2	PRO	89	17.971	40.362	50.514	1.00	0.00	H
ATOM	1407	HD3	PRO	89	16.725	41.584	50.443	1.00	0.00	H
ATOM	1408	CG	PRO	89	16.889	40.364	52.336	1.00	0.00	C
ATOM	1409	HG2	PRO	89	17.528	39.497	52.505	1.00	0.00	H
ATOM	1410	HG3	PRO	89	15.884	39.977	52.160	1.00	0.00	H
ATOM	1411	CB	PRO	89	16.932	41.359	53.510	1.00	0.00	C
ATOM	1412	HB2	PRO	89	16.990	40.847	54.471	1.00	0.00	H
ATOM	1413	HB3	PRO	89	16.056	42.008	53.495	1.00	0.00	H
ATOM	1414	CA	PRO	89	18.283	42.074	53.160	1.00	0.00	C
ATOM	1415	HA	PRO	89	18.122	43.082	53.542	1.00	0.00	H
ATOM	1416	C	PRO	89	19.449	41.427	53.894	1.00	0.00	C
ATOM	1417	O	PRO	89	20.088	40.548	53.295	1.00	0.00	O
ATOM	1418	N	GLY	90	19.707	41.872	55.141	1.00	0.00	N
ATOM	1419	H	GLY	90	19.127	42.557	55.604	1.00	0.00	H
ATOM	1420	CA	GLY	90	20.909	41.521	55.878	1.00	0.00	C
ATOM	1421	HA2	GLY	90	21.736	41.707	55.192	1.00	0.00	H
ATOM	1422	HA3	GLY	90	21.005	42.070	56.815	1.00	0.00	H
ATOM	1423	C	GLY	90	21.058	39.959	56.110	1.00	0.00	C
ATOM	1424	O	GLY	90	22.191	39.478	56.188	1.00	0.00	O
ATOM	1425	N	GLN	91	19.926	39.271	56.071	1.00	0.00	N
ATOM	1426	H	GLN	91	19.040	39.754	56.028	1.00	0.00	H
ATOM	1427	CA	GLN	91	19.852	37.805	56.219	1.00	0.00	C
ATOM	1428	HA	GLN	91	20.356	37.512	57.139	1.00	0.00	H
ATOM	1429	CB	GLN	91	18.244	37.475	56.416	1.00	0.00	C
ATOM	1430	HB2	GLN	91	18.194	36.486	56.870	1.00	0.00	H
ATOM	1431	HB3	GLN	91	17.900	38.239	57.113	1.00	0.00	H
ATOM	1432	CG	GLN	91	17.363	37.523	55.114	1.00	0.00	C
ATOM	1433	HG2	GLN	91	17.515	38.528	54.718	1.00	0.00	H
ATOM	1434	HG3	GLN	91	17.747	36.712	54.495	1.00	0.00	H
ATOM	1435	CD	GLN	91	15.914	37.287	55.410	1.00	0.00	C
ATOM	1436	OE1	GLN	91	15.345	36.272	55.008	1.00	0.00	O
ATOM	1437	NE2	GLN	91	15.264	38.178	56.077	1.00	0.00	N
ATOM	1438	HE21	GLN	91	15.730	38.940	56.547	1.00	0.00	H
ATOM	1439	HE22	GLN	91	14.368	37.797	56.346	1.00	0.00	H
ATOM	1440	C	GLN	91	20.378	36.992	55.033	1.00	0.00	C
ATOM	1441	O	GLN	91	20.344	35.791	55.056	1.00	0.00	O
ATOM	1442	N	THR	92	20.758	37.719	53.963	1.00	0.00	N
ATOM	1443	H	THR	92	20.452	38.681	53.947	1.00	0.00	H
ATOM	1444	CA	THR	92	21.109	37.017	52.701	1.00	0.00	C
ATOM	1445	HA	THR	92	20.374	36.241	52.485	1.00	0.00	H
ATOM	1446	CB	THR	92	21.098	38.002	51.544	1.00	0.00	C
ATOM	1447	HB	THR	92	21.891	38.738	51.671	1.00	0.00	H
ATOM	1448	CG2	THR	92	21.218	37.393	50.249	1.00	0.00	C
ATOM	1449	HG21	THR	92	22.067	36.710	50.278	1.00	0.00	H
ATOM	1450	HG22	THR	92	20.400	36.776	49.878	1.00	0.00	H
ATOM	1451	HG23	THR	92	21.291	38.179	49.496	1.00	0.00	H
ATOM	1452	OG1	THR	92	19.830	38.601	51.534	1.00	0.00	O
ATOM	1453	HG1	THR	92	20.016	39.362	52.088	1.00	0.00	H

ATOM	1454	C	THR	92	22.497	36.336	52.808	1.00	0.00	C
ATOM	1455	O	THR	92	23.456	36.960	53.149	1.00	0.00	O
ATOM	1456	N	GLN	93	22.572	35.007	52.570	1.00	0.00	N
ATOM	1457	H	GLN	93	21.771	34.465	52.282	1.00	0.00	H
ATOM	1458	CA	GLN	93	23.881	34.346	52.431	1.00	0.00	C
ATOM	1459	HA	GLN	93	24.556	34.748	53.187	1.00	0.00	H
ATOM	1460	CB	GLN	93	23.816	32.778	52.596	1.00	0.00	C
ATOM	1461	HB2	GLN	93	23.222	32.343	53.399	1.00	0.00	H
ATOM	1462	HB3	GLN	93	23.248	32.359	51.764	1.00	0.00	H
ATOM	1463	CG	GLN	93	25.180	32.200	52.749	1.00	0.00	C
ATOM	1464	HG2	GLN	93	25.050	31.122	52.647	1.00	0.00	H
ATOM	1465	HG3	GLN	93	25.800	32.553	51.925	1.00	0.00	H
ATOM	1466	CD	GLN	93	25.909	32.419	54.069	1.00	0.00	C
ATOM	1467	OE1	GLN	93	25.324	32.951	55.035	1.00	0.00	O
ATOM	1468	NE2	GLN	93	27.080	31.913	54.225	1.00	0.00	N
ATOM	1469	HE21	GLN	93	27.517	31.358	53.503	1.00	0.00	H
ATOM	1470	HE22	GLN	93	27.474	32.167	55.120	1.00	0.00	H
ATOM	1471	C	GLN	93	24.452	34.748	51.037	1.00	0.00	C
ATOM	1472	O	GLN	93	23.701	34.724	50.036	1.00	0.00	O
ATOM	1473	N	PHE	94	25.737	34.903	50.987	1.00	0.00	N
ATOM	1474	H	PHE	94	26.212	34.670	51.846	1.00	0.00	H
ATOM	1475	CA	PHE	94	26.510	35.194	49.785	1.00	0.00	C
ATOM	1476	HA	PHE	94	25.846	35.099	48.926	1.00	0.00	H
ATOM	1477	CB	PHE	94	27.198	36.593	49.890	1.00	0.00	C
ATOM	1478	HB2	PHE	94	27.769	36.690	50.813	1.00	0.00	H
ATOM	1479	HB3	PHE	94	27.887	36.708	49.053	1.00	0.00	H
ATOM	1480	CG	PHE	94	26.197	37.747	49.854	1.00	0.00	C
ATOM	1481	CD1	PHE	94	25.997	38.590	48.753	1.00	0.00	C
ATOM	1482	HD1	PHE	94	26.494	38.332	47.829	1.00	0.00	H
ATOM	1483	CE1	PHE	94	25.113	39.646	48.777	1.00	0.00	C
ATOM	1484	HE1	PHE	94	24.887	40.290	47.939	1.00	0.00	H
ATOM	1485	CZ	PHE	94	24.567	40.052	50.032	1.00	0.00	C
ATOM	1486	HZ	PHE	94	23.879	40.882	50.101	1.00	0.00	H
ATOM	1487	CE2	PHE	94	24.872	39.343	51.160	1.00	0.00	C
ATOM	1488	HE2	PHE	94	24.460	39.676	52.101	1.00	0.00	H
ATOM	1489	CD2	PHE	94	25.641	38.150	51.084	1.00	0.00	C
ATOM	1490	HD2	PHE	94	25.870	37.517	51.929	1.00	0.00	H
ATOM	1491	C	PHE	94	27.643	34.224	49.358	1.00	0.00	C
ATOM	1492	O	PHE	94	28.133	33.373	50.171	1.00	0.00	O
ATOM	1493	N	TYR	95	27.996	34.100	48.064	1.00	0.00	N
ATOM	1494	H	TYR	95	27.622	34.775	47.411	1.00	0.00	H
ATOM	1495	CA	TYR	95	28.943	33.105	47.568	1.00	0.00	C
ATOM	1496	HA	TYR	95	29.502	32.703	48.412	1.00	0.00	H
ATOM	1497	CB	TYR	95	28.246	31.902	46.959	1.00	0.00	C
ATOM	1498	HB2	TYR	95	27.470	31.578	47.654	1.00	0.00	H
ATOM	1499	HB3	TYR	95	27.713	32.282	46.087	1.00	0.00	H
ATOM	1500	CG	TYR	95	29.141	30.723	46.739	1.00	0.00	C
ATOM	1501	CD1	TYR	95	29.894	30.642	45.573	1.00	0.00	C
ATOM	1502	HD1	TYR	95	29.660	31.285	44.737	1.00	0.00	H
ATOM	1503	CE1	TYR	95	30.759	29.560	45.396	1.00	0.00	C
ATOM	1504	HE1	TYR	95	31.308	29.414	44.477	1.00	0.00	H
ATOM	1505	CZ	TYR	95	30.943	28.594	46.458	1.00	0.00	C
ATOM	1506	OH	TYR	95	31.613	27.488	46.095	1.00	0.00	O
ATOM	1507	HH	TYR	95	31.847	27.512	45.165	1.00	0.00	H
ATOM	1508	CE2	TYR	95	30.303	28.782	47.722	1.00	0.00	C
ATOM	1509	HE2	TYR	95	30.394	28.001	48.463	1.00	0.00	H
ATOM	1510	CD2	TYR	95	29.290	29.763	47.810	1.00	0.00	C
ATOM	1511	HD2	TYR	95	28.743	29.806	48.740	1.00	0.00	H
ATOM	1512	C	TYR	95	29.968	33.653	46.511	1.00	0.00	C
ATOM	1513	O	TYR	95	29.538	34.412	45.609	1.00	0.00	O
ATOM	1514	N	VAL	96	31.212	33.272	46.677	1.00	0.00	N
ATOM	1515	H	VAL	96	31.330	32.506	47.326	1.00	0.00	H
ATOM	1516	CA	VAL	96	32.390	33.914	45.989	1.00	0.00	C

ATOM	1517	HA	VAL	96	32.040	34.760	45.396	1.00	0.00	H
ATOM	1518	CB	VAL	96	33.358	34.584	46.960	1.00	0.00	C
ATOM	1519	HB	VAL	96	33.572	33.790	47.676	1.00	0.00	H
ATOM	1520	CG1	VAL	96	34.627	35.118	46.303	1.00	0.00	C
ATOM	1521	HG11	VAL	96	34.287	35.934	45.667	1.00	0.00	H
ATOM	1522	HG12	VAL	96	35.399	35.435	47.003	1.00	0.00	H
ATOM	1523	HG13	VAL	96	35.153	34.312	45.791	1.00	0.00	H
ATOM	1524	CG2	VAL	96	32.656	35.689	47.765	1.00	0.00	C
ATOM	1525	HG21	VAL	96	32.041	36.238	47.050	1.00	0.00	H
ATOM	1526	HG22	VAL	96	31.902	35.300	48.448	1.00	0.00	H
ATOM	1527	HG23	VAL	96	33.380	36.243	48.364	1.00	0.00	H
ATOM	1528	C	VAL	96	33.090	32.948	45.078	1.00	0.00	C
ATOM	1529	O	VAL	96	33.200	31.766	45.410	1.00	0.00	O
ATOM	1530	N	ILE	97	33.538	33.528	43.925	1.00	0.00	N
ATOM	1531	H	ILE	97	33.315	34.467	43.626	1.00	0.00	H
ATOM	1532	CA	ILE	97	34.454	32.845	42.945	1.00	0.00	C
ATOM	1533	HA	ILE	97	34.737	31.865	43.331	1.00	0.00	H
ATOM	1534	CB	ILE	97	33.694	32.552	41.578	1.00	0.00	C
ATOM	1535	HB	ILE	97	33.648	33.501	41.043	1.00	0.00	H
ATOM	1536	CG2	ILE	97	34.383	31.416	40.754	1.00	0.00	C
ATOM	1537	HG21	ILE	97	35.259	31.879	40.301	1.00	0.00	H
ATOM	1538	HG22	ILE	97	34.621	30.593	41.428	1.00	0.00	H
ATOM	1539	HG23	ILE	97	33.736	31.185	39.909	1.00	0.00	H
ATOM	1540	CG1	ILE	97	32.230	32.043	41.715	1.00	0.00	C
ATOM	1541	HG12	ILE	97	31.901	31.442	40.868	1.00	0.00	H
ATOM	1542	HG13	ILE	97	32.132	31.417	42.603	1.00	0.00	H
ATOM	1543	CD1	ILE	97	31.207	33.157	41.840	1.00	0.00	C
ATOM	1544	HD11	ILE	97	30.233	32.797	42.174	1.00	0.00	H
ATOM	1545	HD12	ILE	97	31.509	33.843	42.631	1.00	0.00	H
ATOM	1546	HD13	ILE	97	31.126	33.633	40.863	1.00	0.00	H
ATOM	1547	C	ILE	97	35.739	33.742	42.765	1.00	0.00	C
ATOM	1548	O	ILE	97	35.622	34.908	42.405	1.00	0.00	O
ATOM	1549	N	GLU	98	36.931	33.152	42.750	1.00	0.00	N
ATOM	1550	H	GLU	98	37.023	32.248	43.188	1.00	0.00	H
ATOM	1551	CA	GLU	98	38.239	33.747	42.302	1.00	0.00	C
ATOM	1552	HA	GLU	98	38.137	34.662	41.717	1.00	0.00	H
ATOM	1553	CB	GLU	98	39.055	34.125	43.566	1.00	0.00	C
ATOM	1554	HB2	GLU	98	38.497	34.977	43.954	1.00	0.00	H
ATOM	1555	HB3	GLU	98	39.019	33.314	44.293	1.00	0.00	H
ATOM	1556	CG	GLU	98	40.491	34.594	43.351	1.00	0.00	C
ATOM	1557	HG2	GLU	98	41.038	33.828	42.801	1.00	0.00	H
ATOM	1558	HG3	GLU	98	40.433	35.453	42.683	1.00	0.00	H
ATOM	1559	CD	GLU	98	41.273	34.956	44.627	1.00	0.00	C
ATOM	1560	OE1	GLU	98	41.816	36.082	44.740	1.00	0.00	O
ATOM	1561	OE2	GLU	98	41.368	34.093	45.488	1.00	0.00	O
ATOM	1562	C	GLU	98	38.987	32.826	41.334	1.00	0.00	C
ATOM	1563	O	GLU	98	39.025	31.610	41.525	1.00	0.00	O
ATOM	1564	N	PHE	99	39.420	33.359	40.113	1.00	0.00	N
ATOM	1565	H	PHE	99	39.179	34.338	40.065	1.00	0.00	H
ATOM	1566	CA	PHE	99	39.728	32.510	38.989	1.00	0.00	C
ATOM	1567	HA	PHE	99	40.038	31.507	39.285	1.00	0.00	H
ATOM	1568	CB	PHE	99	38.357	32.297	38.290	1.00	0.00	C
ATOM	1569	HB2	PHE	99	38.533	31.803	37.333	1.00	0.00	H
ATOM	1570	HB3	PHE	99	37.896	31.502	38.878	1.00	0.00	H
ATOM	1571	CG	PHE	99	37.370	33.514	38.051	1.00	0.00	C
ATOM	1572	CD1	PHE	99	37.471	34.253	36.886	1.00	0.00	C
ATOM	1573	HD1	PHE	99	38.166	33.813	36.186	1.00	0.00	H
ATOM	1574	CE1	PHE	99	36.649	35.359	36.606	1.00	0.00	C
ATOM	1575	HE1	PHE	99	36.686	35.912	35.679	1.00	0.00	H
ATOM	1576	CZ	PHE	99	35.735	35.771	37.532	1.00	0.00	C
ATOM	1577	HZ	PHE	99	34.997	36.551	37.408	1.00	0.00	H
ATOM	1578	CE2	PHE	99	35.608	35.128	38.764	1.00	0.00	C
ATOM	1579	HE2	PHE	99	34.923	35.552	39.483	1.00	0.00	H
ATOM	1580	CD2	PHE	99	36.452	34.018	39.051	1.00	0.00	C

```
ATOM   1581  HD2 PHE    99      36.413  33.566  40.031  1.00  0.00           H
ATOM   1582  C   PHE    99      40.770  32.894  37.984  1.00  0.00           C
ATOM   1583  O   PHE    99      40.985  34.124  37.918  1.00  0.00           O
ATOM   1584  N   LYS   100      41.497  32.023  37.298  1.00  0.00           N
ATOM   1585  H   LYS   100      41.478  31.069  37.628  1.00  0.00           H
ATOM   1586  CA  LYS   100      42.549  32.390  36.395  1.00  0.00           C
ATOM   1587  HA  LYS   100      42.429  33.436  36.114  1.00  0.00           H
ATOM   1588  CB  LYS   100      43.903  32.150  37.145  1.00  0.00           C
ATOM   1589  HB2 LYS   100      43.970  31.102  37.440  1.00  0.00           H
ATOM   1590  HB3 LYS   100      44.697  32.534  36.505  1.00  0.00           H
ATOM   1591  CG  LYS   100      43.844  32.862  38.515  1.00  0.00           C
ATOM   1592  HG2 LYS   100      43.863  33.939  38.353  1.00  0.00           H
ATOM   1593  HG3 LYS   100      43.102  32.461  39.206  1.00  0.00           H
ATOM   1594  CD  LYS
   100      45.149  32.642  39.223  1.00  0.00           C
ATOM   1595  HD2 LYS   100      45.061  31.672  39.714  1.00  0.00           H
ATOM   1596  HD3 LYS   100      45.963  32.703  38.501  1.00  0.00           H
ATOM   1597  CE  LYS   100      45.273  33.755  40.259  1.00  0.00           C
ATOM   1598  HE2 LYS   100      46.251  33.542  40.691  1.00  0.00           H
ATOM   1599  HE3 LYS   100      45.318  34.778  39.883  1.00  0.00           H
ATOM   1600  NZ  LYS   100      44.229  33.544  41.342  1.00  0.00           N
ATOM   1601  HZ1 LYS   100      43.272  33.740  41.085  1.00  0.00           H
ATOM   1602  HZ2 LYS   100      44.348  32.630  41.757  1.00  0.00           H
ATOM   1603  HZ3 LYS   100      44.319  34.237  42.071  1.00  0.00           H
ATOM   1604  C   LYS   100      42.617  31.537  35.076  1.00  0.00           C
ATOM   1605  O   LYS   100      42.682  30.297  35.112  1.00  0.00           O
ATOM   1606  N   CYX   101      42.674  32.347  33.929  1.00  0.00           N
ATOM   1607  H   CYX   101      42.537  33.344  34.015  1.00  0.00           H
ATOM   1608  CA  CYX   101      43.089  31.760  32.633  1.00  0.00           C
ATOM   1609  HA  CYX   101      42.420  30.915  32.479  1.00  0.00           H
ATOM   1610  CB  CYX   101      42.905  32.669  31.394  1.00  0.00           C
ATOM   1611  HB2 CYX   101      41.855  32.963  31.378  1.00  0.00           H
ATOM   1612  HB3 CYX   101      43.470  33.569  31.634  1.00  0.00           H
ATOM   1613  SG  CYX   101      43.432  31.996  29.804  1.00  0.00           S
ATOM   1614  C   CYX   101      44.527  31.077  32.621  1.00  0.00           C
ATOM   1615  O   CYX   101      45.402  31.547  33.402  1.00  0.00           O
ATOM   1616  N   LEU   102      44.715  29.901  31.987  1.00  0.00           N
ATOM   1617  H   LEU   102      43.861  29.523  31.602  1.00  0.00           H
ATOM   1618  CA  LEU   102      45.867  29.006  32.017  1.00  0.00           C
ATOM   1619  HA  LEU   102      46.547  29.251  32.834  1.00  0.00           H
ATOM   1620  CB  LEU   102      45.449  27.538  32.270  1.00  0.00           C
ATOM   1621  HB2 LEU   102      45.000  27.131  31.364  1.00  0.00           H
ATOM   1622  HB3 LEU   102      46.307  26.957  32.611  1.00  0.00           H
ATOM   1623  CG  LEU   102      44.441  27.233  33.344  1.00  0.00           C
ATOM   1624  HG  LEU   102      43.459  27.684  33.204  1.00  0.00           H
ATOM   1625  CD1 LEU   102      44.162  25.741  33.459  1.00  0.00           C
ATOM   1626 HD11 LEU   102      43.924  25.294  32.493  1.00  0.00           H
ATOM   1627 HD12 LEU   102      45.050  25.252  33.859  1.00  0.00           H
ATOM   1628 HD13 LEU   102      43.247  25.674  34.047  1.00  0.00           H
ATOM   1629  CD2 LEU   102      44.873  27.806  34.691  1.00  0.00           C
ATOM   1630 HD21 LEU   102      45.812  27.434  35.100  1.00  0.00           H
ATOM   1631 HD22 LEU   102      44.866  28.869  34.452  1.00  0.00           H
ATOM   1632 HD23 LEU   102      44.017  27.554  35.316  1.00  0.00           H
ATOM   1633  C   LEU   102      46.745  29.276  30.863  1.00  0.00           C
ATOM   1634  O   LEU   102      47.883  29.710  30.938  1.00  0.00           O
ATOM   1635  OXT LEU   102      46.288  28.880  29.711  1.00  0.00           O
HETATM 1637  N   LIG   103      29.885  40.877  38.352  1.00  0.00           N
HETATM 1638  C   LIG   103      29.805  39.610  38.247  1.00  0.00           C
HETATM 1639  O   LIG   103      29.439  42.845  39.388  1.00  0.00           O
HETATM 1640  C5' LIG   103      27.014  40.794  44.753  1.00  0.00           C
HETATM 1641  O5' LIG   103      26.573  39.565  44.134  1.00  0.00           O
HETATM 1642  C4' LIG   103      27.201  41.733  43.552  1.00  0.00           C
HETATM 1643  O4' LIG   103      28.017  41.146  42.594  1.00  0.00           O
HETATM 1644  C3' LIG   103      25.935  42.169  42.810  1.00  0.00           C
```

```
HETATM 1645  O3' LIG   103       25.613  43.520  43.145  1.00  0.00           O
HETATM 1646  C2' LIG   103       26.247  42.001  41.294  1.00  0.00           C
HETATM 1647  O2' LIG   103       25.917  43.167  40.562  1.00  0.00           O
HETATM 1648  C1' LIG   103       27.744  41.726  41.316  1.00  0.00           C
HETATM 1649  N1  LIG   103       28.385  40.988  40.156  1.00  0.00           N
HETATM 1650  O1  LIG   103       29.628  36.903  37.149  1.00  0.00           O
HETATM 1651  N2  LIG   103       30.603  38.932  37.322  1.00  0.00           N
HETATM 1652  C6  LIG   103       29.223  41.623  39.327  1.00  0.00           C
HETATM 1653  C7  LIG   103       28.168  39.690  40.005  1.00  0.00           C
HETATM 1654  C8  LIG   103       28.819  38.956  39.073  1.00  0.00           C
HETATM 1655  C9  LIG   103       30.523  37.680  36.865  1.00  0.00           C
HETATM 1656  C10 LIG   103       31.704  37.206  36.014  1.00  0.00           C
HETATM 1657  H   LIG   103       31.314  39.507  36.886  1.00  0.00           H
HETATM 1658  H1  LIG   103       28.641  37.889  39.024  1.00  0.00           H
HETATM 1659  H4  LIG   103       28.331  42.646  41.407  1.00  0.00           H
HETATM 1660  H6  LIG   103       27.534  42.725  43.884  1.00  0.00           H
HETATM 1661  H7  LIG   103       25.100  41.515  43.089  1.00  0.00           H
HETATM 1662  H8  LIG   103       25.787  41.091  40.890  1.00  0.00           H
HETATM 1663  H9  LIG   103       26.155  41.093  45.365  1.00  0.00           H
HETATM 1664  H10 LIG   103       27.921  40.671  45.356  1.00  0.00           H
HETATM 1665  H11 LIG   103       31.329  36.607  35.175  1.00  0.00           H
HETATM 1666  H12 LIG   103       32.419  36.616  36.602  1.00  0.00           H
HETATM 1667  H13 LIG   103       32.301  38.006  35.560  1.00  0.00           H
HETATM 1668  H14 LIG   103       27.435  39.357  40.727  1.00  0.00           H
HETATM 1669  H2' LIG   103       26.130  43.063  39.619  1.00  0.00           H
HETATM 1670  H3' LIG   103       25.351  43.970  42.323  1.00  0.00           H
HETATM 1671  H5' LIG   103       27.368  39.368  43.609  1.00  0.00           H
ENDMDL
```
